# Splicing-related genes are alternatively spliced upon changes in ambient temperatures in plants

**DOI:** 10.1371/journal.pone.0172950

**Published:** 2017-03-03

**Authors:** Leonie Verhage, Edouard I. Severing, Johan Bucher, Michiel Lammers, Jacqueline Busscher-Lange, Guusje Bonnema, Nicole Rodenburg, Marcel C. G. Proveniers, Gerco C. Angenent, Richard G. H. Immink

**Affiliations:** 1 Laboratory of Molecular Biology, Wageningen University and Research, PB Wageningen, The Netherlands; 2 Bioscience, Wageningen Plant Research, Wageningen University and Research, PB Wageningen, The Netherlands; 3 Max Planck Institute for Plant Breeding Research, Köln, Germany; 4 Wageningen UR Plant Breeding, Wageningen University and Research, PB Wageningen, The Netherlands; 5 Molecular Plant Physiology, Institute of Environmental Biology, Utrecht University, CH Utrecht, The Netherlands; Instituto de Biologia Molecular y Celular de Plantas, SPAIN

## Abstract

Plants adjust their development and architecture to small variations in ambient temperature. In a time in which temperatures are rising world-wide, the mechanism by which plants are able to sense temperature fluctuations and adapt to it, is becoming of special interest. By performing RNA-sequencing on two Arabidopsis accession and one Brassica species exposed to temperature alterations, we showed that alternative splicing is an important mechanism in ambient temperature sensing and adaptation. We found that amongst the differentially alternatively spliced genes, splicing related genes are enriched, suggesting that the splicing machinery itself is targeted for alternative splicing when temperature changes. Moreover, we showed that many different components of the splicing machinery are targeted for ambient temperature regulated alternative splicing. Mutant analysis of a splicing related gene that was differentially spliced in two of the genotypes showed an altered flowering time response to different temperatures. We propose a two-step mechanism where temperature directly influences alternative splicing of the splicing machinery genes, followed by a second step where the altered splicing machinery affects splicing of downstream genes involved in the adaptation to altered temperatures.

## Introduction

As a consequence of a sessile lifestyle, plants are continuously facing fluctuating environmental conditions. In order to both benefit maximally and to protect them from the environment, plants evolved ways to sense and respond to many environmental cues.

Ambient temperature is one of these signals that plants sense and adapt to in order to enhance their chance of survival and reproduction. Small changes in ambient temperature can have major effects on plant architecture and development [1]. One of these adaptations is the moment of flowering, which is an important event in the life cycle of a plant, since reproductive success depends on it. For the widely-used model plant *Arabidopsis thaliana* Col-0, it is known that it flowers earlier when the ambient temperature is higher [2].

The mechanism of how a plant senses temperature and how this affects its phenotype are hardly understood. It has been suggested that alternative splicing (AS)—the phenomenon that one gene produces more than one form of messenger RNA (mRNA)—plays an important role in temperature sensing, since environmental changes trigger differential AS [3–6]. Moreover, various studies showed the impact of environmentally induced AS. For example, genes encoding the components of the circadian clock are prone to AS upon temperature fluctuations. One of them, *CIRCADIAN CLOCK ASSOCIATED 1* (*CCA1*), is besides temperature responsive, also alternatively spliced upon high light intensity [3] and is proposed to regulate the period of the clock through this mechanism [7]. Another example is *FLOWERING LOCUS M (FLM)*, which produces different ratios of splicing variants upon shifting ambient temperature, and in this way regulates floral timing [2, 8, 9].

**Fig 1:**
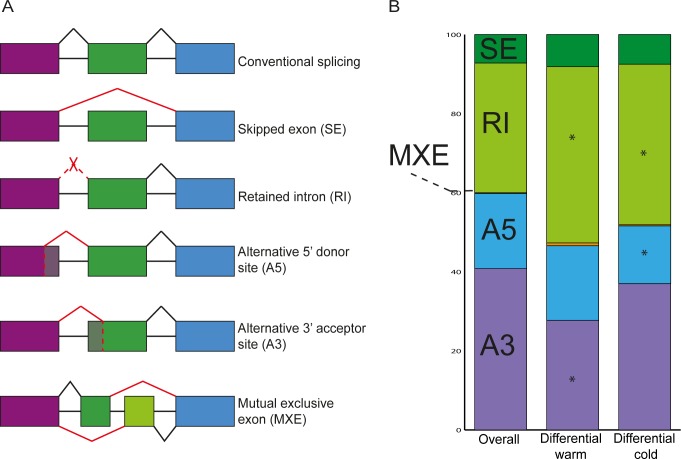
Splicing events. **(A)** Overview of splicing events that can occur. Alternative events are depicted in red, conventional events are depicted in black. Boxes represent exons, lines represent introns. **(B)** Distribution of the differential splicing events upon shifts to higher or lower ambient temperature, compared to the events in the total dataset. The asterisk * indicates a significant difference of the abundance of the event compared to the overall abundance (Using Pearson’s Chi-square test, for data used for significance test, see S2 Table).

An intron-containing gene can potentially produce several to numerous different splice forms, depending on the intron-exon structure. This is achieved by combining conventional splicing, in which all introns are spliced out at the exon-intron border, with alternative selection of splice sites, leading to retention of introns (RI), skipping or mutual exclusion of exons (SE or MXE), or alternative splice site selection at the 5’ or 3’ end (A5 or A3), rendering different transcripts (as reviewed by [10]) (Fig 1A). The driving force behind gene splicing is the spliceosome, a large cellular machinery that catalyses the splicing reaction. The spliceosome is a ribonucleoprotein complex with a highly dynamic structure that assembles de novo at each splice site in a stepwise manner. Many different proteins and riboproteins orchestrate splice-site selection, exon and intron definition, two catalytic splicing steps, and extensive quality control (as reviewed by [11, 12]). The spliceosome recognizes splicing signals located at exon-intron boundaries, but also numerous *cis*-regulatory sequences that act as splicing enhancers or silencers (as reviewed by [13]).

As mentioned above, numerous studies report differential AS upon environmental changes, at gene-specific and transcriptome-wide levels. Although small-scale studies already showed that slight changes in temperature can have a significant effect on AS [14], transcriptome-wide studies in relation to AS have mainly focussed on extreme stresses, such as high light intensity, salt, dehydration, cold and heat [3–5], and much less on ambient conditions like ambient temperature. Moreover, these studies only used a single accession of the model species *Arabidopsis thaliana*, and therefore do not provide information about the conservation of the response or the underlying mechanism. In this study we analysed ambient temperature-directed AS in two accessions of *A*. *thaliana* and in one genotype of *Brassica oleracea* ssp. *botrytis*. We show that splicing related genes are overrepresented amongst the genes that exhibit a different splicing pattern upon ambient temperature changes. Because many different classes of splicing related genes were affected, it suggests that the whole spliceosome is sensitive to ambient temperature fluctuations. Furthermore, when comparing the three different genotypes we used in this study, we demonstrated that although we could not find proof for conservation of the temperature-dependent splicing response at the single gene level, splicing related genes are prominent amongst the conserved cases. Analysis of a mutant of a splicing-related gene in *A*. *thaliana* Col-0, for which alternative splicing was observed in this accession and for its orthologous gene in cauliflower, showed an altered flowering time response under different ambient temperatures. This suggests that AS of splicing-related genes functions as a key molecular mechanism in the plant’s temperature response.

## Results

### The effect of ambient temperature on alternative splicing

We performed ambient temperature shifts on *Arabidopsis thaliana* Col-0 towards both higher and lower temperatures (23°C to 27°C and 23°C to 16°C, respectively) and harvested temperature treated plants together with the non-treated control plants 24 hours after the shift. We used paired-end Illumina HiSeq RNA sequencing and reads were mapped onto the Arabidopsis genome (The Arabidopsis Information Resource 10 (TAIR10)). Detection of AS was performed for each splicing event, rather than whole isoform level, in order to facilitate a better quantitation analysis. The TAIR10 transcriptome was used for annotation-guided splice site detection, complemented with *de novo* assembly since the TAIR10 transcriptome is not exhaustive. To validate the generated data and bioinformatics analysis, qRT-PCR was performed on a selection of splicing events. By normalizing each tested event to an internal control, a region in the mRNA that is present in all transcripts of the respective gene, we were able to show that both the prediction of splicing events as well as the quantification of these events under different ambient temperatures was highly accurate (S1 Fig).

Comparing the overall distribution of splicing events with the distribution of events that were differential upon a shift to high or low ambient temperature, we noticed some changes (Fig 1B). The high temperature shift induced significantly less A3 events whereas low temperature induced significantly less A5 events. Moreover, both temperature shifts induce more RI events.

**Fig 2:**
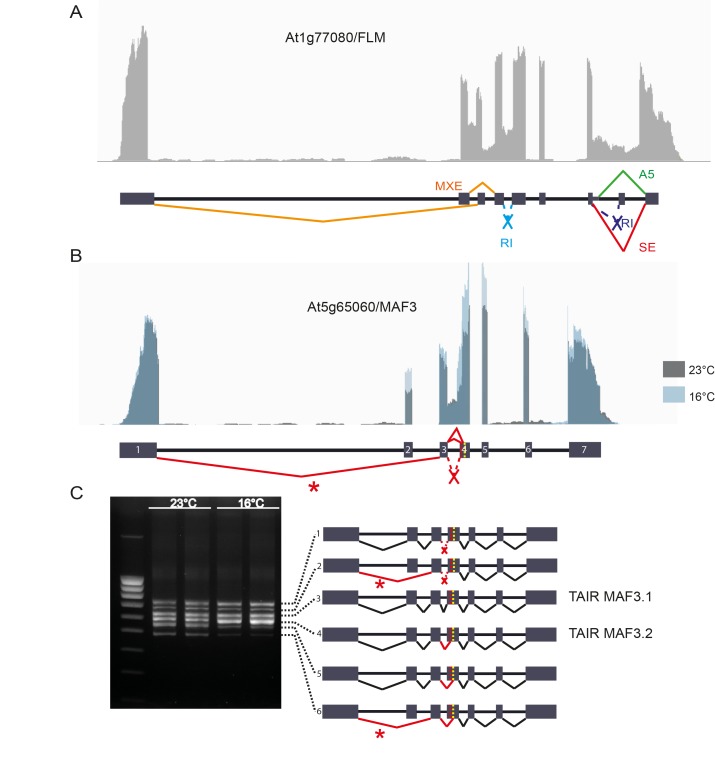
Alternative splicing in *FLM* and *MAF3*. **(A)** Alternative splicing events in *FLM*. Top: raw reads in Integrative Genomics Viewer (IGV). Bottom: intron/exon structure of *FLM* with all detected splicing events. **(B)** Alternative splicing response of *MAF3* upon low ambient temperature in the IGV browser. Up: raw RNAseq reads of *MAF3* visualised in the IGV browser and the intron/exon structure of *MAF3* with all alternative splicing events. In grey the reads of the control temperature of 23°C, in blue the reads of the 16°C temperature treatment. The skipping of exon 2 is differential between the two temperatures, indicated by the asterisk (*). **(C)** RT-PCR on the same samples with *MAF3* primers at the start and stop codons and loaded on a 2% agarose gel. On the left the control temperature of 23° C, on the right the 16° C temperature treatment, each temperature with two biological replicates. The four different splicing events result in six different splice forms, as depicted next to the gel and was confirmed by Sanger sequencing (S2 Fig). Note that isoforms 3 and 4 are the only splice forms annotated in TAIR10. (N = 2 (16 plants per sample).

In addition to identifying splicing events, we determined the relative abundance of splicing events for each alternatively spliced gene, and compared this abundance between the control and temperature-treated plants. We observed differential splicing of around 140 and 290 genes (S3, S4 and S5 Tables) upon the shift to higher or lower ambient temperature, respectively. 40 splicing events showed a reciprocal splicing patterns in the higher versus the lower ambient temperature, i.e. becoming more abundant upon the higher temperature treatment and less abundant upon the shift to lower temperature or vice versa (S3 Table). When comparing our results to previous small-scale studies, we could reproduce several described cases, supporting the quality of our dataset. For example, we observed differential alternative splicing of *PSEUDO-REPONSE REGULATOR 7 (PRR7)* and *CIRCADIAN CLOCK ASSOCIATED 1 (CCA1)*, two genes that have been shown to mediate the response of the circadian clock to cold treatment (4°C) through AS [3, 15]. Also *FLOWERING LOCUS M (FLM)*, a flowering time gene for which ambient temperature dependent AS was discovered [2, 8], displayed a similar response in our transcriptome-wide data set. Like for many genes, we could identify several different AS events in *FLM* (Fig 2A). The two most abundant splice forms of *FLM* that regulate flowering time, *FLMβ* and *FLMδ*, distinguish from each other by a mutually exclusive exon (MXE) event. We found this event to be differential in both the high and low ambient temperature and in a reciprocal fashion. This confirms the data from the original paper describing temperature-dependent regulation of flowering by antagonistic FLM variants [8], Also two genes closely related to *FLM*, *MADS AFFECTING FLOWERING 2* (*MAF2*) and *MAF3*, were found to be differentially spliced upon ambient temperature changes in our dataset. *MAF2* has been shown to regulate flowering time by ambient temperature-directed AS in a slightly different manner than *FLM* [16, 17]. Here we show that also *MAF3* is differentially spliced upon ambient temperature changes (Fig 2B). *MAF3* has been shown to act as a regulator of flowering time as well [18], and our findings suggest that this occurs through temperature-directed alternative splicing comparable to *FLM* and *MAF2*. *MAF3* undergoes several splicing events, but only one event, the skipping of exon 2, shows temperature sensitivity in our dataset. Fig 2B displays *MAF3* sequencing reads at 23°C and 16°C, showing the difference in exon 2 abundance between the two temperatures. When we performed RT-PCR on *MAF3* on the same RNA samples, we could detect six different splice forms of which two isoforms undergo the differential skipped exon (SE) event (Fig 2C and S2 Fig)). Interestingly, from this semi-quantitative gel it becomes clear that the two isoforms undergoing the SE event (isoform 2 and 6) do not respond to temperature in a similar fashion: the intensity of the fragment representing isoform 2 shows hardly any difference upon the temperature shift, whereas the fragment representing isoform 6 is clearly reduced. This suggests that temperature influences the abundance of splicing events in the context of the whole transcript isoform, and that this isoform and/or other events of this isoform influence the final splicing outcome, at least for *MAF3*.

### Splicing-related genes are overrepresented

**Fig 3:**
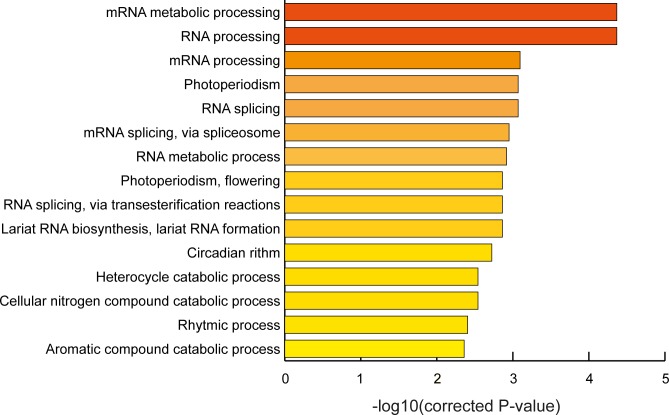
GO-term enrichment of differentially spliced Arabidopsis Col-0 genes upon ambient temperature treatment. GO terms with a corrected P-value < 0.005.–log10(corrected P-value) is shown for visualisation purposes.

To get insight into the biological processes targeted by ambient temperature directed alternative splicing, we performed a gene ontology (GO) analysis on all genes that show differential splicing after lower or higher ambient temperature treatment. We tested for overrepresentation of biological functions amongst all intron-containing genes that were expressed in our dataset (≥ 1 read) and corrected for multiple testing. The results showed clear enrichment of GO terms describing genes involved in the circadian clock (corrected P<0.005) (Fig 3, S3 Table), which confirms earlier reports on AS of clock genes by temperature fluctuations [7, 15]. Related to this, we found enrichment of the GO term “Photoperiodism, flowering”, the term that describes flowering time genes that react to changes in photoperiodism. Because alteration of flowering time is one of the responses of the plant to changes in ambient temperature, it is an interesting finding that this group of flowering time genes are highly affected in splicing pattern upon ambient temperature shifts. For the flowering time gene *FLM* it was already shown that changes in splicing pattern enables the plant to modify flowering time in response to ambient temperature shifts [8], but our results suggest that AS occurs for numerous flowering time genes and hence is a more general mechanism of the plant to regulate flowering time under different ambient temperatures.

Surprisingly, the GO term analysis also revealed a strong enrichment for several categories representing RNA processing and splicing-related genes (corrected P<0.005) (Fig 3, S3 Table). This implies that shifts in ambient temperature change the splicing pattern of splicing-related genes, the genes that encode the spliceosome and its accessory proteins. Although this might at first appear as an unexpected outcome, splicing of splicing-related genes can potentially influence the composition of the spliceosome, and subsequently alter the splicing of many downstream targets, like genes that function in the temperature response of the plant. To get a better insight into the differentially spliced splicing-related genes in our dataset, we made use of the Arabidopsis Splicing Related Gene Database, which contains a comprehensive list of almost 400 splicing-related protein-coding genes [19]. Comparison with our dataset showed that about 8.5% of the differentially spliced genes could be classified as splicing related genes (S3 Table). Chi-square testing showed that this is a highly significant enrichment (P<0.01, taking into account all intron containing genes that showed expression in our dataset). Analysis of the individual cool and warm datasets revealed that splicing-related genes represent 8.3% and 10% of the total differentially spliced genes respectively, showing that both lower and higher ambient temperature shifts induce differential splicing of splicing-related genes to the same extent, suggesting an important role for these genes in temperature sensing.

### The spliceosome is target of ambient temperature-induced alternative splicing

**Fig 4:**
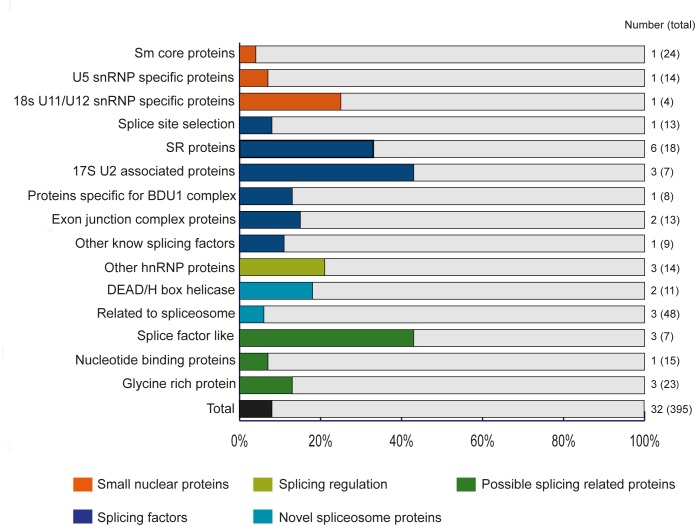
Classes of splicing related Arabidopsis Col-0 genes that show differential splicing of at least one of the class members. Bars represent percentage of genes that is differentially spliced in each class. Total numbers of differentially spliced genes and all genes in each class (between brackets) are given at the end of each bar.

The spliceosome is a large cellular machinery, comprising many different factors. Therefore, we categorized the differentially spliced genes according to the classification of the Splicing Related Gene Database [19]. As it turns out, many classes of splicing related genes were represented in our dataset (Fig 4, S3 Table). For example, three out of the seven annotated 17S U2 associated protein coding genes, six out of 18 serine/arginine-rich proteins (SR proteins) and two out of 11 DEAD/H box helicases are differentially spliced in our dataset. Thus, many genes involved in the splicing process seem to be targeted for ambient temperature induced splicing.

### Alternative splicing of splicing-related genes in other genetic backgrounds

To get a better idea about the importance and the level of conservation of our finding that many splicing-related genes are differentially spliced upon ambient temperature treatment, we performed temperature shift experiments followed by RNAseq on another Arabidopsis accession (*A*. *thaliana* Gy-0) and on *Brassica oleracea* var. *botrytis* (cauliflower). *A*. *thaliana* Col-0 is known to be sensitive for ambient temperature shifts and responds by changing its architecture and developmental program, amongst which adapting floral timing. Col-0 originates from Columbia, Missouri, in the mid-west of the USA, which is a region with sharp seasonal temperature differences. On the other hand, Gy-0 originates from a region in northern central France, in which the climate can be classified as milder, with a narrower annual range of temperatures. However, for the Gy-0 accession of *A*. *thaliana*, nothing was known about the sensitivity for ambient temperature fluctuations. Therefore, before performing RNAseq on temperature-treated plants, we first performed an analysis on the effect of different temperatures in this accession, and it turned out that Gy-0 responded to a higher ambient temperature by later flowering (S3 Fig), but reacted similar to Col-0 regarding hypocotyl- and petiole elongation and leaf hyponasty. For *B*. *oleracea*, an early study showed that it also reacts to higher ambient temperature by adjusting flowering time, in a similar way as the *A*. *thaliana* Gy-0 accession, flowering earlier at lower temperature [20].

Since both cool and warm treatments showed a significant effect on AS of splicing-related genes in *A*. *thaliana* Col-0, we decided to focus only on high temperature treatment in these two other genetic backgrounds. In the Gy-0 accession, we detected differential splicing of ~100 genes upon a shift to higher ambient temperature, which is about the same number as in the Col-0 accession (S3 and S6 Tables). Strikingly, comparing Gy-0 with Col-0 revealed that only 10.9% of the genes that are differentially spliced in the Col-0 accession are also differentially alternatively spliced in Gy-0. Apparently, the ambient temperature directed AS response is not very conserved, not even between these two accessions. Nevertheless, two out of the 15 conserved genes, *SR34* and *MEE5*, are listed in the splicing-related gene database, and a third gene, *AtRH3*, is a DEAD-box RNA helicase that has recently been shown to play an important role in intron splicing, but has not been listed in the database as of now [21].

The cauliflower temperature treatment (21°C → 27°C) rendered a comparable number of differentially spliced genes (156 genes, S3 and S7 Tables). However, as could be expected taking the Gy-0 results into account, the number of orthologous genes being differentially spliced upon the temperature shift that overlapped with the *A*. *thaliana* Col-0 accession or Gy-0 accession was even lower, being 2.2% and 1% respectively. Again, from the three genes overlapping between cauliflower and the Col-0 accession, one gene was a splicing-related gene (*SCL30a*). The same is true for the only gene overlapping between the Gy-0 accession and cauliflower (*RS40*).

Moreover, when we assessed all genes that were alternatively spliced in the Gy-0 accession, 11.5% could be classified as splicing-related genes. In cauliflower, this was ~ 9.5% (taking into account all differentially spliced genes for which we could identify an Arabidopsis orthologue). This is very comparable with the 10% in the Col-0 accession treated with high ambient temperature. Thus, despite the low overlap of the temperature-directed splicing response at the individual gene level, the effect on splicing of splicing factors appears to be a conserved mechanism. This indicates that AS of splicing-related genes might be an important process in the temperature response of the plant.

### A role for the differentially spliced splicing-related gene *ATU2AF65A* in thermosensitive floral timing

**Fig 5:**
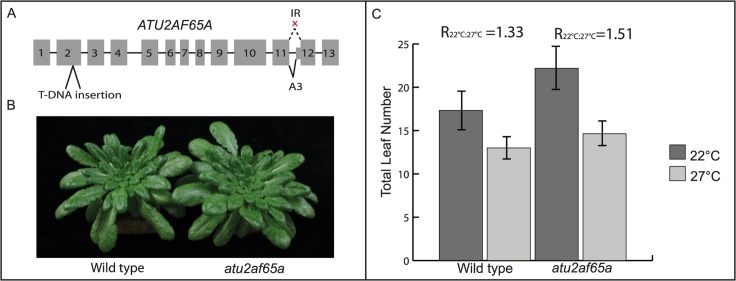
*atu2af65a* mutant has a flowering time phenotype and adapts differently to ambient temperature differences. **(A)** Gene structure of *ATU2AF65A* showing the position of the T-DNA insertion in SALK_144790, used for mutant analysis, and the position of the temperature-sensitive splicing events. **(B)** wild type and *atu2af65a* plants grown at 16°C. Besides the flowering time phenotype shown in panel A, *atu2af65a* has a wild type appearance. **(C)** compared to wild type, *atu2af65a* showed an increased flowering time response upon higher ambient temperature, as calculated by the Total Leaf Number (TLN, including rosette and cauline leaves) at 22°C divided by the TLN at 27°C (presented as the ratio (R)_22°C:27°C_). N = 21 plants (wild type 22°C, 27°C and *atu2af65a* 22°C) and 22 plant (*atu2af65a* 27°C). Error bars represent standard deviations.

An interesting case of a splicing gene that is alternatively spliced in our dataset is *ATU2AF65A*, which encodes a U2 small nuclear ribonucleoprotein (snRNP) auxiliary factor that is part of the spliceosome. We found this gene to be alternatively spliced in *A*. *thaliana* Col-0 upon the shift to lower ambient temperature, whereas in cauliflower we found the orthologous gene to be alternatively spliced upon the higher ambient temperature shift. In *A*. *thaliana* Col-0, the cooler ambient temperature treatment suppresses the production of a transcript that retains the second-last intron of the coding sequence, as well as a transcript that has an alternative 3’ splice site of the second-last exon. In contrast, the warmer ambient temperature treatment in cauliflower shows the exact opposite for the orthologous gene. These outcomes strongly suggest a biological function for the ambient temperature dependent splicing of *ATU2AF65A*. Therefore, an Arabidopsis T-DNA insertion line (SALK_144790) was ordered to test for temperature responsiveness. This line harbours a T-DNA insertion in the second exon, far upstream of the temperature-sensitive splicing events (Fig 5A). *atu2af65a* homozygous plants were grown together with the wild type that segregated from the heterozygous T-DNA insertion line at two different ambient temperatures. Initially, we grew plants under short day at 16°C and 25°C. In the vegetative state, *atu2af65a* plants did not show any morphological difference compared to wild type plants, and the mutant plants showed a similar response to the high ambient temperature regarding petiole and hypocotyl elongation and leaf hyponasty (Fig 5B). However, the *atu2af65a* mutant turned out to be late flowering. Since this complicated the counting of the number of leaves due to decomposition of the leaves, we repeated the experiment under long day, at 22°C and 27°C. By counting Total Leaf Number (TLN, including rosette and cauline leaves) and calculating the ratio of the TLN at 22°C to the TLN at 27°C, we found that at 27°C, wild type plants flowered 1.33 times faster than at 22°C, whereas *atu2af65a* mutant plants showed an acceleration of 1.51 times, showing that the mutant reacts differently on ambient temperature (Fig 5C).

## Discussion

### The splicing machinery as a target for regulation

Our results show that plants that undergo variation in ambient temperature, display changes in AS. GO-enrichment analysis shows that targets of this response are the splicing related genes themselves. In Arabidopsis, almost 400 different splicing related genes have been identified [19], and although the overall structure and dynamics of the spliceosome are relatively well understood, the exact function of all these different factors remains enigmatic. Nevertheless, most of the identified factors can be attributed to different components of the spliceosome. We detected differential splicing in many of these classes. Our study unveiled that, upon small temperature changes, spliceosomal genes are overrepresented amongst the differentially spliced genes, and moreover, that this included many classes of splicing related genes. The relevance of the regulation of the splicing machinery by AS is supported by a recent study on AS in different genotypes of grapevine [22]. The authors showed that there is considerable variability in splicing between different genotypes. When they performed a principle component analysis (PCA) on the ratio’s between the first two highly expressed isoforms in different tissues of the genotypes, the different tissues cluster together with the first two components, but taking into account the third component, the samples further separate on genotype. They speculated that the differences amongst genotypes might be partly caused by differences in the general splicing program, which supports the idea that evolutionary adaptation might be achieved by modifications of the splicing machinery.

### The splicing response in different genotypes

The above mentioned study might also partly explain why we find a very low overlap between the genes that are differentially spliced upon ambient temperature treatment between the different genotypes we have analysed. The Gy-0 accession of Arabidopsis and the cauliflower cultivar showed a high degree of differential splicing in the splicing related genes, although there is hardly overlap between the individual genes. However, we showed that the Gy-0 accession did not respond the same way to different temperatures as the Arabidopsis Col-0 accession for all developmental aspects, since Gy-0 flowered later when grown under higher ambient temperature, whereas Col-0 is known to flower earlier when subjected to higher ambient temperatures. Also cauliflower is known to flower later when ambient temperature are higher [20]. Moreover, the two accessions of Arabidopsis originate from regions with different climate conditions. Col-0 originates from Columbia, Missouri, in the mid-west of the USA, which is a region with sharp seasonal temperature differences. On the other hand, Gy-0 originates from a region in northern central France, in which the climate can be classified as milder, with a narrower annual range of temperatures. Hence, the difference in the differential splicing response between the three genotypes might reflect the different environmental regimes in which they evolve, suggesting that the splicing machinery co-adapts to facilitate optimal survival. Mechanistically, this could in part be due to SNPs in splice sites or splicing regulatory regions, as shown in a small-scale study on splicing in the *A*. *thaliana* C24 and Col-2 ecotypes [23].

### *ATU2AF65A* is involved in ambient temperature directed floral timing

The mutant for *ATU2AF65A*, a gene that showed differential splicing in *A*. *thaliana* Col-0 in our temperature experiments, displayed an altered flowering time response when subjected to different temperatures, compared to wild type. Since this gene did not show any significant differences in expression in our datasets, these outcomes strongly suggest a biological function for the ambient temperature directed splicing of *ATU2AF65A*, although we cannot exclude other mechanisms of regulation as yet. As mentioned above, ATU2AF65A is a U2 small nuclear ribonucleoprotein (snRNP) auxiliary factor, and part of the spliceosome. In mammals, its homologue U2AF^65^ binds to polypyrimidine (Py) tracts to promote the assembly of the spliceosome [24, 25]. Recently it was shown that this protein can promote the exclusion of alternative exons, and regulation of U2AF^65^ leads to differential alternative gene splicing in human cells [26]. For *A*. *thaliana* ATU2AF65A, limited knowledge is available. Structurally, it seems to closely resemble its mammalian counterpart, comprising three RNA recognition motifs. Moreover, like in mammals, the third RNA recognition motif (RRM) is able to interact with (an *A*. *thaliana* homolog of) splicing factor 1 (SF1) [26, 27]. Thus, a similar function in AS of *ATU2AF65A* in Arabidopsis is not unlikely. However, further investigation, like complementation of the mutant with genomic *ATU2AF65A* and the different splice forms, is necessary to get more insight in the function of this gene in the ambient temperature response of the plant.

### Temperature sensing through alternative splicing: A two-step model

**Fig 6:**
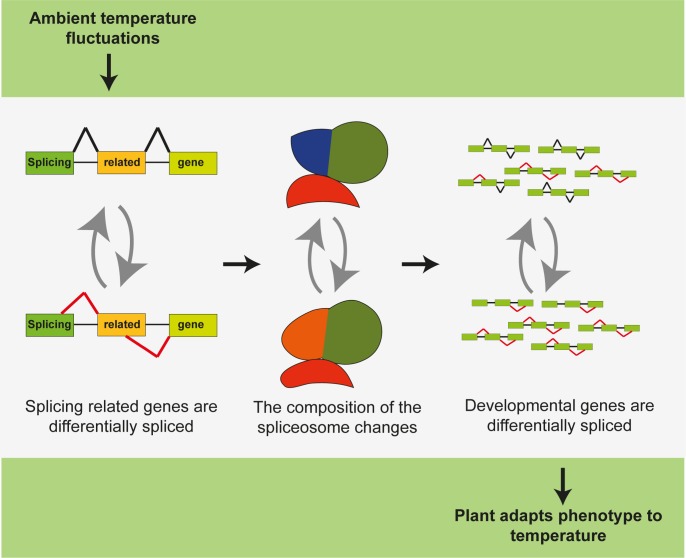
Two-step model showing how ambient temperature may regulate alternative splicing. Upon fluctuations in ambient temperature, splicing related genes are changed in their splicing pattern. This alters the composition of the spliceosome, which in turn affects splicing of many downstream genes.

Because variation in ambient temperatures eventually causes differences in development and architecture of the plant, the question arises how AS of splicing related genes can achieve this. A plausible scenario is that the differential splicing of splicing related genes results in an altered spliceosomal composition. Consequently, this could establish an altered specificity of the spliceosome for splice sites of target genes that control developmental pathways of the plant. Due to this changed specificity, the developmental genes become differentially spliced, which results in a temperature-adapted phenotype (Fig 6). Besides our results on *ATU2AF65A*, our proposition that alternative splicing of splicing related genes leads to differential splicing of downstream genes that control the temperature response of the plant is supported by emerging findings on splicing factors that are essential for certain developmental processes. For example, several studies overexpressing or knocking out SR proteins in Arabidopsis showed a range of developmental and growth phenotypes (as summarized by [28]). Despite the pleiotropic effect of most of these mutations, it seems that distinct functions can be attributed to different splicing factors. For example, SR45 was shown to negatively regulate glucose and abscisic acid signalling during early seed development [29], and ectopic expression of *RSZ33* resulted in cell expansion and a changed polarization of cell elongation and division [30]. Overexpression of a partial cDNA of *SRL1*, a putative splicing factor, conferred increased tolerance to high salt [31]. For *SR45*, it was even shown that two different splicing isoforms have distinct roles during plant development [32]. Moreover, in human cells, different SR proteins are shown to have distinct functions in AS *in vitro*, and display diverse sequence-specific RNA binding abilities, as summarized in [33]. Thus, there is a growing body of evidence that the different splicing factors possess functional specificity, also in plants. An illustrative example is cyclin-dependent kinase G (CDKG1), a novel spliceosomal protein, which encoding gene is differentially spliced in our Col-0 low temperature treatment. Cyclin-dependent protein kinases were originally characterized as cell cycle regulators, but recent data shows that CDKG1 is also involved in splicing [34]. Moreover, the authors showed that it regulates pollen wall formation through alternative splicing of *CALLOSE SYNTHASE5* (*CalS5*). In addition, another publication showed that CDKG1 is essential for synapsis and male meiosis at high ambient temperature. How ambient temperature is able to direct CDKG1 functioning has not been uncovered yet, but the fact that we find *CDKG1* differentially spliced upon ambient temperature variations, it is tempting to speculate that AS is the mode of action by which CDKG1 confers the ability for synapsis and male meiosis at higher ambient temperatures. Nevertheless, deeper investigation like mutant analysis and splice-form specific complementation is necessary to prove this hypothesis.

In conclusion, we have shown that ambient temperature fluctuations have a pivotal effect on gene splicing and based on our detailed analyses, we propose that ambient temperature perception through alternative splicing works at least partially as a two-step model: first the splicing genes, such as *ATU2AF65A*, are changed in their splicing pattern, followed by AS of downstream genes (Fig 6).

## Materials and methods

### Plant material and growth conditions

For RNAseq experiments, *Arabidopsis thaliana* Col-0 and Gy-0 plants were sown on rockwool blocks and stratified for 2–3 days at 4°C and subsequently transferred to 23°C SD conditions in controlled climate cabinets for 3 (in case of the Col-0 and Gy-0 high temperature experiment) or 6 (in case of the Col-0 low temperature experiment) weeks. Plants in vegetative state were transferred to 27°C (Col-0 and Gy-0) or 16°C (Col-0) for 24 hours, after which all above-ground tissue of both control (23°C) and temperature-treated seedlings were harvested and flash-frozen in liquid nitrogen. Each sample contained 18 plants and two biological replicates were collected for each sample.

*Brassica oleracea* var. *botrytis* “Lindurian” F1-hybrids were sown on sowing soil in sowing boxes, and transferred to the greenhouse under long day conditions (day: 16 hours at 21°C/night: 8 hours at 16°C). After two weeks, seedlings were transferred to bigger pots. After five weeks, half of the plants were transferred to higher ambient temperature (day/night: 27°C /22°C) and after 24 hours, meristem-enriched tissue was harvested. Each sample contained tissue from five plants and two biological replicates were collected for each sample.

SALK_144790 (segregating) was ordered from NASC. Seeds were sown and zygosity was tested by PCR (for primer sequences, see S1 Table). RT-PCR was used to test whether the T-DNA insert is indeed present and abolishing expression of the wild type transcript, which turned out to be the case. The T-DNA insert is in the exon coding for the RS domain of the protein, which, in the mammalian orthologue U265, is necessary for interaction with the so-called Prp19 complex, and this interaction is required for RNA polymerase II-coupled splicing activation [35]. A homozygous mutant line and a WT line were selected from the segregating population for seed production.

For flowering time analysis of Gy-0 (CS76139, ecotype id HapMap: 8214) and *atu2af65a*, seeds were sown on soil, cold-imbibed for 4 days at 4°C and then transferred to LD conditions (16h light, 8h dark) at 22°C or 27°C (n = 10 per treatment). Light conditions were standard white light (Sylvania, Luxline Plus, Cool white; 150 μmol m^-2^ s^-1^). We determined flowering time by counting the number of rosette leaves produced at the moment the plants started to flower.

For *atu2af65a* morphology analysis, SALK_144790 seeds were sown on rockwool, cold-imbibed for 2 days at 4°C and then transferred to SD conditions (8h light, 16 h dark) under LED lights. Seedlings were grown for 5 weeks at 16°C, after which half of the plants were transferred to 25°C.

### RNA extraction

For *A*. *thaliana*, samples were ground in liquid nitrogen, and total RNA was isolated from seedlings using the InviTrap Spin Plant RNA Mini Kit (Catalog No. 1064100300, Stratec Molecular) and treated with TURBO DNA-*free*™ Kit (Catalog No. AM1907, Ambion) in solution/DNAse I on column (Catalog No. 18068–015, Invitrogen) to remove DNA contaminations.

For cauliflower, RNA was isolated using the Qiagen RNeasy Mini Kit (catalog No. 74104, Qiagen) and treated with DNAse I (Catalog No. 18068–015, Invitrogen) to remove DNA contaminations. Samples were consequently purified with the above-mentioned Qiagen Mini Kit.

### RNA sequencing

Paired-end libraries were constructed using the Illumina Paired-End DNA Sample Kit (Catalog No. PE-102-1001, PE-102-1002, Illumina) according to the manufacturer’s protocol (in case of the Col-0 cool treatment, Gy-0 and cauliflower) or the modified Fasteris protocol (Fasteris SA) (in case of the Col-0 warm treatment) and paired-end 100 bp reads were generated using Illumina HiSeq 2000. All data were submitted to the National Centre for Biology Institute SRA database (BioProject PRJNA328771).

### RNAseq analysis

RNA-seq reads of Arabidopsis Col-0 and Gy-0 plants were mapped against the *Arabidopsis thaliana* genome version TAIR10 (www.arabidopsis.org) using TopHat [36]. *B*. *oleracea* reads were aligned against the *B*. *oleracea* genome version 1.0 (www.ocri-genomics.org/bolbase/index.html). Reference based full-length transcript isoform reconstruction was performed for each sample separately using Cufflinks [37]. Cuffmerge, which is part of the cufflinks package, was finally used for merging the individual cufflinks results into an overall set of full-length transcripts. For prediction of homologous *A*. *thaliana* genes in *B*. *oleracea*, the predicted proteins from *A*. *thaliana* were searched against the predicted proteome of *B*.*oleracaea* using blastp [38]. Blast alignments were discarded if the aligned sequence fragments were less than 40% identical. All alignments that included less than 75% of the *B*.*oleracaea* protein were also removed.

Custom python scripts were used for detecting the following type of alternative splicing events (Fig 1): retained intron (IR), skipped exon (ES), alternative 5’donor site (A5), alternative 3’ acceptor site (A3) and mutually exclusive exons (MXE). MISO [39] was used to quantify AS-events (PSI values) in each individual sample and in pooled samples that were generated by merging the replicates of each condition.

MISO, which was also used for the differential splicing analyses, does not have built-in methods for analysing experimental replicates. As suggested by the authors of MISO, we used the pooled samples for the actual differential splicing test and the individual replicates for filtering AS events based on the following 2 rules: 1. Only those AS events supported by at least 20 isoform-specific reads in all the replicates of the conditions under comparison were considered. 2, the within condition PSI differences were required to be smaller than the between condition PSI differences. Finally, AS events that met the criteria and for which the compare-sample module of MISO returned a Bayes factor of at least 5 were considered significant.

### RT-qPCR and RT-PCR

Col-0 RNA samples from the 23°C to 16°C temperature experiment were used for cDNA synthesis using the Bio-Rad iScript™ cDNA Synthesis kit (Catalog No. 1708891, Bio-Rad) or Invitrogen SuperScript® II Reverse Transcriptase (Catalog number 18064–014, Thermo Fisher Scientific) according to the manufacturer’s protocol.

qPCR was performed in 20ul reaction volume using the iQ™ SYBR^®^ Green Supermix (Catalog No. 170–8885, Bio-Rad) according to the manufacturer’s protocol. qPCR primers were designed to amplify the splicing event or a region included in all isoforms (S1 Table). The latter were used as an internal reference in order to be able to calculate the ratio change between transcripts containing a specific splicing event versus all transcripts of a selected gene. Primers were tested for efficiency and the Pfaffl method [40] was used to calculate the ratio changes.

RT-PCR on *MAF3* was executed using primers on the start and stop codon (S1 Table), using Q5^®^ high-fidelity polymerase (Catalog No. M0491S, NEB) and the manufacturer’s protocol with an annealing temperature of 67°C and 35 PCR cycles. Samples were run on a 2% agarose gel. Fragments were cut out and extracted from gel using the nucleospin gel and PCR clean-up kit (Catalog No. 740609.250, Macherey-Nagel) and cloned into a pGEM®-T vector using the pGEM®-T Vector System I (Catalog No. A3600, Promega) according to the manufacturer’s manual. Vectors were introduced into DH5α competent cells by electroporation. Plasmids were extracted using the NucleoSpin® Plasmid kit (catalog No. 740588.250, Macherey-Nagel) and sequences were obtained using the Macrogen EZ-seq service (for primer sequence, see S1 Table).

### Statistical methods and GO analysis

For splicing event distribution of differential events upon warm or cold treatment, significant difference of event frequency between total events and differential events was tested using the Chi-square test. For data used for testing, see S2 Table. GO analysis was performed using the Cytoscape plugin BiNGO [41] using the most recent go-basic ontology file and *A*. *thaliana* gene association file from The Gene Ontology Consortium [42]. A custom reference list was created containing intron-containing genes that showed expression in our dataset (≥ 1 read). Genes containing only cryptic introns or genes belonging to the chloroplast were no taken into account in this analysis. Overrepresentation was tested using a hypergeometric statistical test and the Benjamini & Hochberg False Discovery Rate correction was used to correct for multiple testing. Enrichment analysis for splicing-related genes was conducted using Chi-square testing, with a P-value of <0.01. The same set of reference genes that was used for GO analysis was used for these calculations.

## Supporting information

S1 Table Oligo’s used in this study. (DOCX)

S2 Table Detected splicing event counts. (DOCX)

S3 Table Overview of all differential splicing events in this study, reciprocally spliced genes, genes in BiNGO analysis, spliced splicing genes. (XLSX)

S4 Table Alignments of all differential events *A. thaliana* Col-0 high temperature. (PDF)

S5 Table Alignments of all differential events *A. thaliana* Col-0 low temperature. (PDF)

S6 Table Alignments of all differential events *A. thaliana* Gy-0. (PDF)

S7 Table Alignments of all differential events *B. oleracea*. (PDF)

S1 Fig Validation of RNAseq results by qPCR (DOCX)

S2 Fig Sequence alignment of *MAF3* isoforms (DOCX)

S3 Fig Flowering time analysis of *A. thaliana* Gy-0. (DOCX)
