# Supplementary material for: Splicing-related genes are alternatively spliced upon changes in ambient temperatures in plants
Source: PLoS One. 2017 Mar 3;12(3):e0172950. doi: 10.1371/journal.pone.0172950 (PMC5336241; doi:10.1371/journal.pone.0172950)
Supplement: S1 Table — (DOCX) [file pone.0172950.s001.docx]

**S1 Table:** Oligo’s used in this study

| **qPCR primers for RNAseq verification** | | | | | |
| --- | --- | --- | --- | --- | --- |
| **Locus/**  **description** | **Splicing event** | | **Primers on event** | | **Primers on all isoforms** |
| AT4G25500/ *ATRSP35* | RI-AT4G25500-XLOC_021789-10123 | | FW: TCTCGTCTTCATTCCATCCA | | FW: TCTCGGTGGAGTATGCTGTG |
|  |  | | RV: CCAGGTGAATGTGGTCAATG | | RV: GTGACCTATCACGGCGTCTT |
| AT4G36690/ *ATU2AF65A* | RI-AT4G36690-XLOC_024897-8160 | | FW: GCGCAATATGGTCTATTTTGAA | | FW: TACAGTGAGACGTGCCAACC |
|  |  | | RV: AGGCTGTAAAACAGCACAACC | | RV: GCTGCTGTGCATGCAATAAT |
| AT1G02090/ *FUS5* | RI-AT1G02090-XLOC_004204-1143 | | FW: GGGGATGGGAGAGACTTGAT | | FW: TGAATGCATGTATGCGGGTA |
|  |  | | RV: GGCTGATCGATTCGTTTGTT | | RV: AGATCCCTACCAGCTGCAAA |
| AT1G30970/ *SUF4* | RI-AT1G30970-XLOC_005753-4663 | | FW: TGGTGATTGGTTCCACAAGA | | FW: CGGATGGCGTTTTAGAATTT |
|  |  | | RV: GCAGTGAGATTGTGCAAAGG | | RV: TGTTGTAACAAAGCCTTCATCC |
| AT3G61600/ *POB1* | RI-AT3G61600-XLOC_016687-2054 | | FW: CCGTTTACTTCTCAATTGTCACTG | | FW: GTCTGCCCTGCTCTATCTCG |
| **Primers on start and stop of *MAF3*** | | | | | |
| FW *MAF3* on start | | ATGGGAAGAAGAAAAGTCGAGATCA | | | |
| RV *MAF3* on stop | | TGATTACTTGAGCAGCGAAAGAGTCTCC | | | |
| **Primers for testing SALK_144790** | | | | | |
| LP for SALK_144790 | | | | CACAAAGACCACCAAACAACC | |
| RP for SALK_144790 | | | | GCGAAAAGATGTCTGAATTCG | |
| LBb1.3 (on T-DNA) | | | | ATTTTGCCGATTTCGGAAC | |
