# Supplementary material for: Splicing-related genes are alternatively spliced upon changes in ambient temperatures in plants
Source: PLoS One. 2017 Mar 3;12(3):e0172950. doi: 10.1371/journal.pone.0172950 (PMC5336241; doi:10.1371/journal.pone.0172950)
Supplement: S2 Table — (DOCX) [file pone.0172950.s002.docx]

**S2 Table:** Detected splicing event counts

|  | Col-0 cold total | % | Col-0 cold differential | % |
| --- | --- | --- | --- | --- |
| A3 | 4276 | 40,6 | 134 | 37,0 |
| A5 | 1979 | 18,8 | 53 | 14,6 |
| MXE | 25 | 0,2 | 1 | 0,3 |
| RI | 3487 | 33,1 | 147 | 40,6 |
| SE | 760 | 7,2 | 27 | 7,5 |
|  |  |  |  |  |
| Total | 10527 | 100,0 | 362 | 100,0 |

|  | Col-0 warm total | % | Col-0 warm differential | % |
| --- | --- | --- | --- | --- |
| A3 | 3767 | 40,1 | 41 | 27,7 |
| A5 | 1743 | 18,6 | 28 | 18,9 |
| MXE | 23 | 0,2 | 1 | 0,7 |
| RI | 3184 | 33,9 | 66 | 44,6 |
| SE | 668 | 7,1 | 12 | 8,1 |
|  |  |  |  |  |
| Total | 9385 | 100.0 | 148 | 100,0 |

|  | Col-0 Overall (warm+cold) total | % |  |  |
| --- | --- | --- | --- | --- |
| A3 | 4621 | 40,8 |  |  |
| A5 | 2153 | 19,0 |  |  |
| MXE | 26 | 0,2 |  |  |
| RI | 3715 | 32,8 |  |  |
| SE | 820 | 7,2 |  |  |
|  |  |  |  |  |
| Total | 11335 | 100,0 |  |  |

|  | Gy-0 total | % | Gy-0 differential | % |
| --- | --- | --- | --- | --- |
| A3 | 2101 | 36,4 | 53 | 42,1 |
| A5 | 1136 | 19,7 | 18 | 14,3 |
| MXE | 21 | 0,4 | 0 | 0,0 |
| RI | 1885 | 32,7 | 43 | 34,1 |
| SE | 630 | 10,9 | 12 | 9,5 |
|  |  |  |  |  |
| Total | 5773 | 100.0 | 126 | 100.0 |

|  | B.ol total | % | B.ol differential | % |
| --- | --- | --- | --- | --- |
| A3 | 4946 | 37,2 | 53 | 29,4 |
| A5 | 3004 | 22,6 | 40 | 22,2 |
| MXE | 61 | 0,5 | 1 | 0,6 |
| RI | 3753 | 28,2 | 72 | 40,0 |
| SE | 1537 | 11,6 | 14 | 7,8 |
|  |  |  |  |  |
| Total | 13301 | 100.0 | 180 | 100.0 |
