# Supplementary material for: Splicing-related genes are alternatively spliced upon changes in ambient temperatures in plants
Source: PLoS One. 2017 Mar 3;12(3):e0172950. doi: 10.1371/journal.pone.0172950 (PMC5336241; doi:10.1371/journal.pone.0172950)
Supplement: S4 Table — (PDF) [file pone.0172950.s004.pdf]

alignment for event: RI-AT4G35090-XLOC_024813-8010

RI-AT4G35090-XLOC_024813-8010-0
GCAAGAACGATTCATCCAGAGATGGATTGATGCCCTATCCGACCCACGCA
RI-AT4G35090-XLOC_024813-8010-1
GCAAGAACGATTCATCCAGAGATGGATTGATGCCCTATCCGACCCACGCA
CONSENSUS
GCAAGAACGATTCATCCAGAGATGGATTGATGCCCTATCCGACCCACGCA

RI-AT4G35090-XLOC_024813-8010-0
TCACGCATGAAATCCGCAGTATCTGGATCTCTTACTGGTCTCAGGTCTGA
RI-AT4G35090-XLOC_024813-8010-1
TCACGCATGAAATCCGCAGTATCTGGATCTCTTACTGGTCTCA-------
CONSENSUS
TCACGCATGAAATCCGCAGTATCTGGATCTCTTACTGGTCTCA.......

RI-AT4G35090-XLOC_024813-8010-0
AATCTTGATCTCTGTTACTGTCATTGTCTCGTTCTTCAAGTCTCAGCAGT
RI-AT4G35090-XLOC_024813-8010-1

CONSENSUS
....

RI-AT4G35090-XLOC_024813-8010-0
TGAAAAGTTGTCAAGTTTTAAACATTTTTGACTGATTGGTTAAAAATGTG
RI-AT4G35090-XLOC_024813-8010-1

CONSENSUS
....

RI-AT4G35090-XLOC_024813-8010-0
TGAAACAGGCTGATAAGTCTTTGGGACAGAAGCTGGCAAGCCGTCTGAAC
RI-AT4G35090-XLOC_024813-8010-1 --------
GCTGATAAGTCTTTGGGACAGAAGCTGGCAAGCCGTCTGAAC
CONSENSUS
........GCTGATAAGTCTTTGGGACAGAAGCTGGCAAGCCGTCTGAAC

RI-AT4G35090-XLOC_024813-8010-0
GTGAGACCAAGCATCTAAAACGAGTGAAAGAATTCTTGATTGGCCTTTCT
RI-AT4G35090-XLOC_024813-8010-1
GTGAGACCAAGCATCTAAAACGAGTGAAAGAATTCTTGATTGGCCTTTCT
CONSENSUS
GTGAGACCAAGCATCTAAAACGAGTGAAAGAATTCTTGATTGGCCTTTCT

RI-AT4G35090-XLOC_024813-8010-0
GTGGTTGTTAATTAGCAGAGAAAAGAAAATGGGAAGAGATCAAAGTTGCG
RI-AT4G35090-XLOC_024813-8010-1
GTGGTTGTTAATTAGCAGAGAAAAGAAAATGGGAAGAGATCAAAGTTGCG
CONSENSUS
GTGGTTGTTAATTAGCAGAGAAAAGAAAATGGGAAGAGATCAAAGTTGCG

RI-AT4G35090-XLOC_024813-8010-0
ATAATACAATCTTCATCATGTGGATCTTCTGTGCTATGTTATAATAGTAT
RI-AT4G35090-XLOC_024813-8010-1
ATAATACAATCTTCATCATGTGGATCTTCTGTGCTATGTTATAATAGTAT
CONSENSUS

ATAATACAATCTTCATCATGTGGATCTTCTGTGCTATGTTATAATAGTAT

RI-AT4G35090-XLOC_024813-8010-0
CTGTTTAAAGACCAAAAGAACCTTGTGTGTTGTTGTGTCTCTCGTTTATC
RI-AT4G35090-XLOC_024813-8010-1
CTGTTTAAAGACCAAAAGAACCTTGTGTGTTGTTGTGTCTCTCGTTTATC
CONSENSUS
CTGTTTAAAGACCAAAAGAACCTTGTGTGTTGTTGTGTCTCTCGTTTATC

RI-AT4G35090-XLOC_024813-8010-0
AGAATAATACATTATACATTGAAGATTTATAATCCGTGGTGTTACTTGAT
RI-AT4G35090-XLOC_024813-8010-1
AGAATAATACATTATACATTGAAGATTTATAATCCGTGGTGTTACTTGAT
CONSENSUS
AGAATAATACATTATACATTGAAGATTTATAATCCGTGGTGTTACTTGAT

RI-AT4G35090-XLOC_024813-8010-0
GAAAGCTCCTTCTTCCTTGAAAGATTGTGACATATTCTTATATATTTCTC
RI-AT4G35090-XLOC_024813-8010-1
GAAAGCTCCTTCTTCCTTGAAAGATTGTGACATATTCTTATATATTTCTC
CONSENSUS
GAAAGCTCCTTCTTCCTTGAAAGATTGTGACATATTCTTATATATTTCTC

RI-AT4G35090-XLOC_024813-8010-0
ATATATTAACGAATGTAATCCCGTAAGAGCTGTCGACAAAGACGTGATTT
RI-AT4G35090-XLOC_024813-8010-1
ATATATTAACGAATGTAATCCCGTAAGAGCTGTCGACAAAGACGTGATTT
CONSENSUS
ATATATTAACGAATGTAATCCCGTAAGAGCTGTCGACAAAGACGTGATTT

RI-AT4G35090-XLOC_024813-8010-0
TTAGTCTGCATGTTCTGGACGAAAGGGAATAGCAAAGTATGAAACGAAAA
RI-AT4G35090-XLOC_024813-8010-1
TTAGTCTGCATGTTCTGGACGAAAGGGAATAGCAAAGTATGAAACGAAAA
CONSENSUS
TTAGTCTGCATGTTCTGGACGAAAGGGAATAGCAAAGTATGAAACGAAAA

RI-AT4G35090-XLOC_024813-8010-0
AGACAAGAAAATCCATAAATTCTTATGTGTGGGTATTAGGGAATTTACAA
RI-AT4G35090-XLOC_024813-8010-1
AGACAAGAAAATCCATAAATTCTTATGTGTGGGTATTAGGGAATTTACAA
CONSENSUS
AGACAAGAAAATCCATAAATTCTTATGTGTGGGTATTAGGGAATTTACAA

RI-AT4G35090-XLOC_024813-8010-0
TAGTTGTCAATATGTGTTGTAACAGTGGCCACATTTGTGTATAACGTCGT
RI-AT4G35090-XLOC_024813-8010-1
TAGTTGTCAATATGTGTTGTAACAGTGGCCACATTTGTGTATAACGTCGT
CONSENSUS
TAGTTGTCAATATGTGTTGTAACAGTGGCCACATTTGTGTATAACGTCGT

RI-AT4G35090-XLOC_024813-8010-0
TGTATCAAGTGTGGGGTGTCGAGAGTCTTTAGATTTGGTGTGAATAATCT
RI-AT4G35090-XLOC_024813-8010-1
TGTATCAAGTGTGGGGTGTCGAGAGTCTTTAGATTTGGTGTGAATAATCT
CONSENSUS

TGTATCAAGTGTGGGGTGTCGAGAGTCTTTAGATTTGGTGTGAATAATCT

RI-AT4G35090-XLOC_024813-8010-0
GACAATTTGGATTTGAACTCTGCTTTGACATCCTGACATTAGAAAATAAA
RI-AT4G35090-XLOC_024813-8010-1
GACAATTTGGATTTGAACTCTGCTTTGACATCCTGACATTAGAAAATAAA
CONSENSUS
GACAATTTGGATTTGAACTCTGCTTTGACATCCTGACATTAGAAAATAAA

RI-AT4G35090-XLOC_024813-8010-0 CTTGGTTTTGACA
RI-AT4G35090-XLOC_024813-8010-1 CTTGGTTTTGACA
CONSENSUS CTTGGTTTTGACA

alignment for event: A3-AT4G07510.1-XLOC_020634-845

A3-AT4G07510.1-XLOC_020634-845-0
ATGGTTTAGTAGATTACCCCATTTGCTAGGTTGAAGATCCAAAGTGGAGA
A3-AT4G07510.1-XLOC_020634-845-1
ATGGTTTAGTAGATTACCCCATTTGCTAGGTTGAAGATCCAAAGTGGAGA
CONSENSUS
ATGGTTTAGTAGATTACCCCATTTGCTAGGTTGAAGATCCAAAGTGGAGA

A3-AT4G07510.1-XLOC_020634-845-0
GCTTAATCCATATATGTTAGTAGGTAAATCTCTATCAATGAAACTATTAT
A3-AT4G07510.1-XLOC_020634-845-1
GCTTAATCCATATATGTTAGTAGGTAAATCTCTATCAATGAAACTATTAT
CONSENSUS
GCTTAATCCATATATGTTAGTAGGTAAATCTCTATCAATGAAACTATTAT

A3-AT4G07510.1-XLOC_020634-845-0
TCTTTTTAAAATATTTTTTAAATATACTATTGTTTTTCAGTTTGTTATGA
A3-AT4G07510.1-XLOC_020634-845-1
TCTTTTTAAAATATTTTTTAAATATACTATTGTTTTTCAGTTTGTTATGA
CONSENSUS
TCTTTTTAAAATATTTTTTAAATATACTATTGTTTTTCAGTTTGTTATGA

A3-AT4G07510.1-XLOC_020634-845-0
ACTAACACCTACGATCTTTATGTTATAGATGTCATTAGTCAAATTGTTTC
A3-AT4G07510.1-XLOC_020634-845-1
ACTAACACCTACGATCTTTATGTTATAGATGTCATTAGTCAAATTGTTTC
CONSENSUS
ACTAACACCTACGATCTTTATGTTATAGATGTCATTAGTCAAATTGTTTC

A3-AT4G07510.1-XLOC_020634-845-0
GATTGGAGAGCTGGAAAACTTAGAAGCAAGTAACAAACCAACAACCAAAA
A3-AT4G07510.1-XLOC_020634-845-1
GATTGGAGAGCTGGAAAACTTAGAAGCAAGTAACAAACCAACAACCAAAA
CONSENSUS
GATTGGAGAGCTGGAAAACTTAGAAGCAAGTAACAAACCAACAACCAAAA

A3-AT4G07510.1-XLOC_020634-845-0
TCGAATTTGAAATTCGTGATGAAACGTTTGTTTTCATCACTATTATATTT
A3-AT4G07510.1-XLOC_020634-845-1
TCGAATTTGAAATTCGTGATGAAACGTTTGTTTTCATCACTATTATATTT

CONSENSUS
TCGAATTTGAAATTCGTGATGAAACGTTTGTTTTCATCACTATTATATTT

A3-AT4G07510.1-XLOC_020634-845-0
CAGTTTATACACTTTTAGCCATATACTAAACTTGATCTCATCAATCACTT
A3-AT4G07510.1-XLOC_020634-845-1
CAGTTTATACACTTTTAGCCATATACTAAACTTGATCTCATCAATCACTT
CONSENSUS
CAGTTTATACACTTTTAGCCATATACTAAACTTGATCTCATCAATCACTT

A3-AT4G07510.1-XLOC_020634-845-0
GATTTTCTTCCCACAGTCATGAGAGGATTGGTTGTACTTTGTGGGGTACA
A3-AT4G07510.1-XLOC_020634-845-1
GATTTTCTTCCCACAGTCATGAGAGGATTGGTTGTACTTTGTGGGGTACA
CONSENSUS
GATTTTCTTCCCACAGTCATGAGAGGATTGGTTGTACTTTGTGGGGTACA

A3-AT4G07510.1-XLOC_020634-845-0
ATTGCTAAACAGACTTTTAGGGCATGTCAAGACTCTAATCGTGGAATGGT
A3-AT4G07510.1-XLOC_020634-845-1
ATTGCTAAACAGACTTTTAGGGCATGTCAAGACTCTAATCGTGGAATGGT
CONSENSUS
ATTGCTAAACAGACTTTTAGGGCATGTCAAGACTCTAATCGTGGAATGGT

A3-AT4G07510.1-XLOC_020634-845-0
GATTTGTATCCTCAGGTTTGTCAAAATCAAGGCTTACAAAGATACAAAAT
A3-AT4G07510.1-XLOC_020634-845-1
GATTTGTATCCTCAGGTTTGTCAAAATCAAGGCTTACAAAGATACAAAAT
CONSENSUS
GATTTGTATCCTCAGGTTTGTCAAAATCAAGGCTTACAAAGATACAAAAT

A3-AT4G07510.1-XLOC_020634-845-0
TTCTATGTGTTTTTTGTATGCTAACATATAAATTCAGTTAATCACTACAA
A3-AT4G07510.1-XLOC_020634-845-1
TTCTATGTGTTTTTTGTATGCTAACATATAAATTCAGTTAATCACTACAA
CONSENSUS
TTCTATGTGTTTTTTGTATGCTAACATATAAATTCAGTTAATCACTACAA

A3-AT4G07510.1-XLOC_020634-845-0
GAAATATGAACATTATTAGCACATTACGATAGCGCAGTTTTCGTAACTGC
A3-AT4G07510.1-XLOC_020634-845-1
GAAATATGAACATTATTAGCACATTACGATAGCGCAGTTTTCGTAACTGC
CONSENSUS
GAAATATGAACATTATTAGCACATTACGATAGCGCAGTTTTCGTAACTGC

A3-AT4G07510.1-XLOC_020634-845-0
TATTAAAAGTGAAACTCGGAATTTAGTAACTATATTATAGCGTTTCAGAA
A3-AT4G07510.1-XLOC_020634-845-1
TATTAAAAGTGAAACTCGGAATTTAGTAACTATATTATAGCGTTTCAGAA
CONSENSUS
TATTAAAAGTGAAACTCGGAATTTAGTAACTATATTATAGCGTTTCAGAA

A3-AT4G07510.1-XLOC_020634-845-0
ACACTAAACCTAATAATAGCATGGTAGAAAACTAAAATCACTTTTGACCG
A3-AT4G07510.1-XLOC_020634-845-1
ACACTAAACCTAATAATAGCATGGTAGAAAACTAAAATCACTTTTGACCG

CONSENSUS
ACACTAAACCTAATAATAGCATGGTAGAAAACTAAAATCACTTTTGACCG

A3-AT4G07510.1-XLOC_020634-845-0
CCAAGCCAAAATTTTGCCAAAACCGCCAACACTTAGCCAAATATCAGTTT
A3-AT4G07510.1-XLOC_020634-845-1
CCAAGCCAAAATTTTGCCAAAACCGCCAACACTTAGCCAAATATCAGTTT
CONSENSUS
CCAAGCCAAAATTTTGCCAAAACCGCCAACACTTAGCCAAATATCAGTTT

A3-AT4G07510.1-XLOC_020634-845-0
TACACTATACGCCCATTATTTATCCCAAAGAAAAAAAAATTCTTATCAAC
A3-AT4G07510.1-XLOC_020634-845-1
TACACTATACGCCCATTATTTATCCCAAAGAAAAAAAAATTCTTATCAAC
CONSENSUS
TACACTATACGCCCATTATTTATCCCAAAGAAAAAAAAATTCTTATCAAC

A3-AT4G07510.1-XLOC_020634-845-0
CAAGCAAAAACTAATTAATCTATTTCTTGGATTTACTCTTTTGCTCTCAG
A3-AT4G07510.1-XLOC_020634-845-1
CAAGCAAAAACTAATTAATCTATTTCTTGGATTTACTCTTTTGCTCTCAG
CONSENSUS
CAAGCAAAAACTAATTAATCTATTTCTTGGATTTACTCTTTTGCTCTCAG

A3-AT4G07510.1-XLOC_020634-845-0
TCGCATCCTAACATATATTCTCCTCATCGTCTTCTTCGTCGTGACGTCTC
A3-AT4G07510.1-XLOC_020634-845-1
TCGCATCCTAACATATATTCTCCTCATCGTCTTCTTCGTCGTGACGTCTC
CONSENSUS
TCGCATCCTAACATATATTCTCCTCATCGTCTTCTTCGTCGTGACGTCTC

A3-AT4G07510.1-XLOC_020634-845-0
TCCTCCATCATCGTCACTTGCAAATCCAGCTCTCCATTTCAACTCCACAT
A3-AT4G07510.1-XLOC_020634-845-1
TCCTCCATCATCGTCACTTGCAAATCCAGCTCTCCATTTCAACTCCACAT
CONSENSUS
TCCTCCATCATCGTCACTTGCAAATCCAGCTCTCCATTTCAACTCCACAT

A3-AT4G07510.1-XLOC_020634-845-0
CTTCTTCTTCTTTAGATCCCTCATCTTTCTCTCCTTCTCCTCCTTCCTCT
A3-AT4G07510.1-XLOC_020634-845-1
CTTCTTCTTCTTTAGATCCCTCATCTTTCTCTCCTTCTCCTCCTTCCTCT
CONSENSUS
CTTCTTCTTCTTTAGATCCCTCATCTTTCTCTCCTTCTCCTCCTTCCTCT

A3-AT4G07510.1-XLOC_020634-845-0
CATTCTTCTACTTCGTCTTTTCCTTTGTTCATTTCTCTCTCTCTCTCTAG
A3-AT4G07510.1-XLOC_020634-845-1
CATTCTTCTACTTCGTCTTTTCCTTTGTTCATTTCTCTCTCTCTCTCTAG
CONSENSUS
CATTCTTCTACTTCGTCTTTTCCTTTGTTCATTTCTCTCTCTCTCTCTAG

A3-AT4G07510.1-XLOC_020634-845-0
CTTCCACCTTCACTTTAACTTCTCATTACCCTAATTTTTCAAGACATATC
A3-AT4G07510.1-XLOC_020634-845-1
CTTCCACCTTCACTTTAACTTCTCATTACCCTAATTTTTCAAGACATATC

CONSENSUS
      CTTCCACCTTCACTTTAACTTCTCATTACCCTAATTTTTCAAGACATATC

A3-AT4G07510.1-XLOC_020634-845-0
      AATTGAAATTGAAAAGTTGCTCAGAACTCTAATCATCCCCTTCGTTCTCA
A3-AT4G07510.1-XLOC_020634-845-1
      AATTGAAATTGAAAAGTTGCTCAGAACTCTAATCATCCCCTTCGTTCTCA
CONSENSUS
      AATTGAAATTGAAAAGTTGCTCAGAACTCTAATCATCCCCTTCGTTCTCA

A3-AT4G07510.1-XLOC_020634-845-0
      GTTCTTCCAATCTTCAATTTCATAAAAACCCTAATTTTAACTGGTTTGTT
A3-AT4G07510.1-XLOC_020634-845-1
      GTTCTTCCAATCTTCAATTTCATAAAAACCCTAATTTTAACTGGTTTGTT
CONSENSUS
      GTTCTTCCAATCTTCAATTTCATAAAAACCCTAATTTTAACTGGTTTGTT

A3-AT4G07510.1-XLOC_020634-845-0
      TTTTGGAAATTGGATTTGTCTACGTTTTGGAATTGGCTGGAAACGCAGCG
A3-AT4G07510.1-XLOC_020634-845-1
      TTTTGGAAATTGGATTTGTCTACGTTTTGGAATTGGCTGGAAACGCAGCG
CONSENSUS
      TTTTGGAAATTGGATTTGTCTACGTTTTGGAATTGGCTGGAAACGCAGCG

A3-AT4G07510.1-XLOC_020634-845-0
      AAGGATAACAAGAAGACTAGGCTTGTGCCACATCACATACATCTTGCAGT
A3-AT4G07510.1-XLOC_020634-845-1
      AAGGATAACAAGAAGACTAGGCTTGTGCCACATCACATACATCTTGCAGT
CONSENSUS
      AAGGATAACAAGAAGACTAGGCTTGTGCCACATCACATACATCTTGCAGT

A3-AT4G07510.1-XLOC_020634-845-0
      GAGAAACGATGAGGAGCCGAGGAAGTTGCTTGGTGATGTGATGATTGCAA
A3-AT4G07510.1-XLOC_020634-845-1
      GAGAAACGATGAGGAGCCGAGGAAGTTGCTTGGTGATGTGATGATTGCAA
CONSENSUS
      GAGAAACGATGAGGAGCCGAGGAAGTTGCTTGGTGATGTGATGATTGCAA

A3-AT4G07510.1-XLOC_020634-845-0
      AGAAAGCATTGTAAGAAATCTTAGGGGTTCTATTTTTAGAGTTATTTACG
A3-AT4G07510.1-XLOC_020634-845-1
      AG--------------------------------------AGTTATTTACG
CONSENSUS
      AG......................................AGTTATTTACG

A3-AT4G07510.1-XLOC_020634-845-0
      CACACAAGGTGTTTGATGAATTGACGGTGAGAGAGAAATCAAGAAACGAC
A3-AT4G07510.1-XLOC_020634-845-1
      CACACAAGGTGTTTGATGAATTGACGGTGAGAGAGAAATCAAGAAACGAC
CONSENSUS
      CACACAAGGTGTTTGATGAATTGACGGTGAGAGAGAAATCAAGAAACGAC

A3-AT4G07510.1-XLOC_020634-845-0
      TCTCCCAACATGTCTTACGCGATGTTTGAAGAAGCCTAATAGGAAATGTG
A3-AT4G07510.1-XLOC_020634-845-1
      TCTCCCAACATGTCTTACGCGATGTTTGAAGAAGCCTAATAGGAAATGTG

CONSENSUS
      TCTCCCAACATGTCTTACGCGATGTTTGAAGAAGCCTAATAGGAAATGTG

A3-AT4G07510.1-XLOC_020634-845-0 ACTAGACTTTATAGAAG
A3-AT4G07510.1-XLOC_020634-845-1 ACTAGACTTTATAGAAG
CONSENSUS ACTAGACTTTATAGAAG

alignment for event: A3-AT4G31550-XLOC_024617-9872

A3-AT4G31550-XLOC_024617-9872-0
      ATCACAACCATTTCTCTCTCTCGTCTTCGTCTTCTTCTTCTTCAACGTTC
A3-AT4G31550-XLOC_024617-9872-1
      ATCACAACCATTTCTCTCTCTCGTCTTCGTCTTCTTCTTCTTCAACGTTC
CONSENSUS
      ATCACAACCATTTCTCTCTCTCGTCTTCGTCTTCTTCTTCTTCAACGTTC

A3-AT4G31550-XLOC_024617-9872-0
      CTCTCCAAAATCCTCAGACCAAGAAATCATCATGGCCGTCGATCTAATGC
A3-AT4G31550-XLOC_024617-9872-1
      CTCTCCAAAATCCTCAGACCAAGAAATCATCATGGCCGTCGATCTAATGC
CONSENSUS
      CTCTCCAAAATCCTCAGACCAAGAAATCATCATGGCCGTCGATCTAATGC

A3-AT4G31550-XLOC_024617-9872-0
      GTTTCCCTAAGATAGATGATCAAACGGCTATTCAGGAAGCTGCATCGCAA
A3-AT4G31550-XLOC_024617-9872-1
      GTTTCCCTAAGATAGATGATCAAACGGCTATTCAGGAAGCTGCATCGCAA
CONSENSUS
      GTTTCCCTAAGATAGATGATCAAACGGCTATTCAGGAAGCTGCATCGCAA

A3-AT4G31550-XLOC_024617-9872-0
      GGTTTACAAAGTATGGAACATCTGATCCGTGTCCTCTCTAACCGTCCCGA
A3-AT4G31550-XLOC_024617-9872-1
      GGTTTACAAAGTATGGAACATCTGATCCGTGTCCTCTCTAACCGTCCCGA
CONSENSUS
      GGTTTACAAAGTATGGAACATCTGATCCGTGTCCTCTCTAACCGTCCCGA

A3-AT4G31550-XLOC_024617-9872-0
      ACAACAACACAACGTTGACTGCTCCGAGATCACTGACTTCACCGTTTCTA
A3-AT4G31550-XLOC_024617-9872-1
      ACAACAACACAACGTTGACTGCTCCGAGATCACTGACTTCACCGTTTCTA
CONSENSUS
      ACAACAACACAACGTTGACTGCTCCGAGATCACTGACTTCACCGTTTCTA

A3-AT4G31550-XLOC_024617-9872-0
      AATTCAAAACCGTCATTTCTCTCCTTAACCGTACTGGTCACGCTCGGTTC
A3-AT4G31550-XLOC_024617-9872-1
      AATTCAAAACCGTCATTTCTCTCCTTAACCGTACTGGTCACGCTCGGTTC
CONSENSUS
      AATTCAAAACCGTCATTTCTCTCCTTAACCGTACTGGTCACGCTCGGTTC

A3-AT4G31550-XLOC_024617-9872-0
      AGACGCGGACCGGTTCACTCCACTTCCTCTGCCGCATCTCAGAAACTACA
A3-AT4G31550-XLOC_024617-9872-1

AGACGCGGACCGGTTCACTCCACTTCCTCTGCCGCATCTCAGAAACTACA
CONSENSUS
AGACGCGGACCGGTTCACTCCACTTCCTCTGCCGCATCTCAGAAACTACA

A3-AT4G31550-XLOC_024617-9872-0
GAGTCAGATCGTTAAAAATACTCAACCTGAGGCTCCGATAGTGAGAACAA
A3-AT4G31550-XLOC_024617-9872-1
GAGTCAGATCGTTAAAAATACTCAACCTGAGGCTCCGATAGTGAGAACAA
CONSENSUS
GAGTCAGATCGTTAAAAATACTCAACCTGAGGCTCCGATAGTGAGAACAA

A3-AT4G31550-XLOC_024617-9872-0
CTACGAATCACCCTCAAATCGTTCCTCCACCGTCTAGTGTAACACTCGAT
A3-AT4G31550-XLOC_024617-9872-1
CTACGAATCACCCTCAAATCGTTCCTCCACCGTCTAGTGTAACACTCGAT
CONSENSUS
CTACGAATCACCCTCAAATCGTTCCTCCACCGTCTAGTGTAACACTCGAT

A3-AT4G31550-XLOC_024617-9872-0
TTCTCTAAACCAAGCATCTTCGGCACCAAAGCTAAGAGCGCCGAGCTGGA
A3-AT4G31550-XLOC_024617-9872-1
TTCTCTAAACCAAGCATCTTCGGCACCAAAGCTAAGAGCGCCGAGCTGGA
CONSENSUS
TTCTCTAAACCAAGCATCTTCGGCACCAAAGCTAAGAGCGCCGAGCTGGA

A3-AT4G31550-XLOC_024617-9872-0
ATTCTCCAAAGAAAACTTCAGTGTTTCTTTAAACTCCTCATTCATGTCGT
A3-AT4G31550-XLOC_024617-9872-1
ATTCTCCAAAGAAAACTTCAGTGTTTCTTTAAACTCCTCATTCATGTCGT
CONSENSUS
ATTCTCCAAAGAAAACTTCAGTGTTTCTTTAAACTCCTCATTCATGTCGT

A3-AT4G31550-XLOC_024617-9872-0
CGGCGATAACCGGAGACGGCAGCGTCTCCAATGGAAAAATCTTCCTTGCT
A3-AT4G31550-XLOC_024617-9872-1
CGGCGATAACCGGAGACGGCAGCGTCTCCAATGGAAAAATCTTCCTTGCT
CONSENSUS
CGGCGATAACCGGAGACGGCAGCGTCTCCAATGGAAAAATCTTCCTTGCT

A3-AT4G31550-XLOC_024617-9872-0
TCTGCTCCGTTGCAGCCTGTTAACTCTTCCGGAAAACCACCGTTGGCTGG
A3-AT4G31550-XLOC_024617-9872-1
TCTGCTCCGTTGCAGCCTGTTAACTCTTCCGGAAAACCACCGTTGGCTGG
CONSENSUS
TCTGCTCCGTTGCAGCCTGTTAACTCTTCCGGAAAACCACCGTTGGCTGG

A3-AT4G31550-XLOC_024617-9872-0
TCATCCTTACAGAAAGAGATGTCTCGAGCATGAGCACTCAGAGAGTTTCT
A3-AT4G31550-XLOC_024617-9872-1
TCATCCTTACAGAAAGAGATGTCTCGAGCATGAGCACTCAGAGAGTTTCT
CONSENSUS
TCATCCTTACAGAAAGAGATGTCTCGAGCATGAGCACTCAGAGAGTTTCT

A3-AT4G31550-XLOC_024617-9872-0
CCGGAAAAGTCTCCGGCTCCGCCTACGGAAAGTGCCATTGCAAGAAAAG-
A3-AT4G31550-XLOC_024617-9872-1

CCGGAAAAGTCTCCGGCTCCGCCTACGGAAAGTGCCATTGCAAGAAAAGC
CONSENSUS
CCGGAAAAGTCTCCGGCTCCGCCTACGGAAAGTGCCATTGCAAGAAAAG.

A3-AT4G31550-XLOC_024617-9872-0 --
GAAAAATCGGATGAAGAGAACCGTGAGAGTACCGGCGATAAGTGCAAA
A3-AT4G31550-XLOC_024617-9872-1
AGGAAAAATCGGATGAAGAGAACCGTGAGAGTACCGGCGATAAGTGCAAA
CONSENSUS
..GAAAAATCGGATGAAGAGAACCGTGAGAGTACCGGCGATAAGTGCAAA

A3-AT4G31550-XLOC_024617-9872-0
GATCGCCGATATTCCACCGGACGAATATTCGTGGAGGAAGTACGGACAAA
A3-AT4G31550-XLOC_024617-9872-1
GATCGCCGATATTCCACCGGACGAATATTCGTGGAGGAAGTACGGACAAA
CONSENSUS
GATCGCCGATATTCCACCGGACGAATATTCGTGGAGGAAGTACGGACAAA

A3-AT4G31550-XLOC_024617-9872-0 AACCGATCAAGGGCTCACCACACCCACG
A3-AT4G31550-XLOC_024617-9872-1 AACCGATCAAGGGCTCACCACACCCACG
CONSENSUS AACCGATCAAGGGCTCACCACACCCACG

alignment for event: A3-AT4G22890-XLOC_021647-12594

A3-AT4G22890-XLOC_021647-12594-0
ATCTCCTAATTTCTTCCCTCCAAATTTATAATAAAACCAAAAAAAAAAAA
A3-AT4G22890-XLOC_021647-12594-1
ATCTCCTAATTTCTTCCCTCCAAATTTATAATAAAACCAAAAAAAAAAAA
CONSENSUS
ATCTCCTAATTTCTTCCCTCCAAATTTATAATAAAACCAAAAAAAAAAAA

A3-AT4G22890-XLOC_021647-12594-0
AAAAAAAAAAATCAGAAGAAACCTGAGAAGCTCACAGTAAACACATCTTC
A3-AT4G22890-XLOC_021647-12594-1
AAAAAAAAAAATCAGAAGAAACCTGAGAAGCTCACAGTAAACACATCTTC
CONSENSUS
AAAAAAAAAAATCAGAAGAAACCTGAGAAGCTCACAGTAAACACATCTTC

A3-AT4G22890-XLOC_021647-12594-0
AACCACAGGTTCATACTTACTGAAAAAAACAGAGGAAAAAAAGGAGCTCC
A3-AT4G22890-XLOC_021647-12594-1
AACCACAGGTTCATACTTACTGAAAAAAACAGAGGAAAAAAAGGAGCTCC
CONSENSUS
AACCACAGGTTCATACTTACTGAAAAAAACAGAGGAAAAAAAGGAGCTCC

A3-AT4G22890-XLOC_021647-12594-0
CTTTTCTATCTCTAAGGGAAAATGGGTAGCAAGATGTTGTTTAGTTTGAC
A3-AT4G22890-XLOC_021647-12594-1
CTTTTCTATCTCTAAGGGAAAATGGGTAGCAAGATGTTGTTTAGTTTGAC
CONSENSUS
CTTTTCTATCTCTAAGGGAAAATGGGTAGCAAGATGTTGTTTAGTTTGAC

A3-AT4G22890-XLOC_021647-12594-0
AAGTCCTCGACTTTTCTCCGCCGTTTCTCGCAAACCTTCCTCTTCTTTCT

A3-AT4G22890-XLOC_021647-12594-1
      AAGTCCTCGACTTTTCTCCGCCGTTTCTCGCAAACCTTCCTCTTCTTTCT
CONSENSUS
      AAGTCCTCGACTTTTCTCCGCCGTTTCTCGCAAACCTTCCTCTTCTTTCT

A3-AT4G22890-XLOC_021647-12594-0
      CTCCTTCTCCTCCGTCGCCGTCTTCGAGGACTCAATGGACTCAGCTCAGC
A3-AT4G22890-XLOC_021647-12594-1
      CTCCTTCTCCTCCGTCGCCGTCTTCGAGGACTCAATGGACTCAGCTCAGC
CONSENSUS
      CTCCTTCTCCTCCGTCGCCGTCTTCGAGGACTCAATGGACTCAGCTCAGC

A3-AT4G22890-XLOC_021647-12594-0
      CCTGGAAAATCGATTTCTTTGAGAAGAAGAGTCTTCTTGTTGCCTGCTAA
A3-AT4G22890-XLOC_021647-12594-1
      CCTGGAAAATCGATTTCTTTGAGAAGAAGAGTCTTCTTGTTGCCTGCTAA
CONSENSUS
      CCTGGAAAATCGATTTCTTTGAGAAGAAGAGTCTTCTTGTTGCCTGCTAA

A3-AT4G22890-XLOC_021647-12594-0
      AGCCACAACAGAGCAATCAGTAGGAGGAGACAACGTCGATAGCAATGTTT
A3-AT4G22890-XLOC_021647-12594-1 AGCCACAACAGAGCAATCAG---
GAGGAGACAACGTCGATAGCAATGTTT
CONSENSUS
      AGCCACAACAGAGCAATCAG...GAGGAGACAACGTCGATAGCAATGTTT

A3-AT4G22890-XLOC_021647-12594-0
      TGCCCTATTGTAGCATCAACAAGGCTGAGAAGAAAACAATTGGTGAAATG
A3-AT4G22890-XLOC_021647-12594-1
      TGCCCTATTGTAGCATCAACAAGGCTGAGAAGAAAACAATTGGTGAAATG
CONSENSUS
      TGCCCTATTGTAGCATCAACAAGGCTGAGAAGAAAACAATTGGTGAAATG

A3-AT4G22890-XLOC_021647-12594-0 GAACAAGAGTTTCTCCAAGCGTTGCAA
A3-AT4G22890-XLOC_021647-12594-1 GAACAAGAGTTTCTCCAAGCGTTGCAA
CONSENSUS   GAACAAGAGTTTCTCCAAGCGTTGCAA

alignment for event: A5-AT4G31877-XLOC_024638-2053

A5-AT4G31877-XLOC_024638-2053-0
      TAAGACTCTTCTTGAAGAGAGTGAGAGACAGAGAGAGATAAGGTTTTTTG
A5-AT4G31877-XLOC_024638-2053-1
      TAAGACTCTTCTTGAAGAGAGTGAGAGACAGAGAGAGATAAGGTTTTTTG
CONSENSUS
      TAAGACTCTTCTTGAAGAGAGTGAGAGACAGAGAGAGATAAGGTTTTTTG

A5-AT4G31877-XLOC_024638-2053-0
      TTTCCTTCTTTAACAGATTCTTCCTCTCTTCTCCTCTCCTCTTATTAATC
A5-AT4G31877-XLOC_024638-2053-1
      TTTCCTTCTTTAACAGATTCTTCCTCTCTTCTCCTCTCCTCTTATTAATC
CONSENSUS
      TTTCCTTCTTTAACAGATTCTTCCTCTCTTCTCCTCTCCTCTTATTAATC

A5-AT4G31877-XLOC_024638-2053-0

TAATCCTCCTCCCCGAATATTTCTCTGCCTTTAGTTCTTTCTTTTTTGGT
A5-AT4G31877-XLOC_024638-2053-1
TAATCCTCCTCCCCGAATATTTCTCTGCCTTTAGTTCTTTCTTTTTTGGT
CONSENSUS
TAATCCTCCTCCCCGAATATTTCTCTGCCTTTAGTTCTTTCTTTTTTGGT

A5-AT4G31877-XLOC_024638-2053-0
AATATATTTATTTTTCGTTACGATTTGGTCAAAACCCTAGATTTGTTTTC
A5-AT4G31877-XLOC_024638-2053-1
AATATATTTATTTTTCGTTACGATTTGGTCAAAACCCTAGATTTGTTTTC
CONSENSUS
AATATATTTATTTTTCGTTACGATTTGGTCAAAACCCTAGATTTGTTTTC

A5-AT4G31877-XLOC_024638-2053-0
CAAAAGCATATCTGAAAATGAAGGACAACTTTCCTCTTCTCCTTCGGTTA
A5-AT4G31877-XLOC_024638-2053-1
CAAAAGCATATCTGAAAATGAAGGACAACTTTCCTCTTCTCCTTCGGTTA
CONSENSUS
CAAAAGCATATCTGAAAATGAAGGACAACTTTCCTCTTCTCCTTCGGTTA

A5-AT4G31877-XLOC_024638-2053-0
TAAATATTCTCTCCGGTTTTGCTTGTTTAACCTAAAAGCCTCAGATCTAA
A5-AT4G31877-XLOC_024638-2053-1
TAAATATTCTCTCCGGTTTTGCTTGTTTAACCTAAAAGCCTCAGATCTAA
CONSENSUS
TAAATATTCTCTCCGGTTTTGCTTGTTTAACCTAAAAGCCTCAGATCTAA

A5-AT4G31877-XLOC_024638-2053-0
CTCCAACACCTTCAAAGTCTGCCTCCTTTCCAATCTTCTTTCTTCTGTTC
A5-AT4G31877-XLOC_024638-2053-1
CTCCAACACCTTCAAAGTCTGCCTCCTTTCCAATCTTCTTTCTTCTGTTC
CONSENSUS
CTCCAACACCTTCAAAGTCTGCCTCCTTTCCAATCTTCTTTCTTCTGTTC

A5-AT4G31877-XLOC_024638-2053-0
GATCTCTAATCTCAGAATTTGTGTCGGTAAGGTAAAGGTGATAATGAGTG
A5-AT4G31877-XLOC_024638-2053-1
GATCTCTAATCTCAGAATTTGTGTCGGTAAGGTAAAGGTGATAATGAGTG
CONSENSUS
GATCTCTAATCTCAGAATTTGTGTCGGTAAGGTAAAGGTGATAATGAGTG

A5-AT4G31877-XLOC_024638-2053-0
ATGACTGATGAGGGAGTTTTGGGACAAATTTTAAGAGAAACGCATAGAAA
A5-AT4G31877-XLOC_024638-2053-1
ATGACTGATGAGGGAGTTTTGGGACAAATTTTAAGAGAAACGCATAGAAA
CONSENSUS
ATGACTGATGAGGGAGTTTTGGGACAAATTTTAAGAGAAACGCATAGAAA

A5-AT4G31877-XLOC_024638-2053-0
CTGACAGAAGAGAGTGAGCACACAAAGGCACTTTGCATGTTCGATGCATT
A5-AT4G31877-XLOC_024638-2053-1
CTGACAGAAGAGAGTGAGCACACAAAGGCACTTTGCATGTTCGATGCATT
CONSENSUS
CTGACAGAAGAGAGTGAGCACACAAAGGCACTTTGCATGTTCGATGCATT

A5-AT4G31877-XLOC_024638-2053-0

TGCTTCTCTTGCGTGCTCACTGCTCTATCTGTCAGATTCCGGCTCCGATT
A5-AT4G31877-XLOC_024638-2053-1
TGCTTCTCTTGCGTGCTCACTGCTCTATCTGTCAGATTCCGGCTCCGATT
CONSENSUS
TGCTTCTCTTGCGTGCTCACTGCTCTATCTGTCAGATTCCGGCTCCGATT

A5-AT4G31877-XLOC_024638-2053-0
CGGTCCCGGTCACGTTTTCTTCTTCTAATTGTGTTCCCATCTCTCACTTT
A5-AT4G31877-XLOC_024638-2053-1
CGGTCCCGGTCACGTTTTCTTCTTCTAATTGTGTTCCCATCTCTCACTTT
CONSENSUS
CGGTCCCGGTCACGTTTTCTTCTTCTAATTGTGTTCCCATCTCTCACTTT

A5-AT4G31877-XLOC_024638-2053-0
CTCTCTCATGTGTTCTTCATCTCTCAAAGGTAAATTAAATACGATCTGAT
A5-AT4G31877-XLOC_024638-2053-1
CTCTCTCATGTGTTCTTCATCTCTCAAAG---------------------
CONSENSUS
CTCTCTCATGTGTTCTTCATCTCTCAAAG.....................

A5-AT4G31877-XLOC_024638-2053-0
AATATCTGTATGAAGATTGTTCTTATTTGCTGCCATTGATTTGGTTCCCA
A5-AT4G31877-XLOC_024638-2053-1

CONSENSUS
....

A5-AT4G31877-XLOC_024638-2053-0
ATTGCATGCGAGTTTGCTTTTTGTATTTTCCTTTGTTGAACTTTATATTT
A5-AT4G31877-XLOC_024638-2053-1

CONSENSUS
....

A5-AT4G31877-XLOC_024638-2053-0
TATGAGATCATTAGTATCGAAAGCCTAATCTATGAGTTTAAGGCGTGGTT
A5-AT4G31877-XLOC_024638-2053-1

CONSENSUS
....

A5-AT4G31877-XLOC_024638-2053-0
AAGTGAGATTAACATGGGACCATTAACGTTAACCTAGTTTTCTTAGGCTG
A5-AT4G31877-XLOC_024638-2053-1

CONSENSUS
....

A5-AT4G31877-XLOC_024638-2053-0
GTCCAAACACTTTTGAATCCCTTTATTTCCTCGATCCAAGTTAATATTAA
A5-AT4G31877-XLOC_024638-2053-1

CONSENSUS
....

A5-AT4G31877-XLOC_024638-2053-0

ATCGTTTATCTTTTTCTTTTATCTCGTTTCTCATTAGCCCTTTTAAATCG
A5-AT4G31877-XLOC_024638-2053-1

CONSENSUS
....

A5-AT4G31877-XLOC_024638-2053-0
TGATTTCTAGTCGTGATCTCTTTATAAATATCTGTAGATATTATTTGCTC
A5-AT4G31877-XLOC_024638-2053-1

CONSENSUS
....

A5-AT4G31877-XLOC_024638-2053-0
CATACGATCATATCTATGTGTATAGTCATGGATCTTGCAAAGTACTGTTC
A5-AT4G31877-XLOC_024638-2053-1

CONSENSUS
....

A5-AT4G31877-XLOC_024638-2053-0
ATGATCTTTGAACTGCTAGTGATTAGTCGTTTGTTGTGCAGATTATTTAT
A5-AT4G31877-XLOC_024638-2053-1

CONSENSUS
....

A5-AT4G31877-XLOC_024638-2053-0
ATTGCATGCTCGTACGTTTTGATTCCATTTATGTAATTATGTTTCTAATT
A5-AT4G31877-XLOC_024638-2053-1

CONSENSUS
....

A5-AT4G31877-XLOC_024638-2053-0
TCGTCTATTATATATAGTTTATTGTGTTTTGTACTACTTGTAGATGGTAT
A5-AT4G31877-XLOC_024638-2053-1

CONSENSUS
....

A5-AT4G31877-XLOC_024638-2053-0
CTAATTTATATATCATTGATCCATGTGATCACGTTGTGAATCTTGTGAGA
A5-AT4G31877-XLOC_024638-2053-1

CONSENSUS
....

A5-AT4G31877-XLOC_024638-2053-0
ATATTCTCGTGGGTTTGTTTCTTGATTTAAGAACATGCTATTTTGGTTTG
A5-AT4G31877-XLOC_024638-2053-1

CONSENSUS
....

A5-AT4G31877-XLOC_024638-2053-0

TTTCTTGATTTGAGAACATGTTGTTTGCAGGTCCTCAAAACGACGTTTCA
A5-AT4G31877-XLOC_024638-2053-1

CONSENSUS
....

A5-AT4G31877-XLOC_024638-2053-0
GACAGTGTTTGAAATTTGTTTGTTGTTTTTTTACCAAAATCTTCAAGTGA
A5-AT4G31877-XLOC_024638-2053-1

CONSENSUS
....

A5-AT4G31877-XLOC_024638-2053-0
TTTGAACCTGGTAGGGAATTTTTGGATCTAGGAAGCGATTTGTCATTTGC
A5-AT4G31877-XLOC_024638-2053-1

CONSENSUS
....

A5-AT4G31877-XLOC_024638-2053-0
AGTATCATGATTAAGGAATTTCCAGTATTTGACTGCTTTATGTATGAAAT
A5-AT4G31877-XLOC_024638-2053-1
---GTATGAAAT
CONSENSUS
....GTATGAAAT

A5-AT4G31877-XLOC_024638-2053-0
GTTATCTTGGGGTTTTCTTCTGGGGGAAGAGGTGAGACATATTCTACAAA
A5-AT4G31877-XLOC_024638-2053-1
GTTATCTTGGGGTTTTCTTCTGGGGGAAGAGGTGAGACATATTCTACAAA
CONSENSUS
GTTATCTTGGGGTTTTCTTCTGGGGGAAGAGGTGAGACATATTCTACAAA

A5-AT4G31877-XLOC_024638-2053-0
TCAATAAAAACATCTAACATTCATCGTGTCTATTGTATTGCTAGATGTCT
A5-AT4G31877-XLOC_024638-2053-1
TCAATAAAAACATCTAACATTCATCGTGTCTATTGTATTGCTAGATGTCT
CONSENSUS
TCAATAAAAACATCTAACATTCATCGTGTCTATTGTATTGCTAGATGTCT

A5-AT4G31877-XLOC_024638-2053-0
ATTGTCATACGTTTGTGTTGTTCTTGCTACTGATTTGTTTCTTGCTATTG
A5-AT4G31877-XLOC_024638-2053-1
ATTGTCATACGTTTGTGTTGTTCTTGCTACTGATTTGTTTCTTGCTATTG
CONSENSUS
ATTGTCATACGTTTGTGTTGTTCTTGCTACTGATTTGTTTCTTGCTATTG

A5-AT4G31877-XLOC_024638-2053-0
TATTGTTCCCATGTTTTTCATTGTTTACAAACCGACGAAAAGAAGCCAGA
A5-AT4G31877-XLOC_024638-2053-1
TATTGTTCCCATGTTTTTCATTGTTTACAAACCGACGAAAAGAAGCCAGA
CONSENSUS
TATTGTTCCCATGTTTTTCATTGTTTACAAACCGACGAAAAGAAGCCAGA

A5-AT4G31877-XLOC_024638-2053-0

TGAGATGCAAACTTTTGTTCTTGCAATAATTGACAACAATTGGTTTTTAA
A5-AT4G31877-XLOC_024638-2053-1
TGAGATGCAAACTTTTGTTCTTGCAATAATTGACAACAATTGGTTTTTAA
CONSENSUS
TGAGATGCAAACTTTTGTTCTTGCAATAATTGACAACAATTGGTTTTTAA

A5-AT4G31877-XLOC_024638-2053-0
TTTCTTCAAGAGAAATAGTATAGTGTTTTCTTGATGCTTGTGAAATGAGT
A5-AT4G31877-XLOC_024638-2053-1
TTTCTTCAAGAGAAATAGTATAGTGTTTTCTTGATGCTTGTGAAATGAGT
CONSENSUS
TTTCTTCAAGAGAAATAGTATAGTGTTTTCTTGATGCTTGTGAAATGAGT

A5-AT4G31877-XLOC_024638-2053-0
TAGCATTAATCATTCATCGATAATCCTCAAACGCCATGTCTAAAAGAACG
A5-AT4G31877-XLOC_024638-2053-1
TAGCATTAATCATTCATCGATAATCCTCAAACGCCATGTCTAAAAGAACG
CONSENSUS
TAGCATTAATCATTCATCGATAATCCTCAAACGCCATGTCTAAAAGAACG

A5-AT4G31877-XLOC_024638-2053-0
ATATGTAAGTTACAAGTTCTCTCGTTATTCTAAAGTTAGTCTGATGGAAA
A5-AT4G31877-XLOC_024638-2053-1
ATATGTAAGTTACAAGTTCTCTCGTTATTCTAAAGTTAGTCTGATGGAAA
CONSENSUS
ATATGTAAGTTACAAGTTCTCTCGTTATTCTAAAGTTAGTCTGATGGAAA

A5-AT4G31877-XLOC_024638-2053-0
TGTCTCATGATACTAAGCCATCATTTGAAATGGAATATATATGGTTTGCA
A5-AT4G31877-XLOC_024638-2053-1
TGTCTCATGATACTAAGCCATCATTTGAAATGGAATATATATGGTTTGCA
CONSENSUS
TGTCTCATGATACTAAGCCATCATTTGAAATGGAATATATATGGTTTGCA

A5-AT4G31877-XLOC_024638-2053-0
GAGCTCATATGTATACTTAGCTTGCATCACATTATTGCTGTGATTAAGAT
A5-AT4G31877-XLOC_024638-2053-1
GAGCTCATATGTATACTTAGCTTGCATCACATTATTGCTGTGATTAAGAT
CONSENSUS
GAGCTCATATGTATACTTAGCTTGCATCACATTATTGCTGTGATTAAGAT

A5-AT4G31877-XLOC_024638-2053-0
GTCACTAAGGTTTTGTTTTAGAATGACATAGAGCATTTTTGGTGAGCTAG
A5-AT4G31877-XLOC_024638-2053-1
GTCACTAAGGTTTTGTTTTAGAATGACATAGAGCATTTTTGGTGAGCTAG
CONSENSUS
GTCACTAAGGTTTTGTTTTAGAATGACATAGAGCATTTTTGGTGAGCTAG

A5-AT4G31877-XLOC_024638-2053-0
GAAGACAGATCAAAGAGGCTCTAAGTTTGTCAAAG
A5-AT4G31877-XLOC_024638-2053-1
GAAGACAGATCAAAGAGGCTCTAAGTTTGTCAAAG
CONSENSUS
GAAGACAGATCAAAGAGGCTCTAAGTTTGTCAAAG

alignment for event: RI-AT4G01915-XLOC_020222-9045

RI-AT4G01915-XLOC_020222-9045-0
      GTCCCGTTCTTGCTTGGCTTATTTTACTGTACAGTATGTTTACTACAACT
RI-AT4G01915-XLOC_020222-9045-1
      GTCCCGTTCTTGCTTGGCTTATTTTACTGTACAGTATGTTTACTACAACT
CONSENSUS
      GTCCCGTTCTTGCTTGGCTTATTTTACTGTACAGTATGTTTACTACAACT

RI-AT4G01915-XLOC_020222-9045-0
      CCTCTTGGATGATAGAACCTCTATAAAGCCTCCACGTTGCAACCTGTGAC
RI-AT4G01915-XLOC_020222-9045-1
      CCTCTTGGATGATAGAACCTCTATAAAGCCTCCACGTTGCAACCTGTGAC
CONSENSUS
      CCTCTTGGATGATAGAACCTCTATAAAGCCTCCACGTTGCAACCTGTGAC

RI-AT4G01915-XLOC_020222-9045-0
      AAAACCATAATTTCGCTCTTGTGAGTATGTATTGTTGTGTAGAAGTATAA
RI-AT4G01915-XLOC_020222-9045-1
      AAAACCATAATTTCGCTCTT------------------------------
CONSENSUS
      AAAACCATAATTTCGCTCTT..............................

RI-AT4G01915-XLOC_020222-9045-0
      CTATTTTGGGGACAGTGGAAGTGAAACTCTATATGGTTTGGTGAATGAAA
RI-AT4G01915-XLOC_020222-9045-1

CONSENSUS
      ....

RI-AT4G01915-XLOC_020222-9045-0
      CAGAGCACTGGTGTTGTTCTTAGAGCTAAGTTCTGAAATCTACAAAGCTG
RI-AT4G01915-XLOC_020222-9045-1 ---
AGCACTGGTGTTGTTCTTAGAGCTAAGTTCTGAAATCTACAAAGCTG
CONSENSUS
      ...AGCACTGGTGTTGTTCTTAGAGCTAAGTTCTGAAATCTACAAAGCTG

RI-AT4G01915-XLOC_020222-9045-0
      GAGTTTCAGACAACATAGAAGCTTACATTCTATTTGCCTTAGCAGAATTT
RI-AT4G01915-XLOC_020222-9045-1
      GAGTTTCAGACAACATAGAAGCTTACATTCTATTTGCCTTAGCAGAATTT
CONSENSUS
      GAGTTTCAGACAACATAGAAGCTTACATTCTATTTGCCTTAGCAGAATTT

RI-AT4G01915-XLOC_020222-9045-0
      ATTTGGTTCTCTTTGGATAAACTTGGATTGGCTTCACCATTGCCACTCTC
RI-AT4G01915-XLOC_020222-9045-1
      ATTTGGTTCTCTTTGGATAAACTTGGATTGGCTTCACCATTGCCACTCTC
CONSENSUS
      ATTTGGTTCTCTTTGGATAAACTTGGATTGGCTTCACCATTGCCACTCTC

RI-AT4G01915-XLOC_020222-9045-0
      CTTGGTGTCGCCTGTCCTTTAGCCGAAATCTTCATAATGCA
RI-AT4G01915-XLOC_020222-9045-1
      CTTGGTGTCGCCTGTCCTTTAGCCGAAATCTTCATAATGCA

CONSENSUS
      CTTGGTGTCGCCTGTCCTTTAGCCGAAATCTTCATAATGCA

alignment for event: RI-AT4G14550-XLOC_023653-7241

RI-AT4G14550-XLOC_023653-7241-0
      GGAGTTATGGAGCACAAGGGATGATAGATTTCATGAACGAGAGTAAAGTG
RI-AT4G14550-XLOC_023653-7241-1
      GGAGTTATGGAGCACAAGGGATGATAGATTTCATGAACGAGAGTAAAGTG
CONSENSUS
      GGAGTTATGGAGCACAAGGGATGATAGATTTCATGAACGAGAGTAAAGTG

RI-AT4G14550-XLOC_023653-7241-0
      ATGGATCTGTTGAACAGTTCTGAGTATGTTCCAAGCTACGAGGACAAAGA
RI-AT4G14550-XLOC_023653-7241-1
      ATGGATCTGTTGAACAGTTCTGAGTATGTTCCAAGCTACGAGGACAAAGA
CONSENSUS
      ATGGATCTGTTGAACAGTTCTGAGTATGTTCCAAGCTACGAGGACAAAGA

RI-AT4G14550-XLOC_023653-7241-0
      TGGTGACTGGATGCTCGTTGGTGATGTCCCCTGGCCGTGAGTTTCCTCAT
RI-AT4G14550-XLOC_023653-7241-1
      TGGTGACTGGATGCTCGTTGGTGATGTCCCCTGGCC--------------
CONSENSUS
      TGGTGACTGGATGCTCGTTGGTGATGTCCCCTGGCC..............

RI-AT4G14550-XLOC_023653-7241-0
      TCTTCTTGCTTTCATTATTATGACCAAAATTATTCTCTAAACAAAAAAAA
RI-AT4G14550-XLOC_023653-7241-1

CONSENSUS
      ....

RI-AT4G14550-XLOC_023653-7241-0
      CAATATTCTCTAAAGCATTATTATTGATATTACTTATCAAAAAAATACAC
RI-AT4G14550-XLOC_023653-7241-1

CONSENSUS
      ....

RI-AT4G14550-XLOC_023653-7241-0
      AAAATGATAATCAATATCCATGTGTTATAAACACGCACAGCCATCTTTTG
RI-AT4G14550-XLOC_023653-7241-1

CONSENSUS
      ....

RI-AT4G14550-XLOC_023653-7241-0
      GTTGGCATGGGACAGAACTCAGAGACAGAGAAGATGTTTATATATAAATA
RI-AT4G14550-XLOC_023653-7241-1

CONSENSUS
      ....

RI-AT4G14550-XLOC_023653-7241-0
CTAACTCATCAATATGTTACCTCATTTGTAGCTGGCACATATTCTTTCAC
RI-AT4G14550-XLOC_023653-7241-1

CONSENSUS
....

RI-AT4G14550-XLOC_023653-7241-0
TTTCAATAGATTTCTAAATTTAGTCACCAACCCAAATCCCGATTTCAGGA
RI-AT4G14550-XLOC_023653-7241-1
--GA
CONSENSUS
....GA

RI-AT4G14550-XLOC_023653-7241-0
TGTTTGTCGAGTCATGCAAACGTTTGCGCATAATGAAAGGATCCGAAGCA
RI-AT4G14550-XLOC_023653-7241-1
TGTTTGTCGAGTCATGCAAACGTTTGCGCATAATGAAAGGATCCGAAGCA
CONSENSUS
TGTTTGTCGAGTCATGCAAACGTTTGCGCATAATGAAAGGATCCGAAGCA

RI-AT4G14550-XLOC_023653-7241-0 ATTGGACTTG
RI-AT4G14550-XLOC_023653-7241-1 ATTGGACTTG
CONSENSUS ATTGGACTTG

alignment for event: A3-AT4G18120-XLOC_021368-9757

A3-AT4G18120-XLOC_021368-9757-0
CGAAGAAGCAGAATTAATCAAAGTTCAGAGCTTTCACTATGAATATCCCA
A3-AT4G18120-XLOC_021368-9757-1
CGAAGAAGCAGAATTAATCAAAGTTCAGAGCTTTCACTATGAATATCCCA
CONSENSUS
CGAAGAAGCAGAATTAATCAAAGTTCAGAGCTTTCACTATGAATATCCCA

A3-AT4G18120-XLOC_021368-9757-0
AGTGGAACCTTCTCAAGATCTGATCATTTCCATGCTTCAAGTGATGCTTC
A3-AT4G18120-XLOC_021368-9757-1
AGTGGAACCTTCTCAAGATCTGATCATTTCCATGCTTCAAGTGATGCTTC
CONSENSUS
AGTGGAACCTTCTCAAGATCTGATCATTTCCATGCTTCAAGTGATGCTTC

A3-AT4G18120-XLOC_021368-9757-0
TCTCTTTTCAAGCTCTCTTCCTCTTATTCAACACCAAAACATCAACCCTC
A3-AT4G18120-XLOC_021368-9757-1
TCTCTTTTCAAGCTCTCTTCCTCTTATTCAACACCAAAACA---------
CONSENSUS
TCTCTTTTCAAGCTCTCTTCCTCTTATTCAACACCAAAACA.........

A3-AT4G18120-XLOC_021368-9757-0
GTGACAGCTACCATCAGTCTGTTGATGAAATGGCTTCTGGCTTAGACCAT
A3-AT4G18120-XLOC_021368-9757-1 -------
CTACCATCAGTCTGTTGATGAAATGGCTTCTGGCTTAGACCAT
CONSENSUS
.......CTACCATCAGTCTGTTGATGAAATGGCTTCTGGCTTAGACCAT

A3-AT4G18120-XLOC_021368-9757-0
TTTAGTGGAGGCATTGGCAATATGCTTGATGATGGTGACTCACATCCAAT
A3-AT4G18120-XLOC_021368-9757-1
TTTAGTGGAGGCATTGGCAATATGCTTGATGATGGTGACTCACATCCAAT
CONSENSUS
TTTAGTGGAGGCATTGGCAATATGCTTGATGATGGTGACTCACATCCAAT

A3-AT4G18120-XLOC_021368-9757-0
CGGAAACATGCTTCCTGATGATGAGGAAGAGCTTTTCTCTGGTCTAATGG
A3-AT4G18120-XLOC_021368-9757-1
CGGAAACATGCTTCCTGATGATGAGGAAGAGCTTTTCTCTGGTCTAATGG
CONSENSUS
CGGAAACATGCTTCCTGATGATGAGGAAGAGCTTTTCTCTGGTCTAATGG

A3-AT4G18120-XLOC_021368-9757-0
ATGATTTAAACTTAAGTTCCTTGCCAGCTACGTTGGATGACTTGGAAGAT
A3-AT4G18120-XLOC_021368-9757-1
ATGATTTAAACTTAAGTTCCTTGCCAGCTACGTTGGATGACTTGGAAGAT
CONSENSUS
ATGATTTAAACTTAAGTTCCTTGCCAGCTACGTTGGATGACTTGGAAGAT

A3-AT4G18120-XLOC_021368-9757-0
TATGATTTATTCGGTAGCGGAGGAGGTCTTGAATTGGAGACTGATCCATA
A3-AT4G18120-XLOC_021368-9757-1
TATGATTTATTCGGTAGCGGAGGAGGTCTTGAATTGGAGACTGATCCATA
CONSENSUS
TATGATTTATTCGGTAGCGGAGGAGGTCTTGAATTGGAGACTGATCCATA

A3-AT4G18120-XLOC_021368-9757-0
TGACAGTCTAAACAAGGGCTTCTCAAGAATGGGTTTTGCTGATTCTAACG
A3-AT4G18120-XLOC_021368-9757-1
TGACAGTCTAAACAAGGGCTTCTCAAGAATGGGTTTTGCTGATTCTAACG
CONSENSUS
TGACAGTCTAAACAAGGGCTTCTCAAGAATGGGTTTTGCTGATTCTAACG

A3-AT4G18120-XLOC_021368-9757-0
TTGACAATGTTATGCCTCAAAACATTTTCCAAAATGGAGTGGGATCGATT
A3-AT4G18120-XLOC_021368-9757-1
TTGACAATGTTATGCCTCAAAACATTTTCCAAAATGGAGTGGGATCGATT
CONSENSUS
TTGACAATGTTATGCCTCAAAACATTTTCCAAAATGGAGTGGGATCGATT

A3-AT4G18120-XLOC_021368-9757-0
GCTGGCGAACATCCTTATGGTGAACATCCTTCAAGGACTTTGTTTGTTCG
A3-AT4G18120-XLOC_021368-9757-1
GCTGGCGAACATCCTTATGGTGAACATCCTTCAAGGACTTTGTTTGTTCG
CONSENSUS
GCTGGCGAACATCCTTATGGTGAACATCCTTCAAGGACTTTGTTTGTTCG

A3-AT4G18120-XLOC_021368-9757-0
GAATATTAACAGTAACGTAGAGGATTCTGAATTGCAAGCTCTTTTTGAG
A3-AT4G18120-XLOC_021368-9757-1
GAATATTAACAGTAACGTAGAGGATTCTGAATTGCAAGCTCTTTTTGAG
CONSENSUS
GAATATTAACAGTAACGTAGAGGATTCTGAATTGCAAGCTCTTTTTGAG

alignment for event: A5-AT4G11960-XLOC_023465-5958

A5-AT4G11960-XLOC_023465-5958-0
GTCAGGTGGGTGGGGAAGAAGTTGATAGCAAGATTTTACCTTATTGTAGC
A5-AT4G11960-XLOC_023465-5958-1
GTCAGGTGGGTGGGGAAGAAGTTGATAGCAAGATTTTACCTTATTGTAGC
CONSENSUS
GTCAGGTGGGTGGGGAAGAAGTTGATAGCAAGATTTTACCTTATTGTAGC

A5-AT4G11960-XLOC_023465-5958-0
ATCAACAAGAATGAGAAGAGAACTATCGGTGAAATGGAACAAGAGTTCCT
A5-AT4G11960-XLOC_023465-5958-1
ATCAACAAGAATGAGAAGAGAACTATCGGTGAAATGGAACAAGAGTTCCT
CONSENSUS
ATCAACAAGAATGAGAAGAGAACTATCGGTGAAATGGAACAAGAGTTCCT

A5-AT4G11960-XLOC_023465-5958-0
CCAAGCGATGCAATCGTTTTATTACGAAGGCAAAGCGATTATGTCTAATG
A5-AT4G11960-XLOC_023465-5958-1 CCAAGCGAT----
TCGTTTTATTACGAAGGCAAAGCGATTATGTCTAATG
CONSENSUS
CCAAGCGAT....TCGTTTTATTACGAAGGCAAAGCGATTATGTCTAATG

A5-AT4G11960-XLOC_023465-5958-0
AAGAGTTTGATAACCTTAAAGAAGAGTTGATGTGGGAAGGAAGCAGTGTT
A5-AT4G11960-XLOC_023465-5958-1
AAGAGTTTGATAACCTTAAAGAAGAGTTGATGTGGGAAGGAAGCAGTGTT
CONSENSUS
AAGAGTTTGATAACCTTAAAGAAGAGTTGATGTGGGAAGGAAGCAGTGTT

A5-AT4G11960-XLOC_023465-5958-0 GTCATGCTAA
A5-AT4G11960-XLOC_023465-5958-1 GTCATGCTAA
CONSENSUS GTCATGCTAA

alignment for event: RI-AT4G12560-XLOC_021029-8451

RI-AT4G12560-XLOC_021029-8451-0
GGATGATTTCCTGTCCAAAGGATTCAAGCTGGTCTTATAAGCCAAAAACA
RI-AT4G12560-XLOC_021029-8451-1
GGATGATTTCCTGTCCAAAGGATTCAAGCTGGTCTTATAAGCCAAAAACA
CONSENSUS
GGATGATTTCCTGTCCAAAGGATTCAAGCTGGTCTTATAAGCCAAAAACA

RI-AT4G12560-XLOC_021029-8451-0
AGAGACAGGAGCTAAAGCTGCACAAGGTAAGTGAAAAAGTTGACCGTTCT
RI-AT4G12560-XLOC_021029-8451-1
AGAGACAGGAGCTAAAGCTGCACAAGGTAAGTGAAAAAGTTGACCGTTCT
CONSENSUS
AGAGACAGGAGCTAAAGCTGCACAAGGTAAGTGAAAAAGTTGACCGTTCT

RI-AT4G12560-XLOC_021029-8451-0

TAATGGGTCTAGTTTGCTGCTGCATTCTTATTGCATTTCATTCTTTTCTG
RI-AT4G12560-XLOC_021029-8451-1
TAATGGGTCTAGTTTGCTGCTGCATTCTTATTGCATTTCATTCTTTTCTG
CONSENSUS
TAATGGGTCTAGTTTGCTGCTGCATTCTTATTGCATTTCATTCTTTTCTG

RI-AT4G12560-XLOC_021029-8451-0
TCTCTGCTCTGTTTTGTCGCGGACGTTCATATCCGGTGTCTGTTTTGTGG
RI-AT4G12560-XLOC_021029-8451-1
TCTCTGCTCTGTTTTGTCGCGGACGTTCATATCCGGTGTCTGTTTTGTGG
CONSENSUS
TCTCTGCTCTGTTTTGTCGCGGACGTTCATATCCGGTGTCTGTTTTGTGG

RI-AT4G12560-XLOC_021029-8451-0
CGGACGAGCAATTTTCATATTCGGGTTGGGACAAGAGTGAAAGTCACAGT
RI-AT4G12560-XLOC_021029-8451-1
CGGACGAGCAATTTTCATATTCGGGTTGGGACAAGAGTGAAAGTCACAGT
CONSENSUS
CGGACGAGCAATTTTCATATTCGGGTTGGGACAAGAGTGAAAGTCACAGT

RI-AT4G12560-XLOC_021029-8451-0
ATGTCATAGGTATTGAGTGCTTAGAAGTTAGAACATTCCCTAGAAAATGT
RI-AT4G12560-XLOC_021029-8451-1
ATGTCATAG---
CONSENSUS
ATGTCATAG...

RI-AT4G12560-XLOC_021029-8451-0
TATGGAGTCATCATCAGGATAATTACTTTAGCATATGACCATATTCAAGT
RI-AT4G12560-XLOC_021029-8451-1

CONSENSUS
....

RI-AT4G12560-XLOC_021029-8451-0
GTGGTTTTTTCACATCAATCTATCTGCAAAACAATCATTGGGTATGATTT
RI-AT4G12560-XLOC_021029-8451-1

CONSENSUS
....

RI-AT4G12560-XLOC_021029-8451-0
TTTCAGGGAGGAGAAACGAGAAGGGGAACAAACAATAACTTCAACCAAAA
RI-AT4G12560-XLOC_021029-8451-1 ------
GGAGGAGAAACGAGAAGGGGAACAAACAATAACTTCAACCAAAA
CONSENSUS
......GGAGGAGAAACGAGAAGGGGAACAAACAATAACTTCAACCAAAA

RI-AT4G12560-XLOC_021029-8451-0
AGCTCTAATATACCAAAGGAGAATAATATACAACTTACCTACGTATATGT
RI-AT4G12560-XLOC_021029-8451-1
AGCTCTAATATACCAAAGGAGAATAATATACAACTTACCTACGTATATGT
CONSENSUS
AGCTCTAATATACCAAAGGAGAATAATATACAACTTACCTACGTATATGT

RI-AT4G12560-XLOC_021029-8451-0

GCTTATAGAAGAGAGGACAAATGTTTTTGGTTTGTAAATAACTCTTTTTA
RI-AT4G12560-XLOC_021029-8451-1
GCTTATAGAAGAGAGGACAAATGTTTTTGGTTTGTAAATAACTCTTTTTA
CONSENSUS
GCTTATAGAAGAGAGGACAAATGTTTTTGGTTTGTAAATAACTCTTTTTA

RI-AT4G12560-XLOC_021029-8451-0
AGTTTGATATATATAACTTACTAGTACTAACGCTTTGTATACTTGCTTTT
RI-AT4G12560-XLOC_021029-8451-1
AGTTTGATATATATAACTTACTAGTACTAACGCTTTGTATACTTGCTTTT
CONSENSUS
AGTTTGATATATATAACTTACTAGTACTAACGCTTTGTATACTTGCTTTT

RI-AT4G12560-XLOC_021029-8451-0
TTAAGTTTCTGCATAATATGAGATGTTAAATAAATTA
RI-AT4G12560-XLOC_021029-8451-1
TTAAGTTTCTGCATAATATGAGATGTTAAATAAATTA
CONSENSUS
TTAAGTTTCTGCATAATATGAGATGTTAAATAAATTA

alignment for event: A5-AT4G13670-XLOC_021094-9474

A5-AT4G13670-XLOC_021094-9474-0
ATCTTCCCGCCATGTGTATGTGGTAACTAACAAAAGCTTCGATTACTTTA
A5-AT4G13670-XLOC_021094-9474-1
ATCTTCCCGCCATGTGTATGTGGTAACTAACAAAAGCTTCGATTACTTTA
CONSENSUS
ATCTTCCCGCCATGTGTATGTGGTAACTAACAAAAGCTTCGATTACTTTA

A5-AT4G13670-XLOC_021094-9474-0
TCCTCTCACTCTCTAATGGCTTCTTCTTCTCTACCTCTTTCTCTTCCGTT
A5-AT4G13670-XLOC_021094-9474-1
TCCTCTCACTCTCTAATGGCTTCTTCTTCTCTACCTCTTTCTCTTCCGTT
CONSENSUS
TCCTCTCACTCTCTAATGGCTTCTTCTTCTCTACCTCTTTCTCTTCCGTT

A5-AT4G13670-XLOC_021094-9474-0
TCCACTCCGATCTCTTACTAGTACCACTCGATCTCTACCATTTCAATGTT
A5-AT4G13670-XLOC_021094-9474-1
TCCACTCCGATCTCTTACTAGTACCACTCGATCTCTACCATTTCAATGTT
CONSENSUS
TCCACTCCGATCTCTTACTAGTACCACTCGATCTCTACCATTTCAATGTT

A5-AT4G13670-XLOC_021094-9474-0
CTCCTCTCTTTTTCTCTATTCCTTCTTCAATCGTTTGCTTCTCCACTCAA
A5-AT4G13670-XLOC_021094-9474-1
CTCCTCTCTTTTTCTCTATTCCTTCTTCAATCGTTTGCTTCTCCACTCAA
CONSENSUS
CTCCTCTCTTTTTCTCTATTCCTTCTTCAATCGTTTGCTTCTCCACTCAA

A5-AT4G13670-XLOC_021094-9474-0
AATCCCGACCGCGAAGAGGTCCGGTGGCTCCGGGAAGAGCAGAGATGGAT
A5-AT4G13670-XLOC_021094-9474-1
AATCCCGACCGCGAAGAGGTCCGGTGGCTCCGGGAAGAGCAGAGATGGAT

CONSENSUS
AATCCCGACCGCGAAGAGGTCCGGTGGCTCCGGGAAGAGCAGAGATGGAT

A5-AT4G13670-XLOC_021094-9474-0
TCGCGAGGAGCAACGATGGATTCGTGAAGAACAGAGATGGATACGCGAAC
A5-AT4G13670-XLOC_021094-9474-1
TCGCGAGGAGCAACGATGGATTCGTGAAGAACAGAGATGGATACGCGAAC
CONSENSUS
TCGCGAGGAGCAACGATGGATTCGTGAAGAACAGAGATGGATACGCGAAC

A5-AT4G13670-XLOC_021094-9474-0
GTGAATCGCTTCTACAAGAGATTTCGGATCTACAGCTCAGAATTCAATCC
A5-AT4G13670-XLOC_021094-9474-1
GTGAATCGCTTCTACAAGAGATTTCGGATCTACAGCTCAGAATTCAATCC
CONSENSUS
GTGAATCGCTTCTACAAGAGATTTCGGATCTACAGCTCAGAATTCAATCC

A5-AT4G13670-XLOC_021094-9474-0
CTAGAGTCACGAAATTCGCAATTGGGGAATTCTATTCCCGATACGATTTC
A5-AT4G13670-XLOC_021094-9474-1
CTAGAGTCACGAAATTCGCAATTGGGGAATTCTATTCCCGATACGATTTC
CONSENSUS
CTAGAGTCACGAAATTCGCAATTGGGGAATTCTATTCCCGATACGATTTC

A5-AT4G13670-XLOC_021094-9474-0
GAATATCGCTGCTTTGCTTCAGGTTTTGAAGGAGAAGAATCGGATTTCTG
A5-AT4G13670-XLOC_021094-9474-1
GAATATCGCTGCTTTGCTTCAGGTTTTGAAGGAGAAGAATCGGATTTCTG
CONSENSUS
GAATATCGCTGCTTTGCTTCAGGTTTTGAAGGAGAAGAATCGGATTTCTG

A5-AT4G13670-XLOC_021094-9474-0
AGAGTGGATTGAGCGCAACGCCGATGGTATTGGAGAGTACGAGAGAACAA
A5-AT4G13670-XLOC_021094-9474-1
AGAGTGGATTGAGCGCAACGCCGATGGTATTGGAGAGTACGAGAGAACAA
CONSENSUS
AGAGTGGATTGAGCGCAACGCCGATGGTATTGGAGAGTACGAGAGAACAA

A5-AT4G13670-XLOC_021094-9474-0
ATTGTTGAGGAGGTGGAAGAAGAAGAGAAGCGAGTGATTATTGCTGAAGA
A5-AT4G13670-XLOC_021094-9474-1
ATTGTTGAGGAGGTGGAAGAAGAAGAGAAGCGAGTGATTATTGCTGAAGA
CONSENSUS
ATTGTTGAGGAGGTGGAAGAAGAAGAGAAGCGAGTGATTATTGCTGAAGA

A5-AT4G13670-XLOC_021094-9474-0
GAAAGTTAGGGTTTCGGAGCCGGTGAAGAAGATCAAGAGGAGGATATTGA
A5-AT4G13670-XLOC_021094-9474-1
GAAAGTTAGGGTTTCGGAGCCGGTGAAGAAGATCAAGAGGAGGATATTGA
CONSENSUS
GAAAGTTAGGGTTTCGGAGCCGGTGAAGAAGATCAAGAGGAGGATATTGA

A5-AT4G13670-XLOC_021094-9474-0
AAGTTGGAAGCGAAGGCGACGATGTTCAAGCTTTGCAG------------
A5-AT4G13670-XLOC_021094-9474-1
AAGTTGGAAGCGAAGGCGACGATGTTCAAGCTTTGCAGGTCTGCTCTCAG

```
CONSENSUS
      AAGTTGGAAGCGAAGGCGACGATGTTCAAGCTTTGCAG............

A5-AT4G13670-XLOC_021094-9474-0  -------------
GAAGCTCTGTTGAAATTAGGATTCTATTCGGGCGAAG
A5-AT4G13670-XLOC_021094-9474-1
      TTTTAAACCAAAGGAAGCTCTGTTGAAATTAGGATTCTATTCGGGCGAAG
CONSENSUS
      .............GAAGCTCTGTTGAAATTAGGATTCTATTCGGGCGAAG

A5-AT4G13670-XLOC_021094-9474-0
      AGGATATGGAGTTCTCGAGCTTTTCAAGTGGGACTGCAAGTGCTGTTAAG
A5-AT4G13670-XLOC_021094-9474-1
      AGGATATGGAGTTCTCGAGCTTTTCAAGTGGGACTGCAAGTGCTGTTAAG
CONSENSUS
      AGGATATGGAGTTCTCGAGCTTTTCAAGTGGGACTGCAAGTGCTGTTAAG

A5-AT4G13670-XLOC_021094-9474-0  ACTTGGCAA
A5-AT4G13670-XLOC_021094-9474-1  ACTTGGCAA
CONSENSUS                        ACTTGGCAA



alignment for event: RI-AT4G14740-XLOC_023663-5847

RI-AT4G14740-XLOC_023663-5847-0
      ACATTGTGCTTGATGTGATCAAGAATGTCCCGGCCTGGCCTGGACGACAT
RI-AT4G14740-XLOC_023663-5847-1
      ACATTGTGCTTGATGTGATCAAGAATGTCCCGGCCTGGCCTGGACGACAT
CONSENSUS
      ACATTGTGCTTGATGTGATCAAGAATGTCCCGGCCTGGCCTGGACGACAT

RI-AT4G14740-XLOC_023663-5847-0
      TTGCTAGAGGGAGGAGATGATCTAAGATACTTCGGTTTGAAGACGGTTAT
RI-AT4G14740-XLOC_023663-5847-1
      TTGCTAGAGGGAGGAGATGATCTAAGATACTTCGGTTTGAAGACGGTTAT
CONSENSUS
      TTGCTAGAGGGAGGAGATGATCTAAGATACTTCGGTTTGAAGACGGTTAT

RI-AT4G14740-XLOC_023663-5847-0
      GCGAGGTGATGTTGAATTCGAGGTCAAGAGCCAAAGGGAATATGAAATGT
RI-AT4G14740-XLOC_023663-5847-1
      GCGAGGTGATGTTGAATTCGAGGTCAAGAGCCAAAGGGAATATGAAATGT
CONSENSUS
      GCGAGGTGATGTTGAATTCGAGGTCAAGAGCCAAAGGGAATATGAAATGT

RI-AT4G14740-XLOC_023663-5847-0
      GGACACAAGGTGTCTCAAGGCTTCTTGTTCTTGCTGCTGAGAGGAAGTTT
RI-AT4G14740-XLOC_023663-5847-1
      GGACACAAGGTGTCTCAAGGCTTCTTGTTCTTGCTGCTGAGAGGAAGTTT
CONSENSUS
      GGACACAAGGTGTCTCAAGGCTTCTTGTTCTTGCTGCTGAGAGGAAGTTT

RI-AT4G14740-XLOC_023663-5847-0
      AGGATGTGAATAAACGTTCAATGGCTGCTTGGTTTAAGTGTGAGTTTTTT
RI-AT4G14740-XLOC_023663-5847-1
```

AGGATGTGAATAAACGTTCAATGGCTGCTTGGTTTAAGT-----------
CONSENSUS
AGGATGTGAATAAACGTTCAATGGCTGCTTGGTTTAAGT...........

RI-AT4G14740-XLOC_023663-5847-0
TTTAACTTATGTGGTCAAATTTCATTAGTAGGGGTTCTTTTAAGGTAATG
RI-AT4G14740-XLOC_023663-5847-1

CONSENSUS
....

RI-AT4G14740-XLOC_023663-5847-0
GTTTTTTGGGTTGGGTATAGGATAAAATGGACCTACCAGTCAAGGTGAGG
RI-AT4G14740-XLOC_023663-5847-1 --------------------
GATAAAATGGACCTACCAGTCAAGGTGAGG
CONSENSUS
....................GATAAAATGGACCTACCAGTCAAGGTGAGG

RI-AT4G14740-XLOC_023663-5847-0
AAGCATTTGGGTAAACAAAACTTAGTGGGGGTGATCTGTAATATCTATGT
RI-AT4G14740-XLOC_023663-5847-1
AAGCATTTGGGTAAACAAAACTTAGTGGGGGTGATCTGTAATATCTATGT
CONSENSUS
AAGCATTTGGGTAAACAAAACTTAGTGGGGGTGATCTGTAATATCTATGT

RI-AT4G14740-XLOC_023663-5847-0
TCTTAGTTTTTTTTTGGTTGTTGGTGGTCTTTTTGTATAAAAAAACAAAG
RI-AT4G14740-XLOC_023663-5847-1
TCTTAGTTTTTTTTTGGTTGTTGGTGGTCTTTTTGTATAAAAAAACAAAG
CONSENSUS
TCTTAGTTTTTTTTTGGTTGTTGGTGGTCTTTTTGTATAAAAAAACAAAG

RI-AT4G14740-XLOC_023663-5847-0
TTGAAGTAATAGATATATAGTATGTTTTAATTTTAAA
RI-AT4G14740-XLOC_023663-5847-1
TTGAAGTAATAGATATATAGTATGTTTTAATTTTAAA
CONSENSUS
TTGAAGTAATAGATATATAGTATGTTTTAATTTTAAA

alignment for event: A3-AT4G26555-XLOC_024343-9338

A3-AT4G26555-XLOC_024343-9338-0
CGCGGGAAAATCAAGGAACAGCGTTTCTAGGATTAGTCGTGTTGGATTCT
A3-AT4G26555-XLOC_024343-9338-1
CGCGGGAAAATCAAGGAACAGCGTTTCTAGGATTAGTCGTGTTGGATTCT
CONSENSUS
CGCGGGAAAATCAAGGAACAGCGTTTCTAGGATTAGTCGTGTTGGATTCT

A3-AT4G26555-XLOC_024343-9338-0
CTTCGGTCTCGGCTGTACATGTACCCAGAAGGATGTTTATGCAGCTGTCT
A3-AT4G26555-XLOC_024343-9338-1
CTTCGGTCTCGGCTGTACATGTACCCAGAAGGATGTTTATGCAGCTGTCT
CONSENSUS
CTTCGGTCTCGGCTGTACATGTACCCAGAAGGATGTTTATGCAGCTGTCT

A3-AT4G26555-XLOC_024343-9338-0
      GGATTTGGTTCGGTCTTGACGCTTCTGGATTTTCCTAGTTTAGCGGCGCC
A3-AT4G26555-XLOC_024343-9338-1
      GGATTTGGTTCGGTCTTGACGCTTCTGGATTTTCCTAGTTTAGCGGCGCC
CONSENSUS
      GGATTTGGTTCGGTCTTGACGCTTCTGGATTTTCCTAGTTTAGCGGCGCC

A3-AT4G26555-XLOC_024343-9338-0
      GGTTCCTCAGATGAAGGAACCTGAAGTGATCAG-------GACATTGAAA
A3-AT4G26555-XLOC_024343-9338-1
      GGTTCCTCAGATGAAGGAACCTGAAGTGATCAGAATGCAGGACATTGAAA
CONSENSUS
      GGTTCCTCAGATGAAGGAACCTGAAGTGATCAG.......GACATTGAAA

A3-AT4G26555-XLOC_024343-9338-0 CTCCCGAGTGGCGTGAGGTATCAAG
A3-AT4G26555-XLOC_024343-9338-1 CTCCCGAGTGGCGTGAGGTATCAAG
CONSENSUS   CTCCCGAGTGGCGTGAGGTATCAAG

alignment for event: A3-AT4G26555-XLOC_024343-9339

A3-AT4G26555-XLOC_024343-9339-0
      AGATAATTGAAGGTGAAGGACGAGAAGCTCATGAAGGGGACCTGGTCGAA
A3-AT4G26555-XLOC_024343-9339-1
      AGATAATTGAAGGTGAAGGACGAGAAGCTCATGAAGGGGACCTGGTCGAA
CONSENSUS
      AGATAATTGAAGGTGAAGGACGAGAAGCTCATGAAGGGGACCTGGTCGAA

A3-AT4G26555-XLOC_024343-9339-0
      CTAAACTATGTGTGTCGGCGTGCAAATGGATATTTCGTTCATAG------
A3-AT4G26555-XLOC_024343-9339-1
      CTAAACTATGTGTGTCGGCGTGCAAATGGATATTTCGTTCATAGTTTCTT
CONSENSUS
      CTAAACTATGTGTGTCGGCGTGCAAATGGATATTTCGTTCATAG......

A3-AT4G26555-XLOC_024343-9339-0 -------
CACGGTGGATCAATTCAGTGGTGAAAGCTCTCCTGTCAAACTT
A3-AT4G26555-XLOC_024343-9339-1
      GATTCAGCACGGTGGATCAATTCAGTGGTGAAAGCTCTCCTGTCAAACTT
CONSENSUS
      .......CACGGTGGATCAATTCAGTGGTGAAAGCTCTCCTGTCAAACTT

A3-AT4G26555-XLOC_024343-9339-0 ATTCTCGATGAAAACGAC
A3-AT4G26555-XLOC_024343-9339-1 ATTCTCGATGAAAACGAC
CONSENSUS   ATTCTCGATGAAAACGAC

alignment for event: SE-AT4G07990-XLOC_023196-10282

SE-AT4G07990-XLOC_023196-10282-0
      AAAAAAAAAAATCAAAATCCAATTTGATCTTCAAAGTAGTGAAGAAAGAA
SE-AT4G07990-XLOC_023196-10282-1
      AAAAAAAAAAATCAAAATCCAATTTGATCTTCAAAGTAGTGAAGAAAGAA

CONSENSUS
AAAAAAAAAAAATCAAAATCCAATTTGATCTTCAAAGTAGTGAAGAAAGAA

SE-AT4G07990-XLOC_023196-10282-0
GAGAAGCAATCGAAATTCTCTGACGATGTTGTTACAACCCCAAAGATCCT
SE-AT4G07990-XLOC_023196-10282-1
GAGAAGCAATCGAAATTCTCTGACGATGTTGTTACAACCCCAAAGATCCT
CONSENSUS
GAGAAGCAATCGAAATTCTCTGACGATGTTGTTACAACCCCAAAGATCCT

SE-AT4G07990-XLOC_023196-10282-0
CTTATCGATTATCGTAATTCGTAGAAAACGAAGCAGAGAGAAAATGGGAG
SE-AT4G07990-XLOC_023196-10282-1
CTTATCGATTATCGTAATTCGTAGAAAACGAAGCAGAGAGAAAATGGGAG
CONSENSUS
CTTATCGATTATCGTAATTCGTAGAAAACGAAGCAGAGAGAAAATGGGAG

SE-AT4G07990-XLOC_023196-10282-0
GCGAAGATGATAAAGATAAGAAATGGAATCCTCCACCACCGCAGCAGAAG
SE-AT4G07990-XLOC_023196-10282-1
GCGAAGATGATAAAGATAAGAAATGGAATCCTCCACCACCGCAGCAGAAG
CONSENSUS
GCGAAGATGATAAAGATAAGAAATGGAATCCTCCACCACCGCAGCAGAAG

SE-AT4G07990-XLOC_023196-10282-0
CCGGACTCAAGGCCTTGGGAAGTCCTCGCGGCTTTTGTAATCTGCGCAAC
SE-AT4G07990-XLOC_023196-10282-1
CCGGACTCAAGGCCTTGGGAAGTCCTCGCGGCTTTTGTAATCTGCGCAAC
CONSENSUS
CCGGACTCAAGGCCTTGGGAAGTCCTCGCGGCTTTTGTAATCTGCGCAAC

SE-AT4G07990-XLOC_023196-10282-0
CGCTACTACTTTCGCC----------------------------------
SE-AT4G07990-XLOC_023196-10282-1
CGCTACTACTTTCGCCGTTCATCAGCTGCGAAGAAATTTTGATTGGGTCT
CONSENSUS
CGCTACTACTTTCGCC..................................

SE-AT4G07990-XLOC_023196-10282-0 --------
CTAACTAGAACACCGTCGGCGGGAAGAGGAACTTTCCGAACA
SE-AT4G07990-XLOC_023196-10282-1
ATACCCAGCTAACTAGAACACCGTCGGCGGGAAGAGGAACTTTCCGAACA
CONSENSUS
........CTAACTAGAACACCGTCGGCGGGAAGAGGAACTTTCCGAACA

SE-AT4G07990-XLOC_023196-10282-0
TCTTTTCAGGAGGAAGCATGGAGAAGGTACAATAAACGGATGCAAGAGGA
SE-AT4G07990-XLOC_023196-10282-1
TCTTTTCAGGAGGAAGCATGGAGAAGGTACAATAAACGGATGCAAGAGGA
CONSENSUS
TCTTTTCAGGAGGAAGCATGGAGAAGGTACAATAAACGGATGCAAGAGGA

SE-AT4G07990-XLOC_023196-10282-0 GTATGAGGATGAGTTGGAGAGAGTG
SE-AT4G07990-XLOC_023196-10282-1 GTATGAGGATGAGTTGGAGAGAGTG
CONSENSUS GTATGAGGATGAGTTGGAGAGAGTG

alignment for event: RI-AT4G32440-XLOC_022188-5254

RI-AT4G32440-XLOC_022188-5254-0
ATATCTGGTTCTCTGAAGTCATCCACACTGACTGGATCAGACGTACATCA
RI-AT4G32440-XLOC_022188-5254-1
ATATCTGGTTCTCTGAAGTCATCCACACTGACTGGATCAGACGTACATCA
CONSENSUS
ATATCTGGTTCTCTGAAGTCATCCACACTGACTGGATCAGACGTACATCA

RI-AT4G32440-XLOC_022188-5254-0
GAAGCTACAGCCCCACAGGAACAGCATGCCTCTCCACGAGCCTAGCGTGG
RI-AT4G32440-XLOC_022188-5254-1
GAAGCTACAGCCCCACAGGAACAGCATGCCTCTCCACGAGCCTAGCGTGG
CONSENSUS
GAAGCTACAGCCCCACAGGAACAGCATGCCTCTCCACGAGCCTAGCGTGG

RI-AT4G32440-XLOC_022188-5254-0
TCTCTGCTAGATTGTTAAAGAGGCCGTCACCTTACAACTGGTCTGAATGT
RI-AT4G32440-XLOC_022188-5254-1
TCTCTGCTAGATTGTTAAAGAGGCCGTCACCTTACAACTGGTCTGAATGT
CONSENSUS
TCTCTGCTAGATTGTTAAAGAGGCCGTCACCTTACAACTGGTCTGAATGT

RI-AT4G32440-XLOC_022188-5254-0
GCTGAATCATGTACAGGAAACCCTAAGAAGATGCGTTCATTGGAAAAAGA
RI-AT4G32440-XLOC_022188-5254-1
GCTGAATCATGTACAGGAAACCCTAAGAAGATGCGTTCATTGGAAAAAGA
CONSENSUS
GCTGAATCATGTACAGGAAACCCTAAGAAGATGCGTTCATTGGAAAAAGA

RI-AT4G32440-XLOC_022188-5254-0
AGGACAGCAGCAAAAGGTAGATGCCATTTCTTGCCGACCAGAAAACAGGG
RI-AT4G32440-XLOC_022188-5254-1
AGGACAGCAGCAAAAGGTAGATGCCATTTCTTGCCGACCAGAAAACAGGG
CONSENSUS
AGGACAGCAGCAAAAGGTAGATGCCATTTCTTGCCGACCAGAAAACAGGG

RI-AT4G32440-XLOC_022188-5254-0
GTGGTAAATCTCACGTGCAAGCTTCCTTAAACAATCACAAAACTGGTTAC
RI-AT4G32440-XLOC_022188-5254-1
GTGGTAAATCTCACGTGCAAGCTTCCTTAAACAATCACAAAACTGGTTAC
CONSENSUS
GTGGTAAATCTCACGTGCAAGCTTCCTTAAACAATCACAAAACTGGTTAC

RI-AT4G32440-XLOC_022188-5254-0
TGTCAAATTGTCAGGGTAAGATCAAAAGGGTTTAGTGAGAGTGTTCGTGC
RI-AT4G32440-XLOC_022188-5254-1
TGTCAAATTGTCAGG-----------------------------------
CONSENSUS
TGTCAAATTGTCAGG...................................

RI-AT4G32440-XLOC_022188-5254-0
TGATGATTGTTCTGATAGCGATGTATGCTCAGTTGGTAGTTGTAGTGCTA
RI-AT4G32440-XLOC_022188-5254-1

CONSENSUS
      ....

RI-AT4G32440-XLOC_022188-5254-0
      CTAGTTATGATGAGAGTAACATGCCACCTTGTATGCTAGATGGCTCTACT
RI-AT4G32440-XLOC_022188-5254-1
      ---------------------------------------ATGGCTCTACT
CONSENSUS
      ....ATGGCTCTACT

RI-AT4G32440-XLOC_022188-5254-0
      CAACAGGCAGACTCATGTAGCAGCGATGCTGAATCTTCTTGTGGCCTGGG
RI-AT4G32440-XLOC_022188-5254-1
      CAACAGGCAGACTCATGTAGCAGCGATGCTGAATCTTCTTGTGGCCTGGG
CONSENSUS
      CAACAGGCAGACTCATGTAGCAGCGATGCTGAATCTTCTTGTGGCCTGGG

RI-AT4G32440-XLOC_022188-5254-0
      GGAAGAACCAAGGTGGAAACATTCATCAGTTGGTGATGGAGCAAGAAATT
RI-AT4G32440-XLOC_022188-5254-1
      GGAAGAACCAAGGTGGAAACATTCATCAGTTGGTGATGGAGCAAGAAATT
CONSENSUS
      GGAAGAACCAAGGTGGAAACATTCATCAGTTGGTGATGGAGCAAGAAATT

RI-AT4G32440-XLOC_022188-5254-0
      CTTGTAGGTCGGAACTATATTCTTACCGCAGTACTCTGGGGGAATTATTT
RI-AT4G32440-XLOC_022188-5254-1
      CTTGTAGGTCGGAACTATATTCTTACCGCAGTACTCTGGGGGAATTATTT
CONSENSUS
      CTTGTAGGTCGGAACTATATTCTTACCGCAGTACTCTGGGGGAATTATTT

RI-AT4G32440-XLOC_022188-5254-0
      TCTTCTGGTCCCCTAAGTTGGGAGCAAGAAGCATCATTAACTGATCTTCG
RI-AT4G32440-XLOC_022188-5254-1
      TCTTCTGGTCCCCTAAGTTGGGAGCAAGAAGCATCATTAACTGATCTTCG
CONSENSUS
      TCTTCTGGTCCCCTAAGTTGGGAGCAAGAAGCATCATTAACTGATCTTCG

RI-AT4G32440-XLOC_022188-5254-0
      TCTTTCTCTTAATATATCAGATGATGAACATTTGATGGAG
RI-AT4G32440-XLOC_022188-5254-1
      TCTTTCTCTTAATATATCAGATGATGAACATTTGATGGAG
CONSENSUS
      TCTTTCTCTTAATATATCAGATGATGAACATTTGATGGAG

alignment for event: RI-AT4G32060-XLOC_022165-448

RI-AT4G32060-XLOC_022165-448-0
      GTATATATTCTTTGTGACATTACTCAGCATTCCTGAATCAAGCTTCGCAG
RI-AT4G32060-XLOC_022165-448-1
      GTATATATTCTTTGTGACATTACTCAGCATTCCTGAATCAAGCTTCGCAG
CONSENSUS
      GTATATATTCTTTGTGACATTACTCAGCATTCCTGAATCAAGCTTCGCAG

RI-AT4G32060-XLOC_022165-448-0
TGGCTTTCAAAATGTTTGATACTGACAACAACGGGTGAGTAGTCGTTCAC
RI-AT4G32060-XLOC_022165-448-1
TGGCTTTCAAAATGTTTGATACTGACAACAACGG----------------
CONSENSUS
TGGCTTTCAAAATGTTTGATACTGACAACAACGG................

RI-AT4G32060-XLOC_022165-448-0
ATATGTAAATAGATAAATCATGCTTTTGGTAACTTTTTTTCTGGTCCGTA
RI-AT4G32060-XLOC_022165-448-1

CONSENSUS
....

RI-AT4G32060-XLOC_022165-448-0
TTCCCATATTGTGATCCTCCAAACGGACGGGTTGTGGGAATCAGTGGAGT
RI-AT4G32060-XLOC_022165-448-1

CONSENSUS
....

RI-AT4G32060-XLOC_022165-448-0
CTCTTTTTGAGTTTACGCTGAATCATCTGGTCAGTGCTTCTATCCTTTGT
RI-AT4G32060-XLOC_022165-448-1

CONSENSUS
....

RI-AT4G32060-XLOC_022165-448-0
AACTTCTATTTCCTCGAGATGGTACTGACGAATGATTGTTTTAGGGAGAT
RI-AT4G32060-XLOC_022165-448-1
--GGAGAT
CONSENSUS
....GGAGAT

RI-AT4G32060-XLOC_022165-448-0
TGACAAAGAAGAGTTTAAGACAGTGATGAGTCTGATGCGATCTCAGCATA
RI-AT4G32060-XLOC_022165-448-1
TGACAAAGAAGAGTTTAAGACAGTGATGAGTCTGATGCGATCTCAGCATA
CONSENSUS
TGACAAAGAAGAGTTTAAGACAGTGATGAGTCTGATGCGATCTCAGCATA

RI-AT4G32060-XLOC_022165-448-0
GACAAGGAGTTGGCCACAGAGATGGCCTTCGAACAGGGTTACATATGACC
RI-AT4G32060-XLOC_022165-448-1
GACAAGGAGTTGGCCACAGAGATGGCCTTCGAACAGGGTTACATATGACC
CONSENSUS
GACAAGGAGTTGGCCACAGAGATGGCCTTCGAACAGGGTTACATATGACC

RI-AT4G32060-XLOC_022165-448-0
GGTTCTGTCGAGGATGGAGGATTGGTAGAATACTTCTTTGGGAAAGATGG
RI-AT4G32060-XLOC_022165-448-1
GGTTCTGTCGAGGATGGAGGATTGGTAGAATACTTCTTTGGGAAAGATGG
CONSENSUS
GGTTCTGTCGAGGATGGAGGATTGGTAGAATACTTCTTTGGGAAAGATGG

RI-AT4G32060-XLOC_022165-448-0
      CAGTCAAAAACTGAAACACGACAAATTCACTAAGTTTATGAAAGATCTAA
RI-AT4G32060-XLOC_022165-448-1
      CAGTCAAAAACTGAAACACGACAAATTCACTAAGTTTATGAAAGATCTAA
CONSENSUS
      CAGTCAAAAACTGAAACACGACAAATTCACTAAGTTTATGAAAGATCTAA

RI-AT4G32060-XLOC_022165-448-0   CTGAAGAG
RI-AT4G32060-XLOC_022165-448-1   CTGAAGAG
CONSENSUS   CTGAAGAG

alignment for event: RI-AT4G03140-XLOC_020298-10414

RI-AT4G03140-XLOC_020298-10414-0
      AAAAAACGCATTATTCAAGAATGTGAGCAAGAACTTCCTTATCAAGGGAA
RI-AT4G03140-XLOC_020298-10414-1
      AAAAAACGCATTATTCAAGAATGTGAGCAAGAACTTCCTTATCAAGGGAA
CONSENSUS
      AAAAAACGCATTATTCAAGAATGTGAGCAAGAACTTCCTTATCAAGGGAA

RI-AT4G03140-XLOC_020298-10414-0
      TATCCTCATCCTCATCATCTCATTCAACTTCAAGGCAAGTACCATCTTAA
RI-AT4G03140-XLOC_020298-10414-1
      TATCCTCATCCTCATCATCTCATTCAACTTCAAG----------------
CONSENSUS
      TATCCTCATCCTCATCATCTCATTCAACTTCAAG................

RI-AT4G03140-XLOC_020298-10414-0
      TCTCAAAATCTGAATAAAAATATTCATTGTAAGTTGGGATAAAGGCCATA
RI-AT4G03140-XLOC_020298-10414-1

CONSENSUS
      ....

RI-AT4G03140-XLOC_020298-10414-0
      TGTATTCTGTTTATATGTGAATGACAGGGATATACAAAGTAATTTGCTAT
RI-AT4G03140-XLOC_020298-10414-1

CONSENSUS
      ....

RI-AT4G03140-XLOC_020298-10414-0
      TACAAGTTGGTTTCTCTGATTTGTCTTCTTCCACAAATCTTATCTTGAAG
RI-AT4G03140-XLOC_020298-10414-1

CONSENSUS
      ....

RI-AT4G03140-XLOC_020298-10414-0
      TTAGAAAGCTTTTACTCTTTCCACCAGCTAAGAAATGTCAAATATGAGAG
RI-AT4G03140-XLOC_020298-10414-1

CONSENSUS

....

RI-AT4G03140-XLOC_020298-10414-0
TCTTACCAAATACTAATACCTAATCTATAGTTGTTATGTTATAGGAAGCT
RI-AT4G03140-XLOC_020298-10414-1
--GAAGCT
CONSENSUS
....GAAGCT

RI-AT4G03140-XLOC_020298-10414-0
AGAAGGTAAAGTAGCACTCATCACTGGAGGAGCAAGTGGGATTGGCAAAG
RI-AT4G03140-XLOC_020298-10414-1
AGAAGGTAAAGTAGCACTCATCACTGGAGGAGCAAGTGGGATTGGCAAAG
CONSENSUS
AGAAGGTAAAGTAGCACTCATCACTGGAGGAGCAAGTGGGATTGGCAAAG

RI-AT4G03140-XLOC_020298-10414-0
CAACAGCCGGAAAATTCATCAGTCATGGAGCCAAAGTTATCATTGCCGAT
RI-AT4G03140-XLOC_020298-10414-1
CAACAGCCGGAAAATTCATCAGTCATGGAGCCAAAGTTATCATTGCCGAT
CONSENSUS
CAACAGCCGGAAAATTCATCAGTCATGGAGCCAAAGTTATCATTGCCGAT

RI-AT4G03140-XLOC_020298-10414-0
ATCCAACCGCAGATTGGGCGAGAAACCGAGCAAGAACTCGGTCCCAGTTG
RI-AT4G03140-XLOC_020298-10414-1
ATCCAACCGCAGATTGGGCGAGAAACCGAGCAAGAACTCGGTCCCAGTTG
CONSENSUS
ATCCAACCGCAGATTGGGCGAGAAACCGAGCAAGAACTCGGTCCCAGTTG

RI-AT4G03140-XLOC_020298-10414-0
TGCTTACTTCCCATGCGATGTGACCAAAGAATCAGACATTGCTAACGCAG
RI-AT4G03140-XLOC_020298-10414-1
TGCTTACTTCCCATGCGATGTGACCAAAGAATCAGACATTGCTAACGCAG
CONSENSUS
TGCTTACTTCCCATGCGATGTGACCAAAGAATCAGACATTGCTAACGCAG

RI-AT4G03140-XLOC_020298-10414-0
TTGACTTCGCTGTCTCGCTCCATACAAAGCTCGACATTATGTACAACAAT
RI-AT4G03140-XLOC_020298-10414-1
TTGACTTCGCTGTCTCGCTCCATACAAAGCTCGACATTATGTACAACAAT
CONSENSUS
TTGACTTCGCTGTCTCGCTCCATACAAAGCTCGACATTATGTACAACAAT

RI-AT4G03140-XLOC_020298-10414-0
GCTGGTATTCCCTGCAAAACGCCTCCTAGTATCGTTGATCTTGATCTCAA
RI-AT4G03140-XLOC_020298-10414-1
GCTGGTATTCCCTGCAAAACGCCTCCTAGTATCGTTGATCTTGATCTCAA
CONSENSUS
GCTGGTATTCCCTGCAAAACGCCTCCTAGTATCGTTGATCTTGATCTCAA

RI-AT4G03140-XLOC_020298-10414-0 TGTTTTCGACAAG
RI-AT4G03140-XLOC_020298-10414-1 TGTTTTCGACAAG
CONSENSUS TGTTTTCGACAAG

alignment for event: RI-AT4G17310-XLOC_023816-5977

RI-AT4G17310-XLOC_023816-5977-0
GCACATAATTTCACCATTACCATGTGGACCCTCTTGCATCTTTATCAAGT
RI-AT4G17310-XLOC_023816-5977-1
GCACATAATTTCACCATTACCATGTGGACCCTCTTGCATCTTTATCAAGT
CONSENSUS
GCACATAATTTCACCATTACCATGTGGACCCTCTTGCATCTTTATCAAGT

RI-AT4G17310-XLOC_023816-5977-0
ACGAACCTTTGTCATAGCTCCAATAGTTGAAACGAAATCAGGGAAATTAA
RI-AT4G17310-XLOC_023816-5977-1
ACGAACCTTTGTCATAGCTCCAATAGTTGAAACGAAATCAGGGAAATTAA
CONSENSUS
ACGAACCTTTGTCATAGCTCCAATAGTTGAAACGAAATCAGGGAAATTAA

RI-AT4G17310-XLOC_023816-5977-0
GGATTGGGGTAACTAATGTGGGGTAAGTCACTGAAAACGTGTACAACTTA
RI-AT4G17310-XLOC_023816-5977-1
GGATTGGGGTAACTAATGTGGG----------------------------
CONSENSUS
GGATTGGGGTAACTAATGTGGG............................

RI-AT4G17310-XLOC_023816-5977-0
GATTGGAAACTTAGTAAACATACTGTCAAGTTGCATCACATCTGCTCATT
RI-AT4G17310-XLOC_023816-5977-1

CONSENSUS
....

RI-AT4G17310-XLOC_023816-5977-0
GTATTTCCTTCTTTTGTTTTTTTAACTGGAATGCCGGTTTCTGCAAGTTA
RI-AT4G17310-XLOC_023816-5977-1

CONSENSUS
....

RI-AT4G17310-XLOC_023816-5977-0
GTTCGTGCTCTGTGCATAAGAGTGTCACACAGTAACATAGTAATGTACAA
RI-AT4G17310-XLOC_023816-5977-1

CONSENSUS
....

RI-AT4G17310-XLOC_023816-5977-0
TGCTCTTTGTCCAGATGGAGTCGATGGCACGTGAGATGTGTTTGCAGTTT
RI-AT4G17310-XLOC_023816-5977-1

CONSENSUS
....

RI-AT4G17310-XLOC_023816-5977-0
TTTTTAAAGGTGGGTTCTGTGAATAATGATCTCTGACAAGTTAGTTTTGG
RI-AT4G17310-XLOC_023816-5977-1

CONSENSUS
 ....

RI-AT4G17310-XLOC_023816-5977-0
 GAGAAGTTATTTAAGACGAGATTTTATTGTCTAGTTTTAAGGTAGCATTG
RI-AT4G17310-XLOC_023816-5977-1

CONSENSUS
 ....

RI-AT4G17310-XLOC_023816-5977-0
 CTTTGTCAAGCTAAATTATTATAGTAGATTCATTAAGCCATTTGCATCCA
RI-AT4G17310-XLOC_023816-5977-1

CONSENSUS
 ....

RI-AT4G17310-XLOC_023816-5977-0
 TCTTTTGTTTCTTCACCTCATTCAGTGCCCTAATTTAATCGTGGTGATCA
RI-AT4G17310-XLOC_023816-5977-1

CONSENSUS
 ....

RI-AT4G17310-XLOC_023816-5977-0
 TTTATGTTTTGTTGCTCTAGAACTTGGCCTTTCAGTTCCAAGGTGACTCA
RI-AT4G17310-XLOC_023816-5977-1 --------------------
AACTTGGCCTTTCAGTTCCAAGGTGACTCA
CONSENSUS
 ....................AACTTGGCCTTTCAGTTCCAAGGTGACTCA

RI-AT4G17310-XLOC_023816-5977-0
 AAAACCCTCATGAAGGTGAACCATGGAATTATGAAAGGGGCAGGATCACA
RI-AT4G17310-XLOC_023816-5977-1
 AAAACCCTCATGAAGGTGAACCATGGAATTATGAAAGGGGCAGGATCACA
CONSENSUS
 AAAACCCTCATGAAGGTGAACCATGGAATTATGAAAGGGGCAGGATCACA

RI-AT4G17310-XLOC_023816-5977-0
 AAGAGTTGTTTCTTTTCTTCCCTTTGTCAAACTTATAGAAAAGAAGTGTC
RI-AT4G17310-XLOC_023816-5977-1
 AAGAGTTGTTTCTTTTCTTCCCTTTGTCAAACTTATAGAAAAGAAGTGTC
CONSENSUS
 AAGAGTTGTTTCTTTTCTTCCCTTTGTCAAACTTATAGAAAAGAAGTGTC

RI-AT4G17310-XLOC_023816-5977-0
 TGTCATTCTTTTTGTGACATATAAGTATTATTGATTACACAATCTCATGT
RI-AT4G17310-XLOC_023816-5977-1
 TGTCATTCTTTTTGTGACATATAAGTATTATTGATTACACAATCTCATGT
CONSENSUS
 TGTCATTCTTTTTGTGACATATAAGTATTATTGATTACACAATCTCATGT

RI-AT4G17310-XLOC_023816-5977-0
 ACTTTGATTTATGATGAGAGAAATATTATATACAAAATATTTTGTGATAA
RI-AT4G17310-XLOC_023816-5977-1
 ACTTTGATTTATGATGAGAGAAATATTATATACAAAATATTTTGTGATAA

CONSENSUS
      ACTTTGATTTATGATGAGAGAAATATTATATACAAAATATTTTGTGATAA

RI-AT4G17310-XLOC_023816-5977-0
      GTATACTTTATTTTGTTACTAATATTATGTAGATAACTATTTTGTTACAA
RI-AT4G17310-XLOC_023816-5977-1
      GTATACTTTATTTTGTTACTAATATTATGTAGATAACTATTTTGTTACAA
CONSENSUS
      GTATACTTTATTTTGTTACTAATATTATGTAGATAACTATTTTGTTACAA

RI-AT4G17310-XLOC_023816-5977-0 TTTCAC
RI-AT4G17310-XLOC_023816-5977-1 TTTCAC
CONSENSUS   TTTCAC

alignment for event: A3-AT4G15510-XLOC_021214-6273

A3-AT4G15510-XLOC_021214-6273-0
      CTTTTGTTCTCCAGACCCATCTCTTCCGGTCCTAAATACCAATCTGCCAA
A3-AT4G15510-XLOC_021214-6273-1
      CTTTTGTTCTCCAGACCCATCTCTTCCGGTCCTAAATACCAATCTGCCAA
CONSENSUS
      CTTTTGTTCTCCAGACCCATCTCTTCCGGTCCTAAATACCAATCTGCCAA

A3-AT4G15510-XLOC_021214-6273-0
      GTCCGCTAAACCGGAATCTCCAGTCGCCATAAACTGCTTAACCGATGCCA
A3-AT4G15510-XLOC_021214-6273-1
      GTCCGCTAAACCGGAATCTCCAGTCGCCATAAACTGCTTAACCGATGCCA
CONSENSUS
      GTCCGCTAAACCGGAATCTCCAGTCGCCATAAACTGCTTAACCGATGCCA

A3-AT4G15510-XLOC_021214-6273-0
      AACAGGTTTGTGCAGTTGGGAGGAGGAAGAGCATGATGATGGGCTTGCTC
A3-AT4G15510-XLOC_021214-6273-1 AACAG----------
TTGGGAGGAGGAAGAGCATGATGATGGGCTTGCTC
CONSENSUS
      AACAG..........TTGGGAGGAGGAAGAGCATGATGATGGGCTTGCTC

A3-AT4G15510-XLOC_021214-6273-0
      ATGTCTGGTTTAATAGTTTCACAAGCCAATCTTCCAACAGCATTTGCTTC
A3-AT4G15510-XLOC_021214-6273-1
      ATGTCTGGTTTAATAGTTTCACAAGCCAATCTTCCAACAGCATTTGCTTC
CONSENSUS
      ATGTCTGGTTTAATAGTTTCACAAGCCAATCTTCCAACAGCATTTGCTTC

A3-AT4G15510-XLOC_021214-6273-0
      AACTCCAGTGTTTAGAGAATACATAGATACATTTGATGGATACTCCTTCA
A3-AT4G15510-XLOC_021214-6273-1
      AACTCCAGTGTTTAGAGAATACATAGATACATTTGATGGATACTCCTTCA
CONSENSUS
      AACTCCAGTGTTTAGAGAATACATAGATACATTTGATGGATACTCCTTCA

A3-AT4G15510-XLOC_021214-6273-0
      AGTACCCTCAAAATTGGATCCAAGTCCGAGGAGCTGGTGCTGATATATTC
A3-AT4G15510-XLOC_021214-6273-1

AGTACCCTCAAAATTGGATCCAAGTCCGAGGAGCTGGTGCTGATATATTC
CONSENSUS
AGTACCCTCAAAATTGGATCCAAGTCCGAGGAGCTGGTGCTGATATATTC

A3-AT4G15510-XLOC_021214-6273-0
TTTAGAGACCCTGTTGTCCTCGACGAGAACCTTTCAGTCGAGTTTTCTTC
A3-AT4G15510-XLOC_021214-6273-1
TTTAGAGACCCTGTTGTCCTCGACGAGAACCTTTCAGTCGAGTTTTCTTC
CONSENSUS
TTTAGAGACCCTGTTGTCCTCGACGAGAACCTTTCAGTCGAGTTTTCTTC

A3-AT4G15510-XLOC_021214-6273-0
GCCTTCTTCCTCAAATTACACGTCACTTGAAGACTTGGGATCCCCTGAAG
A3-AT4G15510-XLOC_021214-6273-1
GCCTTCTTCCTCAAATTACACGTCACTTGAAGACTTGGGATCCCCTGAAG
CONSENSUS
GCCTTCTTCCTCAAATTACACGTCACTTGAAGACTTGGGATCCCCTGAAG

A3-AT4G15510-XLOC_021214-6273-0
AAGTAGGAAAGAGAGTACTTAGACAGTACTTGACTGAGTTTATGTCCACT
A3-AT4G15510-XLOC_021214-6273-1
AAGTAGGAAAGAGAGTACTTAGACAGTACTTGACTGAGTTTATGTCCACT
CONSENSUS
AAGTAGGAAAGAGAGTACTTAGACAGTACTTGACTGAGTTTATGTCCACT

A3-AT4G15510-XLOC_021214-6273-0
AGACTCGGGGTTAAGCGCCAGGCCAACATTCTAAGCACTTCCTCTAGAGT
A3-AT4G15510-XLOC_021214-6273-1
AGACTCGGGGTTAAGCGCCAGGCCAACATTCTAAGCACTTCCTCTAGAGT
CONSENSUS
AGACTCGGGGTTAAGCGCCAGGCCAACATTCTAAGCACTTCCTCTAGAGT

A3-AT4G15510-XLOC_021214-6273-0
TGCAGATGATGGTAAACTCTACTACCAAGTTGAG
A3-AT4G15510-XLOC_021214-6273-1
TGCAGATGATGGTAAACTCTACTACCAAGTTGAG
CONSENSUS
TGCAGATGATGGTAAACTCTACTACCAAGTTGAG

alignment for event: A3-AT4G34900-XLOC_024804-12748

A3-AT4G34900-XLOC_024804-12748-0
GTTCCTAATGCGTCACCTACTGCTGCTTCTGCGAGTTCTGATATGTATGG
A3-AT4G34900-XLOC_024804-12748-1
GTTCCTAATGCGTCACCTACTGCTGCTTCTGCGAGTTCTGATATGTATGG
CONSENSUS
GTTCCTAATGCGTCACCTACTGCTGCTTCTGCGAGTTCTGATATGTATGG

A3-AT4G34900-XLOC_024804-12748-0
TGCTGCAGTTTTAGACGCTTGTGAGCAGATTATAGCAAGAATGGAGCCTG
A3-AT4G34900-XLOC_024804-12748-1
TGCTGCAGTTTTAGACGCTTGTGAGCAGATTATAGCAAGAATGGAGCCTG
CONSENSUS
TGCTGCAGTTTTAGACGCTTGTGAGCAGATTATAGCAAGAATGGAGCCTG

```
A3-AT4G34900-XLOC_024804-12748-0
      TTGCATCTAAACACAATTTCAACACATTCTCTGAG---------------
A3-AT4G34900-XLOC_024804-12748-1
      TTGCATCTAAACACAATTTCAACACATTCTCTGAGCTAGCAAGTGCCTGC
CONSENSUS
      TTGCATCTAAACACAATTTCAACACATTCTCTGAG...............

A3-AT4G34900-XLOC_024804-12748-0
      -------------------------------------------------A
A3-AT4G34900-XLOC_024804-12748-1
      TACTTTCAACGTATAGACCTATCAGCTCACGGTTTTCACATTGTTCCAGA
CONSENSUS
      .................................................A

A3-AT4G34900-XLOC_024804-12748-0
      ACTTGAATTTGATTGGGTATCTGGAAAAGGGAACGCATATAGATATTACA
A3-AT4G34900-XLOC_024804-12748-1
      ACTTGAATTTGATTGGGTATCTGGAAAAGGGAACGCATATAGATATTACA
CONSENSUS
      ACTTGAATTTGATTGGGTATCTGGAAAAGGGAACGCATATAGATATTACA

A3-AT4G34900-XLOC_024804-12748-0
      CATATGGAGCTGCCTTTGCTGAAGTTGAGATAGATACATTGACTGGTGAT
A3-AT4G34900-XLOC_024804-12748-1
      CATATGGAGCTGCCTTTGCTGAAGTTGAGATAGATACATTGACTGGTGAT
CONSENSUS
      CATATGGAGCTGCCTTTGCTGAAGTTGAGATAGATACATTGACTGGTGAT

A3-AT4G34900-XLOC_024804-12748-0
      TTTCACACAAGAAAAGCAGACATAATGTTGGATCTCGGATATTCTCTTAA
A3-AT4G34900-XLOC_024804-12748-1
      TTTCACACAAGAAAAGCAGACATAATGTTGGATCTCGGATATTCTCTTAA
CONSENSUS
      TTTCACACAAGAAAAGCAGACATAATGTTGGATCTCGGATATTCTCTTAA

A3-AT4G34900-XLOC_024804-12748-0 CCCAACCATTGATATTGGACAA
A3-AT4G34900-XLOC_024804-12748-1 CCCAACCATTGATATTGGACAA
CONSENSUS                        CCCAACCATTGATATTGGACAA
```

alignment for event: RI-AT4G16765-XLOC_023784-8673

```
RI-AT4G16765-XLOC_023784-8673-0
      GTCCGTGGGTAGAAAATTGCTTGGCTTGATTGCCTTAGCATTGGATTTAG
RI-AT4G16765-XLOC_023784-8673-1
      GTCCGTGGGTAGAAAATTGCTTGGCTTGATTGCCTTAGCATTGGATTTAG
CONSENSUS
      GTCCGTGGGTAGAAAATTGCTTGGCTTGATTGCCTTAGCATTGGATTTAG

RI-AT4G16765-XLOC_023784-8673-0
      ATGAGGACTTCTTCGAAAAAGTTGGAGCCTTGAATGATCCTACAGCAGTT
RI-AT4G16765-XLOC_023784-8673-1
      ATGAGGACTTCTTCGAAAAAGTTGGAGCCTTGAATGATCCTACAGCAGTT
CONSENSUS
```

```
      ATGAGGACTTCTTCGAAAAAGTTGGAGCCTTGAATGATCCTACAGCAGTT

RI-AT4G16765-XLOC_023784-8673-0
      GTTCGCCTCTTACGATATCCAGGTGTGTCTATGTACATCAAAAAGTTCTG
RI-AT4G16765-XLOC_023784-8673-1
      GTTCGCCTCTTACGATATCCAG----------------------------
CONSENSUS
      GTTCGCCTCTTACGATATCCAG............................

RI-AT4G16765-XLOC_023784-8673-0
      GTTGGCTTGATATTAATCTTAATGAACTTTGGTCAGAGTTAGATCGTAAC
RI-AT4G16765-XLOC_023784-8673-1
      --------------------------------------------------
CONSENSUS
      ..................................................

RI-AT4G16765-XLOC_023784-8673-0
      ATATGTAATCAGTGGAAACTTAAGCCACATTCCGGGAGTTTGAGTCAGGT
RI-AT4G16765-XLOC_023784-8673-1
      --------------------------------------------------
CONSENSUS
      ..................................................

RI-AT4G16765-XLOC_023784-8673-0
      TTTGATCTCTGCACTTGTTATGATTATAGGTGAAGTGATTTCGTCAGATG
RI-AT4G16765-XLOC_023784-8673-1  -----------------------------
GTGAAGTGATTTCGTCAGATG
CONSENSUS
      .............................GTGAAGTGATTTCGTCAGATG

RI-AT4G16765-XLOC_023784-8673-0
      TTGAAACGTATGGTGCCTCAGCTCACTCAGATTATGGAATGGTCACTCTT
RI-AT4G16765-XLOC_023784-8673-1
      TTGAAACGTATGGTGCCTCAGCTCACTCAGATTATGGAATGGTCACTCTT
CONSENSUS
      TTGAAACGTATGGTGCCTCAGCTCACTCAGATTATGGAATGGTCACTCTT

RI-AT4G16765-XLOC_023784-8673-0  CTTTTGACTGATGGAGTTCCAGGACTTCAG
RI-AT4G16765-XLOC_023784-8673-1  CTTTTGACTGATGGAGTTCCAGGACTTCAG
CONSENSUS                        CTTTTGACTGATGGAGTTCCAGGACTTCAG


alignment for event: RI-AT4G03520-XLOC_022839-9097

RI-AT4G03520-XLOC_022839-9097-0
      TTCAAGTGGTCAATGACTCGACATGGGACTCTCTAGTTCTTAAAGCAACT
RI-AT4G03520-XLOC_022839-9097-1
      TTCAAGTGGTCAATGACTCGACATGGGACTCTCTAGTTCTTAAAGCAACT
CONSENSUS
      TTCAAGTGGTCAATGACTCGACATGGGACTCTCTAGTTCTTAAAGCAACT

RI-AT4G03520-XLOC_022839-9097-0
      GGGCCTGTAGTTGTCGACTTTTGGGCACCATGGTGTGGACCTTGCAAGAT
RI-AT4G03520-XLOC_022839-9097-1
      GGGCCTGTAGTTGTCGACTTTTGGGCACCATGGTGTGGACCTTGCAAGAT
```

CONSENSUS
GGGCCTGTAGTTGTCGACTTTTGGGCACCATGGTGTGGACCTTGCAAGAT

RI-AT4G03520-XLOC_022839-9097-0
GATTGATCCGCTTGTAAACGATCTAGCACAGCATTACACTGGGAAGATCA
RI-AT4G03520-XLOC_022839-9097-1
GATTGATCCGCTTGTAAACGATCTAGCACAGCATTACACTGGGAAGATCA
CONSENSUS
GATTGATCCGCTTGTAAACGATCTAGCACAGCATTACACTGGGAAGATCA

RI-AT4G03520-XLOC_022839-9097-0
AGTTCTACAAATTAAACACTGATGAATCTCCTAATACTCCCGGCCAGTAC
RI-AT4G03520-XLOC_022839-9097-1
AGTTCTACAAATTAAACACTGATGAATCTCCTAATACTCCCGGCCAGTAC
CONSENSUS
AGTTCTACAAATTAAACACTGATGAATCTCCTAATACTCCCGGCCAGTAC

RI-AT4G03520-XLOC_022839-9097-0
GGAGTTAGAAGCATCCCAACGATCATGATCTTTGTCGGTGGCGAGAAGAA
RI-AT4G03520-XLOC_022839-9097-1
GGAGTTAGAAGCATCCCAACGATCATGATCTTTGTCGGTGGCGAGAAGAA
CONSENSUS
GGAGTTAGAAGCATCCCAACGATCATGATCTTTGTCGGTGGCGAGAAGAA

RI-AT4G03520-XLOC_022839-9097-0
GGATACAATCATAGGTGCAGTGCCCAAAACCACACTCACATCTAGCCTCG
RI-AT4G03520-XLOC_022839-9097-1
GGATACAATCATAGGTGCAGTGCCCAAAACCACACTCACATCTAGCCTCG
CONSENSUS
GGATACAATCATAGGTGCAGTGCCCAAAACCACACTCACATCTAGCCTCG

RI-AT4G03520-XLOC_022839-9097-0
ACAAGTTCTTGCCATGAAATATCACTTGTGGAGTCTGTAAGATCTCGTAT
RI-AT4G03520-XLOC_022839-9097-1
ACAAGTTCTTGCCATGAAATATCACTTGTGGAGTCT--------------
CONSENSUS
ACAAGTTCTTGCCATGAAATATCACTTGTGGAGTCT..............

RI-AT4G03520-XLOC_022839-9097-0
CTCTCTGTCTCTCGTTCTTGTTCTCATTATCTCTGTAACTCATTATCACT
RI-AT4G03520-XLOC_022839-9097-1

CONSENSUS
....

RI-AT4G03520-XLOC_022839-9097-0
GAACATCTAGACCTGGATAAATAATATGATGTCATGTCTTATGTCATTTT
RI-AT4G03520-XLOC_022839-9097-1

CONSENSUS
....

RI-AT4G03520-XLOC_022839-9097-0
TACTGGTTTGAAGATGTTTTGAGATGCGGAACTAAAGGTATCAAAGAACA
RI-AT4G03520-XLOC_022839-9097-1 -------------
ATGTTTTGAGATGCGGAACTAAAGGTATCAAAGAACA

CONSENSUS
.............ATGTTTTGAGATGCGGAACTAAAGGTATCAAAGAACA

RI-AT4G03520-XLOC_022839-9097-0
ATCTTGGACTAGACTAATGGAACCACATTAAAATGATTCCACC
RI-AT4G03520-XLOC_022839-9097-1
ATCTTGGACTAGACTAATGGAACCACATTAAAATGATTCCACC
CONSENSUS
ATCTTGGACTAGACTAATGGAACCACATTAAAATGATTCCACC

alignment for event: A3-AT4G12460-XLOC_021026-12123

A3-AT4G12460-XLOC_021026-12123-0
GTTAATGGTTTTGTAGAAGATGTAACTGGAAAAAAGGCTGCGACGGTATT
A3-AT4G12460-XLOC_021026-12123-1
GTTAATGGTTTTGTAGAAGATGTAACTGGAAAAAAGGCTGCGACGGTATT
CONSENSUS
GTTAATGGTTTTGTAGAAGATGTAACTGGAAAAAAGGCTGCGACGGTATT

A3-AT4G12460-XLOC_021026-12123-0
TGGTAAATGGAATGATAGCCTTTATTATGTTGCTGGTGATGGAATCAACA
A3-AT4G12460-XLOC_021026-12123-1
TGGTAAATGGAATGATAGCCTTTATTATGTTGCTGGTGATGGAATCAACA
CONSENSUS
TGGTAAATGGAATGATAGCCTTTATTATGTTGCTGGTGATGGAATCAACA

A3-AT4G12460-XLOC_021026-12123-0
AGGCAAGCGCCTCATTACTGTGGAAAGCGACAAAGGCACCACCTAATGTC
A3-AT4G12460-XLOC_021026-12123-1
AGGCAAGCGCCTCATTACTGTGGAAAGCGACAAAGGCACCACCTAATGTC
CONSENSUS
AGGCAAGCGCCTCATTACTGTGGAAAGCGACAAAGGCACCACCTAATGTC

A3-AT4G12460-XLOC_021026-12123-0
ACCAGATACAACTTTACATCATTCGCGATGACGCTAAATGAATTGATACC
A3-AT4G12460-XLOC_021026-12123-1
ACCAGATACAACTTTACATCATTCGCGATGACGCTAAATGAATTGATACC
CONSENSUS
ACCAGATACAACTTTACATCATTCGCGATGACGCTAAATGAATTGATACC

A3-AT4G12460-XLOC_021026-12123-0
TGGTTTGGAGGAAAAGCTGCCTCCAACAGATTCTAGGCTCCGACCAGATC
A3-AT4G12460-XLOC_021026-12123-1 TGGTTTGGAG------
CTGCCTCCAACAGATTCTAGGCTCCGACCAGATC
CONSENSUS
TGGTTTGGAG......CTGCCTCCAACAGATTCTAGGCTCCGACCAGATC

A3-AT4G12460-XLOC_021026-12123-0
AACGACACTTGGAGAACGGTGAATATGAGAAGGCAAATGAAGAGAAACAA
A3-AT4G12460-XLOC_021026-12123-1
AACGACACTTGGAGAACGGTGAATATGAGAAGGCAAATGAAGAGAAACAA
CONSENSUS
AACGACACTTGGAGAACGGTGAATATGAGAAGGCAAATGAAGAGAAACAA

A3-AT4G12460-XLOC_021026-12123-0 AGATTAGAGAGAAGACAAAGAATG
A3-AT4G12460-XLOC_021026-12123-1 AGATTAGAGAGAAGACAAAGAATG
CONSENSUS AGATTAGAGAGAAGACAAAGAATG

alignment for event: A3-AT4G32330-XLOC_022179-11969

A3-AT4G32330-XLOC_022179-11969-0
GGAGAAGGTGAAACCAAAGTCTCAAAAGAAACAAGCCCATGAGACATCTG
A3-AT4G32330-XLOC_022179-11969-1
GGAGAAGGTGAAACCAAAGTCTCAAAAGAAACAAGCCCATGAGACATCTG
CONSENSUS
GGAGAAGGTGAAACCAAAGTCTCAAAAGAAACAAGCCCATGAGACATCTG

A3-AT4G32330-XLOC_022179-11969-0
AAGATGATACTCAGTCTTCTAATAGTCCGAAAGCAGACGATGGAAAACCT
A3-AT4G32330-XLOC_022179-11969-1 AAGATGATACTCAGTCTTCTAA---
TCCGAAAGCAGACGATGGAAAACCT
CONSENSUS
AAGATGATACTCAGTCTTCTAA...TCCGAAAGCAGACGATGGAAAACCT

A3-AT4G32330-XLOC_022179-11969-0
CGTAAAGTTGGTGCACTTCCAAATTATGGATTCAGTTTCAAATGTGACCA
A3-AT4G32330-XLOC_022179-11969-1
CGTAAAGTTGGTGCACTTCCAAATTATGGATTCAGTTTCAAATGTGACCA
CONSENSUS
CGTAAAGTTGGTGCACTTCCAAATTATGGATTCAGTTTCAAATGTGACCA

A3-AT4G32330-XLOC_022179-11969-0 ACGGGCTGAAAAGAGAAAAGAG
A3-AT4G32330-XLOC_022179-11969-1 ACGGGCTGAAAAGAGAAAAGAG
CONSENSUS ACGGGCTGAAAAGAGAAAAGAG

alignment for event: A5-AT4G33050-XLOC_024702-12464

A5-AT4G33050-XLOC_024702-12464-0
ATGGAGAGAGAAGCATATGAAGTGATAGTAGAAGATGGGAGACTAATGTA
A5-AT4G33050-XLOC_024702-12464-1
ATGGAGAGAGAAGCATATGAAGTGATAGTAGAAGATGGGAGACTAATGTA
CONSENSUS
ATGGAGAGAGAAGCATATGAAGTGATAGTAGAAGATGGGAGACTAATGTA

A5-AT4G33050-XLOC_024702-12464-0
TAAACAGGGCATGACTCTGATCAATTCAACAGAGGAAGCCAAGTCGATTT
A5-AT4G33050-XLOC_024702-12464-1
TAAACAGGGCATGACTCTGATCAATTCAACAGAGGAAGCCAAGTCGATTT
CONSENSUS
TAAACAGGGCATGACTCTGATCAATTCAACAGAGGAAGCCAAGTCGATTT

A5-AT4G33050-XLOC_024702-12464-0
TTGTACTTAGTACTACTAGAAACTTATACGTAGGGATTAAAAAGAAAGGT
A5-AT4G33050-XLOC_024702-12464-1
TTGTACTTAGTACTACTAGAAACTTATACGTAGGGATTAAAAAGAAAGGT
CONSENSUS

TTGTACTTAGTACTACTAGAAACTTATACGTAGGGATTAAAAAGAAAGGT

A5-AT4G33050-XLOC_024702-12464-0
CTTTTCCAGCACTCTAGTTTCTTATCTGGAGGTGCCACAACCGCAGCAGG
A5-AT4G33050-XLOC_024702-12464-1
CTTTTCCAGCACTCTAGTTTCTTATCTGGAGGTGCCACAACCGCAGCAGG
CONSENSUS
CTTTTCCAGCACTCTAGTTTCTTATCTGGAGGTGCCACAACCGCAGCAGG

A5-AT4G33050-XLOC_024702-12464-0
AAGGTTAGTCGCCCGCGATGGGATCCTTGAGGCTATATGGCCATATAGTG
A5-AT4G33050-XLOC_024702-12464-1
AAG---------------------------GCTATATGGCCATATAGTG
CONSENSUS
AAG...........................GCTATATGGCCATATAGTG

A5-AT4G33050-XLOC_024702-12464-0
GACATTATCTCCCAACAGAAGACAACTTCAAGGAGTTCATAAGTTTCTTA
A5-AT4G33050-XLOC_024702-12464-1
GACATTATCTCCCAACAGAAGACAACTTCAAGGAGTTCATAAGTTTCTTA
CONSENSUS
GACATTATCTCCCAACAGAAGACAACTTCAAGGAGTTCATAAGTTTCTTA

A5-AT4G33050-XLOC_024702-12464-0
GAGGAGCACAATGTTGATCTCACCAATGTTAAG
A5-AT4G33050-XLOC_024702-12464-1
GAGGAGCACAATGTTGATCTCACCAATGTTAAG
CONSENSUS
GAGGAGCACAATGTTGATCTCACCAATGTTAAG

alignment for event: A3-AT2G41680-XLOC_013204-12963

A3-AT2G41680-XLOC_013204-12963-0
GTCAAGTGCCATAGTATTATATATGCCACTGGCGCTACAGCAAGGAGGTT
A3-AT2G41680-XLOC_013204-12963-1
GTCAAGTGCCATAGTATTATATATGCCACTGGCGCTACAGCAAGGAGGTT
CONSENSUS
GTCAAGTGCCATAGTATTATATATGCCACTGGCGCTACAGCAAGGAGGTT

A3-AT2G41680-XLOC_013204-12963-0
AAGGTTACCTCGAGAGGAAGAATTCTGGAGTAGGGGGATAAGTGCTTGTG
A3-AT2G41680-XLOC_013204-12963-1
AAGGTTACCTCGAGAGGAAGAATTCTGGAGTAGGGGGATAAGTGCTTGTG
CONSENSUS
AAGGTTACCTCGAGAGGAAGAATTCTGGAGTAGGGGGATAAGTGCTTGTG

A3-AT2G41680-XLOC_013204-12963-0
CTATCTGTGATGGAGCTTCGCCTTTATTTAAGGGGCAAGTACTTGCCGTG
A3-AT2G41680-XLOC_013204-12963-1
CTATCTGTGATGGAGCTTCGCCTTTATTTAAGGGGCAAGTACTTGCCGTG
CONSENSUS
CTATCTGTGATGGAGCTTCGCCTTTATTTAAGGGGCAAGTACTTGCCGTG

A3-AT2G41680-XLOC_013204-12963-0

GTTGGAGGAGGAGATACGGCTACAGAGGAAGCCTTGTATCTCACGAAATA
A3-AT2G41680-XLOC_013204-12963-1
GTTGGAGGAGGAGATACGGCTACAGAGGAAGCCTTGTATCTCACGAAATA
CONSENSUS
GTTGGAGGAGGAGATACGGCTACAGAGGAAGCCTTGTATCTCACGAAATA

A3-AT2G41680-XLOC_013204-12963-0
TGCCCGTCATGTTCATTTGCTTGTTCGCAGAGATCAGTTGAGAGCTTCCA
A3-AT2G41680-XLOC_013204-12963-1
TGCCCGTCATGTTCATTTGCTTGTTCGCAGAGATCAGTTGAGAGCTTCCA
CONSENSUS
TGCCCGTCATGTTCATTTGCTTGTTCGCAGAGATCAGTTGAGAGCTTCCA

A3-AT2G41680-XLOC_013204-12963-0
AGGCTATGCAAGATAG----------------------AGTGATCAACA
A3-AT2G41680-XLOC_013204-12963-1
AGGCTATGCAAGATAGGTTGGTTATGGCGAATGTTTCAGAGTGATCAACA
CONSENSUS
AGGCTATGCAAGATAG.......................AGTGATCAACA

A3-AT2G41680-XLOC_013204-12963-0
ATCCAAACATCACAGTGCATTACAACACGGAAACCGTGGACGTATTGAGC
A3-AT2G41680-XLOC_013204-12963-1
ATCCAAACATCACAGTGCATTACAACACGGAAACCGTGGACGTATTGAGC
CONSENSUS
ATCCAAACATCACAGTGCATTACAACACGGAAACCGTGGACGTATTGAGC

A3-AT2G41680-XLOC_013204-12963-0
AACACCAAGGGACAGATGTCTGGCATTCTACTCAGAAGACTTGATACGGG
A3-AT2G41680-XLOC_013204-12963-1
AACACCAAGGGACAGATGTCTGGCATTCTACTCAGAAGACTTGATACGGG
CONSENSUS
AACACCAAGGGACAGATGTCTGGCATTCTACTCAGAAGACTTGATACGGG

A3-AT2G41680-XLOC_013204-12963-0
TGAAGAAACTGAGCTGGAGGCAAAAGGATTGTTTTATGGAATAGGGCATT
A3-AT2G41680-XLOC_013204-12963-1
TGAAGAAACTGAGCTGGAGGCAAAAGGATTGTTTTATGGAATAGGGCATT
CONSENSUS
TGAAGAAACTGAGCTGGAGGCAAAAGGATTGTTTTATGGAATAGGGCATT

A3-AT2G41680-XLOC_013204-12963-0
CGCCAAACAGTCAGTTATTGGAAGGCCAAGTCGAACTCGACAGCTCCGGG
A3-AT2G41680-XLOC_013204-12963-1
CGCCAAACAGTCAGTTATTGGAAGGCCAAGTCGAACTCGACAGCTCCGGG
CONSENSUS
CGCCAAACAGTCAGTTATTGGAAGGCCAAGTCGAACTCGACAGCTCCGGG

A3-AT2G41680-XLOC_013204-12963-0
TACGTCTTGGTTCGGGAAGGAACATCAAATACATCAGTTGAAGGTGTATT
A3-AT2G41680-XLOC_013204-12963-1
TACGTCTTGGTTCGGGAAGGAACATCAAATACATCAGTTGAAGGTGTATT
CONSENSUS
TACGTCTTGGTTCGGGAAGGAACATCAAATACATCAGTTGAAGGTGTATT

A3-AT2G41680-XLOC_013204-12963-0 TGCTGCAGGAGATGTGCAG

```
A3-AT2G41680-XLOC_013204-12963-1 TGCTGCAGGAGATGTGCAG
CONSENSUS                        TGCTGCAGGAGATGTGCAG



alignment for event: RI-AT2G41870-XLOC_013220-12146

RI-AT2G41870-XLOC_013220-12146-0
     GTCTCGTTTGTTTTGCTCTTTAGAAATTACCGATTTACGCTGAAAAAATG
RI-AT2G41870-XLOC_013220-12146-1
     GTCTCGTTTGTTTTGCTCTTTAGAAATTACCGATTTACGCTGAAAAAATG
CONSENSUS
     GTCTCGTTTGTTTTGCTCTTTAGAAATTACCGATTTACGCTGAAAAAATG

RI-AT2G41870-XLOC_013220-12146-0
     TGAAAAATACAATCGATCGCATTATCTTTATCCCTAGCTAATCATTCATG
RI-AT2G41870-XLOC_013220-12146-1
     TGAAAAATACAATCGATCGCATTATCTTTATCCCTAGCTAATCATTCATG
CONSENSUS
     TGAAAAATACAATCGATCGCATTATCTTTATCCCTAGCTAATCATTCATG

RI-AT2G41870-XLOC_013220-12146-0
     TACAAGCATGTCTCCGAAGGTTAAAAGCAGTCGCTATTTACCGGACCAAC
RI-AT2G41870-XLOC_013220-12146-1
     TACAAGCATGTCTCCGAAGGTTAAAAGCAGTCGCTATTTACCGGACCAAC
CONSENSUS
     TACAAGCATGTCTCCGAAGGTTAAAAGCAGTCGCTATTTACCGGACCAAC

RI-AT2G41870-XLOC_013220-12146-0
     GTAGTTTTCTCGAAGTGGTGGTCCGTTGTCATATTTTAAATTTATCACCT
RI-AT2G41870-XLOC_013220-12146-1
     GTAGTTTTCTCGAAGTGGTG------------------------------
CONSENSUS
     GTAGTTTTCTCGAAGTGGTG..............................

RI-AT2G41870-XLOC_013220-12146-0
     TCTTGAGAATTCCACATTTTTATCCTTTTTGTCATGTAGTGTATATTTTT
RI-AT2G41870-XLOC_013220-12146-1
     --------------------------------------------------
CONSENSUS
     ..................................................

RI-AT2G41870-XLOC_013220-12146-0
     TCCTCTAACCTAATTAAAATCAAAACAAAATCCTTTGACCCAATTAGCTT
RI-AT2G41870-XLOC_013220-12146-1
     --------------------------------------------------
CONSENSUS
     ..................................................

RI-AT2G41870-XLOC_013220-12146-0
     CGCGATATATCAGAAGAGATCAAACTACTTTGATCAGACCATGATCTTCT
RI-AT2G41870-XLOC_013220-12146-1
     --------------------------------------------------
CONSENSUS
     ..................................................
```

RI-AT2G41870-XLOC_013220-12146-0
TCTTCTTCTTCTTCTTCTTCTTCTTCTTTTTAGACGATCACAATTCCTAA
RI-AT2G41870-XLOC_013220-12146-1

CONSENSUS
....

RI-AT2G41870-XLOC_013220-12146-0
ACCCTATTTCTCAGATTATGCTGACTCTTTACCATCAAGAAAGGTCACCG
RI-AT2G41870-XLOC_013220-12146-1 --------------
ATTATGCTGACTCTTTACCATCAAGAAAGGTCACCG
CONSENSUS
..............ATTATGCTGACTCTTTACCATCAAGAAAGGTCACCG

RI-AT2G41870-XLOC_013220-12146-0
GACGCCACAAGTAATGATCGCGATGAGACGCCAGAGACTGTGGTTAGAGA
RI-AT2G41870-XLOC_013220-12146-1
GACGCCACAAGTAATGATCGCGATGAGACGCCAGAGACTGTGGTTAGAGA
CONSENSUS
GACGCCACAAGTAATGATCGCGATGAGACGCCAGAGACTGTGGTTAGAGA

RI-AT2G41870-XLOC_013220-12146-0
AGTCCACGCGCTAACTCCAGCGCCGGAGGATAATTCCCGGACGATGACGG
RI-AT2G41870-XLOC_013220-12146-1
AGTCCACGCGCTAACTCCAGCGCCGGAGGATAATTCCCGGACGATGACGG
CONSENSUS
AGTCCACGCGCTAACTCCAGCGCCGGAGGATAATTCCCGGACGATGACGG

RI-AT2G41870-XLOC_013220-12146-0
CGACGCTACCTCCACCGCCTGCTTTCCGAGGCTATTTTTCTCCTCCAAGG
RI-AT2G41870-XLOC_013220-12146-1
CGACGCTACCTCCACCGCCTGCTTTCCGAGGCTATTTTTCTCCTCCAAGG
CONSENSUS
CGACGCTACCTCCACCGCCTGCTTTCCGAGGCTATTTTTCTCCTCCAAGG

RI-AT2G41870-XLOC_013220-12146-0
TCAGCGACGACGATGAGCGAAGGAGAGAACTTCACAACTATAAGCAGAGA
RI-AT2G41870-XLOC_013220-12146-1
TCAGCGACGACGATGAGCGAAGGAGAGAACTTCACAACTATAAGCAGAGA
CONSENSUS
TCAGCGACGACGATGAGCGAAGGAGAGAACTTCACAACTATAAGCAGAGA

RI-AT2G41870-XLOC_013220-12146-0
GTTCAACGCTCTAGTCATCGCCGGATCCTCCATGGAGAACAACGAACTAA
RI-AT2G41870-XLOC_013220-12146-1
GTTCAACGCTCTAGTCATCGCCGGATCCTCCATGGAGAACAACGAACTAA
CONSENSUS
GTTCAACGCTCTAGTCATCGCCGGATCCTCCATGGAGAACAACGAACTAA

RI-AT2G41870-XLOC_013220-12146-0
TGACTCGTGACGTCACGCAGCGTGAAGATGAGAGACAAGACGAGTTGATG
RI-AT2G41870-XLOC_013220-12146-1
TGACTCGTGACGTCACGCAGCGTGAAGATGAGAGACAAGACGAGTTGATG
CONSENSUS
TGACTCGTGACGTCACGCAGCGTGAAGATGAGAGACAAGACGAGTTGATG

RI-AT2G41870-XLOC_013220-12146-0
AGAATCCACGAGGACACGGATCATGAAGAGGAAACGAATCCTTTAGCAAT
RI-AT2G41870-XLOC_013220-12146-1
AGAATCCACGAGGACACGGATCATGAAGAGGAAACGAATCCTTTAGCAAT
CONSENSUS
AGAATCCACGAGGACACGGATCATGAAGAGGAAACGAATCCTTTAGCAAT

RI-AT2G41870-XLOC_013220-12146-0
CGTGCCGGATCAGTATCCTGGTTCGGGTTTGGATCCTGGAAGTGATAATG
RI-AT2G41870-XLOC_013220-12146-1
CGTGCCGGATCAGTATCCTGGTTCGGGTTTGGATCCTGGAAGTGATAATG
CONSENSUS
CGTGCCGGATCAGTATCCTGGTTCGGGTTTGGATCCTGGAAGTGATAATG

RI-AT2G41870-XLOC_013220-12146-0
GGCCGGGTCAGAGTCGGGTTGGGTCGACGGTGCAAAGAGTTAAGAGGGAA
RI-AT2G41870-XLOC_013220-12146-1
GGCCGGGTCAGAGTCGGGTTGGGTCGACGGTGCAAAGAGTTAAGAGGGAA
CONSENSUS
GGCCGGGTCAGAGTCGGGTTGGGTCGACGGTGCAAAGAGTTAAGAGGGAA

RI-AT2G41870-XLOC_013220-12146-0
GAGGTGGAAGCGAAGATAACGGCGTGGCAGACGGCAAAACTGGCTAAGAT
RI-AT2G41870-XLOC_013220-12146-1
GAGGTGGAAGCGAAGATAACGGCGTGGCAGACGGCAAAACTGGCTAAGAT
CONSENSUS
GAGGTGGAAGCGAAGATAACGGCGTGGCAGACGGCAAAACTGGCTAAGAT

RI-AT2G41870-XLOC_013220-12146-0
TAATAACAGGTTTAAGAGGGAAGACGCCGTTATTAACGGTTGGTTTAATG
RI-AT2G41870-XLOC_013220-12146-1
TAATAACAGGTTTAAGAGGGAAGACGCCGTTATTAACGGTTGGTTTAATG
CONSENSUS
TAATAACAGGTTTAAGAGGGAAGACGCCGTTATTAACGGTTGGTTTAATG

RI-AT2G41870-XLOC_013220-12146-0
AACAAGTTAACAAGGCCAACTCTTGGATGAAGAAAATTGAG
RI-AT2G41870-XLOC_013220-12146-1
AACAAGTTAACAAGGCCAACTCTTGGATGAAGAAAATTGAG
CONSENSUS
AACAAGTTAACAAGGCCAACTCTTGGATGAAGAAAATTGAG

alignment for event: A3-AT2G32000-XLOC_012657-11484

A3-AT2G32000-XLOC_012657-11484-0
ATGTCTACAAGGAGAGGCAGTACAGAGGTGCATGAATTTGATGGCATGTT
A3-AT2G32000-XLOC_012657-11484-1
ATGTCTACAAGGAGAGGCAGTACAGAGGTGCATGAATTTGATGGCATGTT
CONSENSUS
ATGTCTACAAGGAGAGGCAGTACAGAGGTGCATGAATTTGATGGCATGTT

A3-AT2G32000-XLOC_012657-11484-0
TCGAGGCTTCAAAGCACATTATAGAGTTACATCTGTTATCGGTCATGTTT
A3-AT2G32000-XLOC_012657-11484-1

```
      TCGAGGCTTCAAAGCACATTATAGAGTTACATCTGTTATCGGTCATGTTT
CONSENSUS
      TCGAGGCTTCAAAGCACATTATAGAGTTACATCTGTTATCGGTCATGTTT

A3-AT2G32000-XLOC_012657-11484-0 TCAG-----
ATTTCCCAGAAAAATATCAGAATTGGGCAACTATTGATCCA
A3-AT2G32000-XLOC_012657-11484-1
      TCAGTGTAGATTTCCCAGAAAAATATCAGAATTGGGCAACTATTGATCCA
CONSENSUS
      TCAG.....ATTTCCCAGAAAAATATCAGAATTGGGCAACTATTGATCCA

A3-AT2G32000-XLOC_012657-11484-0
      CAAGATCTTTTCGACGCTCCAATTATTAAAAAGGAATCAAACCCAAAG
A3-AT2G32000-XLOC_012657-11484-1
      CAAGATCTTTTCGACGCTCCAATTATTAAAAAGGAATCAAACCCAAAG
CONSENSUS
      CAAGATCTTTTCGACGCTCCAATTATTAAAAAGGAATCAAACCCAAAG



alignment for event: A5-AT2G04690-XLOC_008382-11921

A5-AT2G04690-XLOC_008382-11921-0
      ATTGGCCTAAGGATCATGATTTCCGCTTCTTTAAACTCGAGATCATCGAT
A5-AT2G04690-XLOC_008382-11921-1
      ATTGGCCTAAGGATCATGATTTCCGCTTCTTTAAACTCGAGATCATCGAT
CONSENSUS
      ATTGGCCTAAGGATCATGATTTCCGCTTCTTTAAACTCGAGATCATCGAT

A5-AT2G04690-XLOC_008382-11921-0
      ATATTTCTCATCAATTGGTATGGTGGAGCTAAACCTATCACTGTAGATGA
A5-AT2G04690-XLOC_008382-11921-1
      ATATTTCTCATCAATTGGTATGGTGGAGCTAAACCTATCACTGTAGATGA
CONSENSUS
      ATATTTCTCATCAATTGGTATGGTGGAGCTAAACCTATCACTGTAGATGA

A5-AT2G04690-XLOC_008382-11921-0
      ATACCTTCATGCCAAGTC--------------------------------
A5-AT2G04690-XLOC_008382-11921-1
      ATACCTTCATGCCAAGTCGTAAGTACATTGCGTGAACCCTTTTAAAAACA
CONSENSUS
      ATACCTTCATGCCAAGTC................................

A5-AT2G04690-XLOC_008382-11921-0 ---
GATCAAACTCGCTTCCTTTTTATAATAACTCACAAGAATCAAGCAAA
A5-AT2G04690-XLOC_008382-11921-1
      TCGGATCAAACTCGCTTCCTTTTTATAATAACTCACAAGAATCAAGCAAA
CONSENSUS
      ...GATCAAACTCGCTTCCTTTTTATAATAACTCACAAGAATCAAGCAAA

A5-AT2G04690-XLOC_008382-11921-0
      TGGTGAAGCAAGAAAATGTATATTGGATGAACAATAAAAAGTGTGTAACT
A5-AT2G04690-XLOC_008382-11921-1
      TGGTGAAGCAAGAAAATGTATATTGGATGAACAATAAAAAGTGTGTAACT
CONSENSUS
      TGGTGAAGCAAGAAAATGTATATTGGATGAACAATAAAAAGTGTGTAACT
```

```
A5-AT2G04690-XLOC_008382-11921-0
      GTTTCTGTAAATAACTCTTTGTTTTGGACCATAATAGATATTAAAATCTC
A5-AT2G04690-XLOC_008382-11921-1
      GTTTCTGTAAATAACTCTTTGTTTTGGACCATAATAGATATTAAAATCTC
CONSENSUS
      GTTTCTGTAAATAACTCTTTGTTTTGGACCATAATAGATATTAAAATCTC

A5-AT2G04690-XLOC_008382-11921-0
      TTGTAAATTACCTCAAAGCAATTTCAAAGGTATCCAAAATACTTTTCAAT
A5-AT2G04690-XLOC_008382-11921-1
      TTGTAAATTACCTCAAAGCAATTTCAAAGGTATCCAAAATACTTTTCAAT
CONSENSUS
      TTGTAAATTACCTCAAAGCAATTTCAAAGGTATCCAAAATACTTTTCAAT

A5-AT2G04690-XLOC_008382-11921-0 TTAT
A5-AT2G04690-XLOC_008382-11921-1 TTAT
CONSENSUS                        TTAT



alignment for event: RI-AT2G04540-XLOC_011089-3089

RI-AT2G04540-XLOC_011089-3089-0
      AGGCTGCGGCGGCTTAGTCCGTTTTTCATTCCAAAAATATTGGTAAACAT
RI-AT2G04540-XLOC_011089-3089-1
      AGGCTGCGGCGGCTTAGTCCGTTTTTCATTCCAAAAATATTGGTAAACAT
CONSENSUS
      AGGCTGCGGCGGCTTAGTCCGTTTTTCATTCCAAAAATATTGGTAAACAT

RI-AT2G04540-XLOC_011089-3089-0
      GGCATCTGGTCATGTGAGCATGAAGTATGGATTTCAGGTGTGTTGAATTT
RI-AT2G04540-XLOC_011089-3089-1
      GGCATCTGGTCATGTGAGCATGAAGTATGGATTTCAG-------------
CONSENSUS
      GGCATCTGGTCATGTGAGCATGAAGTATGGATTTCAG.............

RI-AT2G04540-XLOC_011089-3089-0
      TCCAGTTTTCTTTTATGCAAAAGAGTATGAACATCTTAGACTATCCAATG
RI-AT2G04540-XLOC_011089-3089-1
      --------------------------------------------------
CONSENSUS
      ..................................................

RI-AT2G04540-XLOC_011089-3089-0
      GTGGTTGATTTGGATTTTTTTAATGAGAGTTTTAATTATGCAACTCTTTC
RI-AT2G04540-XLOC_011089-3089-1
      --------------------------------------------------
CONSENSUS
      ..................................................

RI-AT2G04540-XLOC_011089-3089-0
      TCATTGTGATAATGGGGTGATAATGATATGGGATTTAAACATACTTTTTA
RI-AT2G04540-XLOC_011089-3089-1
      --------------------------------------------------
CONSENSUS
```

....

RI-AT2G04540-XLOC_011089-3089-0
TTAACGTGTCTAGAATAGTCCTCTGATGTTCTTAAGTAGTAAACCGTTTG
RI-AT2G04540-XLOC_011089-3089-1

CONSENSUS
....

RI-AT2G04540-XLOC_011089-3089-0
CATAGCATTGCCATGCTTTTGAATATAGGGGCCAAATCATGCTGCTGTGA
RI-AT2G04540-XLOC_011089-3089-1 ----------------------------
GGGCCAAATCATGCTGCTGTGA
CONSENSUS
............................GGGCCAAATCATGCTGCTGTGA

RI-AT2G04540-XLOC_011089-3089-0
CAGCTTGCGCAACTGGTGCACATTCTATAGGCGATGCCACTAGGATGATT
RI-AT2G04540-XLOC_011089-3089-1
CAGCTTGCGCAACTGGTGCACATTCTATAGGCGATGCCACTAGGATGATT
CONSENSUS
CAGCTTGCGCAACTGGTGCACATTCTATAGGCGATGCCACTAGGATGATT

RI-AT2G04540-XLOC_011089-3089-0
CAATTTGGAGATGCAGATGTTATGGTGGCTGGTGGAACTGAGTCTAGCAT
RI-AT2G04540-XLOC_011089-3089-1
CAATTTGGAGATGCAGATGTTATGGTGGCTGGTGGAACTGAGTCTAGCAT
CONSENSUS
CAATTTGGAGATGCAGATGTTATGGTGGCTGGTGGAACTGAGTCTAGCAT

RI-AT2G04540-XLOC_011089-3089-0 TGATGCTCTGTCCGTAGCTGGATTCTCTAG
RI-AT2G04540-XLOC_011089-3089-1 TGATGCTCTGTCCGTAGCTGGATTCTCTAG
CONSENSUS TGATGCTCTGTCCGTAGCTGGATTCTCTAG

alignment for event: RI-AT2G02570-XLOC_010981-1731

RI-AT2G02570-XLOC_010981-1731-0
GTATGATGCGACCATTGAGGCACATACTGCAAATGGCTATTTTGTTGCTT
RI-AT2G02570-XLOC_010981-1731-1
GTATGATGCGACCATTGAGGCACATACTGCAAATGGCTATTTTGTTGCTT
CONSENSUS
GTATGATGCGACCATTGAGGCACATACTGCAAATGGCTATTTTGTTGCTT

RI-AT2G02570-XLOC_010981-1731-0
ATGATGAGTGGGGAAACAAGGAAGAGGTAATGCTTCTATTCCAGTTTTAT
RI-AT2G02570-XLOC_010981-1731-1
ATGATGAGTGGGGAAACAAGGAAGAG------------------------
CONSENSUS
ATGATGAGTGGGGAAACAAGGAAGAG........................

RI-AT2G02570-XLOC_010981-1731-0
TTTGTTTTTAAAACACTAACTAATATTGAGATTGCCTTGTTATAATCTAG
RI-AT2G02570-XLOC_010981-1731-1

```
CONSENSUS
      ...............................................

RI-AT2G02570-XLOC_010981-1731-0
      GTGGATCCAGATAATGTGAGGCCAATCGAGCAAAATGCTATTGTCGAAGC
RI-AT2G02570-XLOC_010981-1731-1
      GTGGATCCAGATAATGTGAGGCCAATCGAGCAAAATGCTATTGTCGAAGC
CONSENSUS
      GTGGATCCAGATAATGTGAGGCCAATCGAGCAAAATGCTATTGTCGAAGC

RI-AT2G02570-XLOC_010981-1731-0
      TGAGAGATTAGCTCAAGCTACCAAAAATGCTCTCAAAAGAAAGATTGAGA
RI-AT2G02570-XLOC_010981-1731-1
      TGAGAGATTAGCTCAAGCTACCAAAAATGCTCTCAAAAGAAAGATTGAGA
CONSENSUS
      TGAGAGATTAGCTCAAGCTACCAAAAATGCTCTCAAAAGAAAGATTGAGA

RI-AT2G02570-XLOC_010981-1731-0
      AAGCTGCGAGTTCTGATTATCAGACAAAAACTCTACCAGCAAAGCTCAAA
RI-AT2G02570-XLOC_010981-1731-1
      AAGCTGCGAGTTCTGATTATCAGACAAAAACTCTACCAGCAAAGCTCAAA
CONSENSUS
      AAGCTGCGAGTTCTGATTATCAGACAAAAACTCTACCAGCAAAGCTCAAA

RI-AT2G02570-XLOC_010981-1731-0  ATCGATCCTAATGATCCCGAGGATGTA
RI-AT2G02570-XLOC_010981-1731-1  ATCGATCCTAATGATCCCGAGGATGTA
CONSENSUS                        ATCGATCCTAATGATCCCGAGGATGTA


alignment for event: A5-AT2G03140-XLOC_008280-5672

A5-AT2G03140-XLOC_008280-5672-0
      GGCTGTGGGCTTTATTGGCATGGTGCTCGTTTTATGGTCACCGGTTGTGA
A5-AT2G03140-XLOC_008280-5672-1
      GGCTGTGGGCTTTATTGGCATGGTGCTCGTTTTATGGTCACCGGTTGTGA
CONSENSUS
      GGCTGTGGGCTTTATTGGCATGGTGCTCGTTTTATGGTCACCGGTTGTGA

A5-AT2G03140-XLOC_008280-5672-0
      TTCCATTGCTTCCAACACTTCTCCAGAACTGGTCTACAAGTAATCCTTCT
A5-AT2G03140-XLOC_008280-5672-1
      TTCCATTGCTTCCAACACTTCTCCAGAACTGGTCTACAAGTAATCCTTCT
CONSENSUS
      TTCCATTGCTTCCAACACTTCTCCAGAACTGGTCTACAAGTAATCCTTCT

A5-AT2G03140-XLOC_008280-5672-0
      AGAGTGGCAGAACTAGCTAGTGTAGTCGGTCTCTATGTTGCTGTATTCAT
A5-AT2G03140-XLOC_008280-5672-1
      AGAGTGGCAGAACTAGCTAGTGTAGTCGGTCTCTATGTTGCTGTATTCAT
CONSENSUS
      AGAGTGGCAGAACTAGCTAGTGTAGTCGGTCTCTATGTTGCTGTATTCAT

A5-AT2G03140-XLOC_008280-5672-0
      TCTTGTTATGCTGTGGGGAAAGAGAGTACGGAAGTACGAAAACCCATTCA
A5-AT2G03140-XLOC_008280-5672-1
```

TCTTGTTATGCTGTGGGGAAAGAGAGTACGGAAGTACGAAAACCCATTCA
CONSENSUS
TCTTGTTATGCTGTGGGGAAAGAGAGTACGGAAGTACGAAAACCCATTCA

A5-AT2G03140-XLOC_008280-5672-0
AGCAATATGGGCTTGATTTAAAGGCATCAAACAAAGAAAAG---------
A5-AT2G03140-XLOC_008280-5672-1
AGCAATATGGGCTTGATTTAAAGGCATCAAACAAAGAAAAGGTAAAACCT
CONSENSUS
AGCAATATGGGCTTGATTTAAAGGCATCAAACAAAGAAAAG.........

A5-AT2G03140-XLOC_008280-5672-0

A5-AT2G03140-XLOC_008280-5672-1
TTAATGTAACACGGAGATTAGAATATTAGAATAGTTTGGTTTTCGTTGCA
CONSENSUS
....

A5-AT2G03140-XLOC_008280-5672-0

A5-AT2G03140-XLOC_008280-5672-1
CTGCTGAAGAAAATTTCTCGACCGTCAATACTTCATTAGCCACTAGACTA
CONSENSUS
....

A5-AT2G03140-XLOC_008280-5672-0

A5-AT2G03140-XLOC_008280-5672-1
ACCTTATTCAACTAAGAACTTGGTGGACGTTTTTCAAACAGCAACAGGAG
CONSENSUS
....

A5-AT2G03140-XLOC_008280-5672-0 -----------
ATTCAAGAGTTTTTAAAGGCCTTTGCAGGGGGCATCACG
A5-AT2G03140-XLOC_008280-5672-1
ATGGCTTAGTTATTCAAGAGTTTTTAAAGGCCTTTGCAGGGGGCATCACG
CONSENSUS
...........ATTCAAGAGTTTTTAAAGGCCTTTGCAGGGGGCATCACG

A5-AT2G03140-XLOC_008280-5672-0
GTCGTTCTACTAATTCAGTTCATAAATGCAATATCAGGAGCTGCGTTTCT
A5-AT2G03140-XLOC_008280-5672-1
GTCGTTCTACTAATTCAGTTCATAAATGCAATATCAGGAGCTGCGTTTCT
CONSENSUS
GTCGTTCTACTAATTCAGTTCATAAATGCAATATCAGGAGCTGCGTTTCT

A5-AT2G03140-XLOC_008280-5672-0
TTCTCGGCCACCATATTTTCCACATTCTTTTGATGCCATGAAGTGGCTTA
A5-AT2G03140-XLOC_008280-5672-1
TTCTCGGCCACCATATTTTCCACATTCTTTTGATGCCATGAAGTGGCTTA
CONSENSUS
TTCTCGGCCACCATATTTTCCACATTCTTTTGATGCCATGAAGTGGCTTA

A5-AT2G03140-XLOC_008280-5672-0
AGGGATGTGGGCAATTTCTTCTGCTGATAATTAGAGGATTTACAGCTGCG
A5-AT2G03140-XLOC_008280-5672-1

```
      AGGGATGTGGGCAATTTCTTCTGCTGATAATTAGAGGATTTACAGCTGCG
CONSENSUS
      AGGGATGTGGGCAATTTCTTCTGCTGATAATTAGAGGATTTACAGCTGCG

A5-AT2G03140-XLOC_008280-5672-0
      ACATTTGTAGTGCTTGTGGAAGAGTTGCTCTTCAGATCTTGGATGCCTGC
A5-AT2G03140-XLOC_008280-5672-1
      ACATTTGTAGTGCTTGTGGAAGAGTTGCTCTTCAGATCTTGGATGCCTGC
CONSENSUS
      ACATTTGTAGTGCTTGTGGAAGAGTTGCTCTTCAGATCTTGGATGCCTGC

A5-AT2G03140-XLOC_008280-5672-0
      CGAGATTGCTATAGATCTGGGCTACCATCAAAGCATTATCATTACAGGAC
A5-AT2G03140-XLOC_008280-5672-1
      CGAGATTGCTATAGATCTGGGCTACCATCAAAGCATTATCATTACAGGAC
CONSENSUS
      CGAGATTGCTATAGATCTGGGCTACCATCAAAGCATTATCATTACAGGAC

A5-AT2G03140-XLOC_008280-5672-0  TCATATTTGCCTTGTTCCAGAG
A5-AT2G03140-XLOC_008280-5672-1  TCATATTTGCCTTGTTCCAGAG
CONSENSUS                        TCATATTTGCCTTGTTCCAGAG
```

alignment for event: RI-AT2G32690-XLOC_012689-12499

```
RI-AT2G32690-XLOC_012689-12499-0
      CAACTACTGTCTTCGATCTCTCTTTCATATACACCATATAAAACGAACCA
RI-AT2G32690-XLOC_012689-12499-1
      CAACTACTGTCTTCGATCTCTCTTTCATATACACCATATAAAACGAACCA
CONSENSUS
      CAACTACTGTCTTCGATCTCTCTTTCATATACACCATATAAAACGAACCA

RI-AT2G32690-XLOC_012689-12499-0
      GTTAATTATGGGTTTAATTTCCGGGAAGGTGTGTGTGTTTATCTTTGTAT
RI-AT2G32690-XLOC_012689-12499-1
      GTTAATTATGGGTTTAATTTCCGGGAAGGTGTGTGTGTTTATCTTTGTAT
CONSENSUS
      GTTAATTATGGGTTTAATTTCCGGGAAGGTGTGTGTGTTTATCTTTGTAT

RI-AT2G32690-XLOC_012689-12499-0
      TCGCTCTAGTCGCTGAATTTTCGTTCGGAAATGTTGAGGTTAACGACGAC
RI-AT2G32690-XLOC_012689-12499-1
      TCGCTCTAGTCGCTGAATTTTCGTTCGGAAATGTTGAGGTTAACGACGAC
CONSENSUS
      TCGCTCTAGTCGCTGAATTTTCGTTCGGAAATGTTGAGGTTAACGACGAC

RI-AT2G32690-XLOC_012689-12499-0
      AAACACTTTTTCCACAAACCTCGTCCATTTTTACACAAACCTCGTCCATT
RI-AT2G32690-XLOC_012689-12499-1
      AAACACTTTTTCCACAAACCTCGTCCATTTTTACACAAACCTCGTCCATT
CONSENSUS
      AAACACTTTTTCCACAAACCTCGTCCATTTTTACACAAACCTCGTCCATT

RI-AT2G32690-XLOC_012689-12499-0
      CCTCCACAAGCATGGCATTTACAAGAAAGGTTTCGGTAAGGGTTTGGGCG
```

RI-AT2G32690-XLOC_012689-12499-1
      CCTCCACAAGCATGGCATTTACAAGAAAGGTTTCGGTAAGGGTTTGGGCG
CONSENSUS
      CCTCCACAAGCATGGCATTTACAAGAAAGGTTTCGGTAAGGGTTTGGGCG

RI-AT2G32690-XLOC_012689-12499-0
      GTGGAGGCGGTCTAGGCGGCGGAGGTGGTCTAGGAGGCGGTGGTGGCCTA
RI-AT2G32690-XLOC_012689-12499-1
      GTGGAGGCGGTCTAGGCGGCGGAGGTGGTCTAGGAGGCGGTGGTGGCCTA
CONSENSUS
      GTGGAGGCGGTCTAGGCGGCGGAGGTGGTCTAGGAGGCGGTGGTGGCCTA

RI-AT2G32690-XLOC_012689-12499-0
      GGAGGCGGTGGAGGTTTAGGCGGTGGTGGAGGTTTAGGCGGCGGTGGTGG
RI-AT2G32690-XLOC_012689-12499-1
      GGAGGCGGTGGAGGTTTAGGCGGTGGTGGAGGTTTAGGCGGCGGTGGTGG
CONSENSUS
      GGAGGCGGTGGAGGTTTAGGCGGTGGTGGAGGTTTAGGCGGCGGTGGTGG

RI-AT2G32690-XLOC_012689-12499-0
      TCTAGGAGGCGGTAGCGGTTTGGGAGGAGGAGGGGGTCTAGGAGGCGGTA
RI-AT2G32690-XLOC_012689-12499-1
      TCTAGGAGGCGGTAGCGGTTTGGGAGGAGGAGGGGGTCTAGGAGGCGGTA
CONSENSUS
      TCTAGGAGGCGGTAGCGGTTTGGGAGGAGGAGGGGGTCTAGGAGGCGGTA

RI-AT2G32690-XLOC_012689-12499-0
      GCGGTTTGGGAGGAGGAGGGGGTCTAGGAGGTGGTGGTGGTGGTGGTTTA
RI-AT2G32690-XLOC_012689-12499-1
      GCGGTTTGGGAGGAGGAGGGGGTCTAGGAG--------------------
CONSENSUS
      GCGGTTTGGGAGGAGGAGGGGGTCTAGGAG....................

RI-AT2G32690-XLOC_012689-12499-0
      GGAGGAGGAGGTGGATTGGGTGGTGGAGCTGGAGGAGGATACGGTGGTGG
RI-AT2G32690-XLOC_012689-12499-1 ----------
GTGGATTGGGTGGTGGAGCTGGAGGAGGATACGGTGGTGG
CONSENSUS
      ..........GTGGATTGGGTGGTGGAGCTGGAGGAGGATACGGTGGTGG

RI-AT2G32690-XLOC_012689-12499-0
      TGCTGGAGGAGGACTTGGAGGCGGAGGTGGAATTGGAGGAGGAGGAGGCT
RI-AT2G32690-XLOC_012689-12499-1
      TGCTGGAGGAGGACTTGGAGGCGGAGGTGGAATTGGAGGAGGAGGAGGCT
CONSENSUS
      TGCTGGAGGAGGACTTGGAGGCGGAGGTGGAATTGGAGGAGGAGGAGGCT

RI-AT2G32690-XLOC_012689-12499-0
      TTGGCGGTGGAGGGGGAGGAGGATTCGGGGGCGGAGCCGGTGGTGGATTT
RI-AT2G32690-XLOC_012689-12499-1
      TTGGCGGTGGAGGGGGAGGAGGATTCGGGGGCGGAGCCGGTGGTGGATTT
CONSENSUS
      TTGGCGGTGGAGGGGGAGGAGGATTCGGGGGCGGAGCCGGTGGTGGATTT

RI-AT2G32690-XLOC_012689-12499-0
      GGTAAAGGCATTGGTGGTGGAGGAGGTCTTGGAGGAGGTTATGTTGGTGG

RI-AT2G32690-XLOC_012689-12499-1
GGTAAAGGCATTGGTGGTGGAGGAGGTCTTGGAGGAGGTTATGTTGGTGG
CONSENSUS
GGTAAAGGCATTGGTGGTGGAGGAGGTCTTGGAGGAGGTTATGTTGGTGG

RI-AT2G32690-XLOC_012689-12499-0
TGGCCATCACTGATGAGTGGTGAGGCTCATGCTCATGCATTCGTAACGTT
RI-AT2G32690-XLOC_012689-12499-1
TGGCCATCACTGATGAGTGGTGAGGCTCATGCTCATGCATTCGTAACGTT
CONSENSUS
TGGCCATCACTGATGAGTGGTGAGGCTCATGCTCATGCATTCGTAACGTT

RI-AT2G32690-XLOC_012689-12499-0
GTTATATTATTTAATTAAATGGAAAATGATAAATTAATTATTGCAATAAA
RI-AT2G32690-XLOC_012689-12499-1
GTTATATTATTTAATTAAATGGAAAATGATAAATTAATTATTGCAATAAA
CONSENSUS
GTTATATTATTTAATTAAATGGAAAATGATAAATTAATTATTGCAATAAA

RI-AT2G32690-XLOC_012689-12499-0
ATGATACATTAGCAATTGTGTATGTCGTTATTTTTATTTTGAGTCGCATT
RI-AT2G32690-XLOC_012689-12499-1
ATGATACATTAGCAATTGTGTATGTCGTTATTTTTATTTTGAGTCGCATT
CONSENSUS
ATGATACATTAGCAATTGTGTATGTCGTTATTTTTATTTTGAGTCGCATT

RI-AT2G32690-XLOC_012689-12499-0
GTATGCTGATTCCATATGTTCGGCTTTGGTCGGTTTCAAGTGTGGTTAAT
RI-AT2G32690-XLOC_012689-12499-1
GTATGCTGATTCCATATGTTCGGCTTTGGTCGGTTTCAAGTGTGGTTAAT
CONSENSUS
GTATGCTGATTCCATATGTTCGGCTTTGGTCGGTTTCAAGTGTGGTTAAT

RI-AT2G32690-XLOC_012689-12499-0 AAAATGGTTATTTTACTTTTGCCAACAGTT
RI-AT2G32690-XLOC_012689-12499-1 AAAATGGTTATTTTACTTTTGCCAACAGTT
CONSENSUS AAAATGGTTATTTTACTTTTGCCAACAGTT

alignment for event: SE-AT2G26810-XLOC_012348-1097

SE-AT2G26810-XLOC_012348-1097-0
CTGATTTTGATTTAACAGGGCAGCTGGTTTGGCCTGGTGCGATGCTTATG
SE-AT2G26810-XLOC_012348-1097-1
CTGATTTTGATTTAACAGGGCAGCTGGTTTGGCCTGGTGCGATGCTTATG
CONSENSUS
CTGATTTTGATTTAACAGGGCAGCTGGTTTGGCCTGGTGCGATGCTTATG

SE-AT2G26810-XLOC_012348-1097-0
AATGGTTATCTCTCAGAAAATGCTGACATTCTCCAGGGATGTTCAGTTTT
SE-AT2G26810-XLOC_012348-1097-1
AATGGTTATCTCTCAGAAAATGCTGACATTCTCCAGGGATGTTCAGTTTT
CONSENSUS
AATGGTTATCTCTCAGAAAATGCTGACATTCTCCAGGGATGTTCAGTTTT

SE-AT2G26810-XLOC_012348-1097-0

GGAGTTGGGATCTGGCGTTG------------------------------
SE-AT2G26810-XLOC_012348-1097-1
GGAGTTGGGATCTGGCGTTGTTAGAGATGTTATGTATTGTAACATCATCA
CONSENSUS
GGAGTTGGGATCTGGCGTTG..............................

SE-AT2G26810-XLOC_012348-1097-0

SE-AT2G26810-XLOC_012348-1097-1
CTCCCTGTTAGATATGCTGTATATACAAATATGGTCTTTGCATACTCTGC
CONSENSUS
....

SE-AT2G26810-XLOC_012348-1097-0 ---------------------------
GTATAACTGGAGTCCTATGTAGCA
SE-AT2G26810-XLOC_012348-1097-1
TTGGCTGACTTCACCATCATTCACATGTATAACTGGAGTCCTATGTAGCA
CONSENSUS
..........................GTATAACTGGAGTCCTATGTAGCA

SE-AT2G26810-XLOC_012348-1097-0
AATTTTGCCGTAAAGTTATTTTTACTGACCACAACGATGAAGTGCTCAAG
SE-AT2G26810-XLOC_012348-1097-1
AATTTTGCCGTAAAGTTATTTTTACTGACCACAACGATGAAGTGCTCAAG
CONSENSUS
AATTTTGCCGTAAAGTTATTTTTACTGACCACAACGATGAAGTGCTCAAG

alignment for event: RI-AT2G32690-XLOC_012689-12501

RI-AT2G32690-XLOC_012689-12501-0
CAACTACTGTCTTCGATCTCTCTTTCATATACACCATATAAAACGAACCA
RI-AT2G32690-XLOC_012689-12501-1
CAACTACTGTCTTCGATCTCTCTTTCATATACACCATATAAAACGAACCA
CONSENSUS
CAACTACTGTCTTCGATCTCTCTTTCATATACACCATATAAAACGAACCA

RI-AT2G32690-XLOC_012689-12501-0
GTTAATTATGGGTTTAATTTCCGGGAAGGTGTGTGTGTTTATCTTTGTAT
RI-AT2G32690-XLOC_012689-12501-1
GTTAATTATGGGTTTAATTTCCGGGAAGGTGTGTGTGTTTATCTTTGTAT
CONSENSUS
GTTAATTATGGGTTTAATTTCCGGGAAGGTGTGTGTGTTTATCTTTGTAT

RI-AT2G32690-XLOC_012689-12501-0
TCGCTCTAGTCGCTGAATTTTCGTTCGGAAATGTTGAGGTTAACGACGAC
RI-AT2G32690-XLOC_012689-12501-1
TCGCTCTAGTCGCTGAATTTTCGTTCGGAAATGTTGAGGTTAACGACGAC
CONSENSUS
TCGCTCTAGTCGCTGAATTTTCGTTCGGAAATGTTGAGGTTAACGACGAC

RI-AT2G32690-XLOC_012689-12501-0
AAACACTTTTTCCACAAACCTCGTCCATTTTTACACAAACCTCGTCCATT
RI-AT2G32690-XLOC_012689-12501-1
AAACACTTTTTCCACAAACCTCGTCCATTTTTACACAAACCTCGTCCATT

CONSENSUS
AAACACTTTTTCCACAAACCTCGTCCATTTTTACACAAACCTCGTCCATT

RI-AT2G32690-XLOC_012689-12501-0
CCTCCACAAGCATGGCATTTACAAGAAAGGTTTCGGTAAGGGTTTGGGCG
RI-AT2G32690-XLOC_012689-12501-1
CCTCCACAAGCATGGCATTTACAAGAAAGGTTTCGGTAAGGGTTTGGGCG
CONSENSUS
CCTCCACAAGCATGGCATTTACAAGAAAGGTTTCGGTAAGGGTTTGGGCG

RI-AT2G32690-XLOC_012689-12501-0
GTGGAGGCGGTCTAGGCGGCGGAGGTGGTCTAGGAGGCGGTGGTGGCCTA
RI-AT2G32690-XLOC_012689-12501-1
GTGGAGGCGGTCTAGGCGGCGGAGGTGGTCTAGGAGGCGGTGGTGGCCTA
CONSENSUS
GTGGAGGCGGTCTAGGCGGCGGAGGTGGTCTAGGAGGCGGTGGTGGCCTA

RI-AT2G32690-XLOC_012689-12501-0
GGAGGCGGTGGAGGTTTAGGCGGTGGTGGAGGTTTAGGCGGCGGTGGTGG
RI-AT2G32690-XLOC_012689-12501-1
GGAGGCGGTGGAGGTTTAGGCGGTGGTGGAGGTTTAGGCGGCGGTGGTGG
CONSENSUS
GGAGGCGGTGGAGGTTTAGGCGGTGGTGGAGGTTTAGGCGGCGGTGGTGG

RI-AT2G32690-XLOC_012689-12501-0
TCTAGGAGGCGGTAGCGGTTTGGGAGGAGGAGGGGGTCTAGGAGGCGGTA
RI-AT2G32690-XLOC_012689-12501-1
TCTAGGAGGCGGTAGCGGTTTGGGAGGAGGAGGGGGTCTAGGAGGCGGTA
CONSENSUS
TCTAGGAGGCGGTAGCGGTTTGGGAGGAGGAGGGGGTCTAGGAGGCGGTA

RI-AT2G32690-XLOC_012689-12501-0
GCGGTTTGGGAGGAGGAGGGGGTCTAGGAGGTGGTGGTGGTGGTGGTTTA
RI-AT2G32690-XLOC_012689-12501-1
GCGGTTTGGGAGGAGGAGGGGGTCTAGGAGGTGGTGGTGGTGGTGGTTTA
CONSENSUS
GCGGTTTGGGAGGAGGAGGGGGTCTAGGAGGTGGTGGTGGTGGTGGTTTA

RI-AT2G32690-XLOC_012689-12501-0
GGAGGAGGAGGTGGATTGGGTGGTGGAGCTGGAGGAGGATACGGTGGTGG
RI-AT2G32690-XLOC_012689-12501-1
GGAGGAGGAG------------------------GAGGATACGGTGGTGG
CONSENSUS
GGAGGAGGAG........................GAGGATACGGTGGTGG

RI-AT2G32690-XLOC_012689-12501-0
TGCTGGAGGAGGACTTGGAGGCGGAGGTGGAATTGGAGGAGGAGGAGGCT
RI-AT2G32690-XLOC_012689-12501-1
TGCTGGAGGAGGACTTGGAGGCGGAGGTGGAATTGGAGGAGGAGGAGGCT
CONSENSUS
TGCTGGAGGAGGACTTGGAGGCGGAGGTGGAATTGGAGGAGGAGGAGGCT

RI-AT2G32690-XLOC_012689-12501-0
TTGGCGGTGGAGGGGGAGGAGGATTCGGGGGCGGAGCCGGTGGTGGATTT
RI-AT2G32690-XLOC_012689-12501-1
TTGGCGGTGGAGGGGGAGGAGGATTCGGGGGCGGAGCCGGTGGTGGATTT

CONSENSUS
      TTGGCGGTGGAGGGGGAGGAGGATTCGGGGGCGGAGCCGGTGGTGGATTT

RI-AT2G32690-XLOC_012689-12501-0
      GGTAAAGGCATTGGTGGTGGAGGAGGTCTTGGAGGAGGTTATGTTGGTGG
RI-AT2G32690-XLOC_012689-12501-1
      GGTAAAGGCATTGGTGGTGGAGGAGGTCTTGGAGGAGGTTATGTTGGTGG
CONSENSUS
      GGTAAAGGCATTGGTGGTGGAGGAGGTCTTGGAGGAGGTTATGTTGGTGG

RI-AT2G32690-XLOC_012689-12501-0
      TGGCCATCACTGATGAGTGGTGAGGCTCATGCTCATGCATTCGTAACGTT
RI-AT2G32690-XLOC_012689-12501-1
      TGGCCATCACTGATGAGTGGTGAGGCTCATGCTCATGCATTCGTAACGTT
CONSENSUS
      TGGCCATCACTGATGAGTGGTGAGGCTCATGCTCATGCATTCGTAACGTT

RI-AT2G32690-XLOC_012689-12501-0
      GTTATATTATTTAATTAAATGGAAAATGATAAATTAATTATTGCAATAAA
RI-AT2G32690-XLOC_012689-12501-1
      GTTATATTATTTAATTAAATGGAAAATGATAAATTAATTATTGCAATAAA
CONSENSUS
      GTTATATTATTTAATTAAATGGAAAATGATAAATTAATTATTGCAATAAA

RI-AT2G32690-XLOC_012689-12501-0
      ATGATACATTAGCAATTGTGTATGTCGTTATTTTTATTTTGAGTCGCATT
RI-AT2G32690-XLOC_012689-12501-1
      ATGATACATTAGCAATTGTGTATGTCGTTATTTTTATTTTGAGTCGCATT
CONSENSUS
      ATGATACATTAGCAATTGTGTATGTCGTTATTTTTATTTTGAGTCGCATT

RI-AT2G32690-XLOC_012689-12501-0
      GTATGCTGATTCCATATGTTCGGCTTTGGTCGGTTTCAAGTGTGGTTAAT
RI-AT2G32690-XLOC_012689-12501-1
      GTATGCTGATTCCATATGTTCGGCTTTGGTCGGTTTCAAGTGTGGTTAAT
CONSENSUS
      GTATGCTGATTCCATATGTTCGGCTTTGGTCGGTTTCAAGTGTGGTTAAT

RI-AT2G32690-XLOC_012689-12501-0 AAAATGGTTATTTTACTTTTGCCAACAGTT
RI-AT2G32690-XLOC_012689-12501-1 AAAATGGTTATTTTACTTTTGCCAACAGTT
CONSENSUS   AAAATGGTTATTTTACTTTTGCCAACAGTT

alignment for event: RI-AT2G25590-XLOC_009626-4783

RI-AT2G25590-XLOC_009626-4783-0
      GAATAAAGTAAACTATCATGAGATTCAGAAGAGGAAGTAGAGTTGAGGTG
RI-AT2G25590-XLOC_009626-4783-1
      GAATAAAGTAAACTATCATGAGATTCAGAAGAGGAAGTAGAGTTGAGGTG
CONSENSUS
      GAATAAAGTAAACTATCATGAGATTCAGAAGAGGAAGTAGAGTTGAGGTG

RI-AT2G25590-XLOC_009626-4783-0
      TTTAGCATCAAAGAAGCCTCTTATGGTGTGTGGCGATCAGCGGAGATTAT
RI-AT2G25590-XLOC_009626-4783-1

TTTAGCATCAAAGAAGCCTCTTATGGTGTGTGGCGATCAGCGGAGATTAT
CONSENSUS
TTTAGCATCAAAGAAGCCTCTTATGGTGTGTGGCGATCAGCGGAGATTAT

RI-AT2G25590-XLOC_009626-4783-0
ATCTGGTAATGGTCACACTTACAATGTTAGATATTACTCTTTTGAAATTG
RI-AT2G25590-XLOC_009626-4783-1
ATCTG---
CONSENSUS
ATCTG...

RI-AT2G25590-XLOC_009626-4783-0
CTAACAATGAGGTTGTTGAGGATAGAGTTCCAAGGAAGATAATTAGGCCG
RI-AT2G25590-XLOC_009626-4783-1 -------------------------
AGTTCCAAGGAAGATAATTAGGCCG
CONSENSUS
.........................AGTTCCAAGGAAGATAATTAGGCCG

RI-AT2G25590-XLOC_009626-4783-0
TGTCCTCCGCAAGTAGATGTTGATAGATGGGAAGCTGGTGAATTGGTTGA
RI-AT2G25590-XLOC_009626-4783-1
TGTCCTCCGCAAGTAGATGTTGATAGATGGGAAGCTGGTGAATTGGTTGA
CONSENSUS
TGTCCTCCGCAAGTAGATGTTGATAGATGGGAAGCTGGTGAATTGGTTGA

RI-AT2G25590-XLOC_009626-4783-0
GGTTCTTGATAATAATATTTCATGGAAAACTGCTACTGTCTTGGAGGTGT
RI-AT2G25590-XLOC_009626-4783-1
GGTTCTTGATAATAATATTTCATGGAAAACTGCTACTGTCTTGGAGGTGT
CONSENSUS
GGTTCTTGATAATAATATTTCATGGAAAACTGCTACTGTCTTGGAGGTGT

RI-AT2G25590-XLOC_009626-4783-0
TATCTGGAAGATATTATGTTGTTCGGTTACTTGGTGCTAAAGCAGAACTC
RI-AT2G25590-XLOC_009626-4783-1
TATCTGGAAGATATTATGTTGTTCGGTTACTTGGTGCTAAAGCAGAACTC
CONSENSUS
TATCTGGAAGATATTATGTTGTTCGGTTACTTGGTGCTAAAGCAGAACTC

RI-AT2G25590-XLOC_009626-4783-0
ACGGTTCACAAAGTTTACCTCAGGGCTAGACAATCTTGGCAAGATGAAAG
RI-AT2G25590-XLOC_009626-4783-1
ACGGTTCACAAAGTTTACCTCAGGGCTAGACAATCTTGGCAAGATGAAAG
CONSENSUS
ACGGTTCACAAAGTTTACCTCAGGGCTAGACAATCTTGGCAAGATGAAAG

RI-AT2G25590-XLOC_009626-4783-0 ATGGGTTATGATTGGAAAG
RI-AT2G25590-XLOC_009626-4783-1 ATGGGTTATGATTGGAAAG
CONSENSUS ATGGGTTATGATTGGAAAG

alignment for event: A3-AT2G33820-XLOC_012757-10912

A3-AT2G33820-XLOC_012757-10912-0
AATGCAGATCCAAGGAACGGATTCTCTGGTTCCTAACTTCCGTAGATACA

A3-AT2G33820-XLOC_012757-10912-1
      AATGCAGATCCAAGGAACGGATTCTCTGGTTCCTAACTTCCGTAGATACA
CONSENSUS
      AATGCAGATCCAAGGAACGGATTCTCTGGTTCCTAACTTCCGTAGATACA

A3-AT2G33820-XLOC_012757-10912-0
      ACAGTCCTCTTGATTGTGCCGTTCAGACCGTTAAAAATGATGGG------
A3-AT2G33820-XLOC_012757-10912-1
      ACAGTCCTCTTGATTGTGCCGTTCAGACCGTTAAAAATGATGGGGTAACA
CONSENSUS
      ACAGTCCTCTTGATTGTGCCGTTCAGACCGTTAAAAATGATGGG......

A3-AT2G33820-XLOC_012757-10912-0 -
GTATCTTTCGTGGTGGTTCGGCAACCTTATTAAGAGAATGTACGGGAAA
A3-AT2G33820-XLOC_012757-10912-1
      GGTATCTTTCGTGGTGGTTCGGCAACCTTATTAAGAGAATGTACGGGAAA
CONSENSUS
      .GTATCTTTCGTGGTGGTTCGGCAACCTTATTAAGAGAATGTACGGGAAA

A3-AT2G33820-XLOC_012757-10912-0
      TGCTGTCTTTTTTACTGTCTATGAGTACTTACGGTATCATATCCACTCGA
A3-AT2G33820-XLOC_012757-10912-1
      TGCTGTCTTTTTTACTGTCTATGAGTACTTACGGTATCATATCCACTCGA
CONSENSUS
      TGCTGTCTTTTTTACTGTCTATGAGTACTTACGGTATCATATCCACTCGA

A3-AT2G33820-XLOC_012757-10912-0
      GATTGGAGGATTCTAAGCTGAAAGATGGTTACTTGGTTGACATGGGGATA
A3-AT2G33820-XLOC_012757-10912-1
      GATTGGAGGATTCTAAGCTGAAAGATGGTTACTTGGTTGACATGGGGATA
CONSENSUS
      GATTGGAGGATTCTAAGCTGAAAGATGGTTACTTGGTTGACATGGGGATA

A3-AT2G33820-XLOC_012757-10912-0
      GGAGTTCTCACTGGTGGTCTTGGAGGCATAGCT
A3-AT2G33820-XLOC_012757-10912-1
      GGAGTTCTCACTGGTGGTCTTGGAGGCATAGCT
CONSENSUS
      GGAGTTCTCACTGGTGGTCTTGGAGGCATAGCT

alignment for event: RI-AT2G24690-XLOC_012227-11123

RI-AT2G24690-XLOC_012227-11123-0
      CGGCTTCCGTTGCAATTCATGACGGAGAATAGCATGAACAAGCCCGGGGA
RI-AT2G24690-XLOC_012227-11123-1
      CGGCTTCCGTTGCAATTCATGACGGAGAATAGCATGAACAAGCCCGGGGA
CONSENSUS
      CGGCTTCCGTTGCAATTCATGACGGAGAATAGCATGAACAAGCCCGGGGA

RI-AT2G24690-XLOC_012227-11123-0
      GATAACTCTTTTAGGTACAGATGGTGCAAAGTGGATGGCAAGTCTTCTAC
RI-AT2G24690-XLOC_012227-11123-1
      GATAACTCTTTTAGGTACAGATGGTGCAAAGTGGATGGCAAGTCTTCTAC
CONSENSUS

GATAACTCTTTTAGGTACAGATGGTGCAAAGTGGATGGCAAGTCTTCTAC

RI-AT2G24690-XLOC_012227-11123-0
TAGAAAAAAAGGGAAGAATGAGTTTGGGAAAGGGTTGGAAAGATTTTGCT
RI-AT2G24690-XLOC_012227-11123-1
TAGAAAAAAAGGGAAGAATGAGTTTGGGAAAGGGTTGGAAAGATTTTGCT
CONSENSUS
TAGAAAAAAAGGGAAGAATGAGTTTGGGAAAGGGTTGGAAAGATTTTGCT

RI-AT2G24690-XLOC_012227-11123-0
AAAGCAAATGGCTTAAAGACGGGTGATTCCATTACATTGGAGCCAATATG
RI-AT2G24690-XLOC_012227-11123-1
AAAGCAAATGGCTTAAAGACGGGTGATTCCATTACATTGGAGCCAATATG
CONSENSUS
AAAGCAAATGGCTTAAAGACGGGTGATTCCATTACATTGGAGCCAATATG

RI-AT2G24690-XLOC_012227-11123-0
GGAAGACAGGACTCCTGTGCTCAGTATAAAGTCTAGCAGTGGTAAAGGGC
RI-AT2G24690-XLOC_012227-11123-1
GGAAGACAGGACTCCTGTGCTCAGTATAAAGTCTAGCAGTGGTAAAGGGC
CONSENSUS
GGAAGACAGGACTCCTGTGCTCAGTATAAAGTCTAGCAGTGGTAAAGGGC

RI-AT2G24690-XLOC_012227-11123-0
AAAGTGAGTTTTCCAAGGAATCTCTTTCCATAAAACCTAGTAGTGGAAAC
RI-AT2G24690-XLOC_012227-11123-1
AAAGTGAGTTTTCCAAGGAATCTCTTTCCATAAAACCTAGTAGTGGAAAC
CONSENSUS
AAAGTGAGTTTTCCAAGGAATCTCTTTCCATAAAACCTAGTAGTGGAAAC

RI-AT2G24690-XLOC_012227-11123-0
ATGACTAAGAAAGTAGAGAACAACAGAGAAGCAAGCAGAAAGTATCCTCC
RI-AT2G24690-XLOC_012227-11123-1
ATGACTAAGAAAGTAGAGAACAACAGAGAAGCAAGCAGAAAGTATCCTCC
CONSENSUS
ATGACTAAGAAAGTAGAGAACAACAGAGAAGCAAGCAGAAAGTATCCTCC

RI-AT2G24690-XLOC_012227-11123-0
TAGAAGTAGAGAATCATCTTCAGCAATCCAAAACCAATTCATGGCATTAA
RI-AT2G24690-XLOC_012227-11123-1
TAGAAGTAGAGAATCATCTTCAGCAATCCAAAACCAATTCATGGCATTAA
CONSENSUS
TAGAAGTAGAGAATCATCTTCAGCAATCCAAAACCAATTCATGGCATTAA

RI-AT2G24690-XLOC_012227-11123-0
CACCTCTACGTGATATAGTTAGCCAGGTTGCTCATGATCTTAGCATCGGT
RI-AT2G24690-XLOC_012227-11123-1
CACCTCTACGTGATATAGTTAGCCAGGTTGCTCATGATCTTAGCATCGGT
CONSENSUS
CACCTCTACGTGATATAGTTAGCCAGGTTGCTCATGATCTTAGCATCGGT

RI-AT2G24690-XLOC_012227-11123-0
GAGGTCATCAATTTCAGACATAAAGGAGATAATATGTTGCGGGTCTCAGA
RI-AT2G24690-XLOC_012227-11123-1
GAGGTCATCAATTTCAGACATAAAGGAGATAATATGTTGCGGGTCTCAGA
CONSENSUS

```
      GAGGTCATCAATTTCAGACATAAAGGAGATAATATGTTGCGGGTCTCAGA

RI-AT2G24690-XLOC_012227-11123-0
      TTTGGGATCCAATTGTTGTGGGGTTCAAGATTTACTGGCTCCAAGCAGTA
RI-AT2G24690-XLOC_012227-11123-1
      TTTGGGATCCAATTGTTGTGGGGTTCAAGATTTACTGGCTCCAAGCAGTA
CONSENSUS
      TTTGGGATCCAATTGTTGTGGGGTTCAAGATTTACTGGCTCCAAGCAGTA

RI-AT2G24690-XLOC_012227-11123-0
      ACTATGATCATGACAACATCAGTAAGTTTGATGATTTAAAGCTGTAACGT
RI-AT2G24690-XLOC_012227-11123-1
      ACTATGATCATGACAACATCA-----------------------------
CONSENSUS
      ACTATGATCATGACAACATCA.............................

RI-AT2G24690-XLOC_012227-11123-0
      TGACAAGATTGTTAAATGCAGTCTGAGGATATTCTCTTATACAGGTAACA
RI-AT2G24690-XLOC_012227-11123-1
      --------------------------------------------GTAACA
CONSENSUS
      ............................................GTAACA

RI-AT2G24690-XLOC_012227-11123-0
      TTTCGATGAAAATAAATCCGCATATAAGAAAAGAAGCTGTAACATTTTCG
RI-AT2G24690-XLOC_012227-11123-1
      TTTCGATGAAAATAAATCCGCATATAAGAAAAGAAGCTGTAACATTTTCG
CONSENSUS
      TTTCGATGAAAATAAATCCGCATATAAGAAAAGAAGCTGTAACATTTTCG

RI-AT2G24690-XLOC_012227-11123-0
      TCATATGATGGTTATGCTCATGACAACTTTG
RI-AT2G24690-XLOC_012227-11123-1
      TCATATGATGGTTATGCTCATGACAACTTTG
CONSENSUS
      TCATATGATGGTTATGCTCATGACAACTTTG



alignment for event: A5-AT2G46270-XLOC_010775-7640

A5-AT2G46270-XLOC_010775-7640-0
      ATGGGAAACAATTGGTTCAAGCTAGCTCATTTCATTCTGTTTCTCCGTCA
A5-AT2G46270-XLOC_010775-7640-1
      ATGGGAAACAATTGGTTCAAGCTAGCTCATTTCATTCTGTTTCTCCGTCA
CONSENSUS
      ATGGGAAACAATTGGTTCAAGCTAGCTCATTTCATTCTGTTTCTCCGTCA

A5-AT2G46270-XLOC_010775-7640-0
      AGTGGTGATACCGGCGTAAAACTCATTCAAGGATCTGGAGCTATACTCTC
A5-AT2G46270-XLOC_010775-7640-1
      AGTGGTGATACCGGCGTAAAACTCATTCAAGGATCTGGAGCTATACTCTC
CONSENSUS
      AGTGGTGATACCGGCGTAAAACTCATTCAAGGATCTGGAGCTATACTCTC

A5-AT2G46270-XLOC_010775-7640-0
```

```
      TCCTGGT-----------------------------------------
A5-AT2G46270-XLOC_010775-7640-1
      TCCTGGTGTAAGTGCAAATTCCAACCCCTTCATGTCACAATCTTTAGCCA
CONSENSUS
      TCCTGGT.........................................

A5-AT2G46270-XLOC_010775-7640-0  -------------------------
AACGAGAGAGAACTGAAACGGGAG
A5-AT2G46270-XLOC_010775-7640-1
      TGGTTCCTCCTGAAACTTGGCTTCAGAACGAGAGAGAACTGAAACGGGAG
CONSENSUS
      ..........................AACGAGAGAGAACTGAAACGGGAG

A5-AT2G46270-XLOC_010775-7640-0
      CGAAGGAAACAGTCTAATAGAGAATCTGCTAGAAGGTCAAGATTAAGGAA
A5-AT2G46270-XLOC_010775-7640-1
      CGAAGGAAACAGTCTAATAGAGAATCTGCTAGAAGGTCAAGATTAAGGAA
CONSENSUS
      CGAAGGAAACAGTCTAATAGAGAATCTGCTAGAAGGTCAAGATTAAGGAA

A5-AT2G46270-XLOC_010775-7640-0  ACAG
A5-AT2G46270-XLOC_010775-7640-1  ACAG
CONSENSUS                        ACAG



alignment for event: A5-AT2G38880-XLOC_010355-5542

A5-AT2G38880-XLOC_010355-5542-0
      AGCTGGTAAAAGAAGTTGCAAGTAGTGATTAAGAACAATCGCCAAATGAT
A5-AT2G38880-XLOC_010355-5542-1
      AGCTGGTAAAAGAAGTTGCAAGTAGTGATTAAGAACAATCGCCAAATGAT
CONSENSUS
      AGCTGGTAAAAGAAGTTGCAAGTAGTGATTAAGAACAATCGCCAAATGAT

A5-AT2G38880-XLOC_010355-5542-0
      CAA-----------------------------------------------
A5-AT2G38880-XLOC_010355-5542-1
      CAAGTAATTCTTCTCTCTATCACACGGCCTCATGTACACAAATGAAGAGC
CONSENSUS
      CAA...............................................

A5-AT2G38880-XLOC_010355-5542-0  ---------------
GGGAAATTAGAGATCAGTGAGTTGTTTATAGTTGA
A5-AT2G38880-XLOC_010355-5542-1
      ATAGTCTATATGCATGGGAAATTAGAGATCAGTGAGTTGTTTATAGTTGA
CONSENSUS
      ...............GGGAAATTAGAGATCAGTGAGTTGTTTATAGTTGA

A5-AT2G38880-XLOC_010355-5542-0
      GCTGATCGACAACTATTTCGGGTTTACTCTCAATTTCGGTTATGTTAGTT
A5-AT2G38880-XLOC_010355-5542-1
      GCTGATCGACAACTATTTCGGGTTTACTCTCAATTTCGGTTATGTTAGTT
CONSENSUS
      GCTGATCGACAACTATTTCGGGTTTACTCTCAATTTCGGTTATGTTAGTT
```

A5-AT2G38880-XLOC_010355-5542-0
      TGAACGTTTGGTTTATTGTTTCCGGTTTAGTTGGTTGTATTTAAAGATTT
A5-AT2G38880-XLOC_010355-5542-1
      TGAACGTTTGGTTTATTGTTTCCGGTTTAGTTGGTTGTATTTAAAGATTT
CONSENSUS
      TGAACGTTTGGTTTATTGTTTCCGGTTTAGTTGGTTGTATTTAAAGATTT

A5-AT2G38880-XLOC_010355-5542-0
      CTCTGTTAGATGTTGAGAACACTTGAATGAAGGAAAAATTTGTCCACATC
A5-AT2G38880-XLOC_010355-5542-1
      CTCTGTTAGATGTTGAGAACACTTGAATGAAGGAAAAATTTGTCCACATC
CONSENSUS
      CTCTGTTAGATGTTGAGAACACTTGAATGAAGGAAAAATTTGTCCACATC

A5-AT2G38880-XLOC_010355-5542-0
      CTGTTGTTATTTTCGATTCACTTTCGGAATTTCATAGCTAATTTATTCTC
A5-AT2G38880-XLOC_010355-5542-1
      CTGTTGTTATTTTCGATTCACTTTCGGAATTTCATAGCTAATTTATTCTC
CONSENSUS
      CTGTTGTTATTTTCGATTCACTTTCGGAATTTCATAGCTAATTTATTCTC

A5-AT2G38880-XLOC_010355-5542-0 ATTTAATACAAAATCCTTAAATTAA
A5-AT2G38880-XLOC_010355-5542-1 ATTTAATACAAAATCCTTAAATTAA
CONSENSUS   ATTTAATACAAAATCCTTAAATTAA

alignment for event: A3-AT2G33820-XLOC_012757-10913

A3-AT2G33820-XLOC_012757-10913-0
      TGTAGAATGCAGATCCAAGGAACGGATTCTCTGGTTCCTAACTTCCGTAG
A3-AT2G33820-XLOC_012757-10913-1
      TGTAGAATGCAGATCCAAGGAACGGATTCTCTGGTTCCTAACTTCCGTAG
CONSENSUS
      TGTAGAATGCAGATCCAAGGAACGGATTCTCTGGTTCCTAACTTCCGTAG

A3-AT2G33820-XLOC_012757-10913-0
      ATACAACAGTCCTCTTGATTGTGCCGTTCAGACCGTTAAAAATGATGGG-
A3-AT2G33820-XLOC_012757-10913-1
      ATACAACAGTCCTCTTGATTGTGCCGTTCAGACCGTTAAAAATGATGGGG
CONSENSUS
      ATACAACAGTCCTCTTGATTGTGCCGTTCAGACCGTTAAAAATGATGGG.

A3-AT2G33820-XLOC_012757-10913-0 ------
GTATCTTTCGTGGTGGTTCGGCAACCTTATTAAGAGAATGTACG
A3-AT2G33820-XLOC_012757-10913-1
      TAACAGGTATCTTTCGTGGTGGTTCGGCAACCTTATTAAGAGAATGTACG
CONSENSUS
      ......GTATCTTTCGTGGTGGTTCGGCAACCTTATTAAGAGAATGTACG

A3-AT2G33820-XLOC_012757-10913-0
      GGAAATGCTGTCTTTTTTACTGTCTATGAGTACTTACGGTATCATATCCA
A3-AT2G33820-XLOC_012757-10913-1
      GGAAATGCTGTCTTTTTTACTGTCTATGAGTACTTACGGTATCATATCCA
CONSENSUS
      GGAAATGCTGTCTTTTTTACTGTCTATGAGTACTTACGGTATCATATCCA

A3-AT2G33820-XLOC_012757-10913-0
CTCGAGATTGGAGGATTCTAAGCTGAAAGATGGTTACTTGGTTGACATGG
A3-AT2G33820-XLOC_012757-10913-1
CTCGAGATTGGAGGATTCTAAGCTGAAAGATGGTTACTTGGTTGACATGG
CONSENSUS
CTCGAGATTGGAGGATTCTAAGCTGAAAGATGGTTACTTGGTTGACATGG

A3-AT2G33820-XLOC_012757-10913-0
GGATAGGAGTTCTCACTGGTGGTCTTGGAGGCATAGCT
A3-AT2G33820-XLOC_012757-10913-1
GGATAGGAGTTCTCACTGGTGGTCTTGGAGGCATAGCT
CONSENSUS
GGATAGGAGTTCTCACTGGTGGTCTTGGAGGCATAGCT

alignment for event: RI-AT2G32690-XLOC_012689-12500

RI-AT2G32690-XLOC_012689-12500-0
CAACTACTGTCTTCGATCTCTCTTTCATATACACCATATAAAACGAACCA
RI-AT2G32690-XLOC_012689-12500-1
CAACTACTGTCTTCGATCTCTCTTTCATATACACCATATAAAACGAACCA
CONSENSUS
CAACTACTGTCTTCGATCTCTCTTTCATATACACCATATAAAACGAACCA

RI-AT2G32690-XLOC_012689-12500-0
GTTAATTATGGGTTTAATTTCCGGGAAGGTGTGTGTGTTTATCTTTGTAT
RI-AT2G32690-XLOC_012689-12500-1
GTTAATTATGGGTTTAATTTCCGGGAAGGTGTGTGTGTTTATCTTTGTAT
CONSENSUS
GTTAATTATGGGTTTAATTTCCGGGAAGGTGTGTGTGTTTATCTTTGTAT

RI-AT2G32690-XLOC_012689-12500-0
TCGCTCTAGTCGCTGAATTTTCGTTCGGAAATGTTGAGGTTAACGACGAC
RI-AT2G32690-XLOC_012689-12500-1
TCGCTCTAGTCGCTGAATTTTCGTTCGGAAATGTTGAGGTTAACGACGAC
CONSENSUS
TCGCTCTAGTCGCTGAATTTTCGTTCGGAAATGTTGAGGTTAACGACGAC

RI-AT2G32690-XLOC_012689-12500-0
AAACACTTTTTCCACAAACCTCGTCCATTTTTACACAAACCTCGTCCATT
RI-AT2G32690-XLOC_012689-12500-1
AAACACTTTTTCCACAAACCTCGTCCATTTTTACACAAACCTCGTCCATT
CONSENSUS
AAACACTTTTTCCACAAACCTCGTCCATTTTTACACAAACCTCGTCCATT

RI-AT2G32690-XLOC_012689-12500-0
CCTCCACAAGCATGGCATTTACAAGAAAGGTTTCGGTAAGGGTTTGGGCG
RI-AT2G32690-XLOC_012689-12500-1
CCTCCACAAGCATGGCATTTACAAGAAAGGTTTCGGTAAGGGTTTGGGCG
CONSENSUS
CCTCCACAAGCATGGCATTTACAAGAAAGGTTTCGGTAAGGGTTTGGGCG

RI-AT2G32690-XLOC_012689-12500-0
GTGGAGGCGGTCTAGGCGGCGGAGGTGGTCTAGGAGGCGGTGGTGGCCTA

RI-AT2G32690-XLOC_012689-12500-1
GTGGAGGCGGTCTAGGCGGCGGAGGTGGTCTAGGAGGCGGTGGTGGCCTA
CONSENSUS
GTGGAGGCGGTCTAGGCGGCGGAGGTGGTCTAGGAGGCGGTGGTGGCCTA

RI-AT2G32690-XLOC_012689-12500-0
GGAGGCGGTGGAGGTTTAGGCGGTGGTGGAGGTTTAGGCGGCGGTGGTGG
RI-AT2G32690-XLOC_012689-12500-1
GGAGGCGGTGGAGGTTTAGGCGGTGGTGGAGGTTTAGGCGGCGGTGGTGG
CONSENSUS
GGAGGCGGTGGAGGTTTAGGCGGTGGTGGAGGTTTAGGCGGCGGTGGTGG

RI-AT2G32690-XLOC_012689-12500-0
TCTAGGAGGCGGTAGCGGTTTGGGAGGAGGAGGGGGTCTAGGAGGCGGTA
RI-AT2G32690-XLOC_012689-12500-1
TCTAGGAGGCGGTAGCGGTTTGGGAGGAGGAGGGGGTCTAGGAGGCGGTA
CONSENSUS
TCTAGGAGGCGGTAGCGGTTTGGGAGGAGGAGGGGGTCTAGGAGGCGGTA

RI-AT2G32690-XLOC_012689-12500-0
GCGGTTTGGGAGGAGGAGGGGGTCTAGGAGGTGGTGGTGGTGGTGGTTTA
RI-AT2G32690-XLOC_012689-12500-1
GCGGTTTGGGAGGAGGAGGGGGTCTAGGAGGTGGTGGTGGTGGTGGTTTA
CONSENSUS
GCGGTTTGGGAGGAGGAGGGGGTCTAGGAGGTGGTGGTGGTGGTGGTTTA

RI-AT2G32690-XLOC_012689-12500-0
GGAGGAGGAGGTGGATTGGGTGGTGGAGCTGGAGGAGGATACGGTGGTGG
RI-AT2G32690-XLOC_012689-12500-1
GGAGGAGGAG--
CONSENSUS
GGAGGAGGAG..

RI-AT2G32690-XLOC_012689-12500-0
TGCTGGAGGAGGACTTGGAGGCGGAGGTGGAATTGGAGGAGGAGGAGGCT
RI-AT2G32690-XLOC_012689-12500-1
---GCT
CONSENSUS
....GCT

RI-AT2G32690-XLOC_012689-12500-0
TTGGCGGTGGAGGGGGAGGAGGATTCGGGGGCGGAGCCGGTGGTGGATTT
RI-AT2G32690-XLOC_012689-12500-1
TTGGCGGTGGAGGGGGAGGAGGATTCGGGGGCGGAGCCGGTGGTGGATTT
CONSENSUS
TTGGCGGTGGAGGGGGAGGAGGATTCGGGGGCGGAGCCGGTGGTGGATTT

RI-AT2G32690-XLOC_012689-12500-0
GGTAAAGGCATTGGTGGTGGAGGAGGTCTTGGAGGAGGTTATGTTGGTGG
RI-AT2G32690-XLOC_012689-12500-1
GGTAAAGGCATTGGTGGTGGAGGAGGTCTTGGAGGAGGTTATGTTGGTGG
CONSENSUS
GGTAAAGGCATTGGTGGTGGAGGAGGTCTTGGAGGAGGTTATGTTGGTGG

RI-AT2G32690-XLOC_012689-12500-0
TGGCCATCACTGATGAGTGGTGAGGCTCATGCTCATGCATTCGTAACGTT

RI-AT2G32690-XLOC_012689-12500-1
      TGGCCATCACTGATGAGTGGTGAGGCTCATGCTCATGCATTCGTAACGTT
CONSENSUS
      TGGCCATCACTGATGAGTGGTGAGGCTCATGCTCATGCATTCGTAACGTT

RI-AT2G32690-XLOC_012689-12500-0
      GTTATATTATTTAATTAAATGGAAAATGATAAATTAATTATTGCAATAAA
RI-AT2G32690-XLOC_012689-12500-1
      GTTATATTATTTAATTAAATGGAAAATGATAAATTAATTATTGCAATAAA
CONSENSUS
      GTTATATTATTTAATTAAATGGAAAATGATAAATTAATTATTGCAATAAA

RI-AT2G32690-XLOC_012689-12500-0
      ATGATACATTAGCAATTGTGTATGTCGTTATTTTTATTTTGAGTCGCATT
RI-AT2G32690-XLOC_012689-12500-1
      ATGATACATTAGCAATTGTGTATGTCGTTATTTTTATTTTGAGTCGCATT
CONSENSUS
      ATGATACATTAGCAATTGTGTATGTCGTTATTTTTATTTTGAGTCGCATT

RI-AT2G32690-XLOC_012689-12500-0
      GTATGCTGATTCCATATGTTCGGCTTTGGTCGGTTTCAAGTGTGGTTAAT
RI-AT2G32690-XLOC_012689-12500-1
      GTATGCTGATTCCATATGTTCGGCTTTGGTCGGTTTCAAGTGTGGTTAAT
CONSENSUS
      GTATGCTGATTCCATATGTTCGGCTTTGGTCGGTTTCAAGTGTGGTTAAT

RI-AT2G32690-XLOC_012689-12500-0 AAAATGGTTATTTTACTTTTGCCAACAGTT
RI-AT2G32690-XLOC_012689-12500-1 AAAATGGTTATTTTACTTTTGCCAACAGTT
CONSENSUS AAAATGGTTATTTTACTTTTGCCAACAGTT

alignment for event: A5-AT2G25970-XLOC_012303-2787

A5-AT2G25970-XLOC_012303-2787-0
      GTTATTCCTTTACATTTGCCCCCTGGAGACCCAACGCCAGAACGGACTTT
A5-AT2G25970-XLOC_012303-2787-1
      GTTATTCCTTTACATTTGCCCCCTGGAGACCCAACGCCAGAACGGACTTT
CONSENSUS
      GTTATTCCTTTACATTTGCCCCCTGGAGACCCAACGCCAGAACGGACTTT

A5-AT2G25970-XLOC_012303-2787-0
      GCAGATTGATGGGATAACCGAACAGATTGAACATGCTAAACAATTAGTTA
A5-AT2G25970-XLOC_012303-2787-1
      GCAGATTGATGGGATAACCGAACAGATTGAACATGCTAAACAATTAGTTA
CONSENSUS
      GCAGATTGATGGGATAACCGAACAGATTGAACATGCTAAACAATTAGTTA

A5-AT2G25970-XLOC_012303-2787-0
      ATGAAATCATCAGTGGCGAGGTATGTTTGATTGTTGTGTTTGTTTATAGA
A5-AT2G25970-XLOC_012303-2787-1
      ATGAAATCATCAGTGGCGAG------------------------------
CONSENSUS
      ATGAAATCATCAGTGGCGAG..............................

A5-AT2G25970-XLOC_012303-2787-0

TTATGACAGGCTTCTGCTGTGTCTATTTTTATACATGAATTTGATATTGT
A5-AT2G25970-XLOC_012303-2787-1

CONSENSUS
....

A5-AT2G25970-XLOC_012303-2787-0
GTTGTGATTACATTATTTAGTTGTCATTAGCTTTAGGTCTATCTAGTATA
A5-AT2G25970-XLOC_012303-2787-1

CONSENSUS
....

A5-AT2G25970-XLOC_012303-2787-0
CGTCATACTCTATTATTCAGATAATAGGTGATGTAGGTTATACTAGTTTC
A5-AT2G25970-XLOC_012303-2787-1

CONSENSUS
....

A5-AT2G25970-XLOC_012303-2787-0
ACTTAGTCAATATATTAGCTGCTAAAACCTATATACTCTTCTTATGCCTT
A5-AT2G25970-XLOC_012303-2787-1

CONSENSUS
....

A5-AT2G25970-XLOC_012303-2787-0
CATTTCCTTTGATATTTGCTCCACGATAATTGCATCTGCATTGGATTGCA
A5-AT2G25970-XLOC_012303-2787-1

CONSENSUS
....

A5-AT2G25970-XLOC_012303-2787-0
TTGTTCTTCTCTTCCGCTTATTTGCAGGGGAAGGTTCTTTATTTTATTTT
A5-AT2G25970-XLOC_012303-2787-1

CONSENSUS
....

A5-AT2G25970-XLOC_012303-2787-0
AAACCACGTGAAGATATTTTTTGTGATTTTCTTAACTTACAAAAAATATT
A5-AT2G25970-XLOC_012303-2787-1

CONSENSUS
....

A5-AT2G25970-XLOC_012303-2787-0
CCATCTTGATATATCTTACTTTGTTATTGCATTGTAAGATTATATGTTGC
A5-AT2G25970-XLOC_012303-2787-1

CONSENSUS
....

A5-AT2G25970-XLOC_012303-2787-0

TTCTTCTTCCTCTGCTCATGCTCATTTTGTTGCCGCTACCTTCCTACACG
A5-AT2G25970-XLOC_012303-2787-1

CONSENSUS
....

A5-AT2G25970-XLOC_012303-2787-0
AGTTTCATGTTTCTGTTACTATCTTATTGTGGCAGTTTTTATGGGCATTC
A5-AT2G25970-XLOC_012303-2787-1

CONSENSUS
....

A5-AT2G25970-XLOC_012303-2787-0
GCTTATATCTACTTTTCATCTTTTTCAATATCCAATTTACAGTGTGCTTA
A5-AT2G25970-XLOC_012303-2787-1

CONSENSUS
....

A5-AT2G25970-XLOC_012303-2787-0
GCATGATTCTTCATAACTCCCATTGCTACCAAGAAATATTCTTTAGCATA
A5-AT2G25970-XLOC_012303-2787-1

CONSENSUS
....

A5-AT2G25970-XLOC_012303-2787-0
TTCACTTTTCAAATGCTATTTTCATAGTAGAAATCTTGTTGTCTATTTTT
A5-AT2G25970-XLOC_012303-2787-1

CONSENSUS
....

A5-AT2G25970-XLOC_012303-2787-0
GGAGTATATATGCAGACATTTTCATCGCTTCTGGTCACAGCCAAGGTGGC
A5-AT2G25970-XLOC_012303-2787-1

CONSENSUS
....

A5-AT2G25970-XLOC_012303-2787-0
CAATCCTACATTGGTTTGGGATGCCTTTGTGAGGCTGCTGACTAGTTTCA
A5-AT2G25970-XLOC_012303-2787-1

CONSENSUS
....

A5-AT2G25970-XLOC_012303-2787-0
GTCCAACCTGCTGTGATAGTGTTTCTAAACTGTTTTATACAATACTGTTT
A5-AT2G25970-XLOC_012303-2787-1

CONSENSUS
....

A5-AT2G25970-XLOC_012303-2787-0

TTGTTACATTGTGTTCATCATTTCTTGGGTCAACATAACTTCCGTTCAGC
A5-AT2G25970-XLOC_012303-2787-1

CONSENSUS
....

A5-AT2G25970-XLOC_012303-2787-0
TAGTACTTCTAGTTTCAATCATCAAGGTACCACCAAAGGTGGGCGACTGA
A5-AT2G25970-XLOC_012303-2787-1

CONSENSUS
....

A5-AT2G25970-XLOC_012303-2787-0
CATTTGAAGACCTATTAGCCGTTGTGTTGTGTCAATAATTTGCTAACCGA
A5-AT2G25970-XLOC_012303-2787-1

CONSENSUS
....

A5-AT2G25970-XLOC_012303-2787-0
GTAACTGTTATTATTCTGCCTTGGTACTTTCCCCAATTTTTCTTAAATCC
A5-AT2G25970-XLOC_012303-2787-1

CONSENSUS
....

A5-AT2G25970-XLOC_012303-2787-0
GTTTTTCTTTACCTTCACCATTATATTCTGGTAATATACTTCCATGAGTA
A5-AT2G25970-XLOC_012303-2787-1

CONSENSUS
....

A5-AT2G25970-XLOC_012303-2787-0
TTGACATTGTTGAACACTAGACTCTTTAGTTGGTATTAGGTTATTTTGCT
A5-AT2G25970-XLOC_012303-2787-1

CONSENSUS
....

A5-AT2G25970-XLOC_012303-2787-0
GCACAACCCTTTGAGAAAATAGGTTCCATGTGTATATATATGTTGCTTTT
A5-AT2G25970-XLOC_012303-2787-1

CONSENSUS
....

A5-AT2G25970-XLOC_012303-2787-0
GTCTGCAAAAGCTTCTATAGTATTCGTTGTTTTTTCTTGCACCCATTTAT
A5-AT2G25970-XLOC_012303-2787-1

CONSENSUS
....

A5-AT2G25970-XLOC_012303-2787-0

CTCGAATAGGGAAAGGGATGGGCTTCTGCAGGAGTAACCCAATAATTTCA
A5-AT2G25970-XLOC_012303-2787-1

CONSENSUS
....

A5-AT2G25970-XLOC_012303-2787-0
CCAATCAGTACGAACCGTATGAGAAACTCAGCAATGGGTGGAGGCTATCC
A5-AT2G25970-XLOC_012303-2787-1 ------------
AACCGTATGAGAAACTCAGCAATGGGTGGAGGCTATCC
CONSENSUS
............AACCGTATGAGAAACTCAGCAATGGGTGGAGGCTATCC

A5-AT2G25970-XLOC_012303-2787-0
ACAACAAGGTGGTTATCAAGCCCGCCCACCCTCAAGCTGGGCACCACCTG
A5-AT2G25970-XLOC_012303-2787-1
ACAACAAGGTGGTTATCAAGCCCGCCCACCCTCAAGCTGGGCACCACCTG
CONSENSUS
ACAACAAGGTGGTTATCAAGCCCGCCCACCCTCAAGCTGGGCACCACCTG

A5-AT2G25970-XLOC_012303-2787-0
GTGGTCCGCCAGCACAACCTGGTTATGGTGGTTACATGCAACCAGGAGCA
A5-AT2G25970-XLOC_012303-2787-1
GTGGTCCGCCAGCACAACCTGGTTATGGTGGTTACATGCAACCAGGAGCA
CONSENSUS
GTGGTCCGCCAGCACAACCTGGTTATGGTGGTTACATGCAACCAGGAGCA

A5-AT2G25970-XLOC_012303-2787-0
TATCCAGGTCCACCTCAGTATGGTCAATCACCTTACGGAAGTTACCCTCA
A5-AT2G25970-XLOC_012303-2787-1
TATCCAGGTCCACCTCAGTATGGTCAATCACCTTACGGAAGTTACCCTCA
CONSENSUS
TATCCAGGTCCACCTCAGTATGGTCAATCACCTTACGGAAGTTACCCTCA

A5-AT2G25970-XLOC_012303-2787-0
ACAAACTTCAGCTGGTTACTATGATCAGTCCTCTGTGCCACCATCCCAGC
A5-AT2G25970-XLOC_012303-2787-1
ACAAACTTCAGCTGGTTACTATGATCAGTCCTCTGTGCCACCATCCCAGC
CONSENSUS
ACAAACTTCAGCTGGTTACTATGATCAGTCCTCTGTGCCACCATCCCAGC

A5-AT2G25970-XLOC_012303-2787-0
AGAGCGCGCAAGGTGAGTATGATTATTACGGTCAGCAACAGTCTCAGCAA
A5-AT2G25970-XLOC_012303-2787-1
AGAGCGCGCAAGGTGAGTATGATTATTACGGTCAGCAACAGTCTCAGCAA
CONSENSUS
AGAGCGCGCAAGGTGAGTATGATTATTACGGTCAGCAACAGTCTCAGCAA

A5-AT2G25970-XLOC_012303-2787-0
CCAAGCAGTGGTGGTAGCTCAGCCCCACCAACAGATACCACAGGGTACAA
A5-AT2G25970-XLOC_012303-2787-1
CCAAGCAGTGGTGGTAGCTCAGCCCCACCAACAGATACCACAGGGTACAA
CONSENSUS
CCAAGCAGTGGTGGTAGCTCAGCCCCACCAACAGATACCACAGGGTACAA

A5-AT2G25970-XLOC_012303-2787-0

TTACTACCAGCATGCTTCTGGTTATGGCCAAGCTGGTCAGGGATACCAGC
A5-AT2G25970-XLOC_012303-2787-1
TTACTACCAGCATGCTTCTGGTTATGGCCAAGCTGGTCAGGGATACCAGC
CONSENSUS
TTACTACCAGCATGCTTCTGGTTATGGCCAAGCTGGTCAGGGATACCAGC

A5-AT2G25970-XLOC_012303-2787-0
AAGATGGGTATGGAGCTTACAATGCCTCGCAGCAATCGGGATATGGTCAA
A5-AT2G25970-XLOC_012303-2787-1
AAGATGGGTATGGAGCTTACAATGCCTCGCAGCAATCGGGATATGGTCAA
CONSENSUS
AAGATGGGTATGGAGCTTACAATGCCTCGCAGCAATCGGGATATGGTCAA

A5-AT2G25970-XLOC_012303-2787-0
GCTGCTGGGTATGATCAACAGGGTGGTTACGGCAGCACCACTAATCCAAG
A5-AT2G25970-XLOC_012303-2787-1
GCTGCTGGGTATGATCAACAGGGTGGTTACGGCAGCACCACTAATCCAAG
CONSENSUS
GCTGCTGGGTATGATCAACAGGGTGGTTACGGCAGCACCACTAATCCAAG

A5-AT2G25970-XLOC_012303-2787-0
TCAAGAGGAAGATGCATCTCAAGCCGCTCCACCATCGTCAGCTCAGTCTG
A5-AT2G25970-XLOC_012303-2787-1
TCAAGAGGAAGATGCATCTCAAGCCGCTCCACCATCGTCAGCTCAGTCTG
CONSENSUS
TCAAGAGGAAGATGCATCTCAAGCCGCTCCACCATCGTCAGCTCAGTCTG

A5-AT2G25970-XLOC_012303-2787-0
GACAGGCTGGGTATGGTACAACTGGTCAACAGCCGCCTGCTCAAGGTAGT
A5-AT2G25970-XLOC_012303-2787-1
GACAGGCTGGGTATGGTACAACTGGTCAACAGCCGCCTGCTCAAGGTAGT
CONSENSUS
GACAGGCTGGGTATGGTACAACTGGTCAACAGCCGCCTGCTCAAGGTAGT

A5-AT2G25970-XLOC_012303-2787-0
ACTGGTCAGGCAGGGTATGGAGCTCCTCCAACTTCTCAGGCTGGTTACAG
A5-AT2G25970-XLOC_012303-2787-1
ACTGGTCAGGCAGGGTATGGAGCTCCTCCAACTTCTCAGGCTGGTTACAG
CONSENSUS
ACTGGTCAGGCAGGGTATGGAGCTCCTCCAACTTCTCAGGCTGGTTACAG

A5-AT2G25970-XLOC_012303-2787-0
CAGCCAGCCAGCAGCAGCTTACAATTCTGGGTATGGAGCACCACCACCTG
A5-AT2G25970-XLOC_012303-2787-1
CAGCCAGCCAGCAGCAGCTTACAATTCTGGGTATGGAGCACCACCACCTG
CONSENSUS
CAGCCAGCCAGCAGCAGCTTACAATTCTGGGTATGGAGCACCACCACCTG

A5-AT2G25970-XLOC_012303-2787-0
CTTCAAAGCCACCGACTTATGGCCAGAGCCAGCAGTCTCCAGGTGCTCCT
A5-AT2G25970-XLOC_012303-2787-1
CTTCAAAGCCACCGACTTATGGCCAGAGCCAGCAGTCTCCAGGTGCTCCT
CONSENSUS
CTTCAAAGCCACCGACTTATGGCCAGAGCCAGCAGTCTCCAGGTGCTCCT

A5-AT2G25970-XLOC_012303-2787-0

GGGAGCTATGGTAGTCAGTCTGGGTATGCCCAACCAGCAGCTTCAGGGTA
A5-AT2G25970-XLOC_012303-2787-1
GGGAGCTATGGTAGTCAGTCTGGGTATGCCCAACCAGCAGCTTCAGGGTA
CONSENSUS
GGGAGCTATGGTAGTCAGTCTGGGTATGCCCAACCAGCAGCTTCAGGGTA

A5-AT2G25970-XLOC_012303-2787-0
TGGACAACCTCCAGCGTATGGGTATGGTCAAGCGCCACAGGGATATGGGT
A5-AT2G25970-XLOC_012303-2787-1
TGGACAACCTCCAGCGTATGGGTATGGTCAAGCGCCACAGGGATATGGGT
CONSENSUS
TGGACAACCTCCAGCGTATGGGTATGGTCAAGCGCCACAGGGATATGGGT

A5-AT2G25970-XLOC_012303-2787-0
CTTATGGAGGATACACACAACCTGCTGCTGGTGGAGGTTACTCTTCAGAC
A5-AT2G25970-XLOC_012303-2787-1
CTTATGGAGGATACACACAACCTGCTGCTGGTGGAGGTTACTCTTCAGAC
CONSENSUS
CTTATGGAGGATACACACAACCTGCTGCTGGTGGAGGTTACTCTTCAGAC

A5-AT2G25970-XLOC_012303-2787-0
GGGTCTGCTGGAGCCACTGCTGGTGGTGGTGGTGGTACACCAGCTTCACA
A5-AT2G25970-XLOC_012303-2787-1
GGGTCTGCTGGAGCCACTGCTGGTGGTGGTGGTGGTACACCAGCTTCACA
CONSENSUS
GGGTCTGCTGGAGCCACTGCTGGTGGTGGTGGTGGTACACCAGCTTCACA

A5-AT2G25970-XLOC_012303-2787-0
GAGTGCTGCTCCACCTGCTGGACCGCCCAAAGCATCCCCGAAAAGTTGAT
A5-AT2G25970-XLOC_012303-2787-1
GAGTGCTGCTCCACCTGCTGGACCGCCCAAAGCATCCCCGAAAAGTTGAT
CONSENSUS
GAGTGCTGCTCCACCTGCTGGACCGCCCAAAGCATCCCCGAAAAGTTGAT

A5-AT2G25970-XLOC_012303-2787-0
ATGTGTATGGATAAATTTCTACTGTTATACAAAACATGCTTCCATGCTCT
A5-AT2G25970-XLOC_012303-2787-1
ATGTGTATGGATAAATTTCTACTGTTATACAAAACATGCTTCCATGCTCT
CONSENSUS
ATGTGTATGGATAAATTTCTACTGTTATACAAAACATGCTTCCATGCTCT

A5-AT2G25970-XLOC_012303-2787-0
TATCTTTTTCTTGAGGATTTATCAATGTTTGTGTTTCACATTTTAGCTCT
A5-AT2G25970-XLOC_012303-2787-1
TATCTTTTTCTTGAGGATTTATCAATGTTTGTGTTTCACATTTTAGCTCT
CONSENSUS
TATCTTTTTCTTGAGGATTTATCAATGTTTGTGTTTCACATTTTAGCTCT

A5-AT2G25970-XLOC_012303-2787-0
TGTCTGTCAACTCTCTTTATATTAAAGATAATGTTACGTTTTAGCCTGTT
A5-AT2G25970-XLOC_012303-2787-1
TGTCTGTCAACTCTCTTTATATTAAAGATAATGTTACGTTTTAGCCTGTT
CONSENSUS
TGTCTGTCAACTCTCTTTATATTAAAGATAATGTTACGTTTTAGCCTGTT

A5-AT2G25970-XLOC_012303-2787-0

ATTCTTCTTACAATGATTCAAATTCACCAAAATTTTAAATCC
A5-AT2G25970-XLOC_012303-2787-1
ATTCTTCTTACAATGATTCAAATTCACCAAAATTTTAAATCC
CONSENSUS
ATTCTTCTTACAATGATTCAAATTCACCAAAATTTTAAATCC

alignment for event: RI-AT2G37050-XLOC_010258-6667

RI-AT2G37050-XLOC_010258-6667-0
GCGTGGTACCACGTGACCGGAGAATCAGTTGGATTAAAAGGCTTGAGATA
RI-AT2G37050-XLOC_010258-6667-1
GCGTGGTACCACGTGACCGGAGAATCAGTTGGATTAAAAGGCTTGAGATA
CONSENSUS
GCGTGGTACCACGTGACCGGAGAATCAGTTGGATTAAAAGGCTTGAGATA

RI-AT2G37050-XLOC_010258-6667-0
GCTGAAGATGCAGCCCGAGGTCCGTGAATTGTACAACAAAACAGAAAGCC
RI-AT2G37050-XLOC_010258-6667-1
GCTGAAGATGCAGCCCGAG-------------------------------
CONSENSUS
GCTGAAGATGCAGCCCGAG...............................

RI-AT2G37050-XLOC_010258-6667-0
ATTTTTTCGATATTTGATTTTGTTGGTTTTGATTGTTTCTGTAGGGATCG
RI-AT2G37050-XLOC_010258-6667-1
--GGATCG
CONSENSUS
....GGATCG

RI-AT2G37050-XLOC_010258-6667-0
AGTATCTTCACACAGGGTGTGTTCCAGCAATCATACATAGAGATCTCAAG
RI-AT2G37050-XLOC_010258-6667-1
AGTATCTTCACACAGGGTGTGTTCCAGCAATCATACATAGAGATCTCAAG
CONSENSUS
AGTATCTTCACACAGGGTGTGTTCCAGCAATCATACATAGAGATCTCAAG

RI-AT2G37050-XLOC_010258-6667-0
ACTAGTAACATCCTACTTGACAAACACATGAGGGCAAAGGTTTCAGATTT
RI-AT2G37050-XLOC_010258-6667-1
ACTAGTAACATCCTACTTGACAAACACATGAGGGCAAAGGTTTCAGATTT
CONSENSUS
ACTAGTAACATCCTACTTGACAAACACATGAGGGCAAAGGTTTCAGATTT

RI-AT2G37050-XLOC_010258-6667-0
CGGTTTGTCGAAATTCGCAGTTGACGGGACCTCACATGTCTCCAGCATTG
RI-AT2G37050-XLOC_010258-6667-1
CGGTTTGTCGAAATTCGCAGTTGACGGGACCTCACATGTCTCCAGCATTG
CONSENSUS
CGGTTTGTCGAAATTCGCAGTTGACGGGACCTCACATGTCTCCAGCATTG

RI-AT2G37050-XLOC_010258-6667-0
TCCGTGGCACAGTTGGATATCTTGACCCCGA
RI-AT2G37050-XLOC_010258-6667-1
TCCGTGGCACAGTTGGATATCTTGACCCCGA

```
CONSENSUS
      TCCGTGGCACAGTTGGATATCTTGACCCCGA



alignment for event: A5-AT2G37585-XLOC_010286-6760

A5-AT2G37585-XLOC_010286-6760-0
      ATATTTTGCATATATTCTCATACTTGCCACGCTACCTGAACTTTATCGAG
A5-AT2G37585-XLOC_010286-6760-1
      ATATTTTGCATATATTCTCATACTTGCCACGCTACCTGAACTTTATCGAG
CONSENSUS
      ATATTTTGCATATATTCTCATACTTGCCACGCTACCTGAACTTTATCGAG

A5-AT2G37585-XLOC_010286-6760-0
      CACACAAGCAACATCGGTTGGAAAGA-------------------GAACC
A5-AT2G37585-XLOC_010286-6760-1
      CACACAAGCAACATCGGTTGGAAAGAGTGAGGGAGCTAATATTATGAACC
CONSENSUS
      CACACAAGCAACATCGGTTGGAAAGA...................GAACC

A5-AT2G37585-XLOC_010286-6760-0
      AAAGAGCAAGGCCTATCATTATTGATCCTGGTTTTTACCATTTGAAGAAA
A5-AT2G37585-XLOC_010286-6760-1
      AAAGAGCAAGGCCTATCATTATTGATCCTGGTTTTTACCATTTGAAGAAA
CONSENSUS
      AAAGAGCAAGGCCTATCATTATTGATCCTGGTTTTTACCATTTGAAGAAA

A5-AT2G37585-XLOC_010286-6760-0
      TCTGGTGTCTTTTGGGCTAAAGAACGGAGATCTTTGCCTGCTTCATTCAA
A5-AT2G37585-XLOC_010286-6760-1
      TCTGGTGTCTTTTGGGCTAAAGAACGGAGATCTTTGCCTGCTTCATTCAA
CONSENSUS
      TCTGGTGTCTTTTGGGCTAAAGAACGGAGATCTTTGCCTGCTTCATTCAA

A5-AT2G37585-XLOC_010286-6760-0  ACTTTTCATGG
A5-AT2G37585-XLOC_010286-6760-1  ACTTTTCATGG
CONSENSUS                        ACTTTTCATGG



alignment for event: A5-AT2G46830-XLOC_010808-13518

A5-AT2G46830-XLOC_010808-13518-0
      AACATGTAGCAACAAAAACTGCTGTCCAGATAAGAAGTCACGCTCAGAAA
A5-AT2G46830-XLOC_010808-13518-1
      AACATGTAGCAACAAAAACTGCTGTCCAGATAAGAAGTCACGCTCAGAAA
CONSENSUS
      AACATGTAGCAACAAAAACTGCTGTCCAGATAAGAAGTCACGCTCAGAAA

A5-AT2G46830-XLOC_010808-13518-0
      TTTTTCTCCAAG--------------------------------------
A5-AT2G46830-XLOC_010808-13518-1
      TTTTTCTCCAAGGTAAAATCGGTTAATTTTGAAATGATGTTCTCATCTTC
CONSENSUS
      TTTTTCTCCAAG......................................
```

A5-AT2G46830-XLOC_010808-13518-0

A5-AT2G46830-XLOC_010808-13518-1
ATTGGCTTAATGCTTAAGACTTATTGAAAGCCAGGCAAGTTTTCTGCTTC
CONSENSUS
....

A5-AT2G46830-XLOC_010808-13518-0

A5-AT2G46830-XLOC_010808-13518-1
TTTTGCTTCTTAGTCAGGAGATAGATAGATTACGTTTTTAGAGTTTAGTA
CONSENSUS
....

A5-AT2G46830-XLOC_010808-13518-0

A5-AT2G46830-XLOC_010808-13518-1
ATGAGCAATAAGTCTTAAAATAGTTGGAGAAATGACGAGATGTAATCGTT
CONSENSUS
....

A5-AT2G46830-XLOC_010808-13518-0

A5-AT2G46830-XLOC_010808-13518-1
TTCTTTTGTTTATGCCTATATCTTGTTAATCCACAAACATGTACATAGAT
CONSENSUS
....

A5-AT2G46830-XLOC_010808-13518-0

A5-AT2G46830-XLOC_010808-13518-1
TCTTCAGAAGAATGTTAGTTTCTTTAGATTCTTCAGATAAACTTGTGTCT
CONSENSUS
....

A5-AT2G46830-XLOC_010808-13518-0

A5-AT2G46830-XLOC_010808-13518-1
TCTTACCGATTCTGAGGTAGTGGCAAAAGTGGGCTGAGTGCTAGAAATTT
CONSENSUS
....

A5-AT2G46830-XLOC_010808-13518-0 ----------------------------
GTAGAGAAAGAGGCTGAAGCT
A5-AT2G46830-XLOC_010808-13518-1
TTGAATGTTCCTTGTGATAAGCCATAGAGGTAGAGAAAGAGGCTGAAGCT
CONSENSUS
.............................GTAGAGAAAGAGGCTGAAGCT

A5-AT2G46830-XLOC_010808-13518-0
AAAGGTGTAGCTATGGGTCAAGCGCTAGACATAGCTATTCCTCCTCCACG
A5-AT2G46830-XLOC_010808-13518-1
AAAGGTGTAGCTATGGGTCAAGCGCTAGACATAGCTATTCCTCCTCCACG
CONSENSUS
AAAGGTGTAGCTATGGGTCAAGCGCTAGACATAGCTATTCCTCCTCCACG

A5-AT2G46830-XLOC_010808-13518-0
GCCTAAGCGTAAACCAAACAATCCTTATCCTCGAAAGACGGGAAGTGGAA
A5-AT2G46830-XLOC_010808-13518-1
GCCTAAGCGTAAACCAAACAATCCTTATCCTCGAAAGACGGGAAGTGGAA
CONSENSUS
GCCTAAGCGTAAACCAAACAATCCTTATCCTCGAAAGACGGGAAGTGGAA

A5-AT2G46830-XLOC_010808-13518-0
CGATCCTTATGTCAAAAACGGGTGTGAATGATGGAAAAGAGTCCCTTGGA
A5-AT2G46830-XLOC_010808-13518-1
CGATCCTTATGTCAAAAACGGGTGTGAATGATGGAAAAGAGTCCCTTGGA
CONSENSUS
CGATCCTTATGTCAAAAACGGGTGTGAATGATGGAAAAGAGTCCCTTGGA

A5-AT2G46830-XLOC_010808-13518-0 TCAGAAAAAGTGTCGCATCCTGAG
A5-AT2G46830-XLOC_010808-13518-1 TCAGAAAAAGTGTCGCATCCTGAG
CONSENSUS TCAGAAAAAGTGTCGCATCCTGAG

alignment for event: RI-AT2G35360-XLOC_012844-13903

RI-AT2G35360-XLOC_012844-13903-0
ATTCGTGATTTGAGAAATGCAATTGCGGAAAAGGGTAAATTTCCCGTTTC
RI-AT2G35360-XLOC_012844-13903-1
ATTCGTGATTTGAGAAATGCAATTGCGGAAAAGGGTAAATTTCCCGTTTC
CONSENSUS
ATTCGTGATTTGAGAAATGCAATTGCGGAAAAGGGTAAATTTCCCGTTTC

RI-AT2G35360-XLOC_012844-13903-0
AACTTTAAGGATGATTCTTCGTGGAAAGGCATTGCAAGACGAAGAAGATG
RI-AT2G35360-XLOC_012844-13903-1
AACTTTAAGGATGATTCTTCGTGGAAAGGCATTGCAAGACGAAGAAGATG
CONSENSUS
AACTTTAAGGATGATTCTTCGTGGAAAGGCATTGCAAGACGAAGAAGATG

RI-AT2G35360-XLOC_012844-13903-0
GAGATGATTTATACGTTACGCTCAAGGACCAAGGTAAACAGGTTCTGCTG
RI-AT2G35360-XLOC_012844-13903-1
GAGATGATTTATACGTTACGCTCAAGGACCAAG-----------------
CONSENSUS
GAGATGATTTATACGTTACGCTCAAGGACCAAG.................

RI-AT2G35360-XLOC_012844-13903-0
AAAATGTTATAAGCATCAATGCTTATCTGTGTTTGTTAAATGCGTCTTTA
RI-AT2G35360-XLOC_012844-13903-1

CONSENSUS
....

RI-AT2G35360-XLOC_012844-13903-0
GGTTATGCACAAGTCTTGATTTTATTGTGTAGATTCCTTTATTGTTGCTG
RI-AT2G35360-XLOC_012844-13903-1
--------------------------------ATTCCTTTATTGTTGCTG
CONSENSUS

.................................ATTCCTTTATTGTTGCTG

RI-AT2G35360-XLOC_012844-13903-0
TAATACCAAATCCACCAGCTGGAGTTGAGTCATTTGATGATGATGATGAT
RI-AT2G35360-XLOC_012844-13903-1
TAATACCAAATCCACCAGCTGGAGTTGAGTCATTTGATGATGATGATGAT
CONSENSUS
TAATACCAAATCCACCAGCTGGAGTTGAGTCATTTGATGATGATGATGAT

RI-AT2G35360-XLOC_012844-13903-0 GATGATTTG
RI-AT2G35360-XLOC_012844-13903-1 GATGATTTG
CONSENSUS GATGATTTG

alignment for event: RI-AT3G10030-XLOC_017295-13020

RI-AT3G10030-XLOC_017295-13020-0
TCAATGCGGAGGCAGTGGTGAAAGGAACAAACGTTGATGGTGTTTATGAC
RI-AT3G10030-XLOC_017295-13020-1
TCAATGCGGAGGCAGTGGTGAAAGGAACAAACGTTGATGGTGTTTATGAC
CONSENSUS
TCAATGCGGAGGCAGTGGTGAAAGGAACAAACGTTGATGGTGTTTATGAC

RI-AT3G10030-XLOC_017295-13020-0
TGTCATTCACAAGATAGTAACGTGACATTTGAGCACATATCATTTCAAGA
RI-AT3G10030-XLOC_017295-13020-1
TGTCATTCACAAGATAGTAACGTGACATTTGAGCACATATCATTTCAAGA
CONSENSUS
TGTCATTCACAAGATAGTAACGTGACATTTGAGCACATATCATTTCAAGA

RI-AT3G10030-XLOC_017295-13020-0
TTTGGCTTCTAGAGGTCTTACGTCAATGGATACAATGGCCCTTAACTTTT
RI-AT3G10030-XLOC_017295-13020-1
TTTGGCTTCTAGAGGTCTTACGTCAATGGATACAATGGCCCTTAACTTTT
CONSENSUS
TTTGGCTTCTAGAGGTCTTACGTCAATGGATACAATGGCCCTTAACTTTT

RI-AT3G10030-XLOC_017295-13020-0
GTGAAGAGAACAGCATTCCAGGTCGAGATTCTTCTTCTCTCTCTTATCAT
RI-AT3G10030-XLOC_017295-13020-1
GTGAAGAGAACAGCATTCCAG-----------------------------
CONSENSUS
GTGAAGAGAACAGCATTCCAG.............................

RI-AT3G10030-XLOC_017295-13020-0
GTTTAGCTAATCCGTTTTAGATTACTTAGATCTGGCTGTGAGATAAATGG
RI-AT3G10030-XLOC_017295-13020-1

CONSENSUS
....

RI-AT3G10030-XLOC_017295-13020-0
TTAAACTTAGTGACCTAGACAGAATTATACAAATGGTTTACTCGAGATGG
RI-AT3G10030-XLOC_017295-13020-1

CONSENSUS
    ....

RI-AT3G10030-XLOC_017295-13020-0
    GACCTCTAGAATCCTGATTTCCATCAGTGGATAATAGATCAATACCCTTA
RI-AT3G10030-XLOC_017295-13020-1

CONSENSUS
    ....

RI-AT3G10030-XLOC_017295-13020-0
    TACTTGTCCCGCAATGAGTGCTTAGCACCTGATGATAGAATCGAACGTTG
RI-AT3G10030-XLOC_017295-13020-1

CONSENSUS
    ....

RI-AT3G10030-XLOC_017295-13020-0
    AATGTAAATGACCTGATTCCTTGTCCTCTGGATTAAAAAAAAACAGTTGT
RI-AT3G10030-XLOC_017295-13020-1
    --TTGT
CONSENSUS
    ....TTGT

RI-AT3G10030-XLOC_017295-13020-0
    GGTATTTAACTTTCTGGAAGCTGGGAACATCACAAAAGCTTTGTGCGGAG
RI-AT3G10030-XLOC_017295-13020-1
    GGTATTTAACTTTCTGGAAGCTGGGAACATCACAAAAGCTTTGTGCGGAG
CONSENSUS
    GGTATTTAACTTTCTGGAAGCTGGGAACATCACAAAAGCTTTGTGCGGAG

RI-AT3G10030-XLOC_017295-13020-0
    AACAAGTGGGTACTTTGATCGATAGAAGCGGAAGGGGCGTGAGTTAGTAG
RI-AT3G10030-XLOC_017295-13020-1
    AACAAGTGGGTACTTTGATCGATAGAAGCGGAAGGGGCGTGAGTTAGTAG
CONSENSUS
    AACAAGTGGGTACTTTGATCGATAGAAGCGGAAGGGGCGTGAGTTAGTAG

RI-AT3G10030-XLOC_017295-13020-0
    TATATGTGTGAATTCATAACACTTTGTAAACACGTTGTTTTTTCCGTAAG
RI-AT3G10030-XLOC_017295-13020-1
    TATATGTGTGAATTCATAACACTTTGTAAACACGTTGTTTTTTCCGTAAG
CONSENSUS
    TATATGTGTGAATTCATAACACTTTGTAAACACGTTGTTTTTTCCGTAAG

RI-AT3G10030-XLOC_017295-13020-0
    TAATTATATTTCATAGATGAGTTGACAAGAAAAAGGAGAGTTGCTCCATT
RI-AT3G10030-XLOC_017295-13020-1
    TAATTATATTTCATAGATGAGTTGACAAGAAAAAGGAGAGTTGCTCCATT
CONSENSUS
    TAATTATATTTCATAGATGAGTTGACAAGAAAAAGGAGAGTTGCTCCATT

RI-AT3G10030-XLOC_017295-13020-0
    CACAAGTGAAGACGTTGTAATATTGAAGATGTTGACACGTTTGAGAGATT
RI-AT3G10030-XLOC_017295-13020-1
    CACAAGTGAAGACGTTGTAATATTGAAGATGTTGACACGTTTGAGAGATT

```
CONSENSUS
      CACAAGTGAAGACGTTGTAATATTGAAGATGTTGACACGTTTGAGAGATT

RI-AT3G10030-XLOC_017295-13020-0
      TTCTTTCTCCATCTTCGTTGACTGACTTCTTCTTTCTGAATGATTGTATT
RI-AT3G10030-XLOC_017295-13020-1
      TTCTTTCTCCATCTTCGTTGACTGACTTCTTCTTTCTGAATGATTGTATT
CONSENSUS
      TTCTTTCTCCATCTTCGTTGACTGACTTCTTCTTTCTGAATGATTGTATT

RI-AT3G10030-XLOC_017295-13020-0
      TACGATTTTGTTTGTAATGCTTTAATACTATTAGAGCAAATGTCAAAATC
RI-AT3G10030-XLOC_017295-13020-1
      TACGATTTTGTTTGTAATGCTTTAATACTATTAGAGCAAATGTCAAAATC
CONSENSUS
      TACGATTTTGTTTGTAATGCTTTAATACTATTAGAGCAAATGTCAAAATC

RI-AT3G10030-XLOC_017295-13020-0 T
RI-AT3G10030-XLOC_017295-13020-1 T
CONSENSUS                        T



alignment for event: RI-AT3G46385-XLOC_019097-764

RI-AT3G46385-XLOC_019097-764-0
      ATTGTTTTGTCCGAAATCGATTTTCTTTCTCTCTCGCGCGACCGGTGAAC
RI-AT3G46385-XLOC_019097-764-1
      ATTGTTTTGTCCGAAATCGATTTTCTTTCTCTCTCGCGCGACCGGTGAAC
CONSENSUS
      ATTGTTTTGTCCGAAATCGATTTTCTTTCTCTCTCGCGCGACCGGTGAAC

RI-AT3G46385-XLOC_019097-764-0
      TCTTGATAAGCCGATGAATTGACGATGGTGATTGTTTGTTGGCGTGTTAT
RI-AT3G46385-XLOC_019097-764-1
      TCTTGATAAGCCGATGAATTGACGATGGTGATTGTTTGTTGGCGTGTTAT
CONSENSUS
      TCTTGATAAGCCGATGAATTGACGATGGTGATTGTTTGTTGGCGTGTTAT

RI-AT3G46385-XLOC_019097-764-0
      CTGAGGTTTCCAACGCCGGATTCATCAACGCTAATGACCATCGGCAGAAG
RI-AT3G46385-XLOC_019097-764-1
      CTGAGGTTTCCAACGCCGGATTCATCAACGCTAATGACCATCGGCAGAAG
CONSENSUS
      CTGAGGTTTCCAACGCCGGATTCATCAACGCTAATGACCATCGGCAGAAG

RI-AT3G46385-XLOC_019097-764-0
      TTGGGTGAAATCGCAGAATAACAAATCTGATTTGTAGAGTAATGGTTGAC
RI-AT3G46385-XLOC_019097-764-1
      TTGGGTGAAATCGCAGAATAACAAATCTGATTTGTAGAGTAATGGTTGAC
CONSENSUS
      TTGGGTGAAATCGCAGAATAACAAATCTGATTTGTAGAGTAATGGTTGAC

RI-AT3G46385-XLOC_019097-764-0
      GGCATAGCAAATCGGCTATAGTTTGCTTTGTATGATTACGTTTAAGGTTT
RI-AT3G46385-XLOC_019097-764-1
```

```
      GGCATAGCAAATCGGCTATAGTTTGCTTTGTATGATTACGTTTAAG----
CONSENSUS
      GGCATAGCAAATCGGCTATAGTTTGCTTTGTATGATTACGTTTAAG....

RI-AT3G46385-XLOC_019097-764-0
      ATAGGTTTGTATGATTACGTTTTGGCAATATTGATAGATATATTAGTTTG
RI-AT3G46385-XLOC_019097-764-1
      --------------------------------------------------
CONSENSUS
      ..................................................

RI-AT3G46385-XLOC_019097-764-0
      AATCGTTTTGACTTTCTCGGATTTGGCTTGAATTTAATCTGTTCTGTTTT
RI-AT3G46385-XLOC_019097-764-1
      --------------------------------------------------
CONSENSUS
      ..................................................

RI-AT3G46385-XLOC_019097-764-0
      CAATCATAAAAATCAGTCGTGATTTGTTTGCATATGTTATGTGATTGAAT
RI-AT3G46385-XLOC_019097-764-1
      --------------------------------------------------
CONSENSUS
      ..................................................

RI-AT3G46385-XLOC_019097-764-0
      CAATTTAGGAGCACTTGTTCATTTCCATCTCTATTTTGTTATCATCGAAT
RI-AT3G46385-XLOC_019097-764-1
      --------------------------------------------------
CONSENSUS
      ..................................................

RI-AT3G46385-XLOC_019097-764-0
      ACAGACTAATGGAGCTGAAAATGTTGTATTTGTCTGTAATATGATAATGG
RI-AT3G46385-XLOC_019097-764-1
      --------------------------------------------------
CONSENSUS
      ..................................................

RI-AT3G46385-XLOC_019097-764-0
      CCAGTGATCAAGCTCTGGTCAAATGCTGGTAACGGTGGAGCACCATGGTG
RI-AT3G46385-XLOC_019097-764-1   ----
TGATCAAGCTCTGGTCAAATGCTGGTAACGGTGGAGCACCATGGTG
CONSENSUS
      ....TGATCAAGCTCTGGTCAAATGCTGGTAACGGTGGAGCACCATGGTG

RI-AT3G46385-XLOC_019097-764-0   GACTTATTGGAGTGTCGGCATTACTA
RI-AT3G46385-XLOC_019097-764-1   GACTTATTGGAGTGTCGGCATTACTA
CONSENSUS                        GACTTATTGGAGTGTCGGCATTACTA


alignment for event: RI-AT3G12012-XLOC_014129-6042

RI-AT3G12012-XLOC_014129-6042-0
      GCAATTTCCGCTAGCAGCTCAGACAGGACGTCTCTTCTAGAATTCCTGCA
```

RI-AT3G12012-XLOC_014129-6042-1
      GCAATTTCCGCTAGCAGCTCAGACAGGACGTCTCTTCTAGAATTCCTGCA
CONSENSUS
      GCAATTTCCGCTAGCAGCTCAGACAGGACGTCTCTTCTAGAATTCCTGCA

RI-AT3G12012-XLOC_014129-6042-0
      GCGAAGGAAGTTGGAAGCAAATAAGGTCCAAACTTTCTGTTAATCTGTGT
RI-AT3G12012-XLOC_014129-6042-1
      GCGAAGGAAGTTGGAAGCAAATAAG-------------------------
CONSENSUS
      GCGAAGGAAGTTGGAAGCAAATAAG.........................

RI-AT3G12012-XLOC_014129-6042-0
      TTTAGATAGGAGTTTAAAATTTTGCCCAAAAAACAAGATATGAGTCTAAA
RI-AT3G12012-XLOC_014129-6042-1

CONSENSUS
      ....

RI-AT3G12012-XLOC_014129-6042-0
      ATAAACATGATGAAGCACCTGTTTGTAGCAGCAAACCGTTTGAGGAATAA
RI-AT3G12012-XLOC_014129-6042-1

CONSENSUS
      ....

RI-AT3G12012-XLOC_014129-6042-0
      ATCATAGACAATTGATAAAGAATCTTGGAACTTTAGTTATGGTTTTTGCG
RI-AT3G12012-XLOC_014129-6042-1

CONSENSUS
      ....

RI-AT3G12012-XLOC_014129-6042-0
      CAGTTAGTGAAGTTATATGCAAGTCTAACTTAGGAAATAACTAGTGCTCG
RI-AT3G12012-XLOC_014129-6042-1

CONSENSUS
      ....

RI-AT3G12012-XLOC_014129-6042-0
      TTTCCATATTTAATGGTTTGTAGCGGTTGATGACTCTTTTTCTATGATTA
RI-AT3G12012-XLOC_014129-6042-1

CONSENSUS
      ....

RI-AT3G12012-XLOC_014129-6042-0
      CGTGAATTTATAGTGTCTCCAGGAAAAGTGGTTCACGAAAAGTGTTCGGT
RI-AT3G12012-XLOC_014129-6042-1

CONSENSUS
      ....

RI-AT3G12012-XLOC_014129-6042-0
      GTTTGTAGTAATCTTGGTTGGATCTTTTGATGGTGTTGATTACGTTATTG

```
RI-AT3G12012-XLOC_014129-6042-1
      --------------------------------------------------
CONSENSUS
      ..................................................

RI-AT3G12012-XLOC_014129-6042-0
      CAGGCCAAACAATTGTGCTTGGGGATAGCAAGGGCTATTATTTTGGAACG
RI-AT3G12012-XLOC_014129-6042-1  ---
GCCAAACAATTGTGCTTGGGGATAGCAAGGGCTATTATTTTGGAACG
CONSENSUS
      ...GCCAAACAATTGTGCTTGGGGATAGCAAGGGCTATTATTTTGGAACG

RI-AT3G12012-XLOC_014129-6042-0
      TCGACCAGCAACCCAGGTAACTCAGGCAATCGATGTACTAGTCACAGCAT
RI-AT3G12012-XLOC_014129-6042-1
      TCGACCAGCAACCCAGGTAACTCAGGCAATCGATGTACTAGTCACAGCAT
CONSENSUS
      TCGACCAGCAACCCAGGTAACTCAGGCAATCGATGTACTAGTCACAGCAT

RI-AT3G12012-XLOC_014129-6042-0
      ATTCCTACTCGGTTAAAGCCGGTACATACAAGGAAATAAAGAACGAAAAC
RI-AT3G12012-XLOC_014129-6042-1
      ATTCCTACTCGGTTAAAGCCGGTACATACAAGGAAATAAAGAACGAAAAC
CONSENSUS
      ATTCCTACTCGGTTAAAGCCGGTACATACAAGGAAATAAAGAACGAAAAC

RI-AT3G12012-XLOC_014129-6042-0
      GCCACTGCATCTACTCCAACCCCTGGTGCATCTCCTG
RI-AT3G12012-XLOC_014129-6042-1
      GCCACTGCATCTACTCCAACCCCTGGTGCATCTCCTG
CONSENSUS
      GCCACTGCATCTACTCCAACCCCTGGTGCATCTCCTG



alignment for event: RI-AT3G05030-XLOC_017015-1861

RI-AT3G05030-XLOC_017015-1861-0
      TTTACAAGATCAGGGCACACAGAATTGCGCGGGAATGCAATCATGATTAC
RI-AT3G05030-XLOC_017015-1861-1
      TTTACAAGATCAGGGCACACAGAATTGCGCGGGAATGCAATCATGATTAC
CONSENSUS
      TTTACAAGATCAGGGCACACAGAATTGCGCGGGAATGCAATCATGATTAC

RI-AT3G05030-XLOC_017015-1861-0
      CAGTACAATAACCGTCTGTCTTTTTAGCACCATGGTAAGATATCTCCCTC
RI-AT3G05030-XLOC_017015-1861-1
      CAGTACAATAACCGTCTGTCTTTTTAGCACCATG----------------
CONSENSUS
      CAGTACAATAACCGTCTGTCTTTTTAGCACCATG................

RI-AT3G05030-XLOC_017015-1861-0
      TCAGGTTTGGACTACTAAATGTATTTTCTCTACAGAGATGTTGTCAATGA
RI-AT3G05030-XLOC_017015-1861-1
      --------------------------------------------------
CONSENSUS
```

....

RI-AT3G05030-XLOC_017015-1861-0
GGATAAACCAATGCTGAGCCGTGAGACGGATTTGCATTTTCTATATCCGG
RI-AT3G05030-XLOC_017015-1861-1

CONSENSUS
....

RI-AT3G05030-XLOC_017015-1861-0
AAAACTGTTCCCTCCTATTTGATTTGTTATTCCAATGTTTTCTGGATCGC
RI-AT3G05030-XLOC_017015-1861-1

CONSENSUS
....

RI-AT3G05030-XLOC_017015-1861-0
TAACACTGAACCCTGATACCGGACATTTAACATATGTTTTTTTTTTTCCA
RI-AT3G05030-XLOC_017015-1861-1

CONSENSUS
....

RI-AT3G05030-XLOC_017015-1861-0
CGGCTTTCAGGTGTTTGGTATGCTAACCAAACCACTGATTAGATACCTAA
RI-AT3G05030-XLOC_017015-1861-1 ----------
GTGTTTGGTATGCTAACCAAACCACTGATTAGATACCTAA
CONSENSUS
..........GTGTTTGGTATGCTAACCAAACCACTGATTAGATACCTAA

RI-AT3G05030-XLOC_017015-1861-0
TGCCACACCAAAAAGCGACCACCAGTACCACGAGTATGTTATCGGACGAT
RI-AT3G05030-XLOC_017015-1861-1
TGCCACACCAAAAAGCGACCACCAGTACCACGAGTATGTTATCGGACGAT
CONSENSUS
TGCCACACCAAAAAGCGACCACCAGTACCACGAGTATGTTATCGGACGAT

RI-AT3G05030-XLOC_017015-1861-0
AGCACTCCGAAATCAATCCACATTCCGCTCCTCGATGGTGAACAGCTAGA
RI-AT3G05030-XLOC_017015-1861-1
AGCACTCCGAAATCAATCCACATTCCGCTCCTCGATGGTGAACAGCTAGA
CONSENSUS
AGCACTCCGAAATCAATCCACATTCCGCTCCTCGATGGTGAACAGCTAGA

RI-AT3G05030-XLOC_017015-1861-0
TTCATTTGAGTTACCTGGGAGCCACCAGGACGTGCCACGACCAAACAGCC
RI-AT3G05030-XLOC_017015-1861-1
TTCATTTGAGTTACCTGGGAGCCACCAGGACGTGCCACGACCAAACAGCC
CONSENSUS
TTCATTTGAGTTACCTGGGAGCCACCAGGACGTGCCACGACCAAACAGCC

RI-AT3G05030-XLOC_017015-1861-0
TTCGAGGTTTCCTCATGCGCCCCACACGGACTGTCCACTATTACTGGAGA
RI-AT3G05030-XLOC_017015-1861-1
TTCGAGGTTTCCTCATGCGCCCCACACGGACTGTCCACTATTACTGGAGA
CONSENSUS

TTCGAGGTTTCCTCATGCGCCCCACACGGACTGTCCACTATTACTGGAGA

RI-AT3G05030-XLOC_017015-1861-0
CAGTTTGATGATGCCTTCATGCGTCCTGTGTTTGGTGGTCGCGGATTCGT
RI-AT3G05030-XLOC_017015-1861-1
CAGTTTGATGATGCCTTCATGCGTCCTGTGTTTGGTGGTCGCGGATTCGT
CONSENSUS
CAGTTTGATGATGCCTTCATGCGTCCTGTGTTTGGTGGTCGCGGATTCGT

RI-AT3G05030-XLOC_017015-1861-0
TCCCTTTGTCCCTGGTTCTCCGACTGAGAGAAGCAGCCATGATCTTAGTA
RI-AT3G05030-XLOC_017015-1861-1
TCCCTTTGTCCCTGGTTCTCCGACTGAGAGAAGCAGCCATGATCTTAGTA
CONSENSUS
TCCCTTTGTCCCTGGTTCTCCGACTGAGAGAAGCAGCCATGATCTTAGTA

RI-AT3G05030-XLOC_017015-1861-0
AACCTTGAGGAGAAAGATATATAGAAACTTAACCAAAAAACTTCTTCTTG
RI-AT3G05030-XLOC_017015-1861-1
AACCTTGAGGAGAAAGATATATAGAAACTTAACCAAAAAACTTCTTCTTG
CONSENSUS
AACCTTGAGGAGAAAGATATATAGAAACTTAACCAAAAAACTTCTTCTTG

RI-AT3G05030-XLOC_017015-1861-0
CTCTTCCCTCTTATGGTGACTAGTATTGGTGATGTAAATGTATTTTTCGT
RI-AT3G05030-XLOC_017015-1861-1
CTCTTCCCTCTTATGGTGACTAGTATTGGTGATGTAAATGTATTTTTCGT
CONSENSUS
CTCTTCCCTCTTATGGTGACTAGTATTGGTGATGTAAATGTATTTTTCGT

RI-AT3G05030-XLOC_017015-1861-0
TCTTCAAATTTACATATTCTTCTGTAAATTTGTTATTATTCGATGATGAA
RI-AT3G05030-XLOC_017015-1861-1
TCTTCAAATTTACATATTCTTCTGTAAATTTGTTATTATTCGATGATGAA
CONSENSUS
TCTTCAAATTTACATATTCTTCTGTAAATTTGTTATTATTCGATGATGAA

RI-AT3G05030-XLOC_017015-1861-0
GAAGCTTCTTACGTTTTTGAGAGACGTGTGGG
RI-AT3G05030-XLOC_017015-1861-1
GAAGCTTCTTACGTTTTTGAGAGACGTGTGGG
CONSENSUS
GAAGCTTCTTACGTTTTTGAGAGACGTGTGGG

alignment for event: A5-AT3G17740-XLOC_017744-12416

A5-AT3G17740-XLOC_017744-12416-0
GATCTACTACTTTGTGGAGTTTATGCACAGAGAGAAGCTGCTTCTGGAAA
A5-AT3G17740-XLOC_017744-12416-1
GATCTACTACTTTGTGGAGTTTATGCACAGAGAGAAGCTGCTTCTGGAAA
CONSENSUS
GATCTACTACTTTGTGGAGTTTATGCACAGAGAGAAGCTGCTTCTGGAAA

A5-AT3G17740-XLOC_017744-12416-0

TATGAAACATGCAAGGCGAGTCTTCGACATGGCACTGACTTCTATCTGTG
A5-AT3G17740-XLOC_017744-12416-1
TATGAAACATGCAAGGCGAGTCTTCGACATGGCACTGACTTCTATCTGTG
CONSENSUS
TATGAAACATGCAAGGCGAGTCTTCGACATGGCACTGACTTCTATCTGTG

A5-AT3G17740-XLOC_017744-12416-0
GCCTCCCTAAGGTTAGTTTCAGATCTTTTGGTTATATTGTTACTTGTTAG
A5-AT3G17740-XLOC_017744-12416-1
GCCTCCCTAAG---------------------------------------
CONSENSUS
GCCTCCCTAAG.......................................

A5-AT3G17740-XLOC_017744-12416-0
CATATGATACAAAATTTCAGGTTTCCACGTATGTTTCTAATTCCAAGGAA
A5-AT3G17740-XLOC_017744-12416-1

CONSENSUS
....

A5-AT3G17740-XLOC_017744-12416-0
CTTCTGGATCAGTAACCCTGGTTTTGTGTTTATTTCTGATTCAAATTATC
A5-AT3G17740-XLOC_017744-12416-1

CONSENSUS
....

A5-AT3G17740-XLOC_017744-12416-0
GCGTACTGGTCCCAAAAACTGGGGCACTGCCACTTGAGTTTGGACAAGAT
A5-AT3G17740-XLOC_017744-12416-1

CONSENSUS
....

A5-AT3G17740-XLOC_017744-12416-0
AATCTCTCTCATGGACGGATCATAGTAGAGTTTGTTATAGAACAGTCTCT
A5-AT3G17740-XLOC_017744-12416-1

CONSENSUS
....

A5-AT3G17740-XLOC_017744-12416-0
TCTTGGAATTACAGTGTAACACTCCTTTGCTATGTTTATGGTATGCTGAA
A5-AT3G17740-XLOC_017744-12416-1 -----
GAATTACAGTGTAACACTCCTTTGCTATGTTTATGGTATGCTGAA
CONSENSUS
.....GAATTACAGTGTAACACTCCTTTGCTATGTTTATGGTATGCTGAA

A5-AT3G17740-XLOC_017744-12416-0
TCGGAAGTAGCCAATAGTAGCGGCAGTGGTAGGGATACAGAATCATCATC
A5-AT3G17740-XLOC_017744-12416-1
TCGGAAGTAGCCAATAGTAGCGGCAGTGGTAGGGATACAGAATCATCATC
CONSENSUS
TCGGAAGTAGCCAATAGTAGCGGCAGTGGTAGGGATACAGAATCATCATC

A5-AT3G17740-XLOC_017744-12416-0

TCGTGCTATGCATATTCTGTGCTACCTGGGAAGTGGTCTAGCTTATAGTC
A5-AT3G17740-XLOC_017744-12416-1
TCGTGCTATGCATATTCTGTGCTACCTGGGAAGTGGTCTAGCTTATAGTC
CONSENSUS
TCGTGCTATGCATATTCTGTGCTACCTGGGAAGTGGTCTAGCTTATAGTC

A5-AT3G17740-XLOC_017744-12416-0
CTTATACTTCCCAGTCTTCAAGCATGCAAATTCTTAGAGCACGCCAGGGG
A5-AT3G17740-XLOC_017744-12416-1
CTTATACTTCCCAGTCTTCAAGCATGCAAATTCTTAGAGCACGCCAGGGG
CONSENSUS
CTTATACTTCCCAGTCTTCAAGCATGCAAATTCTTAGAGCACGCCAGGGG

A5-AT3G17740-XLOC_017744-12416-0
TTCAGAGAGAAGCTTAAGAAGATACAATCAACATGGTCTCATGGTGTCAC
A5-AT3G17740-XLOC_017744-12416-1
TTCAGAGAGAAGCTTAAGAAGATACAATCAACATGGTCTCATGGTGTCAC
CONSENSUS
TTCAGAGAGAAGCTTAAGAAGATACAATCAACATGGTCTCATGGTGTCAC

A5-AT3G17740-XLOC_017744-12416-0
AGATGATCAATCAGCAGCGCTTGTTTGTTCGGCAGCTCTATTTGAGGAGT
A5-AT3G17740-XLOC_017744-12416-1
AGATGATCAATCAGCAGCGCTTGTTTGTTCGGCAGCTCTATTTGAGGAGT
CONSENSUS
AGATGATCAATCAGCAGCGCTTGTTTGTTCGGCAGCTCTATTTGAGGAGT

A5-AT3G17740-XLOC_017744-12416-0
TAACAAATGACCTTCCTGGTGCTCTTGAGATCTTAGAGCACATGTTCAGT
A5-AT3G17740-XLOC_017744-12416-1
TAACAAATGACCTTCCTGGTGCTCTTGAGATCTTAGAGCACATGTTCAGT
CONSENSUS
TAACAAATGACCTTCCTGGTGCTCTTGAGATCTTAGAGCACATGTTCAGT

A5-AT3G17740-XLOC_017744-12416-0 TCTGTTCTTCCAG
A5-AT3G17740-XLOC_017744-12416-1 TCTGTTCTTCCAG
CONSENSUS TCTGTTCTTCCAG

alignment for event: RI-AT3G29185-XLOC_018419-8822

RI-AT3G29185-XLOC_018419-8822-0
GTTATATATAAAGCAGCCTACATCAGCTACTTCAGTTTCCGAAGAGGAGG
RI-AT3G29185-XLOC_018419-8822-1
GTTATATATAAAGCAGCCTACATCAGCTACTTCAGTTTCCGAAGAGGAGG
CONSENSUS
GTTATATATAAAGCAGCCTACATCAGCTACTTCAGTTTCCGAAGAGGAGG

RI-AT3G29185-XLOC_018419-8822-0
AAGAGGAACCGGAATGGGTAGAGTACAAAATTAAAGAAACCAATATGTTC
RI-AT3G29185-XLOC_018419-8822-1
AAGAGGAACCGGAATGGGTAGAGTACAAAATTAAAGAAACCAATATGTTC
CONSENSUS
AAGAGGAACCGGAATGGGTAGAGTACAAAATTAAAGAAACCAATATGTTC

```
RI-AT3G29185-XLOC_018419-8822-0
      ACTGTGGACAAGTATCAGCAGGTGAGAGTAGCTCTGTTTATCTCTATTAG
RI-AT3G29185-XLOC_018419-8822-1
      ACTGTGGACAAGTATCAGCAG-----------------------------
CONSENSUS
      ACTGTGGACAAGTATCAGCAG.............................

RI-AT3G29185-XLOC_018419-8822-0
      TCAAGATACTAAGTTTGAGTTTTGTGATTTGTCTCTTAAGTATTTGATGA
RI-AT3G29185-XLOC_018419-8822-1
      --------------------------------------------------
CONSENSUS
      ..................................................

RI-AT3G29185-XLOC_018419-8822-0
      CTATCTTGATATCCATCATCAGTAGTCTGCAGATTGGATTTTTCCCAAAG
RI-AT3G29185-XLOC_018419-8822-1
      --------------------------------ATTGGATTTTTCCCAAAG
CONSENSUS
      ................................ATTGGATTTTTCCCAAAG

RI-AT3G29185-XLOC_018419-8822-0
      GAGAGAGCGTTTTCGCTGAGGTACCAAACCGCTGGAATGTTGGATACGAC
RI-AT3G29185-XLOC_018419-8822-1
      GAGAGAGCGTTTTCGCTGAGGTACCAAACCGCTGGAATGTTGGATACGAC
CONSENSUS
      GAGAGAGCGTTTTCGCTGAGGTACCAAACCGCTGGAATGTTGGATACGAC

RI-AT3G29185-XLOC_018419-8822-0
      GTTAAGGCAGGGCGTGCTCGGGGAGGATGACACGGGAGAAGAATCACCAA
RI-AT3G29185-XLOC_018419-8822-1
      GTTAAGGCAGGGCGTGCTCGGGGAGGATGACACGGGAGAAGAATCACCAA
CONSENSUS
      GTTAAGGCAGGGCGTGCTCGGGGAGGATGACACGGGAGAAGAATCACCAA

RI-AT3G29185-XLOC_018419-8822-0  G
RI-AT3G29185-XLOC_018419-8822-1  G
CONSENSUS                        G


alignment for event: RI-AT3G13060-XLOC_014175-11534

RI-AT3G13060-XLOC_014175-11534-0
      GTAAATGCGAGCTCTCAATTCTGTGGTGTAGCAGAGATGGTTGGACCTGT
RI-AT3G13060-XLOC_014175-11534-1
      GTAAATGCGAGCTCTCAATTCTGTGGTGTAGCAGAGATGGTTGGACCTGT
CONSENSUS
      GTAAATGCGAGCTCTCAATTCTGTGGTGTAGCAGAGATGGTTGGACCTGT

RI-AT3G13060-XLOC_014175-11534-0
      TGATTTTGAGAAGAGCGTTGATTACTGGCAACAAGATAAATGGAGTGGAC
RI-AT3G13060-XLOC_014175-11534-1
      TGATTTTGAGAAGAGCGTTGATTACTGGCAACAAGATAAATGGAGTGGAC
CONSENSUS
      TGATTTTGAGAAGAGCGTTGATTACTGGCAACAAGATAAATGGAGTGGAC
```

```
RI-AT3G13060-XLOC_014175-11534-0
      AGTTCCCAGTGAAGTGGCACATTATCAAAGATGTTCCAAACAGCCAGTTC
RI-AT3G13060-XLOC_014175-11534-1
      AGTTCCCAGTGAAGTGGCACATTATCAAAGATGTTCCAAACAGCCAGTTC
CONSENSUS
      AGTTCCCAGTGAAGTGGCACATTATCAAAGATGTTCCAAACAGCCAGTTC

RI-AT3G13060-XLOC_014175-11534-0
      CGCCACATTATATTGGAAAACAATGACAATAAGCCTGTGACTAATAGTCG
RI-AT3G13060-XLOC_014175-11534-1
      CGCCACATTATATTGGAAAACAATGACAATAAGCCTGTGACTAATAGTCG
CONSENSUS
      CGCCACATTATATTGGAAAACAATGACAATAAGCCTGTGACTAATAGTCG

RI-AT3G13060-XLOC_014175-11534-0
      AGACACCCAAGAAGTAAGTAGCAATTTGTGCCTATCAAGCTTTCTTGTCC
RI-AT3G13060-XLOC_014175-11534-1
      AGACACCCAAGAA-------------------------------------
CONSENSUS
      AGACACCCAAGAA.....................................

RI-AT3G13060-XLOC_014175-11534-0
      ATAAGATACCTCTTTTAAGCCACACCATAACCACAACCAAAAACTACAGG
RI-AT3G13060-XLOC_014175-11534-1
      -------------------------------------------------G
CONSENSUS
      .................................................G

RI-AT3G13060-XLOC_014175-11534-0
      TAAAACTGGAACAGGGCATCGAGATGCTGAAGATCTTCAAGAACTACGAC
RI-AT3G13060-XLOC_014175-11534-1
      TAAAACTGGAACAGGGCATCGAGATGCTGAAGATCTTCAAGAACTACGAC
CONSENSUS
      TAAAACTGGAACAGGGCATCGAGATGCTGAAGATCTTCAAGAACTACGAC

RI-AT3G13060-XLOC_014175-11534-0
      GCTGACACGTCAATCTTGGATGATTTCGGGTTTTATGAAGAGCGGGAGAA
RI-AT3G13060-XLOC_014175-11534-1
      GCTGACACGTCAATCTTGGATGATTTCGGGTTTTATGAAGAGCGGGAGAA
CONSENSUS
      GCTGACACGTCAATCTTGGATGATTTCGGGTTTTATGAAGAGCGGGAGAA

RI-AT3G13060-XLOC_014175-11534-0
      AATTATACAAGACCGGAAGGCAAGAAGACAGCCAAGCTTGCCATCTGCAG
RI-AT3G13060-XLOC_014175-11534-1
      AATTATACAAGACCGGAAGGCAAGAAGACAGCCAAGCTTGCCATCTGCAG
CONSENSUS
      AATTATACAAGACCGGAAGGCAAGAAGACAGCCAAGCTTGCCATCTGCAG

RI-AT3G13060-XLOC_014175-11534-0
      GAGTAGTAGCAGGAGAAAATGAACATAAACCTGCTTCTGCTGCATTACCC
RI-AT3G13060-XLOC_014175-11534-1
      GAGTAGTAGCAGGAGAAAATGAACATAAACCTGCTTCTGCTGCATTACCC
CONSENSUS
      GAGTAGTAGCAGGAGAAAATGAACATAAACCTGCTTCTGCTGCATTACCC
```

RI-AT3G13060-XLOC_014175-11534-0
ACAGACTTCATGAAGAACATGTCCAAGAGTTTTGCGCAAGTTGTCCGCTT
RI-AT3G13060-XLOC_014175-11534-1
ACAGACTTCATGAAGAACATGTCCAAGAGTTTTGCGCAAGTTGTCCGCTT
CONSENSUS
ACAGACTTCATGAAGAACATGTCCAAGAGTTTTGCGCAAGTTGTCCGCTT

RI-AT3G13060-XLOC_014175-11534-0
GGACGAAGGCAGCAAAGAAGCAGTCAAGGCCAGTTCATCTCCAGATGCAA
RI-AT3G13060-XLOC_014175-11534-1
GGACGAAGGCAGCAAAGAAGCAGTCAAGGCCAGTTCATCTCCAGATGCAA
CONSENSUS
GGACGAAGGCAGCAAAGAAGCAGTCAAGGCCAGTTCATCTCCAGATGCAA

RI-AT3G13060-XLOC_014175-11534-0
TTACAACAGCAGCAGTCTCTTCTGGTCAATCCAACTAGTAAGTAAAAGAC
RI-AT3G13060-XLOC_014175-11534-1
TTACAACAGCAGCAGTCTCTTCTGGTCAATCCAACTAGTAAGTAAAAGAC
CONSENSUS
TTACAACAGCAGCAGTCTCTTCTGGTCAATCCAACTAGTAAGTAAAAGAC

RI-AT3G13060-XLOC_014175-11534-0
ATCAATTAGCAAAGACTATGTTTTCAAATTCCATAATATGATCTTTTGTT
RI-AT3G13060-XLOC_014175-11534-1
ATCAATTAGCAAAGACTATGTTTTCAAATTCCATAATATGATCTTTTGTT
CONSENSUS
ATCAATTAGCAAAGACTATGTTTTCAAATTCCATAATATGATCTTTTGTT

RI-AT3G13060-XLOC_014175-11534-0
TGTTCTAAGAAGGTATTTCAAATGAAATTTTACGTCTGACCGGGTCTGGT
RI-AT3G13060-XLOC_014175-11534-1
TGTTCTAAGAAGGTATTTCAAATGAAATTTTACGTCTGACCGGGTCTGGT
CONSENSUS
TGTTCTAAGAAGGTATTTCAAATGAAATTTTACGTCTGACCGGGTCTGGT

RI-AT3G13060-XLOC_014175-11534-0
TTTTGTGTAAAGTAAGCCTCTTTCCTCCGCCCTTTTCTGCTTCAGGGGTA
RI-AT3G13060-XLOC_014175-11534-1
TTTTGTGTAAAGTAAGCCTCTTTCCTCCGCCCTTTTCTGCTTCAGGGGTA
CONSENSUS
TTTTGTGTAAAGTAAGCCTCTTTCCTCCGCCCTTTTCTGCTTCAGGGGTA

RI-AT3G13060-XLOC_014175-11534-0
TTTAGCCAAAATTTGATTCATTTCCCTCTTTTCAATGTTCTATAATACCC
RI-AT3G13060-XLOC_014175-11534-1
TTTAGCCAAAATTTGATTCATTTCCCTCTTTTCAATGTTCTATAATACCC
CONSENSUS
TTTAGCCAAAATTTGATTCATTTCCCTCTTTTCAATGTTCTATAATACCC

RI-AT3G13060-XLOC_014175-11534-0
TCACTCTTCTGAGTTGATAGAAGAAGAAGATGAAGAAGATAATGTATGAA
RI-AT3G13060-XLOC_014175-11534-1
TCACTCTTCTGAGTTGATAGAAGAAGAAGATGAAGAAGATAATGTATGAA
CONSENSUS
TCACTCTTCTGAGTTGATAGAAGAAGAAGATGAAGAAGATAATGTATGAA

RI-AT3G13060-XLOC_014175-11534-0
AGACAATATAATGTTTCTCTTGTAAAGTTGGCCTCATTTATCTTTGCAGG
RI-AT3G13060-XLOC_014175-11534-1
AGACAATATAATGTTTCTCTTGTAAAGTTGGCCTCATTTATCTTTGCAGG
CONSENSUS
AGACAATATAATGTTTCTCTTGTAAAGTTGGCCTCATTTATCTTTGCAGG

RI-AT3G13060-XLOC_014175-11534-0
CAGTTTTTCAAAATAAAAATGCAGATTACAC
RI-AT3G13060-XLOC_014175-11534-1
CAGTTTTTCAAAATAAAAATGCAGATTACAC
CONSENSUS
CAGTTTTTCAAAATAAAAATGCAGATTACAC

alignment for event: RI-AT3G20470-XLOC_017883-12810

RI-AT3G20470-XLOC_017883-12810-0
CATGCCAAATGGTCTATATAAACACCTTATTGGCTGATAGAGTTCCTCAT
RI-AT3G20470-XLOC_017883-12810-1
CATGCCAAATGGTCTATATAAACACCTTATTGGCTGATAGAGTTCCTCAT
CONSENSUS
CATGCCAAATGGTCTATATAAACACCTTATTGGCTGATAGAGTTCCTCAT

RI-AT3G20470-XLOC_017883-12810-0
CATCACATACGCAAAACACAAAAATCGACATGGCTTCCAAGTCACTCTTT
RI-AT3G20470-XLOC_017883-12810-1
CATCACATACGCAAAACACAAAAATCGACATGGCTTCCAAGTCACTCTTT
CONSENSUS
CATCACATACGCAAAACACAAAAATCGACATGGCTTCCAAGTCACTCTTT

RI-AT3G20470-XLOC_017883-12810-0
CTTGTTGCCTTACTTGTCGGCTCTTTTGCTTTCACTTCATTTGCCAGTGT
RI-AT3G20470-XLOC_017883-12810-1
CTTGTTGCCTTACTTGTCGGCTCTTTTGCTTTCACTTCATTTGCCAGTGT
CONSENSUS
CTTGTTGCCTTACTTGTCGGCTCTTTTGCTTTCACTTCATTTGCCAGTGT

RI-AT3G20470-XLOC_017883-12810-0
CGCTAACAGGAAGCTGAAGAGTGGTCTCGAGGACCAAAAGACATTCTTCC
RI-AT3G20470-XLOC_017883-12810-1
CGCTAACAGGAAGCTGAAGAGTGGTCTCGAGGACCAAAAGACATTCTTCC
CONSENSUS
CGCTAACAGGAAGCTGAAGAGTGGTCTCGAGGACCAAAAGACATTCTTCC

RI-AT3G20470-XLOC_017883-12810-0
ACCATCCAGGCGGAGGTCTCGGTGGAGGAGGCGGTATTGGTGGAGGCAGT
RI-AT3G20470-XLOC_017883-12810-1
ACCATCCAGGCGGAGGTCTCGGTGGAGGAGGCGGTATTGGTGGAGGCAGT
CONSENSUS
ACCATCCAGGCGGAGGTCTCGGTGGAGGAGGCGGTATTGGTGGAGGCAGT

RI-AT3G20470-XLOC_017883-12810-0
GGTCTTGGTGGAGGAGGCGGCTTTGGTGGAGGAGGCGGTCTTGGCGGAGG

RI-AT3G20470-XLOC_017883-12810-1
GGTCTTGGTGGAGGAGGCGGCTTTGGTGGAGGAGGCGGTCTTGGCGGAGG
CONSENSUS
GGTCTTGGTGGAGGAGGCGGCTTTGGTGGAGGAGGCGGTCTTGGCGGAGG

RI-AT3G20470-XLOC_017883-12810-0
AGCTGGAGGCGGTGGCGGTTTAGGAGGAGGAGCTGGTGGTGGAGCTGGAG
RI-AT3G20470-XLOC_017883-12810-1
AGCTGGAGGCGGTGGCGGTTTAGGAGGAGGAGCTGGTGGTGGAGCTGGAG
CONSENSUS
AGCTGGAGGCGGTGGCGGTTTAGGAGGAGGAGCTGGTGGTGGAGCTGGAG

RI-AT3G20470-XLOC_017883-12810-0
GAGGATTTGGTGGAGGAGCTGGGAGCGGTGGTGGTCTTGGTGGGGGTGGT
RI-AT3G20470-XLOC_017883-12810-1
GAGGATTTGGTGGAGGAGCTGGGAGCGGTGGTG-----------------
CONSENSUS
GAGGATTTGGTGGAGGAGCTGGGAGCGGTGGTG.................

RI-AT3G20470-XLOC_017883-12810-0
GGTGCTGGTGGAGGATTTGGCGGTGGGGCTGGTGGTGGTTCAGGTGGAGG
RI-AT3G20470-XLOC_017883-12810-1
---G
CONSENSUS
....G

RI-AT3G20470-XLOC_017883-12810-0
GTTTGGAGGAGGAGCTGGTGCTGGGGGTGGACTCGGAGGAGGAGGTGGAG
RI-AT3G20470-XLOC_017883-12810-1
GTTTGGAGGAGGAGCTGGTGCTGGGGGTGGACTCGGAGGAGGAGGTGGAG
CONSENSUS
GTTTGGAGGAGGAGCTGGTGCTGGGGGTGGACTCGGAGGAGGAGGTGGAG

RI-AT3G20470-XLOC_017883-12810-0
CTGGTGGCGGTGGTGGGTTTGGAGGAGGAGGCGGAAGCGGTATTGGTGGT
RI-AT3G20470-XLOC_017883-12810-1
CTGGTGGCGGTGGTGGGTTTGGAGGAGGAGGCGGAAGCGGTATTGGTGGT
CONSENSUS
CTGGTGGCGGTGGTGGGTTTGGAGGAGGAGGCGGAAGCGGTATTGGTGGT

RI-AT3G20470-XLOC_017883-12810-0
GGTTTCGGTGGGGGAGCCGGTGCCGGAGGTGGTTTCGGTGGTGGACATCA
RI-AT3G20470-XLOC_017883-12810-1
GGTTTCGGTGGGGGAGCCGGTGCCGGAGGTGGTTTCGGTGGTGGACATCA
CONSENSUS
GGTTTCGGTGGGGGAGCCGGTGCCGGAGGTGGTTTCGGTGGTGGACATCA

RI-AT3G20470-XLOC_017883-12810-0
TTGATGAGAAGCTATAAGATCTTGACGTTAAACCATGGAATGGAATGCAT
RI-AT3G20470-XLOC_017883-12810-1
TTGATGAGAAGCTATAAGATCTTGACGTTAAACCATGGAATGGAATGCAT
CONSENSUS
TTGATGAGAAGCTATAAGATCTTGACGTTAAACCATGGAATGGAATGCAT

RI-AT3G20470-XLOC_017883-12810-0
GGCAATGGGTTTTACGTACCAAAGTCTTATGAAAAAAGATTAATATATTA

RI-AT3G20470-XLOC_017883-12810-1
      GGCAATGGGTTTTACGTACCAAAGTCTTATGAAAAAAGATTAATATATTA
CONSENSUS
      GGCAATGGGTTTTACGTACCAAAGTCTTATGAAAAAAGATTAATATATTA

RI-AT3G20470-XLOC_017883-12810-0
      TTCATCTAAAGTGATTCGGTCCATGATTTTGTGTTATTATCGGCCTTTGA
RI-AT3G20470-XLOC_017883-12810-1
      TTCATCTAAAGTGATTCGGTCCATGATTTTGTGTTATTATCGGCCTTTGA
CONSENSUS
      TTCATCTAAAGTGATTCGGTCCATGATTTTGTGTTATTATCGGCCTTTGA

RI-AT3G20470-XLOC_017883-12810-0
      GGAAATAAATGAATGAATTAGAAAAAATAATCGAGCTCT
RI-AT3G20470-XLOC_017883-12810-1
      GGAAATAAATGAATGAATTAGAAAAAATAATCGAGCTCT
CONSENSUS
      GGAAATAAATGAATGAATTAGAAAAAATAATCGAGCTCT

alignment for event: A5-AT3G46490-XLOC_015866-1295

A5-AT3G46490-XLOC_015866-1295-0
      ACGTTTTGCCCGGATGGCGGGAGACCATGGAGAAATATTATCAAGAAGCA
A5-AT3G46490-XLOC_015866-1295-1
      ACGTTTTGCCCGGATGGCGGGAGACCATGGAGAAATATTATCAAGAAGCA
CONSENSUS
      ACGTTTTGCCCGGATGGCGGGAGACCATGGAGAAATATTATCAAGAAGCA

A5-AT3G46490-XLOC_015866-1295-0
      TT--
A5-AT3G46490-XLOC_015866-1295-1
      TTGTAAGTGTCATGTTTGTTTAGAAAATATTTAAATAGGGAAATCAAGTG
CONSENSUS
      TT..

A5-AT3G46490-XLOC_015866-1295-0

A5-AT3G46490-XLOC_015866-1295-1
      GGAAAATTTGCTAATGACTTAAAGGATAAAAATTTAGTTGGAAATTAGGT
CONSENSUS
      ....

A5-AT3G46490-XLOC_015866-1295-0

A5-AT3G46490-XLOC_015866-1295-1
      GGAAAGTTTGATTTAAAACAACTTGACTCTTTTTCTCCCAATGAATGTGA
CONSENSUS
      ....

A5-AT3G46490-XLOC_015866-1295-0

A5-AT3G46490-XLOC_015866-1295-1
      CATCTCTCTCTCCACATTAGTTATAAACAAAAATATTCCACATATAGAAT
CONSENSUS

....

A5-AT3G46490-XLOC_015866-1295-0

A5-AT3G46490-XLOC_015866-1295-1
TCCCTCAGAGCATCCACATTGGACATACCTTTGTAGGTGTTTCAAAAGTA
CONSENSUS
....

A5-AT3G46490-XLOC_015866-1295-0

A5-AT3G46490-XLOC_015866-1295-1
AATTAAGATTAAATTTAAAGACATCTTCTATTTTGGGGGTCTGAAATGTC
CONSENSUS
....

A5-AT3G46490-XLOC_015866-1295-0

A5-AT3G46490-XLOC_015866-1295-1
TTTCTAAATACTCTATTACACATTTAAAGACACACCTTCTTGTTTCTTCT
CONSENSUS
....

A5-AT3G46490-XLOC_015866-1295-0

A5-AT3G46490-XLOC_015866-1295-1
ATTATTTTTAACTAGATGAATTCTCACGGTACGCGTGGGTTTGTTTTAAA
CONSENSUS
....

A5-AT3G46490-XLOC_015866-1295-0

A5-AT3G46490-XLOC_015866-1295-1
AATTGTTAATAAAAATATGTTAATTGTTTTAATCGCTAATTTATGTTTAT
CONSENSUS
....

A5-AT3G46490-XLOC_015866-1295-0

A5-AT3G46490-XLOC_015866-1295-1
TTTTTTGTTTATTATTGTATAACTAATAGAATTAGACTCATAATAGTATT
CONSENSUS
....

A5-AT3G46490-XLOC_015866-1295-0

A5-AT3G46490-XLOC_015866-1295-1
TACGTTTCGACAGTTGTTCCAAATACCACCACCTCAGTGATAATTTAAGT
CONSENSUS
....

A5-AT3G46490-XLOC_015866-1295-0

A5-AT3G46490-XLOC_015866-1295-1
GAGTGTATATTTAATGTTATGTATATCGATATTGTGAACTTGAGTTGTAT
CONSENSUS

....

A5-AT3G46490-XLOC_015866-1295-0

A5-AT3G46490-XLOC_015866-1295-1
CTAATGTTTTCATTTTTTAATTTCCGTATAGGTTATACATGATGATGGTT
CONSENSUS
....

A5-AT3G46490-XLOC_015866-1295-0

A5-AT3G46490-XLOC_015866-1295-1
TTGTATCCTATTTATGGTACTTGTCCCTTGGTAAACATTTACTGATGTTG
CONSENSUS
....

A5-AT3G46490-XLOC_015866-1295-0

A5-AT3G46490-XLOC_015866-1295-1
TTTGAAACACATATAAAAATAAATTGGTTATGATGTTGTCCCGTGGTAAA
CONSENSUS
....

A5-AT3G46490-XLOC_015866-1295-0

A5-AT3G46490-XLOC_015866-1295-1
CAATGTCTGTTTGAATATTTATAGAGCTTATAGTAGTGAAAGCTTGTAAT
CONSENSUS
....

A5-AT3G46490-XLOC_015866-1295-0

A5-AT3G46490-XLOC_015866-1295-1
CCCATAAGCTGGGAGTGTGAATATGTTTTAGTGAAATAAATTGTGCATCT
CONSENSUS
....

A5-AT3G46490-XLOC_015866-1295-0

A5-AT3G46490-XLOC_015866-1295-1
ATTTAAAAGACAGTTACAGCTGCTTGTTCCTTGATTCAGTTCTGTGTAGC
CONSENSUS
....

A5-AT3G46490-XLOC_015866-1295-0
---------------------------------GAGGGTTTGTAAGTCTA
A5-AT3G46490-XLOC_015866-1295-1
TTCTACTGTGGCATCCATTCTGGCAGTTACAAGGAGGGTTTGTAAGTCTA
CONSENSUS
.................................GAGGGTTTGTAAGTCTA

A5-AT3G46490-XLOC_015866-1295-0
TTGCGAAAATAATGGCTTTGGCGCTCGACTTGGATGTGGATTACTTTAAT
A5-AT3G46490-XLOC_015866-1295-1
TTGCGAAAATAATGGCTTTGGCGCTCGACTTGGATGTGGATTACTTTAAT
CONSENSUS

TTGCGAAAATAATGGCTTTGGCGCTCGACTTGGATGTGGATTACTTTAAT

A5-AT3G46490-XLOC_015866-1295-0
ACACCAGAGATGCTGGGAAATCCAATTGCAGATATGGTCTTGTTTCACTA
A5-AT3G46490-XLOC_015866-1295-1
ACACCAGAGATGCTGGGAAATCCAATTGCAGATATGGTCTTGTTTCACTA
CONSENSUS
ACACCAGAGATGCTGGGAAATCCAATTGCAGATATGGTCTTGTTTCACTA

A5-AT3G46490-XLOC_015866-1295-0 TGAAG
A5-AT3G46490-XLOC_015866-1295-1 TGAAG
CONSENSUS TGAAG

alignment for event: RI-AT3G52050-XLOC_016174-7838

RI-AT3G52050-XLOC_016174-7838-0
AGAAGATTTTGTTCCGAGGAAGACACGGATACGCAGAAAGCGGTAAAATC
RI-AT3G52050-XLOC_016174-7838-1
AGAAGATTTTGTTCCGAGGAAGACACGGATACGCAGAAAGCGGTAAAATC
CONSENSUS
AGAAGATTTTGTTCCGAGGAAGACACGGATACGCAGAAAGCGGTAAAATC

RI-AT3G52050-XLOC_016174-7838-0
CTCCTTCTTCGTCGTCCTTGTAAAAACCCTTTCTCATTTTTGGAAGGTTC
RI-AT3G52050-XLOC_016174-7838-1
CTCCTTCTTCGTCGTCCTTGTAAAAACCCTTTCTCATTTTTGGAAG----
CONSENSUS
CTCCTTCTTCGTCGTCCTTGTAAAAACCCTTTCTCATTTTTGGAAG....

RI-AT3G52050-XLOC_016174-7838-0
TTTCAAAATCCTTTCTTCGATTGCTTTCGGATTCTCGCTCGCTTCTTCGA
RI-AT3G52050-XLOC_016174-7838-1

CONSENSUS
....

RI-AT3G52050-XLOC_016174-7838-0
TTTTTGGTTTTGATTCATGGTGAATGTGTTTCCTCCGCTAAGGTTAAACG
RI-AT3G52050-XLOC_016174-7838-1

CONSENSUS
....

RI-AT3G52050-XLOC_016174-7838-0
CTCTTCTTCTTCATGACAGTACTTTCAGTGACGTCATGACGCTGCATCTG
RI-AT3G52050-XLOC_016174-7838-1 -------------------
TACTTTCAGTGACGTCATGACGCTGCATCTG
CONSENSUS
...................TACTTTCAGTGACGTCATGACGCTGCATCTG

RI-AT3G52050-XLOC_016174-7838-0
TATCATCACTCCCGTTTTCTATGGAGGAATCTCTGTTTTCCTCGGAGTGT
RI-AT3G52050-XLOC_016174-7838-1
TATCATCACTCCCGTTTTCTATGGAGGAATCTCTGTTTTCCTCGGAGTGT

CONSENSUS
      TATCATCACTCCCGTTTTCTATGGAGGAATCTCTGTTTTCCTCGGAGTGT

RI-AT3G52050-XLOC_016174-7838-0
      TGGAAACTTGTGTAATCGGAACTGTTCTCTCATCTCTCCTTCCCTTGCAC
RI-AT3G52050-XLOC_016174-7838-1
      TGGAAACTTGTGTAATCGGAACTGTTCTCTCATCTCTCCTTCCCTTGCAC
CONSENSUS
      TGGAAACTTGTGTAATCGGAACTGTTCTCTCATCTCTCCTTCCCTTGCAC

RI-AT3G52050-XLOC_016174-7838-0 GCTCTGCGAAG
RI-AT3G52050-XLOC_016174-7838-1 GCTCTGCGAAG
CONSENSUS   GCTCTGCGAAG

alignment for event: A3-AT3G59060-XLOC_019832-194

A3-AT3G59060-XLOC_019832-194-0
      ACAGATAAAGCTTCGATATTGGATGAAGCAATTGATTACTTAAAATCACT
A3-AT3G59060-XLOC_019832-194-1
      ACAGATAAAGCTTCGATATTGGATGAAGCAATTGATTACTTAAAATCACT
CONSENSUS
      ACAGATAAAGCTTCGATATTGGATGAAGCAATTGATTACTTAAAATCACT

A3-AT3G59060-XLOC_019832-194-0
      TCAAATGCAACTCCAAGTGATGTGGATGGGAAGTGGAATGGCGGCGGCGG
A3-AT3G59060-XLOC_019832-194-1
      TCAAATGCAACTCCAAGTGATGTGGATGGGAAGTGGAATGGCGGCGGCGG
CONSENSUS
      TCAAATGCAACTCCAAGTGATGTGGATGGGAAGTGGAATGGCGGCGGCGG

A3-AT3G59060-XLOC_019832-194-0
      CAGCAGCAGCAGCAAGTCCGATGATGTTTCCCGGGGTACAATCATCTCCA
A3-AT3G59060-XLOC_019832-194-1
      CAGCAGCAGCAGCAAGTCCGATGATGTTTCCCGGGGTACAATCATCTCCA
CONSENSUS
      CAGCAGCAGCAGCAAGTCCGATGATGTTTCCCGGGGTACAATCATCTCCA

A3-AT3G59060-XLOC_019832-194-0
      TACATTAATCAGATGGCTATGCAAAGTCAGATGCAATTGTCTCAATTCCC
A3-AT3G59060-XLOC_019832-194-1
      TACATTAATCAGATGGCTATGCAAAGTCAGATGCAATTGTCTCAATTCCC
CONSENSUS
      TACATTAATCAGATGGCTATGCAAAGTCAGATGCAATTGTCTCAATTCCC

A3-AT3G59060-XLOC_019832-194-0
      GGTTATGAACCGGTCCGCTCCGCAGAACCATCCCGGTTTAGTATGTCAAA
A3-AT3G59060-XLOC_019832-194-1
      GGTTATGAACCGGTCCGCTCCGCAGAACCATCCCGGTTTAGTATGTCAAA
CONSENSUS
      GGTTATGAACCGGTCCGCTCCGCAGAACCATCCCGGTTTAGTATGTCAAA

A3-AT3G59060-XLOC_019832-194-0
      ACCCGGTACAGTTGCAGCTCCAAGCACAGAACCAAATCTTATCGGAGCAG
A3-AT3G59060-XLOC_019832-194-1

ACCCGGTACAGTTGCAGCTCCAAGCACAGAACCAAATCTTATCGGAGCAG
CONSENSUS
ACCCGGTACAGTTGCAGCTCCAAGCACAGAACCAAATCTTATCGGAGCAG

A3-AT3G59060-XLOC_019832-194-0
CTCGCTAGGTACATGGGCGGGATTCCCCAGATGCCGCCGGCGGGAAATCA
A3-AT3G59060-XLOC_019832-194-1
CTCGCTAGGTACATGGGCGGGATTCCCCAGATGCCGCCGGCGGGAAATCA
CONSENSUS
CTCGCTAGGTACATGGGCGGGATTCCCCAGATGCCGCCGGCGGGAAATCA

A3-AT3G59060-XLOC_019832-194-0 G------
ACCGTGCAACAACAACCAGCGGACATGTTGGGATTTGGATCTC
A3-AT3G59060-XLOC_019832-194-1
GATGCAGACCGTGCAACAACAACCAGCGGACATGTTGGGATTTGGATCTC
CONSENSUS
G......ACCGTGCAACAACAACCAGCGGACATGTTGGGATTTGGATCTC

A3-AT3G59060-XLOC_019832-194-0
CGGCGGGACCGCAAAGTCAACTGTCGGCACCGGCGACCACCGACAGTCTT
A3-AT3G59060-XLOC_019832-194-1
CGGCGGGACCGCAAAGTCAACTGTCGGCACCGGCGACCACCGACAGTCTT
CONSENSUS
CGGCGGGACCGCAAAGTCAACTGTCGGCACCGGCGACCACCGACAGTCTT

A3-AT3G59060-XLOC_019832-194-0
CATATGGGTAAAATAGGCTGACTTGGCATATAGTTTTCCTCCGAAATTAT
A3-AT3G59060-XLOC_019832-194-1
CATATGGGTAAAATAGGCTGACTTGGCATATAGTTTTCCTCCGAAATTAT
CONSENSUS
CATATGGGTAAAATAGGCTGACTTGGCATATAGTTTTCCTCCGAAATTAT

A3-AT3G59060-XLOC_019832-194-0
TCTTCTTACAGTTGGTGATTGTTATTTATTTTTGGTCGCCTAAGCAAGCA
A3-AT3G59060-XLOC_019832-194-1
TCTTCTTACAGTTGGTGATTGTTATTTATTTTTGGTCGCCTAAGCAAGCA
CONSENSUS
TCTTCTTACAGTTGGTGATTGTTATTTATTTTTGGTCGCCTAAGCAAGCA

A3-AT3G59060-XLOC_019832-194-0
TAAAAGCTAAGTCAAATGTATTATAGAGATCTAATAAGTTAGTCTCATAC
A3-AT3G59060-XLOC_019832-194-1
TAAAAGCTAAGTCAAATGTATTATAGAGATCTAATAAGTTAGTCTCATAC
CONSENSUS
TAAAAGCTAAGTCAAATGTATTATAGAGATCTAATAAGTTAGTCTCATAC

A3-AT3G59060-XLOC_019832-194-0
TTATAACTTATTTTTAAACAGTTGAATTATAGTATCAATCAAGTGTTGGG
A3-AT3G59060-XLOC_019832-194-1
TTATAACTTATTTTTAAACAGTTGAATTATAGTATCAATCAAGTGTTGGG
CONSENSUS
TTATAACTTATTTTTAAACAGTTGAATTATAGTATCAATCAAGTGTTGGG

A3-AT3G59060-XLOC_019832-194-0
AACCTAAAGATCATACATGTGTCAATACTTTT
A3-AT3G59060-XLOC_019832-194-1

AACCTAAAGATCATACATGTGTCAATACTTTT
CONSENSUS
AACCTAAAGATCATACATGTGTCAATACTTTT

alignment for event: A3-AT3G03350-XLOC_013688-13141

A3-AT3G03350-XLOC_013688-13141-0
GTTTTGGGGTTATTTCTCAAATCGATTCCTGTCACCAAAATCAAAATCTG
A3-AT3G03350-XLOC_013688-13141-1
GTTTTGGGGTTATTTCTCAAATCGATTCCTGTCACCAAAATCAAAATCTG
CONSENSUS
GTTTTGGGGTTATTTCTCAAATCGATTCCTGTCACCAAAATCAAAATCTG

A3-AT3G03350-XLOC_013688-13141-0
AAAGCAAATGGTTGATGAGGATCACTTGGAAGATTTTGTCTGTGTTTGAC
A3-AT3G03350-XLOC_013688-13141-1
AAAGCAAATGGTTGATGAGGATCACTTGGAAGATTTTGTCTGTGTTTGAC
CONSENSUS
AAAGCAAATGGTTGATGAGGATCACTTGGAAGATTTTGTCTGTGTTTGAC

A3-AT3G03350-XLOC_013688-13141-0
TATGGAGTTGCACATAAATGGTTAGGGATCAGAGGAATAAGTTGACTCAG
A3-AT3G03350-XLOC_013688-13141-1
TATGGAGTTGCACATAAATGGTTAGGGATCAGAGGAATAAGTTGACTCAG
CONSENSUS
TATGGAGTTGCACATAAATGGTTAGGGATCAGAGGAATAAGTTGACTCAG

A3-AT3G03350-XLOC_013688-13141-0
GAAGTTTCTTCTGCTTTCAGCGGCAAGCTCAACATCCTTGCAAGATCTGT
A3-AT3G03350-XLOC_013688-13141-1
GAAGTTTCTTCTGCTTTCAGCGGCAAGCTCAACATCCTT-----------
CONSENSUS
GAAGTTTCTTCTGCTTTCAGCGGCAAGCTCAACATCCTT...........

A3-AT3G03350-XLOC_013688-13141-0
TATATGTTAACATAGTTAAGATTACCAGCACCGGAGGAAATCGCGTCACT
A3-AT3G03350-XLOC_013688-13141-1

CONSENSUS
....

A3-AT3G03350-XLOC_013688-13141-0
GGTTGCTTTTCTTTGCCTTCCTGCAGCCTCCTACACTACAAGACAGGAAG
A3-AT3G03350-XLOC_013688-13141-1 --------------------------
CCTCCTACACTACAAGACAGGAAG
CONSENSUS
..........................CCTCCTACACTACAAGACAGGAAG

A3-AT3G03350-XLOC_013688-13141-0
AGCTAAAAGCATGCTTAAATGATTGCAAATCCAGTGGTTTAGTGGTGTCT
A3-AT3G03350-XLOC_013688-13141-1
AGCTAAAAGCATGCTTAAATGATTGCAAATCCAGTGGTTTAGTGGTGTCT
CONSENSUS
AGCTAAAAGCATGCTTAAATGATTGCAAATCCAGTGGTTTAGTGGTGTCT

A3-AT3G03350-XLOC_013688-13141-0
      GGTTCGGTTAGAGATCAGAGGGATAAGTTGATTCAGTGGCAAGCTCAACA
A3-AT3G03350-XLOC_013688-13141-1
      GGTTCGGTTAGAGATCAGAGGGATAAGTTGATTCAGTGGCAAGCTCAACA
CONSENSUS
      GGTTCGGTTAGAGATCAGAGGGATAAGTTGATTCAGTGGCAAGCTCAACA

A3-AT3G03350-XLOC_013688-13141-0 TTCTT
A3-AT3G03350-XLOC_013688-13141-1 TTCTT
CONSENSUS   TTCTT

alignment for event: A3-AT3G01480-XLOC_013581-5094

A3-AT3G01480-XLOC_013581-5094-0
      AATGTGAGGCAGGCAAGTAGAACATTGCAGCAAGGGAAAAGTATAATTGT
A3-AT3G01480-XLOC_013581-5094-1
      AATGTGAGGCAGGCAAGTAGAACATTGCAGCAAGGGAAAAGTATAATTGT
CONSENSUS
      AATGTGAGGCAGGCAAGTAGAACATTGCAGCAAGGGAAAAGTATAATTGT

A3-AT3G01480-XLOC_013581-5094-0
      GGCAGGTTTTGCTGAATCGAAGAAGGATCATGGTAATGAAATGATTGAAA
A3-AT3G01480-XLOC_013581-5094-1
      GGCAGGTTTTGCTGAATCGAAGAAGGATCATGGTAATGAAATGATTGAAA
CONSENSUS
      GGCAGGTTTTGCTGAATCGAAGAAGGATCATGGTAATGAAATGATTGAAA

A3-AT3G01480-XLOC_013581-5094-0
      AGTTGGAAGCTGGGATGCAAGATATGCTTAAGATAGTGGAAGATCGAAAA
A3-AT3G01480-XLOC_013581-5094-1
      AGTTGGAAGCTGGGATGCAAGATATGCTTAAGATAGTGGAAGATCGAAAA
CONSENSUS
      AGTTGGAAGCTGGGATGCAAGATATGCTTAAGATAGTGGAAGATCGAAAA

A3-AT3G01480-XLOC_013581-5094-0
      AGAGACGCAGTTGCTCCAAAACAGAAAGAAATTCTCAAATATGTTGGCGG
A3-AT3G01480-XLOC_013581-5094-1
      AGAGACGCAGTTGCTCCAAAACAGAAAGAAATTCTCAAATATGTTGGCGG
CONSENSUS
      AGAGACGCAGTTGCTCCAAAACAGAAAGAAATTCTCAAATATGTTGGCGG

A3-AT3G01480-XLOC_013581-5094-0
      CTGAGATCTTCATGTGGGGCATTCGCAGAATAGAAGAGGATATGGTTGAT
A3-AT3G01480-XLOC_013581-5094-1 ---------------------------
AATAGAAGAGGATATGGTTGAT
CONSENSUS
      ...........................AATAGAAGAGGATATGGTTGAT

A3-AT3G01480-XLOC_013581-5094-0
      GGCTTTCCATATGAAGTGCCGGAAGAGTATCGGAACATGCCTCTCCTCAA
A3-AT3G01480-XLOC_013581-5094-1
      GGCTTTCCATATGAAGTGCCGGAAGAGTATCGGAACATGCCTCTCCTCAA
CONSENSUS

GGCTTTCCATATGAAGTGCCGGAAGAGTATCGGAACATGCCTCTCCTCAA

A3-AT3G01480-XLOC_013581-5094-0
GGGAAGAGCTAGTGTGGACATGAAGGTCAAGATCAAGGACAATCCCAACA
A3-AT3G01480-XLOC_013581-5094-1
GGGAAGAGCTAGTGTGGACATGAAGGTCAAGATCAAGGACAATCCCAACA
CONSENSUS
GGGAAGAGCTAGTGTGGACATGAAGGTCAAGATCAAGGACAATCCCAACA

A3-AT3G01480-XLOC_013581-5094-0
TCGAGGACTGTGTGTTCCGCATTGTTCTTGATGGTTATAACGCCCCTGTT
A3-AT3G01480-XLOC_013581-5094-1
TCGAGGACTGTGTGTTCCGCATTGTTCTTGATGGTTATAACGCCCCTGTT
CONSENSUS
TCGAGGACTGTGTGTTCCGCATTGTTCTTGATGGTTATAACGCCCCTGTT

A3-AT3G01480-XLOC_013581-5094-0
ACCGCCGGAAACTTTGTGGACTTGGTAGAGAGGCATTTCTACGATGGCAT
A3-AT3G01480-XLOC_013581-5094-1
ACCGCCGGAAACTTTGTGGACTTGGTAGAGAGGCATTTCTACGATGGCAT
CONSENSUS
ACCGCCGGAAACTTTGTGGACTTGGTAGAGAGGCATTTCTACGATGGCAT

A3-AT3G01480-XLOC_013581-5094-0 GGAGATCCAGAGAT
A3-AT3G01480-XLOC_013581-5094-1 GGAGATCCAGAGAT
CONSENSUS GGAGATCCAGAGAT

alignment for event: SE-AT3G61860-XLOC_019993-10448

SE-AT3G61860-XLOC_019993-10448-0
GAATTGCGAATTAAGATAAAGATGAGGCCAGTGTTCGTCGGCAATTTCGA
SE-AT3G61860-XLOC_019993-10448-1
GAATTGCGAATTAAGATAAAGATGAGGCCAGTGTTCGTCGGCAATTTCGA
CONSENSUS
GAATTGCGAATTAAGATAAAGATGAGGCCAGTGTTCGTCGGCAATTTCGA

SE-AT3G61860-XLOC_019993-10448-0
GTATGAAACTCGCCAGTCGGATCTGGAACGGTTGTTCGACAAGTATGGGA
SE-AT3G61860-XLOC_019993-10448-1
GTATGAAACTCGCCAGTCGGATCTGGAACGGTTGTTCGACAAGTATGGGA
CONSENSUS
GTATGAAACTCGCCAGTCGGATCTGGAACGGTTGTTCGACAAGTATGGGA

SE-AT3G61860-XLOC_019993-10448-0
GAGTCGACCGAGTGGACATGAAATCTG-----------------------
SE-AT3G61860-XLOC_019993-10448-1
GAGTCGACCGAGTGGACATGAAATCTGGAACTTTTCTTCAAAGGAACAAG
CONSENSUS
GAGTCGACCGAGTGGACATGAAATCTG.......................

SE-AT3G61860-XLOC_019993-10448-0

SE-AT3G61860-XLOC_019993-10448-1
ATTTGCCATCTTCTTCTTCTTCCTGCAAAATCATTTCTACATTTTCATAC

CONSENSUS
 ....

SE-AT3G61860-XLOC_019993-10448-0

SE-AT3G61860-XLOC_019993-10448-1
 CTGCCTTTATCATCTTGTGCCTACCAATCTTCACTGACTCTAGAGAAACT
CONSENSUS
 ....

SE-AT3G61860-XLOC_019993-10448-0

SE-AT3G61860-XLOC_019993-10448-1
 TTGGAATTTGTCTGTCTATTACCAATCATCGATGCTTCAGATGGAAGTCT
CONSENSUS
 ....

SE-AT3G61860-XLOC_019993-10448-0

SE-AT3G61860-XLOC_019993-10448-1
 ATTGAATCCATCATTGCCATGCCTTGCCTTCCATATCTGAACTCCTCCAC
CONSENSUS
 ....

SE-AT3G61860-XLOC_019993-10448-0

SE-AT3G61860-XLOC_019993-10448-1
 TCAGAATGTTTAAGCATGCCTGGCAAATCACTCAGATTTTTTAATATAAA
CONSENSUS
 ....

SE-AT3G61860-XLOC_019993-10448-0

SE-AT3G61860-XLOC_019993-10448-1
 ATTGGTGTTTTTAAAAATAAAAATCCTGAATTCCTGTGTTATGCTACAAA
CONSENSUS
 ....

SE-AT3G61860-XLOC_019993-10448-0

SE-AT3G61860-XLOC_019993-10448-1
 TCTTGTTTTTCTACTACATCATGGAATGCCTCTACATCTCTCATATTCAC
CONSENSUS
 ....

SE-AT3G61860-XLOC_019993-10448-0 --------------------
GATATGCTTTTGTGTACTTTGAGGATGAAC
SE-AT3G61860-XLOC_019993-10448-1
 CAGCCTTCACTTATATGAAGGATATGCTTTTGTGTACTTTGAGGATGAAC
CONSENSUS
 ....................GATATGCTTTTGTGTACTTTGAGGATGAAC

SE-AT3G61860-XLOC_019993-10448-0
 GTGATGCTGAAGACGCTATTCGCAAACTCGACAATTTTCCTTTTGGATAT
SE-AT3G61860-XLOC_019993-10448-1
 GTGATGCTGAAGACGCTATTCGCAAACTCGACAATTTTCCTTTTGGATAT

CONSENSUS
      GTGATGCTGAAGACGCTATTCGCAAACTCGACAATTTTCCTTTTGGATAT

SE-AT3G61860-XLOC_019993-10448-0
      GAGAAACGCAGGTTATCAGTTGAATGGGCAAAG
SE-AT3G61860-XLOC_019993-10448-1
      GAGAAACGCAGGTTATCAGTTGAATGGGCAAAG
CONSENSUS
      GAGAAACGCAGGTTATCAGTTGAATGGGCAAAG

alignment for event: A3-AT3G09350-XLOC_013981-1100

A3-AT3G09350-XLOC_013981-1100-0
      AAGATTTTTAAGAATGTTGTAGTAGCTTCGCGAATAATCTCAGGTCTTCT
A3-AT3G09350-XLOC_013981-1100-1
      AAGATTTTTAAGAATGTTGTAGTAGCTTCGCGAATAATCTCAGGTCTTCT
CONSENSUS
      AAGATTTTTAAGAATGTTGTAGTAGCTTCGCGAATAATCTCAGGTCTTCT

A3-AT3G09350-XLOC_013981-1100-0
      ATAAAACCAAACTAGCGATTAGCCGACTCTTCCTCTTTATTATTACAAAG
A3-AT3G09350-XLOC_013981-1100-1
      ATAAAACCAAACTAGCGATTAGCCGACTCTTCCTCTTTATTATTACAAAG
CONSENSUS
      ATAAAACCAAACTAGCGATTAGCCGACTCTTCCTCTTTATTATTACAAAG

A3-AT3G09350-XLOC_013981-1100-0
      ACGAAAAGGCTTCGAGTTTTGTAATTTCTTCTTCTGTTACAAGTTCTTTT
A3-AT3G09350-XLOC_013981-1100-1
      ACGAAAAGGCTTCGAGTTTTGTAATTTCTTCTTCTGTTACAAGTTCTTTT
CONSENSUS
      ACGAAAAGGCTTCGAGTTTTGTAATTTCTTCTTCTGTTACAAGTTCTTTT

A3-AT3G09350-XLOC_013981-1100-0
      CACTGTGAAAGTAATTCTTCAATGGCGAAAGACGGACCTAATTGGGATGG
A3-AT3G09350-XLOC_013981-1100-1
      CACTGTGAAAGTAATTCTTCAATGGCGAAAGACGGACCTAATTGGGATGG
CONSENSUS
      CACTGTGAAAGTAATTCTTCAATGGCGAAAGACGGACCTAATTGGGATGG

A3-AT3G09350-XLOC_013981-1100-0
      TTTGCTTAAGTGGAGTCTCTCTCACGCCGACGGTACTCGACCCACTCGCC
A3-AT3G09350-XLOC_013981-1100-1
      TTTGCTTAAGTGGAGTCTCTCTCACGCCGACGGTACTCGACCCACTCGCC
CONSENSUS
      TTTGCTTAAGTGGAGTCTCTCTCACGCCGACGGTACTCGACCCACTCGCC

A3-AT3G09350-XLOC_013981-1100-0
      AGTTGAGGTATCTGTGTGGCTATGATTGAGCTTTCACTTGAGGAGATGAA
A3-AT3G09350-XLOC_013981-1100-1
      AGTTGAG---
CONSENSUS
      AGTTGAG...

A3-AT3G09350-XLOC_013981-1100-0
      TCTTATGAGTGAATTCGAATCTGGGTTTGTTTAAAGTAGTTTTCTTTTTT
A3-AT3G09350-XLOC_013981-1100-1

CONSENSUS
      ....

A3-AT3G09350-XLOC_013981-1100-0
      GAAGATATCTGTTCAGTTCATCGGTTTGGTTAAGATTCAATTATCGAATT
A3-AT3G09350-XLOC_013981-1100-1

CONSENSUS
      ....

A3-AT3G09350-XLOC_013981-1100-0
      CTGCTGTTATGTAGGTGAAAGGTTATCTATTTTCTCGTTTTTACATGAAA
A3-AT3G09350-XLOC_013981-1100-1

CONSENSUS
      ....

A3-AT3G09350-XLOC_013981-1100-0
      CTCTGTTAATTTTTTTTAAGGGAATTCACGATTTGTTCATGTCAAATCAT
A3-AT3G09350-XLOC_013981-1100-1

CONSENSUS
      ....

A3-AT3G09350-XLOC_013981-1100-0
      TCTGTATGTGTTTTATTGTGGATTGTTTTGTTGATTTGAGTTGTTTTAAT
A3-AT3G09350-XLOC_013981-1100-1

CONSENSUS
      ....

A3-AT3G09350-XLOC_013981-1100-0
      GAGACAGTGAGGAGGATCGAAAATGGTTTATGGAGGCTATGCAATCGCAG
A3-AT3G09350-XLOC_013981-1100-1 -------
TGAGGAGGATCGAAAATGGTTTATGGAGGCTATGCAATCGCAG
CONSENSUS
      .......TGAGGAGGATCGAAAATGGTTTATGGAGGCTATGCAATCGCAG

A3-AT3G09350-XLOC_013981-1100-0
      ACTGTAGATGTAGTCAAACGCATGAAGGAGATTACACTAGTTATGCAAAC
A3-AT3G09350-XLOC_013981-1100-1
      ACTGTAGATGTAGTCAAACGCATGAAGGAGATTACACTAGTTATGCAAAC
CONSENSUS
      ACTGTAGATGTAGTCAAACGCATGAAGGAGATTACACTAGTTATGCAAAC

A3-AT3G09350-XLOC_013981-1100-0
      ACCTGAACAAGTTTTGGTGGAACATGGAGTAACACCTGAAGATATCCAAG
A3-AT3G09350-XLOC_013981-1100-1
      ACCTGAACAAGTTTTGGTGGAACATGGAGTAACACCTGAAGATATCCAAG
CONSENSUS
      ACCTGAACAAGTTTTGGTGGAACATGGAGTAACACCTGAAGATATCCAAG

alignment for event: A3-AT3G51550-XLOC_019400-1449

A3-AT3G51550-XLOC_019400-1449-0
ACTCATTAATTGATTTTCTCTCTCTCTCCCCCAAAATATCTCTGTCTTCT
A3-AT3G51550-XLOC_019400-1449-1
ACTCATTAATTGATTTTCTCTCTCTCTCCCCCAAAATATCTCTGTCTTCT
CONSENSUS
ACTCATTAATTGATTTTCTCTCTCTCTCCCCCAAAATATCTCTGTCTTCT

A3-AT3G51550-XLOC_019400-1449-0
CCAAAAACTCTCTCCGATTTCATCGCTTAGGGTTTCTTCCCCGATTCTTC
A3-AT3G51550-XLOC_019400-1449-1
CCAAAAACTCTCTCCGATTTCATCGCTTAGGGTTTCTTCCCCGATTCTTC
CONSENSUS
CCAAAAACTCTCTCCGATTTCATCGCTTAGGGTTTCTTCCCCGATTCTTC

A3-AT3G51550-XLOC_019400-1449-0
AG--
A3-AT3G51550-XLOC_019400-1449-1
AGATCTGAGAAGAAGATCTTCCCGGAGAAGTGCTCTTGATCGATGAAGAT
CONSENSUS
AG..

A3-AT3G51550-XLOC_019400-1449-0

A3-AT3G51550-XLOC_019400-1449-1
CACAGAGGGACGATTCCGTCTCTCTCTTCTTCTTCTTCTTCTTCTCATAT
CONSENSUS
....

A3-AT3G51550-XLOC_019400-1449-0

A3-AT3G51550-XLOC_019400-1449-1
CTGCAGCAACTTTAATCTCAGCTGCTGATTACTCTCCAACAGAGAAAATC
CONSENSUS
....

A3-AT3G51550-XLOC_019400-1449-0

A3-AT3G51550-XLOC_019400-1449-1
CTATTGAATTGCGGTGGTGGTGCTTCTAATCTAACCGACACAGATAACCG
CONSENSUS
....

A3-AT3G51550-XLOC_019400-1449-0

A3-AT3G51550-XLOC_019400-1449-1
TATATGGATCTCCGATGTCAAATCAAAATTCTTATCATCTTCCTCTGAAG
CONSENSUS
....

A3-AT3G51550-XLOC_019400-1449-0

A3-AT3G51550-XLOC_019400-1449-1

ACTCTAAAACATCACCAGCGTTAACACAAGATCCTTCCGTTCCCGAAGTT
CONSENSUS
....

A3-AT3G51550-XLOC_019400-1449-0

A3-AT3G51550-XLOC_019400-1449-1
CCTTACATGACGGCGAGAGTTTTCCGATCTCCTTTCACTTACACTTTCCC
CONSENSUS
....

A3-AT3G51550-XLOC_019400-1449-0

A3-AT3G51550-XLOC_019400-1449-1
TGTAGCATCAGGTCGTAAATTCGTGCGTCTCTACTTCTACCCAAACTCGT
CONSENSUS
....

A3-AT3G51550-XLOC_019400-1449-0

A3-AT3G51550-XLOC_019400-1449-1
ACGACGGTCTCAACGCTACCAACTCGTTATTCTCCGTCTCCTTTGGTCCT
CONSENSUS
....

A3-AT3G51550-XLOC_019400-1449-0

A3-AT3G51550-XLOC_019400-1449-1
TACACTCTTCTCAAGAATTTCAGTGCTTCTCAGACGGCGGAGGCGTTGAC
CONSENSUS
....

A3-AT3G51550-XLOC_019400-1449-0

A3-AT3G51550-XLOC_019400-1449-1
TTACGCTTTCATCATCAAGGAGTTTGTTGTCAACGTTGAAGGTGGAACGT
CONSENSUS
....

A3-AT3G51550-XLOC_019400-1449-0

A3-AT3G51550-XLOC_019400-1449-1
TGAACATGACGTTTACACCGGAATCAGCTCCGTCTAATGCGTATGCGTTT
CONSENSUS
....

A3-AT3G51550-XLOC_019400-1449-0

A3-AT3G51550-XLOC_019400-1449-1
GTTAATGGGATTGAGGTTACTTCAATGCCTGATATGTATAGTAGTACTGA
CONSENSUS
....

A3-AT3G51550-XLOC_019400-1449-0

A3-AT3G51550-XLOC_019400-1449-1

TGGGACTTTGACTATGGTTGGATCATCTGGCTCTGTTACTATTGATAACA
CONSENSUS
....

A3-AT3G51550-XLOC_019400-1449-0

A3-AT3G51550-XLOC_019400-1449-1
GTACTGCTCTTGAGAATGTGTATAGGCTCAATGTTGGAGGGAATGATATC
CONSENSUS
....

A3-AT3G51550-XLOC_019400-1449-0

A3-AT3G51550-XLOC_019400-1449-1
TCGCCTTCCGCGGATACGGGTTTGTATAGGTCGTGGTATGATGATCAGCC
CONSENSUS
....

A3-AT3G51550-XLOC_019400-1449-0 -----------------
GACTTGGTATTCCAGAGACTGCTGATCCCAACA
A3-AT3G51550-XLOC_019400-1449-1
TTATATATTTGGTGCAGGACTTGGTATTCCAGAGACTGCTGATCCCAACA
CONSENSUS
.................GACTTGGTATTCCAGAGACTGCTGATCCCAACA

A3-AT3G51550-XLOC_019400-1449-0
TGACGATTAAGTATCCTACGGGGACTCCTACTTATGTTGCTCCTGTGGAT
A3-AT3G51550-XLOC_019400-1449-1
TGACGATTAAGTATCCTACGGGGACTCCTACTTATGTTGCTCCTGTGGAT
CONSENSUS
TGACGATTAAGTATCCTACGGGGACTCCTACTTATGTTGCTCCTGTGGAT

A3-AT3G51550-XLOC_019400-1449-0
GTTTATTCAACCGCGAGGTCTATGGGTCCAACAGCTCAGATCAATCTCAA
A3-AT3G51550-XLOC_019400-1449-1
GTTTATTCAACCGCGAGGTCTATGGGTCCAACAGCTCAGATCAATCTCAA
CONSENSUS
GTTTATTCAACCGCGAGGTCTATGGGTCCAACAGCTCAGATCAATCTCAA

A3-AT3G51550-XLOC_019400-1449-0
CTACAATCTTACTTGGATTTTCAGCATTGACTCTGGTTTCACTTACCTTG
A3-AT3G51550-XLOC_019400-1449-1
CTACAATCTTACTTGGATTTTCAGCATTGACTCTGGTTTCACTTACCTTG
CONSENSUS
CTACAATCTTACTTGGATTTTCAGCATTGACTCTGGTTTCACTTACCTTG

A3-AT3G51550-XLOC_019400-1449-0
TTAGACTTCATTTCTGTGAGGTTTCTTCGAATATCACTAAGATCAACCAA
A3-AT3G51550-XLOC_019400-1449-1
TTAGACTTCATTTCTGTGAGGTTTCTTCGAATATCACTAAGATCAACCAA
CONSENSUS
TTAGACTTCATTTCTGTGAGGTTTCTTCGAATATCACTAAGATCAACCAA

A3-AT3G51550-XLOC_019400-1449-0
CGGGTGTTTACAATCTACCTCAACAATCAAACTGCTGAGCCTGAAGCTGA
A3-AT3G51550-XLOC_019400-1449-1

CGGGTGTTTACAATCTACCTCAACAATCAAACTGCTGAGCCTGAAGCTGA
CONSENSUS
CGGGTGTTTACAATCTACCTCAACAATCAAACTGCTGAGCCTGAAGCTGA

A3-AT3G51550-XLOC_019400-1449-0
TGTGATTGCTTGGACTAGTTCAAACGGGGTTCCGTTTCACAAGGATTACG
A3-AT3G51550-XLOC_019400-1449-1
TGTGATTGCTTGGACTAGTTCAAACGGGGTTCCGTTTCACAAGGATTACG
CONSENSUS
TGTGATTGCTTGGACTAGTTCAAACGGGGTTCCGTTTCACAAGGATTACG

A3-AT3G51550-XLOC_019400-1449-0
TGGTGAATCCTCCAGAGGGAAATGGACAGCAAGATTTGTGGCTTGCTCTT
A3-AT3G51550-XLOC_019400-1449-1
TGGTGAATCCTCCAGAGGGAAATGGACAGCAAGATTTGTGGCTTGCTCTT
CONSENSUS
TGGTGAATCCTCCAGAGGGAAATGGACAGCAAGATTTGTGGCTTGCTCTT

A3-AT3G51550-XLOC_019400-1449-0
CATCCTAACCCAGTTAACAAGCCGGAGTATTATGATTCTCTTCTTAATGG
A3-AT3G51550-XLOC_019400-1449-1
CATCCTAACCCAGTTAACAAGCCGGAGTATTATGATTCTCTTCTTAATGG
CONSENSUS
CATCCTAACCCAGTTAACAAGCCGGAGTATTATGATTCTCTTCTTAATGG

A3-AT3G51550-XLOC_019400-1449-0
AGTGGAGATATTCAAGATGAATACTTCTGATGGTAATCTGGCTGGTACCA
A3-AT3G51550-XLOC_019400-1449-1
AGTGGAGATATTCAAGATGAATACTTCTGATGGTAATCTGGCTGGTACCA
CONSENSUS
AGTGGAGATATTCAAGATGAATACTTCTGATGGTAATCTGGCTGGTACCA

A3-AT3G51550-XLOC_019400-1449-0
ATCCTATACCTGGTCCACAGGTGACTGCTGATCCATCTAAAGTCCTACGC
A3-AT3G51550-XLOC_019400-1449-1
ATCCTATACCTGGTCCACAGGTGACTGCTGATCCATCTAAAGTCCTACGC
CONSENSUS
ATCCTATACCTGGTCCACAGGTGACTGCTGATCCATCTAAAGTCCTACGC

A3-AT3G51550-XLOC_019400-1449-0
CCGACTACTAGGAAATCGAAAAGCAATACGGCTATTATTGCAGGCGCAGC
A3-AT3G51550-XLOC_019400-1449-1
CCGACTACTAGGAAATCGAAAAGCAATACGGCTATTATTGCAGGCGCAGC
CONSENSUS
CCGACTACTAGGAAATCGAAAAGCAATACGGCTATTATTGCAGGCGCAGC

A3-AT3G51550-XLOC_019400-1449-0
CAGTGGTGCAGTTGTTCTGGCCCTTATCATTGGGTTTTGTGTGTTTGGTG
A3-AT3G51550-XLOC_019400-1449-1
CAGTGGTGCAGTTGTTCTGGCCCTTATCATTGGGTTTTGTGTGTTTGGTG
CONSENSUS
CAGTGGTGCAGTTGTTCTGGCCCTTATCATTGGGTTTTGTGTGTTTGGTG

A3-AT3G51550-XLOC_019400-1449-0
CTTACCGCAGACGTAAGCGTGGTGATTACCAGCCTGCTAGTGATGCAACA
A3-AT3G51550-XLOC_019400-1449-1

CTTACCGCAGACGTAAGCGTGGTGATTACCAGCCTGCTAGTGATGCAACA
CONSENSUS
CTTACCGCAGACGTAAGCGTGGTGATTACCAGCCTGCTAGTGATGCAACA

A3-AT3G51550-XLOC_019400-1449-0
TCAGGGTGGCTTCCACTATCTCTGTATGGAAACTCACATTCTGCTGGCTC
A3-AT3G51550-XLOC_019400-1449-1
TCAGGGTGGCTTCCACTATCTCTGTATGGAAACTCACATTCTGCTGGCTC
CONSENSUS
TCAGGGTGGCTTCCACTATCTCTGTATGGAAACTCACATTCTGCTGGCTC

A3-AT3G51550-XLOC_019400-1449-0
GGCGAAGACAAACACAACAGGAAGTTATGCCTCGTCCCTTCCGTCAAATC
A3-AT3G51550-XLOC_019400-1449-1
GGCGAAGACAAACACAACAGGAAGTTATGCCTCGTCCCTTCCGTCAAATC
CONSENSUS
GGCGAAGACAAACACAACAGGAAGTTATGCCTCGTCCCTTCCGTCAAATC

A3-AT3G51550-XLOC_019400-1449-0
TTTGCCGTCACTTCTCGTTTGCTGAGATCAAAGCTGCCACTAAAAACTTT
A3-AT3G51550-XLOC_019400-1449-1
TTTGCCGTCACTTCTCGTTTGCTGAGATCAAAGCTGCCACTAAAAACTTT
CONSENSUS
TTTGCCGTCACTTCTCGTTTGCTGAGATCAAAGCTGCCACTAAAAACTTT

A3-AT3G51550-XLOC_019400-1449-0
GATGAGTCCCGGGTGCTTGGTGTTGGTGGTTTCGGCAAGGTTTACAGAGG
A3-AT3G51550-XLOC_019400-1449-1
GATGAGTCCCGGGTGCTTGGTGTTGGTGGTTTCGGCAAGGTTTACAGAGG
CONSENSUS
GATGAGTCCCGGGTGCTTGGTGTTGGTGGTTTCGGCAAGGTTTACAGAGG

A3-AT3G51550-XLOC_019400-1449-0
AGAGATTGATGGCGGAACTACAAAGGTAGCCATCAAGAGAGGCAACCCAA
A3-AT3G51550-XLOC_019400-1449-1
AGAGATTGATGGCGGAACTACAAAGGTAGCCATCAAGAGAGGCAACCCAA
CONSENSUS
AGAGATTGATGGCGGAACTACAAAGGTAGCCATCAAGAGAGGCAACCCAA

A3-AT3G51550-XLOC_019400-1449-0
TGTCCGAGCAAGGTGTACATGAGTTCCAGACTGAGATTGAAATGCTTTCA
A3-AT3G51550-XLOC_019400-1449-1
TGTCCGAGCAAGGTGTACATGAGTTCCAGACTGAGATTGAAATGCTTTCA
CONSENSUS
TGTCCGAGCAAGGTGTACATGAGTTCCAGACTGAGATTGAAATGCTTTCA

A3-AT3G51550-XLOC_019400-1449-0
AAGCTTAGACATCGTCATCTTGTGTCCTTGATTGGATACTGTGAAGAGAA
A3-AT3G51550-XLOC_019400-1449-1
AAGCTTAGACATCGTCATCTTGTGTCCTTGATTGGATACTGTGAAGAGAA
CONSENSUS
AAGCTTAGACATCGTCATCTTGTGTCCTTGATTGGATACTGTGAAGAGAA

A3-AT3G51550-XLOC_019400-1449-0
CTGCGAAATGATCTTAGTCTATGATTACATGGCTCATGGTACAATGAGGG
A3-AT3G51550-XLOC_019400-1449-1

CTGCGAAATGATCTTAGTCTATGATTACATGGCTCATGGTACAATGAGGG
CONSENSUS
CTGCGAAATGATCTTAGTCTATGATTACATGGCTCATGGTACAATGAGGG

A3-AT3G51550-XLOC_019400-1449-0
AGCATCTCTACAAAACCCAGAATCCTTCTCTTCCATGGAAGCAACGTCTT
A3-AT3G51550-XLOC_019400-1449-1
AGCATCTCTACAAAACCCAGAATCCTTCTCTTCCATGGAAGCAACGTCTT
CONSENSUS
AGCATCTCTACAAAACCCAGAATCCTTCTCTTCCATGGAAGCAACGTCTT

A3-AT3G51550-XLOC_019400-1449-0
GAGATATGCATTGGAGCAGCCCGAGGTTTACACTATCTACACACTGGTGC
A3-AT3G51550-XLOC_019400-1449-1
GAGATATGCATTGGAGCAGCCCGAGGTTTACACTATCTACACACTGGTGC
CONSENSUS
GAGATATGCATTGGAGCAGCCCGAGGTTTACACTATCTACACACTGGTGC

A3-AT3G51550-XLOC_019400-1449-0
AAAACACACAATCATCCATAGAGATGTGAAGACAACAAACATTCTATTGG
A3-AT3G51550-XLOC_019400-1449-1
AAAACACACAATCATCCATAGAGATGTGAAGACAACAAACATTCTATTGG
CONSENSUS
AAAACACACAATCATCCATAGAGATGTGAAGACAACAAACATTCTATTGG

A3-AT3G51550-XLOC_019400-1449-0
ATGAGAAATGGGTGGCCAAGGTCTCTGATTTTGGTCTATCGAAGACTGGT
A3-AT3G51550-XLOC_019400-1449-1
ATGAGAAATGGGTGGCCAAGGTCTCTGATTTTGGTCTATCGAAGACTGGT
CONSENSUS
ATGAGAAATGGGTGGCCAAGGTCTCTGATTTTGGTCTATCGAAGACTGGT

A3-AT3G51550-XLOC_019400-1449-0
CCTACACTAGACCACACACACGTAAGCACAGTTGTGAAAGGAAGTTTCGG
A3-AT3G51550-XLOC_019400-1449-1
CCTACACTAGACCACACACACGTAAGCACAGTTGTGAAAGGAAGTTTCGG
CONSENSUS
CCTACACTAGACCACACACACGTAAGCACAGTTGTGAAAGGAAGTTTCGG

A3-AT3G51550-XLOC_019400-1449-0
TTATCTTGACCCAGAGTATTTCAGACGGCAGCAACTGACTGAGAAATCCG
A3-AT3G51550-XLOC_019400-1449-1
TTATCTTGACCCAGAGTATTTCAGACGGCAGCAACTGACTGAGAAATCCG
CONSENSUS
TTATCTTGACCCAGAGTATTTCAGACGGCAGCAACTGACTGAGAAATCCG

A3-AT3G51550-XLOC_019400-1449-0
ATGTCTACTCCTTTGGCGTTGTTCTATTCGAAGCTCTATGCGCTCGTCCA
A3-AT3G51550-XLOC_019400-1449-1
ATGTCTACTCCTTTGGCGTTGTTCTATTCGAAGCTCTATGCGCTCGTCCA
CONSENSUS
ATGTCTACTCCTTTGGCGTTGTTCTATTCGAAGCTCTATGCGCTCGTCCA

A3-AT3G51550-XLOC_019400-1449-0
GCCTTGAACCCAACACTTGCAAAGGAACAAGTGAGCTTAGCTGAGTGGGC
A3-AT3G51550-XLOC_019400-1449-1

GCCTTGAACCCAACACTTGCAAAGGAACAAGTGAGCTTAGCTGAGTGGGC
CONSENSUS
GCCTTGAACCCAACACTTGCAAAGGAACAAGTGAGCTTAGCTGAGTGGGC

A3-AT3G51550-XLOC_019400-1449-0
ACCATACTGCTACAAGAAAGGCATGCTAGATCAAATCGTTGATCCCTACC
A3-AT3G51550-XLOC_019400-1449-1
ACCATACTGCTACAAGAAAGGCATGCTAGATCAAATCGTTGATCCCTACC
CONSENSUS
ACCATACTGCTACAAGAAAGGCATGCTAGATCAAATCGTTGATCCCTACC

A3-AT3G51550-XLOC_019400-1449-0
TCAAGGGCAAGATCACACCAGAATGCTTCAAAAAGTTTGCTGAAACCGCG
A3-AT3G51550-XLOC_019400-1449-1
TCAAGGGCAAGATCACACCAGAATGCTTCAAAAAGTTTGCTGAAACCGCG
CONSENSUS
TCAAGGGCAAGATCACACCAGAATGCTTCAAAAAGTTTGCTGAAACCGCG

A3-AT3G51550-XLOC_019400-1449-0
ATGAAGTGTGTTCTAGACCAGGGCATTGAGAGACCATCAATGGGAGATGT
A3-AT3G51550-XLOC_019400-1449-1
ATGAAGTGTGTTCTAGACCAGGGCATTGAGAGACCATCAATGGGAGATGT
CONSENSUS
ATGAAGTGTGTTCTAGACCAGGGCATTGAGAGACCATCAATGGGAGATGT

A3-AT3G51550-XLOC_019400-1449-0
TCTGTGGAACTTAGAATTTGCGTTGCAGCTCCAGGAAAGCGCAGAAGAGA
A3-AT3G51550-XLOC_019400-1449-1
TCTGTGGAACTTAGAATTTGCGTTGCAGCTCCAGGAAAGCGCAGAAGAGA
CONSENSUS
TCTGTGGAACTTAGAATTTGCGTTGCAGCTCCAGGAAAGCGCAGAAGAGA

A3-AT3G51550-XLOC_019400-1449-0
ACGGAAAAGGAGTATGCGGTGACATGGACATGGATGAGATTAAGTACGAT
A3-AT3G51550-XLOC_019400-1449-1
ACGGAAAAGGAGTATGCGGTGACATGGACATGGATGAGATTAAGTACGAT
CONSENSUS
ACGGAAAAGGAGTATGCGGTGACATGGACATGGATGAGATTAAGTACGAT

A3-AT3G51550-XLOC_019400-1449-0
GATGGAAACTGTAAAGGAAAGAACGACAAGAGTTCTGATGTGTATGAAGG
A3-AT3G51550-XLOC_019400-1449-1
GATGGAAACTGTAAAGGAAAGAACGACAAGAGTTCTGATGTGTATGAAGG
CONSENSUS
GATGGAAACTGTAAAGGAAAGAACGACAAGAGTTCTGATGTGTATGAAGG

A3-AT3G51550-XLOC_019400-1449-0
GAATGTGACGGACTCGAGGAGCAGTGGAATAGATATGAGCATCGGTGGTA
A3-AT3G51550-XLOC_019400-1449-1
GAATGTGACGGACTCGAGGAGCAGTGGAATAGATATGAGCATCGGTGGTA
CONSENSUS
GAATGTGACGGACTCGAGGAGCAGTGGAATAGATATGAGCATCGGTGGTA

A3-AT3G51550-XLOC_019400-1449-0
GGAGTTTGGCCAGCGAAGATTCAGATGGACTCACTCCAAGTGCTGTGTTT
A3-AT3G51550-XLOC_019400-1449-1

GGAGTTTGGCCAGCGAAGATTCAGATGGACTCACTCCAAGTGCTGTGTTT
CONSENSUS
GGAGTTTGGCCAGCGAAGATTCAGATGGACTCACTCCAAGTGCTGTGTTT

A3-AT3G51550-XLOC_019400-1449-0
TCTCAGATCATGAATCCAAAGGGACGTTAGAGAAGTCTTATAACACGGTA
A3-AT3G51550-XLOC_019400-1449-1
TCTCAGATCATGAATCCAAAGGGACGTTAGAGAAGTCTTATAACACGGTA
CONSENSUS
TCTCAGATCATGAATCCAAAGGGACGTTAGAGAAGTCTTATAACACGGTA

A3-AT3G51550-XLOC_019400-1449-0
CAACACTACTACCTTTCTAAACCGGCTCCATCCAAAGGAGACCGGTTCGT
A3-AT3G51550-XLOC_019400-1449-1
CAACACTACTACCTTTCTAAACCGGCTCCATCCAAAGGAGACCGGTTCGT
CONSENSUS
CAACACTACTACCTTTCTAAACCGGCTCCATCCAAAGGAGACCGGTTCGT

A3-AT3G51550-XLOC_019400-1449-0
TTCTCTTTTTTATATTTTTTTTCCTCTTAACTATTTATTATTATTCTCTT
A3-AT3G51550-XLOC_019400-1449-1
TTCTCTTTTTTATATTTTTTTTCCTCTTAACTATTTATTATTATTCTCTT
CONSENSUS
TTCTCTTTTTTATATTTTTTTTCCTCTTAACTATTTATTATTATTCTCTT

A3-AT3G51550-XLOC_019400-1449-0
TGCTGTTATTTATTTAGAATTTTGGATTTGTGTATTTGATGCCACGATGG
A3-AT3G51550-XLOC_019400-1449-1
TGCTGTTATTTATTTAGAATTTTGGATTTGTGTATTTGATGCCACGATGG
CONSENSUS
TGCTGTTATTTATTTAGAATTTTGGATTTGTGTATTTGATGCCACGATGG

A3-AT3G51550-XLOC_019400-1449-0
AGAGTGTAAAAAATAATGTATTTTTTTTTCTTCTTTCTACAAAAACATTA
A3-AT3G51550-XLOC_019400-1449-1
AGAGTGTAAAAAATAATGTATTTTTTTTTCTTCTTTCTACAAAAACATTA
CONSENSUS
AGAGTGTAAAAAATAATGTATTTTTTTTTCTTCTTTCTACAAAAACATTA

A3-AT3G51550-XLOC_019400-1449-0
AACATGAATCGCGTCAATGTACTATTTGGTTTGTTATCTATAACGTTTTT
A3-AT3G51550-XLOC_019400-1449-1
AACATGAATCGCGTCAATGTACTATTTGGTTTGTTATCTATAACGTTTTT
CONSENSUS
AACATGAATCGCGTCAATGTACTATTTGGTTTGTTATCTATAACGTTTTT

A3-AT3G51550-XLOC_019400-1449-0 GGTAATTGTTGCATCTTTACTCT
A3-AT3G51550-XLOC_019400-1449-1 GGTAATTGTTGCATCTTTACTCT
CONSENSUS GGTAATTGTTGCATCTTTACTCT

alignment for event: RI-AT3G53100-XLOC_019494-3571

RI-AT3G53100-XLOC_019494-3571-0
CCGAGTACCTTGGTTTCTCTTCATACCCACCTGCATTCCTAAGCCGGGAA

RI-AT3G53100-XLOC_019494-3571-1
      CCGAGTACCTTGGTTTCTCTTCATACCCACCTGCATTCCTAAGCCGGGAA
CONSENSUS
      CCGAGTACCTTGGTTTCTCTTCATACCCACCTGCATTCCTAAGCCGGGAA

RI-AT3G53100-XLOC_019494-3571-0
      GCAAGCAACGAGAATATCCTGATCGGTGCCAACTTTGCATCAGCTTCTTC
RI-AT3G53100-XLOC_019494-3571-1
      GCAAGCAACGAGAATATCCTGATCGGTGCCAACTTTGCATCAGCTTCTTC
CONSENSUS
      GCAAGCAACGAGAATATCCTGATCGGTGCCAACTTTGCATCAGCTTCTTC

RI-AT3G53100-XLOC_019494-3571-0
      AGGATACTATGATGCCACATCTGTTCCATTTGTAAAGGAGACAACACAGT
RI-AT3G53100-XLOC_019494-3571-1
      AGGATACTATGATGCCACATCTGTTCCATTT-------------------
CONSENSUS
      AGGATACTATGATGCCACATCTGTTCCATTT...................

RI-AT3G53100-XLOC_019494-3571-0
      CAAACATCATTACACACATACCCAAGAAATGATCCATAAGGTTTTACTCT
RI-AT3G53100-XLOC_019494-3571-1

CONSENSUS
      ....

RI-AT3G53100-XLOC_019494-3571-0
      TCCTTTTGCAGGGCTCGATCTCCTTAACACGACAGCTGAGCTATTACAGG
RI-AT3G53100-XLOC_019494-3571-1 -----------
GGCTCGATCTCCTTAACACGACAGCTGAGCTATTACAGG
CONSENSUS
      ...........GGCTCGATCTCCTTAACACGACAGCTGAGCTATTACAGG

RI-AT3G53100-XLOC_019494-3571-0
      GCGTATCAAAATAGAGTAACGAGAATGATCGGGAGGGGGAACGCGCGCAT
RI-AT3G53100-XLOC_019494-3571-1
      GCGTATCAAAATAGAGTAACGAGAATGATCGGGAGGGGGAACGCGCGCAT
CONSENSUS
      GCGTATCAAAATAGAGTAACGAGAATGATCGGGAGGGGGAACGCGCGCAT

RI-AT3G53100-XLOC_019494-3571-0
      ACTGTTCTCTAGAGGTATCCACATTTTGAGTGCGGGATCTAGCGACTTTC
RI-AT3G53100-XLOC_019494-3571-1
      ACTGTTCTCTAGAGGTATCCACATTTTGAGTGCGGGATCTAGCGACTTTC
CONSENSUS
      ACTGTTCTCTAGAGGTATCCACATTTTGAGTGCGGGATCTAGCGACTTTC

RI-AT3G53100-XLOC_019494-3571-0
      TTCAGAATTATTACATCAATCCTCTACTCAACATCCTAAATACACCTGAT
RI-AT3G53100-XLOC_019494-3571-1
      TTCAGAATTATTACATCAATCCTCTACTCAACATCCTAAATACACCTGAT
CONSENSUS
      TTCAGAATTATTACATCAATCCTCTACTCAACATCCTAAATACACCTGAT

RI-AT3G53100-XLOC_019494-3571-0
      CAGTTCGCTGACATTCTCCTGAGATCCTTCTCAGAATTCATCCAG

RI-AT3G53100-XLOC_019494-3571-1
      CAGTTCGCTGACATTCTCCTGAGATCCTTCTCAGAATTCATCCAG
CONSENSUS
      CAGTTCGCTGACATTCTCCTGAGATCCTTCTCAGAATTCATCCAG

alignment for event: A5-AT3G52560-XLOC_019461-11442

A5-AT3G52560-XLOC_019461-11442-0
      TTCCGAGGAATTTTCGGTTGTTGGAGGAGCTTGAACGTGGGGAGAAAGGT
A5-AT3G52560-XLOC_019461-11442-1
      TTCCGAGGAATTTTCGGTTGTTGGAGGAGCTTGAACGTGGGGAGAAAGGT
CONSENSUS
      TTCCGAGGAATTTTCGGTTGTTGGAGGAGCTTGAACGTGGGGAGAAAGGT

A5-AT3G52560-XLOC_019461-11442-0
      ATTGGGGATGGAACTGTGAGCTATGGAATGGATGATGGAGATGACATCTA
A5-AT3G52560-XLOC_019461-11442-1
      ATTGGGGATGGAACTGTGAGCTATGGAATGGATGATGGAGATGACATCTA
CONSENSUS
      ATTGGGGATGGAACTGTGAGCTATGGAATGGATGATGGAGATGACATCTA

A5-AT3G52560-XLOC_019461-11442-0
      TATGCGTTCTTGGACTGGCACCATCATCGGTCCTCACAACGTAACTGTTC
A5-AT3G52560-XLOC_019461-11442-1
      TATGCGTTCTTGGACTGGCACCATCATCGGTCCTCACAAC---ACTGTTC
CONSENSUS
      TATGCGTTCTTGGACTGGCACCATCATCGGTCCTCACAAC...ACTGTTC

A5-AT3G52560-XLOC_019461-11442-0
      ATGAAGGTAGAATCTATCAGTTGAAGCTCTTTTGTGACAAAGATTACCCG
A5-AT3G52560-XLOC_019461-11442-1
      ATGAAGGTAGAATCTATCAGTTGAAGCTCTTTTGTGACAAAGATTACCCG
CONSENSUS
      ATGAAGGTAGAATCTATCAGTTGAAGCTCTTTTGTGACAAAGATTACCCG

A5-AT3G52560-XLOC_019461-11442-0
      GAGAAACCTCCAACTGTCCGATTCCATTCACGTGTCAACATGGCTTGTGT
A5-AT3G52560-XLOC_019461-11442-1
      GAGAAACCTCCAACTGTCCGATTCCATTCACGTGTCAACATGGCTTGTGT
CONSENSUS
      GAGAAACCTCCAACTGTCCGATTCCATTCACGTGTCAACATGGCTTGTGT

A5-AT3G52560-XLOC_019461-11442-0 CAACCACGAAACAGGAGTG
A5-AT3G52560-XLOC_019461-11442-1 CAACCACGAAACAGGAGTG
CONSENSUS   CAACCACGAAACAGGAGTG

alignment for event: RI-AT3G54500-XLOC_019571-12894

RI-AT3G54500-XLOC_019571-12894-0
      AAAAAAAACTCTTTTTTCTTTTTTCCACTGTAACTCCTTTCTCTTCCCAT
RI-AT3G54500-XLOC_019571-12894-1
      AAAAAAAACTCTTTTTTCTTTTTTCCACTGTAACTCCTTTCTCTTCCCAT

CONSENSUS
AAAAAAAACTCTTTTTTCTTTTTTCCACTGTAACTCCTTTCTCTTCCCAT

RI-AT3G54500-XLOC_019571-12894-0
ATCCTTTGAGCACGAAGGAGGATTCTGCTGAATCAAGGTTTTTCAAAGCT
RI-AT3G54500-XLOC_019571-12894-1
ATCCTTTGAGCACGAAGGAGGATTCTGCTGAATCAAG-------------
CONSENSUS
ATCCTTTGAGCACGAAGGAGGATTCTGCTGAATCAAG.............

RI-AT3G54500-XLOC_019571-12894-0
TGTTGCTTTTTCAGTTTAATATTCTTTTATTTATCGTTTTTTCCACTTTA
RI-AT3G54500-XLOC_019571-12894-1

CONSENSUS
....

RI-AT3G54500-XLOC_019571-12894-0
TTATTGTGTGTGGATTTACGTTATCCGATTTGTTGATCTTGTTGTTTTTT
RI-AT3G54500-XLOC_019571-12894-1

CONSENSUS
....

RI-AT3G54500-XLOC_019571-12894-0
CCTGGATTTTTTTCGTTGTGGAGCTGGCGATGTGTGGAGCAATCAGGGAG
RI-AT3G54500-XLOC_019571-12894-1 -----------------------
CTGGCGATGTGTGGAGCAATCAGGGAG
CONSENSUS
.......................CTGGCGATGTGTGGAGCAATCAGGGAG

RI-AT3G54500-XLOC_019571-12894-0
CTTGATTTTGGGTCTGTGATTCGATTTTGTTTCTCCAGTCATGTTTGATT
RI-AT3G54500-XLOC_019571-12894-1
CTTGATTTTGGGTCTGTGATTCGATTTTGTTTCTCCAGTCATGTTTGATT
CONSENSUS
CTTGATTTTGGGTCTGTGATTCGATTTTGTTTCTCCAGTCATGTTTGATT

RI-AT3G54500-XLOC_019571-12894-0 GGGAAGAAGAAGAG
RI-AT3G54500-XLOC_019571-12894-1 GGGAAGAAGAAGAG
CONSENSUS GGGAAGAAGAAGAG

alignment for event: A3-AT3G50240-XLOC_019322-2953

A3-AT3G50240-XLOC_019322-2953-0
GAAGATGGTGTAATTTTTTCAAAAGATGATGGCCTTAAAAGGGGTTTCGA
A3-AT3G50240-XLOC_019322-2953-1
GAAGATGGTGTAATTTTTTCAAAAGATGATGGCCTTAAAAGGGGTTTCGA
CONSENSUS
GAAGATGGTGTAATTTTTTCAAAAGATGATGGCCTTAAAAGGGGTTTCGA

A3-AT3G50240-XLOC_019322-2953-0
AAGCATGGATTCAGATTATGAAATGAGTGAAGCAACATCAG---------
A3-AT3G50240-XLOC_019322-2953-1

AAGCATGGATTCAGATTATGAAATGAGTGAAGCAACATCAGGTGGAATTT
CONSENSUS
AAGCATGGATTCAGATTATGAAATGAGTGAAGCAACATCAG.........

A3-AT3G50240-XLOC_019322-2953-0 ------
ATATTGGTGCAGCAGAAGAGTGGGAACATGCACTTAGGCAGAAT
A3-AT3G50240-XLOC_019322-2953-1
CTGAAGATATTGGTGCAGCAGAAGAGTGGGAACATGCACTTAGGCAGAAT
CONSENSUS
......ATATTGGTGCAGCAGAAGAGTGGGAACATGCACTTAGGCAGAAT

A3-AT3G50240-XLOC_019322-2953-0
AGCATGGGCAAGGAACTAAATGAACTAAGTAAACGTCTTGAGGAGAAAGA
A3-AT3G50240-XLOC_019322-2953-1
AGCATGGGCAAGGAACTAAATGAACTAAGTAAACGTCTTGAGGAGAAAGA
CONSENSUS
AGCATGGGCAAGGAACTAAATGAACTAAGTAAACGTCTTGAGGAGAAAGA

A3-AT3G50240-XLOC_019322-2953-0 G
A3-AT3G50240-XLOC_019322-2953-1 G
CONSENSUS   G

alignment for event: A5-AT3G15354-XLOC_017622-5884

A5-AT3G15354-XLOC_017622-5884-0
ATATGGGATGTTGCAAGAAGCCAGTTGGTTACAGAGATGAAGGAGCACAA
A5-AT3G15354-XLOC_017622-5884-1
ATATGGGATGTTGCAAGAAGCCAGTTGGTTACAGAGATGAAGGAGCACAA
CONSENSUS
ATATGGGATGTTGCAAGAAGCCAGTTGGTTACAGAGATGAAGGAGCACAA

A5-AT3G15354-XLOC_017622-5884-0
GAAGCGAGTATGGTCCATCGATATTTCATCAGCAGACCCGACTTTGCTGG
A5-AT3G15354-XLOC_017622-5884-1
GAAGCGAGTATGGTCCATCGATATTTCATCAGCAGACCCGACTTTGCTGG
CONSENSUS
GAAGCGAGTATGGTCCATCGATATTTCATCAGCAGACCCGACTTTGCTGG

A5-AT3G15354-XLOC_017622-5884-0
CTAGCGGAAGCGATGATGGAACCGTTAAGCTCTGGAGTATCAATCAGGCA
A5-AT3G15354-XLOC_017622-5884-1
CTAGCGGAAGCGATGATGGAACCGTTAAGCTCTGGAGTATCAATCAG---
CONSENSUS
CTAGCGGAAGCGATGATGGAACCGTTAAGCTCTGGAGTATCAATCAG...

A5-AT3G15354-XLOC_017622-5884-0
ATTCTAATTTGAAATGGAGTTAGCATTGGGACCATCAAGACAAAGGCCAA
A5-AT3G15354-XLOC_017622-5884-1 ---------------
GGAGTTAGCATTGGGACCATCAAGACAAAGGCCAA
CONSENSUS
...............GGAGTTAGCATTGGGACCATCAAGACAAAGGCCAA

A5-AT3G15354-XLOC_017622-5884-0
TGTATGCTGTGTCCAGTTTCCATCAGACTCCGGACGGTCTCTAGCATTTG

A5-AT3G15354-XLOC_017622-5884-1
      TGTATGCTGTGTCCAGTTTCCATCAGACTCCGGACGGTCTCTAGCATTTG
CONSENSUS
      TGTATGCTGTGTCCAGTTTCCATCAGACTCCGGACGGTCTCTAGCATTTG

A5-AT3G15354-XLOC_017622-5884-0
      GTTCTGCAGATCATAAAGTGTATTACTACGATCTTCGAAACCCCAAGATT
A5-AT3G15354-XLOC_017622-5884-1
      GTTCTGCAGATCATAAAGTGTATTACTACGATCTTCGAAACCCCAAGATT
CONSENSUS
      GTTCTGCAGATCATAAAGTGTATTACTACGATCTTCGAAACCCCAAGATT

A5-AT3G15354-XLOC_017622-5884-0
      CCTCTGTGCACAATGATTGGTCATAGCAAGACAGTGAGTTATGTCAAGTT
A5-AT3G15354-XLOC_017622-5884-1
      CCTCTGTGCACAATGATTGGTCATAGCAAGACAGTGAGTTATGTCAAGTT
CONSENSUS
      CCTCTGTGCACAATGATTGGTCATAGCAAGACAGTGAGTTATGTCAAGTT

A5-AT3G15354-XLOC_017622-5884-0
      TGTAGATTCATCCACTCTTGTGTCCTCTTCTACTGATAACACACTGAAGC
A5-AT3G15354-XLOC_017622-5884-1
      TGTAGATTCATCCACTCTTGTGTCCTCTTCTACTGATAACACACTGAAGC
CONSENSUS
      TGTAGATTCATCCACTCTTGTGTCCTCTTCTACTGATAACACACTGAAGC

A5-AT3G15354-XLOC_017622-5884-0
      TTTGGGACTTGTCGATGTCTGCTTCTGGGATTAATGAATCCCCTCTTCAC
A5-AT3G15354-XLOC_017622-5884-1
      TTTGGGACTTGTCGATGTCTGCTTCTGGGATTAATGAATCCCCTCTTCAC
CONSENSUS
      TTTGGGACTTGTCGATGTCTGCTTCTGGGATTAATGAATCCCCTCTTCAC

A5-AT3G15354-XLOC_017622-5884-0 TCATTCACTGGACACACTAATTTAAAG
A5-AT3G15354-XLOC_017622-5884-1 TCATTCACTGGACACACTAATTTAAAG
CONSENSUS   TCATTCACTGGACACACTAATTTAAAG

alignment for event: RI-AT3G23450-XLOC_014830-1194

RI-AT3G23450-XLOC_014830-1194-0
      GATCATCGGGCAGTGCACTCTGCAAATTAACAAACATACTTCAAAAAAAT
RI-AT3G23450-XLOC_014830-1194-1
      GATCATCGGGCAGTGCACTCTGCAAATTAACAAACATACTTCAAAAAAAT
CONSENSUS
      GATCATCGGGCAGTGCACTCTGCAAATTAACAAACATACTTCAAAAAAAT

RI-AT3G23450-XLOC_014830-1194-0
      ATGGGGCGTCTCGTTAGTGGTGCTACTCTTTTGGCTTTGTTATGTTTCCA
RI-AT3G23450-XLOC_014830-1194-1
      ATGGGGCGTCTCGTTAGTGGTGCTACTCTTTTGGCTTTGTTATGTTTCCA
CONSENSUS
      ATGGGGCGTCTCGTTAGTGGTGCTACTCTTTTGGCTTTGTTATGTTTCCA

RI-AT3G23450-XLOC_014830-1194-0

TGTTTTTGTGGTGAATGTTGTAGCGAGAGATGTGAGCTCGGGAAGAGATG
RI-AT3G23450-XLOC_014830-1194-1
TGTTTTTGTGGTGAATGTTGTAGCGAGAGATGTGAGCTCGGGAAGAGATG
CONSENSUS
TGTTTTTGTGGTGAATGTTGTAGCGAGAGATGTGAGCTCGGGAAGAGATG

RI-AT3G23450-XLOC_014830-1194-0
AGGATGAGAAGACGTTAGTTGGAGGAGGAAAGGGTGGTGGCTTTGGCGGT
RI-AT3G23450-XLOC_014830-1194-1
AGGATGAGAAGACGTTAGTTGGAGGAGGAAAGGGTGGTG-----------
CONSENSUS
AGGATGAGAAGACGTTAGTTGGAGGAGGAAAGGGTGGTG...........

RI-AT3G23450-XLOC_014830-1194-0
GGATTTGGAGGAGGAGCCGGTGGTGGAGTTGGAGGAGGAGCCGGTGGTGG
RI-AT3G23450-XLOC_014830-1194-1

CONSENSUS
....

RI-AT3G23450-XLOC_014830-1194-0
CTTTGGAGGAGGAGCAGGCGGTGGGTTTGGAGGAGGTGGTGGTGGAGGTG
RI-AT3G23450-XLOC_014830-1194-1

CONSENSUS
....

RI-AT3G23450-XLOC_014830-1194-0
GAGGTGGAGGTGGTGGTGGTGGAGGAGGCTTTGGTGGTGGAGGAGGTTTT
RI-AT3G23450-XLOC_014830-1194-1
---GTTTT
CONSENSUS
....GTTTT

RI-AT3G23450-XLOC_014830-1194-0
GGTGGTGGACATGGTGGAGGTGTTGGTGGTGGAGTTGGCGGTGGACATGG
RI-AT3G23450-XLOC_014830-1194-1
GGTGGTGGACATGGTGGAGGTGTTGGTGGTGGAGTTGGCGGTGGACATGG
CONSENSUS
GGTGGTGGACATGGTGGAGGTGTTGGTGGTGGAGTTGGCGGTGGACATGG

RI-AT3G23450-XLOC_014830-1194-0
CGGAGGAGTCGGTGGTGGGTTTGGAAAAGGTGGAGGGATCGGAGGTGGAA
RI-AT3G23450-XLOC_014830-1194-1
CGGAGGAGTCGGTGGTGGGTTTGGAAAAGGTGGAGGGATCGGAGGTGGAA
CONSENSUS
CGGAGGAGTCGGTGGTGGGTTTGGAAAAGGTGGAGGGATCGGAGGTGGAA

RI-AT3G23450-XLOC_014830-1194-0
TTGGAAAAGGTGGAGGAGTTGGCGGTGGCATCGGTAAAGGCGGCGGCATT
RI-AT3G23450-XLOC_014830-1194-1
TTGGAAAAGGTGGAGGAGTTGGCGGTGGCATCGGTAAAGGCGGCGGCATT
CONSENSUS
TTGGAAAAGGTGGAGGAGTTGGCGGTGGCATCGGTAAAGGCGGCGGCATT

RI-AT3G23450-XLOC_014830-1194-0

GGTGGTGGAATAGGCAAAGGTGGAGGAGTTGGCGGCGGTATCGGTAAAGG
RI-AT3G23450-XLOC_014830-1194-1
GGTGGTGGAATAGGCAAAGGTGGAGGAGTTGGCGGCGGTATCGGTAAAGG
CONSENSUS
GGTGGTGGAATAGGCAAAGGTGGAGGAGTTGGCGGCGGTATCGGTAAAGG

RI-AT3G23450-XLOC_014830-1194-0
TGGAGGTATCGGTGGCGGGATCGGCAAGGGCGGAGGTATCGGTGGCGGGA
RI-AT3G23450-XLOC_014830-1194-1
TGGAGGTATCGGTGGCGGGATCGGCAAGGGCGGAGGTATCGGTGGCGGGA
CONSENSUS
TGGAGGTATCGGTGGCGGGATCGGCAAGGGCGGAGGTATCGGTGGCGGGA

RI-AT3G23450-XLOC_014830-1194-0
TCGGCAAGGGTGGAGGTATCGGTGGCGGGATCGGCAAGGGTGGAGGTATT
RI-AT3G23450-XLOC_014830-1194-1
TCGGCAAGGGTGGAGGTATCGGTGGCGGGATCGGCAAGGGTGGAGGTATT
CONSENSUS
TCGGCAAGGGTGGAGGTATCGGTGGCGGGATCGGCAAGGGTGGAGGTATT

RI-AT3G23450-XLOC_014830-1194-0
GGTGGTGGGATCGGCAAAGGTGGAGGAGTTGGCGGTGGTTTTGGTAAAGG
RI-AT3G23450-XLOC_014830-1194-1
GGTGGTGGGATCGGCAAAGGTGGAGGAGTTGGCGGTGGTTTTGGTAAAGG
CONSENSUS
GGTGGTGGGATCGGCAAAGGTGGAGGAGTTGGCGGTGGTTTTGGTAAAGG

RI-AT3G23450-XLOC_014830-1194-0
CGGAGGTGTTGGCGGTGGAATTGGCAAAGGCGGAGGAGTTGGCGGTGGTT
RI-AT3G23450-XLOC_014830-1194-1
CGGAGGTGTTGGCGGTGGAATTGGCAAAGGCGGAGGAGTTGGCGGTGGTT
CONSENSUS
CGGAGGTGTTGGCGGTGGAATTGGCAAAGGCGGAGGAGTTGGCGGTGGTT

RI-AT3G23450-XLOC_014830-1194-0
TCGGCAAAGGCGGAGGAGTTGGTGGTGGAATTGGCAAAGGTGGAGGTATT
RI-AT3G23450-XLOC_014830-1194-1
TCGGCAAAGGCGGAGGAGTTGGTGGTGGAATTGGCAAAGGTGGAGGTATT
CONSENSUS
TCGGCAAAGGCGGAGGAGTTGGTGGTGGAATTGGCAAAGGTGGAGGTATT

RI-AT3G23450-XLOC_014830-1194-0
GGCGGTGGCATTGGCAAAGGTGGTGGAATTGGTGGGGGCATTGGCAAAGG
RI-AT3G23450-XLOC_014830-1194-1
GGCGGTGGCATTGGCAAAGGTGGTGGAATTGGTGGGGGCATTGGCAAAGG
CONSENSUS
GGCGGTGGCATTGGCAAAGGTGGTGGAATTGGTGGGGGCATTGGCAAAGG

RI-AT3G23450-XLOC_014830-1194-0
CGGAGGCATTGGTGGGGGAATAGGTAAAGGCGGTGGAATTGGTGGCGGCA
RI-AT3G23450-XLOC_014830-1194-1
CGGAGGCATTGGTGGGGGAATAGGTAAAGGCGGTGGAATTGGTGGCGGCA
CONSENSUS
CGGAGGCATTGGTGGGGGAATAGGTAAAGGCGGTGGAATTGGTGGCGGCA

RI-AT3G23450-XLOC_014830-1194-0

TTGGCAAAGGCGGAGGGATTGGTGGTGGAATAGGCAAAGGCGGTGGAATT
RI-AT3G23450-XLOC_014830-1194-1
TTGGCAAAGGCGGAGGGATTGGTGGTGGAATAGGCAAAGGCGGTGGAATT
CONSENSUS
TTGGCAAAGGCGGAGGGATTGGTGGTGGAATAGGCAAAGGCGGTGGAATT

RI-AT3G23450-XLOC_014830-1194-0
GGTGGCGGCATTGGCAAAGGCGGGGGCATTGGCGGCGGAATTGGCAAAGG
RI-AT3G23450-XLOC_014830-1194-1
GGTGGCGGCATTGGCAAAGGCGGGGGCATTGGCGGCGGAATTGGCAAAGG
CONSENSUS
GGTGGCGGCATTGGCAAAGGCGGGGGCATTGGCGGCGGAATTGGCAAAGG

RI-AT3G23450-XLOC_014830-1194-0
CGGAGGCATTGGTGGTGGAGGTGGATTTGGTAAGGGTGGAGGCATTGGTG
RI-AT3G23450-XLOC_014830-1194-1
CGGAGGCATTGGTGGTGGAGGTGGATTTGGTAAGGGTGGAGGCATTGGTG
CONSENSUS
CGGAGGCATTGGTGGTGGAGGTGGATTTGGTAAGGGTGGAGGCATTGGTG

RI-AT3G23450-XLOC_014830-1194-0
GTGGTATTGGTAAAGGTGGGGGTATTGGCGGAGGAGGTGGATTTGGTAAG
RI-AT3G23450-XLOC_014830-1194-1
GTGGTATTGGTAAAGGTGGGGGTATTGGCGGAGGAGGTGGATTTGGTAAG
CONSENSUS
GTGGTATTGGTAAAGGTGGGGGTATTGGCGGAGGAGGTGGATTTGGTAAG

RI-AT3G23450-XLOC_014830-1194-0
GGTGGAGGCATTGGAGGCGGTATTGGCAAAGGCGGAGGCATTGGGGGCGG
RI-AT3G23450-XLOC_014830-1194-1
GGTGGAGGCATTGGAGGCGGTATTGGCAAAGGCGGAGGCATTGGGGGCGG
CONSENSUS
GGTGGAGGCATTGGAGGCGGTATTGGCAAAGGCGGAGGCATTGGGGGCGG

RI-AT3G23450-XLOC_014830-1194-0
TTTCGGCAAAGGTGGAGGCATTGGTGGTGGCATTGGAGGTGGAGGAGGCT
RI-AT3G23450-XLOC_014830-1194-1
TTTCGGCAAAGGTGGAGGCATTGGTGGTGGCATTGGAGGTGGAGGAGGCT
CONSENSUS
TTTCGGCAAAGGTGGAGGCATTGGTGGTGGCATTGGAGGTGGAGGAGGCT

RI-AT3G23450-XLOC_014830-1194-0
TTGGTGGAGGAGGTGGATTTGGAAAAGGTGGAGGAATCGGTGGTGGAATA
RI-AT3G23450-XLOC_014830-1194-1
TTGGTGGAGGAGGTGGATTTGGAAAAGGTGGAGGAATCGGTGGTGGAATA
CONSENSUS
TTGGTGGAGGAGGTGGATTTGGAAAAGGTGGAGGAATCGGTGGTGGAATA

RI-AT3G23450-XLOC_014830-1194-0
GGCAAAGGAGGAGGTTTCGGCGGAGGAGGTGGATTTGGGAAAGGTGGAGG
RI-AT3G23450-XLOC_014830-1194-1
GGCAAAGGAGGAGGTTTCGGCGGAGGAGGTGGATTTGGGAAAGGTGGAGG
CONSENSUS
GGCAAAGGAGGAGGTTTCGGCGGAGGAGGTGGATTTGGGAAAGGTGGAGG

RI-AT3G23450-XLOC_014830-1194-0

TATTGGTGGAGGAGGTGGATTTGGTAAGGGCGGAGGATTTGGCGGTGGAG
RI-AT3G23450-XLOC_014830-1194-1
TATTGGTGGAGGAGGTGGATTTGGTAAGGGCGGAGGATTTGGCGGTGGAG
CONSENSUS
TATTGGTGGAGGAGGTGGATTTGGTAAGGGCGGAGGATTTGGCGGTGGAG

RI-AT3G23450-XLOC_014830-1194-0
GCTTCGGAGGTGGTGGCGGAGGTGGAGGCGGAGGAGGTGGCGGAATCGGA
RI-AT3G23450-XLOC_014830-1194-1
GCTTCGGAGGTGGTGGCGGAGGTGGAGGCGGAGGAGGTGGCGGAATCGGA
CONSENSUS
GCTTCGGAGGTGGTGGCGGAGGTGGAGGCGGAGGAGGTGGCGGAATCGGA

RI-AT3G23450-XLOC_014830-1194-0
CACCACTAAACAAAAACACATGAAGAAGTTGCATTGAGTGCATGTCTCAC
RI-AT3G23450-XLOC_014830-1194-1
CACCACTAAACAAAAACACATGAAGAAGTTGCATTGAGTGCATGTCTCAC
CONSENSUS
CACCACTAAACAAAAACACATGAAGAAGTTGCATTGAGTGCATGTCTCAC

RI-AT3G23450-XLOC_014830-1194-0
GTCACGTACAACATGGTTTACAAGTTTGAGAGTGTTGTTACCAAATATAG
RI-AT3G23450-XLOC_014830-1194-1
GTCACGTACAACATGGTTTACAAGTTTGAGAGTGTTGTTACCAAATATAG
CONSENSUS
GTCACGTACAACATGGTTTACAAGTTTGAGAGTGTTGTTACCAAATATAG

RI-AT3G23450-XLOC_014830-1194-0
TACTAGTTTTATCTTTTTATGTACTTTGTCTCTATTTGAATTACTAGCTA
RI-AT3G23450-XLOC_014830-1194-1
TACTAGTTTTATCTTTTTATGTACTTTGTCTCTATTTGAATTACTAGCTA
CONSENSUS
TACTAGTTTTATCTTTTTATGTACTTTGTCTCTATTTGAATTACTAGCTA

RI-AT3G23450-XLOC_014830-1194-0
TGAGTTTTGTTAGACGTACGCACTCTAATAATATTCATTTTATCATGTAA
RI-AT3G23450-XLOC_014830-1194-1
TGAGTTTTGTTAGACGTACGCACTCTAATAATATTCATTTTATCATGTAA
CONSENSUS
TGAGTTTTGTTAGACGTACGCACTCTAATAATATTCATTTTATCATGTAA

RI-AT3G23450-XLOC_014830-1194-0
AACGCTAGGACTGTATTTGGTTGTAAGTCAATGTTCTTAAAAAAATATTG
RI-AT3G23450-XLOC_014830-1194-1
AACGCTAGGACTGTATTTGGTTGTAAGTCAATGTTCTTAAAAAAATATTG
CONSENSUS
AACGCTAGGACTGTATTTGGTTGTAAGTCAATGTTCTTAAAAAAATATTG

RI-AT3G23450-XLOC_014830-1194-0
ATGAAGCAATGTTACAAATATATAGCAACATGAAATGCAAAATTTGTTAA
RI-AT3G23450-XLOC_014830-1194-1
ATGAAGCAATGTTACAAATATATAGCAACATGAAATGCAAAATTTGTTAA
CONSENSUS
ATGAAGCAATGTTACAAATATATAGCAACATGAAATGCAAAATTTGTTAA

RI-AT3G23450-XLOC_014830-1194-0

```
      GAAATGGAAATAAACAGATACAAAAAGAGTATAAGAGTTTCACGGATAAA
RI-AT3G23450-XLOC_014830-1194-1
      GAAATGGAAATAAACAGATACAAAAAGAGTATAAGAGTTTCACGGATAAA
CONSENSUS
      GAAATGGAAATAAACAGATACAAAAAGAGTATAAGAGTTTCACGGATAAA

RI-AT3G23450-XLOC_014830-1194-0  TGTTACATTCG
RI-AT3G23450-XLOC_014830-1194-1  TGTTACATTCG
CONSENSUS                        TGTTACATTCG
```

alignment for event: SE-AT3G03690-XLOC_016932-2411

```
SE-AT3G03690-XLOC_016932-2411-0
      ATCTGATCTATGCATTTTCTGAGTTGCCTAGAGATCTCAACTTCATACAG
SE-AT3G03690-XLOC_016932-2411-1
      ATCTGATCTATGCATTTTCTGAGTTGCCTAGAGATCTCAACTTCATACAG
CONSENSUS
      ATCTGATCTATGCATTTTCTGAGTTGCCTAGAGATCTCAACTTCATACAG

SE-AT3G03690-XLOC_016932-2411-0
      CACACGAGCCGATTAGGATGGAAAAT------------------------
SE-AT3G03690-XLOC_016932-2411-1
      CACACGAGCCGATTAGGATGGAAAATGAACAAAAGAGGGAAACCAATAAT
CONSENSUS
      CACACGAGCCGATTAGGATGGAAAAT........................

SE-AT3G03690-XLOC_016932-2411-0
      --------------------------------------------------
SE-AT3G03690-XLOC_016932-2411-1
      AATAGATCCAGGGCTTTATAGCCTCAACAAATCAGAGATTTGGTGGGTTA
CONSENSUS
      ..................................................

SE-AT3G03690-XLOC_016932-2411-0
      ------------------------------------------GTTCTGCT
SE-AT3G03690-XLOC_016932-2411-1
      GTAATCAGCGAAGCCTCCCTACTTCTTTCAAGCTCTTCACAGGTTCTGCT
CONSENSUS
      ..........................................GTTCTGCT

SE-AT3G03690-XLOC_016932-2411-0
      TGGACATTCCTATCAAGACCGTTCGCAGAATATTGTATAATAGGTTATGA
SE-AT3G03690-XLOC_016932-2411-1
      TGGACATTCCTATCAAGACCGTTCGCAGAATATTGTATAATAGGTTATGA
CONSENSUS
      TGGACATTCCTATCAAGACCGTTCGCAGAATATTGTATAATAGGTTATGA

SE-AT3G03690-XLOC_016932-2411-0
      CAACTTACCAAGAACACTGCTTCTCTACTACACAAACTTCGTTTCATCCC
SE-AT3G03690-XLOC_016932-2411-1
      CAACTTACCAAGAACACTGCTTCTCTACTACACAAACTTCGTTTCATCCC
CONSENSUS
      CAACTTACCAAGAACACTGCTTCTCTACTACACAAACTTCGTTTCATCCC
```

SE-AT3G03690-XLOC_016932-2411-0
CGGAAGGCTATTTTCAGACGCTTATATGCAACTCGGATGAGTTCAAGAAC
SE-AT3G03690-XLOC_016932-2411-1
CGGAAGGCTATTTTCAGACGCTTATATGCAACTCGGATGAGTTCAAGAAC
CONSENSUS
CGGAAGGCTATTTTCAGACGCTTATATGCAACTCGGATGAGTTCAAGAAC

SE-AT3G03690-XLOC_016932-2411-0
ACGACCGTGAACCACGACCTCCATTACATCGCGTGGGACAATCCGCCAAA
SE-AT3G03690-XLOC_016932-2411-1
ACGACCGTGAACCACGACCTCCATTACATCGCGTGGGACAATCCGCCAAA
CONSENSUS
ACGACCGTGAACCACGACCTCCATTACATCGCGTGGGACAATCCGCCAAA

SE-AT3G03690-XLOC_016932-2411-0
GCAGCACCCCAAGATATTAGGGACAAGGGATTACAGAAAGATGGTAATGA
SE-AT3G03690-XLOC_016932-2411-1
GCAGCACCCCAAGATATTAGGGACAAGGGATTACAGAAAGATGGTAATGA
CONSENSUS
GCAGCACCCCAAGATATTAGGGACAAGGGATTACAGAAAGATGGTAATGA

SE-AT3G03690-XLOC_016932-2411-0
GCAACCGACCATTCGCTAGGAAGTTCAAGAGCAATGACCCCGTTCTCAAT
SE-AT3G03690-XLOC_016932-2411-1
GCAACCGACCATTCGCTAGGAAGTTCAAGAGCAATGACCCCGTTCTCAAT
CONSENSUS
GCAACCGACCATTCGCTAGGAAGTTCAAGAGCAATGACCCCGTTCTCAAT

SE-AT3G03690-XLOC_016932-2411-0
AGGATAGACCGGGAGATTCTGAGAAGGAAACGGAAACTTGGGTCGAAACC
SE-AT3G03690-XLOC_016932-2411-1
AGGATAGACCGGGAGATTCTGAGAAGGAAACGGAAACTTGGGTCGAAACC
CONSENSUS
AGGATAGACCGGGAGATTCTGAGAAGGAAACGGAAACTTGGGTCGAAACC

SE-AT3G03690-XLOC_016932-2411-0
CGATCTTGGTCCTGGTCCGGGGGCTAGAAGATTGAAGAGTTTACTAATGA
SE-AT3G03690-XLOC_016932-2411-1
CGATCTTGGTCCTGGTCCGGGGGCTAGAAGATTGAAGAGTTTACTAATGA
CONSENSUS
CGATCTTGGTCCTGGTCCGGGGGCTAGAAGATTGAAGAGTTTACTAATGA

SE-AT3G03690-XLOC_016932-2411-0
GGCTTTTGTTGAGAAGAAACTTTGTCAATAGACAATGTAGATAGGAAGTG
SE-AT3G03690-XLOC_016932-2411-1
GGCTTTTGTTGAGAAGAAACTTTGTCAATAGACAATGTAGATAGGAAGTG
CONSENSUS
GGCTTTTGTTGAGAAGAAACTTTGTCAATAGACAATGTAGATAGGAAGTG

SE-AT3G03690-XLOC_016932-2411-0
TGACTTACACAGCTCTATATGGAGATACTTTTTATTTTGATCTTATTCTT
SE-AT3G03690-XLOC_016932-2411-1
TGACTTACACAGCTCTATATGGAGATACTTTTTATTTTGATCTTATTCTT
CONSENSUS
TGACTTACACAGCTCTATATGGAGATACTTTTTATTTTGATCTTATTCTT

SE-AT3G03690-XLOC_016932-2411-0
TCTTGAATATTTTTTTGGGGGGTTGGTGTTTGTTCTTTAGTGATTCATAA
SE-AT3G03690-XLOC_016932-2411-1
TCTTGAATATTTTTTTGGGGGGTTGGTGTTTGTTCTTTAGTGATTCATAA
CONSENSUS
TCTTGAATATTTTTTTGGGGGGTTGGTGTTTGTTCTTTAGTGATTCATAA

SE-AT3G03690-XLOC_016932-2411-0
GGGAATAAATAACGAAGAATTTACATATAATGTGTTGGCCTGGTTTGGCT
SE-AT3G03690-XLOC_016932-2411-1
GGGAATAAATAACGAAGAATTTACATATAATGTGTTGGCCTGGTTTGGCT
CONSENSUS
GGGAATAAATAACGAAGAATTTACATATAATGTGTTGGCCTGGTTTGGCT

SE-AT3G03690-XLOC_016932-2411-0
TTGGACTTCAATCAAAGTCATTAGCATGTTTAGAATGTCTGGTGGGCTAG
SE-AT3G03690-XLOC_016932-2411-1
TTGGACTTCAATCAAAGTCATTAGCATGTTTAGAATGTCTGGTGGGCTAG
CONSENSUS
TTGGACTTCAATCAAAGTCATTAGCATGTTTAGAATGTCTGGTGGGCTAG

SE-AT3G03690-XLOC_016932-2411-0
ATTGTGATACTCCATAACCATGTTATAAAGCTTTTCCCCAATTTGATCTT
SE-AT3G03690-XLOC_016932-2411-1
ATTGTGATACTCCATAACCATGTTATAAAGCTTTTCCCCAATTTGATCTT
CONSENSUS
ATTGTGATACTCCATAACCATGTTATAAAGCTTTTCCCCAATTTGATCTT

alignment for event: RI-AT3G07360-XLOC_013904-6504

RI-AT3G07360-XLOC_013904-6504-0
ACATACGACAAGTTATTTATCCAGAAATGGTTGAGCTCAGGGAACAGAAC
RI-AT3G07360-XLOC_013904-6504-1
ACATACGACAAGTTATTTATCCAGAAATGGTTGAGCTCAGGGAACAGAAC
CONSENSUS
ACATACGACAAGTTATTTATCCAGAAATGGTTGAGCTCAGGGAACAGAAC

RI-AT3G07360-XLOC_013904-6504-0
ATGTCCCAAGACTCAGCAAGTTCTGCCTCACACGGCTTTAACACCTAATC
RI-AT3G07360-XLOC_013904-6504-1
ATGTCCCAAGACTCAGCAAGTTCTGCCTCACACGGCTTTAACACCTAATC
CONSENSUS
ATGTCCCAAGACTCAGCAAGTTCTGCCTCACACGGCTTTAACACCTAATC

RI-AT3G07360-XLOC_013904-6504-0
TCTTAATCCGTGAAATGATCTCGAAATGGTGCAAGAAGAACGGGCTTGAG
RI-AT3G07360-XLOC_013904-6504-1
TCTTAATCCGTGAAATGATCTCGAAATGGTGCAAGAAGAACGGGCTTGAG
CONSENSUS
TCTTAATCCGTGAAATGATCTCGAAATGGTGCAAGAAGAACGGGCTTGAG

RI-AT3G07360-XLOC_013904-6504-0
ACGAAGAGCCAATATCATCCCAACCTTGTAAATGAAGATGAAACTGTGAC
RI-AT3G07360-XLOC_013904-6504-1

ACGAAGAGCCAATATCATCCCAACCTTGTAAATGAAGATGAAACTGTGAC
CONSENSUS
ACGAAGAGCCAATATCATCCCAACCTTGTAAATGAAGATGAAACTGTGAC

RI-AT3G07360-XLOC_013904-6504-0
AAGATCAGATCGTGAGATTTTCAATTCCTTGCTCTGTAAAGTCTCTTCTT
RI-AT3G07360-XLOC_013904-6504-1
AAGATCAGATCGTGAGATTTTCAATTCCTTGCTCTGTAAAGTCTCTTCTT
CONSENSUS
AAGATCAGATCGTGAGATTTTCAATTCCTTGCTCTGTAAAGTCTCTTCTT

RI-AT3G07360-XLOC_013904-6504-0
CGAACCTTCAAGATCAAAAATCAGCTGCCAAGGAGCTAAGACTTCTGACC
RI-AT3G07360-XLOC_013904-6504-1
CGAACCTTCAAGATCAAAAATCAGCTGCCAAGGAGCTAAGACTTCTGACC
CONSENSUS
CGAACCTTCAAGATCAAAAATCAGCTGCCAAGGAGCTAAGACTTCTGACC

RI-AT3G07360-XLOC_013904-6504-0
AGGAAAGGCACTGAGTTCCGAGCTCTTTTTGGCGAATCTCCGGATGAGAT
RI-AT3G07360-XLOC_013904-6504-1
AGGAAAGGCACTGAGTTCCGAGCTCTTTTTGGCGAATCTCCGGATGAGAT
CONSENSUS
AGGAAAGGCACTGAGTTCCGAGCTCTTTTTGGCGAATCTCCGGATGAGAT

RI-AT3G07360-XLOC_013904-6504-0
CACCAGGTTGGTGAATCCCTTGTTACACGGGTCAAACCCAGATGAGAAGC
RI-AT3G07360-XLOC_013904-6504-1
CACCAGGTTGGTGAATCCCTTGTTACACGGGTCAAACCCAGATGAGAAGC
CONSENSUS
CACCAGGTTGGTGAATCCCTTGTTACACGGGTCAAACCCAGATGAGAAGC

RI-AT3G07360-XLOC_013904-6504-0
TTCAAGAAGATGTGGTTACAACATTGTTGAACATATCAATACATGATGAC
RI-AT3G07360-XLOC_013904-6504-1
TTCAAGAAGATGTGGTTACAACATTGTTGAACATATCAATACATGATGAC
CONSENSUS
TTCAAGAAGATGTGGTTACAACATTGTTGAACATATCAATACATGATGAC

RI-AT3G07360-XLOC_013904-6504-0
AGCAACAAGAAGCTCGTCTGCGAAAATCCTAATGTGATTCCTCTCCTTAT
RI-AT3G07360-XLOC_013904-6504-1
AGCAACAAGAAGCTCGTCTGCGAAAATCCTAATGTGATTCCTCTCCTTAT
CONSENSUS
AGCAACAAGAAGCTCGTCTGCGAAAATCCTAATGTGATTCCTCTCCTTAT

RI-AT3G07360-XLOC_013904-6504-0
CGATGCATTGAGGCGTGGAACAGTCGCCACGAGAAGCAATGCAGCTGCAG
RI-AT3G07360-XLOC_013904-6504-1
CGATGCATTGAGGCGTGGAACAGTCGCCACGAGAAGCAATGCAGCTGCAG
CONSENSUS
CGATGCATTGAGGCGTGGAACAGTCGCCACGAGAAGCAATGCAGCTGCAG

RI-AT3G07360-XLOC_013904-6504-0
CGATCTTCACTCTGTCAGCTCTCGATTCAAACAAAGTACTTATAGGGAAG
RI-AT3G07360-XLOC_013904-6504-1

CGATCTTCACTCTGTCAGCTCTCGATTCAAACAAAGTACTTATAGGGAAG
CONSENSUS
CGATCTTCACTCTGTCAGCTCTCGATTCAAACAAAGTACTTATAGGGAAG

RI-AT3G07360-XLOC_013904-6504-0
TCCGGAATCCTGAAACCGCTTATCGATCTCCTAGAAGAAGGGAATCCATT
RI-AT3G07360-XLOC_013904-6504-1
TCCGGAATCCTGAAACCGCTTATCGATCTCCTAGAAGAAGGGAATCCATT
CONSENSUS
TCCGGAATCCTGAAACCGCTTATCGATCTCCTAGAAGAAGGGAATCCATT

RI-AT3G07360-XLOC_013904-6504-0
AGCTATCAAAGACGTAGCTGCAGCGATCTTCACTCTTTGTATAGCCCATG
RI-AT3G07360-XLOC_013904-6504-1
AGCTATCAAAGACGTAGCTGCAGCGATCTTCACTCTTTGTATAGCCCATG
CONSENSUS
AGCTATCAAAGACGTAGCTGCAGCGATCTTCACTCTTTGTATAGCCCATG

RI-AT3G07360-XLOC_013904-6504-0
AGAACAGGAGTAGAGCTGTGAGAGACGGAGCTGTTAGGGTTTTAGGTAAG
RI-AT3G07360-XLOC_013904-6504-1
AGAACAGGAGTAGAGCTGTGAGAGACGGAGCTGTTAGGGTTTTAGGTAAG
CONSENSUS
AGAACAGGAGTAGAGCTGTGAGAGACGGAGCTGTTAGGGTTTTAGGTAAG

RI-AT3G07360-XLOC_013904-6504-0
AAAATCTCGAATGGGTTGTACGTTGATGAGCTTTTAGCTATATTGGCAAT
RI-AT3G07360-XLOC_013904-6504-1
AAAATCTCGAATGGGTTGTACGTTGATGAGCTTTTAGCTATATTGGCAAT
CONSENSUS
AAAATCTCGAATGGGTTGTACGTTGATGAGCTTTTAGCTATATTGGCAAT

RI-AT3G07360-XLOC_013904-6504-0
GCTTGTTACTCACTGGAAGGCTGTGGAGGAATTGGGTGAGCTCGGTGGGG
RI-AT3G07360-XLOC_013904-6504-1
GCTTGTTACTCACTGGAAGGCTGTGGAGGAATTGGGTGAGCTCGGTGGGG
CONSENSUS
GCTTGTTACTCACTGGAAGGCTGTGGAGGAATTGGGTGAGCTCGGTGGGG

RI-AT3G07360-XLOC_013904-6504-0
TTTCATGGTTGCTGAAGATAACTCGAGAGAGCGAGTGCAAGCGAAACAAA
RI-AT3G07360-XLOC_013904-6504-1
TTTCATGGTTGCTGAAGATAACTCGAGAGAGCGAGTGCAAGCGAAACAAA
CONSENSUS
TTTCATGGTTGCTGAAGATAACTCGAGAGAGCGAGTGCAAGCGAAACAAA

RI-AT3G07360-XLOC_013904-6504-0
GAGAATGCGATAGTGATACTGCATACTATATGTTTCAGCGACAGGACAAA
RI-AT3G07360-XLOC_013904-6504-1
GAGAATGCGATAGTGATACTGCATACTATATGTTTCAGCGACAGGACAAA
CONSENSUS
GAGAATGCGATAGTGATACTGCATACTATATGTTTCAGCGACAGGACAAA

RI-AT3G07360-XLOC_013904-6504-0
GTGGAAGGAGATCAAAGAAGAGGAGAATGCTCATGGAACGATAACAAAGC
RI-AT3G07360-XLOC_013904-6504-1

GTGGAAGGAGATCAAAGAAGAGGAGAATGCTCATGGAACGATAACAAAGC
CONSENSUS
GTGGAAGGAGATCAAAGAAGAGGAGAATGCTCATGGAACGATAACAAAGC

RI-AT3G07360-XLOC_013904-6504-0
TTTCGCGTGAAGGAACTTCAAGGGCGCAGAGGAAAGCAAACGGGATATTG
RI-AT3G07360-XLOC_013904-6504-1
TTTCGCGTGAAGGAACTTCAAGGGCGCAGAGGAAAGCAAACGGGATATTG
CONSENSUS
TTTCGCGTGAAGGAACTTCAAGGGCGCAGAGGAAAGCAAACGGGATATTG

RI-AT3G07360-XLOC_013904-6504-0
GACAGACTGAGAAAAGCTATGAATCTCACTCATACAGCCTGAGAAAAGCC
RI-AT3G07360-XLOC_013904-6504-1
GACAGACTGAGAAAAGCTATGAATCTCACTCATACAGCCTGAGAAAAGCC
CONSENSUS
GACAGACTGAGAAAAGCTATGAATCTCACTCATACAGCCTGAGAAAAGCC

RI-AT3G07360-XLOC_013904-6504-0
TTGAAATCTGCAATGTAAATTACCTAAAATCTTGTTTCTTTTTTTTACTT
RI-AT3G07360-XLOC_013904-6504-1
TTGAAATCTGCAATGTAAATTACCTAAAATCTTGTTTCTTTTTTTTACTT
CONSENSUS
TTGAAATCTGCAATGTAAATTACCTAAAATCTTGTTTCTTTTTTTTACTT

RI-AT3G07360-XLOC_013904-6504-0
TATTTGGCTGTAAGAATTTTTGTAAATTATTCATATCTTTAGGATCTGAA
RI-AT3G07360-XLOC_013904-6504-1
TATTTGGCTGTAAGAATTTTTGTAAATTATTCATATCTTTAGGATCTGAA
CONSENSUS
TATTTGGCTGTAAGAATTTTTGTAAATTATTCATATCTTTAGGATCTGAA

RI-AT3G07360-XLOC_013904-6504-0
TGGTTATTGGTTTTCGTATGTGAGATGTAAGTAGAAATCTGTGTGAATAG
RI-AT3G07360-XLOC_013904-6504-1
TGGTTATTGGTTTTCGTATGTGAGATGTAAGTAGAAATCT----------
CONSENSUS
TGGTTATTGGTTTTCGTATGTGAGATGTAAGTAGAAATCT..........

RI-AT3G07360-XLOC_013904-6504-0
TTTTTGCGTTTGATGTTTCTGGAGAAATCAATCAATAAAATCAATGTTGG
RI-AT3G07360-XLOC_013904-6504-1

CONSENSUS
....

RI-AT3G07360-XLOC_013904-6504-0
TGGCTTTATTCAGCTGTGAAAGCCTTGGTCAAC
RI-AT3G07360-XLOC_013904-6504-1 -------------
CTGTGAAAGCCTTGGTCAAC
CONSENSUS
.............CTGTGAAAGCCTTGGTCAAC

alignment for event: A3-AT3G52660-XLOC_016209-2002

A3-AT3G52660-XLOC_016209-2002-0
GTGACTACACCTCCTTGATAGTCTCGCTTAGGCATCTTTTAGGTCATATC
A3-AT3G52660-XLOC_016209-2002-1
GTGACTACACCTCCTTGATAGTCTCGCTTAGGCATCTTTTAGGTCATATC
CONSENSUS
GTGACTACACCTCCTTGATAGTCTCGCTTAGGCATCTTTTAGGTCATATC

A3-AT3G52660-XLOC_016209-2002-0
AATACAACATACACATATACATATTTTTCAACACCTGAGGTGCGCGAAGA
A3-AT3G52660-XLOC_016209-2002-1
AATACAACATACACATATACATATTTTTCAACACCTGAGGTGCGCGAAGA
CONSENSUS
AATACAACATACACATATACATATTTTTCAACACCTGAGGTGCGCGAAGA

A3-AT3G52660-XLOC_016209-2002-0
TAGACACTTCTTCATCCTCTCCGTAATTCTCAAAATTAGGGTTTTCTTCC
A3-AT3G52660-XLOC_016209-2002-1
TAGACACTTCTTCATCCTCTCCGTAATTCTCAAAATTAGGGTTTTCTTCC
CONSENSUS
TAGACACTTCTTCATCCTCTCCGTAATTCTCAAAATTAGGGTTTTCTTCC

A3-AT3G52660-XLOC_016209-2002-0
GGCAGCTCTCTCAGGTTAGATTGGCAGCATAAAAAATATCACTTGCAAAT
A3-AT3G52660-XLOC_016209-2002-1
GGCAGCTCTCTCAG------------------------------------
CONSENSUS
GGCAGCTCTCTCAG....................................

A3-AT3G52660-XLOC_016209-2002-0
TCTGAACAATGTCTGTTGTGGGAGATTCTAACATGGAGCTGGAGTTTTCT
A3-AT3G52660-XLOC_016209-2002-1

CONSENSUS
....

A3-AT3G52660-XLOC_016209-2002-0
AAGCAAAGATACTATTTTGACTTTAGCCCTTCTTATCTTTCTTGTTGCCA
A3-AT3G52660-XLOC_016209-2002-1

CONSENSUS
....

A3-AT3G52660-XLOC_016209-2002-0
TGAATATTTTAACCTTCTTTGCATCTAACCAAGGCCTATGATATCATGGT
A3-AT3G52660-XLOC_016209-2002-1

CONSENSUS
....

A3-AT3G52660-XLOC_016209-2002-0
TTTTATCCGCTCTCAGGGGTCATTTCGTTTCTTATCTTTTTTTGTTTCAC
A3-AT3G52660-XLOC_016209-2002-1

CONSENSUS
....

A3-AT3G52660-XLOC_016209-2002-0
CTGTTGAGCATTTCATCGCCCTCTTCTCTCTTTGTCTATTTGTTTGTCAC
A3-AT3G52660-XLOC_016209-2002-1

CONSENSUS
....

A3-AT3G52660-XLOC_016209-2002-0
CTTTTTAGCCGCATTATGGCAAAGCGTCAATTCGAACTTCTTATTCTTCT
A3-AT3G52660-XLOC_016209-2002-1

CONSENSUS
....

A3-AT3G52660-XLOC_016209-2002-0
TTTCTATTGGCCTCTTATTGTTTCTTTTCCAACCTTTCCTCGGTTTTATT
A3-AT3G52660-XLOC_016209-2002-1

CONSENSUS
....

A3-AT3G52660-XLOC_016209-2002-0
CTCTCTCCCCACTTGCTTCTTGCTTCTGTGTCTCCTCCACAGTTGTACTG
A3-AT3G52660-XLOC_016209-2002-1
--TTGTACTG
CONSENSUS
....TTGTACTG

A3-AT3G52660-XLOC_016209-2002-0
CAAGCATTCTTTTCTAGATATTTAAGATGTCAAGGACGAGGACTGCTGCT
A3-AT3G52660-XLOC_016209-2002-1
CAAGCATTCTTTTCTAGATATTTAAGATGTCAAGGACGAGGACTGCTGCT
CONSENSUS
CAAGCATTCTTTTCTAGATATTTAAGATGTCAAGGACGAGGACTGCTGCT

A3-AT3G52660-XLOC_016209-2002-0
TCTGAGGCTCATGACTCCATGGAATCTGAGGAAAGGGTAGACCTTGATGG
A3-AT3G52660-XLOC_016209-2002-1
TCTGAGGCTCATGACTCCATGGAATCTGAGGAAAGGGTAGACCTTGATGG
CONSENSUS
TCTGAGGCTCATGACTCCATGGAATCTGAGGAAAGGGTAGACCTTGATGG

A3-AT3G52660-XLOC_016209-2002-0
TGACAATGATCCTGAGGAGATTCTGGAGGAGGAAGTTGAATACGAAGAAG
A3-AT3G52660-XLOC_016209-2002-1
TGACAATGATCCTGAGGAGATTCTGGAGGAGGAAGTTGAATACGAAGAAG
CONSENSUS
TGACAATGATCCTGAGGAGATTCTGGAGGAGGAAGTTGAATACGAAGAAG

A3-AT3G52660-XLOC_016209-2002-0
TTGAAGAGGAGGAGATTGAAGAGATAGAAGAGGAGATAGAGGAGGAGGTT
A3-AT3G52660-XLOC_016209-2002-1
TTGAAGAGGAGGAGATTGAAGAGATAGAAGAGGAGATAGAGGAGGAGGTT
CONSENSUS
TTGAAGAGGAGGAGATTGAAGAGATAGAAGAGGAGATAGAGGAGGAGGTT

A3-AT3G52660-XLOC_016209-2002-0
GAAGTGGAAGAAGAGGAGGAGGAGGAGGATGCTGTTGCAACGGAAGAGGA
A3-AT3G52660-XLOC_016209-2002-1
GAAGTGGAAGAAGAGGAGGAGGAGGAGGATGCTGTTGCAACGGAAGAGGA
CONSENSUS
GAAGTGGAAGAAGAGGAGGAGGAGGAGGATGCTGTTGCAACGGAAGAGGA

A3-AT3G52660-XLOC_016209-2002-0
AGAAGAAAAGAAAAGGCATGTTGAACTTCTTGCACTTCCTCCACATGGTT
A3-AT3G52660-XLOC_016209-2002-1
AGAAGAAAAGAAAAGGCATGTTGAACTTCTTGCACTTCCTCCACATGGTT
CONSENSUS
AGAAGAAAAGAAAAGGCATGTTGAACTTCTTGCACTTCCTCCACATGGTT

A3-AT3G52660-XLOC_016209-2002-0
CAGAGGTTTATCTTGGAGGGATTCCTACTGATGCTACTGAAGGGGACTTA
A3-AT3G52660-XLOC_016209-2002-1
CAGAGGTTTATCTTGGAGGGATTCCTACTGATGCTACTGAAGGGGACTTA
CONSENSUS
CAGAGGTTTATCTTGGAGGGATTCCTACTGATGCTACTGAAGGGGACTTA

A3-AT3G52660-XLOC_016209-2002-0
AAGGGCTTCTGTGGATCTATAGGAGAAGTTACTGAG
A3-AT3G52660-XLOC_016209-2002-1
AAGGGCTTCTGTGGATCTATAGGAGAAGTTACTGAG
CONSENSUS
AAGGGCTTCTGTGGATCTATAGGAGAAGTTACTGAG

alignment for event: RI-AT3G19720-XLOC_017850-2567

RI-AT3G19720-XLOC_017850-2567-0
AACCGAGCTGGTCTACGTCAATTCTTGGACTCATTTGGTGGAACAGAGCA
RI-AT3G19720-XLOC_017850-2567-1
AACCGAGCTGGTCTACGTCAATTCTTGGACTCATTTGGTGGAACAGAGCA
CONSENSUS
AACCGAGCTGGTCTACGTCAATTCTTGGACTCATTTGGTGGAACAGAGCA

RI-AT3G19720-XLOC_017850-2567-0
TAATACGACATCAGGTAATGCCATAGGATTTAGTCTTCCCCAAGATGCAT
RI-AT3G19720-XLOC_017850-2567-1
TAATACGACATCAG------------------------------------
CONSENSUS
TAATACGACATCAG....................................

RI-AT3G19720-XLOC_017850-2567-0
TAGGTGGCACAACAGACACCAAGTCAAGATCAGATGTAAAGCTAAGCCAT
RI-AT3G19720-XLOC_017850-2567-1

CONSENSUS
....

RI-AT3G19720-XLOC_017850-2567-0
CTCGCCTCAAACATCGATTCAGGTTCCAGTATTCAGACAACAGAAATGCG

RI-AT3G19720-XLOC_017850-2567-1 ---------------------
GTTCCAGTATTCAGACAACAGAAATGCG
CONSENSUS
.....................GTTCCAGTATTCAGACAACAGAAATGCG

RI-AT3G19720-XLOC_017850-2567-0
GTTGGCTGATCTTCTAGATAGCACACTTTGGAACCGCAAGCTTGCTCCTT
RI-AT3G19720-XLOC_017850-2567-1
GTTGGCTGATCTTCTAGATAGCACACTTTGGAACCGCAAGCTTGCTCCTT
CONSENSUS
GTTGGCTGATCTTCTAGATAGCACACTTTGGAACCGCAAGCTTGCTCCTT

RI-AT3G19720-XLOC_017850-2567-0
CCTCTGAGAGAATTGTGTACGCATTGGTCCAACAGATATTCCAGGGCATA
RI-AT3G19720-XLOC_017850-2567-1
CCTCTGAGAGAATTGTGTACGCATTGGTCCAACAGATATTCCAGGGCATA
CONSENSUS
CCTCTGAGAGAATTGTGTACGCATTGGTCCAACAGATATTCCAGGGCATA

RI-AT3G19720-XLOC_017850-2567-0
CGAGAGTACTTTCTCGCCTCTGCTGAGTTAAAG
RI-AT3G19720-XLOC_017850-2567-1
CGAGAGTACTTTCTCGCCTCTGCTGAGTTAAAG
CONSENSUS
CGAGAGTACTTTCTCGCCTCTGCTGAGTTAAAG

alignment for event: A5-AT3G13570-XLOC_017517-9103

A5-AT3G13570-XLOC_017517-9103-0
GCAAGAAGACCTCAGGAGGCCATTTGAGCAGTTTGGTCCCGTCAAGGACA
A5-AT3G13570-XLOC_017517-9103-1
GCAAGAAGACCTCAGGAGGCCATTTGAGCAGTTTGGTCCCGTCAAGGACA
CONSENSUS
GCAAGAAGACCTCAGGAGGCCATTTGAGCAGTTTGGTCCCGTCAAGGACA

A5-AT3G13570-XLOC_017517-9103-0
TCTACCTTCCTAGGGATTACTATACTGGGTGAGGATGCTCTGTAGATAAG
A5-AT3G13570-XLOC_017517-9103-1
TCTACCTTCCTAGGGATTACTATACTGG----------------------
CONSENSUS
TCTACCTTCCTAGGGATTACTATACTGG......................

A5-AT3G13570-XLOC_017517-9103-0
ACATTGAATGTTTATACATCCAGGGCATAATTTTACATTACTTCAATTAG
A5-AT3G13570-XLOC_017517-9103-1

CONSENSUS
....

A5-AT3G13570-XLOC_017517-9103-0
ACTATGTCTCTTTCTTATTGTTTTCTTTTGCTTCGTAAACCACCTTTTGT
A5-AT3G13570-XLOC_017517-9103-1

CONSENSUS

....

A5-AT3G13570-XLOC_017517-9103-0
GAATCCTACATTCCATCGTATGCAGCTCATGAATTTGTTAGATTTATAAG
A5-AT3G13570-XLOC_017517-9103-1

CONSENSUS
....

A5-AT3G13570-XLOC_017517-9103-0
AATTTTCTGAAAGGTCTATGTCAAACATGTCCGGTTTTATATCCAATCTT
A5-AT3G13570-XLOC_017517-9103-1

CONSENSUS
....

A5-AT3G13570-XLOC_017517-9103-0
CCCAAAATCACATTTGGAGCCTTGAAACGTTTGTTTTATCTACACTTAGG
A5-AT3G13570-XLOC_017517-9103-1

CONSENSUS
....

A5-AT3G13570-XLOC_017517-9103-0
TTTTCAGCTTAAGTCTAGTACACCTAAAACTTCGCCCTCTTCGAGTCACT
A5-AT3G13570-XLOC_017517-9103-1

CONSENSUS
....

A5-AT3G13570-XLOC_017517-9103-0
TTAGGGAAAGAATACAGTGATATGTTCCATGTTCGGTCACACTTCGAGTC
A5-AT3G13570-XLOC_017517-9103-1

CONSENSUS
....

A5-AT3G13570-XLOC_017517-9103-0
TGTTTATTAAAGTTGTTGAGGTTTAACAGTGAATCTAGAGAGTTGAAGAG
A5-AT3G13570-XLOC_017517-9103-1

CONSENSUS
....

A5-AT3G13570-XLOC_017517-9103-0
ACCAATGAAGTAATTAGAGTTCTTTGGAAGATGTTCTAAATGGTAGTGAA
A5-AT3G13570-XLOC_017517-9103-1

CONSENSUS
....

A5-AT3G13570-XLOC_017517-9103-0
GGTTGGAAGGAAGTTGGGTTTGAATTCAATTTGAAGAGATATCAAGTCTT
A5-AT3G13570-XLOC_017517-9103-1

CONSENSUS

```
      ..................................................

A5-AT3G13570-XLOC_017517-9103-0
      GGAGAAGTCATCTTTATACTGAAACTTGCAAGGTTTGCAGAGATCCAAGG
A5-AT3G13570-XLOC_017517-9103-1
      ----------------------------------------AGATCCAAGG
CONSENSUS
      ........................................AGATCCAAGG

A5-AT3G13570-XLOC_017517-9103-0
      GGGTTTGGATTCATTCAGTTTATGGATCCTGCTGATGCTGCTGAGGCTAA
A5-AT3G13570-XLOC_017517-9103-1
      GGGTTTGGATTCATTCAGTTTATGGATCCTGCTGATGCTGCTGAGGCTAA
CONSENSUS
      GGGTTTGGATTCATTCAGTTTATGGATCCTGCTGATGCTGCTGAGGCTAA

A5-AT3G13570-XLOC_017517-9103-0
      ACATCAAATGGATGGTTATCTTCTTCTTGGTCGTGAGTTGACTGTCGTAT
A5-AT3G13570-XLOC_017517-9103-1
      ACATCAAATGGATGGTTATCTTCTTCTTGGTCGTGAGTTGACTGTCGTAT
CONSENSUS
      ACATCAAATGGATGGTTATCTTCTTCTTGGTCGTGAGTTGACTGTCGTAT

A5-AT3G13570-XLOC_017517-9103-0
      TTGCTGAAGAAAACCGGAAGAAGCCAACTGAGATGAGAACAAGGGATCGA
A5-AT3G13570-XLOC_017517-9103-1
      TTGCTGAAGAAAACCGGAAGAAGCCAACTGAGATGAGAACAAGGGATCGA
CONSENSUS
      TTGCTGAAGAAAACCGGAAGAAGCCAACTGAGATGAGAACAAGGGATCGA

A5-AT3G13570-XLOC_017517-9103-0  GGTGGAAG
A5-AT3G13570-XLOC_017517-9103-1  GGTGGAAG
CONSENSUS                        GGTGGAAG


alignment for event: A3-AT3G54500-XLOC_019571-12901

A3-AT3G54500-XLOC_019571-12901-0
      CTGGCGATGTGTGGAGCAATCAGGGAGCTTGATTTTGGGTCTGTGATTCG
A3-AT3G54500-XLOC_019571-12901-1
      CTGGCGATGTGTGGAGCAATCAGGGAGCTTGATTTTGGGTCTGTGATTCG
CONSENSUS
      CTGGCGATGTGTGGAGCAATCAGGGAGCTTGATTTTGGGTCTGTGATTCG

A3-AT3G54500-XLOC_019571-12901-0
      ATTTTGTTTCTCCAGTCATGTTTGATTGGGAAGAAGAAGAG---------
A3-AT3G54500-XLOC_019571-12901-1
      ATTTTGTTTCTCCAGTCATGTTTGATTGGGAAGAAGAAGAGCTTACTAAT
CONSENSUS
      ATTTTGTTTCTCCAGTCATGTTTGATTGGGAAGAAGAAGAG.........

A3-AT3G54500-XLOC_019571-12901-0 ----------------------------
GCGACCATATTGTGCCTTTTAA
A3-AT3G54500-XLOC_019571-12901-1
      ATGATATGGGGTGATGATGCTGAGACAGGCGACCATATTGTGCCTTTTAA
```

CONSENSUS
..............................GCGACCATATTGTGCCTTTTAA

A3-AT3G54500-XLOC_019571-12901-0
AGTCAGAAGTGAACAACTTAACAAAAAGGAACAGATTGAGGAATCTAAGA
A3-AT3G54500-XLOC_019571-12901-1
AGTCAGAAGTGAACAACTTAACAAAAAGGAACAGATTGAGGAATCTAAGA
CONSENSUS
AGTCAGAAGTGAACAACTTAACAAAAAGGAACAGATTGAGGAATCTAAGA

A3-AT3G54500-XLOC_019571-12901-0
CAGCTGAGCAAAAGATAACTGGGACTAAAATTGACCTCCATGATAAAAAT
A3-AT3G54500-XLOC_019571-12901-1
CAGCTGAGCAAAAGATAACTGGGACTAAAATTGACCTCCATGATAAAAAT
CONSENSUS
CAGCTGAGCAAAAGATAACTGGGACTAAAATTGACCTCCATGATAAAAAT

A3-AT3G54500-XLOC_019571-12901-0
TTGGGGAGCAGTTCGAGCCATAATGTTGATGAGGGGCTTCCTCAGCCAGA
A3-AT3G54500-XLOC_019571-12901-1
TTGGGGAGCAGTTCGAGCCATAATGTTGATGAGGGGCTTCCTCAGCCAGA
CONSENSUS
TTGGGGAGCAGTTCGAGCCATAATGTTGATGAGGGGCTTCCTCAGCCAGA

A3-AT3G54500-XLOC_019571-12901-0
TTTCTGTATGAGCTCATGGCCTGACACGTCGCTAACTAATGCTACAAAGG
A3-AT3G54500-XLOC_019571-12901-1
TTTCTGTATGAGCTCATGGCCTGACACGTCGCTAACTAATGCTACAAAGG
CONSENSUS
TTTCTGTATGAGCTCATGGCCTGACACGTCGCTAACTAATGCTACAAAGG

A3-AT3G54500-XLOC_019571-12901-0
TTGATCAAGATTTGAGTGCGACTGAACTTTCAAAATGCTTAGCTGAGCCA
A3-AT3G54500-XLOC_019571-12901-1
TTGATCAAGATTTGAGTGCGACTGAACTTTCAAAATGCTTAGCTGAGCCA
CONSENSUS
TTGATCAAGATTTGAGTGCGACTGAACTTTCAAAATGCTTAGCTGAGCCA

A3-AT3G54500-XLOC_019571-12901-0 GTCAGATATGATTCAACAAGAGGTG
A3-AT3G54500-XLOC_019571-12901-1 GTCAGATATGATTCAACAAGAGGTG
CONSENSUS GTCAGATATGATTCAACAAGAGGTG

alignment for event: A3-AT3G07300-XLOC_017168-787

A3-AT3G07300-XLOC_017168-787-0
GGTCTCCTATTATCAATTCCGTTTCTCTCTCGCCGGAAGATTTTTGCTAC
A3-AT3G07300-XLOC_017168-787-1
GGTCTCCTATTATCAATTCCGTTTCTCTCTCGCCGGAAGATTTTTGCTAC
CONSENSUS
GGTCTCCTATTATCAATTCCGTTTCTCTCTCGCCGGAAGATTTTTGCTAC

A3-AT3G07300-XLOC_017168-787-0
TTCATCTCCTTATCGCCCCACCACCGTCACATATTCACTG----------
A3-AT3G07300-XLOC_017168-787-1

TTCATCTCCTTATCGCCCCACCACCGTCACATATTCACTGGTTTCGCATT
CONSENSUS
TTCATCTCCTTATCGCCCCACCACCGTCACATATTCACTG..........

A3-AT3G07300-XLOC_017168-787-0 --------
TTTCTCGTTAATTTCGGTTAAAGAAGATGCCAGACGTTCAAT
A3-AT3G07300-XLOC_017168-787-1
TTCTCAAGTTTCTCGTTAATTTCGGTTAAAGAAGATGCCAGACGTTCAAT
CONSENSUS
........TTTCTCGTTAATTTCGGTTAAAGAAGATGCCAGACGTTCAAT

A3-AT3G07300-XLOC_017168-787-0
CAACGGTGGTGGAATTTGTTAACAAGCTCAGAAAGCG
A3-AT3G07300-XLOC_017168-787-1
CAACGGTGGTGGAATTTGTTAACAAGCTCAGAAAGCG
CONSENSUS
CAACGGTGGTGGAATTTGTTAACAAGCTCAGAAAGCG

alignment for event: A3-AT3G47910-XLOC_019181-9223

A3-AT3G47910-XLOC_019181-9223-0
GAGAAAAGGAATGGACGACTTGATGACCTGGAAGGAGCCAGTGTGAATAC
A3-AT3G47910-XLOC_019181-9223-1
GAGAAAAGGAATGGACGACTTGATGACCTGGAAGGAGCCAGTGTGAATAC
CONSENSUS
GAGAAAAGGAATGGACGACTTGATGACCTGGAAGGAGCCAGTGTGAATAC

A3-AT3G47910-XLOC_019181-9223-0
AAATGGAGTTTTCCCGTCAACAAATCATTCTGCGATATCTGATACTGCAA
A3-AT3G47910-XLOC_019181-9223-1
AAATGGAGTTTTCCCGTCAACAAATCATTCTGCGATATCTGATACTGCAA
CONSENSUS
AAATGGAGTTTTCCCGTCAACAAATCATTCTGCGATATCTGATACTGCAA

A3-AT3G47910-XLOC_019181-9223-0 AGGTGCAGAATGTAAAATCCCAAAAAG---
TGCCTAACGGCACAGCTATG
A3-AT3G47910-XLOC_019181-9223-1
AGGTGCAGAATGTAAAATCCCAAAAAGTAGTGCCTAACGGCACAGCTATG
CONSENSUS
AGGTGCAGAATGTAAAATCCCAAAAAG...TGCCTAACGGCACAGCTATG

A3-AT3G47910-XLOC_019181-9223-0
CAAGCTGGTATTTTCCAATCTGATCAACGAACTGGGAGGAGAACTAGACG
A3-AT3G47910-XLOC_019181-9223-1
CAAGCTGGTATTTTCCAATCTGATCAACGAACTGGGAGGAGAACTAGACG
CONSENSUS
CAAGCTGGTATTTTCCAATCTGATCAACGAACTGGGAGGAGAACTAGACG

A3-AT3G47910-XLOC_019181-9223-0
CCAAAAAGCTTCAAACAAGTTAGCTGATGGAAAATATCCGGTCACACCTC
A3-AT3G47910-XLOC_019181-9223-1
CCAAAAAGCTTCAAACAAGTTAGCTGATGGAAAATATCCGGTCACACCTC
CONSENSUS
CCAAAAAGCTTCAAACAAGTTAGCTGATGGAAAATATCCGGTCACACCTC

```
A3-AT3G47910-XLOC_019181-9223-0
      CTGAGACCGAAAATAGTAAATCTCAGTTGTCAGGCACCAACGGCGAGAGA
A3-AT3G47910-XLOC_019181-9223-1
      CTGAGACCGAAAATAGTAAATCTCAGTTGTCAGGCACCAACGGCGAGAGA
CONSENSUS
      CTGAGACCGAAAATAGTAAATCTCAGTTGTCAGGCACCAACGGCGAGAGA

A3-AT3G47910-XLOC_019181-9223-0  CATTCTGAAACTCTACGTAACAATGGTG
A3-AT3G47910-XLOC_019181-9223-1  CATTCTGAAACTCTACGTAACAATGGTG
CONSENSUS                        CATTCTGAAACTCTACGTAACAATGGTG
```

alignment for event: RI-AT5G49710-XLOC_027769-9162

```
RI-AT5G49710-XLOC_027769-9162-0
      GACTTGACTCCTGAGCAGCTTCAGAAAGGTATTCTCGACTCTTCTGTCAA
RI-AT5G49710-XLOC_027769-9162-1
      GACTTGACTCCTGAGCAGCTTCAGAAAG----------------------
CONSENSUS
      GACTTGACTCCTGAGCAGCTTCAGAAAG......................

RI-AT5G49710-XLOC_027769-9162-0
      CCTTCAAATGCTGTGTATCTCAATGATAAGTAGACATAATAGTTGGCTGT
RI-AT5G49710-XLOC_027769-9162-1
      --------------------------------------------------
CONSENSUS
      ..................................................

RI-AT5G49710-XLOC_027769-9162-0
      AACATAATAGCCACGTCACTGTTGCTAACACTCAAATTCAGAACAGATAG
RI-AT5G49710-XLOC_027769-9162-1
      --------------------------------------------------
CONSENSUS
      ..................................................

RI-AT5G49710-XLOC_027769-9162-0
      ATCTTTAGAGGAAATACGGCATTGGACAGACTGGTAAAGGGCATTGATTG
RI-AT5G49710-XLOC_027769-9162-1
      --------------------------------------------------
CONSENSUS
      ..................................................

RI-AT5G49710-XLOC_027769-9162-0
      TTCTTGGGAATCGGTTTTGGGGTTTGAGAGGGCATAGGAAAGTAGGGGAG
RI-AT5G49710-XLOC_027769-9162-1
      --------------------------------------------------
CONSENSUS
      ..................................................

RI-AT5G49710-XLOC_027769-9162-0
      GCTCAAGGATTCATCCATAGATTATTCTGTGGTTAGATATGTGTTCTACA
RI-AT5G49710-XLOC_027769-9162-1
      --------------------------------------------------
CONSENSUS
```

....

RI-AT5G49710-XLOC_027769-9162-0
ATTCAGTAAGAAATAGAGTTTACCGGTTCCAATCTTAAAACTCAACTAGG
RI-AT5G49710-XLOC_027769-9162-1

CONSENSUS
....

RI-AT5G49710-XLOC_027769-9162-0
AAGCTTGTTATTGCGCTGACACAAACAAAGACCATTAAGTCGCCAAGTTA
RI-AT5G49710-XLOC_027769-9162-1

CONSENSUS
....

RI-AT5G49710-XLOC_027769-9162-0
TTTTCTTTGATGTTGATTATGTGTGAAGTTGATCAGTGGTGGCATATCAG
RI-AT5G49710-XLOC_027769-9162-1

CONSENSUS
....

RI-AT5G49710-XLOC_027769-9162-0
ATTAGTAGTAAAACTGTAACATTAGTGTTTGAATTGTAATGTAGCACTTA
RI-AT5G49710-XLOC_027769-9162-1
--CACTTA
CONSENSUS
....CACTTA

RI-AT5G49710-XLOC_027769-9162-0
GCAGGATGTTCTCAGTGAAGAATAGAAAGGGGAAACTTCGGAAAGCCTGG
RI-AT5G49710-XLOC_027769-9162-1
GCAGGATGTTCTCAGTGAAGAATAGAAAGGGGAAACTTCGGAAAGCCTGG
CONSENSUS
GCAGGATGTTCTCAGTGAAGAATAGAAAGGGGAAACTTCGGAAAGCCTGG

RI-AT5G49710-XLOC_027769-9162-0
GATGGAAGTAAGGTTGCTTACAACGTAGCGTCATGGAGCGCAACTGTTAT
RI-AT5G49710-XLOC_027769-9162-1
GATGGAAGTAAGGTTGCTTACAACGTAGCGTCATGGAGCGCAACTGTTAT
CONSENSUS
GATGGAAGTAAGGTTGCTTACAACGTAGCGTCATGGAGCGCAACTGTTAT

RI-AT5G49710-XLOC_027769-9162-0 AGG
RI-AT5G49710-XLOC_027769-9162-1 AGG
CONSENSUS   AGG

alignment for event: A3-AT5G48330-XLOC_027687-13546

A3-AT5G48330-XLOC_027687-13546-0
ACTCAAGATGCCGGAATCATTTACACAATGAAAACTTTTCTTTCTCCGGC
A3-AT5G48330-XLOC_027687-13546-1
ACTCAAGATGCCGGAATCATTTACACAATGAAAACTTTTCTTTCTCCGGC

CONSENSUS
ACTCAAGATGCCGGAATCATTTACACAATGAAAACTTTTCTTTCTCCGGC

A3-AT5G48330-XLOC_027687-13546-0
GATATGCTTCGATCATGGTCGATCGCGAAACTACAAATCAGTAAAGTTTA
A3-AT5G48330-XLOC_027687-13546-1
GATATGCTTCGATCATGGTCGATCGCGAAACTACAAATCAGTAAAGTTTA
CONSENSUS
GATATGCTTCGATCATGGTCGATCGCGAAACTACAAATCAGTAAAGTTTA

A3-AT5G48330-XLOC_027687-13546-0
TTCATCCGATTCTCGCTTTTGCACACACGTTCTCTTCAATGGAGTTGTCA
A3-AT5G48330-XLOC_027687-13546-1
TTCATCCGATTCTCGCTTTTGCACACACGTTCTCTTCAATGGAGTTGTCA
CONSENSUS
TTCATCCGATTCTCGCTTTTGCACACACGTTCTCTTCAATGGAGTTGTCA

A3-AT5G48330-XLOC_027687-13546-0
GACGACAATTCACGAGCACTAGCGGCGAAAGAAGAACAGTGATGAGCTTC
A3-AT5G48330-XLOC_027687-13546-1
GACGACAATTCACGAGCACTAGCGGCGAAAGAAGAACAGTGATGAGCTTC
CONSENSUS
GACGACAATTCACGAGCACTAGCGGCGAAAGAAGAACAGTGATGAGCTTC

A3-AT5G48330-XLOC_027687-13546-0
GGTGACGGAAACCTTGGCGCTCTTGGTCTTTCATCATCGTCGATTCCAGG
A3-AT5G48330-XLOC_027687-13546-1
GGTGACGGAAACCTTGGCGCTCTTGGTCTTTCATCATCGTCGATTCCAGG
CONSENSUS
GGTGACGGAAACCTTGGCGCTCTTGGTCTTTCATCATCGTCGATTCCAGG

A3-AT5G48330-XLOC_027687-13546-0
CATGGGAATGGACGCTTACGAACCCACCGTAGTTTCTAATTTGCCTTCCG
A3-AT5G48330-XLOC_027687-13546-1
CATGGGAATGGACGCTTACGAACCCACCGTAGTTTCTAATTTGCCTTCCG
CONSENSUS
CATGGGAATGGACGCTTACGAACCCACCGTAGTTTCTAATTTGCCTTCCG

A3-AT5G48330-XLOC_027687-13546-0
ATATCTCTTCCATATCCGCCGGACATTACCATTCTCTTGCCGTTACATCT
A3-AT5G48330-XLOC_027687-13546-1
ATATCTCTTCCATATCCGCCGGACATTACCATTCTCTTGCCGTTACATCT
CONSENSUS
ATATCTCTTCCATATCCGCCGGACATTACCATTCTCTTGCCGTTACATCT

A3-AT5G48330-XLOC_027687-13546-0
AGCGGTGAGATTTGGGCTTGGGGTCGCAACGACGAAGGCCAGCTCGGTCG
A3-AT5G48330-XLOC_027687-13546-1
AGCGGTGAGATTTGGGCTTGGGGTCGCAACGACGAAGGCCAGCTCGGTCG
CONSENSUS
AGCGGTGAGATTTGGGCTTGGGGTCGCAACGACGAAGGCCAGCTCGGTCG

A3-AT5G48330-XLOC_027687-13546-0
AATCGTAATTGATTCTAGTAGAGATTCAAGGAGTGAACCTAAGAGAGTGG
A3-AT5G48330-XLOC_027687-13546-1 AATCGTAATTGATTCTAG---
AGATTCAAGGAGTGAACCTAAGAGAGTGG

CONSENSUS
      AATCGTAATTGATTCTAG...AGATTCAAGGAGTGAACCTAAGAGAGTGG

A3-AT5G48330-XLOC_027687-13546-0
      AAGGTCTAGAGAATGTGAATGTCCAGGCAGCGTTTGCGTCTGGAGTTGTT
A3-AT5G48330-XLOC_027687-13546-1
      AAGGTCTAGAGAATGTGAATGTCCAGGCAGCGTTTGCGTCTGGAGTTGTT
CONSENSUS
      AAGGTCTAGAGAATGTGAATGTCCAGGCAGCGTTTGCGTCTGGAGTTGTT

A3-AT5G48330-XLOC_027687-13546-0
      TCAGCTGCTATTGGAGATGATGGCTCTTTGTGGGTTTGGGGAAGATCTAA
A3-AT5G48330-XLOC_027687-13546-1
      TCAGCTGCTATTGGAGATGATGGCTCTTTGTGGGTTTGGGGAAGATCTAA
CONSENSUS
      TCAGCTGCTATTGGAGATGATGGCTCTTTGTGGGTTTGGGGAAGATCTAA

A3-AT5G48330-XLOC_027687-13546-0
      AAGAGGACAGCTTGGTCTTGGAAATGGTATCATTGAAGCTCGAGTTCCTT
A3-AT5G48330-XLOC_027687-13546-1
      AAGAGGACAGCTTGGTCTTGGAAATGGTATCATTGAAGCTCGAGTTCCTT
CONSENSUS
      AAGAGGACAGCTTGGTCTTGGAAATGGTATCATTGAAGCTCGAGTTCCTT

A3-AT5G48330-XLOC_027687-13546-0
      CGAGAGTTGAAAATTTAGCTGCAGAGCACGTTGTTAAG
A3-AT5G48330-XLOC_027687-13546-1
      CGAGAGTTGAAAATTTAGCTGCAGAGCACGTTGTTAAG
CONSENSUS
      CGAGAGTTGAAAATTTAGCTGCAGAGCACGTTGTTAAG

alignment for event: RI-AT5G63460-XLOC_032162-3443

RI-AT5G63460-XLOC_032162-3443-0
      GGAAGCTTGCGGGTCTACATTTGTGAGCATGACACATCTCCCCCAGGTAA
RI-AT5G63460-XLOC_032162-3443-1
      GGAAGCTTGCGGGTCTACATTTGTGAGCATGACACATCTCCCCCAG----
CONSENSUS
      GGAAGCTTGCGGGTCTACATTTGTGAGCATGACACATCTCCCCCAG....

RI-AT5G63460-XLOC_032162-3443-0
      TTCTTTTCTTTTTTCGTTTTGTTCCACGAGTTGTTTTCTTAGAGACAGTT
RI-AT5G63460-XLOC_032162-3443-1

CONSENSUS
      ....

RI-AT5G63460-XLOC_032162-3443-0
      TCTTGTAGCAATCTCATAAAACAATTCCTTTTCATTTTCTCTCTGGGTGA
RI-AT5G63460-XLOC_032162-3443-1

CONSENSUS
      ....

```
RI-AT5G63460-XLOC_032162-3443-0
      GAATTGGCTACACTGGAAAAGTAATTGGGAGTCTCAGGATTATCAGTTTC
RI-AT5G63460-XLOC_032162-3443-1
      --------------------------------------------------
CONSENSUS
      ..................................................

RI-AT5G63460-XLOC_032162-3443-0
      AGAATAAGCTGCGTTTCAGTAATAAGTCTTTGTTTCCTGTAGAATTTTGT
RI-AT5G63460-XLOC_032162-3443-1
      --------------------------------------------------
CONSENSUS
      ..................................................

RI-AT5G63460-XLOC_032162-3443-0
      CTTACTAACAACTCCTAACTTATTTTGTTTGTCATATATCCAGAAGGACA
RI-AT5G63460-XLOC_032162-3443-1
      -------------------------------------------AAGGACA
CONSENSUS
      ...........................................AAGGACA

RI-AT5G63460-XLOC_032162-3443-0
      GCTTATCAAAACAAACCAGCAAAATATACTAATCAGATCTCTCTTGTTAA
RI-AT5G63460-XLOC_032162-3443-1
      GCTTATCAAAACAAACCAGCAAAATATACTAATCAGATCTCTCTTGTTAA
CONSENSUS
      GCTTATCAAAACAAACCAGCAAAATATACTAATCAGATCTCTCTTGTTAA

RI-AT5G63460-XLOC_032162-3443-0
      AGAAGCAAAAAGGCGAATCTAGTTCTAAAGACTCAAAAGGAACTGCTGAA
RI-AT5G63460-XLOC_032162-3443-1
      AGAAGCAAAAAGGCGAATCTAGTTCTAAAGACTCAAAAGGAACTGCTGAA
CONSENSUS
      AGAAGCAAAAAGGCGAATCTAGTTCTAAAGACTCAAAAGGAACTGCTGAA

RI-AT5G63460-XLOC_032162-3443-0  GATGGTCCTAAAAAGAG
RI-AT5G63460-XLOC_032162-3443-1  GATGGTCCTAAAAAGAG
CONSENSUS                        GATGGTCCTAAAAAGAG


alignment for event: RI-AT5G02810-XLOC_028866-12011

RI-AT5G02810-XLOC_028866-12011-0
      TTGTTGAGGCGTCAAATGGGATACAAGCTTGGAAGGTGTTAGAAGATCTA
RI-AT5G02810-XLOC_028866-12011-1
      TTGTTGAGGCGTCAAATGGGATACAAGCTTGGAAGGTGTTAGAAGATCTA
CONSENSUS
      TTGTTGAGGCGTCAAATGGGATACAAGCTTGGAAGGTGTTAGAAGATCTA

RI-AT5G02810-XLOC_028866-12011-0
      AACAATCATATTGATATTGTGCTAACAGAGGTGATCATGCCTTACTTATC
RI-AT5G02810-XLOC_028866-12011-1
      AACAATCATATTGATATTGTGCTAACAGAGGTGATCATGCCTTACTTATC
CONSENSUS
      AACAATCATATTGATATTGTGCTAACAGAGGTGATCATGCCTTACTTATC
```

```
RI-AT5G02810-XLOC_028866-12011-0
      TGGTATCGGTCTCTTGTGCAAGATTTTGAACCACAAATCTCGTCGGAACA
RI-AT5G02810-XLOC_028866-12011-1
      TGGTATCGGTCTCTTGTGCAAGATTTTGAACCACAAATCTCGTCGGAACA
CONSENSUS
      TGGTATCGGTCTCTTGTGCAAGATTTTGAACCACAAATCTCGTCGGAACA

RI-AT5G02810-XLOC_028866-12011-0
      TCCCTGTCATCAGTGAGTTCTTTTTCCTTGGTCGTTTTACATTGAGCTCT
RI-AT5G02810-XLOC_028866-12011-1
      TCCCTGTCATCA--------------------------------------
CONSENSUS
      TCCCTGTCATCA......................................

RI-AT5G02810-XLOC_028866-12011-0
      TTCTTTTGAAGTTACACGATTTGTTGAGTCTTCTCTAGCGTATGTTGGAA
RI-AT5G02810-XLOC_028866-12011-1
      --------------------------------------------------
CONSENSUS
      ..................................................

RI-AT5G02810-XLOC_028866-12011-0
      AGTAGATGCTTTTAACTACATTCCCCTGTGAGATTTGTGTTGCAGTGATG
RI-AT5G02810-XLOC_028866-12011-1
      ---------------------------------------------TGATG
CONSENSUS
      .............................................TGATG

RI-AT5G02810-XLOC_028866-12011-0
      TCATCTCATGACTCAATGGGGCTGGTCTTTAAGTGCTTATCGAAAGGAGC
RI-AT5G02810-XLOC_028866-12011-1
      TCATCTCATGACTCAATGGGGCTGGTCTTTAAGTGCTTATCGAAAGGAGC
CONSENSUS
      TCATCTCATGACTCAATGGGGCTGGTCTTTAAGTGCTTATCGAAAGGAGC

RI-AT5G02810-XLOC_028866-12011-0
      TGTTGACTTTCTTGTTAAGCCAATAAGAAAAAATGAGCTTAAGATCCTTT
RI-AT5G02810-XLOC_028866-12011-1
      TGTTGACTTTCTTGTTAAGCCAATAAGAAAAAATGAGCTTAAGATCCTTT
CONSENSUS
      TGTTGACTTTCTTGTTAAGCCAATAAGAAAAAATGAGCTTAAGATCCTTT

RI-AT5G02810-XLOC_028866-12011-0 GGCAGCATGTTTGGAGAAGATGCCAAAGT
RI-AT5G02810-XLOC_028866-12011-1 GGCAGCATGTTTGGAGAAGATGCCAAAGT
CONSENSUS                        GGCAGCATGTTTGGAGAAGATGCCAAAGT


alignment for event: A5-AT5G22300-XLOC_026181-7769

A5-AT5G22300-XLOC_026181-7769-0
      ATAAGGCAGAGAGACTGCTTTCTGAGGCAGCGGAGAATGGATCTCAGCTA
A5-AT5G22300-XLOC_026181-7769-1
      ATAAGGCAGAGAGACTGCTTTCTGAGGCAGCGGAGAATGGATCTCAGCTA
CONSENSUS
```

```
      ATAAGGCAGAGAGACTGCTTTCTGAGGCAGCGGAGAATGGATCTCAGCTA

A5-AT5G22300-XLOC_026181-7769-0
      GTGGTGTTCCCGGAGGCTTTCATCGGTGGATATCCACGTGGCTCTACCTT
A5-AT5G22300-XLOC_026181-7769-1
      GTGGTGTTCCCGGAGGCTTTCATCGGTGGATATCCACGTGGCTCTACCTT
CONSENSUS
      GTGGTGTTCCCGGAGGCTTTCATCGGTGGATATCCACGTGGCTCTACCTT

A5-AT5G22300-XLOC_026181-7769-0
      TGAATTGGCTATTGGTTCTCGTACCGCTAAAGGACGAGATGACTTTCGCA
A5-AT5G22300-XLOC_026181-7769-1
      TGAATTGGCTATTGGTTCTCGTACCGCTAAAGGACGAGATGACTTTCGCA
CONSENSUS
      TGAATTGGCTATTGGTTCTCGTACCGCTAAAGGACGAGATGACTTTCGCA

A5-AT5G22300-XLOC_026181-7769-0
      AGTACCATGCTTCTGCCATTGATGTTCCTG--------------------
A5-AT5G22300-XLOC_026181-7769-1
      AGTACCATGCTTCTGCCATTGATGTTCCTGGTAATAAAGTCCTCCACCAT
CONSENSUS
      AGTACCATGCTTCTGCCATTGATGTTCCTG....................

A5-AT5G22300-XLOC_026181-7769-0
      --------------------------------------------------
A5-AT5G22300-XLOC_026181-7769-1
      ATCTCCGATTATGTTCTCACTTTTTTTCTTTCGATTTTGGATCTTAGCAT
CONSENSUS
      ..................................................

A5-AT5G22300-XLOC_026181-7769-0
      --------------------------------------------------
A5-AT5G22300-XLOC_026181-7769-1
      GAATACCAGGGCACGCACTTATTACCACATAAATCCATTGTTCTCTAACT
CONSENSUS
      ..................................................

A5-AT5G22300-XLOC_026181-7769-0
      --------------------------------------------------
A5-AT5G22300-XLOC_026181-7769-1
      AGCTACCAACATAGAACAACTAGGCAACCCTCTTTTATATTCACTTAGGT
CONSENSUS
      ..................................................

A5-AT5G22300-XLOC_026181-7769-0
      --------------------------------------------------
A5-AT5G22300-XLOC_026181-7769-1
      CGCGTGTTCGATTCACGTTCACTGCACCATTTGATAAGTTTGAAGCTATG
CONSENSUS
      ..................................................

A5-AT5G22300-XLOC_026181-7769-0
      --------------------------------------------------
A5-AT5G22300-XLOC_026181-7769-1
      AACTCTTATCTTTTATCACTTCTCATTCCAAGATACAAACTCAAGAACTA
CONSENSUS
```

```
       .................................................

A5-AT5G22300-XLOC_026181-7769-0
       -------------------------------------------------
A5-AT5G22300-XLOC_026181-7769-1
      CCCTAACAATGGAGATTTTTTCAAGAGATCTCCTAACTCTCCTCCATAAA
CONSENSUS
       .................................................

A5-AT5G22300-XLOC_026181-7769-0
       -------------------------------------------------
A5-AT5G22300-XLOC_026181-7769-1
      ACCATACGTTTGTTAAAAACACATCAATACATGCATAACTTCATCAATTC
CONSENSUS
       .................................................

A5-AT5G22300-XLOC_026181-7769-0
       -------------------------------------------------
A5-AT5G22300-XLOC_026181-7769-1
      TCTTGACAAGAGGTTACAAAATCTGTTTCTTCTTGCTCTGTTTTAACTCC
CONSENSUS
       .................................................

A5-AT5G22300-XLOC_026181-7769-0
       -------------------------------------------------
A5-AT5G22300-XLOC_026181-7769-1
      TTTTCACATGACTTCTATGCTTCTCTTTGCTTATTCACGTGTCTCATAAC
CONSENSUS
       .................................................

A5-AT5G22300-XLOC_026181-7769-0
       -------------------------------------------------
A5-AT5G22300-XLOC_026181-7769-1
      CTCCTTTTCAGCTACCACACGTATCTCCTAGCTTATCACACTTAAGATCT
CONSENSUS
       .................................................

A5-AT5G22300-XLOC_026181-7769-0
       -------------------------------------------------
A5-AT5G22300-XLOC_026181-7769-1
      AATAAATTCTCACCAAAGCCGTCTTTAAAACAGCTTCTGTTCTACTACGA
CONSENSUS
       .................................................

A5-AT5G22300-XLOC_026181-7769-0
       -------------------------------------------------
A5-AT5G22300-XLOC_026181-7769-1
      CACTGTAACAGAAACTGCAACAAACAACCAAGGTGGTTCTTGTTTTTTTT
CONSENSUS
       .................................................

A5-AT5G22300-XLOC_026181-7769-0
       -------------------------------------------------
A5-AT5G22300-XLOC_026181-7769-1
      TGGTATCAAGGTGGTTCTTGTTGCTTTGAGGGAGATGCCTCTTCCTTCTT
CONSENSUS
```

....

A5-AT5G22300-XLOC_026181-7769-0

A5-AT5G22300-XLOC_026181-7769-1
ACATGAGAGCTTGTAGCATCACCAAGATCTGACCAGTTCATCAAGAGACG
CONSENSUS
....

A5-AT5G22300-XLOC_026181-7769-0

A5-AT5G22300-XLOC_026181-7769-1
TAGCTTGGTTGCTCCACCCGTGACTCCACAACAAGCTTCATTCAAGGGAG
CONSENSUS
....

A5-AT5G22300-XLOC_026181-7769-0 -----------------
GCCCTGAAGTGGAACGATTAGCGTTAATGGCCA
A5-AT5G22300-XLOC_026181-7769-1
GCACAGATGCTCCAAGAGCCCTGAAGTGGAACGATTAGCGTTAATGGCCA
CONSENSUS
.................GCCCTGAAGTGGAACGATTAGCGTTAATGGCCA

A5-AT5G22300-XLOC_026181-7769-0
AGAAGTACAAAGTATACTTGGTTATGGGTGTGATAGAGAGGGAAGGCTAC
A5-AT5G22300-XLOC_026181-7769-1
AGAAGTACAAAGTATACTTGGTTATGGGTGTGATAGAGAGGGAAGGCTAC
CONSENSUS
AGAAGTACAAAGTATACTTGGTTATGGGTGTGATAGAGAGGGAAGGCTAC

A5-AT5G22300-XLOC_026181-7769-0
ACGCTATACTGCACCGTTCTTTTCTTCGATTCACAAGGTCTGTTCTTAGG
A5-AT5G22300-XLOC_026181-7769-1
ACGCTATACTGCACCGTTCTTTTCTTCGATTCACAAGGTCTGTTCTTAGG
CONSENSUS
ACGCTATACTGCACCGTTCTTTTCTTCGATTCACAAGGTCTGTTCTTAGG

A5-AT5G22300-XLOC_026181-7769-0
TAAGCACCGCAAACTCATGCCTACAGCTCTTGAACGTTGCATTTGGGGAT
A5-AT5G22300-XLOC_026181-7769-1
TAAGCACCGCAAACTCATGCCTACAGCTCTTGAACGTTGCATTTGGGGAT
CONSENSUS
TAAGCACCGCAAACTCATGCCTACAGCTCTTGAACGTTGCATTTGGGGAT

A5-AT5G22300-XLOC_026181-7769-0
TTGGAGATGGATCAACCATCCCTGTGTTCGATACTCCTATTGGGAAAATC
A5-AT5G22300-XLOC_026181-7769-1
TTGGAGATGGATCAACCATCCCTGTGTTCGATACTCCTATTGGGAAAATC
CONSENSUS
TTGGAGATGGATCAACCATCCCTGTGTTCGATACTCCTATTGGGAAAATC

A5-AT5G22300-XLOC_026181-7769-0
GGTGCTGCTATTTGTTGGGAAAATAGGATGCCTTCTTTGAGAACCGCAAT
A5-AT5G22300-XLOC_026181-7769-1
GGTGCTGCTATTTGTTGGGAAAATAGGATGCCTTCTTTGAGAACCGCAAT
CONSENSUS

GGTGCTGCTATTTGTTGGGAAAATAGGATGCCTTCTTTGAGAACCGCAAT

A5-AT5G22300-XLOC_026181-7769-0 GTATGCCAAAG
A5-AT5G22300-XLOC_026181-7769-1 GTATGCCAAAG
CONSENSUS   GTATGCCAAAG

alignment for event: RI-AT5G67610-XLOC_032399-2625

RI-AT5G67610-XLOC_032399-2625-0
GAATTTGTCGCGTGGAATTGGAATGTGCCCTCATAGTCGATGGGAGAAAG
RI-AT5G67610-XLOC_032399-2625-1
GAATTTGTCGCGTGGAATTGGAATGTGCCCTCATAGTCGATGGGAGAAAG
CONSENSUS
GAATTTGTCGCGTGGAATTGGAATGTGCCCTCATAGTCGATGGGAGAAAG

RI-AT5G67610-XLOC_032399-2625-0
CCTCTAAAGGTTCATGGGTTCAGACAATGTCACCCTTCGACCATAAAATC
RI-AT5G67610-XLOC_032399-2625-1
CCTCTAAAGGTTCATGGGTTCAGACAATGTCACCCTTCGACCATAAAATC
CONSENSUS
CCTCTAAAGGTTCATGGGTTCAGACAATGTCACCCTTCGACCATAAAATC

RI-AT5G67610-XLOC_032399-2625-0
CTTGATGTTAGAGTACCTAGCTCTAACAAGGTCTCATTGGAAGTGTCTGC
RI-AT5G67610-XLOC_032399-2625-1
CTTGATGTTAGAGTACCTAGCTCTAACAAGGTCTCATTGGAAGTGTCTGC
CONSENSUS
CTTGATGTTAGAGTACCTAGCTCTAACAAGGTCTCATTGGAAGTGTCTGC

RI-AT5G67610-XLOC_032399-2625-0
TGTAGAAGGTGCCTTTTCTGTTTCCTTTTATTGCATTTTCTTTTCTCTTT
RI-AT5G67610-XLOC_032399-2625-1
TGTAGAAG--
CONSENSUS
TGTAGAAG..

RI-AT5G67610-XLOC_032399-2625-0
TCTAAGCTATCTATTTTCTCTTTGCAGAATTATTTATGCATCGCATAGTA
RI-AT5G67610-XLOC_032399-2625-1 --------------------------
AATTATTTATGCATCGCATAGTA
CONSENSUS
...........................AATTATTTATGCATCGCATAGTA

RI-AT5G67610-XLOC_032399-2625-0
TTCTTACTCCTTGGTGCTGTATTACTGGCTTCGGCTTCTACACTGAGCCA
RI-AT5G67610-XLOC_032399-2625-1
TTCTTACTCCTTGGTGCTGTATTACTGGCTTCGGCTTCTACACTGAGCCA
CONSENSUS
TTCTTACTCCTTGGTGCTGTATTACTGGCTTCGGCTTCTACACTGAGCCA

RI-AT5G67610-XLOC_032399-2625-0
ATCTTTAGCGTTTTACTACAGTAGTGCAATGGCTGTTGGTATTATCCTTG
RI-AT5G67610-XLOC_032399-2625-1
ATCTTTAGCGTTTTACTACAGTAGTGCAATGGCTGTTGGTATTATCCTTG

```
CONSENSUS
      ATCTTTAGCGTTTTACTACAGTAGTGCAATGGCTGTTGGTATTATCCTTG

RI-AT5G67610-XLOC_032399-2625-0  TCGTGCTGCTTGTTCTCTTTCAG
RI-AT5G67610-XLOC_032399-2625-1  TCGTGCTGCTTGTTCTCTTTCAG
CONSENSUS                        TCGTGCTGCTTGTTCTCTTTCAG



alignment for event: A3-AT5G26742-XLOC_030103-4451

A3-AT5G26742-XLOC_030103-4451-0
      TTACCTCCACTTCAAGATGACGGACCATCTAGTGATAACTACGGACGGTT
A3-AT5G26742-XLOC_030103-4451-1
      TTACCTCCACTTCAAGATGACGGACCATCTAGTGATAACTACGGACGGTT
CONSENSUS
      TTACCTCCACTTCAAGATGACGGACCATCTAGTGATAACTACGGACGGTT

A3-AT5G26742-XLOC_030103-4451-0
      CTCTAGCAGAGACAGGATGCCTAGAGGAGGAGGAGGTTCTAGAGGGTCAA
A3-AT5G26742-XLOC_030103-4451-1
      CTCTAGCAGAGACAGGATGCCTAGAGGAGGAGGAGGTTCTAGAGGGTCAA
CONSENSUS
      CTCTAGCAGAGACAGGATGCCTAGAGGAGGAGGAGGTTCTAGAGGGTCAA

A3-AT5G26742-XLOC_030103-4451-0
      GAGGCGGTAGAGGAGGATCATCACGAGGCCGTGATAGTTGGGGAGGTGAT
A3-AT5G26742-XLOC_030103-4451-1
      GAGGCGGTAGAGGAGGATCATCACGAGGCCGTGATAGTTGGGGAGGTGAT
CONSENSUS
      GAGGCGGTAGAGGAGGATCATCACGAGGCCGTGATAGTTGGGGAGGTGAT

A3-AT5G26742-XLOC_030103-4451-0
      GATGACAGAGGTAGTAGAAGGAGCAGTGGTGGAGGAAGCAGCTGGTCCCG
A3-AT5G26742-XLOC_030103-4451-1
      GATGACAGAGGTAGTAGAAGGAGCAGTGGTGGAGGAAGCAGCTGGTCCCG
CONSENSUS
      GATGACAGAGGTAGTAGAAGGAGCAGTGGTGGAGGAAGCAGCTGGTCCCG

A3-AT5G26742-XLOC_030103-4451-0
      AGGTGGTAGTAGTTCCAGAGGAAGTTCTGATGATTGGTTGATCGGTGGCA
A3-AT5G26742-XLOC_030103-4451-1
      AGGTGGTAGTAGTTCCAGAGGAAGTTCTGATGATTGGTTGATCGGTGGCA
CONSENSUS
      AGGTGGTAGTAGTTCCAGAGGAAGTTCTGATGATTGGTTGATCGGTGGCA

A3-AT5G26742-XLOC_030103-4451-0
      GAAGTTCATCAAGCAGCAGAGCTCCTTCGCGGGAGAG---TTTTGGAGGT
A3-AT5G26742-XLOC_030103-4451-1
      GAAGTTCATCAAGCAGCAGAGCTCCTTCGCGGGAGAGAAGTTTTGGAGGT
CONSENSUS
      GAAGTTCATCAAGCAGCAGAGCTCCTTCGCGGGAGAG...TTTTGGAGGT

A3-AT5G26742-XLOC_030103-4451-0
      TCATGCTTCATTTGTGGGAAATCTGGACACAGGGCAACAGATTGTCCTGA
A3-AT5G26742-XLOC_030103-4451-1
```

TCATGCTTCATTTGTGGGAAATCTGGACACAGGGCAACAGATTGTCCTGA
CONSENSUS
TCATGCTTCATTTGTGGGAAATCTGGACACAGGGCAACAGATTGTCCTGA

A3-AT5G26742-XLOC_030103-4451-0
TAAGAGAGGATTTTAGAGTTATCGTAGCAAATGGTTTCTGCTCTTGGGAG
A3-AT5G26742-XLOC_030103-4451-1
TAAGAGAGGATTTTAGAGTTATCGTAGCAAATGGTTTCTGCTCTTGGGAG
CONSENSUS
TAAGAGAGGATTTTAGAGTTATCGTAGCAAATGGTTTCTGCTCTTGGGAG

A3-AT5G26742-XLOC_030103-4451-0
AAAGATCCTTGTCCTTTTTGGTGTTCTTAATGCGAATCTTTTCGTGCTTG
A3-AT5G26742-XLOC_030103-4451-1
AAAGATCCTTGTCCTTTTTGGTGTTCTTAATGCGAATCTTTTCGTGCTTG
CONSENSUS
AAAGATCCTTGTCCTTTTTGGTGTTCTTAATGCGAATCTTTTCGTGCTTG

A3-AT5G26742-XLOC_030103-4451-0
CTTCAACGGTTTGTTTCAGACGGTAGAGAAGCAATCAGGGAGGAGGATAC
A3-AT5G26742-XLOC_030103-4451-1
CTTCAACGGTTTGTTTCAGACGGTAGAGAAGCAATCAGGGAGGAGGATAC
CONSENSUS
CTTCAACGGTTTGTTTCAGACGGTAGAGAAGCAATCAGGGAGGAGGATAC

A3-AT5G26742-XLOC_030103-4451-0
ATGCTTCGGACAAGGTAGAGCAGAAATAAATAAGAGAGAGGCAATAGCAA
A3-AT5G26742-XLOC_030103-4451-1
ATGCTTCGGACAAGGTAGAGCAGAAATAAATAAGAGAGAGGCAATAGCAA
CONSENSUS
ATGCTTCGGACAAGGTAGAGCAGAAATAAATAAGAGAGAGGCAATAGCAA

A3-AT5G26742-XLOC_030103-4451-0
AAGTAAAGGAGAAGGTTTTCCTCAAATTGGCATTTTCAGAGTTTGCGTGA
A3-AT5G26742-XLOC_030103-4451-1
AAGTAAAGGAGAAGGTTTTCCTCAAATTGGCATTTTCAGAGTTTGCGTGA
CONSENSUS
AAGTAAAGGAGAAGGTTTTCCTCAAATTGGCATTTTCAGAGTTTGCGTGA

A3-AT5G26742-XLOC_030103-4451-0
GAAACAAGGGAAAGCTGATGGATCTTTTTATGTTAGGGTTATTATAATAG
A3-AT5G26742-XLOC_030103-4451-1
GAAACAAGGGAAAGCTGATGGATCTTTTTATGTTAGGGTTATTATAATAG
CONSENSUS
GAAACAAGGGAAAGCTGATGGATCTTTTTATGTTAGGGTTATTATAATAG

A3-AT5G26742-XLOC_030103-4451-0
TCTTAATGATCAGTTTCCATGTTTCTAAGGTTTTGTTTGATTTAAGTGGT
A3-AT5G26742-XLOC_030103-4451-1
TCTTAATGATCAGTTTCCATGTTTCTAAGGTTTTGTTTGATTTAAGTGGT
CONSENSUS
TCTTAATGATCAGTTTCCATGTTTCTAAGGTTTTGTTTGATTTAAGTGGT

A3-AT5G26742-XLOC_030103-4451-0
TATTAGTCAGTCTACTTACACTACTTTTATTTTCATTTTTGTACGTTTTC
A3-AT5G26742-XLOC_030103-4451-1

TATTAGTCAGTCTACTTACACTACTTTTATTTTCATTTTTGTACGTTTTC
CONSENSUS
TATTAGTCAGTCTACTTACACTACTTTTATTTTCATTTTTGTACGTTTTC

A3-AT5G26742-XLOC_030103-4451-0
TATTGTTATATGTGAATTCGGATCACATGTGTTTGGACAGAA
A3-AT5G26742-XLOC_030103-4451-1
TATTGTTATATGTGAATTCGGATCACATGTGTTTGGACAGAA
CONSENSUS
TATTGTTATATGTGAATTCGGATCACATGTGTTTGGACAGAA

alignment for event: RI-AT5G58470-XLOC_031899-1310

RI-AT5G58470-XLOC_031899-1310-0
AGGTGGGGGTAGAGGAGGAGGAGGTGGTGGCTATGGTGGTGGTGGGGGAG
RI-AT5G58470-XLOC_031899-1310-1
AGGTGGGGGTAGAGGAGGAGGAGGTGGTGGCTATGGTGGTGGTGGGGGAG
CONSENSUS
AGGTGGGGGTAGAGGAGGAGGAGGTGGTGGCTATGGTGGTGGTGGGGGAG

RI-AT5G58470-XLOC_031899-1310-0
ACAGAAGAAGAGACAACTACAGTTCAGGGCCAGACAGAAACCACCATGGG
RI-AT5G58470-XLOC_031899-1310-1
ACAGAAGAAGAGACAACTACAGTTCAGGGCCAGACAGAAACCACCATGGG
CONSENSUS
ACAGAAGAAGAGACAACTACAGTTCAGGGCCAGACAGAAACCACCATGGG

RI-AT5G58470-XLOC_031899-1310-0
GGAAACCGGTCTCGTCCATATTGAGAGAAAGAGAGGAATGAATGAATCAG
RI-AT5G58470-XLOC_031899-1310-1
GGAAACCGGTCTCGTCCATATTGAGAGAAAGAGAGGAATGAATGAATCAG
CONSENSUS
GGAAACCGGTCTCGTCCATATTGAGAGAAAGAGAGGAATGAATGAATCAG

RI-AT5G58470-XLOC_031899-1310-0
CTGTTATGTGTTCTAAGAGTTTTGTGTGTGTTAAGGAGGGAATTTTAGTA
RI-AT5G58470-XLOC_031899-1310-1
CTGTTATGTGTTCTAAGAGTTTTGTGTGTGTTAAGGAGGGAATTTTAGTA
CONSENSUS
CTGTTATGTGTTCTAAGAGTTTTGTGTGTGTTAAGGAGGGAATTTTAGTA

RI-AT5G58470-XLOC_031899-1310-0
AAATGTTCTATGAATCCTCATATTGGAGATACAAAAAAAGAGGTAGGTTT
RI-AT5G58470-XLOC_031899-1310-1
AAATGTTCTATGAATCCTCATATTGGAGATACAAAAAAAGAG--------
CONSENSUS
AAATGTTCTATGAATCCTCATATTGGAGATACAAAAAAAGAG........

RI-AT5G58470-XLOC_031899-1310-0
TTGTTCACTTTGACCTTGAGCAAAATTACCCCTTTTTTCTCCCTACTTGT
RI-AT5G58470-XLOC_031899-1310-1

CONSENSUS
....

RI-AT5G58470-XLOC_031899-1310-0
TCTGTTTCTTTGGGGATCTATTATTCATGCATCTCCGGTTTATTTTTAGT
RI-AT5G58470-XLOC_031899-1310-1

CONSENSUS
....

RI-AT5G58470-XLOC_031899-1310-0
TTGTTTTTCACGATGTGAAATGTTTGTTTTTTGTTTTATTGAAGGAAGCT
RI-AT5G58470-XLOC_031899-1310-1
--GAAGCT
CONSENSUS
....GAAGCT

RI-AT5G58470-XLOC_031899-1310-0
GATATTTAACTGCAAAATGCATTGCAAATTGGTGCTATGATGATTTCAGG
RI-AT5G58470-XLOC_031899-1310-1
GATATTTAACTGCAAAATGCATTGCAAATTGGTGCTATGATGATTTCAGG
CONSENSUS
GATATTTAACTGCAAAATGCATTGCAAATTGGTGCTATGATGATTTCAGG

RI-AT5G58470-XLOC_031899-1310-0
TGAGTGCATTCATCTTGATTACTTCTTTCAACACCAGAAGTCCCTTTTTT
RI-AT5G58470-XLOC_031899-1310-1
TGAGTGCATTCATCTTGATTACTTCTTTCAACACCAGAAGTCCCTTTTTT
CONSENSUS
TGAGTGCATTCATCTTGATTACTTCTTTCAACACCAGAAGTCCCTTTTTT

RI-AT5G58470-XLOC_031899-1310-0
GCTTCTCTTCCTTCTTTTTTTGACTCTGTTCAACACAAACAAAACAGTCG
RI-AT5G58470-XLOC_031899-1310-1
GCTTCTCTTCCTTCTTTTTTTGACTCTGTTCAACACAAACAAAACAGTCG
CONSENSUS
GCTTCTCTTCCTTCTTTTTTTGACTCTGTTCAACACAAACAAAACAGTCG

RI-AT5G58470-XLOC_031899-1310-0
TATGTTTTCATGTTGTATTGTGTAATGCATTCATTTAGGCAACCACACTT
RI-AT5G58470-XLOC_031899-1310-1
TATGTTTTCATGTTGTATTGTGTAATGCATTCATTTAGGCAACCACACTT
CONSENSUS
TATGTTTTCATGTTGTATTGTGTAATGCATTCATTTAGGCAACCACACTT

RI-AT5G58470-XLOC_031899-1310-0
CTCTTTACAATTTTCGGAGTTGTTATCGTTTTTTCTCGCAAACTTCTTTT
RI-AT5G58470-XLOC_031899-1310-1
CTCTTTACAATTTTCGGAGTTGTTATCGTTTTTTCTCGCAAACTTCTTTT
CONSENSUS
CTCTTTACAATTTTCGGAGTTGTTATCGTTTTTTCTCGCAAACTTCTTTT

RI-AT5G58470-XLOC_031899-1310-0
ATAGCAACATGTGTTCCTCGAGAATTGGCAGGAGTGGGGGATGGCTAGGC
RI-AT5G58470-XLOC_031899-1310-1
ATAGCAACATGTGTTCCTCGAGAATTGGCAGGAGTGGGGGATGGCTAGGC
CONSENSUS
ATAGCAACATGTGTTCCTCGAGAATTGGCAGGAGTGGGGGATGGCTAGGC

RI-AT5G58470-XLOC_031899-1310-0
ACTTGTGTCTGATCAGTTGATCTGAAGTATGCAGTGACCTTTCAAAGATC
RI-AT5G58470-XLOC_031899-1310-1
ACTTGTGTCTGATCAGTTGATCTGAAGTATGCAGTGACCTTTCAAAGATC
CONSENSUS
ACTTGTGTCTGATCAGTTGATCTGAAGTATGCAGTGACCTTTCAAAGATC

RI-AT5G58470-XLOC_031899-1310-0
AACTTGATGATTTTATTGCCAACTTCACTCACTGGGGCTATGATTAGGAT
RI-AT5G58470-XLOC_031899-1310-1
AACTTGATGATTTTATTGCCAACTTCACTCACTGGGGCTATGATTAGGAT
CONSENSUS
AACTTGATGATTTTATTGCCAACTTCACTCACTGGGGCTATGATTAGGAT

RI-AT5G58470-XLOC_031899-1310-0
TAGGGTTTTACAAATGAGTGGCCTTTTTTTGGATAAAAGACGTTGTTAAT
RI-AT5G58470-XLOC_031899-1310-1
TAGGGTTTTACAAATGAGTGGCCTTTTTTTGGATAAAAGACGTTGTTAAT
CONSENSUS
TAGGGTTTTACAAATGAGTGGCCTTTTTTTGGATAAAAGACGTTGTTAAT

RI-AT5G58470-XLOC_031899-1310-0
AAATAGTTAATCGTTCAATCTGGATCCCAAGGACTAAAGAGAAGGGTGTC
RI-AT5G58470-XLOC_031899-1310-1
AAATAGTTAATCGTTCAATCTGGATCCCAAGGACTAAAGAGAAGGGTGTC
CONSENSUS
AAATAGTTAATCGTTCAATCTGGATCCCAAGGACTAAAGAGAAGGGTGTC

RI-AT5G58470-XLOC_031899-1310-0
TCGTAAAGTTCTCTATGTGTATATGTCATGACCTTTTGGTCAAGTGCCGG
RI-AT5G58470-XLOC_031899-1310-1
TCGTAAAGTTCTCTATGTGTATATGTCATGACCTTTTGGTCAAGTGCCGG
CONSENSUS
TCGTAAAGTTCTCTATGTGTATATGTCATGACCTTTTGGTCAAGTGCCGG

RI-AT5G58470-XLOC_031899-1310-0
ATTGGCCAACTCTTTAATGTGTTACATGTAACG
RI-AT5G58470-XLOC_031899-1310-1
ATTGGCCAACTCTTTAATGTGTTACATGTAACG
CONSENSUS
ATTGGCCAACTCTTTAATGTGTTACATGTAACG

alignment for event: A3-AT5G07740-XLOC_029125-4623

A3-AT5G07740-XLOC_029125-4623-0
GAAGAGTGTATCCTAGTGAAATTAGATATACAGTGCCGCGTTCAAGGGGA
A3-AT5G07740-XLOC_029125-4623-1
GAAGAGTGTATCCTAGTGAAATTAGATATACAGTGCCGCGTTCAAGGGGA
CONSENSUS
GAAGAGTGTATCCTAGTGAAATTAGATATACAGTGCCGCGTTCAAGGGGA

A3-AT5G07740-XLOC_029125-4623-0
TGTTGTGCTGGAATGTATACATTTGCATGATGATTTGGTGAGTGAGGAGA

A3-AT5G07740-XLOC_029125-4623-1
TGTTGTGCTGGAATGTATACATTTGCATGATGATTTGGTGAGTGAGGAGA
CONSENSUS
TGTTGTGCTGGAATGTATACATTTGCATGATGATTTGGTGAGTGAGGAGA

A3-AT5G07740-XLOC_029125-4623-0
TGGTTTTTAGGATCATGTTCCACACAGCGTTTGTGCGTGCTAATATTTTA
A3-AT5G07740-XLOC_029125-4623-1
TGGTTTTTAGGATCATGTTCCACACAGCGTTTGTGCGTGCTAATATTTTA
CONSENSUS
TGGTTTTTAGGATCATGTTCCACACAGCGTTTGTGCGTGCTAATATTTTA

A3-AT5G07740-XLOC_029125-4623-0
ATGCTCCAACGCGATGAGATGGATATACTCTGGGATGTCAAGGACCAGTT
A3-AT5G07740-XLOC_029125-4623-1
ATGCTCCAACGCGATGAGATGGATATACTCTGGGATGTCAAGGACCAGTT
CONSENSUS
ATGCTCCAACGCGATGAGATGGATATACTCTGGGATGTCAAGGACCAGTT

A3-AT5G07740-XLOC_029125-4623-0
CCCAAAGGAATTTAAAGCAGAG----------------------------
A3-AT5G07740-XLOC_029125-4623-1
CCCAAAGGAATTTAAAGCAGAGGTACTTTTCTCTGGCGCTGATGCCGTGG
CONSENSUS
CCCAAAGGAATTTAAAGCAGAG............................

A3-AT5G07740-XLOC_029125-4623-0 ------------------------------
ATGATGAGAACGATTTTGAT
A3-AT5G07740-XLOC_029125-4623-1
TGCCTCCTATTACAACGTCTACATTATCAGATGATGAGAACGATTTTGAT
CONSENSUS
..............................ATGATGAGAACGATTTTGAT

A3-AT5G07740-XLOC_029125-4623-0
ATGACTTCACCTGAAGAATTTTTTGAGGTGGAGGAGATCTTTAGCGATGT
A3-AT5G07740-XLOC_029125-4623-1
ATGACTTCACCTGAAGAATTTTTTGAGGTGGAGGAGATCTTTAGCGATGT
CONSENSUS
ATGACTTCACCTGAAGAATTTTTTGAGGTGGAGGAGATCTTTAGCGATGT

A3-AT5G07740-XLOC_029125-4623-0
GATTGATGGACCTGACCATAAGAGAGACTCGGATAGTTTTGTGGTTGTTG
A3-AT5G07740-XLOC_029125-4623-1
GATTGATGGACCTGACCATAAGAGAGACTCGGATAGTTTTGTGGTTGTTG
CONSENSUS
GATTGATGGACCTGACCATAAGAGAGACTCGGATAGTTTTGTGGTTGTTG

A3-AT5G07740-XLOC_029125-4623-0
ATACTGCTTCAGATGATTCTGAGGGTAAAGAGGTGTGGAAGGGGGATGTG
A3-AT5G07740-XLOC_029125-4623-1
ATACTGCTTCAGATGATTCTGAGGGTAAAGAGGTGTGGAAGGGGGATGTG
CONSENSUS
ATACTGCTTCAGATGATTCTGAGGGTAAAGAGGTGTGGAAGGGGGATGTG

A3-AT5G07740-XLOC_029125-4623-0
GAACCCAATGCGTTTCTAGATTGTGCATCAGATGATTCAAACCATAAACA

A3-AT5G07740-XLOC_029125-4623-1
GAACCCAATGCGTTTCTAGATTGTGCATCAGATGATTCAAACCATAAACA
CONSENSUS
GAACCCAATGCGTTTCTAGATTGTGCATCAGATGATTCAAACCATAAACA

A3-AT5G07740-XLOC_029125-4623-0
TGATATGCATGCAGAGACTAGCACGGATCCAGTCAAAGATATCACTGTGG
A3-AT5G07740-XLOC_029125-4623-1
TGATATGCATGCAGAGACTAGCACGGATCCAGTCAAAGATATCACTGTGG
CONSENSUS
TGATATGCATGCAGAGACTAGCACGGATCCAGTCAAAGATATCACTGTGG

A3-AT5G07740-XLOC_029125-4623-0
ATGATGTACAGTATAGATCGGATGGGAAGGCAGATTCTAATATCGACTCA
A3-AT5G07740-XLOC_029125-4623-1
ATGATGTACAGTATAGATCGGATGGGAAGGCAGATTCTAATATCGACTCA
CONSENSUS
ATGATGTACAGTATAGATCGGATGGGAAGGCAGATTCTAATATCGACTCA

A3-AT5G07740-XLOC_029125-4623-0
GTGAAGGATATAGGAATAGATGATGGTGATGAGCAGCGAAAGAGAAGAAC
A3-AT5G07740-XLOC_029125-4623-1
GTGAAGGATATAGGAATAGATGATGGTGATGAGCAGCGAAAGAGAAGAAC
CONSENSUS
GTGAAGGATATAGGAATAGATGATGGTGATGAGCAGCGAAAGAGAAGAAC

A3-AT5G07740-XLOC_029125-4623-0
AGTGGAAGCAAAGGAAAATGATTCTAGCACAGTACAGACTCAAAGCAAAG
A3-AT5G07740-XLOC_029125-4623-1
AGTGGAAGCAAAGGAAAATGATTCTAGCACAGTACAGACTCAAAGCAAAG
CONSENSUS
AGTGGAAGCAAAGGAAAATGATTCTAGCACAGTACAGACTCAAAGCAAAG

A3-AT5G07740-XLOC_029125-4623-0
GTGATGAAGAAAGTAACGATTTGGAATCTATGAGTCAGAAAACAAATACT
A3-AT5G07740-XLOC_029125-4623-1
GTGATGAAGAAAGTAACGATTTGGAATCTATGAGTCAGAAAACAAATACT
CONSENSUS
GTGATGAAGAAAGTAACGATTTGGAATCTATGAGTCAGAAAACAAATACT

A3-AT5G07740-XLOC_029125-4623-0
AGTCTCAACAAACCCATATCTGAAAAACCACAAGCTACACTAAGGAAACA
A3-AT5G07740-XLOC_029125-4623-1
AGTCTCAACAAACCCATATCTGAAAAACCACAAGCTACACTAAGGAAACA
CONSENSUS
AGTCTCAACAAACCCATATCTGAAAAACCACAAGCTACACTAAGGAAACA

A3-AT5G07740-XLOC_029125-4623-0
GGTCGGAGCAAATGCAAAACCGGCTGCTGCTGGAGATTCCCTTAAACCGA
A3-AT5G07740-XLOC_029125-4623-1
GGTCGGAGCAAATGCAAAACCGGCTGCTGCTGGAGATTCCCTTAAACCGA
CONSENSUS
GGTCGGAGCAAATGCAAAACCGGCTGCTGCTGGAGATTCCCTTAAACCGA

A3-AT5G07740-XLOC_029125-4623-0
AGTCTAAGCAGCAAGAAACTCAGGGTCCTAATGTAAGAATGGCTAAGCCT

A3-AT5G07740-XLOC_029125-4623-1
AGTCTAAGCAGCAAGAAACTCAGGGTCCTAATGTAAGAATGGCTAAGCCT
CONSENSUS
AGTCTAAGCAGCAAGAAACTCAGGGTCCTAATGTAAGAATGGCTAAGCCT

A3-AT5G07740-XLOC_029125-4623-0
AACGCAGTCTCTCGATGGATTCCTTCAAATAAGGGCTCATATAAAGATTC
A3-AT5G07740-XLOC_029125-4623-1
AACGCAGTCTCTCGATGGATTCCTTCAAATAAGGGCTCATATAAAGATTC
CONSENSUS
AACGCAGTCTCTCGATGGATTCCTTCAAATAAGGGCTCATATAAAGATTC

A3-AT5G07740-XLOC_029125-4623-0
TATGCATGTGGCTTATCCCCCGACAAGAATTAACAGTGCTCCTGCCTCAA
A3-AT5G07740-XLOC_029125-4623-1
TATGCATGTGGCTTATCCCCCGACAAGAATTAACAGTGCTCCTGCCTCAA
CONSENSUS
TATGCATGTGGCTTATCCCCCGACAAGAATTAACAGTGCTCCTGCCTCAA

A3-AT5G07740-XLOC_029125-4623-0
TTACCACTTCTCTCAAGGATGGTAAAAGAGCTACATCACCTGATGGCGTG
A3-AT5G07740-XLOC_029125-4623-1
TTACCACTTCTCTCAAGGATGGTAAAAGAGCTACATCACCTGATGGCGTG
CONSENSUS
TTACCACTTCTCTCAAGGATGGTAAAAGAGCTACATCACCTGATGGCGTG

A3-AT5G07740-XLOC_029125-4623-0
ATTCCAAAGGATGCTAAGACTAAATATTTGAGAGCATCTGTTTCTTCACC
A3-AT5G07740-XLOC_029125-4623-1
ATTCCAAAGGATGCTAAGACTAAATATTTGAGAGCATCTGTTTCTTCACC
CONSENSUS
ATTCCAAAGGATGCTAAGACTAAATATTTGAGAGCATCTGTTTCTTCACC

A3-AT5G07740-XLOC_029125-4623-0
GGATATGAGGAGTCGAGCTCCAATTTGTTCATCACCAGATTCTAGCCCGA
A3-AT5G07740-XLOC_029125-4623-1
GGATATGAGGAGTCGAGCTCCAATTTGTTCATCACCAGATTCTAGCCCGA
CONSENSUS
GGATATGAGGAGTCGAGCTCCAATTTGTTCATCACCAGATTCTAGCCCGA

A3-AT5G07740-XLOC_029125-4623-0
AGGAAACGCCTTCTTCTCTACCTCCTGCCTCTCCACATCAGGCTCCTCCA
A3-AT5G07740-XLOC_029125-4623-1
AGGAAACGCCTTCTTCTCTACCTCCTGCCTCTCCACATCAGGCTCCTCCA
CONSENSUS
AGGAAACGCCTTCTTCTCTACCTCCTGCCTCTCCACATCAGGCTCCTCCA

A3-AT5G07740-XLOC_029125-4623-0
CCCCTGCCTTCTTTAACCAGTGAGGCCAAAACAGTTTTACACTCTTCACA
A3-AT5G07740-XLOC_029125-4623-1
CCCCTGCCTTCTTTAACCAGTGAGGCCAAAACAGTTTTACACTCTTCACA
CONSENSUS
CCCCTGCCTTCTTTAACCAGTGAGGCCAAAACAGTTTTACACTCTTCACA

A3-AT5G07740-XLOC_029125-4623-0
AGCAGTTGCATCACCTCCTCCTCCTCCGCCCCCACCACCATTGCCAACTT

A3-AT5G07740-XLOC_029125-4623-1
AGCAGTTGCATCACCTCCTCCTCCTCCGCCCCCACCACCATTGCCAACTT
CONSENSUS
AGCAGTTGCATCACCTCCTCCTCCTCCGCCCCCACCACCATTGCCAACTT

A3-AT5G07740-XLOC_029125-4623-0
ACAGCCACTATCAAACATCTCAGCTCCCACCACCACCTCCGCCTCCTCCT
A3-AT5G07740-XLOC_029125-4623-1
ACAGCCACTATCAAACATCTCAGCTCCCACCACCACCTCCGCCTCCTCCT
CONSENSUS
ACAGCCACTATCAAACATCTCAGCTCCCACCACCACCTCCGCCTCCTCCT

A3-AT5G07740-XLOC_029125-4623-0
CCTTTTTCATCTGAGAGACCAAACAGTGGAACTGTGTTGCCTCCACCACC
A3-AT5G07740-XLOC_029125-4623-1
CCTTTTTCATCTGAGAGACCAAACAGTGGAACTGTGTTGCCTCCACCACC
CONSENSUS
CCTTTTTCATCTGAGAGACCAAACAGTGGAACTGTGTTGCCTCCACCACC

A3-AT5G07740-XLOC_029125-4623-0
ACCGCCTCCTCCTCCTTTTTCATCTGAGAGACCAAACAGTGGAACTGTGT
A3-AT5G07740-XLOC_029125-4623-1
ACCGCCTCCTCCTCCTTTTTCATCTGAGAGACCAAACAGTGGAACTGTGT
CONSENSUS
ACCGCCTCCTCCTCCTTTTTCATCTGAGAGACCAAACAGTGGAACTGTGT

A3-AT5G07740-XLOC_029125-4623-0
TGCCTCCACCACCACCACCACCTCTTCCTTTTTCATCTGAGAGACCAAAC
A3-AT5G07740-XLOC_029125-4623-1
TGCCTCCACCACCACCACCACCTCTTCCTTTTTCATCTGAGAGACCAAAC
CONSENSUS
TGCCTCCACCACCACCACCACCTCTTCCTTTTTCATCTGAGAGACCAAAC

A3-AT5G07740-XLOC_029125-4623-0
AGTGGAACTGTATTGCCTCCCCCACCATCTCCACCTTGGAAGTCTGTCTA
A3-AT5G07740-XLOC_029125-4623-1
AGTGGAACTGTATTGCCTCCCCCACCATCTCCACCTTGGAAGTCTGTCTA
CONSENSUS
AGTGGAACTGTATTGCCTCCCCCACCATCTCCACCTTGGAAGTCTGTCTA

A3-AT5G07740-XLOC_029125-4623-0
TGCTTCAGCTTTGGCGATTCCTGCAATATGTTCTACATCTCAAGCCCCTA
A3-AT5G07740-XLOC_029125-4623-1
TGCTTCAGCTTTGGCGATTCCTGCAATATGTTCTACATCTCAAGCCCCTA
CONSENSUS
TGCTTCAGCTTTGGCGATTCCTGCAATATGTTCTACATCTCAAGCCCCTA

A3-AT5G07740-XLOC_029125-4623-0
CGTCTTCACCAACTCCACCACCACCTCCGCCTGCATACTATTCAGTAGGT
A3-AT5G07740-XLOC_029125-4623-1
CGTCTTCACCAACTCCACCACCACCTCCGCCTGCATACTATTCAGTAGGT
CONSENSUS
CGTCTTCACCAACTCCACCACCACCTCCGCCTGCATACTATTCAGTAGGT

A3-AT5G07740-XLOC_029125-4623-0
CAAAAGAGCAGTGACTTGCAAACATCTCAGCTACCATCGCCGCCGCCTCC

A3-AT5G07740-XLOC_029125-4623-1
CAAAAGAGCAGTGACTTGCAAACATCTCAGCTACCATCGCCGCCGCCTCC
CONSENSUS
CAAAAGAGCAGTGACTTGCAAACATCTCAGCTACCATCGCCGCCGCCTCC

A3-AT5G07740-XLOC_029125-4623-0
TCCTCCACCACCTCCGTTTGCATCTGTGAGGCGAAACAGTGAAACTTTGT
A3-AT5G07740-XLOC_029125-4623-1
TCCTCCACCACCTCCGTTTGCATCTGTGAGGCGAAACAGTGAAACTTTGT
CONSENSUS
TCCTCCACCACCTCCGTTTGCATCTGTGAGGCGAAACAGTGAAACTTTGT

A3-AT5G07740-XLOC_029125-4623-0
TGCCTCCACCTCCACCTCCTCCACCACCTCCGTTTGCATCTGTGAGGCGA
A3-AT5G07740-XLOC_029125-4623-1
TGCCTCCACCTCCACCTCCTCCACCACCTCCGTTTGCATCTGTGAGGCGA
CONSENSUS
TGCCTCCACCTCCACCTCCTCCACCACCTCCGTTTGCATCTGTGAGGCGA

A3-AT5G07740-XLOC_029125-4623-0
AACAGTGAAACTTTGTTGCCTCCACCTCCACCTCCACCACCTTGGAAGTC
A3-AT5G07740-XLOC_029125-4623-1
AACAGTGAAACTTTGTTGCCTCCACCTCCACCTCCACCACCTTGGAAGTC
CONSENSUS
AACAGTGAAACTTTGTTGCCTCCACCTCCACCTCCACCACCTTGGAAGTC

A3-AT5G07740-XLOC_029125-4623-0
TCTGTATGCTTCGACTTTTGAAACTCATGAGGCGTGCTCTACATCTTCTT
A3-AT5G07740-XLOC_029125-4623-1
TCTGTATGCTTCGACTTTTGAAACTCATGAGGCGTGCTCTACATCTTCTT
CONSENSUS
TCTGTATGCTTCGACTTTTGAAACTCATGAGGCGTGCTCTACATCTTCTT

A3-AT5G07740-XLOC_029125-4623-0
CCCCACCACCACCACCACCACCTCCTCCTTTTAGCCCATTAAATACAACA
A3-AT5G07740-XLOC_029125-4623-1
CCCCACCACCACCACCACCACCTCCTCCTTTTAGCCCATTAAATACAACA
CONSENSUS
CCCCACCACCACCACCACCACCTCCTCCTTTTAGCCCATTAAATACAACA

A3-AT5G07740-XLOC_029125-4623-0
AAAGCAAATGATTACATCCTCCCTCCTCCCCCTCTACCTTACACTAGTAT
A3-AT5G07740-XLOC_029125-4623-1
AAAGCAAATGATTACATCCTCCCTCCTCCCCCTCTACCTTACACTAGTAT
CONSENSUS
AAAGCAAATGATTACATCCTCCCTCCTCCCCCTCTACCTTACACTAGTAT

A3-AT5G07740-XLOC_029125-4623-0
TGCACCTTCACCATCAGTCAAGATCCTTCCTTTACATGGTATTTCCAGTG
A3-AT5G07740-XLOC_029125-4623-1
TGCACCTTCACCATCAGTCAAGATCCTTCCTTTACATGGTATTTCCAGTG
CONSENSUS
TGCACCTTCACCATCAGTCAAGATCCTTCCTTTACATGGTATTTCCAGTG

A3-AT5G07740-XLOC_029125-4623-0
CTCCATCTCCACCTGTTAAAACAGCTCCTCCACCACCTCCACCTCCTCCT

A3-AT5G07740-XLOC_029125-4623-1
CTCCATCTCCACCTGTTAAAACAGCTCCTCCACCACCTCCACCTCCTCCT
CONSENSUS
CTCCATCTCCACCTGTTAAAACAGCTCCTCCACCACCTCCACCTCCTCCT

A3-AT5G07740-XLOC_029125-4623-0
TTTAGTAATGCACATTCAGTACTTTCCCCTCCTCCACCGAGCTATGGATC
A3-AT5G07740-XLOC_029125-4623-1
TTTAGTAATGCACATTCAGTACTTTCCCCTCCTCCACCGAGCTATGGATC
CONSENSUS
TTTAGTAATGCACATTCAGTACTTTCCCCTCCTCCACCGAGCTATGGATC

A3-AT5G07740-XLOC_029125-4623-0
TCCACCCCCACCACCGCCTCCTCCACCGAGCTATGGATCTCCACCCCCAC
A3-AT5G07740-XLOC_029125-4623-1
TCCACCCCCACCACCGCCTCCTCCACCGAGCTATGGATCTCCACCCCCAC
CONSENSUS
TCCACCCCCACCACCGCCTCCTCCACCGAGCTATGGATCTCCACCCCCAC

A3-AT5G07740-XLOC_029125-4623-0
CACCTCCTCCTCCACCGAGCTATGGATCTCCACCCCCACCACCTCCTCCT
A3-AT5G07740-XLOC_029125-4623-1
CACCTCCTCCTCCACCGAGCTATGGATCTCCACCCCCACCACCTCCTCCT
CONSENSUS
CACCTCCTCCTCCACCGAGCTATGGATCTCCACCCCCACCACCTCCTCCT

A3-AT5G07740-XLOC_029125-4623-0
CCACCGGGCTATGGATCTCCACCCCCACCCCCACCTCCTCCACCAAGTTA
A3-AT5G07740-XLOC_029125-4623-1
CCACCGGGCTATGGATCTCCACCCCCACCCCCACCTCCTCCACCAAGTTA
CONSENSUS
CCACCGGGCTATGGATCTCCACCCCCACCCCCACCTCCTCCACCAAGTTA

A3-AT5G07740-XLOC_029125-4623-0
TGGATCTCCACCCCCACCCCCACCTCCTCCTTTCAGTCATGTAAGCTCAA
A3-AT5G07740-XLOC_029125-4623-1
TGGATCTCCACCCCCACCCCCACCTCCTCCTTTCAGTCATGTAAGCTCAA
CONSENSUS
TGGATCTCCACCCCCACCCCCACCTCCTCCTTTCAGTCATGTAAGCTCAA

A3-AT5G07740-XLOC_029125-4623-0
TTCCACCGCCGCCACCGCCTCCCCCTATGCATGGAGGAGCTCCACCGCCG
A3-AT5G07740-XLOC_029125-4623-1
TTCCACCGCCGCCACCGCCTCCCCCTATGCATGGAGGAGCTCCACCGCCG
CONSENSUS
TTCCACCGCCGCCACCGCCTCCCCCTATGCATGGAGGAGCTCCACCGCCG

A3-AT5G07740-XLOC_029125-4623-0
CCACCGCCTCCCCCTATGCATGGAGGAGCTCCACCACCGCCGCCGCCTCC
A3-AT5G07740-XLOC_029125-4623-1
CCACCGCCTCCCCCTATGCATGGAGGAGCTCCACCACCGCCGCCGCCTCC
CONSENSUS
CCACCGCCTCCCCCTATGCATGGAGGAGCTCCACCACCGCCGCCGCCTCC

A3-AT5G07740-XLOC_029125-4623-0
CCCTATGCATGGAGGAGCTCCACCGCCGCCGCCGCCTCCCCCTATGCATG

A3-AT5G07740-XLOC_029125-4623-1
CCCTATGCATGGAGGAGCTCCACCGCCGCCGCCGCCTCCCCCTATGCATG
CONSENSUS
CCCTATGCATGGAGGAGCTCCACCGCCGCCGCCGCCTCCCCCTATGCATG

A3-AT5G07740-XLOC_029125-4623-0
GAGGAGCTCCACCGCCACCGCCGCCCCCTATGTTTGGAGGAGCCCAACCG
A3-AT5G07740-XLOC_029125-4623-1
GAGGAGCTCCACCGCCACCGCCGCCCCCTATGTTTGGAGGAGCCCAACCG
CONSENSUS
GAGGAGCTCCACCGCCACCGCCGCCCCCTATGTTTGGAGGAGCCCAACCG

A3-AT5G07740-XLOC_029125-4623-0
CCGCCGCCTCCCCCTATGCGTGGAGGAGCTCCACCGCCGCCGCCTCCCCC
A3-AT5G07740-XLOC_029125-4623-1
CCGCCGCCTCCCCCTATGCGTGGAGGAGCTCCACCGCCGCCGCCTCCCCC
CONSENSUS
CCGCCGCCTCCCCCTATGCGTGGAGGAGCTCCACCGCCGCCGCCTCCCCC

A3-AT5G07740-XLOC_029125-4623-0
GATGCGTGGAGGAGCCCCACCGCCGCCACCTCCCCCTATGCGTGGAGGAG
A3-AT5G07740-XLOC_029125-4623-1
GATGCGTGGAGGAGCCCCACCGCCGCCACCTCCCCCTATGCGTGGAGGAG
CONSENSUS
GATGCGTGGAGGAGCCCCACCGCCGCCACCTCCCCCTATGCGTGGAGGAG

A3-AT5G07740-XLOC_029125-4623-0
CCCCACCACCGCCGCCTCCCCCGATGCATGGAGGAGCTCCACCGCCGCCG
A3-AT5G07740-XLOC_029125-4623-1
CCCCACCACCGCCGCCTCCCCCGATGCATGGAGGAGCTCCACCGCCGCCG
CONSENSUS
CCCCACCACCGCCGCCTCCCCCGATGCATGGAGGAGCTCCACCGCCGCCG

A3-AT5G07740-XLOC_029125-4623-0
CCTCCCCCGATGCGTGGAGGAGCCCCGCCACCACCTCCGCCTCCTGGCGG
A3-AT5G07740-XLOC_029125-4623-1
CCTCCCCCGATGCGTGGAGGAGCCCCGCCACCACCTCCGCCTCCTGGCGG
CONSENSUS
CCTCCCCCGATGCGTGGAGGAGCCCCGCCACCACCTCCGCCTCCTGGCGG

A3-AT5G07740-XLOC_029125-4623-0
TAGAGGGCCTGGTGCACCGCCGCCTCCACCACCTCCTGGTGGCCGTGCTC
A3-AT5G07740-XLOC_029125-4623-1
TAGAGGGCCTGGTGCACCGCCGCCTCCACCACCTCCTGGTGGCCGTGCTC
CONSENSUS
TAGAGGGCCTGGTGCACCGCCGCCTCCACCACCTCCTGGTGGCCGTGCTC

A3-AT5G07740-XLOC_029125-4623-0
CTGGTCCACCCCCACCTCCTGGACCAAGACCTCCGGGTGGTGGACCTCCT
A3-AT5G07740-XLOC_029125-4623-1
CTGGTCCACCCCCACCTCCTGGACCAAGACCTCCGGGTGGTGGACCTCCT
CONSENSUS
CTGGTCCACCCCCACCTCCTGGACCAAGACCTCCGGGTGGTGGACCTCCT

A3-AT5G07740-XLOC_029125-4623-0
CCACCTCCAATGCTTGGTGCCAGAGGAGCTGCAGTTGATCCTAGAGGTGC

A3-AT5G07740-XLOC_029125-4623-1
CCACCTCCAATGCTTGGTGCCAGAGGAGCTGCAGTTGATCCTAGAGGTGC
CONSENSUS
CCACCTCCAATGCTTGGTGCCAGAGGAGCTGCAGTTGATCCTAGAGGTGC

A3-AT5G07740-XLOC_029125-4623-0
AGGAAGAGGGCGTGGTCTTCCTCGTCCAGGTTTTGGGTCTGCAGCTCAAA
A3-AT5G07740-XLOC_029125-4623-1
AGGAAGAGGGCGTGGTCTTCCTCGTCCAGGTTTTGGGTCTGCAGCTCAAA
CONSENSUS
AGGAAGAGGGCGTGGTCTTCCTCGTCCAGGTTTTGGGTCTGCAGCTCAAA

A3-AT5G07740-XLOC_029125-4623-0
AAAAGTCTTCCCTGAAGCCATTACACTGGGTTAAAGTAACAAGGGCCTTG
A3-AT5G07740-XLOC_029125-4623-1
AAAAGTCTTCCCTGAAGCCATTACACTGGGTTAAAGTAACAAGGGCCTTG
CONSENSUS
AAAAGTCTTCCCTGAAGCCATTACACTGGGTTAAAGTAACAAGGGCCTTG

A3-AT5G07740-XLOC_029125-4623-0
CAAGGGAGCTTATGGGATGAGTTACAGAGACATGGAGAATCACAAAC
A3-AT5G07740-XLOC_029125-4623-1
CAAGGGAGCTTATGGGATGAGTTACAGAGACATGGAGAATCACAAAC
CONSENSUS
CAAGGGAGCTTATGGGATGAGTTACAGAGACATGGAGAATCACAAAC

alignment for event: SE-AT5G20450-XLOC_029790-6543

SE-AT5G20450-XLOC_029790-6543-0
GCTTCGCTTCAGTCTGAACGACAAGCAGCAGAAGACTTGAGAAATGCTTT
SE-AT5G20450-XLOC_029790-6543-1
GCTTCGCTTCAGTCTGAACGACAAGCAGCAGAAGACTTGAGAAATGCTTT
CONSENSUS
GCTTCGCTTCAGTCTGAACGACAAGCAGCAGAAGACTTGAGAAATGCTTT

SE-AT5G20450-XLOC_029790-6543-0
CTCAGAGGCAGAGGCTCGAAATTCGGAGCTGGCCACAAATCTTGAAAATG
SE-AT5G20450-XLOC_029790-6543-1
CTCAGAGGCAGAGGCTCGAAATTCGGAGCTGGCCACAAATCTTGAAAATG
CONSENSUS
CTCAGAGGCAGAGGCTCGAAATTCGGAGCTGGCCACAAATCTTGAAAATG

SE-AT5G20450-XLOC_029790-6543-0
TGACAAGAAGAGTAGATCAGCTTTGTGAATCG------------------
SE-AT5G20450-XLOC_029790-6543-1
TGACAAGAAGAGTAGATCAGCTTTGTGAATCGGCTTCGCTCCAGTCTGAA
CONSENSUS
TGACAAGAAGAGTAGATCAGCTTTGTGAATCG..................

SE-AT5G20450-XLOC_029790-6543-0

SE-AT5G20450-XLOC_029790-6543-1
CAACAAGCAGCAGAAGACTTGAGAAAAGCTTTGTCCCTGGCAGAGGCTAG
CONSENSUS

....

SE-AT5G20450-XLOC_029790-6543-0

SE-AT5G20450-XLOC_029790-6543-1
AAATTTGGAACTGACCACAAAACTTGAAAATGTGACAAGAAGAGTAGATC
CONSENSUS
....

SE-AT5G20450-XLOC_029790-6543-0 --------------
GAAAGCCAGGAAGTACTAGTGAAGTGCATCTCACAA
SE-AT5G20450-XLOC_029790-6543-1
AGCTTTGTGAATCGGAAAGCCAGGAAGTACTAGTGAAGTGCATCTCACAA
CONSENSUS
..............GAAAGCCAGGAAGTACTAGTGAAGTGCATCTCACAA

SE-AT5G20450-XLOC_029790-6543-0
AACCTTGGATATGATGGAGGCAAGCCTGTTGCTGCATGTGTCATTTACAA
SE-AT5G20450-XLOC_029790-6543-1
AACCTTGGATATGATGGAGGCAAGCCTGTTGCTGCATGTGTCATTTACAA
CONSENSUS
AACCTTGGATATGATGGAGGCAAGCCTGTTGCTGCATGTGTCATTTACAA

SE-AT5G20450-XLOC_029790-6543-0
ATGTCTCCTTCACTGGAGATCTTTTGAAGTCGAAAGAACCAACATCTTTG
SE-AT5G20450-XLOC_029790-6543-1
ATGTCTCCTTCACTGGAGATCTTTTGAAGTCGAAAGAACCAACATCTTTG
CONSENSUS
ATGTCTCCTTCACTGGAGATCTTTTGAAGTCGAAAGAACCAACATCTTTG

SE-AT5G20450-XLOC_029790-6543-0
ACCGTATCGTGAAAATAATAGCCTCCGCCATTGAA
SE-AT5G20450-XLOC_029790-6543-1
ACCGTATCGTGAAAATAATAGCCTCCGCCATTGAA
CONSENSUS
ACCGTATCGTGAAAATAATAGCCTCCGCCATTGAA

alignment for event: RI-AT5G41000-XLOC_027244-8696

RI-AT5G41000-XLOC_027244-8696-0
ATAAAAGTAAGACCTCAGTATTAATGCGGGAGAAGAAGAAAAGAGATATC
RI-AT5G41000-XLOC_027244-8696-1
ATAAAAGTAAGACCTCAGTATTAATGCGGGAGAAGAAGAAAAGAGATATC
CONSENSUS
ATAAAAGTAAGACCTCAGTATTAATGCGGGAGAAGAAGAAAAGAGATATC

RI-AT5G41000-XLOC_027244-8696-0
ATATTTCTCAAGGACCGTATACCCCTCGAGTTTGCGGTTTCTGGTTATGT
RI-AT5G41000-XLOC_027244-8696-1
ATATTTCTCAAGGACCGTATACCCCTCGAGTTTGCGGTTTCTGGTTATGT
CONSENSUS
ATATTTCTCAAGGACCGTATACCCCTCGAGTTTGCGGTTTCTGGTTATGT

RI-AT5G41000-XLOC_027244-8696-0

GGGTCTTGCAGCTATTTCAACCGCTATAATCCCGTTGATATTCCCACCAT
RI-AT5G41000-XLOC_027244-8696-1
GGGTCTTGCAGCTATTTCAACCGCTATAATCCCGTTGATATTCCCACCAT
CONSENSUS
GGGTCTTGCAGCTATTTCAACCGCTATAATCCCGTTGATATTCCCACCAT

RI-AT5G41000-XLOC_027244-8696-0
TGAAATGGTACTTTGTCCTCTGTTCATACTTAGTTGCCCCGGGTCTTGCT
RI-AT5G41000-XLOC_027244-8696-1
TGAAATG---
CONSENSUS
TGAAATG...

RI-AT5G41000-XLOC_027244-8696-0
TTTTGCAATTCTTATGGAGCCGGGCTCACAGATATGAGCATGCCTTCAAC
RI-AT5G41000-XLOC_027244-8696-1

CONSENSUS
....

RI-AT5G41000-XLOC_027244-8696-0
CTACGGAAAGACTGGTCTTTTCATCGTTGCTTCAATTGTAGGAAATAATG
RI-AT5G41000-XLOC_027244-8696-1
--GAAATAATG
CONSENSUS
....GAAATAATG

RI-AT5G41000-XLOC_027244-8696-0
GTGGAGTCATTGCCGGTTTAGCAGCATGTGGCATTATGATGTCAATCGTC
RI-AT5G41000-XLOC_027244-8696-1
GTGGAGTCATTGCCGGTTTAGCAGCATGTGGCATTATGATGTCAATCGTC
CONSENSUS
GTGGAGTCATTGCCGGTTTAGCAGCATGTGGCATTATGATGTCAATCGTC

RI-AT5G41000-XLOC_027244-8696-0
TCAACTGCAGCGGATCTCATGCAGGATTTTAAAACAGGTTACCTCACACT
RI-AT5G41000-XLOC_027244-8696-1
TCAACTGCAGCGGATCTCATGCAGGATTTTAAAACAGGTTACCTCACACT
CONSENSUS
TCAACTGCAGCGGATCTCATGCAGGATTTTAAAACAGGTTACCTCACACT

RI-AT5G41000-XLOC_027244-8696-0
ATCATCTGCAAAATCCATGTTTGTAACTCAGCTTTTGGGTACAGCAATGG
RI-AT5G41000-XLOC_027244-8696-1
ATCATCTGCAAAATCCATGTTTGTAACTCAGCTTTTGGGTACAGCAATGG
CONSENSUS
ATCATCTGCAAAATCCATGTTTGTAACTCAGCTTTTGGGTACAGCAATGG

RI-AT5G41000-XLOC_027244-8696-0
GTTGCATAATCGCTCCTCTCACGTTCTGGCTGTTTTGGACTGCTTTTGAT
RI-AT5G41000-XLOC_027244-8696-1
GTTGCATAATCGCTCCTCTCACGTTCTGGCTGTTTTGGACTGCTTTTGAT
CONSENSUS
GTTGCATAATCGCTCCTCTCACGTTCTGGCTGTTTTGGACTGCTTTTGAT

RI-AT5G41000-XLOC_027244-8696-0

ATTGGAGATCCTGATGGTCTATACAAAGCACCTTACGCGGTCATCTACCG
RI-AT5G41000-XLOC_027244-8696-1
ATTGGAGATCCTGATGGTCTATACAAAGCACCTTACGCGGTCATCTACCG
CONSENSUS
ATTGGAGATCCTGATGGTCTATACAAAGCACCTTACGCGGTCATCTACCG

RI-AT5G41000-XLOC_027244-8696-0
CGAAATGGCTATTCTCGGGGTCGAAGGCTTTGCCAAACTGCCCAAACACT
RI-AT5G41000-XLOC_027244-8696-1
CGAAATGGCTATTCTCGGGGTCGAAGGCTTTGCCAAACTGCCCAAACACT
CONSENSUS
CGAAATGGCTATTCTCGGGGTCGAAGGCTTTGCCAAACTGCCCAAACACT

RI-AT5G41000-XLOC_027244-8696-0
GTTTGGCACTTTGTTGTGGATTCTTCATCGCTGCTCTGATTGTAAATCTA
RI-AT5G41000-XLOC_027244-8696-1
GTTTGGCACTTTGTTGTGGATTCTTCATCGCTGCTCTGATTGTAAATCTA
CONSENSUS
GTTTGGCACTTTGTTGTGGATTCTTCATCGCTGCTCTGATTGTAAATCTA

RI-AT5G41000-XLOC_027244-8696-0
ATTAGAGACATGACACCACCAAAGATCTCTAAGTTGATACCACTTCCAAT
RI-AT5G41000-XLOC_027244-8696-1
ATTAGAGACATGACACCACCAAAGATCTCTAAGTTGATACCACTTCCAAT
CONSENSUS
ATTAGAGACATGACACCACCAAAGATCTCTAAGTTGATACCACTTCCAAT

RI-AT5G41000-XLOC_027244-8696-0
GGCAATGGCTGGTCCATTCTACATAGGAGCTTACTTCGCCATCGACATGT
RI-AT5G41000-XLOC_027244-8696-1
GGCAATGGCTGGTCCATTCTACATAGGAGCTTACTTCGCCATCGACATGT
CONSENSUS
GGCAATGGCTGGTCCATTCTACATAGGAGCTTACTTCGCCATCGACATGT

RI-AT5G41000-XLOC_027244-8696-0
TCGTAGGAACCGTGATAATGCTCGTATGGGAACGGATGAATAAGAAAGAC
RI-AT5G41000-XLOC_027244-8696-1
TCGTAGGAACCGTGATAATGCTCGTATGGGAACGGATGAATAAGAAAGAC
CONSENSUS
TCGTAGGAACCGTGATAATGCTCGTATGGGAACGGATGAATAAGAAAGAC

RI-AT5G41000-XLOC_027244-8696-0
GCAGATGATTACTCGGGTGCAGTAGCTTCAGGTCTGATCTGCGGAGATGG
RI-AT5G41000-XLOC_027244-8696-1
GCAGATGATTACTCGGGTGCAGTAGCTTCAGGTCTGATCTGCGGAGATGG
CONSENSUS
GCAGATGATTACTCGGGTGCAGTAGCTTCAGGTCTGATCTGCGGAGATGG

RI-AT5G41000-XLOC_027244-8696-0
AATTTGGACCATCCCATCCGCAATTCTTTCTATATTAAGAATCAATCCAC
RI-AT5G41000-XLOC_027244-8696-1
AATTTGGACCATCCCATCCGCAATTCTTTCTATATTAAGAATCAATCCAC
CONSENSUS
AATTTGGACCATCCCATCCGCAATTCTTTCTATATTAAGAATCAATCCAC

RI-AT5G41000-XLOC_027244-8696-0

CCATTTGTATGTACTTTAGACCATCCTAGACATTGTTATCATGTTAAAAA
RI-AT5G41000-XLOC_027244-8696-1
CCATTTGTATGTACTTTAGACCATCCTAGACATTGTTATCATGTTAAAAA
CONSENSUS
CCATTTGTATGTACTTTAGACCATCCTAGACATTGTTATCATGTTAAAAA

RI-AT5G41000-XLOC_027244-8696-0
TTTGTTGCATTATGGCAGATAATAGAGTTTTTATATCTCACTCTAGAACA
RI-AT5G41000-XLOC_027244-8696-1
TTTGTTGCATTATGGCAGATAATAGAGTTTTTATATCTCACTCTAGAACA
CONSENSUS
TTTGTTGCATTATGGCAGATAATAGAGTTTTTATATCTCACTCTAGAACA

RI-AT5G41000-XLOC_027244-8696-0
CAAAGTGCCAATAGCAACAAGTATGTGGCATAAAGTCAAACAGACAAGGT
RI-AT5G41000-XLOC_027244-8696-1
CAAAGTGCCAATAGCAACAAGTATGTGGCATAAAGTCAAACAGACAAGGT
CONSENSUS
CAAAGTGCCAATAGCAACAAGTATGTGGCATAAAGTCAAACAGACAAGGT

RI-AT5G41000-XLOC_027244-8696-0 TGACCAACGAAA
RI-AT5G41000-XLOC_027244-8696-1 TGACCAACGAAA
CONSENSUS TGACCAACGAAA

alignment for event: A3-AT5G43470-XLOC_031072-12450

A3-AT5G43470-XLOC_031072-12450-0
TAAGGCTTGATGAAGAAATGGAAGCCATGGGAAAGGAAATGGTCACACAT
A3-AT5G43470-XLOC_031072-12450-1
TAAGGCTTGATGAAGAAATGGAAGCCATGGGAAAGGAAATGGTCACACAT
CONSENSUS
TAAGGCTTGATGAAGAAATGGAAGCCATGGGAAAGGAAATGGTCACACAT

A3-AT5G43470-XLOC_031072-12450-0
TGTGGAGGACTACCATTAGCTGTTAAAGCGTTGGGAGGCTTGTTAGCTAA
A3-AT5G43470-XLOC_031072-12450-1
TGTGGAGGACTACCATTAGCTGTTAAAGCGTTGGGAGGCTTGTTAGCTAA
CONSENSUS
TGTGGAGGACTACCATTAGCTGTTAAAGCGTTGGGAGGCTTGTTAGCTAA

A3-AT5G43470-XLOC_031072-12450-0
TAAACATACTGTTCCTGAGTGGAAAAGAGTTTTTGACAATATTGGATCTC
A3-AT5G43470-XLOC_031072-12450-1
TAAACATACTGTTCCTGAGTGGAAAAGAGTTTTTGACAATATTGGATCTC
CONSENSUS
TAAACATACTGTTCCTGAGTGGAAAAGAGTTTTTGACAATATTGGATCTC

A3-AT5G43470-XLOC_031072-12450-0
AAATAGTAGGAGGATCGTGGTTAGATGACAACAGTCTCAATTCGGTTTAC
A3-AT5G43470-XLOC_031072-12450-1
AAATAGTAGGAGGATCGTGGTTAGATGACAACAGTCTCAATTCGGTTTAC
CONSENSUS
AAATAGTAGGAGGATCGTGGTTAGATGACAACAGTCTCAATTCGGTTTAC

A3-AT5G43470-XLOC_031072-12450-0
CGAATATTGTCTCTGAGTTATGAAGATTTGCCAACGCATTTAAAGCATTG
A3-AT5G43470-XLOC_031072-12450-1
CGAATATTGTCTCTGAGTTATGAAGATTTGCCAACGCATTTAAAGCATTG
CONSENSUS
CGAATATTGTCTCTGAGTTATGAAGATTTGCCAACGCATTTAAAGCATTG

A3-AT5G43470-XLOC_031072-12450-0
CTTCCTTAACCTAGCCCATTTCCCCGAAGATTCCGAGATATCTACGTATA
A3-AT5G43470-XLOC_031072-12450-1
CTTCCTTAACCTAGCCCATTTCCCCGAAGATTCCGAGATATCTACGTATA
CONSENSUS
CTTCCTTAACCTAGCCCATTTCCCCGAAGATTCCGAGATATCTACGTATA

A3-AT5G43470-XLOC_031072-12450-0
GTTTGTTCTATTACTGGGCTGCAGAAGGGATTTACGATGGATCAACCATC
A3-AT5G43470-XLOC_031072-12450-1
GTTTGTTCTATTACTGGGCTGCAGAAGGGATTTACGATGGATCAACCATC
CONSENSUS
GTTTGTTCTATTACTGGGCTGCAGAAGGGATTTACGATGGATCAACCATC

A3-AT5G43470-XLOC_031072-12450-0
GAAGATAGTGGAGAATACTACCTAGAAGAGCTAGTGAGGAGAAATTTGGT
A3-AT5G43470-XLOC_031072-12450-1
GAAGATAGTGGAGAATACTACCTAGAAGAGCTAGTGAGGAGAAATTTGGT
CONSENSUS
GAAGATAGTGGAGAATACTACCTAGAAGAGCTAGTGAGGAGAAATTTGGT

A3-AT5G43470-XLOC_031072-12450-0
TATTGCAGACGACAACTATTTGAGTTGGCAATCTAAATATTGTCAAATGC
A3-AT5G43470-XLOC_031072-12450-1
TATTGCAGACGACAACTATTTGAGTTGGCAATCTAAATATTGTCAAATGC
CONSENSUS
TATTGCAGACGACAACTATTTGAGTTGGCAATCTAAATATTGTCAAATGC

A3-AT5G43470-XLOC_031072-12450-0
ATGACATGATGAGAGAAGTATGTTTATCTAAAGCCAAAGAAGAGAACTTC
A3-AT5G43470-XLOC_031072-12450-1
ATGACATGATGAGAGAAGTATGTTTATCTAAAGCCAAAGAAGAGAACTTC
CONSENSUS
ATGACATGATGAGAGAAGTATGTTTATCTAAAGCCAAAGAAGAGAACTTC

A3-AT5G43470-XLOC_031072-12450-0
CTACAAATTATCATAGACCCTACTTGCACCTCTACCATCAATGCTCAATC
A3-AT5G43470-XLOC_031072-12450-1
CTACAAATTATCATAGACCCTACTTGCACCTCTACCATCAATGCTCAATC
CONSENSUS
CTACAAATTATCATAGACCCTACTTGCACCTCTACCATCAATGCTCAATC

A3-AT5G43470-XLOC_031072-12450-0
TCCTAGTAGATCTCGCAGACTCAGCATACATAGTGGTAAAGCCTTTCATA
A3-AT5G43470-XLOC_031072-12450-1
TCCTAGTAGATCTCGCAGACTCAGCATACATAGTGGTAAAGCCTTTCATA
CONSENSUS
TCCTAGTAGATCTCGCAGACTCAGCATACATAGTGGTAAAGCCTTTCATA

A3-AT5G43470-XLOC_031072-12450-0
TATTGGGACACAAAAACAAGACAAAGGTGAGATCTCTTATAGTTCCGAGA
A3-AT5G43470-XLOC_031072-12450-1
TATTGGGACACAAAAACAAGACAAAGGTGAGATCTCTTATAGTTCCGAGA
CONSENSUS
TATTGGGACACAAAAACAAGACAAAGGTGAGATCTCTTATAGTTCCGAGA

A3-AT5G43470-XLOC_031072-12450-0
TTCGAGGAAGACTATTGGATACGATCAGCTTCAGTCTTCCACAACTTAAC
A3-AT5G43470-XLOC_031072-12450-1
TTCGAGGAAGACTATTGGATACGATCAGCTTCAGTCTTCCACAACTTAAC
CONSENSUS
TTCGAGGAAGACTATTGGATACGATCAGCTTCAGTCTTCCACAACTTAAC

A3-AT5G43470-XLOC_031072-12450-0
ATTGCTCAGGGTGTTGGATCTTTCTTGGGTAAAGTTTGAAGGAGGGAAGT
A3-AT5G43470-XLOC_031072-12450-1
ATTGCTCAGGGTGTTGGATCTTTCTTGGGTAAAGTTTGAAGGAGGGAAGT
CONSENSUS
ATTGCTCAGGGTGTTGGATCTTTCTTGGGTAAAGTTTGAAGGAGGGAAGT

A3-AT5G43470-XLOC_031072-12450-0
TACCTTGTAGCATTGGAGGGCTCATCCACTTGAGATACTTGAGTTTATAC
A3-AT5G43470-XLOC_031072-12450-1
TACCTTGTAGCATTGGAGGGCTCATCCACTTGAGATACTTGAGTTTATAC
CONSENSUS
TACCTTGTAGCATTGGAGGGCTCATCCACTTGAGATACTTGAGTTTATAC

A3-AT5G43470-XLOC_031072-12450-0
GAGGCTAAGGTATCTCATCTACCTTCTACTATGCGGAACCTAAAGCTTCT
A3-AT5G43470-XLOC_031072-12450-1
GAGGCTAAGGTATCTCATCTACCTTCTACTATGCGGAACCTAAAGCTTCT
CONSENSUS
GAGGCTAAGGTATCTCATCTACCTTCTACTATGCGGAACCTAAAGCTTCT

A3-AT5G43470-XLOC_031072-12450-0
GCTCTATTTGAACTTACGTGTTGATACCGAGGAACCGATTCACGTGCCAA
A3-AT5G43470-XLOC_031072-12450-1
GCTCTATTTGAACTTACGTGTTGATACCGAGGAACCGATTCACGTGCCAA
CONSENSUS
GCTCTATTTGAACTTACGTGTTGATACCGAGGAACCGATTCACGTGCCAA

A3-AT5G43470-XLOC_031072-12450-0
ATGTTTTGAAAGAGATGATACAGTTGAGGTACCTTTCCCTACCTCTTAAA
A3-AT5G43470-XLOC_031072-12450-1
ATGTTTTGAAAGAGATGATACAGTTGAGGTACCTTTCCCTACCTCTTAAA
CONSENSUS
ATGTTTTGAAAGAGATGATACAGTTGAGGTACCTTTCCCTACCTCTTAAA

A3-AT5G43470-XLOC_031072-12450-0
ATGGATGATAAAACCAAGTTGGAATTGGGTGATCTAGTGAACCTGGAGTA
A3-AT5G43470-XLOC_031072-12450-1
ATGGATGATAAAACCAAGTTGGAATTGGGTGATCTAGTGAACCTGGAGTA
CONSENSUS
ATGGATGATAAAACCAAGTTGGAATTGGGTGATCTAGTGAACCTGGAGTA

A3-AT5G43470-XLOC_031072-12450-0
CTTGTATGGTTTCTCAACGCAACACAGTAGTGTGACAGACCTCCTCCGTA
A3-AT5G43470-XLOC_031072-12450-1
CTTGTATGGTTTCTCAACGCAACACAGTAGTGTGACAGACCTCCTCCGTA
CONSENSUS
CTTGTATGGTTTCTCAACGCAACACAGTAGTGTGACAGACCTCCTCCGTA

A3-AT5G43470-XLOC_031072-12450-0
TGACTAAGCTCCGGTATCTCGCTGTATCTTTGAGCGAAAGGTGCAATTTT
A3-AT5G43470-XLOC_031072-12450-1
TGACTAAGCTCCGGTATCTCGCTGTATCTTTGAGCGAAAGGTGCAATTTT
CONSENSUS
TGACTAAGCTCCGGTATCTCGCTGTATCTTTGAGCGAAAGGTGCAATTTT

A3-AT5G43470-XLOC_031072-12450-0
GAAACTCTATCGTCATCTCTCCGCGAATTGCGAAACCTCGAGACGCTTAA
A3-AT5G43470-XLOC_031072-12450-1
GAAACTCTATCGTCATCTCTCCGCGAATTGCGAAACCTCGAGACGCTTAA
CONSENSUS
GAAACTCTATCGTCATCTCTCCGCGAATTGCGAAACCTCGAGACGCTTAA

A3-AT5G43470-XLOC_031072-12450-0
TTTCCTTTTTTCGCTCGAAACATATATGGTTGATTATATGGGAGAGTTTG
A3-AT5G43470-XLOC_031072-12450-1
TTTCCTTTTTTCGCTCGAAACATATATGGTTGATTATATGGGAGAGTTTG
CONSENSUS
TTTCCTTTTTTCGCTCGAAACATATATGGTTGATTATATGGGAGAGTTTG

A3-AT5G43470-XLOC_031072-12450-0
TTCTGGATCATTTTATTCATCTAAAACAGTTGGGATTGGCAGTACGTATG
A3-AT5G43470-XLOC_031072-12450-1
TTCTGGATCATTTTATTCATCTAAAACAGTTGGGATTGGCAGTACGTATG
CONSENSUS
TTCTGGATCATTTTATTCATCTAAAACAGTTGGGATTGGCAGTACGTATG

A3-AT5G43470-XLOC_031072-12450-0
TCAAAGATTCCTGATCAACATCAATTCCCTCCCCACCTTGTACACTTATT
A3-AT5G43470-XLOC_031072-12450-1
TCAAAGATTCCTGATCAACATCAATTCCCTCCCCACCTTGTACACTTATT
CONSENSUS
TCAAAGATTCCTGATCAACATCAATTCCCTCCCCACCTTGTACACTTATT

A3-AT5G43470-XLOC_031072-12450-0
TCTAATTTATTGCGGCATGGAAGAGGATCCAATGCCAATTCTAGAAAAGT
A3-AT5G43470-XLOC_031072-12450-1
TCTAATTTATTGCGGCATGGAAGAGGATCCAATGCCAATTCTAGAAAAGT
CONSENSUS
TCTAATTTATTGCGGCATGGAAGAGGATCCAATGCCAATTCTAGAAAAGT

A3-AT5G43470-XLOC_031072-12450-0
TGCTTCATTTGAAGTCGGTTCGTTTAGCACGTAAGGCATTCCTAGGGAGC
A3-AT5G43470-XLOC_031072-12450-1
TGCTTCATTTGAAGTCGGTTCGTTTAGCACGTAAGGCATTCCTAGGGAGC
CONSENSUS
TGCTTCATTTGAAGTCGGTTCGTTTAGCACGTAAGGCATTCCTAGGGAGC

A3-AT5G43470-XLOC_031072-12450-0
AGAATGGTGTGCTCAAAAGGCGGATTTCCTCAATTATGTGTTATAGAAAT
A3-AT5G43470-XLOC_031072-12450-1
AGAATGGTGTGCTCAAAAGGCGGATTTCCTCAATTATGTGTTATAGAAAT
CONSENSUS
AGAATGGTGTGCTCAAAAGGCGGATTTCCTCAATTATGTGTTATAGAAAT

A3-AT5G43470-XLOC_031072-12450-0
ATCTAAAGAATCAGAGTTGGAAGAGTGGATAGTAGAAGAAGGGTCGATGC
A3-AT5G43470-XLOC_031072-12450-1
ATCTAAAGAATCAGAGTTGGAAGAGTGGATAGTAGAAGAAGGGTCGATGC
CONSENSUS
ATCTAAAGAATCAGAGTTGGAAGAGTGGATAGTAGAAGAAGGGTCGATGC

A3-AT5G43470-XLOC_031072-12450-0
CATGTCTTCGTACTTTGACTATAGATGATTGCAAAAAGTTGAAGGAACTT
A3-AT5G43470-XLOC_031072-12450-1
CATGTCTTCGTACTTTGACTATAGATGATTGCAAAAAGTTGAAGGAACTT
CONSENSUS
CATGTCTTCGTACTTTGACTATAGATGATTGCAAAAAGTTGAAGGAACTT

A3-AT5G43470-XLOC_031072-12450-0
CCAGATGGACTCAAGTACATAACATCCTTAAAGGAACTGAAGATTGAAGG
A3-AT5G43470-XLOC_031072-12450-1
CCAGATGGACTCAAGTACATAACATCCTTAAAGGAACTGAAGATTGAAGG
CONSENSUS
CCAGATGGACTCAAGTACATAACATCCTTAAAGGAACTGAAGATTGAAGG

A3-AT5G43470-XLOC_031072-12450-0
AATGAAGAGGGAGTGGAAGGAGAAACTGGTACCAGGTGGAGAAGATTACT
A3-AT5G43470-XLOC_031072-12450-1
AATGAAGAGGGAGTGGAAGGAGAAACTGGTACCAGGTGGAGAAGATTACT
CONSENSUS
AATGAAGAGGGAGTGGAAGGAGAAACTGGTACCAGGTGGAGAAGATTACT

A3-AT5G43470-XLOC_031072-12450-0
ACAAAGTCCAACACATTCCCGATGTTCAATTTATCAACTGTGACCAGTAG
A3-AT5G43470-XLOC_031072-12450-1
ACAAAGTCCAACACATTCCCGATGTTCAATTTATCAACTGTGACCAGTAG
CONSENSUS
ACAAAGTCCAACACATTCCCGATGTTCAATTTATCAACTGTGACCAGTAG

A3-AT5G43470-XLOC_031072-12450-0 CAG------
GAAAGACCAAGAAGCGAGCCAAATTCTCTTATATTGGGAGA
A3-AT5G43470-XLOC_031072-12450-1
CAGATGCAGGAAAGACCAAGAAGCGAGCCAAATTCTCTTATATTGGGAGA
CONSENSUS
CAG......GAAAGACCAAGAAGCGAGCCAAATTCTCTTATATTGGGAGA

A3-AT5G43470-XLOC_031072-12450-0
TATAGACGCTGCTTCAACAGAGTCATCTGCTGATCAACAGGTTTTTCCGA
A3-AT5G43470-XLOC_031072-12450-1
TATAGACGCTGCTTCAACAGAGTCATCTGCTGATCAACAGGTTTTTCCGA
CONSENSUS
TATAGACGCTGCTTCAACAGAGTCATCTGCTGATCAACAGGTTTTTCCGA

A3-AT5G43470-XLOC_031072-12450-0
AGAATATCTGGTATTGCTTATGAAGAATACAGATTCTCAAGTATTAACGA
A3-AT5G43470-XLOC_031072-12450-1
AGAATATCTGGTATTGCTTATGAAGAATACAGATTCTCAAGTATTAACGA
CONSENSUS
AGAATATCTGGTATTGCTTATGAAGAATACAGATTCTCAAGTATTAACGA

A3-AT5G43470-XLOC_031072-12450-0
TTATGTTTCCAGTCTTGGAGCTCTCGCTTTTTCAGTGCATCGTTATGTTG
A3-AT5G43470-XLOC_031072-12450-1
TTATGTTTCCAGTCTTGGAGCTCTCGCTTTTTCAGTGCATCGTTATGTTG
CONSENSUS
TTATGTTTCCAGTCTTGGAGCTCTCGCTTTTTCAGTGCATCGTTATGTTG

A3-AT5G43470-XLOC_031072-12450-0
TGCGGCTCAGAACTATATTATAGAAACAATGCTTATATTGTTGTTTGTTT
A3-AT5G43470-XLOC_031072-12450-1
TGCGGCTCAGAACTATATTATAGAAACAATGCTTATATTGTTGTTTGTTT
CONSENSUS
TGCGGCTCAGAACTATATTATAGAAACAATGCTTATATTGTTGTTTGTTT

A3-AT5G43470-XLOC_031072-12450-0
TTGATGTGGATGCATGGAACATGCTTGAGAACTCTTTTACAAAATGGAGA
A3-AT5G43470-XLOC_031072-12450-1
TTGATGTGGATGCATGGAACATGCTTGAGAACTCTTTTACAAAATGGAGA
CONSENSUS
TTGATGTGGATGCATGGAACATGCTTGAGAACTCTTTTACAAAATGGAGA

A3-AT5G43470-XLOC_031072-12450-0
AACGAAACTTGTAAAATTTGGATATTTTATGTTTGTTGTAAAATTTGGAT
A3-AT5G43470-XLOC_031072-12450-1
AACGAAACTTGTAAAATTTGGATATTTTATGTTTGTTGTAAAATTTGGAT
CONSENSUS
AACGAAACTTGTAAAATTTGGATATTTTATGTTTGTTGTAAAATTTGGAT

A3-AT5G43470-XLOC_031072-12450-0
ATTTTGTGTTTGTGTAAAATTTGGATATTATATGTTTTGCTTTCTCGTTT
A3-AT5G43470-XLOC_031072-12450-1
ATTTTGTGTTTGTGTAAAATTTGGATATTATATGTTTTGCTTTCTCGTTT
CONSENSUS
ATTTTGTGTTTGTGTAAAATTTGGATATTATATGTTTTGCTTTCTCGTTT

A3-AT5G43470-XLOC_031072-12450-0
GTGATTTAACACAGTTCGTTTTGTAAGCTTTATTTACAGTTTACACCATC
A3-AT5G43470-XLOC_031072-12450-1
GTGATTTAACACAGTTCGTTTTGTAAGCTTTATTTACAGTTTACACCATC
CONSENSUS
GTGATTTAACACAGTTCGTTTTGTAAGCTTTATTTACAGTTTACACCATC

A3-AT5G43470-XLOC_031072-12450-0 AAATCTTGTC
A3-AT5G43470-XLOC_031072-12450-1 AAATCTTGTC
CONSENSUS AAATCTTGTC

alignment for event: SE-AT5G64170-XLOC_032202-7094

SE-AT5G64170-XLOC_032202-7094-0
GTCAAAAAAAAAAAAAATAGGAAGCCTTCATCGTCTTCTGCCGAACATCTC
SE-AT5G64170-XLOC_032202-7094-1
GTCAAAAAAAAAAAAAATAGGAAGCCTTCATCGTCTTCTGCCGAACATCTC
CONSENSUS
GTCAAAAAAAAAAAAAATAGGAAGCCTTCATCGTCTTCTGCCGAACATCTC

SE-AT5G64170-XLOC_032202-7094-0
TAGAAAGTCCTACACAGTTTTTTTTTTCGATCTAGCTTCGATGAATTGAT
SE-AT5G64170-XLOC_032202-7094-1
TAGAAAGTCCTACACAGTTTTTTTTTTCGATCTAGCTTCGATGAATTGAT
CONSENSUS
TAGAAAGTCCTACACAGTTTTTTTTTTCGATCTAGCTTCGATGAATTGAT

SE-AT5G64170-XLOC_032202-7094-0
ACAATCTCTTCTATCTGCTGAGTTTTTTGTTTTCTGGTAAACAATTTTAT
SE-AT5G64170-XLOC_032202-7094-1
ACAATCTCTTCTATCTGCTGAGTTTTTTGTTTTCTGGTAAACAATTTTAT
CONSENSUS
ACAATCTCTTCTATCTGCTGAGTTTTTTGTTTTCTGGTAAACAATTTTAT

SE-AT5G64170-XLOC_032202-7094-0
CTGTGGAGTTTTCTGGAGTTGCTAGTGGAAGAATCTGTCTCACAG-----
SE-AT5G64170-XLOC_032202-7094-1
CTGTGGAGTTTTCTGGAGTTGCTAGTGGAAGAATCTGTCTCACAGAACCA
CONSENSUS
CTGTGGAGTTTTCTGGAGTTGCTAGTGGAAGAATCTGTCTCACAG.....

SE-AT5G64170-XLOC_032202-7094-0

SE-AT5G64170-XLOC_032202-7094-1
GAAATAGTGTTTGTGGGAGTTACATGTTTTGCAATAGAGATGACATATCG
CONSENSUS
....

SE-AT5G64170-XLOC_032202-7094-0

SE-AT5G64170-XLOC_032202-7094-1
GGGTAATGTGCATGATTTTAGTGGTGATTAAAGAGTCTGAAGTTGGGAGG
CONSENSUS
....

SE-AT5G64170-XLOC_032202-7094-0 -------------------------
CTAGGCGATTATCTTTCGGATGAAT
SE-AT5G64170-XLOC_032202-7094-1
AATGTCGGACTTGTACATTCATGAGCTAGGCGATTATCTTTCGGATGAAT
CONSENSUS
.........................CTAGGCGATTATCTTTCGGATGAAT

SE-AT5G64170-XLOC_032202-7094-0
TTCATGGGAACGATGATGGTATAGTGCCAGACTCAGCGTATGAGGATGGA
SE-AT5G64170-XLOC_032202-7094-1
TTCATGGGAACGATGATGGTATAGTGCCAGACTCAGCGTATGAGGATGGA
CONSENSUS
TTCATGGGAACGATGATGGTATAGTGCCAGACTCAGCGTATGAGGATGGA

SE-AT5G64170-XLOC_032202-7094-0
GGTCAGTTTCCAATTCTAGTTAGTAACAGGAAGAAACGAAGAAATGATGA
SE-AT5G64170-XLOC_032202-7094-1
GGTCAGTTTCCAATTCTAGTTAGTAACAGGAAGAAACGAAGAAATGATGA
CONSENSUS
GGTCAGTTTCCAATTCTAGTTAGTAACAGGAAGAAACGAAGAAATGATGA

SE-AT5G64170-XLOC_032202-7094-0
TATGGGTAGTGGAACAAACCATCTAAAGAGTAATACTTTTATCAAGAGAG
SE-AT5G64170-XLOC_032202-7094-1
TATGGGTAGTGGAACAAACCATCTAAAGAGTAATACTTTTATCAAGAGAG
CONSENSUS
TATGGGTAGTGGAACAAACCATCTAAAGAGTAATACTTTTATCAAGAGAG

SE-AT5G64170-XLOC_032202-7094-0
AGGCAAACATGTTAGGAAAAAATCCATGGCCTGAGAAAGATAGTGGTGGC
SE-AT5G64170-XLOC_032202-7094-1
AGGCAAACATGTTAGGAAAAAATCCATGGCCTGAGAAAGATAGTGGTGGC
CONSENSUS
AGGCAAACATGTTAGGAAAAAATCCATGGCCTGAGAAAGATAGTGGTGGC

SE-AT5G64170-XLOC_032202-7094-0
TCTTCGGTTTCTCGTGATACGGGAACAGGAAAAGATGTTCAGGATATGAC
SE-AT5G64170-XLOC_032202-7094-1
TCTTCGGTTTCTCGTGATACGGGAACAGGAAAAGATGTTCAGGATATGAC
CONSENSUS
TCTTCGGTTTCTCGTGATACGGGAACAGGAAAAGATGTTCAGGATATGAC

SE-AT5G64170-XLOC_032202-7094-0
ATTGGAGGATACAAATACTTCAGATCATGGTTTCAATGGTGGCCACGTAG
SE-AT5G64170-XLOC_032202-7094-1
ATTGGAGGATACAAATACTTCAGATCATGGTTTCAATGGTGGCCACGTAG
CONSENSUS
ATTGGAGGATACAAATACTTCAGATCATGGTTTCAATGGTGGCCACGTAG

SE-AT5G64170-XLOC_032202-7094-0
ATGTGGTTGAAAATTTCTCCACTGGGGATCCCATGTTGTGTGACACTTCC
SE-AT5G64170-XLOC_032202-7094-1
ATGTGGTTGAAAATTTCTCCACTGGGGATCCCATGTTGTGTGACACTTCC
CONSENSUS
ATGTGGTTGAAAATTTCTCCACTGGGGATCCCATGTTGTGTGACACTTCC

SE-AT5G64170-XLOC_032202-7094-0
GCTGCAACGAATGATGGCGTATATAATTATTCCCTCAACAGCATTCCGGA
SE-AT5G64170-XLOC_032202-7094-1
GCTGCAACGAATGATGGCGTATATAATTATTCCCTCAACAGCATTCCGGA
CONSENSUS
GCTGCAACGAATGATGGCGTATATAATTATTCCCTCAACAGCATTCCGGA

SE-AT5G64170-XLOC_032202-7094-0
TGCTGAAAATGATCTTAGCTTTTTCGACAATGGAGATAAAGAAAAAAATG
SE-AT5G64170-XLOC_032202-7094-1
TGCTGAAAATGATCTTAGCTTTTTCGACAATGGAGATAAAGAAAAAAATG
CONSENSUS
TGCTGAAAATGATCTTAGCTTTTTCGACAATGGAGATAAAGAAAAAAATG

SE-AT5G64170-XLOC_032202-7094-0
      ATCTCTTCTATGGCTGGGGTGACATAGGAAATTTTGAGGATGTGGACAAC
SE-AT5G64170-XLOC_032202-7094-1
      ATCTCTTCTATGGCTGGGGTGACATAGGAAATTTTGAGGATGTGGACAAC
CONSENSUS
      ATCTCTTCTATGGCTGGGGTGACATAGGAAATTTTGAGGATGTGGACAAC

SE-AT5G64170-XLOC_032202-7094-0 ATGCTTAG
SE-AT5G64170-XLOC_032202-7094-1 ATGCTTAG
CONSENSUS   ATGCTTAG

alignment for event: A3-AT5G02810-XLOC_028866-12013

A3-AT5G02810-XLOC_028866-12013-0
      TCTAGTGGTAGTGGAAGTGAGAGCGGAACGCATCAAACTCAAAAGTCTGT
A3-AT5G02810-XLOC_028866-12013-1
      TCTAGTGGTAGTGGAAGTGAGAGCGGAACGCATCAAACTCAAAAGTCTGT
CONSENSUS
      TCTAGTGGTAGTGGAAGTGAGAGCGGAACGCATCAAACTCAAAAGTCTGT

A3-AT5G02810-XLOC_028866-12013-0
      GAAATCGAAAAGTATTAAAAAATCTGATCAAGATTCAGGAAGCAGTGATG
A3-AT5G02810-XLOC_028866-12013-1
      GAAATCGAAAAGTATTAAAAAATCTGATCAAGATTCAGGAAGCAGTGATG
CONSENSUS
      GAAATCGAAAAGTATTAAAAAATCTGATCAAGATTCAGGAAGCAGTGATG

A3-AT5G02810-XLOC_028866-12013-0
      AGAATGAAAATGGGAGCATTGGCCTGAATGCTAGTGATGGAAGTAGTGAT
A3-AT5G02810-XLOC_028866-12013-1
      AGAATGAAAATGGGAGCATTGGCCTGAATGCTAGTGATGGAAGTAGTGAT
CONSENSUS
      AGAATGAAAATGGGAGCATTGGCCTGAATGCTAGTGATGGAAGTAGTGAT

A3-AT5G02810-XLOC_028866-12013-0
      GGGAGTGGCGCTCAG-----------------------------------
A3-AT5G02810-XLOC_028866-12013-1
      GGGAGTGGCGCTCAGGGGTCGGATGATCGAACTTCGCATGTGCACTCAGA
CONSENSUS
      GGGAGTGGCGCTCAG...................................

A3-AT5G02810-XLOC_028866-12013-0

A3-AT5G02810-XLOC_028866-12013-1
      TGCTTATAAATGGTGGAAGCGCTTAACTAACCAATTGTAGAACTCAATGA
CONSENSUS
      ....

A3-AT5G02810-XLOC_028866-12013-0

A3-AT5G02810-XLOC_028866-12013-1
      TGTTTAACTTAGATCTATTCAGTGATAAACTGGGTGAAAATTTCTAGTTC
CONSENSUS

....

A3-AT5G02810-XLOC_028866-12013-0

A3-AT5G02810-XLOC_028866-12013-1
ATTTTGAAGTTTTATTTTGCACATGGTTTATCTCCAAAACCTAGGTTGAT
CONSENSUS
....

A3-AT5G02810-XLOC_028866-12013-0 -----------------------
AGCTCTTGGACGAAAAAAGCTGTGGA
A3-AT5G02810-XLOC_028866-12013-1
TTTGACATACCTTTTTTTGTTCAGAGCTCTTGGACGAAAAAAGCTGTGGA
CONSENSUS
........................AGCTCTTGGACGAAAAAAGCTGTGGA

A3-AT5G02810-XLOC_028866-12013-0
TGTTGATGACAGTCCACGAGCGGTATCTCTATGGGACCGAGTTGATAGCA
A3-AT5G02810-XLOC_028866-12013-1
TGTTGATGACAGTCCACGAGCGGTATCTCTATGGGACCGAGTTGATAGCA
CONSENSUS
TGTTGATGACAGTCCACGAGCGGTATCTCTATGGGACCGAGTTGATAGCA

A3-AT5G02810-XLOC_028866-12013-0
CTTGCGCCCAAGTGGTACATTCTAACCCTGAGTTTCCAAGTAATCAGTTG
A3-AT5G02810-XLOC_028866-12013-1
CTTGCGCCCAAGTGGTACATTCTAACCCTGAGTTTCCAAGTAATCAGTTG
CONSENSUS
CTTGCGCCCAAGTGGTACATTCTAACCCTGAGTTTCCAAGTAATCAGTTG

A3-AT5G02810-XLOC_028866-12013-0
GTTGCACCACCTGCTGAGAAGGAGACTCAAGAACATGATGATAAATTTG
A3-AT5G02810-XLOC_028866-12013-1
GTTGCACCACCTGCTGAGAAGGAGACTCAAGAACATGATGATAAATTTG
CONSENSUS
GTTGCACCACCTGCTGAGAAGGAGACTCAAGAACATGATGATAAATTTG

alignment for event: A3-AT5G13730-XLOC_029420-9277

A3-AT5G13730-XLOC_029420-9277-0
GAAGGAGCAAAACTTGAAAATTTGGGAACAAGTGTTGAAGAGAATGAAAT
A3-AT5G13730-XLOC_029420-9277-1
GAAGGAGCAAAACTTGAAAATTTGGGAACAAGTGTTGAAGAGAATGAAAT
CONSENSUS
GAAGGAGCAAAACTTGAAAATTTGGGAACAAGTGTTGAAGAGAATGAAAT

A3-AT5G13730-XLOC_029420-9277-0
GGTATCAGTTTTGTTAGCCAGTGGCAGAGGGAAGAAGAAGCGTAGTGCAA
A3-AT5G13730-XLOC_029420-9277-1
GGTATCAGTTTTGTTAGCCAGTGGCAGAGGGAAGAAGAAGCGTAGTGCAA
CONSENSUS
GGTATCAGTTTTGTTAGCCAGTGGCAGAGGGAAGAAGAAGCGTAGTGCAA

A3-AT5G13730-XLOC_029420-9277-0

ATGAAATACTATGCCGTAGAAAAGAAGCGAGAGAAAAGATTACTCGTTGT
A3-AT5G13730-XLOC_029420-9277-1
ATGAAATACTATGCCGTAGAAAAGAAGCGAGAGAAAAGATTACTCGTTGT
CONSENSUS
ATGAAATACTATGCCGTAGAAAAGAAGCGAGAGAAAAGATTACTCGTTGT

A3-AT5G13730-XLOC_029420-9277-0
TACCGTAGGTTGGTTGTTTCTATTGCAACAGGGTACCAAGGCAAAGGTTT
A3-AT5G13730-XLOC_029420-9277-1
TACCGTAGGTTGGTTGTTTCTATTGCAACAGGGTACCAAGGCAAAGGTTT
CONSENSUS
TACCGTAGGTTGGTTGTTTCTATTGCAACAGGGTACCAAGGCAAAGGTTT

A3-AT5G13730-XLOC_029420-9277-0
GAATTTGCAAGACCTTATTCAGGAAGGAAGCATAGGGCTGCTTCGTGGAG
A3-AT5G13730-XLOC_029420-9277-1
GAATTTGCAAGACCTTATTCAGGAAGGAAGCATAGGGCTGCTTCGTGGAG
CONSENSUS
GAATTTGCAAGACCTTATTCAGGAAGGAAGCATAGGGCTGCTTCGTGGAG

A3-AT5G13730-XLOC_029420-9277-0
CTGAGAGATTTGATCCCGACCGAGGATACAAACTATCAACTTATGTCTAC
A3-AT5G13730-XLOC_029420-9277-1
CTGAGAGATTTGATCCCGACCGAGGATACAAACTATCAACTTATGTCTAC
CONSENSUS
CTGAGAGATTTGATCCCGACCGAGGATACAAACTATCAACTTATGTCTAC

A3-AT5G13730-XLOC_029420-9277-0
TGGTGGATCAAACAGGCCATCTTAAGGGCTATAGCGCATAAATCTAGACT
A3-AT5G13730-XLOC_029420-9277-1
TGGTGGATCAAACAGGCCATCTTAAGGGCTATAGCGCATAAATCTAGACT
CONSENSUS
TGGTGGATCAAACAGGCCATCTTAAGGGCTATAGCGCATAAATCTAGACT

A3-AT5G13730-XLOC_029420-9277-0 TGTCAAATTGCCG--------------
GGAAGCATGTGGGAGTTAACGGC
A3-AT5G13730-XLOC_029420-9277-1
TGTCAAATTGCCGCTTGTTGTGTGTAGGGAAGCATGTGGGAGTTAACGGC
CONSENSUS
TGTCAAATTGCCG..............GGAAGCATGTGGGAGTTAACGGC

A3-AT5G13730-XLOC_029420-9277-0
AAAAGTTGCAGAAGCTAGTAATGTGTTGACCAGAAAACTAAGAAGGCAAC
A3-AT5G13730-XLOC_029420-9277-1
AAAAGTTGCAGAAGCTAGTAATGTGTTGACCAGAAAACTAAGAAGGCAAC
CONSENSUS
AAAAGTTGCAGAAGCTAGTAATGTGTTGACCAGAAAACTAAGAAGGCAAC

A3-AT5G13730-XLOC_029420-9277-0
CAAGCTGTGAAGAGATTGCAGAGCACCTAAACCTCAATGTATCCGCGGTT
A3-AT5G13730-XLOC_029420-9277-1
CAAGCTGTGAAGAGATTGCAGAGCACCTAAACCTCAATGTATCCGCGGTT
CONSENSUS
CAAGCTGTGAAGAGATTGCAGAGCACCTAAACCTCAATGTATCCGCGGTT

A3-AT5G13730-XLOC_029420-9277-0

AGACTAGCTGTGGAGCGTAGCAGATCCCCTGTTTCGTTGGACCGAGTCGC
A3-AT5G13730-XLOC_029420-9277-1
AGACTAGCTGTGGAGCGTAGCAGATCCCCTGTTTCGTTGGACCGAGTCGC
CONSENSUS
AGACTAGCTGTGGAGCGTAGCAGATCCCCTGTTTCGTTGGACCGAGTCGC

A3-AT5G13730-XLOC_029420-9277-0 GTCTCAAAATGGCCGCATGACATTGCAG
A3-AT5G13730-XLOC_029420-9277-1 GTCTCAAAATGGCCGCATGACATTGCAG
CONSENSUS   GTCTCAAAATGGCCGCATGACATTGCAG

alignment for event: A3-AT5G14440-XLOC_029464-4828

A3-AT5G14440-XLOC_029464-4828-0
GTGAATTTGATGAAGATGGAGAGATTAGTATGGAAGAGTCCATACTAATA
A3-AT5G14440-XLOC_029464-4828-1
GTGAATTTGATGAAGATGGAGAGATTAGTATGGAAGAGTCCATACTAATA
CONSENSUS
GTGAATTTGATGAAGATGGAGAGATTAGTATGGAAGAGTCCATACTAATA

A3-AT5G14440-XLOC_029464-4828-0
GGTGAAGTTGATGAAGATGGCAAGATTGTTTTGGATGACACTCATGCCAG
A3-AT5G14440-XLOC_029464-4828-1
GGTGAAGTTGATGAAGATGGCAAGATTGTTTTGGATGACACTCATGCCAG
CONSENSUS
GGTGAAGTTGATGAAGATGGCAAGATTGTTTTGGATGACACTCATGCCAG

A3-AT5G14440-XLOC_029464-4828-0 ---
CAACAAGAGGAAACATGAAGAACTCGGTTCCAGTGACCTTCCTTCGA
A3-AT5G14440-XLOC_029464-4828-1
CAGCAACAAGAGGAAACATGAAGAACTCGGTTCCAGTGACCTTCCTTCGA
CONSENSUS
...CAACAAGAGGAAACATGAAGAACTCGGTTCCAGTGACCTTCCTTCGA

A3-AT5G14440-XLOC_029464-4828-0
AGAAGAAGAATAAGAAGAAGAAAAAGAAGAAGAATGTCGAAGCAACATCG
A3-AT5G14440-XLOC_029464-4828-1
AGAAGAAGAATAAGAAGAAGAAAAAGAAGAAGAATGTCGAAGCAACATCG
CONSENSUS
AGAAGAAGAATAAGAAGAAGAAAAAGAAGAAGAATGTCGAAGCAACATCG

A3-AT5G14440-XLOC_029464-4828-0
TCGTCATATTAAGTCTGATATGTTTTGTATTTCACTTTGTACTTGCTCTA
A3-AT5G14440-XLOC_029464-4828-1
TCGTCATATTAAGTCTGATATGTTTTGTATTTCACTTTGTACTTGCTCTA
CONSENSUS
TCGTCATATTAAGTCTGATATGTTTTGTATTTCACTTTGTACTTGCTCTA

A3-AT5G14440-XLOC_029464-4828-0
TTTCAGCTTGTGTGCTCAGTTTTGTTATAATTGGTGATGGAAAATGGTGA
A3-AT5G14440-XLOC_029464-4828-1
TTTCAGCTTGTGTGCTCAGTTTTGTTATAATTGGTGATGGAAAATGGTGA
CONSENSUS
TTTCAGCTTGTGTGCTCAGTTTTGTTATAATTGGTGATGGAAAATGGTGA

A3-AT5G14440-XLOC_029464-4828-0
      TTTACATATTAATGAAGCAATTTATTTTTCTCACAAAAGCAGATTTTAAT
A3-AT5G14440-XLOC_029464-4828-1
      TTTACATATTAATGAAGCAATTTATTTTTCTCACAAAAGCAGATTTTAAT
CONSENSUS
      TTTACATATTAATGAAGCAATTTATTTTTCTCACAAAAGCAGATTTTAAT

A3-AT5G14440-XLOC_029464-4828-0 TATAAAGAGGAACATGAAAATCGC
A3-AT5G14440-XLOC_029464-4828-1 TATAAAGAGGAACATGAAAATCGC
CONSENSUS   TATAAAGAGGAACATGAAAATCGC

alignment for event: SE-AT5G22620-XLOC_029881-8529

SE-AT5G22620-XLOC_029881-8529-0
      ACACCTAATTCGCCTCTAGCTGGTGGAAGTTCTGGTGGCCGGAAAGCTAG
SE-AT5G22620-XLOC_029881-8529-1
      ACACCTAATTCGCCTCTAGCTGGTGGAAGTTCTGGTGGCCGGAAAGCTAG
CONSENSUS
      ACACCTAATTCGCCTCTAGCTGGTGGAAGTTCTGGTGGCCGGAAAGCTAG

SE-AT5G22620-XLOC_029881-8529-0
      TAAGCAGATTATACTTGTCTGCCATGGTCAGGGGAATAATGAG-------
SE-AT5G22620-XLOC_029881-8529-1
      TAAGCAGATTATACTTGTCTGCCATGGTCAGGGGAATAATGAGGATTCTG
CONSENSUS
      TAAGCAGATTATACTTGTCTGCCATGGTCAGGGGAATAATGAG.......

SE-AT5G22620-XLOC_029881-8529-0

SE-AT5G22620-XLOC_029881-8529-1
      CTGTTATTAACCAAGCAGCTAATAATGATCAGGCAATGAACATGCTTGGT
CONSENSUS
      ....

SE-AT5G22620-XLOC_029881-8529-0 ---------
TCACAGAAAACCGCAGAGCTTCTACTTGATCTAAGGGTTAG
SE-AT5G22620-XLOC_029881-8529-1
      GTGATACATTCACAGAAAACCGCAGAGCTTCTACTTGATCTAAGGGTTAG
CONSENSUS
      .........TCACAGAAAACCGCAGAGCTTCTACTTGATCTAAGGGTTAG

SE-AT5G22620-XLOC_029881-8529-0
      TTCAATAGTTTGCAGCCCTAAAACAGCCTCCATTGAGTCTTCTGGAGTAA
SE-AT5G22620-XLOC_029881-8529-1
      TTCAATAGTTTGCAGCCCTAAAACAGCCTCCATTGAGTCTTCTGGAGTAA
CONSENSUS
      TTCAATAGTTTGCAGCCCTAAAACAGCCTCCATTGAGTCTTCTGGAGTAA

SE-AT5G22620-XLOC_029881-8529-0 TATCCCGG
SE-AT5G22620-XLOC_029881-8529-1 TATCCCGG
CONSENSUS   TATCCCGG

alignment for event: RI-AT5G04510-XLOC_025315-1932

RI-AT5G04510-XLOC_025315-1932-0
TCTCAGACAGCATCTCCCGAGAGGGATGACACACATGGTTCTCCATGGAA
RI-AT5G04510-XLOC_025315-1932-1
TCTCAGACAGCATCTCCCGAGAGGGATGACACACATGGTTCTCCATGGAA
CONSENSUS
TCTCAGACAGCATCTCCCGAGAGGGATGACACACATGGTTCTCCATGGAA

RI-AT5G04510-XLOC_025315-1932-0
CCTGACACATATTGGAGATTCTTTAGCCACACAGAACGAGGGGCACAGTG
RI-AT5G04510-XLOC_025315-1932-1
CCTGACACATATTGGAGATTCTTTAGCCACACAGAACGAGGGGCACAGTG
CONSENSUS
CCTGACACATATTGGAGATTCTTTAGCCACACAGAACGAGGGGCACAGTG

RI-AT5G04510-XLOC_025315-1932-0
CTCCTCCTACATCTTCTGAATCATCGGGTTCCATAACTCGACTTGCTTCA
RI-AT5G04510-XLOC_025315-1932-1
CTCCTCCTACATCTTCTGAATCATCGGGTTCCATAACTCGACTTGCTTCA
CONSENSUS
CTCCTCCTACATCTTCTGAATCATCGGGTTCCATAACTCGACTTGCTTCA

RI-AT5G04510-XLOC_025315-1932-0
ATAGACTCTTTTGATTCAAGATGGTGGGGGCATTTAAGCTTCATTTGTTT
RI-AT5G04510-XLOC_025315-1932-1
ATAGACTCTTTTGATTCAAGATG---------------------------
CONSENSUS
ATAGACTCTTTTGATTCAAGATG...........................

RI-AT5G04510-XLOC_025315-1932-0
TCTGGGCAGCAACCTTGTTCAAAGCTCCTAAAACTATACTTCAGTTAGAC
RI-AT5G04510-XLOC_025315-1932-1

CONSENSUS
....

RI-AT5G04510-XLOC_025315-1932-0
AATCTGCTAATTTTTTTCTTGTGAATATTTGATAAACAGGCAACAGTTTT
RI-AT5G04510-XLOC_025315-1932-1
--GCAACAGTTTT
CONSENSUS
....GCAACAGTTTT

RI-AT5G04510-XLOC_025315-1932-0
TAGAGCCAGGAGAATCGGTTCTGATGATATCAGCGGTGAAGAAGCTTCAG
RI-AT5G04510-XLOC_025315-1932-1
TAGAGCCAGGAGAATCGGTTCTGATGATATCAGCGGTGAAGAAGCTTCAG
CONSENSUS
TAGAGCCAGGAGAATCGGTTCTGATGATATCAGCGGTGAAGAAGCTTCAG

RI-AT5G04510-XLOC_025315-1932-0
AAAATAACGAGCAAGAAGGTGCAGCTAATACTCACCAACAAACCCAAGCT
RI-AT5G04510-XLOC_025315-1932-1
AAAATAACGAGCAAGAAGGTGCAGCTAATACTCACCAACAAACCCAAGCT
CONSENSUS

AAAATAACGAGCAAGAAGGTGCAGCTAATACTCACCAACAAACCCAAGCT

RI-AT5G04510-XLOC_025315-1932-0
GATCTATGTCGACCCGTCAAAACTAGTTGTGAAAGGAAACATTATATGGT
RI-AT5G04510-XLOC_025315-1932-1
GATCTATGTCGACCCGTCAAAACTAGTTGTGAAAGGAAACATTATATGGT
CONSENSUS
GATCTATGTCGACCCGTCAAAACTAGTTGTGAAAGGAAACATTATATGGT

RI-AT5G04510-XLOC_025315-1932-0
CTGATAACTCGAATGACCTCAACGTTGTAGTCACTAGCCCTTCACATTTC
RI-AT5G04510-XLOC_025315-1932-1
CTGATAACTCGAATGACCTCAACGTTGTAGTCACTAGCCCTTCACATTTC
CONSENSUS
CTGATAACTCGAATGACCTCAACGTTGTAGTCACTAGCCCTTCACATTTC

RI-AT5G04510-XLOC_025315-1932-0 AAGATTTGCACG
RI-AT5G04510-XLOC_025315-1932-1 AAGATTTGCACG
CONSENSUS AAGATTTGCACG

alignment for event: RI-AT5G46470-XLOC_027583-8128

RI-AT5G46470-XLOC_027583-8128-0
GGATAACGAAGCAAGCATGATCGAAGAAATCGCCAATGATATTTTGGGTA
RI-AT5G46470-XLOC_027583-8128-1
GGATAACGAAGCAAGCATGATCGAAGAAATCGCCAATGATATTTTGGGTA
CONSENSUS
GGATAACGAAGCAAGCATGATCGAAGAAATCGCCAATGATATTTTGGGTA

RI-AT5G46470-XLOC_027583-8128-0
AAATGAATATATCTCCATCAAATGATTTTGAGGACTTGGTCGGTATTGAA
RI-AT5G46470-XLOC_027583-8128-1
AAATGAATATATCTCCATCAAATGATTTTGAGGACTTGGTCGGTATTGAA
CONSENSUS
AAATGAATATATCTCCATCAAATGATTTTGAGGACTTGGTCGGTATTGAA

RI-AT5G46470-XLOC_027583-8128-0
GATCATATCACAAAGATGAGTTCATTGCTGCACTTGGAATCTGAGGAAGT
RI-AT5G46470-XLOC_027583-8128-1
GATCATATCACAAAGATGAGTTCATTGCTGCACTTGGAATCTGAGGAAGT
CONSENSUS
GATCATATCACAAAGATGAGTTCATTGCTGCACTTGGAATCTGAGGAAGT

RI-AT5G46470-XLOC_027583-8128-0
GAGGATGGTCGGGATATGGGGTCCCTCGGGAATTGGCAAAACGACCATTG
RI-AT5G46470-XLOC_027583-8128-1
GAGGATGGTCGGGATATGGGGTCCCTCGGGAATTGGCAAAACGACCATTG
CONSENSUS
GAGGATGGTCGGGATATGGGGTCCCTCGGGAATTGGCAAAACGACCATTG

RI-AT5G46470-XLOC_027583-8128-0
CAAGAGCTCTATTTAGTCGACTCTCTTGTCAGTTTCAAAGTAGTGTTTTC
RI-AT5G46470-XLOC_027583-8128-1
CAAGAGCTCTATTTAGTCGACTCTCTTGTCAGTTTCAAAGTAGTGTTTTC

CONSENSUS
CAAGAGCTCTATTTAGTCGACTCTCTTGTCAGTTTCAAAGTAGTGTTTTC

RI-AT5G46470-XLOC_027583-8128-0
ATTGACAAGGTTTTCATATCTAAGAGTATGGAAGTTTACAGTGGAGCTAA
RI-AT5G46470-XLOC_027583-8128-1
ATTGACAAGGTTTTCATATCTAAGAGTATGGAAGTTTACAGTGGAGCTAA
CONSENSUS
ATTGACAAGGTTTTCATATCTAAGAGTATGGAAGTTTACAGTGGAGCTAA

RI-AT5G46470-XLOC_027583-8128-0
TCTTGTTGACTATAACATGAAGTTGCACTTGCAAAGAGCCTTTCTAGCTG
RI-AT5G46470-XLOC_027583-8128-1
TCTTGTTGACTATAACATGAAGTTGCACTTGCAAAGAGCCTTTCTAGCTG
CONSENSUS
TCTTGTTGACTATAACATGAAGTTGCACTTGCAAAGAGCCTTTCTAGCTG

RI-AT5G46470-XLOC_027583-8128-0
AAATTTTTGACAAAAAGGACATAAAGATACATGTAGGTGCAATGGAAAAG
RI-AT5G46470-XLOC_027583-8128-1
AAATTTTTGACAAAAAGGACATAAAGATACATGTAGGTGCAATGGAAAAG
CONSENSUS
AAATTTTTGACAAAAAGGACATAAAGATACATGTAGGTGCAATGGAAAAG

RI-AT5G46470-XLOC_027583-8128-0
ATGGTAAAGCACCGGAAAGCTCTCATCGTTATTGATGATTTAGATGATCA
RI-AT5G46470-XLOC_027583-8128-1
ATGGTAAAGCACCGGAAAGCTCTCATCGTTATTGATGATTTAGATGATCA
CONSENSUS
ATGGTAAAGCACCGGAAAGCTCTCATCGTTATTGATGATTTAGATGATCA

RI-AT5G46470-XLOC_027583-8128-0
AGATGTGCTAGATGCTTTAGCGGATCAAACTCAATGGTTTGGAAGTGGGA
RI-AT5G46470-XLOC_027583-8128-1
AGATGTGCTAGATGCTTTAGCGGATCAAACTCAATGGTTTGGAAGTGGGA
CONSENSUS
AGATGTGCTAGATGCTTTAGCGGATCAAACTCAATGGTTTGGAAGTGGGA

RI-AT5G46470-XLOC_027583-8128-0
GTAGAATCATTGTGGTTACAGAAAATAAGCATTTTTTAAGGGCCAATCGG
RI-AT5G46470-XLOC_027583-8128-1
GTAGAATCATTGTGGTTACAGAAAATAAGCATTTTTTAAGGGCCAATCGG
CONSENSUS
GTAGAATCATTGTGGTTACAGAAAATAAGCATTTTTTAAGGGCCAATCGG

RI-AT5G46470-XLOC_027583-8128-0
ATTGATCACATTTACAAGGTCTGTCTCCCATCTAATGCGCTGGCTCTTGA
RI-AT5G46470-XLOC_027583-8128-1
ATTGATCACATTTACAAGGTCTGTCTCCCATCTAATGCGCTGGCTCTTGA
CONSENSUS
ATTGATCACATTTACAAGGTCTGTCTCCCATCTAATGCGCTGGCTCTTGA

RI-AT5G46470-XLOC_027583-8128-0
GATGTTTTGTCGTTCTGCTTTTAAGAAGAATTCTCCTCCTGATGACTTTT
RI-AT5G46470-XLOC_027583-8128-1
GATGTTTTGTCGTTCTGCTTTTAAGAAGAATTCTCCTCCTGATGACTTTT

CONSENSUS
GATGTTTTGTCGTTCTGCTTTTAAGAAGAATTCTCCTCCTGATGACTTTT

RI-AT5G46470-XLOC_027583-8128-0
TGGAGCTTTCTTCTGAAGTTGCATTGCGTGCTGGTAATCTTCCTTTGGGC
RI-AT5G46470-XLOC_027583-8128-1
TGGAGCTTTCTTCTGAAGTTGCATTGCGTGCTGGTAATCTTCCTTTGGGC
CONSENSUS
TGGAGCTTTCTTCTGAAGTTGCATTGCGTGCTGGTAATCTTCCTTTGGGC

RI-AT5G46470-XLOC_027583-8128-0
CTGAACGTATTGGGTTCAAATTTACGGGGCATAAACAAAGGTTACTGGAT
RI-AT5G46470-XLOC_027583-8128-1
CTGAACGTATTGGGTTCAAATTTACGGGGCATAAACAAAGGTTACTGGAT
CONSENSUS
CTGAACGTATTGGGTTCAAATTTACGGGGCATAAACAAAGGTTACTGGAT

RI-AT5G46470-XLOC_027583-8128-0
AGATATGCTACCGAGGCTTCAAGGTTTGGATGGAAAAATAGGGAAAACAC
RI-AT5G46470-XLOC_027583-8128-1
AGATATGCTACCGAGGCTTCAAGGTTTGGATGGAAAAATAGGGAAAACAC
CONSENSUS
AGATATGCTACCGAGGCTTCAAGGTTTGGATGGAAAAATAGGGAAAACAC

RI-AT5G46470-XLOC_027583-8128-0
TAAGAGTCAGCTATGATGGGTTGAATAACAGAAAAGATGAAGCAATATTT
RI-AT5G46470-XLOC_027583-8128-1
TAAGAGTCAGCTATGATGGGTTGAATAACAGAAAAGATGAAGCAATATTT
CONSENSUS
TAAGAGTCAGCTATGATGGGTTGAATAACAGAAAAGATGAAGCAATATTT

RI-AT5G46470-XLOC_027583-8128-0
CGTCACATTGCATGTATTTTCAATGGTGAGAAAGTCAGTGACATCAAACT
RI-AT5G46470-XLOC_027583-8128-1
CGTCACATTGCATGTATTTTCAATG-------------------------
CONSENSUS
CGTCACATTGCATGTATTTTCAATG.........................

RI-AT5G46470-XLOC_027583-8128-0
ATTACTAGCAAATAGTAACTTGGATGTTAATATCGGGCTAAAAAACCTTG
RI-AT5G46470-XLOC_027583-8128-1

CONSENSUS
....

RI-AT5G46470-XLOC_027583-8128-0
TTGATAGATCCCTTATCTGTGAAAGATTCAATACTCTGGAGATGCACTCT
RI-AT5G46470-XLOC_027583-8128-1 -------
ATCCCTTATCTGTGAAAGATTCAATACTCTGGAGATGCACTCT
CONSENSUS
.......ATCCCTTATCTGTGAAAGATTCAATACTCTGGAGATGCACTCT

RI-AT5G46470-XLOC_027583-8128-0
TTGCTACAAGAATTGGGTAAGGAGATTGTCCGTACTCAGTCCAATCAGCC
RI-AT5G46470-XLOC_027583-8128-1
TTGCTACAAGAATTGGGTAAGGAGATTGTCCGTACTCAGTCCAATCAGCC

CONSENSUS
      TTGCTACAAGAATTGGGTAAGGAGATTGTCCGTACTCAGTCCAATCAGCC

RI-AT5G46470-XLOC_027583-8128-0
      TGGAGAACGGGAGTTCCTTGTGGATTTGAAGGATATTTGCGATGTACTTG
RI-AT5G46470-XLOC_027583-8128-1
      TGGAGAACGGGAGTTCCTTGTGGATTTGAAGGATATTTGCGATGTACTTG
CONSENSUS
      TGGAGAACGGGAGTTCCTTGTGGATTTGAAGGATATTTGCGATGTACTTG

RI-AT5G46470-XLOC_027583-8128-0 AACATAACACT
RI-AT5G46470-XLOC_027583-8128-1 AACATAACACT
CONSENSUS   AACATAACACT

alignment for event: A5-AT5G04480-XLOC_028956-7405

A5-AT5G04480-XLOC_028956-7405-0
      ATGGCTGATGAAGTTCATGGAATATTTTTTCGAAGAAACGATCCTGATGC
A5-AT5G04480-XLOC_028956-7405-1
      ATGGCTGATGAAGTTCATGGAATATTTTTTCGAAGAAACGATCCTGATGC
CONSENSUS
      ATGGCTGATGAAGTTCATGGAATATTTTTTCGAAGAAACGATCCTGATGC

A5-AT5G04480-XLOC_028956-7405-0
      ATTGTTGAAGGCTTTCTCACCCTTGATATCAGATGGAAGGCTCTCTAAAT
A5-AT5G04480-XLOC_028956-7405-1
      ATTGTTGAAGGCTTTCTCACCCTTGATATCAGATGGAAGGCTCTCTAAAT
CONSENSUS
      ATTGTTGAAGGCTTTCTCACCCTTGATATCAGATGGAAGGCTCTCTAAAT

A5-AT5G04480-XLOC_028956-7405-0
      TTGCTCAAACAATTGCTTCTTCAGGAAGACTCTTAACTAAGAACTTGATG
A5-AT5G04480-XLOC_028956-7405-1
      TTGCTCAAACAATTGCTTCTTCAGGAAGACTCTTAACTAAGAACTTGATG
CONSENSUS
      TTGCTCAAACAATTGCTTCTTCAGGAAGACTCTTAACTAAGAACTTGATG

A5-AT5G04480-XLOC_028956-7405-0
      GCAACAGAATGCATTACTGGTTATGCTCGGCTTTTGGAGAACATGCTCCA
A5-AT5G04480-XLOC_028956-7405-1
      GCAACAGAATGCATTACTGGTTATGCTCGGCTTTTGGAGAACATGCTCCA
CONSENSUS
      GCAACAGAATGCATTACTGGTTATGCTCGGCTTTTGGAGAACATGCTCCA

A5-AT5G04480-XLOC_028956-7405-0
      TTTCCCATCTGATACTTTCTTGCCAGGTTCCATTTCTCAACTTCAAGTGG
A5-AT5G04480-XLOC_028956-7405-1
      TTTCCCATCTGATACTTTCTTGCCAGGTTCCATTTCTCAACTTCAAGTGG
CONSENSUS
      TTTCCCATCTGATACTTTCTTGCCAGGTTCCATTTCTCAACTTCAAGTGG

A5-AT5G04480-XLOC_028956-7405-0
      CAGCATGGGAGTGGAATTTCTTCCGGAGCGAATTAGAGCAGCCAAAAAGT
A5-AT5G04480-XLOC_028956-7405-1

CAGCATGGGAGTGGAATTTCTTCCGGAGCGAATTAGAGCAGCCAAAAAGT
CONSENSUS
CAGCATGGGAGTGGAATTTCTTCCGGAGCGAATTAGAGCAGCCAAAAAGT

A5-AT5G04480-XLOC_028956-7405-0
TTCATCTTGGACTCCGCTTATGCTTTCATTGGAAAATCTGGAATTGTTTT
A5-AT5G04480-XLOC_028956-7405-1
TTCATCTTGGACTCCGCTTATGCTTTCATTGGAAAATCTGGAATTGTTTT
CONSENSUS
TTCATCTTGGACTCCGCTTATGCTTTCATTGGAAAATCTGGAATTGTTTT

A5-AT5G04480-XLOC_028956-7405-0
CCAAGTCGAAGAGAAGTTCATGGGTGTTATTGAGTCAACAAACCCTGTTG
A5-AT5G04480-XLOC_028956-7405-1
CCAAGTCGAAGAGAAGTTCATGGGTGTTATTGAGTCAACAAACCCTGTTG
CONSENSUS
CCAAGTCGAAGAGAAGTTCATGGGTGTTATTGAGTCAACAAACCCTGTTG

A5-AT5G04480-XLOC_028956-7405-0
ACAACAATACCCTGTTTGTTTCCGATGAGCTTCCTTCCAAATTAGACTGG
A5-AT5G04480-XLOC_028956-7405-1
ACAACAATACCCTGTTTGTTTCCGATGAGCTTCCTTCCAAATTAGACTGG
CONSENSUS
ACAACAATACCCTGTTTGTTTCCGATGAGCTTCCTTCCAAATTAGACTGG

A5-AT5G04480-XLOC_028956-7405-0
GATGTTCTTGAAGAGATTGAGGGAGCTGAAGAGTATGAGAAAGTAGAATC
A5-AT5G04480-XLOC_028956-7405-1
GATGTTCTTGAAGAGATTGAGGGAGCTGAAGAGTATGAGAAAGTAGAATC
CONSENSUS
GATGTTCTTGAAGAGATTGAGGGAGCTGAAGAGTATGAGAAAGTAGAATC

A5-AT5G04480-XLOC_028956-7405-0
TGAGGAGGTATCAAATTTTGCTGCCATCAGACATATTTCTAGCTAGAGGA
A5-AT5G04480-XLOC_028956-7405-1
TGAGGAG-----------------------------------CTAGAGGA
CONSENSUS
TGAGGAG...................................CTAGAGGA

A5-AT5G04480-XLOC_028956-7405-0
CAGAATGGAAAGAGATGTTGAGGATTGGGAGGAAATATACCGGAATGCTC
A5-AT5G04480-XLOC_028956-7405-1
CAGAATGGAAAGAGATGTTGAGGATTGGGAGGAAATATACCGGAATGCTC
CONSENSUS
CAGAATGGAAAGAGATGTTGAGGATTGGGAGGAAATATACCGGAATGCTC

A5-AT5G04480-XLOC_028956-7405-0
GCAAATCGGAGAAGCTCAAATTTGAGGTGAATGAACGGGATGAAGGAGAG
A5-AT5G04480-XLOC_028956-7405-1
GCAAATCGGAGAAGCTCAAATTTGAGGTGAATGAACGGGATGAAGGAGAG
CONSENSUS
GCAAATCGGAGAAGCTCAAATTTGAGGTGAATGAACGGGATGAAGGAGAG

A5-AT5G04480-XLOC_028956-7405-0
CTAGAACGAACAGGCGAGCCTTTATGTATTTATGAGATTTACAATGGCGC
A5-AT5G04480-XLOC_028956-7405-1

CTAGAACGAACAGGCGAGCCTTTATGTATTTATGAGATTTACAATGGCGC
CONSENSUS
CTAGAACGAACAGGCGAGCCTTTATGTATTTATGAGATTTACAATGGCGC

A5-AT5G04480-XLOC_028956-7405-0
TGGGGCTTGGCCCTTTCTGCATCATGGCTCTCTGTACCGTGGTTTGAGCC
A5-AT5G04480-XLOC_028956-7405-1
TGGGGCTTGGCCCTTTCTGCATCATGGCTCTCTGTACCGTGGTTTGAGCC
CONSENSUS
TGGGGCTTGGCCCTTTCTGCATCATGGCTCTCTGTACCGTGGTTTGAGCC

A5-AT5G04480-XLOC_028956-7405-0 TG
A5-AT5G04480-XLOC_028956-7405-1 TG
CONSENSUS TG

alignment for event: A5-AT5G63120-XLOC_032139-11618

A5-AT5G63120-XLOC_032139-11618-0
GCTCCTTACACTACTTAAACAGTTAATGGATGGGAGTAAAATCCTAATTT
A5-AT5G63120-XLOC_032139-11618-1
GCTCCTTACACTACTTAAACAGTTAATGGATGGGAGTAAAATCCTAATTT
CONSENSUS
GCTCCTTACACTACTTAAACAGTTAATGGATGGGAGTAAAATCCTAATTT

A5-AT5G63120-XLOC_032139-11618-0
TTGTGGAGACAAAGAGAGGGTGTGATCAAGTGACTAGACAATTGAGAATG
A5-AT5G63120-XLOC_032139-11618-1
TTGTGGAGACAAAGAGAGGGTGTGATCAAGTGACTAGACAATTGAGAATG
CONSENSUS
TTGTGGAGACAAAGAGAGGGTGTGATCAAGTGACTAGACAATTGAGAATG

A5-AT5G63120-XLOC_032139-11618-0
GACGGATGGCCAGCTCTTGCCATACATGGTGACAAGACCCAATCGGAAAG
A5-AT5G63120-XLOC_032139-11618-1
GACGGATGGCCAGCTCTTGCCATACATGGTGACAAGACCCAATCGGAAAG
CONSENSUS
GACGGATGGCCAGCTCTTGCCATACATGGTGACAAGACCCAATCGGAAAG

A5-AT5G63120-XLOC_032139-11618-0
AGACCGAGTCTTGGCAGAATTTAAGAGTGGACGAAGCCCGATAATGACTG
A5-AT5G63120-XLOC_032139-11618-1
AGACCGAGTCTTGGCAGAATTTAAGAGTGGACGAAGCCCGATAATGACTG
CONSENSUS
AGACCGAGTCTTGGCAGAATTTAAGAGTGGACGAAGCCCGATAATGACTG

A5-AT5G63120-XLOC_032139-11618-0
CCACTGATGTAGCAGCAAGGGGACTTGGTAGGATTAACTGTGATACACAA
A5-AT5G63120-XLOC_032139-11618-1
CCACTGATGTAGCAGCAAGGGGACTTG-----------------------
CONSENSUS
CCACTGATGTAGCAGCAAGGGGACTTG.......................

A5-AT5G63120-XLOC_032139-11618-0
TAGGAGAGATTATACTTAGAGCATTTCGTTTTCTTGGGGTTTTAAAGATA

A5-AT5G63120-XLOC_032139-11618-1

CONSENSUS
....

A5-AT5G63120-XLOC_032139-11618-0
ATAAAAAAAGTCGCCTGGCTGCCGCTTTCCACAGTTTCCTCTGGTATTAG
A5-AT5G63120-XLOC_032139-11618-1

CONSENSUS
....

A5-AT5G63120-XLOC_032139-11618-0
GGAAAAAAGCACATAGAAAGAAAAAAGGAGCTTCTTTGAAGAATTTGTTT
A5-AT5G63120-XLOC_032139-11618-1

CONSENSUS
....

A5-AT5G63120-XLOC_032139-11618-0
CCTGCATCCATCTCCCGGGCATGGAGCTGATTGGTTTTTGAGCAGCCTGG
A5-AT5G63120-XLOC_032139-11618-1

CONSENSUS
....

A5-AT5G63120-XLOC_032139-11618-0
GCGACTGACACCTGCATGCATTAATAGTGTTATAACGCCGTCCCAAACAC
A5-AT5G63120-XLOC_032139-11618-1

CONSENSUS
....

A5-AT5G63120-XLOC_032139-11618-0
TCCTTATCCCTGCAAGTTAAGATGTGAAGGACATTAAATGTGTGGTTAAT
A5-AT5G63120-XLOC_032139-11618-1 --------------------
ATGTGAAGGACATTAAATGTGTGGTTAAT
CONSENSUS
.....................ATGTGAAGGACATTAAATGTGTGGTTAAT

A5-AT5G63120-XLOC_032139-11618-0
TATGATTTCCCAAATACATTGGAGGATTACATCCATAGGATTGGTCGAAC
A5-AT5G63120-XLOC_032139-11618-1
TATGATTTCCCAAATACATTGGAGGATTACATCCATAGGATTGGTCGAAC
CONSENSUS
TATGATTTCCCAAATACATTGGAGGATTACATCCATAGGATTGGTCGAAC

A5-AT5G63120-XLOC_032139-11618-0
CGGGCGTGCAGGAGCTAAAGGAATGGCCTTTACATTCTTCACACATGACA
A5-AT5G63120-XLOC_032139-11618-1
CGGGCGTGCAGGAGCTAAAGGAATGGCCTTTACATTCTTCACACATGACA
CONSENSUS
CGGGCGTGCAGGAGCTAAAGGAATGGCCTTTACATTCTTCACACATGACA

A5-AT5G63120-XLOC_032139-11618-0
ATGCTAAGTTTGCGAGAGAGCTTGTCAAGATCCTTCAAGAAGCTGGTCAA

A5-AT5G63120-XLOC_032139-11618-1
ATGCTAAGTTTGCGAGAGAGCTTGTCAAGATCCTTCAAGAAGCTGGTCAA
CONSENSUS
ATGCTAAGTTTGCGAGAGAGCTTGTCAAGATCCTTCAAGAAGCTGGTCAA

A5-AT5G63120-XLOC_032139-11618-0
GTTGTACCTCCTACTCTCTCCGCACTAGTCCGATCATCTGGTTCTGGTTA
A5-AT5G63120-XLOC_032139-11618-1
GTTGTACCTCCTACTCTCTCCGCACTAGTCCGATCATCTGGTTCTGGTTA
CONSENSUS
GTTGTACCTCCTACTCTCTCCGCACTAGTCCGATCATCTGGTTCTGGTTA

A5-AT5G63120-XLOC_032139-11618-0 TGGAG
A5-AT5G63120-XLOC_032139-11618-1 TGGAG
CONSENSUS TGGAG

alignment for event: RI-AT5G19090-XLOC_026045-7482

RI-AT5G19090-XLOC_026045-7482-0
GTGTTTTCACGACAAAGATAGACGCAGAGCTAGGGAAAGTGACTGTATCT
RI-AT5G19090-XLOC_026045-7482-1
GTGTTTTCACGACAAAGATAGACGCAGAGCTAGGGAAAGTGACTGTATCT
CONSENSUS
GTGTTTTCACGACAAAGATAGACGCAGAGCTAGGGAAAGTGACTGTATCT

RI-AT5G19090-XLOC_026045-7482-0
GGAAACGTCGACCCATCAGTTCTTATCAAGAAGCTCTTGAAATCTGGTAA
RI-AT5G19090-XLOC_026045-7482-1
GGAAACGTCGACCCATCAGTTCTTATCAAGAAGCTCTTGAAATCTGGTAA
CONSENSUS
GGAAACGTCGACCCATCAGTTCTTATCAAGAAGCTCTTGAAATCTGGTAA

RI-AT5G19090-XLOC_026045-7482-0
ACATGCCGAAATCTGGGGAGCTCCCAAGGGAGGATCTAACAACAATCAGA
RI-AT5G19090-XLOC_026045-7482-1
ACATGCCGAAATCTGGGGAGCTCCCAAGGGAGGATCTAACAACAATCAGA
CONSENSUS
ACATGCCGAAATCTGGGGAGCTCCCAAGGGAGGATCTAACAACAATCAGA

RI-AT5G19090-XLOC_026045-7482-0
ACCAACCCAACTTGGCCAATCAGTTCAAAGCCATGCAGATTGATCATGGC
RI-AT5G19090-XLOC_026045-7482-1
ACCAACCCAACTTGGCCAATCAGTTCAAAGCCATGCAGATTGATCATGGC
CONSENSUS
ACCAACCCAACTTGGCCAATCAGTTCAAAGCCATGCAGATTGATCATGGC

RI-AT5G19090-XLOC_026045-7482-0
GGCAAAGGCGGTGGCGGTGGTGGTGGAGGCCCTGCTAACAATAACAAAGG
RI-AT5G19090-XLOC_026045-7482-1
GGCAAAGGCGGTGGCGGTGGTGGTGGAGGCCCTGCTAACAATAACAAAGG
CONSENSUS
GGCAAAGGCGGTGGCGGTGGTGGTGGAGGCCCTGCTAACAATAACAAAGG

RI-AT5G19090-XLOC_026045-7482-0

CCAAAAGATCGGTGGAGGCGGTGGTGGAGGAGGAGGCGGCGGCGGAGGTG
RI-AT5G19090-XLOC_026045-7482-1
CCAAAAGATCGGTGGAGGCGGTGGTGGAGGAGGAGGCGGCGGCGGAGGTG
CONSENSUS
CCAAAAGATCGGTGGAGGCGGTGGTGGAGGAGGAGGCGGCGGCGGAGGTG

RI-AT5G19090-XLOC_026045-7482-0
GTGGTGGCGGACCACCGAAGATGGTTATTCCACAGTTAACACCACAACAG
RI-AT5G19090-XLOC_026045-7482-1
GTGGTGGCGGACCACCGAAGATGGTTATTCCACAGTTAACACCACAACAG
CONSENSUS
GTGGTGGCGGACCACCGAAGATGGTTATTCCACAGTTAACACCACAACAG

RI-AT5G19090-XLOC_026045-7482-0
ATGCAGCAGTTGAATCCTCAACAGCTTCAGCAGCTACAACAACTTCAGCA
RI-AT5G19090-XLOC_026045-7482-1
ATGCAGCAGTTGAATCCTCAACAGCTTCAGCAGCTACAACAACTTCAGCA
CONSENSUS
ATGCAGCAGTTGAATCCTCAACAGCTTCAGCAGCTACAACAACTTCAGCA

RI-AT5G19090-XLOC_026045-7482-0
GATGAAAGGGTTTCAAGATCTGAAGCTTCCTCCTCAGTTAAAGGGTGGTC
RI-AT5G19090-XLOC_026045-7482-1
GATGAAAGGGTTTCAAGATCTGAAGCTTCCTCCTCAGTTAAAGGGTGGTC
CONSENSUS
GATGAAAGGGTTTCAAGATCTGAAGCTTCCTCCTCAGTTAAAGGGTGGTC

RI-AT5G19090-XLOC_026045-7482-0
CAGGTCCGGGACCTGGCTCTGTTCCTATGAACAAGAATCCTCAGATGCCT
RI-AT5G19090-XLOC_026045-7482-1
CAGGTCCGGGACCTGGCTCTGTTCCTATGAACAAGAATCCTCAGATGCCT
CONSENSUS
CAGGTCCGGGACCTGGCTCTGTTCCTATGAACAAGAATCCTCAGATGCCT

RI-AT5G19090-XLOC_026045-7482-0
AATAATCCTAATCAGAAGGCTGTGAAGTTCAATGTTCCTGATGATGATGA
RI-AT5G19090-XLOC_026045-7482-1
AATAATCCTAATCAGAAGGCTGTGAAGTTCAATGTTCCTGATGATGATGA
CONSENSUS
AATAATCCTAATCAGAAGGCTGTGAAGTTCAATGTTCCTGATGATGATGA

RI-AT5G19090-XLOC_026045-7482-0
TGAGGAAGATTTTAGTGATGAATTCGATGATGAGTTTGATGAAGATGACG
RI-AT5G19090-XLOC_026045-7482-1
TGAGGAAGATTTTAGTGATGAATTCGATGATGAGTTTGATGAAGATGACG
CONSENSUS
TGAGGAAGATTTTAGTGATGAATTCGATGATGAGTTTGATGAAGATGACG

RI-AT5G19090-XLOC_026045-7482-0
ACGAGTTTGATGATGATTTGGAAGATGATGAGTTTGACGACCACCCACCT
RI-AT5G19090-XLOC_026045-7482-1
ACGAGTTTGATGATGATTTGGAAGATGATGAGTTTGACGACCACCCACCT
CONSENSUS
ACGAGTTTGATGATGATTTGGAAGATGATGAGTTTGACGACCACCCACCT

RI-AT5G19090-XLOC_026045-7482-0

CCGCCTAACAAAATGAAGCCTATGATGGGTGGTGGTAACATGATCATGCC
RI-AT5G19090-XLOC_026045-7482-1
CCGCCTAACAAAATGAAGCCTATGATGGGTGGTGGTAACATGATCATGCC
CONSENSUS
CCGCCTAACAAAATGAAGCCTATGATGGGTGGTGGTAACATGATCATGCC

RI-AT5G19090-XLOC_026045-7482-0
TAATAACATGATGCCTAACATGATGATGCCTAACGCTCAACAAATGTTAA
RI-AT5G19090-XLOC_026045-7482-1
TAATAACATGATGCCTAACATGATGATGCCTAACGCTCAACAAATGTTAA
CONSENSUS
TAATAACATGATGCCTAACATGATGATGCCTAACGCTCAACAAATGTTAA

RI-AT5G19090-XLOC_026045-7482-0
ACGCTCATAAGAATGGTGGTGGTCCTGGACCCGCCGGAGGTAAAATTGAG
RI-AT5G19090-XLOC_026045-7482-1
ACGCTCATAAGAATGGTG--------------------------------
CONSENSUS
ACGCTCATAAGAATGGTG................................

RI-AT5G19090-XLOC_026045-7482-0
GGTAAAGGTATGCCTTTTCCGGTTCAAATGGGCGGCGGTGGTGGAGGTCC
RI-AT5G19090-XLOC_026045-7482-1

CONSENSUS
....

RI-AT5G19090-XLOC_026045-7482-0
CGGTGGTAAGAAGGGAGGTCCCGGTGGAGGCGGGGGTAATATGGGGAATC
RI-AT5G19090-XLOC_026045-7482-1

CONSENSUS
....

RI-AT5G19090-XLOC_026045-7482-0
AGAACCAAGGAGGAGGAGGCAAGAACGGTGGAAAAGGCGGTGGTGGACAT
RI-AT5G19090-XLOC_026045-7482-1

CONSENSUS
....

RI-AT5G19090-XLOC_026045-7482-0
CCGTTAGATGGAAAAATGGGCGGCGGAGGTGGCGGTCCCAACGGTAATAA
RI-AT5G19090-XLOC_026045-7482-1

CONSENSUS
....

RI-AT5G19090-XLOC_026045-7482-0
AGGAGGTGGCGGAGTCCAGATGAACGGAGGTCCCAACGGTGGGAAAAAGG
RI-AT5G19090-XLOC_026045-7482-1

CONSENSUS
....

RI-AT5G19090-XLOC_026045-7482-0

GTGGTGGTGGAGGCGGCGGCGGAGGAGGAGGACCCATGAGTGGAGGCCTC
RI-AT5G19090-XLOC_026045-7482-1

CONSENSUS
....

RI-AT5G19090-XLOC_026045-7482-0
CCGCCAGGTTTCCGGCCAATGGGAGGCGGTGGTGGTGGAGGGGGAGGACC
RI-AT5G19090-XLOC_026045-7482-1
-----------------------------------GTGGAGGGGGAGGACC
CONSENSUS
...................................GTGGAGGGGGAGGACC

RI-AT5G19090-XLOC_026045-7482-0
TCAGAGCATGAGTATGCCAATGGGCGGTGCAATGGGTGGTCCAATGGGAA
RI-AT5G19090-XLOC_026045-7482-1
TCAGAGCATGAGTATGCCAATGGGCGGTGCAATGGGTGGTCCAATGGGAA
CONSENSUS
TCAGAGCATGAGTATGCCAATGGGCGGTGCAATGGGTGGTCCAATGGGAA

RI-AT5G19090-XLOC_026045-7482-0
GTCTACCACAAATGGGTGGTGGTCCCGGTCCAATGAGTAATAATATGCAG
RI-AT5G19090-XLOC_026045-7482-1
GTCTACCACAAATGGGTGGTGGTCCCGGTCCAATGAGTAATAATATGCAG
CONSENSUS
GTCTACCACAAATGGGTGGTGGTCCCGGTCCAATGAGTAATAATATGCAG

RI-AT5G19090-XLOC_026045-7482-0
GCGGTTCAAGGATTACCCGCAATGGGTCCAGGAGGTGGTGGTGGCGGTGG
RI-AT5G19090-XLOC_026045-7482-1
GCGGTTCAAGGATTACCCGCAATGGGTCCAGGAGGTGGTGGTGGCGGTGG
CONSENSUS
GCGGTTCAAGGATTACCCGCAATGGGTCCAGGAGGTGGTGGTGGCGGTGG

RI-AT5G19090-XLOC_026045-7482-0
CCCATCCGCAGAAGCACCACCAGGATATTTCCAAGGCCAAGTCTCCGGAA
RI-AT5G19090-XLOC_026045-7482-1
CCCATCCGCAGAAGCACCACCAGGATATTTCCAAGGCCAAGTCTCCGGAA
CONSENSUS
CCCATCCGCAGAAGCACCACCAGGATATTTCCAAGGCCAAGTCTCCGGAA

RI-AT5G19090-XLOC_026045-7482-0
ACGGCGGAGGCGGACAAGACTCAATGCCGGGAAACCCCTACTTACAACAG
RI-AT5G19090-XLOC_026045-7482-1
ACGGCGGAGGCGGACAAGACTCAATGCCGGGAAACCCCTACTTACAACAG
CONSENSUS
ACGGCGGAGGCGGACAAGACTCAATGCCGGGAAACCCCTACTTACAACAG

RI-AT5G19090-XLOC_026045-7482-0
CAGCAACAGCAACAACAACAACAATACTTAGCGGCGGTTATGAACCAGCA
RI-AT5G19090-XLOC_026045-7482-1
CAGCAACAGCAACAACAACAACAATACTTAGCGGCGGTTATGAACCAGCA
CONSENSUS
CAGCAACAGCAACAACAACAACAATACTTAGCGGCGGTTATGAACCAGCA

RI-AT5G19090-XLOC_026045-7482-0

ACGATCCATGGGAAACGAACGGTTCCAGCCGATGATGTACGCTAGACCAC
RI-AT5G19090-XLOC_026045-7482-1
ACGATCCATGGGAAACGAACGGTTCCAGCCGATGATGTACGCTAGACCAC
CONSENSUS
ACGATCCATGGGAAACGAACGGTTCCAGCCGATGATGTACGCTAGACCAC

RI-AT5G19090-XLOC_026045-7482-0
CGCCAGCTGTCAACTATATGCCACCACAACCGCAACCGCACCAACAACAT
RI-AT5G19090-XLOC_026045-7482-1
CGCCAGCTGTCAACTATATGCCACCACAACCGCAACCGCACCAACAACAT
CONSENSUS
CGCCAGCTGTCAACTATATGCCACCACAACCGCAACCGCACCAACAACAT

RI-AT5G19090-XLOC_026045-7482-0
CCATACCCGTATCCATATCCGTATCCACCCCAGTATCCGCCTCACAATGG
RI-AT5G19090-XLOC_026045-7482-1
CCATACCCGTATCCATATCCGTATCCACCCCAGTATCCGCCTCACAATGG
CONSENSUS
CCATACCCGTATCCATATCCGTATCCACCCCAGTATCCGCCTCACAATGG

RI-AT5G19090-XLOC_026045-7482-0
TGACCAGTATTCTGATTACTTCAATGACGAGAACACATCAAGCTGCAATA
RI-AT5G19090-XLOC_026045-7482-1
TGACCAGTATTCTGATTACTTCAATGACGAGAACACATCAAGCTGCAATA
CONSENSUS
TGACCAGTATTCTGATTACTTCAATGACGAGAACACATCAAGCTGCAATA

RI-AT5G19090-XLOC_026045-7482-0
TTATGTGAACAACAACAACAAAACTTTCCATAGTTTGTGTCCGGTCTGTG
RI-AT5G19090-XLOC_026045-7482-1
TTATGTGAACAACAACAACAAAACTTTCCATAGTTTGTGTCCGGTCTGTG
CONSENSUS
TTATGTGAACAACAACAACAAAACTTTCCATAGTTTGTGTCCGGTCTGTG

RI-AT5G19090-XLOC_026045-7482-0
ATTATACATGGAAATGTTTTTTTTTTCATAATTATTGGGGGTTCTTAGAA
RI-AT5G19090-XLOC_026045-7482-1
ATTATACATGGAAATGTTTTTTTTTTCATAATTATTGGGGGTTCTTAGAA
CONSENSUS
ATTATACATGGAAATGTTTTTTTTTTCATAATTATTGGGGGTTCTTAGAA

RI-AT5G19090-XLOC_026045-7482-0
TCCTTTTTTTTCCTCCAACGGATCAAATCCTAAATTATATTAGGATTTTT
RI-AT5G19090-XLOC_026045-7482-1
TCCTTTTTTTTCCTCCAACGGATCAAATCCTAAATTATATTAGGATTTTT
CONSENSUS
TCCTTTTTTTTCCTCCAACGGATCAAATCCTAAATTATATTAGGATTTTT

RI-AT5G19090-XLOC_026045-7482-0 TTTTATAATTTGTGATAGTTCTTAAGT
RI-AT5G19090-XLOC_026045-7482-1 TTTTATAATTTGTGATAGTTCTTAAGT
CONSENSUS   TTTTATAATTTGTGATAGTTCTTAAGT

alignment for event: A5-AT5G14060-XLOC_025779-13615

A5-AT5G14060-XLOC_025779-13615-0
      GTGTTGCATCCACTGTCTATGAGGCCAGCAAGAGATGGCGACATTCCTGT
A5-AT5G14060-XLOC_025779-13615-1
      GTGTTGCATCCACTGTCTATGAGGCCAGCAAGAGATGGCGACATTCCTGT
CONSENSUS
      GTGTTGCATCCACTGTCTATGAGGCCAGCAAGAGATGGCGACATTCCTGT

A5-AT5G14060-XLOC_025779-13615-0
      GAGGGTTAAGAACTCGTACAACCCCACTGCTCCAGGAACTGTCATCACCA
A5-AT5G14060-XLOC_025779-13615-1
      GAGGGTTAAGAACTCGTACAACCCCACTGCTCCAGGAACTGTCATCACCA
CONSENSUS
      GAGGGTTAAGAACTCGTACAACCCCACTGCTCCAGGAACTGTCATCACCA

A5-AT5G14060-XLOC_025779-13615-0 GATCAAGAGACATGA-----
GCTGTGCTAACCAGCATTGTTCTGAAACGT
A5-AT5G14060-XLOC_025779-13615-1
      GATCAAGAGACATGAGTAAGGCTGTGCTAACCAGCATTGTTCTGAAACGT
CONSENSUS
      GATCAAGAGACATGA.....GCTGTGCTAACCAGCATTGTTCTGAAACGT

A5-AT5G14060-XLOC_025779-13615-0
      AATGTGACCATGTTGGACATAGCAAGCACCCGTATGCTTGGCCAATATGG
A5-AT5G14060-XLOC_025779-13615-1
      AATGTGACCATGTTGGACATAGCAAGCACCCGTATGCTTGGCCAATATGG
CONSENSUS
      AATGTGACCATGTTGGACATAGCAAGCACCCGTATGCTTGGCCAATATGG

A5-AT5G14060-XLOC_025779-13615-0 TTTCCTTGCCAAG
A5-AT5G14060-XLOC_025779-13615-1 TTTCCTTGCCAAG
CONSENSUS TTTCCTTGCCAAG

alignment for event: A5-AT1G51690-XLOC_002583-13606

A5-AT1G51690-XLOC_002583-13606-0
      GTTCAAGACAAGAAGATCAAGAAAATCTGTGATATGAATTCAGATCCTTC
A5-AT1G51690-XLOC_002583-13606-1
      GTTCAAGACAAGAAGATCAAGAAAATCTGTGATATGAATTCAGATCCTTC
CONSENSUS
      GTTCAAGACAAGAAGATCAAGAAAATCTGTGATATGAATTCAGATCCTTC

A5-AT1G51690-XLOC_002583-13606-0
      AAGAACTGTAGGAAACGGAACCGTTGCAAGCTCGAGCAATTCAAACATTA
A5-AT1G51690-XLOC_002583-13606-1
      AAGAACTGTAGGAAACGGAACCGTTGCAAGCTCGAGCAATTCAAACATTA
CONSENSUS
      AAGAACTGTAGGAAACGGAACCGTTGCAAGCTCGAGCAATTCAAACATTA

A5-AT1G51690-XLOC_002583-13606-0
      CAAACTCATGCCTTGTGAATGGAGGAGTATCTGAAGTGAACAACTCCTTA
A5-AT1G51690-XLOC_002583-13606-1
      CAAACTCATGCCTTGTGAATGGAGGAGTATCTGAAGTGAACAACTCCTTA
CONSENSUS
      CAAACTCATGCCTTGTGAATGGAGGAGTATCTGAAGTGAACAACTCCTTA

A5-AT1G51690-XLOC_002583-13606-0
TGTAATGACTTCTCATTGCCAGCAGGAGGCATCTCTTCGCTGCGATTACC
A5-AT1G51690-XLOC_002583-13606-1
TGTAATGACTTCTCATTGCCAGCAGGAGGCATCTCTTCGCTGCGATTACC
CONSENSUS
TGTAATGACTTCTCATTGCCAGCAGGAGGCATCTCTTCGCTGCGATTACC

A5-AT1G51690-XLOC_002583-13606-0 AGTG---
GTAACTAGCCATGAGTCGAGCCCTGTGGCTAGATGTCGAAGAG
A5-AT1G51690-XLOC_002583-13606-1
AGTGGTAGTAACTAGCCATGAGTCGAGCCCTGTGGCTAGATGTCGAAGAG
CONSENSUS
AGTG...GTAACTAGCCATGAGTCGAGCCCTGTGGCTAGATGTCGAAGAG

A5-AT1G51690-XLOC_002583-13606-0
TATATGCTCATGCTCATGATTATCATATTAATTCAATCTCAAATAACAG
A5-AT1G51690-XLOC_002583-13606-1
TATATGCTCATGCTCATGATTATCATATTAATTCAATCTCAAATAACAG
CONSENSUS
TATATGCTCATGCTCATGATTATCATATTAATTCAATCTCAAATAACAG

alignment for event: A5-AT1G80245-XLOC_004111-11152

A5-AT1G80245-XLOC_004111-11152-0
GGAGCAAATTTAGGGTTTATCGTTTGCATCGGTCTCGCGTCTCGTTTCCC
A5-AT1G80245-XLOC_004111-11152-1
GGAGCAAATTTAGGGTTTATCGTTTGCATCGGTCTCGCGTCTCGTTTCCC
CONSENSUS
GGAGCAAATTTAGGGTTTATCGTTTGCATCGGTCTCGCGTCTCGTTTCCC

A5-AT1G80245-XLOC_004111-11152-0
ACAGACACATCTCTCGAATCTTTTGAAGGCCTTCATTGCAATAGATTGCT
A5-AT1G80245-XLOC_004111-11152-1
ACAGACACATCTCTCGAATCTTTTGAAGGCCTTCATTGCAATAGATTGCT
CONSENSUS
ACAGACACATCTCTCGAATCTTTTGAAGGCCTTCATTGCAATAGATTGCT

A5-AT1G80245-XLOC_004111-11152-0
CGTAAGGGATAG--------------------------------------
A5-AT1G80245-XLOC_004111-11152-1
CGTAAGGGATAGGTAGGCCTTTATGTTCTGCCGCTTAATTTCTTCGCCGG
CONSENSUS
CGTAAGGGATAG......................................

A5-AT1G80245-XLOC_004111-11152-0 ----------------------
GAATTAGGATGGAGGAATTAACCCAAAT
A5-AT1G80245-XLOC_004111-11152-1
ATTCTGTTTGATTCATTTTAAGGAATTAGGATGGAGGAATTAACCCAAAT
CONSENSUS
......................GAATTAGGATGGAGGAATTAACCCAAAT

A5-AT1G80245-XLOC_004111-11152-0
CTTGAAGAACAATAGGACAGATGATCTCACTTGGTTTTGCTCTCTCTCTG

A5-AT1G80245-XLOC_004111-11152-1
      CTTGAAGAACAATAGGACAGATGATCTCACTTGGTTTTGCTCTCTCTCTG
CONSENSUS
      CTTGAAGAACAATAGGACAGATGATCTCACTTGGTTTTGCTCTCTCTCTG

A5-AT1G80245-XLOC_004111-11152-0 AATCTGAACTG
A5-AT1G80245-XLOC_004111-11152-1 AATCTGAACTG
CONSENSUS   AATCTGAACTG

alignment for event: RI-AT1G79790-XLOC_008095-9018

RI-AT1G79790-XLOC_008095-9018-0
      GGCTTAAGGAGTGCATGAGGTCAGGTTACTCCTACTTAGATGGCATGCAA
RI-AT1G79790-XLOC_008095-9018-1
      GGCTTAAGGAGTGCATGAGGTCAGGTTACTCCTACTTAGATGGCATGCAA
CONSENSUS
      GGCTTAAGGAGTGCATGAGGTCAGGTTACTCCTACTTAGATGGCATGCAA

RI-AT1G79790-XLOC_008095-9018-0
      GAGCTCCTTCAAACTTTGGCAGCTGACGACTTCGAGATTCATGCTTTCAC
RI-AT1G79790-XLOC_008095-9018-1
      GAGCTCCTTCAAACTTTGGCAGCTGACGACTTCGAGATTCATGCTTTCAC
CONSENSUS
      GAGCTCCTTCAAACTTTGGCAGCTGACGACTTCGAGATTCATGCTTTCAC

RI-AT1G79790-XLOC_008095-9018-0
      TAACTATCCCATCTGGTTTCCATCCCTTGCCTTTCACTCTTTGAAACGGA
RI-AT1G79790-XLOC_008095-9018-1
      TAACTATCCCATCTG-----------------------------------
CONSENSUS
      TAACTATCCCATCTG...................................

RI-AT1G79790-XLOC_008095-9018-0
      GCTTCATGTCTCTTTGTTTACTCTTGTCGTGTTTCCATAGGTACAACATT
RI-AT1G79790-XLOC_008095-9018-1
      --GTACAACATT
CONSENSUS
      ....GTACAACATT

RI-AT1G79790-XLOC_008095-9018-0
      ATCGAAGACAAGTTAAAGCTTTCTGCCTACTTATCATGGACGTTTTGCTC
RI-AT1G79790-XLOC_008095-9018-1
      ATCGAAGACAAGTTAAAGCTTTCTGCCTACTTATCATGGACGTTTTGCTC
CONSENSUS
      ATCGAAGACAAGTTAAAGCTTTCTGCCTACTTATCATGGACGTTTTGCTC

RI-AT1G79790-XLOC_008095-9018-0 CTGTATTGCTG
RI-AT1G79790-XLOC_008095-9018-1 CTGTATTGCTG
CONSENSUS   CTGTATTGCTG

alignment for event: SE-AT1G06220-XLOC_000279-13450

SE-AT1G06220-XLOC_000279-13450-0
AGAGTCTTCCAATCAAAACCTAAATCTAAATTTTTCATTCCCTTGTTCCG
SE-AT1G06220-XLOC_000279-13450-1
AGAGTCTTCCAATCAAAACCTAAATCTAAATTTTTCATTCCCTTGTTCCG
CONSENSUS
AGAGTCTTCCAATCAAAACCTAAATCTAAATTTTTCATTCCCTTGTTCCG

SE-AT1G06220-XLOC_000279-13450-0
GCGATAACAGTCTCAGTGGCGACAAACCCTATTGCCGAGCCAACTCGCAT
SE-AT1G06220-XLOC_000279-13450-1
GCGATAACAGTCTCAGTGGCGACAAACCCTATTGCCGAGCCAACTCGCAT
CONSENSUS
GCGATAACAGTCTCAGTGGCGACAAACCCTATTGCCGAGCCAACTCGCAT

SE-AT1G06220-XLOC_000279-13450-0
TTTCCTCTAGCTATCGCAACGCCTCTCGCTCTCTCTCCCCACCG------
SE-AT1G06220-XLOC_000279-13450-1
TTTCCTCTAGCTATCGCAACGCCTCTCGCTCTCTCTCCCCACCGCTTTTT
CONSENSUS
TTTCCTCTAGCTATCGCAACGCCTCTCGCTCTCTCTCCCCACCG......

SE-AT1G06220-XLOC_000279-13450-0

SE-AT1G06220-XLOC_000279-13450-1
CATGGTGTTCTCTTGGACTTGGTGTTGAGCATTAGAGGAGATTACTCGCC
CONSENSUS
....

SE-AT1G06220-XLOC_000279-13450-0

SE-AT1G06220-XLOC_000279-13450-1
AATGGGTTAAATAAAATATGTTGCAGAATTGCTATAGTAAATAACCCAGA
CONSENSUS
....

SE-AT1G06220-XLOC_000279-13450-0 ----------------------------
GTTTACATAAAGATGGAAAGTAG
SE-AT1G06220-XLOC_000279-13450-1
TAGATCTATCAAAAGCTTGATTTTGTGGTTTACATAAAGATGGAAAGTAG
CONSENSUS
...........................GTTTACATAAAGATGGAAAGTAG

SE-AT1G06220-XLOC_000279-13450-0
CTTGTATGATGAGTTTGGTAACTATGTTGGACCTGAGATTGAGTCTGACA
SE-AT1G06220-XLOC_000279-13450-1
CTTGTATGATGAGTTTGGTAACTATGTTGGACCTGAGATTGAGTCTGACA
CONSENSUS
CTTGTATGATGAGTTTGGTAACTATGTTGGACCTGAGATTGAGTCTGACA

SE-AT1G06220-XLOC_000279-13450-0
GAGATAGTGATGATGAAGTAGAAGATGAAGATCTCCAAGATAAGCATCTT
SE-AT1G06220-XLOC_000279-13450-1
GAGATAGTGATGATGAAGTAGAAGATGAAGATCTCCAAGATAAGCATCTT
CONSENSUS
GAGATAGTGATGATGAAGTAGAAGATGAAGATCTCCAAGATAAGCATCTT

SE-AT1G06220-XLOC_000279-13450-0
GAAGAAAATGGTTCCGATGGCGAACAAGGGCCTGGGGGTTCTAATGGCTG
SE-AT1G06220-XLOC_000279-13450-1
GAAGAAAATGGTTCCGATGGCGAACAAGGGCCTGGGGGTTCTAATGGCTG
CONSENSUS
GAAGAAAATGGTTCCGATGGCGAACAAGGGCCTGGGGGTTCTAATGGCTG

SE-AT1G06220-XLOC_000279-13450-0
GATTACCACTATCAATGATGTTGAGATGGAGAACCAAATTGTTCTTCCTG
SE-AT1G06220-XLOC_000279-13450-1
GATTACCACTATCAATGATGTTGAGATGGAGAACCAAATTGTTCTTCCTG
CONSENSUS
GATTACCACTATCAATGATGTTGAGATGGAGAACCAAATTGTTCTTCCTG

SE-AT1G06220-XLOC_000279-13450-0
AGGATAAGAAGTACTACCCTACTGCTGAGGAGGTTTATGGTGAGGATGTT
SE-AT1G06220-XLOC_000279-13450-1
AGGATAAGAAGTACTACCCTACTGCTGAGGAGGTTTATGGTGAGGATGTT
CONSENSUS
AGGATAAGAAGTACTACCCTACTGCTGAGGAGGTTTATGGTGAGGATGTT

SE-AT1G06220-XLOC_000279-13450-0
GAGACCTTGGTTATGGATGAAGATGAGCAGCCTCTTGAGCAACCCATTAT
SE-AT1G06220-XLOC_000279-13450-1
GAGACCTTGGTTATGGATGAAGATGAGCAGCCTCTTGAGCAACCCATTAT
CONSENSUS
GAGACCTTGGTTATGGATGAAGATGAGCAGCCTCTTGAGCAACCCATTAT

SE-AT1G06220-XLOC_000279-13450-0
CAAACCTGTTAGAGATATTAGATTTGAGGTGGGGGTCAAAGATCAAGCAA
SE-AT1G06220-XLOC_000279-13450-1
CAAACCTGTTAGAGATATTAGATTTGAGGTGGGGGTCAAAGATCAAGCAA
CONSENSUS
CAAACCTGTTAGAGATATTAGATTTGAGGTGGGGGTCAAAGATCAAGCAA

SE-AT1G06220-XLOC_000279-13450-0
CGTATGTGTCAACACAGTTTCTTATCGGCCTCATGTCTAATCCTGCGCTT
SE-AT1G06220-XLOC_000279-13450-1
CGTATGTGTCAACACAGTTTCTTATCGGCCTCATGTCTAATCCTGCGCTT
CONSENSUS
CGTATGTGTCAACACAGTTTCTTATCGGCCTCATGTCTAATCCTGCGCTT

SE-AT1G06220-XLOC_000279-13450-0
GTGAGGAATGTTGCTCTTGTGGGGCATCTACAGCACGGCAAAACTGTCTT
SE-AT1G06220-XLOC_000279-13450-1
GTGAGGAATGTTGCTCTTGTGGGGCATCTACAGCACGGCAAAACTGTCTT
CONSENSUS
GTGAGGAATGTTGCTCTTGTGGGGCATCTACAGCACGGCAAAACTGTCTT

SE-AT1G06220-XLOC_000279-13450-0
CATGGATATGTTGGTAGAGCAGACGCATCATATGTCTACTTTTAATGCTA
SE-AT1G06220-XLOC_000279-13450-1
CATGGATATGTTGGTAGAGCAGACGCATCATATGTCTACTTTTAATGCTA
CONSENSUS
CATGGATATGTTGGTAGAGCAGACGCATCATATGTCTACTTTTAATGCTA

SE-AT1G06220-XLOC_000279-13450-0
AAAACGAGAAGCATATGAAATATACAGACACACGAGTCGATGAGCAGGAG
SE-AT1G06220-XLOC_000279-13450-1
AAAACGAGAAGCATATGAAATATACAGACACACGAGTCGATGAGCAGGAG
CONSENSUS
AAAACGAGAAGCATATGAAATATACAGACACACGAGTCGATGAGCAGGAG

SE-AT1G06220-XLOC_000279-13450-0
AGAAATATATCAATCAAGGCGGTTCCAATGTCTCTTGTCCTTGAGGACAG
SE-AT1G06220-XLOC_000279-13450-1
AGAAATATATCAATCAAGGCGGTTCCAATGTCTCTTGTCCTTGAGGACAG
CONSENSUS
AGAAATATATCAATCAAGGCGGTTCCAATGTCTCTTGTCCTTGAGGACAG

SE-AT1G06220-XLOC_000279-13450-0
TAGATCCAAATCATACCTGTGCAATATCATGGATACCCCTGGACATGTCA
SE-AT1G06220-XLOC_000279-13450-1
TAGATCCAAATCATACCTGTGCAATATCATGGATACCCCTGGACATGTCA
CONSENSUS
TAGATCCAAATCATACCTGTGCAATATCATGGATACCCCTGGACATGTCA

SE-AT1G06220-XLOC_000279-13450-0
ATTTCTCTGACGAAATGACTGCTTCTTTAAGACTTGCTGATGGTGCTGTT
SE-AT1G06220-XLOC_000279-13450-1
ATTTCTCTGACGAAATGACTGCTTCTTTAAGACTTGCTGATGGTGCTGTT
CONSENSUS
ATTTCTCTGACGAAATGACTGCTTCTTTAAGACTTGCTGATGGTGCTGTT

SE-AT1G06220-XLOC_000279-13450-0 CTGATTGTTGATGCTGCTGAAGGAGTGATG
SE-AT1G06220-XLOC_000279-13450-1 CTGATTGTTGATGCTGCTGAAGGAGTGATG
CONSENSUS CTGATTGTTGATGCTGCTGAAGGAGTGATG

alignment for event: RI-AT1G28530-XLOC_005630-2042

RI-AT1G28530-XLOC_005630-2042-0
AAGTTATATGCATCTCCGCTCTTCAAAGCACCGAGAAGCATTTCCTCGAT
RI-AT1G28530-XLOC_005630-2042-1
AAGTTATATGCATCTCCGCTCTTCAAAGCACCGAGAAGCATTTCCTCGAT
CONSENSUS
AAGTTATATGCATCTCCGCTCTTCAAAGCACCGAGAAGCATTTCCTCGAT

RI-AT1G28530-XLOC_005630-2042-0
GATTATCTGGGAAAAGCTAAGTTTCCGTTTATGAAGTGGTTATCTAAACG
RI-AT1G28530-XLOC_005630-2042-1
GATTATCTGGGAAAAGCTAAGTTTCCGTTTATGAAGTGGTTATCTAAACG
CONSENSUS
GATTATCTGGGAAAAGCTAAGTTTCCGTTTATGAAGTGGTTATCTAAACG

RI-AT1G28530-XLOC_005630-2042-0
CAGGAGGATTGCTTCAAGAGACTGCTCGGTTGTCCTGCATAAGTTATTTG
RI-AT1G28530-XLOC_005630-2042-1
CAGGAGGATTGCTTCAAGAGACTGCTCGGTTGTCCTGCATAAGTTATTTG
CONSENSUS
CAGGAGGATTGCTTCAAGAGACTGCTCGGTTGTCCTGCATAAGTTATTTG

RI-AT1G28530-XLOC_005630-2042-0
ATGATGAGCAGAATACCAAACTTTTGCTTGAGTATTACCAGTCAAGGAAG
RI-AT1G28530-XLOC_005630-2042-1
ATGATGAGCAGAATACCAAACTTTTGCTTGAGTATTACCAGTCAAGGAAG
CONSENSUS
ATGATGAGCAGAATACCAAACTTTTGCTTGAGTATTACCAGTCAAGGAAG

RI-AT1G28530-XLOC_005630-2042-0
GAAAATTTCAAACTTGCAGACACAAAGCAAAGAAGCCGGTGGTGGGACTT
RI-AT1G28530-XLOC_005630-2042-1
GAAAATTTCAAACTTGCAGACACAAAGCAAAGAAGCCGGTGGTGGGACTT
CONSENSUS
GAAAATTTCAAACTTGCAGACACAAAGCAAAGAAGCCGGTGGTGGGACTT

RI-AT1G28530-XLOC_005630-2042-0
ATCTGCCAATTCCAAGTTGGAAAAAAATTGGTGGCCCTGGATTTAGTAGTT
RI-AT1G28530-XLOC_005630-2042-1
ATCTGCCAATTCCAAGTTGGAAAAAAATTGGTGGCCCTGGATTTAGTAGTT
CONSENSUS
ATCTGCCAATTCCAAGTTGGAAAAAAATTGGTGGCCCTGGATTTAGTAGTT

RI-AT1G28530-XLOC_005630-2042-0
GGGCAAGTGAATATTTACCCGCATATCGACTGGAAATGGATAGTACAATA
RI-AT1G28530-XLOC_005630-2042-1
GGGCAAGTGAATATTTACCCGCATATCGACTGGAAATGGATAGTACAATA
CONSENSUS
GGGCAAGTGAATATTTACCCGCATATCGACTGGAAATGGATAGTACAATA

RI-AT1G28530-XLOC_005630-2042-0
CTTGCAGACTTAAAGCTTGAGGGCTGGAGAAAGTCCAGTGAGAATAAGTG
RI-AT1G28530-XLOC_005630-2042-1
CTTGCAGACTTAAAGCTTGAGGGCTGGAGAAAGTCCAGTGAGAATAAGTG
CONSENSUS
CTTGCAGACTTAAAGCTTGAGGGCTGGAGAAAGTCCAGTGAGAATAAGTG

RI-AT1G28530-XLOC_005630-2042-0
GGAAGTTCTTCTAACTCACTCCCAAATGGTATGACGTTTTCTCTTTATTT
RI-AT1G28530-XLOC_005630-2042-1
GGAAGTTCTTCTAACTCACTCCCAAATG----------------------
CONSENSUS
GGAAGTTCTTCTAACTCACTCCCAAATG......................

RI-AT1G28530-XLOC_005630-2042-0
ATGTAACCTACCCGTCTATGCTTTTGCTGTGTGTAAGACTAGAAATTGCA
RI-AT1G28530-XLOC_005630-2042-1

CONSENSUS
....

RI-AT1G28530-XLOC_005630-2042-0
ATGTTGCTCTCGCTCTATATATTTGTCTGGCTAGCAGTCTAGGTTGTTGC
RI-AT1G28530-XLOC_005630-2042-1

CONSENSUS
....

RI-AT1G28530-XLOC_005630-2042-0
TGCTATATGATAACATGGAGCTATGTTATATTGTTCTGTTTAGTATAGAT
RI-AT1G28530-XLOC_005630-2042-1

CONSENSUS
....

RI-AT1G28530-XLOC_005630-2042-0
AAAGACGTCGAATGTTGTTCAAAGTTTATGGATAGACCGTCTTTAGACAA
RI-AT1G28530-XLOC_005630-2042-1

CONSENSUS
....

RI-AT1G28530-XLOC_005630-2042-0
TGTTGAATCCGTCATAAGAGTTGTCATTCCCATTTACCTATACACTTAAC
RI-AT1G28530-XLOC_005630-2042-1

CONSENSUS
....

RI-AT1G28530-XLOC_005630-2042-0
GAGATGCTGATATGTTGTGTGGGAATGAACCTAGCAAGATGAAAGAATAT
RI-AT1G28530-XLOC_005630-2042-1

CONSENSUS
....

RI-AT1G28530-XLOC_005630-2042-0
TATCAATCATGGGAATAAAATTTCCTGTACTGCTGCATTTTGTGTGCTCT
RI-AT1G28530-XLOC_005630-2042-1

CONSENSUS
....

RI-AT1G28530-XLOC_005630-2042-0
GTGGTGACTAAGGTGTCTGTTTTTGTGCATGTTATTTCTTGAGGCCTCTA
RI-AT1G28530-XLOC_005630-2042-1

CONSENSUS
....

RI-AT1G28530-XLOC_005630-2042-0
CATTCCTGAGACTTGTATAAAACTCTTAGGTTGGATTAGCTGAAGCATTG
RI-AT1G28530-XLOC_005630-2042-1 -----------------------------
GTTGGATTAGCTGAAGCATTG
CONSENSUS
.............................GTTGGATTAGCTGAAGCATTG

RI-AT1G28530-XLOC_005630-2042-0
GATATTTACTTTGAAGACACATATTCACTTCCCAGGAAACAGCTACCATG
RI-AT1G28530-XLOC_005630-2042-1
GATATTTACTTTGAAGACACATATTCACTTCCCAGGAAACAGCTACCATG
CONSENSUS
GATATTTACTTTGAAGACACATATTCACTTCCCAGGAAACAGCTACCATG

RI-AT1G28530-XLOC_005630-2042-0
      TGATGTTCCTGGAAATTATGCAAACTTACCCAATGAAAAG
RI-AT1G28530-XLOC_005630-2042-1
      TGATGTTCCTGGAAATTATGCAAACTTACCCAATGAAAAG
CONSENSUS
      TGATGTTCCTGGAAATTATGCAAACTTACCCAATGAAAAG

alignment for event: A5-AT1G47550-XLOC_002341-5726

A5-AT1G47550-XLOC_002341-5726-0
      GAGGATTTCTATGCTGTTGTTGACTGGGCATATAAAATAGACCCCCTACG
A5-AT1G47550-XLOC_002341-5726-1
      GAGGATTTCTATGCTGTTGTTGACTGGGCATATAAAATAGACCCCCTACG
CONSENSUS
      GAGGATTTCTATGCTGTTGTTGACTGGGCATATAAAATAGACCCCCTACG

A5-AT1G47550-XLOC_002341-5726-0
      GTGTATTTCAATGCATGGTATAACTGAACGTTATCTATCTGGTCAGAAAG
A5-AT1G47550-XLOC_002341-5726-1
      GTGTATTTCAATGCATGGTATAACTGAACGTTATCTATCTGGTCAGAAAG
CONSENSUS
      GTGTATTTCAATGCATGGTATAACTGAACGTTATCTATCTGGTCAGAAAG

A5-AT1G47550-XLOC_002341-5726-0
      CTGATGCTGCAGGATTTGTTCGCCTTTTGCTTGGAGATCTGGAGTCGAGA
A5-AT1G47550-XLOC_002341-5726-1
      CTGATGCTGCAGGATTTGTTCGCCTTTTGCTTGGAGATCTGGAGTCGAGA
CONSENSUS
      CTGATGCTGCAGGATTTGTTCGCCTTTTGCTTGGAGATCTGGAGTCGAGA

A5-AT1G47550-XLOC_002341-5726-0 GTTTCTATGCAATTCAGCCGT---
TTTGTGGATGAAGCTTGTCACCAAAT
A5-AT1G47550-XLOC_002341-5726-1
      GTTTCTATGCAATTCAGCCGTGTATTTGTGGATGAAGCTTGTCACCAAAT
CONSENSUS
      GTTTCTATGCAATTCAGCCGT...TTTGTGGATGAAGCTTGTCACCAAAT

A5-AT1G47550-XLOC_002341-5726-0
      TGAAAGAAATGAACGTAATGTAAGACAGATGGGTGTCTTGCCATATATTC
A5-AT1G47550-XLOC_002341-5726-1
      TGAAAGAAATGAACGTAATGTAAGACAGATGGGTGTCTTGCCATATATTC
CONSENSUS
      TGAAAGAAATGAACGTAATGTAAGACAGATGGGTGTCTTGCCATATATTC

A5-AT1G47550-XLOC_002341-5726-0 CAAG
A5-AT1G47550-XLOC_002341-5726-1 CAAG
CONSENSUS   CAAG

alignment for event: RI-AT1G05135-XLOC_004382-2334

RI-AT1G05135-XLOC_004382-2334-0

AATGTTCAAAACTAGAAAGATAGAAAGCTACTAAAAATCTAAACAAAGTG
RI-AT1G05135-XLOC_004382-2334-1
AATGTTCAAAACTAGAAAGATAGAAAGCTACTAAAAATCTAAACAAAGTG
CONSENSUS
AATGTTCAAAACTAGAAAGATAGAAAGCTACTAAAAATCTAAACAAAGTG

RI-AT1G05135-XLOC_004382-2334-0
TAGATCTATTTTGACTTAGTCGAAGAAAACGTTCATTAAATGGTTCCCGC
RI-AT1G05135-XLOC_004382-2334-1
TAGATCTATTTTGACTTAGTCGAAGAAAACGTTCATTAAATGGTTCCCGC
CONSENSUS
TAGATCTATTTTGACTTAGTCGAAGAAAACGTTCATTAAATGGTTCCCGC

RI-AT1G05135-XLOC_004382-2334-0
CAATGGCCCAACGGCCCCAAACGCGTAGAACGTAACATGCATGGGGATCC
RI-AT1G05135-XLOC_004382-2334-1
CAATGGCCCAACGGCCCCAAACGCGTAGAACGTAACATGCATGGGGATCC
CONSENSUS
CAATGGCCCAACGGCCCCAAACGCGTAGAACGTAACATGCATGGGGATCC

RI-AT1G05135-XLOC_004382-2334-0
ATATTAATGGCTTCTTCATCCCACTTGCTTATTTCTTTCGACTTTGGAGG
RI-AT1G05135-XLOC_004382-2334-1
ATATTAATGGCTTCTTCATCCCACTTGCTTATTTCTTTCGACTTTGGAGG
CONSENSUS
ATATTAATGGCTTCTTCATCCCACTTGCTTATTTCTTTCGACTTTGGAGG

RI-AT1G05135-XLOC_004382-2334-0
TTTCATTTACCTATTATATAAGGGAATGTTCAGTTCCAGTGACTGAGATA
RI-AT1G05135-XLOC_004382-2334-1
TTTCATTTACCTATTATATAAGGGAATGTTCAGTTCCAGTGACTGAGATA
CONSENSUS
TTTCATTTACCTATTATATAAGGGAATGTTCAGTTCCAGTGACTGAGATA

RI-AT1G05135-XLOC_004382-2334-0
TCACAAGCAAACAAGAGAAGACATTTTATTGTTATTTCTCTCTCTCTCTC
RI-AT1G05135-XLOC_004382-2334-1
TCACAAGCAAACAAGAGAAGACATTTTATTGTTATTTCTCTCTCTCTCTC
CONSENSUS
TCACAAGCAAACAAGAGAAGACATTTTATTGTTATTTCTCTCTCTCTCTC

RI-AT1G05135-XLOC_004382-2334-0
CTTGTAGATGGAACTTAAGGGCGTTACTTGTTTGCTTCTTTCTTTGGTTT
RI-AT1G05135-XLOC_004382-2334-1
CTTGTAGATGGAACTTAAGGGCGTTACTTGTTTGCTTCTTTCTTTGGTTT
CONSENSUS
CTTGTAGATGGAACTTAAGGGCGTTACTTGTTTGCTTCTTTCTTTGGTTT

RI-AT1G05135-XLOC_004382-2334-0
TGCTTAATTCGTGTGTGGAATGTGTTTTGGGGGATGGATCTGTGGTGGGT
RI-AT1G05135-XLOC_004382-2334-1
TGCTTAATTCGTGTGTGGAATGTGTTTTGGGGGATGGATCTGTGGTGGGT
CONSENSUS
TGCTTAATTCGTGTGTGGAATGTGTTTTGGGGGATGGATCTGTGGTGGGT

RI-AT1G05135-XLOC_004382-2334-0

CCTGCGAGGTTTAGAGACGATGATTGTAGGTGGGGCCGGAGGTGTGCTGG
RI-AT1G05135-XLOC_004382-2334-1
CCTGCGAGGTTTAGAGACGATGATTGTAGGTGGGGCCGGAGGTGTGCTGG
CONSENSUS
CCTGCGAGGTTTAGAGACGATGATTGTAGGTGGGGCCGGAGGTGTGCTGG

RI-AT1G05135-XLOC_004382-2334-0
ACGTGGCCGGTTTGGACGCGGTGGTGGTGGAGGGTTCGGCGGTGGGAGAG
RI-AT1G05135-XLOC_004382-2334-1
ACGTGGCCGGTTTGGACGCGGTGGTGGTGGAGGGTTCGGCGGTGGGAGAG
CONSENSUS
ACGTGGCCGGTTTGGACGCGGTGGTGGTGGAGGGTTCGGCGGTGGGAGAG

RI-AT1G05135-XLOC_004382-2334-0
GAAGTGGTGGCGGTATAGGCGGTGGTGGTGGACAAGGTGGAGGGTTTGGA
RI-AT1G05135-XLOC_004382-2334-1
GAAGTGGTGGCGGTATAGGCGGTGGTGGTGGACAAGGTGGAGGGTTTGGA
CONSENSUS
GAAGTGGTGGCGGTATAGGCGGTGGTGGTGGACAAGGTGGAGGGTTTGGA

RI-AT1G05135-XLOC_004382-2334-0
GCCGGAGGAGGAGTTGGTGGCGGAGCTGGAGGAGGACTTGGTGGTGGAGG
RI-AT1G05135-XLOC_004382-2334-1
GCCGGAGGAGGAGTTGGTGGCGGAGCTGGAGGAGGACTTGGTGGTGGAGG
CONSENSUS
GCCGGAGGAGGAGTTGGTGGCGGAGCTGGAGGAGGACTTGGTGGTGGAGG

RI-AT1G05135-XLOC_004382-2334-0
CGGAGCTGGTGGTGGTGGTGGAGGAGGTATTGGTGGTGGTTCCGGTCATG
RI-AT1G05135-XLOC_004382-2334-1
CGGAGCTGGTGGTGGTGGTGGAGGAGGTATTGGTGGTGGTTCCGGTCATG
CONSENSUS
CGGAGCTGGTGGTGGTGGTGGAGGAGGTATTGGTGGTGGTTCCGGTCATG

RI-AT1G05135-XLOC_004382-2334-0
GTGGTGGGTTTGGAGCCGGCGGAGGAGTTGGTGGGGGTGCTGGTGGAGGA
RI-AT1G05135-XLOC_004382-2334-1
GTGGTGGGTTTGGAGCCGGCGGAGGAGTTGGTGGGGGTGCTGGTGGAGGA
CONSENSUS
GTGGTGGGTTTGGAGCCGGCGGAGGAGTTGGTGGGGGTGCTGGTGGAGGA

RI-AT1G05135-XLOC_004382-2334-0
ATTGGTGGGGGAGGCGGAGCTGGTGGAGGCGGAGGAGGAGGTGTTGGCGG
RI-AT1G05135-XLOC_004382-2334-1
ATTGGTGGGGGAGGCGGAGCTGGTGGAGGCGGAGGAGGAG----------
CONSENSUS
ATTGGTGGGGGAGGCGGAGCTGGTGGAGGCGGAGGAGGAG..........

RI-AT1G05135-XLOC_004382-2334-0
TGGTTCTGGTCACGGTGGTGGGTTTGGAGCCGGAGGAGGTGTAGGCGGCG
RI-AT1G05135-XLOC_004382-2334-1

CONSENSUS
....

RI-AT1G05135-XLOC_004382-2334-0

GTGCTGGAGGAATTGGTGGTGGAGGGGGAGCTGGTGGGGGCGGAGGGGGA
RI-AT1G05135-XLOC_004382-2334-1

CONSENSUS
....

RI-AT1G05135-XLOC_004382-2334-0
GGTGTTGGCGGTGGTTCCGGTCATGGCAGTGGGTTTGGAGCCGGAGGAGG
RI-AT1G05135-XLOC_004382-2334-1 -
GTGTTGGCGGTGGTTCCGGTCATGGCAGTGGGTTTGGAGCCGGAGGAGG
CONSENSUS
.GTGTTGGCGGTGGTTCCGGTCATGGCAGTGGGTTTGGAGCCGGAGGAGG

RI-AT1G05135-XLOC_004382-2334-0
GATTGGTGGTGGAGCTGGCGGAGGAGTTGGCGGTGGAGGTGGAGGTGGAG
RI-AT1G05135-XLOC_004382-2334-1
GATTGGTGGTGGAGCTGGCGGAGGAGTTGGCGGTGGAGGTGGAGGTGGAG
CONSENSUS
GATTGGTGGTGGAGCTGGCGGAGGAGTTGGCGGTGGAGGTGGAGGTGGAG

RI-AT1G05135-XLOC_004382-2334-0
GTGGGGGCGGCGGAGGCGGTGGTGCCAATGGTGGATCCGGTCACGGTAGT
RI-AT1G05135-XLOC_004382-2334-1
GTGGGGGCGGCGGAGGCGGTGGTGCCAATGGTGGATCCGGTCACGGTAGT
CONSENSUS
GTGGGGGCGGCGGAGGCGGTGGTGCCAATGGTGGATCCGGTCACGGTAGT

RI-AT1G05135-XLOC_004382-2334-0 GGTTTTGGTGCTGGAGGTGGAGTAGGAG
RI-AT1G05135-XLOC_004382-2334-1 GGTTTTGGTGCTGGAGGTGGAGTAGGAG
CONSENSUS GGTTTTGGTGCTGGAGGTGGAGTAGGAG

alignment for event: A3-AT1G10890-XLOC_000523-8253

A3-AT1G10890-XLOC_000523-8253-0
TTTCTTCCCGTTCCCACGGGAGGGGAAATGAGCAGCTGATGGATTGCTCT
A3-AT1G10890-XLOC_000523-8253-1
TTTCTTCCCGTTCCCACGGGAGGGGAAATGAGCAGCTGATGGATTGCTCT
CONSENSUS
TTTCTTCCCGTTCCCACGGGAGGGGAAATGAGCAGCTGATGGATTGCTCT

A3-AT1G10890-XLOC_000523-8253-0
TAGGTGTTCTTTCATTGATCCCTTGTCTCGTTAGCTTCTCTTCGTGTGAT
A3-AT1G10890-XLOC_000523-8253-1
TAGGTGTTCTTTCATTGATCCCTTGTCTCGTTAGCTTCTCTTCGTGTGAT
CONSENSUS
TAGGTGTTCTTTCATTGATCCCTTGTCTCGTTAGCTTCTCTTCGTGTGAT

A3-AT1G10890-XLOC_000523-8253-0
AAAAGTTGGAGCTGATAATTCTTTACAGTGTATGAGGTAACTGCACATAC
A3-AT1G10890-XLOC_000523-8253-1
AAAAGTTGGAGCTGATAATTCTTTACAGTGTATGAGGTAACTGCACATAC
CONSENSUS
AAAAGTTGGAGCTGATAATTCTTTACAGTGTATGAGGTAACTGCACATAC

A3-AT1G10890-XLOC_000523-8253-0
TTTTGTTTTCTTTATGTGTAACTGTGGGCTACCTGATGCCTAGTCTTCAC
A3-AT1G10890-XLOC_000523-8253-1
TTTTGTTTTCTTTATGTGTAACTGTGGGCTACCTGATGCCTAGTCTTCAC
CONSENSUS
TTTTGTTTTCTTTATGTGTAACTGTGGGCTACCTGATGCCTAGTCTTCAC

A3-AT1G10890-XLOC_000523-8253-0
GTGCGTGGTACTTCATTAGTTCCATCTTTTTGCTTACTACTTTATTTTGG
A3-AT1G10890-XLOC_000523-8253-1
GTGCGTGGTACTTCATTAGTTCCATCTTTTTGCTTACTACTTTATTTTGG
CONSENSUS
GTGCGTGGTACTTCATTAGTTCCATCTTTTTGCTTACTACTTTATTTTGG

A3-AT1G10890-XLOC_000523-8253-0
GTAATGTTCAGGCGAAAAAGTCGTTCTATTTCTCCTAGGCGCCATCGAAG
A3-AT1G10890-XLOC_000523-8253-1
GTAATGTTCAGGCGAAAAAGTCGTTCTATTTCTCCTAGGCGCCATCGAAG
CONSENSUS
GTAATGTTCAGGCGAAAAAGTCGTTCTATTTCTCCTAGGCGCCATCGAAG

A3-AT1G10890-XLOC_000523-8253-0
TCGATCTGTTACTCCTAAGAGACGTTCTCCAACCCCAAAACGTTACAAAA
A3-AT1G10890-XLOC_000523-8253-1
TCGATCTGTTACTCCTAAGAGACGTTCTCCAACCCCAAAACGTTACAAAA
CONSENSUS
TCGATCTGTTACTCCTAAGAGACGTTCTCCAACCCCAAAACGTTACAAAA

A3-AT1G10890-XLOC_000523-8253-0
GACAAAAGAGTAGGAGTTCAACTCCATCTCCTGCAAAAAGATCTCCCGCC
A3-AT1G10890-XLOC_000523-8253-1
GACAAAAGAGTAGGAGTTCAACTCCATCTCCTGCAAAAAGATCTCCCGCC
CONSENSUS
GACAAAAGAGTAGGAGTTCAACTCCATCTCCTGCAAAAAGATCTCCCGCC

A3-AT1G10890-XLOC_000523-8253-0
GCAACCCTTGAGTCAGCCAAAAATAGGAATGGAGAAAAACTTAAAAGAGA
A3-AT1G10890-XLOC_000523-8253-1
GCAACCCTTGAGTCAGCCAAAAATAGGAATGGAGAAAAACTTAAAAGAGA
CONSENSUS
GCAACCCTTGAGTCAGCCAAAAATAGGAATGGAGAAAAACTTAAAAGAGA

A3-AT1G10890-XLOC_000523-8253-0
AGAGGAAGAACGAAAAAGGTAAATCAATACAAAATTTTGAAGTAAGATGG
A3-AT1G10890-XLOC_000523-8253-1
AGAGGAAGAACGAAAAAGGTAAATCAATACAAAATTTTGAAGTAAGATGG
CONSENSUS
AGAGGAAGAACGAAAAAGGTAAATCAATACAAAATTTTGAAGTAAGATGG

A3-AT1G10890-XLOC_000523-8253-0
ATGTTATGGTGAAATTAAGGTTTTTTGTTCTCAAATGTTATTTTAGTGTA
A3-AT1G10890-XLOC_000523-8253-1
ATGTTATGGTGAAATTAAGGTTTTTTGTTCTCAAATGTTATTTTAGTGTA
CONSENSUS
ATGTTATGGTGAAATTAAGGTTTTTTGTTCTCAAATGTTATTTTAGTGTA

A3-AT1G10890-XLOC_000523-8253-0
GTGTGGAAGTCCTTGATTGTTAGTCTCAAAAGTGCGACAGGTTTTATGGT
A3-AT1G10890-XLOC_000523-8253-1
GTGTGGAAGTCCTTGATTGTTAGTCTCAAAAGTGCGACAGGTTTTATGGT
CONSENSUS
GTGTGGAAGTCCTTGATTGTTAGTCTCAAAAGTGCGACAGGTTTTATGGT

A3-AT1G10890-XLOC_000523-8253-0
ATTCTCTCACAGGCTTTGCTTATGGAAGATAAGCTATATTTCGATTTGTT
A3-AT1G10890-XLOC_000523-8253-1
ATTCTCTCACAGGCTTTGCTTATGGAAGATAAGCTATATTTCGATTTGTT
CONSENSUS
ATTCTCTCACAGGCTTTGCTTATGGAAGATAAGCTATATTTCGATTTGTT

A3-AT1G10890-XLOC_000523-8253-0
TATATTTGATCCTCTGGCACAAATGTTCGTACTATCTCGATTTGATGAGT
A3-AT1G10890-XLOC_000523-8253-1
TATATTTGATCCTCTGGCACAAATGTTCGTACTATCTCGATTTGATGAGT
CONSENSUS
TATATTTGATCCTCTGGCACAAATGTTCGTACTATCTCGATTTGATGAGT

A3-AT1G10890-XLOC_000523-8253-0
TACCGTTTGTCATTACATGGATCATGATTGACTTAGAGATGTAAATATGT
A3-AT1G10890-XLOC_000523-8253-1
TACCGTTTGTCATTACATGGATCATGATTGACTTAGAGATGTAAATATGT
CONSENSUS
TACCGTTTGTCATTACATGGATCATGATTGACTTAGAGATGTAAATATGT

A3-AT1G10890-XLOC_000523-8253-0
CCAAAGCACAAGAATAATCTTGGTGAAGGTTTGCTTGTGTTATGGTGACA
A3-AT1G10890-XLOC_000523-8253-1
CCAAAGCACAAGAATAATCTTGGTGAAGGTTTGCTTGTGTTATGGTGACA
CONSENSUS
CCAAAGCACAAGAATAATCTTGGTGAAGGTTTGCTTGTGTTATGGTGACA

A3-AT1G10890-XLOC_000523-8253-0
AAAAAGAAATTTCTTCACGAAATTTACCATTGGTATTGGGAGGAGTGAAA
A3-AT1G10890-XLOC_000523-8253-1
AAAAAGAAATTTCTTCACGAAATTTACCATTGGTATTGGGAGGAGTGAAA
CONSENSUS
AAAAAGAAATTTCTTCACGAAATTTACCATTGGTATTGGGAGGAGTGAAA

A3-AT1G10890-XLOC_000523-8253-0
ATGGAAACGATCTCAACATTTTTTACGGATTTGCAATAAAATTTTGGTCT
A3-AT1G10890-XLOC_000523-8253-1
ATGGAAACGATCTCAACATTTTTTACGGATTTGCAATAAAATTTTGGTCT
CONSENSUS
ATGGAAACGATCTCAACATTTTTTACGGATTTGCAATAAAATTTTGGTCT

A3-AT1G10890-XLOC_000523-8253-0
TTTGCTGTTCAAGATTGAAGACATCAGGAAATGAGAAAGTTCAGGTGATA
A3-AT1G10890-XLOC_000523-8253-1
TTTGCTGTTCAAGATTGAAGACATCAGGAAATGAGAAAGTTCAGGTGATA
CONSENSUS
TTTGCTGTTCAAGATTGAAGACATCAGGAAATGAGAAAGTTCAGGTGATA

A3-AT1G10890-XLOC_000523-8253-0
TTCAATATTCTGAACCTGCTGTGGATGTCCTCTAATTTTTCACTGTATTT
A3-AT1G10890-XLOC_000523-8253-1
TTCAATATTCTGAACCTGCTGTGGATGTCCTCTAATTTTTCACTGTATTT
CONSENSUS
TTCAATATTCTGAACCTGCTGTGGATGTCCTCTAATTTTTCACTGTATTT

A3-AT1G10890-XLOC_000523-8253-0
GCTACCAGGCGACAGCGTGAAGCAGAACTGAAGCTAATAGAGGAAGAAAC
A3-AT1G10890-XLOC_000523-8253-1
GCTACCAGGCGACAGCGTGAAGCAGAACTGAAGCTAATAGAGGAAGAAAC
CONSENSUS
GCTACCAGGCGACAGCGTGAAGCAGAACTGAAGCTAATAGAGGAAGAAAC

A3-AT1G10890-XLOC_000523-8253-0
TGTGAAACGGGTTGAAGAAGCTATTCGAAAGAAGGTCGAAGAAAGCTTAC
A3-AT1G10890-XLOC_000523-8253-1
TGTGAAACGGGTTGAAGAAGCTATTCGAAAGAAGGTCGAAGAAAGCTTAC
CONSENSUS
TGTGAAACGGGTTGAAGAAGCTATTCGAAAGAAGGTCGAAGAAAGCTTAC

A3-AT1G10890-XLOC_000523-8253-0
AGTCTGAGAAAATCAAAATGGAAATTCTAACGCTGTTGGAGGAAGGGCGA
A3-AT1G10890-XLOC_000523-8253-1
AGTCTGAGAAAATCAAAATGGAAATTCTAACGCTGTTGGAGGAAGGGCGA
CONSENSUS
AGTCTGAGAAAATCAAAATGGAAATTCTAACGCTGTTGGAGGAAGGGCGA

A3-AT1G10890-XLOC_000523-8253-0
AAGAGACTTAATGAAGAAGTCGCGGCTCAACTTGAGGAGGAGAAAGAGGC
A3-AT1G10890-XLOC_000523-8253-1
AAGAGACTTAATGAAGAAGTCGCGGCTCAACTTGAGGAGGAGAAAGAGGC
CONSENSUS
AAGAGACTTAATGAAGAAGTCGCGGCTCAACTTGAGGAGGAGAAAGAGGC

A3-AT1G10890-XLOC_000523-8253-0
TTCTCTTATTGAGGCTAAAGAAAAAGAGGGTGTTATGCGGTGTTTGTCAC
A3-AT1G10890-XLOC_000523-8253-1
TTCTCTTATTGAGGCTAAAGAAAAAGAG----------------------
CONSENSUS
TTCTCTTATTGAGGCTAAAGAAAAAGAG......................

A3-AT1G10890-XLOC_000523-8253-0
AGGAAAGAGAGCAACAAGAGAAAGAAGAGAGGGAGAGAATAGCAGAGGAG
A3-AT1G10890-XLOC_000523-8253-1 --
GAAAGAGAGCAACAAGAGAAAGAAGAGAGGGAGAGAATAGCAGAGGAG
CONSENSUS
..GAAAGAGAGCAACAAGAGAAAGAAGAGAGGGAGAGAATAGCAGAGGAG

A3-AT1G10890-XLOC_000523-8253-0
AACCTAAAGAGAGTGGAAGAAGCTCAGAGAAAAGAAGCAATGGAGAGGCA
A3-AT1G10890-XLOC_000523-8253-1
AACCTAAAGAGAGTGGAAGAAGCTCAGAGAAAAGAAGCAATGGAGAGGCA
CONSENSUS
AACCTAAAGAGAGTGGAAGAAGCTCAGAGAAAAGAAGCAATGGAGAGGCA

A3-AT1G10890-XLOC_000523-8253-0
AAGGAAAGAGGAGGAACGGTATCGAGAGCTAGAGGAGCTGCAACGACAGA
A3-AT1G10890-XLOC_000523-8253-1
AAGGAAAGAGGAGGAACGGTATCGAGAGCTAGAGGAGCTGCAACGACAGA
CONSENSUS
AAGGAAAGAGGAGGAACGGTATCGAGAGCTAGAGGAGCTGCAACGACAGA

A3-AT1G10890-XLOC_000523-8253-0
AAGAAGAAGCGATGCGAAGGAAGAAAGCTGAAGAGGAAGAAGAACGTCTC
A3-AT1G10890-XLOC_000523-8253-1
AAGAAGAAGCGATGCGAAGGAAGAAAGCTGAAGAGGAAGAAGAACGTCTC
CONSENSUS
AAGAAGAAGCGATGCGAAGGAAGAAAGCTGAAGAGGAAGAAGAACGTCTC

A3-AT1G10890-XLOC_000523-8253-0
AAACAGATGAAACTGTTGGGTAAAAACAAATCACGGCCTAAATTATCCTT
A3-AT1G10890-XLOC_000523-8253-1
AAACAGATGAAACTGTTGGGTAAAAACAAATCACGGCCTAAATTATCCTT
CONSENSUS
AAACAGATGAAACTGTTGGGTAAAAACAAATCACGGCCTAAATTATCCTT

A3-AT1G10890-XLOC_000523-8253-0
TGCCTTAAGCTCCAAGTAAATGCGTGCATGCATGAAGATAAAAGGATTGA
A3-AT1G10890-XLOC_000523-8253-1
TGCCTTAAGCTCCAAGTAAATGCGTGCATGCATGAAGATAAAAGGATTGA
CONSENSUS
TGCCTTAAGCTCCAAGTAAATGCGTGCATGCATGAAGATAAAAGGATTGA

A3-AT1G10890-XLOC_000523-8253-0
TGTGATGGATGATGATATGCATCTTCTTCCTCTCAAAGATGCTTTATGAT
A3-AT1G10890-XLOC_000523-8253-1
TGTGATGGATGATGATATGCATCTTCTTCCTCTCAAAGATGCTTTATGAT
CONSENSUS
TGTGATGGATGATGATATGCATCTTCTTCCTCTCAAAGATGCTTTATGAT

A3-AT1G10890-XLOC_000523-8253-0
TATTGTTATTAGTGCTTCTTGTTGGAGCTTAAACTCTTTTATGGCTTTTA
A3-AT1G10890-XLOC_000523-8253-1
TATTGTTATTAGTGCTTCTTGTTGGAGCTTAAACTCTTTTATGGCTTTTA
CONSENSUS
TATTGTTATTAGTGCTTCTTGTTGGAGCTTAAACTCTTTTATGGCTTTTA

A3-AT1G10890-XLOC_000523-8253-0
ATTTTTTGTAATTCTATTTTTCTCGTTTTGTAATTTTACGTTAGGTTAAT
A3-AT1G10890-XLOC_000523-8253-1
ATTTTTTGTAATTCTATTTTTCTCGTTTTGTAATTTTACGTTAGGTTAAT
CONSENSUS
ATTTTTTGTAATTCTATTTTTCTCGTTTTGTAATTTTACGTTAGGTTAAT

A3-AT1G10890-XLOC_000523-8253-0
GGTGATGAATGATAATATAGCAATGATTCAGAAAATTTA
A3-AT1G10890-XLOC_000523-8253-1
GGTGATGAATGATAATATAGCAATGATTCAGAAAATTTA
CONSENSUS
GGTGATGAATGATAATATAGCAATGATTCAGAAAATTTA

alignment for event: A3-AT1G32230-XLOC_001707-10045

A3-AT1G32230-XLOC_001707-10045-0
GTAATTTGATTGCTAAGCGTGATAACTCAGGGGTCACTTTGGAAGGACCT
A3-AT1G32230-XLOC_001707-10045-1
GTAATTTGATTGCTAAGCGTGATAACTCAGGGGTCACTTTGGAAGGACCT
CONSENSUS
GTAATTTGATTGCTAAGCGTGATAACTCAGGGGTCACTTTGGAAGGACCT

A3-AT1G32230-XLOC_001707-10045-0
AAGGATCTTCCTCCTCAATTGGAGTCAAACCAGGGAGCAAGAGGTTCAGG
A3-AT1G32230-XLOC_001707-10045-1
AAGGATCTTCCTCCTCAATTGGAGTCAAAC---GGAGCAAGAGGTTCAGG
CONSENSUS
AAGGATCTTCCTCCTCAATTGGAGTCAAAC...GGAGCAAGAGGTTCAGG

A3-AT1G32230-XLOC_001707-10045-0
AAGTGCAAACAGTGTTGGTTCAAGCACTACAAGACCCAAATCTCCATGGA
A3-AT1G32230-XLOC_001707-10045-1
AAGTGCAAACAGTGTTGGTTCAAGCACTACAAGACCCAAATCTCCATGGA
CONSENSUS
AAGTGCAAACAGTGTTGGTTCAAGCACTACAAGACCCAAATCTCCATGGA

A3-AT1G32230-XLOC_001707-10045-0
TGCCATTTCCTACTCTGTTTGCAGCAATCTCACATAAGGTTGCAGAGAAC
A3-AT1G32230-XLOC_001707-10045-1
TGCCATTTCCTACTCTGTTTGCAGCAATCTCACATAAGGTTGCAGAGAAC
CONSENSUS
TGCCATTTCCTACTCTGTTTGCAGCAATCTCACATAAGGTTGCAGAGAAC

A3-AT1G32230-XLOC_001707-10045-0
GACATGTTGTTGATCAATGCTGACTACCAACAACTGAGG
A3-AT1G32230-XLOC_001707-10045-1
GACATGTTGTTGATCAATGCTGACTACCAACAACTGAGG
CONSENSUS
GACATGTTGTTGATCAATGCTGACTACCAACAACTGAGG

alignment for event: SE-AT1G67900-XLOC_003455-2402

SE-AT1G67900-XLOC_003455-2402-0
TTTATATATATTTATTCTTTGTTCACCTTCATTTCAACTCATATTTCTCC
SE-AT1G67900-XLOC_003455-2402-1
TTTATATATATTTATTCTTTGTTCACCTTCATTTCAACTCATATTTCTCC
CONSENSUS
TTTATATATATTTATTCTTTGTTCACCTTCATTTCAACTCATATTTCTCC

SE-AT1G67900-XLOC_003455-2402-0
TCATTTGGTAATAAAAACAAATATCAGCTTTCGTCTTTCTTCTACATCTT
SE-AT1G67900-XLOC_003455-2402-1
TCATTTGGTAATAAAAACAAATATCAGCTTTCGTCTTTCTTCTACATCTT
CONSENSUS
TCATTTGGTAATAAAAACAAATATCAGCTTTCGTCTTTCTTCTACATCTT

SE-AT1G67900-XLOC_003455-2402-0
    TCTCTTTGTAACTTCGTTTTTGGATAATTGAGGAATTTCGGGCCGATACA
SE-AT1G67900-XLOC_003455-2402-1
    TCTCTTTGTAACTTCGTTTTTGGATAATTGAGGAATTTCGGGCCGATACA
CONSENSUS
    TCTCTTTGTAACTTCGTTTTTGGATAATTGAGGAATTTCGGGCCGATACA

SE-AT1G67900-XLOC_003455-2402-0
    TCTTTTCATCGGACGAATTCCTTCTCAAGAAAGTTTTGAACCATAGTTTA
SE-AT1G67900-XLOC_003455-2402-1
    TCTTTTCATCGGACGAATTCCTTCTCAAGAAAGTTTTGAACCATAGTTTA
CONSENSUS
    TCTTTTCATCGGACGAATTCCTTCTCAAGAAAGTTTTGAACCATAGTTTA

SE-AT1G67900-XLOC_003455-2402-0
    TGGAAGATAAGCTTTTTTCGGAAGAAAACAACTACCAATAGAGCTACATA
SE-AT1G67900-XLOC_003455-2402-1
    TGGAAGATAAGCTTTTTTCGGAAGAAAACAACTACCAATAGAGCTACATA
CONSENSUS
    TGGAAGATAAGCTTTTTTCGGAAGAAAACAACTACCAATAGAGCTACATA

SE-AT1G67900-XLOC_003455-2402-0
    CGCTTGATCATT--------------------------------------
SE-AT1G67900-XLOC_003455-2402-1
    CGCTTGATCATTTCTTTGAATGATTGGTGAAGAGTGTTTTCTGGGAACTT
CONSENSUS
    CGCTTGATCATT......................................

SE-AT1G67900-XLOC_003455-2402-0

SE-AT1G67900-XLOC_003455-2402-1
    GCCTTAAGCTATAGCTAGAAAGATACACAAAGATATATCCATCTCAAAGC
CONSENSUS
    ....

SE-AT1G67900-XLOC_003455-2402-0
    --GAGATATTGT
SE-AT1G67900-XLOC_003455-2402-1
    TCCTGATGTGAAGAGTGTTTGTTCTGTTTTTCTAATCCAAGAGATATTGT
CONSENSUS
    ....GAGATATTGT

SE-AT1G67900-XLOC_003455-2402-0
    CCGGAACTGTTCTTCAGTGTTTGGTGTCTTAGAAGAAAGAAACACTTTTG
SE-AT1G67900-XLOC_003455-2402-1
    CCGGAACTGTTCTTCAGTGTTTGGTGTCTTAGAAGAAAGAAACACTTTTG
CONSENSUS
    CCGGAACTGTTCTTCAGTGTTTGGTGTCTTAGAAGAAAGAAACACTTTTG

SE-AT1G67900-XLOC_003455-2402-0
    GAGCATTATGCATAGGAGTTTCTGAAGATTTCAAGCTAATTACTATCATT
SE-AT1G67900-XLOC_003455-2402-1
    GAGCATTATGCATAGGAGTTTCTGAAGATTTCAAGCTAATTACTATCATT
CONSENSUS
    GAGCATTATGCATAGGAGTTTCTGAAGATTTCAAGCTAATTACTATCATT

SE-AT1G67900-XLOC_003455-2402-0
      AATCAGCTATGAAGTTTATGAAACTTGGATCTCGTCCTGACACTTTCTAC
SE-AT1G67900-XLOC_003455-2402-1
      AATCAGCTATGAAGTTTATGAAACTTGGATCTCGTCCTGACACTTTCTAC
CONSENSUS
      AATCAGCTATGAAGTTTATGAAACTTGGATCTCGTCCTGACACTTTCTAC

SE-AT1G67900-XLOC_003455-2402-0 ACTTCTGAAGATTTAAG
SE-AT1G67900-XLOC_003455-2402-1 ACTTCTGAAGATTTAAG
CONSENSUS   ACTTCTGAAGATTTAAG

alignment for event: A5-AT1G26850-XLOC_005542-4756

A5-AT1G26850-XLOC_005542-4756-0
      CAAGCTATCAGCTTTTCATTTTCTTTATTTTTTTCCTTTTTATTTTTTAT
A5-AT1G26850-XLOC_005542-4756-1
      CAAGCTATCAGCTTTTCATTTTCTTTATTTTTTTCCTTTTTATTTTTTAT
CONSENSUS
      CAAGCTATCAGCTTTTCATTTTCTTTATTTTTTTCCTTTTTATTTTTTAT

A5-AT1G26850-XLOC_005542-4756-0
      TTATTTTATCAAATTTTTCTTAGCTTTTATTAATTTTATAATTTTTCCGC
A5-AT1G26850-XLOC_005542-4756-1
      TTATTTTATCAAATTTTTCTTAGCTTTTATTAATTTTATAATTTTTCCGC
CONSENSUS
      TTATTTTATCAAATTTTTCTTAGCTTTTATTAATTTTATAATTTTTCCGC

A5-AT1G26850-XLOC_005542-4756-0
      CGATTTTAGGTTAAAGAAAAAAATAAAAATACGAAAATATCAGACGTCGA
A5-AT1G26850-XLOC_005542-4756-1
      CGATTTTAGGTTAAAGAAAAAAATAAAAATACGAAAATATCAGACGTCGA
CONSENSUS
      CGATTTTAGGTTAAAGAAAAAAATAAAAATACGAAAATATCAGACGTCGA

A5-AT1G26850-XLOC_005542-4756-0
      TTGAATCTACAAAGTTTCATCTCCTCTTCTCTCTCTCTCAATCTCAATGG
A5-AT1G26850-XLOC_005542-4756-1
      TTGAATCTACAAAGTTTCATCTCCTCTTCTCTCTCTCTCAATCTCAATGG
CONSENSUS
      TTGAATCTACAAAGTTTCATCTCCTCTTCTCTCTCTCTCAATCTCAATGG

A5-AT1G26850-XLOC_005542-4756-0
      CTCTGTTTTAAAAAGGGCAAATTAGAGAGATTTCTCCTTCAATAACAAAA
A5-AT1G26850-XLOC_005542-4756-1
      CTCTGTTTTAAAAAGGGCAAATTAGAGAGATTTCTCCTTCAATAACAAAA
CONSENSUS
      CTCTGTTTTAAAAAGGGCAAATTAGAGAGATTTCTCCTTCAATAACAAAA

A5-AT1G26850-XLOC_005542-4756-0
      AATCGCCAGCTTTATCTCTCTCTTTTTCACATCCCATGGCTAGATGGTGA
A5-AT1G26850-XLOC_005542-4756-1
      AATCGCCAGCTTTATCTCTCTCTTTTTCACATCCCATGGCTAGATGGTGA
CONSENSUS

AATCGCCAGCTTTATCTCTCTCTTTTTCACATCCCATGGCTAGATGGTGA

A5-AT1G26850-XLOC_005542-4756-0
CGACGATTCACGCATAAACTAGGGTTCCCCCACAAATCATCTTACACGTA
A5-AT1G26850-XLOC_005542-4756-1
CGACGATTCACGCATAAACTAGGGTTCCCCCACAAATCATCTTACACGTA
CONSENSUS
CGACGATTCACGCATAAACTAGGGTTCCCCCACAAATCATCTTACACGTA

A5-AT1G26850-XLOC_005542-4756-0
TCCTCTATTCCTTCCTTCCTTTCTCCGTCATCATCATTTTTCCCACCGAC
A5-AT1G26850-XLOC_005542-4756-1
TCCTCTATTCCTTCCTTCCTTTCTCCGTCATCATCATTTTTCCCACCGAC
CONSENSUS
TCCTCTATTCCTTCCTTCCTTTCTCCGTCATCATCATTTTTCCCACCGAC

A5-AT1G26850-XLOC_005542-4756-0
AACAATCTCTCGGAGTTTGGGTATTATTACTTCTCTCATCAACCAATTGT
A5-AT1G26850-XLOC_005542-4756-1
AACAATCTCTCGGAGTTTGG------------------------------
CONSENSUS
AACAATCTCTCGGAGTTTGG..............................

A5-AT1G26850-XLOC_005542-4756-0
ACCACTAGATCTCCCAGATTCCTTCATTATCTCTATCAATAATGGCGTTG
A5-AT1G26850-XLOC_005542-4756-1
----------------------------------ATCAATAATGGCGTTG
CONSENSUS
..................................ATCAATAATGGCGTTG

A5-AT1G26850-XLOC_005542-4756-0
AAGTCTAGTTCTGCTGATGGTAAAACCAGAAGCTCTGTTCAGATCTTCAT
A5-AT1G26850-XLOC_005542-4756-1
AAGTCTAGTTCTGCTGATGGTAAAACCAGAAGCTCTGTTCAGATCTTCAT
CONSENSUS
AAGTCTAGTTCTGCTGATGGTAAAACCAGAAGCTCTGTTCAGATCTTCAT

A5-AT1G26850-XLOC_005542-4756-0
TGTGTTTAGTTTATGTTGCTTCTTTTACATTTTGGGAGCATGGCAACGAA
A5-AT1G26850-XLOC_005542-4756-1
TGTGTTTAGTTTATGTTGCTTCTTTTACATTTTGGGAGCATGGCAACGAA
CONSENSUS
TGTGTTTAGTTTATGTTGCTTCTTTTACATTTTGGGAGCATGGCAACGAA

A5-AT1G26850-XLOC_005542-4756-0
GTGGTTTTGGTAAAGGAGACAGTATTGCTCTTGAGATGACCAACAGTGGA
A5-AT1G26850-XLOC_005542-4756-1
GTGGTTTTGGTAAAGGAGACAGTATTGCTCTTGAGATGACCAACAGTGGA
CONSENSUS
GTGGTTTTGGTAAAGGAGACAGTATTGCTCTTGAGATGACCAACAGTGGA

A5-AT1G26850-XLOC_005542-4756-0
GCTGATTGCAACATTGTTCCTAGCTTGAATTTCGAGACTCATCACGCTGG
A5-AT1G26850-XLOC_005542-4756-1
GCTGATTGCAACATTGTTCCTAGCTTGAATTTCGAGACTCATCACGCTGG
CONSENSUS

GCTGATTGCAACATTGTTCCTAGCTTGAATTTCGAGACTCATCACGCTGG

A5-AT1G26850-XLOC_005542-4756-0
CGAATCAAGTCTTGTTGGTGCTTCTGAAGCTGCAAAGGTCAAGGCTTTCG
A5-AT1G26850-XLOC_005542-4756-1
CGAATCAAGTCTTGTTGGTGCTTCTGAAGCTGCAAAGGTCAAGGCTTTCG
CONSENSUS
CGAATCAAGTCTTGTTGGTGCTTCTGAAGCTGCAAAGGTCAAGGCTTTCG

A5-AT1G26850-XLOC_005542-4756-0
AGCCCTGTGATGGTCGTTACACGGATTACACTCCTTGTCAAGATCAGAGA
A5-AT1G26850-XLOC_005542-4756-1
AGCCCTGTGATGGTCGTTACACGGATTACACTCCTTGTCAAGATCAGAGA
CONSENSUS
AGCCCTGTGATGGTCGTTACACGGATTACACTCCTTGTCAAGATCAGAGA

A5-AT1G26850-XLOC_005542-4756-0
CGTGCCATGACTTTCCCGAGAGATAGTATGATTTACCGAGAAAGGCATTG
A5-AT1G26850-XLOC_005542-4756-1
CGTGCCATGACTTTCCCGAGAGATAGTATGATTTACCGAGAAAGGCATTG
CONSENSUS
CGTGCCATGACTTTCCCGAGAGATAGTATGATTTACCGAGAAAGGCATTG

A5-AT1G26850-XLOC_005542-4756-0
CGCTCCTGAGAATGAGAAGCTCCATTGTCTTATACCGGCTCCTAAAGGAT
A5-AT1G26850-XLOC_005542-4756-1
CGCTCCTGAGAATGAGAAGCTCCATTGTCTTATACCGGCTCCTAAAGGAT
CONSENSUS
CGCTCCTGAGAATGAGAAGCTCCATTGTCTTATACCGGCTCCTAAAGGAT

A5-AT1G26850-XLOC_005542-4756-0
ATGTGACACCTTTCTCTTGGCCTAAGAGTCGAGACTATGTGCCTTATGCT
A5-AT1G26850-XLOC_005542-4756-1
ATGTGACACCTTTCTCTTGGCCTAAGAGTCGAGACTATGTGCCTTATGCT
CONSENSUS
ATGTGACACCTTTCTCTTGGCCTAAGAGTCGAGACTATGTGCCTTATGCT

A5-AT1G26850-XLOC_005542-4756-0
AATGCGCCGTATAAAGCATTGACTGTTGAGAAGGCTATTCAGAATTGGAT
A5-AT1G26850-XLOC_005542-4756-1
AATGCGCCGTATAAAGCATTGACTGTTGAGAAGGCTATTCAGAATTGGAT
CONSENSUS
AATGCGCCGTATAAAGCATTGACTGTTGAGAAGGCTATTCAGAATTGGAT

A5-AT1G26850-XLOC_005542-4756-0
TCAGTATGAGGGAGACGTTTTTAGATTCCCTGGTGGTGGAACTCAGTTCC
A5-AT1G26850-XLOC_005542-4756-1
TCAGTATGAGGGAGACGTTTTTAGATTCCCTGGTGGTGGAACTCAGTTCC
CONSENSUS
TCAGTATGAGGGAGACGTTTTTAGATTCCCTGGTGGTGGAACTCAGTTCC

A5-AT1G26850-XLOC_005542-4756-0
CTCAAGGTGCTGATAAGTATATCGATCAGCTTGCTTCCGTGATACCTATG
A5-AT1G26850-XLOC_005542-4756-1
CTCAAGGTGCTGATAAGTATATCGATCAGCTTGCTTCCGTGATACCTATG
CONSENSUS

CTCAAGGTGCTGATAAGTATATCGATCAGCTTGCTTCCGTGATACCTATG

A5-AT1G26850-XLOC_005542-4756-0
GAGAACGGAACTGTTAGGACTGCTTTGGACACTGGTTGTGGG
A5-AT1G26850-XLOC_005542-4756-1
GAGAACGGAACTGTTAGGACTGCTTTGGACACTGGTTGTGGG
CONSENSUS
GAGAACGGAACTGTTAGGACTGCTTTGGACACTGGTTGTGGG

alignment for event: A3-AT1G67325-XLOC_007426-9840

A3-AT1G67325-XLOC_007426-9840-0
ATTCAACTCGCGATAATGATTGGACATGTCCGAATTGTGGTAATGTAAAC
A3-AT1G67325-XLOC_007426-9840-1
ATTCAACTCGCGATAATGATTGGACATGTCCGAATTGTGGTAATGTAAAC
CONSENSUS
ATTCAACTCGCGATAATGATTGGACATGTCCGAATTGTGGTAATGTAAAC

A3-AT1G67325-XLOC_007426-9840-0
TTCTCATTCAGAACTGTATGTAACATGAGGAAGTGCAACACTCCAAAGCC
A3-AT1G67325-XLOC_007426-9840-1
TTCTCATTCAGAACTGTATGTAACATGAGGAAGTGCAACACTCCAAAGCC
CONSENSUS
TTCTCATTCAGAACTGTATGTAACATGAGGAAGTGCAACACTCCAAAGCC

A3-AT1G67325-XLOC_007426-9840-0 TGGTTCTCAG---
GGTGGAAGCTCAGATAAAATATCCA
A3-AT1G67325-XLOC_007426-9840-1
TGGTTCTCAGCAGGGTGGAAGCTCAGATAAAATATCCA
CONSENSUS
TGGTTCTCAG...GGTGGAAGCTCAGATAAAATATCCA

alignment for event: RI-AT1G08230-XLOC_004571-1256

RI-AT1G08230-XLOC_004571-1256-0
GGACATGGTGGCACTGCGGATTCCACTTGACGACGTCTATAGTGGCGCCT
RI-AT1G08230-XLOC_004571-1256-1
GGACATGGTGGCACTGCGGATTCCACTTGACGACGTCTATAGTGGCGCCT
CONSENSUS
GGACATGGTGGCACTGCGGATTCCACTTGACGACGTCTATAGTGGCGCCT

RI-AT1G08230-XLOC_004571-1256-0
GCGCTACTGAGTCTACCGTACGCCTTCAAGTTCTTAGGATGGGCTGCCGG
RI-AT1G08230-XLOC_004571-1256-1
GCGCTACTGAGTCTACCGTACGCCTTCAAGTTCTTAGGATGGGCTGCCGG
CONSENSUS
GCGCTACTGAGTCTACCGTACGCCTTCAAGTTCTTAGGATGGGCTGCCGG

RI-AT1G08230-XLOC_004571-1256-0
GATATCTTGTCTGGTCGGAGGAGCAGCCGTTACTTTCTACTCGTACACTC
RI-AT1G08230-XLOC_004571-1256-1
GATATCTTGTCTGGTCGGAGGAGCAGCCGTTACTTTCTACTCGTACACTC

CONSENSUS
GATATCTTGTCTGGTCGGAGGAGCAGCCGTTACTTTCTACTCGTACACTC

RI-AT1G08230-XLOC_004571-1256-0
TTCTTTCTCTTACTCTCGAACACCACGCTTCTCTCGGCAACCGTTACCTC
RI-AT1G08230-XLOC_004571-1256-1
TTCTTTCTCTTACTCTCGAACACCACGCTTCTCTCGGCAACCGTTACCTC
CONSENSUS
TTCTTTCTCTTACTCTCGAACACCACGCTTCTCTCGGCAACCGTTACCTC

RI-AT1G08230-XLOC_004571-1256-0
CGCTTCCGTGACATGGCTCATCACATCCTCAGTAATTCTTTTTCTTTTTA
RI-AT1G08230-XLOC_004571-1256-1
CGCTTCCGTGACATGGCTCATCACATCCTCA-------------------
CONSENSUS
CGCTTCCGTGACATGGCTCATCACATCCTCA...................

RI-AT1G08230-XLOC_004571-1256-0
CATTTGCTCTTTCCTATAATCAAAGCTGCGACAAATTTACCTTAAGAGAA
RI-AT1G08230-XLOC_004571-1256-1

CONSENSUS
....

RI-AT1G08230-XLOC_004571-1256-0
TTTCATTCAAGATTGAGATGTATAGTTGTACGTAGACATACGTGTCTATA
RI-AT1G08230-XLOC_004571-1256-1

CONSENSUS
....

RI-AT1G08230-XLOC_004571-1256-0
TGAACTTCTATACGTAATAATTGACACACGGCCGGTACCACTCCAATAAT
RI-AT1G08230-XLOC_004571-1256-1

CONSENSUS
....

RI-AT1G08230-XLOC_004571-1256-0
TTCATCCACGCAATAAAGGAAGGTACATGTCCTACGTAATAATTTGTTTG
RI-AT1G08230-XLOC_004571-1256-1

CONSENSUS
....

RI-AT1G08230-XLOC_004571-1256-0
AACAAAAAAAAAAGTTAATTTTTGTGTGAAAACGCGAGATGGAATACTTA
RI-AT1G08230-XLOC_004571-1256-1

CONSENSUS
....

RI-AT1G08230-XLOC_004571-1256-0
TAATCACGTTTGCTATTACGAACAACGGAATTTTATAAGAAACCAAGTAA
RI-AT1G08230-XLOC_004571-1256-1

CONSENSUS
 ....

RI-AT1G08230-XLOC_004571-1256-0
 ACATTGGTATGAGCTCAACTTTTGTTCGTGGTTGGTTAATATCACAATAT
RI-AT1G08230-XLOC_004571-1256-1

CONSENSUS
 ....

RI-AT1G08230-XLOC_004571-1256-0
 ATGTATTGTGTAAACCTATGATTTTTAAAAAAATACTTTTCTATACTGTT
RI-AT1G08230-XLOC_004571-1256-1

CONSENSUS
 ....

RI-AT1G08230-XLOC_004571-1256-0
 TGGTCTTGTTTCGAATTATAAGAAGAAAAAAAACTAATCATATGGAATTC
RI-AT1G08230-XLOC_004571-1256-1

CONSENSUS
 ....

RI-AT1G08230-XLOC_004571-1256-0
 ATATTATAGTGAATTTTTTGATATGGCTTGCTTCGGTTCAATTTTGTTGG
RI-AT1G08230-XLOC_004571-1256-1

CONSENSUS
 ....

RI-AT1G08230-XLOC_004571-1256-0
 GATCCGGATCGAAGTGTTGACCATTCCACATTTCCACTCATACCACTGTT
RI-AT1G08230-XLOC_004571-1256-1

CONSENSUS
 ....

RI-AT1G08230-XLOC_004571-1256-0
 AAGTTTCAACTTTTTCATGCGTTAGATAGCTGTCTCTTTCCGTGTCTTGT
RI-AT1G08230-XLOC_004571-1256-1

CONSENSUS
 ....

RI-AT1G08230-XLOC_004571-1256-0
 TTCATTTTTGATGCAGAAATTTGTCTTTCAAAACTAATTTCAATTTTTAA
RI-AT1G08230-XLOC_004571-1256-1

CONSENSUS
 ....

RI-AT1G08230-XLOC_004571-1256-0
 AAATATGTGCGTGTCAGAAGAGTATTCATAAAACGTTGGTGCCGACCAAA
RI-AT1G08230-XLOC_004571-1256-1

CONSENSUS
      ....

RI-AT1G08230-XLOC_004571-1256-0
      ATTTAATCTTATATAAGTAAAGTTGGATAGGTTCGATCTGCTTTCAAAAG
RI-AT1G08230-XLOC_004571-1256-1

CONSENSUS
      ....

RI-AT1G08230-XLOC_004571-1256-0
      TAGAATATTCTAAATTTCAGATCCTACATAGGATTCGATCCAAATTAGGA
RI-AT1G08230-XLOC_004571-1256-1

CONSENSUS
      ....

RI-AT1G08230-XLOC_004571-1256-0
      TAGTTACGCTTTGACTGTCGACTACCAAGGAAATCCACATGTCAACAAAA
RI-AT1G08230-XLOC_004571-1256-1

CONSENSUS
      ....

RI-AT1G08230-XLOC_004571-1256-0
      CTTTTAAGACAAAATTTAACGTATTTATCTATGCAAAAACTACAAAATGA
RI-AT1G08230-XLOC_004571-1256-1

CONSENSUS
      ....

RI-AT1G08230-XLOC_004571-1256-0
      TGAGAAAAAAATTGATTGAAATTTTTTATCTTACTGGATAATTCTGTTTTA
RI-AT1G08230-XLOC_004571-1256-1

CONSENSUS
      ....

RI-AT1G08230-XLOC_004571-1256-0
      GTAGCTAATATGAAACTAAATCCATTATGCATCGAATCATCGATTTGGAT
RI-AT1G08230-XLOC_004571-1256-1

CONSENSUS
      ....

RI-AT1G08230-XLOC_004571-1256-0
      TTTAGTTTTCATTAGAGTACACCGCTAATCTGAAGGATTTATAGGAATTC
RI-AT1G08230-XLOC_004571-1256-1

CONSENSUS
      ....

RI-AT1G08230-XLOC_004571-1256-0
      GATTTAGCCCACTTTTTCGGGACTGTATACAACTTTAATTAGGATAAGGT
RI-AT1G08230-XLOC_004571-1256-1

CONSENSUS
      ....

RI-AT1G08230-XLOC_004571-1256-0
      AAAGTATAATACAGGTTCCGAATGTTTGATTAGTTAAGATCAGTTTGGTT
RI-AT1G08230-XLOC_004571-1256-1

CONSENSUS
      ....

RI-AT1G08230-XLOC_004571-1256-0
      TTCCTCTTAACAATTATTGGGGTGTGTTTGATAAGGTCCGAAATGGGGAA
RI-AT1G08230-XLOC_004571-1256-1
      -----------------------------------GTCCGAAATGGGGAA
CONSENSUS
      ...................................GTCCGAAATGGGGAA

RI-AT1G08230-XLOC_004571-1256-0
      GGTACTACGTTGGACCAATACAAATGGCGGTGTGCTATGGTGTGGTTATT
RI-AT1G08230-XLOC_004571-1256-1
      GGTACTACGTTGGACCAATACAAATGGCGGTGTGCTATGGTGTGGTTATT
CONSENSUS
      GGTACTACGTTGGACCAATACAAATGGCGGTGTGCTATGGTGTGGTTATT

RI-AT1G08230-XLOC_004571-1256-0
      GCCAATGCGCTCTTGGGAGGGCAATGCCTAAAGGCAATGTACTTGGTAGT
RI-AT1G08230-XLOC_004571-1256-1
      GCCAATGCGCTCTTGGGAGGGCAATGCCTAAAGGCAATGTACTTGGTAGT
CONSENSUS
      GCCAATGCGCTCTTGGGAGGGCAATGCCTAAAGGCAATGTACTTGGTAGT

RI-AT1G08230-XLOC_004571-1256-0
      GCAGCCAAATGGGGAAATGAAACTCTTTGAGTTTGTGATTATTTTTGGAT
RI-AT1G08230-XLOC_004571-1256-1
      GCAGCCAAATGGGGAAATGAAACTCTTTGAGTTTGTGATTATTTTTGGAT
CONSENSUS
      GCAGCCAAATGGGGAAATGAAACTCTTTGAGTTTGTGATTATTTTTGGAT

RI-AT1G08230-XLOC_004571-1256-0
      GCTTACTTTTGGTTTTGGCACAATTCCCATCCTTTCACTCTCTACGATAC
RI-AT1G08230-XLOC_004571-1256-1
      GCTTACTTTTGGTTTTGGCACAATTCCCATCCTTTCACTCTCTACGATAC
CONSENSUS
      GCTTACTTTTGGTTTTGGCACAATTCCCATCCTTTCACTCTCTACGATAC

RI-AT1G08230-XLOC_004571-1256-0
      ATCAACTCACTGTCTCTCCTCCTCTGCCTCCTCTACAGTGCTTCTGCCGC
RI-AT1G08230-XLOC_004571-1256-1
      ATCAACTCACTGTCTCTCCTCCTCTGCCTCCTCTACAGTGCTTCTGCCGC
CONSENSUS
      ATCAACTCACTGTCTCTCCTCCTCTGCCTCCTCTACAGTGCTTCTGCCGC

RI-AT1G08230-XLOC_004571-1256-0 TGCTGCATCCATCTACATTG
RI-AT1G08230-XLOC_004571-1256-1 TGCTGCATCCATCTACATTG
CONSENSUS   TGCTGCATCCATCTACATTG

alignment for event: RI-AT1G02160-XLOC_000065-13703

RI-AT1G02160-XLOC_000065-13703-0
GTCTTGAAGAATTTGGATCAGACAAGAGTAAATGCCAGGATCATTTTGAT
RI-AT1G02160-XLOC_000065-13703-1
GTCTTGAAGAATTTGGATCAGACAAGAGTAAATGCCAGGATCATTTTGAT
CONSENSUS
GTCTTGAAGAATTTGGATCAGACAAGAGTAAATGCCAGGATCATTTTGAT

RI-AT1G02160-XLOC_000065-13703-0
GTGTACAAGGAATGCAAGAAGAAAGAGGTTGTTGTTGTGAATGAATATTT
RI-AT1G02160-XLOC_000065-13703-1
GTGTACAAGGAATGCAAGAAGAAAGAG-----------------------
CONSENSUS
GTGTACAAGGAATGCAAGAAGAAAGAG.......................

RI-AT1G02160-XLOC_000065-13703-0
AGGCTTTTGGCGTTTCCAACTTCTTTGCTGCTTTACCTATGTGTTATTTT
RI-AT1G02160-XLOC_000065-13703-1

CONSENSUS
....

RI-AT1G02160-XLOC_000065-13703-0
GTTTCTCAGAGGGAAGCTCGACTGGAACGCAATAAGACACGGTCGTTGTT
RI-AT1G02160-XLOC_000065-13703-1 ---------
AGGGAAGCTCGACTGGAACGCAATAAGACACGGTCGTTGTT
CONSENSUS
.........AGGGAAGCTCGACTGGAACGCAATAAGACACGGTCGTTGTT

RI-AT1G02160-XLOC_000065-13703-0
CTCGTGAATGCAGATCCTCGAATACCAATGATGTCTCAGAACATCACCTA
RI-AT1G02160-XLOC_000065-13703-1
CTCGTGAATGCAGATCCTCGAATACCAATGATGTCTCAGAACATCACCTA
CONSENSUS
CTCGTGAATGCAGATCCTCGAATACCAATGATGTCTCAGAACATCACCTA

RI-AT1G02160-XLOC_000065-13703-0
GCTAGTAGTATCCTGTTGTTTCATTTGCAATGGCTGTGTTTGTATGATCT
RI-AT1G02160-XLOC_000065-13703-1
GCTAGTAGTATCCTGTTGTTTCATTTGCAATGGCTGTGTTTGTATGATCT
CONSENSUS
GCTAGTAGTATCCTGTTGTTTCATTTGCAATGGCTGTGTTTGTATGATCT

RI-AT1G02160-XLOC_000065-13703-0
ATCTAAGTAAACAAGTGGAAAGTGTTTGTTAATGTTACTTTTTACTCCCC
RI-AT1G02160-XLOC_000065-13703-1
ATCTAAGTAAACAAGTGGAAAGTGTTTGTTAATGTTACTTTTTACTCCCC
CONSENSUS
ATCTAAGTAAACAAGTGGAAAGTGTTTGTTAATGTTACTTTTTACTCCCC

RI-AT1G02160-XLOC_000065-13703-0
ATTGGTGAATCATGGTGTATACCTGTTAAACTTTGAGAGCAAGTGGTTAT
RI-AT1G02160-XLOC_000065-13703-1

ATTGGTGAATCATGGTGTATACCTGTTAAACTTTGAGAGCAAGTGGTTAT
CONSENSUS
ATTGGTGAATCATGGTGTATACCTGTTAAACTTTGAGAGCAAGTGGTTAT

RI-AT1G02160-XLOC_000065-13703-0 TTATTTAGTTAGAGAGTTAGA
RI-AT1G02160-XLOC_000065-13703-1 TTATTTAGTTAGAGAGTTAGA
CONSENSUS TTATTTAGTTAGAGAGTTAGA

alignment for event: A3-AT1G71220-XLOC_003644-13374

A3-AT1G71220-XLOC_003644-13374-0
GTCATTGCGCGGAAAAAGATCATGAGGCTCCTCGTGGCCTTCAGCTGATT
A3-AT1G71220-XLOC_003644-13374-1
GTCATTGCGCGGAAAAAGATCATGAGGCTCCTCGTGGCCTTCAGCTGATT
CONSENSUS
GTCATTGCGCGGAAAAAGATCATGAGGCTCCTCGTGGCCTTCAGCTGATT

A3-AT1G71220-XLOC_003644-13374-0
CTGGGAACCAAAAATAGACCGCATTTGGTTGATACCCTTGTAATGGCCAA
A3-AT1G71220-XLOC_003644-13374-1
CTGGGAACCAAAAATAGACCGCATTTGGTTGATACCCTTGTAATGGCCAA
CONSENSUS
CTGGGAACCAAAAATAGACCGCATTTGGTTGATACCCTTGTAATGGCCAA

A3-AT1G71220-XLOC_003644-13374-0
TTTGGGTTATTGGCAGATGAAAGTATCCCCAGGGGTTTGGTATTTGCAAC
A3-AT1G71220-XLOC_003644-13374-1
TTTGGGTTATTGGCAGATGAAAGTATCCCCAGGGGTTTGGTATTTGCAAC
CONSENSUS
TTTGGGTTATTGGCAGATGAAAGTATCCCCAGGGGTTTGGTATTTGCAAC

A3-AT1G71220-XLOC_003644-13374-0
TTGCTCCGGGTAGAAGTTCGGAGCTATACGCTTTGAAAGGAGGAAATGAT
A3-AT1G71220-XLOC_003644-13374-1
TTGCTCCGGGTAGAAGTTCGGAGCTATACGCTTTGAAAGGAGGAAATGAT
CONSENSUS
TTGCTCCGGGTAGAAGTTCGGAGCTATACGCTTTGAAAGGAGGAAATGAT

A3-AT1G71220-XLOC_003644-13374-0
GGGAGTCAAGATCAATCCTCGCTGAAACGTATAACTATCGATGATCTGCG
A3-AT1G71220-XLOC_003644-13374-1
GGGAGTCAAGATCAATCCTCGCTGAAACGTATAACTATCGATGATCTGCG
CONSENSUS
GGGAGTCAAGATCAATCCTCGCTGAAACGTATAACTATCGATGATCTGCG

A3-AT1G71220-XLOC_003644-13374-0
TGGTAAAGTTGTTCATCTAGAAGTAGTTAAGAGAAAGGGTAAGGAGCATG
A3-AT1G71220-XLOC_003644-13374-1
TGGTAAAGTTGTTCATCTAGAAGTAGTTAAGAGAAAGGGTAAGGAGCATG
CONSENSUS
TGGTAAAGTTGTTCATCTAGAAGTAGTTAAGAGAAAGGGTAAGGAGCATG

A3-AT1G71220-XLOC_003644-13374-0
AAAAGTTGCTAGTTCCTTCAGACGGTGATGATGCTGTGCAACAAAACAAG

A3-AT1G71220-XLOC_003644-13374-1
      AAAAGTTGCTAGTTCCTTCAGACGGTGATGATGCTGTGCAACAAAACAAG
CONSENSUS
      AAAAGTTGCTAGTTCCTTCAGACGGTGATGATGCTGTGCAACAAAACAAG

A3-AT1G71220-XLOC_003644-13374-0
      GAACAGGGAAGCTGGAACTCAAATTTCTTGAAATGGGCGTCTGGTTTTGT
A3-AT1G71220-XLOC_003644-13374-1 GAA---
GGAAGCTGGAACTCAAATTTCTTGAAATGGGCGTCTGGTTTTGT
CONSENSUS
      GAA...GGAAGCTGGAACTCAAATTTCTTGAAATGGGCGTCTGGTTTTGT

A3-AT1G71220-XLOC_003644-13374-0
      TGGTGGTCGTCAACAATCAATGAAGGGAGGTCCTGACAAA
A3-AT1G71220-XLOC_003644-13374-1
      TGGTGGTCGTCAACAATCAATGAAGGGAGGTCCTGACAAA
CONSENSUS
      TGGTGGTCGTCAACAATCAATGAAGGGAGGTCCTGACAAA

alignment for event: A5-AT1G77080-XLOC_003953-9827

A5-AT1G77080-XLOC_003953-9827-0
      GAGAAATTGCTGAGAGAAGAGAACCAGGTTCTGGCTAGCCAG--------
A5-AT1G77080-XLOC_003953-9827-1
      GAGAAATTGCTGAGAGAAGAGAACCAGGTTCTGGCTAGCCAGGTAACAAT
CONSENSUS
      GAGAAATTGCTGAGAGAAGAGAACCAGGTTCTGGCTAGCCAG........

A5-AT1G77080-XLOC_003953-9827-0

A5-AT1G77080-XLOC_003953-9827-1
      GACCACAATATCTTCTGCTCTTGAAGCTAATTAATCACTTTATACGTCCC
CONSENSUS
      ....

A5-AT1G77080-XLOC_003953-9827-0

A5-AT1G77080-XLOC_003953-9827-1
      CGTTATAGAGAGATACACATATACACGTACATGAAAACTAAAAGTTGAAG
CONSENSUS
      ....

A5-AT1G77080-XLOC_003953-9827-0

A5-AT1G77080-XLOC_003953-9827-1
      GACTTTGATGGATACTAGACAATTATAGTGAAACCCTAAATATGTGATAA
CONSENSUS
      ....

A5-AT1G77080-XLOC_003953-9827-0

A5-AT1G77080-XLOC_003953-9827-1
      GTGATAACAAAATGCTTTTAAAATCTATCTTTCTTGTTAATTTAGTAGCT
CONSENSUS

....

A5-AT1G77080-XLOC_003953-9827-0
--AT
A5-AT1G77080-XLOC_003953-9827-1
GTCAGAGAAGAAAGGTATGTCTCACCGATGAAAGATACTCAAAACCCGAT
CONSENSUS
....AT

A5-AT1G77080-XLOC_003953-9827-0
GGGAAAGAATACGTTGCTGGCAACAGATGATGAGAGAGGAATGTTTCCGG
A5-AT1G77080-XLOC_003953-9827-1
GGGAAAGAATACGTTGCTGGCAACAGATGATGAGAGAGGAATGTTTCCGG
CONSENSUS
GGGAAAGAATACGTTGCTGGCAACAGATGATGAGAGAGGAATGTTTCCGG

A5-AT1G77080-XLOC_003953-9827-0
GAAGTAGCTCCGGCAACAAAATACCGGAGACTCTCCCGCTGCTCAATTAG
A5-AT1G77080-XLOC_003953-9827-1
GAAGTAGCTCCGGCAACAAAATACCGGAGACTCTCCCGCTGCTCAATTAG
CONSENSUS
GAAGTAGCTCCGGCAACAAAATACCGGAGACTCTCCCGCTGCTCAATTAG

A5-AT1G77080-XLOC_003953-9827-0
CCACCATCATCAACGGCTGAGTTTTCACCTTAAACTCAAAGCCTGATTCA
A5-AT1G77080-XLOC_003953-9827-1
CCACCATCATCAACGGCTGAGTTTTCACCTTAAACTCAAAGCCTGATTCA
CONSENSUS
CCACCATCATCAACGGCTGAGTTTTCACCTTAAACTCAAAGCCTGATTCA

A5-AT1G77080-XLOC_003953-9827-0
TAATTAAGAGAATAAATTTGTATATTATAAAAAGCTGTGTAATCTCAAAC
A5-AT1G77080-XLOC_003953-9827-1
TAATTAAGAGAATAAATTTGTATATTATAAAAAGCTGTGTAATCTCAAAC
CONSENSUS
TAATTAAGAGAATAAATTTGTATATTATAAAAAGCTGTGTAATCTCAAAC

A5-AT1G77080-XLOC_003953-9827-0
CTTTTATCTTCCTCTAGTGTGGAATTTAAGGTCAAAAAGAAAACGAGAAA
A5-AT1G77080-XLOC_003953-9827-1
CTTTTATCTTCCTCTAGTGTGGAATTTAAGGTCAAAAAGAAAACGAGAAA
CONSENSUS
CTTTTATCTTCCTCTAGTGTGGAATTTAAGGTCAAAAAGAAAACGAGAAA

A5-AT1G77080-XLOC_003953-9827-0
GTATGGATCAGTGTTGTACCTCCTTCGGAGACAAGATCAGAGTTTGTGTG
A5-AT1G77080-XLOC_003953-9827-1
GTATGGATCAGTGTTGTACCTCCTTCGGAGACAAGATCAGAGTTTGTGTG
CONSENSUS
GTATGGATCAGTGTTGTACCTCCTTCGGAGACAAGATCAGAGTTTGTGTG

A5-AT1G77080-XLOC_003953-9827-0
TTTGTGTCTGAATGTACGGATTGGATTTTTAAAGTTGTGCTTTCTTTCTT
A5-AT1G77080-XLOC_003953-9827-1
TTTGTGTCTGAATGTACGGATTGGATTTTTAAAGTTGTGCTTTCTTTCTT
CONSENSUS

TTTGTGTCTGAATGTACGGATTGGATTTTTAAAGTTGTGCTTTCTTTCTT

A5-AT1G77080-XLOC_003953-9827-0 C
A5-AT1G77080-XLOC_003953-9827-1 C
CONSENSUS   C

alignment for event: A3-AT1G48410-XLOC_006423-1560

A3-AT1G48410-XLOC_006423-1560-0
ACGAGAAAGGGAATTTAAAGTTGTGATCAAGCTAGTTGCACGTGCTGATC
A3-AT1G48410-XLOC_006423-1560-1
ACGAGAAAGGGAATTTAAAGTTGTGATCAAGCTAGTTGCACGTGCTGATC
CONSENSUS
ACGAGAAAGGGAATTTAAAGTTGTGATCAAGCTAGTTGCACGTGCTGATC

A3-AT1G48410-XLOC_006423-1560-0
TGCATCACCTAGGAATGTTTTTGGAGGGGAAACAATCAGATGCCCCACAG
A3-AT1G48410-XLOC_006423-1560-1
TGCATCACCTAGGAATGTTTTTGGAGGGGAAACAATCAGATGCCCCACAG
CONSENSUS
TGCATCACCTAGGAATGTTTTTGGAGGGGAAACAATCAGATGCCCCACAG

A3-AT1G48410-XLOC_006423-1560-0
GAAGCTCTGCAGGTTCTTGACATTGTTCTTCGTGAGCTGCCGACCTCTAG
A3-AT1G48410-XLOC_006423-1560-1
GAAGCTCTGCAGGTTCTTGACATTGTTCTTCGTGAGCTGCCGACCTCTAG
CONSENSUS
GAAGCTCTGCAGGTTCTTGACATTGTTCTTCGTGAGCTGCCGACCTCTAG

A3-AT1G48410-XLOC_006423-1560-0 ------
GTATATTCCGGTGGGCCGGTCCTTTTATTCCCCTGATATAGGAA
A3-AT1G48410-XLOC_006423-1560-1
AATCAGGTATATTCCGGTGGGCCGGTCCTTTTATTCCCCTGATATAGGAA
CONSENSUS
......GTATATTCCGGTGGGCCGGTCCTTTTATTCCCCTGATATAGGAA

A3-AT1G48410-XLOC_006423-1560-0
AAAAACAATCATTGGGGGATGGCTTGGAGAGCTGGCGTGGATTCTACCAA
A3-AT1G48410-XLOC_006423-1560-1
AAAAACAATCATTGGGGGATGGCTTGGAGAGCTGGCGTGGATTCTACCAA
CONSENSUS
AAAAACAATCATTGGGGGATGGCTTGGAGAGCTGGCGTGGATTCTACCAA

A3-AT1G48410-XLOC_006423-1560-0
AGCATTCGTCCTACACAGATGGGCTTATCACTCAATATTG
A3-AT1G48410-XLOC_006423-1560-1
AGCATTCGTCCTACACAGATGGGCTTATCACTCAATATTG
CONSENSUS
AGCATTCGTCCTACACAGATGGGCTTATCACTCAATATTG

alignment for event: RI-AT1G05940-XLOC_004432-1277

RI-AT1G05940-XLOC_004432-1277-0
ACTGGGTATTCAGTTGTTGCGGCTTGTGTTGTGGCATTGAGACTGAATGA
RI-AT1G05940-XLOC_004432-1277-1
ACTGGGTATTCAGTTGTTGCGGCTTGTGTTGTGGCATTGAGACTGAATGA
CONSENSUS
ACTGGGTATTCAGTTGTTGCGGCTTGTGTTGTGGCATTGAGACTGAATGA

RI-AT1G05940-XLOC_004432-1277-0
TAAAAAAGATAGAGAGTCGTCCAATAGATGGACATCAAGTTGGCAGGAAG
RI-AT1G05940-XLOC_004432-1277-1
TAAAAAAGATAGAGAGTCGTCCAATAGATGGACATCAAGTTGGCAGGAAG
CONSENSUS
TAAAAAAGATAGAGAGTCGTCCAATAGATGGACATCAAGTTGGCAGGAAG

RI-AT1G05940-XLOC_004432-1277-0
GCGTCATTTGCCTTGTTATAATTGCATGCAGTGGTTTTGGTGCTGGCGTA
RI-AT1G05940-XLOC_004432-1277-1
GCGTCATTTGCCTTGTTATAATTGCATGCAGTGGTTTTGGTGCTGGCGTA
CONSENSUS
GCGTCATTTGCCTTGTTATAATTGCATGCAGTGGTTTTGGTGCTGGCGTA

RI-AT1G05940-XLOC_004432-1277-0
TTTTACCGTTTCAGTGCTTCAGTTATATTTATTCTCCTCTCAGTTGGTGT
RI-AT1G05940-XLOC_004432-1277-1
TTTTACCGTTTCAGTGCTTCAGTTATATTTATTCTCCTCTCAGTTGGTGT
CONSENSUS
TTTTACCGTTTCAGTGCTTCAGTTATATTTATTCTCCTCTCAGTTGGTGT

RI-AT1G05940-XLOC_004432-1277-0
GGCAGTTGTTGCTTCAGCAGTTCTTCATTATCGCCAAGTAAGTAGCCTTA
RI-AT1G05940-XLOC_004432-1277-1
GGCAGTTGTTGCTTCAGCAGTTCTTCATTATCGCCAAGTAAGTAGCCTTA
CONSENSUS
GGCAGTTGTTGCTTCAGCAGTTCTTCATTATCGCCAAGTAAGTAGCCTTA

RI-AT1G05940-XLOC_004432-1277-0
AGTATAAGCCTTAATTCCATAGCACACTCTTCTTAATTTCATAGCGCCAA
RI-AT1G05940-XLOC_004432-1277-1
AGTATAAGCCTTAATTCCATAGCACACTCTTCTTAATTTCATAGCGCCAA
CONSENSUS
AGTATAAGCCTTAATTCCATAGCACACTCTTCTTAATTTCATAGCGCCAA

RI-AT1G05940-XLOC_004432-1277-0
AAAAAATAAAATAAAAAAACCAAGTTTCACCCCTGTTAAAATGCTAGATA
RI-AT1G05940-XLOC_004432-1277-1
AAAAAATAAAATAAAAAAACCAAGTTTCACCCCTGTTAAAATGCTAGATA
CONSENSUS
AAAAAATAAAATAAAAAAACCAAGTTTCACCCCTGTTAAAATGCTAGATA

RI-AT1G05940-XLOC_004432-1277-0
CTAAGTCTTTTACTGATACAGCATAAAATAGAGACCACCACTAGCATGTT
RI-AT1G05940-XLOC_004432-1277-1
CTAAGTCTTTTACTGATACAGCATAAAATAGAGACCACCACTAGCATGTT
CONSENSUS
CTAAGTCTTTTACTGATACAGCATAAAATAGAGACCACCACTAGCATGTT

RI-AT1G05940-XLOC_004432-1277-0
CTGGCACTTAGAACTCTACAGTGATACTATGAAAGTCTGTGACTATTGAA
RI-AT1G05940-XLOC_004432-1277-1
CTGGCACTTAGAACTCTACAGTGATACTATGAAAGTCTGTGACTATTGAA
CONSENSUS
CTGGCACTTAGAACTCTACAGTGATACTATGAAAGTCTGTGACTATTGAA

RI-AT1G05940-XLOC_004432-1277-0
GTCTTTGCATTCTTGGTTTAGAGGAGAAGTTTGTTAACATCAATTAGGAA
RI-AT1G05940-XLOC_004432-1277-1
GTCTTTGCATTCTTGGTTTAGAGGAGAAGTTTGTTAACATCAATTAGGAA
CONSENSUS
GTCTTTGCATTCTTGGTTTAGAGGAGAAGTTTGTTAACATCAATTAGGAA

RI-AT1G05940-XLOC_004432-1277-0
ATATACCCTCATGGTTGTCTCCACAAATCAGGCAAATGCCTTGCTAGCTA
RI-AT1G05940-XLOC_004432-1277-1
ATATACCCTCATGGTTGTCTCCACAAATCAGGCAAATGCCTTGCTAGCTA
CONSENSUS
ATATACCCTCATGGTTGTCTCCACAAATCAGGCAAATGCCTTGCTAGCTA

RI-AT1G05940-XLOC_004432-1277-0
TGCTTCATGTTAAACCCCTCAGCTTTTCGCTTCCAGGCTTATGCTCTGCC
RI-AT1G05940-XLOC_004432-1277-1
TGCTTCATGTTAAACCCCTCAGCTTTTCGCTTCCAGGCTTATGCTCTGCC
CONSENSUS
TGCTTCATGTTAAACCCCTCAGCTTTTCGCTTCCAGGCTTATGCTCTGCC

RI-AT1G05940-XLOC_004432-1277-0
TCTAGGTTCTGGGTTTAGCTGTCCTGGTGTACCCATTGTGCCATCCGTTT
RI-AT1G05940-XLOC_004432-1277-1
TCTAGGTTCTGGGTTTAGCTGTCCTGGTGTACCCATTGTGCCATCCGTTT
CONSENSUS
TCTAGGTTCTGGGTTTAGCTGTCCTGGTGTACCCATTGTGCCATCCGTTT

RI-AT1G05940-XLOC_004432-1277-0
GCATTTTCTTCAACATCTTCTTATTTGCTCAGGTACACGCTAAGAACTAA
RI-AT1G05940-XLOC_004432-1277-1
GCATTTTCTTCAACATCTTCTTATTTGCTCAG------------------
CONSENSUS
GCATTTTCTTCAACATCTTCTTATTTGCTCAG..................

RI-AT1G05940-XLOC_004432-1277-0
CTTTATACTAATTTCACATACCATATGATCTACCTTTGATTTTTCCGCTA
RI-AT1G05940-XLOC_004432-1277-1

CONSENSUS
....

RI-AT1G05940-XLOC_004432-1277-0
CTGTGCAGTTGCATTATGAAGCATGGATAAGATTTGTTGTTGTCAGTGTG
RI-AT1G05940-XLOC_004432-1277-1 --------
TTGCATTATGAAGCATGGATAAGATTTGTTGTTGTCAGTGTG
CONSENSUS
........TTGCATTATGAAGCATGGATAAGATTTGTTGTTGTCAGTGTG

RI-AT1G05940-XLOC_004432-1277-0
CTTGCAACTGCCGTTTATGCCTTGTATGGGCAGTACCACGCAGATCCGAG
RI-AT1G05940-XLOC_004432-1277-1
CTTGCAACTGCCGTTTATGCCTTGTATGGGCAGTACCACGCAGATCCGAG
CONSENSUS
CTTGCAACTGCCGTTTATGCCTTGTATGGGCAGTACCACGCAGATCCGAG

RI-AT1G05940-XLOC_004432-1277-0
CATGTTGGATTATCAGAGGGCACCTGAAACTGAAAGCGACGCTTAGTAAT
RI-AT1G05940-XLOC_004432-1277-1
CATGTTGGATTATCAGAGGGCACCTGAAACTGAAAGCGACGCTTAGTAAT
CONSENSUS
CATGTTGGATTATCAGAGGGCACCTGAAACTGAAAGCGACGCTTAGTAAT

RI-AT1G05940-XLOC_004432-1277-0
GCTACATTATAGTTGTGTTACTTAAAGCCATTTGTAGAGTTGATAGCAAG
RI-AT1G05940-XLOC_004432-1277-1
GCTACATTATAGTTGTGTTACTTAAAGCCATTTGTAGAGTTGATAGCAAG
CONSENSUS
GCTACATTATAGTTGTGTTACTTAAAGCCATTTGTAGAGTTGATAGCAAG

RI-AT1G05940-XLOC_004432-1277-0
TTTCTGCGTTTCGTGAGAGACATACTGTATGTCACATACATATTCAGGTC
RI-AT1G05940-XLOC_004432-1277-1
TTTCTGCGTTTCGTGAGAGACATACTGTATGTCACATACATATTCAGGTC
CONSENSUS
TTTCTGCGTTTCGTGAGAGACATACTGTATGTCACATACATATTCAGGTC

RI-AT1G05940-XLOC_004432-1277-0
GAGTTTAAGGATTGGATGGGATAAGGCAGTTAAACATTATTGTTGTAAAG
RI-AT1G05940-XLOC_004432-1277-1
GAGTTTAAGGATTGGATGGGATAAGGCAGTTAAACATTATTGTTGTAAAG
CONSENSUS
GAGTTTAAGGATTGGATGGGATAAGGCAGTTAAACATTATTGTTGTAAAG

RI-AT1G05940-XLOC_004432-1277-0
AAGTTAATAAAAGTATGCTTGTGCATTTCAACTAAAGCCTTGAATGACT
RI-AT1G05940-XLOC_004432-1277-1
AAGTTAATAAAAGTATGCTTGTGCATTTCAACTAAAGCCTTGAATGACT
CONSENSUS
AAGTTAATAAAAGTATGCTTGTGCATTTCAACTAAAGCCTTGAATGACT

alignment for event: RI-AT1G05135-XLOC_004382-2333

RI-AT1G05135-XLOC_004382-2333-0
AATGTTCAAAACTAGAAAGATAGAAAGCTACTAAAAATCTAAACAAAGTG
RI-AT1G05135-XLOC_004382-2333-1
AATGTTCAAAACTAGAAAGATAGAAAGCTACTAAAAATCTAAACAAAGTG
CONSENSUS
AATGTTCAAAACTAGAAAGATAGAAAGCTACTAAAAATCTAAACAAAGTG

RI-AT1G05135-XLOC_004382-2333-0
TAGATCTATTTTGACTTAGTCGAAGAAAACGTTCATTAAATGGTTCCCGC
RI-AT1G05135-XLOC_004382-2333-1

TAGATCTATTTTGACTTAGTCGAAGAAAACGTTCATTAAATGGTTCCCGC
CONSENSUS
TAGATCTATTTTGACTTAGTCGAAGAAAACGTTCATTAAATGGTTCCCGC

RI-AT1G05135-XLOC_004382-2333-0
CAATGGCCCAACGGCCCCAAACGCGTAGAACGTAACATGCATGGGGATCC
RI-AT1G05135-XLOC_004382-2333-1
CAATGGCCCAACGGCCCCAAACGCGTAGAACGTAACATGCATGGGGATCC
CONSENSUS
CAATGGCCCAACGGCCCCAAACGCGTAGAACGTAACATGCATGGGGATCC

RI-AT1G05135-XLOC_004382-2333-0
ATATTAATGGCTTCTTCATCCCACTTGCTTATTTCTTTCGACTTTGGAGG
RI-AT1G05135-XLOC_004382-2333-1
ATATTAATGGCTTCTTCATCCCACTTGCTTATTTCTTTCGACTTTGGAGG
CONSENSUS
ATATTAATGGCTTCTTCATCCCACTTGCTTATTTCTTTCGACTTTGGAGG

RI-AT1G05135-XLOC_004382-2333-0
TTTCATTTACCTATTATATAAGGGAATGTTCAGTTCCAGTGACTGAGATA
RI-AT1G05135-XLOC_004382-2333-1
TTTCATTTACCTATTATATAAGGGAATGTTCAGTTCCAGTGACTGAGATA
CONSENSUS
TTTCATTTACCTATTATATAAGGGAATGTTCAGTTCCAGTGACTGAGATA

RI-AT1G05135-XLOC_004382-2333-0
TCACAAGCAAACAAGAGAAGACATTTTATTGTTATTTCTCTCTCTCTCTC
RI-AT1G05135-XLOC_004382-2333-1
TCACAAGCAAACAAGAGAAGACATTTTATTGTTATTTCTCTCTCTCTCTC
CONSENSUS
TCACAAGCAAACAAGAGAAGACATTTTATTGTTATTTCTCTCTCTCTCTC

RI-AT1G05135-XLOC_004382-2333-0
CTTGTAGATGGAACTTAAGGGCGTTACTTGTTTGCTTCTTTCTTTGGTTT
RI-AT1G05135-XLOC_004382-2333-1
CTTGTAGATGGAACTTAAGGGCGTTACTTGTTTGCTTCTTTCTTTGGTTT
CONSENSUS
CTTGTAGATGGAACTTAAGGGCGTTACTTGTTTGCTTCTTTCTTTGGTTT

RI-AT1G05135-XLOC_004382-2333-0
TGCTTAATTCGTGTGTGGAATGTGTTTTGGGGGATGGATCTGTGGTGGGT
RI-AT1G05135-XLOC_004382-2333-1
TGCTTAATTCGTGTGTGGAATGTGTTTTGGGGGATGGATCTGTGGTGGGT
CONSENSUS
TGCTTAATTCGTGTGTGGAATGTGTTTTGGGGGATGGATCTGTGGTGGGT

RI-AT1G05135-XLOC_004382-2333-0
CCTGCGAGGTTTAGAGACGATGATTGTAGGTGGGGCCGGAGGTGTGCTGG
RI-AT1G05135-XLOC_004382-2333-1
CCTGCGAGGTTTAGAGACGATGATTGTAGGTGGGGCCGGAGGTGTGCTGG
CONSENSUS
CCTGCGAGGTTTAGAGACGATGATTGTAGGTGGGGCCGGAGGTGTGCTGG

RI-AT1G05135-XLOC_004382-2333-0
ACGTGGCCGGTTTGGACGCGGTGGTGGTGGAGGGTTCGGCGGTGGGAGAG
RI-AT1G05135-XLOC_004382-2333-1

ACGTGGCCGGTTTGGACGCGGTGGTGGTGGAGGGTTCGGCGGTGGGAGAG
CONSENSUS
ACGTGGCCGGTTTGGACGCGGTGGTGGTGGAGGGTTCGGCGGTGGGAGAG

RI-AT1G05135-XLOC_004382-2333-0
GAAGTGGTGGCGGTATAGGCGGTGGTGGTGGACAAGGTGGAGGGTTTGGA
RI-AT1G05135-XLOC_004382-2333-1
GAAGTGGTGGCGGTATAGGCGGTGGTGGTGGACAAGGTGGAGGGTTTGGA
CONSENSUS
GAAGTGGTGGCGGTATAGGCGGTGGTGGTGGACAAGGTGGAGGGTTTGGA

RI-AT1G05135-XLOC_004382-2333-0
GCCGGAGGAGGAGTTGGTGGCGGAGCTGGAGGAGGACTTGGTGGTGGAGG
RI-AT1G05135-XLOC_004382-2333-1
GCCGGAGGAGGAGTTGGTGGCGGAGCTGGAGGAGGACTTGGTGGTGGAGG
CONSENSUS
GCCGGAGGAGGAGTTGGTGGCGGAGCTGGAGGAGGACTTGGTGGTGGAGG

RI-AT1G05135-XLOC_004382-2333-0
CGGAGCTGGTGGTGGTGGTGGAGGAGGTATTGGTGGTGGTTCCGGTCATG
RI-AT1G05135-XLOC_004382-2333-1
CGGAGCTGGTGGTG------------------------------------
CONSENSUS
CGGAGCTGGTGGTG....................................

RI-AT1G05135-XLOC_004382-2333-0
GTGGTGGGTTTGGAGCCGGCGGAGGAGTTGGTGGGGGTGCTGGTGGAGGA
RI-AT1G05135-XLOC_004382-2333-1

CONSENSUS
....

RI-AT1G05135-XLOC_004382-2333-0
ATTGGTGGGGGAGGCGGAGCTGGTGGAGGCGGAGGAGGAG
RI-AT1G05135-XLOC_004382-2333-1 ----------------------------
GCGGAGGAGGAG
CONSENSUS
............................GCGGAGGAGGAG

alignment for event: RI-AT1G28960-XLOC_005651-5870

RI-AT1G28960-XLOC_005651-5870-0
GATGAGAATCGAAGATCTGAAGAGAGAGAGTGGATGGGTGAAAAGTATTT
RI-AT1G28960-XLOC_005651-5870-1
GATGAGAATCGAAGATCTGAAGAGAGAGAGTGGATGGGTGAAAAGTATTT
CONSENSUS
GATGAGAATCGAAGATCTGAAGAGAGAGAGTGGATGGGTGAAAAGTATTT

RI-AT1G28960-XLOC_005651-5870-0
GATCCATTACTTTGACTACAGAACAGGAGATAAGGATTATATGATATGGG
RI-AT1G28960-XLOC_005651-5870-1
GATCCATTACTTTGACTACAGAACAGGAGATAAGGATTATATGATATGGG
CONSENSUS
GATCCATTACTTTGACTACAGAACAGGAGATAAGGATTATATGATATGGG

RI-AT1G28960-XLOC_005651-5870-0
GTTTAACTGCTGGGATTTTGATCAGAGCTGCATCTGTGACTTATGAAAGA
RI-AT1G28960-XLOC_005651-5870-1
GTTTAACTGCTGGGATTTTGATCAGAGCTGCATCTGTGACTTATGAAAGA
CONSENSUS
GTTTAACTGCTGGGATTTTGATCAGAGCTGCATCTGTGACTTATGAAAGA

RI-AT1G28960-XLOC_005651-5870-0
CCACCTGCTTTTATCGAGCAGTGCCCGAAGTTTAAGTACCCTAAAATGGT
RI-AT1G28960-XLOC_005651-5870-1
CCACCTGCTTTTATCGAGCAGTGCCCGAAGTTTAAGTACCCTAAAATG--
CONSENSUS
CCACCTGCTTTTATCGAGCAGTGCCCGAAGTTTAAGTACCCTAAAATG..

RI-AT1G28960-XLOC_005651-5870-0
AGAAAAACATACTTGTATGCCTTAATGAACATAATTTTTCGAATGATGCT
RI-AT1G28960-XLOC_005651-5870-1

CONSENSUS
....

RI-AT1G28960-XLOC_005651-5870-0
TTCCTAGACTAGTCTTCTTCCACTATTTGATAACTTGTACCAGACAACAA
RI-AT1G28960-XLOC_005651-5870-1

CONSENSUS
....

RI-AT1G28960-XLOC_005651-5870-0
GACAATAACATTCCATACAACTGATTCTCTGTACCAGTGAATGGCTGAAT
RI-AT1G28960-XLOC_005651-5870-1
-------------------------------------TGAATGGCTGAAT
CONSENSUS
.....................................TGAATGGCTGAAT

RI-AT1G28960-XLOC_005651-5870-0
GCAGAACTTGGCTACTTGCTTATATATATGCAGACGCAGTAGCTGAAGCT
RI-AT1G28960-XLOC_005651-5870-1
GCAGAACTTGGCTACTTGCTTATATATATGCAGACGCAGTAGCTGAAGCT
CONSENSUS
GCAGAACTTGGCTACTTGCTTATATATATGCAGACGCAGTAGCTGAAGCT

RI-AT1G28960-XLOC_005651-5870-0
CCAATTCATTTCCTGCTGGGGCATCTCATAACAGGTTTCCATGACTCTTC
RI-AT1G28960-XLOC_005651-5870-1
CCAATTCATTTCCTGCTGGGGCATCTCATAACAGGTTTCCATGACTCTTC
CONSENSUS
CCAATTCATTTCCTGCTGGGGCATCTCATAACAGGTTTCCATGACTCTTC

RI-AT1G28960-XLOC_005651-5870-0
TGTTTGTCATTAGATAATTGGAGGGGCTTCTATTTCATTATGATCTAG
RI-AT1G28960-XLOC_005651-5870-1
TGTTTGTCATTAGATAATTGGAGGGGCTTCTATTTCATTATGATCTAG
CONSENSUS
TGTTTGTCATTAGATAATTGGAGGGGCTTCTATTTCATTATGATCTAG

alignment for event: A5-AT1G16710-XLOC_000858-9986

A5-AT1G16710-XLOC_000858-9986-0
      GAGGATTACATGAACCGGTCAACCCTTGAATCTCGTATTACAAGCCTAAT
A5-AT1G16710-XLOC_000858-9986-1
      GAGGATTACATGAACCGGTCAACCCTTGAATCTCGTATTACAAGCCTAAT
CONSENSUS
      GAGGATTACATGAACCGGTCAACCCTTGAATCTCGTATTACAAGCCTAAT

A5-AT1G16710-XLOC_000858-9986-0
      AAAAGGAAGACAGATAAATAACTACAATCAGCGACATGCTAATTCTTCTT
A5-AT1G16710-XLOC_000858-9986-1
      AAAAGGAAGACAGATAAATAACTACAATCAGCGACATGCTAATTCTTCTT
CONSENSUS
      AAAAGGAAGACAGATAAATAACTACAATCAGCGACATGCTAATTCTTCTT

A5-AT1G16710-XLOC_000858-9986-0
      CAGTTGGAACGATGATACCTACTCCAGGATTATCACAGACTGCGG-----
A5-AT1G16710-XLOC_000858-9986-1
      CAGTTGGAACGATGATACCTACTCCAGGATTATCACAGACTGCGGGTAAT
CONSENSUS
      CAGTTGGAACGATGATACCTACTCCAGGATTATCACAGACTGCGG.....

A5-AT1G16710-XLOC_000858-9986-0

A5-AT1G16710-XLOC_000858-9986-1
      CCTAATTTGATGGTTACATCCTCTGTTGATGCTACTATAGTTGGCAACAC
CONSENSUS
      ....

A5-AT1G16710-XLOC_000858-9986-0

A5-AT1G16710-XLOC_000858-9986-1
      CAACATCACAAGCACTGCTTTGAACACTGGAAACCCTCTAATTGCTGGTG
CONSENSUS
      ....

A5-AT1G16710-XLOC_000858-9986-0 ------------
GTAATATGTCTAATGGATATCAGCACTCGTCGAGAAAC
A5-AT1G16710-XLOC_000858-9986-1
      GCATGCATGGAGGTAATATGTCTAATGGATATCAGCACTCGTCGAGAAAC
CONSENSUS
      ............GTAATATGTCTAATGGATATCAGCACTCGTCGAGAAAC

A5-AT1G16710-XLOC_000858-9986-0
      TTTTCTCTTGGTTCTGGAGGGAGCATGACATCCATGGGTGCTCAAAGAAG
A5-AT1G16710-XLOC_000858-9986-1
      TTTTCTCTTGGTTCTGGAGGGAGCATGACATCCATGGGTGCTCAAAGAAG
CONSENSUS
      TTTTCTCTTGGTTCTGGAGGGAGCATGACATCCATGGGTGCTCAAAGAAG

A5-AT1G16710-XLOC_000858-9986-0
      TACAGCCCAAATGATTCCTACACCTGGCTTCGTTAACAGTGTTACTAACA

A5-AT1G16710-XLOC_000858-9986-1
TACAGCCCAAATGATTCCTACACCTGGCTTCGTTAACAGTGTTACTAACA
CONSENSUS
TACAGCCCAAATGATTCCTACACCTGGCTTCGTTAACAGTGTTACTAACA

A5-AT1G16710-XLOC_000858-9986-0
ACAACAGTGGTGGATTTTCTGCTGAGCCCACAATTGTTCCTCAGTCTCAG
A5-AT1G16710-XLOC_000858-9986-1
ACAACAGTGGTGGATTTTCTGCTGAGCCCACAATTGTTCCTCAGTCTCAG
CONSENSUS
ACAACAGTGGTGGATTTTCTGCTGAGCCCACAATTGTTCCTCAGTCTCAG

A5-AT1G16710-XLOC_000858-9986-0
CAACAACAACAAAGGCAACATACTGGTGGTCAAAACAGTCACATGTTGTC
A5-AT1G16710-XLOC_000858-9986-1
CAACAACAACAAAGGCAACATACTGGTGGTCAAAACAGTCACATGTTGTC
CONSENSUS
CAACAACAACAAAGGCAACATACTGGTGGTCAAAACAGTCACATGTTGTC

A5-AT1G16710-XLOC_000858-9986-0
CAACCATATGGCTGCTGGCGTTAGACCCGACATGCAATCAAAGCCGTCGG
A5-AT1G16710-XLOC_000858-9986-1
CAACCATATGGCTGCTGGCGTTAGACCCGACATGCAATCAAAGCCGTCGG
CONSENSUS
CAACCATATGGCTGCTGGCGTTAGACCCGACATGCAATCAAAGCCGTCGG

A5-AT1G16710-XLOC_000858-9986-0
GTGCGGCCAACAGCTCTGTAAATGGTGATGTTGGAGCGAACGAAAAAATT
A5-AT1G16710-XLOC_000858-9986-1
GTGCGGCCAACAGCTCTGTAAATGGTGATGTTGGAGCGAACGAAAAAATT
CONSENSUS
GTGCGGCCAACAGCTCTGTAAATGGTGATGTTGGAGCGAACGAAAAAATT

A5-AT1G16710-XLOC_000858-9986-0
GTAGACTCAGGTTCATCTTACACCAATGCGTCCAAAAAATTACAGCAGGG
A5-AT1G16710-XLOC_000858-9986-1
GTAGACTCAGGTTCATCTTACACCAATGCGTCCAAAAAATTACAGCAGGG
CONSENSUS
GTAGACTCAGGTTCATCTTACACCAATGCGTCCAAAAAATTACAGCAGGG

A5-AT1G16710-XLOC_000858-9986-0
TAACTTCTCTCTTCTTTCCTTCTGTCCGGATGATCTCATTTCAGGTCAGC
A5-AT1G16710-XLOC_000858-9986-1
TAACTTCTCTCTTCTTTCCTTCTGTCCGGATGATCTCATTTCAGGTCAGC
CONSENSUS
TAACTTCTCTCTTCTTTCCTTCTGTCCGGATGATCTCATTTCAGGTCAGC

A5-AT1G16710-XLOC_000858-9986-0
ATATTGAAAGCACGTTTCATATTTCAGGGGAAGGTTATAGCACAACAAAT
A5-AT1G16710-XLOC_000858-9986-1
ATATTGAAAGCACGTTTCATATTTCAGGGGAAGGTTATAGCACAACAAAT
CONSENSUS
ATATTGAAAGCACGTTTCATATTTCAGGGGAAGGTTATAGCACAACAAAT

A5-AT1G16710-XLOC_000858-9986-0
CCTGACCCCTTCGATGGAGCTATAACATCGGCTGGGACAGGGACAAAGGC

A5-AT1G16710-XLOC_000858-9986-1
      CCTGACCCCTTCGATGGAGCTATAACATCGGCTGGGACAGGGACAAAGGC
CONSENSUS
      CCTGACCCCTTCGATGGAGCTATAACATCGGCTGGGACAGGGACAAAGGC

A5-AT1G16710-XLOC_000858-9986-0
      TCATAATATCAATACAGCAAGTTTCCAGCCTGTGTCCAGAGTTAACTCTT
A5-AT1G16710-XLOC_000858-9986-1
      TCATAATATCAATACAGCAAGTTTCCAGCCTGTGTCCAGAGTTAACTCTT
CONSENSUS
      TCATAATATCAATACAGCAAGTTTCCAGCCTGTGTCCAGAGTTAACTCTT

A5-AT1G16710-XLOC_000858-9986-0 CTCTG
A5-AT1G16710-XLOC_000858-9986-1 CTCTG
CONSENSUS   CTCTG

alignment for event: SE-AT1G59218-XLOC_003003-8781

SE-AT1G59218-XLOC_003003-8781-0
      GTCGGAAGATAGTTGCAGGTAGGAAGCTAATCAAACAAGACACCTTATTT
SE-AT1G59218-XLOC_003003-8781-1
      GTCGGAAGATAGTTGCAGGTAGGAAGCTAATCAAACAAGACACCTTATTT
CONSENSUS
      GTCGGAAGATAGTTGCAGGTAGGAAGCTAATCAAACAAGACACCTTATTT

SE-AT1G59218-XLOC_003003-8781-0
      TTCACGACGAGATGTTGAACCCCTGAAGAATGTTGGACACCATTTTGATG
SE-AT1G59218-XLOC_003003-8781-1
      TTCACGACGAGATGTTGAACCCCTGAAGAATGTTGGACACCATTTTGATG
CONSENSUS
      TTCACGACGAGATGTTGAACCCCTGAAGAATGTTGGACACCATTTTGATG

SE-AT1G59218-XLOC_003003-8781-0
      GATAAAACATTTTCATAGGAGAAATGAT----------------------
SE-AT1G59218-XLOC_003003-8781-1
      GATAAAACATTTTCATAGGAGAAATGATAGAGTCGAGGTGAAAAGGTGTC
CONSENSUS
      GATAAAACATTTTCATAGGAGAAATGAT......................

SE-AT1G59218-XLOC_003003-8781-0

SE-AT1G59218-XLOC_003003-8781-1
      TAGCTTTTGAAGCTTTGCTGTGTACTAAGTGGACAACTTTCTTATTCTCT
CONSENSUS
      ....

SE-AT1G59218-XLOC_003003-8781-0 -----------
ACTTTATGGGAGTGTTGCGGCGAAGCTACTGAACCTTTT
SE-AT1G59218-XLOC_003003-8781-1
      ATCTGTTTTTGACTTTATGGGAGTGTTGCGGCGAAGCTACTGAACCTTTT
CONSENSUS
      ...........ACTTTATGGGAGTGTTGCGGCGAAGCTACTGAACCTTTT

SE-AT1G59218-XLOC_003003-8781-0

TGAAAGCGGTGTTGCCTATCTTTAAGAGCGTCGGAGGTGATATAACTGGG
SE-AT1G59218-XLOC_003003-8781-1
TGAAAGCGGTGTTGCCTATCTTTAAGAGCGTCGGAGGTGATATAACTGGG
CONSENSUS
TGAAAGCGGTGTTGCCTATCTTTAAGAGCGTCGGAGGTGATATAACTGGG

SE-AT1G59218-XLOC_003003-8781-0
AGACTCTTGATGAGATGTTGGCCTATCTGGAATACTTCCCAGTTGGGAGA
SE-AT1G59218-XLOC_003003-8781-1
AGACTCTTGATGAGATGTTGGCCTATCTGGAATACTTCCCAGTTGGGAGA
CONSENSUS
AGACTCTTGATGAGATGTTGGCCTATCTGGAATACTTCCCAGTTGGGAGA

SE-AT1G59218-XLOC_003003-8781-0
CCAACGGAGATGAATACCGTCAACAATTCACCAACGACCCATCTCAAGAG
SE-AT1G59218-XLOC_003003-8781-1
CCAACGGAGATGAATACCGTCAACAATTCACCAACGACCCATCTCAAGAG
CONSENSUS
CCAACGGAGATGAATACCGTCAACAATTCACCAACGACCCATCTCAAGAG

SE-AT1G59218-XLOC_003003-8781-0
CTTGGACACGAGGGAGAAGGTTGGTGCCTGGTTCAAACAGTGGTGCATCC
SE-AT1G59218-XLOC_003003-8781-1
CTTGGACACGAGGGAGAAGGTTGGTGCCTGGTTCAAACAGTGGTGCATCC
CONSENSUS
CTTGGACACGAGGGAGAAGGTTGGTGCCTGGTTCAAACAGTGGTGCATCC

SE-AT1G59218-XLOC_003003-8781-0
AAAACGCAGGCCGTGTAGCATTTTGTTTCGGGTTCTACATGGCCATGGCG
SE-AT1G59218-XLOC_003003-8781-1
AAAACGCAGGCCGTGTAGCATTTTGTTTCGGGTTCTACATGGCCATGGCG
CONSENSUS
AAAACGCAGGCCGTGTAGCATTTTGTTTCGGGTTCTACATGGCCATGGCG

SE-AT1G59218-XLOC_003003-8781-0
AAGAAGGGTGACCAGTTGGAAGGTGCATACACCCTAAATACTATAAAGGT
SE-AT1G59218-XLOC_003003-8781-1
AAGAAGGGTGACCAGTTGGAAGGTGCATACACCCTAAATACTATAAAGGT
CONSENSUS
AAGAAGGGTGACCAGTTGGAAGGTGCATACACCCTAAATACTATAAAGGT

SE-AT1G59218-XLOC_003003-8781-0
TTATAGAAGTGATTGATTTATTCCATTCAACGATTTACAATATATATACA
SE-AT1G59218-XLOC_003003-8781-1
TTATAGAAGTGATTGATTTATTCCATTCAACGATTTACAATATATATACA
CONSENSUS
TTATAGAAGTGATTGATTTATTCCATTCAACGATTTACAATATATATACA

SE-AT1G59218-XLOC_003003-8781-0 CAAGAGAGACTTGCTGAGC
SE-AT1G59218-XLOC_003003-8781-1 CAAGAGAGACTTGCTGAGC
CONSENSUS   CAAGAGAGACTTGCTGAGC

alignment for event: A3-AT1G57870-XLOC_006911-9031

A3-AT1G57870-XLOC_006911-9031-0
      TGATTGATCAAGGGAACATAAGCAGCTAGAGACCACACCACTCAGTTATT
A3-AT1G57870-XLOC_006911-9031-1
      TGATTGATCAAGGGAACATAAGCAGCTAGAGACCACACCACTCAGTTATT
CONSENSUS
      TGATTGATCAAGGGAACATAAGCAGCTAGAGACCACACCACTCAGTTATT

A3-AT1G57870-XLOC_006911-9031-0
      TCTATCTCATAGTGCTTGTGGATGTTCAAGCTATAAGAATGTGCTCGGTG
A3-AT1G57870-XLOC_006911-9031-1
      TCTATCTCATAGTGCTTGTGGATGTTCAAGCTATAAGAATGTGCTCGGTG
CONSENSUS
      TCTATCTCATAGTGCTTGTGGATGTTCAAGCTATAAGAATGTGCTCGGTG

A3-AT1G57870-XLOC_006911-9031-0 GTTCTATGAATATATAG----
ATTGGTGTTTTGATGGGATGGGACACCAA
A3-AT1G57870-XLOC_006911-9031-1
      GTTCTATGAATATATAGGAAGATTGGTGTTTTGATGGGATGGGACACCAA
CONSENSUS
      GTTCTATGAATATATAG....ATTGGTGTTTTGATGGGATGGGACACCAA

A3-AT1G57870-XLOC_006911-9031-0
      ATTGCTAAGGTTTATGGTGTTATCAATTACTAGTACGTGTTATCTTCCGA
A3-AT1G57870-XLOC_006911-9031-1
      ATTGCTAAGGTTTATGGTGTTATCAATTACTAGTACGTGTTATCTTCCGA
CONSENSUS
      ATTGCTAAGGTTTATGGTGTTATCAATTACTAGTACGTGTTATCTTCCGA

A3-AT1G57870-XLOC_006911-9031-0
      ACAACCACCACCATGGAATCTCATCTGGGAAATGGAGTAGGCAGTTCGAG
A3-AT1G57870-XLOC_006911-9031-1
      ACAACCACCACCATGGAATCTCATCTGGGAAATGGAGTAGGCAGTTCGAG
CONSENSUS
      ACAACCACCACCATGGAATCTCATCTGGGAAATGGAGTAGGCAGTTCGAG

A3-AT1G57870-XLOC_006911-9031-0
      ATCTGCCAAAAATACAAAGAACACTTCTAGTTCAGTTGATTGGTTGAGCA
A3-AT1G57870-XLOC_006911-9031-1
      ATCTGCCAAAAATACAAAGAACACTTCTAGTTCAGTTGATTGGTTGAGCA
CONSENSUS
      ATCTGCCAAAAATACAAAGAACACTTCTAGTTCAGTTGATTGGTTGAGCA

A3-AT1G57870-XLOC_006911-9031-0
      GAGATATGCTTGAGATGAAGATAAGGGACAAGACAGAGGCTGATGAGGAG
A3-AT1G57870-XLOC_006911-9031-1
      GAGATATGCTTGAGATGAAGATAAGGGACAAGACAGAGGCTGATGAGGAG
CONSENSUS
      GAGATATGCTTGAGATGAAGATAAGGGACAAGACAGAGGCTGATGAGGAG

A3-AT1G57870-XLOC_006911-9031-0 AGA
A3-AT1G57870-XLOC_006911-9031-1 AGA
CONSENSUS   AGA

alignment for event: A5-AT1G03160-XLOC_000119-13744

A5-AT1G03160-XLOC_000119-13744-0
GTTGCGTTTCTCCGGTACACACAGCAGTGGAAAAAGAAATTTGTGTTTAT
A5-AT1G03160-XLOC_000119-13744-1
GTTGCGTTTCTCCGGTACACACAGCAGTGGAAAAAGAAATTTGTGTTTAT
CONSENSUS
GTTGCGTTTCTCCGGTACACACAGCAGTGGAAAAAGAAATTTGTGTTTAT

A5-AT1G03160-XLOC_000119-13744-0
TCTGAATAAATCTGATATCTATCGTGATGCTC-----CTTGAGGAAGCTA
A5-AT1G03160-XLOC_000119-13744-1
TCTGAATAAATCTGATATCTATCGTGATGCTCGTGAGCTTGAGGAAGCTA
CONSENSUS
TCTGAATAAATCTGATATCTATCGTGATGCTC.....CTTGAGGAAGCTA

A5-AT1G03160-XLOC_000119-13744-0
TTTCATTTGTTAAAGAGAATACACGGAAGTTGCTTAATACAGAAAATGTG
A5-AT1G03160-XLOC_000119-13744-1
TTTCATTTGTTAAAGAGAATACACGGAAGTTGCTTAATACAGAAAATGTG
CONSENSUS
TTTCATTTGTTAAAGAGAATACACGGAAGTTGCTTAATACAGAAAATGTG

A5-AT1G03160-XLOC_000119-13744-0
ATATTGTATCCGGTGTCCGCACGGTCTGCTCTTGAGGCGAAGCTTTCAAC
A5-AT1G03160-XLOC_000119-13744-1
ATATTGTATCCGGTGTCCGCACGGTCTGCTCTTGAGGCGAAGCTTTCAAC
CONSENSUS
ATATTGTATCCGGTGTCCGCACGGTCTGCTCTTGAGGCGAAGCTTTCAAC

A5-AT1G03160-XLOC_000119-13744-0
AGCTTCTTTGGTTGGCAGAGATGATCTTGAGATCGCAGATCCTGGTTCTA
A5-AT1G03160-XLOC_000119-13744-1
AGCTTCTTTGGTTGGCAGAGATGATCTTGAGATCGCAGATCCTGGTTCTA
CONSENSUS
AGCTTCTTTGGTTGGCAGAGATGATCTTGAGATCGCAGATCCTGGTTCTA

A5-AT1G03160-XLOC_000119-13744-0
ATTGGAGAGTCCAGAGCTTCAATGAACTTGAGAAATTTCTTTATAGCTTC
A5-AT1G03160-XLOC_000119-13744-1
ATTGGAGAGTCCAGAGCTTCAATGAACTTGAGAAATTTCTTTATAGCTTC
CONSENSUS
ATTGGAGAGTCCAGAGCTTCAATGAACTTGAGAAATTTCTTTATAGCTTC

A5-AT1G03160-XLOC_000119-13744-0
TTGGATAGCTCAACAGCTACCGGGATGGAGAGAATAAGGCTTAAATTGGA
A5-AT1G03160-XLOC_000119-13744-1
TTGGATAGCTCAACAGCTACCGGGATGGAGAGAATAAGGCTTAAATTGGA
CONSENSUS
TTGGATAGCTCAACAGCTACCGGGATGGAGAGAATAAGGCTTAAATTGGA

A5-AT1G03160-XLOC_000119-13744-0
GACACCCATGGCGATTGCTGAGCGTCTCCTTTCTTCTGTGGAAGCTCTTG
A5-AT1G03160-XLOC_000119-13744-1
GACACCCATGGCGATTGCTGAGCGTCTCCTTTCTTCTGTGGAAGCTCTTG
CONSENSUS
GACACCCATGGCGATTGCTGAGCGTCTCCTTTCTTCTGTGGAAGCTCTTG

A5-AT1G03160-XLOC_000119-13744-0
TGAGACAAGATTGCCTAGCTGCTAGGGAAGACTTGGCTTCAGCAGACAAG
A5-AT1G03160-XLOC_000119-13744-1
TGAGACAAGATTGCCTAGCTGCTAGGGAAGACTTGGCTTCAGCAGACAAG
CONSENSUS
TGAGACAAGATTGCCTAGCTGCTAGGGAAGACTTGGCTTCAGCAGACAAG

A5-AT1G03160-XLOC_000119-13744-0
ATTATCAGTCGAACTAAAGAATACGCGCTTAAGATGGAATATGAGAGCAT
A5-AT1G03160-XLOC_000119-13744-1
ATTATCAGTCGAACTAAAGAATACGCGCTTAAGATGGAATATGAGAGCAT
CONSENSUS
ATTATCAGTCGAACTAAAGAATACGCGCTTAAGATGGAATATGAGAGCAT

A5-AT1G03160-XLOC_000119-13744-0 TTCTTGGAGAAGGCAGGCTCTCTCGTTG
A5-AT1G03160-XLOC_000119-13744-1 TTCTTGGAGAAGGCAGGCTCTCTCGTTG
CONSENSUS TTCTTGGAGAAGGCAGGCTCTCTCGTTG

alignment for event: SE-AT1G45249-XLOC_006343-6997

SE-AT1G45249-XLOC_006343-6997-0
ATCCAGTTATTAGGTGGAAGCAGATTTTGTAGAAAAATGGATGGTAGTAT
SE-AT1G45249-XLOC_006343-6997-1
ATCCAGTTATTAGGTGGAAGCAGATTTTGTAGAAAAATGGATGGTAGTAT
CONSENSUS
ATCCAGTTATTAGGTGGAAGCAGATTTTGTAGAAAAATGGATGGTAGTAT

SE-AT1G45249-XLOC_006343-6997-0
GAATTTGGGGAATGAGCCACCAGGAGATGGTGGTGGAGGTGGAGGGTTGA
SE-AT1G45249-XLOC_006343-6997-1
GAATTTGGGGAATGAGCCACCAGGAGATGGTGGTGGAGGTGGAGGGTTGA
CONSENSUS
GAATTTGGGGAATGAGCCACCAGGAGATGGTGGTGGAGGTGGAGGGTTGA

SE-AT1G45249-XLOC_006343-6997-0
CTAGACAAGGTTCGATATACTCGTTGACGTTTGATGAGTTTCAGAGCAGT
SE-AT1G45249-XLOC_006343-6997-1
CTAGACAAGGTTCGATATACTCGTTGACGTTTGATGAGTTTCAGAGCAGT
CONSENSUS
CTAGACAAGGTTCGATATACTCGTTGACGTTTGATGAGTTTCAGAGCAGT

SE-AT1G45249-XLOC_006343-6997-0
GTAGGGAAAGATTTTGGGTCAATGAACATGGATGAGTTGTTAAAGAATAT
SE-AT1G45249-XLOC_006343-6997-1
GTAGGGAAAGATTTTGGGTCAATGAACATGGATGAGTTGTTAAAGAATAT
CONSENSUS
GTAGGGAAAGATTTTGGGTCAATGAACATGGATGAGTTGTTAAAGAATAT

SE-AT1G45249-XLOC_006343-6997-0
ATGGAGTGCTGAAGAAACACAAGCCATGGCTAGTGGTGTGGTTCCAGTTC
SE-AT1G45249-XLOC_006343-6997-1
ATGGAGTGCTGAAGAAACACAAGCCATGGCTAGTGGTGTGGTTCCAGTTC
CONSENSUS

ATGGAGTGCTGAAGAAACACAAGCCATGGCTAGTGGTGTGGTTCCAGTTC

SE-AT1G45249-XLOC_006343-6997-0
TTGGTGGAGGTCAAGAGGGTTTGCAGCTGCAGAGGCAAGGCTCGTTGACT
SE-AT1G45249-XLOC_006343-6997-1
TTGGTGGAGGTCAAGAGGGTTTGCAGCTGCAGAGGCAAGGCTCGTTGACT
CONSENSUS
TTGGTGGAGGTCAAGAGGGTTTGCAGCTGCAGAGGCAAGGCTCGTTGACT

SE-AT1G45249-XLOC_006343-6997-0
CTGCCTCGAACGCTTAGTCAGAAGACGGTTGATCAAGTTTGGAAAGATCT
SE-AT1G45249-XLOC_006343-6997-1
CTGCCTCGAACGCTTAGTCAGAAGACGGTTGATCAAGTTTGGAAAGATCT
CONSENSUS
CTGCCTCGAACGCTTAGTCAGAAGACGGTTGATCAAGTTTGGAAAGATCT

SE-AT1G45249-XLOC_006343-6997-0
ATCCAAAGTTGGAAGTAGTGGAGTAGGGGGAAGTAACTTGTCTCAGGTGG
SE-AT1G45249-XLOC_006343-6997-1
ATCCAAAGTTGGAAGTAGTGGAGTAGGGGGAAGTAACTTGTCTCAGGTGG
CONSENSUS
ATCCAAAGTTGGAAGTAGTGGAGTAGGGGGAAGTAACTTGTCTCAGGTGG

SE-AT1G45249-XLOC_006343-6997-0
CTCAGGCTCAGAGTCAGAGTCAGAGTCAGAGGCAGCAAACATTAGGTGAA
SE-AT1G45249-XLOC_006343-6997-1
CTCAGGCTCAGAGTCAGAGTCAGAGTCAGAGGCAGCAAACATTAGGTGAA
CONSENSUS
CTCAGGCTCAGAGTCAGAGTCAGAGTCAGAGGCAGCAAACATTAGGTGAA

SE-AT1G45249-XLOC_006343-6997-0
GTAACTTTGGAGGAGTTTTTGGTTCGTGCTGGTGTTGTGAGAGAGGAAGC
SE-AT1G45249-XLOC_006343-6997-1
GTAACTTTGGAGGAGTTTTTGGTTCGTGCTGGTGTTGTGAGAGAGGAAGC
CONSENSUS
GTAACTTTGGAGGAGTTTTTGGTTCGTGCTGGTGTTGTGAGAGAGGAAGC

SE-AT1G45249-XLOC_006343-6997-0
TCAGGTTGCTGCAAGAGCTCAGATTGCTGAGAACAATAAAGGCGGTTACT
SE-AT1G45249-XLOC_006343-6997-1
TCAGGTTGCTGCAAGAGCTCAGATTGCTGAGAACAATAAAGGCGGTTACT
CONSENSUS
TCAGGTTGCTGCAAGAGCTCAGATTGCTGAGAACAATAAAGGCGGTTACT

SE-AT1G45249-XLOC_006343-6997-0
TTGGTAATGATGCCAACACAGGTTTCTCTGTCGAGTTTCAGCAGCCTTCT
SE-AT1G45249-XLOC_006343-6997-1
TTGGTAATGATGCCAACACAGGTTTCTCTGTCGAGTTTCAGCAGCCTTCT
CONSENSUS
TTGGTAATGATGCCAACACAGGTTTCTCTGTCGAGTTTCAGCAGCCTTCT

SE-AT1G45249-XLOC_006343-6997-0
CCACGAGTTGTTGCCGCTGGTGTAATGGGAAATCTTGGTGCAGAGACTGC
SE-AT1G45249-XLOC_006343-6997-1
CCACGAGTTGTTGCCGCTGGTGTAATGGGAAATCTTGGTGCAGAGACTGC
CONSENSUS

CCACGAGTTGTTGCCGCTGGTGTAATGGGAAATCTTGGTGCAGAGACTGC

SE-AT1G45249-XLOC_006343-6997-0
AAATTCTTTGCAGGTTCAAGGTTCTAGTTTGCCTCTGAATGTGAATGGAG
SE-AT1G45249-XLOC_006343-6997-1
AAATTCTTTGCAGGTTCAAGGTTCTAGTTTGCCTCTGAATGTGAATGGAG
CONSENSUS
AAATTCTTTGCAGGTTCAAGGTTCTAGTTTGCCTCTGAATGTGAATGGAG

SE-AT1G45249-XLOC_006343-6997-0
CTAGAACAACATACCAGCAATCGCAACAGCAACAGCCAATCATGCCTAAG
SE-AT1G45249-XLOC_006343-6997-1
CTAGAACAACATACCAGCAATCGCAACAGCAACAGCCAATCATGCCTAAG
CONSENSUS
CTAGAACAACATACCAGCAATCGCAACAGCAACAGCCAATCATGCCTAAG

SE-AT1G45249-XLOC_006343-6997-0
CAGCCTGGTTTTGGTTATGGAACACAAATGGGTCAGCTTAATAGTCCTGG
SE-AT1G45249-XLOC_006343-6997-1
CAGCCTGGTTTTGGTTATGGAACACAAATGGGTCAGCTTAATAGTCCTGG
CONSENSUS
CAGCCTGGTTTTGGTTATGGAACACAAATGGGTCAGCTTAATAGTCCTGG

SE-AT1G45249-XLOC_006343-6997-0
GATAAGAGGTGGTGGTCTTGTGGGACTTGGAGATCAGTCTTTAACGAACA
SE-AT1G45249-XLOC_006343-6997-1
GATAAGAGGTGGTGGTCTTGTGGGACTTGGAGATCAGTCTTTAACGAACA
CONSENSUS
GATAAGAGGTGGTGGTCTTGTGGGACTTGGAGATCAGTCTTTAACGAACA

SE-AT1G45249-XLOC_006343-6997-0
ATGTGGGCTTTGTCCAAGGTGCTTCTGCTGCAATTCCTGGAGCTTTAGGC
SE-AT1G45249-XLOC_006343-6997-1
ATGTGGGCTTTGTCCAAGGTGCTTCTGCTGCAATTCCTGGAGCTTTAGGC
CONSENSUS
ATGTGGGCTTTGTCCAAGGTGCTTCTGCTGCAATTCCTGGAGCTTTAGGC

SE-AT1G45249-XLOC_006343-6997-0
GTTGGTGCTGTGTCGCCTGTTACGCCATTGTCATCAGAAGGGATAGGGAA
SE-AT1G45249-XLOC_006343-6997-1
GTTGGTGCTGTGTCGCCTGTTACGCCATTGTCATCAGAAGGGATAGGGAA
CONSENSUS
GTTGGTGCTGTGTCGCCTGTTACGCCATTGTCATCAGAAGGGATAGGGAA

SE-AT1G45249-XLOC_006343-6997-0
GAGTAATGGTGATTCTTCATCACTCTCTCCGTCTCCTTACATGTTTAATG
SE-AT1G45249-XLOC_006343-6997-1
GAGTAATGGTGATTCTTCATCACTCTCTCCGTCTCCTTACATGTTTAATG
CONSENSUS
GAGTAATGGTGATTCTTCATCACTCTCTCCGTCTCCTTACATGTTTAATG

SE-AT1G45249-XLOC_006343-6997-0
GTGGTGTGAGAGGTAGAAAGAGTGGCACTGTGGAGAAAGTTGTAGAGAGA
SE-AT1G45249-XLOC_006343-6997-1
GTGGTGTGAGAGGTAGAAAGAGTGGCACTGTGGAGAAAGTTGTAGAGAGA
CONSENSUS

GTGGTGTGAGAGGTAGAAAGAGTGGCACTGTGGAGAAAGTTGTAGAGAGA

SE-AT1G45249-XLOC_006343-6997-0
AGGCAAAGGAGAATGATAAAGAACCGAGAATCAGCTGCAAGGTCCCGGGC
SE-AT1G45249-XLOC_006343-6997-1
AGGCAAAGGAGAATGATAAAGAACCGAGAATCAGCTGCAAGGTCCCGGGC
CONSENSUS
AGGCAAAGGAGAATGATAAAGAACCGAGAATCAGCTGCAAGGTCCCGGGC

SE-AT1G45249-XLOC_006343-6997-0
CAGGAAACAG--
SE-AT1G45249-XLOC_006343-6997-1
CAGGAAACAGATATTCACGGCAGACCAAACAATATACTGGAATGTAATTT
CONSENSUS
CAGGAAACAG..

SE-AT1G45249-XLOC_006343-6997-0

SE-AT1G45249-XLOC_006343-6997-1
GCTAATGTCATGAGGTGGCTCAGAGTTTAAATGAGTAGTGAGGGTTCATC
CONSENSUS
....

SE-AT1G45249-XLOC_006343-6997-0
---GCTTA
SE-AT1G45249-XLOC_006343-6997-1
GTCAAAACCTGAGTAGTGGCGACATGGTTTTTCTTTTGGGGCAAGGCTTA
CONSENSUS
....GCTTA

SE-AT1G45249-XLOC_006343-6997-0
CACCGTGGAGCTTGAAGCTGAAGTTGCAAAGTTAAAGGAAGAGAATGACG
SE-AT1G45249-XLOC_006343-6997-1
CACCGTGGAGCTTGAAGCTGAAGTTGCAAAGTTAAAGGAAGAGAATGACG
CONSENSUS
CACCGTGGAGCTTGAAGCTGAAGTTGCAAAGTTAAAGGAAGAGAATGACG

SE-AT1G45249-XLOC_006343-6997-0 AGTTACAACGAAAGCAG
SE-AT1G45249-XLOC_006343-6997-1 AGTTACAACGAAAGCAG
CONSENSUS AGTTACAACGAAAGCAG

alignment for event: RI-AT1G16825-XLOC_000865-2197

RI-AT1G16825-XLOC_000865-2197-0
GGTACCTGTGGATCCATTAGAGTGGCAAATATCACAAGACACAGCCTGTA
RI-AT1G16825-XLOC_000865-2197-1
GGTACCTGTGGATCCATTAGAGTGGCAAATATCACAAGACACAGCCTGTA
CONSENSUS
GGTACCTGTGGATCCATTAGAGTGGCAAATATCACAAGACACAGCCTGTA

RI-AT1G16825-XLOC_000865-2197-0
ACATTGTGGCGCGCTTAGCTAATACTGTTGGAGCAGCTGAATCCGTTCTG
RI-AT1G16825-XLOC_000865-2197-1
ACATTGTGGCGCGCTTAGCTAATACTGTTGGAGCAGCTGAATCCGTTCTG

CONSENSUS
ACATTGTGGCGCGCTTAGCTAATACTGTTGGAGCAGCTGAATCCGTTCTG

RI-AT1G16825-XLOC_000865-2197-0
CGGGTTGCAGCAACAGGACATGACAAGAGGCTCTTTGTTAAGGCAAGTGC
RI-AT1G16825-XLOC_000865-2197-1
CGGGTTGCAGCAACAGGACATGACAAGAGGCTCTTTGTTAAG--------
CONSENSUS
CGGGTTGCAGCAACAGGACATGACAAGAGGCTCTTTGTTAAG........

RI-AT1G16825-XLOC_000865-2197-0
ATTCTGGTTTCTTCCTTGTTTGCATTACAAGAGGTAGTTTCTTCGATCAC
RI-AT1G16825-XLOC_000865-2197-1

CONSENSUS
....

RI-AT1G16825-XLOC_000865-2197-0
CCGGTGCGGTGCCTGCATGAATAGGAAATGTGTATAACAACATTCAAGCA
RI-AT1G16825-XLOC_000865-2197-1

CONSENSUS
....

RI-AT1G16825-XLOC_000865-2197-0
TTTATTGATGAAGACAAAGATGGTTAAGACCATTGACTGAAATTCCACAG
RI-AT1G16825-XLOC_000865-2197-1

CONSENSUS
....

RI-AT1G16825-XLOC_000865-2197-0
AACCTGTCAGTTGTGGTTTTAGCCAATTACCACTCCGATGCAATGCAGTT
RI-AT1G16825-XLOC_000865-2197-1

CONSENSUS
....

RI-AT1G16825-XLOC_000865-2197-0
CTCTGGTTTTATGCTTGGTGGCAGAATTCTAAACTGAACTTTTTTTATCT
RI-AT1G16825-XLOC_000865-2197-1

CONSENSUS
....

RI-AT1G16825-XLOC_000865-2197-0
TCGCCTGTTTCTGTCTCTCAGGTTGTGATCTGTCTTTACTTCTTGGCAGC
RI-AT1G16825-XLOC_000865-2197-1 --------------------
GTTGTGATCTGTCTTTACTTCTTGGCAGC
CONSENSUS
.....................GTTGTGATCTGTCTTTACTTCTTGGCAGC

RI-AT1G16825-XLOC_000865-2197-0
TCTAGGACGAATCATATCGGGTGTGACCATTGCCTATGCAG
RI-AT1G16825-XLOC_000865-2197-1
TCTAGGACGAATCATATCGGGTGTGACCATTGCCTATGCAG

CONSENSUS
      TCTAGGACGAATCATATCGGGTGTGACCATTGCCTATGCAG

alignment for event: A3-AT1G02840-XLOC_000103-11645

A3-AT1G02840-XLOC_000103-11645-0
      GTTAGAGAATATGATTCAAGGAAGGATTCTAGGAGTCCTAGCCGGGGAAG
A3-AT1G02840-XLOC_000103-11645-1
      GTTAGAGAATATGATTCAAGGAAGGATTCTAGGAGTCCTAGCCGGGGAAG
CONSENSUS
      GTTAGAGAATATGATTCAAGGAAGGATTCTAGGAGTCCTAGCCGGGGAAG

A3-AT1G02840-XLOC_000103-11645-0
      ATCCTATTCTAAGAGCCGCAGCCGCAGCCGTGGACGAAGCGTGAGCCGAA
A3-AT1G02840-XLOC_000103-11645-1
      ATCCTATTCTAAGAGCCGCAGCCGCAGCCGTGGACGAAGCGTGAGCCGAA
CONSENSUS
      ATCCTATTCTAAGAGCCGCAGCCGCAGCCGTGGACGAAGCGTGAGCCGAA

A3-AT1G02840-XLOC_000103-11645-0
      GCAGGAGCAGAAGCAGGAGCAGGAGCAGAAGTCCCAAGGCAAAGTCTTCA
A3-AT1G02840-XLOC_000103-11645-1
      GCAGGAGCAGAAGCAGGAGCAGGAGCAGAAGTCCCAAGGCAAAGTCTTCA
CONSENSUS
      GCAGGAGCAGAAGCAGGAGCAGGAGCAGAAGTCCCAAGGCAAAGTCTTCA

A3-AT1G02840-XLOC_000103-11645-0
      CGTAGGTCCCCTGCAAAATCTACATCAAGATCTCCTGGCCCCCGCTCGAA
A3-AT1G02840-XLOC_000103-11645-1
      CGTAGGTCCCCTGCAAAATCTACATCAAGATCTCCTGGCCCCCGCTCGAA
CONSENSUS
      CGTAGGTCCCCTGCAAAATCTACATCAAGATCTCCTGGCCCCCGCTCGAA

A3-AT1G02840-XLOC_000103-11645-0
      GTCAAGGTCACCGTCTCCAAGAAGATGGATAACAGTGGAGACATTGGATC
A3-AT1G02840-XLOC_000103-11645-1
      GTCAAGGTCACCGTCTCCAAGAAG--------------------------
CONSENSUS
      GTCAAGGTCACCGTCTCCAAGAAG..........................

A3-AT1G02840-XLOC_000103-11645-0
      ACTTGGATCACAATATTATATCGGGATTTCTGTAAAACTATATTGGCTCG
A3-AT1G02840-XLOC_000103-11645-1

CONSENSUS
      ....

A3-AT1G02840-XLOC_000103-11645-0
      ATGGATTGACAATATGGAATCTGGGTTCTCTTGGGACGTCCGTGGCTCAT
A3-AT1G02840-XLOC_000103-11645-1

CONSENSUS
      ....

A3-AT1G02840-XLOC_000103-11645-0
TTGGCAACACAAGTTTTTTTGGCCACATGGCTTATAAAACCTCTGTCCTA
A3-AT1G02840-XLOC_000103-11645-1

CONSENSUS
....

A3-AT1G02840-XLOC_000103-11645-0
TCACCTATGTTTTAACTAAGTAGCAGAATAGTTTGGTTTATGTTTCTTTT
A3-AT1G02840-XLOC_000103-11645-1

CONSENSUS
....

A3-AT1G02840-XLOC_000103-11645-0
TTTTTATTTGTTGCAACTTCTTAATCTCTGTGAGATAGAAGGAGAGGCTC
A3-AT1G02840-XLOC_000103-11645-1

CONSENSUS
....

A3-AT1G02840-XLOC_000103-11645-0
CAGGACCTTGCTGAACAGTATAAAACACAACATGTTTGGATTTTTGAATC
A3-AT1G02840-XLOC_000103-11645-1

CONSENSUS
....

A3-AT1G02840-XLOC_000103-11645-0
TGAGTTTCTTTTCTTGGACTTTTGCAGATCGCGTTCAAGATCAAGATCTC
A3-AT1G02840-XLOC_000103-11645-1 ---------------------------
ATCGCGTTCAAGATCAAGATCTC
CONSENSUS
...........................ATCGCGTTCAAGATCAAGATCTC

A3-AT1G02840-XLOC_000103-11645-0 CTCTACCTTCT
A3-AT1G02840-XLOC_000103-11645-1 CTCTACCTTCT
CONSENSUS CTCTACCTTCT

alignment for event: SE-AT1G55310-XLOC_002795-1799

SE-AT1G55310-XLOC_002795-1799-0
GCAAGAAGATCTCAGGAAGTCGTTTGAGCAGTTTGGTCCTGTCAAGGACA
SE-AT1G55310-XLOC_002795-1799-1
GCAAGAAGATCTCAGGAAGTCGTTTGAGCAGTTTGGTCCTGTCAAGGACA
CONSENSUS
GCAAGAAGATCTCAGGAAGTCGTTTGAGCAGTTTGGTCCTGTCAAGGACA

SE-AT1G55310-XLOC_002795-1799-0
TTTACCTGCCAAGGGATTATTATACCGG----------------------
SE-AT1G55310-XLOC_002795-1799-1
TTTACCTGCCAAGGGATTATTATACCGGTGAATCTAAAGAATTGAAGACA
CONSENSUS
TTTACCTGCCAAGGGATTATTATACCGG......................

SE-AT1G55310-XLOC_002795-1799-0

SE-AT1G55310-XLOC_002795-1799-1
      TCAAAGAAGTAATTAGAGTTCTTATGAAGATGTTCTATATGGTAGTGAAG
CONSENSUS
      ....

SE-AT1G55310-XLOC_002795-1799-0

SE-AT1G55310-XLOC_002795-1799-1
      AATTGAAGTGAAGTTGAGTTTGTATTCTATGTGAAGATGAATCAAGTCTT
CONSENSUS
      ....

SE-AT1G55310-XLOC_002795-1799-0
      --AGATCCGCGA
SE-AT1G55310-XLOC_002795-1799-1
      CAAGAAGTCATCTTTGTACTGACACTTGCAAGGCTAGCAGAGATCCGCGA
CONSENSUS
      ....AGATCCGCGA

SE-AT1G55310-XLOC_002795-1799-0
      GGGTTTGGGTTCGTTCAATTTATGGACCCTGCTGATGCTGCTGATGCAAA
SE-AT1G55310-XLOC_002795-1799-1
      GGGTTTGGGTTCGTTCAATTTATGGACCCTGCTGATGCTGCTGATGCAAA
CONSENSUS
      GGGTTTGGGTTCGTTCAATTTATGGACCCTGCTGATGCTGCTGATGCAAA

SE-AT1G55310-XLOC_002795-1799-0
      ACATCACATGGATGGTTATCTTCTTCTTGGCCGTGAGTTGACTGTCGTGT
SE-AT1G55310-XLOC_002795-1799-1
      ACATCACATGGATGGTTATCTTCTTCTTGGCCGTGAGTTGACTGTCGTGT
CONSENSUS
      ACATCACATGGATGGTTATCTTCTTCTTGGCCGTGAGTTGACTGTCGTGT

SE-AT1G55310-XLOC_002795-1799-0
      TTGCAGAAGAGAACAGAAAGAAACCGACTGAAATGAGAGCAAGGGAGCGT
SE-AT1G55310-XLOC_002795-1799-1
      TTGCAGAAGAGAACAGAAAGAAACCGACTGAAATGAGAGCAAGGGAGCGT
CONSENSUS
      TTGCAGAAGAGAACAGAAAGAAACCGACTGAAATGAGAGCAAGGGAGCGT

SE-AT1G55310-XLOC_002795-1799-0 GGTGGAGGAAG
SE-AT1G55310-XLOC_002795-1799-1 GGTGGAGGAAG
CONSENSUS   GGTGGAGGAAG

alignment for event: RI-AT1G28960-XLOC_005651-5872

RI-AT1G28960-XLOC_005651-5872-0
      GATGAGAATCGAAGATCTGAAGAGAGAGAGTGGATGGGTGAAAAGTATTT
RI-AT1G28960-XLOC_005651-5872-1
      GATGAGAATCGAAGATCTGAAGAGAGAGAGTGGATGGGTGAAAAGTATTT
CONSENSUS

GATGAGAATCGAAGATCTGAAGAGAGAGAGTGGATGGGTGAAAAGTATTT

RI-AT1G28960-XLOC_005651-5872-0
GATCCATTACTTTGACTACAGAACAGGAGATAAGGATTATATGATATGGG
RI-AT1G28960-XLOC_005651-5872-1
GATCCATTACTTTGACTACAGAACAGGAGATAAGGATTATATGATATGGG
CONSENSUS
GATCCATTACTTTGACTACAGAACAGGAGATAAGGATTATATGATATGGG

RI-AT1G28960-XLOC_005651-5872-0
GTTTAACTGCTGGGATTTTGATCAGAGCTGCATCTGTGACTTATGAAAGA
RI-AT1G28960-XLOC_005651-5872-1
GTTTAACTGCTGGGATTTTGATCAGAGCTGCATCTGTGACTTATGAAAGA
CONSENSUS
GTTTAACTGCTGGGATTTTGATCAGAGCTGCATCTGTGACTTATGAAAGA

RI-AT1G28960-XLOC_005651-5872-0
CCACCTGCTTTTATCGAGCAGTGCCCGAAGTTTAAGTACCCTAAAATGGT
RI-AT1G28960-XLOC_005651-5872-1
CCACCTGCTTTTATCGAGCAGTGCCCGAAGTTTAAGTACCCTAAAATG--
CONSENSUS
CCACCTGCTTTTATCGAGCAGTGCCCGAAGTTTAAGTACCCTAAAATG..

RI-AT1G28960-XLOC_005651-5872-0
AGAAAAACATACTTGTATGCCTTAATGAACATAATTTTTCGAATGATGCT
RI-AT1G28960-XLOC_005651-5872-1

CONSENSUS
....

RI-AT1G28960-XLOC_005651-5872-0
TTCCTAGACTAGTCTTCTTCCACTATTTGATAACTTGTACCAGACAACAA
RI-AT1G28960-XLOC_005651-5872-1

CONSENSUS
....

RI-AT1G28960-XLOC_005651-5872-0
GACAATAACATTCCATACAACTGATTCTCTGTACCAGTGAATGGCTGAAT
RI-AT1G28960-XLOC_005651-5872-1
-------------------------------------TGAATGGCTGAAT
CONSENSUS
.....................................TGAATGGCTGAAT

RI-AT1G28960-XLOC_005651-5872-0
GCAGAACTTGGCTACTTGCTTATATATATGCAGACGCAGTAGCTGAAGCT
RI-AT1G28960-XLOC_005651-5872-1
GCAGAACTTGGCTACTTGCTTATATATATGCAGACGCAGTAGCTGAAGCT
CONSENSUS
GCAGAACTTGGCTACTTGCTTATATATATGCAGACGCAGTAGCTGAAGCT

RI-AT1G28960-XLOC_005651-5872-0
CCAATTCATTTCCTGCTGGGGCATCTCATAACAG
RI-AT1G28960-XLOC_005651-5872-1
CCAATTCATTTCCTGCTGGGGCATCTCATAACAG
CONSENSUS

CCAATTCATTTCCTGCTGGGGCATCTCATAACAG

alignment for event: RI-AT1G72770-XLOC_003729-13469

RI-AT1G72770-XLOC_003729-13469-0
CCAGATAGAGAGGATGAATATGCAAGAATAGAAAATGCTGGAGGCAAAGT
RI-AT1G72770-XLOC_003729-13469-1
CCAGATAGAGAGGATGAATATGCAAGAATAGAAAATGCTGGAGGCAAAGT
CONSENSUS
CCAGATAGAGAGGATGAATATGCAAGAATAGAAAATGCTGGAGGCAAAGT

RI-AT1G72770-XLOC_003729-13469-0
TATACAATGGCAAGGCGCACGTGTTTTTGGTGTTCTCGCCATGTCTAGGT
RI-AT1G72770-XLOC_003729-13469-1
TATACAATGGCAAGGCGCACGTGTTTTTGGTGTTCTCGCCATGTCTAGGT
CONSENSUS
TATACAATGGCAAGGCGCACGTGTTTTTGGTGTTCTCGCCATGTCTAGGT

RI-AT1G72770-XLOC_003729-13469-0
CCATCGGTAAGCATTGCTTCTTTTGATGACCAGTAATGCACATCACACAA
RI-AT1G72770-XLOC_003729-13469-1
CCATCG--
CONSENSUS
CCATCG..

RI-AT1G72770-XLOC_003729-13469-0
ACGTGGCTTGATAATGTAATTACTCCCCTACATGAAACATTAGATCCTCC
RI-AT1G72770-XLOC_003729-13469-1

CONSENSUS
....

RI-AT1G72770-XLOC_003729-13469-0
GGTTTCTTTCCTCCTCTGGAACTATGCAGGTGACAGATATCTGAAGCCAT
RI-AT1G72770-XLOC_003729-13469-1 -----------------------------
GTGACAGATATCTGAAGCCAT
CONSENSUS
.............................GTGACAGATATCTGAAGCCAT

RI-AT1G72770-XLOC_003729-13469-0
ATGTGATCCCAGAACCGGAAGTGACATTCATGCCTCGGTCAAGAGAAGAC
RI-AT1G72770-XLOC_003729-13469-1
ATGTGATCCCAGAACCGGAAGTGACATTCATGCCTCGGTCAAGAGAAGAC
CONSENSUS
ATGTGATCCCAGAACCGGAAGTGACATTCATGCCTCGGTCAAGAGAAGAC

RI-AT1G72770-XLOC_003729-13469-0
GAGTGTCTCATACTAGCCAGTGACGGTCTTTGGGATGTAATGAACAACCA
RI-AT1G72770-XLOC_003729-13469-1
GAGTGTCTCATACTAGCCAGTGACGGTCTTTGGGATGTAATGAACAACCA
CONSENSUS
GAGTGTCTCATACTAGCCAGTGACGGTCTTTGGGATGTAATGAACAACCA

RI-AT1G72770-XLOC_003729-13469-0

AGAAGTCTGCGAAATAGCAAGGAGACGGATATTGATGTGGCACAAGAAGA
RI-AT1G72770-XLOC_003729-13469-1
AGAAGTCTGCGAAATAGCAAGGAGACGGATATTGATGTGGCACAAGAAGA
CONSENSUS
AGAAGTCTGCGAAATAGCAAGGAGACGGATATTGATGTGGCACAAGAAGA

RI-AT1G72770-XLOC_003729-13469-0
ACGGTGCACCGCCTCTAGCAGAGAGAGGCAAAGGAATAGATCCAGCTTGC
RI-AT1G72770-XLOC_003729-13469-1
ACGGTGCACCGCCTCTAGCAGAGAGAGGCAAAGGAATAGATCCAGCTTGC
CONSENSUS
ACGGTGCACCGCCTCTAGCAGAGAGAGGCAAAGGAATAGATCCAGCTTGC

RI-AT1G72770-XLOC_003729-13469-0
CAAGCCGCAGCTGACTACCTCTCAATGCTTGCTCTACAAAAAGGAAGTAA
RI-AT1G72770-XLOC_003729-13469-1
CAAGCCGCAGCTGACTACCTCTCAATGCTTGCTCTACAAAAAGGAAGTAA
CONSENSUS
CAAGCCGCAGCTGACTACCTCTCAATGCTTGCTCTACAAAAAGGAAGTAA

RI-AT1G72770-XLOC_003729-13469-0
AGACAACATCTCCATCATTGTGATTGACTTGAAAGCTCAAAGAAAGTTCA
RI-AT1G72770-XLOC_003729-13469-1
AGACAACATCTCCATCATTGTGATTGACTTGAAAGCTCAAAGAAAGTTCA
CONSENSUS
AGACAACATCTCCATCATTGTGATTGACTTGAAAGCTCAAAGAAAGTTCA

RI-AT1G72770-XLOC_003729-13469-0
AGACCAGAACCTGAAGCTTAATTACAAGCAGTACGCTTACTTACTTTTTT
RI-AT1G72770-XLOC_003729-13469-1
AGACCAGAACCTGAAGCTTAATTACAAGCAGTACGCTTACTTACTTTTTT
CONSENSUS
AGACCAGAACCTGAAGCTTAATTACAAGCAGTACGCTTACTTACTTTTTT

RI-AT1G72770-XLOC_003729-13469-0
TTTTACTGGGGGTAAGTTTCGAGTTTTCGTATAATTTAGATCGTTAAGGA
RI-AT1G72770-XLOC_003729-13469-1
TTTTACTGGGGGTAAGTTTCGAGTTTTCGTATAATTTAGATCGTTAAGGA
CONSENSUS
TTTTACTGGGGGTAAGTTTCGAGTTTTCGTATAATTTAGATCGTTAAGGA

RI-AT1G72770-XLOC_003729-13469-0
TCTAATTTCGACAGGTTTTTACATGTTAATTACTCAATATTGTTAGAAGG
RI-AT1G72770-XLOC_003729-13469-1
TCTAATTTCGACAGGTTTTTACATGTTAATTACTCAATATTGTTAGAAGG
CONSENSUS
TCTAATTTCGACAGGTTTTTACATGTTAATTACTCAATATTGTTAGAAGG

RI-AT1G72770-XLOC_003729-13469-0
GAGAAAAAATAAAGAAAAAAAATGGAAATGCGGGTTGGGGAGGAGAAGTA
RI-AT1G72770-XLOC_003729-13469-1
GAGAAAAAATAAAGAAAAAAAATGGAAATGCGGGTTGGGGAGGAGAAGTA
CONSENSUS
GAGAAAAAATAAAGAAAAAAAATGGAAATGCGGGTTGGGGAGGAGAAGTA

RI-AT1G72770-XLOC_003729-13469-0

AAGACTAAAGACTGAAGATTAAGAAGATGATGCATAGCTAATTATAAGTA
RI-AT1G72770-XLOC_003729-13469-1
AAGACTAAAGACTGAAGATTAAGAAGATGATGCATAGCTAATTATAAGTA
CONSENSUS
AAGACTAAAGACTGAAGATTAAGAAGATGATGCATAGCTAATTATAAGTA

RI-AT1G72770-XLOC_003729-13469-0
CATTCCTTTTTTTCTCATGGTACTTGAATTCGTATGTATAAGAAGAAATA
RI-AT1G72770-XLOC_003729-13469-1
CATTCCTTTTTTTCTCATGGTACTTGAATTCGTATGTATAAGAAGAAATA
CONSENSUS
CATTCCTTTTTTTCTCATGGTACTTGAATTCGTATGTATAAGAAGAAATA

RI-AT1G72770-XLOC_003729-13469-0
GGCAGAGAAATGCTTTTTTTTTTTGGGGTATTGAAATAGTTGAATATGTT
RI-AT1G72770-XLOC_003729-13469-1
GGCAGAGAAATGCTTTTTTTTTTTGGGGTATTGAAATAGTTGAATATGTT
CONSENSUS
GGCAGAGAAATGCTTTTTTTTTTTGGGGTATTGAAATAGTTGAATATGTT

RI-AT1G72770-XLOC_003729-13469-0
GTAATAGTTTGAGAGATGTGGCAAATGAAATTGTAATTGAAACTATGTCA
RI-AT1G72770-XLOC_003729-13469-1
GTAATAGTTTGAGAGATGTGGCAAATGAAATTGTAATTGAAACTATGTCA
CONSENSUS
GTAATAGTTTGAGAGATGTGGCAAATGAAATTGTAATTGAAACTATGTCA

RI-AT1G72770-XLOC_003729-13469-0
CATTAACCTTCTTGCATTTGTCTTTTCATGTTGTGTTCTTCGCT
RI-AT1G72770-XLOC_003729-13469-1
CATTAACCTTCTTGCATTTGTCTTTTCATGTTGTGTTCTTCGCT
CONSENSUS
CATTAACCTTCTTGCATTTGTCTTTTCATGTTGTGTTCTTCGCT

alignment for event: RI-AT1G73960-XLOC_007768-1696

RI-AT1G73960-XLOC_007768-1696-0
GATCACATCTGTAAACTTATTGAACCTTTCCGGAATTCGGATACAATATT
RI-AT1G73960-XLOC_007768-1696-1
GATCACATCTGTAAACTTATTGAACCTTTCCGGAATTCGGATACAATATT
CONSENSUS
GATCACATCTGTAAACTTATTGAACCTTTCCGGAATTCGGATACAATATT

RI-AT1G73960-XLOC_007768-1696-0
GCAAATTCGTATAGAAGGAAGTAGGGCACTTCTTGACATCGAGTACCAAT
RI-AT1G73960-XLOC_007768-1696-1
GCAAATTCGTATAGAAGGAAGTAGGGCACTTCTTGACATCGAGTACCAAT
CONSENSUS
GCAAATTCGTATAGAAGGAAGTAGGGCACTTCTTGACATCGAGTACCAAT

RI-AT1G73960-XLOC_007768-1696-0
CCAAAGGCATAAGTTCCGCACTTTTGTTGTTTATGAAATATTTGGTAGAA
RI-AT1G73960-XLOC_007768-1696-1
CCAAAGGCATAAGTTCCGCACTTTTGTTGTTTATGAAATATTTGGTAGAA

CONSENSUS
      CCAAAGGCATAAGTTCCGCACTTTTGTTGTTTATGAAATATTTGGTAGAA

RI-AT1G73960-XLOC_007768-1696-0
      GAATCATCTTTGAGAGGTTGGTTATATTCTCATGCTGGAACATAGCAGCT
RI-AT1G73960-XLOC_007768-1696-1
      GAATCATCTTTGAGAG----------------------------------
CONSENSUS
      GAATCATCTTTGAGAG..................................

RI-AT1G73960-XLOC_007768-1696-0
      TTCTATTTCTAATAGAGTTCATTTATTGCTTTTATATCTTTGGCCCTTGA
RI-AT1G73960-XLOC_007768-1696-1

CONSENSUS
      ....

RI-AT1G73960-XLOC_007768-1696-0
      TTACAGGGCAGGTAAAATTGTGTGTACACACAATGCGGTTATGTCAAATA
RI-AT1G73960-XLOC_007768-1696-1 ------
GGCAGGTAAAATTGTGTGTACACACAATGCGGTTATGTCAAATA
CONSENSUS
      ......GGCAGGTAAAATTGTGTGTACACACAATGCGGTTATGTCAAATA

RI-AT1G73960-XLOC_007768-1696-0
      GCAGTTGGATGTGACTCAGACGATTGTGTCGACACTGTTACTCTTCTGGA
RI-AT1G73960-XLOC_007768-1696-1
      GCAGTTGGATGTGACTCAGACGATTGTGTCGACACTGTTACTCTTCTGGA
CONSENSUS
      GCAGTTGGATGTGACTCAGACGATTGTGTCGACACTGTTACTCTTCTGGA

RI-AT1G73960-XLOC_007768-1696-0
      TTTGCTGCATCTATTCAAGAGCCATGTAGTATTTAACAATGAACTTCTTC
RI-AT1G73960-XLOC_007768-1696-1
      TTTGCTGCATCTATTCAAGAGCCATGTAGTATTTAACAATGAACTTCTTC
CONSENSUS
      TTTGCTGCATCTATTCAAGAGCCATGTAGTATTTAACAATGAACTTCTTC

RI-AT1G73960-XLOC_007768-1696-0
      GCTACTACTTGTTCTGCATTTTTCAAATCCTCGCTGGAAG
RI-AT1G73960-XLOC_007768-1696-1
      GCTACTACTTGTTCTGCATTTTTCAAATCCTCGCTGGAAG
CONSENSUS
      GCTACTACTTGTTCTGCATTTTTCAAATCCTCGCTGGAAG

alignment for event: A3-AT1G80490-XLOC_008129-10788

A3-AT1G80490-XLOC_008129-10788-0
      CCTTTTCAACCAACACCTTCTCCGGTTCCGACACCTCTTGCTGGTTGGAT
A3-AT1G80490-XLOC_008129-10788-1
      CCTTTTCAACCAACACCTTCTCCGGTTCCGACACCTCTTGCTGGTTGGAT
CONSENSUS
      CCTTTTCAACCAACACCTTCTCCGGTTCCGACACCTCTTGCTGGTTGGAT

A3-AT1G80490-XLOC_008129-10788-0
GTCTAGTCCTTCCTCTGTCCCACATCCAGCTGTGTCTGGAGGACCCATTG
A3-AT1G80490-XLOC_008129-10788-1
GTCTAGTCCTTCCTCTGTCCCACATCCAGCTGTGTCTGGAGGACCCATTG
CONSENSUS
GTCTAGTCCTTCCTCTGTCCCACATCCAGCTGTGTCTGGAGGACCCATTG

A3-AT1G80490-XLOC_008129-10788-0 CTCTTGGTGCTCCATCCATCCAAG---
CCTTGAAACACCCGAGAACTCCT
A3-AT1G80490-XLOC_008129-10788-1
CTCTTGGTGCTCCATCCATCCAAGCAGCCTTGAAACACCCGAGAACTCCT
CONSENSUS
CTCTTGGTGCTCCATCCATCCAAG...CCTTGAAACACCCGAGAACTCCT

A3-AT1G80490-XLOC_008129-10788-0
CCTTCTAATTCCGCTGTAGACTATCCATCAGGTGACTCAGACCATGTCTC
A3-AT1G80490-XLOC_008129-10788-1
CCTTCTAATTCCGCTGTAGACTATCCATCAGGTGACTCAGACCATGTCTC
CONSENSUS
CCTTCTAATTCCGCTGTAGACTATCCATCAGGTGACTCAGACCATGTCTC

A3-AT1G80490-XLOC_008129-10788-0
AAAGCGAACCAGACCTATGGGAATCTCTGACGAG
A3-AT1G80490-XLOC_008129-10788-1
AAAGCGAACCAGACCTATGGGAATCTCTGACGAG
CONSENSUS
AAAGCGAACCAGACCTATGGGAATCTCTGACGAG

alignment for event: A3-AT1G67140-XLOC_007412-4573

A3-AT1G67140-XLOC_007412-4573-0
TGCAGGAGGAATGGCATTGTCGTCCTTAGTACCTGCTACTGTTAATTCAG
A3-AT1G67140-XLOC_007412-4573-1
TGCAGGAGGAATGGCATTGTCGTCCTTAGTACCTGCTACTGTTAATTCAG
CONSENSUS
TGCAGGAGGAATGGCATTGTCGTCCTTAGTACCTGCTACTGTTAATTCAG

A3-AT1G67140-XLOC_007412-4573-0
TCTCATCTTTGACCAAAACCTCTGTTCTTGGTCTTAAGATATGGGCCTTG
A3-AT1G67140-XLOC_007412-4573-1
TCTCATCTTTGACCAAAACCTCTGTTCTTGGTCTTAAGATATGGGCCTTG
CONSENSUS
TCTCATCTTTGACCAAAACCTCTGTTCTTGGTCTTAAGATATGGGCCTTG

A3-AT1G67140-XLOC_007412-4573-0
CATGGGCTTCTTTTGACCATTGAAGCTGCTGGTTTATCATTCGTATCTCA
A3-AT1G67140-XLOC_007412-4573-1
CATGGGCTTCTTTTGACCATTGAAGCTGCTGGTTTATCATTCGTATCTCA
CONSENSUS
CATGGGCTTCTTTTGACCATTGAAGCTGCTGGTTTATCATTCGTATCTCA

A3-AT1G67140-XLOC_007412-4573-0 TGTTCAG----
CATTAGGACTTGCCTTGGACATTTTGTTGACTGAAGAAA
A3-AT1G67140-XLOC_007412-4573-1

TGTTCAGGCAGCATTAGGACTTGCCTTGGACATTTTGTTGACTGAAGAAA
CONSENSUS
TGTTCAG....CATTAGGACTTGCCTTGGACATTTTGTTGACTGAAGAAA

A3-AT1G67140-XLOC_007412-4573-0
GTGGATGGATCGATCTTTCTCAAGGCATTGGACGCCTTATTAATGCTATT
A3-AT1G67140-XLOC_007412-4573-1
GTGGATGGATCGATCTTTCTCAAGGCATTGGACGCCTTATTAATGCTATT
CONSENSUS
GTGGATGGATCGATCTTTCTCAAGGCATTGGACGCCTTATTAATGCTATT

A3-AT1G67140-XLOC_007412-4573-0
GTTGCTGTCCTTGGCCCTGAGCTTTCTCCTGGCAGCATTCTGTTTTCACG
A3-AT1G67140-XLOC_007412-4573-1
GTTGCTGTCCTTGGCCCTGAGCTTTCTCCTGGCAGCATTCTGTTTTCACG
CONSENSUS
GTTGCTGTCCTTGGCCCTGAGCTTTCTCCTGGCAGCATTCTGTTTTCACG

A3-AT1G67140-XLOC_007412-4573-0 CTGCAAG
A3-AT1G67140-XLOC_007412-4573-1 CTGCAAG
CONSENSUS CTGCAAG

alignment for event: RI-AT1G50300-XLOC_006519-1343

RI-AT1G50300-XLOC_006519-1343-0
GTATTGATAAAGATGGAAGAGAGAGAAGCAGAGACAGGCAAAGAGACCGT
RI-AT1G50300-XLOC_006519-1343-1
GTATTGATAAAGATGGAAGAGAGAGAAGCAGAGACAGGCAAAGAGACCGT
CONSENSUS
GTATTGATAAAGATGGAAGAGAGAGAAGCAGAGACAGGCAAAGAGACCGT

RI-AT1G50300-XLOC_006519-1343-0
GGTAGAGATCATCACTACGATAAGGATAGACGCAGAAGCAGAAGCCGAGA
RI-AT1G50300-XLOC_006519-1343-1
GGTAGAGATCATCACTACGATAAGGATAGACGCAGAAGCAGAAGCCGAGA
CONSENSUS
GGTAGAGATCATCACTACGATAAGGATAGACGCAGAAGCAGAAGCCGAGA

RI-AT1G50300-XLOC_006519-1343-0
GAGGGAAAGAGGCAAGGAGCGTGACTATGACTATGACCATGATCGGGACA
RI-AT1G50300-XLOC_006519-1343-1
GAGGGAAAGAGGCAAGGAGCGTGACTATGACTATGACCATGATCGGGACA
CONSENSUS
GAGGGAAAGAGGCAAGGAGCGTGACTATGACTATGACCATGATCGGGACA

RI-AT1G50300-XLOC_006519-1343-0
GAGACAGAGACTACGGTCGCGAACGTGGAAGCAGGTACCGTAACTGAGAG
RI-AT1G50300-XLOC_006519-1343-1
GAGACAGAGACTACGGTCGCGAACGTGGAAGCAG----------------
CONSENSUS
GAGACAGAGACTACGGTCGCGAACGTGGAAGCAG................

RI-AT1G50300-XLOC_006519-1343-0
ATTACTCATAAGTGTTAGGGTTTAGTTTCTCATCTGTTTTGTCGACTTAC

RI-AT1G50300-XLOC_006519-1343-1

CONSENSUS
....

RI-AT1G50300-XLOC_006519-1343-0
TTTTACAACGGTATCATTACAATGTCTTCGCCTTTTGTTTACTTATTGTG
RI-AT1G50300-XLOC_006519-1343-1

CONSENSUS
....

RI-AT1G50300-XLOC_006519-1343-0
TGAAGTGAAACTCATTTTGGTTTGAATCAATGGCTATTGTTTTTCACTTG
RI-AT1G50300-XLOC_006519-1343-1

CONSENSUS
....

RI-AT1G50300-XLOC_006519-1343-0
TTTTGGTCTGATTATGTTCTGAGAATACTCCATGTTTATCGTTTCGAAAA
RI-AT1G50300-XLOC_006519-1343-1

CONSENSUS
....

RI-AT1G50300-XLOC_006519-1343-0
TTGGTAATTATGGCAAAACATGGTTAGCGACGTCAATTGCTAATTTAGAC
RI-AT1G50300-XLOC_006519-1343-1

CONSENSUS
....

RI-AT1G50300-XLOC_006519-1343-0
GTGTATTAAGTATTATTTTGTCTTTTGTAGTATTATTTTTAGTTGTCCCT
RI-AT1G50300-XLOC_006519-1343-1

CONSENSUS
....

RI-AT1G50300-XLOC_006519-1343-0
TTGTTTTTTTTTCCATCGCCAATCATTTCATCGCTGTATGATTTTAATGG
RI-AT1G50300-XLOC_006519-1343-1

CONSENSUS
....

RI-AT1G50300-XLOC_006519-1343-0
GAATAAAAAAAATAGAACAAGAAGATTTTTGTGGACAATATAGTTTCTGA
RI-AT1G50300-XLOC_006519-1343-1

CONSENSUS
....

RI-AT1G50300-XLOC_006519-1343-0
AACTATAATAGGATAAGAAAAAATTAAGCTTATTTGTGGGGATATATATT

RI-AT1G50300-XLOC_006519-1343-1

CONSENSUS
      ....

RI-AT1G50300-XLOC_006519-1343-0
      AATGTCATATCATTGTCATAAACATGGTCGGCAAAACTTGGGACGGCAGT
RI-AT1G50300-XLOC_006519-1343-1
      ---T
CONSENSUS
      ....T

RI-AT1G50300-XLOC_006519-1343-0
      TTACCAGAATAATCCGGTCGACAGAATTCAACAGTTCCTCTCTCTAAAAG
RI-AT1G50300-XLOC_006519-1343-1
      TTACCAGAATAATCCGGTCGACAGAATTCAACAGTTCCTCTCTCTAAAAG
CONSENSUS
      TTACCAGAATAATCCGGTCGACAGAATTCAACAGTTCCTCTCTCTAAAAG

RI-AT1G50300-XLOC_006519-1343-0
      ATATGAAGGAGATTAATTTTGGCTTTGTTAGCAAAGATGGCCGGTCCTCT
RI-AT1G50300-XLOC_006519-1343-1
      ATATGAAGGAGATTAATTTTGGCTTTGTTAGCAAAGATGGCCGGTCCTCT
CONSENSUS
      ATATGAAGGAGATTAATTTTGGCTTTGTTAGCAAAGATGGCCGGTCCTCT

RI-AT1G50300-XLOC_006519-1343-0
      TCTGGCAAATCCGGCTTTTACAAGCGGTTTTGGCCGGCTTTTATCCTTCC
RI-AT1G50300-XLOC_006519-1343-1
      TCTGGCAAATCCGGCTTTTACAAGCGGTTTTGGCCGGCTTTTATCCTTCC
CONSENSUS
      TCTGGCAAATCCGGCTTTTACAAGCGGTTTTGGCCGGCTTTTATCCTTCC

RI-AT1G50300-XLOC_006519-1343-0
      TCCGCCGTTCGATCGGTTGTTCTTACCGATTGCTTTAGTTTCTTTTTCAG
RI-AT1G50300-XLOC_006519-1343-1
      TCCGCCGTTCGATCGGTTGTTCTTACCGATTGCTTTAGTTTCTTTTTCAG
CONSENSUS
      TCCGCCGTTCGATCGGTTGTTCTTACCGATTGCTTTAGTTTCTTTTTCAG

RI-AT1G50300-XLOC_006519-1343-0
      TTTTTCTTTCAATAATTTCTAAAGATTTCAATTGGAGATTAATTTTAGGT
RI-AT1G50300-XLOC_006519-1343-1
      TTTTTCTTTCAATAATTTCTAAAGATTTCAATTGGAGATTAATTTTAGGT
CONSENSUS
      TTTTTCTTTCAATAATTTCTAAAGATTTCAATTGGAGATTAATTTTAGGT

RI-AT1G50300-XLOC_006519-1343-0
      CATAATAGCCTAAGATTAAGTTTTCGGATTTGTATCTTTAGAAAGTTTGA
RI-AT1G50300-XLOC_006519-1343-1
      CATAATAGCCTAAGATTAAGTTTTCGGATTTGTATCTTTAGAAAGTTTGA
CONSENSUS
      CATAATAGCCTAAGATTAAGTTTTCGGATTTGTATCTTTAGAAAGTTTGA

RI-AT1G50300-XLOC_006519-1343-0
      TTTGCAGAATTACCTCAAGTATGAAAGGAAGTTTTGTTTCCAGCATATTG

RI-AT1G50300-XLOC_006519-1343-1
      TTTGCAGAATTACCTCAAGTATGAAAGGAAGTTTTGTTTCCAGCATATTG
CONSENSUS
      TTTGCAGAATTACCTCAAGTATGAAAGGAAGTTTTGTTTCCAGCATATTG

RI-AT1G50300-XLOC_006519-1343-0
      GGATTGCCTGTAGCCGTTTCTACATGTTTATGGAGCTTCGATCGATCAAA
RI-AT1G50300-XLOC_006519-1343-1
      GGATTGCCTGTAGCCGTTTCTACATGTTTATGGAGCTTCGATCGATCAAA
CONSENSUS
      GGATTGCCTGTAGCCGTTTCTACATGTTTATGGAGCTTCGATCGATCAAA

RI-AT1G50300-XLOC_006519-1343-0
      TTTGAGGAATACTCAGATTTTTCTTTCCTCATTACCTGTTTTTGCCAAAT
RI-AT1G50300-XLOC_006519-1343-1
      TTTGAGGAATACTCAGATTTTTCTTTCCTCATTACCTGTTTTTGCCAAAT
CONSENSUS
      TTTGAGGAATACTCAGATTTTTCTTTCCTCATTACCTGTTTTTGCCAAAT

RI-AT1G50300-XLOC_006519-1343-0
      CTCTTGATTGATGATTTTGGAATTGAATTCTCTGTTTGTGTATCCTCCCT
RI-AT1G50300-XLOC_006519-1343-1
      CTCTTGATTGATGATTTTGGAATTGAATTCTCTGTTTGTGTATCCTCCCT
CONSENSUS
      CTCTTGATTGATGATTTTGGAATTGAATTCTCTGTTTGTGTATCCTCCCT

RI-AT1G50300-XLOC_006519-1343-0
      TCTCTGTTTAGCAGTTTCCGGTGAGAAAAAAACAAAAATCGATGTTTGCC
RI-AT1G50300-XLOC_006519-1343-1
      TCTCTGTTTAGCAGTTTCCGGTGAGAAAAAAACAAAAATCGATGTTTGCC
CONSENSUS
      TCTCTGTTTAGCAGTTTCCGGTGAGAAAAAAACAAAAATCGATGTTTGCC

RI-AT1G50300-XLOC_006519-1343-0
      CTTTTTCGACGTTGATTGTGTTCACCGGATCGGAGTCTTTGACGGCACAG
RI-AT1G50300-XLOC_006519-1343-1
      CTTTTTCGACGTTGATTGTGTTCACCGGATCGGAGTCTTTGACGGCACAG
CONSENSUS
      CTTTTTCGACGTTGATTGTGTTCACCGGATCGGAGTCTTTGACGGCACAG

RI-AT1G50300-XLOC_006519-1343-0
      GATGACAGGAACGTTTCATCGCTTCTCTTGCAACACGTGTCTTGTTATAC
RI-AT1G50300-XLOC_006519-1343-1
      GATGACAGGAACGTTTCATCGCTTCTCTTGCAACACGTGTCTTGTTATAC
CONSENSUS
      GATGACAGGAACGTTTCATCGCTTCTCTTGCAACACGTGTCTTGTTATAC

RI-AT1G50300-XLOC_006519-1343-0
      GATCGTGTTTTGTTAAAGAGATATCCTTCCTTTAAAGAAATGTAACTAAG
RI-AT1G50300-XLOC_006519-1343-1
      GATCGTGTTTTGTTAAAGAGATATCCTTCCTTTAAAGAAATGTAACTAAG
CONSENSUS
      GATCGTGTTTTGTTAAAGAGATATCCTTCCTTTAAAGAAATGTAACTAAG

RI-AT1G50300-XLOC_006519-1343-0
      TGGACCTTTGTTGGGCTGTATTCTGTGGATCATTAATAAACATAATCTTT

```
RI-AT1G50300-XLOC_006519-1343-1
      TGGACCTTTGTTGGGCTGTATTCTGTGGATCATTAATAAACATAATCTTT
CONSENSUS
      TGGACCTTTGTTGGGCTGTATTCTGTGGATCATTAATAAACATAATCTTT

RI-AT1G50300-XLOC_006519-1343-0  CCAGTG
RI-AT1G50300-XLOC_006519-1343-1  CCAGTG
CONSENSUS                        CCAGTG
```

alignment for event: RI-AT1G64430-XLOC_003266-6563

```
RI-AT1G64430-XLOC_003266-6563-0
      AAATGGAAAATACAGGCTGAAGCAAACGACGAGGCCGAAAGACTACTTAG
RI-AT1G64430-XLOC_003266-6563-1
      AAATGGAAAATACAGGCTGAAGCAAACGACGAGGCCGAAAGACTACTTAG
CONSENSUS
      AAATGGAAAATACAGGCTGAAGCAAACGACGAGGCCGAAAGACTACTTAG

RI-AT1G64430-XLOC_003266-6563-0
      CTCCCAACCTTAAGCACTCACACACTTGTCAACAAACCCGGTTAGTTAAT
RI-AT1G64430-XLOC_003266-6563-1
      CTCCCAACCTTAAGCACTCACACACTTGTCAACAAACCCG----------
CONSENSUS
      CTCCCAACCTTAAGCACTCACACACTTGTCAACAAACCCG..........

RI-AT1G64430-XLOC_003266-6563-0
      CATTATACATCACACATCATACATCATCATCATCATTAATTCATTATCAT
RI-AT1G64430-XLOC_003266-6563-1
      --------------------------------------------------
CONSENSUS
      ..................................................

RI-AT1G64430-XLOC_003266-6563-0
      CATCATGTAAAGCAATTATCTTTCGGCGGCATATGTATGACATTAACAAT
RI-AT1G64430-XLOC_003266-6563-1
      --------------------------------------------------
CONSENSUS
      ..................................................

RI-AT1G64430-XLOC_003266-6563-0
      GTCTGCTGTCAAAACTGCAACGTAGATTCGTCAATGATATGAAAATGTGG
RI-AT1G64430-XLOC_003266-6563-1
      --------------------------------------------------
CONSENSUS
      ..................................................

RI-AT1G64430-XLOC_003266-6563-0
      ACGACTGTTCTGGCAATTACACTTTTAAGTACAATATTTATTGAATTTTA
RI-AT1G64430-XLOC_003266-6563-1
      --------------------------------------------------
CONSENSUS
      ..................................................

RI-AT1G64430-XLOC_003266-6563-0
```

GTTCCCTTTTTAAGAATCTTTTTCTTTCTCCAGAGCTGCATTGGGATTTT
RI-AT1G64430-XLOC_003266-6563-1

CONSENSUS
....

RI-AT1G64430-XLOC_003266-6563-0
TGACACTGTAAACATAATACTTTTTGAAAATTTTAGTAATGGAAATTGTG
RI-AT1G64430-XLOC_003266-6563-1

CONSENSUS
....

RI-AT1G64430-XLOC_003266-6563-0
ATCATCTTGTATCTGGTACAGGTCAAAAACTCTGGAGCATCGCTAAAAGT
RI-AT1G64430-XLOC_003266-6563-1 ---------------------
GTCAAAAACTCTGGAGCATCGCTAAAAGT
CONSENSUS
.....................GTCAAAAACTCTGGAGCATCGCTAAAAGT

RI-AT1G64430-XLOC_003266-6563-0
TAAGTTTCTTTTGTCGGTCGCTATAAGTTAATGTTGATAAATTTGTTTGT
RI-AT1G64430-XLOC_003266-6563-1
TAAGTTTCTTTTGTCGGTCGCTATAAGTTAATGTTGATAAATTTGTTTGT
CONSENSUS
TAAGTTTCTTTTGTCGGTCGCTATAAGTTAATGTTGATAAATTTGTTTGT

RI-AT1G64430-XLOC_003266-6563-0
AATACTATAGTTTAATAATCACCGAAATCATTAATTTCTTGTTTCTTGTC
RI-AT1G64430-XLOC_003266-6563-1
AATACTATAGTTTAATAATCACCGAAATCATTAATTTCTTGTTTCTTGTC
CONSENSUS
AATACTATAGTTTAATAATCACCGAAATCATTAATTTCTTGTTTCTTGTC

RI-AT1G64430-XLOC_003266-6563-0
CATTTGTAATCTTTTTTTCTTAAATGCAAGCATTTGTTAGTTAAATTATT
RI-AT1G64430-XLOC_003266-6563-1
CATTTGTAATCTTTTTTTCTTAAATGCAAGCATTTGTTAGTTAAATTATT
CONSENSUS
CATTTGTAATCTTTTTTTCTTAAATGCAAGCATTTGTTAGTTAAATTATT

RI-AT1G64430-XLOC_003266-6563-0
AGTAGTTGCTAAACACACAAAGTGACACTTTTCTCCAGAGAATTGTGACA
RI-AT1G64430-XLOC_003266-6563-1
AGTAGTTGCTAAACACACAAAGTGACACTTTTCTCCAGAGAATTGTGACA
CONSENSUS
AGTAGTTGCTAAACACACAAAGTGACACTTTTCTCCAGAGAATTGTGACA

RI-AT1G64430-XLOC_003266-6563-0
TTAATTACTTAATTGAAGTATTAATTAATAAGGTATAAACAAAAAAGCCA
RI-AT1G64430-XLOC_003266-6563-1
TTAATTACTTAATTGAAGTATTAATTAATAAGGTATAAACAAAAAAGCCA
CONSENSUS
TTAATTACTTAATTGAAGTATTAATTAATAAGGTATAAACAAAAAAGCCA

RI-AT1G64430-XLOC_003266-6563-0

CGTGGCTTCTCTTCTCCACTCCTCTTTTTATTCCTTCCCAAACAAGAAGG
RI-AT1G64430-XLOC_003266-6563-1
CGTGGCTTCTCTTCTCCACTCCTCTTTTTATTCCTTCCCAAACAAGAAGG
CONSENSUS
CGTGGCTTCTCTTCTCCACTCCTCTTTTTATTCCTTCCCAAACAAGAAGG

RI-AT1G64430-XLOC_003266-6563-0
TTAGTTATTATTATTTCCAGAGAAGCTTCTCTTTTGATTCTCTCGCTCTC
RI-AT1G64430-XLOC_003266-6563-1
TTAGTTATTATTATTTCCAGAGAAGCTTCTCTTTTGATTCTCTCGCTCTC
CONSENSUS
TTAGTTATTATTATTTCCAGAGAAGCTTCTCTTTTGATTCTCTCGCTCTC

RI-AT1G64430-XLOC_003266-6563-0
TCTTTCTTCATCGCCTCTTCGAGCTTCGATGGTTGGGAATATTCTGGTGA
RI-AT1G64430-XLOC_003266-6563-1
TCTTTCTTCATCGCCTCTTCGAGCTTCGATGGTTGGGAATATTCTGGTGA
CONSENSUS
TCTTTCTTCATCGCCTCTTCGAGCTTCGATGGTTGGGAATATTCTGGTGA

RI-AT1G64430-XLOC_003266-6563-0
CCGGTGGTGCTGGTTACATCGGAAGTCACACGGTTCTTCAGCTTCTTCTC
RI-AT1G64430-XLOC_003266-6563-1
CCGGTGGTGCTGGTTACATCGGAAGTCACACGGTTCTTCAGCTTCTTCTC
CONSENSUS
CCGGTGGTGCTGGTTACATCGGAAGTCACACGGTTCTTCAGCTTCTTCTC

RI-AT1G64430-XLOC_003266-6563-0
GGAGGCTATAACACCGTCGTTATAGACAACCTCGACAATTCCTCTCTCGT
RI-AT1G64430-XLOC_003266-6563-1
GGAGGCTATAACACCGTCGTTATAGACAACCTCGACAATTCCTCTCTCGT
CONSENSUS
GGAGGCTATAACACCGTCGTTATAGACAACCTCGACAATTCCTCTCTCGT

RI-AT1G64430-XLOC_003266-6563-0
TTCGATCCAACGCGTCAAGGATCTCGCCGGAGATCATGGACAAAATCTCA
RI-AT1G64430-XLOC_003266-6563-1
TTCGATCCAACGCGTCAAGGATCTCGCCGGAGATCATGGACAAAATCTCA
CONSENSUS
TTCGATCCAACGCGTCAAGGATCTCGCCGGAGATCATGGACAAAATCTCA

RI-AT1G64430-XLOC_003266-6563-0 CCGTCCACCAG
RI-AT1G64430-XLOC_003266-6563-1 CCGTCCACCAG
CONSENSUS   CCGTCCACCAG

alignment for event: RI-AT1G08510-XLOC_004583-11928

RI-AT1G08510-XLOC_004583-11928-0
GAAACGGCGCTTAATCATGTTAAGACTGCTGGATTGCTTGGAGATGGGTT
RI-AT1G08510-XLOC_004583-11928-1
GAAACGGCGCTTAATCATGTTAAGACTGCTGGATTGCTTGGAGATGGGTT
CONSENSUS
GAAACGGCGCTTAATCATGTTAAGACTGCTGGATTGCTTGGAGATGGGTT

RI-AT1G08510-XLOC_004583-11928-0
TGGCTCTACACCTGAGATGTTTAAGAAGAACTTGATATGGGTTGTCACTC
RI-AT1G08510-XLOC_004583-11928-1
TGGCTCTACACCTGAGATGTTTAAGAAGAACTTGATATGGGTTGTCACTC
CONSENSUS
TGGCTCTACACCTGAGATGTTTAAGAAGAACTTGATATGGGTTGTCACTC

RI-AT1G08510-XLOC_004583-11928-0
GTATGCAGGTTGTGGTTGATAAATATCCTACTTGGTAAGCTATCCTCTTG
RI-AT1G08510-XLOC_004583-11928-1
GTATGCAGGTTGTGGTTGATAAATATCCTACTTG----------------
CONSENSUS
GTATGCAGGTTGTGGTTGATAAATATCCTACTTG................

RI-AT1G08510-XLOC_004583-11928-0
CATAAACCTGGTTCTGCAGGTTCATGCTCTCACTCTTTTTAACCAGGTTT
RI-AT1G08510-XLOC_004583-11928-1

CONSENSUS
....

RI-AT1G08510-XLOC_004583-11928-0
GGGAAAAAATGATGTGTATTTCGTTTTTTCAGTTGATACTGCTTTTACAGT
RI-AT1G08510-XLOC_004583-11928-1

CONSENSUS
....

RI-AT1G08510-XLOC_004583-11928-0
ACGAGATATATGCTCATATGACTAATGACTTCTTGCACCCTGAATTATAT
RI-AT1G08510-XLOC_004583-11928-1

CONSENSUS
....

RI-AT1G08510-XLOC_004583-11928-0
GCTCTGCATGCATATTATATTGCATCATAACTCATTTGCTTATTCAATAT
RI-AT1G08510-XLOC_004583-11928-1

CONSENSUS
....

RI-AT1G08510-XLOC_004583-11928-0
ATGCCTCACAGGGGAGATGTTGTTGAAGTAGACACCTGGGTCAGTCAGTC
RI-AT1G08510-XLOC_004583-11928-1 -----------
GGGAGATGTTGTTGAAGTAGACACCTGGGTCAGTCAGTC
CONSENSUS
...........GGGAGATGTTGTTGAAGTAGACACCTGGGTCAGTCAGTC

RI-AT1G08510-XLOC_004583-11928-0
TGGAAAGAATGGTATGCGTCGTGATTGGCTAGTTCGGGACTGTAATACTG
RI-AT1G08510-XLOC_004583-11928-1
TGGAAAGAATGGTATGCGTCGTGATTGGCTAGTTCGGGACTGTAATACTG
CONSENSUS
TGGAAAGAATGGTATGCGTCGTGATTGGCTAGTTCGGGACTGTAATACTG

RI-AT1G08510-XLOC_004583-11928-0 GAGAAACCTTAACACGAGCATCAAG
RI-AT1G08510-XLOC_004583-11928-1 GAGAAACCTTAACACGAGCATCAAG
CONSENSUS GAGAAACCTTAACACGAGCATCAAG

alignment for event: A3-AT1G28320-XLOC_005621-6

A3-AT1G28320-XLOC_005621-6-0
GAATGGAAGGGGCTCCGGTGTTTGCTAAGAATGGGCACTTAATTGGCATT
A3-AT1G28320-XLOC_005621-6-1
GAATGGAAGGGGCTCCGGTGTTTGCTAAGAATGGGCACTTAATTGGCATT
CONSENSUS
GAATGGAAGGGGCTCCGGTGTTTGCTAAGAATGGGCACTTAATTGGCATT

A3-AT1G28320-XLOC_005621-6-0
TTGATTAGACCACTAAGGCAAAAGAATAGCGGCGTTGAAATTCAG-----
A3-AT1G28320-XLOC_005621-6-1
TTGATTAGACCACTAAGGCAAAAGAATAGCGGCGTTGAAATTCAGCCTTC
CONSENSUS
TTGATTAGACCACTAAGGCAAAAGAATAGCGGCGTTGAAATTCAG.....

A3-AT1G28320-XLOC_005621-6-0 --------
CTGGTGGTTCCATGGGGAGCAATCACAACTGCTTGCAGCCAC
A3-AT1G28320-XLOC_005621-6-1
GGTAGCAGCTGGTGGTTCCATGGGGAGCAATCACAACTGCTTGCAGCCAC
CONSENSUS
........CTGGTGGTTCCATGGGGAGCAATCACAACTGCTTGCAGCCAC

A3-AT1G28320-XLOC_005621-6-0
TTGCTGCTTGAGGAACCATCTGTAGAAGGAAAAGCAAGTCAGTGGGGGAG
A3-AT1G28320-XLOC_005621-6-1
TTGCTGCTTGAGGAACCATCTGTAGAAGGAAAAGCAAGTCAGTGGGGGAG
CONSENSUS
TTGCTGCTTGAGGAACCATCTGTAGAAGGAAAAGCAAGTCAGTGGGGGAG

A3-AT1G28320-XLOC_005621-6-0
CGAAGTCCTAAGTGTTAAATCAGATGCTAGTATTCCTGCACAAGTGGCTA
A3-AT1G28320-XLOC_005621-6-1
CGAAGTCCTAAGTGTTAAATCAGATGCTAGTATTCCTGCACAAGTGGCTA
CONSENSUS
CGAAGTCCTAAGTGTTAAATCAGATGCTAGTATTCCTGCACAAGTGGCTA

A3-AT1G28320-XLOC_005621-6-0
TTGAGAAGGCGATGGAATCAGTTTGTCTTATTACGGTCAATGATGGTGTT
A3-AT1G28320-XLOC_005621-6-1
TTGAGAAGGCGATGGAATCAGTTTGTCTTATTACGGTCAATGATGGTGTT
CONSENSUS
TTGAGAAGGCGATGGAATCAGTTTGTCTTATTACGGTCAATGATGGTGTT

A3-AT1G28320-XLOC_005621-6-0
TGGGCTTCTGGTATTATTCTTAACGAACATGGTCTCATACTAACAAATGC
A3-AT1G28320-XLOC_005621-6-1
TGGGCTTCTGGTATTATTCTTAACGAACATGGTCTCATACTAACAAATGC
CONSENSUS
TGGGCTTCTGGTATTATTCTTAACGAACATGGTCTCATACTAACAAATGC

A3-AT1G28320-XLOC_005621-6-0
TCACCTGCTTGAGCCGTGGAGGTATGGAAAAGGTGGTGTATATGGTGAAG
A3-AT1G28320-XLOC_005621-6-1
TCACCTGCTTGAGCCGTGGAGGTATGGAAAAGGTGGTGTATATGGTGAAG
CONSENSUS
TCACCTGCTTGAGCCGTGGAGGTATGGAAAAGGTGGTGTATATGGTGAAG

A3-AT1G28320-XLOC_005621-6-0
GATTTAAACCCTATGTCTTAGGAGCCGAGGAATTTTCTTCCACGGGAAGT
A3-AT1G28320-XLOC_005621-6-1
GATTTAAACCCTATGTCTTAGGAGCCGAGGAATTTTCTTCCACGGGAAGT
CONSENSUS
GATTTAAACCCTATGTCTTAGGAGCCGAGGAATTTTCTTCCACGGGAAGT

A3-AT1G28320-XLOC_005621-6-0
AAATTTTGGGAACAGAAGAGTCAAACATTGCCACGGAAAGCTCCACGAAA
A3-AT1G28320-XLOC_005621-6-1
AAATTTTGGGAACAGAAGAGTCAAACATTGCCACGGAAAGCTCCACGAAA
CONSENSUS
AAATTTTGGGAACAGAAGAGTCAAACATTGCCACGGAAAGCTCCACGAAA

A3-AT1G28320-XLOC_005621-6-0
TCATTATTCGTCTGTTGGAGAGAACATCAGGGAATACAAACACAATTTCC
A3-AT1G28320-XLOC_005621-6-1
TCATTATTCGTCTGTTGGAGAGAACATCAGGGAATACAAACACAATTTCC
CONSENSUS
TCATTATTCGTCTGTTGGAGAGAACATCAGGGAATACAAACACAATTTCC

A3-AT1G28320-XLOC_005621-6-0
TTCAGACTGGGCATAGAGACATACGTGTGCGTTTGTGTCACCTAGATTCT
A3-AT1G28320-XLOC_005621-6-1
TTCAGACTGGGCATAGAGACATACGTGTGCGTTTGTGTCACCTAGATTCT
CONSENSUS
TTCAGACTGGGCATAGAGACATACGTGTGCGTTTGTGTCACCTAGATTCT

A3-AT1G28320-XLOC_005621-6-0
TGGACTTGGTGTCCTGCAAACGTGGTCTATATTTGCAAGGAACAATTAGA
A3-AT1G28320-XLOC_005621-6-1
TGGACTTGGTGTCCTGCAAACGTGGTCTATATTTGCAAGGAACAATTAGA
CONSENSUS
TGGACTTGGTGTCCTGCAAACGTGGTCTATATTTGCAAGGAACAATTAGA

A3-AT1G28320-XLOC_005621-6-0
TATTGCCTTACTGCAGCTAGAATATGTCCCTGGAAAGCTCCAACCTATTA
A3-AT1G28320-XLOC_005621-6-1
TATTGCCTTACTGCAGCTAGAATATGTCCCTGGAAAGCTCCAACCTATTA
CONSENSUS
TATTGCCTTACTGCAGCTAGAATATGTCCCTGGAAAGCTCCAACCTATTA

A3-AT1G28320-XLOC_005621-6-0
CTGCCAATTTTTCTTCTCCTCCTTTGGGTACAACAGCGCATGTTGTTGGA
A3-AT1G28320-XLOC_005621-6-1
CTGCCAATTTTTCTTCTCCTCCTTTGGGTACAACAGCGCATGTTGTTGGA
CONSENSUS
CTGCCAATTTTTCTTCTCCTCCTTTGGGTACAACAGCGCATGTTGTTGGA

A3-AT1G28320-XLOC_005621-6-0   CATGGACTCTTCGGACCAAGATGTG
A3-AT1G28320-XLOC_005621-6-1   CATGGACTCTTCGGACCAAGATGTG
CONSENSUS   CATGGACTCTTCGGACCAAGATGTG

alignment for event: RI-AT1G60270-XLOC_007003-1360

RI-AT1G60270-XLOC_007003-1360-0
GATAAGCAGGGTGGTTCCATAGGCTTTGGCTTATATTTAATGGGGTTGAC
RI-AT1G60270-XLOC_007003-1360-1
GATAAGCAGGGTGGTTCCATAGGCTTTGGCTTATATTTAATGGGGTTGAC
CONSENSUS
GATAAGCAGGGTGGTTCCATAGGCTTTGGCTTATATTTAATGGGGTTGAC

RI-AT1G60270-XLOC_007003-1360-0
TCCTTCTACAAGCTCCAAGGATGATGCCATTGCAACTCAAAGAGCCAAAG
RI-AT1G60270-XLOC_007003-1360-1
TCCTTCTACAAGCTCCAAGGATGATGCCATTGCAACTCAAAGAGCCAAAG
CONSENSUS
TCCTTCTACAAGCTCCAAGGATGATGCCATTGCAACTCAAAGAGCCAAAG

RI-AT1G60270-XLOC_007003-1360-0
ATTTCTATTTCGGTTGGTGAGTCAGGCACAAACAGTTCTAAAAGGTTTTT
RI-AT1G60270-XLOC_007003-1360-1
ATTTCTATTTCGGTTG----------------------------------
CONSENSUS
ATTTCTATTTCGGTTG..................................

RI-AT1G60270-XLOC_007003-1360-0
CAAATTGGGCAACATGTGCAAGATCTTAAGCTGAAAAGAAAGAAAATCTG
RI-AT1G60270-XLOC_007003-1360-1

CONSENSUS
....

RI-AT1G60270-XLOC_007003-1360-0
CTCTTTAAAACTCAGGTTCCTTGGACCTCTTATATTTGGAGACTATCCAG
RI-AT1G60270-XLOC_007003-1360-1 ---------------
GTTCCTTGGACCTCTTATATTTGGAGACTATCCAG
CONSENSUS
...............GTTCCTTGGACCTCTTATATTTGGAGACTATCCAG

RI-AT1G60270-XLOC_007003-1360-0
ATACAATGAAAAGAACCATTGGATCAAGACTGCCAGTTTTCTCAGAGGAA
RI-AT1G60270-XLOC_007003-1360-1
ATACAATGAAAAGAACCATTGGATCAAGACTGCCAGTTTTCTCAGAGGAA
CONSENSUS
ATACAATGAAAAGAACCATTGGATCAAGACTGCCAGTTTTCTCAGAGGAA

RI-AT1G60270-XLOC_007003-1360-0
GAATCAGAACAAGTTAAAGGCTCATCTGACTTCATAGGAATCAATCATTA
RI-AT1G60270-XLOC_007003-1360-1
GAATCAGAACAAGTTAAAGGCTCATCTGACTTCATAGGAATCAATCATTA
CONSENSUS

GAATCAGAACAAGTTAAAGGCTCATCTGACTTCATAGGAATCAATCATTA

RI-AT1G60270-XLOC_007003-1360-0
GTTTGCGGCTTCTGTCACAAACATCAAATTCAAACCTTCTATTTCTGGAA
RI-AT1G60270-XLOC_007003-1360-1
GTTTGCGGCTTCTGTCACAAACATCAAATTCAAACCTTCTATTTCTGGAA
CONSENSUS
GTTTGCGGCTTCTGTCACAAACATCAAATTCAAACCTTCTATTTCTGGAA

RI-AT1G60270-XLOC_007003-1360-0
ACCCGGACTTCTACTCAGACATGGGCGCATATGTGACTT
RI-AT1G60270-XLOC_007003-1360-1
ACCCGGACTTCTACTCAGACATGGGCGCATATGTGACTT
CONSENSUS
ACCCGGACTTCTACTCAGACATGGGCGCATATGTGACTT

alignment for event: RI-AT1G02050-XLOC_004202-11568

RI-AT1G02050-XLOC_004202-11568-0
GTCTCTATGGACGTTGCAACTCTCTGTTCCTAACTTTCACCTTCATGGTA
RI-AT1G02050-XLOC_004202-11568-1
GTCTCTATGGACGTTGCAACTCTCTGTTCCTAACTTTCACCTTCATG---
CONSENSUS
GTCTCTATGGACGTTGCAACTCTCTGTTCCTAACTTTCACCTTCATG...

RI-AT1G02050-XLOC_004202-11568-0
CCTATTTCCTAAGTAGATTAGGCTTATATTACTTATCCCGGGAAAATAGG
RI-AT1G02050-XLOC_004202-11568-1

CONSENSUS
....

RI-AT1G02050-XLOC_004202-11568-0
TTTCATGGTGTTTTTTGCATTTCTAACCAACACAGGCCAAAGAATCACCA
RI-AT1G02050-XLOC_004202-11568-1

CONSENSUS
....

RI-AT1G02050-XLOC_004202-11568-0
AACCCTAATAAAATAACCGTTTCTCTCTTTACATGATCTTCACTCTTCAG
RI-AT1G02050-XLOC_004202-11568-1

CONSENSUS
....

RI-AT1G02050-XLOC_004202-11568-0
CACCAAACTCTCCCCAAGAAAACCCTCAACCCTTCCTTTATAATCCTTTG
RI-AT1G02050-XLOC_004202-11568-1

CONSENSUS
....

RI-AT1G02050-XLOC_004202-11568-0

CCATTTGCTGATATTCTGCATCCACCATCTTTCTTCCCAATATCTAATTG
RI-AT1G02050-XLOC_004202-11568-1

CONSENSUS
....

RI-AT1G02050-XLOC_004202-11568-0
AGTTTTTTCCTTCTGCGCTTGAAAAGAAATGTCGAATTCTAGGATGAATG
RI-AT1G02050-XLOC_004202-11568-1 -------------------------
AAATGTCGAATTCTAGGATGAATG
CONSENSUS
..........................AAATGTCGAATTCTAGGATGAATG

RI-AT1G02050-XLOC_004202-11568-0
GTGTTGAGAAGCTAAGCAGCAAATCCACAAGGCGTGTCGCAAATGCCGGA
RI-AT1G02050-XLOC_004202-11568-1
GTGTTGAGAAGCTAAGCAGCAAATCCACAAGGCGTGTCGCAAATGCCGGA
CONSENSUS
GTGTTGAGAAGCTAAGCAGCAAATCCACAAGGCGTGTCGCAAATGCCGGA

RI-AT1G02050-XLOC_004202-11568-0
AAGGCAACACTGCTTGCTCTTGGCAAAGCCTTCCCAAGCCAAGTCGTTCC
RI-AT1G02050-XLOC_004202-11568-1
AAGGCAACACTGCTTGCTCTTGGCAAAGCCTTCCCAAGCCAAGTCGTTCC
CONSENSUS
AAGGCAACACTGCTTGCTCTTGGCAAAGCCTTCCCAAGCCAAGTCGTTCC

RI-AT1G02050-XLOC_004202-11568-0
TCAGGAGAATCTAGTGGAAGGATTTCTACGAGACACCAAATGTGATGACG
RI-AT1G02050-XLOC_004202-11568-1
TCAGGAGAATCTAGTGGAAGGATTTCTACGAGACACCAAATGTGATGACG
CONSENSUS
TCAGGAGAATCTAGTGGAAGGATTTCTACGAGACACCAAATGTGATGACG

RI-AT1G02050-XLOC_004202-11568-0 CGTTTATCAAAGAAAAGCTGGAACACTTGT
RI-AT1G02050-XLOC_004202-11568-1 CGTTTATCAAAGAAAAGCTGGAACACTTGT
CONSENSUS CGTTTATCAAAGAAAAGCTGGAACACTTGT

alignment for event: RI-AT1G15240-XLOC_000770-4996

RI-AT1G15240-XLOC_000770-4996-0
ATTTCGACATTTTGAGGAGCTTCACCGACGGCTAAAAGTGTTTCCTGAGT
RI-AT1G15240-XLOC_000770-4996-1
ATTTCGACATTTTGAGGAGCTTCACCGACGGCTAAAAGTGTTTCCTGAGT
CONSENSUS
ATTTCGACATTTTGAGGAGCTTCACCGACGGCTAAAAGTGTTTCCTGAGT

RI-AT1G15240-XLOC_000770-4996-0
ACAAACTTCATTTACCGCCAAAGCATTTCCTATCAACAGGCGTGGACATA
RI-AT1G15240-XLOC_000770-4996-1
ACAAACTTCATTTACCGCCAAAGCATTTCCTATCAACAGGCGTGGACATA
CONSENSUS
ACAAACTTCATTTACCGCCAAAGCATTTCCTATCAACAGGCGTGGACATA

RI-AT1G15240-XLOC_000770-4996-0
CCTGTTATCCAAGAGCGATGTGTACTACTTGACGAGTATATCAAGGTCAA
RI-AT1G15240-XLOC_000770-4996-1
CCTGTTATCCAAGAGCGATGTGTACTACTTGACGAGTATATCAAG-----
CONSENSUS
CCTGTTATCCAAGAGCGATGTGTACTACTTGACGAGTATATCAAG.....

RI-AT1G15240-XLOC_000770-4996-0
ACGCCATGTCTCTTGTTTTTAACTATCTGTTCATGCTTTACATGGTGTAT
RI-AT1G15240-XLOC_000770-4996-1

CONSENSUS
....

RI-AT1G15240-XLOC_000770-4996-0
TGTATATGGAGTATGTTTTATTTTTCTACTGGATTTGTTTTTTTGATGAG
RI-AT1G15240-XLOC_000770-4996-1

CONSENSUS
....

RI-AT1G15240-XLOC_000770-4996-0
TAGTTTACCGTGAATTAGTTTTTGGTTTTCATACTCTGGTAGCTACTTTG
RI-AT1G15240-XLOC_000770-4996-1

CONSENSUS
....

RI-AT1G15240-XLOC_000770-4996-0
GATTTCTGAGAAGCTAATACTGAATGTTCTTATGTATGAAACTTCACCAT
RI-AT1G15240-XLOC_000770-4996-1

CONSENSUS
....

RI-AT1G15240-XLOC_000770-4996-0
CTACTGCTCTGTAATTTTACTCTGAAGTCTGAAGTTTAGTCAGGCTTGCT
RI-AT1G15240-XLOC_000770-4996-1

CONSENSUS
....

RI-AT1G15240-XLOC_000770-4996-0
TTGATCTACTGAAGTTATGTACCTTTGTAGTTTGATCTGCTCAAGTAGCT
RI-AT1G15240-XLOC_000770-4996-1

CONSENSUS
....

RI-AT1G15240-XLOC_000770-4996-0
AAGGCTGATAGTTCTAATGCTTGAACTTTTGTACACAGTTGCTTTTCAAC
RI-AT1G15240-XLOC_000770-4996-1

CONSENSUS
....

RI-AT1G15240-XLOC_000770-4996-0
GCCTTCTATTCTTCCATGTTAACTAAATGTGAATGTTTGCTTTGCATTGA
RI-AT1G15240-XLOC_000770-4996-1

CONSENSUS
....

RI-AT1G15240-XLOC_000770-4996-0
AGTGATTCTGCTGTTTTCCTTATTCTGCGTATTATCCTTTCTCTACTTTG
RI-AT1G15240-XLOC_000770-4996-1

CONSENSUS
....

RI-AT1G15240-XLOC_000770-4996-0
CTCAGAAGCTTTTGCAGCTACAACGAATTTCAGGATCAATTGAAGTTTGG
RI-AT1G15240-XLOC_000770-4996-1 -----
AAGCTTTTGCAGCTACAACGAATTTCAGGATCAATTGAAGTTTGG
CONSENSUS
.....AAGCTTTTGCAGCTACAACGAATTTCAGGATCAATTGAAGTTTGG

RI-AT1G15240-XLOC_000770-4996-0 GACTTCCTCAGTGTGGATTCTCAG
RI-AT1G15240-XLOC_000770-4996-1 GACTTCCTCAGTGTGGATTCTCAG
CONSENSUS GACTTCCTCAGTGTGGATTCTCAG

alignment for event: RI-AT1G30970-XLOC_005753-4663

RI-AT1G30970-XLOC_005753-4663-0
GAGGAAAGAAGAATGTCCTTACCAAAATACAAAGTGCACGATGAAACCAG
RI-AT1G30970-XLOC_005753-4663-1
GAGGAAAGAAGAATGTCCTTACCAAAATACAAAGTGCACGATGAAACCAG
CONSENSUS
GAGGAAAGAAGAATGTCCTTACCAAAATACAAAGTGCACGATGAAACCAG

RI-AT1G30970-XLOC_005753-4663-0
CCAGGTAAGTTCTGATTTACCCTTTTTAAAGTTAAGAAGAGTTTGCTTTT
RI-AT1G30970-XLOC_005753-4663-1
CCAG--
CONSENSUS
CCAG..

RI-AT1G30970-XLOC_005753-4663-0
GCAAGTCTAACCCATTCGGTAGGCTTTATTTATACATTTCTGTTCCAGAT
RI-AT1G30970-XLOC_005753-4663-1

CONSENSUS
....

RI-AT1G30970-XLOC_005753-4663-0
CCTATTGTTTCTGTGCTAAGAGGTACCACCGTATTATTCAGTTTATAATT
RI-AT1G30970-XLOC_005753-4663-1

CONSENSUS
....

RI-AT1G30970-XLOC_005753-4663-0
TCTTCCCTTAGAATATGATAAGAAGGACTTAGGCAAGAAACTTGAGCATT
RI-AT1G30970-XLOC_005753-4663-1

CONSENSUS
....

RI-AT1G30970-XLOC_005753-4663-0
TTGAATCAATGTTCGAAAGGTATCATTTGTAGCTTGCAATAGTAGGTAGT
RI-AT1G30970-XLOC_005753-4663-1

CONSENSUS
....

RI-AT1G30970-XLOC_005753-4663-0
GAGAAACATAGCGTTTGTATCTGTTTGTTTTTCATTATATTTCCTGTCTA
RI-AT1G30970-XLOC_005753-4663-1

CONSENSUS
....

RI-AT1G30970-XLOC_005753-4663-0
TTGTATTGTATTAAAAATGTGGTGATTGGTTCCACAAGATCATACTCTAC
RI-AT1G30970-XLOC_005753-4663-1

CONSENSUS
....

RI-AT1G30970-XLOC_005753-4663-0
AGGTTTGGTTTCAAACCCAGGTCAGCTCTTTTTATATTCTTAACTCTGCA
RI-AT1G30970-XLOC_005753-4663-1

CONSENSUS
....

RI-AT1G30970-XLOC_005753-4663-0
AAGTTTGGTACCTTTGCACAATCTCACTGCCTTGGGGAACCAAAACAATA
RI-AT1G30970-XLOC_005753-4663-1

CONSENSUS
....

RI-AT1G30970-XLOC_005753-4663-0
CGCACTTAACTCTTTCAAAATAAAGCATCCAACTTCTTAACTCAACACCA
RI-AT1G30970-XLOC_005753-4663-1

CONSENSUS
....

RI-AT1G30970-XLOC_005753-4663-0
AGTTTGTTTCTTTTGGTTTGCAGATGAACTCGATAAATGCAGCGATAGAC
RI-AT1G30970-XLOC_005753-4663-1 ----------------------
ATGAACTCGATAAATGCAGCGATAGAC
CONSENSUS
......................ATGAACTCGATAAATGCAGCGATAGAC

RI-AT1G30970-XLOC_005753-4663-0
AGACGAATCTCAGAGAGTAGGCTTGCTGGGCGGATGGCGTTTTAGAATTT
RI-AT1G30970-XLOC_005753-4663-1
AGACGAATCTCAGAGAGTAGGCTTGCTGGGCGGATGGCGTTTTAGAATTT
CONSENSUS
AGACGAATCTCAGAGAGTAGGCTTGCTGGGCGGATGGCGTTTTAGAATTT

RI-AT1G30970-XLOC_005753-4663-0
GGCACCAAACCAAACCAGCGTCAAAGAAAAGGCGATATGGATGAAGGCTT
RI-AT1G30970-XLOC_005753-4663-1
GGCACCAAACCAAACCAGCGTCAAAGAAAAGGCGATATGGATGAAGGCTT
CONSENSUS
GGCACCAAACCAAACCAGCGTCAAAGAAAAGGCGATATGGATGAAGGCTT

RI-AT1G30970-XLOC_005753-4663-0
TGTTACAACAACAAGTCTTCACTCACTTTTGTTCAGATACTGAATTTATG
RI-AT1G30970-XLOC_005753-4663-1
TGTTACAACAACAAGTCTTCACTCACTTTTGTTCAGATACTGAATTTATG
CONSENSUS
TGTTACAACAACAAGTCTTCACTCACTTTTGTTCAGATACTGAATTTATG

RI-AT1G30970-XLOC_005753-4663-0
ATCTGATTTTAAGTTATTAACTTTCCATCTGTTGTATTCTTTAGCCAGGT
RI-AT1G30970-XLOC_005753-4663-1
ATCTGATTTTAAGTTATTAACTTTCCATCTGTTGTATTCTTTAGCCAGGT
CONSENSUS
ATCTGATTTTAAGTTATTAACTTTCCATCTGTTGTATTCTTTAGCCAGGT

RI-AT1G30970-XLOC_005753-4663-0
GAGTTAGTTTACCATTACATTGTTTAGGGATCTCAGAGATGTTTTTTCTC
RI-AT1G30970-XLOC_005753-4663-1
GAGTTAGTTTACCATTACATTGTTTAGGGATCTCAGAGATGTTTTTTCTC
CONSENSUS
GAGTTAGTTTACCATTACATTGTTTAGGGATCTCAGAGATGTTTTTTCTC

RI-AT1G30970-XLOC_005753-4663-0
AGAAGTTTAGTCCTTTTTTTTTTTGGTCGTTGGAGACTTGGAGGAAGAAC
RI-AT1G30970-XLOC_005753-4663-1
AGAAGTTTAGTCCTTTTTTTTTTTGGTCGTTGGAGACTTGGAGGAAGAAC
CONSENSUS
AGAAGTTTAGTCCTTTTTTTTTTTGGTCGTTGGAGACTTGGAGGAAGAAC

RI-AT1G30970-XLOC_005753-4663-0
TTTGTCTCCTTTTCTTCTGAATTGATCAAAGTATTATATTATACAGATTC
RI-AT1G30970-XLOC_005753-4663-1
TTTGTCTCCTTTTCTTCTGAATTGATCAAAGTATTATATTATACAGATTC
CONSENSUS
TTTGTCTCCTTTTCTTCTGAATTGATCAAAGTATTATATTATACAGATTC

RI-AT1G30970-XLOC_005753-4663-0 ACCCAAAATGTTTGCACCATGTTGTTC
RI-AT1G30970-XLOC_005753-4663-1 ACCCAAAATGTTTGCACCATGTTGTTC
CONSENSUS ACCCAAAATGTTTGCACCATGTTGTTC

alignment for event: A5-AT1G73750-XLOC_003779-6988

A5-AT1G73750-XLOC_003779-6988-0
GTTCCAAGATCCTTGGGATGCAAGACCGTCTATCGAAGAGAGCGGGAGAT
A5-AT1G73750-XLOC_003779-6988-1
GTTCCAAGATCCTTGGGATGCAAGACCGTCTATCGAAGAGAGCGGGAGAT
CONSENSUS
GTTCCAAGATCCTTGGGATGCAAGACCGTCTATCGAAGAGAGCGGGAGAT

A5-AT1G73750-XLOC_003779-6988-0
TTCAAGCAGCGGTTTGAACTTATCCCTCATTACAATTGGGATTTTGATAA
A5-AT1G73750-XLOC_003779-6988-1
TTCAAGCAGCGGTTTGAACTTATCCCTCATTACAATTGGGATTTTGATAA
CONSENSUS
TTCAAGCAGCGGTTTGAACTTATCCCTCATTACAATTGGGATTTTGATAA

A5-AT1G73750-XLOC_003779-6988-0
CTATCTAGAAGAAGATGTTCCTTCTGCG-----ATGGACTATGTAAGGAC
A5-AT1G73750-XLOC_003779-6988-1
CTATCTAGAAGAAGATGTTCCTTCTGCGGTGAGATGGACTATGTAAGGAC
CONSENSUS
CTATCTAGAAGAAGATGTTCCTTCTGCG.....ATGGACTATGTAAGGAC

A5-AT1G73750-XLOC_003779-6988-0
TCAAACCAAGTCAAAAGATGGAAAGTTGCTAGCAGTTGGTCACTCAATGG
A5-AT1G73750-XLOC_003779-6988-1
TCAAACCAAGTCAAAAGATGGAAAGTTGCTAGCAGTTGGTCACTCAATGG
CONSENSUS
TCAAACCAAGTCAAAAGATGGAAAGTTGCTAGCAGTTGGTCACTCAATGG

A5-AT1G73750-XLOC_003779-6988-0
GTGGTATCTTGTTATATGCCTTGCTCTCAAGATGTG
A5-AT1G73750-XLOC_003779-6988-1
GTGGTATCTTGTTATATGCCTTGCTCTCAAGATGTG
CONSENSUS
GTGGTATCTTGTTATATGCCTTGCTCTCAAGATGTG

alignment for event: RI-AT1G67060-XLOC_003407-10131

RI-AT1G67060-XLOC_003407-10131-0
CTATGGAGGACATTTCTATTGACTCCATTTAAACTCATTACGGTGTTTTT
RI-AT1G67060-XLOC_003407-10131-1
CTATGGAGGACATTTCTATTGACTCCATTTAAACTCATTACGGTGTTTTT
CONSENSUS
CTATGGAGGACATTTCTATTGACTCCATTTAAACTCATTACGGTGTTTTT

RI-AT1G67060-XLOC_003407-10131-0
GCACGAAGCTAGTCATGCCGTTGCTTGCAAGCTTACATGTGGAGATGTAA
RI-AT1G67060-XLOC_003407-10131-1
GCACGAAGCTAGTCATGCCGTTGCTTGCAAGCTTACATGTGGAGAT----
CONSENSUS
GCACGAAGCTAGTCATGCCGTTGCTTGCAAGCTTACATGTGGAGAT....

RI-AT1G67060-XLOC_003407-10131-0

GGAGCTTTCTCACTTCAACTACTAGTCTTCAAATGAACTTAAAAGGGTAT
RI-AT1G67060-XLOC_003407-10131-1

CONSENSUS
....

RI-AT1G67060-XLOC_003407-10131-0
AAGATTGATTTTAGGTCACTTGAACCCTTGATTTAGTTTGCAAACATGAA
RI-AT1G67060-XLOC_003407-10131-1

CONSENSUS
....

RI-AT1G67060-XLOC_003407-10131-0
TGTGTTTCCTTCCATTTGTAAAATGTTGCTGTGTAAGAGCAGAGAAGATC
RI-AT1G67060-XLOC_003407-10131-1

CONSENSUS
....

RI-AT1G67060-XLOC_003407-10131-0
TTCTGGTTTCCTTAGATAGAAAGATTTTTACTTTAGCAATATGTCAGTTC
RI-AT1G67060-XLOC_003407-10131-1

CONSENSUS
....

RI-AT1G67060-XLOC_003407-10131-0
ATTCTTCTCTCTGGACATGACTGTTAGTATAGCCATAGATAAGAGGTTGC
RI-AT1G67060-XLOC_003407-10131-1

CONSENSUS
....

RI-AT1G67060-XLOC_003407-10131-0
AGTATCCTTATATGTCTTGCTATCTGTTTTATCCGTAATCATATTTTTAG
RI-AT1G67060-XLOC_003407-10131-1

CONSENSUS
....

RI-AT1G67060-XLOC_003407-10131-0
ACGATTCTTGCTTGCTTCCATTGTTAATGTGTAAACTACTAGCTTCTGTC
RI-AT1G67060-XLOC_003407-10131-1

CONSENSUS
....

RI-AT1G67060-XLOC_003407-10131-0
ATTAGTTTAAGAACACACAGTGAGTCTACTTGAATAAGTGCAGCTGCTCA
RI-AT1G67060-XLOC_003407-10131-1

CONSENSUS
....

RI-AT1G67060-XLOC_003407-10131-0

AGCCTTAGCTGTAGGTCTACATTAACTTCCCTAGGAGTGAATGTAACAGA
RI-AT1G67060-XLOC_003407-10131-1

CONSENSUS
....

RI-AT1G67060-XLOC_003407-10131-0
TTATAGCTCTTTTGTGTTGGTTAGAAAGCTTTTAGTGAGGACTGGTTTTA
RI-AT1G67060-XLOC_003407-10131-1

CONSENSUS
....

RI-AT1G67060-XLOC_003407-10131-0
TAAAGATGTCTCTGCAGATAGTTAGGGTGTAGATTTGATGGCGATTTTGG
RI-AT1G67060-XLOC_003407-10131-1

CONSENSUS
....

RI-AT1G67060-XLOC_003407-10131-0
TTCTGTAAGAGTAGGAGTGTTCAGCTTCAGGTCTTAGTCTTACTATACTT
RI-AT1G67060-XLOC_003407-10131-1

CONSENSUS
....

RI-AT1G67060-XLOC_003407-10131-0
CTGCTTGTGTTTCTTCTAGATTGATTGATGCATATCACAATTTTACCTTA
RI-AT1G67060-XLOC_003407-10131-1

CONSENSUS
....

RI-AT1G67060-XLOC_003407-10131-0
TGCAAGTATTGCAGTGACAATGTGAGATCTACAAACTTTCCTTTGCAAGT
RI-AT1G67060-XLOC_003407-10131-1

CONSENSUS
....

RI-AT1G67060-XLOC_003407-10131-0
ACTTCCTGTATGAAACTTATCTACCAGCTTCTCTCTATGAGTCTTTATGT
RI-AT1G67060-XLOC_003407-10131-1

CONSENSUS
....

RI-AT1G67060-XLOC_003407-10131-0
TTTACCCCCTCATAAGAATTATCTTGAATAGGCTATGTAAAGGTTGAACA
RI-AT1G67060-XLOC_003407-10131-1

CONSENSUS
....

RI-AT1G67060-XLOC_003407-10131-0

```
      TAAATACCACCAAATGTGTGCACCATCTCTCTGTGATTCTGCAACTAACA
RI-AT1G67060-XLOC_003407-10131-1
      --------------------------------------------------
CONSENSUS
      ..................................................

RI-AT1G67060-XLOC_003407-10131-0
      GTCGCATATAATGTGGATAATGTGTTTCTACTAACAAATTGTCACTCGTT
RI-AT1G67060-XLOC_003407-10131-1
      --------------------------------------------------
CONSENSUS
      ..................................................

RI-AT1G67060-XLOC_003407-10131-0
      TTGGTAAATCAACTTCTTATTGAATCAAAAGGTAGAGGGGATGGAGGTGA
RI-AT1G67060-XLOC_003407-10131-1
      -------------------------------GTAGAGGGGATGGAGGTGA
CONSENSUS
      ...............................GTAGAGGGGATGGAGGTGA

RI-AT1G67060-XLOC_003407-10131-0
      ATGCAAATGAAGGGGGTTCGACCACAACACGTGGTGGCATTTATTGGTTG
RI-AT1G67060-XLOC_003407-10131-1
      ATGCAAATGAAGGGGGTTCGACCACAACACGTGGTGGCATTTATTGGTTG
CONSENSUS
      ATGCAAATGAAGGGGGTTCGACCACAACACGTGGTGGCATTTATTGGTTG

RI-AT1G67060-XLOC_003407-10131-0 ATCTTACCTGCTGGCT
RI-AT1G67060-XLOC_003407-10131-1 ATCTTACCTGCTGGCT
CONSENSUS                        ATCTTACCTGCTGGCT


alignment for event: RI-AT1G05135-XLOC_004382-2332

RI-AT1G05135-XLOC_004382-2332-0
      AATGTTCAAAACTAGAAAGATAGAAAGCTACTAAAAATCTAAACAAAGTG
RI-AT1G05135-XLOC_004382-2332-1
      AATGTTCAAAACTAGAAAGATAGAAAGCTACTAAAAATCTAAACAAAGTG
CONSENSUS
      AATGTTCAAAACTAGAAAGATAGAAAGCTACTAAAAATCTAAACAAAGTG

RI-AT1G05135-XLOC_004382-2332-0
      TAGATCTATTTTGACTTAGTCGAAGAAAACGTTCATTAAATGGTTCCCGC
RI-AT1G05135-XLOC_004382-2332-1
      TAGATCTATTTTGACTTAGTCGAAGAAAACGTTCATTAAATGGTTCCCGC
CONSENSUS
      TAGATCTATTTTGACTTAGTCGAAGAAAACGTTCATTAAATGGTTCCCGC

RI-AT1G05135-XLOC_004382-2332-0
      CAATGGCCCAACGGCCCCAAACGCGTAGAACGTAACATGCATGGGGATCC
RI-AT1G05135-XLOC_004382-2332-1
      CAATGGCCCAACGGCCCCAAACGCGTAGAACGTAACATGCATGGGGATCC
CONSENSUS
      CAATGGCCCAACGGCCCCAAACGCGTAGAACGTAACATGCATGGGGATCC
```

RI-AT1G05135-XLOC_004382-2332-0
ATATTAATGGCTTCTTCATCCCACTTGCTTATTTCTTTCGACTTTGGAGG
RI-AT1G05135-XLOC_004382-2332-1
ATATTAATGGCTTCTTCATCCCACTTGCTTATTTCTTTCGACTTTGGAGG
CONSENSUS
ATATTAATGGCTTCTTCATCCCACTTGCTTATTTCTTTCGACTTTGGAGG

RI-AT1G05135-XLOC_004382-2332-0
TTTCATTTACCTATTATATAAGGGAATGTTCAGTTCCAGTGACTGAGATA
RI-AT1G05135-XLOC_004382-2332-1
TTTCATTTACCTATTATATAAGGGAATGTTCAGTTCCAGTGACTGAGATA
CONSENSUS
TTTCATTTACCTATTATATAAGGGAATGTTCAGTTCCAGTGACTGAGATA

RI-AT1G05135-XLOC_004382-2332-0
TCACAAGCAAACAAGAGAAGACATTTTATTGTTATTTCTCTCTCTCTCTC
RI-AT1G05135-XLOC_004382-2332-1
TCACAAGCAAACAAGAGAAGACATTTTATTGTTATTTCTCTCTCTCTCTC
CONSENSUS
TCACAAGCAAACAAGAGAAGACATTTTATTGTTATTTCTCTCTCTCTCTC

RI-AT1G05135-XLOC_004382-2332-0
CTTGTAGATGGAACTTAAGGGCGTTACTTGTTTGCTTCTTTCTTTGGTTT
RI-AT1G05135-XLOC_004382-2332-1
CTTGTAGATGGAACTTAAGGGCGTTACTTGTTTGCTTCTTTCTTTGGTTT
CONSENSUS
CTTGTAGATGGAACTTAAGGGCGTTACTTGTTTGCTTCTTTCTTTGGTTT

RI-AT1G05135-XLOC_004382-2332-0
TGCTTAATTCGTGTGTGGAATGTGTTTTGGGGGATGGATCTGTGGTGGGT
RI-AT1G05135-XLOC_004382-2332-1
TGCTTAATTCGTGTGTGGAATGTGTTTTGGGGGATGGATCTGTGGTGGGT
CONSENSUS
TGCTTAATTCGTGTGTGGAATGTGTTTTGGGGGATGGATCTGTGGTGGGT

RI-AT1G05135-XLOC_004382-2332-0
CCTGCGAGGTTTAGAGACGATGATTGTAGGTGGGGCCGGAGGTGTGCTGG
RI-AT1G05135-XLOC_004382-2332-1
CCTGCGAGGTTTAGAGACGATGATTGTAGGTGGGGCCGGAGGTGTGCTGG
CONSENSUS
CCTGCGAGGTTTAGAGACGATGATTGTAGGTGGGGCCGGAGGTGTGCTGG

RI-AT1G05135-XLOC_004382-2332-0
ACGTGGCCGGTTTGGACGCGGTGGTGGTGGAGGGTTCGGCGGTGGGAGAG
RI-AT1G05135-XLOC_004382-2332-1
ACGTGGCCGGTTTGGACGCGGTGGTGGTGGAGGGTTCGGCGGTGGGAGAG
CONSENSUS
ACGTGGCCGGTTTGGACGCGGTGGTGGTGGAGGGTTCGGCGGTGGGAGAG

RI-AT1G05135-XLOC_004382-2332-0
GAAGTGGTGGCGGTATAGGCGGTGGTGGTGGACAAGGTGGAGGGTTTGGA
RI-AT1G05135-XLOC_004382-2332-1
GAAGTGGTGGCGGTATAGGCGGTGGTGGTGGACAAGGTGGAGGGTTTGGA
CONSENSUS
GAAGTGGTGGCGGTATAGGCGGTGGTGGTGGACAAGGTGGAGGGTTTGGA

RI-AT1G05135-XLOC_004382-2332-0
GCCGGAGGAGGAGTTGGTGGCGGAGCTGGAGGAGGACTTGGTGGTGGAGG
RI-AT1G05135-XLOC_004382-2332-1
GCCGGAGGAGGAGTTGGTGGCGGAGCTGGAGGAGGACTTGGTGGTGGAG-
CONSENSUS
GCCGGAGGAGGAGTTGGTGGCGGAGCTGGAGGAGGACTTGGTGGTGGAG.

RI-AT1G05135-XLOC_004382-2332-0
CGGAGCTGGTGGTGGTGGTGGAGGAGGTATTGGTGGTGGTTCCGGTCATG
RI-AT1G05135-XLOC_004382-2332-1

CONSENSUS
....

RI-AT1G05135-XLOC_004382-2332-0
GTGGTGGGTTTGGAGCCGGCGGAGGAGTTGGTGGGGGTGCTGGTGGAGGA
RI-AT1G05135-XLOC_004382-2332-1

CONSENSUS
....

RI-AT1G05135-XLOC_004382-2332-0
ATTGGTGGGGGAGGCGGAGCTGGTGGAGGCGGAGGAGGAG
RI-AT1G05135-XLOC_004382-2332-1 -------------
GCGGAGCTGGTGGAGGCGGAGGAGGAG
CONSENSUS
.............GCGGAGCTGGTGGAGGCGGAGGAGGAG

alignment for event: RI-AT1G35340-XLOC_001902-2562

RI-AT1G35340-XLOC_001902-2562-0
GAGCTTAACGGAGAATCCCTGAATCCGATTACTGCGATTCACATTTTCCC
RI-AT1G35340-XLOC_001902-2562-1
GAGCTTAACGGAGAATCCCTGAATCCGATTACTGCGATTCACATTTTCCC
CONSENSUS
GAGCTTAACGGAGAATCCCTGAATCCGATTACTGCGATTCACATTTTCCC

RI-AT1G35340-XLOC_001902-2562-0
GATGACTTCCACTCTAATCTCCTCAAACCTCTCTTCCTCTTTCTTCCCAA
RI-AT1G35340-XLOC_001902-2562-1
GATGACTTCCACTCTAATCTCCTCAAACCTCTCTTCCTCTTTCTTCCCAA
CONSENSUS
GATGACTTCCACTCTAATCTCCTCAAACCTCTCTTCCTCTTTCTTCCCAA

RI-AT1G35340-XLOC_001902-2562-0
CCCAAAATATCCACCGAATTCGAATCCCGACGACTTCAATTCCCGGAAGT
RI-AT1G35340-XLOC_001902-2562-1
CCCAAAATATCCACCGAATTCGAATCCCGACGACTTCAATTCCCGGAAGT
CONSENSUS
CCCAAAATATCCACCGAATTCGAATCCCGACGACTTCAATTCCCGGAAGT

RI-AT1G35340-XLOC_001902-2562-0
TTCAACATTCGCGCACGCCGTTCAAAAATCGTCGCCAAATCTCTCGATCT
RI-AT1G35340-XLOC_001902-2562-1

```
      TTCAACATTCGCGCACGCCGTTCAAAAATCGTCGCCAAATCTCTCGATCT
CONSENSUS
      TTCAACATTCGCGCACGCCGTTCAAAAATCGTCGCCAAATCTCTCGATCT

RI-AT1G35340-XLOC_001902-2562-0
      TCCTCTTCTTCCTTTTAGCATGAGCGAGGTTATCACCATGAATCTCACCT
RI-AT1G35340-XLOC_001902-2562-1
      TCCTCTTCTTCCTTTTAGCATGAGCGAG----------------------
CONSENSUS
      TCCTCTTCTTCCTTTTAGCATGAGCGAG......................

RI-AT1G35340-XLOC_001902-2562-0
      TAACCTTCCTTAGCTGATTTTTGATTTGAGAAATTCTTACACAGGTTCTT
RI-AT1G35340-XLOC_001902-2562-1
      --------------------------------------------------
CONSENSUS
      ..................................................

RI-AT1G35340-XLOC_001902-2562-0
      GTACCAACGGAGAGTAAAACTTTGCATTTATATGAAGCTAGGTACTTAGC
RI-AT1G35340-XLOC_001902-2562-1
      -----------------------------------------GTACTTAGC
CONSENSUS
      .........................................GTACTTAGC

RI-AT1G35340-XLOC_001902-2562-0  TTTACTCGAAGAG
RI-AT1G35340-XLOC_001902-2562-1  TTTACTCGAAGAG
CONSENSUS                        TTTACTCGAAGAG


alignment for event: MXE-AT1G77080-XLOC_003953-9829

MXE-AT1G77080-XLOC_003953-9829-0
      AGGATTAAATTAGGGCATAACCCTTATCGGAGATTTGAAGCCATGGGAAG
MXE-AT1G77080-XLOC_003953-9829-1
      AGGATTAAATTAGGGCATAACCCTTATCGGAGATTTGAAGCCATGGGAAG
CONSENSUS
      AGGATTAAATTAGGGCATAACCCTTATCGGAGATTTGAAGCCATGGGAAG

MXE-AT1G77080-XLOC_003953-9829-0
      AAGAAAAAATCGAGATCAAGCGAATCGAGAACAAAAGCAGTCGACAAGTCA
MXE-AT1G77080-XLOC_003953-9829-1
      AAGAAAAAATCGAGATCAAGCGAATCGAGAACAAAAGCAGTCGACAAGTCA
CONSENSUS
      AAGAAAAAATCGAGATCAAGCGAATCGAGAACAAAAGCAGTCGACAAGTCA

MXE-AT1G77080-XLOC_003953-9829-0
      CTTTCTCCAAACGACGCAATGGTCTCATCGACAAAGCTCGACAACTTTCG
MXE-AT1G77080-XLOC_003953-9829-1
      CTTTCTCCAAACGACGCAATGGTCTCATCGACAAAGCTCGACAACTTTCG
CONSENSUS
      CTTTCTCCAAACGACGCAATGGTCTCATCGACAAAGCTCGACAACTTTCG

MXE-AT1G77080-XLOC_003953-9829-0
      ATTCTCTGTGAATCCTCCGTCGCTGTTGTCGTCGTATCTGCCTCCGGAAA
```

MXE-AT1G77080-XLOC_003953-9829-1
      ATTCTCTGTGAATCCTCCGTCGCTGTTGTCGTCGTATCTGCCTCCGGAAA
CONSENSUS
      ATTCTCTGTGAATCCTCCGTCGCTGTTGTCGTCGTATCTGCCTCCGGAAA

MXE-AT1G77080-XLOC_003953-9829-0
      ACTCTATGACTCTTCCTCCGGTGACGACATTTCCAAGATCATTGATCGTT
MXE-AT1G77080-XLOC_003953-9829-1
      ACTCTATGACTCTTCCTCCGGTGACGA-----------------------
CONSENSUS
      ACTCTATGACTCTTCCTCCGGTGACGA.......................

MXE-AT1G77080-XLOC_003953-9829-0
      ATGAAATACAACATGCTGATGAACTTAGAGCCTTA---------------
MXE-AT1G77080-XLOC_003953-9829-1
      -----------------------------------GATAGAAGCGCTGTT
CONSENSUS
      ....

MXE-AT1G77080-XLOC_003953-9829-0
      -------------------------------GATCTTGAAGAAAAAATTC
MXE-AT1G77080-XLOC_003953-9829-1
      CAAGCCGGAGAAACCTCAATGTTTTGAACTCGATCTTGAAGAAAAAATTC
CONSENSUS
      ...............................GATCTTGAAGAAAAAATTC

MXE-AT1G77080-XLOC_003953-9829-0
      AGAATTATCTTCCACACAAGGAGTTACTAGAAACAGTCCAAAG
MXE-AT1G77080-XLOC_003953-9829-1
      AGAATTATCTTCCACACAAGGAGTTACTAGAAACAGTCCAAAG
CONSENSUS
      AGAATTATCTTCCACACAAGGAGTTACTAGAAACAGTCCAAAG

alignment for event: RI-AT1G05870-XLOC_004430-8333

RI-AT1G05870-XLOC_004430-8333-0
      TGCTGAGAGACTTTGTGTTTATGAGAGCTATCACTAACTGGTTATGGACC
RI-AT1G05870-XLOC_004430-8333-1
      TGCTGAGAGACTTTGTGTTTATGAGAGCTATCACTAACTGGTTATGGACC
CONSENSUS
      TGCTGAGAGACTTTGTGTTTATGAGAGCTATCACTAACTGGTTATGGACC

RI-AT1G05870-XLOC_004430-8333-0
      TCCACTTGCTGAGGTAACTAATCTTAACTTGGTAACTAAATCTACAACCC
RI-AT1G05870-XLOC_004430-8333-1
      TCCACTTGCTGAG-------------------------------------
CONSENSUS
      TCCACTTGCTGAG.....................................

RI-AT1G05870-XLOC_004430-8333-0
      TCTTTATGAAGGTACTGACATTACTACTGTCCTCTGACAGTCTTTAACTC
RI-AT1G05870-XLOC_004430-8333-1
      --TCTTTAACTC
CONSENSUS

.....................................TCTTTAACTC

RI-AT1G05870-XLOC_004430-8333-0
ATTCCATTTTTGTGAGTGTGTGTTGAATCTACAAACCTTGTGGCTAGATA
RI-AT1G05870-XLOC_004430-8333-1
ATTCCATTTTTGTGAGTGTGTGTTGAATCTACAAACCTTGTGGCTAGATA
CONSENSUS
ATTCCATTTTTGTGAGTGTGTGTTGAATCTACAAACCTTGTGGCTAGATA

RI-AT1G05870-XLOC_004430-8333-0
ACCTAATTTGGTGATGCTCCTTAATCATTTCATGAAGAATCGAGTGGGAC
RI-AT1G05870-XLOC_004430-8333-1
ACCTAATTTGGTGATGCTCCTTAATCATTTCATGAAGAATCGAGTGGGAC
CONSENSUS
ACCTAATTTGGTGATGCTCCTTAATCATTTCATGAAGAATCGAGTGGGAC

RI-AT1G05870-XLOC_004430-8333-0
GAAGAGAAAGATGATAGACGCCAAATATTATGTGTTTCCATCTTTTTCGT
RI-AT1G05870-XLOC_004430-8333-1
GAAGAGAAAGATGATAGACGCCAAATATTATGTGTTTCCATCTTTTTCGT
CONSENSUS
GAAGAGAAAGATGATAGACGCCAAATATTATGTGTTTCCATCTTTTTCGT

RI-AT1G05870-XLOC_004430-8333-0
TCTTCATCAACTAAAAGATGATGGCTGATTACAGTCCTAGATGCTCTATA
RI-AT1G05870-XLOC_004430-8333-1
TCTTCATCAACTAAAAGATGATGGCTGATTACAGTCCTAGATGCTCTATA
CONSENSUS
TCTTCATCAACTAAAAGATGATGGCTGATTACAGTCCTAGATGCTCTATA

RI-AT1G05870-XLOC_004430-8333-0
TGGCGACTGAAGATGAGTTATTTTCTGGAATTTAAGAGTGAGTGTAAATA
RI-AT1G05870-XLOC_004430-8333-1
TGGCGACTGAAGATGAGTTATTTTCTGGAATTTAAGAGTGAGTGTAAATA
CONSENSUS
TGGCGACTGAAGATGAGTTATTTTCTGGAATTTAAGAGTGAGTGTAAATA

RI-AT1G05870-XLOC_004430-8333-0
TAAGCTTAAACAGTTTCTGGTGACTGTTCTGTAATACATGTTAACGACTC
RI-AT1G05870-XLOC_004430-8333-1
TAAGCTTAAACAGTTTCTGGTGACTGTTCTGTAATACATGTTAACGACTC
CONSENSUS
TAAGCTTAAACAGTTTCTGGTGACTGTTCTGTAATACATGTTAACGACTC

RI-AT1G05870-XLOC_004430-8333-0
CACTCAACAATAAGTCTTGAGAGAACCTAATCAATGAAATTCTGAACATC
RI-AT1G05870-XLOC_004430-8333-1
CACTCAACAATAAGTCTTGAGAGAACCTAATCAATGAAATTCTGAACATC
CONSENSUS
CACTCAACAATAAGTCTTGAGAGAACCTAATCAATGAAATTCTGAACATC

RI-AT1G05870-XLOC_004430-8333-0 AAAC
RI-AT1G05870-XLOC_004430-8333-1 AAAC
CONSENSUS AAAC
