# Supplementary material for: Splicing-related genes are alternatively spliced upon changes in ambient temperatures in plants
Source: PLoS One. 2017 Mar 3;12(3):e0172950. doi: 10.1371/journal.pone.0172950 (PMC5336241; doi:10.1371/journal.pone.0172950)
Supplement: S5 Table — (PDF) [file pone.0172950.s005.pdf]

alignment for event: A5-AT4G10970-XLOC_023413-13725

A5-AT4G10970-XLOC_023413-13725-0
GGTGCTTTTGCTAAGAAGAGGTCTAATTTCCAAGGAAACCAGTTTCCTGT
A5-AT4G10970-XLOC_023413-13725-1
GGTGCTTTTGCTAAGAAGAGGTCTAATTTCCAAGGAAACCAGTTTCCTGT
CONSENSUS
GGTGCTTTTGCTAAGAAGAGGTCTAATTTCCAAGGAAACCAGTTTCCTGT

A5-AT4G10970-XLOC_023413-13725-0
AACAACAACCGTTGCTCGTAAAGCCGCTTCTGCTACTCCGCGTGGTAGAC
A5-AT4G10970-XLOC_023413-13725-1
AACAACAACCGTTGCTCGTAAAGCCGCTTCTGCTACTCCGCGTGGTAGAC
CONSENSUS
AACAACAACCGTTGCTCGTAAAGCCGCTTCTGCTACTCCGCGTGGTAGAC

A5-AT4G10970-XLOC_023413-13725-0
CTTATAATGGTGGAAGGATGACTAATACGAATCAATCAAGGTCTAGATTG
A5-AT4G10970-XLOC_023413-13725-1
CTTATAATGGTGGAAGGATGACTAATACGAATCAATCAAG----------
CONSENSUS
CTTATAATGGTGGAAGGATGACTAATACGAATCAATCAAG..........

A5-AT4G10970-XLOC_023413-13725-0
CTACAGATAATTCTCTGATCTGTTTGTTAACATGGGCTCTTTTTATGGTC
A5-AT4G10970-XLOC_023413-13725-1

CONSENSUS
....

A5-AT4G10970-XLOC_023413-13725-0
TTCGATTATTTCTGCTACTAGGAGAAGCTTCTAAGCTGCTTATCATAGGG
A5-AT4G10970-XLOC_023413-13725-1

CONSENSUS
....

A5-AT4G10970-XLOC_023413-13725-0
TGTGTCTATGGAAGGAAACACGGGTTTGTATACTTGTGTTTCAAACCCGT
A5-AT4G10970-XLOC_023413-13725-1

CONSENSUS
....

A5-AT4G10970-XLOC_023413-13725-0
GTTTCAAACATAGTAATTCCCTTCCTGGATGATGGATGCTCATCAGATTG
A5-AT4G10970-XLOC_023413-13725-1

CONSENSUS
....

A5-AT4G10970-XLOC_023413-13725-0
ATGACTGTATAACCCTGGCTCAAAATAGCGAGTCAAAACCATCTAGTTGA
A5-AT4G10970-XLOC_023413-13725-1

CONSENSUS

....

A5-AT4G10970-XLOC_023413-13725-0
GTTTAAGGAGGATATTTGGAGGAGGAGTTAAACTTTAATGGGGTGGTAGG
A5-AT4G10970-XLOC_023413-13725-1

CONSENSUS
....

A5-AT4G10970-XLOC_023413-13725-0
TTTGACATAAAGGAAGGAGTTTAAGCTCATTCAGATTGAGGAGAGACTTT
A5-AT4G10970-XLOC_023413-13725-1

CONSENSUS
....

A5-AT4G10970-XLOC_023413-13725-0
TCTGGTCATTGGTTTTGGAGTTTGCTTGAAGTCAGTTTTAATTATCAGAT
A5-AT4G10970-XLOC_023413-13725-1

CONSENSUS
....

A5-AT4G10970-XLOC_023413-13725-0
TTAGCCAATTGGTGCAATATGTTATCGTGCTTAATTCTTAAAACTCAGTT
A5-AT4G10970-XLOC_023413-13725-1

CONSENSUS
....

A5-AT4G10970-XLOC_023413-13725-0
GCATTCATGTCAGCTTCCTAAATGTTGAGTTCTGTCTTCTTGTTGTGACC
A5-AT4G10970-XLOC_023413-13725-1

CONSENSUS
....

A5-AT4G10970-XLOC_023413-13725-0
TAAAGAGTATAGCTTGAGTCAGTTGGTCTATTGTTGGGAGATTGAAATGG
A5-AT4G10970-XLOC_023413-13725-1

CONSENSUS
....

A5-AT4G10970-XLOC_023413-13725-0
GTAGATGCACGGTTTATTGCTCCACCAGCTCAGAATAGAGCTTCACAAAG
A5-AT4G10970-XLOC_023413-13725-1 -----------
GTTTATTGCTCCACCAGCTCAGAATAGAGCTTCACAAAG
CONSENSUS
...........GTTTATTGCTCCACCAGCTCAGAATAGAGCTTCACAAAG

A5-AT4G10970-XLOC_023413-13725-0 AGGGTTTGTCGGAAAG
A5-AT4G10970-XLOC_023413-13725-1 AGGGTTTGTCGGAAAG
CONSENSUS AGGGTTTGTCGGAAAG

```
alignment for event: A3-AT4G21160-XLOC_021545-9946

A3-AT4G21160-XLOC_021545-9946-0
      CAACTTGAAGGCATGGTTGAGTTTATTGGATTGTTGAAGGTGACTATTAA
A3-AT4G21160-XLOC_021545-9946-1
      CAACTTGAAGGCATGGTTGAGTTTATTGGATTGTTGAAGGTGACTATTAA
CONSENSUS
      CAACTTGAAGGCATGGTTGAGTTTATTGGATTGTTGAAGGTGACTATTAA

A3-AT4G21160-XLOC_021545-9946-0
      AAAGGGTACCAATATGGCCATCCGAGATATGATGTCAAGTGATCCCTATG
A3-AT4G21160-XLOC_021545-9946-1
      AAAGGGTACCAATATGGCCATCCGAGATATGATGTCAAGTGATCCCTATG
CONSENSUS
      AAAGGGTACCAATATGGCCATCCGAGATATGATGTCAAGTGATCCCTATG

A3-AT4G21160-XLOC_021545-9946-0
      TTGTGTTGACTCTAGGACAACAGAAGGCTCAATCTACTGTAGTGAAGAGC
A3-AT4G21160-XLOC_021545-9946-1  TTGTGTTGACTCTAGGACAACAG---
GCTCAATCTACTGTAGTGAAGAGC
CONSENSUS
      TTGTGTTGACTCTAGGACAACAG...GCTCAATCTACTGTAGTGAAGAGC

A3-AT4G21160-XLOC_021545-9946-0
      AACTTAAACCCTGTCTGGAATGAGGAACTCATGCTCTCTGTTCCTCATAA
A3-AT4G21160-XLOC_021545-9946-1
      AACTTAAACCCTGTCTGGAATGAGGAACTCATGCTCTCTGTTCCTCATAA
CONSENSUS
      AACTTAAACCCTGTCTGGAATGAGGAACTCATGCTCTCTGTTCCTCATAA

A3-AT4G21160-XLOC_021545-9946-0  CTATGGCTCAGTGAAATTG
A3-AT4G21160-XLOC_021545-9946-1  CTATGGCTCAGTGAAATTG
CONSENSUS                        CTATGGCTCAGTGAAATTG


alignment for event: A3-AT4G37480-XLOC_022464-8165

A3-AT4G37480-XLOC_022464-8165-0
      GTGAGACATTTGCACTGGTACAAAATTGGGGAAAAGGTTTCCATTTGTGA
A3-AT4G37480-XLOC_022464-8165-1
      GTGAGACATTTGCACTGGTACAAAATTGGGGAAAAGGTTTCCATTTGTGA
CONSENSUS
      GTGAGACATTTGCACTGGTACAAAATTGGGGAAAAGGTTTCCATTTGTGA

A3-AT4G37480-XLOC_022464-8165-0
      ATGTAGATGCAGTAGAGCGAAGTTACCACCAAGCAATGACATGAAACCAT
A3-AT4G37480-XLOC_022464-8165-1
      ATGTAGATGCAGTAGAGCGAAGTTACCACCAAGCAA--------------
CONSENSUS
      ATGTAGATGCAGTAGAGCGAAGTTACCACCAAGCAA..............

A3-AT4G37480-XLOC_022464-8165-0
      AATTGCATGGATTTGAAAACAGGTTTTGGTTGTTTGAGCCTCGTTGTGGT
A3-AT4G37480-XLOC_022464-8165-1  ---------------------
```

GTTTTGGTTGTTTGAGCCTCGTTGTGGT
CONSENSUS
      ......................GTTTTGGTTGTTTGAGCCTCGTTGTGGT

A3-AT4G37480-XLOC_022464-8165-0
      TTGCATGATGTCGGAGGATGGTATGTTGAAACTTATGGGAAAGATAAAAA
A3-AT4G37480-XLOC_022464-8165-1
      TTGCATGATGTCGGAGGATGGTATGTTGAAACTTATGGGAAAGATAAAAA
CONSENSUS
      TTGCATGATGTCGGAGGATGGTATGTTGAAACTTATGGGAAAGATAAAAA

A3-AT4G37480-XLOC_022464-8165-0
      GAGCAGGACGGTCCTGGCACAAAGATTCTGGGATGGCCTGGAAGAGGGGA
A3-AT4G37480-XLOC_022464-8165-1
      GAGCAGGACGGTCCTGGCACAAAGATTCTGGGATGGCCTGGAAGAGGGGA
CONSENSUS
      GAGCAGGACGGTCCTGGCACAAAGATTCTGGGATGGCCTGGAAGAGGGGA

A3-AT4G37480-XLOC_022464-8165-0 GTACACTGGATGG
A3-AT4G37480-XLOC_022464-8165-1 GTACACTGGATGG
CONSENSUS   GTACACTGGATGG

alignment for event: A3-AT4G31550-XLOC_024617-9872

A3-AT4G31550-XLOC_024617-9872-0
      ATCACAACCATTTCTCTCTCTCGTCTTCGTCTTCTTCTTCTTCAACGTTC
A3-AT4G31550-XLOC_024617-9872-1
      ATCACAACCATTTCTCTCTCTCGTCTTCGTCTTCTTCTTCTTCAACGTTC
CONSENSUS
      ATCACAACCATTTCTCTCTCTCGTCTTCGTCTTCTTCTTCTTCAACGTTC

A3-AT4G31550-XLOC_024617-9872-0
      CTCTCCAAAATCCTCAGACCAAGAAATCATCATGGCCGTCGATCTAATGC
A3-AT4G31550-XLOC_024617-9872-1
      CTCTCCAAAATCCTCAGACCAAGAAATCATCATGGCCGTCGATCTAATGC
CONSENSUS
      CTCTCCAAAATCCTCAGACCAAGAAATCATCATGGCCGTCGATCTAATGC

A3-AT4G31550-XLOC_024617-9872-0
      GTTTCCCTAAGATAGATGATCAAACGGCTATTCAGGAAGCTGCATCGCAA
A3-AT4G31550-XLOC_024617-9872-1
      GTTTCCCTAAGATAGATGATCAAACGGCTATTCAGGAAGCTGCATCGCAA
CONSENSUS
      GTTTCCCTAAGATAGATGATCAAACGGCTATTCAGGAAGCTGCATCGCAA

A3-AT4G31550-XLOC_024617-9872-0
      GGTTTACAAAGTATGGAACATCTGATCCGTGTCCTCTCTAACCGTCCCGA
A3-AT4G31550-XLOC_024617-9872-1
      GGTTTACAAAGTATGGAACATCTGATCCGTGTCCTCTCTAACCGTCCCGA
CONSENSUS
      GGTTTACAAAGTATGGAACATCTGATCCGTGTCCTCTCTAACCGTCCCGA

A3-AT4G31550-XLOC_024617-9872-0
      ACAACAACACAACGTTGACTGCTCCGAGATCACTGACTTCACCGTTTCTA

A3-AT4G31550-XLOC_024617-9872-1
ACAACAACACAACGTTGACTGCTCCGAGATCACTGACTTCACCGTTTCTA
CONSENSUS
ACAACAACACAACGTTGACTGCTCCGAGATCACTGACTTCACCGTTTCTA

A3-AT4G31550-XLOC_024617-9872-0
AATTCAAAACCGTCATTTCTCTCCTTAACCGTACTGGTCACGCTCGGTTC
A3-AT4G31550-XLOC_024617-9872-1
AATTCAAAACCGTCATTTCTCTCCTTAACCGTACTGGTCACGCTCGGTTC
CONSENSUS
AATTCAAAACCGTCATTTCTCTCCTTAACCGTACTGGTCACGCTCGGTTC

A3-AT4G31550-XLOC_024617-9872-0
AGACGCGGACCGGTTCACTCCACTTCCTCTGCCGCATCTCAGAAACTACA
A3-AT4G31550-XLOC_024617-9872-1
AGACGCGGACCGGTTCACTCCACTTCCTCTGCCGCATCTCAGAAACTACA
CONSENSUS
AGACGCGGACCGGTTCACTCCACTTCCTCTGCCGCATCTCAGAAACTACA

A3-AT4G31550-XLOC_024617-9872-0
GAGTCAGATCGTTAAAAATACTCAACCTGAGGCTCCGATAGTGAGAACAA
A3-AT4G31550-XLOC_024617-9872-1
GAGTCAGATCGTTAAAAATACTCAACCTGAGGCTCCGATAGTGAGAACAA
CONSENSUS
GAGTCAGATCGTTAAAAATACTCAACCTGAGGCTCCGATAGTGAGAACAA

A3-AT4G31550-XLOC_024617-9872-0
CTACGAATCACCCTCAAATCGTTCCTCCACCGTCTAGTGTAACACTCGAT
A3-AT4G31550-XLOC_024617-9872-1
CTACGAATCACCCTCAAATCGTTCCTCCACCGTCTAGTGTAACACTCGAT
CONSENSUS
CTACGAATCACCCTCAAATCGTTCCTCCACCGTCTAGTGTAACACTCGAT

A3-AT4G31550-XLOC_024617-9872-0
TTCTCTAAACCAAGCATCTTCGGCACCAAAGCTAAGAGCGCCGAGCTGGA
A3-AT4G31550-XLOC_024617-9872-1
TTCTCTAAACCAAGCATCTTCGGCACCAAAGCTAAGAGCGCCGAGCTGGA
CONSENSUS
TTCTCTAAACCAAGCATCTTCGGCACCAAAGCTAAGAGCGCCGAGCTGGA

A3-AT4G31550-XLOC_024617-9872-0
ATTCTCCAAAGAAAACTTCAGTGTTTCTTTAAACTCCTCATTCATGTCGT
A3-AT4G31550-XLOC_024617-9872-1
ATTCTCCAAAGAAAACTTCAGTGTTTCTTTAAACTCCTCATTCATGTCGT
CONSENSUS
ATTCTCCAAAGAAAACTTCAGTGTTTCTTTAAACTCCTCATTCATGTCGT

A3-AT4G31550-XLOC_024617-9872-0
CGGCGATAACCGGAGACGGCAGCGTCTCCAATGGAAAAATCTTCCTTGCT
A3-AT4G31550-XLOC_024617-9872-1
CGGCGATAACCGGAGACGGCAGCGTCTCCAATGGAAAAATCTTCCTTGCT
CONSENSUS
CGGCGATAACCGGAGACGGCAGCGTCTCCAATGGAAAAATCTTCCTTGCT

A3-AT4G31550-XLOC_024617-9872-0
TCTGCTCCGTTGCAGCCTGTTAACTCTTCCGGAAAACCACCGTTGGCTGG

A3-AT4G31550-XLOC_024617-9872-1
      TCTGCTCCGTTGCAGCCTGTTAACTCTTCCGGAAAACCACCGTTGGCTGG
CONSENSUS
      TCTGCTCCGTTGCAGCCTGTTAACTCTTCCGGAAAACCACCGTTGGCTGG

A3-AT4G31550-XLOC_024617-9872-0
      TCATCCTTACAGAAAGAGATGTCTCGAGCATGAGCACTCAGAGAGTTTCT
A3-AT4G31550-XLOC_024617-9872-1
      TCATCCTTACAGAAAGAGATGTCTCGAGCATGAGCACTCAGAGAGTTTCT
CONSENSUS
      TCATCCTTACAGAAAGAGATGTCTCGAGCATGAGCACTCAGAGAGTTTCT

A3-AT4G31550-XLOC_024617-9872-0
      CCGGAAAAGTCTCCGGCTCCGCCTACGGAAAGTGCCATTGCAAGAAAAG-
A3-AT4G31550-XLOC_024617-9872-1
      CCGGAAAAGTCTCCGGCTCCGCCTACGGAAAGTGCCATTGCAAGAAAAGC
CONSENSUS
      CCGGAAAAGTCTCCGGCTCCGCCTACGGAAAGTGCCATTGCAAGAAAAG.

A3-AT4G31550-XLOC_024617-9872-0 --
GAAAAATCGGATGAAGAGAACCGTGAGAGTACCGGCGATAAGTGCAAA
A3-AT4G31550-XLOC_024617-9872-1
      AGGAAAAATCGGATGAAGAGAACCGTGAGAGTACCGGCGATAAGTGCAAA
CONSENSUS
      ..GAAAAATCGGATGAAGAGAACCGTGAGAGTACCGGCGATAAGTGCAAA

A3-AT4G31550-XLOC_024617-9872-0
      GATCGCCGATATTCCACCGGACGAATATTCGTGGAGGAAGTACGGACAAA
A3-AT4G31550-XLOC_024617-9872-1
      GATCGCCGATATTCCACCGGACGAATATTCGTGGAGGAAGTACGGACAAA
CONSENSUS
      GATCGCCGATATTCCACCGGACGAATATTCGTGGAGGAAGTACGGACAAA

A3-AT4G31550-XLOC_024617-9872-0 AACCGATCAAGGGCTCACCACACCCACG
A3-AT4G31550-XLOC_024617-9872-1 AACCGATCAAGGGCTCACCACACCCACG
CONSENSUS   AACCGATCAAGGGCTCACCACACCCACG

alignment for event: A5-AT4G24440-XLOC_021734-4019

A5-AT4G24440-XLOC_021734-4019-0
      ATAAAAGCCCCTAATTTTTTCTCCGCCCAAGAGACAAAAATCCTTCGCAA
A5-AT4G24440-XLOC_021734-4019-1
      ATAAAAGCCCCTAATTTTTTCTCCGCCCAAGAGACAAAAATCCTTCGCAA
CONSENSUS
      ATAAAAGCCCCTAATTTTTTCTCCGCCCAAGAGACAAAAATCCTTCGCAA

A5-AT4G24440-XLOC_021734-4019-0
      TCGAGCAACGGAAGCTCCTGCATCCACCGTCCAAATCTCTTCTAACGTCG
A5-AT4G24440-XLOC_021734-4019-1
      TCGAGCAACGGAAGCTCCTGCATCCACCGTCCAAATCTCTTCTAACGTCG
CONSENSUS
      TCGAGCAACGGAAGCTCCTGCATCCACCGTCCAAATCTCTTCTAACGTCG

A5-AT4G24440-XLOC_021734-4019-0

CCGGAGATCG---------------------------------------A
A5-AT4G24440-XLOC_021734-4019-1
CCGGAGATCGGTAAACCGGTTCTTCATCCCGTATTCATCTCTTATTGAGA
CONSENSUS
CCGGAGATCG.......................................A

A5-AT4G24440-XLOC_021734-4019-0
TCGTTGGGGAAGTAGAGAATGGCGACGTTTGAGCTGTACAGGAGATCGAC
A5-AT4G24440-XLOC_021734-4019-1
TCGTTGGGGAAGTAGAGAATGGCGACGTTTGAGCTGTACAGGAGATCGAC
CONSENSUS
TCGTTGGGGAAGTAGAGAATGGCGACGTTTGAGCTGTACAGGAGATCGAC

A5-AT4G24440-XLOC_021734-4019-0
GATCGGGATGTGTCTGACGGAGACTTTGGACGAGATGGTTCAGAGCGGTA
A5-AT4G24440-XLOC_021734-4019-1
GATCGGGATGTGTCTGACGGAGACTTTGGACGAGATGGTTCAGAGCGGTA
CONSENSUS
GATCGGGATGTGTCTGACGGAGACTTTGGACGAGATGGTTCAGAGCGGTA

A5-AT4G24440-XLOC_021734-4019-0
CGCTGAGCCCTGAGCTAGCTATCCAAGTCCTTGTTCAATTTGACAAG
A5-AT4G24440-XLOC_021734-4019-1
CGCTGAGCCCTGAGCTAGCTATCCAAGTCCTTGTTCAATTTGACAAG
CONSENSUS
CGCTGAGCCCTGAGCTAGCTATCCAAGTCCTTGTTCAATTTGACAAG

alignment for event: A3-AT4G22890-XLOC_021647-12594

A3-AT4G22890-XLOC_021647-12594-0
ATCTCCTAATTTCTTCCCTCCAAATTTATAATAAAACCAAAAAAAAAAAA
A3-AT4G22890-XLOC_021647-12594-1
ATCTCCTAATTTCTTCCCTCCAAATTTATAATAAAACCAAAAAAAAAAAA
CONSENSUS
ATCTCCTAATTTCTTCCCTCCAAATTTATAATAAAACCAAAAAAAAAAAA

A3-AT4G22890-XLOC_021647-12594-0
AAAAAAAAAAATCAGAAGAAACCTGAGAAGCTCACAGTAAACACATCTTC
A3-AT4G22890-XLOC_021647-12594-1
AAAAAAAAAAATCAGAAGAAACCTGAGAAGCTCACAGTAAACACATCTTC
CONSENSUS
AAAAAAAAAAATCAGAAGAAACCTGAGAAGCTCACAGTAAACACATCTTC

A3-AT4G22890-XLOC_021647-12594-0
AACCACAGGTTCATACTTACTGAAAAAAACAGAGGAAAAAAAGGAGCTCC
A3-AT4G22890-XLOC_021647-12594-1
AACCACAGGTTCATACTTACTGAAAAAAACAGAGGAAAAAAAGGAGCTCC
CONSENSUS
AACCACAGGTTCATACTTACTGAAAAAAACAGAGGAAAAAAAGGAGCTCC

A3-AT4G22890-XLOC_021647-12594-0
CTTTTCTATCTCTAAGGGAAAATGGGTAGCAAGATGTTGTTTAGTTTGAC
A3-AT4G22890-XLOC_021647-12594-1
CTTTTCTATCTCTAAGGGAAAATGGGTAGCAAGATGTTGTTTAGTTTGAC

CONSENSUS
CTTTTCTATCTCTAAGGGAAAATGGGTAGCAAGATGTTGTTTAGTTTGAC

A3-AT4G22890-XLOC_021647-12594-0
AAGTCCTCGACTTTTCTCCGCCGTTTCTCGCAAACCTTCCTCTTCTTTCT
A3-AT4G22890-XLOC_021647-12594-1
AAGTCCTCGACTTTTCTCCGCCGTTTCTCGCAAACCTTCCTCTTCTTTCT
CONSENSUS
AAGTCCTCGACTTTTCTCCGCCGTTTCTCGCAAACCTTCCTCTTCTTTCT

A3-AT4G22890-XLOC_021647-12594-0
CTCCTTCTCCTCCGTCGCCGTCTTCGAGGACTCAATGGACTCAGCTCAGC
A3-AT4G22890-XLOC_021647-12594-1
CTCCTTCTCCTCCGTCGCCGTCTTCGAGGACTCAATGGACTCAGCTCAGC
CONSENSUS
CTCCTTCTCCTCCGTCGCCGTCTTCGAGGACTCAATGGACTCAGCTCAGC

A3-AT4G22890-XLOC_021647-12594-0
CCTGGAAAATCGATTTCTTTGAGAAGAAGAGTCTTCTTGTTGCCTGCTAA
A3-AT4G22890-XLOC_021647-12594-1
CCTGGAAAATCGATTTCTTTGAGAAGAAGAGTCTTCTTGTTGCCTGCTAA
CONSENSUS
CCTGGAAAATCGATTTCTTTGAGAAGAAGAGTCTTCTTGTTGCCTGCTAA

A3-AT4G22890-XLOC_021647-12594-0
AGCCACAACAGAGCAATCAGTAGGAGGAGACAACGTCGATAGCAATGTTT
A3-AT4G22890-XLOC_021647-12594-1 AGCCACAACAGAGCAATCAG---
GAGGAGACAACGTCGATAGCAATGTTT
CONSENSUS
AGCCACAACAGAGCAATCAG...GAGGAGACAACGTCGATAGCAATGTTT

A3-AT4G22890-XLOC_021647-12594-0
TGCCCTATTGTAGCATCAACAAGGCTGAGAAGAAAACAATTGGTGAAATG
A3-AT4G22890-XLOC_021647-12594-1
TGCCCTATTGTAGCATCAACAAGGCTGAGAAGAAAACAATTGGTGAAATG
CONSENSUS
TGCCCTATTGTAGCATCAACAAGGCTGAGAAGAAAACAATTGGTGAAATG

A3-AT4G22890-XLOC_021647-12594-0 GAACAAGAGTTTCTCCAAGCGTTGCAA
A3-AT4G22890-XLOC_021647-12594-1 GAACAAGAGTTTCTCCAAGCGTTGCAA
CONSENSUS GAACAAGAGTTTCTCCAAGCGTTGCAA

alignment for event: SE-AT4G38960-XLOC_022539-6912

SE-AT4G38960-XLOC_022539-6912-0
CAAAGTATGCTTATTTGATACTCTCGGCGCGTGGATTTGGTCGTCGTCTC
SE-AT4G38960-XLOC_022539-6912-1
CAAAGTATGCTTATTTGATACTCTCGGCGCGTGGATTTGGTCGTCGTCTC
CONSENSUS
CAAAGTATGCTTATTTGATACTCTCGGCGCGTGGATTTGGTCGTCGTCTC

SE-AT4G38960-XLOC_022539-6912-0
TTCTGGTCTCTCAAGCTTCTCCTCCATCGTAGAAAAAGATTTTAAAGGAG
SE-AT4G38960-XLOC_022539-6912-1

TTCTGGTCTCTCAAGCTTCTCCTCCATCGTAGAAAAAGATTTTAAAGGAG
CONSENSUS
TTCTGGTCTCTCAAGCTTCTCCTCCATCGTAGAAAAAGATTTTAAAGGAG

SE-AT4G38960-XLOC_022539-6912-0
CGATTTTTATAATCTTAAATAATTTCTTCCCTTTCTTCTTCTTCTTCTAG
SE-AT4G38960-XLOC_022539-6912-1
CGATTTTTATAATCTTAAATAATTTCTTCCCTTTCTTCTTCTTCTTCTAG
CONSENSUS
CGATTTTTATAATCTTAAATAATTTCTTCCCTTTCTTCTTCTTCTTCTAG

SE-AT4G38960-XLOC_022539-6912-0
TTCCTTGATACCAACGCGTAATCCTAACCCTTAATCTACTTCCTCCTTAA
SE-AT4G38960-XLOC_022539-6912-1
TTCCTTGATACCAACGCGTAATCCTAACCCTTAATCTACTTCCTCCTTAA
CONSENSUS
TTCCTTGATACCAACGCGTAATCCTAACCCTTAATCTACTTCCTCCTTAA

SE-AT4G38960-XLOC_022539-6912-0
TTAATCAGAGGTTTCTACTTATTATTTCCACTTCTCATCTCTTTCAATCG
SE-AT4G38960-XLOC_022539-6912-1
TTAATCAGAGGTTTCTACTTATTATTTCCACTTCTCATCTCTTTCAATCG
CONSENSUS
TTAATCAGAGGTTTCTACTTATTATTTCCACTTCTCATCTCTTTCAATCG

SE-AT4G38960-XLOC_022539-6912-0
CAATCTGAGTTCTGGCTAAAGATTTTGTCTTGAAGGTGAGATTCATTGTT
SE-AT4G38960-XLOC_022539-6912-1
CAATCTGAGTTCTGGCTAAAGATTTTGTCTTGAAGGTGAGATTCATTGTT
CONSENSUS
CAATCTGAGTTCTGGCTAAAGATTTTGTCTTGAAGGTGAGATTCATTGTT

SE-AT4G38960-XLOC_022539-6912-0
CCGAATAGAGTGAGAAAAAGACTTATAAGATTGAAAAAGCAAGATGCGGA
SE-AT4G38960-XLOC_022539-6912-1
CCGAATAGAGTGAGAAAAAGACTTATAAGATTGAAAAAGCAAGATGCGGA
CONSENSUS
CCGAATAGAGTGAGAAAAAGACTTATAAGATTGAAAAAGCAAGATGCGGA

SE-AT4G38960-XLOC_022539-6912-0
TTTTGTGCGATGCTTGCGAGAACGCAGCCGCAATCATCTTTTGCGCCGCC
SE-AT4G38960-XLOC_022539-6912-1
TTTTGTGCGATGCTTGCGAGAACGCAGCCGCAATCATCTTTTGCGCCGCC
CONSENSUS
TTTTGTGCGATGCTTGCGAGAACGCAGCCGCAATCATCTTTTGCGCCGCC

SE-AT4G38960-XLOC_022539-6912-0
GATGAAGCTGCCCTTTGTCGCCCCTGCGATGAAAAA-------------
SE-AT4G38960-XLOC_022539-6912-1
GATGAAGCTGCCCTTTGTCGCCCCTGCGATGAAAAAGGAACGTTTTGTCT
CONSENSUS
GATGAAGCTGCCCTTTGTCGCCCCTGCGATGAAAAA..............

SE-AT4G38960-XLOC_022539-6912-0

SE-AT4G38960-XLOC_022539-6912-1

CCAGAGTTTGCATTTAGTAGTATTAACTTCTGGAGAAGAAAATAATCGAT
CONSENSUS
....

SE-AT4G38960-XLOC_022539-6912-0
---G
SE-AT4G38960-XLOC_022539-6912-1
TTTCTGCTCTTTGCGCTTAATCCTTACTTCAGTTAGATGACTCTTAAGGG
CONSENSUS
....G

SE-AT4G38960-XLOC_022539-6912-0
TTCATATGTGCAACAAGCTAGCTAGTCGGCATGTACGTGTTGGTTTAGCT
SE-AT4G38960-XLOC_022539-6912-1
TTCATATGTGCAACAAGCTAGCTAGTCGGCATGTACGTGTTGGTTTAGCT
CONSENSUS
TTCATATGTGCAACAAGCTAGCTAGTCGGCATGTACGTGTTGGTTTAGCT

SE-AT4G38960-XLOC_022539-6912-0
GAACCAAGCAATGCCCCATGCTGTGATATATGCGAAAATGCACCTG
SE-AT4G38960-XLOC_022539-6912-1
GAACCAAGCAATGCCCCATGCTGTGATATATGCGAAAATGCACCTG
CONSENSUS
GAACCAAGCAATGCCCCATGCTGTGATATATGCGAAAATGCACCTG

alignment for event: RI-AT4G36690-XLOC_024897-8160

RI-AT4G36690-XLOC_024897-8160-0
AGGGTTATGTTACAGCCTGGTGCAGTGGCAACCACTGTGGTGTGTCTGAC
RI-AT4G36690-XLOC_024897-8160-1
AGGGTTATGTTACAGCCTGGTGCAGTGGCAACCACTGTGGTGTGTCTGAC
CONSENSUS
AGGGTTATGTTACAGCCTGGTGCAGTGGCAACCACTGTGGTGTGTCTGAC

RI-AT4G36690-XLOC_024897-8160-0
TCAAGTTGTTACTGAGGATGAGCTTAGAGATGATGAGGAGTATGGAGATA
RI-AT4G36690-XLOC_024897-8160-1
TCAAGTTGTTACTGAGGATGAGCTTAGAGATGATGAGGAGTATGGAGATA
CONSENSUS
TCAAGTTGTTACTGAGGATGAGCTTAGAGATGATGAGGAGTATGGAGATA

RI-AT4G36690-XLOC_024897-8160-0
TAATGGAAGACATGAGACAGGAAGGCGGAAAGTTTGGTAAGAGACCTCTT
RI-AT4G36690-XLOC_024897-8160-1
TAATGGAAGACATGAGACAGGAAGGCGGAAAGTTTG--------------
CONSENSUS
TAATGGAAGACATGAGACAGGAAGGCGGAAAGTTTG..............

RI-AT4G36690-XLOC_024897-8160-0
AATTGCGCAATATGGTCTATTTTGAAATATAAGATTAAAAGCATATTGAT
RI-AT4G36690-XLOC_024897-8160-1

CONSENSUS
....

```
RI-AT4G36690-XLOC_024897-8160-0
      CTGTCTCTCTGTTTTCTTGGTTGTGCTGTTTTACAGCCTTTTGCTATAAA
RI-AT4G36690-XLOC_024897-8160-1
      --------------------------------------------------
CONSENSUS
      ..................................................

RI-AT4G36690-XLOC_024897-8160-0
      GAATCAGCCCTAACATACACAGACAGGAGACTGCATAAACCTCCTAATCT
RI-AT4G36690-XLOC_024897-8160-1
      --------------------------------------------------
CONSENSUS
      ..................................................

RI-AT4G36690-XLOC_024897-8160-0
      ATTTATCACCAATGGCCACTATTTCTTAAAAGAAAAAACTGACTTGTTTT
RI-AT4G36690-XLOC_024897-8160-1
      --------------------------------------------------
CONSENSUS
      ..................................................

RI-AT4G36690-XLOC_024897-8160-0
      TGTCTGTGTTTTCTTGTCTTGTATTCGAAATGTTTTGTTCTTTAACTCTT
RI-AT4G36690-XLOC_024897-8160-1
      --------------------------------------------------
CONSENSUS
      ..................................................

RI-AT4G36690-XLOC_024897-8160-0
      AAAATGCAGGTGCTTTGACCAATGTTGTGATTCCGCGTCCAAGCCCCAAT
RI-AT4G36690-XLOC_024897-8160-1  ---------
GTGCTTTGACCAATGTTGTGATTCCGCGTCCAAGCCCCAAT
CONSENSUS
      .........GTGCTTTGACCAATGTTGTGATTCCGCGTCCAAGCCCCAAT

RI-AT4G36690-XLOC_024897-8160-0  GGTGAGCCAGTGGCAGGCCTTGGCAAG
RI-AT4G36690-XLOC_024897-8160-1  GGTGAGCCAGTGGCAGGCCTTGGCAAG
CONSENSUS                        GGTGAGCCAGTGGCAGGCCTTGGCAAG


alignment for event: RI-AT4G12990-XLOC_023528-3924

RI-AT4G12990-XLOC_023528-3924-0
      AGCAAAATCGATGAGCTGATCAAGAGTCTCTTGGATTTGTGCTTCTGTAC
RI-AT4G12990-XLOC_023528-3924-1
      AGCAAAATCGATGAGCTGATCAAGAGTCTCTTGGATTTGTGCTTCTGTAC
CONSENSUS
      AGCAAAATCGATGAGCTGATCAAGAGTCTCTTGGATTTGTGCTTCTGTAC

RI-AT4G12990-XLOC_023528-3924-0
      TTCTTACGAGGAAGGTGTGTTGACTTTTCGACTTGTTCTTTGTGTTCTTG
RI-AT4G12990-XLOC_023528-3924-1
      TTCTTACGAGGAAG------------------------------------
CONSENSUS
```

TTCTTACGAGGAAG..................................

RI-AT4G12990-XLOC_023528-3924-0
GATACATGTTCTTTTCAACTTCTATAACATTGTTTTCTTTAGCAAGGTAT
RI-AT4G12990-XLOC_023528-3924-1

CONSENSUS
....

RI-AT4G12990-XLOC_023528-3924-0
TGATTTTTCTTTTTCAGATTTAGAGATTGTGGAAAACAATTTGAAACATT
RI-AT4G12990-XLOC_023528-3924-1 -----------------
ATTTAGAGATTGTGGAAAACAATTTGAAACATT
CONSENSUS
.................ATTTAGAGATTGTGGAAAACAATTTGAAACATT

RI-AT4G12990-XLOC_023528-3924-0
TTGAAGAGAACCCCACTTGAGAGATACTACCATTGGATTGTGAAATTTTT
RI-AT4G12990-XLOC_023528-3924-1
TTGAAGAGAACCCCACTTGAGAGATACTACCATTGGATTGTGAAATTTTT
CONSENSUS
TTGAAGAGAACCCCACTTGAGAGATACTACCATTGGATTGTGAAATTTTT

RI-AT4G12990-XLOC_023528-3924-0
TTCAAAAAAATATACAAGCTCTCTTCTTACAAATTTTCACAATTAATAAT
RI-AT4G12990-XLOC_023528-3924-1
TTCAAAAAAATATACAAGCTCTCTTCTTACAAATTTTCACAATTAATAAT
CONSENSUS
TTCAAAAAAATATACAAGCTCTCTTCTTACAAATTTTCACAATTAATAAT

RI-AT4G12990-XLOC_023528-3924-0
AAAGAATTGGAGAGAGCCCATGAGAGGGCTCTCACCATTGGAGATGGTTT
RI-AT4G12990-XLOC_023528-3924-1
AAAGAATTGGAGAGAGCCCATGAGAGGGCTCTCACCATTGGAGATGGTTT
CONSENSUS
AAAGAATTGGAGAGAGCCCATGAGAGGGCTCTCACCATTGGAGATGGTTT

RI-AT4G12990-XLOC_023528-3924-0
ATATTGTACATGTCACAAGAAGAGTATGTGAAAAAAAACTATTGGAGAAG
RI-AT4G12990-XLOC_023528-3924-1
ATATTGTACATGTCACAAGAAGAGTATGTGAAAAAAAACTATTGGAGAAG
CONSENSUS
ATATTGTACATGTCACAAGAAGAGTATGTGAAAAAAAACTATTGGAGAAG

RI-AT4G12990-XLOC_023528-3924-0
TCTGATATGGGTAACACAAAACTGGTTATTACTCCCCATGCGAGCCAATT
RI-AT4G12990-XLOC_023528-3924-1
TCTGATATGGGTAACACAAAACTGGTTATTACTCCCCATGCGAGCCAATT
CONSENSUS
TCTGATATGGGTAACACAAAACTGGTTATTACTCCCCATGCGAGCCAATT

RI-AT4G12990-XLOC_023528-3924-0
TTTGCTATTCAGAAATCGATAACCAATAAGTGAACATGAGAAGGCG
RI-AT4G12990-XLOC_023528-3924-1
TTTGCTATTCAGAAATCGATAACCAATAAGTGAACATGAGAAGGCG
CONSENSUS

TTTGCTATTCAGAAATCGATAACCAATAAGTGAACATGAGAAGGCG

alignment for event: RI-AT4G36520-XLOC_024883-981

RI-AT4G36520-XLOC_024883-981-0
GCGAATCGTGGTTTTGCCAGTTACGTATTTAAAGGGGCAGTCTTTTACAC
RI-AT4G36520-XLOC_024883-981-1
GCGAATCGTGGTTTTGCCAGTTACGTATTTAAAGGGGCAGTCTTTTACAC
CONSENSUS
GCGAATCGTGGTTTTGCCAGTTACGTATTTAAAGGGGCAGTCTTTTACAC

RI-AT4G36520-XLOC_024883-981-0
GTCATACGCCATGAGTATGTAATTCGTAGCCGGGAAGAAGATCATCATGC
RI-AT4G36520-XLOC_024883-981-1
GTCATACGCCATGAGTATGTAATTCGTAGCCGGGAAGAAGATCATCATGC
CONSENSUS
GTCATACGCCATGAGTATGTAATTCGTAGCCGGGAAGAAGATCATCATGC

RI-AT4G36520-XLOC_024883-981-0
TTCTGCTTCTGGTTCCCTTGGCGAGGTAAAAGAAAGAAAATAGTTTGTTT
RI-AT4G36520-XLOC_024883-981-1
TTCTGCTTCTGGTTCCCTTGGCGAG-------------------------
CONSENSUS
TTCTGCTTCTGGTTCCCTTGGCGAG.........................

RI-AT4G36520-XLOC_024883-981-0
TTGGATTTTATTTTCAAAACCCACTAGTTGGATCTTCATGTATCAACATG
RI-AT4G36520-XLOC_024883-981-1

CONSENSUS
....

RI-AT4G36520-XLOC_024883-981-0
ATCATCGTTTCAGTCGTCGACGACTTCCCAAGACAAGAGAGAATGAAGGA
RI-AT4G36520-XLOC_024883-981-1 -------------
TCGTCGACGACTTCCCAAGACAAGAGAGAATGAAGGA
CONSENSUS
.............TCGTCGACGACTTCCCAAGACAAGAGAGAATGAAGGA

RI-AT4G36520-XLOC_024883-981-0
ATCAATCGTAATGGCTTTTGCTTGTTTCTTTCGCTTTTTATGTATTTATG
RI-AT4G36520-XLOC_024883-981-1
ATCAATCGTAATGGCTTTTGCTTGTTTCTTTCGCTTTTTATGTATTTATG
CONSENSUS
ATCAATCGTAATGGCTTTTGCTTGTTTCTTTCGCTTTTTATGTATTTATG

RI-AT4G36520-XLOC_024883-981-0
TTCTTACCTGAGTTGGGATCTGTAGAACTAACTTCCCATAATTGTATCTC
RI-AT4G36520-XLOC_024883-981-1
TTCTTACCTGAGTTGGGATCTGTAGAACTAACTTCCCATAATTGTATCTC
CONSENSUS
TTCTTACCTGAGTTGGGATCTGTAGAACTAACTTCCCATAATTGTATCTC

RI-AT4G36520-XLOC_024883-981-0

ACGAACGTGATGTAGTAATCTGAGTAAATCTAAAACTATCTCGTGGAATT
RI-AT4G36520-XLOC_024883-981-1
ACGAACGTGATGTAGTAATCTGAGTAAATCTAAAACTATCTCGTGGAATT
CONSENSUS
ACGAACGTGATGTAGTAATCTGAGTAAATCTAAAACTATCTCGTGGAATT

RI-AT4G36520-XLOC_024883-981-0
GCAACAACTATCTCAAGTCTCAACTCTTCTTAACTGCAAATCCACTTGTA
RI-AT4G36520-XLOC_024883-981-1
GCAACAACTATCTCAAGTCTCAACTCTTCTTAACTGCAAATCCACTTGTA
CONSENSUS
GCAACAACTATCTCAAGTCTCAACTCTTCTTAACTGCAAATCCACTTGTA

RI-AT4G36520-XLOC_024883-981-0 TGATTTCACA
RI-AT4G36520-XLOC_024883-981-1 TGATTTCACA
CONSENSUS TGATTTCACA

alignment for event: RI-AT4G14550-XLOC_023653-7241

RI-AT4G14550-XLOC_023653-7241-0
GGAGTTATGGAGCACAAGGGATGATAGATTTCATGAACGAGAGTAAAGTG
RI-AT4G14550-XLOC_023653-7241-1
GGAGTTATGGAGCACAAGGGATGATAGATTTCATGAACGAGAGTAAAGTG
CONSENSUS
GGAGTTATGGAGCACAAGGGATGATAGATTTCATGAACGAGAGTAAAGTG

RI-AT4G14550-XLOC_023653-7241-0
ATGGATCTGTTGAACAGTTCTGAGTATGTTCCAAGCTACGAGGACAAAGA
RI-AT4G14550-XLOC_023653-7241-1
ATGGATCTGTTGAACAGTTCTGAGTATGTTCCAAGCTACGAGGACAAAGA
CONSENSUS
ATGGATCTGTTGAACAGTTCTGAGTATGTTCCAAGCTACGAGGACAAAGA

RI-AT4G14550-XLOC_023653-7241-0
TGGTGACTGGATGCTCGTTGGTGATGTCCCCTGGCCGTGAGTTTCCTCAT
RI-AT4G14550-XLOC_023653-7241-1
TGGTGACTGGATGCTCGTTGGTGATGTCCCCTGGCC--------------
CONSENSUS
TGGTGACTGGATGCTCGTTGGTGATGTCCCCTGGCC..............

RI-AT4G14550-XLOC_023653-7241-0
TCTTCTTGCTTTCATTATTATGACCAAAATTATTCTCTAAACAAAAAAAA
RI-AT4G14550-XLOC_023653-7241-1

CONSENSUS
....

RI-AT4G14550-XLOC_023653-7241-0
CAATATTCTCTAAAGCATTATTATTGATATTACTTATCAAAAAAATACAC
RI-AT4G14550-XLOC_023653-7241-1

CONSENSUS
....

RI-AT4G14550-XLOC_023653-7241-0
    AAAATGATAATCAATATCCATGTGTTATAAACACGCACAGCCATCTTTTG
RI-AT4G14550-XLOC_023653-7241-1

CONSENSUS
    ....

RI-AT4G14550-XLOC_023653-7241-0
    GTTGGCATGGGACAGAACTCAGAGACAGAGAAGATGTTTATATATAAATA
RI-AT4G14550-XLOC_023653-7241-1

CONSENSUS
    ....

RI-AT4G14550-XLOC_023653-7241-0
    CTAACTCATCAATATGTTACCTCATTTGTAGCTGGCACATATTCTTTCAC
RI-AT4G14550-XLOC_023653-7241-1

CONSENSUS
    ....

RI-AT4G14550-XLOC_023653-7241-0
    TTTCAATAGATTTCTAAATTTAGTCACCAACCCAAATCCCGATTTCAGGA
RI-AT4G14550-XLOC_023653-7241-1
    --GA
CONSENSUS
    ....GA

RI-AT4G14550-XLOC_023653-7241-0
    TGTTTGTCGAGTCATGCAAACGTTTGCGCATAATGAAAGGATCCGAAGCA
RI-AT4G14550-XLOC_023653-7241-1
    TGTTTGTCGAGTCATGCAAACGTTTGCGCATAATGAAAGGATCCGAAGCA
CONSENSUS
    TGTTTGTCGAGTCATGCAAACGTTTGCGCATAATGAAAGGATCCGAAGCA

RI-AT4G14550-XLOC_023653-7241-0 ATTGGACTTG
RI-AT4G14550-XLOC_023653-7241-1 ATTGGACTTG
CONSENSUS   ATTGGACTTG

alignment for event: RI-AT4G21410-XLOC_024048-1037

RI-AT4G21410-XLOC_024048-1037-0
    GTGTGGAGAAGCTGGAGAGAAGACACTATACTAAGCGTGATCGATCCGAG
RI-AT4G21410-XLOC_024048-1037-1
    GTGTGGAGAAGCTGGAGAGAAGACACTATACTAAGCGTGATCGATCCGAG
CONSENSUS
    GTGTGGAGAAGCTGGAGAGAAGACACTATACTAAGCGTGATCGATCCGAG

RI-AT4G21410-XLOC_024048-1037-0
    TTTAACCGCAGGATCAAGAAATGAGATCTTGAGATGCATACACATTGGTC
RI-AT4G21410-XLOC_024048-1037-1
    TTTAACCGCAGGATCAAGAAATGAGATCTTGAGATGCATACACATTGGTC
CONSENSUS
    TTTAACCGCAGGATCAAGAAATGAGATCTTGAGATGCATACACATTGGTC

RI-AT4G21410-XLOC_024048-1037-0
TTTTATGTGTGCAAGAAAGTGCAGCGACTAGACCAACAATGGCTACGGTT
RI-AT4G21410-XLOC_024048-1037-1
TTTTATGTGTGCAAGAAAGTGCAGCGACTAGACCAACAATGGCTACGGTT
CONSENSUS
TTTTATGTGTGCAAGAAAGTGCAGCGACTAGACCAACAATGGCTACGGTT

RI-AT4G21410-XLOC_024048-1037-0
TCTCTCATGCTCAATAGCTATTCTTTTACCCTCCCGACGCCTTTGAGGCC
RI-AT4G21410-XLOC_024048-1037-1
TCTCTCATGCTCAATAGCTATTCTTTTACCCTCCCGACGCCTTTGAGGCC
CONSENSUS
TCTCTCATGCTCAATAGCTATTCTTTTACCCTCCCGACGCCTTTGAGGCC

RI-AT4G21410-XLOC_024048-1037-0
TGCGTTTGTGTTGGAGAGTGTCGTCATACCTTCAAATGTTTCTTCTTCAA
RI-AT4G21410-XLOC_024048-1037-1
TGCGTTTGTGTTGGAGAGTGTCGTCATACCTTCAAATGTTTCTTCTTCAA
CONSENSUS
TGCGTTTGTGTTGGAGAGTGTCGTCATACCTTCAAATGTTTCTTCTTCAA

RI-AT4G21410-XLOC_024048-1037-0
CGGAAGGGTTACAAATGTCGTCAAATGATGTTACTGTTTCTGAGTTTTCT
RI-AT4G21410-XLOC_024048-1037-1
CGGAAGGGTTACAAATGTCGTCAAATGATGTTACTGTTTCTGAGTTTTCT
CONSENSUS
CGGAAGGGTTACAAATGTCGTCAAATGATGTTACTGTTTCTGAGTTTTCT

RI-AT4G21410-XLOC_024048-1037-0
CCTCGTTAATCTGTAAATGTTTTGATTCTTCTCTGAAAACCTTTGAAATG
RI-AT4G21410-XLOC_024048-1037-1
CCTCGTTAATCTGTAAATGTTTTGATTCTTCTCTGAAAACCTTTGAAATG
CONSENSUS
CCTCGTTAATCTGTAAATGTTTTGATTCTTCTCTGAAAACCTTTGAAATG

RI-AT4G21410-XLOC_024048-1037-0
GGTTTTGTTTGTGTGATTTTTCAGGCAGCCTGGCTTGATCTTGTTGTGAA
RI-AT4G21410-XLOC_024048-1037-1
GGTTTTGTTTGTGTGATTTTTCAGGCAGCCTGGCTTGATCTTGTTGTGAA
CONSENSUS
GGTTTTGTTTGTGTGATTTTTCAGGCAGCCTGGCTTGATCTTGTTGTGAA

RI-AT4G21410-XLOC_024048-1037-0
TAAAACATGAATGTGAAGCTGTGAGATACTTTTTCCTTTGTCTCATTTTT
RI-AT4G21410-XLOC_024048-1037-1
TAAAACATGAATGTGAAGCT------------------------------
CONSENSUS
TAAAACATGAATGTGAAGCT..............................

RI-AT4G21410-XLOC_024048-1037-0
TTTCTTCCAGTTTATCGTTACTTGATTTCGTTTTAAGGTACAATTTGATA
RI-AT4G21410-XLOC_024048-1037-1

CONSENSUS
....

RI-AT4G21410-XLOC_024048-1037-0
GAAGTATATATATATGTCAGTAAGTTTAGAAAATTGGATAAGAGATTTAT
RI-AT4G21410-XLOC_024048-1037-1

CONSENSUS
....

RI-AT4G21410-XLOC_024048-1037-0
ATTGAGTCCACATATATAAATATGAGAGTTGAAAGATGATCCACTAATCA
RI-AT4G21410-XLOC_024048-1037-1

CONSENSUS
....

RI-AT4G21410-XLOC_024048-1037-0
TCTACAGAAGTAAAAGAAATTGTAAAATAAATGGCTTAGACGAAATTAGA
RI-AT4G21410-XLOC_024048-1037-1

CONSENSUS
....

RI-AT4G21410-XLOC_024048-1037-0
TGGTTCCTAGTTAGTCTTATATTTTCTAGTAGGTTTGAAAAGTATTCAAA
RI-AT4G21410-XLOC_024048-1037-1

CONSENSUS
....

RI-AT4G21410-XLOC_024048-1037-0
CTCTTTAGTTTTCTTTCCTGGTACTTGTGGGGAAAGAAGTGAGCAATGAA
RI-AT4G21410-XLOC_024048-1037-1

CONSENSUS
....

RI-AT4G21410-XLOC_024048-1037-0
CACTTCTAATTTAAATAACGTTTTCACACCAAGTCGTTAGTTTCATATTG
RI-AT4G21410-XLOC_024048-1037-1

CONSENSUS
....

RI-AT4G21410-XLOC_024048-1037-0
TGAGTCTTTGAATCTACTACCATCATGGACCCCCACAACCTCATAAAGAC
RI-AT4G21410-XLOC_024048-1037-1

CONSENSUS
....

RI-AT4G21410-XLOC_024048-1037-0
TCCACTTGTGTTTGCTACTGGGTTTTATAATTGTTATTGATCAACAAGTT
RI-AT4G21410-XLOC_024048-1037-1

CONSENSUS
....

RI-AT4G21410-XLOC_024048-1037-0
TTGTTCTTTCTGCTGTTGGATATTGATTTAGTTCAAAGACCATACTGCTT
RI-AT4G21410-XLOC_024048-1037-1

CONSENSUS
....

RI-AT4G21410-XLOC_024048-1037-0
TTGAATAATTCCAAGGTCAAGTTAGATTGACTTCTTCCATTTTTAGACTC
RI-AT4G21410-XLOC_024048-1037-1

CONSENSUS
....

RI-AT4G21410-XLOC_024048-1037-0
GTTATTACATTCTGGGGTTTTCTCATATAGATTACAGAAGAAGAAAAAAG
RI-AT4G21410-XLOC_024048-1037-1

CONSENSUS
....

RI-AT4G21410-XLOC_024048-1037-0
CTTCAAGCGTCCATGGAACATGTCAGAGTTATCTTTTTCTTCTTTGCTTG
RI-AT4G21410-XLOC_024048-1037-1

CONSENSUS
....

RI-AT4G21410-XLOC_024048-1037-0
TGTCCTAAAGATTGTACCATTTATCTGCTTAGCACAGAAGGATAAATATG
RI-AT4G21410-XLOC_024048-1037-1

CONSENSUS
....

RI-AT4G21410-XLOC_024048-1037-0
AGTTTCCTCCAGGTTTCAACTGTGTAGCTAGTGGAGGCAATTTCACGGCC
RI-AT4G21410-XLOC_024048-1037-1 ------------
GTTTCAACTGTGTAGCTAGTGGAGGCAATTTCACGGCC
CONSENSUS
............GTTTCAACTGTGTAGCTAGTGGAGGCAATTTCACGGCC

RI-AT4G21410-XLOC_024048-1037-0
AACAGCTCTTTCGCTGGTAATCTCAACGGCCTTGTCTCCTCTCTCTCGTC
RI-AT4G21410-XLOC_024048-1037-1
AACAGCTCTTTCGCTGGTAATCTCAACGGCCTTGTCTCCTCTCTCTCGTC
CONSENSUS
AACAGCTCTTTCGCTGGTAATCTCAACGGCCTTGTCTCCTCTCTCTCGTC

RI-AT4G21410-XLOC_024048-1037-0
ACTCACATCCAAACCTTATGGCTTCTACAACCTCTCTTCTGGAGATTCAT
RI-AT4G21410-XLOC_024048-1037-1
ACTCACATCCAAACCTTATGGCTTCTACAACCTCTCTTCTGGAGATTCAT
CONSENSUS
ACTCACATCCAAACCTTATGGCTTCTACAACCTCTCTTCTGGAGATTCAT

RI-AT4G21410-XLOC_024048-1037-0
CTGGAGAAAGAGCTTATGCAATTGGTCTTTGTAGAAGAGAAGTAAAAAGA
RI-AT4G21410-XLOC_024048-1037-1
CTGGAGAAAGAGCTTATGCAATTGGTCTTTGTAGAAGAGAAGTAAAAAGA
CONSENSUS
CTGGAGAAAGAGCTTATGCAATTGGTCTTTGTAGAAGAGAAGTAAAAAGA

RI-AT4G21410-XLOC_024048-1037-0
GATGATTGTCTCAGCTGCATTCAGATAGCTGCAAGAAACCTCATCGAGCA
RI-AT4G21410-XLOC_024048-1037-1
GATGATTGTCTCAGCTGCATTCAGATAGCTGCAAGAAACCTCATCGAGCA
CONSENSUS
GATGATTGTCTCAGCTGCATTCAGATAGCTGCAAGAAACCTCATCGAGCA

RI-AT4G21410-XLOC_024048-1037-0
GTGTCCACTGACAAATCAAGCTGTTGTGTGGTACACACACTGTATGTTTC
RI-AT4G21410-XLOC_024048-1037-1
GTGTCCACTGACAAATCAAGCTGTTGTGTGGTACACACACTGTATGTTTC
CONSENSUS
GTGTCCACTGACAAATCAAGCTGTTGTGTGGTACACACACTGTATGTTTC

RI-AT4G21410-XLOC_024048-1037-0
GTTACTCGAACATGATAATCTATGGAAGAAAAGAGACGACCCCAACTCTG
RI-AT4G21410-XLOC_024048-1037-1
GTTACTCGAACATGATAATCTATGGAAGAAAAGAGACGACCCCAACTCTG
CONSENSUS
GTTACTCGAACATGATAATCTATGGAAGAAAAGAGACGACCCCAACTCTG

RI-AT4G21410-XLOC_024048-1037-0
TCTTTTCAAGCCGGTAAAAATATATCAGCAAACAGAGATGAGTTTGATCG
RI-AT4G21410-XLOC_024048-1037-1
TCTTTTCAAGCCGGTAAAAATATATCAGCAAACAGAGATGAGTTTGATCG
CONSENSUS
TCTTTTCAAGCCGGTAAAAATATATCAGCAAACAGAGATGAGTTTGATCG

RI-AT4G21410-XLOC_024048-1037-0
TCTGCAGATAGAACTATTGGACAGACTCAAAGGGATTGCTGCAGCTGGTG
RI-AT4G21410-XLOC_024048-1037-1
TCTGCAGATAGAACTATTGGACAGACTCAAAGGGATTGCTGCAGCTGGTG
CONSENSUS
TCTGCAGATAGAACTATTGGACAGACTCAAAGGGATTGCTGCAGCTGGTG

RI-AT4G21410-XLOC_024048-1037-0
GGCCAAATAGAAAATACGCTCAAGGGAGCGGTTCGGGTGTGGCAGGGTAC
RI-AT4G21410-XLOC_024048-1037-1
GGCCAAATAGAAAATACGCTCAAGGGAGCGGTTCGGGTGTGGCAGGGTAC
CONSENSUS
GGCCAAATAGAAAATACGCTCAAGGGAGCGGTTCGGGTGTGGCAGGGTAC

RI-AT4G21410-XLOC_024048-1037-0
CCGCAATTCTACGGAAGTGCACACTGTACGCCGGATTTGTCTGAACAGGA
RI-AT4G21410-XLOC_024048-1037-1
CCGCAATTCTACGGAAGTGCACACTGTACGCCGGATTTGTCTGAACAGGA
CONSENSUS
CCGCAATTCTACGGAAGTGCACACTGTACGCCGGATTTGTCTGAACAGGA

RI-AT4G21410-XLOC_024048-1037-0
TTGTAATGACTGTCTAGTCTTTGGTTTTGAAAAGATCCCAGGTTGTTGTG
RI-AT4G21410-XLOC_024048-1037-1
TTGTAATGACTGTCTAGTCTTTGGTTTTGAAAAGATCCCAGGTTGTTGTG
CONSENSUS
TTGTAATGACTGTCTAGTCTTTGGTTTTGAAAAGATCCCAGGTTGTTGTG

RI-AT4G21410-XLOC_024048-1037-0
CTGGTCAGGTTGGTCTTAGGTGGTTTTTTCCTAGTTGTAGCTACAGATTT
RI-AT4G21410-XLOC_024048-1037-1
CTGGTCAGGTTGGTCTTAGGTGGTTTTTTCCTAGTTGTAGCTACAGATTT
CONSENSUS
CTGGTCAGGTTGGTCTTAGGTGGTTTTTTCCTAGTTGTAGCTACAGATTT

RI-AT4G21410-XLOC_024048-1037-0
GAGACCTGGCGATTCTACGAGTTCGATGCCGATCTAGAGCCTGATCCACC
RI-AT4G21410-XLOC_024048-1037-1
GAGACCTGGCGATTCTACGAGTTCGATGCCGATCTAGAGCCTGATCCACC
CONSENSUS
GAGACCTGGCGATTCTACGAGTTCGATGCCGATCTAGAGCCTGATCCACC

RI-AT4G21410-XLOC_024048-1037-0
TGCTATTCAGCCTGCTGACTCCCCAACATCAGCTGCAAGAACTGAGAGAA
RI-AT4G21410-XLOC_024048-1037-1
TGCTATTCAGCCTGCTGACTCCCCAACATCAGCTGCAAGAACTGAGAGAA
CONSENSUS
TGCTATTCAGCCTGCTGACTCCCCAACATCAGCTGCAAGAACTGAGAGAA

RI-AT4G21410-XLOC_024048-1037-0 CAG
RI-AT4G21410-XLOC_024048-1037-1 CAG
CONSENSUS CAG

alignment for event: SE-AT4G34265-XLOC_022285-2308

SE-AT4G34265-XLOC_022285-2308-0
GAATTTGGGAGAGCTAGGATGCACAGAGTCATTCTTGCCTCTGTACAATG
SE-AT4G34265-XLOC_022285-2308-1
GAATTTGGGAGAGCTAGGATGCACAGAGTCATTCTTGCCTCTGTACAATG
CONSENSUS
GAATTTGGGAGAGCTAGGATGCACAGAGTCATTCTTGCCTCTGTACAATG

SE-AT4G34265-XLOC_022285-2308-0
TTGTGGCTGCAGCGAGACTCACATCTCACCTTAATGTTAATCTGCGAGCT
SE-AT4G34265-XLOC_022285-2308-1
TTGTGGCTGCAGCGAGACTCACATCTCACCTTAATGTTAATCTGCGAGCT
CONSENSUS
TTGTGGCTGCAGCGAGACTCACATCTCACCTTAATGTTAATCTGCGAGCT

SE-AT4G34265-XLOC_022285-2308-0
TTCTGCGAACTCTCTAACG-------------------------------
SE-AT4G34265-XLOC_022285-2308-1
TTCTGCGAACTCTCTAACGGGAATGGAAAAGATGGGTGATGGAAGTGGAT
CONSENSUS

TTCTGCGAACTCTCTAACG..............................

SE-AT4G34265-XLOC_022285-2308-0 --------------------
GTACTTGAATGCACAAAGTGGATGCATCA
SE-AT4G34265-XLOC_022285-2308-1
TTTCAGTCGCTGGCAAGAGAGGTACTTGAATGCACAAAGTGGATGCATCA
CONSENSUS
....................GTACTTGAATGCACAAAGTGGATGCATCA

SE-AT4G34265-XLOC_022285-2308-0
GTTATCTTGAAGCAGTTGGAGACTTGAGAAAAGGGGTGTACCAAGAAGAA
SE-AT4G34265-XLOC_022285-2308-1
GTTATCTTGAAGCAGTTGGAGACTTGAGAAAAGGGGTGTACCAAGAAGAA
CONSENSUS
GTTATCTTGAAGCAGTTGGAGACTTGAGAAAAGGGGTGTACCAAGAAGAA

SE-AT4G34265-XLOC_022285-2308-0
CTCATTGGTTGTTTTGTGTTCCATTAGGAACCAAAAACTATATACATTAT
SE-AT4G34265-XLOC_022285-2308-1
CTCATTGGTTGTTTTGTGTTCCATTAGGAACCAAAAACTATATACATTAT
CONSENSUS
CTCATTGGTTGTTTTGTGTTCCATTAGGAACCAAAAACTATATACATTAT

SE-AT4G34265-XLOC_022285-2308-0
TATCGATCTTCCTTCGCTTTTGTCAATTTCCAGTTTTAGAATATCACTGG
SE-AT4G34265-XLOC_022285-2308-1
TATCGATCTTCCTTCGCTTTTGTCAATTTCCAGTTTTAGAATATCACTGG
CONSENSUS
TATCGATCTTCCTTCGCTTTTGTCAATTTCCAGTTTTAGAATATCACTGG

SE-AT4G34265-XLOC_022285-2308-0
ATTTCTCTTCATATTTCTTGTTGTTTAGCCCAATGGCTTTAAATACTTGA
SE-AT4G34265-XLOC_022285-2308-1
ATTTCTCTTCATATTTCTTGTTGTTTAGCCCAATGGCTTTAAATACTTGA
CONSENSUS
ATTTCTCTTCATATTTCTTGTTGTTTAGCCCAATGGCTTTAAATACTTGA

SE-AT4G34265-XLOC_022285-2308-0
GGGTAGATTCAGCAAATTCTTGAAGAACCATTGTTTCATCGTTGTATTTA
SE-AT4G34265-XLOC_022285-2308-1
GGGTAGATTCAGCAAATTCTTGAAGAACCATTGTTTCATCGTTGTATTTA
CONSENSUS
GGGTAGATTCAGCAAATTCTTGAAGAACCATTGTTTCATCGTTGTATTTA

SE-AT4G34265-XLOC_022285-2308-0
TAATGTTATGAACTCCCTTCGGGTTTCCTGAAAGGAATTTAAAGAAAACT
SE-AT4G34265-XLOC_022285-2308-1
TAATGTTATGAACTCCCTTCGGGTTTCCTGAAAGGAATTTAAAGAAAACT
CONSENSUS
TAATGTTATGAACTCCCTTCGGGTTTCCTGAAAGGAATTTAAAGAAAACT

SE-AT4G34265-XLOC_022285-2308-0
TTCTTATAGTGAAAAGAGTTCGAGAGTCACCGTCTCTTCC
SE-AT4G34265-XLOC_022285-2308-1
TTCTTATAGTGAAAAGAGTTCGAGAGTCACCGTCTCTTCC
CONSENSUS

TTCTTATAGTGAAAAGAGTTCGAGAGTCACCGTCTCTTCC

alignment for event: A5-AT4G26070-XLOC_021824-11396

A5-AT4G26070-XLOC_021824-11396-0
GTCATTCAATTGAACACAGAAGAATCAACATGTCGGGCGATTTCTCAGGA
A5-AT4G26070-XLOC_021824-11396-1
GTCATTCAATTGAACACAGAAGAATCAACATGTCGGGCGATTTCTCAGGA
CONSENSUS
GTCATTCAATTGAACACAGAAGAATCAACATGTCGGGCGATTTCTCAGGA

A5-AT4G26070-XLOC_021824-11396-0
GCTGAGAATAAACTTGAGCTCGCAATGTCCATATCTTGTCTCATGTTATC
A5-AT4G26070-XLOC_021824-11396-1
GCTGAGAATAAACTTGAGCTCGCAATGTCCATATCTTGTCTCATGTTATC
CONSENSUS
GCTGAGAATAAACTTGAGCTCGCAATGTCCATATCTTGTCTCATGTTATC

A5-AT4G26070-XLOC_021824-11396-0
AATCTTTCTACCACAACGGTCTTGTTTCAATCATATTGGAATTCATGGAT
A5-AT4G26070-XLOC_021824-11396-1
AATCTTTCTACCACAACGGTCTTGTTTCAATCATATTGGAATTCATGGAT
CONSENSUS
AATCTTTCTACCACAACGGTCTTGTTTCAATCATATTGGAATTCATGGAT

A5-AT4G26070-XLOC_021824-11396-0
GGTGGATCCCTTGCAGACTTGTTAAAGAAAGTCGGAAAAGTTCCTGAAAA
A5-AT4G26070-XLOC_021824-11396-1
GGTGGATCCCTTGCAGACTTGTTAAAGAAAGTCGGAAAAGTTCCTGAAAA
CONSENSUS
GGTGGATCCCTTGCAGACTTGTTAAAGAAAGTCGGAAAAGTTCCTGAAAA

A5-AT4G26070-XLOC_021824-11396-0 CATGCTATCTGCCATCTGCAA----
GTTCTTCGAGGTCTTTGTTATATTC
A5-AT4G26070-XLOC_021824-11396-1
CATGCTATCTGCCATCTGCAAGCGAGTTCTTCGAGGTCTTTGTTATATTC
CONSENSUS
CATGCTATCTGCCATCTGCAA....GTTCTTCGAGGTCTTTGTTATATTC

A5-AT4G26070-XLOC_021824-11396-0
ATCATGAGAGGCGAATCATTCATCGGGACTTAAAGCCTTCAAACTTGCTA
A5-AT4G26070-XLOC_021824-11396-1
ATCATGAGAGGCGAATCATTCATCGGGACTTAAAGCCTTCAAACTTGCTA
CONSENSUS
ATCATGAGAGGCGAATCATTCATCGGGACTTAAAGCCTTCAAACTTGCTA

A5-AT4G26070-XLOC_021824-11396-0
ATCAATCATAGAGGTGAAGTCAAGATCACAGACTTTGGTGTCAGCAAGAT
A5-AT4G26070-XLOC_021824-11396-1
ATCAATCATAGAGGTGAAGTCAAGATCACAGACTTTGGTGTCAGCAAGAT
CONSENSUS
ATCAATCATAGAGGTGAAGTCAAGATCACAGACTTTGGTGTCAGCAAGAT

A5-AT4G26070-XLOC_021824-11396-0

CTTGACAAGCACAAGTAGTCTTGCTAATTCTTTCGTGGGCACATACCCTT
A5-AT4G26070-XLOC_021824-11396-1
CTTGACAAGCACAAGTAGTCTTGCTAATTCTTTCGTGGGCACATACCCTT
CONSENSUS
CTTGACAAGCACAAGTAGTCTTGCTAATTCTTTCGTGGGCACATACCCTT

A5-AT4G26070-XLOC_021824-11396-0 ATATGTCT
A5-AT4G26070-XLOC_021824-11396-1 ATATGTCT
CONSENSUS ATATGTCT

alignment for event: A5-AT4G11960-XLOC_023465-5958

A5-AT4G11960-XLOC_023465-5958-0
GTCAGGTGGGTGGGGAAGAAGTTGATAGCAAGATTTTACCTTATTGTAGC
A5-AT4G11960-XLOC_023465-5958-1
GTCAGGTGGGTGGGGAAGAAGTTGATAGCAAGATTTTACCTTATTGTAGC
CONSENSUS
GTCAGGTGGGTGGGGAAGAAGTTGATAGCAAGATTTTACCTTATTGTAGC

A5-AT4G11960-XLOC_023465-5958-0
ATCAACAAGAATGAGAAGAGAACTATCGGTGAAATGGAACAAGAGTTCCT
A5-AT4G11960-XLOC_023465-5958-1
ATCAACAAGAATGAGAAGAGAACTATCGGTGAAATGGAACAAGAGTTCCT
CONSENSUS
ATCAACAAGAATGAGAAGAGAACTATCGGTGAAATGGAACAAGAGTTCCT

A5-AT4G11960-XLOC_023465-5958-0
CCAAGCGATGCAATCGTTTTATTACGAAGGCAAAGCGATTATGTCTAATG
A5-AT4G11960-XLOC_023465-5958-1 CCAAGCGAT----
TCGTTTTATTACGAAGGCAAAGCGATTATGTCTAATG
CONSENSUS
CCAAGCGAT....TCGTTTTATTACGAAGGCAAAGCGATTATGTCTAATG

A5-AT4G11960-XLOC_023465-5958-0
AAGAGTTTGATAACCTTAAAGAAGAGTTGATGTGGGAAGGAAGCAGTGTT
A5-AT4G11960-XLOC_023465-5958-1
AAGAGTTTGATAACCTTAAAGAAGAGTTGATGTGGGAAGGAAGCAGTGTT
CONSENSUS
AAGAGTTTGATAACCTTAAAGAAGAGTTGATGTGGGAAGGAAGCAGTGTT

A5-AT4G11960-XLOC_023465-5958-0 GTCATGCTAA
A5-AT4G11960-XLOC_023465-5958-1 GTCATGCTAA
CONSENSUS GTCATGCTAA

alignment for event: RI-AT4G23330-XLOC_021667-5299

RI-AT4G23330-XLOC_021667-5299-0
GTTAGTAGGAGTTTGCTGGTTATTAAGACTTTAGTCTGAATCCAGGCCTG
RI-AT4G23330-XLOC_021667-5299-1
GTTAGTAGGAGTTTGCTGGTTATTAAGACTTTAGTCTGAATCCAGGCCTG
CONSENSUS
GTTAGTAGGAGTTTGCTGGTTATTAAGACTTTAGTCTGAATCCAGGCCTG

RI-AT4G23330-XLOC_021667-5299-0
      GCTCTTGGGGTTTGCTCTGTTGCTAGTGCACAGGGTCCAACCCTGTCTGA
RI-AT4G23330-XLOC_021667-5299-1
      GCTCTTGGGGTTTGCTCTGTTGCTAGTGCACAGGGTCCAACCCTGTCTGA
CONSENSUS
      GCTCTTGGGGTTTGCTCTGTTGCTAGTGCACAGGGTCCAACCCTGTCTGA

RI-AT4G23330-XLOC_021667-5299-0
      ATCATGGCGATTTTCACCAAACCTGAGAGTGCATTGAAGCGTACAGATGG
RI-AT4G23330-XLOC_021667-5299-1
      ATCATGGCGATTTTCACCAAACCTGAGAGTGCATTGAAGCGTACAGATG-
CONSENSUS
      ATCATGGCGATTTTCACCAAACCTGAGAGTGCATTGAAGCGTACAGATG.

RI-AT4G23330-XLOC_021667-5299-0
      TAACATTGCAAATTCTTACCTTTTTGACTCATGCTTTGATGTATGAATGT
RI-AT4G23330-XLOC_021667-5299-1

CONSENSUS
      ....

RI-AT4G23330-XLOC_021667-5299-0
      TGCGTGTTTGCCGAGCTTATCAATGTTGGGCAGAGACAAGATGCTCTCAT
RI-AT4G23330-XLOC_021667-5299-1

CONSENSUS
      ....

RI-AT4G23330-XLOC_021667-5299-0
      GGTGCTTTCTGATCTTTTTACCTGCTAACGAGAAAGTTGAGCAGGCTTGT
RI-AT4G23330-XLOC_021667-5299-1
      --GCTTGT
CONSENSUS
      ....GCTTGT

RI-AT4G23330-XLOC_021667-5299-0
      AGTCAGCGGATGCTCTGGAGGAGGAGGCCCTTGATGTCGATGACTTTGAA
RI-AT4G23330-XLOC_021667-5299-1
      AGTCAGCGGATGCTCTGGAGGAGGAGGCCCTTGATGTCGATGACTTTGAA
CONSENSUS
      AGTCAGCGGATGCTCTGGAGGAGGAGGCCCTTGATGTCGATGACTTTGAA

RI-AT4G23330-XLOC_021667-5299-0
      GCAGATAAAAAGCCTGAAGACTTGCAGCCAAGTGTTGTATATCAGCAACG
RI-AT4G23330-XLOC_021667-5299-1
      GCAGATAAAAAGCCTGAAGACTTGCAGCCAAGTGTTGTATATCAGCAACG
CONSENSUS
      GCAGATAAAAAGCCTGAAGACTTGCAGCCAAGTGTTGTATATCAGCAACG

RI-AT4G23330-XLOC_021667-5299-0
      ACTGCTAGAGGAAAGGAAAATTTATGAAAATGAGCAACAG
RI-AT4G23330-XLOC_021667-5299-1
      ACTGCTAGAGGAAAGGAAAATTTATGAAAATGAGCAACAG
CONSENSUS
      ACTGCTAGAGGAAAGGAAAATTTATGAAAATGAGCAACAG

alignment for event: RI-AT4G12560-XLOC_021029-8451

RI-AT4G12560-XLOC_021029-8451-0
GGATGATTTCCTGTCCAAAGGATTCAAGCTGGTCTTATAAGCCAAAAACA
RI-AT4G12560-XLOC_021029-8451-1
GGATGATTTCCTGTCCAAAGGATTCAAGCTGGTCTTATAAGCCAAAAACA
CONSENSUS
GGATGATTTCCTGTCCAAAGGATTCAAGCTGGTCTTATAAGCCAAAAACA

RI-AT4G12560-XLOC_021029-8451-0
AGAGACAGGAGCTAAAGCTGCACAAGGTAAGTGAAAAAGTTGACCGTTCT
RI-AT4G12560-XLOC_021029-8451-1
AGAGACAGGAGCTAAAGCTGCACAAGGTAAGTGAAAAAGTTGACCGTTCT
CONSENSUS
AGAGACAGGAGCTAAAGCTGCACAAGGTAAGTGAAAAAGTTGACCGTTCT

RI-AT4G12560-XLOC_021029-8451-0
TAATGGGTCTAGTTTGCTGCTGCATTCTTATTGCATTTCATTCTTTTCTG
RI-AT4G12560-XLOC_021029-8451-1
TAATGGGTCTAGTTTGCTGCTGCATTCTTATTGCATTTCATTCTTTTCTG
CONSENSUS
TAATGGGTCTAGTTTGCTGCTGCATTCTTATTGCATTTCATTCTTTTCTG

RI-AT4G12560-XLOC_021029-8451-0
TCTCTGCTCTGTTTTGTCGCGGACGTTCATATCCGGTGTCTGTTTTGTGG
RI-AT4G12560-XLOC_021029-8451-1
TCTCTGCTCTGTTTTGTCGCGGACGTTCATATCCGGTGTCTGTTTTGTGG
CONSENSUS
TCTCTGCTCTGTTTTGTCGCGGACGTTCATATCCGGTGTCTGTTTTGTGG

RI-AT4G12560-XLOC_021029-8451-0
CGGACGAGCAATTTTCATATTCGGGTTGGGACAAGAGTGAAAGTCACAGT
RI-AT4G12560-XLOC_021029-8451-1
CGGACGAGCAATTTTCATATTCGGGTTGGGACAAGAGTGAAAGTCACAGT
CONSENSUS
CGGACGAGCAATTTTCATATTCGGGTTGGGACAAGAGTGAAAGTCACAGT

RI-AT4G12560-XLOC_021029-8451-0
ATGTCATAGGTATTGAGTGCTTAGAAGTTAGAACATTCCCTAGAAAATGT
RI-AT4G12560-XLOC_021029-8451-1
ATGTCATAG---
CONSENSUS
ATGTCATAG...

RI-AT4G12560-XLOC_021029-8451-0
TATGGAGTCATCATCAGGATAATTACTTTAGCATATGACCATATTCAAGT
RI-AT4G12560-XLOC_021029-8451-1

CONSENSUS
....

RI-AT4G12560-XLOC_021029-8451-0
GTGGTTTTTTCACATCAATCTATCTGCAAAACAATCATTGGGTATGATTT

RI-AT4G12560-XLOC_021029-8451-1

CONSENSUS
      ....

RI-AT4G12560-XLOC_021029-8451-0
      TTTCAGGGAGGAGAAACGAGAAGGGGAACAAACAATAACTTCAACCAAAA
RI-AT4G12560-XLOC_021029-8451-1 ------
GGAGGAGAAACGAGAAGGGGAACAAACAATAACTTCAACCAAAA
CONSENSUS
      ......GGAGGAGAAACGAGAAGGGGAACAAACAATAACTTCAACCAAAA

RI-AT4G12560-XLOC_021029-8451-0
      AGCTCTAATATACCAAAGGAGAATAATATACAACTTACCTACGTATATGT
RI-AT4G12560-XLOC_021029-8451-1
      AGCTCTAATATACCAAAGGAGAATAATATACAACTTACCTACGTATATGT
CONSENSUS
      AGCTCTAATATACCAAAGGAGAATAATATACAACTTACCTACGTATATGT

RI-AT4G12560-XLOC_021029-8451-0
      GCTTATAGAAGAGAGGACAAATGTTTTTGGTTTGTAAATAACTCTTTTTA
RI-AT4G12560-XLOC_021029-8451-1
      GCTTATAGAAGAGAGGACAAATGTTTTTGGTTTGTAAATAACTCTTTTTA
CONSENSUS
      GCTTATAGAAGAGAGGACAAATGTTTTTGGTTTGTAAATAACTCTTTTTA

RI-AT4G12560-XLOC_021029-8451-0
      AGTTTGATATATATAACTTACTAGTACTAACGCTTTGTATACTTGCTTTT
RI-AT4G12560-XLOC_021029-8451-1
      AGTTTGATATATATAACTTACTAGTACTAACGCTTTGTATACTTGCTTTT
CONSENSUS
      AGTTTGATATATATAACTTACTAGTACTAACGCTTTGTATACTTGCTTTT

RI-AT4G12560-XLOC_021029-8451-0
      TTAAGTTTCTGCATAATATGAGATGTTAAATAAATTA
RI-AT4G12560-XLOC_021029-8451-1
      TTAAGTTTCTGCATAATATGAGATGTTAAATAAATTA
CONSENSUS
      TTAAGTTTCTGCATAATATGAGATGTTAAATAAATTA

alignment for event: SE-AT4G35785-XLOC_024844-4195

SE-AT4G35785-XLOC_024844-4195-0
      GTTGCGTCTTGTTTTCTTGTGATGGAGCCGCGCACCCGTGTGTCTCGTGG
SE-AT4G35785-XLOC_024844-4195-1
      GTTGCGTCTTGTTTTCTTGTGATGGAGCCGCGCACCCGTGTGTCTCGTGG
CONSENSUS
      GTTGCGTCTTGTTTTCTTGTGATGGAGCCGCGCACCCGTGTGTCTCGTGG

SE-AT4G35785-XLOC_024844-4195-0
      TTTTGCCTTTGTTACAATGAGCAGCCTTAAAGATGCTGAGCGGTGCATTA
SE-AT4G35785-XLOC_024844-4195-1
      TTTTGCCTTTGTTACAATGAGCAGCCTTAAAGATGCTGAGCGGTGCATTA
CONSENSUS

TTTTGCCTTTGTTACAATGAGCAGCCTTAAAGATGCTGAGCGGTGCATTA

SE-AT4G35785-XLOC_024844-4195-0
AATATCTCAACCAGTCTGTACTAGAAGGCCGATACATAACTGTGGAAAGG
SE-AT4G35785-XLOC_024844-4195-1
AATATCTCAACCAGTCTGTACTAGAAGGCCGATACATAACTGTGGAAAGG
CONSENSUS
AATATCTCAACCAGTCTGTACTAGAAGGCCGATACATAACTGTGGAAAGG

SE-AT4G35785-XLOC_024844-4195-0
--------------------------------------TCCAGGAGAAAGC
SE-AT4G35785-XLOC_024844-4195-1
AAAATATTGCATCAAGCAACAATCAGAGATGATAAGATCCAGGAGAAAGC
CONSENSUS
......................................TCCAGGAGAAAGC

SE-AT4G35785-XLOC_024844-4195-0
GCCCGAGAACTCCCACCCCAGGCCACTATCTTGGCTTGAAAAGCTCCAGA
SE-AT4G35785-XLOC_024844-4195-1
GCCCGAGAACTCCCACCCCAGGCCACTATCTTGGCTTGAAAAGCTCCAGA
CONSENSUS
GCCCGAGAACTCCCACCCCAGGCCACTATCTTGGCTTGAAAAGCTCCAGA

SE-AT4G35785-XLOC_024844-4195-0 GACAGCG
SE-AT4G35785-XLOC_024844-4195-1 GACAGCG
CONSENSUS GACAGCG

alignment for event: RI-AT4G08470-XLOC_023255-4046

RI-AT4G08470-XLOC_023255-4046-0
GCGTCAAAGTTCAACGACATTATGTCTTGCAAGGGAACATTATTTTGGAT
RI-AT4G08470-XLOC_023255-4046-1
GCGTCAAAGTTCAACGACATTATGTCTTGCAAGGGAACATTATTTTGGAT
CONSENSUS
GCGTCAAAGTTCAACGACATTATGTCTTGCAAGGGAACATTATTTTGGAT

RI-AT4G08470-XLOC_023255-4046-0
GGCTCCGGAGGTAATTGTGTTGGGATCCTTCTCATTGTTTTGGATTGTGT
RI-AT4G08470-XLOC_023255-4046-1
GGCTCCGGAG--
CONSENSUS
GGCTCCGGAG..

RI-AT4G08470-XLOC_023255-4046-0
GAATACAATGATAGACAACATATCATACATCAATAGCAATGTTCTCGGCA
RI-AT4G08470-XLOC_023255-4046-1

CONSENSUS
....

RI-AT4G08470-XLOC_023255-4046-0
TTGATAACCTTAAAAAAACCCGATTATGTTTCCTTCAAGGTATTTGGTTA
RI-AT4G08470-XLOC_023255-4046-1

```
CONSENSUS
      ..................................................

RI-AT4G08470-XLOC_023255-4046-0
      AATTTTTGTTTATGGTGAGTTGGTAACTGTTAGTATGTTTATACTCGAGG
RI-AT4G08470-XLOC_023255-4046-1
      --------------------------------------------------
CONSENSUS
      ..................................................

RI-AT4G08470-XLOC_023255-4046-0
      CTATACTTGTATGGTTATTTCTAAATAGAACATAACCATTTGATCAGGTT
RI-AT4G08470-XLOC_023255-4046-1
      -----------------------------------------------GTT
CONSENSUS
      ...............................................GTT

RI-AT4G08470-XLOC_023255-4046-0
      ATTAACCGAAAGGATAGTGATGGCAATGGAAGTCCAGCTGATATATGGAG
RI-AT4G08470-XLOC_023255-4046-1
      ATTAACCGAAAGGATAGTGATGGCAATGGAAGTCCAGCTGATATATGGAG
CONSENSUS
      ATTAACCGAAAGGATAGTGATGGCAATGGAAGTCCAGCTGATATATGGAG

RI-AT4G08470-XLOC_023255-4046-0
      CCTTGGGTGCACTGTGCTGGAAATGTGTACTGGTCAGATCCCCTACTCCG
RI-AT4G08470-XLOC_023255-4046-1
      CCTTGGGTGCACTGTGCTGGAAATGTGTACTGGTCAGATCCCCTACTCCG
CONSENSUS
      CCTTGGGTGCACTGTGCTGGAAATGTGTACTGGTCAGATCCCCTACTCCG

RI-AT4G08470-XLOC_023255-4046-0  ATCTAAAACCC
RI-AT4G08470-XLOC_023255-4046-1  ATCTAAAACCC
CONSENSUS                        ATCTAAAACCC


alignment for event: A3-AT4G18740-XLOC_023907-7338

A3-AT4G18740-XLOC_023907-7338-0
      ATTTGTTTCTTTTTTGTTGTGAGGTGAAGAAAGAGGCCAAAGTGAGAACT
A3-AT4G18740-XLOC_023907-7338-1
      ATTTGTTTCTTTTTTGTTGTGAGGTGAAGAAAGAGGCCAAAGTGAGAACT
CONSENSUS
      ATTTGTTTCTTTTTTGTTGTGAGGTGAAGAAAGAGGCCAAAGTGAGAACT

A3-AT4G18740-XLOC_023907-7338-0
      GTCGGAAGACAATCTCTGATTTTCAGGGAAAGTCAGGAATTAAAGCTATG
A3-AT4G18740-XLOC_023907-7338-1
      GTCGGAAGACAATCTCTGATTTTCAGGGAAAGTCAGGAATTAAAGCTATG
CONSENSUS
      GTCGGAAGACAATCTCTGATTTTCAGGGAAAGTCAGGAATTAAAGCTATG

A3-AT4G18740-XLOC_023907-7338-0
      GACTTGGCTCTTCATTGTCATTGTGCTTACCCGTTGATCAAACTAGGATT
A3-AT4G18740-XLOC_023907-7338-1
```

GACTTGGCTCTTCATTGTCATTGTGCTTACCCGTTGATCAAACTAGGATT
CONSENSUS
GACTTGGCTCTTCATTGTCATTGTGCTTACCCGTTGATCAAACTAGGATT

A3-AT4G18740-XLOC_023907-7338-0
CATGAGAGCTAAATCAGCCAATCATCTTCATGTTCGAG------------
A3-AT4G18740-XLOC_023907-7338-1
CATGAGAGCTAAATCAGCCAATCATCTTCATGTTCGAGATTGCAGATACT
CONSENSUS
CATGAGAGCTAAATCAGCCAATCATCTTCATGTTCGAG............

A3-AT4G18740-XLOC_023907-7338-0
---------------------------------AGTCTTTCGAGAACAAC
A3-AT4G18740-XLOC_023907-7338-1
CATTCACTGCCGTTTGCGTTGCGCTTTGAGCAGAGTCTTTCGAGAACAAC
CONSENSUS
.................................AGTCTTTCGAGAACAAC

A3-AT4G18740-XLOC_023907-7338-0
GATTAATGGAGATAGAAGTATTTGGTTTCAAGAGAAAGGTGGTTCTTCTT
A3-AT4G18740-XLOC_023907-7338-1
GATTAATGGAGATAGAAGTATTTGGTTTCAAGAGAAAGGTGGTTCTTCTT
CONSENSUS
GATTAATGGAGATAGAAGTATTTGGTTTCAAGAGAAAGGTGGTTCTTCTT

A3-AT4G18740-XLOC_023907-7338-0
ATACTTCAATGGGAAGATCCAAGAAAGGATGTGTTTGTTGCAAGAAACCG
A3-AT4G18740-XLOC_023907-7338-1
ATACTTCAATGGGAAGATCCAAGAAAGGATGTGTTTGTTGCAAGAAACCG
CONSENSUS
ATACTTCAATGGGAAGATCCAAGAAAGGATGTGTTTGTTGCAAGAAACCG

A3-AT4G18740-XLOC_023907-7338-0
TCTGATAGACGAACGTCAAACCCAAGTAAATCGAACCAAGAGGAGATCAT
A3-AT4G18740-XLOC_023907-7338-1
TCTGATAGACGAACGTCAAACCCAAGTAAATCGAACCAAGAGGAGATCAT
CONSENSUS
TCTGATAGACGAACGTCAAACCCAAGTAAATCGAACCAAGAGGAGATCAT

A3-AT4G18740-XLOC_023907-7338-0
TTCGCTCTTGAAACGGATTCAATCTTCGATCTCTAAAGGAGAGTCTCGAG
A3-AT4G18740-XLOC_023907-7338-1
TTCGCTCTTGAAACGGATTCAATCTTCGATCTCTAAAGGAGAGTCTCGAG
CONSENSUS
TTCGCTCTTGAAACGGATTCAATCTTCGATCTCTAAAGGAGAGTCTCGAG

A3-AT4G18740-XLOC_023907-7338-0
GAGTCGAGGAAGAGAAGAACAGCGATGAGTCTTCTAAGGAAAAGCCGCTG
A3-AT4G18740-XLOC_023907-7338-1
GAGTCGAGGAAGAGAAGAACAGCGATGAGTCTTCTAAGGAAAAGCCGCTG
CONSENSUS
GAGTCGAGGAAGAGAAGAACAGCGATGAGTCTTCTAAGGAAAAGCCGCTG

A3-AT4G18740-XLOC_023907-7338-0
ACCAAAGCTATTCTTGACGTTCTTGAGAAATCAAGAAAAAAAACTGAGG
A3-AT4G18740-XLOC_023907-7338-1

ACCAAAGCTATTCTTGACGTTCTTGAGAAATCAAGAAAAAAAAACTGAGG
CONSENSUS
ACCAAAGCTATTCTTGACGTTCTTGAGAAATCAAGAAAAAAAAACTGAGG

alignment for event: RI-AT4G25500-XLOC_021789-10123

RI-AT4G25500-XLOC_021789-10123-0
GAAGCATGAAGCCAGTCTTCTGTGGGAACTTTGAGTATGATGCGCGCGAA
RI-AT4G25500-XLOC_021789-10123-1
GAAGCATGAAGCCAGTCTTCTGTGGGAACTTTGAGTATGATGCGCGCGAA
CONSENSUS
GAAGCATGAAGCCAGTCTTCTGTGGGAACTTTGAGTATGATGCGCGCGAA

RI-AT4G25500-XLOC_021789-10123-0
GGTGACCTGGAACGACTATTCAGGAAATACGGCAAGGTTGAGAGGGTTGA
RI-AT4G25500-XLOC_021789-10123-1
GGTGACCTGGAACGACTATTCAGGAAATACGGCAAGGTTGAGAGGGTTGA
CONSENSUS
GGTGACCTGGAACGACTATTCAGGAAATACGGCAAGGTTGAGAGGGTTGA

RI-AT4G25500-XLOC_021789-10123-0
TATGAAAGCTGGTGAGCCTTTTTTTTCTTTCTTTCTTTCTTCTGCCTCTT
RI-AT4G25500-XLOC_021789-10123-1
TATGAAAGCTG---------------------------------------
CONSENSUS
TATGAAAGCTG.......................................

RI-AT4G25500-XLOC_021789-10123-0
ACTACTCTGTCTTTGTGGTTAACCTGAATGAATTATAATAAGCAGTATTG
RI-AT4G25500-XLOC_021789-10123-1

CONSENSUS
....

RI-AT4G25500-XLOC_021789-10123-0
TTATCCACTAAGTACAATGAAAAAGCGAAATTCAAATGCATTTCATGAAA
RI-AT4G25500-XLOC_021789-10123-1

CONSENSUS
....

RI-AT4G25500-XLOC_021789-10123-0
TCCACTCTCGTCTTCATTCCATCCACGAGAAGGCTCTGGTGGACTATCTC
RI-AT4G25500-XLOC_021789-10123-1

CONSENSUS
....

RI-AT4G25500-XLOC_021789-10123-0
CCTTTGGAAGGAGTACTAAACATTGACCACATTCACCTGGCCACCTGATG
RI-AT4G25500-XLOC_021789-10123-1

CONSENSUS
....

RI-AT4G25500-XLOC_021789-10123-0
CAGCCTGCTTGAACTTTGGAAAGTACTTCAGAGGAATAAGATTTTTAGAA
RI-AT4G25500-XLOC_021789-10123-1

CONSENSUS
....

RI-AT4G25500-XLOC_021789-10123-0
AAAAAATTAGTGTAAAACCGCATTTTCTTTGACGCAGGATGTGTTTGATA
RI-AT4G25500-XLOC_021789-10123-1

CONSENSUS
....

RI-AT4G25500-XLOC_021789-10123-0
ATCTTGGGACGCCTATACTCACACCTGGCCATGAATGGACCATAGGGAAA
RI-AT4G25500-XLOC_021789-10123-1

CONSENSUS
....

RI-AT4G25500-XLOC_021789-10123-0
TCCATCTCTTATCCCTCAAAATCAGTCACTTTCCATCCGCATTAGCACTG
RI-AT4G25500-XLOC_021789-10123-1

CONSENSUS
....

RI-AT4G25500-XLOC_021789-10123-0
CCATCTTAATTGCATTTCATTCCTCATCACTTTGCACACTTGGACATGCC
RI-AT4G25500-XLOC_021789-10123-1

CONSENSUS
....

RI-AT4G25500-XLOC_021789-10123-0
TCTGCAATGGAGAAGGCTCTCTATTTTCATTCATATCCCCGTCTGACTTC
RI-AT4G25500-XLOC_021789-10123-1

CONSENSUS
....

RI-AT4G25500-XLOC_021789-10123-0
CACATTTTCAGTTGTTCCTTGACATATTAATTCCATAATGCAAGGTCTGT
RI-AT4G25500-XLOC_021789-10123-1

CONSENSUS
....

RI-AT4G25500-XLOC_021789-10123-0
AACTGCTTCACTGGTCATTTGGAGTTTTTTTAAGGTTCTAGGAATGTTCA
RI-AT4G25500-XLOC_021789-10123-1

CONSENSUS
....

```
RI-AT4G25500-XLOC_021789-10123-0
      AAATTTAATATGTTCTAGGAGTTAAGTTAGGCGTTGAAGGAGGGAGGACA
RI-AT4G25500-XLOC_021789-10123-1
      --------------------------------------------------
CONSENSUS
      ..................................................

RI-AT4G25500-XLOC_021789-10123-0
      GTGTTCGTGTATGTTGAAGGTTCTTAATCTGGTCCACTTCCTCACTGTTA
RI-AT4G25500-XLOC_021789-10123-1
      --------------------------------------------------
CONSENSUS
      ..................................................

RI-AT4G25500-XLOC_021789-10123-0
      ATGTACTCAGATCTTTGCTTTTGGCTTTTTCTTTTTTCTCCAGGGTTTGC
RI-AT4G25500-XLOC_021789-10123-1
      -------------------------------------------GGTTTGC
CONSENSUS
      ...........................................GGTTTGC

RI-AT4G25500-XLOC_021789-10123-0
      TTTTGTATACATGGAAGATGAAAGGGATGCGGAAGATGCCATCCGAGCAC
RI-AT4G25500-XLOC_021789-10123-1
      TTTTGTATACATGGAAGATGAAAGGGATGCGGAAGATGCCATCCGAGCAC
CONSENSUS
      TTTTGTATACATGGAAGATGAAAGGGATGCGGAAGATGCCATCCGAGCAC

RI-AT4G25500-XLOC_021789-10123-0
      TTGACCGCTTTGAATTTGGGCGTAAGGGACGCAGACTTCGTGTTGAATGG
RI-AT4G25500-XLOC_021789-10123-1
      TTGACCGCTTTGAATTTGGGCGTAAGGGACGCAGACTTCGTGTTGAATGG
CONSENSUS
      TTGACCGCTTTGAATTTGGGCGTAAGGGACGCAGACTTCGTGTTGAATGG

RI-AT4G25500-XLOC_021789-10123-0 ACAAAG
RI-AT4G25500-XLOC_021789-10123-1 ACAAAG
CONSENSUS                        ACAAAG


alignment for event: A3-AT4G20260-XLOC_021487-8829

A3-AT4G20260-XLOC_021487-8829-0
      TTAATCAGTTTCGTGTGTTTGGAGAAGAAGAAGAACAGATCAAATACGAG
A3-AT4G20260-XLOC_021487-8829-1
      TTAATCAGTTTCGTGTGTTTGGAGAAGAAGAAGAACAGATCAAATACGAG
CONSENSUS
      TTAATCAGTTTCGTGTGTTTGGAGAAGAAGAAGAACAGATCAAATACGAG

A3-AT4G20260-XLOC_021487-8829-0
      GAGAGATCTCTAAAGAGATTTATCGTTTCAATTTAATTTAGATTAAACAA
A3-AT4G20260-XLOC_021487-8829-1
      GAGAGATCTCTAAAGAGATTTATCGTTTCAA----------ATTAAACAA
CONSENSUS
```

```
      GAGAGATCTCTAAAGAGATTTATCGTTTCAA..........ATTAAACAA

A3-AT4G20260-XLOC_021487-8829-0
      CAAAAAGATGGGTTACTGGAATTCCAAGGTTGTTCCAAAATTCAAGAAGT
A3-AT4G20260-XLOC_021487-8829-1
      CAAAAAGATGGGTTACTGGAATTCCAAGGTTGTTCCAAAATTCAAGAAGT
CONSENSUS
      CAAAAAGATGGGTTACTGGAATTCCAAGGTTGTTCCAAAATTCAAGAAGT

A3-AT4G20260-XLOC_021487-8829-0
      TATTCGAGAAAAATAGTGCTAAGAAGGCTGCTGCTGCTGAAGCTACCAAG
A3-AT4G20260-XLOC_021487-8829-1
      TATTCGAGAAAAATAGTGCTAAGAAGGCTGCTGCTGCTGAAGCTACCAAG
CONSENSUS
      TATTCGAGAAAAATAGTGCTAAGAAGGCTGCTGCTGCTGAAGCTACCAAG

A3-AT4G20260-XLOC_021487-8829-0  ACCTTTGATGAATCTAAG
A3-AT4G20260-XLOC_021487-8829-1  ACCTTTGATGAATCTAAG
CONSENSUS                        ACCTTTGATGAATCTAAG



alignment for event: A3-AT4G31050-XLOC_022105-12135

A3-AT4G31050-XLOC_022105-12135-0
      GTGCCTGTGTTTTGATCTTTACGACAAATTGGTTCCATACAAGAAAGCTT
A3-AT4G31050-XLOC_022105-12135-1
      GTGCCTGTGTTTTGATCTTTACGACAAATTGGTTCCATACAAGAAAGCTT
CONSENSUS
      GTGCCTGTGTTTTGATCTTTACGACAAATTGGTTCCATACAAGAAAGCTT

A3-AT4G31050-XLOC_022105-12135-0
      GGTCATGGCAGAAGAGCATTGTCGAGGAGAAAAAGACATTGATTGATAGA
A3-AT4G31050-XLOC_022105-12135-1
      GGTCATGGCAGAAGAGCATTGTCGAGGAGAAAAAGACATTGATTGATAGA
CONSENSUS
      GGTCATGGCAGAAGAGCATTGTCGAGGAGAAAAAGACATTGATTGATAGA

A3-AT4G31050-XLOC_022105-12135-0
      AACCAAGACTGCGCTGATACAGTGATTTTGTTACAACACTCTCCTGTTTA
A3-AT4G31050-XLOC_022105-12135-1
      AACCAAGACTGCGCTGATACAGTGATTTTGTTACAACACTCTCCTGTTTA
CONSENSUS
      AACCAAGACTGCGCTGATACAGTGATTTTGTTACAACACTCTCCTGTTTA

A3-AT4G31050-XLOC_022105-12135-0
      CACAATGGGAACTGCTAGTACAGAGGATTATCTCAATTTCGATATAAAGG
A3-AT4G31050-XLOC_022105-12135-1
      CACAATGGGAACTGCTAGTACAGAGGATTATCTCAATTTCGATATAAAGG
CONSENSUS
      CACAATGGGAACTGCTAGTACAGAGGATTATCTCAATTTCGATATAAAGG

A3-AT4G31050-XLOC_022105-12135-0
      ATGCTCCTTTCAATGTTTATCGAACTGAACGTGGTGGCGAAGTCACTTAC
A3-AT4G31050-XLOC_022105-12135-1
      ATGCTCCTTTCAATGTTTATCGAACTGAACGTGGTGGCGAAGTCACTTAC
```

CONSENSUS
      ATGCTCCTTTCAATGTTTATCGAACTGAACGTGGTGGCGAAGTCACTTAC

A3-AT4G31050-XLOC_022105-12135-0
      CATGGCCCTGGTCAGTTATGTATCCTATAATCAATCTTCGAAATCACGAG
A3-AT4G31050-XLOC_022105-12135-1
      CATGGCCCTGGTCAG-----------------------------------
CONSENSUS
      CATGGCCCTGGTCAG...................................

A3-AT4G31050-XLOC_022105-12135-0
      ATGGATCTTCATTGGTACCTCAGGATGCTTGAAGAGATTGTCATCCGTGT
A3-AT4G31050-XLOC_022105-12135-1 -----------------------
GATGCTTGAAGAGATTGTCATCCGTGT
CONSENSUS
      .......................GATGCTTGAAGAGATTGTCATCCGTGT

A3-AT4G31050-XLOC_022105-12135-0
      GCTTTCCTCAACTTTCTCCATTAAAGCTTCTCGTCTTGACGGTTTAACTG
A3-AT4G31050-XLOC_022105-12135-1
      GCTTTCCTCAACTTTCTCCATTAAAGCTTCTCGTCTTGACGGTTTAACTG
CONSENSUS
      GCTTTCCTCAACTTTCTCCATTAAAGCTTCTCGTCTTGACGGTTTAACTG

A3-AT4G31050-XLOC_022105-12135-0 GAGTTTGGGTTG
A3-AT4G31050-XLOC_022105-12135-1 GAGTTTGGGTTG
CONSENSUS GAGTTTGGGTTG

alignment for event: RI-AT4G06634-XLOC_023086-3428

RI-AT4G06634-XLOC_023086-3428-0
      GGTACCACAAGATATTGTTGCTACAGGTGGAAAGTGTCATCTACACAAAT
RI-AT4G06634-XLOC_023086-3428-1
      GGTACCACAAGATATTGTTGCTACAGGTGGAAAGTGTCATCTACACAAAT
CONSENSUS
      GGTACCACAAGATATTGTTGCTACAGGTGGAAAGTGTCATCTACACAAAT

RI-AT4G06634-XLOC_023086-3428-0
      GGGTCACAGGTGAAATTGTGATGTTTTAATCTAATTTTCATGTTTGTGAA
RI-AT4G06634-XLOC_023086-3428-1
      GGGTCACAG---
CONSENSUS
      GGGTCACAG...

RI-AT4G06634-XLOC_023086-3428-0
      TGTTAAATTGTATCATTGTGTTGTTGTTATTACCAGAGGATACTTTTAGC
RI-AT4G06634-XLOC_023086-3428-1
      ------------------------------------AGGATACTTTTAGC
CONSENSUS
      ....................................AGGATACTTTTAGC

RI-AT4G06634-XLOC_023086-3428-0
      AGACTTAAAGAAAAAGAGAAAGAGCCTGATGTTCCTGAGCCTGAACCTGA
RI-AT4G06634-XLOC_023086-3428-1

AGACTTAAAGAAAAAGAGAAAGAGCCTGATGTTCCTGAGCCTGAACCTGA
CONSENSUS
AGACTTAAAGAAAAAGAGAAAGAGCCTGATGTTCCTGAGCCTGAACCTGA

RI-AT4G06634-XLOC_023086-3428-0
ACCAACTACAGAGATTTTGTTTCTCTGTAGTTATGATGGTTGCGGGAAGA
RI-AT4G06634-XLOC_023086-3428-1
ACCAACTACAGAGATTTTGTTTCTCTGTAGTTATGATGGTTGCGGGAAGA
CONSENSUS
ACCAACTACAGAGATTTTGTTTCTCTGTAGTTATGATGGTTGCGGGAAGA

RI-AT4G06634-XLOC_023086-3428-0
CTTTCTTTGATGTTAGTGCATTGAGGAAACATTCTCATATCCATGGAGAA
RI-AT4G06634-XLOC_023086-3428-1
CTTTCTTTGATGTTAGTGCATTGAGGAAACATTCTCATATCCATGGAGAA
CONSENSUS
CTTTCTTTGATGTTAGTGCATTGAGGAAACATTCTCATATCCATGGAGAA

RI-AT4G06634-XLOC_023086-3428-0
AGACAATATGTTTGTGATCAGGAAGGATGTGGAAAG
RI-AT4G06634-XLOC_023086-3428-1
AGACAATATGTTTGTGATCAGGAAGGATGTGGAAAG
CONSENSUS
AGACAATATGTTTGTGATCAGGAAGGATGTGGAAAG

alignment for event: A3-AT4G35140-XLOC_024816-9738

A3-AT4G35140-XLOC_024816-9738-0
TTTGATCTGAGGACTGAAGCCCCTACAGAACTTTTCACTTGCCGATCAGT
A3-AT4G35140-XLOC_024816-9738-1
TTTGATCTGAGGACTGAAGCCCCTACAGAACTTTTCACTTGCCGATCAGT
CONSENSUS
TTTGATCTGAGGACTGAAGCCCCTACAGAACTTTTCACTTGCCGATCAGT

A3-AT4G35140-XLOC_024816-9738-0
TGATCCTAGGAGGAGGAACATGGACGCCATCCAGCTAAATGCGATTGCGA
A3-AT4G35140-XLOC_024816-9738-1
TGATCCTAGGAGGAGGAACATGGACGCCATCCAGCTAAATGCGATTGCGA
CONSENSUS
TGATCCTAGGAGGAGGAACATGGACGCCATCCAGCTAAATGCGATTGCGA

A3-AT4G35140-XLOC_024816-9738-0
TCGACCCGAGAAATTCTAACCTCTTTGCTGTTGGGGGAATGGAAGAGTAC
A3-AT4G35140-XLOC_024816-9738-1
TCGACCCGAGAAATTCTAACCTCTTTGCTGTTGGGGGAATGGAAGAGTAC
CONSENSUS
TCGACCCGAGAAATTCTAACCTCTTTGCTGTTGGGGGAATGGAAGAGTAC

A3-AT4G35140-XLOC_024816-9738-0
GCTCGTCTTTATGATATCCGTAGATTTCAGGGTGAGGGTTTAAATGGTTT
A3-AT4G35140-XLOC_024816-9738-1
GCTCGTCTTTATGATATCCGTAGATTTCAGGGTGAGGGTTTAAATGGTTT
CONSENSUS
GCTCGTCTTTATGATATCCGTAGATTTCAGGGTGAGGGTTTAAATGGTTT

A3-AT4G35140-XLOC_024816-9738-0
TACCCGAGCTGCAGATCACTTTTGTCCTCCACATCTTATTGGCAATGAAG
A3-AT4G35140-XLOC_024816-9738-1
TACCCGAGCTGCAGATCACTTTTGTCCTCCACATCTTATTGGCAATGAAG
CONSENSUS
TACCCGAGCTGCAGATCACTTTTGTCCTCCACATCTTATTGGCAATGAAG

A3-AT4G35140-XLOC_024816-9738-0
ATGTTGGAATAACGGGGTTGGCTTTCTCAGAGCAAAGCGAGCTTCTTGTT
A3-AT4G35140-XLOC_024816-9738-1
ATGTTGGAATAACGGGGTTGGCTTTCTCAGAGCAAAGCGAGCTTCTTGTT
CONSENSUS
ATGTTGGAATAACGGGGTTGGCTTTCTCAGAGCAAAGCGAGCTTCTTGTT

A3-AT4G35140-XLOC_024816-9738-0
TCATACAATGATGAGTTCATCTATCTCTTCACACCCGGTATGGGATTAGG
A3-AT4G35140-XLOC_024816-9738-1
TCATACAATGATGAGTTCATCTATCTCTTCACACCCGGTATGGGATTAGG
CONSENSUS
TCATACAATGATGAGTTCATCTATCTCTTCACACCCGGTATGGGATTAGG

A3-AT4G35140-XLOC_024816-9738-0
GTCTAACCCTATCCCATCCTCCCCGATATCCAAGAGTCCTGTATCAAAAT
A3-AT4G35140-XLOC_024816-9738-1
GTCTAACCCTATCCCATCCTCCCCGATATCCAAGAGTCCTGTATCAAAAT
CONSENSUS
GTCTAACCCTATCCCATCCTCCCCGATATCCAAGAGTCCTGTATCAAAAT

A3-AT4G35140-XLOC_024816-9738-0
CAGAATCATCTTCTTCCCCAAAAGATGAGAACGAACACTCAGTTTCTTTG
A3-AT4G35140-XLOC_024816-9738-1
CAGAATCATCTTCTTCCCCAAAAGATGAGAACGAACACTCAGTTTCTTTG
CONSENSUS
CAGAATCATCTTCTTCCCCAAAAGATGAGAACGAACACTCAGTTTCTTTG

A3-AT4G35140-XLOC_024816-9738-0
GTTTATAAGGGGCACAAGAACTGTGAGACAGTTAAGGGTGTGAACTTTTT
A3-AT4G35140-XLOC_024816-9738-1
GTTTATAAGGGGCACAAGAACTGTGAGACAGTTAAGGGTGTGAACTTTTT
CONSENSUS
GTTTATAAGGGGCACAAGAACTGTGAGACAGTTAAGGGTGTGAACTTTTT

A3-AT4G35140-XLOC_024816-9738-0
TGGACCTCGGTCTGAATATGTGGTTAGTGGGTCAGACTGTGGGAGGATAT
A3-AT4G35140-XLOC_024816-9738-1
TGGACCTCGGTCTGAATATGTGGTTAGTGGGTCAGACTGTGGGAGGATAT
CONSENSUS
TGGACCTCGGTCTGAATATGTGGTTAGTGGGTCAGACTGTGGGAGGATAT

A3-AT4G35140-XLOC_024816-9738-0
TTATTTGGAGGAAAAAGGGTGGGGAGCTTATCCGTGTTATGGAAGCAGAT
A3-AT4G35140-XLOC_024816-9738-1
TTATTTGGAGGAAAAAGGGTGGGGAGCTTATCCGTGTTATGGAAGCAGAT
CONSENSUS
TTATTTGGAGGAAAAAGGGTGGGGAGCTTATCCGTGTTATGGAAGCAGAT

A3-AT4G35140-XLOC_024816-9738-0
AGGCATGTAGTTAACTGTATTGAGCCTCATCCACATATTCCTGTGCTTGC
A3-AT4G35140-XLOC_024816-9738-1
AGGCATGTAGTTAACTGTATTGAGCCTCATCCACATATTCCTGTGCTTGC
CONSENSUS
AGGCATGTAGTTAACTGTATTGAGCCTCATCCACATATTCCTGTGCTTGC

A3-AT4G35140-XLOC_024816-9738-0
TAGCAGTGGAATTGAAAGTGACATTAAGGTGTGGACGTCAAAGGCAGCTG
A3-AT4G35140-XLOC_024816-9738-1
TAGCAGTGGAATTGAAAGTGACATTAAGGTGTGGACGTCAAAGGCAGCTG
CONSENSUS
TAGCAGTGGAATTGAAAGTGACATTAAGGTGTGGACGTCAAAGGCAGCTG

A3-AT4G35140-XLOC_024816-9738-0
AGAGAGCTACGTTACCTGAAAACATCGAACTG------------------
A3-AT4G35140-XLOC_024816-9738-1
AGAGAGCTACGTTACCTGAAAACATCGAACTGTTACCGAGTCGGTTTCGC
CONSENSUS
AGAGAGCTACGTTACCTGAAAACATCGAACTG..................

A3-AT4G35140-XLOC_024816-9738-0

A3-AT4G35140-XLOC_024816-9738-1
ATTCCTTGGTTATCGTTTCTTAGCTTCCATGATTATGACGACGAATTATT
CONSENSUS
....

A3-AT4G35140-XLOC_024816-9738-0

A3-AT4G35140-XLOC_024816-9738-1
TGGTAATGGTATGGATATTGGTATTGATGGGAACGAGGGGGAAGATGAAT
CONSENSUS
....

A3-AT4G35140-XLOC_024816-9738-0

A3-AT4G35140-XLOC_024816-9738-1
CAATTGATGATGATGCTGAGGATAATGATGATGATTCAGATTATAGCAGT
CONSENSUS
....

A3-AT4G35140-XLOC_024816-9738-0

A3-AT4G35140-XLOC_024816-9738-1
GGAGTAGATCTAGATGATAATGATTCTGATGATGACATGGATAGTGATGA
CONSENSUS
....

A3-AT4G35140-XLOC_024816-9738-0

A3-AT4G35140-XLOC_024816-9738-1
TGATGATGTTGATGACATGGATAGTGATGATGAATGTTTTATTGAAATTG
CONSENSUS
....

A3-AT4G35140-XLOC_024816-9738-0

A3-AT4G35140-XLOC_024816-9738-1
      ATAACAACAATATGGATAATAATGGTGATGGTGGTAGTGAGACGAATGTT
CONSENSUS
      ....

A3-AT4G35140-XLOC_024816-9738-0

A3-AT4G35140-XLOC_024816-9738-1
      GATAGTATTAGTGGCAGTCATCAAGATGATGATGTTGATGATGATGATTG
CONSENSUS
      ....

A3-AT4G35140-XLOC_024816-9738-0 ----------------------------
CGCAAGCGAACGCCTAGGGGAT
A3-AT4G35140-XLOC_024816-9738-1
      AATATTGTTTGAAATTTGTGTGTTCCAGCGCAAGCGAACGCCTAGGGGAT
CONSENSUS
      ............................CGCAAGCGAACGCCTAGGGGAT

A3-AT4G35140-XLOC_024816-9738-0
      GGATGTATCGTGTCTCTTCACCGCATGAACTATTGGCGCAACTCTTCTCG
A3-AT4G35140-XLOC_024816-9738-1
      GGATGTATCGTGTCTCTTCACCGCATGAACTATTGGCGCAACTCTTCTCG
CONSENSUS
      GGATGTATCGTGTCTCTTCACCGCATGAACTATTGGCGCAACTCTTCTCG

A3-AT4G35140-XLOC_024816-9738-0
      CTGCAAAACCGAAGCAGTAGTAGTCCAGAGAGAGAAGGAGAATCTTCTTC
A3-AT4G35140-XLOC_024816-9738-1
      CTGCAAAACCGAAGCAGTAGTAGTCCAGAGAGAGAAGGAGAATCTTCTTC
CONSENSUS
      CTGCAAAACCGAAGCAGTAGTAGTCCAGAGAGAGAAGGAGAATCTTCTTC

A3-AT4G35140-XLOC_024816-9738-0
      AGCCACTGGAAGAGAACTTCTTGATCTTATCCTCACCTTCAATGATCAGA
A3-AT4G35140-XLOC_024816-9738-1
      AGCCACTGGAAGAGAACTTCTTGATCTTATCCTCACCTTCAATGATCAGA
CONSENSUS
      AGCCACTGGAAGAGAACTTCTTGATCTTATCCTCACCTTCAATGATCAGA

A3-AT4G35140-XLOC_024816-9738-0
      GCGACGATGAAAATGCAACTGATGATGATGATGGTAATAGCCATGAAGAC
A3-AT4G35140-XLOC_024816-9738-1
      GCGACGATGAAAATGCAACTGATGATGATGATGGTAATAGCCATGAAGAC
CONSENSUS
      GCGACGATGAAAATGCAACTGATGATGATGATGGTAATAGCCATGAAGAC

A3-AT4G35140-XLOC_024816-9738-0
      TTCTTCTCTTAAGAGAAAACACTCATCTGGAAGAATTTGTAAATATTTTT
A3-AT4G35140-XLOC_024816-9738-1
      TTCTTCTCTTAAGAGAAAACACTCATCTGGAAGAATTTGTAAATATTTTT
CONSENSUS
      TTCTTCTCTTAAGAGAAAACACTCATCTGGAAGAATTTGTAAATATTTTT

```
A3-AT4G35140-XLOC_024816-9738-0
      CAGTCCCTTTAAAACCTTTTTTGTATTTTGATTTACCACATGGTTTTTCA
A3-AT4G35140-XLOC_024816-9738-1
      CAGTCCCTTTAAAACCTTTTTTGTATTTTGATTTACCACATGGTTTTTCA
CONSENSUS
      CAGTCCCTTTAAAACCTTTTTTGTATTTTGATTTACCACATGGTTTTTCA

A3-AT4G35140-XLOC_024816-9738-0
      TTGTTCAATAAAGTCTCAACATTATATGTTCTTGTAATTATACATACACG
A3-AT4G35140-XLOC_024816-9738-1
      TTGTTCAATAAAGTCTCAACATTATATGTTCTTGTAATTATACATACACG
CONSENSUS
      TTGTTCAATAAAGTCTCAACATTATATGTTCTTGTAATTATACATACACG

A3-AT4G35140-XLOC_024816-9738-0  TTCATCATCATAATTCGTTGC
A3-AT4G35140-XLOC_024816-9738-1  TTCATCATCATAATTCGTTGC
CONSENSUS                        TTCATCATCATAATTCGTTGC



alignment for event: A3-AT4G03110-XLOC_022802-9839

A3-AT4G03110-XLOC_022802-9839-0
      GGTTCAACTGTTCCTTTAGTTGTCAAATGGGCAGACACAGAACGAGAAAG
A3-AT4G03110-XLOC_022802-9839-1
      GGTTCAACTGTTCCTTTAGTTGTCAAATGGGCAGACACAGAACGAGAAAG
CONSENSUS
      GGTTCAACTGTTCCTTTAGTTGTCAAATGGGCAGACACAGAACGAGAAAG

A3-AT4G03110-XLOC_022802-9839-0
      ACACACAAGAAGACTTCAAAAGGCTCAATCTCACATCGCCCGACTAGGGA
A3-AT4G03110-XLOC_022802-9839-1
      ACACACAAGAAGACTTCAAAAGGCTCAATCTCACATCGCCCGACTAGGGA
CONSENSUS
      ACACACAAGAAGACTTCAAAAGGCTCAATCTCACATCGCCCGACTAGGGA

A3-AT4G03110-XLOC_022802-9839-0
      ACGGTGATCCAACAAATCCCTCATTGTTTGGAGCATTACCTATGGGTTAT
A3-AT4G03110-XLOC_022802-9839-1
      ACGGTGATCCAACAAATCCCTCATTGTTTGGAGCATTACCTATGGGTTAT
CONSENSUS
      ACGGTGATCCAACAAATCCCTCATTGTTTGGAGCATTACCTATGGGTTAT

A3-AT4G03110-XLOC_022802-9839-0
      GTACCACCATATAATGGATATGGTTATCAT---CCTCCTGGAACTTATGG
A3-AT4G03110-XLOC_022802-9839-1
      GTACCACCATATAATGGATATGGTTATCATCAGCCTCCTGGAACTTATGG
CONSENSUS
      GTACCACCATATAATGGATATGGTTATCAT...CCTCCTGGAACTTATGG

A3-AT4G03110-XLOC_022802-9839-0
      TTACATGCTACCACCAATTCAGAACCAAGCTGCATTTTCCAATATGATTG
A3-AT4G03110-XLOC_022802-9839-1
      TTACATGCTACCACCAATTCAGAACCAAGCTGCATTTTCCAATATGATTG
CONSENSUS
```

TTACATGCTACCACCAATTCAGAACCAAGCTGCATTTTCCAATATGATTG

A3-AT4G03110-XLOC_022802-9839-0
CACAACCAAACCAAGGTAATAACAATGCATTGCAAGGAACATCGCCTGAC
A3-AT4G03110-XLOC_022802-9839-1
CACAACCAAACCAAGGTAATAACAATGCATTGCAAGGAACATCGCCTGAC
CONSENSUS
CACAACCAAACCAAGGTAATAACAATGCATTGCAAGGAACATCGCCTGAC

A3-AT4G03110-XLOC_022802-9839-0
TCTGTGCCGCCTCGTTTGGCCCGTAGAAACTTTCCTATGCCTCCTGGAAA
A3-AT4G03110-XLOC_022802-9839-1
TCTGTGCCGCCTCGTTTGGCCCGTAGAAACTTTCCTATGCCTCCTGGAAA
CONSENSUS
TCTGTGCCGCCTCGTTTGGCCCGTAGAAACTTTCCTATGCCTCCTGGAAA

A3-AT4G03110-XLOC_022802-9839-0
CTACATGGGATCTGGTTATCCTGCTATGCGAGGTCATCCTTTTCCATTTG
A3-AT4G03110-XLOC_022802-9839-1
CTACATGGGATCTGGTTATCCTGCTATGCGAGGTCATCCTTTTCCATTTG
CONSENSUS
CTACATGGGATCTGGTTATCCTGCTATGCGAGGTCATCCTTTTCCATTTG

A3-AT4G03110-XLOC_022802-9839-0
CTTATCCCAGAGGAATTGTTAGTCCTCGCCCTCTAAGTAGCTCTCCTGGA
A3-AT4G03110-XLOC_022802-9839-1
CTTATCCCAGAGGAATTGTTAGTCCTCGCCCTCTAAGTAGCTCTCCTGGA
CONSENSUS
CTTATCCCAGAGGAATTGTTAGTCCTCGCCCTCTAAGTAGCTCTCCTGGA

A3-AT4G03110-XLOC_022802-9839-0
TCTATATCACCTGGCATGTCAACACCTTTAGGCATTGGTTTGAGTTCTGT
A3-AT4G03110-XLOC_022802-9839-1
TCTATATCACCTGGCATGTCAACACCTTTAGGCATTGGTTTGAGTTCTGT
CONSENSUS
TCTATATCACCTGGCATGTCAACACCTTTAGGCATTGGTTTGAGTTCTGT

A3-AT4G03110-XLOC_022802-9839-0 GGTTCAAACCGAAG
A3-AT4G03110-XLOC_022802-9839-1 GGTTCAAACCGAAG
CONSENSUS GGTTCAAACCGAAG

alignment for event: A3-AT4G34100-XLOC_022274-10054

A3-AT4G34100-XLOC_022274-10054-0
TGTATCCGACCCATTCACTGAGATTCCCGCCGACATGCTTCTGTTTCAAA
A3-AT4G34100-XLOC_022274-10054-1
TGTATCCGACCCATTCACTGAGATTCCCGCCGACATGCTTCTGTTTCAAA
CONSENSUS
TGTATCCGACCCATTCACTGAGATTCCCGCCGACATGCTTCTGTTTCAAA

A3-AT4G34100-XLOC_022274-10054-0
TATGCATTCCATTTATCATCGAGCATTTCAGACTTCGGACCACAATTAAG
A3-AT4G34100-XLOC_022274-10054-1
TATGCATTCCATTTATCATCGAGCATTTCAGACTTCGGACCACAATTAAG

CONSENSUS
TATGCATTCCATTTATCATCGAGCATTTCAGACTTCGGACCACAATTAAG

A3-AT4G34100-XLOC_022274-10054-0
TCCCTTCTACGCTGCTGGTTCACTGGGGTTGGTTGGGCGCTTGGTTTAAC
A3-AT4G34100-XLOC_022274-10054-1
TCCCTTCTACGCTGCTGGTTCACTGGGGTTGGTTGGGCGCTTGGTTTAAC
CONSENSUS
TCCCTTCTACGCTGCTGGTTCACTGGGGTTGGTTGGGCGCTTGGTTTAAC

A3-AT4G34100-XLOC_022274-10054-0
AGACTTCCTCTTGCCAAGACCAGAAGACAACATTGGTCAGGACAATGGAA
A3-AT4G34100-XLOC_022274-10054-1
AGACTTCCTCTTGCCAAGACCAGAAGACAACATTGGTCAGGACAATGGAA
CONSENSUS
AGACTTCCTCTTGCCAAGACCAGAAGACAACATTGGTCAGGACAATGGAA

A3-AT4G34100-XLOC_022274-10054-0
ATGGTGAACCAGGAAGGCAGAACAGAGCACAAGTGTTGCAGGTCGGAGGA
A3-AT4G34100-XLOC_022274-10054-1
ATGGTGAACCAGGAAGGCAGAACAGAGCACAAGTGTTGCAGGTCGGAGGA
CONSENSUS
ATGGTGAACCAGGAAGGCAGAACAGAGCACAAGTGTTGCAGGTCGGAGGA

A3-AT4G34100-XLOC_022274-10054-0
CCTGACAGGGCCATGGCTGCGCTTCCAGTAGCTGATGACCCTAACAGAAG
A3-AT4G34100-XLOC_022274-10054-1
CCTGACAGGGCCATGGCTGCGCTTCCAGTAGCTGATGACCCTAACAGAAG
CONSENSUS
CCTGACAGGGCCATGGCTGCGCTTCCAGTAGCTGATGACCCTAACAGAAG

A3-AT4G34100-XLOC_022274-10054-0
TCGCCTCCGTGCTGGGAATGTCAACACGGGTGAGGAATATGAAGATGATG
A3-AT4G34100-XLOC_022274-10054-1
TCGCCTCCGTGCTGGGAATGTCAACACGGGTGAGGAATATGAAGATGATG
CONSENSUS
TCGCCTCCGTGCTGGGAATGTCAACACGGGTGAGGAATATGAAGATGATG

A3-AT4G34100-XLOC_022274-10054-0
ACGAACAATCTGATTCTGACAGGTACAACTTTGTGGTCCGGATAATTCTC
A3-AT4G34100-XLOC_022274-10054-1 ACGAACAATCTGATTCTGA---
GTACAACTTTGTGGTCCGGATAATTCTC
CONSENSUS
ACGAACAATCTGATTCTGA...GTACAACTTTGTGGTCCGGATAATTCTC

A3-AT4G34100-XLOC_022274-10054-0
CTTCTACTTGTTGCATGGGTGACTCTTCTTCTCTTCAATTCAGCATTGAT
A3-AT4G34100-XLOC_022274-10054-1
CTTCTACTTGTTGCATGGGTGACTCTTCTTCTCTTCAATTCAGCATTGAT
CONSENSUS
CTTCTACTTGTTGCATGGGTGACTCTTCTTCTCTTCAATTCAGCATTGAT

A3-AT4G34100-XLOC_022274-10054-0
AGTTGTGCCAGTTTCGCTGGGACGTGCTCTATTCAGTGCCATTCCCATTC
A3-AT4G34100-XLOC_022274-10054-1
AGTTGTGCCAGTTTCGCTGGGACGTGCTCTATTCAGTGCCATTCCCATTC

CONSENSUS
AGTTGTGCCAGTTTCGCTGGGACGTGCTCTATTCAGTGCCATTCCCATTC

A3-AT4G34100-XLOC_022274-10054-0 TTCCAATAACTCATGGCATCAAGTGCAATG
A3-AT4G34100-XLOC_022274-10054-1 TTCCAATAACTCATGGCATCAAGTGCAATG
CONSENSUS TTCCAATAACTCATGGCATCAAGTGCAATG

alignment for event: A3-AT4G26555-XLOC_024343-9338

A3-AT4G26555-XLOC_024343-9338-0
CGCGGGAAAATCAAGGAACAGCGTTTCTAGGATTAGTCGTGTTGGATTCT
A3-AT4G26555-XLOC_024343-9338-1
CGCGGGAAAATCAAGGAACAGCGTTTCTAGGATTAGTCGTGTTGGATTCT
CONSENSUS
CGCGGGAAAATCAAGGAACAGCGTTTCTAGGATTAGTCGTGTTGGATTCT

A3-AT4G26555-XLOC_024343-9338-0
CTTCGGTCTCGGCTGTACATGTACCCAGAAGGATGTTTATGCAGCTGTCT
A3-AT4G26555-XLOC_024343-9338-1
CTTCGGTCTCGGCTGTACATGTACCCAGAAGGATGTTTATGCAGCTGTCT
CONSENSUS
CTTCGGTCTCGGCTGTACATGTACCCAGAAGGATGTTTATGCAGCTGTCT

A3-AT4G26555-XLOC_024343-9338-0
GGATTTGGTTCGGTCTTGACGCTTCTGGATTTTCCTAGTTTAGCGGCGCC
A3-AT4G26555-XLOC_024343-9338-1
GGATTTGGTTCGGTCTTGACGCTTCTGGATTTTCCTAGTTTAGCGGCGCC
CONSENSUS
GGATTTGGTTCGGTCTTGACGCTTCTGGATTTTCCTAGTTTAGCGGCGCC

A3-AT4G26555-XLOC_024343-9338-0
GGTTCCTCAGATGAAGGAACCTGAAGTGATCAG-------GACATTGAAA
A3-AT4G26555-XLOC_024343-9338-1
GGTTCCTCAGATGAAGGAACCTGAAGTGATCAGAATGCAGGACATTGAAA
CONSENSUS
GGTTCCTCAGATGAAGGAACCTGAAGTGATCAG.......GACATTGAAA

A3-AT4G26555-XLOC_024343-9338-0 CTCCCGAGTGGCGTGAGGTATCAAG
A3-AT4G26555-XLOC_024343-9338-1 CTCCCGAGTGGCGTGAGGTATCAAG
CONSENSUS CTCCCGAGTGGCGTGAGGTATCAAG

alignment for event: A3-AT4G01880-XLOC_022725-889

A3-AT4G01880-XLOC_022725-889-0
GTTTTGTGGAAACCATAGCCAGAGATTGGAAGGTCAATGGATTCCATGCC
A3-AT4G01880-XLOC_022725-889-1
GTTTTGTGGAAACCATAGCCAGAGATTGGAAGGTCAATGGATTCCATGCC
CONSENSUS
GTTTTGTGGAAACCATAGCCAGAGATTGGAAGGTCAATGGATTCCATGCC

A3-AT4G01880-XLOC_022725-889-0 CTATTGATCCTTCTCA-------------
GAGAATCTCGAATGGCATGTA

A3-AT4G01880-XLOC_022725-889-1
      CTATTGATCCTTCTCACTCTGTGTTGCAGGAGAATCTCGAATGGCATGTA
CONSENSUS
      CTATTGATCCTTCTCA.............GAGAATCTCGAATGGCATGTA

A3-AT4G01880-XLOC_022725-889-0
      AAGAGATGTCCTTTACTTAAACAAACTGTAGAATTGTCTGGTCAACGATT
A3-AT4G01880-XLOC_022725-889-1
      AAGAGATGTCCTTTACTTAAACAAACTGTAGAATTGTCTGGTCAACGATT
CONSENSUS
      AAGAGATGTCCTTTACTTAAACAAACTGTAGAATTGTCTGGTCAACGATT

A3-AT4G01880-XLOC_022725-889-0
      TTATCAGAAGGGTATTAATGCTGGGAACGACGAAGAAGAAGAAAAGATGG
A3-AT4G01880-XLOC_022725-889-1
      TTATCAGAAGGGTATTAATGCTGGGAACGACGAAGAAGAAGAAAAGATGG
CONSENSUS
      TTATCAGAAGGGTATTAATGCTGGGAACGACGAAGAAGAAGAAAAGATGG

A3-AT4G01880-XLOC_022725-889-0
      GATCTTGTTCTCTTGTTACTTCAGAGATGAAGAGAAATCTGCTCTATAGT
A3-AT4G01880-XLOC_022725-889-1
      GATCTTGTTCTCTTGTTACTTCAGAGATGAAGAGAAATCTGCTCTATAGT
CONSENSUS
      GATCTTGTTCTCTTGTTACTTCAGAGATGAAGAGAAATCTGCTCTATAGT

A3-AT4G01880-XLOC_022725-889-0
      ATGAGTGTTTCTAAGTTTCATCAGCTTATTAAGAAAATTGAGGATGTTCA
A3-AT4G01880-XLOC_022725-889-1
      ATGAGTGTTTCTAAGTTTCATCAGCTTATTAAGAAAATTGAGGATGTTCA
CONSENSUS
      ATGAGTGTTTCTAAGTTTCATCAGCTTATTAAGAAAATTGAGGATGTTCA

A3-AT4G01880-XLOC_022725-889-0
      TGGCGGTATTTGTAAAGATATCGAAGATTCGTATTTGTCACCTGAAGCTT
A3-AT4G01880-XLOC_022725-889-1
      TGGCGGTATTTGTAAAGATATCGAAGATTCGTATTTGTCACCTGAAGCTT
CONSENSUS
      TGGCGGTATTTGTAAAGATATCGAAGATTCGTATTTGTCACCTGAAGCTT

A3-AT4G01880-XLOC_022725-889-0
      GTAACATATGGTTTAATAAGGAGATAGATAG
A3-AT4G01880-XLOC_022725-889-1
      GTAACATATGGTTTAATAAGGAGATAGATAG
CONSENSUS
      GTAACATATGGTTTAATAAGGAGATAGATAG

alignment for event: A5-AT4G32900-XLOC_024691-11410

A5-AT4G32900-XLOC_024691-11410-0
      TGACCGATACCTTCTGCGGCGATGGGAGGAAAATGGGCAGCCAAAGATAG
A5-AT4G32900-XLOC_024691-11410-1
      TGACCGATACCTTCTGCGGCGATGGGAGGAAAATGGGCAGCCAAAGATAG
CONSENSUS

TGACCGATACCTTCTGCGGCGATGGGAGGAAAATGGGCAGCCAAAGATAG

A5-AT4G32900-XLOC_024691-11410-0
TCGTCACTTGCAAAAACCAGCAAGAAATGTAAGCAGGAATAAGATCACAG
A5-AT4G32900-XLOC_024691-11410-1
TCGTCACTTGCAAAAACCAGCAAGAAAT--------GAATAAGATCACAG
CONSENSUS
TCGTCACTTGCAAAAACCAGCAAGAAAT........GAATAAGATCACAG

A5-AT4G32900-XLOC_024691-11410-0
AGGCTGCTGAGAGCGTTGGCCTCCCGACTTTTGTTGTAGCTGATGCTGGA
A5-AT4G32900-XLOC_024691-11410-1
AGGCTGCTGAGAGCGTTGGCCTCCCGACTTTTGTTGTAGCTGATGCTGGA
CONSENSUS
AGGCTGCTGAGAGCGTTGGCCTCCCGACTTTTGTTGTAGCTGATGCTGGA

A5-AT4G32900-XLOC_024691-11410-0 AGAACTGAG
A5-AT4G32900-XLOC_024691-11410-1 AGAACTGAG
CONSENSUS AGAACTGAG

alignment for event: A3-AT4G25080-XLOC_021769-5434

A3-AT4G25080-XLOC_021769-5434-0
GCAAAGGCACAACTACCATCAGAGAATTTACCAAAATTTGAGGTGAATGA
A3-AT4G25080-XLOC_021769-5434-1
GCAAAGGCACAACTACCATCAGAGAATTTACCAAAATTTGAGGTGAATGA
CONSENSUS
GCAAAGGCACAACTACCATCAGAGAATTTACCAAAATTTGAGGTGAATGA

A3-AT4G25080-XLOC_021769-5434-0
TTTGGAGAGCCTAACTGGGAAGTATGATACCGTTGTATGTCTCGACGTGT
A3-AT4G25080-XLOC_021769-5434-1
TTTGGAGAGCCTAACTGGGAAGTATGATACCGTTGTATGTCTCGACGTGT
CONSENSUS
TTTGGAGAGCCTAACTGGGAAGTATGATACCGTTGTATGTCTCGACGTGT

A3-AT4G25080-XLOC_021769-5434-0
TGATACATTACCCGCAGAACAAAGCAGACGGAATGATCGCACATCTTGCT
A3-AT4G25080-XLOC_021769-5434-1
TGATACATTACCCGCAGAACAAAGCAGACGGAATGATCGCACATCTTGCT
CONSENSUS
TGATACATTACCCGCAGAACAAAGCAGACGGAATGATCGCACATCTTGCT

A3-AT4G25080-XLOC_021769-5434-0
TCTTTAGCAGAGAAGAGAGTGATTCTGAGTTTTGCTCCAAAGACTTTTTA
A3-AT4G25080-XLOC_021769-5434-1
TCTTTAGCAGAGAAGAGAGTGATTCTGAGTTTTGCTCCAAAGACTTTTTA
CONSENSUS
TCTTTAGCAGAGAAGAGAGTGATTCTGAGTTTTGCTCCAAAGACTTTTTA

A3-AT4G25080-XLOC_021769-5434-0
CTATGATATCTTAAAGAGAATTGGAGAGCTTTTCCCAGGGCCATCAAAAG
A3-AT4G25080-XLOC_021769-5434-1
CTATGATATCTTAAAGAGAATTGGAGAGCTTTTCCCAGGGCCATCAAAAG

CONSENSUS
CTATGATATCTTAAAGAGAATTGGAGAGCTTTTCCCAGGGCCATCAAAAG

A3-AT4G25080-XLOC_021769-5434-0
CTACAAGGGCGTATCTACACTCGGAGGCGGATGTGGAAAGAGCGTTGGGT
A3-AT4G25080-XLOC_021769-5434-1
CTACAAGGGCGTATCTACACTCGGAGGCGGATGTGGAAAGAGCGTTGGGT
CONSENSUS
CTACAAGGGCGTATCTACACTCGGAGGCGGATGTGGAAAGAGCGTTGGGT

A3-AT4G25080-XLOC_021769-5434-0
AAAGTCGGCTGGAAAATCAGCAAGAGAGGACTCACTACCACACAGTTCTA
A3-AT4G25080-XLOC_021769-5434-1
AAAGTCGGCTGGAAAATCAGCAAGAGAGGACTCACTACCACACAGTTCTA
CONSENSUS
AAAGTCGGCTGGAAAATCAGCAAGAGAGGACTCACTACCACACAGTTCTA

A3-AT4G25080-XLOC_021769-5434-0
CTTCTCTAGGCTCATCGAAGCTGTTCCAATGTAGACCAATTTGAAAACTA
A3-AT4G25080-XLOC_021769-5434-1
CTTCTCTAGGCTCATCGAAGCTGTTCCAATGTAGACCAATTTGAAAACTA
CONSENSUS
CTTCTCTAGGCTCATCGAAGCTGTTCCAATGTAGACCAATTTGAAAACTA

A3-AT4G25080-XLOC_021769-5434-0
GAAATCAAATTTGACGGTTAGCTTTCCGTTTGGAACTTTTGCAGTAGATC
A3-AT4G25080-XLOC_021769-5434-1
G--ATC
CONSENSUS
G..ATC

A3-AT4G25080-XLOC_021769-5434-0
TCTGTATAGCATATTCCGAGAAGGATTTTCGTGGAAGACATGCAGATTCT
A3-AT4G25080-XLOC_021769-5434-1
TCTGTATAGCATATTCCGAGAAGGATTTTCGTGGAAGACATGCAGATTCT
CONSENSUS
TCTGTATAGCATATTCCGAGAAGGATTTTCGTGGAAGACATGCAGATTCT

A3-AT4G25080-XLOC_021769-5434-0
CCTAAATTCGCTGAAGAGATTCTCATAAACTCTTCTTATATACTAGGAGG
A3-AT4G25080-XLOC_021769-5434-1
CCTAAATTCGCTGAAGAGATTCTCATAAACTCTTCTTATATACTAGGAGG
CONSENSUS
CCTAAATTCGCTGAAGAGATTCTCATAAACTCTTCTTATATACTAGGAGG

A3-AT4G25080-XLOC_021769-5434-0
ACGGTCTGTACAAACTTGCACTTCTGCAGACCCTAATTGATAATTGGCCA
A3-AT4G25080-XLOC_021769-5434-1
ACGGTCTGTACAAACTTGCACTTCTGCAGACCCTAATTGATAATTGGCCA
CONSENSUS
ACGGTCTGTACAAACTTGCACTTCTGCAGACCCTAATTGATAATTGGCCA

A3-AT4G25080-XLOC_021769-5434-0
AAATGTTCAATGTAATCAACGTTATTTGTTGTGCTGCTTAACTGTGTCCA
A3-AT4G25080-XLOC_021769-5434-1
AAATGTTCAATGTAATCAACGTTATTTGTTGTGCTGCTTAACTGTGTCCA

```
CONSENSUS
      AAATGTTCAATGTAATCAACGTTATTTGTTGTGCTGCTTAACTGTGTCCA

A3-AT4G25080-XLOC_021769-5434-0
      AAAACATGTCTATACTTTTCTACCCATTCTTGTGCATAGTTTAAAATAAA
A3-AT4G25080-XLOC_021769-5434-1
      AAAACATGTCTATACTTTTCTACCCATTCTTGTGCATAGTTTAAAATAAA
CONSENSUS
      AAAACATGTCTATACTTTTCTACCCATTCTTGTGCATAGTTTAAAATAAA

A3-AT4G25080-XLOC_021769-5434-0  AAGGAAATTGAAAT
A3-AT4G25080-XLOC_021769-5434-1  AAGGAAATTGAAAT
CONSENSUS                        AAGGAAATTGAAAT



alignment for event: A3-AT4G16360-XLOC_021277-2761

A3-AT4G16360-XLOC_021277-2761-0
      GTTCCGGTGCTTCCACTTCAAAGACCTGATGAAATTCATATCCCTAATCC
A3-AT4G16360-XLOC_021277-2761-1
      GTTCCGGTGCTTCCACTTCAAAGACCTGATGAAATTCATATCCCTAATCC
CONSENSUS
      GTTCCGGTGCTTCCACTTCAAAGACCTGATGAAATTCATATCCCTAATCC

A3-AT4G16360-XLOC_021277-2761-0
      TTCGTGGATGCAATCGCCATCTTCGTTGTATGAAGAAGCTTCTAACGAAC
A3-AT4G16360-XLOC_021277-2761-1
      TTCGTGGATGCAATCGCCATCTTCGTTGTATGAAGAAGCTTCTAACGAAC
CONSENSUS
      TTCGTGGATGCAATCGCCATCTTCGTTGTATGAAGAAGCTTCTAACGAAC

A3-AT4G16360-XLOC_021277-2761-0
      AAGGAATCCCTACGATGATCACTTGGTGTCATGGAGGCAAGGAGATTGCT
A3-AT4G16360-XLOC_021277-2761-1
      AAGGAATCCCTACGATGATCACTTGGTGTCATGGAGGCAAGGAGATTGCT
CONSENSUS
      AAGGAATCCCTACGATGATCACTTGGTGTCATGGAGGCAAGGAGATTGCT

A3-AT4G16360-XLOC_021277-2761-0
      GTGGAGGGATCATGGGATAATTGGAAGACAAGAAGTCGGCTGCAGAGATC
A3-AT4G16360-XLOC_021277-2761-1
      GTGGAGGGATCATGGGATAATTGGAAGACAAG---TCGGCTGCAGAGATC
CONSENSUS
      GTGGAGGGATCATGGGATAATTGGAAGACAAG...TCGGCTGCAGAGATC

A3-AT4G16360-XLOC_021277-2761-0
      TGGGAAGGACTTCACTATCATGAAAGTGTTACCTTCAGGAGTCTATGAGT
A3-AT4G16360-XLOC_021277-2761-1
      TGGGAAGGACTTCACTATCATGAAAGTGTTACCTTCAGGAGTCTATGAGT
CONSENSUS
      TGGGAAGGACTTCACTATCATGAAAGTGTTACCTTCAGGAGTCTATGAGT

A3-AT4G16360-XLOC_021277-2761-0
      ACAGGTTCATTGTGGATGGACAGTGGAGGCATGCCCCTGAGCTCCCTTTA
A3-AT4G16360-XLOC_021277-2761-1
```

ACAGGTTCATTGTGGATGGACAGTGGAGGCATGCCCCTGAGCTCCCTTTA
CONSENSUS
ACAGGTTCATTGTGGATGGACAGTGGAGGCATGCCCCTGAGCTCCCTTTA

A3-AT4G16360-XLOC_021277-2761-0
GCTAGAGATGATGCTGGGAACACTTTCAACATTTTGGATCTTCAG
A3-AT4G16360-XLOC_021277-2761-1
GCTAGAGATGATGCTGGGAACACTTTCAACATTTTGGATCTTCAG
CONSENSUS
GCTAGAGATGATGCTGGGAACACTTTCAACATTTTGGATCTTCAG

alignment for event: A3-AT4G21540-XLOC_021570-6649

A3-AT4G21540-XLOC_021570-6649-0
GGTCTTCAGAGGATAATATGCTTAAGACAATACCATGGACGAATTCTATT
A3-AT4G21540-XLOC_021570-6649-1
GGTCTTCAGAGGATAATATGCTTAAGACAATACCATGGACGAATTCTATT
CONSENSUS
GGTCTTCAGAGGATAATATGCTTAAGACAATACCATGGACGAATTCTATT

A3-AT4G21540-XLOC_021570-6649-0
TGTGCCAGCTCCTGGGTTCGAAAGCTATGGGCAACGAGCCAGTTGCAGTA
A3-AT4G21540-XLOC_021570-6649-1
TGTGCCAGCTCCTGGGTTCGAAAGCTATGGGCAACGAGCCAGTTGCAGTA
CONSENSUS
TGTGCCAGCTCCTGGGTTCGAAAGCTATGGGCAACGAGCCAGTTGCAGTA

A3-AT4G21540-XLOC_021570-6649-0
TAGATAAAGAGCCATCTGGTAGTGATAAGACACTCGTATACCAAGGACCT
A3-AT4G21540-XLOC_021570-6649-1
TAGATAAAGAGCCATCTGGTAGTGATAAGACACTCGTATACCAAGGACCT
CONSENSUS
TAGATAAAGAGCCATCTGGTAGTGATAAGACACTCGTATACCAAGGACCT

A3-AT4G21540-XLOC_021570-6649-0
GATAGTAAACTTGAAAATCTGGATTGGAGAGAAATGAAAGGCCCATTTGT
A3-AT4G21540-XLOC_021570-6649-1
GATAGTAAACTTGAAAATCTGGATTGGAGAGAAATGAAAGGCCCATTTGT
CONSENSUS
GATAGTAAACTTGAAAATCTGGATTGGAGAGAAATGAAAGGCCCATTTGT

A3-AT4G21540-XLOC_021570-6649-0
TTCAGTATGGCTTCATAATGTTCCCTGGGGTGCTGAGAACACTTTGGCTG
A3-AT4G21540-XLOC_021570-6649-1
TTCAGTATGGCTTCATAATGTTCCCTGGGGTGCTGAGAACACTTTGGCTG
CONSENSUS
TTCAGTATGGCTTCATAATGTTCCCTGGGGTGCTGAGAACACTTTGGCTG

A3-AT4G21540-XLOC_021570-6649-0
CTCCTGACGCTAAGTTTTCTGATGGCTTCCTGGATTTGATTGTCATGAAA
A3-AT4G21540-XLOC_021570-6649-1
CTCCTGACGCTAAG------------------------------------
CONSENSUS
CTCCTGACGCTAAG....................................

A3-AT4G21540-XLOC_021570-6649-0
GACTGTCCTAAACTAGCCTTGCTATCACTTATGACAAAGTTGAGTGATGG
A3-AT4G21540-XLOC_021570-6649-1 -
ACTGTCCTAAACTAGCCTTGCTATCACTTATGACAAAGTTGAGTGATGG
CONSENSUS
.ACTGTCCTAAACTAGCCTTGCTATCACTTATGACAAAGTTGAGTGATGG

A3-AT4G21540-XLOC_021570-6649-0
AACCCATGTCCAATCACCATATGCATCATATCTGAAG
A3-AT4G21540-XLOC_021570-6649-1
AACCCATGTCCAATCACCATATGCATCATATCTGAAG
CONSENSUS
AACCCATGTCCAATCACCATATGCATCATATCTGAAG

alignment for event: RI-AT4G32272-XLOC_024666-8544

RI-AT4G32272-XLOC_024666-8544-0
CTAGCCTCTATGGCATCTGTTAGAGGGGTAAATGTTCCCATGTATACCAC
RI-AT4G32272-XLOC_024666-8544-1
CTAGCCTCTATGGCATCTGTTAGAGGGGTAAATGTTCCCATGTATACCAC
CONSENSUS
CTAGCCTCTATGGCATCTGTTAGAGGGGTAAATGTTCCCATGTATACCAC

RI-AT4G32272-XLOC_024666-8544-0
GCTTAGGCGTACTACTGTGGCATTTACTATGGTGATTGAGTACATGCTCA
RI-AT4G32272-XLOC_024666-8544-1
GCTTAGGCGTACTACTGTGGCATTTACTATGGTGATTGAGTACATGCTCA
CONSENSUS
GCTTAGGCGTACTACTGTGGCATTTACTATGGTGATTGAGTACATGCTCA

RI-AT4G32272-XLOC_024666-8544-0
CAGGTCAGAGATATACACGGTCTATCATTGGAAGGTAAATCACTTTTTTT
RI-AT4G32272-XLOC_024666-8544-1
CAGGTCAGAGATATACACGGTCTATCATTGGAAG----------------
CONSENSUS
CAGGTCAGAGATATACACGGTCTATCATTGGAAG................

RI-AT4G32272-XLOC_024666-8544-0
TTCTTTCTTCCATACTGTAGTTTCTGTTCGTGCAAAATTGATATCTTTTC
RI-AT4G32272-XLOC_024666-8544-1

CONSENSUS
....

RI-AT4G32272-XLOC_024666-8544-0
ACTGTTGAATTTGATAATTTCCTGAAGTATTCAGATTCTGGGTTTAATAG
RI-AT4G32272-XLOC_024666-8544-1

CONSENSUS
....

RI-AT4G32272-XLOC_024666-8544-0
TATCTCTTAGGAAGAAATAACTCCTTATGTTTGATCAAAATAGACAAGAA

RI-AT4G32272-XLOC_024666-8544-1

CONSENSUS
      ....

RI-AT4G32272-XLOC_024666-8544-0
      TTAGGATTTAGGAGTTTGCGAGAGATAATTGTAGCTCTACGTTTATTCTG
RI-AT4G32272-XLOC_024666-8544-1

CONSENSUS
      ....

RI-AT4G32272-XLOC_024666-8544-0
      TATGTTACATGCCCCAGAGGCTTATGTGGTATGGTGCTTTAAGTCCCTCC
RI-AT4G32272-XLOC_024666-8544-1

CONSENSUS
      ....

RI-AT4G32272-XLOC_024666-8544-0
      ATGAAAAAGTATGCACATGTTATTACCTCTAAAAGGAGAATCATGCTCCT
RI-AT4G32272-XLOC_024666-8544-1

CONSENSUS
      ....

RI-AT4G32272-XLOC_024666-8544-0
      TGACTCTTACATGCACAACTAAACTCTTGTAAAATTGATGTCAGTGTTGG
RI-AT4G32272-XLOC_024666-8544-1
      --TGTTGG
CONSENSUS
      ....TGTTGG

RI-AT4G32272-XLOC_024666-8544-0
      TATTATCTTGCTTGGCGCATTTTTTGCTGGAGCTAGGGACTTGTCATTTG
RI-AT4G32272-XLOC_024666-8544-1
      TATTATCTTGCTTGGCGCATTTTTTGCTGGAGCTAGGGACTTGTCATTTG
CONSENSUS
      TATTATCTTGCTTGGCGCATTTTTTGCTGGAGCTAGGGACTTGTCATTTG

RI-AT4G32272-XLOC_024666-8544-0
      ACTTTTATGGATATGGTGTTGTTTTCTTAGCCAACATATCAACAGCAGTA
RI-AT4G32272-XLOC_024666-8544-1
      ACTTTTATGGATATGGTGTTGTTTTCTTAGCCAACATATCAACAGCAGTA
CONSENSUS
      ACTTTTATGGATATGGTGTTGTTTTCTTAGCCAACATATCAACAGCAGTA

RI-AT4G32272-XLOC_024666-8544-0 TATCTAGCAACCATTGCCCGTACTG
RI-AT4G32272-XLOC_024666-8544-1 TATCTAGCAACCATTGCCCGTACTG
CONSENSUS   TATCTAGCAACCATTGCCCGTACTG

alignment for event: SE-AT4G35785-XLOC_024844-4196

SE-AT4G35785-XLOC_024844-4196-0

GTTGCGTCTTGTTTTCTTGTGATGGAGCCGCGCACCCGTGTGTCTCGTGG
SE-AT4G35785-XLOC_024844-4196-1
GTTGCGTCTTGTTTTCTTGTGATGGAGCCGCGCACCCGTGTGTCTCGTGG
CONSENSUS
GTTGCGTCTTGTTTTCTTGTGATGGAGCCGCGCACCCGTGTGTCTCGTGG

SE-AT4G35785-XLOC_024844-4196-0
TTTTGCCTTTGTTACAATGAGCAGCCTTAAAGATGCTGAGCGGTGCATTA
SE-AT4G35785-XLOC_024844-4196-1
TTTTGCCTTTGTTACAATGAGCAGCCTTAAAGATGCTGAGCGGTGCATTA
CONSENSUS
TTTTGCCTTTGTTACAATGAGCAGCCTTAAAGATGCTGAGCGGTGCATTA

SE-AT4G35785-XLOC_024844-4196-0
AATATCTCAACCAGTCTGTACTAGAAGGCCGATACATAACTGTGGAAAGG
SE-AT4G35785-XLOC_024844-4196-1
AATATCTCAACCAGTCTGTACTAGAAGGCCGATACATAACTGTGGAAAGG
CONSENSUS
AATATCTCAACCAGTCTGTACTAGAAGGCCGATACATAACTGTGGAAAGG

SE-AT4G35785-XLOC_024844-4196-0

SE-AT4G35785-XLOC_024844-4196-1
AAAATATTGCATCAAGCAACAATCAGAGATGATAAGAGTACTCATCCTTG
CONSENSUS
....

SE-AT4G35785-XLOC_024844-4196-0

SE-AT4G35785-XLOC_024844-4196-1
CTCAACATCATTAAGTGAAACACAGCTTGATCAATCGAATCGCCAGCTTA
CONSENSUS
....

SE-AT4G35785-XLOC_024844-4196-0

SE-AT4G35785-XLOC_024844-4196-1
AACATAATCATAAGCAACTATATTGCCTCAACTCTCAACTCTCAACTAGT
CONSENSUS
....

SE-AT4G35785-XLOC_024844-4196-0 ----------------
TCCAGGAGAAAGCGCCCGAGAACTCCCACCCCAG
SE-AT4G35785-XLOC_024844-4196-1
CAAAGGACATTATCAATCCAGGAGAAAGCGCCCGAGAACTCCCACCCCAG
CONSENSUS
................TCCAGGAGAAAGCGCCCGAGAACTCCCACCCCAG

SE-AT4G35785-XLOC_024844-4196-0
GCCACTATCTTGGCTTGAAAAGCTCCAGAGACAGCG
SE-AT4G35785-XLOC_024844-4196-1
GCCACTATCTTGGCTTGAAAAGCTCCAGAGACAGCG
CONSENSUS
GCCACTATCTTGGCTTGAAAAGCTCCAGAGACAGCG

alignment for event: RI-AT4G03935-XLOC_022870-2971

RI-AT4G03935-XLOC_022870-2971-0
ACATTGGAGCTCTTGGGATTTCTCTTTCCCTCAAGCAGTCTCCATGTCTT
RI-AT4G03935-XLOC_022870-2971-1
ACATTGGAGCTCTTGGGATTTCTCTTTCCCTCAAGCAGTCTCCATGTCTT
CONSENSUS
ACATTGGAGCTCTTGGGATTTCTCTTTCCCTCAAGCAGTCTCCATGTCTT

RI-AT4G03935-XLOC_022870-2971-0
CTCCGCATCTTCTCGGTGAGCCTCCCTCACGTCTCTTCCGGTTGTCGCTG
RI-AT4G03935-XLOC_022870-2971-1
CTCCGCATCTTCTCGGTGAGCCTCCCTCACGTCTCTTCCGGTTGTCGCTG
CONSENSUS
CTCCGCATCTTCTCGGTGAGCCTCCCTCACGTCTCTTCCGGTTGTCGCTG

RI-AT4G03935-XLOC_022870-2971-0
CAACCTTCTTCAGCTTCCTCTCTCTCAGGTAAGCTTCTCTTCTCCTCTTC
RI-AT4G03935-XLOC_022870-2971-1
CAACCTTCTTCAGCTTCCTCTCTCTCAG----------------------
CONSENSUS
CAACCTTCTTCAGCTTCCTCTCTCTCAG......................

RI-AT4G03935-XLOC_022870-2971-0
TTGGTGTCCACCTTTGAACACCTCCTTCTTTACTTTGATTCTTAGAACCA
RI-AT4G03935-XLOC_022870-2971-1

CONSENSUS
....

RI-AT4G03935-XLOC_022870-2971-0
TCACTTTAGCTTTAGCTTCAGAATGTTTGACTCGAACCATCACTCGACTT
RI-AT4G03935-XLOC_022870-2971-1

CONSENSUS
....

RI-AT4G03935-XLOC_022870-2971-0
TCTCTAACCGTAGTCTATTTGGCTTCTCCTTGAGTCATCACCTTAGCCTT
RI-AT4G03935-XLOC_022870-2971-1

CONSENSUS
....

RI-AT4G03935-XLOC_022870-2971-0
AGCCGGTTGGCTCTGTAGAGAACACGAAACTGAACGGTTGCTTGTAACAG
RI-AT4G03935-XLOC_022870-2971-1

CONSENSUS
....

RI-AT4G03935-XLOC_022870-2971-0
AAACAAACACAGAAATTGATAGCCAAGTTCACGGCTTAACCGCTATGTCT
RI-AT4G03935-XLOC_022870-2971-1

CONSENSUS
    ....

RI-AT4G03935-XLOC_022870-2971-0
    GAGGAGAGATCTCTTGCTCCCAAAGCTTCACTATATCAAAGAGATTACAA
RI-AT4G03935-XLOC_022870-2971-1

CONSENSUS
    ....

RI-AT4G03935-XLOC_022870-2971-0
    ACACCACTTGACTTGTGATGTTGTCTTTGGTTTCAGGTACAATCTCAAGA
RI-AT4G03935-XLOC_022870-2971-1
    ------------------------------------GTACAATCTCAAGA
CONSENSUS
    ....................................GTACAATCTCAAGA

RI-AT4G03935-XLOC_022870-2971-0
    AGAACAAAGATCAAACACAAAGAACAAAGAAGGAGAAAGCTTCTTGAAGA
RI-AT4G03935-XLOC_022870-2971-1
    AGAACAAAGATCAAACACAAAGAACAAAGAAGGAGAAAGCTTCTTGAAGA
CONSENSUS
    AGAACAAAGATCAAACACAAAGAACAAAGAAGGAGAAAGCTTCTTGAAGA

RI-AT4G03935-XLOC_022870-2971-0
    ATAAACTCTCGTTTCTTCTCTTTCTCTCTAGAACTCTCTTTGTGTCTTGT
RI-AT4G03935-XLOC_022870-2971-1
    ATAAACTCTCGTTTCTTCTCTTTCTCTCTAGAACTCTCTTTGTGTCTTGT
CONSENSUS
    ATAAACTCTCGTTTCTTCTCTTTCTCTCTAGAACTCTCTTTGTGTCTTGT

RI-AT4G03935-XLOC_022870-2971-0
    CTCACACCTACATCCACATATATATATATATATATATATATATATGTGTG
RI-AT4G03935-XLOC_022870-2971-1
    CTCACACCTACATCCACATATATATATATATATATATATATATATGTGTG
CONSENSUS
    CTCACACCTACATCCACATATATATATATATATATATATATATATGTGTG

RI-AT4G03935-XLOC_022870-2971-0
    TGATGGACCACTTTTGTTCTTCTTTTCTTTATATTAACTCCGTCTGGCCC
RI-AT4G03935-XLOC_022870-2971-1
    TGATGGACCACTTTTGTTCTTCTTTTCTTTATATTAACTCCGTCTGGCCC
CONSENSUS
    TGATGGACCACTTTTGTTCTTCTTTTCTTTATATTAACTCCGTCTGGCCC

RI-AT4G03935-XLOC_022870-2971-0
    AATAAAAGCCCAATGTTGTGTGTTTTGGTCAAACCCAATAACAATCTCCA
RI-AT4G03935-XLOC_022870-2971-1
    AATAAAAGCCCAATGTTGTGTGTTTTGGTCAAACCCAATAACAATCTCCA
CONSENSUS
    AATAAAAGCCCAATGTTGTGTGTTTTGGTCAAACCCAATAACAATCTCCA

RI-AT4G03935-XLOC_022870-2971-0
    CCTTTTGACAACAACACACTTCCTCGACGACATCGACATCTCCGACAACC
RI-AT4G03935-XLOC_022870-2971-1
    CCTTTTGACAACAACACACTTCCTCGACGACATCGACATCTCCGACAACC

CONSENSUS
CCTTTTGACAACAACACACTTCCTCGACGACATCGACATCTCCGACAACC

RI-AT4G03935-XLOC_022870-2971-0
AACGTCATCTCTTTCTCATATCGTGAAACCAACACCACTGCCAGACTCCA
RI-AT4G03935-XLOC_022870-2971-1
AACGTCATCTCTTTCTCATATCGTGAAACCAACACCACTGCCAGACTCCA
CONSENSUS
AACGTCATCTCTTTCTCATATCGTGAAACCAACACCACTGCCAGACTCCA

RI-AT4G03935-XLOC_022870-2971-0
ACGACCTCCGGCGAACATACGAATTTTTCCCCGTCAGCCATCGTCTCCTC
RI-AT4G03935-XLOC_022870-2971-1
ACGACCTCCGGCGAACATACGAATTTTTCCCCGTCAGCCATCGTCTCCTC
CONSENSUS
ACGACCTCCGGCGAACATACGAATTTTTCCCCGTCAGCCATCGTCTCCTC

RI-AT4G03935-XLOC_022870-2971-0
CTTTCCCCACCGAGCTCTCTTGTTCTCGACATGGAGAAGATGGAGCTTGC
RI-AT4G03935-XLOC_022870-2971-1
CTTTCCCCACCGAGCTCTCTTGTTCTCGACATGGAGAAGATGGAGCTTGC
CONSENSUS
CTTTCCCCACCGAGCTCTCTTGTTCTCGACATGGAGAAGATGGAGCTTGC

RI-AT4G03935-XLOC_022870-2971-0
CTTTCAATGACAAAATCAAACTCAGATTCCGACTACTTTGATGGCTACCT
RI-AT4G03935-XLOC_022870-2971-1
CTTTCAATGACAAAATCAAACTCAGATTCCGACTACTTTGATGGCTACCT
CONSENSUS
CTTTCAATGACAAAATCAAACTCAGATTCCGACTACTTTGATGGCTACCT

RI-AT4G03935-XLOC_022870-2971-0
CCGGCGAAAGCAACTGATAAGCCCTGATATGAAAAGAACAGAAAGAAATG
RI-AT4G03935-XLOC_022870-2971-1
CCGGCGAAAGCAACTGATAAGCCCTGATATGAAAAGAACAGAAAGAAATG
CONSENSUS
CCGGCGAAAGCAACTGATAAGCCCTGATATGAAAAGAACAGAAAGAAATG

RI-AT4G03935-XLOC_022870-2971-0
GAGGGAGAGAAGTATTGCACCTTTAAGGTCGAGAAGAAGAGAGATAAGAG
RI-AT4G03935-XLOC_022870-2971-1
GAGGGAGAGAAGTATTGCACCTTTAAGGTCGAGAAGAAGAGAGATAAGAG
CONSENSUS
GAGGGAGAGAAGTATTGCACCTTTAAGGTCGAGAAGAAGAGAGATAAGAG

RI-AT4G03935-XLOC_022870-2971-0
AGATTTTAGAAAGTTTGTTTGATTGATCTTCATAAGATAAAAACCCTAAT
RI-AT4G03935-XLOC_022870-2971-1
AGATTTTAGAAAGTTTGTTTGATTGATCTTCATAAGATAAAAACCCTAAT
CONSENSUS
AGATTTTAGAAAGTTTGTTTGATTGATCTTCATAAGATAAAAACCCTAAT

RI-AT4G03935-XLOC_022870-2971-0
AATGCGGAAGCAGTGCTTAAATACACCCACAAGCCTAAAACGACACTAAG
RI-AT4G03935-XLOC_022870-2971-1
AATGCGGAAGCAGTGCTTAAATACACCCACAAGCCTAAAACGACACTAAG

CONSENSUS
AATGCGGAAGCAGTGCTTAAATACACCCACAAGCCTAAAACGACACTAAG

RI-AT4G03935-XLOC_022870-2971-0
ATAGTAGCTAAGACCCTCTCTTACAATAAGAGTCTTTCTCCCTTGTCTCA
RI-AT4G03935-XLOC_022870-2971-1
ATAGTAGCTAAGACCCTCTCTTACAATAAGAGTCTTTCTCCCTTGTCTCA
CONSENSUS
ATAGTAGCTAAGACCCTCTCTTACAATAAGAGTCTTTCTCCCTTGTCTCA

RI-AT4G03935-XLOC_022870-2971-0
TTCTTCGACACTTAGTCTCACACCAGCTTCTTCTTCTTCTTTATAAAGGT
RI-AT4G03935-XLOC_022870-2971-1
TTCTTCGACACTTAGTCTCACACCAGCTTCTTCTTCTTCTTTATAAAGGT
CONSENSUS
TTCTTCGACACTTAGTCTCACACCAGCTTCTTCTTCTTCTTTATAAAGGT

RI-AT4G03935-XLOC_022870-2971-0
AATGGGAATCCATAACCTATCCGCAACTGCAAACCTTCACCTTTTGTTGG
RI-AT4G03935-XLOC_022870-2971-1
AATGGGAATCCATAACCTATCCGCAACTGCAAACCTTCACCTTTTGTTGG
CONSENSUS
AATGGGAATCCATAACCTATCCGCAACTGCAAACCTTCACCTTTTGTTGG

RI-AT4G03935-XLOC_022870-2971-0
TTGCTCTTCGTTCTGGTGAAACAAGAGACTTCTCTTCGTCTCTCATCCAG
RI-AT4G03935-XLOC_022870-2971-1
TTGCTCTTCGTTCTGGTGAAACAAGAGACTTCTCTTCGTCTCTCATCCAG
CONSENSUS
TTGCTCTTCGTTCTGGTGAAACAAGAGACTTCTCTTCGTCTCTCATCCAG

RI-AT4G03935-XLOC_022870-2971-0
GCCTCTCGTCCGTGACACATCACAACATCACAGTCATCTTCACCATCAAT
RI-AT4G03935-XLOC_022870-2971-1
GCCTCTCGTCCGTGACACATCACAACATCACAGTCATCTTCACCATCAAT
CONSENSUS
GCCTCTCGTCCGTGACACATCACAACATCACAGTCATCTTCACCATCAAT

RI-AT4G03935-XLOC_022870-2971-0
TAACTCCGGCTGCGTTTAACTCCGACGAATCAGACCGCCACCATCACCGC
RI-AT4G03935-XLOC_022870-2971-1
TAACTCCGGCTGCGTTTAACTCCGACGAATCAGACCGCCACCATCACCGC
CONSENSUS
TAACTCCGGCTGCGTTTAACTCCGACGAATCAGACCGCCACCATCACCGC

RI-AT4G03935-XLOC_022870-2971-0 TCAACTCATG
RI-AT4G03935-XLOC_022870-2971-1 TCAACTCATG
CONSENSUS TCAACTCATG

alignment for event: A3-AT4G36690-XLOC_024897-8161

A3-AT4G36690-XLOC_024897-8161-0
AGGGTTATGTTACAGCCTGGTGCAGTGGCAACCACTGTGGTGTGTCTGAC
A3-AT4G36690-XLOC_024897-8161-1

AGGGTTATGTTACAGCCTGGTGCAGTGGCAACCACTGTGGTGTGTCTGAC
CONSENSUS
AGGGTTATGTTACAGCCTGGTGCAGTGGCAACCACTGTGGTGTGTCTGAC

A3-AT4G36690-XLOC_024897-8161-0
TCAAGTTGTTACTGAGGATGAGCTTAGAGATGATGAGGAGTATGGAGATA
A3-AT4G36690-XLOC_024897-8161-1
TCAAGTTGTTACTGAGGATGAGCTTAGAGATGATGAGGAGTATGGAGATA
CONSENSUS
TCAAGTTGTTACTGAGGATGAGCTTAGAGATGATGAGGAGTATGGAGATA

A3-AT4G36690-XLOC_024897-8161-0
TAATGGAAGACATGAGACAGGAAGGCGGAAAGTTTG--------------
A3-AT4G36690-XLOC_024897-8161-1
TAATGGAAGACATGAGACAGGAAGGCGGAAAGTTTGCCTTTTGCTATAAA
CONSENSUS
TAATGGAAGACATGAGACAGGAAGGCGGAAAGTTTG..............

A3-AT4G36690-XLOC_024897-8161-0

A3-AT4G36690-XLOC_024897-8161-1
GAATCAGCCCTAACATACACAGACAGGAGACTGCATAAACCTCCTAATCT
CONSENSUS
....

A3-AT4G36690-XLOC_024897-8161-0

A3-AT4G36690-XLOC_024897-8161-1
ATTTATCACCAATGGCCACTATTTCTTAAAAGAAAAAACTGACTTGTTTT
CONSENSUS
....

A3-AT4G36690-XLOC_024897-8161-0

A3-AT4G36690-XLOC_024897-8161-1
TGTCTGTGTTTTCTTGTCTTGTATTCGAAATGTTTTGTTCTTTAACTCTT
CONSENSUS
....

A3-AT4G36690-XLOC_024897-8161-0 ---------
GTGCTTTGACCAATGTTGTGATTCCGCGTCCAAGCCCCAAT
A3-AT4G36690-XLOC_024897-8161-1
AAAATGCAGGTGCTTTGACCAATGTTGTGATTCCGCGTCCAAGCCCCAAT
CONSENSUS
.........GTGCTTTGACCAATGTTGTGATTCCGCGTCCAAGCCCCAAT

A3-AT4G36690-XLOC_024897-8161-0 GGTGAGCCAGTGGCAGGCCTTGGCAAG
A3-AT4G36690-XLOC_024897-8161-1 GGTGAGCCAGTGGCAGGCCTTGGCAAG
CONSENSUS GGTGAGCCAGTGGCAGGCCTTGGCAAG

alignment for event: RI-AT4G26140-XLOC_021828-70

RI-AT4G26140-XLOC_021828-70-0
GTAATGAGACTTCAAATTACCGTATTCATCAAGAGGAGCATCATAGTCAT

RI-AT4G26140-XLOC_021828-70-1
GTAATGAGACTTCAAATTACCGTATTCATCAAGAGGAGCATCATAGTCAT
CONSENSUS
GTAATGAGACTTCAAATTACCGTATTCATCAAGAGGAGCATCATAGTCAT

RI-AT4G26140-XLOC_021828-70-0
AGCTGGTGGCGATGAATTCACCGGCTGTTCTGTCGAAGTTGGTTCCTCCA
RI-AT4G26140-XLOC_021828-70-1
AGCTGGTGGCGATGAATTCACCGGCTGTTCTGTCGAAGTTGGTTCCTCCA
CONSENSUS
AGCTGGTGGCGATGAATTCACCGGCTGTTCTGTCGAAGTTGGTTCCTCCA

RI-AT4G26140-XLOC_021828-70-0
TGGTACTGTTTAACAACATTTCCTCACTCCATGAATAATCAAATCTAGTA
RI-AT4G26140-XLOC_021828-70-1
TG--
CONSENSUS
TG..

RI-AT4G26140-XLOC_021828-70-0
TTTAAAGTTTCAACTATAGAGAAGCTGAATGAAGTTACCATATAATAGTT
RI-AT4G26140-XLOC_021828-70-1
--TT
CONSENSUS
....TT

RI-AT4G26140-XLOC_021828-70-0
GATGAAAGAGCCACCGTTCTGTATGAAGCGAGCCACGGATAAGGCAATGT
RI-AT4G26140-XLOC_021828-70-1
GATGAAAGAGCCACCGTTCTGTATGAAGCGAGCCACGGATAAGGCAATGT
CONSENSUS
GATGAAAGAGCCACCGTTCTGTATGAAGCGAGCCACGGATAAGGCAATGT

RI-AT4G26140-XLOC_021828-70-0
CTTCGGCTGGCCTGTATGGTACTGCACCTCCAAACTCTGTAAACCTGATA
RI-AT4G26140-XLOC_021828-70-1
CTTCGGCTGGCCTGTATGGTACTGCACCTCCAAACTCTGTAAACCTGATA
CONSENSUS
CTTCGGCTGGCCTGTATGGTACTGCACCTCCAAACTCTGTAAACCTGATA

RI-AT4G26140-XLOC_021828-70-0
AATTATTTGCAGATTCACAAATAAAAAAGTTATTTCGTACAAAAATAAAA
RI-AT4G26140-XLOC_021828-70-1
AATTATTTGCAGATTCACAAATAAAAAAGTTATTTCGTACAAAAATAAAA
CONSENSUS
AATTATTTGCAGATTCACAAATAAAAAAGTTATTTCGTACAAAAATAAAA

RI-AT4G26140-XLOC_021828-70-0
GATACATTAAATTTTTATTTTCTTGGTCTTAAAGATTTGTATTGAATAGT
RI-AT4G26140-XLOC_021828-70-1
GATACATTAAATTTTTATTTTCTTGGTCTTAAAGATTTGTATTGAATAGT
CONSENSUS
GATACATTAAATTTTTATTTTCTTGGTCTTAAAGATTTGTATTGAATAGT

RI-AT4G26140-XLOC_021828-70-0
AATATTTTTGGAAGGGACAATTGTGTTGTGTGACTTACCAACCAGTCCAG

RI-AT4G26140-XLOC_021828-70-1
      AATATTTTTGGAAGGGACAATTGTGTTGTGTGACTTACCAACCAGTCCAG
CONSENSUS
      AATATTTTTGGAAGGGACAATTGTGTTGTGTGACTTACCAACCAGTCCAG

RI-AT4G26140-XLOC_021828-70-0
      TTCTCTGTCCACATTTTCGGCTTATTGTCCGAGTTTGGTTTGAAATTCTC
RI-AT4G26140-XLOC_021828-70-1
      TTCTCTGTCCACATTTTCGGCTTATTGTCCGAGTTTGGTTTGAAATTCTC
CONSENSUS
      TTCTCTGTCCACATTTTCGGCTTATTGTCCGAGTTTGGTTTGAAATTCTC

RI-AT4G26140-XLOC_021828-70-0   AC
RI-AT4G26140-XLOC_021828-70-1   AC
CONSENSUS   AC

alignment for event: A3-AT4G00335-XLOC_020130-2017

A3-AT4G00335-XLOC_020130-2017-0
      AGGATTACAGTATAATTACTTACTATGATGAGGTTATTCGACTAGAGAAA
A3-AT4G00335-XLOC_020130-2017-1
      AGGATTACAGTATAATTACTTACTATGATGAGGTTATTCGACTAGAGAAA
CONSENSUS
      AGGATTACAGTATAATTACTTACTATGATGAGGTTATTCGACTAGAGAAA

A3-AT4G00335-XLOC_020130-2017-0
      AGGAGCTCTGTGTGTCGAAACCCTCCGTCGCCAGCATTTATTCTTCTCAC
A3-AT4G00335-XLOC_020130-2017-1
      AGGAGCTCTGTGTGTCGAAACCCTCCGTCGCCAGCATTTATTCTTCTCAC
CONSENSUS
      AGGAGCTCTGTGTGTCGAAACCCTCCGTCGCCAGCATTTATTCTTCTCAC

A3-AT4G00335-XLOC_020130-2017-0
      TTTTCTTCACCGGAGAATCACGACAACATCAAATTCTCCGGCGACATCTC
A3-AT4G00335-XLOC_020130-2017-1
      TTTTCTTCACCGGAGAATCACGACAACATCAAATTCTCCGGCGACATCTC
CONSENSUS
      TTTTCTTCACCGGAGAATCACGACAACATCAAATTCTCCGGCGACATCTC

A3-AT4G00335-XLOC_020130-2017-0
      AGTTCACTCTTTTCCTGCAGTGGCAGTGCATCTGTAAAATGCTTCAAGGA
A3-AT4G00335-XLOC_020130-2017-1 AG------------------
TGGCAGTGCATCTGTAAAATGCTTCAAGGA
CONSENSUS
      AG..................TGGCAGTGCATCTGTAAAATGCTTCAAGGA

A3-AT4G00335-XLOC_020130-2017-0
      CTGTTGTTCGCTACTCTTGCTTAATTATTACTATAGCTGGTGAAAATGGG
A3-AT4G00335-XLOC_020130-2017-1
      CTGTTGTTCGCTACTCTTGCTTAATTATTACTATAGCTGGTGAAAATGGG
CONSENSUS
      CTGTTGTTCGCTACTCTTGCTTAATTATTACTATAGCTGGTGAAAATGGG

A3-AT4G00335-XLOC_020130-2017-0

AGGTTGCTGTAGTTCTTCGAGGAAGTCACATCTTGTTGGAACACCTGTTT
A3-AT4G00335-XLOC_020130-2017-1
AGGTTGCTGTAGTTCTTCGAGGAAGTCACATCTTGTTGGAACACCTGTTT
CONSENSUS
AGGTTGCTGTAGTTCTTCGAGGAAGTCACATCTTGTTGGAACACCTGTTT

A3-AT4G00335-XLOC_020130-2017-0
ACTATTATGTCAGTAATCCTTTTCACCATCTTGCATTTGTGTTGCTTTGC
A3-AT4G00335-XLOC_020130-2017-1
ACTATTATGTCAGTAATCCTTTTCACCATCTTGCATTTGTGTTGCTTTGC
CONSENSUS
ACTATTATGTCAGTAATCCTTTTCACCATCTTGCATTTGTGTTGCTTTGC

A3-AT4G00335-XLOC_020130-2017-0
ACTACTGACTATCTATAGATTATGATTTTAGTTAAAACAAATCGGGACAA
A3-AT4G00335-XLOC_020130-2017-1
ACTACTGACTATCTATAGATTATGATTTTAGTTAAAACAAATCGGGACAA
CONSENSUS
ACTACTGACTATCTATAGATTATGATTTTAGTTAAAACAAATCGGGACAA

A3-AT4G00335-XLOC_020130-2017-0
GAGCATGATTTTAGAATCTCAACAATTTCTTGTAACAATCTCGCTTGAGT
A3-AT4G00335-XLOC_020130-2017-1
GAGCATGATTTTAGAATCTCAACAATTTCTTGTAACAATCTCGCTTGAGT
CONSENSUS
GAGCATGATTTTAGAATCTCAACAATTTCTTGTAACAATCTCGCTTGAGT

A3-AT4G00335-XLOC_020130-2017-0
GGTCGATAGTAGACTACTAGAGGTCGTGAGTGAGCTGTGTTTGAAAAATG
A3-AT4G00335-XLOC_020130-2017-1
GGTCGATAGTAGACTACTAGAGGTCGTGAGTGAGCTGTGTTTGAAAAATG
CONSENSUS
GGTCGATAGTAGACTACTAGAGGTCGTGAGTGAGCTGTGTTTGAAAAATG

A3-AT4G00335-XLOC_020130-2017-0
TCTTCTGAATTATCTACAGTTATTTGATTGTGACACTCTACAAAGTTTCT
A3-AT4G00335-XLOC_020130-2017-1
TCTTCTGAATTATCTACAGTTATTTGATTGTGACACTCTACAAAGTTTCT
CONSENSUS
TCTTCTGAATTATCTACAGTTATTTGATTGTGACACTCTACAAAGTTTCT

A3-AT4G00335-XLOC_020130-2017-0
ATTTATTAAAAGTTTGTTACGCAGTGTCCAGAATCTTTTGAAGAGCTTGT
A3-AT4G00335-XLOC_020130-2017-1
ATTTATTAAAAGTTTGTTACGCAGTGTCCAGAATCTTTTGAAGAGCTTGT
CONSENSUS
ATTTATTAAAAGTTTGTTACGCAGTGTCCAGAATCTTTTGAAGAGCTTGT

A3-AT4G00335-XLOC_020130-2017-0
CCCTTCAGGAACCCGTGCTGGTGTGGGATCAGCATTCACCACAGGCCTCT
A3-AT4G00335-XLOC_020130-2017-1
CCCTTCAGGAACCCGTGCTGGTGTGGGATCAGCATTCACCACAGGCCTCT
CONSENSUS
CCCTTCAGGAACCCGTGCTGGTGTGGGATCAGCATTCACCACAGGCCTCT

A3-AT4G00335-XLOC_020130-2017-0

TGGTTGATATAGGTTTGGAGACATCTATACCTGACACCTTTTGTGCTCCT
A3-AT4G00335-XLOC_020130-2017-1
TGGTTGATATAGGTTTGGAGACATCTATACCTGACACCTTTTGTGCTCCT
CONSENSUS
TGGTTGATATAGGTTTGGAGACATCTATACCTGACACCTTTTGTGCTCCT

A3-AT4G00335-XLOC_020130-2017-0
GCTCCTCTTCCATATGATTTGCTTTTGGGGCGACCACAATGCACAGATTC
A3-AT4G00335-XLOC_020130-2017-1
GCTCCTCTTCCATATGATTTGCTTTTGGGGCGACCACAATGCACAGATTC
CONSENSUS
GCTCCTCTTCCATATGATTTGCTTTTGGGGCGACCACAATGCACAGATTC

A3-AT4G00335-XLOC_020130-2017-0
TGAGTCCATCAAAGGAAGGATGAGTGGAAGCAGTTTTGAAACGCTAGCGA
A3-AT4G00335-XLOC_020130-2017-1
TGAGTCCATCAAAGGAAGGATGAGTGGAAGCAGTTTTGAAACGCTAGCGA
CONSENSUS
TGAGTCCATCAAAGGAAGGATGAGTGGAAGCAGTTTTGAAACGCTAGCGA

A3-AT4G00335-XLOC_020130-2017-0
CGTGTGAAGATCTTGGGGAATCAGACTGTAAAACTCTAGCCAGCTCAGTC
A3-AT4G00335-XLOC_020130-2017-1
CGTGTGAAGATCTTGGGGAATCAGACTGTAAAACTCTAGCCAGCTCAGTC
CONSENSUS
CGTGTGAAGATCTTGGGGAATCAGACTGTAAAACTCTAGCCAGCTCAGTC

A3-AT4G00335-XLOC_020130-2017-0
ATTCTTTCACCGAGGAAATCAGATTTCTCGAAACATCAAGGATTGAAGAT
A3-AT4G00335-XLOC_020130-2017-1
ATTCTTTCACCGAGGAAATCAGATTTCTCGAAACATCAAGGATTGAAGAT
CONSENSUS
ATTCTTTCACCGAGGAAATCAGATTTCTCGAAACATCAAGGATTGAAGAT

A3-AT4G00335-XLOC_020130-2017-0
ATTGGTTGATGAAGAGGAGGACTGTTGTCCTATTTGCTTTGAAG
A3-AT4G00335-XLOC_020130-2017-1
ATTGGTTGATGAAGAGGAGGACTGTTGTCCTATTTGCTTTGAAG
CONSENSUS
ATTGGTTGATGAAGAGGAGGACTGTTGTCCTATTTGCTTTGAAG

alignment for event: RI-AT4G26640-XLOC_024350-5602

RI-AT4G26640-XLOC_024350-5602-0
CCAGAACCTTCCCCTACTACTGGTTCTTTGTTCAAGCCTCGACCAGTGCA
RI-AT4G26640-XLOC_024350-5602-1
CCAGAACCTTCCCCTACTACTGGTTCTTTGTTCAAGCCTCGACCAGTGCA
CONSENSUS
CCAGAACCTTCCCCTACTACTGGTTCTTTGTTCAAGCCTCGACCAGTGCA

RI-AT4G26640-XLOC_024350-5602-0
CATTTCTGCTAGCTCAAGTTCTTATACAGGCAGGGGGTTCCATCAGAACA
RI-AT4G26640-XLOC_024350-5602-1
CATTTCTGCTAGCTCAAGTTCTTATACAGGCAGGGGGTTCCATCAGAACA

CONSENSUS
CATTTCTGCTAGCTCAAGTTCTTATACAGGCAGGGGGTTCCATCAGAACA

RI-AT4G26640-XLOC_024350-5602-0
CCTTTACTGAGCAGAAGTCCAGTGAATTTGAGTTCAGACCTCCTGCATCA
RI-AT4G26640-XLOC_024350-5602-1
CCTTTACTGAGCAGAAGTCCAGTGAATTTGAGTTCAGACCTCCTGCATCA
CONSENSUS
CCTTTACTGAGCAGAAGTCCAGTGAATTTGAGTTCAGACCTCCTGCATCA

RI-AT4G26640-XLOC_024350-5602-0
AATATGGTATTGTCCAAAACAATGCTGTTTGTTATGCTTTGCATGTAATG
RI-AT4G26640-XLOC_024350-5602-1
AATATG--
CONSENSUS
AATATG..

RI-AT4G26640-XLOC_024350-5602-0
TACTGTTCAGATAGTCGACGACATATGAACCCCGAACAGTGGCTGACCTA
RI-AT4G26640-XLOC_024350-5602-1

CONSENSUS
....

RI-AT4G26640-XLOC_024350-5602-0
ATTTTTCTGCACGAAGTCTATTTATTCAGCGCTTGGATTGCTTGTAAATC
RI-AT4G26640-XLOC_024350-5602-1

CONSENSUS
....

RI-AT4G26640-XLOC_024350-5602-0
TCAAAATCTTTCGCCTTTGCATACATATAATACCATGCCAGCCTTAAATT
RI-AT4G26640-XLOC_024350-5602-1

CONSENSUS
....

RI-AT4G26640-XLOC_024350-5602-0
GTTTCATAAGGAGCCTTGTATCCAAAAGCTCACAGTTTATAAGTTACTTG
RI-AT4G26640-XLOC_024350-5602-1

CONSENSUS
....

RI-AT4G26640-XLOC_024350-5602-0
TATATTGTTGGGGGATGACTTTGTCTAAACATCTTTATTGATTTCAAGGT
RI-AT4G26640-XLOC_024350-5602-1
--GT
CONSENSUS
....GT

RI-AT4G26640-XLOC_024350-5602-0
ATATGCAGAGCTTGGCAAGATTAGAAGTGAGCCACCAGTACATTTTCAAG
RI-AT4G26640-XLOC_024350-5602-1
ATATGCAGAGCTTGGCAAGATTAGAAGTGAGCCACCAGTACATTTTCAAG

CONSENSUS
ATATGCAGAGCTTGGCAAGATTAGAAGTGAGCCACCAGTACATTTTCAAG

RI-AT4G26640-XLOC_024350-5602-0
GCCAGGGCCATGGATCCTCACACTCACCTTCTTCGATCAGTGATGCTGCA
RI-AT4G26640-XLOC_024350-5602-1
GCCAGGGCCATGGATCCTCACACTCACCTTCTTCGATCAGTGATGCTGCA
CONSENSUS
GCCAGGGCCATGGATCCTCACACTCACCTTCTTCGATCAGTGATGCTGCA

RI-AT4G26640-XLOC_024350-5602-0
GGTTCCTCAAGTGAGCTAAGCCGGCCAACTCCTCCTTGTCAGATGACACC
RI-AT4G26640-XLOC_024350-5602-1
GGTTCCTCAAGTGAGCTAAGCCGGCCAACTCCTCCTTGTCAGATGACACC
CONSENSUS
GGTTCCTCAAGTGAGCTAAGCCGGCCAACTCCTCCTTGTCAGATGACACC

RI-AT4G26640-XLOC_024350-5602-0
AACGAGCTCAGATATTCCGGCTGGATCTGATCAAGAGGAATCAATCCAGA
RI-AT4G26640-XLOC_024350-5602-1
AACGAGCTCAGATATTCCGGCTGGATCTGATCAAGAGGAATCAATCCAGA
CONSENSUS
AACGAGCTCAGATATTCCGGCTGGATCTGATCAAGAGGAATCAATCCAGA

RI-AT4G26640-XLOC_024350-5602-0
CTTCCCAAAATGACTCCAGAGGAAGCACTCCATCCATCTTGGCTGATGAT
RI-AT4G26640-XLOC_024350-5602-1
CTTCCCAAAATGACTCCAGAGGAAGCACTCCATCCATCTTGGCTGATGAT
CONSENSUS
CTTCCCAAAATGACTCCAGAGGAAGCACTCCATCCATCTTGGCTGATGAT

RI-AT4G26640-XLOC_024350-5602-0
GGTTATAACTGGAGAAAATATGGTCAAAAGCATGTCAAAGGGAGTGAATT
RI-AT4G26640-XLOC_024350-5602-1
GGTTATAACTGGAGAAAATATGGTCAAAAGCATGTCAAAGGGAGTGAATT
CONSENSUS
GGTTATAACTGGAGAAAATATGGTCAAAAGCATGTCAAAGGGAGTGAATT

RI-AT4G26640-XLOC_024350-5602-0
TCCCCGGAGCTATTATAAATGTACACATCCTAATTGTGAAGTGAAAAAGT
RI-AT4G26640-XLOC_024350-5602-1
TCCCCGGAGCTATTATAAATGTACACATCCTAATTGTGAAGTGAAAAAGT
CONSENSUS
TCCCCGGAGCTATTATAAATGTACACATCCTAATTGTGAAGTGAAAAAGT

RI-AT4G26640-XLOC_024350-5602-0
TATTTGAAAGATCTCATGATGGGCAGATCACCGATATTATATACAAGGGT
RI-AT4G26640-XLOC_024350-5602-1
TATTTGAAAGATCTCATGATGGGCAGATCACCGATATTATATACAAGGGT
CONSENSUS
TATTTGAAAGATCTCATGATGGGCAGATCACCGATATTATATACAAGGGT

RI-AT4G26640-XLOC_024350-5602-0
ACACATGACCATCCTAAACCTCAACCTGGTCGCCGAAACTCTGGTGGTAT
RI-AT4G26640-XLOC_024350-5602-1
ACACATGACCATCCTAAACCTCAACCTGGTCGCCGAAACTCTGGTGGTAT

CONSENSUS
ACACATGACCATCCTAAACCTCAACCTGGTCGCCGAAACTCTGGTGGTAT

RI-AT4G26640-XLOC_024350-5602-0
GGCTGCACAAGAAGAAAGGCTAGACAAGTATCCTTCTTCAACTGGCCGAG
RI-AT4G26640-XLOC_024350-5602-1
GGCTGCACAAGAAGAAAGGCTAGACAAGTATCCTTCTTCAACTGGCCGAG
CONSENSUS
GGCTGCACAAGAAGAAAGGCTAGACAAGTATCCTTCTTCAACTGGCCGAG

RI-AT4G26640-XLOC_024350-5602-0 ATG
RI-AT4G26640-XLOC_024350-5602-1 ATG
CONSENSUS ATG

alignment for event: A3-AT4G25570-XLOC_024287-13737

A3-AT4G25570-XLOC_024287-13737-0
CCATCATAAGTTACAAATCGCTTCCGCTGGAGAAACCAGTGAAGAAGTTG
A3-AT4G25570-XLOC_024287-13737-1
CCATCATAAGTTACAAATCGCTTCCGCTGGAGAAACCAGTGAAGAAGTTG
CONSENSUS
CCATCATAAGTTACAAATCGCTTCCGCTGGAGAAACCAGTGAAGAAGTTG

A3-AT4G25570-XLOC_024287-13737-0
ATCCACCTTATACTCCATGCCATTGCTCTGGCTCTTGGGATATTTGGCAT
A3-AT4G25570-XLOC_024287-13737-1
ATCCACCTTATACTCCATGCCATTGCTCTGGCTCTTGGGATATTTGGCAT
CONSENSUS
ATCCACCTTATACTCCATGCCATTGCTCTGGCTCTTGGGATATTTGGCAT

A3-AT4G25570-XLOC_024287-13737-0
CTGTGCAGCCTTTAAGAACCACAATGAAAGCCATATCCCTAATCTCTACA
A3-AT4G25570-XLOC_024287-13737-1
CTGTGCAGCCTTTAAGAACCACAATGAAAGCCATATCCCTAATCTCTACA
CONSENSUS
CTGTGCAGCCTTTAAGAACCACAATGAAAGCCATATCCCTAATCTCTACA

A3-AT4G25570-XLOC_024287-13737-0
GTCTCCATTCCTGGATTGGTATTGGAGTCATTTCTCTTTATGGCTTCCAG
A3-AT4G25570-XLOC_024287-13737-1
GTCTCCATTCCTGGATTGGTATTGGAGTCATTTCTCTTTATGGCTTCCAG
CONSENSUS
GTCTCCATTCCTGGATTGGTATTGGAGTCATTTCTCTTTATGGCTTCCAG

A3-AT4G25570-XLOC_024287-13737-0 -----------
CTTCATAGTGTTCTTCTTCCCAGGAGGATCAACAAATTT
A3-AT4G25570-XLOC_024287-13737-1
TGGGTGTACAGCTTCATAGTGTTCTTCTTCCCAGGAGGATCAACAAATTT
CONSENSUS
...........CTTCATAGTGTTCTTCTTCCCAGGAGGATCAACAAATTT

A3-AT4G25570-XLOC_024287-13737-0
GAAAAGCGGATTGCTTCCGTGGCACGCAATGCTTGGCCTGTTTGTTTACA
A3-AT4G25570-XLOC_024287-13737-1

GAAAAGCGGATTGCTTCCGTGGCACGCAATGCTTGGCCTGTTTGTTTACA
CONSENSUS
GAAAAGCGGATTGCTTCCGTGGCACGCAATGCTTGGCCTGTTTGTTTACA

A3-AT4G25570-XLOC_024287-13737-0
TACTTGCTGTCGGGAATGCAGCTTTAGGGTTTCTGGAAAAGCTGACTTTC
A3-AT4G25570-XLOC_024287-13737-1
TACTTGCTGTCGGGAATGCAGCTTTAGGGTTTCTGGAAAAGCTGACTTTC
CONSENSUS
TACTTGCTGTCGGGAATGCAGCTTTAGGGTTTCTGGAAAAGCTGACTTTC

A3-AT4G25570-XLOC_024287-13737-0
TTGGAGAATGGAGGGCTTGACAAGTATGGATCCGAAGCATTTCTTATCAA
A3-AT4G25570-XLOC_024287-13737-1
TTGGAGAATGGAGGGCTTGACAAGTATGGATCCGAAGCATTTCTTATCAA
CONSENSUS
TTGGAGAATGGAGGGCTTGACAAGTATGGATCCGAAGCATTTCTTATCAA

A3-AT4G25570-XLOC_024287-13737-0
CTTCACGGCCATTATCACTATTCTCTTTGGTGCCTTTGTGGTACTCACTG
A3-AT4G25570-XLOC_024287-13737-1
CTTCACGGCCATTATCACTATTCTCTTTGGTGCCTTTGTGGTACTCACTG
CONSENSUS
CTTCACGGCCATTATCACTATTCTCTTTGGTGCCTTTGTGGTACTCACTG

A3-AT4G25570-XLOC_024287-13737-0
CTTCTGCTGAGTCTCCTTCTCCTTCTCCTTCCGTCTCCAATGATGATAGT
A3-AT4G25570-XLOC_024287-13737-1
CTTCTGCTGAGTCTCCTTCTCCTTCTCCTTCCGTCTCCAATGATGATAGT
CONSENSUS
CTTCTGCTGAGTCTCCTTCTCCTTCTCCTTCCGTCTCCAATGATGATAGT

A3-AT4G25570-XLOC_024287-13737-0
GTTGACTTCAGTTATTCTGCTATATAAAACACTTTCTACATTCCACGTTA
A3-AT4G25570-XLOC_024287-13737-1
GTTGACTTCAGTTATTCTGCTATATAAAACACTTTCTACATTCCACGTTA
CONSENSUS
GTTGACTTCAGTTATTCTGCTATATAAAACACTTTCTACATTCCACGTTA

A3-AT4G25570-XLOC_024287-13737-0
CATCTGTATCGCCCCTTGTACTCTCAAAACTCCATTTATGTATGATGCAT
A3-AT4G25570-XLOC_024287-13737-1
CATCTGTATCGCCCCTTGTACTCTCAAAACTCCATTTATGTATGATGCAT
CONSENSUS
CATCTGTATCGCCCCTTGTACTCTCAAAACTCCATTTATGTATGATGCAT

A3-AT4G25570-XLOC_024287-13737-0
TTGTAGCGAAAAGCTTTGTGTGTTACGGCTTTGCTACTTCTGGTGTGTAA
A3-AT4G25570-XLOC_024287-13737-1
TTGTAGCGAAAAGCTTTGTGTGTTACGGCTTTGCTACTTCTGGTGTGTAA
CONSENSUS
TTGTAGCGAAAAGCTTTGTGTGTTACGGCTTTGCTACTTCTGGTGTGTAA

A3-AT4G25570-XLOC_024287-13737-0
AATCTTCAATGATATTGATGAATCTTTTAAATCATTTTCTTTTCTTTTCT
A3-AT4G25570-XLOC_024287-13737-1

AATCTTCAATGATATTGATGAATCTTTTAAATCATTTTCTTTTCTTTTCT
CONSENSUS
AATCTTCAATGATATTGATGAATCTTTTAAATCATTTTCTTTTCTTTTCT

A3-AT4G25570-XLOC_024287-13737-0 TTTCTTTCATATTGCCATTCCTTTAACC
A3-AT4G25570-XLOC_024287-13737-1 TTTCTTTCATATTGCCATTCCTTTAACC
CONSENSUS TTTCTTTCATATTGCCATTCCTTTAACC

alignment for event: RI-AT4G32060-XLOC_022165-448

RI-AT4G32060-XLOC_022165-448-0
GTATATATTCTTTGTGACATTACTCAGCATTCCTGAATCAAGCTTCGCAG
RI-AT4G32060-XLOC_022165-448-1
GTATATATTCTTTGTGACATTACTCAGCATTCCTGAATCAAGCTTCGCAG
CONSENSUS
GTATATATTCTTTGTGACATTACTCAGCATTCCTGAATCAAGCTTCGCAG

RI-AT4G32060-XLOC_022165-448-0
TGGCTTTCAAAATGTTTGATACTGACAACAACGGGTGAGTAGTCGTTCAC
RI-AT4G32060-XLOC_022165-448-1
TGGCTTTCAAAATGTTTGATACTGACAACAACGG----------------
CONSENSUS
TGGCTTTCAAAATGTTTGATACTGACAACAACGG................

RI-AT4G32060-XLOC_022165-448-0
ATATGTAAATAGATAAATCATGCTTTTGGTAACTTTTTTTCTGGTCCGTA
RI-AT4G32060-XLOC_022165-448-1

CONSENSUS
....

RI-AT4G32060-XLOC_022165-448-0
TTCCCATATTGTGATCCTCCAAACGGACGGGTTGTGGGAATCAGTGGAGT
RI-AT4G32060-XLOC_022165-448-1

CONSENSUS
....

RI-AT4G32060-XLOC_022165-448-0
CTCTTTTTGAGTTTACGCTGAATCATCTGGTCAGTGCTTCTATCCTTTGT
RI-AT4G32060-XLOC_022165-448-1

CONSENSUS
....

RI-AT4G32060-XLOC_022165-448-0
AACTTCTATTTCCTCGAGATGGTACTGACGAATGATTGTTTTAGGGAGAT
RI-AT4G32060-XLOC_022165-448-1
--GGAGAT
CONSENSUS
....GGAGAT

RI-AT4G32060-XLOC_022165-448-0
TGACAAAGAAGAGTTTAAGACAGTGATGAGTCTGATGCGATCTCAGCATA

RI-AT4G32060-XLOC_022165-448-1
TGACAAAGAAGAGTTTAAGACAGTGATGAGTCTGATGCGATCTCAGCATA
CONSENSUS
TGACAAAGAAGAGTTTAAGACAGTGATGAGTCTGATGCGATCTCAGCATA

RI-AT4G32060-XLOC_022165-448-0
GACAAGGAGTTGGCCACAGAGATGGCCTTCGAACAGGGTTACATATGACC
RI-AT4G32060-XLOC_022165-448-1
GACAAGGAGTTGGCCACAGAGATGGCCTTCGAACAGGGTTACATATGACC
CONSENSUS
GACAAGGAGTTGGCCACAGAGATGGCCTTCGAACAGGGTTACATATGACC

RI-AT4G32060-XLOC_022165-448-0
GGTTCTGTCGAGGATGGAGGATTGGTAGAATACTTCTTTGGGAAAGATGG
RI-AT4G32060-XLOC_022165-448-1
GGTTCTGTCGAGGATGGAGGATTGGTAGAATACTTCTTTGGGAAAGATGG
CONSENSUS
GGTTCTGTCGAGGATGGAGGATTGGTAGAATACTTCTTTGGGAAAGATGG

RI-AT4G32060-XLOC_022165-448-0
CAGTCAAAAACTGAAACACGACAAATTCACTAAGTTTATGAAAGATCTAA
RI-AT4G32060-XLOC_022165-448-1
CAGTCAAAAACTGAAACACGACAAATTCACTAAGTTTATGAAAGATCTAA
CONSENSUS
CAGTCAAAAACTGAAACACGACAAATTCACTAAGTTTATGAAAGATCTAA

RI-AT4G32060-XLOC_022165-448-0 CTGAAGAG
RI-AT4G32060-XLOC_022165-448-1 CTGAAGAG
CONSENSUS CTGAAGAG

alignment for event: RI-AT4G11110-XLOC_020942-12787

RI-AT4G11110-XLOC_020942-12787-0
TTGCTGAGCCAATTTCAGTGTGAAAGAGCTCGTGAGGCTGCAATGTCTGA
RI-AT4G11110-XLOC_020942-12787-1
TTGCTGAGCCAATTTCAGTGTGAAAGAGCTCGTGAGGCTGCAATGTCTGA
CONSENSUS
TTGCTGAGCCAATTTCAGTGTGAAAGAGCTCGTGAGGCTGCAATGTCTGA

RI-AT4G11110-XLOC_020942-12787-0
TATCCGACACCGCATTCTTCCTCCCAAATTTCTGTCGGAGAATCCAAAGG
RI-AT4G11110-XLOC_020942-12787-1
TATCCGACACCGCATTCTTCCTCCCAAATTTCTGTCGGAGAATCCAAAGG
CONSENSUS
TATCCGACACCGCATTCTTCCTCCCAAATTTCTGTCGGAGAATCCAAAGG

RI-AT4G11110-XLOC_020942-12787-0
AAGCTGGGTTTTGTCTTTGGTTACTTCATCCAGAATCTTCATGCCGACCA
RI-AT4G11110-XLOC_020942-12787-1
AAGCTGGGTTTTGTCTTTGGTTACTTCATCCAGAATCTTCATGCCGACCA
CONSENSUS
AAGCTGGGTTTTGTCTTTGGTTACTTCATCCAGAATCTTCATGCCGACCA

RI-AT4G11110-XLOC_020942-12787-0

TCAACCAGGCAATGCAAAATCTTTAGCTACTATAATTCTCAAATGATATA
RI-AT4G11110-XLOC_020942-12787-1
TCAACCAG--
CONSENSUS
TCAACCAG..

RI-AT4G11110-XLOC_020942-12787-0
AAGCGTGTTATTGAACTTTTGTATGCTTGTTTCTGTTGATAGTTTAAGGC
RI-AT4G11110-XLOC_020942-12787-1

CONSENSUS
....

RI-AT4G11110-XLOC_020942-12787-0
CATGAATCCCTAGTGGGTCTATTGGAAGATAATCTTGTTGTATATCTAGA
RI-AT4G11110-XLOC_020942-12787-1

CONSENSUS
....

RI-AT4G11110-XLOC_020942-12787-0
AATGTACATATGCACCAAATGTGGAAGTTTTGTTCTCCTGGCTGTAAACG
RI-AT4G11110-XLOC_020942-12787-1

CONSENSUS
....

RI-AT4G11110-XLOC_020942-12787-0
GGAGGACGATTGTCTCGTCAACAGTTTCACATAGGATTGTTAGGTTCTAC
RI-AT4G11110-XLOC_020942-12787-1

CONSENSUS
....

RI-AT4G11110-XLOC_020942-12787-0
TCTGAAAAATAATTAGTCTTTGTACTAACTGAGCTCCCTTTATGTGTTGA
RI-AT4G11110-XLOC_020942-12787-1

CONSENSUS
....

RI-AT4G11110-XLOC_020942-12787-0
TAGTCTCCTTCCTGTCATAATTTATCTGTCAAGAATGTGAGTTGGCTTAA
RI-AT4G11110-XLOC_020942-12787-1

CONSENSUS
....

RI-AT4G11110-XLOC_020942-12787-0
TCTCTAAATAAGTTTGATGTTGTTCTGTATTAGGGACATCTTACAATCTG
RI-AT4G11110-XLOC_020942-12787-1
----------------------------------GGACATCTTACAATCTG
CONSENSUS
..................................GGACATCTTACAATCTG

RI-AT4G11110-XLOC_020942-12787-0

AAGTAGTTAATGGAATCCCGGATTTGTATGCTGAAGGCTTATCTTTATCC
RI-AT4G11110-XLOC_020942-12787-1
AAGTAGTTAATGGAATCCCGGATTTGTATGCTGAAGGCTTATCTTTATCC
CONSENSUS
AAGTAGTTAATGGAATCCCGGATTTGTATGCTGAAGGCTTATCTTTATCC

RI-AT4G11110-XLOC_020942-12787-0
ATTGAACAAGAGGACACAGAGTCTGAACTCTTGCAGCATTTCCTTTTCTT
RI-AT4G11110-XLOC_020942-12787-1
ATTGAACAAGAGGACACAGAGTCTGAACTCTTGCAGCATTTCCTTTTCTT
CONSENSUS
ATTGAACAAGAGGACACAGAGTCTGAACTCTTGCAGCATTTCCTTTTCTT

RI-AT4G11110-XLOC_020942-12787-0
GTCCCAAGAGAAAAGGCAGAAGCATGCTGGAAATTTGATGGAAGAAATTG
RI-AT4G11110-XLOC_020942-12787-1
GTCCCAAGAGAAAAGGCAGAAGCATGCTGGAAATTTGATGGAAGAAATTG
CONSENSUS
GTCCCAAGAGAAAAGGCAGAAGCATGCTGGAAATTTGATGGAAGAAATTG

RI-AT4G11110-XLOC_020942-12787-0
CGTCCGTAGAAGCAGATATCGAGGAAATTGTCAAGAGACGTTGTGCCATA
RI-AT4G11110-XLOC_020942-12787-1
CGTCCGTAGAAGCAGATATCGAGGAAATTGTCAAGAGACGTTGTGCCATA
CONSENSUS
CGTCCGTAGAAGCAGATATCGAGGAAATTGTCAAGAGACGTTGTGCCATA

RI-AT4G11110-XLOC_020942-12787-0
GGGCCTCCGTCCCTGGAAGAAGCTTCTAGCTCATCTCCTGCTTCTAGTGT
RI-AT4G11110-XLOC_020942-12787-1
GGGCCTCCGTCCCTGGAAGAAGCTTCTAGCTCATCTCCTGCTTCTAGTGT
CONSENSUS
GGGCCTCCGTCCCTGGAAGAAGCTTCTAGCTCATCTCCTGCTTCTAGTGT

RI-AT4G11110-XLOC_020942-12787-0
ACCTGAAATGAGACTGATCAGGAACATTAATCAGCTTGAAAGCGCTTATT
RI-AT4G11110-XLOC_020942-12787-1
ACCTGAAATGAGACTGATCAGGAACATTAATCAGCTTGAAAGCGCTTATT
CONSENSUS
ACCTGAAATGAGACTGATCAGGAACATTAATCAGCTTGAAAGCGCTTATT

RI-AT4G11110-XLOC_020942-12787-0
TCGCTGCAAGGATAGACGCACATCTCCCTGAAGCACGTTATAGGTTACGC
RI-AT4G11110-XLOC_020942-12787-1
TCGCTGCAAGGATAGACGCACATCTCCCTGAAGCACGTTATAGGTTACGC
CONSENSUS
TCGCTGCAAGGATAGACGCACATCTCCCTGAAGCACGTTATAGGTTACGC

RI-AT4G11110-XLOC_020942-12787-0
CCAGATAGAGATCTGCTAAGAAATAGTGATAATACTGTAGCAGAAGTAGA
RI-AT4G11110-XLOC_020942-12787-1
CCAGATAGAGATCTGCTAAGAAATAGTGATAATACTGTAGCAGAAGTAGA
CONSENSUS
CCAGATAGAGATCTGCTAAGAAATAGTGATAATACTGTAGCAGAAGTAGA

RI-AT4G11110-XLOC_020942-12787-0

AAACAGTGAGACTTGGAGCTCAGATGATCGTGTTGGAGCGTTCTTTGATG
RI-AT4G11110-XLOC_020942-12787-1
AAACAGTGAGACTTGGAGCTCAGATGATCGTGTTGGAGCGTTCTTTGATG
CONSENSUS
AAACAGTGAGACTTGGAGCTCAGATGATCGTGTTGGAGCGTTCTTTGATG

RI-AT4G11110-XLOC_020942-12787-0
GGTTATGCAAATATGCTCGATATAGTAAGTTTGAAACTCGGGGTGTGCTG
RI-AT4G11110-XLOC_020942-12787-1
GGTTATGCAAATATGCTCGATATAGTAAGTTTGAAACTCGGGGTGTGCTG
CONSENSUS
GGTTATGCAAATATGCTCGATATAGTAAGTTTGAAACTCGGGGTGTGCTG

RI-AT4G11110-XLOC_020942-12787-0
AGGACCAGTGAGTTGAACAACACTTCAAATGTGATTTGTTCTCTGGGTTT
RI-AT4G11110-XLOC_020942-12787-1
AGGACCAGTGAGTTGAACAACACTTCAAATGTGATTTGTTCTCTGGGTTT
CONSENSUS
AGGACCAGTGAGTTGAACAACACTTCAAATGTGATTTGTTCTCTGGGTTT

RI-AT4G11110-XLOC_020942-12787-0
TGATCGGGACGAGGATTATTTTGCCACAGCTGGTGTGTCGAAAAAAATTA
RI-AT4G11110-XLOC_020942-12787-1
TGATCGGGACGAGGATTATTTTGCCACAGCTGGTGTGTCGAAAAAAATTA
CONSENSUS
TGATCGGGACGAGGATTATTTTGCCACAGCTGGTGTGTCGAAAAAAATTA

RI-AT4G11110-XLOC_020942-12787-0
AGATATATGAGTTCAATTCCCTTTTCAACGAATCTGTTGACATTCATTAT
RI-AT4G11110-XLOC_020942-12787-1
AGATATATGAGTTCAATTCCCTTTTCAACGAATCTGTTGACATTCATTAT
CONSENSUS
AGATATATGAGTTCAATTCCCTTTTCAACGAATCTGTTGACATTCATTAT

RI-AT4G11110-XLOC_020942-12787-0
CCAGCCATAGAAATGCCAAATAGATCAAAGCTTAGCGGCGTTTGCTGGAA
RI-AT4G11110-XLOC_020942-12787-1
CCAGCCATAGAAATGCCAAATAGATCAAAGCTTAGCGGCGTTTGCTGGAA
CONSENSUS
CCAGCCATAGAAATGCCAAATAGATCAAAGCTTAGCGGCGTTTGCTGGAA

RI-AT4G11110-XLOC_020942-12787-0
CAACTACATTAGGAACTACCTAGCTTCCTCTGATTATGATGGTATTGTCA
RI-AT4G11110-XLOC_020942-12787-1
CAACTACATTAGGAACTACCTAGCTTCCTCTGATTATGATGGTATTGTCA
CONSENSUS
CAACTACATTAGGAACTACCTAGCTTCCTCTGATTATGATGGTATTGTCA

RI-AT4G11110-XLOC_020942-12787-0 AG
RI-AT4G11110-XLOC_020942-12787-1 AG
CONSENSUS AG

alignment for event: RI-AT4G27040-XLOC_024372-1532

RI-AT4G27040-XLOC_024372-1532-0
      TGCAAGACGACAAGTGATGAAAAGATAGAGACAAAGAAAGGCTTCAAGTT
RI-AT4G27040-XLOC_024372-1532-1
      TGCAAGACGACAAGTGATGAAAAGATAGAGACAAAGAAAGGCTTCAAGTT
CONSENSUS
      TGCAAGACGACAAGTGATGAAAAGATAGAGACAAAGAAAGGCTTCAAGTT

RI-AT4G27040-XLOC_024372-1532-0
      TGTTGATAACTGCTGTCTAAAGAGTTCGTTCCCTTGAGAGATTCTGACTC
RI-AT4G27040-XLOC_024372-1532-1
      TGTTGATAACTGCTGTCTAAAGAGTTCGTTCCCTTGAGAGATTCTGACTC
CONSENSUS
      TGTTGATAACTGCTGTCTAAAGAGTTCGTTCCCTTGAGAGATTCTGACTC

RI-AT4G27040-XLOC_024372-1532-0
      TGACATAAGAGGTAAAAAAGTTCAAATACTCAACACTTACTTGGAATTTA
RI-AT4G27040-XLOC_024372-1532-1
      TGACATAAGAG---------------------------------------
CONSENSUS
      TGACATAAGAG.......................................

RI-AT4G27040-XLOC_024372-1532-0
      AAAGAACACTGATTTGTTGATATTATAAGGTATAAAAATATTTCGTCTTC
RI-AT4G27040-XLOC_024372-1532-1 -----------------------------
GTATAAAAATATTTCGTCTTC
CONSENSUS
      .............................GTATAAAAATATTTCGTCTTC

RI-AT4G27040-XLOC_024372-1532-0
      TCAAAATCCAATTCTTCATCATATTATAAGTTCCCCCAACCGGAAGAGAA
RI-AT4G27040-XLOC_024372-1532-1
      TCAAAATCCAATTCTTCATCATATTATAAGTTCCCCCAACCGGAAGAGAA
CONSENSUS
      TCAAAATCCAATTCTTCATCATATTATAAGTTCCCCCAACCGGAAGAGAA

RI-AT4G27040-XLOC_024372-1532-0
      ATTCAAAGATCAGAAAACGGTGAAAGATGCGACGACGACCAGGAATTGGA
RI-AT4G27040-XLOC_024372-1532-1
      ATTCAAAGATCAGAAAACGGTGAAAGATGCGACGACGACCAGGAATTGGA
CONSENSUS
      ATTCAAAGATCAGAAAACGGTGAAAGATGCGACGACGACCAGGAATTGGA

RI-AT4G27040-XLOC_024372-1532-0 GGATTACAAAAGGCCGCAGCTGCTAGG
RI-AT4G27040-XLOC_024372-1532-1 GGATTACAAAAGGCCGCAGCTGCTAGG
CONSENSUS   GGATTACAAAAGGCCGCAGCTGCTAGG

alignment for event: SE-AT4G20440-XLOC_024010-9127

SE-AT4G20440-XLOC_024010-9127-0
      TTTGCACAGCCAAACCCCCCCAATTTTGGCCTCTCGATTTAGTTTTTTGA
SE-AT4G20440-XLOC_024010-9127-1
      TTTGCACAGCCAAACCCCCCCAATTTTGGCCTCTCGATTTAGTTTTTTGA
CONSENSUS
      TTTGCACAGCCAAACCCCCCCAATTTTGGCCTCTCGATTTAGTTTTTTGA

SE-AT4G20440-XLOC_024010-9127-0
AACCCTAAGCTCGATTTCGATCCTCCGGCATACTCGGTTTCGCCGCCGAA
SE-AT4G20440-XLOC_024010-9127-1
AACCCTAAGCTCGATTTCGATCCTCCGGCATACTCGGTTTCGCCGCCGAA
CONSENSUS
AACCCTAAGCTCGATTTCGATCCTCCGGCATACTCGGTTTCGCCGCCGAA

SE-AT4G20440-XLOC_024010-9127-0
TCTCTGCTTGTAGCGAGTGTTACTATTCAATCGGCGAGAATGTCGATGTC
SE-AT4G20440-XLOC_024010-9127-1
TCTCTGCTTGTAGCGAGTGTTACTATTCAATCGGCGAGAATGTCGATGTC
CONSENSUS
TCTCTGCTTGTAGCGAGTGTTACTATTCAATCGGCGAGAATGTCGATGTC

SE-AT4G20440-XLOC_024010-9127-0
GAAGAGTTCAAAGATGCTTCAGTTCATCAACTACAGGATGCGAGTGACGA
SE-AT4G20440-XLOC_024010-9127-1
GAAGAGTTCAAAGATGCTTCAGTTCATCAACTACAGGATGCGAGTGACGA
CONSENSUS
GAAGAGTTCAAAGATGCTTCAGTTCATCAACTACAGGATGCGAGTGACGA

SE-AT4G20440-XLOC_024010-9127-0
TCCAAGACGGAAGACAGCTCGTTGGGAAGTTCATGGCGTTTGACCGTCAC
SE-AT4G20440-XLOC_024010-9127-1
TCCAAGACGGAAGACAGCTCGTTGGGAAGTTCATGGCGTTTGACCGTCAC
CONSENSUS
TCCAAGACGGAAGACAGCTCGTTGGGAAGTTCATGGCGTTTGACCGTCAC

SE-AT4G20440-XLOC_024010-9127-0
ATGAACCTCGTTCTCGGCGATTGCGAGGAGTTTCGTAAGCTTCCACCAGC
SE-AT4G20440-XLOC_024010-9127-1
ATGAACCTCGTTCTCGGCGATTGCGAGGAGTTTCGTAAGCTTCCACCAGC
CONSENSUS
ATGAACCTCGTTCTCGGCGATTGCGAGGAGTTTCGTAAGCTTCCACCAGC

SE-AT4G20440-XLOC_024010-9127-0
TAAAGGGAAGAAGATCAACGAAGAGCGTGAAGATCGCCGTACGCTAGGTT
SE-AT4G20440-XLOC_024010-9127-1
TAAAGGGAAGAAGATCAACGAAGAGCGTGAAGATCGCCGTACGCTAGGTT
CONSENSUS
TAAAGGGAAGAAGATCAACGAAGAGCGTGAAGATCGCCGTACGCTAGGTT

SE-AT4G20440-XLOC_024010-9127-0
TAGTGTTGCTTAGAGGTGAAGAAGTTATCTCAATGACTGTCGAAGGACCA
SE-AT4G20440-XLOC_024010-9127-1
TAGTGTTGCTTAGAGGTGAAGAAGTTATCTCAATGACTGTCGAAGGACCA
CONSENSUS
TAGTGTTGCTTAGAGGTGAAGAAGTTATCTCAATGACTGTCGAAGGACCA

SE-AT4G20440-XLOC_024010-9127-0
CCTCCTCCTGAAGAATCTCGTGCTAAAGCTGGCTCTGCAGCTGCTGTTGC
SE-AT4G20440-XLOC_024010-9127-1
CCTCCTCCTGAAGAATCTCGTGCTAAAGCTGGCTCTGCAGCTGCTGTTGC
CONSENSUS
CCTCCTCCTGAAGAATCTCGTGCTAAAGCTGGCTCTGCAGCTGCTGTTGC

SE-AT4G20440-XLOC_024010-9127-0
TGGTCCAGGAATTGGCCGTGCCGCTGGGCGTGGAGTTCCTACTGGTCCGT
SE-AT4G20440-XLOC_024010-9127-1
TGGTCCAGGAATTGGCCGTGCCGCTGGGCGTGGAGTTCCTACTGGTCCGT
CONSENSUS
TGGTCCAGGAATTGGCCGTGCCGCTGGGCGTGGAGTTCCTACTGGTCCGT

SE-AT4G20440-XLOC_024010-9127-0
TAGTTCAAGCTCAGCCTGGTTTATCTGGTCCTGTTCGTGGTGTTGGTGGA
SE-AT4G20440-XLOC_024010-9127-1
TAGTTCAAGCTCAGCCTGGTTTATCTGGTCCTGTTCGTGGTGTTGGTGGA
CONSENSUS
TAGTTCAAGCTCAGCCTGGTTTATCTGGTCCTGTTCGTGGTGTTGGTGGA

SE-AT4G20440-XLOC_024010-9127-0
CCTGCTCCGGGAATGATGCAGCCTCAGATCTCTCGTCCACCACAGCTTTC
SE-AT4G20440-XLOC_024010-9127-1
CCTGCTCCGGGAATGATGCAGCCTCAGATCTCTCGTCCACCACAGCTTTC
CONSENSUS
CCTGCTCCGGGAATGATGCAGCCTCAGATCTCTCGTCCACCACAGCTTTC

SE-AT4G20440-XLOC_024010-9127-0
AGCTCCTCCGATTATTCGACCTCCGGGACAGATGTTGCCACCGCCTCCTT
SE-AT4G20440-XLOC_024010-9127-1
AGCTCCTCCGATTATTCGACCTCCGGGACAGATGTTGCCACCGCCTCCTT
CONSENSUS
AGCTCCTCCGATTATTCGACCTCCGGGACAGATGTTGCCACCGCCTCCTT

SE-AT4G20440-XLOC_024010-9127-0
TTGGTGGTCAAGGTCCTCCTATGGGAAGAGGTCCTCCACCGCCTTATGGT
SE-AT4G20440-XLOC_024010-9127-1
TTGGTGGTCAAGGTCCTCCTATGGGAAGAGGTCCTCCACCGCCTTATGGT
CONSENSUS
TTGGTGGTCAAGGTCCTCCTATGGGAAGAGGTCCTCCACCGCCTTATGGT

SE-AT4G20440-XLOC_024010-9127-0
ATGAGGCCACCGCCACAGCAGTTTTCTGGACCACCACCGCCTCAGTATGG
SE-AT4G20440-XLOC_024010-9127-1
ATGAGGCCACCGCCACAGCAGTTTTCTGGACCACCACCGCCTCAGTATGG
CONSENSUS
ATGAGGCCACCGCCACAGCAGTTTTCTGGACCACCACCGCCTCAGTATGG

SE-AT4G20440-XLOC_024010-9127-0
GCAAAGGCCAATGATTCCTCCTCCTGGTGGTATGATGAGAGGACCTCCTC
SE-AT4G20440-XLOC_024010-9127-1
GCAAAGGCCAATGATTCCTCCTCCTGGTGGTATGATGAGAGGACCTCCTC
CONSENSUS
GCAAAGGCCAATGATTCCTCCTCCTGGTGGTATGATGAGAGGACCTCCTC

SE-AT4G20440-XLOC_024010-9127-0
CTCCACCTCATGGAATGCAAGGACCTCCTCCGCCTCGCCCTGGAATGCCT
SE-AT4G20440-XLOC_024010-9127-1
CTCCACCTCATGGAATGCAAGGACCTCCTCCGCCTCGCCCTGGAATGCCT
CONSENSUS
CTCCACCTCATGGAATGCAAGGACCTCCTCCGCCTCGCCCTGGAATGCCT

```
SE-AT4G20440-XLOC_024010-9127-0
      CCTGCTCCTGGTGGTTTTGCTCCACCGCGTCCTGGCATGCCACCACATAA
SE-AT4G20440-XLOC_024010-9127-1
      CCTGCTCCTGGTGGTTTTGCTCCACCGCGTCCTGGCATGCCACCACATAA
CONSENSUS
      CCTGCTCCTGGTGGTTTTGCTCCACCGCGTCCTGGCATGCCACCACATAA

SE-AT4G20440-XLOC_024010-9127-0
      TCAGCAGCAGTGATTAG---------------------------------
SE-AT4G20440-XLOC_024010-9127-1
      TCAGCAGCAGTGATTAGGTTTACGGTATCGTGCTATCATGTTGCCTTTCA
CONSENSUS
      TCAGCAGCAGTGATTAG.................................

SE-AT4G20440-XLOC_024010-9127-0
      --------------------------------------------------
SE-AT4G20440-XLOC_024010-9127-1
      TTGACTTTAGCCGCAATTTCTCATCTATCTTAGCTTTCAAAGTTTGTAGA
CONSENSUS
      ..................................................

SE-AT4G20440-XLOC_024010-9127-0
      --------------------------------------------------
SE-AT4G20440-XLOC_024010-9127-1
      TTTGTTGACGAGTGAGTTGTCTTTGCCTTATCTTCTTGTGTTTTCTTTTA
CONSENSUS
      ..................................................

SE-AT4G20440-XLOC_024010-9127-0
      -------------------------------------GTAGAAGTCAAAA
SE-AT4G20440-XLOC_024010-9127-1
      AGAACGAATCCATTAAACTTTGTTATGTCTTCTTAAGGTAGAAGTCAAAA
CONSENSUS
      .....................................GTAGAAGTCAAAA

SE-AT4G20440-XLOC_024010-9127-0
      CCTTGTTTAGGGAGCTATGGATGTTGTGATGAATCGTGGATACATTCAGC
SE-AT4G20440-XLOC_024010-9127-1
      CCTTGTTTAGGGAGCTATGGATGTTGTGATGAATCGTGGATACATTCAGC
CONSENSUS
      CCTTGTTTAGGGAGCTATGGATGTTGTGATGAATCGTGGATACATTCAGC

SE-AT4G20440-XLOC_024010-9127-0
      TATTTGTGATGAAGAACTCGTCGATCTATAAAAAGATTAACAAGTCACAT
SE-AT4G20440-XLOC_024010-9127-1
      TATTTGTGATGAAGAACTCGTCGATCTATAAAAAGATTAACAAGTCACAT
CONSENSUS
      TATTTGTGATGAAGAACTCGTCGATCTATAAAAAGATTAACAAGTCACAT

SE-AT4G20440-XLOC_024010-9127-0
      TACCTTGCAATTTTGGATATAGTTCAAATCTAACCAAAATTCTGTTGGTT
SE-AT4G20440-XLOC_024010-9127-1
      TACCTTGCAATTTTGGATATAGTTCAAATCTAACCAAAATTCTGTTGGTT
CONSENSUS
      TACCTTGCAATTTTGGATATAGTTCAAATCTAACCAAAATTCTGTTGGTT
```

```
SE-AT4G20440-XLOC_024010-9127-0
      CAGTTTGGTTGGGTTTCACATTCTTACATATGTTAATTTTGATGAAATGA
SE-AT4G20440-XLOC_024010-9127-1
      CAGTTTGGTTGGGTTTCACATTCTTACATATGTTAATTTTGATGAAATGA
CONSENSUS
      CAGTTTGGTTGGGTTTCACATTCTTACATATGTTAATTTTGATGAAATGA

SE-AT4G20440-XLOC_024010-9127-0
      AATTGTGAATTAGTGTGGTTAGTTGAATGGTAAGTAATACTTGTTTATAG
SE-AT4G20440-XLOC_024010-9127-1
      AATTGTGAATTAGTGTGGTTAGTTGAATGGTAAGTAATACTTGTTTATAG
CONSENSUS
      AATTGTGAATTAGTGTGGTTAGTTGAATGGTAAGTAATACTTGTTTATAG

SE-AT4G20440-XLOC_024010-9127-0  TGAATTATTTTTCAGAACTTTC
SE-AT4G20440-XLOC_024010-9127-1  TGAATTATTTTTCAGAACTTTC
CONSENSUS                        TGAATTATTTTTCAGAACTTTC


alignment for event: A3-AT4G08470-XLOC_023255-4050

A3-AT4G08470-XLOC_023255-4050-0
      GTGTCAAAGTTAAACGACATTAAGTCCCGCAAGGAAACTCTATTTTGGAT
A3-AT4G08470-XLOC_023255-4050-1
      GTGTCAAAGTTAAACGACATTAAGTCCCGCAAGGAAACTCTATTTTGGAT
CONSENSUS
      GTGTCAAAGTTAAACGACATTAAGTCCCGCAAGGAAACTCTATTTTGGAT

A3-AT4G08470-XLOC_023255-4050-0
      GGCTCCAGAG--------------------------------AAGTCCAG
A3-AT4G08470-XLOC_023255-4050-1
      GGCTCCAGAGGTTATTAACCGGAAGGATAATGATGGTTACAGAAGTCCAG
CONSENSUS
      GGCTCCAGAG................................AAGTCCAG

A3-AT4G08470-XLOC_023255-4050-0
      CTGATATATGGAGCCTCGGGTGCACTGTGCTGGAAATGTGTACTGGTCAG
A3-AT4G08470-XLOC_023255-4050-1
      CTGATATATGGAGCCTCGGGTGCACTGTGCTGGAAATGTGTACTGGTCAG
CONSENSUS
      CTGATATATGGAGCCTCGGGTGCACTGTGCTGGAAATGTGTACTGGTCAG

A3-AT4G08470-XLOC_023255-4050-0  ATCCCATACTCTGATCTAGAACCC
A3-AT4G08470-XLOC_023255-4050-1  ATCCCATACTCTGATCTAGAACCC
CONSENSUS                        ATCCCATACTCTGATCTAGAACCC


alignment for event: RI-AT4G02500-XLOC_020260-4302

RI-AT4G02500-XLOC_020260-4302-0
      GTGTACCTAGAAAGTGGATACTATCTTCACGGTTACTGGGGGATTCTTGT
RI-AT4G02500-XLOC_020260-4302-1
      GTGTACCTAGAAAGTGGATACTATCTTCACGGTTACTGGGGGATTCTTGT
```

CONSENSUS
GTGTACCTAGAAAGTGGATACTATCTTCACGGTTACTGGGGGATTCTTGT

RI-AT4G02500-XLOC_020260-4302-0
GGACAGATATGAAGAAATGATAGAAAACTACCACCCGGGTCTAGGAGATC
RI-AT4G02500-XLOC_020260-4302-1
GGACAGATATGAAGAAATGATAGAAAACTACCACCCGGGTCTAGGAGATC
CONSENSUS
GGACAGATATGAAGAAATGATAGAAAACTACCACCCGGGTCTAGGAGATC

RI-AT4G02500-XLOC_020260-4302-0
ACAGATGGCCATTGGTGACTCACTTTGTCGGTTGCAAACCGTGTGGGAAA
RI-AT4G02500-XLOC_020260-4302-1
ACAGATGGCCATTGGTGACTCACTTTGTCGGTTGCAAACCGTGTGGGAAA
CONSENSUS
ACAGATGGCCATTGGTGACTCACTTTGTCGGTTGCAAACCGTGTGGGAAA

RI-AT4G02500-XLOC_020260-4302-0
TTTGGTGATTACCCGGTGGAACGGTGTCTAAAACAAATGGACAGAGCCTT
RI-AT4G02500-XLOC_020260-4302-1
TTTGGTGATTACCCGGTGGAACGGTGTCTAAAACAAATGGACAGAGCCTT
CONSENSUS
TTTGGTGATTACCCGGTGGAACGGTGTCTAAAACAAATGGACAGAGCCTT

RI-AT4G02500-XLOC_020260-4302-0
TAACTTTGGAGATAACCAGATTCTGCAAATCTATGGTTTCACTCACAAAT
RI-AT4G02500-XLOC_020260-4302-1
TAACTTTGGAGATAACCAGATTCTGCAAATCTATGGTTTCACTCACAAAT
CONSENSUS
TAACTTTGGAGATAACCAGATTCTGCAAATCTATGGTTTCACTCACAAAT

RI-AT4G02500-XLOC_020260-4302-0
CTTTGGCTAGTCGTAAAGTCAAGAGAGTGCGGAACGAGACTAGCAATCCG
RI-AT4G02500-XLOC_020260-4302-1
CTTTGGCTAGTCGTAAAGTCAAGAGAGTGCGGAACGAGACTAGCAATCCG
CONSENSUS
CTTTGGCTAGTCGTAAAGTCAAGAGAGTGCGGAACGAGACTAGCAATCCG

RI-AT4G02500-XLOC_020260-4302-0
TTGGAGATGAAAGACGAGCTCGGGTTGCTTCATCCGGCGTTTAAGGCGGT
RI-AT4G02500-XLOC_020260-4302-1
TTGGAGATGAAAGACGAGCTCGGGTTGCTTCATCCGGCGTTTAAGGCGGT
CONSENSUS
TTGGAGATGAAAGACGAGCTCGGGTTGCTTCATCCGGCGTTTAAGGCGGT

RI-AT4G02500-XLOC_020260-4302-0
TAAGGTACAAACCAATCAAGTTTGAATCGGTTTTGCGTTTAGGATTTGTG
RI-AT4G02500-XLOC_020260-4302-1
TAAG--
CONSENSUS
TAAG..

RI-AT4G02500-XLOC_020260-4302-0
TTATGTTTCTCGCTATATTTATGTCTTGTGTCTTTGCTTGTTGTTGTACT
RI-AT4G02500-XLOC_020260-4302-1

CONSENSUS
      ....

RI-AT4G02500-XLOC_020260-4302-0
      AAGATGGATGATAAAGTATTGTGCAGAGACATCAGTTAATTCTTTGGTTT
RI-AT4G02500-XLOC_020260-4302-1 ---
ATGGATGATAAAGTATTGTGCAGAGACATCAGTTAATTCTTTGGTTT
CONSENSUS
      ...ATGGATGATAAAGTATTGTGCAGAGACATCAGTTAATTCTTTGGTTT

RI-AT4G02500-XLOC_020260-4302-0
      TACTTTGTCTCTACGTCTAGAGTTCTATTACGAAATCAAAATGTTGTTTT
RI-AT4G02500-XLOC_020260-4302-1
      TACTTTGTCTCTACGTCTAGAGTTCTATTACGAAATCAAAATGTTGTTTT
CONSENSUS
      TACTTTGTCTCTACGTCTAGAGTTCTATTACGAAATCAAAATGTTGTTTT

RI-AT4G02500-XLOC_020260-4302-0
      TCTTCCTTTTTGTTCTTTGTAATCCATTAGAACTTGAGATTTTGTAGCTT
RI-AT4G02500-XLOC_020260-4302-1
      TCTTCCTTTTTGTTCTTTGTAATCCATTAGAACTTGAGATTTTGTAGCTT
CONSENSUS
      TCTTCCTTTTTGTTCTTTGTAATCCATTAGAACTTGAGATTTTGTAGCTT

RI-AT4G02500-XLOC_020260-4302-0
      CATGGATCAATAATCTGTGTTAATTCAAGAACTTTTTTTTAACCTGAT
RI-AT4G02500-XLOC_020260-4302-1
      CATGGATCAATAATCTGTGTTAATTCAAGAACTTTTTTTTAACCTGAT
CONSENSUS
      CATGGATCAATAATCTGTGTTAATTCAAGAACTTTTTTTTAACCTGAT

alignment for event: A3-AT4G39100-XLOC_025034-4450

A3-AT4G39100-XLOC_025034-4450-0
      GTTCTGCAAGTGTGAGATGCCGTATAACCCAGATGACTTGATGGTGCAAT
A3-AT4G39100-XLOC_025034-4450-1
      GTTCTGCAAGTGTGAGATGCCGTATAACCCAGATGACTTGATGGTGCAAT
CONSENSUS
      GTTCTGCAAGTGTGAGATGCCGTATAACCCAGATGACTTGATGGTGCAAT

A3-AT4G39100-XLOC_025034-4450-0
      GCGAGGAGTGTTCTGAGTG--------------------GAACAACAATA
A3-AT4G39100-XLOC_025034-4450-1
      GCGAGGAGTGTTCTGAGTGGTTTCATCCTTCTTGTATAGGAACAACAATA
CONSENSUS
      GCGAGGAGTGTTCTGAGTG....................GAACAACAATA

A3-AT4G39100-XLOC_025034-4450-0
      GAGGAAGCTAAAAAGCCAGATAACTTCTACTGCGAAGAGTGTTCCCCACA
A3-AT4G39100-XLOC_025034-4450-1
      GAGGAAGCTAAAAAGCCAGATAACTTCTACTGCGAAGAGTGTTCCCCACA
CONSENSUS
      GAGGAAGCTAAAAAGCCAGATAACTTCTACTGCGAAGAGTGTTCCCCACA

```
A3-AT4G39100-XLOC_025034-4450-0
      ACAGCAGAATTTGCACAACTCTAATTCAACTTCCAATAACAGAGATGCTA
A3-AT4G39100-XLOC_025034-4450-1
      ACAGCAGAATTTGCACAACTCTAATTCAACTTCCAATAACAGAGATGCTA
CONSENSUS
      ACAGCAGAATTTGCACAACTCTAATTCAACTTCCAATAACAGAGATGCTA

A3-AT4G39100-XLOC_025034-4450-0  AG
A3-AT4G39100-XLOC_025034-4450-1  AG
CONSENSUS                        AG



alignment for event: A3-AT4G34900-XLOC_024804-12748

A3-AT4G34900-XLOC_024804-12748-0
      GTTCCTAATGCGTCACCTACTGCTGCTTCTGCGAGTTCTGATATGTATGG
A3-AT4G34900-XLOC_024804-12748-1
      GTTCCTAATGCGTCACCTACTGCTGCTTCTGCGAGTTCTGATATGTATGG
CONSENSUS
      GTTCCTAATGCGTCACCTACTGCTGCTTCTGCGAGTTCTGATATGTATGG

A3-AT4G34900-XLOC_024804-12748-0
      TGCTGCAGTTTTAGACGCTTGTGAGCAGATTATAGCAAGAATGGAGCCTG
A3-AT4G34900-XLOC_024804-12748-1
      TGCTGCAGTTTTAGACGCTTGTGAGCAGATTATAGCAAGAATGGAGCCTG
CONSENSUS
      TGCTGCAGTTTTAGACGCTTGTGAGCAGATTATAGCAAGAATGGAGCCTG

A3-AT4G34900-XLOC_024804-12748-0
      TTGCATCTAAACACAATTTCAACACATTCTCTGAG---------------
A3-AT4G34900-XLOC_024804-12748-1
      TTGCATCTAAACACAATTTCAACACATTCTCTGAGCTAGCAAGTGCCTGC
CONSENSUS
      TTGCATCTAAACACAATTTCAACACATTCTCTGAG...............

A3-AT4G34900-XLOC_024804-12748-0
      -------------------------------------------------A
A3-AT4G34900-XLOC_024804-12748-1
      TACTTTCAACGTATAGACCTATCAGCTCACGGTTTTCACATTGTTCCAGA
CONSENSUS
      .................................................A

A3-AT4G34900-XLOC_024804-12748-0
      ACTTGAATTTGATTGGGTATCTGGAAAAGGGAACGCATATAGATATTACA
A3-AT4G34900-XLOC_024804-12748-1
      ACTTGAATTTGATTGGGTATCTGGAAAAGGGAACGCATATAGATATTACA
CONSENSUS
      ACTTGAATTTGATTGGGTATCTGGAAAAGGGAACGCATATAGATATTACA

A3-AT4G34900-XLOC_024804-12748-0
      CATATGGAGCTGCCTTTGCTGAAGTTGAGATAGATACATTGACTGGTGAT
A3-AT4G34900-XLOC_024804-12748-1
      CATATGGAGCTGCCTTTGCTGAAGTTGAGATAGATACATTGACTGGTGAT
CONSENSUS
      CATATGGAGCTGCCTTTGCTGAAGTTGAGATAGATACATTGACTGGTGAT
```

A3-AT4G34900-XLOC_024804-12748-0
      TTTCACACAAGAAAAGCAGACATAATGTTGGATCTCGGATATTCTCTTAA
A3-AT4G34900-XLOC_024804-12748-1
      TTTCACACAAGAAAAGCAGACATAATGTTGGATCTCGGATATTCTCTTAA
CONSENSUS
      TTTCACACAAGAAAAGCAGACATAATGTTGGATCTCGGATATTCTCTTAA

A3-AT4G34900-XLOC_024804-12748-0 CCCAACCATTGATATTGGACAA
A3-AT4G34900-XLOC_024804-12748-1 CCCAACCATTGATATTGGACAA
CONSENSUS   CCCAACCATTGATATTGGACAA

alignment for event: A3-AT4G02260-XLOC_020246-3506

A3-AT4G02260-XLOC_020246-3506-0
      CATCTACAAAGCTGCTCTCAAATCGAAAGGATCAATTAATGATTACAACC
A3-AT4G02260-XLOC_020246-3506-1
      CATCTACAAAGCTGCTCTCAAATCGAAAGGATCAATTAATGATTACAACC
CONSENSUS
      CATCTACAAAGCTGCTCTCAAATCGAAAGGATCAATTAATGATTACAACC

A3-AT4G02260-XLOC_020246-3506-0
      AGATTGCTCAGCAGTTACGGATTGTTGTAAAGCCAAAACCATCTGTAGGG
A3-AT4G02260-XLOC_020246-3506-1 AGATTGCTCAG---
TTACGGATTGTTGTAAAGCCAAAACCATCTGTAGGG
CONSENSUS
      AGATTGCTCAG...TTACGGATTGTTGTAAAGCCAAAACCATCTGTAGGG

A3-AT4G02260-XLOC_020246-3506-0 GTCGGGCCTTTGTGCAGTCCACAACAG
A3-AT4G02260-XLOC_020246-3506-1 GTCGGGCCTTTGTGCAGTCCACAACAG
CONSENSUS   GTCGGGCCTTTGTGCAGTCCACAACAG

alignment for event: RI-AT4G10970-XLOC_023413-13721

RI-AT4G10970-XLOC_023413-13721-0
      GGTGCTTTTGCTAAGAAGAGGTCTAATTTCCAAGGAAACCAGTTTCCTGT
RI-AT4G10970-XLOC_023413-13721-1
      GGTGCTTTTGCTAAGAAGAGGTCTAATTTCCAAGGAAACCAGTTTCCTGT
CONSENSUS
      GGTGCTTTTGCTAAGAAGAGGTCTAATTTCCAAGGAAACCAGTTTCCTGT

RI-AT4G10970-XLOC_023413-13721-0
      AACAACAACCGTTGCTCGTAAAGCCGCTTCTGCTACTCCGCGTGGTAGAC
RI-AT4G10970-XLOC_023413-13721-1
      AACAACAACCGTTGCTCGTAAAGCCGCTTCTGCTACTCCGCGTGGTAGAC
CONSENSUS
      AACAACAACCGTTGCTCGTAAAGCCGCTTCTGCTACTCCGCGTGGTAGAC

RI-AT4G10970-XLOC_023413-13721-0
      CTTATAATGGTGGAAGGATGACTAATACGAATCAATCAAGGTCTAGATTG
RI-AT4G10970-XLOC_023413-13721-1
      CTTATAATGGTGGAAGGATGACTAATACGAATCAATCAAG----------

CONSENSUS
      CTTATAATGGTGGAAGGATGACTAATACGAATCAATCAAG..........

RI-AT4G10970-XLOC_023413-13721-0
      CTACAGATAATTCTCTGATCTGTTTGTTAACATGGGCTCTTTTTATGGTC
RI-AT4G10970-XLOC_023413-13721-1

CONSENSUS
      ....

RI-AT4G10970-XLOC_023413-13721-0
      TTCGATTATTTCTGCTACTAGGAGAAGCTTCTAAGCTGCTTATCATAGGG
RI-AT4G10970-XLOC_023413-13721-1

CONSENSUS
      ....

RI-AT4G10970-XLOC_023413-13721-0
      TGTGTCTATGGAAGGAAACACGGGTTTGTATACTTGTGTTTCAAACCCGT
RI-AT4G10970-XLOC_023413-13721-1

CONSENSUS
      ....

RI-AT4G10970-XLOC_023413-13721-0
      GTTTCAAACATAGTAATTCCCTTCCTGGATGATGGATGCTCATCAGATTG
RI-AT4G10970-XLOC_023413-13721-1

CONSENSUS
      ....

RI-AT4G10970-XLOC_023413-13721-0
      ATGACTGTATAACCCTGGCTCAAAATAGCGAGTCAAAACCATCTAGTTGA
RI-AT4G10970-XLOC_023413-13721-1

CONSENSUS
      ....

RI-AT4G10970-XLOC_023413-13721-0
      GTTTAAGGAGGATATTTGGAGGAGGAGTTAAACTTTAATGGGGTGGTAGG
RI-AT4G10970-XLOC_023413-13721-1

CONSENSUS
      ....

RI-AT4G10970-XLOC_023413-13721-0
      TTTGACATAAAGGAAGGAGTTTAAGCTCATTCAGATTGAGGAGAGACTTT
RI-AT4G10970-XLOC_023413-13721-1

CONSENSUS
      ....

RI-AT4G10970-XLOC_023413-13721-0
      TCTGGTCATTGGTTTTGGAGTTTGCTTGAAGTCAGTTTTAATTATCAGAT
RI-AT4G10970-XLOC_023413-13721-1

CONSENSUS
....

RI-AT4G10970-XLOC_023413-13721-0
TTAGCCAATTGGTGCAATATGTTATCGTGCTTAATTCTTAAAACTCAGTT
RI-AT4G10970-XLOC_023413-13721-1

CONSENSUS
....

RI-AT4G10970-XLOC_023413-13721-0
GCATTCATGTCAGCTTCCTAAATGTTGAGTTCTGTCTTCTTGTTGTGACC
RI-AT4G10970-XLOC_023413-13721-1

CONSENSUS
....

RI-AT4G10970-XLOC_023413-13721-0
TAAAGAGTATAGCTTGAGTCAGTTGGTCTATTGTTGGGAGATTGAAATGG
RI-AT4G10970-XLOC_023413-13721-1

CONSENSUS
....

RI-AT4G10970-XLOC_023413-13721-0
GTAGATGCACGGTTACTAAGACAGCATGAGGATTCAGATGCTCAGATCTT
RI-AT4G10970-XLOC_023413-13721-1

CONSENSUS
....

RI-AT4G10970-XLOC_023413-13721-0
CATGATTGAACATGCGACGGATCATTCTTCGAGCTGTCTGCTCTTAATTC
RI-AT4G10970-XLOC_023413-13721-1

CONSENSUS
....

RI-AT4G10970-XLOC_023413-13721-0
CTGATAAAGTCTTAAAGAAGTTGCATTGTCCGGACTCTTGACCAATTAAC
RI-AT4G10970-XLOC_023413-13721-1

CONSENSUS
....

RI-AT4G10970-XLOC_023413-13721-0
TACTAAATCATTTGGAAAATGCAAGAATTGTCCTTTTTTCGTGTAGCTAC
RI-AT4G10970-XLOC_023413-13721-1

CONSENSUS
....

RI-AT4G10970-XLOC_023413-13721-0
CGGGAAACTGGAGTTAGTGTCTTAGTATTCTTTCATTCAATCCTGCATGA
RI-AT4G10970-XLOC_023413-13721-1

CONSENSUS
      ....

RI-AT4G10970-XLOC_023413-13721-0
      GCCGGCTACATACTTTTTGTGCACTGAGGGCATTAGGATCATGAAGTGTT
RI-AT4G10970-XLOC_023413-13721-1

CONSENSUS
      ....

RI-AT4G10970-XLOC_023413-13721-0
      TCTTTTTCTATTATCAAAGGCTCTTTCTTTAACCTGTTAGTGGCTTTCTT
RI-AT4G10970-XLOC_023413-13721-1

CONSENSUS
      ....

RI-AT4G10970-XLOC_023413-13721-0
      CTTAGATCCTTCTATGGTGGTAAGAAGTTAAATGTTTAATATCAAGAACT
RI-AT4G10970-XLOC_023413-13721-1

CONSENSUS
      ....

RI-AT4G10970-XLOC_023413-13721-0
      TGGTATCAGAACGATGATCTATTGGTGCAAATTCTCAGCTGATATACTCT
RI-AT4G10970-XLOC_023413-13721-1

CONSENSUS
      ....

RI-AT4G10970-XLOC_023413-13721-0
      AGGCTCCTGATGTTCTTTATAAGTGGCTTGTTCCTGCAGATCTTCATCAG
RI-AT4G10970-XLOC_023413-13721-1

CONSENSUS
      ....

RI-AT4G10970-XLOC_023413-13721-0
      TTTTTCAGCTTTTTTTTTCTTTCTGTGCAGGTTTATTGCTCCACCAGCTC
RI-AT4G10970-XLOC_023413-13721-1 ------------------------------
GTTTATTGCTCCACCAGCTC
CONSENSUS
      ............................GTTTATTGCTCCACCAGCTC

RI-AT4G10970-XLOC_023413-13721-0
      AGAATAGAGCTTCACAAAGAGGGTTTGTCGGAAAG
RI-AT4G10970-XLOC_023413-13721-1
      AGAATAGAGCTTCACAAAGAGGGTTTGTCGGAAAG
CONSENSUS
      AGAATAGAGCTTCACAAAGAGGGTTTGTCGGAAAG

alignment for event: RI-AT4G11830-XLOC_023455-10223

RI-AT4G11830-XLOC_023455-10223-0
TTCTTTCGAGCCTCGAGTTTCGTATCGTGGTGTTGCTGCTGGAATAGACG
RI-AT4G11830-XLOC_023455-10223-1
TTCTTTCGAGCCTCGAGTTTCGTATCGTGGTGTTGCTGCTGGAATAGACG
CONSENSUS
TTCTTTCGAGCCTCGAGTTTCGTATCGTGGTGTTGCTGCTGGAATAGACG

RI-AT4G11830-XLOC_023455-10223-0
ATGATTACCTTGGTGCTATACGGAAAATGTCTGCGGATGGAAACTTCTAA
RI-AT4G11830-XLOC_023455-10223-1
ATGATTACCTTGGTGCTATACGGAAAATGTCTGCGGATGGAAACTTCTAA
CONSENSUS
ATGATTACCTTGGTGCTATACGGAAAATGTCTGCGGATGGAAACTTCTAA

RI-AT4G11830-XLOC_023455-10223-0
AGGGTAACATCATCTTTAATATGATTTCATTTAAAGAAAATGTCATGCTT
RI-AT4G11830-XLOC_023455-10223-1
AGGGTAACATCATCTTTAATATGATTTCATTTAAAGAAAATGTCATGCTT
CONSENSUS
AGGGTAACATCATCTTTAATATGATTTCATTTAAAGAAAATGTCATGCTT

RI-AT4G11830-XLOC_023455-10223-0
TATTTATTAATTACACTACTAAATGATATAATTTAGTGATTATGTAGAAT
RI-AT4G11830-XLOC_023455-10223-1
TATTTATTAATTACACTACTAAATGATATAATTTAGTGATTATGTAGAAT
CONSENSUS
TATTTATTAATTACACTACTAAATGATATAATTTAGTGATTATGTAGAAT

RI-AT4G11830-XLOC_023455-10223-0
GTTTAATTTCTTGTTGATATATAAGAATAGTAATATCACCAATCAAAATG
RI-AT4G11830-XLOC_023455-10223-1
GTTTAATTTCTTGTTGATATATAAGAATAGTAATATCACCAATCAAAATG
CONSENSUS
GTTTAATTTCTTGTTGATATATAAGAATAGTAATATCACCAATCAAAATG

RI-AT4G11830-XLOC_023455-10223-0
AAGTTGCATCAAAAAAGGGCCAGAGAGAAAGAGCTTTGTGAGCAGCCAAA
RI-AT4G11830-XLOC_023455-10223-1
AAGTTGCATCAAAAAAGGGCCAGAGAGAAAGAGCTTTGTGAGCAGCCAAA
CONSENSUS
AAGTTGCATCAAAAAAGGGCCAGAGAGAAAGAGCTTTGTGAGCAGCCAAA

RI-AT4G11830-XLOC_023455-10223-0
AACCAAAGTGGGCAAGTCTTCTTTCTTAGTCGTCGTTACTAAAACACAAA
RI-AT4G11830-XLOC_023455-10223-1
AACCAAAGTGGGCAAGTCTTCTTTCTTAGTCGTCGTTACTAAAACACAAA
CONSENSUS
AACCAAAGTGGGCAAGTCTTCTTTCTTAGTCGTCGTTACTAAAACACAAA

RI-AT4G11830-XLOC_023455-10223-0
TTGTTTATTCGGCATTTGTGTTTAATTAAGTTCTTTAATTAATGATTAAA
RI-AT4G11830-XLOC_023455-10223-1
TTGTTTATTCGGCATTTGTGTTTAATTAAGTTCTTTAATTAATGATTAAA
CONSENSUS
TTGTTTATTCGGCATTTGTGTTTAATTAAGTTCTTTAATTAATGATTAAA

RI-AT4G11830-XLOC_023455-10223-0
GATCTTAAATTTGTGTTATTAATGGCTAAAGTTAAACGATTCAACAACGG
RI-AT4G11830-XLOC_023455-10223-1
GATCTTAAATTTGTGTTATTAATGGCTAAAGTTAAACGATTCAACAACGG
CONSENSUS
GATCTTAAATTTGTGTTATTAATGGCTAAAGTTAAACGATTCAACAACGG

RI-AT4G11830-XLOC_023455-10223-0
CATCCAAAATCTTTAAATTTTTCCGATTCTTCCGTTGCAAGTCAACGTTT
RI-AT4G11830-XLOC_023455-10223-1
CATCCAAAATCTTTAAATTTTTCCGATTCTTCCGTTGCAAGTCAACGTTT
CONSENSUS
CATCCAAAATCTTTAAATTTTTCCGATTCTTCCGTTGCAAGTCAACGTTT

RI-AT4G11830-XLOC_023455-10223-0
TCCTTCGCTTCCTCCTACCTTCTTCTTCTTCTTCACAAAAATCTGAACTT
RI-AT4G11830-XLOC_023455-10223-1
TCCTTCGCTTCCTCCTACCTTCTTCTTCTTCTTCACAAAAATCTGAACTT
CONSENSUS
TCCTTCGCTTCCTCCTACCTTCTTCTTCTTCTTCACAAAAATCTGAACTT

RI-AT4G11830-XLOC_023455-10223-0
TAAATTTGAATTTGCCCATATTTTTAGACCCACATTTATTCATATAGATT
RI-AT4G11830-XLOC_023455-10223-1
TAAATTTGAATTTGCCCATATTTTTAGACCCACATTTATTCATATAGATT
CONSENSUS
TAAATTTGAATTTGCCCATATTTTTAGACCCACATTTATTCATATAGATT

RI-AT4G11830-XLOC_023455-10223-0
TGATCTGAGTTTGTATTATCTCAAAGTTTTATCCTTTTGTTGAATCTTGA
RI-AT4G11830-XLOC_023455-10223-1
TGATCTGAGTTTGTATTATCTCAAAGTTTTATCCTTTTGTTGAATCTTGA
CONSENSUS
TGATCTGAGTTTGTATTATCTCAAAGTTTTATCCTTTTGTTGAATCTTGA

RI-AT4G11830-XLOC_023455-10223-0
TCTTTCGATTAGTCCCCACCAAGCACCAAGTGATCGATCTTTTGTCTCAC
RI-AT4G11830-XLOC_023455-10223-1
TCTTTCGATTAGTCCCCACCAAGCACCAAGTGATCGATCTTTTGTCTCAC
CONSENSUS
TCTTTCGATTAGTCCCCACCAAGCACCAAGTGATCGATCTTTTGTCTCAC

RI-AT4G11830-XLOC_023455-10223-0
TGAACTTGGTTATGCTTGAAATTTGATCTGACAACTAAAAGTTTCCTGCT
RI-AT4G11830-XLOC_023455-10223-1
TGAACTTGGTTATGCTTGAAATTTGATCTGACAACTAAAAGTTTCCTGCT
CONSENSUS
TGAACTTGGTTATGCTTGAAATTTGATCTGACAACTAAAAGTTTCCTGCT

RI-AT4G11830-XLOC_023455-10223-0
TTGGAGGTTTTTGATTTTGTATCATCCAGTTTATAACGAGACTATGTCAA
RI-AT4G11830-XLOC_023455-10223-1
TTGGAGGTTTTTGATTTTGTATCATCCAGTTTATAACGAGACTATGTCAA
CONSENSUS
TTGGAGGTTTTTGATTTTGTATCATCCAGTTTATAACGAGACTATGTCAA

RI-AT4G11830-XLOC_023455-10223-0
TGGGAGGAGGGTCAAACCACGAGTTTGGCCAGTGGCTTGACCAGCAACTC
RI-AT4G11830-XLOC_023455-10223-1
TGGGAGGAGGGTCAAACCACGAGTTTGGCCAGTGGCTTGACCAGCAACTC
CONSENSUS
TGGGAGGAGGGTCAAACCACGAGTTTGGCCAGTGGCTTGACCAGCAACTC

RI-AT4G11830-XLOC_023455-10223-0
GTTCCGTTAGCTACGAGTAGTGGCTCTTTGATGGTTGAATTGTTACATGG
RI-AT4G11830-XLOC_023455-10223-1
GTTCCGTTAGCTACGAGTAGTGGCTCTTTGATGGTTGAATTGTTACATG-
CONSENSUS
GTTCCGTTAGCTACGAGTAGTGGCTCTTTGATGGTTGAATTGTTACATG.

RI-AT4G11830-XLOC_023455-10223-0
TAACTTAGACATTTGGGTTAAGGAAGCTAAACATCTTCCTAACATGATAT
RI-AT4G11830-XLOC_023455-10223-1

CONSENSUS
....

RI-AT4G11830-XLOC_023455-10223-0
GTTACCGTAACAAGCTTGTTGGTGGGATTTCGTTTTCTGAGTTAGGTCGG
RI-AT4G11830-XLOC_023455-10223-1
---GTCGG
CONSENSUS
....GTCGG

RI-AT4G11830-XLOC_023455-10223-0
AGGATTCGTAAAGTGGATGGTGAGAAGTCTTCTAAGTTCACAAGTGATCC
RI-AT4G11830-XLOC_023455-10223-1
AGGATTCGTAAAGTGGATGGTGAGAAGTCTTCTAAGTTCACAAGTGATCC
CONSENSUS
AGGATTCGTAAAGTGGATGGTGAGAAGTCTTCTAAGTTCACAAGTGATCC

RI-AT4G11830-XLOC_023455-10223-0
TTATGTTACTGTCTCTATCTCTGGTGCTGTCATTGGTAGAACTTTTGTTA
RI-AT4G11830-XLOC_023455-10223-1
TTATGTTACTGTCTCTATCTCTGGTGCTGTCATTGGTAGAACTTTTGTTA
CONSENSUS
TTATGTTACTGTCTCTATCTCTGGTGCTGTCATTGGTAGAACTTTTGTTA

RI-AT4G11830-XLOC_023455-10223-0
TTAGCAATAGTGAGAATCCTGTGTGGATGCAGCATTTCGATGTACCCGTT
RI-AT4G11830-XLOC_023455-10223-1
TTAGCAATAGTGAGAATCCTGTGTGGATGCAGCATTTCGATGTACCCGTT
CONSENSUS
TTAGCAATAGTGAGAATCCTGTGTGGATGCAGCATTTCGATGTACCCGTT

RI-AT4G11830-XLOC_023455-10223-0
GCTCATAGTGCTGCTGAAGTACATTTTGTTGTGAAAGACAATGATCCTAT
RI-AT4G11830-XLOC_023455-10223-1
GCTCATAGTGCTGCTGAAGTACATTTTGTTGTGAAAGACAATGATCCTAT
CONSENSUS
GCTCATAGTGCTGCTGAAGTACATTTTGTTGTGAAAGACAATGATCCTAT

RI-AT4G11830-XLOC_023455-10223-0
TGGATCAAAGATCATAGGAGTTGTTGGAATACCAACCAAGCAGTTGTGTT
RI-AT4G11830-XLOC_023455-10223-1
TGGATCAAAGATCATAGGAGTTGTTGGAATACCAACCAAGCAGTTGTGTT
CONSENSUS
TGGATCAAAGATCATAGGAGTTGTTGGAATACCAACCAAGCAGTTGTGTT

RI-AT4G11830-XLOC_023455-10223-0
CCGGGAATAGAATCGAAGGGCTGTTTCCGATACTTAACAGTAGTGGGAAG
RI-AT4G11830-XLOC_023455-10223-1
CCGGGAATAGAATCGAAGGGCTGTTTCCGATACTTAACAGTAGTGGGAAG
CONSENSUS
CCGGGAATAGAATCGAAGGGCTGTTTCCGATACTTAACAGTAGTGGGAAG

RI-AT4G11830-XLOC_023455-10223-0
CCTTGTAGAAAAGGTGCTATGTTGAGTCTGTCTATTCAGTATACTCCAAT
RI-AT4G11830-XLOC_023455-10223-1
CCTTGTAGAAAAGGTGCTATGTTGAGTCTGTCTATTCAGTATACTCCAAT
CONSENSUS
CCTTGTAGAAAAGGTGCTATGTTGAGTCTGTCTATTCAGTATACTCCAAT

RI-AT4G11830-XLOC_023455-10223-0
GGAAAGAATGAGACTTTACCAAAAGGGTGTTGGTTCTGGTGTTGAGTGTG
RI-AT4G11830-XLOC_023455-10223-1
GGAAAGAATGAGACTTTACCAAAAGGGTGTTGGTTCTGGTGTTGAGTGTG
CONSENSUS
GGAAAGAATGAGACTTTACCAAAAGGGTGTTGGTTCTGGTGTTGAGTGTG

RI-AT4G11830-XLOC_023455-10223-0
TAGGAGTTCCCGGTACATACTTCCCTTTGAGGAAAGGCGGTAGGGTTACT
RI-AT4G11830-XLOC_023455-10223-1
TAGGAGTTCCCGGTACATACTTCCCTTTGAGGAAAGGCGGTAGGGTTACT
CONSENSUS
TAGGAGTTCCCGGTACATACTTCCCTTTGAGGAAAGGCGGTAGGGTTACT

RI-AT4G11830-XLOC_023455-10223-0
CTTTATCAGGATGCTCATGTCGATGACGGTACTCTTCCGAGTGTACATCT
RI-AT4G11830-XLOC_023455-10223-1
CTTTATCAGGATGCTCATGTCGATGACGGTACTCTTCCGAGTGTACATCT
CONSENSUS
CTTTATCAGGATGCTCATGTCGATGACGGTACTCTTCCGAGTGTACATCT

RI-AT4G11830-XLOC_023455-10223-0
TGATGGTGGGATTCAGTATAGACATGGAAAATGCTGGGAGGATATGGCTG
RI-AT4G11830-XLOC_023455-10223-1
TGATGGTGGGATTCAGTATAGACATGGAAAATGCTGGGAGGATATGGCTG
CONSENSUS
TGATGGTGGGATTCAGTATAGACATGGAAAATGCTGGGAGGATATGGCTG

RI-AT4G11830-XLOC_023455-10223-0
ATGCGATACGACGGGCAAGGAGGCTGATTTATATCACAGGTTGGTCAGTT
RI-AT4G11830-XLOC_023455-10223-1
ATGCGATACGACGGGCAAGGAGGCTGATTTATATCACAGGTTGGTCAGTT
CONSENSUS
ATGCGATACGACGGGCAAGGAGGCTGATTTATATCACAGGTTGGTCAGTT

RI-AT4G11830-XLOC_023455-10223-0
TTCCATCCGGTTAGGCTGGTTCGTCGTAACAATGATCCGACCGAAGGTAC
RI-AT4G11830-XLOC_023455-10223-1
TTCCATCCGGTTAGGCTGGTTCGTCGTAACAATGATCCGACCGAAGGTAC
CONSENSUS
TTCCATCCGGTTAGGCTGGTTCGTCGTAACAATGATCCGACCGAAGGTAC

RI-AT4G11830-XLOC_023455-10223-0
ATTAGGGGAGTTACTTAAAGTCAAATCTCAAGAAGGTGTTAGAGTGTTGG
RI-AT4G11830-XLOC_023455-10223-1
ATTAGGGGAGTTACTTAAAGTCAAATCTCAAGAAGGTGTTAGAGTGTTGG
CONSENSUS
ATTAGGGGAGTTACTTAAAGTCAAATCTCAAGAAGGTGTTAGAGTGTTGG

RI-AT4G11830-XLOC_023455-10223-0
TTTTGGTGTGGGATGATCCAACTTCAATGAGTTTTCCGGGATTCAGTACA
RI-AT4G11830-XLOC_023455-10223-1
TTTTGGTGTGGGATGATCCAACTTCAATGAGTTTTCCGGGATTCAGTACA
CONSENSUS
TTTTGGTGTGGGATGATCCAACTTCAATGAGTTTTCCGGGATTCAGTACA

alignment for event: A3-AT4G23710-XLOC_021682-11866

A3-AT4G23710-XLOC_021682-11866-0
ATTCATTCTTCTGTATTCAATAATTTTTAAGTTTTTCTCCGGTCTTCTCT
A3-AT4G23710-XLOC_021682-11866-1
ATTCATTCTTCTGTATTCAATAATTTTTAAGTTTTTCTCCGGTCTTCTCT
CONSENSUS
ATTCATTCTTCTGTATTCAATAATTTTTAAGTTTTTCTCCGGTCTTCTCT

A3-AT4G23710-XLOC_021682-11866-0
GTCTCTCTGTCCATTTTCGTGGCGAAGAAGATTTTTTAAGGTCTTTTCAA
A3-AT4G23710-XLOC_021682-11866-1
GTCTCTCTGTCCATTTTCGTGGCGAAGAAG----------GTCTTTTCAA
CONSENSUS
GTCTCTCTGTCCATTTTCGTGGCGAAGAAG..........GTCTTTTCAA

A3-AT4G23710-XLOC_021682-11866-0
GAAATTATGGAATCTGCAGGGATCCAGCAACTGCTTGCTGCTGAACGTGA
A3-AT4G23710-XLOC_021682-11866-1
GAAATTATGGAATCTGCAGGGATCCAGCAACTGCTTGCTGCTGAACGTGA
CONSENSUS
GAAATTATGGAATCTGCAGGGATCCAGCAACTGCTTGCTGCTGAACGTGA

A3-AT4G23710-XLOC_021682-11866-0
AGCTCAGCAAATTGTCAATGCCGCTAGGACCG
A3-AT4G23710-XLOC_021682-11866-1
AGCTCAGCAAATTGTCAATGCCGCTAGGACCG
CONSENSUS
AGCTCAGCAAATTGTCAATGCCGCTAGGACCG

alignment for event: A3-AT4G32330-XLOC_022179-11969

A3-AT4G32330-XLOC_022179-11969-0
      GGAGAAGGTGAAACCAAAGTCTCAAAAGAAACAAGCCCATGAGACATCTG
A3-AT4G32330-XLOC_022179-11969-1
      GGAGAAGGTGAAACCAAAGTCTCAAAAGAAACAAGCCCATGAGACATCTG
CONSENSUS
      GGAGAAGGTGAAACCAAAGTCTCAAAAGAAACAAGCCCATGAGACATCTG

A3-AT4G32330-XLOC_022179-11969-0
      AAGATGATACTCAGTCTTCTAATAGTCCGAAAGCAGACGATGGAAAACCT
A3-AT4G32330-XLOC_022179-11969-1 AAGATGATACTCAGTCTTCTAA---
TCCGAAAGCAGACGATGGAAAACCT
CONSENSUS
      AAGATGATACTCAGTCTTCTAA...TCCGAAAGCAGACGATGGAAAACCT

A3-AT4G32330-XLOC_022179-11969-0
      CGTAAAGTTGGTGCACTTCCAAATTATGGATTCAGTTTCAAATGTGACCA
A3-AT4G32330-XLOC_022179-11969-1
      CGTAAAGTTGGTGCACTTCCAAATTATGGATTCAGTTTCAAATGTGACCA
CONSENSUS
      CGTAAAGTTGGTGCACTTCCAAATTATGGATTCAGTTTCAAATGTGACCA

A3-AT4G32330-XLOC_022179-11969-0 ACGGGCTGAAAAGAGAAAAGAG
A3-AT4G32330-XLOC_022179-11969-1 ACGGGCTGAAAAGAGAAAAGAG
CONSENSUS   ACGGGCTGAAAAGAGAAAAGAG

alignment for event: SE-AT4G27620-XLOC_024406-7352

SE-AT4G27620-XLOC_024406-7352-0
      GAAGAAAAAAAAAAAGAAGCTTCAACGGCCTTTGAGTAGTTGTTTCTGCG
SE-AT4G27620-XLOC_024406-7352-1
      GAAGAAAAAAAAAAAGAAGCTTCAACGGCCTTTGAGTAGTTGTTTCTGCG
CONSENSUS
      GAAGAAAAAAAAAAAGAAGCTTCAACGGCCTTTGAGTAGTTGTTTCTGCG

SE-AT4G27620-XLOC_024406-7352-0
      CCGCGCTGTCTCTCTAAAACAGCCTG-----------------------
SE-AT4G27620-XLOC_024406-7352-1
      CCGCGCTGTCTCTCTAAAACAGCCTGCAACCCTAATTTCTGGGAGAATCG
CONSENSUS
      CCGCGCTGTCTCTCTAAAACAGCCTG........................

SE-AT4G27620-XLOC_024406-7352-0 ----------------------------
TGTTAAGGTTGGATGATAGAAG
SE-AT4G27620-XLOC_024406-7352-1
      TCCCTGCAGTATTACCCATCCCGATGAGTGTTAAGGTTGGATGATAGAAG
CONSENSUS
      ..........................TGTTAAGGTTGGATGATAGAAG

SE-AT4G27620-XLOC_024406-7352-0
      ACATTTTTTGACCTTCTCTTACAATGACTACACGAAGAAATGGCGTTTCC
SE-AT4G27620-XLOC_024406-7352-1
      ACATTTTTTGACCTTCTCTTACAATGACTACACGAAGAAATGGCGTTTCC
CONSENSUS

ACATTTTTTGACCTTCTCTTACAATGACTACACGAAGAAATGGCGTTTCC

SE-AT4G27620-XLOC_024406-7352-0
AGGCATCAGCGGTTTGAGAGTTTTCGCGGTGAAGGACCAAATTGGATCTT
SE-AT4G27620-XLOC_024406-7352-1
AGGCATCAGCGGTTTGAGAGTTTTCGCGGTGAAGGACCAAATTGGATCTT
CONSENSUS
AGGCATCAGCGGTTTGAGAGTTTTCGCGGTGAAGGACCAAATTGGATCTT

SE-AT4G27620-XLOC_024406-7352-0
AATTGCTGGGGGTGCTTTGTTGAGTACATTGTCCATTCGTTTTGGCTACA
SE-AT4G27620-XLOC_024406-7352-1
AATTGCTGGGGGTGCTTTGTTGAGTACATTGTCCATTCGTTTTGGCTACA
CONSENSUS
AATTGCTGGGGGTGCTTTGTTGAGTACATTGTCCATTCGTTTTGGCTACA

SE-AT4G27620-XLOC_024406-7352-0
AATTAAAGCAGTCTATTGACTCCAAACCTCCTCATTCCAATGCCACTGGT
SE-AT4G27620-XLOC_024406-7352-1
AATTAAAGCAGTCTATTGACTCCAAACCTCCTCATTCCAATGCCACTGGT
CONSENSUS
AATTAAAGCAGTCTATTGACTCCAAACCTCCTCATTCCAATGCCACTGGT

SE-AT4G27620-XLOC_024406-7352-0 GGATTAAAAC
SE-AT4G27620-XLOC_024406-7352-1 GGATTAAAAC
CONSENSUS GGATTAAAAC

alignment for event: RI-AT4G34650-XLOC_022311-7590

RI-AT4G34650-XLOC_022311-7590-0
GTGGTACAAAAGAGTATAAACTTCTAATGGACCAATTTCACCATGTTTCT
RI-AT4G34650-XLOC_022311-7590-1
GTGGTACAAAAGAGTATAAACTTCTAATGGACCAATTTCACCATGTTTCT
CONSENSUS
GTGGTACAAAAGAGTATAAACTTCTAATGGACCAATTTCACCATGTTTCT

RI-AT4G34650-XLOC_022311-7590-0
GCAGCTTTTCTGAAACTTGAAAAAGGGTTAATTTGCTATATACAAATTCT
RI-AT4G34650-XLOC_022311-7590-1
GCAGCTTTTCTGAAACTTGAAAAAGG------------------------
CONSENSUS
GCAGCTTTTCTGAAACTTGAAAAAGG........................

RI-AT4G34650-XLOC_022311-7590-0
GTTCTGTTTTTTATAAATGTTCTTGCTCATTTATTGATTGATCTACTGAA
RI-AT4G34650-XLOC_022311-7590-1

CONSENSUS
....

RI-AT4G34650-XLOC_022311-7590-0
TTTGGTGATACTAATTTTATGTTTTGTTTTACTAACTTATATATACAAAA
RI-AT4G34650-XLOC_022311-7590-1

```
CONSENSUS
      ..................................................

RI-AT4G34650-XLOC_022311-7590-0
      AAGGTATCAAGAGGCTATTGAAGATATAACTAAAAGAATGGGTGCAGGAA
RI-AT4G34650-XLOC_022311-7590-1  ---
GTATCAAGAGGCTATTGAAGATATAACTAAAAGAATGGGTGCAGGAA
CONSENSUS
      ...GTATCAAGAGGCTATTGAAGATATAACTAAAAGAATGGGTGCAGGAA

RI-AT4G34650-XLOC_022311-7590-0  TGGCCAAGTTCATTTGCAAGGAG
RI-AT4G34650-XLOC_022311-7590-1  TGGCCAAGTTCATTTGCAAGGAG
CONSENSUS                        TGGCCAAGTTCATTTGCAAGGAG



alignment for event: SE-AT4G25500-XLOC_021789-10124

SE-AT4G25500-XLOC_021789-10124-0
      GAAGCATGAAGCCAGTCTTCTGTGGGAACTTTGAGTATGATGCGCGCGAA
SE-AT4G25500-XLOC_021789-10124-1
      GAAGCATGAAGCCAGTCTTCTGTGGGAACTTTGAGTATGATGCGCGCGAA
CONSENSUS
      GAAGCATGAAGCCAGTCTTCTGTGGGAACTTTGAGTATGATGCGCGCGAA

SE-AT4G25500-XLOC_021789-10124-0
      GGTGACCTGGAACGACTATTCAGGAAATACGGCAAGGTTGAGAGGGTTGA
SE-AT4G25500-XLOC_021789-10124-1
      GGTGACCTGGAACGACTATTCAGGAAATACGGCAAGGTTGAGAGGGTTGA
CONSENSUS
      GGTGACCTGGAACGACTATTCAGGAAATACGGCAAGGTTGAGAGGGTTGA

SE-AT4G25500-XLOC_021789-10124-0
      TATGAAAGCTG---------------------------------------
SE-AT4G25500-XLOC_021789-10124-1
      TATGAAAGCTGGATGTGTTTGATAATCTTGGGACGCCTATACTCACACCT
CONSENSUS
      TATGAAAGCTG.......................................

SE-AT4G25500-XLOC_021789-10124-0
      --------------------------------------------------
SE-AT4G25500-XLOC_021789-10124-1
      GGCCATGAATGGACCATAGGGAAATCCATCTCTTATCCCTCAAAATCAGT
CONSENSUS
      ..................................................

SE-AT4G25500-XLOC_021789-10124-0
      --------------------------------------------------
SE-AT4G25500-XLOC_021789-10124-1
      CACTTTCCATCCGCATTAGCACTGCCATCTTAATTGCATTTCATTCCTCA
CONSENSUS
      ..................................................

SE-AT4G25500-XLOC_021789-10124-0
      --------------------------------------------------
SE-AT4G25500-XLOC_021789-10124-1
```

TCACTTTGCACACTTGGACATGCCTCTGCAATGGAGAAGGCTCTCTATTT
CONSENSUS
....

SE-AT4G25500-XLOC_021789-10124-0

SE-AT4G25500-XLOC_021789-10124-1
TCATTCATATCCCCGTCTGACTTCCACATTTTCAGTTGTTCCTTGACATA
CONSENSUS
....

SE-AT4G25500-XLOC_021789-10124-0 ------------------
GGTTTGCTTTTGTATACATGGAAGATGAAAGG
SE-AT4G25500-XLOC_021789-10124-1
TTAATTCCATAATGCAAGGGTTTGCTTTTGTATACATGGAAGATGAAAGG
CONSENSUS
..................GGTTTGCTTTTGTATACATGGAAGATGAAAGG

SE-AT4G25500-XLOC_021789-10124-0
GATGCGGAAGATGCCATCCGAGCACTTGACCGCTTTGAATTTGGGCGTAA
SE-AT4G25500-XLOC_021789-10124-1
GATGCGGAAGATGCCATCCGAGCACTTGACCGCTTTGAATTTGGGCGTAA
CONSENSUS
GATGCGGAAGATGCCATCCGAGCACTTGACCGCTTTGAATTTGGGCGTAA

SE-AT4G25500-XLOC_021789-10124-0
GGGACGCAGACTTCGTGTTGAATGGACAAAG
SE-AT4G25500-XLOC_021789-10124-1
GGGACGCAGACTTCGTGTTGAATGGACAAAG
CONSENSUS
GGGACGCAGACTTCGTGTTGAATGGACAAAG

alignment for event: A3-AT2G45380-XLOC_013402-5511

A3-AT2G45380-XLOC_013402-5511-0
GAGTATTTTGGTCTGAGTGGTTCTTTAGGAGAAAGGATCTTTGATATGGT
A3-AT2G45380-XLOC_013402-5511-1
GAGTATTTTGGTCTGAGTGGTTCTTTAGGAGAAAGGATCTTTGATATGGT
CONSENSUS
GAGTATTTTGGTCTGAGTGGTTCTTTAGGAGAAAGGATCTTTGATATGGT

A3-AT2G45380-XLOC_013402-5511-0
TACTCAACACAGGAAAGATGATAAAATGACTTTTGAAGATCTTGTTATTG
A3-AT2G45380-XLOC_013402-5511-1
TACTCAACACAGGAAAGATGATAAAATGACTTTTGAAGATCTTGTTATTG
CONSENSUS
TACTCAACACAGGAAAGATGATAAAATGACTTTTGAAGATCTTGTTATTG

A3-AT2G45380-XLOC_013402-5511-0
CTAAAATGACTTTTGAAGATCTTGTTATTGCTAAG---------------
A3-AT2G45380-XLOC_013402-5511-1
CTAAAATGACTTTTGAAGATCTTGTTATTGCTAAGAAAATTCGCAAGGAT
CONSENSUS
CTAAAATGACTTTTGAAGATCTTGTTATTGCTAAG...............

A3-AT2G45380-XLOC_013402-5511-0

A3-AT2G45380-XLOC_013402-5511-1
      GAAGAATGATGAAGAAGCTTCTAGGTTATGGTAACATGACACGTGTATCA
CONSENSUS
      ....

A3-AT2G45380-XLOC_013402-5511-0

A3-AT2G45380-XLOC_013402-5511-1
      ATCTCAGACGTCCTTTTTTGTTATAATTGAGGTTATATAAAATCAGAGTG
CONSENSUS
      ....

A3-AT2G45380-XLOC_013402-5511-0 -------------------------
AGTACTCTGCCTCTGGAATTTCTGG
A3-AT2G45380-XLOC_013402-5511-1
      ATGTTATTTTGTCTTTTGAATTAAGAGTACTCTGCCTCTGGAATTTCTGG
CONSENSUS
      .........................AGTACTCTGCCTCTGGAATTTCTGG

A3-AT2G45380-XLOC_013402-5511-0 GTTCTTCTCGGATTCTCTCAAAG
A3-AT2G45380-XLOC_013402-5511-1 GTTCTTCTCGGATTCTCTCAAAG
CONSENSUS   GTTCTTCTCGGATTCTCTCAAAG

alignment for event: A3-AT2G44750-XLOC_010686-9801

A3-AT2G44750-XLOC_010686-9801-0
      GGAACTAAGGTTATAGATGAATCTCATGATCAAGATACCACTGATCTTGA
A3-AT2G44750-XLOC_010686-9801-1
      GGAACTAAGGTTATAGATGAATCTCATGATCAAGATACCACTGATCTTGA
CONSENSUS
      GGAACTAAGGTTATAGATGAATCTCATGATCAAGATACCACTGATCTTGA

A3-AT2G44750-XLOC_010686-9801-0
      TAAATGCATTTCGTATATCCGTCACTCTACTTTGAATCAGGAGAGTTCCA
A3-AT2G44750-XLOC_010686-9801-1
      TAAATGCATTTCGTATATCCGTCACTCTACTTTGAATCAGGAGAGTTCCA
CONSENSUS
      TAAATGCATTTCGTATATCCGTCACTCTACTTTGAATCAGGAGAGTTCCA

A3-AT2G44750-XLOC_010686-9801-0
      GACTCCAGATTCTTGCCACTGGAGCACTCGGGGGAAGATTCGATCATGAA
A3-AT2G44750-XLOC_010686-9801-1 GA------
ATTCTTGCCACTGGAGCACTCGGGGGAAGATTCGATCATGAA
CONSENSUS
      GA......ATTCTTGCCACTGGAGCACTCGGGGGAAGATTCGATCATGAA

A3-AT2G44750-XLOC_010686-9801-0
      GCCGGTAATCTCAACGTCTTATATCGATATCCAGACACAAGGATAGTCCT
A3-AT2G44750-XLOC_010686-9801-1
      GCCGGTAATCTCAACGTCTTATATCGATATCCAGACACAAGGATAGTCCT
CONSENSUS

GCCGGTAATCTCAACGTCTTATATCGATATCCAGACACAAGGATAGTCCT

A3-AT2G44750-XLOC_010686-9801-0
TTTATCTGATGATTGTCTCATCCAACTCCTTCCAAAGACTCATCGACATG
A3-AT2G44750-XLOC_010686-9801-1
TTTATCTGATGATTGTCTCATCCAACTCCTTCCAAAGACTCATCGACATG
CONSENSUS
TTTATCTGATGATTGTCTCATCCAACTCCTTCCAAAGACTCATCGACATG

A3-AT2G44750-XLOC_010686-9801-0
AAATACATATTCACTCTTCTCTTCAAGGACCTCACTGTGGACTTATACCC
A3-AT2G44750-XLOC_010686-9801-1
AAATACATATTCACTCTTCTCTTCAAGGACCTCACTGTGGACTTATACCC
CONSENSUS
AAATACATATTCACTCTTCTCTTCAAGGACCTCACTGTGGACTTATACCC

A3-AT2G44750-XLOC_010686-9801-0
ATTGGAACTCCATCTGCCAATACCACTACCTCAGGGCTTAAATGGGATCT
A3-AT2G44750-XLOC_010686-9801-1
ATTGGAACTCCATCTGCCAATACCACTACCTCAGGGCTTAAATGGGATCT
CONSENSUS
ATTGGAACTCCATCTGCCAATACCACTACCTCAGGGCTTAAATGGGATCT

A3-AT2G44750-XLOC_010686-9801-0 CA
A3-AT2G44750-XLOC_010686-9801-1 CA
CONSENSUS CA

alignment for event: RI-AT2G11240.1-XLOC_011450-10489

RI-AT2G11240.1-XLOC_011450-10489-0
AAATTTTAATGATGCCTTGCTTGCAAAGTTGAGTTGGCGTATTGTGCAAT
RI-AT2G11240.1-XLOC_011450-10489-1
AAATTTTAATGATGCCTTGCTTGCAAAGTTGAGTTGGCGTATTGTGCAAT
CONSENSUS
AAATTTTAATGATGCCTTGCTTGCAAAGTTGAGTTGGCGTATTGTGCAAT

RI-AT2G11240.1-XLOC_011450-10489-0
CTCCATCGTGTGTTTTGGTTAGAATTCTGCTAGGAAAGTATTGTCGAACC
RI-AT2G11240.1-XLOC_011450-10489-1
CTCCATCGTGTGTTTTGGTTAGAATTCTGCTAGGAAAGTATTGTCGAACC
CONSENSUS
CTCCATCGTGTGTTTTGGTTAGAATTCTGCTAGGAAAGTATTGTCGAACC

RI-AT2G11240.1-XLOC_011450-10489-0
TCGTCCTTCCTAGACTGCTCAGTCACTGCTGCTTCTTCTCACGGTTGGAG
RI-AT2G11240.1-XLOC_011450-10489-1
TCGTCCTTCCTAGACTGCTCAGTCACTGCTGCTTCTTCTCACGGTTGGAG
CONSENSUS
TCGTCCTTCCTAGACTGCTCAGTCACTGCTGCTTCTTCTCACGGTTGGAG

RI-AT2G11240.1-XLOC_011450-10489-0
AGGCATTTGTACGGGCAAAGATCTAATCAAATCTCAGCTGGGGAAAGTGA
RI-AT2G11240.1-XLOC_011450-10489-1
AGGCATTTGTACGGGCAAAGATCTAATCAAATCTCAGCTGGGGAAAGTGA

CONSENSUS
      AGGCATTTGTACGGGCAAAGATCTAATCAAATCTCAGCTGGGGAAAGTGA

RI-AT2G11240.1-XLOC_011450-10489-0
      TTGGATCGGGCTTGGATACTTTGGTTTGGAACGAACCGTGGCTATCTCTT
RI-AT2G11240.1-XLOC_011450-10489-1
      TTGGATCGGGCTTGGATACTTTGGTTTGGAACGAACCGTGGCTATCTCTT
CONSENSUS
      TTGGATCGGGCTTGGATACTTTGGTTTGGAACGAACCGTGGCTATCTCTT

RI-AT2G11240.1-XLOC_011450-10489-0
      TCAACCTCCTCAACTCCAATGGGTCCTGCACTTGAACAATTCAAATCAAT
RI-AT2G11240.1-XLOC_011450-10489-1
      TCAACCTCCTCAACTCCAATGGGTC-------------------------
CONSENSUS
      TCAACCTCCTCAACTCCAATGGGTC.........................

RI-AT2G11240.1-XLOC_011450-10489-0
      GACAGTAGCTCAGCTTATTTGTCAAACCACAAAGTCTTGGGATAGAGAGA
RI-AT2G11240.1-XLOC_011450-10489-1

CONSENSUS
      ....

RI-AT2G11240.1-XLOC_011450-10489-0
      AAGTAAGATTGTTACTTCCCAGCTATGAAAAGGAGATCTTCCTTCTAAGA
RI-AT2G11240.1-XLOC_011450-10489-1

CONSENSUS
      ....

RI-AT2G11240.1-XLOC_011450-10489-0
      CCCAGCAAATTTGGAGCCAGAGATCGATATGTTTGGTTACCTAATAAGAC
RI-AT2G11240.1-XLOC_011450-10489-1
      --CTAATAAGAC
CONSENSUS
      ....CTAATAAGAC

RI-AT2G11240.1-XLOC_011450-10489-0
      TAGAGACTACTCGGTCAAGACAGGGTATCACGCAGCTGATGCTCTTTCTC
RI-AT2G11240.1-XLOC_011450-10489-1
      TAGAGACTACTCGGTCAAGACAGGGTATCACGCAGCTGATGCTCTTTCTC
CONSENSUS
      TAGAGACTACTCGGTCAAGACAGGGTATCACGCAGCTGATGCTCTTTCTC

RI-AT2G11240.1-XLOC_011450-10489-0
      ACCAAGGTACTCAACTAGAAGAACCTCCATTGGACTTTAACTGAAATAAG
RI-AT2G11240.1-XLOC_011450-10489-1
      ACCAAGGTACTCAACTAGAAGAACCTCCATTGGACTTTAACTGAAATAAG
CONSENSUS
      ACCAAGGTACTCAACTAGAAGAACCTCCATTGGACTTTAACTGAAATAAG

RI-AT2G11240.1-XLOC_011450-10489-0
      GAGATATGGACCAAACACTGCCCCCCAAAATTGAAAATTTTCCTCTGGAA
RI-AT2G11240.1-XLOC_011450-10489-1
      GAGATATGGACCAAACACTGCCCCCCAAAATTGAAAATTTTCCTCTGGAA

CONSENSUS
GAGATATGGACCAAACACTGCCCCCCAAAATTGAAAATTTTCCTCTGGAA

RI-AT2G11240.1-XLOC_011450-10489-0
AGCCATGAAGAATGCTCTCCCAGTTGGTGAAAACCTGAGGCTGCGTGGTA
RI-AT2G11240.1-XLOC_011450-10489-1
AGCCATGAAGAATGCTCTCCCAGTTGGTGAAAACCTGAGGCTGCGTGGTA
CONSENSUS
AGCCATGAAGAATGCTCTCCCAGTTGGTGAAAACCTGAGGCTGCGTGGTA

RI-AT2G11240.1-XLOC_011450-10489-0
TAAATCCGCTTGCTCTCTGTCCTCACTGCGACGAGGCTGAATCAATTCTC
RI-AT2G11240.1-XLOC_011450-10489-1
TAAATCCGCTTGCTCTCTGTCCTCACTGCGACGAGGCTGAATCAATTCTC
CONSENSUS
TAAATCCGCTTGCTCTCTGTCCTCACTGCGACGAGGCTGAATCAATTCTC

RI-AT2G11240.1-XLOC_011450-10489-0
CATCTGCTTTTCCTCTGCCCATTTGCTTCTAAGGTCTGGGATCTTTCTCC
RI-AT2G11240.1-XLOC_011450-10489-1
CATCTGCTTTTCCTCTGCCCATTTGCTTCTAAGGTCTGGGATCTTTCTCC
CONSENSUS
CATCTGCTTTTCCTCTGCCCATTTGCTTCTAAGGTCTGGGATCTTTCTCC

RI-AT2G11240.1-XLOC_011450-10489-0
TTTCAAAACCACGCTTCAAGCTTCCCGGATCACAAGTATGAAACAGGGTT
RI-AT2G11240.1-XLOC_011450-10489-1
TTTCAAAACCACGCTTCAAGCTTCCCGGATCACAAGTATGAAACAGGGTT
CONSENSUS
TTTCAAAACCACGCTTCAAGCTTCCCGGATCACAAGTATGAAACAGGGTT

RI-AT2G11240.1-XLOC_011450-10489-0
TGGAAGTCTCAAAGCTCCTCGTTACCCTTCCACCAATCGGAATTGGACAA
RI-AT2G11240.1-XLOC_011450-10489-1
TGGAAGTCTCAAAGCTCCTCGTTACCCTTCCACCAATCGGAATTGGACAA
CONSENSUS
TGGAAGTCTCAAAGCTCCTCGTTACCCTTCCACCAATCGGAATTGGACAA

RI-AT2G11240.1-XLOC_011450-10489-0
GGACAACTTCCCATTTGGATCTTGTGGAACCTATGGAATTGCAGGAACAA
RI-AT2G11240.1-XLOC_011450-10489-1
GGACAACTTCCCATTTGGATCTTGTGGAACCTATGGAATTGCAGGAACAA
CONSENSUS
GGACAACTTCCCATTTGGATCTTGTGGAACCTATGGAATTGCAGGAACAA

RI-AT2G11240.1-XLOC_011450-10489-0
GCTAATTTTTGAGCAAAAACACATCTCGAGTATGGATCTCATATCCCAAT
RI-AT2G11240.1-XLOC_011450-10489-1
GCTAATTTTTGAGCAAAAACACATCTCGAGTATGGATCTCATATCCCAAT
CONSENSUS
GCTAATTTTTGAGCAAAAACACATCTCGAGTATGGATCTCATATCCCAAT

RI-AT2G11240.1-XLOC_011450-10489-0
CTATTTCTCAATCCACAGAATGGTTAGGAGCTCAGATCCAAGCATCGAAA
RI-AT2G11240.1-XLOC_011450-10489-1
CTATTTCTCAATCCACAGAATGGTTAGGAGCTCAGATCCAAGCATCGAAA

CONSENSUS
CTATTTCTCAATCCACAGAATGGTTAGGAGCTCAGATCCAAGCATCGAAA

RI-AT2G11240.1-XLOC_011450-10489-0
TCTAAGATCGTGATTCCAGGTATATCTCCCTCAGAGATCGATTTAGACAC
RI-AT2G11240.1-XLOC_011450-10489-1
TCTAAGATCGTGATTCCAGGTATATCTCCCTCAGAGATCGATTTAGACAC
CONSENSUS
TCTAAGATCGTGATTCCAGGTATATCTCCCTCAGAGATCGATTTAGACAC

RI-AT2G11240.1-XLOC_011450-10489-0
GATTCAATGTTCTACAGATGCTTCGTGGAGAGAAGAGACCTTACAGGCTG
RI-AT2G11240.1-XLOC_011450-10489-1
GATTCAATGTTCTACAGATGCTTCGTGGAGAGAAGAGACCTTACAGGCTG
CONSENSUS
GATTCAATGTTCTACAGATGCTTCGTGGAGAGAAGAGACCTTACAGGCTG

RI-AT2G11240.1-XLOC_011450-10489-0
GTTTCGGTTGGGTTTTTGTGGACCATTCGAACCATTTGGAATCCCATCAT
RI-AT2G11240.1-XLOC_011450-10489-1
GTTTCGGTTGGGTTTTTGTGGACCATTCGAACCATTTGGAATCCCATCAT
CONSENSUS
GTTTCGGTTGGGTTTTTGTGGACCATTCGAACCATTTGGAATCCCATCAT

RI-AT2G11240.1-XLOC_011450-10489-0
AAAGCTGCAGCGATGAACATCAGATCTCCCTTATTGGCAAAAGCATCGGC
RI-AT2G11240.1-XLOC_011450-10489-1
AAAGCTGCAGCGATGAACATCAGATCTCCCTTATTGGCAAAAGCATCGGC
CONSENSUS
AAAGCTGCAGCGATGAACATCAGATCTCCCTTATTGGCAAAAGCATCGGC

RI-AT2G11240.1-XLOC_011450-10489-0
TCTCTCTCTCGCTATCCAACACGCAGCAGATCTTGGATTCAAAAAACTTG
RI-AT2G11240.1-XLOC_011450-10489-1
TCTCTCTCTCGCTATCCAACACGCAGCAGATCTTGGATTCAAAAAACTTG
CONSENSUS
TCTCTCTCTCGCTATCCAACACGCAGCAGATCTTGGATTCAAAAAACTTG

RI-AT2G11240.1-XLOC_011450-10489-0
TTGTAGCTTCAGACTCGCAGCAGCTAGTCAAAGTACTAAATGGTGAGCCT
RI-AT2G11240.1-XLOC_011450-10489-1
TTGTAGCTTCAGACTCGCAGCAGCTAGTCAAAGTACTAAATGGTGAGCCT
CONSENSUS
TTGTAGCTTCAGACTCGCAGCAGCTAGTCAAAGTACTAAATGGTGAGCCT

RI-AT2G11240.1-XLOC_011450-10489-0
CATCCAATGGAGCTCCATGGGATAGTCTTCGACATCTCAGTTCTCTCCCT
RI-AT2G11240.1-XLOC_011450-10489-1
CATCCAATGGAGCTCCATGGGATAGTCTTCGACATCTCAGTTCTCTCCCT
CONSENSUS
CATCCAATGGAGCTCCATGGGATAGTCTTCGACATCTCAGTTCTCTCCCT

RI-AT2G11240.1-XLOC_011450-10489-0
AAACTTTGAAGAAAATTCATTTTCTTTTGTTAAGAGAGAAAACAATTCTA
RI-AT2G11240.1-XLOC_011450-10489-1
AAACTTTGAAGAAAATTCATTTTCTTTTGTTAAGAGAGAAAACAATTCTA

CONSENSUS
      AAACTTTGAAGAAAATTCATTTTCTTTTGTTAAGAGAGAAAACAATTCTA

RI-AT2G11240.1-XLOC_011450-10489-0
      AAGCCGATGCACTTGCTAAGGCCGCTTTGCTTTTAATTCCAGCTGTACCG
RI-AT2G11240.1-XLOC_011450-10489-1
      AAGCCGATGCACTTGCTAAGGCCGCTTTGCTTTTAATTCCAGCTGTACCG
CONSENSUS
      AAGCCGATGCACTTGCTAAGGCCGCTTTGCTTTTAATTCCAGCTGTACCG

RI-AT2G11240.1-XLOC_011450-10489-0   GTTCTCAATT
RI-AT2G11240.1-XLOC_011450-10489-1   GTTCTCAATT
CONSENSUS   GTTCTCAATT

alignment for event: RI-AT2G24600-XLOC_012218-5611

RI-AT2G24600-XLOC_012218-5611-0
      CTTATACGTTACATTGTTGGTAAGAAAATAATAGATATCAGAGACAGAAA
RI-AT2G24600-XLOC_012218-5611-1
      CTTATACGTTACATTGTTGGTAAGAAAATAATAGATATCAGAGACAGAAA
CONSENSUS
      CTTATACGTTACATTGTTGGTAAGAAAATAATAGATATCAGAGACAGAAA

RI-AT2G24600-XLOC_012218-5611-0
      CAATATGGGTTATCGAGCTTATCACCTTCTCCCTCGACAAGCTCAAGACT
RI-AT2G24600-XLOC_012218-5611-1
      CAATATGGGTTATCGAGCTTATCACCTTCTCCCTCGACAAGCTCAAGACT
CONSENSUS
      CAATATGGGTTATCGAGCTTATCACCTTCTCCCTCGACAAGCTCAAGACT

RI-AT2G24600-XLOC_012218-5611-0
      ATGAGTTTATATCAAGCTACCTGAGGTGTGACACCAAGACTTCAGAAGAA
RI-AT2G24600-XLOC_012218-5611-1
      ATGAGTTTATATCAAGCTACCTGAGGTGTGACACCAAGACTTCAGAAGAA
CONSENSUS
      ATGAGTTTATATCAAGCTACCTGAGGTGTGACACCAAGACTTCAGAAGAA

RI-AT2G24600-XLOC_012218-5611-0
      GTGGACTCTAAAAAGGCTGAGAGAAATGAACCACACATAGGGCATTCTGA
RI-AT2G24600-XLOC_012218-5611-1
      GTGGACTCTAAAAAGGCTGAGAGAAATGAACCACACATAGGGCATTCTGA
CONSENSUS
      GTGGACTCTAAAAAGGCTGAGAGAAATGAACCACACATAGGGCATTCTGA

RI-AT2G24600-XLOC_012218-5611-0
      GGTAATAAGGCTGCTTAAGCTAATCGAAATAAGCACATCAGAGATAGCAG
RI-AT2G24600-XLOC_012218-5611-1
      GGTAATAAGGCTGCTTAAGCTAATCGAAATAAGCACATCAGAGATAGCAG
CONSENSUS
      GGTAATAAGGCTGCTTAAGCTAATCGAAATAAGCACATCAGAGATAGCAG

RI-AT2G24600-XLOC_012218-5611-0
      AGAGAAAGAAAAGCAAGAAACATCATGTTAAAAGAGGTCATAAGAGCTTG
RI-AT2G24600-XLOC_012218-5611-1

AGAGAAAGAAAAGCAAGAAACATCATGTTAAAAGAGGTCATAAGAGCTTG
CONSENSUS
AGAGAAAGAAAAGCAAGAAACATCATGTTAAAAGAGGTCATAAGAGCTTG

RI-AT2G24600-XLOC_012218-5611-0
GAACATGAGATGCATATAGAAGCATTACAAAATGCAAGAAATACGATCGC
RI-AT2G24600-XLOC_012218-5611-1
GAACATGAGATGCATATAGAAGCATTACAAAATGCAAGAAATACGATCGC
CONSENSUS
GAACATGAGATGCATATAGAAGCATTACAAAATGCAAGAAATACGATCGC

RI-AT2G24600-XLOC_012218-5611-0
GATAGTGGCAGTCTTGATTGCTTCAGTTTCTTATGCCGGTGGGATAAACC
RI-AT2G24600-XLOC_012218-5611-1
GATAGTGGCAGTCTTGATTGCTTCAGTTTCTTATGCCGGTGGGATAAACC
CONSENSUS
GATAGTGGCAGTCTTGATTGCTTCAGTTTCTTATGCCGGTGGGATAAACC

RI-AT2G24600-XLOC_012218-5611-0
CGCCGGGCGGTGTTTACCAAGATGGGCCATGGAAAGGGAAATCGCTTGTA
RI-AT2G24600-XLOC_012218-5611-1
CGCCGGGCGGTGTTTACCAAGATGGGCCATGGAAAGGGAAATCGCTTGTA
CONSENSUS
CGCCGGGCGGTGTTTACCAAGATGGGCCATGGAAAGGGAAATCGCTTGTA

RI-AT2G24600-XLOC_012218-5611-0
GGTAATACGGCAGCATTTAAAGTCTTTGCAATATGTAACAACATTGCATT
RI-AT2G24600-XLOC_012218-5611-1
G---
CONSENSUS
G...

RI-AT2G24600-XLOC_012218-5611-0
GTTCACGTCCTTGTGCATTGTTATTCTTCTCGTTAGCATCATACCTTACC
RI-AT2G24600-XLOC_012218-5611-1

CONSENSUS
....

RI-AT2G24600-XLOC_012218-5611-0
AGAGGAAACCCTTGAAGAAATTATTGGTGGCCACTCACAGGATGATGTGG
RI-AT2G24600-XLOC_012218-5611-1

CONSENSUS
....

RI-AT2G24600-XLOC_012218-5611-0
GTTTCTGTAGGGTTTATGGCAACGGCTTATGTTGCGGCATCTTTGGTGAC
RI-AT2G24600-XLOC_012218-5611-1 ----------
GGTTTATGGCAACGGCTTATGTTGCGGCATCTTTGGTGAC
CONSENSUS
..........GGTTTATGGCAACGGCTTATGTTGCGGCATCTTTGGTGAC

RI-AT2G24600-XLOC_012218-5611-0
CATACCGCATTTTCCCGGAACTCGATGGTTATTTCCGGTTATTATCTCTG
RI-AT2G24600-XLOC_012218-5611-1

CATACCGCATTTTCCCGGAACTCGATGGTTATTTCCGGTTATTATCTCTG
CONSENSUS
CATACCGCATTTTCCCGGAACTCGATGGTTATTTCCGGTTATTATCTCTG

RI-AT2G24600-XLOC_012218-5611-0
TAGCTGGTGGATCATTGACGGTACTCTTTTCCTATCTAGGAGTTGAGACC
RI-AT2G24600-XLOC_012218-5611-1
TAGCTGGTGGATCATTGACGGTACTCTTTTCCTATCTAGGAGTTGAGACC
CONSENSUS
TAGCTGGTGGATCATTGACGGTACTCTTTTCCTATCTAGGAGTTGAGACC

RI-AT2G24600-XLOC_012218-5611-0
ATCAGTCACTGGTTTAAGAAGATGAATCGTGTAGGGAGAGGACTACCTAT
RI-AT2G24600-XLOC_012218-5611-1
ATCAGTCACTGGTTTAAGAAGATGAATCGTGTAGGGAGAGGACTACCTAT
CONSENSUS
ATCAGTCACTGGTTTAAGAAGATGAATCGTGTAGGGAGAGGACTACCTAT

RI-AT2G24600-XLOC_012218-5611-0
TTATTTTATCAAAAATAACCGTGTAGAAGATATACCTGCCATTGCAAAAA
RI-AT2G24600-XLOC_012218-5611-1
TTATTTTATCAAAAATAACCGTGTAGAAGATATACCTGCCATTGCAAAAA
CONSENSUS
TTATTTTATCAAAAATAACCGTGTAGAAGATATACCTGCCATTGCAAAAA

RI-AT2G24600-XLOC_012218-5611-0
ATGAAGGTGAAATGCCTTCCTTAGCAAGGACCAACTCAGACTTGGCCGCC
RI-AT2G24600-XLOC_012218-5611-1
ATGAAGGTGAAATGCCTTCCTTAGCAAGGACCAACTCAGACTTGGCCGCC
CONSENSUS
ATGAAGGTGAAATGCCTTCCTTAGCAAGGACCAACTCAGACTTGGCCGCC

RI-AT2G24600-XLOC_012218-5611-0
TCAGAAGGGTCAGGCTATTTCACCTATTGAATCTTTAAGGTAAATTTTCT
RI-AT2G24600-XLOC_012218-5611-1
TCAGAAGGGTCAGGCTATTTCACCTATTGAATCTTTAAGGTAAATTTTCT
CONSENSUS
TCAGAAGGGTCAGGCTATTTCACCTATTGAATCTTTAAGGTAAATTTTCT

RI-AT2G24600-XLOC_012218-5611-0
ACGAGACTACTCTCTCTGGTGCTCGAGCTTTTACCGTGGTGTTTGCAGCC
RI-AT2G24600-XLOC_012218-5611-1
ACGAGACTACTCTCTCTGGTGCTCGAGCTTTTACCGTGGTGTTTGCAGCC
CONSENSUS
ACGAGACTACTCTCTCTGGTGCTCGAGCTTTTACCGTGGTGTTTGCAGCC

RI-AT2G24600-XLOC_012218-5611-0
TATCTTATGGGTTTTGAGTGTCTAAAGTGATTTTCTTGATGAGCTTCTTC
RI-AT2G24600-XLOC_012218-5611-1
TATCTTATGGGTTTTGAGTGTCTAAAGTGATTTTCTTGATGAGCTTCTTC
CONSENSUS
TATCTTATGGGTTTTGAGTGTCTAAAGTGATTTTCTTGATGAGCTTCTTC

RI-AT2G24600-XLOC_012218-5611-0
TCCTCACAGGGACCGGCACAAGATTTACTTGAAGCAGATTTATATTGCCA
RI-AT2G24600-XLOC_012218-5611-1

TCCTCACAGGGACCGGCACAAGATTTACTTGAAGCAGATTTATATTGCCA
CONSENSUS
TCCTCACAGGGACCGGCACAAGATTTACTTGAAGCAGATTTATATTGCCA

RI-AT2G24600-XLOC_012218-5611-0
TTGTATGATGCATCCCTACTTAAGGAGTTTCGTGTAAACTGGTCTCACCT
RI-AT2G24600-XLOC_012218-5611-1
TTGTATGATGCATCCCTACTTAAGGAGTTTCGTGTAAACTGGTCTCACCT
CONSENSUS
TTGTATGATGCATCCCTACTTAAGGAGTTTCGTGTAAACTGGTCTCACCT

RI-AT2G24600-XLOC_012218-5611-0
ATGTTATCTCTATCATTTAAAGTCCAAAAAGAATGAAAAATGTTGTCAAT
RI-AT2G24600-XLOC_012218-5611-1
ATGTTATCTCTATCATTTAAAGTCCAAAAAGAATGAAAAATGTTGTCAAT
CONSENSUS
ATGTTATCTCTATCATTTAAAGTCCAAAAAGAATGAAAAATGTTGTCAAT

RI-AT2G24600-XLOC_012218-5611-0
GATGTTTTACATGTTTTATATCAATACTCAGTACATGTTTCATC
RI-AT2G24600-XLOC_012218-5611-1
GATGTTTTACATGTTTTATATCAATACTCAGTACATGTTTCATC
CONSENSUS
GATGTTTTACATGTTTTATATCAATACTCAGTACATGTTTCATC

alignment for event: RI-AT2G24600-XLOC_012218-5610

RI-AT2G24600-XLOC_012218-5610-0
GGTTTATGGCAACGGCTTATGTTGCGGCATCTTTGGTGACCATACCGCAT
RI-AT2G24600-XLOC_012218-5610-1
GGTTTATGGCAACGGCTTATGTTGCGGCATCTTTGGTGACCATACCGCAT
CONSENSUS
GGTTTATGGCAACGGCTTATGTTGCGGCATCTTTGGTGACCATACCGCAT

RI-AT2G24600-XLOC_012218-5610-0
TTTCCCGGAACTCGATGGTTATTTCCGGTTATTATCTCTGTAGCTGGTGG
RI-AT2G24600-XLOC_012218-5610-1
TTTCCCGGAACTCGATGGTTATTTCCGGTTATTATCTCTGTAGCTGGTGG
CONSENSUS
TTTCCCGGAACTCGATGGTTATTTCCGGTTATTATCTCTGTAGCTGGTGG

RI-AT2G24600-XLOC_012218-5610-0
ATCATTGACGGTACTCTTTTCCTATCTAGGAGTTGAGACCATCAGTCACT
RI-AT2G24600-XLOC_012218-5610-1
ATCATTGACGGTACTCTTTTCCTATCTAGGAGTTGAGACCATCAGTCACT
CONSENSUS
ATCATTGACGGTACTCTTTTCCTATCTAGGAGTTGAGACCATCAGTCACT

RI-AT2G24600-XLOC_012218-5610-0
GGTTTAAGAAGATGAATCGTGTAGGGAGAGGACTACCTATTTATTTTATC
RI-AT2G24600-XLOC_012218-5610-1
GGTTTAAGAAGATGAATCGTGTAGGGAGAGGACTACCTATTTATTTTATC
CONSENSUS
GGTTTAAGAAGATGAATCGTGTAGGGAGAGGACTACCTATTTATTTTATC

RI-AT2G24600-XLOC_012218-5610-0
AAAAATAACCGTGTAGAAGATATACCTGCCATTGCAAAAAATGAAGGTGA
RI-AT2G24600-XLOC_012218-5610-1
AAAAATAACCGTGTAGAAGATATACCTGCCATTGCAAAAAATGAAGGTGA
CONSENSUS
AAAAATAACCGTGTAGAAGATATACCTGCCATTGCAAAAAATGAAGGTGA

RI-AT2G24600-XLOC_012218-5610-0
AATGCCTTCCTTAGCAAGGACCAACTCAGACTTGGCCGCCTCAGAAGGGT
RI-AT2G24600-XLOC_012218-5610-1
AATGCCTTCCTTAGCAAGGACCAACTCAGACTTGGCCGCCTCAGAAGGGT
CONSENSUS
AATGCCTTCCTTAGCAAGGACCAACTCAGACTTGGCCGCCTCAGAAGGGT

RI-AT2G24600-XLOC_012218-5610-0
CAGGCTATTTCACCTATTGAATCTTTAAGGTAAATTTTCTACGAGACTAC
RI-AT2G24600-XLOC_012218-5610-1
CAGGCTATTTCACCTATTGAATCTTTAAG---------------------
CONSENSUS
CAGGCTATTTCACCTATTGAATCTTTAAG.....................

RI-AT2G24600-XLOC_012218-5610-0
TCTCTCTGGTGCTCGAGCTTTTACCGTGGTGTTTGCAGCCTATCTTATGG
RI-AT2G24600-XLOC_012218-5610-1

CONSENSUS
....

RI-AT2G24600-XLOC_012218-5610-0
GTTTTGAGTGTCTAAAGTGATTTTCTTGATGAGCTTCTTCTCCTCACAGG
RI-AT2G24600-XLOC_012218-5610-1
---G
CONSENSUS
....G

RI-AT2G24600-XLOC_012218-5610-0
GACCGGCACAAGATTTACTTGAAGCAGATTTATATTGCCATTGTATGATG
RI-AT2G24600-XLOC_012218-5610-1
GACCGGCACAAGATTTACTTGAAGCAGATTTATATTGCCATTGTATGATG
CONSENSUS
GACCGGCACAAGATTTACTTGAAGCAGATTTATATTGCCATTGTATGATG

RI-AT2G24600-XLOC_012218-5610-0
CATCCCTACTTAAGGAGTTTCGTGTAAACTGGTCTCACCTATGTTATCTC
RI-AT2G24600-XLOC_012218-5610-1
CATCCCTACTTAAGGAGTTTCGTGTAAACTGGTCTCACCTATGTTATCTC
CONSENSUS
CATCCCTACTTAAGGAGTTTCGTGTAAACTGGTCTCACCTATGTTATCTC

RI-AT2G24600-XLOC_012218-5610-0
TATCATTTAAAGTCCAAAAAGAATGAAAAATGTTGTCAATGATGTTTTAC
RI-AT2G24600-XLOC_012218-5610-1
TATCATTTAAAGTCCAAAAAGAATGAAAAATGTTGTCAATGATGTTTTAC
CONSENSUS
TATCATTTAAAGTCCAAAAAGAATGAAAAATGTTGTCAATGATGTTTTAC

RI-AT2G24600-XLOC_012218-5610-0
      ATGTTTTATATCAATACTCAGTACATGTTTCATC
RI-AT2G24600-XLOC_012218-5610-1
      ATGTTTTATATCAATACTCAGTACATGTTTCATC
CONSENSUS
      ATGTTTTATATCAATACTCAGTACATGTTTCATC

alignment for event: RI-AT2G18440-XLOC_011870-10761

RI-AT2G18440-XLOC_011870-10761-0
      ATCTAGATCTGGATCGTCCTTCGCGGCTTCTTCACATGCAAGATTCTGTG
RI-AT2G18440-XLOC_011870-10761-1
      ATCTAGATCTGGATCGTCCTTCGCGGCTTCTTCACATGCAAGATTCTGTG
CONSENSUS
      ATCTAGATCTGGATCGTCCTTCGCGGCTTCTTCACATGCAAGATTCTGTG

RI-AT2G18440-XLOC_011870-10761-0
      AATTGTTCAAGTTGAGGCATCATTTCTGGATTACTAGGAGACGAATCTGT
RI-AT2G18440-XLOC_011870-10761-1
      AATTGTTCAAGTTGAGGCATCATTTCTGGATTACTAGGAGACGAATCTGT
CONSENSUS
      AATTGTTCAAGTTGAGGCATCATTTCTGGATTACTAGGAGACGAATCTGT

RI-AT2G18440-XLOC_011870-10761-0
      TGCAGACGGATGTGTGTGTGTTGGATTGAATTGAGATTAGGGTGTAGAAG
RI-AT2G18440-XLOC_011870-10761-1
      TGCAGACGGATGTGTGTGTGTTGGATTGAATTGAGATTAGGGTGTAGAAG
CONSENSUS
      TGCAGACGGATGTGTGTGTGTTGGATTGAATTGAGATTAGGGTGTAGAAG

RI-AT2G18440-XLOC_011870-10761-0
      ATGTTTTGTGGATAGCTAATAGCTTCCTGATTGCATCTCTGTCATCCGAC
RI-AT2G18440-XLOC_011870-10761-1
      ATGTTTTGTGGATAGCTAATAGCTTCCTGATTGCATCTCTGTCATCCGAC
CONSENSUS
      ATGTTTTGTGGATAGCTAATAGCTTCCTGATTGCATCTCTGTCATCCGAC

RI-AT2G18440-XLOC_011870-10761-0
      CTTTGCCATGTCAGGTGCGCTTGCATGGCAGGTCAAAAAACTGATCCTCA
RI-AT2G18440-XLOC_011870-10761-1
      CTTTGCCATGTCAGGTGCGCTTGCATGGCAGGTCAAAAAACTGATCCTCA
CONSENSUS
      CTTTGCCATGTCAGGTGCGCTTGCATGGCAGGTCAAAAAACTGATCCTCA

RI-AT2G18440-XLOC_011870-10761-0
      ATAAAAAAAAGATTTTGTGGGTTTTGGAGAGGAGGTCGCACGGGTTATTA
RI-AT2G18440-XLOC_011870-10761-1
      ATAAAAAAAAGATTTTGTGGGTTTTGGAGAGGAGGTCGCACGGGTTATTA
CONSENSUS
      ATAAAAAAAAGATTTTGTGGGTTTTGGAGAGGAGGTCGCACGGGTTATTA

RI-AT2G18440-XLOC_011870-10761-0
      TTTTTTCCCGGGATCTCTTCTCCTCTGTGTGTGTCGTCTTGCCTCTGCTC

RI-AT2G18440-XLOC_011870-10761-1
      TTTTTTCCCGGGATCTCTTCTCCTCTGTGTGTGTCGTCTTGCCTCTGCTC
CONSENSUS
      TTTTTTCCCGGGATCTCTTCTCCTCTGTGTGTGTCGTCTTGCCTCTGCTC

RI-AT2G18440-XLOC_011870-10761-0
      TATCTCTCTCCCTGCTCTTTCACATTTCATATCTTTCTTAAATGCTCATA
RI-AT2G18440-XLOC_011870-10761-1
      TATCTCTCTCCCTGCTCTTTCACATTTCATATCTTTCTTAAATGCTCATA
CONSENSUS
      TATCTCTCTCCCTGCTCTTTCACATTTCATATCTTTCTTAAATGCTCATA

RI-AT2G18440-XLOC_011870-10761-0
      TACACTCAAAAACCGATCATAAGCAGAGTTTGTAACCAATATGGAGCAGT
RI-AT2G18440-XLOC_011870-10761-1
      TACACTCAAAAACCGATCATAAGCAGAGTTTGTAACCAATATGGAGCAGT
CONSENSUS
      TACACTCAAAAACCGATCATAAGCAGAGTTTGTAACCAATATGGAGCAGT

RI-AT2G18440-XLOC_011870-10761-0
      GGGCGATTACAAATCTTCTTGCCCAACAACCTCGAGAGTTAAATCAGGTA
RI-AT2G18440-XLOC_011870-10761-1
      GGGCGATTACAAATCTTCTTGCCCAACAACCTCGAGAGTTAAATCA----
CONSENSUS
      GGGCGATTACAAATCTTCTTGCCCAACAACCTCGAGAGTTAAATCA....

RI-AT2G18440-XLOC_011870-10761-0
      CTCATATCCATATTAAATCGAATTCTTAATTAGCATAATAAGGTAAACAT
RI-AT2G18440-XLOC_011870-10761-1

CONSENSUS
      ....

RI-AT2G18440-XLOC_011870-10761-0
      AATCTGCAAGAGGAATTCTGGATTTAAATAAACCATAATCCGGTTGCCCT
RI-AT2G18440-XLOC_011870-10761-1

CONSENSUS
      ....

RI-AT2G18440-XLOC_011870-10761-0
      AATTGTTAACTTGTATCAACGCTTATGCGAATCATGAAATTTTAATTTTA
RI-AT2G18440-XLOC_011870-10761-1

CONSENSUS
      ....

RI-AT2G18440-XLOC_011870-10761-0
      TTGGTTTTGTTATTTATCTTATTGATCAATTATTGTTTTACAAGTTGTCA
RI-AT2G18440-XLOC_011870-10761-1

CONSENSUS
      ....

RI-AT2G18440-XLOC_011870-10761-0
      TTTAATCTATTGAAAATGATGGTATTAAGAATTGCTGTTAACAATTTTG

RI-AT2G18440-XLOC_011870-10761-1

CONSENSUS
      ....

RI-AT2G18440-XLOC_011870-10761-0
      CGGGTTCTTTGTATGTATCATTGGTATTGTTTTATTAGTTCTATCCGTTT
RI-AT2G18440-XLOC_011870-10761-1

CONSENSUS
      ....

RI-AT2G18440-XLOC_011870-10761-0
      AGAAGCTTTTTAGAATATGGTAAAGAACATTTTTATTTGCTCGTAGTTTG
RI-AT2G18440-XLOC_011870-10761-1

CONSENSUS
      ....

RI-AT2G18440-XLOC_011870-10761-0
      TATATGTCTGTAACCACCAGTTCCTGTAGTAACTATGAAGCTGGTAGACG
RI-AT2G18440-XLOC_011870-10761-1 -----------------------------
TAACTATGAAGCTGGTAGACG
CONSENSUS
      .............................TAACTATGAAGCTGGTAGACG

RI-AT2G18440-XLOC_011870-10761-0
      ACGGAATTGAAGCTATGCAAAGCTGACGTTGGTTTTTTCTGAAGTTGATT
RI-AT2G18440-XLOC_011870-10761-1
      ACGGAATTGAAGCTATGCAAAGCTGACGTTGGTTTTTTCTGAAGTTGATT
CONSENSUS
      ACGGAATTGAAGCTATGCAAAGCTGACGTTGGTTTTTTCTGAAGTTGATT

RI-AT2G18440-XLOC_011870-10761-0 TGTTCTTGAAAG
RI-AT2G18440-XLOC_011870-10761-1 TGTTCTTGAAAG
CONSENSUS TGTTCTTGAAAG

alignment for event: RI-AT2G25930-XLOC_009647-12881

RI-AT2G25930-XLOC_009647-12881-0
      CCTTGTGGTGTGGAAAGAAACTTATCTGTCCAGCATCTTGATTCTTCAGC
RI-AT2G25930-XLOC_009647-12881-1
      CCTTGTGGTGTGGAAAGAAACTTATCTGTCCAGCATCTTGATTCTTCAGC
CONSENSUS
      CCTTGTGGTGTGGAAAGAAACTTATCTGTCCAGCATCTTGATTCTTCAGC

RI-AT2G25930-XLOC_009647-12881-0
      CGCAAACCAAGCAACTGAGAAGTTTGTCTCCCAAATGTCCTTCATGGAAA
RI-AT2G25930-XLOC_009647-12881-1
      CGCAAACCAAGCAACTGAGAAGTTTGTCTCCCAAATGTCCTTCATGGAAA
CONSENSUS
      CGCAAACCAAGCAACTGAGAAGTTTGTCTCCCAAATGTCCTTCATGGAAA

RI-AT2G25930-XLOC_009647-12881-0

ATGTGAGATCTTCGGCACAGCATGATCAGAGGAAAATGGTGAGAGAGGAA
RI-AT2G25930-XLOC_009647-12881-1
ATGTGAGATCTTCGGCACAGCATGATCAGAGGAAAATGGTGAGAGAGGAA
CONSENSUS
ATGTGAGATCTTCGGCACAGCATGATCAGAGGAAAATGGTGAGAGAGGAA

RI-AT2G25930-XLOC_009647-12881-0
GAAGATTTTGCAGTTCCAGTATATATTAACTCAAGAAGATCTCAGTCTCA
RI-AT2G25930-XLOC_009647-12881-1
GAAGATTTTGCAGTTCCAGTATATATTAACTCAAGAAGATCTCAGTCTCA
CONSENSUS
GAAGATTTTGCAGTTCCAGTATATATTAACTCAAGAAGATCTCAGTCTCA

RI-AT2G25930-XLOC_009647-12881-0
TGGCAGAACCAAGAGTGGTATTGAGAAGGAAAAACACACCCCAATGGTGG
RI-AT2G25930-XLOC_009647-12881-1
TGGCAGAACCAAGAGTGGTATTGAGAAGGAAAAACACACCCCAATGGTGG
CONSENSUS
TGGCAGAACCAAGAGTGGTATTGAGAAGGAAAAACACACCCCAATGGTGG

RI-AT2G25930-XLOC_009647-12881-0
CACCTAGCTCTCATCACTCCATTCGATTTCAAGAAGTGAATCAGACAGGC
RI-AT2G25930-XLOC_009647-12881-1
CACCTAGCTCTCATCACTCCATTCGATTTCAAGAAGTGAATCAGACAGGC
CONSENSUS
CACCTAGCTCTCATCACTCCATTCGATTTCAAGAAGTGAATCAGACAGGC

RI-AT2G25930-XLOC_009647-12881-0
TCAAAGCAAAACGTATGTTTGGCTACTTGTTCAAAACCTGAAGTTAGGGA
RI-AT2G25930-XLOC_009647-12881-1
TCAAAGCAAAACGTATGTTTGGCTACTTGTTCAAAACCTGAAGTTAGGGA
CONSENSUS
TCAAAGCAAAACGTATGTTTGGCTACTTGTTCAAAACCTGAAGTTAGGGA

RI-AT2G25930-XLOC_009647-12881-0
TCAGGTCAAGGCGAATGCAAGGTCAGGTGGCTTTGTAATCTCTTTAGATG
RI-AT2G25930-XLOC_009647-12881-1
TCAGGTCAAGGCGAATGCAAGGTCAGGTGGCTTTGTAATCTCTTTAGATG
CONSENSUS
TCAGGTCAAGGCGAATGCAAGGTCAGGTGGCTTTGTAATCTCTTTAGATG

RI-AT2G25930-XLOC_009647-12881-0
TATCAGTCACAGAGGAGATTGATCTCGAAAAATCAGCATCAAGTCATGAT
RI-AT2G25930-XLOC_009647-12881-1
TATCAGTCACAGAGGAGATTGATCTCGAAAAATCAGCATCAAGTCATGAT
CONSENSUS
TATCAGTCACAGAGGAGATTGATCTCGAAAAATCAGCATCAAGTCATGAT

RI-AT2G25930-XLOC_009647-12881-0
AGAGTAAATGATTATAATGCTTCCTTGAGACAAGAGTCTAGAAATCGGTT
RI-AT2G25930-XLOC_009647-12881-1
AGAGTAAATGATTATAATGCTTCCTTGAGACAAGAGTCTAGAAATCGGTT
CONSENSUS
AGAGTAAATGATTATAATGCTTCCTTGAGACAAGAGTCTAGAAATCGGTT

RI-AT2G25930-XLOC_009647-12881-0

ATACCGAGATGGTGGCAAAACTCGTCTGAAGGACACTGATAATGGAGCTG
RI-AT2G25930-XLOC_009647-12881-1
ATACCGAGATGGTGGCAAAACTCGTCTGAAGGACACTGATAATGGAGCTG
CONSENSUS
ATACCGAGATGGTGGCAAAACTCGTCTGAAGGACACTGATAATGGAGCTG

RI-AT2G25930-XLOC_009647-12881-0
AATCTCACTTGGCAACGGAAAATCATTCACAAGAGGGTCATGGCAGTCCT
RI-AT2G25930-XLOC_009647-12881-1
AATCTCACTTGGCAACGGAAAATCATTCACAAGAGGGTCATGGCAGTCCT
CONSENSUS
AATCTCACTTGGCAACGGAAAATCATTCACAAGAGGGTCATGGCAGTCCT

RI-AT2G25930-XLOC_009647-12881-0
GAAGACATTGATAATGATCGTGAATACAGCAAAAGCAGAGCATGCGCCTC
RI-AT2G25930-XLOC_009647-12881-1
GAAGACATTGATAATGATCGTGAATACAGCAAAAGCAGAGCATGCGCCTC
CONSENSUS
GAAGACATTGATAATGATCGTGAATACAGCAAAAGCAGAGCATGCGCCTC

RI-AT2G25930-XLOC_009647-12881-0
TCTGCAGCAGATAAATGAAGAGGCAAGTGATGACGTTTCTGATGATTCGA
RI-AT2G25930-XLOC_009647-12881-1
TCTGCAGCAGATAAATGAAGAGGCAAGTGATGACGTTTCTGATGATTCGA
CONSENSUS
TCTGCAGCAGATAAATGAAGAGGCAAGTGATGACGTTTCTGATGATTCGA

RI-AT2G25930-XLOC_009647-12881-0
TGGTGGATTCTATATCCAGCATAGATGTCTCTCCCGATGATGTTGTGGGT
RI-AT2G25930-XLOC_009647-12881-1
TGGTGGATTCTATATCCAGCATAGATGTCTCTCCCGATGATGTTGTGGGT
CONSENSUS
TGGTGGATTCTATATCCAGCATAGATGTCTCTCCCGATGATGTTGTGGGT

RI-AT2G25930-XLOC_009647-12881-0
ATATTAGGTCAAAAACGTTTCTGGAGAGCAAGGAAAGCCATTGCCAAGTA
RI-AT2G25930-XLOC_009647-12881-1
ATATTAGGTCAAAAACGTTTCTGGAGAGCAAGGAAAGCCATTGCCAA---
CONSENSUS
ATATTAGGTCAAAAACGTTTCTGGAGAGCAAGGAAAGCCATTGCCAA...

RI-AT2G25930-XLOC_009647-12881-0
AGTTCACTAGAAATTTACAGTTTGGTTATTTATTCTCCGCTCTTTCTATT
RI-AT2G25930-XLOC_009647-12881-1

CONSENSUS
....

RI-AT2G25930-XLOC_009647-12881-0
TATCTCCTTCTTTGATACCAACATTTTTTGCTTGAAAGAAGTTAATATTT
RI-AT2G25930-XLOC_009647-12881-1

CONSENSUS
....

RI-AT2G25930-XLOC_009647-12881-0

```
      AAGCATTGTTCCGTAGTCTTACTGAAGCTTTTTCCTCTGTTGTTTTTTGC
RI-AT2G25930-XLOC_009647-12881-1
      --------------------------------------------------
CONSENSUS
      ..................................................

RI-AT2G25930-XLOC_009647-12881-0
      TATTTTCATTGAGGACTGTGGTAGGGCATATTTCACTATCACCAAATTTC
RI-AT2G25930-XLOC_009647-12881-1
      --------------------------------------------------
CONSENSUS
      ..................................................

RI-AT2G25930-XLOC_009647-12881-0
      AAATTTCTAGAACACTCTCCTTCATATTTTTTTTCATGATTAATGCTGCA
RI-AT2G25930-XLOC_009647-12881-1
      --------------------------------------------------
CONSENSUS
      ..................................................

RI-AT2G25930-XLOC_009647-12881-0
      ATTGATTGCTGATATACATATATGACTATAACTCAGTTTCATATTCTGTC
RI-AT2G25930-XLOC_009647-12881-1
      --------------------------------------------------
CONSENSUS
      ..................................................

RI-AT2G25930-XLOC_009647-12881-0
      TCATTTTGGGAGAAAGAGATTTCAGGTTTATGCTTGAGAAGTGATGGTTC
RI-AT2G25930-XLOC_009647-12881-1
      --------------------------------------------------
CONSENSUS
      ..................................................

RI-AT2G25930-XLOC_009647-12881-0
      TATAGTTGAGAGGCCCCTGATTCATCTAAAATGGTCCTATTATGTGTTTA
RI-AT2G25930-XLOC_009647-12881-1
      --------------------------------------------------
CONSENSUS
      ..................................................

RI-AT2G25930-XLOC_009647-12881-0
      GTTGTAGAGTCCTCGGTAGAATATTAACGCGTTTAACACGTTGGATCATG
RI-AT2G25930-XLOC_009647-12881-1
      --------------------------------------------------
CONSENSUS
      ..................................................

RI-AT2G25930-XLOC_009647-12881-0
      TTATAGCAGGGAGGGACATTCTCTGTTGACCTATATTGTGCAAG
RI-AT2G25930-XLOC_009647-12881-1 ---------
GGAGGGACATTCTCTGTTGACCTATATTGTGCAAG
CONSENSUS
      .........GGAGGGACATTCTCTGTTGACCTATATTGTGCAAG
```

alignment for event: RI-AT2G41870-XLOC_013220-12146

RI-AT2G41870-XLOC_013220-12146-0
GTCTCGTTTGTTTTGCTCTTTAGAAATTACCGATTTACGCTGAAAAAATG
RI-AT2G41870-XLOC_013220-12146-1
GTCTCGTTTGTTTTGCTCTTTAGAAATTACCGATTTACGCTGAAAAAATG
CONSENSUS
GTCTCGTTTGTTTTGCTCTTTAGAAATTACCGATTTACGCTGAAAAAATG

RI-AT2G41870-XLOC_013220-12146-0
TGAAAAATACAATCGATCGCATTATCTTTATCCCTAGCTAATCATTCATG
RI-AT2G41870-XLOC_013220-12146-1
TGAAAAATACAATCGATCGCATTATCTTTATCCCTAGCTAATCATTCATG
CONSENSUS
TGAAAAATACAATCGATCGCATTATCTTTATCCCTAGCTAATCATTCATG

RI-AT2G41870-XLOC_013220-12146-0
TACAAGCATGTCTCCGAAGGTTAAAAGCAGTCGCTATTTACCGGACCAAC
RI-AT2G41870-XLOC_013220-12146-1
TACAAGCATGTCTCCGAAGGTTAAAAGCAGTCGCTATTTACCGGACCAAC
CONSENSUS
TACAAGCATGTCTCCGAAGGTTAAAAGCAGTCGCTATTTACCGGACCAAC

RI-AT2G41870-XLOC_013220-12146-0
GTAGTTTTCTCGAAGTGGTGGTCCGTTGTCATATTTTAAATTTATCACCT
RI-AT2G41870-XLOC_013220-12146-1
GTAGTTTTCTCGAAGTGGTG------------------------------
CONSENSUS
GTAGTTTTCTCGAAGTGGTG..............................

RI-AT2G41870-XLOC_013220-12146-0
TCTTGAGAATTCCACATTTTTATCCTTTTTGTCATGTAGTGTATATTTTT
RI-AT2G41870-XLOC_013220-12146-1

CONSENSUS
....

RI-AT2G41870-XLOC_013220-12146-0
TCCTCTAACCTAATTAAAATCAAAACAAAATCCTTTGACCCAATTAGCTT
RI-AT2G41870-XLOC_013220-12146-1

CONSENSUS
....

RI-AT2G41870-XLOC_013220-12146-0
CGCGATATATCAGAAGAGATCAAACTACTTTGATCAGACCATGATCTTCT
RI-AT2G41870-XLOC_013220-12146-1

CONSENSUS
....

RI-AT2G41870-XLOC_013220-12146-0
TCTTCTTCTTCTTCTTCTTCTTCTTCTTTTTAGACGATCACAATTCCTAA
RI-AT2G41870-XLOC_013220-12146-1

CONSENSUS
      ....

RI-AT2G41870-XLOC_013220-12146-0
      ACCCTATTTCTCAGATTATGCTGACTCTTTACCATCAAGAAAGGTCACCG
RI-AT2G41870-XLOC_013220-12146-1 --------------
ATTATGCTGACTCTTTACCATCAAGAAAGGTCACCG
CONSENSUS
      ..............ATTATGCTGACTCTTTACCATCAAGAAAGGTCACCG

RI-AT2G41870-XLOC_013220-12146-0
      GACGCCACAAGTAATGATCGCGATGAGACGCCAGAGACTGTGGTTAGAGA
RI-AT2G41870-XLOC_013220-12146-1
      GACGCCACAAGTAATGATCGCGATGAGACGCCAGAGACTGTGGTTAGAGA
CONSENSUS
      GACGCCACAAGTAATGATCGCGATGAGACGCCAGAGACTGTGGTTAGAGA

RI-AT2G41870-XLOC_013220-12146-0
      AGTCCACGCGCTAACTCCAGCGCCGGAGGATAATTCCCGGACGATGACGG
RI-AT2G41870-XLOC_013220-12146-1
      AGTCCACGCGCTAACTCCAGCGCCGGAGGATAATTCCCGGACGATGACGG
CONSENSUS
      AGTCCACGCGCTAACTCCAGCGCCGGAGGATAATTCCCGGACGATGACGG

RI-AT2G41870-XLOC_013220-12146-0
      CGACGCTACCTCCACCGCCTGCTTTCCGAGGCTATTTTTCTCCTCCAAGG
RI-AT2G41870-XLOC_013220-12146-1
      CGACGCTACCTCCACCGCCTGCTTTCCGAGGCTATTTTTCTCCTCCAAGG
CONSENSUS
      CGACGCTACCTCCACCGCCTGCTTTCCGAGGCTATTTTTCTCCTCCAAGG

RI-AT2G41870-XLOC_013220-12146-0
      TCAGCGACGACGATGAGCGAAGGAGAGAACTTCACAACTATAAGCAGAGA
RI-AT2G41870-XLOC_013220-12146-1
      TCAGCGACGACGATGAGCGAAGGAGAGAACTTCACAACTATAAGCAGAGA
CONSENSUS
      TCAGCGACGACGATGAGCGAAGGAGAGAACTTCACAACTATAAGCAGAGA

RI-AT2G41870-XLOC_013220-12146-0
      GTTCAACGCTCTAGTCATCGCCGGATCCTCCATGGAGAACAACGAACTAA
RI-AT2G41870-XLOC_013220-12146-1
      GTTCAACGCTCTAGTCATCGCCGGATCCTCCATGGAGAACAACGAACTAA
CONSENSUS
      GTTCAACGCTCTAGTCATCGCCGGATCCTCCATGGAGAACAACGAACTAA

RI-AT2G41870-XLOC_013220-12146-0
      TGACTCGTGACGTCACGCAGCGTGAAGATGAGAGACAAGACGAGTTGATG
RI-AT2G41870-XLOC_013220-12146-1
      TGACTCGTGACGTCACGCAGCGTGAAGATGAGAGACAAGACGAGTTGATG
CONSENSUS
      TGACTCGTGACGTCACGCAGCGTGAAGATGAGAGACAAGACGAGTTGATG

RI-AT2G41870-XLOC_013220-12146-0
      AGAATCCACGAGGACACGGATCATGAAGAGGAAACGAATCCTTTAGCAAT
RI-AT2G41870-XLOC_013220-12146-1
      AGAATCCACGAGGACACGGATCATGAAGAGGAAACGAATCCTTTAGCAAT

CONSENSUS
AGAATCCACGAGGACACGGATCATGAAGAGGAAACGAATCCTTTAGCAAT

RI-AT2G41870-XLOC_013220-12146-0
CGTGCCGGATCAGTATCCTGGTTCGGGTTTGGATCCTGGAAGTGATAATG
RI-AT2G41870-XLOC_013220-12146-1
CGTGCCGGATCAGTATCCTGGTTCGGGTTTGGATCCTGGAAGTGATAATG
CONSENSUS
CGTGCCGGATCAGTATCCTGGTTCGGGTTTGGATCCTGGAAGTGATAATG

RI-AT2G41870-XLOC_013220-12146-0
GGCCGGGTCAGAGTCGGGTTGGGTCGACGGTGCAAAGAGTTAAGAGGGAA
RI-AT2G41870-XLOC_013220-12146-1
GGCCGGGTCAGAGTCGGGTTGGGTCGACGGTGCAAAGAGTTAAGAGGGAA
CONSENSUS
GGCCGGGTCAGAGTCGGGTTGGGTCGACGGTGCAAAGAGTTAAGAGGGAA

RI-AT2G41870-XLOC_013220-12146-0
GAGGTGGAAGCGAAGATAACGGCGTGGCAGACGGCAAAACTGGCTAAGAT
RI-AT2G41870-XLOC_013220-12146-1
GAGGTGGAAGCGAAGATAACGGCGTGGCAGACGGCAAAACTGGCTAAGAT
CONSENSUS
GAGGTGGAAGCGAAGATAACGGCGTGGCAGACGGCAAAACTGGCTAAGAT

RI-AT2G41870-XLOC_013220-12146-0
TAATAACAGGTTTAAGAGGGAAGACGCCGTTATTAACGGTTGGTTTAATG
RI-AT2G41870-XLOC_013220-12146-1
TAATAACAGGTTTAAGAGGGAAGACGCCGTTATTAACGGTTGGTTTAATG
CONSENSUS
TAATAACAGGTTTAAGAGGGAAGACGCCGTTATTAACGGTTGGTTTAATG

RI-AT2G41870-XLOC_013220-12146-0
AACAAGTTAACAAGGCCAACTCTTGGATGAAGAAAATTGAG
RI-AT2G41870-XLOC_013220-12146-1
AACAAGTTAACAAGGCCAACTCTTGGATGAAGAAAATTGAG
CONSENSUS
AACAAGTTAACAAGGCCAACTCTTGGATGAAGAAAATTGAG

alignment for event: RI-AT2G25730-XLOC_012292-5814

RI-AT2G25730-XLOC_012292-5814-0
AAAGGTGCTGGCTTGTGTGGTATGTGGCCGCCTGAAAAGCGCATTCCAGA
RI-AT2G25730-XLOC_012292-5814-1
AAAGGTGCTGGCTTGTGTGGTATGTGGCCGCCTGAAAAGCGCATTCCAGA
CONSENSUS
AAAGGTGCTGGCTTGTGTGGTATGTGGCCGCCTGAAAAGCGCATTCCAGA

RI-AT2G25730-XLOC_012292-5814-0
TTGCATCTAAAAGCGGAAGCGTGGCTGATGTTCAATATGTAGCTCATCAA
RI-AT2G25730-XLOC_012292-5814-1
TTGCATCTAAAAGCGGAAGCGTGGCTGATGTTCAATATGTAGCTCATCAA
CONSENSUS
TTGCATCTAAAAGCGGAAGCGTGGCTGATGTTCAATATGTAGCTCATCAA

RI-AT2G25730-XLOC_012292-5814-0
GCCTTACATGCCAATTCGCACACAGTACTCGATATGTGCAAGCAATGGCT
RI-AT2G25730-XLOC_012292-5814-1
GCCTTACATGCCAATTCGCACACAGTACTCGATATGTGCAAGCAATGGCT
CONSENSUS
GCCTTACATGCCAATTCGCACACAGTACTCGATATGTGCAAGCAATGGCT

RI-AT2G25730-XLOC_012292-5814-0
AGCTAAATACATGTAAAGCCCTGTAATCTACACGATAATAGAAGCCAACA
RI-AT2G25730-XLOC_012292-5814-1
AGCTAAATACATGTAAAGCCCTGTAATCTACACGATAATAGAAGCCAACA
CONSENSUS
AGCTAAATACATGTAAAGCCCTGTAATCTACACGATAATAGAAGCCAACA

RI-AT2G25730-XLOC_012292-5814-0
TAAAAACTCGAGGTATACATCTTTTATTTTTGGCAGATTAGCGATTGTTC
RI-AT2G25730-XLOC_012292-5814-1
TAAAAACTCGAG--------------------------------------
CONSENSUS
TAAAAACTCGAG......................................

RI-AT2G25730-XLOC_012292-5814-0
TTGCCTCGTATTTGTTCCTGGCTAAATTTTTCTTGCAAAAAAAAAGGAAA
RI-AT2G25730-XLOC_012292-5814-1
--GAAA
CONSENSUS
....GAAA

RI-AT2G25730-XLOC_012292-5814-0
AACACACTGCCTTGTGAGGTTTTTGCTTGAAACTTTGGGAGCAGATTGTT
RI-AT2G25730-XLOC_012292-5814-1
AACACACTGCCTTGTGAGGTTTTTGCTTGAAACTTTGGGAGCAGATTGTT
CONSENSUS
AACACACTGCCTTGTGAGGTTTTTGCTTGAAACTTTGGGAGCAGATTGTT

RI-AT2G25730-XLOC_012292-5814-0
TAAAAGAACATGGAGGGAAATGGTTCAGTCTCTCATAGGAAGATCATTAA
RI-AT2G25730-XLOC_012292-5814-1
TAAAAGAACATGGAGGGAAATGGTTCAGTCTCTCATAGGAAGATCATTAA
CONSENSUS
TAAAAGAACATGGAGGGAAATGGTTCAGTCTCTCATAGGAAGATCATTAA

RI-AT2G25730-XLOC_012292-5814-0
CTGATGATTCGCACATGTGAGATTATGGGTTTTGTGTGTTGTAACACATG
RI-AT2G25730-XLOC_012292-5814-1
CTGATGATTCGCACATGTGAGATTATGGGTTTTGTGTGTTGTAACACATG
CONSENSUS
CTGATGATTCGCACATGTGAGATTATGGGTTTTGTGTGTTGTAACACATG

RI-AT2G25730-XLOC_012292-5814-0
TCTACTTGTAAAAATTTATATGGTTGGTATATTTACTATTGAATGTATGA
RI-AT2G25730-XLOC_012292-5814-1
TCTACTTGTAAAAATTTATATGGTTGGTATATTTACTATTGAATGTATGA
CONSENSUS
TCTACTTGTAAAAATTTATATGGTTGGTATATTTACTATTGAATGTATGA

```
RI-AT2G25730-XLOC_012292-5814-0
      GGTTGATAACTTGCGGCTTTTTTGCAGTTGAGTTTCATAGTTAATCCTAT
RI-AT2G25730-XLOC_012292-5814-1
      GGTTGATAACTTGCGGCTTTTTTGCAGTTGAGTTTCATAGTTAATCCTAT
CONSENSUS
      GGTTGATAACTTGCGGCTTTTTTGCAGTTGAGTTTCATAGTTAATCCTAT

RI-AT2G25730-XLOC_012292-5814-0
      ATTGTTGTTAAAACTATATTGGAATTTGACACATATGTAAGTGTTGTATA
RI-AT2G25730-XLOC_012292-5814-1
      ATTGTTGTTAAAACTATATTGGAATTTGACACATATGTAAGTGTTGTATA
CONSENSUS
      ATTGTTGTTAAAACTATATTGGAATTTGACACATATGTAAGTGTTGTATA

RI-AT2G25730-XLOC_012292-5814-0  AAAATTCTTACTTTTACCGGGAC
RI-AT2G25730-XLOC_012292-5814-1  AAAATTCTTACTTTTACCGGGAC
CONSENSUS                        AAAATTCTTACTTTTACCGGGAC



alignment for event: RI-AT2G45380-XLOC_013402-5508

RI-AT2G45380-XLOC_013402-5508-0
      GAGTATTTTGGTCTGAGTGGTTCTTTAGGAGAAAGGATCTTTGATATGGT
RI-AT2G45380-XLOC_013402-5508-1
      GAGTATTTTGGTCTGAGTGGTTCTTTAGGAGAAAGGATCTTTGATATGGT
CONSENSUS
      GAGTATTTTGGTCTGAGTGGTTCTTTAGGAGAAAGGATCTTTGATATGGT

RI-AT2G45380-XLOC_013402-5508-0
      TACTCAACACAGGAAAGATGATAAAATGACTTTTGAAGATCTTGTTATTG
RI-AT2G45380-XLOC_013402-5508-1
      TACTCAACACAGGAAAGATGATAAAATGACTTTTGAAGATCTTGTTATTG
CONSENSUS
      TACTCAACACAGGAAAGATGATAAAATGACTTTTGAAGATCTTGTTATTG

RI-AT2G45380-XLOC_013402-5508-0
      CTAAAATGACTTTTGAAGATCTTGTTATTGCTAAGGTACACACTTAAGTC
RI-AT2G45380-XLOC_013402-5508-1
      CTAAAATGACTTTTGAAGATCTTGTTATTGCTAAG---------------
CONSENSUS
      CTAAAATGACTTTTGAAGATCTTGTTATTGCTAAG...............

RI-AT2G45380-XLOC_013402-5508-0
      TTTTCATCCTCTTAATCATTGGTTTCTTCTTCTGAGCTTTTGAAGATCTT
RI-AT2G45380-XLOC_013402-5508-1
      --------------------------------------------------
CONSENSUS
      ..................................................

RI-AT2G45380-XLOC_013402-5508-0
      AAAATGCAGAGTTCAATTTTGTAATCTCCATATGAAAAGTGATTGTAGTT
RI-AT2G45380-XLOC_013402-5508-1
      --------------------------------------------------
CONSENSUS
      ..................................................
```

RI-AT2G45380-XLOC_013402-5508-0
      ATAAATGTAGAACCAGTCTCACGTTGAATAGAATCCTTCCTCAGGTTAAG
RI-AT2G45380-XLOC_013402-5508-1

CONSENSUS
      ....

RI-AT2G45380-XLOC_013402-5508-0
      TCACTGATTTTTCTGAAAAGTCTATTCCTTTTTGTTAAAACCCTGGTTTC
RI-AT2G45380-XLOC_013402-5508-1

CONSENSUS
      ....

RI-AT2G45380-XLOC_013402-5508-0
      AATGCTTGCCATTGGAATTTTTGTGGTAAAATAAAGAAAATTCGCAAGGA
RI-AT2G45380-XLOC_013402-5508-1

CONSENSUS
      ....

RI-AT2G45380-XLOC_013402-5508-0
      TGAAGAATGATGAAGAAGCTTCTAGGTTATGGTAACATGACACGTGTATC
RI-AT2G45380-XLOC_013402-5508-1

CONSENSUS
      ....

RI-AT2G45380-XLOC_013402-5508-0
      AATCTCAGACGTCCTTTTTTGTTATAATTGAGGTTATATAAAATCAGAGT
RI-AT2G45380-XLOC_013402-5508-1

CONSENSUS
      ....

RI-AT2G45380-XLOC_013402-5508-0
      GATGTTATTTTGTCTTTTGAATTAAGAGTACTCTGCCTCTGGAATTTCTG
RI-AT2G45380-XLOC_013402-5508-1 -------------------------
AGTACTCTGCCTCTGGAATTTCTG
CONSENSUS
      .........................AGTACTCTGCCTCTGGAATTTCTG

RI-AT2G45380-XLOC_013402-5508-0 GGTTCTTCTCGGATTCTCTCAAAG
RI-AT2G45380-XLOC_013402-5508-1 GGTTCTTCTCGGATTCTCTCAAAG
CONSENSUS   GGTTCTTCTCGGATTCTCTCAAAG

alignment for event: RI-AT2G39090-XLOC_013055-9224

RI-AT2G39090-XLOC_013055-9224-0
      GGAAATCTACTTTTACAAGCAAAACGACCAGAAGCTGCAGCGATCGCCTT
RI-AT2G39090-XLOC_013055-9224-1
      GGAAATCTACTTTTACAAGCAAAACGACCAGAAGCTGCAGCGATCGCCTT
CONSENSUS

GGAAATCTACTTTTACAAGCAAAACGACCAGAAGCTGCAGCGATCGCCTT

RI-AT2G39090-XLOC_013055-9224-0
CAGGGCTGCCCAGAATTTGAGGTCCGATCTTCGTTCATATCAAGGTCTGG
RI-AT2G39090-XLOC_013055-9224-1
CAGGGCTGCCCAGAATTTGAGGTCCGATCTTCGTTCATATCAAG------
CONSENSUS
CAGGGCTGCCCAGAATTTGAGGTCCGATCTTCGTTCATATCAAG......

RI-AT2G39090-XLOC_013055-9224-0
TTCACGAGGGAAAAAGATCTGATATACACTTCCACTGTTGTACTGTTTTG
RI-AT2G39090-XLOC_013055-9224-1

CONSENSUS
....

RI-AT2G39090-XLOC_013055-9224-0
GTGGTATAAACCTTTAACAATCATGAAAAGGTTGGAGTGTGTAATTAACT
RI-AT2G39090-XLOC_013055-9224-1

CONSENSUS
....

RI-AT2G39090-XLOC_013055-9224-0
TGCGCTATTGAGGTCATTTCAGGCTTAGTCCATTCTTATCTTGCATTTGG
RI-AT2G39090-XLOC_013055-9224-1 ---------------------
GCTTAGTCCATTCTTATCTTGCATTTGG
CONSENSUS
.....................GCTTAGTCCATTCTTATCTTGCATTTGG

RI-AT2G39090-XLOC_013055-9224-0
TAAAACCAAAGAAGCATTGTATACCGCCAGGGAAGCAATGAATGCAATGC
RI-AT2G39090-XLOC_013055-9224-1
TAAAACCAAAGAAGCATTGTATACCGCCAGGGAAGCAATGAATGCAATGC
CONSENSUS
TAAAACCAAAGAAGCATTGTATACCGCCAGGGAAGCAATGAATGCAATGC

RI-AT2G39090-XLOC_013055-9224-0
CTCAATCCGCGAAGGCTCTGAAATTAGTTGGTGATGTTCATGCGTTTACA
RI-AT2G39090-XLOC_013055-9224-1
CTCAATCCGCGAAGGCTCTGAAATTAGTTGGTGATGTTCATGCGTTTACA
CONSENSUS
CTCAATCCGCGAAGGCTCTGAAATTAGTTGGTGATGTTCATGCGTTTACA

RI-AT2G39090-XLOC_013055-9224-0 TCAAGTGGCAGGGAAAAG
RI-AT2G39090-XLOC_013055-9224-1 TCAAGTGGCAGGGAAAAG
CONSENSUS TCAAGTGGCAGGGAAAAG

alignment for event: A5-AT2G04690-XLOC_008382-11921

A5-AT2G04690-XLOC_008382-11921-0
ATTGGCCTAAGGATCATGATTTCCGCTTCTTTAAACTCGAGATCATCGAT
A5-AT2G04690-XLOC_008382-11921-1
ATTGGCCTAAGGATCATGATTTCCGCTTCTTTAAACTCGAGATCATCGAT

CONSENSUS
      ATTGGCCTAAGGATCATGATTTCCGCTTCTTTAAACTCGAGATCATCGAT

A5-AT2G04690-XLOC_008382-11921-0
      ATATTTCTCATCAATTGGTATGGTGGAGCTAAACCTATCACTGTAGATGA
A5-AT2G04690-XLOC_008382-11921-1
      ATATTTCTCATCAATTGGTATGGTGGAGCTAAACCTATCACTGTAGATGA
CONSENSUS
      ATATTTCTCATCAATTGGTATGGTGGAGCTAAACCTATCACTGTAGATGA

A5-AT2G04690-XLOC_008382-11921-0
      ATACCTTCATGCCAAGTC--------------------------------
A5-AT2G04690-XLOC_008382-11921-1
      ATACCTTCATGCCAAGTCGTAAGTACATTGCGTGAACCCTTTTAAAAACA
CONSENSUS
      ATACCTTCATGCCAAGTC................................

A5-AT2G04690-XLOC_008382-11921-0 ---
GATCAAACTCGCTTCCTTTTTATAATAACTCACAAGAATCAAGCAAA
A5-AT2G04690-XLOC_008382-11921-1
      TCGGATCAAACTCGCTTCCTTTTTATAATAACTCACAAGAATCAAGCAAA
CONSENSUS
      ...GATCAAACTCGCTTCCTTTTTATAATAACTCACAAGAATCAAGCAAA

A5-AT2G04690-XLOC_008382-11921-0
      TGGTGAAGCAAGAAAATGTATATTGGATGAACAATAAAAAGTGTGTAACT
A5-AT2G04690-XLOC_008382-11921-1
      TGGTGAAGCAAGAAAATGTATATTGGATGAACAATAAAAAGTGTGTAACT
CONSENSUS
      TGGTGAAGCAAGAAAATGTATATTGGATGAACAATAAAAAGTGTGTAACT

A5-AT2G04690-XLOC_008382-11921-0
      GTTTCTGTAAATAACTCTTTGTTTTGGACCATAATAGATATTAAAATCTC
A5-AT2G04690-XLOC_008382-11921-1
      GTTTCTGTAAATAACTCTTTGTTTTGGACCATAATAGATATTAAAATCTC
CONSENSUS
      GTTTCTGTAAATAACTCTTTGTTTTGGACCATAATAGATATTAAAATCTC

A5-AT2G04690-XLOC_008382-11921-0
      TTGTAAATTACCTCAAAGCAATTTCAAAGGTATCCAAAATACTTTTCAAT
A5-AT2G04690-XLOC_008382-11921-1
      TTGTAAATTACCTCAAAGCAATTTCAAAGGTATCCAAAATACTTTTCAAT
CONSENSUS
      TTGTAAATTACCTCAAAGCAATTTCAAAGGTATCCAAAATACTTTTCAAT

A5-AT2G04690-XLOC_008382-11921-0 TTAT
A5-AT2G04690-XLOC_008382-11921-1 TTAT
CONSENSUS   TTAT

alignment for event: A3-AT2G32700-XLOC_010004-7200

A3-AT2G32700-XLOC_010004-7200-0
      AACAACCGCAAAAGAAAAGGGCCTTCCTCTTCTGGTCCTGCTAACAGCAC
A3-AT2G32700-XLOC_010004-7200-1

AACAACCGCAAAAGAAAAGGGCCTTCCTCTTCTGGTCCTGCTAACAGCAC
CONSENSUS
AACAACCGCAAAAGAAAAGGGCCTTCCTCTTCTGGTCCTGCTAACAGCAC

A3-AT2G32700-XLOC_010004-7200-0
AGGGACAGGAAACACTGTTGGCCCATCCAACTCACAGCCATCGACTCCGT
A3-AT2G32700-XLOC_010004-7200-1
AGGGACAGGAAACACTGTTGGCCCATCCAACTCACAGCCATCGACTCCGT
CONSENSUS
AGGGACAGGAAACACTGTTGGCCCATCCAACTCACAGCCATCGACTCCGT

A3-AT2G32700-XLOC_010004-7200-0
CAACGCATACCCCTGTTGATGGAGTTGCTATAGCTGGTAACATGCACCAT
A3-AT2G32700-XLOC_010004-7200-1
CAACGCATACCCCTGTTGATGGAGTTGCTATAGCTGGTAACATGCACCAT
CONSENSUS
CAACGCATACCCCTGTTGATGGAGTTGCTATAGCTGGTAACATGCACCAT

A3-AT2G32700-XLOC_010004-7200-0
GTGAATAGCATGCCAAAAGGGCCAATGATGTATGGTTCTGATGGGATCGG
A3-AT2G32700-XLOC_010004-7200-1
GTGAATAGCATGCCAAAAGGGCCAATGATGTATGGTTCTGATGGGATCGG
CONSENSUS
GTGAATAGCATGCCAAAAGGGCCAATGATGTATGGTTCTGATGGGATCGG

A3-AT2G32700-XLOC_010004-7200-0
TGGTCTTGCATCATCAGCAAATCAACTGCTGCAGGATGACATGGACCAGT
A3-AT2G32700-XLOC_010004-7200-1
TGGTCTTGCATCATCAGCAAATCAACTG------GATGACATGGACCAGT
CONSENSUS
TGGTCTTGCATCATCAGCAAATCAACTG......GATGACATGGACCAGT

A3-AT2G32700-XLOC_010004-7200-0
TTGGAGATGTGGGAGCTCTAGAAGATAATGTAGAATCATTTTTGTCCCAA
A3-AT2G32700-XLOC_010004-7200-1
TTGGAGATGTGGGAGCTCTAGAAGATAATGTAGAATCATTTTTGTCCCAA
CONSENSUS
TTGGAGATGTGGGAGCTCTAGAAGATAATGTAGAATCATTTTTGTCCCAA

A3-AT2G32700-XLOC_010004-7200-0
GATGATGGAGACGGAGGAAGCTTGTTTGGCACCCTAAAGCGGAACTCTTC
A3-AT2G32700-XLOC_010004-7200-1
GATGATGGAGACGGAGGAAGCTTGTTTGGCACCCTAAAGCGGAACTCTTC
CONSENSUS
GATGATGGAGACGGAGGAAGCTTGTTTGGCACCCTAAAGCGGAACTCTTC

A3-AT2G32700-XLOC_010004-7200-0 TGTGCATACCGAAACCTCAAAGC
A3-AT2G32700-XLOC_010004-7200-1 TGTGCATACCGAAACCTCAAAGC
CONSENSUS TGTGCATACCGAAACCTCAAAGC

alignment for event: RI-AT2G18440-XLOC_011870-10762

RI-AT2G18440-XLOC_011870-10762-0
ATCTAGATCTGGATCGTCCTTCGCGGCTTCTTCACATGCAAGATTCTGTG

RI-AT2G18440-XLOC_011870-10762-1
ATCTAGATCTGGATCGTCCTTCGCGGCTTCTTCACATGCAAGATTCTGTG
CONSENSUS
ATCTAGATCTGGATCGTCCTTCGCGGCTTCTTCACATGCAAGATTCTGTG

RI-AT2G18440-XLOC_011870-10762-0
AATTGTTCAAGTTGAGGCATCATTTCTGGATTACTAGGAGACGAATCTGT
RI-AT2G18440-XLOC_011870-10762-1
AATTGTTCAAGTTGAGGCATCATTTCTGGATTACTAGGAGACGAATCTGT
CONSENSUS
AATTGTTCAAGTTGAGGCATCATTTCTGGATTACTAGGAGACGAATCTGT

RI-AT2G18440-XLOC_011870-10762-0
TGCAGACGGATGTGTGTGTGTTGGATTGAATTGAGATTAGGGTGTAGAAG
RI-AT2G18440-XLOC_011870-10762-1
TGCAGACGGATGTGTGTGTGTTGGATTGAATTGAGATTAGGGTGTAGAAG
CONSENSUS
TGCAGACGGATGTGTGTGTGTTGGATTGAATTGAGATTAGGGTGTAGAAG

RI-AT2G18440-XLOC_011870-10762-0
ATGTTTTGTGGATAGCTAATAGCTTCCTGATTGCATCTCTGTCATCCGAC
RI-AT2G18440-XLOC_011870-10762-1
ATGTTTTGTGGATAGCTAATAGCTTCCTGATTGCATCTCTGTCATCCGAC
CONSENSUS
ATGTTTTGTGGATAGCTAATAGCTTCCTGATTGCATCTCTGTCATCCGAC

RI-AT2G18440-XLOC_011870-10762-0
CTTTGCCATGTCAGGTGCGCTTGCATGGCAGGTCAAAAAACTGATCCTCA
RI-AT2G18440-XLOC_011870-10762-1
CTTTGCCATGTCAGGTGCGCTTGCATGGCAGGTCAAAAAACTGATCCTCA
CONSENSUS
CTTTGCCATGTCAGGTGCGCTTGCATGGCAGGTCAAAAAACTGATCCTCA

RI-AT2G18440-XLOC_011870-10762-0
ATAAAAAAAAGATTTTGTGGGTTTTGGAGAGGAGGTCGCACGGGTTATTA
RI-AT2G18440-XLOC_011870-10762-1
ATAAAAAAAAGATTTTGTGGGTTTTGGAGAGGAGGTCGCACGGGTTATTA
CONSENSUS
ATAAAAAAAAGATTTTGTGGGTTTTGGAGAGGAGGTCGCACGGGTTATTA

RI-AT2G18440-XLOC_011870-10762-0
TTTTTTCCCGGGATCTCTTCTCCTCTGTGTGTGTCGTCTTGCCTCTGCTC
RI-AT2G18440-XLOC_011870-10762-1
TTTTTTCCCGGGATCTCTTCTCCTCTGTGTGTGTCGTCTTGCCTCTGCTC
CONSENSUS
TTTTTTCCCGGGATCTCTTCTCCTCTGTGTGTGTCGTCTTGCCTCTGCTC

RI-AT2G18440-XLOC_011870-10762-0
TATCTCTCTCCCTGCTCTTTCACATTTCATATCTTTCTTAAATGCTCATA
RI-AT2G18440-XLOC_011870-10762-1
TATCTCTCTCCCTGCTCTTTCACATTTCATATCTTTCTTAAATGCTCATA
CONSENSUS
TATCTCTCTCCCTGCTCTTTCACATTTCATATCTTTCTTAAATGCTCATA

RI-AT2G18440-XLOC_011870-10762-0
TACACTCAAAAACCGATCATAAGCAGAGTTTGTAACCAATATGGAGCAGT

RI-AT2G18440-XLOC_011870-10762-1
TACACTCAAAAACCGATCATAAGCAGAGTTTGTAACCAATATGGAGCAGT
CONSENSUS
TACACTCAAAAACCGATCATAAGCAGAGTTTGTAACCAATATGGAGCAGT

RI-AT2G18440-XLOC_011870-10762-0
GGGCGATTACAAATCTTCTTGCCCAACAACCTCGAGAGTTAAATCAGGTA
RI-AT2G18440-XLOC_011870-10762-1
GGGCGATTACAAATCTTCTTGCCCAACAACCTCGAGAGTTAAATCAG---
CONSENSUS
GGGCGATTACAAATCTTCTTGCCCAACAACCTCGAGAGTTAAATCAG...

RI-AT2G18440-XLOC_011870-10762-0
CTCATATCCATATTAAATCGAATTCTTAATTAGCATAATAAGGTAAACAT
RI-AT2G18440-XLOC_011870-10762-1

CONSENSUS
....

RI-AT2G18440-XLOC_011870-10762-0
AATCTGCAAGAGGAATTCTGGATTTAAATAAACCATAATCCGGTTGCCCT
RI-AT2G18440-XLOC_011870-10762-1

CONSENSUS
....

RI-AT2G18440-XLOC_011870-10762-0
AATTGTTAACTTGTATCAACGCTTATGCGAATCATGAAATTTTAATTTTA
RI-AT2G18440-XLOC_011870-10762-1

CONSENSUS
....

RI-AT2G18440-XLOC_011870-10762-0
TTGGTTTTGTTATTTATCTTATTGATCAATTATTGTTTTACAAGTTGTCA
RI-AT2G18440-XLOC_011870-10762-1

CONSENSUS
....

RI-AT2G18440-XLOC_011870-10762-0
TTTAATCTATTGAAAATGATGGTATTAAGAATTGCTGTTAAACAATTTTG
RI-AT2G18440-XLOC_011870-10762-1

CONSENSUS
....

RI-AT2G18440-XLOC_011870-10762-0
CGGGTTCTTTGTATGTATCATTGGTATTGTTTTATTAGTTCTATCCGTTT
RI-AT2G18440-XLOC_011870-10762-1

CONSENSUS
....

RI-AT2G18440-XLOC_011870-10762-0
AGAAGCTTTTTAGAATATGGTAAAGAACATTTTTATTTGCTCGTAGTTTG

RI-AT2G18440-XLOC_011870-10762-1

CONSENSUS
      ....

RI-AT2G18440-XLOC_011870-10762-0
      TATATGTCTGTAACCACCAGTTCCTGTAGTAACTATGAAGCTGGTAGACG
RI-AT2G18440-XLOC_011870-10762-1 ------------------------------
TAACTATGAAGCTGGTAGACG
CONSENSUS
      ..............................TAACTATGAAGCTGGTAGACG

RI-AT2G18440-XLOC_011870-10762-0
      ACGGAATTGAAGCTATGCAAAGCTGACGTTGGTTTTTTCTGAAGTTGATT
RI-AT2G18440-XLOC_011870-10762-1
      ACGGAATTGAAGCTATGCAAAGCTGACGTTGGTTTTTTCTGAAGTTGATT
CONSENSUS
      ACGGAATTGAAGCTATGCAAAGCTGACGTTGGTTTTTTCTGAAGTTGATT

RI-AT2G18440-XLOC_011870-10762-0 TGTTCTTGAAAG
RI-AT2G18440-XLOC_011870-10762-1 TGTTCTTGAAAG
CONSENSUS   TGTTCTTGAAAG

alignment for event: RI-AT2G01100-XLOC_008170-7880

RI-AT2G01100-XLOC_008170-7880-0
      ATTCGTAGTCTGTTGTGCGCTTGCCTCTCCTCTTTTGGATTAGCATACCT
RI-AT2G01100-XLOC_008170-7880-1
      ATTCGTAGTCTGTTGTGCGCTTGCCTCTCCTCTTTTGGATTAGCATACCT
CONSENSUS
      ATTCGTAGTCTGTTGTGCGCTTGCCTCTCCTCTTTTGGATTAGCATACCT

RI-AT2G01100-XLOC_008170-7880-0
      CTCACTCGCATCAGGTAAGAAGTTTGGTTCAGATTTAGTGAATCTCATTG
RI-AT2G01100-XLOC_008170-7880-1
      CTCACTCGCATCAG------------------------------------
CONSENSUS
      CTCACTCGCATCAG....................................

RI-AT2G01100-XLOC_008170-7880-0
      CTTTAGTTTCAAAGCTTATTAGCTCTCTGTGTTTTTGCGTTGGTTATTAC
RI-AT2G01100-XLOC_008170-7880-1

CONSENSUS
      ....

RI-AT2G01100-XLOC_008170-7880-0
      GTTGGAATACTTAACCCTTATAGCAACTACTGGAATTTTGCTGAAACATA
RI-AT2G01100-XLOC_008170-7880-1

CONSENSUS
      ....

RI-AT2G01100-XLOC_008170-7880-0

GTTAGTCTCTATGCTTTGTTTGGATTGAATTGATTATGATGGAATGCAAC
RI-AT2G01100-XLOC_008170-7880-1

CONSENSUS
....

RI-AT2G01100-XLOC_008170-7880-0
ATAGTTATCGCTGTGCCTTGTTTGGATGGAATGTTTTACCCTAATAGCAA
RI-AT2G01100-XLOC_008170-7880-1

CONSENSUS
....

RI-AT2G01100-XLOC_008170-7880-0
CAACTGTGATTTAGCTGAAAGTGTTTTTTTTATTCCTTCTGTATTAGCAT
RI-AT2G01100-XLOC_008170-7880-1

CONSENSUS
....

RI-AT2G01100-XLOC_008170-7880-0
TGTGTAGTGTTCTGTATTACCATCATGTAGATACTCTCTGTGCTTTGGCT
RI-AT2G01100-XLOC_008170-7880-1

CONSENSUS
....

RI-AT2G01100-XLOC_008170-7880-0
GGACTGATTGATGGAAGAGCTAACATTGTTTTTTCTTCTCTTTTGTGAGA
RI-AT2G01100-XLOC_008170-7880-1
---A
CONSENSUS
....A

RI-AT2G01100-XLOC_008170-7880-0
TGCACTTTTGGTTATCCTCATACCAAAATGCAGATTTTCAGTTGCTTTTT
RI-AT2G01100-XLOC_008170-7880-1
TGCACTTTTGGTTATCCTCATACCAAAATGCAGATTTTCAGTTGCTTTTT
CONSENSUS
TGCACTTTTGGTTATCCTCATACCAAAATGCAGATTTTCAGTTGCTTTTT

RI-AT2G01100-XLOC_008170-7880-0
CTATCCAAATGGACTGCAAGAAGTTCATCCAGATGGTCGAGGAGAAGAAA
RI-AT2G01100-XLOC_008170-7880-1
CTATCCAAATGGACTGCAAGAAGTTCATCCAGATGGTCGAGGAGAAGAAA
CONSENSUS
CTATCCAAATGGACTGCAAGAAGTTCATCCAGATGGTCGAGGAGAAGAAA

RI-AT2G01100-XLOC_008170-7880-0
CGAAGAGTTCTTGAGAAGCAAGAAGCTCCTTTGAAATGGGAGCAGAAGCT
RI-AT2G01100-XLOC_008170-7880-1
CGAAGAGTTCTTGAGAAGCAAGAAGCTCCTTTGAAATGGGAGCAGAAGCT
CONSENSUS
CGAAGAGTTCTTGAGAAGCAAGAAGCTCCTTTGAAATGGGAGCAGAAGCT

RI-AT2G01100-XLOC_008170-7880-0

AGAGGCGGCTGCGAATGCCAAAGCAGACACAGAAACTAAAGTGAAGAGAT
RI-AT2G01100-XLOC_008170-7880-1
AGAGGCGGCTGCGAATGCCAAAGCAGACACAGAAACTAAAGTGAAGAGAT
CONSENSUS
AGAGGCGGCTGCGAATGCCAAAGCAGACACAGAAACTAAAGTGAAGAGAT

RI-AT2G01100-XLOC_008170-7880-0
CAAAGGGCCCTAAGAGAAAACAAAGGGCTGCGTCTGAATCTAGCTCAGAA
RI-AT2G01100-XLOC_008170-7880-1
CAAAGGGCCCTAAGAGAAAACAAAGGGCTGCGTCTGAATCTAGCTCAGAA
CONSENSUS
CAAAGGGCCCTAAGAGAAAACAAAGGGCTGCGTCTGAATCTAGCTCAGAA

RI-AT2G01100-XLOC_008170-7880-0
AGTGATAGTAGCTCTGAGGTGAGAAGAAAGTCTAGAAGATCTCACAATAA
RI-AT2G01100-XLOC_008170-7880-1
AGTGATAGTAGCTCTGAGGTGAGAAGAAAGTCTAGAAGATCTCACAATAA
CONSENSUS
AGTGATAGTAGCTCTGAGGTGAGAAGAAAGTCTAGAAGATCTCACAATAA

RI-AT2G01100-XLOC_008170-7880-0
GCACCGAAGACATGCACACTCTGATTCAGATGACAGTGATAGGAGGAAAG
RI-AT2G01100-XLOC_008170-7880-1
GCACCGAAGACATGCACACTCTGATTCAGATGACAGTGATAGGAGGAAAG
CONSENSUS
GCACCGAAGACATGCACACTCTGATTCAGATGACAGTGATAGGAGGAAAG

RI-AT2G01100-XLOC_008170-7880-0
AGAAGAAATCCAGGAGGCAGAAAAGAAGGTCCTTGAGTCCAAGCGATGAT
RI-AT2G01100-XLOC_008170-7880-1
AGAAGAAATCCAGGAGGCAGAAAAGAAGGTCCTTGAGTCCAAGCGATGAT
CONSENSUS
AGAAGAAATCCAGGAGGCAGAAAAGAAGGTCCTTGAGTCCAAGCGATGAT

RI-AT2G01100-XLOC_008170-7880-0
AGCACTGGTGATTATGAAAGTGGGTCAGAGGATGAGCTGAGGATGAAGAT
RI-AT2G01100-XLOC_008170-7880-1
AGCACTGGTGATTATGAAAGTGGGTCAGAGGATGAGCTGAGGATGAAGAT
CONSENSUS
AGCACTGGTGATTATGAAAGTGGGTCAGAGGATGAGCTGAGGATGAAGAT

RI-AT2G01100-XLOC_008170-7880-0
AAAGCACCATCGGAGGCACAAGTGGCATAGCTCAAGAAAGACTTGCGATG
RI-AT2G01100-XLOC_008170-7880-1
AAAGCACCATCGGAGGCACAAGTGGCATAGCTCAAGAAAGACTTGCGATG
CONSENSUS
AAAGCACCATCGGAGGCACAAGTGGCATAGCTCAAGAAAGACTTGCGATG

RI-AT2G01100-XLOC_008170-7880-0
ATGACAGTACCGAAGATGTGAGAAGAAAACATTTAAAGCATCACAGGCGC
RI-AT2G01100-XLOC_008170-7880-1
ATGACAGTACCGAAGATGTGAGAAGAAAACATTTAAAGCATCACAGGCGC
CONSENSUS
ATGACAGTACCGAAGATGTGAGAAGAAAACATTTAAAGCATCACAGGCGC

RI-AT2G01100-XLOC_008170-7880-0

AGTGAGGTGGTCACTTCAAGTGATAGTGAGGAAGAGAGTGGAAGAAGAAG
RI-AT2G01100-XLOC_008170-7880-1
AGTGAGGTGGTCACTTCAAGTGATAGTGAGGAAGAGAGTGGAAGAAGAAG
CONSENSUS
AGTGAGGTGGTCACTTCAAGTGATAGTGAGGAAGAGAGTGGAAGAAGAAG

RI-AT2G01100-XLOC_008170-7880-0
GCGAGGCAAATATCACAGGCACAACAGAGGTTCAGCCTCCTCGAGTGGCT
RI-AT2G01100-XLOC_008170-7880-1
GCGAGGCAAATATCACAGGCACAACAGAGGTTCAGCCTCCTCGAGTGGCT
CONSENSUS
GCGAGGCAAATATCACAGGCACAACAGAGGTTCAGCCTCCTCGAGTGGCT

RI-AT2G01100-XLOC_008170-7880-0
CGGAAGAAGATAGTGGGAAAAGTATGAAGAGAAGGCAACATAAAAGGCAT
RI-AT2G01100-XLOC_008170-7880-1
CGGAAGAAGATAGTGGGAAAAGTATGAAGAGAAGGCAACATAAAAGGCAT
CONSENSUS
CGGAAGAAGATAGTGGGAAAAGTATGAAGAGAAGGCAACATAAAAGGCAT

RI-AT2G01100-XLOC_008170-7880-0
CGTTTGGCTGAGTCTTCAAGTGAGGAAGATGGGGCAATGAGAAGGACTAG
RI-AT2G01100-XLOC_008170-7880-1
CGTTTGGCTGAGTCTTCAAGTGAGGAAGATGGGGCAATGAGAAGGACTAG
CONSENSUS
CGTTTGGCTGAGTCTTCAAGTGAGGAAGATGGGGCAATGAGAAGGACTAG

RI-AT2G01100-XLOC_008170-7880-0
GCATCATAAACATGGCAGAGATTCAGCATCTGAGTCTGATGGAAGAAGGT
RI-AT2G01100-XLOC_008170-7880-1
GCATCATAAACATGGCAGAGATTCAGCATCTGAGTCTGATGGAAGAAGGT
CONSENSUS
GCATCATAAACATGGCAGAGATTCAGCATCTGAGTCTGATGGAAGAAGGT

RI-AT2G01100-XLOC_008170-7880-0
AATCAGATGAGAAAAGAGAGCACTACTTCGACAAATGAAAATGGAAAATG
RI-AT2G01100-XLOC_008170-7880-1
AATCAGATGAGAAAAGAGAGCACTACTTCGACAAATGAAAATGGAAAATG
CONSENSUS
AATCAGATGAGAAAAGAGAGCACTACTTCGACAAATGAAAATGGAAAATG

RI-AT2G01100-XLOC_008170-7880-0
TCTTTGTTTAATTCACATCATAAGAACTGAATCTTGTGGTAAACCATCAG
RI-AT2G01100-XLOC_008170-7880-1
TCTTTGTTTAATTCACATCATAAGAACTGAATCTTGTGGTAAACCATCAG
CONSENSUS
TCTTTGTTTAATTCACATCATAAGAACTGAATCTTGTGGTAAACCATCAG

RI-AT2G01100-XLOC_008170-7880-0
CACATGATTACTACCCAGATGTTGGGACCCAAATACGATGAATATTGTGT
RI-AT2G01100-XLOC_008170-7880-1
CACATGATTACTACCCAGATGTTGGGACCCAAATACGATGAATATTGTGT
CONSENSUS
CACATGATTACTACCCAGATGTTGGGACCCAAATACGATGAATATTGTGT

RI-AT2G01100-XLOC_008170-7880-0

TGAATTACTATGGTGGCACTCTGTTCTTAGATTTTCATTTTTACATTTGT
RI-AT2G01100-XLOC_008170-7880-1
TGAATTACTATGGTGGCACTCTGTTCTTAGATTTTCATTTTTACATTTGT
CONSENSUS
TGAATTACTATGGTGGCACTCTGTTCTTAGATTTTCATTTTTACATTTGT

RI-AT2G01100-XLOC_008170-7880-0
AGCAAGAATGGTACACAACCAATTGAACTCT
RI-AT2G01100-XLOC_008170-7880-1
AGCAAGAATGGTACACAACCAATTGAACTCT
CONSENSUS
AGCAAGAATGGTACACAACCAATTGAACTCT

alignment for event: RI-AT2G25640-XLOC_009631-1720

RI-AT2G25640-XLOC_009631-1720-0
TGGCGAAAAGACTACTACAAAAGAGTGGCCTATTTTGCTCGAGATTAAGG
RI-AT2G25640-XLOC_009631-1720-1
TGGCGAAAAGACTACTACAAAAGAGTGGCCTATTTTGCTCGAGATTAAGG
CONSENSUS
TGGCGAAAAGACTACTACAAAAGAGTGGCCTATTTTGCTCGAGATTAAGG

RI-AT2G25640-XLOC_009631-1720-0
GTAGAGTCAGATTAGATGCATTTGAGAAGTTTGTTCGAGAGCTCCCAAAT
RI-AT2G25640-XLOC_009631-1720-1
GTAGAGTCAGATTAGATGCATTTGAGAAGTTTGTTCGAGAGCTCCCAAAT
CONSENSUS
GTAGAGTCAGATTAGATGCATTTGAGAAGTTTGTTCGAGAGCTCCCAAAT

RI-AT2G25640-XLOC_009631-1720-0
TCCAGGAGTCGTGCTGTAATGGTATTTGTCTACACGTTTACCTCTATCAT
RI-AT2G25640-XLOC_009631-1720-1
TCCAGGAGTCGTGCTGTAATG-----------------------------
CONSENSUS
TCCAGGAGTCGTGCTGTAATG.............................

RI-AT2G25640-XLOC_009631-1720-0
TAATGTACATGTTTTATATACGCATTCTGTGTTTGAGCCCCTGCAAAAGC
RI-AT2G25640-XLOC_009631-1720-1

CONSENSUS
....

RI-AT2G25640-XLOC_009631-1720-0
TATTAACTGTGACGTTTAACCAAGCTTAAGTAGAAGCATTTAGGAAAGAA
RI-AT2G25640-XLOC_009631-1720-1

CONSENSUS
....

RI-AT2G25640-XLOC_009631-1720-0
GAGGAGAGATTAACGGCCAATACTGATTTGTGAATGATTGAGAAAAATAC
RI-AT2G25640-XLOC_009631-1720-1

```
CONSENSUS
      ..................................................

RI-AT2G25640-XLOC_009631-1720-0
      ATTACATCTGTGAATCACAATGGAGATAGATGGACTTACAAAAATATTTT
RI-AT2G25640-XLOC_009631-1720-1
      --------------------------------------------------
CONSENSUS
      ..................................................

RI-AT2G25640-XLOC_009631-1720-0
      CACCAGAACAATTGCATAGCTTATGTTTCTTGAATAGGCCTCCATATTCC
RI-AT2G25640-XLOC_009631-1720-1
      --------------------------------------------------
CONSENSUS
      ..................................................

RI-AT2G25640-XLOC_009631-1720-0
      AATAAAACTAAGACCTGTTTGTTCTCGATTCTATATGTATGTGATGAACT
RI-AT2G25640-XLOC_009631-1720-1
      --------------------------------------------------
CONSENSUS
      ..................................................

RI-AT2G25640-XLOC_009631-1720-0
      CGTTTATCCATCATGGTACTTCTGAAACCTTGCTTTCGTGTTACAGGTTA
RI-AT2G25640-XLOC_009631-1720-1
      ----------------------------------------------GTTA
CONSENSUS
      ..............................................GTTA

RI-AT2G25640-XLOC_009631-1720-0
      TGTGTTTCGTCTGCAAAGAAGAGTGCTCAAAGACAGAACAAGAGAACATT
RI-AT2G25640-XLOC_009631-1720-1
      TGTGTTTCGTCTGCAAAGAAGAGTGCTCAAAGACAGAACAAGAGAACATT
CONSENSUS
      TGTGTTTCGTCTGCAAAGAAGAGTGCTCAAAGACAGAACAAGAGAACATT

RI-AT2G25640-XLOC_009631-1720-0  TCCGAG
RI-AT2G25640-XLOC_009631-1720-1  TCCGAG
CONSENSUS                        TCCGAG


alignment for event: A3-AT2G44530-XLOC_010677-9288

A3-AT2G44530-XLOC_010677-9288-0
      GAATGAGATTTAAAGAATCTGAATTACAGTTATATTTTTAACGGAAATCG
A3-AT2G44530-XLOC_010677-9288-1
      GAATGAGATTTAAAGAATCTGAATTACAGTTATATTTTTAACGGAAATCG
CONSENSUS
      GAATGAGATTTAAAGAATCTGAATTACAGTTATATTTTTAACGGAAATCG

A3-AT2G44530-XLOC_010677-9288-0
      TTACGGTTAGTTAAAAACTTAGAAATGAGTTTTTGAGTATATAAAGGAAG
A3-AT2G44530-XLOC_010677-9288-1
```

TTACGGTTAGTTAAAAACTTAGAAATGAGTTTTTGAGTATATAAAGGAAG
CONSENSUS
TTACGGTTAGTTAAAAACTTAGAAATGAGTTTTTGAGTATATAAAGGAAG

A3-AT2G44530-XLOC_010677-9288-0
AAGAAGAAGAGAGAGTCATTTTCTTGTAGTCAAATTCGTATCTCGCGGGG
A3-AT2G44530-XLOC_010677-9288-1
AAGAAGAAGAGAGAGTCATTTTCTTGTAGTCAAATTCGTATCTCGCGGGG
CONSENSUS
AAGAAGAAGAGAGAGTCATTTTCTTGTAGTCAAATTCGTATCTCGCGGGG

A3-AT2G44530-XLOC_010677-9288-0
AAGAAGAAGAAAAGACTGCTTTTTCTCAAAACCCTAAAATCTCTCTCTCC
A3-AT2G44530-XLOC_010677-9288-1
AAGAAGAAGAAAAGACTGCTTTTTCTCAAAACCCTAAAATCTCTCTCTCC
CONSENSUS
AAGAAGAAGAAAAGACTGCTTTTTCTCAAAACCCTAAAATCTCTCTCTCC

A3-AT2G44530-XLOC_010677-9288-0
GGATCCATTTCTCGAACATGGCGTCTATTGTTCAACCATCGCCTACTTTC
A3-AT2G44530-XLOC_010677-9288-1
GGATCCATTTCTCGAACATGGCGTCTATTGTTCAACCATCGCCTACTTTC
CONSENSUS
GGATCCATTTCTCGAACATGGCGTCTATTGTTCAACCATCGCCTACTTTC

A3-AT2G44530-XLOC_010677-9288-0
CCGGCGCTAAATCTCCGGCGTTCTTCTCTGATTCGTCCGCCTTCTTCCGT
A3-AT2G44530-XLOC_010677-9288-1
CCGGCGCTAAATCTCCGGCGTTCTTCTCTGATTCGTCCGCCTTCTTCCGT
CONSENSUS
CCGGCGCTAAATCTCCGGCGTTCTTCTCTGATTCGTCCGCCTTCTTCCGT

A3-AT2G44530-XLOC_010677-9288-0
TCGATTTCCTCTTAAGTGTAACGCGGCGGATCCGTACAAGTTCGACGGCG
A3-AT2G44530-XLOC_010677-9288-1 TCGATTTCCTCTT---
TGTAACGCGGCGGATCCGTACAAGTTCGACGGCG
CONSENSUS
TCGATTTCCTCTT...TGTAACGCGGCGGATCCGTACAAGTTCGACGGCG

A3-AT2G44530-XLOC_010677-9288-0
GAAACTCTGCCGGTTTCCATCTGCTTACTGGCGACACCGTTCCGGCTAGC
A3-AT2G44530-XLOC_010677-9288-1
GAAACTCTGCCGGTTTCCATCTGCTTACTGGCGACACCGTTCCGGCTAGC
CONSENSUS
GAAACTCTGCCGGTTTCCATCTGCTTACTGGCGACACCGTTCCGGCTAGC

A3-AT2G44530-XLOC_010677-9288-0
TTTTCGAGGACACGTTTGGAAGATTCGATTTATCAGAACACCACACGACT
A3-AT2G44530-XLOC_010677-9288-1
TTTTCGAGGACACGTTTGGAAGATTCGATTTATCAGAACACCACACGACT
CONSENSUS
TTTTCGAGGACACGTTTGGAAGATTCGATTTATCAGAACACCACACGACT

A3-AT2G44530-XLOC_010677-9288-0
TCGTATCTTTTCCGGCACTGCTAATCCTATTTTGGCTCAG
A3-AT2G44530-XLOC_010677-9288-1

TCGTATCTTTTCCGGCACTGCTAATCCTATTTTGGCTCAG
CONSENSUS
TCGTATCTTTTCCGGCACTGCTAATCCTATTTTGGCTCAG

alignment for event: A5-AT2G39730-XLOC_013098-5080

A5-AT2G39730-XLOC_013098-5080-0
ATTTCTTCGGTGCTTTGAGGGCGAGAGTGTACGATGATGAAGTGAGGAAG
A5-AT2G39730-XLOC_013098-5080-1
ATTTCTTCGGTGCTTTGAGGGCGAGAGTGTACGATGATGAAGTGAGGAAG
CONSENSUS
ATTTCTTCGGTGCTTTGAGGGCGAGAGTGTACGATGATGAAGTGAGGAAG

A5-AT2G39730-XLOC_013098-5080-0
TTCGTTGAGAGCCTTGGAGTTGAGAAGATCGGAAAGAGGCTGGTTAACTC
A5-AT2G39730-XLOC_013098-5080-1
TTCGTTGAGAGCCTTGGAGTTGAGAAGATCGGAAAGAGGCTGGTTAACTC
CONSENSUS
TTCGTTGAGAGCCTTGGAGTTGAGAAGATCGGAAAGAGGCTGGTTAACTC

A5-AT2G39730-XLOC_013098-5080-0
AAGGGAAGGACCTCCCGTGTTCGAGCAACCCGAGATGACTTATGAGAAGC
A5-AT2G39730-XLOC_013098-5080-1
AAGGGAAGGACCTCCCGTGTTCGAGCAACCCGAGATGACTTATGAGAAGC
CONSENSUS
AAGGGAAGGACCTCCCGTGTTCGAGCAACCCGAGATGACTTATGAGAAGC

A5-AT2G39730-XLOC_013098-5080-0
TTATGGAATACGGAAACATGCTTGTGATGGAACAAGAGAATGTCAAGAGA
A5-AT2G39730-XLOC_013098-5080-1
TTATGGAATACGGAAACATGCTTGTGATGGAACAAGAGAATGTCAAGAGA
CONSENSUS
TTATGGAATACGGAAACATGCTTGTGATGGAACAAGAGAATGTCAAGAGA

A5-AT2G39730-XLOC_013098-5080-0
GTCCAACTTGCCGAGACCTACCTCAGCCAGGCTGCTTTGGGAGACGCAAA
A5-AT2G39730-XLOC_013098-5080-1
GTCCAACTTGCCGAGACCTACCTCAGCCAGGCTGCTTTGGGAGACGCAAA
CONSENSUS
GTCCAACTTGCCGAGACCTACCTCAGCCAGGCTGCTTTGGGAGACGCAAA

A5-AT2G39730-XLOC_013098-5080-0
CGCTGACGCCATCGGCCGCGGAACTTTCTACGGTAAAACAGAGGAAAAGG
A5-AT2G39730-XLOC_013098-5080-1
CGCTGACGCCATCGGCCGCGGAACTTTCTACG-----------GAAAAGG
CONSENSUS
CGCTGACGCCATCGGCCGCGGAACTTTCTACG...........GAAAAGG

A5-AT2G39730-XLOC_013098-5080-0
AGCCCAGCAAGTAAACCTGCCAGTTCCTGAAGGGTGTACTGATCCTGTGG
A5-AT2G39730-XLOC_013098-5080-1
AGCCCAGCAAGTAAACCTGCCAGTTCCTGAAGGGTGTACTGATCCTGTGG
CONSENSUS
AGCCCAGCAAGTAAACCTGCCAGTTCCTGAAGGGTGTACTGATCCTGTGG

A5-AT2G39730-XLOC_013098-5080-0
CTGAAAACTTTGATCCAACGGCTAGAAGTGACGATGGAACCTGTGTCTAC
A5-AT2G39730-XLOC_013098-5080-1
CTGAAAACTTTGATCCAACGGCTAGAAGTGACGATGGAACCTGTGTCTAC
CONSENSUS
CTGAAAACTTTGATCCAACGGCTAGAAGTGACGATGGAACCTGTGTCTAC

A5-AT2G39730-XLOC_013098-5080-0
AACTTTTGAGCAATATTATCCTGCTTATTAATTTGCTGTTTTACTCCTAT
A5-AT2G39730-XLOC_013098-5080-1
AACTTTTGAGCAATATTATCCTGCTTATTAATTTGCTGTTTTACTCCTAT
CONSENSUS
AACTTTTGAGCAATATTATCCTGCTTATTAATTTGCTGTTTTACTCCTAT

A5-AT2G39730-XLOC_013098-5080-0
TGTCTCTTTTGGTTTATTTTTCTCCTTTGTGTAATTGTGGATTGGATCTT
A5-AT2G39730-XLOC_013098-5080-1
TGTCTCTTTTGGTTTATTTTTCTCCTTTGTGTAATTGTGGATTGGATCTT
CONSENSUS
TGTCTCTTTTGGTTTATTTTTCTCCTTTGTGTAATTGTGGATTGGATCTT

A5-AT2G39730-XLOC_013098-5080-0
GTCCTCTTTTGTTCCCTTTTTTTTTTTTTATGATGTACAACACATTGGTA
A5-AT2G39730-XLOC_013098-5080-1
GTCCTCTTTTGTTCCCTTTTTTTTTTTTTATGATGTACAACACATTGGTA
CONSENSUS
GTCCTCTTTTGTTCCCTTTTTTTTTTTTTATGATGTACAACACATTGGTA

A5-AT2G39730-XLOC_013098-5080-0
ATTTAAAATTGCCTTGTCATAAACTACACTTCTTATTCCAATGTTGCACA
A5-AT2G39730-XLOC_013098-5080-1
ATTTAAAATTGCCTTGTCATAAACTACACTTCTTATTCCAATGTTGCACA
CONSENSUS
ATTTAAAATTGCCTTGTCATAAACTACACTTCTTATTCCAATGTTGCACA

A5-AT2G39730-XLOC_013098-5080-0 CTAGGTAAATGCTT
A5-AT2G39730-XLOC_013098-5080-1 CTAGGTAAATGCTT
CONSENSUS CTAGGTAAATGCTT

alignment for event: A5-AT2G25930-XLOC_009647-12888

A5-AT2G25930-XLOC_009647-12888-0
CCTTGTGGTGTGGAAAGAAACTTATCTGTCCAGCATCTTGATTCTTCAGC
A5-AT2G25930-XLOC_009647-12888-1
CCTTGTGGTGTGGAAAGAAACTTATCTGTCCAGCATCTTGATTCTTCAGC
CONSENSUS
CCTTGTGGTGTGGAAAGAAACTTATCTGTCCAGCATCTTGATTCTTCAGC

A5-AT2G25930-XLOC_009647-12888-0
CGCAAACCAAGCAACTGAGAAGTTTGTCTCCCAAATGTCCTTCATGGAAA
A5-AT2G25930-XLOC_009647-12888-1
CGCAAACCAAGCAACTGAGAAGTTTGTCTCCCAAATGTCCTTCATGGAAA
CONSENSUS

CGCAAACCAAGCAACTGAGAAGTTTGTCTCCCAAATGTCCTTCATGGAAA

A5-AT2G25930-XLOC_009647-12888-0
ATGTGAGATCTTCGGCACAGCATGATCAGAGGAAAATGGTGAGAGAGGAA
A5-AT2G25930-XLOC_009647-12888-1
ATGTGAGATCTTCGGCACAGCATGATCAGAGGAAAATGGTGAGAGAGGAA
CONSENSUS
ATGTGAGATCTTCGGCACAGCATGATCAGAGGAAAATGGTGAGAGAGGAA

A5-AT2G25930-XLOC_009647-12888-0
GAAGATTTTGCAGTTCCAGTATATATTAACTCAAGAAGATCTCAGTCTCA
A5-AT2G25930-XLOC_009647-12888-1
GAAGATTTTGCAGTTCCAGTATATATTAACTCAAGAAGATCTCAGTCTCA
CONSENSUS
GAAGATTTTGCAGTTCCAGTATATATTAACTCAAGAAGATCTCAGTCTCA

A5-AT2G25930-XLOC_009647-12888-0
TGGCAGAACCAAGAGTGGTATTGAGAAGGAAAAACACACCCCAATGGTGG
A5-AT2G25930-XLOC_009647-12888-1
TGGCAGAACCAAGAGTGGTATTGAGAAGGAAAAACACACCCCAATGGTGG
CONSENSUS
TGGCAGAACCAAGAGTGGTATTGAGAAGGAAAAACACACCCCAATGGTGG

A5-AT2G25930-XLOC_009647-12888-0
CACCTAGCTCTCATCACTCCATTCGATTTCAAGAAGTGAATCAGACAGGC
A5-AT2G25930-XLOC_009647-12888-1
CACCTAGCTCTCATCACTCCATTCGATTTCAAGAAGTGAATCAGACAGGC
CONSENSUS
CACCTAGCTCTCATCACTCCATTCGATTTCAAGAAGTGAATCAGACAGGC

A5-AT2G25930-XLOC_009647-12888-0
TCAAAGCAAAACGTATGTTTGGCTACTTGTTCAAAACCTGAAGTTAGGGA
A5-AT2G25930-XLOC_009647-12888-1
TCAAAGCAAAACGTATGTTTGGCTACTTGTTCAAAACCTGAAGTTAGGGA
CONSENSUS
TCAAAGCAAAACGTATGTTTGGCTACTTGTTCAAAACCTGAAGTTAGGGA

A5-AT2G25930-XLOC_009647-12888-0
TCAGGTCAAGGCGAATGCAAGGTCAGGTGGCTTTGTAATCTCTTTAGATG
A5-AT2G25930-XLOC_009647-12888-1
TCAGGTCAAGGCGAATGCAAGGTCAGGTGGCTTTGTAATCTCTTTAGATG
CONSENSUS
TCAGGTCAAGGCGAATGCAAGGTCAGGTGGCTTTGTAATCTCTTTAGATG

A5-AT2G25930-XLOC_009647-12888-0
TATCAGTCACAGAGGAGATTGATCTCGAAAAATCAGCATCAAGTCATGAT
A5-AT2G25930-XLOC_009647-12888-1
TATCAGTCACAGAGGAGATTGATCTCGAAAAATCAGCATCAAGTCATGAT
CONSENSUS
TATCAGTCACAGAGGAGATTGATCTCGAAAAATCAGCATCAAGTCATGAT

A5-AT2G25930-XLOC_009647-12888-0
AGAGTAAATGATTATAATGCTTCCTTGAGACAAGAGTCTAGAAATCGGTT
A5-AT2G25930-XLOC_009647-12888-1
AGAGTAAATGATTATAATGCTTCCTTGAGACAAGAGTCTAGAAATCGGTT
CONSENSUS

AGAGTAAATGATTATAATGCTTCCTTGAGACAAGAGTCTAGAAATCGGTT

A5-AT2G25930-XLOC_009647-12888-0
ATACCGAGATGGTGGCAAAACTCGTCTGAAGGACACTGATAATGGAGCTG
A5-AT2G25930-XLOC_009647-12888-1
ATACCGAGATGGTGGCAAAACTCGTCTGAAGGACACTGATAATGGAGCTG
CONSENSUS
ATACCGAGATGGTGGCAAAACTCGTCTGAAGGACACTGATAATGGAGCTG

A5-AT2G25930-XLOC_009647-12888-0
AATCTCACTTGGCAACGGAAAATCATTCACAAGAGGGTCATGGCAGTCCT
A5-AT2G25930-XLOC_009647-12888-1
AATCTCACTTGGCAACGGAAAATCATTCACAAGAGGGTCATGGCAGTCCT
CONSENSUS
AATCTCACTTGGCAACGGAAAATCATTCACAAGAGGGTCATGGCAGTCCT

A5-AT2G25930-XLOC_009647-12888-0
GAAGACATTGATAATGATCGTGAATACAGCAAAAGCAGAGCATGCGCCTC
A5-AT2G25930-XLOC_009647-12888-1
GAAGACATTGATAATGATCGTGAATACAGCAAAAGCAGAGCATGCGCCTC
CONSENSUS
GAAGACATTGATAATGATCGTGAATACAGCAAAAGCAGAGCATGCGCCTC

A5-AT2G25930-XLOC_009647-12888-0
TCTGCAGCAGATAAATGAAGAGGCAAGTGATGACGTTTCTGATGATTCGA
A5-AT2G25930-XLOC_009647-12888-1
TCTGCAGCAGATAAATGAAGAGGCAAGTGATGACGTTTCTGATGATTCGA
CONSENSUS
TCTGCAGCAGATAAATGAAGAGGCAAGTGATGACGTTTCTGATGATTCGA

A5-AT2G25930-XLOC_009647-12888-0
TGGTGGATTCTATATCCAGCATAGATGTCTCTCCCGATGATGTTGTGGGT
A5-AT2G25930-XLOC_009647-12888-1
TGGTGGATTCTATATCCAGCATAGATGTCTCTCCCGATGATGTTGTGGGT
CONSENSUS
TGGTGGATTCTATATCCAGCATAGATGTCTCTCCCGATGATGTTGTGGGT

A5-AT2G25930-XLOC_009647-12888-0
ATATTAGGTCAAAAACGTTTCTGGAGAGCAAGGAAAGCCATTGCCAA---
A5-AT2G25930-XLOC_009647-12888-1
ATATTAGGTCAAAAACGTTTCTGGAGAGCAAGGAAAGCCATTGCCAAGTA
CONSENSUS
ATATTAGGTCAAAAACGTTTCTGGAGAGCAAGGAAAGCCATTGCCAA...

A5-AT2G25930-XLOC_009647-12888-0

A5-AT2G25930-XLOC_009647-12888-1
AGTTCACTAGAAATTTACAGTTTGGTTATTTATTCTCCGCTCTTTCTATT
CONSENSUS
....

A5-AT2G25930-XLOC_009647-12888-0

A5-AT2G25930-XLOC_009647-12888-1
TATCTCCTTCTTTGATACCAACATTTTTTGCTTGAAAGAAGTTAATATTT
CONSENSUS

....

A5-AT2G25930-XLOC_009647-12888-0

A5-AT2G25930-XLOC_009647-12888-1
AAGCATTGTTCCGTAGTCTTACTGAAGCTTTTTCCTCTGTTGTTTTTTGC
CONSENSUS
....

A5-AT2G25930-XLOC_009647-12888-0

A5-AT2G25930-XLOC_009647-12888-1
TATTTTCATTGAGGACTGTGGTAGGGCATATTTCACTATCACCAAATTTC
CONSENSUS
....

A5-AT2G25930-XLOC_009647-12888-0

A5-AT2G25930-XLOC_009647-12888-1
AAATTTCTAGAACACTCTCCTTCATATTTTTTTCATGATTAATGCTGCA
CONSENSUS
....

A5-AT2G25930-XLOC_009647-12888-0

A5-AT2G25930-XLOC_009647-12888-1
ATTGATTGCTGATATACATATATGACTATAACTCAGTTTCATATTCTGTC
CONSENSUS
....

A5-AT2G25930-XLOC_009647-12888-0

A5-AT2G25930-XLOC_009647-12888-1
TCATTTTGGGAGAAAGAGATTTCAGGTTTATGCTTGAGAAGTGATGGTTC
CONSENSUS
....

A5-AT2G25930-XLOC_009647-12888-0

A5-AT2G25930-XLOC_009647-12888-1
TATAGTTGAGAGGCCCCTGATTCATCTAAAATGGTCCTATTATGTGTTTA
CONSENSUS
....

A5-AT2G25930-XLOC_009647-12888-0

A5-AT2G25930-XLOC_009647-12888-1
GTTGTAGAGTCCTCGGTAGAATATTAACGCGTTTAACACGTTGGATCATG
CONSENSUS
....

A5-AT2G25930-XLOC_009647-12888-0

A5-AT2G25930-XLOC_009647-12888-1
TTATAGCAGGGAGGGACATTCTCTGTTGACCTATATTGTGCAAGGTGCCC
CONSENSUS

```
      ..................................................

A5-AT2G25930-XLOC_009647-12888-0
      --------------------------------------------------
A5-AT2G25930-XLOC_009647-12888-1
      GCCGATGGCTTTATTACTATACCTTCTTTGCATCTGGTTGTTGGAACATG
CONSENSUS
      ..................................................

A5-AT2G25930-XLOC_009647-12888-0
      --------------------------------------------------
A5-AT2G25930-XLOC_009647-12888-1
      TCCCTGTCTCGGTTTGGTATTGCTTTTATTCTGCACTGTCGTCTTGGGCA
CONSENSUS
      ..................................................

A5-AT2G25930-XLOC_009647-12888-0
      ----------------------------------TCAACAAAGAGTATTT
A5-AT2G25930-XLOC_009647-12888-1
      TTTTCCCTACTTGTCATTCAAGGGGTTGAACCAGTCAACAAAGAGTATTT
CONSENSUS
      ..................................TCAACAAAGAGTATTT

A5-AT2G25930-XLOC_009647-12888-0
      GCTGTTCAACTATTTGAGTTGCACAGACTGATTAAG
A5-AT2G25930-XLOC_009647-12888-1
      GCTGTTCAACTATTTGAGTTGCACAGACTGATTAAG
CONSENSUS
      GCTGTTCAACTATTTGAGTTGCACAGACTGATTAAG



alignment for event: A5-AT2G41880-XLOC_010523-8982

A5-AT2G41880-XLOC_010523-8982-0
      AGGAACCGAGACAGAGGAGCAAATTCAAAAGCGGCTTAGAAATGCTGAGG
A5-AT2G41880-XLOC_010523-8982-1
      AGGAACCGAGACAGAGGAGCAAATTCAAAAGCGGCTTAGAAATGCTGAGG
CONSENSUS
      AGGAACCGAGACAGAGGAGCAAATTCAAAAGCGGCTTAGAAATGCTGAGG

A5-AT2G41880-XLOC_010523-8982-0
      CAGAGATCAAAGAAGGGATATCCTCGGGTATTTTCGGTCTCATTTTGTAT
A5-AT2G41880-XLOC_010523-8982-1
      CAGAGATCAAAGAAGGGATATCCTCGGGTATTTTCGGTCTCATTTTGTAT
CONSENSUS
      CAGAGATCAAAGAAGGGATATCCTCGGGTATTTTCGGTCTCATTTTGTAT

A5-AT2G41880-XLOC_010523-8982-0
      AATGACAACCTTGAGGAATGCTACAAGAAGCTCAAG--------------
A5-AT2G41880-XLOC_010523-8982-1
      AATGACAACCTTGAGGAATGCTACAAGAAGCTCAAGGTGAACCACCTTTT
CONSENSUS
      AATGACAACCTTGAGGAATGCTACAAGAAGCTCAAG..............

A5-AT2G41880-XLOC_010523-8982-0
```

```
      ---------------------------------------------------
A5-AT2G41880-XLOC_010523-8982-1
      GTTAATTGCTCAAAATGTTGTCATGTTCTGAATGCATAACATTACCGGAT
CONSENSUS
      ..................................................

A5-AT2G41880-XLOC_010523-8982-0
      ---------------------------------------------------
A5-AT2G41880-XLOC_010523-8982-1
      CTGTTTCCAGAATCTCTTGGGGCTAGATGGACTCGCTCATGTCAATGGTG
CONSENSUS
      ..................................................

A5-AT2G41880-XLOC_010523-8982-0  ------
TAGAGGGGATCAATCTTCCCATTGAGTACGCAGTATCTAAAATG
A5-AT2G41880-XLOC_010523-8982-1
      TAGAAATAGAGGGGATCAATCTTCCCATTGAGTACGCAGTATCTAAAATG
CONSENSUS
      ......TAGAGGGGATCAATCTTCCCATTGAGTACGCAGTATCTAAAATG

A5-AT2G41880-XLOC_010523-8982-0
      GAAGATAAGATCATTATTCAAGAAACAGGAAAAGAAACAAGGAATAA
A5-AT2G41880-XLOC_010523-8982-1
      GAAGATAAGATCATTATTCAAGAAACAGGAAAAGAAACAAGGAATAA
CONSENSUS
      GAAGATAAGATCATTATTCAAGAAACAGGAAAAGAAACAAGGAATAA



alignment for event: A3-AT2G26210-XLOC_009661-13838

A3-AT2G26210-XLOC_009661-13838-0
      CAAAATAGTTCTCAATCAGTTGGATCATCTTTTTATTCCTAAATAAATAA
A3-AT2G26210-XLOC_009661-13838-1
      CAAAATAGTTCTCAATCAGTTGGATCATCTTTTTATTCCTAAATAAATAA
CONSENSUS
      CAAAATAGTTCTCAATCAGTTGGATCATCTTTTTATTCCTAAATAAATAA

A3-AT2G26210-XLOC_009661-13838-0
      ACTCCAAAAGCATCTCTCTGTTTCTTCTCCTCTCTTCAAGCTTTTTGTTT
A3-AT2G26210-XLOC_009661-13838-1
      ACTCCAAAAGCATCTCTCTGTTTCTTCTCCTCTCTTCAAGCTTTTTGTTT
CONSENSUS
      ACTCCAAAAGCATCTCTCTGTTTCTTCTCCTCTCTTCAAGCTTTTTGTTT

A3-AT2G26210-XLOC_009661-13838-0
      CGTCTCAGTCGTCGTCATCATCTCTGACAGGTTATTCACATAGGCAAAGA
A3-AT2G26210-XLOC_009661-13838-1
      CGTCTCAGTCGTCGTCATCATCTCTGACAG--------------------
CONSENSUS
      CGTCTCAGTCGTCGTCATCATCTCTGACAG....................

A3-AT2G26210-XLOC_009661-13838-0
      AGTCATTCTTTTGCATGCTGTTTTCCAATAAAGTTAATTCTCTAACAGAA
A3-AT2G26210-XLOC_009661-13838-1
      ------------------------------------------------AA
```

CONSENSUS
      ....AA

A3-AT2G26210-XLOC_009661-13838-0
      CAAGTTTCGAGATTGGATATTGACGACAACGATATGGGACTTGGAGGTAG
A3-AT2G26210-XLOC_009661-13838-1
      CAAGTTTCGAGATTGGATATTGACGACAACGATATGGGACTTGGAGGTAG
CONSENSUS
      CAAGTTTCGAGATTGGATATTGACGACAACGATATGGGACTTGGAGGTAG

A3-AT2G26210-XLOC_009661-13838-0
      TGAAACAATGGAATGCAAGTGTGGTATGCCTCTGTGTATCTGTGTAGCTC
A3-AT2G26210-XLOC_009661-13838-1
      TGAAACAATGGAATGCAAGTGTGGTATGCCTCTGTGTATCTGTGTAGCTC
CONSENSUS
      TGAAACAATGGAATGCAAGTGTGGTATGCCTCTGTGTATCTGTGTAGCTC

A3-AT2G26210-XLOC_009661-13838-0
      CTCCCAAATCAACTGATAAACCAAACCCACCT
A3-AT2G26210-XLOC_009661-13838-1
      CTCCCAAATCAACTGATAAACCAAACCCACCT
CONSENSUS
      CTCCCAAATCAACTGATAAACCAAACCCACCT

alignment for event: A3-AT2G39000-XLOC_013051-2306

A3-AT2G39000-XLOC_013051-2306-0
      GAGCTTGCAATGCAAGTCAAATAGTTGATCTTTTTCCAGCGGTTTCACCT
A3-AT2G39000-XLOC_013051-2306-1
      GAGCTTGCAATGCAAGTCAAATAGTTGATCTTTTTCCAGCGGTTTCACCT
CONSENSUS
      GAGCTTGCAATGCAAGTCAAATAGTTGATCTTTTTCCAGCGGTTTCACCT

A3-AT2G39000-XLOC_013051-2306-0
      GAAATCGTTGTTCGTGAAGCACGGTTAGAGGACTGTTGGGAGGTGGCAGA
A3-AT2G39000-XLOC_013051-2306-1
      GAAATCGTTGTTCGTGAAGCACGGTTAGAGGACTGTTGGGAGGTGGCAGA
CONSENSUS
      GAAATCGTTGTTCGTGAAGCACGGTTAGAGGACTGTTGGGAGGTGGCAGA

A3-AT2G39000-XLOC_013051-2306-0
      GACTCATTGCAGCTCTTTCTTCCCGGGATATTCGTTCCCGCTTGATGTTG
A3-AT2G39000-XLOC_013051-2306-1
      GACTCATTGCAGCTCTTTCTTCCCGGGATATTCGTTCCCGCTTGATGTTG
CONSENSUS
      GACTCATTGCAGCTCTTTCTTCCCGGGATATTCGTTCCCGCTTGATGTTG

A3-AT2G39000-XLOC_013051-2306-0
      TTCTAAGAGTAGATAGGTTAATGGCAATGGTGATGGGATTTTCTATTCCA
A3-AT2G39000-XLOC_013051-2306-1
      TTCTAAGAGTAGATAGGTTAATGGCAATGGTGATGGGATTTTCTATTCCA
CONSENSUS
      TTCTAAGAGTAGATAGGTTAATGGCAATGGTGATGGGATTTTCTATTCCA

A3-AT2G39000-XLOC_013051-2306-0
CCTGGGTGCCAGAGGACTTGTTTAGTGGCTGTGATAGGTAGTTCAGTAGA
A3-AT2G39000-XLOC_013051-2306-1
CCTGGGTGCCAGAGGACTTGTTTAGTGGCTGTGATAGGTAGTTCAGTAGA
CONSENSUS
CCTGGGTGCCAGAGGACTTGTTTAGTGGCTGTGATAGGTAGTTCAGTAGA

A3-AT2G39000-XLOC_013051-2306-0
TGAAACCATCTGCTTTGGAAGTGATGATTTCAAAATTGGAGCTTTTGATG
A3-AT2G39000-XLOC_013051-2306-1
TGAAACCATCTGCTTTGGAAGTGATGATTTCAAAATTGGAGCTTTTGATG
CONSENSUS
TGAAACCATCTGCTTTGGAAGTGATGATTTCAAAATTGGAGCTTTTGATG

A3-AT2G39000-XLOC_013051-2306-0
CAAAGATCAGCTTAAACAAAGGTTATGTTGCTGGAATCTTGACTGTTGAT
A3-AT2G39000-XLOC_013051-2306-1
CAAAGATCAGCTTAAACAAAGGTTATGTTGCTGGAATCTTGACTGTTGAT
CONSENSUS
CAAAGATCAGCTTAAACAAAGGTTATGTTGCTGGAATCTTGACTGTTGAT

A3-AT2G39000-XLOC_013051-2306-0
ACTGTGGCTGATTACCTTCCAAGAAAAGGACCTCTCCGCCAGAGAAG---
A3-AT2G39000-XLOC_013051-2306-1
ACTGTGGCTGATTACCTTCCAAGAAAAGGACCTCTCCGCCAGAGAAGCCA
CONSENSUS
ACTGTGGCTGATTACCTTCCAAGAAAAGGACCTCTCCGCCAGAGAAG...

A3-AT2G39000-XLOC_013051-2306-0 -
GACCGGGATCGCTTATGTATCAAATGTGGCAGTTCGAGAGAATTTCCGG
A3-AT2G39000-XLOC_013051-2306-1
GGACCGGGATCGCTTATGTATCAAATGTGGCAGTTCGAGAGAATTTCCGG
CONSENSUS
.GACCGGGATCGCTTATGTATCAAATGTGGCAGTTCGAGAGAATTTCCGG

A3-AT2G39000-XLOC_013051-2306-0
CGCAAAGGAATAGCCAAGAGACTCATATGGAAAGCAGAGGCTTTAGCCAA
A3-AT2G39000-XLOC_013051-2306-1
CGCAAAGGAATAGCCAAGAGACTCATATGGAAAGCAGAGGCTTTAGCCAA
CONSENSUS
CGCAAAGGAATAGCCAAGAGACTCATATGGAAAGCAGAGGCTTTAGCCAA

A3-AT2G39000-XLOC_013051-2306-0
GAACTGGGGATGTAGAGCTATCGGCCTTCACTGTGATCTCAACAACTTAG
A3-AT2G39000-XLOC_013051-2306-1
GAACTGGGGATGTAGAGCTATCGGCCTTCACTGTGATCTCAACAACTTAG
CONSENSUS
GAACTGGGGATGTAGAGCTATCGGCCTTCACTGTGATCTCAACAACTTAG

A3-AT2G39000-XLOC_013051-2306-0
GAGCAACTAAACTTTACAAAGATCAAGGTTTCAGATCCATCAAGATCCCT
A3-AT2G39000-XLOC_013051-2306-1
GAGCAACTAAACTTTACAAAGATCAAGGTTTCAGATCCATCAAGATCCCT
CONSENSUS
GAGCAACTAAACTTTACAAAGATCAAGGTTTCAGATCCATCAAGATCCCT

A3-AT2G39000-XLOC_013051-2306-0
      GAAGGAGCGACTTGGCCTCAACCGAAGACATCTCCTGACACCAGGTTTAC
A3-AT2G39000-XLOC_013051-2306-1
      GAAGGAGCGACTTGGCCTCAACCGAAGACATCTCCTGACACCAGGTTTAC
CONSENSUS
      GAAGGAGCGACTTGGCCTCAACCGAAGACATCTCCTGACACCAGGTTTAC

A3-AT2G39000-XLOC_013051-2306-0
      CTTCATGATGAAGCTAGTGAACAACAACAATACACAAGCTCTTGAACAGT
A3-AT2G39000-XLOC_013051-2306-1
      CTTCATGATGAAGCTAGTGAACAACAACAATACACAAGCTCTTGAACAGT
CONSENSUS
      CTTCATGATGAAGCTAGTGAACAACAACAATACACAAGCTCTTGAACAGT

A3-AT2G39000-XLOC_013051-2306-0
      TTCGGTAAATCCGAGGAAAAAGGAAATTTGAATTCCGTTTGTTGTTGAAA
A3-AT2G39000-XLOC_013051-2306-1
      TTCGGTAAATCCGAGGAAAAAGGAAATTTGAATTCCGTTTGTTGTTGAAA
CONSENSUS
      TTCGGTAAATCCGAGGAAAAAGGAAATTTGAATTCCGTTTGTTGTTGAAA

A3-AT2G39000-XLOC_013051-2306-0
      TATAGCTCTGATTATGTAATTGTAAATATGGTTGCAGTCTTGTGTAAATG
A3-AT2G39000-XLOC_013051-2306-1
      TATAGCTCTGATTATGTAATTGTAAATATGGTTGCAGTCTTGTGTAAATG
CONSENSUS
      TATAGCTCTGATTATGTAATTGTAAATATGGTTGCAGTCTTGTGTAAATG

A3-AT2G39000-XLOC_013051-2306-0 CTATTGTCTATAATATTTCGACC
A3-AT2G39000-XLOC_013051-2306-1 CTATTGTCTATAATATTTCGACC
CONSENSUS   CTATTGTCTATAATATTTCGACC

alignment for event: SE-AT2G26810-XLOC_012348-1097

SE-AT2G26810-XLOC_012348-1097-0
      CTGATTTTGATTTAACAGGGCAGCTGGTTTGGCCTGGTGCGATGCTTATG
SE-AT2G26810-XLOC_012348-1097-1
      CTGATTTTGATTTAACAGGGCAGCTGGTTTGGCCTGGTGCGATGCTTATG
CONSENSUS
      CTGATTTTGATTTAACAGGGCAGCTGGTTTGGCCTGGTGCGATGCTTATG

SE-AT2G26810-XLOC_012348-1097-0
      AATGGTTATCTCTCAGAAAATGCTGACATTCTCCAGGGATGTTCAGTTTT
SE-AT2G26810-XLOC_012348-1097-1
      AATGGTTATCTCTCAGAAAATGCTGACATTCTCCAGGGATGTTCAGTTTT
CONSENSUS
      AATGGTTATCTCTCAGAAAATGCTGACATTCTCCAGGGATGTTCAGTTTT

SE-AT2G26810-XLOC_012348-1097-0
      GGAGTTGGGATCTGGCGTTG------------------------------
SE-AT2G26810-XLOC_012348-1097-1
      GGAGTTGGGATCTGGCGTTGTTAGAGATGTTATGTATTGTAACATCATCA
CONSENSUS
      GGAGTTGGGATCTGGCGTTG..............................

SE-AT2G26810-XLOC_012348-1097-0

SE-AT2G26810-XLOC_012348-1097-1
      CTCCCTGTTAGATATGCTGTATATACAAATATGGTCTTTGCATACTCTGC
CONSENSUS
      ....

SE-AT2G26810-XLOC_012348-1097-0 -------------------------
GTATAACTGGAGTCCTATGTAGCA
SE-AT2G26810-XLOC_012348-1097-1
      TTGGCTGACTTCACCATCATTCACATGTATAACTGGAGTCCTATGTAGCA
CONSENSUS
      ..........................GTATAACTGGAGTCCTATGTAGCA

SE-AT2G26810-XLOC_012348-1097-0
      AATTTTGCCGTAAAGTTATTTTTACTGACCACAACGATGAAGTGCTCAAG
SE-AT2G26810-XLOC_012348-1097-1
      AATTTTGCCGTAAAGTTATTTTTACTGACCACAACGATGAAGTGCTCAAG
CONSENSUS
      AATTTTGCCGTAAAGTTATTTTTACTGACCACAACGATGAAGTGCTCAAG

alignment for event: RI-AT2G14170-XLOC_011616-12762

RI-AT2G14170-XLOC_011616-12762-0
      ATCGGTATTAATGTTCCAATCCCGGTTCCATTACCGTTCTTTTCCTTCAC
RI-AT2G14170-XLOC_011616-12762-1
      ATCGGTATTAATGTTCCAATCCCGGTTCCATTACCGTTCTTTTCCTTCAC
CONSENSUS
      ATCGGTATTAATGTTCCAATCCCGGTTCCATTACCGTTCTTTTCCTTCAC

RI-AT2G14170-XLOC_011616-12762-0
      CGGGAACAAGGCCTCATTCGCAGGAGATCTTAACTTCTATGGTAAAATTC
RI-AT2G14170-XLOC_011616-12762-1
      CGGGAACAAGGCCTCATTCGCAGGAGATCTTAACTTCTATG---------
CONSENSUS
      CGGGAACAAGGCCTCATTCGCAGGAGATCTTAACTTCTATG.........

RI-AT2G14170-XLOC_011616-12762-0
      TTGTCACAACTAAACAAAAGAAGATCCATTCTCCTTTCTCTTTGCTTATA
RI-AT2G14170-XLOC_011616-12762-1

CONSENSUS
      ....

RI-AT2G14170-XLOC_011616-12762-0
      TATATACTTTGAGTGAACTATTTTTGCAGGCAAAGCAGGAGTGGACTTTT
RI-AT2G14170-XLOC_011616-12762-1 ------------------------------
GCAAAGCAGGAGTGGACTTTT
CONSENSUS
      .............................GCAAAGCAGGAGTGGACTTTT

RI-AT2G14170-XLOC_011616-12762-0
      TCACTCAGATCAAAACTGTTACACAACAGTGGAAAGATATTCCAACTTCA

RI-AT2G14170-XLOC_011616-12762-1
TCACTCAGATCAAAACTGTTACACAACAGTGGAAAGATATTCCAACTTCA
CONSENSUS
TCACTCAGATCAAAACTGTTACACAACAGTGGAAAGATATTCCAACTTCA

RI-AT2G14170-XLOC_011616-12762-0
GTATCTCTGGCAATGCCAACGTCTCAAAAGCAATAATGAGACCTTAACTT
RI-AT2G14170-XLOC_011616-12762-1
GTATCTCTGGCAATGCCAACGTCTCAAAAGCAATAATGAGACCTTAACTT
CONSENSUS
GTATCTCTGGCAATGCCAACGTCTCAAAAGCAATAATGAGACCTTAACTT

RI-AT2G14170-XLOC_011616-12762-0
CAAATACTTTTTTTACTTTTGGATTATCGAATCAGCTTCAAGAAGAACCC
RI-AT2G14170-XLOC_011616-12762-1
CAAATACTTTTTTTACTTTTGGATTATCGAATCAGCTTCAAGAAGAACCC
CONSENSUS
CAAATACTTTTTTTACTTTTGGATTATCGAATCAGCTTCAAGAAGAACCC

RI-AT2G14170-XLOC_011616-12762-0
TTTGTTTATTTTTCAGCTCTCTAGTATTTTCTCATGCTTAGTTTCCTTCT
RI-AT2G14170-XLOC_011616-12762-1
TTTGTTTATTTTTCAGCTCTCTAGTATTTTCTCATGCTTAGTTTCCTTCT
CONSENSUS
TTTGTTTATTTTTCAGCTCTCTAGTATTTTCTCATGCTTAGTTTCCTTCT

RI-AT2G14170-XLOC_011616-12762-0
TTTAATCACTATGGTGACTAGAACTCTAGAAGTAACCATGGGAATAAGAA
RI-AT2G14170-XLOC_011616-12762-1
TTTAATCACTATGGTGACTAGAACTCTAGAAGTAACCATGGGAATAAGAA
CONSENSUS
TTTAATCACTATGGTGACTAGAACTCTAGAAGTAACCATGGGAATAAGAA

RI-AT2G14170-XLOC_011616-12762-0
AAAGTAAAAATCTCAAATTTGCCTAATTTGTCAAATAAAACCCCTAAATG
RI-AT2G14170-XLOC_011616-12762-1
AAAGTAAAAATCTCAAATTTGCCTAATTTGTCAAATAAAACCCCTAAATG
CONSENSUS
AAAGTAAAAATCTCAAATTTGCCTAATTTGTCAAATAAAACCCCTAAATG

RI-AT2G14170-XLOC_011616-12762-0
ACATTCTTTGAGTCGTAAACTCATAAAAGTCAAAGTGAAACTTATATATA
RI-AT2G14170-XLOC_011616-12762-1
ACATTCTTTGAGTCGTAAACTCATAAAAGTCAAAGTGAAACTTATATATA
CONSENSUS
ACATTCTTTGAGTCGTAAACTCATAAAAGTCAAAGTGAAACTTATATATA

RI-AT2G14170-XLOC_011616-12762-0
CAAGTTATTATGTGTCTTCATTCCCAATTGAAACCCAAAAATCCCTGAAT
RI-AT2G14170-XLOC_011616-12762-1
CAAGTTATTATGTGTCTTCATTCCCAATTGAAACCCAAAAATCCCTGAAT
CONSENSUS
CAAGTTATTATGTGTCTTCATTCCCAATTGAAACCCAAAAATCCCTGAAT

RI-AT2G14170-XLOC_011616-12762-0
TCAATAAACTCGAATTACGAAATTTTGAGTTTTATTTTGACTGTTTTTTA

RI-AT2G14170-XLOC_011616-12762-1
      TCAATAAACTCGAATTACGAAATTTTGAGTTTTATTTTGACTGTTTTTTA
CONSENSUS
      TCAATAAACTCGAATTACGAAATTTTGAGTTTTATTTTGACTGTTTTTTA

RI-AT2G14170-XLOC_011616-12762-0 ATTATCA
RI-AT2G14170-XLOC_011616-12762-1 ATTATCA
CONSENSUS ATTATCA

alignment for event: RI-AT2G25930-XLOC_009647-12884

RI-AT2G25930-XLOC_009647-12884-0
      CCTTGTGGTGTGGAAAGAAACTTATCTGTCCAGCATCTTGATTCTTCAGC
RI-AT2G25930-XLOC_009647-12884-1
      CCTTGTGGTGTGGAAAGAAACTTATCTGTCCAGCATCTTGATTCTTCAGC
CONSENSUS
      CCTTGTGGTGTGGAAAGAAACTTATCTGTCCAGCATCTTGATTCTTCAGC

RI-AT2G25930-XLOC_009647-12884-0
      CGCAAACCAAGCAACTGAGAAGTTTGTCTCCCAAATGTCCTTCATGGAAA
RI-AT2G25930-XLOC_009647-12884-1
      CGCAAACCAAGCAACTGAGAAGTTTGTCTCCCAAATGTCCTTCATGGAAA
CONSENSUS
      CGCAAACCAAGCAACTGAGAAGTTTGTCTCCCAAATGTCCTTCATGGAAA

RI-AT2G25930-XLOC_009647-12884-0
      ATGTGAGATCTTCGGCACAGCATGATCAGAGGAAAATGGTGAGAGAGGAA
RI-AT2G25930-XLOC_009647-12884-1
      ATGTGAGATCTTCGGCACAGCATGATCAGAGGAAAATGGTGAGAGAGGAA
CONSENSUS
      ATGTGAGATCTTCGGCACAGCATGATCAGAGGAAAATGGTGAGAGAGGAA

RI-AT2G25930-XLOC_009647-12884-0
      GAAGATTTTGCAGTTCCAGTATATATTAACTCAAGAAGATCTCAGTCTCA
RI-AT2G25930-XLOC_009647-12884-1
      GAAGATTTTGCAGTTCCAGTATATATTAACTCAAGAAGATCTCAGTCTCA
CONSENSUS
      GAAGATTTTGCAGTTCCAGTATATATTAACTCAAGAAGATCTCAGTCTCA

RI-AT2G25930-XLOC_009647-12884-0
      TGGCAGAACCAAGAGTGGTATTGAGAAGGAAAAACACACCCCAATGGTGG
RI-AT2G25930-XLOC_009647-12884-1
      TGGCAGAACCAAGAGTGGTATTGAGAAGGAAAAACACACCCCAATGGTGG
CONSENSUS
      TGGCAGAACCAAGAGTGGTATTGAGAAGGAAAAACACACCCCAATGGTGG

RI-AT2G25930-XLOC_009647-12884-0
      CACCTAGCTCTCATCACTCCATTCGATTTCAAGAAGTGAATCAGACAGGC
RI-AT2G25930-XLOC_009647-12884-1
      CACCTAGCTCTCATCACTCCATTCGATTTCAAGAAGTGAATCAGACAGGC
CONSENSUS
      CACCTAGCTCTCATCACTCCATTCGATTTCAAGAAGTGAATCAGACAGGC

RI-AT2G25930-XLOC_009647-12884-0

TCAAAGCAAAACGTATGTTTGGCTACTTGTTCAAAACCTGAAGTTAGGGA
RI-AT2G25930-XLOC_009647-12884-1
TCAAAGCAAAACGTATGTTTGGCTACTTGTTCAAAACCTGAAGTTAGGGA
CONSENSUS
TCAAAGCAAAACGTATGTTTGGCTACTTGTTCAAAACCTGAAGTTAGGGA

RI-AT2G25930-XLOC_009647-12884-0
TCAGGTCAAGGCGAATGCAAGGTCAGGTGGCTTTGTAATCTCTTTAGATG
RI-AT2G25930-XLOC_009647-12884-1
TCAGGTCAAGGCGAATGCAAGGTCAGGTGGCTTTGTAATCTCTTTAGATG
CONSENSUS
TCAGGTCAAGGCGAATGCAAGGTCAGGTGGCTTTGTAATCTCTTTAGATG

RI-AT2G25930-XLOC_009647-12884-0
TATCAGTCACAGAGGAGATTGATCTCGAAAAATCAGCATCAAGTCATGAT
RI-AT2G25930-XLOC_009647-12884-1
TATCAGTCACAGAGGAGATTGATCTCGAAAAATCAGCATCAAGTCATGAT
CONSENSUS
TATCAGTCACAGAGGAGATTGATCTCGAAAAATCAGCATCAAGTCATGAT

RI-AT2G25930-XLOC_009647-12884-0
AGAGTAAATGATTATAATGCTTCCTTGAGACAAGAGTCTAGAAATCGGTT
RI-AT2G25930-XLOC_009647-12884-1
AGAGTAAATGATTATAATGCTTCCTTGAGACAAGAGTCTAGAAATCGGTT
CONSENSUS
AGAGTAAATGATTATAATGCTTCCTTGAGACAAGAGTCTAGAAATCGGTT

RI-AT2G25930-XLOC_009647-12884-0
ATACCGAGATGGTGGCAAAACTCGTCTGAAGGACACTGATAATGGAGCTG
RI-AT2G25930-XLOC_009647-12884-1
ATACCGAGATGGTGGCAAAACTCGTCTGAAGGACACTGATAATGGAGCTG
CONSENSUS
ATACCGAGATGGTGGCAAAACTCGTCTGAAGGACACTGATAATGGAGCTG

RI-AT2G25930-XLOC_009647-12884-0
AATCTCACTTGGCAACGGAAAATCATTCACAAGAGGGTCATGGCAGTCCT
RI-AT2G25930-XLOC_009647-12884-1
AATCTCACTTGGCAACGGAAAATCATTCACAAGAGGGTCATGGCAGTCCT
CONSENSUS
AATCTCACTTGGCAACGGAAAATCATTCACAAGAGGGTCATGGCAGTCCT

RI-AT2G25930-XLOC_009647-12884-0
GAAGACATTGATAATGATCGTGAATACAGCAAAAGCAGAGCATGCGCCTC
RI-AT2G25930-XLOC_009647-12884-1
GAAGACATTGATAATGATCGTGAATACAGCAAAAGCAGAGCATGCGCCTC
CONSENSUS
GAAGACATTGATAATGATCGTGAATACAGCAAAAGCAGAGCATGCGCCTC

RI-AT2G25930-XLOC_009647-12884-0
TCTGCAGCAGATAAATGAAGAGGCAAGTGATGACGTTTCTGATGATTCGA
RI-AT2G25930-XLOC_009647-12884-1
TCTGCAGCAGATAAATGAAGAGGCAAGTGATGACGTTTCTGATGATTCGA
CONSENSUS
TCTGCAGCAGATAAATGAAGAGGCAAGTGATGACGTTTCTGATGATTCGA

RI-AT2G25930-XLOC_009647-12884-0

TGGTGGATTCTATATCCAGCATAGATGTCTCTCCCGATGATGTTGTGGGT
RI-AT2G25930-XLOC_009647-12884-1
TGGTGGATTCTATATCCAGCATAGATGTCTCTCCCGATGATGTTGTGGGT
CONSENSUS
TGGTGGATTCTATATCCAGCATAGATGTCTCTCCCGATGATGTTGTGGGT

RI-AT2G25930-XLOC_009647-12884-0
ATATTAGGTCAAAAACGTTTCTGGAGAGCAAGGAAAGCCATTGCCAAGTA
RI-AT2G25930-XLOC_009647-12884-1
ATATTAGGTCAAAAACGTTTCTGGAGAGCAAGGAAAGCCATTGCCAA---
CONSENSUS
ATATTAGGTCAAAAACGTTTCTGGAGAGCAAGGAAAGCCATTGCCAA...

RI-AT2G25930-XLOC_009647-12884-0
AGTTCACTAGAAATTTACAGTTTGGTTATTTATTCTCCGCTCTTTCTATT
RI-AT2G25930-XLOC_009647-12884-1

CONSENSUS
....

RI-AT2G25930-XLOC_009647-12884-0
TATCTCCTTCTTTGATACCAACATTTTTTGCTTGAAAGAAGTTAATATTT
RI-AT2G25930-XLOC_009647-12884-1

CONSENSUS
....

RI-AT2G25930-XLOC_009647-12884-0
AAGCATTGTTCCGTAGTCTTACTGAAGCTTTTTCCTCTGTTGTTTTTTGC
RI-AT2G25930-XLOC_009647-12884-1

CONSENSUS
....

RI-AT2G25930-XLOC_009647-12884-0
TATTTTCATTGAGGACTGTGGTAGGGCATATTTCACTATCACCAAATTTC
RI-AT2G25930-XLOC_009647-12884-1

CONSENSUS
....

RI-AT2G25930-XLOC_009647-12884-0
AAATTTCTAGAACACTCTCCTTCATATTTTTTTTCATGATTAATGCTGCA
RI-AT2G25930-XLOC_009647-12884-1

CONSENSUS
....

RI-AT2G25930-XLOC_009647-12884-0
ATTGATTGCTGATATACATATATGACTATAACTCAGTTTCATATTCTGTC
RI-AT2G25930-XLOC_009647-12884-1

CONSENSUS
....

RI-AT2G25930-XLOC_009647-12884-0

TCATTTTGGGAGAAAGAGATTTCAGGTTTATGCTTGAGAAGTGATGGTTC
RI-AT2G25930-XLOC_009647-12884-1

CONSENSUS
....

RI-AT2G25930-XLOC_009647-12884-0
TATAGTTGAGAGGCCCCTGATTCATCTAAAATGGTCCTATTATGTGTTTA
RI-AT2G25930-XLOC_009647-12884-1

CONSENSUS
....

RI-AT2G25930-XLOC_009647-12884-0
GTTGTAGAGTCCTCGGTAGAATATTAACGCGTTTAACACGTTGGATCATG
RI-AT2G25930-XLOC_009647-12884-1

CONSENSUS
....

RI-AT2G25930-XLOC_009647-12884-0
TTATAGCAGGGAGGGACATTCTCTGTTGACCTATATTGTGCAAGGTGCCC
RI-AT2G25930-XLOC_009647-12884-1 ---------
GGAGGGACATTCTCTGTTGACCTATATTGTGCAAGGTGCCC
CONSENSUS
.........GGAGGGACATTCTCTGTTGACCTATATTGTGCAAGGTGCCC

RI-AT2G25930-XLOC_009647-12884-0
GCCGATGGCTTTATTACTATACCTTCTTTGCATCTGGTTGTTGGAACATG
RI-AT2G25930-XLOC_009647-12884-1
GCCGATGGCTTTATTACTATACCTTCTTTGCATCTGGTTGTTGGAACATG
CONSENSUS
GCCGATGGCTTTATTACTATACCTTCTTTGCATCTGGTTGTTGGAACATG

RI-AT2G25930-XLOC_009647-12884-0
TCCCTGTCTCGGTTTGGTATTGCTTTTATTCTGCACTGTCGTCTTGGGCA
RI-AT2G25930-XLOC_009647-12884-1
TCCCTGTCTCGGTTTGGTATTGCTTTTATTCTGCACTGTCGTCTTGGGCA
CONSENSUS
TCCCTGTCTCGGTTTGGTATTGCTTTTATTCTGCACTGTCGTCTTGGGCA

RI-AT2G25930-XLOC_009647-12884-0
TTTTCCCTACTTGTCATTCAAGGGGTTGAACCAGGTAGGGAAATGTTTTT
RI-AT2G25930-XLOC_009647-12884-1
TTTTCCCTACTTGTCATTCAAGGGGTTGAACCAGGTAGGGAAATGTTTTT
CONSENSUS
TTTTCCCTACTTGTCATTCAAGGGGTTGAACCAGGTAGGGAAATGTTTTT

RI-AT2G25930-XLOC_009647-12884-0
CCGAGGACCCCAGGATCTAAATTTTAGTTAACCATACGTAAAGTTAGTTT
RI-AT2G25930-XLOC_009647-12884-1
CCGAGGACCCCAGGATCTAAATTTTAGTTAACCATACGTAAAGTTAGTTT
CONSENSUS
CCGAGGACCCCAGGATCTAAATTTTAGTTAACCATACGTAAAGTTAGTTT

RI-AT2G25930-XLOC_009647-12884-0

TGAGTCTTATGACGATGCAGAATTATAGTTTCTTCTTACTACTGCTTAAG
RI-AT2G25930-XLOC_009647-12884-1
TGAGTCTTATGACGATGCAGAATTATAGTTTCTTCTTACTACTGCTTAAG
CONSENSUS
TGAGTCTTATGACGATGCAGAATTATAGTTTCTTCTTACTACTGCTTAAG

RI-AT2G25930-XLOC_009647-12884-0
AGGATCCTTAGTGTGGTTGTGAACTACAGAGTTTTTATGATTGTAGGCTT
RI-AT2G25930-XLOC_009647-12884-1
AGGATCCTTAGTGTGGTTGTGAACTACAGAGTTTTTATGATTGTAGGCTT
CONSENSUS
AGGATCCTTAGTGTGGTTGTGAACTACAGAGTTTTTATGATTGTAGGCTT

RI-AT2G25930-XLOC_009647-12884-0
CATGACTTAACTTTTAAGGTTCAATGTACTCTAATCCATATGGTAAGGTA
RI-AT2G25930-XLOC_009647-12884-1
CATGACTTAACTTTTAAGGTTCAATGTACTCTAATCCATATGGTAAGGTA
CONSENSUS
CATGACTTAACTTTTAAGGTTCAATGTACTCTAATCCATATGGTAAGGTA

RI-AT2G25930-XLOC_009647-12884-0
TCGGATTCACGACCAATGCAAATAATAAGATTTTTATTTCTTGCTTCTTG
RI-AT2G25930-XLOC_009647-12884-1
TCGGATTCACGACCAATGCAAATAATAAGATTTTTATTTCTTGCTTCTTG
CONSENSUS
TCGGATTCACGACCAATGCAAATAATAAGATTTTTATTTCTTGCTTCTTG

RI-AT2G25930-XLOC_009647-12884-0
TTAAATATCTGACATCTCATTTTGCAGAGGATAAGCTGCGCTGTAAGCTA
RI-AT2G25930-XLOC_009647-12884-1
TTAAATATCTGACATCTCATTTTGCAGAGGATAAGCTGCGCTGTAAGCTA
CONSENSUS
TTAAATATCTGACATCTCATTTTGCAGAGGATAAGCTGCGCTGTAAGCTA

RI-AT2G25930-XLOC_009647-12884-0
GATTTCAATAAGCCCGTCCTTTGCATTGTTATCTATGCTTTAATATGTCA
RI-AT2G25930-XLOC_009647-12884-1
GATTTCAATAAGCCCGTCCTTTGCATTGTTATCTATGCTTTAATATGTCA
CONSENSUS
GATTTCAATAAGCCCGTCCTTTGCATTGTTATCTATGCTTTAATATGTCA

RI-AT2G25930-XLOC_009647-12884-0
TTGGACCCATTGATTTGGTTTTCTTCTATCTTTTTTGATTGGCTATGTAT
RI-AT2G25930-XLOC_009647-12884-1
TTGGACCCATTGATTTGGTTTTCTTCTATCTTTTTTGATTGGCTATGTAT
CONSENSUS
TTGGACCCATTGATTTGGTTTTCTTCTATCTTTTTTGATTGGCTATGTAT

RI-AT2G25930-XLOC_009647-12884-0
TCTTGTTTCTTTTTTCCTATCTCATTTCGATCGTATTGTTCCATTAGCTG
RI-AT2G25930-XLOC_009647-12884-1
TCTTGTTTCTTTTTTCCTATCTCATTTCGATCGTATTGTTCCATTAGCTG
CONSENSUS
TCTTGTTTCTTTTTTCCTATCTCATTTCGATCGTATTGTTCCATTAGCTG

RI-AT2G25930-XLOC_009647-12884-0

TTCAACCTAAACTATGTCTCTCTTTGTTGAACTTTTGATGGATAATCTTC
RI-AT2G25930-XLOC_009647-12884-1
TTCAACCTAAACTATGTCTCTCTTTGTTGAACTTTTGATGGATAATCTTC
CONSENSUS
TTCAACCTAAACTATGTCTCTCTTTGTTGAACTTTTGATGGATAATCTTC

RI-AT2G25930-XLOC_009647-12884-0
TTAATGTGACTCTGTTTCTCATTACAGTCAACAAAGAGTATTTGCTGTTC
RI-AT2G25930-XLOC_009647-12884-1
TTAATGTGACTCTGTTTCTCATTACAGTCAACAAAGAGTATTTGCTGTTC
CONSENSUS
TTAATGTGACTCTGTTTCTCATTACAGTCAACAAAGAGTATTTGCTGTTC

RI-AT2G25930-XLOC_009647-12884-0 AACTATTTGAGTTGCACAGACTGATTAAG
RI-AT2G25930-XLOC_009647-12884-1 AACTATTTGAGTTGCACAGACTGATTAAG
CONSENSUS AACTATTTGAGTTGCACAGACTGATTAAG

alignment for event: SE-AT2G30600-XLOC_009886-10151

SE-AT2G30600-XLOC_009886-10151-0
AGATCTGACTCACTATTACGTGTCACGCACAGTTCATTAG----------
SE-AT2G30600-XLOC_009886-10151-1
AGATCTGACTCACTATTACGTGTCACGCACAGTTCATTAGCTTCTCAAAT
CONSENSUS
AGATCTGACTCACTATTACGTGTCACGCACAGTTCATTAG..........

SE-AT2G30600-XLOC_009886-10151-0

SE-AT2G30600-XLOC_009886-10151-1
CTTTTGTCTTTATTCGATTTAAAAGTTGATGTACTCTGCATCTGATTGTT
CONSENSUS
....

SE-AT2G30600-XLOC_009886-10151-0

SE-AT2G30600-XLOC_009886-10151-1
TTGCTCGTGTTTTATTACCACGTTGTGGTTGAAATTTGACGGCGGAAGTT
CONSENSUS
....

SE-AT2G30600-XLOC_009886-10151-0 ---------
GTGAAGGCAAGAGATTTTATGAAGAAAGATGGTTGCGGCGA
SE-AT2G30600-XLOC_009886-10151-1
CATGGTGGGGTGAAGGCAAGAGATTTTATGAAGAAAGATGGTTGCGGCGA
CONSENSUS
.........GTGAAGGCAAGAGATTTTATGAAGAAAGATGGTTGCGGCGA

SE-AT2G30600-XLOC_009886-10151-0
AAGAGAACAAGTTTCTGACAGTGGCACCTTTTGAGTGTGCTTGGAGTGAT
SE-AT2G30600-XLOC_009886-10151-1
AAGAGAACAAGTTTCTGACAGTGGCACCTTTTGAGTGTGCTTGGAGTGAT
CONSENSUS
AAGAGAACAAGTTTCTGACAGTGGCACCTTTTGAGTGTGCTTGGAGTGAT

SE-AT2G30600-XLOC_009886-10151-0
GATCTGAAGTTCCGGGAAGCGGGAAGAGGTTGTGTTGCGTTTGATGCGTT
SE-AT2G30600-XLOC_009886-10151-1
GATCTGAAGTTCCGGGAAGCGGGAAGAGGTTGTGTTGCGTTTGATGCGTT
CONSENSUS
GATCTGAAGTTCCGGGAAGCGGGAAGAGGTTGTGTTGCGTTTGATGCGTT

SE-AT2G30600-XLOC_009886-10151-0
TGCTCACAATGATGTCACGGTGGTGTTTAGAGAGAATGTGGGGACTCAAC
SE-AT2G30600-XLOC_009886-10151-1
TGCTCACAATGATGTCACGGTGGTGTTTAGAGAGAATGTGGGGACTCAAC
CONSENSUS
TGCTCACAATGATGTCACGGTGGTGTTTAGAGAGAATGTGGGGACTCAAC

SE-AT2G30600-XLOC_009886-10151-0
ATTACCATTATAAGAAAGATAATAGTCCTCACTACATTGTTATCATTGGT
SE-AT2G30600-XLOC_009886-10151-1
ATTACCATTATAAGAAAGATAATAGTCCTCACTACATTGTTATCATTGGT
CONSENSUS
ATTACCATTATAAGAAAGATAATAGTCCTCACTACATTGTTATCATTGGT

SE-AT2G30600-XLOC_009886-10151-0
AGCAATAGGAATCGTAGGTTGAAGATTCAGGTAGATGGAAAATCTGTGGT
SE-AT2G30600-XLOC_009886-10151-1
AGCAATAGGAATCGTAGGTTGAAGATTCAGGTAGATGGAAAATCTGTGGT
CONSENSUS
AGCAATAGGAATCGTAGGTTGAAGATTCAGGTAGATGGAAAATCTGTGGT

SE-AT2G30600-XLOC_009886-10151-0
CGATGAGGAAGCTTCTGATCTTTGTCGTTGTTCTTTGGAGTTTCAGAGTT
SE-AT2G30600-XLOC_009886-10151-1
CGATGAGGAAGCTTCTGATCTTTGTCGTTGTTCTTTGGAGTTTCAGAGTT
CONSENSUS
CGATGAGGAAGCTTCTGATCTTTGTCGTTGTTCTTTGGAGTTTCAGAGTT

SE-AT2G30600-XLOC_009886-10151-0
ACTGGATTAGTATCTATGATGGGTTGATTAGCATTGGTAAAGGTCGGTAT
SE-AT2G30600-XLOC_009886-10151-1
ACTGGATTAGTATCTATGATGGGTTGATTAGCATTGGTAAAGGTCGGTAT
CONSENSUS
ACTGGATTAGTATCTATGATGGGTTGATTAGCATTGGTAAAGGTCGGTAT

SE-AT2G30600-XLOC_009886-10151-0
CCGTTTCAGAACCTGGTATTTAAGTGGCAAGACCCCAAGCCCAATTGTAA
SE-AT2G30600-XLOC_009886-10151-1
CCGTTTCAGAACCTGGTATTTAAGTGGCAAGACCCCAAGCCCAATTGTAA
CONSENSUS
CCGTTTCAGAACCTGGTATTTAAGTGGCAAGACCCCAAGCCCAATTGTAA

SE-AT2G30600-XLOC_009886-10151-0
TGTTCAGTATGTTGGTCTGAGCAGCTGGGATAAACATGTTGGATATAGAA
SE-AT2G30600-XLOC_009886-10151-1
TGTTCAGTATGTTGGTCTGAGCAGCTGGGATAAACATGTTGGATATAGAA
CONSENSUS
TGTTCAGTATGTTGGTCTGAGCAGCTGGGATAAACATGTTGGATATAGAA

SE-AT2G30600-XLOC_009886-10151-0
ACGTGAGTGTGTTTCCTGTGACACATAATCATATCTTGCTGTGGAAGCAA
SE-AT2G30600-XLOC_009886-10151-1
ACGTGAGTGTGTTTCCTGTGACACATAATCATATCTTGCTGTGGAAGCAA
CONSENSUS
ACGTGAGTGTGTTTCCTGTGACACATAATCATATCTTGCTGTGGAAGCAA

SE-AT2G30600-XLOC_009886-10151-0
GTGGATTGCCGTGAAGTTAGAGGAGATGAGTCTGGTGACGAGAAGGTTGT
SE-AT2G30600-XLOC_009886-10151-1
GTGGATTGCCGTGAAGTTAGAGGAGATGAGTCTGGTGACGAGAAGGTTGT
CONSENSUS
GTGGATTGCCGTGAAGTTAGAGGAGATGAGTCTGGTGACGAGAAGGTTGT

SE-AT2G30600-XLOC_009886-10151-0
GGAGGAAGGGACTGGTTATGATTATGAACAATGGGGACTTGGGAATTTCT
SE-AT2G30600-XLOC_009886-10151-1
GGAGGAAGGGACTGGTTATGATTATGAACAATGGGGACTTGGGAATTTCT
CONSENSUS
GGAGGAAGGGACTGGTTATGATTATGAACAATGGGGACTTGGGAATTTCT

SE-AT2G30600-XLOC_009886-10151-0
TGGAGAGTTGGCAATTATCTGACACAGTCTTCCTTGTTGGTGAAGAGGAA
SE-AT2G30600-XLOC_009886-10151-1
TGGAGAGTTGGCAATTATCTGACACAGTCTTCCTTGTTGGTGAAGAGGAA
CONSENSUS
TGGAGAGTTGGCAATTATCTGACACAGTCTTCCTTGTTGGTGAAGAGGAA

SE-AT2G30600-XLOC_009886-10151-0
ATGGATGTCCCTGCTCACAAGGTTATATTACAAGCATCAGGTAATTTTCC
SE-AT2G30600-XLOC_009886-10151-1
ATGGATGTCCCTGCTCACAAGGTTATATTACAAGCATCAGGTAATTTTCC
CONSENSUS
ATGGATGTCCCTGCTCACAAGGTTATATTACAAGCATCAGGTAATTTTCC

SE-AT2G30600-XLOC_009886-10151-0
TTTGAGATCATCTGATGGGGATGTCATTCAACTTCGTGGAGTGTCGTACC
SE-AT2G30600-XLOC_009886-10151-1
TTTGAGATCATCTGATGGGGATGTCATTCAACTTCGTGGAGTGTCGTACC
CONSENSUS
TTTGAGATCATCTGATGGGGATGTCATTCAACTTCGTGGAGTGTCGTACC

SE-AT2G30600-XLOC_009886-10151-0
CGATTCTTCATGCTCTTCTTCAATATATCTATACTGGACGAACTCAG
SE-AT2G30600-XLOC_009886-10151-1
CGATTCTTCATGCTCTTCTTCAATATATCTATACTGGACGAACTCAG
CONSENSUS
CGATTCTTCATGCTCTTCTTCAATATATCTATACTGGACGAACTCAG

alignment for event: RI-AT2G24600-XLOC_012218-5612

RI-AT2G24600-XLOC_012218-5612-0
CTTATACGTTACATTGTTGGTAAGAAAATAATAGATATCAGAGACAGAAA
RI-AT2G24600-XLOC_012218-5612-1

CTTATACGTTACATTGTTGGTAAGAAAATAATAGATATCAGAGACAGAAA
CONSENSUS
CTTATACGTTACATTGTTGGTAAGAAAATAATAGATATCAGAGACAGAAA

RI-AT2G24600-XLOC_012218-5612-0
CAATATGGGTTATCGAGCTTATCACCTTCTCCCTCGACAAGCTCAAGACT
RI-AT2G24600-XLOC_012218-5612-1
CAATATGGGTTATCGAGCTTATCACCTTCTCCCTCGACAAGCTCAAGACT
CONSENSUS
CAATATGGGTTATCGAGCTTATCACCTTCTCCCTCGACAAGCTCAAGACT

RI-AT2G24600-XLOC_012218-5612-0
ATGAGTTTATATCAAGCTACCTGAGGTGTGACACCAAGACTTCAGAAGAA
RI-AT2G24600-XLOC_012218-5612-1
ATGAGTTTATATCAAGCTACCTGAGGTGTGACACCAAGACTTCAGAAGAA
CONSENSUS
ATGAGTTTATATCAAGCTACCTGAGGTGTGACACCAAGACTTCAGAAGAA

RI-AT2G24600-XLOC_012218-5612-0
GTGGACTCTAAAAAGGCTGAGAGAAATGAACCACACATAGGGCATTCTGA
RI-AT2G24600-XLOC_012218-5612-1
GTGGACTCTAAAAAGGCTGAGAGAAATGAACCACACATAGGGCATTCTGA
CONSENSUS
GTGGACTCTAAAAAGGCTGAGAGAAATGAACCACACATAGGGCATTCTGA

RI-AT2G24600-XLOC_012218-5612-0
GGTAATAAGGCTGCTTAAGCTAATCGAAATAAGCACATCAGAGATAGCAG
RI-AT2G24600-XLOC_012218-5612-1
GGTAATAAGGCTGCTTAAGCTAATCGAAATAAGCACATCAGAGATAGCAG
CONSENSUS
GGTAATAAGGCTGCTTAAGCTAATCGAAATAAGCACATCAGAGATAGCAG

RI-AT2G24600-XLOC_012218-5612-0
AGAGAAAGAAAAGCAAGAAACATCATGTTAAAAGAGGTCATAAGAGCTTG
RI-AT2G24600-XLOC_012218-5612-1
AGAGAAAGAAAAGCAAGAAACATCATGTTAAAAGAGGTCATAAGAGCTTG
CONSENSUS
AGAGAAAGAAAAGCAAGAAACATCATGTTAAAAGAGGTCATAAGAGCTTG

RI-AT2G24600-XLOC_012218-5612-0
GAACATGAGATGCATATAGAAGCATTACAAAATGCAAGAAATACGATCGC
RI-AT2G24600-XLOC_012218-5612-1
GAACATGAGATGCATATAGAAGCATTACAAAATGCAAGAAATACGATCGC
CONSENSUS
GAACATGAGATGCATATAGAAGCATTACAAAATGCAAGAAATACGATCGC

RI-AT2G24600-XLOC_012218-5612-0
GATAGTGGCAGTCTTGATTGCTTCAGTTTCTTATGCCGGTGGGATAAACC
RI-AT2G24600-XLOC_012218-5612-1
GATAGTGGCAGTCTTGATTGCTTCAGTTTCTTATGCCGGTGGGATAAACC
CONSENSUS
GATAGTGGCAGTCTTGATTGCTTCAGTTTCTTATGCCGGTGGGATAAACC

RI-AT2G24600-XLOC_012218-5612-0
CGCCGGGCGGTGTTTACCAAGATGGGCCATGGAAAGGGAAATCGCTTGTA
RI-AT2G24600-XLOC_012218-5612-1

CGCCGGGCGGTGTTTACCAAGATGGGCCATGGAAAGGGAAATCGCTTGTA
CONSENSUS
CGCCGGGCGGTGTTTACCAAGATGGGCCATGGAAAGGGAAATCGCTTGTA

RI-AT2G24600-XLOC_012218-5612-0
GGTAATACGGCAGCATTTAAAGTCTTTGCAATATGTAACAACATTGCATT
RI-AT2G24600-XLOC_012218-5612-1
G---
CONSENSUS
G...

RI-AT2G24600-XLOC_012218-5612-0
GTTCACGTCCTTGTGCATTGTTATTCTTCTCGTTAGCATCATACCTTACC
RI-AT2G24600-XLOC_012218-5612-1

CONSENSUS
....

RI-AT2G24600-XLOC_012218-5612-0
AGAGGAAACCCTTGAAGAAATTATTGGTGGCCACTCACAGGATGATGTGG
RI-AT2G24600-XLOC_012218-5612-1

CONSENSUS
....

RI-AT2G24600-XLOC_012218-5612-0
GTTTCTGTAGGGTTTATGGCAACGGCTTATGTTGCGGCATCTTTGGTGAC
RI-AT2G24600-XLOC_012218-5612-1 ----------
GGTTTATGGCAACGGCTTATGTTGCGGCATCTTTGGTGAC
CONSENSUS
..........GGTTTATGGCAACGGCTTATGTTGCGGCATCTTTGGTGAC

RI-AT2G24600-XLOC_012218-5612-0
CATACCGCATTTTCCCGGAACTCGATGGTTATTTCCGGTTATTATCTCTG
RI-AT2G24600-XLOC_012218-5612-1
CATACCGCATTTTCCCGGAACTCGATGGTTATTTCCGGTTATTATCTCTG
CONSENSUS
CATACCGCATTTTCCCGGAACTCGATGGTTATTTCCGGTTATTATCTCTG

RI-AT2G24600-XLOC_012218-5612-0
TAGCTGGTGGATCATTGACGGTACTCTTTTCCTATCTAGGAGTTGAGACC
RI-AT2G24600-XLOC_012218-5612-1
TAGCTGGTGGATCATTGACGGTACTCTTTTCCTATCTAGGAGTTGAGACC
CONSENSUS
TAGCTGGTGGATCATTGACGGTACTCTTTTCCTATCTAGGAGTTGAGACC

RI-AT2G24600-XLOC_012218-5612-0
ATCAGTCACTGGTTTAAGAAGATGAATCGTGTAGGGAGAGGACTACCTAT
RI-AT2G24600-XLOC_012218-5612-1
ATCAGTCACTGGTTTAAGAAGATGAATCGTGTAGGGAGAGGACTACCTAT
CONSENSUS
ATCAGTCACTGGTTTAAGAAGATGAATCGTGTAGGGAGAGGACTACCTAT

RI-AT2G24600-XLOC_012218-5612-0
TTATTTTATCAAAAATAACCGTGTAGAAGATATACCTGCCATTGCAAAAA
RI-AT2G24600-XLOC_012218-5612-1

TTATTTTATCAAAAATAACCGTGTAGAAGATATACCTGCCATTGCAAAAA
CONSENSUS
TTATTTTATCAAAAATAACCGTGTAGAAGATATACCTGCCATTGCAAAAA

RI-AT2G24600-XLOC_012218-5612-0
ATGAAGGTGAAATGCCTTCCTTAGCAAGGACCAACTCAGACTTGGCCGCC
RI-AT2G24600-XLOC_012218-5612-1
ATGAAGGTGAAATGCCTTCCTTAGCAAGGACCAACTCAGACTTGGCCGCC
CONSENSUS
ATGAAGGTGAAATGCCTTCCTTAGCAAGGACCAACTCAGACTTGGCCGCC

RI-AT2G24600-XLOC_012218-5612-0
TCAGAAGGGTCAGGCTATTTCACCTATTGAATCTTTAAG
RI-AT2G24600-XLOC_012218-5612-1
TCAGAAGGGTCAGGCTATTTCACCTATTGAATCTTTAAG
CONSENSUS
TCAGAAGGGTCAGGCTATTTCACCTATTGAATCTTTAAG

alignment for event: RI-AT2G25930-XLOC_009647-12883

RI-AT2G25930-XLOC_009647-12883-0
CCTTGTGGTGTGGAAAGAAACTTATCTGTCCAGCATCTTGATTCTTCAGC
RI-AT2G25930-XLOC_009647-12883-1
CCTTGTGGTGTGGAAAGAAACTTATCTGTCCAGCATCTTGATTCTTCAGC
CONSENSUS
CCTTGTGGTGTGGAAAGAAACTTATCTGTCCAGCATCTTGATTCTTCAGC

RI-AT2G25930-XLOC_009647-12883-0
CGCAAACCAAGCAACTGAGAAGTTTGTCTCCCAAATGTCCTTCATGGAAA
RI-AT2G25930-XLOC_009647-12883-1
CGCAAACCAAGCAACTGAGAAGTTTGTCTCCCAAATGTCCTTCATGGAAA
CONSENSUS
CGCAAACCAAGCAACTGAGAAGTTTGTCTCCCAAATGTCCTTCATGGAAA

RI-AT2G25930-XLOC_009647-12883-0
ATGTGAGATCTTCGGCACAGCATGATCAGAGGAAAATGGTGAGAGAGGAA
RI-AT2G25930-XLOC_009647-12883-1
ATGTGAGATCTTCGGCACAGCATGATCAGAGGAAAATGGTGAGAGAGGAA
CONSENSUS
ATGTGAGATCTTCGGCACAGCATGATCAGAGGAAAATGGTGAGAGAGGAA

RI-AT2G25930-XLOC_009647-12883-0
GAAGATTTTGCAGTTCCAGTATATATTAACTCAAGAAGATCTCAGTCTCA
RI-AT2G25930-XLOC_009647-12883-1
GAAGATTTTGCAGTTCCAGTATATATTAACTCAAGAAGATCTCAGTCTCA
CONSENSUS
GAAGATTTTGCAGTTCCAGTATATATTAACTCAAGAAGATCTCAGTCTCA

RI-AT2G25930-XLOC_009647-12883-0
TGGCAGAACCAAGAGTGGTATTGAGAAGGAAAAACACACCCCAATGGTGG
RI-AT2G25930-XLOC_009647-12883-1
TGGCAGAACCAAGAGTGGTATTGAGAAGGAAAAACACACCCCAATGGTGG
CONSENSUS
TGGCAGAACCAAGAGTGGTATTGAGAAGGAAAAACACACCCCAATGGTGG

RI-AT2G25930-XLOC_009647-12883-0
CACCTAGCTCTCATCACTCCATTCGATTTCAAGAAGTGAATCAGACAGGC
RI-AT2G25930-XLOC_009647-12883-1
CACCTAGCTCTCATCACTCCATTCGATTTCAAGAAGTGAATCAGACAGGC
CONSENSUS
CACCTAGCTCTCATCACTCCATTCGATTTCAAGAAGTGAATCAGACAGGC

RI-AT2G25930-XLOC_009647-12883-0
TCAAAGCAAAACGTATGTTTGGCTACTTGTTCAAAACCTGAAGTTAGGGA
RI-AT2G25930-XLOC_009647-12883-1
TCAAAGCAAAACGTATGTTTGGCTACTTGTTCAAAACCTGAAGTTAGGGA
CONSENSUS
TCAAAGCAAAACGTATGTTTGGCTACTTGTTCAAAACCTGAAGTTAGGGA

RI-AT2G25930-XLOC_009647-12883-0
TCAGGTCAAGGCGAATGCAAGGTCAGGTGGCTTTGTAATCTCTTTAGATG
RI-AT2G25930-XLOC_009647-12883-1
TCAGGTCAAGGCGAATGCAAGGTCAGGTGGCTTTGTAATCTCTTTAGATG
CONSENSUS
TCAGGTCAAGGCGAATGCAAGGTCAGGTGGCTTTGTAATCTCTTTAGATG

RI-AT2G25930-XLOC_009647-12883-0
TATCAGTCACAGAGGAGATTGATCTCGAAAAATCAGCATCAAGTCATGAT
RI-AT2G25930-XLOC_009647-12883-1
TATCAGTCACAGAGGAGATTGATCTCGAAAAATCAGCATCAAGTCATGAT
CONSENSUS
TATCAGTCACAGAGGAGATTGATCTCGAAAAATCAGCATCAAGTCATGAT

RI-AT2G25930-XLOC_009647-12883-0
AGAGTAAATGATTATAATGCTTCCTTGAGACAAGAGTCTAGAAATCGGTT
RI-AT2G25930-XLOC_009647-12883-1
AGAGTAAATGATTATAATGCTTCCTTGAGACAAGAGTCTAGAAATCGGTT
CONSENSUS
AGAGTAAATGATTATAATGCTTCCTTGAGACAAGAGTCTAGAAATCGGTT

RI-AT2G25930-XLOC_009647-12883-0
ATACCGAGATGGTGGCAAAACTCGTCTGAAGGACACTGATAATGGAGCTG
RI-AT2G25930-XLOC_009647-12883-1
ATACCGAGATGGTGGCAAAACTCGTCTGAAGGACACTGATAATGGAGCTG
CONSENSUS
ATACCGAGATGGTGGCAAAACTCGTCTGAAGGACACTGATAATGGAGCTG

RI-AT2G25930-XLOC_009647-12883-0
AATCTCACTTGGCAACGGAAAATCATTCACAAGAGGGTCATGGCAGTCCT
RI-AT2G25930-XLOC_009647-12883-1
AATCTCACTTGGCAACGGAAAATCATTCACAAGAGGGTCATGGCAGTCCT
CONSENSUS
AATCTCACTTGGCAACGGAAAATCATTCACAAGAGGGTCATGGCAGTCCT

RI-AT2G25930-XLOC_009647-12883-0
GAAGACATTGATAATGATCGTGAATACAGCAAAAGCAGAGCATGCGCCTC
RI-AT2G25930-XLOC_009647-12883-1
GAAGACATTGATAATGATCGTGAATACAGCAAAAGCAGAGCATGCGCCTC
CONSENSUS
GAAGACATTGATAATGATCGTGAATACAGCAAAAGCAGAGCATGCGCCTC

RI-AT2G25930-XLOC_009647-12883-0
TCTGCAGCAGATAAATGAAGAGGCAAGTGATGACGTTTCTGATGATTCGA
RI-AT2G25930-XLOC_009647-12883-1
TCTGCAGCAGATAAATGAAGAGGCAAGTGATGACGTTTCTGATGATTCGA
CONSENSUS
TCTGCAGCAGATAAATGAAGAGGCAAGTGATGACGTTTCTGATGATTCGA

RI-AT2G25930-XLOC_009647-12883-0
TGGTGGATTCTATATCCAGCATAGATGTCTCTCCCGATGATGTTGTGGGT
RI-AT2G25930-XLOC_009647-12883-1
TGGTGGATTCTATATCCAGCATAGATGTCTCTCCCGATGATGTTGTGGGT
CONSENSUS
TGGTGGATTCTATATCCAGCATAGATGTCTCTCCCGATGATGTTGTGGGT

RI-AT2G25930-XLOC_009647-12883-0
ATATTAGGTCAAAAACGTTTCTGGAGAGCAAGGAAAGCCATTGCCAAGTA
RI-AT2G25930-XLOC_009647-12883-1
ATATTAGGTCAAAAACGTTTCTGGAGAGCAAGGAAAGCCATTGCCAA---
CONSENSUS
ATATTAGGTCAAAAACGTTTCTGGAGAGCAAGGAAAGCCATTGCCAA...

RI-AT2G25930-XLOC_009647-12883-0
AGTTCACTAGAAATTTACAGTTTGGTTATTTATTCTCCGCTCTTTCTATT
RI-AT2G25930-XLOC_009647-12883-1

CONSENSUS
....

RI-AT2G25930-XLOC_009647-12883-0
TATCTCCTTCTTTGATACCAACATTTTTTGCTTGAAAGAAGTTAATATTT
RI-AT2G25930-XLOC_009647-12883-1

CONSENSUS
....

RI-AT2G25930-XLOC_009647-12883-0
AAGCATTGTTCCGTAGTCTTACTGAAGCTTTTTCCTCTGTTGTTTTTTGC
RI-AT2G25930-XLOC_009647-12883-1

CONSENSUS
....

RI-AT2G25930-XLOC_009647-12883-0
TATTTTCATTGAGGACTGTGGTAGGGCATATTTCACTATCACCAAATTTC
RI-AT2G25930-XLOC_009647-12883-1

CONSENSUS
....

RI-AT2G25930-XLOC_009647-12883-0
AAATTTCTAGAACACTCTCCTTCATATTTTTTTTCATGATTAATGCTGCA
RI-AT2G25930-XLOC_009647-12883-1

CONSENSUS
....

RI-AT2G25930-XLOC_009647-12883-0
ATTGATTGCTGATATACATATATGACTATAACTCAGTTTCATATTCTGTC
RI-AT2G25930-XLOC_009647-12883-1

CONSENSUS
....

RI-AT2G25930-XLOC_009647-12883-0
TCATTTTGGGAGAAAGAGATTTCAGGTTTATGCTTGAGAAGTGATGGTTC
RI-AT2G25930-XLOC_009647-12883-1

CONSENSUS
....

RI-AT2G25930-XLOC_009647-12883-0
TATAGTTGAGAGGCCCCTGATTCATCTAAAATGGTCCTATTATGTGTTTA
RI-AT2G25930-XLOC_009647-12883-1

CONSENSUS
....

RI-AT2G25930-XLOC_009647-12883-0
GTTGTAGAGTCCTCGGTAGAATATTAACGCGTTTAACACGTTGGATCATG
RI-AT2G25930-XLOC_009647-12883-1

CONSENSUS
....

RI-AT2G25930-XLOC_009647-12883-0
TTATAGCAGGGAGGGACATTCTCTGTTGACCTATATTGTGCAAGGTGCCC
RI-AT2G25930-XLOC_009647-12883-1 ------
CAGGGAGGGACATTCTCTGTTGACCTATATTGTGCAAGGTGCCC
CONSENSUS
......CAGGGAGGGACATTCTCTGTTGACCTATATTGTGCAAGGTGCCC

RI-AT2G25930-XLOC_009647-12883-0
GCCGATGGCTTTATTACTATACCTTCTTTGCATCTGGTTGTTGGAACATG
RI-AT2G25930-XLOC_009647-12883-1
GCCGATGGCTTTATTACTATACCTTCTTTGCATCTGGTTGTTGGAACATG
CONSENSUS
GCCGATGGCTTTATTACTATACCTTCTTTGCATCTGGTTGTTGGAACATG

RI-AT2G25930-XLOC_009647-12883-0
TCCCTGTCTCGGTTTGGTATTGCTTTTATTCTGCACTGTCGTCTTGGGCA
RI-AT2G25930-XLOC_009647-12883-1
TCCCTGTCTCGGTTTGGTATTGCTTTTATTCTGCACTGTCGTCTTGGGCA
CONSENSUS
TCCCTGTCTCGGTTTGGTATTGCTTTTATTCTGCACTGTCGTCTTGGGCA

RI-AT2G25930-XLOC_009647-12883-0
TTTTCCCTACTTGTCATTCAAGGGGTTGAACCAGGTAGGGAAATGTTTTT
RI-AT2G25930-XLOC_009647-12883-1
TTTTCCCTACTTGTCATTCAAGGGGTTGAACCAGGTAGGGAAATGTTTTT
CONSENSUS
TTTTCCCTACTTGTCATTCAAGGGGTTGAACCAGGTAGGGAAATGTTTTT

RI-AT2G25930-XLOC_009647-12883-0
CCGAGGACCCCAGGATCTAAATTTTAGTTAACCATACGTAAAGTTAGTTT
RI-AT2G25930-XLOC_009647-12883-1
CCGAGGACCCCAGGATCTAAATTTTAGTTAACCATACGTAAAGTTAGTTT
CONSENSUS
CCGAGGACCCCAGGATCTAAATTTTAGTTAACCATACGTAAAGTTAGTTT

RI-AT2G25930-XLOC_009647-12883-0
TGAGTCTTATGACGATGCAGAATTATAGTTTCTTCTTACTACTGCTTAAG
RI-AT2G25930-XLOC_009647-12883-1
TGAGTCTTATGACGATGCAGAATTATAGTTTCTTCTTACTACTGCTTAAG
CONSENSUS
TGAGTCTTATGACGATGCAGAATTATAGTTTCTTCTTACTACTGCTTAAG

RI-AT2G25930-XLOC_009647-12883-0
AGGATCCTTAGTGTGGTTGTGAACTACAGAGTTTTTATGATTGTAGGCTT
RI-AT2G25930-XLOC_009647-12883-1
AGGATCCTTAGTGTGGTTGTGAACTACAGAGTTTTTATGATTGTAGGCTT
CONSENSUS
AGGATCCTTAGTGTGGTTGTGAACTACAGAGTTTTTATGATTGTAGGCTT

RI-AT2G25930-XLOC_009647-12883-0
CATGACTTAACTTTTAAGGTTCAATGTACTCTAATCCATATGGTAAGGTA
RI-AT2G25930-XLOC_009647-12883-1
CATGACTTAACTTTTAAGGTTCAATGTACTCTAATCCATATGGTAAGGTA
CONSENSUS
CATGACTTAACTTTTAAGGTTCAATGTACTCTAATCCATATGGTAAGGTA

RI-AT2G25930-XLOC_009647-12883-0
TCGGATTCACGACCAATGCAAATAATAAGATTTTTATTTCTTGCTTCTTG
RI-AT2G25930-XLOC_009647-12883-1
TCGGATTCACGACCAATGCAAATAATAAGATTTTTATTTCTTGCTTCTTG
CONSENSUS
TCGGATTCACGACCAATGCAAATAATAAGATTTTTATTTCTTGCTTCTTG

RI-AT2G25930-XLOC_009647-12883-0
TTAAATATCTGACATCTCATTTTGCAGAGGATAAGCTGCGCTGTAAGCTA
RI-AT2G25930-XLOC_009647-12883-1
TTAAATATCTGACATCTCATTTTGCAGAGGATAAGCTGCGCTGTAAGCTA
CONSENSUS
TTAAATATCTGACATCTCATTTTGCAGAGGATAAGCTGCGCTGTAAGCTA

RI-AT2G25930-XLOC_009647-12883-0
GATTTCAATAAGCCCGTCCTTTGCATTGTTATCTATGCTTTAATATGTCA
RI-AT2G25930-XLOC_009647-12883-1
GATTTCAATAAGCCCGTCCTTTGCATTGTTATCTATGCTTTAATATGTCA
CONSENSUS
GATTTCAATAAGCCCGTCCTTTGCATTGTTATCTATGCTTTAATATGTCA

RI-AT2G25930-XLOC_009647-12883-0
TTGGACCCATTGATTTGGTTTTCTTCTATCTTTTTTGATTGGCTATGTAT
RI-AT2G25930-XLOC_009647-12883-1
TTGGACCCATTGATTTGGTTTTCTTCTATCTTTTTTGATTGGCTATGTAT
CONSENSUS
TTGGACCCATTGATTTGGTTTTCTTCTATCTTTTTTGATTGGCTATGTAT

RI-AT2G25930-XLOC_009647-12883-0
TCTTGTTTCTTTTTTCCTATCTCATTTCGATCGTATTGTTCCATTAGCTG
RI-AT2G25930-XLOC_009647-12883-1
TCTTGTTTCTTTTTTCCTATCTCATTTCGATCGTATTGTTCCATTAGCTG
CONSENSUS
TCTTGTTTCTTTTTTCCTATCTCATTTCGATCGTATTGTTCCATTAGCTG

RI-AT2G25930-XLOC_009647-12883-0
TTCAACCTAAACTATGTCTCTCTTTGTTGAACTTTTGATGGATAATCTTC
RI-AT2G25930-XLOC_009647-12883-1
TTCAACCTAAACTATGTCTCTCTTTGTTGAACTTTTGATGGATAATCTTC
CONSENSUS
TTCAACCTAAACTATGTCTCTCTTTGTTGAACTTTTGATGGATAATCTTC

RI-AT2G25930-XLOC_009647-12883-0
TTAATGTGACTCTGTTTCTCATTACAGTCAACAAAGAGTATTTGCTGTTC
RI-AT2G25930-XLOC_009647-12883-1
TTAATGTGACTCTGTTTCTCATTACAGTCAACAAAGAGTATTTGCTGTTC
CONSENSUS
TTAATGTGACTCTGTTTCTCATTACAGTCAACAAAGAGTATTTGCTGTTC

RI-AT2G25930-XLOC_009647-12883-0 AACTATTTGAGTTGCACAGACTGATTAAG
RI-AT2G25930-XLOC_009647-12883-1 AACTATTTGAGTTGCACAGACTGATTAAG
CONSENSUS AACTATTTGAGTTGCACAGACTGATTAAG

alignment for event: RI-AT2G44090-XLOC_013343-2022

RI-AT2G44090-XLOC_013343-2022-0
GTTTGGATGCTTCTGTTGACTACGTTTTGCTAAAATTCAGACCCTTTTCG
RI-AT2G44090-XLOC_013343-2022-1
GTTTGGATGCTTCTGTTGACTACGTTTTGCTAAAATTCAGACCCTTTTCG
CONSENSUS
GTTTGGATGCTTCTGTTGACTACGTTTTGCTAAAATTCAGACCCTTTTCG

RI-AT2G44090-XLOC_013343-2022-0
CTCTCGTGAATCGATTCTACCGCATTTTCTGAACGAAATCTCTAGTCTCT
RI-AT2G44090-XLOC_013343-2022-1
CTCTCGTGAATCGATTCTACCGCATTTTCTGAACGAAATCTCTAGTCTCT
CONSENSUS
CTCTCGTGAATCGATTCTACCGCATTTTCTGAACGAAATCTCTAGTCTCT

RI-AT2G44090-XLOC_013343-2022-0
TTTTTCTTTTCTTCCTGGGTTTGATTTCTTGGAGTGGTTGATTGCGCAAG
RI-AT2G44090-XLOC_013343-2022-1
TTTTTCTTTTCTTCCTGGGTTTGATTTCTTGGAGTGGTTGATTGCGCAAG
CONSENSUS
TTTTTCTTTTCTTCCTGGGTTTGATTTCTTGGAGTGGTTGATTGCGCAAG

RI-AT2G44090-XLOC_013343-2022-0
TCTGGATTTTGATTTTGGTACAAGACATTTGCTTGTACAAGGAGGAAGGG
RI-AT2G44090-XLOC_013343-2022-1
TCTGGATTTTGATTTTGGTACAAGACATTTGCTTGTACAAGGAGGAAGGG
CONSENSUS

TCTGGATTTTGATTTTGGTACAAGACATTTGCTTGTACAAGGAGGAAGGG

RI-AT2G44090-XLOC_013343-2022-0
ATTTGTTGGTATACAGTATTGTTATTGCAAAGGTTTTTCGAATGTCAAAA
RI-AT2G44090-XLOC_013343-2022-1
ATTTGTTGGTATACAGTATTGTTATTGCAAAGGTTTTTCGAATGTCAAAA
CONSENSUS
ATTTGTTGGTATACAGTATTGTTATTGCAAAGGTTTTTCGAATGTCAAAA

RI-AT2G44090-XLOC_013343-2022-0
CAAGTTTTTGGATTATTGATTTTGGAAATCTGAGTGGATGGAGAACGATG
RI-AT2G44090-XLOC_013343-2022-1
CAAGTTTTTGGATTATTGATTTTGGAAATCTGAGTGGATGGAGAACGATG
CONSENSUS
CAAGTTTTTGGATTATTGATTTTGGAAATCTGAGTGGATGGAGAACGATG

RI-AT2G44090-XLOC_013343-2022-0
ACATGTATCCTGATGAGCCTGTAGGTTCTGACTTGGAACCTCAAGAGAGT
RI-AT2G44090-XLOC_013343-2022-1
ACATGTATCCTGATGAGCCTGTAGGTTCTGACTTGGAACCTCAAGAGAGT
CONSENSUS
ACATGTATCCTGATGAGCCTGTAGGTTCTGACTTGGAACCTCAAGAGAGT

RI-AT2G44090-XLOC_013343-2022-0
GATGTTGTTTTTGCAAGAGAGTCACCTCTTGTAGGTATCATCACTCAACC
RI-AT2G44090-XLOC_013343-2022-1
GATGTTGTTTTTGCAAGAGAGTCACCTCTTGTAGGTATCATCACTCAACC
CONSENSUS
GATGTTGTTTTTGCAAGAGAGTCACCTCTTGTAGGTATCATCACTCAACC

RI-AT2G44090-XLOC_013343-2022-0
GACCAAATGCTCTGGATCTACTGCAAAGATACTGAAATGCAGGGGTCTTG
RI-AT2G44090-XLOC_013343-2022-1
GACCAAATGCTCTGGATCTACTGCAAAGATACTGAAATGCAGGGGTCTTG
CONSENSUS
GACCAAATGCTCTGGATCTACTGCAAAGATACTGAAATGCAGGGGTCTTG

RI-AT2G44090-XLOC_013343-2022-0
AGCAACAGAAGAAGCTTAATAGACAGGATAGAATCGAATTGGGACGGCTT
RI-AT2G44090-XLOC_013343-2022-1
AGCAACAGAAGAAGCTTAATAGACAGGATAGAATCGAATTGGGACGGCTT
CONSENSUS
AGCAACAGAAGAAGCTTAATAGACAGGATAGAATCGAATTGGGACGGCTT

RI-AT2G44090-XLOC_013343-2022-0
TTCCAAGGTGCTGTTAGCTCACAGGATTGGCAACTTTCAGAGAGATTTAT
RI-AT2G44090-XLOC_013343-2022-1
TTCCAAGGTGCTGTTAGCTCACAGGATTGGCAACTTTCAGAGAGATTTAT
CONSENSUS
TTCCAAGGTGCTGTTAGCTCACAGGATTGGCAACTTTCAGAGAGATTTAT

RI-AT2G44090-XLOC_013343-2022-0
TCAGCTGGCTGATCCACAAACTTTGAATGATCTGTTGTGTATTAGTTTAG
RI-AT2G44090-XLOC_013343-2022-1
TCAGCTGGCTGATCCACAAACTTTGAATGATCTGTTGTGTATTAGTTTAG
CONSENSUS

TCAGCTGGCTGATCCACAAACTTTGAATGATCTGTTGTGTATTAGTTTAG

RI-AT2G44090-XLOC_013343-2022-0
ATTCTATTTGGTTCTTGAGCACAGAGCATGAGTTACGGGGAATCACTGAA
RI-AT2G44090-XLOC_013343-2022-1
ATTCTATTTGGTTCTTGAGCACAGAGCATGAGTTACGGGGAATCACTGAA
CONSENSUS
ATTCTATTTGGTTCTTGAGCACAGAGCATGAGTTACGGGGAATCACTGAA

RI-AT2G44090-XLOC_013343-2022-0
TTGATTGCTAAGATCATATGTCATGGTGCTCAGGACTATACTAGAGCTAC
RI-AT2G44090-XLOC_013343-2022-1
TTGATTGCTAAGATCATATGTCATGGTGCTCAGGACTATACTAGAGCTAC
CONSENSUS
TTGATTGCTAAGATCATATGTCATGGTGCTCAGGACTATACTAGAGCTAC

RI-AT2G44090-XLOC_013343-2022-0
ACTTAGGACTTCCTTTCTTGCTTCTTGTGTTTCATCTTGCCATAGCCGGA
RI-AT2G44090-XLOC_013343-2022-1
ACTTAGGACTTCCTTTCTTGCTTCTTGTGTTTCATCTTGCCATAGCCGGA
CONSENSUS
ACTTAGGACTTCCTTTCTTGCTTCTTGTGTTTCATCTTGCCATAGCCGGA

RI-AT2G44090-XLOC_013343-2022-0
CTGTGAGTCTTGCTGATACTGTAACTGTTATGGCTCAAAGGTACACATTT
RI-AT2G44090-XLOC_013343-2022-1
CTGTGAGTCTTGCTGATACTGTAACTGTTATGGCTCAAAG----------
CONSENSUS
CTGTGAGTCTTGCTGATACTGTAACTGTTATGGCTCAAAG..........

RI-AT2G44090-XLOC_013343-2022-0
GATACTTTCTCTTATAAGATGTTTCCCCTTTTTCTCAGTATAATTATGGT
RI-AT2G44090-XLOC_013343-2022-1

CONSENSUS
....

RI-AT2G44090-XLOC_013343-2022-0
AAAACACTTTTGGATTATTGGAGAAGTTGGTTTCTGTTATGATACCTTGT
RI-AT2G44090-XLOC_013343-2022-1

CONSENSUS
....

RI-AT2G44090-XLOC_013343-2022-0
TTTTGCTTTATATGATCACTACATTGTGTCTTACATGTTACATTGGTTGG
RI-AT2G44090-XLOC_013343-2022-1

CONSENSUS
....

RI-AT2G44090-XLOC_013343-2022-0
TTACATCTGCAAGTGTTTTAGTGTTGTTAACTTTTCTTAGAAATCGCTTT
RI-AT2G44090-XLOC_013343-2022-1

CONSENSUS

....

RI-AT2G44090-XLOC_013343-2022-0
TTGTTAAATCTATAGTATCTCTTATGTTCTTATTCCTCCTGGTTTATCTT
RI-AT2G44090-XLOC_013343-2022-1

CONSENSUS
....

RI-AT2G44090-XLOC_013343-2022-0
TGGTTAGCTGGGAAGGGTGAGATACCCTTTCTATATAAATCCATTTTTTC
RI-AT2G44090-XLOC_013343-2022-1

CONSENSUS
....

RI-AT2G44090-XLOC_013343-2022-0
CCATCTCGGCGTTTCCAGGTTGCACGAGCGTCTCCAAGAGTGTAACGGTG
RI-AT2G44090-XLOC_013343-2022-1 ------------------
GTTGCACGAGCGTCTCCAAGAGTGTAACGGTG
CONSENSUS
..................GTTGCACGAGCGTCTCCAAGAGTGTAACGGTG

RI-AT2G44090-XLOC_013343-2022-0
ATGAGGTCCTCAAAGCAGAAGCTGGTGCTAAAGTTCAAAAGTTTACTGAA
RI-AT2G44090-XLOC_013343-2022-1
ATGAGGTCCTCAAAGCAGAAGCTGGTGCTAAAGTTCAAAAGTTTACTGAA
CONSENSUS
ATGAGGTCCTCAAAGCAGAAGCTGGTGCTAAAGTTCAAAAGTTTACTGAA

RI-AT2G44090-XLOC_013343-2022-0
TGGGCACTCAAATGTATAGGTTTTCACTCCCGATGCCAGGGCCCTAGAGA
RI-AT2G44090-XLOC_013343-2022-1
TGGGCACTCAAATGTATAGGTTTTCACTCCCGATGCCAGGGCCCTAGAGA
CONSENSUS
TGGGCACTCAAATGTATAGGTTTTCACTCCCGATGCCAGGGCCCTAGAGA

RI-AT2G44090-XLOC_013343-2022-0
TAAAGCTAACCAAGACTCAGCTGCTGAGATCCAACTCCAGCTTTCTGCTT
RI-AT2G44090-XLOC_013343-2022-1
TAAAGCTAACCAAGACTCAGCTGCTGAGATCCAACTCCAGCTTTCTGCTT
CONSENSUS
TAAAGCTAACCAAGACTCAGCTGCTGAGATCCAACTCCAGCTTTCTGCTT

RI-AT2G44090-XLOC_013343-2022-0
TCAAGATGTTCTTAGATCTTGCAGGAAACCATCTATCAGGAAAGGATTTC
RI-AT2G44090-XLOC_013343-2022-1
TCAAGATGTTCTTAGATCTTGCAGGAAACCATCTATCAGGAAAGGATTTC
CONSENSUS
TCAAGATGTTCTTAGATCTTGCAGGAAACCATCTATCAGGAAAGGATTTC

RI-AT2G44090-XLOC_013343-2022-0
ACAGAAGCCTTTGATGCAGCTTGCTTCCCGCTTACGCTGTTCTCTACCTC
RI-AT2G44090-XLOC_013343-2022-1
ACAGAAGCCTTTGATGCAGCTTGCTTCCCGCTTACGCTGTTCTCTACCTC
CONSENSUS

ACAGAAGCCTTTGATGCAGCTTGCTTCCCGCTTACGCTGTTCTCTACCTC

RI-AT2G44090-XLOC_013343-2022-0
GTTTAATCCTGGCTGGGCTTCTGGAATTTCAGCTACTGTCATACATGGAT
RI-AT2G44090-XLOC_013343-2022-1
GTTTAATCCTGGCTGGGCTTCTGGAATTTCAGCTACTGTCATACATGGAT
CONSENSUS
GTTTAATCCTGGCTGGGCTTCTGGAATTTCAGCTACTGTCATACATGGAT

RI-AT2G44090-XLOC_013343-2022-0
TGCTCGGTATGCTGGTGGAAGGAGGCGCAGATAATGTAAATCAGTGCTTC
RI-AT2G44090-XLOC_013343-2022-1
TGCTCGGTATGCTGGTGGAAGGAGGCGCAGATAATGTAAATCAGTGCTTC
CONSENSUS
TGCTCGGTATGCTGGTGGAAGGAGGCGCAGATAATGTAAATCAGTGCTTC

RI-AT2G44090-XLOC_013343-2022-0
CTTGAAGCTTCTCGTTTTGGGAGCACAGAACTTGTGCGCATCTTGTTGCA
RI-AT2G44090-XLOC_013343-2022-1
CTTGAAGCTTCTCGTTTTGGGAGCACAGAACTTGTGCGCATCTTGTTGCA
CONSENSUS
CTTGAAGCTTCTCGTTTTGGGAGCACAGAACTTGTGCGCATCTTGTTGCA

RI-AT2G44090-XLOC_013343-2022-0 G
RI-AT2G44090-XLOC_013343-2022-1 G
CONSENSUS G

alignment for event: A3-AT2G26210-XLOC_009661-13837

A3-AT2G26210-XLOC_009661-13837-0
CAAAATAGTTCTCAATCAGTTGGATCATCTTTTTATTCCTAAATAAATAA
A3-AT2G26210-XLOC_009661-13837-1
CAAAATAGTTCTCAATCAGTTGGATCATCTTTTTATTCCTAAATAAATAA
CONSENSUS
CAAAATAGTTCTCAATCAGTTGGATCATCTTTTTATTCCTAAATAAATAA

A3-AT2G26210-XLOC_009661-13837-0
ACTCCAAAAGCATCTCTCTGTTTCTTCTCCTCTCTTCAAGCTTTTTGTTT
A3-AT2G26210-XLOC_009661-13837-1
ACTCCAAAAGCATCTCTCTGTTTCTTCTCCTCTCTTCAAGCTTTTTGTTT
CONSENSUS
ACTCCAAAAGCATCTCTCTGTTTCTTCTCCTCTCTTCAAGCTTTTTGTTT

A3-AT2G26210-XLOC_009661-13837-0
CGTCTCAGTCGTCGTCATCATCTCTGACAGCAAGGTTATTCACATAGGCA
A3-AT2G26210-XLOC_009661-13837-1
CGTCTCAGTCGTCGTCATCATCTCTGACAG--------------------
CONSENSUS
CGTCTCAGTCGTCGTCATCATCTCTGACAG....................

A3-AT2G26210-XLOC_009661-13837-0
AAGAAGTCATTCTTTTGCATGCTGTTTTCCAATAAAGTTAATTCTCTAAC
A3-AT2G26210-XLOC_009661-13837-1

CONSENSUS
      ....

A3-AT2G26210-XLOC_009661-13837-0
      AGAACAAGTTTCGAGATTGGATATTGACGACAACGATATGGGACTTGGAG
A3-AT2G26210-XLOC_009661-13837-1 --
AACAAGTTTCGAGATTGGATATTGACGACAACGATATGGGACTTGGAG
CONSENSUS
      ..AACAAGTTTCGAGATTGGATATTGACGACAACGATATGGGACTTGGAG

A3-AT2G26210-XLOC_009661-13837-0
      GTAGTGAAACAATGGAATGCAAGTGTGGTATGCCTCTGTGTATCTGTGTA
A3-AT2G26210-XLOC_009661-13837-1
      GTAGTGAAACAATGGAATGCAAGTGTGGTATGCCTCTGTGTATCTGTGTA
CONSENSUS
      GTAGTGAAACAATGGAATGCAAGTGTGGTATGCCTCTGTGTATCTGTGTA

A3-AT2G26210-XLOC_009661-13837-0
      GCTCCTCCCAAATCAACTGATAAACCAAACCCACCT
A3-AT2G26210-XLOC_009661-13837-1
      GCTCCTCCCAAATCAACTGATAAACCAAACCCACCT
CONSENSUS
      GCTCCTCCCAAATCAACTGATAAACCAAACCCACCT

alignment for event: RI-AT2G24600-XLOC_012218-5613

RI-AT2G24600-XLOC_012218-5613-0
      CTTATACGTTACATTGTTGGTAAGAAAATAATAGATATCAGAGACAGAAA
RI-AT2G24600-XLOC_012218-5613-1
      CTTATACGTTACATTGTTGGTAAGAAAATAATAGATATCAGAGACAGAAA
CONSENSUS
      CTTATACGTTACATTGTTGGTAAGAAAATAATAGATATCAGAGACAGAAA

RI-AT2G24600-XLOC_012218-5613-0
      CAATATGGGTTATCGAGCTTATCACCTTCTCCCTCGACAAGCTCAAGACT
RI-AT2G24600-XLOC_012218-5613-1
      CAATATGGGTTATCGAGCTTATCACCTTCTCCCTCGACAAGCTCAAGACT
CONSENSUS
      CAATATGGGTTATCGAGCTTATCACCTTCTCCCTCGACAAGCTCAAGACT

RI-AT2G24600-XLOC_012218-5613-0
      ATGAGTTTATATCAAGCTACCTGAGGTGTGACACCAAGACTTCAGAAGAA
RI-AT2G24600-XLOC_012218-5613-1
      ATGAGTTTATATCAAGCTACCTGAGGTGTGACACCAAGACTTCAGAAGAA
CONSENSUS
      ATGAGTTTATATCAAGCTACCTGAGGTGTGACACCAAGACTTCAGAAGAA

RI-AT2G24600-XLOC_012218-5613-0
      GTGGACTCTAAAAAGGCTGAGAGAAATGAACCACACATAGGGCATTCTGA
RI-AT2G24600-XLOC_012218-5613-1
      GTGGACTCTAAAAAGGCTGAGAGAAATGAACCACACATAGGGCATTCTGA
CONSENSUS
      GTGGACTCTAAAAAGGCTGAGAGAAATGAACCACACATAGGGCATTCTGA

RI-AT2G24600-XLOC_012218-5613-0
GGTAATAAGGCTGCTTAAGCTAATCGAAATAAGCACATCAGAGATAGCAG
RI-AT2G24600-XLOC_012218-5613-1
GGTAATAAGGCTGCTTAAGCTAATCGAAATAAGCACATCAGAGATAGCAG
CONSENSUS
GGTAATAAGGCTGCTTAAGCTAATCGAAATAAGCACATCAGAGATAGCAG

RI-AT2G24600-XLOC_012218-5613-0
AGAGAAAGAAAAGCAAGAAACATCATGTTAAAAGAGGTCATAAGAGCTTG
RI-AT2G24600-XLOC_012218-5613-1
AGAGAAAGAAAAGCAAGAAACATCATGTTAAAAGAGGTCATAAGAGCTTG
CONSENSUS
AGAGAAAGAAAAGCAAGAAACATCATGTTAAAAGAGGTCATAAGAGCTTG

RI-AT2G24600-XLOC_012218-5613-0
GAACATGAGATGCATATAGAAGCATTACAAAATGCAAGAAATACGATCGC
RI-AT2G24600-XLOC_012218-5613-1
GAACATGAGATGCATATAGAAGCATTACAAAATGCAAGAAATACGATCGC
CONSENSUS
GAACATGAGATGCATATAGAAGCATTACAAAATGCAAGAAATACGATCGC

RI-AT2G24600-XLOC_012218-5613-0
GATAGTGGCAGTCTTGATTGCTTCAGTTTCTTATGCCGGTGGGATAAACC
RI-AT2G24600-XLOC_012218-5613-1
GATAGTGGCAGTCTTGATTGCTTCAGTTTCTTATGCCGGTGGGATAAACC
CONSENSUS
GATAGTGGCAGTCTTGATTGCTTCAGTTTCTTATGCCGGTGGGATAAACC

RI-AT2G24600-XLOC_012218-5613-0
CGCCGGGCGGTGTTTACCAAGATGGGCCATGGAAAGGGAAATCGCTTGTA
RI-AT2G24600-XLOC_012218-5613-1
CGCCGGGCGGTGTTTACCAAGATGGGCCATGGAAAGGGAAATCGCTTGTA
CONSENSUS
CGCCGGGCGGTGTTTACCAAGATGGGCCATGGAAAGGGAAATCGCTTGTA

RI-AT2G24600-XLOC_012218-5613-0
GGTAATACGGCAGCATTTAAAGTCTTTGCAATATGTAACAACATTGCATT
RI-AT2G24600-XLOC_012218-5613-1
GGTAATACGGCAGCATTTAAAGTCTTTGCAATATGTAACAACATTGCATT
CONSENSUS
GGTAATACGGCAGCATTTAAAGTCTTTGCAATATGTAACAACATTGCATT

RI-AT2G24600-XLOC_012218-5613-0
GTTCACGTCCTTGTGCATTGTTATTCTTCTCGTTAGCATCATACCTTACC
RI-AT2G24600-XLOC_012218-5613-1
GTTCACGTCCTTGTGCATTGTTATTCTTCTCGTTAGCATCATACCTTACC
CONSENSUS
GTTCACGTCCTTGTGCATTGTTATTCTTCTCGTTAGCATCATACCTTACC

RI-AT2G24600-XLOC_012218-5613-0
AGAGGAAACCCTTGAAGAAATTATTGGTGGCCACTCACAGGATGATGTGG
RI-AT2G24600-XLOC_012218-5613-1
AGAGGAAACCCTTGAAGAAATTATTGGTGGCCACTCACAGGATGATGTGG
CONSENSUS
AGAGGAAACCCTTGAAGAAATTATTGGTGGCCACTCACAGGATGATGTGG

RI-AT2G24600-XLOC_012218-5613-0
GTTTCTGTAGGGTTTATGGCAACGGCTTATGTTGCGGCATCTTTGGTGAC
RI-AT2G24600-XLOC_012218-5613-1
GTTTCTGTAGGGTTTATGGCAACGGCTTATGTTGCGGCATCTTTGGTGAC
CONSENSUS
GTTTCTGTAGGGTTTATGGCAACGGCTTATGTTGCGGCATCTTTGGTGAC

RI-AT2G24600-XLOC_012218-5613-0
CATACCGCATTTTCCCGGAACTCGATGGTTATTTCCGGTTATTATCTCTG
RI-AT2G24600-XLOC_012218-5613-1
CATACCGCATTTTCCCGGAACTCGATGGTTATTTCCGGTTATTATCTCTG
CONSENSUS
CATACCGCATTTTCCCGGAACTCGATGGTTATTTCCGGTTATTATCTCTG

RI-AT2G24600-XLOC_012218-5613-0
TAGCTGGTGGATCATTGACGGTACTCTTTTCCTATCTAGGAGTTGAGACC
RI-AT2G24600-XLOC_012218-5613-1
TAGCTGGTGGATCATTGACGGTACTCTTTTCCTATCTAGGAGTTGAGACC
CONSENSUS
TAGCTGGTGGATCATTGACGGTACTCTTTTCCTATCTAGGAGTTGAGACC

RI-AT2G24600-XLOC_012218-5613-0
ATCAGTCACTGGTTTAAGAAGATGAATCGTGTAGGGAGAGGACTACCTAT
RI-AT2G24600-XLOC_012218-5613-1
ATCAGTCACTGGTTTAAGAAGATGAATCGTGTAGGGAGAGGACTACCTAT
CONSENSUS
ATCAGTCACTGGTTTAAGAAGATGAATCGTGTAGGGAGAGGACTACCTAT

RI-AT2G24600-XLOC_012218-5613-0
TTATTTTATCAAAAATAACCGTGTAGAAGATATACCTGCCATTGCAAAAA
RI-AT2G24600-XLOC_012218-5613-1
TTATTTTATCAAAAATAACCGTGTAGAAGATATACCTGCCATTGCAAAAA
CONSENSUS
TTATTTTATCAAAAATAACCGTGTAGAAGATATACCTGCCATTGCAAAAA

RI-AT2G24600-XLOC_012218-5613-0
ATGAAGGTGAAATGCCTTCCTTAGCAAGGACCAACTCAGACTTGGCCGCC
RI-AT2G24600-XLOC_012218-5613-1
ATGAAGGTGAAATGCCTTCCTTAGCAAGGACCAACTCAGACTTGGCCGCC
CONSENSUS
ATGAAGGTGAAATGCCTTCCTTAGCAAGGACCAACTCAGACTTGGCCGCC

RI-AT2G24600-XLOC_012218-5613-0
TCAGAAGGGTCAGGCTATTTCACCTATTGAATCTTTAAGGTAAATTTTCT
RI-AT2G24600-XLOC_012218-5613-1
TCAGAAGGGTCAGGCTATTTCACCTATTGAATCTTTAAG-----------
CONSENSUS
TCAGAAGGGTCAGGCTATTTCACCTATTGAATCTTTAAG...........

RI-AT2G24600-XLOC_012218-5613-0
ACGAGACTACTCTCTCTGGTGCTCGAGCTTTTACCGTGGTGTTTGCAGCC
RI-AT2G24600-XLOC_012218-5613-1

CONSENSUS
....

RI-AT2G24600-XLOC_012218-5613-0
      TATCTTATGGGTTTTGAGTGTCTAAAGTGATTTTCTTGATGAGCTTCTTC
RI-AT2G24600-XLOC_012218-5613-1

CONSENSUS
      ....

RI-AT2G24600-XLOC_012218-5613-0
      TCCTCACAGGGACCGGCACAAGATTTACTTGAAGCAGATTTATATTGCCA
RI-AT2G24600-XLOC_012218-5613-1 ---------
GGACCGGCACAAGATTTACTTGAAGCAGATTTATATTGCCA
CONSENSUS
      .........GGACCGGCACAAGATTTACTTGAAGCAGATTTATATTGCCA

RI-AT2G24600-XLOC_012218-5613-0
      TTGTATGATGCATCCCTACTTAAGGAGTTTCGTGTAAACTGGTCTCACCT
RI-AT2G24600-XLOC_012218-5613-1
      TTGTATGATGCATCCCTACTTAAGGAGTTTCGTGTAAACTGGTCTCACCT
CONSENSUS
      TTGTATGATGCATCCCTACTTAAGGAGTTTCGTGTAAACTGGTCTCACCT

RI-AT2G24600-XLOC_012218-5613-0
      ATGTTATCTCTATCATTTAAAGTCCAAAAAGAATGAAAAATGTTGTCAAT
RI-AT2G24600-XLOC_012218-5613-1
      ATGTTATCTCTATCATTTAAAGTCCAAAAAGAATGAAAAATGTTGTCAAT
CONSENSUS
      ATGTTATCTCTATCATTTAAAGTCCAAAAAGAATGAAAAATGTTGTCAAT

RI-AT2G24600-XLOC_012218-5613-0
      GATGTTTTACATGTTTTATATCAATACTCAGTACATGTTTCATC
RI-AT2G24600-XLOC_012218-5613-1
      GATGTTTTACATGTTTTATATCAATACTCAGTACATGTTTCATC
CONSENSUS
      GATGTTTTACATGTTTTATATCAATACTCAGTACATGTTTCATC

alignment for event: A5-AT2G25930-XLOC_009647-12886

A5-AT2G25930-XLOC_009647-12886-0
      CCTTGTGGTGTGGAAAGAAACTTATCTGTCCAGCATCTTGATTCTTCAGC
A5-AT2G25930-XLOC_009647-12886-1
      CCTTGTGGTGTGGAAAGAAACTTATCTGTCCAGCATCTTGATTCTTCAGC
CONSENSUS
      CCTTGTGGTGTGGAAAGAAACTTATCTGTCCAGCATCTTGATTCTTCAGC

A5-AT2G25930-XLOC_009647-12886-0
      CGCAAACCAAGCAACTGAGAAGTTTGTCTCCCAAATGTCCTTCATGGAAA
A5-AT2G25930-XLOC_009647-12886-1
      CGCAAACCAAGCAACTGAGAAGTTTGTCTCCCAAATGTCCTTCATGGAAA
CONSENSUS
      CGCAAACCAAGCAACTGAGAAGTTTGTCTCCCAAATGTCCTTCATGGAAA

A5-AT2G25930-XLOC_009647-12886-0
      ATGTGAGATCTTCGGCACAGCATGATCAGAGGAAAATGGTGAGAGAGGAA
A5-AT2G25930-XLOC_009647-12886-1

ATGTGAGATCTTCGGCACAGCATGATCAGAGGAAAATGGTGAGAGAGGAA
CONSENSUS
ATGTGAGATCTTCGGCACAGCATGATCAGAGGAAAATGGTGAGAGAGGAA

A5-AT2G25930-XLOC_009647-12886-0
GAAGATTTTGCAGTTCCAGTATATATTAACTCAAGAAGATCTCAGTCTCA
A5-AT2G25930-XLOC_009647-12886-1
GAAGATTTTGCAGTTCCAGTATATATTAACTCAAGAAGATCTCAGTCTCA
CONSENSUS
GAAGATTTTGCAGTTCCAGTATATATTAACTCAAGAAGATCTCAGTCTCA

A5-AT2G25930-XLOC_009647-12886-0
TGGCAGAACCAAGAGTGGTATTGAGAAGGAAAAACACACCCCAATGGTGG
A5-AT2G25930-XLOC_009647-12886-1
TGGCAGAACCAAGAGTGGTATTGAGAAGGAAAAACACACCCCAATGGTGG
CONSENSUS
TGGCAGAACCAAGAGTGGTATTGAGAAGGAAAAACACACCCCAATGGTGG

A5-AT2G25930-XLOC_009647-12886-0
CACCTAGCTCTCATCACTCCATTCGATTTCAAGAAGTGAATCAGACAGGC
A5-AT2G25930-XLOC_009647-12886-1
CACCTAGCTCTCATCACTCCATTCGATTTCAAGAAGTGAATCAGACAGGC
CONSENSUS
CACCTAGCTCTCATCACTCCATTCGATTTCAAGAAGTGAATCAGACAGGC

A5-AT2G25930-XLOC_009647-12886-0
TCAAAGCAAAACGTATGTTTGGCTACTTGTTCAAAACCTGAAGTTAGGGA
A5-AT2G25930-XLOC_009647-12886-1
TCAAAGCAAAACGTATGTTTGGCTACTTGTTCAAAACCTGAAGTTAGGGA
CONSENSUS
TCAAAGCAAAACGTATGTTTGGCTACTTGTTCAAAACCTGAAGTTAGGGA

A5-AT2G25930-XLOC_009647-12886-0
TCAGGTCAAGGCGAATGCAAGGTCAGGTGGCTTTGTAATCTCTTTAGATG
A5-AT2G25930-XLOC_009647-12886-1
TCAGGTCAAGGCGAATGCAAGGTCAGGTGGCTTTGTAATCTCTTTAGATG
CONSENSUS
TCAGGTCAAGGCGAATGCAAGGTCAGGTGGCTTTGTAATCTCTTTAGATG

A5-AT2G25930-XLOC_009647-12886-0
TATCAGTCACAGAGGAGATTGATCTCGAAAAATCAGCATCAAGTCATGAT
A5-AT2G25930-XLOC_009647-12886-1
TATCAGTCACAGAGGAGATTGATCTCGAAAAATCAGCATCAAGTCATGAT
CONSENSUS
TATCAGTCACAGAGGAGATTGATCTCGAAAAATCAGCATCAAGTCATGAT

A5-AT2G25930-XLOC_009647-12886-0
AGAGTAAATGATTATAATGCTTCCTTGAGACAAGAGTCTAGAAATCGGTT
A5-AT2G25930-XLOC_009647-12886-1
AGAGTAAATGATTATAATGCTTCCTTGAGACAAGAGTCTAGAAATCGGTT
CONSENSUS
AGAGTAAATGATTATAATGCTTCCTTGAGACAAGAGTCTAGAAATCGGTT

A5-AT2G25930-XLOC_009647-12886-0
ATACCGAGATGGTGGCAAAACTCGTCTGAAGGACACTGATAATGGAGCTG
A5-AT2G25930-XLOC_009647-12886-1

ATACCGAGATGGTGGCAAAACTCGTCTGAAGGACACTGATAATGGAGCTG
CONSENSUS
ATACCGAGATGGTGGCAAAACTCGTCTGAAGGACACTGATAATGGAGCTG

A5-AT2G25930-XLOC_009647-12886-0
AATCTCACTTGGCAACGGAAAATCATTCACAAGAGGGTCATGGCAGTCCT
A5-AT2G25930-XLOC_009647-12886-1
AATCTCACTTGGCAACGGAAAATCATTCACAAGAGGGTCATGGCAGTCCT
CONSENSUS
AATCTCACTTGGCAACGGAAAATCATTCACAAGAGGGTCATGGCAGTCCT

A5-AT2G25930-XLOC_009647-12886-0
GAAGACATTGATAATGATCGTGAATACAGCAAAAGCAGAGCATGCGCCTC
A5-AT2G25930-XLOC_009647-12886-1
GAAGACATTGATAATGATCGTGAATACAGCAAAAGCAGAGCATGCGCCTC
CONSENSUS
GAAGACATTGATAATGATCGTGAATACAGCAAAAGCAGAGCATGCGCCTC

A5-AT2G25930-XLOC_009647-12886-0
TCTGCAGCAGATAAATGAAGAGGCAAGTGATGACGTTTCTGATGATTCGA
A5-AT2G25930-XLOC_009647-12886-1
TCTGCAGCAGATAAATGAAGAGGCAAGTGATGACGTTTCTGATGATTCGA
CONSENSUS
TCTGCAGCAGATAAATGAAGAGGCAAGTGATGACGTTTCTGATGATTCGA

A5-AT2G25930-XLOC_009647-12886-0
TGGTGGATTCTATATCCAGCATAGATGTCTCTCCCGATGATGTTGTGGGT
A5-AT2G25930-XLOC_009647-12886-1
TGGTGGATTCTATATCCAGCATAGATGTCTCTCCCGATGATGTTGTGGGT
CONSENSUS
TGGTGGATTCTATATCCAGCATAGATGTCTCTCCCGATGATGTTGTGGGT

A5-AT2G25930-XLOC_009647-12886-0
ATATTAGGTCAAAAACGTTTCTGGAGAGCAAGGAAAGCCATTGCCAA---
A5-AT2G25930-XLOC_009647-12886-1
ATATTAGGTCAAAAACGTTTCTGGAGAGCAAGGAAAGCCATTGCCAAGTA
CONSENSUS
ATATTAGGTCAAAAACGTTTCTGGAGAGCAAGGAAAGCCATTGCCAA...

A5-AT2G25930-XLOC_009647-12886-0

A5-AT2G25930-XLOC_009647-12886-1
AGTTCACTAGAAATTTACAGTTTGGTTATTTATTCTCCGCTCTTTCTATT
CONSENSUS
....

A5-AT2G25930-XLOC_009647-12886-0

A5-AT2G25930-XLOC_009647-12886-1
TATCTCCTTCTTTGATACCAACATTTTTTGCTTGAAAGAAGTTAATATTT
CONSENSUS
....

A5-AT2G25930-XLOC_009647-12886-0

A5-AT2G25930-XLOC_009647-12886-1

AAGCATTGTTCCGTAGTCTTACTGAAGCTTTTTCCTCTGTTGTTTTTTGC
CONSENSUS
....

A5-AT2G25930-XLOC_009647-12886-0

A5-AT2G25930-XLOC_009647-12886-1
TATTTTCATTGAGGACTGTGGTAGGGCATATTTCACTATCACCAAATTTC
CONSENSUS
....

A5-AT2G25930-XLOC_009647-12886-0

A5-AT2G25930-XLOC_009647-12886-1
AAATTTCTAGAACACTCTCCTTCATATTTTTTTTCATGATTAATGCTGCA
CONSENSUS
....

A5-AT2G25930-XLOC_009647-12886-0

A5-AT2G25930-XLOC_009647-12886-1
ATTGATTGCTGATATACATATATGACTATAACTCAGTTTCATATTCTGTC
CONSENSUS
....

A5-AT2G25930-XLOC_009647-12886-0

A5-AT2G25930-XLOC_009647-12886-1
TCATTTTGGGAGAAAGAGATTTCAGGTTTATGCTTGAGAAGTGATGGTTC
CONSENSUS
....

A5-AT2G25930-XLOC_009647-12886-0

A5-AT2G25930-XLOC_009647-12886-1
TATAGTTGAGAGGCCCCTGATTCATCTAAAATGGTCCTATTATGTGTTTA
CONSENSUS
....

A5-AT2G25930-XLOC_009647-12886-0

A5-AT2G25930-XLOC_009647-12886-1
GTTGTAGAGTCCTCGGTAGAATATTAACGCGTTTAACACGTTGGATCATG
CONSENSUS
....

A5-AT2G25930-XLOC_009647-12886-0
--TCAACA
A5-AT2G25930-XLOC_009647-12886-1
TTATAGCAGGGAGGGACATTCTCTGTTGACCTATATTGTGCAAGTCAACA
CONSENSUS
....TCAACA

A5-AT2G25930-XLOC_009647-12886-0
AAGAGTATTTGCTGTTCAACTATTTGAGTTGCACAGACTGATTAAG
A5-AT2G25930-XLOC_009647-12886-1

AAGAGTATTTGCTGTTCAACTATTTGAGTTGCACAGACTGATTAAG
CONSENSUS
AAGAGTATTTGCTGTTCAACTATTTGAGTTGCACAGACTGATTAAG

alignment for event: RI-AT2G32415-XLOC_012677-118

RI-AT2G32415-XLOC_012677-118-0
CGTGAAGATTGGAGACAGCGTCCTCTGTCCGAAGAGATGGTGCGATATGC
RI-AT2G32415-XLOC_012677-118-1
CGTGAAGATTGGAGACAGCGTCCTCTGTCCGAAGAGATGGTGCGATATGC
CONSENSUS
CGTGAAGATTGGAGACAGCGTCCTCTGTCCGAAGAGATGGTGCGATATGC

RI-AT2G32415-XLOC_012677-118-0
TAGAACAGATGCACACTATCTGCTTTATATTGCAGATAGTTTGACAACTG
RI-AT2G32415-XLOC_012677-118-1
TAGAACAGATGCACACTATCTGCTTTATATTGCAGATAGTTTGACAACTG
CONSENSUS
TAGAACAGATGCACACTATCTGCTTTATATTGCAGATAGTTTGACAACTG

RI-AT2G32415-XLOC_012677-118-0
AACTCAAACAATTAGCCACTGGTAGGCATCTTTGCTATGGAGAAACATTT
RI-AT2G32415-XLOC_012677-118-1
AACTCAAACAATTAGCCACTG-----------------------------
CONSENSUS
AACTCAAACAATTAGCCACTG.............................

RI-AT2G32415-XLOC_012677-118-0
TAGTATATGATGGAAAAGTAGAGCTAACCCGGCAACAATGTTAGCGATAT
RI-AT2G32415-XLOC_012677-118-1

CONSENSUS
....

RI-AT2G32415-XLOC_012677-118-0
TGTTTTATTCTTGCAACAAAGTTCTAAAATGTGAAATGTATCAGAAGATT
RI-AT2G32415-XLOC_012677-118-1
--AAGATT
CONSENSUS
....AAGATT

RI-AT2G32415-XLOC_012677-118-0
CATCTAGCCCCGATGACAGATTCCATTTTCTTCTCGAGGCTAGTAGGCGG
RI-AT2G32415-XLOC_012677-118-1
CATCTAGCCCCGATGACAGATTCCATTTTCTTCTCGAGGCTAGTAGGCGG
CONSENSUS
CATCTAGCCCCGATGACAGATTCCATTTTCTTCTCGAGGCTAGTAGGCGG

RI-AT2G32415-XLOC_012677-118-0
TCAAACATGACCTGTTTGCAATTGTACACGAAAGAAACTGAAGATTTTCC
RI-AT2G32415-XLOC_012677-118-1
TCAAACATGACCTGTTTGCAATTGTACACGAAAGAAACTGAAGATTTTCC
CONSENSUS
TCAAACATGACCTGTTTGCAATTGTACACGAAAGAAACTGAAGATTTTCC

RI-AT2G32415-XLOC_012677-118-0
TGGTAGTGCTGCGTCTTCCTCAATAATTTATCGGCATTTAAACGGACACG
RI-AT2G32415-XLOC_012677-118-1
TGGTAGTGCTGCGTCTTCCTCAATAATTTATCGGCATTTAAACGGACACG
CONSENSUS
TGGTAGTGCTGCGTCTTCCTCAATAATTTATCGGCATTTAAACGGACACG

RI-AT2G32415-XLOC_012677-118-0
GAGATAAGTCTAACATCTCCTTGAACGCAGAA
RI-AT2G32415-XLOC_012677-118-1
GAGATAAGTCTAACATCTCCTTGAACGCAGAA
CONSENSUS
GAGATAAGTCTAACATCTCCTTGAACGCAGAA

alignment for event: A3-AT2G20585-XLOC_009319-9790

A3-AT2G20585-XLOC_009319-9790-0
AAATCCTGTGGAACTGAGCTTCTGTGTGGAGTCATTGTTACCATATCACT
A3-AT2G20585-XLOC_009319-9790-1
AAATCCTGTGGAACTGAGCTTCTGTGTGGAGTCATTGTTACCATATCACT
CONSENSUS
AAATCCTGTGGAACTGAGCTTCTGTGTGGAGTCATTGTTACCATATCACT

A3-AT2G20585-XLOC_009319-9790-0
CGGCTACAGCTTCAGCGCTAATGACTTCAAAGCTTTCTATCTCTGGCCAA
A3-AT2G20585-XLOC_009319-9790-1
CGGCTACAGCTTCAGCGCTAATGACTTCAAAGCTTTCTATCTCTGGCCAA
CONSENSUS
CGGCTACAGCTTCAGCGCTAATGACTTCAAAGCTTTCTATCTCTGGCCAA

A3-AT2G20585-XLOC_009319-9790-0
ACCTATGGCTGGCTCTCTGACGGCTGACACAAGTGTAGATGGAGACAACG
A3-AT2G20585-XLOC_009319-9790-1
ACCTATGGCTGGCTCTCTGACG----------------------------
CONSENSUS
ACCTATGGCTGGCTCTCTGACG............................

A3-AT2G20585-XLOC_009319-9790-0
AAGCCAAGATCTGGGTTTTCATGCAAATAATATAAACGATTAGAACGGGT
A3-AT2G20585-XLOC_009319-9790-1

CONSENSUS
....

A3-AT2G20585-XLOC_009319-9790-0
TTCAGGCAATAAGATAGGCTTTAGATACACATCAAGCAATGGTTGATGCT
A3-AT2G20585-XLOC_009319-9790-1

CONSENSUS
....

A3-AT2G20585-XLOC_009319-9790-0
GCATTGTGTTTTAAAAGAACTGGTTCTTACATATCTTCTTAAAAAAAATA

A3-AT2G20585-XLOC_009319-9790-1

CONSENSUS
      ....

A3-AT2G20585-XLOC_009319-9790-0
      CATGTACCCGGAAACGTGTCTTCTTTTCTTGTTGGTTATAGCATTTGAGT
A3-AT2G20585-XLOC_009319-9790-1

CONSENSUS
      ....

A3-AT2G20585-XLOC_009319-9790-0
      TATTACTGATTGGTCTTATACTCCCAGCTTGCAATGATGATGTGTGATGA
A3-AT2G20585-XLOC_009319-9790-1 ---------------------------
CTTGCAATGATGATGTGTGATGA
CONSENSUS
      ...........................CTTGCAATGATGATGTGTGATGA

A3-AT2G20585-XLOC_009319-9790-0
      GTTAGCCAGAGGAACAATGAAGCTACAGTTTAT
A3-AT2G20585-XLOC_009319-9790-1
      GTTAGCCAGAGGAACAATGAAGCTACAGTTTAT
CONSENSUS
      GTTAGCCAGAGGAACAATGAAGCTACAGTTTAT

alignment for event: A3-AT2G47250-XLOC_013504-13502

A3-AT2G47250-XLOC_013504-13502-0
      GTATTTGACTGATGGTATGCTTTTGAGAGAGGCAATGGCGGATCCGCTTT
A3-AT2G47250-XLOC_013504-13502-1
      GTATTTGACTGATGGTATGCTTTTGAGAGAGGCAATGGCGGATCCGCTTT
CONSENSUS
      GTATTTGACTGATGGTATGCTTTTGAGAGAGGCAATGGCGGATCCGCTTT

A3-AT2G47250-XLOC_013504-13502-0
      TAGAGAGATACAAAGTTATTATTCTCGATGAAGCTCATGAAAGGACTCTA
A3-AT2G47250-XLOC_013504-13502-1
      TAGAGAGATACAAAGTTATTATTCTCGATGAAGCTCATGAAAGGACTCTA
CONSENSUS
      TAGAGAGATACAAAGTTATTATTCTCGATGAAGCTCATGAAAGGACTCTA

A3-AT2G47250-XLOC_013504-13502-0
      GCCACGGATGTGCTCTTTGGTCTTCTCAAAGAGGTCTTGAGGAATAGGCC
A3-AT2G47250-XLOC_013504-13502-1
      GCCACGGATGTGCTCTTTGGTCTTCTCAAAGAGGTCTTGAGGAATAGGCC
CONSENSUS
      GCCACGGATGTGCTCTTTGGTCTTCTCAAAGAGGTCTTGAGGAATAGGCC

A3-AT2G47250-XLOC_013504-13502-0
      TGATCTTAAGCTAGTTGTCATGAGTGCAACTTTAGAAGCTGAAAAGTTTC
A3-AT2G47250-XLOC_013504-13502-1
      TGATCTTAAGCTAGTTGTCATGAGTGCAACTTTAGAAGCTGAAAAGTTTC
CONSENSUS

TGATCTTAAGCTAGTTGTCATGAGTGCAACTTTAGAAGCTGAAAAGTTTC

A3-AT2G47250-XLOC_013504-13502-0
AGGAATATTTTAGCGGTGCTCCTCTTATGAAAGTCCCTGGTAGGCTTCAT
A3-AT2G47250-XLOC_013504-13502-1
AGGAATATTTTAGCGGTGCTCCTCTTATGAAAGTCCCTGGTAGGCTTCAT
CONSENSUS
AGGAATATTTTAGCGGTGCTCCTCTTATGAAAGTCCCTGGTAGGCTTCAT

A3-AT2G47250-XLOC_013504-13502-0
CCTGTTGAGATCTTCTATACACAGGAACCTGAGAGGGATTATCTCGAGGC
A3-AT2G47250-XLOC_013504-13502-1
CCTGTTGAGATCTTCTATACACAGGAACCTGAGAGGGATTATCTCGAGGC
CONSENSUS
CCTGTTGAGATCTTCTATACACAGGAACCTGAGAGGGATTATCTCGAGGC

A3-AT2G47250-XLOC_013504-13502-0
TGCTATAAGGACTGTTGTTCAGATACACATGTGCGAGCCACCTGGAGATA
A3-AT2G47250-XLOC_013504-13502-1
TGCTATAAGGACTGTTGTTCAGATACACATGTGCGAGCCACCTGGAGATA
CONSENSUS
TGCTATAAGGACTGTTGTTCAGATACACATGTGCGAGCCACCTGGAGATA

A3-AT2G47250-XLOC_013504-13502-0
TTCTTGTTTTCTTAACTGGAGAGGAGGAAATAGAAGATGCTTGCCGTAAA
A3-AT2G47250-XLOC_013504-13502-1
TTCTTGTTTTCTTAACTGGAGAGGAGGAAATAGAAGATGCTTGCCGTAAA
CONSENSUS
TTCTTGTTTTCTTAACTGGAGAGGAGGAAATAGAAGATGCTTGCCGTAAA

A3-AT2G47250-XLOC_013504-13502-0
ATCAATAAAGAGGTCAGCAATCTTGGAGATCAAGTGGGTCCTGTCAAAGT
A3-AT2G47250-XLOC_013504-13502-1
ATCAATAAAGAGGTCAGCAATCTTGGAGATCAAGTGGGTCCTGTCAAAGT
CONSENSUS
ATCAATAAAGAGGTCAGCAATCTTGGAGATCAAGTGGGTCCTGTCAAAGT

A3-AT2G47250-XLOC_013504-13502-0
TGTGCCTTTGTATTCTACTCTTCCACCTGCGATGCAGCAGAAGATTTTCG
A3-AT2G47250-XLOC_013504-13502-1
TGTGCCTTTGTATTCTACTCTTCCACCTGCGATGCAGCAGAAGATTTTCG
CONSENSUS
TGTGCCTTTGTATTCTACTCTTCCACCTGCGATGCAGCAGAAGATTTTCG

A3-AT2G47250-XLOC_013504-13502-0
ACCCTGCTCCAGTGCCGTTAACAGAAGGTGGTCCTGCTGGACGAAAGATT
A3-AT2G47250-XLOC_013504-13502-1
ACCCTGCTCCAGTGCCGTTAACAGAAGGTGGTCCTGCTGGACGAAAGATT
CONSENSUS
ACCCTGCTCCAGTGCCGTTAACAGAAGGTGGTCCTGCTGGACGAAAGATT

A3-AT2G47250-XLOC_013504-13502-0
GTTGTCTCAACCAACATTGCTGAAACCTCTCTAACCATTGATGGGATTGT
A3-AT2G47250-XLOC_013504-13502-1
GTTGTCTCAACCAACATTGCTGAAACCTCTCTAACCATTGATGGGATTGT
CONSENSUS

GTTGTCTCAACCAACATTGCTGAAACCTCTCTAACCATTGATGGGATTGT

A3-AT2G47250-XLOC_013504-13502-0
TTATGTTATTGACCCTGGTTTTGCTAAGCAGAAAGTCTACAACCCACGGA
A3-AT2G47250-XLOC_013504-13502-1
TTATGTTATTGACCCTGGTTTTGCTAAGCAGAAAGTCTACAACCCACGGA
CONSENSUS
TTATGTTATTGACCCTGGTTTTGCTAAGCAGAAAGTCTACAACCCACGGA

A3-AT2G47250-XLOC_013504-13502-0
TTCGAGTTGAGTCATTGTTGGTGTCCCCAATATCAAAGGCAAGTGCTCAC
A3-AT2G47250-XLOC_013504-13502-1
TTCGAGTTGAGTCATTGTTGGTGTCCCCAATATCAAAGGCAAGTGCTCAC
CONSENSUS
TTCGAGTTGAGTCATTGTTGGTGTCCCCAATATCAAAGGCAAGTGCTCAC

A3-AT2G47250-XLOC_013504-13502-0
CAGAGATCAGGTCGTGCTGGTAGAACTCGCCCTGGAAAATGTTTTAGGCT
A3-AT2G47250-XLOC_013504-13502-1
CAGAGATCAGGTCGTGCTGGTAGAACTCGCCCTGGAAAATGTTTTAGGCT
CONSENSUS
CAGAGATCAGGTCGTGCTGGTAGAACTCGCCCTGGAAAATGTTTTAGGCT

A3-AT2G47250-XLOC_013504-13502-0
CTACACAGAGAAGAGTTTCAACAATGACCTGCAGCCACAGACATATCCTG
A3-AT2G47250-XLOC_013504-13502-1
CTACACAGAGAAGAGTTTCAACAATGACCTGCAGCCACAGACATATCCTG
CONSENSUS
CTACACAGAGAAGAGTTTCAACAATGACCTGCAGCCACAGACATATCCTG

A3-AT2G47250-XLOC_013504-13502-0
AGATATTGAGATCAAACCTTGCAAATACAGTCCTGACATTGAAAAAACTT
A3-AT2G47250-XLOC_013504-13502-1
AGATATTGAGATCAAACCTTGCAAATACAGTCCTGACATTGAAAAAACTT
CONSENSUS
AGATATTGAGATCAAACCTTGCAAATACAGTCCTGACATTGAAAAAACTT

A3-AT2G47250-XLOC_013504-13502-0
GGCATTGATGACTTGGTGCACTTTGATTTCATGGATCCTCCTGCTCCTGA
A3-AT2G47250-XLOC_013504-13502-1
GGCATTGATGACTTGGTGCACTTTGATTTCATGGATCCTCCTGCTCCTGA
CONSENSUS
GGCATTGATGACTTGGTGCACTTTGATTTCATGGATCCTCCTGCTCCTGA

A3-AT2G47250-XLOC_013504-13502-0
GACACTGATGCGGGCATTAGAGGTTCTGAATTATTTGGGAGCACTTGATG
A3-AT2G47250-XLOC_013504-13502-1
GACACTGATGCGGGCATTAGAGGTTCTGAATTATTTGGGAGCACTTGATG
CONSENSUS
GACACTGATGCGGGCATTAGAGGTTCTGAATTATTTGGGAGCACTTGATG

A3-AT2G47250-XLOC_013504-13502-0
ATGAAGGTAACTTGACAAAGACGGGTGAAATAATGAGTGAATTTCCCTTG
A3-AT2G47250-XLOC_013504-13502-1
ATGAAGGTAACTTGACAAAGACGGGTGAAATAATGAGTGAATTTCCCTTG
CONSENSUS

ATGAAGGTAACTTGACAAAGACGGGTGAAATAATGAGTGAATTTCCCTTG

A3-AT2G47250-XLOC_013504-13502-0
GATCCACAAATGTCAAAGATGCTCATAGTCAGTCCTGAATTCAACTGTTC
A3-AT2G47250-XLOC_013504-13502-1
GATCCACAAATGTCAAAGATGCTCATAGTCAGTCCTGAATTCAACTGTTC
CONSENSUS
GATCCACAAATGTCAAAGATGCTCATAGTCAGTCCTGAATTCAACTGTTC

A3-AT2G47250-XLOC_013504-13502-0
CAATGAGATTCTCTCGGTTTCTGCAATGTTATCAG---------------
A3-AT2G47250-XLOC_013504-13502-1
CAATGAGATTCTCTCGGTTTCTGCAATGTTATCAGTTGCAGTTTCTCAGT
CONSENSUS
CAATGAGATTCTCTCGGTTTCTGCAATGTTATCAG...............

A3-AT2G47250-XLOC_013504-13502-0

A3-AT2G47250-XLOC_013504-13502-1
ATTCTCACAAGCAAATGGTATCGTCTCTCGCCTCTATGCATCTGCTGACC
CONSENSUS
....

A3-AT2G47250-XLOC_013504-13502-0
--TACCGA
A3-AT2G47250-XLOC_013504-13502-1
TCTTCCATGGGCATATTCTGTTATAAAAACAATTCTTTCTCTAGTACCGA
CONSENSUS
....TACCGA

A3-AT2G47250-XLOC_013504-13502-0
ATTGCTTTGTCCGGCCTAGAGAGGCTCAAAAAGCAGCAGATGAAGCTAAA
A3-AT2G47250-XLOC_013504-13502-1
ATTGCTTTGTCCGGCCTAGAGAGGCTCAAAAAGCAGCAGATGAAGCTAAA
CONSENSUS
ATTGCTTTGTCCGGCCTAGAGAGGCTCAAAAAGCAGCAGATGAAGCTAAA

A3-AT2G47250-XLOC_013504-13502-0
GCTAGGTTTGGACACATTGATGGAGATCACCTGACGTTGCTAAACGTGTA
A3-AT2G47250-XLOC_013504-13502-1
GCTAGGTTTGGACACATTGATGGAGATCACCTGACGTTGCTAAACGTGTA
CONSENSUS
GCTAGGTTTGGACACATTGATGGAGATCACCTGACGTTGCTAAACGTGTA

A3-AT2G47250-XLOC_013504-13502-0 CCACGCCTACAAGCAAAACA
A3-AT2G47250-XLOC_013504-13502-1 CCACGCCTACAAGCAAAACA
CONSENSUS CCACGCCTACAAGCAAAACA

alignment for event: A3-AT2G13650-XLOC_011587-1247

A3-AT2G13650-XLOC_011587-1247-0
GTCTTAGCTTTGCAATGAAATTGTACGAACACGATGGAGTTGATTTAGAA
A3-AT2G13650-XLOC_011587-1247-1
GTCTTAGCTTTGCAATGAAATTGTACGAACACGATGGAGTTGATTTAGAA

CONSENSUS
GTCTTAGCTTTGCAATGAAATTGTACGAACACGATGGAGTTGATTTAGAA

A3-AT2G13650-XLOC_011587-1247-0
GATGGGAAGACAGTAAAATCCGGAGGAGATAAACCAATTCCAAGAAAGAT
A3-AT2G13650-XLOC_011587-1247-1
GATGGGAAGACAGTAAAATCCGGAGGAGATAAACCAATTCCAAGAAAGAT
CONSENSUS
GATGGGAAGACAGTAAAATCCGGAGGAGATAAACCAATTCCAAGAAAGAT

A3-AT2G13650-XLOC_011587-1247-0
ACATAACCGGGCTTTGTTATCCGGTTTAGCTTATTGCATTTCATCATGCA
A3-AT2G13650-XLOC_011587-1247-1
ACATAACCGGGCTTTGTTATCCGGTTTAGCTTATTGCATTTCATCATGCA
CONSENSUS
ACATAACCGGGCTTTGTTATCCGGTTTAGCTTATTGCATTTCATCATGCA

A3-AT2G13650-XLOC_011587-1247-0
GTATGATACTTGTCAACAAGTTTGTTCTCTCCAGCTACAACTTCAATGCT
A3-AT2G13650-XLOC_011587-1247-1
GTATGATACTTGTCAACAAGTTTGTTCTCTCCAGCTACAACTTCAATGCT
CONSENSUS
GTATGATACTTGTCAACAAGTTTGTTCTCTCCAGCTACAACTTCAATGCT

A3-AT2G13650-XLOC_011587-1247-0
GGGATCTTCCTTATGTTATACCAGAACTTTGTCTCGGTGATCATTGTGGT
A3-AT2G13650-XLOC_011587-1247-1
GGGATCTTCCTTATGTTATACCAGAACTTTGTCTCGGTGATCATTGTGGT
CONSENSUS
GGGATCTTCCTTATGTTATACCAGAACTTTGTCTCGGTGATCATTGTGGT

A3-AT2G13650-XLOC_011587-1247-0
TGGTTTGAGTTTAATGGGTCTAATAACTACTGAACCACTTACTTTGAGGT
A3-AT2G13650-XLOC_011587-1247-1
TGGTTTGAGTTTAATGGGTCTAATAACTACTGAACCACTTACTTTGAGGT
CONSENSUS
TGGTTTGAGTTTAATGGGTCTAATAACTACTGAACCACTTACTTTGAGGT

A3-AT2G13650-XLOC_011587-1247-0
TGATGAAGGTCTGGTTTCCAGTGAATGTCATCTTTGTTGGTATGCTTATC
A3-AT2G13650-XLOC_011587-1247-1
TGATGAAGGTCTGGTTTCCAGTGAATGTCATCTTTGTTGGTATGCTTATC
CONSENSUS
TGATGAAGGTCTGGTTTCCAGTGAATGTCATCTTTGTTGGTATGCTTATC

A3-AT2G13650-XLOC_011587-1247-0
ACAAGCATGTTTAG--------------------CAATGGTCACTGTCCT
A3-AT2G13650-XLOC_011587-1247-1
ACAAGCATGTTTAGTTTGAAATACATCAATGTAGCAATGGTCACTGTCCT
CONSENSUS
ACAAGCATGTTTAG....................CAATGGTCACTGTCCT

A3-AT2G13650-XLOC_011587-1247-0
GAAGAATGTCACTAATGTGATAACTGCAGTTGGTGAGATGTATCTGTTCA
A3-AT2G13650-XLOC_011587-1247-1
GAAGAATGTCACTAATGTGATAACTGCAGTTGGTGAGATGTATCTGTTCA

CONSENSUS
GAAGAATGTCACTAATGTGATAACTGCAGTTGGTGAGATGTATCTGTTCA

A3-AT2G13650-XLOC_011587-1247-0
ACAAGCAACATGACAACAGAGTGTGGGCTGCTCTCTTCTTAATG
A3-AT2G13650-XLOC_011587-1247-1
ACAAGCAACATGACAACAGAGTGTGGGCTGCTCTCTTCTTAATG
CONSENSUS
ACAAGCAACATGACAACAGAGTGTGGGCTGCTCTCTTCTTAATG

alignment for event: A3-AT2G44530-XLOC_010677-9289

A3-AT2G44530-XLOC_010677-9289-0
AGAAATGCTGCATCAACGTACAACATTCTGAATGAAGAAGGAAGAGTCGT
A3-AT2G44530-XLOC_010677-9289-1
AGAAATGCTGCATCAACGTACAACATTCTGAATGAAGAAGGAAGAGTCGT
CONSENSUS
AGAAATGCTGCATCAACGTACAACATTCTGAATGAAGAAGGAAGAGTCGT

A3-AT2G44530-XLOC_010677-9289-0
TGCTGCGGCATTGCTTCCATATGGAGTTACATCCTAAAGAAAACTAACAA
A3-AT2G44530-XLOC_010677-9289-1
TGCTGCGGCATTGCTTCCATATGGAGTTACATCCTAAAGAAAACTAACAA
CONSENSUS
TGCTGCGGCATTGCTTCCATATGGAGTTACATCCTAAAGAAAACTAACAA

A3-AT2G44530-XLOC_010677-9289-0
CTCGAGCTCTGGCTTTCTAAACTGTAAATGGTTTTTGAGACCTTCCGAAA
A3-AT2G44530-XLOC_010677-9289-1
CTCGAGCTCTGGCTTTCTAAACTGTAAATGGTTTTTGAGACCTTCCGAAA
CONSENSUS
CTCGAGCTCTGGCTTTCTAAACTGTAAATGGTTTTTGAGACCTTCCGAAA

A3-AT2G44530-XLOC_010677-9289-0
ATAAACCGAATTTCATCGGAGTTTTTATTGCGTTTGAGCTATATAACAAA
A3-AT2G44530-XLOC_010677-9289-1
ATAAACCGAATTTCATCGGAGTTTTTATTGCGTTTGAGCTATATAACAAA
CONSENSUS
ATAAACCGAATTTCATCGGAGTTTTTATTGCGTTTGAGCTATATAACAAA

A3-AT2G44530-XLOC_010677-9289-0
CGTTGTAACATGGTGCGACTTTATCTCGCATTTCTCATCCTTCCAAAGAT
A3-AT2G44530-XLOC_010677-9289-1
CGTTGTAACATGGTGCGACTTTATCTCGCATTTCTCATCCTTCCAAAGAT
CONSENSUS
CGTTGTAACATGGTGCGACTTTATCTCGCATTTCTCATCCTTCCAAAGAT

A3-AT2G44530-XLOC_010677-9289-0
ATAATATCTCCAGAGACTATGTTGTATCAATGAATGAGATTTAAAGAATC
A3-AT2G44530-XLOC_010677-9289-1
ATAATATCTCCAGAGACTATGTTGTATCAATGAATGAGATTTAAAGAATC
CONSENSUS
ATAATATCTCCAGAGACTATGTTGTATCAATGAATGAGATTTAAAGAATC

A3-AT2G44530-XLOC_010677-9289-0
TGAATTACAGTTATATTTTTAACGGAAATCGTTACGGTTAGTTAAAAACT
A3-AT2G44530-XLOC_010677-9289-1
TGAATTACAGTTATATTTTTAACGGAAATCGTTACGGTTAGTTAAAAACT
CONSENSUS
TGAATTACAGTTATATTTTTAACGGAAATCGTTACGGTTAGTTAAAAACT

A3-AT2G44530-XLOC_010677-9289-0
TAGAAATGAGTTTTTGAGTATATAAAGGAAGAAGAAGAAGAGAGAGTCAT
A3-AT2G44530-XLOC_010677-9289-1
TAGAAATGAGTTTTTGAGTATATAAAGGAAGAAGAAGAAGAGAGAGTCAT
CONSENSUS
TAGAAATGAGTTTTTGAGTATATAAAGGAAGAAGAAGAAGAGAGAGTCAT

A3-AT2G44530-XLOC_010677-9289-0
TTTCTTGTAGTCAAATTCGTATCTCGCGGGGAAGAAGAAGAAAAGACTGC
A3-AT2G44530-XLOC_010677-9289-1
TTTCTTGTAGTCAAATTCGTATCTCGCGGGGAAGAAGAAGAAAAGACTGC
CONSENSUS
TTTCTTGTAGTCAAATTCGTATCTCGCGGGGAAGAAGAAGAAAAGACTGC

A3-AT2G44530-XLOC_010677-9289-0
TTTTTCTCAAAACCCTAAAATCTCTCTCTCCGGATCCATTTCTCGAACAT
A3-AT2G44530-XLOC_010677-9289-1
TTTTTCTCAAAACCCTAAAATCTCTCTCTCCGGATCCATTTCTCGAACAT
CONSENSUS
TTTTTCTCAAAACCCTAAAATCTCTCTCTCCGGATCCATTTCTCGAACAT

A3-AT2G44530-XLOC_010677-9289-0
GGCGTCTATTGTTCAACCATCGCCTACTTTCCCGGCGCTAAATCTCCGGC
A3-AT2G44530-XLOC_010677-9289-1
GGCGTCTATTGTTCAACCATCGCCTACTTTCCCGGCGCTAAATCTCCGGC
CONSENSUS
GGCGTCTATTGTTCAACCATCGCCTACTTTCCCGGCGCTAAATCTCCGGC

A3-AT2G44530-XLOC_010677-9289-0
GTTCTTCTCTGATTCGTCCGCCTTCTTCCGTTCGATTTCCTCTTAAGTGT
A3-AT2G44530-XLOC_010677-9289-1
GTTCTTCTCTGATTCGTCCGCCTTCTTCCGTTCGATTTCCTCTT---TGT
CONSENSUS
GTTCTTCTCTGATTCGTCCGCCTTCTTCCGTTCGATTTCCTCTT...TGT

A3-AT2G44530-XLOC_010677-9289-0
AACGCGGCGGATCCGTACAAGTTCGACGGCGGAAACTCTGCCGGTTTCCA
A3-AT2G44530-XLOC_010677-9289-1
AACGCGGCGGATCCGTACAAGTTCGACGGCGGAAACTCTGCCGGTTTCCA
CONSENSUS
AACGCGGCGGATCCGTACAAGTTCGACGGCGGAAACTCTGCCGGTTTCCA

A3-AT2G44530-XLOC_010677-9289-0
TCTGCTTACTGGCGACACCGTTCCGGCTAGCTTTTCGAGGACACGTTTGG
A3-AT2G44530-XLOC_010677-9289-1
TCTGCTTACTGGCGACACCGTTCCGGCTAGCTTTTCGAGGACACGTTTGG
CONSENSUS
TCTGCTTACTGGCGACACCGTTCCGGCTAGCTTTTCGAGGACACGTTTGG

A3-AT2G44530-XLOC_010677-9289-0
      AAGATTCGATTTATCAGAACACCACACGACTTCGTATCTTTTCCGGCACT
A3-AT2G44530-XLOC_010677-9289-1
      AAGATTCGATTTATCAGAACACCACACGACTTCGTATCTTTTCCGGCACT
CONSENSUS
      AAGATTCGATTTATCAGAACACCACACGACTTCGTATCTTTTCCGGCACT

A3-AT2G44530-XLOC_010677-9289-0 GCTAATCCTATTTTGGCTCAG
A3-AT2G44530-XLOC_010677-9289-1 GCTAATCCTATTTTGGCTCAG
CONSENSUS   GCTAATCCTATTTTGGCTCAG

alignment for event: A3-AT2G47250-XLOC_013504-13503

A3-AT2G47250-XLOC_013504-13503-0
      GTATTTGACTGATGGTATGCTTTTGAGAGAGGCAATGGCGGATCCGCTTT
A3-AT2G47250-XLOC_013504-13503-1
      GTATTTGACTGATGGTATGCTTTTGAGAGAGGCAATGGCGGATCCGCTTT
CONSENSUS
      GTATTTGACTGATGGTATGCTTTTGAGAGAGGCAATGGCGGATCCGCTTT

A3-AT2G47250-XLOC_013504-13503-0
      TAGAGAGATACAAAGTTATTATTCTCGATGAAGCTCATGAAAGGACTCTA
A3-AT2G47250-XLOC_013504-13503-1
      TAGAGAGATACAAAGTTATTATTCTCGATGAAGCTCATGAAAGGACTCTA
CONSENSUS
      TAGAGAGATACAAAGTTATTATTCTCGATGAAGCTCATGAAAGGACTCTA

A3-AT2G47250-XLOC_013504-13503-0
      GCCACGGATGTGCTCTTTGGTCTTCTCAAAGAGGTCTTGAGGAATAGGCC
A3-AT2G47250-XLOC_013504-13503-1
      GCCACGGATGTGCTCTTTGGTCTTCTCAAAGAGGTCTTGAGGAATAGGCC
CONSENSUS
      GCCACGGATGTGCTCTTTGGTCTTCTCAAAGAGGTCTTGAGGAATAGGCC

A3-AT2G47250-XLOC_013504-13503-0
      TGATCTTAAGCTAGTTGTCATGAGTGCAACTTTAGAAGCTGAAAAGTTTC
A3-AT2G47250-XLOC_013504-13503-1
      TGATCTTAAGCTAGTTGTCATGAGTGCAACTTTAGAAGCTGAAAAGTTTC
CONSENSUS
      TGATCTTAAGCTAGTTGTCATGAGTGCAACTTTAGAAGCTGAAAAGTTTC

A3-AT2G47250-XLOC_013504-13503-0
      AGGAATATTTTAGCGGTGCTCCTCTTATGAAAGTCCCTGGTAGGCTTCAT
A3-AT2G47250-XLOC_013504-13503-1
      AGGAATATTTTAGCGGTGCTCCTCTTATGAAAGTCCCTGGTAGGCTTCAT
CONSENSUS
      AGGAATATTTTAGCGGTGCTCCTCTTATGAAAGTCCCTGGTAGGCTTCAT

A3-AT2G47250-XLOC_013504-13503-0
      CCTGTTGAGATCTTCTATACACAGGAACCTGAGAGGGATTATCTCGAGGC
A3-AT2G47250-XLOC_013504-13503-1
      CCTGTTGAGATCTTCTATACACAGGAACCTGAGAGGGATTATCTCGAGGC
CONSENSUS
      CCTGTTGAGATCTTCTATACACAGGAACCTGAGAGGGATTATCTCGAGGC

A3-AT2G47250-XLOC_013504-13503-0
TGCTATAAGGACTGTTGTTCAGATACACATGTGCGAGCCACCTGGAGATA
A3-AT2G47250-XLOC_013504-13503-1
TGCTATAAGGACTGTTGTTCAGATACACATGTGCGAGCCACCTGGAGATA
CONSENSUS
TGCTATAAGGACTGTTGTTCAGATACACATGTGCGAGCCACCTGGAGATA

A3-AT2G47250-XLOC_013504-13503-0
TTCTTGTTTTCTTAACTGGAGAGGAGGAAATAGAAGATGCTTGCCGTAAA
A3-AT2G47250-XLOC_013504-13503-1
TTCTTGTTTTCTTAACTGGAGAGGAGGAAATAGAAGATGCTTGCCGTAAA
CONSENSUS
TTCTTGTTTTCTTAACTGGAGAGGAGGAAATAGAAGATGCTTGCCGTAAA

A3-AT2G47250-XLOC_013504-13503-0
ATCAATAAAGAGGTCAGCAATCTTGGAGATCAAGTGGGTCCTGTCAAAGT
A3-AT2G47250-XLOC_013504-13503-1
ATCAATAAAGAGGTCAGCAATCTTGGAGATCAAGTGGGTCCTGTCAAAGT
CONSENSUS
ATCAATAAAGAGGTCAGCAATCTTGGAGATCAAGTGGGTCCTGTCAAAGT

A3-AT2G47250-XLOC_013504-13503-0
TGTGCCTTTGTATTCTACTCTTCCACCTGCGATGCAGCAGAAGATTTTCG
A3-AT2G47250-XLOC_013504-13503-1
TGTGCCTTTGTATTCTACTCTTCCACCTGCGATGCAGCAGAAGATTTTCG
CONSENSUS
TGTGCCTTTGTATTCTACTCTTCCACCTGCGATGCAGCAGAAGATTTTCG

A3-AT2G47250-XLOC_013504-13503-0
ACCCTGCTCCAGTGCCGTTAACAGAAGGTGGTCCTGCTGGACGAAAGATT
A3-AT2G47250-XLOC_013504-13503-1
ACCCTGCTCCAGTGCCGTTAACAGAAGGTGGTCCTGCTGGACGAAAGATT
CONSENSUS
ACCCTGCTCCAGTGCCGTTAACAGAAGGTGGTCCTGCTGGACGAAAGATT

A3-AT2G47250-XLOC_013504-13503-0
GTTGTCTCAACCAACATTGCTGAAACCTCTCTAACCATTGATGGGATTGT
A3-AT2G47250-XLOC_013504-13503-1
GTTGTCTCAACCAACATTGCTGAAACCTCTCTAACCATTGATGGGATTGT
CONSENSUS
GTTGTCTCAACCAACATTGCTGAAACCTCTCTAACCATTGATGGGATTGT

A3-AT2G47250-XLOC_013504-13503-0
TTATGTTATTGACCCTGGTTTTGCTAAGCAGAAAGTCTACAACCCACGGA
A3-AT2G47250-XLOC_013504-13503-1
TTATGTTATTGACCCTGGTTTTGCTAAGCAGAAAGTCTACAACCCACGGA
CONSENSUS
TTATGTTATTGACCCTGGTTTTGCTAAGCAGAAAGTCTACAACCCACGGA

A3-AT2G47250-XLOC_013504-13503-0
TTCGAGTTGAGTCATTGTTGGTGTCCCCAATATCAAAGGCAAGTGCTCAC
A3-AT2G47250-XLOC_013504-13503-1
TTCGAGTTGAGTCATTGTTGGTGTCCCCAATATCAAAGGCAAGTGCTCAC
CONSENSUS
TTCGAGTTGAGTCATTGTTGGTGTCCCCAATATCAAAGGCAAGTGCTCAC

A3-AT2G47250-XLOC_013504-13503-0
CAGAGATCAGGTCGTGCTGGTAGAACTCGCCCTGGAAAATGTTTTAGGCT
A3-AT2G47250-XLOC_013504-13503-1
CAGAGATCAGGTCGTGCTGGTAGAACTCGCCCTGGAAAATGTTTTAGGCT
CONSENSUS
CAGAGATCAGGTCGTGCTGGTAGAACTCGCCCTGGAAAATGTTTTAGGCT

A3-AT2G47250-XLOC_013504-13503-0
CTACACAGAGAAGAGTTTCAACAATGACCTGCAGCCACAGACATATCCTG
A3-AT2G47250-XLOC_013504-13503-1
CTACACAGAGAAGAGTTTCAACAATGACCTGCAGCCACAGACATATCCTG
CONSENSUS
CTACACAGAGAAGAGTTTCAACAATGACCTGCAGCCACAGACATATCCTG

A3-AT2G47250-XLOC_013504-13503-0
AGATATTGAGATCAAACCTTGCAAATACAGTCCTGACATTGAAAAAACTT
A3-AT2G47250-XLOC_013504-13503-1
AGATATTGAGATCAAACCTTGCAAATACAGTCCTGACATTGAAAAAACTT
CONSENSUS
AGATATTGAGATCAAACCTTGCAAATACAGTCCTGACATTGAAAAAACTT

A3-AT2G47250-XLOC_013504-13503-0
GGCATTGATGACTTGGTGCACTTTGATTTCATGGATCCTCCTGCTCCTGA
A3-AT2G47250-XLOC_013504-13503-1
GGCATTGATGACTTGGTGCACTTTGATTTCATGGATCCTCCTGCTCCTGA
CONSENSUS
GGCATTGATGACTTGGTGCACTTTGATTTCATGGATCCTCCTGCTCCTGA

A3-AT2G47250-XLOC_013504-13503-0
GACACTGATGCGGGCATTAGAGGTTCTGAATTATTTGGGAGCACTTGATG
A3-AT2G47250-XLOC_013504-13503-1
GACACTGATGCGGGCATTAGAGGTTCTGAATTATTTGGGAGCACTTGATG
CONSENSUS
GACACTGATGCGGGCATTAGAGGTTCTGAATTATTTGGGAGCACTTGATG

A3-AT2G47250-XLOC_013504-13503-0
ATGAAGGTAACTTGACAAAGACGGGTGAAATAATGAGTGAATTTCCCTTG
A3-AT2G47250-XLOC_013504-13503-1
ATGAAGGTAACTTGACAAAGACGGGTGAAATAATGAGTGAATTTCCCTTG
CONSENSUS
ATGAAGGTAACTTGACAAAGACGGGTGAAATAATGAGTGAATTTCCCTTG

A3-AT2G47250-XLOC_013504-13503-0
GATCCACAAATGTCAAAGATGCTCATAGTCAGTCCTGAATTCAACTGTTC
A3-AT2G47250-XLOC_013504-13503-1
GATCCACAAATGTCAAAGATGCTCATAGTCAGTCCTGAATTCAACTGTTC
CONSENSUS
GATCCACAAATGTCAAAGATGCTCATAGTCAGTCCTGAATTCAACTGTTC

A3-AT2G47250-XLOC_013504-13503-0
CAATGAGATTCTCTCGGTTTCTGCAATGTTATCAG------TTTCTCAGT
A3-AT2G47250-XLOC_013504-13503-1
CAATGAGATTCTCTCGGTTTCTGCAATGTTATCAGTTGCAGTTTCTCAGT
CONSENSUS
CAATGAGATTCTCTCGGTTTCTGCAATGTTATCAG......TTTCTCAGT

```
A3-AT2G47250-XLOC_013504-13503-0
      ATTCTCACAAGCAAATGGTATCGTCTCTCGCCTCTATGCATCTGCTGACC
A3-AT2G47250-XLOC_013504-13503-1
      ATTCTCACAAGCAAATGGTATCGTCTCTCGCCTCTATGCATCTGCTGACC
CONSENSUS
      ATTCTCACAAGCAAATGGTATCGTCTCTCGCCTCTATGCATCTGCTGACC

A3-AT2G47250-XLOC_013504-13503-0
      TCTTCCATGGGCATATTCTGTTATAAAAACAATTCTTTCTCTAGTACCGA
A3-AT2G47250-XLOC_013504-13503-1
      TCTTCCATGGGCATATTCTGTTATAAAAACAATTCTTTCTCTAGTACCGA
CONSENSUS
      TCTTCCATGGGCATATTCTGTTATAAAAACAATTCTTTCTCTAGTACCGA

A3-AT2G47250-XLOC_013504-13503-0
      ATTGCTTTGTCCGGCCTAGAGAGGCTCAAAAAGCAGCAGATGAAGCTAAA
A3-AT2G47250-XLOC_013504-13503-1
      ATTGCTTTGTCCGGCCTAGAGAGGCTCAAAAAGCAGCAGATGAAGCTAAA
CONSENSUS
      ATTGCTTTGTCCGGCCTAGAGAGGCTCAAAAAGCAGCAGATGAAGCTAAA

A3-AT2G47250-XLOC_013504-13503-0
      GCTAGGTTTGGACACATTGATGGAGATCACCTGACGTTGCTAAACGTGTA
A3-AT2G47250-XLOC_013504-13503-1
      GCTAGGTTTGGACACATTGATGGAGATCACCTGACGTTGCTAAACGTGTA
CONSENSUS
      GCTAGGTTTGGACACATTGATGGAGATCACCTGACGTTGCTAAACGTGTA

A3-AT2G47250-XLOC_013504-13503-0 CCACGCCTACAAGCAAAACA
A3-AT2G47250-XLOC_013504-13503-1 CCACGCCTACAAGCAAAACA
CONSENSUS                         CCACGCCTACAAGCAAAACA


alignment for event: A5-AT2G36060-XLOC_012887-4066

A5-AT2G36060-XLOC_012887-4066-0
      TTCCGAGGAATTTCCGGTTGCTGGAGGAGCTTGAACGTGGAGAGAAAGGT
A5-AT2G36060-XLOC_012887-4066-1
      TTCCGAGGAATTTCCGGTTGCTGGAGGAGCTTGAACGTGGAGAGAAAGGT
CONSENSUS
      TTCCGAGGAATTTCCGGTTGCTGGAGGAGCTTGAACGTGGAGAGAAAGGT

A5-AT2G36060-XLOC_012887-4066-0
      ATTGGAGATGGAACTGTGAGCTATGGAATGGATGATGGAGATGACATTTA
A5-AT2G36060-XLOC_012887-4066-1
      ATTGGAGATGGAACTGTGAGCTATGGAATGGATGATGGAGATGACATTTA
CONSENSUS
      ATTGGAGATGGAACTGTGAGCTATGGAATGGATGATGGAGATGACATTTA

A5-AT2G36060-XLOC_012887-4066-0
      TATGCGCTCTTGGACTGGCACTATCATCGGTCCTCACAACGTAACTGTAC
A5-AT2G36060-XLOC_012887-4066-1
      TATGCGCTCTTGGACTGGCACTATCATCGGTCCTCACAAC---ACTGTAC
CONSENSUS
```

```
      TATGCGCTCTTGGACTGGCACTATCATCGGTCCTCACAAC...ACTGTAC

A5-AT2G36060-XLOC_012887-4066-0
      ATGAGGGTCGGATTTATCAGTTGAAGCTCTTCTGTGACAAAGATTACCCT
A5-AT2G36060-XLOC_012887-4066-1
      ATGAGGGTCGGATTTATCAGTTGAAGCTCTTCTGTGACAAAGATTACCCT
CONSENSUS
      ATGAGGGTCGGATTTATCAGTTGAAGCTCTTCTGTGACAAAGATTACCCT

A5-AT2G36060-XLOC_012887-4066-0
      GAGAAACCTCCGACTGTTCGGTTCCATTCGCGTATCAACATGACTTGTGT
A5-AT2G36060-XLOC_012887-4066-1
      GAGAAACCTCCGACTGTTCGGTTCCATTCGCGTATCAACATGACTTGTGT
CONSENSUS
      GAGAAACCTCCGACTGTTCGGTTCCATTCGCGTATCAACATGACTTGTGT

A5-AT2G36060-XLOC_012887-4066-0  CAACCATGATACCGGCGTG
A5-AT2G36060-XLOC_012887-4066-1  CAACCATGATACCGGCGTG
CONSENSUS                        CAACCATGATACCGGCGTG



alignment for event: SE-AT2G26350-XLOC_012323-7069

SE-AT2G26350-XLOC_012323-7069-0
      GGAGAGGTTTACCTGTTTTAAATGAAGAGGGGAATTTGATAACTTCGGAA
SE-AT2G26350-XLOC_012323-7069-1
      GGAGAGGTTTACCTGTTTTAAATGAAGAGGGGAATTTGATAACTTCGGAA
CONSENSUS
      GGAGAGGTTTACCTGTTTTAAATGAAGAGGGGAATTTGATAACTTCGGAA

SE-AT2G26350-XLOC_012323-7069-0
      GCTGAAAAGGGAAACTGGTCTACCTCCGATTCAACTTCAACGGAG-----
SE-AT2G26350-XLOC_012323-7069-1
      GCTGAAAAGGGAAACTGGTCTACCTCCGATTCAACTTCAACGGAGGTAAT
CONSENSUS
      GCTGAAAAGGGAAACTGGTCTACCTCCGATTCAACTTCAACGGAG.....

SE-AT2G26350-XLOC_012323-7069-0
      --------------------------------------------------
SE-AT2G26350-XLOC_012323-7069-1
      AATATACTTGGAAGAGAGGCACATAACTCTGAGCAAAATGTATTATGCTC
CONSENSUS
      ..................................................

SE-AT2G26350-XLOC_012323-7069-0
      --------------------------------------------------
SE-AT2G26350-XLOC_012323-7069-1
      TTACGCAAAGGTTATGTTGTATAAGTGTGATTAAGAAAACCAAAATGATA
CONSENSUS
      ..................................................

SE-AT2G26350-XLOC_012323-7069-0
      --------------------------------------------------
SE-AT2G26350-XLOC_012323-7069-1
      GTTTACTCTGTTGGTATGAGTATCTGATAAGAGTAGATGGCCACTCGACT
```

CONSENSUS
      ....

SE-AT2G26350-XLOC_012323-7069-0
      -----------------------------------GCAGTAGGGAAATGC
SE-AT2G26350-XLOC_012323-7069-1
      GATCCCGCTCTACATATAGTTTCTAGCGAGACAAGGCAGTAGGGAAATGC
CONSENSUS
      ...................................GCAGTAGGGAAATGC

SE-AT2G26350-XLOC_012323-7069-0
      ACTCTCTGCTTAAGCACCCGTCAGCACCCAACGGCCACTCCTTGTGGTCA
SE-AT2G26350-XLOC_012323-7069-1
      ACTCTCTGCTTAAGCACCCGTCAGCACCCAACGGCCACTCCTTGTGGTCA
CONSENSUS
      ACTCTCTGCTTAAGCACCCGTCAGCACCCAACGGCCACTCCTTGTGGTCA

SE-AT2G26350-XLOC_012323-7069-0 TGTGTTTTGTTG
SE-AT2G26350-XLOC_012323-7069-1 TGTGTTTTGTTG
CONSENSUS   TGTGTTTTGTTG

alignment for event: RI-AT2G25930-XLOC_009647-12879

RI-AT2G25930-XLOC_009647-12879-0
      CCTTGTGGTGTGGAAAGAAACTTATCTGTCCAGCATCTTGATTCTTCAGC
RI-AT2G25930-XLOC_009647-12879-1
      CCTTGTGGTGTGGAAAGAAACTTATCTGTCCAGCATCTTGATTCTTCAGC
CONSENSUS
      CCTTGTGGTGTGGAAAGAAACTTATCTGTCCAGCATCTTGATTCTTCAGC

RI-AT2G25930-XLOC_009647-12879-0
      CGCAAACCAAGCAACTGAGAAGTTTGTCTCCCAAATGTCCTTCATGGAAA
RI-AT2G25930-XLOC_009647-12879-1
      CGCAAACCAAGCAACTGAGAAGTTTGTCTCCCAAATGTCCTTCATGGAAA
CONSENSUS
      CGCAAACCAAGCAACTGAGAAGTTTGTCTCCCAAATGTCCTTCATGGAAA

RI-AT2G25930-XLOC_009647-12879-0
      ATGTGAGATCTTCGGCACAGCATGATCAGAGGAAAATGGTGAGAGAGGAA
RI-AT2G25930-XLOC_009647-12879-1
      ATGTGAGATCTTCGGCACAGCATGATCAGAGGAAAATGGTGAGAGAGGAA
CONSENSUS
      ATGTGAGATCTTCGGCACAGCATGATCAGAGGAAAATGGTGAGAGAGGAA

RI-AT2G25930-XLOC_009647-12879-0
      GAAGATTTTGCAGTTCCAGTATATATTAACTCAAGAAGATCTCAGTCTCA
RI-AT2G25930-XLOC_009647-12879-1
      GAAGATTTTGCAGTTCCAGTATATATTAACTCAAGAAGATCTCAGTCTCA
CONSENSUS
      GAAGATTTTGCAGTTCCAGTATATATTAACTCAAGAAGATCTCAGTCTCA

RI-AT2G25930-XLOC_009647-12879-0
      TGGCAGAACCAAGAGTGGTATTGAGAAGGAAAAACACACCCCAATGGTGG
RI-AT2G25930-XLOC_009647-12879-1

TGGCAGAACCAAGAGTGGTATTGAGAAGGAAAAACACACCCCAATGGTGG
CONSENSUS
TGGCAGAACCAAGAGTGGTATTGAGAAGGAAAAACACACCCCAATGGTGG

RI-AT2G25930-XLOC_009647-12879-0
CACCTAGCTCTCATCACTCCATTCGATTTCAAGAAGTGAATCAGACAGGC
RI-AT2G25930-XLOC_009647-12879-1
CACCTAGCTCTCATCACTCCATTCGATTTCAAGAAGTGAATCAGACAGGC
CONSENSUS
CACCTAGCTCTCATCACTCCATTCGATTTCAAGAAGTGAATCAGACAGGC

RI-AT2G25930-XLOC_009647-12879-0
TCAAAGCAAAACGTATGTTTGGCTACTTGTTCAAAACCTGAAGTTAGGGA
RI-AT2G25930-XLOC_009647-12879-1
TCAAAGCAAAACGTATGTTTGGCTACTTGTTCAAAACCTGAAGTTAGGGA
CONSENSUS
TCAAAGCAAAACGTATGTTTGGCTACTTGTTCAAAACCTGAAGTTAGGGA

RI-AT2G25930-XLOC_009647-12879-0
TCAGGTCAAGGCGAATGCAAGGTCAGGTGGCTTTGTAATCTCTTTAGATG
RI-AT2G25930-XLOC_009647-12879-1
TCAGGTCAAGGCGAATGCAAGGTCAGGTGGCTTTGTAATCTCTTTAGATG
CONSENSUS
TCAGGTCAAGGCGAATGCAAGGTCAGGTGGCTTTGTAATCTCTTTAGATG

RI-AT2G25930-XLOC_009647-12879-0
TATCAGTCACAGAGGAGATTGATCTCGAAAAATCAGCATCAAGTCATGAT
RI-AT2G25930-XLOC_009647-12879-1
TATCAGTCACAGAGGAGATTGATCTCGAAAAATCAGCATCAAGTCATGAT
CONSENSUS
TATCAGTCACAGAGGAGATTGATCTCGAAAAATCAGCATCAAGTCATGAT

RI-AT2G25930-XLOC_009647-12879-0
AGAGTAAATGATTATAATGCTTCCTTGAGACAAGAGTCTAGAAATCGGTT
RI-AT2G25930-XLOC_009647-12879-1
AGAGTAAATGATTATAATGCTTCCTTGAGACAAGAGTCTAGAAATCGGTT
CONSENSUS
AGAGTAAATGATTATAATGCTTCCTTGAGACAAGAGTCTAGAAATCGGTT

RI-AT2G25930-XLOC_009647-12879-0
ATACCGAGATGGTGGCAAAACTCGTCTGAAGGACACTGATAATGGAGCTG
RI-AT2G25930-XLOC_009647-12879-1
ATACCGAGATGGTGGCAAAACTCGTCTGAAGGACACTGATAATGGAGCTG
CONSENSUS
ATACCGAGATGGTGGCAAAACTCGTCTGAAGGACACTGATAATGGAGCTG

RI-AT2G25930-XLOC_009647-12879-0
AATCTCACTTGGCAACGGAAAATCATTCACAAGAGGGTCATGGCAGTCCT
RI-AT2G25930-XLOC_009647-12879-1
AATCTCACTTGGCAACGGAAAATCATTCACAAGAGGGTCATGGCAGTCCT
CONSENSUS
AATCTCACTTGGCAACGGAAAATCATTCACAAGAGGGTCATGGCAGTCCT

RI-AT2G25930-XLOC_009647-12879-0
GAAGACATTGATAATGATCGTGAATACAGCAAAAGCAGAGCATGCGCCTC
RI-AT2G25930-XLOC_009647-12879-1

GAAGACATTGATAATGATCGTGAATACAGCAAAAGCAGAGCATGCGCCTC
CONSENSUS
GAAGACATTGATAATGATCGTGAATACAGCAAAAGCAGAGCATGCGCCTC

RI-AT2G25930-XLOC_009647-12879-0
TCTGCAGCAGATAAATGAAGAGGCAAGTGATGACGTTTCTGATGATTCGA
RI-AT2G25930-XLOC_009647-12879-1
TCTGCAGCAGATAAATGAAGAGGCAAGTGATGACGTTTCTGATGATTCGA
CONSENSUS
TCTGCAGCAGATAAATGAAGAGGCAAGTGATGACGTTTCTGATGATTCGA

RI-AT2G25930-XLOC_009647-12879-0
TGGTGGATTCTATATCCAGCATAGATGTCTCTCCCGATGATGTTGTGGGT
RI-AT2G25930-XLOC_009647-12879-1
TGGTGGATTCTATATCCAGCATAGATGTCTCTCCCGATGATGTTGTGGGT
CONSENSUS
TGGTGGATTCTATATCCAGCATAGATGTCTCTCCCGATGATGTTGTGGGT

RI-AT2G25930-XLOC_009647-12879-0
ATATTAGGTCAAAAACGTTTCTGGAGAGCAAGGAAAGCCATTGCCAAGTA
RI-AT2G25930-XLOC_009647-12879-1
ATATTAGGTCAAAAACGTTTCTGGAGAGCAAGGAAAGCCATTGCCAA---
CONSENSUS
ATATTAGGTCAAAAACGTTTCTGGAGAGCAAGGAAAGCCATTGCCAA...

RI-AT2G25930-XLOC_009647-12879-0
AGTTCACTAGAAATTTACAGTTTGGTTATTTATTCTCCGCTCTTTCTATT
RI-AT2G25930-XLOC_009647-12879-1

CONSENSUS
....

RI-AT2G25930-XLOC_009647-12879-0
TATCTCCTTCTTTGATACCAACATTTTTTGCTTGAAAGAAGTTAATATTT
RI-AT2G25930-XLOC_009647-12879-1

CONSENSUS
....

RI-AT2G25930-XLOC_009647-12879-0
AAGCATTGTTCCGTAGTCTTACTGAAGCTTTTTCCTCTGTTGTTTTTTGC
RI-AT2G25930-XLOC_009647-12879-1

CONSENSUS
....

RI-AT2G25930-XLOC_009647-12879-0
TATTTTCATTGAGGACTGTGGTAGGGCATATTTCACTATCACCAAATTTC
RI-AT2G25930-XLOC_009647-12879-1

CONSENSUS
....

RI-AT2G25930-XLOC_009647-12879-0
AAATTTCTAGAACACTCTCCTTCATATTTTTTTTCATGATTAATGCTGCA
RI-AT2G25930-XLOC_009647-12879-1

CONSENSUS
....

RI-AT2G25930-XLOC_009647-12879-0
ATTGATTGCTGATATACATATATGACTATAACTCAGTTTCATATTCTGTC
RI-AT2G25930-XLOC_009647-12879-1

CONSENSUS
....

RI-AT2G25930-XLOC_009647-12879-0
TCATTTTGGGAGAAAGAGATTTCAGGTTTATGCTTGAGAAGTGATGGTTC
RI-AT2G25930-XLOC_009647-12879-1

CONSENSUS
....

RI-AT2G25930-XLOC_009647-12879-0
TATAGTTGAGAGGCCCCTGATTCATCTAAAATGGTCCTATTATGTGTTTA
RI-AT2G25930-XLOC_009647-12879-1

CONSENSUS
....

RI-AT2G25930-XLOC_009647-12879-0
GTTGTAGAGTCCTCGGTAGAATATTAACGCGTTTAACACGTTGGATCATG
RI-AT2G25930-XLOC_009647-12879-1

CONSENSUS
....

RI-AT2G25930-XLOC_009647-12879-0
TTATAGCAGGGAGGGACATTCTCTGTTGACCTATATTGTGCAAGGTGCCC
RI-AT2G25930-XLOC_009647-12879-1

CONSENSUS
....

RI-AT2G25930-XLOC_009647-12879-0
GCCGATGGCTTTATTACTATACCTTCTTTGCATCTGGTTGTTGGAACATG
RI-AT2G25930-XLOC_009647-12879-1

CONSENSUS
....

RI-AT2G25930-XLOC_009647-12879-0
TCCCTGTCTCGGTTTGGTATTGCTTTTATTCTGCACTGTCGTCTTGGGCA
RI-AT2G25930-XLOC_009647-12879-1

CONSENSUS
....

RI-AT2G25930-XLOC_009647-12879-0
TTTTCCCTACTTGTCATTCAAGGGGTTGAACCAGGTAGGGAAATGTTTTT
RI-AT2G25930-XLOC_009647-12879-1

CONSENSUS
....

RI-AT2G25930-XLOC_009647-12879-0
CCGAGGACCCCAGGATCTAAATTTTAGTTAACCATACGTAAAGTTAGTTT
RI-AT2G25930-XLOC_009647-12879-1

CONSENSUS
....

RI-AT2G25930-XLOC_009647-12879-0
TGAGTCTTATGACGATGCAGAATTATAGTTTCTTCTTACTACTGCTTAAG
RI-AT2G25930-XLOC_009647-12879-1

CONSENSUS
....

RI-AT2G25930-XLOC_009647-12879-0
AGGATCCTTAGTGTGGTTGTGAACTACAGAGTTTTTATGATTGTAGGCTT
RI-AT2G25930-XLOC_009647-12879-1

CONSENSUS
....

RI-AT2G25930-XLOC_009647-12879-0
CATGACTTAACTTTTAAGGTTCAATGTACTCTAATCCATATGGTAAGGTA
RI-AT2G25930-XLOC_009647-12879-1

CONSENSUS
....

RI-AT2G25930-XLOC_009647-12879-0
TCGGATTCACGACCAATGCAAATAATAAGATTTTTATTTCTTGCTTCTTG
RI-AT2G25930-XLOC_009647-12879-1

CONSENSUS
....

RI-AT2G25930-XLOC_009647-12879-0
TTAAATATCTGACATCTCATTTTGCAGAGGATAAGCTGCGCTGTAAGCTA
RI-AT2G25930-XLOC_009647-12879-1

CONSENSUS
....

RI-AT2G25930-XLOC_009647-12879-0
GATTTCAATAAGCCCGTCCTTTGCATTGTTATCTATGCTTTAATATGTCA
RI-AT2G25930-XLOC_009647-12879-1

CONSENSUS
....

RI-AT2G25930-XLOC_009647-12879-0
TTGGACCCATTGATTTGGTTTTCTTCTATCTTTTTTGATTGGCTATGTAT
RI-AT2G25930-XLOC_009647-12879-1

CONSENSUS
....

RI-AT2G25930-XLOC_009647-12879-0
TCTTGTTTCTTTTTTCCTATCTCATTTCGATCGTATTGTTCCATTAGCTG
RI-AT2G25930-XLOC_009647-12879-1

CONSENSUS
....

RI-AT2G25930-XLOC_009647-12879-0
TTCAACCTAAACTATGTCTCTCTTTGTTGAACTTTTGATGGATAATCTTC
RI-AT2G25930-XLOC_009647-12879-1

CONSENSUS
....

RI-AT2G25930-XLOC_009647-12879-0
TTAATGTGACTCTGTTTCTCATTACAGTCAACAAAGAGTATTTGCTGTTC
RI-AT2G25930-XLOC_009647-12879-1 ---------------------------
TCAACAAAGAGTATTTGCTGTTC
CONSENSUS
...........................TCAACAAAGAGTATTTGCTGTTC

RI-AT2G25930-XLOC_009647-12879-0 AACTATTTGAGTTGCACAGACTGATTAAG
RI-AT2G25930-XLOC_009647-12879-1 AACTATTTGAGTTGCACAGACTGATTAAG
CONSENSUS AACTATTTGAGTTGCACAGACTGATTAAG

alignment for event: RI-AT2G35510-XLOC_012854-3336

RI-AT2G35510-XLOC_012854-3336-0
TGCTAGATACTGTGATATTGACGATAATGGTGTGCGGCACATGGTTTTGT
RI-AT2G35510-XLOC_012854-3336-1
TGCTAGATACTGTGATATTGACGATAATGGTGTGCGGCACATGGTTTTGT
CONSENSUS
TGCTAGATACTGTGATATTGACGATAATGGTGTGCGGCACATGGTTTTGT

RI-AT2G35510-XLOC_012854-3336-0
GCCGCGTAATAATGGGGAATATGGAGCCTCTTCGTGGTGATAACACGCAG
RI-AT2G35510-XLOC_012854-3336-1
GCCGCGTAATAATGGGGAATATGGAGCCTCTTCGTGGTGATAACACGCAG
CONSENSUS
GCCGCGTAATAATGGGGAATATGGAGCCTCTTCGTGGTGATAACACGCAG

RI-AT2G35510-XLOC_012854-3336-0
TACTTTACTGGTGGAGAAGAGTATGATAATGGGGTTGATGATGTTGAGAG
RI-AT2G35510-XLOC_012854-3336-1
TACTTTACTGGTGGAGAAGAGTATGATAATGGGGTTGATGATGTTGAGAG
CONSENSUS
TACTTTACTGGTGGAGAAGAGTATGATAATGGGGTTGATGATGTTGAGAG

RI-AT2G35510-XLOC_012854-3336-0
TCCAAAGCATTACCTTATCTGGAACATGAATATGAACACTCACATTTACC

RI-AT2G35510-XLOC_012854-3336-1
TCCAAAGCATTACCTTATCTGGAACATGAATATGAACACTCACATTTACC
CONSENSUS
TCCAAAGCATTACCTTATCTGGAACATGAATATGAACACTCACATTTACC

RI-AT2G35510-XLOC_012854-3336-0
CAGAATTTGTAGTTAGCTTCAAGCTGTCTATCCCCAATGCTGAAGGTGAT
RI-AT2G35510-XLOC_012854-3336-1
CAGAATTTGTAGTTAGCTTCAAGCTGTCTATCCCCAATGCTGAAG-----
CONSENSUS
CAGAATTTGTAGTTAGCTTCAAGCTGTCTATCCCCAATGCTGAAG.....

RI-AT2G35510-XLOC_012854-3336-0
TACCATTTGTTATTTCTCCTGTAATTTCCATAAAAATCAACTGATGACGC
RI-AT2G35510-XLOC_012854-3336-1

CONSENSUS
....

RI-AT2G35510-XLOC_012854-3336-0
ATTGAATGGGAGACAGCATATAGATCTTACTGAAGATCAGCTATTTGGAT
RI-AT2G35510-XLOC_012854-3336-1

CONSENSUS
....

RI-AT2G35510-XLOC_012854-3336-0
CCTGAAATCTTCCTCTATAAGTTAGAAAAGGATCCACATCACCTTGTGAC
RI-AT2G35510-XLOC_012854-3336-1

CONSENSUS
....

RI-AT2G35510-XLOC_012854-3336-0
CTCTGGGGGTGGTAAACATACTTCATCATCCCAAGGGTCCAGTGAGATAT
RI-AT2G35510-XLOC_012854-3336-1

CONSENSUS
....

RI-AT2G35510-XLOC_012854-3336-0
CCATGGCTAATATATATGGGTATTTCGATGATCACATTTGTTTTCTGTAG
RI-AT2G35510-XLOC_012854-3336-1

CONSENSUS
....

RI-AT2G35510-XLOC_012854-3336-0
TATTCGAAACGGGATGACATGTTTTGTTTGTGCAGGGAATATTCTTCCTA
RI-AT2G35510-XLOC_012854-3336-1
-----------------------------------GGAATATTCTTCCTA
CONSENSUS
...................................GGAATATTCTTCCTA

RI-AT2G35510-XLOC_012854-3336-0
CTACTCAGAGTAGGCACGAGAGTTCGGGACTCACCTTGGAAGGACCCAAA

RI-AT2G35510-XLOC_012854-3336-1
      CTACTCAGAGTAGGCACGAGAGTTCGGGACTCACCTTGGAAGGACCCAAA
CONSENSUS
      CTACTCAGAGTAGGCACGAGAGTTCGGGACTCACCTTGGAAGGACCCAAA

RI-AT2G35510-XLOC_012854-3336-0 GGTTCTCCCTCAAAT
RI-AT2G35510-XLOC_012854-3336-1 GGTTCTCCCTCAAAT
CONSENSUS   GGTTCTCCCTCAAAT

alignment for event: A3-AT2G42950-XLOC_010576-6020

A3-AT2G42950-XLOC_010576-6020-0
      ACATTCATCTCTCTCTCTCAGTCAAATTGTTGTTTTCTTTCTTCGAATCG
A3-AT2G42950-XLOC_010576-6020-1
      ACATTCATCTCTCTCTCTCAGTCAAATTGTTGTTTTCTTTCTTCGAATCG
CONSENSUS
      ACATTCATCTCTCTCTCTCAGTCAAATTGTTGTTTTCTTTCTTCGAATCG

A3-AT2G42950-XLOC_010576-6020-0
      GTGCAGAAAATTCAGGGAAGTTCTGGGGAAGGTTGTTGCGTTTGACTCCT
A3-AT2G42950-XLOC_010576-6020-1
      GTGCAGAAAATTCAGGGAAGTTCTGGGGAAGGTTGTTGCGTTTGACTCCT
CONSENSUS
      GTGCAGAAAATTCAGGGAAGTTCTGGGGAAGGTTGTTGCGTTTGACTCCT

A3-AT2G42950-XLOC_010576-6020-0
      TTGGCTTAGTTTTCTTTCGAATTCCGTGCTTCCTGATGATCTTACGTGAA
A3-AT2G42950-XLOC_010576-6020-1
      TTGGCTTAGTTTTCTTTCGAATTCCGTGCTTCCTGATGATCTTACGTGAA
CONSENSUS
      TTGGCTTAGTTTTCTTTCGAATTCCGTGCTTCCTGATGATCTTACGTGAA

A3-AT2G42950-XLOC_010576-6020-0
      ATTGCAGCCTAAAATTTCGAGATTGTTTTTTTTACTCAGAAAACGAGATT
A3-AT2G42950-XLOC_010576-6020-1
      ATTGCAGCCTAAAATTTCGAGATTGTTTTTTTTACTCAGAAAACGAGATT
CONSENSUS
      ATTGCAGCCTAAAATTTCGAGATTGTTTTTTTTACTCAGAAAACGAGATT

A3-AT2G42950-XLOC_010576-6020-0
      TGACTGATATGAATCGAAAATCTGTGATTTAAAGTGAAGCATGAATGCGA
A3-AT2G42950-XLOC_010576-6020-1
      TGACTGATATGAATCGAAAATCTGTGATTTAAAGTGAAGCATGAATGCGA
CONSENSUS
      TGACTGATATGAATCGAAAATCTGTGATTTAAAGTGAAGCATGAATGCGA

A3-AT2G42950-XLOC_010576-6020-0
      ATCACAAGAACGGGAATCCAGGTTACCATAATCTCTGGACCAATGGACTT
A3-AT2G42950-XLOC_010576-6020-1
      ATCACAAGAACGGGAATCCAGGTTACCATAATCTCTGGACCAATGGACTT
CONSENSUS
      ATCACAAGAACGGGAATCCAGGTTACCATAATCTCTGGACCAATGGACTT

A3-AT2G42950-XLOC_010576-6020-0

ATCTGTGCTTTCGAGTTTTGTCAAGGTCGTCGTAAGAATAACACAAGTGT
A3-AT2G42950-XLOC_010576-6020-1
ATCTGTGCTTTCGAGTTTTGTCAAGGTCGTCGTAAGAATAACACAAGTGT
CONSENSUS
ATCTGTGCTTTCGAGTTTTGTCAAGGTCGTCGTAAGAATAACACAAGTGT

A3-AT2G42950-XLOC_010576-6020-0
CCATGGAGATTCGTCTCTCAGAATAAAGAAACAGGAATGTGAAACAGATC
A3-AT2G42950-XLOC_010576-6020-1 CCATGGAGATTCGTCTCTCAGAAT----AAACAGGAATGTGAAACAGATC
CONSENSUS
CCATGGAGATTCGTCTCTCAGAAT....AAACAGGAATGTGAAACAGATC

A3-AT2G42950-XLOC_010576-6020-0
AATTTGGTACTGGTGAAGACGAAGAACATTCTAGGAGTTATTGGCGAGGA
A3-AT2G42950-XLOC_010576-6020-1
AATTTGGTACTGGTGAAGACGAAGAACATTCTAGGAGTTATTGGCGAGGA
CONSENSUS
AATTTGGTACTGGTGAAGACGAAGAACATTCTAGGAGTTATTGGCGAGGA

A3-AT2G42950-XLOC_010576-6020-0
ATTGGATGGGATAGGATCTCTGAGCTTGTTCAGACTGTTCAAGTTGATAA
A3-AT2G42950-XLOC_010576-6020-1
ATTGGATGGGATAGGATCTCTGAGCTTGTTCAGACTGTTCAAGTTGATAA
CONSENSUS
ATTGGATGGGATAGGATCTCTGAGCTTGTTCAGACTGTTCAAGTTGATAA

A3-AT2G42950-XLOC_010576-6020-0
TAATTGGGATTTGCGAAAGATTGACTTAGATGAGGATGAAGCCACTGTTG
A3-AT2G42950-XLOC_010576-6020-1
TAATTGGGATTTGCGAAAGATTGACTTAGATGAGGATGAAGCCACTGTTG
CONSENSUS
TAATTGGGATTTGCGAAAGATTGACTTAGATGAGGATGAAGCCACTGTTG

A3-AT2G42950-XLOC_010576-6020-0
CTGAGCTTGCTGCTCCTTACTGGGAACGACCACTTGCTGGTCCAACTTGG
A3-AT2G42950-XLOC_010576-6020-1
CTGAGCTTGCTGCTCCTTACTGGGAACGACCACTTGCTGGTCCAACTTGG
CONSENSUS
CTGAGCTTGCTGCTCCTTACTGGGAACGACCACTTGCTGGTCCAACTTGG

A3-AT2G42950-XLOC_010576-6020-0
TGGTGTCATCTTGATGCTACCCATCATGGTATTGCCTCGTGGTTACGTAA
A3-AT2G42950-XLOC_010576-6020-1
TGGTGTCATCTTGATGCTACCCATCATGGTATTGCCTCGTGGTTACGTAA
CONSENSUS
TGGTGTCATCTTGATGCTACCCATCATGGTATTGCCTCGTGGTTACGTAA

A3-AT2G42950-XLOC_010576-6020-0
TGCGCATTGGTTACATCCCGCGGTCAGTCTTGCTCTGCGGGACGAAAGCA
A3-AT2G42950-XLOC_010576-6020-1
TGCGCATTGGTTACATCCCGCGGTCAGTCTTGCTCTGCGGGACGAAAGCA
CONSENSUS
TGCGCATTGGTTACATCCCGCGGTCAGTCTTGCTCTGCGGGACGAAAGCA

A3-AT2G42950-XLOC_010576-6020-0

AACTGATTAGTGAACGAATGAAACATATTTTCTATGAG
A3-AT2G42950-XLOC_010576-6020-1
AACTGATTAGTGAACGAATGAAACATATTTTCTATGAG
CONSENSUS
AACTGATTAGTGAACGAATGAAACATATTTTCTATGAG

alignment for event: A3-AT2G01460-XLOC_008183-13632

A3-AT2G01460-XLOC_008183-13632-0
TTTTATTGAGATGTACCTCAGACCACCTTCTGCAAGCGAAGAGGCACGGA
A3-AT2G01460-XLOC_008183-13632-1
TTTTATTGAGATGTACCTCAGACCACCTTCTGCAAGCGAAGAGGCACGGA
CONSENSUS
TTTTATTGAGATGTACCTCAGACCACCTTCTGCAAGCGAAGAGGCACGGA

A3-AT2G01460-XLOC_008183-13632-0
TAAATGACTGGATTAAAGTTCGTCAAGCTGGTATAAGGTACTATCTGTCA
A3-AT2G01460-XLOC_008183-13632-1
TAAATGACTGGATTAAAGTTCGTCAAGCTGGTATAAGGTACTATCTGTCA
CONSENSUS
TAAATGACTGGATTAAAGTTCGTCAAGCTGGTATAAGGTACTATCTGTCA

A3-AT2G01460-XLOC_008183-13632-0
CTTGGAGACCAAAGGATTGTTGACAAGCATTTCATTATCCGGCCTAAAGC
A3-AT2G01460-XLOC_008183-13632-1
CTTGGAGACCAAAGGATTGTTGACAAGCATTTCATTATCCGGCCTAAAGC
CONSENSUS
CTTGGAGACCAAAGGATTGTTGACAAGCATTTCATTATCCGGCCTAAAGC

A3-AT2G01460-XLOC_008183-13632-0
TGAGTTTGAGAAGAGGCATGCCTATATTTGTGGAATCTGGATTCTGGTCA
A3-AT2G01460-XLOC_008183-13632-1
TGAGTTTGAG--
CONSENSUS
TGAGTTTGAG..

A3-AT2G01460-XLOC_008183-13632-0
AGATGACGGGCCGCTTGCACACATCCTATTAAAAGATACCCCTTACAATC
A3-AT2G01460-XLOC_008183-13632-1

CONSENSUS
....

A3-AT2G01460-XLOC_008183-13632-0
CTTCTGTATGTGACTTAATTTCTAATGTCTAGCAGGTTGGACGGATGACA
A3-AT2G01460-XLOC_008183-13632-1
-----------------------------------GTTGGACGGATGACA
CONSENSUS
...................................GTTGGACGGATGACA

A3-AT2G01460-XLOC_008183-13632-0
CTTGGAGGGTTGCTAGCTTTAGGCTATAATGTTGTTGTGAGTTACAAGAG
A3-AT2G01460-XLOC_008183-13632-1
CTTGGAGGGTTGCTAGCTTTAGGCTATAATGTTGTTGTGAGTTACAAGAG

CONSENSUS
CTTGGAGGGTTGCTAGCTTTAGGCTATAATGTTGTTGTGAGTTACAAGAG

A3-AT2G01460-XLOC_008183-13632-0
AGCTTCAACTGCTGTCAGTTATGGTAATTTATCCCTATCACGTGAAACGA
A3-AT2G01460-XLOC_008183-13632-1
AGCTTCAACTGCTGTCAGTTATGGTAATTTATCCCTATCACGTGAAACGA
CONSENSUS
AGCTTCAACTGCTGTCAGTTATGGTAATTTATCCCTATCACGTGAAACGA

A3-AT2G01460-XLOC_008183-13632-0
TTGATACTCTTGGAGAGACTTTCCTGGTTCTGAGAGGGACAGATAGGAAG
A3-AT2G01460-XLOC_008183-13632-1
TTGATACTCTTGGAGAGACTTTCCTGGTTCTGAGAGGGACAGATAGGAAG
CONSENSUS
TTGATACTCTTGGAGAGACTTTCCTGGTTCTGAGAGGGACAGATAGGAAG

alignment for event: A5-AT2G18440-XLOC_011870-10764

A5-AT2G18440-XLOC_011870-10764-0
ATCTAGATCTGGATCGTCCTTCGCGGCTTCTTCACATGCAAGATTCTGTG
A5-AT2G18440-XLOC_011870-10764-1
ATCTAGATCTGGATCGTCCTTCGCGGCTTCTTCACATGCAAGATTCTGTG
CONSENSUS
ATCTAGATCTGGATCGTCCTTCGCGGCTTCTTCACATGCAAGATTCTGTG

A5-AT2G18440-XLOC_011870-10764-0
AATTGTTCAAGTTGAGGCATCATTTCTGGATTACTAGGAGACGAATCTGT
A5-AT2G18440-XLOC_011870-10764-1
AATTGTTCAAGTTGAGGCATCATTTCTGGATTACTAGGAGACGAATCTGT
CONSENSUS
AATTGTTCAAGTTGAGGCATCATTTCTGGATTACTAGGAGACGAATCTGT

A5-AT2G18440-XLOC_011870-10764-0
TGCAGACGGATGTGTGTGTGTTGGATTGAATTGAGATTAGGGTGTAGAAG
A5-AT2G18440-XLOC_011870-10764-1
TGCAGACGGATGTGTGTGTGTTGGATTGAATTGAGATTAGGGTGTAGAAG
CONSENSUS
TGCAGACGGATGTGTGTGTGTTGGATTGAATTGAGATTAGGGTGTAGAAG

A5-AT2G18440-XLOC_011870-10764-0
ATGTTTTGTGGATAGCTAATAGCTTCCTGATTGCATCTCTGTCATCCGAC
A5-AT2G18440-XLOC_011870-10764-1
ATGTTTTGTGGATAGCTAATAGCTTCCTGATTGCATCTCTGTCATCCGAC
CONSENSUS
ATGTTTTGTGGATAGCTAATAGCTTCCTGATTGCATCTCTGTCATCCGAC

A5-AT2G18440-XLOC_011870-10764-0
CTTTGCCATGTCAGGTGCGCTTGCATGGCAGGTCAAAAAACTGATCCTCA
A5-AT2G18440-XLOC_011870-10764-1
CTTTGCCATGTCAGGTGCGCTTGCATGGCAGGTCAAAAAACTGATCCTCA
CONSENSUS
CTTTGCCATGTCAGGTGCGCTTGCATGGCAGGTCAAAAAACTGATCCTCA

A5-AT2G18440-XLOC_011870-10764-0
    ATAAAAAAAAGATTTTGTGGGTTTTGGAGAGGAGGTCGCACGGGTTATTA
A5-AT2G18440-XLOC_011870-10764-1
    ATAAAAAAAAGATTTTGTGGGTTTTGGAGAGGAGGTCGCACGGGTTATTA
CONSENSUS
    ATAAAAAAAAGATTTTGTGGGTTTTGGAGAGGAGGTCGCACGGGTTATTA

A5-AT2G18440-XLOC_011870-10764-0
    TTTTTTCCCGGGATCTCTTCTCCTCTGTGTGTGTCGTCTTGCCTCTGCTC
A5-AT2G18440-XLOC_011870-10764-1
    TTTTTTCCCGGGATCTCTTCTCCTCTGTGTGTGTCGTCTTGCCTCTGCTC
CONSENSUS
    TTTTTTCCCGGGATCTCTTCTCCTCTGTGTGTGTCGTCTTGCCTCTGCTC

A5-AT2G18440-XLOC_011870-10764-0
    TATCTCTCTCCCTGCTCTTTCACATTTCATATCTTTCTTAAATGCTCATA
A5-AT2G18440-XLOC_011870-10764-1
    TATCTCTCTCCCTGCTCTTTCACATTTCATATCTTTCTTAAATGCTCATA
CONSENSUS
    TATCTCTCTCCCTGCTCTTTCACATTTCATATCTTTCTTAAATGCTCATA

A5-AT2G18440-XLOC_011870-10764-0
    TACACTCAAAAACCGATCATAAGCAGAGTTTGTAACCAATATGGAGCAGT
A5-AT2G18440-XLOC_011870-10764-1
    TACACTCAAAAACCGATCATAAGCAGAGTTTGTAACCAATATGGAGCAGT
CONSENSUS
    TACACTCAAAAACCGATCATAAGCAGAGTTTGTAACCAATATGGAGCAGT

A5-AT2G18440-XLOC_011870-10764-0
    GGGCGATTACAAATCTTCTTGCCCAACAACCTCGAGAGTTAAATCAGGTA
A5-AT2G18440-XLOC_011870-10764-1
    GGGCGATTACAAATCTTCTTGCCCAACAACCTCGAGAGTTAAATCAG---
CONSENSUS
    GGGCGATTACAAATCTTCTTGCCCAACAACCTCGAGAGTTAAATCAG...

A5-AT2G18440-XLOC_011870-10764-0
    CTCATATCCATATTAAATCGAATTCTTAATTAGCATAATAAGGTAAACAT
A5-AT2G18440-XLOC_011870-10764-1

CONSENSUS
    ....

A5-AT2G18440-XLOC_011870-10764-0
    AATCTGCAAGAGGAATTCTGGATTTAAATAAACCATAATCCGGTTGCCCT
A5-AT2G18440-XLOC_011870-10764-1

CONSENSUS
    ....

A5-AT2G18440-XLOC_011870-10764-0
    AATTGTTAACTTGTATCAACGCTTATGCGAATCATGAAATTTTAATTTTA
A5-AT2G18440-XLOC_011870-10764-1

CONSENSUS
    ....

A5-AT2G18440-XLOC_011870-10764-0
TTGGTTTTGTTATTTATCTTATTGATCAATTATTGTTTTACAAGTTGTCA
A5-AT2G18440-XLOC_011870-10764-1

CONSENSUS
....

A5-AT2G18440-XLOC_011870-10764-0
TTTAATCTATTGAAAATGATGGTATTAAGAATTGCTGTTAAACAATTTTG
A5-AT2G18440-XLOC_011870-10764-1

CONSENSUS
....

A5-AT2G18440-XLOC_011870-10764-0
CGGGTTCTTTGTATGTATCATTGGTATTGTTTTATTAGTTCTATCCGTTT
A5-AT2G18440-XLOC_011870-10764-1

CONSENSUS
....

A5-AT2G18440-XLOC_011870-10764-0
AGAAGCTTTTTAGAATATGGTAAAGAACATTTTTATTTGCTCGTAGTTTG
A5-AT2G18440-XLOC_011870-10764-1

CONSENSUS
....

A5-AT2G18440-XLOC_011870-10764-0
TATATGTCTGTAACCACCAGTTCCTGTAGTAACTATGAAGCTGGTAGACG
A5-AT2G18440-XLOC_011870-10764-1

CONSENSUS
....

A5-AT2G18440-XLOC_011870-10764-0
ACGGAATTGAAGCTATGCAAAGCTGACGTTGGTTTTTTCTGAAGTTGATT
A5-AT2G18440-XLOC_011870-10764-1

CONSENSUS
....

A5-AT2G18440-XLOC_011870-10764-0
TGTTCTTGAAAGGATTGTCCCACACTGAACTCTGGAAGATGTACTATTTG
A5-AT2G18440-XLOC_011870-10764-1 ------------
GATTGTCCCACACTGAACTCTGGAAGATGTACTATTTG
CONSENSUS
............GATTGTCCCACACTGAACTCTGGAAGATGTACTATTTG

A5-AT2G18440-XLOC_011870-10764-0
CTTAACGTGACTAGTGTTGTGTTGCTTTTGAATTGCGGATTGTGCAAGCG
A5-AT2G18440-XLOC_011870-10764-1
CTTAACGTGACTAGTGTTGTGTTGCTTTTGAATTGCGGATTGTGCAAGCG
CONSENSUS
CTTAACGTGACTAGTGTTGTGTTGCTTTTGAATTGCGGATTGTGCAAGCG

A5-AT2G18440-XLOC_011870-10764-0
ACGGGGAATTTAGGAAAGAGAAGATGGGAGTTTATGATACATGGGGTTTG
A5-AT2G18440-XLOC_011870-10764-1
ACGGGGAATTTAGGAAAGAGAAGATGGGAGTTTATGATACATGGGGTTTG
CONSENSUS
ACGGGGAATTTAGGAAAGAGAAGATGGGAGTTTATGATACATGGGGTTTG

A5-AT2G18440-XLOC_011870-10764-0
ATTGTTTCGACGGTATCTTGCTTTTTTGTCCGGTTTGATCTTGAATATGT
A5-AT2G18440-XLOC_011870-10764-1
ATTGTTTCGACGGTATCTTGCTTTTTTGTCCGGTTTGATCTTGAATATGT
CONSENSUS
ATTGTTTCGACGGTATCTTGCTTTTTTGTCCGGTTTGATCTTGAATATGT

A5-AT2G18440-XLOC_011870-10764-0
TACATTACACTGCAGTATACTGTTGAAGATATGATTGATGCTTCTTTTGT
A5-AT2G18440-XLOC_011870-10764-1
TACATTACACTGCAGTATACTGTTGAAGATATGATTGATGCTTCTTTTGT
CONSENSUS
TACATTACACTGCAGTATACTGTTGAAGATATGATTGATGCTTCTTTTGT

A5-AT2G18440-XLOC_011870-10764-0
TGAAGCTCATGTTATATAGAATATAGAATAATAGTTGAACGATT
A5-AT2G18440-XLOC_011870-10764-1
TGAAGCTCATGTTATATAGAATATAGAATAATAGTTGAACGATT
CONSENSUS
TGAAGCTCATGTTATATAGAATATAGAATAATAGTTGAACGATT

alignment for event: A5-AT2G33620-XLOC_010054-10765

A5-AT2G33620-XLOC_010054-10765-0
AACCTTAATGCGCGATCCGCTTTTCTAAAGTTTTGTGAGAGAGAAGAGAT
A5-AT2G33620-XLOC_010054-10765-1
AACCTTAATGCGCGATCCGCTTTTCTAAAGTTTTGTGAGAGAGAAGAGAT
CONSENSUS
AACCTTAATGCGCGATCCGCTTTTCTAAAGTTTTGTGAGAGAGAAGAGAT

A5-AT2G33620-XLOC_010054-10765-0
CTAAAAAAATCCACAATTTTGTTCAAATCTTGGAGTTAAATGCTGAATTT
A5-AT2G33620-XLOC_010054-10765-1
CTAAAAAAATCCACAATTTTGTTCAAATCTTGGAGTTAAATGCTGAATTT
CONSENSUS
CTAAAAAAATCCACAATTTTGTTCAAATCTTGGAGTTAAATGCTGAATTT

A5-AT2G33620-XLOC_010054-10765-0
TAGGCCTTGTTGCTTAGATTTATGGCTTAAAGTTTCAAACTTTTCATTGG
A5-AT2G33620-XLOC_010054-10765-1
TAGGCCTTGTTGCTTAGATTTATGGCTTAAAGTTTCAAACTTTTCATTGG
CONSENSUS
TAGGCCTTGTTGCTTAGATTTATGGCTTAAAGTTTCAAACTTTTCATTGG

A5-AT2G33620-XLOC_010054-10765-0
ATATG---
A5-AT2G33620-XLOC_010054-10765-1

ATATGGTACAAATCTTGTAGTTAAACACTGAATTGTGTCCCTCGTGGGCG
CONSENSUS
ATATG...

A5-AT2G33620-XLOC_010054-10765-0
---TGAGAAG
A5-AT2G33620-XLOC_010054-10765-1
TTGTTGCTTTGATTTATGGCCAAAAGTTTCAAACTTGGATATGTGAGAAG
CONSENSUS
....TGAGAAG

A5-AT2G33620-XLOC_010054-10765-0
AAAATGTCAGGATCTGAGACGGGTTTAATGGCGGCGACCAGAGAATCAAT
A5-AT2G33620-XLOC_010054-10765-1
AAAATGTCAGGATCTGAGACGGGTTTAATGGCGGCGACCAGAGAATCAAT
CONSENSUS
AAAATGTCAGGATCTGAGACGGGTTTAATGGCGGCGACCAGAGAATCAAT

A5-AT2G33620-XLOC_010054-10765-0
GCAATTTACAATGGCTCTCCACCAGCAGCAGCAACACAGTCAAGCTCAAC
A5-AT2G33620-XLOC_010054-10765-1
GCAATTTACAATGGCTCTCCACCAGCAGCAGCAACACAGTCAAGCTCAAC
CONSENSUS
GCAATTTACAATGGCTCTCCACCAGCAGCAGCAACACAGTCAAGCTCAAC

A5-AT2G33620-XLOC_010054-10765-0
CTCAGCAGTCTCAGAACAGGCCATTGTCATTCGGTGGAGACGACGGAACT
A5-AT2G33620-XLOC_010054-10765-1
CTCAGCAGTCTCAGAACAGGCCATTGTCATTCGGTGGAGACGACGGAACT
CONSENSUS
CTCAGCAGTCTCAGAACAGGCCATTGTCATTCGGTGGAGACGACGGAACT

A5-AT2G33620-XLOC_010054-10765-0
GCTCTTTACAAGCAGCCGATGAGATCAGTATCACCACCGCAGCAGTACCA
A5-AT2G33620-XLOC_010054-10765-1
GCTCTTTACAAGCAGCCGATGAGATCAGTATCACCACCGCAGCAGTACCA
CONSENSUS
GCTCTTTACAAGCAGCCGATGAGATCAGTATCACCACCGCAGCAGTACCA

A5-AT2G33620-XLOC_010054-10765-0
ACCCAACTCAGCTGGTGAGAATTCTGTCTTGAACATGAACTTGCCCGGAG
A5-AT2G33620-XLOC_010054-10765-1
ACCCAACTCAGCTGGTGAGAATTCTGTCTTGAACATGAACTTGCCCGGAG
CONSENSUS
ACCCAACTCAGCTGGTGAGAATTCTGTCTTGAACATGAACTTGCCCGGAG

A5-AT2G33620-XLOC_010054-10765-0
GTGAGTCTGGAGGCATGACTGGAACTGGAAGTGAGCCAGTGAAAAAGAGG
A5-AT2G33620-XLOC_010054-10765-1
GTGAGTCTGGAGGCATGACTGGAACTGGAAGTGAGCCAGTGAAAAAGAGG
CONSENSUS
GTGAGTCTGGAGGCATGACTGGAACTGGAAGTGAGCCAGTGAAAAAGAGG

A5-AT2G33620-XLOC_010054-10765-0
AGAGGTAGACCGAGGAAATATGGGCCTGATAGTGGTGAAATGTCACTTGG
A5-AT2G33620-XLOC_010054-10765-1

AGAGGTAGACCGAGGAAATATGGGCCTGATAGTGGTGAAATGTCACTTGG
CONSENSUS
AGAGGTAGACCGAGGAAATATGGGCCTGATAGTGGTGAAATGTCACTTGG

A5-AT2G33620-XLOC_010054-10765-0
TTTGAATCCTGGAGCTCCTTCTTTCACTGTCAGCCAACCTAGTAGCGGCG
A5-AT2G33620-XLOC_010054-10765-1
TTTGAATCCTGGAGCTCCTTCTTTCACTGTCAGCCAACCTAGTAGCGGCG
CONSENSUS
TTTGAATCCTGGAGCTCCTTCTTTCACTGTCAGCCAACCTAGTAGCGGCG

A5-AT2G33620-XLOC_010054-10765-0
GCGATGGAGGAGAGAAGAAGAGAGGAAGACCTCCTGGTTCTTCTAGCAAA
A5-AT2G33620-XLOC_010054-10765-1
GCGATGGAGGAGAGAAGAAGAGAGGAAGACCTCCTGGTTCTTCTAGCAAA
CONSENSUS
GCGATGGAGGAGAGAAGAAGAGAGGAAGACCTCCTGGTTCTTCTAGCAAA

A5-AT2G33620-XLOC_010054-10765-0 AGGCTCAAGCTTCAAGCTTTAG
A5-AT2G33620-XLOC_010054-10765-1 AGGCTCAAGCTTCAAGCTTTAG
CONSENSUS AGGCTCAAGCTTCAAGCTTTAG

alignment for event: RI-AT2G30460-XLOC_012563-11417

RI-AT2G30460-XLOC_012563-11417-0
ATCATCGTAGATCGTGATAGAAGAAGCGAGCAAATATGAGCGATGCCCAG
RI-AT2G30460-XLOC_012563-11417-1
ATCATCGTAGATCGTGATAGAAGAAGCGAGCAAATATGAGCGATGCCCAG
CONSENSUS
ATCATCGTAGATCGTGATAGAAGAAGCGAGCAAATATGAGCGATGCCCAG

RI-AT2G30460-XLOC_012563-11417-0
AAGTTCCAGCTTGGAACAATCGGCGCTTTGAGTTTATCCGTTGTGTCCTC
RI-AT2G30460-XLOC_012563-11417-1
AAGTTCCAGCTTGGAACAATCGGCGCTTTGAGTTTATCCGTTGTGTCCTC
CONSENSUS
AAGTTCCAGCTTGGAACAATCGGCGCTTTGAGTTTATCCGTTGTGTCCTC

RI-AT2G30460-XLOC_012563-11417-0
TGTTTCGATCGTGATCTGTAACAAGGCCCTTATTAGCACCCTTGGTTTCA
RI-AT2G30460-XLOC_012563-11417-1
TGTTTCGATCGTGATCTGTAACAAGGCCCTTATTAGCACCCTTGGTTTCA
CONSENSUS
TGTTTCGATCGTGATCTGTAACAAGGCCCTTATTAGCACCCTTGGTTTCA

RI-AT2G30460-XLOC_012563-11417-0
CATTTGGTAAGATTATGTGTCTACTAATTCTGGTTTGATTCCACAGGAAA
RI-AT2G30460-XLOC_012563-11417-1
CATTTG--
CONSENSUS
CATTTG..

RI-AT2G30460-XLOC_012563-11417-0
CTATTCGAGGTATCTAGTGGCTTTGAATCATACTTTTTGTTTGTGTGTCC

RI-AT2G30460-XLOC_012563-11417-1

CONSENSUS
      ....

RI-AT2G30460-XLOC_012563-11417-0
      CAGAAATAAGGAAACTTTTTGCTTCTTTATTGATTGAATCTCTACTTTAG
RI-AT2G30460-XLOC_012563-11417-1

CONSENSUS
      ....

RI-AT2G30460-XLOC_012563-11417-0
      TTCTTCTTTACCATGGGTGAACTCCATTCATGATAACAGTTTATGCATGG
RI-AT2G30460-XLOC_012563-11417-1

CONSENSUS
      ....

RI-AT2G30460-XLOC_012563-11417-0
      AATCTCATAGTCTATCTGTGTGTAATCATTTATTACTTTATATTGAACTT
RI-AT2G30460-XLOC_012563-11417-1

CONSENSUS
      ....

RI-AT2G30460-XLOC_012563-11417-0
      ACAGCAACTACTTTGACAAGCTGGCATCTTTTGGTGACATTTTGTTCACT
RI-AT2G30460-XLOC_012563-11417-1 ----
CAACTACTTTGACAAGCTGGCATCTTTTGGTGACATTTTGTTCACT
CONSENSUS
      ....CAACTACTTTGACAAGCTGGCATCTTTTGGTGACATTTTGTTCACT

RI-AT2G30460-XLOC_012563-11417-0
      TCATGTGGCATTATGGATGAAGTTTTTTGAGCACAAGCCTTTTGATCCAC
RI-AT2G30460-XLOC_012563-11417-1
      TCATGTGGCATTATGGATGAAGTTTTTTGAGCACAAGCCTTTTGATCCAC
CONSENSUS
      TCATGTGGCATTATGGATGAAGTTTTTTGAGCACAAGCCTTTTGATCCAC

RI-AT2G30460-XLOC_012563-11417-0
      GAGCTGTCCTGGGATTTGGTGTATTAAATGGTATATCCATTGGATTATTA
RI-AT2G30460-XLOC_012563-11417-1
      GAGCTGTCCTGGGATTTGGTGTATTAAATGGTATATCCATTGGATTATTA
CONSENSUS
      GAGCTGTCCTGGGATTTGGTGTATTAAATGGTATATCCATTGGATTATTA

RI-AT2G30460-XLOC_012563-11417-0
      AATCTCAGCTTGGGTTTTAATTCTGTTGGTTTTTACCAG
RI-AT2G30460-XLOC_012563-11417-1
      AATCTCAGCTTGGGTTTTAATTCTGTTGGTTTTTACCAG
CONSENSUS
      AATCTCAGCTTGGGTTTTAATTCTGTTGGTTTTTACCAG

alignment for event: RI-AT2G32850-XLOC_012697-7224

RI-AT2G32850-XLOC_012697-7224-0
AGAGATAAGATGGAAGGAACAATGTGGGAACTTCAACAGGACAGATCTAA
RI-AT2G32850-XLOC_012697-7224-1
AGAGATAAGATGGAAGGAACAATGTGGGAACTTCAACAGGACAGATCTAA
CONSENSUS
AGAGATAAGATGGAAGGAACAATGTGGGAACTTCAACAGGACAGATCTAA

RI-AT2G32850-XLOC_012697-7224-0
CTGGTCAACTGGCAGCTCTGATACAAATTCATGGCAACCTTTTAGTGATG
RI-AT2G32850-XLOC_012697-7224-1
CTGGTCAACTGGCAGCTCTGATACAAATTCATGGCAACCTTTTAGTGATG
CONSENSUS
CTGGTCAACTGGCAGCTCTGATACAAATTCATGGCAACCTTTTAGTGATG

RI-AT2G32850-XLOC_012697-7224-0
AAGCGAAACCCGTGATGGAATCTGCATCAAAGGGTAACAATAATACGATT
RI-AT2G32850-XLOC_012697-7224-1
AAGCGAAACCCGTGATGGAATCTGCATCAAAGG-----------------
CONSENSUS
AAGCGAAACCCGTGATGGAATCTGCATCAAAGG.................

RI-AT2G32850-XLOC_012697-7224-0
AATCAGTCTGTGAGAACCAGAAGTAAACCAGCTTCTGCTGCAGGTACTCA
RI-AT2G32850-XLOC_012697-7224-1
---GTACTCA
CONSENSUS
....GTACTCA

RI-AT2G32850-XLOC_012697-7224-0
AGGTTTTGAGCCATGGGGTTTCGAGACAGAATCCTTTAGAGCCGCTGCAA
RI-AT2G32850-XLOC_012697-7224-1
AGGTTTTGAGCCATGGGGTTTCGAGACAGAATCCTTTAGAGCCGCTGCAA
CONSENSUS
AGGTTTTGAGCCATGGGGTTTCGAGACAGAATCCTTTAGAGCCGCTGCAA

RI-AT2G32850-XLOC_012697-7224-0
CGTCTGCAGCTGCTACTTCTGCATCTGGAACACAAAGATCTATGGGCTCT
RI-AT2G32850-XLOC_012697-7224-1
CGTCTGCAGCTGCTACTTCTGCATCTGGAACACAAAGATCTATGGGCTCT
CONSENSUS
CGTCTGCAGCTGCTACTTCTGCATCTGGAACACAAAGATCTATGGGCTCT

RI-AT2G32850-XLOC_012697-7224-0
GGAAACAGCACTTCACAGAGATATGGGAACTCAAAGATGAGAGAAAACCA
RI-AT2G32850-XLOC_012697-7224-1
GGAAACAGCACTTCACAGAGATATGGGAACTCAAAGATGAGAGAAAACCA
CONSENSUS
GGAAACAGCACTTCACAGAGATATGGGAACTCAAAGATGAGAGAAAACCA

RI-AT2G32850-XLOC_012697-7224-0
GAAAACAGCTCAACCTGCTGGATGGGCCGGCTTCTAAAACTCACTCAGAT
RI-AT2G32850-XLOC_012697-7224-1
GAAAACAGCTCAACCTGCTGGATGGGCCGGCTTCTAAAACTCACTCAGAT
CONSENSUS

GAAAACAGCTCAACCTGCTGGATGGGCCGGCTTCTAAAACTCACTCAGAT

RI-AT2G32850-XLOC_012697-7224-0
GCGCATTGCTATTTCTTCCTCATGCAACAACAACAGCTTCATGTTGCTGC
RI-AT2G32850-XLOC_012697-7224-1
GCGCATTGCTATTTCTTCCTCATGCAACAACAACAGCTTCATGTTGCTGC
CONSENSUS
GCGCATTGCTATTTCTTCCTCATGCAACAACAACAGCTTCATGTTGCTGC

RI-AT2G32850-XLOC_012697-7224-0
CATTACAGAATCTTGAATCTTTTTGTAGATGATGATCATCCCCTGTTTTT
RI-AT2G32850-XLOC_012697-7224-1
CATTACAGAATCTTGAATCTTTTTGTAGATGATGATCATCCCCTGTTTTT
CONSENSUS
CATTACAGAATCTTGAATCTTTTTGTAGATGATGATCATCCCCTGTTTTT

RI-AT2G32850-XLOC_012697-7224-0
GTGCTGCTGCTAAATATACCCCACCCATATCCATTATGTTTTATGTAGAG
RI-AT2G32850-XLOC_012697-7224-1
GTGCTGCTGCTAAATATACCCCACCCATATCCATTATGTTTTATGTAGAG
CONSENSUS
GTGCTGCTGCTAAATATACCCCACCCATATCCATTATGTTTTATGTAGAG

RI-AT2G32850-XLOC_012697-7224-0
TCTTAAACTTTACACATTCTCTGAGCTGTGGCCAGATATCTCAGCTTATG
RI-AT2G32850-XLOC_012697-7224-1
TCTTAAACTTTACACATTCTCTGAGCTGTGGCCAGATATCTCAGCTTATG
CONSENSUS
TCTTAAACTTTACACATTCTCTGAGCTGTGGCCAGATATCTCAGCTTATG

RI-AT2G32850-XLOC_012697-7224-0
GATTATTATTGGAAAAATAAATAATTCAAGATTCAGACAGCTTACACTT
RI-AT2G32850-XLOC_012697-7224-1
GATTATTATTGGAAAAATAAATAATTCAAGATTCAGACAGCTTACACTT
CONSENSUS
GATTATTATTGGAAAAATAAATAATTCAAGATTCAGACAGCTTACACTT

alignment for event: SE-AT2G13650-XLOC_011587-1248

SE-AT2G13650-XLOC_011587-1248-0
TTTGAAATACATCAATGTAGCAATGGTCACTGTCCTGAAGAATGTCACTA
SE-AT2G13650-XLOC_011587-1248-1
TTTGAAATACATCAATGTAGCAATGGTCACTGTCCTGAAGAATGTCACTA
CONSENSUS
TTTGAAATACATCAATGTAGCAATGGTCACTGTCCTGAAGAATGTCACTA

SE-AT2G13650-XLOC_011587-1248-0
ATGTGATAACTGCAGTTGGTGAGATGTATCTGTTCAACAAGCAACATGAC
SE-AT2G13650-XLOC_011587-1248-1
ATGTGATAACTGCAGTTGGTGAGATGTATCTGTTCAACAAGCAACATGAC
CONSENSUS
ATGTGATAACTGCAGTTGGTGAGATGTATCTGTTCAACAAGCAACATGAC

SE-AT2G13650-XLOC_011587-1248-0

AACAGAGTGTGGGCTGCTCTCTTCTTAATG-------------------
SE-AT2G13650-XLOC_011587-1248-1
AACAGAGTGTGGGCTGCTCTCTTCTTAATGTTACACTTGTCGTAGCGGCT
CONSENSUS
AACAGAGTGTGGGCTGCTCTCTTCTTAATG....................

SE-AT2G13650-XLOC_011587-1248-0

SE-AT2G13650-XLOC_011587-1248-1
TTCAGTGGTGTGGCATAGCCTAACCTTTCAATACTGTTGGAGATACATGT
CONSENSUS
....

SE-AT2G13650-XLOC_011587-1248-0
------------------------------------ATAATTTCCGCAGT
SE-AT2G13650-XLOC_011587-1248-1
TTTTAACTGCTTCCACAATTAGTCTGCCAGGGAAATATAATTTCCGCAGT
CONSENSUS
....................................ATAATTTCCGCAGT

SE-AT2G13650-XLOC_011587-1248-0
TTCTGGAGGAATAACAGACCTATCATTCAATGCTGTTGGCTATGCTTGGC
SE-AT2G13650-XLOC_011587-1248-1
TTCTGGAGGAATAACAGACCTATCATTCAATGCTGTTGGCTATGCTTGGC
CONSENSUS
TTCTGGAGGAATAACAGACCTATCATTCAATGCTGTTGGCTATGCTTGGC

SE-AT2G13650-XLOC_011587-1248-0
AGATTGCTAATTGCTTCTTAACTGCATCGTACTCG
SE-AT2G13650-XLOC_011587-1248-1
AGATTGCTAATTGCTTCTTAACTGCATCGTACTCG
CONSENSUS
AGATTGCTAATTGCTTCTTAACTGCATCGTACTCG

alignment for event: RI-AT2G02570-XLOC_010981-1728

RI-AT2G02570-XLOC_010981-1728-0
GTAGGGTTCTTCACAGGGAGGAAGAAAGAGAGTATATTTAAATCACCTGA
RI-AT2G02570-XLOC_010981-1728-1
GTAGGGTTCTTCACAGGGAGGAAGAAAGAGAGTATATTTAAATCACCTGA
CONSENSUS
GTAGGGTTCTTCACAGGGAGGAAGAAAGAGAGTATATTTAAATCACCTGA

RI-AT2G02570-XLOC_010981-1728-0
GGATCCATTTGGAAAAGTGGGTGTGACTGGAAGTGGGAAAGGTTTGACAG
RI-AT2G02570-XLOC_010981-1728-1
GGATCCATTTGGAAAAGTGGGTGTGACTGGAAGTGGGAAAGGTTTGACAG
CONSENSUS
GGATCCATTTGGAAAAGTGGGTGTGACTGGAAGTGGGAAAGGTTTGACAG

RI-AT2G02570-XLOC_010981-1728-0
ATTTCCAAAAGCGAGAGAAGCATCTTCATCTCAAGTCTGGTAATGCTGAG
RI-AT2G02570-XLOC_010981-1728-1
ATTTCCAAAAGCGAGAGAAGCATCTTCATCTCAAGTCTGGTAATGCTGAG

CONSENSUS
      ATTTCCAAAAGCGAGAGAAGCATCTTCATCTCAAGTCTGGTAATGCTGAG

RI-AT2G02570-XLOC_010981-1728-0
      GGCACTGATGAATGAACGAGAGATTTTTATGCTTTTGTAAGGATTTTCAG
RI-AT2G02570-XLOC_010981-1728-1
      GGCACTGATGAATGAACGAGAGATTTTTATGCTTTTGTAAGGATTTTCAG
CONSENSUS
      GGCACTGATGAATGAACGAGAGATTTTTATGCTTTTGTAAGGATTTTCAG

RI-AT2G02570-XLOC_010981-1728-0
      GTACTTAAAAAGTCTTTCTTCTGTCTCCAGTAACCTCTAAAAACAAAAGG
RI-AT2G02570-XLOC_010981-1728-1

CONSENSUS
      ....

RI-AT2G02570-XLOC_010981-1728-0
      ACCTAAACCCGCAATTTTTTTGTGGACTTCTGCTCTTTTGTAAGATGAAT
RI-AT2G02570-XLOC_010981-1728-1

CONSENSUS
      ....

RI-AT2G02570-XLOC_010981-1728-0
      TAAAGATTAGTTTTATCTTTCTATTTCTTTGTAGTGACTATGATGAATAG
RI-AT2G02570-XLOC_010981-1728-1
      ----------------------------------TGACTATGATGAATAG
CONSENSUS
      ..................................TGACTATGATGAATAG

RI-AT2G02570-XLOC_010981-1728-0
      AGCCACAGCCAAAACGGCATTACCGTTAACATTGTTGTTGTTTCTTCTTC
RI-AT2G02570-XLOC_010981-1728-1
      AGCCACAGCCAAAACGGCATTACCGTTAACATTGTTGTTGTTTCTTCTTC
CONSENSUS
      AGCCACAGCCAAAACGGCATTACCGTTAACATTGTTGTTGTTTCTTCTTC

RI-AT2G02570-XLOC_010981-1728-0
      GGATGGTAAGTTGTCTATTGATGAATCACTCTCACTGTCTCATATTGAAC
RI-AT2G02570-XLOC_010981-1728-1
      GGATGGTAAGTTGTCTATTGATGAATCACTCTCACTGTCTCATATTGAAC
CONSENSUS
      GGATGGTAAGTTGTCTATTGATGAATCACTCTCACTGTCTCATATTGAAC

RI-AT2G02570-XLOC_010981-1728-0
      TGTGCCGCATCATACGGTTTATGTTTAAAATACTAATTCAGAAAAACTTT
RI-AT2G02570-XLOC_010981-1728-1
      TGTGCCGCATCATACGGTTTATGTTTAAAATACTAATTCAGAAAAACTTT
CONSENSUS
      TGTGCCGCATCATACGGTTTATGTTTAAAATACTAATTCAGAAAAACTTT

RI-AT2G02570-XLOC_010981-1728-0
      AGACCCACCATTTTAATGTATTTGTATCAAGTTTCATAATGAAAAATGGT
RI-AT2G02570-XLOC_010981-1728-1
      AGACCCACCATTTTAATGTATTTGTATCAAGTTTCATAATGAAAAATGGT

CONSENSUS
AGACCCACCATTTTAATGTATTTGTATCAAGTTTCATAATGAAAAATGGT

RI-AT2G02570-XLOC_010981-1728-0
GTTAACTGTTAAGTATATATTTTCCATGGCGTAGTTTTTGTTCCTTTTGA
RI-AT2G02570-XLOC_010981-1728-1
GTTAACTGTTAAGTATATATTTTCCATGGCGTAGTTTTTGTTCCTTTTGA
CONSENSUS
GTTAACTGTTAAGTATATATTTTCCATGGCGTAGTTTTTGTTCCTTTTGA

RI-AT2G02570-XLOC_010981-1728-0
TTAAAATTGAGTATTTTTCTATGTTGTATGGGG
RI-AT2G02570-XLOC_010981-1728-1
TTAAAATTGAGTATTTTTCTATGTTGTATGGGG
CONSENSUS
TTAAAATTGAGTATTTTTCTATGTTGTATGGGG

alignment for event: A3-AT2G46450-XLOC_010784-3543

A3-AT2G46450-XLOC_010784-3543-0
ATTTTAATCATTATTTTAGTAGACCAAAACTGTTTAGTGTTGAAACTTGT
A3-AT2G46450-XLOC_010784-3543-1
ATTTTAATCATTATTTTAGTAGACCAAAACTGTTTAGTGTTGAAACTTGT
CONSENSUS
ATTTTAATCATTATTTTAGTAGACCAAAACTGTTTAGTGTTGAAACTTGT

A3-AT2G46450-XLOC_010784-3543-0
GCATCCACATGAGTGTTGGTTAAAGGTTGAGAACATCGATTTGCCAACCA
A3-AT2G46450-XLOC_010784-3543-1
GCATCCACATGAGTGTTGGTTAAAGGTTGAGAACATCGATTTGCCAACCA
CONSENSUS
GCATCCACATGAGTGTTGGTTAAAGGTTGAGAACATCGATTTGCCAACCA

A3-AT2G46450-XLOC_010784-3543-0
TATCTCACAGCCACAGCCATTTTTATTCTCTCTCGGTCTCTCTTCTCAGT
A3-AT2G46450-XLOC_010784-3543-1
TATCTCACAGCCACAGCCATTTTTATTCTCTCTCGGTCTCTCTTCTCAGT
CONSENSUS
TATCTCACAGCCACAGCCATTTTTATTCTCTCTCGGTCTCTCTTCTCAGT

A3-AT2G46450-XLOC_010784-3543-0
CTCTCACTACCACAGCCTTAAGTTCGAGAAGACCGAAGTGGACTTCATTT
A3-AT2G46450-XLOC_010784-3543-1
CTCTCACTACCACAGCCTTAAGTTCGAGAAGACCGAAGTGGACTTCATTT
CONSENSUS
CTCTCACTACCACAGCCTTAAGTTCGAGAAGACCGAAGTGGACTTCATTT

A3-AT2G46450-XLOC_010784-3543-0
TCTTGTACGGGAATTAAAATATTGCAGATTGACGACATATACTAACATGA
A3-AT2G46450-XLOC_010784-3543-1
TCTTGTACGGGAATTAAAATATTGCAGATTGACGACATATACTAACATGA
CONSENSUS
TCTTGTACGGGAATTAAAATATTGCAGATTGACGACATATACTAACATGA

A3-AT2G46450-XLOC_010784-3543-0
ATCATCGGAGGAGTAAATTTGCCAGGATAGATAGTATGGGAGTTGATGGA
A3-AT2G46450-XLOC_010784-3543-1 ATCATCGGAGGAGTAAATTTGCCAG-----
ATAGTATGGGAGTTGATGGA
CONSENSUS
ATCATCGGAGGAGTAAATTTGCCAG.....ATAGTATGGGAGTTGATGGA

A3-AT2G46450-XLOC_010784-3543-0
AAATTGAAAAGTGTTAGAGGACGCTTGAAGAAGGTTTACGGGAAGATGAA
A3-AT2G46450-XLOC_010784-3543-1
AAATTGAAAAGTGTTAGAGGACGCTTGAAGAAGGTTTACGGGAAGATGAA
CONSENSUS
AAATTGAAAAGTGTTAGAGGACGCTTGAAGAAGGTTTACGGGAAGATGAA

A3-AT2G46450-XLOC_010784-3543-0
AACACTCGAAAACTGGAGGAAGACCGTCTTGTTAGCTTGCGTGGTTGCTT
A3-AT2G46450-XLOC_010784-3543-1
AACACTCGAAAACTGGAGGAAGACCGTCTTGTTAGCTTGCGTGGTTGCTT
CONSENSUS
AACACTCGAAAACTGGAGGAAGACCGTCTTGTTAGCTTGCGTGGTTGCTT

A3-AT2G46450-XLOC_010784-3543-0
TGGCTATTGATCCTTTGTTTCTGTTTATCCCTTTGATTGATTCTCAGAGA
A3-AT2G46450-XLOC_010784-3543-1
TGGCTATTGATCCTTTGTTTCTGTTTATCCCTTTGATTGATTCTCAGAGA
CONSENSUS
TGGCTATTGATCCTTTGTTTCTGTTTATCCCTTTGATTGATTCTCAGAGA

A3-AT2G46450-XLOC_010784-3543-0
TTTTGCTTCACTTTCGACAAGACACTTGTAGCAGTA
A3-AT2G46450-XLOC_010784-3543-1
TTTTGCTTCACTTTCGACAAGACACTTGTAGCAGTA
CONSENSUS
TTTTGCTTCACTTTCGACAAGACACTTGTAGCAGTA

alignment for event: A3-AT2G16900-XLOC_011787-2642

A3-AT2G16900-XLOC_011787-2642-0
TTCTTGTTTCTGATTGAATCTAGATATTGATTAAGCTATGGATGTCAATC
A3-AT2G16900-XLOC_011787-2642-1
TTCTTGTTTCTGATTGAATCTAGATATTGATTAAGCTATGGATGTCAATC
CONSENSUS
TTCTTGTTTCTGATTGAATCTAGATATTGATTAAGCTATGGATGTCAATC

A3-AT2G16900-XLOC_011787-2642-0
GGAAAGCTCACCCGGATTGTCGATATTCTTCAAATCCTTTCCATGAATGT
A3-AT2G16900-XLOC_011787-2642-1
GGAAAGCTCACCCGGATTGTCGATATTCTTCAAATCCTTTCCATGAATGT
CONSENSUS
GGAAAGCTCACCCGGATTGTCGATATTCTTCAAATCCTTTCCATGAATGT

A3-AT2G16900-XLOC_011787-2642-0
GCTTCAGATTGTTTAGAGAAGATCTCTCAAGGCCGTGGAAATAAGAATTC
A3-AT2G16900-XLOC_011787-2642-1

GCTTCAGATTGTTTAGAGAAGATCTCTCAAGGCCGTGGAAATAAGAATTC
CONSENSUS
GCTTCAGATTGTTTAGAGAAGATCTCTCAAGGCCGTGGAAATAAGAATTC

A3-AT2G16900-XLOC_011787-2642-0
AAAGAAGCAAG---------------------------------------
A3-AT2G16900-XLOC_011787-2642-1
AAAGAAGCAAGGTATTGCTTCAAGGATAATTCTTGGAGGGTTGTCTGTTA
CONSENSUS
AAAGAAGCAAG.......................................

A3-AT2G16900-XLOC_011787-2642-0

A3-AT2G16900-XLOC_011787-2642-1
TGACAGTTATAGTTTGATCAAGGCTTTTTAGCTTTCGATGGATCTGCAAA
CONSENSUS
....

A3-AT2G16900-XLOC_011787-2642-0
---GTTCAAAGA
A3-AT2G16900-XLOC_011787-2642-1
GTTGTTTCCTTTCTTTTCTCATTGTGATTTGGTTGTTACAGGTTCAAAGA
CONSENSUS
....GTTCAAAGA

A3-AT2G16900-XLOC_011787-2642-0
TTCTTAGCCTTCCGGGGAGTTTTGGTAAGAAAAAGACGGAGTCACAGCCA
A3-AT2G16900-XLOC_011787-2642-1
TTCTTAGCCTTCCGGGGAGTTTTGGTAAGAAAAAGACGGAGTCACAGCCA
CONSENSUS
TTCTTAGCCTTCCGGGGAGTTTTGGTAAGAAAAAGACGGAGTCACAGCCA

A3-AT2G16900-XLOC_011787-2642-0
CCGTCACCCCTGAGTACAAGGAACTATCAGAATGGTGCTGCTAATACCCC
A3-AT2G16900-XLOC_011787-2642-1
CCGTCACCCCTGAGTACAAGGAACTATCAGAATGGTGCTGCTAATACCCC
CONSENSUS
CCGTCACCCCTGAGTACAAGGAACTATCAGAATGGTGCTGCTAATACCCC

A3-AT2G16900-XLOC_011787-2642-0
AAAGGTTCGTCAATCAAGACCTTCACCAGTAGCTATGAAGAAGACGCCAG
A3-AT2G16900-XLOC_011787-2642-1
AAAGGTTCGTCAATCAAGACCTTCACCAGTAGCTATGAAGAAGACGCCAG
CONSENSUS
AAAGGTTCGTCAATCAAGACCTTCACCAGTAGCTATGAAGAAGACGCCAG

A3-AT2G16900-XLOC_011787-2642-0
TTCCAGAAGCGAACAAATCTTTACACTCTTTATCGTCTGATGGAATCTCT
A3-AT2G16900-XLOC_011787-2642-1
TTCCAGAAGCGAACAAATCTTTACACTCTTTATCGTCTGATGGAATCTCT
CONSENSUS
TTCCAGAAGCGAACAAATCTTTACACTCTTTATCGTCTGATGGAATCTCT

A3-AT2G16900-XLOC_011787-2642-0
ATTGACCTCAACGGTCAGAATGATTCTTTTAACCATAAGCAGGAGAAGCC
A3-AT2G16900-XLOC_011787-2642-1

ATTGACCTCAACGGTCAGAATGATTCTTTTAACCATAAGCAGGAGAAGCC
CONSENSUS
ATTGACCTCAACGGTCAGAATGATTCTTTTAACCATAAGCAGGAGAAGCC

A3-AT2G16900-XLOC_011787-2642-0
ATCGCGAACTGTTCCTCTATCCCCAAACAGCATG
A3-AT2G16900-XLOC_011787-2642-1
ATCGCGAACTGTTCCTCTATCCCCAAACAGCATG
CONSENSUS
ATCGCGAACTGTTCCTCTATCCCCAAACAGCATG

alignment for event: A5-AT2G46830-XLOC_010808-13518

A5-AT2G46830-XLOC_010808-13518-0
AACATGTAGCAACAAAAACTGCTGTCCAGATAAGAAGTCACGCTCAGAAA
A5-AT2G46830-XLOC_010808-13518-1
AACATGTAGCAACAAAAACTGCTGTCCAGATAAGAAGTCACGCTCAGAAA
CONSENSUS
AACATGTAGCAACAAAAACTGCTGTCCAGATAAGAAGTCACGCTCAGAAA

A5-AT2G46830-XLOC_010808-13518-0
TTTTTCTCCAAG--------------------------------------
A5-AT2G46830-XLOC_010808-13518-1
TTTTTCTCCAAGGTAAAATCGGTTAATTTTGAAATGATGTTCTCATCTTC
CONSENSUS
TTTTTCTCCAAG......................................

A5-AT2G46830-XLOC_010808-13518-0

A5-AT2G46830-XLOC_010808-13518-1
ATTGGCTTAATGCTTAAGACTTATTGAAAGCCAGGCAAGTTTTCTGCTTC
CONSENSUS
....

A5-AT2G46830-XLOC_010808-13518-0

A5-AT2G46830-XLOC_010808-13518-1
TTTTGCTTCTTAGTCAGGAGATAGATAGATTACGTTTTTAGAGTTTAGTA
CONSENSUS
....

A5-AT2G46830-XLOC_010808-13518-0

A5-AT2G46830-XLOC_010808-13518-1
ATGAGCAATAAGTCTTAAAATAGTTGGAGAAATGACGAGATGTAATCGTT
CONSENSUS
....

A5-AT2G46830-XLOC_010808-13518-0

A5-AT2G46830-XLOC_010808-13518-1
TTCTTTTGTTTATGCCTATATCTTGTTAATCCACAAACATGTACATAGAT
CONSENSUS
....

A5-AT2G46830-XLOC_010808-13518-0

A5-AT2G46830-XLOC_010808-13518-1
      TCTTCAGAAGAATGTTAGTTTCTTTAGATTCTTCAGATAAACTTGTGTCT
CONSENSUS
      ....

A5-AT2G46830-XLOC_010808-13518-0

A5-AT2G46830-XLOC_010808-13518-1
      TCTTACCGATTCTGAGGTAGTGGCAAAAGTGGGCTGAGTGCTAGAAATTT
CONSENSUS
      ....

A5-AT2G46830-XLOC_010808-13518-0 ----------------------------
GTAGAGAAAGAGGCTGAAGCT
A5-AT2G46830-XLOC_010808-13518-1
      TTGAATGTTCCTTGTGATAAGCCATAGAGGTAGAGAAAGAGGCTGAAGCT
CONSENSUS
      .............................GTAGAGAAAGAGGCTGAAGCT

A5-AT2G46830-XLOC_010808-13518-0
      AAAGGTGTAGCTATGGGTCAAGCGCTAGACATAGCTATTCCTCCTCCACG
A5-AT2G46830-XLOC_010808-13518-1
      AAAGGTGTAGCTATGGGTCAAGCGCTAGACATAGCTATTCCTCCTCCACG
CONSENSUS
      AAAGGTGTAGCTATGGGTCAAGCGCTAGACATAGCTATTCCTCCTCCACG

A5-AT2G46830-XLOC_010808-13518-0
      GCCTAAGCGTAAACCAAACAATCCTTATCCTCGAAAGACGGGAAGTGGAA
A5-AT2G46830-XLOC_010808-13518-1
      GCCTAAGCGTAAACCAAACAATCCTTATCCTCGAAAGACGGGAAGTGGAA
CONSENSUS
      GCCTAAGCGTAAACCAAACAATCCTTATCCTCGAAAGACGGGAAGTGGAA

A5-AT2G46830-XLOC_010808-13518-0
      CGATCCTTATGTCAAAAACGGGTGTGAATGATGGAAAAGAGTCCCTTGGA
A5-AT2G46830-XLOC_010808-13518-1
      CGATCCTTATGTCAAAAACGGGTGTGAATGATGGAAAAGAGTCCCTTGGA
CONSENSUS
      CGATCCTTATGTCAAAAACGGGTGTGAATGATGGAAAAGAGTCCCTTGGA

A5-AT2G46830-XLOC_010808-13518-0 TCAGAAAAAGTGTCGCATCCTGAG
A5-AT2G46830-XLOC_010808-13518-1 TCAGAAAAAGTGTCGCATCCTGAG
CONSENSUS   TCAGAAAAAGTGTCGCATCCTGAG

alignment for event: SE-AT2G39780-XLOC_010399-10442

SE-AT2G39780-XLOC_010399-10442-0
      ATTTCAACGTTGATGGATGGTCTTGAGAAGTACTGGCCTAGTCTCAGTTG
SE-AT2G39780-XLOC_010399-10442-1
      ATTTCAACGTTGATGGATGGTCTTGAGAAGTACTGGCCTAGTCTCAGTTG
CONSENSUS

ATTTCAACGTTGATGGATGGTCTTGAGAAGTACTGGCCTAGTCTCAGTTG

SE-AT2G39780-XLOC_010399-10442-0
TGGTTCTCCATCATCATGCAATGGTGGGAAAGGGTCATTTTGGGGCCACG
SE-AT2G39780-XLOC_010399-10442-1
TGGTTCTCCATCATCATGCAATGGTGGGAAAGGGTCATTTTGGGGCCACG
CONSENSUS
TGGTTCTCCATCATCATGCAATGGTGGGAAAGGGTCATTTTGGGGCCACG

SE-AT2G39780-XLOC_010399-10442-0 AG----
AGAAACATGGGACTTGTTCTTCTCCTGTTTTTCATGATGAGTAT
SE-AT2G39780-XLOC_010399-10442-1
AGTGGGAGAAACATGGGACTTGTTCTTCTCCTGTTTTTCATGATGAGTAT
CONSENSUS
AG....AGAAACATGGGACTTGTTCTTCTCCTGTTTTTCATGATGAGTAT

SE-AT2G39780-XLOC_010399-10442-0
AATTACTTCCTTACCACACTTAATCTCTACTTGAAGCATAATGTCACG
SE-AT2G39780-XLOC_010399-10442-1
AATTACTTCCTTACCACACTTAATCTCTACTTGAAGCATAATGTCACG
CONSENSUS
AATTACTTCCTTACCACACTTAATCTCTACTTGAAGCATAATGTCACG

alignment for event: RI-AT2G47250-XLOC_013504-13501

RI-AT2G47250-XLOC_013504-13501-0
GTATTTGACTGATGGTATGCTTTTGAGAGAGGCAATGGCGGATCCGCTTT
RI-AT2G47250-XLOC_013504-13501-1
GTATTTGACTGATGGTATGCTTTTGAGAGAGGCAATGGCGGATCCGCTTT
CONSENSUS
GTATTTGACTGATGGTATGCTTTTGAGAGAGGCAATGGCGGATCCGCTTT

RI-AT2G47250-XLOC_013504-13501-0
TAGAGAGATACAAAGTTATTATTCTCGATGAAGCTCATGAAAGGACTCTA
RI-AT2G47250-XLOC_013504-13501-1
TAGAGAGATACAAAGTTATTATTCTCGATGAAGCTCATGAAAGGACTCTA
CONSENSUS
TAGAGAGATACAAAGTTATTATTCTCGATGAAGCTCATGAAAGGACTCTA

RI-AT2G47250-XLOC_013504-13501-0
GCCACGGATGTGCTCTTTGGTCTTCTCAAAGAGGTCTTGAGGAATAGGCC
RI-AT2G47250-XLOC_013504-13501-1
GCCACGGATGTGCTCTTTGGTCTTCTCAAAGAGGTCTTGAGGAATAGGCC
CONSENSUS
GCCACGGATGTGCTCTTTGGTCTTCTCAAAGAGGTCTTGAGGAATAGGCC

RI-AT2G47250-XLOC_013504-13501-0
TGATCTTAAGCTAGTTGTCATGAGTGCAACTTTAGAAGCTGAAAAGTTTC
RI-AT2G47250-XLOC_013504-13501-1
TGATCTTAAGCTAGTTGTCATGAGTGCAACTTTAGAAGCTGAAAAGTTTC
CONSENSUS
TGATCTTAAGCTAGTTGTCATGAGTGCAACTTTAGAAGCTGAAAAGTTTC

RI-AT2G47250-XLOC_013504-13501-0

AGGAATATTTTAGCGGTGCTCCTCTTATGAAAGTCCCTGGTAGGCTTCAT
RI-AT2G47250-XLOC_013504-13501-1
AGGAATATTTTAGCGGTGCTCCTCTTATGAAAGTCCCTGGTAGGCTTCAT
CONSENSUS
AGGAATATTTTAGCGGTGCTCCTCTTATGAAAGTCCCTGGTAGGCTTCAT

RI-AT2G47250-XLOC_013504-13501-0
CCTGTTGAGATCTTCTATACACAGGAACCTGAGAGGGATTATCTCGAGGC
RI-AT2G47250-XLOC_013504-13501-1
CCTGTTGAGATCTTCTATACACAGGAACCTGAGAGGGATTATCTCGAGGC
CONSENSUS
CCTGTTGAGATCTTCTATACACAGGAACCTGAGAGGGATTATCTCGAGGC

RI-AT2G47250-XLOC_013504-13501-0
TGCTATAAGGACTGTTGTTCAGATACACATGTGCGAGCCACCTGGAGATA
RI-AT2G47250-XLOC_013504-13501-1
TGCTATAAGGACTGTTGTTCAGATACACATGTGCGAGCCACCTGGAGATA
CONSENSUS
TGCTATAAGGACTGTTGTTCAGATACACATGTGCGAGCCACCTGGAGATA

RI-AT2G47250-XLOC_013504-13501-0
TTCTTGTTTTCTTAACTGGAGAGGAGGAAATAGAAGATGCTTGCCGTAAA
RI-AT2G47250-XLOC_013504-13501-1
TTCTTGTTTTCTTAACTGGAGAGGAGGAAATAGAAGATGCTTGCCGTAAA
CONSENSUS
TTCTTGTTTTCTTAACTGGAGAGGAGGAAATAGAAGATGCTTGCCGTAAA

RI-AT2G47250-XLOC_013504-13501-0
ATCAATAAAGAGGTCAGCAATCTTGGAGATCAAGTGGGTCCTGTCAAAGT
RI-AT2G47250-XLOC_013504-13501-1
ATCAATAAAGAGGTCAGCAATCTTGGAGATCAAGTGGGTCCTGTCAAAGT
CONSENSUS
ATCAATAAAGAGGTCAGCAATCTTGGAGATCAAGTGGGTCCTGTCAAAGT

RI-AT2G47250-XLOC_013504-13501-0
TGTGCCTTTGTATTCTACTCTTCCACCTGCGATGCAGCAGAAGATTTTCG
RI-AT2G47250-XLOC_013504-13501-1
TGTGCCTTTGTATTCTACTCTTCCACCTGCGATGCAGCAGAAGATTTTCG
CONSENSUS
TGTGCCTTTGTATTCTACTCTTCCACCTGCGATGCAGCAGAAGATTTTCG

RI-AT2G47250-XLOC_013504-13501-0
ACCCTGCTCCAGTGCCGTTAACAGAAGGTGGTCCTGCTGGACGAAAGATT
RI-AT2G47250-XLOC_013504-13501-1
ACCCTGCTCCAGTGCCGTTAACAGAAGGTGGTCCTGCTGGACGAAAGATT
CONSENSUS
ACCCTGCTCCAGTGCCGTTAACAGAAGGTGGTCCTGCTGGACGAAAGATT

RI-AT2G47250-XLOC_013504-13501-0
GTTGTCTCAACCAACATTGCTGAAACCTCTCTAACCATTGATGGGATTGT
RI-AT2G47250-XLOC_013504-13501-1
GTTGTCTCAACCAACATTGCTGAAACCTCTCTAACCATTGATGGGATTGT
CONSENSUS
GTTGTCTCAACCAACATTGCTGAAACCTCTCTAACCATTGATGGGATTGT

RI-AT2G47250-XLOC_013504-13501-0

TTATGTTATTGACCCTGGTTTTGCTAAGCAGAAAGTCTACAACCCACGGA
RI-AT2G47250-XLOC_013504-13501-1
TTATGTTATTGACCCTGGTTTTGCTAAGCAGAAAGTCTACAACCCACGGA
CONSENSUS
TTATGTTATTGACCCTGGTTTTGCTAAGCAGAAAGTCTACAACCCACGGA

RI-AT2G47250-XLOC_013504-13501-0
TTCGAGTTGAGTCATTGTTGGTGTCCCCAATATCAAAGGCAAGTGCTCAC
RI-AT2G47250-XLOC_013504-13501-1
TTCGAGTTGAGTCATTGTTGGTGTCCCCAATATCAAAGGCAAGTGCTCAC
CONSENSUS
TTCGAGTTGAGTCATTGTTGGTGTCCCCAATATCAAAGGCAAGTGCTCAC

RI-AT2G47250-XLOC_013504-13501-0
CAGAGATCAGGTCGTGCTGGTAGAACTCGCCCTGGAAAATGTTTTAGGCT
RI-AT2G47250-XLOC_013504-13501-1
CAGAGATCAGGTCGTGCTGGTAGAACTCGCCCTGGAAAATGTTTTAGGCT
CONSENSUS
CAGAGATCAGGTCGTGCTGGTAGAACTCGCCCTGGAAAATGTTTTAGGCT

RI-AT2G47250-XLOC_013504-13501-0
CTACACAGAGAAGAGTTTCAACAATGACCTGCAGCCACAGACATATCCTG
RI-AT2G47250-XLOC_013504-13501-1
CTACACAGAGAAGAGTTTCAACAATGACCTGCAGCCACAGACATATCCTG
CONSENSUS
CTACACAGAGAAGAGTTTCAACAATGACCTGCAGCCACAGACATATCCTG

RI-AT2G47250-XLOC_013504-13501-0
AGATATTGAGATCAAACCTTGCAAATACAGTCCTGACATTGAAAAAACTT
RI-AT2G47250-XLOC_013504-13501-1
AGATATTGAGATCAAACCTTGCAAATACAGTCCTGACATTGAAAAAACTT
CONSENSUS
AGATATTGAGATCAAACCTTGCAAATACAGTCCTGACATTGAAAAAACTT

RI-AT2G47250-XLOC_013504-13501-0
GGCATTGATGACTTGGTGCACTTTGATTTCATGGATCCTCCTGCTCCTGA
RI-AT2G47250-XLOC_013504-13501-1
GGCATTGATGACTTGGTGCACTTTGATTTCATGGATCCTCCTGCTCCTGA
CONSENSUS
GGCATTGATGACTTGGTGCACTTTGATTTCATGGATCCTCCTGCTCCTGA

RI-AT2G47250-XLOC_013504-13501-0
GACACTGATGCGGGCATTAGAGGTTCTGAATTATTTGGGAGCACTTGATG
RI-AT2G47250-XLOC_013504-13501-1
GACACTGATGCGGGCATTAGAGGTTCTGAATTATTTGGGAGCACTTGATG
CONSENSUS
GACACTGATGCGGGCATTAGAGGTTCTGAATTATTTGGGAGCACTTGATG

RI-AT2G47250-XLOC_013504-13501-0
ATGAAGGTAACTTGACAAAGACGGGTGAAATAATGAGTGAATTTCCCTTG
RI-AT2G47250-XLOC_013504-13501-1
ATGAAGGTAACTTGACAAAGACGGGTGAAATAATGAGTGAATTTCCCTTG
CONSENSUS
ATGAAGGTAACTTGACAAAGACGGGTGAAATAATGAGTGAATTTCCCTTG

RI-AT2G47250-XLOC_013504-13501-0

GATCCACAAATGTCAAAGATGCTCATAGTCAGTCCTGAATTCAACTGTTC
RI-AT2G47250-XLOC_013504-13501-1
GATCCACAAATGTCAAAGATGCTCATAGTCAGTCCTGAATTCAACTGTTC
CONSENSUS
GATCCACAAATGTCAAAGATGCTCATAGTCAGTCCTGAATTCAACTGTTC

RI-AT2G47250-XLOC_013504-13501-0
CAATGAGATTCTCTCGGTTTCTGCAATGTTATCAGGTACATCTTCTACTC
RI-AT2G47250-XLOC_013504-13501-1
CAATGAGATTCTCTCGGTTTCTGCAATGTTATCAG---------------
CONSENSUS
CAATGAGATTCTCTCGGTTTCTGCAATGTTATCAG...............

RI-AT2G47250-XLOC_013504-13501-0
GTAAGTATGGTTTGTCTTCTCCAAAGTTCATTATAGATGTCGGTTTACTT
RI-AT2G47250-XLOC_013504-13501-1

CONSENSUS
....

RI-AT2G47250-XLOC_013504-13501-0
GAGCGTACTATGTTTCACTTCACTTGAATTTACGGAAATAAGTCTGTGTG
RI-AT2G47250-XLOC_013504-13501-1

CONSENSUS
....

RI-AT2G47250-XLOC_013504-13501-0
TATGTGAACGCATCTATAAGTATGTCATACACATGAACATCTACTGCATC
RI-AT2G47250-XLOC_013504-13501-1

CONSENSUS
....

RI-AT2G47250-XLOC_013504-13501-0
CCATGTGTCTTCTTAAAATTCACTAGTGTCTGTGTTTGCCATTCAATTTT
RI-AT2G47250-XLOC_013504-13501-1

CONSENSUS
....

RI-AT2G47250-XLOC_013504-13501-0
AATCTTAGAGTTAGGTTGTCATAAGCAGCTCACTAGTCACTAGTTTGGGG
RI-AT2G47250-XLOC_013504-13501-1

CONSENSUS
....

RI-AT2G47250-XLOC_013504-13501-0
TCTCCATGTCCATGGTTGGGGAGTTCTAAGTAAAACCTGATGCATCTAGT
RI-AT2G47250-XLOC_013504-13501-1

CONSENSUS
....

RI-AT2G47250-XLOC_013504-13501-0

TGCAGTTTCTCAGTATTCTCACAAGCAAATGGTATCGTCTCTCGCCTCTA
RI-AT2G47250-XLOC_013504-13501-1

CONSENSUS
....

RI-AT2G47250-XLOC_013504-13501-0
TGCATCTGCTGACCTCTTCCATGGGCATATTCTGTTATAAAAACAATTCT
RI-AT2G47250-XLOC_013504-13501-1

CONSENSUS
....

RI-AT2G47250-XLOC_013504-13501-0
TTCTCTAGTACCGAATTGCTTTGTCCGGCCTAGAGAGGCTCAAAAAGCAG
RI-AT2G47250-XLOC_013504-13501-1 --------
TACCGAATTGCTTTGTCCGGCCTAGAGAGGCTCAAAAAGCAG
CONSENSUS
........TACCGAATTGCTTTGTCCGGCCTAGAGAGGCTCAAAAAGCAG

RI-AT2G47250-XLOC_013504-13501-0
CAGATGAAGCTAAAGCTAGGTTTGGACACATTGATGGAGATCACCTGACG
RI-AT2G47250-XLOC_013504-13501-1
CAGATGAAGCTAAAGCTAGGTTTGGACACATTGATGGAGATCACCTGACG
CONSENSUS
CAGATGAAGCTAAAGCTAGGTTTGGACACATTGATGGAGATCACCTGACG

RI-AT2G47250-XLOC_013504-13501-0
TTGCTAAACGTGTACCACGCCTACAAGCAAAACA
RI-AT2G47250-XLOC_013504-13501-1
TTGCTAAACGTGTACCACGCCTACAAGCAAAACA
CONSENSUS
TTGCTAAACGTGTACCACGCCTACAAGCAAAACA

alignment for event: RI-AT2G21830-XLOC_009392-8985

RI-AT2G21830-XLOC_009392-8985-0
GTTCATGATATCAACCATAATCATGTGGTGACATCGATTGATATGTGTGA
RI-AT2G21830-XLOC_009392-8985-1
GTTCATGATATCAACCATAATCATGTGGTGACATCGATTGATATGTGTGA
CONSENSUS
GTTCATGATATCAACCATAATCATGTGGTGACATCGATTGATATGTGTGA

RI-AT2G21830-XLOC_009392-8985-0
GCTTGAAGGCAAAGAACATTGTCACATATGTGAAAAAGAAATACTTGGTA
RI-AT2G21830-XLOC_009392-8985-1
GCTTGAAGGCAAAGAACATTGTCACATATGTGAAAAAGAAATACTTGGTA
CONSENSUS
GCTTGAAGGCAAAGAACATTGTCACATATGTGAAAAAGAAATACTTGGTA

RI-AT2G21830-XLOC_009392-8985-0
ATCCATGGAAATGCGAGACTTGCAACTTTGTGACACATAACTTCTGTGTG
RI-AT2G21830-XLOC_009392-8985-1
ATCCATGGAAATGCGAGACTTGCAACTTTGTGACACATAACTTCTGTGTG

CONSENSUS
ATCCATGGAAATGCGAGACTTGCAACTTTGTGACACATAACTTCTGTGTG

RI-AT2G21830-XLOC_009392-8985-0
GAGCTAGGGAAACCATCAAGACATCGGTTTCATTGGAACCATCTTTTGAC
RI-AT2G21830-XLOC_009392-8985-1
GAGCTAGGGAAACCATCAAGACATCGGTTTCATTGGAACCATCTTTTGAC
CONSENSUS
GAGCTAGGGAAACCATCAAGACATCGGTTTCATTGGAACCATCTTTTGAC

RI-AT2G21830-XLOC_009392-8985-0
ACTCATGCCAAAACCAGTTGCTACGGATATGACGAGTTGTAAAAGCTGCA
RI-AT2G21830-XLOC_009392-8985-1
ACTCATGCCAAAACCAGTTGCTACGGATATGACGAGTTGTAAAAGCTGCA
CONSENSUS
ACTCATGCCAAAACCAGTTGCTACGGATATGACGAGTTGTAAAAGCTGCA

RI-AT2G21830-XLOC_009392-8985-0
GAGAGGATATAAAAGGGTTCAATCTATTTTGTCGAATATGCAATTTTATT
RI-AT2G21830-XLOC_009392-8985-1
GAGAGGATATAAAAGG----------------------------------
CONSENSUS
GAGAGGATATAAAAGG..................................

RI-AT2G21830-XLOC_009392-8985-0
ATCCACGTCAGTTGCGCCATGAAAGGTAAACGTTTTCTTGGGATGACAGG
RI-AT2G21830-XLOC_009392-8985-1
---G
CONSENSUS
....G

RI-AT2G21830-XLOC_009392-8985-0
ACCGAAAGTCGTTGGAACTTGGAGAGGACGTTGTTTGGGAGGCAAGCATA
RI-AT2G21830-XLOC_009392-8985-1
ACCGAAAGTCGTTGGAACTTGGAGAGGACGTTGTTTGGGAGGCAAGCATA
CONSENSUS
ACCGAAAGTCGTTGGAACTTGGAGAGGACGTTGTTTGGGAGGCAAGCATA

RI-AT2G21830-XLOC_009392-8985-0
GAATGGTCCAAGTTATGTTCCCGAGGTCATATCAAAAAGTTTGTATTATT
RI-AT2G21830-XLOC_009392-8985-1
GAATGGTCCAAGTTATGTTCCCGAGGTCATATCAAAAAGTTTGTATTATT
CONSENSUS
GAATGGTCCAAGTTATGTTCCCGAGGTCATATCAAAAAGTTTGTATTATT

RI-AT2G21830-XLOC_009392-8985-0
TGTCATGAGAGGGTGCTTGGGAAGGCTGTGTCATGTATGGAGTGTGAAAC
RI-AT2G21830-XLOC_009392-8985-1
TGTCATGAGAGGGTGCTTGGGAAGGCTGTGTCATGTATGGAGTGTGAAAC
CONSENSUS
TGTCATGAGAGGGTGCTTGGGAAGGCTGTGTCATGTATGGAGTGTGAAAC

RI-AT2G21830-XLOC_009392-8985-0
GATTTATCATCTTCGGTGTATTGACCGACATCGTATCAAAGACCGTGATT
RI-AT2G21830-XLOC_009392-8985-1
GATTTATCATCTTCGGTGTATTGACCGACATCGTATCAAAGACCGTGATT

CONSENSUS
GATTTATCATCTTCGGTGTATTGACCGACATCGTATCAAAGACCGTGATT

RI-AT2G21830-XLOC_009392-8985-0
CTTCGTAGTTAGGTTGTTCCTCCTCTGAAACTAAGTTCTGCAACTTCTTG
RI-AT2G21830-XLOC_009392-8985-1
CTTCGTAGTTAGGTTGTTCCTCCTCTGAAACTAAGTTCTGCAACTTCTTG
CONSENSUS
CTTCGTAGTTAGGTTGTTCCTCCTCTGAAACTAAGTTCTGCAACTTCTTG

RI-AT2G21830-XLOC_009392-8985-0
TTATGTTATATACCTCTAAAATTTTGTCGGTTAGTAAATCATGTTTGTAA
RI-AT2G21830-XLOC_009392-8985-1
TTATGTTATATACCTCTAAAATTTTGTCGGTTAGTAAATCATGTTTGTAA
CONSENSUS
TTATGTTATATACCTCTAAAATTTTGTCGGTTAGTAAATCATGTTTGTAA

RI-AT2G21830-XLOC_009392-8985-0
TATGTTTATTTGCTCTGTTTATTTCTTGTTCTGTTTTCTTTGTTTTTGGC
RI-AT2G21830-XLOC_009392-8985-1
TATGTTTATTTGCTCTGTTTATTTCTTGTTCTGTTTTCTTTGTTTTTGGC
CONSENSUS
TATGTTTATTTGCTCTGTTTATTTCTTGTTCTGTTTTCTTTGTTTTTGGC

RI-AT2G21830-XLOC_009392-8985-0
ACACTATGTATTTTCAATCCTCTGTTTTGGATTCCATTATCGTTTTTCCA
RI-AT2G21830-XLOC_009392-8985-1
ACACTATGTATTTTCAATCCTCTGTTTTGGATTCCATTATCGTTTTTCCA
CONSENSUS
ACACTATGTATTTTCAATCCTCTGTTTTGGATTCCATTATCGTTTTTCCA

RI-AT2G21830-XLOC_009392-8985-0 GTTCC
RI-AT2G21830-XLOC_009392-8985-1 GTTCC
CONSENSUS GTTCC

alignment for event: RI-AT3G53500-XLOC_019516-11512

RI-AT3G53500-XLOC_019516-11512-0
CTTGACAATGCCTCGCTATGATGATCGCTATGGAAACACTCGCCTCTATG
RI-AT3G53500-XLOC_019516-11512-1
CTTGACAATGCCTCGCTATGATGATCGCTATGGAAACACTCGCCTCTATG
CONSENSUS
CTTGACAATGCCTCGCTATGATGATCGCTATGGAAACACTCGCCTCTATG

RI-AT3G53500-XLOC_019516-11512-0
TTGGTCGCTTATCATCTAGAACTCGTACCAGAGACCTTGAGCGTCTCTTT
RI-AT3G53500-XLOC_019516-11512-1
TTGGTCGCTTATCATCTAGAACTCGTACCAGAGACCTTGAGCGTCTCTTT
CONSENSUS
TTGGTCGCTTATCATCTAGAACTCGTACCAGAGACCTTGAGCGTCTCTTT

RI-AT3G53500-XLOC_019516-11512-0
AGCAGATACGGAAGGTAAAGAGTCATATTCCTTTGTCTTGACTGACCTAA
RI-AT3G53500-XLOC_019516-11512-1

AGCAGATACGGAAG------------------------------------
CONSENSUS
AGCAGATACGGAAG....................................

RI-AT3G53500-XLOC_019516-11512-0
TTTGACTTTTGTATTCTTGGGGAGATAAGTGTTTGGATTTTTTGTTAATT
RI-AT3G53500-XLOC_019516-11512-1

CONSENSUS
....

RI-AT3G53500-XLOC_019516-11512-0
GTTCTTTGGTGTGAATCTGGAATGATTCCTCTTTGATGTTGTGCAGGGGA
RI-AT3G53500-XLOC_019516-11512-1

CONSENSUS
....

RI-AT3G53500-XLOC_019516-11512-0
TATATACCTAAAGTTTAACATTAGAATCAAAGAGGGTAGTTTTTACTTAT
RI-AT3G53500-XLOC_019516-11512-1

CONSENSUS
....

RI-AT3G53500-XLOC_019516-11512-0
AACGTTGCATTGGTACATGTGGGAAAATTGTGGAGCTAGGTTTAGTGGAT
RI-AT3G53500-XLOC_019516-11512-1

CONSENSUS
....

RI-AT3G53500-XLOC_019516-11512-0
TTTTTTTTCTTTAACTTGAATGATCTTGTTGAAGGTTTCTGGGGGTTTGC
RI-AT3G53500-XLOC_019516-11512-1

CONSENSUS
....

RI-AT3G53500-XLOC_019516-11512-0
CTAACCTGGAGATGTTTGATGAATGGGTGGACTGTGGTGTAATTGGTGGA
RI-AT3G53500-XLOC_019516-11512-1

CONSENSUS
....

RI-AT3G53500-XLOC_019516-11512-0
AATTCTCGTCTGGTGTATAGAGGGTGCATCGGCGATTTTGTTTGTTGATT
RI-AT3G53500-XLOC_019516-11512-1

CONSENSUS
....

RI-AT3G53500-XLOC_019516-11512-0
TATTGCATGTTTTTATTGGAAATGGCCTCAAATCTCAAAAACTGGTGTTT
RI-AT3G53500-XLOC_019516-11512-1

CONSENSUS
....

RI-AT3G53500-XLOC_019516-11512-0
GGAAGTTCCTTAAGTAATGATGGCCTTGATTTTCTGGCAACAATTTACGC
RI-AT3G53500-XLOC_019516-11512-1

CONSENSUS
....

RI-AT3G53500-XLOC_019516-11512-0
AGAGTACGAGATGTGGATATGAAGCGTGATTATGCCTTTGTT
RI-AT3G53500-XLOC_019516-11512-1 --
AGTACGAGATGTGGATATGAAGCGTGATTATGCCTTTGTT
CONSENSUS
..AGTACGAGATGTGGATATGAAGCGTGATTATGCCTTTGTT

alignment for event: A3-AT3G61010-XLOC_019935-387

A3-AT3G61010-XLOC_019935-387-0
GGCTTTGGTTACCATGTTTCACTCCAAGGTCTCCAACAGTCAGATTGTTG
A3-AT3G61010-XLOC_019935-387-1
GGCTTTGGTTACCATGTTTCACTCCAAGGTCTCCAACAGTCAGATTGTTG
CONSENSUS
GGCTTTGGTTACCATGTTTCACTCCAAGGTCTCCAACAGTCAGATTGTTG

A3-AT3G61010-XLOC_019935-387-0
CTTTTAGTTTTATTCTTCACAGATGCCCCATGGTACAACATTTCTGCCAA
A3-AT3G61010-XLOC_019935-387-1
CTTTTAGTTTTATTCTTCACAGATGCCCCATGGTACAACATTTCTGCCAA
CONSENSUS
CTTTTAGTTTTATTCTTCACAGATGCCCCATGGTACAACATTTCTGCCAA

A3-AT3G61010-XLOC_019935-387-0
AGTCTTCAG---
A3-AT3G61010-XLOC_019935-387-1
AGTCTTCAGTTGATCTTCTTGATCTGGGGGTCTCTCTCCTCTCATCTGAT
CONSENSUS
AGTCTTCAG...

A3-AT3G61010-XLOC_019935-387-0 ------------------------------
CCTCTCCTAGAACTCAATGAA
A3-AT3G61010-XLOC_019935-387-1
CTCCGTCTTTGTTATATGCTTCACATTAGCCTCTCCTAGAACTCAATGAA
CONSENSUS
.............................CCTCTCCTAGAACTCAATGAA

A3-AT3G61010-XLOC_019935-387-0
GACAACAAGGATGTCATTCAGGCCACTCTTGAGTGAGTTCTGGCCCTAAT
A3-AT3G61010-XLOC_019935-387-1
GACAACAAGGATGTCATTCAGGCCACTCTTGAGTGAGTTCTGGCCCTAAT
CONSENSUS
GACAACAAGGATGTCATTCAGGCCACTCTTGAGTGAGTTCTGGCCCTAAT

A3-AT3G61010-XLOC_019935-387-0
ACTATCATCATATTTATTTCGGCAAAGCCAAAAAGGGAAAAATGTTTTCG
A3-AT3G61010-XLOC_019935-387-1
ACTATCATCATATTTATTTCGGCAAAGCCAAAAAGGGAAAAATGTTTTCG
CONSENSUS
ACTATCATCATATTTATTTCGGCAAAGCCAAAAAGGGAAAAATGTTTTCG

A3-AT3G61010-XLOC_019935-387-0
ACATTTCTGAGCAAACATTTAAACAATAAGTACTATGAGTCTGATAGTAC
A3-AT3G61010-XLOC_019935-387-1
ACATTTCTGAGCAAACATTTAAACAATAAGTACTATGAGTCTGATAGTAC
CONSENSUS
ACATTTCTGAGCAAACATTTAAACAATAAGTACTATGAGTCTGATAGTAC

A3-AT3G61010-XLOC_019935-387-0
TCGAGAGGCATCTTTTAACGGAGGAGACTACATCACTTTTAGAGGAAAAC
A3-AT3G61010-XLOC_019935-387-1
TCGAGAGGCATCTTTTAACGGAGGAGACTACATCACTTTTAGAGGAAAAC
CONSENSUS
TCGAGAGGCATCTTTTAACGGAGGAGACTACATCACTTTTAGAGGAAAAC

A3-AT3G61010-XLOC_019935-387-0
TCGAGGGAGATGCGTATTTCACAACAAGGCTCTTCAAATCCCATCTTCAT
A3-AT3G61010-XLOC_019935-387-1
TCGAGGGAGATGCGTATTTCACAACAAGGCTCTTCAAATCCCATCTTCAT
CONSENSUS
TCGAGGGAGATGCGTATTTCACAACAAGGCTCTTCAAATCCCATCTTCAT

A3-AT3G61010-XLOC_019935-387-0
CTTTCATCTTCCCCCATCACAATTTCTTTCTCT
A3-AT3G61010-XLOC_019935-387-1
CTTTCATCTTCCCCCATCACAATTTCTTTCTCT
CONSENSUS
CTTTCATCTTCCCCCATCACAATTTCTTTCTCT

alignment for event: RI-AT3G53920-XLOC_019537-4871

RI-AT3G53920-XLOC_019537-4871-0
GCAGGGTATGTGGGGGTGTTACAAGGAGCAGAGAGGTTTGATCACACAAG
RI-AT3G53920-XLOC_019537-4871-1
GCAGGGTATGTGGGGGTGTTACAAGGAGCAGAGAGGTTTGATCACACAAG
CONSENSUS
GCAGGGTATGTGGGGGTGTTACAAGGAGCAGAGAGGTTTGATCACACAAG

RI-AT3G53920-XLOC_019537-4871-0
GGGTTACAAGTTTTCAACATATGTGCAGTATTGGATAAGAAAATCAATGT
RI-AT3G53920-XLOC_019537-4871-1
GGGTTACAAGTTTTCAACATATGTGCAGTATTGGATAAGAAAATCAATGT
CONSENSUS
GGGTTACAAGTTTTCAACATATGTGCAGTATTGGATAAGAAAATCAATGT

RI-AT3G53920-XLOC_019537-4871-0
CTACGATGGTGTCACGGCATGCAAGAGGCGTCCATATTCCTGTTTGTGCA

RI-AT3G53920-XLOC_019537-4871-1
CTACGATGGTGTCACGGCATGCAAGAGGCGTCCATATTCCT---------
CONSENSUS
CTACGATGGTGTCACGGCATGCAAGAGGCGTCCATATTCCT.........

RI-AT3G53920-XLOC_019537-4871-0
TTTTGATTTTTGGCCCTTTTTGTCTCATTTTCAACAAAGCTAGAACCTCA
RI-AT3G53920-XLOC_019537-4871-1

CONSENSUS
....

RI-AT3G53920-XLOC_019537-4871-0
CTTGTTTTCCTTTATTGTTTCTGTTTTTTTGGCAGTCATCAATAATCCGA
RI-AT3G53920-XLOC_019537-4871-1
------------------------------------TCATCAATAATCCGA
CONSENSUS
....................................TCATCAATAATCCGA

RI-AT3G53920-XLOC_019537-4871-0
ACTATCAATCATATACAAAAGGCTCGTAAGACCCTGAAAACGAGCCATGG
RI-AT3G53920-XLOC_019537-4871-1
ACTATCAATCATATACAAAAGGCTCGTAAGACCCTGAAAACGAGCCATGG
CONSENSUS
ACTATCAATCATATACAAAAGGCTCGTAAGACCCTGAAAACGAGCCATGG

RI-AT3G53920-XLOC_019537-4871-0
TATAAAATATGCGGCTGATGAGGAGATTGCAAAACTAACAGGCCACTCGG
RI-AT3G53920-XLOC_019537-4871-1
TATAAAATATGCGGCTGATGAGGAGATTGCAAAACTAACAGGCCACTCGG
CONSENSUS
TATAAAATATGCGGCTGATGAGGAGATTGCAAAACTAACAGGCCACTCGG

RI-AT3G53920-XLOC_019537-4871-0
TGAAGAAGATTCGAGCAGCTAACCAATGCCTAAAAGTGGTCGGTTCAATT
RI-AT3G53920-XLOC_019537-4871-1
TGAAGAAGATTCGAGCAGCTAACCAATGCCTAAAAGTGGTCGGTTCAATT
CONSENSUS
TGAAGAAGATTCGAGCAGCTAACCAATGCCTAAAAGTGGTCGGTTCAATT

RI-AT3G53920-XLOC_019537-4871-0
GACAAGAAAGTTGGGGATTGCTTTACTACAAAATTCCTG
RI-AT3G53920-XLOC_019537-4871-1
GACAAGAAAGTTGGGGATTGCTTTACTACAAAATTCCTG
CONSENSUS
GACAAGAAAGTTGGGGATTGCTTTACTACAAAATTCCTG

alignment for event: A5-AT3G46210-XLOC_019084-5739

A5-AT3G46210-XLOC_019084-5739-0
GTGGTTTCAGTTGTTCTTCCAGCTTTCAGTTCCGTAGCTGTGTATTCTCT
A5-AT3G46210-XLOC_019084-5739-1
GTGGTTTCAGTTGTTCTTCCAGCTTTCAGTTCCGTAGCTGTGTATTCTCT
CONSENSUS

GTGGTTTCAGTTGTTCTTCCAGCTTTCAGTTCCGTAGCTGTGTATTCTCT

A5-AT3G46210-XLOC_019084-5739-0
CTTTCGTTAGTCTTCTTGTTTGATACGGAATCGTCTCTGCTTCTAGCTTC
A5-AT3G46210-XLOC_019084-5739-1
CTTTCGTTAGTCTTCTTGTTTGATACGGAATCGTCTCTGCTTCTAGCTTC
CONSENSUS
CTTTCGTTAGTCTTCTTGTTTGATACGGAATCGTCTCTGCTTCTAGCTTC

A5-AT3G46210-XLOC_019084-5739-0
CTCAAAATTAGGTTCTTAGTCTAGCTCTGTGTTGATTCGTGTGTCCTTTT
A5-AT3G46210-XLOC_019084-5739-1
CTCAAAATTAG---------------------------------------
CONSENSUS
CTCAAAATTAG.......................................

A5-AT3G46210-XLOC_019084-5739-0
GTTCATACATCGTCTTTTATGTGCTAGATAACGAGTATTGTAGAATTGAA
A5-AT3G46210-XLOC_019084-5739-1

CONSENSUS
....

A5-AT3G46210-XLOC_019084-5739-0
AGAACGATTTTTTTGTCTCAATTAACAAAAAAACTACTCGGATGTTGGTC
A5-AT3G46210-XLOC_019084-5739-1

CONSENSUS
....

A5-AT3G46210-XLOC_019084-5739-0
AGAACCATTAAAATACACCTACTGTTGAGCTAGAAGCAGTTCAAATTTTG
A5-AT3G46210-XLOC_019084-5739-1

CONSENSUS
....

A5-AT3G46210-XLOC_019084-5739-0
TTTTCAGTGAAGTATTGTAGCAATGGAGATTGATAGAGAAGATGGAAGAA
A5-AT3G46210-XLOC_019084-5739-1 ---------
AAGTATTGTAGCAATGGAGATTGATAGAGAAGATGGAAGAA
CONSENSUS
.........AAGTATTGTAGCAATGGAGATTGATAGAGAAGATGGAAGAA

A5-AT3G46210-XLOC_019084-5739-0
CACCAAATCAGCTGAGACCTCTTGCTTGTTCCCGTAATATCCTTCATCGT
A5-AT3G46210-XLOC_019084-5739-1
CACCAAATCAGCTGAGACCTCTTGCTTGTTCCCGTAATATCCTTCATCGT
CONSENSUS
CACCAAATCAGCTGAGACCTCTTGCTTGTTCCCGTAATATCCTTCATCGT

A5-AT3G46210-XLOC_019084-5739-0 CCTCATGGCTCTGCTAGTTGGTCACAAG
A5-AT3G46210-XLOC_019084-5739-1 CCTCATGGCTCTGCTAGTTGGTCACAAG
CONSENSUS CCTCATGGCTCTGCTAGTTGGTCACAAG

alignment for event: A3-AT3G27460-XLOC_018293-9934

A3-AT3G27460-XLOC_018293-9934-0
CGTTTCAAATATTCTGCTTACTCAGTTGGATTCTCTGCTTCCATCTGGAC
A3-AT3G27460-XLOC_018293-9934-1
CGTTTCAAATATTCTGCTTACTCAGTTGGATTCTCTGCTTCCATCTGGAC
CONSENSUS
CGTTTCAAATATTCTGCTTACTCAGTTGGATTCTCTGCTTCCATCTGGAC

A3-AT3G27460-XLOC_018293-9934-0
CAACTGGGCAACAACGCAGAAAATTAG---------GCAACGAACAGAAG
A3-AT3G27460-XLOC_018293-9934-1
CAACTGGGCAACAACGCAGAAAATTAGTGGCAGAAGGCAACGAACAGAAG
CONSENSUS
CAACTGGGCAACAACGCAGAAAATTAG.........GCAACGAACAGAAG

A3-AT3G27460-XLOC_018293-9934-0
AGAAAGAGAATGAAAGTAGATACAGATGTAACAAGAGTTTCTCCTTCCAT
A3-AT3G27460-XLOC_018293-9934-1
AGAAAGAGAATGAAAGTAGATACAGATGTAACAAGAGTTTCTCCTTCCAT
CONSENSUS
AGAAAGAGAATGAAAGTAGATACAGATGTAACAAGAGTTTCTCCTTCCAT

A3-AT3G27460-XLOC_018293-9934-0
GAGAAATCAAATCGAGGCATATGCTAGTCTAAAGGGTGAACAG
A3-AT3G27460-XLOC_018293-9934-1
GAGAAATCAAATCGAGGCATATGCTAGTCTAAAGGGTGAACAG
CONSENSUS
GAGAAATCAAATCGAGGCATATGCTAGTCTAAAGGGTGAACAG

alignment for event: A3-AT3G63400-XLOC_016772-976

A3-AT3G63400-XLOC_016772-976-0
ATCCAAAACATAATTAGTATAACGATACTACTACTAACCGACTGGACTAG
A3-AT3G63400-XLOC_016772-976-1
ATCCAAAACATAATTAGTATAACGATACTACTACTAACCGACTGGACTAG
CONSENSUS
ATCCAAAACATAATTAGTATAACGATACTACTACTAACCGACTGGACTAG

A3-AT3G63400-XLOC_016772-976-0
ATTATTCTACTTGATATATCCTCTCGAGAACTATTGCTGCCGCTACTCGT
A3-AT3G63400-XLOC_016772-976-1
ATTATTCTACTTGATATATCCTCTCGAGAACTATTGCTGCCGCTACTCGT
CONSENSUS
ATTATTCTACTTGATATATCCTCTCGAGAACTATTGCTGCCGCTACTCGT

A3-AT3G63400-XLOC_016772-976-0
TTCATATCTCCGTCTCCGGCTCGATCCGCCAAGTAGAGTAAGCAGCACAT
A3-AT3G63400-XLOC_016772-976-1
TTCATATCTCCGTCTCCGGCTCGATCCGCCAAGTAGAGTAAGCAG-----
CONSENSUS
TTCATATCTCCGTCTCCGGCTCGATCCGCCAAGTAGAGTAAGCAG.....

A3-AT3G63400-XLOC_016772-976-0
      CATCTGCACTGCAGCAATGACTAAAAAGAAGAATCCTAATGTTTTCTTGG
A3-AT3G63400-XLOC_016772-976-1   --------------
CAATGACTAAAAAGAAGAATCCTAATGTTTTCTTGG
CONSENSUS
      ..............CAATGACTAAAAAGAAGAATCCTAATGTTTTCTTGG

A3-AT3G63400-XLOC_016772-976-0
      ATGTATCAATTGGTGGGGATCCCGTTCAACGGATTGTCATTGAG
A3-AT3G63400-XLOC_016772-976-1
      ATGTATCAATTGGTGGGGATCCCGTTCAACGGATTGTCATTGAG
CONSENSUS
      ATGTATCAATTGGTGGGGATCCCGTTCAACGGATTGTCATTGAG

alignment for event: A5-AT3G61010-XLOC_019935-377

A5-AT3G61010-XLOC_019935-377-0
      ATGGTGGTCACTTGTCGAGAAGTCATGTGGAATAGTCCAAACTATCCACA
A5-AT3G61010-XLOC_019935-377-1
      ATGGTGGTCACTTGTCGAGAAGTCATGTGGAATAGTCCAAACTATCCACA
CONSENSUS
      ATGGTGGTCACTTGTCGAGAAGTCATGTGGAATAGTCCAAACTATCCACA

A5-AT3G61010-XLOC_019935-377-0
      AGTCTTCCCTTTTTACTCGGATTTCAATCAGGTAAGGGCTTTGGTTACCA
A5-AT3G61010-XLOC_019935-377-1
      AGTCTTCCCTTTTTACTCGGATTTCAATCAG-----GGCTTTGGTTACCA
CONSENSUS
      AGTCTTCCCTTTTTACTCGGATTTCAATCAG.....GGCTTTGGTTACCA

A5-AT3G61010-XLOC_019935-377-0
      TGTTTCACTCCAAGGTCTCCAACAGTCAGATTGTTGCTTTTAGTTTTATT
A5-AT3G61010-XLOC_019935-377-1
      TGTTTCACTCCAAGGTCTCCAACAGTCAGATTGTTGCTTTTAGTTTTATT
CONSENSUS
      TGTTTCACTCCAAGGTCTCCAACAGTCAGATTGTTGCTTTTAGTTTTATT

A5-AT3G61010-XLOC_019935-377-0
      CTTCACAGATGCCCCATGGTACAACATTTCTGCCAAAGTCTTCAG
A5-AT3G61010-XLOC_019935-377-1
      CTTCACAGATGCCCCATGGTACAACATTTCTGCCAAAGTCTTCAG
CONSENSUS
      CTTCACAGATGCCCCATGGTACAACATTTCTGCCAAAGTCTTCAG

alignment for event: RI-AT3G13300-XLOC_014189-8500

RI-AT3G13300-XLOC_014189-8500-0
      AAAAGTTCTCTACCTGTTCTCACTTTCAAGTCATCATCGTCTCTCTCTCT
RI-AT3G13300-XLOC_014189-8500-1
      AAAAGTTCTCTACCTGTTCTCACTTTCAAGTCATCATCGTCTCTCTCTCT
CONSENSUS
      AAAAGTTCTCTACCTGTTCTCACTTTCAAGTCATCATCGTCTCTCTCTCT

RI-AT3G13300-XLOC_014189-8500-0
    CTATTGTTGCTCTCTCTCTCGCTTTTTATGTGACTTTCAGATCCGCCGAG
RI-AT3G13300-XLOC_014189-8500-1
    CTATTGTTGCTCTCTCTCTCGCTTTTTATGTGACTTTCAGATCCGCCGAG
CONSENSUS
    CTATTGTTGCTCTCTCTCTCGCTTTTTATGTGACTTTCAGATCCGCCGAG

RI-AT3G13300-XLOC_014189-8500-0
    TCTTCTTTTTGTGGAATCTTTTCAAGGAGCTCAGTATTCTATATACACCT
RI-AT3G13300-XLOC_014189-8500-1
    TCTTCTTTTTGTGGAATCTTTTCAAGGAGCTCAGTATTCTATATACACCT
CONSENSUS
    TCTTCTTTTTGTGGAATCTTTTCAAGGAGCTCAGTATTCTATATACACCT

RI-AT3G13300-XLOC_014189-8500-0
    CTCTTTGTTTGATCGATTTCACAAACCCTAGCTTCGGCGATCTTAAATCT
RI-AT3G13300-XLOC_014189-8500-1
    CTCTTTGTTTGATCGATTTCACAAACCCTAGCTTCGGCGATCTTAAATCT
CONSENSUS
    CTCTTTGTTTGATCGATTTCACAAACCCTAGCTTCGGCGATCTTAAATCT

RI-AT3G13300-XLOC_014189-8500-0
    GCAAAAAATCTATGGCGTCTTCTCCTGGTAATACTAACCCTCACAATACT
RI-AT3G13300-XLOC_014189-8500-1
    GCAAAAAATCTATGGCGTCTTCTCCTG-----------------------
CONSENSUS
    GCAAAAAATCTATGGCGTCTTCTCCTG.......................

RI-AT3G13300-XLOC_014189-8500-0
    CCTCCGTTTGATCTCGGTATTCTCTTCAAACCTTCATCCAATCCTTATCC
RI-AT3G13300-XLOC_014189-8500-1

CONSENSUS
    ....

RI-AT3G13300-XLOC_014189-8500-0
    GCCACCGGCGGCATCTTATCCTCCGCCTACAGGTCCTTTTCTTCACAACC
RI-AT3G13300-XLOC_014189-8500-1
    --------------------------------GTCCTTTTCTTCACAACC
CONSENSUS
    ................................GTCCTTTTCTTCACAACC

RI-AT3G13300-XLOC_014189-8500-0
    AGTACGATCAGCAGCACTACGCTCCGCCTGGTATCTCCGCTCAACCATCT
RI-AT3G13300-XLOC_014189-8500-1
    AGTACGATCAGCAGCACTACGCTCCGCCTGGTATCTCCGCTCAACCATCT
CONSENSUS
    AGTACGATCAGCAGCACTACGCTCCGCCTGGTATCTCCGCTCAACCATCT

RI-AT3G13300-XLOC_014189-8500-0
    CCGGTGACTCAGCAGCAACAGGATGTGTCTTCCTCTTCCGCCGCGACTAA
RI-AT3G13300-XLOC_014189-8500-1
    CCGGTGACTCAGCAGCAACAGGATGTGTCTTCCTCTTCCGCCGCGACTAA
CONSENSUS
    CCGGTGACTCAGCAGCAACAGGATGTGTCTTCCTCTTCCGCCGCGACTAA

RI-AT3G13300-XLOC_014189-8500-0
CTTGCATCCTCAGAGAACGCTGTCTTATCCCACGCCACCTCTCAATCTAC
RI-AT3G13300-XLOC_014189-8500-1
CTTGCATCCTCAGAGAACGCTGTCTTATCCCACGCCACCTCTCAATCTAC
CONSENSUS
CTTGCATCCTCAGAGAACGCTGTCTTATCCCACGCCACCTCTCAATCTAC

RI-AT3G13300-XLOC_014189-8500-0
AATCCCCCCGTTCCAATCACAATCCCGGTACGCACATCCTCGCTCTCCTT
RI-AT3G13300-XLOC_014189-8500-1
AATCCCCCCGTTCCAATCACAATCCCGGTACGCACATCCTCGCTCTCCTT
CONSENSUS
AATCCCCCCGTTCCAATCACAATCCCGGTACGCACATCCTCGCTCTCCTT

RI-AT3G13300-XLOC_014189-8500-0
AACAATACCAACAACGGAGCCCCCGTGGCTAACCAAGAGCCGTCGCATCA
RI-AT3G13300-XLOC_014189-8500-1
AACAATACCAACAACGGAGCCCCCGTGGCTAACCAAGAGCCGTCGCATCA
CONSENSUS
AACAATACCAACAACGGAGCCCCCGTGGCTAACCAAGAGCCGTCGCATCA

RI-AT3G13300-XLOC_014189-8500-0
GCTCCCAGTAGTCAATCACAACGAGATCGCTCGCTCTTTTCCCGGCGGTT
RI-AT3G13300-XLOC_014189-8500-1
GCTCCCAGTAGTCAATCACAACGAGATCGCTCGCTCTTTTCCCGGCGGTT
CONSENSUS
GCTCCCAGTAGTCAATCACAACGAGATCGCTCGCTCTTTTCCCGGCGGTT

RI-AT3G13300-XLOC_014189-8500-0
CAGGTCCTATTCGTGTACCGAGCTGTAAATTGCCCAAGGGAAGGCGATTG
RI-AT3G13300-XLOC_014189-8500-1
CAGGTCCTATTCGTGTACCGAGCTGTAAATTGCCCAAGGGAAGGCGATTG
CONSENSUS
CAGGTCCTATTCGTGTACCGAGCTGTAAATTGCCCAAGGGAAGGCGATTG

RI-AT3G13300-XLOC_014189-8500-0
ATTGGTGAACACGCTGTGTACGATGTTGATGTGAGATTACAAGGTGAGAT
RI-AT3G13300-XLOC_014189-8500-1
ATTGGTGAACACGCTGTGTACGATGTTGATGTGAGATTACAAGGTGAGAT
CONSENSUS
ATTGGTGAACACGCTGTGTACGATGTTGATGTGAGATTACAAGGTGAGAT

RI-AT3G13300-XLOC_014189-8500-0
TCAGCCGCAGCTGGAGGTGACTCCGATTACCAAATACGGGTCGGATCCTC
RI-AT3G13300-XLOC_014189-8500-1
TCAGCCGCAGCTGGAGGTGACTCCGATTACCAAATACGGGTCGGATCCTC
CONSENSUS
TCAGCCGCAGCTGGAGGTGACTCCGATTACCAAATACGGGTCGGATCCTC

RI-AT3G13300-XLOC_014189-8500-0
AGCTCGTAGTGGGTAGGCAAATCGCCGTGAATAAGGTTTACATTTGCTAT
RI-AT3G13300-XLOC_014189-8500-1
AGCTCGTAGTGGGTAGGCAAATCGCCGTGAATAAGGTTTACATTTGCTAT
CONSENSUS
AGCTCGTAGTGGGTAGGCAAATCGCCGTGAATAAGGTTTACATTTGCTAT

RI-AT3G13300-XLOC_014189-8500-0
      GGATTGAAAGGAGGAAACATTAGGGTTCTCAATATCAACACAGCATTGAG
RI-AT3G13300-XLOC_014189-8500-1
      GGATTGAAAGGAGGAAACATTAGGGTTCTCAATATCAACACAGCATTGAG
CONSENSUS
      GGATTGAAAGGAGGAAACATTAGGGTTCTCAATATCAACACAGCATTGAG

RI-AT3G13300-XLOC_014189-8500-0 GTCTCTGTTCCGTGGCCATTCACAG
RI-AT3G13300-XLOC_014189-8500-1 GTCTCTGTTCCGTGGCCATTCACAG
CONSENSUS   GTCTCTGTTCCGTGGCCATTCACAG

alignment for event: RI-AT3G05970-XLOC_017081-663

RI-AT3G05970-XLOC_017081-663-0
      GAAAGAGGTGATTGATGAAGATGGATGGCTTCACACTGGAGATATAGGTC
RI-AT3G05970-XLOC_017081-663-1
      GAAAGAGGTGATTGATGAAGATGGATGGCTTCACACTGGAGATATAGGTC
CONSENSUS
      GAAAGAGGTGATTGATGAAGATGGATGGCTTCACACTGGAGATATAGGTC

RI-AT3G05970-XLOC_017081-663-0
      TGTGGCTGCCGGGAGGACGTCTAAAAATTATTGACAGGTTTTCCAAAAAA
RI-AT3G05970-XLOC_017081-663-1
      TGTGGCTGCCGGGAGGACGTCTAAAAATTATTGACAG-------------
CONSENSUS
      TGTGGCTGCCGGGAGGACGTCTAAAAATTATTGACAG.............

RI-AT3G05970-XLOC_017081-663-0
      TCACTTGATTGTTATCAGCTGTTTCTTCACTAAAATCAAATCTCTTGGCT
RI-AT3G05970-XLOC_017081-663-1

CONSENSUS
      ....

RI-AT3G05970-XLOC_017081-663-0
      CACAAAGAATAAGCCTACTTCTTACTGATGATACAAACACTAAGAAATAA
RI-AT3G05970-XLOC_017081-663-1

CONSENSUS
      ....

RI-AT3G05970-XLOC_017081-663-0
      CATCTGCAAAAGTCTAGGGAGTGCAAAGCTCTAGTCTTGGGTTACATTTT
RI-AT3G05970-XLOC_017081-663-1

CONSENSUS
      ....

RI-AT3G05970-XLOC_017081-663-0
      GGGGACTTGTTGGTATCTTTTGAATCGCGAATCATCTGAGTGAAATTTTC
RI-AT3G05970-XLOC_017081-663-1

CONSENSUS

....

RI-AT3G05970-XLOC_017081-663-0
TGATCCTTTGAATAGAAAGAAGAACATCTTCAAATTGGCGCAGGGGGAGT
RI-AT3G05970-XLOC_017081-663-1 ---------------
AAAGAAGAACATCTTCAAATTGGCGCAGGGGGAGT
CONSENSUS
...............AAAGAAGAACATCTTCAAATTGGCGCAGGGGGAGT

RI-AT3G05970-XLOC_017081-663-0
ATATAGCTCCAGAGAAAATTGAAAACGTCTATGCCAAATGCAAATTTGTG
RI-AT3G05970-XLOC_017081-663-1
ATATAGCTCCAGAGAAAATTGAAAACGTCTATGCCAAATGCAAATTTGTG
CONSENSUS
ATATAGCTCCAGAGAAAATTGAAAACGTCTATGCCAAATGCAAATTTGTG

RI-AT3G05970-XLOC_017081-663-0 GGCCAGTGCTTCATATATG
RI-AT3G05970-XLOC_017081-663-1 GGCCAGTGCTTCATATATG
CONSENSUS GGCCAGTGCTTCATATATG

alignment for event: RI-AT3G61210-XLOC_019956-3758

RI-AT3G61210-XLOC_019956-3758-0
GTCGCTGAGTACTACCAGAAAGTGGTGGCAACTGATATAAATGAATCACA
RI-AT3G61210-XLOC_019956-3758-1
GTCGCTGAGTACTACCAGAAAGTGGTGGCAACTGATATAAATGAATCACA
CONSENSUS
GTCGCTGAGTACTACCAGAAAGTGGTGGCAACTGATATAAATGAATCACA

RI-AT3G61210-XLOC_019956-3758-0
ACTCCAACGTGCAATGAAGCACCCAAAAGTCACTTACTATCACACTCCAT
RI-AT3G61210-XLOC_019956-3758-1
ACTCCAACGTGCAATGAAGCACCCAAAAGTCACTTACTATCACACTCCAT
CONSENSUS
ACTCCAACGTGCAATGAAGCACCCAAAAGTCACTTACTATCACACTCCAT

RI-AT3G61210-XLOC_019956-3758-0
CATCAATGTCCGACGACGATCTAGTGACTCTACTCGGTGGAGAAAACTCC
RI-AT3G61210-XLOC_019956-3758-1
CATCAATGTCCGACGACGATCTAGTGACTCTACTCGGTGGAGAAAACTCC
CONSENSUS
CATCAATGTCCGACGACGATCTAGTGACTCTACTCGGTGGAGAAAACTCC

RI-AT3G61210-XLOC_019956-3758-0
ATAGATATCATAATAGCCGCTCAAGCTCTTCACTACTTCGACCTAAAGAG
RI-AT3G61210-XLOC_019956-3758-1
ATAGATATCATAATAGCCGCTCAAGCTCTTCACTACTTCGACCTAAAGAG
CONSENSUS
ATAGATATCATAATAGCCGCTCAAGCTCTTCACTACTTCGACCTAAAGAG

RI-AT3G61210-XLOC_019956-3758-0
ATTCTACCCCATAGTGAAACGTGTTCTTCGTAAACAAGGCGGTATCATCG
RI-AT3G61210-XLOC_019956-3758-1
ATTCTACCCCATAGTGAAACGTGTTCTTCGTAAACAAGGCGGTATCATCG

CONSENSUS
ATTCTACCCCATAGTGAAACGTGTTCTTCGTAAACAAGGCGGTATCATCG

RI-AT3G61210-XLOC_019956-3758-0
TAGTTTGGGTTTACAACGATCTCATCATCACCCCAAAGGTTGATTCCATC
RI-AT3G61210-XLOC_019956-3758-1
TAGTTTGGGTTTACAACGATCTCATCATCACCCCAAAGGTTGATTCCATC
CONSENSUS
TAGTTTGGGTTTACAACGATCTCATCATCACCCCAAAGGTTGATTCCATC

RI-AT3G61210-XLOC_019956-3758-0
ATGAAACGTCTTGTGGATTCAACATTACCTTACAGAAACCCGACTATGAA
RI-AT3G61210-XLOC_019956-3758-1
ATGAAACGTCTTGTGGATTCAACATTACCTTACAGAAACCCGACTATGAA
CONSENSUS
ATGAAACGTCTTGTGGATTCAACATTACCTTACAGAAACCCGACTATGAA

RI-AT3G61210-XLOC_019956-3758-0
TTTGGCGTTTGATGGTTATAAAACAATAGAGTTTCCTTTTAAAAACATTA
RI-AT3G61210-XLOC_019956-3758-1
TTTGGCGTTTGATGGTTATAAAACAATAGAGTTTCCTTTTAAAAACATTA
CONSENSUS
TTTGGCGTTTGATGGTTATAAAACAATAGAGTTTCCTTTTAAAAACATTA

RI-AT3G61210-XLOC_019956-3758-0
GGATGGGGACTCAAGGAAGGCCTAAAGCTCTTGACATCCCACACATGCTT
RI-AT3G61210-XLOC_019956-3758-1
GGATGGGGACTCAAGGAAGGCCTAAAGCTCTTGACATCCCACACATGCTT
CONSENSUS
GGATGGGGACTCAAGGAAGGCCTAAAGCTCTTGACATCCCACACATGCTT

RI-AT3G61210-XLOC_019956-3758-0
TCGCTAGATGGATTTCTAGGGTTCTTAAAATCATGGCAGCCTCTAGTGAA
RI-AT3G61210-XLOC_019956-3758-1
TCGCTAGATGGATTTCTAGGGTTCTTAAAATCATGGCAGCCTCTAGTGAA
CONSENSUS
TCGCTAGATGGATTTCTAGGGTTCTTAAAATCATGGCAGCCTCTAGTGAA

RI-AT3G61210-XLOC_019956-3758-0
AGCAAAGGAGCAAGGAGAGGATCTTTTAACTTCTTATATGATTGATGAGT
RI-AT3G61210-XLOC_019956-3758-1
AGCAAAGGAGCAAGGAGAGGATCTTTTAACTTCTTATATGATTGATGAGT
CONSENSUS
AGCAAAGGAGCAAGGAGAGGATCTTTTAACTTCTTATATGATTGATGAGT

RI-AT3G61210-XLOC_019956-3758-0
TTAAGGAAGCTTGGGGTGATGATGAACAAGTCAAGGATGTTCACTACAAG
RI-AT3G61210-XLOC_019956-3758-1
TTAAGGAAGCTTGGGGTGATGATGAACAAGTCAAGGATGTTCACTACAAG
CONSENSUS
TTAAGGAAGCTTGGGGTGATGATGAACAAGTCAAGGATGTTCACTACAAG

RI-AT3G61210-XLOC_019956-3758-0
GCATTTATGCTTGCAGGGAAACTTTAGGTAATTAATATATGTTCCCCTTT
RI-AT3G61210-XLOC_019956-3758-1
GCATTTATGCTTGCAGGGAAACTTTAG-----------------------

CONSENSUS
      GCATTTATGCTTGCAGGGAAACTTTAG.......................

RI-AT3G61210-XLOC_019956-3758-0
      GAGATATATATTAACTATATGTCATACACGCATGTCATCAATGATCAATC
RI-AT3G61210-XLOC_019956-3758-1

CONSENSUS
      ....

RI-AT3G61210-XLOC_019956-3758-0
      GAATTAGGATTGAAAACTTATGTTTAACAGGTCTTGAGAGCAACCTGGTT
RI-AT3G61210-XLOC_019956-3758-1 ------------------------------
GTCTTGAGAGCAACCTGGTT
CONSENSUS
      ..............................GTCTTGAGAGCAACCTGGTT

RI-AT3G61210-XLOC_019956-3758-0
      GATTTAAATGCGGATCATTACTTTGTATAACTTTCGTTTCTGTATAAATT
RI-AT3G61210-XLOC_019956-3758-1
      GATTTAAATGCGGATCATTACTTTGTATAACTTTCGTTTCTGTATAAATT
CONSENSUS
      GATTTAAATGCGGATCATTACTTTGTATAACTTTCGTTTCTGTATAAATT

RI-AT3G61210-XLOC_019956-3758-0 AATATATTAATTAATATATTTATAGCACTG
RI-AT3G61210-XLOC_019956-3758-1 AATATATTAATTAATATATTTATAGCACTG
CONSENSUS   AATATATTAATTAATATATTTATAGCACTG

alignment for event: RI-AT3G14205-XLOC_017561-13878

RI-AT3G14205-XLOC_017561-13878-0
      GATTCATAAGGTTCTTGGGACCTTATTACATGCTGATAATCACTAAAAGA
RI-AT3G14205-XLOC_017561-13878-1
      GATTCATAAGGTTCTTGGGACCTTATTACATGCTGATAATCACTAAAAGA
CONSENSUS
      GATTCATAAGGTTCTTGGGACCTTATTACATGCTGATAATCACTAAAAGA

RI-AT3G14205-XLOC_017561-13878-0
      AAGAAGCTTGGTGAAATTTGTGGGCACACTGTTTATGGTGTTGCGAAGAG
RI-AT3G14205-XLOC_017561-13878-1
      AAGAAGCTTGGTGAAATTTGTGGGCACACTGTTTATGGTGTTGCGAAGAG
CONSENSUS
      AAGAAGCTTGGTGAAATTTGTGGGCACACTGTTTATGGTGTTGCGAAGAG

RI-AT3G14205-XLOC_017561-13878-0
      CAAGATCATCACGATTCCACATGCCTCTGTGCTATCCAATGTGGCTTATT
RI-AT3G14205-XLOC_017561-13878-1
      CAAGATCATCACGATTCCACATGCCTCTGTGCTATCCAATGTGGCTTATT
CONSENSUS
      CAAGATCATCACGATTCCACATGCCTCTGTGCTATCCAATGTGGCTTATT

RI-AT3G14205-XLOC_017561-13878-0
      CTAAGGACGAGAAAAGGTTTTCTTATGTTACTTCTGCTTGTAAAATGGAA
RI-AT3G14205-XLOC_017561-13878-1

CTAAGGACGAGAAAAG----------------------------------
CONSENSUS
CTAAGGACGAGAAAAG..................................

RI-AT3G14205-XLOC_017561-13878-0
AACCTGTACCTTGCTACCTTTATCCTTACATTACTTTTCGAAATTATAAA
RI-AT3G14205-XLOC_017561-13878-1

CONSENSUS
....

RI-AT3G14205-XLOC_017561-13878-0
GCTTTATCACTTGTATTTAGATTTTAAGACCATTCTGCTATCTTTTTTTT
RI-AT3G14205-XLOC_017561-13878-1

CONSENSUS
....

RI-AT3G14205-XLOC_017561-13878-0
TCTCTCTATTGTGACCCTCTTTGATCTGCACAATAATAGTGTCACAGGTT
RI-AT3G14205-XLOC_017561-13878-1

CONSENSUS
....

RI-AT3G14205-XLOC_017561-13878-0
TCTATTAGACAAGTTAACAGTTGTTGAGGTAACTGTGATTGAATTCGTTA
RI-AT3G14205-XLOC_017561-13878-1

CONSENSUS
....

RI-AT3G14205-XLOC_017561-13878-0
CATGTTCCTCTTTACTTTGTCTATCGCTGTTGGAGATAATCCAGCAGCTT
RI-AT3G14205-XLOC_017561-13878-1

CONSENSUS
....

RI-AT3G14205-XLOC_017561-13878-0
AGAGTTTCAAATCTTATTTAGTCTGTAGTGGGACAATTTTTTGTTGCGCA
RI-AT3G14205-XLOC_017561-13878-1

CONSENSUS
....

RI-AT3G14205-XLOC_017561-13878-0
TTTTTGCTTTTTACTATTTCTAGGTATTCTTCTCCTCTGTTTGTACAGTG
RI-AT3G14205-XLOC_017561-13878-1

CONSENSUS
....

RI-AT3G14205-XLOC_017561-13878-0
GACACACTTCTATTTGTTGTATTTATCTTGATATGTTATATGACAGAGGT
RI-AT3G14205-XLOC_017561-13878-1

CONSENSUS
....

RI-AT3G14205-XLOC_017561-13878-0
ACTATCAAGAAAGGGAGAGCTCATTTTTATTCTCTCCTACTGTCATTTAA
RI-AT3G14205-XLOC_017561-13878-1

CONSENSUS
....

RI-AT3G14205-XLOC_017561-13878-0
TCTGTTATATTGTGAACTTTGTATTATCTTGTTAACTTCCTTCTTTTTCA
RI-AT3G14205-XLOC_017561-13878-1

CONSENSUS
....

RI-AT3G14205-XLOC_017561-13878-0
GATACTGATCCAGGTTCTAAATCATGTTTGGTTTTTGTTTTCTTCTGTCT
RI-AT3G14205-XLOC_017561-13878-1

CONSENSUS
....

RI-AT3G14205-XLOC_017561-13878-0
TCCATGGAAATTTTGTAGGTACAAGAGGCTGCTCTGCACTGTTGATCTCA
RI-AT3G14205-XLOC_017561-13878-1 ------------------
GTACAAGAGGCTGCTCTGCACTGTTGATCTCA
CONSENSUS
..................GTACAAGAGGCTGCTCTGCACTGTTGATCTCA

RI-AT3G14205-XLOC_017561-13878-0
CAAAGGACTTCTTCTTCAGCTATTCCTATCACATCATGCATACACTTCAG
RI-AT3G14205-XLOC_017561-13878-1
CAAAGGACTTCTTCTTCAGCTATTCCTATCACATCATGCATACACTTCAG
CONSENSUS
CAAAGGACTTCTTCTTCAGCTATTCCTATCACATCATGCATACACTTCAG

RI-AT3G14205-XLOC_017561-13878-0
AGGAATCTGTCTAACAATGTGGAAGGACACACTTACTACGAATCGATGTT
RI-AT3G14205-XLOC_017561-13878-1
AGGAATCTGTCTAACAATGTGGAAGGACACACTTACTACGAATCGATGTT
CONSENSUS
AGGAATCTGTCTAACAATGTGGAAGGACACACTTACTACGAATCGATGTT

RI-AT3G14205-XLOC_017561-13878-0
TGTCTGGAACGAATACTTAACTCGAAGGATTCGGAATAACGTGAAGGACT
RI-AT3G14205-XLOC_017561-13878-1
TGTCTGGAACGAATACTTAACTCGAAGGATTCGGAATAACGTGAAGGACT
CONSENSUS
TGTCTGGAACGAATACTTAACTCGAAGGATTCGGAATAACGTGAAGGACT

RI-AT3G14205-XLOC_017561-13878-0
GTATGTGGACAGTTGCCTTGGTATATGGATTTTTCAAACAG
RI-AT3G14205-XLOC_017561-13878-1

GTATGTGGACAGTTGCCTTGGTATATGGATTTTTCAAACAG
CONSENSUS
GTATGTGGACAGTTGCCTTGGTATATGGATTTTTCAAACAG

alignment for event: RI-AT3G05030-XLOC_017015-1861

RI-AT3G05030-XLOC_017015-1861-0
TTTACAAGATCAGGGCACACAGAATTGCGCGGGAATGCAATCATGATTAC
RI-AT3G05030-XLOC_017015-1861-1
TTTACAAGATCAGGGCACACAGAATTGCGCGGGAATGCAATCATGATTAC
CONSENSUS
TTTACAAGATCAGGGCACACAGAATTGCGCGGGAATGCAATCATGATTAC

RI-AT3G05030-XLOC_017015-1861-0
CAGTACAATAACCGTCTGTCTTTTTAGCACCATGGTAAGATATCTCCCTC
RI-AT3G05030-XLOC_017015-1861-1
CAGTACAATAACCGTCTGTCTTTTTAGCACCATG----------------
CONSENSUS
CAGTACAATAACCGTCTGTCTTTTTAGCACCATG................

RI-AT3G05030-XLOC_017015-1861-0
TCAGGTTTGGACTACTAAATGTATTTTCTCTACAGAGATGTTGTCAATGA
RI-AT3G05030-XLOC_017015-1861-1

CONSENSUS
....

RI-AT3G05030-XLOC_017015-1861-0
GGATAAACCAATGCTGAGCCGTGAGACGGATTTGCATTTTCTATATCCGG
RI-AT3G05030-XLOC_017015-1861-1

CONSENSUS
....

RI-AT3G05030-XLOC_017015-1861-0
AAAACTGTTCCCTCCTATTTGATTTGTTATTCCAATGTTTTCTGGATCGC
RI-AT3G05030-XLOC_017015-1861-1

CONSENSUS
....

RI-AT3G05030-XLOC_017015-1861-0
TAACACTGAACCCTGATACCGGACATTTAACATATGTTTTTTTTTTTCCA
RI-AT3G05030-XLOC_017015-1861-1

CONSENSUS
....

RI-AT3G05030-XLOC_017015-1861-0
CGGCTTTCAGGTGTTTGGTATGCTAACCAAACCACTGATTAGATACCTAA
RI-AT3G05030-XLOC_017015-1861-1 ----------
GTGTTTGGTATGCTAACCAAACCACTGATTAGATACCTAA
CONSENSUS
..........GTGTTTGGTATGCTAACCAAACCACTGATTAGATACCTAA

RI-AT3G05030-XLOC_017015-1861-0
TGCCACACCAAAAAGCGACCACCAGTACCACGAGTATGTTATCGGACGAT
RI-AT3G05030-XLOC_017015-1861-1
TGCCACACCAAAAAGCGACCACCAGTACCACGAGTATGTTATCGGACGAT
CONSENSUS
TGCCACACCAAAAAGCGACCACCAGTACCACGAGTATGTTATCGGACGAT

RI-AT3G05030-XLOC_017015-1861-0
AGCACTCCGAAATCAATCCACATTCCGCTCCTCGATGGTGAACAGCTAGA
RI-AT3G05030-XLOC_017015-1861-1
AGCACTCCGAAATCAATCCACATTCCGCTCCTCGATGGTGAACAGCTAGA
CONSENSUS
AGCACTCCGAAATCAATCCACATTCCGCTCCTCGATGGTGAACAGCTAGA

RI-AT3G05030-XLOC_017015-1861-0
TTCATTTGAGTTACCTGGGAGCCACCAGGACGTGCCACGACCAAACAGCC
RI-AT3G05030-XLOC_017015-1861-1
TTCATTTGAGTTACCTGGGAGCCACCAGGACGTGCCACGACCAAACAGCC
CONSENSUS
TTCATTTGAGTTACCTGGGAGCCACCAGGACGTGCCACGACCAAACAGCC

RI-AT3G05030-XLOC_017015-1861-0
TTCGAGGTTTCCTCATGCGCCCCACACGGACTGTCCACTATTACTGGAGA
RI-AT3G05030-XLOC_017015-1861-1
TTCGAGGTTTCCTCATGCGCCCCACACGGACTGTCCACTATTACTGGAGA
CONSENSUS
TTCGAGGTTTCCTCATGCGCCCCACACGGACTGTCCACTATTACTGGAGA

RI-AT3G05030-XLOC_017015-1861-0
CAGTTTGATGATGCCTTCATGCGTCCTGTGTTTGGTGGTCGCGGATTCGT
RI-AT3G05030-XLOC_017015-1861-1
CAGTTTGATGATGCCTTCATGCGTCCTGTGTTTGGTGGTCGCGGATTCGT
CONSENSUS
CAGTTTGATGATGCCTTCATGCGTCCTGTGTTTGGTGGTCGCGGATTCGT

RI-AT3G05030-XLOC_017015-1861-0
TCCCTTTGTCCCTGGTTCTCCGACTGAGAGAAGCAGCCATGATCTTAGTA
RI-AT3G05030-XLOC_017015-1861-1
TCCCTTTGTCCCTGGTTCTCCGACTGAGAGAAGCAGCCATGATCTTAGTA
CONSENSUS
TCCCTTTGTCCCTGGTTCTCCGACTGAGAGAAGCAGCCATGATCTTAGTA

RI-AT3G05030-XLOC_017015-1861-0
AACCTTGAGGAGAAAGATATATAGAAACTTAACCAAAAAACTTCTTCTTG
RI-AT3G05030-XLOC_017015-1861-1
AACCTTGAGGAGAAAGATATATAGAAACTTAACCAAAAAACTTCTTCTTG
CONSENSUS
AACCTTGAGGAGAAAGATATATAGAAACTTAACCAAAAAACTTCTTCTTG

RI-AT3G05030-XLOC_017015-1861-0
CTCTTCCCTCTTATGGTGACTAGTATTGGTGATGTAAATGTATTTTTCGT
RI-AT3G05030-XLOC_017015-1861-1
CTCTTCCCTCTTATGGTGACTAGTATTGGTGATGTAAATGTATTTTTCGT
CONSENSUS
CTCTTCCCTCTTATGGTGACTAGTATTGGTGATGTAAATGTATTTTTCGT

RI-AT3G05030-XLOC_017015-1861-0
TCTTCAAATTTACATATTCTTCTGTAAATTTGTTATTATTCGATGATGAA
RI-AT3G05030-XLOC_017015-1861-1
TCTTCAAATTTACATATTCTTCTGTAAATTTGTTATTATTCGATGATGAA
CONSENSUS
TCTTCAAATTTACATATTCTTCTGTAAATTTGTTATTATTCGATGATGAA

RI-AT3G05030-XLOC_017015-1861-0
GAAGCTTCTTACGTTTTTGAGAGACGTGTGGG
RI-AT3G05030-XLOC_017015-1861-1
GAAGCTTCTTACGTTTTTGAGAGACGTGTGGG
CONSENSUS
GAAGCTTCTTACGTTTTTGAGAGACGTGTGGG

alignment for event: A3-AT3G62310-XLOC_020022-9256

A3-AT3G62310-XLOC_020022-9256-0
GTACTTGACTGATGGTATGCTCCTGAGAGAAGCAATGGCGGACCCTCTTC
A3-AT3G62310-XLOC_020022-9256-1
GTACTTGACTGATGGTATGCTCCTGAGAGAAGCAATGGCGGACCCTCTTC
CONSENSUS
GTACTTGACTGATGGTATGCTCCTGAGAGAAGCAATGGCGGACCCTCTTC

A3-AT3G62310-XLOC_020022-9256-0
TAGAGAGATACAAAGTCATTATTCTTGATGAGGCCCACGAAAGGACTCTA
A3-AT3G62310-XLOC_020022-9256-1
TAGAGAGATACAAAGTCATTATTCTTGATGAGGCCCACGAAAGGACTCTA
CONSENSUS
TAGAGAGATACAAAGTCATTATTCTTGATGAGGCCCACGAAAGGACTCTA

A3-AT3G62310-XLOC_020022-9256-0
GCCACAGATGTGCTGTTTGGTCTTCTGAAAGAAGTGTTGAAAAATAGACC
A3-AT3G62310-XLOC_020022-9256-1
GCCACAGATGTGCTGTTTGGTCTTCTGAAAGAAGTGTTGAAAAATAGACC
CONSENSUS
GCCACAGATGTGCTGTTTGGTCTTCTGAAAGAAGTGTTGAAAAATAGACC

A3-AT3G62310-XLOC_020022-9256-0
TGATCTTAAGTTAGTTGTGATGAGTGCAACTCTAGAAGCTGAAAAGTTTC
A3-AT3G62310-XLOC_020022-9256-1
TGATCTTAAGTTAGTTGTGATGAGTGCAACTCTAGAAGCTGAAAAGTTTC
CONSENSUS
TGATCTTAAGTTAGTTGTGATGAGTGCAACTCTAGAAGCTGAAAAGTTTC

A3-AT3G62310-XLOC_020022-9256-0
AGGATTATTTTAGCGGTGCTCCTCTGATGAAAGTTCCTGGTAGGCTTCAT
A3-AT3G62310-XLOC_020022-9256-1
AGGATTATTTTAGCGGTGCTCCTCTGATGAAAGTTCCTGGTAGGCTTCAT
CONSENSUS
AGGATTATTTTAGCGGTGCTCCTCTGATGAAAGTTCCTGGTAGGCTTCAT

A3-AT3G62310-XLOC_020022-9256-0
CCAGTTGAGATCTTTTACACTCAGGAGCCTGAGAGGGACTATCTTGAGGC

A3-AT3G62310-XLOC_020022-9256-1
CCAGTTGAGATCTTTTACACTCAGGAGCCTGAGAGGGACTATCTTGAGGC
CONSENSUS
CCAGTTGAGATCTTTTACACTCAGGAGCCTGAGAGGGACTATCTTGAGGC

A3-AT3G62310-XLOC_020022-9256-0
TGCTATTAGAACTGTTGTCCAGATTCACATGTGTGAGCCACCTGGCGATA
A3-AT3G62310-XLOC_020022-9256-1
TGCTATTAGAACTGTTGTCCAGATTCACATGTGTGAGCCACCTGGCGATA
CONSENSUS
TGCTATTAGAACTGTTGTCCAGATTCACATGTGTGAGCCACCTGGCGATA

A3-AT3G62310-XLOC_020022-9256-0
TTCTTGTTTTCTTAACTGGGGAGGAGGAGATTGAAGATGCATGCCGCAAA
A3-AT3G62310-XLOC_020022-9256-1
TTCTTGTTTTCTTAACTGGGGAGGAGGAGATTGAAGATGCATGCCGCAAA
CONSENSUS
TTCTTGTTTTCTTAACTGGGGAGGAGGAGATTGAAGATGCATGCCGCAAA

A3-AT3G62310-XLOC_020022-9256-0
ATCAATAAAGAGGTTGGTAATCTTGGGGATCAAGTGGGGCCTATTAAAGT
A3-AT3G62310-XLOC_020022-9256-1
ATCAATAAAGAGGTTGGTAATCTTGGGGATCAAGTGGGGCCTATTAAAGT
CONSENSUS
ATCAATAAAGAGGTTGGTAATCTTGGGGATCAAGTGGGGCCTATTAAAGT

A3-AT3G62310-XLOC_020022-9256-0
AGTGCCACTGTATTCTACTCTTCCACCTGCGATGCAGCAGAAGATATTTG
A3-AT3G62310-XLOC_020022-9256-1
AGTGCCACTGTATTCTACTCTTCCACCTGCGATGCAGCAGAAGATATTTG
CONSENSUS
AGTGCCACTGTATTCTACTCTTCCACCTGCGATGCAGCAGAAGATATTTG

A3-AT3G62310-XLOC_020022-9256-0
ATCCTGCTCCAGAGCCTGTAACAGAAGGTGGTCCTCCTGGGAGAAAGATT
A3-AT3G62310-XLOC_020022-9256-1
ATCCTGCTCCAGAGCCTGTAACAGAAGGTGGTCCTCCTGGGAGAAAGATT
CONSENSUS
ATCCTGCTCCAGAGCCTGTAACAGAAGGTGGTCCTCCTGGGAGAAAGATT

A3-AT3G62310-XLOC_020022-9256-0
GTTGTCTCCACTAACATTGCTGAGACTTCTCTCACCATAGATGGGATTGT
A3-AT3G62310-XLOC_020022-9256-1
GTTGTCTCCACTAACATTGCTGAGACTTCTCTCACCATAGATGGGATTGT
CONSENSUS
GTTGTCTCCACTAACATTGCTGAGACTTCTCTCACCATAGATGGGATTGT

A3-AT3G62310-XLOC_020022-9256-0
CTACGTCATTGACCCTGGTTTTGCTAAGCAAAAAGTCTATAACCCTCGAA
A3-AT3G62310-XLOC_020022-9256-1
CTACGTCATTGACCCTGGTTTTGCTAAGCAAAAAGTCTATAACCCTCGAA
CONSENSUS
CTACGTCATTGACCCTGGTTTTGCTAAGCAAAAAGTCTATAACCCTCGAA

A3-AT3G62310-XLOC_020022-9256-0
TCCGTGTTGAGTCTTTGTTGGTGTCCCCAATTTCAAAGGCAAGTGCTCAC

A3-AT3G62310-XLOC_020022-9256-1
TCCGTGTTGAGTCTTTGTTGGTGTCCCCAATTTCAAAGGCAAGTGCTCAC
CONSENSUS
TCCGTGTTGAGTCTTTGTTGGTGTCCCCAATTTCAAAGGCAAGTGCTCAC

A3-AT3G62310-XLOC_020022-9256-0
CAGAGATCTGGTCGTGCCGGTAGAACACGGCCTGGAAAATGCTTCAGGCT
A3-AT3G62310-XLOC_020022-9256-1
CAGAGATCTGGTCGTGCCGGTAGAACACGGCCTGGAAAATGCTTCAGGCT
CONSENSUS
CAGAGATCTGGTCGTGCCGGTAGAACACGGCCTGGAAAATGCTTCAGGCT

A3-AT3G62310-XLOC_020022-9256-0
TTACACAGAGAAGAGTTTTAATAATGACTTGCAGCCGCAGACGTATCCTG
A3-AT3G62310-XLOC_020022-9256-1
TTACACAGAGAAGAGTTTTAATAATGACTTGCAGCCGCAGACGTATCCTG
CONSENSUS
TTACACAGAGAAGAGTTTTAATAATGACTTGCAGCCGCAGACGTATCCTG

A3-AT3G62310-XLOC_020022-9256-0
AAATATTGAGATCAAACCTTGCAAATACAGTTCTGACCTTGAAAAAACTG
A3-AT3G62310-XLOC_020022-9256-1
AAATATTGAGATCAAACCTTGCAAATACAGTTCTGACCTTGAAAAAACTG
CONSENSUS
AAATATTGAGATCAAACCTTGCAAATACAGTTCTGACCTTGAAAAAACTG

A3-AT3G62310-XLOC_020022-9256-0
GGTATTGATGACTTGGTGCACTTTGATTTTATGGATCCCCCTGCTCCCGA
A3-AT3G62310-XLOC_020022-9256-1
GGTATTGATGACTTGGTGCACTTTGATTTTATGGATCCCCCTGCTCCCGA
CONSENSUS
GGTATTGATGACTTGGTGCACTTTGATTTTATGGATCCCCCTGCTCCCGA

A3-AT3G62310-XLOC_020022-9256-0
AACACTGATGCGAGCCTTAGAAGTTTTGAATTACTTGGGAGCACTGGATG
A3-AT3G62310-XLOC_020022-9256-1
AACACTGATGCGAGCCTTAGAAGTTTTGAATTACTTGGGAGCACTGGATG
CONSENSUS
AACACTGATGCGAGCCTTAGAAGTTTTGAATTACTTGGGAGCACTGGATG

A3-AT3G62310-XLOC_020022-9256-0
ATGATGGTAACTTGACAAAGACAGGTGAGATCATGAGTGAGTTCCCCTTG
A3-AT3G62310-XLOC_020022-9256-1
ATGATGGTAACTTGACAAAGACAGGTGAGATCATGAGTGAGTTCCCCTTG
CONSENSUS
ATGATGGTAACTTGACAAAGACAGGTGAGATCATGAGTGAGTTCCCCTTG

A3-AT3G62310-XLOC_020022-9256-0
GATCCACAGATGGCAAAGATGCTCATAGTCAGTCCTGAATTCAACTGCTC
A3-AT3G62310-XLOC_020022-9256-1
GATCCACAGATGGCAAAGATGCTCATAGTCAGTCCTGAATTCAACTGCTC
CONSENSUS
GATCCACAGATGGCAAAGATGCTCATAGTCAGTCCTGAATTCAACTGCTC

A3-AT3G62310-XLOC_020022-9256-0
AAACGAGATTCTCTCAGTTTCAGCTATGCTATCAG---------------

A3-AT3G62310-XLOC_020022-9256-1
      AAACGAGATTCTCTCAGTTTCAGCTATGCTATCAGTTGCAGTTTCTCAGT
CONSENSUS
      AAACGAGATTCTCTCAGTTTCAGCTATGCTATCAG...............

A3-AT3G62310-XLOC_020022-9256-0

A3-AT3G62310-XLOC_020022-9256-1
      TATTCTCTCTCATGAAGCAAATGGTATCAGTCTCATCGCCTCTATGCATC
CONSENSUS
      ....

A3-AT3G62310-XLOC_020022-9256-0

A3-AT3G62310-XLOC_020022-9256-1
      TGCTGACCTCTTCCCTGGACCTCTTCTGTTATAATTACCAATTCTTTCTC
CONSENSUS
      ....

A3-AT3G62310-XLOC_020022-9256-0 ---
TACCGAATTGCTTTATTCGGCCTAGAGAGGCTCAAAAAGCAGCAGAT
A3-AT3G62310-XLOC_020022-9256-1
      TAGTACCGAATTGCTTTATTCGGCCTAGAGAGGCTCAAAAAGCAGCAGAT
CONSENSUS
      ...TACCGAATTGCTTTATTCGGCCTAGAGAGGCTCAAAAAGCAGCAGAT

A3-AT3G62310-XLOC_020022-9256-0
      GAAGCTAAAGCTAGGTTTGGACACATTGAGGGAGATCACCTCACATTGTT
A3-AT3G62310-XLOC_020022-9256-1
      GAAGCTAAAGCTAGGTTTGGACACATTGAGGGAGATCACCTCACATTGTT
CONSENSUS
      GAAGCTAAAGCTAGGTTTGGACACATTGAGGGAGATCACCTCACATTGTT

A3-AT3G62310-XLOC_020022-9256-0 GAATGTCTACCACGCTTTCAAGCAAAACA
A3-AT3G62310-XLOC_020022-9256-1 GAATGTCTACCACGCTTTCAAGCAAAACA
CONSENSUS   GAATGTCTACCACGCTTTCAAGCAAAACA

alignment for event: A5-AT3G05030-XLOC_017015-1863

A5-AT3G05030-XLOC_017015-1863-0
      GTGCAATTCAGTTCTTTAAGAAATTAGACATTGGGACCTTTGACTTGGGC
A5-AT3G05030-XLOC_017015-1863-1
      GTGCAATTCAGTTCTTTAAGAAATTAGACATTGGGACCTTTGACTTGGGC
CONSENSUS
      GTGCAATTCAGTTCTTTAAGAAATTAGACATTGGGACCTTTGACTTGGGC

A5-AT3G05030-XLOC_017015-1863-0
      GATTTTCTTGGTAATGTGTTAAGGAGTTTCTTTTATCGATTTTTTGTTCT
A5-AT3G05030-XLOC_017015-1863-1
      GATTTTCTTG--
CONSENSUS
      GATTTTCTTG..

A5-AT3G05030-XLOC_017015-1863-0

AGAAGACTTGATTTTTTTTTTGCTTACTTAATCAGCAATCGGCGCCATAT
A5-AT3G05030-XLOC_017015-1863-1

CONSENSUS
....

A5-AT3G05030-XLOC_017015-1863-0
TTGCTGCAACCGACTCTGTATGCACACTACAGGTTCTCAATCAAGATGAG
A5-AT3G05030-XLOC_017015-1863-1
--------------------------------GTTCTCAATCAAGATGAG
CONSENSUS
................................GTTCTCAATCAAGATGAG

A5-AT3G05030-XLOC_017015-1863-0
ACACCTTTGCTTTACAGTCTTGTATTTGGAGAGGGCGTTGTGAATGATGC
A5-AT3G05030-XLOC_017015-1863-1
ACACCTTTGCTTTACAGTCTTGTATTTGGAGAGGGCGTTGTGAATGATGC
CONSENSUS
ACACCTTTGCTTTACAGTCTTGTATTTGGAGAGGGCGTTGTGAATGATGC

A5-AT3G05030-XLOC_017015-1863-0
CACATCTGTTGTGCTCTTCAATGCTATTCAGAGTTTTGACCTCACCCACC
A5-AT3G05030-XLOC_017015-1863-1
CACATCTGTTGTGCTCTTCAATGCTATTCAGAGTTTTGACCTCACCCACC
CONSENSUS
CACATCTGTTGTGCTCTTCAATGCTATTCAGAGTTTTGACCTCACCCACC

A5-AT3G05030-XLOC_017015-1863-0
TTAACCATGAAGCAGCTTTTCAATTTCTTGGGAACTTTTTTTATCTGTTT
A5-AT3G05030-XLOC_017015-1863-1
TTAACCATGAAGCAGCTTTTCAATTTCTTGGGAACTTTTTTTATCTGTTT
CONSENSUS
TTAACCATGAAGCAGCTTTTCAATTTCTTGGGAACTTTTTTTATCTGTTT

A5-AT3G05030-XLOC_017015-1863-0 CTCTTGAGCACCGGACTTGGTGTCGCA
A5-AT3G05030-XLOC_017015-1863-1 CTCTTGAGCACCGGACTTGGTGTCGCA
CONSENSUS   CTCTTGAGCACCGGACTTGGTGTCGCA

alignment for event: A3-AT3G24120-XLOC_018081-5248

A3-AT3G24120-XLOC_018081-5248-0
GGGTTACCAAGTCACCGAAGCTCTACGTGCTCAGATGGAAGTCCAAAGAA
A3-AT3G24120-XLOC_018081-5248-1
GGGTTACCAAGTCACCGAAGCTCTACGTGCTCAGATGGAAGTCCAAAGAA
CONSENSUS
GGGTTACCAAGTCACCGAAGCTCTACGTGCTCAGATGGAAGTCCAAAGAA

A3-AT3G24120-XLOC_018081-5248-0 GACTACACGATCAATTGGAG---------
GTGCAACGGAGGCTCCAGCTG
A3-AT3G24120-XLOC_018081-5248-1
GACTACACGATCAATTGGAGTATGGGCAGGTGCAACGGAGGCTCCAGCTG
CONSENSUS
GACTACACGATCAATTGGAG.........GTGCAACGGAGGCTCCAGCTG

A3-AT3G24120-XLOC_018081-5248-0
AGGATAGAGGCACAAGGAAAATACCTGCAATCGATTCTTGAAAAAGCTTG
A3-AT3G24120-XLOC_018081-5248-1
AGGATAGAGGCACAAGGAAAATACCTGCAATCGATTCTTGAAAAAGCTTG
CONSENSUS
AGGATAGAGGCACAAGGAAAATACCTGCAATCGATTCTTGAAAAAGCTTG

A3-AT3G24120-XLOC_018081-5248-0
CAAGGCCTTTGACGAGCAAGCTGCTACTTTTGCTGGACTTGAGGCTGCTA
A3-AT3G24120-XLOC_018081-5248-1
CAAGGCCTTTGACGAGCAAGCTGCTACTTTTGCTGGACTTGAGGCTGCTA
CONSENSUS
CAAGGCCTTTGACGAGCAAGCTGCTACTTTTGCTGGACTTGAGGCTGCTA

A3-AT3G24120-XLOC_018081-5248-0
GGGAAGAGCTATCAGAGCTAGCCATCAAAGTCTCCAATAGCTCTCAAGGA
A3-AT3G24120-XLOC_018081-5248-1
GGGAAGAGCTATCAGAGCTAGCCATCAAAGTCTCCAATAGCTCTCAAGGA
CONSENSUS
GGGAAGAGCTATCAGAGCTAGCCATCAAAGTCTCCAATAGCTCTCAAGGA

A3-AT3G24120-XLOC_018081-5248-0
ACATCAGTCCCGTACTTCGATGCAACAAAGATGATGATGATGCCATCGTT
A3-AT3G24120-XLOC_018081-5248-1
ACATCAGTCCCGTACTTCGATGCAACAAAGATGATGATGATGCCATCGTT
CONSENSUS
ACATCAGTCCCGTACTTCGATGCAACAAAGATGATGATGATGCCATCGTT

A3-AT3G24120-XLOC_018081-5248-0
GTCAGAGCTTGCAGTAGCAATAGACAACAAAAACAACATCACAACCAACT
A3-AT3G24120-XLOC_018081-5248-1
GTCAGAGCTTGCAGTAGCAATAGACAACAAAAACAACATCACAACCAACT
CONSENSUS
GTCAGAGCTTGCAGTAGCAATAGACAACAAAAACAACATCACAACCAACT

A3-AT3G24120-XLOC_018081-5248-0
GTTCAGTAGAAAGCTCTCTGACTTCCATCACACATGGGAGCTCTATATCT
A3-AT3G24120-XLOC_018081-5248-1
GTTCAGTAGAAAGCTCTCTGACTTCCATCACACATGGGAGCTCTATATCT
CONSENSUS
GTTCAGTAGAAAGCTCTCTGACTTCCATCACACATGGGAGCTCTATATCT

A3-AT3G24120-XLOC_018081-5248-0
GCTGCATCAATGAAGAAGCGTCAACGTGGAGACAATTTGGGCGTAGGGTA
A3-AT3G24120-XLOC_018081-5248-1
GCTGCATCAATGAAGAAGCGTCAACGTGGAGACAATTTGGGCGTAGGGTA
CONSENSUS
GCTGCATCAATGAAGAAGCGTCAACGTGGAGACAATTTGGGCGTAGGGTA

A3-AT3G24120-XLOC_018081-5248-0
TGAATCAGGCTGGATTATGCCTAGTAGCACCATTGGATAAAGTTTAGGAG
A3-AT3G24120-XLOC_018081-5248-1
TGAATCAGGCTGGATTATGCCTAGTAGCACCATTGGATAAAGTTTAGGAG
CONSENSUS
TGAATCAGGCTGGATTATGCCTAGTAGCACCATTGGATAAAGTTTAGGAG

A3-AT3G24120-XLOC_018081-5248-0
AGGGAAAAAGTTCATTATGGGAAAGGTAGAGATAAGATTTAACTCTTCTT
A3-AT3G24120-XLOC_018081-5248-1
AGGGAAAAAGTTCATTATGGGAAAGGTAGAGATAAGATTTAACTCTTCTT
CONSENSUS
AGGGAAAAAGTTCATTATGGGAAAGGTAGAGATAAGATTTAACTCTTCTT

A3-AT3G24120-XLOC_018081-5248-0
TACTTGCTTTGAGGGGCCTGTATTATTATTCGATACAAACTTGTCATCAC
A3-AT3G24120-XLOC_018081-5248-1
TACTTGCTTTGAGGGGCCTGTATTATTATTCGATACAAACTTGTCATCAC
CONSENSUS
TACTTGCTTTGAGGGGCCTGTATTATTATTCGATACAAACTTGTCATCAC

A3-AT3G24120-XLOC_018081-5248-0
ATTTTCATCTTAGCCTACTCATGTATATATATTTCAATGTTGAAGCTGAA
A3-AT3G24120-XLOC_018081-5248-1
ATTTTCATCTTAGCCTACTCATGTATATATATTTCAATGTTGAAGCTGAA
CONSENSUS
ATTTTCATCTTAGCCTACTCATGTATATATATTTCAATGTTGAAGCTGAA

A3-AT3G24120-XLOC_018081-5248-0
GCTTTAGTGTTTCCTTTAACCTTTGCCTCTAATTATTCTTGTATTCAAGA
A3-AT3G24120-XLOC_018081-5248-1
GCTTTAGTGTTTCCTTTAACCTTTGCCTCTAATTATTCTTGTATTCAAGA
CONSENSUS
GCTTTAGTGTTTCCTTTAACCTTTGCCTCTAATTATTCTTGTATTCAAGA

A3-AT3G24120-XLOC_018081-5248-0
GAAAGGTTTAAAGGTACAAACTATAAAGACATAAGAAAAAGATCTATTGA
A3-AT3G24120-XLOC_018081-5248-1
GAAAGGTTTAAAGGTACAAACTATAAAGACATAAGAAAAAGATCTATTGA
CONSENSUS
GAAAGGTTTAAAGGTACAAACTATAAAGACATAAGAAAAAGATCTATTGA

A3-AT3G24120-XLOC_018081-5248-0
TAATCTATAATAAACATTGAAATGATGAAAGGGCCACCAAAGTTTAAGCC
A3-AT3G24120-XLOC_018081-5248-1
TAATCTATAATAAACATTGAAATGATGAAAGGGCCACCAAAGTTTAAGCC
CONSENSUS
TAATCTATAATAAACATTGAAATGATGAAAGGGCCACCAAAGTTTAAGCC

A3-AT3G24120-XLOC_018081-5248-0
TTTAACTTTGATACTATTCGTAATGCCTTTCTTCATTACTCTTTATGCTT
A3-AT3G24120-XLOC_018081-5248-1
TTTAACTTTGATACTATTCGTAATGCCTTTCTTCATTACTCTTTATGCTT
CONSENSUS
TTTAACTTTGATACTATTCGTAATGCCTTTCTTCATTACTCTTTATGCTT

A3-AT3G24120-XLOC_018081-5248-0
TACTTTATCTACTTACGCCTTGTTCCTCTCATCCATCTTTTCAACAAACA
A3-AT3G24120-XLOC_018081-5248-1
TACTTTATCTACTTACGCCTTGTTCCTCTCATCCATCTTTTCAACAAACA
CONSENSUS
TACTTTATCTACTTACGCCTTGTTCCTCTCATCCATCTTTTCAACAAACA

A3-AT3G24120-XLOC_018081-5248-0 TCAGAATTAACATGA
A3-AT3G24120-XLOC_018081-5248-1 TCAGAATTAACATGA
CONSENSUS   TCAGAATTAACATGA

alignment for event: A5-AT3G24760-XLOC_014906-10936

A5-AT3G24760-XLOC_014906-10936-0
GACTCTCGTTTGCTTACTTGAATTCTTGTAATGGTATGCTTGTTCTTGGT
A5-AT3G24760-XLOC_014906-10936-1
GACTCTCGTTTGCTTACTTGAATTCTTGTAATGGTATGCTTGTTCTTGGT
CONSENSUS
GACTCTCGTTTGCTTACTTGAATTCTTGTAATGGTATGCTTGTTCTTGGT

A5-AT3G24760-XLOC_014906-10936-0
GGAATGTGTGGGTTCTCGTTTAATCTTTGGAGTATTGAAGAAGGCTCGAT
A5-AT3G24760-XLOC_014906-10936-1
GGAATGTGTGGGTTCTCGTTTAATCTTTGGAGTATTGAAGAAGGCTCGAT
CONSENSUS
GGAATGTGTGGGTTCTCGTTTAATCTTTGGAGTATTGAAGAAGGCTCGAT

A5-AT3G24760-XLOC_014906-10936-0
GGAATTTAGCGAAATCGCGGTTATGCCTGAGGATTTGTTGTTTGGATTAG
A5-AT3G24760-XLOC_014906-10936-1
GGAATTTAGCGAAATCGCGGTTATGCCTGAGGATTTGTTGTTTGGATTAG
CONSENSUS
GGAATTTAGCGAAATCGCGGTTATGCCTGAGGATTTGTTGTTTGGATTAG

A5-AT3G24760-XLOC_014906-10936-0
TTGATAGTGATGATGAGGATGACAAGTTTAGGAGTTTGAAATGTGCTGGC
A5-AT3G24760-XLOC_014906-10936-1
TTGATAGTGATGATGAGGATGACAAGTTTAGGAGTTTGAAATGTGCTGGC
CONSENSUS
TTGATAGTGATGATGAGGATGACAAGTTTAGGAGTTTGAAATGTGCTGGC

A5-AT3G24760-XLOC_014906-10936-0
TCTGGGAATCTTGTTTATGTGTTTAATGATGATTGTCATAAGAAATTTCC
A5-AT3G24760-XLOC_014906-10936-1
TCTGGGAATCTTGTTTATGTGTTTAATGATGATTGTCATAAGAAATTTCC
CONSENSUS
TCTGGGAATCTTGTTTATGTGTTTAATGATGATTGTCATAAGAAATTTCC

A5-AT3G24760-XLOC_014906-10936-0
AGCTTGTGTTTGTGAGATTGGTGGTGGTGAGAATGGGATATGTAGTTGGA
A5-AT3G24760-XLOC_014906-10936-1
AGCTTGTGTTTGTGAGATTGGTGGTGGTGAGAATGGGATATGTAGTTGGA
CONSENSUS
AGCTTGTGTTTGTGAGATTGGTGGTGGTGAGAATGGGATATGTAGTTGGA

A5-AT3G24760-XLOC_014906-10936-0
GAAGAGTTCCTTGTTTGCCTTCTCCGGTTAATAAGTTTCATAAAGTTGTT
A5-AT3G24760-XLOC_014906-10936-1
GAAGAGTTCCTTGTTTGCCTTCTCCGGTTAATAAGTTTCATAAAGTTGTT
CONSENSUS
GAAGAGTTCCTTGTTTGCCTTCTCCGGTTAATAAGTTTCATAAAGTTGTT

A5-AT3G24760-XLOC_014906-10936-0
AGTTTCTGCTCAACGGTGTCTATTACCGATGTTTTCCACCCCGAGGAAGC
A5-AT3G24760-XLOC_014906-10936-1
AGTTTCTGCTCAACGGTGTCTATTACCGATGTTTTCCACCCCGAGGAAGC
CONSENSUS
AGTTTCTGCTCAACGGTGTCTATTACCGATGTTTTCCACCCCGAGGAAGC

A5-AT3G24760-XLOC_014906-10936-0
TCGGATTGGCGGTTCGATCCGTGGTTGAGTTTGCTTAG-----------T
A5-AT3G24760-XLOC_014906-10936-1
TCGGATTGGCGGTTCGATCCGTGGTTGAGTTTGCTTAGGTATGTTCCTGT
CONSENSUS
TCGGATTGGCGGTTCGATCCGTGGTTGAGTTTGCTTAG...........T

A5-AT3G24760-XLOC_014906-10936-0
GCTAATGTAAAGTGGAGATGAAGGGAGAAGATTTAATAATGAAGTGTATA
A5-AT3G24760-XLOC_014906-10936-1
GCTAATGTAAAGTGGAGATGAAGGGAGAAGATTTAATAATGAAGTGTATA
CONSENSUS
GCTAATGTAAAGTGGAGATGAAGGGAGAAGATTTAATAATGAAGTGTATA

A5-AT3G24760-XLOC_014906-10936-0
AAAATGGTTATCAATTTATTATGGTAATCATGTAATTAGACGTAAACTGG
A5-AT3G24760-XLOC_014906-10936-1
AAAATGGTTATCAATTTATTATGGTAATCATGTAATTAGACGTAAACTGG
CONSENSUS
AAAATGGTTATCAATTTATTATGGTAATCATGTAATTAGACGTAAACTGG

A5-AT3G24760-XLOC_014906-10936-0
TTTATAATTCTTCGTTGTATGAATCATTTACATACATACACATATCTACA
A5-AT3G24760-XLOC_014906-10936-1
TTTATAATTCTTCGTTGTATGAATCATTTACATACATACACATATCTACA
CONSENSUS
TTTATAATTCTTCGTTGTATGAATCATTTACATACATACACATATCTACA

A5-AT3G24760-XLOC_014906-10936-0
GAATCATTAAGTATTGTTGTTTGTTTATAAATGAGTGTGTTTTCACAATC
A5-AT3G24760-XLOC_014906-10936-1
GAATCATTAAGTATTGTTGTTTGTTTATAAATGAGTGTGTTTTCACAATC
CONSENSUS
GAATCATTAAGTATTGTTGTTTGTTTATAAATGAGTGTGTTTTCACAATC

A5-AT3G24760-XLOC_014906-10936-0
TCTTATACCAAAGCAAGCTAACATTTATACGTTCTAATGAATTCTATTAA
A5-AT3G24760-XLOC_014906-10936-1
TCTTATACCAAAGCAAGCTAACATTTATACGTTCTAATGAATTCTATTAA
CONSENSUS
TCTTATACCAAAGCAAGCTAACATTTATACGTTCTAATGAATTCTATTAA

A5-AT3G24760-XLOC_014906-10936-0
GTATTGTTGTTATTTTTTGGTGAACATATAATGAAAATGTTGATCAGCAA
A5-AT3G24760-XLOC_014906-10936-1
GTATTGTTGTTATTTTTTGGTGAACATATAATGAAAATGTTGATCAGCAA
CONSENSUS
GTATTGTTGTTATTTTTTGGTGAACATATAATGAAAATGTTGATCAGCAA

A5-AT3G24760-XLOC_014906-10936-0 TC
A5-AT3G24760-XLOC_014906-10936-1 TC
CONSENSUS TC

alignment for event: A5-AT3G02280-XLOC_013628-7923

A5-AT3G02280-XLOC_013628-7923-0
TTTGTAGCAAAGAAGCTAGACAAAAGATTATCAGATCTTGGGGCCACAAC
A5-AT3G02280-XLOC_013628-7923-1
TTTGTAGCAAAGAAGCTAGACAAAAGATTATCAGATCTTGGGGCCACAAC
CONSENSUS
TTTGTAGCAAAGAAGCTAGACAAAAGATTATCAGATCTTGGGGCCACAAC

A5-AT3G02280-XLOC_013628-7923-0
CATCATTGAAAAAGGTCTAGGAGATGATCAACACCCATCAGG--------
A5-AT3G02280-XLOC_013628-7923-1
CATCATTGAAAAAGGTCTAGGAGATGATCAACACCCATCAGGGTATGATA
CONSENSUS
CATCATTGAAAAAGGTCTAGGAGATGATCAACACCCATCAGG........

A5-AT3G02280-XLOC_013628-7923-0 --
GTATGAAGGAACTCTTGATCCGTGGATGCTTTCTTTGTGGAGGACATT
A5-AT3G02280-XLOC_013628-7923-1
CGGTATGAAGGAACTCTTGATCCGTGGATGCTTTCTTTGTGGAGGACATT
CONSENSUS
..GTATGAAGGAACTCTTGATCCGTGGATGCTTTCTTTGTGGAGGACATT

A5-AT3G02280-XLOC_013628-7923-0
ATATCAGATCAATCCAAAATACTTTCCTAAAGGTCCAGATGTGAAAATTC
A5-AT3G02280-XLOC_013628-7923-1
ATATCAGATCAATCCAAAATACTTTCCTAAAGGTCCAGATGTGAAAATTC
CONSENSUS
ATATCAGATCAATCCAAAATACTTTCCTAAAGGTCCAGATGTGAAAATTC

A5-AT3G02280-XLOC_013628-7923-0
CTCAAGATGAAGTAATTGATAAGCCTAAATATAGGATTTTATTTCATAAG
A5-AT3G02280-XLOC_013628-7923-1
CTCAAGATGAAGTAATTGATAAGCCTAAATATAGGATTTTATTTCATAAG
CONSENSUS
CTCAAGATGAAGTAATTGATAAGCCTAAATATAGGATTTTATTTCATAAG

A5-AT3G02280-XLOC_013628-7923-0
CAGGAGAAATTGGAGCCTAAATTATTGTCAGATTCAG
A5-AT3G02280-XLOC_013628-7923-1
CAGGAGAAATTGGAGCCTAAATTATTGTCAGATTCAG
CONSENSUS
CAGGAGAAATTGGAGCCTAAATTATTGTCAGATTCAG

alignment for event: A3-AT3G49645-XLOC_019281-197

A3-AT3G49645-XLOC_019281-197-0

GTTTTCTTCAACGCCCTCCAATAGTTACTTCGTCCAAGTTGATTGAGAAC
A3-AT3G49645-XLOC_019281-197-1
GTTTTCTTCAACGCCCTCCAATAGTTACTTCGTCCAAGTTGATTGAGAAC
CONSENSUS
GTTTTCTTCAACGCCCTCCAATAGTTACTTCGTCCAAGTTGATTGAGAAC

A3-AT3G49645-XLOC_019281-197-0
ATTATCAAGAAGAATGAAACAAGGCGGCTTAAATCATATATCGAGGCAGG
A3-AT3G49645-XLOC_019281-197-1
ATTATCAAGAAGAATGAAACAAGGCGGCTTAAATCATATATCGAGGCAGG
CONSENSUS
ATTATCAAGAAGAATGAAACAAGGCGGCTTAAATCATATATCGAGGCAGG

A3-AT3G49645-XLOC_019281-197-0
ATGTATTAATATCCATGATGCTGCACAAAGCACAAGAGCTT---TGCATA
A3-AT3G49645-XLOC_019281-197-1
ATGTATTAATATCCATGATGCTGCACAAAGCACAAGAGCTTAAGTGCATA
CONSENSUS
ATGTATTAATATCCATGATGCTGCACAAAGCACAAGAGCTT...TGCATA

A3-AT3G49645-XLOC_019281-197-0
CATCTCTATCAGGACTTTCTGACCACCTAATCAAAG
A3-AT3G49645-XLOC_019281-197-1
CATCTCTATCAGGACTTTCTGACCACCTAATCAAAG
CONSENSUS
CATCTCTATCAGGACTTTCTGACCACCTAATCAAAG

alignment for event: A3-AT3G53170-XLOC_019497-1284

A3-AT3G53170-XLOC_019497-1284-0
ATATAGGCAAGAAGAGGCTTACGGAGTGATGCTTTAGTTTGTGAATGAGC
A3-AT3G53170-XLOC_019497-1284-1
ATATAGGCAAGAAGAGGCTTACGGAGTGATGCTTTAGTTTGTGAATGAGC
CONSENSUS
ATATAGGCAAGAAGAGGCTTACGGAGTGATGCTTTAGTTTGTGAATGAGC

A3-AT3G53170-XLOC_019497-1284-0
AAATCTAATGCAGAAGGAATTTAGAAGTTGTGAATTGTTCCAACTAAGAG
A3-AT3G53170-XLOC_019497-1284-1
AAATCTAATGCAGAAGGAATTTAGAAGTTGTGAATTGTTCCAACTAAGAG
CONSENSUS
AAATCTAATGCAGAAGGAATTTAGAAGTTGTGAATTGTTCCAACTAAGAG

A3-AT3G53170-XLOC_019497-1284-0 AATCACTTGAG-----------
GTGTTTGGAGCGTTGGTTAGCAAGATCC
A3-AT3G53170-XLOC_019497-1284-1
AATCACTTGAGTGTTTGTTCAGGTGTTTGGAGCGTTGGTTAGCAAGATCC
CONSENSUS
AATCACTTGAG...........GTGTTTGGAGCGTTGGTTAGCAAGATCC

A3-AT3G53170-XLOC_019497-1284-0
CAAGAGAGGCATGGCCATTACTAATGAGAATTTGGGTCGGTTCTGAAATA
A3-AT3G53170-XLOC_019497-1284-1
CAAGAGAGGCATGGCCATTACTAATGAGAATTTGGGTCGGTTCTGAAATA

CONSENSUS
      CAAGAGAGGCATGGCCATTACTAATGAGAATTTGGGTCGGTTCTGAAATA

A3-AT3G53170-XLOC_019497-1284-0
      ACCAAATCAGCATTGGGGAGAAAACGTAATGATTCCAGTCAAT
A3-AT3G53170-XLOC_019497-1284-1
      ACCAAATCAGCATTGGGGAGAAAACGTAATGATTCCAGTCAAT
CONSENSUS
      ACCAAATCAGCATTGGGGAGAAAACGTAATGATTCCAGTCAAT

alignment for event: A5-AT3G01310-XLOC_016797-4932

A5-AT3G01310-XLOC_016797-4932-0
      AGAGAAGAACACTGCGACGTCGTTAAAAGTAGGTTGAGAGCGAGAAAGGC
A5-AT3G01310-XLOC_016797-4932-1
      AGAGAAGAACACTGCGACGTCGTTAAAAGTAGGTTGAGAGCGAGAAAGGC
CONSENSUS
      AGAGAAGAACACTGCGACGTCGTTAAAAGTAGGTTGAGAGCGAGAAAGGC

A5-AT3G01310-XLOC_016797-4932-0
      CGAGAGAATGGAGATGGAAGAAGGAGCAAGTGGTGTTGGTGAGAAGATAA
A5-AT3G01310-XLOC_016797-4932-1
      CGAGAGAATGGAGATGGAAGAAGGAGCAAGTGGTGTTGGTGAGAAGATAA
CONSENSUS
      CGAGAGAATGGAGATGGAAGAAGGAGCAAGTGGTGTTGGTGAGAAGATAA

A5-AT3G01310-XLOC_016797-4932-0
      AGATTGGAGTCTGCGTCATGGAAAAGAAGGTGAAATGCGGCTCCGAGGTT
A5-AT3G01310-XLOC_016797-4932-1
      AGATTGGAGTCTGCGTCATGGAAAAGAAG------------------GTT
CONSENSUS
      AGATTGGAGTCTGCGTCATGGAAAAGAAG..................GTT

A5-AT3G01310-XLOC_016797-4932-0
      TTCTCAGCTCCCATGGGGGAAATTCTCGACAGACTCCAGTCTTTTGGTGA
A5-AT3G01310-XLOC_016797-4932-1
      TTCTCAGCTCCCATGGGGGAAATTCTCGACAGACTCCAGTCTTTTGGTGA
CONSENSUS
      TTCTCAGCTCCCATGGGGGAAATTCTCGACAGACTCCAGTCTTTTGGTGA

A5-AT3G01310-XLOC_016797-4932-0 ATTTGAG
A5-AT3G01310-XLOC_016797-4932-1 ATTTGAG
CONSENSUS   ATTTGAG

alignment for event: RI-AT3G26690-XLOC_018236-3815

RI-AT3G26690-XLOC_018236-3815-0
      CAACAAACCAAAACCGTTCCTTTCTTTTTCCATTTTTCTTGTTTTTTTTT
RI-AT3G26690-XLOC_018236-3815-1
      CAACAAACCAAAACCGTTCCTTTCTTTTTCCATTTTTCTTGTTTTTTTTT
CONSENSUS
      CAACAAACCAAAACCGTTCCTTTCTTTTTCCATTTTTCTTGTTTTTTTTT

RI-AT3G26690-XLOC_018236-3815-0
      GTTTTGTTTTTGGAGTTGTGTTTCACCTGTTGTTGGTTCTTTAGTTCGTC
RI-AT3G26690-XLOC_018236-3815-1
      GTTTTGTTTTTGGAGTTGTGTTTCACCTGTTGTTGGTTCTTTAGTTCGTC
CONSENSUS
      GTTTTGTTTTTGGAGTTGTGTTTCACCTGTTGTTGGTTCTTTAGTTCGTC

RI-AT3G26690-XLOC_018236-3815-0
      TTCTGGGTTAAACAAAGAAGATCGGTTCTGTTTTTATTTTTATTTTGAAA
RI-AT3G26690-XLOC_018236-3815-1
      TTCTGGGTTAAACAAAGAAGATCGGTTCTGTTTTTATTTTTATTTTGAAA
CONSENSUS
      TTCTGGGTTAAACAAAGAAGATCGGTTCTGTTTTTATTTTTATTTTGAAA

RI-AT3G26690-XLOC_018236-3815-0
      CAAGAAGAAGAAAGCAAGCTACGATGATGTTTCTTGCTGACGCCAATAGA
RI-AT3G26690-XLOC_018236-3815-1
      CAAGAAGAAGAAAGCAAGCTACGATGATGTTTCTTGCTGACGCCAATAGA
CONSENSUS
      CAAGAAGAAGAAAGCAAGCTACGATGATGTTTCTTGCTGACGCCAATAGA

RI-AT3G26690-XLOC_018236-3815-0
      TAAAGACACGACTTTTACAAAGCTTAGGACCTGCATTCTCACCGTAATTA
RI-AT3G26690-XLOC_018236-3815-1
      TAAAGACACGACTTTTACAAAGCTTAGGACCTGCATTCTCACC-------
CONSENSUS
      TAAAGACACGACTTTTACAAAGCTTAGGACCTGCATTCTCACC.......

RI-AT3G26690-XLOC_018236-3815-0
      GCTCTTCTTCTCAATTTCTCCAAATAAATTTGTGTGTTTTTAATGTGTGT
RI-AT3G26690-XLOC_018236-3815-1

CONSENSUS
      ....

RI-AT3G26690-XLOC_018236-3815-0
      GTTGCAGGTTATCAAAAATGTCGAATCTTTCTGCAAGAACAGGACGAGAC
RI-AT3G26690-XLOC_018236-3815-1 -------
GTTATCAAAAATGTCGAATCTTTCTGCAAGAACAGGACGAGAC
CONSENSUS
      .......GTTATCAAAAATGTCGAATCTTTCTGCAAGAACAGGACGAGAC

RI-AT3G26690-XLOC_018236-3815-0
      CATCAACGTTATGACAACAACTTCCGTCTTGTTTCTGG
RI-AT3G26690-XLOC_018236-3815-1
      CATCAACGTTATGACAACAACTTCCGTCTTGTTTCTGG
CONSENSUS
      CATCAACGTTATGACAACAACTTCCGTCTTGTTTCTGG

alignment for event: A3-AT3G01540-XLOC_016824-4701

A3-AT3G01540-XLOC_016824-4701-0
      GGGGGCCAAGTACCACCACCTCTAATGTCCTTTGAAGCTACTGGTTTTCC

A3-AT3G01540-XLOC_016824-4701-1
      GGGGGCCAAGTACCACCACCTCTAATGTCCTTTGAAGCTACTGGTTTTCC
CONSENSUS
      GGGGGCCAAGTACCACCACCTCTAATGTCCTTTGAAGCTACTGGTTTTCC

A3-AT3G01540-XLOC_016824-4701-0
      ACCTGAGCTTCTGCGGGAG-------------------------------
A3-AT3G01540-XLOC_016824-4701-1
      ACCTGAGCTTCTGCGGGAGGTGCGATGTTTGCTCCTGGGAGGAGCCAATG
CONSENSUS
      ACCTGAGCTTCTGCGGGAG...............................

A3-AT3G01540-XLOC_016824-4701-0

A3-AT3G01540-XLOC_016824-4701-1
      TCGTACACTGTGCCACTTCTTTGAGGTCCTTGCAAATTATTTGGAGTGCC
CONSENSUS
      ....

A3-AT3G01540-XLOC_016824-4701-0

A3-AT3G01540-XLOC_016824-4701-1
      CTATATGGTGGACCAAATTTTTTGGAAAGGGCTTCCCAAATCATCCAAGC
CONSENSUS
      ....

A3-AT3G01540-XLOC_016824-4701-0

A3-AT3G01540-XLOC_016824-4701-1
      TTGTGGCAGGGCCTGTTAATGGTGTGATGATTCATTTCACCACCGGGTCC
CONSENSUS
      ....

A3-AT3G01540-XLOC_016824-4701-0

A3-AT3G01540-XLOC_016824-4701-1
      AGACAGAAGAAGAGGCGTCCTCCTGCTGCAGGTTTCATCAGTAACCTTTT
CONSENSUS
      ....

A3-AT3G01540-XLOC_016824-4701-0

A3-AT3G01540-XLOC_016824-4701-1
      GCCACCTGCAAATGGGGGATGGTGCTTCTTCTGACATGAGCCACCATTCC
CONSENSUS
      ....

A3-AT3G01540-XLOC_016824-4701-0

A3-AT3G01540-XLOC_016824-4701-1
      ATAAGCCCAAAAGAACCTTGGTGTTCCCCTTCTCATAGGACAGTGTGGTT
CONSENSUS
      ....

A3-AT3G01540-XLOC_016824-4701-0 --------------
GTACTCAGTGCAGGTTTCTCTGCTCCAACTCCAATT

A3-AT3G01540-XLOC_016824-4701-1
      TTCTGTTTTCTTAGGTACTCAGTGCAGGTTTCTCTGCTCCAACTCCAATT
CONSENSUS
      ..............GTACTCAGTGCAGGTTTCTCTGCTCCAACTCCAATT

A3-AT3G01540-XLOC_016824-4701-0
      CAAGCTCAGTCATGGCCCATTGCTATGCAAGGTAGGGACATAGTAGCCAT
A3-AT3G01540-XLOC_016824-4701-1
      CAAGCTCAGTCATGGCCCATTGCTATGCAAGGTAGGGACATAGTAGCCAT
CONSENSUS
      CAAGCTCAGTCATGGCCCATTGCTATGCAAGGTAGGGACATAGTAGCCAT

A3-AT3G01540-XLOC_016824-4701-0
      TGCTAAAACTGGCTCGGGAAAAACTTTGGGTTACTTGATTCCTGGATTTT
A3-AT3G01540-XLOC_016824-4701-1
      TGCTAAAACTGGCTCGGGAAAAACTTTGGGTTACTTGATTCCTGGATTTT
CONSENSUS
      TGCTAAAACTGGCTCGGGAAAAACTTTGGGTTACTTGATTCCTGGATTTT

A3-AT3G01540-XLOC_016824-4701-0
      TGCATCTTCAACGTATCCGAAATGATTCGCGAATGGGCCCAACAATCTTG
A3-AT3G01540-XLOC_016824-4701-1
      TGCATCTTCAACGTATCCGAAATGATTCGCGAATGGGCCCAACAATCTTG
CONSENSUS
      TGCATCTTCAACGTATCCGAAATGATTCGCGAATGGGCCCAACAATCTTG

A3-AT3G01540-XLOC_016824-4701-0
      GTATTGTCTCCAACGAGAGAGCTGGCCACACAAATCCAAGAAGAAGCTGT
A3-AT3G01540-XLOC_016824-4701-1
      GTATTGTCTCCAACGAGAGAGCTGGCCACACAAATCCAAGAAGAAGCTGT
CONSENSUS
      GTATTGTCTCCAACGAGAGAGCTGGCCACACAAATCCAAGAAGAAGCTGT

A3-AT3G01540-XLOC_016824-4701-0
      TAAATTTGGGAGGTCATCAAGAATTTCGTGTACG
A3-AT3G01540-XLOC_016824-4701-1
      TAAATTTGGGAGGTCATCAAGAATTTCGTGTACG
CONSENSUS
      TAAATTTGGGAGGTCATCAAGAATTTCGTGTACG

alignment for event: A5-AT3G60250-XLOC_019895-13432

A5-AT3G60250-XLOC_019895-13432-0
      ACTGAGAAGTACAAGAACTGTGATTTCGGGAGATGCCCGAGAGTTTTCTG
A5-AT3G60250-XLOC_019895-13432-1
      ACTGAGAAGTACAAGAACTGTGATTTCGGGAGATGCCCGAGAGTTTTCTG
CONSENSUS
      ACTGAGAAGTACAAGAACTGTGATTTCGGGAGATGCCCGAGAGTTTTCTG

A5-AT3G60250-XLOC_019895-13432-0
      TTGCGGTCAGTCTTGTCTTCCAGTTGGACAATCCGATATCCCGAGATCGA
A5-AT3G60250-XLOC_019895-13432-1
      TTGCGGTCAGTCTTGTCTTCCAGTTGGACAATCCGATATCCCGAGATCGA
CONSENSUS

TTGCGGTCAGTCTTGTCTTCCAGTTGGACAATCCGATATCCCGAGATCGA

A5-AT3G60250-XLOC_019895-13432-0
GTACTGTGAAGATATACTGCCCTAAATGCGAGGATATATCTTACCCGCGA
A5-AT3G60250-XLOC_019895-13432-1
GTACTGTGAAGATATACTGCCCTAAATGCGAGGATATATCTTACCCGCGA
CONSENSUS
GTACTGTGAAGATATACTGCCCTAAATGCGAGGATATATCTTACCCGCGA

A5-AT3G60250-XLOC_019895-13432-0
TCTAAATTCCAAGGCAATATTGATGGAGCGTACTTTGGAACCACATTCCC
A5-AT3G60250-XLOC_019895-13432-1 TCTAAATTCCAAG---
ATATTGATGGAGCGTACTTTGGAACCACATTCCC
CONSENSUS
TCTAAATTCCAAG...ATATTGATGGAGCGTACTTTGGAACCACATTCCC

A5-AT3G60250-XLOC_019895-13432-0
TCACTTGTTCTTGATGACTTACGGGAACTTAAAGCCGCAGAAGCCTACTC
A5-AT3G60250-XLOC_019895-13432-1
TCACTTGTTCTTGATGACTTACGGGAACTTAAAGCCGCAGAAGCCTACTC
CONSENSUS
TCACTTGTTCTTGATGACTTACGGGAACTTAAAGCCGCAGAAGCCTACTC

A5-AT3G60250-XLOC_019895-13432-0
AAAGCTATGTCCCAAAAATCTTTGGCTTCAAGGTACACAAACCATGATAC
A5-AT3G60250-XLOC_019895-13432-1
AAAGCTATGTCCCAAAAATCTTTGGCTTCAAGGTACACAAACCATGATAC
CONSENSUS
AAAGCTATGTCCCAAAAATCTTTGGCTTCAAGGTACACAAACCATGATAC

A5-AT3G60250-XLOC_019895-13432-0
TAGTGCTCTGCATTCTCAATGGTGATACATTTAGTGGCTCTGTAATTGCA
A5-AT3G60250-XLOC_019895-13432-1
TAGTGCTCTGCATTCTCAATGGTGATACATTTAGTGGCTCTGTAATTGCA
CONSENSUS
TAGTGCTCTGCATTCTCAATGGTGATACATTTAGTGGCTCTGTAATTGCA

A5-AT3G60250-XLOC_019895-13432-0
TCCGGATGAGCAACTGAAACGATAGCTGCGGTGACTGGAGCATACATCAA
A5-AT3G60250-XLOC_019895-13432-1
TCCGGATGAGCAACTGAAACGATAGCTGCGGTGACTGGAGCATACATCAA
CONSENSUS
TCCGGATGAGCAACTGAAACGATAGCTGCGGTGACTGGAGCATACATCAA

A5-AT3G60250-XLOC_019895-13432-0
CCATTGGGTTAGAGGATGAGAGCTGAAGTTGAAAGATGGAATCTTGAAAG
A5-AT3G60250-XLOC_019895-13432-1
CCATTGGGTTAGAGGATGAGAGCTGAAGTTGAAAGATGGAATCTTGAAAG
CONSENSUS
CCATTGGGTTAGAGGATGAGAGCTGAAGTTGAAAGATGGAATCTTGAAAG

A5-AT3G60250-XLOC_019895-13432-0
AATCTCATGAAGTTCCAAGAAAACTTAGAAGTGCAACAGCCTGGCTCATT
A5-AT3G60250-XLOC_019895-13432-1
AATCTCATGAAGTTCCAAGAAAACTTAGAAGTGCAACAGCCTGGCTCATT
CONSENSUS

AATCTCATGAAGTTCCAAGAAAACTTAGAAGTGCAACAGCCTGGCTCATT

A5-AT3G60250-XLOC_019895-13432-0
GGGGGAAAAGAACGACACACTTGGGTCTTGTTGTTGTCTCCGAAAAGATT
A5-AT3G60250-XLOC_019895-13432-1
GGGGGAAAAGAACGACACACTTGGGTCTTGTTGTTGTCTCCGAAAAGATT
CONSENSUS
GGGGGAAAAGAACGACACACTTGGGTCTTGTTGTTGTCTCCGAAAAGATT

A5-AT3G60250-XLOC_019895-13432-0
TTCCCCAGTTAGTTGGATTTTGTTTTGAACTGTTGGATTATTATTCGTTT
A5-AT3G60250-XLOC_019895-13432-1
TTCCCCAGTTAGTTGGATTTTGTTTTGAACTGTTGGATTATTATTCGTTT
CONSENSUS
TTCCCCAGTTAGTTGGATTTTGTTTTGAACTGTTGGATTATTATTCGTTT

A5-AT3G60250-XLOC_019895-13432-0
TTCTAATTTCATGTAAAACTCATAAATTTCAACTGAAGTATAACTTAGAT
A5-AT3G60250-XLOC_019895-13432-1
TTCTAATTTCATGTAAAACTCATAAATTTCAACTGAAGTATAACTTAGAT
CONSENSUS
TTCTAATTTCATGTAAAACTCATAAATTTCAACTGAAGTATAACTTAGAT

A5-AT3G60250-XLOC_019895-13432-0 CAATCAATTTCATCAAAGATCATA
A5-AT3G60250-XLOC_019895-13432-1 CAATCAATTTCATCAAAGATCATA
CONSENSUS CAATCAATTTCATCAAAGATCATA

alignment for event: A3-AT3G61420-XLOC_019966-11071

A3-AT3G61420-XLOC_019966-11071-0
GACCACGGGATCCAGCGCGATGGCACCAATGACATTATTGAACCACAGAA
A3-AT3G61420-XLOC_019966-11071-1
GACCACGGGATCCAGCGCGATGGCACCAATGACATTATTGAACCACAGAA
CONSENSUS
GACCACGGGATCCAGCGCGATGGCACCAATGACATTATTGAACCACAGAA

A3-AT3G61420-XLOC_019966-11071-0
TGACCAGCTTAAGAGGTCACTTTTACAAGATCTTAATCGTCATGCTGCTG
A3-AT3G61420-XLOC_019966-11071-1
TGACCAGCTTAAGAGGTCACTTTTACAAGATCTTAATCGTCATGCTGCTG
CONSENSUS
TGACCAGCTTAAGAGGTCACTTTTACAAGATCTTAATCGTCATGCTGCTG

A3-AT3G61420-XLOC_019966-11071-0
TTGTATTGGAAGGAAGATGCATCAGTAAACACTTTCAACTCTGATTGTTT
A3-AT3G61420-XLOC_019966-11071-1
TTGTATTGGAAGGAAGATGCATCAGTAAACACTTTCAACTCTGATTGTTT
CONSENSUS
TTGTATTGGAAGGAAGATGCATCAGTAAACACTTTCAACTCTGATTGTTT

A3-AT3G61420-XLOC_019966-11071-0
CATCTGGTGTTTTGTGTAACAGTATAAAAAATAGCCATCAAATGACTTCC
A3-AT3G61420-XLOC_019966-11071-1
CATCTGGTGTTTTGTGTAACAGTATAAAAAATAGCCATCAAATGACTTCC

CONSENSUS
      CATCTGGTGTTTTGTGTAACAGTATAAAAAATAGCCATCAAATGACTTCC

A3-AT3G61420-XLOC_019966-11071-0
      CAACAGATAGTTGATGAGGGATAAACATGTATTTTGGCCCTCTGCTTTTA
A3-AT3G61420-XLOC_019966-11071-1
      CAACAGATAGTTGATGAGGGATAAACATGTATTTTGGCCCTCTGCTTTTA
CONSENSUS
      CAACAGATAGTTGATGAGGGATAAACATGTATTTTGGCCCTCTGCTTTTA

A3-AT3G61420-XLOC_019966-11071-0
      GTTCTTGGTGGTAAAGTATACAATGATACTCACTTATTCTTTTATACTGG
A3-AT3G61420-XLOC_019966-11071-1
      GTTCTTGGTGGTAAAGTATACAATGATACTCACTTATTCTTTTATACTGG
CONSENSUS
      GTTCTTGGTGGTAAAGTATACAATGATACTCACTTATTCTTTTATACTGG

A3-AT3G61420-XLOC_019966-11071-0
      AAAGTAGATGTTCAGTCTGAAGATACGAGAATTGTTGCAGAGGCTCTCAC
A3-AT3G61420-XLOC_019966-11071-1
      AAAGTAGATGTTCAGTCTGAAGATACGAGAATTGTTGCAGAGGCTCTCAC
CONSENSUS
      AAAGTAGATGTTCAGTCTGAAGATACGAGAATTGTTGCAGAGGCTCTCAC

A3-AT3G61420-XLOC_019966-11071-0
      ACGGGCGAAACAAG-------------------------TAAGCAAAGC
A3-AT3G61420-XLOC_019966-11071-1
      ACGGGCGAAACAAGGTATCCATGTCTATTACCCCTGGTAGTAAGCAAAGC
CONSENSUS
      ACGGGCGAAACAAG.........................TAAGCAAAGC

A3-AT3G61420-XLOC_019966-11071-0
      TGATGGTGAAATCACCAAGGACGCTAATCAGGAGAGGTTGGAGAGAATGT
A3-AT3G61420-XLOC_019966-11071-1
      TGATGGTGAAATCACCAAGGACGCTAATCAGGAGAGGTTGGAGAGAATGT
CONSENSUS
      TGATGGTGAAATCACCAAGGACGCTAATCAGGAGAGGTTGGAGAGAATGT

A3-AT3G61420-XLOC_019966-11071-0
      CCCGTGCAACAGAAATGGAGGATCTTCAAGCACCACAAAACTTCCCATTA
A3-AT3G61420-XLOC_019966-11071-1
      CCCGTGCAACAGAAATGGAGGATCTTCAAGCACCACAAAACTTCCCATTA
CONSENSUS
      CCCGTGCAACAGAAATGGAGGATCTTCAAGCACCACAAAACTTCCCATTA

A3-AT3G61420-XLOC_019966-11071-0 GCACCACTCTCTATCAAG
A3-AT3G61420-XLOC_019966-11071-1 GCACCACTCTCTATCAAG
CONSENSUS   GCACCACTCTCTATCAAG

alignment for event: RI-AT3G62310-XLOC_020022-9254

RI-AT3G62310-XLOC_020022-9254-0
      GTACTTGACTGATGGTATGCTCCTGAGAGAAGCAATGGCGGACCCTCTTC
RI-AT3G62310-XLOC_020022-9254-1

GTACTTGACTGATGGTATGCTCCTGAGAGAAGCAATGGCGGACCCTCTTC
CONSENSUS
GTACTTGACTGATGGTATGCTCCTGAGAGAAGCAATGGCGGACCCTCTTC

RI-AT3G62310-XLOC_020022-9254-0
TAGAGAGATACAAAGTCATTATTCTTGATGAGGCCCACGAAAGGACTCTA
RI-AT3G62310-XLOC_020022-9254-1
TAGAGAGATACAAAGTCATTATTCTTGATGAGGCCCACGAAAGGACTCTA
CONSENSUS
TAGAGAGATACAAAGTCATTATTCTTGATGAGGCCCACGAAAGGACTCTA

RI-AT3G62310-XLOC_020022-9254-0
GCCACAGATGTGCTGTTTGGTCTTCTGAAAGAAGTGTTGAAAAATAGACC
RI-AT3G62310-XLOC_020022-9254-1
GCCACAGATGTGCTGTTTGGTCTTCTGAAAGAAGTGTTGAAAAATAGACC
CONSENSUS
GCCACAGATGTGCTGTTTGGTCTTCTGAAAGAAGTGTTGAAAAATAGACC

RI-AT3G62310-XLOC_020022-9254-0
TGATCTTAAGTTAGTTGTGATGAGTGCAACTCTAGAAGCTGAAAAGTTTC
RI-AT3G62310-XLOC_020022-9254-1
TGATCTTAAGTTAGTTGTGATGAGTGCAACTCTAGAAGCTGAAAAGTTTC
CONSENSUS
TGATCTTAAGTTAGTTGTGATGAGTGCAACTCTAGAAGCTGAAAAGTTTC

RI-AT3G62310-XLOC_020022-9254-0
AGGATTATTTTAGCGGTGCTCCTCTGATGAAAGTTCCTGGTAGGCTTCAT
RI-AT3G62310-XLOC_020022-9254-1
AGGATTATTTTAGCGGTGCTCCTCTGATGAAAGTTCCTGGTAGGCTTCAT
CONSENSUS
AGGATTATTTTAGCGGTGCTCCTCTGATGAAAGTTCCTGGTAGGCTTCAT

RI-AT3G62310-XLOC_020022-9254-0
CCAGTTGAGATCTTTTACACTCAGGAGCCTGAGAGGGACTATCTTGAGGC
RI-AT3G62310-XLOC_020022-9254-1
CCAGTTGAGATCTTTTACACTCAGGAGCCTGAGAGGGACTATCTTGAGGC
CONSENSUS
CCAGTTGAGATCTTTTACACTCAGGAGCCTGAGAGGGACTATCTTGAGGC

RI-AT3G62310-XLOC_020022-9254-0
TGCTATTAGAACTGTTGTCCAGATTCACATGTGTGAGCCACCTGGCGATA
RI-AT3G62310-XLOC_020022-9254-1
TGCTATTAGAACTGTTGTCCAGATTCACATGTGTGAGCCACCTGGCGATA
CONSENSUS
TGCTATTAGAACTGTTGTCCAGATTCACATGTGTGAGCCACCTGGCGATA

RI-AT3G62310-XLOC_020022-9254-0
TTCTTGTTTTCTTAACTGGGGAGGAGGAGATTGAAGATGCATGCCGCAAA
RI-AT3G62310-XLOC_020022-9254-1
TTCTTGTTTTCTTAACTGGGGAGGAGGAGATTGAAGATGCATGCCGCAAA
CONSENSUS
TTCTTGTTTTCTTAACTGGGGAGGAGGAGATTGAAGATGCATGCCGCAAA

RI-AT3G62310-XLOC_020022-9254-0
ATCAATAAAGAGGTTGGTAATCTTGGGGATCAAGTGGGGCCTATTAAAGT
RI-AT3G62310-XLOC_020022-9254-1

ATCAATAAAGAGGTTGGTAATCTTGGGGATCAAGTGGGGCCTATTAAAGT
CONSENSUS
ATCAATAAAGAGGTTGGTAATCTTGGGGATCAAGTGGGGCCTATTAAAGT

RI-AT3G62310-XLOC_020022-9254-0
AGTGCCACTGTATTCTACTCTTCCACCTGCGATGCAGCAGAAGATATTTG
RI-AT3G62310-XLOC_020022-9254-1
AGTGCCACTGTATTCTACTCTTCCACCTGCGATGCAGCAGAAGATATTTG
CONSENSUS
AGTGCCACTGTATTCTACTCTTCCACCTGCGATGCAGCAGAAGATATTTG

RI-AT3G62310-XLOC_020022-9254-0
ATCCTGCTCCAGAGCCTGTAACAGAAGGTGGTCCTCCTGGGAGAAAGATT
RI-AT3G62310-XLOC_020022-9254-1
ATCCTGCTCCAGAGCCTGTAACAGAAGGTGGTCCTCCTGGGAGAAAGATT
CONSENSUS
ATCCTGCTCCAGAGCCTGTAACAGAAGGTGGTCCTCCTGGGAGAAAGATT

RI-AT3G62310-XLOC_020022-9254-0
GTTGTCTCCACTAACATTGCTGAGACTTCTCTCACCATAGATGGGATTGT
RI-AT3G62310-XLOC_020022-9254-1
GTTGTCTCCACTAACATTGCTGAGACTTCTCTCACCATAGATGGGATTGT
CONSENSUS
GTTGTCTCCACTAACATTGCTGAGACTTCTCTCACCATAGATGGGATTGT

RI-AT3G62310-XLOC_020022-9254-0
CTACGTCATTGACCCTGGTTTTGCTAAGCAAAAAGTCTATAACCCTCGAA
RI-AT3G62310-XLOC_020022-9254-1
CTACGTCATTGACCCTGGTTTTGCTAAGCAAAAAGTCTATAACCCTCGAA
CONSENSUS
CTACGTCATTGACCCTGGTTTTGCTAAGCAAAAAGTCTATAACCCTCGAA

RI-AT3G62310-XLOC_020022-9254-0
TCCGTGTTGAGTCTTTGTTGGTGTCCCCAATTTCAAAGGCAAGTGCTCAC
RI-AT3G62310-XLOC_020022-9254-1
TCCGTGTTGAGTCTTTGTTGGTGTCCCCAATTTCAAAGGCAAGTGCTCAC
CONSENSUS
TCCGTGTTGAGTCTTTGTTGGTGTCCCCAATTTCAAAGGCAAGTGCTCAC

RI-AT3G62310-XLOC_020022-9254-0
CAGAGATCTGGTCGTGCCGGTAGAACACGGCCTGGAAAATGCTTCAGGCT
RI-AT3G62310-XLOC_020022-9254-1
CAGAGATCTGGTCGTGCCGGTAGAACACGGCCTGGAAAATGCTTCAGGCT
CONSENSUS
CAGAGATCTGGTCGTGCCGGTAGAACACGGCCTGGAAAATGCTTCAGGCT

RI-AT3G62310-XLOC_020022-9254-0
TTACACAGAGAAGAGTTTTAATAATGACTTGCAGCCGCAGACGTATCCTG
RI-AT3G62310-XLOC_020022-9254-1
TTACACAGAGAAGAGTTTTAATAATGACTTGCAGCCGCAGACGTATCCTG
CONSENSUS
TTACACAGAGAAGAGTTTTAATAATGACTTGCAGCCGCAGACGTATCCTG

RI-AT3G62310-XLOC_020022-9254-0
AAATATTGAGATCAAACCTTGCAAATACAGTTCTGACCTTGAAAAAACTG
RI-AT3G62310-XLOC_020022-9254-1

AAATATTGAGATCAAACCTTGCAAATACAGTTCTGACCTTGAAAAAACTG
CONSENSUS
AAATATTGAGATCAAACCTTGCAAATACAGTTCTGACCTTGAAAAAACTG

RI-AT3G62310-XLOC_020022-9254-0
GGTATTGATGACTTGGTGCACTTTGATTTTATGGATCCCCCTGCTCCCGA
RI-AT3G62310-XLOC_020022-9254-1
GGTATTGATGACTTGGTGCACTTTGATTTTATGGATCCCCCTGCTCCCGA
CONSENSUS
GGTATTGATGACTTGGTGCACTTTGATTTTATGGATCCCCCTGCTCCCGA

RI-AT3G62310-XLOC_020022-9254-0
AACACTGATGCGAGCCTTAGAAGTTTTGAATTACTTGGGAGCACTGGATG
RI-AT3G62310-XLOC_020022-9254-1
AACACTGATGCGAGCCTTAGAAGTTTTGAATTACTTGGGAGCACTGGATG
CONSENSUS
AACACTGATGCGAGCCTTAGAAGTTTTGAATTACTTGGGAGCACTGGATG

RI-AT3G62310-XLOC_020022-9254-0
ATGATGGTAACTTGACAAAGACAGGTGAGATCATGAGTGAGTTCCCCTTG
RI-AT3G62310-XLOC_020022-9254-1
ATGATGGTAACTTGACAAAGACAGGTGAGATCATGAGTGAGTTCCCCTTG
CONSENSUS
ATGATGGTAACTTGACAAAGACAGGTGAGATCATGAGTGAGTTCCCCTTG

RI-AT3G62310-XLOC_020022-9254-0
GATCCACAGATGGCAAAGATGCTCATAGTCAGTCCTGAATTCAACTGCTC
RI-AT3G62310-XLOC_020022-9254-1
GATCCACAGATGGCAAAGATGCTCATAGTCAGTCCTGAATTCAACTGCTC
CONSENSUS
GATCCACAGATGGCAAAGATGCTCATAGTCAGTCCTGAATTCAACTGCTC

RI-AT3G62310-XLOC_020022-9254-0
AAACGAGATTCTCTCAGTTTCAGCTATGCTATCAGGTACGCCCTTTACCT
RI-AT3G62310-XLOC_020022-9254-1
AAACGAGATTCTCTCAGTTTCAGCTATGCTATCAG---------------
CONSENSUS
AAACGAGATTCTCTCAGTTTCAGCTATGCTATCAG...............

RI-AT3G62310-XLOC_020022-9254-0
TTATCTTTTTGTTTTTGCTACCTTGGGTTAGGTATGTAGTTAAGGGTAGG
RI-AT3G62310-XLOC_020022-9254-1

CONSENSUS
....

RI-AT3G62310-XLOC_020022-9254-0
CGTAAACTTGCTGGCAGTTTCCATTCTGCATATATTCTAATGTGCATTAG
RI-AT3G62310-XLOC_020022-9254-1

CONSENSUS
....

RI-AT3G62310-XLOC_020022-9254-0
ATCTCATCTTTTATCGTTCATGTGTATAATTGTCATTTAGAATTAAGTAA
RI-AT3G62310-XLOC_020022-9254-1

CONSENSUS
....

RI-AT3G62310-XLOC_020022-9254-0
TAGAGTTAGGTTGTCATCAGCTGTCTCTAGTGTGGATTCTGCATATCCAT
RI-AT3G62310-XLOC_020022-9254-1

CONSENSUS
....

RI-AT3G62310-XLOC_020022-9254-0
GGTTAGGGGGTGTCCTAAGCAATTGTTGATGCAAATAGTTGCAGTTTCTC
RI-AT3G62310-XLOC_020022-9254-1

CONSENSUS
....

RI-AT3G62310-XLOC_020022-9254-0
AGTTATTCTCTCTCATGAAGCAAATGGTATCAGTCTCATCGCCTCTATGC
RI-AT3G62310-XLOC_020022-9254-1

CONSENSUS
....

RI-AT3G62310-XLOC_020022-9254-0
ATCTGCTGACCTCTTCCCTGGACCTCTTCTGTTATAATTACCAATTCTTT
RI-AT3G62310-XLOC_020022-9254-1

CONSENSUS
....

RI-AT3G62310-XLOC_020022-9254-0
CTCTAGTACCGAATTGCTTTATTCGGCCTAGAGAGGCTCAAAAAGCAGCA
RI-AT3G62310-XLOC_020022-9254-1 ------
TACCGAATTGCTTTATTCGGCCTAGAGAGGCTCAAAAAGCAGCA
CONSENSUS
......TACCGAATTGCTTTATTCGGCCTAGAGAGGCTCAAAAAGCAGCA

RI-AT3G62310-XLOC_020022-9254-0
GATGAAGCTAAAGCTAGGTTTGGACACATTGAGGGAGATCACCTCACATT
RI-AT3G62310-XLOC_020022-9254-1
GATGAAGCTAAAGCTAGGTTTGGACACATTGAGGGAGATCACCTCACATT
CONSENSUS
GATGAAGCTAAAGCTAGGTTTGGACACATTGAGGGAGATCACCTCACATT

RI-AT3G62310-XLOC_020022-9254-0
GTTGAATGTCTACCACGCTTTCAAGCAAAACA
RI-AT3G62310-XLOC_020022-9254-1
GTTGAATGTCTACCACGCTTTCAAGCAAAACA
CONSENSUS
GTTGAATGTCTACCACGCTTTCAAGCAAAACA

alignment for event: A5-AT3G01540-XLOC_016824-4697

A5-AT3G01540-XLOC_016824-4697-0
GGGGGCCAAGTACCACCACCTCTAATGTCCTTTGAAGCTACTGGTTTTCC
A5-AT3G01540-XLOC_016824-4697-1
GGGGGCCAAGTACCACCACCTCTAATGTCCTTTGAAGCTACTGGTTTTCC
CONSENSUS
GGGGGCCAAGTACCACCACCTCTAATGTCCTTTGAAGCTACTGGTTTTCC

A5-AT3G01540-XLOC_016824-4697-0
ACCTGAGCTTCTGCGGGAGGTATGATTGGTTAATAGTAAACTGAAATGAC
A5-AT3G01540-XLOC_016824-4697-1
ACCTGAGCTTCTGCGGGAG-------------------------------
CONSENSUS
ACCTGAGCTTCTGCGGGAG...............................

A5-AT3G01540-XLOC_016824-4697-0
TTAGGAACCCCTCAAACCTGATAATTGTTCTACCAATAAGCGGAATCTAT
A5-AT3G01540-XLOC_016824-4697-1

CONSENSUS
....

A5-AT3G01540-XLOC_016824-4697-0
AACCTTATAATTGTTCTACCAATAAGCGGAATCTGTATTGAGTGTTTTTG
A5-AT3G01540-XLOC_016824-4697-1

CONSENSUS
....

A5-AT3G01540-XLOC_016824-4697-0
CTAAAGTCTGATTTATTAACATAGCTTTGTTTTTCCAGTCTCAGACTTTT
A5-AT3G01540-XLOC_016824-4697-1

CONSENSUS
....

A5-AT3G01540-XLOC_016824-4697-0
CTGTCACTTAGCTAAAATCGACTTAGGTATAACATTTAGAGTCAAAGTTT
A5-AT3G01540-XLOC_016824-4697-1

CONSENSUS
....

A5-AT3G01540-XLOC_016824-4697-0
TATGCCATACGCACCCTTTTCTCTTTTTTTCCCAGGTGCGATGTTTGCTC
A5-AT3G01540-XLOC_016824-4697-1

CONSENSUS
....

A5-AT3G01540-XLOC_016824-4697-0
CTGGGAGGAGCCAATGTCGTACACTGTGCCACTTCTTTGAGGTCCTTGCA
A5-AT3G01540-XLOC_016824-4697-1

CONSENSUS
....

A5-AT3G01540-XLOC_016824-4697-0
AATTATTTGGAGTGCCCTATATGGTGGACCAAATTTTTTGGAAAGGGCTT
A5-AT3G01540-XLOC_016824-4697-1

CONSENSUS
....

A5-AT3G01540-XLOC_016824-4697-0
CCCAAATCATCCAAGCTTGTGGCAGGGCCTGTTAATGGTGTGATGATTCA
A5-AT3G01540-XLOC_016824-4697-1

CONSENSUS
....

A5-AT3G01540-XLOC_016824-4697-0
TTTCACCACCGGGTCCAGACAGAAGAAGAGGCGTCCTCCTGCTGCAGGTA
A5-AT3G01540-XLOC_016824-4697-1
---GTA
CONSENSUS
....GTA

A5-AT3G01540-XLOC_016824-4697-0
CTCAGTGCAGGTTTCTCTGCTCCAACTCCAATTCAAGCTCAGTCATGGCC
A5-AT3G01540-XLOC_016824-4697-1
CTCAGTGCAGGTTTCTCTGCTCCAACTCCAATTCAAGCTCAGTCATGGCC
CONSENSUS
CTCAGTGCAGGTTTCTCTGCTCCAACTCCAATTCAAGCTCAGTCATGGCC

A5-AT3G01540-XLOC_016824-4697-0
CATTGCTATGCAAGGTAGGGACATAGTAGCCATTGCTAAAACTGGCTCGG
A5-AT3G01540-XLOC_016824-4697-1
CATTGCTATGCAAGGTAGGGACATAGTAGCCATTGCTAAAACTGGCTCGG
CONSENSUS
CATTGCTATGCAAGGTAGGGACATAGTAGCCATTGCTAAAACTGGCTCGG

A5-AT3G01540-XLOC_016824-4697-0
GAAAAACTTTGGGTTACTTGATTCCTGGATTTTTGCATCTTCAACGTATC
A5-AT3G01540-XLOC_016824-4697-1
GAAAAACTTTGGGTTACTTGATTCCTGGATTTTTGCATCTTCAACGTATC
CONSENSUS
GAAAAACTTTGGGTTACTTGATTCCTGGATTTTTGCATCTTCAACGTATC

A5-AT3G01540-XLOC_016824-4697-0
CGAAATGATTCGCGAATGGGCCCAACAATCTTGGTATTGTCTCCAACGAG
A5-AT3G01540-XLOC_016824-4697-1
CGAAATGATTCGCGAATGGGCCCAACAATCTTGGTATTGTCTCCAACGAG
CONSENSUS
CGAAATGATTCGCGAATGGGCCCAACAATCTTGGTATTGTCTCCAACGAG

A5-AT3G01540-XLOC_016824-4697-0
AGAGCTGGCCACACAAATCCAAGAAGAAGCTGTTAAATTTGGGAGGTCAT
A5-AT3G01540-XLOC_016824-4697-1
AGAGCTGGCCACACAAATCCAAGAAGAAGCTGTTAAATTTGGGAGGTCAT
CONSENSUS
AGAGCTGGCCACACAAATCCAAGAAGAAGCTGTTAAATTTGGGAGGTCAT

A5-AT3G01540-XLOC_016824-4697-0 CAAGAATTTCGTGTACG
A5-AT3G01540-XLOC_016824-4697-1 CAAGAATTTCGTGTACG
CONSENSUS   CAAGAATTTCGTGTACG

alignment for event: A5-AT3G57050-XLOC_019708-10492

A5-AT3G57050-XLOC_019708-10492-0
GGTTTGATCAGTAAGAACTCTCCGACCAGTGTTTCCATTTCCAAGGTTCC
A5-AT3G57050-XLOC_019708-10492-1
GGTTTGATCAGTAAGAACTCTCCGACCAGTGTTTCCATTTCCAAGGTTCC
CONSENSUS
GGTTTGATCAGTAAGAACTCTCCGACCAGTGTTTCCATTTCCAAGGTTCC

A5-AT3G57050-XLOC_019708-10492-0
AACTTGGGAGAAGAAGCAGATCTCAAATCGGAACTCTTTCAAGCTGAATT
A5-AT3G57050-XLOC_019708-10492-1
AACTTGGGAGAAGAAGCAGATCTCAAATCGGAACTCTTTCAAGCTGAATT
CONSENSUS
AACTTGGGAGAAGAAGCAGATCTCAAATCGGAACTCTTTCAAGCTGAATT

A5-AT3G57050-XLOC_019708-10492-0
GCGTGATGGAGAAAAGTGTCGATGGTCAAACTCATTCTACCGTTAACAAT
A5-AT3G57050-XLOC_019708-10492-1
GCGTGATGGAGAAAAGTGTCGATG--------------------------
CONSENSUS
GCGTGATGGAGAAAAGTGTCGATG..........................

A5-AT3G57050-XLOC_019708-10492-0
ACCACTGATAGCTTAAATACTATGAATATCAAAGAAGAAGCTAGCGTCTC
A5-AT3G57050-XLOC_019708-10492-1 -------------------
CTATGAATATCAAAGAAGAAGCTAGCGTCTC
CONSENSUS
...................CTATGAATATCAAAGAAGAAGCTAGCGTCTC

A5-AT3G57050-XLOC_019708-10492-0
TACATTATTGGTGAACTTGGATAATAAATTTGATCCCTTTGATGCAATGA
A5-AT3G57050-XLOC_019708-10492-1
TACATTATTGGTGAACTTGGATAATAAATTTGATCCCTTTGATGCAATGA
CONSENSUS
TACATTATTGGTGAACTTGGATAATAAATTTGATCCCTTTGATGCAATGA

A5-AT3G57050-XLOC_019708-10492-0
GCACTCCGCTTTACCAAACTGCTACTTTTAAGCAG
A5-AT3G57050-XLOC_019708-10492-1
GCACTCCGCTTTACCAAACTGCTACTTTTAAGCAG
CONSENSUS
GCACTCCGCTTTACCAAACTGCTACTTTTAAGCAG

alignment for event: RI-AT3G62310-XLOC_020022-9252

RI-AT3G62310-XLOC_020022-9252-0

GTACTTGACTGATGGTATGCTCCTGAGAGAAGCAATGGCGGACCCTCTTC
RI-AT3G62310-XLOC_020022-9252-1
GTACTTGACTGATGGTATGCTCCTGAGAGAAGCAATGGCGGACCCTCTTC
CONSENSUS
GTACTTGACTGATGGTATGCTCCTGAGAGAAGCAATGGCGGACCCTCTTC

RI-AT3G62310-XLOC_020022-9252-0
TAGAGAGATACAAAGTCATTATTCTTGATGAGGCCCACGAAAGGACTCTA
RI-AT3G62310-XLOC_020022-9252-1
TAGAGAGATACAAAGTCATTATTCTTGATGAGGCCCACGAAAGGACTCTA
CONSENSUS
TAGAGAGATACAAAGTCATTATTCTTGATGAGGCCCACGAAAGGACTCTA

RI-AT3G62310-XLOC_020022-9252-0
GCCACAGATGTGCTGTTTGGTCTTCTGAAAGAAGTGTTGAAAAATAGACC
RI-AT3G62310-XLOC_020022-9252-1
GCCACAGATGTGCTGTTTGGTCTTCTGAAAGAAGTGTTGAAAAATAGACC
CONSENSUS
GCCACAGATGTGCTGTTTGGTCTTCTGAAAGAAGTGTTGAAAAATAGACC

RI-AT3G62310-XLOC_020022-9252-0
TGATCTTAAGTTAGTTGTGATGAGTGCAACTCTAGAAGCTGAAAAGTTTC
RI-AT3G62310-XLOC_020022-9252-1
TGATCTTAAGTTAGTTGTGATGAGTGCAACTCTAGAAGCTGAAAAGTTTC
CONSENSUS
TGATCTTAAGTTAGTTGTGATGAGTGCAACTCTAGAAGCTGAAAAGTTTC

RI-AT3G62310-XLOC_020022-9252-0
AGGATTATTTTAGCGGTGCTCCTCTGATGAAAGTTCCTGGTAGGCTTCAT
RI-AT3G62310-XLOC_020022-9252-1
AGGATTATTTTAGCGGTGCTCCTCTGATGAAAGTTCCTGGTAGGCTTCAT
CONSENSUS
AGGATTATTTTAGCGGTGCTCCTCTGATGAAAGTTCCTGGTAGGCTTCAT

RI-AT3G62310-XLOC_020022-9252-0
CCAGTTGAGATCTTTTACACTCAGGAGCCTGAGAGGGACTATCTTGAGGC
RI-AT3G62310-XLOC_020022-9252-1
CCAGTTGAGATCTTTTACACTCAGGAGCCTGAGAGGGACTATCTTGAGGC
CONSENSUS
CCAGTTGAGATCTTTTACACTCAGGAGCCTGAGAGGGACTATCTTGAGGC

RI-AT3G62310-XLOC_020022-9252-0
TGCTATTAGAACTGTTGTCCAGATTCACATGTGTGAGCCACCTGGCGATA
RI-AT3G62310-XLOC_020022-9252-1
TGCTATTAGAACTGTTGTCCAGATTCACATGTGTGAGCCACCTGGCGATA
CONSENSUS
TGCTATTAGAACTGTTGTCCAGATTCACATGTGTGAGCCACCTGGCGATA

RI-AT3G62310-XLOC_020022-9252-0
TTCTTGTTTTCTTAACTGGGGAGGAGGAGATTGAAGATGCATGCCGCAAA
RI-AT3G62310-XLOC_020022-9252-1
TTCTTGTTTTCTTAACTGGGGAGGAGGAGATTGAAGATGCATGCCGCAAA
CONSENSUS
TTCTTGTTTTCTTAACTGGGGAGGAGGAGATTGAAGATGCATGCCGCAAA

RI-AT3G62310-XLOC_020022-9252-0

ATCAATAAAGAGGTTGGTAATCTTGGGGATCAAGTGGGGCCTATTAAAGT
RI-AT3G62310-XLOC_020022-9252-1
ATCAATAAAGAGGTTGGTAATCTTGGGGATCAAGTGGGGCCTATTAAAGT
CONSENSUS
ATCAATAAAGAGGTTGGTAATCTTGGGGATCAAGTGGGGCCTATTAAAGT

RI-AT3G62310-XLOC_020022-9252-0
AGTGCCACTGTATTCTACTCTTCCACCTGCGATGCAGCAGAAGATATTTG
RI-AT3G62310-XLOC_020022-9252-1
AGTGCCACTGTATTCTACTCTTCCACCTGCGATGCAGCAGAAGATATTTG
CONSENSUS
AGTGCCACTGTATTCTACTCTTCCACCTGCGATGCAGCAGAAGATATTTG

RI-AT3G62310-XLOC_020022-9252-0
ATCCTGCTCCAGAGCCTGTAACAGAAGGTGGTCCTCCTGGGAGAAAGATT
RI-AT3G62310-XLOC_020022-9252-1
ATCCTGCTCCAGAGCCTGTAACAGAAGGTGGTCCTCCTGGGAGAAAGATT
CONSENSUS
ATCCTGCTCCAGAGCCTGTAACAGAAGGTGGTCCTCCTGGGAGAAAGATT

RI-AT3G62310-XLOC_020022-9252-0
GTTGTCTCCACTAACATTGCTGAGACTTCTCTCACCATAGATGGGATTGT
RI-AT3G62310-XLOC_020022-9252-1
GTTGTCTCCACTAACATTGCTGAGACTTCTCTCACCATAGATGGGATTGT
CONSENSUS
GTTGTCTCCACTAACATTGCTGAGACTTCTCTCACCATAGATGGGATTGT

RI-AT3G62310-XLOC_020022-9252-0
CTACGTCATTGACCCTGGTTTTGCTAAGCAAAAAGTCTATAACCCTCGAA
RI-AT3G62310-XLOC_020022-9252-1
CTACGTCATTGACCCTGGTTTTGCTAAGCAAAAAGTCTATAACCCTCGAA
CONSENSUS
CTACGTCATTGACCCTGGTTTTGCTAAGCAAAAAGTCTATAACCCTCGAA

RI-AT3G62310-XLOC_020022-9252-0
TCCGTGTTGAGTCTTTGTTGGTGTCCCCAATTTCAAAGGCAAGTGCTCAC
RI-AT3G62310-XLOC_020022-9252-1
TCCGTGTTGAGTCTTTGTTGGTGTCCCCAATTTCAAAGGCAAGTGCTCAC
CONSENSUS
TCCGTGTTGAGTCTTTGTTGGTGTCCCCAATTTCAAAGGCAAGTGCTCAC

RI-AT3G62310-XLOC_020022-9252-0
CAGAGATCTGGTCGTGCCGGTAGAACACGGCCTGGAAAATGCTTCAGGCT
RI-AT3G62310-XLOC_020022-9252-1
CAGAGATCTGGTCGTGCCGGTAGAACACGGCCTGGAAAATGCTTCAGGCT
CONSENSUS
CAGAGATCTGGTCGTGCCGGTAGAACACGGCCTGGAAAATGCTTCAGGCT

RI-AT3G62310-XLOC_020022-9252-0
TTACACAGAGAAGAGTTTTAATAATGACTTGCAGCCGCAGACGTATCCTG
RI-AT3G62310-XLOC_020022-9252-1
TTACACAGAGAAGAGTTTTAATAATGACTTGCAGCCGCAGACGTATCCTG
CONSENSUS
TTACACAGAGAAGAGTTTTAATAATGACTTGCAGCCGCAGACGTATCCTG

RI-AT3G62310-XLOC_020022-9252-0

AAATATTGAGATCAAACCTTGCAAATACAGTTCTGACCTTGAAAAAACTG
RI-AT3G62310-XLOC_020022-9252-1
AAATATTGAGATCAAACCTTGCAAATACAGTTCTGACCTTGAAAAAACTG
CONSENSUS
AAATATTGAGATCAAACCTTGCAAATACAGTTCTGACCTTGAAAAAACTG

RI-AT3G62310-XLOC_020022-9252-0
GGTATTGATGACTTGGTGCACTTTGATTTTATGGATCCCCCTGCTCCCGA
RI-AT3G62310-XLOC_020022-9252-1
GGTATTGATGACTTGGTGCACTTTGATTTTATGGATCCCCCTGCTCCCGA
CONSENSUS
GGTATTGATGACTTGGTGCACTTTGATTTTATGGATCCCCCTGCTCCCGA

RI-AT3G62310-XLOC_020022-9252-0
AACACTGATGCGAGCCTTAGAAGTTTTGAATTACTTGGGAGCACTGGATG
RI-AT3G62310-XLOC_020022-9252-1
AACACTGATGCGAGCCTTAGAAGTTTTGAATTACTTGGGAGCACTGGATG
CONSENSUS
AACACTGATGCGAGCCTTAGAAGTTTTGAATTACTTGGGAGCACTGGATG

RI-AT3G62310-XLOC_020022-9252-0
ATGATGGTAACTTGACAAAGACAGGTGAGATCATGAGTGAGTTCCCCTTG
RI-AT3G62310-XLOC_020022-9252-1
ATGATGGTAACTTGACAAAGACAGGTGAGATCATGAGTGAGTTCCCCTTG
CONSENSUS
ATGATGGTAACTTGACAAAGACAGGTGAGATCATGAGTGAGTTCCCCTTG

RI-AT3G62310-XLOC_020022-9252-0
GATCCACAGATGGCAAAGATGCTCATAGTCAGTCCTGAATTCAACTGCTC
RI-AT3G62310-XLOC_020022-9252-1
GATCCACAGATGGCAAAGATGCTCATAGTCAGTCCTGAATTCAACTGCTC
CONSENSUS
GATCCACAGATGGCAAAGATGCTCATAGTCAGTCCTGAATTCAACTGCTC

RI-AT3G62310-XLOC_020022-9252-0
AAACGAGATTCTCTCAGTTTCAGCTATGCTATCAGGTACGCCCTTTACCT
RI-AT3G62310-XLOC_020022-9252-1
AAACGAGATTCTCTCAGTTTCAGCTATGCTATCAG---------------
CONSENSUS
AAACGAGATTCTCTCAGTTTCAGCTATGCTATCAG...............

RI-AT3G62310-XLOC_020022-9252-0
TTATCTTTTTGTTTTTGCTACCTTGGGTTAGGTATGTAGTTAAGGGTAGG
RI-AT3G62310-XLOC_020022-9252-1

CONSENSUS
....

RI-AT3G62310-XLOC_020022-9252-0
CGTAAACTTGCTGGCAGTTTCCATTCTGCATATATTCTAATGTGCATTAG
RI-AT3G62310-XLOC_020022-9252-1

CONSENSUS
....

RI-AT3G62310-XLOC_020022-9252-0

ATCTCATCTTTTATCGTTCATGTGTATAATTGTCATTTAGAATTAAGTAA
RI-AT3G62310-XLOC_020022-9252-1

CONSENSUS
....

RI-AT3G62310-XLOC_020022-9252-0
TAGAGTTAGGTTGTCATCAGCTGTCTCTAGTGTGGATTCTGCATATCCAT
RI-AT3G62310-XLOC_020022-9252-1

CONSENSUS
....

RI-AT3G62310-XLOC_020022-9252-0
GGTTAGGGGGTGTCCTAAGCAATTGTTGATGCAAATAGTTGCAGTTTCTC
RI-AT3G62310-XLOC_020022-9252-1
--TTTCTC
CONSENSUS
....TTTCTC

RI-AT3G62310-XLOC_020022-9252-0
AGTTATTCTCTCTCATGAAGCAAATGGTATCAGTCTCATCGCCTCTATGC
RI-AT3G62310-XLOC_020022-9252-1
AGTTATTCTCTCTCATGAAGCAAATGGTATCAGTCTCATCGCCTCTATGC
CONSENSUS
AGTTATTCTCTCTCATGAAGCAAATGGTATCAGTCTCATCGCCTCTATGC

RI-AT3G62310-XLOC_020022-9252-0
ATCTGCTGACCTCTTCCCTGGACCTCTTCTGTTATAATTACCAATTCTTT
RI-AT3G62310-XLOC_020022-9252-1
ATCTGCTGACCTCTTCCCTGGACCTCTTCTGTTATAATTACCAATTCTTT
CONSENSUS
ATCTGCTGACCTCTTCCCTGGACCTCTTCTGTTATAATTACCAATTCTTT

RI-AT3G62310-XLOC_020022-9252-0
CTCTAGTACCGAATTGCTTTATTCGGCCTAGAGAGGCTCAAAAAGCAGCA
RI-AT3G62310-XLOC_020022-9252-1
CTCTAGTACCGAATTGCTTTATTCGGCCTAGAGAGGCTCAAAAAGCAGCA
CONSENSUS
CTCTAGTACCGAATTGCTTTATTCGGCCTAGAGAGGCTCAAAAAGCAGCA

RI-AT3G62310-XLOC_020022-9252-0
GATGAAGCTAAAGCTAGGTTTGGACACATTGAGGGAGATCACCTCACATT
RI-AT3G62310-XLOC_020022-9252-1
GATGAAGCTAAAGCTAGGTTTGGACACATTGAGGGAGATCACCTCACATT
CONSENSUS
GATGAAGCTAAAGCTAGGTTTGGACACATTGAGGGAGATCACCTCACATT

RI-AT3G62310-XLOC_020022-9252-0
GTTGAATGTCTACCACGCTTTCAAGCAAAACA
RI-AT3G62310-XLOC_020022-9252-1
GTTGAATGTCTACCACGCTTTCAAGCAAAACA
CONSENSUS
GTTGAATGTCTACCACGCTTTCAAGCAAAACA

alignment for event: A3-AT3G55080-XLOC_019607-2702

A3-AT3G55080-XLOC_019607-2702-0
GTCACTGTTCGCCTCCAAAGTTATCTATGCTGGTGACTGCATGTTGAAAG
A3-AT3G55080-XLOC_019607-2702-1
GTCACTGTTCGCCTCCAAAGTTATCTATGCTGGTGACTGCATGTTGAAAG
CONSENSUS
GTCACTGTTCGCCTCCAAAGTTATCTATGCTGGTGACTGCATGTTGAAAG

A3-AT3G55080-XLOC_019607-2702-0 TCCCTTTTAATGCG-------------
ATGAGCTCCCCTCGGATATTAGA
A3-AT3G55080-XLOC_019607-2702-1
TCCCTTTTAATGCGCAAATAACTCCAGATGAGCTCCCCTCGGATATTAGA
CONSENSUS
TCCCTTTTAATGCG.............ATGAGCTCCCCTCGGATATTAGA

A3-AT3G55080-XLOC_019607-2702-0
GTTTTATTGAGCAATGAAGTTGGGAATATCGGAATGCTTGCTGCTGTTCT
A3-AT3G55080-XLOC_019607-2702-1
GTTTTATTGAGCAATGAAGTTGGGAATATCGGAATGCTTGCTGCTGTTCT
CONSENSUS
GTTTTATTGAGCAATGAAGTTGGGAATATCGGAATGCTTGCTGCTGTTCT

A3-AT3G55080-XLOC_019607-2702-0 AATAAGAGAGAAGAAAATGGGTCAA
A3-AT3G55080-XLOC_019607-2702-1 AATAAGAGAGAAGAAAATGGGTCAA
CONSENSUS AATAAGAGAGAAGAAAATGGGTCAA

alignment for event: SE-AT3G54380-XLOC_019563-11694

SE-AT3G54380-XLOC_019563-11694-0
AATGTTCTAGATTCTTACTTGAGTTGAATTTTGAATGCTGCATTTGTAGA
SE-AT3G54380-XLOC_019563-11694-1
AATGTTCTAGATTCTTACTTGAGTTGAATTTTGAATGCTGCATTTGTAGA
CONSENSUS
AATGTTCTAGATTCTTACTTGAGTTGAATTTTGAATGCTGCATTTGTAGA

SE-AT3G54380-XLOC_019563-11694-0
AATGCAAATTTGTGTACAAGTCAGAACCGGTGTAAATTAAATTGACCCGG
SE-AT3G54380-XLOC_019563-11694-1
AATGCAAATTTGTGTACAAGTCAGAACCGGTGTAAATTAAATTGACCCGG
CONSENSUS
AATGCAAATTTGTGTACAAGTCAGAACCGGTGTAAATTAAATTGACCCGG

SE-AT3G54380-XLOC_019563-11694-0
TTGCCGCCAGATTTAATTCGCTGAGAGTACTGTACCGGATTTGGTTCTCT
SE-AT3G54380-XLOC_019563-11694-1
TTGCCGCCAGATTTAATTCGCTGAGAGTACTGTACCGGATTTGGTTCTCT
CONSENSUS
TTGCCGCCAGATTTAATTCGCTGAGAGTACTGTACCGGATTTGGTTCTCT

SE-AT3G54380-XLOC_019563-11694-0
TCCGGTTTATTCCAAATAATAGTTCGGTAACAGCTTTGATTGGTTCGTTT
SE-AT3G54380-XLOC_019563-11694-1

TCCGGTTTATTCCAAATAATAGTTCGGTAACAGCTTTGATTGGTTCGTTT
CONSENSUS
TCCGGTTTATTCCAAATAATAGTTCGGTAACAGCTTTGATTGGTTCGTTT

SE-AT3G54380-XLOC_019563-11694-0
CTTCAGAGTCTGGTTTGGGACTTGGGAGGGAAGAAGATCTCGTTCCCTCT
SE-AT3G54380-XLOC_019563-11694-1
CTTCAGAGTCTGGTTTGGGACTTGGGAGGGAAGAAGATCTCGTTCCCTCT
CONSENSUS
CTTCAGAGTCTGGTTTGGGACTTGGGAGGGAAGAAGATCTCGTTCCCTCT

SE-AT3G54380-XLOC_019563-11694-0
AAGCCATGAATCGTCGTAATCGTGGCTCTTCTTCTTCTTCTTCTAGGGTT
SE-AT3G54380-XLOC_019563-11694-1
AAGCCATGAATCGTCGTAATCGTGGCTCTTCTTCTTCTTCTTCTAGGGTT
CONSENSUS
AAGCCATGAATCGTCGTAATCGTGGCTCTTCTTCTTCTTCTTCTAGGGTT

SE-AT3G54380-XLOC_019563-11694-0
TCGAATACTTATGGGAACCGCCAGTTCTCTGATAACCCGAGGACTGGATC
SE-AT3G54380-XLOC_019563-11694-1
TCGAATACTTATGGGAACCGCCAGTTCTCTGATAACCCGAGGACTGGATC
CONSENSUS
TCGAATACTTATGGGAACCGCCAGTTCTCTGATAACCCGAGGACTGGATC

SE-AT3G54380-XLOC_019563-11694-0
CGGCGGCGGAGTTAATGAATCCTTTCAGAGAAGATCCGATGCACCGCACA
SE-AT3G54380-XLOC_019563-11694-1
CGGCGGCGGAGTTAATGAATCCTTTCAGAGAAGATCCGATGCACCGCACA
CONSENSUS
CGGCGGCGGAGTTAATGAATCCTTTCAGAGAAGATCCGATGCACCGCACA

SE-AT3G54380-XLOC_019563-11694-0
AACGAAACAATGAAAAAGACGAATCAAAACATAAAGACGAAGATCCTGCT
SE-AT3G54380-XLOC_019563-11694-1
AACGAAACAATGAAAAAGACGAATCAAAACATAAAGACGAAGATCCTGCT
CONSENSUS
AACGAAACAATGAAAAAGACGAATCAAAACATAAAGACGAAGATCCTGCT

SE-AT3G54380-XLOC_019563-11694-0
GACGTTTCATTGATTGTAGGAACTTGTTCTTCTATGTGCCCAG-------
SE-AT3G54380-XLOC_019563-11694-1
GACGTTTCATTGATTGTAGGAACTTGTTCTTCTATGTGCCCAGTTCTTAG
CONSENSUS
GACGTTTCATTGATTGTAGGAACTTGTTCTTCTATGTGCCCAG.......

SE-AT3G54380-XLOC_019563-11694-0

SE-AT3G54380-XLOC_019563-11694-1
GTCAATTAGGTACTTTCCCTTCATCAATTCATGATCCTTCCGTGCATCGG
CONSENSUS
....

SE-AT3G54380-XLOC_019563-11694-0
---------------------------------------AGAGGGAAAGA
SE-AT3G54380-XLOC_019563-11694-1

CAGTTCAGAACAAAGAATTATCTTTTGCTAGGGGTTTAGAGAGGGAAAGA
CONSENSUS
....AGAGGGAAAGA

SE-AT3G54380-XLOC_019563-11694-0
GTCACAAGAGAACGCCTGCGTGATCTTGCAGTGTTTGAGAGGCTTTATGG
SE-AT3G54380-XLOC_019563-11694-1
GTCACAAGAGAACGCCTGCGTGATCTTGCAGTGTTTGAGAGGCTTTATGG
CONSENSUS
GTCACAAGAGAACGCCTGCGTGATCTTGCAGTGTTTGAGAGGCTTTATGG

SE-AT3G54380-XLOC_019563-11694-0
AAACCCCTCAAAATCATCCACAGAGATAGCTGTCAAAAAG
SE-AT3G54380-XLOC_019563-11694-1
AAACCCCTCAAAATCATCCACAGAGATAGCTGTCAAAAAG
CONSENSUS
AAACCCCTCAAAATCATCCACAGAGATAGCTGTCAAAAAG

alignment for event: A3-AT3G51830-XLOC_016161-2470

A3-AT3G51830-XLOC_016161-2470-0
ATATGTCTCCTTTGACTTCCATCAAGTCTGTGGAACTACAAACTTCGACA
A3-AT3G51830-XLOC_016161-2470-1
ATATGTCTCCTTTGACTTCCATCAAGTCTGTGGAACTACAAACTTCGACA
CONSENSUS
ATATGTCTCCTTTGACTTCCATCAAGTCTGTGGAACTACAAACTTCGACA

A3-AT3G51830-XLOC_016161-2470-0
ACCTGGGGGTACTGTATGAGCAGATTGGGGATGAATTTGAAAAGCAAGGA
A3-AT3G51830-XLOC_016161-2470-1
ACCTGGGGGTACTGTATGAGCAGATTGGGGATGAATTTGAAAAGCAAGG-
CONSENSUS
ACCTGGGGGTACTGTATGAGCAGATTGGGGATGAATTTGAAAAGCAAGG.

A3-AT3G51830-XLOC_016161-2470-0
TATTTCCTTGTAGACGCAGACGAGAACATCCTAGAAGAGCAGAAAGGAGT
A3-AT3G51830-XLOC_016161-2470-1 -------------
ACGCAGACGAGAACATCCTAGAAGAGCAGAAAGGAGT
CONSENSUS
.............ACGCAGACGAGAACATCCTAGAAGAGCAGAAAGGAGT

A3-AT3G51830-XLOC_016161-2470-0
TATCAGATCTAACTGCATCGACTGTCTTGACCGCACAAATGTTACCCAG
A3-AT3G51830-XLOC_016161-2470-1
TATCAGATCTAACTGCATCGACTGTCTTGACCGCACAAATGTTACCCAG
CONSENSUS
TATCAGATCTAACTGCATCGACTGTCTTGACCGCACAAATGTTACCCAG

alignment for event: RI-AT3G19900-XLOC_017860-12389

RI-AT3G19900-XLOC_017860-12389-0
GAGTTCATGGGGAAGAATCTTCTTGCAATGAATGTCATGTGGAAGTACAG

RI-AT3G19900-XLOC_017860-12389-1
GAGTTCATGGGGAAGAATCTTCTTGCAATGAATGTCATGTGGAAGTACAG
CONSENSUS
GAGTTCATGGGGAAGAATCTTCTTGCAATGAATGTCATGTGGAAGTACAG

RI-AT3G19900-XLOC_017860-12389-0
AGAACAGGTAAGAGAATCCTTTGCTGTATACTTGATCACAGAAGCCTAAT
RI-AT3G19900-XLOC_017860-12389-1
AGAACAG---
CONSENSUS
AGAACAG...

RI-AT3G19900-XLOC_017860-12389-0
GATATGATAGTTCAATTGATTTTAGTGTACTTGTTTGGACTTTAGATAAT
RI-AT3G19900-XLOC_017860-12389-1

CONSENSUS
....

RI-AT3G19900-XLOC_017860-12389-0
TATGATCTTCGTTTTCCATATCATTCAGATTTGGTATGAGAATCTTTGCC
RI-AT3G19900-XLOC_017860-12389-1

CONSENSUS
....

RI-AT3G19900-XLOC_017860-12389-0
ACATAGAAAGATGAAGTGTATATCCATATTTTGTTGGTATAGTTCTTGCT
RI-AT3G19900-XLOC_017860-12389-1

CONSENSUS
....

RI-AT3G19900-XLOC_017860-12389-0
TTTGCAAGTCAATCCTTGTAAGCTAATTCACCTTTATATATTTCTTGCTT
RI-AT3G19900-XLOC_017860-12389-1

CONSENSUS
....

RI-AT3G19900-XLOC_017860-12389-0
CTTCTTCAACGCCTAGAGGTCTTTTCCTCTAACCGAAGAAGAATATATAC
RI-AT3G19900-XLOC_017860-12389-1 ----------------
AGGTCTTTTCCTCTAACCGAAGAAGAATATATAC
CONSENSUS
................AGGTCTTTTCCTCTAACCGAAGAAGAATATATAC

RI-AT3G19900-XLOC_017860-12389-0
TTAGACTTGACGATGTAGCCAACATGTTGAAATGCTGGGGAGCAGTCTCA
RI-AT3G19900-XLOC_017860-12389-1
TTAGACTTGACGATGTAGCCAACATGTTGAAATGCTGGGGAGCAGTCTCA
CONSENSUS
TTAGACTTGACGATGTAGCCAACATGTTGAAATGCTGGGGAGCAGTCTCA

RI-AT3G19900-XLOC_017860-12389-0
CATATCCGTAGCAGCCTAGCAAAGTCGAAGGAGCGACCTCGGATAGGGAA

RI-AT3G19900-XLOC_017860-12389-1
      CATATCCGTAGCAGCCTAGCAAAGTCGAAGGAGCGACCTCGGATAGGGAA
CONSENSUS
      CATATCCGTAGCAGCCTAGCAAAGTCGAAGGAGCGACCTCGGATAGGGAA

RI-AT3G19900-XLOC_017860-12389-0 G
RI-AT3G19900-XLOC_017860-12389-1 G
CONSENSUS G

alignment for event: RI-AT3G61010-XLOC_019935-360

RI-AT3G61010-XLOC_019935-360-0
      GGCTTTGGTTACCATGTTTCACTCCAAGGTCTCCAACAGTCAGATTGTTG
RI-AT3G61010-XLOC_019935-360-1
      GGCTTTGGTTACCATGTTTCACTCCAAGGTCTCCAACAGTCAGATTGTTG
CONSENSUS
      GGCTTTGGTTACCATGTTTCACTCCAAGGTCTCCAACAGTCAGATTGTTG

RI-AT3G61010-XLOC_019935-360-0
      CTTTTAGTTTTATTCTTCACAGATGCCCCATGGTACAACATTTCTGCCAA
RI-AT3G61010-XLOC_019935-360-1
      CTTTTAGTTTTATTCTTCACAGATGCCCCATGGTACAACATTTCTGCCAA
CONSENSUS
      CTTTTAGTTTTATTCTTCACAGATGCCCCATGGTACAACATTTCTGCCAA

RI-AT3G61010-XLOC_019935-360-0
      AGTCTTCAGGTTCAACCATCTTACTGTCTTTGATTTTACTGCACACATCT
RI-AT3G61010-XLOC_019935-360-1
      AGTCTTCAG---
CONSENSUS
      AGTCTTCAG...

RI-AT3G61010-XLOC_019935-360-0
      AATGTTTACATGAACCCCATTTCCTGCCACAACATGACAGTTGATCTTCT
RI-AT3G61010-XLOC_019935-360-1

CONSENSUS
      ....

RI-AT3G61010-XLOC_019935-360-0
      TGATCTGGGGGTCTCTCTCCTCTCATCTGATCTCCGTCTTTGTTATATGC
RI-AT3G61010-XLOC_019935-360-1

CONSENSUS
      ....

RI-AT3G61010-XLOC_019935-360-0
      TTCACATTAGCCTCTCCTAGAACTCAATGAAGACAACAAGGATGTCATTC
RI-AT3G61010-XLOC_019935-360-1 ----------
CCTCTCCTAGAACTCAATGAAGACAACAAGGATGTCATTC
CONSENSUS
      ..........CCTCTCCTAGAACTCAATGAAGACAACAAGGATGTCATTC

RI-AT3G61010-XLOC_019935-360-0

AGGCCACTCTTGAGTGAGTTCTGGCCCTAATACTATCATCATATTTATTT
RI-AT3G61010-XLOC_019935-360-1
AGGCCACTCTTGAGTGAGTTCTGGCCCTAATACTATCATCATATTTATTT
CONSENSUS
AGGCCACTCTTGAGTGAGTTCTGGCCCTAATACTATCATCATATTTATTT

RI-AT3G61010-XLOC_019935-360-0
CGGCAAAGCCAAAAAGGGAAAAATGTTTTCGACATTTCTGAGCAAACATT
RI-AT3G61010-XLOC_019935-360-1
CGGCAAAGCCAAAAAGGGAAAAATGTTTTCGACATTTCTGAGCAAACATT
CONSENSUS
CGGCAAAGCCAAAAAGGGAAAAATGTTTTCGACATTTCTGAGCAAACATT

RI-AT3G61010-XLOC_019935-360-0
TAAACAATAAGTACTATGAGTCTGATAGTACTCGAGAGGCATCTTTTAAC
RI-AT3G61010-XLOC_019935-360-1
TAAACAATAAGTACTATGAGTCTGATAGTACTCGAGAGGCATCTTTTAAC
CONSENSUS
TAAACAATAAGTACTATGAGTCTGATAGTACTCGAGAGGCATCTTTTAAC

RI-AT3G61010-XLOC_019935-360-0
GGAGGAGACTACATCACTTTTAGAGGAAAACTCGAGGGAGATGCGTATTT
RI-AT3G61010-XLOC_019935-360-1
GGAGGAGACTACATCACTTTTAGAGGAAAACTCGAGGGAGATGCGTATTT
CONSENSUS
GGAGGAGACTACATCACTTTTAGAGGAAAACTCGAGGGAGATGCGTATTT

RI-AT3G61010-XLOC_019935-360-0
CACAACAAGGCTCTTCAAATCCCATCTTCATCTTTCATCTTCCCCCATCA
RI-AT3G61010-XLOC_019935-360-1
CACAACAAGGCTCTTCAAATCCCATCTTCATCTTTCATCTTCCCCCATCA
CONSENSUS
CACAACAAGGCTCTTCAAATCCCATCTTCATCTTTCATCTTCCCCCATCA

RI-AT3G61010-XLOC_019935-360-0   CAATTTCTTTCTCT
RI-AT3G61010-XLOC_019935-360-1   CAATTTCTTTCTCT
CONSENSUS   CAATTTCTTTCTCT

alignment for event: RI-AT3G61010-XLOC_019935-374

RI-AT3G61010-XLOC_019935-374-0
GTGACATCAGATGAAACCTCTAAACATGGAATCCTGCTTTCTTTCTCATC
RI-AT3G61010-XLOC_019935-374-1
GTGACATCAGATGAAACCTCTAAACATGGAATCCTGCTTTCTTTCTCATC
CONSENSUS
GTGACATCAGATGAAACCTCTAAACATGGAATCCTGCTTTCTTTCTCATC

RI-AT3G61010-XLOC_019935-374-0
TCCATCACACGAGACGAAATCCATTCTCGTTTCACGACAAGAATCCATCT
RI-AT3G61010-XLOC_019935-374-1
TCCATCACACGAGACGAAATCCATTCTCGTTTCACGACAAGAATCCATCT
CONSENSUS
TCCATCACACGAGACGAAATCCATTCTCGTTTCACGACAAGAATCCATCT

RI-AT3G61010-XLOC_019935-374-0
GTAGATTCAACAACATGTTCTTACAGTGTCTCGCCACGTCAGCGCAGACT
RI-AT3G61010-XLOC_019935-374-1
GTAGATTCAACAACATGTTCTTACAGTGTCTCGCCACGTCAGCGCAGACT
CONSENSUS
GTAGATTCAACAACATGTTCTTACAGTGTCTCGCCACGTCAGCGCAGACT

RI-AT3G61010-XLOC_019935-374-0
GTATCCGAGTGGACAGTACAGGAGACAAGCCTTGTCCTGGATGGTCACAG
RI-AT3G61010-XLOC_019935-374-1
GTATCCGAGTGGACAGTACAGGAGACAAGCCTTGTCCTGGATGGTCACAG
CONSENSUS
GTATCCGAGTGGACAGTACAGGAGACAAGCCTTGTCCTGGATGGTCACAG

RI-AT3G61010-XLOC_019935-374-0
ACTTACTGAAATCTCTGCATTTTGCTACAGACCAGAGAATTTGACAAAGA
RI-AT3G61010-XLOC_019935-374-1
ACTTACTGAAATCTCTGCATTTTGCTACAGACCAGAGAATTTGACAAAGA
CONSENSUS
ACTTACTGAAATCTCTGCATTTTGCTACAGACCAGAGAATTTGACAAAGA

RI-AT3G61010-XLOC_019935-374-0
GAACAGAATATGTTGCATTGCTCGGACACTTAAGATCATGTTCAGTACCA
RI-AT3G61010-XLOC_019935-374-1
GAACAGAATATGTTGCATTGCTCGGACACTTAAGATCATGTTCAGTACCA
CONSENSUS
GAACAGAATATGTTGCATTGCTCGGACACTTAAGATCATGTTCAGTACCA

RI-AT3G61010-XLOC_019935-374-0
GCAGAAACCCGAGACTTTACTTCCGGCATCACCGTTGGTCATTGAAGCTC
RI-AT3G61010-XLOC_019935-374-1
GCAGAAACCCGAGACTTTACTTCCGGCATCACCGTTGGTCATTGAAGCTC
CONSENSUS
GCAGAAACCCGAGACTTTACTTCCGGCATCACCGTTGGTCATTGAAGCTC

RI-AT3G61010-XLOC_019935-374-0
ATAACATGGAGCTTGTACCCGGTTATTCTGGTTCCAAGAGCCTCAGGGTT
RI-AT3G61010-XLOC_019935-374-1
ATAACATGGAGCTTGTACCCGGTTATTCTGGTTCCAAGAGCCTCAGGGTT
CONSENSUS
ATAACATGGAGCTTGTACCCGGTTATTCTGGTTCCAAGAGCCTCAGGGTT

RI-AT3G61010-XLOC_019935-374-0
AAGCTAGAATGGAGACAGAAAGACCTTGAAGATTCTGCATTCCCAAGGTA
RI-AT3G61010-XLOC_019935-374-1
AAGCTAGAATGGAGACAGAAAGACCTTGAAGATTCTGCATTCCCAAGGTA
CONSENSUS
AAGCTAGAATGGAGACAGAAAGACCTTGAAGATTCTGCATTCCCAAGGTA

RI-AT3G61010-XLOC_019935-374-0
CAATGTGTATGCGGAGAATGTAAAGTCTACTGATCTAAGACCGAGGAAGG
RI-AT3G61010-XLOC_019935-374-1
CAATGTGTATGCGGAGAATGTAAAGTCTACTGATCTAAGACCGAGGAAGG
CONSENSUS
CAATGTGTATGCGGAGAATGTAAAGTCTACTGATCTAAGACCGAGGAAGG

RI-AT3G61010-XLOC_019935-374-0
      TTCTAGAGAAGCCGAGAAGCGAAACAGTGTTTCTCGGAGTCGCTCACGTA
RI-AT3G61010-XLOC_019935-374-1
      TTCTAGAGAAGCCGAGAAGCGAAACAGTGTTTCTCGGAGTCGCTCACGTA
CONSENSUS
      TTCTAGAGAAGCCGAGAAGCGAAACAGTGTTTCTCGGAGTCGCTCACGTA

RI-AT3G61010-XLOC_019935-374-0
      CCATCCTATTACATAGCAGAACTGGTGGTAGAATCAGACGTGAAAGGAGT
RI-AT3G61010-XLOC_019935-374-1
      CCATCCTATTACATAGCAGAACTGGTGGTAGAATCAGACGTGAAAGGAGT
CONSENSUS
      CCATCCTATTACATAGCAGAACTGGTGGTAGAATCAGACGTGAAAGGAGT

RI-AT3G61010-XLOC_019935-374-0
      CCGCTTTGTGTTCAAGCCTGTGCTAAAGATGGTTCATGGGGCAAGCTGGA
RI-AT3G61010-XLOC_019935-374-1
      CCGCTTTGTGTTCAAGCCTGTGCTAAAGATG-------------------
CONSENSUS
      CCGCTTTGTGTTCAAGCCTGTGCTAAAGATG...................

RI-AT3G61010-XLOC_019935-374-0
      TTTTTATTTATTTTACACCATCATAATCGTCTCATTTGATTTATTTTTGT
RI-AT3G61010-XLOC_019935-374-1

CONSENSUS
      ....

RI-AT3G61010-XLOC_019935-374-0
      TTGTTCGGTAATCTCTTTCATAATTGATTTATGGGTACTTTTCTACTGAT
RI-AT3G61010-XLOC_019935-374-1

CONSENSUS
      ....

RI-AT3G61010-XLOC_019935-374-0
      TCAGCTACGATTCACTTATCATGGTGGGGCTGGTCGGAATCGCAATTGTC
RI-AT3G61010-XLOC_019935-374-1   ----
CTACGATTCACTTATCATGGTGGGGCTGGTCGGAATCGCAATTGTC
CONSENSUS
      ....CTACGATTCACTTATCATGGTGGGGCTGGTCGGAATCGCAATTGTC

RI-AT3G61010-XLOC_019935-374-0
      ATTTCTTGCTTTCAATGATTTTGCTTGTGGGAATCGA
RI-AT3G61010-XLOC_019935-374-1
      ATTTCTTGCTTTCAATGATTTTGCTTGTGGGAATCGA
CONSENSUS
      ATTTCTTGCTTTCAATGATTTTGCTTGTGGGAATCGA

alignment for event: A3-AT3G02750-XLOC_016876-11169

A3-AT3G02750-XLOC_016876-11169-0
      TGACCCATCTCCATTACTGTTTCTATCTCTCTCTTCTCCATTCTCTCTCT
A3-AT3G02750-XLOC_016876-11169-1

TGACCCATCTCCATTACTGTTTCTATCTCTCTCTTCTCCATTCTCTCTCT
CONSENSUS
TGACCCATCTCCATTACTGTTTCTATCTCTCTCTTCTCCATTCTCTCTCT

A3-AT3G02750-XLOC_016876-11169-0
CTATAGAGACACACAAAAGTAGAAAGACGAAGAAGAAGAGAGAACGGGTT
A3-AT3G02750-XLOC_016876-11169-1
CTATAGAGACACACAAAAGTAGAAAGACGAAGAAGAAGAGAGAACGGGTT
CONSENSUS
CTATAGAGACACACAAAAGTAGAAAGACGAAGAAGAAGAGAGAACGGGTT

A3-AT3G02750-XLOC_016876-11169-0
TGTAGTTGGTGGTTGCGTTTCTTTCTCGTTTATATTCTTCTTTCTTTTTT
A3-AT3G02750-XLOC_016876-11169-1
TGTAGTTGGTGGTTGCGTTTCTTTCTCGTTTATATTCTTCTTTCTTTTTT
CONSENSUS
TGTAGTTGGTGGTTGCGTTTCTTTCTCGTTTATATTCTTCTTTCTTTTTT

A3-AT3G02750-XLOC_016876-11169-0
GTTTTCATCTTTCAATTCCTTTTTTTCTTTTTTGTTTATAATCTAATCGA
A3-AT3G02750-XLOC_016876-11169-1
GTTTTCATCTTTCAATTCCTTTTTTTCTTTTTTGTTTATAATCTAATCGA
CONSENSUS
GTTTTCATCTTTCAATTCCTTTTTTTCTTTTTTGTTTATAATCTAATCGA

A3-AT3G02750-XLOC_016876-11169-0
CGCACGAACAGAGCAATCTCAAGCTCTTCTTCTTCCCCACAATTGAATCT
A3-AT3G02750-XLOC_016876-11169-1
CGCACGAACAGAGCAATCTCAAGCTCTTCTTCTTCCCCACAATTGAATCT
CONSENSUS
CGCACGAACAGAGCAATCTCAAGCTCTTCTTCTTCCCCACAATTGAATCT

A3-AT3G02750-XLOC_016876-11169-0
GATTCTGAGATCCTCTTACCTTCTGTTTGATCTCTCTGTTTCCTG-----
A3-AT3G02750-XLOC_016876-11169-1
GATTCTGAGATCCTCTTACCTTCTGTTTGATCTCTCTGTTTCCTGACGAA
CONSENSUS
GATTCTGAGATCCTCTTACCTTCTGTTTGATCTCTCTGTTTCCTG.....

A3-AT3G02750-XLOC_016876-11169-0

A3-AT3G02750-XLOC_016876-11169-1
GGAGATATTGTAGAGAAAGAAAGGGGTTCTCATGATTCTCGTGTTTTGAG
CONSENSUS
....

A3-AT3G02750-XLOC_016876-11169-0

A3-AT3G02750-XLOC_016876-11169-1
TCTCTTTCCAGTATAAATTAAAACTTACACTTGTCTTTCTTTTTCTTTTT
CONSENSUS
....

A3-AT3G02750-XLOC_016876-11169-0

A3-AT3G02750-XLOC_016876-11169-1

GGTGGAAATCTACGTAGATCTGTTCCTTTTTTCTCATAAAACTTTTGGGT
CONSENSUS
....

A3-AT3G02750-XLOC_016876-11169-0

A3-AT3G02750-XLOC_016876-11169-1
TTAAATTTCTGTACAAATAAAATATTTGTAAGAAGAAAAAAACGTTTTTT
CONSENSUS
....

A3-AT3G02750-XLOC_016876-11169-0

A3-AT3G02750-XLOC_016876-11169-1
TAAACAAGACTCGAAAAGGGTCAGACCCAGAAGTCTCATCTTTTACCTTT
CONSENSUS
....

A3-AT3G02750-XLOC_016876-11169-0

A3-AT3G02750-XLOC_016876-11169-1
TTTCTTGGTCAGCTTTGAAAGGCTTCATCTTTTTTTTATTCCGTTTTTTT
CONSENSUS
....

A3-AT3G02750-XLOC_016876-11169-0

A3-AT3G02750-XLOC_016876-11169-1
TTTCTTCATTCAATTTTGACGATTTGGGTTTAAAAATCACTGCTTGTGTA
CONSENSUS
....

A3-AT3G02750-XLOC_016876-11169-0 -----------------------------
ATTTTGCTCAAAAGGGTTCTT
A3-AT3G02750-XLOC_016876-11169-1
TGTATGTATACTGGTTGTTGACGAATTAGATTTTGCTCAAAAGGGTTCTT
CONSENSUS
.............................ATTTTGCTCAAAAGGGTTCTT

A3-AT3G02750-XLOC_016876-11169-0 GCACAAAGTAGCTGGAACTAAATTTTGAG
A3-AT3G02750-XLOC_016876-11169-1 GCACAAAGTAGCTGGAACTAAATTTTGAG
CONSENSUS GCACAAAGTAGCTGGAACTAAATTTTGAG

alignment for event: RI-AT3G10820-XLOC_014066-7411

RI-AT3G10820-XLOC_014066-7411-0
GATACTGAAATTGGAAAGGCTGTTAATGGTCTGAGGAGACATAGTTCCGA
RI-AT3G10820-XLOC_014066-7411-1
GATACTGAAATTGGAAAGGCTGTTAATGGTCTGAGGAGACATAGTTCCGA
CONSENSUS
GATACTGAAATTGGAAAGGCTGTTAATGGTCTGAGGAGACATAGTTCCGA

RI-AT3G10820-XLOC_014066-7411-0
TAAGATTAGCAAACTTGCAAAGACTCTTTTCGCGTATGAGTTTCATCATT

RI-AT3G10820-XLOC_014066-7411-1
TAAGATTAGCAAACTTGCAAAGACTCTTTTCGC----------------
CONSENSUS
TAAGATTAGCAAACTTGCAAAGACTCTTTTCGC................

RI-AT3G10820-XLOC_014066-7411-0
CTCATTGTGTGCTTAGTTTCATTTGTTCTCATACTCTCGCATCTGTGATG
RI-AT3G10820-XLOC_014066-7411-1

CONSENSUS
....

RI-AT3G10820-XLOC_014066-7411-0
TGAATTAAACTTTGGCAAAATGTTCCAAAAAAATTGAACCTCCACGGTTT
RI-AT3G10820-XLOC_014066-7411-1

CONSENSUS
....

RI-AT3G10820-XLOC_014066-7411-0
GATTTAGATGCAAACCAGTTGCCTTGTAGGATGTCTCAAATTGTAAAATG
RI-AT3G10820-XLOC_014066-7411-1

CONSENSUS
....

RI-AT3G10820-XLOC_014066-7411-0
TCATTATAAAAGCCTTGGTTGATCATTAGGGAGCTGCTCTTGTGTTAGCT
RI-AT3G10820-XLOC_014066-7411-1

CONSENSUS
....

RI-AT3G10820-XLOC_014066-7411-0
CAATACTTAGAACATTTTACTCTTCCTTACTTTCTGGCTCCCGAGTATAA
RI-AT3G10820-XLOC_014066-7411-1

CONSENSUS
....

RI-AT3G10820-XLOC_014066-7411-0
AGATTGTGTTTTTATATATGGGATGCACAGAGAGTGGAAGAGGCTGGTGG
RI-AT3G10820-XLOC_014066-7411-1 ------------------------------
AGAGTGGAAGAGGCTGGTGG
CONSENSUS
..............................AGAGTGGAAGAGGCTGGTGG

RI-AT3G10820-XLOC_014066-7411-0
ACCAGTGGATGAACACCCCAGAGGAAATGGCTG
RI-AT3G10820-XLOC_014066-7411-1
ACCAGTGGATGAACACCCCAGAGGAAATGGCTG
CONSENSUS
ACCAGTGGATGAACACCCCAGAGGAAATGGCTG

alignment for event: RI-AT3G51240-XLOC_016131-10886

RI-AT3G51240-XLOC_016131-10886-0
TCTGAAGTTAAAAACAGATGTAGTTAGTTGAGTAAATTGTGTTCTAGAAA
RI-AT3G51240-XLOC_016131-10886-1
TCTGAAGTTAAAAACAGATGTAGTTAGTTGAGTAAATTGTGTTCTAGAAA
CONSENSUS
TCTGAAGTTAAAAACAGATGTAGTTAGTTGAGTAAATTGTGTTCTAGAAA

RI-AT3G51240-XLOC_016131-10886-0
GAGAAGAGAGAGCAGTAGTACCGTGGTAGGTAGCTAGCGACCTCTTCGTT
RI-AT3G51240-XLOC_016131-10886-1
GAGAAGAGAGAGCAGTAGTACCGTG-------------------------
CONSENSUS
GAGAAGAGAGAGCAGTAGTACCGTG.........................

RI-AT3G51240-XLOC_016131-10886-0
CGTCAGTCATCACAAGCTTTGAAAGATTTTCAGCTACCACTCTCTCCTTT
RI-AT3G51240-XLOC_016131-10886-1

CONSENSUS
....

RI-AT3G51240-XLOC_016131-10886-0
ATATATTCATTACACATCTCTTCTTTCTATATCTCTCTTAATTTAGTCTT
RI-AT3G51240-XLOC_016131-10886-1

CONSENSUS
....

RI-AT3G51240-XLOC_016131-10886-0
TTGTCTTCGTAATTACAATGGCTCCAGGAACTTTGACTGAGCTAGCCGGA
RI-AT3G51240-XLOC_016131-10886-1 ---------------------------
GAACTTTGACTGAGCTAGCCGGA
CONSENSUS
...........................GAACTTTGACTGAGCTAGCCGGA

RI-AT3G51240-XLOC_016131-10886-0
GAGTCTAAGCTCAACTCTAAATTTGTCAGGGACGAAGATGAACGGCCCAA
RI-AT3G51240-XLOC_016131-10886-1
GAGTCTAAGCTCAACTCTAAATTTGTCAGGGACGAAGATGAACGGCCCAA
CONSENSUS
GAGTCTAAGCTCAACTCTAAATTTGTCAGGGACGAAGATGAACGGCCCAA

RI-AT3G51240-XLOC_016131-10886-0
AGTCGCTTACAATGTGTTTAGCGACGAAATCCCGGTGATCTCTCTCGCCG
RI-AT3G51240-XLOC_016131-10886-1
AGTCGCTTACAATGTGTTTAGCGACGAAATCCCGGTGATCTCTCTCGCCG
CONSENSUS
AGTCGCTTACAATGTGTTTAGCGACGAAATCCCGGTGATCTCTCTCGCCG

RI-AT3G51240-XLOC_016131-10886-0
GTATCGATGACGTCGATGGAAAAAGAGGAGAGATCTGCCGTCAGATCGTT
RI-AT3G51240-XLOC_016131-10886-1
GTATCGATGACGTCGATGGAAAAAGAGGAGAGATCTGCCGTCAGATCGTT
CONSENSUS

GTATCGATGACGTCGATGGAAAAAGAGGAGAGATCTGCCGTCAGATCGTT

RI-AT3G51240-XLOC_016131-10886-0
GAGGCTTGTGAGAATTGGGGCATCTTCCAAGTGGTCGATCACGGCGTCGA
RI-AT3G51240-XLOC_016131-10886-1
GAGGCTTGTGAGAATTGGGGCATCTTCCAAGTGGTCGATCACGGCGTCGA
CONSENSUS
GAGGCTTGTGAGAATTGGGGCATCTTCCAAGTGGTCGATCACGGCGTCGA

RI-AT3G51240-XLOC_016131-10886-0
TACTAACTTAGTGGCGGATATGACTCGTCTCGCTCGTGACTTCTTTGCTT
RI-AT3G51240-XLOC_016131-10886-1
TACTAACTTAGTGGCGGATATGACTCGTCTCGCTCGTGACTTCTTTGCTT
CONSENSUS
TACTAACTTAGTGGCGGATATGACTCGTCTCGCTCGTGACTTCTTTGCTT

RI-AT3G51240-XLOC_016131-10886-0
TACCTCCGGAAGACAAGCTCCGTTTCGACATGTCCGGTGGTAAAAAAGGA
RI-AT3G51240-XLOC_016131-10886-1
TACCTCCGGAAGACAAGCTCCGTTTCGACATGTCCGGTGGTAAAAAAGGA
CONSENSUS
TACCTCCGGAAGACAAGCTCCGTTTCGACATGTCCGGTGGTAAAAAAGGA

RI-AT3G51240-XLOC_016131-10886-0 GGATTCATCGTCTCTAGTCACCTCCAG
RI-AT3G51240-XLOC_016131-10886-1 GGATTCATCGTCTCTAGTCACCTCCAG
CONSENSUS GGATTCATCGTCTCTAGTCACCTCCAG

alignment for event: RI-AT3G01540-XLOC_016824-4694

RI-AT3G01540-XLOC_016824-4694-0
GGGGGCCAAGTACCACCACCTCTAATGTCCTTTGAAGCTACTGGTTTTCC
RI-AT3G01540-XLOC_016824-4694-1
GGGGGCCAAGTACCACCACCTCTAATGTCCTTTGAAGCTACTGGTTTTCC
CONSENSUS
GGGGGCCAAGTACCACCACCTCTAATGTCCTTTGAAGCTACTGGTTTTCC

RI-AT3G01540-XLOC_016824-4694-0
ACCTGAGCTTCTGCGGGAGGTATGATTGGTTAATAGTAAACTGAAATGAC
RI-AT3G01540-XLOC_016824-4694-1
ACCTGAGCTTCTGCGGGAGGTATGATTGGTTAATAGTAAACTGAAATGAC
CONSENSUS
ACCTGAGCTTCTGCGGGAGGTATGATTGGTTAATAGTAAACTGAAATGAC

RI-AT3G01540-XLOC_016824-4694-0
TTAGGAACCCCTCAAACCTGATAATTGTTCTACCAATAAGCGGAATCTAT
RI-AT3G01540-XLOC_016824-4694-1
TTAGGAACCCCTCAAACCTGATAATTGTTCTACCAATAAGCGGAATCTAT
CONSENSUS
TTAGGAACCCCTCAAACCTGATAATTGTTCTACCAATAAGCGGAATCTAT

RI-AT3G01540-XLOC_016824-4694-0
AACCTTATAATTGTTCTACCAATAAGCGGAATCTGTATTGAGTGTTTTTG
RI-AT3G01540-XLOC_016824-4694-1
AACCTTATAATTGTTCTACCAATAAGCGGAATCTGTATTGAGTGTTTTTG

CONSENSUS
AACCTTATAATTGTTCTACCAATAAGCGGAATCTGTATTGAGTGTTTTTG

RI-AT3G01540-XLOC_016824-4694-0
CTAAAGTCTGATTTATTAACATAGCTTTGTTTTTCCAGTCTCAGACTTTT
RI-AT3G01540-XLOC_016824-4694-1
CTAAAGTCTGATTTATTAACATAGCTTTGTTTTTCCAGTCTCAGACTTTT
CONSENSUS
CTAAAGTCTGATTTATTAACATAGCTTTGTTTTTCCAGTCTCAGACTTTT

RI-AT3G01540-XLOC_016824-4694-0
CTGTCACTTAGCTAAAATCGACTTAGGTATAACATTTAGAGTCAAAGTTT
RI-AT3G01540-XLOC_016824-4694-1
CTGTCACTTAGCTAAAATCGACTTAGGTATAACATTTAGAGTCAAAGTTT
CONSENSUS
CTGTCACTTAGCTAAAATCGACTTAGGTATAACATTTAGAGTCAAAGTTT

RI-AT3G01540-XLOC_016824-4694-0
TATGCCATACGCACCCTTTTCTCTTTTTTTCCCAGGTGCGATGTTTGCTC
RI-AT3G01540-XLOC_016824-4694-1
TATGCCATACGCACCCTTTTCTCTTTTTTTCCCAGGTGCGATGTTTGCTC
CONSENSUS
TATGCCATACGCACCCTTTTCTCTTTTTTTCCCAGGTGCGATGTTTGCTC

RI-AT3G01540-XLOC_016824-4694-0
CTGGGAGGAGCCAATGTCGTACACTGTGCCACTTCTTTGAGGTCCTTGCA
RI-AT3G01540-XLOC_016824-4694-1
CTGGGAGGAGCCAATGTCGTACACTGTGCCACTTCTTTGAGGTCCTTGCA
CONSENSUS
CTGGGAGGAGCCAATGTCGTACACTGTGCCACTTCTTTGAGGTCCTTGCA

RI-AT3G01540-XLOC_016824-4694-0
AATTATTTGGAGTGCCCTATATGGTGGACCAAATTTTTTGGAAAGGGCTT
RI-AT3G01540-XLOC_016824-4694-1
AATTATTTGGAGTGCCCTATATGGTGGACCAAATTTTTTGGAAAGGGCTT
CONSENSUS
AATTATTTGGAGTGCCCTATATGGTGGACCAAATTTTTTGGAAAGGGCTT

RI-AT3G01540-XLOC_016824-4694-0
CCCAAATCATCCAAGCTTGTGGCAGGGCCTGTTAATGGTGTGATGATTCA
RI-AT3G01540-XLOC_016824-4694-1
CCCAAATCATCCAAGCTTGTGGCAGGGCCTGTTAATGGTGTGATGATTCA
CONSENSUS
CCCAAATCATCCAAGCTTGTGGCAGGGCCTGTTAATGGTGTGATGATTCA

RI-AT3G01540-XLOC_016824-4694-0
TTTCACCACCGGGTCCAGACAGAAGAAGAGGCGTCCTCCTGCTGCAGGTT
RI-AT3G01540-XLOC_016824-4694-1
TTTCACCACCGGGTCCAGACAGAAGAAGAGGCGTCCTCCTGCTGCAG---
CONSENSUS
TTTCACCACCGGGTCCAGACAGAAGAAGAGGCGTCCTCCTGCTGCAG...

RI-AT3G01540-XLOC_016824-4694-0
TCATCAGTAACCTTTTGCCACCTGCAAATGGGGGATGGTGCTTCTTCTGA
RI-AT3G01540-XLOC_016824-4694-1

CONSENSUS
      ....

RI-AT3G01540-XLOC_016824-4694-0
      CATGAGCCACCATTCCATAAGCCCAAAAGAACCTTGGTGTTCCCCTTCTC
RI-AT3G01540-XLOC_016824-4694-1

CONSENSUS
      ....

RI-AT3G01540-XLOC_016824-4694-0
      ATAGGACAGTGTGGTTTTCTGTTTTCTTAGGTACTCAGTGCAGGTTTCTC
RI-AT3G01540-XLOC_016824-4694-1 ------------------------------
GTACTCAGTGCAGGTTTCTC
CONSENSUS
      ..............................GTACTCAGTGCAGGTTTCTC

RI-AT3G01540-XLOC_016824-4694-0
      TGCTCCAACTCCAATTCAAGCTCAGTCATGGCCCATTGCTATGCAAGGTA
RI-AT3G01540-XLOC_016824-4694-1
      TGCTCCAACTCCAATTCAAGCTCAGTCATGGCCCATTGCTATGCAAGGTA
CONSENSUS
      TGCTCCAACTCCAATTCAAGCTCAGTCATGGCCCATTGCTATGCAAGGTA

RI-AT3G01540-XLOC_016824-4694-0
      GGGACATAGTAGCCATTGCTAAAACTGGCTCGGGAAAAACTTTGGGTTAC
RI-AT3G01540-XLOC_016824-4694-1
      GGGACATAGTAGCCATTGCTAAAACTGGCTCGGGAAAAACTTTGGGTTAC
CONSENSUS
      GGGACATAGTAGCCATTGCTAAAACTGGCTCGGGAAAAACTTTGGGTTAC

RI-AT3G01540-XLOC_016824-4694-0
      TTGATTCCTGGATTTTTGCATCTTCAACGTATCCGAAATGATTCGCGAAT
RI-AT3G01540-XLOC_016824-4694-1
      TTGATTCCTGGATTTTTGCATCTTCAACGTATCCGAAATGATTCGCGAAT
CONSENSUS
      TTGATTCCTGGATTTTTGCATCTTCAACGTATCCGAAATGATTCGCGAAT

RI-AT3G01540-XLOC_016824-4694-0
      GGGCCCAACAATCTTGGTATTGTCTCCAACGAGAGAGCTGGCCACACAAA
RI-AT3G01540-XLOC_016824-4694-1
      GGGCCCAACAATCTTGGTATTGTCTCCAACGAGAGAGCTGGCCACACAAA
CONSENSUS
      GGGCCCAACAATCTTGGTATTGTCTCCAACGAGAGAGCTGGCCACACAAA

RI-AT3G01540-XLOC_016824-4694-0
      TCCAAGAAGAAGCTGTTAAATTTGGGAGGTCATCAAGAATTTCGTGTACG
RI-AT3G01540-XLOC_016824-4694-1
      TCCAAGAAGAAGCTGTTAAATTTGGGAGGTCATCAAGAATTTCGTGTACG
CONSENSUS
      TCCAAGAAGAAGCTGTTAAATTTGGGAGGTCATCAAGAATTTCGTGTACG

alignment for event: A3-AT3G20330-XLOC_017875-11231

A3-AT3G20330-XLOC_017875-11231-0
GCGTTTCGTTTTTTCCCTCCCCTAATATTCCAAAACGTCTTGAATTCATC
A3-AT3G20330-XLOC_017875-11231-1
GCGTTTCGTTTTTTCCCTCCCCTAATATTCCAAAACGTCTTGAATTCATC
CONSENSUS
GCGTTTCGTTTTTTCCCTCCCCTAATATTCCAAAACGTCTTGAATTCATC

A3-AT3G20330-XLOC_017875-11231-0
ATTACTAGTTTCCTTTCCTTAGTAAAACCCTACATTTCTCCGCCCGCCTT
A3-AT3G20330-XLOC_017875-11231-1
ATTACTAGTTTCCTTTCCTTAGTAAAACCCTACATTTCTCCGCCCGCCTT
CONSENSUS
ATTACTAGTTTCCTTTCCTTAGTAAAACCCTACATTTCTCCGCCCGCCTT

A3-AT3G20330-XLOC_017875-11231-0
TGATTTCTCCCCGGCCGTTCCCGCCGCCGGTCTCCGATTTAACCAGGATT
A3-AT3G20330-XLOC_017875-11231-1
TGATTTCTCCCCGGCCGTTCCCGCCGCCGGTCTCCGATTTAACCAGGATT
CONSENSUS
TGATTTCTCCCCGGCCGTTCCCGCCGCCGGTCTCCGATTTAACCAGGATT

A3-AT3G20330-XLOC_017875-11231-0
GAGTCTAATTCCTTCTTTTTGTGCTCATTGCTGTCGGATTCTCTACCGGC
A3-AT3G20330-XLOC_017875-11231-1
GAGTCTAATTCCTTCTTTTTGTGCTCATTGCTGTCGGATTCTCTACCGGC
CONSENSUS
GAGTCTAATTCCTTCTTTTTGTGCTCATTGCTGTCGGATTCTCTACCGGC

A3-AT3G20330-XLOC_017875-11231-0
GTTACATCGACCAGCCAGACTCTTCCCCATTTGACTGCTTTACTGATTCA
A3-AT3G20330-XLOC_017875-11231-1
GTTACATCGACCAGCCAGACTCTTCCCCATTTGACTGCTTTACTGATTCA
CONSENSUS
GTTACATCGACCAGCCAGACTCTTCCCCATTTGACTGCTTTACTGATTCA

A3-AT3G20330-XLOC_017875-11231-0 ATCGCTCTTTTAG---
ATTCTGGAAGTTGTGACTCAGTATCTCCCCTTCA
A3-AT3G20330-XLOC_017875-11231-1
ATCGCTCTTTTAGCAGATTCTGGAAGTTGTGACTCAGTATCTCCCCTTCA
CONSENSUS
ATCGCTCTTTTAG...ATTCTGGAAGTTGTGACTCAGTATCTCCCCTTCA

A3-AT3G20330-XLOC_017875-11231-0
ACGAATTAGCATCCTGCGCAAGAATGTCTATTGCATCATCACTTACTTCA
A3-AT3G20330-XLOC_017875-11231-1
ACGAATTAGCATCCTGCGCAAGAATGTCTATTGCATCATCACTTACTTCA
CONSENSUS
ACGAATTAGCATCCTGCGCAAGAATGTCTATTGCATCATCACTTACTTCA

A3-AT3G20330-XLOC_017875-11231-0
GCCACACTTTGCGGCGCCTCAGTTTTTCCTAAAGCATTAGCCTGCAGCTC
A3-AT3G20330-XLOC_017875-11231-1
GCCACACTTTGCGGCGCCTCAGTTTTTCCTAAAGCATTAGCCTGCAGCTC
CONSENSUS
GCCACACTTTGCGGCGCCTCAGTTTTTCCTAAAGCATTAGCCTGCAGCTC

A3-AT3G20330-XLOC_017875-11231-0
TGAGTTCCCTATCAATCTTCCCAGCCCTTTTGAAAGTTCAAAGATTTGTT
A3-AT3G20330-XLOC_017875-11231-1
TGAGTTCCCTATCAATCTTCCCAGCCCTTTTGAAAGTTCAAAGATTTGTT
CONSENSUS
TGAGTTCCCTATCAATCTTCCCAGCCCTTTTGAAAGTTCAAAGATTTGTT

A3-AT3G20330-XLOC_017875-11231-0
TGACTTCGTTTCCTGCCTCTAGGGATCTCAAGAAAAATGCTACTTTGAAT
A3-AT3G20330-XLOC_017875-11231-1
TGACTTCGTTTCCTGCCTCTAGGGATCTCAAGAAAAATGCTACTTTGAAT
CONSENSUS
TGACTTCGTTTCCTGCCTCTAGGGATCTCAAGAAAAATGCTACTTTGAAT

A3-AT3G20330-XLOC_017875-11231-0
CTGACTCGAAATGTTGGTCCAGTCAGGTGTCATGCTATGCAAGCTGGGAC
A3-AT3G20330-XLOC_017875-11231-1
CTGACTCGAAATGTTGGTCCAGTCAGGTGTCATGCTATGCAAGCTGGGAC
CONSENSUS
CTGACTCGAAATGTTGGTCCAGTCAGGTGTCATGCTATGCAAGCTGGGAC

A3-AT3G20330-XLOC_017875-11231-0
GAGGGAGTTGAAGAAATTTGAACTTAGTGATGTGATTGAAGGGAAACAGT
A3-AT3G20330-XLOC_017875-11231-1
GAGGGAGTTGAAGAAATTTGAACTTAGTGATGTGATTGAAGGGAAACAGT
CONSENSUS
GAGGGAGTTGAAGAAATTTGAACTTAGTGATGTGATTGAAGGGAAACAGT

A3-AT3G20330-XLOC_017875-11231-0
TTGATAGAGAGATGCTAAGCGCTATATTCGATGTTGCACGCGAAATGGAA
A3-AT3G20330-XLOC_017875-11231-1
TTGATAGAGAGATGCTAAGCGCTATATTCGATGTTGCACGCGAAATGGAA
CONSENSUS
TTGATAGAGAGATGCTAAGCGCTATATTCGATGTTGCACGCGAAATGGAA

A3-AT3G20330-XLOC_017875-11231-0
AAGATAGAAAAGAGCTCTTCACAAAGTGAAATCCTCAAGGGTTATTTAAT
A3-AT3G20330-XLOC_017875-11231-1
AAGATAGAAAAGAGCTCTTCACAAAGTGAAATCCTCAAGGGTTATTTAAT
CONSENSUS
AAGATAGAAAAGAGCTCTTCACAAAGTGAAATCCTCAAGGGTTATTTAAT

A3-AT3G20330-XLOC_017875-11231-0
GGCTACCCTCTTTTATGAGCCTTCTACCCGTACCAGGCTTTCATTTGAAT
A3-AT3G20330-XLOC_017875-11231-1
GGCTACCCTCTTTTATGAGCCTTCTACCCGTACCAGGCTTTCATTTGAAT
CONSENSUS
GGCTACCCTCTTTTATGAGCCTTCTACCCGTACCAGGCTTTCATTTGAAT

A3-AT3G20330-XLOC_017875-11231-0
CTGCTATGAAACGCCTTGGAGGTGAAGTCTTAACTACTGAGAACGCTAGA
A3-AT3G20330-XLOC_017875-11231-1
CTGCTATGAAACGCCTTGGAGGTGAAGTCTTAACTACTGAGAACGCTAGA
CONSENSUS
CTGCTATGAAACGCCTTGGAGGTGAAGTCTTAACTACTGAGAACGCTAGA

A3-AT3G20330-XLOC_017875-11231-0
      GAGTTTTCGTCTGCCGCGAAAGGGGAAACACTTGAAG
A3-AT3G20330-XLOC_017875-11231-1
      GAGTTTTCGTCTGCCGCGAAAGGGGAAACACTTGAAG
CONSENSUS
      GAGTTTTCGTCTGCCGCGAAAGGGGAAACACTTGAAG

alignment for event: RI-AT3G59800-XLOC_019865-7599

RI-AT3G59800-XLOC_019865-7599-0
      AGTTTCTTCTGTTCTCGTCTCTCTCTCGTTTGCTTTCTCTACAGATCCCT
RI-AT3G59800-XLOC_019865-7599-1
      AGTTTCTTCTGTTCTCGTCTCTCTCTCGTTTGCTTTCTCTACAGATCCCT
CONSENSUS
      AGTTTCTTCTGTTCTCGTCTCTCTCTCGTTTGCTTTCTCTACAGATCCCT

RI-AT3G59800-XLOC_019865-7599-0
      AAATCCTCTCCCTTTGGACCGTTAATCCCCCAAACCCTAGATTTTACAAA
RI-AT3G59800-XLOC_019865-7599-1
      AAATCCTCTCCCTTTGGACCGTTAATCCCCCAAACCCTAGATTTTACAAA
CONSENSUS
      AAATCCTCTCCCTTTGGACCGTTAATCCCCCAAACCCTAGATTTTACAAA

RI-AT3G59800-XLOC_019865-7599-0
      CAATTCCTCTCAATCTCTCTAAATTCTCTTTCTCCTGCAAGGAATTTGCG
RI-AT3G59800-XLOC_019865-7599-1
      CAATTCCTCTCAATCTCTCTAAATTCTCTTTCTCCTGCAAGGAATTTGCG
CONSENSUS
      CAATTCCTCTCAATCTCTCTAAATTCTCTTTCTCCTGCAAGGAATTTGCG

RI-AT3G59800-XLOC_019865-7599-0
      GACTGTTTCTTGTGGGGTAAATCTTCTATTAGCTATGGGGAAGAATCAAG
RI-AT3G59800-XLOC_019865-7599-1
      GACTGTTTCTTGTGGGGTAAATCTTCTATTAGCTATGGGGAAGAATCAAG
CONSENSUS
      GACTGTTTCTTGTGGGGTAAATCTTCTATTAGCTATGGGGAAGAATCAAG

RI-AT3G59800-XLOC_019865-7599-0
      CTTACAAGGCTATGCAGAGATCTAGGGTTGGTTCCAGCTCCGCCCAGCCG
RI-AT3G59800-XLOC_019865-7599-1
      CTTACAAGGCTATGCAGAGATCTAGGGTTGGTTCCAGCTCCGCCCAGCCG
CONSENSUS
      CTTACAAGGCTATGCAGAGATCTAGGGTTGGTTCCAGCTCCGCCCAGCCG

RI-AT3G59800-XLOC_019865-7599-0
      GATGAGGTTGAAGACGGAATGGTTTGTACCTTTTCATACTCTCATCTTCT
RI-AT3G59800-XLOC_019865-7599-1
      GATGAGGTTGAAGACGGAATG-----------------------------
CONSENSUS
      GATGAGGTTGAAGACGGAATG.............................

RI-AT3G59800-XLOC_019865-7599-0
      TCATTGCAAATTGATTCTGTTTTTTTTTACTTCTCCAAACAAAATTCCAA
RI-AT3G59800-XLOC_019865-7599-1

CONSENSUS
....

RI-AT3G59800-XLOC_019865-7599-0
TTTGGTTGATTTACTTATGGTTTTGTTGATCTTTTGTGTTGTTGGGTTAT
RI-AT3G59800-XLOC_019865-7599-1

CONSENSUS
....

RI-AT3G59800-XLOC_019865-7599-0
CGAGCTGCATTTAGAATTAAGCCTACAAGAGATCCTTTATGAGTCCCAAT
RI-AT3G59800-XLOC_019865-7599-1

CONSENSUS
....

RI-AT3G59800-XLOC_019865-7599-0
TTTGGGGGAAGAACTTGTATCTAATACCTGCTAGGTCTTGTAGACGATAT
RI-AT3G59800-XLOC_019865-7599-1

CONSENSUS
....

RI-AT3G59800-XLOC_019865-7599-0
CAAGTCCATGTTTAAATGTGATTTCGTAAAAGTGTAGCATCAGAGTTTGT
RI-AT3G59800-XLOC_019865-7599-1

CONSENSUS
....

RI-AT3G59800-XLOC_019865-7599-0
GTTACAGTGATTCAAGAGTCTTTTTATTGTTGTTGTAAGCTGTGAACACA
RI-AT3G59800-XLOC_019865-7599-1

CONSENSUS
....

RI-AT3G59800-XLOC_019865-7599-0
AAGAAGCATTTGGATTAGTCAAACTTTGATATTTTGCATTGGTACCGGAA
RI-AT3G59800-XLOC_019865-7599-1

CONSENSUS
....

RI-AT3G59800-XLOC_019865-7599-0
CATTTCTGATGAGGATTGTCTCAGGTGGATGGTTCATTTCATACACCAGA
RI-AT3G59800-XLOC_019865-7599-1 ------------------------
GTGGATGGTTCATTTCATACACCAGA
CONSENSUS
........................GTGGATGGTTCATTTCATACACCAGA

RI-AT3G59800-XLOC_019865-7599-0
GTGGCATGCGGCTCGTTTGGCTAGCCTCAAGACTACACATACTATTACCT
RI-AT3G59800-XLOC_019865-7599-1

GTGGCATGCGGCTCGTTTGGCTAGCCTCAAGACTACACATACTATTACCT
CONSENSUS
GTGGCATGCGGCTCGTTTGGCTAGCCTCAAGACTACACATACTATTACCT

RI-AT3G59800-XLOC_019865-7599-0 GGGAAGAGTATAAGCAGAAGCAAAAG
RI-AT3G59800-XLOC_019865-7599-1 GGGAAGAGTATAAGCAGAAGCAAAAG
CONSENSUS GGGAAGAGTATAAGCAGAAGCAAAAG

alignment for event: RI-AT3G29390-XLOC_015187-11093

RI-AT3G29390-XLOC_015187-11093-0
GGTTTCCTCAAGCAAGGTGTATGGTGCAGTACCACCACCACAGCAACTGA
RI-AT3G29390-XLOC_015187-11093-1
GGTTTCCTCAAGCAAGGTGTATGGTGCAGTACCACCACCACAGCAACTGA
CONSENSUS
GGTTTCCTCAAGCAAGGTGTATGGTGCAGTACCACCACCACAGCAACTGA

RI-AT3G29390-XLOC_015187-11093-0
TTTCTGGAGCTCCGGGTTCTGACCAGGAGAACCAAAATTTGATTTCAACG
RI-AT3G29390-XLOC_015187-11093-1
TTTCTGGAGCTCCGGGTTCTGACCAGGAGAACCAAAATTTGATTTCAACG
CONSENSUS
TTTCTGGAGCTCCGGGTTCTGACCAGGAGAACCAAAATTTGATTTCAACG

RI-AT3G29390-XLOC_015187-11093-0
TATGGTTTGATGACATCGATACCCATCACAGCACCACCATACGCTGTTAG
RI-AT3G29390-XLOC_015187-11093-1
TATGGTTTGATGACATCGATACCCATCACAGCACCACCATACGCTGTTAG
CONSENSUS
TATGGTTTGATGACATCGATACCCATCACAGCACCACCATACGCTGTTAG

RI-AT3G29390-XLOC_015187-11093-0
TTCGTTTCCAGTTACTCCAGCAACAAGTCTTTATCCTCAGTTTCCAGTAA
RI-AT3G29390-XLOC_015187-11093-1
TTCGTTTCCAGTTACTCCAGCAACAAGTCTTTATCCTCAGTTTCCAGTAA
CONSENSUS
TTCGTTTCCAGTTACTCCAGCAACAAGTCTTTATCCTCAGTTTCCAGTAA

RI-AT3G29390-XLOC_015187-11093-0
TGCAATCTCTAGGAATATCAAATGGTGGCCCCTCGCAGCCCGTGGCTGGA
RI-AT3G29390-XLOC_015187-11093-1
TGCAATCTCTAGGAATATCAAATGGTGGCCCCTCGCAGCCCGTGGCTGGA
CONSENSUS
TGCAATCTCTAGGAATATCAAATGGTGGCCCCTCGCAGCCCGTGGCTGGA

RI-AT3G29390-XLOC_015187-11093-0
GGAACTAGCTATAGTGGGTATGCTGGAATATACCCTCAAGCCACACCATT
RI-AT3G29390-XLOC_015187-11093-1
GGAACTAGCTATAGTGGGTATGCTGGAATATACCCTCAAGCCACACCATT
CONSENSUS
GGAACTAGCTATAGTGGGTATGCTGGAATATACCCTCAAGCCACACCATT

RI-AT3G29390-XLOC_015187-11093-0
GCAACAAGTTGCTCAAGTCCTTAAGCAATCAATTTCTCCTGTTATCTCTA

RI-AT3G29390-XLOC_015187-11093-1
GCAACAAGTTGCTCAAGTCCTTAAGCAATCAATTTCTCCTGTTATCTCTA
CONSENSUS
GCAACAAGTTGCTCAAGTCCTTAAGCAATCAATTTCTCCTGTTATCTCTA

RI-AT3G29390-XLOC_015187-11093-0
CTGTGCCCCCTACTATGTTGACAGCTACGTCCTTATCGATCCCAAGTGAT
RI-AT3G29390-XLOC_015187-11093-1
CTGTGCCCCCTACTATGTTGACAGCTACGTCCTTATCGATCCCAAGTGAT
CONSENSUS
CTGTGCCCCCTACTATGTTGACAGCTACGTCCTTATCGATCCCAAGTGAT

RI-AT3G29390-XLOC_015187-11093-0
AATGCAAGTAATGAAATGGAAAGGCGTCCACCCCGGAAGCGAAAGTTTCA
RI-AT3G29390-XLOC_015187-11093-1
AATGCAAGTAATGAAATGGAAAGGCGTCCACCCCGGAAGCGAAAGTTTCA
CONSENSUS
AATGCAAGTAATGAAATGGAAAGGCGTCCACCCCGGAAGCGAAAGTTTCA

RI-AT3G29390-XLOC_015187-11093-0
GGAACTTCCAGCTGATTGTAAGGTTCCAGAAAAAGACAAACAGGTAAAAA
RI-AT3G29390-XLOC_015187-11093-1
GGAACTTCCAGCTGATTGTAAGGTTCCAGAAAAAGACAAACAG-------
CONSENSUS
GGAACTTCCAGCTGATTGTAAGGTTCCAGAAAAAGACAAACAG.......

RI-AT3G29390-XLOC_015187-11093-0
ACATTCTGTATCCGCGGTGATTTTGACGAAGAAATACATGGATTGAACCC
RI-AT3G29390-XLOC_015187-11093-1

CONSENSUS
....

RI-AT3G29390-XLOC_015187-11093-0
TGGGGTCTTCCTTAACTAAGTAACCATGATAAAAATTTGTGCTTAGGGTT
RI-AT3G29390-XLOC_015187-11093-1

CONSENSUS
....

RI-AT3G29390-XLOC_015187-11093-0
GTTTGTAAATAACTGGGGTCTTCCATAAGTAAGTAACAATGAGAAAACTT
RI-AT3G29390-XLOC_015187-11093-1

CONSENSUS
....

RI-AT3G29390-XLOC_015187-11093-0
GTGCTTAGGGTTGTTTGAAATAAAATTTTGCCAGTATTGTCAGGTAAGTG
RI-AT3G29390-XLOC_015187-11093-1

CONSENSUS
....

RI-AT3G29390-XLOC_015187-11093-0
TTTTTCATAGACAAATGATTAAATACTTTGGGTAGAATGAGAAAAGGTAA

RI-AT3G29390-XLOC_015187-11093-1

CONSENSUS
....

RI-AT3G29390-XLOC_015187-11093-0
AGTTTTTTGCTACCCTCACTAGGCTTAGCATGCATCTTCTTAAGCATGTC
RI-AT3G29390-XLOC_015187-11093-1

CONSENSUS
....

RI-AT3G29390-XLOC_015187-11093-0
ATATGTGCACATCTGCGTCTAACAGTATTAAACCTGTTTATGAAATATTT
RI-AT3G29390-XLOC_015187-11093-1

CONSENSUS
....

RI-AT3G29390-XLOC_015187-11093-0
TTACTGGGGATTCAAAATATATCATGAGATTGTAATGAGGATCATGAAAA
RI-AT3G29390-XLOC_015187-11093-1

CONSENSUS
....

RI-AT3G29390-XLOC_015187-11093-0
TTCATGGTTTCTAATACTTTCTTTCAGTTTCCGTGTCATGTATTTTATCT
RI-AT3G29390-XLOC_015187-11093-1

CONSENSUS
....

RI-AT3G29390-XLOC_015187-11093-0
TGTGCTGATTTTTTCCCCTTACTATTCCAGGTATTTTTCTGAACTTCTAA
RI-AT3G29390-XLOC_015187-11093-1

CONSENSUS
....

RI-AT3G29390-XLOC_015187-11093-0
GAAGCTAACTAGCTGATTCTGGACCCGTGGGCCATTGGTTTGCATTTTTC
RI-AT3G29390-XLOC_015187-11093-1

CONSENSUS
....

RI-AT3G29390-XLOC_015187-11093-0
ACAGGGTATTTTTGATATTGGAGGCACCATGTGGTTTGGAGTCATTTTCG
RI-AT3G29390-XLOC_015187-11093-1 ----
GGTATTTTTGATATTGGAGGCACCATGTGGTTTGGAGTCATTTTCG
CONSENSUS
....GGTATTTTTGATATTGGAGGCACCATGTGGTTTGGAGTCATTTTCG

RI-AT3G29390-XLOC_015187-11093-0
CTTCAACTTGGCGATCAGAATCAAATTTTATTCAGACTTTTAACGACTTG

RI-AT3G29390-XLOC_015187-11093-1
CTTCAACTTGGCGATCAGAATCAAATTTTATTCAGACTTTTAACGACTTG
CONSENSUS
CTTCAACTTGGCGATCAGAATCAAATTTTATTCAGACTTTTAACGACTTG

RI-AT3G29390-XLOC_015187-11093-0
TCGTGTCCCTTGTGTGCATTGCATAATCTAGTGTATCTGCACTTTAGTAC
RI-AT3G29390-XLOC_015187-11093-1
TCGTGTCCCTTGTGTGCATTGCATAATCTAGTGTATCTGCACTTTAGTAC
CONSENSUS
TCGTGTCCCTTGTGTGCATTGCATAATCTAGTGTATCTGCACTTTAGTAC

RI-AT3G29390-XLOC_015187-11093-0
CTTGCATTTAAGTTGGCATTACTGACATCTACTATTACCCTTATCCATTG
RI-AT3G29390-XLOC_015187-11093-1
CTTGCATTTAAGTTGGCATTACTGACATCTACTATTACCCTTATCCATTG
CONSENSUS
CTTGCATTTAAGTTGGCATTACTGACATCTACTATTACCCTTATCCATTG

RI-AT3G29390-XLOC_015187-11093-0
TGTAATAGCCTCATTCTTTTCCGTAGATGGAATTCTGTGCTTGATATAGC
RI-AT3G29390-XLOC_015187-11093-1
TGTAATAGCCTCATTCTTTTCCGTAGATGGAATTCTGTGCTTGATATAGC
CONSENSUS
TGTAATAGCCTCATTCTTTTCCGTAGATGGAATTCTGTGCTTGATATAGC

RI-AT3G29390-XLOC_015187-11093-0
AGTCACATGTCTCTTAATGCATGTAGTTAGAGAGAATAATTAACATTTGA
RI-AT3G29390-XLOC_015187-11093-1
AGTCACATGTCTCTTAATGCATGTAGTTAGAGAGAATAATTAACATTTGA
CONSENSUS
AGTCACATGTCTCTTAATGCATGTAGTTAGAGAGAATAATTAACATTTGA

RI-AT3G29390-XLOC_015187-11093-0
TTGACCAGTATAAACATATCTTAGGCTGTCGTTAGTACCGAGCAGCTATG
RI-AT3G29390-XLOC_015187-11093-1
TTGACCAGTATAAACATATCTTAGGCTGTCGTTAGTACCGAGCAGCTATG
CONSENSUS
TTGACCAGTATAAACATATCTTAGGCTGTCGTTAGTACCGAGCAGCTATG

RI-AT3G29390-XLOC_015187-11093-0
TATCTTCTGATCTTGTAGGATATTAGTGAGCTTGTGACTGTTTGAATGAA
RI-AT3G29390-XLOC_015187-11093-1
TATCTTCTGATCTTGTAGGATATTAGTGAGCTTGTGACTGTTTGAATGAA
CONSENSUS
TATCTTCTGATCTTGTAGGATATTAGTGAGCTTGTGACTGTTTGAATGAA

RI-AT3G29390-XLOC_015187-11093-0
AATGTTTGTTTGAATTAATTTTGGGCATGAATGTTCTTGTAAATGTTTAC
RI-AT3G29390-XLOC_015187-11093-1
AATGTTTGTTTGAATTAATTTTGGGCATGAATGTTCTTGTAAATGTTTAC
CONSENSUS
AATGTTTGTTTGAATTAATTTTGGGCATGAATGTTCTTGTAAATGTTTAC

RI-AT3G29390-XLOC_015187-11093-0
ATTTACCAAACTCGTCTCCATGATTTAGCATTCGGGTACATTTTTCTTGG

RI-AT3G29390-XLOC_015187-11093-1
ATTTACCAAACTCGTCTCCATGATTTAGCATTCGGGTACATTTTTCTTGG
CONSENSUS
ATTTACCAAACTCGTCTCCATGATTTAGCATTCGGGTACATTTTTCTTGG

RI-AT3G29390-XLOC_015187-11093-0
ATTTGAAAGTGTTCACATTACTCTCTAGGCTTTTAGGAATACCATCTGTT
RI-AT3G29390-XLOC_015187-11093-1
ATTTGAAAGTGTTCACATTACTCTCTAGGCTTTTAGGAATACCATCTGTT
CONSENSUS
ATTTGAAAGTGTTCACATTACTCTCTAGGCTTTTAGGAATACCATCTGTT

RI-AT3G29390-XLOC_015187-11093-0
CATAGTTTATATATATGCAGTCACTTTTATCCGCCAAAACGTCTAACTGT
RI-AT3G29390-XLOC_015187-11093-1
CATAGTTTATATATATGCAGTCACTTTTATCCGCCAAAACGTCTAACTGT
CONSENSUS
CATAGTTTATATATATGCAGTCACTTTTATCCGCCAAAACGTCTAACTGT

RI-AT3G29390-XLOC_015187-11093-0
TGTAGTGTATATGCCGATGAATGTTTTAATTCTATCATATGTTGCAGCAA
RI-AT3G29390-XLOC_015187-11093-1
TGTAGTGTATATGCCGATGAATGTTTTAATTCTATCATATGTTGCAGCAA
CONSENSUS
TGTAGTGTATATGCCGATGAATGTTTTAATTCTATCATATGTTGCAGCAA

RI-AT3G29390-XLOC_015187-11093-0
TCGGAGTTAGCAATGACAGGTGATGTTACTCCATCAGCAAATAGAGTGCG
RI-AT3G29390-XLOC_015187-11093-1
TCGGAGTTAGCAATGACAGGTGATGTTACTCCATCAGCAAATAGAGTGCG
CONSENSUS
TCGGAGTTAGCAATGACAGGTGATGTTACTCCATCAGCAAATAGAGTGCG

RI-AT3G29390-XLOC_015187-11093-0
GTCGCCGCCTTCACCAAGATCTGTAATGCCTCCTCCTCCACCAAAGACCA
RI-AT3G29390-XLOC_015187-11093-1
GTCGCCGCCTTCACCAAGATCTGTAATGCCTCCTCCTCCACCAAAGACCA
CONSENSUS
GTCGCCGCCTTCACCAAGATCTGTAATGCCTCCTCCTCCACCAAAGACCA

RI-AT3G29390-XLOC_015187-11093-0
TCGCACCACCGCCTTCTAAGACCATGTCTCCTCCATCATCAAAAAGCATG
RI-AT3G29390-XLOC_015187-11093-1
TCGCACCACCGCCTTCTAAGACCATGTCTCCTCCATCATCAAAAAGCATG
CONSENSUS
TCGCACCACCGCCTTCTAAGACCATGTCTCCTCCATCATCAAAAAGCATG

RI-AT3G29390-XLOC_015187-11093-0
CTTCCTCCACCACCACGTTCTAAGACCATGTCTCCTCTATCATCAAAAAG
RI-AT3G29390-XLOC_015187-11093-1
CTTCCTCCACCACCACGTTCTAAGACCATGTCTCCTCTATCATCAAAAAG
CONSENSUS
CTTCCTCCACCACCACGTTCTAAGACCATGTCTCCTCTATCATCAAAAAG

RI-AT3G29390-XLOC_015187-11093-0
CATGCTTCCTCCACCACCGCGATTTACACTGACAACTCAACGTTCAAGAT

RI-AT3G29390-XLOC_015187-11093-1
      CATGCTTCCTCCACCACCGCGATTTACACTGACAACTCAACGTTCAAGAT
CONSENSUS
      CATGCTTCCTCCACCACCGCGATTTACACTGACAACTCAACGTTCAAGAT

RI-AT3G29390-XLOC_015187-11093-0
      TACAGGACAACCACATCAGTGTAAAGAAACCAAATCCAGTTCCAG
RI-AT3G29390-XLOC_015187-11093-1
      TACAGGACAACCACATCAGTGTAAAGAAACCAAATCCAGTTCCAG
CONSENSUS
      TACAGGACAACCACATCAGTGTAAAGAAACCAAATCCAGTTCCAG

alignment for event: A3-AT3G20810-XLOC_014648-11306

A3-AT3G20810-XLOC_014648-11306-0
      ATAAATGAACTGAGAGATGATATATGTATTCCTGATTACTGTTTTGTCGG
A3-AT3G20810-XLOC_014648-11306-1
      ATAAATGAACTGAGAGATGATATATGTATTCCTGATTACTGTTTTGTCGG
CONSENSUS
      ATAAATGAACTGAGAGATGATATATGTATTCCTGATTACTGTTTTGTCGG

A3-AT3G20810-XLOC_014648-11306-0
      TGGTGGGGAACTCCAATCACTTAATGCATGGTTTGGCCCGGCTGGGACAG
A3-AT3G20810-XLOC_014648-11306-1
      TGGTGGGGAACTCCAATCACTTAATGCATGGTTTGGCCCGGCTGGGACAG
CONSENSUS
      TGGTGGGGAACTCCAATCACTTAATGCATGGTTTGGCCCGGCTGGGACAG

A3-AT3G20810-XLOC_014648-11306-0
      TTACTCCGTTACACCATGATCCACATCATAATATACTTGCTCAGCCTGAT
A3-AT3G20810-XLOC_014648-11306-1
      TTACTCCGTTACACCATGATCCACATCATAATATACTTGCTCAG------
CONSENSUS
      TTACTCCGTTACACCATGATCCACATCATAATATACTTGCTCAG......

A3-AT3G20810-XLOC_014648-11306-0
      CTTCTCGCTGGATTGAAGTTTGATGACTCTTTGACTAACCAAAGAAATCG
A3-AT3G20810-XLOC_014648-11306-1

CONSENSUS
      ....

A3-AT3G20810-XLOC_014648-11306-0
      TTTGGCCTGCAATTCATATGTTTCTAGTTTACGGAAACTTCTTTACTTAG
A3-AT3G20810-XLOC_014648-11306-1

CONSENSUS
      ....

A3-AT3G20810-XLOC_014648-11306-0
      ATGCTTTCTCCTTCTACTGAAGTTTTGATATCATCTGTTCTTCAGGTTGT
A3-AT3G20810-XLOC_014648-11306-1
      ---GTTGT
CONSENSUS

....GTTGT

A3-AT3G20810-XLOC_014648-11306-0
TGGCAAGAAGTATATAAGGCTTTACCCATCCTTCCTGCAAGACGAACTTT
A3-AT3G20810-XLOC_014648-11306-1
TGGCAAGAAGTATATAAGGCTTTACCCATCCTTCCTGCAAGACGAACTTT
CONSENSUS
TGGCAAGAAGTATATAAGGCTTTACCCATCCTTCCTGCAAGACGAACTTT

A3-AT3G20810-XLOC_014648-11306-0
ACCCTTACTCTGAGACAATGCTCTGCAACTCTAGTCAG
A3-AT3G20810-XLOC_014648-11306-1
ACCCTTACTCTGAGACAATGCTCTGCAACTCTAGTCAG
CONSENSUS
ACCCTTACTCTGAGACAATGCTCTGCAACTCTAGTCAG

alignment for event: A5-AT3G57470-XLOC_019730-3664

A5-AT3G57470-XLOC_019730-3664-0
TGTGCTGCTTCAATGAACGTTAGCGTTGGATCGTTTACGGACCCAGAAGG
A5-AT3G57470-XLOC_019730-3664-1
TGTGCTGCTTCAATGAACGTTAGCGTTGGATCGTTTACGGACCCAGAAGG
CONSENSUS
TGTGCTGCTTCAATGAACGTTAGCGTTGGATCGTTTACGGACCCAGAAGG

A5-AT3G57470-XLOC_019730-3664-0
ATTGGAAGGGCTAGCTCATTTCCTTGGTACTTTATATTTATTATTCCCGT
A5-AT3G57470-XLOC_019730-3664-1
ATTGGAAGGGCTAGCTCATTTCCTTG------------------------
CONSENSUS
ATTGGAAGGGCTAGCTCATTTCCTTG........................

A5-AT3G57470-XLOC_019730-3664-0
TTTAATTCTTCATTGCAGGATTCAATCACTGAGCTGAGGCTAAAGTCTAA
A5-AT3G57470-XLOC_019730-3664-1

CONSENSUS
....

A5-AT3G57470-XLOC_019730-3664-0
ACTGTCTCTATAAACAGTAGCATATGCTGTTTTATGCAAGTGAAAAATAC
A5-AT3G57470-XLOC_019730-3664-1 ------------------
AGCATATGCTGTTTTATGCAAGTGAAAAATAC
CONSENSUS
..................AGCATATGCTGTTTTATGCAAGTGAAAAATAC

A5-AT3G57470-XLOC_019730-3664-0
CCTGAGGAAGATAGTTACTCCAAGTACATCACAGAG
A5-AT3G57470-XLOC_019730-3664-1
CCTGAGGAAGATAGTTACTCCAAGTACATCACAGAG
CONSENSUS
CCTGAGGAAGATAGTTACTCCAAGTACATCACAGAG

alignment for event: A5-AT3G61010-XLOC_019935-376

A5-AT3G61010-XLOC_019935-376-0
GTGACATCAGATGAAACCTCTAAACATGGAATCCTGCTTTCTTTCTCATC
A5-AT3G61010-XLOC_019935-376-1
GTGACATCAGATGAAACCTCTAAACATGGAATCCTGCTTTCTTTCTCATC
CONSENSUS
GTGACATCAGATGAAACCTCTAAACATGGAATCCTGCTTTCTTTCTCATC

A5-AT3G61010-XLOC_019935-376-0
TCCATCACACGAGACGAAATCCATTCTCGTTTCACGACAAGAATCCATCT
A5-AT3G61010-XLOC_019935-376-1
TCCATCACACGAGACGAAATCCATTCTCGTTTCACGACAAGAATCCATCT
CONSENSUS
TCCATCACACGAGACGAAATCCATTCTCGTTTCACGACAAGAATCCATCT

A5-AT3G61010-XLOC_019935-376-0
GTAGATTCAACAACATGTTCTTACAGTGTCTCGCCACGTCAGCGCAGACT
A5-AT3G61010-XLOC_019935-376-1
GTAGATTCAACAACATGTTCTTACAGTGTCTCGCCACGTCAGCGCAGACT
CONSENSUS
GTAGATTCAACAACATGTTCTTACAGTGTCTCGCCACGTCAGCGCAGACT

A5-AT3G61010-XLOC_019935-376-0
GTATCCGAGTGGACAGTACAGGAGACAAGCCTTGTCCTGGATGGTCACAG
A5-AT3G61010-XLOC_019935-376-1
GTATCCGAGTGGACAGTACAGGAGACAAGCCTTGTCCTGGATGGTCACAG
CONSENSUS
GTATCCGAGTGGACAGTACAGGAGACAAGCCTTGTCCTGGATGGTCACAG

A5-AT3G61010-XLOC_019935-376-0
ACTTACTGAAATCTCTGCATTTTGCTACAGACCAGAGAATTTGACAAAGA
A5-AT3G61010-XLOC_019935-376-1
ACTTACTGAAATCTCTGCATTTTGCTACAGACCAGAGAATTTGACAAAGA
CONSENSUS
ACTTACTGAAATCTCTGCATTTTGCTACAGACCAGAGAATTTGACAAAGA

A5-AT3G61010-XLOC_019935-376-0
GAACAGAATATGTTGCATTGCTCGGACACTTAAGATCATGTTCAGTACCA
A5-AT3G61010-XLOC_019935-376-1
GAACAGAATATGTTGCATTGCTCGGACACTTAAGATCATGTTCAGTACCA
CONSENSUS
GAACAGAATATGTTGCATTGCTCGGACACTTAAGATCATGTTCAGTACCA

A5-AT3G61010-XLOC_019935-376-0
GCAGAAACCCGAGACTTTACTTCCGGCATCACCGTTGGTCATTGAAGCTC
A5-AT3G61010-XLOC_019935-376-1
GCAGAAACCCGAGACTTTACTTCCGGCATCACCGTTGGTCATTGAAGCTC
CONSENSUS
GCAGAAACCCGAGACTTTACTTCCGGCATCACCGTTGGTCATTGAAGCTC

A5-AT3G61010-XLOC_019935-376-0
ATAACATGGAGCTTGTACCCGGTTATTCTGGTTCCAAGAGCCTCAGGGTT
A5-AT3G61010-XLOC_019935-376-1
ATAACATGGAGCTTGTACCCGGTTATTCTGGTTCCAAGAGCCTCAGGGTT

CONSENSUS
ATAACATGGAGCTTGTACCCGGTTATTCTGGTTCCAAGAGCCTCAGGGTT

A5-AT3G61010-XLOC_019935-376-0
AAGCTAGAATGGAGACAGAAAGACCTTGAAGATTCTGCATTCCCAAGGTA
A5-AT3G61010-XLOC_019935-376-1
AAGCTAGAATGGAGACAGAAAGACCTTGAAGATTCTGCATTCCCAAG---
CONSENSUS
AAGCTAGAATGGAGACAGAAAGACCTTGAAGATTCTGCATTCCCAAG...

A5-AT3G61010-XLOC_019935-376-0
CAATGTGTATGCGGAGAATGTAAAGTCTACTGATCTAAGACCGAGGAAGG
A5-AT3G61010-XLOC_019935-376-1

CONSENSUS
....

A5-AT3G61010-XLOC_019935-376-0
TTCTAGAGAAGCCGAGAAGCGAAACAGTGTTTCTCGGAGTCGCTCACGTA
A5-AT3G61010-XLOC_019935-376-1

CONSENSUS
....

A5-AT3G61010-XLOC_019935-376-0
CCATCCTATTACATAGCAGAACTGGTGGTAGAATCAGACGTGAAAGGAGT
A5-AT3G61010-XLOC_019935-376-1

CONSENSUS
....

A5-AT3G61010-XLOC_019935-376-0
CCGCTTTGTGTTCAAGCCTGTGCTAAAGATGGTTCATGGGGCAAGCTGGA
A5-AT3G61010-XLOC_019935-376-1

CONSENSUS
....

A5-AT3G61010-XLOC_019935-376-0
TTTTTATTTATTTTACACCATCATAATCGTCTCATTTGATTTATTTTTGT
A5-AT3G61010-XLOC_019935-376-1

CONSENSUS
....

A5-AT3G61010-XLOC_019935-376-0
TTGTTCGGTAATCTCTTTCATAATTGATTTATGGGTACTTTTCTACTGAT
A5-AT3G61010-XLOC_019935-376-1

CONSENSUS
....

A5-AT3G61010-XLOC_019935-376-0
TCAGCTACGATTCACTTATCATGGTGGGGCTGGTCGGAATCGCAATTGTC
A5-AT3G61010-XLOC_019935-376-1

CONSENSUS
      ....

A5-AT3G61010-XLOC_019935-376-0
      ATTTCTTGCTTTCAATGATTTTGCTTGTGGGAATCGACGTGGAGTACAAT
A5-AT3G61010-XLOC_019935-376-1
      -------------------------------------CGTGGAGTACAAT
CONSENSUS
      .....................................CGTGGAGTACAAT

A5-AT3G61010-XLOC_019935-376-0
      GTCTCCTATGTCTACCATGCCCTAGATGCCTACATCGAGAGAGACAATGT
A5-AT3G61010-XLOC_019935-376-1
      GTCTCCTATGTCTACCATGCCCTAGATGCCTACATCGAGAGAGACAATGT
CONSENSUS
      GTCTCCTATGTCTACCATGCCCTAGATGCCTACATCGAGAGAGACAATGT

A5-AT3G61010-XLOC_019935-376-0   CGGCTTGAAAGGTTTCACCAA
A5-AT3G61010-XLOC_019935-376-1   CGGCTTGAAAGGTTTCACCAA
CONSENSUS   CGGCTTGAAAGGTTTCACCAA

alignment for event: A3-AT3G58050-XLOC_016502-13784

A3-AT3G58050-XLOC_016502-13784-0
      ATTCGACAGCAAGAGATTTTGTAGAGACTGCAGACGAAACGTTATTCGTG
A3-AT3G58050-XLOC_016502-13784-1
      ATTCGACAGCAAGAGATTTTGTAGAGACTGCAGACGAAACGTTATTCGTG
CONSENSUS
      ATTCGACAGCAAGAGATTTTGTAGAGACTGCAGACGAAACGTTATTCGTG

A3-AT3G58050-XLOC_016502-13784-0
      AGTTCAAAGAGCTTAAGGAACTGAAACGAATGCGGAGAGAACCTCGGTGT
A3-AT3G58050-XLOC_016502-13784-1
      AGTTCAAAGAGCTTAAGGAACTGAAACGAATGCGGAGAGAACCTCGGTGT
CONSENSUS
      AGTTCAAAGAGCTTAAGGAACTGAAACGAATGCGGAGAGAACCTCGGTGT

A3-AT3G58050-XLOC_016502-13784-0
      ACCACATGGTTTTGTGTTGCCAATACAACATTCCAATATGAGGTATCCAT
A3-AT3G58050-XLOC_016502-13784-1
      ACCACATGGTTTTGTGTTGCCAATACAACATTCCAATATGAG--------
CONSENSUS
      ACCACATGGTTTTGTGTTGCCAATACAACATTCCAATATGAG........

A3-AT3G58050-XLOC_016502-13784-0
      TGATTCGGTGAAAGCAGACTGGCGGGAAACTTTTTCTGAGAATGCTGGAA
A3-AT3G58050-XLOC_016502-13784-1 -----------------
ACTGGCGGGAAACTTTTTCTGAGAATGCTGGAA
CONSENSUS
      .................ACTGGCGGGAAACTTTTTCTGAGAATGCTGGAA

A3-AT3G58050-XLOC_016502-13784-0
      AGTACCATCACTTTGAGTGGGCAATTGGATCCGGAGAAGGAAAATGTGAT
A3-AT3G58050-XLOC_016502-13784-1

AGTACCATCACTTTGAGTGGGCAATTGGATCCGGAGAAGGAAAATGTGAT
CONSENSUS
AGTACCATCACTTTGAGTGGGCAATTGGATCCGGAGAAGGAAAATGTGAT

A3-AT3G58050-XLOC_016502-13784-0
ATCCTCAAGTTTGAAAATGTTGGCATGAATGGGAGAGTTCAAGTCAATGG
A3-AT3G58050-XLOC_016502-13784-1
ATCCTCAAGTTTGAAAATGTTGGCATGAATGGGAGAGTTCAAGTCAATGG
CONSENSUS
ATCCTCAAGTTTGAAAATGTTGGCATGAATGGGAGAGTTCAAGTCAATGG

A3-AT3G58050-XLOC_016502-13784-0
CCTAAACCTTCGTGGTTTAAATTCATGCTACATTACCCTTAGAGCTTATA
A3-AT3G58050-XLOC_016502-13784-1
CCTAAACCTTCGTGGTTTAAATTCATGCTACATTACCCTTAGAGCTTATA
CONSENSUS
CCTAAACCTTCGTGGTTTAAATTCATGCTACATTACCCTTAGAGCTTATA

A3-AT3G58050-XLOC_016502-13784-0
AATTAGATGGCCGCTGGTCTGAAGTATCCGCCAAAGCCCATGCATTAAAA
A3-AT3G58050-XLOC_016502-13784-1
AATTAGATGGCCGCTGGTCTGAAGTATCCGCCAAAGCCCATGCATTAAAA
CONSENSUS
AATTAGATGGCCGCTGGTCTGAAGTATCCGCCAAAGCCCATGCATTAAAA

A3-AT3G58050-XLOC_016502-13784-0
GGTCAAAACTGTGTTCACGGCAGGCTAGTAGTTGGGGATGGCTTTGTTTC
A3-AT3G58050-XLOC_016502-13784-1
GGTCAAAACTGTGTTCACGGCAGGCTAGTAGTTGGGGATGGCTTTGTTTC
CONSENSUS
GGTCAAAACTGTGTTCACGGCAGGCTAGTAGTTGGGGATGGCTTTGTTTC

A3-AT3G58050-XLOC_016502-13784-0
AATCAAGAGAGGTGAAAGCATCCGAAGGTTTTTTGAGCATGCTGAAGAGG
A3-AT3G58050-XLOC_016502-13784-1
AATCAAGAGAGGTGAAAGCATCCGAAGGTTTTTTGAGCATGCTGAAGAGG
CONSENSUS
AATCAAGAGAGGTGAAAGCATCCGAAGGTTTTTTGAGCATGCTGAAGAGG

A3-AT3G58050-XLOC_016502-13784-0 CTGAGGAAGAAGAG
A3-AT3G58050-XLOC_016502-13784-1 CTGAGGAAGAAGAG
CONSENSUS CTGAGGAAGAAGAG

alignment for event: RI-AT3G07020-XLOC_017150-6411

RI-AT3G07020-XLOC_017150-6411-0
GTAAAGAGCAGTGCAGAGACACTAGCAAAGGCGATGAAGGACGAGGATGG
RI-AT3G07020-XLOC_017150-6411-1
GTAAAGAGCAGTGCAGAGACACTAGCAAAGGCGATGAAGGACGAGGATGG
CONSENSUS
GTAAAGAGCAGTGCAGAGACACTAGCAAAGGCGATGAAGGACGAGGATGG

RI-AT3G07020-XLOC_017150-6411-0
TGTGGCTGGAGCCGTGAAGGCCTTCTTTAAACATCTTCCAAGTGCAAAAC

RI-AT3G07020-XLOC_017150-6411-1
      TGTGGCTGGAGCCGTGAAGGCCTTCTTTAAACATCTTCCAAGTGCAAAAC
CONSENSUS
      TGTGGCTGGAGCCGTGAAGGCCTTCTTTAAACATCTTCCAAGTGCAAAAC

RI-AT3G07020-XLOC_017150-6411-0
      AGAATATCTCGGATCCGATCCCAGAACCTTCTGGATTTCTCTCTTTCAGG
RI-AT3G07020-XLOC_017150-6411-1
      AGAATATCTCGGATCCGATCCCAGAACCTTCTGGATTTCTCTCTTTCAGG
CONSENSUS
      AGAATATCTCGGATCCGATCCCAGAACCTTCTGGATTTCTCTCTTTCAGG

RI-AT3G07020-XLOC_017150-6411-0
      AAATGCTTTGGCTGTTCGTAACTTTCTTCTCTCCCTCCAGAATCTCCTCT
RI-AT3G07020-XLOC_017150-6411-1
      AAATGCTTTGGCT-------------------------------------
CONSENSUS
      AAATGCTTTGGCT.....................................

RI-AT3G07020-XLOC_017150-6411-0
      TTTCTCTTTTGTATTGTTGTCTCTTGTAATGTTTTTCTTCTTCGGTTTTG
RI-AT3G07020-XLOC_017150-6411-1

CONSENSUS
      ....

RI-AT3G07020-XLOC_017150-6411-0
      GCTATACAACAACTTGCTTAGGAAAAGTTTTAACATTTGTGAAGTGCTTG
RI-AT3G07020-XLOC_017150-6411-1

CONSENSUS
      ....

RI-AT3G07020-XLOC_017150-6411-0
      GGAAATTTGCTGTTCTAGGGGATGCATATATTATAAAATTGTTATAAGCA
RI-AT3G07020-XLOC_017150-6411-1

CONSENSUS
      ....

RI-AT3G07020-XLOC_017150-6411-0
      GCAAAAAAAAAAAAAAAAAAAATTTCTGAAGATGTGCAGATTAGTGAACA
RI-AT3G07020-XLOC_017150-6411-1

CONSENSUS
      ....

RI-AT3G07020-XLOC_017150-6411-0
      TTGTTGTATCGAGTTTTAATATTATGACATATTTTGTTTCAGTTTCTTGA
RI-AT3G07020-XLOC_017150-6411-1
      --TTTCTTGA
CONSENSUS
      ....TTTCTTGA

RI-AT3G07020-XLOC_017150-6411-0
      GCTGCAACTTCAATGGAATGGTGTTGTACAGAAACAACAACAAATCTCTA

RI-AT3G07020-XLOC_017150-6411-1
      GCTGCAACTTCAATGGAATGGTGTTGTACAGAAACAACAACAAATCTCTA
CONSENSUS
      GCTGCAACTTCAATGGAATGGTGTTGTACAGAAACAACAACAAATCTCTA

RI-AT3G07020-XLOC_017150-6411-0
      TTTTTTTTTATTAATTTGTTGCTTCTTTTGTTTTGGTCATGAATTGATCC
RI-AT3G07020-XLOC_017150-6411-1
      TTTTTTTTTATTAATTTGTTGCTTCTTTTGTTTTGGTCATGAATTGATCC
CONSENSUS
      TTTTTTTTTATTAATTTGTTGCTTCTTTTGTTTTGGTCATGAATTGATCC

RI-AT3G07020-XLOC_017150-6411-0
      ATGTAAGAGCATGCTTGATTGCTTCTTGTTGTTGTTGTTGTTGTATAATT
RI-AT3G07020-XLOC_017150-6411-1
      ATGTAAGAGCATGCTTGATTGCTTCTTGTTGTTGTTGTTGTTGTATAATT
CONSENSUS
      ATGTAAGAGCATGCTTGATTGCTTCTTGTTGTTGTTGTTGTTGTATAATT

RI-AT3G07020-XLOC_017150-6411-0
      GATACGGCCGATAAATGAATATCCTAATGTGGTGAGCTATTTTTGTTTGT
RI-AT3G07020-XLOC_017150-6411-1
      GATACGGCCGATAAATGAATATCCTAATGTGGTGAGCTATTTTTGTTTGT
CONSENSUS
      GATACGGCCGATAAATGAATATCCTAATGTGGTGAGCTATTTTTGTTTGT

RI-AT3G07020-XLOC_017150-6411-0 TAATCTCTTCCTTTCTTTC
RI-AT3G07020-XLOC_017150-6411-1 TAATCTCTTCCTTTCTTTC
CONSENSUS   TAATCTCTTCCTTTCTTTC

alignment for event: RI-AT3G12570-XLOC_014152-11270

RI-AT3G12570-XLOC_014152-11270-0
      GTGGGATTGTAGCTCTCCATGTGTTGTACTAGTGCCTCTTAGGACAATGT
RI-AT3G12570-XLOC_014152-11270-1
      GTGGGATTGTAGCTCTCCATGTGTTGTACTAGTGCCTCTTAGGACAATGT
CONSENSUS
      GTGGGATTGTAGCTCTCCATGTGTTGTACTAGTGCCTCTTAGGACAATGT

RI-AT3G12570-XLOC_014152-11270-0
      TTTGGAGCTTATAGTCAAATTCTGTGAAAACTTTGAGGTGGGAATTGTGA
RI-AT3G12570-XLOC_014152-11270-1
      TTTGGAGCTTATAGTCAAATTCTGTGAAAACTTTGAG-------------
CONSENSUS
      TTTGGAGCTTATAGTCAAATTCTGTGAAAACTTTGAG.............

RI-AT3G12570-XLOC_014152-11270-0
      GATCCTGTATGATGAAGATATCCTTGAATTGCATATATGGCTTTGTTGAT
RI-AT3G12570-XLOC_014152-11270-1

CONSENSUS
      ....

RI-AT3G12570-XLOC_014152-11270-0

TGATGTTCTCATCTCTTGCCACCTAAGGGTTTTGATTCGGCCACTGTATC
RI-AT3G12570-XLOC_014152-11270-1 ---------------------------
GGTTTTGATTCGGCCACTGTATC
CONSENSUS
..........................GGTTTTGATTCGGCCACTGTATC

RI-AT3G12570-XLOC_014152-11270-0
TGCTAGGGGTTCGCTTCTGGAAGAAATATGGGTGAGACTCTCATCACCAC
RI-AT3G12570-XLOC_014152-11270-1
TGCTAGGGGTTCGCTTCTGGAAGAAATATGGGTGAGACTCTCATCACCAC
CONSENSUS
TGCTAGGGGTTCGCTTCTGGAAGAAATATGGGTGAGACTCTCATCACCAC

RI-AT3G12570-XLOC_014152-11270-0
CCTGTCTATGGAGAACTATCACCCGTCAACACTGCTATCCATGGATTCAG
RI-AT3G12570-XLOC_014152-11270-1
CCTGTCTATGGAGAACTATCACCCGTCAACACTGCTATCCATGGATTCAG
CONSENSUS
CCTGTCTATGGAGAACTATCACCCGTCAACACTGCTATCCATGGATTCAG

RI-AT3G12570-XLOC_014152-11270-0
GTGCTTTTACTCATGAAGAATCAGAGAGGGATATGAATGGGTCTCTCATA
RI-AT3G12570-XLOC_014152-11270-1
GTGCTTTTACTCATGAAGAATCAGAGAGGGATATGAATGGGTCTCTCATA
CONSENSUS
GTGCTTTTACTCATGAAGAATCAGAGAGGGATATGAATGGGTCTCTCATA

RI-AT3G12570-XLOC_014152-11270-0
CTTACTGGACCGCCTGATATTAATCTACCACTCTCTTCTGAAGCTAGGCC
RI-AT3G12570-XLOC_014152-11270-1
CTTACTGGACCGCCTGATATTAATCTACCACTCTCTTCTGAAGCTAGGCC
CONSENSUS
CTTACTGGACCGCCTGATATTAATCTACCACTCTCTTCTGAAGCTAGGCC

RI-AT3G12570-XLOC_014152-11270-0
ATCTCTGCTTTTGTGGAACGAACACTGTGACATCTTAGACGTTGGACTTG
RI-AT3G12570-XLOC_014152-11270-1
ATCTCTGCTTTTGTGGAACGAACACTGTGACATCTTAGACGTTGGACTTG
CONSENSUS
ATCTCTGCTTTTGTGGAACGAACACTGTGACATCTTAGACGTTGGACTTG

RI-AT3G12570-XLOC_014152-11270-0
GACCTCAGCTATATGAGCCTGAGGCTGTTGTTCATGTGCCTAAGGTTGCT
RI-AT3G12570-XLOC_014152-11270-1
GACCTCAGCTATATGAGCCTGAGGCTGTTGTTCATGTGCCTAAGGTTGCT
CONSENSUS
GACCTCAGCTATATGAGCCTGAGGCTGTTGTTCATGTGCCTAAGGTTGCT

RI-AT3G12570-XLOC_014152-11270-0
AAGAAGTACAACAAGCGTGTTGATAGTGCATGGGGTGCTTGGCTTTTCTT
RI-AT3G12570-XLOC_014152-11270-1
AAGAAGTACAACAAGCGTGTTGATAGTGCATGGGGTGCTTGGCTTTTCTT
CONSENSUS
AAGAAGTACAACAAGCGTGTTGATAGTGCATGGGGTGCTTGGCTTTTCTT

RI-AT3G12570-XLOC_014152-11270-0

CAGTTTCTACTTCAAACCTGTTTTGGATGAGAAGTCTAAAAGTAAGTTAA
RI-AT3G12570-XLOC_014152-11270-1
CAGTTTCTACTTCAAACCTGTTTTGGATGAGAAGTCTAAAAGTAAGTTAA
CONSENSUS
CAGTTTCTACTTCAAACCTGTTTTGGATGAGAAGTCTAAAAGTAAGTTAA

RI-AT3G12570-XLOC_014152-11270-0
CGAGGGACAGCAATGGCTTGTCTGGGTATGATAAATCTGATTTGCAGCTC
RI-AT3G12570-XLOC_014152-11270-1
CGAGGGACAGCAATGGCTTGTCTGGGTATGATAAATCTGATTTGCAGCTC
CONSENSUS
CGAGGGACAGCAATGGCTTGTCTGGGTATGATAAATCTGATTTGCAGCTC

RI-AT3G12570-XLOC_014152-11270-0
GACTCGTTCTTGGTTCAGCATGACATGGAGAATATGTACATGTGGGTGTT
RI-AT3G12570-XLOC_014152-11270-1
GACTCGTTCTTGGTTCAGCATGACATGGAGAATATGTACATGTGGGTGTT
CONSENSUS
GACTCGTTCTTGGTTCAGCATGACATGGAGAATATGTACATGTGGGTGTT

RI-AT3G12570-XLOC_014152-11270-0
CAAGGAAAAGCCTGAAAATGCCCTTGGCAAGATGCAGCTGCGGAGCTACA
RI-AT3G12570-XLOC_014152-11270-1
CAAGGAAAAGCCTGAAAATGCCCTTGGCAAGATGCAGCTGCGGAGCTACA
CONSENSUS
CAAGGAAAAGCCTGAAAATGCCCTTGGCAAGATGCAGCTGCGGAGCTACA

RI-AT3G12570-XLOC_014152-11270-0
TGAACGGACACTCACGCGAGGGTGAGCGTCCTTTCCCTTTTAGTGTGGAC
RI-AT3G12570-XLOC_014152-11270-1
TGAACGGACACTCACGCGAGGGTGAGCGTCCTTTCCCTTTTAGTGTGGAC
CONSENSUS
TGAACGGACACTCACGCGAGGGTGAGCGTCCTTTCCCTTTTAGTGTGGAC

RI-AT3G12570-XLOC_014152-11270-0
AAAGGTTTTGTCCGCTCTCATAGGATGCAGAGGAAACATTACCGTGGTCT
RI-AT3G12570-XLOC_014152-11270-1
AAAGGTTTTGTCCGCTCTCATAGGATGCAGAGGAAACATTACCGTGGTCT
CONSENSUS
AAAGGTTTTGTCCGCTCTCATAGGATGCAGAGGAAACATTACCGTGGTCT

RI-AT3G12570-XLOC_014152-11270-0
CTCTAACCCACAGTGCCTCCATGGAATCGAAGTTGTTTATTCACCCAACC
RI-AT3G12570-XLOC_014152-11270-1
CTCTAACCCACAGTGCCTCCATGGAATCGAAGTTGTTTATTCACCCAACC
CONSENSUS
CTCTAACCCACAGTGCCTCCATGGAATCGAAGTTGTTTATTCACCCAACC

RI-AT3G12570-XLOC_014152-11270-0
TTTCTGTACTCAGCGAGGATGAAAAGAAGAAGTGGACAGAACTCACAGGT
RI-AT3G12570-XLOC_014152-11270-1
TTTCTGTACTCAGCGAGGATGAAAAGAAGAAGTGGACAGAACTCACAGGT
CONSENSUS
TTTCTGTACTCAGCGAGGATGAAAAGAAGAAGTGGACAGAACTCACAGGT

RI-AT3G12570-XLOC_014152-11270-0

CGAGATGTGAACTTTGCTATTCCAGCTGAAGCTAGTGATTACGGTTCATG
RI-AT3G12570-XLOC_014152-11270-1
CGAGATGTGAACTTTGCTATTCCAGCTGAAGCTAGTGATTACGGTTCATG
CONSENSUS
CGAGATGTGAACTTTGCTATTCCAGCTGAAGCTAGTGATTACGGTTCATG

RI-AT3G12570-XLOC_014152-11270-0
GAGGAATCTCCCAAACACCGAATTTGAGGCTGAGAGACCTCTTCCTCTGG
RI-AT3G12570-XLOC_014152-11270-1
GAGGAATCTCCCAAACACCGAATTTGAGGCTGAGAGACCTCTTCCTCTGG
CONSENSUS
GAGGAATCTCCCAAACACCGAATTTGAGGCTGAGAGACCTCTTCCTCTGG

RI-AT3G12570-XLOC_014152-11270-0
CTAAAGCTAACGGGCACACCCACCTGAAAAAGCTGAATGGTACCTGTCTA
RI-AT3G12570-XLOC_014152-11270-1
CTAAAGCTAACGGGCACACCCACCTGAAAAAGCTGAATGGTACCTGTCTA
CONSENSUS
CTAAAGCTAACGGGCACACCCACCTGAAAAAGCTGAATGGTACCTGTCTA

RI-AT3G12570-XLOC_014152-11270-0
AACCTGTCTACACATTCCCCAGACCATGCAGTTGATACAGTGGAAGTTCA
RI-AT3G12570-XLOC_014152-11270-1
AACCTGTCTACACATTCCCCAGACCATGCAGTTGATACAGTGGAAGTTCA
CONSENSUS
AACCTGTCTACACATTCCCCAGACCATGCAGTTGATACAGTGGAAGTTCA

RI-AT3G12570-XLOC_014152-11270-0
ACTCGCTGGTAGCAATAAACGCAAAAGGGATTGTCTTGCTCTAGGAAACT
RI-AT3G12570-XLOC_014152-11270-1
ACTCGCTGGTAGCAATAAACGCAAAAGGGATTGTCTTGCTCTAGGAAACT
CONSENSUS
ACTCGCTGGTAGCAATAAACGCAAAAGGGATTGTCTTGCTCTAGGAAACT

RI-AT3G12570-XLOC_014152-11270-0
GCGATGACTCGAGCTCAAGTGAGAAATCTCTGGACATGAAAATCCATGCA
RI-AT3G12570-XLOC_014152-11270-1
GCGATGACTCGAGCTCAAGTGAGAAATCTCTGGACATGAAAATCCATGCA
CONSENSUS
GCGATGACTCGAGCTCAAGTGAGAAATCTCTGGACATGAAAATCCATGCA

RI-AT3G12570-XLOC_014152-11270-0
ACGGAGCTGCCTTGGTCAAATGATTTTAGCGGGGTGATGAAGAATGTGTA
RI-AT3G12570-XLOC_014152-11270-1
ACGGAGCTGCCTTGGTCAAATGATTTTAGCGGGGTGATGAAGAATGTGTA
CONSENSUS
ACGGAGCTGCCTTGGTCAAATGATTTTAGCGGGGTGATGAAGAATGTGTA

RI-AT3G12570-XLOC_014152-11270-0
CGGTCCAGTCACAGCCGCAAAAACGATATATGAAGACGACAGAGGGTTCT
RI-AT3G12570-XLOC_014152-11270-1
CGGTCCAGTCACAGCCGCAAAAACGATATATGAAGACGACAGAGGGTTCT
CONSENSUS
CGGTCCAGTCACAGCCGCAAAAACGATATATGAAGACGACAGAGGGTTCT

RI-AT3G12570-XLOC_014152-11270-0

TGATAGTTATGAGCCTGCCATTTGTTGATTCCGGAAGGGTGAAAGTGACA
RI-AT3G12570-XLOC_014152-11270-1
TGATAGTTATGAGCCTGCCATTTGTTGATTCCGGAAGGGTGAAAGTGACA
CONSENSUS
TGATAGTTATGAGCCTGCCATTTGTTGATTCCGGAAGGGTGAAAGTGACA

RI-AT3G12570-XLOC_014152-11270-0
TGGAGGAATACACCAGCACATGGAATAGTGAAGATATCATGTGTAAGTAC
RI-AT3G12570-XLOC_014152-11270-1
TGGAGGAATACACCAGCACATGGAATAGTGAAGATATCATGTGTAAGTAC
CONSENSUS
TGGAGGAATACACCAGCACATGGAATAGTGAAGATATCATGTGTAAGTAC

RI-AT3G12570-XLOC_014152-11270-0
AGCATGTGAGCCATTCATCAAGAGACATGATAGAACATTTAAGCTAACAG
RI-AT3G12570-XLOC_014152-11270-1
AGCATGTGAGCCATTCATCAAGAGACATGATAGAACATTTAAGCTAACAG
CONSENSUS
AGCATGTGAGCCATTCATCAAGAGACATGATAGAACATTTAAGCTAACAG

RI-AT3G12570-XLOC_014152-11270-0
ATCCAACACCAGAGCATTGCCCACCGGGGGAATTTGTCCGCGAAGTCTCC
RI-AT3G12570-XLOC_014152-11270-1
ATCCAACACCAGAGCATTGCCCACCGGGGGAATTTGTCCGCGAAGTCTCC
CONSENSUS
ATCCAACACCAGAGCATTGCCCACCGGGGGAATTTGTCCGCGAAGTCTCC

RI-AT3G12570-XLOC_014152-11270-0
CTGCCGAACAGGATTCCAGATGACGCCAAGCTTGAAGCTTACCGGGACGA
RI-AT3G12570-XLOC_014152-11270-1
CTGCCGAACAGGATTCCAGATGACGCCAAGCTTGAAGCTTACCGGGACGA
CONSENSUS
CTGCCGAACAGGATTCCAGATGACGCCAAGCTTGAAGCTTACCGGGACGA

RI-AT3G12570-XLOC_014152-11270-0
AACAGGAACAACACTAGAGGTTTTAGTGCCTAAACACCGAATGGGACCAG
RI-AT3G12570-XLOC_014152-11270-1
AACAGGAACAACACTAGAGGTTTTAGTGCCTAAACACCGAATGGGACCAG
CONSENSUS
AACAGGAACAACACTAGAGGTTTTAGTGCCTAAACACCGAATGGGACCAG

RI-AT3G12570-XLOC_014152-11270-0
AGGAGCATGAGGTTCGCGTCTGTCTCCGTCCGTTCGTGCTAGAGTGAGAT
RI-AT3G12570-XLOC_014152-11270-1
AGGAGCATGAGGTTCGCGTCTGTCTCCGTCCGTTCGTGCTAGAGTGAGAT
CONSENSUS
AGGAGCATGAGGTTCGCGTCTGTCTCCGTCCGTTCGTGCTAGAGTGAGAT

RI-AT3G12570-XLOC_014152-11270-0
TGTAGCGCATAACTTCTTAAAAGAATTTTATTGTTCATGTATCATTGGAT
RI-AT3G12570-XLOC_014152-11270-1
TGTAGCGCATAACTTCTTAAAAGAATTTTATTGTTCATGTATCATTGGAT
CONSENSUS
TGTAGCGCATAACTTCTTAAAAGAATTTTATTGTTCATGTATCATTGGAT

RI-AT3G12570-XLOC_014152-11270-0

TATTCATATTTAATCATATTTACTCCATTTCATTGTGTTCTTTTAACCTC
RI-AT3G12570-XLOC_014152-11270-1
TATTCATATTTAATCATATTTACTCCATTTCATTGTGTTCTTTTAACCTC
CONSENSUS
TATTCATATTTAATCATATTTACTCCATTTCATTGTGTTCTTTTAACCTC

RI-AT3G12570-XLOC_014152-11270-0
TTAATTCCTTATTTGTTCATATGCTTGAATTTGTGTTACGCAATATGTGA
RI-AT3G12570-XLOC_014152-11270-1
TTAATTCCTTATTTGTTCATATGCTTGAATTTGTGTTACGCAATATGTGA
CONSENSUS
TTAATTCCTTATTTGTTCATATGCTTGAATTTGTGTTACGCAATATGTGA

RI-AT3G12570-XLOC_014152-11270-0 CAACATTCAATGGAAGACTCTTT
RI-AT3G12570-XLOC_014152-11270-1 CAACATTCAATGGAAGACTCTTT
CONSENSUS CAACATTCAATGGAAGACTCTTT

alignment for event: A3-AT3G25150-XLOC_018144-5264

A3-AT3G25150-XLOC_018144-5264-0
GCTATTGACAAGAAGATAATGGCGCTTGGTTACGGTGTAATCAGTGCAGA
A3-AT3G25150-XLOC_018144-5264-1
GCTATTGACAAGAAGATAATGGCGCTTGGTTACGGTGTAATCAGTGCAGA
CONSENSUS
GCTATTGACAAGAAGATAATGGCGCTTGGTTACGGTGTAATCAGTGCAGA

A3-AT3G25150-XLOC_018144-5264-0
GATAGCTACTGTGGACACACAAGAATCTCATGGAGGTGGTTATATTGTAC
A3-AT3G25150-XLOC_018144-5264-1
GATAGCTACTGTGGACACACAAGAATCTCATGGAGGTGGTTATATTGTAC
CONSENSUS
GATAGCTACTGTGGACACACAAGAATCTCATGGAGGTGGTTATATTGTAC

A3-AT3G25150-XLOC_018144-5264-0
TGGTGACTGGGTATTTGACGGGAAAAGACAGTGTCAGGAGGACGTTTAGT
A3-AT3G25150-XLOC_018144-5264-1
TGGTGACTGGGTATTTGACGGGAAAAGACAGTGTCAGGAGGACGTTTAGT
CONSENSUS
TGGTGACTGGGTATTTGACGGGAAAAGACAGTGTCAGGAGGACGTTTAGT

A3-AT3G25150-XLOC_018144-5264-0
CAGACCTTCTTCCTTGCTCCACAGGAGACAGGATACTTTGTCTTGAATGA
A3-AT3G25150-XLOC_018144-5264-1
CAGACCTTCTTCCTTGCTCCACAGGAGACAGGATACTTTGTCTTGAATGA
CONSENSUS
CAGACCTTCTTCCTTGCTCCACAGGAGACAGGATACTTTGTCTTGAATGA

A3-AT3G25150-XLOC_018144-5264-0
TATGTTTCGATTCATTGATGAAGGCACTGTCGTACATGGAAATCAGATTC
A3-AT3G25150-XLOC_018144-5264-1
TATGTTTCGATTCATTGATGAAGGCACTGTCGTACATGGAAATCAGATTC
CONSENSUS
TATGTTTCGATTCATTGATGAAGGCACTGTCGTACATGGAAATCAGATTC

A3-AT3G25150-XLOC_018144-5264-0
      CAGTGAACAACGTCCAAGCTCCTGTCAACACTTACCAGG------CTGCT
A3-AT3G25150-XLOC_018144-5264-1
      CAGTGAACAACGTCCAAGCTCCTGTCAACACTTACCAGGACACAGCTGCT
CONSENSUS
      CAGTGAACAACGTCCAAGCTCCTGTCAACACTTACCAGG......CTGCT

A3-AT3G25150-XLOC_018144-5264-0
      GCGAAGGAAATTCCAGATGACTTTGTTCAGGAGAAATATGTCCAAGAGAA
A3-AT3G25150-XLOC_018144-5264-1
      GCGAAGGAAATTCCAGATGACTTTGTTCAGGAGAAATATGTCCAAGAGAA
CONSENSUS
      GCGAAGGAAATTCCAGATGACTTTGTTCAGGAGAAATATGTCCAAGAGAA

A3-AT3G25150-XLOC_018144-5264-0
      TCATGCTGTTAAGCAAACCGAGGTGTTGTCCAAGAGCATTAATGAGCCTG
A3-AT3G25150-XLOC_018144-5264-1
      TCATGCTGTTAAGCAAACCGAGGTGTTGTCCAAGAGCATTAATGAGCCTG
CONSENSUS
      TCATGCTGTTAAGCAAACCGAGGTGTTGTCCAAGAGCATTAATGAGCCTG

A3-AT3G25150-XLOC_018144-5264-0
      AAAAAGTGTTCACGCCCTCTGAAGATGAACAAGTATCAGCTGCAGAAGAA
A3-AT3G25150-XLOC_018144-5264-1
      AAAAAGTGTTCACGCCCTCTGAAGATGAACAAGTATCAGCTGCAGAAGAA
CONSENSUS
      AAAAAGTGTTCACGCCCTCTGAAGATGAACAAGTATCAGCTGCAGAAGAA

A3-AT3G25150-XLOC_018144-5264-0
      GCTCTGGTGACTGAAACAGTTAATGAAGCACCAATTGAAGTGCAAAAGGT
A3-AT3G25150-XLOC_018144-5264-1
      GCTCTGGTGACTGAAACAGTTAATGAAGCACCAATTGAAGTGCAAAAGGT
CONSENSUS
      GCTCTGGTGACTGAAACAGTTAATGAAGCACCAATTGAAGTGCAAAAGGT

A3-AT3G25150-XLOC_018144-5264-0
      TGGAGAATCTGATTCTAGGACTGGCGAAATTCCAAAGAGATCTTATGCAT
A3-AT3G25150-XLOC_018144-5264-1
      TGGAGAATCTGATTCTAGGACTGGCGAAATTCCAAAGAGATCTTATGCAT
CONSENSUS
      TGGAGAATCTGATTCTAGGACTGGCGAAATTCCAAAGAGATCTTATGCAT

A3-AT3G25150-XLOC_018144-5264-0 CAATT
A3-AT3G25150-XLOC_018144-5264-1 CAATT
CONSENSUS   CAATT

alignment for event: RI-AT3G25570-XLOC_018162-9920

RI-AT3G25570-XLOC_018162-9920-0
      ATCACTGGAAAACCATCCTGATATTCCTCAATACGTTTTCTTCTTAACCA
RI-AT3G25570-XLOC_018162-9920-1
      ATCACTGGAAAACCATCCTGATATTCCTCAATACGTTTTCTTCTTAACCA
CONSENSUS
      ATCACTGGAAAACCATCCTGATATTCCTCAATACGTTTTCTTCTTAACCA

RI-AT3G25570-XLOC_018162-9920-0
CTTACTAACTGTGGTTGATCAATGTTCGCGATAAATCTTCGTTTTTTCTT
RI-AT3G25570-XLOC_018162-9920-1
CTTACTAACTGTGGTTGATCAAT---------------------------
CONSENSUS
CTTACTAACTGTGGTTGATCAAT...........................

RI-AT3G25570-XLOC_018162-9920-0
GTTTTGTTCTCTTTCAAAACTCATTTTTCGATAGTCTCTTCGCCTTTTGA
RI-AT3G25570-XLOC_018162-9920-1

CONSENSUS
....

RI-AT3G25570-XLOC_018162-9920-0
AAAATGGCGGTCTCTGCTACAGGTTTCGAAGGATTTGAGAAAAGGCTCGA
RI-AT3G25570-XLOC_018162-9920-1 ----------------------
GTTTCGAAGGATTTGAGAAAAGGCTCGA
CONSENSUS
......................GTTTCGAAGGATTTGAGAAAAGGCTCGA

RI-AT3G25570-XLOC_018162-9920-0
AATCTCATTCTTTGAGACTACTGACTTTCTTGATCCCCAAGGAAAGAGTC
RI-AT3G25570-XLOC_018162-9920-1
AATCTCATTCTTTGAGACTACTGACTTTCTTGATCCCCAAGGAAAGAGTC
CONSENSUS
AATCTCATTCTTTGAGACTACTGACTTTCTTGATCCCCAAGGAAAGAGTC

RI-AT3G25570-XLOC_018162-9920-0
TTCGTTCTCTTACCAAATCCCAGTTAGATGAGATCTTGACTCCAGCAGAG
RI-AT3G25570-XLOC_018162-9920-1
TTCGTTCTCTTACCAAATCCCAGTTAGATGAGATCTTGACTCCAGCAGAG
CONSENSUS
TTCGTTCTCTTACCAAATCCCAGTTAGATGAGATCTTGACTCCAGCAGAG

RI-AT3G25570-XLOC_018162-9920-0
TGCACAATTGTTTCCTCTCTCACAAACTCTTTCGTTGACTCTTACGTGCT
RI-AT3G25570-XLOC_018162-9920-1
TGCACAATTGTTTCCTCTCTCACAAACTCTTTCGTTGACTCTTACGTGCT
CONSENSUS
TGCACAATTGTTTCCTCTCTCACAAACTCTTTCGTTGACTCTTACGTGCT

RI-AT3G25570-XLOC_018162-9920-0
CTCTGAATCTAGTCTCTTTGTTTATCCTTACAAGATCATCATCAAAACAT
RI-AT3G25570-XLOC_018162-9920-1
CTCTGAATCTAGTCTCTTTGTTTATCCTTACAAGATCATCATCAAAACAT
CONSENSUS
CTCTGAATCTAGTCTCTTTGTTTATCCTTACAAGATCATCATCAAAACAT

RI-AT3G25570-XLOC_018162-9920-0
GCGGGACTACTAAGCTTCTCTTGTCGATCCCGCATATCCTTAGGCTGGCT
RI-AT3G25570-XLOC_018162-9920-1
GCGGGACTACTAAGCTTCTCTTGTCGATCCCGCATATCCTTAGGCTGGCT
CONSENSUS
GCGGGACTACTAAGCTTCTCTTGTCGATCCCGCATATCCTTAGGCTGGCT

RI-AT3G25570-XLOC_018162-9920-0
GATTCACTTTGCCTCACTGTCAAATCTGTTCGCTACACTCGTGGTAGCTT
RI-AT3G25570-XLOC_018162-9920-1
GATTCACTTTGCCTCACTGTCAAATCTGTTCGCTACACTCGTGGTAGCTT
CONSENSUS
GATTCACTTTGCCTCACTGTCAAATCTGTTCGCTACACTCGTGGTAGCTT

RI-AT3G25570-XLOC_018162-9920-0
TATTTTTCCGGGAGCTCAATCTTACCCTCACCGTAGCTTCTCAGAGGAAG
RI-AT3G25570-XLOC_018162-9920-1
TATTTTTCCGGGAGCTCAATCTTACCCTCACCGTAGCTTCTCAGAGGAAG
CONSENSUS
TATTTTTCCGGGAGCTCAATCTTACCCTCACCGTAGCTTCTCAGAGGAAG

RI-AT3G25570-XLOC_018162-9920-0
TTGCTTTACTTGATGACTATTTTGGTAAGCTCAATGCAGGAAGCAAAGCT
RI-AT3G25570-XLOC_018162-9920-1
TTGCTTTACTTGATGACTATTTTGGTAAGCTCAATGCAGGAAGCAAAGCT
CONSENSUS
TTGCTTTACTTGATGACTATTTTGGTAAGCTCAATGCAGGAAGCAAAGCT

RI-AT3G25570-XLOC_018162-9920-0
TTTGTGATGGGAGGTTCAGATAATAACCCTCAGAGATGGCATGTTTACTC
RI-AT3G25570-XLOC_018162-9920-1
TTTGTGATGGGAGGTTCAGATAATAACCCTCAGAGATGGCATGTTTACTC
CONSENSUS
TTTGTGATGGGAGGTTCAGATAATAACCCTCAGAGATGGCATGTTTACTC

RI-AT3G25570-XLOC_018162-9920-0
TGCTTCTTCTACTGAAGAATCTGCGGTTTGCGATAAACCCGTGTACACAT
RI-AT3G25570-XLOC_018162-9920-1
TGCTTCTTCTACTGAAGAATCTGCGGTTTGCGATAAACCCGTGTACACAT
CONSENSUS
TGCTTCTTCTACTGAAGAATCTGCGGTTTGCGATAAACCCGTGTACACAT

RI-AT3G25570-XLOC_018162-9920-0
TGGAGATGTGTATGACTGGTCTAGACAATATAAAAGCTTCGGTGTTCTTT
RI-AT3G25570-XLOC_018162-9920-1
TGGAGATGTGTATGACTGGTCTAGACAATATAAAAGCTTCGGTGTTCTTT
CONSENSUS
TGGAGATGTGTATGACTGGTCTAGACAATATAAAAGCTTCGGTGTTCTTT

RI-AT3G25570-XLOC_018162-9920-0
AAAACCAACTCTGTTTCAGCTTCTGAGATGACTATTAGCTCTGGGATCAG
RI-AT3G25570-XLOC_018162-9920-1
AAAACCAACTCTGTTTCAGCTTCTGAGATGACTATTAGCTCTGGGATCAG
CONSENSUS
AAAACCAACTCTGTTTCAGCTTCTGAGATGACTATTAGCTCTGGGATCAG

RI-AT3G25570-XLOC_018162-9920-0
AAACATCCTTCCAGGCTCTGAAATCTGTGATTTTAACTTTGAACCGTGCG
RI-AT3G25570-XLOC_018162-9920-1
AAACATCCTTCCAGGCTCTGAAATCTGTGATTTTAACTTTGAACCGTGCG
CONSENSUS
AAACATCCTTCCAGGCTCTGAAATCTGTGATTTTAACTTTGAACCGTGCG

RI-AT3G25570-XLOC_018162-9920-0
GTTACTCGATGAATTCGATTGAAGGGGATGCGGTTTCTACCATCCATGTG
RI-AT3G25570-XLOC_018162-9920-1
GTTACTCGATGAATTCGATTGAAGGGGATGCGGTTTCTACCATCCATGTG
CONSENSUS
GTTACTCGATGAATTCGATTGAAGGGGATGCGGTTTCTACCATCCATGTG

RI-AT3G25570-XLOC_018162-9920-0
ACCCCGGAAGATGGTTTTAGCTATGCAAGCTTTGAAACAGTTGGTTATGA
RI-AT3G25570-XLOC_018162-9920-1
ACCCCGGAAGATGGTTTTAGCTATGCAAGCTTTGAAACAGTTGGTTATGA
CONSENSUS
ACCCCGGAAGATGGTTTTAGCTATGCAAGCTTTGAAACAGTTGGTTATGA

RI-AT3G25570-XLOC_018162-9920-0
TCTAAAGGCTTTGAACTTTAAGGAGTTGGTGGATAGGGTTCTGGTTTGTT
RI-AT3G25570-XLOC_018162-9920-1
TCTAAAGGCTTTGAACTTTAAGGAGTTGGTGGATAGGGTTCTGGTTTGTT
CONSENSUS
TCTAAAGGCTTTGAACTTTAAGGAGTTGGTGGATAGGGTTCTGGTTTGTT

RI-AT3G25570-XLOC_018162-9920-0
TTGGACCGGAAGAATTTTCTGTAGCGGTGCATGCTAATCTTGGAACCGAG
RI-AT3G25570-XLOC_018162-9920-1
TTGGACCGGAAGAATTTTCTGTAGCGGTGCATGCTAATCTTGGAACCGAG
CONSENSUS
TTGGACCGGAAGAATTTTCTGTAGCGGTGCATGCTAATCTTGGAACCGAG

RI-AT3G25570-XLOC_018162-9920-0
GTATTGGCGTCTGATTGTGTAGCTGATGTGAACGGATACTTTAGCCAAGA
RI-AT3G25570-XLOC_018162-9920-1
GTATTGGCGTCTGATTGTGTAGCTGATGTGAACGGATACTTTAGCCAAGA
CONSENSUS
GTATTGGCGTCTGATTGTGTAGCTGATGTGAACGGATACTTTAGCCAAGA

RI-AT3G25570-XLOC_018162-9920-0
GAGAGAACTAGAGGAGCTTGGACTTGGAGGTTCGGTTCTATACCAGAGAT
RI-AT3G25570-XLOC_018162-9920-1
GAGAGAACTAGAGGAGCTTGGACTTGGAGGTTCGGTTCTATACCAGAGAT
CONSENSUS
GAGAGAACTAGAGGAGCTTGGACTTGGAGGTTCGGTTCTATACCAGAGAT

RI-AT3G25570-XLOC_018162-9920-0
TTGTCAAGACTGTTGAATGTTGCTCACCAAAATCTACTCTTGGGTTCTGT
RI-AT3G25570-XLOC_018162-9920-1
TTGTCAAGACTGTTGAATGTTGCTCACCAAAATCTACTCTTGGGTTCTGT
CONSENSUS
TTGTCAAGACTGTTGAATGTTGCTCACCAAAATCTACTCTTGGGTTCTGT

RI-AT3G25570-XLOC_018162-9920-0
TAGAAGAAGTGATTAAAGAAGAAGAGTGATCTATTTCTGGATTTATAGTT
RI-AT3G25570-XLOC_018162-9920-1
TAGAAGAAGTGATTAAAGAAGAAGAGTGATCTATTTCTGGATTTATAGTT
CONSENSUS
TAGAAGAAGTGATTAAAGAAGAAGAGTGATCTATTTCTGGATTTATAGTT

RI-AT3G25570-XLOC_018162-9920-0
TTTTTTTTTTGTAAAGATCTTTTGTGTTTTGTTTTCGAATAAGAAAGAAC
RI-AT3G25570-XLOC_018162-9920-1
TTTTTTTTTTGTAAAGATCTTTTGTGTTTTGTTTTCGAATAAGAAAGAAC
CONSENSUS
TTTTTTTTTTGTAAAGATCTTTTGTGTTTTGTTTTCGAATAAGAAAGAAC

RI-AT3G25570-XLOC_018162-9920-0
ATGTGATGTTCTGATGAAAGCATTTCTTGTTTATGTTCTCTGGTTGCTGC
RI-AT3G25570-XLOC_018162-9920-1
ATGTGATGTTCTGATGAAAGCATTTCTTGTTTATGTTCTCTGGTTGCTGC
CONSENSUS
ATGTGATGTTCTGATGAAAGCATTTCTTGTTTATGTTCTCTGGTTGCTGC

RI-AT3G25570-XLOC_018162-9920-0
ACCATAAATATTTTATGTTCTATGCTTTGTTGTGTTTAAGCTTTGGATAA
RI-AT3G25570-XLOC_018162-9920-1
ACCATAAATATTTTATGTTCTATGCTTTGTTGTGTTTAAGCTTTGGATAA
CONSENSUS
ACCATAAATATTTTATGTTCTATGCTTTGTTGTGTTTAAGCTTTGGATAA

RI-AT3G25570-XLOC_018162-9920-0
CTTTTAATCTTGTGTGCTTATAAATTTTAATCTTCTAT
RI-AT3G25570-XLOC_018162-9920-1
CTTTTAATCTTGTGTGCTTATAAATTTTAATCTTCTAT
CONSENSUS
CTTTTAATCTTGTGTGCTTATAAATTTTAATCTTCTAT

alignment for event: A3-AT3G61010-XLOC_019935-384

A3-AT3G61010-XLOC_019935-384-0
GGCTTTGGTTACCATGTTTCACTCCAAGGTCTCCAACAGTCAGATTGTTG
A3-AT3G61010-XLOC_019935-384-1
GGCTTTGGTTACCATGTTTCACTCCAAGGTCTCCAACAGTCAGATTGTTG
CONSENSUS
GGCTTTGGTTACCATGTTTCACTCCAAGGTCTCCAACAGTCAGATTGTTG

A3-AT3G61010-XLOC_019935-384-0
CTTTTAGTTTTATTCTTCACAGATGCCCCATG------------------
A3-AT3G61010-XLOC_019935-384-1
CTTTTAGTTTTATTCTTCACAGATGCCCCATGTTGATCTTCTTGATCTGG
CONSENSUS
CTTTTAGTTTTATTCTTCACAGATGCCCCATG..................

A3-AT3G61010-XLOC_019935-384-0

A3-AT3G61010-XLOC_019935-384-1
GGGTCTCTCTCCTCTCATCTGATCTCCGTCTTTGTTATATGCTTCACATT
CONSENSUS
....

A3-AT3G61010-XLOC_019935-384-0 --
CCTCTCCTAGAACTCAATGAAGACAACAAGGATGTCATTCAGGCCACT

A3-AT3G61010-XLOC_019935-384-1
      AGCCTCTCCTAGAACTCAATGAAGACAACAAGGATGTCATTCAGGCCACT
CONSENSUS
      ..CCTCTCCTAGAACTCAATGAAGACAACAAGGATGTCATTCAGGCCACT

A3-AT3G61010-XLOC_019935-384-0
      CTTGAGTGAGTTCTGGCCCTAATACTATCATCATATTTATTTCGGCAAAG
A3-AT3G61010-XLOC_019935-384-1
      CTTGAGTGAGTTCTGGCCCTAATACTATCATCATATTTATTTCGGCAAAG
CONSENSUS
      CTTGAGTGAGTTCTGGCCCTAATACTATCATCATATTTATTTCGGCAAAG

A3-AT3G61010-XLOC_019935-384-0
      CCAAAAAGGGAAAAATGTTTTCGACATTTCTGAGCAAACATTTAAACAAT
A3-AT3G61010-XLOC_019935-384-1
      CCAAAAAGGGAAAAATGTTTTCGACATTTCTGAGCAAACATTTAAACAAT
CONSENSUS
      CCAAAAAGGGAAAAATGTTTTCGACATTTCTGAGCAAACATTTAAACAAT

A3-AT3G61010-XLOC_019935-384-0
      AAGTACTATGAGTCTGATAGTACTCGAGAGGCATCTTTTAACGGAGGAGA
A3-AT3G61010-XLOC_019935-384-1
      AAGTACTATGAGTCTGATAGTACTCGAGAGGCATCTTTTAACGGAGGAGA
CONSENSUS
      AAGTACTATGAGTCTGATAGTACTCGAGAGGCATCTTTTAACGGAGGAGA

A3-AT3G61010-XLOC_019935-384-0
      CTACATCACTTTTAGAGGAAAACTCGAGGGAGATGCGTATTTCACAACAA
A3-AT3G61010-XLOC_019935-384-1
      CTACATCACTTTTAGAGGAAAACTCGAGGGAGATGCGTATTTCACAACAA
CONSENSUS
      CTACATCACTTTTAGAGGAAAACTCGAGGGAGATGCGTATTTCACAACAA

A3-AT3G61010-XLOC_019935-384-0
      GGCTCTTCAAATCCCATCTTCATCTTTCATCTTCCCCCATCACAATTTCT
A3-AT3G61010-XLOC_019935-384-1
      GGCTCTTCAAATCCCATCTTCATCTTTCATCTTCCCCCATCACAATTTCT
CONSENSUS
      GGCTCTTCAAATCCCATCTTCATCTTTCATCTTCCCCCATCACAATTTCT

A3-AT3G61010-XLOC_019935-384-0   TTCTCT
A3-AT3G61010-XLOC_019935-384-1   TTCTCT
CONSENSUS   TTCTCT

alignment for event: RI-AT3G07860-XLOC_013931-1613

RI-AT3G07860-XLOC_013931-1613-0
      GTGACTTTCATGCCATATGTGATGAAGAAGGGTCGAGGAAGACACTCCAA
RI-AT3G07860-XLOC_013931-1613-1
      GTGACTTTCATGCCATATGTGATGAAGAAGGGTCGAGGAAGACACTCCAA
CONSENSUS
      GTGACTTTCATGCCATATGTGATGAAGAAGGGTCGAGGAAGACACTCCAA

RI-AT3G07860-XLOC_013931-1613-0

GAGGAAGAAGCATCGTCTCTTTCGTTCTCTCCACAAGACTTCTTCTTGAA
RI-AT3G07860-XLOC_013931-1613-1
GAGGAAGAAGCATCGTCTCTTTCGTTCTCTCCACAAGACTTCTTCTTGAA
CONSENSUS
GAGGAAGAAGCATCGTCTCTTTCGTTCTCTCCACAAGACTTCTTCTTGAA

RI-AT3G07860-XLOC_013931-1613-0
GCCTTTTTATAATTTGGTAACCAAAACCAGTCTCTCAGCTTTATTCACAT
RI-AT3G07860-XLOC_013931-1613-1
GCCTTTTTATAATTTG----------------------------------
CONSENSUS
GCCTTTTTATAATTTG..................................

RI-AT3G07860-XLOC_013931-1613-0
GTTAGATAGCATTGGTAGGTTTGATTCGACTAAACTTAGGTATATCACTC
RI-AT3G07860-XLOC_013931-1613-1

CONSENSUS
....

RI-AT3G07860-XLOC_013931-1613-0
AACACTTGAGCTAAGAACCCTGAGACATGGCAGGATATCAAATCATTATA
RI-AT3G07860-XLOC_013931-1613-1

CONSENSUS
....

RI-AT3G07860-XLOC_013931-1613-0
TACTTGTCAAGAATGACTTGGGGTTTTACTTAAGTATTGATCAAATGATT
RI-AT3G07860-XLOC_013931-1613-1

CONSENSUS
....

RI-AT3G07860-XLOC_013931-1613-0
TGAGACATTAGGTCTGATATGTTTTCGAAACTAAGTTAAGATTCCCAGTG
RI-AT3G07860-XLOC_013931-1613-1

CONSENSUS
....

RI-AT3G07860-XLOC_013931-1613-0
ATGAACATTTTAGTAATCTGGAACGGTGATAAAATGGGGCTCCCTCTTGG
RI-AT3G07860-XLOC_013931-1613-1

CONSENSUS
....

RI-AT3G07860-XLOC_013931-1613-0
ATTATTTCCGTGGAGGTTTTAGAGGAACCATTGTTGAAGATACTTGGCAA
RI-AT3G07860-XLOC_013931-1613-1

CONSENSUS
....

RI-AT3G07860-XLOC_013931-1613-0

CTTCTGAGGGTCTCTTCGCAAAATGAAATCTGTTATTTTTCTTACTGTTT
RI-AT3G07860-XLOC_013931-1613-1

CONSENSUS
....

RI-AT3G07860-XLOC_013931-1613-0
TGTTGGGATTCTGAAACATTGCACTACACGCTGATTTCACTTTTTTCCAT
RI-AT3G07860-XLOC_013931-1613-1

CONSENSUS
....

RI-AT3G07860-XLOC_013931-1613-0
TCATCATTGCTACTTTTGTTACTATCACAAGTGGTGAATTTTGATCAGGT
RI-AT3G07860-XLOC_013931-1613-1
--GT
CONSENSUS
....GT

RI-AT3G07860-XLOC_013931-1613-0
TGGAGTGGACAAAGTCGTCTCAGCACGGATGAAATCAAAACTAACAGATT
RI-AT3G07860-XLOC_013931-1613-1
TGGAGTGGACAAAGTCGTCTCAGCACGGATGAAATCAAAACTAACAGATT
CONSENSUS
TGGAGTGGACAAAGTCGTCTCAGCACGGATGAAATCAAAACTAACAGATT

RI-AT3G07860-XLOC_013931-1613-0
CCGTCTGTGGTTTATCCCACCTTGGAATACACCAGCAGCACAACACGCAG
RI-AT3G07860-XLOC_013931-1613-1
CCGTCTGTGGTTTATCCCACCTTGGAATACACCAGCAGCACAACACGCAG
CONSENSUS
CCGTCTGTGGTTTATCCCACCTTGGAATACACCAGCAGCACAACACGCAG

RI-AT3G07860-XLOC_013931-1613-0
CAGCAACTACTTGTGAATTCTGTGATAGACTTGTGGGAGTTGGTTTATCA
RI-AT3G07860-XLOC_013931-1613-1
CAGCAACTACTTGTGAATTCTGTGATAGACTTGTGGGAGTTGGTTTATCA
CONSENSUS
CAGCAACTACTTGTGAATTCTGTGATAGACTTGTGGGAGTTGGTTTATCA

RI-AT3G07860-XLOC_013931-1613-0
CAAGTAGTCAGCTTAGGATTGATCTTTAATAGACTTGTGGGAGTTGGTTT
RI-AT3G07860-XLOC_013931-1613-1
CAAGTAGTCAGCTTAGGATTGATCTTTAATAGACTTGTGGGAGTTGGTTT
CONSENSUS
CAAGTAGTCAGCTTAGGATTGATCTTTAATAGACTTGTGGGAGTTGGTTT

RI-AT3G07860-XLOC_013931-1613-0
AGCTATATTTGTATTCATCCGGTTCAGTGTATAAGCAGTAAAATTATTGG
RI-AT3G07860-XLOC_013931-1613-1
AGCTATATTTGTATTCATCCGGTTCAGTGTATAAGCAGTAAAATTATTGG
CONSENSUS
AGCTATATTTGTATTCATCCGGTTCAGTGTATAAGCAGTAAAATTATTGG

RI-AT3G07860-XLOC_013931-1613-0

```
      TGGCAATGTGAATAACAGAGCAGAAAACTGCTAATATTTTCATTATTAAA
RI-AT3G07860-XLOC_013931-1613-1
      TGGCAATGTGAATAACAGAGCAGAAAACTGCTAATATTTTCATTATTAAA
CONSENSUS
      TGGCAATGTGAATAACAGAGCAGAAAACTGCTAATATTTTCATTATTAAA

RI-AT3G07860-XLOC_013931-1613-0
      TAAAACAGGAAACCGAAAGGAATTTGAGCTTCAATACATTTTTTTACTTT
RI-AT3G07860-XLOC_013931-1613-1
      TAAAACAGGAAACCGAAAGGAATTTGAGCTTCAATACATTTTTTTACTTT
CONSENSUS
      TAAAACAGGAAACCGAAAGGAATTTGAGCTTCAATACATTTTTTTACTTT

RI-AT3G07860-XLOC_013931-1613-0  AG
RI-AT3G07860-XLOC_013931-1613-1  AG
CONSENSUS                        AG



alignment for event: A3-AT3G04500-XLOC_016978-11219

A3-AT3G04500-XLOC_016978-11219-0
      ATGACTATCGTCTGTTCTGTGGTGATCTTGGGAACGAAGTGAATGATGAT
A3-AT3G04500-XLOC_016978-11219-1
      ATGACTATCGTCTGTTCTGTGGTGATCTTGGGAACGAAGTGAATGATGAT
CONSENSUS
      ATGACTATCGTCTGTTCTGTGGTGATCTTGGGAACGAAGTGAATGATGAT

A3-AT3G04500-XLOC_016978-11219-0
      GTTCTTTCCAAAGCATTTGCTAGATTCCCCACCTTCAATATGGCCAAG--
A3-AT3G04500-XLOC_016978-11219-1
      GTTCTTTCCAAAGCATTTGCTAGATTCCCCACCTTCAATATGGCCAAGTC
CONSENSUS
      GTTCTTTCCAAAGCATTTGCTAGATTCCCCACCTTCAATATGGCCAAG..

A3-AT3G04500-XLOC_016978-11219-0
      --------------------------------------------------
A3-AT3G04500-XLOC_016978-11219-1
      AACTGTGATGCATTTGCAAACCAAAACACATAGTATGTTCAGAACAGAGC
CONSENSUS
      ..................................................

A3-AT3G04500-XLOC_016978-11219-0
      --------------------------------------------------
A3-AT3G04500-XLOC_016978-11219-1
      ATGGTTGACCTTAGATCATACGGATCATTCTTACTCGAATCACAAAGATA
CONSENSUS
      ..................................................

A3-AT3G04500-XLOC_016978-11219-0
      --------------------------------------------------
A3-AT3G04500-XLOC_016978-11219-1
      AGTATTCAGTGTTAGTTTTGTGGTCAAACTCGTACCAGAAAAATAAGAGT
CONSENSUS
      ..................................................
```

A3-AT3G04500-XLOC_016978-11219-0 -----------------
GTCATTAGAGATAAGCGGACTGGTAAAACCAAG
A3-AT3G04500-XLOC_016978-11219-1
CTTATTTACCATGACAGGTCATTAGAGATAAGCGGACTGGTAAAACCAAG
CONSENSUS
.................GTCATTAGAGATAAGCGGACTGGTAAAACCAAG

A3-AT3G04500-XLOC_016978-11219-0
GGTTATGGGTTTGTGAGTTTCTTAAATCCTGCGGATCTAGCAGCAGCCTT
A3-AT3G04500-XLOC_016978-11219-1
GGTTATGGGTTTGTGAGTTTCTTAAATCCTGCGGATCTAGCAGCAGCCTT
CONSENSUS
GGTTATGGGTTTGTGAGTTTCTTAAATCCTGCGGATCTAGCAGCAGCCTT

A3-AT3G04500-XLOC_016978-11219-0 AAAAGAAATGAATG
A3-AT3G04500-XLOC_016978-11219-1 AAAAGAAATGAATG
CONSENSUS AAAAGAAATGAATG

alignment for event: RI-AT3G56680-XLOC_016429-13763

RI-AT3G56680-XLOC_016429-13763-0
GCACAGTTCGTTCAACAACCCTTGAGCTTCGACTACATGCAGAACCGCTA
RI-AT3G56680-XLOC_016429-13763-1
GCACAGTTCGTTCAACAACCCTTGAGCTTCGACTACATGCAGAACCGCTA
CONSENSUS
GCACAGTTCGTTCAACAACCCTTGAGCTTCGACTACATGCAGAACCGCTA

RI-AT3G56680-XLOC_016429-13763-0
GGTGCTTGCCTCTTTGTCGATCTTTATCTCTGTTTACCATTTTTTTTTGT
RI-AT3G56680-XLOC_016429-13763-1
GGTGCTTGCCTCTTTGTCGATCTTTATCTCTGTTTACCATTTTTTTTTGT
CONSENSUS
GGTGCTTGCCTCTTTGTCGATCTTTATCTCTGTTTACCATTTTTTTTTGT

RI-AT3G56680-XLOC_016429-13763-0
AGAACCTAGGAACTGTTGTAATGGAAATTTTGGAGTTGTAATGTCAAAAT
RI-AT3G56680-XLOC_016429-13763-1
AGAACCTAGGAA---------------------------------------
CONSENSUS
AGAACCTAGGAA......................................

RI-AT3G56680-XLOC_016429-13763-0
TCAGAATGAAGAGACCTTTTGTTCCTTTTACAATTATCAGATTCTGTCTA
RI-AT3G56680-XLOC_016429-13763-1

CONSENSUS
....

RI-AT3G56680-XLOC_016429-13763-0
TTATGGAAAACCAATGAACTACCTGTGGGTCAAATCTTCATCGAAATTCT
RI-AT3G56680-XLOC_016429-13763-1 ----------------------
CTGTGGGTCAAATCTTCATCGAAATTCT
CONSENSUS
......................CTGTGGGTCAAATCTTCATCGAAATTCT

RI-AT3G56680-XLOC_016429-13763-0
      CTGTTTTGAATTGAATGTCTTCCAAATCTGCA
RI-AT3G56680-XLOC_016429-13763-1
      CTGTTTTGAATTGAATGTCTTCCAAATCTGCA
CONSENSUS
      CTGTTTTGAATTGAATGTCTTCCAAATCTGCA

alignment for event: A5-AT3G52560-XLOC_019461-11442

A5-AT3G52560-XLOC_019461-11442-0
      TTCCGAGGAATTTTCGGTTGTTGGAGGAGCTTGAACGTGGGGAGAAAGGT
A5-AT3G52560-XLOC_019461-11442-1
      TTCCGAGGAATTTTCGGTTGTTGGAGGAGCTTGAACGTGGGGAGAAAGGT
CONSENSUS
      TTCCGAGGAATTTTCGGTTGTTGGAGGAGCTTGAACGTGGGGAGAAAGGT

A5-AT3G52560-XLOC_019461-11442-0
      ATTGGGGATGGAACTGTGAGCTATGGAATGGATGATGGAGATGACATCTA
A5-AT3G52560-XLOC_019461-11442-1
      ATTGGGGATGGAACTGTGAGCTATGGAATGGATGATGGAGATGACATCTA
CONSENSUS
      ATTGGGGATGGAACTGTGAGCTATGGAATGGATGATGGAGATGACATCTA

A5-AT3G52560-XLOC_019461-11442-0
      TATGCGTTCTTGGACTGGCACCATCATCGGTCCTCACAACGTAACTGTTC
A5-AT3G52560-XLOC_019461-11442-1
      TATGCGTTCTTGGACTGGCACCATCATCGGTCCTCACAAC---ACTGTTC
CONSENSUS
      TATGCGTTCTTGGACTGGCACCATCATCGGTCCTCACAAC...ACTGTTC

A5-AT3G52560-XLOC_019461-11442-0
      ATGAAGGTAGAATCTATCAGTTGAAGCTCTTTTGTGACAAAGATTACCCG
A5-AT3G52560-XLOC_019461-11442-1
      ATGAAGGTAGAATCTATCAGTTGAAGCTCTTTTGTGACAAAGATTACCCG
CONSENSUS
      ATGAAGGTAGAATCTATCAGTTGAAGCTCTTTTGTGACAAAGATTACCCG

A5-AT3G52560-XLOC_019461-11442-0
      GAGAAACCTCCAACTGTCCGATTCCATTCACGTGTCAACATGGCTTGTGT
A5-AT3G52560-XLOC_019461-11442-1
      GAGAAACCTCCAACTGTCCGATTCCATTCACGTGTCAACATGGCTTGTGT
CONSENSUS
      GAGAAACCTCCAACTGTCCGATTCCATTCACGTGTCAACATGGCTTGTGT

A5-AT3G52560-XLOC_019461-11442-0 CAACCACGAAACAGGAGTG
A5-AT3G52560-XLOC_019461-11442-1 CAACCACGAAACAGGAGTG
CONSENSUS   CAACCACGAAACAGGAGTG

alignment for event: RI-AT3G04450-XLOC_013743-8731

RI-AT3G04450-XLOC_013743-8731-0

ATTGTCTACACATCCGTGTTTGTCTAGCTGATGTTCCAAGTTAGCCATCA
RI-AT3G04450-XLOC_013743-8731-1
ATTGTCTACACATCCGTGTTTGTCTAGCTGATGTTCCAAGTTAGCCATCA
CONSENSUS
ATTGTCTACACATCCGTGTTTGTCTAGCTGATGTTCCAAGTTAGCCATCA

RI-AT3G04450-XLOC_013743-8731-0
GATTTTCTTCCCCTTTATGTTCACTATCAGGAGCGATTCTCAGCTGTAAG
RI-AT3G04450-XLOC_013743-8731-1
GATTTTCTTCCCCTTTATGTTCACTATCAGGAGCGATTCTCAGCT-----
CONSENSUS
GATTTTCTTCCCCTTTATGTTCACTATCAGGAGCGATTCTCAGCT.....

RI-AT3G04450-XLOC_013743-8731-0
TCGAACTTGGCAAGTGTTTTTCGACCCTTTGCTATTTTGGAGTAATGATG
RI-AT3G04450-XLOC_013743-8731-1

CONSENSUS
....

RI-AT3G04450-XLOC_013743-8731-0
TAAGAAGACCATATCATTGCCTAAATGATGATCTGAATGGACCTTCTTAT
RI-AT3G04450-XLOC_013743-8731-1

CONSENSUS
....

RI-AT3G04450-XLOC_013743-8731-0
GGTACAGTGGTGGATTTTTTAAGCGACAAGGGAGAACCAAAGATCACGAG
RI-AT3G04450-XLOC_013743-8731-1 -------
TGGTGGATTTTTTAAGCGACAAGGGAGAACCAAAGATCACGAG
CONSENSUS
.......TGGTGGATTTTTTAAGCGACAAGGGAGAACCAAAGATCACGAG

RI-AT3G04450-XLOC_013743-8731-0 GAG
RI-AT3G04450-XLOC_013743-8731-1 GAG
CONSENSUS GAG

alignment for event: A5-AT3G15354-XLOC_017622-5884

A5-AT3G15354-XLOC_017622-5884-0
ATATGGGATGTTGCAAGAAGCCAGTTGGTTACAGAGATGAAGGAGCACAA
A5-AT3G15354-XLOC_017622-5884-1
ATATGGGATGTTGCAAGAAGCCAGTTGGTTACAGAGATGAAGGAGCACAA
CONSENSUS
ATATGGGATGTTGCAAGAAGCCAGTTGGTTACAGAGATGAAGGAGCACAA

A5-AT3G15354-XLOC_017622-5884-0
GAAGCGAGTATGGTCCATCGATATTTCATCAGCAGACCCGACTTTGCTGG
A5-AT3G15354-XLOC_017622-5884-1
GAAGCGAGTATGGTCCATCGATATTTCATCAGCAGACCCGACTTTGCTGG
CONSENSUS
GAAGCGAGTATGGTCCATCGATATTTCATCAGCAGACCCGACTTTGCTGG

A5-AT3G15354-XLOC_017622-5884-0
CTAGCGGAAGCGATGATGGAACCGTTAAGCTCTGGAGTATCAATCAGGCA
A5-AT3G15354-XLOC_017622-5884-1
CTAGCGGAAGCGATGATGGAACCGTTAAGCTCTGGAGTATCAATCAG---
CONSENSUS
CTAGCGGAAGCGATGATGGAACCGTTAAGCTCTGGAGTATCAATCAG...

A5-AT3G15354-XLOC_017622-5884-0
ATTCTAATTTGAAATGGAGTTAGCATTGGGACCATCAAGACAAAGGCCAA
A5-AT3G15354-XLOC_017622-5884-1 ---------------
GGAGTTAGCATTGGGACCATCAAGACAAAGGCCAA
CONSENSUS
...............GGAGTTAGCATTGGGACCATCAAGACAAAGGCCAA

A5-AT3G15354-XLOC_017622-5884-0
TGTATGCTGTGTCCAGTTTCCATCAGACTCCGGACGGTCTCTAGCATTTG
A5-AT3G15354-XLOC_017622-5884-1
TGTATGCTGTGTCCAGTTTCCATCAGACTCCGGACGGTCTCTAGCATTTG
CONSENSUS
TGTATGCTGTGTCCAGTTTCCATCAGACTCCGGACGGTCTCTAGCATTTG

A5-AT3G15354-XLOC_017622-5884-0
GTTCTGCAGATCATAAAGTGTATTACTACGATCTTCGAAACCCCAAGATT
A5-AT3G15354-XLOC_017622-5884-1
GTTCTGCAGATCATAAAGTGTATTACTACGATCTTCGAAACCCCAAGATT
CONSENSUS
GTTCTGCAGATCATAAAGTGTATTACTACGATCTTCGAAACCCCAAGATT

A5-AT3G15354-XLOC_017622-5884-0
CCTCTGTGCACAATGATTGGTCATAGCAAGACAGTGAGTTATGTCAAGTT
A5-AT3G15354-XLOC_017622-5884-1
CCTCTGTGCACAATGATTGGTCATAGCAAGACAGTGAGTTATGTCAAGTT
CONSENSUS
CCTCTGTGCACAATGATTGGTCATAGCAAGACAGTGAGTTATGTCAAGTT

A5-AT3G15354-XLOC_017622-5884-0
TGTAGATTCATCCACTCTTGTGTCCTCTTCTACTGATAACACACTGAAGC
A5-AT3G15354-XLOC_017622-5884-1
TGTAGATTCATCCACTCTTGTGTCCTCTTCTACTGATAACACACTGAAGC
CONSENSUS
TGTAGATTCATCCACTCTTGTGTCCTCTTCTACTGATAACACACTGAAGC

A5-AT3G15354-XLOC_017622-5884-0
TTTGGGACTTGTCGATGTCTGCTTCTGGGATTAATGAATCCCCTCTTCAC
A5-AT3G15354-XLOC_017622-5884-1
TTTGGGACTTGTCGATGTCTGCTTCTGGGATTAATGAATCCCCTCTTCAC
CONSENSUS
TTTGGGACTTGTCGATGTCTGCTTCTGGGATTAATGAATCCCCTCTTCAC

A5-AT3G15354-XLOC_017622-5884-0 TCATTCACTGGACACACTAATTTAAAG
A5-AT3G15354-XLOC_017622-5884-1 TCATTCACTGGACACACTAATTTAAAG
CONSENSUS TCATTCACTGGACACACTAATTTAAAG

alignment for event: A3-AT3G53500-XLOC_019516-11514

A3-AT3G53500-XLOC_019516-11514-0
      CTTGACAATGCCTCGCTATGATGATCGCTATGGAAACACTCGCCTCTATG
A3-AT3G53500-XLOC_019516-11514-1
      CTTGACAATGCCTCGCTATGATGATCGCTATGGAAACACTCGCCTCTATG
CONSENSUS
      CTTGACAATGCCTCGCTATGATGATCGCTATGGAAACACTCGCCTCTATG

A3-AT3G53500-XLOC_019516-11514-0
      TTGGTCGCTTATCATCTAGAACTCGTACCAGAGACCTTGAGCGTCTCTTT
A3-AT3G53500-XLOC_019516-11514-1
      TTGGTCGCTTATCATCTAGAACTCGTACCAGAGACCTTGAGCGTCTCTTT
CONSENSUS
      TTGGTCGCTTATCATCTAGAACTCGTACCAGAGACCTTGAGCGTCTCTTT

A3-AT3G53500-XLOC_019516-11514-0
      AGCAGATACGGAAG------------------------------------
A3-AT3G53500-XLOC_019516-11514-1
      AGCAGATACGGAAGGTTTCTGGGGGTTTGCCTAACCTGGAGATGTTTGAT
CONSENSUS
      AGCAGATACGGAAG....................................

A3-AT3G53500-XLOC_019516-11514-0

A3-AT3G53500-XLOC_019516-11514-1
      GAATGGGTGGACTGTGGTGTAATTGGTGGAAATTCTCGTCTGGTGTATAG
CONSENSUS
      ....

A3-AT3G53500-XLOC_019516-11514-0

A3-AT3G53500-XLOC_019516-11514-1
      AGGGTGCATCGGCGATTTTGTTTGTTGATTTATTGCATGTTTTTATTGGA
CONSENSUS
      ....

A3-AT3G53500-XLOC_019516-11514-0

A3-AT3G53500-XLOC_019516-11514-1
      AATGGCCTCAAATCTCAAAAACTGGTGTTTGGAAGTTCCTTAAGTAATGA
CONSENSUS
      ....

A3-AT3G53500-XLOC_019516-11514-0
      --------------------------------AGTACGAGATGTGGATAT
A3-AT3G53500-XLOC_019516-11514-1
      TGGCCTTGATTTTCTGGCAACAATTTACGCAGAGTACGAGATGTGGATAT
CONSENSUS
      ................................AGTACGAGATGTGGATAT

A3-AT3G53500-XLOC_019516-11514-0 GAAGCGTGATTATGCCTTTGTT
A3-AT3G53500-XLOC_019516-11514-1 GAAGCGTGATTATGCCTTTGTT
CONSENSUS   GAAGCGTGATTATGCCTTTGTT

alignment for event: RI-AT3G22190-XLOC_017981-1371

RI-AT3G22190-XLOC_017981-1371-0
CATTCAATCAATCGTATCTGAGAGAGGAGAGGGAAAACGAAACGATCAAT
RI-AT3G22190-XLOC_017981-1371-1
CATTCAATCAATCGTATCTGAGAGAGGAGAGGGAAAACGAAACGATCAAT
CONSENSUS
CATTCAATCAATCGTATCTGAGAGAGGAGAGGGAAAACGAAACGATCAAT

RI-AT3G22190-XLOC_017981-1371-0
CAAGTCAATTCCTATCTATCTGTATATGTTCGTTCACACACATATCCCCT
RI-AT3G22190-XLOC_017981-1371-1
CAAGTCAATTCCTATCTATCTGTATATGTTCGTTCACACACATATCCCCT
CONSENSUS
CAAGTCAATTCCTATCTATCTGTATATGTTCGTTCACACACATATCCCCT

RI-AT3G22190-XLOC_017981-1371-0
AGAGAGAGAATAAAGTATGGACCTTTTTCTTTTTGTAGAGTAGAGAGAGA
RI-AT3G22190-XLOC_017981-1371-1
AGAGAGAGAATAAAGTATGGACCTTTTTCTTTTTGTAGAGTAGAGAGAGA
CONSENSUS
AGAGAGAGAATAAAGTATGGACCTTTTTCTTTTTGTAGAGTAGAGAGAGA

RI-AT3G22190-XLOC_017981-1371-0
GAGGAAAAAGTAAAGTAGCCGAGAAAGACGCTCACTGACTGAAGAAGAAA
RI-AT3G22190-XLOC_017981-1371-1
GAGGAAAAAGTAAAGTAGCCGAGAAAGACGCTCACTGACTGAAGAAGAAA
CONSENSUS
GAGGAAAAAGTAAAGTAGCCGAGAAAGACGCTCACTGACTGAAGAAGAAA

RI-AT3G22190-XLOC_017981-1371-0
AGATCACGAAGTCGCCACTCACTCTCTTTAAAGATCTCATCGGCGATCTT
RI-AT3G22190-XLOC_017981-1371-1
AGATCACGAAGTCGCCACTCACTCTCTTTAAAGATCTCATCGGCGATCTT
CONSENSUS
AGATCACGAAGTCGCCACTCACTCTCTTTAAAGATCTCATCGGCGATCTT

RI-AT3G22190-XLOC_017981-1371-0
TCCTTCGCCGCCGGTGTAAAGTTTTTGAATTTTGCTTCTCCTCGATGTGT
RI-AT3G22190-XLOC_017981-1371-1
TCCTTCGCCGCCGGT-----------------------------------
CONSENSUS
TCCTTCGCCGCCGGT...................................

RI-AT3G22190-XLOC_017981-1371-0
CTCAATTTTGCAAAGTTTGGATTTTTTGTTAGTGTTCATTACTAAATTTC
RI-AT3G22190-XLOC_017981-1371-1

CONSENSUS
....

RI-AT3G22190-XLOC_017981-1371-0
TTGGTTTTAATATTAAGGATCCAACATTGTCAGGAGAATTTAGTGAACTC
RI-AT3G22190-XLOC_017981-1371-1

CONSENSUS

```
      ..................................................

RI-AT3G22190-XLOC_017981-1371-0
      AATTAAAGTTTCCATTTTTGTCTATTTTGTTATCAAAAAGGAGAAAGCTT
RI-AT3G22190-XLOC_017981-1371-1
      --------------------------------------------------
CONSENSUS
      ..................................................

RI-AT3G22190-XLOC_017981-1371-0
      TACTGTTTTGAATAATTTAGTTGCTAAATTGGCAATTTTGCAGGTTTTGT
RI-AT3G22190-XLOC_017981-1371-1
      -------------------------------------------GTTTTGT
CONSENSUS
      ...........................................GTTTTGT

RI-AT3G22190-XLOC_017981-1371-0
      GTTGTTTTATGTTGCTGATCGTTGGAATTGAGATAGAATGGGAGCTTCAG
RI-AT3G22190-XLOC_017981-1371-1
      GTTGTTTTATGTTGCTGATCGTTGGAATTGAGATAGAATGGGAGCTTCAG
CONSENSUS
      GTTGTTTTATGTTGCTGATCGTTGGAATTGAGATAGAATGGGAGCTTCAG

RI-AT3G22190-XLOC_017981-1371-0
      GGAGATGGATTAAAGCATTGGTTGGTTTTACTAAATCTGATAAGTCTAGG
RI-AT3G22190-XLOC_017981-1371-1
      GGAGATGGATTAAAGCATTGGTTGGTTTTACTAAATCTGATAAGTCTAGG
CONSENSUS
      GGAGATGGATTAAAGCATTGGTTGGTTTTACTAAATCTGATAAGTCTAGG

RI-AT3G22190-XLOC_017981-1371-0  TCTTCCAAGAAAGATGAAAAT
RI-AT3G22190-XLOC_017981-1371-1  TCTTCCAAGAAAGATGAAAAT
CONSENSUS                        TCTTCCAAGAAAGATGAAAAT


alignment for event: A3-AT3G59780-XLOC_016578-4269

A3-AT3G59780-XLOC_016578-4269-0
      GTATCTGTTGCCATTGGAGGTTTGGAGAGAAATATCGGTTTGGATCCAGA
A3-AT3G59780-XLOC_016578-4269-1
      GTATCTGTTGCCATTGGAGGTTTGGAGAGAAATATCGGTTTGGATCCAGA
CONSENSUS
      GTATCTGTTGCCATTGGAGGTTTGGAGAGAAATATCGGTTTGGATCCAGA

A3-AT3G59780-XLOC_016578-4269-0
      TGACCCAATCCTTCACCTTTTTCTCTTCGTAGGCACCACAGGAACCTTTT
A3-AT3G59780-XLOC_016578-4269-1
      TGACCCAATCCTTCACCTTTTTCTCTTCGTAGGCACCACAGGAACCTTTT
CONSENSUS
      TGACCCAATCCTTCACCTTTTTCTCTTCGTAGGCACCACAGGAACCTTTT

A3-AT3G59780-XLOC_016578-4269-0
      GATATTTGCAGGGTTCTATATCGGGTTTGGACATATGGTGGATATGCTGG
A3-AT3G59780-XLOC_016578-4269-1  G----------
GGTTCTATATCGGGTTTGGACATATGGTGGATATGCTGG
```

CONSENSUS
      G..........GGTTCTATATCGGGTTTGGACATATGGTGGATATGCTGG

A3-AT3G59780-XLOC_016578-4269-0
      AGATTTGTCTCCCAAGTCAACTCTGGACCTTTTAAAATCAAGAGACAAGT
A3-AT3G59780-XLOC_016578-4269-1
      AGATTTGTCTCCCAAGTCAACTCTGGACCTTTTAAAATCAAGAGACAAGT
CONSENSUS
      AGATTTGTCTCCCAAGTCAACTCTGGACCTTTTAAAATCAAGAGACAAGT

A3-AT3G59780-XLOC_016578-4269-0 CAGTGCTCATAGATGTCAGGCCTGAA
A3-AT3G59780-XLOC_016578-4269-1 CAGTGCTCATAGATGTCAGGCCTGAA
CONSENSUS   CAGTGCTCATAGATGTCAGGCCTGAA

alignment for event: A5-AT3G02300-XLOC_013630-10990

A5-AT3G02300-XLOC_013630-10990-0
      GCTATAATGAACAAGGCCAACTTGGTAGAGGAGTCACTTGTGAAGGACTA
A5-AT3G02300-XLOC_013630-10990-1
      GCTATAATGAACAAGGCCAACTTGGTAGAGGAGTCACTTGTGAAGGACTA
CONSENSUS
      GCTATAATGAACAAGGCCAACTTGGTAGAGGAGTCACTTGTGAAGGACTA

A5-AT3G02300-XLOC_013630-10990-0
      CAAGCACCTCGTGTGATAAATGCTTATGCGAAGTTCCTTGATGAAGCACC
A5-AT3G02300-XLOC_013630-10990-1
      CAAGCACCTCGTGTGATAAATGCTTATGCGAAGTTCCTTGATGAAGCACC
CONSENSUS
      CAAGCACCTCGTGTGATAAATGCTTATGCGAAGTTCCTTGATGAAGCACC

A5-AT3G02300-XLOC_013630-10990-0
      CGAGCTTGTGAAGATTATGCAACTTTCATGTGGAGAATACCATACTGCTG
A5-AT3G02300-XLOC_013630-10990-1
      CGAGCTTGTGAAGATTATGCAACTTTCATGTGGAGAATACCATACTGCTG
CONSENSUS
      CGAGCTTGTGAAGATTATGCAACTTTCATGTGGAGAATACCATACTGCTG

A5-AT3G02300-XLOC_013630-10990-0
      CTCTTTCTGATGCAGGCGAGGT----------------------------
A5-AT3G02300-XLOC_013630-10990-1
      CTCTTTCTGATGCAGGCGAGGTGTAAGTTATCTAAATGCCTTCTCTTGAA
CONSENSUS
      CTCTTTCTGATGCAGGCGAGGT............................

A5-AT3G02300-XLOC_013630-10990-0

A5-AT3G02300-XLOC_013630-10990-1
      AAGTTTGTGGTTATTCACATGAATGGGTAGCTAATTCTCTGTGCAATGTT
CONSENSUS
      ....

A5-AT3G02300-XLOC_013630-10990-0

A5-AT3G02300-XLOC_013630-10990-1

CCGAGGAAGGAAATCCTTTTGATGGAGATATTGGTCTCATCACATCCTAG
CONSENSUS
....

A5-AT3G02300-XLOC_013630-10990-0
--TTACACTTGG
A5-AT3G02300-XLOC_013630-10990-1
CAGATGGGAATGCTTGAGGAAGCAAATGAATTCCTGACTTTTACACTTGG
CONSENSUS
....TTACACTTGG

A5-AT3G02300-XLOC_013630-10990-0
GGATTAGGAAGCATGGGTCAACTTGGGCATGTTTCTCTTCAATCCGGGGA
A5-AT3G02300-XLOC_013630-10990-1
GGATTAGGAAGCATGGGTCAACTTGGGCATGTTTCTCTTCAATCCGGGGA
CONSENSUS
GGATTAGGAAGCATGGGTCAACTTGGGCATGTTTCTCTTCAATCCGGGGA

A5-AT3G02300-XLOC_013630-10990-0
TAAGGAGTTAATACCAAGGAGAGTCGTTGGTCTCGATGGTGTGTCCATGA
A5-AT3G02300-XLOC_013630-10990-1
TAAGGAGTTAATACCAAGGAGAGTCGTTGGTCTCGATGGTGTGTCCATGA
CONSENSUS
TAAGGAGTTAATACCAAGGAGAGTCGTTGGTCTCGATGGTGTGTCCATGA

A5-AT3G02300-XLOC_013630-10990-0
AAGAAGTTGCTTGTGGTGGTGTACACACTTGTGCTTTATCTTTGGAAGGA
A5-AT3G02300-XLOC_013630-10990-1
AAGAAGTTGCTTGTGGTGGTGTACACACTTGTGCTTTATCTTTGGAAGGA
CONSENSUS
AAGAAGTTGCTTGTGGTGGTGTACACACTTGTGCTTTATCTTTGGAAGGA

A5-AT3G02300-XLOC_013630-10990-0
GCACTTTATGCTTGGGGTGGTGGCCAAGCAGGACAGCTAGGACTTGGTCC
A5-AT3G02300-XLOC_013630-10990-1
GCACTTTATGCTTGGGGTGGTGGCCAAGCAGGACAGCTAGGACTTGGTCC
CONSENSUS
GCACTTTATGCTTGGGGTGGTGGCCAAGCAGGACAGCTAGGACTTGGTCC

A5-AT3G02300-XLOC_013630-10990-0
TCAATCTGGTTTCTTTTTTAGTGTCTCTAATGGAAGCGAAATGCTTCTAC
A5-AT3G02300-XLOC_013630-10990-1
TCAATCTGGTTTCTTTTTTAGTGTCTCTAATGGAAGCGAAATGCTTCTAC
CONSENSUS
TCAATCTGGTTTCTTTTTTAGTGTCTCTAATGGAAGCGAAATGCTTCTAC

A5-AT3G02300-XLOC_013630-10990-0
GAAATGTCCCGGTTTTAGTCATCCCAACTGATGTCAGGCTTGTTGCGTGT
A5-AT3G02300-XLOC_013630-10990-1
GAAATGTCCCGGTTTTAGTCATCCCAACTGATGTCAGGCTTGTTGCGTGT
CONSENSUS
GAAATGTCCCGGTTTTAGTCATCCCAACTGATGTCAGGCTTGTTGCGTGT

A5-AT3G02300-XLOC_013630-10990-0
GGACATTCTCACACTCTCGTTTATATGAGAGAGGGACGAATTTGTGGATG
A5-AT3G02300-XLOC_013630-10990-1

GGACATTCTCACACTCTCGTTTATATGAGAGAGGGACGAATTTGTGGATG
CONSENSUS
GGACATTCTCACACTCTCGTTTATATGAGAGAGGGACGAATTTGTGGATG

A5-AT3G02300-XLOC_013630-10990-0
GGGTTATAATAGTTATGGTCAAGCAGCAAATGAGAAATCGTCATATGCTT
A5-AT3G02300-XLOC_013630-10990-1
GGGTTATAATAGTTATGGTCAAGCAGCAAATGAGAAATCGTCATATGCTT
CONSENSUS
GGGTTATAATAGTTATGGTCAAGCAGCAAATGAGAAATCGTCATATGCTT

A5-AT3G02300-XLOC_013630-10990-0 GGTACCCATCGCCTGTAGACTG
A5-AT3G02300-XLOC_013630-10990-1 GGTACCCATCGCCTGTAGACTG
CONSENSUS GGTACCCATCGCCTGTAGACTG

alignment for event: RI-AT3G04500-XLOC_016978-11218

RI-AT3G04500-XLOC_016978-11218-0
AAAAAAAGAATAAACAAAATTTGAGGTTTCGATTTTGCGATTCGGCGATT
RI-AT3G04500-XLOC_016978-11218-1
AAAAAAAGAATAAACAAAATTTGAGGTTTCGATTTTGCGATTCGGCGATT
CONSENSUS
AAAAAAAGAATAAACAAAATTTGAGGTTTCGATTTTGCGATTCGGCGATT

RI-AT3G04500-XLOC_016978-11218-0
TGCTTCTCCGATGTCAATCCCACCATCTTCAGGTTCTTCTTCTTCATCTT
RI-AT3G04500-XLOC_016978-11218-1
TGCTTCTCCGATGTCAATCCCACCATCTTCAG------------------
CONSENSUS
TGCTTCTCCGATGTCAATCCCACCATCTTCAG..................

RI-AT3G04500-XLOC_016978-11218-0
CCTCGTCTCAGTACACATACGCAGCTAATTCTTACTATTCCGCTCCGTAT
RI-AT3G04500-XLOC_016978-11218-1

CONSENSUS
....

RI-AT3G04500-XLOC_016978-11218-0
CAGCCTCCACAGCCTTACGCGGCGGCGCCTTCGCCTGCTGTACCGGCGCC
RI-AT3G04500-XLOC_016978-11218-1 ---
CCTCCACAGCCTTACGCGGCGGCGCCTTCGCCTGCTGTACCGGCGCC
CONSENSUS
...CCTCCACAGCCTTACGCGGCGGCGCCTTCGCCTGCTGTACCGGCGCC

RI-AT3G04500-XLOC_016978-11218-0
TGTGGCATCCATTCCCGGAGCTACGGTCTATCCTCAGCCTGTTGGACCGG
RI-AT3G04500-XLOC_016978-11218-1
TGTGGCATCCATTCCCGGAGCTACGGTCTATCCTCAGCCTGTTGGACCGG
CONSENSUS
TGTGGCATCCATTCCCGGAGCTACGGTCTATCCTCAGCCTGTTGGACCGG

RI-AT3G04500-XLOC_016978-11218-0
TTCCTGCCGTCTACGCTTACCCTCAGTACCAACAG

RI-AT3G04500-XLOC_016978-11218-1
      TTCCTGCCGTCTACGCTTACCCTCAGTACCAACAG
CONSENSUS
      TTCCTGCCGTCTACGCTTACCCTCAGTACCAACAG

alignment for event: A5-AT3G13570-XLOC_017517-9103

A5-AT3G13570-XLOC_017517-9103-0
      GCAAGAAGACCTCAGGAGGCCATTTGAGCAGTTTGGTCCCGTCAAGGACA
A5-AT3G13570-XLOC_017517-9103-1
      GCAAGAAGACCTCAGGAGGCCATTTGAGCAGTTTGGTCCCGTCAAGGACA
CONSENSUS
      GCAAGAAGACCTCAGGAGGCCATTTGAGCAGTTTGGTCCCGTCAAGGACA

A5-AT3G13570-XLOC_017517-9103-0
      TCTACCTTCCTAGGGATTACTATACTGGGTGAGGATGCTCTGTAGATAAG
A5-AT3G13570-XLOC_017517-9103-1
      TCTACCTTCCTAGGGATTACTATACTGG----------------------
CONSENSUS
      TCTACCTTCCTAGGGATTACTATACTGG......................

A5-AT3G13570-XLOC_017517-9103-0
      ACATTGAATGTTTATACATCCAGGGCATAATTTTACATTACTTCAATTAG
A5-AT3G13570-XLOC_017517-9103-1

CONSENSUS
      ....

A5-AT3G13570-XLOC_017517-9103-0
      ACTATGTCTCTTTCTTATTGTTTTCTTTTGCTTCGTAAACCACCTTTTGT
A5-AT3G13570-XLOC_017517-9103-1

CONSENSUS
      ....

A5-AT3G13570-XLOC_017517-9103-0
      GAATCCTACATTCCATCGTATGCAGCTCATGAATTTGTTAGATTTATAAG
A5-AT3G13570-XLOC_017517-9103-1

CONSENSUS
      ....

A5-AT3G13570-XLOC_017517-9103-0
      AATTTTCTGAAAGGTCTATGTCAAACATGTCCGGTTTTATATCCAATCTT
A5-AT3G13570-XLOC_017517-9103-1

CONSENSUS
      ....

A5-AT3G13570-XLOC_017517-9103-0
      CCCAAAATCACATTTGGAGCCTTGAAACGTTTGTTTTATCTACACTTAGG
A5-AT3G13570-XLOC_017517-9103-1

CONSENSUS

....

A5-AT3G13570-XLOC_017517-9103-0
TTTTCAGCTTAAGTCTAGTACACCTAAAACTTCGCCCTCTTCGAGTCACT
A5-AT3G13570-XLOC_017517-9103-1

CONSENSUS
....

A5-AT3G13570-XLOC_017517-9103-0
TTAGGGAAAGAATACAGTGATATGTTCCATGTTCGGTCACACTTCGAGTC
A5-AT3G13570-XLOC_017517-9103-1

CONSENSUS
....

A5-AT3G13570-XLOC_017517-9103-0
TGTTTATTAAAGTTGTTGAGGTTTAACAGTGAATCTAGAGAGTTGAAGAG
A5-AT3G13570-XLOC_017517-9103-1

CONSENSUS
....

A5-AT3G13570-XLOC_017517-9103-0
ACCAATGAAGTAATTAGAGTTCTTTGGAAGATGTTCTAAATGGTAGTGAA
A5-AT3G13570-XLOC_017517-9103-1

CONSENSUS
....

A5-AT3G13570-XLOC_017517-9103-0
GGTTGGAAGGAAGTTGGGTTTGAATTCAATTTGAAGAGATATCAAGTCTT
A5-AT3G13570-XLOC_017517-9103-1

CONSENSUS
....

A5-AT3G13570-XLOC_017517-9103-0
GGAGAAGTCATCTTTATACTGAAACTTGCAAGGTTTGCAGAGATCCAAGG
A5-AT3G13570-XLOC_017517-9103-1
--AGATCCAAGG
CONSENSUS
....AGATCCAAGG

A5-AT3G13570-XLOC_017517-9103-0
GGGTTTGGATTCATTCAGTTTATGGATCCTGCTGATGCTGCTGAGGCTAA
A5-AT3G13570-XLOC_017517-9103-1
GGGTTTGGATTCATTCAGTTTATGGATCCTGCTGATGCTGCTGAGGCTAA
CONSENSUS
GGGTTTGGATTCATTCAGTTTATGGATCCTGCTGATGCTGCTGAGGCTAA

A5-AT3G13570-XLOC_017517-9103-0
ACATCAAATGGATGGTTATCTTCTTCTTGGTCGTGAGTTGACTGTCGTAT
A5-AT3G13570-XLOC_017517-9103-1
ACATCAAATGGATGGTTATCTTCTTCTTGGTCGTGAGTTGACTGTCGTAT
CONSENSUS

ACATCAAATGGATGGTTATCTTCTTCTTGGTCGTGAGTTGACTGTCGTAT

A5-AT3G13570-XLOC_017517-9103-0
TTGCTGAAGAAAACCGGAAGAAGCCAACTGAGATGAGAACAAGGGATCGA
A5-AT3G13570-XLOC_017517-9103-1
TTGCTGAAGAAAACCGGAAGAAGCCAACTGAGATGAGAACAAGGGATCGA
CONSENSUS
TTGCTGAAGAAAACCGGAAGAAGCCAACTGAGATGAGAACAAGGGATCGA

A5-AT3G13570-XLOC_017517-9103-0 GGTGGAAG
A5-AT3G13570-XLOC_017517-9103-1 GGTGGAAG
CONSENSUS GGTGGAAG

alignment for event: A5-AT3G44310-XLOC_015736-3338

A5-AT3G44310-XLOC_015736-3338-0
ATCTTGGTGATATAGCAAGAGCCAAGTTATACTTCGATTCGGTTGGACAT
A5-AT3G44310-XLOC_015736-3338-1
ATCTTGGTGATATAGCAAGAGCCAAGTTATACTTCGATTCGGTTGGACAT
CONSENSUS
ATCTTGGTGATATAGCAAGAGCCAAGTTATACTTCGATTCGGTTGGACAT

A5-AT3G44310-XLOC_015736-3338-0
TACTCGAGACCAGATGTTTTACACTTGACCGTAAATGAGCACCCGAGGAA
A5-AT3G44310-XLOC_015736-3338-1
TACTCGAGACCAGATGTTTTACACTTGACCGTAAATGAGCACCCGAGGAA
CONSENSUS
TACTCGAGACCAGATGTTTTACACTTGACCGTAAATGAGCACCCGAGGAA

A5-AT3G44310-XLOC_015736-3338-0
ATCGGTTACATTCGTGACGAAGGTGGAGAAAGCTGAGGATGACTCAAACA
A5-AT3G44310-XLOC_015736-3338-1
ATCGGTTACATTCGTGACGAAGGTGGAGAAAGCTGAGGATGACTCAAACA
CONSENSUS
ATCGGTTACATTCGTGACGAAGGTGGAGAAAGCTGAGGATGACTCAAACA

A5-AT3G44310-XLOC_015736-3338-0
AATA--
A5-AT3G44310-XLOC_015736-3338-1
AATAGTAAGAGACTTGAAGTTCGTATCTGCTGGAGTTATGTCAATCGTAT
CONSENSUS
AATA..

A5-AT3G44310-XLOC_015736-3338-0

A5-AT3G44310-XLOC_015736-3338-1
GGAGTCAAGTCCAAAATGTTCTGTTGCGTTTTCATTTTATGTTCAAGTTT
CONSENSUS
....

A5-AT3G44310-XLOC_015736-3338-0
--GA
A5-AT3G44310-XLOC_015736-3338-1
ATTTATCTTTCTCTTCAATGGTAAGATCTATGGAGTCAAGTAATAATGGA

CONSENSUS
      ....GA

A5-AT3G44310-XLOC_015736-3338-0 AGTTCTGGATCGAAACTTGTAAT
A5-AT3G44310-XLOC_015736-3338-1 AGTTCTGGATCGAAACTTGTAAT
CONSENSUS   AGTTCTGGATCGAAACTTGTAAT

alignment for event: A3-AT3G07300-XLOC_017168-787

A3-AT3G07300-XLOC_017168-787-0
      GGTCTCCTATTATCAATTCCGTTTCTCTCTCGCCGGAAGATTTTTGCTAC
A3-AT3G07300-XLOC_017168-787-1
      GGTCTCCTATTATCAATTCCGTTTCTCTCTCGCCGGAAGATTTTTGCTAC
CONSENSUS
      GGTCTCCTATTATCAATTCCGTTTCTCTCTCGCCGGAAGATTTTTGCTAC

A3-AT3G07300-XLOC_017168-787-0
      TTCATCTCCTTATCGCCCCACCACCGTCACATATTCACTG----------
A3-AT3G07300-XLOC_017168-787-1
      TTCATCTCCTTATCGCCCCACCACCGTCACATATTCACTGGTTTCGCATT
CONSENSUS
      TTCATCTCCTTATCGCCCCACCACCGTCACATATTCACTG..........

A3-AT3G07300-XLOC_017168-787-0   --------
TTTCTCGTTAATTTCGGTTAAAGAAGATGCCAGACGTTCAAT
A3-AT3G07300-XLOC_017168-787-1
      TTCTCAAGTTTCTCGTTAATTTCGGTTAAAGAAGATGCCAGACGTTCAAT
CONSENSUS
      ........TTTCTCGTTAATTTCGGTTAAAGAAGATGCCAGACGTTCAAT

A3-AT3G07300-XLOC_017168-787-0
      CAACGGTGGTGGAATTTGTTAACAAGCTCAGAAAGCG
A3-AT3G07300-XLOC_017168-787-1
      CAACGGTGGTGGAATTTGTTAACAAGCTCAGAAAGCG
CONSENSUS
      CAACGGTGGTGGAATTTGTTAACAAGCTCAGAAAGCG

alignment for event: RI-AT3G01540-XLOC_016824-4695

RI-AT3G01540-XLOC_016824-4695-0
      GGGGGCCAAGTACCACCACCTCTAATGTCCTTTGAAGCTACTGGTTTTCC
RI-AT3G01540-XLOC_016824-4695-1
      GGGGGCCAAGTACCACCACCTCTAATGTCCTTTGAAGCTACTGGTTTTCC
CONSENSUS
      GGGGGCCAAGTACCACCACCTCTAATGTCCTTTGAAGCTACTGGTTTTCC

RI-AT3G01540-XLOC_016824-4695-0
      ACCTGAGCTTCTGCGGGAGGTATGATTGGTTAATAGTAAACTGAAATGAC
RI-AT3G01540-XLOC_016824-4695-1
      ACCTGAGCTTCTGCGGGAG-------------------------------
CONSENSUS
      ACCTGAGCTTCTGCGGGAG...............................

RI-AT3G01540-XLOC_016824-4695-0
TTAGGAACCCCTCAAACCTGATAATTGTTCTACCAATAAGCGGAATCTAT
RI-AT3G01540-XLOC_016824-4695-1

CONSENSUS
....

RI-AT3G01540-XLOC_016824-4695-0
AACCTTATAATTGTTCTACCAATAAGCGGAATCTGTATTGAGTGTTTTTG
RI-AT3G01540-XLOC_016824-4695-1

CONSENSUS
....

RI-AT3G01540-XLOC_016824-4695-0
CTAAAGTCTGATTTATTAACATAGCTTTGTTTTTCCAGTCTCAGACTTTT
RI-AT3G01540-XLOC_016824-4695-1

CONSENSUS
....

RI-AT3G01540-XLOC_016824-4695-0
CTGTCACTTAGCTAAAATCGACTTAGGTATAACATTTAGAGTCAAAGTTT
RI-AT3G01540-XLOC_016824-4695-1

CONSENSUS
....

RI-AT3G01540-XLOC_016824-4695-0
TATGCCATACGCACCCTTTTCTCTTTTTTTCCCAGGTGCGATGTTTGCTC
RI-AT3G01540-XLOC_016824-4695-1

CONSENSUS
....

RI-AT3G01540-XLOC_016824-4695-0
CTGGGAGGAGCCAATGTCGTACACTGTGCCACTTCTTTGAGGTCCTTGCA
RI-AT3G01540-XLOC_016824-4695-1

CONSENSUS
....

RI-AT3G01540-XLOC_016824-4695-0
AATTATTTGGAGTGCCCTATATGGTGGACCAAATTTTTTGGAAAGGGCTT
RI-AT3G01540-XLOC_016824-4695-1

CONSENSUS
....

RI-AT3G01540-XLOC_016824-4695-0
CCCAAATCATCCAAGCTTGTGGCAGGGCCTGTTAATGGTGTGATGATTCA
RI-AT3G01540-XLOC_016824-4695-1

CONSENSUS
....

RI-AT3G01540-XLOC_016824-4695-0
      TTTCACCACCGGGTCCAGACAGAAGAAGAGGCGTCCTCCTGCTGCAGGTT
RI-AT3G01540-XLOC_016824-4695-1

CONSENSUS
      ....

RI-AT3G01540-XLOC_016824-4695-0
      TCATCAGTAACCTTTTGCCACCTGCAAATGGGGGATGGTGCTTCTTCTGA
RI-AT3G01540-XLOC_016824-4695-1

CONSENSUS
      ....

RI-AT3G01540-XLOC_016824-4695-0
      CATGAGCCACCATTCCATAAGCCCAAAAGAACCTTGGTGTTCCCCTTCTC
RI-AT3G01540-XLOC_016824-4695-1

CONSENSUS
      ....

RI-AT3G01540-XLOC_016824-4695-0
      ATAGGACAGTGTGGTTTTCTGTTTTCTTAGGTACTCAGTGCAGGTTTCTC
RI-AT3G01540-XLOC_016824-4695-1 ------------------------------
GTACTCAGTGCAGGTTTCTC
CONSENSUS
      ..............................GTACTCAGTGCAGGTTTCTC

RI-AT3G01540-XLOC_016824-4695-0
      TGCTCCAACTCCAATTCAAGCTCAGTCATGGCCCATTGCTATGCAAGGTA
RI-AT3G01540-XLOC_016824-4695-1
      TGCTCCAACTCCAATTCAAGCTCAGTCATGGCCCATTGCTATGCAAGGTA
CONSENSUS
      TGCTCCAACTCCAATTCAAGCTCAGTCATGGCCCATTGCTATGCAAGGTA

RI-AT3G01540-XLOC_016824-4695-0
      GGGACATAGTAGCCATTGCTAAAACTGGCTCGGGAAAAACTTTGGGTTAC
RI-AT3G01540-XLOC_016824-4695-1
      GGGACATAGTAGCCATTGCTAAAACTGGCTCGGGAAAAACTTTGGGTTAC
CONSENSUS
      GGGACATAGTAGCCATTGCTAAAACTGGCTCGGGAAAAACTTTGGGTTAC

RI-AT3G01540-XLOC_016824-4695-0
      TTGATTCCTGGATTTTTGCATCTTCAACGTATCCGAAATGATTCGCGAAT
RI-AT3G01540-XLOC_016824-4695-1
      TTGATTCCTGGATTTTTGCATCTTCAACGTATCCGAAATGATTCGCGAAT
CONSENSUS
      TTGATTCCTGGATTTTTGCATCTTCAACGTATCCGAAATGATTCGCGAAT

RI-AT3G01540-XLOC_016824-4695-0
      GGGCCCAACAATCTTGGTATTGTCTCCAACGAGAGAGCTGGCCACACAAA
RI-AT3G01540-XLOC_016824-4695-1
      GGGCCCAACAATCTTGGTATTGTCTCCAACGAGAGAGCTGGCCACACAAA
CONSENSUS
      GGGCCCAACAATCTTGGTATTGTCTCCAACGAGAGAGCTGGCCACACAAA

RI-AT3G01540-XLOC_016824-4695-0
      TCCAAGAAGAAGCTGTTAAATTTGGGAGGTCATCAAGAATTTCGTGTACG
RI-AT3G01540-XLOC_016824-4695-1
      TCCAAGAAGAAGCTGTTAAATTTGGGAGGTCATCAAGAATTTCGTGTACG
CONSENSUS
      TCCAAGAAGAAGCTGTTAAATTTGGGAGGTCATCAAGAATTTCGTGTACG

alignment for event: RI-AT3G52180-XLOC_019437-4852

RI-AT3G52180-XLOC_019437-4852-0
      CTTACAGGACTCAAGAGGAAGACTGTTACTCTGACACTGAAAGATAAGGG
RI-AT3G52180-XLOC_019437-4852-1
      CTTACAGGACTCAAGAGGAAGACTGTTACTCTGACACTGAAAGATAAGGG
CONSENSUS
      CTTACAGGACTCAAGAGGAAGACTGTTACTCTGACACTGAAAGATAAGGG

RI-AT3G52180-XLOC_019437-4852-0
      GTTCTCCAGAGTAGAAATTTCTGGCCTTGACATTGGATGGGGACAGGTAA
RI-AT3G52180-XLOC_019437-4852-1
      GTTCTCCAGAGTAGAAATTTCTGGCCTTGACATTGGATGGGGACAG----
CONSENSUS
      GTTCTCCAGAGTAGAAATTTCTGGCCTTGACATTGGATGGGGACAG....

RI-AT3G52180-XLOC_019437-4852-0
      ATATATTTTATCTTAAACCAATTTATTTATTATGACACCTTGCTCTTAAA
RI-AT3G52180-XLOC_019437-4852-1

CONSENSUS
      ....

RI-AT3G52180-XLOC_019437-4852-0
      TCTATGGTGTGCCATATAAGCTTTAAAGCCAGGATGCACTTTTGTCACTG
RI-AT3G52180-XLOC_019437-4852-1

CONSENSUS
      ....

RI-AT3G52180-XLOC_019437-4852-0
      CTATTAAAAGAATGGCCTCTTTGATAAGAAGTGGAAGAGATGAGTGTTGG
RI-AT3G52180-XLOC_019437-4852-1

CONSENSUS
      ....

RI-AT3G52180-XLOC_019437-4852-0
      ATTTCTGAAAATTATTGTCTTATGATTGTGCAAAACTATTTGGACGTATA
RI-AT3G52180-XLOC_019437-4852-1

CONSENSUS
      ....

RI-AT3G52180-XLOC_019437-4852-0
      TATATAAAAGGTAATGTATTTCTTTAGCTCTTTTCGTTTTTACCATTTTT

RI-AT3G52180-XLOC_019437-4852-1

CONSENSUS
  ....

RI-AT3G52180-XLOC_019437-4852-0
  CTCTTTATGTAGAGGATACCTCTAACACTGGACAAGGGAACAGGATTCTG
RI-AT3G52180-XLOC_019437-4852-1 ------------
AGGATACCTCTAACACTGGACAAGGGAACAGGATTCTG
CONSENSUS
  ............AGGATACCTCTAACACTGGACAAGGGAACAGGATTCTG

RI-AT3G52180-XLOC_019437-4852-0 GATCCTAAAGAGAGAACTGCCT
RI-AT3G52180-XLOC_019437-4852-1 GATCCTAAAGAGAGAACTGCCT
CONSENSUS GATCCTAAAGAGAGAACTGCCT

alignment for event: RI-AT3G49590-XLOC_016042-98

RI-AT3G49590-XLOC_016042-98-0
  AAACTCTTTTTGTTTATGTTGATCATCATCATCGTCATCTGCGTGTAAAA
RI-AT3G49590-XLOC_016042-98-1
  AAACTCTTTTTGTTTATGTTGATCATCATCATCGTCATCTGCGTGTAAAA
CONSENSUS
  AAACTCTTTTTGTTTATGTTGATCATCATCATCGTCATCTGCGTGTAAAA

RI-AT3G49590-XLOC_016042-98-0
  CACACTCACGCCATCGTTGACGTCTCAGATCTTCACATAAAAGCTTCTAT
RI-AT3G49590-XLOC_016042-98-1
  CACACTCACGCCATCGTTGACGTCTCAGATCTTCACATAAAAGCTTCTAT
CONSENSUS
  CACACTCACGCCATCGTTGACGTCTCAGATCTTCACATAAAAGCTTCTAT

RI-AT3G49590-XLOC_016042-98-0
  TATCAAACTCTCTTCCTCACGGTAACCCTAACTATCTGTAATTTATCTCT
RI-AT3G49590-XLOC_016042-98-1
  TATCAAACTCTCTTCCTCACG-----------------------------
CONSENSUS
  TATCAAACTCTCTTCCTCACG.............................

RI-AT3G49590-XLOC_016042-98-0
  CTACACTTTCTTCATCTCTTAATCACCTTAATCATCTTAAGCTTTGTATT
RI-AT3G49590-XLOC_016042-98-1

CONSENSUS
  ....

RI-AT3G49590-XLOC_016042-98-0
  TCTAGATCCTTCTCCCAAAACAACGATTCGTGATCTCCGTTCTCATAGAT
RI-AT3G49590-XLOC_016042-98-1 -----
ATCCTTCTCCCAAAACAACGATTCGTGATCTCCGTTCTCATAGAT
CONSENSUS
  .....ATCCTTCTCCCAAAACAACGATTCGTGATCTCCGTTCTCATAGAT

RI-AT3G49590-XLOC_016042-98-0

CAGTTTCGGATTTGGATATTTCTTAGGGATTTGTGTGAGATATTTTTAGA
RI-AT3G49590-XLOC_016042-98-1
CAGTTTCGGATTTGGATATTTCTTAGGGATTTGTGTGAGATATTTTTAGA
CONSENSUS
CAGTTTCGGATTTGGATATTTCTTAGGGATTTGTGTGAGATATTTTTAGA

RI-AT3G49590-XLOC_016042-98-0
ATTGTTTCGATTTAGGGATTTGTGTGAAGAAGATGGATTTTCCAGAGAAT
RI-AT3G49590-XLOC_016042-98-1
ATTGTTTCGATTTAGGGATTTGTGTGAAGAAGATGGATTTTCCAGAGAAT
CONSENSUS
ATTGTTTCGATTTAGGGATTTGTGTGAAGAAGATGGATTTTCCAGAGAAT

RI-AT3G49590-XLOC_016042-98-0
TTGCCTTCAGATATAGGAAGATTAGAGCAGATTGTTTCTCATTTCTTCCC
RI-AT3G49590-XLOC_016042-98-1
TTGCCTTCAGATATAGGAAGATTAGAGCAGATTGTTTCTCATTTCTTCCC
CONSENSUS
TTGCCTTCAGATATAGGAAGATTAGAGCAGATTGTTTCTCATTTCTTCCC

RI-AT3G49590-XLOC_016042-98-0
AAAAGCATTACACATAGTTCTTAATTCCAGAATCCCTTCGTTGCAATCTC
RI-AT3G49590-XLOC_016042-98-1
AAAAGCATTACACATAGTTCTTAATTCCAGAATCCCTTCGTTGCAATCTC
CONSENSUS
AAAAGCATTACACATAGTTCTTAATTCCAGAATCCCTTCGTTGCAATCTC

RI-AT3G49590-XLOC_016042-98-0
GTGGTCGTACTCGTGAGCGTTTATCAGGTCTTAACGTTAGAAAAAGTGAC
RI-AT3G49590-XLOC_016042-98-1
GTGGTCGTACTCGTGAGCGTTTATCAGGTCTTAACGTTAGAAAAAGTGAC
CONSENSUS
GTGGTCGTACTCGTGAGCGTTTATCAGGTCTTAACGTTAGAAAAAGTGAC

RI-AT3G49590-XLOC_016042-98-0
AAATGGTTTAATCTTGTGATGGGAGATCGTCCTGCTGCGTTGGAGAAATT
RI-AT3G49590-XLOC_016042-98-1
AAATGGTTTAATCTTGTGATGGGAGATCGTCCTGCTGCGTTGGAGAAATT
CONSENSUS
AAATGGTTTAATCTTGTGATGGGAGATCGTCCTGCTGCGTTGGAGAAATT

RI-AT3G49590-XLOC_016042-98-0
GCATTCTTGGCATAGGAATATTCTGGATTCTATGATTATTGATATCATTC
RI-AT3G49590-XLOC_016042-98-1
GCATTCTTGGCATAGGAATATTCTGGATTCTATGATTATTGATATCATTC
CONSENSUS
GCATTCTTGGCATAGGAATATTCTGGATTCTATGATTATTGATATCATTC

RI-AT3G49590-XLOC_016042-98-0
TTGTCCATCCGATTTCAAACGATAATTTGGATGATGATGATGATCATAGT
RI-AT3G49590-XLOC_016042-98-1
TTGTCCATCCGATTTCAAACGATAATTTGGATGATGATGATGATCATAGT
CONSENSUS
TTGTCCATCCGATTTCAAACGATAATTTGGATGATGATGATGATCATAGT

RI-AT3G49590-XLOC_016042-98-0

GATTCTGTTGTTAGATCAGCTGAGACTGTGATTGAGCGTTGGGTTGTTCA
RI-AT3G49590-XLOC_016042-98-1
GATTCTGTTGTTAGATCAGCTGAGACTGTGATTGAGCGTTGGGTTGTTCA
CONSENSUS
GATTCTGTTGTTAGATCAGCTGAGACTGTGATTGAGCGTTGGGTTGTTCA

RI-AT3G49590-XLOC_016042-98-0
ATATGAGAATCCTTTGATTATGTCTCCTCAGAGTTCTGACTCTGCAACGC
RI-AT3G49590-XLOC_016042-98-1
ATATGAGAATCCTTTGATTATGTCTCCTCAGAGTTCTGACTCTGCAACGC
CONSENSUS
ATATGAGAATCCTTTGATTATGTCTCCTCAGAGTTCTGACTCTGCAACGC

RI-AT3G49590-XLOC_016042-98-0
GTTACCAGAAGGTTTACAAGAAATCTATCATTCTGTTGCGGTCTCTTTAT
RI-AT3G49590-XLOC_016042-98-1
GTTACCAGAAGGTTTACAAGAAATCTATCATTCTGTTGCGGTCTCTTTAT
CONSENSUS
GTTACCAGAAGGTTTACAAGAAATCTATCATTCTGTTGCGGTCTCTTTAT

RI-AT3G49590-XLOC_016042-98-0
GCACAGACTCGGCTTCTTCCTGCTTATCGTGTCTCTAGGCAGCTGAGTTC
RI-AT3G49590-XLOC_016042-98-1
GCACAGACTCGGCTTCTTCCTGCTTATCGTGTCTCTAGGCAGCTGAGTTC
CONSENSUS
GCACAGACTCGGCTTCTTCCTGCTTATCGTGTCTCTAGGCAGCTGAGTTC

RI-AT3G49590-XLOC_016042-98-0
TTCTCTTGCTTCTTCTGGTTATGATTTGATTTACAAGGTTTCATCTTTCA
RI-AT3G49590-XLOC_016042-98-1
TTCTCTTGCTTCTTCTGGTTATGATTTGATTTACAAGGTTTCATCTTTCA
CONSENSUS
TTCTCTTGCTTCTTCTGGTTATGATTTGATTTACAAGGTTTCATCTTTCA

RI-AT3G49590-XLOC_016042-98-0
GTGACATATTCTCTGGTCCTGTGACGGAAACAATGAAAGAGTTTCGTTTT
RI-AT3G49590-XLOC_016042-98-1
GTGACATATTCTCTGGTCCTGTGACGGAAACAATGAAAGAGTTTCGTTTT
CONSENSUS
GTGACATATTCTCTGGTCCTGTGACGGAAACAATGAAAGAGTTTCGTTTT

RI-AT3G49590-XLOC_016042-98-0
GCTCCTGTTGAAGTGCCTCCTGGTCGTCTTTGTGCATCGGTTACTTACCG
RI-AT3G49590-XLOC_016042-98-1
GCTCCTGTTGAAGTGCCTCCTGGTCGTCTTTGTGCATCGGTTACTTACCG
CONSENSUS
GCTCCTGTTGAAGTGCCTCCTGGTCGTCTTTGTGCATCGGTTACTTACCG

RI-AT3G49590-XLOC_016042-98-0
TTCTGACTTATCTGATTTTAATCTCGGTGCTCATATCACATTGCCTCCGA
RI-AT3G49590-XLOC_016042-98-1
TTCTGACTTATCTGATTTTAATCTCGGTGCTCATATCACATTGCCTCCGA
CONSENSUS
TTCTGACTTATCTGATTTTAATCTCGGTGCTCATATCACATTGCCTCCGA

RI-AT3G49590-XLOC_016042-98-0

GGATTATAACTGATTATGTGGGAAGTCCTGCAACAGATCCTATGAGGTTT
RI-AT3G49590-XLOC_016042-98-1
GGATTATAACTGATTATGTGGGAAGTCCTGCAACAGATCCTATGAGGTTT
CONSENSUS
GGATTATAACTGATTATGTGGGAAGTCCTGCAACAGATCCTATGAGGTTT

RI-AT3G49590-XLOC_016042-98-0
TTCCCTTCTCCAGGGAGAAGTGTTGAAGGCCATTCCTTTACTGGTAGAGC
RI-AT3G49590-XLOC_016042-98-1
TTCCCTTCTCCAGGGAGAAGTGTTGAAGGCCATTCCTTTACTGGTAGAGC
CONSENSUS
TTCCCTTCTCCAGGGAGAAGTGTTGAAGGCCATTCCTTTACTGGTAGAGC

RI-AT3G49590-XLOC_016042-98-0
TGGTCGGCCTCCATTGACTGGTTCATCTGCAGAACGTCCGCATAGCTGGA
RI-AT3G49590-XLOC_016042-98-1
TGGTCGGCCTCCATTGACTGGTTCATCTGCAGAACGTCCGCATAGCTGGA
CONSENSUS
TGGTCGGCCTCCATTGACTGGTTCATCTGCAGAACGTCCGCATAGCTGGA

RI-AT3G49590-XLOC_016042-98-0
CTAGCGGCTTTCACAGACCTCCAGCTCAATTCGCAACACCGAACCAATCT
RI-AT3G49590-XLOC_016042-98-1
CTAGCGGCTTTCACAGACCTCCAGCTCAATTCGCAACACCGAACCAATCT
CONSENSUS
CTAGCGGCTTTCACAGACCTCCAGCTCAATTCGCAACACCGAACCAATCT

RI-AT3G49590-XLOC_016042-98-0
TTTTCGCCAGCTCAGTCTCATCAGTTGTCACCTGGGTTACACGATTTCCA
RI-AT3G49590-XLOC_016042-98-1
TTTTCGCCAGCTCAGTCTCATCAGTTGTCACCTGGGTTACACGATTTCCA
CONSENSUS
TTTTCGCCAGCTCAGTCTCATCAGTTGTCACCTGGGTTACACGATTTCCA

RI-AT3G49590-XLOC_016042-98-0
CTGGTCACGTACAGATGCTTTTGGTGACAATCACCAGCTTTCACCTCCCT
RI-AT3G49590-XLOC_016042-98-1
CTGGTCACGTACAGATGCTTTTGGTGACAATCACCAGCTTTCACCTCCCT
CONSENSUS
CTGGTCACGTACAGATGCTTTTGGTGACAATCACCAGCTTTCACCTCCCT

RI-AT3G49590-XLOC_016042-98-0
TTTCACCATCGGGTTCTCCATCTACTCCAAGATACATATCTGGGGGTAAC
RI-AT3G49590-XLOC_016042-98-1
TTTCACCATCGGGTTCTCCATCTACTCCAAGATACATATCTGGGGGTAAC
CONSENSUS
TTTCACCATCGGGTTCTCCATCTACTCCAAGATACATATCTGGGGGTAAC

RI-AT3G49590-XLOC_016042-98-0
AGTCCGCGCATTAATGTGAGACCAGGGACTGCTCCAGTGACCATTCCTTC
RI-AT3G49590-XLOC_016042-98-1
AGTCCGCGCATTAATGTGAGACCAGGGACTGCTCCAGTGACCATTCCTTC
CONSENSUS
AGTCCGCGCATTAATGTGAGACCAGGGACTGCTCCAGTGACCATTCCTTC

RI-AT3G49590-XLOC_016042-98-0

TTCAGCCACGTTAAATAGATACGTTTCATCTAACTTCTCTGAGCCAGGTA
RI-AT3G49590-XLOC_016042-98-1
TTCAGCCACGTTAAATAGATACGTTTCATCTAACTTCTCTGAGCCAGGTA
CONSENSUS
TTCAGCCACGTTAAATAGATACGTTTCATCTAACTTCTCTGAGCCAGGTA

RI-AT3G49590-XLOC_016042-98-0
GGAATCCACTTCCTCCTTTTTCCCCCAAAAGCACAAGACGCTCCCCTTCA
RI-AT3G49590-XLOC_016042-98-1
GGAATCCACTTCCTCCTTTTTCCCCCAAAAGCACAAGACGCTCCCCTTCA
CONSENSUS
GGAATCCACTTCCTCCTTTTTCCCCCAAAAGCACAAGACGCTCCCCTTCA

RI-AT3G49590-XLOC_016042-98-0
TCGCAGGACTCTTTGCCTGGGATTGCGTTGTACAGGAGTTCTAGAAGCGG
RI-AT3G49590-XLOC_016042-98-1
TCGCAGGACTCTTTGCCTGGGATTGCGTTGTACAGGAGTTCTAGAAGCGG
CONSENSUS
TCGCAGGACTCTTTGCCTGGGATTGCGTTGTACAGGAGTTCTAGAAGCGG

RI-AT3G49590-XLOC_016042-98-0
AGAGTCTCCTTCTGGATTAATGAACCAGTACCCTACTCAAAAG
RI-AT3G49590-XLOC_016042-98-1
AGAGTCTCCTTCTGGATTAATGAACCAGTACCCTACTCAAAAG
CONSENSUS
AGAGTCTCCTTCTGGATTAATGAACCAGTACCCTACTCAAAAG

alignment for event: RI-AT3G61010-XLOC_019935-367

RI-AT3G61010-XLOC_019935-367-0
GTGACATCAGATGAAACCTCTAAACATGGAATCCTGCTTTCTTTCTCATC
RI-AT3G61010-XLOC_019935-367-1
GTGACATCAGATGAAACCTCTAAACATGGAATCCTGCTTTCTTTCTCATC
CONSENSUS
GTGACATCAGATGAAACCTCTAAACATGGAATCCTGCTTTCTTTCTCATC

RI-AT3G61010-XLOC_019935-367-0
TCCATCACACGAGACGAAATCCATTCTCGTTTCACGACAAGAATCCATCT
RI-AT3G61010-XLOC_019935-367-1
TCCATCACACGAGACGAAATCCATTCTCGTTTCACGACAAGAATCCATCT
CONSENSUS
TCCATCACACGAGACGAAATCCATTCTCGTTTCACGACAAGAATCCATCT

RI-AT3G61010-XLOC_019935-367-0
GTAGATTCAACAACATGTTCTTACAGTGTCTCGCCACGTCAGCGCAGACT
RI-AT3G61010-XLOC_019935-367-1
GTAGATTCAACAACATGTTCTTACAGTGTCTCGCCACGTCAGCGCAGACT
CONSENSUS
GTAGATTCAACAACATGTTCTTACAGTGTCTCGCCACGTCAGCGCAGACT

RI-AT3G61010-XLOC_019935-367-0
GTATCCGAGTGGACAGTACAGGAGACAAGCCTTGTCCTGGATGGTCACAG
RI-AT3G61010-XLOC_019935-367-1
GTATCCGAGTGGACAGTACAGGAGACAAGCCTTGTCCTGGATGGTCACAG

CONSENSUS
      GTATCCGAGTGGACAGTACAGGAGACAAGCCTTGTCCTGGATGGTCACAG

RI-AT3G61010-XLOC_019935-367-0
      ACTTACTGAAATCTCTGCATTTTGCTACAGACCAGAGAATTTGACAAAGA
RI-AT3G61010-XLOC_019935-367-1
      ACTTACTGAAATCTCTGCATTTTGCTACAGACCAGAGAATTTGACAAAGA
CONSENSUS
      ACTTACTGAAATCTCTGCATTTTGCTACAGACCAGAGAATTTGACAAAGA

RI-AT3G61010-XLOC_019935-367-0
      GAACAGAATATGTTGCATTGCTCGGACACTTAAGATCATGTTCAGTACCA
RI-AT3G61010-XLOC_019935-367-1
      GAACAGAATATGTTGCATTGCTCGGACACTTAAGATCATGTTCAGTACCA
CONSENSUS
      GAACAGAATATGTTGCATTGCTCGGACACTTAAGATCATGTTCAGTACCA

RI-AT3G61010-XLOC_019935-367-0
      GCAGAAACCCGAGACTTTACTTCCGGCATCACCGTTGGTCATTGAAGCTC
RI-AT3G61010-XLOC_019935-367-1
      GCAGAAACCCGAGACTTTACTTCCGGCATCACCGTTGGTCATTGAAGCTC
CONSENSUS
      GCAGAAACCCGAGACTTTACTTCCGGCATCACCGTTGGTCATTGAAGCTC

RI-AT3G61010-XLOC_019935-367-0
      ATAACATGGAGCTTGTACCCGGTTATTCTGGTTCCAAGAGCCTCAGGGTT
RI-AT3G61010-XLOC_019935-367-1
      ATAACATGGAGCTTGTACCCGGTTATTCTGGTTCCAAGAGCCTCAGGGTT
CONSENSUS
      ATAACATGGAGCTTGTACCCGGTTATTCTGGTTCCAAGAGCCTCAGGGTT

RI-AT3G61010-XLOC_019935-367-0
      AAGCTAGAATGGAGACAGAAAGACCTTGAAGATTCTGCATTCCCAAGGTA
RI-AT3G61010-XLOC_019935-367-1
      AAGCTAGAATGGAGACAGAAAGACCTTGAAGATTCTGCATTCCCAAG---
CONSENSUS
      AAGCTAGAATGGAGACAGAAAGACCTTGAAGATTCTGCATTCCCAAG...

RI-AT3G61010-XLOC_019935-367-0
      CAATGTGTATGCGGAGAATGTAAAGTCTACTGATCTAAGACCGAGGAAGG
RI-AT3G61010-XLOC_019935-367-1

CONSENSUS
      ....

RI-AT3G61010-XLOC_019935-367-0
      TTCTAGAGAAGCCGAGAAGCGAAACAGTGTTTCTCGGAGTCGCTCACGTA
RI-AT3G61010-XLOC_019935-367-1

CONSENSUS
      ....

RI-AT3G61010-XLOC_019935-367-0
      CCATCCTATTACATAGCAGAACTGGTGGTAGAATCAGACGTGAAAGGAGT
RI-AT3G61010-XLOC_019935-367-1

CONSENSUS
      ....

RI-AT3G61010-XLOC_019935-367-0
      CCGCTTTGTGTTCAAGCCTGTGCTAAAGATGGTTCATGGGGCAAGCTGGA
RI-AT3G61010-XLOC_019935-367-1

CONSENSUS
      ....

RI-AT3G61010-XLOC_019935-367-0
      TTTTTATTTATTTTACACCATCATAATCGTCTCATTTGATTTATTTTTGT
RI-AT3G61010-XLOC_019935-367-1

CONSENSUS
      ....

RI-AT3G61010-XLOC_019935-367-0
      TTGTTCGGTAATCTCTTTCATAATTGATTTATGGGTACTTTTCTACTGAT
RI-AT3G61010-XLOC_019935-367-1

CONSENSUS
      ....

RI-AT3G61010-XLOC_019935-367-0
      TCAGCTACGATTCACTTATCATGGTGGGGCTGGTCGGAATCGCAATTGTC
RI-AT3G61010-XLOC_019935-367-1

CONSENSUS
      ....

RI-AT3G61010-XLOC_019935-367-0
      ATTTCTTGCTTTCAATGATTTTGCTTGTGGGAATCGAGTTAGCTAAGTTA
RI-AT3G61010-XLOC_019935-367-1

CONSENSUS
      ....

RI-AT3G61010-XLOC_019935-367-0
      ACACGTGACGATGTGTCTGAATCTTAACATTCATTTTCTTATTTGCTTTT
RI-AT3G61010-XLOC_019935-367-1

CONSENSUS
      ....

RI-AT3G61010-XLOC_019935-367-0
      TGTGAATCTTAGCGTGGAGTACAATGTCTCCTATGTCTACCATGCCCTAG
RI-AT3G61010-XLOC_019935-367-1   ------------
CGTGGAGTACAATGTCTCCTATGTCTACCATGCCCTAG
CONSENSUS
      ............CGTGGAGTACAATGTCTCCTATGTCTACCATGCCCTAG

RI-AT3G61010-XLOC_019935-367-0
      ATGCCTACATCGAGAGAGACAATGTCGGCTTGAAAGGTTTCACCAAGTCA
RI-AT3G61010-XLOC_019935-367-1
      ATGCCTACATCGAGAGAGACAATGTCGGCTTGAAAGGTTTCACCAAGTCA

CONSENSUS
      ATGCCTACATCGAGAGAGACAATGTCGGCTTGAAAGGTTTCACCAAGTCA

RI-AT3G61010-XLOC_019935-367-0
      GTTTCTTTAGTCTAAAGGAAAACCGTATTTGTGTCTCTTCAGCTGGTGGA
RI-AT3G61010-XLOC_019935-367-1
      GTTTCTTTAGTCTAAAGGAAAACCGTATTTGTGTCTCTTCAGCTGGTGGA
CONSENSUS
      GTTTCTTTAGTCTAAAGGAAAACCGTATTTGTGTCTCTTCAGCTGGTGGA

RI-AT3G61010-XLOC_019935-367-0
      TCATCTTTTTGTTATTGTTGAGGGTTTAACGCTAATAGGTTCTTTAACGA
RI-AT3G61010-XLOC_019935-367-1
      TCATCTTTTTGTTATTGTTGAGGGTTTAACGCTAATAGGTTCTTTAACGA
CONSENSUS
      TCATCTTTTTGTTATTGTTGAGGGTTTAACGCTAATAGGTTCTTTAACGA

RI-AT3G61010-XLOC_019935-367-0
      TTCAAGTCTTGAAGAACGAGGTTATGCTGAGAAGTTTATGGAGTATCAGA
RI-AT3G61010-XLOC_019935-367-1
      TTCAAGTCTTGAAGAACGAGGTTATGCTGAGAAGTTTATGGAGTATCAGA
CONSENSUS
      TTCAAGTCTTGAAGAACGAGGTTATGCTGAGAAGTTTATGGAGTATCAGA

RI-AT3G61010-XLOC_019935-367-0   TGCATTGTTTG
RI-AT3G61010-XLOC_019935-367-1   TGCATTGTTTG
CONSENSUS   TGCATTGTTTG

alignment for event: RI-AT3G61600-XLOC_016687-2054

RI-AT3G61600-XLOC_016687-2054-0
      GTTTCATGAGGAGGTAATGTCTCTACCTTTGGCTGGAATTGAGGCAATTC
RI-AT3G61600-XLOC_016687-2054-1
      GTTTCATGAGGAGGTAATGTCTCTACCTTTGGCTGGAATTGAGGCAATTC
CONSENSUS
      GTTTCATGAGGAGGTAATGTCTCTACCTTTGGCTGGAATTGAGGCAATTC

RI-AT3G61600-XLOC_016687-2054-0
      TATCAAGCGATGAACTCCAAATTGCATCAGAGGATGCAGTTTATGATTTT
RI-AT3G61600-XLOC_016687-2054-1
      TATCAAGCGATGAACTCCAAATTGCATCAGAGGATGCAGTTTATGATTTT
CONSENSUS
      TATCAAGCGATGAACTCCAAATTGCATCAGAGGATGCAGTTTATGATTTT

RI-AT3G61600-XLOC_016687-2054-0
      ATCTTGAAGTGGGCAAGGGCGCAATACCCTTGTTTGGAAGAGCGAAGAGA
RI-AT3G61600-XLOC_016687-2054-1
      ATCTTGAAGTGGGCAAGGGCGCAATACCCTTGTTTGGAAGAGCGAAGAGA
CONSENSUS
      ATCTTGAAGTGGGCAAGGGCGCAATACCCTTGTTTGGAAGAGCGAAGAGA

RI-AT3G61600-XLOC_016687-2054-0
      GATTCTCGGGTCACGCCTTGCACTCTCCATCCGCTTCCCATTCATGACAT
RI-AT3G61600-XLOC_016687-2054-1

GATTCTCGGGTCACGCCTTGCACTCTCCATCCGCTTCCCATTCATGACAT
CONSENSUS
GATTCTCGGGTCACGCCTTGCACTCTCCATCCGCTTCCCATTCATGACAT

RI-AT3G61600-XLOC_016687-2054-0
GCCGAAAGCTGAAGAAAGTGCTGACTTGCAGTGACTTTGAGCATGAAATA
RI-AT3G61600-XLOC_016687-2054-1
GCCGAAAGCTGAAGAAAGTGCTGACTTGCAGTGACTTTGAGCATGAAATA
CONSENSUS
GCCGAAAGCTGAAGAAAGTGCTGACTTGCAGTGACTTTGAGCATGAAATA

RI-AT3G61600-XLOC_016687-2054-0
GCATCAAAGCTTGTTCTAGAAGCTCTTTTCTTCAAAGCAGAAGCCCCACA
RI-AT3G61600-XLOC_016687-2054-1
GCATCAAAGCTTGTTCTAGAAGCTCTTTTCTTCAAAGCAGAAGCCCCACA
CONSENSUS
GCATCAAAGCTTGTTCTAGAAGCTCTTTTCTTCAAAGCAGAAGCCCCACA

RI-AT3G61600-XLOC_016687-2054-0
CAGACAACGTAGCCTAGCCTCCGAAGAATCTGCATCCCTGAACCGCCGCC
RI-AT3G61600-XLOC_016687-2054-1
CAGACAACGTAGCCTAGCCTCCGAAGAATCTGCATCCCTGAACCGCCGCC
CONSENSUS
CAGACAACGTAGCCTAGCCTCCGAAGAATCTGCATCCCTGAACCGCCGCC

RI-AT3G61600-XLOC_016687-2054-0
TGATAGAGAGGGCTTACAAATACAGACCCGTCAAAGTGGTCGAGTTTGAG
RI-AT3G61600-XLOC_016687-2054-1
TGATAGAGAGGGCTTACAAATACAGACCCGTCAAAGTGGTCGAGTTTGAG
CONSENSUS
TGATAGAGAGGGCTTACAAATACAGACCCGTCAAAGTGGTCGAGTTTGAG

RI-AT3G61600-XLOC_016687-2054-0
CTTCCTAGACCGCAGTGTGTAGTCTACCTAGACTTGAAAAGAGAAGAATG
RI-AT3G61600-XLOC_016687-2054-1
CTTCCTAGACCGCAGTGTGTAGTCTACCTAGACTTGAAAAGAGAAGAATG
CONSENSUS
CTTCCTAGACCGCAGTGTGTAGTCTACCTAGACTTGAAAAGAGAAGAATG

RI-AT3G61600-XLOC_016687-2054-0
TGGGGGACTGTTCCCGTCGGGTAGAGTGTATTCGCAGGCCTTTCACTTGG
RI-AT3G61600-XLOC_016687-2054-1
TGGGGGACTGTTCCCGTCGGGTAGAGTGTATTCGCAGGCCTTTCACTTGG
CONSENSUS
TGGGGGACTGTTCCCGTCGGGTAGAGTGTATTCGCAGGCCTTTCACTTGG

RI-AT3G61600-XLOC_016687-2054-0
GAGGTCAAGGGTTTTTCCTGTCAGCTCACTGCAACATGGACCAACAGAGC
RI-AT3G61600-XLOC_016687-2054-1
GAGGTCAAGGGTTTTTCCTGTCAGCTCACTGCAACATGGACCAACAGAGC
CONSENSUS
GAGGTCAAGGGTTTTTCCTGTCAGCTCACTGCAACATGGACCAACAGAGC

RI-AT3G61600-XLOC_016687-2054-0
TCGTTCCACTGTTTCGGGCTGTTCCTAGGGATGCAGGAGAAAGGGTCGGT
RI-AT3G61600-XLOC_016687-2054-1

TCGTTCCACTGTTTCGGGCTGTTCCTAGGGATGCAGGAGAAAGGGTCGGT
CONSENSUS
TCGTTCCACTGTTTCGGGCTGTTCCTAGGGATGCAGGAGAAAGGGTCGGT

RI-AT3G61600-XLOC_016687-2054-0
GAGTTTCGGAGTGGACTATGAATTCTCGGCAAGGTCAAAGCCCGCAGAGG
RI-AT3G61600-XLOC_016687-2054-1
GAGTTTCGGAGTGGACTATGAATTCTCGGCAAGGTCAAAGCCCGCAGAGG
CONSENSUS
GAGTTTCGGAGTGGACTATGAATTCTCGGCAAGGTCAAAGCCCGCAGAGG

RI-AT3G61600-XLOC_016687-2054-0
ATTTCATAAGCAAATACAAAGGGAACTACACATTCACAGGAGGGAAAGCA
RI-AT3G61600-XLOC_016687-2054-1
ATTTCATAAGCAAATACAAAGGGAACTACACATTCACAGGAGGGAAAGCA
CONSENSUS
ATTTCATAAGCAAATACAAAGGGAACTACACATTCACAGGAGGGAAAGCA

RI-AT3G61600-XLOC_016687-2054-0
GTAGGTTACAGAAACCTGTTTGGGGTCCCATGGACGTCTTTTATAGCGGA
RI-AT3G61600-XLOC_016687-2054-1
GTAGGTTACAGAAACCTGTTTGGGGTCCCATGGACGTCTTTTATAGCGGA
CONSENSUS
GTAGGTTACAGAAACCTGTTTGGGGTCCCATGGACGTCTTTTATAGCGGA

RI-AT3G61600-XLOC_016687-2054-0
AGATAGTCAATACTTCATCAATGGCATTCTCCATCTCAGAGCAGAGCTTA
RI-AT3G61600-XLOC_016687-2054-1
AGATAGTCAATACTTCATCAATGGCATTCTCCATCTCAGAGCAGAGCTTA
CONSENSUS
AGATAGTCAATACTTCATCAATGGCATTCTCCATCTCAGAGCAGAGCTTA

RI-AT3G61600-XLOC_016687-2054-0
CCATCAAAAGGTCTACAGATCCTTAGTGACCGTTTACTTCTCAATTGTCA
RI-AT3G61600-XLOC_016687-2054-1
CCATCAAAAG--
CONSENSUS
CCATCAAAAG..

RI-AT3G61600-XLOC_016687-2054-0
CTGTCTCTCTTTCCTTTACCTGCTGCTAAATCTCTCGCTCTCTCTAACTT
RI-AT3G61600-XLOC_016687-2054-1

CONSENSUS
....

RI-AT3G61600-XLOC_016687-2054-0
GCCTTTTTTTTCTTTTCTTTTATAAGGCTTGAAGAAGACTAATAAAGCTT
RI-AT3G61600-XLOC_016687-2054-1 --------------------------
GCTTGAAGAAGACTAATAAAGCTT
CONSENSUS
..........................GCTTGAAGAAGACTAATAAAGCTT

RI-AT3G61600-XLOC_016687-2054-0
ATAAAAGTAAGGTTAAAAACGTTTCGACGACAACGTTATAAGAAGAATGT
RI-AT3G61600-XLOC_016687-2054-1

ATAAAAGTAAGGTTAAAAACGTTTCGACGACAACGTTATAAGAAGAATGT
CONSENSUS
ATAAAAGTAAGGTTAAAAACGTTTCGACGACAACGTTATAAGAAGAATGT

RI-AT3G61600-XLOC_016687-2054-0
AAATGCGTTCTTTTTTTCTATCTACCCTTACTCTTTTGGCTTTGGATTAT
RI-AT3G61600-XLOC_016687-2054-1
AAATGCGTTCTTTTTTTCTATCTACCCTTACTCTTTTGGCTTTGGATTAT
CONSENSUS
AAATGCGTTCTTTTTTTCTATCTACCCTTACTCTTTTGGCTTTGGATTAT

RI-AT3G61600-XLOC_016687-2054-0
AAGTTTAACTCTTCAATGAAATTCTTAGCGTAGAGATTCTTGTATTATGA
RI-AT3G61600-XLOC_016687-2054-1
AAGTTTAACTCTTCAATGAAATTCTTAGCGTAGAGATTCTTGTATTATGA
CONSENSUS
AAGTTTAACTCTTCAATGAAATTCTTAGCGTAGAGATTCTTGTATTATGA

RI-AT3G61600-XLOC_016687-2054-0
ACGAAGTTGTGAACAGAAAAGAACGAACGTAACTCTCTCGTCTCATTTAA
RI-AT3G61600-XLOC_016687-2054-1
ACGAAGTTGTGAACAGAAAAGAACGAACGTAACTCTCTCGTCTCATTTAA
CONSENSUS
ACGAAGTTGTGAACAGAAAAGAACGAACGTAACTCTCTCGTCTCATTTAA

RI-AT3G61600-XLOC_016687-2054-0
CTAATTTATCTAGTTGGATTGAAATAGACATGACTTTTCGATT
RI-AT3G61600-XLOC_016687-2054-1
CTAATTTATCTAGTTGGATTGAAATAGACATGACTTTTCGATT
CONSENSUS
CTAATTTATCTAGTTGGATTGAAATAGACATGACTTTTCGATT

alignment for event: RI-AT3G01540-XLOC_016824-4696

RI-AT3G01540-XLOC_016824-4696-0
GGGGGCCAAGTACCACCACCTCTAATGTCCTTTGAAGCTACTGGTTTTCC
RI-AT3G01540-XLOC_016824-4696-1
GGGGGCCAAGTACCACCACCTCTAATGTCCTTTGAAGCTACTGGTTTTCC
CONSENSUS
GGGGGCCAAGTACCACCACCTCTAATGTCCTTTGAAGCTACTGGTTTTCC

RI-AT3G01540-XLOC_016824-4696-0
ACCTGAGCTTCTGCGGGAGGTATGATTGGTTAATAGTAAACTGAAATGAC
RI-AT3G01540-XLOC_016824-4696-1
ACCTGAGCTTCTGCGGGAG-------------------------------
CONSENSUS
ACCTGAGCTTCTGCGGGAG...............................

RI-AT3G01540-XLOC_016824-4696-0
TTAGGAACCCCTCAAACCTGATAATTGTTCTACCAATAAGCGGAATCTAT
RI-AT3G01540-XLOC_016824-4696-1

CONSENSUS
....

RI-AT3G01540-XLOC_016824-4696-0
AACCTTATAATTGTTCTACCAATAAGCGGAATCTGTATTGAGTGTTTTTG
RI-AT3G01540-XLOC_016824-4696-1

CONSENSUS
....

RI-AT3G01540-XLOC_016824-4696-0
CTAAAGTCTGATTTATTAACATAGCTTTGTTTTTCCAGTCTCAGACTTTT
RI-AT3G01540-XLOC_016824-4696-1

CONSENSUS
....

RI-AT3G01540-XLOC_016824-4696-0
CTGTCACTTAGCTAAAATCGACTTAGGTATAACATTTAGAGTCAAAGTTT
RI-AT3G01540-XLOC_016824-4696-1

CONSENSUS
....

RI-AT3G01540-XLOC_016824-4696-0
TATGCCATACGCACCCTTTTCTCTTTTTTTCCCAGGTGCGATGTTTGCTC
RI-AT3G01540-XLOC_016824-4696-1
-----------------------------------GTGCGATGTTTGCTC
CONSENSUS
...................................GTGCGATGTTTGCTC

RI-AT3G01540-XLOC_016824-4696-0
CTGGGAGGAGCCAATGTCGTACACTGTGCCACTTCTTTGAGGTCCTTGCA
RI-AT3G01540-XLOC_016824-4696-1
CTGGGAGGAGCCAATGTCGTACACTGTGCCACTTCTTTGAGGTCCTTGCA
CONSENSUS
CTGGGAGGAGCCAATGTCGTACACTGTGCCACTTCTTTGAGGTCCTTGCA

RI-AT3G01540-XLOC_016824-4696-0
AATTATTTGGAGTGCCCTATATGGTGGACCAAATTTTTTGGAAAGGGCTT
RI-AT3G01540-XLOC_016824-4696-1
AATTATTTGGAGTGCCCTATATGGTGGACCAAATTTTTTGGAAAGGGCTT
CONSENSUS
AATTATTTGGAGTGCCCTATATGGTGGACCAAATTTTTTGGAAAGGGCTT

RI-AT3G01540-XLOC_016824-4696-0
CCCAAATCATCCAAGCTTGTGGCAGGGCCTGTTAATGGTGTGATGATTCA
RI-AT3G01540-XLOC_016824-4696-1
CCCAAATCATCCAAGCTTGTGGCAGGGCCTGTTAATGGTGTGATGATTCA
CONSENSUS
CCCAAATCATCCAAGCTTGTGGCAGGGCCTGTTAATGGTGTGATGATTCA

RI-AT3G01540-XLOC_016824-4696-0
TTTCACCACCGGGTCCAGACAGAAGAAGAGGCGTCCTCCTGCTGCAGGTT
RI-AT3G01540-XLOC_016824-4696-1
TTTCACCACCGGGTCCAGACAGAAGAAGAGGCGTCCTCCTGCTGCAGGTT
CONSENSUS
TTTCACCACCGGGTCCAGACAGAAGAAGAGGCGTCCTCCTGCTGCAGGTT

RI-AT3G01540-XLOC_016824-4696-0
TCATCAGTAACCTTTTGCCACCTGCAAATGGGGGATGGTGCTTCTTCTGA
RI-AT3G01540-XLOC_016824-4696-1
TCATCAGTAACCTTTTGCCACCTGCAAATGGGGGATGGTGCTTCTTCTGA
CONSENSUS
TCATCAGTAACCTTTTGCCACCTGCAAATGGGGGATGGTGCTTCTTCTGA

RI-AT3G01540-XLOC_016824-4696-0
CATGAGCCACCATTCCATAAGCCCAAAAGAACCTTGGTGTTCCCCTTCTC
RI-AT3G01540-XLOC_016824-4696-1
CATGAGCCACCATTCCATAAGCCCAAAAGAACCTTGGTGTTCCCCTTCTC
CONSENSUS
CATGAGCCACCATTCCATAAGCCCAAAAGAACCTTGGTGTTCCCCTTCTC

RI-AT3G01540-XLOC_016824-4696-0
ATAGGACAGTGTGGTTTTCTGTTTTCTTAGGTACTCAGTGCAGGTTTCTC
RI-AT3G01540-XLOC_016824-4696-1
ATAGGACAGTGTGGTTTTCTGTTTTCTTAGGTACTCAGTGCAGGTTTCTC
CONSENSUS
ATAGGACAGTGTGGTTTTCTGTTTTCTTAGGTACTCAGTGCAGGTTTCTC

RI-AT3G01540-XLOC_016824-4696-0
TGCTCCAACTCCAATTCAAGCTCAGTCATGGCCCATTGCTATGCAAGGTA
RI-AT3G01540-XLOC_016824-4696-1
TGCTCCAACTCCAATTCAAGCTCAGTCATGGCCCATTGCTATGCAAGGTA
CONSENSUS
TGCTCCAACTCCAATTCAAGCTCAGTCATGGCCCATTGCTATGCAAGGTA

RI-AT3G01540-XLOC_016824-4696-0
GGGACATAGTAGCCATTGCTAAAACTGGCTCGGGAAAAACTTTGGGTTAC
RI-AT3G01540-XLOC_016824-4696-1
GGGACATAGTAGCCATTGCTAAAACTGGCTCGGGAAAAACTTTGGGTTAC
CONSENSUS
GGGACATAGTAGCCATTGCTAAAACTGGCTCGGGAAAAACTTTGGGTTAC

RI-AT3G01540-XLOC_016824-4696-0
TTGATTCCTGGATTTTTGCATCTTCAACGTATCCGAAATGATTCGCGAAT
RI-AT3G01540-XLOC_016824-4696-1
TTGATTCCTGGATTTTTGCATCTTCAACGTATCCGAAATGATTCGCGAAT
CONSENSUS
TTGATTCCTGGATTTTTGCATCTTCAACGTATCCGAAATGATTCGCGAAT

RI-AT3G01540-XLOC_016824-4696-0
GGGCCCAACAATCTTGGTATTGTCTCCAACGAGAGAGCTGGCCACACAAA
RI-AT3G01540-XLOC_016824-4696-1
GGGCCCAACAATCTTGGTATTGTCTCCAACGAGAGAGCTGGCCACACAAA
CONSENSUS
GGGCCCAACAATCTTGGTATTGTCTCCAACGAGAGAGCTGGCCACACAAA

RI-AT3G01540-XLOC_016824-4696-0
TCCAAGAAGAAGCTGTTAAATTTGGGAGGTCATCAAGAATTTCGTGTACG
RI-AT3G01540-XLOC_016824-4696-1
TCCAAGAAGAAGCTGTTAAATTTGGGAGGTCATCAAGAATTTCGTGTACG
CONSENSUS
TCCAAGAAGAAGCTGTTAAATTTGGGAGGTCATCAAGAATTTCGTGTACG

alignment for event: A3-AT3G47910-XLOC_019181-9223

A3-AT3G47910-XLOC_019181-9223-0
      GAGAAAAGGAATGGACGACTTGATGACCTGGAAGGAGCCAGTGTGAATAC
A3-AT3G47910-XLOC_019181-9223-1
      GAGAAAAGGAATGGACGACTTGATGACCTGGAAGGAGCCAGTGTGAATAC
CONSENSUS
      GAGAAAAGGAATGGACGACTTGATGACCTGGAAGGAGCCAGTGTGAATAC

A3-AT3G47910-XLOC_019181-9223-0
      AAATGGAGTTTTCCCGTCAACAAATCATTCTGCGATATCTGATACTGCAA
A3-AT3G47910-XLOC_019181-9223-1
      AAATGGAGTTTTCCCGTCAACAAATCATTCTGCGATATCTGATACTGCAA
CONSENSUS
      AAATGGAGTTTTCCCGTCAACAAATCATTCTGCGATATCTGATACTGCAA

A3-AT3G47910-XLOC_019181-9223-0 AGGTGCAGAATGTAAAATCCCAAAAAG---
TGCCTAACGGCACAGCTATG
A3-AT3G47910-XLOC_019181-9223-1
      AGGTGCAGAATGTAAAATCCCAAAAAGTAGTGCCTAACGGCACAGCTATG
CONSENSUS
      AGGTGCAGAATGTAAAATCCCAAAAAG...TGCCTAACGGCACAGCTATG

A3-AT3G47910-XLOC_019181-9223-0
      CAAGCTGGTATTTTCCAATCTGATCAACGAACTGGGAGGAGAACTAGACG
A3-AT3G47910-XLOC_019181-9223-1
      CAAGCTGGTATTTTCCAATCTGATCAACGAACTGGGAGGAGAACTAGACG
CONSENSUS
      CAAGCTGGTATTTTCCAATCTGATCAACGAACTGGGAGGAGAACTAGACG

A3-AT3G47910-XLOC_019181-9223-0
      CCAAAAAGCTTCAAACAAGTTAGCTGATGGAAAATATCCGGTCACACCTC
A3-AT3G47910-XLOC_019181-9223-1
      CCAAAAAGCTTCAAACAAGTTAGCTGATGGAAAATATCCGGTCACACCTC
CONSENSUS
      CCAAAAAGCTTCAAACAAGTTAGCTGATGGAAAATATCCGGTCACACCTC

A3-AT3G47910-XLOC_019181-9223-0
      CTGAGACCGAAAATAGTAAATCTCAGTTGTCAGGCACCAACGGCGAGAGA
A3-AT3G47910-XLOC_019181-9223-1
      CTGAGACCGAAAATAGTAAATCTCAGTTGTCAGGCACCAACGGCGAGAGA
CONSENSUS
      CTGAGACCGAAAATAGTAAATCTCAGTTGTCAGGCACCAACGGCGAGAGA

A3-AT3G47910-XLOC_019181-9223-0 CATTCTGAAACTCTACGTAACAATGGTG
A3-AT3G47910-XLOC_019181-9223-1 CATTCTGAAACTCTACGTAACAATGGTG
CONSENSUS   CATTCTGAAACTCTACGTAACAATGGTG

alignment for event: A3-AT3G53170-XLOC_019497-1286

A3-AT3G53170-XLOC_019497-1286-0

GCAAGAAGAGGCTTACGGAGTGATGCTTTAGTTTGTGAATGAGCAAATCT
A3-AT3G53170-XLOC_019497-1286-1
GCAAGAAGAGGCTTACGGAGTGATGCTTTAGTTTGTGAATGAGCAAATCT
CONSENSUS
GCAAGAAGAGGCTTACGGAGTGATGCTTTAGTTTGTGAATGAGCAAATCT

A3-AT3G53170-XLOC_019497-1286-0
AATGCAGAAGGAATTTAGAAGTTGTGAATTGTTCCAACTAAGAGAATCAC
A3-AT3G53170-XLOC_019497-1286-1
AATGCAGAAGGAATTTAGAAGTTGTGAATTGTTCCAACTAAGAGAATCAC
CONSENSUS
AATGCAGAAGGAATTTAGAAGTTGTGAATTGTTCCAACTAAGAGAATCAC

A3-AT3G53170-XLOC_019497-1286-0 TTGAG-----------
GTGTTTGGAGCGTTGGTTAGCAAGATCCCAAGAG
A3-AT3G53170-XLOC_019497-1286-1
TTGAGTGTTTGTTCAGGTGTTTGGAGCGTTGGTTAGCAAGATCCCAAGAG
CONSENSUS
TTGAG...........GTGTTTGGAGCGTTGGTTAGCAAGATCCCAAGAG

A3-AT3G53170-XLOC_019497-1286-0
AGGCATGGCCATTACTAATGAGAATTTGGGTCGGTTCTGAAATAACCAAA
A3-AT3G53170-XLOC_019497-1286-1
AGGCATGGCCATTACTAATGAGAATTTGGGTCGGTTCTGAAATAACCAAA
CONSENSUS
AGGCATGGCCATTACTAATGAGAATTTGGGTCGGTTCTGAAATAACCAAA

A3-AT3G53170-XLOC_019497-1286-0
TCAGCATTGGGGAGAAAACGTAATGATTCCAGTCAATGTAAGTAATAAAA
A3-AT3G53170-XLOC_019497-1286-1
TCAGCATTGGGGAGAAAACGTAATGATTCCAGTCAATGTAAGTAATAAAA
CONSENSUS
TCAGCATTGGGGAGAAAACGTAATGATTCCAGTCAATGTAAGTAATAAAA

A3-AT3G53170-XLOC_019497-1286-0
GCCATCACTAGTCTAAATGTTATGGTCTATTTTTCACATGTATTAGAAAA
A3-AT3G53170-XLOC_019497-1286-1
GCCATCACTAGTCTAAATGTTATGGTCTATTTTTCACATGTATTAGAAAA
CONSENSUS
GCCATCACTAGTCTAAATGTTATGGTCTATTTTTCACATGTATTAGAAAA

A3-AT3G53170-XLOC_019497-1286-0
TATTATCACATATTGTGTCTGTATATTTTGGATTTGTTTTCATCGTATAG
A3-AT3G53170-XLOC_019497-1286-1
TATTATCACATATTGTGTCTGTATATTTTGGATTTGTTTTCATCGTATAG
CONSENSUS
TATTATCACATATTGTGTCTGTATATTTTGGATTTGTTTTCATCGTATAG

A3-AT3G53170-XLOC_019497-1286-0
GTTGGGACAGCTGTCTAGAACCAAAACTCAATTCAAAGTTTTCTTGGTAA
A3-AT3G53170-XLOC_019497-1286-1
GTTGGGACAGCTGTCTAGAACCAAAACTCAATTCAAAGTTTTCTTGGTAA
CONSENSUS
GTTGGGACAGCTGTCTAGAACCAAAACTCAATTCAAAGTTTTCTTGGTAA

A3-AT3G53170-XLOC_019497-1286-0

AGTAAAATATAATTTTCTTACGCAGGCTCTTTAGACGGGTCAAAACAAAC
A3-AT3G53170-XLOC_019497-1286-1
AGTAAAATATAATTTTCTTACGCAGGCTCTTTAGACGGGTCAAAACAAAC
CONSENSUS
AGTAAAATATAATTTTCTTACGCAGGCTCTTTAGACGGGTCAAAACAAAC

A3-AT3G53170-XLOC_019497-1286-0
TGTGATCTAAAACTTTATCGTAGCTATATACACATAAATTATTTTCCAAC
A3-AT3G53170-XLOC_019497-1286-1
TGTGATCTAAAACTTTATCGTAGCTATATACACATAAATTATTTTCCAAC
CONSENSUS
TGTGATCTAAAACTTTATCGTAGCTATATACACATAAATTATTTTCCAAC

A3-AT3G53170-XLOC_019497-1286-0
ATCGTATTATATATATCATTTGTAAAGAGTGGCCTGCTTTTCAAGCCTTA
A3-AT3G53170-XLOC_019497-1286-1
ATCGTATTATATATATCATTTGTAAAGAGTGGCCTGCTTTTCAAGCCTTA
CONSENSUS
ATCGTATTATATATATCATTTGTAAAGAGTGGCCTGCTTTTCAAGCCTTA

A3-AT3G53170-XLOC_019497-1286-0
AAAGATCTTTTTTTTTTTTCCTTGTAAGATCTTCTACAATGTGTTCTCAT
A3-AT3G53170-XLOC_019497-1286-1
AAAGATCTTTTTTTTTTTTCCTTGTAAGATCTTCTACAATGTGTTCTCAT
CONSENSUS
AAAGATCTTTTTTTTTTTTCCTTGTAAGATCTTCTACAATGTGTTCTCAT

A3-AT3G53170-XLOC_019497-1286-0
GATCCTCTTCACTTCGTCGTAATACCCTTTATGGCCCAAGGCCATATGAT
A3-AT3G53170-XLOC_019497-1286-1
GATCCTCTTCACTTCGTCGTAATACCCTTTATGGCCCAAGGCCATATGAT
CONSENSUS
GATCCTCTTCACTTCGTCGTAATACCCTTTATGGCCCAAGGCCATATGAT

A3-AT3G53170-XLOC_019497-1286-0
CCCATTGGTCGACATCTCTAGGCTCTTGTCCCAGCGCCAAGGCGTGACTG
A3-AT3G53170-XLOC_019497-1286-1
CCCATTGGTCGACATCTCTAGGCTCTTGTCCCAGCGCCAAGGCGTGACTG
CONSENSUS
CCCATTGGTCGACATCTCTAGGCTCTTGTCCCAGCGCCAAGGCGTGACTG

A3-AT3G53170-XLOC_019497-1286-0
TCTGCATCATCACAACTACTCAAAATGTAGCCAAGATCAAGACTTCACTC
A3-AT3G53170-XLOC_019497-1286-1
TCTGCATCATCACAACTACTCAAAATGTAGCCAAGATCAAGACTTCACTC
CONSENSUS
TCTGCATCATCACAACTACTCAAAATGTAGCCAAGATCAAGACTTCACTC

A3-AT3G53170-XLOC_019497-1286-0
TCATTTTCCTCTTTGTTTGCGACTATCAACATCGTTGAAGTTAAGTTTCT
A3-AT3G53170-XLOC_019497-1286-1
TCATTTTCCTCTTTGTTTGCGACTATCAACATCGTTGAAGTTAAGTTTCT
CONSENSUS
TCATTTTCCTCTTTGTTTGCGACTATCAACATCGTTGAAGTTAAGTTTCT

A3-AT3G53170-XLOC_019497-1286-0

GTCTCAACAAACGGGTTTGCCAGAAGGGTGCGAGAGTTTAGATATGTTGG
A3-AT3G53170-XLOC_019497-1286-1
GTCTCAACAAACGGGTTTGCCAGAAGGGTGCGAGAGTTTAGATATGTTGG
CONSENSUS
GTCTCAACAAACGGGTTTGCCAGAAGGGTGCGAGAGTTTAGATATGTTGG

A3-AT3G53170-XLOC_019497-1286-0
CTTCAATGGGCGATATGGTGAAGTTCTTTGATGCTGCCAACTCACTTGAG
A3-AT3G53170-XLOC_019497-1286-1
CTTCAATGGGCGATATGGTGAAGTTCTTTGATGCTGCCAACTCACTTGAG
CONSENSUS
CTTCAATGGGCGATATGGTGAAGTTCTTTGATGCTGCCAACTCACTTGAG

A3-AT3G53170-XLOC_019497-1286-0
GAGCAAGTTGAGAAAGCTATGGAAGAGATGGTTCAGCCGCGGCCAAGCTG
A3-AT3G53170-XLOC_019497-1286-1
GAGCAAGTTGAGAAAGCTATGGAAGAGATGGTTCAGCCGCGGCCAAGCTG
CONSENSUS
GAGCAAGTTGAGAAAGCTATGGAAGAGATGGTTCAGCCGCGGCCAAGCTG

A3-AT3G53170-XLOC_019497-1286-0
CATCATTGGAGACATGAGCCTTCCTTTCACTTCAAGACTTGCCAAGAAAT
A3-AT3G53170-XLOC_019497-1286-1
CATCATTGGAGACATGAGCCTTCCTTTCACTTCAAGACTTGCCAAGAAAT
CONSENSUS
CATCATTGGAGACATGAGCCTTCCTTTCACTTCAAGACTTGCCAAGAAAT

A3-AT3G53170-XLOC_019497-1286-0
TCAAGATCCCCAAACTTATCTTCCATGGGTTTTCTTGTTTCAGCCTCATG
A3-AT3G53170-XLOC_019497-1286-1
TCAAGATCCCCAAACTTATCTTCCATGGGTTTTCTTGTTTCAGCCTCATG
CONSENSUS
TCAAGATCCCCAAACTTATCTTCCATGGGTTTTCTTGTTTCAGCCTCATG

A3-AT3G53170-XLOC_019497-1286-0
TCTATACAAGTGGTTCGAGAAAGCGGGATCTTGAAAATGATAGAATCAAA
A3-AT3G53170-XLOC_019497-1286-1
TCTATACAAGTGGTTCGAGAAAGCGGGATCTTGAAAATGATAGAATCAAA
CONSENSUS
TCTATACAAGTGGTTCGAGAAAGCGGGATCTTGAAAATGATAGAATCAAA

A3-AT3G53170-XLOC_019497-1286-0
CGACGAGTATTTTGATTTGCCCGGCTTGCCTGACAAAGTTGAGTTCACGA
A3-AT3G53170-XLOC_019497-1286-1
CGACGAGTATTTTGATTTGCCCGGCTTGCCTGACAAAGTTGAGTTCACGA
CONSENSUS
CGACGAGTATTTTGATTTGCCCGGCTTGCCTGACAAAGTTGAGTTCACGA

A3-AT3G53170-XLOC_019497-1286-0
AACCTCAGGTCTCTGTGTTGCAACCTGTTGAAGGAAATATGAAAGAGAGT
A3-AT3G53170-XLOC_019497-1286-1
AACCTCAGGTCTCTGTGTTGCAACCTGTTGAAGGAAATATGAAAGAGAGT
CONSENSUS
AACCTCAGGTCTCTGTGTTGCAACCTGTTGAAGGAAATATGAAAGAGAGT

A3-AT3G53170-XLOC_019497-1286-0

ACGGCCAAGATTATTGAAGCTGATAATGACTCTTATGGTGTTATTGTGAA
A3-AT3G53170-XLOC_019497-1286-1
ACGGCCAAGATTATTGAAGCTGATAATGACTCTTATGGTGTTATTGTGAA
CONSENSUS
ACGGCCAAGATTATTGAAGCTGATAATGACTCTTATGGTGTTATTGTGAA

A3-AT3G53170-XLOC_019497-1286-0
CACTTTTGAAGAGTTAGAGGTTGATTATGCAAGAGAATATAGGAAAGCAA
A3-AT3G53170-XLOC_019497-1286-1
CACTTTTGAAGAGTTAGAGGTTGATTATGCAAGAGAATATAGGAAAGCAA
CONSENSUS
CACTTTTGAAGAGTTAGAGGTTGATTATGCAAGAGAATATAGGAAAGCAA

A3-AT3G53170-XLOC_019497-1286-0
GGGCTGGAAAAGTTTGGTGCGTTGGACCTGTTTCCTTGTGCAATAGGTTA
A3-AT3G53170-XLOC_019497-1286-1
GGGCTGGAAAAGTTTGGTGCGTTGGACCTGTTTCCTTGTGCAATAGGTTA
CONSENSUS
GGGCTGGAAAAGTTTGGTGCGTTGGACCTGTTTCCTTGTGCAATAGGTTA

A3-AT3G53170-XLOC_019497-1286-0
GGGTTAGACAAAGCTAAAAGAGGAGATAAGGCTTCTATTGGTCAAGACCA
A3-AT3G53170-XLOC_019497-1286-1
GGGTTAGACAAAGCTAAAAGAGGAGATAAGGCTTCTATTGGTCAAGACCA
CONSENSUS
GGGTTAGACAAAGCTAAAAGAGGAGATAAGGCTTCTATTGGTCAAGACCA

A3-AT3G53170-XLOC_019497-1286-0
ATGTCTTCAATGGCTTGACTCTCAAGAAACTGGTTCAGTGCTCTACGTTT
A3-AT3G53170-XLOC_019497-1286-1
ATGTCTTCAATGGCTTGACTCTCAAGAAACTGGTTCAGTGCTCTACGTTT
CONSENSUS
ATGTCTTCAATGGCTTGACTCTCAAGAAACTGGTTCAGTGCTCTACGTTT

A3-AT3G53170-XLOC_019497-1286-0
GCCTTGGAAGTCTATGTAATCTTCCCTTGGCTCAGCTCAAAGAGCTGGGA
A3-AT3G53170-XLOC_019497-1286-1
GCCTTGGAAGTCTATGTAATCTTCCCTTGGCTCAGCTCAAAGAGCTGGGA
CONSENSUS
GCCTTGGAAGTCTATGTAATCTTCCCTTGGCTCAGCTCAAAGAGCTGGGA

A3-AT3G53170-XLOC_019497-1286-0
CTAGGCCTTGAGGCATCTAATAAACCTTTCATATGGGTTATAAGAGAATG
A3-AT3G53170-XLOC_019497-1286-1
CTAGGCCTTGAGGCATCTAATAAACCTTTCATATGGGTTATAAGAGAATG
CONSENSUS
CTAGGCCTTGAGGCATCTAATAAACCTTTCATATGGGTTATAAGAGAATG

A3-AT3G53170-XLOC_019497-1286-0
GGGAAAATATGGAGATTTAGCAAATTGGATGCAACAAAGCGGATTTGAAG
A3-AT3G53170-XLOC_019497-1286-1
GGGAAAATATGGAGATTTAGCAAATTGGATGCAACAAAGCGGATTTGAAG
CONSENSUS
GGGAAAATATGGAGATTTAGCAAATTGGATGCAACAAAGCGGATTTGAAG

A3-AT3G53170-XLOC_019497-1286-0

AGCGGATCAAAGATAGAGGACTGGTGATCAAAGGTTGGGCGCCGCAAGTT
A3-AT3G53170-XLOC_019497-1286-1
AGCGGATCAAAGATAGAGGACTGGTGATCAAAGGTTGGGCGCCGCAAGTT
CONSENSUS
AGCGGATCAAAGATAGAGGACTGGTGATCAAAGGTTGGGCGCCGCAAGTT

A3-AT3G53170-XLOC_019497-1286-0
TTCATCCTCTCACACGCATCCATTGGAGGGTTTTTGACTCACTGTGGATG
A3-AT3G53170-XLOC_019497-1286-1
TTCATCCTCTCACACGCATCCATTGGAGGGTTTTTGACTCACTGTGGATG
CONSENSUS
TTCATCCTCTCACACGCATCCATTGGAGGGTTTTTGACTCACTGTGGATG

A3-AT3G53170-XLOC_019497-1286-0
GAACTCGACACTAGAAGGAATTACTGCAGGAGTTCCATTATTGACATGGC
A3-AT3G53170-XLOC_019497-1286-1
GAACTCGACACTAGAAGGAATTACTGCAGGAGTTCCATTATTGACATGGC
CONSENSUS
GAACTCGACACTAGAAGGAATTACTGCAGGAGTTCCATTATTGACATGGC

A3-AT3G53170-XLOC_019497-1286-0
CTTTGTTTGCTGAACAATTCTTGAATGAGAAGTTAGTTGTGCAGATACTA
A3-AT3G53170-XLOC_019497-1286-1
CTTTGTTTGCTGAACAATTCTTGAATGAGAAGTTAGTTGTGCAGATACTA
CONSENSUS
CTTTGTTTGCTGAACAATTCTTGAATGAGAAGTTAGTTGTGCAGATACTA

A3-AT3G53170-XLOC_019497-1286-0
AAAGCAGGGTTAAAGATAGGAGTAGAGAAATTGATGAAATATGGAAAAGA
A3-AT3G53170-XLOC_019497-1286-1
AAAGCAGGGTTAAAGATAGGAGTAGAGAAATTGATGAAATATGGAAAAGA
CONSENSUS
AAAGCAGGGTTAAAGATAGGAGTAGAGAAATTGATGAAATATGGAAAAGA

A3-AT3G53170-XLOC_019497-1286-0
AGAGGAGATAGGAGCGATGGTGAGCAGAGAATGTGTGAGAAAAGCTGTGG
A3-AT3G53170-XLOC_019497-1286-1
AGAGGAGATAGGAGCGATGGTGAGCAGAGAATGTGTGAGAAAAGCTGTGG
CONSENSUS
AGAGGAGATAGGAGCGATGGTGAGCAGAGAATGTGTGAGAAAAGCTGTGG

A3-AT3G53170-XLOC_019497-1286-0
ATGAGCTAATGGGTGATAGTGAAGAAGCAGAAGAGAGAAGAAGAAAAGTT
A3-AT3G53170-XLOC_019497-1286-1
ATGAGCTAATGGGTGATAGTGAAGAAGCAGAAGAGAGAAGAAGAAAAGTT
CONSENSUS
ATGAGCTAATGGGTGATAGTGAAGAAGCAGAAGAGAGAAGAAGAAAAGTT

A3-AT3G53170-XLOC_019497-1286-0
ACAGAACTTAGTGACTTGGCAAATAAGGCTTTGGAAAAAGGAGGATCTTC
A3-AT3G53170-XLOC_019497-1286-1
ACAGAACTTAGTGACTTGGCAAATAAGGCTTTGGAAAAAGGAGGATCTTC
CONSENSUS
ACAGAACTTAGTGACTTGGCAAATAAGGCTTTGGAAAAAGGAGGATCTTC

A3-AT3G53170-XLOC_019497-1286-0

AGATTCTAATATCACATTGCTCATTCAAGATATTATGGAGCAATCACAAA
A3-AT3G53170-XLOC_019497-1286-1
AGATTCTAATATCACATTGCTCATTCAAGATATTATGGAGCAATCACAAA
CONSENSUS
AGATTCTAATATCACATTGCTCATTCAAGATATTATGGAGCAATCACAAA

A3-AT3G53170-XLOC_019497-1286-0
ATCAATTTTAAATTTTGGTATTTGGCACCAGAGAAAAACAAAATATATGC
A3-AT3G53170-XLOC_019497-1286-1
ATCAATTTTAAATTTTGGTATTTGGCACCAGAGAAAAACAAAATATATGC
CONSENSUS
ATCAATTTTAAATTTTGGTATTTGGCACCAGAGAAAAACAAAATATATGC

A3-AT3G53170-XLOC_019497-1286-0
TTATAAAATAGTGTTTTATTTTCCATGTTAGATTAACAATGTTTCATGTG
A3-AT3G53170-XLOC_019497-1286-1
TTATAAAATAGTGTTTTATTTTCCATGTTAGATTAACAATGTTTCATGTG
CONSENSUS
TTATAAAATAGTGTTTTATTTTCCATGTTAGATTAACAATGTTTCATGTG

A3-AT3G53170-XLOC_019497-1286-0
TCAAAGGTGCTTTTGTGCTGTATATATCCACAATATCCAGACAGAATTTG
A3-AT3G53170-XLOC_019497-1286-1
TCAAAGGTGCTTTTGTGCTGTATATATCCACAATATCCAGACAGAATTTG
CONSENSUS
TCAAAGGTGCTTTTGTGCTGTATATATCCACAATATCCAGACAGAATTTG

A3-AT3G53170-XLOC_019497-1286-0 TT
A3-AT3G53170-XLOC_019497-1286-1 TT
CONSENSUS   TT

alignment for event: A5-AT5G53850-XLOC_031648-8184

A5-AT5G53850-XLOC_031648-8184-0
GTGTTCAAAAGGAGAGGATGCAACCTGAGGATATGTACATCTTATCTGCT
A5-AT5G53850-XLOC_031648-8184-1
GTGTTCAAAAGGAGAGGATGCAACCTGAGGATATGTACATCTTATCTGCT
CONSENSUS
GTGTTCAAAAGGAGAGGATGCAACCTGAGGATATGTACATCTTATCTGCT

A5-AT5G53850-XLOC_031648-8184-0
AATGGATCCATCATATCTACACCCTCTCCAAAGCCATACCCAAATAAGCC
A5-AT5G53850-XLOC_031648-8184-1
AATGGATCCATCATATCTACACCCTCTCCAAAGCCATACCCAAATAAGCC
CONSENSUS
AATGGATCCATCATATCTACACCCTCTCCAAAGCCATACCCAAATAAGCC

A5-AT5G53850-XLOC_031648-8184-0
TCCCAAGTGTACCGATTGTGCTCCACTTTTCATGAAGGTATGGTTTTGTC
A5-AT5G53850-XLOC_031648-8184-1
TCCCAAGTGTACCGATTGTGCTCCACTTTTCATGAAG-------------
CONSENSUS
TCCCAAGTGTACCGATTGTGCTCCACTTTTCATGAAG.............

A5-AT5G53850-XLOC_031648-8184-0
      GCTTTCAAGGTAGTAATACTTGATTCTGCGTTAGGGATTTCTGTATACGT
A5-AT5G53850-XLOC_031648-8184-1

CONSENSUS
      ....

A5-AT5G53850-XLOC_031648-8184-0
      AGCTTGATTATCACTTTGCTATTGGCCTTCTTTATCTTATGGTATTTTCT
A5-AT5G53850-XLOC_031648-8184-1

CONSENSUS
      ....

A5-AT5G53850-XLOC_031648-8184-0
      TTGATGATTGTGCATTTTTTGTTTTCTCAAATTGTTGAGCAATGTTGGAT
A5-AT5G53850-XLOC_031648-8184-1

CONSENSUS
      ....

A5-AT5G53850-XLOC_031648-8184-0
      TTCGTCAAAGTGTTGAGCAGCAGTTACGGATTTCAAGTTATAATGTTTTC
A5-AT5G53850-XLOC_031648-8184-1

CONSENSUS
      ....

A5-AT5G53850-XLOC_031648-8184-0
      TGTTAAGAAGGATTTTTCTTAGAGCACATGGTTATGAACGAAATATGTTA
A5-AT5G53850-XLOC_031648-8184-1

CONSENSUS
      ....

A5-AT5G53850-XLOC_031648-8184-0
      AGAGCTCGTCGCTCTTTTCTAGTGAACAGGCGACTTTCTTTGGGGAAACG
A5-AT5G53850-XLOC_031648-8184-1 ------------------------
AACAGGCGACTTTCTTTGGGGAAACG
CONSENSUS
      ........................AACAGGCGACTTTCTTTGGGGAAACG

A5-AT5G53850-XLOC_031648-8184-0 CTTGCGAAAACTCGGTTACAAG
A5-AT5G53850-XLOC_031648-8184-1 CTTGCGAAAACTCGGTTACAAG
CONSENSUS   CTTGCGAAAACTCGGTTACAAG

alignment for event: A3-AT5G36890-XLOC_030673-8078

A3-AT5G36890-XLOC_030673-8078-0
      GTATTCAGCCGTATGTAACTCTGTACCATTGGGATCTCCCATCACATCTC
A3-AT5G36890-XLOC_030673-8078-1
      GTATTCAGCCGTATGTAACTCTGTACCATTGGGATCTCCCATCACATCTC
CONSENSUS
      GTATTCAGCCGTATGTAACTCTGTACCATTGGGATCTCCCATCACATCTC

A3-AT5G36890-XLOC_030673-8078-0
      CAGGAAGCAATCGGAGGTTGGACAAATAGGAAAATTGT------------
A3-AT5G36890-XLOC_030673-8078-1
      CAGGAAGCAATCGGAGGTTGGACAAATAGGAAAATTGTCGATTATTTTGG
CONSENSUS
      CAGGAAGCAATCGGAGGTTGGACAAATAGGAAAATTGT............

A3-AT5G36890-XLOC_030673-8078-0 -----------
ATGCTTGTTTTGCCAATTTTGGTGATAGAGTGAAGCACT
A3-AT5G36890-XLOC_030673-8078-1
      CCTCTATGCAGATGCTTGTTTTGCCAATTTTGGTGATAGAGTGAAGCACT
CONSENSUS
      ...........ATGCTTGTTTTGCCAATTTTGGTGATAGAGTGAAGCACT

A3-AT5G36890-XLOC_030673-8078-0
      GGATCACATTAAATGAACCTCTTCAGACCTCGGTGAATGGACACTGTATT
A3-AT5G36890-XLOC_030673-8078-1
      GGATCACATTAAATGAACCTCTTCAGACCTCGGTGAATGGACACTGTATT
CONSENSUS
      GGATCACATTAAATGAACCTCTTCAGACCTCGGTGAATGGACACTGTATT

A3-AT5G36890-XLOC_030673-8078-0
      GGTATATTTGCACCTGGAAGAAATGAGAAGCCCTTGATCGAACCATATTT
A3-AT5G36890-XLOC_030673-8078-1
      GGTATATTTGCACCTGGAAGAAATGAGAAGCCCTTGATCGAACCATATTT
CONSENSUS
      GGTATATTTGCACCTGGAAGAAATGAGAAGCCCTTGATCGAACCATATTT

A3-AT5G36890-XLOC_030673-8078-0
      GGTCTCACATCATCAGGTTCTGGCCCATGCAACTGCTGTATCCATATATA
A3-AT5G36890-XLOC_030673-8078-1
      GGTCTCACATCATCAGGTTCTGGCCCATGCAACTGCTGTATCCATATATA
CONSENSUS
      GGTCTCACATCATCAGGTTCTGGCCCATGCAACTGCTGTATCCATATATA

A3-AT5G36890-XLOC_030673-8078-0 GAAGCAAGTACAAG
A3-AT5G36890-XLOC_030673-8078-1 GAAGCAAGTACAAG
CONSENSUS   GAAGCAAGTACAAG

alignment for event: SE-AT5G65060-XLOC_028632-12325

SE-AT5G65060-XLOC_028632-12325-0
      AAAGAAGAAGATAGAAACGAAGAAAAAAAGCAAACACATTTTGGGTCCCC
SE-AT5G65060-XLOC_028632-12325-1
      AAAGAAGAAGATAGAAACGAAGAAAAAAAGCAAACACATTTTGGGTCCCC
CONSENSUS
      AAAGAAGAAGATAGAAACGAAGAAAAAAAGCAAACACATTTTGGGTCCCC

SE-AT5G65060-XLOC_028632-12325-0
      GGTGGTTAGGATCAAATTAGGGCACAAACCTTATCGGAGAAAGAAGCCAT
SE-AT5G65060-XLOC_028632-12325-1
      GGTGGTTAGGATCAAATTAGGGCACAAACCTTATCGGAGAAAGAAGCCAT
CONSENSUS

GGTGGTTAGGATCAAATTAGGGCACAAACCTTATCGGAGAAAGAAGCCAT

SE-AT5G65060-XLOC_028632-12325-0
GGGAAGAAGAAAAGTCGAGATCAAGCGAATCGAGAACAAAAGCAGTCGAC
SE-AT5G65060-XLOC_028632-12325-1
GGGAAGAAGAAAAGTCGAGATCAAGCGAATCGAGAACAAAAGCAGTCGAC
CONSENSUS
GGGAAGAAGAAAAGTCGAGATCAAGCGAATCGAGAACAAAAGCAGTCGAC

SE-AT5G65060-XLOC_028632-12325-0
AAGTCACTTTCTCCAAACGACGCAAAGGTCTCATCGAAAAAGCTCGACAA
SE-AT5G65060-XLOC_028632-12325-1
AAGTCACTTTCTCCAAACGACGCAAAGGTCTCATCGAAAAAGCTCGACAA
CONSENSUS
AAGTCACTTTCTCCAAACGACGCAAAGGTCTCATCGAAAAAGCTCGACAA

SE-AT5G65060-XLOC_028632-12325-0
CTTTCAATTCTCTGTGAATCTTCCATCGCTGTTGTCGCCGTCTCCGGTTC
SE-AT5G65060-XLOC_028632-12325-1
CTTTCAATTCTCTGTGAATCTTCCATCGCTGTTGTCGCCGTCTCCGGTTC
CONSENSUS
CTTTCAATTCTCTGTGAATCTTCCATCGCTGTTGTCGCCGTCTCCGGTTC

SE-AT5G65060-XLOC_028632-12325-0
CGGAAAACTCTACGACTCTGCCTCCGGTGACAA-----------------
SE-AT5G65060-XLOC_028632-12325-1
CGGAAAACTCTACGACTCTGCCTCCGGTGACAACATGTCAAAGATCATTG
CONSENSUS
CGGAAAACTCTACGACTCTGCCTCCGGTGACAA.................

SE-AT5G65060-XLOC_028632-12325-0
---GATCTTGCA
SE-AT5G65060-XLOC_028632-12325-1
ATCGTTATGAAATACATCATGCTGATGAACTTAAAGCCTTAGATCTTGCA
CONSENSUS
....GATCTTGCA

SE-AT5G65060-XLOC_028632-12325-0
GAAAAAATTCGGAATTATCTTCCACACAAGGAGTTACTAGAAATAGTCCA
SE-AT5G65060-XLOC_028632-12325-1
GAAAAAATTCGGAATTATCTTCCACACAAGGAGTTACTAGAAATAGTCCA
CONSENSUS
GAAAAAATTCGGAATTATCTTCCACACAAGGAGTTACTAGAAATAGTCCA

SE-AT5G65060-XLOC_028632-12325-0 AAG
SE-AT5G65060-XLOC_028632-12325-1 AAG
CONSENSUS AAG

alignment for event: RI-AT5G37350-XLOC_027024-12471

RI-AT5G37350-XLOC_027024-12471-0
GCTCTTGATCCAAGGACGCGTATGGTATTGTTTAGAATGCTTAATAGGGG
RI-AT5G37350-XLOC_027024-12471-1
GCTCTTGATCCAAGGACGCGTATGGTATTGTTTAGAATGCTTAATAGGGG

CONSENSUS
      GCTCTTGATCCAAGGACGCGTATGGTATTGTTTAGAATGCTTAATAGGGG

RI-AT5G37350-XLOC_027024-12471-0
      TGTGTTTAACGATGTTAATGGCTGTATCTCGACAGGCAAAGAAGTATGAT
RI-AT5G37350-XLOC_027024-12471-1
      TGTGTTTAACGATGTTAATGGCTGTATCTCGACAGGCAAAGAA-------
CONSENSUS
      TGTGTTTAACGATGTTAATGGCTGTATCTCGACAGGCAAAGAA.......

RI-AT5G37350-XLOC_027024-12471-0
      ACATTTCACAACTCTCATATTTGAGATTATCTGTTTTTATTCTTTTTTGT
RI-AT5G37350-XLOC_027024-12471-1

CONSENSUS
      ....

RI-AT5G37350-XLOC_027024-12471-0
      TGAAAGCACTCTCTTTAATTATTGTCTTTCAGGCAAATGTTTATCATGCC
RI-AT5G37350-XLOC_027024-12471-1
      --------------------------------GCAAATGTTTATCATGCC
CONSENSUS
      ................................GCAAATGTTTATCATGCC

RI-AT5G37350-XLOC_027024-12471-0
      ACAAAATCTGACGGTTCGGAACTTGCAATAAAAGTGTACAAGACATCTGT
RI-AT5G37350-XLOC_027024-12471-1
      ACAAAATCTGACGGTTCGGAACTTGCAATAAAAGTGTACAAGACATCTGT
CONSENSUS
      ACAAAATCTGACGGTTCGGAACTTGCAATAAAAGTGTACAAGACATCTGT

RI-AT5G37350-XLOC_027024-12471-0 TCTGGTTTTCAA
RI-AT5G37350-XLOC_027024-12471-1 TCTGGTTTTCAA
CONSENSUS TCTGGTTTTCAA

alignment for event: RI-AT5G25520-XLOC_026355-9924

RI-AT5G25520-XLOC_026355-9924-0
      AGACTAGGCTTCTCATTAGCTAAAACATGGAACCCAGAAAGCAAAAAAGC
RI-AT5G25520-XLOC_026355-9924-1
      AGACTAGGCTTCTCATTAGCTAAAACATGGAACCCAGAAAGCAAAAAAGC
CONSENSUS
      AGACTAGGCTTCTCATTAGCTAAAACATGGAACCCAGAAAGCAAAAAAGC

RI-AT5G25520-XLOC_026355-9924-0
      TCACTGTTGATCTACATCTGTGAAACAGGACCGGGGCAGTAATAAAAAAA
RI-AT5G25520-XLOC_026355-9924-1
      TCACTGTTGATCTACATCTGTGAAACAGGACCGGGGCAGTAATAAAAAAA
CONSENSUS
      TCACTGTTGATCTACATCTGTGAAACAGGACCGGGGCAGTAATAAAAAAA

RI-AT5G25520-XLOC_026355-9924-0
      AGGCCTGATTGACCAGAGAATCAATCAACTGTGGATTGAATCTGAAATTT
RI-AT5G25520-XLOC_026355-9924-1

AGGCCTGATTGACCAGAGAATCAATCAACTGTGGATTGAATCTGAAATTT
CONSENSUS
AGGCCTGATTGACCAGAGAATCAATCAACTGTGGATTGAATCTGAAATTT

RI-AT5G25520-XLOC_026355-9924-0
TGTACAAAATCATGAAATTTTGTATGCCAAATCACTTGTAGATGTATTGT
RI-AT5G25520-XLOC_026355-9924-1
TGTACAAAATCATGAAATTTTGTATGCCAAATCACTTGTAGATGTATTGT
CONSENSUS
TGTACAAAATCATGAAATTTTGTATGCCAAATCACTTGTAGATGTATTGT

RI-AT5G25520-XLOC_026355-9924-0
TAACTAGGTAGGATATAGAATCATCAAAAATGTTACCAAGAATCTCTTAC
RI-AT5G25520-XLOC_026355-9924-1
TAACTAG---
CONSENSUS
TAACTAG...

RI-AT5G25520-XLOC_026355-9924-0
AGAGAGAGAGTTACAAGACCACAAACACCAGAACTTGGTCCATTAACTTA
RI-AT5G25520-XLOC_026355-9924-1

CONSENSUS
....

RI-AT5G25520-XLOC_026355-9924-0
CGTGCAGATCATGATTCATGACCCAAAATAGAATCTTTCGAAACATATAA
RI-AT5G25520-XLOC_026355-9924-1

CONSENSUS
....

RI-AT5G25520-XLOC_026355-9924-0
CTTTGTCTTATAGGAAAAAGTTTCTACTTTTATGCATTTTTCGTAAGTAG
RI-AT5G25520-XLOC_026355-9924-1

CONSENSUS
....

RI-AT5G25520-XLOC_026355-9924-0
TGTAAGTAATTAAGTTGGTACATCAAATTGCAGGTAATGAACGTGGTTTG
RI-AT5G25520-XLOC_026355-9924-1
---------------------------------GTAATGAACGTGGTTTG
CONSENSUS
.................................GTAATGAACGTGGTTTG

RI-AT5G25520-XLOC_026355-9924-0
CAAGAATGGTATCTCTCAGAGCCAACGTGATAGCCTAATTGAG
RI-AT5G25520-XLOC_026355-9924-1
CAAGAATGGTATCTCTCAGAGCCAACGTGATAGCCTAATTGAG
CONSENSUS
CAAGAATGGTATCTCTCAGAGCCAACGTGATAGCCTAATTGAG

alignment for event: SE-AT5G58370-XLOC_028274-4021

SE-AT5G58370-XLOC_028274-4021-0
ATGATGGTGAGCTCAAGATCAGGAGCAGGTATAGGAAGTTTAAGAACTGC
SE-AT5G58370-XLOC_028274-4021-1
ATGATGGTGAGCTCAAGATCAGGAGCAGGTATAGGAAGTTTAAGAACTGC
CONSENSUS
ATGATGGTGAGCTCAAGATCAGGAGCAGGTATAGGAAGTTTAAGAACTGC

SE-AT5G58370-XLOC_028274-4021-0
CCTTGCTAAAATTGCTAGGTTTGCAAAGTTTTAGGTTCTCAAAGCAATCC
SE-AT5G58370-XLOC_028274-4021-1
CCTTGCTAAAATTGCTAGGTTTGCAAAGTTTTAGGTTCTCAAAGCAATCC
CONSENSUS
CCTTGCTAAAATTGCTAGGTTTGCAAAGTTTTAGGTTCTCAAAGCAATCC

SE-AT5G58370-XLOC_028274-4021-0
TCAAAACCAAG---------------------------------------
SE-AT5G58370-XLOC_028274-4021-1
TCAAAACCAAGGATAAGGAAGAAGTCAAAATGTTGAGATAAAGAAGAATG
CONSENSUS
TCAAAACCAAG.......................................

SE-AT5G58370-XLOC_028274-4021-0 ------------------------
CTCCTCTATCTCTCTCTTTCCTGATT
SE-AT5G58370-XLOC_028274-4021-1
GTGTGACGATGATGATCCGACTAACTCCTCTATCTCTCTCTTTCCTGATT
CONSENSUS
........................CTCCTCTATCTCTCTCTTTCCTGATT

SE-AT5G58370-XLOC_028274-4021-0
GTCTTCATCAAAAACTGGAAAAAGCAAATCAAGAAAAGAGAAGAAGAAGC
SE-AT5G58370-XLOC_028274-4021-1
GTCTTCATCAAAAACTGGAAAAAGCAAATCAAGAAAAGAGAAGAAGAAGC
CONSENSUS
GTCTTCATCAAAAACTGGAAAAAGCAAATCAAGAAAAGAGAAGAAGAAGC

SE-AT5G58370-XLOC_028274-4021-0
AGATGTGTCCGTTAAGGCTTATCCTCATATTCCTTTCAGCGACTCTCGCT
SE-AT5G58370-XLOC_028274-4021-1
AGATGTGTCCGTTAAGGCTTATCCTCATATTCCTTTCAGCGACTCTCGCT
CONSENSUS
AGATGTGTCCGTTAAGGCTTATCCTCATATTCCTTTCAGCGACTCTCGCT

SE-AT5G58370-XLOC_028274-4021-0
GGCTTCTTCGTCCTCCAGAAGCTCAACTCTACCTCTGACGATCCTCTCGA
SE-AT5G58370-XLOC_028274-4021-1
GGCTTCTTCGTCCTCCAGAAGCTCAACTCTACCTCTGACGATCCTCTCGA
CONSENSUS
GGCTTCTTCGTCCTCCAGAAGCTCAACTCTACCTCTGACGATCCTCTCGA

SE-AT5G58370-XLOC_028274-4021-0
TGATTCCTTCACCGACGCTGAACACGCCGACGACGATTCAGTCTCTGGAT
SE-AT5G58370-XLOC_028274-4021-1
TGATTCCTTCACCGACGCTGAACACGCCGACGACGATTCAGTCTCTGGAT
CONSENSUS
TGATTCCTTCACCGACGCTGAACACGCCGACGACGATTCAGTCTCTGGAT

SE-AT5G58370-XLOC_028274-4021-0 TCTCCAAG
SE-AT5G58370-XLOC_028274-4021-1 TCTCCAAG
CONSENSUS   TCTCCAAG

alignment for event: A3-AT5G25360-XLOC_030042-5324

A3-AT5G25360-XLOC_030042-5324-0
      GATTGTGAGACTTCTCTTACCTGGAATCAGGGTTTTGATTAGAAGATCTC
A3-AT5G25360-XLOC_030042-5324-1
      GATTGTGAGACTTCTCTTACCTGGAATCAGGGTTTTGATTAGAAGATCTC
CONSENSUS
      GATTGTGAGACTTCTCTTACCTGGAATCAGGGTTTTGATTAGAAGATCTC

A3-AT5G25360-XLOC_030042-5324-0
      TAATTTGGTTGTTGTAGATGCATCTCCGTGAAATCATTCCCTCTTGGATC
A3-AT5G25360-XLOC_030042-5324-1
      TAATTTGGTTGTTGTAGATGCATCTCCGTGAAATCATTCCCTCTTGGATC
CONSENSUS
      TAATTTGGTTGTTGTAGATGCATCTCCGTGAAATCATTCCCTCTTGGATC

A3-AT5G25360-XLOC_030042-5324-0 TACCAGTTATTTGGTTGCATGGG-----
AGGTTGTTTTGGATGCTGCAAT
A3-AT5G25360-XLOC_030042-5324-1
      TACCAGTTATTTGGTTGCATGGGTACAGAGGTTGTTTTGGATGCTGCAAT
CONSENSUS
      TACCAGTTATTTGGTTGCATGGG.....AGGTTGTTTTGGATGCTGCAAT

A3-AT5G25360-XLOC_030042-5324-0
      AAACCACCGCTTATAGTTGCAGTGGATGAGCCGTCTAAAGGGTTAAGGAT
A3-AT5G25360-XLOC_030042-5324-1
      AAACCACCGCTTATAGTTGCAGTGGATGAGCCGTCTAAAGGGTTAAGGAT
CONSENSUS
      AAACCACCGCTTATAGTTGCAGTGGATGAGCCGTCTAAAGGGTTAAGGAT

A3-AT5G25360-XLOC_030042-5324-0
      TCAAGGTCGTTTAGTGAAGAAGCCAAGCGTATCAGAGGACTTCTGGAGCA
A3-AT5G25360-XLOC_030042-5324-1
      TCAAGGTCGTTTAGTGAAGAAGCCAAGCGTATCAGAGGACTTCTGGAGCA
CONSENSUS
      TCAAGGTCGTTTAGTGAAGAAGCCAAGCGTATCAGAGGACTTCTGGAGCA

A3-AT5G25360-XLOC_030042-5324-0
      CAAGCACCTGCGAGATGGATAATAGTACACTTCAGTCACAGAGAAGCATG
A3-AT5G25360-XLOC_030042-5324-1
      CAAGCACCTGCGAGATGGATAATAGTACACTTCAGTCACAGAGAAGCATG
CONSENSUS
      CAAGCACCTGCGAGATGGATAATAGTACACTTCAGTCACAGAGAAGCATG

A3-AT5G25360-XLOC_030042-5324-0
      TCTTCTATTAGCTTCACTAACAATACTTCTACTTCTGCAAGTACAAGCAA
A3-AT5G25360-XLOC_030042-5324-1
      TCTTCTATTAGCTTCACTAACAATACTTCTACTTCTGCAAGTACAAGCAA
CONSENSUS

TCTTCTATTAGCTTCACTAACAATACTTCTACTTCTGCAAGTACAAGCAA

A3-AT5G25360-XLOC_030042-5324-0 CCCCACAGAATTTGTAAACCACG
A3-AT5G25360-XLOC_030042-5324-1 CCCCACAGAATTTGTAAACCACG
CONSENSUS   CCCCACAGAATTTGTAAACCACG

alignment for event: A3-AT5G53490-XLOC_031634-1299

A3-AT5G53490-XLOC_031634-1299-0
AAAAATCTGAAACACACTTCCATGGCTTCGCTTCCTGTTCAATTCACGAG
A3-AT5G53490-XLOC_031634-1299-1
AAAAATCTGAAACACACTTCCATGGCTTCGCTTCCTGTTCAATTCACGAG
CONSENSUS
AAAAATCTGAAACACACTTCCATGGCTTCGCTTCCTGTTCAATTCACGAG

A3-AT5G53490-XLOC_031634-1299-0
GAATCAAATCTCTTCACCGTTTTTCTCTGTAAACCTCCGCCGTGAGCCTA
A3-AT5G53490-XLOC_031634-1299-1
GAATCAAATCTCTTCACCGTTTTTCTCTGTAAACCTCCGCCGTGAGCCTA
CONSENSUS
GAATCAAATCTCTTCACCGTTTTTCTCTGTAAACCTCCGCCGTGAGCCTA

A3-AT5G53490-XLOC_031634-1299-0 GATCTTTAGTAACAGTACACTGCTCCG---
GAGAAAACAGAGAAAATGGT
A3-AT5G53490-XLOC_031634-1299-1
GATCTTTAGTAACAGTACACTGCTCCGCAGGAGAAAACAGAGAAAATGGT
CONSENSUS
GATCTTTAGTAACAGTACACTGCTCCG...GAGAAAACAGAGAAAATGGT

A3-AT5G53490-XLOC_031634-1299-0
GAAGGTGTAAAGAAGAGTCTCTTTCCTCTTAAGGAGCTTGGATCTATCGC
A3-AT5G53490-XLOC_031634-1299-1
GAAGGTGTAAAGAAGAGTCTCTTTCCTCTTAAGGAGCTTGGATCTATCGC
CONSENSUS
GAAGGTGTAAAGAAGAGTCTCTTTCCTCTTAAGGAGCTTGGATCTATCGC

A3-AT5G53490-XLOC_031634-1299-0
TTGCGCAGCTCTCTGTGCTTGCACTCTTACAATAGCTTCTCCTGTTATTG
A3-AT5G53490-XLOC_031634-1299-1
TTGCGCAGCTCTCTGTGCTTGCACTCTTACAATAGCTTCTCCTGTTATTG
CONSENSUS
TTGCGCAGCTCTCTGTGCTTGCACTCTTACAATAGCTTCTCCTGTTATTG

A3-AT5G53490-XLOC_031634-1299-0 CTGCTAACCAG
A3-AT5G53490-XLOC_031634-1299-1 CTGCTAACCAG
CONSENSUS   CTGCTAACCAG

alignment for event: RI-AT5G46470-XLOC_027583-8127

RI-AT5G46470-XLOC_027583-8127-0
ATCAGTTTCCTTTGGACTCTTCCTCGGTGGACGCCGTTTTAGCCATTTCC
RI-AT5G46470-XLOC_027583-8127-1

ATCAGTTTCCTTTGGACTCTTCCTCGGTGGACGCCGTTTTAGCCATTTCC
CONSENSUS
ATCAGTTTCCTTTGGACTCTTCCTCGGTGGACGCCGTTTTAGCCATTTCC

RI-AT5G46470-XLOC_027583-8127-0
AAAACATCTGGTTTCCCAGTGATAAAATATGTGGTCAAGTATCTTGAATG
RI-AT5G46470-XLOC_027583-8127-1
AAAACATCTGGTTTCCCAGTGATAAAATATGTGGTCAAGTATCTTGAATG
CONSENSUS
AAAACATCTGGTTTCCCAGTGATAAAATATGTGGTCAAGTATCTTGAATG

RI-AT5G46470-XLOC_027583-8127-0
GCACTGATTTTGTGTGCAAACATTTGGAGGGTGAAATGCAACGGGTTTGT
RI-AT5G46470-XLOC_027583-8127-1
GCACTGATTTTGTGTGCAAACATTTGGAGGGTGAAATGCAACGGGTTTGT
CONSENSUS
GCACTGATTTTGTGTGCAAACATTTGGAGGGTGAAATGCAACGGGTTTGT

RI-AT5G46470-XLOC_027583-8127-0
CTCGTGCATCTGGTCTATGTAATGGGTTTATTTGACCTCTTATAATGGCA
RI-AT5G46470-XLOC_027583-8127-1
CTCGTGCATCTGGTCTATGTAATGGGTTTATTTGACCTCTTATAATGGCA
CONSENSUS
CTCGTGCATCTGGTCTATGTAATGGGTTTATTTGACCTCTTATAATGGCA

RI-AT5G46470-XLOC_027583-8127-0
TCTTCCTTGCAGACCATTCAAAGGAGAGTGGCGTTGGCTGGTTTTCTGGA
RI-AT5G46470-XLOC_027583-8127-1
TCTTCCTTGCAGACCATTCAAAGGAGAGTGGCGTTGGCTGGTTTTCTGGA
CONSENSUS
TCTTCCTTGCAGACCATTCAAAGGAGAGTGGCGTTGGCTGGTTTTCTGGA

RI-AT5G46470-XLOC_027583-8127-0
GCCACAGTGTCTCTATTTGAAATCTATTAAGTTGTCTACATTCAGTTTAT
RI-AT5G46470-XLOC_027583-8127-1
GCCACAGTGTCTCTATTTGAAATCTATTAAGTTGTCTACATTCAGTTTAT
CONSENSUS
GCCACAGTGTCTCTATTTGAAATCTATTAAGTTGTCTACATTCAGTTTAT

RI-AT5G46470-XLOC_027583-8127-0
CCTTTGGGGTTAGCATTTTTACACTTCTTGTCTGTCTTCTCTTTCTTTTA
RI-AT5G46470-XLOC_027583-8127-1
CCTTTGGG--
CONSENSUS
CCTTTGGG..

RI-AT5G46470-XLOC_027583-8127-0
GTATGAGGAAACGTTTCTTCTTCCCCCAACATATGTATAAAAACATGTTC
RI-AT5G46470-XLOC_027583-8127-1

CONSENSUS
....

RI-AT5G46470-XLOC_027583-8127-0
TTTGAGTTCAGGCCATTCTAGGCCTTTAACATGAAGGATAAAAGATCTTC
RI-AT5G46470-XLOC_027583-8127-1 -----------

GCCATTCTAGGCCTTTAACATGAAGGATAAAAGATCTTC
CONSENSUS
...........GCCATTCTAGGCCTTTAACATGAAGGATAAAAGATCTTC

RI-AT5G46470-XLOC_027583-8127-0
ATTCTACTTATCTGTCAAACTTGGATATCAGGGGACAAG
RI-AT5G46470-XLOC_027583-8127-1
ATTCTACTTATCTGTCAAACTTGGATATCAGGGGACAAG
CONSENSUS
ATTCTACTTATCTGTCAAACTTGGATATCAGGGGACAAG

alignment for event: SE-AT5G06770-XLOC_029075-3853

SE-AT5G06770-XLOC_029075-3853-0
ATTTATATTGTTGAGCTGTGTGTGTGGAGATGATGATGATGATGATAAAC
SE-AT5G06770-XLOC_029075-3853-1
ATTTATATTGTTGAGCTGTGTGTGTGGAGATGATGATGATGATGATAAAC
CONSENSUS
ATTTATATTGTTGAGCTGTGTGTGTGGAGATGATGATGATGATGATAAAC

SE-AT5G06770-XLOC_029075-3853-0
CCAATAGAATTTTAAAAGGATAAGAATCGCAAAGCTCATATCACAAAACT
SE-AT5G06770-XLOC_029075-3853-1
CCAATAGAATTTTAAAAGGATAAGAATCGCAAAGCTCATATCACAAAACT
CONSENSUS
CCAATAGAATTTTAAAAGGATAAGAATCGCAAAGCTCATATCACAAAACT

SE-AT5G06770-XLOC_029075-3853-0
TTGAGATCTCATTTCATCTTTCTTCGATTCTACCTACTCCGATTCAATCG
SE-AT5G06770-XLOC_029075-3853-1
TTGAGATCTCATTTCATCTTTCTTCGATTCTACCTACTCCGATTCAATCG
CONSENSUS
TTGAGATCTCATTTCATCTTTCTTCGATTCTACCTACTCCGATTCAATCG

SE-AT5G06770-XLOC_029075-3853-0
ATCTTATCGAGACATGGATGCTCGTAAGAGAGGACGCCCTGAAGCTGCTG
SE-AT5G06770-XLOC_029075-3853-1
ATCTTATCGAGACATGGATGCTCGTAAGAGAGGACGCCCTGAAGCTGCTG
CONSENSUS
ATCTTATCGAGACATGGATGCTCGTAAGAGAGGACGCCCTGAAGCTGCTG

SE-AT5G06770-XLOC_029075-3853-0
CCTCTCACAACTCCAATGGCGGATTCAAGAGGTCTAAGCAAG--------
SE-AT5G06770-XLOC_029075-3853-1
CCTCTCACAACTCCAATGGCGGATTCAAGAGGTCTAAGCAAGAGATGGAA
CONSENSUS
CCTCTCACAACTCCAATGGCGGATTCAAGAGGTCTAAGCAAG........

SE-AT5G06770-XLOC_029075-3853-0

SE-AT5G06770-XLOC_029075-3853-1
TCAATTTCAACTGGTTTAGGAAGCAAATCCAAGCCATGCACTAAATTTTT
CONSENSUS
....

SE-AT5G06770-XLOC_029075-3853-0 ---
CACTTCTGGATGTCCATTTGGTGACAATTGCCACTTCTTGCACTATG
SE-AT5G06770-XLOC_029075-3853-1
CAGCACTTCTGGATGTCCATTTGGTGACAATTGCCACTTCTTGCACTATG
CONSENSUS
...CACTTCTGGATGTCCATTTGGTGACAATTGCCACTTCTTGCACTATG

SE-AT5G06770-XLOC_029075-3853-0
TGCCCGGTGGGTACAATGCTGCAGCGCAGATGACAAATCTCCGACCACCG
SE-AT5G06770-XLOC_029075-3853-1
TGCCCGGTGGGTACAATGCTGCAGCGCAGATGACAAATCTCCGACCACCG
CONSENSUS
TGCCCGGTGGGTACAATGCTGCAGCGCAGATGACAAATCTCCGACCACCG

SE-AT5G06770-XLOC_029075-3853-0
GTTTCTCAAGTTTCCAGAAATATGCAAGGATCTGGTGGTCCCGGCGGCAG
SE-AT5G06770-XLOC_029075-3853-1
GTTTCTCAAGTTTCCAGAAATATGCAAGGATCTGGTGGTCCCGGCGGCAG
CONSENSUS
GTTTCTCAAGTTTCCAGAAATATGCAAGGATCTGGTGGTCCCGGCGGCAG

SE-AT5G06770-XLOC_029075-3853-0
ATTCTCAGGGAGAGGAGATCCAGGATCAGGCCCTGTTTCAATCTTTGGTG
SE-AT5G06770-XLOC_029075-3853-1
ATTCTCAGGGAGAGGAGATCCAGGATCAGGCCCTGTTTCAATCTTTGGTG
CONSENSUS
ATTCTCAGGGAGAGGAGATCCAGGATCAGGCCCTGTTTCAATCTTTGGTG

SE-AT5G06770-XLOC_029075-3853-0
CTTCTACTTCCAAGATCAGTGTAGATGCTTCTTTAGCCGGTGCCATCATT
SE-AT5G06770-XLOC_029075-3853-1
CTTCTACTTCCAAGATCAGTGTAGATGCTTCTTTAGCCGGTGCCATCATT
CONSENSUS
CTTCTACTTCCAAGATCAGTGTAGATGCTTCTTTAGCCGGTGCCATCATT

SE-AT5G06770-XLOC_029075-3853-0
GGAAAAGGTGGAATCCATTCCAAACAGATATGCCGTGAAACAGGAGCAAA
SE-AT5G06770-XLOC_029075-3853-1
GGAAAAGGTGGAATCCATTCCAAACAGATATGCCGTGAAACAGGAGCAAA
CONSENSUS
GGAAAAGGTGGAATCCATTCCAAACAGATATGCCGTGAAACAGGAGCAAA

SE-AT5G06770-XLOC_029075-3853-0
ATTATCGATTAAAGATCATGAAAGAGACCCAAACTTGAAGATTATCGAGC
SE-AT5G06770-XLOC_029075-3853-1
ATTATCGATTAAAGATCATGAAAGAGACCCAAACTTGAAGATTATCGAGC
CONSENSUS
ATTATCGATTAAAGATCATGAAAGAGACCCAAACTTGAAGATTATCGAGC

SE-AT5G06770-XLOC_029075-3853-0
TGGAAGGAACATTTGAACAGATCAATGTAGCGAGTGGGATGGTGAGAGAG
SE-AT5G06770-XLOC_029075-3853-1
TGGAAGGAACATTTGAACAGATCAATGTAGCGAGTGGGATGGTGAGAGAG
CONSENSUS
TGGAAGGAACATTTGAACAGATCAATGTAGCGAGTGGGATGGTGAGAGAG

SE-AT5G06770-XLOC_029075-3853-0
CTTATAGGGAGGCTTGGATCAGTGAAGAAACCTCAAGGGATTGGTGGTCC
SE-AT5G06770-XLOC_029075-3853-1
CTTATAGGGAGGCTTGGATCAGTGAAGAAACCTCAAGGGATTGGTGGTCC
CONSENSUS
CTTATAGGGAGGCTTGGATCAGTGAAGAAACCTCAAGGGATTGGTGGTCC

SE-AT5G06770-XLOC_029075-3853-0
TGAAGGGAAACCACATCCTGGGAGCAACTACAAAACCAAGATCTGTGATA
SE-AT5G06770-XLOC_029075-3853-1
TGAAGGGAAACCACATCCTGGGAGCAACTACAAAACCAAGATCTGTGATA
CONSENSUS
TGAAGGGAAACCACATCCTGGGAGCAACTACAAAACCAAGATCTGTGATA

SE-AT5G06770-XLOC_029075-3853-0
GGTACTCTAAAGGGAACTGTACATATGGAGATAGATGCCATTTTGCTCAT
SE-AT5G06770-XLOC_029075-3853-1
GGTACTCTAAAGGGAACTGTACATATGGAGATAGATGCCATTTTGCTCAT
CONSENSUS
GGTACTCTAAAGGGAACTGTACATATGGAGATAGATGCCATTTTGCTCAT

SE-AT5G06770-XLOC_029075-3853-0
GGTGAATCTGAGCTGCGCAGGTCAGGAATCGCTTAGTTATGTCTTTAGAC
SE-AT5G06770-XLOC_029075-3853-1
GGTGAATCTGAGCTGCGCAGGTCAGGAATCGCTTAGTTATGTCTTTAGAC
CONSENSUS
GGTGAATCTGAGCTGCGCAGGTCAGGAATCGCTTAGTTATGTCTTTAGAC

SE-AT5G06770-XLOC_029075-3853-0
TCTTGAGAACAGATTATGCATTGTTAGCTCCATTATCATTGTGACTTTTT
SE-AT5G06770-XLOC_029075-3853-1
TCTTGAGAACAGATTATGCATTGTTAGCTCCATTATCATTGTGACTTTTT
CONSENSUS
TCTTGAGAACAGATTATGCATTGTTAGCTCCATTATCATTGTGACTTTTT

SE-AT5G06770-XLOC_029075-3853-0
GCTCTCTCTTTTTGTTTTATCAATTTGTTTTATGCGACTCGCTTTGAAAA
SE-AT5G06770-XLOC_029075-3853-1
GCTCTCTCTTTTTGTTTTATCAATTTGTTTTATGCGACTCGCTTTGAAAA
CONSENSUS
GCTCTCTCTTTTTGTTTTATCAATTTGTTTTATGCGACTCGCTTTGAAAA

SE-AT5G06770-XLOC_029075-3853-0
CTTTAGCCCATTCTGTATTGAGCTCTGAAGATTCGACGAGTTCTGTAAGT
SE-AT5G06770-XLOC_029075-3853-1
CTTTAGCCCATTCTGTATTGAGCTCTGAAGATTCGACGAGTTCTGTAAGT
CONSENSUS
CTTTAGCCCATTCTGTATTGAGCTCTGAAGATTCGACGAGTTCTGTAAGT

SE-AT5G06770-XLOC_029075-3853-0
TACCGATCACAGTTAAAAGACTTTGATATGTTAAAACCCTTATATTACAG
SE-AT5G06770-XLOC_029075-3853-1
TACCGATCACAGTTAAAAGACTTTGATATGTTAAAACCCTTATATTACAG
CONSENSUS
TACCGATCACAGTTAAAAGACTTTGATATGTTAAAACCCTTATATTACAG

```
SE-AT5G06770-XLOC_029075-3853-0  CTACATACTATTTTTGTCTT
SE-AT5G06770-XLOC_029075-3853-1  CTACATACTATTTTTGTCTT
CONSENSUS                        CTACATACTATTTTTGTCTT


alignment for event: A5-AT5G58410-XLOC_028275-2475

A5-AT5G58410-XLOC_028275-2475-0
     ACATTTGAGTACAAGAAGCTGATACTGGACTACAGTCGAGATGTTCGGCG
A5-AT5G58410-XLOC_028275-2475-1
     ACATTTGAGTACAAGAAGCTGATACTGGACTACAGTCGAGATGTTCGGCG
CONSENSUS
     ACATTTGAGTACAAGAAGCTGATACTGGACTACAGTCGAGATGTTCGGCG

A5-AT5G58410-XLOC_028275-2475-0
     AGCTACTCATGACGTCATGACTAATGTTGTCACTGGTGCCGG--------
A5-AT5G58410-XLOC_028275-2475-1
     AGCTACTCATGACGTCATGACTAATGTTGTCACTGGTGCCGGGTTTGACT
CONSENSUS
     AGCTACTCATGACGTCATGACTAATGTTGTCACTGGTGCCGG........

A5-AT5G58410-XLOC_028275-2475-0
     --------------------------------------------------
A5-AT5G58410-XLOC_028275-2475-1
     CTCAATTTTCTAATGCATGCCCTTTTTCTTGCCGTGATGCAGTGCCTCAT
CONSENSUS
     ..................................................

A5-AT5G58410-XLOC_028275-2475-0
     --------------------------------------------------
A5-AT5G58410-XLOC_028275-2475-1
     TTACTTCTTCTTCTTTTAGTATCATTCACCTGGATATATGTAGTTTTCTT
CONSENSUS
     ..................................................

A5-AT5G58410-XLOC_028275-2475-0
     --------------------------------------------------
A5-AT5G58410-XLOC_028275-2475-1
     GTGTCATGAGTGATATGATTTAGCTTTGAGGTTATCCTAACATCAATATC
CONSENSUS
     ..................................................

A5-AT5G58410-XLOC_028275-2475-0
     --------------------------------------------------
A5-AT5G58410-XLOC_028275-2475-1
     AAAATTATCATCAATATTTTGTTGGAATGATTCGTTTGCTAAGTGCTGTA
CONSENSUS
     ..................................................

A5-AT5G58410-XLOC_028275-2475-0
     --------------------------------------------------
A5-AT5G58410-XLOC_028275-2475-1
     GAAGCTTGTCTATATTGAGCGTGTCATGTAGGAACTATGTCTTGATGATA
CONSENSUS
```

```
      .............................................

A5-AT5G58410-XLOC_028275-2475-0  ------------
GAGAGATATAGCTCCTCATCTAAAGTCTATAATGGGGC
A5-AT5G58410-XLOC_028275-2475-1
      TTGTCTTAGCATGAGAGATATAGCTCCTCATCTAAAGTCTATAATGGGGC
CONSENSUS
      ............GAGAGATATAGCTCCTCATCTAAAGTCTATAATGGGGC

A5-AT5G58410-XLOC_028275-2475-0
      CCTGGTGGTTTTCGCAATTTGATTTGGCATCTGAAGTTTCTCAAGCAGCA
A5-AT5G58410-XLOC_028275-2475-1
      CCTGGTGGTTTTCGCAATTTGATTTGGCATCTGAAGTTTCTCAAGCAGCA
CONSENSUS
      CCTGGTGGTTTTCGCAATTTGATTTGGCATCTGAAGTTTCTCAAGCAGCA

A5-AT5G58410-XLOC_028275-2475-0  AAGTCGTCTTTCCAG
A5-AT5G58410-XLOC_028275-2475-1  AAGTCGTCTTTCCAG
CONSENSUS                        AAGTCGTCTTTCCAG



alignment for event: RI-AT5G02810-XLOC_028866-12011

RI-AT5G02810-XLOC_028866-12011-0
      TTGTTGAGGCGTCAAATGGGATACAAGCTTGGAAGGTGTTAGAAGATCTA
RI-AT5G02810-XLOC_028866-12011-1
      TTGTTGAGGCGTCAAATGGGATACAAGCTTGGAAGGTGTTAGAAGATCTA
CONSENSUS
      TTGTTGAGGCGTCAAATGGGATACAAGCTTGGAAGGTGTTAGAAGATCTA

RI-AT5G02810-XLOC_028866-12011-0
      AACAATCATATTGATATTGTGCTAACAGAGGTGATCATGCCTTACTTATC
RI-AT5G02810-XLOC_028866-12011-1
      AACAATCATATTGATATTGTGCTAACAGAGGTGATCATGCCTTACTTATC
CONSENSUS
      AACAATCATATTGATATTGTGCTAACAGAGGTGATCATGCCTTACTTATC

RI-AT5G02810-XLOC_028866-12011-0
      TGGTATCGGTCTCTTGTGCAAGATTTTGAACCACAAATCTCGTCGGAACA
RI-AT5G02810-XLOC_028866-12011-1
      TGGTATCGGTCTCTTGTGCAAGATTTTGAACCACAAATCTCGTCGGAACA
CONSENSUS
      TGGTATCGGTCTCTTGTGCAAGATTTTGAACCACAAATCTCGTCGGAACA

RI-AT5G02810-XLOC_028866-12011-0
      TCCCTGTCATCAGTGAGTTCTTTTTCCTTGGTCGTTTTACATTGAGCTCT
RI-AT5G02810-XLOC_028866-12011-1
      TCCCTGTCATCA--------------------------------------
CONSENSUS
      TCCCTGTCATCA......................................

RI-AT5G02810-XLOC_028866-12011-0
      TTCTTTTGAAGTTACACGATTTGTTGAGTCTTCTCTAGCGTATGTTGGAA
RI-AT5G02810-XLOC_028866-12011-1
      --------------------------------------------------
```

CONSENSUS
      ....

RI-AT5G02810-XLOC_028866-12011-0
      AGTAGATGCTTTTAACTACATTCCCCTGTGAGATTTGTGTTGCAGTGATG
RI-AT5G02810-XLOC_028866-12011-1
      ---TGATG
CONSENSUS
      ....TGATG

RI-AT5G02810-XLOC_028866-12011-0
      TCATCTCATGACTCAATGGGGCTGGTCTTTAAGTGCTTATCGAAAGGAGC
RI-AT5G02810-XLOC_028866-12011-1
      TCATCTCATGACTCAATGGGGCTGGTCTTTAAGTGCTTATCGAAAGGAGC
CONSENSUS
      TCATCTCATGACTCAATGGGGCTGGTCTTTAAGTGCTTATCGAAAGGAGC

RI-AT5G02810-XLOC_028866-12011-0
      TGTTGACTTTCTTGTTAAGCCAATAAGAAAAAATGAGCTTAAGATCCTTT
RI-AT5G02810-XLOC_028866-12011-1
      TGTTGACTTTCTTGTTAAGCCAATAAGAAAAAATGAGCTTAAGATCCTTT
CONSENSUS
      TGTTGACTTTCTTGTTAAGCCAATAAGAAAAAATGAGCTTAAGATCCTTT

RI-AT5G02810-XLOC_028866-12011-0 GGCAGCATGTTTGGAGAAGATGCCAAAGT
RI-AT5G02810-XLOC_028866-12011-1 GGCAGCATGTTTGGAGAAGATGCCAAAGT
CONSENSUS GGCAGCATGTTTGGAGAAGATGCCAAAGT

alignment for event: A5-AT5G19130-XLOC_029722-13391

A5-AT5G19130-XLOC_029722-13391-0
      GCTCTGCACGGTCTATGCTCTCTAATCGAGATGTTTCTGATGGAAGCAAA
A5-AT5G19130-XLOC_029722-13391-1
      GCTCTGCACGGTCTATGCTCTCTAATCGAGATGTTTCTGATGGAAGCAAA
CONSENSUS
      GCTCTGCACGGTCTATGCTCTCTAATCGAGATGTTTCTGATGGAAGCAAA

A5-AT5G19130-XLOC_029722-13391-0
      CTGGTGAAGGACATAAAGAACTTTAGGTTGAATCATGAAGGCCAGGGTGT
A5-AT5G19130-XLOC_029722-13391-1
      CTGGTGAAGGACATAAAGAACTTTAGGTTGAATCATGAAGGCCAGG----
CONSENSUS
      CTGGTGAAGGACATAAAGAACTTTAGGTTGAATCATGAAGGCCAGG....

A5-AT5G19130-XLOC_029722-13391-0
      TGAAGTCCAAAAGCTCATTGGAAAATACATGTCAGATATGGGTGCAGAAG
A5-AT5G19130-XLOC_029722-13391-1
      TGAAGTCCAAAAGCTCATTGGAAAATACATGTCAGATATGGGTGCAGAAG
CONSENSUS
      TGAAGTCCAAAAGCTCATTGGAAAATACATGTCAGATATGGGTGCAGAAG

A5-AT5G19130-XLOC_029722-13391-0
      TTTCTTATCAAAAGTTCCATCCTGAAGGGAATCAATTTCACCCCCTGCAC
A5-AT5G19130-XLOC_029722-13391-1

TTTCTTATCAAAAGTTCCATCCTGAAGGGAATCAATTTCACCCCCTGCAC
CONSENSUS
TTTCTTATCAAAAGTTCCATCCTGAAGGGAATCAATTTCACCCCCTGCAC

A5-AT5G19130-XLOC_029722-13391-0
TTTTTCTCCGGTCCAGATTCATATACATTGCTGGAGAATGTCAGTTGTGC
A5-AT5G19130-XLOC_029722-13391-1
TTTTTCTCCGGTCCAGATTCATATACATTGCTGGAGAATGTCAGTTGTGC
CONSENSUS
TTTTTCTCCGGTCCAGATTCATATACATTGCTGGAGAATGTCAGTTGTGC

A5-AT5G19130-XLOC_029722-13391-0
TTCTTATGGGGTCAATGTTGCGGGGATTATAAGAGCCCCTCGTGGTGATG
A5-AT5G19130-XLOC_029722-13391-1
TTCTTATGGGGTCAATGTTGCGGGGATTATAAGAGCCCCTCGTGGTGATG
CONSENSUS
TTCTTATGGGGTCAATGTTGCGGGGATTATAAGAGCCCCTCGTGGTGATG

A5-AT5G19130-XLOC_029722-13391-0
GAAAAGAGTCTATTGTGCTGGTTACTCCCTATGATTTTATAAATGGTGGA
A5-AT5G19130-XLOC_029722-13391-1
GAAAAGAGTCTATTGTGCTGGTTACTCCCTATGATTTTATAAATGGTGGA
CONSENSUS
GAAAAGAGTCTATTGTGCTGGTTACTCCCTATGATTTTATAAATGGTGGA

A5-AT5G19130-XLOC_029722-13391-0
GACTATGAGGATTTGTCTTTAGGAATTGTCAGTTCTCTTTTTTCCTTGCT
A5-AT5G19130-XLOC_029722-13391-1
GACTATGAGGATTTGTCTTTAGGAATTGTCAGTTCTCTTTTTTCCTTGCT
CONSENSUS
GACTATGAGGATTTGTCTTTAGGAATTGTCAGTTCTCTTTTTTCCTTGCT

A5-AT5G19130-XLOC_029722-13391-0
CTCTAGAGTTACCTGGCTTTCCAAAGACATAATATGGCTTGTCGCTGATT
A5-AT5G19130-XLOC_029722-13391-1
CTCTAGAGTTACCTGGCTTTCCAAAGACATAATATGGCTTGTCGCTGATT
CONSENSUS
CTCTAGAGTTACCTGGCTTTCCAAAGACATAATATGGCTTGTCGCTGATT

A5-AT5G19130-XLOC_029722-13391-0
CTCGTTATGGAGACTATAGGCCTGTTGCTGCGTGGTTAACTGAATACCAC
A5-AT5G19130-XLOC_029722-13391-1
CTCGTTATGGAGACTATAGGCCTGTTGCTGCGTGGTTAACTGAATACCAC
CONSENSUS
CTCGTTATGGAGACTATAGGCCTGTTGCTGCGTGGTTAACTGAATACCAC

A5-AT5G19130-XLOC_029722-13391-0
TCACCTTCATTTAAGGTCTCGGATCTTCTGAAGTGTGACGAGCAAAATAC
A5-AT5G19130-XLOC_029722-13391-1
TCACCTTCATTTAAGGTCTCGGATCTTCTGAAGTGTGACGAGCAAAATAC
CONSENSUS
TCACCTTCATTTAAGGTCTCGGATCTTCTGAAGTGTGACGAGCAAAATAC

A5-AT5G19130-XLOC_029722-13391-0
AGCTGATAACTTCAGACGAGCTGGAACAATGGCTGCTGCTTTGGTTTTGA
A5-AT5G19130-XLOC_029722-13391-1

AGCTGATAACTTCAGACGAGCTGGAACAATGGCTGCTGCTTTGGTTTTGA
CONSENSUS
AGCTGATAACTTCAGACGAGCTGGAACAATGGCTGCTGCTTTGGTTTTGA

A5-AT5G19130-XLOC_029722-13391-0
AGGTTGATGGTAGAAGTGAAAAGTTTGAGGACACACTAAGTATCTATGCA
A5-AT5G19130-XLOC_029722-13391-1
AGGTTGATGGTAGAAGTGAAAAGTTTGAGGACACACTAAGTATCTATGCA
CONSENSUS
AGGTTGATGGTAGAAGTGAAAAGTTTGAGGACACACTAAGTATCTATGCA

A5-AT5G19130-XLOC_029722-13391-0
GAGGCATCTAATGGGCAGATGCCAAATCTCGACCTCATCAATGTTGTAAA
A5-AT5G19130-XLOC_029722-13391-1
GAGGCATCTAATGGGCAGATGCCAAATCTCGACCTCATCAATGTTGTAAA
CONSENSUS
GAGGCATCTAATGGGCAGATGCCAAATCTCGACCTCATCAATGTTGTAAA

A5-AT5G19130-XLOC_029722-13391-0
TTACTTAGCGGTGCACAGGCAGGGGTTTTATGTCAAGGTTGAGAAGGTTG
A5-AT5G19130-XLOC_029722-13391-1
TTACTTAGCGGTGCACAGGCAGGGGTTTTATGTCAAGGTTGAGAAGGTTG
CONSENSUS
TTACTTAGCGGTGCACAGGCAGGGGTTTTATGTCAAGGTTGAGAAGGTTG

A5-AT5G19130-XLOC_029722-13391-0
TATCTTTACTTTCCTCTAGTTGGCTAAAGATTTTTGGGGAAATATTTGAA
A5-AT5G19130-XLOC_029722-13391-1
TATCTTTACTTTCCTCTAGTTGGCTAAAGATTTTTGGGGAAATATTTGAA
CONSENSUS
TATCTTTACTTTCCTCTAGTTGGCTAAAGATTTTTGGGGAAATATTTGAA

A5-AT5G19130-XLOC_029722-13391-0
GCTGTTGGGAAATTGGCGCATATGTTAAATCCGGACTGGAATTTTGGTAT
A5-AT5G19130-XLOC_029722-13391-1
GCTGTTGGGAAATTGGCGCATATGTTAAATCCGGACTGGAATTTTGGTAT
CONSENSUS
GCTGTTGGGAAATTGGCGCATATGTTAAATCCGGACTGGAATTTTGGTAT

A5-AT5G19130-XLOC_029722-13391-0
CCCAGCTGCAGACTATCTTGAAGGCAGTGCTACCCTTGCAAGTTCACTGT
A5-AT5G19130-XLOC_029722-13391-1
CCCAGCTGCAGACTATCTTGAAGGCAGTGCTACCCTTGCAAGTTCACTGT
CONSENSUS
CCCAGCTGCAGACTATCTTGAAGGCAGTGCTACCCTTGCAAGTTCACTGT

A5-AT5G19130-XLOC_029722-13391-0 ACTCCCAG
A5-AT5G19130-XLOC_029722-13391-1 ACTCCCAG
CONSENSUS ACTCCCAG

alignment for event: A5-AT5G22300-XLOC_026181-7769

A5-AT5G22300-XLOC_026181-7769-0
ATAAGGCAGAGAGACTGCTTTCTGAGGCAGCGGAGAATGGATCTCAGCTA

A5-AT5G22300-XLOC_026181-7769-1
      ATAAGGCAGAGAGACTGCTTTCTGAGGCAGCGGAGAATGGATCTCAGCTA
CONSENSUS
      ATAAGGCAGAGAGACTGCTTTCTGAGGCAGCGGAGAATGGATCTCAGCTA

A5-AT5G22300-XLOC_026181-7769-0
      GTGGTGTTCCCGGAGGCTTTCATCGGTGGATATCCACGTGGCTCTACCTT
A5-AT5G22300-XLOC_026181-7769-1
      GTGGTGTTCCCGGAGGCTTTCATCGGTGGATATCCACGTGGCTCTACCTT
CONSENSUS
      GTGGTGTTCCCGGAGGCTTTCATCGGTGGATATCCACGTGGCTCTACCTT

A5-AT5G22300-XLOC_026181-7769-0
      TGAATTGGCTATTGGTTCTCGTACCGCTAAAGGACGAGATGACTTTCGCA
A5-AT5G22300-XLOC_026181-7769-1
      TGAATTGGCTATTGGTTCTCGTACCGCTAAAGGACGAGATGACTTTCGCA
CONSENSUS
      TGAATTGGCTATTGGTTCTCGTACCGCTAAAGGACGAGATGACTTTCGCA

A5-AT5G22300-XLOC_026181-7769-0
      AGTACCATGCTTCTGCCATTGATGTTCCTG--------------------
A5-AT5G22300-XLOC_026181-7769-1
      AGTACCATGCTTCTGCCATTGATGTTCCTGGTAATAAAGTCCTCCACCAT
CONSENSUS
      AGTACCATGCTTCTGCCATTGATGTTCCTG....................

A5-AT5G22300-XLOC_026181-7769-0

A5-AT5G22300-XLOC_026181-7769-1
      ATCTCCGATTATGTTCTCACTTTTTTTCTTTCGATTTTGGATCTTAGCAT
CONSENSUS
      ....

A5-AT5G22300-XLOC_026181-7769-0

A5-AT5G22300-XLOC_026181-7769-1
      GAATACCAGGGCACGCACTTATTACCACATAAATCCATTGTTCTCTAACT
CONSENSUS
      ....

A5-AT5G22300-XLOC_026181-7769-0

A5-AT5G22300-XLOC_026181-7769-1
      AGCTACCAACATAGAACAACTAGGCAACCCTCTTTTATATTCACTTAGGT
CONSENSUS
      ....

A5-AT5G22300-XLOC_026181-7769-0

A5-AT5G22300-XLOC_026181-7769-1
      CGCGTGTTCGATTCACGTTCACTGCACCATTTGATAAGTTTGAAGCTATG
CONSENSUS
      ....

A5-AT5G22300-XLOC_026181-7769-0

A5-AT5G22300-XLOC_026181-7769-1
AACTCTTATCTTTTATCACTTCTCATTCCAAGATACAAACTCAAGAACTA
CONSENSUS
....

A5-AT5G22300-XLOC_026181-7769-0

A5-AT5G22300-XLOC_026181-7769-1
CCCTAACAATGGAGATTTTTTCAAGAGATCTCCTAACTCTCCTCCATAAA
CONSENSUS
....

A5-AT5G22300-XLOC_026181-7769-0

A5-AT5G22300-XLOC_026181-7769-1
ACCATACGTTTGTTAAAAACACATCAATACATGCATAACTTCATCAATTC
CONSENSUS
....

A5-AT5G22300-XLOC_026181-7769-0

A5-AT5G22300-XLOC_026181-7769-1
TCTTGACAAGAGGTTACAAAATCTGTTTCTTCTTGCTCTGTTTTAACTCC
CONSENSUS
....

A5-AT5G22300-XLOC_026181-7769-0

A5-AT5G22300-XLOC_026181-7769-1
TTTTCACATGACTTCTATGCTTCTCTTTGCTTATTCACGTGTCTCATAAC
CONSENSUS
....

A5-AT5G22300-XLOC_026181-7769-0

A5-AT5G22300-XLOC_026181-7769-1
CTCCTTTTCAGCTACCACACGTATCTCCTAGCTTATCACACTTAAGATCT
CONSENSUS
....

A5-AT5G22300-XLOC_026181-7769-0

A5-AT5G22300-XLOC_026181-7769-1
AATAAATTCTCACCAAAGCCGTCTTTAAAACAGCTTCTGTTCTACTACGA
CONSENSUS
....

A5-AT5G22300-XLOC_026181-7769-0

A5-AT5G22300-XLOC_026181-7769-1
CACTGTAACAGAAACTGCAACAAACAACCAAGGTGGTTCTTGTTTTTTTT
CONSENSUS
....

A5-AT5G22300-XLOC_026181-7769-0

A5-AT5G22300-XLOC_026181-7769-1
TGGTATCAAGGTGGTTCTTGTTGCTTTGAGGGAGATGCCTCTTCCTTCTT
CONSENSUS
....

A5-AT5G22300-XLOC_026181-7769-0

A5-AT5G22300-XLOC_026181-7769-1
ACATGAGAGCTTGTAGCATCACCAAGATCTGACCAGTTCATCAAGAGACG
CONSENSUS
....

A5-AT5G22300-XLOC_026181-7769-0

A5-AT5G22300-XLOC_026181-7769-1
TAGCTTGGTTGCTCCACCCGTGACTCCACAACAAGCTTCATTCAAGGGAG
CONSENSUS
....

A5-AT5G22300-XLOC_026181-7769-0 -----------------
GCCCTGAAGTGGAACGATTAGCGTTAATGGCCA
A5-AT5G22300-XLOC_026181-7769-1
GCACAGATGCTCCAAGAGCCCTGAAGTGGAACGATTAGCGTTAATGGCCA
CONSENSUS
.................GCCCTGAAGTGGAACGATTAGCGTTAATGGCCA

A5-AT5G22300-XLOC_026181-7769-0
AGAAGTACAAAGTATACTTGGTTATGGGTGTGATAGAGAGGGAAGGCTAC
A5-AT5G22300-XLOC_026181-7769-1
AGAAGTACAAAGTATACTTGGTTATGGGTGTGATAGAGAGGGAAGGCTAC
CONSENSUS
AGAAGTACAAAGTATACTTGGTTATGGGTGTGATAGAGAGGGAAGGCTAC

A5-AT5G22300-XLOC_026181-7769-0
ACGCTATACTGCACCGTTCTTTTCTTCGATTCACAAGGTCTGTTCTTAGG
A5-AT5G22300-XLOC_026181-7769-1
ACGCTATACTGCACCGTTCTTTTCTTCGATTCACAAGGTCTGTTCTTAGG
CONSENSUS
ACGCTATACTGCACCGTTCTTTTCTTCGATTCACAAGGTCTGTTCTTAGG

A5-AT5G22300-XLOC_026181-7769-0
TAAGCACCGCAAACTCATGCCTACAGCTCTTGAACGTTGCATTTGGGGAT
A5-AT5G22300-XLOC_026181-7769-1
TAAGCACCGCAAACTCATGCCTACAGCTCTTGAACGTTGCATTTGGGGAT
CONSENSUS
TAAGCACCGCAAACTCATGCCTACAGCTCTTGAACGTTGCATTTGGGGAT

A5-AT5G22300-XLOC_026181-7769-0
TTGGAGATGGATCAACCATCCCTGTGTTCGATACTCCTATTGGGAAAATC
A5-AT5G22300-XLOC_026181-7769-1
TTGGAGATGGATCAACCATCCCTGTGTTCGATACTCCTATTGGGAAAATC
CONSENSUS
TTGGAGATGGATCAACCATCCCTGTGTTCGATACTCCTATTGGGAAAATC

A5-AT5G22300-XLOC_026181-7769-0
GGTGCTGCTATTTGTTGGGAAAATAGGATGCCTTCTTTGAGAACCGCAAT

A5-AT5G22300-XLOC_026181-7769-1
      GGTGCTGCTATTTGTTGGGAAAATAGGATGCCTTCTTTGAGAACCGCAAT
CONSENSUS
      GGTGCTGCTATTTGTTGGGAAAATAGGATGCCTTCTTTGAGAACCGCAAT

A5-AT5G22300-XLOC_026181-7769-0 GTATGCCAAAG
A5-AT5G22300-XLOC_026181-7769-1 GTATGCCAAAG
CONSENSUS   GTATGCCAAAG

alignment for event: A3-AT5G22860-XLOC_029899-7440

A3-AT5G22860-XLOC_029899-7440-0
      CATAGGTATTATGGGGAGACGATGCCATTTGGATCAGCAGAAGAAGCACT
A3-AT5G22860-XLOC_029899-7440-1
      CATAGGTATTATGGGGAGACGATGCCATTTGGATCAGCAGAAGAAGCACT
CONSENSUS
      CATAGGTATTATGGGGAGACGATGCCATTTGGATCAGCAGAAGAAGCACT

A3-AT5G22860-XLOC_029899-7440-0
      AAAGAACGCAAGTACGTTGGGGTATTTGAACGCAGCACAAGCCCTAGCAG
A3-AT5G22860-XLOC_029899-7440-1
      AAAGAACGCAAGTACGTTGGGGTATTTGAACGCAGCACAAGCCCTAGCAG
CONSENSUS
      AAAGAACGCAAGTACGTTGGGGTATTTGAACGCAGCACAAGCCCTAGCAG

A3-AT5G22860-XLOC_029899-7440-0
      ACTATGCCGCGATTCTCTTGCACGTTAAGGAGAAGTACTCCACCAATCAC
A3-AT5G22860-XLOC_029899-7440-1
      ACTATGCCGCGATTCTCTTGCACGTTAAGGAGAAGTACTCCACCAATCAC
CONSENSUS
      ACTATGCCGCGATTCTCTTGCACGTTAAGGAGAAGTACTCCACCAATCAC

A3-AT5G22860-XLOC_029899-7440-0
      AGCCCTATCATTGTAATTGGAGGATCCTATGGTGGAA-------------
A3-AT5G22860-XLOC_029899-7440-1
      AGCCCTATCATTGTAATTGGAGGATCCTATGGTGGAATGTTAGCGGCATG
CONSENSUS
      AGCCCTATCATTGTAATTGGAGGATCCTATGGTGGAA.............

A3-AT5G22860-XLOC_029899-7440-0 ------
GCTAAAATATCCACACATAGCACTCGGAGCATTAGCATCTTCAG
A3-AT5G22860-XLOC_029899-7440-1
      GTTCAGGCTAAAATATCCACACATAGCACTCGGAGCATTAGCATCTTCAG
CONSENSUS
      ......GCTAAAATATCCACACATAGCACTCGGAGCATTAGCATCTTCAG

A3-AT5G22860-XLOC_029899-7440-0
      CTCCTCTTCTCTACTTTGAAGATACTCGTCCTAAATTTGGTTATTATTAT
A3-AT5G22860-XLOC_029899-7440-1
      CTCCTCTTCTCTACTTTGAAGATACTCGTCCTAAATTTGGTTATTATTAT
CONSENSUS
      CTCCTCTTCTCTACTTTGAAGATACTCGTCCTAAATTTGGTTATTATTAT

A3-AT5G22860-XLOC_029899-7440-0 ATTGTAACCAAAGTTTTCAAG

A3-AT5G22860-XLOC_029899-7440-1 ATTGTAACCAAAGTTTTCAAG
CONSENSUS   ATTGTAACCAAAGTTTTCAAG

alignment for event: A3-AT5G20380-XLOC_029787-3201

A3-AT5G20380-XLOC_029787-3201-0
      AAAAGTGCTTGAGATTGGTGTCTTTACATGGTCTTTCGCTACAGCTCTTG
A3-AT5G20380-XLOC_029787-3201-1
      AAAAGTGCTTGAGATTGGTGTCTTTACATGGTCTTTCGCTACAGCTCTTG
CONSENSUS
      AAAAGTGCTTGAGATTGGTGTCTTTACATGGTCTTTCGCTACAGCTCTTG

A3-AT5G20380-XLOC_029787-3201-0
      TTCCACTACTTGCTGGATTTATGCCCGGTTTGATCTTTTCTCGAATTTTG
A3-AT5G20380-XLOC_029787-3201-1
      TTCCACTACTTGCTGGATTTATGCCCGGTTTGATCTTTTCTCGAATTTTG
CONSENSUS
      TTCCACTACTTGCTGGATTTATGCCCGGTTTGATCTTTTCTCGAATTTTG

A3-AT5G20380-XLOC_029787-3201-0 ----------------
GTGGGAATAGGAGAAGGTGTTTCCCCATCGGCTG
A3-AT5G20380-XLOC_029787-3201-1
      AGATTTGTAACTCCAGGTGGGAATAGGAGAAGGTGTTTCCCCATCGGCTG
CONSENSUS
      ................GTGGGAATAGGAGAAGGTGTTTCCCCATCGGCTG

A3-AT5G20380-XLOC_029787-3201-0 CGACAGACCTTATTGCCAG
A3-AT5G20380-XLOC_029787-3201-1 CGACAGACCTTATTGCCAG
CONSENSUS   CGACAGACCTTATTGCCAG

alignment for event: A3-AT5G26742-XLOC_030103-4451

A3-AT5G26742-XLOC_030103-4451-0
      TTACCTCCACTTCAAGATGACGGACCATCTAGTGATAACTACGGACGGTT
A3-AT5G26742-XLOC_030103-4451-1
      TTACCTCCACTTCAAGATGACGGACCATCTAGTGATAACTACGGACGGTT
CONSENSUS
      TTACCTCCACTTCAAGATGACGGACCATCTAGTGATAACTACGGACGGTT

A3-AT5G26742-XLOC_030103-4451-0
      CTCTAGCAGAGACAGGATGCCTAGAGGAGGAGGAGGTTCTAGAGGGTCAA
A3-AT5G26742-XLOC_030103-4451-1
      CTCTAGCAGAGACAGGATGCCTAGAGGAGGAGGAGGTTCTAGAGGGTCAA
CONSENSUS
      CTCTAGCAGAGACAGGATGCCTAGAGGAGGAGGAGGTTCTAGAGGGTCAA

A3-AT5G26742-XLOC_030103-4451-0
      GAGGCGGTAGAGGAGGATCATCACGAGGCCGTGATAGTTGGGGAGGTGAT
A3-AT5G26742-XLOC_030103-4451-1
      GAGGCGGTAGAGGAGGATCATCACGAGGCCGTGATAGTTGGGGAGGTGAT
CONSENSUS
      GAGGCGGTAGAGGAGGATCATCACGAGGCCGTGATAGTTGGGGAGGTGAT

A3-AT5G26742-XLOC_030103-4451-0
GATGACAGAGGTAGTAGAAGGAGCAGTGGTGGAGGAAGCAGCTGGTCCCG
A3-AT5G26742-XLOC_030103-4451-1
GATGACAGAGGTAGTAGAAGGAGCAGTGGTGGAGGAAGCAGCTGGTCCCG
CONSENSUS
GATGACAGAGGTAGTAGAAGGAGCAGTGGTGGAGGAAGCAGCTGGTCCCG

A3-AT5G26742-XLOC_030103-4451-0
AGGTGGTAGTAGTTCCAGAGGAAGTTCTGATGATTGGTTGATCGGTGGCA
A3-AT5G26742-XLOC_030103-4451-1
AGGTGGTAGTAGTTCCAGAGGAAGTTCTGATGATTGGTTGATCGGTGGCA
CONSENSUS
AGGTGGTAGTAGTTCCAGAGGAAGTTCTGATGATTGGTTGATCGGTGGCA

A3-AT5G26742-XLOC_030103-4451-0
GAAGTTCATCAAGCAGCAGAGCTCCTTCGCGGGAGAG---TTTTGGAGGT
A3-AT5G26742-XLOC_030103-4451-1
GAAGTTCATCAAGCAGCAGAGCTCCTTCGCGGGAGAGAAGTTTTGGAGGT
CONSENSUS
GAAGTTCATCAAGCAGCAGAGCTCCTTCGCGGGAGAG...TTTTGGAGGT

A3-AT5G26742-XLOC_030103-4451-0
TCATGCTTCATTTGTGGGAAATCTGGACACAGGGCAACAGATTGTCCTGA
A3-AT5G26742-XLOC_030103-4451-1
TCATGCTTCATTTGTGGGAAATCTGGACACAGGGCAACAGATTGTCCTGA
CONSENSUS
TCATGCTTCATTTGTGGGAAATCTGGACACAGGGCAACAGATTGTCCTGA

A3-AT5G26742-XLOC_030103-4451-0
TAAGAGAGGATTTTAGAGTTATCGTAGCAAATGGTTTCTGCTCTTGGGAG
A3-AT5G26742-XLOC_030103-4451-1
TAAGAGAGGATTTTAGAGTTATCGTAGCAAATGGTTTCTGCTCTTGGGAG
CONSENSUS
TAAGAGAGGATTTTAGAGTTATCGTAGCAAATGGTTTCTGCTCTTGGGAG

A3-AT5G26742-XLOC_030103-4451-0
AAAGATCCTTGTCCTTTTTGGTGTTCTTAATGCGAATCTTTTCGTGCTTG
A3-AT5G26742-XLOC_030103-4451-1
AAAGATCCTTGTCCTTTTTGGTGTTCTTAATGCGAATCTTTTCGTGCTTG
CONSENSUS
AAAGATCCTTGTCCTTTTTGGTGTTCTTAATGCGAATCTTTTCGTGCTTG

A3-AT5G26742-XLOC_030103-4451-0
CTTCAACGGTTTGTTTCAGACGGTAGAGAAGCAATCAGGGAGGAGGATAC
A3-AT5G26742-XLOC_030103-4451-1
CTTCAACGGTTTGTTTCAGACGGTAGAGAAGCAATCAGGGAGGAGGATAC
CONSENSUS
CTTCAACGGTTTGTTTCAGACGGTAGAGAAGCAATCAGGGAGGAGGATAC

A3-AT5G26742-XLOC_030103-4451-0
ATGCTTCGGACAAGGTAGAGCAGAAATAAATAAGAGAGAGGCAATAGCAA
A3-AT5G26742-XLOC_030103-4451-1
ATGCTTCGGACAAGGTAGAGCAGAAATAAATAAGAGAGAGGCAATAGCAA
CONSENSUS
ATGCTTCGGACAAGGTAGAGCAGAAATAAATAAGAGAGAGGCAATAGCAA

A3-AT5G26742-XLOC_030103-4451-0
AAGTAAAGGAGAAGGTTTTCCTCAAATTGGCATTTTCAGAGTTTGCGTGA
A3-AT5G26742-XLOC_030103-4451-1
AAGTAAAGGAGAAGGTTTTCCTCAAATTGGCATTTTCAGAGTTTGCGTGA
CONSENSUS
AAGTAAAGGAGAAGGTTTTCCTCAAATTGGCATTTTCAGAGTTTGCGTGA

A3-AT5G26742-XLOC_030103-4451-0
GAAACAAGGGAAAGCTGATGGATCTTTTTATGTTAGGGTTATTATAATAG
A3-AT5G26742-XLOC_030103-4451-1
GAAACAAGGGAAAGCTGATGGATCTTTTTATGTTAGGGTTATTATAATAG
CONSENSUS
GAAACAAGGGAAAGCTGATGGATCTTTTTATGTTAGGGTTATTATAATAG

A3-AT5G26742-XLOC_030103-4451-0
TCTTAATGATCAGTTTCCATGTTTCTAAGGTTTTGTTTGATTTAAGTGGT
A3-AT5G26742-XLOC_030103-4451-1
TCTTAATGATCAGTTTCCATGTTTCTAAGGTTTTGTTTGATTTAAGTGGT
CONSENSUS
TCTTAATGATCAGTTTCCATGTTTCTAAGGTTTTGTTTGATTTAAGTGGT

A3-AT5G26742-XLOC_030103-4451-0
TATTAGTCAGTCTACTTACACTACTTTTATTTTCATTTTTGTACGTTTTC
A3-AT5G26742-XLOC_030103-4451-1
TATTAGTCAGTCTACTTACACTACTTTTATTTTCATTTTTGTACGTTTTC
CONSENSUS
TATTAGTCAGTCTACTTACACTACTTTTATTTTCATTTTTGTACGTTTTC

A3-AT5G26742-XLOC_030103-4451-0
TATTGTTATATGTGAATTCGGATCACATGTGTTTGGACAGAA
A3-AT5G26742-XLOC_030103-4451-1
TATTGTTATATGTGAATTCGGATCACATGTGTTTGGACAGAA
CONSENSUS
TATTGTTATATGTGAATTCGGATCACATGTGTTTGGACAGAA

alignment for event: A5-AT5G61910-XLOC_032083-2847

A5-AT5G61910-XLOC_032083-2847-0
GTTATGTCTCTGTTATCATTGTTTCGTTCATTTACGTCTCCAGAACTCGA
A5-AT5G61910-XLOC_032083-2847-1
GTTATGTCTCTGTTATCATTGTTTCGTTCATTTACGTCTCCAGAACTCGA
CONSENSUS
GTTATGTCTCTGTTATCATTGTTTCGTTCATTTACGTCTCCAGAACTCGA

A5-AT5G61910-XLOC_032083-2847-0
TTTGTTGCCTCATAGGTTGGCTTCCAGAGCTTCAGCACCTAGGACTCTAT
A5-AT5G61910-XLOC_032083-2847-1
TTTGTTGCCTCATAGGTTGGCTTCCAGAGCTTCAGCACCTAGGACTCTAT
CONSENSUS
TTTGTTGCCTCATAGGTTGGCTTCCAGAGCTTCAGCACCTAGGACTCTAT

A5-AT5G61910-XLOC_032083-2847-0
CGTTTGAGGAAAGGTTCATAGCGGCTACACATCTTAGAAATGCTTCATCG

A5-AT5G61910-XLOC_032083-2847-1
CGTTTGAGGAAAGGTTCATAGCGGCTACACATCTTAGAAATGCTTCATCG
CONSENSUS
CGTTTGAGGAAAGGTTCATAGCGGCTACACATCTTAGAAATGCTTCATCG

A5-AT5G61910-XLOC_032083-2847-0
GTTTTAGATCCTCTGTCTGCTCGTCATGTAGAACCACGACTTGGCTCTGT
A5-AT5G61910-XLOC_032083-2847-1
GTTTTAGATCCTCTGTCTGCTCGTCATGTAGAACCACGACTTGGCTCTGT
CONSENSUS
GTTTTAGATCCTCTGTCTGCTCGTCATGTAGAACCACGACTTGGCTCTGT

A5-AT5G61910-XLOC_032083-2847-0
TATGGCCCATCAGCCTGTTCCCCGCACATCTCTGCTTCAACATAGTTACT
A5-AT5G61910-XLOC_032083-2847-1
TATGGCCCATCAGCCTGTTCCCCGCACATCTCTGCTTCAACATAGTTACT
CONSENSUS
TATGGCCCATCAGCCTGTTCCCCGCACATCTCTGCTTCAACATAGTTACT

A5-AT5G61910-XLOC_032083-2847-0
TTAGACAAGACGACTACACGACCCCACCTAGAGAAAGTCTGTCAAATTTG
A5-AT5G61910-XLOC_032083-2847-1
TTAGACAAGACGACTACACGACCCCACCTAGAGAAAGTCTGTCAAATTTG
CONSENSUS
TTAGACAAGACGACTACACGACCCCACCTAGAGAAAGTCTGTCAAATTTG

A5-AT5G61910-XLOC_032083-2847-0
AATCAGCCTTATTATCCTACAGAAGCTCGGCAACTACGGTTGCTCGGAGA
A5-AT5G61910-XLOC_032083-2847-1
AATCAGCCTTATTATCCTACAGAAGCTCGGCAACTACGGTTGCTCGGAGA
CONSENSUS
AATCAGCCTTATTATCCTACAGAAGCTCGGCAACTACGGTTGCTCGGAGA

A5-AT5G61910-XLOC_032083-2847-0
CCCTTCTCGCTCAGATTCTCCTCGGTCCGAACCTCCGCGGTCATCTATTC
A5-AT5G61910-XLOC_032083-2847-1
CCCTTCTCGCTCAGATTCTCCTCGGTCCGAACCTCCGCGGTCATCTATTC
CONSENSUS
CCCTTCTCGCTCAGATTCTCCTCGGTCCGAACCTCCGCGGTCATCTATTC

A5-AT5G61910-XLOC_032083-2847-0
AGGATCCTCAACTCAAATACCTCACCATTTTATCTAATATTCGGAGGTAC
A5-AT5G61910-XLOC_032083-2847-1
AGGATCCTCAACTCAAATACCTCACCATTTTATCTAATATTCGGAG----
CONSENSUS
AGGATCCTCAACTCAAATACCTCACCATTTTATCTAATATTCGGAG....

A5-AT5G61910-XLOC_032083-2847-0
CTATGGTGAACTTTTATCTTTGCCCAAATATGTCGATCATAGCTCACTGA
A5-AT5G61910-XLOC_032083-2847-1

CONSENSUS
....

A5-AT5G61910-XLOC_032083-2847-0
AGACCTGAAGTTGTAGCCATCAGATGTTTAAAATCATAGCTAAACCTGAG

A5-AT5G61910-XLOC_032083-2847-1

CONSENSUS
      ....

A5-AT5G61910-XLOC_032083-2847-0
      AACTCTACTAGATATGGATCTGCATCAGATCGCTTGGCCTCAGAAAATGA
A5-AT5G61910-XLOC_032083-2847-1 -----------
ATATGGATCTGCATCAGATCGCTTGGCCTCAGAAAATGA
CONSENSUS
      ...........ATATGGATCTGCATCAGATCGCTTGGCCTCAGAAAATGA

A5-AT5G61910-XLOC_032083-2847-0
      ATATCATCCGGCAACACCATCAGAGAAGGACCAGTTTGCTGTACCTTATT
A5-AT5G61910-XLOC_032083-2847-1
      ATATCATCCGGCAACACCATCAGAGAAGGACCAGTTTGCTGTACCTTATT
CONSENSUS
      ATATCATCCGGCAACACCATCAGAGAAGGACCAGTTTGCTGTACCTTATT

A5-AT5G61910-XLOC_032083-2847-0
      CTGATAATAAAAATTACCCTTCGACTTTATCTGGGAGTGAGCATCCTTCT
A5-AT5G61910-XLOC_032083-2847-1
      CTGATAATAAAAATTACCCTTCGACTTTATCTGGGAGTGAGCATCCTTCT
CONSENSUS
      CTGATAATAAAAATTACCCTTCGACTTTATCTGGGAGTGAGCATCCTTCT

A5-AT5G61910-XLOC_032083-2847-0
      GCTTCTGCAGCTAATGGGAGTGTGTATAGAAGTGAGTTCTATAATTCAGC
A5-AT5G61910-XLOC_032083-2847-1
      GCTTCTGCAGCTAATGGGAGTGTGTATAGAAGTGAGTTCTATAATTCAGC
CONSENSUS
      GCTTCTGCAGCTAATGGGAGTGTGTATAGAAGTGAGTTCTATAATTCAGC

A5-AT5G61910-XLOC_032083-2847-0
      ATCACAAAAGGAAGGGGAAGCTAGTCAGCAGCATGAGATCCCTGCTGGGA
A5-AT5G61910-XLOC_032083-2847-1
      ATCACAAAAGGAAGGGGAAGCTAGTCAGCAGCATGAGATCCCTGCTGGGA
CONSENSUS
      ATCACAAAAGGAAGGGGAAGCTAGTCAGCAGCATGAGATCCCTGCTGGGA

A5-AT5G61910-XLOC_032083-2847-0
      CTTACCATCACCCAGAAGCATCAACAGTTTCCAATACTACAAAGTCCATG
A5-AT5G61910-XLOC_032083-2847-1
      CTTACCATCACCCAGAAGCATCAACAGTTTCCAATACTACAAAGTCCATG
CONSENSUS
      CTTACCATCACCCAGAAGCATCAACAGTTTCCAATACTACAAAGTCCATG

A5-AT5G61910-XLOC_032083-2847-0
      CAACCAGATATGCAGGCAGTTAGTGTTGCTCAATCTCATACCGAGACAGC
A5-AT5G61910-XLOC_032083-2847-1
      CAACCAGATATGCAGGCAGTTAGTGTTGCTCAATCTCATACCGAGACAGC
CONSENSUS
      CAACCAGATATGCAGGCAGTTAGTGTTGCTCAATCTCATACCGAGACAGC

A5-AT5G61910-XLOC_032083-2847-0
      TGGCTATCCTACACCGGCACATGGTGAAGCTTCACAACCACCTGCTGGAG

A5-AT5G61910-XLOC_032083-2847-1
TGGCTATCCTACACCGGCACATGGTGAAGCTTCACAACCACCTGCTGGAG
CONSENSUS
TGGCTATCCTACACCGGCACATGGTGAAGCTTCACAACCACCTGCTGGAG

A5-AT5G61910-XLOC_032083-2847-0
CAATTGGTTACACACACCAACCTCAAAGTGTAGCGGGTAATTATAGCACG
A5-AT5G61910-XLOC_032083-2847-1
CAATTGGTTACACACACCAACCTCAAAGTGTAGCGGGTAATTATAGCACG
CONSENSUS
CAATTGGTTACACACACCAACCTCAAAGTGTAGCGGGTAATTATAGCACG

A5-AT5G61910-XLOC_032083-2847-0
CATTCACAGCCTGGGAATGTGGAAGAAAGTACACAATCATATGCTGGAAC
A5-AT5G61910-XLOC_032083-2847-1
CATTCACAGCCTGGGAATGTGGAAGAAAGTACACAATCATATGCTGGAAC
CONSENSUS
CATTCACAGCCTGGGAATGTGGAAGAAAGTACACAATCATATGCTGGAAC

A5-AT5G61910-XLOC_032083-2847-0
AGATAGTTACTCACAACAGCAATATTATGCAGCTATGGGGCCTACTACTC
A5-AT5G61910-XLOC_032083-2847-1
AGATAGTTACTCACAACAGCAATATTATGCAGCTATGGGGCCTACTACTC
CONSENSUS
AGATAGTTACTCACAACAGCAATATTATGCAGCTATGGGGCCTACTACTC

A5-AT5G61910-XLOC_032083-2847-0
AACTCCATGCTGGTGGATATATTCAAAAACCTCATGAAATTGGGTACTCT
A5-AT5G61910-XLOC_032083-2847-1
AACTCCATGCTGGTGGATATATTCAAAAACCTCATGAAATTGGGTACTCT
CONSENSUS
AACTCCATGCTGGTGGATATATTCAAAAACCTCATGAAATTGGGTACTCT

A5-AT5G61910-XLOC_032083-2847-0
CAGCAACCTCATGATGCGGCAACCGGGTACTCTCAGCAACCTCATGATGC
A5-AT5G61910-XLOC_032083-2847-1
CAGCAACCTCATGATGCGGCAACCGGGTACTCTCAGCAACCTCATGATGC
CONSENSUS
CAGCAACCTCATGATGCGGCAACCGGGTACTCTCAGCAACCTCATGATGC

A5-AT5G61910-XLOC_032083-2847-0
AGCAACCGGGTACTCTCAGCAACCTCATGATGCAGCAACCGGGTACTCTC
A5-AT5G61910-XLOC_032083-2847-1
AGCAACCGGGTACTCTCAGCAACCTCATGATGCAGCAACCGGGTACTCTC
CONSENSUS
AGCAACCGGGTACTCTCAGCAACCTCATGATGCAGCAACCGGGTACTCTC

A5-AT5G61910-XLOC_032083-2847-0
AGCAACCTCATGCCGCATCAACCGGGTACTCTCAGCAAACCTATGCTGCA
A5-AT5G61910-XLOC_032083-2847-1
AGCAACCTCATGCCGCATCAACCGGGTACTCTCAGCAAACCTATGCTGCA
CONSENSUS
AGCAACCTCATGCCGCATCAACCGGGTACTCTCAGCAAACCTATGCTGCA

A5-AT5G61910-XLOC_032083-2847-0
GCAACCGGGTACACTCAGCAACCCCATGCGGCGGCAGCCGGGTACACTCA

A5-AT5G61910-XLOC_032083-2847-1
GCAACCGGGTACACTCAGCAACCCCATGCGGCGGCAGCCGGGTACACTCA
CONSENSUS
GCAACCGGGTACACTCAGCAACCCCATGCGGCGGCAGCCGGGTACACTCA

A5-AT5G61910-XLOC_032083-2847-0
GCAACCCCATGCGGCGGCAACCGGGTATTCTCAGCAACCCCATGCTGCAG
A5-AT5G61910-XLOC_032083-2847-1
GCAACCCCATGCGGCGGCAACCGGGTATTCTCAGCAACCCCATGCTGCAG
CONSENSUS
GCAACCCCATGCGGCGGCAACCGGGTATTCTCAGCAACCCCATGCTGCAG

A5-AT5G61910-XLOC_032083-2847-0
CTACCGCGCACGCTCAGCAACCCTATGCTGCAGCGACCGCGCACGCTCAG
A5-AT5G61910-XLOC_032083-2847-1
CTACCGCGCACGCTCAGCAACCCTATGCTGCAGCGACCGCGCACGCTCAG
CONSENSUS
CTACCGCGCACGCTCAGCAACCCTATGCTGCAGCGACCGCGCACGCTCAG

A5-AT5G61910-XLOC_032083-2847-0
CAACTCCATGCTGTAGCTACTGGGTATGCTCTGCAACTCCATGCTGCAGC
A5-AT5G61910-XLOC_032083-2847-1
CAACTCCATGCTGTAGCTACTGGGTATGCTCTGCAACTCCATGCTGCAGC
CONSENSUS
CAACTCCATGCTGTAGCTACTGGGTATGCTCTGCAACTCCATGCTGCAGC

A5-AT5G61910-XLOC_032083-2847-0
TACCGGGTACGCTCAGCAACCCCATGCTGCCGCAACTGGGTATGCTTTGC
A5-AT5G61910-XLOC_032083-2847-1
TACCGGGTACGCTCAGCAACCCCATGCTGCCGCAACTGGGTATGCTTTGC
CONSENSUS
TACCGGGTACGCTCAGCAACCCCATGCTGCCGCAACTGGGTATGCTTTGC

A5-AT5G61910-XLOC_032083-2847-0
AACCACATGCTCAAGCTGTTGAATACACAATGCAACCACATGCTCAAGCT
A5-AT5G61910-XLOC_032083-2847-1
AACCACATGCTCAAGCTGTTGAATACACAATGCAACCACATGCTCAAGCT
CONSENSUS
AACCACATGCTCAAGCTGTTGAATACACAATGCAACCACATGCTCAAGCT

A5-AT5G61910-XLOC_032083-2847-0
GTTGGCTACATGCCGCAATACCATGCTCACGCTGTTGTATACAGTCAACA
A5-AT5G61910-XLOC_032083-2847-1
GTTGGCTACATGCCGCAATACCATGCTCACGCTGTTGTATACAGTCAACA
CONSENSUS
GTTGGCTACATGCCGCAATACCATGCTCACGCTGTTGTATACAGTCAACA

A5-AT5G61910-XLOC_032083-2847-0
AGGTGTGACGCAAGGTTCTGTACCAAGGGCTCCTGGGACAACTGATTGTA
A5-AT5G61910-XLOC_032083-2847-1
AGGTGTGACGCAAGGTTCTGTACCAAGGGCTCCTGGGACAACTGATTGTA
CONSENSUS
AGGTGTGACGCAAGGTTCTGTACCAAGGGCTCCTGGGACAACTGATTGTA

A5-AT5G61910-XLOC_032083-2847-0
ATGCTGCAAACCAGGCATATTCTGCAACAGGAGATTGGAATGCAGTGCAT

A5-AT5G61910-XLOC_032083-2847-1
      ATGCTGCAAACCAGGCATATTCTGCAACAGGAGATTGGAATGCAGTGCAT
CONSENSUS
      ATGCTGCAAACCAGGCATATTCTGCAACAGGAGATTGGAATGCAGTGCAT

A5-AT5G61910-XLOC_032083-2847-0
      CAGTCTTATTACCCTCAGACAGCAGATGCAACCACAACGTATTATCAAAC
A5-AT5G61910-XLOC_032083-2847-1
      CAGTCTTATTACCCTCAGACAGCAGATGCAACCACAACGTATTATCAAAC
CONSENSUS
      CAGTCTTATTACCCTCAGACAGCAGATGCAACCACAACGTATTATCAAAC

A5-AT5G61910-XLOC_032083-2847-0
      ATCTTAAAACGTTAAAGCAAAGTGTTGCTACTCTG
A5-AT5G61910-XLOC_032083-2847-1
      ATCTTAAAACGTTAAAGCAAAGTGTTGCTACTCTG
CONSENSUS
      ATCTTAAAACGTTAAAGCAAAGTGTTGCTACTCTG

alignment for event: RI-AT5G39790-XLOC_030855-13647

RI-AT5G39790-XLOC_030855-13647-0
      GAACATGAGATAACTAACATCAAAACTGAGCTTGCACTTATGGAGCTCGA
RI-AT5G39790-XLOC_030855-13647-1
      GAACATGAGATAACTAACATCAAAACTGAGCTTGCACTTATGGAGCTCGA
CONSENSUS
      GAACATGAGATAACTAACATCAAAACTGAGCTTGCACTTATGGAGCTCGA

RI-AT5G39790-XLOC_030855-13647-0
      AGTTCAGGTCTTTATTTCTACTTTTGATTTTTCAATGTCACGGTCTCACT
RI-AT5G39790-XLOC_030855-13647-1
      AGTTCAG---
CONSENSUS
      AGTTCAG...

RI-AT5G39790-XLOC_030855-13647-0
      ATCTCATTGCCTTAAGCTGATTTGGTTTAGTTGTCATTATCCCATGTCTA
RI-AT5G39790-XLOC_030855-13647-1

CONSENSUS
      ....

RI-AT5G39790-XLOC_030855-13647-0
      CTGCCAATCAAATACATATTTTTGTTGGTTATAATATTGGGAAATATTTA
RI-AT5G39790-XLOC_030855-13647-1

CONSENSUS
      ....

RI-AT5G39790-XLOC_030855-13647-0
      GCTTCAGATTTTTGTATGCACAAGCACATATGTATAGTTTCTTTAGTAGA
RI-AT5G39790-XLOC_030855-13647-1

CONSENSUS

....

RI-AT5G39790-XLOC_030855-13647-0
AATGTTTCATGATATCACTTTCTGGAGGAAATAGGCTCTGGTGAAACTGG
RI-AT5G39790-XLOC_030855-13647-1
----------------------------------GCTCTGGTGAAACTGG
CONSENSUS
..................................GCTCTGGTGAAACTGG

RI-AT5G39790-XLOC_030855-13647-0
CGGAAGAAATAGCAAACCTTGGTATCCCACAAGGTTCTAGAAAAATCAGT
RI-AT5G39790-XLOC_030855-13647-1
CGGAAGAAATAGCAAACCTTGGTATCCCACAAGGTTCTAGAAAAATCAGT
CONSENSUS
CGGAAGAAATAGCAAACCTTGGTATCCCACAAGGTTCTAGAAAAATCAGT

RI-AT5G39790-XLOC_030855-13647-0
GGAAAGTACATTCAATCGCACCTTCTCTCTCGTTTAGACG
RI-AT5G39790-XLOC_030855-13647-1
GGAAAGTACATTCAATCGCACCTTCTCTCTCGTTTAGACG
CONSENSUS
GGAAAGTACATTCAATCGCACCTTCTCTCTCGTTTAGACG

alignment for event: SE-AT5G53850-XLOC_031648-8186

SE-AT5G53850-XLOC_031648-8186-0
GTGTTCAAAAGGAGAGGATGCAACCTGAGGATATGTACATCTTATCTGCT
SE-AT5G53850-XLOC_031648-8186-1
GTGTTCAAAAGGAGAGGATGCAACCTGAGGATATGTACATCTTATCTGCT
CONSENSUS
GTGTTCAAAAGGAGAGGATGCAACCTGAGGATATGTACATCTTATCTGCT

SE-AT5G53850-XLOC_031648-8186-0
AATGGATCCATCATATCTACACCCTCTCCAAAGCCATACCCAAATAAGCC
SE-AT5G53850-XLOC_031648-8186-1
AATGGATCCATCATATCTACACCCTCTCCAAAGCCATACCCAAATAAGCC
CONSENSUS
AATGGATCCATCATATCTACACCCTCTCCAAAGCCATACCCAAATAAGCC

SE-AT5G53850-XLOC_031648-8186-0
TCCCAAGTGTACCGATTGTGCTCCACTTTTCATGAAG-------------
SE-AT5G53850-XLOC_031648-8186-1
TCCCAAGTGTACCGATTGTGCTCCACTTTTCATGAAGAACAGGCGACTTT
CONSENSUS
TCCCAAGTGTACCGATTGTGCTCCACTTTTCATGAAG.............

SE-AT5G53850-XLOC_031648-8186-0
-----------------------------------GCATATGAGATGCGA
SE-AT5G53850-XLOC_031648-8186-1
CTTTGGGGAAACGCTTGCGAAAACTCGGTTACAAGGCATATGAGATGCGA
CONSENSUS
...................................GCATATGAGATGCGA

SE-AT5G53850-XLOC_031648-8186-0

AATGCTGGAGCTGTTATTCACAGTCATGGCATGGAATCTTGTCTTGTGAC
SE-AT5G53850-XLOC_031648-8186-1
AATGCTGGAGCTGTTATTCACAGTCATGGCATGGAATCTTGTCTTGTGAC
CONSENSUS
AATGCTGGAGCTGTTATTCACAGTCATGGCATGGAATCTTGTCTTGTGAC

SE-AT5G53850-XLOC_031648-8186-0
GATGCTGAATCCGCAAGCCAAAGAATTCCGT
SE-AT5G53850-XLOC_031648-8186-1
GATGCTGAATCCGCAAGCCAAAGAATTCCGT
CONSENSUS
GATGCTGAATCCGCAAGCCAAAGAATTCCGT

alignment for event: A3-AT5G04430-XLOC_028954-4693

A3-AT5G04430-XLOC_028954-4693-0
GTCATTCATTGAGGAATCTAAAGCTGGTATTAAGATATCCCCTCTGGATA
A3-AT5G04430-XLOC_028954-4693-1
GTCATTCATTGAGGAATCTAAAGCTGGTATTAAGATATCCCCTCTGGATA
CONSENSUS
GTCATTCATTGAGGAATCTAAAGCTGGTATTAAGATATCCCCTCTGGATA

A3-AT5G04430-XLOC_028954-4693-0
ATACCTTTTATGGGTTGAGCGATAGGTTAGTGACATTATCTGGGACCTTC
A3-AT5G04430-XLOC_028954-4693-1
ATACCTTTTATGGGTTGAGCGATAGGTTAGTGACATTATCTGGGACCTTC
CONSENSUS
ATACCTTTTATGGGTTGAGCGATAGGTTAGTGACATTATCTGGGACCTTC

A3-AT5G04430-XLOC_028954-4693-0
GAGGAGCAGATGCGGGCAATCGATTTGATTTTGGCTAAGCTTACTGAGGA
A3-AT5G04430-XLOC_028954-4693-1
GAGGAGCAGATGCGGGCAATCGATTTGATTTTGGCTAAGCTTACTGAGGA
CONSENSUS
GAGGAGCAGATGCGGGCAATCGATTTGATTTTGGCTAAGCTTACTGAGGA

A3-AT5G04430-XLOC_028954-4693-0
CGATCATTACTCCCAGAATGTGCATTCCCCATATTCATATGCAG------
A3-AT5G04430-XLOC_028954-4693-1
CGATCATTACTCCCAGAATGTGCATTCCCCATATTCATATGCAGGTCTTT
CONSENSUS
CGATCATTACTCCCAGAATGTGCATTCCCCATATTCATATGCAG......

A3-AT5G04430-XLOC_028954-4693-0

A3-AT5G04430-XLOC_028954-4693-1
TCTACTCTGGTTTTCATGGTCCTCCATATGCGTATGCGCTTCCTTCTGTT
CONSENSUS
....

A3-AT5G04430-XLOC_028954-4693-0 -------
CGGGATACAATTCGGTTAACTACGCACCCAACGGTTCTGGAGG
A3-AT5G04430-XLOC_028954-4693-1
GCAACAGCGGGATACAATTCGGTTAACTACGCACCCAACGGTTCTGGAGG

CONSENSUS
.......CGGGATACAATTCGGTTAACTACGCACCCAACGGTTCTGGAGG

A3-AT5G04430-XLOC_028954-4693-0 CAAGTATCAAAACCACAAG
A3-AT5G04430-XLOC_028954-4693-1 CAAGTATCAAAACCACAAG
CONSENSUS   CAAGTATCAAAACCACAAG

alignment for event: A3-AT5G14530-XLOC_029471-13037

A3-AT5G14530-XLOC_029471-13037-0
GGTTGTATCACTTTGTATGTCTCCTATAAATGATAGCTTCATGTCTGGTT
A3-AT5G14530-XLOC_029471-13037-1
GGTTGTATCACTTTGTATGTCTCCTATAAATGATAGCTTCATGTCTGGTT
CONSENSUS
GGTTGTATCACTTTGTATGTCTCCTATAAATGATAGCTTCATGTCTGGTT

A3-AT5G14530-XLOC_029471-13037-0
CTCTCGACCGAAGTGTTAGACTCTGGGATCTTCGTGTAAATGCCTGCCAG
A3-AT5G14530-XLOC_029471-13037-1
CTCTCGACCGAAGTGTTAGACTCTGGGATCTTCGTGTAAATGCCTGCCAG
CONSENSUS
CTCTCGACCGAAGTGTTAGACTCTGGGATCTTCGTGTAAATGCCTGCCAG

A3-AT5G14530-XLOC_029471-13037-0 -------
GGAATTCTACATCTACGTGGTAGACCTGCAGTTGCGTATGACC
A3-AT5G14530-XLOC_029471-13037-1
TCAATAGGGAATTCTACATCTACGTGGTAGACCTGCAGTTGCGTATGACC
CONSENSUS
.......GGAATTCTACATCTACGTGGTAGACCTGCAGTTGCGTATGACC

A3-AT5G14530-XLOC_029471-13037-0
AACAAGGCCTTGTGTTTGCAATTGCAATGGAAGGAGGTGCTGTTAAATTA
A3-AT5G14530-XLOC_029471-13037-1
AACAAGGCCTTGTGTTTGCAATTGCAATGGAAGGAGGTGCTGTTAAATTA
CONSENSUS
AACAAGGCCTTGTGTTTGCAATTGCAATGGAAGGAGGTGCTGTTAAATTA

A3-AT5G14530-XLOC_029471-13037-0 TTTGATTCCAGGTGTTATGACAAG
A3-AT5G14530-XLOC_029471-13037-1 TTTGATTCCAGGTGTTATGACAAG
CONSENSUS   TTTGATTCCAGGTGTTATGACAAG

alignment for event: RI-AT5G52310-XLOC_027910-1992

RI-AT5G52310-XLOC_027910-1992-0
AAACTGGAGGAGTACCGGAGATTGCTGAGTCTTTTGGTAATATGGAAGTG
RI-AT5G52310-XLOC_027910-1992-1
AAACTGGAGGAGTACCGGAGATTGCTGAGTCTTTTGGTAATATGGAAGTG
CONSENSUS
AAACTGGAGGAGTACCGGAGATTGCTGAGTCTTTTGGTAATATGGAAGTG

RI-AT5G52310-XLOC_027910-1992-0
ACTGATGAGTCTCCTGATCAGAAGCCAGGACAATTTGAAAGAGACTTGTC

RI-AT5G52310-XLOC_027910-1992-1
ACTGATGAGTCTCCTGATCAGAAGCCAGGACAATTTGAAAGAGACTTGTC
CONSENSUS
ACTGATGAGTCTCCTGATCAGAAGCCAGGACAATTTGAAAGAGACTTGTC

RI-AT5G52310-XLOC_027910-1992-0
GACGAGAAGCAAAGAATTCAAAGAGTTTGATCAGGACTTTGACTCTGTTC
RI-AT5G52310-XLOC_027910-1992-1
GACGAGAAGCAAAGAATTCAAAGAGTTTGATCAGGACTTTGACTCTGTTC
CONSENSUS
GACGAGAAGCAAAGAATTCAAAGAGTTTGATCAGGACTTTGACTCTGTTC

RI-AT5G52310-XLOC_027910-1992-0
TCGGTAAGGATTCGCCGGCGAAATTTCCAGGTGAATCAGGAGTTGTTTTC
RI-AT5G52310-XLOC_027910-1992-1
TCGGTAAGGATTCGCCGGCGAAATTTCCAGGTGAATCAGGAGTTGTTTTC
CONSENSUS
TCGGTAAGGATTCGCCGGCGAAATTTCCAGGTGAATCAGGAGTTGTTTTC

RI-AT5G52310-XLOC_027910-1992-0
CCGGTGGGCTTTGGTGACGAGTCAGGAGCTGAGCTGGAAAAAGATTTTCC
RI-AT5G52310-XLOC_027910-1992-1
CCGGTGGGCTTTGGTGACGAGTCAGGAGCTGAGCTGGAAAAAGATTTTCC
CONSENSUS
CCGGTGGGCTTTGGTGACGAGTCAGGAGCTGAGCTGGAAAAAGATTTTCC

RI-AT5G52310-XLOC_027910-1992-0
GACGAGAAGTCATGATTTTGATATGAAGACTGAAACTGGAATGGACACGA
RI-AT5G52310-XLOC_027910-1992-1
GACGAGAAGTCATGATTTTGATATGAAGACTGAAACTGGAATGGACACGA
CONSENSUS
GACGAGAAGTCATGATTTTGATATGAAGACTGAAACTGGAATGGACACGA

RI-AT5G52310-XLOC_027910-1992-0
ATTCTCCATCAAGAAGCCATGAATTTGATCTGAAGACTGAATCTGGAAAC
RI-AT5G52310-XLOC_027910-1992-1
ATTCTCCATCAAGAAGCCATGAATTTGATCTGAAGACTGAATCTGGAAAC
CONSENSUS
ATTCTCCATCAAGAAGCCATGAATTTGATCTGAAGACTGAATCTGGAAAC

RI-AT5G52310-XLOC_027910-1992-0
GACAAGAATTCTCCGATGGGCTTTGGTAGTGAATCAGGAGCTGAGCTGGA
RI-AT5G52310-XLOC_027910-1992-1
GACAAGAATTCTCCGATGGGCTTTGGTAGTGAATCAGGAGCTGAGCTGGA
CONSENSUS
GACAAGAATTCTCCGATGGGCTTTGGTAGTGAATCAGGAGCTGAGCTGGA

RI-AT5G52310-XLOC_027910-1992-0
AAAAGAATTTGATCAGAAGAACGATTCTGGAAGAAACGAGTATTCGCCGG
RI-AT5G52310-XLOC_027910-1992-1
AAAAGAATTTGATCAGAAGAACGATTCTGGAAGAAACGAGTATTCGCCGG
CONSENSUS
AAAAGAATTTGATCAGAAGAACGATTCTGGAAGAAACGAGTATTCGCCGG

RI-AT5G52310-XLOC_027910-1992-0
AATCTGACGGCGGTTTAGGAGCTCCGTTGGGAGGAAATTTTCCGGTGAGA

RI-AT5G52310-XLOC_027910-1992-1
AATCTGACGGCGGTTTAGGAGCTCCGTTGGGAGGAAATTTTCCGGTGAGA
CONSENSUS
AATCTGACGGCGGTTTAGGAGCTCCGTTGGGAGGAAATTTTCCGGTGAGA

RI-AT5G52310-XLOC_027910-1992-0
AGTCATGAGTTGGATCTGAAGAACGAATCTGATATCGACAAGGATGTGCC
RI-AT5G52310-XLOC_027910-1992-1
AGTCATGAGTTGGATCTGAAGAACGAATCTGATATCGACAAGGATGTGCC
CONSENSUS
AGTCATGAGTTGGATCTGAAGAACGAATCTGATATCGACAAGGATGTGCC

RI-AT5G52310-XLOC_027910-1992-0
GACGGGATTTGACGGAGAACCAGATTTTCTGGCGAAGGGAAGACCTGGAT
RI-AT5G52310-XLOC_027910-1992-1
GACGGGATTTGACGGAGAACCAGATTTTCTGGCGAAGGGAAGACCTGGAT
CONSENSUS
GACGGGATTTGACGGAGAACCAGATTTTCTGGCGAAGGGAAGACCTGGAT

RI-AT5G52310-XLOC_027910-1992-0
ACGGTGAGGCATCAGAAGAGGATAAATTTCCGGCGAGAAGTGATGATGTG
RI-AT5G52310-XLOC_027910-1992-1
ACGGTGAGGCATCAGAAGAGGATAAATTTCCGGCGAGAAGTGATGATGTG
CONSENSUS
ACGGTGAGGCATCAGAAGAGGATAAATTTCCGGCGAGAAGTGATGATGTG

RI-AT5G52310-XLOC_027910-1992-0
GAAGTAGAGACTGAGCTGGGAAGAGACCCAAAGACGGAGACTCTTGATCA
RI-AT5G52310-XLOC_027910-1992-1
GAAGTAGAGACTGAGCTGGGAAGAGACCCAAAGACGGAGACTCTTGATCA
CONSENSUS
GAAGTAGAGACTGAGCTGGGAAGAGACCCAAAGACGGAGACTCTTGATCA

RI-AT5G52310-XLOC_027910-1992-0
ATTCTCACCGGAACTTTCTCATCCTAAAGAAAGAGATGAGTTTAAGGAGT
RI-AT5G52310-XLOC_027910-1992-1
ATTCTCACCGGAACTTTCTCATCCTAAAGAAAGAGATGAGTTTAAGGAGT
CONSENSUS
ATTCTCACCGGAACTTTCTCATCCTAAAGAAAGAGATGAGTTTAAGGAGT

RI-AT5G52310-XLOC_027910-1992-0
CCAGAGATGATTTTGAGGAGACGAGAGATGAGAAAACAGAGGAGCCAAAA
RI-AT5G52310-XLOC_027910-1992-1
CCAGAGATGATTTTGAGGAGACGAGAGATGAGAAAACAGAGGAGCCAAAA
CONSENSUS
CCAGAGATGATTTTGAGGAGACGAGAGATGAGAAAACAGAGGAGCCAAAA

RI-AT5G52310-XLOC_027910-1992-0
CAGAGCACTTACACAGAGAAGTTTGCTTCAATGCTAGGTTACTCCGGAGA
RI-AT5G52310-XLOC_027910-1992-1
CAGAGCACTTACACAGAGAAGTTTGCTTCAATGCTAGGTTACTCCGGAGA
CONSENSUS
CAGAGCACTTACACAGAGAAGTTTGCTTCAATGCTAGGTTACTCCGGAGA

RI-AT5G52310-XLOC_027910-1992-0
AATTCCGGTGGGAGATCAAACTCAAGTGGCGGGAACTGTTGATGAGAAGT

RI-AT5G52310-XLOC_027910-1992-1
AATTCCGGTGGGAGATCAAACTCAAGTGGCGGGAACTGTTGATGAGAAGT
CONSENSUS
AATTCCGGTGGGAGATCAAACTCAAGTGGCGGGAACTGTTGATGAGAAGT

RI-AT5G52310-XLOC_027910-1992-0
TGACTCCGGTCAATGAGAAGGATCAAGAAACAGAGTCTGCCGTGACGACG
RI-AT5G52310-XLOC_027910-1992-1
TGACTCCGGTCAATGAGAAGGATCAAGAAACAGAGTCTGCCGTGACGACG
CONSENSUS
TGACTCCGGTCAATGAGAAGGATCAAGAAACAGAGTCTGCCGTGACGACG

RI-AT5G52310-XLOC_027910-1992-0
AAGTTACCTATCTCCGGAGGTGGAAGTGGAGTAGAGGAGCAACGAGGGGA
RI-AT5G52310-XLOC_027910-1992-1
AAGTTACCTATCTCCGGAGGTGGAAGTGGAGTAGAGGAGCAACGAGGGGA
CONSENSUS
AAGTTACCTATCTCCGGAGGTGGAAGTGGAGTAGAGGAGCAACGAGGGGA

RI-AT5G52310-XLOC_027910-1992-0
AGATAAAAGTGTGTCGGGTAGAGATTATGTGGCGGAGAAACTGACAACTG
RI-AT5G52310-XLOC_027910-1992-1
AGATAAAAGTGTGTCGGGTAGAGATTATGTGGCGGAGAAACTGACAACTG
CONSENSUS
AGATAAAAGTGTGTCGGGTAGAGATTATGTGGCGGAGAAACTGACAACTG

RI-AT5G52310-XLOC_027910-1992-0
AAGAAGAAGACAAAGCCTTTTCTGATATGGTTGCCGAGAAACTTCAGATT
RI-AT5G52310-XLOC_027910-1992-1
AAGAAGAAGACAAAGCCTTTTCTGATATGGTTGCCGAGAAACTTCAGATT
CONSENSUS
AAGAAGAAGACAAAGCCTTTTCTGATATGGTTGCCGAGAAACTTCAGATT

RI-AT5G52310-XLOC_027910-1992-0
GGAGGAGAAGAAGAGAAGAAGGAAACGACGACAAAGGAAGTGGAGAAGAT
RI-AT5G52310-XLOC_027910-1992-1
GGAGGAGAAGAAGAGAAGAAGGAAACGACGACAAAGGAAGTGGAGAAGAT
CONSENSUS
GGAGGAGAAGAAGAGAAGAAGGAAACGACGACAAAGGAAGTGGAGAAGAT

RI-AT5G52310-XLOC_027910-1992-0
CTCTACCGAGAAGGCAGCATCGGAGGAGGGTGAGGCGGTGGAAGAGGAAG
RI-AT5G52310-XLOC_027910-1992-1
CTCTACCGAGAAGGCAGCATCGGAGGAGGGTGAGGCGGTGGAAGAGGAAG
CONSENSUS
CTCTACCGAGAAGGCAGCATCGGAGGAGGGTGAGGCGGTGGAAGAGGAAG

RI-AT5G52310-XLOC_027910-1992-0
TGAAAGGAGGAGGAGGAATGGTTGGGAGGATTAAAGGATGGTTCGGTGGT
RI-AT5G52310-XLOC_027910-1992-1
TGAAAGGAGGAGGAGGAATGGTTGGGAGGATTAAAGGATGGTTCGGTGGT
CONSENSUS
TGAAAGGAGGAGGAGGAATGGTTGGGAGGATTAAAGGATGGTTCGGTGGT

RI-AT5G52310-XLOC_027910-1992-0
GGTGCGACTGATGAGGTGAAGCCAGAATCGCCACATTCTGTTGAAGAGGC

RI-AT5G52310-XLOC_027910-1992-1
      GGTGCGACTGATGAGGTGAAGCCAGAATCGCCACATTCTGTTGAAGAG--
CONSENSUS
      GGTGCGACTGATGAGGTGAAGCCAGAATCGCCACATTCTGTTGAAGAG..

RI-AT5G52310-XLOC_027910-1992-0
      TCCAAAATCATCTGGCTGGTTTGGTGGTGGTGCGACGGAGGAGGTGAAGC
RI-AT5G52310-XLOC_027910-1992-1

CONSENSUS
      ....

RI-AT5G52310-XLOC_027910-1992-0
      CAAAATCGCCTCATTCCGTTGAAGAGTCTCCACAATCACTTGGCTCCACT
RI-AT5G52310-XLOC_027910-1992-1 -------------------------
TCTCCACAATCACTTGGCTCCACT
CONSENSUS
      ..........................TCTCCACAATCACTTGGCTCCACT

RI-AT5G52310-XLOC_027910-1992-0
      GTTGTTCCGGTGCAGAAGGAGCTTTAAGAATATGAGAACTGAGATTTTCA
RI-AT5G52310-XLOC_027910-1992-1
      GTTGTTCCGGTGCAGAAGGAGCTTTAAGAATATGAGAACTGAGATTTTCA
CONSENSUS
      GTTGTTCCGGTGCAGAAGGAGCTTTAAGAATATGAGAACTGAGATTTTCA

RI-AT5G52310-XLOC_027910-1992-0
      AGTTTCACTTTGGATGTTTATGTGTGTTTTGTTTGACGTCTTTGATGTAT
RI-AT5G52310-XLOC_027910-1992-1
      AGTTTCACTTTGGATGTTTATGTGTGTTTTGTTTGACGTCTTTGATGTAT
CONSENSUS
      AGTTTCACTTTGGATGTTTATGTGTGTTTTGTTTGACGTCTTTGATGTAT

RI-AT5G52310-XLOC_027910-1992-0
      TATGGTATAATTCCTTGTTTGTGTGAAAAAAGGACATTTGGTTAATAAAT
RI-AT5G52310-XLOC_027910-1992-1
      TATGGTATAATTCCTTGTTTGTGTGAAAAAAGGACATTTGGTTAATAAAT
CONSENSUS
      TATGGTATAATTCCTTGTTTGTGTGAAAAAAGGACATTTGGTTAATAAAT

RI-AT5G52310-XLOC_027910-1992-0
      TGTTCGGCTTTGGATTAAGAAGTTCCTCCATACCAGCTACTAGGTCTAAA
RI-AT5G52310-XLOC_027910-1992-1
      TGTTCGGCTTTGGATTAAGAAGTTCCTCCATACCAGCTACTAGGTCTAAA
CONSENSUS
      TGTTCGGCTTTGGATTAAGAAGTTCCTCCATACCAGCTACTAGGTCTAAA

RI-AT5G52310-XLOC_027910-1992-0
      GTGGGTAAAATCATTGGATTTATTCCCTTCAAAGTTCTTAGAATTATTCA
RI-AT5G52310-XLOC_027910-1992-1
      GTGGGTAAAATCATTGGATTTATTCCCTTCAAAGTTCTTAGAATTATTCA
CONSENSUS
      GTGGGTAAAATCATTGGATTTATTCCCTTCAAAGTTCTTAGAATTATTCA

RI-AT5G52310-XLOC_027910-1992-0
      CAGGATTTTACATTATGAGCTAGTAGTGTGACTTGTTGAGGTGTTGTCTC

RI-AT5G52310-XLOC_027910-1992-1
      CAGGATTTTACATTATGAGCTAGTAGTGTGACTTGTTGAGGTGTTGTCTC
CONSENSUS
      CAGGATTTTACATTATGAGCTAGTAGTGTGACTTGTTGAGGTGTTGTCTC

RI-AT5G52310-XLOC_027910-1992-0 TATCGTTAAAGTTC
RI-AT5G52310-XLOC_027910-1992-1 TATCGTTAAAGTTC
CONSENSUS   TATCGTTAAAGTTC

alignment for event: A3-AT5G46490-XLOC_027584-7423

A3-AT5G46490-XLOC_027584-7423-0
      CCGCTTACGCCCTTCCTGGCTATGATGTTGTCTCCCACTTTGACGAGCCA
A3-AT5G46490-XLOC_027584-7423-1
      CCGCTTACGCCCTTCCTGGCTATGATGTTGTCTCCCACTTTGACGAGCCA
CONSENSUS
      CCGCTTACGCCCTTCCTGGCTATGATGTTGTCTCCCACTTTGACGAGCCA

A3-AT5G46490-XLOC_027584-7423-0
      TTGCAGCAGGCTAAAATGTGTATCCCTACACATTTCTAAACTGAAACATC
A3-AT5G46490-XLOC_027584-7423-1
      TTGCAGCAGGCTAAAATGTGTATCCCTACACATTTCTAAACTGAAACATC
CONSENSUS
      TTGCAGCAGGCTAAAATGTGTATCCCTACACATTTCTAAACTGAAACATC

A3-AT5G46490-XLOC_027584-7423-0
      TTGAGGACGCTTTGTTTCCAGCTTGTGGGGCATTAAATCGAGTTGAATTG
A3-AT5G46490-XLOC_027584-7423-1
      TTGAGGACGCTTTGTTTCCAGCTTGTGGGGCATTAAATCGAGTTGAATTG
CONSENSUS
      TTGAGGACGCTTTGTTTCCAGCTTGTGGGGCATTAAATCGAGTTGAATTG

A3-AT5G46490-XLOC_027584-7423-0
      AGTGGTTCTTCTAGTGGGATGAAAGCAGACAATATTGACACAGCCTCCTC
A3-AT5G46490-XLOC_027584-7423-1
      AGTGGTTCTTCTAGTGGGATGAAAGCAGACAATATTGACACAGCCTCCTC
CONSENSUS
      AGTGGTTCTTCTAGTGGGATGAAAGCAGACAATATTGACACAGCCTCCTC

A3-AT5G46490-XLOC_027584-7423-0
      TTCTCTTCCTCAAGTCGAACTTGATTTCAGGGAGTGCTTCAACTTGGATC
A3-AT5G46490-XLOC_027584-7423-1
      TTCTCTTCCTCAAGTCGAACTTGATTTCAGGGAGTGCTTCAACTTGGATC
CONSENSUS
      TTCTCTTCCTCAAGTCGAACTTGATTTCAGGGAGTGCTTCAACTTGGATC

A3-AT5G46490-XLOC_027584-7423-0
      CAGAAACTGTCCTTCACCAAGAATCAATTATATTCAAGTACATGTTATTT
A3-AT5G46490-XLOC_027584-7423-1
      CAGAAACTGTCCTTCACCAAGAATCAATTATATTCAAGTACATGTTATTT
CONSENSUS
      CAGAAACTGTCCTTCACCAAGAATCAATTATATTCAAGTACATGTTATTT

A3-AT5G46490-XLOC_027584-7423-0

CCAGGGAAAGAAGAAGTGCCATCATATTTCACTTACCGTACTACTGGAGT
A3-AT5G46490-XLOC_027584-7423-1
CCAGGGAAAGAAGAAGTGCCATCATATTTCACTTACCGTACTACTGGAGT
CONSENSUS
CCAGGGAAAGAAGAAGTGCCATCATATTTCACTTACCGTACTACTGGAGT

A3-AT5G46490-XLOC_027584-7423-0
CTCCTCTCTGACCATTCCTCTACTTCACCTCCCTCTCTCCCAACCATTCT
A3-AT5G46490-XLOC_027584-7423-1
CTCCTCTCTGACCATTCCTCTACTTCACCTCCCTCTCTCCCAACCATTCT
CONSENSUS
CTCCTCTCTGACCATTCCTCTACTTCACCTCCCTCTCTCCCAACCATTCT

A3-AT5G46490-XLOC_027584-7423-0
TCAGATTTAGGGTTGGCGCATTGGTAACTAATGTTAAGCATGGAAAAAAT
A3-AT5G46490-XLOC_027584-7423-1
TCAGATTTAGGGTTGGCGCATTGGTAACTAATGTTAAGCATGGAAAAAAT
CONSENSUS
TCAGATTTAGGGTTGGCGCATTGGTAACTAATGTTAAGCATGGAAAAAAT

A3-AT5G46490-XLOC_027584-7423-0
ATAAAGGTAAAATGTGAGTTCAAAGACAGATTTGGGAACAGCTTTCATGT
A3-AT5G46490-XLOC_027584-7423-1
ATAAAGGTAAAATGTGAGTTCAAAGACAGATTTGGGAACAGCTTTCATGT
CONSENSUS
ATAAAGGTAAAATGTGAGTTCAAAGACAGATTTGGGAACAGCTTTCATGT

A3-AT5G46490-XLOC_027584-7423-0
TGGCTCGGATGATTTCTACGTTTATCTACTTTTCACGAAAAGTCAGAAGG
A3-AT5G46490-XLOC_027584-7423-1
TGGCTCGGATGATTTCTACGTTTATCTACTTTTCACGAAAAGTCAGAAGG
CONSENSUS
TGGCTCGGATGATTTCTACGTTTATCTACTTTTCACGAAAAGTCAGAAGG

A3-AT5G46490-XLOC_027584-7423-0
GTAGTCAAATGTTAACTATATTGGACTGTTGTATCCCTCTAAACGAAGGT
A3-AT5G46490-XLOC_027584-7423-1
GTAGTCAAATGTTAACTATATTGGACTGTTGTATCCCTCTAAACGAAGGT
CONSENSUS
GTAGTCAAATGTTAACTATATTGGACTGTTGTATCCCTCTAAACGAAGGT

A3-AT5G46490-XLOC_027584-7423-0
AATGCTTCTCTAGCTCAAGGGAACTACTACGATCATGTGGATATAAATAT
A3-AT5G46490-XLOC_027584-7423-1
AATGCTTCTCTAGCTCAAGGGAACTACTACGATCATGTGGATATAAATAT
CONSENSUS
AATGCTTCTCTAGCTCAAGGGAACTACTACGATCATGTGGATATAAATAT

A3-AT5G46490-XLOC_027584-7423-0
TCATATAACAGAATCTCTTGGATCTTTTGGATCTACGTCTGAATTAAAAG
A3-AT5G46490-XLOC_027584-7423-1
TCATATAACAGAATCTCTTGGATCTTTTGGATCTACGTCTGAATTAAAAG
CONSENSUS
TCATATAACAGAATCTCTTGGATCTTTTGGATCTACGTCTGAATTAAAAG

A3-AT5G46490-XLOC_027584-7423-0

AATGGGGTATACGACTCTTAGAGGAAGACTCTTCATCAGCAGAGAACCAA
A3-AT5G46490-XLOC_027584-7423-1
AATGGGGTATACGACTCTTAGAGGAAGACTCTTCATCAGCAGAGAACCAA
CONSENSUS
AATGGGGTATACGACTCTTAGAGGAAGACTCTTCATCAGCAGAGAACCAA

A3-AT5G46490-XLOC_027584-7423-0
CTTGGTAATCCGAACAGTACTCTTCCACATGTTTCTGAAGCCGAAGAAGG
A3-AT5G46490-XLOC_027584-7423-1
CTTGGTAATCCGAACAGTACTCTTCCACATGTTTCTGAAGCCGAAGAAGG
CONSENSUS
CTTGGTAATCCGAACAGTACTCTTCCACATGTTTCTGAAGCCGAAGAAGG

A3-AT5G46490-XLOC_027584-7423-0
CAATATGGGGTATTATACACCTGTTCAAGGACTTGTTAATGAGATTGAAC
A3-AT5G46490-XLOC_027584-7423-1
CAATATGGGGTATTATACACCTGTTCAAGGACTTGTTAATGAGATTGAAC
CONSENSUS
CAATATGGGGTATTATACACCTGTTCAAGGACTTGTTAATGAGATTGAAC

A3-AT5G46490-XLOC_027584-7423-0
ACAATGGAGAGTCTGGAGATAACAATGTAGAGACTGAGAGAAGCACGAAG
A3-AT5G46490-XLOC_027584-7423-1
ACAATGGAGAGTCTGGAGATAACAATGTAGAGACTGAGAGAAGCACGAAG
CONSENSUS
ACAATGGAGAGTCTGGAGATAACAATGTAGAGACTGAGAGAAGCACGAAG

A3-AT5G46490-XLOC_027584-7423-0
CATGCGGCTCAACGGAACTACTACGATCATGTGGATATAGATATTGAGCA
A3-AT5G46490-XLOC_027584-7423-1
CATGCGG---------------------------------ATATTGAGCA
CONSENSUS
CATGCGG.................................ATATTGAGCA

A3-AT5G46490-XLOC_027584-7423-0
AATGGATAGTGATGATGATATTGAAGAATGGGGTAGACACTTAGAGGACT
A3-AT5G46490-XLOC_027584-7423-1
AATGGATAGTGATGATGATATTGAAGAATGGGGTAGACACTTAGAGGACT
CONSENSUS
AATGGATAGTGATGATGATATTGAAGAATGGGGTAGACACTTAGAGGACT

A3-AT5G46490-XLOC_027584-7423-0
GTCATCAGCGAAGAATCGACATGGTAATCCAAACACTATGCAGTATGTTT
A3-AT5G46490-XLOC_027584-7423-1
GTCATCAGCGAAGAATCGACATGGTAATCCAAACACTATGCAGTATGTTT
CONSENSUS
GTCATCAGCGAAGAATCGACATGGTAATCCAAACACTATGCAGTATGTTT

A3-AT5G46490-XLOC_027584-7423-0
GTGAAGCCGATGAAGACAATGATGGATGCCATTAGACTGATCACAACGAA
A3-AT5G46490-XLOC_027584-7423-1
GTGAAGCCGATGAAGACAATGATGGATGCCATTAGACTGATCACAACGAA
CONSENSUS
GTGAAGCCGATGAAGACAATGATGGATGCCATTAGACTGATCACAACGAA

A3-AT5G46490-XLOC_027584-7423-0

GAGCGAGGAGACAGTGATGAATAGTGATATAGGCAGGGAGACTGATCACT
A3-AT5G46490-XLOC_027584-7423-1
GAGCGAGGAGACAGTGATGAATAGTGATATAGGCAGGGAGACTGATCACT
CONSENSUS
GAGCGAGGAGACAGTGATGAATAGTGATATAGGCAGGGAGACTGATCACT

A3-AT5G46490-XLOC_027584-7423-0
TTGAAGAGTGCGAAGATAGTGATATAAGCAATGAGAGTGATCAGAGTGAA
A3-AT5G46490-XLOC_027584-7423-1
TTGAAGAGTGCGAAGATAGTGATATAAGCAATGAGAGTGATCAGAGTGAA
CONSENSUS
TTGAAGAGTGCGAAGATAGTGATATAAGCAATGAGAGTGATCAGAGTGAA

A3-AT5G46490-XLOC_027584-7423-0
GAGCGTGGGGATAGTGACGATGATGGTATAAACAATGAGACTGATCTGTT
A3-AT5G46490-XLOC_027584-7423-1
GAGCGTGGGGATAGTGACGATGATGGTATAAACAATGAGACTGATCTGTT
CONSENSUS
GAGCGTGGGGATAGTGACGATGATGGTATAAACAATGAGACTGATCTGTT

A3-AT5G46490-XLOC_027584-7423-0
CGAAGAGTGTGGAAACAGTGATTAGGCAATGAGCTTGATCACAATGAAGA
A3-AT5G46490-XLOC_027584-7423-1
CGAAGAGTGTGGAAACAGTGATTAGGCAATGAGCTTGATCACAATGAAGA
CONSENSUS
CGAAGAGTGTGGAAACAGTGATTAGGCAATGAGCTTGATCACAATGAAGA

A3-AT5G46490-XLOC_027584-7423-0
GAGTTGAGACAGAGAGAACCAATACACAATACAAG
A3-AT5G46490-XLOC_027584-7423-1
GAGTTGAGACAGAGAGAACCAATACACAATACAAG
CONSENSUS
GAGTTGAGACAGAGAGAACCAATACACAATACAAG

alignment for event: A3-AT5G61910-XLOC_032083-2850

A3-AT5G61910-XLOC_032083-2850-0
CGTGACCTTCAGCTTCCTTCTCTGAAAGTCATCTTCTTCTCCCGACTTTT
A3-AT5G61910-XLOC_032083-2850-1
CGTGACCTTCAGCTTCCTTCTCTGAAAGTCATCTTCTTCTCCCGACTTTT
CONSENSUS
CGTGACCTTCAGCTTCCTTCTCTGAAAGTCATCTTCTTCTCCCGACTTTT

A3-AT5G61910-XLOC_032083-2850-0
TTGAACAAGGGAAGAAGAATCCAACTTCATCAATTCAGATTCGCACGCTT
A3-AT5G61910-XLOC_032083-2850-1
TTGAACAAGGGAAGAAGAATCCAACTTCATCAATTCAGATTCGCACGCTT
CONSENSUS
TTGAACAAGGGAAGAAGAATCCAACTTCATCAATTCAGATTCGCACGCTT

A3-AT5G61910-XLOC_032083-2850-0
GAAATTATCTGAATAAAAATCGAAAGCTAATAATGAGCTGATCGCTAAAA
A3-AT5G61910-XLOC_032083-2850-1
GAAATTATCTGAATAAAAATCGAAAGCTAATAATGAGCTGATCGCTAAAA

CONSENSUS
GAAATTATCTGAATAAAAATCGAAAGCTAATAATGAGCTGATCGCTAAAA

A3-AT5G61910-XLOC_032083-2850-0
ATATCTCTCGAATTCAATCTTCCTGATTTTCAATTTCTGTCTCTAGGGTT
A3-AT5G61910-XLOC_032083-2850-1
ATATCTCTCGAATTCAATCTTCCTGATTTTCAATTTCTGTCTCTAGGGTT
CONSENSUS
ATATCTCTCGAATTCAATCTTCCTGATTTTCAATTTCTGTCTCTAGGGTT

A3-AT5G61910-XLOC_032083-2850-0
TCAATATCCGATAA------------------------------------
A3-AT5G61910-XLOC_032083-2850-1
TCAATATCCGATAATGTCATCTTCGTACTTGTACTGAAATGCATTGAAAA
CONSENSUS
TCAATATCCGATAA....................................

A3-AT5G61910-XLOC_032083-2850-0 ------
AAGCGATGGAGACGGAGATGGATTTCTCAGATGGTGAACAAACT
A3-AT5G61910-XLOC_032083-2850-1
TGGCAGAAGCGATGGAGACGGAGATGGATTTCTCAGATGGTGAACAAACT
CONSENSUS
......AAGCGATGGAGACGGAGATGGATTTCTCAGATGGTGAACAAACT

A3-AT5G61910-XLOC_032083-2850-0
AATGGTAATTCACATGTTACTGCCTCCCAATACTTTGCTCCTCCGGGTTA
A3-AT5G61910-XLOC_032083-2850-1
AATGGTAATTCACATGTTACTGCCTCCCAATACTTTGCTCCTCCGGGTTA
CONSENSUS
AATGGTAATTCACATGTTACTGCCTCCCAATACTTTGCTCCTCCGGGTTA

A3-AT5G61910-XLOC_032083-2850-0
TAATAGAAGTCTTGTGGCTGCTTATGGGAATGGAAACACAACCATTGGAT
A3-AT5G61910-XLOC_032083-2850-1
TAATAGAAGTCTTGTGGCTGCTTATGGGAATGGAAACACAACCATTGGAT
CONSENSUS
TAATAGAAGTCTTGTGGCTGCTTATGGGAATGGAAACACAACCATTGGAT

A3-AT5G61910-XLOC_032083-2850-0
TAGAAAAAGGAATAGAAAGGAGACTAGATCATCATGAGCAACTACCTGGT
A3-AT5G61910-XLOC_032083-2850-1
TAGAAAAAGGAATAGAAAGGAGACTAGATCATCATGAGCAACTACCTGGT
CONSENSUS
TAGAAAAAGGAATAGAAAGGAGACTAGATCATCATGAGCAACTACCTGGT

A3-AT5G61910-XLOC_032083-2850-0
TATATCTTTATGTGTAATGGGAGAACGAAAACCGATTGCTACCGTTACCG
A3-AT5G61910-XLOC_032083-2850-1
TATATCTTTATGTGTAATGGGAGAACGAAAACCGATTGCTACCGTTACCG
CONSENSUS
TATATCTTTATGTGTAATGGGAGAACGAAAACCGATTGCTACCGTTACCG

A3-AT5G61910-XLOC_032083-2850-0
TGTTTTCGGGATTCCAAGAGGAGGAAAAGACGTTGTTGAGAGTATAAAGC
A3-AT5G61910-XLOC_032083-2850-1
TGTTTTCGGGATTCCAAGAGGAGGAAAAGACGTTGTTGAGAGTATAAAGC

CONSENSUS
      TGTTTTCGGGATTCCAAGAGGAGGAAAAGACGTTGTTGAGAGTATAAAGC

A3-AT5G61910-XLOC_032083-2850-0
      CAGGCATGAAGCTTTTCCTCTATGACTTTGAGAAGAGACTTCTTTATGGT
A3-AT5G61910-XLOC_032083-2850-1
      CAGGCATGAAGCTTTTCCTCTATGACTTTGAGAAGAGACTTCTTTATGGT
CONSENSUS
      CAGGCATGAAGCTTTTCCTCTATGACTTTGAGAAGAGACTTCTTTATGGT

A3-AT5G61910-XLOC_032083-2850-0
      GTGTATGAAGCTACTGTTGGTGGCAGGTTGGATATAGAACCTGAAGCTTT
A3-AT5G61910-XLOC_032083-2850-1
      GTGTATGAAGCTACTGTTGGTGGCAGGTTGGATATAGAACCTGAAGCTTT
CONSENSUS
      GTGTATGAAGCTACTGTTGGTGGCAGGTTGGATATAGAACCTGAAGCTTT

A3-AT5G61910-XLOC_032083-2850-0 TGAAGGGAAATATCCAGCTCAA
A3-AT5G61910-XLOC_032083-2850-1 TGAAGGGAAATATCCAGCTCAA
CONSENSUS   TGAAGGGAAATATCCAGCTCAA

alignment for event: RI-AT5G53900-XLOC_031651-6827

RI-AT5G53900-XLOC_031651-6827-0
      GATGTTGATGTGGGAAGATGGGTTTTGTGGTGGAGGAAGAAGTGAAGATC
RI-AT5G53900-XLOC_031651-6827-1
      GATGTTGATGTGGGAAGATGGGTTTTGTGGTGGAGGAAGAAGTGAAGATC
CONSENSUS
      GATGTTGATGTGGGAAGATGGGTTTTGTGGTGGAGGAAGAAGTGAAGATC

RI-AT5G53900-XLOC_031651-6827-0
      TTTGTCTGGAAACAGACATTGAAGGTCATGAAGAAGATCTTGTCAGAAAA
RI-AT5G53900-XLOC_031651-6827-1
      TTTGTCTGGAAACAGACATTGAAGGTCATGAAGAAGATCTTGTCAGAAAA
CONSENSUS
      TTTGTCTGGAAACAGACATTGAAGGTCATGAAGAAGATCTTGTCAGAAAA

RI-AT5G53900-XLOC_031651-6827-0
      GCTTTCAGCAAGATGTCCATTCAGTTATATAATTATGGAGAAGGGTGGGT
RI-AT5G53900-XLOC_031651-6827-1
      GCTTTCAGCAAGATGTCCATTCAGTTATATAATTATGGAGAAGG------
CONSENSUS
      GCTTTCAGCAAGATGTCCATTCAGTTATATAATTATGGAGAAGG......

RI-AT5G53900-XLOC_031651-6827-0
      ACTGGGTAGGTGTTATATATAAGTAATTATGGACCAAAAGATAATTAGAT
RI-AT5G53900-XLOC_031651-6827-1

CONSENSUS
      ....

RI-AT5G53900-XLOC_031651-6827-0
      TATATAAACACACCCTAATCACTTAACTTATGATTAAAGATATAATTATT
RI-AT5G53900-XLOC_031651-6827-1

CONSENSUS
....

RI-AT5G53900-XLOC_031651-6827-0
TACAACACAAGTTATTATGATCTTGTGATGTTCATGTGATATATCTGCAG
RI-AT5G53900-XLOC_031651-6827-1

CONSENSUS
....

RI-AT5G53900-XLOC_031651-6827-0
ATTGATGGGTAAAGTTGCTTCTGATAAATGTCACAAGTGGGTTTTCAAAG
RI-AT5G53900-XLOC_031651-6827-1
ATTGATGGGTAAAGTTGCTTCTGATAAATGTCACAAGTGGGTTTTCAAAG
CONSENSUS
ATTGATGGGTAAAGTTGCTTCTGATAAATGTCACAAGTGGGTTTTCAAAG

RI-AT5G53900-XLOC_031651-6827-0
AACCATCTGAATCTGAACCAAATCTTGCTAATTACTGGCAAAGTTCTTTT
RI-AT5G53900-XLOC_031651-6827-1
AACCATCTGAATCTGAACCAAATCTTGCTAATTACTGGCAAAGTTCTTTT
CONSENSUS
AACCATCTGAATCTGAACCAAATCTTGCTAATTACTGGCAAAGTTCTTTT

RI-AT5G53900-XLOC_031651-6827-0 GATGCT
RI-AT5G53900-XLOC_031651-6827-1 GATGCT
CONSENSUS GATGCT

alignment for event: RI-AT5G22690-XLOC_026204-13495

RI-AT5G22690-XLOC_026204-13495-0
CCGCTTACATGCCTCAGGGAGATGCAGTTGTGGGGATCAAAAAAACTGAA
RI-AT5G22690-XLOC_026204-13495-1
CCGCTTACATGCCTCAGGGAGATGCAGTTGTGGGGATCAAAAAAACTGAA
CONSENSUS
CCGCTTACATGCCTCAGGGAGATGCAGTTGTGGGGATCAAAAAAACTGAA

RI-AT5G22690-XLOC_026204-13495-0
AGAAATCCCAGATCTCTCGTTGGCCACCAATCTTGAAACACTTTATCTCA
RI-AT5G22690-XLOC_026204-13495-1
AGAAATCCCAGATCTCTCGTTGGCCACCAATCTTGAAACACTTTATCTCA
CONSENSUS
AGAAATCCCAGATCTCTCGTTGGCCACCAATCTTGAAACACTTTATCTCA

RI-AT5G22690-XLOC_026204-13495-0
ACGATTGCTCGAGTTTAGTGGAGCTTCCTTCCTCTATCAAGAATCTGAAT
RI-AT5G22690-XLOC_026204-13495-1
ACGATTGCTCGAGTTTAGTGGAGCTTCCTTCCTCTATCAAGAATCTGAAT
CONSENSUS
ACGATTGCTCGAGTTTAGTGGAGCTTCCTTCCTCTATCAAGAATCTGAAT

RI-AT5G22690-XLOC_026204-13495-0
AAACTGTGGGACTTGGGTATGAAAGGATGTGAAAAGCTGGAGCTTCTTCC

RI-AT5G22690-XLOC_026204-13495-1
AAACTGTGGGACTTGGGTATGAAAGGATGTGAAAAGCTGGAGCTTCTTCC
CONSENSUS
AAACTGTGGGACTTGGGTATGAAAGGATGTGAAAAGCTGGAGCTTCTTCC

RI-AT5G22690-XLOC_026204-13495-0
AACCGACATCAACCTCAAATCTCTCTATCGCCTTGATCTTGGTAGATGCT
RI-AT5G22690-XLOC_026204-13495-1
AACCGACATCAACCTCAAATCTCTCTATCGCCTTGATCTTGGTAGATGCT
CONSENSUS
AACCGACATCAACCTCAAATCTCTCTATCGCCTTGATCTTGGTAGATGCT

RI-AT5G22690-XLOC_026204-13495-0
CACGGTTGAAGAGTTTTCCTGATATCTCAAGTAACATTTCAGAGCTCTAT
RI-AT5G22690-XLOC_026204-13495-1
CACGGTTGAAGAGTTTTCCTGATATCTCAAGTAACATTTCAGAGCTCTAT
CONSENSUS
CACGGTTGAAGAGTTTTCCTGATATCTCAAGTAACATTTCAGAGCTCTAT

RI-AT5G22690-XLOC_026204-13495-0
CTGAATCGAACAGCGATTGAAGAAGTTCCTTGGTGGATCCAGAAATTCTC
RI-AT5G22690-XLOC_026204-13495-1
CTGAATCGAACAGCGATTGAAGAAGTTCCTTGGTGGATCCAGAAATTCTC
CONSENSUS
CTGAATCGAACAGCGATTGAAGAAGTTCCTTGGTGGATCCAGAAATTCTC

RI-AT5G22690-XLOC_026204-13495-0
CAGGCTTAAACGTTTACGTATGAGGGAATGCAAAAAGCTCAAATGTATAT
RI-AT5G22690-XLOC_026204-13495-1
CAGGCTTAAACGTTTACGTATGAGGGAATGCAAAAAGCTCAAATGTATAT
CONSENSUS
CAGGCTTAAACGTTTACGTATGAGGGAATGCAAAAAGCTCAAATGTATAT

RI-AT5G22690-XLOC_026204-13495-0
CTCCAAACATCTCCAAACTGAAACATCTTGAGATGCTCGACTTTTCAAAC
RI-AT5G22690-XLOC_026204-13495-1
CTCCAAACATCTCCAAACTGAAACATCTTGAGATGCTCGACTTTTCAAAC
CONSENSUS
CTCCAAACATCTCCAAACTGAAACATCTTGAGATGCTCGACTTTTCAAAC

RI-AT5G22690-XLOC_026204-13495-0
TGCATAGCAACTACAGAAGAAGAAGCTCTTGTTCAACAACAATCAGTTTT
RI-AT5G22690-XLOC_026204-13495-1
TGCATAGCAACTACAGAAGAAGAAGCTCTTGTTCAACAACAATCAGTTTT
CONSENSUS
TGCATAGCAACTACAGAAGAAGAAGCTCTTGTTCAACAACAATCAGTTTT

RI-AT5G22690-XLOC_026204-13495-0
GAAGTATCTCATATTTCCAGGAGGACAAGTGCCTTTGTATTTCACTTACC
RI-AT5G22690-XLOC_026204-13495-1
GAAGTATCTCATATTTCCAGGAGGACAAGTGCCTTTGTATTTCACTTACC
CONSENSUS
GAAGTATCTCATATTTCCAGGAGGACAAGTGCCTTTGTATTTCACTTACC

RI-AT5G22690-XLOC_026204-13495-0
AAGCTACTGGAAGCTCTTTGGCCATCCCTTTGTCATTACATCAGAGCTCT

RI-AT5G22690-XLOC_026204-13495-1
      AAGCTACTGGAAGCTCTTTGGCCATCCCTTTGTCATTACATCAGAGCTCT
CONSENSUS
      AAGCTACTGGAAGCTCTTTGGCCATCCCTTTGTCATTACATCAGAGCTCT

RI-AT5G22690-XLOC_026204-13495-0
      CTCTCTCAACAATTATTGGGATTTAGGGCTTGCGTCGTGCTTGATGCTGA
RI-AT5G22690-XLOC_026204-13495-1
      CTCTCTCAACAATTATTGGGATTTAGGGCTTGCGTCGTGCTTGATGCTGA
CONSENSUS
      CTCTCTCAACAATTATTGGGATTTAGGGCTTGCGTCGTGCTTGATGCTGA

RI-AT5G22690-XLOC_026204-13495-0
      ATCTATGTCCTCCGAGTTATACGTAATTGATATCAAGGTATGTTGTCGCT
RI-AT5G22690-XLOC_026204-13495-1
      ATCTATGTCCTCCGAGTTATACGTAATTGATATCAAG-------------
CONSENSUS
      ATCTATGTCCTCCGAGTTATACGTAATTGATATCAAG.............

RI-AT5G22690-XLOC_026204-13495-0
      TGAGTGGTAAACGCAGCAACCTCTTTGATTCCGCTGACTGTCGGGATGCC
RI-AT5G22690-XLOC_026204-13495-1

CONSENSUS
      ....

RI-AT5G22690-XLOC_026204-13495-0
      TTCTTTACACCTCAGATGGATAGTCATTTGGTTATATTTGATTGTTGTTT
RI-AT5G22690-XLOC_026204-13495-1

CONSENSUS
      ....

RI-AT5G22690-XLOC_026204-13495-0
      CCCTCTAAACCAAGATAACGTTCGTCTAGCTGAACTGAACAATGATAAGG
RI-AT5G22690-XLOC_026204-13495-1

CONSENSUS
      ....

RI-AT5G22690-XLOC_026204-13495-0
      TGGTCACAGAGTTTCATTTCACTAGTATTTCTCGCTGCAAAATAACAGGA
RI-AT5G22690-XLOC_026204-13495-1

CONSENSUS
      ....

RI-AT5G22690-XLOC_026204-13495-0
      GTCGGTGTACGCTTCCTCAGGGACTGTTCATTACCAGAGAACCATCATAA
RI-AT5G22690-XLOC_026204-13495-1 --------------------
GGACTGTTCATTACCAGAGAACCATCATAA
CONSENSUS
      ....................GGACTGTTCATTACCAGAGAACCATCATAA

RI-AT5G22690-XLOC_026204-13495-0
      TGATCCAAATATTCTTGCACCTAATTGTGGATGCCCTGAGACTGAACACT

RI-AT5G22690-XLOC_026204-13495-1
      TGATCCAAATATTCTTGCACCTAATTGTGGATGCCCTGAGACTGAACACT
CONSENSUS
      TGATCCAAATATTCTTGCACCTAATTGTGGATGCCCTGAGACTGAACACT

RI-AT5G22690-XLOC_026204-13495-0
      CTGATGAGTATGGAGAATTCGGTGTAGAGACGAAAAGAAGTAGGAAGCGA
RI-AT5G22690-XLOC_026204-13495-1
      CTGATGAGTATGGAGAATTCGGTGTAGAGACGAAAAGAAGTAGGAAGCGA
CONSENSUS
      CTGATGAGTATGGAGAATTCGGTGTAGAGACGAAAAGAAGTAGGAAGCGA

RI-AT5G22690-XLOC_026204-13495-0 AAGCGG
RI-AT5G22690-XLOC_026204-13495-1 AAGCGG
CONSENSUS AAGCGG

alignment for event: A3-AT5G16300-XLOC_025900-9397

A3-AT5G16300-XLOC_025900-9397-0
      GTACGAGCCTTATCTTTGGGAGAACGAAAAGCAGTCGTACCTACGCCATG
A3-AT5G16300-XLOC_025900-9397-1
      GTACGAGCCTTATCTTTGGGAGAACGAAAAGCAGTCGTACCTACGCCATG
CONSENSUS
      GTACGAGCCTTATCTTTGGGAGAACGAAAAGCAGTCGTACCTACGCCATG

A3-AT5G16300-XLOC_025900-9397-0
      CTGTACTCTTTGGTTTCTTTGTGCAACTAAACCGAATGTACACAGATACT
A3-AT5G16300-XLOC_025900-9397-1
      CTGTACTCTTTGGTTTCTTTGTGCAACTAAACCGAATGTACACAGATACT
CONSENSUS
      CTGTACTCTTTGGTTTCTTTGTGCAACTAAACCGAATGTACACAGATACT

A3-AT5G16300-XLOC_025900-9397-0
      GCGCAGAAACTATCAATTAATATAGAATCAAATATCATGCCATGTTCCAC
A3-AT5G16300-XLOC_025900-9397-1
      GCGCAGAAACTATCAATTAATATAGAATCAAATATCATGCCATGTTCCAC
CONSENSUS
      GCGCAGAAACTATCAATTAATATAGAATCAAATATCATGCCATGTTCCAC

A3-AT5G16300-XLOC_025900-9397-0
      AGTTCCCCGCTTCAAATACCTTCCCATAAGTTTCTGATATCAGAAGTCTT
A3-AT5G16300-XLOC_025900-9397-1
      AGTTCCCCGCTTCAAATACCTTCCCATAAG--------------------
CONSENSUS
      AGTTCCCCGCTTCAAATACCTTCCCATAAG....................

A3-AT5G16300-XLOC_025900-9397-0
      CGTTTCGGGTCAAATGCAGCGCTCCAGCTCTGTCATCTAGAAGTACAAAT
A3-AT5G16300-XLOC_025900-9397-1 -------------------
CGCTCCAGCTCTGTCATCTAGAAGTACAAAT
CONSENSUS
      ...................CGCTCCAGCTCTGTCATCTAGAAGTACAAAT

A3-AT5G16300-XLOC_025900-9397-0

AAGGTCTCTATTCCAGTTACATCAAATGATGCTTCGGCAAGAAACTCATG
A3-AT5G16300-XLOC_025900-9397-1
AAGGTCTCTATTCCAGTTACATCAAATGATGCTTCGGCAAGAAACTCATG
CONSENSUS
AAGGTCTCTATTCCAGTTACATCAAATGATGCTTCGGCAAGAAACTCATG

A3-AT5G16300-XLOC_025900-9397-0
GAAGGCATTTACAAACGGCGAGCAATCTCAAACAAGTGATTTAGAAGAAA
A3-AT5G16300-XLOC_025900-9397-1
GAAGGCATTTACAAACGGCGAGCAATCTCAAACAAGTGATTTAGAAGAAA
CONSENSUS
GAAGGCATTTACAAACGGCGAGCAATCTCAAACAAGTGATTTAGAAGAAA

A3-AT5G16300-XLOC_025900-9397-0
ATTCCAATTTCGGCGTGGCTTTTAAGTCTTTCATGCAGGTAAAG
A3-AT5G16300-XLOC_025900-9397-1
ATTCCAATTTCGGCGTGGCTTTTAAGTCTTTCATGCAGGTAAAG
CONSENSUS
ATTCCAATTTCGGCGTGGCTTTTAAGTCTTTCATGCAGGTAAAG

alignment for event: RI-AT5G37590-XLOC_030720-10980

RI-AT5G37590-XLOC_030720-10980-0
GATAGCAAAGGACGTTCTGCATAAGCTTAAAAACCAAAAGAGCAAAGCGC
RI-AT5G37590-XLOC_030720-10980-1
GATAGCAAAGGACGTTCTGCATAAGCTTAAAAACCAAAAGAGCAAAGCGC
CONSENSUS
GATAGCAAAGGACGTTCTGCATAAGCTTAAAAACCAAAAGAGCAAAGCGC

RI-AT5G37590-XLOC_030720-10980-0
AGAAAGACGAAAAATCCAGTGCAGCTCTTAGGAATTATGAACACGCGGCT
RI-AT5G37590-XLOC_030720-10980-1
AGAAAGACGAAAAATCCAGTGCAGCTCTTAGGAATTATGAACACGCGGCT
CONSENSUS
AGAAAGACGAAAAATCCAGTGCAGCTCTTAGGAATTATGAACACGCGGCT

RI-AT5G37590-XLOC_030720-10980-0
TTAGTCATACTGGTAAGTCTCTATAGAAGCAATACTTGGGAATTATATGC
RI-AT5G37590-XLOC_030720-10980-1
TTAGTCATACTG--------------------------------------
CONSENSUS
TTAGTCATACTG......................................

RI-AT5G37590-XLOC_030720-10980-0
TCTTAAGAACAATTCGTTTGGGACCTTTTCTATTCGTTGCAGCTGCAATC
RI-AT5G37590-XLOC_030720-10980-1
--CTGCAATC
CONSENSUS
....CTGCAATC

RI-AT5G37590-XLOC_030720-10980-0
TCTTGAGAGTCTTGCAGCTTTGGAGATGAGCAAAAATGAGATCCATGAGC
RI-AT5G37590-XLOC_030720-10980-1
TCTTGAGAGTCTTGCAGCTTTGGAGATGAGCAAAAATGAGATCCATGAGC

CONSENSUS
      TCTTGAGAGTCTTGCAGCTTTGGAGATGAGCAAAAATGAGATCCATGAGC

RI-AT5G37590-XLOC_030720-10980-0 CAAAG
RI-AT5G37590-XLOC_030720-10980-1 CAAAG
CONSENSUS   CAAAG

alignment for event: A3-AT5G14550-XLOC_029473-3497

A3-AT5G14550-XLOC_029473-3497-0
      GTAGATTGGGGTGAATCAACCATGATTGAAGCAGAACGTGTATTGCTTAG
A3-AT5G14550-XLOC_029473-3497-1
      GTAGATTGGGGTGAATCAACCATGATTGAAGCAGAACGTGTATTGCTTAG
CONSENSUS
      GTAGATTGGGGTGAATCAACCATGATTGAAGCAGAACGTGTATTGCTTAG

A3-AT5G14550-XLOC_029473-3497-0
      ACATGCACTTAGAGATTCATTTAATCACCGCTTTGTTTTTCTTTCTGATA
A3-AT5G14550-XLOC_029473-3497-1
      ACATGCACTTAGAGATTCATTTAATCACCGCTTTGTTTTTCTTTCTGATA
CONSENSUS
      ACATGCACTTAGAGATTCATTTAATCACCGCTTTGTTTTTCTTTCTGATA

A3-AT5G14550-XLOC_029473-3497-0 G--------------------
CTGCATACCTCTGTACAGTTTCAGCTACA
A3-AT5G14550-XLOC_029473-3497-1
      GTTTTTTTGGTTTCTTCTCAGCTGCATACCTCTGTACAGTTTCAGCTACA
CONSENSUS
      G....................CTGCATACCTCTGTACAGTTTCAGCTACA

A3-AT5G14550-XLOC_029473-3497-0
      CGTATAACTACATCATGTCAACACCAACTAGTTTCGTTGATAG
A3-AT5G14550-XLOC_029473-3497-1
      CGTATAACTACATCATGTCAACACCAACTAGTTTCGTTGATAG
CONSENSUS
      CGTATAACTACATCATGTCAACACCAACTAGTTTCGTTGATAG

alignment for event: SE-AT5G52070-XLOC_027898-13817

SE-AT5G52070-XLOC_027898-13817-0
      TTCTCATCTTGGAAAAAAAAATGACTCAAATCAATTTGCCCTTCCAAATT
SE-AT5G52070-XLOC_027898-13817-1
      TTCTCATCTTGGAAAAAAAAATGACTCAAATCAATTTGCCCTTCCAAATT
CONSENSUS
      TTCTCATCTTGGAAAAAAAAATGACTCAAATCAATTTGCCCTTCCAAATT

SE-AT5G52070-XLOC_027898-13817-0
      GGGCAAATTGTGGAGGTTAAATCTTTTATCAGAGGATACCGTGGAGCTTG
SE-AT5G52070-XLOC_027898-13817-1
      GGGCAAATTGTGGAGGTTAAATCTTTTATCAGAGGATACCGTGGAGCTTG
CONSENSUS
      GGGCAAATTGTGGAGGTTAAATCTTTTATCAGAGGATACCGTGGAGCTTG

```
SE-AT5G52070-XLOC_027898-13817-0
      GTTTCGATGCAAG-------------------------------------
SE-AT5G52070-XLOC_027898-13817-1
      GTTTCGATGCAAGATATTGGATATAAGCAATGAAAAGGGTGAACTAAGAT
CONSENSUS
      GTTTCGATGCAAG.....................................

SE-AT5G52070-XLOC_027898-13817-0
      ------------------------------------CTTTAGATAATGCACTG
SE-AT5G52070-XLOC_027898-13817-1
      ACAAGTTTAAGTATCTCGACTTTCCAGATGAACCTTTAGATAATGCACTG
CONSENSUS
      .................................CTTTAGATAATGCACTG

SE-AT5G52070-XLOC_027898-13817-0
      GTCTTTGAAACACCTAAAGGTGGGACAGAAAGTCAGCTAATGCTTCGTCC
SE-AT5G52070-XLOC_027898-13817-1
      GTCTTTGAAACACCTAAAGGTGGGACAGAAAGTCAGCTAATGCTTCGTCC
CONSENSUS
      GTCTTTGAAACACCTAAAGGTGGGACAGAAAGTCAGCTAATGCTTCGTCC

SE-AT5G52070-XLOC_027898-13817-0
      AAAATATCCACCACTTTATCTTGAAAGTGAATATCTTAATCTCGAGAGTG
SE-AT5G52070-XLOC_027898-13817-1
      AAAATATCCACCACTTTATCTTGAAAGTGAATATCTTAATCTCGAGAGTG
CONSENSUS
      AAAATATCCACCACTTTATCTTGAAAGTGAATATCTTAATCTCGAGAGTG

SE-AT5G52070-XLOC_027898-13817-0
      ACAACGTAGAACCTCTTGTCGTCGTTCATGATTCTTGGAAAGTTGGAGAT
SE-AT5G52070-XLOC_027898-13817-1
      ACAACGTAGAACCTCTTGTCGTCGTTCATGATTCTTGGAAAGTTGGAGAT
CONSENSUS
      ACAACGTAGAACCTCTTGTCGTCGTTCATGATTCTTGGAAAGTTGGAGAT

SE-AT5G52070-XLOC_027898-13817-0
      TTAGTTGATTGGTTGAGAGATGACATTTATTGGTCTGGAGAAATTGTGGA
SE-AT5G52070-XLOC_027898-13817-1
      TTAGTTGATTGGTTGAGAGATGACATTTATTGGTCTGGAGAAATTGTGGA
CONSENSUS
      TTAGTTGATTGGTTGAGAGATGACATTTATTGGTCTGGAGAAATTGTGGA

SE-AT5G52070-XLOC_027898-13817-0 AATGAGAGGTAGGAGAGCATGTCAG
SE-AT5G52070-XLOC_027898-13817-1 AATGAGAGGTAGGAGAGCATGTCAG
CONSENSUS                        AATGAGAGGTAGGAGAGCATGTCAG


alignment for event: RI-AT5G02880-XLOC_025216-3047

RI-AT5G02880-XLOC_025216-3047-0
      GTAAATTTGGATAACCTCGAGGAATATATCAAGGGTATTGTCAATGCCAC
RI-AT5G02880-XLOC_025216-3047-1
      GTAAATTTGGATAACCTCGAGGAATATATCAAGGGTATTGTCAATGCCAC
CONSENSUS
```

GTAAATTTGGATAACCTCGAGGAATATATCAAGGGTATTGTCAATGCCAC

RI-AT5G02880-XLOC_025216-3047-0
AGTATGTAATGGGATCCAAAAACAAGTGGAAGCATTTCGGTCTGGATTTA
RI-AT5G02880-XLOC_025216-3047-1
AGTATGTAATGGGATCCAAAAACAAGTGGAAGCATTTCGGTCTGGATTTA
CONSENSUS
AGTATGTAATGGGATCCAAAAACAAGTGGAAGCATTTCGGTCTGGATTTA

RI-AT5G02880-XLOC_025216-3047-0
ATCAGGTAAATTCTTTAGCTTTACTCTTACCAATAGATTTAACTATAGAT
RI-AT5G02880-XLOC_025216-3047-1
ATCAG---
CONSENSUS
ATCAG...

RI-AT5G02880-XLOC_025216-3047-0
TAAATCACTCCTTCTTTTATGCATCCTTCTTCAGGTTTTCTCTATTGAAC
RI-AT5G02880-XLOC_025216-3047-1
----------------------------------GTTTTCTCTATTGAAC
CONSENSUS
..................................GTTTTCTCTATTGAAC

RI-AT5G02880-XLOC_025216-3047-0
ATCTTCGGATATTCAACGAAGAGGAGCTGGAAACTATGCTGTGTGGAGAA
RI-AT5G02880-XLOC_025216-3047-1
ATCTTCGGATATTCAACGAAGAGGAGCTGGAAACTATGCTGTGTGGAGAA
CONSENSUS
ATCTTCGGATATTCAACGAAGAGGAGCTGGAAACTATGCTGTGTGGAGAA

RI-AT5G02880-XLOC_025216-3047-0 TGTGATCTCTTTAGT
RI-AT5G02880-XLOC_025216-3047-1 TGTGATCTCTTTAGT
CONSENSUS TGTGATCTCTTTAGT

alignment for event: A3-AT5G02810-XLOC_028866-12013

A3-AT5G02810-XLOC_028866-12013-0
TCTAGTGGTAGTGGAAGTGAGAGCGGAACGCATCAAACTCAAAAGTCTGT
A3-AT5G02810-XLOC_028866-12013-1
TCTAGTGGTAGTGGAAGTGAGAGCGGAACGCATCAAACTCAAAAGTCTGT
CONSENSUS
TCTAGTGGTAGTGGAAGTGAGAGCGGAACGCATCAAACTCAAAAGTCTGT

A3-AT5G02810-XLOC_028866-12013-0
GAAATCGAAAAGTATTAAAAAATCTGATCAAGATTCAGGAAGCAGTGATG
A3-AT5G02810-XLOC_028866-12013-1
GAAATCGAAAAGTATTAAAAAATCTGATCAAGATTCAGGAAGCAGTGATG
CONSENSUS
GAAATCGAAAAGTATTAAAAAATCTGATCAAGATTCAGGAAGCAGTGATG

A3-AT5G02810-XLOC_028866-12013-0
AGAATGAAAATGGGAGCATTGGCCTGAATGCTAGTGATGGAAGTAGTGAT
A3-AT5G02810-XLOC_028866-12013-1
AGAATGAAAATGGGAGCATTGGCCTGAATGCTAGTGATGGAAGTAGTGAT

CONSENSUS
AGAATGAAAATGGGAGCATTGGCCTGAATGCTAGTGATGGAAGTAGTGAT

A3-AT5G02810-XLOC_028866-12013-0
GGGAGTGGCGCTCAG-----------------------------------
A3-AT5G02810-XLOC_028866-12013-1
GGGAGTGGCGCTCAGGGGTCGGATGATCGAACTTCGCATGTGCACTCAGA
CONSENSUS
GGGAGTGGCGCTCAG...................................

A3-AT5G02810-XLOC_028866-12013-0

A3-AT5G02810-XLOC_028866-12013-1
TGCTTATAAATGGTGGAAGCGCTTAACTAACCAATTGTAGAACTCAATGA
CONSENSUS
....

A3-AT5G02810-XLOC_028866-12013-0

A3-AT5G02810-XLOC_028866-12013-1
TGTTTAACTTAGATCTATTCAGTGATAAACTGGGTGAAAATTTCTAGTTC
CONSENSUS
....

A3-AT5G02810-XLOC_028866-12013-0

A3-AT5G02810-XLOC_028866-12013-1
ATTTTGAAGTTTTATTTTGCACATGGTTTATCTCCAAAACCTAGGTTGAT
CONSENSUS
....

A3-AT5G02810-XLOC_028866-12013-0 ------------------------
AGCTCTTGGACGAAAAAAGCTGTGGA
A3-AT5G02810-XLOC_028866-12013-1
TTTGACATACCTTTTTTTGTTCAGAGCTCTTGGACGAAAAAAGCTGTGGA
CONSENSUS
........................AGCTCTTGGACGAAAAAAGCTGTGGA

A3-AT5G02810-XLOC_028866-12013-0
TGTTGATGACAGTCCACGAGCGGTATCTCTATGGGACCGAGTTGATAGCA
A3-AT5G02810-XLOC_028866-12013-1
TGTTGATGACAGTCCACGAGCGGTATCTCTATGGGACCGAGTTGATAGCA
CONSENSUS
TGTTGATGACAGTCCACGAGCGGTATCTCTATGGGACCGAGTTGATAGCA

A3-AT5G02810-XLOC_028866-12013-0
CTTGCGCCCAAGTGGTACATTCTAACCCTGAGTTTCCAAGTAATCAGTTG
A3-AT5G02810-XLOC_028866-12013-1
CTTGCGCCCAAGTGGTACATTCTAACCCTGAGTTTCCAAGTAATCAGTTG
CONSENSUS
CTTGCGCCCAAGTGGTACATTCTAACCCTGAGTTTCCAAGTAATCAGTTG

A3-AT5G02810-XLOC_028866-12013-0
GTTGCACCACCTGCTGAGAAGGAGACTCAAGAACATGATGATAAATTTG
A3-AT5G02810-XLOC_028866-12013-1
GTTGCACCACCTGCTGAGAAGGAGACTCAAGAACATGATGATAAATTTG

CONSENSUS
GTTGCACCACCTGCTGAGAAGGAGACTCAAGAACATGATGATAAATTTG

alignment for event: A3-AT5G49960-XLOC_031452-13802

A3-AT5G49960-XLOC_031452-13802-0
GTGCTAGAAGCTCTGCTTGCGCCTGGATCTGAGTTATGGATGTTTAACGA
A3-AT5G49960-XLOC_031452-13802-1
GTGCTAGAAGCTCTGCTTGCGCCTGGATCTGAGTTATGGATGTTTAACGA
CONSENSUS
GTGCTAGAAGCTCTGCTTGCGCCTGGATCTGAGTTATGGATGTTTAACGA

A3-AT5G49960-XLOC_031452-13802-0
GGTTCCAGATCAAGAAAGAGAGAAAAAGTTGACAGATGCAGGACTAAATA
A3-AT5G49960-XLOC_031452-13802-1
GGTTCCAGATCAAGAAAGAGAGAAAAAGTTGACAGATGCAGGACTAAATA
CONSENSUS
GGTTCCAGATCAAGAAAGAGAGAAAAAGTTGACAGATGCAGGACTAAATA

A3-AT5G49960-XLOC_031452-13802-0
TATCCAAATTGGTGAATATAAAACTTGTGCATAGGCAGGGAAATGCAGTG
A3-AT5G49960-XLOC_031452-13802-1
TATCCAAATTGGTGAATATAAAACTTGTGCATAGGCAGGGAAATGCAGTG
CONSENSUS
TATCCAAATTGGTGAATATAAAACTTGTGCATAGGCAGGGAAATGCAGTG

A3-AT5G49960-XLOC_031452-13802-0
ATCAGGCGTCATTTAGAGAGTCTCCCTCTAGAAACTTTTGATTCA-----
A3-AT5G49960-XLOC_031452-13802-1
ATCAGGCGTCATTTAGAGAGTCTCCCTCTAGAAACTTTTGATTCAATCTT
CONSENSUS
ATCAGGCGTCATTTAGAGAGTCTCCCTCTAGAAACTTTTGATTCA.....

A3-AT5G49960-XLOC_031452-13802-0 -----------
AACAGTCACTAGAGAACTCTATTGTTCATTCAGACTCTC
A3-AT5G49960-XLOC_031452-13802-1
AATTCTTGCAGAACAGTCACTAGAGAACTCTATTGTTCATTCAGACTCTC
CONSENSUS
...........AACAGTCACTAGAGAACTCTATTGTTCATTCAGACTCTC

A3-AT5G49960-XLOC_031452-13802-0
GATCTCTTGCCACGCTTCTCCTTATTCGGGATATACAG
A3-AT5G49960-XLOC_031452-13802-1
GATCTCTTGCCACGCTTCTCCTTATTCGGGATATACAG
CONSENSUS
GATCTCTTGCCACGCTTCTCCTTATTCGGGATATACAG

alignment for event: RI-AT5G02810-XLOC_028866-12012

RI-AT5G02810-XLOC_028866-12012-0
GGGTCGGATGATCGAACTTCGCATGTGCACTCAGATGCTTATAAATGGTG
RI-AT5G02810-XLOC_028866-12012-1

GGGTCGGATGATCGAACTTCGCATGTGCACTCAGATGCTTATAAATGGTG
CONSENSUS
GGGTCGGATGATCGAACTTCGCATGTGCACTCAGATGCTTATAAATGGTG

RI-AT5G02810-XLOC_028866-12012-0
GAAGCGCTTAACTAACCAATTGTAGAACTCAATGATGTTTAACTTAGATC
RI-AT5G02810-XLOC_028866-12012-1
GAAGCGCTTAACTAACCAATTGTAGAACTCAATGATGTTTAACTTAGATC
CONSENSUS
GAAGCGCTTAACTAACCAATTGTAGAACTCAATGATGTTTAACTTAGATC

RI-AT5G02810-XLOC_028866-12012-0
TATTCAGTGATAAACTGGGTGAAAATTTCTAGTTCATTTTGAAGTTTTAT
RI-AT5G02810-XLOC_028866-12012-1
TATTCAGTGATAAACTGG--------------------------------
CONSENSUS
TATTCAGTGATAAACTGG................................

RI-AT5G02810-XLOC_028866-12012-0
TTTGCACATGGTTTATCTCCAAAACCTAGGTTGATTTTGACATACCTTTT
RI-AT5G02810-XLOC_028866-12012-1

CONSENSUS
....

RI-AT5G02810-XLOC_028866-12012-0
TTTGTTCAGAGCTCTTGGACGAAAAAAGCTGTGGATGTTGATGACAGTCC
RI-AT5G02810-XLOC_028866-12012-1 ---------
AGCTCTTGGACGAAAAAAGCTGTGGATGTTGATGACAGTCC
CONSENSUS
.........AGCTCTTGGACGAAAAAAGCTGTGGATGTTGATGACAGTCC

RI-AT5G02810-XLOC_028866-12012-0
ACGAGCGGTATCTCTATGGGACCGAGTTGATAGCACTTGCGCCCAAGTGG
RI-AT5G02810-XLOC_028866-12012-1
ACGAGCGGTATCTCTATGGGACCGAGTTGATAGCACTTGCGCCCAAGTGG
CONSENSUS
ACGAGCGGTATCTCTATGGGACCGAGTTGATAGCACTTGCGCCCAAGTGG

RI-AT5G02810-XLOC_028866-12012-0
TACATTCTAACCCTGAGTTTCCAAGTAATCAGTTGGTTGCACCACCTGCT
RI-AT5G02810-XLOC_028866-12012-1
TACATTCTAACCCTGAGTTTCCAAGTAATCAGTTGGTTGCACCACCTGCT
CONSENSUS
TACATTCTAACCCTGAGTTTCCAAGTAATCAGTTGGTTGCACCACCTGCT

RI-AT5G02810-XLOC_028866-12012-0
GAGAAGGAGACTCAAGAACATGATGATAAATTTG
RI-AT5G02810-XLOC_028866-12012-1
GAGAAGGAGACTCAAGAACATGATGATAAATTTG
CONSENSUS
GAGAAGGAGACTCAAGAACATGATGATAAATTTG

alignment for event: RI-AT5G35210-XLOC_030529-4360

RI-AT5G35210-XLOC_030529-4360-0
AGTGGTTCCACACTGAAGCTGTTAAGCTCAAAGATTCACAAATTCCTGAA
RI-AT5G35210-XLOC_030529-4360-1
AGTGGTTCCACACTGAAGCTGTTAAGCTCAAAGATTCACAAATTCCTGAA
CONSENSUS
AGTGGTTCCACACTGAAGCTGTTAAGCTCAAAGATTCACAAATTCCTGAA

RI-AT5G35210-XLOC_030529-4360-0
GTTGTTGGGTTCAAATGTTGCAAATGTCGACGTATACGATCCCCTGATTG
RI-AT5G35210-XLOC_030529-4360-1
GTTGTTGGGTTCAAATGTTGCAAATGTCGACGTATACGATCCCCTGATTG
CONSENSUS
GTTGTTGGGTTCAAATGTTGCAAATGTCGACGTATACGATCCCCTGATTG

RI-AT5G35210-XLOC_030529-4360-0
CCCTTACATGGATCCCAAACTCAAGGAACAGAAGCAGATCAAAAGAATAG
RI-AT5G35210-XLOC_030529-4360-1
CCCTTACATGGATCCCAAACTCAAGGAACAGAAGCAGATCAAAAGAATAG
CONSENSUS
CCCTTACATGGATCCCAAACTCAAGGAACAGAAGCAGATCAAAAGAATAG

RI-AT5G35210-XLOC_030529-4360-0
TCTTCACGAACCAGAAACAGAGGCAAGGAAATTCTGGGTTGGATTCTGAT
RI-AT5G35210-XLOC_030529-4360-1
TCTTCACGAACCAGAAACAGAGGCAAGGAAATTCTGGGTTGGATTCTGAT
CONSENSUS
TCTTCACGAACCAGAAACAGAGGCAAGGAAATTCTGGGTTGGATTCTGAT

RI-AT5G35210-XLOC_030529-4360-0
TCTGAAAGAATGTCTGAACAAAAAGACTCGAAACCTTCTACTCCGTTACC
RI-AT5G35210-XLOC_030529-4360-1
TCTGAAAGAATGTCTGAACAAAAAGACTCGAAACCTTCTACTCCGTTACC
CONSENSUS
TCTGAAAGAATGTCTGAACAAAAAGACTCGAAACCTTCTACTCCGTTACC

RI-AT5G35210-XLOC_030529-4360-0
TGCCACTCCTTTATATCCTCCTGACGATGTATTTATTCCAGAAGATGATC
RI-AT5G35210-XLOC_030529-4360-1
TGCCACTCCTTTATATCCTCCTGACGATGTATTTATTCCAGAAGATGATC
CONSENSUS
TGCCACTCCTTTATATCCTCCTGACGATGTATTTATTCCAGAAGATGATC

RI-AT5G35210-XLOC_030529-4360-0
CTCTCCTGGTATCAGTTTCCAAAGTCAAACAAATCACACCCAGTAGTTTC
RI-AT5G35210-XLOC_030529-4360-1
CTCTCCTGGTATCAGTTTCCAAAGTCAAACAAATCACACCCAGTAGTTTC
CONSENSUS
CTCTCCTGGTATCAGTTTCCAAAGTCAAACAAATCACACCCAGTAGTTTC

RI-AT5G35210-XLOC_030529-4360-0
GATCTTGAATGGAGCACCACTGCTTTTGCGCCTGGACCCCAAAAGCTACC
RI-AT5G35210-XLOC_030529-4360-1
GATCTTGAATGGAGCACCACTGCTTTTGCGCCTGGACCCCAAAAGCTACC
CONSENSUS
GATCTTGAATGGAGCACCACTGCTTTTGCGCCTGGACCCCAAAAGCTACC

RI-AT5G35210-XLOC_030529-4360-0
AGTTAGAAGGCAAGTGAAACGAGAAGATTCTGATGCAGCCTATCCTGAAC
RI-AT5G35210-XLOC_030529-4360-1
AGTTAGAAGGCAAGTGAAACGAGAAGATTCTGATGCAGCCTATCCTGAAC
CONSENSUS
AGTTAGAAGGCAAGTGAAACGAGAAGATTCTGATGCAGCCTATCCTGAAC

RI-AT5G35210-XLOC_030529-4360-0
TGCATCCTATTGTGAAACCCGAGGCAGAGGAGCAAGCTTTACCTGTTCTA
RI-AT5G35210-XLOC_030529-4360-1
TGCATCCTATTGTGAAACCCGAGGCAGAGGAGCAAGCTTTACCTGTTCTA
CONSENSUS
TGCATCCTATTGTGAAACCCGAGGCAGAGGAGCAAGCTTTACCTGTTCTA

RI-AT5G35210-XLOC_030529-4360-0
ACAGAATGGGATTTGTCTGGTGAGCTACTTTTCGACTACGAGGACATGGA
RI-AT5G35210-XLOC_030529-4360-1
ACAGAATGGGATTTGTCTGGTGAGCTACTTTTCGACTACGAGGACATGGA
CONSENSUS
ACAGAATGGGATTTGTCTGGTGAGCTACTTTTCGACTACGAGGACATGGA

RI-AT5G35210-XLOC_030529-4360-0
ATTTGAACCGCAAACTTATTTCTCGTTGACCGAGCTACTGACAGCCGATG
RI-AT5G35210-XLOC_030529-4360-1
ATTTGAACCGCAAACTTATTTCTCGTTGACCGAGCTACTGACAGCCGATG
CONSENSUS
ATTTGAACCGCAAACTTATTTCTCGTTGACCGAGCTACTGACAGCCGATG

RI-AT5G35210-XLOC_030529-4360-0
ATAGTGGTGGTGGTCAGTACCAGGAAAATGGTGATATGGTTGTTTCAGGA
RI-AT5G35210-XLOC_030529-4360-1
ATAGTGGTGGTGGTCAGTACCAGGAAAATGGTGATATGGTTGTTTCAGGA
CONSENSUS
ATAGTGGTGGTGGTCAGTACCAGGAAAATGGTGATATGGTTGTTTCAGGA

RI-AT5G35210-XLOC_030529-4360-0
AACCCTCAGTTCGAACCAACAGAAAAAGAAGAGTGTGAAGATGATATGGG
RI-AT5G35210-XLOC_030529-4360-1
AACCCTCAGTTCGAACCAACAGAAAAAGAAGAGTGTGAAGATGATATGGG
CONSENSUS
AACCCTCAGTTCGAACCAACAGAAAAAGAAGAGTGTGAAGATGATATGGG

RI-AT5G35210-XLOC_030529-4360-0
TCCATGTCAGAGATGTTTGCAAATGGATCCAGCGCCTGATCTTTTGTGCA
RI-AT5G35210-XLOC_030529-4360-1
TCCATGTCAGAGATGTTTGCAAATGGATCCAGCGCCTGATCTTTTGTGCA
CONSENSUS
TCCATGTCAGAGATGTTTGCAAATGGATCCAGCGCCTGATCTTTTGTGCA

RI-AT5G35210-XLOC_030529-4360-0
CGGTTTGTGGATTGCTTATACATTCTCACTGTTCTCCATGGTCAGCCTTA
RI-AT5G35210-XLOC_030529-4360-1
CG--
CONSENSUS
CG..

RI-AT5G35210-XLOC_030529-4360-0
CCCGGAAGTAGCTGGAGCTGTGGTCAATGCCGTGAGTGGCAGTAGCTGGA
RI-AT5G35210-XLOC_030529-4360-1

CONSENSUS
....

RI-AT5G35210-XLOC_030529-4360-0
CCCTTTGATTTCAAGCCCCTTCCCAAATTTTAACTTCCACATTCTTTGAC
RI-AT5G35210-XLOC_030529-4360-1

CONSENSUS
....

RI-AT5G35210-XLOC_030529-4360-0
AATGAATTTGAGGGAAAATTGTATCCTCCTTTGTTTGGCTTATAATGGCA
RI-AT5G35210-XLOC_030529-4360-1

CONSENSUS
....

RI-AT5G35210-XLOC_030529-4360-0
AATGAGCTGTAATTTCTAGTTTTTTTTTAGGTTTGTCTTCGTCTATTTTA
RI-AT5G35210-XLOC_030529-4360-1

CONSENSUS
....

RI-AT5G35210-XLOC_030529-4360-0
TTTATCTTCGTTTATAGAGTGGATACATGTTTGCTTTCAAATTCATACCA
RI-AT5G35210-XLOC_030529-4360-1

CONSENSUS
....

RI-AT5G35210-XLOC_030529-4360-0
AGCAGGGATGCTTACTTGGTGGAATGATTAACTTTCATGTATTGGTGATA
RI-AT5G35210-XLOC_030529-4360-1

CONSENSUS
....

RI-AT5G35210-XLOC_030529-4360-0
TCTGATTTTCTTATTTTGCTCTCAGTGCTTCGTTTAGAGAGTTATATCTA
RI-AT5G35210-XLOC_030529-4360-1

CONSENSUS
....

RI-AT5G35210-XLOC_030529-4360-0
TGACTTGTTTATTTGCAGGAATTAGGGCACTAGGAAGCATTACACTAGGA
RI-AT5G35210-XLOC_030529-4360-1 ------------------
GAATTAGGGCACTAGGAAGCATTACACTAGGA
CONSENSUS
..................GAATTAGGGCACTAGGAAGCATTACACTAGGA

```
RI-AT5G35210-XLOC_030529-4360-0  AGCTTTGGTGCAATCACACAG
RI-AT5G35210-XLOC_030529-4360-1  AGCTTTGGTGCAATCACACAG
CONSENSUS                        AGCTTTGGTGCAATCACACAG
```

alignment for event: A3-AT5G04360-XLOC_025304-1491

```
A3-AT5G04360-XLOC_025304-1491-0
     GCTCTTAATTGTACTGGTCTTAATGTCGTCTTAGATGTTGTTTACAACCA
A3-AT5G04360-XLOC_025304-1491-1
     GCTCTTAATTGTACTGGTCTTAATGTCGTCTTAGATGTTGTTTACAACCA
CONSENSUS
     GCTCTTAATTGTACTGGTCTTAATGTCGTCTTAGATGTTGTTTACAACCA

A3-AT5G04360-XLOC_025304-1491-0
     CTTGCATGCAAGTGGGCCACATGACAAAGAATCTGTTCTTGATAAGATAG
A3-AT5G04360-XLOC_025304-1491-1
     CTTGCATGCAAGTGGGCCACATGACAAAGAATCTGTTCTTGATAAG----
CONSENSUS
     CTTGCATGCAAGTGGGCCACATGACAAAGAATCTGTTCTTGATAAG....

A3-AT5G04360-XLOC_025304-1491-0
     TTCCAGGTTACTATTTGAGAAGGAACAGTGATGGTTTTATTGAAAACAGT
A3-AT5G04360-XLOC_025304-1491-1
     TTCCAGGTTACTATTTGAGAAGGAACAGTGATGGTTTTATTGAAAACAGT
CONSENSUS
     TTCCAGGTTACTATTTGAGAAGGAACAGTGATGGTTTTATTGAAAACAGT

A3-AT5G04360-XLOC_025304-1491-0
     ACATGCGTAAACAACACTGCTAGCGAGCACTATATGGTTGATCGTCTCAT
A3-AT5G04360-XLOC_025304-1491-1
     ACATGCGTAAACAACACTGCTAGCGAGCACTATATGGTTGATCGTCTCAT
CONSENSUS
     ACATGCGTAAACAACACTGCTAGCGAGCACTATATGGTTGATCGTCTCAT

A3-AT5G04360-XLOC_025304-1491-0
     ACGGGATGATCTATTAAATTGGGTTGTTAACTATAAG
A3-AT5G04360-XLOC_025304-1491-1
     ACGGGATGATCTATTAAATTGGGTTGTTAACTATAAG
CONSENSUS
     ACGGGATGATCTATTAAATTGGGTTGTTAACTATAAG
```

alignment for event: RI-AT5G27970-XLOC_026502-11515

```
RI-AT5G27970-XLOC_026502-11515-0
     GTCAGGAATTCAGCTGTCAGGACATTTTTTCAGATTTTGGGAAGTCATGG
RI-AT5G27970-XLOC_026502-11515-1
     GTCAGGAATTCAGCTGTCAGGACATTTTTTCAGATTTTGGGAAGTCATGG
CONSENSUS
     GTCAGGAATTCAGCTGTCAGGACATTTTTTCAGATTTTGGGAAGTCATGG

RI-AT5G27970-XLOC_026502-11515-0
```

AAACAAACTTTCAAAAAGCATGTGGGAGGATTGTCTATGGAACTATATCT
RI-AT5G27970-XLOC_026502-11515-1
AAACAAACTTTCAAAAAGCATGTGGGAGGATTGTCTATGGAACTATATCT
CONSENSUS
AAACAAACTTTCAAAAAGCATGTGGGAGGATTGTCTATGGAACTATATCT

RI-AT5G27970-XLOC_026502-11515-0
TCCCAATGTTGGATGGCGCCTCTCACAAGGTGAGGAAATAGGTTCAATAG
RI-AT5G27970-XLOC_026502-11515-1
TCCCAATGTTGGATGGCGCCTCTCACAAG---------------------
CONSENSUS
TCCCAATGTTGGATGGCGCCTCTCACAAG.....................

RI-AT5G27970-XLOC_026502-11515-0
ATTCATCTTAGAAGACCCTTGTGACAAGTGAATCTTTTTTGTAGGCTGCA
RI-AT5G27970-XLOC_026502-11515-1
--GCTGCA
CONSENSUS
....GCTGCA

RI-AT5G27970-XLOC_026502-11515-0
ACATCATCGAAGGACGAATGGCAAGGGAAAGAAATCGGTACTCGAGGAGG
RI-AT5G27970-XLOC_026502-11515-1
ACATCATCGAAGGACGAATGGCAAGGGAAAGAAATCGGTACTCGAGGAGG
CONSENSUS
ACATCATCGAAGGACGAATGGCAAGGGAAAGAAATCGGTACTCGAGGAGG

RI-AT5G27970-XLOC_026502-11515-0 GAAAGCTGTGCACATGCTTATACATCATAG
RI-AT5G27970-XLOC_026502-11515-1 GAAAGCTGTGCACATGCTTATACATCATAG
CONSENSUS GAAAGCTGTGCACATGCTTATACATCATAG

alignment for event: A3-AT5G14440-XLOC_029464-4828

A3-AT5G14440-XLOC_029464-4828-0
GTGAATTTGATGAAGATGGAGAGATTAGTATGGAAGAGTCCATACTAATA
A3-AT5G14440-XLOC_029464-4828-1
GTGAATTTGATGAAGATGGAGAGATTAGTATGGAAGAGTCCATACTAATA
CONSENSUS
GTGAATTTGATGAAGATGGAGAGATTAGTATGGAAGAGTCCATACTAATA

A3-AT5G14440-XLOC_029464-4828-0
GGTGAAGTTGATGAAGATGGCAAGATTGTTTTGGATGACACTCATGCCAG
A3-AT5G14440-XLOC_029464-4828-1
GGTGAAGTTGATGAAGATGGCAAGATTGTTTTGGATGACACTCATGCCAG
CONSENSUS
GGTGAAGTTGATGAAGATGGCAAGATTGTTTTGGATGACACTCATGCCAG

A3-AT5G14440-XLOC_029464-4828-0 ---
CAACAAGAGGAAACATGAAGAACTCGGTTCCAGTGACCTTCCTTCGA
A3-AT5G14440-XLOC_029464-4828-1
CAGCAACAAGAGGAAACATGAAGAACTCGGTTCCAGTGACCTTCCTTCGA
CONSENSUS
...CAACAAGAGGAAACATGAAGAACTCGGTTCCAGTGACCTTCCTTCGA

```
A3-AT5G14440-XLOC_029464-4828-0
      AGAAGAAGAATAAGAAGAAGAAAAAGAAGAAGAATGTCGAAGCAACATCG
A3-AT5G14440-XLOC_029464-4828-1
      AGAAGAAGAATAAGAAGAAGAAAAAGAAGAAGAATGTCGAAGCAACATCG
CONSENSUS
      AGAAGAAGAATAAGAAGAAGAAAAAGAAGAAGAATGTCGAAGCAACATCG

A3-AT5G14440-XLOC_029464-4828-0
      TCGTCATATTAAGTCTGATATGTTTTGTATTTCACTTTGTACTTGCTCTA
A3-AT5G14440-XLOC_029464-4828-1
      TCGTCATATTAAGTCTGATATGTTTTGTATTTCACTTTGTACTTGCTCTA
CONSENSUS
      TCGTCATATTAAGTCTGATATGTTTTGTATTTCACTTTGTACTTGCTCTA

A3-AT5G14440-XLOC_029464-4828-0
      TTTCAGCTTGTGTGCTCAGTTTTGTTATAATTGGTGATGGAAAATGGTGA
A3-AT5G14440-XLOC_029464-4828-1
      TTTCAGCTTGTGTGCTCAGTTTTGTTATAATTGGTGATGGAAAATGGTGA
CONSENSUS
      TTTCAGCTTGTGTGCTCAGTTTTGTTATAATTGGTGATGGAAAATGGTGA

A3-AT5G14440-XLOC_029464-4828-0
      TTTACATATTAATGAAGCAATTTATTTTTCTCACAAAAGCAGATTTTAAT
A3-AT5G14440-XLOC_029464-4828-1
      TTTACATATTAATGAAGCAATTTATTTTTCTCACAAAAGCAGATTTTAAT
CONSENSUS
      TTTACATATTAATGAAGCAATTTATTTTTCTCACAAAAGCAGATTTTAAT

A3-AT5G14440-XLOC_029464-4828-0  TATAAAGAGGAACATGAAAATCGC
A3-AT5G14440-XLOC_029464-4828-1  TATAAAGAGGAACATGAAAATCGC
CONSENSUS                        TATAAAGAGGAACATGAAAATCGC


alignment for event: SE-AT5G22620-XLOC_029881-8529

SE-AT5G22620-XLOC_029881-8529-0
      ACACCTAATTCGCCTCTAGCTGGTGGAAGTTCTGGTGGCCGGAAAGCTAG
SE-AT5G22620-XLOC_029881-8529-1
      ACACCTAATTCGCCTCTAGCTGGTGGAAGTTCTGGTGGCCGGAAAGCTAG
CONSENSUS
      ACACCTAATTCGCCTCTAGCTGGTGGAAGTTCTGGTGGCCGGAAAGCTAG

SE-AT5G22620-XLOC_029881-8529-0
      TAAGCAGATTATACTTGTCTGCCATGGTCAGGGGAATAATGAG-------
SE-AT5G22620-XLOC_029881-8529-1
      TAAGCAGATTATACTTGTCTGCCATGGTCAGGGGAATAATGAGGATTCTG
CONSENSUS
      TAAGCAGATTATACTTGTCTGCCATGGTCAGGGGAATAATGAG.......

SE-AT5G22620-XLOC_029881-8529-0
      --------------------------------------------------
SE-AT5G22620-XLOC_029881-8529-1
      CTGTTATTAACCAAGCAGCTAATAATGATCAGGCAATGAACATGCTTGGT
CONSENSUS
      ..................................................
```

SE-AT5G22620-XLOC_029881-8529-0 ---------
TCACAGAAAACCGCAGAGCTTCTACTTGATCTAAGGGTTAG
SE-AT5G22620-XLOC_029881-8529-1
      GTGATACATTCACAGAAAACCGCAGAGCTTCTACTTGATCTAAGGGTTAG
CONSENSUS
      .........TCACAGAAAACCGCAGAGCTTCTACTTGATCTAAGGGTTAG

SE-AT5G22620-XLOC_029881-8529-0
      TTCAATAGTTTGCAGCCCTAAAACAGCCTCCATTGAGTCTTCTGGAGTAA
SE-AT5G22620-XLOC_029881-8529-1
      TTCAATAGTTTGCAGCCCTAAAACAGCCTCCATTGAGTCTTCTGGAGTAA
CONSENSUS
      TTCAATAGTTTGCAGCCCTAAAACAGCCTCCATTGAGTCTTCTGGAGTAA

SE-AT5G22620-XLOC_029881-8529-0 TATCCCGG
SE-AT5G22620-XLOC_029881-8529-1 TATCCCGG
CONSENSUS   TATCCCGG

alignment for event: A3-AT5G67540-XLOC_032395-10758

A3-AT5G67540-XLOC_032395-10758-0
      AGAAAAAAAAAAGAAAAAAAAATCAGAGGAAGAAGAAGACTAGAATCGTC
A3-AT5G67540-XLOC_032395-10758-1
      AGAAAAAAAAAAGAAAAAAAAATCAGAGGAAGAAGAAGACTAGAATCGTC
CONSENSUS
      AGAAAAAAAAAAGAAAAAAAAATCAGAGGAAGAAGAAGACTAGAATCGTC

A3-AT5G67540-XLOC_032395-10758-0
      TTCATTAGTCTCGTCTTCGTTCACACGTCTGAATCGTCAATAGAACTTTG
A3-AT5G67540-XLOC_032395-10758-1
      TTCATTAGTCTCGTCTTCGTTCACACGTCTGAATCGTCAATAGAACTTTG
CONSENSUS
      TTCATTAGTCTCGTCTTCGTTCACACGTCTGAATCGTCAATAGAACTTTG

A3-AT5G67540-XLOC_032395-10758-0
      ATAGATCCATTTCAACTAGACTTCTCTTTACTCACTTCTGAATCTCCCCC
A3-AT5G67540-XLOC_032395-10758-1
      ATAGATCCATTTCAACTAGACTTCTCTTTACTCACTTCTGAATCTCCCCC
CONSENSUS
      ATAGATCCATTTCAACTAGACTTCTCTTTACTCACTTCTGAATCTCCCCC

A3-AT5G67540-XLOC_032395-10758-0
      AAAAAAAAGAAAGAATCTAGCTCTGAATTTCTCGATTCAGTTTCCAGACC
A3-AT5G67540-XLOC_032395-10758-1
      AAAAAAAAGAAAGAATCTAGCTCTGAATTTCTCGATTCAGTTTCCAGACC
CONSENSUS
      AAAAAAAAGAAAGAATCTAGCTCTGAATTTCTCGATTCAGTTTCCAGACC

A3-AT5G67540-XLOC_032395-10758-0
      AAGCTTCGCATCAGACAGAACAAAGTGTTTCGGATTTGGTGTTTGCTCCG
A3-AT5G67540-XLOC_032395-10758-1
      AAGCTTCGCATCAGACAGAACAAAGTGTTTCGGATTTGGTGTTTGCTCCG
CONSENSUS

AAGCTTCGCATCAGACAGAACAAAGTGTTTCGGATTTGGTGTTTGCTCCG

A3-AT5G67540-XLOC_032395-10758-0
GAGAAAGCGAAGAGAGTTGAGAATTTTTTGTATAATTTCTGAGATTATAT
A3-AT5G67540-XLOC_032395-10758-1
GAGAAAGCGAAGAGAGTTGAGAATTTTTTGTATAATTTCTGAGATTATAT
CONSENSUS
GAGAAAGCGAAGAGAGTTGAGAATTTTTTGTATAATTTCTGAGATTATAT

A3-AT5G67540-XLOC_032395-10758-0
ACATGTGATGTCGGGATATTCGTCTTCTGCTGGCCTCCGAGGATTTGCAG
A3-AT5G67540-XLOC_032395-10758-1
ACATGTGATGTCGGGATATTCGTCTTCTGCTGGCCTCCGAGGATTTGCAG
CONSENSUS
ACATGTGATGTCGGGATATTCGTCTTCTGCTGGCCTCCGAGGATTTGCAG

A3-AT5G67540-XLOC_032395-10758-0

A3-AT5G67540-XLOC_032395-10758-1
TGTTTTAGAAGACATAAAGAGGATAATGAAGAAGAACAACAAATACAATA
CONSENSUS
....

A3-AT5G67540-XLOC_032395-10758-0
----------------------------------GAGGTTGCAGATATTCA
A3-AT5G67540-XLOC_032395-10758-1
AGAAATCAACATCTTTGCATTGCAATGATGCAGGAGGTTGCAGATATTCA
CONSENSUS
..................................GAGGTTGCAGATATTCA

A3-AT5G67540-XLOC_032395-10758-0
TTGCTTACTATTGTTTGGACCGTCGTCGGGTTTTTCCTGGTGGCTCATCT
A3-AT5G67540-XLOC_032395-10758-1
TTGCTTACTATTGTTTGGACCGTCGTCGGGTTTTTCCTGGTGGCTCATCT
CONSENSUS
TTGCTTACTATTGTTTGGACCGTCGTCGGGTTTTTCCTGGTGGCTCATCT

A3-AT5G67540-XLOC_032395-10758-0
TATCTCTCTGTATAGCAGAAAAGATAACAACATCCATCAACAAGTTTCTT
A3-AT5G67540-XLOC_032395-10758-1
TATCTCTCTGTATAGCAGAAAAGATAACAACATCCATCAACAAGTTTCTT
CONSENSUS
TATCTCTCTGTATAGCAGAAAAGATAACAACATCCATCAACAAGTTTCTT

A3-AT5G67540-XLOC_032395-10758-0
CAGATCAGTTACAAGTAGTACATCATCTTGCTCATCCTATTGTTCGTGAA
A3-AT5G67540-XLOC_032395-10758-1
CAGATCAGTTACAAGTAGTACATCATCTTGCTCATCCTATTGTTCGTGAA
CONSENSUS
CAGATCAGTTACAAGTAGTACATCATCTTGCTCATCCTATTGTTCGTGAA

A3-AT5G67540-XLOC_032395-10758-0
CTTATAAGAGTTGAAGAAGAGGTCCTGAGAATGCCGCCACCTAGGAAGCG
A3-AT5G67540-XLOC_032395-10758-1
CTTATAAGAGTTGAAGAAGAGGTCCTGAGAATGCCGCCACCTAGGAAGCG
CONSENSUS

CTTATAAGAGTTGAAGAAGAGGTCCTGAGAATGCCGCCACCTAGGAAGCG

A3-AT5G67540-XLOC_032395-10758-0
TTCTCCTCGTACTAGTAAACGGAGATCAAGAAAACCGATCCCTCTCGTTG
A3-AT5G67540-XLOC_032395-10758-1
TTCTCCTCGTACTAGTAAACGGAGATCAAGAAAACCGATCCCTCTCGTTG
CONSENSUS
TTCTCCTCGTACTAGTAAACGGAGATCAAGAAAACCGATCCCTCTCGTTG

A3-AT5G67540-XLOC_032395-10758-0
AGGAGTTTCTTGACGACAAGTCACCAATTCGACATCTTTTCTTTCCCGGT
A3-AT5G67540-XLOC_032395-10758-1
AGGAGTTTCTTGACGACAAGTCACCAATTCGACATCTTTTCTTTCCCGGT
CONSENSUS
AGGAGTTTCTTGACGACAAGTCACCAATTCGACATCTTTTCTTTCCCGGT

A3-AT5G67540-XLOC_032395-10758-0
ATCAAAACCGCCGCTTTTGGTCCCACCAAGGATATGGGAAACGAGACATC
A3-AT5G67540-XLOC_032395-10758-1
ATCAAAACCGCCGCTTTTGGTCCCACCAAGGATATGGGAAACGAGACATC
CONSENSUS
ATCAAAACCGCCGCTTTTGGTCCCACCAAGGATATGGGAAACGAGACATC

A3-AT5G67540-XLOC_032395-10758-0
GTATTACTTCCCCGGGAAAATTTGGATGGACACACAAGGGAATCCAATTC
A3-AT5G67540-XLOC_032395-10758-1
GTATTACTTCCCCGGGAAAATTTGGATGGACACACAAGGGAATCCAATTC
CONSENSUS
GTATTACTTCCCCGGGAAAATTTGGATGGACACACAAGGGAATCCAATTC

A3-AT5G67540-XLOC_032395-10758-0
AAGCACATGGCGGAGGGATTCTGCTTGACGTGAAATCTAACACTTACTAT
A3-AT5G67540-XLOC_032395-10758-1
AAGCACATGGCGGAGGGATTCTGCTTGACGTGAAATCTAACACTTACTAT
CONSENSUS
AAGCACATGGCGGAGGGATTCTGCTTGACGTGAAATCTAACACTTACTAT

A3-AT5G67540-XLOC_032395-10758-0
TGGTATGGAGAGTATAAAGATGGACCAACTTACCATGCTCACAAGAAAGG
A3-AT5G67540-XLOC_032395-10758-1
TGGTATGGAGAGTATAAAGATGGACCAACTTACCATGCTCACAAGAAAGG
CONSENSUS
TGGTATGGAGAGTATAAAGATGGACCAACTTACCATGCTCACAAGAAAGG

A3-AT5G67540-XLOC_032395-10758-0 ACCAGCTAGA
A3-AT5G67540-XLOC_032395-10758-1 ACCAGCTAGA
CONSENSUS ACCAGCTAGA

alignment for event: RI-AT5G39790-XLOC_030855-13648

RI-AT5G39790-XLOC_030855-13648-0
CTTTAAAACACAGGAACATGAGATAACTAACATCAAAACTGAGCTTGCAC
RI-AT5G39790-XLOC_030855-13648-1
CTTTAAAACACAGGAACATGAGATAACTAACATCAAAACTGAGCTTGCAC

CONSENSUS
      CTTTAAAACACAGGAACATGAGATAACTAACATCAAAACTGAGCTTGCAC

RI-AT5G39790-XLOC_030855-13648-0
      TTATGGAGCTCGAAGTTCAGGTCTTTATTTCTACTTTTGATTTTTCAATG
RI-AT5G39790-XLOC_030855-13648-1
      TTATGGAGCTCGAAGTTCAG------------------------------
CONSENSUS
      TTATGGAGCTCGAAGTTCAG..............................

RI-AT5G39790-XLOC_030855-13648-0
      TCACGGTCTCACTATCTCATTGCCTTAAGCTGATTTGGTTTAGTTGTCAT
RI-AT5G39790-XLOC_030855-13648-1

CONSENSUS
      ....

RI-AT5G39790-XLOC_030855-13648-0
      TATCCCATGTCTACTGCCAATCAAATACATATTTTTGTTGGTTATAATAT
RI-AT5G39790-XLOC_030855-13648-1

CONSENSUS
      ....

RI-AT5G39790-XLOC_030855-13648-0
      TGGGAAATATTTAGCTTCAGATTTTTGTATGCACAAGCACATATGTATAG
RI-AT5G39790-XLOC_030855-13648-1

CONSENSUS
      ....

RI-AT5G39790-XLOC_030855-13648-0
      TTTCTTTAGTAGAAATGTTTCATGATATCACTTTCTGGAGGAAATAGGCT
RI-AT5G39790-XLOC_030855-13648-1
      ---GCT
CONSENSUS
      ....GCT

RI-AT5G39790-XLOC_030855-13648-0
      CTGGTGAAACTGGCGGAAGAAATAGCAAACCTTGGTATCCCACAAGGTTC
RI-AT5G39790-XLOC_030855-13648-1
      CTGGTGAAACTGGCGGAAGAAATAGCAAACCTTGGTATCCCACAAGGTTC
CONSENSUS
      CTGGTGAAACTGGCGGAAGAAATAGCAAACCTTGGTATCCCACAAGGTTC

RI-AT5G39790-XLOC_030855-13648-0
      TAGAAAAATCAGTGGAAAGTACATTCAATCGCACCTTCTCTCTCGTTTAG
RI-AT5G39790-XLOC_030855-13648-1
      TAGAAAAATCAGTGGAAAGTACATTCAATCGCACCTTCTCTCTCGTTTAG
CONSENSUS
      TAGAAAAATCAGTGGAAAGTACATTCAATCGCACCTTCTCTCTCGTTTAG

RI-AT5G39790-XLOC_030855-13648-0 ACG
RI-AT5G39790-XLOC_030855-13648-1 ACG
CONSENSUS   ACG

alignment for event: A5-AT5G35210-XLOC_030529-4361

A5-AT5G35210-XLOC_030529-4361-0
AGTGGTTCCACACTGAAGCTGTTAAGCTCAAAGATTCACAAATTCCTGAA
A5-AT5G35210-XLOC_030529-4361-1
AGTGGTTCCACACTGAAGCTGTTAAGCTCAAAGATTCACAAATTCCTGAA
CONSENSUS
AGTGGTTCCACACTGAAGCTGTTAAGCTCAAAGATTCACAAATTCCTGAA

A5-AT5G35210-XLOC_030529-4361-0
GTTGTTGGGTTCAAATGTTGCAAATGTCGACGTATACGATCCCCTGATTG
A5-AT5G35210-XLOC_030529-4361-1
GTTGTTGGGTTCAAATGTTGCAAATGTCGACGTATACGATCCCCTGATTG
CONSENSUS
GTTGTTGGGTTCAAATGTTGCAAATGTCGACGTATACGATCCCCTGATTG

A5-AT5G35210-XLOC_030529-4361-0
CCCTTACATGGATCCCAAACTCAAGGAACAGAAGCAGATCAAAAGAATAG
A5-AT5G35210-XLOC_030529-4361-1
CCCTTACATGGATCCCAAACTCAAGGAACAGAAGCAGATCAAAAGAATAG
CONSENSUS
CCCTTACATGGATCCCAAACTCAAGGAACAGAAGCAGATCAAAAGAATAG

A5-AT5G35210-XLOC_030529-4361-0
TCTTCACGAACCAGAAACAGAGGCAAGGAAATTCTGGGTTGGATTCTGAT
A5-AT5G35210-XLOC_030529-4361-1
TCTTCACGAACCAGAAACAGAGGCAAGGAAATTCTGGGTTGGATTCTGAT
CONSENSUS
TCTTCACGAACCAGAAACAGAGGCAAGGAAATTCTGGGTTGGATTCTGAT

A5-AT5G35210-XLOC_030529-4361-0
TCTGAAAGAATGTCTGAACAAAAAGACTCGAAACCTTCTACTCCGTTACC
A5-AT5G35210-XLOC_030529-4361-1
TCTGAAAGAATGTCTGAACAAAAAGACTCGAAACCTTCTACTCCGTTACC
CONSENSUS
TCTGAAAGAATGTCTGAACAAAAAGACTCGAAACCTTCTACTCCGTTACC

A5-AT5G35210-XLOC_030529-4361-0
TGCCACTCCTTTATATCCTCCTGACGATGTATTTATTCCAGAAGATGATC
A5-AT5G35210-XLOC_030529-4361-1
TGCCACTCCTTTATATCCTCCTGACGATGTATTTATTCCAGAAGATGATC
CONSENSUS
TGCCACTCCTTTATATCCTCCTGACGATGTATTTATTCCAGAAGATGATC

A5-AT5G35210-XLOC_030529-4361-0
CTCTCCTGGTATCAGTTTCCAAAGTCAAACAAATCACACCCAGTAGTTTC
A5-AT5G35210-XLOC_030529-4361-1
CTCTCCTGGTATCAGTTTCCAAAGTCAAACAAATCACACCCAGTAGTTTC
CONSENSUS
CTCTCCTGGTATCAGTTTCCAAAGTCAAACAAATCACACCCAGTAGTTTC

A5-AT5G35210-XLOC_030529-4361-0
GATCTTGAATGGAGCACCACTGCTTTTGCGCCTGGACCCCAAAAGCTACC
A5-AT5G35210-XLOC_030529-4361-1

GATCTTGAATGGAGCACCACTGCTTTTGCGCCTGGACCCCAAAAGCTACC
CONSENSUS
GATCTTGAATGGAGCACCACTGCTTTTGCGCCTGGACCCCAAAAGCTACC

A5-AT5G35210-XLOC_030529-4361-0
AGTTAGAAGGCAAGTGAAACGAGAAGATTCTGATGCAGCCTATCCTGAAC
A5-AT5G35210-XLOC_030529-4361-1
AGTTAGAAGGCAAGTGAAACGAGAAGATTCTGATGCAGCCTATCCTGAAC
CONSENSUS
AGTTAGAAGGCAAGTGAAACGAGAAGATTCTGATGCAGCCTATCCTGAAC

A5-AT5G35210-XLOC_030529-4361-0
TGCATCCTATTGTGAAACCCGAGGCAGAGGAGCAAGCTTTACCTGTTCTA
A5-AT5G35210-XLOC_030529-4361-1
TGCATCCTATTGTGAAACCCGAGGCAGAGGAGCAAGCTTTACCTGTTCTA
CONSENSUS
TGCATCCTATTGTGAAACCCGAGGCAGAGGAGCAAGCTTTACCTGTTCTA

A5-AT5G35210-XLOC_030529-4361-0
ACAGAATGGGATTTGTCTGGTGAGCTACTTTTCGACTACGAGGACATGGA
A5-AT5G35210-XLOC_030529-4361-1
ACAGAATGGGATTTGTCTGGTGAGCTACTTTTCGACTACGAGGACATGGA
CONSENSUS
ACAGAATGGGATTTGTCTGGTGAGCTACTTTTCGACTACGAGGACATGGA

A5-AT5G35210-XLOC_030529-4361-0
ATTTGAACCGCAAACTTATTTCTCGTTGACCGAGCTACTGACAGCCGATG
A5-AT5G35210-XLOC_030529-4361-1
ATTTGAACCGCAAACTTATTTCTCGTTGACCGAGCTACTGACAGCCGATG
CONSENSUS
ATTTGAACCGCAAACTTATTTCTCGTTGACCGAGCTACTGACAGCCGATG

A5-AT5G35210-XLOC_030529-4361-0
ATAGTGGTGGTGGTCAGTACCAGGAAAATGGTGATATGGTTGTTTCAGGA
A5-AT5G35210-XLOC_030529-4361-1
ATAGTGGTGGTGGTCAGTACCAGGAAAATGGTGATATGGTTGTTTCAGGA
CONSENSUS
ATAGTGGTGGTGGTCAGTACCAGGAAAATGGTGATATGGTTGTTTCAGGA

A5-AT5G35210-XLOC_030529-4361-0
AACCCTCAGTTCGAACCAACAGAAAAAGAAGAGTGTGAAGATGATATGGG
A5-AT5G35210-XLOC_030529-4361-1
AACCCTCAGTTCGAACCAACAGAAAAAGAAGAGTGTGAAGATGATATGGG
CONSENSUS
AACCCTCAGTTCGAACCAACAGAAAAAGAAGAGTGTGAAGATGATATGGG

A5-AT5G35210-XLOC_030529-4361-0
TCCATGTCAGAGATGTTTGCAAATGGATCCAGCGCCTGATCTTTTGTGCA
A5-AT5G35210-XLOC_030529-4361-1
TCCATGTCAGAGATGTTTGCAAATGGATCCAGCGCCTGATCTTTTGTGCA
CONSENSUS
TCCATGTCAGAGATGTTTGCAAATGGATCCAGCGCCTGATCTTTTGTGCA

A5-AT5G35210-XLOC_030529-4361-0
CGGTTTGTGGATTGCTTATACATTCTCACTGTTCTCCATGGTCAGCCTTA
A5-AT5G35210-XLOC_030529-4361-1

CG---
CONSENSUS
CG...

A5-AT5G35210-XLOC_030529-4361-0
CCCGGAAGTAGCTGGAGCTGTGGTCAATGCCGTGAGTGGCAGTAGCTGGA
A5-AT5G35210-XLOC_030529-4361-1

CONSENSUS
....

A5-AT5G35210-XLOC_030529-4361-0
CCCTTTGATTTCAAGCCCCTTCCCAAATTTTAACTTCCACATTCTTTGAC
A5-AT5G35210-XLOC_030529-4361-1

CONSENSUS
....

A5-AT5G35210-XLOC_030529-4361-0
AATGAATTTGAGGGAAAATTGTATCCTCCTTTGTTTGGCTTATAATGGCA
A5-AT5G35210-XLOC_030529-4361-1

CONSENSUS
....

A5-AT5G35210-XLOC_030529-4361-0
AATGAGCTGTAATTTCTAGTTTTTTTTTAGGAATTAGGGCACTAGGAAGC
A5-AT5G35210-XLOC_030529-4361-1 ------------------------------
GAATTAGGGCACTAGGAAGC
CONSENSUS
..............................GAATTAGGGCACTAGGAAGC

A5-AT5G35210-XLOC_030529-4361-0
ATTACACTAGGAAGCTTTGGTGCAATCACACAG
A5-AT5G35210-XLOC_030529-4361-1
ATTACACTAGGAAGCTTTGGTGCAATCACACAG
CONSENSUS
ATTACACTAGGAAGCTTTGGTGCAATCACACAG

alignment for event: A3-AT5G05570-XLOC_025371-5110

A3-AT5G05570-XLOC_025371-5110-0
ATGTTAGGTCTTGATTGGTCTTCTGGTATGGGAGGTCTGAAATGTGTTGG
A3-AT5G05570-XLOC_025371-5110-1
ATGTTAGGTCTTGATTGGTCTTCTGGTATGGGAGGTCTGAAATGTGTTGG
CONSENSUS
ATGTTAGGTCTTGATTGGTCTTCTGGTATGGGAGGTCTGAAATGTGTTGG

A3-AT5G05570-XLOC_025371-5110-0
CCGTGCTGACCTGACCCTTAGTGGTTCATTTGCTGATATGGTCTTGTCTC
A3-AT5G05570-XLOC_025371-5110-1
CCGTGCTGACCTGACCCTTAGTGGTTCATTTGCTGATATGGTCTTGTCTC
CONSENSUS
CCGTGCTGACCTGACCCTTAGTGGTTCATTTGCTGATATGGTCTTGTCTC

A3-AT5G05570-XLOC_025371-5110-0
CAATTGCCAGTTCAAGGCAGAGTGGGGTGTTTTTATTTCTGTTGACAAAT
A3-AT5G05570-XLOC_025371-5110-1
CAATTGCCAGTTCAAGGCAGAGTGGGGTGTTTTTATTTCTGTTGACAAAT
CONSENSUS
CAATTGCCAGTTCAAGGCAGAGTGGGGTGTTTTTATTTCTGTTGACAAAT

A3-AT5G05570-XLOC_025371-5110-0
CCAGGACAACTGCAAGCATATGACGATACCTCTCTGGCTTCTCTGATGTC
A3-AT5G05570-XLOC_025371-5110-1
CCAGGACAACTGCAAGCATATGACGATACCTCTCTGGCTTCTCTGATGTC
CONSENSUS
CCAGGACAACTGCAAGCATATGACGATACCTCTCTGGCTTCTCTGATGTC

A3-AT5G05570-XLOC_025371-5110-0
TCAGAAAGAGAATAAGATTTCTGTTTCTCCACTGCCATATCCAATGGTTG
A3-AT5G05570-XLOC_025371-5110-1
TCAGAAAGAGAATAAGATTTCTGTTTCTCCACTGCCATATCCAATGGTTG
CONSENSUS
TCAGAAAGAGAATAAGATTTCTGTTTCTCCACTGCCATATCCAATGGTTG

A3-AT5G05570-XLOC_025371-5110-0
TACCAACGATGGATCCACATATGACAGTAGCAACGTTTTCTGCATTAAAT
A3-AT5G05570-XLOC_025371-5110-1
TACCAACGATGGATCCACATATGACAGTAGCAACGTTTTCTGCATTAAAT
CONSENSUS
TACCAACGATGGATCCACATATGACAGTAGCAACGTTTTCTGCATTAAAT

A3-AT5G05570-XLOC_025371-5110-0
GTGAATGATAAAACGTCATTAGCTCTTTCTGAGATAGTCTTAGCTGCAAA
A3-AT5G05570-XLOC_025371-5110-1
GTGAATGATAAAACGTCATTAGCTCTTTCTGAG----TCTTAGCTGCAAA
CONSENSUS
GTGAATGATAAAACGTCATTAGCTCTTTCTGAG....TCTTAGCTGCAAA

A3-AT5G05570-XLOC_025371-5110-0
AGCTCGAACCCCTCGTACCCCATCCGGAGAAAGTGCACAATGGCCTCTGA
A3-AT5G05570-XLOC_025371-5110-1
AGCTCGAACCCCTCGTACCCCATCCGGAGAAAGTGCACAATGGCCTCTGA
CONSENSUS
AGCTCGAACCCCTCGTACCCCATCCGGAGAAAGTGCACAATGGCCTCTGA

A3-AT5G05570-XLOC_025371-5110-0
CAGGTGGTGTTCCAAGCCACGTTGATGACTACAAGCTTGAAAGACTTTAC
A3-AT5G05570-XLOC_025371-5110-1
CAGGTGGTGTTCCAAGCCACGTTGATGACTACAAGCTTGAAAGACTTTAC
CONSENSUS
CAGGTGGTGTTCCAAGCCACGTTGATGACTACAAGCTTGAAAGACTTTAC

A3-AT5G05570-XLOC_025371-5110-0
ATGGCAGGATATCAAGATGGGTCTATGCGAATATGGGATGCAACCTATCC
A3-AT5G05570-XLOC_025371-5110-1
ATGGCAGGATATCAAGATGGGTCTATGCGAATATGGGATGCAACCTATCC
CONSENSUS
ATGGCAGGATATCAAGATGGGTCTATGCGAATATGGGATGCAACCTATCC

A3-AT5G05570-XLOC_025371-5110-0
ATGTCTGTCCCTTATTTATATTTTGGAGCCAAAG
A3-AT5G05570-XLOC_025371-5110-1
ATGTCTGTCCCTTATTTATATTTTGGAGCCAAAG
CONSENSUS
ATGTCTGTCCCTTATTTATATTTTGGAGCCAAAG

alignment for event: A5-AT5G35210-XLOC_030529-4362

A5-AT5G35210-XLOC_030529-4362-0
AGTGGTTCCACACTGAAGCTGTTAAGCTCAAAGATTCACAAATTCCTGAA
A5-AT5G35210-XLOC_030529-4362-1
AGTGGTTCCACACTGAAGCTGTTAAGCTCAAAGATTCACAAATTCCTGAA
CONSENSUS
AGTGGTTCCACACTGAAGCTGTTAAGCTCAAAGATTCACAAATTCCTGAA

A5-AT5G35210-XLOC_030529-4362-0
GTTGTTGGGTTCAAATGTTGCAAATGTCGACGTATACGATCCCCTGATTG
A5-AT5G35210-XLOC_030529-4362-1
GTTGTTGGGTTCAAATGTTGCAAATGTCGACGTATACGATCCCCTGATTG
CONSENSUS
GTTGTTGGGTTCAAATGTTGCAAATGTCGACGTATACGATCCCCTGATTG

A5-AT5G35210-XLOC_030529-4362-0
CCCTTACATGGATCCCAAACTCAAGGAACAGAAGCAGATCAAAAGAATAG
A5-AT5G35210-XLOC_030529-4362-1
CCCTTACATGGATCCCAAACTCAAGGAACAGAAGCAGATCAAAAGAATAG
CONSENSUS
CCCTTACATGGATCCCAAACTCAAGGAACAGAAGCAGATCAAAAGAATAG

A5-AT5G35210-XLOC_030529-4362-0
TCTTCACGAACCAGAAACAGAGGCAAGGAAATTCTGGGTTGGATTCTGAT
A5-AT5G35210-XLOC_030529-4362-1
TCTTCACGAACCAGAAACAGAGGCAAGGAAATTCTGGGTTGGATTCTGAT
CONSENSUS
TCTTCACGAACCAGAAACAGAGGCAAGGAAATTCTGGGTTGGATTCTGAT

A5-AT5G35210-XLOC_030529-4362-0
TCTGAAAGAATGTCTGAACAAAAAGACTCGAAACCTTCTACTCCGTTACC
A5-AT5G35210-XLOC_030529-4362-1
TCTGAAAGAATGTCTGAACAAAAAGACTCGAAACCTTCTACTCCGTTACC
CONSENSUS
TCTGAAAGAATGTCTGAACAAAAAGACTCGAAACCTTCTACTCCGTTACC

A5-AT5G35210-XLOC_030529-4362-0
TGCCACTCCTTTATATCCTCCTGACGATGTATTTATTCCAGAAGATGATC
A5-AT5G35210-XLOC_030529-4362-1
TGCCACTCCTTTATATCCTCCTGACGATGTATTTATTCCAGAAGATGATC
CONSENSUS
TGCCACTCCTTTATATCCTCCTGACGATGTATTTATTCCAGAAGATGATC

A5-AT5G35210-XLOC_030529-4362-0
CTCTCCTGGTATCAGTTTCCAAAGTCAAACAAATCACACCCAGTAGTTTC

A5-AT5G35210-XLOC_030529-4362-1
CTCTCCTGGTATCAGTTTCCAAAGTCAAACAAATCACACCCAGTAGTTTC
CONSENSUS
CTCTCCTGGTATCAGTTTCCAAAGTCAAACAAATCACACCCAGTAGTTTC

A5-AT5G35210-XLOC_030529-4362-0
GATCTTGAATGGAGCACCACTGCTTTTGCGCCTGGACCCCAAAAGCTACC
A5-AT5G35210-XLOC_030529-4362-1
GATCTTGAATGGAGCACCACTGCTTTTGCGCCTGGACCCCAAAAGCTACC
CONSENSUS
GATCTTGAATGGAGCACCACTGCTTTTGCGCCTGGACCCCAAAAGCTACC

A5-AT5G35210-XLOC_030529-4362-0
AGTTAGAAGGCAAGTGAAACGAGAAGATTCTGATGCAGCCTATCCTGAAC
A5-AT5G35210-XLOC_030529-4362-1
AGTTAGAAGGCAAGTGAAACGAGAAGATTCTGATGCAGCCTATCCTGAAC
CONSENSUS
AGTTAGAAGGCAAGTGAAACGAGAAGATTCTGATGCAGCCTATCCTGAAC

A5-AT5G35210-XLOC_030529-4362-0
TGCATCCTATTGTGAAACCCGAGGCAGAGGAGCAAGCTTTACCTGTTCTA
A5-AT5G35210-XLOC_030529-4362-1
TGCATCCTATTGTGAAACCCGAGGCAGAGGAGCAAGCTTTACCTGTTCTA
CONSENSUS
TGCATCCTATTGTGAAACCCGAGGCAGAGGAGCAAGCTTTACCTGTTCTA

A5-AT5G35210-XLOC_030529-4362-0
ACAGAATGGGATTTGTCTGGTGAGCTACTTTTCGACTACGAGGACATGGA
A5-AT5G35210-XLOC_030529-4362-1
ACAGAATGGGATTTGTCTGGTGAGCTACTTTTCGACTACGAGGACATGGA
CONSENSUS
ACAGAATGGGATTTGTCTGGTGAGCTACTTTTCGACTACGAGGACATGGA

A5-AT5G35210-XLOC_030529-4362-0
ATTTGAACCGCAAACTTATTTCTCGTTGACCGAGCTACTGACAGCCGATG
A5-AT5G35210-XLOC_030529-4362-1
ATTTGAACCGCAAACTTATTTCTCGTTGACCGAGCTACTGACAGCCGATG
CONSENSUS
ATTTGAACCGCAAACTTATTTCTCGTTGACCGAGCTACTGACAGCCGATG

A5-AT5G35210-XLOC_030529-4362-0
ATAGTGGTGGTGGTCAGTACCAGGAAAATGGTGATATGGTTGTTTCAGGA
A5-AT5G35210-XLOC_030529-4362-1
ATAGTGGTGGTGGTCAGTACCAGGAAAATGGTGATATGGTTGTTTCAGGA
CONSENSUS
ATAGTGGTGGTGGTCAGTACCAGGAAAATGGTGATATGGTTGTTTCAGGA

A5-AT5G35210-XLOC_030529-4362-0
AACCCTCAGTTCGAACCAACAGAAAAAGAAGAGTGTGAAGATGATATGGG
A5-AT5G35210-XLOC_030529-4362-1
AACCCTCAGTTCGAACCAACAGAAAAAGAAGAGTGTGAAGATGATATGGG
CONSENSUS
AACCCTCAGTTCGAACCAACAGAAAAAGAAGAGTGTGAAGATGATATGGG

A5-AT5G35210-XLOC_030529-4362-0
TCCATGTCAGAGATGTTTGCAAATGGATCCAGCGCCTGATCTTTTGTGCA

A5-AT5G35210-XLOC_030529-4362-1
      TCCATGTCAGAGATGTTTGCAAATGGATCCAGCGCCTGATCTTTTGTGCA
CONSENSUS
      TCCATGTCAGAGATGTTTGCAAATGGATCCAGCGCCTGATCTTTTGTGCA

A5-AT5G35210-XLOC_030529-4362-0
      CGGTTTGTGGATTGCTTATACATTCTCACTGTTCTCCATGGTCAGCCTTA
A5-AT5G35210-XLOC_030529-4362-1
      CG--
CONSENSUS
      CG..

A5-AT5G35210-XLOC_030529-4362-0
      CCCGGAAGTAGCTGGAGCTGTGGTCAATGCCGTGAGTGGCAGTAGCTGGA
A5-AT5G35210-XLOC_030529-4362-1

CONSENSUS
      ....

A5-AT5G35210-XLOC_030529-4362-0
      CCCTTTGATTTCAAGCCCCTTCCCAAATTTTAACTTCCACATTCTTTGAC
A5-AT5G35210-XLOC_030529-4362-1

CONSENSUS
      ....

A5-AT5G35210-XLOC_030529-4362-0
      AATGAATTTGAGGGAAAATTGTATCCTCCTTTGTTTGGCTTATAATGGCA
A5-AT5G35210-XLOC_030529-4362-1

CONSENSUS
      ....

A5-AT5G35210-XLOC_030529-4362-0
      AATGAGCTGTAATTTCTAGTTTTTTTTTAGGTTTGTCTTCGTCTATTTTA
A5-AT5G35210-XLOC_030529-4362-1

CONSENSUS
      ....

A5-AT5G35210-XLOC_030529-4362-0
      TTTATCTTCGTTTATAGAGTGGATACATGTTTGCTTTCAAATTCATACCA
A5-AT5G35210-XLOC_030529-4362-1

CONSENSUS
      ....

A5-AT5G35210-XLOC_030529-4362-0
      AGCAGGGATGCTTACTTGGTGGAATGATTAACTTTCATGAATTAGGGCAC
A5-AT5G35210-XLOC_030529-4362-1
      ---------------------------------------GAATTAGGGCAC
CONSENSUS
      ....GAATTAGGGCAC

A5-AT5G35210-XLOC_030529-4362-0
      TAGGAAGCATTACACTAGGAAGCTTTGGTGCAATCACACAG

A5-AT5G35210-XLOC_030529-4362-1
TAGGAAGCATTACACTAGGAAGCTTTGGTGCAATCACACAG
CONSENSUS
TAGGAAGCATTACACTAGGAAGCTTTGGTGCAATCACACAG

alignment for event: RI-AT5G46470-XLOC_027583-8128

RI-AT5G46470-XLOC_027583-8128-0
GGATAACGAAGCAAGCATGATCGAAGAAATCGCCAATGATATTTTGGGTA
RI-AT5G46470-XLOC_027583-8128-1
GGATAACGAAGCAAGCATGATCGAAGAAATCGCCAATGATATTTTGGGTA
CONSENSUS
GGATAACGAAGCAAGCATGATCGAAGAAATCGCCAATGATATTTTGGGTA

RI-AT5G46470-XLOC_027583-8128-0
AAATGAATATATCTCCATCAAATGATTTTGAGGACTTGGTCGGTATTGAA
RI-AT5G46470-XLOC_027583-8128-1
AAATGAATATATCTCCATCAAATGATTTTGAGGACTTGGTCGGTATTGAA
CONSENSUS
AAATGAATATATCTCCATCAAATGATTTTGAGGACTTGGTCGGTATTGAA

RI-AT5G46470-XLOC_027583-8128-0
GATCATATCACAAAGATGAGTTCATTGCTGCACTTGGAATCTGAGGAAGT
RI-AT5G46470-XLOC_027583-8128-1
GATCATATCACAAAGATGAGTTCATTGCTGCACTTGGAATCTGAGGAAGT
CONSENSUS
GATCATATCACAAAGATGAGTTCATTGCTGCACTTGGAATCTGAGGAAGT

RI-AT5G46470-XLOC_027583-8128-0
GAGGATGGTCGGGATATGGGGTCCCTCGGGAATTGGCAAAACGACCATTG
RI-AT5G46470-XLOC_027583-8128-1
GAGGATGGTCGGGATATGGGGTCCCTCGGGAATTGGCAAAACGACCATTG
CONSENSUS
GAGGATGGTCGGGATATGGGGTCCCTCGGGAATTGGCAAAACGACCATTG

RI-AT5G46470-XLOC_027583-8128-0
CAAGAGCTCTATTTAGTCGACTCTCTTGTCAGTTTCAAAGTAGTGTTTTC
RI-AT5G46470-XLOC_027583-8128-1
CAAGAGCTCTATTTAGTCGACTCTCTTGTCAGTTTCAAAGTAGTGTTTTC
CONSENSUS
CAAGAGCTCTATTTAGTCGACTCTCTTGTCAGTTTCAAAGTAGTGTTTTC

RI-AT5G46470-XLOC_027583-8128-0
ATTGACAAGGTTTTCATATCTAAGAGTATGGAAGTTTACAGTGGAGCTAA
RI-AT5G46470-XLOC_027583-8128-1
ATTGACAAGGTTTTCATATCTAAGAGTATGGAAGTTTACAGTGGAGCTAA
CONSENSUS
ATTGACAAGGTTTTCATATCTAAGAGTATGGAAGTTTACAGTGGAGCTAA

RI-AT5G46470-XLOC_027583-8128-0
TCTTGTTGACTATAACATGAAGTTGCACTTGCAAAGAGCCTTTCTAGCTG
RI-AT5G46470-XLOC_027583-8128-1
TCTTGTTGACTATAACATGAAGTTGCACTTGCAAAGAGCCTTTCTAGCTG
CONSENSUS

TCTTGTTGACTATAACATGAAGTTGCACTTGCAAAGAGCCTTTCTAGCTG

RI-AT5G46470-XLOC_027583-8128-0
AAATTTTTGACAAAAAGGACATAAAGATACATGTAGGTGCAATGGAAAAG
RI-AT5G46470-XLOC_027583-8128-1
AAATTTTTGACAAAAAGGACATAAAGATACATGTAGGTGCAATGGAAAAG
CONSENSUS
AAATTTTTGACAAAAAGGACATAAAGATACATGTAGGTGCAATGGAAAAG

RI-AT5G46470-XLOC_027583-8128-0
ATGGTAAAGCACCGGAAAGCTCTCATCGTTATTGATGATTTAGATGATCA
RI-AT5G46470-XLOC_027583-8128-1
ATGGTAAAGCACCGGAAAGCTCTCATCGTTATTGATGATTTAGATGATCA
CONSENSUS
ATGGTAAAGCACCGGAAAGCTCTCATCGTTATTGATGATTTAGATGATCA

RI-AT5G46470-XLOC_027583-8128-0
AGATGTGCTAGATGCTTTAGCGGATCAAACTCAATGGTTTGGAAGTGGGA
RI-AT5G46470-XLOC_027583-8128-1
AGATGTGCTAGATGCTTTAGCGGATCAAACTCAATGGTTTGGAAGTGGGA
CONSENSUS
AGATGTGCTAGATGCTTTAGCGGATCAAACTCAATGGTTTGGAAGTGGGA

RI-AT5G46470-XLOC_027583-8128-0
GTAGAATCATTGTGGTTACAGAAAATAAGCATTTTTTAAGGGCCAATCGG
RI-AT5G46470-XLOC_027583-8128-1
GTAGAATCATTGTGGTTACAGAAAATAAGCATTTTTTAAGGGCCAATCGG
CONSENSUS
GTAGAATCATTGTGGTTACAGAAAATAAGCATTTTTTAAGGGCCAATCGG

RI-AT5G46470-XLOC_027583-8128-0
ATTGATCACATTTACAAGGTCTGTCTCCCATCTAATGCGCTGGCTCTTGA
RI-AT5G46470-XLOC_027583-8128-1
ATTGATCACATTTACAAGGTCTGTCTCCCATCTAATGCGCTGGCTCTTGA
CONSENSUS
ATTGATCACATTTACAAGGTCTGTCTCCCATCTAATGCGCTGGCTCTTGA

RI-AT5G46470-XLOC_027583-8128-0
GATGTTTTGTCGTTCTGCTTTTAAGAAGAATTCTCCTCCTGATGACTTTT
RI-AT5G46470-XLOC_027583-8128-1
GATGTTTTGTCGTTCTGCTTTTAAGAAGAATTCTCCTCCTGATGACTTTT
CONSENSUS
GATGTTTTGTCGTTCTGCTTTTAAGAAGAATTCTCCTCCTGATGACTTTT

RI-AT5G46470-XLOC_027583-8128-0
TGGAGCTTTCTTCTGAAGTTGCATTGCGTGCTGGTAATCTTCCTTTGGGC
RI-AT5G46470-XLOC_027583-8128-1
TGGAGCTTTCTTCTGAAGTTGCATTGCGTGCTGGTAATCTTCCTTTGGGC
CONSENSUS
TGGAGCTTTCTTCTGAAGTTGCATTGCGTGCTGGTAATCTTCCTTTGGGC

RI-AT5G46470-XLOC_027583-8128-0
CTGAACGTATTGGGTTCAAATTTACGGGGCATAAACAAAGGTTACTGGAT
RI-AT5G46470-XLOC_027583-8128-1
CTGAACGTATTGGGTTCAAATTTACGGGGCATAAACAAAGGTTACTGGAT
CONSENSUS

CTGAACGTATTGGGTTCAAATTTACGGGGCATAAACAAAGGTTACTGGAT

RI-AT5G46470-XLOC_027583-8128-0
AGATATGCTACCGAGGCTTCAAGGTTTGGATGGAAAAATAGGGAAAACAC
RI-AT5G46470-XLOC_027583-8128-1
AGATATGCTACCGAGGCTTCAAGGTTTGGATGGAAAAATAGGGAAAACAC
CONSENSUS
AGATATGCTACCGAGGCTTCAAGGTTTGGATGGAAAAATAGGGAAAACAC

RI-AT5G46470-XLOC_027583-8128-0
TAAGAGTCAGCTATGATGGGTTGAATAACAGAAAAGATGAAGCAATATTT
RI-AT5G46470-XLOC_027583-8128-1
TAAGAGTCAGCTATGATGGGTTGAATAACAGAAAAGATGAAGCAATATTT
CONSENSUS
TAAGAGTCAGCTATGATGGGTTGAATAACAGAAAAGATGAAGCAATATTT

RI-AT5G46470-XLOC_027583-8128-0
CGTCACATTGCATGTATTTTCAATGGTGAGAAAGTCAGTGACATCAAACT
RI-AT5G46470-XLOC_027583-8128-1
CGTCACATTGCATGTATTTTCAATG-------------------------
CONSENSUS
CGTCACATTGCATGTATTTTCAATG.........................

RI-AT5G46470-XLOC_027583-8128-0
ATTACTAGCAAATAGTAACTTGGATGTTAATATCGGGCTAAAAAACCTTG
RI-AT5G46470-XLOC_027583-8128-1

CONSENSUS
....

RI-AT5G46470-XLOC_027583-8128-0
TTGATAGATCCCTTATCTGTGAAAGATTCAATACTCTGGAGATGCACTCT
RI-AT5G46470-XLOC_027583-8128-1 -------
ATCCCTTATCTGTGAAAGATTCAATACTCTGGAGATGCACTCT
CONSENSUS
.......ATCCCTTATCTGTGAAAGATTCAATACTCTGGAGATGCACTCT

RI-AT5G46470-XLOC_027583-8128-0
TTGCTACAAGAATTGGGTAAGGAGATTGTCCGTACTCAGTCCAATCAGCC
RI-AT5G46470-XLOC_027583-8128-1
TTGCTACAAGAATTGGGTAAGGAGATTGTCCGTACTCAGTCCAATCAGCC
CONSENSUS
TTGCTACAAGAATTGGGTAAGGAGATTGTCCGTACTCAGTCCAATCAGCC

RI-AT5G46470-XLOC_027583-8128-0
TGGAGAACGGGAGTTCCTTGTGGATTTGAAGGATATTTGCGATGTACTTG
RI-AT5G46470-XLOC_027583-8128-1
TGGAGAACGGGAGTTCCTTGTGGATTTGAAGGATATTTGCGATGTACTTG
CONSENSUS
TGGAGAACGGGAGTTCCTTGTGGATTTGAAGGATATTTGCGATGTACTTG

RI-AT5G46470-XLOC_027583-8128-0 AACATAACACT
RI-AT5G46470-XLOC_027583-8128-1 AACATAACACT
CONSENSUS AACATAACACT

alignment for event: A3-AT5G16030-XLOC_025888-10744

A3-AT5G16030-XLOC_025888-10744-0
      AAGAGATTGACCCATCAACATATTCCTTCACCAATGCACTCAAAGCAGCA
A3-AT5G16030-XLOC_025888-10744-1
      AAGAGATTGACCCATCAACATATTCCTTCACCAATGCACTCAAAG---CA
CONSENSUS
      AAGAGATTGACCCATCAACATATTCCTTCACCAATGCACTCAAAG...CA

A3-AT5G16030-XLOC_025888-10744-0
      TTACAAGCAAAGACAATGTACAATAACAGGGAATGGTTAGCACAAGAAGG
A3-AT5G16030-XLOC_025888-10744-1
      TTACAAGCAAAGACAATGTACAATAACAGGGAATGGTTAGCACAAGAAGG
CONSENSUS
      TTACAAGCAAAGACAATGTACAATAACAGGGAATGGTTAGCACAAGAAGG

A3-AT5G16030-XLOC_025888-10744-0
      GTTTGCATTGAATTCGAAGTGGAACGAAGCAGAGAAATATATCTGCAATC
A3-AT5G16030-XLOC_025888-10744-1
      GTTTGCATTGAATTCGAAGTGGAACGAAGCAGAGAAATATATCTGCAATC
CONSENSUS
      GTTTGCATTGAATTCGAAGTGGAACGAAGCAGAGAAATATATCTGCAATC

A3-AT5G16030-XLOC_025888-10744-0
      CATTGTCTGGAGAAGTACCAATGGAGTGTTTGTCTGCTAAAACATTAAGT
A3-AT5G16030-XLOC_025888-10744-1
      CATTGTCTGGAGAAGTACCAATGGAGTGTTTGTCTGCTAAAACATTAAGT
CONSENSUS
      CATTGTCTGGAGAAGTACCAATGGAGTGTTTGTCTGCTAAAACATTAAGT

A3-AT5G16030-XLOC_025888-10744-0
      GCAAGATCCTTCAGAAACTTATCAACCATGTCTGCTCCTCTTCACTTCCC
A3-AT5G16030-XLOC_025888-10744-1
      GCAAGATCCTTCAGAAACTTATCAACCATGTCTGCTCCTCTTCACTTCCC
CONSENSUS
      GCAAGATCCTTCAGAAACTTATCAACCATGTCTGCTCCTCTTCACTTCCC

A3-AT5G16030-XLOC_025888-10744-0
      TAGTCCCAATCCATTGATGAATAATATTGCTCAAAACAAACCAAATAATA
A3-AT5G16030-XLOC_025888-10744-1
      TAGTCCCAATCCATTGATGAATAATATTGCTCAAAACAAACCAAATAATA
CONSENSUS
      TAGTCCCAATCCATTGATGAATAATATTGCTCAAAACAAACCAAATAATA

A3-AT5G16030-XLOC_025888-10744-0
      ATCCTAACGTTAGAGTCATTCACGAGGATCTTTATGCTCCTGATCCTGAA
A3-AT5G16030-XLOC_025888-10744-1
      ATCCTAACGTTAGAGTCATTCACGAGGATCTTTATGCTCCTGATCCTGAA
CONSENSUS
      ATCCTAACGTTAGAGTCATTCACGAGGATCTTTATGCTCCTGATCCTGAA

A3-AT5G16030-XLOC_025888-10744-0 CTTCTTGCTCTAG
A3-AT5G16030-XLOC_025888-10744-1 CTTCTTGCTCTAG
CONSENSUS   CTTCTTGCTCTAG

alignment for event: A3-AT5G11170-XLOC_025653-5520

A3-AT5G11170-XLOC_025653-5520-0
ACATGCGAAGGGATGTGCAGGAGATTTTCAAGATGACTCCTCATGACAAA
A3-AT5G11170-XLOC_025653-5520-1
ACATGCGAAGGGATGTGCAGGAGATTTTCAAGATGACTCCTCATGACAAA
CONSENSUS
ACATGCGAAGGGATGTGCAGGAGATTTTCAAGATGACTCCTCATGACAAA

A3-AT5G11170-XLOC_025653-5520-0
CAAGTAATGATGTTCTCAGCAACGCTCAGCAAAGAGATACGCCCAGTCTG
A3-AT5G11170-XLOC_025653-5520-1
CAAGTAATGATGTTCTCAGCAACGCTCAGCAAAGAGATACGCCCAGTCTG
CONSENSUS
CAAGTAATGATGTTCTCAGCAACGCTCAGCAAAGAGATACGCCCAGTCTG

A3-AT5G11170-XLOC_025653-5520-0
CAAAAAATTTATGCAAGATTGCTGGGTGGGTTTTGGGTGGGGTACTGTGT
A3-AT5G11170-XLOC_025653-5520-1
CAAAAAATTTATGCAAGAT-------------------------------
CONSENSUS
CAAAAAATTTATGCAAGAT...............................

A3-AT5G11170-XLOC_025653-5520-0
TGTCCCAAGGTTTCATTGTGATTGTATGGGCCTTAATGTTCCGAGCAATA
A3-AT5G11170-XLOC_025653-5520-1

CONSENSUS
....

A3-AT5G11170-XLOC_025653-5520-0
TCGCTGTATCATAGCAAAACTCACATCTATGAAGAGAACCTGGTGGACGA
A3-AT5G11170-XLOC_025653-5520-1

CONSENSUS
....

A3-AT5G11170-XLOC_025653-5520-0
GGATCTCAGATCAGGGGTTTTACATCCATCTTCACTTTTGTAGTGTAAAT
A3-AT5G11170-XLOC_025653-5520-1

CONSENSUS
....

A3-AT5G11170-XLOC_025653-5520-0
CATTTCCTGAGAAAAGCTTGCTAATTATTACCTGATATCTATTCCTTTCA
A3-AT5G11170-XLOC_025653-5520-1

CONSENSUS
....

A3-AT5G11170-XLOC_025653-5520-0
GCCAATGGAAATATATGTCGATGATGAAGCCAAGTTGACTCTTCATGGGC
A3-AT5G11170-XLOC_025653-5520-1 -

CCAATGGAAATATATGTCGATGATGAAGCCAAGTTGACTCTTCATGGGC
CONSENSUS
.CCAATGGAAATATATGTCGATGATGAAGCCAAGTTGACTCTTCATGGGC

A3-AT5G11170-XLOC_025653-5520-0 TTGTCCAG
A3-AT5G11170-XLOC_025653-5520-1 TTGTCCAG
CONSENSUS TTGTCCAG

alignment for event: A3-AT5G24430-XLOC_029989-10827

A3-AT5G24430-XLOC_029989-10827-0
ATGCAACCTCTGGCGCAAAAGAAGCTTGACTTTGAAGAGTTCTGTGCGGC
A3-AT5G24430-XLOC_029989-10827-1
ATGCAACCTCTGGCGCAAAAGAAGCTTGACTTTGAAGAGTTCTGTGCGGC
CONSENSUS
ATGCAACCTCTGGCGCAAAAGAAGCTTGACTTTGAAGAGTTCTGTGCGGC

A3-AT5G24430-XLOC_029989-10827-0
TGCGGTCAGTGTTTACCAACTGGAGGCTCTTGAAGAATGGGAACAGATTG
A3-AT5G24430-XLOC_029989-10827-1
TGCGGTCAGTGTTTACCAACTGGAGGCTCTTGAAGAATGGGAACAGATTG
CONSENSUS
TGCGGTCAGTGTTTACCAACTGGAGGCTCTTGAAGAATGGGAACAGATTG

A3-AT5G24430-XLOC_029989-10827-0
CAACTTCAGCATTTGAGCACTTTGAACATGAAGGAAACCGAATCATATCT
A3-AT5G24430-XLOC_029989-10827-1
CAACTTCAGCATTTGAGCACTTTGAACATGAAGGAAACCGAATCATATCT
CONSENSUS
CAACTTCAGCATTTGAGCACTTTGAACATGAAGGAAACCGAATCATATCT

A3-AT5G24430-XLOC_029989-10827-0 GTCCAAGAACTCGCCGGG---
GAGATGAGCGTGGGACCGAGTGCATATCC
A3-AT5G24430-XLOC_029989-10827-1
GTCCAAGAACTCGCCGGGCAGGAGATGAGCGTGGGACCGAGTGCATATCC
CONSENSUS
GTCCAAGAACTCGCCGGG...GAGATGAGCGTGGGACCGAGTGCATATCC

A3-AT5G24430-XLOC_029989-10827-0
TCTGCTCAAGGATTGGATCCGAAGTTCGGATGGAAAGCTGAGTTTCTTGG
A3-AT5G24430-XLOC_029989-10827-1
TCTGCTCAAGGATTGGATCCGAAGTTCGGATGGAAAGCTGAGTTTCTTGG
CONSENSUS
TCTGCTCAAGGATTGGATCCGAAGTTCGGATGGAAAGCTGAGTTTCTTGG

A3-AT5G24430-XLOC_029989-10827-0
GGTACGCTAAGTTCTTGCATGGTGTGACCGTACGAAGCTCAAGCTCGAGA
A3-AT5G24430-XLOC_029989-10827-1
GGTACGCTAAGTTCTTGCATGGTGTGACCGTACGAAGCTCAAGCTCGAGA
CONSENSUS
GGTACGCTAAGTTCTTGCATGGTGTGACCGTACGAAGCTCAAGCTCGAGA

A3-AT5G24430-XLOC_029989-10827-0
CCTAGGTGACACACCATGATGTAAAAAGTCTTCTAACATGTGAATTGAGT

A3-AT5G24430-XLOC_029989-10827-1
      CCTAGGTGACACACCATGATGTAAAAAGTCTTCTAACATGTGAATTGAGT
CONSENSUS
      CCTAGGTGACACACCATGATGTAAAAAGTCTTCTAACATGTGAATTGAGT

A3-AT5G24430-XLOC_029989-10827-0
      TTCAAGAAACATCCTAAACAATGTCGTCGTCAGAGACCTTGGATTCAAAG
A3-AT5G24430-XLOC_029989-10827-1
      TTCAAGAAACATCCTAAACAATGTCGTCGTCAGAGACCTTGGATTCAAAG
CONSENSUS
      TTCAAGAAACATCCTAAACAATGTCGTCGTCAGAGACCTTGGATTCAAAG

A3-AT5G24430-XLOC_029989-10827-0
      ATTAAAAAAAAAAAACTTGAAATGGAAGTGAAGTCAGGTAGGTATTTCCTT
A3-AT5G24430-XLOC_029989-10827-1
      ATTAAAAAAAAAAAACTTGAAATGGAAGTGAAGTCAGGTAGGTATTTCCTT
CONSENSUS
      ATTAAAAAAAAAAAACTTGAAATGGAAGTGAAGTCAGGTAGGTATTTCCTT

A3-AT5G24430-XLOC_029989-10827-0
      TGTAAATGATTCTTTGAGCATTTTGTTTTCGAAACAAACTAAAAAAGGAA
A3-AT5G24430-XLOC_029989-10827-1
      TGTAAATGATTCTTTGAGCATTTTGTTTTCGAAACAAACTAAAAAAGGAA
CONSENSUS
      TGTAAATGATTCTTTGAGCATTTTGTTTTCGAAACAAACTAAAAAAGGAA

A3-AT5G24430-XLOC_029989-10827-0
      AAACATATTATATATGTTATAGAAGGAATTGGAGAGGTGTTGTTCTTCTG
A3-AT5G24430-XLOC_029989-10827-1
      AAACATATTATATATGTTATAGAAGGAATTGGAGAGGTGTTGTTCTTCTG
CONSENSUS
      AAACATATTATATATGTTATAGAAGGAATTGGAGAGGTGTTGTTCTTCTG

A3-AT5G24430-XLOC_029989-10827-0 GTTTT
A3-AT5G24430-XLOC_029989-10827-1 GTTTT
CONSENSUS GTTTT

alignment for event: A3-AT5G47690-XLOC_027655-8739

A3-AT5G47690-XLOC_027655-8739-0
      GCCAAAAGGAAAAAGAACTCCAAAGAAAAACTTAAAACAGTTACACCCAA
A3-AT5G47690-XLOC_027655-8739-1
      GCCAAAAGGAAAAAGAACTCCAAAGAAAAACTTAAAACAGTTACACCCAA
CONSENSUS
      GCCAAAAGGAAAAAGAACTCCAAAGAAAAACTTAAAACAGTTACACCCAA

A3-AT5G47690-XLOC_027655-8739-0
      AAGATACACCAAAAAGTCTTTCTTTAGAACATGAGAAAGTAGAGAGCAGA
A3-AT5G47690-XLOC_027655-8739-1
      AAGATACACCAAAAAGTCTTTCTTTAGAACATGAGAAAGTAGAGAGCAGA
CONSENSUS
      AAGATACACCAAAAAGTCTTTCTTTAGAACATGAGAAAGTAGAGAGCAGA

A3-AT5G47690-XLOC_027655-8739-0

AACAAGAAAAGACGATCTTCTGCTTTGCCAATAGAGACAGAATATAGTGG
A3-AT5G47690-XLOC_027655-8739-1
AACAAGAAAAGACGATCTTCTGCTTTGCCAA---AGACAGAATATAGTGG
CONSENSUS
AACAAGAAAAGACGATCTTCTGCTTTGCCAA...AGACAGAATATAGTGG

A3-AT5G47690-XLOC_027655-8739-0
GGAGGCTGGCGAAGAAAAATCAGAGTCTGAAGGAAAGTCGTTGAAAGAAG
A3-AT5G47690-XLOC_027655-8739-1
GGAGGCTGGCGAAGAAAAATCAGAGTCTGAAGGAAAGTCGTTGAAAGAAG
CONSENSUS
GGAGGCTGGCGAAGAAAAATCAGAGTCTGAAGGAAAGTCGTTGAAAGAAG

A3-AT5G47690-XLOC_027655-8739-0
GTGAGGATGATGAGGAAGTTGTAAACAAGGAAGAAGATTTACAGGAGGCA
A3-AT5G47690-XLOC_027655-8739-1
GTGAGGATGATGAGGAAGTTGTAAACAAGGAAGAAGATTTACAGGAGGCA
CONSENSUS
GTGAGGATGATGAGGAAGTTGTAAACAAGGAAGAAGATTTACAGGAGGCA

A3-AT5G47690-XLOC_027655-8739-0
AAAACAGAGTCGAGTGGGGACGCTGAGGGGAAAGAAGCTGAACATGACGA
A3-AT5G47690-XLOC_027655-8739-1
AAAACAGAGTCGAGTGGGGACGCTGAGGGGAAAGAAGCTGAACATGACGA
CONSENSUS
AAAACAGAGTCGAGTGGGGACGCTGAGGGGAAAGAAGCTGAACATGACGA

A3-AT5G47690-XLOC_027655-8739-0
CTCAGATACTGAAGGGAAACAAGAAAACAATGAAATGGAGAGAGAAGCTG
A3-AT5G47690-XLOC_027655-8739-1
CTCAGATACTGAAGGGAAACAAGAAAACAATGAAATGGAGAGAGAAGCTG
CONSENSUS
CTCAGATACTGAAGGGAAACAAGAAAACAATGAAATGGAGAGAGAAGCTG

A3-AT5G47690-XLOC_027655-8739-0
AAGAGAATGCTGAGACTTCTGACAATGAGACTCTT
A3-AT5G47690-XLOC_027655-8739-1
AAGAGAATGCTGAGACTTCTGACAATGAGACTCTT
CONSENSUS
AAGAGAATGCTGAGACTTCTGACAATGAGACTCTT

alignment for event: A3-AT5G42900-XLOC_031025-2810

A3-AT5G42900-XLOC_031025-2810-0
TTCAAGGTTCTTCATGATGGTTTCTGGCAGAAGATTAATGTGAAACAACC
A3-AT5G42900-XLOC_031025-2810-1
TTCAAGGTTCTTCATGATGGTTTCTGGCAGAAGATTAATGTGAAACAACC
CONSENSUS
TTCAAGGTTCTTCATGATGGTTTCTGGCAGAAGATTAATGTGAAACAACC

A3-AT5G42900-XLOC_031025-2810-0
TGAACATCGGATTAACGGAAGGCACGGTGGTAATTCTCATGAGTTTCTTA
A3-AT5G42900-XLOC_031025-2810-1
TGAACATCGGATTAACGGAAGGCACGGTGGTAATTCTCATGAGTTTCTTA

CONSENSUS
TGAACATCGGATTAACGGAAGGCACGGTGGTAATTCTCATGAGTTTCTTA

A3-AT5G42900-XLOC_031025-2810-0
GGAGTCCATGGATTAAGCATTATAAACCTTTAGTAAAGACACAAATCCCG
A3-AT5G42900-XLOC_031025-2810-1
GGAGTCCATGGATTAAGCATTATAAACCTTTAGTAAAGACACAAATCCCG
CONSENSUS
GGAGTCCATGGATTAAGCATTATAAACCTTTAGTAAAGACACAAATCCCG

A3-AT5G42900-XLOC_031025-2810-0
GTAACGGATGAGCCCGAAAATCAAGTTGTTAGCAGCTCTAATGGGAAGAA
A3-AT5G42900-XLOC_031025-2810-1
GTAACGGATGAGCCCGAAAATCAAGTTGTTAGCAGCTCTAATGGGAAGAA
CONSENSUS
GTAACGGATGAGCCCGAAAATCAAGTTGTTAGCAGCTCTAATGGGAAGAA

A3-AT5G42900-XLOC_031025-2810-0
GGGAATATGCAGCTCTGGCTCAGCCTCTAGTCTCAAGCAGCTAAGCTCTC
A3-AT5G42900-XLOC_031025-2810-1
GGGAATATGCAGCTCTGGCTCAGCCTCTAGTCTCAAGCAGCTAAGCTCTC
CONSENSUS
GGGAATATGCAGCTCTGGCTCAGCCTCTAGTCTCAAGCAGCTAAGCTCTC

A3-AT5G42900-XLOC_031025-2810-0
ATTCGCGTGACCACGACCAAATCAGCGTTGGAGAAG---AGGTATCGGAT
A3-AT5G42900-XLOC_031025-2810-1
ATTCGCGTGACCACGACCAAATCAGCGTTGGAGAAGCAGAGGTATCGGAT
CONSENSUS
ATTCGCGTGACCACGACCAAATCAGCGTTGGAGAAG...AGGTATCGGAT

A3-AT5G42900-XLOC_031025-2810-0
CAGAACTTTGTTAACGAAGGAATAAAAGGCGAAAACGGAAGCTCGAAGAA
A3-AT5G42900-XLOC_031025-2810-1
CAGAACTTTGTTAACGAAGGAATAAAAGGCGAAAACGGAAGCTCGAAGAA
CONSENSUS
CAGAACTTTGTTAACGAAGGAATAAAAGGCGAAAACGGAAGCTCGAAGAA

A3-AT5G42900-XLOC_031025-2810-0
GATGAAGACGGTGATGATGAGTGAATCGTCGAGTACCGATCAG
A3-AT5G42900-XLOC_031025-2810-1
GATGAAGACGGTGATGATGAGTGAATCGTCGAGTACCGATCAG
CONSENSUS
GATGAAGACGGTGATGATGAGTGAATCGTCGAGTACCGATCAG

alignment for event: A5-AT5G63120-XLOC_032139-11618

A5-AT5G63120-XLOC_032139-11618-0
GCTCCTTACACTACTTAAACAGTTAATGGATGGGAGTAAAATCCTAATTT
A5-AT5G63120-XLOC_032139-11618-1
GCTCCTTACACTACTTAAACAGTTAATGGATGGGAGTAAAATCCTAATTT
CONSENSUS
GCTCCTTACACTACTTAAACAGTTAATGGATGGGAGTAAAATCCTAATTT

A5-AT5G63120-XLOC_032139-11618-0
TTGTGGAGACAAAGAGAGGGTGTGATCAAGTGACTAGACAATTGAGAATG
A5-AT5G63120-XLOC_032139-11618-1
TTGTGGAGACAAAGAGAGGGTGTGATCAAGTGACTAGACAATTGAGAATG
CONSENSUS
TTGTGGAGACAAAGAGAGGGTGTGATCAAGTGACTAGACAATTGAGAATG

A5-AT5G63120-XLOC_032139-11618-0
GACGGATGGCCAGCTCTTGCCATACATGGTGACAAGACCCAATCGGAAAG
A5-AT5G63120-XLOC_032139-11618-1
GACGGATGGCCAGCTCTTGCCATACATGGTGACAAGACCCAATCGGAAAG
CONSENSUS
GACGGATGGCCAGCTCTTGCCATACATGGTGACAAGACCCAATCGGAAAG

A5-AT5G63120-XLOC_032139-11618-0
AGACCGAGTCTTGGCAGAATTTAAGAGTGGACGAAGCCCGATAATGACTG
A5-AT5G63120-XLOC_032139-11618-1
AGACCGAGTCTTGGCAGAATTTAAGAGTGGACGAAGCCCGATAATGACTG
CONSENSUS
AGACCGAGTCTTGGCAGAATTTAAGAGTGGACGAAGCCCGATAATGACTG

A5-AT5G63120-XLOC_032139-11618-0
CCACTGATGTAGCAGCAAGGGGACTTGGTAGGATTAACTGTGATACACAA
A5-AT5G63120-XLOC_032139-11618-1
CCACTGATGTAGCAGCAAGGGGACTTG-----------------------
CONSENSUS
CCACTGATGTAGCAGCAAGGGGACTTG.......................

A5-AT5G63120-XLOC_032139-11618-0
TAGGAGAGATTATACTTAGAGCATTTCGTTTTCTTGGGGTTTTAAAGATA
A5-AT5G63120-XLOC_032139-11618-1

CONSENSUS
....

A5-AT5G63120-XLOC_032139-11618-0
ATAAAAAAAGTCGCCTGGCTGCCGCTTTCCACAGTTTCCTCTGGTATTAG
A5-AT5G63120-XLOC_032139-11618-1

CONSENSUS
....

A5-AT5G63120-XLOC_032139-11618-0
GGAAAAAAGCACATAGAAAGAAAAAAGGAGCTTCTTTGAAGAATTTGTTT
A5-AT5G63120-XLOC_032139-11618-1

CONSENSUS
....

A5-AT5G63120-XLOC_032139-11618-0
CCTGCATCCATCTCCCGGGCATGGAGCTGATTGGTTTTTGAGCAGCCTGG
A5-AT5G63120-XLOC_032139-11618-1

CONSENSUS
....

A5-AT5G63120-XLOC_032139-11618-0
GCGACTGACACCTGCATGCATTAATAGTGTTATAACGCCGTCCCAAACAC
A5-AT5G63120-XLOC_032139-11618-1

CONSENSUS
....

A5-AT5G63120-XLOC_032139-11618-0
TCCTTATCCCTGCAAGTTAAGATGTGAAGGACATTAAATGTGTGGTTAAT
A5-AT5G63120-XLOC_032139-11618-1 ---------------------
ATGTGAAGGACATTAAATGTGTGGTTAAT
CONSENSUS
.....................ATGTGAAGGACATTAAATGTGTGGTTAAT

A5-AT5G63120-XLOC_032139-11618-0
TATGATTTCCCAAATACATTGGAGGATTACATCCATAGGATTGGTCGAAC
A5-AT5G63120-XLOC_032139-11618-1
TATGATTTCCCAAATACATTGGAGGATTACATCCATAGGATTGGTCGAAC
CONSENSUS
TATGATTTCCCAAATACATTGGAGGATTACATCCATAGGATTGGTCGAAC

A5-AT5G63120-XLOC_032139-11618-0
CGGGCGTGCAGGAGCTAAAGGAATGGCCTTTACATTCTTCACACATGACA
A5-AT5G63120-XLOC_032139-11618-1
CGGGCGTGCAGGAGCTAAAGGAATGGCCTTTACATTCTTCACACATGACA
CONSENSUS
CGGGCGTGCAGGAGCTAAAGGAATGGCCTTTACATTCTTCACACATGACA

A5-AT5G63120-XLOC_032139-11618-0
ATGCTAAGTTTGCGAGAGAGCTTGTCAAGATCCTTCAAGAAGCTGGTCAA
A5-AT5G63120-XLOC_032139-11618-1
ATGCTAAGTTTGCGAGAGAGCTTGTCAAGATCCTTCAAGAAGCTGGTCAA
CONSENSUS
ATGCTAAGTTTGCGAGAGAGCTTGTCAAGATCCTTCAAGAAGCTGGTCAA

A5-AT5G63120-XLOC_032139-11618-0
GTTGTACCTCCTACTCTCTCCGCACTAGTCCGATCATCTGGTTCTGGTTA
A5-AT5G63120-XLOC_032139-11618-1
GTTGTACCTCCTACTCTCTCCGCACTAGTCCGATCATCTGGTTCTGGTTA
CONSENSUS
GTTGTACCTCCTACTCTCTCCGCACTAGTCCGATCATCTGGTTCTGGTTA

A5-AT5G63120-XLOC_032139-11618-0 TGGAG
A5-AT5G63120-XLOC_032139-11618-1 TGGAG
CONSENSUS TGGAG

alignment for event: RI-AT5G66210-XLOC_032314-12933

RI-AT5G66210-XLOC_032314-12933-0
GCACTTGCCAAAGATCTTCCTTGGAAACTGAAAGACTCACGAGTTGCTGA
RI-AT5G66210-XLOC_032314-12933-1
GCACTTGCCAAAGATCTTCCTTGGAAACTGAAAGACTCACGAGTTGCTGA
CONSENSUS
GCACTTGCCAAAGATCTTCCTTGGAAACTGAAAGACTCACGAGTTGCTGA

RI-AT5G66210-XLOC_032314-12933-0
GATCCTTGAAGCGGTAGTTATATTTTTGTTTTCCATTGCAATTGCATCTC
RI-AT5G66210-XLOC_032314-12933-1
GATCCTTGAAGCG-------------------------------------
CONSENSUS
GATCCTTGAAGCG.....................................

RI-AT5G66210-XLOC_032314-12933-0
TGGGGGTATCAGAGGGAGACGTAGTTTCATAATGAGCCGTTGTGTACTTG
RI-AT5G66210-XLOC_032314-12933-1

CONSENSUS
....

RI-AT5G66210-XLOC_032314-12933-0
TATCTTTGCTGGTCACAGATTGATAGCAACACTGATGGGTTAGTGGACTT
RI-AT5G66210-XLOC_032314-12933-1 ------------------
ATTGATAGCAACACTGATGGGTTAGTGGACTT
CONSENSUS
..................ATTGATAGCAACACTGATGGGTTAGTGGACTT

RI-AT5G66210-XLOC_032314-12933-0
CACAGAGTTTGTAGCAGCAGCTCTACATGTTCATCAACTAGAAGAACATG
RI-AT5G66210-XLOC_032314-12933-1
CACAGAGTTTGTAGCAGCAGCTCTACATGTTCATCAACTAGAAGAACATG
CONSENSUS
CACAGAGTTTGTAGCAGCAGCTCTACATGTTCATCAACTAGAAGAACATG

RI-AT5G66210-XLOC_032314-12933-0
ATTCAGAGAAATGGCAGCTAAGGTCAAGAGCAGCTTTTGAGAAATTCGAC
RI-AT5G66210-XLOC_032314-12933-1
ATTCAGAGAAATGGCAGCTAAGGTCAAGAGCAGCTTTTGAGAAATTCGAC
CONSENSUS
ATTCAGAGAAATGGCAGCTAAGGTCAAGAGCAGCTTTTGAGAAATTCGAC

RI-AT5G66210-XLOC_032314-12933-0
CTAGACAAAGACGGGTACATAACGCCTGAGGAACTTCGAATG
RI-AT5G66210-XLOC_032314-12933-1
CTAGACAAAGACGGGTACATAACGCCTGAGGAACTTCGAATG
CONSENSUS
CTAGACAAAGACGGGTACATAACGCCTGAGGAACTTCGAATG

alignment for event: RI-AT5G48657-XLOC_031374-11254

RI-AT5G48657-XLOC_031374-11254-0
AATTCTTCATTCTGGCTGCTTGACATTCAGAAATATGGCAGTAAGTTTTT
RI-AT5G48657-XLOC_031374-11254-1
AATTCTTCATTCTGGCTGCTTGACATTCAGAAATATGGCA----------
CONSENSUS
AATTCTTCATTCTGGCTGCTTGACATTCAGAAATATGGCA..........

RI-AT5G48657-XLOC_031374-11254-0
TTTTTTCTTTTGGGTGTGGTTTTAGTCTTCTTTTTATTTTGATTTTCTAA

RI-AT5G48657-XLOC_031374-11254-1

CONSENSUS
....

RI-AT5G48657-XLOC_031374-11254-0
AGCTTATTATCCTACAAGTGTAGTATTTATTATACCCACAATCTCTGAGT
RI-AT5G48657-XLOC_031374-11254-1

CONSENSUS
....

RI-AT5G48657-XLOC_031374-11254-0
TTTATTCCGTGAGAATGGAGAGTAATTGTCAAATATTTGTAGTTCTAAAT
RI-AT5G48657-XLOC_031374-11254-1

CONSENSUS
....

RI-AT5G48657-XLOC_031374-11254-0
ATGGATCCTATATCATCCTGTTTAGGACATATAGTGTTTTGTTTTAGAAT
RI-AT5G48657-XLOC_031374-11254-1

CONSENSUS
....

RI-AT5G48657-XLOC_031374-11254-0
CGGCGGTATCATGAGATTTTCTTTTGTTTGACATTTATGCGTAGGAAGAT
RI-AT5G48657-XLOC_031374-11254-1

CONSENSUS
....

RI-AT5G48657-XLOC_031374-11254-0
CACCTAATTCTTTTTGGTCATCTTCTGAACATCAATATAATTCATATGTA
RI-AT5G48657-XLOC_031374-11254-1

CONSENSUS
....

RI-AT5G48657-XLOC_031374-11254-0
ATGAAATTATACTTAGATTCTACAGATGAGGTTTCTGTTCAATCAAAACC
RI-AT5G48657-XLOC_031374-11254-1

CONSENSUS
....

RI-AT5G48657-XLOC_031374-11254-0
AAAAAAAAAGAGATAAGGTTTATGTTTCTTGCACCTTAAGATTCAGCTCT
RI-AT5G48657-XLOC_031374-11254-1

CONSENSUS
....

RI-AT5G48657-XLOC_031374-11254-0
AAACATTTAGCCAAAAACAAAGTTTCAACTGTAAATCTCTGGCATCAACT

RI-AT5G48657-XLOC_031374-11254-1

CONSENSUS
....

RI-AT5G48657-XLOC_031374-11254-0
CCAAAATACCGTATATATGAGATTTCGATTTTCTAATATTTATGTCTCCT
RI-AT5G48657-XLOC_031374-11254-1

CONSENSUS
....

RI-AT5G48657-XLOC_031374-11254-0
ACTTTCAATTTAAATGCAAGCCTATGTTTCTCAAAATCTTTTTTTTTTGG
RI-AT5G48657-XLOC_031374-11254-1

CONSENSUS
....

RI-AT5G48657-XLOC_031374-11254-0
TTCTTGTGGTTATAGAATCGTCCACACGTGCCCAAATTCGGAGACTGGAC
RI-AT5G48657-XLOC_031374-11254-1 ---------------
AATCGTCCACACGTGCCCAAATTCGGAGACTGGAC
CONSENSUS
...............AATCGTCCACACGTGCCCAAATTCGGAGACTGGAC

RI-AT5G48657-XLOC_031374-11254-0
CGAAGATGCTCCATTCACGGTCGTGTTCGAAAAAGCAAGCAAGAGCAAGA
RI-AT5G48657-XLOC_031374-11254-1
CGAAGATGCTCCATTCACGGTCGTGTTCGAAAAAGCAAGCAAGAGCAAGA
CONSENSUS
CGAAGATGCTCCATTCACGGTCGTGTTCGAAAAAGCAAGCAAGAGCAAGA

RI-AT5G48657-XLOC_031374-11254-0
AAAATATGAACGTGGCCAACCCGAATGAATATCCAGATATGAATCCAAAC
RI-AT5G48657-XLOC_031374-11254-1
AAAATATGAACGTGGCCAACCCGAATGAATATCCAGATATGAATCCAAAC
CONSENSUS
AAAATATGAACGTGGCCAACCCGAATGAATATCCAGATATGAATCCAAAC

RI-AT5G48657-XLOC_031374-11254-0
GCTGCACAAAATCGAAATATGTCTAGGCCTGACCAACAACCACCAAACCA
RI-AT5G48657-XLOC_031374-11254-1
GCTGCACAAAATCGAAATATGTCTAGGCCTGACCAACAACCACCAAACCA
CONSENSUS
GCTGCACAAAATCGAAATATGTCTAGGCCTGACCAACAACCACCAAACCA

RI-AT5G48657-XLOC_031374-11254-0
TAATGTTAGACCAAGACATGAAAGATTCAATAGCAGAGACGAAACCGAAT
RI-AT5G48657-XLOC_031374-11254-1
TAATGTTAGACCAAGACATGAAAGATTCAATAGCAGAGACGAAACCGAAT
CONSENSUS
TAATGTTAGACCAAGACATGAAAGATTCAATAGCAGAGACGAAACCGAAT

RI-AT5G48657-XLOC_031374-11254-0
TCAGACCGTCTCCTGCACACAATGAAAGAAACAACAGAGTTAGATCAGTT

RI-AT5G48657-XLOC_031374-11254-1
      TCAGACCGTCTCCTGCACACAATGAAAGAAACAACAGAGTTAGATCAGTT
CONSENSUS
      TCAGACCGTCTCCTGCACACAATGAAAGAAACAACAGAGTTAGATCAGTT

RI-AT5G48657-XLOC_031374-11254-0
      CCTCCCACACCAGAAACATACAACCATCAAACATATGGTGGAGGTGGCAG
RI-AT5G48657-XLOC_031374-11254-1
      CCTCCCACACCAGAAACATACAACCATCAAACATATGGTGGAGGTGGCAG
CONSENSUS
      CCTCCCACACCAGAAACATACAACCATCAAACATATGGTGGAGGTGGCAG

RI-AT5G48657-XLOC_031374-11254-0
      ATCAATGGGAAATCCAACAGAAATAAATAGACGGCAATCACGTGATCATG
RI-AT5G48657-XLOC_031374-11254-1
      ATCAATGGGAAATCCAACAGAAATAAATAGACGGCAATCACGTGATCATG
CONSENSUS
      ATCAATGGGAAATCCAACAGAAATAAATAGACGGCAATCACGTGATCATG

RI-AT5G48657-XLOC_031374-11254-0
      TCCCGGCGCGGCCAATACGCAATCTTAGAGGGCAAAGTAGTGAAAGG
RI-AT5G48657-XLOC_031374-11254-1
      TCCCGGCGCGGCCAATACGCAATCTTAGAGGGCAAAGTAGTGAAAGG
CONSENSUS
      TCCCGGCGCGGCCAATACGCAATCTTAGAGGGCAAAGTAGTGAAAGG

alignment for event: A3-AT5G45940-XLOC_031221-8775

A3-AT5G45940-XLOC_031221-8775-0
      AAGGGAATGTCGGTAGCACCAGTTATCGGTTTTCTACACGATAAGAAAGC
A3-AT5G45940-XLOC_031221-8775-1
      AAGGGAATGTCGGTAGCACCAGTTATCGGTTTTCTACACGATAAGAAAGC
CONSENSUS
      AAGGGAATGTCGGTAGCACCAGTTATCGGTTTTCTACACGATAAGAAAGC

A3-AT5G45940-XLOC_031221-8775-0
      GTTTAAACAGTTACCAAATCCAGCTGAAGTGGAAGAGATCTTTGATGTGC
A3-AT5G45940-XLOC_031221-8775-1
      GTTTAAACAGTTACCAAATCCAGCTGAAGTGGAAGAGATCTTTGATGTGC
CONSENSUS
      GTTTAAACAGTTACCAAATCCAGCTGAAGTGGAAGAGATCTTTGATGTGC

A3-AT5G45940-XLOC_031221-8775-0 CATTAGAGATGTTCCTCAAG-----
AAACAGGCGAGCAGAGGAACGAGAG
A3-AT5G45940-XLOC_031221-8775-1
      CATTAGAGATGTTCCTCAAGGACAGAAACAGGCGAGCAGAGGAACGAGAG
CONSENSUS
      CATTAGAGATGTTCCTCAAG.....AAACAGGCGAGCAGAGGAACGAGAG

A3-AT5G45940-XLOC_031221-8775-0
      CATGAAGGAGAGAGATATCTTCTTCAATACTTTGATTACTATTCCGAAGA
A3-AT5G45940-XLOC_031221-8775-1
      CATGAAGGAGAGAGATATCTTCTTCAATACTTTGATTACTATTCCGAAGA
CONSENSUS

CATGAAGGAGAGAGATATCTTCTTCAATACTTTGATTACTATTCCGAAGA

A3-AT5G45940-XLOC_031221-8775-0
TAAAGAGAGAAGCTTTATCATATGGGCACTCACTGCTGGTATTCTGATCA
A3-AT5G45940-XLOC_031221-8775-1
TAAAGAGAGAAGCTTTATCATATGGGCACTCACTGCTGGTATTCTGATCA
CONSENSUS
TAAAGAGAGAAGCTTTATCATATGGGCACTCACTGCTGGTATTCTGATCA

A3-AT5G45940-XLOC_031221-8775-0
GAGTTGCCTCCATTGTTTATCAGAGATTACCGGAGTTTCAAGAACGTAAA
A3-AT5G45940-XLOC_031221-8775-1
GAGTTGCCTCCATTGTTTATCAGAGATTACCGGAGTTTCAAGAACGTAAA
CONSENSUS
GAGTTGCCTCCATTGTTTATCAGAGATTACCGGAGTTTCAAGAACGTAAA

A3-AT5G45940-XLOC_031221-8775-0
CCGAGTTTCTGGAATCAGCCTAACTGATTCTTGACTTGATCTGATTGAGA
A3-AT5G45940-XLOC_031221-8775-1
CCGAGTTTCTGGAATCAGCCTAACTGATTCTTGACTTGATCTGATTGAGA
CONSENSUS
CCGAGTTTCTGGAATCAGCCTAACTGATTCTTGACTTGATCTGATTGAGA

A3-AT5G45940-XLOC_031221-8775-0
AAATTGAAACTTTTATGTATAATAAGCGATACAGATTGAATCTTCTTTAA
A3-AT5G45940-XLOC_031221-8775-1
AAATTGAAACTTTTATGTATAATAAGCGATACAGATTGAATCTTCTTTAA
CONSENSUS
AAATTGAAACTTTTATGTATAATAAGCGATACAGATTGAATCTTCTTTAA

A3-AT5G45940-XLOC_031221-8775-0 ATGTGTTTCAAGAATCTATCTTTACTCTAA
A3-AT5G45940-XLOC_031221-8775-1 ATGTGTTTCAAGAATCTATCTTTACTCTAA
CONSENSUS ATGTGTTTCAAGAATCTATCTTTACTCTAA

alignment for event: A5-AT5G35210-XLOC_030529-4365

A5-AT5G35210-XLOC_030529-4365-0
AGTGGTTCCACACTGAAGCTGTTAAGCTCAAAGATTCACAAATTCCTGAA
A5-AT5G35210-XLOC_030529-4365-1
AGTGGTTCCACACTGAAGCTGTTAAGCTCAAAGATTCACAAATTCCTGAA
CONSENSUS
AGTGGTTCCACACTGAAGCTGTTAAGCTCAAAGATTCACAAATTCCTGAA

A5-AT5G35210-XLOC_030529-4365-0
GTTGTTGGGTTCAAATGTTGCAAATGTCGACGTATACGATCCCCTGATTG
A5-AT5G35210-XLOC_030529-4365-1
GTTGTTGGGTTCAAATGTTGCAAATGTCGACGTATACGATCCCCTGATTG
CONSENSUS
GTTGTTGGGTTCAAATGTTGCAAATGTCGACGTATACGATCCCCTGATTG

A5-AT5G35210-XLOC_030529-4365-0
CCCTTACATGGATCCCAAACTCAAGGAACAGAAGCAGATCAAAAGAATAG
A5-AT5G35210-XLOC_030529-4365-1
CCCTTACATGGATCCCAAACTCAAGGAACAGAAGCAGATCAAAAGAATAG

CONSENSUS
CCCTTACATGGATCCCAAACTCAAGGAACAGAAGCAGATCAAAAGAATAG

A5-AT5G35210-XLOC_030529-4365-0
TCTTCACGAACCAGAAACAGAGGCAAGGAAATTCTGGGTTGGATTCTGAT
A5-AT5G35210-XLOC_030529-4365-1
TCTTCACGAACCAGAAACAGAGGCAAGGAAATTCTGGGTTGGATTCTGAT
CONSENSUS
TCTTCACGAACCAGAAACAGAGGCAAGGAAATTCTGGGTTGGATTCTGAT

A5-AT5G35210-XLOC_030529-4365-0
TCTGAAAGAATGTCTGAACAAAAAGACTCGAAACCTTCTACTCCGTTACC
A5-AT5G35210-XLOC_030529-4365-1
TCTGAAAGAATGTCTGAACAAAAAGACTCGAAACCTTCTACTCCGTTACC
CONSENSUS
TCTGAAAGAATGTCTGAACAAAAAGACTCGAAACCTTCTACTCCGTTACC

A5-AT5G35210-XLOC_030529-4365-0
TGCCACTCCTTTATATCCTCCTGACGATGTATTTATTCCAGAAGATGATC
A5-AT5G35210-XLOC_030529-4365-1
TGCCACTCCTTTATATCCTCCTGACGATGTATTTATTCCAGAAGATGATC
CONSENSUS
TGCCACTCCTTTATATCCTCCTGACGATGTATTTATTCCAGAAGATGATC

A5-AT5G35210-XLOC_030529-4365-0
CTCTCCTGGTATCAGTTTCCAAAGTCAAACAAATCACACCCAGTAGTTTC
A5-AT5G35210-XLOC_030529-4365-1
CTCTCCTGGTATCAGTTTCCAAAGTCAAACAAATCACACCCAGTAGTTTC
CONSENSUS
CTCTCCTGGTATCAGTTTCCAAAGTCAAACAAATCACACCCAGTAGTTTC

A5-AT5G35210-XLOC_030529-4365-0
GATCTTGAATGGAGCACCACTGCTTTTGCGCCTGGACCCCAAAAGCTACC
A5-AT5G35210-XLOC_030529-4365-1
GATCTTGAATGGAGCACCACTGCTTTTGCGCCTGGACCCCAAAAGCTACC
CONSENSUS
GATCTTGAATGGAGCACCACTGCTTTTGCGCCTGGACCCCAAAAGCTACC

A5-AT5G35210-XLOC_030529-4365-0
AGTTAGAAGGCAAGTGAAACGAGAAGATTCTGATGCAGCCTATCCTGAAC
A5-AT5G35210-XLOC_030529-4365-1
AGTTAGAAGGCAAGTGAAACGAGAAGATTCTGATGCAGCCTATCCTGAAC
CONSENSUS
AGTTAGAAGGCAAGTGAAACGAGAAGATTCTGATGCAGCCTATCCTGAAC

A5-AT5G35210-XLOC_030529-4365-0
TGCATCCTATTGTGAAACCCGAGGCAGAGGAGCAAGCTTTACCTGTTCTA
A5-AT5G35210-XLOC_030529-4365-1
TGCATCCTATTGTGAAACCCGAGGCAGAGGAGCAAGCTTTACCTGTTCTA
CONSENSUS
TGCATCCTATTGTGAAACCCGAGGCAGAGGAGCAAGCTTTACCTGTTCTA

A5-AT5G35210-XLOC_030529-4365-0
ACAGAATGGGATTTGTCTGGTGAGCTACTTTTCGACTACGAGGACATGGA
A5-AT5G35210-XLOC_030529-4365-1
ACAGAATGGGATTTGTCTGGTGAGCTACTTTTCGACTACGAGGACATGGA

CONSENSUS
    ACAGAATGGGATTTGTCTGGTGAGCTACTTTTCGACTACGAGGACATGGA

A5-AT5G35210-XLOC_030529-4365-0
    ATTTGAACCGCAAACTTATTTCTCGTTGACCGAGCTACTGACAGCCGATG
A5-AT5G35210-XLOC_030529-4365-1
    ATTTGAACCGCAAACTTATTTCTCGTTGACCGAGCTACTGACAGCCGATG
CONSENSUS
    ATTTGAACCGCAAACTTATTTCTCGTTGACCGAGCTACTGACAGCCGATG

A5-AT5G35210-XLOC_030529-4365-0
    ATAGTGGTGGTGGTCAGTACCAGGAAAATGGTGATATGGTTGTTTCAGGA
A5-AT5G35210-XLOC_030529-4365-1
    ATAGTGGTGGTGGTCAGTACCAGGAAAATGGTGATATGGTTGTTTCAGGA
CONSENSUS
    ATAGTGGTGGTGGTCAGTACCAGGAAAATGGTGATATGGTTGTTTCAGGA

A5-AT5G35210-XLOC_030529-4365-0
    AACCCTCAGTTCGAACCAACAGAAAAAGAAGAGTGTGAAGATGATATGGG
A5-AT5G35210-XLOC_030529-4365-1
    AACCCTCAGTTCGAACCAACAGAAAAAGAAGAGTGTGAAGATGATATGGG
CONSENSUS
    AACCCTCAGTTCGAACCAACAGAAAAAGAAGAGTGTGAAGATGATATGGG

A5-AT5G35210-XLOC_030529-4365-0
    TCCATGTCAGAGATGTTTGCAAATGGATCCAGCGCCTGATCTTTTGTGCA
A5-AT5G35210-XLOC_030529-4365-1
    TCCATGTCAGAGATGTTTGCAAATGGATCCAGCGCCTGATCTTTTGTGCA
CONSENSUS
    TCCATGTCAGAGATGTTTGCAAATGGATCCAGCGCCTGATCTTTTGTGCA

A5-AT5G35210-XLOC_030529-4365-0
    CGGTTTGTGGATTGCTTATACATTCTCACTGTTCTCCATGGTCAGCCTTA
A5-AT5G35210-XLOC_030529-4365-1
    CGGTTTGTGGATTGCTTATACATTCTCACTGTTCTCCATGGTCAGCCTTA
CONSENSUS
    CGGTTTGTGGATTGCTTATACATTCTCACTGTTCTCCATGGTCAGCCTTA

A5-AT5G35210-XLOC_030529-4365-0
    CCCGGAAGTAGCTGGAGCTGTGGTCAATGCCGTGAGTGGCAGTAGCTGGA
A5-AT5G35210-XLOC_030529-4365-1
    CCCGGAAGTAGCTGGAGCTGTGGTCAATGCC-------------------
CONSENSUS
    CCCGGAAGTAGCTGGAGCTGTGGTCAATGCC...................

A5-AT5G35210-XLOC_030529-4365-0
    CCCTTTGATTTCAAGCCCCTTCCCAAATTTTAACTTCCACATTCTTTGAC
A5-AT5G35210-XLOC_030529-4365-1

CONSENSUS
    ....

A5-AT5G35210-XLOC_030529-4365-0
    AATGAATTTGAGGGAAAATTGTATCCTCCTTTGTTTGGCTTATAATGGCA
A5-AT5G35210-XLOC_030529-4365-1

CONSENSUS
      ....

A5-AT5G35210-XLOC_030529-4365-0
      AATGAGCTGTAATTTCTAGTTTTTTTTTAGGTTTGTCTTCGTCTATTTTA
A5-AT5G35210-XLOC_030529-4365-1

CONSENSUS
      ....

A5-AT5G35210-XLOC_030529-4365-0
      TTTATCTTCGTTTATAGAGTGGATACATGTTTGCTTTCAAATTCATACCA
A5-AT5G35210-XLOC_030529-4365-1

CONSENSUS
      ....

A5-AT5G35210-XLOC_030529-4365-0
      AGCAGGGATGCTTACTTGGTGGAATGATTAACTTTCATGAATTAGGGCAC
A5-AT5G35210-XLOC_030529-4365-1
      --------------------------------------GAATTAGGGCAC
CONSENSUS
      ......................................GAATTAGGGCAC

A5-AT5G35210-XLOC_030529-4365-0
      TAGGAAGCATTACACTAGGAAGCTTTGGTGCAATCACACAG
A5-AT5G35210-XLOC_030529-4365-1
      TAGGAAGCATTACACTAGGAAGCTTTGGTGCAATCACACAG
CONSENSUS
      TAGGAAGCATTACACTAGGAAGCTTTGGTGCAATCACACAG

alignment for event: RI-AT5G04360-XLOC_025304-1487

RI-AT5G04360-XLOC_025304-1487-0
      GCTTTCAGCAGATGGGAGGAAAACTTTTCTGGTTAATCTTGATTCTGATG
RI-AT5G04360-XLOC_025304-1487-1
      GCTTTCAGCAGATGGGAGGAAAACTTTTCTGGTTAATCTTGATTCTGATG
CONSENSUS
      GCTTTCAGCAGATGGGAGGAAAACTTTTCTGGTTAATCTTGATTCTGATG

RI-AT5G04360-XLOC_025304-1487-0
      ATCTAAAACCTGAAGGATGGGACAACTTAGCAGATAAGAAACCATGCCTA
RI-AT5G04360-XLOC_025304-1487-1
      ATCTAAAACCTGAAGGATGGGACAACTTAGCAGATAAGAAACCATGCCTA
CONSENSUS
      ATCTAAAACCTGAAGGATGGGACAACTTAGCAGATAAGAAACCATGCCTA

RI-AT5G04360-XLOC_025304-1487-0
      AGATCCTTCTCAGATATAAGTATTTATGAGCTGCATGTGAGGGATTTCAG
RI-AT5G04360-XLOC_025304-1487-1
      AGATCCTTCTCAGATATAAGTATTTATGAGCTGCATGTGAGGGATTTCAG
CONSENSUS
      AGATCCTTCTCAGATATAAGTATTTATGAGCTGCATGTGAGGGATTTCAG

RI-AT5G04360-XLOC_025304-1487-0
GTAATTATACTCAATTTCCAGATTCATGGTTTACTTTTTACCATGGGGCA
RI-AT5G04360-XLOC_025304-1487-1

CONSENSUS
....

RI-AT5G04360-XLOC_025304-1487-0
AGACTTAGCAGGTAATTTTCGTTACAGATATACGTACTCTTTCTTACCAA
RI-AT5G04360-XLOC_025304-1487-1

CONSENSUS
....

RI-AT5G04360-XLOC_025304-1487-0
TTATCTTCCTCTCTTCCCGCAACCAAGAAACAGTAACTTCTTGAATAGAC
RI-AT5G04360-XLOC_025304-1487-1

CONSENSUS
....

RI-AT5G04360-XLOC_025304-1487-0
AAAGAAAATATATATTTTTCTAGCCTATATTTGTTTGGTCAATCTTTGAA
RI-AT5G04360-XLOC_025304-1487-1

CONSENSUS
....

RI-AT5G04360-XLOC_025304-1487-0
TCACATTATTGAAATTGTAGAATCATTTTACTTGCTCGTGCATATTTCCC
RI-AT5G04360-XLOC_025304-1487-1

CONSENSUS
....

RI-AT5G04360-XLOC_025304-1487-0
TTTTCTTCTCGTTTGTCTAATTTTTACCTGCTATTTCAGTGCCAATGATG
RI-AT5G04360-XLOC_025304-1487-1
---------------------------------------TGCCAATGATG
CONSENSUS
....TGCCAATGATG

RI-AT5G04360-XLOC_025304-1487-0
AGACTGTTGAGCCTGAAAATCGTGGTGGATATCTGGCTTTCACTTCAAAG
RI-AT5G04360-XLOC_025304-1487-1
AGACTGTTGAGCCTGAAAATCGTGGTGGATATCTGGCTTTCACTTCAAAG
CONSENSUS
AGACTGTTGAGCCTGAAAATCGTGGTGGATATCTGGCTTTCACTTCAAAG

alignment for event: RI-AT5G63370-XLOC_032156-2593

RI-AT5G63370-XLOC_032156-2593-0
AAACAAAGGGTAAAGTAATAATAGAGCCACTTGACAAAATTGAAAAACGC
RI-AT5G63370-XLOC_032156-2593-1

AAACAAAGGGTAAAGTAATAATAGAGCCACTTGACAAAATTGAAAAACGC
CONSENSUS
AAACAAAGGGTAAAGTAATAATAGAGCCACTTGACAAAATTGAAAAACGC

RI-AT5G63370-XLOC_032156-2593-0
GTCTCCGAGAGGAAAATTTAGAGAAAAGCTTCTCGCGATCGCATCGGGCT
RI-AT5G63370-XLOC_032156-2593-1
GTCTCCGAGAGGAAAATTTAGAGAAAAGCTTCTCGCGATCGCATCGGGCT
CONSENSUS
GTCTCCGAGAGGAAAATTTAGAGAAAAGCTTCTCGCGATCGCATCGGGCT

RI-AT5G63370-XLOC_032156-2593-0
CGTAGATCGATCTCCTCCTCCATCCAATCTTCCGTTTTCGTATTCACCGT
RI-AT5G63370-XLOC_032156-2593-1
CGTAGATCGATCTCCTCCTCCATCCAATCTTCCGTTTTCGTATTCACCGT
CONSENSUS
CGTAGATCGATCTCCTCCTCCATCCAATCTTCCGTTTTCGTATTCACCGT

RI-AT5G63370-XLOC_032156-2593-0
CCGGTAAGACCTTCCGGTACTTGTGAATCGGAGAAGAGGAAGGAGAAAAA
RI-AT5G63370-XLOC_032156-2593-1
CCGGTAAGACCTTCCGGTACTTGTGAATCGGAGAAGAGGAAGGAGAAAAA
CONSENSUS
CCGGTAAGACCTTCCGGTACTTGTGAATCGGAGAAGAGGAAGGAGAAAAA

RI-AT5G63370-XLOC_032156-2593-0
TTATCGCGTTCCTGGAAATTGAATTTCTAGGGTTTCGTTTTTTTCTGAAT
RI-AT5G63370-XLOC_032156-2593-1
TTATCGCGTTCCTGGAAATTGAATTTCTAGGGTTTCGTTTTTTTCTGAAT
CONSENSUS
TTATCGCGTTCCTGGAAATTGAATTTCTAGGGTTTCGTTTTTTTCTGAAT

RI-AT5G63370-XLOC_032156-2593-0
TATTCTGATTCTGATTTCGTCAAGGGTGTTTTTCTTAATTGGTTGATTGA
RI-AT5G63370-XLOC_032156-2593-1
TATTCTGATTCTGATTTCGTCAAGGGTGTTTTTCTTAATTGGTTGATTGA
CONSENSUS
TATTCTGATTCTGATTTCGTCAAGGGTGTTTTTCTTAATTGGTTGATTGA

RI-AT5G63370-XLOC_032156-2593-0
TCGTCTTTCTAGTTCAATTGTTGAAAAGTAATTGAACATCTATTGAAATC
RI-AT5G63370-XLOC_032156-2593-1
TCGTCTTTCTAGTTCAATTGTTGAAAAGTAATTGAACATCTATTGAAATC
CONSENSUS
TCGTCTTTCTAGTTCAATTGTTGAAAAGTAATTGAACATCTATTGAAATC

RI-AT5G63370-XLOC_032156-2593-0
TGCTTCTCGTGATTTGATTCTGGGTTTCGTTGATTGGTTTTTTTCGAGGG
RI-AT5G63370-XLOC_032156-2593-1
TGCTTCTCGTGATTTGATTCTGGGTTTCGTTGATTGGTTTTTTTCGAGGG
CONSENSUS
TGCTTCTCGTGATTTGATTCTGGGTTTCGTTGATTGGTTTTTTTCGAGGG

RI-AT5G63370-XLOC_032156-2593-0
GTTTGTTTGTTTTATCCTTTTTTATTTCTATAAGATCAATTTCTCATTGA
RI-AT5G63370-XLOC_032156-2593-1

GTTTGTTTGTTTTATCCTTTTTTATTTCTATAAGATCAATTTCTCATTGA
CONSENSUS
GTTTGTTTGTTTTATCCTTTTTTATTTCTATAAGATCAATTTCTCATTGA

RI-AT5G63370-XLOC_032156-2593-0
GAAGAATACGATGGCAGCAGGGGGTGTTGATGTTTCCAGAAGTTCCGTTG
RI-AT5G63370-XLOC_032156-2593-1
GAAGAATACGATGGCAGCAGGGGGTGTTGATGTTTCCAGAAGTTCCGTTG
CONSENSUS
GAAGAATACGATGGCAGCAGGGGGTGTTGATGTTTCCAGAAGTTCCGTTG

RI-AT5G63370-XLOC_032156-2593-0
CTGTCAAAAAAGACTACGACTTTTACAGGAATGGTTCTCGTGATGTGTAT
RI-AT5G63370-XLOC_032156-2593-1
CTGTCAAAAAAGACTACGACTTTTACAGGAATGGTTCTCGTGATGTGTAT
CONSENSUS
CTGTCAAAAAAGACTACGACTTTTACAGGAATGGTTCTCGTGATGTGTAT

RI-AT5G63370-XLOC_032156-2593-0
GTTCGACAGAGTGGTAGAGATGACGAGAGGCGTCAAATCAAAAGGCCTAG
RI-AT5G63370-XLOC_032156-2593-1
GTTCGACAGAGTGGTAGAGATGACGAGAGGCGTCAAATCAAAAGGCCTAG
CONSENSUS
GTTCGACAGAGTGGTAGAGATGACGAGAGGCGTCAAATCAAAAGGCCTAG

RI-AT5G63370-XLOC_032156-2593-0
TGACCATGATCTCAGGAGGAATGATGGGCGTCACCGATCACGGTTGGCAT
RI-AT5G63370-XLOC_032156-2593-1
TGACCATGATCTCAGGAGGAATGATGGGCGTCACCGATCACGGTTGGCAT
CONSENSUS
TGACCATGATCTCAGGAGGAATGATGGGCGTCACCGATCACGGTTGGCAT

RI-AT5G63370-XLOC_032156-2593-0
ATGAGAAAGGGGAACTACGGGAGGAAGCAGAGGTTCAGAGACCCTCTGAG
RI-AT5G63370-XLOC_032156-2593-1
ATGAGAAAGGGGAACTACGGGAGGAAGCAGAGGTTCAGAGACCCTCTGAG
CONSENSUS
ATGAGAAAGGGGAACTACGGGAGGAAGCAGAGGTTCAGAGACCCTCTGAG

RI-AT5G63370-XLOC_032156-2593-0
AAAAGGAGGAAGTTTTCACCCATCGTGTGGAATGCAGAAAAAGTGGGTAG
RI-AT5G63370-XLOC_032156-2593-1
AAAAGGAGGAAGTTTTCACCCATCGTGTGGAATGCAGAAAAAGTGGGTAG
CONSENSUS
AAAAGGAGGAAGTTTTCACCCATCGTGTGGAATGCAGAAAAAGTGGGTAG

RI-AT5G63370-XLOC_032156-2593-0
AGCTCCATCTAGGGAGAAGACCAAGTCTCCTTTCCCTGTTCCGACTACAA
RI-AT5G63370-XLOC_032156-2593-1
AGCTCCATCTAGGGAGAAGACCAAGTCTCCTTTCCCTGTTCCGACTACAA
CONSENSUS
AGCTCCATCTAGGGAGAAGACCAAGTCTCCTTTCCCTGTTCCGACTACAA

RI-AT5G63370-XLOC_032156-2593-0
CTGTGATATCCAATCAGGCTGTTGCTGGTAAGACTACTTCAAACGATCAA
RI-AT5G63370-XLOC_032156-2593-1

CTGTGATATCCAATCAGGCTGTTGCTGGTAAGACTACTTCAAACGATCAA
CONSENSUS
CTGTGATATCCAATCAGGCTGTTGCTGGTAAGACTACTTCAAACGATCAA

RI-AT5G63370-XLOC_032156-2593-0
GTGAATGCCTTGATGTCGCCAGAACCTAGTTATCTTGCTCCAGTGCAGCC
RI-AT5G63370-XLOC_032156-2593-1
GTGAATGCCTTGATGTCGCCAGAACCTAGTTATCTTGCTCCAGTGCAGCC
CONSENSUS
GTGAATGCCTTGATGTCGCCAGAACCTAGTTATCTTGCTCCAGTGCAGCC

RI-AT5G63370-XLOC_032156-2593-0
TTCAGAAGCCCTGCTGGCTGTGAAACATCCTGTTGATGATTTGGAGGAGG
RI-AT5G63370-XLOC_032156-2593-1
TTCAGAAGCCCTGCTGGCTGTGAAACATCCTGTTGATGATTTGGAGGAGG
CONSENSUS
TTCAGAAGCCCTGCTGGCTGTGAAACATCCTGTTGATGATTTGGAGGAGG

RI-AT5G63370-XLOC_032156-2593-0
GTCAGTTGGAGGAAGAACAGGTGATGCAGGAGGATGTAAAGGAGGGTCTG
RI-AT5G63370-XLOC_032156-2593-1
GTCAGTTGGAGGAAGAACAGGTGATGCAGGAGGATGTAAAGGAGGGTCTG
CONSENSUS
GTCAGTTGGAGGAAGAACAGGTGATGCAGGAGGATGTAAAGGAGGGTCTG

RI-AT5G63370-XLOC_032156-2593-0
TTGGAGGAAGAACAAGTGATGCAGGAACCAAATATAAAGACATCTAGGTG
RI-AT5G63370-XLOC_032156-2593-1
TTGGAGGAAGAACAAGTGATGCAGGAACCAAATATAAAGACATCTAGGTG
CONSENSUS
TTGGAGGAAGAACAAGTGATGCAGGAACCAAATATAAAGACATCTAGGTG

RI-AT5G63370-XLOC_032156-2593-0
GGGGACAGGTTTGACTTCCCCAAAGGAGGAGTTAATATCTGTTAACGTTT
RI-AT5G63370-XLOC_032156-2593-1
GGGGACAGGTTTGACTTCCCCAAAGGAGGAGTTAATATCTGTTAACGTTT
CONSENSUS
GGGGACAGGTTTGACTTCCCCAAAGGAGGAGTTAATATCTGTTAACGTTT

RI-AT5G63370-XLOC_032156-2593-0
CCAAGACTAATAGATGGAACAGGAGCAGTTTGACACCGGAGTGCGAGGAA
RI-AT5G63370-XLOC_032156-2593-1
CCAAGACTAATAGATGGAACAGGAGCAGTTTGACACCGGAGTGCGAGGAA
CONSENSUS
CCAAGACTAATAGATGGAACAGGAGCAGTTTGACACCGGAGTGCGAGGAA

RI-AT5G63370-XLOC_032156-2593-0
GTAATGGTGTCTGAAGAACAGCAGTGCTATTCGTCTGGATCTGGCAGTGG
RI-AT5G63370-XLOC_032156-2593-1
GTAATGGTGTCTGAAGAACAGCAGTGCTATTCGTCTGGATCTGGCAGTGG
CONSENSUS
GTAATGGTGTCTGAAGAACAGCAGTGCTATTCGTCTGGATCTGGCAGTGG

RI-AT5G63370-XLOC_032156-2593-0
ACATCTCAGCGTAGAGAAGCTTAGTGCAGATGGAAATTCTGGTCGTGAGT
RI-AT5G63370-XLOC_032156-2593-1

ACATCTCAGCGTAGAGAAGCTTAGTGCAGATGGAAATTCTGGTCGTGAGT
CONSENSUS
ACATCTCAGCGTAGAGAAGCTTAGTGCAGATGGAAATTCTGGTCGTGAGT

RI-AT5G63370-XLOC_032156-2593-0
ATTATAGTTCTGATCATGATGAGTTAGAACATGAAGATCAAGATTCTTTA
RI-AT5G63370-XLOC_032156-2593-1
ATTATAGTTCTGATCATGATGAGTTAGAACATGAAGATCAAGATTCTTTA
CONSENSUS
ATTATAGTTCTGATCATGATGAGTTAGAACATGAAGATCAAGATTCTTTA

RI-AT5G63370-XLOC_032156-2593-0
ACTCCGGGAGAGATGAACATGATGTTTGGGAGCAGGTCTGTGAATGAATT
RI-AT5G63370-XLOC_032156-2593-1
ACTCCGGGAGAGATGAACATGATGTTTGGGAGCAGGTCTGTGAATGAATT
CONSENSUS
ACTCCGGGAGAGATGAACATGATGTTTGGGAGCAGGTCTGTGAATGAATT

RI-AT5G63370-XLOC_032156-2593-0
TCAGAAGCTAAACAAGATAAATGAAGGAACATATGGAATTGTTTACAAAG
RI-AT5G63370-XLOC_032156-2593-1
TCAGAAGCTAAACAAGATAAATGAAGGAACATATGGAATTGTTTACAAAG
CONSENSUS
TCAGAAGCTAAACAAGATAAATGAAGGAACATATGGAATTGTTTACAAAG

RI-AT5G63370-XLOC_032156-2593-0
CAAGGGATGAGAAAACAAAAGAGATTGTTGCGCTCAAAAAGATCAAGATG
RI-AT5G63370-XLOC_032156-2593-1
CAAGGGATGAGAAAACAAAAGAGATTGTTGCGCTCAAAAAGATCAAGATG
CONSENSUS
CAAGGGATGAGAAAACAAAAGAGATTGTTGCGCTCAAAAAGATCAAGATG

RI-AT5G63370-XLOC_032156-2593-0
AAGGAAGATAGGTTCGAAGAAGAGTATGGATTCCCTTTGACATCATTGAG
RI-AT5G63370-XLOC_032156-2593-1
AAGGAAGATAGGTTCGAAGAAGAGTATGGATTCCCTTTGACATCATTGAG
CONSENSUS
AAGGAAGATAGGTTCGAAGAAGAGTATGGATTCCCTTTGACATCATTGAG

RI-AT5G63370-XLOC_032156-2593-0
GGAAATTAACATACTTTTGTCATGCAATCACCCTGCGATTGTGAACGTGA
RI-AT5G63370-XLOC_032156-2593-1
GGAAATTAACATACTTTTGTCATGCAATCACCCTGCGATTGTGAACGTGA
CONSENSUS
GGAAATTAACATACTTTTGTCATGCAATCACCCTGCGATTGTGAACGTGA

RI-AT5G63370-XLOC_032156-2593-0
AGGAGGTTGTGGTTGGAGGGAAAAACGATAATGATGTTTATATGGTCATG
RI-AT5G63370-XLOC_032156-2593-1
AGGAGGTTGTGGTTGGAGGGAAAAACGATAATGATGTTTATATGGTCATG
CONSENSUS
AGGAGGTTGTGGTTGGAGGGAAAAACGATAATGATGTTTATATGGTCATG

RI-AT5G63370-XLOC_032156-2593-0
GAACACTTAGAACACGACCTGAGGGGAGTAATGGATAGAAGGAAGGAACC
RI-AT5G63370-XLOC_032156-2593-1

GAACACTTAGAACACGACCTGAGGGGAGTAATGGATAGAAGGAAGGAACC
CONSENSUS
GAACACTTAGAACACGACCTGAGGGGAGTAATGGATAGAAGGAAGGAACC

RI-AT5G63370-XLOC_032156-2593-0
TTTTAGCACTAGCGAAGTCAAGTGCTTGATGATGCAGCTGTTGGATGGTT
RI-AT5G63370-XLOC_032156-2593-1
TTTTAGCACTAGCGAAGTCAAGTGCTTGATGATGCAGCTGTTGGATGGTT
CONSENSUS
TTTTAGCACTAGCGAAGTCAAGTGCTTGATGATGCAGCTGTTGGATGGTT

RI-AT5G63370-XLOC_032156-2593-0
TGAAATACCTCCACACAAATTGGATTATCCACAGGGATCTGAAGCCATCT
RI-AT5G63370-XLOC_032156-2593-1
TGAAATACCTCCACACAAATTGGATTATCCACAGGGATCTGAAGCCATCT
CONSENSUS
TGAAATACCTCCACACAAATTGGATTATCCACAGGGATCTGAAGCCATCT

RI-AT5G63370-XLOC_032156-2593-0
AATCTTCTGATGAACAATTGTGGGGAGTTAAAAATATGTGATTTTGGGAT
RI-AT5G63370-XLOC_032156-2593-1
AATCTTCTGATGAACAATTGTGGGGAGTTAAAAATATGTGATTTTGGGAT
CONSENSUS
AATCTTCTGATGAACAATTGTGGGGAGTTAAAAATATGTGATTTTGGGAT

RI-AT5G63370-XLOC_032156-2593-0
GGCGCGCCAGTATGGGAGCCCTATCAAGCCTTACACCCAGATGGTTATTA
RI-AT5G63370-XLOC_032156-2593-1
GGCGCGCCAGTATGGGAGCCCTATCAAGCCTTACACCCAGATGGTTATTA
CONSENSUS
GGCGCGCCAGTATGGGAGCCCTATCAAGCCTTACACCCAGATGGTTATTA

RI-AT5G63370-XLOC_032156-2593-0
CCCAGTGGTACAGGCCACCTGAACTTCTTCTAGGAGCAAAAGAGTACTCT
RI-AT5G63370-XLOC_032156-2593-1
CCCAGTGGTACAGGCCACCTGAACTTCTTCTAGGAGCAAAAGAGTACTCT
CONSENSUS
CCCAGTGGTACAGGCCACCTGAACTTCTTCTAGGAGCAAAAGAGTACTCT

RI-AT5G63370-XLOC_032156-2593-0
ACAGCAGTTGATATGTGGTCAGTGGGTTGCATTATGGCTGAACTGTTGTC
RI-AT5G63370-XLOC_032156-2593-1
ACAGCAGTTGATATGTGGTCAGTGGGTTGCATTATGGCTGAACTGTTGTC
CONSENSUS
ACAGCAGTTGATATGTGGTCAGTGGGTTGCATTATGGCTGAACTGTTGTC

RI-AT5G63370-XLOC_032156-2593-0
TCAAAAGCCTTTGTTCCCGGGTAAGAGTGAGCTTGACCAACTTCAGAAGA
RI-AT5G63370-XLOC_032156-2593-1
TCAAAAGCCTTTGTTCCCGGGTAAGAGTGAGCTTGACCAACTTCAGAAGA
CONSENSUS
TCAAAAGCCTTTGTTCCCGGGTAAGAGTGAGCTTGACCAACTTCAGAAGA

RI-AT5G63370-XLOC_032156-2593-0
TCTTTGCGGTCCTTGGAACACCAAACGAAGCAATATGGCCTGGGTTCTCA
RI-AT5G63370-XLOC_032156-2593-1

TCTTTGCGGTCCTTGGAACACCAAACGAAGCAATATGGCCTGGGTTCTCA
CONSENSUS
TCTTTGCGGTCCTTGGAACACCAAACGAAGCAATATGGCCTGGGTTCTCA

RI-AT5G63370-XLOC_032156-2593-0
TCATTTCCGAATGCTAAAGCCAAGTTTCCTACACAGCCTTACAATATGTT
RI-AT5G63370-XLOC_032156-2593-1
TCATTTCCGAATGCTAAAGCCAAGTTTCCTACACAGCCTTACAATATGTT
CONSENSUS
TCATTTCCGAATGCTAAAGCCAAGTTTCCTACACAGCCTTACAATATGTT

RI-AT5G63370-XLOC_032156-2593-0
ACGTAAGAAGTTTCCAGCTATTTCATTTGTAGGTGGTCAAATTCTTTCTG
RI-AT5G63370-XLOC_032156-2593-1
AC--
CONSENSUS
AC..

RI-AT5G63370-XLOC_032156-2593-0
AACGTGGATTTGATTTGCTGAACAGTCTACTAACTTTGGACCCTGAGAAA
RI-AT5G63370-XLOC_032156-2593-1 -------------------------
TCTACTAACTTTGGACCCTGAGAAA
CONSENSUS
.........................TCTACTAACTTTGGACCCTGAGAAA

RI-AT5G63370-XLOC_032156-2593-0
CGTCTAACAGTGGAAGATGCTCTCAACCATGGTTGGTTCCATGAAGTCCC
RI-AT5G63370-XLOC_032156-2593-1
CGTCTAACAGTGGAAGATGCTCTCAACCATGGTTGGTTCCATGAAGTCCC
CONSENSUS
CGTCTAACAGTGGAAGATGCTCTCAACCATGGTTGGTTCCATGAAGTCCC

RI-AT5G63370-XLOC_032156-2593-0
GTTACCAAAATCCAAAGATTTCATGCCGACATATCCTCCAAAGCGGTAAC
RI-AT5G63370-XLOC_032156-2593-1
GTTACCAAAATCCAAAGATTTCATGCCGACATATCCTCCAAAGCGGTAAC
CONSENSUS
GTTACCAAAATCCAAAGATTTCATGCCGACATATCCTCCAAAGCGGTAAC

RI-AT5G63370-XLOC_032156-2593-0
TGAGAAAAAACGTGCTATCATTTGGACGAGAGGATGTCAACAAATACACA
RI-AT5G63370-XLOC_032156-2593-1
TGAGAAAAAACGTGCTATCATTTGGACGAGAGGATGTCAACAAATACACA
CONSENSUS
TGAGAAAAAACGTGCTATCATTTGGACGAGAGGATGTCAACAAATACACA

RI-AT5G63370-XLOC_032156-2593-0
GGGAAGTCGACTGGTCGTATTTTGGTATTTTCCTTTTTACTCAAAAGACT
RI-AT5G63370-XLOC_032156-2593-1
GGGAAGTCGACTGGTCGTATTTTGGTATTTTCCTTTTTACTCAAAAGACT
CONSENSUS
GGGAAGTCGACTGGTCGTATTTTGGTATTTTCCTTTTTACTCAAAAGACT

RI-AT5G63370-XLOC_032156-2593-0
TGCTTTCATCTATTCTTAACTCAGTTGCGTTGCTTTGTTGCCTGGAGATT
RI-AT5G63370-XLOC_032156-2593-1

TGCTTTCATCTATTCTTAACTCAGTTGCGTTGCTTTGTTGCCTGGAGATT
CONSENSUS
TGCTTTCATCTATTCTTAACTCAGTTGCGTTGCTTTGTTGCCTGGAGATT

RI-AT5G63370-XLOC_032156-2593-0
CAGGGCATTCTAGTGTACCATTTATTCGGATTCATGTGTAAATATGAGTG
RI-AT5G63370-XLOC_032156-2593-1
CAGGGCATTCTAGTGTACCATTTATTCGGATTCATGTGTAAATATGAGTG
CONSENSUS
CAGGGCATTCTAGTGTACCATTTATTCGGATTCATGTGTAAATATGAGTG

RI-AT5G63370-XLOC_032156-2593-0
GTCCGTAAAACTGAAAGGTGTGTTTCTCTCTTTTCGTTAGGACCCTAATG
RI-AT5G63370-XLOC_032156-2593-1
GTCCGTAAAACTGAAAGGTGTGTTTCTCTCTTTTCGTTAGGACCCTAATG
CONSENSUS
GTCCGTAAAACTGAAAGGTGTGTTTCTCTCTTTTCGTTAGGACCCTAATG

RI-AT5G63370-XLOC_032156-2593-0
TTATTGCTATCAAGAGTTGTACACACTATTGACTCACATGCTTTAATGGA
RI-AT5G63370-XLOC_032156-2593-1
TTATTGCTATCAAGAGTTGTACACACTATTGACTCACATGCTTTAATGGA
CONSENSUS
TTATTGCTATCAAGAGTTGTACACACTATTGACTCACATGCTTTAATGGA

RI-AT5G63370-XLOC_032156-2593-0
AGAGATCTCAGTATTCACTTTCTCTCTCTAGTAATTGGGTTGGGATGGTT
RI-AT5G63370-XLOC_032156-2593-1
AGAGATCTCAGTATTCACTTTCTCTCTCTAGTAATTGGGTTGGGATGGTT
CONSENSUS
AGAGATCTCAGTATTCACTTTCTCTCTCTAGTAATTGGGTTGGGATGGTT

RI-AT5G63370-XLOC_032156-2593-0
ATTGTTTATGTCCTCATATTTCTTCTGTGCATTGTTTTTATTCACGAGGT
RI-AT5G63370-XLOC_032156-2593-1
ATTGTTTATGTCCTCATATTTCTTCTGTGCATTGTTTTTATTCACGAGGT
CONSENSUS
ATTGTTTATGTCCTCATATTTCTTCTGTGCATTGTTTTTATTCACGAGGT

RI-AT5G63370-XLOC_032156-2593-0
AAACGCAAAGAGTTTATCACAAACGTGAGAATGGAAGTTGACATCAGTAA
RI-AT5G63370-XLOC_032156-2593-1
AAACGCAAAGAGTTTATCACAAACGTGAGAATGGAAGTTGACATCAGTAA
CONSENSUS
AAACGCAAAGAGTTTATCACAAACGTGAGAATGGAAGTTGACATCAGTAA

RI-AT5G63370-XLOC_032156-2593-0
GTTTATTTTGATTACTTATTTACCACGCTCTGTTATCAATGTTGGTTTGT
RI-AT5G63370-XLOC_032156-2593-1
GTTTATTTTGATTACTTATTTACCACGCTCTGTTATCAATGTTGGTTTGT
CONSENSUS
GTTTATTTTGATTACTTATTTACCACGCTCTGTTATCAATGTTGGTTTGT

RI-AT5G63370-XLOC_032156-2593-0
TGTTCGACGTCCCTTTGTCTTTGAGGTGCTCGTATTTGGTCTGTTGGCAG
RI-AT5G63370-XLOC_032156-2593-1

TGTTCGACGTCCCTTTGTCTTTGAGGTGCTCGTATTTGGTCTGTTGGCAG
CONSENSUS
TGTTCGACGTCCCTTTGTCTTTGAGGTGCTCGTATTTGGTCTGTTGGCAG

RI-AT5G63370-XLOC_032156-2593-0
TGAGAAAAAGATGATTCATGATGAGTTTCTGTGGACATAGGAG
RI-AT5G63370-XLOC_032156-2593-1
TGAGAAAAAGATGATTCATGATGAGTTTCTGTGGACATAGGAG
CONSENSUS
TGAGAAAAAGATGATTCATGATGAGTTTCTGTGGACATAGGAG

alignment for event: SE-AT5G02810-XLOC_028866-12014

SE-AT5G02810-XLOC_028866-12014-0
TCTAGTGGTAGTGGAAGTGAGAGCGGAACGCATCAAACTCAAAAGTCTGT
SE-AT5G02810-XLOC_028866-12014-1
TCTAGTGGTAGTGGAAGTGAGAGCGGAACGCATCAAACTCAAAAGTCTGT
CONSENSUS
TCTAGTGGTAGTGGAAGTGAGAGCGGAACGCATCAAACTCAAAAGTCTGT

SE-AT5G02810-XLOC_028866-12014-0
GAAATCGAAAAGTATTAAAAAATCTGATCAAGATTCAGGAAGCAGTGATG
SE-AT5G02810-XLOC_028866-12014-1
GAAATCGAAAAGTATTAAAAAATCTGATCAAGATTCAGGAAGCAGTGATG
CONSENSUS
GAAATCGAAAAGTATTAAAAAATCTGATCAAGATTCAGGAAGCAGTGATG

SE-AT5G02810-XLOC_028866-12014-0
AGAATGAAAATGGGAGCATTGGCCTGAATGCTAGTGATGGAAGTAGTGAT
SE-AT5G02810-XLOC_028866-12014-1
AGAATGAAAATGGGAGCATTGGCCTGAATGCTAGTGATGGAAGTAGTGAT
CONSENSUS
AGAATGAAAATGGGAGCATTGGCCTGAATGCTAGTGATGGAAGTAGTGAT

SE-AT5G02810-XLOC_028866-12014-0
GGGAGTGGCGCTCAG-----------------------------------
SE-AT5G02810-XLOC_028866-12014-1
GGGAGTGGCGCTCAGGGGTCGGATGATCGAACTTCGCATGTGCACTCAGA
CONSENSUS
GGGAGTGGCGCTCAG...................................

SE-AT5G02810-XLOC_028866-12014-0

SE-AT5G02810-XLOC_028866-12014-1
TGCTTATAAATGGTGGAAGCGCTTAACTAACCAATTGTAGAACTCAATGA
CONSENSUS
....

SE-AT5G02810-XLOC_028866-12014-0
----------------------------------AGCTCTTGGACGAAAAA
SE-AT5G02810-XLOC_028866-12014-1
TGTTTAACTTAGATCTATTCAGTGATAAACTGGAGCTCTTGGACGAAAAA
CONSENSUS
..................................AGCTCTTGGACGAAAAA

SE-AT5G02810-XLOC_028866-12014-0
AGCTGTGGATGTTGATGACAGTCCACGAGCGGTATCTCTATGGGACCGAG
SE-AT5G02810-XLOC_028866-12014-1
AGCTGTGGATGTTGATGACAGTCCACGAGCGGTATCTCTATGGGACCGAG
CONSENSUS
AGCTGTGGATGTTGATGACAGTCCACGAGCGGTATCTCTATGGGACCGAG

SE-AT5G02810-XLOC_028866-12014-0
TTGATAGCACTTGCGCCCAAGTGGTACATTCTAACCCTGAGTTTCCAAGT
SE-AT5G02810-XLOC_028866-12014-1
TTGATAGCACTTGCGCCCAAGTGGTACATTCTAACCCTGAGTTTCCAAGT
CONSENSUS
TTGATAGCACTTGCGCCCAAGTGGTACATTCTAACCCTGAGTTTCCAAGT

SE-AT5G02810-XLOC_028866-12014-0
AATCAGTTGGTTGCACCACCTGCTGAGAAGGAGACTCAAGAACATGATGA
SE-AT5G02810-XLOC_028866-12014-1
AATCAGTTGGTTGCACCACCTGCTGAGAAGGAGACTCAAGAACATGATGA
CONSENSUS
AATCAGTTGGTTGCACCACCTGCTGAGAAGGAGACTCAAGAACATGATGA

SE-AT5G02810-XLOC_028866-12014-0 TAAATTTG
SE-AT5G02810-XLOC_028866-12014-1 TAAATTTG
CONSENSUS TAAATTTG

alignment for event: RI-AT5G40930-XLOC_027238-13211

RI-AT5G40930-XLOC_027238-13211-0
GGTACTGGATCTATCATTGGTATATCATTTGATATGTCCAAGATCGGGGA
RI-AT5G40930-XLOC_027238-13211-1
GGTACTGGATCTATCATTGGTATATCATTTGATATGTCCAAGATCGGGGA
CONSENSUS
GGTACTGGATCTATCATTGGTATATCATTTGATATGTCCAAGATCGGGGA

RI-AT5G40930-XLOC_027238-13211-0
GTTCTCTATAAGGAAACGAGTCTTTGAAGGGATGCATAATCTCAAATTCT
RI-AT5G40930-XLOC_027238-13211-1
GTTCTCTATAAGGAAACGAGTCTTTGAAGGGATGCATAATCTCAAATTCT
CONSENSUS
GTTCTCTATAAGGAAACGAGTCTTTGAAGGGATGCATAATCTCAAATTCT

RI-AT5G40930-XLOC_027238-13211-0
TAAAATTCTACAATGGAAATGTTAGCTTGTTAGAGGACATGAAATATCTA
RI-AT5G40930-XLOC_027238-13211-1
TAAAATTCTACAATGGAAATGTTAGCTTGTTAGAGGACATGAAATATCTA
CONSENSUS
TAAAATTCTACAATGGAAATGTTAGCTTGTTAGAGGACATGAAATATCTA

RI-AT5G40930-XLOC_027238-13211-0
CCTCGTCTAAGGTTACTTCATTGGGATTCATACCCGAGAAAAAGGCTTCC
RI-AT5G40930-XLOC_027238-13211-1
CCTCGTCTAAGGTTACTTCATTGGGATTCATACCCGAGAAAAAGGCTTCC
CONSENSUS

CCTCGTCTAAGGTTACTTCATTGGGATTCATACCCGAGAAAAAGGCTTCC

RI-AT5G40930-XLOC_027238-13211-0
TCTGACATTTCAGCCAGAATGTCTCGTCGAACTCTATTTGGTATCCAGCA
RI-AT5G40930-XLOC_027238-13211-1
TCTGACATTTCAGCCAGAATGTCTCGTCGAACTCTATTTGGTATCCAGCA
CONSENSUS
TCTGACATTTCAGCCAGAATGTCTCGTCGAACTCTATTTGGTATCCAGCA

RI-AT5G40930-XLOC_027238-13211-0
AGTTGGAGAAGCTATGGGGAGGAATCCAGGTTGGTATTTGGTACTTGTGA
RI-AT5G40930-XLOC_027238-13211-1
AGTTGGAGAAGCTATGGGGAGGAATCCAG---------------------
CONSENSUS
AGTTGGAGAAGCTATGGGGAGGAATCCAG.....................

RI-AT5G40930-XLOC_027238-13211-0
TTGTGAATACGTTTGTTTACTAATGATAATTAAACTTAGTATGATATTCG
RI-AT5G40930-XLOC_027238-13211-1

CONSENSUS
....

RI-AT5G40930-XLOC_027238-13211-0
TCATCTTTGTGACATGCAGCCCCTCACAAATCTAAAGAAAATAAATTTGG
RI-AT5G40930-XLOC_027238-13211-1 -------------------
CCCCTCACAAATCTAAAGAAAATAAATTTGG
CONSENSUS
...................CCCCTCACAAATCTAAAGAAAATAAATTTGG

RI-AT5G40930-XLOC_027238-13211-0
AATACTCCTCTAATTTGAAAGAAATCCCAAATCTTTCGAAAGCCACTAAT
RI-AT5G40930-XLOC_027238-13211-1
AATACTCCTCTAATTTGAAAGAAATCCCAAATCTTTCGAAAGCCACTAAT
CONSENSUS
AATACTCCTCTAATTTGAAAGAAATCCCAAATCTTTCGAAAGCCACTAAT

RI-AT5G40930-XLOC_027238-13211-0
CTCGAGACGTTGAGACTTACCGGTTGTGAGAGTTTGATGGAGATTCCGTC
RI-AT5G40930-XLOC_027238-13211-1
CTCGAGACGTTGAGACTTACCGGTTGTGAGAGTTTGATGGAGATTCCGTC
CONSENSUS
CTCGAGACGTTGAGACTTACCGGTTGTGAGAGTTTGATGGAGATTCCGTC

RI-AT5G40930-XLOC_027238-13211-0
CTCTATTTCGAATCTACACAAATTGGAAGTACTGGATGCGTCAGGGTGCT
RI-AT5G40930-XLOC_027238-13211-1
CTCTATTTCGAATCTACACAAATTGGAAGTACTGGATGCGTCAGGGTGCT
CONSENSUS
CTCTATTTCGAATCTACACAAATTGGAAGTACTGGATGCGTCAGGGTGCT

RI-AT5G40930-XLOC_027238-13211-0
CTAAGCTACATGTTATTCCCACCAAGATCAACTTATCATCTCTAAAAATG
RI-AT5G40930-XLOC_027238-13211-1
CTAAGCTACATGTTATTCCCACCAAGATCAACTTATCATCTCTAAAAATG
CONSENSUS

CTAAGCTACATGTTATTCCCACCAAGATCAACTTATCATCTCTAAAAATG

RI-AT5G40930-XLOC_027238-13211-0
GTCGGCATGGATGATTGCTCACGACTGAGAAGTTTTCCAGATATTTCGAC
RI-AT5G40930-XLOC_027238-13211-1
GTCGGCATGGATGATTGCTCACGACTGAGAAGTTTTCCAGATATTTCGAC
CONSENSUS
GTCGGCATGGATGATTGCTCACGACTGAGAAGTTTTCCAGATATTTCGAC

RI-AT5G40930-XLOC_027238-13211-0
TAACATCAAGATACTCAGTATAAGAGGCACAAAGATTAAAGAATTTCCTG
RI-AT5G40930-XLOC_027238-13211-1
TAACATCAAGATACTCAGTATAAGAGGCACAAAGATTAAAGAATTTCCTG
CONSENSUS
TAACATCAAGATACTCAGTATAAGAGGCACAAAGATTAAAGAATTTCCTG

RI-AT5G40930-XLOC_027238-13211-0
CGTCCATTGTTGGAGGTCTTGGTATTCTTCTGATAGGCAGCAGAAGCCTC
RI-AT5G40930-XLOC_027238-13211-1
CGTCCATTGTTGGAGGTCTTGGTATTCTTCTGATAGGCAGCAGAAGCCTC
CONSENSUS
CGTCCATTGTTGGAGGTCTTGGTATTCTTCTGATAGGCAGCAGAAGCCTC

RI-AT5G40930-XLOC_027238-13211-0
AAGAGATTAACACATGTCCCAGAGAGTGTATCGTATTTAGACCTAAGCCA
RI-AT5G40930-XLOC_027238-13211-1
AAGAGATTAACACATGTCCCAGAGAGTGTATCGTATTTAGACCTAAGCCA
CONSENSUS
AAGAGATTAACACATGTCCCAGAGAGTGTATCGTATTTAGACCTAAGCCA

RI-AT5G40930-XLOC_027238-13211-0
TTCTGATATAAAGATGATTCCAGATTACGTCATAGGTCTCCCGCATCTAC
RI-AT5G40930-XLOC_027238-13211-1
TTCTGATATAAAGATGATTCCAGATTACGTCATAGGTCTCCCGCATCTAC
CONSENSUS
TTCTGATATAAAGATGATTCCAGATTACGTCATAGGTCTCCCGCATCTAC

RI-AT5G40930-XLOC_027238-13211-0
AACATCTGACCATCGGAAACTGCAGAAAACTGGTGTCTATTGAGGGTCAT
RI-AT5G40930-XLOC_027238-13211-1
AACATCTGACCATCGGAAACTGCAGAAAACTGGTGTCTATTGAGGGTCAT
CONSENSUS
AACATCTGACCATCGGAAACTGCAGAAAACTGGTGTCTATTGAGGGTCAT

RI-AT5G40930-XLOC_027238-13211-0
TCCCCTTCGCTGGAGTCCATAGTTGCATACCGCTGTATATCACTCGAGAG
RI-AT5G40930-XLOC_027238-13211-1
TCCCCTTCGCTGGAGTCCATAGTTGCATACCGCTGTATATCACTCGAGAG
CONSENSUS
TCCCCTTCGCTGGAGTCCATAGTTGCATACCGCTGTATATCACTCGAGAG

RI-AT5G40930-XLOC_027238-13211-0
CATGTGTTGCTCTTTCCACAGGCCAATCTTGAAACTGGAGTTCTACAACT
RI-AT5G40930-XLOC_027238-13211-1
CATGTGTTGCTCTTTCCACAGGCCAATCTTGAAACTGGAGTTCTACAACT
CONSENSUS

CATGTGTTGCTCTTTCCACAGGCCAATCTTGAAACTGGAGTTCTACAACT

RI-AT5G40930-XLOC_027238-13211-0
GTCTGAAACTGGATAATGAATCAAAAAGACGAATCATACTACACTCGGGT
RI-AT5G40930-XLOC_027238-13211-1
GTCTGAAACTGGATAATGAATCAAAAAGACGAATCATACTACACTCGGGT
CONSENSUS
GTCTGAAACTGGATAATGAATCAAAAAGACGAATCATACTACACTCGGGT

RI-AT5G40930-XLOC_027238-13211-0
CACAGAATTATTTTTTTGACAGGTAATGAAGTCCCTGCACAGTTCACTCA
RI-AT5G40930-XLOC_027238-13211-1
CACAGAATTATTTTTTTGACAGGTAATGAAGTCCCTGCACAGTTCACTCA
CONSENSUS
CACAGAATTATTTTTTTGACAGGTAATGAAGTCCCTGCACAGTTCACTCA

RI-AT5G40930-XLOC_027238-13211-0
CCAAACTAGAGGGAACTCGATAACCATCTCTCTGTCTCCAGGTGGTGAGG
RI-AT5G40930-XLOC_027238-13211-1
CCAAACTAGAGGGAACTCGATAACCATCTCTCTGTCTCCAGGTGGTGAGG
CONSENSUS
CCAAACTAGAGGGAACTCGATAACCATCTCTCTGTCTCCAGGTGGTGAGG

RI-AT5G40930-XLOC_027238-13211-0
AGTCTTTCTCTGTGTCCTCAAGATTTAGGGCTTGCCTTGTGCTTTCGCCA
RI-AT5G40930-XLOC_027238-13211-1
AGTCTTTCTCTGTGTCCTCAAGATTTAGGGCTTGCCTTGTGCTTTCGCCA
CONSENSUS
AGTCTTTCTCTGTGTCCTCAAGATTTAGGGCTTGCCTTGTGCTTTCGCCA

RI-AT5G40930-XLOC_027238-13211-0
AGCAAGAATTCCCCATATTCTGACATAAATTGTTTTCTAAGAACCAAACA
RI-AT5G40930-XLOC_027238-13211-1
AGCAAGAATTCCCCATATTCTGACATAAATTGTTTTCTAAGAACCAAACA
CONSENSUS
AGCAAGAATTCCCCATATTCTGACATAAATTGTTTTCTAAGAACCAAACA

RI-AT5G40930-XLOC_027238-13211-0
AGGTGTCGAAATTAACAGCACTGCAAAGTCCATATATTCAAGTCCCCCTA
RI-AT5G40930-XLOC_027238-13211-1
AGGTGTCGAAATTAACAGCACTGCAAAGTCCATATATTCAAGTCCCCCTA
CONSENSUS
AGGTGTCGAAATTAACAGCACTGCAAAGTCCATATATTCAAGTCCCCCTA

RI-AT5G40930-XLOC_027238-13211-0
ACAGATCTCTATCAGAATATCTGTTAATTTTTTTTGGCGACATATTTCCT
RI-AT5G40930-XLOC_027238-13211-1
ACAGATCTCTATCAGAATATCTGTTAATTTTTTTTGGCGACATATTTCCT
CONSENSUS
ACAGATCTCTATCAGAATATCTGTTAATTTTTTTTGGCGACATATTTCCT

RI-AT5G40930-XLOC_027238-13211-0
GAAGCAAACAGATGCCTTATGGACGTGACCCCGAACGAAATTGTGTTTGA
RI-AT5G40930-XLOC_027238-13211-1
GAAGCAAACAGATGCCTTATGGACGTGACCCCGAACGAAATTGTGTTTGA
CONSENSUS

GAAGCAAACAGATGCCTTATGGACGTGACCCCGAACGAAATTGTGTTTGA

RI-AT5G40930-XLOC_027238-13211-0
ATTCAGCTCCTCTAGTGCTAAGACTATGGAATGTGGTGTACAGATCTTGG
RI-AT5G40930-XLOC_027238-13211-1
ATTCAGCTCCTCTAGTGCTAAGACTATGGAATGTGGTGTACAGATCTTGG
CONSENSUS
ATTCAGCTCCTCTAGTGCTAAGACTATGGAATGTGGTGTACAGATCTTGG

RI-AT5G40930-XLOC_027238-13211-0
CAGAAGGAGGACAGAATTGTAGTGTCGTCGAAATGGGCCACTCTGAAACT
RI-AT5G40930-XLOC_027238-13211-1
CAGAAGGAGGACAGAATTGTAGTGTCGTCGAAATGGGCCACTCTGAAACT
CONSENSUS
CAGAAGGAGGACAGAATTGTAGTGTCGTCGAAATGGGCCACTCTGAAACT

RI-AT5G40930-XLOC_027238-13211-0
GGAGGCAACAGAAACCACCTCACAGATGTTTTAAAGGTATCTCAAGTCGA
RI-AT5G40930-XLOC_027238-13211-1
GGAGGCAACAGAAACCACCTCACAGATGTTTTAAAGGTATCTCAAGTCGA
CONSENSUS
GGAGGCAACAGAAACCACCTCACAGATGTTTTAAAGGTATCTCAAGTCGA

RI-AT5G40930-XLOC_027238-13211-0
AACCATCAAAAACAGTAATAATACAGGTCACTGGAGTTGGCTTCTAGGGA
RI-AT5G40930-XLOC_027238-13211-1
AACCATCAAAAACAGTAATAATACAGGTCACTGGAGTTGGCTTCTAGGGA
CONSENSUS
AACCATCAAAAACAGTAATAATACAGGTCACTGGAGTTGGCTTCTAGGGA

RI-AT5G40930-XLOC_027238-13211-0
AGAAGAAGACAGAGCTTAGTTCGACATTAGTCTCAGGATCCAGTGATGAT
RI-AT5G40930-XLOC_027238-13211-1
AGAAGAAGACAGAGCTTAGTTCGACATTAGTCTCAGGATCCAGTGATGAT
CONSENSUS
AGAAGAAGACAGAGCTTAGTTCGACATTAGTCTCAGGATCCAGTGATGAT

RI-AT5G40930-XLOC_027238-13211-0
ACCTTGATGAGGCATCATGAGCCTGAAGCTGTTCAGCTCAGTAATGATGA
RI-AT5G40930-XLOC_027238-13211-1
ACCTTGATGAGGCATCATGAGCCTGAAGCTGTTCAGCTCAGTAATGATGA
CONSENSUS
ACCTTGATGAGGCATCATGAGCCTGAAGCTGTTCAGCTCAGTAATGATGA

RI-AT5G40930-XLOC_027238-13211-0
AAACACCAGAAATCTTTCGCGTTTGCTATGTATTGTTTCCCTTGTGATCT
RI-AT5G40930-XLOC_027238-13211-1
AAACACCAGAAATCTTTCGCGTTTGCTATGTATTGTTTCCCTTGTGATCT
CONSENSUS
AAACACCAGAAATCTTTCGCGTTTGCTATGTATTGTTTCCCTTGTGATCT

RI-AT5G40930-XLOC_027238-13211-0
CTGTTTTGTTACTTTTCCATTGCTTTTTTCTTCAAATGCAATGACCTGAT
RI-AT5G40930-XLOC_027238-13211-1
CTGTTTTGTTACTTTTCCATTGCTTTTTTCTTCAAATGCAATGACCTGAT
CONSENSUS

CTGTTTTGTTACTTTTCCATTGCTTTTTTCTTCAAATGCAATGACCTGAT

RI-AT5G40930-XLOC_027238-13211-0
GCATTACACTACAGATCCTGTTGTGGTTAGACGTAATAGTTGTTGTTGTA
RI-AT5G40930-XLOC_027238-13211-1
GCATTACACTACAGATCCTGTTGTGGTTAGACGTAATAGTTGTTGTTGTA
CONSENSUS
GCATTACACTACAGATCCTGTTGTGGTTAGACGTAATAGTTGTTGTTGTA

RI-AT5G40930-XLOC_027238-13211-0
TTGTTTGGTGTTTTTTTTGTTGTTACTAGTTGTTGTATGATTGTGTGAAA
RI-AT5G40930-XLOC_027238-13211-1
TTGTTTGGTGTTTTTTTTGTTGTTACTAGTTGTTGTATGATTGTGTGAAA
CONSENSUS
TTGTTTGGTGTTTTTTTTGTTGTTACTAGTTGTTGTATGATTGTGTGAAA

RI-AT5G40930-XLOC_027238-13211-0
AAAAAAGGGTTTAGTTTTCTTAGATATTGTTGGGCTGACTTTAGCAAAAG
RI-AT5G40930-XLOC_027238-13211-1
AAAAAAGGGTTTAGTTTTCTTAGATATTGTTGGGCTGACTTTAGCAAAAG
CONSENSUS
AAAAAAGGGTTTAGTTTTCTTAGATATTGTTGGGCTGACTTTAGCAAAAG

RI-AT5G40930-XLOC_027238-13211-0
CCCATAAGGCCTTTTAAGCCCACTTCCCGGACGAGAAGAGCGAACCGACT
RI-AT5G40930-XLOC_027238-13211-1
CCCATAAGGCCTTTTAAGCCCACTTCCCGGACGAGAAGAGCGAACCGACT
CONSENSUS
CCCATAAGGCCTTTTAAGCCCACTTCCCGGACGAGAAGAGCGAACCGACT

RI-AT5G40930-XLOC_027238-13211-0
TATATACGAGGATTTATAGCTCGTCTTCCTTAAATTATCGTTTGTGACGG
RI-AT5G40930-XLOC_027238-13211-1
TATATACGAGGATTTATAGCTCGTCTTCCTTAAATTATCGTTTGTGACGG
CONSENSUS
TATATACGAGGATTTATAGCTCGTCTTCCTTAAATTATCGTTTGTGACGG

RI-AT5G40930-XLOC_027238-13211-0
AAGAAGAATCAAAACAATTAATCGCGAGGCTTGAGAATCAATCAATGGAT
RI-AT5G40930-XLOC_027238-13211-1
AAGAAGAATCAAAACAATTAATCGCGAGGCTTGAGAATCAATCAATGGAT
CONSENSUS
AAGAAGAATCAAAACAATTAATCGCGAGGCTTGAGAATCAATCAATGGAT

RI-AT5G40930-XLOC_027238-13211-0
ATGCAGAATGAAAACGAGAGATTGATGGTCTTCGAACATGCTCGCAAAGT
RI-AT5G40930-XLOC_027238-13211-1
ATGCAGAATGAAAACGAGAGATTGATGGTCTTCGAACATGCTCGCAAAGT
CONSENSUS
ATGCAGAATGAAAACGAGAGATTGATGGTCTTCGAACATGCTCGCAAAGT

RI-AT5G40930-XLOC_027238-13211-0
AGCAGAAGCAACCTACGTCAAAAACCCTTTAGATGCCGAG
RI-AT5G40930-XLOC_027238-13211-1
AGCAGAAGCAACCTACGTCAAAAACCCTTTAGATGCCGAG
CONSENSUS

AGCAGAAGCAACCTACGTCAAAAACCCTTTAGATGCCGAG

alignment for event: RI-AT5G17010-XLOC_029600-6263

RI-AT5G17010-XLOC_029600-6263-0
GAACTGCTGGGAGCTGGGATACTGTTTTGTGGGTTTGGTGTGATATGTGT
RI-AT5G17010-XLOC_029600-6263-1
GAACTGCTGGGAGCTGGGATACTGTTTTGTGGGTTTGGTGTGATATGTGT
CONSENSUS
GAACTGCTGGGAGCTGGGATACTGTTTTGTGGGTTTGGTGTGATATGTGT

RI-AT5G17010-XLOC_029600-6263-0
ATTGTCTCTGGTATTCATATTCTTCATTGTGCCAGAGACAAAGGGTCTCA
RI-AT5G17010-XLOC_029600-6263-1
ATTGTCTCTGGTATTCATATTCTTCATTGTGCCAGAGACAAAGGGTCTCA
CONSENSUS
ATTGTCTCTGGTATTCATATTCTTCATTGTGCCAGAGACAAAGGGTCTCA

RI-AT5G17010-XLOC_029600-6263-0
CTCTTGAGGAAATTGAAGCCAAATGTCTCTAAAAAAGAGGTCTGTTCTTT
RI-AT5G17010-XLOC_029600-6263-1
CTCTTGAGGAAATTGAAGCCAAATGTCTCTAAAAAAGAG-----------
CONSENSUS
CTCTTGAGGAAATTGAAGCCAAATGTCTCTAAAAAAGAG...........

RI-AT5G17010-XLOC_029600-6263-0
GCTTAGAAACCACAAAGTCGTGTGCTTCCTCACATGATTTTGATTCATAT
RI-AT5G17010-XLOC_029600-6263-1

CONSENSUS
....

RI-AT5G17010-XLOC_029600-6263-0
TGTTAATCAGTGAGGAAAATAATAGTGCAGGTTCAGAAACAAATAAACAT
RI-AT5G17010-XLOC_029600-6263-1 -------------------------------
GTTCAGAAACAAATAAACAT
CONSENSUS
..............................GTTCAGAAACAAATAAACAT

RI-AT5G17010-XLOC_029600-6263-0
ATGAATCTGCCGCACAAGACGGGAAATGAATCTTCAGAGACCACTAAGTT
RI-AT5G17010-XLOC_029600-6263-1
ATGAATCTGCCGCACAAGACGGGAAATGAATCTTCAGAGACCACTAAGTT
CONSENSUS
ATGAATCTGCCGCACAAGACGGGAAATGAATCTTCAGAGACCACTAAGTT

RI-AT5G17010-XLOC_029600-6263-0
ATTTGAAGCAATGTTACTTCAAAGGCTCGGTTGTTTCTAGCAAAATACAT
RI-AT5G17010-XLOC_029600-6263-1
ATTTGAAGCAATGTTACTTCAAAGGCTCGGTTGTTTCTAGCAAAATACAT
CONSENSUS
ATTTGAAGCAATGTTACTTCAAAGGCTCGGTTGTTTCTAGCAAAATACAT

RI-AT5G17010-XLOC_029600-6263-0

GTACGAGAATTCATAAATACAGAAATCTTTGTAATGATTATTAGCGCTCT
RI-AT5G17010-XLOC_029600-6263-1
GTACGAGAATTCATAAATACAGAAATCTTTGTAATGATTATTAGCGCTCT
CONSENSUS
GTACGAGAATTCATAAATACAGAAATCTTTGTAATGATTATTAGCGCTCT

RI-AT5G17010-XLOC_029600-6263-0
GATGAAGTTAGAAAATAAAAAAAGAAAACATCATAGAAGAATTTAAATTT
RI-AT5G17010-XLOC_029600-6263-1
GATGAAGTTAGAAAATAAAAAAAGAAAACATCATAGAAGAATTTAAATTT
CONSENSUS
GATGAAGTTAGAAAATAAAAAAAGAAAACATCATAGAAGAATTTAAATTT

RI-AT5G17010-XLOC_029600-6263-0
GTAGAATATGTCCTAACCAGTGATGTTTCGAAATCCGAAGGTTTCTCAAA
RI-AT5G17010-XLOC_029600-6263-1
GTAGAATATGTCCTAACCAGTGATGTTTCGAAATCCGAAGGTTTCTCAAA
CONSENSUS
GTAGAATATGTCCTAACCAGTGATGTTTCGAAATCCGAAGGTTTCTCAAA

RI-AT5G17010-XLOC_029600-6263-0
GTTTGTATTTTTTTTAAACGATTCCACGATTCTGCAATGCTGCATTATGA
RI-AT5G17010-XLOC_029600-6263-1
GTTTGTATTTTTTTTAAACGATTCCACGATTCTGCAATGCTGCATTATGA
CONSENSUS
GTTTGTATTTTTTTTAAACGATTCCACGATTCTGCAATGCTGCATTATGA

RI-AT5G17010-XLOC_029600-6263-0
TATAGAACATTATGCTGAATAGAAGATATTTTTCGGGATTTGTAAGACTT
RI-AT5G17010-XLOC_029600-6263-1
TATAGAACATTATGCTGAATAGAAGATATTTTTCGGGATTTGTAAGACTT
CONSENSUS
TATAGAACATTATGCTGAATAGAAGATATTTTTCGGGATTTGTAAGACTT

RI-AT5G17010-XLOC_029600-6263-0
GATGTGATATAGTATAATGGAACATTGTGGTC
RI-AT5G17010-XLOC_029600-6263-1
GATGTGATATAGTATAATGGAACATTGTGGTC
CONSENSUS
GATGTGATATAGTATAATGGAACATTGTGGTC

alignment for event: SE-AT5G27410-XLOC_026458-5127

SE-AT5G27410-XLOC_026458-5127-0
GCTGCGAATGGTAACGGATCATACGCGAAGAAGTTACTGAAGAAACTCAT
SE-AT5G27410-XLOC_026458-5127-1
GCTGCGAATGGTAACGGATCATACGCGAAGAAGTTACTGAAGAAACTCAT
CONSENSUS
GCTGCGAATGGTAACGGATCATACGCGAAGAAGTTACTGAAGAAACTCAT

SE-AT5G27410-XLOC_026458-5127-0
AAATGAAGTTGAGTTAGAGAATGGTGAAGTATTGGATGAAGTGTATGAAG
SE-AT5G27410-XLOC_026458-5127-1
AAATGAAGTTGAGTTAGAGAATGGTGAAGTATTGGATGAAGTGTATGAAG

CONSENSUS
AAATGAAGTTGAGTTAGAGAATGGTGAAGTATTGGATGAAGTGTATGAAG

SE-AT5G27410-XLOC_026458-5127-0
AGTATGCTCTTTACATGTTAACTTCTAAG---------------------
SE-AT5G27410-XLOC_026458-5127-1
AGTATGCTCTTTACATGTTAACTTCTAAGGAAGATATATTGGTGAAAGAA
CONSENSUS
AGTATGCTCTTTACATGTTAACTTCTAAG.....................

SE-AT5G27410-XLOC_026458-5127-0
---GTTCGTA
SE-AT5G27410-XLOC_026458-5127-1
AACATAAGGATAACCAAATTCATTTCTTTTCTTTTCCCTGAAGGTTCGTA
CONSENSUS
....GTTCGTA

SE-AT5G27410-XLOC_026458-5127-0
CAAACACCCGAGTTGTCCAAGGTCGAGGAAATTAGTTATTCCGCTGCATT
SE-AT5G27410-XLOC_026458-5127-1
CAAACACCCGAGTTGTCCAAGGTCGAGGAAATTAGTTATTCCGCTGCATT
CONSENSUS
CAAACACCCGAGTTGTCCAAGGTCGAGGAAATTAGTTATTCCGCTGCATT

SE-AT5G27410-XLOC_026458-5127-0
GTTCGTTGAACATGCTTGAAGGAGATACCGG
SE-AT5G27410-XLOC_026458-5127-1
GTTCGTTGAACATGCTTGAAGGAGATACCGG
CONSENSUS
GTTCGTTGAACATGCTTGAAGGAGATACCGG

alignment for event: A5-AT5G41760-XLOC_027292-13434

A5-AT5G41760-XLOC_027292-13434-0
GTTAAGGGTTGTGGAGAAGCTTCTTGTGATTCTTTATTCACGGCACCAAT
A5-AT5G41760-XLOC_027292-13434-1
GTTAAGGGTTGTGGAGAAGCTTCTTGTGATTCTTTATTCACGGCACCAAT
CONSENSUS
GTTAAGGGTTGTGGAGAAGCTTCTTGTGATTCTTTATTCACGGCACCAAT

A5-AT5G41760-XLOC_027292-13434-0
ACAAGGTTATTTGCTGGGCATCCTCTCAGCTGGTTTGTCCGCGCTAGCTG
A5-AT5G41760-XLOC_027292-13434-1
ACAAGGTTATTTGCTGGGCATCCTCTCAGCTGGTTTGTCCGCGCTAGCTG
CONSENSUS
ACAAGGTTATTTGCTGGGCATCCTCTCAGCTGGTTTGTCCGCGCTAGCTG

A5-AT5G41760-XLOC_027292-13434-0
GAATCTACACAGAGTTTCTGATGAAGAGAAACAATGACACCTTATACTGG
A5-AT5G41760-XLOC_027292-13434-1
GAATCTACACAGAGTTTCTGATGAAGAGAAACAATGACACCTTATACTGG
CONSENSUS
GAATCTACACAGAGTTTCTGATGAAGAGAAACAATGACACCTTATACTGG

A5-AT5G41760-XLOC_027292-13434-0 CAAAACTTGCAGTTATATAC----GTTTGGTTCACTTTTCAACGTAGCTC
A5-AT5G41760-XLOC_027292-13434-1
CAAAACTTGCAGTTATATACGTAGGTTTGGTTCACTTTTCAACGTAGCTC
CONSENSUS
CAAAACTTGCAGTTATATAC....GTTTGGTTCACTTTTCAACGTAGCTC

A5-AT5G41760-XLOC_027292-13434-0
GACTTATAGCAGATGATTTCAGACACGGGTTTGAAAAGGGTCCTTGGTGG
A5-AT5G41760-XLOC_027292-13434-1
GACTTATAGCAGATGATTTCAGACACGGGTTTGAAAAGGGTCCTTGGTGG
CONSENSUS
GACTTATAGCAGATGATTTCAGACACGGGTTTGAAAAGGGTCCTTGGTGG

A5-AT5G41760-XLOC_027292-13434-0
CAACGTATCTTTGATGGGTACAGCATCACCACTTGGCTGGTAGTTCTGAA
A5-AT5G41760-XLOC_027292-13434-1
CAACGTATCTTTGATGGGTACAGCATCACCACTTGGCTGGTAGTTCTGAA
CONSENSUS
CAACGTATCTTTGATGGGTACAGCATCACCACTTGGCTGGTAGTTCTGAA

A5-AT5G41760-XLOC_027292-13434-0
TCTCGGATCCACTGGCTTACTGGTCTCGTGGTTAATGAAGTATGCAGACA
A5-AT5G41760-XLOC_027292-13434-1
TCTCGGATCCACTGGCTTACTGGTCTCGTGGTTAATGAAGTATGCAGACA
CONSENSUS
TCTCGGATCCACTGGCTTACTGGTCTCGTGGTTAATGAAGTATGCAGACA

A5-AT5G41760-XLOC_027292-13434-0 ACATCGTCAAG
A5-AT5G41760-XLOC_027292-13434-1 ACATCGTCAAG
CONSENSUS ACATCGTCAAG

alignment for event: RI-ATCG00720-XLOC_032438-11444

RI-ATCG00720-XLOC_032438-11444-0
AAAGTTTATGATTGGTTCGAAGAACGTCTTGAGATTCAGGCGATTGCAGA
RI-ATCG00720-XLOC_032438-11444-1
AAAGTTTATGATTGGTTCGAAGAACGTCTTGAGATTCAGGCGATTGCAGA
CONSENSUS
AAAGTTTATGATTGGTTCGAAGAACGTCTTGAGATTCAGGCGATTGCAGA

RI-ATCG00720-XLOC_032438-11444-0
TGATATAACTAGTAAATATGTTCCTCCGCATGTCAACATATTTTATTGTC
RI-ATCG00720-XLOC_032438-11444-1
TGATATAACTAGTAAATATGTTCCTCCGCATGTCAACATATTTTATTGTC
CONSENSUS
TGATATAACTAGTAAATATGTTCCTCCGCATGTCAACATATTTTATTGTC

RI-ATCG00720-XLOC_032438-11444-0
TAGGCGGAATTACCCTTACTTGTTTTTTAGTACAAGTAGCTACGGGATTT
RI-ATCG00720-XLOC_032438-11444-1
TAGGCGGAATTACCCTTACTTGTTTTTTAGTACAAGTAGCTACGGGATTT
CONSENSUS
TAGGCGGAATTACCCTTACTTGTTTTTTAGTACAAGTAGCTACGGGATTT

RI-ATCG00720-XLOC_032438-11444-0
GCTATGACTTTTTATTACCGTCCAACCGTTACTGAAGCTTTTGCTTCTGT
RI-ATCG00720-XLOC_032438-11444-1
GCTATGACTTTTTATTACCGTCCAACCGTTACTGAAGCTTTTGCTTCTGT
CONSENSUS
GCTATGACTTTTTATTACCGTCCAACCGTTACTGAAGCTTTTGCTTCTGT

RI-ATCG00720-XLOC_032438-11444-0
TCAATATATAATGACTGAAGCTAACTTTGGTTGGTTAATCCGATCAGTTC
RI-ATCG00720-XLOC_032438-11444-1
TCAATATATAATGACTGAAGCTAACTTTGGTTGGTTAATCCGATCAGTTC
CONSENSUS
TCAATATATAATGACTGAAGCTAACTTTGGTTGGTTAATCCGATCAGTTC

RI-ATCG00720-XLOC_032438-11444-0
ATCGATGGTCGGCAAGTATGATGGTCCTAATGATGATCCTGCACGTATTT
RI-ATCG00720-XLOC_032438-11444-1
ATCGATGGTCGGCAAGTATGATGGTCCTAATGATGATCCTGCACGTATTT
CONSENSUS
ATCGATGGTCGGCAAGTATGATGGTCCTAATGATGATCCTGCACGTATTT

RI-ATCG00720-XLOC_032438-11444-0
CGTGTATACCTCACCGGTGGTTTTAAAAAACCTCGCGAATTAACTTGGGT
RI-ATCG00720-XLOC_032438-11444-1
CGTGTATACCTCACCGGTGGTTTTAAAAAACCTCGCGAATTAACTTGGGT
CONSENSUS
CGTGTATACCTCACCGGTGGTTTTAAAAAACCTCGCGAATTAACTTGGGT

RI-ATCG00720-XLOC_032438-11444-0
TACTGGTGTGGTTCTGGGTGTATTGACCGCATCTTTTGGTGTAACAGGTT
RI-ATCG00720-XLOC_032438-11444-1
TACTGGTGTGGTTCTGGGTGTATTGACCGCATCTTTTGGTGTAACAGGTT
CONSENSUS
TACTGGTGTGGTTCTGGGTGTATTGACCGCATCTTTTGGTGTAACAGGTT

RI-ATCG00720-XLOC_032438-11444-0
ATTCTTTACCTTGGGATCAAATTGGTTATTGGGCGGTCAAAATTGTAACA
RI-ATCG00720-XLOC_032438-11444-1
ATTCTTTACCTTGGGATCAAATTGGTTATTGGGCGGTCAAAATTGTAACA
CONSENSUS
ATTCTTTACCTTGGGATCAAATTGGTTATTGGGCGGTCAAAATTGTAACA

RI-ATCG00720-XLOC_032438-11444-0
GGTGTACCTGACGCTATTCCGGTAATAGGATCACCTCTTGTAGAATTATT
RI-ATCG00720-XLOC_032438-11444-1
GGTGTACCTGACGCTATTCCGGTAATAGGATCACCTCTTGTAGAATTATT
CONSENSUS
GGTGTACCTGACGCTATTCCGGTAATAGGATCACCTCTTGTAGAATTATT

RI-ATCG00720-XLOC_032438-11444-0
ACGCGGAAGTGCTAGTGTTGGACAATCCACTTTGACTCGTTTTTATAGTT
RI-ATCG00720-XLOC_032438-11444-1
ACGCGGAAGTGCTAGTGTTGGACAATCCACTTTGACTCGTTTTTATAGTT
CONSENSUS
ACGCGGAAGTGCTAGTGTTGGACAATCCACTTTGACTCGTTTTTATAGTT

RI-ATCG00720-XLOC_032438-11444-0
TACACACTTTTGTATTACCTCTTCTTACGGCCGTCTTTATGTTAATGCAT
RI-ATCG00720-XLOC_032438-11444-1
TACACACTTTTGTATTACCTCTTCTTACGGCCGTCTTTATGTTAATGCAT
CONSENSUS
TACACACTTTTGTATTACCTCTTCTTACGGCCGTCTTTATGTTAATGCAT

RI-ATCG00720-XLOC_032438-11444-0
TTCCTAATGATACGTAAGCAAGGTATTTCTGGTCCCTTATAAATAATATA
RI-ATCG00720-XLOC_032438-11444-1
TTCCTAATGATACGTAAGCAAGGTATTTCTGGTCCCTTATAAATAATATA
CONSENSUS
TTCCTAATGATACGTAAGCAAGGTATTTCTGGTCCCTTATAAATAATATA

RI-ATCG00720-XLOC_032438-11444-0
GATTCTAGGTATTTGTACTTACTTATTACTTGGTGAAGGAACGATAGTAT
RI-ATCG00720-XLOC_032438-11444-1
GATTCTAGGTATTTGTACTTACTTATTACTTGGTGAAGGAACGATAGTAT
CONSENSUS
GATTCTAGGTATTTGTACTTACTTATTACTTGGTGAAGGAACGATAGTAT

RI-ATCG00720-XLOC_032438-11444-0
TTTATTGCTATAAATATGGATTATTAAAAAAATAAAACATGTATTTGGAT
RI-ATCG00720-XLOC_032438-11444-1
TTTATTGCTATAAATATGGATTATTAAAAAAATAAAACATGTATTTGGAT
CONSENSUS
TTTATTGCTATAAATATGGATTATTAAAAAAATAAAACATGTATTTGGAT

RI-ATCG00720-XLOC_032438-11444-0
ATTTCCCTTCAACTCCACAATATTTTATTATTTTTTTGACATAAAAAGTT
RI-ATCG00720-XLOC_032438-11444-1
ATTTCCCTTCAACTCCACAATATTTTATTATTTTTTTGACATAAAAAGTT
CONSENSUS
ATTTCCCTTCAACTCCACAATATTTTATTATTTTTTTGACATAAAAAGTT

RI-ATCG00720-XLOC_032438-11444-0
GAAGGGAATTCTATGAAGAGATAATGGATTATGGGAGTGTGTGACTTGAA
RI-ATCG00720-XLOC_032438-11444-1
GAAGGGAATTCTATGAAGAGATAATGGATTATGGGAGT------------
CONSENSUS
GAAGGGAATTCTATGAAGAGATAATGGATTATGGGAGT............

RI-ATCG00720-XLOC_032438-11444-0
CTATTGATCGGGCCGTGCAGAAATATTACTTTATCTGCTACATTGGAATT
RI-ATCG00720-XLOC_032438-11444-1

CONSENSUS
....

RI-ATCG00720-XLOC_032438-11444-0
CACAACCAAATGTGTCTTTGTTCCAACCACTGTGTAAGCCCCATACAGGG
RI-ATCG00720-XLOC_032438-11444-1

CONSENSUS
....

RI-ATCG00720-XLOC_032438-11444-0
GATAGGCTGGTTCACTTGAAGAGAATCTTTTCTATGATCGTACCCGACGC
RI-ATCG00720-XLOC_032438-11444-1

CONSENSUS
....

RI-ATCG00720-XLOC_032438-11444-0
TGTCGTGGATGAGTGGGCTCCGTAAAATCCAGTAGATTAAGGGATGGAAC
RI-ATCG00720-XLOC_032438-11444-1

CONSENSUS
....

RI-ATCG00720-XLOC_032438-11444-0
AAAATCCTGATTATGTTTTTAGTTATTTTGGACTAAAAAAAAAAAAGAAA
RI-ATCG00720-XLOC_032438-11444-1

CONSENSUS
....

RI-ATCG00720-XLOC_032438-11444-0
ATAATTATTAATAGTAATAGTATGTAAATGCATTCATTTTCTCTGCATCG
RI-ATCG00720-XLOC_032438-11444-1

CONSENSUS
....

RI-ATCG00720-XLOC_032438-11444-0
ACTCGATTTATGATACTATCGGAGTGAATACAGGATCTAATGAAGAGTAG
RI-ATCG00720-XLOC_032438-11444-1

CONSENSUS
....

RI-ATCG00720-XLOC_032438-11444-0
AGGGTAGACTCCATTAGTAACAAGTAAATCCTTTGTATTTGAAAAATCTC
RI-ATCG00720-XLOC_032438-11444-1

CONSENSUS
....

RI-ATCG00720-XLOC_032438-11444-0
GATATAATTGTTGAGATTAAGGACGAATTTATAAGGTATGAGACGACCCA
RI-ATCG00720-XLOC_032438-11444-1

CONSENSUS
....

RI-ATCG00720-XLOC_032438-11444-0
GAAAGCACTTAATCATGATAAACTTTTTAAGCTTACGTGGGTGTTGAGCA
RI-ATCG00720-XLOC_032438-11444-1

CONSENSUS
....

RI-ATCG00720-XLOC_032438-11444-0
TTTACCTGTAAGAATGGAATTTCTTGAATCTTTAGTTGCAATAACTTTGG
RI-ATCG00720-XLOC_032438-11444-1

CONSENSUS
....

RI-ATCG00720-XLOC_032438-11444-0
AATCGGATAATTAATTTTTACATATTAAATTATATTTTTTGTATGTATTC
RI-ATCG00720-XLOC_032438-11444-1

CONSENSUS
....

RI-ATCG00720-XLOC_032438-11444-0
ATTTAGTTTGGTTAATTCTTGCTCGAGCCGGATGATAAAAAATTATCATG
RI-ATCG00720-XLOC_032438-11444-1

CONSENSUS
....

RI-ATCG00720-XLOC_032438-11444-0
TCCGGTTCCCTCGGGGGATGGATCCATAAGAATTCACCTATCCCAATAAC
RI-ATCG00720-XLOC_032438-11444-1
---AAC
CONSENSUS
....AAC

RI-ATCG00720-XLOC_032438-11444-0
AAAAAAACCAGATTTGAATGATCCTGTATTACGAGCTAAATTAGCTAAAG
RI-ATCG00720-XLOC_032438-11444-1
AAAAAAACCAGATTTGAATGATCCTGTATTACGAGCTAAATTAGCTAAAG
CONSENSUS
AAAAAAACCAGATTTGAATGATCCTGTATTACGAGCTAAATTAGCTAAAG

RI-ATCG00720-XLOC_032438-11444-0
GTATGGGTCACAATTATTACGGGGAACCCGCATGGCCCAACGACCTTTTA
RI-ATCG00720-XLOC_032438-11444-1
GTATGGGTCACAATTATTACGGGGAACCCGCATGGCCCAACGACCTTTTA
CONSENSUS
GTATGGGTCACAATTATTACGGGGAACCCGCATGGCCCAACGACCTTTTA

RI-ATCG00720-XLOC_032438-11444-0
TATATTTTTCCAGTAGTTATTCTTGGTACCATTGCCTGTAACGTAGGCTT
RI-ATCG00720-XLOC_032438-11444-1
TATATTTTTCCAGTAGTTATTCTTGGTACCATTGCCTGTAACGTAGGCTT
CONSENSUS
TATATTTTTCCAGTAGTTATTCTTGGTACCATTGCCTGTAACGTAGGCTT

RI-ATCG00720-XLOC_032438-11444-0
AGCGGTTTTAGAACCATCAATGATTGGTGAACCTGCGGATCCTTTTGCAA
RI-ATCG00720-XLOC_032438-11444-1
AGCGGTTTTAGAACCATCAATGATTGGTGAACCTGCGGATCCTTTTGCAA
CONSENSUS
AGCGGTTTTAGAACCATCAATGATTGGTGAACCTGCGGATCCTTTTGCAA

RI-ATCG00720-XLOC_032438-11444-0
CTCCTTTGGAAATATTACCTGAATGGTATTTCTTTCCTGTATTTCAAATA
RI-ATCG00720-XLOC_032438-11444-1
CTCCTTTGGAAATATTACCTGAATGGTATTTCTTTCCTGTATTTCAAATA
CONSENSUS
CTCCTTTGGAAATATTACCTGAATGGTATTTCTTTCCTGTATTTCAAATA

RI-ATCG00720-XLOC_032438-11444-0
CTTCGTACAGTGCCTAACAAATTATTGGGTGTTCTTTTAATGGTTTCAGT
RI-ATCG00720-XLOC_032438-11444-1
CTTCGTACAGTGCCTAACAAATTATTGGGTGTTCTTTTAATGGTTTCAGT
CONSENSUS
CTTCGTACAGTGCCTAACAAATTATTGGGTGTTCTTTTAATGGTTTCAGT

RI-ATCG00720-XLOC_032438-11444-0
ACCAGCGGGATTATTAACCGTACCCTTTTTGGAAAATGTTAATAAGTTTC
RI-ATCG00720-XLOC_032438-11444-1
ACCAGCGGGATTATTAACCGTACCCTTTTTGGAAAATGTTAATAAGTTTC
CONSENSUS
ACCAGCGGGATTATTAACCGTACCCTTTTTGGAAAATGTTAATAAGTTTC

RI-ATCG00720-XLOC_032438-11444-0
AAAATCCATTTCGTCGTCCAGTAGCGACAACCGTCTTTTTGATTGGCACC
RI-ATCG00720-XLOC_032438-11444-1
AAAATCCATTTCGTCGTCCAGTAGCGACAACCGTCTTTTTGATTGGCACC
CONSENSUS
AAAATCCATTTCGTCGTCCAGTAGCGACAACCGTCTTTTTGATTGGCACC

RI-ATCG00720-XLOC_032438-11444-0
GCGGCGGCCCTGTGGTTAGGTATTGGAGCAACATTACCGATTGATAAATC
RI-ATCG00720-XLOC_032438-11444-1
GCGGCGGCCCTGTGGTTAGGTATTGGAGCAACATTACCGATTGATAAATC
CONSENSUS
GCGGCGGCCCTGTGGTTAGGTATTGGAGCAACATTACCGATTGATAAATC

RI-ATCG00720-XLOC_032438-11444-0
TCTAACTTTAGGTCTTTTTTAATTCAATTTATTCAATTGTAAAATAAACG
RI-ATCG00720-XLOC_032438-11444-1
TCTAACTTTAGGTCTTTTTTAATTCAATTTATTCAATTGTAAAATAAACG
CONSENSUS
TCTAACTTTAGGTCTTTTTTAATTCAATTTATTCAATTGTAAAATAAACG

RI-ATCG00720-XLOC_032438-11444-0
ACGTGGGTATCTAGGGAGGTATCTAGGGAGTAGTCATTTCCAAATGAATT
RI-ATCG00720-XLOC_032438-11444-1
ACGTGGGTATCTAGGGAGGTATCTAGGGAGTAGTCATTTCCAAATGAATT
CONSENSUS
ACGTGGGTATCTAGGGAGGTATCTAGGGAGTAGTCATTTCCAAATGAATT

RI-ATCG00720-XLOC_032438-11444-0
CTCCCTAGATACATATCTAATTCTAATTAATTTAATTAATTAATTAAAAT
RI-ATCG00720-XLOC_032438-11444-1
CTCCCTAGATACATATCTAATTCTAATTAATTTAATTAATTAATTAAAAT
CONSENSUS
CTCCCTAGATACATATCTAATTCTAATTAATTTAATTAATTAATTAAAAT

RI-ATCG00720-XLOC_032438-11444-0
GGGTTCGACTGGAAAATAGAAATTACGTTGAAATTTGAAAATCCATTTCA
RI-ATCG00720-XLOC_032438-11444-1
GGGTTCGACTGGAAAATAGAAATTACGTTGAAATTTGAAAATCCATTTCA
CONSENSUS
GGGTTCGACTGGAAAATAGAAATTACGTTGAAATTTGAAAATCCATTTCA

RI-ATCG00720-XLOC_032438-11444-0
ATTTCAAATTGACTTTTTAGTCTTAGTCAATTTTTTTTGAAATGTTTTTT
RI-ATCG00720-XLOC_032438-11444-1
ATTTCAAATTGACTTTTTAGTCTTAGTCAATTTTTTTTGAAATGTTTTTT
CONSENSUS
ATTTCAAATTGACTTTTTAGTCTTAGTCAATTTTTTTTGAAATGTTTTTT

RI-ATCG00720-XLOC_032438-11444-0
CTATTTTTTTTCTAGAATGTCTAATATCTTTTTTACATCTTCTACGTGAA
RI-ATCG00720-XLOC_032438-11444-1
CTATTTTTTTTCTAGAATGTCTAATATCTTTTTTACATCTTCTACGTGAA
CONSENSUS
CTATTTTTTTTCTAGAATGTCTAATATCTTTTTTACATCTTCTACGTGAA

RI-ATCG00720-XLOC_032438-11444-0
AATGTTCAATTTTGATAAGGTCTTCTTGACTGTTATTCAAAAGGTCCA
RI-ATCG00720-XLOC_032438-11444-1
AATGTTCAATTTTGATAAGGTCTTCTTGACTGTTATTCAAAAGGTCCA
CONSENSUS
AATGTTCAATTTTGATAAGGTCTTCTTGACTGTTATTCAAAAGGTCCA

alignment for event: RI-ATCG00670-XLOC_032465-5637

RI-ATCG00670-XLOC_032465-5637-0
CAACCGACTTTATCGAGAAAGATTATTTTTTTTAGGCCAAGAGGTTGATA
RI-ATCG00670-XLOC_032465-5637-1
CAACCGACTTTATCGAGAAAGATTATTTTTTTTAGGCCAAGAGGTTGATA
CONSENSUS
CAACCGACTTTATCGAGAAAGATTATTTTTTTTAGGCCAAGAGGTTGATA

RI-ATCG00670-XLOC_032465-5637-0
CCGAAATCTCGAATCAACTTATTAGTCTTATGATATATCTCAGTATAGAA
RI-ATCG00670-XLOC_032465-5637-1
CCGAAATCTCGAATCAACTTATTAGTCTTATGATATATCTCAGTATAGAA
CONSENSUS
CCGAAATCTCGAATCAACTTATTAGTCTTATGATATATCTCAGTATAGAA

RI-ATCG00670-XLOC_032465-5637-0
AAGGATACCAAAGATCTTTATTTGTTTATAAACTCTCCTGGTGGATGGGT
RI-ATCG00670-XLOC_032465-5637-1
AAGGATACCAAAGATCTTTATTTGTTTATAAACTCTCCTGGTGGATGGGT
CONSENSUS
AAGGATACCAAAGATCTTTATTTGTTTATAAACTCTCCTGGTGGATGGGT

RI-ATCG00670-XLOC_032465-5637-0
AATATCTGGAATGGCTATTTATGATACTATGCAATTTGTGCGACCCGATG

RI-ATCG00670-XLOC_032465-5637-1
AATATCTGGAATGGCTATTTATGATACTATGCAATTTGTGCGACCCGATG
CONSENSUS
AATATCTGGAATGGCTATTTATGATACTATGCAATTTGTGCGACCCGATG

RI-ATCG00670-XLOC_032465-5637-0
TACAGACAATATGCATGGGATTGGCCGCTTCAATAGCATCCTTTATCCTA
RI-ATCG00670-XLOC_032465-5637-1
TACAGACAATATGCATGGGATTGGCCGCTTCAATAGCATCCTTTATCCTA
CONSENSUS
TACAGACAATATGCATGGGATTGGCCGCTTCAATAGCATCCTTTATCCTA

RI-ATCG00670-XLOC_032465-5637-0
GTCGGAGGAGCAATTACCAAACGTATAGCATTCCCTCACGCTTGGCGCCA
RI-ATCG00670-XLOC_032465-5637-1
GTCGGAGGAGCAATTACCAAACGTATAGCATTCCCTCACGCT--------
CONSENSUS
GTCGGAGGAGCAATTACCAAACGTATAGCATTCCCTCACGCT........

RI-ATCG00670-XLOC_032465-5637-0
ATGAGTTTTTTTATTTTCGAGAAAAAAATACTATGCCTTCGCCATTTGAA
RI-ATCG00670-XLOC_032465-5637-1

CONSENSUS
....

RI-ATCG00670-XLOC_032465-5637-0
ATATGAATTAGTTAAGTAATAATAGCATGGCACTTCGAATTCAATATGAA
RI-ATCG00670-XLOC_032465-5637-1

CONSENSUS
....

RI-ATCG00670-XLOC_032465-5637-0
ATTTTTTAGATTAAAAAAAAATTCGATTATATATTGAAAGAGTAGTATGA
RI-ATCG00670-XLOC_032465-5637-1

CONSENSUS
....

RI-ATCG00670-XLOC_032465-5637-0
GATAAGGAAGAGGTTTTTCAAATGATATCTTACCTATTCGGGCACATTTC
RI-ATCG00670-XLOC_032465-5637-1

CONSENSUS
....

RI-ATCG00670-XLOC_032465-5637-0
AGCGTCACAAACTTTGTTTTCACACCGTAAAAAAAAAAAAAGACACTTTG
RI-ATCG00670-XLOC_032465-5637-1

CONSENSUS
....

RI-ATCG00670-XLOC_032465-5637-0
GGATTGCTGAATCATCGACGAATCAAAACAATGATATATAAAGCAACGGA

RI-ATCG00670-XLOC_032465-5637-1

CONSENSUS
      ....

RI-ATCG00670-XLOC_032465-5637-0
      ACCATCATAGTATTTTTTTAACTCCTACAAAAAAAAGAAGGATGGTAATT
RI-ATCG00670-XLOC_032465-5637-1

CONSENSUS
      ....

RI-ATCG00670-XLOC_032465-5637-0
      GGATGATTTAAGGAAAGGTCAAAAAAGTCAATTCCATTGTGGAGCCGTAT
RI-ATCG00670-XLOC_032465-5637-1

CONSENSUS
      ....

RI-ATCG00670-XLOC_032465-5637-0
      GCAATGCACAAAAAAAGCCTGTACGGTTATTCAAATTTATCTATTTTTTT
RI-ATCG00670-XLOC_032465-5637-1

CONSENSUS
      ....

RI-ATCG00670-XLOC_032465-5637-0
      TTTTTTGTTATCCCGTCTCATTCTGCGAAATAGAAAAACCTTTTCTATTA
RI-ATCG00670-XLOC_032465-5637-1

CONSENSUS
      ....

RI-ATCG00670-XLOC_032465-5637-0
      TATCATCAGGGTAATGATCCATCAACCCGCTAGTTCGTTTTATGAGGCAC
RI-ATCG00670-XLOC_032465-5637-1 -------
AGGGTAATGATCCATCAACCCGCTAGTTCGTTTTATGAGGCAC
CONSENSUS
      .......AGGGTAATGATCCATCAACCCGCTAGTTCGTTTTATGAGGCAC

RI-ATCG00670-XLOC_032465-5637-0
      AAACGGGAGAATTTATCTTGGAAGCGGAAGAATTACTTAAACTTCGCGAA
RI-ATCG00670-XLOC_032465-5637-1
      AAACGGGAGAATTTATCTTGGAAGCGGAAGAATTACTTAAACTTCGCGAA
CONSENSUS
      AAACGGGAGAATTTATCTTGGAAGCGGAAGAATTACTTAAACTTCGCGAA

RI-ATCG00670-XLOC_032465-5637-0
      ACCATCACAAGGGTTTATGTACAAAGAACGGGCAAACCTATATGGGTTAT
RI-ATCG00670-XLOC_032465-5637-1
      ACCATCACAAGGGTTTATGTACAAAGAACGGGCAAACCTATATGGGTTAT
CONSENSUS
      ACCATCACAAGGGTTTATGTACAAAGAACGGGCAAACCTATATGGGTTAT

RI-ATCG00670-XLOC_032465-5637-0
      ATCCGAAGACATGGAACGGGATGTTTTTATGTCAGCAACAGAAGCCCAAG

RI-ATCG00670-XLOC_032465-5637-1
      ATCCGAAGACATGGAACGGGATGTTTTTATGTCAGCAACAGAAGCCCAAG
CONSENSUS
      ATCCGAAGACATGGAACGGGATGTTTTTATGTCAGCAACAGAAGCCCAAG

RI-ATCG00670-XLOC_032465-5637-0
      CTCATGGAATTGTTGATCTTGTAGCGGTTCAATAAAAAATAGGAGCGATT
RI-ATCG00670-XLOC_032465-5637-1
      CTCATGGAATTGTTGATCTTGTAGCGGTTCAATAAAAAATAGGAGCGATT
CONSENSUS
      CTCATGGAATTGTTGATCTTGTAGCGGTTCAATAAAAAATAGGAGCGATT

RI-ATCG00670-XLOC_032465-5637-0
      TCATGCAAATCTACAATTTAGAATTTTATATCTTTTTAGATTAAAGGTTT
RI-ATCG00670-XLOC_032465-5637-1
      TCATGCAAATCTACAATTTAGAATTTTATATCTTTTTAGATTAAAGGTTT
CONSENSUS
      TCATGCAAATCTACAATTTAGAATTTTATATCTTTTTAGATTAAAGGTTT

RI-ATCG00670-XLOC_032465-5637-0
      AGTTTAATATTCTCTTCAATATAAAAATAAAAAAATATTGAAGAGAATCT
RI-ATCG00670-XLOC_032465-5637-1
      AGTTTAATATTCTCTTCAATATAAAAATAAAAAAATATTGAAGAGAATCT
CONSENSUS
      AGTTTAATATTCTCTTCAATATAAAAATAAAAAAATATTGAAGAGAATCT

RI-ATCG00670-XLOC_032465-5637-0
      TGAAGAGAAGTAATTCAGAATTAGCCGGTTAGAACTAATCTAAACCAGCC
RI-ATCG00670-XLOC_032465-5637-1
      TGAAGAGAAGTAATTCAGAATTAGCCGGTTAGAACTAATCTAAACCAGCC
CONSENSUS
      TGAAGAGAAGTAATTCAGAATTAGCCGGTTAGAACTAATCTAAACCAGCC

RI-ATCG00670-XLOC_032465-5637-0
      CATTATTTATATGATTCCGTATGCCAACCATTAAACAACTTATTAGAAAT
RI-ATCG00670-XLOC_032465-5637-1
      CATTATTTATATGATTCCGTATGCCAACCATTAAACAACTTATTAGAAAT
CONSENSUS
      CATTATTTATATGATTCCGTATGCCAACCATTAAACAACTTATTAGAAAT

RI-ATCG00670-XLOC_032465-5637-0
      ACAAGACAGCCAATCCGAAACGTCACGAAATCCCCAGCGCTTCGGGGATG
RI-ATCG00670-XLOC_032465-5637-1
      ACAAGACAGCCAATCCGAAACGTCACGAAATCCCCAGCGCTTCGGGGATG
CONSENSUS
      ACAAGACAGCCAATCCGAAACGTCACGAAATCCCCAGCGCTTCGGGGATG

RI-ATCG00670-XLOC_032465-5637-0
      CCCTCAGCGACGAGGAACATGTACTCGGGTGTATGTGCGACTCGTTTAGA
RI-ATCG00670-XLOC_032465-5637-1
      CCCTCAGCGACGAGGAACATGTACTCGGGTGTATGTGCGACTCGTTTAGA
CONSENSUS
      CCCTCAGCGACGAGGAACATGTACTCGGGTGTATGTGCGACTCGTTTAGA

RI-ATCG00670-XLOC_032465-5637-0
      TCATAGACTGAGACATAAAAAGGAAATTCTTTTTGAAATATCACGGATCA

RI-ATCG00670-XLOC_032465-5637-1
TCATAGACTGAGACATAAAAAGGAAATTCTTTTTGAAATATCACGGATCA
CONSENSUS
TCATAGACTGAGACATAAAAAGGAAATTCTTTTTGAAATATCACGGATCA

RI-ATCG00670-XLOC_032465-5637-0
GTACCTATGAATAAAATAGAATGGACGAACTCGATTCATTATTTACAAAA
RI-ATCG00670-XLOC_032465-5637-1
GTACCTATGAATAAAATAGAATGGACGAACTCGATTCATTATTTACAAAA
CONSENSUS
GTACCTATGAATAAAATAGAATGGACGAACTCGATTCATTATTTACAAAA

RI-ATCG00670-XLOC_032465-5637-0
TATAGATCGTTCATATACTCTATTGTGTCTGAAACTTATGGTTTACATTG
RI-ATCG00670-XLOC_032465-5637-1
TATAGATCGTTCATATACTCTATTGTGTCTGAAACTTATGGTTTACATTG
CONSENSUS
TATAGATCGTTCATATACTCTATTGTGTCTGAAACTTATGGTTTACATTG

RI-ATCG00670-XLOC_032465-5637-0
GTGCAAATCCAATCAACTCCATTTTTTAGAAAACCCCCAATGATAACAAA
RI-ATCG00670-XLOC_032465-5637-1
GTGCAAATCCAATCAACTCCATTTTTTAGAAAACCCCCAATGATAACAAA
CONSENSUS
GTGCAAATCCAATCAACTCCATTTTTTAGAAAACCCCCAATGATAACAAA

RI-ATCG00670-XLOC_032465-5637-0
TGAGTATCCATTAAGCGGGGGAAATTCCATTTTTTAGAAAAAAGATTTCA
RI-ATCG00670-XLOC_032465-5637-1
TGAGTATCCATTAAGCGGGGGAAATTCCATTTTTTAGAAAAAAGATTTCA
CONSENSUS
TGAGTATCCATTAAGCGGGGGAAATTCCATTTTTTAGAAAAAAGATTTCA

RI-ATCG00670-XLOC_032465-5637-0
CTCTTGAAAGAAAAGAACGGACTAACAGGGTAAATGACCTAATCAATTTT
RI-ATCG00670-XLOC_032465-5637-1
CTCTTGAAAGAAAAGAACGGACTAACAGGGTAAATGACCTAATCAATTTT
CONSENSUS
CTCTTGAAAGAAAAGAACGGACTAACAGGGTAAATGACCTAATCAATTTT

RI-ATCG00670-XLOC_032465-5637-0
CAAAATGAAATACCGTTACTGTATAAAGGGGATCTCATTGTGAAAGACCT
RI-ATCG00670-XLOC_032465-5637-1
CAAAATGAAATACCGTTACTGTATAAAGGGGATCTCATTGTGAAAGACCT
CONSENSUS
CAAAATGAAATACCGTTACTGTATAAAGGGGATCTCATTGTGAAAGACCT

RI-ATCG00670-XLOC_032465-5637-0
ATTACTGGAATATTCATGGGGTAGAGCCAAAGAATGTGAATTGTACAAGT
RI-ATCG00670-XLOC_032465-5637-1
ATTACTGGAATATTCATGGGGTAGAGCCAAAGAATGTGAATTGTACAAGT
CONSENSUS
ATTACTGGAATATTCATGGGGTAGAGCCAAAGAATGTGAATTGTACAAGT

RI-ATCG00670-XLOC_032465-5637-0
TACCAATAGTATTGATTAATTAAAAGAAGGAGCTCCGGTGTATAGAGAGG

RI-ATCG00670-XLOC_032465-5637-1
TACCAATAGTATTGATTAATTAAAAGAAGGAGCTCCGGTGTATAGAGAGG
CONSENSUS
TACCAATAGTATTGATTAATTAAAAGAAGGAGCTCCGGTGTATAGAGAGG

RI-ATCG00670-XLOC_032465-5637-0
ACCTCACCGTTTTAGAAGTAACCATAGAAACGATGGAACCCACTATTTAT
RI-ATCG00670-XLOC_032465-5637-1
ACCTCACCGTTTTAGAAGTAACCATAGAAACGATGGAACCCACTATTTAT
CONSENSUS
ACCTCACCGTTTTAGAAGTAACCATAGAAACGATGGAACCCACTATTTAT

RI-ATCG00670-XLOC_032465-5637-0
CCAATTCTATTTACTACGTTTTGTAGTTATTCTATTTTTTTATATCAAGG
RI-ATCG00670-XLOC_032465-5637-1
CCAATTCTATTTACTACGTTTTGTAGTTATTCTATTTTTTTATATCAAGG
CONSENSUS
CCAATTCTATTTACTACGTTTTGTAGTTATTCTATTTTTTTATATCAAGG

RI-ATCG00670-XLOC_032465-5637-0
CCTTTCAAAGTAAAGGAAAATACCTAGCAAAAAATAGTGGTGGGGAAGGT
RI-ATCG00670-XLOC_032465-5637-1
CCTTTCAAAGTAAAGGAAAATACCTAGCAAAAAATAGTGGTGGGGAAGGT
CONSENSUS
CCTTTCAAAGTAAAGGAAAATACCTAGCAAAAAATAGTGGTGGGGAAGGT

RI-ATCG00670-XLOC_032465-5637-0
TAGAGTAGCAAAAGCCATTGGAATTTTTTTTATACATTGGAATAATTCGT
RI-ATCG00670-XLOC_032465-5637-1
TAGAGTAGCAAAAGCCATTGGAATTTTTTTTATACATTGGAATAATTCGT
CONSENSUS
TAGAGTAGCAAAAGCCATTGGAATTTTTTTTATACATTGGAATAATTCGT

RI-ATCG00670-XLOC_032465-5637-0
TTGGTTATTAATAGACTAGTAAAGGGGAAAAGAATAACTAAAAAAAGAAT
RI-ATCG00670-XLOC_032465-5637-1
TTGGTTATTAATAGACTAGTAAAGGGGAAAAGAATAACTAAAAAAAGAAT
CONSENSUS
TTGGTTATTAATAGACTAGTAAAGGGGAAAAGAATAACTAAAAAAAGAAT

RI-ATCG00670-XLOC_032465-5637-0
TAGTTATTCATCAAAGTTTGATTTATTCAATGACTAGAATTAAACGCGGA
RI-ATCG00670-XLOC_032465-5637-1
TAGTTATTCATCAAAGTTTGATTTATTCAATGACTAGAATTAAACGCGGA
CONSENSUS
TAGTTATTCATCAAAGTTTGATTTATTCAATGACTAGAATTAAACGCGGA

RI-ATCG00670-XLOC_032465-5637-0
TATATAGCTCGGAGGCGTAGAACAAAACTTCGTTTATTTGCATCAAGCTT
RI-ATCG00670-XLOC_032465-5637-1
TATATAGCTCGGAGGCGTAGAACAAAACTTCGTTTATTTGCATCAAGCTT
CONSENSUS
TATATAGCTCGGAGGCGTAGAACAAAACTTCGTTTATTTGCATCAAGCTT

RI-ATCG00670-XLOC_032465-5637-0
TCGAGGGGCTCATTCACGGCTTACACGAACTATGACTCAACAGAGAATAA

RI-ATCG00670-XLOC_032465-5637-1
TCGAGGGGCTCATTCACGGCTTACACGAACTATGACTCAACAGAGAATAA
CONSENSUS
TCGAGGGGCTCATTCACGGCTTACACGAACTATGACTCAACAGAGAATAA

RI-ATCG00670-XLOC_032465-5637-0
GAGCTTTAGTTTCGGCTCATCGGGATAGGGGTAAAAGAAAAAGAGATTTT
RI-ATCG00670-XLOC_032465-5637-1
GAGCTTTAGTTTCGGCTCATCGGGATAGGGGTAAAAGAAAAAGAGATTTT
CONSENSUS
GAGCTTTAGTTTCGGCTCATCGGGATAGGGGTAAAAGAAAAAGAGATTTT

RI-ATCG00670-XLOC_032465-5637-0
CGCCGTTTATGGATCACTCGAATAAATGCCGTAATTCACGAAATGGGGGT
RI-ATCG00670-XLOC_032465-5637-1
CGCCGTTTATGGATCACTCGAATAAATGCCGTAATTCACGAAATGGGGGT
CONSENSUS
CGCCGTTTATGGATCACTCGAATAAATGCCGTAATTCACGAAATGGGGGT

RI-ATCG00670-XLOC_032465-5637-0
ATTCTATAGTTATAATGAATTCATACACAATCTATACAAGAAGCAATTAC
RI-ATCG00670-XLOC_032465-5637-1
ATTCTATAGTTATAATGAATTCATACACAATCTATACAAGAAGCAATTAC
CONSENSUS
ATTCTATAGTTATAATGAATTCATACACAATCTATACAAGAAGCAATTAC

RI-ATCG00670-XLOC_032465-5637-0
TTCTTAATCGGAAAATACTTGCACAAATAGCTCTATTAAATAGGAGTTGT
RI-ATCG00670-XLOC_032465-5637-1
TTCTTAATCGGAAAATACTTGCACAAATAGCTCTATTAAATAGGAGTTGT
CONSENSUS
TTCTTAATCGGAAAATACTTGCACAAATAGCTCTATTAAATAGGAGTTGT

RI-ATCG00670-XLOC_032465-5637-0
CTTTATACAATTTCGAATGACATAAAAAAATAAGGGGATTGGAAGAAATC
RI-ATCG00670-XLOC_032465-5637-1
CTTTATACAATTTCGAATGACATAAAAAAATAAGGGGATTGGAAGAAATC
CONSENSUS
CTTTATACAATTTCGAATGACATAAAAAAATAAGGGGATTGGAAGAAATC

RI-ATCG00670-XLOC_032465-5637-0
CACGAAAATATTTGAAATAGAGTTCTAAGGAGAATGAGCTCGGGGAAGGT
RI-ATCG00670-XLOC_032465-5637-1
CACGAAAATATTTGAAATAGAGTTCTAAGGAGAATGAGCTCGGGGAAGGT
CONSENSUS
CACGAAAATATTTGAAATAGAGTTCTAAGGAGAATGAGCTCGGGGAAGGT

RI-ATCG00670-XLOC_032465-5637-0
AGAGTAGAAATTAGTATTAAAAAAAAAGTCGTCAAAACAAAACGAGAGTA
RI-ATCG00670-XLOC_032465-5637-1
AGAGTAGAAATTAGTATTAAAAAAAAAGTCGTCAAAACAAAACGAGAGTA
CONSENSUS
AGAGTAGAAATTAGTATTAAAAAAAAAGTCGTCAAAACAAAACGAGAGTA

RI-ATCG00670-XLOC_032465-5637-0
TATAGTTTCGAAAAAACGAGTTATGCTTTTCGACGATTCTTTTCTTTTTT

RI-ATCG00670-XLOC_032465-5637-1
TATAGTTTCGAAAAAACGAGTTATGCTTTTCGACGATTCTTTTCTTTTTT
CONSENSUS
TATAGTTTCGAAAAAACGAGTTATGCTTTTCGACGATTCTTTTCTTTTTT

RI-ATCG00670-XLOC_032465-5637-0
TTTTTATAATAGACACAGACGTAACAATCAAATTTTTATTCTTGATTGGA
RI-ATCG00670-XLOC_032465-5637-1
TTTTTATAATAGACACAGACGTAACAATCAAATTTTTATTCTTGATTGGA
CONSENSUS
TTTTTATAATAGACACAGACGTAACAATCAAATTTTTATTCTTGATTGGA

RI-ATCG00670-XLOC_032465-5637-0
TTTTTTGAACAAAACATTAATCTCTTTTTTAATTCCTTCAGATTGGAATT
RI-ATCG00670-XLOC_032465-5637-1
TTTTTTGAACAAAACATTAATCTCTTTTTTAATTCCTTCAGATTGGAATT
CONSENSUS
TTTTTTGAACAAAACATTAATCTCTTTTTTAATTCCTTCAGATTGGAATT

RI-ATCG00670-XLOC_032465-5637-0
GTTATTCAATTGAAAAATAAGTCTATTTTTTTCTGGTTCTAAGACTAGTA
RI-ATCG00670-XLOC_032465-5637-1
GTTATTCAATTGAAAAATAAGTCTATTTTTTTCTGGTTCTAAGACTAGTA
CONSENSUS
GTTATTCAATTGAAAAATAAGTCTATTTTTTTCTGGTTCTAAGACTAGTA

RI-ATCG00670-XLOC_032465-5637-0
GTTCTAGGAGTCGACTCACTTCTTTCAAATTGTTTCTGATTATTAAGAAA
RI-ATCG00670-XLOC_032465-5637-1
GTTCTAGGAGTCGACTCACTTCTTTCAAATTGTTTCTGATTATTAAGAAA
CONSENSUS
GTTCTAGGAGTCGACTCACTTCTTTCAAATTGTTTCTGATTATTAAGAAA

RI-ATCG00670-XLOC_032465-5637-0 AGGTAACAAAGATAAAAT
RI-ATCG00670-XLOC_032465-5637-1 AGGTAACAAAGATAAAAT
CONSENSUS AGGTAACAAAGATAAAAT

alignment for event: RI-ATCG00180-XLOC_032457-8103

RI-ATCG00180-XLOC_032457-8103-0
TTTTCTTTTGCTAGGCCCATAACTAAAAAACCTACTTTCTTACGATTACG
RI-ATCG00180-XLOC_032457-8103-1
TTTTCTTTTGCTAGGCCCATAACTAAAAAACCTACTTTCTTACGATTACG
CONSENSUS
TTTTCTTTTGCTAGGCCCATAACTAAAAAACCTACTTTCTTACGATTACG

RI-ATCG00180-XLOC_032457-8103-0
AGGTTCATTTGAATATGAAATTCAATCCTGGAAATACAGCATCCCACTTT
RI-ATCG00180-XLOC_032457-8103-1
AGGTTCATTTGAATATGAAATTCAATCCTGGAAATACAGCATCCCACTTT
CONSENSUS
AGGTTCATTTGAATATGAAATTCAATCCTGGAAATACAGCATCCCACTTT

RI-ATCG00180-XLOC_032457-8103-0

TTTTTACTACTCAAGGTTTCGATATATTTCGAAATCGAGAAATTTCTACT
RI-ATCG00180-XLOC_032457-8103-1
TTTTTACTACTCAAGGTTTCGATATATTTCGAAATCGAGAAATTTCTACT
CONSENSUS
TTTTTACTACTCAAGGTTTCGATATATTTCGAAATCGAGAAATTTCTACT

RI-ATCG00180-XLOC_032457-8103-0
GGGGCGGGTGCTATCCGAGAACAATTAGCCGATTTAGATTTGCGAATTAT
RI-ATCG00180-XLOC_032457-8103-1
GGGGCGGGTGCTATCCGAGAACAATTAGCCGATTTAGATTTGCGAATTAT
CONSENSUS
GGGGCGGGTGCTATCCGAGAACAATTAGCCGATTTAGATTTGCGAATTAT

RI-ATCG00180-XLOC_032457-8103-0
TATAGAAAATTCGTTGGTAGAATGGAAACAATTAGGAGAAGAAGGGCCTA
RI-ATCG00180-XLOC_032457-8103-1
TATAGAAAATTCGTTGGTAGAATGGAAACAATTAGGAGAAGAAGGGCCTA
CONSENSUS
TATAGAAAATTCGTTGGTAGAATGGAAACAATTAGGAGAAGAAGGGCCTA

RI-ATCG00180-XLOC_032457-8103-0
CGGGGAATGAATGGGAAGATCGAAAAATTGTAAGAAGAAAAGATTTTTTA
RI-ATCG00180-XLOC_032457-8103-1
CGGGGAATGAATGGGAAGATCGAAAAATTGTAAGAAGAAAAGATTTTTTA
CONSENSUS
CGGGGAATGAATGGGAAGATCGAAAAATTGTAAGAAGAAAAGATTTTTTA

RI-ATCG00180-XLOC_032457-8103-0
GTTAGACGTATGGAATTAGCTAAGCATTTTATTCGAACAAATATAGAACC
RI-ATCG00180-XLOC_032457-8103-1
GTTAGACGTATGGAATTAGCTAAGCATTTTATTCGAACAAATATAGAACC
CONSENSUS
GTTAGACGTATGGAATTAGCTAAGCATTTTATTCGAACAAATATAGAACC

RI-ATCG00180-XLOC_032457-8103-0
CGAATGGATGGTTTTATGTCTCTTACCGGTTCTTCCTCCCGAGTTGAGAC
RI-ATCG00180-XLOC_032457-8103-1
CGAATGGATGGTTTTATGTCTCTTACCGGTTCTTCCTCCCGAGTTGAGAC
CONSENSUS
CGAATGGATGGTTTTATGTCTCTTACCGGTTCTTCCTCCCGAGTTGAGAC

RI-ATCG00180-XLOC_032457-8103-0
CCATCATTCAGATAGAAGGGGGTAAACTAATGAGTTCAGATATTAATGAA
RI-ATCG00180-XLOC_032457-8103-1
CCATCATTCAGATAGAAGGGGGTAAACTAATGAGTTCAGATATTAATGAA
CONSENSUS
CCATCATTCAGATAGAAGGGGGTAAACTAATGAGTTCAGATATTAATGAA

RI-ATCG00180-XLOC_032457-8103-0
CTCTATAGAAGAGTTATCTATCGGAACAATACTCTTACCGATCTATTAAC
RI-ATCG00180-XLOC_032457-8103-1
CTCTATAGAAGAGTTATCTATCGGAACAATACTCTTACCGATCTATTAAC
CONSENSUS
CTCTATAGAAGAGTTATCTATCGGAACAATACTCTTACCGATCTATTAAC

RI-ATCG00180-XLOC_032457-8103-0

AACAAGTAGATCTACACCGGGGGAATTAGTAATGTGTCAGGAAAAATTGG
RI-ATCG00180-XLOC_032457-8103-1
AACAAGTAGATCTACACCGGGGGAATTAGTAATGTGTCAGGAAAAATTGG
CONSENSUS
AACAAGTAGATCTACACCGGGGGAATTAGTAATGTGTCAGGAAAAATTGG

RI-ATCG00180-XLOC_032457-8103-0
TACAAGAAGCCGTGGATACACTTCTTGATAATGGAATCCGTGGACAACCC
RI-ATCG00180-XLOC_032457-8103-1
TACAAGAAGCCGTGGATACACTTCTTGATAATGGAATCCGTGGACAACCC
CONSENSUS
TACAAGAAGCCGTGGATACACTTCTTGATAATGGAATCCGTGGACAACCC

RI-ATCG00180-XLOC_032457-8103-0
ATGAGGGATGGTCATAATAAGGTTTACAAGTCATTTTCAGATGTAATTGA
RI-ATCG00180-XLOC_032457-8103-1
ATGAGGGATGGTCATAATAAGGTTTACAAGTCATTTTCAGATGTAATTGA
CONSENSUS
ATGAGGGATGGTCATAATAAGGTTTACAAGTCATTTTCAGATGTAATTGA

RI-ATCG00180-XLOC_032457-8103-0
AGGCAAAGAGGGAAGATTTCGCGAGACTCTGCTTGGGAAACGGGTCGATT
RI-ATCG00180-XLOC_032457-8103-1
AGGCAAAGAGGGAAGATTTCGCGAGACTCTGCTTGGGAAACGGGTCGATT
CONSENSUS
AGGCAAAGAGGGAAGATTTCGCGAGACTCTGCTTGGGAAACGGGTCGATT

RI-ATCG00180-XLOC_032457-8103-0
ATTCGGGGCGCTCGGTGATTGTCGTTGGACCTTCACTTTCATTACATCGC
RI-ATCG00180-XLOC_032457-8103-1
ATTCGGGGCGCTCGGTGATTGTCGTTGGACCTTCACTTTCATTACATCGC
CONSENSUS
ATTCGGGGCGCTCGGTGATTGTCGTTGGACCTTCACTTTCATTACATCGC

RI-ATCG00180-XLOC_032457-8103-0
TGTGGATTGCCTCGCGAAATAGCAATAGAGCTCTTCCAGACATTTGTAAT
RI-ATCG00180-XLOC_032457-8103-1
TGTGGATTGCCTCGCGAAATAGCAATAGAGCTCTTCCAGACATTTGTAAT
CONSENSUS
TGTGGATTGCCTCGCGAAATAGCAATAGAGCTCTTCCAGACATTTGTAAT

RI-ATCG00180-XLOC_032457-8103-0
TCGTGGTTTAATTAGACAACACCTGGCTTCGAACATAGGAGTTGCTAAGA
RI-ATCG00180-XLOC_032457-8103-1
TCGTGGTTTAATTAGACAACACCTGGCTTCGAACATAGGAGTTGCTAAGA
CONSENSUS
TCGTGGTTTAATTAGACAACACCTGGCTTCGAACATAGGAGTTGCTAAGA

RI-ATCG00180-XLOC_032457-8103-0
GTCAAATTCGTGAAAAAAAGCCGATTGTCTGGGAAATCCTTCAAGAAGTC
RI-ATCG00180-XLOC_032457-8103-1
GTCAAATTCGTGAAAAAAAGCCGATTGTCTGGGAAATCCTTCAAGAAGTC
CONSENSUS
GTCAAATTCGTGAAAAAAAGCCGATTGTCTGGGAAATCCTTCAAGAAGTC

RI-ATCG00180-XLOC_032457-8103-0

ATGCAGGGGCATCCCGTATTACTGAATAGAGCACCTACCCTACATAGATT
RI-ATCG00180-XLOC_032457-8103-1
ATGCAGGGGCATCCCGTATTACTGAATAGAGCACCTACCCTACATAGATT
CONSENSUS
ATGCAGGGGCATCCCGTATTACTGAATAGAGCACCTACCCTACATAGATT

RI-ATCG00180-XLOC_032457-8103-0
AGGCATACAGTCATTCCAACCTATTTTAGTGGAAGGACGCACTATTTGTT
RI-ATCG00180-XLOC_032457-8103-1
AGGCATACAGTCATTCCAACCTATTTTAGTGGAAGGACGCACTATTTGTT
CONSENSUS
AGGCATACAGTCATTCCAACCTATTTTAGTGGAAGGACGCACTATTTGTT

RI-ATCG00180-XLOC_032457-8103-0
TACATCCATTAGTTTGTAAGGGGTTCAATGCAGACTTTGATGGGGATCAA
RI-ATCG00180-XLOC_032457-8103-1
TACATCCATTAGTTTGTAAGGGGTTCAATGCAGACTTTGATGGGGATCAA
CONSENSUS
TACATCCATTAGTTTGTAAGGGGTTCAATGCAGACTTTGATGGGGATCAA

RI-ATCG00180-XLOC_032457-8103-0
ATGGCTGTTCATGTGCCTTTATCTTTAGAGGCTCAAGCAGAGGCTCGTTT
RI-ATCG00180-XLOC_032457-8103-1
ATGGCTGTTCATGTGCCTTTATCTTTAGAGGCTCAAGCAGAGGCTCGTTT
CONSENSUS
ATGGCTGTTCATGTGCCTTTATCTTTAGAGGCTCAAGCAGAGGCTCGTTT

RI-ATCG00180-XLOC_032457-8103-0
ACTTATGTTTTCTCATATGAATCTCTTATCTCCAGCTATTGGAGATCCCA
RI-ATCG00180-XLOC_032457-8103-1
ACTTATGTTTTCTCATATGAATCTCTTATCTCCAGCTATTGGAGATCCCA
CONSENSUS
ACTTATGTTTTCTCATATGAATCTCTTATCTCCAGCTATTGGAGATCCCA

RI-ATCG00180-XLOC_032457-8103-0
TTTCGGTACCGACTCAAGATATGCTTATTGGACTCTATGTATTAACGAGC
RI-ATCG00180-XLOC_032457-8103-1
TTTCGGTACCGACTCAAGATATGCTTATTGGACTCTATGTATTAACGAGC
CONSENSUS
TTTCGGTACCGACTCAAGATATGCTTATTGGACTCTATGTATTAACGAGC

RI-ATCG00180-XLOC_032457-8103-0
GGCACTCGTCGAGGTATTTGTGCAAACAGATATAATCCATGTAATCGAAA
RI-ATCG00180-XLOC_032457-8103-1
GGCACTCGTCGAGGTATTTGTGCAAACAGATATAATCCATGTAATCGAAA
CONSENSUS
GGCACTCGTCGAGGTATTTGTGCAAACAGATATAATCCATGTAATCGAAA

RI-ATCG00180-XLOC_032457-8103-0
AAACTATCAAAATGAAAGAATTTACGAAACAAACTATAAGTATACGAAAG
RI-ATCG00180-XLOC_032457-8103-1
AAACTATCAAAATGAAAGAATTTACGAAACAAACTATAAGTATACGAAAG
CONSENSUS
AAACTATCAAAATGAAAGAATTTACGAAACAAACTATAAGTATACGAAAG

RI-ATCG00180-XLOC_032457-8103-0

AACCCTTTTTTTGCAATTCCTATGATGCAATTGGAGCTTATCGACAGAAA
RI-ATCG00180-XLOC_032457-8103-1
AACCCTTTTTTTGCAATTCCTATGATGCAATTGGAGCTTATCGACAGAAA
CONSENSUS
AACCCTTTTTTTGCAATTCCTATGATGCAATTGGAGCTTATCGACAGAAA

RI-ATCG00180-XLOC_032457-8103-0
AAAATCAATTTAGATAGTCCTTTGTGGCTTCGGTGGCAATTAGATCAACG
RI-ATCG00180-XLOC_032457-8103-1
AAAATCAATTTAGATAGTCCTTTGTGGCTTCGGTGGCAATTAGATCAACG
CONSENSUS
AAAATCAATTTAGATAGTCCTTTGTGGCTTCGGTGGCAATTAGATCAACG

RI-ATCG00180-XLOC_032457-8103-0
CGTTATTGCTTCAAGAGAAGTTCCTATCGAAGTTCACTATGAATCTTTTG
RI-ATCG00180-XLOC_032457-8103-1
CGTTATTGCTTCAAGAGAAGTTCCTATCGAAGTTCACTATGAATCTTTTG
CONSENSUS
CGTTATTGCTTCAAGAGAAGTTCCTATCGAAGTTCACTATGAATCTTTTG

RI-ATCG00180-XLOC_032457-8103-0
GTAACTATCATGAGATTTATGCACACTATCTAATAGTAAGAAGTGTAAAA
RI-ATCG00180-XLOC_032457-8103-1
GTAACTATCATGAGATTTATGCACACTATCTAATAGTAAGAAGTGTAAAA
CONSENSUS
GTAACTATCATGAGATTTATGCACACTATCTAATAGTAAGAAGTGTAAAA

RI-ATCG00180-XLOC_032457-8103-0
AAAGAAAATTTTTGTATATATATTCGAACCACAGTTGGTCATATTTCTTT
RI-ATCG00180-XLOC_032457-8103-1
AAAGAAAATTTTTGTATATATATTCGAACCACAGTTGGTCATATTTCTTT
CONSENSUS
AAAGAAAATTTTTGTATATATATTCGAACCACAGTTGGTCATATTTCTTT

RI-ATCG00180-XLOC_032457-8103-0
TTATCGAGAAATCGAGGAAGCTATACAAGGTTTTTCTCAAGCCTGTTCAT
RI-ATCG00180-XLOC_032457-8103-1
TTATCGAGAAATCGAGGAAGCTATACAAGGTTTTTCTCAAGCCTGTTCAT
CONSENSUS
TTATCGAGAAATCGAGGAAGCTATACAAGGTTTTTCTCAAGCCTGTTCAT

RI-ATCG00180-XLOC_032457-8103-0
ATGATACCTAATAATATGCTATTAAAAAAAAGGAATTCTAGGACACCCGT
RI-ATCG00180-XLOC_032457-8103-1
ATGATACCTAATAATATGCTATTAAAAAAAAGGAATTCTAGGACACCCGT
CONSENSUS
ATGATACCTAATAATATGCTATTAAAAAAAAGGAATTCTAGGACACCCGT

RI-ATCG00180-XLOC_032457-8103-0
GTGAATTCGGACCTCTCCGATTCAAATCGGACCATAGTTCCTGACCTAAA
RI-ATCG00180-XLOC_032457-8103-1
GTGAATTCGGACCTCTCCGATTCAAATCGGACCATAGTTCCTGACCTAAA
CONSENSUS
GTGAATTCGGACCTCTCCGATTCAAATCGGACCATAGTTCCTGACCTAAA

RI-ATCG00180-XLOC_032457-8103-0

ATTCTACGCAAATCAAGATCGAGAAAAGGAAGTTTTGCAATCATTGACTC
RI-ATCG00180-XLOC_032457-8103-1
ATTCTACGCAAATCAAGATCGAGAAAAGGAAGTTTTGCAATCATTGACTC
CONSENSUS
ATTCTACGCAAATCAAGATCGAGAAAAGGAAGTTTTGCAATCATTGACTC

RI-ATCG00180-XLOC_032457-8103-0
AAACTCATTGTCGAATCCCATTCAGCAGAATATGGAGGTACTTATGGCGG
RI-ATCG00180-XLOC_032457-8103-1
AAACTCATTGTCGAATCCCATTCAGCAGAATATGGAGGTACTTATGGCGG
CONSENSUS
AAACTCATTGTCGAATCCCATTCAGCAGAATATGGAGGTACTTATGGCGG

RI-ATCG00180-XLOC_032457-8103-0
AACGGGCCAATCTGGTATTTCACAATAAAGTGATAGATGGAACTGCTATT
RI-ATCG00180-XLOC_032457-8103-1
AACGGGCCAATCTGGTATTTCACAATAAAGTGATAGATGGAACTGCTATT
CONSENSUS
AACGGGCCAATCTGGTATTTCACAATAAAGTGATAGATGGAACTGCTATT

RI-ATCG00180-XLOC_032457-8103-0
AAACGACTTATTAGCCGATTAATAGATCACTTCGGGATGGCATATACATC
RI-ATCG00180-XLOC_032457-8103-1
AAACGACTTATTAGCCGATTAATAGATCACTTCGGGATGGCATATACATC
CONSENSUS
AAACGACTTATTAGCCGATTAATAGATCACTTCGGGATGGCATATACATC

RI-ATCG00180-XLOC_032457-8103-0
ACACATCCTAGATCAAGTAAAGACTCTGGGTTTCCAGCAAGCCACTGCTA
RI-ATCG00180-XLOC_032457-8103-1
ACACATCCTAGATCAAGTAAAGACTCTGGGTTTCCAGCAAGCCACTGCTA
CONSENSUS
ACACATCCTAGATCAAGTAAAGACTCTGGGTTTCCAGCAAGCCACTGCTA

RI-ATCG00180-XLOC_032457-8103-0
CATCCATTTCATTAGGAATTGATGATCTTTTAACGATACCTTCTAAGGGC
RI-ATCG00180-XLOC_032457-8103-1
CATCCATTTCATTAGGAATTGATGATCTTTTAACGATACCTTCTAAGGGC
CONSENSUS
CATCCATTTCATTAGGAATTGATGATCTTTTAACGATACCTTCTAAGGGC

RI-ATCG00180-XLOC_032457-8103-0
TGGCTTGTCCAAGATGCTGAACAACAAAGTTGGATTTTGGAAAAACACCA
RI-ATCG00180-XLOC_032457-8103-1
TGGCTTGTCCAAGATGCTGAACAACAAAGTTGGATTTTGGAAAAACACCA
CONSENSUS
TGGCTTGTCCAAGATGCTGAACAACAAAGTTGGATTTTGGAAAAACACCA

RI-ATCG00180-XLOC_032457-8103-0
TCATTATGGGAATGTACATGCGGTAGAAAAATTACGCCAATCTATTGAGA
RI-ATCG00180-XLOC_032457-8103-1
TCATTATGGGAATGTACATGCGGTAGAAAAATTACGCCAATCTATTGAGA
CONSENSUS
TCATTATGGGAATGTACATGCGGTAGAAAAATTACGCCAATCTATTGAGA

RI-ATCG00180-XLOC_032457-8103-0

TATGGTATGCTACAAGTGAATATTTGCGACAAGAAATGAATCCTAATTTT
RI-ATCG00180-XLOC_032457-8103-1
TATGGTATGCTACAAGTGAATATTTGCGACAAGAAATGAATCCTAATTTT
CONSENSUS
TATGGTATGCTACAAGTGAATATTTGCGACAAGAAATGAATCCTAATTTT

RI-ATCG00180-XLOC_032457-8103-0
AGGATGACGGACCCCTTCAATCCAGTCCATATGATGTCTTTTTCGGGAGC
RI-ATCG00180-XLOC_032457-8103-1
AGGATGACGGACCCCTTCAATCCAGTCCATATGATGTCTTTTTCGGGAGC
CONSENSUS
AGGATGACGGACCCCTTCAATCCAGTCCATATGATGTCTTTTTCGGGAGC

RI-ATCG00180-XLOC_032457-8103-0
TAGGGGAAATGCATCTCAAGTACATCAATTAGTAGGTATGAGAGGATTAA
RI-ATCG00180-XLOC_032457-8103-1
TAGGGGAAATGCATCTCAAGTACATCAATTAGTAGGTATGAGAGGATTAA
CONSENSUS
TAGGGGAAATGCATCTCAAGTACATCAATTAGTAGGTATGAGAGGATTAA

RI-ATCG00180-XLOC_032457-8103-0
TGTCGGATCCCCAAGGACAAATGATTGATTTACCTATTCAAAGCAATTTA
RI-ATCG00180-XLOC_032457-8103-1
TGTCGGATCCCCAAGGACAAATGATTGATTTACCTATTCAAAGCAATTTA
CONSENSUS
TGTCGGATCCCCAAGGACAAATGATTGATTTACCTATTCAAAGCAATTTA

RI-ATCG00180-XLOC_032457-8103-0
CGCGAAGGACTGTCTTTAACAGAATATATTATTTCTTGCTATGGAGCCCG
RI-ATCG00180-XLOC_032457-8103-1
CGCGAAGGACTGTCTTTAACAGAATATATTATTTCTTGCTATGGAGCCCG
CONSENSUS
CGCGAAGGACTGTCTTTAACAGAATATATTATTTCTTGCTATGGAGCCCG

RI-ATCG00180-XLOC_032457-8103-0
TAAAGGAGTTGTAGATACTGCTGTACGCACATCAGATGCTGGATATCTTA
RI-ATCG00180-XLOC_032457-8103-1
TAAAGGAGTTGTAGATACTGCTGTACGCACATCAGATGCTGGATATCTTA
CONSENSUS
TAAAGGAGTTGTAGATACTGCTGTACGCACATCAGATGCTGGATATCTTA

RI-ATCG00180-XLOC_032457-8103-0
CGCGTCGACTTGTTGAAGTAGTTCAACATATTGTTGTACGTAGAACAGAT
RI-ATCG00180-XLOC_032457-8103-1
CGCGTCGACTTGTTGAAGTAGTTCAACATATTGTTGTACGTAGAACAGAT
CONSENSUS
CGCGTCGACTTGTTGAAGTAGTTCAACATATTGTTGTACGTAGAACAGAT

RI-ATCG00180-XLOC_032457-8103-0
TGTGGCACTATCCGAGGGATTTCGGTGAGTCCTCGAAATAAAAATCGGAT
RI-ATCG00180-XLOC_032457-8103-1
TGTGGCACTATCCGAGGGATTTCGGTGAGTCCTCGAAATAAAAATCGGAT
CONSENSUS
TGTGGCACTATCCGAGGGATTTCGGTGAGTCCTCGAAATAAAAATCGGAT

RI-ATCG00180-XLOC_032457-8103-0

GATGTCAGAAAGAATTTTTATCCAAACATTAATTGGTCGTGTCTTAGCAG
RI-ATCG00180-XLOC_032457-8103-1
GATGTCAGAAAGAATTTTTATCCAAACATTAATTGGTCGTGTCTTAGCAG
CONSENSUS
GATGTCAGAAAGAATTTTTATCCAAACATTAATTGGTCGTGTCTTAGCAG

RI-ATCG00180-XLOC_032457-8103-0
ACGATATATATATAGGTTCCCGATGTGTCGCCTTTCGAAATCAAGATCTT
RI-ATCG00180-XLOC_032457-8103-1
ACGATATATATATAGGTTCCCGATGTGTCGCCTTTCGAAATCAAGATCTT
CONSENSUS
ACGATATATATATAGGTTCCCGATGTGTCGCCTTTCGAAATCAAGATCTT

RI-ATCG00180-XLOC_032457-8103-0
GGGATTGGACTTGTCAATCGATTAATAACCTTTGGAACACAATCAATATC
RI-ATCG00180-XLOC_032457-8103-1
GGGATTGGACTTGTCAATCGATTAATAACCTTTGGAACACAATCAATATC
CONSENSUS
GGGATTGGACTTGTCAATCGATTAATAACCTTTGGAACACAATCAATATC

RI-ATCG00180-XLOC_032457-8103-0
CATTCGAACTCCCTTTACTTGTCGGAGTACATCTTGGATCTGTCGATTAT
RI-ATCG00180-XLOC_032457-8103-1
CATTCGAACTCCCTTTACTTGTCGGAGTACATCTTGGATCTGTCGATTAT
CONSENSUS
CATTCGAACTCCCTTTACTTGTCGGAGTACATCTTGGATCTGTCGATTAT

RI-ATCG00180-XLOC_032457-8103-0
GTTATGGTCGGAGTCCCACTCATGGTGACCTCGTTGAATTGGGGGAAGCT
RI-ATCG00180-XLOC_032457-8103-1
GTTATGGTCGGAGTCCCACTCATGGTGACCTCGTTGAATTGGGGGAAGCT
CONSENSUS
GTTATGGTCGGAGTCCCACTCATGGTGACCTCGTTGAATTGGGGGAAGCT

RI-ATCG00180-XLOC_032457-8103-0
GTAGGTATTATCGCGGGTCAATCGATCGGCGAACCGGGGACTCAACTAAC
RI-ATCG00180-XLOC_032457-8103-1
GTAGGTATTATCGCGGGTCAATCGATCGGCGAACCGGGGACTCAACTAAC
CONSENSUS
GTAGGTATTATCGCGGGTCAATCGATCGGCGAACCGGGGACTCAACTAAC

RI-ATCG00180-XLOC_032457-8103-0
ATTAAGAACTTTTCATACCGGCGGAGTATTTACAGGAGGTACTGCCGAAC
RI-ATCG00180-XLOC_032457-8103-1
ATTAAGAACTTTTCATACCGGCGGAGTATTTACAGGAGGTACTGCCGAAC
CONSENSUS
ATTAAGAACTTTTCATACCGGCGGAGTATTTACAGGAGGTACTGCCGAAC

RI-ATCG00180-XLOC_032457-8103-0
ATGTACGAGCCCCTTATAATGGAAAAATCAAATTCAATGAGGATTTGGTT
RI-ATCG00180-XLOC_032457-8103-1
ATGTACGAGCCCCTTATAATGGAAAAATCAAATTCAATGAGGATTTGGTT
CONSENSUS
ATGTACGAGCCCCTTATAATGGAAAAATCAAATTCAATGAGGATTTGGTT

RI-ATCG00180-XLOC_032457-8103-0

CATCCTACACGTACACGTCACGGGCATCCTGCCTTTCTATGTTATATAGA
RI-ATCG00180-XLOC_032457-8103-1
CATCCTACACGTACACGTCACGGGCATCCTGCCTTTCTATGTTATATAGA
CONSENSUS
CATCCTACACGTACACGTCACGGGCATCCTGCCTTTCTATGTTATATAGA

RI-ATCG00180-XLOC_032457-8103-0
CTTGTCTGTAATTATTGAGAGCGAAGATATTATACATAGCGTGACTATTC
RI-ATCG00180-XLOC_032457-8103-1
CTTGTCTGTAATTATTGAGAGCGAAGATATTATACATAGCGTGACTATTC
CONSENSUS
CTTGTCTGTAATTATTGAGAGCGAAGATATTATACATAGCGTGACTATTC

RI-ATCG00180-XLOC_032457-8103-0
CACCAAAAAGTTTTCTTTTAGTTCAAAATGATCAATATGTGGAATCAGAA
RI-ATCG00180-XLOC_032457-8103-1
CACCAAAAAGTTTTCTTTTAGTTCAAAATGATCAATATGTGGAATCAGAA
CONSENSUS
CACCAAAAAGTTTTCTTTTAGTTCAAAATGATCAATATGTGGAATCAGAA

RI-ATCG00180-XLOC_032457-8103-0
CAAGTGATTGCTGAGATTCGCGAGGGAACATACACTTTTCATTTTAAAGA
RI-ATCG00180-XLOC_032457-8103-1
CAAGTGATTGCTGAGATTCGCGAGGGAACATACACTTTTCATTTTAAAGA
CONSENSUS
CAAGTGATTGCTGAGATTCGCGAGGGAACATACACTTTTCATTTTAAAGA

RI-ATCG00180-XLOC_032457-8103-0
GAGGGTTAGAAAATATATTTATTCTGACTCAGAGGGCGAAATGCACTGGA
RI-ATCG00180-XLOC_032457-8103-1
GAGGGTTAGAAAATATATTTATTCTGACTCAGAGGGCGAAATGCACTGGA
CONSENSUS
GAGGGTTAGAAAATATATTTATTCTGACTCAGAGGGCGAAATGCACTGGA

RI-ATCG00180-XLOC_032457-8103-0
GTACTGATGTGTCCCATGCACCCGAATTTACATATAGTAATGTCCACCTC
RI-ATCG00180-XLOC_032457-8103-1
GTACTGATGTGTCCCATGCACCCGAATTTACATATAGTAATGTCCACCTC
CONSENSUS
GTACTGATGTGTCCCATGCACCCGAATTTACATATAGTAATGTCCACCTC

RI-ATCG00180-XLOC_032457-8103-0
TTACCAAAAACAAGTCATTTATGGATATTATCCGGAGGTTCATGTGGATC
RI-ATCG00180-XLOC_032457-8103-1
TTACCAAAAACAAGTCATTTATGGATATTATCCGGAGGTTCATGTGGATC
CONSENSUS
TTACCAAAAACAAGTCATTTATGGATATTATCCGGAGGTTCATGTGGATC

RI-ATCG00180-XLOC_032457-8103-0
TAGTCTAATTCGTTTTTCGATCCATAAAGATCAAGATCAAATGAATATAC
RI-ATCG00180-XLOC_032457-8103-1
TAGTCTAATTCGTTTTTCGATCCATAAAGATCAAGATCAAATGAATATAC
CONSENSUS
TAGTCTAATTCGTTTTTCGATCCATAAAGATCAAGATCAAATGAATATAC

RI-ATCG00180-XLOC_032457-8103-0

CCTTTCTTTCCGCCGAAAGAAAATCTATTTCTAGCCTCTCAGTGAATAAT
RI-ATCG00180-XLOC_032457-8103-1
CCTTTCTTTCCGCCGAAAGAAAATCTATTTCTAGCCTCTCAGTGAATAAT
CONSENSUS
CCTTTCTTTCCGCCGAAAGAAAATCTATTTCTAGCCTCTCAGTGAATAAT

RI-ATCG00180-XLOC_032457-8103-0
GATCAAGTGAGCCAAAAATTTTTTAGTTCCGATTTTGCCGATCCAAAAAA
RI-ATCG00180-XLOC_032457-8103-1
GATCAAGTGAGCCAAAAATTTTTTAGTTCCGATTTTGCCGATCCAAAAAA
CONSENSUS
GATCAAGTGAGCCAAAAATTTTTTAGTTCCGATTTTGCCGATCCAAAAAA

RI-ATCG00180-XLOC_032457-8103-0
ATTAGGGATTTACGATTATTCAGAATTGAATGGAAACTTAGGTACTAGTC
RI-ATCG00180-XLOC_032457-8103-1
ATTAGGGATTTACGATTATTCAGAATTGAATGGAAACTTAGGTACTAGTC
CONSENSUS
ATTAGGGATTTACGATTATTCAGAATTGAATGGAAACTTAGGTACTAGTC

RI-ATCG00180-XLOC_032457-8103-0
ATTATAATTTAATATATTCTGCTATTTTTCATGAGAACTCGGATTTATTA
RI-ATCG00180-XLOC_032457-8103-1
ATTATAATTTAATATATTCTGCTATTTTTCATGAGAACTCGGATTTATTA
CONSENSUS
ATTATAATTTAATATATTCTGCTATTTTTCATGAGAACTCGGATTTATTA

RI-ATCG00180-XLOC_032457-8103-0
GCAAAAAGACGAAGAAATAGATTTCTCATTCCATTCCAATCGATTCAAGA
RI-ATCG00180-XLOC_032457-8103-1
GCAAAAAGACGAAGAAATAGATTTCTCATTCCATTCCAATCGATTCAAGA
CONSENSUS
GCAAAAAGACGAAGAAATAGATTTCTCATTCCATTCCAATCGATTCAAGA

RI-ATCG00180-XLOC_032457-8103-0
GCAAGAGAAAGAATTCATACCGCAGTCAGGTATCTCGGTTGAAATACCCA
RI-ATCG00180-XLOC_032457-8103-1
GCAAGAGAAAGAATTCATACCGCAGTCAGGTATCTCGGTTGAAATACCCA
CONSENSUS
GCAAGAGAAAGAATTCATACCGCAGTCAGGTATCTCGGTTGAAATACCCA

RI-ATCG00180-XLOC_032457-8103-0
TAAATGGTATTTTCCGTAGAAATAGTATTTTTGCTTTTTTTGATGATCCT
RI-ATCG00180-XLOC_032457-8103-1
TAAATGGTATTTTCCGTAGAAATAGTATTTTTGCTTTTTTTGATGATCCT
CONSENSUS
TAAATGGTATTTTCCGTAGAAATAGTATTTTTGCTTTTTTTGATGATCCT

RI-ATCG00180-XLOC_032457-8103-0
AGATACCGAAGAAAGAGTTCCGGAATTCTTAAATATGGGACTCTAAAGGC
RI-ATCG00180-XLOC_032457-8103-1
AGATACCGAAGAAAGAGTTCCGGAATTCTTAAATATGGGACTCTAAAGGC
CONSENSUS
AGATACCGAAGAAAGAGTTCCGGAATTCTTAAATATGGGACTCTAAAGGC

RI-ATCG00180-XLOC_032457-8103-0

GGATTCAATCATCCAAAAAGAGGATATGATTGAGTATCGAGGAGTCCAAA
RI-ATCG00180-XLOC_032457-8103-1
GGATTCAATCATCCAAAAAGAGGATATGATTGAGTATCGAGGAGTCCAAA
CONSENSUS
GGATTCAATCATCCAAAAAGAGGATATGATTGAGTATCGAGGAGTCCAAA

RI-ATCG00180-XLOC_032457-8103-0
AAATTAAGACAAAATACGAAATGAAAGTAGATCGCTTTTTTTTCATTCCT
RI-ATCG00180-XLOC_032457-8103-1
AAATTAAGACAAAATACGAAATGAAAGTAGATCGCTTTTTTTTCATTCCT
CONSENSUS
AAATTAAGACAAAATACGAAATGAAAGTAGATCGCTTTTTTTTCATTCCT

RI-ATCG00180-XLOC_032457-8103-0
GAGGAAGTGCATATTTTACCCGAATCCTCTGCCATAATGGTACAGAACTA
RI-ATCG00180-XLOC_032457-8103-1
GAGGAAGTGCATATTTTACCCGAATCCTCTGCCATAATGGTACAGAACTA
CONSENSUS
GAGGAAGTGCATATTTTACCCGAATCCTCTGCCATAATGGTACAGAACTA

RI-ATCG00180-XLOC_032457-8103-0
TAGTATCATTGGAGTGGATACACGACTCACTTTAAATATAAGAAGCCAAG
RI-ATCG00180-XLOC_032457-8103-1
TAGTATCATTGGAGTGGATACACGACTCACTTTAAATATAAGAAGCCAAG
CONSENSUS
TAGTATCATTGGAGTGGATACACGACTCACTTTAAATATAAGAAGCCAAG

RI-ATCG00180-XLOC_032457-8103-0
TCGGCGGGTTGATCCGAGTAGAGAAAAAAAAAAAAAAGGATTGAACTCAAA
RI-ATCG00180-XLOC_032457-8103-1
TCGGCGGGTTGATCCGAGTAGAGAAAAAAAAAAAAAAGGATTGAACTCAAA
CONSENSUS
TCGGCGGGTTGATCCGAGTAGAGAAAAAAAAAAAAAAGGATTGAACTCAAA

RI-ATCG00180-XLOC_032457-8103-0
ATATTTTCAGGGGATATCCATTTTCCGGACAAGACAGATAAAATATCCCG
RI-ATCG00180-XLOC_032457-8103-1
ATATTTTCAGGGGATATCCATTTTCCGGACAAGACAGATAAAATATCCCG
CONSENSUS
ATATTTTCAGGGGATATCCATTTTCCGGACAAGACAGATAAAATATCCCG

RI-ATCG00180-XLOC_032457-8103-0
ACACAGTGGCATCTTGATACCGCCAGGAAGAGGAAAAAAAAACTCGAAGG
RI-ATCG00180-XLOC_032457-8103-1
ACACAGTGGCATCTTGATACCGCCAGGAAGAGGAAAAAAAAACTCGAAGG
CONSENSUS
ACACAGTGGCATCTTGATACCGCCAGGAAGAGGAAAAAAAAACTCGAAGG

RI-ATCG00180-XLOC_032457-8103-0
AATCCAAAAAAATTTAAAAATTGGATTTATGTCCAACGGATTACACCAACC
RI-ATCG00180-XLOC_032457-8103-1
AATCCAAAAAAATTTAAAAATTGGATTTATGTCCAACGGATTACACCAACC
CONSENSUS
AATCCAAAAAAATTTAAAAATTGGATTTATGTCCAACGGATTACACCAACC

RI-ATCG00180-XLOC_032457-8103-0

AAGAAAAAGTTTTTTGTTTTGGTGCGGCCCGTAGCCACCTATGAGATAGC
RI-ATCG00180-XLOC_032457-8103-1
AAGAAAAAGTTTTTTGTTTTGGTGCGGCCCGTAGCCACCTATGAGATAGC
CONSENSUS
AAGAAAAAGTTTTTTGTTTTGGTGCGGCCCGTAGCCACCTATGAGATAGC

RI-ATCG00180-XLOC_032457-8103-0
GGACAGTATAAATTTGGCAACACTCTTCCCACAAGATCTCTTTCGGGAAA
RI-ATCG00180-XLOC_032457-8103-1
GGACAGTATAAATTTGGCAACACTCTTCCCACAAGATCTCTTTCGGGAAA
CONSENSUS
GGACAGTATAAATTTGGCAACACTCTTCCCACAAGATCTCTTTCGGGAAA

RI-ATCG00180-XLOC_032457-8103-0
AGGATAATATTCAACTTCGAGTTTTCAACTATATCCTTTATGGAAATGGC
RI-ATCG00180-XLOC_032457-8103-1
AGGATAATATTCAACTTCGAGTTTTCAACTATATCCTTTATGGAAATGGC
CONSENSUS
AGGATAATATTCAACTTCGAGTTTTCAACTATATCCTTTATGGAAATGGC

RI-ATCG00180-XLOC_032457-8103-0
AAACCAACTCGAGGAATTTCTGACACAAGTATTCAATTAGTTCGAACTTG
RI-ATCG00180-XLOC_032457-8103-1
AAACCAACTCGAGGAATTTCTGACACAAGTATTCAATTAGTTCGAACTTG
CONSENSUS
AAACCAACTCGAGGAATTTCTGACACAAGTATTCAATTAGTTCGAACTTG

RI-ATCG00180-XLOC_032457-8103-0
TTTAGTCTTGAATTGGGACAAAAATTCTTCTCTCGAGGAGGTCCGTGCTT
RI-ATCG00180-XLOC_032457-8103-1
TTTAGTCTTGAATTGGGACAAAAATTCTTCTCTCGAGGAGGTCCGTGCTT
CONSENSUS
TTTAGTCTTGAATTGGGACAAAAATTCTTCTCTCGAGGAGGTCCGTGCTT

RI-ATCG00180-XLOC_032457-8103-0
TCTTTGTTGAAGTAAGTACAAAGGGTTTGATTCAAGATTTCATAAGAATT
RI-ATCG00180-XLOC_032457-8103-1
TCTTTGTTGAAGTAAGTACAAAGGGTTTGATTCAAGATTTCATAAGAATT
CONSENSUS
TCTTTGTTGAAGTAAGTACAAAGGGTTTGATTCAAGATTTCATAAGAATT

RI-ATCG00180-XLOC_032457-8103-0
GGCTTAGTGAAATCCCATATTTCATATATAAGAAAAAGGAATAATTCGCC
RI-ATCG00180-XLOC_032457-8103-1
GGCTTAGTGAAATCCCATATTTCATATATAAGAAAAAGGAATAATTCGCC
CONSENSUS
GGCTTAGTGAAATCCCATATTTCATATATAAGAAAAAGGAATAATTCGCC

RI-ATCG00180-XLOC_032457-8103-0
AGATTCGGGATTGATCTCTGCAGATCACATGAATCCGTTTTATTCGATTT
RI-ATCG00180-XLOC_032457-8103-1
AGATTCGGGATTGATCTCTGCAGATCACATGAATCCGTTTTATTCGATTT
CONSENSUS
AGATTCGGGATTGATCTCTGCAGATCACATGAATCCGTTTTATTCGATTT

RI-ATCG00180-XLOC_032457-8103-0

CGCCCAAGTCTGGCATTCTTCAACAATCACTTAGACAAAATCACGGAACT
RI-ATCG00180-XLOC_032457-8103-1
CGCCCAAGTCTGGCATTCTTCAACAATCACTTAGACAAAATCACGGAACT
CONSENSUS
CGCCCAAGTCTGGCATTCTTCAACAATCACTTAGACAAAATCACGGAACT

RI-ATCG00180-XLOC_032457-8103-0
ATTCGCATGTTCTTAAATCGAAATAAGGAGTCCCAATCTTTGTTAATTTT
RI-ATCG00180-XLOC_032457-8103-1
ATTCGCATGTTCTTAAATCGAAATAAGGAGTCCCAATCTTTGTTAATTTT
CONSENSUS
ATTCGCATGTTCTTAAATCGAAATAAGGAGTCCCAATCTTTGTTAATTTT

RI-ATCG00180-XLOC_032457-8103-0
ATCATCATCTAATTGTTTTAGAATGGGTCCATTTAATCATGTAAAACATC
RI-ATCG00180-XLOC_032457-8103-1
ATCATCATCTAATTGTTTTAGAATGGGTCCATTTAATCATGTAAAACATC
CONSENSUS
ATCATCATCTAATTGTTTTAGAATGGGTCCATTTAATCATGTAAAACATC

RI-ATCG00180-XLOC_032457-8103-0
ACAATGTGATAAACCAATCAATAAAAAAAAATACTCTAATTACAATTAAA
RI-ATCG00180-XLOC_032457-8103-1
ACAATGTGATAAACCAATCAATAAAAAAAAATACTCTAATTACAATTAAA
CONSENSUS
ACAATGTGATAAACCAATCAATAAAAAAAAATACTCTAATTACAATTAAA

RI-ATCG00180-XLOC_032457-8103-0
AATTCGTCGGGCCCCTTAGGAACAGCTACCCCAATTTCAAATTTTTATTC
RI-ATCG00180-XLOC_032457-8103-1
AATTCGTCGGGCCCCTTAGGAACAGCTACCCCAATTTCAAATTTTTATTC
CONSENSUS
AATTCGTCGGGCCCCTTAGGAACAGCTACCCCAATTTCAAATTTTTATTC

RI-ATCG00180-XLOC_032457-8103-0
GTTTTTGCCTTTACTAACTTATAATCAGATCTCTTTAATTAAATATTTTC
RI-ATCG00180-XLOC_032457-8103-1
GTTTTTGCCTTTACTAACTTATAATCAGATCTCTTTAATTAAATATTTTC
CONSENSUS
GTTTTTGCCTTTACTAACTTATAATCAGATCTCTTTAATTAAATATTTTC

RI-ATCG00180-XLOC_032457-8103-0
AACTTGATAACTTAAAATATATTTTTCAAAAAATTAACTCTTATTTAATC
RI-ATCG00180-XLOC_032457-8103-1
AACTTGATAACTTAAAATATATTTTTCAAAAAATTAACTCTTATTTAATC
CONSENSUS
AACTTGATAACTTAAAATATATTTTTCAAAAAATTAACTCTTATTTAATC

RI-ATCG00180-XLOC_032457-8103-0
GATGAAAACGGAATAATTTTAAATCTCGACCCATATAGTAACGTTGTTTT
RI-ATCG00180-XLOC_032457-8103-1
GATGAAAACGGAATAATTTTAAATCTCGACCCATATAGTAACGTTGTTTT
CONSENSUS
GATGAAAACGGAATAATTTTAAATCTCGACCCATATAGTAACGTTGTTTT

RI-ATCG00180-XLOC_032457-8103-0

GAATCCATTCAAATTGAATTGGTATTTTCTTCATCAAAATTATCATCATA
RI-ATCG00180-XLOC_032457-8103-1
GAATCCATTCAAATTGAATTGGTATTTTCTTCATCAAAATTATCATCATA
CONSENSUS
GAATCCATTCAAATTGAATTGGTATTTTCTTCATCAAAATTATCATCATA

RI-ATCG00180-XLOC_032457-8103-0
ATTATTGTGAGGAAACGTCCACAATAATTAGTCTTGGACAATTTTTTTGT
RI-ATCG00180-XLOC_032457-8103-1
ATTATTGTGAGGAAACGTCCACAATAATTAGTCTTGGACAATTTTTTTGT
CONSENSUS
ATTATTGTGAGGAAACGTCCACAATAATTAGTCTTGGACAATTTTTTTGT

RI-ATCG00180-XLOC_032457-8103-0
GAAAATGTATGTATAGCTAAAAAAGAACCGCACCTAAAATCGGGTCAAGT
RI-ATCG00180-XLOC_032457-8103-1
GAAAATGTATGTATAGCTAAAAAAGAACCGCACCTAAAATCGGGTCAAGT
CONSENSUS
GAAAATGTATGTATAGCTAAAAAAGAACCGCACCTAAAATCGGGTCAAGT

RI-ATCG00180-XLOC_032457-8103-0
TTTAATTGTTCAAAGGGATTCCGCAGTAATAAGATCCGCTAAGCCCTATT
RI-ATCG00180-XLOC_032457-8103-1
TTTAATTGTTCAAAGGGATTCCGCAGTAATAAGATCCGCTAAGCCCTATT
CONSENSUS
TTTAATTGTTCAAAGGGATTCCGCAGTAATAAGATCCGCTAAGCCCTATT

RI-ATCG00180-XLOC_032457-8103-0
TGGCTACTCCGGGAGCAAAAGTTCATGGGCATTACAGCGAAATTCTTTAC
RI-ATCG00180-XLOC_032457-8103-1
TGGCTACTCCGGGAGCAAAAGTTCATGGGCATTACAGCGAAATTCTTTAC
CONSENSUS
TGGCTACTCCGGGAGCAAAAGTTCATGGGCATTACAGCGAAATTCTTTAC

RI-ATCG00180-XLOC_032457-8103-0
GAAGGGGATACATTAGTTACATTTATATATGAAAAATCGAGATCCGGTGA
RI-ATCG00180-XLOC_032457-8103-1
GAAGGGGATACATTAGTTACATTTATATATGAAAAATCGAGATCCGGTGA
CONSENSUS
GAAGGGGATACATTAGTTACATTTATATATGAAAAATCGAGATCCGGTGA

RI-ATCG00180-XLOC_032457-8103-0
TATAACCCAAGGTCTTCCAAAAGTAGAACAAGTTTTAGAAGTCCGCTCGA
RI-ATCG00180-XLOC_032457-8103-1
TATAACCCAAGGTCTTCCAAAAGTAGAACAAGTTTTAGAAGTCCGCTCGA
CONSENSUS
TATAACCCAAGGTCTTCCAAAAGTAGAACAAGTTTTAGAAGTCCGCTCGA

RI-ATCG00180-XLOC_032457-8103-0
TTGATTCAATATCGTTGAACTTAGAAAAGCGGATTAAGGGTTGGAACAAG
RI-ATCG00180-XLOC_032457-8103-1
TTGATTCAATATCGTTGAACTTAGAAAAGCGGATTAAGGGTTGGAACAAG
CONSENSUS
TTGATTCAATATCGTTGAACTTAGAAAAGCGGATTAAGGGTTGGAACAAG

RI-ATCG00180-XLOC_032457-8103-0

TGTATAACAAGAATTCTTGGAATTCCTTGGGGATTCTTGATTGGTGCTGA
RI-ATCG00180-XLOC_032457-8103-1
TGTATAACAAGAATTCTTGGAATTCCTTGGGGATTCTTGATTGGTGCTGA
CONSENSUS
TGTATAACAAGAATTCTTGGAATTCCTTGGGGATTCTTGATTGGTGCTGA

RI-ATCG00180-XLOC_032457-8103-0
GTTAACTATAGTGCAAAGTCGTATTTCTTTGGTTAATAAGATTCAAAAGG
RI-ATCG00180-XLOC_032457-8103-1
GTTAACTATAGTGCAAAGTCGTATTTCTTTGGTTAATAAGATTCAAAAGG
CONSENSUS
GTTAACTATAGTGCAAAGTCGTATTTCTTTGGTTAATAAGATTCAAAAGG

RI-ATCG00180-XLOC_032457-8103-0
TTTATCGATCCCAGGGGGTGCAGATTCATAATAGGCATATCGAAATTATT
RI-ATCG00180-XLOC_032457-8103-1
TTTATCGATCCCAGGGGGTGCAGATTCATAATAGGCATATCGAAATTATT
CONSENSUS
TTTATCGATCCCAGGGGGTGCAGATTCATAATAGGCATATCGAAATTATT

RI-ATCG00180-XLOC_032457-8103-0
GTACGTCAAATAACATCAAAAGTTTTGGTTTCCGAAGAGGGAATGTCTAA
RI-ATCG00180-XLOC_032457-8103-1
GTACGTCAAATAACATCAAAAGTTTTGGTTTCCGAAGAGGGAATGTCTAA
CONSENSUS
GTACGTCAAATAACATCAAAAGTTTTGGTTTCCGAAGAGGGAATGTCTAA

RI-ATCG00180-XLOC_032457-8103-0
TGTTTTTTTACCTGGAGAACTTATTGGATTATTACGAGCAGAACGAACGG
RI-ATCG00180-XLOC_032457-8103-1
TGTTTTTTTACCTGGAGAACTTATTGGATTATTACGAGCAGAACGAACGG
CONSENSUS
TGTTTTTTTACCTGGAGAACTTATTGGATTATTACGAGCAGAACGAACGG

RI-ATCG00180-XLOC_032457-8103-0
GGCGTGCTTTAGAAGAAGCAATCTGTTATCGAGCCGTTTTATTAGGAATA
RI-ATCG00180-XLOC_032457-8103-1
GGCGTGCTTTAGAAGAAGCAATCTGTTATCGAGCCGTTTTATTAGGAATA
CONSENSUS
GGCGTGCTTTAGAAGAAGCAATCTGTTATCGAGCCGTTTTATTAGGAATA

RI-ATCG00180-XLOC_032457-8103-0
ACTCGAGCATCTTTGAATACTCAAAGTTTTATATCCGAAGCAAGTTTTCA
RI-ATCG00180-XLOC_032457-8103-1
ACTCGAGCATCTTTGAATACTCAAAGTTTTATATCCGAAGCAAGTTTTCA
CONSENSUS
ACTCGAGCATCTTTGAATACTCAAAGTTTTATATCCGAAGCAAGTTTTCA

RI-ATCG00180-XLOC_032457-8103-0
AGAAACTGCTCGAGTTTTAGCAAAAGCTGCTCTTCGGGGTCGTATCGATT
RI-ATCG00180-XLOC_032457-8103-1
AGAAACTGCTCGAGTTTTAGCAAAAGCTGCTCTTCGGGGTCGTATCGATT
CONSENSUS
AGAAACTGCTCGAGTTTTAGCAAAAGCTGCTCTTCGGGGTCGTATCGATT

RI-ATCG00180-XLOC_032457-8103-0

GGTTGAAAGGCCTGAAAGAAAATGTTGTTCTAGGGGGGGTGATCCCCGCC
RI-ATCG00180-XLOC_032457-8103-1
GGTTGAAAGGCCTGAAAGAAAATGTTGTTCTAGGGGGGGTGATCCCCGCC
CONSENSUS
GGTTGAAAGGCCTGAAAGAAAATGTTGTTCTAGGGGGGGTGATCCCCGCC

RI-ATCG00180-XLOC_032457-8103-0
GGGACCGGATTCAACAAAGGATTGGTGCATTGTTCACGGCAACATACCAA
RI-ATCG00180-XLOC_032457-8103-1
GGGACCGGATTCAACAAAGGATTGGTGCATTGTTCACGGCAACATACCAA
CONSENSUS
GGGACCGGATTCAACAAAGGATTGGTGCATTGTTCACGGCAACATACCAA

RI-ATCG00180-XLOC_032457-8103-0
CATTATTTTGGAAAAAAAAACAAAGAATTTAGCTTTATTCGAGGGAGATA
RI-ATCG00180-XLOC_032457-8103-1
CATTATTTTGGAAAAAAAAACAAAGAATTTAGCTTTATTCGAGGGAGATA
CONSENSUS
CATTATTTTGGAAAAAAAAACAAAGAATTTAGCTTTATTCGAGGGAGATA

RI-ATCG00180-XLOC_032457-8103-0
TGAGAGATATTTTATTCTACCACAGGGAATTTTGTGACTCTTCTATTTCC
RI-ATCG00180-XLOC_032457-8103-1
TGAGAGATATTTTATTCTACCACAGGGAATTTTGTGACTCTTCTATTTCC
CONSENSUS
TGAGAGATATTTTATTCTACCACAGGGAATTTTGTGACTCTTCTATTTCC

RI-ATCG00180-XLOC_032457-8103-0
AAATCTGACTTTTCTAGGATTTAATGATTTCTAATAGCGGACTATTTAAA
RI-ATCG00180-XLOC_032457-8103-1
AAATCTGACTTTTCTAGGATTTAATGATTTCTAATAGCGGACTATTTAAA
CONSENSUS
AAATCTGACTTTTCTAGGATTTAATGATTTCTAATAGCGGACTATTTAAA

RI-ATCG00180-XLOC_032457-8103-0
ATTTTAGTTTTTGTTGTTTGCTGTAGTCATTTGCTTTCGTAATTTGTTAA
RI-ATCG00180-XLOC_032457-8103-1
ATTTTAGTTTTTGTTGTTTGCTGTAGTCATTTGCTTTCGTAATTTGTTAA
CONSENSUS
ATTTTAGTTTTTGTTGTTTGCTGTAGTCATTTGCTTTCGTAATTTGTTAA

RI-ATCG00180-XLOC_032457-8103-0
CACTAATAAAGATAATAATCAATACAAAATAATGGCTTGGTTCATGCATA
RI-ATCG00180-XLOC_032457-8103-1
CACTAATAAAGATAATAATCAATACAAAATAATGGCTTGGTTCATGCATA
CONSENSUS
CACTAATAAAGATAATAATCAATACAAAATAATGGCTTGGTTCATGCATA

RI-ATCG00180-XLOC_032457-8103-0
AATGGCCATCCCCGGTACAAGAGAAGGTTCCATCGGAACAAAATTTTATT
RI-ATCG00180-XLOC_032457-8103-1
AATGGCCATCCCCGGTACAAGAGAAGGTTCCATCGGAACAAAATTTTATT
CONSENSUS
AATGGCCATCCCCGGTACAAGAGAAGGTTCCATCGGAACAAAATTTTATT

RI-ATCG00180-XLOC_032457-8103-0

TTAGTTTGGGGTGCCCCCCTTTTTAAGTGTGAAAAGAAATGACAAAAAGA
RI-ATCG00180-XLOC_032457-8103-1
TTAGTTTGGGGTGCCCCCCTTTTTAAGTGTGAAAAGAAATGACAAAAAGA
CONSENSUS
TTAGTTTGGGGTGCCCCCCTTTTTAAGTGTGAAAAGAAATGACAAAAAGA

RI-ATCG00180-XLOC_032457-8103-0
TATTGGAACATCGATTTGGAAGAGATGATGAGAGCAGGAGTTCATTTTGG
RI-ATCG00180-XLOC_032457-8103-1
TATTGGAACATCGATTTGGAAGAGATGATGAGAGCAGGAGTTCATTTTGG
CONSENSUS
TATTGGAACATCGATTTGGAAGAGATGATGAGAGCAGGAGTTCATTTTGG

RI-ATCG00180-XLOC_032457-8103-0
ACATGGTACGAGGAAATGGAATCCTAGAATGGCACCTTATATTTCTGCAA
RI-ATCG00180-XLOC_032457-8103-1
ACATGGTACGAGGAAATGGAATCCTAGAATGGCACCTTATATTTCTGCAA
CONSENSUS
ACATGGTACGAGGAAATGGAATCCTAGAATGGCACCTTATATTTCTGCAA

RI-ATCG00180-XLOC_032457-8103-0
AGCGTAAAGGTATTCATATTATAAATCTGACTAGAACTGCTCGTTTTTTA
RI-ATCG00180-XLOC_032457-8103-1
AGCGTAAAGGTATTCATATTATAAATCTGACTAGAACTGCTCGTTTTTTA
CONSENSUS
AGCGTAAAGGTATTCATATTATAAATCTGACTAGAACTGCTCGTTTTTTA

RI-ATCG00180-XLOC_032457-8103-0
TCAGAAGCTTGTGATTTAGTTTTTGATGCGGCAAGTAGGGGAAAACAATT
RI-ATCG00180-XLOC_032457-8103-1
TCAGAAGCTTGTGATTTAGTTTTTGATGCGGCAAGTAGGGGAAAACAATT
CONSENSUS
TCAGAAGCTTGTGATTTAGTTTTTGATGCGGCAAGTAGGGGAAAACAATT

RI-ATCG00180-XLOC_032457-8103-0
CTTAATTGTTGGGACAAAAAATAAAGCAGCTGATTTAGTGTCGCGGGCTG
RI-ATCG00180-XLOC_032457-8103-1
CTTAATTGTTGGGACAAAAAATAAAGCAGCTGATTTAGTGTCGCGGGCTG
CONSENSUS
CTTAATTGTTGGGACAAAAAATAAAGCAGCTGATTTAGTGTCGCGGGCTG

RI-ATCG00180-XLOC_032457-8103-0
CAATACGGGCTCGGTGTCATTATGTTAATAAAAAATGGCTCGGCGGCATG
RI-ATCG00180-XLOC_032457-8103-1
CAATACGGGCTCGGTGTCATTATGTTAATAAAAAATGGCTCGGCGGCATG
CONSENSUS
CAATACGGGCTCGGTGTCATTATGTTAATAAAAAATGGCTCGGCGGCATG

RI-ATCG00180-XLOC_032457-8103-0
TTAACAAATTGGTCCACTACCGAAAAAAGACTTCATAAGTTTAGGGACTT
RI-ATCG00180-XLOC_032457-8103-1
TTAACAAATTGGTCCACTACCGAAAAAAGACTTCATAAGTTTAGGGACTT
CONSENSUS
TTAACAAATTGGTCCACTACCGAAAAAAGACTTCATAAGTTTAGGGACTT

RI-ATCG00180-XLOC_032457-8103-0

GAGAACTGAACAAAAGACAGAGGGATTCAACCGTCTTCCGAAAAGGGATG
RI-ATCG00180-XLOC_032457-8103-1
GAGAACTGAACAAAAGACAGAGGGATTCAACCGTCTTCCGAAAAGGGATG
CONSENSUS
GAGAACTGAACAAAAGACAGAGGGATTCAACCGTCTTCCGAAAAGGGATG

RI-ATCG00180-XLOC_032457-8103-0
CAGCTGTGTTGAAGAGACAATTATCTCGCTTGGAAACATATCTAGGCGGG
RI-ATCG00180-XLOC_032457-8103-1
CAGCTGTGTTGAAGAGACAATTATCTCGCTTGGAAACATATCTAGGCGGG
CONSENSUS
CAGCTGTGTTGAAGAGACAATTATCTCGCTTGGAAACATATCTAGGCGGG

RI-ATCG00180-XLOC_032457-8103-0
ATTAAATATATGACGGGATTGCCTGATATTGTAATCATCCTCGATCAGCA
RI-ATCG00180-XLOC_032457-8103-1
ATTAAATATATGACGGGATTGCCTGATATTGTAATCATCCTCGATCAGCA
CONSENSUS
ATTAAATATATGACGGGATTGCCTGATATTGTAATCATCCTCGATCAGCA

RI-ATCG00180-XLOC_032457-8103-0
AGAAGAATATACGGCTCTTAGAGAATGTATCACTTTGGGAATTCCAACCA
RI-ATCG00180-XLOC_032457-8103-1
AGAAGAATATACGGCTCTTAGAGAATGTATCACTTTGGGAATTCCAACCA
CONSENSUS
AGAAGAATATACGGCTCTTAGAGAATGTATCACTTTGGGAATTCCAACCA

RI-ATCG00180-XLOC_032457-8103-0
TTTCTTTAATCGATACAAATTGTAATCCCGATCTCGCGGATATTTCTATT
RI-ATCG00180-XLOC_032457-8103-1
TTTCTTTAATCGATACAAATTGTAATCCCGATCTCGCGGATATTTCTATT
CONSENSUS
TTTCTTTAATCGATACAAATTGTAATCCCGATCTCGCGGATATTTCTATT

RI-ATCG00180-XLOC_032457-8103-0
CCCGCAAATGATGACGCTATAGCTTCAATTCGATTCATTCTTAACAAATT
RI-ATCG00180-XLOC_032457-8103-1
CCCGCAAATGATGACGCTATAGCTTCAATTCGATTCATTCTTAACAAATT
CONSENSUS
CCCGCAAATGATGACGCTATAGCTTCAATTCGATTCATTCTTAACAAATT

RI-ATCG00180-XLOC_032457-8103-0
AGTATTCGCAATTTGTGAGGGCCGTTCTAGCTATATACAAAATTCTTGAT
RI-ATCG00180-XLOC_032457-8103-1
AGTATTCGCAATTTGTGAGGGCCGTTCTAGCTATATACAAAATTCTTGAT
CONSENSUS
AGTATTCGCAATTTGTGAGGGCCGTTCTAGCTATATACAAAATTCTTGAT

RI-ATCG00180-XLOC_032457-8103-0
TAATAATAAGATAAATAAATCAATTTTTTTTTTAGGGAGGGGCTCCCTGT
RI-ATCG00180-XLOC_032457-8103-1
TAATAATAAGATAAATAAATCAATTTTTTTTTTAGGGAGGGGCTCCCTGT
CONSENSUS
TAATAATAAGATAAATAAATCAATTTTTTTTTTAGGGAGGGGCTCCCTGT

RI-ATCG00180-XLOC_032457-8103-0

ATTTCGATTTCAAAAATCGGTTACTACTCCTGAATTGTAGAAAAGAAAAA
RI-ATCG00180-XLOC_032457-8103-1
ATTTCGATTTCAAAAATCGGTTACTACTCCTGAATTGTAGAAAAGAAAAA
CONSENSUS
ATTTCGATTTCAAAAATCGGTTACTACTCCTGAATTGTAGAAAAGAAAAA

RI-ATCG00180-XLOC_032457-8103-0
GAGAGAAAAAACTGGGGATATTGTGTGATTTGTTTAGTTGGGATCCAAAA
RI-ATCG00180-XLOC_032457-8103-1
GAGAGAAAAAACTGGGGATATTGTGTGATTTGTTTAGTTGGGATCCAAAA
CONSENSUS
GAGAGAAAAAACTGGGGATATTGTGTGATTTGTTTAGTTGGGATCCAAAA

RI-ATCG00180-XLOC_032457-8103-0
CTAAAATATAAAATTTAAGTAAATAAGTAAAAAAAAAGGGGGGGTCTTGA
RI-ATCG00180-XLOC_032457-8103-1
CTAAAATATAAAATTTAAGTAAATAAGTAAAAAAAAAGGGGGGGTCTTGA
CONSENSUS
CTAAAATATAAAATTTAAGTAAATAAGTAAAAAAAAAGGGGGGGTCTTGA

RI-ATCG00180-XLOC_032457-8103-0
ATCAAAATAATTTAAAGTTCTTATTTCTGTCAGAGGGCAATATGAATGTT
RI-ATCG00180-XLOC_032457-8103-1
ATCAAAATAATTTAAAGTTCTTATTTCTGTCAGAGGGCAATATGAATGTT
CONSENSUS
ATCAAAATAATTTAAAGTTCTTATTTCTGTCAGAGGGCAATATGAATGTT

RI-ATCG00180-XLOC_032457-8103-0
TTATCATGTTCCATCAACACACTAATAAAAGAAGGGTTATATGAGATATC
RI-ATCG00180-XLOC_032457-8103-1
TTATCATGTTCCATCAACACACTAATAAAAGAAGGGTTATATGAGATATC
CONSENSUS
TTATCATGTTCCATCAACACACTAATAAAAGAAGGGTTATATGAGATATC

RI-ATCG00180-XLOC_032457-8103-0
TGGTGTAGAAGTAGGCCAACATTTCTATTGGCAAATAGGGGGTTTCCAGG
RI-ATCG00180-XLOC_032457-8103-1
TGGTGTAGAAGTAGGCCAACATTTCTATTGGCAAATAGGGGGTTTCCAGG
CONSENSUS
TGGTGTAGAAGTAGGCCAACATTTCTATTGGCAAATAGGGGGTTTCCAGG

RI-ATCG00180-XLOC_032457-8103-0
TCCATGCCCAAGTTCTTATTACTTCTTGGGTTGTAATTGCTATCTTATTA
RI-ATCG00180-XLOC_032457-8103-1
TCCATGCCCAAGTTCTTATTACTTCTTGGGTTGTAATTGCTATCTTATTA
CONSENSUS
TCCATGCCCAAGTTCTTATTACTTCTTGGGTTGTAATTGCTATCTTATTA

RI-ATCG00180-XLOC_032457-8103-0
GGTTCCGCAGTTCTAGCGATTCGCAATCCACAAACAATTCCAACTGACGG
RI-ATCG00180-XLOC_032457-8103-1
GGTTCCGCAGTTCTAGCGATTCGCAATCCACAAACAATTCCAACTGACGG
CONSENSUS
GGTTCCGCAGTTCTAGCGATTCGCAATCCACAAACAATTCCAACTGACGG

RI-ATCG00180-XLOC_032457-8103-0

CCAAAATTTCTTTGAATTTGTCCTTGAATTCATTCGAGACGTGAGTAAAA
RI-ATCG00180-XLOC_032457-8103-1
CCAAAATTTCTTTGAATTTGTCCTTGAATTCATTCGAGACGTGAGTAAAA
CONSENSUS
CCAAAATTTCTTTGAATTTGTCCTTGAATTCATTCGAGACGTGAGTAAAA

RI-ATCG00180-XLOC_032457-8103-0
CCCAGATTGGAGAAGAATATGGTCCATGGGTTCCCTTTATTGGAACCCTG
RI-ATCG00180-XLOC_032457-8103-1
CCCAGATTGGAGAAGAATATGGTCCATGGGTTCCCTTTATTGGAACCCTG
CONSENSUS
CCCAGATTGGAGAAGAATATGGTCCATGGGTTCCCTTTATTGGAACCCTG

RI-ATCG00180-XLOC_032457-8103-0
TTTTTATTTATTTTTGTTTCTAACTGGTCAGGAGCCCTTTTACCGTGGAA
RI-ATCG00180-XLOC_032457-8103-1
TTTTTATTTATTTTTGTTTCTAACTGGTCAGGAGCCCTTTTACCGTGGAA
CONSENSUS
TTTTTATTTATTTTTGTTTCTAACTGGTCAGGAGCCCTTTTACCGTGGAA

RI-ATCG00180-XLOC_032457-8103-0
AATTATCCAGTTACCTCAAGGGGAGTTAGCAGCACCAACGAATGATATAA
RI-ATCG00180-XLOC_032457-8103-1
AATTATCCAGTTACCTCAAGGGGAGTTAGCAGCACCAACGAATGATATAA
CONSENSUS
AATTATCCAGTTACCTCAAGGGGAGTTAGCAGCACCAACGAATGATATAA

RI-ATCG00180-XLOC_032457-8103-0
ATACGACGGTTGCTTTAGCTTTACTCACATCAGTAGCCTATTTTTATGCG
RI-ATCG00180-XLOC_032457-8103-1
ATACGACGGTTGCTTTAGCTTTACTCACATCAGTAGCCTATTTTTATGCG
CONSENSUS
ATACGACGGTTGCTTTAGCTTTACTCACATCAGTAGCCTATTTTTATGCG

RI-ATCG00180-XLOC_032457-8103-0
GGGCTTAGCAAAAAAGGATTAGGGTATTTCAGTAAATACATTCAACCAAC
RI-ATCG00180-XLOC_032457-8103-1
GGGCTTAGCAAAAAAGGATTAGGGTATTTCAGTAAATACATTCAACCAAC
CONSENSUS
GGGCTTAGCAAAAAAGGATTAGGGTATTTCAGTAAATACATTCAACCAAC

RI-ATCG00180-XLOC_032457-8103-0
CCCAATTCTTTTACCCATTAACATCTTAGAAGATTTTACAAAACCCCTAT
RI-ATCG00180-XLOC_032457-8103-1
CCCAATTCTTTTACCCATTAACATCTTAGAAGATTTTACAAAACCCCTAT
CONSENSUS
CCCAATTCTTTTACCCATTAACATCTTAGAAGATTTTACAAAACCCCTAT

RI-ATCG00180-XLOC_032457-8103-0
CACTTAGTTTTCGACTTTTCGGAAATATATTAGCCGATGAATTAGTCGTT
RI-ATCG00180-XLOC_032457-8103-1
CACTTAGTTTTCGACTTTTCGGAAATATATTAGCCGATGAATTAGTCGTT
CONSENSUS
CACTTAGTTTTCGACTTTTCGGAAATATATTAGCCGATGAATTAGTCGTT

RI-ATCG00180-XLOC_032457-8103-0

GTTGTTCTTGTTTCTTTAGTACCTTTAGTGGTTCCTATACCTGTTATGTT
RI-ATCG00180-XLOC_032457-8103-1
GTTGTTCTTGTTTCTTTAGTACCTTTAGTGGTTCCTATACCTGTTATGTT
CONSENSUS
GTTGTTCTTGTTTCTTTAGTACCTTTAGTGGTTCCTATACCTGTTATGTT

RI-ATCG00180-XLOC_032457-8103-0
CCTTGGATTATTTACAAGCGGGATTCAAGCTCTCATTTTTGCCACTTTAG
RI-ATCG00180-XLOC_032457-8103-1
CCTTGGATTATTTACAAGCGGGATTCAAGCTCTCATTTTTGCCACTTTAG
CONSENSUS
CCTTGGATTATTTACAAGCGGGATTCAAGCTCTCATTTTTGCCACTTTAG

RI-ATCG00180-XLOC_032457-8103-0
CTGCGGCTTATATAGGTGAGTCTATGGAAGGTCATCATTAATGGTTTTTT
RI-ATCG00180-XLOC_032457-8103-1
CTGCGGCTTATATAGGTGAGTCTATGGAAGGTCATCATTAATGGTTTTTT
CONSENSUS
CTGCGGCTTATATAGGTGAGTCTATGGAAGGTCATCATTAATGGTTTTTT

RI-ATCG00180-XLOC_032457-8103-0
TTTTAATATTCTTTTTTAGGTTAGCCCAATTAATTATAGAATAGTTGGTT
RI-ATCG00180-XLOC_032457-8103-1
TTTTAATATTCTTTTTTAGGTTAGCCCAATTAATTATAGAATAGTTGGTT
CONSENSUS
TTTTAATATTCTTTTTTAGGTTAGCCCAATTAATTATAGAATAGTTGGTT

RI-ATCG00180-XLOC_032457-8103-0
TACATTATGTAAGAAACACTTGTATATGTGATATTTGATATTGCCTAGGT
RI-ATCG00180-XLOC_032457-8103-1
TACATTATGTAAGAAACACTTGTATATGTGATATTTGATATTGCCTAGGT
CONSENSUS
TACATTATGTAAGAAACACTTGTATATGTGATATTTGATATTGCCTAGGT

RI-ATCG00180-XLOC_032457-8103-0
ATATATGAGTTAAAGATCTATATAATCTGAATCTGCTCTACTATTTTTGT
RI-ATCG00180-XLOC_032457-8103-1
ATATATGAGTTAAAGATCTATATAATCTGAATCTGCTCTACTATTTTTGT
CONSENSUS
ATATATGAGTTAAAGATCTATATAATCTGAATCTGCTCTACTATTTTTGT

RI-ATCG00180-XLOC_032457-8103-0
GAATTTCTATTTAGATTTAGATAGGGATTCGATTAGAAGTTCTTCCTTTT
RI-ATCG00180-XLOC_032457-8103-1
GAATTTCTATTTAGATTTAGATAGGGATTCGATTAGAAGTTCTTCCTTTT
CONSENSUS
GAATTTCTATTTAGATTTAGATAGGGATTCGATTAGAAGTTCTTCCTTTT

RI-ATCG00180-XLOC_032457-8103-0
TATCTGTGAATTGGCTGAAAAAATGATAAAAAAAGAACGAAGAATTCAAA
RI-ATCG00180-XLOC_032457-8103-1
TATCTGTGAATTGGCTGAAAAAATGATAAAAAAAGAACGAAGAATTCAAA
CONSENSUS
TATCTGTGAATTGGCTGAAAAAATGATAAAAAAAGAACGAAGAATTCAAA

RI-ATCG00180-XLOC_032457-8103-0

GAATGGTTCATAAAAAAAATGGCATAAAAAAGTCGTATATATATTATGGA
RI-ATCG00180-XLOC_032457-8103-1
GAATGGTTCATAAAAAAAATGGCATAAAAAAGTCGTATATATATTATGGA
CONSENSUS
GAATGGTTCATAAAAAAAATGGCATAAAAAAGTCGTATATATATTATGGA

RI-ATCG00180-XLOC_032457-8103-0
TTTTTAAAAATCCCGCGGATAGGAACTACTATCAAAGTAATTCTTATGAT
RI-ATCG00180-XLOC_032457-8103-1
TTTTTAAAAATCCCGCGGATAGGAACTACTATCAAAGTAATTCTTATGAT
CONSENSUS
TTTTTAAAAATCCCGCGGATAGGAACTACTATCAAAGTAATTCTTATGAT

RI-ATCG00180-XLOC_032457-8103-0
TCAATAATTTTATTATATTATTTCATATTTCAATTAAAGTTTTTGGTTTT
RI-ATCG00180-XLOC_032457-8103-1
TCAATAATTTTATTATATTATTTCATATTTCAATTAAAGTTTTTGGTTTT
CONSENSUS
TCAATAATTTTATTATATTATTTCATATTTCAATTAAAGTTTTTGGTTTT

RI-ATCG00180-XLOC_032457-8103-0
TTTGGCTGGATTAATCTTAGCGATTACTTAATTAGAATTACGTCCTAAGT
RI-ATCG00180-XLOC_032457-8103-1
TTTGGCTGGATTAATCTTAGCGATTACTTAATTAGAATTACGTCCTAAGT
CONSENSUS
TTTGGCTGGATTAATCTTAGCGATTACTTAATTAGAATTACGTCCTAAGT

RI-ATCG00180-XLOC_032457-8103-0
CATTGGATGATTGTATCATTAACTATTTCTTTATTTTGGTGTGAGGAACT
RI-ATCG00180-XLOC_032457-8103-1
CATTGGATGATTGTATCATTAACTATTTCTTTATTTTGGTGTGAGGAACT
CONSENSUS
CATTGGATGATTGTATCATTAACTATTTCTTTATTTTGGTGTGAGGAACT

RI-ATCG00180-XLOC_032457-8103-0
TATCATGAATCCACTGGTTTCTGCTGCTTCGGTTATTGCTGCTGGGTTGG
RI-ATCG00180-XLOC_032457-8103-1
TATCATGAATCCACTGGTTTCTGCTGCTTCGGTTATTGCTGCTGGGTTGG
CONSENSUS
TATCATGAATCCACTGGTTTCTGCTGCTTCGGTTATTGCTGCTGGGTTGG

RI-ATCG00180-XLOC_032457-8103-0
CTGTTGGGCTTGCTTCTATTGGACCTGGAGTCGGTCAAGGTACAGCTGCG
RI-ATCG00180-XLOC_032457-8103-1
CTGTTGGGCTTGCTTCTATTGGACCTGGAGTCGGTCAAGGTACAGCTGCG
CONSENSUS
CTGTTGGGCTTGCTTCTATTGGACCTGGAGTCGGTCAAGGTACAGCTGCG

RI-ATCG00180-XLOC_032457-8103-0
GGTCAAGCTGTCGAAGGTATCGCGAGACAACCTGAGGCAGAAGGAAAAAT
RI-ATCG00180-XLOC_032457-8103-1
GGTCAAGCTGTCGAAGGTATCGCGAGACAACCTGAGGCAGAAGGAAAAAT
CONSENSUS
GGTCAAGCTGTCGAAGGTATCGCGAGACAACCTGAGGCAGAAGGAAAAAT

RI-ATCG00180-XLOC_032457-8103-0

ACGAGGTACTTTATTGCTTAGTTTGGCTTTTATGGAAGCTTTAACAATTT
RI-ATCG00180-XLOC_032457-8103-1
ACGAGGTACTTTATTGCTTAGTTTGGCTTTTATGGAAGCTTTAACAATTT
CONSENSUS
ACGAGGTACTTTATTGCTTAGTTTGGCTTTTATGGAAGCTTTAACAATTT

RI-ATCG00180-XLOC_032457-8103-0
ATGGCCTGGTTGTAGCATTAGCGCTTTTATTTGCGAATCCTTTTGTTTAA
RI-ATCG00180-XLOC_032457-8103-1
ATGGCCTGGTTGTAGCATTAGCGCTTTTATTTGCGAATCCTTTTGTTTAA
CONSENSUS
ATGGCCTGGTTGTAGCATTAGCGCTTTTATTTGCGAATCCTTTTGTTTAA

RI-ATCG00180-XLOC_032457-8103-0
GCCCAGAAATCACAAAATTCTGGATTTTTTTTGTAGTTTTTTTAATTCTA
RI-ATCG00180-XLOC_032457-8103-1
GCCCAGAAATCACAAAATTCTGGATTTTTTTTGTAGTTTTTTTAATTCTA
CONSENSUS
GCCCAGAAATCACAAAATTCTGGATTTTTTTTGTAGTTTTTTTAATTCTA

RI-ATCG00180-XLOC_032457-8103-0
TCAAGATTTAACTCCTACAATTATTCATTGAGACAACAATCCTTGGGAAG
RI-ATCG00180-XLOC_032457-8103-1
TCAAGATTTAACTCCTACAATTATTCATTGAGACAACAATCCTTGGGAAG
CONSENSUS
TCAAGATTTAACTCCTACAATTATTCATTGAGACAACAATCCTTGGGAAG

RI-ATCG00180-XLOC_032457-8103-0
GACTAATTTGAGGATGGGGAATTAGCACATCGATTCGCTTTCTTCCTTCC
RI-ATCG00180-XLOC_032457-8103-1
GACTAATTTGAGGATGGGGAATTAGCACATCGATTCGCTTTCTTCCTTCC
CONSENSUS
GACTAATTTGAGGATGGGGAATTAGCACATCGATTCGCTTTCTTCCTTCC

RI-ATCG00180-XLOC_032457-8103-0
CCTTATTCTTTTAGTTTAATAGAAACTTTTTTTTAAGGAGTGTTGCGAAA
RI-ATCG00180-XLOC_032457-8103-1
CCTTATTCTTTTAGTTTAATAGAAACTTTTTTTTAAGGAGTGTTGCGAAA
CONSENSUS
CCTTATTCTTTTAGTTTAATAGAAACTTTTTTTTAAGGAGTGTTGCGAAA

RI-ATCG00180-XLOC_032457-8103-0
AAAGTAGGTTTAGGTTTTTTGAACTTGACATAAAACTTGGTCTCAAATTC
RI-ATCG00180-XLOC_032457-8103-1
AAAGTAGGTTTAGGTTTTTTGAACTTGACATAAAACTTGGTCTCAAATTC
CONSENSUS
AAAGTAGGTTTAGGTTTTTTGAACTTGACATAAAACTTGGTCTCAAATTC

RI-ATCG00180-XLOC_032457-8103-0
TAGCTTAATTCTAATAAGTCTCATTATTATTATTGAAAATTAAAAATTTT
RI-ATCG00180-XLOC_032457-8103-1
TAGCTTAATTCTAATAAGTCTCATTATTATTATTGAAAATTAAAAATTTT
CONSENSUS
TAGCTTAATTCTAATAAGTCTCATTATTATTATTGAAAATTAAAAATTTT

RI-ATCG00180-XLOC_032457-8103-0

GGAAAATACGTTTGTAAATAGAATAGGTTTTTGATTCTGTTTACAAATAT
RI-ATCG00180-XLOC_032457-8103-1
GGAAAATACGTTTGTAAATAGAATAGGTTTTTGATTCTGTTTACAAATAT
CONSENSUS
GGAAAATACGTTTGTAAATAGAATAGGTTTTTGATTCTGTTTACAAATAT

RI-ATCG00180-XLOC_032457-8103-0
CCAAATAGGTAAATTAAATTATTAAATTCAAATTTCTTTTTATTTTCAAT
RI-ATCG00180-XLOC_032457-8103-1
CCAAATAGGTAAATTAAATTATTAAATTCAAATTTCTTTTTATTTTCAAT
CONSENSUS
CCAAATAGGTAAATTAAATTATTAAATTCAAATTTCTTTTTATTTTCAAT

RI-ATCG00180-XLOC_032457-8103-0
AAAAAGAATAAAAAAAAAAAGGACAGAGTTCCTTTTTTATAGTTTAGCTAG
RI-ATCG00180-XLOC_032457-8103-1
AAAAAGAATAAAAAAAAAAAGGACAGAGTTCCTTTTTTATAGTTTAGCTAG
CONSENSUS
AAAAAGAATAAAAAAAAAAAGGACAGAGTTCCTTTTTTATAGTTTAGCTAG

RI-ATCG00180-XLOC_032457-8103-0
AAGAGGAGATTATATGAAAAATTTAACCGATTCTTTCGTTTACTTGGGTC
RI-ATCG00180-XLOC_032457-8103-1
AAGAGGAGATTATATGAAAAATTTAACCGATTCTTTCGTTTACTTGGGTC
CONSENSUS
AAGAGGAGATTATATGAAAAATTTAACCGATTCTTTCGTTTACTTGGGTC

RI-ATCG00180-XLOC_032457-8103-0
ACTGGCCATCCGCCGGGAGTTTCGGATTTAATACCGATATTTTAGCAACA
RI-ATCG00180-XLOC_032457-8103-1
ACTGGCCATCCGCCGGGAGTTTCGGATTTAATACCGATATTTTAGCAACA
CONSENSUS
ACTGGCCATCCGCCGGGAGTTTCGGATTTAATACCGATATTTTAGCAACA

RI-ATCG00180-XLOC_032457-8103-0
AATCCAATAAATCTAAGTGTAGTTTTCGGTGTATTGATCTTTTTTGGAAA
RI-ATCG00180-XLOC_032457-8103-1
AATCCAATAAATCTAAGTGTAGTTTTCGGTGTATTGATCTTTTTTGGAAA
CONSENSUS
AATCCAATAAATCTAAGTGTAGTTTTCGGTGTATTGATCTTTTTTGGAAA

RI-ATCG00180-XLOC_032457-8103-0
GGGAGTGTGTGTGAGTTGTTCATTTCAAGAATAGGCTGGATTCACCCAGT
RI-ATCG00180-XLOC_032457-8103-1
GGGAGTGT--
CONSENSUS
GGGAGTGT..

RI-ATCG00180-XLOC_032457-8103-0
GGCACTATAACTAGGAAAGAGTGCATAATCCCGCGAATTACTTCTGAATA
RI-ATCG00180-XLOC_032457-8103-1

CONSENSUS
....

RI-ATCG00180-XLOC_032457-8103-0

AAAAAATCATATTTGAGAACCATAGCCTTTCGTTATTCTTTGGTAAGTCC
RI-ATCG00180-XLOC_032457-8103-1

CONSENSUS
....

RI-ATCG00180-XLOC_032457-8103-0
GCTTTACTTTGATTCTCTATTAACCAAAAATTGGGACAATTAATTAACAT
RI-ATCG00180-XLOC_032457-8103-1

CONSENSUS
....

RI-ATCG00180-XLOC_032457-8103-0
GGTTAAAGCTAAACCGTTTGAAGTTCAGATGCAACATGGTACTCTTTCTA
RI-ATCG00180-XLOC_032457-8103-1

CONSENSUS
....

RI-ATCG00180-XLOC_032457-8103-0
CTATATATAGTCTAAATGTAGACTAAATAAAAAAATGAATAAAATTTTCA
RI-ATCG00180-XLOC_032457-8103-1

CONSENSUS
....

RI-ATCG00180-XLOC_032457-8103-0
AAAAGAATTGTTATACTTTTTTCGATATAAAACACTCATGTCGATAAAAT
RI-ATCG00180-XLOC_032457-8103-1

CONSENSUS
....

RI-ATCG00180-XLOC_032457-8103-0
TTTTTGAGTCTTTTTTTATAATGCAGAATTGATAACCTACGTATAAAATA
RI-ATCG00180-XLOC_032457-8103-1

CONSENSUS
....

RI-ATCG00180-XLOC_032457-8103-0
ATAAGAATTCTTTGGATTTCAAGAAAAAAAGAAACAACTTTGCTGACAAT
RI-ATCG00180-XLOC_032457-8103-1

CONSENSUS
....

RI-ATCG00180-XLOC_032457-8103-0
TATTAATTTTTCATTGGTCAGAAGAATCCTCCGAATATTTTTATCTTGGA
RI-ATCG00180-XLOC_032457-8103-1

CONSENSUS
....

RI-ATCG00180-XLOC_032457-8103-0

TTGGTGATCTTTTTCGATAGAAATATATATTGAATATGAATTGAATAAAA
RI-ATCG00180-XLOC_032457-8103-1

CONSENSUS
....

RI-ATCG00180-XLOC_032457-8103-0
AAACTCGGGAGAGGGTAGGCTCATTGCATGAAAAAGATGGGAAGGGAAGT
RI-ATCG00180-XLOC_032457-8103-1

CONSENSUS
....

RI-ATCG00180-XLOC_032457-8103-0
CTCATAAATAATTGATTGGAATAAGTGAGCATGAGAGCCAAATGAATCGA
RI-ATCG00180-XLOC_032457-8103-1

CONSENSUS
....

RI-ATCG00180-XLOC_032457-8103-0
AAGATTCATGTTTGGTTCGGGAAGGGATCATAGAACTTTTTTTTTAATGA
RI-ATCG00180-XLOC_032457-8103-1

CONSENSUS
....

RI-ATCG00180-XLOC_032457-8103-0
ATGGAAAGATAATCTACTTTCATTAAATGATTTATTAGATAACCGAAAGC
RI-ATCG00180-XLOC_032457-8103-1 ----------------------
TAAATGATTTATTAGATAACCGAAAGC
CONSENSUS
.......................TAAATGATTTATTAGATAACCGAAAGC

RI-ATCG00180-XLOC_032457-8103-0
AGAGGATATTAAATACTATTCGAAATTCAGAAGAACTGCGTGAAGGAGCT
RI-ATCG00180-XLOC_032457-8103-1
AGAGGATATTAAATACTATTCGAAATTCAGAAGAACTGCGTGAAGGAGCT
CONSENSUS
AGAGGATATTAAATACTATTCGAAATTCAGAAGAACTGCGTGAAGGAGCT

RI-ATCG00180-XLOC_032457-8103-0
ATTCAACAATTAGAAAATGCCCGGGCGCGCTTGCGTAACGTAGAAACGGA
RI-ATCG00180-XLOC_032457-8103-1
ATTCAACAATTAGAAAATGCCCGGGCGCGCTTGCGTAACGTAGAAACGGA
CONSENSUS
ATTCAACAATTAGAAAATGCCCGGGCGCGCTTGCGTAACGTAGAAACGGA

RI-ATCG00180-XLOC_032457-8103-0
AGCGGATAAGTTTCGCGTGAATGGATACTCTGAAATCGAACGAGAAAAAT
RI-ATCG00180-XLOC_032457-8103-1
AGCGGATAAGTTTCGCGTGAATGGATACTCTGAAATCGAACGAGAAAAAT
CONSENSUS
AGCGGATAAGTTTCGCGTGAATGGATACTCTGAAATCGAACGAGAAAAAT

RI-ATCG00180-XLOC_032457-8103-0

TGAATTTGATTAATTCAACTTATAAAACTTTGAAACAATTAGAAAATTAC
RI-ATCG00180-XLOC_032457-8103-1
TGAATTTGATTAATTCAACTTATAAAACTTTGAAACAATTAGAAAATTAC
CONSENSUS
TGAATTTGATTAATTCAACTTATAAAACTTTGAAACAATTAGAAAATTAC

RI-ATCG00180-XLOC_032457-8103-0
AAAAATGAAACCATTCTTTTTGAGCAACAAAGAACAATTAATCAAGTCCG
RI-ATCG00180-XLOC_032457-8103-1
AAAAATGAAACCATTCTTTTTGAGCAACAAAGAACAATTAATCAAGTCCG
CONSENSUS
AAAAATGAAACCATTCTTTTTGAGCAACAAAGAACAATTAATCAAGTCCG

RI-ATCG00180-XLOC_032457-8103-0
CGAACGGGTTTTCCAACAAGCTTTACAAGGAGCTATAGGAACCCTAAATA
RI-ATCG00180-XLOC_032457-8103-1
CGAACGGGTTTTCCAACAAGCTTTACAAGGAGCTATAGGAACCCTAAATA
CONSENSUS
CGAACGGGTTTTCCAACAAGCTTTACAAGGAGCTATAGGAACCCTAAATA

RI-ATCG00180-XLOC_032457-8103-0
GTTGTTTGAGTAATGAGTTACATTTACGTACTATTAATGCAAATATTGGG
RI-ATCG00180-XLOC_032457-8103-1
GTTGTTTGAGTAATGAGTTACATTTACGTACTATTAATGCAAATATTGGG
CONSENSUS
GTTGTTTGAGTAATGAGTTACATTTACGTACTATTAATGCAAATATTGGG

RI-ATCG00180-XLOC_032457-8103-0
ATGTTTGGTACGATGAAAGAAATAACTGATTAATTATTTCCTTACGATTA
RI-ATCG00180-XLOC_032457-8103-1
ATGTTTGGTACGATGAAAGAAATAACTGATTAATTATTTCCTTACGATTA
CONSENSUS
ATGTTTGGTACGATGAAAGAAATAACTGATTAATTATTTCCTTACGATTA

RI-ATCG00180-XLOC_032457-8103-0
TTATAGGCATTATTTTTTTTTCTTCCAACAAAAGAATCAGGACAATACTC
RI-ATCG00180-XLOC_032457-8103-1
TTATAGGCATTATTTTTTTTTCTTCCAACAAAAGAATCAGGACAATACTC
CONSENSUS
TTATAGGCATTATTTTTTTTTCTTCCAACAAAAGAATCAGGACAATACTC

RI-ATCG00180-XLOC_032457-8103-0
ATGGTAACCATTAGAGCCGACGAAATTAGTAATATTATCCGTGAACGTAT
RI-ATCG00180-XLOC_032457-8103-1
ATGGTAACCATTAGAGCCGACGAAATTAGTAATATTATCCGTGAACGTAT
CONSENSUS
ATGGTAACCATTAGAGCCGACGAAATTAGTAATATTATCCGTGAACGTAT

RI-ATCG00180-XLOC_032457-8103-0
TGAGCAATATAATAGAGAAGTAACGATTGTAAATACCGGTACCGTACTTC
RI-ATCG00180-XLOC_032457-8103-1
TGAGCAATATAATAGAGAAGTAACGATTGTAAATACCGGTACCGTACTTC
CONSENSUS
TGAGCAATATAATAGAGAAGTAACGATTGTAAATACCGGTACCGTACTTC

RI-ATCG00180-XLOC_032457-8103-0

AAGTGGGCGATGGCATCGCTCGTATTTATGGTCTTGATGAAGTAATGGCA
RI-ATCG00180-XLOC_032457-8103-1
AAGTGGGCGATGGCATCGCTCGTATTTATGGTCTTGATGAAGTAATGGCA
CONSENSUS
AAGTGGGCGATGGCATCGCTCGTATTTATGGTCTTGATGAAGTAATGGCA

RI-ATCG00180-XLOC_032457-8103-0
GGTGAATTAGTAGAATTTGAGGAGGGTACTATAGGTATTGCCCTTAATTT
RI-ATCG00180-XLOC_032457-8103-1
GGTGAATTAGTAGAATTTGAGGAGGGTACTATAGGTATTGCCCTTAATTT
CONSENSUS
GGTGAATTAGTAGAATTTGAGGAGGGTACTATAGGTATTGCCCTTAATTT

RI-ATCG00180-XLOC_032457-8103-0
AGAATCCAATAATGTTGGTGTTGTATTAATGGGTGACGGTTTGATGATCC
RI-ATCG00180-XLOC_032457-8103-1
AGAATCCAATAATGTTGGTGTTGTATTAATGGGTGACGGTTTGATGATCC
CONSENSUS
AGAATCCAATAATGTTGGTGTTGTATTAATGGGTGACGGTTTGATGATCC

RI-ATCG00180-XLOC_032457-8103-0
AAGAAGGAAGTTCAGTCAAAGCTACGGGAAAAATTGCTCAGATACCGGTG
RI-ATCG00180-XLOC_032457-8103-1
AAGAAGGAAGTTCAGTCAAAGCTACGGGAAAAATTGCTCAGATACCGGTG
CONSENSUS
AAGAAGGAAGTTCAGTCAAAGCTACGGGAAAAATTGCTCAGATACCGGTG

RI-ATCG00180-XLOC_032457-8103-0
AGTGAGGCTTATTTGGGGCGTGTTATAAACGCCTTGGCTAACCCTATTGA
RI-ATCG00180-XLOC_032457-8103-1
AGTGAGGCTTATTTGGGGCGTGTTATAAACGCCTTGGCTAACCCTATTGA
CONSENSUS
AGTGAGGCTTATTTGGGGCGTGTTATAAACGCCTTGGCTAACCCTATTGA

RI-ATCG00180-XLOC_032457-8103-0
TGGTCGAGGTAAGATTTCCGCTTCTGAATCTCGGTTAATTGAATCTCCTG
RI-ATCG00180-XLOC_032457-8103-1
TGGTCGAGGTAAGATTTCCGCTTCTGAATCTCGGTTAATTGAATCTCCTG
CONSENSUS
TGGTCGAGGTAAGATTTCCGCTTCTGAATCTCGGTTAATTGAATCTCCTG

RI-ATCG00180-XLOC_032457-8103-0
CCCCAGGTATTATTTCGAGACGTTCTGTATATGAGCCTCTTCAAACAGGA
RI-ATCG00180-XLOC_032457-8103-1
CCCCAGGTATTATTTCGAGACGTTCTGTATATGAGCCTCTTCAAACAGGA
CONSENSUS
CCCCAGGTATTATTTCGAGACGTTCTGTATATGAGCCTCTTCAAACAGGA

RI-ATCG00180-XLOC_032457-8103-0
CTTATTGCTATTGATTCCATGATCCCTATAGGACGCGGCCAGCGAGAATT
RI-ATCG00180-XLOC_032457-8103-1
CTTATTGCTATTGATTCCATGATCCCTATAGGACGCGGCCAGCGAGAATT
CONSENSUS
CTTATTGCTATTGATTCCATGATCCCTATAGGACGCGGCCAGCGAGAATT

RI-ATCG00180-XLOC_032457-8103-0

AATTATTGGTGACAGACAGACCGGTAAAACAGCAGTAGCCACAGATACAA
RI-ATCG00180-XLOC_032457-8103-1
AATTATTGGTGACAGACAGACCGGTAAAACAGCAGTAGCCACAGATACAA
CONSENSUS
AATTATTGGTGACAGACAGACCGGTAAAACAGCAGTAGCCACAGATACAA

RI-ATCG00180-XLOC_032457-8103-0
TTCTCAATCAACAAGGCCAAAATGTAATATGTGTTTATGTAGCTATTGGT
RI-ATCG00180-XLOC_032457-8103-1
TTCTCAATCAACAAGGCCAAAATGTAATATGTGTTTATGTAGCTATTGGT
CONSENSUS
TTCTCAATCAACAAGGCCAAAATGTAATATGTGTTTATGTAGCTATTGGT

RI-ATCG00180-XLOC_032457-8103-0
CAAAAAGCTTCTTCCGTGGCTCAGGTAGTGACCAGTTTACAGGAACGAGG
RI-ATCG00180-XLOC_032457-8103-1
CAAAAAGCTTCTTCCGTGGCTCAGGTAGTGACCAGTTTACAGGAACGAGG
CONSENSUS
CAAAAAGCTTCTTCCGTGGCTCAGGTAGTGACCAGTTTACAGGAACGAGG

RI-ATCG00180-XLOC_032457-8103-0
GGCAATGGAATACACTATTGTGGTAGCTGAAACGGCCGATTCCCCAGCTA
RI-ATCG00180-XLOC_032457-8103-1
GGCAATGGAATACACTATTGTGGTAGCTGAAACGGCCGATTCCCCAGCTA
CONSENSUS
GGCAATGGAATACACTATTGTGGTAGCTGAAACGGCCGATTCCCCAGCTA

RI-ATCG00180-XLOC_032457-8103-0
CGTTACAATACCTCGCGCCTTATACAGGAGCAGCCTTGGCTGAATATTTT
RI-ATCG00180-XLOC_032457-8103-1
CGTTACAATACCTCGCGCCTTATACAGGAGCAGCCTTGGCTGAATATTTT
CONSENSUS
CGTTACAATACCTCGCGCCTTATACAGGAGCAGCCTTGGCTGAATATTTT

RI-ATCG00180-XLOC_032457-8103-0
ATGTACCGTGAACAACACACTTTAATAATTTATGATGATCTTTCCAAACA
RI-ATCG00180-XLOC_032457-8103-1
ATGTACCGTGAACAACACACTTTAATAATTTATGATGATCTTTCCAAACA
CONSENSUS
ATGTACCGTGAACAACACACTTTAATAATTTATGATGATCTTTCCAAACA

RI-ATCG00180-XLOC_032457-8103-0
AGCACAAGCTTATCGACAAATGTCTCTTCTATTACGAAGACCGCCGGGTC
RI-ATCG00180-XLOC_032457-8103-1
AGCACAAGCTTATCGACAAATGTCTCTTCTATTACGAAGACCGCCGGGTC
CONSENSUS
AGCACAAGCTTATCGACAAATGTCTCTTCTATTACGAAGACCGCCGGGTC

RI-ATCG00180-XLOC_032457-8103-0
GTGAAGCTTATCCAGGAGATGTTTTTTATTTACATTCACGTCTTTTAGAA
RI-ATCG00180-XLOC_032457-8103-1
GTGAAGCTTATCCAGGAGATGTTTTTTATTTACATTCACGTCTTTTAGAA
CONSENSUS
GTGAAGCTTATCCAGGAGATGTTTTTTATTTACATTCACGTCTTTTAGAA

RI-ATCG00180-XLOC_032457-8103-0

AGAGCCGCTAAATTAAGCTCTCAATTAGGTGAAGGGAGTATGACTGCCTT
RI-ATCG00180-XLOC_032457-8103-1
AGAGCCGCTAAATTAAGCTCTCAATTAGGTGAAGGGAGTATGACTGCCTT
CONSENSUS
AGAGCCGCTAAATTAAGCTCTCAATTAGGTGAAGGGAGTATGACTGCCTT

RI-ATCG00180-XLOC_032457-8103-0
ACCAATCGTCGAGACCCAGTCAGGAGATGTTTCAGCTTATATTCCTACTA
RI-ATCG00180-XLOC_032457-8103-1
ACCAATCGTCGAGACCCAGTCAGGAGATGTTTCAGCTTATATTCCTACTA
CONSENSUS
ACCAATCGTCGAGACCCAGTCAGGAGATGTTTCAGCTTATATTCCTACTA

RI-ATCG00180-XLOC_032457-8103-0
ATGTAATTTCCATTACAGATGGACAAATATTCTTATCCGCCGATCTTTTT
RI-ATCG00180-XLOC_032457-8103-1
ATGTAATTTCCATTACAGATGGACAAATATTCTTATCCGCCGATCTTTTT
CONSENSUS
ATGTAATTTCCATTACAGATGGACAAATATTCTTATCCGCCGATCTTTTT

RI-ATCG00180-XLOC_032457-8103-0
AATGCTGGAATCAGACCTGCTATTAATGTAGGGATTTCTGTCTCGAGAGT
RI-ATCG00180-XLOC_032457-8103-1
AATGCTGGAATCAGACCTGCTATTAATGTAGGGATTTCTGTCTCGAGAGT
CONSENSUS
AATGCTGGAATCAGACCTGCTATTAATGTAGGGATTTCTGTCTCGAGAGT

RI-ATCG00180-XLOC_032457-8103-0
AGGATCCGCCGCTCAAATTAAAGCTATGAAACAGGTAGCTGGAAAATTAA
RI-ATCG00180-XLOC_032457-8103-1
AGGATCCGCCGCTCAAATTAAAGCTATGAAACAGGTAGCTGGAAAATTAA
CONSENSUS
AGGATCCGCCGCTCAAATTAAAGCTATGAAACAGGTAGCTGGAAAATTAA

RI-ATCG00180-XLOC_032457-8103-0
AATTGGAATTGGCTCAATTCGCTGAATTAGAAGCCTTTTCCCAATTTTCT
RI-ATCG00180-XLOC_032457-8103-1
AATTGGAATTGGCTCAATTCGCTGAATTAGAAGCCTTTTCCCAATTTTCT
CONSENSUS
AATTGGAATTGGCTCAATTCGCTGAATTAGAAGCCTTTTCCCAATTTTCT

RI-ATCG00180-XLOC_032457-8103-0
TCTGATCTCGATAAAGCTACTCAGAATCAATTGGCAAGAGGTCAACGATT
RI-ATCG00180-XLOC_032457-8103-1
TCTGATCTCGATAAAGCTACTCAGAATCAATTGGCAAGAGGTCAACGATT
CONSENSUS
TCTGATCTCGATAAAGCTACTCAGAATCAATTGGCAAGAGGTCAACGATT

RI-ATCG00180-XLOC_032457-8103-0
GCGTGAGTTACTGAAACAATCCCAATCAGCCCCCCTCACAGTGGAAGAAC
RI-ATCG00180-XLOC_032457-8103-1
GCGTGAGTTACTGAAACAATCCCAATCAGCCCCCCTCACAGTGGAAGAAC
CONSENSUS
GCGTGAGTTACTGAAACAATCCCAATCAGCCCCCCTCACAGTGGAAGAAC

RI-ATCG00180-XLOC_032457-8103-0

AGATAATGACCATTTATACCGGAACAAATGGTTATCTGGATGGATTAGAA
RI-ATCG00180-XLOC_032457-8103-1
AGATAATGACCATTTATACCGGAACAAATGGTTATCTGGATGGATTAGAA
CONSENSUS
AGATAATGACCATTTATACCGGAACAAATGGTTATCTGGATGGATTAGAA

RI-ATCG00180-XLOC_032457-8103-0
ATTGGACAAGTAAGAAAATTTCTCGTTCAATTACGCACTTACTTAAAAAC
RI-ATCG00180-XLOC_032457-8103-1
ATTGGACAAGTAAGAAAATTTCTCGTTCAATTACGCACTTACTTAAAAAC
CONSENSUS
ATTGGACAAGTAAGAAAATTTCTCGTTCAATTACGCACTTACTTAAAAAC

RI-ATCG00180-XLOC_032457-8103-0
GAATAAACCTCAGTTTCAAGAAATCATAGCCTCTACCAAGACATTAACCG
RI-ATCG00180-XLOC_032457-8103-1
GAATAAACCTCAGTTTCAAGAAATCATAGCCTCTACCAAGACATTAACCG
CONSENSUS
GAATAAACCTCAGTTTCAAGAAATCATAGCCTCTACCAAGACATTAACCG

RI-ATCG00180-XLOC_032457-8103-0
CTGAAGCAGAAAGCTTTTTGAAAGAAGGTATTCAAGAGCAACTAGAACGT
RI-ATCG00180-XLOC_032457-8103-1
CTGAAGCAGAAAGCTTTTTGAAAGAAGGTATTCAAGAGCAACTAGAACGT
CONSENSUS
CTGAAGCAGAAAGCTTTTTGAAAGAAGGTATTCAAGAGCAACTAGAACGT

RI-ATCG00180-XLOC_032457-8103-0
TTCCTACTTCAGGAGAAAGTATAAAAAAACGAAACGGATTATTCTTAATTT
RI-ATCG00180-XLOC_032457-8103-1
TTCCTACTTCAGGAGAAAGTATAAAAAAACGAAACGGATTATTCTTAATTT
CONSENSUS
TTCCTACTTCAGGAGAAAGTATAAAAAAACGAAACGGATTATTCTTAATTT

RI-ATCG00180-XLOC_032457-8103-0
TTGATTCTTTAGTCAATTTTACTCTTTAATTTTAATTAATTTTTTAAGAA
RI-ATCG00180-XLOC_032457-8103-1
TTGATTCTTTAGTCAATTTTACTCTTTAATTTTAATTAATTTTTTAAGAA
CONSENSUS
TTGATTCTTTAGTCAATTTTACTCTTTAATTTTAATTAATTTTTTAAGAA

RI-ATCG00180-XLOC_032457-8103-0
ATTCTAGAAATAGAAGTCTATAAGTCAAGTATATATAATAGAAAGAAGTA
RI-ATCG00180-XLOC_032457-8103-1
ATTCTAGAAATAGAAGTCTATAAGTCAAGTATATATAATAGAAAGAAGTA
CONSENSUS
ATTCTAGAAATAGAAGTCTATAAGTCAAGTATATATAATAGAAAGAAGTA

RI-ATCG00180-XLOC_032457-8103-0
TCTAACTAATATCTGTTTAATATTAAATCTTAAAGCATTCAGAATTTCTT
RI-ATCG00180-XLOC_032457-8103-1
TCTAACTAATATCTGTTTAATATTAAATCTTAAAGCATTCAGAATTTCTT
CONSENSUS
TCTAACTAATATCTGTTTAATATTAAATCTTAAAGCATTCAGAATTTCTT

RI-ATCG00180-XLOC_032457-8103-0

TCTTATTCTATTTATTTCTTATCTTTTTTCGAAATAGATATTATATATAA
RI-ATCG00180-XLOC_032457-8103-1
TCTTATTCTATTTATTTCTTATCTTTTTTCGAAATAGATATTATATATAA
CONSENSUS
TCTTATTCTATTTATTTCTTATCTTTTTTCGAAATAGATATTATATATAA

RI-ATCG00180-XLOC_032457-8103-0
TATATAGAAATGGAAATAATAGATAAATATCAATATAGATATATTTATAT
RI-ATCG00180-XLOC_032457-8103-1
TATATAGAAATGGAAATAATAGATAAATATCAATATAGATATATTTATAT
CONSENSUS
TATATAGAAATGGAAATAATAGATAAATATCAATATAGATATATTTATAT

RI-ATCG00180-XLOC_032457-8103-0
TGCGTCCAATAGGATTTGAACCTATACCAAAGGTTTAGAAGACCTATGTC
RI-ATCG00180-XLOC_032457-8103-1
TGCGTCCAATAGGATTTGAACCTATACCAAAGGTTTAGAAGACCTATGTC
CONSENSUS
TGCGTCCAATAGGATTTGAACCTATACCAAAGGTTTAGAAGACCTATGTC

RI-ATCG00180-XLOC_032457-8103-0
CTATCCATTAGACAATGGACGCTTTTCGCTTTTTTTTACTTTCCTATTGT
RI-ATCG00180-XLOC_032457-8103-1
CTATCCATTAGACAATGGACGCTTTTCGCTTTTTTTTACTTTCCTATTGT
CONSENSUS
CTATCCATTAGACAATGGACGCTTTTCGCTTTTTTTTACTTTCCTATTGT

RI-ATCG00180-XLOC_032457-8103-0
TAAAAAAAAAGAATGTGCAAAATCTTTTTAGCAATTGTGTATTGAAGTGA
RI-ATCG00180-XLOC_032457-8103-1
TAAAAAAAAAGAATGTGCAAAATCTTTTTAGCAATTGTGTATTGAAGTGA
CONSENSUS
TAAAAAAAAAGAATGTGCAAAATCTTTTTAGCAATTGTGTATTGAAGTGA

RI-ATCG00180-XLOC_032457-8103-0
ATTCAATACACAATTGCTATTAGACAATTCTAATATAACAAATTTTCTCT
RI-ATCG00180-XLOC_032457-8103-1
ATTCAATACACAATTGCTATTAGACAATTCTAATATAACAAATTTTCTCT
CONSENSUS
ATTCAATACACAATTGCTATTAGACAATTCTAATATAACAAATTTTCTCT

RI-ATCG00180-XLOC_032457-8103-0
AATAAAAAAAAAGGGCAATTGTGAATTTAGAGCGGGTAGCGGGAATCGAA
RI-ATCG00180-XLOC_032457-8103-1
AATAAAAAAAAAGGGCAATTGTGAATTTAGAGCGGGTAGCGGGAATCGAA
CONSENSUS
AATAAAAAAAAAGGGCAATTGTGAATTTAGAGCGGGTAGCGGGAATCGAA

RI-ATCG00180-XLOC_032457-8103-0
CCCGCATCGTTAGCTTGGAAGGCTAAGGGTTTTAGTCGACGTCGATTGAT
RI-ATCG00180-XLOC_032457-8103-1
CCCGCATCGTTAGCTTGGAAGGCTAAGGGTTTTAGTCGACGTCGATTGAT
CONSENSUS
CCCGCATCGTTAGCTTGGAAGGCTAAGGGTTTTAGTCGACGTCGATTGAT

RI-ATCG00180-XLOC_032457-8103-0

CATTTTTATATTTTTAACGTCTCTAATTCAAAACCGAACATGAAACTTTG
RI-ATCG00180-XLOC_032457-8103-1
CATTTTTATATTTTTAACGTCTCTAATTCAAAACCGAACATGAAACTTTG
CONSENSUS
CATTTTTATATTTTTAACGTCTCTAATTCAAAACCGAACATGAAACTTTG

RI-ATCG00180-XLOC_032457-8103-0
GTTTCATTCGGCTCCTTTATGATGGATGGAGAAATTCCCAAATAAAAAAA
RI-ATCG00180-XLOC_032457-8103-1
GTTTCATTCGGCTCCTTTATGATGGATGGAGAAATTCCCAAATAAAAAAA
CONSENSUS
GTTTCATTCGGCTCCTTTATGATGGATGGAGAAATTCCCAAATAAAAAAA

RI-ATCG00180-XLOC_032457-8103-0
GACGGGAACGAAATAAAAATTCGACCCATAACATCTATGTGAGCTTTTTT
RI-ATCG00180-XLOC_032457-8103-1
GACGGGAACGAAATAAAAATTCGACCCATAACATCTATGTGAGCTTTTTT
CONSENSUS
GACGGGAACGAAATAAAAATTCGACCCATAACATCTATGTGAGCTTTTTT

RI-ATCG00180-XLOC_032457-8103-0
CCGTCTGGGTACTTTCACAGAAAATTTTGCTTTCTAGATGATCCTTCTAG
RI-ATCG00180-XLOC_032457-8103-1
CCGTCTGGGTACTTTCACAGAAAATTTTGCTTTCTAGATGATCCTTCTAG
CONSENSUS
CCGTCTGGGTACTTTCACAGAAAATTTTGCTTTCTAGATGATCCTTCTAG

RI-ATCG00180-XLOC_032457-8103-0
AAGATAGATCGGGCCAACTCGAACAATCTTTCTAGTTACTTCGTTCTTTA
RI-ATCG00180-XLOC_032457-8103-1
AAGATAGATCGGGCCAACTCGAACAATCTTTCTAGTTACTTCGTTCTTTA
CONSENSUS
AAGATAGATCGGGCCAACTCGAACAATCTTTCTAGTTACTTCGTTCTTTA

RI-ATCG00180-XLOC_032457-8103-0
TTTCTATTTAACGGAATCCTTAGGAAAAGTATTTGGTTTCTACCGAGCTA
RI-ATCG00180-XLOC_032457-8103-1
TTTCTATTTAACGGAATCCTTAGGAAAAGTATTTGGTTTCTACCGAGCTA
CONSENSUS
TTTCTATTTAACGGAATCCTTAGGAAAAGTATTTGGTTTCTACCGAGCTA

RI-ATCG00180-XLOC_032457-8103-0
AAACAATATAATATGTCGATGTCTTTAGTAAACCAAAGTTCTCGTTTAAT
RI-ATCG00180-XLOC_032457-8103-1
AAACAATATAATATGTCGATGTCTTTAGTAAACCAAAGTTCTCGTTTAAT
CONSENSUS
AAACAATATAATATGTCGATGTCTTTAGTAAACCAAAGTTCTCGTTTAAT

RI-ATCG00180-XLOC_032457-8103-0
AGCTATTTTGCTTCAATTTTTCTATACCAAACAATGAATAGAACTGAAGA
RI-ATCG00180-XLOC_032457-8103-1
AGCTATTTTGCTTCAATTTTTCTATACCAAACAATGAATAGAACTGAAGA
CONSENSUS
AGCTATTTTGCTTCAATTTTTCTATACCAAACAATGAATAGAACTGAAGA

RI-ATCG00180-XLOC_032457-8103-0

TTTAGTTACGATTAGAAAGACACTTTTCTAGCTTTATCCATGGATCCTCT
RI-ATCG00180-XLOC_032457-8103-1
TTTAGTTACGATTAGAAAGACACTTTTCTAGCTTTATCCATGGATCCTCT
CONSENSUS
TTTAGTTACGATTAGAAAGACACTTTTCTAGCTTTATCCATGGATCCTCT

RI-ATCG00180-XLOC_032457-8103-0
TGTTATACTTATTGAATTGTAAATAGTCATCCAATTCAAAAATTATGTTT
RI-ATCG00180-XLOC_032457-8103-1
TGTTATACTTATTGAATTGTAAATAGTCATCCAATTCAAAAATTATGTTT
CONSENSUS
TGTTATACTTATTGAATTGTAAATAGTCATCCAATTCAAAAATTATGTTT

RI-ATCG00180-XLOC_032457-8103-0
CGGAATTTCATAATCCAAATTTACAATTGATTAGAGTCTTTGGATATAAA
RI-ATCG00180-XLOC_032457-8103-1
CGGAATTTCATAATCCAAATTTACAATTGATTAGAGTCTTTGGATATAAA
CONSENSUS
CGGAATTTCATAATCCAAATTTACAATTGATTAGAGTCTTTGGATATAAA

RI-ATCG00180-XLOC_032457-8103-0
TTACGAGAATCTATAATCTTCCTTGAATCTTTCATTGAAAGGGAAAGGAC
RI-ATCG00180-XLOC_032457-8103-1
TTACGAGAATCTATAATCTTCCTTGAATCTTTCATTGAAAGGGAAAGGAC
CONSENSUS
TTACGAGAATCTATAATCTTCCTTGAATCTTTCATTGAAAGGGAAAGGAC

RI-ATCG00180-XLOC_032457-8103-0
TAAATCCTTTTAAGAAATAAAATTTTTGATCGGAAGATTAATCAAACCGA
RI-ATCG00180-XLOC_032457-8103-1
TAAATCCTTTTAAGAAATAAAATTTTTGATCGGAAGATTAATCAAACCGA
CONSENSUS
TAAATCCTTTTAAGAAATAAAATTTTTGATCGGAAGATTAATCAAACCGA

RI-ATCG00180-XLOC_032457-8103-0
GAGACCCTTTAACTATTAAAGGGATTAAAGGAACGAATCACACTTTTACC
RI-ATCG00180-XLOC_032457-8103-1
GAGACCCTTTAACTATTAAAGGGATTAAAGGAACGAATCACACTTTTACC
CONSENSUS
GAGACCCTTTAACTATTAAAGGGATTAAAGGAACGAATCACACTTTTACC

RI-ATCG00180-XLOC_032457-8103-0
ACTAAACTATACCCGCTACAATGCAATTATTGCATATAAATTAACCTTTT
RI-ATCG00180-XLOC_032457-8103-1
ACTAAACTATACCCGCTACAATGCAATTATTGCATATAAATTAACCTTTT
CONSENSUS
ACTAAACTATACCCGCTACAATGCAATTATTGCATATAAATTAACCTTTT

RI-ATCG00180-XLOC_032457-8103-0
GTCGAACAAAGTAATCGGGGGTGTAATAAAAAAATCTGAAAAAATTTAGA
RI-ATCG00180-XLOC_032457-8103-1
GTCGAACAAAGTAATCGGGGGTGTAATAAAAAAATCTGAAAAAATTTAGA
CONSENSUS
GTCGAACAAAGTAATCGGGGGTGTAATAAAAAAATCTGAAAAAATTTAGA

RI-ATCG00180-XLOC_032457-8103-0

AAAAACTAGAGTTGACTTAAGAAAATTAGTAAAAGCGAATTCAATTCCAC
RI-ATCG00180-XLOC_032457-8103-1
AAAAACTAGAGTTGACTTAAGAAAATTAGTAAAAGCGAATTCAATTCCAC
CONSENSUS
AAAAACTAGAGTTGACTTAAGAAAATTAGTAAAAGCGAATTCAATTCCAC

RI-ATCG00180-XLOC_032457-8103-0
TTTTTTTTTTATTTCAAATCTTTTTTTATCTAAAAATCCATCGGATGAGT
RI-ATCG00180-XLOC_032457-8103-1
TTTTTTTTTTATTTCAAATCTTTTTTTATCTAAAAATCCATCGGATGAGT
CONSENSUS
TTTTTTTTTTATTTCAAATCTTTTTTTATCTAAAAATCCATCGGATGAGT

RI-ATCG00180-XLOC_032457-8103-0
CTTTTTAATTTTAACAAAAAAAGGCCCGGCTGGGGACTGACCAGGCCAGG
RI-ATCG00180-XLOC_032457-8103-1
CTTTTTAATTTTAACAAAAAAAGGCCCGGCTGGGGACTGACCAGGCCAGG
CONSENSUS
CTTTTTAATTTTAACAAAAAAAGGCCCGGCTGGGGACTGACCAGGCCAGG

RI-ATCG00180-XLOC_032457-8103-0
CTATTAAAATAAAAAAGATCTTTTACTTGTGTTTTTGTTTTGACGAGACA
RI-ATCG00180-XLOC_032457-8103-1
CTATTAAAATAAAAAAGATCTTTTACTTGTGTTTTTGTTTTGACGAGACA
CONSENSUS
CTATTAAAATAAAAAAGATCTTTTACTTGTGTTTTTGTTTTGACGAGACA

RI-ATCG00180-XLOC_032457-8103-0
AAATAAAAAAAGGATTCTCTATTTCTATGATTATGGTTTTATTTATTTCT
RI-ATCG00180-XLOC_032457-8103-1
AAATAAAAAAAGGATTCTCTATTTCTATGATTATGGTTTTATTTATTTCT
CONSENSUS
AAATAAAAAAAGGATTCTCTATTTCTATGATTATGGTTTTATTTATTTCT

RI-ATCG00180-XLOC_032457-8103-0
TTTTTGAGTTCGCGCCTTTTTTATCTAATCTTTAGCTAAATTTTTAATGT
RI-ATCG00180-XLOC_032457-8103-1
TTTTTGAGTTCGCGCCTTTTTTATCTAATCTTTAGCTAAATTTTTAATGT
CONSENSUS
TTTTTGAGTTCGCGCCTTTTTTATCTAATCTTTAGCTAAATTTTTAATGT

RI-ATCG00180-XLOC_032457-8103-0
AACTAGAATTGCTGAGAAAGTTCTATAAAAACAGTTATATAATAGTAATA
RI-ATCG00180-XLOC_032457-8103-1
AACTAGAATTGCTGAGAAAGTTCTATAAAAACAGTTATATAATAGTAATA
CONSENSUS
AACTAGAATTGCTGAGAAAGTTCTATAAAAACAGTTATATAATAGTAATA

RI-ATCG00180-XLOC_032457-8103-0
TATATATTATATAATATATATTATATATATATTATATATATAATAGAGGA
RI-ATCG00180-XLOC_032457-8103-1
TATATATTATATAATATATATTATATATATATTATATATATAATAGAGGA
CONSENSUS
TATATATTATATAATATATATTATATATATATTATATATATAATAGAGGA

RI-ATCG00180-XLOC_032457-8103-0

TTTATATAATTTATATATAGGATATATATTATATATATATTATATATTTA
RI-ATCG00180-XLOC_032457-8103-1
TTTATATAATTTATATATAGGATATATATTATATATATATTATATATTTA
CONSENSUS
TTTATATAATTTATATATAGGATATATATTATATATATATTATATATTTA

RI-ATCG00180-XLOC_032457-8103-0
AATATAGGATATATATATTTATATAATCAAATAATCAAAAAGAATATATA
RI-ATCG00180-XLOC_032457-8103-1
AATATAGGATATATATATTTATATAATCAAATAATCAAAAAGAATATATA
CONSENSUS
AATATAGGATATATATATTTATATAATCAAATAATCAAAAAGAATATATA

RI-ATCG00180-XLOC_032457-8103-0
TATATATAATAATATAATAAAATATAGAAAAAAATATAGAAAATGAAAAA
RI-ATCG00180-XLOC_032457-8103-1
TATATATAATAATATAATAAAATATAGAAAAAAATATAGAAAATGAAAAA
CONSENSUS
TATATATAATAATATAATAAAATATAGAAAAAAATATAGAAAATGAAAAA

RI-ATCG00180-XLOC_032457-8103-0
AAATATATATATATATAATATAAAGATAAAGAATATCTATAACAAAAAAA
RI-ATCG00180-XLOC_032457-8103-1
AAATATATATATATATAATATAAAGATAAAGAATATCTATAACAAAAAAA
CONSENSUS
AAATATATATATATATAATATAAAGATAAAGAATATCTATAACAAAAAAA

RI-ATCG00180-XLOC_032457-8103-0
GAAAGCTTTTTAAGAATAAAGTGGAGAAGGTTCTTTTTCAAGCCTTTTTT
RI-ATCG00180-XLOC_032457-8103-1
GAAAGCTTTTTAAGAATAAAGTGGAGAAGGTTCTTTTTCAAGCCTTTTTT
CONSENSUS
GAAAGCTTTTTAAGAATAAAGTGGAGAAGGTTCTTTTTCAAGCCTTTTTT

RI-ATCG00180-XLOC_032457-8103-0
TTTTTTTATGGAACCTAATTAAATATCCCTTTTCGATTAGGAGAGATGGC
RI-ATCG00180-XLOC_032457-8103-1
TTTTTTTATGGAACCTAATTAAATATCCCTTTTCGATTAGGAGAGATGGC
CONSENSUS
TTTTTTTATGGAACCTAATTAAATATCCCTTTTCGATTAGGAGAGATGGC

RI-ATCG00180-XLOC_032457-8103-0
TGAGTGGACTAAAGCGTTGGATTGCTAATCCATTGTACGAGTTAATCGTA
RI-ATCG00180-XLOC_032457-8103-1
TGAGTGGACTAAAGCGTTGGATTGCTAATCCATTGTACGAGTTAATCGTA
CONSENSUS
TGAGTGGACTAAAGCGTTGGATTGCTAATCCATTGTACGAGTTAATCGTA

RI-ATCG00180-XLOC_032457-8103-0
CCGAGGGTTCGAATCCCTCTCTTTCCCCTTCTCCTTTTGATGGTGTGTAA
RI-ATCG00180-XLOC_032457-8103-1
CCGAGGGTTCGAATCCCTCTCTTTCCCCTTCTCCTTTTGATGGTGTGTAA
CONSENSUS
CCGAGGGTTCGAATCCCTCTCTTTCCCCTTCTCCTTTTGATGGTGTGTAA

RI-ATCG00180-XLOC_032457-8103-0

TAGATAAAATCCTAATAAAATTCGAGCATTTCCACGAATTAAATAAAGCA
RI-ATCG00180-XLOC_032457-8103-1
TAGATAAAATCCTAATAAAATTCGAGCATTTCCACGAATTAAATAAAGCA
CONSENSUS
TAGATAAAATCCTAATAAAATTCGAGCATTTCCACGAATTAAATAAAGCA

RI-ATCG00180-XLOC_032457-8103-0
ATAAAAAAACTTTCTTTTTTATTCTTCACGTCCCGGATTACGTCCTGGAT
RI-ATCG00180-XLOC_032457-8103-1
ATAAAAAAACTTTCTTTTTTATTCTTCACGTCCCGGATTACGTCCTGGAT
CONSENSUS
ATAAAAAAACTTTCTTTTTTATTCTTCACGTCCCGGATTACGTCCTGGAT

RI-ATCG00180-XLOC_032457-8103-0
CATTAGATAGGAATCCAAATATGAAGAGAGAAACAAAGAATATAACTACA
RI-ATCG00180-XLOC_032457-8103-1
CATTAGATAGGAATCCAAATATGAAGAGAGAAACAAAGAATATAACTACA
CONSENSUS
CATTAGATAGGAATCCAAATATGAAGAGAGAAACAAAGAATATAACTACA

RI-ATCG00180-XLOC_032457-8103-0
GTGTATACAAAAAGTTTGAGAGTAAGCATTACACAATCTCCAAGATCTTA
RI-ATCG00180-XLOC_032457-8103-1
GTGTATACAAAAAGTTTGAGAGTAAGCATTACACAATCTCCAAGATCTTA
CONSENSUS
GTGTATACAAAAAGTTTGAGAGTAAGCATTACACAATCTCCAAGATCTTA

RI-ATCG00180-XLOC_032457-8103-0
CTAAAAAAAAAGAGAATAGATTCTCTATTTTTATATCAAATATCTAATTT
RI-ATCG00180-XLOC_032457-8103-1
CTAAAAAAAAAGAGAATAGATTCTCTATTTTTATATCAAATATCTAATTT
CONSENSUS
CTAAAAAAAAAGAGAATAGATTCTCTATTTTTATATCAAATATCTAATTT

RI-ATCG00180-XLOC_032457-8103-0
TGATACCAATAAAAAAAAAGGTTTCAGAAAGTTATTTGTAATTAAGATAA
RI-ATCG00180-XLOC_032457-8103-1
TGATACCAATAAAAAAAAAGGTTTCAGAAAGTTATTTGTAATTAAGATAA
CONSENSUS
TGATACCAATAAAAAAAAAGGTTTCAGAAAGTTATTTGTAATTAAGATAA

RI-ATCG00180-XLOC_032457-8103-0
TAGTCGAATCTTTCATATTCAAACAGATTTGCATACCAACATCTAGATAT
RI-ATCG00180-XLOC_032457-8103-1
TAGTCGAATCTTTCATATTCAAACAGATTTGCATACCAACATCTAGATAT
CONSENSUS
TAGTCGAATCTTTCATATTCAAACAGATTTGCATACCAACATCTAGATAT

RI-ATCG00180-XLOC_032457-8103-0
TTTTTTGGTGAACTTGAATCTAATTAGGTTCTTTCATTTAGGGAAAAGAG
RI-ATCG00180-XLOC_032457-8103-1
TTTTTTGGTGAACTTGAATCTAATTAGGTTCTTTCATTTAGGGAAAAGAG
CONSENSUS
TTTTTTGGTGAACTTGAATCTAATTAGGTTCTTTCATTTAGGGAAAAGAG

RI-ATCG00180-XLOC_032457-8103-0

GATAAAATTTAGGAAATAGAAATGATCCAGATTTAGTATGGCTACCAAAA
RI-ATCG00180-XLOC_032457-8103-1
GATAAAATTTAGGAAATAGAAATGATCCAGATTTAGTATGGCTACCAAAA
CONSENSUS
GATAAAATTTAGGAAATAGAAATGATCCAGATTTAGTATGGCTACCAAAA

RI-ATCG00180-XLOC_032457-8103-0
AAATTAAATGTTTGAATTGAGAGTTCGTTCATACGTCAAGATTTTTTGGA
RI-ATCG00180-XLOC_032457-8103-1
AAATTAAATGTTTGAATTGAGAGTTCGTTCATACGTCAAGATTTTTTGGA
CONSENSUS
AAATTAAATGTTTGAATTGAGAGTTCGTTCATACGTCAAGATTTTTTGGA

RI-ATCG00180-XLOC_032457-8103-0
TCTTTTGAATTGTTGGAAAAATCAAAATCGTGAATTTGTTTAAGACAGTA
RI-ATCG00180-XLOC_032457-8103-1
TCTTTTGAATTGTTGGAAAAATCAAAATCGTGAATTTGTTTAAGACAGTA
CONSENSUS
TCTTTTGAATTGTTGGAAAAATCAAAATCGTGAATTTGTTTAAGACAGTA

RI-ATCG00180-XLOC_032457-8103-0
TTAAGAATTTTATCGAAAACTTACAGCGGCTTGCCAAACAAAGGCTAAGA
RI-ATCG00180-XLOC_032457-8103-1
TTAAGAATTTTATCGAAAACTTACAGCGGCTTGCCAAACAAAGGCTAAGA
CONSENSUS
TTAAGAATTTTATCGAAAACTTACAGCGGCTTGCCAAACAAAGGCTAAGA

RI-ATCG00180-XLOC_032457-8103-0
GAAGAAAGAAAAGAGGTATTACGGGCATAACATCTACGATTGGATTCAAA
RI-ATCG00180-XLOC_032457-8103-1
GAAGAAAGAAAAGAGGTATTACGGGCATAACATCTACGATTGGATTCAAA
CONSENSUS
GAAGAAAGAAAAGAGGTATTACGGGCATAACATCTACGATTGGATTCAAA

RI-ATCG00180-XLOC_032457-8103-0
AAGGCGTAGGCCTCTGGCAATTTGGCGACTAAAAAAGTGCTTGAAAAAAG
RI-ATCG00180-XLOC_032457-8103-1
AAGGCGTAGGCCTCTGGCAATTTGGCGACTAAAAAAGTGCTTGAAAAAAG
CONSENSUS
AAGGCGTAGGCCTCTGGCAATTTGGCGACTAAAAAAGTGCTTGAAAAAAG

RI-ATCG00180-XLOC_032457-8103-0
GGTAGAATTAAAAAAAAATACAGATCAAATTAAATATATTAAGCATAACAA
RI-ATCG00180-XLOC_032457-8103-1
GGTAGAATTAAAAAAAAATACAGATCAAATTAAATATATTAAGCATAACAA
CONSENSUS
GGTAGAATTAAAAAAAAATACAGATCAAATTAAATATATTAAGCATAACAA

RI-ATCG00180-XLOC_032457-8103-0
AAATTGGTGTTCTTGTGGATAATTTAATTTTGATTGAGTTTTTAATATAT
RI-ATCG00180-XLOC_032457-8103-1
AAATTGGTGTTCTTGTGGATAATTTAATTTTGATTGAGTTTTTAATATAT
CONSENSUS
AAATTGGTGTTCTTGTGGATAATTTAATTTTGATTGAGTTTTTAATATAT

RI-ATCG00180-XLOC_032457-8103-0

TTTTTATATAAGGAAAAAATAAAGAAAGATAGTCTAAAAAGACTTTGTTG
RI-ATCG00180-XLOC_032457-8103-1
TTTTTATATAAGGAAAAAATAAAGAAAGATAGTCTAAAAAGACTTTGTTG
CONSENSUS
TTTTTATATAAGGAAAAAATAAAGAAAGATAGTCTAAAAAGACTTTGTTG

RI-ATCG00180-XLOC_032457-8103-0
AACTTACTCAATCAAAAATCCTTTTATTCTCTTATGAGAATGAAAGAAAT
RI-ATCG00180-XLOC_032457-8103-1
AACTTACTCAATCAAAAATCCTTTTATTCTCTTATGAGAATGAAAGAAAT
CONSENSUS
AACTTACTCAATCAAAAATCCTTTTATTCTCTTATGAGAATGAAAGAAAT

RI-ATCG00180-XLOC_032457-8103-0
AATATTTTCATACAATATCTTAATTGAATTCTATGTAATGATCAATAAAG
RI-ATCG00180-XLOC_032457-8103-1
AATATTTTCATACAATATCTTAATTGAATTCTATGTAATGATCAATAAAG
CONSENSUS
AATATTTTCATACAATATCTTAATTGAATTCTATGTAATGATCAATAAAG

RI-ATCG00180-XLOC_032457-8103-0
TAAGTTTTATTTGCTATCTACACGTGTTAAAACTTTAGCACCAATGTAAT
RI-ATCG00180-XLOC_032457-8103-1
TAAGTTTTATTTGCTATCTACACGTGTTAAAACTTTAGCACCAATGTAAT
CONSENSUS
TAAGTTTTATTTGCTATCTACACGTGTTAAAACTTTAGCACCAATGTAAT

RI-ATCG00180-XLOC_032457-8103-0
CTATATTTCATTTTGAATTGACGAACAACCAATAGGAATATTACTCTTTT
RI-ATCG00180-XLOC_032457-8103-1
CTATATTTCATTTTGAATTGACGAACAACCAATAGGAATATTACTCTTTT
CONSENSUS
CTATATTTCATTTTGAATTGACGAACAACCAATAGGAATATTACTCTTTT

RI-ATCG00180-XLOC_032457-8103-0
AGTAGTCTTGAATAAAAAGCGAAATGGGGCGTAGCCAAGCGGTAAGGCAA
RI-ATCG00180-XLOC_032457-8103-1
AGTAGTCTTGAATAAAAAGCGAAATGGGGCGTAGCCAAGCGGTAAGGCAA
CONSENSUS
AGTAGTCTTGAATAAAAAGCGAAATGGGGCGTAGCCAAGCGGTAAGGCAA

RI-ATCG00180-XLOC_032457-8103-0
CGGGTTTTGGTCCCGCTATTCGGAGGTTCGAATCCTTCCGTCCCAGAGTA
RI-ATCG00180-XLOC_032457-8103-1
CGGGTTTTGGTCCCGCTATTCGGAGGTTCGAATCCTTCCGTCCCAGAGTA
CONSENSUS
CGGGTTTTGGTCCCGCTATTCGGAGGTTCGAATCCTTCCGTCCCAGAGTA

RI-ATCG00180-XLOC_032457-8103-0
CAGTCATTACAGAATCAATCTTCTATTGCCTTTGTACACCATCTTTCTCT
RI-ATCG00180-XLOC_032457-8103-1
CAGTCATTACAGAATCAATCTTCTATTGCCTTTGTACACCATCTTTCTCT
CONSENSUS
CAGTCATTACAGAATCAATCTTCTATTGCCTTTGTACACCATCTTTCTCT

RI-ATCG00180-XLOC_032457-8103-0

TTCTGTTTAAAAATCCAAATTTTTTAAGAATAAAATATTGAAATGAAAAG
RI-ATCG00180-XLOC_032457-8103-1
TTCTGTTTAAAAATCCAAATTTTTTAAGAATAAAATATTGAAATGAAAAG
CONSENSUS
TTCTGTTTAAAAATCCAAATTTTTTAAGAATAAAATATTGAAATGAAAAG

RI-ATCG00180-XLOC_032457-8103-0
AAGAATAAACTTAATTTTTCATTATTTCAACCGAATTCAAAAAAAATCTA
RI-ATCG00180-XLOC_032457-8103-1
AAGAATAAACTTAATTTTTCATTATTTCAACCGAATTCAAAAAAAATCTA
CONSENSUS
AAGAATAAACTTAATTTTTCATTATTTCAACCGAATTCAAAAAAAATCTA

RI-ATCG00180-XLOC_032457-8103-0
TTTGCTTTTTTAATTTGTGTGATGCGGGTAAGTAAGGTAGAACCGCAGAT
RI-ATCG00180-XLOC_032457-8103-1
TTTGCTTTTTTAATTTGTGTGATGCGGGTAAGTAAGGTAGAACCGCAGAT
CONSENSUS
TTTGCTTTTTTAATTTGTGTGATGCGGGTAAGTAAGGTAGAACCGCAGAT

RI-ATCG00180-XLOC_032457-8103-0
TCTAAGAGTTTTAATAAAAAAATCTATATTTATTTATTTATATATAAATA
RI-ATCG00180-XLOC_032457-8103-1
TCTAAGAGTTTTAATAAAAAAATCTATATTTATTTATTTATATATAAATA
CONSENSUS
TCTAAGAGTTTTAATAAAAAAATCTATATTTATTTATTTATATATAAATA

RI-ATCG00180-XLOC_032457-8103-0
TAGATTTCTATTCAAACTAATATGGAATTCATTTTCATTCTGACCGAACC
RI-ATCG00180-XLOC_032457-8103-1
TAGATTTCTATTCAAACTAATATGGAATTCATTTTCATTCTGACCGAACC
CONSENSUS
TAGATTTCTATTCAAACTAATATGGAATTCATTTTCATTCTGACCGAACC

RI-ATCG00180-XLOC_032457-8103-0
TTTACGCGTAAATAAACTATTCCATTCATAGAGAAAGAAAAGGTATAGAT
RI-ATCG00180-XLOC_032457-8103-1
TTTACGCGTAAATAAACTATTCCATTCATAGAGAAAGAAAAGGTATAGAT
CONSENSUS
TTTACGCGTAAATAAACTATTCCATTCATAGAGAAAGAAAAGGTATAGAT

RI-ATCG00180-XLOC_032457-8103-0
TATTATATACTGACTGAACTATGACTATTCATGATTCCATCATTGAATCA
RI-ATCG00180-XLOC_032457-8103-1
TATTATATACTGACTGAACTATGACTATTCATGATTCCATCATTGAATCA
CONSENSUS
TATTATATACTGACTGAACTATGACTATTCATGATTCCATCATTGAATCA

RI-ATCG00180-XLOC_032457-8103-0
ATTACACAAACTAGAGGAATGTTATGGTAAAACTTCGTTTAAAACGATGT
RI-ATCG00180-XLOC_032457-8103-1
ATTACACAAACTAGAGGAATGTTATGGTAAAACTTCGTTTAAAACGATGT
CONSENSUS
ATTACACAAACTAGAGGAATGTTATGGTAAAACTTCGTTTAAAACGATGT

RI-ATCG00180-XLOC_032457-8103-0 GGTAGAAAGCAAC

RI-ATCG00180-XLOC_032457-8103-1 GGTAGAAAGCAAC
CONSENSUS   GGTAGAAAGCAAC

alignment for event: RI-AT1G03410-XLOC_004276-1535

RI-AT1G03410-XLOC_004276-1535-0
AAAAAAAAAGGTCCAGTCCCAATTGGGCTTAGGCCCGGAATTACAATCGT
RI-AT1G03410-XLOC_004276-1535-1
AAAAAAAAAGGTCCAGTCCCAATTGGGCTTAGGCCCGGAATTACAATCGT
CONSENSUS
AAAAAAAAAGGTCCAGTCCCAATTGGGCTTAGGCCCGGAATTACAATCGT

RI-AT1G03410-XLOC_004276-1535-0
CCACAGTTACAAAACAGGAAAAATCCGGTCACCGGAAAAAGAGAACCAGA
RI-AT1G03410-XLOC_004276-1535-1
CCACAGTTACAAAACAGGAAAAATCCGGTCACCGGAAAAAGAGAACCAGA
CONSENSUS
CCACAGTTACAAAACAGGAAAAATCCGGTCACCGGAAAAAGAGAACCAGA

RI-AT1G03410-XLOC_004276-1535-0
GGTGGATTTGGGCAGTGGAACGGAGGCAGCAAATAGATCTATTAGTACCG
RI-AT1G03410-XLOC_004276-1535-1
GGTGGATTTGGGCAGTGGAACGGAGGCAGCAAATAGATCTATTAGTACCG
CONSENSUS
GGTGGATTTGGGCAGTGGAACGGAGGCAGCAAATAGATCTATTAGTACCG

RI-AT1G03410-XLOC_004276-1535-0
TCGTATACAAGGCCCACTACTTTGCAACACAACCACCGGAACGAGATCAT
RI-AT1G03410-XLOC_004276-1535-1
TCGTATACAAGGCCCACTACTTTGCAACACAACCACCGGAACGAGATCAT
CONSENSUS
TCGTATACAAGGCCCACTACTTTGCAACACAACCACCGGAACGAGATCAT

RI-AT1G03410-XLOC_004276-1535-0
TGGCCGAAGTATACGAGTCCTGCCGCAAATCTCTTACCAATGAGCTGGCC
RI-AT1G03410-XLOC_004276-1535-1
TGGCCGAAGTATACGAGTCCTGCCGCAAATCTCTTACCAATGAGCTGGCC
CONSENSUS
TGGCCGAAGTATACGAGTCCTGCCGCAAATCTCTTACCAATGAGCTGGCC

RI-AT1G03410-XLOC_004276-1535-0
GGACCTGCAACAAACCTAACGGAAAAAACCCAGTTTCCGACACTCCGCCA
RI-AT1G03410-XLOC_004276-1535-1
GGACCTGCAACAAACCTAACGGAAAAAACCCAGTTTCCGACACTCCGCCA
CONSENSUS
GGACCTGCAACAAACCTAACGGAAAAAACCCAGTTTCCGACACTCCGCCA

RI-AT1G03410-XLOC_004276-1535-0
CCCAATTTGGTCGAGTCCCCAACCTCGCAAAACGACCACGCATTTCCAAG
RI-AT1G03410-XLOC_004276-1535-1
CCCAATTTGGTCGAGTCCCCAACCTCGCAAAACGACCACGCATTTCCAAG
CONSENSUS
CCCAATTTGGTCGAGTCCCCAACCTCGCAAAACGACCACGCATTTCCAAG

RI-AT1G03410-XLOC_004276-1535-0
CCTCTGACCTCCAAAACCTTAGATCTGAAAATTTTCCAAAAGCAAAGCAA
RI-AT1G03410-XLOC_004276-1535-1
CCTCTGACCTCCAAAACCTTAGATCTGAAAATTTTCCAAAAGCAAAGCAA
CONSENSUS
CCTCTGACCTCCAAAACCTTAGATCTGAAAATTTTCCAAAAGCAAAGCAA

RI-AT1G03410-XLOC_004276-1535-0
CCTTCAATCCGGATTGATGGAGACAAAAAACGGAGGCTAAAGAAGGGTGA
RI-AT1G03410-XLOC_004276-1535-1
CCTTCAATCCGGATTGATGGAGACAAAAAACGGAGGCTAAAGAAGGGTGA
CONSENSUS
CCTTCAATCCGGATTGATGGAGACAAAAAACGGAGGCTAAAGAAGGGTGA

RI-AT1G03410-XLOC_004276-1535-0
TGGCTCAGACAACCCTTCAGCAACCGGAACCCGCAACAGCCATCTCAGAT
RI-AT1G03410-XLOC_004276-1535-1
TGGCTCAGACAACCCTTCAGCAACCGGAACCCGCAACAGCCATCTCAGAT
CONSENSUS
TGGCTCAGACAACCCTTCAGCAACCGGAACCCGCAACAGCCATCTCAGAT

RI-AT1G03410-XLOC_004276-1535-0
CCAGTTTGTTAACCATCAAACACGCACTGAAAACCCACCAATCCACAGCG
RI-AT1G03410-XLOC_004276-1535-1
CCAGTTTGTTAACCATCAAACACGCACTGAAAACCCACCAATCCACAGCG
CONSENSUS
CCAGTTTGTTAACCATCAAACACGCACTGAAAACCCACCAATCCACAGCG

RI-AT1G03410-XLOC_004276-1535-0
TCTTCCGCCACAGCTACAGCGTCTAACCAAGTGGTTGAACCAAGAAAGAC
RI-AT1G03410-XLOC_004276-1535-1
TCTTCCGCCACAGCTACAGCGTCTAACCAAGTGGTTGAACCAAGAAAGAC
CONSENSUS
TCTTCCGCCACAGCTACAGCGTCTAACCAAGTGGTTGAACCAAGAAAGAC

RI-AT1G03410-XLOC_004276-1535-0
TTCACCGATCTCCACCGACGTCCATAACCAGGAAGAAACGGAAGAGAAGC
RI-AT1G03410-XLOC_004276-1535-1
TTCACCGATCTCCACCGACGTCCATAACCAGGAAGAAACGGAAGAGAAGC
CONSENSUS
TTCACCGATCTCCACCGACGTCCATAACCAGGAAGAAACGGAAGAGAAGC

RI-AT1G03410-XLOC_004276-1535-0
CTCTCATCCACCGAGAAAGGGCCACCGCTGCCACGCTTACCGGAAAAACC
RI-AT1G03410-XLOC_004276-1535-1
CTCTCATCCACCGAGAAAGGGCCACCGCTGCCACGCTTACCGGAAAAACC
CONSENSUS
CTCTCATCCACCGAGAAAGGGCCACCGCTGCCACGCTTACCGGAAAAACC

RI-AT1G03410-XLOC_004276-1535-0
ACACCACTCCAATGCCGGAGAAGATAGCTTGCCTTACTCCTGCTTTGTCT
RI-AT1G03410-XLOC_004276-1535-1
ACACCACTCCAATGCCGGAGAAGATAGCTTGCCTTACTCCTGCTTTGTCT
CONSENSUS
ACACCACTCCAATGCCGGAGAAGATAGCTTGCCTTACTCCTGCTTTGTCT

RI-AT1G03410-XLOC_004276-1535-0
TGCAAATTCATAACCTAAGATGAAGAACAGAGGCAGAAAAAACTAGATCT
RI-AT1G03410-XLOC_004276-1535-1
TGCAAATTCATAACCTAAGATGAAGAACAGAGGCAGAAAAAACTAGATCT
CONSENSUS
TGCAAATTCATAACCTAAGATGAAGAACAGAGGCAGAAAAAACTAGATCT

RI-AT1G03410-XLOC_004276-1535-0
GTAAAAACTGGAAAAAACTTCAAAGAGAGAGAAAATGAACAGTAGCGGAA
RI-AT1G03410-XLOC_004276-1535-1
GTAAAAACTGGAAAAAACTTCAAAGAGAGAGAAAATGAACAGTAGCGGAA
CONSENSUS
GTAAAAACTGGAAAAAACTTCAAAGAGAGAGAAAATGAACAGTAGCGGAA

RI-AT1G03410-XLOC_004276-1535-0
CGATTGTCCAAAGTCCTCCAAACGAGATATCTTTAAATGTTGTTTTGCTA
RI-AT1G03410-XLOC_004276-1535-1
CGATTGTCCAAAGTCCTCCAAACGAGATATCTTTAAATGTTGTTTTGCTA
CONSENSUS
CGATTGTCCAAAGTCCTCCAAACGAGATATCTTTAAATGTTGTTTTGCTA

RI-AT1G03410-XLOC_004276-1535-0
ATTAGTGGCGTTTCGAGAGCTAGGCGAGCAACAATGGCGATGAGATTTAA
RI-AT1G03410-XLOC_004276-1535-1
ATTAGTGGCGTTTCGAGAGCTAGGCGAGCAACAATGGCGATGAGATTTAA
CONSENSUS
ATTAGTGGCGTTTCGAGAGCTAGGCGAGCAACAATGGCGATGAGATTTAA

RI-AT1G03410-XLOC_004276-1535-0
TATGGTAAGTGTGTAGTTAATTAACAAAAGTTCAAACCGGATGCTATTCT
RI-AT1G03410-XLOC_004276-1535-1
TATG--
CONSENSUS
TATG..

RI-AT1G03410-XLOC_004276-1535-0
TACTTGTAAAGACAAACGACCAATTAAGAATCCAAATTTTAATATAGGAA
RI-AT1G03410-XLOC_004276-1535-1

CONSENSUS
....

RI-AT1G03410-XLOC_004276-1535-0
TTGAAATAAACTAATGGACCTTGTTTTTGGCGATTCATTTCTTTATTTGT
RI-AT1G03410-XLOC_004276-1535-1

CONSENSUS
....

RI-AT1G03410-XLOC_004276-1535-0
TATCTTAATATGGGTCACGATTCCTTTTGTTATTTAATTGTATTAAGATG
RI-AT1G03410-XLOC_004276-1535-1

CONSENSUS
....

RI-AT1G03410-XLOC_004276-1535-0
TGCATTAAGATGTGGAATAATAGCACTTATGCAAATCTGTGCATTGCAAA
RI-AT1G03410-XLOC_004276-1535-1

CONSENSUS
....

RI-AT1G03410-XLOC_004276-1535-0
AAAAAGAAAGAAGAAGCAAGATGGAGTCAAGTGATCGTTCAAGTCAAGCA
RI-AT1G03410-XLOC_004276-1535-1 ------
AAAGAAGAAGCAAGATGGAGTCAAGTGATCGTTCAAGTCAAGCA
CONSENSUS
......AAAGAAGAAGCAAGATGGAGTCAAGTGATCGTTCAAGTCAAGCA

RI-AT1G03410-XLOC_004276-1535-0
AAAGCTTTCGACGAGACAAAAACCGGCGTGAAAGGGCTTGTGGCTTCGGG
RI-AT1G03410-XLOC_004276-1535-1
AAAGCTTTCGACGAGACAAAAACCGGCGTGAAAGGGCTTGTGGCTTCGGG
CONSENSUS
AAAGCTTTCGACGAGACAAAAACCGGCGTGAAAGGGCTTGTGGCTTCGGG

RI-AT1G03410-XLOC_004276-1535-0
AATCAAAGAGATTCCAGCCATGTTCCATACACCTCCGGATACTCTAACAA
RI-AT1G03410-XLOC_004276-1535-1
AATCAAAGAGATTCCAGCCATGTTCCATACACCTCCGGATACTCTAACAA
CONSENSUS
AATCAAAGAGATTCCAGCCATGTTCCATACACCTCCGGATACTCTAACAA

RI-AT1G03410-XLOC_004276-1535-0
GCCTGAAACAAACAGCACCACCTTCGCAGCAGCTGACGATCCCCACGGTG
RI-AT1G03410-XLOC_004276-1535-1
GCCTGAAACAAACAGCACCACCTTCGCAGCAGCTGACGATCCCCACGGTG
CONSENSUS
GCCTGAAACAAACAGCACCACCTTCGCAGCAGCTGACGATCCCCACGGTG

RI-AT1G03410-XLOC_004276-1535-0
GATCTGAAAGGAGGAAGCATGGATTTGATATCGCGGCGGAGCGTGGTGGA
RI-AT1G03410-XLOC_004276-1535-1
GATCTGAAAGGAGGAAGCATGGATTTGATATCGCGGCGGAGCGTGGTGGA
CONSENSUS
GATCTGAAAGGAGGAAGCATGGATTTGATATCGCGGCGGAGCGTGGTGGA

RI-AT1G03410-XLOC_004276-1535-0
GAAGATTGGAGACGCTGCGGAGAGATGGGGATTCTTCCAGGTGGTGAATC
RI-AT1G03410-XLOC_004276-1535-1
GAAGATTGGAGACGCTGCGGAGAGATGGGGATTCTTCCAGGTGGTGAATC
CONSENSUS
GAAGATTGGAGACGCTGCGGAGAGATGGGGATTCTTCCAGGTGGTGAATC

RI-AT1G03410-XLOC_004276-1535-0
ATGGGATCTCGGTGGAGGTGATGGAGAGGATGAAAGAAGGGATTCGCAGG
RI-AT1G03410-XLOC_004276-1535-1
ATGGGATCTCGGTGGAGGTGATGGAGAGGATGAAAGAAGGGATTCGCAGG
CONSENSUS
ATGGGATCTCGGTGGAGGTGATGGAGAGGATGAAAGAAGGGATTCGCAGG

RI-AT1G03410-XLOC_004276-1535-0
TTTCACGAGCAGGACCCGGAAGTGAAGAAACGGTTCTACTCTAGGGATCA
RI-AT1G03410-XLOC_004276-1535-1
TTTCACGAGCAGGACCCGGAAGTGAAGAAACGGTTCTACTCTAGGGATCA
CONSENSUS
TTTCACGAGCAGGACCCGGAAGTGAAGAAACGGTTCTACTCTAGGGATCA

RI-AT1G03410-XLOC_004276-1535-0
CACTAGAGATGTGCTTTACTACAGCAACATCGATCTCCACACTTGTAATA
RI-AT1G03410-XLOC_004276-1535-1
CACTAGAGATGTGCTTTACTACAGCAACATCGATCTCCACACTTGTAATA
CONSENSUS
CACTAGAGATGTGCTTTACTACAGCAACATCGATCTCCACACTTGTAATA

RI-AT1G03410-XLOC_004276-1535-0
AGGCTGCAAATTGGAGAGATACGCTCGCCTGTTACATGGCCCCCGATCCT
RI-AT1G03410-XLOC_004276-1535-1
AGGCTGCAAATTGGAGAGATACGCTCGCCTGTTACATGGCCCCCGATCCT
CONSENSUS
AGGCTGCAAATTGGAGAGATACGCTCGCCTGTTACATGGCCCCCGATCCT

RI-AT1G03410-XLOC_004276-1535-0
CCCAAGTTACAGGACTTGCCCGCGGTTTGCGG
RI-AT1G03410-XLOC_004276-1535-1
CCCAAGTTACAGGACTTGCCCGCGGTTTGCGG
CONSENSUS
CCCAAGTTACAGGACTTGCCCGCGGTTTGCGG

alignment for event: RI-AT1G75010-XLOC_007827-11322

RI-AT1G75010-XLOC_007827-11322-0
GATCTAAACGCTATTATATGCATTGTGGCTAGTTCGGTACCTCTCCAGAA
RI-AT1G75010-XLOC_007827-11322-1
GATCTAAACGCTATTATATGCATTGTGGCTAGTTCGGTACCTCTCCAGAA
CONSENSUS
GATCTAAACGCTATTATATGCATTGTGGCTAGTTCGGTACCTCTCCAGAA

RI-AT1G75010-XLOC_007827-11322-0
GAAAGACGTGAAAACAATTCTTCGTACTTTCCGTCAAACTATGGAGTACA
RI-AT1G75010-XLOC_007827-11322-1
GAAAGACGTGAAAACAATTCTTCGTACTTTCCGTCAAACTATGGAGTACA
CONSENSUS
GAAAGACGTGAAAACAATTCTTCGTACTTTCCGTCAAACTATGGAGTACA

RI-AT1G75010-XLOC_007827-11322-0
CTGGAGATATTATAGTATCTACTGTTCATGAGCCTGATCTGGAGCCTAAA
RI-AT1G75010-XLOC_007827-11322-1
CTGGAGATATTATAGTATCTACTGTTCATGAGCCTGATCTGGAGCCTAAA
CONSENSUS
CTGGAGATATTATAGTATCTACTGTTCATGAGCCTGATCTGGAGCCTAAA

RI-AT1G75010-XLOC_007827-11322-0
GTGCGTGTCACAACGTTTTTTATTCTAAGGTGTGTCTTCATGTTTAGTGA
RI-AT1G75010-XLOC_007827-11322-1

GTGCGTGTCACAACGTTTTTTATTCTAAG--------------------
CONSENSUS
GTGCGTGTCACAACGTTTTTTATTCTAAG.....................

RI-AT1G75010-XLOC_007827-11322-0
CAGCTAACAATTTCAAGCTCATGCATATGGTATCATATTTAGCTGAAAAT
RI-AT1G75010-XLOC_007827-11322-1

CONSENSUS
....

RI-AT1G75010-XLOC_007827-11322-0
CAACAAAGAAAAGTAAATATATGGTTTCAGCATTTATACCATATATTTTT
RI-AT1G75010-XLOC_007827-11322-1

CONSENSUS
....

RI-AT1G75010-XLOC_007827-11322-0
ATGTGTTTGATTAAGATCTATGATGCTTTTGACCAAGACTCAGATTCTAT
RI-AT1G75010-XLOC_007827-11322-1

CONSENSUS
....

RI-AT1G75010-XLOC_007827-11322-0
TTCTCGTGACCACTTTTAAGCATTAATGCTAGTCTCAAATGTCATCACAG
RI-AT1G75010-XLOC_007827-11322-1

CONSENSUS
....

RI-AT1G75010-XLOC_007827-11322-0
TTCTTCAGAAGTGGAAACATCCAACAAAGGCAACATTTTTTCTGGTCTTG
RI-AT1G75010-XLOC_007827-11322-1
TTCTTCAGAAGTGGAAACATCCAACAAAGGCAACATTTTTTCTGGTCTTG
CONSENSUS
TTCTTCAGAAGTGGAAACATCCAACAAAGGCAACATTTTTTCTGGTCTTG

RI-AT1G75010-XLOC_007827-11322-0
TTCCTTTTGTTTTAAATATCTTCACGAGATATCGTTCACAGCTCCAAAAA
RI-AT1G75010-XLOC_007827-11322-1
TTCCTTTTGTTTTAAATATCTTCACGAGATATCGTTCACAGCTCCAAAAA
CONSENSUS
TTCCTTTTGTTTTAAATATCTTCACGAGATATCGTTCACAGCTCCAAAAA

RI-AT1G75010-XLOC_007827-11322-0
GAAACAAATATTGGACTTGGAGAGACGCCAGTTTCAATTAAAGATTCAGC
RI-AT1G75010-XLOC_007827-11322-1
GAAACAAATATTGGACTTGGAGAGACGCCAGTTTCAATTAAAGATTCAGC
CONSENSUS
GAAACAAATATTGGACTTGGAGAGACGCCAGTTTCAATTAAAGATTCAGC

RI-AT1G75010-XLOC_007827-11322-0
AGACTCTACTGACGTAAAAACTTCCAATCAGAATATTGAGGAATTTGAAA
RI-AT1G75010-XLOC_007827-11322-1

AGACTCTACTGACGTAAAAACTTCCAATCAGAATATTGAGGAATTTGAAA
CONSENSUS
AGACTCTACTGACGTAAAAACTTCCAATCAGAATATTGAGGAATTTGAAA

RI-AT1G75010-XLOC_007827-11322-0
TTGACTCTGAGGACCTCCTGGAAGTTTCTGAGAACGGTGATGATAGTGAA
RI-AT1G75010-XLOC_007827-11322-1
TTGACTCTGAGGACCTCCTGGAAGTTTCTGAGAACGGTGATGATAGTGAA
CONSENSUS
TTGACTCTGAGGACCTCCTGGAAGTTTCTGAGAACGGTGATGATAGTGAA

RI-AT1G75010-XLOC_007827-11322-0
TACCCATTAAAAGAGGGGGAACCATCCAGAAACAGTCGTCTGGATCTTAA
RI-AT1G75010-XLOC_007827-11322-1
TACCCATTAAAAGAGGGGGAACCATCCAGAAACAGTCGTCTGGATCTTAA
CONSENSUS
TACCCATTAAAAGAGGGGGAACCATCCAGAAACAGTCGTCTGGATCTTAA

RI-AT1G75010-XLOC_007827-11322-0 AGATGAAAATGTAGAAG
RI-AT1G75010-XLOC_007827-11322-1 AGATGAAAATGTAGAAG
CONSENSUS AGATGAAAATGTAGAAG

alignment for event: A5-AT1G51690-XLOC_002583-13606

A5-AT1G51690-XLOC_002583-13606-0
GTTCAAGACAAGAAGATCAAGAAAATCTGTGATATGAATTCAGATCCTTC
A5-AT1G51690-XLOC_002583-13606-1
GTTCAAGACAAGAAGATCAAGAAAATCTGTGATATGAATTCAGATCCTTC
CONSENSUS
GTTCAAGACAAGAAGATCAAGAAAATCTGTGATATGAATTCAGATCCTTC

A5-AT1G51690-XLOC_002583-13606-0
AAGAACTGTAGGAAACGGAACCGTTGCAAGCTCGAGCAATTCAAACATTA
A5-AT1G51690-XLOC_002583-13606-1
AAGAACTGTAGGAAACGGAACCGTTGCAAGCTCGAGCAATTCAAACATTA
CONSENSUS
AAGAACTGTAGGAAACGGAACCGTTGCAAGCTCGAGCAATTCAAACATTA

A5-AT1G51690-XLOC_002583-13606-0
CAAACTCATGCCTTGTGAATGGAGGAGTATCTGAAGTGAACAACTCCTTA
A5-AT1G51690-XLOC_002583-13606-1
CAAACTCATGCCTTGTGAATGGAGGAGTATCTGAAGTGAACAACTCCTTA
CONSENSUS
CAAACTCATGCCTTGTGAATGGAGGAGTATCTGAAGTGAACAACTCCTTA

A5-AT1G51690-XLOC_002583-13606-0
TGTAATGACTTCTCATTGCCAGCAGGAGGCATCTCTTCGCTGCGATTACC
A5-AT1G51690-XLOC_002583-13606-1
TGTAATGACTTCTCATTGCCAGCAGGAGGCATCTCTTCGCTGCGATTACC
CONSENSUS
TGTAATGACTTCTCATTGCCAGCAGGAGGCATCTCTTCGCTGCGATTACC

A5-AT1G51690-XLOC_002583-13606-0 AGTG---
GTAACTAGCCATGAGTCGAGCCCTGTGGCTAGATGTCGAAGAG

```
A5-AT1G51690-XLOC_002583-13606-1
      AGTGGTAGTAACTAGCCATGAGTCGAGCCCTGTGGCTAGATGTCGAAGAG
CONSENSUS
      AGTG...GTAACTAGCCATGAGTCGAGCCCTGTGGCTAGATGTCGAAGAG

A5-AT1G51690-XLOC_002583-13606-0
      TATATGCTCATGCTCATGATTATCATATTAATTCAATCTCAAATAACAG
A5-AT1G51690-XLOC_002583-13606-1
      TATATGCTCATGCTCATGATTATCATATTAATTCAATCTCAAATAACAG
CONSENSUS
      TATATGCTCATGCTCATGATTATCATATTAATTCAATCTCAAATAACAG
```

alignment for event: A3-AT1G29850-XLOC_001571-3195

```
A3-AT1G29850-XLOC_001571-3195-0
      GCTGATCCTGAACTAGAAGCTATTAGACAGAGGAGAATGCAAGAGCTCAT
A3-AT1G29850-XLOC_001571-3195-1
      GCTGATCCTGAACTAGAAGCTATTAGACAGAGGAGAATGCAAGAGCTCAT
CONSENSUS
      GCTGATCCTGAACTAGAAGCTATTAGACAGAGGAGAATGCAAGAGCTCAT

A3-AT1G29850-XLOC_001571-3195-0
      GGCTCGACAAGGCATGCAGGGTAAGCAGGGCAATCAGCAGAATCCAGAGC
A3-AT1G29850-XLOC_001571-3195-1  GGCTCGACAAGGCATG---
GGTAAGCAGGGCAATCAGCAGAATCCAGAGC
CONSENSUS
      GGCTCGACAAGGCATG...GGTAAGCAGGGCAATCAGCAGAATCCAGAGC

A3-AT1G29850-XLOC_001571-3195-0  AAGAGAAACAGCAGGAAGATGCTAAAAG
A3-AT1G29850-XLOC_001571-3195-1  AAGAGAAACAGCAGGAAGATGCTAAAAG
CONSENSUS                        AAGAGAAACAGCAGGAAGATGCTAAAAG
```

alignment for event: RI-AT1G79245-XLOC_008063-11625

```
RI-AT1G79245-XLOC_008063-11625-0
      TGGAAGTTCTGTTTCGGCCTGCTTGCCTACATCGCCCTATATCTGTTACA
RI-AT1G79245-XLOC_008063-11625-1
      TGGAAGTTCTGTTTCGGCCTGCTTGCCTACATCGCCCTATATCTGTTACA
CONSENSUS
      TGGAAGTTCTGTTTCGGCCTGCTTGCCTACATCGCCCTATATCTGTTACA

RI-AT1G79245-XLOC_008063-11625-0
      TCATGAGTTTATGAGGTGGCTTATTTCTATTGATATCTATAGTGTTGATT
RI-AT1G79245-XLOC_008063-11625-1
      TCATGAGTTTATGAG-----------------------------------
CONSENSUS
      TCATGAGTTTATGAG...................................

RI-AT1G79245-XLOC_008063-11625-0
      GCCGAGGAAGGAAAAACAAGTTTCAACCGTGGACCGTGGTTGTCTCAACA
RI-AT1G79245-XLOC_008063-11625-1
      --------------------------------------------------
```

CONSENSUS
      ....

RI-AT1G79245-XLOC_008063-11625-0
      GGGATGAACTTGGAGGTTCCATCTCTGGTTGGATTCAGGTGTTTTGAGCT
RI-AT1G79245-XLOC_008063-11625-1 -
GGATGAACTTGGAGGTTCCATCTCTGGTTGGATTCAGGTGTTTTGAGCT
CONSENSUS
      .GGATGAACTTGGAGGTTCCATCTCTGGTTGGATTCAGGTGTTTTGAGCT

RI-AT1G79245-XLOC_008063-11625-0
      AATCTTCTTCTGGGTTTACTTCTCTACCATACTTTCTACCGATTTGAAGA
RI-AT1G79245-XLOC_008063-11625-1
      AATCTTCTTCTGGGTTTACTTCTCTACCATACTTTCTACCGATTTGAAGA
CONSENSUS
      AATCTTCTTCTGGGTTTACTTCTCTACCATACTTTCTACCGATTTGAAGA

RI-AT1G79245-XLOC_008063-11625-0
      TTTGTCTTCTGTACCTTTGATGATGATTCTCAGTTTTGCAGCAATGACTC
RI-AT1G79245-XLOC_008063-11625-1
      TTTGTCTTCTGTACCTTTGATGATGATTCTCAGTTTTGCAGCAATGACTC
CONSENSUS
      TTTGTCTTCTGTACCTTTGATGATGATTCTCAGTTTTGCAGCAATGACTC

RI-AT1G79245-XLOC_008063-11625-0
      AGTTTTTCGCCTTCTAGTCATTAAACTACTTTCCCACAATGAGATACTTA
RI-AT1G79245-XLOC_008063-11625-1
      AGTTTTTCGCCTTCTAGTCATTAAACTACTTTCCCACAATGAGATACTTA
CONSENSUS
      AGTTTTTCGCCTTCTAGTCATTAAACTACTTTCCCACAATGAGATACTTA

RI-AT1G79245-XLOC_008063-11625-0
      GTGTTTTTGTAGGTTCTTTCACACTTGTTAGCAAGGGTATATGTATGTTG
RI-AT1G79245-XLOC_008063-11625-1
      GTGTTTTTGTAGGTTCTTTCACACTTGTTAGCAAGGGTATATGTATGTTG
CONSENSUS
      GTGTTTTTGTAGGTTCTTTCACACTTGTTAGCAAGGGTATATGTATGTTG

RI-AT1G79245-XLOC_008063-11625-0
      CCGTTCTCAGAATTTGGTAGGTATGAATCACAGATGTCATTGTTTATTAT
RI-AT1G79245-XLOC_008063-11625-1
      CCGTTCTCAGAATTTGGTAGGTATGAATCACAGATGTCATTGTTTATTAT
CONSENSUS
      CCGTTCTCAGAATTTGGTAGGTATGAATCACAGATGTCATTGTTTATTAT

RI-AT1G79245-XLOC_008063-11625-0
      TGATAACATAAGTGATTCTTGCTGGTAGTGTGACCTCTTGAGGGCAGGCT
RI-AT1G79245-XLOC_008063-11625-1
      TGATAACATAAGTGATTCTTGCTGGTAGTGTGACCTCTTGAGGGCAGGCT
CONSENSUS
      TGATAACATAAGTGATTCTTGCTGGTAGTGTGACCTCTTGAGGGCAGGCT

RI-AT1G79245-XLOC_008063-11625-0
      CATAGAAGATGAAGAGCCTCTTGGTTTTGATGATAATGCTCCCTACTTCT
RI-AT1G79245-XLOC_008063-11625-1
      CATAGAAGATGAAGAGCCTCTTGGTTTTGATGATAATGCTCCCTACTTCT

CONSENSUS
CATAGAAGATGAAGAGCCTCTTGGTTTTGATGATAATGCTCCCTACTTCT

RI-AT1G79245-XLOC_008063-11625-0
GAGTTCTGAGGTTTCTTTATTCCTGGGTTGTAGCTGGTTTAATGGTTCTG
RI-AT1G79245-XLOC_008063-11625-1
GAGTTCTGAGGTTTCTTTATTCCTGGGTTGTAGCTGGTTTAATGGTTCTG
CONSENSUS
GAGTTCTGAGGTTTCTTTATTCCTGGGTTGTAGCTGGTTTAATGGTTCTG

RI-AT1G79245-XLOC_008063-11625-0
TAGACATTTGATAATGAAGTCTCTCACTTCATTTTGTTTGGGTGATGTAA
RI-AT1G79245-XLOC_008063-11625-1
TAGACATTTGATAATGAAGTCTCTCACTTCATTTTGTTTGGGTGATGTAA
CONSENSUS
TAGACATTTGATAATGAAGTCTCTCACTTCATTTTGTTTGGGTGATGTAA

RI-AT1G79245-XLOC_008063-11625-0
AATGTTGTCAGTGGATTTTAGGTGAGGCTTAATCTCATGGATTATTCTTT
RI-AT1G79245-XLOC_008063-11625-1
AATGTTGTCAGTGGATTTTAGGTGAGGCTTAATCTCATGGATTATTCTTT
CONSENSUS
AATGTTGTCAGTGGATTTTAGGTGAGGCTTAATCTCATGGATTATTCTTT

RI-AT1G79245-XLOC_008063-11625-0
TGAAATCCCCTCAGGTGAATCAGTGAAAATTGAGAAAGTGATGTTCAACT
RI-AT1G79245-XLOC_008063-11625-1
TGAAATCCCCTCAGGTGAATCAGTGAAAATTGAGAAAGTGATGTTCAACT
CONSENSUS
TGAAATCCCCTCAGGTGAATCAGTGAAAATTGAGAAAGTGATGTTCAACT

RI-AT1G79245-XLOC_008063-11625-0
TCTTATCTCTTAATCCTTCATTTGCTCTGTCACACATTGAAGCCTATTTT
RI-AT1G79245-XLOC_008063-11625-1
TCTTATCTCTTAATCCTTCATTTGCTCTGTCACACATTGAAGCCTATTTT
CONSENSUS
TCTTATCTCTTAATCCTTCATTTGCTCTGTCACACATTGAAGCCTATTTT

RI-AT1G79245-XLOC_008063-11625-0
CGGTTGCAGTTTGTTAAGTTTTCATGGGAAGCGTTGTCTTTGGAGTGTTC
RI-AT1G79245-XLOC_008063-11625-1
CGGTTGCAGTTTGTTAAGTTTTCATGGGAAGCGTTGTCTTTGGAGTGTTC
CONSENSUS
CGGTTGCAGTTTGTTAAGTTTTCATGGGAAGCGTTGTCTTTGGAGTGTTC

RI-AT1G79245-XLOC_008063-11625-0
TGAAGTTGACTTTAATCCTGAAAGAAGAAGGATATGCTTTTACTGAAGTT
RI-AT1G79245-XLOC_008063-11625-1
TGAAGTTGACTTTAATCCTGAAAGAAGAAGGATATGCTTTTACTGAAGTT
CONSENSUS
TGAAGTTGACTTTAATCCTGAAAGAAGAAGGATATGCTTTTACTGAAGTT

RI-AT1G79245-XLOC_008063-11625-0
ATCATGGGAAGCACTTTTTGTGGAGTTTACAGGAGTGTCTATGCCGTTGC
RI-AT1G79245-XLOC_008063-11625-1
ATCATGGGAAGCACTTTTTGTGGAGTTTACAGGAGTGTCTATGCCGTTGC

CONSENSUS
ATCATGGGAAGCACTTTTTGTGGAGTTTACAGGAGTGTCTATGCCGTTGC

RI-AT1G79245-XLOC_008063-11625-0
AGTATTCGAGGATCGCTTTGATCTGCCAGTCGTTCCTTGTTACCTTTTTC
RI-AT1G79245-XLOC_008063-11625-1
AGTATTCGAGGATCGCTTTGATCTGCCAGTCGTTCCTTGTTACCTTTTTC
CONSENSUS
AGTATTCGAGGATCGCTTTGATCTGCCAGTCGTTCCTTGTTACCTTTTTC

RI-AT1G79245-XLOC_008063-11625-0
GCTTTTGCAGTTCTTTCTTACTTGCCTTGCTTTTCTCTCGTTCATGTATT
RI-AT1G79245-XLOC_008063-11625-1
GCTTTTGCAGTTCTTTCTTACTTGCCTTGCTTTTCTCTCGTTCATGTATT
CONSENSUS
GCTTTTGCAGTTCTTTCTTACTTGCCTTGCTTTTCTCTCGTTCATGTATT

RI-AT1G79245-XLOC_008063-11625-0
ACTTGATGCAAGTCTTGAAACTTTCCCGATATTGGACCAAACTCTTTTTC
RI-AT1G79245-XLOC_008063-11625-1
ACTTGATGCAAGTCTTGAAACTTTCCCGATATTGGACCAAACTCTTTTTC
CONSENSUS
ACTTGATGCAAGTCTTGAAACTTTCCCGATATTGGACCAAACTCTTTTTC

RI-AT1G79245-XLOC_008063-11625-0
ATTCATGCCTTTTAGGACTACTAAGTATGAACTCCTCTGATGTAGAAGGT
RI-AT1G79245-XLOC_008063-11625-1
ATTCATGCCTTTTAGGACTACTAAGTATGAACTCCTCTGATGTAGAAGGT
CONSENSUS
ATTCATGCCTTTTAGGACTACTAAGTATGAACTCCTCTGATGTAGAAGGT

RI-AT1G79245-XLOC_008063-11625-0 TCGATATGTGCTAATCAGTCTACTG
RI-AT1G79245-XLOC_008063-11625-1 TCGATATGTGCTAATCAGTCTACTG
CONSENSUS TCGATATGTGCTAATCAGTCTACTG

alignment for event: RI-AT1G17590-XLOC_005073-10228

RI-AT1G17590-XLOC_005073-10228-0
GAAAATTGTGAGTGACGGAAATGAAGCATCAAAAGAATACACACACACAC
RI-AT1G17590-XLOC_005073-10228-1
GAAAATTGTGAGTGACGGAAATGAAGCATCAAAAGAATACACACACACAC
CONSENSUS
GAAAATTGTGAGTGACGGAAATGAAGCATCAAAAGAATACACACACACAC

RI-AT1G17590-XLOC_005073-10228-0
ATACACACTGTTGAGTATCTATCTGAGAGAATAAGCAAAAGAGAAAAGAG
RI-AT1G17590-XLOC_005073-10228-1
ATACACACTGTTGAGTATCTATCTGAGAGAATAAGCAAAAGAGAAAAGAG
CONSENSUS
ATACACACTGTTGAGTATCTATCTGAGAGAATAAGCAAAAGAGAAAAGAG

RI-AT1G17590-XLOC_005073-10228-0
ATTTACTTTGTTTTGATAGGCATCCCTTGAGTTCCTGTTCTTCATCTCTT
RI-AT1G17590-XLOC_005073-10228-1

ATTTACTTTGTTTTGATAGGCATCCCTTGAGTTCCTGTTCTTCATCTCTT
CONSENSUS
ATTTACTTTGTTTTGATAGGCATCCCTTGAGTTCCTGTTCTTCATCTCTT

RI-AT1G17590-XLOC_005073-10228-0
CCTTGAATCCGTAAGGTCTCTCTATCTCCTTCTCTTTGAATCCGTCATTT
RI-AT1G17590-XLOC_005073-10228-1
CCTTGAATCC--
CONSENSUS
CCTTGAATCC..

RI-AT1G17590-XLOC_005073-10228-0
CTTCGCTTTTTTTTAGAGTGATGTGCTGATTCGGAAATCTTCTGGAAATT
RI-AT1G17590-XLOC_005073-10228-1

CONSENSUS
....

RI-AT1G17590-XLOC_005073-10228-0
TCTTCCCAAGTCAACGCCTTTTAGGACGAAAGTAATGGTGCTGATTTTAG
RI-AT1G17590-XLOC_005073-10228-1

CONSENSUS
....

RI-AT1G17590-XLOC_005073-10228-0
TTGGTCGGAACTTTACTTTTTTTTCTGCCGCGCTCTTCTAATAGTTTGCC
RI-AT1G17590-XLOC_005073-10228-1

CONSENSUS
....

RI-AT1G17590-XLOC_005073-10228-0
CGAAATAGTTCTGAATGTTGCTCTGTTTCTGGGTTTTTTTCCCTTTTTGG
RI-AT1G17590-XLOC_005073-10228-1

CONSENSUS
....

RI-AT1G17590-XLOC_005073-10228-0
ATTATCCCCTTCTTTGTCTACTACATCGTCAAAGGTTTTGCTGTTTCCAG
RI-AT1G17590-XLOC_005073-10228-1

CONSENSUS
....

RI-AT1G17590-XLOC_005073-10228-0
GAATAAATATCTTTTGAAAGTTTTATCATTTATAAAGATGTTCTGAATTT
RI-AT1G17590-XLOC_005073-10228-1

CONSENSUS
....

RI-AT1G17590-XLOC_005073-10228-0
GGAGCTTCTTTGACAGACACATGTATCATCAATCTTCTCTGTTGAAGCAG
RI-AT1G17590-XLOC_005073-10228-1 ----------------

ACACATGTATCATCAATCTTCTCTGTTGAAGCAG
CONSENSUS
................ACACATGTATCATCAATCTTCTCTGTTGAAGCAG

RI-AT1G17590-XLOC_005073-10228-0
AGAGAGAGAGAGCTAATTGTTGCCTCTGAGTCACATGGATAAGAAAGTTT
RI-AT1G17590-XLOC_005073-10228-1
AGAGAGAGAGAGCTAATTGTTGCCTCTGAGTCACATGGATAAGAAAGTTT
CONSENSUS
AGAGAGAGAGAGCTAATTGTTGCCTCTGAGTCACATGGATAAGAAAGTTT

RI-AT1G17590-XLOC_005073-10228-0
CATTTACTAGCTCTGTGGCACATTCAACTCCACCATACCTTAGTACTTCC
RI-AT1G17590-XLOC_005073-10228-1
CATTTACTAGCTCTGTGGCACATTCAACTCCACCATACCTTAGTACTTCC
CONSENSUS
CATTTACTAGCTCTGTGGCACATTCAACTCCACCATACCTTAGTACTTCC

RI-AT1G17590-XLOC_005073-10228-0
ATCTCATGGGGACTTCCAACCAAATCCAATGGTGTGACTGAATCACTGAG
RI-AT1G17590-XLOC_005073-10228-1
ATCTCATGGGGACTTCCAACCAAATCCAATGGTGTGACTGAATCACTGAG
CONSENSUS
ATCTCATGGGGACTTCCAACCAAATCCAATGGTGTGACTGAATCACTGAG

RI-AT1G17590-XLOC_005073-10228-0
TTTGAAGGTGGTAGATGCAAGACCAGAACGTCTTATAAACACAAAGAATA
RI-AT1G17590-XLOC_005073-10228-1
TTTGAAGGTGGTAGATGCAAGACCAGAACGTCTTATAAACACAAAGAATA
CONSENSUS
TTTGAAGGTGGTAGATGCAAGACCAGAACGTCTTATAAACACAAAGAATA

RI-AT1G17590-XLOC_005073-10228-0
TCAGTTTCCAGGACCAGGATTCATCTTCAACTCTGTCCTCTGCTCAATCT
RI-AT1G17590-XLOC_005073-10228-1
TCAGTTTCCAGGACCAGGATTCATCTTCAACTCTGTCCTCTGCTCAATCT
CONSENSUS
TCAGTTTCCAGGACCAGGATTCATCTTCAACTCTGTCCTCTGCTCAATCT

RI-AT1G17590-XLOC_005073-10228-0
TCTAACGATGTTACAAGTAGTGGAGATGATAACCCCTCAAGACAAATCTC
RI-AT1G17590-XLOC_005073-10228-1
TCTAACGATGTTACAAGTAGTGGAGATGATAACCCCTCAAGACAAATCTC
CONSENSUS
TCTAACGATGTTACAAGTAGTGGAGATGATAACCCCTCAAGACAAATCTC

RI-AT1G17590-XLOC_005073-10228-0 ATTTTTAGCACATTCAG
RI-AT1G17590-XLOC_005073-10228-1 ATTTTTAGCACATTCAG
CONSENSUS ATTTTTAGCACATTCAG

alignment for event: A5-AT1G54180-XLOC_002726-4324

A5-AT1G54180-XLOC_002726-4324-0
CTAAAAGACATGGCAGTAAAGGCTTCAGGGGCATACAAAAACTGCAAACC

A5-AT1G54180-XLOC_002726-4324-1
CTAAAAGACATGGCAGTAAAGGCTTCAGGGGCATACAAAAACTGCAAACC
CONSENSUS
CTAAAAGACATGGCAGTAAAGGCTTCAGGGGCATACAAAAACTGCAAACC

A5-AT1G54180-XLOC_002726-4324-0
GTGTTCTGGGACAACAAACCGGAATCAGAATCGAAACTATGCTGATTCGG
A5-AT1G54180-XLOC_002726-4324-1
GTGTTCTGGGACAACAAACCGGAATCAGAATCGAAACTATGCTGATTCGG
CONSENSUS
GTGTTCTGGGACAACAAACCGGAATCAGAATCGAAACTATGCTGATTCGG

A5-AT1G54180-XLOC_002726-4324-0
ATGCTGCTTCAGATTCTGGAAGATTCCATTATTCATACCAGAGAGCTGGG
A5-AT1G54180-XLOC_002726-4324-1
ATGCTGCTTCAGATTCTGGAAGATTCCATTATTCATACCAGAGAGCTGGG
CONSENSUS
ATGCTGCTTCAGATTCTGGAAGATTCCATTATTCATACCAGAGAGCTGGG

A5-AT1G54180-XLOC_002726-4324-0
ACTGCAACCTCAACTCCTAAGATTTGGGGGAATGAAATGGAGTCAAGGTT
A5-AT1G54180-XLOC_002726-4324-1
ACTGCAACCTCAACTCCTAAGATTTGGGGGAATGAAATGGAGTCAAGGTT
CONSENSUS
ACTGCAACCTCAACTCCTAAGATTTGGGGGAATGAAATGGAGTCAAGGTT

A5-AT1G54180-XLOC_002726-4324-0
AAAAGGGATTTCTAGTGAAGAAGGTACACCTACGTCTATGAGTGGTCGAA
A5-AT1G54180-XLOC_002726-4324-1
AAAAGGGATTTCTAGTGAAGAAGGTACACCTACGTCTATGAGTGGTCGAA
CONSENSUS
AAAAGGGATTTCTAGTGAAGAAGGTACACCTACGTCTATGAGTGGTCGAA

A5-AT1G54180-XLOC_002726-4324-0
CAGAATCCATAGTGTTCATGGAGGATGATGAGGTCAAGGAATGGGTTGCT
A5-AT1G54180-XLOC_002726-4324-1
CAGAATCCATAGTGTTCATGGAGGATGATGAGGTCAAGGAATGGGTTGCT
CONSENSUS
CAGAATCCATAGTGTTCATGGAGGATGATGAGGTCAAGGAATGGGTTGCT

A5-AT1G54180-XLOC_002726-4324-0
CAAGTGGAGCCTGGTGTTCTCATCACATTTGTGTCATTGCCTCAGGGAGG
A5-AT1G54180-XLOC_002726-4324-1
CAAGTGGAGCCTGGTGTTCTCATCACATTTGTGTCATTGCCTCAGGGAGG
CONSENSUS
CAAGTGGAGCCTGGTGTTCTCATCACATTTGTGTCATTGCCTCAGGGAGG

A5-AT1G54180-XLOC_002726-4324-0
GAATGATCTTAAGAGAATTCGGTTCAG-----------------------
A5-AT1G54180-XLOC_002726-4324-1
GAATGATCTTAAGAGAATTCGGTTCAGGTAATCGTTTCTTGCCAATATTG
CONSENSUS
GAATGATCTTAAGAGAATTCGGTTCAG.......................

A5-AT1G54180-XLOC_002726-4324-0

A5-AT1G54180-XLOC_002726-4324-1
      AACTGAATATATGTTTTGTGTTTTCCCATTGATCTGCTTTTACTGTCTTG
CONSENSUS
      ....

A5-AT1G54180-XLOC_002726-4324-0

A5-AT1G54180-XLOC_002726-4324-1
      TTACGGCATGTTAGGTTCTGAATTTGGAAATAGTGGTTCTTTTTCTTCTT
CONSENSUS
      ....

A5-AT1G54180-XLOC_002726-4324-0

A5-AT1G54180-XLOC_002726-4324-1
      CTTTTAACACTCATGAGTAGTACAATTGTTGATGATATTATTTGTGGGAC
CONSENSUS
      ....

A5-AT1G54180-XLOC_002726-4324-0

A5-AT1G54180-XLOC_002726-4324-1
      TACTGTACTGAGAACACTTGTATTGTTTCTGATTCAATTGAAGTCTAGAT
CONSENSUS
      ....

A5-AT1G54180-XLOC_002726-4324-0

A5-AT1G54180-XLOC_002726-4324-1
      GACCCTGAGCTTTAACAAGTCTTTCGTTGGCAATGGTTTTCATGCTATTG
CONSENSUS
      ....

A5-AT1G54180-XLOC_002726-4324-0

A5-AT1G54180-XLOC_002726-4324-1
      CTGAAATTAAGAAGCACCAGGTTTCCATATTACCGAGACCAGCTTCTGTT
CONSENSUS
      ....

A5-AT1G54180-XLOC_002726-4324-0
      ---------------------------------------TCGTGAGATGT
A5-AT1G54180-XLOC_002726-4324-1
      GTGGTGCCGTCAAGGATGGGTATTTTGGCCCCAGAATTGTCGTGAGATGT
CONSENSUS
      ....TCGTGAGATGT

A5-AT1G54180-XLOC_002726-4324-0
      TTAATAAATGGCAAGCTCAAAAATGGTGGGTGGAGAATTTCGAGAAGGTC
A5-AT1G54180-XLOC_002726-4324-1
      TTAATAAATGGCAAGCTCAAAAATGGTGGGTGGAGAATTTCGAGAAGGTC
CONSENSUS
      TTAATAAATGGCAAGCTCAAAAATGGTGGGTGGAGAATTTCGAGAAGGTC

A5-AT1G54180-XLOC_002726-4324-0
      ATGGAGTTATACAACGTGCAGTTCAATCAGCAGAGCGTACCGCTTCAAAC

A5-AT1G54180-XLOC_002726-4324-1
      ATGGAGTTATACAACGTGCAGTTCAATCAGCAGAGCGTACCGCTTCAAAC
CONSENSUS
      ATGGAGTTATACAACGTGCAGTTCAATCAGCAGAGCGTACCGCTTCAAAC

A5-AT1G54180-XLOC_002726-4324-0 TCCTCCTGTATCTGAAGATGGG
A5-AT1G54180-XLOC_002726-4324-1 TCCTCCTGTATCTGAAGATGGG
CONSENSUS   TCCTCCTGTATCTGAAGATGGG

alignment for event: A3-AT1G54180-XLOC_002726-4325

A3-AT1G54180-XLOC_002726-4325-0
      CTAAAAGACATGGCAGTAAAGGCTTCAGGGGCATACAAAAACTGCAAACC
A3-AT1G54180-XLOC_002726-4325-1
      CTAAAAGACATGGCAGTAAAGGCTTCAGGGGCATACAAAAACTGCAAACC
CONSENSUS
      CTAAAAGACATGGCAGTAAAGGCTTCAGGGGCATACAAAAACTGCAAACC

A3-AT1G54180-XLOC_002726-4325-0
      GTGTTCTGGGACAACAAACCGGAATCAGAATCGAAACTATGCTGATTCGG
A3-AT1G54180-XLOC_002726-4325-1
      GTGTTCTGGGACAACAAACCGGAATCAGAATCGAAACTATGCTGATTCGG
CONSENSUS
      GTGTTCTGGGACAACAAACCGGAATCAGAATCGAAACTATGCTGATTCGG

A3-AT1G54180-XLOC_002726-4325-0
      ATGCTGCTTCAGATTCTGGAAGATTCCATTATTCATACCAGAGAGCTGGG
A3-AT1G54180-XLOC_002726-4325-1
      ATGCTGCTTCAGATTCTGGAAGATTCCATTATTCATACCAGAGAGCTGGG
CONSENSUS
      ATGCTGCTTCAGATTCTGGAAGATTCCATTATTCATACCAGAGAGCTGGG

A3-AT1G54180-XLOC_002726-4325-0
      ACTGCAACCTCAACTCCTAAGATTTGGGGGAATGAAATGGAGTCAAGGTT
A3-AT1G54180-XLOC_002726-4325-1
      ACTGCAACCTCAACTCCTAAGATTTGGGGGAATGAAATGGAGTCAAGGTT
CONSENSUS
      ACTGCAACCTCAACTCCTAAGATTTGGGGGAATGAAATGGAGTCAAGGTT

A3-AT1G54180-XLOC_002726-4325-0
      AAAAGGGATTTCTAGTGAAGAAGGTACACCTACGTCTATGAGTGGTCGAA
A3-AT1G54180-XLOC_002726-4325-1
      AAAAGGGATTTCTAGTGAAGAAGGTACACCTACGTCTATGAGTGGTCGAA
CONSENSUS
      AAAAGGGATTTCTAGTGAAGAAGGTACACCTACGTCTATGAGTGGTCGAA

A3-AT1G54180-XLOC_002726-4325-0
      CAGAATCCATAGTGTTCATGGAGGATGATGAGGTCAAGGAATGGGTTGCT
A3-AT1G54180-XLOC_002726-4325-1
      CAGAATCCATAGTGTTCATGGAGGATGATGAGGTCAAGGAATGGGTTGCT
CONSENSUS
      CAGAATCCATAGTGTTCATGGAGGATGATGAGGTCAAGGAATGGGTTGCT

A3-AT1G54180-XLOC_002726-4325-0

CAAGTGGAGCCTGGTGTTCTCATCACATTTGTGTCATTGCCTCAGGGAGG
A3-AT1G54180-XLOC_002726-4325-1
CAAGTGGAGCCTGGTGTTCTCATCACATTTGTGTCATTGCCTCAGGGAGG
CONSENSUS
CAAGTGGAGCCTGGTGTTCTCATCACATTTGTGTCATTGCCTCAGGGAGG

A3-AT1G54180-XLOC_002726-4325-0
GAATGATCTTAAGAGAATTCGGTTCAGTCTAGATGACCCTGAGCTTTAAC
A3-AT1G54180-XLOC_002726-4325-1
GAATGATCTTAAGAGAATTCGGTTCAG-----------------------
CONSENSUS
GAATGATCTTAAGAGAATTCGGTTCAG.......................

A3-AT1G54180-XLOC_002726-4325-0
AAGTCTTTCGTTGGCAATGGTTTTCATGCTATTGCTGAAATTAAGAAGCA
A3-AT1G54180-XLOC_002726-4325-1

CONSENSUS
....

A3-AT1G54180-XLOC_002726-4325-0
CCAGGTTTCCATATTACCGAGACCAGCTTCTGTTGTGGTGCCGTCAAGGA
A3-AT1G54180-XLOC_002726-4325-1

CONSENSUS
....

A3-AT1G54180-XLOC_002726-4325-0
TGGGTATTTTGGCCCCAGAATTGGTATCTCTAGCTCTTGGGGATTTTTTT
A3-AT1G54180-XLOC_002726-4325-1

CONSENSUS
....

A3-AT1G54180-XLOC_002726-4325-0
GTATTCTGCGGATGCTGAGTTTTTTTGTGTTACACGTTAGACCTGTTTCC
A3-AT1G54180-XLOC_002726-4325-1

CONSENSUS
....

A3-AT1G54180-XLOC_002726-4325-0
CTTTGTGGCTAAGCTAAAAGATGTTTTGGTGATTCGAAGCTAGATGGTAA
A3-AT1G54180-XLOC_002726-4325-1

CONSENSUS
....

A3-AT1G54180-XLOC_002726-4325-0
TGTTTTTAAATTGTTACTTCTTGTAGTCGTGAGATGTTTAATAAATGGCA
A3-AT1G54180-XLOC_002726-4325-1 -------------------------
TCGTGAGATGTTTAATAAATGGCA
CONSENSUS
.........................TCGTGAGATGTTTAATAAATGGCA

A3-AT1G54180-XLOC_002726-4325-0

AGCTCAAAAATGGTGGGTGGAGAATTTCGAGAAGGTCATGGAGTTATACA
A3-AT1G54180-XLOC_002726-4325-1
AGCTCAAAAATGGTGGGTGGAGAATTTCGAGAAGGTCATGGAGTTATACA
CONSENSUS
AGCTCAAAAATGGTGGGTGGAGAATTTCGAGAAGGTCATGGAGTTATACA

A3-AT1G54180-XLOC_002726-4325-0
ACGTGCAGTTCAATCAGCAGAGCGTACCGCTTCAAACTCCTCCTGTATCT
A3-AT1G54180-XLOC_002726-4325-1
ACGTGCAGTTCAATCAGCAGAGCGTACCGCTTCAAACTCCTCCTGTATCT
CONSENSUS
ACGTGCAGTTCAATCAGCAGAGCGTACCGCTTCAAACTCCTCCTGTATCT

A3-AT1G54180-XLOC_002726-4325-0 GAAGATGGG
A3-AT1G54180-XLOC_002726-4325-1 GAAGATGGG
CONSENSUS GAAGATGGG

alignment for event: RI-AT1G09270-XLOC_000437-5350

RI-AT1G09270-XLOC_000437-5350-0
ATATTTGGTGACCCAAGGCTGCATCAAACCACTTTGTGATCTTCTAATCT
RI-AT1G09270-XLOC_000437-5350-1
ATATTTGGTGACCCAAGGCTGCATCAAACCACTTTGTGATCTTCTAATCT
CONSENSUS
ATATTTGGTGACCCAAGGCTGCATCAAACCACTTTGTGATCTTCTAATCT

RI-AT1G09270-XLOC_000437-5350-0
GCCCTGATCCAAGGATCGTGACAGTGTGCCTCGAGGGTCTTGAAAACATT
RI-AT1G09270-XLOC_000437-5350-1
GCCCTGATCCAAGGATCGTGACAGTGTGCCTCGAGGGTCTTGAAAACATT
CONSENSUS
GCCCTGATCCAAGGATCGTGACAGTGTGCCTCGAGGGTCTTGAAAACATT

RI-AT1G09270-XLOC_000437-5350-0
CTCAAGGTTGGTGAGGCTGACAAGGAAATGGGATTAAACAGTGGGGTGAA
RI-AT1G09270-XLOC_000437-5350-1
CTCAAGGTTGGTGAGGCTGACAAGGAAATGGGATTAAACAGTGGGGTGAA
CONSENSUS
CTCAAGGTTGGTGAGGCTGACAAGGAAATGGGATTAAACAGTGGGGTGAA

RI-AT1G09270-XLOC_000437-5350-0
TCTCTATGCTCAAATAATAGAAGAATCTGATGGGTTGGACAAAGTCGAAA
RI-AT1G09270-XLOC_000437-5350-1
TCTCTATGCTCAAATAATAGAAGAATCTGATGGGTTGGACAAAGTCGAAA
CONSENSUS
TCTCTATGCTCAAATAATAGAAGAATCTGATGGGTTGGACAAAGTCGAAA

RI-AT1G09270-XLOC_000437-5350-0
ACCTTCAATCCCATGATAACAACGAGATATATGAGAAGGCCGTTAAGATA
RI-AT1G09270-XLOC_000437-5350-1
ACCTTCAATCCCATGATAACAACGAGATATATGAGAAGGCCGTTAAGATA
CONSENSUS
ACCTTCAATCCCATGATAACAACGAGATATATGAGAAGGCCGTTAAGATA

RI-AT1G09270-XLOC_000437-5350-0
TTGGAGAGATATTGGGCTGAAGAAGAGGAAGAACAGATTCTGCAGGACGG
RI-AT1G09270-XLOC_000437-5350-1
TTGGAGAGATATTGGGCTGAAGAAGAGGAAGAACAGATTCTGCAGGACGG
CONSENSUS
TTGGAGAGATATTGGGCTGAAGAAGAGGAAGAACAGATTCTGCAGGACGG

RI-AT1G09270-XLOC_000437-5350-0
CGGGAATGACAATTCACAACAGGCTTTCAATTTTGGGAACAATCCCGCTG
RI-AT1G09270-XLOC_000437-5350-1
CGGGAATGACAATTCACAACAGGCTTTCAATTTTGGGAACAATCCCGCTG
CONSENSUS
CGGGAATGACAATTCACAACAGGCTTTCAATTTTGGGAACAATCCCGCTG

RI-AT1G09270-XLOC_000437-5350-0
CTCCCGTTGGTGGATTCAAATTTGCCTGAAAAAGGTATACAGTAGTACAA
RI-AT1G09270-XLOC_000437-5350-1
CTCCCGTTGGTGGATTCAAATTTGCCTGAAAAAG----------------
CONSENSUS
CTCCCGTTGGTGGATTCAAATTTGCCTGAAAAAG................

RI-AT1G09270-XLOC_000437-5350-0
ATTGAAATCCTTTGGTTTGGGAAACATGAGATTCAGCTTTTGCAACTGTT
RI-AT1G09270-XLOC_000437-5350-1

CONSENSUS
....

RI-AT1G09270-XLOC_000437-5350-0
ATTGACAATTGCCAGGTTCAAGTTCGACTTTTTGAGTTCTGGTGCAAAAA
RI-AT1G09270-XLOC_000437-5350-1 ---------------
GTTCAAGTTCGACTTTTTGAGTTCTGGTGCAAAAA
CONSENSUS
...............GTTCAAGTTCGACTTTTTGAGTTCTGGTGCAAAAA

RI-AT1G09270-XLOC_000437-5350-0
GGCTTATTTTTCTGTTTTTTTTTCTTTTTGTTGTTGATGTTGTCCTTATT
RI-AT1G09270-XLOC_000437-5350-1
GGCTTATTTTTCTGTTTTTTTTTCTTTTTGTTGTTGATGTTGTCCTTATT
CONSENSUS
GGCTTATTTTTCTGTTTTTTTTTCTTTTTGTTGTTGATGTTGTCCTTATT

RI-AT1G09270-XLOC_000437-5350-0
TGTCTGAATCATTCTCTGATTCTGTATCTTAAACTCTGCTATGAATTATG
RI-AT1G09270-XLOC_000437-5350-1
TGTCTGAATCATTCTCTGATTCTGTATCTTAAACTCTGCTATGAATTATG
CONSENSUS
TGTCTGAATCATTCTCTGATTCTGTATCTTAAACTCTGCTATGAATTATG

RI-AT1G09270-XLOC_000437-5350-0
TGTAGTGTGGTTTCTGGTTTAGGATATGGTACACTTTGTATTTGTCACTT
RI-AT1G09270-XLOC_000437-5350-1
TGTAGTGTGGTTTCTGGTTTAGGATATGGTACACTTTGTATTTGTCACTT
CONSENSUS
TGTAGTGTGGTTTCTGGTTTAGGATATGGTACACTTTGTATTTGTCACTT

RI-AT1G09270-XLOC_000437-5350-0
      TAATCTTTGTCGTCGTTATAGTTTAATAACTTTTACTTGGCAAGAGTACA
RI-AT1G09270-XLOC_000437-5350-1
      TAATCTTTGTCGTCGTTATAGTTTAATAACTTTTACTTGGCAAGAGTACA
CONSENSUS
      TAATCTTTGTCGTCGTTATAGTTTAATAACTTTTACTTGGCAAGAGTACA

RI-AT1G09270-XLOC_000437-5350-0
      AGTACAAGAAATATTTGATTGCAAAAGAGTCTTCTTGTCTTGTTAATAAA
RI-AT1G09270-XLOC_000437-5350-1
      AGTACAAGAAATATTTGATTGCAAAAGAGTCTTCTTGTCTTGTTAATAAA
CONSENSUS
      AGTACAAGAAATATTTGATTGCAAAAGAGTCTTCTTGTCTTGTTAATAAA

RI-AT1G09270-XLOC_000437-5350-0 GTCCTCAAATGAATTGGT
RI-AT1G09270-XLOC_000437-5350-1 GTCCTCAAATGAATTGGT
CONSENSUS   GTCCTCAAATGAATTGGT

alignment for event: A3-AT1G30510-XLOC_005726-1251

A3-AT1G30510-XLOC_005726-1251-0
      GCTGGTGCTGTCTCAGTTTCAATTGAAAACCAACGTTCTCTTAGAAGATC
A3-AT1G30510-XLOC_005726-1251-1
      GCTGGTGCTGTCTCAGTTTCAATTGAAAACCAACGTTCTCTTAGAAGATC
CONSENSUS
      GCTGGTGCTGTCTCAGTTTCAATTGAAAACCAACGTTCTCTTAGAAGATC

A3-AT1G30510-XLOC_005726-1251-0 CGTCTTCAAG---
AACAATAGCATAAGCTTCAACAGCAAGTCATGGTCAT
A3-AT1G30510-XLOC_005726-1251-1
      CGTCTTCAAGCAGAACAATAGCATAAGCTTCAACAGCAAGTCATGGTCAT
CONSENSUS
      CGTCTTCAAG...AACAATAGCATAAGCTTCAACAGCAAGTCATGGTCAT

A3-AT1G30510-XLOC_005726-1251-0
      CTTCTTTAGCATTGAACCAGAAGACAACAAGCATAAGAGATGGGAAACGG
A3-AT1G30510-XLOC_005726-1251-1
      CTTCTTTAGCATTGAACCAGAAGACAACAAGCATAAGAGATGGGAAACGG
CONSENSUS
      CTTCTTTAGCATTGAACCAGAAGACAACAAGCATAAGAGATGGGAAACGG

A3-AT1G30510-XLOC_005726-1251-0
      TACCCGAGCACGACAATATGTATGTCGGTTCAACAAACAAGTAGTTCCAA
A3-AT1G30510-XLOC_005726-1251-1
      TACCCGAGCACGACAATATGTATGTCGGTTCAACAAACAAGTAGTTCCAA
CONSENSUS
      TACCCGAGCACGACAATATGTATGTCGGTTCAACAAACAAGTAGTTCCAA

A3-AT1G30510-XLOC_005726-1251-0
      GGTTACTGTCTCTCCTATAGAGTTGGAAGACCCTAAGGATCCTCCTTTGA
A3-AT1G30510-XLOC_005726-1251-1
      GGTTACTGTCTCTCCTATAGAGTTGGAAGACCCTAAGGATCCTCCTTTGA
CONSENSUS
      GGTTACTGTCTCTCCTATAGAGTTGGAAGACCCTAAGGATCCTCCTTTGA

```
A3-AT1G30510-XLOC_005726-1251-0
      ACTTGTACAAACCCAAGGAGTCTTACACCGCTAAGATTGTCTCTGTGGAG
A3-AT1G30510-XLOC_005726-1251-1
      ACTTGTACAAACCCAAGGAGTCTTACACCGCTAAGATTGTCTCTGTGGAG
CONSENSUS
      ACTTGTACAAACCCAAGGAGTCTTACACCGCTAAGATTGTCTCTGTGGAG

A3-AT1G30510-XLOC_005726-1251-0
      CGAGTAGTTGGCCCGAAAGCCCCTGGAGAAACTTGTCATATCGTCATCGA
A3-AT1G30510-XLOC_005726-1251-1
      CGAGTAGTTGGCCCGAAAGCCCCTGGAGAAACTTGTCATATCGTCATCGA
CONSENSUS
      CGAGTAGTTGGCCCGAAAGCCCCTGGAGAAACTTGTCATATCGTCATCGA

A3-AT1G30510-XLOC_005726-1251-0
      TCATGATGGTAACCTTCCTTACTGGGAAGGACAGAGTTACGGTGTGATTC
A3-AT1G30510-XLOC_005726-1251-1
      TCATGATGGTAACCTTCCTTACTGGGAAGGACAGAGTTACGGTGTGATTC
CONSENSUS
      TCATGATGGTAACCTTCCTTACTGGGAAGGACAGAGTTACGGTGTGATTC

A3-AT1G30510-XLOC_005726-1251-0  CTCCA
A3-AT1G30510-XLOC_005726-1251-1  CTCCA
CONSENSUS                        CTCCA


alignment for event: A3-AT1G35320-XLOC_005993-8471

A3-AT1G35320-XLOC_005993-8471-0
      TCTTCCTCATCAACCAAGCAGCAGCCATTTACATCTTCTTCATCACCATC
A3-AT1G35320-XLOC_005993-8471-1
      TCTTCCTCATCAACCAAGCAGCAGCCATTTACATCTTCTTCATCACCATC
CONSENSUS
      TCTTCCTCATCAACCAAGCAGCAGCCATTTACATCTTCTTCATCACCATC

A3-AT1G35320-XLOC_005993-8471-0
      TCTTCTTTATCTCTCATCTCAATCTCAGCCATCATTATCATCATCGTTGT
A3-AT1G35320-XLOC_005993-8471-1
      TCTTCTTTATCTCTCATCTCAATCTCAGCCATCATTATCATCATCGTTGT
CONSENSUS
      TCTTCTTTATCTCTCATCTCAATCTCAGCCATCATTATCATCATCGTTGT

A3-AT1G35320-XLOC_005993-8471-0
      GACATCGGAGTTGACGTCACGTTGTGGTCGTGATTGTTATACGTGGTTGG
A3-AT1G35320-XLOC_005993-8471-1
      GACATCGGAGTTGACGTCACGTTGTGGTCGTGATTGTTATACGTGGTTGG
CONSENSUS
      GACATCGGAGTTGACGTCACGTTGTGGTCGTGATTGTTATACGTGGTTGG

A3-AT1G35320-XLOC_005993-8471-0
      TTTCGTCGTCGTTCATCACCGGATTGTTAG---AAAACAGTTCAAGTGGT
A3-AT1G35320-XLOC_005993-8471-1
      TTTCGTCGTCGTTCATCACCGGATTGTTAGAAGAAAACAGTTCAAGTGGT
CONSENSUS
```

TTTCGTCGTCGTTCATCACCGGATTGTTAG...AAAACAGTTCAAGTGGT

A3-AT1G35320-XLOC_005993-8471-0
GGAATTGGTGATCCGTTTCCCGTCACGTCTGAAGGATTTTGGGCGAGTTC
A3-AT1G35320-XLOC_005993-8471-1
GGAATTGGTGATCCGTTTCCCGTCACGTCTGAAGGATTTTGGGCGAGTTC
CONSENSUS
GGAATTGGTGATCCGTTTCCCGTCACGTCTGAAGGATTTTGGGCGAGTTC

A3-AT1G35320-XLOC_005993-8471-0
TCAACGGTTCTGCGTAGATCTCGTTCCCAAGTTTTCAGAATAGTTTTCGG
A3-AT1G35320-XLOC_005993-8471-1
TCAACGGTTCTGCGTAGATCTCGTTCCCAAGTTTTCAGAATAGTTTTCGG
CONSENSUS
TCAACGGTTCTGCGTAGATCTCGTTCCCAAGTTTTCAGAATAGTTTTCGG

A3-AT1G35320-XLOC_005993-8471-0 ATCTAAAATCGGAG
A3-AT1G35320-XLOC_005993-8471-1 ATCTAAAATCGGAG
CONSENSUS ATCTAAAATCGGAG

alignment for event: RI-AT1G10890-XLOC_000523-8249

RI-AT1G10890-XLOC_000523-8249-0
GACCAATCCGTTCAGAGAGATTAAAGCTGAGCTGAGCTTCTCCTTATAAA
RI-AT1G10890-XLOC_000523-8249-1
GACCAATCCGTTCAGAGAGATTAAAGCTGAGCTGAGCTTCTCCTTATAAA
CONSENSUS
GACCAATCCGTTCAGAGAGATTAAAGCTGAGCTGAGCTTCTCCTTATAAA

RI-AT1G10890-XLOC_000523-8249-0
GGAAGATACTGCTTGTTCTACGGAATTTTGAGAGATGCCTCGGGACTTGT
RI-AT1G10890-XLOC_000523-8249-1
GGAAGATACTGCTTGTTCTACGGAATTTTGAGAGATGCCTCGGGACTTGT
CONSENSUS
GGAAGATACTGCTTGTTCTACGGAATTTTGAGAGATGCCTCGGGACTTGT

RI-AT1G10890-XLOC_000523-8249-0
CAAGATCGAGGTCACCGTCTCCATCACCTTCACGTCGTAGAAAGCACTCG
RI-AT1G10890-XLOC_000523-8249-1
CAAGATCGAGGTCACCGTCTCCATCACCTTCACGTCGTAGAAAGCACTCG
CONSENSUS
CAAGATCGAGGTCACCGTCTCCATCACCTTCACGTCGTAGAAAGCACTCG

RI-AT1G10890-XLOC_000523-8249-0
AGGTCTCCCGTAAGGCAGAGGCATAGCAGGAGGAGTAGAAGAGACAGAAG
RI-AT1G10890-XLOC_000523-8249-1
AGGTCTCCCGTAAGGCAGAGGCATAGCAGGAGGAGTAGAAGAGACAGAAG
CONSENSUS
AGGTCTCCCGTAAGGCAGAGGCATAGCAGGAGGAGTAGAAGAGACAGAAG

RI-AT1G10890-XLOC_000523-8249-0
CCCTTCTCCATACTCATCTCATTCGTATAGCAGGTGATACCCCTAGTGTT
RI-AT1G10890-XLOC_000523-8249-1
CCCTTCTCCATACTCATCTCATTCGTATAGCAG-----------------

CONSENSUS
      CCCTTCTCCATACTCATCTCATTCGTATAGCAG.................

RI-AT1G10890-XLOC_000523-8249-0
      TGTTTTTTCCGCTATTCGTTTGCCTTAGTTATCATTCGTTTATACGAACT
RI-AT1G10890-XLOC_000523-8249-1

CONSENSUS
      ....

RI-AT1G10890-XLOC_000523-8249-0
      GTAAGAAACATTGGTGGAACACGGTTATAGATAGACATGTAAAGTTTACA
RI-AT1G10890-XLOC_000523-8249-1

CONSENSUS
      ....

RI-AT1G10890-XLOC_000523-8249-0
      GACTCAAGAATTGCTTTTAGTAAAGTTCCTACTTATAGATATATTTTCTA
RI-AT1G10890-XLOC_000523-8249-1

CONSENSUS
      ....

RI-AT1G10890-XLOC_000523-8249-0
      CCCTTCTCTAAAAGAGAGTGCAATGTCTGGATGCTGCTGGCTCTGTATCG
RI-AT1G10890-XLOC_000523-8249-1

CONSENSUS
      ....

RI-AT1G10890-XLOC_000523-8249-0
      TAAGCGTTTTTTTGAGTGATGCTGCGTATTGGAAATATATCGTGTTTATT
RI-AT1G10890-XLOC_000523-8249-1

CONSENSUS
      ....

RI-AT1G10890-XLOC_000523-8249-0
      TGGCTGTTGTGATTAAATTTGTTGCAAGAGAATATGTTTTATGACCTGAG
RI-AT1G10890-XLOC_000523-8249-1

CONSENSUS
      ....

RI-AT1G10890-XLOC_000523-8249-0
      CTTTGTGATTTTTAGAATGAAACCTGTATATTCGGTAAAGACATAAGTTA
RI-AT1G10890-XLOC_000523-8249-1

CONSENSUS
      ....

RI-AT1G10890-XLOC_000523-8249-0
      AGCTGGGATAATAGCACTACTGATAGTCTGATCTAGTTTATATTTTAATT
RI-AT1G10890-XLOC_000523-8249-1

CONSENSUS
      ....

RI-AT1G10890-XLOC_000523-8249-0
      GTATCTGTAGATTATTAGTTAGAATCAGTTTTAAGAGCGACTATATTATG
RI-AT1G10890-XLOC_000523-8249-1

CONSENSUS
      ....

RI-AT1G10890-XLOC_000523-8249-0
      TTCTTTTAAATGGAGTTTGTTAGCAATAGTGATACAAAACCACCACTATT
RI-AT1G10890-XLOC_000523-8249-1

CONSENSUS
      ....

RI-AT1G10890-XLOC_000523-8249-0
      CCTTAGGTAAGATAATATAGTAATTTGTTGAGAACATGGCTGAGAAGCTA
RI-AT1G10890-XLOC_000523-8249-1

CONSENSUS
      ....

RI-AT1G10890-XLOC_000523-8249-0
      GGAAAATGATACATGCGCTTACCATTATAAAATAGCTCAGTTTCTTCCCG
RI-AT1G10890-XLOC_000523-8249-1

CONSENSUS
      ....

RI-AT1G10890-XLOC_000523-8249-0
      TTCCCACGGGAGGGGAAATGAGCAGCTGATGGATTGCTCTTAGGTGTTCT
RI-AT1G10890-XLOC_000523-8249-1
      ---GTGTTCT
CONSENSUS
      ....GTGTTCT

RI-AT1G10890-XLOC_000523-8249-0
      TTCATTGATCCCTTGTCTCGTTAGCTTCTCTTCGTGTGATAAAAGTTGGA
RI-AT1G10890-XLOC_000523-8249-1
      TTCATTGATCCCTTGTCTCGTTAGCTTCTCTTCGTGTGATAAAAGTTGGA
CONSENSUS
      TTCATTGATCCCTTGTCTCGTTAGCTTCTCTTCGTGTGATAAAAGTTGGA

RI-AT1G10890-XLOC_000523-8249-0
      GCTGATAATTCTTTACAGTGTATGAGGTAACTGCACATACTTTTGTTTTC
RI-AT1G10890-XLOC_000523-8249-1
      GCTGATAATTCTTTACAGTGTATGAGGTAACTGCACATACTTTTGTTTTC
CONSENSUS
      GCTGATAATTCTTTACAGTGTATGAGGTAACTGCACATACTTTTGTTTTC

RI-AT1G10890-XLOC_000523-8249-0
      TTTATGTGTAACTGTGGGCTACCTGATGCCTAGTCTTCACGTGCGTGGTA
RI-AT1G10890-XLOC_000523-8249-1
      TTTATGTGTAACTGTGGGCTACCTGATGCCTAGTCTTCACGTGCGTGGTA

CONSENSUS
TTTATGTGTAACTGTGGGCTACCTGATGCCTAGTCTTCACGTGCGTGGTA

RI-AT1G10890-XLOC_000523-8249-0
CTTCATTAGTTCCATCTTTTTGCTTACTACTTTATTTTGGGTAATGTTCA
RI-AT1G10890-XLOC_000523-8249-1
CTTCATTAGTTCCATCTTTTTGCTTACTACTTTATTTTGGGTAATGTTCA
CONSENSUS
CTTCATTAGTTCCATCTTTTTGCTTACTACTTTATTTTGGGTAATGTTCA

RI-AT1G10890-XLOC_000523-8249-0
GGCGAAAAAGTCGTTCTATTTCTCCTAGGCGCCATCGAAGTCGATCTGTT
RI-AT1G10890-XLOC_000523-8249-1
GGCGAAAAAGTCGTTCTATTTCTCCTAGGCGCCATCGAAGTCGATCTGTT
CONSENSUS
GGCGAAAAAGTCGTTCTATTTCTCCTAGGCGCCATCGAAGTCGATCTGTT

RI-AT1G10890-XLOC_000523-8249-0
ACTCCTAAGAGACGTTCTCCAACCCCAAAACGTTACAAAAGACAAAAGAG
RI-AT1G10890-XLOC_000523-8249-1
ACTCCTAAGAGACGTTCTCCAACCCCAAAACGTTACAAAAGACAAAAGAG
CONSENSUS
ACTCCTAAGAGACGTTCTCCAACCCCAAAACGTTACAAAAGACAAAAGAG

RI-AT1G10890-XLOC_000523-8249-0
TAGGAGTTCAACTCCATCTCCTGCAAAAAGATCTCCCGCCGCAACCCTTG
RI-AT1G10890-XLOC_000523-8249-1
TAGGAGTTCAACTCCATCTCCTGCAAAAAGATCTCCCGCCGCAACCCTTG
CONSENSUS
TAGGAGTTCAACTCCATCTCCTGCAAAAAGATCTCCCGCCGCAACCCTTG

RI-AT1G10890-XLOC_000523-8249-0
AGTCAGCCAAAAATAGGAATGGAGAAAAACTTAAAAGAGAAGAGGAAGAA
RI-AT1G10890-XLOC_000523-8249-1
AGTCAGCCAAAAATAGGAATGGAGAAAAACTTAAAAGAGAAGAGGAAGAA
CONSENSUS
AGTCAGCCAAAAATAGGAATGGAGAAAAACTTAAAAGAGAAGAGGAAGAA

RI-AT1G10890-XLOC_000523-8249-0
CGAAAAAGGTAAATCAATACAAAATTTTGAAGTAAGATGGATGTTATGGT
RI-AT1G10890-XLOC_000523-8249-1
CGAAAAAGGTAAATCAATACAAAATTTTGAAGTAAGATGGATGTTATGGT
CONSENSUS
CGAAAAAGGTAAATCAATACAAAATTTTGAAGTAAGATGGATGTTATGGT

RI-AT1G10890-XLOC_000523-8249-0
GAAATTAAGGTTTTTTGTTCTCAAATGTTATTTTAGTGTAGTGTGGAAGT
RI-AT1G10890-XLOC_000523-8249-1
GAAATTAAGGTTTTTTGTTCTCAAATGTTATTTTAGTGTAGTGTGGAAGT
CONSENSUS
GAAATTAAGGTTTTTTGTTCTCAAATGTTATTTTAGTGTAGTGTGGAAGT

RI-AT1G10890-XLOC_000523-8249-0
CCTTGATTGTTAGTCTCAAAAGTGCGACAGGTTTTATGGTATTCTCTCAC
RI-AT1G10890-XLOC_000523-8249-1
CCTTGATTGTTAGTCTCAAAAGTGCGACAGGTTTTATGGTATTCTCTCAC

CONSENSUS
CCTTGATTGTTAGTCTCAAAAGTGCGACAGGTTTTATGGTATTCTCTCAC

RI-AT1G10890-XLOC_000523-8249-0
AGGCTTTGCTTATGGAAGATAAGCTATATTTCGATTTGTTTATATTTGAT
RI-AT1G10890-XLOC_000523-8249-1
AGGCTTTGCTTATGGAAGATAAGCTATATTTCGATTTGTTTATATTTGAT
CONSENSUS
AGGCTTTGCTTATGGAAGATAAGCTATATTTCGATTTGTTTATATTTGAT

RI-AT1G10890-XLOC_000523-8249-0
CCTCTGGCACAAATGTTCGTACTATCTCGATTTGATGAGTTACCGTTTGT
RI-AT1G10890-XLOC_000523-8249-1
CCTCTGGCACAAATGTTCGTACTATCTCGATTTGATGAGTTACCGTTTGT
CONSENSUS
CCTCTGGCACAAATGTTCGTACTATCTCGATTTGATGAGTTACCGTTTGT

RI-AT1G10890-XLOC_000523-8249-0
CATTACATGGATCATGATTGACTTAGAGATGTAAATATGTCCAAAGCACA
RI-AT1G10890-XLOC_000523-8249-1
CATTACATGGATCATGATTGACTTAGAGATGTAAATATGTCCAAAGCACA
CONSENSUS
CATTACATGGATCATGATTGACTTAGAGATGTAAATATGTCCAAAGCACA

RI-AT1G10890-XLOC_000523-8249-0
AGAATAATCTTGGTGAAGGTTTGCTTGTGTTATGGTGACAAAAAAGAAAT
RI-AT1G10890-XLOC_000523-8249-1
AGAATAATCTTGGTGAAGGTTTGCTTGTGTTATGGTGACAAAAAAGAAAT
CONSENSUS
AGAATAATCTTGGTGAAGGTTTGCTTGTGTTATGGTGACAAAAAAGAAAT

RI-AT1G10890-XLOC_000523-8249-0
TTCTTCACGAAATTTACCATTGGTATTGGGAGGAGTGAAAATGGAAACGA
RI-AT1G10890-XLOC_000523-8249-1
TTCTTCACGAAATTTACCATTGGTATTGGGAGGAGTGAAAATGGAAACGA
CONSENSUS
TTCTTCACGAAATTTACCATTGGTATTGGGAGGAGTGAAAATGGAAACGA

RI-AT1G10890-XLOC_000523-8249-0
TCTCAACATTTTTTACGGATTTGCAATAAAATTTTGGTCTTTTGCTGTTC
RI-AT1G10890-XLOC_000523-8249-1
TCTCAACATTTTTTACGGATTTGCAATAAAATTTTGGTCTTTTGCTGTTC
CONSENSUS
TCTCAACATTTTTTACGGATTTGCAATAAAATTTTGGTCTTTTGCTGTTC

RI-AT1G10890-XLOC_000523-8249-0
AAGATTGAAGACATCAGGAAATGAGAAAGTTCAGGTGATATTCAATATTC
RI-AT1G10890-XLOC_000523-8249-1
AAGATTGAAGACATCAGGAAATGAGAAAGTTCAGGTGATATTCAATATTC
CONSENSUS
AAGATTGAAGACATCAGGAAATGAGAAAGTTCAGGTGATATTCAATATTC

RI-AT1G10890-XLOC_000523-8249-0
TGAACCTGCTGTGGATGTCCTCTAATTTTTCACTGTATTTGCTACCAGGC
RI-AT1G10890-XLOC_000523-8249-1
TGAACCTGCTGTGGATGTCCTCTAATTTTTCACTGTATTTGCTACCAGGC

CONSENSUS
      TGAACCTGCTGTGGATGTCCTCTAATTTTTCACTGTATTTGCTACCAGGC

RI-AT1G10890-XLOC_000523-8249-0
      GACAGCGTGAAGCAGAACTGAAGCTAATAGAGGAAGAAACTGTGAAACGG
RI-AT1G10890-XLOC_000523-8249-1
      GACAGCGTGAAGCAGAACTGAAGCTAATAGAGGAAGAAACTGTGAAACGG
CONSENSUS
      GACAGCGTGAAGCAGAACTGAAGCTAATAGAGGAAGAAACTGTGAAACGG

RI-AT1G10890-XLOC_000523-8249-0
      GTTGAAGAAGCTATTCGAAAGAAGGTCGAAGAAAGCTTACAGTCTGAGAA
RI-AT1G10890-XLOC_000523-8249-1
      GTTGAAGAAGCTATTCGAAAGAAGGTCGAAGAAAGCTTACAGTCTGAGAA
CONSENSUS
      GTTGAAGAAGCTATTCGAAAGAAGGTCGAAGAAAGCTTACAGTCTGAGAA

RI-AT1G10890-XLOC_000523-8249-0
      AATCAAAATGGAAATTCTAACGCTGTTGGAGGAAGGGCGAAAGAGACTTA
RI-AT1G10890-XLOC_000523-8249-1
      AATCAAAATGGAAATTCTAACGCTGTTGGAGGAAGGGCGAAAGAGACTTA
CONSENSUS
      AATCAAAATGGAAATTCTAACGCTGTTGGAGGAAGGGCGAAAGAGACTTA

RI-AT1G10890-XLOC_000523-8249-0
      ATGAAGAAGTCGCGGCTCAACTTGAGGAGGAGAAAGAGGCTTCTCTTATT
RI-AT1G10890-XLOC_000523-8249-1
      ATGAAGAAGTCGCGGCTCAACTTGAGGAGGAGAAAGAGGCTTCTCTTATT
CONSENSUS
      ATGAAGAAGTCGCGGCTCAACTTGAGGAGGAGAAAGAGGCTTCTCTTATT

RI-AT1G10890-XLOC_000523-8249-0 GAGGCTAAAGAAAAAGAG
RI-AT1G10890-XLOC_000523-8249-1 GAGGCTAAAGAAAAAGAG
CONSENSUS   GAGGCTAAAGAAAAAGAG

alignment for event: A3-AT1G05790-XLOC_000249-6396

A3-AT1G05790-XLOC_000249-6396-0
      TTGAAGTATAAGACCTGGCTTTGGTGGACTCGGTTTGCAATGGTGATTAC
A3-AT1G05790-XLOC_000249-6396-1
      TTGAAGTATAAGACCTGGCTTTGGTGGACTCGGTTTGCAATGGTGATTAC
CONSENSUS
      TTGAAGTATAAGACCTGGCTTTGGTGGACTCGGTTTGCAATGGTGATTAC

A3-AT1G05790-XLOC_000249-6396-0
      TGTATTGCAATTTATTGGTGCCACTTACCTTTTATTCCGTCTCGCCAAAT
A3-AT1G05790-XLOC_000249-6396-1
      TGTATTGCAATTTATTGGTGCCACTTACCTTTTATTCCGTCTCGCCAAAT
CONSENSUS
      TGTATTGCAATTTATTGGTGCCACTTACCTTTTATTCCGTCTCGCCAAAT

A3-AT1G05790-XLOC_000249-6396-0
      ATGTTTCCCGTGATGGATTGCCAAGGAATTGCGTTTTAGTAACTTCTTTG
A3-AT1G05790-XLOC_000249-6396-1

ATGTTTCCCGTGATGGATTGCCAAGGAATTGCGTTTTAG-----------
CONSENSUS
ATGTTTCCCGTGATGGATTGCCAAGGAATTGCGTTTTAG...........

A3-AT1G05790-XLOC_000249-6396-0
CTCAGGGTTATCTCCGGACACTGGTGGGTGGAAGCAAACACTTCAGGTTA
A3-AT1G05790-XLOC_000249-6396-1 -----
GGTTATCTCCGGACACTGGTGGGTGGAAGCAAACACTTCAGGTTA
CONSENSUS
.....GGTTATCTCCGGACACTGGTGGGTGGAAGCAAACACTTCAGGTTA

A3-AT1G05790-XLOC_000249-6396-0
CCTTCTTGATCACTGTTTGCTTTGTTGCGCTGGCACAATGCTTTACGGGA
A3-AT1G05790-XLOC_000249-6396-1
CCTTCTTGATCACTGTTTGCTTTGTTGCGCTGGCACAATGCTTTACGGGA
CONSENSUS
CCTTCTTGATCACTGTTTGCTTTGTTGCGCTGGCACAATGCTTTACGGGA

A3-AT1G05790-XLOC_000249-6396-0
TCAGATATATTGCAATGGCGGTCTTTCTATGCAACCCAAGATGATGCCTG
A3-AT1G05790-XLOC_000249-6396-1
TCAGATATATTGCAATGGCGGTCTTTCTATGCAACCCAAGATGATGCCTG
CONSENSUS
TCAGATATATTGCAATGGCGGTCTTTCTATGCAACCCAAGATGATGCCTG

A3-AT1G05790-XLOC_000249-6396-0
GAAAGCTCACTACCAGGAAGTATTTGACCATGGAATTCGTGAAGTTTTGT
A3-AT1G05790-XLOC_000249-6396-1
GAAAGCTCACTACCAGGAAGTATTTGACCATGGAATTCGTGAAGTTTTGT
CONSENSUS
GAAAGCTCACTACCAGGAAGTATTTGACCATGGAATTCGTGAAGTTTTGT

A3-AT1G05790-XLOC_000249-6396-0 GCTGTCTTGGACGTCGTGAATATAT
A3-AT1G05790-XLOC_000249-6396-1 GCTGTCTTGGACGTCGTGAATATAT
CONSENSUS GCTGTCTTGGACGTCGTGAATATAT

alignment for event: A3-AT1G11330-XLOC_000547-8536

A3-AT1G11330-XLOC_000547-8536-0
AAACACATAGCAATCTAGCAGTTATGATCGCAGCACCTGTGATAGGCGTT
A3-AT1G11330-XLOC_000547-8536-1
AAACACATAGCAATCTAGCAGTTATGATCGCAGCACCTGTGATAGGCGTT
CONSENSUS
AAACACATAGCAATCTAGCAGTTATGATCGCAGCACCTGTGATAGGCGTT

A3-AT1G11330-XLOC_000547-8536-0
ATGTTAATTGCTGCGGTCTGCGTTCTTTTAGCATGCCGGAAATACAAAAA
A3-AT1G11330-XLOC_000547-8536-1
ATGTTAATTGCTGCGGTCTGCGTTCTTTTAGCATGCCGGAAATACAAAAA
CONSENSUS
ATGTTAATTGCTGCGGTCTGCGTTCTTTTAGCATGCCGGAAATACAAAAA

A3-AT1G11330-XLOC_000547-8536-0
GCGTCCAGCAGCTCCAGCGAAAGATAGAAGTGCAGAGCTAATGTTTAAGA

A3-AT1G11330-XLOC_000547-8536-1 GCGTCCAG---
CTCCAGCGAAAGATAGAAGTGCAGAGCTAATGTTTAAGA
CONSENSUS
      GCGTCCAG...CTCCAGCGAAAGATAGAAGTGCAGAGCTAATGTTTAAGA

A3-AT1G11330-XLOC_000547-8536-0
      GAATGGAAGCACTTACAAGTGATAATGAGTCTGCTTCTAACCAAATCAAG
A3-AT1G11330-XLOC_000547-8536-1
      GAATGGAAGCACTTACAAGTGATAATGAGTCTGCTTCTAACCAAATCAAG
CONSENSUS
      GAATGGAAGCACTTACAAGTGATAATGAGTCTGCTTCTAACCAAATCAAG

A3-AT1G11330-XLOC_000547-8536-0
      CTCAAGGAGCTTCCACTCTTTGAGTTTCAAGTGTTAGCTACATCAACTGA
A3-AT1G11330-XLOC_000547-8536-1
      CTCAAGGAGCTTCCACTCTTTGAGTTTCAAGTGTTAGCTACATCAACTGA
CONSENSUS
      CTCAAGGAGCTTCCACTCTTTGAGTTTCAAGTGTTAGCTACATCAACTGA

A3-AT1G11330-XLOC_000547-8536-0
      TAGCTTCTCTCTAAGAAACAAGCTCGGGCAAGGCGGGTTTGGTCCTGTTT
A3-AT1G11330-XLOC_000547-8536-1
      TAGCTTCTCTCTAAGAAACAAGCTCGGGCAAGGCGGGTTTGGTCCTGTTT
CONSENSUS
      TAGCTTCTCTCTAAGAAACAAGCTCGGGCAAGGCGGGTTTGGTCCTGTTT

A3-AT1G11330-XLOC_000547-8536-0 ACAAG
A3-AT1G11330-XLOC_000547-8536-1 ACAAG
CONSENSUS   ACAAG

alignment for event: RI-AT1G68560-XLOC_007495-718

RI-AT1G68560-XLOC_007495-718-0
      GATTTCATCAATGTAGATGGGGATACCATAATCTATCAGTTGTTGAAGAT
RI-AT1G68560-XLOC_007495-718-1
      GATTTCATCAATGTAGATGGGGATACCATAATCTATCAGTTGTTGAAGAT
CONSENSUS
      GATTTCATCAATGTAGATGGGGATACCATAATCTATCAGTTGTTGAAGAT

RI-AT1G68560-XLOC_007495-718-0
      GTGGTGGATAATTACAAAAAGGCTAAGATCCCACTTGATGTGATTTGGAA
RI-AT1G68560-XLOC_007495-718-1
      GTGGTGGATAATTACAAAAAGGCTAAGATCCCACTTGATGTGATTTGGAA
CONSENSUS
      GTGGTGGATAATTACAAAAAGGCTAAGATCCCACTTGATGTGATTTGGAA

RI-AT1G68560-XLOC_007495-718-0
      CGATGATGATCACATGGATGGTCACAAAGATTTCACGTTGAATCCTGTGG
RI-AT1G68560-XLOC_007495-718-1
      CGATGATGATCACATGGATGGTCACAAAGATTTCACGTTGAATCCTGTGG
CONSENSUS
      CGATGATGATCACATGGATGGTCACAAAGATTTCACGTTGAATCCTGTGG

RI-AT1G68560-XLOC_007495-718-0

CTTATCCTCGTGCTAAGCTATTGGCGTTCTTGGACAAAATCCACAAGATT
RI-AT1G68560-XLOC_007495-718-1
CTTATCCTCGTGCTAAGCTATTGGCGTTCTTGGACAAAATCCACAAGATT
CONSENSUS
CTTATCCTCGTGCTAAGCTATTGGCGTTCTTGGACAAAATCCACAAGATT

RI-AT1G68560-XLOC_007495-718-0
GGAATGAAGTATATTGTGATCAATGATCCTGGTATTGGTGTCAACGCTAG
RI-AT1G68560-XLOC_007495-718-1
GGAATGAAGTATATTGTGATCAATGATCCTGGTATTGGTGTCAACGCTAG
CONSENSUS
GGAATGAAGTATATTGTGATCAATGATCCTGGTATTGGTGTCAACGCTAG

RI-AT1G68560-XLOC_007495-718-0
CTACGGTACATTTCAGAGAGCCATGGCTGCTGATGTGTTTATTAAGTATG
RI-AT1G68560-XLOC_007495-718-1
CTACGGTACATTTCAGAGAGCCATGGCTGCTGATGTGTTTATTAAGTATG
CONSENSUS
CTACGGTACATTTCAGAGAGCCATGGCTGCTGATGTGTTTATTAAGTATG

RI-AT1G68560-XLOC_007495-718-0
AAGGAAAGCCATTCTTGGCTCAAGTGTGGCCTGGTCCGGTTTACTTCCCT
RI-AT1G68560-XLOC_007495-718-1
AAGGAAAGCCATTCTTGGCTCAAGTGTGGCCTGGTCCGGTTTACTTCCCT
CONSENSUS
AAGGAAAGCCATTCTTGGCTCAAGTGTGGCCTGGTCCGGTTTACTTCCCT

RI-AT1G68560-XLOC_007495-718-0
GATTTCCTTAACCCGAAGACGGTTTCTTGGTGGGGTGATGAAATCAAACG
RI-AT1G68560-XLOC_007495-718-1
GATTTCCTTAACCCGAAGACGGTTTCTTGGTGGG----------------
CONSENSUS
GATTTCCTTAACCCGAAGACGGTTTCTTGGTGGG................

RI-AT1G68560-XLOC_007495-718-0
GTTCCATGATTTAGTCCCTATTGATGGTTTATGGATTGACATGAACGAGG
RI-AT1G68560-XLOC_007495-718-1

CONSENSUS
....

RI-AT1G68560-XLOC_007495-718-0
TGTCGAACTTTTGTTCTGGTTTATGCACAATCCCTGAAGGAAAGCAGTGT
RI-AT1G68560-XLOC_007495-718-1

CONSENSUS
....

RI-AT1G68560-XLOC_007495-718-0
CCAAGTGGGGAAGGTCCTGGTTGGGTTTGTTGCTTGGACTGCAAGAATAT
RI-AT1G68560-XLOC_007495-718-1

CONSENSUS
....

RI-AT1G68560-XLOC_007495-718-0

AACCAAGACTAGATGGGATGATCCTCCTTACAAGATTAATGCTACTGGTG
RI-AT1G68560-XLOC_007495-718-1 --------------
GGGATGATCCTCCTTACAAGATTAATGCTACTGGTG
CONSENSUS
..............GGGATGATCCTCCTTACAAGATTAATGCTACTGGTG

RI-AT1G68560-XLOC_007495-718-0
TCGTAGCTCCTGTTGGGTTCAAGACTATTGCTACAAGTGCTACTCATTAC
RI-AT1G68560-XLOC_007495-718-1
TCGTAGCTCCTGTTGGGTTCAAGACTATTGCTACAAGTGCTACTCATTAC
CONSENSUS
TCGTAGCTCCTGTTGGGTTCAAGACTATTGCTACAAGTGCTACTCATTAC

RI-AT1G68560-XLOC_007495-718-0
AATGGTGTTCGAGAGTACGATGCTCACAGTATCTACGGATTCTCTGAGAC
RI-AT1G68560-XLOC_007495-718-1
AATGGTGTTCGAGAGTACGATGCTCACAGTATCTACGGATTCTCTGAGAC
CONSENSUS
AATGGTGTTCGAGAGTACGATGCTCACAGTATCTACGGATTCTCTGAGAC

RI-AT1G68560-XLOC_007495-718-0
CATCGCAACTCACAAGGGCTTACTTAATGTCCAAGGCAAACGTCCCTTCA
RI-AT1G68560-XLOC_007495-718-1
CATCGCAACTCACAAGGGCTTACTTAATGTCCAAGGCAAACGTCCCTTCA
CONSENSUS
CATCGCAACTCACAAGGGCTTACTTAATGTCCAAGGCAAACGTCCCTTCA

RI-AT1G68560-XLOC_007495-718-0
TTTTATCCCGGTCTACTTTCGTTGGTTCGGGTCAATATGCGGCTCACTGG
RI-AT1G68560-XLOC_007495-718-1
TTTTATCCCGGTCTACTTTCGTTGGTTCGGGTCAATATGCGGCTCACTGG
CONSENSUS
TTTTATCCCGGTCTACTTTCGTTGGTTCGGGTCAATATGCGGCTCACTGG

RI-AT1G68560-XLOC_007495-718-0
ACCGGAGATAACCAAGGAACATGGCAGAGCTTGCAAGTGTCTATCTCAAC
RI-AT1G68560-XLOC_007495-718-1
ACCGGAGATAACCAAGGAACATGGCAGAGCTTGCAAGTGTCTATCTCAAC
CONSENSUS
ACCGGAGATAACCAAGGAACATGGCAGAGCTTGCAAGTGTCTATCTCAAC

RI-AT1G68560-XLOC_007495-718-0
TATGTTGAACTTCGGTATATTTGGAGTTCCCATGGTTGGTTCAGACATTT
RI-AT1G68560-XLOC_007495-718-1
TATGTTGAACTTCGGTATATTTGGAGTTCCCATGGTTGGTTCAGACATTT
CONSENSUS
TATGTTGAACTTCGGTATATTTGGAGTTCCCATGGTTGGTTCAGACATTT

RI-AT1G68560-XLOC_007495-718-0
GTGGATTCTACCCACAACCAACAGAAGAACTCTGCAACCGTTGGATCGAA
RI-AT1G68560-XLOC_007495-718-1
GTGGATTCTACCCACAACCAACAGAAGAACTCTGCAACCGTTGGATCGAA
CONSENSUS
GTGGATTCTACCCACAACCAACAGAAGAACTCTGCAACCGTTGGATCGAA

RI-AT1G68560-XLOC_007495-718-0

GTAGGCGCGTTTTACCCGTTTTCAAGAGATCACGCCAATTACTACTCACC
RI-AT1G68560-XLOC_007495-718-1
GTAGGCGCGTTTTACCCGTTTTCAAGAGATCACGCCAATTACTACTCACC
CONSENSUS
GTAGGCGCGTTTTACCCGTTTTCAAGAGATCACGCCAATTACTACTCACC

RI-AT1G68560-XLOC_007495-718-0
AAGACAAGAGCTTTACCAATGGGACACAGTTGCAGACTCAGCTCGTAACG
RI-AT1G68560-XLOC_007495-718-1
AAGACAAGAGCTTTACCAATGGGACACAGTTGCAGACTCAGCTCGTAACG
CONSENSUS
AAGACAAGAGCTTTACCAATGGGACACAGTTGCAGACTCAGCTCGTAACG

RI-AT1G68560-XLOC_007495-718-0
CTCTTGGTATGAGATACAAGATCCTTCCTTTCCTCTACACTCTTAACTAC
RI-AT1G68560-XLOC_007495-718-1
CTCTTGGTATGAGATACAAGATCCTTCCTTTCCTCTACACTCTTAACTAC
CONSENSUS
CTCTTGGTATGAGATACAAGATCCTTCCTTTCCTCTACACTCTTAACTAC

RI-AT1G68560-XLOC_007495-718-0
GAAGCGCATATGACTGGTGCACCCATCGCTAGACCGCTCTTCTTCTCATT
RI-AT1G68560-XLOC_007495-718-1
GAAGCGCATATGACTGGTGCACCCATCGCTAGACCGCTCTTCTTCTCATT
CONSENSUS
GAAGCGCATATGACTGGTGCACCCATCGCTAGACCGCTCTTCTTCTCATT

RI-AT1G68560-XLOC_007495-718-0
CCCTGAATACACCGAATGTTACGGCAACAGCAGACAGTTCTTGCTTGGAA
RI-AT1G68560-XLOC_007495-718-1
CCCTGAATACACCGAATGTTACGGCAACAGCAGACAGTTCTTGCTTGGAA
CONSENSUS
CCCTGAATACACCGAATGTTACGGCAACAGCAGACAGTTCTTGCTTGGAA

RI-AT1G68560-XLOC_007495-718-0
GCAGCTTCATGATATCTCCGGTTCTCGAGCAAGGCAAAACCGAAGTAGAA
RI-AT1G68560-XLOC_007495-718-1
GCAGCTTCATGATATCTCCGGTTCTCGAGCAAGGCAAAACCGAAGTAGAA
CONSENSUS
GCAGCTTCATGATATCTCCGGTTCTCGAGCAAGGCAAAACCGAAGTAGAA

RI-AT1G68560-XLOC_007495-718-0
GCATTGTTCCCACCAGGTTCTTGGTACCACATGTTCGACATGACTCAAGC
RI-AT1G68560-XLOC_007495-718-1
GCATTGTTCCCACCAGGTTCTTGGTACCACATGTTCGACATGACTCAAGC
CONSENSUS
GCATTGTTCCCACCAGGTTCTTGGTACCACATGTTCGACATGACTCAAGC

RI-AT1G68560-XLOC_007495-718-0
AGTAGTGTCCAAGAACGGGAAACGTGTAACTCTCCCAGCTCCTTTGAACT
RI-AT1G68560-XLOC_007495-718-1
AGTAGTGTCCAAGAACGGGAAACGTGTAACTCTCCCAGCTCCTTTGAACT
CONSENSUS
AGTAGTGTCCAAGAACGGGAAACGTGTAACTCTCCCAGCTCCTTTGAACT

RI-AT1G68560-XLOC_007495-718-0

TTGTGAACGTTCATCTTTACCAAAACACTATCTTACCAACGCAACAAGGC
RI-AT1G68560-XLOC_007495-718-1
TTGTGAACGTTCATCTTTACCAAAACACTATCTTACCAACGCAACAAGGC
CONSENSUS
TTGTGAACGTTCATCTTTACCAAAACACTATCTTACCAACGCAACAAGGC

RI-AT1G68560-XLOC_007495-718-0
GGGTTGATTTCCAAGGACGCAAGAACAACACCGTTTAGCCTCGTCATTGC
RI-AT1G68560-XLOC_007495-718-1
GGGTTGATTTCCAAGGACGCAAGAACAACACCGTTTAGCCTCGTCATTGC
CONSENSUS
GGGTTGATTTCCAAGGACGCAAGAACAACACCGTTTAGCCTCGTCATTGC

RI-AT1G68560-XLOC_007495-718-0
TTTCCCTGCAGGAGCTTCTGAAGGTTACGCCACCGGGAAACTCTATCTAG
RI-AT1G68560-XLOC_007495-718-1
TTTCCCTGCAGGAGCTTCTGAAGGTTACGCCACCGGGAAACTCTATCTAG
CONSENSUS
TTTCCCTGCAGGAGCTTCTGAAGGTTACGCCACCGGGAAACTCTATCTAG

RI-AT1G68560-XLOC_007495-718-0
ACGAAGACGAGCTTCCGGAAATGAAGCTAGGAAATGGACAGTCCACGTAC
RI-AT1G68560-XLOC_007495-718-1
ACGAAGACGAGCTTCCGGAAATGAAGCTAGGAAATGGACAGTCCACGTAC
CONSENSUS
ACGAAGACGAGCTTCCGGAAATGAAGCTAGGAAATGGACAGTCCACGTAC

RI-AT1G68560-XLOC_007495-718-0
GTTGACTTCTACGCTTCGGTCGGAAACGGGACAATGAAGATGTGGTCACA
RI-AT1G68560-XLOC_007495-718-1
GTTGACTTCTACGCTTCGGTCGGAAACGGGACAATGAAGATGTGGTCACA
CONSENSUS
GTTGACTTCTACGCTTCGGTCGGAAACGGGACAATGAAGATGTGGTCACA

RI-AT1G68560-XLOC_007495-718-0
AGTTAAAGAAGGTAAGTTTGCATTGAGCAAAGGATGGGTGATTGAGAAAG
RI-AT1G68560-XLOC_007495-718-1
AGTTAAAGAAGGTAAGTTTGCATTGAGCAAAGGATGGGTGATTGAGAAAG
CONSENSUS
AGTTAAAGAAGGTAAGTTTGCATTGAGCAAAGGATGGGTGATTGAGAAAG

RI-AT1G68560-XLOC_007495-718-0
TGAGTGTTTTGGGACTTAGAGGAGCAGGACAAGTGTCTGAGATTCAAATC
RI-AT1G68560-XLOC_007495-718-1
TGAGTGTTTTGGGACTTAGAGGAGCAGGACAAGTGTCTGAGATTCAAATC
CONSENSUS
TGAGTGTTTTGGGACTTAGAGGAGCAGGACAAGTGTCTGAGATTCAAATC

RI-AT1G68560-XLOC_007495-718-0
AATGGAAGTCCAATGACAAAGAAGATTGAGGTTAGCTCGAAGGAGCATAC
RI-AT1G68560-XLOC_007495-718-1
AATGGAAGTCCAATGACAAAGAAGATTGAGGTTAGCTCGAAGGAGCATAC
CONSENSUS
AATGGAAGTCCAATGACAAAGAAGATTGAGGTTAGCTCGAAGGAGCATAC

RI-AT1G68560-XLOC_007495-718-0

```
      CTATGTGATTGGTTTGGAAGATGAAGAAGAGAACAAGAGTGTGATGGTTG
RI-AT1G68560-XLOC_007495-718-1
      CTATGTGATTGGTTTGGAAGATGAAGAAGAGAACAAGAGTGTGATGGTTG
CONSENSUS
      CTATGTGATTGGTTTGGAAGATGAAGAAGAGAACAAGAGTGTGATGGTTG

RI-AT1G68560-XLOC_007495-718-0
      AGGTTAGAGGACTTGAGATGCTTGTTGGAAAGGATTTTAACATGTCCTGG
RI-AT1G68560-XLOC_007495-718-1
      AGGTTAGAGGACTTGAGATGCTTGTTGGAAAGGATTTTAACATGTCCTGG
CONSENSUS
      AGGTTAGAGGACTTGAGATGCTTGTTGGAAAGGATTTTAACATGTCCTGG

RI-AT1G68560-XLOC_007495-718-0
      AAAATGGGTATCAATTAATTTACATGTGAGAGTTGGTAAACTATTGAGGA
RI-AT1G68560-XLOC_007495-718-1
      AAAATGGGTATCAATTAATTTACATGTGAGAGTTGGTAAACTATTGAGGA
CONSENSUS
      AAAATGGGTATCAATTAATTTACATGTGAGAGTTGGTAAACTATTGAGGA

RI-AT1G68560-XLOC_007495-718-0
      GGAGAATGAGATTATTTAAAAAAAAAGGGTAATGTTTTGTTCTTCTCCAT
RI-AT1G68560-XLOC_007495-718-1
      GGAGAATGAGATTATTTAAAAAAAAAGGGTAATGTTTTGTTCTTCTCCAT
CONSENSUS
      GGAGAATGAGATTATTTAAAAAAAAAGGGTAATGTTTTGTTCTTCTCCAT

RI-AT1G68560-XLOC_007495-718-0
      TTCGGATTATAAGAAAGAAAAAAAGAATTAGAGGTTTGGTTTTAGATTGT
RI-AT1G68560-XLOC_007495-718-1
      TTCGGATTATAAGAAAGAAAAAAAGAATTAGAGGTTTGGTTTTAGATTGT
CONSENSUS
      TTCGGATTATAAGAAAGAAAAAAAGAATTAGAGGTTTGGTTTTAGATTGT

RI-AT1G68560-XLOC_007495-718-0
      GTCTCTGTCTTTTTATTAAAAGATGCTTTAGTCTGAATAAAATTTGAAGC
RI-AT1G68560-XLOC_007495-718-1
      GTCTCTGTCTTTTTATTAAAAGATGCTTTAGTCTGAATAAAATTTGAAGC
CONSENSUS
      GTCTCTGTCTTTTTATTAAAAGATGCTTTAGTCTGAATAAAATTTGAAGC

RI-AT1G68560-XLOC_007495-718-0
      AGAAGAAGTGTTACTTCATTGGCTTATTTACTTCTTTGTTTTCCATTGGA
RI-AT1G68560-XLOC_007495-718-1
      AGAAGAAGTGTTACTTCATTGGCTTATTTACTTCTTTGTTTTCCATTGGA
CONSENSUS
      AGAAGAAGTGTTACTTCATTGGCTTATTTACTTCTTTGTTTTCCATTGGA

RI-AT1G68560-XLOC_007495-718-0   GTATTATCTTTTGGTTCTCTC
RI-AT1G68560-XLOC_007495-718-1   GTATTATCTTTTGGTTCTCTC
CONSENSUS                        GTATTATCTTTTGGTTCTCTC
```

alignment for event: RI-AT1G60510-XLOC_003054-4426

RI-AT1G60510-XLOC_003054-4426-0
ATGGGAGGTAGTAAGAAGCATGTTGTCACTAGAACCTCTTCTCCTTCTCT
RI-AT1G60510-XLOC_003054-4426-1
ATGGGAGGTAGTAAGAAGCATGTTGTCACTAGAACCTCTTCTCCTTCTCT
CONSENSUS
ATGGGAGGTAGTAAGAAGCATGTTGTCACTAGAACCTCTTCTCCTTCTCT

RI-AT1G60510-XLOC_003054-4426-0
TGCAATTGTCCAGGCGAATCCTCATGACAACAGAGAAGTTGTTCCCATCG
RI-AT1G60510-XLOC_003054-4426-1
TGCAATTGTCCAGGCGAATCCTCATGACAACAGAGAAGTTGTTCCCATCG
CONSENSUS
TGCAATTGTCCAGGCGAATCCTCATGACAACAGAGAAGTTGTTCCCATCG

RI-AT1G60510-XLOC_003054-4426-0
AAGCACCGATTATATCTTCATATAACGATAGGATTAGGCCGTTGCTTGAC
RI-AT1G60510-XLOC_003054-4426-1
AAGCACCGATTATATCTTCATATAACGATAGGATTAGGCCGTTGCTTGAC
CONSENSUS
AAGCACCGATTATATCTTCATATAACGATAGGATTAGGCCGTTGCTTGAC

RI-AT1G60510-XLOC_003054-4426-0
ACGGTTGACCGTCTAAGGAACCTCAATGTGATGAGAGAAGGCATTCAGCT
RI-AT1G60510-XLOC_003054-4426-1
ACGGTTGACCGTCTAAGGAACCTCAATGTGATGAGAGAAGGCATTCAGCT
CONSENSUS
ACGGTTGACCGTCTAAGGAACCTCAATGTGATGAGAGAAGGCATTCAGCT

RI-AT1G60510-XLOC_003054-4426-0
TCCCACCATTGTCGTGGTTGGAGACCAGTCCTCGGGGAAGTCAAGTGTCC
RI-AT1G60510-XLOC_003054-4426-1
TCCCACCATTGTCGTGGTTGGAGACCAGTCCTCGGGGAAGTCAAGTGTCC
CONSENSUS
TCCCACCATTGTCGTGGTTGGAGACCAGTCCTCGGGGAAGTCAAGTGTCC

RI-AT1G60510-XLOC_003054-4426-0
TCGACTCGTTGGCAGGAATCAGTCTACCTCGTGGCCAAGGAATCTGCACT
RI-AT1G60510-XLOC_003054-4426-1
TCGACTCGTTGGCAGGAATCAGTCTACCTCGTGGCCAAGGAATCTGCACT
CONSENSUS
TCGACTCGTTGGCAGGAATCAGTCTACCTCGTGGCCAAGGAATCTGCACT

RI-AT1G60510-XLOC_003054-4426-0
AGGGTTCCTCTTGTCATGCGACTTCAGCGAAGCTCTAGCCCTGTACCTGA
RI-AT1G60510-XLOC_003054-4426-1
AGGGTTCCTCTTGTCATGCGACTTCAGCGAAGCTCTAGCCCTGTACCTGA
CONSENSUS
AGGGTTCCTCTTGTCATGCGACTTCAGCGAAGCTCTAGCCCTGTACCTGA

RI-AT1G60510-XLOC_003054-4426-0
GATATGGCTTGAGTACAGTGACAAGATTGTTCCCACGGATGAGGAGCACA
RI-AT1G60510-XLOC_003054-4426-1
GATATGGCTTGAGTACAGTGACAAGATTGTTCCCACGGATGAGGAGCACA
CONSENSUS
GATATGGCTTGAGTACAGTGACAAGATTGTTCCCACGGATGAGGAGCACA

RI-AT1G60510-XLOC_003054-4426-0
TCGCTGAAGCTATCTGTGCTGCAACAGATGTGATTGCTGGTAAGTTCACT
RI-AT1G60510-XLOC_003054-4426-1
TCGCTGAAGCTATCTGTGCTGCAACAGATGTGATTGCTGGTAAGTTCACT
CONSENSUS
TCGCTGAAGCTATCTGTGCTGCAACAGATGTGATTGCTGGTAAGTTCACT

RI-AT1G60510-XLOC_003054-4426-0
CTCTATCTTGGTATTAAGTGTGTTTAGCTGCAAGAGAGATTACTGAGAGT
RI-AT1G60510-XLOC_003054-4426-1
CTCTATCTTGGTATTAAGTGTGTTTAGCTGCAAGAGAGATTACTGAGAGT
CONSENSUS
CTCTATCTTGGTATTAAGTGTGTTTAGCTGCAAGAGAGATTACTGAGAGT

RI-AT1G60510-XLOC_003054-4426-0
GCGACCTTTAATCTAAAACATGAATATTTGAACGAAGTTGGTTTACTTAC
RI-AT1G60510-XLOC_003054-4426-1
GCGACCTTTAATCTAAAACATGAATATTTGAACGAAGTTGGTTTACTTAC
CONSENSUS
GCGACCTTTAATCTAAAACATGAATATTTGAACGAAGTTGGTTTACTTAC

RI-AT1G60510-XLOC_003054-4426-0
CATAGAATTCCTTGGAGTTAGATGTGTACATATACCTAAATGACAGTGCC
RI-AT1G60510-XLOC_003054-4426-1
CATAGAATTCCTTGGAGTTAGATGTGTACATATACCTAAATGACAGTGCC
CONSENSUS
CATAGAATTCCTTGGAGTTAGATGTGTACATATACCTAAATGACAGTGCC

RI-AT1G60510-XLOC_003054-4426-0
TAGTGATGTGTTTTATATACTCTGAAACTCCTAGTTGGATCTTAATGGTC
RI-AT1G60510-XLOC_003054-4426-1
TAGTGATGTGTTTTATATACTCTGAAACTCCTAGTTGGATCTTAATGGTC
CONSENSUS
TAGTGATGTGTTTTATATACTCTGAAACTCCTAGTTGGATCTTAATGGTC

RI-AT1G60510-XLOC_003054-4426-0
TGGTTTTTTTTCATGCTGTTAATTATACTTTAGCTGTTCACGTTACTTAC
RI-AT1G60510-XLOC_003054-4426-1
TGGTTTTTTTTCATGCTGTTAATTATACTTTAGCTGTTCACGTTACTTAC
CONSENSUS
TGGTTTTTTTTCATGCTGTTAATTATACTTTAGCTGTTCACGTTACTTAC

RI-AT1G60510-XLOC_003054-4426-0
TGTTGAAACTTCATAGTGTTACTTACTACCATTGATGTCCCTAAAATAAG
RI-AT1G60510-XLOC_003054-4426-1
TGTTGAAACTTCATAGTGTTACTTACTACCATTGATGTCCCTAAAATAAG
CONSENSUS
TGTTGAAACTTCATAGTGTTACTTACTACCATTGATGTCCCTAAAATAAG

RI-AT1G60510-XLOC_003054-4426-0
ATGTCTACATGAACCTGAATACAAGTACCTAGATTTGAAATATGATTATG
RI-AT1G60510-XLOC_003054-4426-1
ATGTCTACATGAACCTGAATACAAGTACCTAGATTTGAAATATGATTATG
CONSENSUS
ATGTCTACATGAACCTGAATACAAGTACCTAGATTTGAAATATGATTATG

RI-AT1G60510-XLOC_003054-4426-0
GAAAACCTAGTGTAGTCTGATAACTTTAGTGCTCTTTATACTACTAGTTT
RI-AT1G60510-XLOC_003054-4426-1
GAAAACCTAGTGTAGTCTGATAACTTTAGTGCTCTTTATACTACTAGTTT
CONSENSUS
GAAAACCTAGTGTAGTCTGATAACTTTAGTGCTCTTTATACTACTAGTTT

RI-AT1G60510-XLOC_003054-4426-0
TGCTATTTGAAGGAACTAAAAGTGTTGTGTGTGATGCTAACCAGTTTTGA
RI-AT1G60510-XLOC_003054-4426-1
TGCTATTTGAAGGAACTAAAAGTGTTGTGTGTGATGCTAACCAGTTTTGA
CONSENSUS
TGCTATTTGAAGGAACTAAAAGTGTTGTGTGTGATGCTAACCAGTTTTGA

RI-AT1G60510-XLOC_003054-4426-0
ACTTTTGACTACCTATACTGCAGGATCTGGCAAAGGGGTCTCAGACGCTC
RI-AT1G60510-XLOC_003054-4426-1
ACTTTTGACTACCTATACTGCAGGATCTGGCAAAGGGGTCTCAGACGCTC
CONSENSUS
ACTTTTGACTACCTATACTGCAGGATCTGGCAAAGGGGTCTCAGACGCTC

RI-AT1G60510-XLOC_003054-4426-0
CATTGACCCTCCATGTTAAGAAGGCTGGGGTCCCATATCTTACCATGGTC
RI-AT1G60510-XLOC_003054-4426-1
CATTGACCCTCCATGTTAAGAAGGCTGGGGTCCCATATCTTACCATGGTC
CONSENSUS
CATTGACCCTCCATGTTAAGAAGGCTGGGGTCCCATATCTTACCATGGTC

RI-AT1G60510-XLOC_003054-4426-0
GATCTTCCCGGTATAACTCGAGGCCAGTGAATGGACAACCGGAGAATATT
RI-AT1G60510-XLOC_003054-4426-1
GATCTTCCCGGTATAACTCGAGGCCAGTGAATGGACAACCGGAGAATATT
CONSENSUS
GATCTTCCCGGTATAACTCGAGGCCAGTGAATGGACAACCGGAGAATATT

RI-AT1G60510-XLOC_003054-4426-0
TACGAACAGATTTCTGGGATGATCATGAAGTACATCGAGCCACAAGAATC
RI-AT1G60510-XLOC_003054-4426-1
TACGAACAGATTTCTGGGATGATCATGAAGTACATCGAGCCACAAGAATC
CONSENSUS
TACGAACAGATTTCTGGGATGATCATGAAGTACATCGAGCCACAAGAATC

RI-AT1G60510-XLOC_003054-4426-0
AATAATCCTCAATGTTCTGTCAGCTACAGTCGACTTCACCACCTGTGAAT
RI-AT1G60510-XLOC_003054-4426-1
AATAATCCTCAATGTTCTGTCAGCTACAGTCGACTTCACCACCTGTGAAT
CONSENSUS
AATAATCCTCAATGTTCTGTCAGCTACAGTCGACTTCACCACCTGTGAAT

RI-AT1G60510-XLOC_003054-4426-0
CCATCCGTATGTGAACGGACTCTGGCCGTTGTCACCAAGGCAGACATGGC
RI-AT1G60510-XLOC_003054-4426-1
CCATCCGTATGTGAACGGACTCTGGCCGTTGTCACCAAGGCAGACATGGC
CONSENSUS
CCATCCGTATGTGAACGGACTCTGGCCGTTGTCACCAAGGCAGACATGGC

RI-AT1G60510-XLOC_003054-4426-0
TCCCGAAGGTCTCCTACAGAAAGTAACTGCGGATGATGTGAGTATTGGTC
RI-AT1G60510-XLOC_003054-4426-1
TCCCGAAGGTCTCCTACAGAAAGTAA------------------------
CONSENSUS
TCCCGAAGGTCTCCTACAGAAAGTAA........................

RI-AT1G60510-XLOC_003054-4426-0
TAGGTTACGTCTGTGTCAGAAACCGCATTGGAGAAGAGCGTATGAACAAG
RI-AT1G60510-XLOC_003054-4426-1

CONSENSUS
....

RI-AT1G60510-XLOC_003054-4426-0
CTAGAATGCAAGAAGAGTTGCTCTTCAGGACTCACCCGATGCTGAGCTTG
RI-AT1G60510-XLOC_003054-4426-1
-----------------------------------CCGATGCTGAGCTTG
CONSENSUS
...................................CCGATGCTGAGCTTG

RI-AT1G60510-XLOC_003054-4426-0
ATTGATGAAGACATCGTGGGAATCCCTGTTATAGCTCAGAAGCTAATGCT
RI-AT1G60510-XLOC_003054-4426-1
ATTGATGAAGACATCGTGGGAATCCCTGTTATAGCTCAGAAGCTAATGCT
CONSENSUS
ATTGATGAAGACATCGTGGGAATCCCTGTTATAGCTCAGAAGCTAATGCT

RI-AT1G60510-XLOC_003054-4426-0
TATCCAAGCAACGATGATTTCCCGCTGTTTGCCTGAAATCGTTCGCAAGA
RI-AT1G60510-XLOC_003054-4426-1
TATCCAAGCAACGATGATTTCCCGCTGTTTGCCTGAAATCGTTCGCAAGA
CONSENSUS
TATCCAAGCAACGATGATTTCCCGCTGTTTGCCTGAAATCGTTCGCAAGA

RI-AT1G60510-XLOC_003054-4426-0
TCAATCACAAGATGGAAACTGCTGTCCTGGAGTTGAACAAGCTACCGATG
RI-AT1G60510-XLOC_003054-4426-1
TCAATCACAAGATGGAAACTGCTGTCCTGGAGTTGAACAAGCTACCGATG
CONSENSUS
TCAATCACAAGATGGAAACTGCTGTCCTGGAGTTGAACAAGCTACCGATG

RI-AT1G60510-XLOC_003054-4426-0
GTTATGGCTTCTACCGGAGAAGCATTGATGGCATTGATGGACATCATTGG
RI-AT1G60510-XLOC_003054-4426-1
GTTATGGCTTCTACCGGAGAAGCATTGATGGCATTGATGGACATCATTGG
CONSENSUS
GTTATGGCTTCTACCGGAGAAGCATTGATGGCATTGATGGACATCATTGG

RI-AT1G60510-XLOC_003054-4426-0
TTCTGCCAAGGAGTCCCTCTTCAGAATCCTTGTCCAAGGAGACTTCTCTG
RI-AT1G60510-XLOC_003054-4426-1
TTCTGCCAAGGAGTCCCTCTTCAGAATCCTTGTCCAAGGAGACTTCTCTG
CONSENSUS
TTCTGCCAAGGAGTCCCTCTTCAGAATCCTTGTCCAAGGAGACTTCTCTG

RI-AT1G60510-XLOC_003054-4426-0
AATTCCCGGATGACCAGAACATGCACTGTACTGCTCGTTTGGCTGACATG
RI-AT1G60510-XLOC_003054-4426-1
AATTCCCGGATGACCAGAACATGCACTGTACTGCTCGTTTGGCTGACATG
CONSENSUS
AATTCCCGGATGACCAGAACATGCACTGTACTGCTCGTTTGGCTGACATG

RI-AT1G60510-XLOC_003054-4426-0
TTAAGCCAATTCTCCGATAATCTGCAAGAGAAGCCAAAGGAAGTGACCGA
RI-AT1G60510-XLOC_003054-4426-1
TTAAGCCAATTCTCCGATAATCTGCAAGAGAAGCCAAAGGAAGTGACCGA
CONSENSUS
TTAAGCCAATTCTCCGATAATCTGCAAGAGAAGCCAAAGGAAGTGACCGA

RI-AT1G60510-XLOC_003054-4426-0
GTTCTTGATGAATGAGATCAAGATCCTTGACGAATGCAAGTGTGTTGGAC
RI-AT1G60510-XLOC_003054-4426-1
GTTCTTGATGAATGAGATCAAGATCCTTGACGAATGCAAGTGTGTTGGAC
CONSENSUS
GTTCTTGATGAATGAGATCAAGATCCTTGACGAATGCAAGTGTGTTGGAC

RI-AT1G60510-XLOC_003054-4426-0
TGCCCAATTTCATCCCCAGGTCAGCCTTCTTGGCTATACTATCACAACAC
RI-AT1G60510-XLOC_003054-4426-1
TGCCCAATTTCATCCCCAGGTCAGCCTTCTTGGCTATACTATCACAACAC
CONSENSUS
TGCCCAATTTCATCCCCAGGTCAGCCTTCTTGGCTATACTATCACAACAC

RI-AT1G60510-XLOC_003054-4426-0
GAAGATGCCATACATGTCAAGCCCGTGGAGTTCATCAAGAAGATATGGGA
RI-AT1G60510-XLOC_003054-4426-1
GAAGATGCCATACATGTCAAGCCCGTGGAGTTCATCAAGAAGATATGGGA
CONSENSUS
GAAGATGCCATACATGTCAAGCCCGTGGAGTTCATCAAGAAGATATGGGA

RI-AT1G60510-XLOC_003054-4426-0
CTACATTGAAGTTGTTCTCTCATCCGTCATTGCCAAGTATTCTGAAAACT
RI-AT1G60510-XLOC_003054-4426-1
CTACATTGAAGTTGTTCTCTCATCCGTCATTGCCAAGTATTCTGAAAACT
CONSENSUS
CTACATTGAAGTTGTTCTCTCATCCGTCATTGCCAAGTATTCTGAAAACT

RI-AT1G60510-XLOC_003054-4426-0
TCCCACAGATCCAATCTGCCATCAAACGTGCTGGTCGAAATCTAATCACC
RI-AT1G60510-XLOC_003054-4426-1
TCCCACAGATCCAATCTGCCATCAAACGTGCTGGTCGAAATCTAATCACC
CONSENSUS
TCCCACAGATCCAATCTGCCATCAAACGTGCTGGTCGAAATCTAATCACC

RI-AT1G60510-XLOC_003054-4426-0
AAGATCAAGGAACACTCTGTGGAGCGAGTGTTGGAGATTGTTGAAATGGA
RI-AT1G60510-XLOC_003054-4426-1
AAGATCAAGGAACACTCTGTGGAGCGAGTGTTGGAGATTGTTGAAATGGA
CONSENSUS
AAGATCAAGGAACACTCTGTGGAGCGAGTGTTGGAGATTGTTGAAATGGA

RI-AT1G60510-XLOC_003054-4426-0
GAAGCTGACTGATTACACATGTAACCCTGAGTACATGACGTCTTGGACTC
RI-AT1G60510-XLOC_003054-4426-1
GAAGCTGACTGATTACACATGTAACCCTGAGTACATGACGTCTTGGACTC
CONSENSUS
GAAGCTGACTGATTACACATGTAACCCTGAGTACATGACGTCTTGGACTC

RI-AT1G60510-XLOC_003054-4426-0
AGATGACTGCTGAACAACAAAACTTCATTTATGCTGTGTTGTCCGATGGG
RI-AT1G60510-XLOC_003054-4426-1
AGATGACTGCTGAACAACAAAACTTCATTTATGCTGTGTTGTCCGATGGG
CONSENSUS
AGATGACTGCTGAACAACAAAACTTCATTTATGCTGTGTTGTCCGATGGG

RI-AT1G60510-XLOC_003054-4426-0
AAAAAGCCTGAGCACTTCTCGTTGACTGGATTTGGTGGAATTGTGAAGAT
RI-AT1G60510-XLOC_003054-4426-1
AAAAAGCCTGAGCACTTCTCGTTGACTGGATTTGGTGGAATTGTGAAGAT
CONSENSUS
AAAAAGCCTGAGCACTTCTCGTTGACTGGATTTGGTGGAATTGTGAAGAT

RI-AT1G60510-XLOC_003054-4426-0
CTCTCACCTACGGAAGTATCATGCTCATCTCCTGCAGCAAGCCTTCGATA
RI-AT1G60510-XLOC_003054-4426-1
CTCTCACCTACGGAAGTATCATGCTCATCTCCTGCAGCAAGCCTTCGATA
CONSENSUS
CTCTCACCTACGGAAGTATCATGCTCATCTCCTGCAGCAAGCCTTCGATA

RI-AT1G60510-XLOC_003054-4426-0
TGAAGATGAGGATAGCATCTTACTGGACGATAGTCTTACGAAGGATTGTG
RI-AT1G60510-XLOC_003054-4426-1
TGAAGATGAGGATAGCATCTTACTGGACGATAGTCTTACGAAGGATTGTG
CONSENSUS
TGAAGATGAGGATAGCATCTTACTGGACGATAGTCTTACGAAGGATTGTG

RI-AT1G60510-XLOC_003054-4426-0
GACAGTCTTGCTCTGTATCTTCAGCTGTCAGTGAAGAATCTCGTGAATTA
RI-AT1G60510-XLOC_003054-4426-1
GACAGTCTTGCTCTGTATCTTCAGCTGTCAGTGAAGAATCTCGTGAATTA
CONSENSUS
GACAGTCTTGCTCTGTATCTTCAGCTGTCAGTGAAGAATCTCGTGAATTA

RI-AT1G60510-XLOC_003054-4426-0
TTAGTTTCAGAAGGAGATTGTGGCGGAGATGGTGGATCCAAGAGCTGGCG
RI-AT1G60510-XLOC_003054-4426-1
TTAGTTTCAGAAGGAGATTGTGGCGGAGATGGTGGATCCAAGAGCTGGCG
CONSENSUS
TTAGTTTCAGAAGGAGATTGTGGCGGAGATGGTGGATCCAAGAGCTGGCG

RI-AT1G60510-XLOC_003054-4426-0
GAGGTCTTCAGAGGATGCTAGAGGAGTCGCCGTCGGTGGCGAGCAAGAGA
RI-AT1G60510-XLOC_003054-4426-1
GAGGTCTTCAGAGGATGCTAGAGGAGTCGCCGTCGGTGGCGAGCAAGAGA
CONSENSUS
GAGGTCTTCAGAGGATGCTAGAGGAGTCGCCGTCGGTGGCGAGCAAGAGA

RI-AT1G60510-XLOC_003054-4426-0 GAGAAGCTGAAGAACAGT
RI-AT1G60510-XLOC_003054-4426-1 GAGAAGCTGAAGAACAGT
CONSENSUS   GAGAAGCTGAAGAACAGT

alignment for event: RI-AT1G21000-XLOC_001101-3961

RI-AT1G21000-XLOC_001101-3961-0
      ATACGAAGATCTTCGTACCATAACGTTGTGAGAGTGAATGAGATACAGAA
RI-AT1G21000-XLOC_001101-3961-1
      ATACGAAGATCTTCGTACCATAACGTTGTGAGAGTGAATGAGATACAGAA
CONSENSUS
      ATACGAAGATCTTCGTACCATAACGTTGTGAGAGTGAATGAGATACAGAA

RI-AT1G21000-XLOC_001101-3961-0
      GTTCATAGACATCGCTTGTGTTCAGACATATATTATCAACAGCGCAAAGA
RI-AT1G21000-XLOC_001101-3961-1
      GTTCATAGACATCGCTTGTGTTCAGACATATATTATCAACAGCGCAAAGA
CONSENSUS
      GTTCATAGACATCGCTTGTGTTCAGACATATATTATCAACAGCGCAAAGA

RI-AT1G21000-XLOC_001101-3961-0
      TCGTGTTCCTCAATGAAAGACCTCAGCCTAGAATCGGTAAAGGTGTTACA
RI-AT1G21000-XLOC_001101-3961-1
      TCGTGTTCCTCAATGAAAGACCTCAGCCTAGAATCGGTAAAGGTGTTACA
CONSENSUS
      TCGTGTTCCTCAATGAAAGACCTCAGCCTAGAATCGGTAAAGGTGTTACA

RI-AT1G21000-XLOC_001101-3961-0
      AACACTTGTGAAATCTGTTGCAGAAGTCTCCTTGATTCTTTCAGGTTCTG
RI-AT1G21000-XLOC_001101-3961-1
      AACACTTGTGAAATCTGTTGCAGAAGTCTCCTTGATTCTTTCAGGTTCTG
CONSENSUS
      AACACTTGTGAAATCTGTTGCAGAAGTCTCCTTGATTCTTTCAGGTTCTG

RI-AT1G21000-XLOC_001101-3961-0
      CTCTCTCGGCTGCAAGGTATAACACAAAAAGACTCTCCCTCTGTTTTTTC
RI-AT1G21000-XLOC_001101-3961-1
      CTCTCTCGGCTGCAAG----------------------------------
CONSENSUS
      CTCTCTCGGCTGCAAG..................................

RI-AT1G21000-XLOC_001101-3961-0
      CCCTGTTTTCACCATTTTTGTTTGAAACAATTCATATGGTTTTAACAAAA
RI-AT1G21000-XLOC_001101-3961-1

CONSENSUS
      ....

RI-AT1G21000-XLOC_001101-3961-0
      ATGTAAACTTTCTTAAAACCCATTAACGCATTTCACTTCTTAATGATATC
RI-AT1G21000-XLOC_001101-3961-1

CONSENSUS
      ....

RI-AT1G21000-XLOC_001101-3961-0
TATATCAAATTTTATCCGGTTTTGTTTAGTTTTGTTCGTGATTCATGTGG
RI-AT1G21000-XLOC_001101-3961-1

CONSENSUS
....

RI-AT1G21000-XLOC_001101-3961-0
ATATGCTCTGTTTTTGTAATGTTATTTGTGGAAATAACGATATTGTCCTT
RI-AT1G21000-XLOC_001101-3961-1

CONSENSUS
....

RI-AT1G21000-XLOC_001101-3961-0
TGGTTGTTTTTTGTTCTGTTTAGCTTGGAGGAATGAGGAGAGGAGATTTA
RI-AT1G21000-XLOC_001101-3961-1 -----------------------
CTTGGAGGAATGAGGAGAGGAGATTTA
CONSENSUS
.......................CTTGGAGGAATGAGGAGAGGAGATTTA

RI-AT1G21000-XLOC_001101-3961-0
TCTCTAACCTTCTCTTTGAAAGGAAAGCATGGGAGAGAGTACCTTGGTGG
RI-AT1G21000-XLOC_001101-3961-1
TCTCTAACCTTCTCTTTGAAAGGAAAGCATGGGAGAGAGTACCTTGGTGG
CONSENSUS
TCTCTAACCTTCTCTTTGAAAGGAAAGCATGGGAGAGAGTACCTTGGTGG

RI-AT1G21000-XLOC_001101-3961-0
GTCGGAATCAGATGAAGCTACTACACCGACTAAGATGCGTAAGACCAATG
RI-AT1G21000-XLOC_001101-3961-1
GTCGGAATCAGATGAAGCTACTACACCGACTAAGATGCGTAAGACCAATG
CONSENSUS
GTCGGAATCAGATGAAGCTACTACACCGACTAAGATGCGTAAGACCAATG

RI-AT1G21000-XLOC_001101-3961-0
CTTTCAACCGTCTGATGAGTGGTCTCTCGATCTCAACCGTTAGATTCGAT
RI-AT1G21000-XLOC_001101-3961-1
CTTTCAACCGTCTGATGAGTGGTCTCTCGATCTCAACCGTTAGATTCGAT
CONSENSUS
CTTTCAACCGTCTGATGAGTGGTCTCTCGATCTCAACCGTTAGATTCGAT

RI-AT1G21000-XLOC_001101-3961-0
GACTACGGTCCCAATGGTGATCAAAGGTCTTCAAGCTCTGGTGATGAAGG
RI-AT1G21000-XLOC_001101-3961-1
GACTACGGTCCCAATGGTGATCAAAGGTCTTCAAGCTCTGGTGATGAAGG
CONSENSUS
GACTACGGTCCCAATGGTGATCAAAGGTCTTCAAGCTCTGGTGATGAAGG

RI-AT1G21000-XLOC_001101-3961-0
TGGTTTTAGTTTCTCTCCGGGAACACCTCCGATCTATAATCACCGGAATT
RI-AT1G21000-XLOC_001101-3961-1
TGGTTTTAGTTTCTCTCCGGGAACACCTCCGATCTATAATCACCGGAATT
CONSENSUS
TGGTTTTAGTTTCTCTCCGGGAACACCTCCGATCTATAATCACCGGAATT

RI-AT1G21000-XLOC_001101-3961-0
CAAGCAGACGAAAGGGTGTCCCTCACCGTGCTCCGTTCTAAGAAAAGATA
RI-AT1G21000-XLOC_001101-3961-1
CAAGCAGACGAAAGGGTGTCCCTCACCGTGCTCCGTTCTAAGAAAAGATA
CONSENSUS
CAAGCAGACGAAAGGGTGTCCCTCACCGTGCTCCGTTCTAAGAAAAGATA

RI-AT1G21000-XLOC_001101-3961-0
ATTACTAAATTTCCAACCCCATAGTAATTAATTATATATATATATATATA
RI-AT1G21000-XLOC_001101-3961-1
ATTACTAAATTTCCAACCCCATAGTAATTAATTATATATATATATATATA
CONSENSUS
ATTACTAAATTTCCAACCCCATAGTAATTAATTATATATATATATATATA

RI-AT1G21000-XLOC_001101-3961-0
GATAATGGTATAATACAATGTATAACTTGGGGTGAAATTTAGGATGTAGT
RI-AT1G21000-XLOC_001101-3961-1
GATAATGGTATAATACAATGTATAACTTGGGGTGAAATTTAGGATGTAGT
CONSENSUS
GATAATGGTATAATACAATGTATAACTTGGGGTGAAATTTAGGATGTAGT

RI-AT1G21000-XLOC_001101-3961-0
CATAATGTTGTAAATGTGTATTATTTCTTTTTATAGTTTTTCTAAAAGAT
RI-AT1G21000-XLOC_001101-3961-1
CATAATGTTGTAAATGTGTATTATTTCTTTTTATAGTTTTTCTAAAAGAT
CONSENSUS
CATAATGTTGTAAATGTGTATTATTTCTTTTTATAGTTTTTCTAAAAGAT

RI-AT1G21000-XLOC_001101-3961-0
AAAGGTGAAATAATTAGAACACATACATAAATACATTTTTTATAAGTATA
RI-AT1G21000-XLOC_001101-3961-1
AAAGGTGAAATAATTAGAACACATACATAAATACATTTTTTATAAGTATA
CONSENSUS
AAAGGTGAAATAATTAGAACACATACATAAATACATTTTTTATAAGTATA

RI-AT1G21000-XLOC_001101-3961-0
GAAGAATTAGGTTTTATAGTGGAAGTGTTTGATGTTTAGTTTGTTTAAAA
RI-AT1G21000-XLOC_001101-3961-1
GAAGAATTAGGTTTTATAGTGGAAGTGTTTGATGTTTAGTTTGTTTAAAA
CONSENSUS
GAAGAATTAGGTTTTATAGTGGAAGTGTTTGATGTTTAGTTTGTTTAAAA

RI-AT1G21000-XLOC_001101-3961-0
GGCATGGCTTTGTCTTGTGTTGTAATGTAATGTGTACATTAAATTTTTGT
RI-AT1G21000-XLOC_001101-3961-1
GGCATGGCTTTGTCTTGTGTTGTAATGTAATGTGTACATTAAATTTTTGT
CONSENSUS
GGCATGGCTTTGTCTTGTGTTGTAATGTAATGTGTACATTAAATTTTTGT

RI-AT1G21000-XLOC_001101-3961-0
TCATACAATAATAATATCAAGGTGAATGATATATTATAAAGTTTATGCGT
RI-AT1G21000-XLOC_001101-3961-1
TCATACAATAATAATATCAAGGTGAATGATATATTATAAAGTTTATGCGT
CONSENSUS
TCATACAATAATAATATCAAGGTGAATGATATATTATAAAGTTTATGCGT

RI-AT1G21000-XLOC_001101-3961-0 TGTCT
RI-AT1G21000-XLOC_001101-3961-1 TGTCT
CONSENSUS   TGTCT

alignment for event: A5-AT1G53165-XLOC_002665-441

A5-AT1G53165-XLOC_002665-441-0
      AGAGCGGCCAAAATACCAAGTAAAAGAGGACGAAGAGATTCCAACAAACG
A5-AT1G53165-XLOC_002665-441-1
      AGAGCGGCCAAAATACCAAGTAAAAGAGGACGAAGAGATTCCAACAAACG
CONSENSUS
      AGAGCGGCCAAAATACCAAGTAAAAGAGGACGAAGAGATTCCAACAAACG

A5-AT1G53165-XLOC_002665-441-0
      GTCCAAAAGCTCCGGCTGAATCATCTGGGACTGTTAGAGTGGCTAAAGAT
A5-AT1G53165-XLOC_002665-441-1
      GTCCAAAAGCTCCGGCTGAATCATCTGGGACTGTTAGAGTGGCTAAAGAT
CONSENSUS
      GTCCAAAAGCTCCGGCTGAATCATCTGGGACTGTTAGAGTGGCTAAAGAT

A5-AT1G53165-XLOC_002665-441-0
      GAGAGAGGCCAGGGAACTTCTGGAACTAG------------TTTTCAGGT
A5-AT1G53165-XLOC_002665-441-1
      GAGAGAGGCCAGGGAACTTCTGGAACTAGGTTGGATAAATGTTTTCAGGT
CONSENSUS
      GAGAGAGGCCAGGGAACTTCTGGAACTAG............TTTTCAGGT

A5-AT1G53165-XLOC_002665-441-0
      AAAAACTGTCAGAAATGCGGGGTGGGACTTTAGCATTGGGGGTTCGCAGG
A5-AT1G53165-XLOC_002665-441-1
      AAAAACTGTCAGAAATGCGGGGTGGGACTTTAGCATTGGGGGTTCGCAGG
CONSENSUS
      AAAAACTGTCAGAAATGCGGGGTGGGACTTTAGCATTGGGGGTTCGCAGG

A5-AT1G53165-XLOC_002665-441-0
      GTGCTGGAACTGTTCGAGCTTTAAAACCTCCACAAGCCAGGGAAAGGCGC
A5-AT1G53165-XLOC_002665-441-1
      GTGCTGGAACTGTTCGAGCTTTAAAACCTCCACAAGCCAGGGAAAGGCGC
CONSENSUS
      GTGCTGGAACTGTTCGAGCTTTAAAACCTCCACAAGCCAGGGAAAGGCGC

A5-AT1G53165-XLOC_002665-441-0
      CAAGAAGTTAATTCTAATCAGACTTCTCAAAAAACATCACGAACCAGTGG
A5-AT1G53165-XLOC_002665-441-1
      CAAGAAGTTAATTCTAATCAGACTTCTCAAAAAACATCACGAACCAGTGG
CONSENSUS
      CAAGAAGTTAATTCTAATCAGACTTCTCAAAAAACATCACGAACCAGTGG

A5-AT1G53165-XLOC_002665-441-0
      TAGTCAATTGTCATCCACTTTTGGTGTCCCTGAAATCTCTGAAGGAGGTT
A5-AT1G53165-XLOC_002665-441-1
      TAGTCAATTGTCATCCACTTTTGGTGTCCCTGAAATCTCTGAAGGAGGTT
CONSENSUS

TAGTCAATTGTCATCCACTTTTGGTGTCCCTGAAATCTCTGAAGGAGGTT

A5-AT1G53165-XLOC_002665-441-0
TTAATAAAAGAGATTCTTATCAGAATGACTATCAAGAAGAA
A5-AT1G53165-XLOC_002665-441-1
TTAATAAAAGAGATTCTTATCAGAATGACTATCAAGAAGAA
CONSENSUS
TTAATAAAAGAGATTCTTATCAGAATGACTATCAAGAAGAA

alignment for event: A3-AT1G03080-XLOC_004263-10599

A3-AT1G03080-XLOC_004263-10599-0
GTTACTACTGGATTTGGCTAGTTAATTATTTTTGTCTCGAATTTGGCCTT
A3-AT1G03080-XLOC_004263-10599-1
GTTACTACTGGATTTGGCTAGTTAATTATTTTTGTCTCGAATTTGGCCTT
CONSENSUS
GTTACTACTGGATTTGGCTAGTTAATTATTTTTGTCTCGAATTTGGCCTT

A3-AT1G03080-XLOC_004263-10599-0 CATTGACTTAGTG-----------------
AGGAATTTGGTGTACTTGGC
A3-AT1G03080-XLOC_004263-10599-1
CATTGACTTAGTGTTAGGATTTTTTTACAGAGGAATTTGGTGTACTTGGC
CONSENSUS
CATTGACTTAGTG.................AGGAATTTGGTGTACTTGGC

A3-AT1G03080-XLOC_004263-10599-0
TTTGAGAGAGACTTGGTTCCGGAGTGTTCTAATATCCTCATAATTTTTGT
A3-AT1G03080-XLOC_004263-10599-1
TTTGAGAGAGACTTGGTTCCGGAGTGTTCTAATATCCTCATAATTTTTGT
CONSENSUS
TTTGAGAGAGACTTGGTTCCGGAGTGTTCTAATATCCTCATAATTTTTGT

A3-AT1G03080-XLOC_004263-10599-0
GTGATTTTGCCGGGAAAAATGACTGCTGTTGTGAATGGTAACTCTAAACG
A3-AT1G03080-XLOC_004263-10599-1
GTGATTTTGCCGGGAAAAATGACTGCTGTTGTGAATGGTAACTCTAAACG
CONSENSUS
GTGATTTTGCCGGGAAAAATGACTGCTGTTGTGAATGGTAACTCTAAACG

A3-AT1G03080-XLOC_004263-10599-0
CTATTCTTGGTGGTGGGATAGTCACATAAGCCCCAAGAACTCGAAATGGC
A3-AT1G03080-XLOC_004263-10599-1
CTATTCTTGGTGGTGGGATAGTCACATAAGCCCCAAGAACTCGAAATGGC
CONSENSUS
CTATTCTTGGTGGTGGGATAGTCACATAAGCCCCAAGAACTCGAAATGGC

A3-AT1G03080-XLOC_004263-10599-0 TTCAGGAGAATCTTACAG
A3-AT1G03080-XLOC_004263-10599-1 TTCAGGAGAATCTTACAG
CONSENSUS TTCAGGAGAATCTTACAG

alignment for event: A3-AT1G22750-XLOC_001197-3436

```
A3-AT1G22750-XLOC_001197-3436-0
      CGTTGGCACGAGCGCAGTCATTGTCGTGCAGCTATTCCGTCTGTCTCCTC
A3-AT1G22750-XLOC_001197-3436-1
      CGTTGGCACGAGCGCAGTCATTGTCGTGCAGCTATTCCGTCTGTCTCCTC
CONSENSUS
      CGTTGGCACGAGCGCAGTCATTGTCGTGCAGCTATTCCGTCTGTCTCCTC

A3-AT1G22750-XLOC_001197-3436-0
      TCGACCCGCTCTACCTCGTTCTAGTGAACAACAGCAATCGTTAAAAGTAA
A3-AT1G22750-XLOC_001197-3436-1
      TCGACCCGCTCTACCTCGTTCTAGTGAACAACAGCAATCG----------
CONSENSUS
      TCGACCCGCTCTACCTCGTTCTAGTGAACAACAGCAATCG..........

A3-AT1G22750-XLOC_001197-3436-0
      AGGCTAAGTTAGTGAACTCTCTCATGGGTCAATGGCAGGGCTGGAGATAT
A3-AT1G22750-XLOC_001197-3436-1
      --------------------------------------GGCTGGAGATAT
CONSENSUS
      ......................................GGCTGGAGATAT

A3-AT1G22750-XLOC_001197-3436-0
      GTACGAGCGCACTCATTCTGCGGCTTTGCGCATAATGTGAAGCTTTGTCA
A3-AT1G22750-XLOC_001197-3436-1
      GTACGAGCGCACTCATTCTGCGGCTTTGCGCATAATGTGAAGCTTTGTCA
CONSENSUS
      GTACGAGCGCACTCATTCTGCGGCTTTGCGCATAATGTGAAGCTTTGTCA

A3-AT1G22750-XLOC_001197-3436-0
      TTTCATTTTTAACATTTTACCACAAAAGTTCAAAACATATTTTTATCTAC
A3-AT1G22750-XLOC_001197-3436-1
      TTTCATTTTTAACATTTTACCACAAAAGTTCAAAACATATTTTTATCTAC
CONSENSUS
      TTTCATTTTTAACATTTTACCACAAAAGTTCAAAACATATTTTTATCTAC

A3-AT1G22750-XLOC_001197-3436-0
      ATTTTAAGTTTGTATGTTTCATAGTTTTCTTCTGTATATACTTTCTCTTA
A3-AT1G22750-XLOC_001197-3436-1
      ATTTTAAGTTTGTATGTTTCATAGTTTTCTTCTGTATATACTTTCTCTTA
CONSENSUS
      ATTTTAAGTTTGTATGTTTCATAGTTTTCTTCTGTATATACTTTCTCTTA

A3-AT1G22750-XLOC_001197-3436-0
      TGTGACGACAATTACTTATGTACTTAACTTTTTTGGGTCACGACTTGAAA
A3-AT1G22750-XLOC_001197-3436-1
      TGTGACGACAATTACTTATGTACTTAACTTTTTTGGGTCACGACTTGAAA
CONSENSUS
      TGTGACGACAATTACTTATGTACTTAACTTTTTTGGGTCACGACTTGAAA

A3-AT1G22750-XLOC_001197-3436-0  AACCACATATGGATTGAGACTTTTTGGGCT
A3-AT1G22750-XLOC_001197-3436-1  AACCACATATGGATTGAGACTTTTTGGGCT
CONSENSUS                        AACCACATATGGATTGAGACTTTTTGGGCT
```

alignment for event: A3-AT1G10890-XLOC_000523-8253

A3-AT1G10890-XLOC_000523-8253-0
TTTCTTCCCGTTCCCACGGGAGGGGAAATGAGCAGCTGATGGATTGCTCT
A3-AT1G10890-XLOC_000523-8253-1
TTTCTTCCCGTTCCCACGGGAGGGGAAATGAGCAGCTGATGGATTGCTCT
CONSENSUS
TTTCTTCCCGTTCCCACGGGAGGGGAAATGAGCAGCTGATGGATTGCTCT

A3-AT1G10890-XLOC_000523-8253-0
TAGGTGTTCTTTCATTGATCCCTTGTCTCGTTAGCTTCTCTTCGTGTGAT
A3-AT1G10890-XLOC_000523-8253-1
TAGGTGTTCTTTCATTGATCCCTTGTCTCGTTAGCTTCTCTTCGTGTGAT
CONSENSUS
TAGGTGTTCTTTCATTGATCCCTTGTCTCGTTAGCTTCTCTTCGTGTGAT

A3-AT1G10890-XLOC_000523-8253-0
AAAAGTTGGAGCTGATAATTCTTTACAGTGTATGAGGTAACTGCACATAC
A3-AT1G10890-XLOC_000523-8253-1
AAAAGTTGGAGCTGATAATTCTTTACAGTGTATGAGGTAACTGCACATAC
CONSENSUS
AAAAGTTGGAGCTGATAATTCTTTACAGTGTATGAGGTAACTGCACATAC

A3-AT1G10890-XLOC_000523-8253-0
TTTTGTTTTCTTTATGTGTAACTGTGGGCTACCTGATGCCTAGTCTTCAC
A3-AT1G10890-XLOC_000523-8253-1
TTTTGTTTTCTTTATGTGTAACTGTGGGCTACCTGATGCCTAGTCTTCAC
CONSENSUS
TTTTGTTTTCTTTATGTGTAACTGTGGGCTACCTGATGCCTAGTCTTCAC

A3-AT1G10890-XLOC_000523-8253-0
GTGCGTGGTACTTCATTAGTTCCATCTTTTTGCTTACTACTTTATTTTGG
A3-AT1G10890-XLOC_000523-8253-1
GTGCGTGGTACTTCATTAGTTCCATCTTTTTGCTTACTACTTTATTTTGG
CONSENSUS
GTGCGTGGTACTTCATTAGTTCCATCTTTTTGCTTACTACTTTATTTTGG

A3-AT1G10890-XLOC_000523-8253-0
GTAATGTTCAGGCGAAAAAGTCGTTCTATTTCTCCTAGGCGCCATCGAAG
A3-AT1G10890-XLOC_000523-8253-1
GTAATGTTCAGGCGAAAAAGTCGTTCTATTTCTCCTAGGCGCCATCGAAG
CONSENSUS
GTAATGTTCAGGCGAAAAAGTCGTTCTATTTCTCCTAGGCGCCATCGAAG

A3-AT1G10890-XLOC_000523-8253-0
TCGATCTGTTACTCCTAAGAGACGTTCTCCAACCCCAAAACGTTACAAAA
A3-AT1G10890-XLOC_000523-8253-1
TCGATCTGTTACTCCTAAGAGACGTTCTCCAACCCCAAAACGTTACAAAA
CONSENSUS
TCGATCTGTTACTCCTAAGAGACGTTCTCCAACCCCAAAACGTTACAAAA

A3-AT1G10890-XLOC_000523-8253-0
GACAAAAGAGTAGGAGTTCAACTCCATCTCCTGCAAAAAGATCTCCCGCC
A3-AT1G10890-XLOC_000523-8253-1
GACAAAAGAGTAGGAGTTCAACTCCATCTCCTGCAAAAAGATCTCCCGCC
CONSENSUS
GACAAAAGAGTAGGAGTTCAACTCCATCTCCTGCAAAAAGATCTCCCGCC

A3-AT1G10890-XLOC_000523-8253-0
GCAACCCTTGAGTCAGCCAAAAATAGGAATGGAGAAAAACTTAAAAGAGA
A3-AT1G10890-XLOC_000523-8253-1
GCAACCCTTGAGTCAGCCAAAAATAGGAATGGAGAAAAACTTAAAAGAGA
CONSENSUS
GCAACCCTTGAGTCAGCCAAAAATAGGAATGGAGAAAAACTTAAAAGAGA

A3-AT1G10890-XLOC_000523-8253-0
AGAGGAAGAACGAAAAAGGTAAATCAATACAAAATTTTGAAGTAAGATGG
A3-AT1G10890-XLOC_000523-8253-1
AGAGGAAGAACGAAAAAGGTAAATCAATACAAAATTTTGAAGTAAGATGG
CONSENSUS
AGAGGAAGAACGAAAAAGGTAAATCAATACAAAATTTTGAAGTAAGATGG

A3-AT1G10890-XLOC_000523-8253-0
ATGTTATGGTGAAATTAAGGTTTTTTGTTCTCAAATGTTATTTTAGTGTA
A3-AT1G10890-XLOC_000523-8253-1
ATGTTATGGTGAAATTAAGGTTTTTTGTTCTCAAATGTTATTTTAGTGTA
CONSENSUS
ATGTTATGGTGAAATTAAGGTTTTTTGTTCTCAAATGTTATTTTAGTGTA

A3-AT1G10890-XLOC_000523-8253-0
GTGTGGAAGTCCTTGATTGTTAGTCTCAAAAGTGCGACAGGTTTTATGGT
A3-AT1G10890-XLOC_000523-8253-1
GTGTGGAAGTCCTTGATTGTTAGTCTCAAAAGTGCGACAGGTTTTATGGT
CONSENSUS
GTGTGGAAGTCCTTGATTGTTAGTCTCAAAAGTGCGACAGGTTTTATGGT

A3-AT1G10890-XLOC_000523-8253-0
ATTCTCTCACAGGCTTTGCTTATGGAAGATAAGCTATATTTCGATTTGTT
A3-AT1G10890-XLOC_000523-8253-1
ATTCTCTCACAGGCTTTGCTTATGGAAGATAAGCTATATTTCGATTTGTT
CONSENSUS
ATTCTCTCACAGGCTTTGCTTATGGAAGATAAGCTATATTTCGATTTGTT

A3-AT1G10890-XLOC_000523-8253-0
TATATTTGATCCTCTGGCACAAATGTTCGTACTATCTCGATTTGATGAGT
A3-AT1G10890-XLOC_000523-8253-1
TATATTTGATCCTCTGGCACAAATGTTCGTACTATCTCGATTTGATGAGT
CONSENSUS
TATATTTGATCCTCTGGCACAAATGTTCGTACTATCTCGATTTGATGAGT

A3-AT1G10890-XLOC_000523-8253-0
TACCGTTTGTCATTACATGGATCATGATTGACTTAGAGATGTAAATATGT
A3-AT1G10890-XLOC_000523-8253-1
TACCGTTTGTCATTACATGGATCATGATTGACTTAGAGATGTAAATATGT
CONSENSUS
TACCGTTTGTCATTACATGGATCATGATTGACTTAGAGATGTAAATATGT

A3-AT1G10890-XLOC_000523-8253-0
CCAAAGCACAAGAATAATCTTGGTGAAGGTTTGCTTGTGTTATGGTGACA
A3-AT1G10890-XLOC_000523-8253-1
CCAAAGCACAAGAATAATCTTGGTGAAGGTTTGCTTGTGTTATGGTGACA
CONSENSUS
CCAAAGCACAAGAATAATCTTGGTGAAGGTTTGCTTGTGTTATGGTGACA

A3-AT1G10890-XLOC_000523-8253-0
AAAAAGAAATTTCTTCACGAAATTTACCATTGGTATTGGGAGGAGTGAAA
A3-AT1G10890-XLOC_000523-8253-1
AAAAAGAAATTTCTTCACGAAATTTACCATTGGTATTGGGAGGAGTGAAA
CONSENSUS
AAAAAGAAATTTCTTCACGAAATTTACCATTGGTATTGGGAGGAGTGAAA

A3-AT1G10890-XLOC_000523-8253-0
ATGGAAACGATCTCAACATTTTTTACGGATTTGCAATAAAATTTTGGTCT
A3-AT1G10890-XLOC_000523-8253-1
ATGGAAACGATCTCAACATTTTTTACGGATTTGCAATAAAATTTTGGTCT
CONSENSUS
ATGGAAACGATCTCAACATTTTTTACGGATTTGCAATAAAATTTTGGTCT

A3-AT1G10890-XLOC_000523-8253-0
TTTGCTGTTCAAGATTGAAGACATCAGGAAATGAGAAAGTTCAGGTGATA
A3-AT1G10890-XLOC_000523-8253-1
TTTGCTGTTCAAGATTGAAGACATCAGGAAATGAGAAAGTTCAGGTGATA
CONSENSUS
TTTGCTGTTCAAGATTGAAGACATCAGGAAATGAGAAAGTTCAGGTGATA

A3-AT1G10890-XLOC_000523-8253-0
TTCAATATTCTGAACCTGCTGTGGATGTCCTCTAATTTTTCACTGTATTT
A3-AT1G10890-XLOC_000523-8253-1
TTCAATATTCTGAACCTGCTGTGGATGTCCTCTAATTTTTCACTGTATTT
CONSENSUS
TTCAATATTCTGAACCTGCTGTGGATGTCCTCTAATTTTTCACTGTATTT

A3-AT1G10890-XLOC_000523-8253-0
GCTACCAGGCGACAGCGTGAAGCAGAACTGAAGCTAATAGAGGAAGAAAC
A3-AT1G10890-XLOC_000523-8253-1
GCTACCAGGCGACAGCGTGAAGCAGAACTGAAGCTAATAGAGGAAGAAAC
CONSENSUS
GCTACCAGGCGACAGCGTGAAGCAGAACTGAAGCTAATAGAGGAAGAAAC

A3-AT1G10890-XLOC_000523-8253-0
TGTGAAACGGGTTGAAGAAGCTATTCGAAAGAAGGTCGAAGAAAGCTTAC
A3-AT1G10890-XLOC_000523-8253-1
TGTGAAACGGGTTGAAGAAGCTATTCGAAAGAAGGTCGAAGAAAGCTTAC
CONSENSUS
TGTGAAACGGGTTGAAGAAGCTATTCGAAAGAAGGTCGAAGAAAGCTTAC

A3-AT1G10890-XLOC_000523-8253-0
AGTCTGAGAAAATCAAAATGGAAATTCTAACGCTGTTGGAGGAAGGGCGA
A3-AT1G10890-XLOC_000523-8253-1
AGTCTGAGAAAATCAAAATGGAAATTCTAACGCTGTTGGAGGAAGGGCGA
CONSENSUS
AGTCTGAGAAAATCAAAATGGAAATTCTAACGCTGTTGGAGGAAGGGCGA

A3-AT1G10890-XLOC_000523-8253-0
AAGAGACTTAATGAAGAAGTCGCGGCTCAACTTGAGGAGGAGAAAGAGGC
A3-AT1G10890-XLOC_000523-8253-1
AAGAGACTTAATGAAGAAGTCGCGGCTCAACTTGAGGAGGAGAAAGAGGC
CONSENSUS
AAGAGACTTAATGAAGAAGTCGCGGCTCAACTTGAGGAGGAGAAAGAGGC

A3-AT1G10890-XLOC_000523-8253-0
TTCTCTTATTGAGGCTAAAGAAAAAGAGGGTGTTATGCGGTGTTTGTCAC
A3-AT1G10890-XLOC_000523-8253-1
TTCTCTTATTGAGGCTAAAGAAAAAGAG----------------------
CONSENSUS
TTCTCTTATTGAGGCTAAAGAAAAAGAG......................

A3-AT1G10890-XLOC_000523-8253-0
AGGAAAGAGAGCAACAAGAGAAAGAAGAGAGGGAGAGAATAGCAGAGGAG
A3-AT1G10890-XLOC_000523-8253-1 --
GAAAGAGAGCAACAAGAGAAAGAAGAGAGGGAGAGAATAGCAGAGGAG
CONSENSUS
..GAAAGAGAGCAACAAGAGAAAGAAGAGAGGGAGAGAATAGCAGAGGAG

A3-AT1G10890-XLOC_000523-8253-0
AACCTAAAGAGAGTGGAAGAAGCTCAGAGAAAAGAAGCAATGGAGAGGCA
A3-AT1G10890-XLOC_000523-8253-1
AACCTAAAGAGAGTGGAAGAAGCTCAGAGAAAAGAAGCAATGGAGAGGCA
CONSENSUS
AACCTAAAGAGAGTGGAAGAAGCTCAGAGAAAAGAAGCAATGGAGAGGCA

A3-AT1G10890-XLOC_000523-8253-0
AAGGAAAGAGGAGGAACGGTATCGAGAGCTAGAGGAGCTGCAACGACAGA
A3-AT1G10890-XLOC_000523-8253-1
AAGGAAAGAGGAGGAACGGTATCGAGAGCTAGAGGAGCTGCAACGACAGA
CONSENSUS
AAGGAAAGAGGAGGAACGGTATCGAGAGCTAGAGGAGCTGCAACGACAGA

A3-AT1G10890-XLOC_000523-8253-0
AAGAAGAAGCGATGCGAAGGAAGAAAGCTGAAGAGGAAGAAGAACGTCTC
A3-AT1G10890-XLOC_000523-8253-1
AAGAAGAAGCGATGCGAAGGAAGAAAGCTGAAGAGGAAGAAGAACGTCTC
CONSENSUS
AAGAAGAAGCGATGCGAAGGAAGAAAGCTGAAGAGGAAGAAGAACGTCTC

A3-AT1G10890-XLOC_000523-8253-0
AAACAGATGAAACTGTTGGGTAAAAACAAATCACGGCCTAAATTATCCTT
A3-AT1G10890-XLOC_000523-8253-1
AAACAGATGAAACTGTTGGGTAAAAACAAATCACGGCCTAAATTATCCTT
CONSENSUS
AAACAGATGAAACTGTTGGGTAAAAACAAATCACGGCCTAAATTATCCTT

A3-AT1G10890-XLOC_000523-8253-0
TGCCTTAAGCTCCAAGTAAATGCGTGCATGCATGAAGATAAAAGGATTGA
A3-AT1G10890-XLOC_000523-8253-1
TGCCTTAAGCTCCAAGTAAATGCGTGCATGCATGAAGATAAAAGGATTGA
CONSENSUS
TGCCTTAAGCTCCAAGTAAATGCGTGCATGCATGAAGATAAAAGGATTGA

A3-AT1G10890-XLOC_000523-8253-0
TGTGATGGATGATGATATGCATCTTCTTCCTCTCAAAGATGCTTTATGAT
A3-AT1G10890-XLOC_000523-8253-1
TGTGATGGATGATGATATGCATCTTCTTCCTCTCAAAGATGCTTTATGAT
CONSENSUS
TGTGATGGATGATGATATGCATCTTCTTCCTCTCAAAGATGCTTTATGAT

A3-AT1G10890-XLOC_000523-8253-0
      TATTGTTATTAGTGCTTCTTGTTGGAGCTTAAACTCTTTTATGGCTTTTA
A3-AT1G10890-XLOC_000523-8253-1
      TATTGTTATTAGTGCTTCTTGTTGGAGCTTAAACTCTTTTATGGCTTTTA
CONSENSUS
      TATTGTTATTAGTGCTTCTTGTTGGAGCTTAAACTCTTTTATGGCTTTTA

A3-AT1G10890-XLOC_000523-8253-0
      ATTTTTTGTAATTCTATTTTTCTCGTTTTGTAATTTTACGTTAGGTTAAT
A3-AT1G10890-XLOC_000523-8253-1
      ATTTTTTGTAATTCTATTTTTCTCGTTTTGTAATTTTACGTTAGGTTAAT
CONSENSUS
      ATTTTTTGTAATTCTATTTTTCTCGTTTTGTAATTTTACGTTAGGTTAAT

A3-AT1G10890-XLOC_000523-8253-0
      GGTGATGAATGATAATATAGCAATGATTCAGAAAATTTA
A3-AT1G10890-XLOC_000523-8253-1
      GGTGATGAATGATAATATAGCAATGATTCAGAAAATTTA
CONSENSUS
      GGTGATGAATGATAATATAGCAATGATTCAGAAAATTTA

alignment for event: RI-AT1G61600-XLOC_003109-6930

RI-AT1G61600-XLOC_003109-6930-0
      GAAAGTTTCCGTGTGTTCTAGAGAGATAGAGAAAGGGGTATGTTGTTTTC
RI-AT1G61600-XLOC_003109-6930-1
      GAAAGTTTCCGTGTGTTCTAGAGAGATAGAGAAAGGGGTATGTTGTTTTC
CONSENSUS
      GAAAGTTTCCGTGTGTTCTAGAGAGATAGAGAAAGGGGTATGTTGTTTTC

RI-AT1G61600-XLOC_003109-6930-0
      CAGATATCTTGCATGACAAGAAACCTAAACCTCTGGATCCAAGAAACATA
RI-AT1G61600-XLOC_003109-6930-1
      CAGATATCTTGCATGACAAGAAACCTAAACCT------------------
CONSENSUS
      CAGATATCTTGCATGACAAGAAACCTAAACCT..................

RI-AT1G61600-XLOC_003109-6930-0
      TATCAGACGGTTAAGATCAACCGGCATCACGACCGGACATTCTATGCAAA
RI-AT1G61600-XLOC_003109-6930-1

CONSENSUS
      ....

RI-AT1G61600-XLOC_003109-6930-0
      ATCTGTGGCGCCAGACGGTACACCGCCAACATTTCTCAAGAAGAAAGGAT
RI-AT1G61600-XLOC_003109-6930-1

CONSENSUS
      ....

RI-AT1G61600-XLOC_003109-6930-0
      GGGAGCTACGAACCTCGAGGAGCCTACATCCGAGGAGACCTAGAGAAGCT

RI-AT1G61600-XLOC_003109-6930-1 -------------
CTCGAGGAGCCTACATCCGAGGAGACCTAGAGAAGCT
CONSENSUS
.............CTCGAGGAGCCTACATCCGAGGAGACCTAGAGAAGCT

RI-AT1G61600-XLOC_003109-6930-0
CTTGGCCTAGACGAGGAACTAAGAGCTCGTCTCCCAGCATTTGGATTTCC
RI-AT1G61600-XLOC_003109-6930-1
CTTGGCCTAGACGAGGAACTAAGAGCTCGTCTCCCAGCATTTGGATTTCC
CONSENSUS
CTTGGCCTAGACGAGGAACTAAGAGCTCGTCTCCCAGCATTTGGATTTCC

RI-AT1G61600-XLOC_003109-6930-0
AGTTTCTACAATTCGGTCAGGAAGTGTGATAGTTGGAGAATGGTATTGCC
RI-AT1G61600-XLOC_003109-6930-1
AGTTTCTACAATTCGGTCAGGAAGTGTGATAGTTGGAGAATGGTATTGCC
CONSENSUS
AGTTTCTACAATTCGGTCAGGAAGTGTGATAGTTGGAGAATGGTATTGCC

RI-AT1G61600-XLOC_003109-6930-0
CTTTTATGTTTGTGAAGGAGAATTGTAGTGTAAGTCAACAAATGAGGAAG
RI-AT1G61600-XLOC_003109-6930-1
CTTTTATGTTTGTGAAGGAGAATTGTAGTGTAAGTCAACAAATGAGGAAG
CONSENSUS
CTTTTATGTTTGTGAAGGAGAATTGTAGTGTAAGTCAACAAATGAGGAAG

RI-AT1G61600-XLOC_003109-6930-0
TCAATGTTCTATAGGATCACACTATCCCAATACTGGGAACGAATTTACCA
RI-AT1G61600-XLOC_003109-6930-1
TCAATGTTCTATAGGATCACACTATCCCAATACTGGGAACGAATTTACCA
CONSENSUS
TCAATGTTCTATAGGATCACACTATCCCAATACTGGGAACGAATTTACCA

RI-AT1G61600-XLOC_003109-6930-0
TTGCGGAAACAATGATCTTGATGAAAACAATGATGAAAACGAAGAAGAAG
RI-AT1G61600-XLOC_003109-6930-1
TTGCGGAAACAATGATCTTGATGAAAACAATGATGAAAACGAAGAAGAAG
CONSENSUS
TTGCGGAAACAATGATCTTGATGAAAACAATGATGAAAACGAAGAAGAAG

RI-AT1G61600-XLOC_003109-6930-0
TTGTGAGAGTAGAGGCTAACGTCGTGAGAGAGGCCAACTATGTGAAGGGT
RI-AT1G61600-XLOC_003109-6930-1
TTGTGAGAGTAGAGGCTAACGTCGTGAGAGAGGCCAACTATGTGAAGGGT
CONSENSUS
TTGTGAGAGTAGAGGCTAACGTCGTGAGAGAGGCCAACTATGTGAAGGGT

RI-AT1G61600-XLOC_003109-6930-0
ATGGAGGCGGTTAAGGGAGAGAAAGAAGGGCATGGAGGGTTTTATTGGTA
RI-AT1G61600-XLOC_003109-6930-1
ATGGAGGCGGTTAAGGGAGAGAAAGAAGGGCATGGAGGGTTTTATTGGTA
CONSENSUS
ATGGAGGCGGTTAAGGGAGAGAAAGAAGGGCATGGAGGGTTTTATTGGTA

RI-AT1G61600-XLOC_003109-6930-0
TAGGCAGGTTCAAGGTCCACGGGGACCGGGGGAGAGGAGGAGGAAGACGG

RI-AT1G61600-XLOC_003109-6930-1
TAGGCAGGTTCAAGGTCCACGGGGACCGGGGGAGAGGAGGAGGAAGACGG
CONSENSUS
TAGGCAGGTTCAAGGTCCACGGGGACCGGGGGAGAGGAGGAGGAAGACGG

RI-AT1G61600-XLOC_003109-6930-0
GTTTAAGGTCTCCGGTGGGGCTGAGTTTTGTGGTGGTAGAAAGGATGAGA
RI-AT1G61600-XLOC_003109-6930-1
GTTTAAGGTCTCCGGTGGGGCTGAGTTTTGTGGTGGTAGAAAGGATGAGA
CONSENSUS
GTTTAAGGTCTCCGGTGGGGCTGAGTTTTGTGGTGGTAGAAAGGATGAGA

RI-AT1G61600-XLOC_003109-6930-0
AGGGTAATGGAGGAAGGAGGGTGGGTGGGAGGAGGAAGGAAAGTGGTGAG
RI-AT1G61600-XLOC_003109-6930-1
AGGGTAATGGAGGAAGGAGGGTGGGTGGGAGGAGGAAGGAAAGTGGTGAG
CONSENSUS
AGGGTAATGGAGGAAGGAGGGTGGGTGGGAGGAGGAAGGAAAGTGGTGAG

RI-AT1G61600-XLOC_003109-6930-0
GGTGGAGAGAGATGAACCAATCAGGGTTTGTAGAAGAGATGGTAGGAATA
RI-AT1G61600-XLOC_003109-6930-1
GGTGGAGAGAGATGAACCAATCAGGGTTTGTAGAAGAGATGGTAGGAATA
CONSENSUS
GGTGGAGAGAGATGAACCAATCAGGGTTTGTAGAAGAGATGGTAGGAATA

RI-AT1G61600-XLOC_003109-6930-0
TGAATGGTAACAATGATAGAAATTGGAGGAGATTTGGGTGTTATGTGTTG
RI-AT1G61600-XLOC_003109-6930-1
TGAATGGTAACAATGATAGAAATTGGAGGAGATTTGGGTGTTATGTGTTG
CONSENSUS
TGAATGGTAACAATGATAGAAATTGGAGGAGATTTGGGTGTTATGTGTTG

RI-AT1G61600-XLOC_003109-6930-0
GTGGAGAGTTTTGGGCTGAGAAGAGCAGATGGAGTTTTGTTGGTTAAATG
RI-AT1G61600-XLOC_003109-6930-1
GTGGAGAGTTTTGGGCTGAGAAGAGCAGATGGAGTTTTGTTGGTTAAATG
CONSENSUS
GTGGAGAGTTTTGGGCTGAGAAGAGCAGATGGAGTTTTGTTGGTTAAATG

RI-AT1G61600-XLOC_003109-6930-0
TGTATTTAGACATACTAATAGATTGAGATGTAACTGGGAGTGACACTTCA
RI-AT1G61600-XLOC_003109-6930-1
TGTATTTAGACATACTAATAGATTGAGATGTAACTGGGAGTGACACTTCA
CONSENSUS
TGTATTTAGACATACTAATAGATTGAGATGTAACTGGGAGTGACACTTCA

RI-AT1G61600-XLOC_003109-6930-0 TCTTATGTTATAAAATT
RI-AT1G61600-XLOC_003109-6930-1 TCTTATGTTATAAAATT
CONSENSUS TCTTATGTTATAAAATT

alignment for event: RI-AT1G64355-XLOC_003259-3970

RI-AT1G64355-XLOC_003259-3970-0

TTCCAGCTGGGATTTATGGTAATTTCCTTCAACATTATAAAACCTTAATC
RI-AT1G64355-XLOC_003259-3970-1
TTCCAGCTGGGATTTATG--------------------------------
CONSENSUS
TTCCAGCTGGGATTTATG................................

RI-AT1G64355-XLOC_003259-3970-0
TTCTTCTTGTTGATTTTTAAATTTGTAATGGTTCTTGAAAATTGTTTAAA
RI-AT1G64355-XLOC_003259-3970-1

CONSENSUS
....

RI-AT1G64355-XLOC_003259-3970-0
TGGGGTTTGATAAATGATAATGAATGATTTCAGCTAAGGTGCATTATGGA
RI-AT1G64355-XLOC_003259-3970-1
----------------------------------CTAAGGTGCATTATGGA
CONSENSUS
..................................CTAAGGTGCATTATGGA

RI-AT1G64355-XLOC_003259-3970-0
ACATCGTTGTCGAATGTTGATTGGTTACACGGAGGAGCTGAATCACTTCT
RI-AT1G64355-XLOC_003259-3970-1
ACATCGTTGTCGAATGTTGATTGGTTACACGGAGGAGCTGAATCACTTCT
CONSENSUS
ACATCGTTGTCGAATGTTGATTGGTTACACGGAGGAGCTGAATCACTTCT

RI-AT1G64355-XLOC_003259-3970-0
TGCTCTTACCAATTTGTTTATCGTGTTGGGTCTTAGACAAGCTCTGAGGA
RI-AT1G64355-XLOC_003259-3970-1
TGCTCTTACCAATTTGTTTATCGTGTTGGGTCTTAGACAAGCTCTGAGGA
CONSENSUS
TGCTCTTACCAATTTGTTTATCGTGTTGGGTCTTAGACAAGCTCTGAGGA

RI-AT1G64355-XLOC_003259-3970-0
AGTCTCAAGATGATGATGATGATAAACTTGGTAATGATGATGAAGTTCCA
RI-AT1G64355-XLOC_003259-3970-1
AGTCTCAAGATGATGATGATGATAAACTTGGTAATGATGATGAAGTTCCA
CONSENSUS
AGTCTCAAGATGATGATGATGATAAACTTGGTAATGATGATGAAGTTCCA

RI-AT1G64355-XLOC_003259-3970-0
ACAACTCAAGAACAAGGGAAATCTTCAGTGTAGTAAAACAAATGTAAATT
RI-AT1G64355-XLOC_003259-3970-1
ACAACTCAAGAACAAGGGAAATCTTCAGTGTAGTAAAACAAATGTAAATT
CONSENSUS
ACAACTCAAGAACAAGGGAAATCTTCAGTGTAGTAAAACAAATGTAAATT

RI-AT1G64355-XLOC_003259-3970-0
TTTTAATTATGGAGTTTCACTTGTTTTTTAATTAGATTATATATAGTCGA
RI-AT1G64355-XLOC_003259-3970-1
TTTTAATTATGGAGTTTCACTTGTTTTTTAATTAGATTATATATAGTCGA
CONSENSUS
TTTTAATTATGGAGTTTCACTTGTTTTTTAATTAGATTATATATAGTCGA

RI-AT1G64355-XLOC_003259-3970-0

CGCCCATCTAATTCCCATTTTAGATGCATGAGTTCAACGCTAGTTAGATG
RI-AT1G64355-XLOC_003259-3970-1
CGCCCATCTAATTCCCATTTTAGATGCATGAGTTCAACGCTAGTTAGATG
CONSENSUS
CGCCCATCTAATTCCCATTTTAGATGCATGAGTTCAACGCTAGTTAGATG

RI-AT1G64355-XLOC_003259-3970-0
TGTTTTGTTCTTATTTGGTCTTTTTCTCCTTAATATGCTAAAGTATCATT
RI-AT1G64355-XLOC_003259-3970-1
TGTTTTGTTCTTATTTGGTCTTTTTCTCCTTAATATGCTAAAGTATCATT
CONSENSUS
TGTTTTGTTCTTATTTGGTCTTTTTCTCCTTAATATGCTAAAGTATCATT

RI-AT1G64355-XLOC_003259-3970-0
TTGTGGAGGTATAAATCCGATTGAGAAATGGCATTTAGTTACTATTGGAA
RI-AT1G64355-XLOC_003259-3970-1
TTGTGGAGGTATAAATCCGATTGAGAAATGGCATTTAGTTACTATTGGAA
CONSENSUS
TTGTGGAGGTATAAATCCGATTGAGAAATGGCATTTAGTTACTATTGGAA

RI-AT1G64355-XLOC_003259-3970-0 TATTGTTC
RI-AT1G64355-XLOC_003259-3970-1 TATTGTTC
CONSENSUS TATTGTTC

alignment for event: RI-AT1G77800-XLOC_003990-10546

RI-AT1G77800-XLOC_003990-10546-0
CGAGAGTTGGCTATTTCCTCACATGAAATACTTGCTGCCAAAAGGGATCA
RI-AT1G77800-XLOC_003990-10546-1
CGAGAGTTGGCTATTTCCTCACATGAAATACTTGCTGCCAAAAGGGATCA
CONSENSUS
CGAGAGTTGGCTATTTCCTCACATGAAATACTTGCTGCCAAAAGGGATCA

RI-AT1G77800-XLOC_003990-10546-0
TGCTGCACGGCCATTACATGTCCGTAATCCATTTTCTCCTCCAGAAGTTT
RI-AT1G77800-XLOC_003990-10546-1
TGCTGCACGGCCATTACATGTCCGTAATCCATTTTCTCCTCCAGAAGTTT
CONSENSUS
TGCTGCACGGCCATTACATGTCCGTAATCCATTTTCTCCTCCAGAAGTTT

RI-AT1G77800-XLOC_003990-10546-0
CGTCGGACTCAGCTACAACATCAATAAAAGGCCATCCTGATAGTAATATA
RI-AT1G77800-XLOC_003990-10546-1
CGTCGGACTCAGCTACAACATCAATAAAAGGCCATCCTGATAGTAATATA
CONSENSUS
CGTCGGACTCAGCTACAACATCAATAAAAGGCCATCCTGATAGTAATATA

RI-AT1G77800-XLOC_003990-10546-0
TCTGGCAGTGAAGCAATACAGAGGTCAGATGATATCACTATTGACAGCAC
RI-AT1G77800-XLOC_003990-10546-1
TCTGGCAGTGAAGCAATACAGAGGTCAGATGATATCACTATTGACAGCAC
CONSENSUS
TCTGGCAGTGAAGCAATACAGAGGTCAGATGATATCACTATTGACAGCAC

RI-AT1G77800-XLOC_003990-10546-0
AGTCACTGATAAGCGCAGAGGCAAAGGTCCCTTATTAATGGACACGGATC
RI-AT1G77800-XLOC_003990-10546-1
AGTCACTGATAAGCGCAGAGGCAAAGGTCCCTTATTAATGGACACGGATC
CONSENSUS
AGTCACTGATAAGCGCAGAGGCAAAGGTCCCTTATTAATGGACACGGATC

RI-AT1G77800-XLOC_003990-10546-0
AGAAAACTGATGACAGTGCTACTTCCAAGAGTCGGTTTTCCCGTAAACTA
RI-AT1G77800-XLOC_003990-10546-1
AGAAAACTGATGACAGTGCTACTTCCAAGAGTCGGTTTTCCCGTAAACTA
CONSENSUS
AGAAAACTGATGACAGTGCTACTTCCAAGAGTCGGTTTTCCCGTAAACTA

RI-AT1G77800-XLOC_003990-10546-0
ACAGAAAGACAGATCTTATCTGGGAAAACTGTTCCCCGCAAACATTGTAT
RI-AT1G77800-XLOC_003990-10546-1
ACAGAAAGACAGATCTTATCTGGGAAAACTGTTCCCCGCAAACATTGTAT
CONSENSUS
ACAGAAAGACAGATCTTATCTGGGAAAACTGTTCCCCGCAAACATTGTAT

RI-AT1G77800-XLOC_003990-10546-0
AGTGTCACCTAGTGTTTCAGAGGATGGAGATAACGGGTCAAAGCCCAAGA
RI-AT1G77800-XLOC_003990-10546-1
AGTGTCACCTAGTGTTTCAGAGGATGGAGATAACGGGTCAAAGCCCAAGA
CONSENSUS
AGTGTCACCTAGTGTTTCAGAGGATGGAGATAACGGGTCAAAGCCCAAGA

RI-AT1G77800-XLOC_003990-10546-0
AGGTGAGAAGATTAACTTTGTCGTAAGTTACATGGATTGTTAAGTTCCGA
RI-AT1G77800-XLOC_003990-10546-1
AG--
CONSENSUS
AG..

RI-AT1G77800-XLOC_003990-10546-0
CTCTTTCTGTGCCCCCAACTCAGCACATTCATAGGATCTTACACTTGTTT
RI-AT1G77800-XLOC_003990-10546-1

CONSENSUS
....

RI-AT1G77800-XLOC_003990-10546-0
GTCTTGATAACTGATAATAGCAGCATGTAGAAACATTTGCAAAAGAGCTG
RI-AT1G77800-XLOC_003990-10546-1 --------------------
CAGCATGTAGAAACATTTGCAAAAGAGCTG
CONSENSUS
....................CAGCATGTAGAAACATTTGCAAAAGAGCTG

RI-AT1G77800-XLOC_003990-10546-0
GTGATGACATCAGATGAAGCTTCTTTCAAGAACCGGCGGCTCCCAAAGGG
RI-AT1G77800-XLOC_003990-10546-1
GTGATGACATCAGATGAAGCTTCTTTCAAGAACCGGCGGCTCCCAAAGGG
CONSENSUS
GTGATGACATCAGATGAAGCTTCTTTCAAGAACCGGCGGCTCCCAAAGGG

RI-AT1G77800-XLOC_003990-10546-0
ATACTTCTATGTTCCTGTTGATTGTCTTCAGGAAGACAAACCGGGAAACC
RI-AT1G77800-XLOC_003990-10546-1
ATACTTCTATGTTCCTGTTGATTGTCTTCAGGAAGACAAACCGGGAAACC
CONSENSUS
ATACTTCTATGTTCCTGTTGATTGTCTTCAGGAAGACAAACCGGGAAACC

RI-AT1G77800-XLOC_003990-10546-0
AGAAGCTGGCTTCATCTGACAAGCCAGCGAACCAGAAGACATCTTCAGGT
RI-AT1G77800-XLOC_003990-10546-1
AGAAGCTGGCTTCATCTGACAAGCCAGCGAACCAGAAGACATCTTCAGGT
CONSENSUS
AGAAGCTGGCTTCATCTGACAAGCCAGCGAACCAGAAGACATCTTCAGGT

RI-AT1G77800-XLOC_003990-10546-0
GATCAGTCAGGTAAAGACGACGGGTAAAAGCTGAGAACACACATTTTGGC
RI-AT1G77800-XLOC_003990-10546-1
GATCAGTCAGGTAAAGACGACGGGTAAAAGCTGAGAACACACATTTTGGC
CONSENSUS
GATCAGTCAGGTAAAGACGACGGGTAAAAGCTGAGAACACACATTTTGGC

RI-AT1G77800-XLOC_003990-10546-0
TCATTGCTCCTACCTTAATTAGATACTGGCTATTGGTGGACATTGTCTTC
RI-AT1G77800-XLOC_003990-10546-1
TCATTGCTCCTACCTTAATTAGATACTGGCTATTGGTGGACATTGTCTTC
CONSENSUS
TCATTGCTCCTACCTTAATTAGATACTGGCTATTGGTGGACATTGTCTTC

RI-AT1G77800-XLOC_003990-10546-0
TGCGCCCTAACCAGGATTCACCAACTGGCTTAAAGCAAAAGAACAGATGG
RI-AT1G77800-XLOC_003990-10546-1
TGCGCCCTAACCAGGATTCACCAACTGGCTTAAAGCAAAAGAACAGATGG
CONSENSUS
TGCGCCCTAACCAGGATTCACCAACTGGCTTAAAGCAAAAGAACAGATGG

RI-AT1G77800-XLOC_003990-10546-0
TATTGCGCTACAAAAGAGCTGTGGCTGCAACTGAGAATCAGCTAAAAAAA
RI-AT1G77800-XLOC_003990-10546-1
TATTGCGCTACAAAAGAGCTGTGGCTGCAACTGAGAATCAGCTAAAAAAA
CONSENSUS
TATTGCGCTACAAAAGAGCTGTGGCTGCAACTGAGAATCAGCTAAAAAAA

RI-AT1G77800-XLOC_003990-10546-0
GCTATTGTGAAGCTTTTGGGTACTCGATACAAACTGTGCCTATTGGTAAT
RI-AT1G77800-XLOC_003990-10546-1
GCTATTGTGAAGCTTTTGGGTACTCGATACAAACTGTGCCTATTGGTAAT
CONSENSUS
GCTATTGTGAAGCTTTTGGGTACTCGATACAAACTGTGCCTATTGGTAAT

RI-AT1G77800-XLOC_003990-10546-0
GTTTGAGATTTGAGGGTAACATCTCTATAGGAAAAAGGCTTCAGATTGGA
RI-AT1G77800-XLOC_003990-10546-1
GTTTGAGATTTGAGGGTAACATCTCTATAGGAAAAAGGCTTCAGATTGGA
CONSENSUS
GTTTGAGATTTGAGGGTAACATCTCTATAGGAAAAAGGCTTCAGATTGGA

```
RI-AT1G77800-XLOC_003990-10546-0
      TTCAGGGAAGTGAGAAAAAAGCTCCCTATTACCATATAAAGAAGGATCGG
RI-AT1G77800-XLOC_003990-10546-1
      TTCAGGGAAGTGAGAAAAAAGCTCCCTATTACCATATAAAGAAGGATCGG
CONSENSUS
      TTCAGGGAAGTGAGAAAAAAGCTCCCTATTACCATATAAAGAAGGATCGG

RI-AT1G77800-XLOC_003990-10546-0
      TCACGTGTAATTCTGTTTGTATAAAGAAGCGGAAAAAAAAGAAAAGGGTT
RI-AT1G77800-XLOC_003990-10546-1
      TCACGTGTAATTCTGTTTGTATAAAGAAGCGGAAAAAAAAGAAAAGGGTT
CONSENSUS
      TCACGTGTAATTCTGTTTGTATAAAGAAGCGGAAAAAAAAGAAAAGGGTT

RI-AT1G77800-XLOC_003990-10546-0
      GCATAAGTGTATATATTCATCAATTGTGTCTAAAATCTGACCAAGGGAAT
RI-AT1G77800-XLOC_003990-10546-1
      GCATAAGTGTATATATTCATCAATTGTGTCTAAAATCTGACCAAGGGAAT
CONSENSUS
      GCATAAGTGTATATATTCATCAATTGTGTCTAAAATCTGACCAAGGGAAT

RI-AT1G77800-XLOC_003990-10546-0 AATAGTTGTCTGAAA
RI-AT1G77800-XLOC_003990-10546-1 AATAGTTGTCTGAAA
CONSENSUS                        AATAGTTGTCTGAAA



alignment for event: A3-AT1G32230-XLOC_001707-10045

A3-AT1G32230-XLOC_001707-10045-0
      GTAATTTGATTGCTAAGCGTGATAACTCAGGGGTCACTTTGGAAGGACCT
A3-AT1G32230-XLOC_001707-10045-1
      GTAATTTGATTGCTAAGCGTGATAACTCAGGGGTCACTTTGGAAGGACCT
CONSENSUS
      GTAATTTGATTGCTAAGCGTGATAACTCAGGGGTCACTTTGGAAGGACCT

A3-AT1G32230-XLOC_001707-10045-0
      AAGGATCTTCCTCCTCAATTGGAGTCAAACCAGGGAGCAAGAGGTTCAGG
A3-AT1G32230-XLOC_001707-10045-1
      AAGGATCTTCCTCCTCAATTGGAGTCAAAC---GGAGCAAGAGGTTCAGG
CONSENSUS
      AAGGATCTTCCTCCTCAATTGGAGTCAAAC...GGAGCAAGAGGTTCAGG

A3-AT1G32230-XLOC_001707-10045-0
      AAGTGCAAACAGTGTTGGTTCAAGCACTACAAGACCCAAATCTCCATGGA
A3-AT1G32230-XLOC_001707-10045-1
      AAGTGCAAACAGTGTTGGTTCAAGCACTACAAGACCCAAATCTCCATGGA
CONSENSUS
      AAGTGCAAACAGTGTTGGTTCAAGCACTACAAGACCCAAATCTCCATGGA

A3-AT1G32230-XLOC_001707-10045-0
      TGCCATTTCCTACTCTGTTTGCAGCAATCTCACATAAGGTTGCAGAGAAC
A3-AT1G32230-XLOC_001707-10045-1
      TGCCATTTCCTACTCTGTTTGCAGCAATCTCACATAAGGTTGCAGAGAAC
CONSENSUS
      TGCCATTTCCTACTCTGTTTGCAGCAATCTCACATAAGGTTGCAGAGAAC
```

A3-AT1G32230-XLOC_001707-10045-0
GACATGTTGTTGATCAATGCTGACTACCAACAACTGAGG
A3-AT1G32230-XLOC_001707-10045-1
GACATGTTGTTGATCAATGCTGACTACCAACAACTGAGG
CONSENSUS
GACATGTTGTTGATCAATGCTGACTACCAACAACTGAGG

alignment for event: A3-AT1G33410-XLOC_005885-3069

A3-AT1G33410-XLOC_005885-3069-0
AGCTTCATCAGGTGAAGGATCTTGGAAGGCTTTGCACAGCTTGTCTAAAG
A3-AT1G33410-XLOC_005885-3069-1
AGCTTCATCAGGTGAAGGATCTTGGAAGGCTTTGCACAGCTTGTCTAAAG
CONSENSUS
AGCTTCATCAGGTGAAGGATCTTGGAAGGCTTTGCACAGCTTGTCTAAAG

A3-AT1G33410-XLOC_005885-3069-0 AAGCAGGATTTTCACCTGCCACAACGG---GTCCAAGTATTTTAGATGGT
A3-AT1G33410-XLOC_005885-3069-1
AAGCAGGATTTTCACCTGCCACAACGGCAGGTCCAAGTATTTTAGATGGT
CONSENSUS
AAGCAGGATTTTCACCTGCCACAACGG...GTCCAAGTATTTTAGATGGT

A3-AT1G33410-XLOC_005885-3069-0
TCTACGTCTTCTGCAGCATGGAAACTTCATTACTATGAGTGGGCTATGCA
A3-AT1G33410-XLOC_005885-3069-1
TCTACGTCTTCTGCAGCATGGAAACTTCATTACTATGAGTGGGCTATGCA
CONSENSUS
TCTACGTCTTCTGCAGCATGGAAACTTCATTACTATGAGTGGGCTATGCA

A3-AT1G33410-XLOC_005885-3069-0
AATTTTTGAACGGTACAATATAAGTGAAGGAGCTTGTCAGTTTGCCTATG
A3-AT1G33410-XLOC_005885-3069-1
AATTTTTGAACGGTACAATATAAGTGAAGGAGCTTGTCAGTTTGCCTATG
CONSENSUS
AATTTTTGAACGGTACAATATAAGTGAAGGAGCTTGTCAGTTTGCCTATG

A3-AT1G33410-XLOC_005885-3069-0
CAGCCCTTGAGCAAGTAGATGATGCCTATAATTTCATAGAGATGACTGAG
A3-AT1G33410-XLOC_005885-3069-1
CAGCCCTTGAGCAAGTAGATGATGCCTATAATTTCATAGAGATGACTGAG
CONSENSUS
CAGCCCTTGAGCAAGTAGATGATGCCTATAATTTCATAGAGATGACTGAG

A3-AT1G33410-XLOC_005885-3069-0
GAATTCGATCCAACTAAAGCAGCCACCTACACTAGAGGACGACTGTGGGC
A3-AT1G33410-XLOC_005885-3069-1
GAATTCGATCCAACTAAAGCAGCCACCTACACTAGAGGACGACTGTGGGC
CONSENSUS
GAATTCGATCCAACTAAAGCAGCCACCTACACTAGAGGACGACTGTGGGC

A3-AT1G33410-XLOC_005885-3069-0
AAATGTTTTCAAGTTCACCTTAGATCTCAATCTCTTGAATGATGCCTACT

A3-AT1G33410-XLOC_005885-3069-1
AAATGTTTTCAAGTTCACCTTAGATCTCAATCTCTTGAATGATGCCTACT
CONSENSUS
AAATGTTTTCAAGTTCACCTTAGATCTCAATCTCTTGAATGATGCCTACT

A3-AT1G33410-XLOC_005885-3069-0
GTGCTATAATTTCAAACCCTGATGAGGAGATTAAGCGCATCTGTTTGAGG
A3-AT1G33410-XLOC_005885-3069-1
GTGCTATAATTTCAAACCCTGATGAGGAGATTAAGCGCATCTGTTTGAGG
CONSENSUS
GTGCTATAATTTCAAACCCTGATGAGGAGATTAAGCGCATCTGTTTGAGG

A3-AT1G33410-XLOC_005885-3069-0
CGCTTCATAATAGTTCTATTTGAATGTGGCAAAACGAAG
A3-AT1G33410-XLOC_005885-3069-1
CGCTTCATAATAGTTCTATTTGAATGTGGCAAAACGAAG
CONSENSUS
CGCTTCATAATAGTTCTATTTGAATGTGGCAAAACGAAG

alignment for event: A3-AT1G49720-XLOC_002473-12453

A3-AT1G49720-XLOC_002473-12453-0
AAACACACTTGTCGTCTCTTCTCTCTCTCGCAGCTCTTCTCTATTTTCAG
A3-AT1G49720-XLOC_002473-12453-1
AAACACACTTGTCGTCTCTTCTCTCTCTCGCAGCTCTTCTCTATTTTCAG
CONSENSUS
AAACACACTTGTCGTCTCTTCTCTCTCTCGCAGCTCTTCTCTATTTTCAG

A3-AT1G49720-XLOC_002473-12453-0
CAATCGCCTATAATATTAGGTAAAGCTAAAAGGGTCTGATTCGTTTGTTT
A3-AT1G49720-XLOC_002473-12453-1
CAATCGCCTATAATATTAG-------------------------------
CONSENSUS
CAATCGCCTATAATATTAG...............................

A3-AT1G49720-XLOC_002473-12453-0
TTTCACTGAAGAATTTGGAAGGAAGTGATTCCGTTGTGAAACAGAAAAGA
A3-AT1G49720-XLOC_002473-12453-1 -----------
AATTTGGAAGGAAGTGATTCCGTTGTGAAACAGAAAAGA
CONSENSUS
...........AATTTGGAAGGAAGTGATTCCGTTGTGAAACAGAAAAGA

A3-AT1G49720-XLOC_002473-12453-0
AGTATGGGTACTCACATTGATATCAACAACTTAGGCGGCGATACTTCTAG
A3-AT1G49720-XLOC_002473-12453-1
AGTATGGGTACTCACATTGATATCAACAACTTAGGCGGCGATACTTCTAG
CONSENSUS
AGTATGGGTACTCACATTGATATCAACAACTTAGGCGGCGATACTTCTAG

A3-AT1G49720-XLOC_002473-12453-0
AGGGAATGAGTCAAAGCCATTGGCGAGGCAGTCTTCGTTATATTCCTTAA
A3-AT1G49720-XLOC_002473-12453-1
AGGGAATGAGTCAAAGCCATTGGCGAGGCAGTCTTCGTTATATTCCTTAA
CONSENSUS

AGGGAATGAGTCAAAGCCATTGGCGAGGCAGTCTTCGTTATATTCCTTAA

A3-AT1G49720-XLOC_002473-12453-0
CGTTTGATGAGCTTCAGAGCACATTAGGTGAGCCGGGGAAAGATTTTGGG
A3-AT1G49720-XLOC_002473-12453-1
CGTTTGATGAGCTTCAGAGCACATTAGGTGAGCCGGGGAAAGATTTTGGG
CONSENSUS
CGTTTGATGAGCTTCAGAGCACATTAGGTGAGCCGGGGAAAGATTTTGGG

A3-AT1G49720-XLOC_002473-12453-0
TCTATGAATATGGATGAGTTACTCAAGAACATATGGACTGCTGAGGATAC
A3-AT1G49720-XLOC_002473-12453-1
TCTATGAATATGGATGAGTTACTCAAGAACATATGGACTGCTGAGGATAC
CONSENSUS
TCTATGAATATGGATGAGTTACTCAAGAACATATGGACTGCTGAGGATAC

A3-AT1G49720-XLOC_002473-12453-0
TCAAGCCTTTATGACTACTACATCTTCGGTTGCAGCCCCGGGACCTAGTG
A3-AT1G49720-XLOC_002473-12453-1
TCAAGCCTTTATGACTACTACATCTTCGGTTGCAGCCCCGGGACCTAGTG
CONSENSUS
TCAAGCCTTTATGACTACTACATCTTCGGTTGCAGCCCCGGGACCTAGTG

A3-AT1G49720-XLOC_002473-12453-0
GTTTTGTTCCGGGAGGAAATGGTTTACAGAGGCAAGGCTCCTTGACCTTG
A3-AT1G49720-XLOC_002473-12453-1
GTTTTGTTCCGGGAGGAAATGGTTTACAGAGGCAAGGCTCCTTGACCTTG
CONSENSUS
GTTTTGTTCCGGGAGGAAATGGTTTACAGAGGCAAGGCTCCTTGACCTTG

A3-AT1G49720-XLOC_002473-12453-0
CCTAGAACGCTTAGTCAGAAGACTGTCGATGAAGTCTGGAAATACCTGAA
A3-AT1G49720-XLOC_002473-12453-1
CCTAGAACGCTTAGTCAGAAGACTGTCGATGAAGTCTGGAAATACCTGAA
CONSENSUS
CCTAGAACGCTTAGTCAGAAGACTGTCGATGAAGTCTGGAAATACCTGAA

A3-AT1G49720-XLOC_002473-12453-0
TTCGAAAGAAGGTAGTAATGGGAATACTGGAACGGATGCGCTTGAGAGGC
A3-AT1G49720-XLOC_002473-12453-1
TTCGAAAGAAGGTAGTAATGGGAATACTGGAACGGATGCGCTTGAGAGGC
CONSENSUS
TTCGAAAGAAGGTAGTAATGGGAATACTGGAACGGATGCGCTTGAGAGGC

A3-AT1G49720-XLOC_002473-12453-0
AACAGACTTTAGGGGAAATGACTCTGGAAGATTTCTTACTCCGTGCTGGC
A3-AT1G49720-XLOC_002473-12453-1
AACAGACTTTAGGGGAAATGACTCTGGAAGATTTCTTACTCCGTGCTGGC
CONSENSUS
AACAGACTTTAGGGGAAATGACTCTGGAAGATTTCTTACTCCGTGCTGGC

A3-AT1G49720-XLOC_002473-12453-0
GTTGTTAAAGAAGATAATACTCAGCAGAACGAAAACAGTAGTAGCGGGTT
A3-AT1G49720-XLOC_002473-12453-1
GTTGTTAAAGAAGATAATACTCAGCAGAACGAAAACAGTAGTAGCGGGTT
CONSENSUS

GTTGTTAAAGAAGATAATACTCAGCAGAACGAAAACAGTAGTAGCGGGTT

A3-AT1G49720-XLOC_002473-12453-0
TTATGCTAACAACGGTGCTGCTGGTTTGGAGTTTGGATTTGGTCAGCCGA
A3-AT1G49720-XLOC_002473-12453-1
TTATGCTAACAACGGTGCTGCTGGTTTGGAGTTTGGATTTGGTCAGCCGA
CONSENSUS
TTATGCTAACAACGGTGCTGCTGGTTTGGAGTTTGGATTTGGTCAGCCGA

A3-AT1G49720-XLOC_002473-12453-0
ATCAAAACAGCATATCGTTCAACGGGAACAATAGTTCTATGATCATGAAT
A3-AT1G49720-XLOC_002473-12453-1
ATCAAAACAGCATATCGTTCAACGGGAACAATAGTTCTATGATCATGAAT
CONSENSUS
ATCAAAACAGCATATCGTTCAACGGGAACAATAGTTCTATGATCATGAAT

A3-AT1G49720-XLOC_002473-12453-0
CAAGCACCTGGTTTAGGCCTCAAAGTTGGTGGAACCATGCAGCAGCAGCA
A3-AT1G49720-XLOC_002473-12453-1
CAAGCACCTGGTTTAGGCCTCAAAGTTGGTGGAACCATGCAGCAGCAGCA
CONSENSUS
CAAGCACCTGGTTTAGGCCTCAAAGTTGGTGGAACCATGCAGCAGCAGCA

A3-AT1G49720-XLOC_002473-12453-0
GCAGCCACATCAGCAGCAGTTGCAGCAGCCACATCAGAGACTGCCTCCAA
A3-AT1G49720-XLOC_002473-12453-1
GCAGCCACATCAGCAGCAGTTGCAGCAGCCACATCAGAGACTGCCTCCAA
CONSENSUS
GCAGCCACATCAGCAGCAGTTGCAGCAGCCACATCAGAGACTGCCTCCAA

A3-AT1G49720-XLOC_002473-12453-0
CTATCTTTCCAAAACAAGCGAATGTAACATTTGCGGCGCCTGTAAATATG
A3-AT1G49720-XLOC_002473-12453-1
CTATCTTTCCAAAACAAGCGAATGTAACATTTGCGGCGCCTGTAAATATG
CONSENSUS
CTATCTTTCCAAAACAAGCGAATGTAACATTTGCGGCGCCTGTAAATATG

A3-AT1G49720-XLOC_002473-12453-0
GTCAACAGGGGTTTATTTGAGACTAGCGCAGATGGTCCAGCCAACAGTAA
A3-AT1G49720-XLOC_002473-12453-1
GTCAACAGGGGTTTATTTGAGACTAGCGCAGATGGTCCAGCCAACAGTAA
CONSENSUS
GTCAACAGGGGTTTATTTGAGACTAGCGCAGATGGTCCAGCCAACAGTAA

A3-AT1G49720-XLOC_002473-12453-0
TATGGGAGGAGCAGGGGGTACTGTTACAGCTACTTCTCCTGGGACGAGCA
A3-AT1G49720-XLOC_002473-12453-1
TATGGGAGGAGCAGGGGGTACTGTTACAGCTACTTCTCCTGGGACGAGCA
CONSENSUS
TATGGGAGGAGCAGGGGGTACTGTTACAGCTACTTCTCCTGGGACGAGCA

A3-AT1G49720-XLOC_002473-12453-0
GTGCAGAAAACAATACTTGGTCATCACCAGTTCCTTACGTGTTTGGTCGG
A3-AT1G49720-XLOC_002473-12453-1
GTGCAGAAAACAATACTTGGTCATCACCAGTTCCTTACGTGTTTGGTCGG
CONSENSUS

GTGCAGAAAACAATACTTGGTCATCACCAGTTCCTTACGTGTTTGGTCGG

A3-AT1G49720-XLOC_002473-12453-0
GGAAGAAGAAGCAATACGGGCCTGGAGAAGGTTGTTGAGAGAAGGCAAAA
A3-AT1G49720-XLOC_002473-12453-1
GGAAGAAGAAGCAATACGGGCCTGGAGAAGGTTGTTGAGAGAAGGCAAAA
CONSENSUS
GGAAGAAGAAGCAATACGGGCCTGGAGAAGGTTGTTGAGAGAAGGCAAAA

A3-AT1G49720-XLOC_002473-12453-0
GAGAATGATCAAGAATCGGGAATCCGCTGCTAGATCAAGGGCTCGAAAAC
A3-AT1G49720-XLOC_002473-12453-1
GAGAATGATCAAGAATCGGGAATCCGCTGCTAGATCAAGGGCTCGAAAAC
CONSENSUS
GAGAATGATCAAGAATCGGGAATCCGCTGCTAGATCAAGGGCTCGAAAAC

A3-AT1G49720-XLOC_002473-12453-0 AG
A3-AT1G49720-XLOC_002473-12453-1 AG
CONSENSUS AG

alignment for event: A5-AT1G13350-XLOC_004843-11308

A5-AT1G13350-XLOC_004843-11308-0
ATAACCTGAATGTCTCATGCATTTGTAAGAAATGCAAAAGCAGTTGCACT
A5-AT1G13350-XLOC_004843-11308-1
ATAACCTGAATGTCTCATGCATTTGTAAGAAATGCAAAAGCAGTTGCACT
CONSENSUS
ATAACCTGAATGTCTCATGCATTTGTAAGAAATGCAAAAGCAGTTGCACT

A5-AT1G13350-XLOC_004843-11308-0
AGGCGTTCAATGGAGATGATGGCTTTCCCTTGATTTGTAAGAGCTGGATA
A5-AT1G13350-XLOC_004843-11308-1
AGGCGTTCAATGGAGATGATGGCTTTCCCTTGATTT--------------
CONSENSUS
AGGCGTTCAATGGAGATGATGGCTTTCCCTTGATTT..............

A5-AT1G13350-XLOC_004843-11308-0
CGCGTTTATAACTATCTTTCATATAAATGTCATGGGAGAAATCAGCGGAG
A5-AT1G13350-XLOC_004843-11308-1
----------------------------------GGGAGAAATCAGCGGAG
CONSENSUS
..................................GGGAGAAATCAGCGGAG

A5-AT1G13350-XLOC_004843-11308-0
GGAAAGAGAATGTGAGAAAAGAAAAGAGATAGAGCCTGACCGTGAAAGGA
A5-AT1G13350-XLOC_004843-11308-1
GGAAAGAGAATGTGAGAAAAGAAAAGAGATAGAGCCTGACCGTGAAAGGA
CONSENSUS
GGAAAGAGAATGTGAGAAAAGAAAAGAGATAGAGCCTGACCGTGAAAGGA

A5-AT1G13350-XLOC_004843-11308-0
GAAAAGAGAGGGGAAGCGTTGATAGAGATAGCAGGGGAGACAGGGAAAAA
A5-AT1G13350-XLOC_004843-11308-1
GAAAAGAGAGGGGAAGCGTTGATAGAGATAGCAGGGGAGACAGGGAAAAA

CONSENSUS
      GAAAAGAGAGGGGAAGCGTTGATAGAGATAGCAGGGGAGACAGGGAAAAA

A5-AT1G13350-XLOC_004843-11308-0
      GATTACCTACGGGATAGAGACAACGACAGAGGTAGGAGTAGAGATAAAGC
A5-AT1G13350-XLOC_004843-11308-1
      GATTACCTACGGGATAGAGACAACGACAGAGGTAGGAGTAGAGATAAAGC
CONSENSUS
      GATTACCTACGGGATAGAGACAACGACAGAGGTAGGAGTAGAGATAAAGC

A5-AT1G13350-XLOC_004843-11308-0
      CAGGTATAGTAGTAGAGAGAGGGGGAGGGAGAATGAAAGAGAGAGACGGA
A5-AT1G13350-XLOC_004843-11308-1
      CAGGTATAGTAGTAGAGAGAGGGGGAGGGAGAATGAAAGAGAGAGACGGA
CONSENSUS
      CAGGTATAGTAGTAGAGAGAGGGGGAGGGAGAATGAAAGAGAGAGACGGA

A5-AT1G13350-XLOC_004843-11308-0
      GTGAAAAAGATAGGGATAAAGGACGAGAATTCCAGAGTGATAGAGAGAAG
A5-AT1G13350-XLOC_004843-11308-1
      GTGAAAAAGATAGGGATAAAGGACGAGAATTCCAGAGTGATAGAGAGAAG
CONSENSUS
      GTGAAAAAGATAGGGATAAAGGACGAGAATTCCAGAGTGATAGAGAGAAG

A5-AT1G13350-XLOC_004843-11308-0
      CATAAAAGTCTTGATGATGGATATGGTGAAGTGAGGCATAAACATTCTGG
A5-AT1G13350-XLOC_004843-11308-1
      CATAAAAGTCTTGATGATGGATATGGTGAAGTGAGGCATAAACATTCTGG
CONSENSUS
      CATAAAAGTCTTGATGATGGATATGGTGAAGTGAGGCATAAACATTCTGG

A5-AT1G13350-XLOC_004843-11308-0
      ACACTCAAGACATGATGCGGAAGATGACTTAGAGTTAAGAAGCCCAACTT
A5-AT1G13350-XLOC_004843-11308-1
      ACACTCAAGACATGATGCGGAAGATGACTTAGAGTTAAGAAGCCCAACTT
CONSENSUS
      ACACTCAAGACATGATGCGGAAGATGACTTAGAGTTAAGAAGCCCAACTT

A5-AT1G13350-XLOC_004843-11308-0
      CTGTAAATGGCCATGATCCTAACAGTGGCGATGTCAAAGAAACTCGGGGA
A5-AT1G13350-XLOC_004843-11308-1
      CTGTAAATGGCCATGATCCTAACAGTGGCGATGTCAAAGAAACTCGGGGA
CONSENSUS
      CTGTAAATGGCCATGATCCTAACAGTGGCGATGTCAAAGAAACTCGGGGA

A5-AT1G13350-XLOC_004843-11308-0 AATGTTGAAAG
A5-AT1G13350-XLOC_004843-11308-1 AATGTTGAAAG
CONSENSUS   AATGTTGAAAG

alignment for event: A3-AT1G72640-XLOC_007700-6971

A3-AT1G72640-XLOC_007700-6971-0
      GCGGTCCCAAGCTTAGGAAATGGTATGGAGCTCCTGAACTTCGGCCAAAG
A3-AT1G72640-XLOC_007700-6971-1

GCGGTCCCAAGCTTAGGAAATGGTATGGAGCTCCTGAACTTCGGCCAAAG
CONSENSUS
GCGGTCCCAAGCTTAGGAAATGGTATGGAGCTCCTGAACTTCGGCCAAAG

A3-AT1G72640-XLOC_007700-6971-0
GATGGATCTTTAAGTGGAGACGACGATGAATTTGAAG---AGGAAAAAGA
A3-AT1G72640-XLOC_007700-6971-1
GATGGATCTTTAAGTGGAGACGACGATGAATTTGAAGCAGAGGAAAAAGA
CONSENSUS
GATGGATCTTTAAGTGGAGACGACGATGAATTTGAAG...AGGAAAAAGA

A3-AT1G72640-XLOC_007700-6971-0
AGATGATCTTGATGGAGAGAAAGACGTTGTTTTTGTAACTGATGGAGACA
A3-AT1G72640-XLOC_007700-6971-1
AGATGATCTTGATGGAGAGAAAGACGTTGTTTTTGTAACTGATGGAGACA
CONSENSUS
AGATGATCTTGATGGAGAGAAAGACGTTGTTTTTGTAACTGATGGAGACA

A3-AT1G72640-XLOC_007700-6971-0 GTGATTTGGGTCAG
A3-AT1G72640-XLOC_007700-6971-1 GTGATTTGGGTCAG
CONSENSUS   GTGATTTGGGTCAG

alignment for event: RI-AT1G07870-XLOC_004539-10962

RI-AT1G07870-XLOC_004539-10962-0
GCGATATAAAAAAAGGCGAAGAAATTAAGCTTTCTCTGTTTGTCACTAAG
RI-AT1G07870-XLOC_004539-10962-1
GCGATATAAAAAAAGGCGAAGAAATTAAGCTTTCTCTGTTTGTCACTAAG
CONSENSUS
GCGATATAAAAAAAGGCGAAGAAATTAAGCTTTCTCTGTTTGTCACTAAG

RI-AT1G07870-XLOC_004539-10962-0
CTCTCTCTCTCTCCTTCTTCTTCGACTTTTTTTTTTCTTCCTCTCTTCTC
RI-AT1G07870-XLOC_004539-10962-1
CTCTCTCTCTCTCCTTCTTCTTCGACTTTTTTTTTTCTTCCTCTCTTCTC
CONSENSUS
CTCTCTCTCTCTCCTTCTTCTTCGACTTTTTTTTTTCTTCCTCTCTTCTC

RI-AT1G07870-XLOC_004539-10962-0
TTTGGTTTTTTGGTGTTGGGTTTTATCTGGGTTCCTGATTTCTTCAACGA
RI-AT1G07870-XLOC_004539-10962-1
TTTGGTTTTTTGGTGTTGGGTTTTATCTGGGTTCCTGATTTCTTCAACGA
CONSENSUS
TTTGGTTTTTTGGTGTTGGGTTTTATCTGGGTTCCTGATTTCTTCAACGA

RI-AT1G07870-XLOC_004539-10962-0
GGAAGGAAGGAAGAAGAAGAGAAAAGGGACAAGCGAGAATTACTCTTTTC
RI-AT1G07870-XLOC_004539-10962-1
GGAAGGAAGGAAGAAGAAGAGAAAAGGGACAAGCGAGAATTACTCTTTTC
CONSENSUS
GGAAGGAAGGAAGAAGAAGAGAAAAGGGACAAGCGAGAATTACTCTTTTC

RI-AT1G07870-XLOC_004539-10962-0
TCCTTCAATTTCCGATGGGTTGTTTCGGATGCAGTAAGAAATCGAGCAAA

RI-AT1G07870-XLOC_004539-10962-1
TCCTTCAATTTCCGATGGGTTGTTTCGGATGCAGTAAGAAATCGAGCAAA
CONSENSUS
TCCTTCAATTTCCGATGGGTTGTTTCGGATGCAGTAAGAAATCGAGCAAA

RI-AT1G07870-XLOC_004539-10962-0
CGATCCGAGACTAACAAAGATACTGTCATAAATAGAAAAATTGTCGGTGG
RI-AT1G07870-XLOC_004539-10962-1
CGATCCGAGACTAACAAAGATACTGTCATAAATAGAAAAATTGTCGGTGG
CONSENSUS
CGATCCGAGACTAACAAAGATACTGTCATAAATAGAAAAATTGTCGGTGG

RI-AT1G07870-XLOC_004539-10962-0
TACTACGAGCGTTGCTAAGAGTGATAAGCGAGACGATCAGACTCAGCCCA
RI-AT1G07870-XLOC_004539-10962-1
TACTACGAGCGTTGCTAAGAGTGATAAGCGAGACGATCAGACTCAGCCCA
CONSENSUS
TACTACGAGCGTTGCTAAGAGTGATAAGCGAGACGATCAGACTCAGCCCA

RI-AT1G07870-XLOC_004539-10962-0
GTTCAGGTATCGAGTGATTTTGACTAATTTCGTTATTCTCTCTTCTCCAC
RI-AT1G07870-XLOC_004539-10962-1
GTTCAG--
CONSENSUS
GTTCAG..

RI-AT1G07870-XLOC_004539-10962-0
AAGCTCTCTTTTGGATTTGTTGATTTTTTTTTATCGGATACTTATGTTTA
RI-AT1G07870-XLOC_004539-10962-1

CONSENSUS
....

RI-AT1G07870-XLOC_004539-10962-0
GGTTTTTCTGTATCTGTTACATTTGGGAGAGACATTTAATGGAGATTTTT
RI-AT1G07870-XLOC_004539-10962-1

CONSENSUS
....

RI-AT1G07870-XLOC_004539-10962-0
TGTGATTTTTCCCCGGAGAATTTTGATGCATGTGTACTATTCACCATATA
RI-AT1G07870-XLOC_004539-10962-1

CONSENSUS
....

RI-AT1G07870-XLOC_004539-10962-0
AACGATGTTGGGTTTTTGAAATCGAGCTAGTTTTTTTTGGTTTTGTCTCC
RI-AT1G07870-XLOC_004539-10962-1

CONSENSUS
....

RI-AT1G07870-XLOC_004539-10962-0
TCCATGAGGGGACAAAGTGTCAAAATTGAGCCTTTGGTATCTTGCCGTGT

RI-AT1G07870-XLOC_004539-10962-1

CONSENSUS
      ....

RI-AT1G07870-XLOC_004539-10962-0
      CTGTTTTTGGTTAAGTCACATGATAACTGGTAATCGATTTTGTTACTCGT
RI-AT1G07870-XLOC_004539-10962-1

CONSENSUS
      ....

RI-AT1G07870-XLOC_004539-10962-0
      TCCATGTGTTGTAGATTCAACAAAAGTCAGTCCATATAGAGATGTAAACA
RI-AT1G07870-XLOC_004539-10962-1 --------------
ATTCAACAAAAGTCAGTCCATATAGAGATGTAAACA
CONSENSUS
      ..............ATTCAACAAAAGTCAGTCCATATAGAGATGTAAACA

RI-AT1G07870-XLOC_004539-10962-0
      ATGAAGGAGGAGTTGGTAAGGAAGATCAGTTATCTTTGGATGTGAAGGGT
RI-AT1G07870-XLOC_004539-10962-1
      ATGAAGGAGGAGTTGGTAAGGAAGATCAGTTATCTTTGGATGTGAAGGGT
CONSENSUS
      ATGAAGGAGGAGTTGGTAAGGAAGATCAGTTATCTTTGGATGTGAAGGGT

RI-AT1G07870-XLOC_004539-10962-0
      TTGAATCTAAATGACCAAGTCACAGGGAAGAAAGCACAGACTTTTACTTT
RI-AT1G07870-XLOC_004539-10962-1
      TTGAATCTAAATGACCAAGTCACAGGGAAGAAAGCACAGACTTTTACTTT
CONSENSUS
      TTGAATCTAAATGACCAAGTCACAGGGAAGAAAGCACAGACTTTTACTTT

RI-AT1G07870-XLOC_004539-10962-0
      TCAAGAACTTGCAGAAGCTACGGGAAACTTTAGATCGGATTGTTTTCTTG
RI-AT1G07870-XLOC_004539-10962-1
      TCAAGAACTTGCAGAAGCTACGGGAAACTTTAGATCGGATTGTTTTCTTG
CONSENSUS
      TCAAGAACTTGCAGAAGCTACGGGAAACTTTAGATCGGATTGTTTTCTTG

RI-AT1G07870-XLOC_004539-10962-0
      GTGAAGGAGGTTTCGGAAAAGTTTTCAAGGGAACTATAGAGAAATTGGAT
RI-AT1G07870-XLOC_004539-10962-1
      GTGAAGGAGGTTTCGGAAAAGTTTTCAAGGGAACTATAGAGAAATTGGAT
CONSENSUS
      GTGAAGGAGGTTTCGGAAAAGTTTTCAAGGGAACTATAGAGAAATTGGAT

RI-AT1G07870-XLOC_004539-10962-0 CAG
RI-AT1G07870-XLOC_004539-10962-1 CAG
CONSENSUS CAG

alignment for event: A3-AT1G67840-XLOC_003450-9736

A3-AT1G67840-XLOC_003450-9736-0

GCTAATATAGTGCGCCACAACGAAGAAGCCCTAAAGAAGATAAATAAAAC
A3-AT1G67840-XLOC_003450-9736-1
GCTAATATAGTGCGCCACAACGAAGAAGCCCTAAAGAAGATAAATAAAAC
CONSENSUS
GCTAATATAGTGCGCCACAACGAAGAAGCCCTAAAGAAGATAAATAAAAC

A3-AT1G67840-XLOC_003450-9736-0
ACACAATGAAACAAGGAGATCAAAGTATGAACATAAGGACCCCATTGATG
A3-AT1G67840-XLOC_003450-9736-1
ACACAATGAAACAAGGAGATCAAAGTATGAACATAAGGACCCCATTGATG
CONSENSUS
ACACAATGAAACAAGGAGATCAAAGTATGAACATAAGGACCCCATTGATG

A3-AT1G67840-XLOC_003450-9736-0
GCTCACAAATCTCAAGTACACGCCTTTCCTTGGGTTCTGGATTAGATGAT
A3-AT1G67840-XLOC_003450-9736-1
GCTCACAAATCTCAAGTACACGCCTTTCCTTGGGTTCTGGATTAGATGAT
CONSENSUS
GCTCACAAATCTCAAGTACACGCCTTTCCTTGGGTTCTGGATTAGATGAT

A3-AT1G67840-XLOC_003450-9736-0
TCAGAAATGCCTATGCCACCTCTTGCCCTGGCACCCCTACAAATGCACAG
A3-AT1G67840-XLOC_003450-9736-1
TCAGAAATGCCTATGCCACCTCTTGCCCTGGCACCCCTACAAATGCACAG
CONSENSUS
TCAGAAATGCCTATGCCACCTCTTGCCCTGGCACCCCTACAAATGCACAG

A3-AT1G67840-XLOC_003450-9736-0
TATAAGGAAGACCATGCGACATTTCTAATGTGCTTTTAGATATGGTCGAG
A3-AT1G67840-XLOC_003450-9736-1
TATAAG----------------------------------ATATGGTCGAG
CONSENSUS
TATAAG..................................ATATGGTCGAG

A3-AT1G67840-XLOC_003450-9736-0
ACTGTGAGACCACTAGCACTTACTCAGCAAAGAGTAGTGGAGCTAGGTGA
A3-AT1G67840-XLOC_003450-9736-1
ACTGTGAGACCACTAGCACTTACTCAGCAAAGAGTAGTGGAGCTAGGTGA
CONSENSUS
ACTGTGAGACCACTAGCACTTACTCAGCAAAGAGTAGTGGAGCTAGGTGA

A3-AT1G67840-XLOC_003450-9736-0
GAACTCAGCTTCCCTGCAAGTTGCTGTTGAAGAACCAGCTTTGAGGCAAG
A3-AT1G67840-XLOC_003450-9736-1
GAACTCAGCTTCCCTGCAAGTTGCTGTTGAAGAACCAGCTTTGAGGCAAG
CONSENSUS
GAACTCAGCTTCCCTGCAAGTTGCTGTTGAAGAACCAGCTTTGAGGCAAG

A3-AT1G67840-XLOC_003450-9736-0
CTCTGAGCAACCTCATCGAAGGTGCATTGTTGCGCACTCATGTTGGGGGG
A3-AT1G67840-XLOC_003450-9736-1
CTCTGAGCAACCTCATCGAAGGTGCATTGTTGCGCACTCATGTTGGGGGG
CONSENSUS
CTCTGAGCAACCTCATCGAAGGTGCATTGTTGCGCACTCATGTTGGGGGG

A3-AT1G67840-XLOC_003450-9736-0

AAAGTTGAAATTTTGTCGACCAGAGCTCCTGCTGGCGGTAGTCTTGTAGT
A3-AT1G67840-XLOC_003450-9736-1
AAAGTTGAAATTTTGTCGACCAGAGCTCCTGCTGGCGGTAGTCTTGTAGT
CONSENSUS
AAAGTTGAAATTTTGTCGACCAGAGCTCCTGCTGGCGGTAGTCTTGTAGT

A3-AT1G67840-XLOC_003450-9736-0
AATTGATGATGATGGTCCTGATATGCGCTATATG
A3-AT1G67840-XLOC_003450-9736-1
AATTGATGATGATGGTCCTGATATGCGCTATATG
CONSENSUS
AATTGATGATGATGGTCCTGATATGCGCTATATG

alignment for event: A5-AT1G08660-XLOC_004590-2814

A5-AT1G08660-XLOC_004590-2814-0
ATAATAATCGTAAACTAGATCTTCAGCCGGAAGATATCCAGATCTTGTCA
A5-AT1G08660-XLOC_004590-2814-1
ATAATAATCGTAAACTAGATCTTCAGCCGGAAGATATCCAGATCTTGTCA
CONSENSUS
ATAATAATCGTAAACTAGATCTTCAGCCGGAAGATATCCAGATCTTGTCA

A5-AT1G08660-XLOC_004590-2814-0
GATTTTCAGTCCAGCGTTCAACAGTGCGTGGCTAACAGAGGGCTTGGACT
A5-AT1G08660-XLOC_004590-2814-1 GATTTTCAGTCCAGCGTTCAACA-------
GCTAACAGAGGGCTTGGACT
CONSENSUS
GATTTTCAGTCCAGCGTTCAACA.......GCTAACAGAGGGCTTGGACT

A5-AT1G08660-XLOC_004590-2814-0
CTCTGCCCATATTATCGATCACTGTAATCTTATCCTCAAGTTCCCTGAAG
A5-AT1G08660-XLOC_004590-2814-1
CTCTGCCCATATTATCGATCACTGTAATCTTATCCTCAAGTTCCCTGAAG
CONSENSUS
CTCTGCCCATATTATCGATCACTGTAATCTTATCCTCAAGTTCCCTGAAG

A5-AT1G08660-XLOC_004590-2814-0 GCACTAACAGTACTTGG
A5-AT1G08660-XLOC_004590-2814-1 GCACTAACAGTACTTGG
CONSENSUS   GCACTAACAGTACTTGG

alignment for event: RI-AT1G50300-XLOC_006519-1342

RI-AT1G50300-XLOC_006519-1342-0
GTATTGATAAAGATGGAAGAGAGAGAAGCAGAGACAGGCAAAGAGACCGT
RI-AT1G50300-XLOC_006519-1342-1
GTATTGATAAAGATGGAAGAGAGAGAAGCAGAGACAGGCAAAGAGACCGT
CONSENSUS
GTATTGATAAAGATGGAAGAGAGAGAAGCAGAGACAGGCAAAGAGACCGT

RI-AT1G50300-XLOC_006519-1342-0
GGTAGAGATCATCACTACGATAAGGATAGACGCAGAAGCAGAAGCCGAGA
RI-AT1G50300-XLOC_006519-1342-1

GGTAGAGATCATCACTACGATAAGGATAGACGCAGAAGCAGAAGCCGAGA
CONSENSUS
GGTAGAGATCATCACTACGATAAGGATAGACGCAGAAGCAGAAGCCGAGA

RI-AT1G50300-XLOC_006519-1342-0
GAGGGAAAGAGGCAAGGAGCGTGACTATGACTATGACCATGATCGGGACA
RI-AT1G50300-XLOC_006519-1342-1
GAGGGAAAGAGGCAAGGAGCGTGACTATGACTATGACCATGATCGGGACA
CONSENSUS
GAGGGAAAGAGGCAAGGAGCGTGACTATGACTATGACCATGATCGGGACA

RI-AT1G50300-XLOC_006519-1342-0
GAGACAGAGACTACGGTCGCGAACGTGGAAGCAGGTACCGTAACTGAGAG
RI-AT1G50300-XLOC_006519-1342-1
GAGACAGAGACTACGGTCGCGAACGTGGAAGCAG----------------
CONSENSUS
GAGACAGAGACTACGGTCGCGAACGTGGAAGCAG................

RI-AT1G50300-XLOC_006519-1342-0
ATTACTCATAAGTGTTAGGGTTTAGTTTCTCATCTGTTTTGTCGACTTAC
RI-AT1G50300-XLOC_006519-1342-1

CONSENSUS
....

RI-AT1G50300-XLOC_006519-1342-0
TTTTACAACGGTATCATTACAATGTCTTCGCCTTTTGTTTACTTATTGTG
RI-AT1G50300-XLOC_006519-1342-1

CONSENSUS
....

RI-AT1G50300-XLOC_006519-1342-0
TGAAGTGAAACTCATTTTGGTTTGAATCAATGGCTATTGTTTTTCACTTG
RI-AT1G50300-XLOC_006519-1342-1

CONSENSUS
....

RI-AT1G50300-XLOC_006519-1342-0
TTTTGGTCTGATTATGTTCTGAGAATACTCCATGTTTATCGTTTCGAAAA
RI-AT1G50300-XLOC_006519-1342-1

CONSENSUS
....

RI-AT1G50300-XLOC_006519-1342-0
TTGGTAATTATGGCAAAACATGGTTAGCGACGTCAATTGCTAATTTAGAC
RI-AT1G50300-XLOC_006519-1342-1

CONSENSUS
....

RI-AT1G50300-XLOC_006519-1342-0
GTGTATTAAGTATTATTTTGTCTTTTGTAGTATTATTTTTAGTTGTCCCT
RI-AT1G50300-XLOC_006519-1342-1

CONSENSUS
....

RI-AT1G50300-XLOC_006519-1342-0
TTGTTTTTTTTTCCATCGCCAATCATTTCATCGCTGTATGATTTTAATGG
RI-AT1G50300-XLOC_006519-1342-1

CONSENSUS
....

RI-AT1G50300-XLOC_006519-1342-0
GAATAAAAAAAATAGAACAAGAAGATTTTTGTGGACAATATAGTTTCTGA
RI-AT1G50300-XLOC_006519-1342-1

CONSENSUS
....

RI-AT1G50300-XLOC_006519-1342-0
AACTATAATAGGATAAGAAAAAATTAAGCTTATTTGTGGGGATATATATT
RI-AT1G50300-XLOC_006519-1342-1

CONSENSUS
....

RI-AT1G50300-XLOC_006519-1342-0
AATGTCATATCATTGTCATAAACATGGTCGGCAAAACTTGGGACGGCAGT
RI-AT1G50300-XLOC_006519-1342-1

CONSENSUS
....

RI-AT1G50300-XLOC_006519-1342-0
TTACCAGAATAATCCGGTCGACAGAATTCAACAGTTCCTCTCTCTAAAAG
RI-AT1G50300-XLOC_006519-1342-1

CONSENSUS
....

RI-AT1G50300-XLOC_006519-1342-0
ATATGAAGGAGATTAATTTTGGCTTTGTTAGCAAAGATGGCCGGTCCTCT
RI-AT1G50300-XLOC_006519-1342-1

CONSENSUS
....

RI-AT1G50300-XLOC_006519-1342-0
TCTGGCAAATCCGGCTTTTACAAGCGGTTTTGGCCGGCTTTTATCCTTCC
RI-AT1G50300-XLOC_006519-1342-1

CONSENSUS
....

RI-AT1G50300-XLOC_006519-1342-0
TCCGCCGTTCGATCGGTTGTTCTTACCGATTGCTTTAGTTTCTTTTTCAG
RI-AT1G50300-XLOC_006519-1342-1

CONSENSUS
....

RI-AT1G50300-XLOC_006519-1342-0
TTTTTCTTTCAATAATTTCTAAAGATTTCAATTGGAGATTAATTTTAGGT
RI-AT1G50300-XLOC_006519-1342-1

CONSENSUS
....

RI-AT1G50300-XLOC_006519-1342-0
CATAATAGCCTAAGATTAAGTTTTCGGATTTGTATCTTTAGAAAGTTTGA
RI-AT1G50300-XLOC_006519-1342-1

CONSENSUS
....

RI-AT1G50300-XLOC_006519-1342-0
TTTGCAGAATTACCTCAAGTATGAAAGGAAGTTTTGTTTCCAGCATATTG
RI-AT1G50300-XLOC_006519-1342-1

CONSENSUS
....

RI-AT1G50300-XLOC_006519-1342-0
GGATTGCCTGTAGCCGTTTCTACATGTTTATGGAGCTTCGATCGATCAAA
RI-AT1G50300-XLOC_006519-1342-1

CONSENSUS
....

RI-AT1G50300-XLOC_006519-1342-0
TTTGAGGAATACTCAGATTTTTCTTTCCTCATTACCTGTTTTTGCCAAAT
RI-AT1G50300-XLOC_006519-1342-1

CONSENSUS
....

RI-AT1G50300-XLOC_006519-1342-0
CTCTTGATTGATGATTTTGGAATTGAATTCTCTGTTTGTGTATCCTCCCT
RI-AT1G50300-XLOC_006519-1342-1

CONSENSUS
....

RI-AT1G50300-XLOC_006519-1342-0
TCTCTGTTTAGCAGTTTCCGGTGAGAAAAAAACAAAAATCGATGTTTGCC
RI-AT1G50300-XLOC_006519-1342-1 -----------
CAGTTTCCGGTGAGAAAAAAACAAAAATCGATGTTTGCC
CONSENSUS
...........CAGTTTCCGGTGAGAAAAAAACAAAAATCGATGTTTGCC

RI-AT1G50300-XLOC_006519-1342-0
CTTTTTCGACGTTGATTGTGTTCACCGGATCGGAGTCTTTGACGGCACAG
RI-AT1G50300-XLOC_006519-1342-1

CTTTTTCGACGTTGATTGTGTTCACCGGATCGGAGTCTTTGACGGCACAG
CONSENSUS
CTTTTTCGACGTTGATTGTGTTCACCGGATCGGAGTCTTTGACGGCACAG

RI-AT1G50300-XLOC_006519-1342-0
GATGACAGGAACGTTTCATCGCTTCTCTTGCAACACGTGTCTTGTTATAC
RI-AT1G50300-XLOC_006519-1342-1
GATGACAGGAACGTTTCATCGCTTCTCTTGCAACACGTGTCTTGTTATAC
CONSENSUS
GATGACAGGAACGTTTCATCGCTTCTCTTGCAACACGTGTCTTGTTATAC

RI-AT1G50300-XLOC_006519-1342-0
GATCGTGTTTTGTTAAAGAGATATCCTTCCTTTAAAGAAATGTAACTAAG
RI-AT1G50300-XLOC_006519-1342-1
GATCGTGTTTTGTTAAAGAGATATCCTTCCTTTAAAGAAATGTAACTAAG
CONSENSUS
GATCGTGTTTTGTTAAAGAGATATCCTTCCTTTAAAGAAATGTAACTAAG

RI-AT1G50300-XLOC_006519-1342-0
TGGACCTTTGTTGGGCTGTATTCTGTGGATCATTAATAAACATAATCTTT
RI-AT1G50300-XLOC_006519-1342-1
TGGACCTTTGTTGGGCTGTATTCTGTGGATCATTAATAAACATAATCTTT
CONSENSUS
TGGACCTTTGTTGGGCTGTATTCTGTGGATCATTAATAAACATAATCTTT

RI-AT1G50300-XLOC_006519-1342-0 CCAGTG
RI-AT1G50300-XLOC_006519-1342-1 CCAGTG
CONSENSUS CCAGTG

alignment for event: A3-AT1G67490-XLOC_007435-512

A3-AT1G67490-XLOC_007435-512-0
AACTCCATTGTCCTTGGTAGCTGGCTTGATGTGGCTTGGTGTCAAAGATG
A3-AT1G67490-XLOC_007435-512-1
AACTCCATTGTCCTTGGTAGCTGGCTTGATGTGGCTTGGTGTCAAAGATG
CONSENSUS
AACTCCATTGTCCTTGGTAGCTGGCTTGATGTGGCTTGGTGTCAAAGATG

A3-AT1G67490-XLOC_007435-512-0
AGATGTATGTTATGCGGCATTTCTGTGAAAACTCTGATGATTTAAGTACA
A3-AT1G67490-XLOC_007435-512-1
AGATGTATGTTATGCGGCATTTCTGTGAAAACTCTGATGATTTAAGTACA
CONSENSUS
AGATGTATGTTATGCGGCATTTCTGTGAAAACTCTGATGATTTAAGTACA

A3-AT1G67490-XLOC_007435-512-0
TTTGGCTGGAGAGAACATAATGGACGGGATTATGGTCGGCAAGAGCTGGT
A3-AT1G67490-XLOC_007435-512-1
TTTGGCTGGAGAGAACATAATGGACGGGATTATGGTCGGCAAGAGCTGGT
CONSENSUS
TTTGGCTGGAGAGAACATAATGGACGGGATTATGGTCGGCAAGAGCTGGT

A3-AT1G67490-XLOC_007435-512-0
TGAAAACGATATGGTAATAGAGACGAGTTTTGTGAAGTCCAAGGGAGACG

A3-AT1G67490-XLOC_007435-512-1
TGAAAACGATATGGTAATAGAGACGAGTTTTGTGAAGTCCAAGGGAGACG
CONSENSUS
TGAAAACGATATGGTAATAGAGACGAGTTTTGTGAAGTCCAAGGGAGACG

A3-AT1G67490-XLOC_007435-512-0
GCCTTGGTTATGGTGGGGATTGGGCAGTTCGGATTGATGTAAAAAATAAA
A3-AT1G67490-XLOC_007435-512-1
GCCTTGGTTATGGTGGGGATTGGGCAGTTCGGATTGATGTAAAAAATAAA
CONSENSUS
GCCTTGGTTATGGTGGGGATTGGGCAGTTCGGATTGATGTAAAAAATAAA

A3-AT1G67490-XLOC_007435-512-0
GG--
A3-AT1G67490-XLOC_007435-512-1
GGGTTGAATGATGATGTGAAGAGAAGTGCACATCTCTTCTTTTATTTAGC
CONSENSUS
GG..

A3-AT1G67490-XLOC_007435-512-0 -----------------------------
GCCAAGATGGTTTAGATTTCC
A3-AT1G67490-XLOC_007435-512-1
TGATGAAGGTGGCAATGTTTTAAATTTAGGCCAAGATGGTTTAGATTTCC
CONSENSUS
.............................GCCAAGATGGTTTAGATTTCC

A3-AT1G67490-XLOC_007435-512-0
AAGGGAGCTCTCTCCTGGTTTCGGGGTCACGTGAAGATGTAGGAGACTGG
A3-AT1G67490-XLOC_007435-512-1
AAGGGAGCTCTCTCCTGGTTTCGGGGTCACGTGAAGATGTAGGAGACTGG
CONSENSUS
AAGGGAGCTCTCTCCTGGTTTCGGGGTCACGTGAAGATGTAGGAGACTGG

A3-AT1G67490-XLOC_007435-512-0 CAGATACACTTAAAATCACAG
A3-AT1G67490-XLOC_007435-512-1 CAGATACACTTAAAATCACAG
CONSENSUS CAGATACACTTAAAATCACAG

alignment for event: A3-AT1G53570-XLOC_002689-8923

A3-AT1G53570-XLOC_002689-8923-0
GTTGCTGCGATTTTCAAAATCGGAAACAGTAAAGACACCCCGGAAATACC
A3-AT1G53570-XLOC_002689-8923-1
GTTGCTGCGATTTTCAAAATCGGAAACAGTAAAGACACCCCGGAAATACC
CONSENSUS
GTTGCTGCGATTTTCAAAATCGGAAACAGTAAAGACACCCCGGAAATACC

A3-AT1G53570-XLOC_002689-8923-0
TGATCACCTTTCAAATGATGCAAAGAATTTTATAAGGCTTTGTCTGCAAC
A3-AT1G53570-XLOC_002689-8923-1
TGATCACCTTTCAAATGATGCAAAGAATTTTATAAGGCTTTGTCTGCAAC
CONSENSUS
TGATCACCTTTCAAATGATGCAAAGAATTTTATAAGGCTTTGTCTGCAAC

A3-AT1G53570-XLOC_002689-8923-0

GAAATCCGACAGTACGTCCTACAGCTTCTCAGCTTCTAGAACACCCTTTT
A3-AT1G53570-XLOC_002689-8923-1
GAAATCCGACAGTACGTCCTACAGCTTCTCAGCTTCTAGAACACCCTTTT
CONSENSUS
GAAATCCGACAGTACGTCCTACAGCTTCTCAGCTTCTAGAACACCCTTTT

A3-AT1G53570-XLOC_002689-8923-0
CTACGTAACACAACAAGAGTGGCTAGTACTAGTTTGCCCAAAGACTTCCC
A3-AT1G53570-XLOC_002689-8923-1
CTACGTAACACAACAAGAGTGGCTAGTACTAGTTTGCCCAAAGACTTCCC
CONSENSUS
CTACGTAACACAACAAGAGTGGCTAGTACTAGTTTGCCCAAAGACTTCCC

A3-AT1G53570-XLOC_002689-8923-0
CCCACGTTCCTATGATGGAAACTTCTCACTGCAGCCTACAAGGGAACCCT
A3-AT1G53570-XLOC_002689-8923-1
CCCACGTTCCTATGATGGAAACTTCTCACTG---CCTACAAGGGAACCCT
CONSENSUS
CCCACGTTCCTATGATGGAAACTTCTCACTG...CCTACAAGGGAACCCT

A3-AT1G53570-XLOC_002689-8923-0
ATCCAGGGAGACTGAGCCATGATAATTATGCAAAACAGCCATTGTCTAGA
A3-AT1G53570-XLOC_002689-8923-1
ATCCAGGGAGACTGAGCCATGATAATTATGCAAAACAGCCATTGTCTAGA
CONSENSUS
ATCCAGGGAGACTGAGCCATGATAATTATGCAAAACAGCCATTGTCTAGA

A3-AT1G53570-XLOC_002689-8923-0 ACTATAAAGAGCCCGAG
A3-AT1G53570-XLOC_002689-8923-1 ACTATAAAGAGCCCGAG
CONSENSUS ACTATAAAGAGCCCGAG

alignment for event: RI-AT1G10890-XLOC_000523-8246

RI-AT1G10890-XLOC_000523-8246-0
GACCAATCCGTTCAGAGAGATTAAAGCTGAGCTGAGCTTCTCCTTATAAA
RI-AT1G10890-XLOC_000523-8246-1
GACCAATCCGTTCAGAGAGATTAAAGCTGAGCTGAGCTTCTCCTTATAAA
CONSENSUS
GACCAATCCGTTCAGAGAGATTAAAGCTGAGCTGAGCTTCTCCTTATAAA

RI-AT1G10890-XLOC_000523-8246-0
GGAAGATACTGCTTGTTCTACGGAATTTTGAGAGATGCCTCGGGACTTGT
RI-AT1G10890-XLOC_000523-8246-1
GGAAGATACTGCTTGTTCTACGGAATTTTGAGAGATGCCTCGGGACTTGT
CONSENSUS
GGAAGATACTGCTTGTTCTACGGAATTTTGAGAGATGCCTCGGGACTTGT

RI-AT1G10890-XLOC_000523-8246-0
CAAGATCGAGGTCACCGTCTCCATCACCTTCACGTCGTAGAAAGCACTCG
RI-AT1G10890-XLOC_000523-8246-1
CAAGATCGAGGTCACCGTCTCCATCACCTTCACGTCGTAGAAAGCACTCG
CONSENSUS
CAAGATCGAGGTCACCGTCTCCATCACCTTCACGTCGTAGAAAGCACTCG

RI-AT1G10890-XLOC_000523-8246-0
AGGTCTCCCGTAAGGCAGAGGCATAGCAGGAGGAGTAGAAGAGACAGAAG
RI-AT1G10890-XLOC_000523-8246-1
AGGTCTCCCGTAAGGCAGAGGCATAGCAGGAGGAGTAGAAGAGACAGAAG
CONSENSUS
AGGTCTCCCGTAAGGCAGAGGCATAGCAGGAGGAGTAGAAGAGACAGAAG

RI-AT1G10890-XLOC_000523-8246-0
CCCTTCTCCATACTCATCTCATTCGTATAGCAGGTGATACCCCTAGTGTT
RI-AT1G10890-XLOC_000523-8246-1
CCCTTCTCCATACTCATCTCATTCGTATAGCAG-----------------
CONSENSUS
CCCTTCTCCATACTCATCTCATTCGTATAGCAG.................

RI-AT1G10890-XLOC_000523-8246-0
TGTTTTTTCCGCTATTCGTTTGCCTTAGTTATCATTCGTTTATACGAACT
RI-AT1G10890-XLOC_000523-8246-1

CONSENSUS
....

RI-AT1G10890-XLOC_000523-8246-0
GTAAGAAACATTGGTGGAACACGGTTATAGATAGACATGTAAAGTTTACA
RI-AT1G10890-XLOC_000523-8246-1

CONSENSUS
....

RI-AT1G10890-XLOC_000523-8246-0
GACTCAAGAATTGCTTTTAGTAAAGTTCCTACTTATAGATATATTTTCTA
RI-AT1G10890-XLOC_000523-8246-1

CONSENSUS
....

RI-AT1G10890-XLOC_000523-8246-0
CCCTTCTCTAAAAGAGAGTGCAATGTCTGGATGCTGCTGGCTCTGTATCG
RI-AT1G10890-XLOC_000523-8246-1

CONSENSUS
....

RI-AT1G10890-XLOC_000523-8246-0
TAAGCGTTTTTTTGAGTGATGCTGCGTATTGGAAATATATCGTGTTTATT
RI-AT1G10890-XLOC_000523-8246-1

CONSENSUS
....

RI-AT1G10890-XLOC_000523-8246-0
TGGCTGTTGTGATTAAATTTGTTGCAAGAGAATATGTTTTATGACCTGAG
RI-AT1G10890-XLOC_000523-8246-1

CONSENSUS
....

RI-AT1G10890-XLOC_000523-8246-0
CTTTGTGATTTTTAGAATGAAACCTGTATATTCGGTAAAGACATAAGTTA
RI-AT1G10890-XLOC_000523-8246-1

CONSENSUS
....

RI-AT1G10890-XLOC_000523-8246-0
AGCTGGGATAATAGCACTACTGATAGTCTGATCTAGTTTATATTTTAATT
RI-AT1G10890-XLOC_000523-8246-1

CONSENSUS
....

RI-AT1G10890-XLOC_000523-8246-0
GTATCTGTAGATTATTAGTTAGAATCAGTTTTAAGAGCGACTATATTATG
RI-AT1G10890-XLOC_000523-8246-1

CONSENSUS
....

RI-AT1G10890-XLOC_000523-8246-0
TTCTTTTAAATGGAGTTTGTTAGCAATAGTGATACAAAACCACCACTATT
RI-AT1G10890-XLOC_000523-8246-1

CONSENSUS
....

RI-AT1G10890-XLOC_000523-8246-0
CCTTAGGTAAGATAATATAGTAATTTGTTGAGAACATGGCTGAGAAGCTA
RI-AT1G10890-XLOC_000523-8246-1

CONSENSUS
....

RI-AT1G10890-XLOC_000523-8246-0
GGAAAATGATACATGCGCTTACCATTATAAAATAGCTCAGTTTCTTCCCG
RI-AT1G10890-XLOC_000523-8246-1
--TTTCTTCCCG
CONSENSUS
....TTTCTTCCCG

RI-AT1G10890-XLOC_000523-8246-0
TTCCCACGGGAGGGGAAATGAGCAGCTGATGGATTGCTCTTAGGTGTTCT
RI-AT1G10890-XLOC_000523-8246-1
TTCCCACGGGAGGGGAAATGAGCAGCTGATGGATTGCTCTTAGGTGTTCT
CONSENSUS
TTCCCACGGGAGGGGAAATGAGCAGCTGATGGATTGCTCTTAGGTGTTCT

RI-AT1G10890-XLOC_000523-8246-0
TTCATTGATCCCTTGTCTCGTTAGCTTCTCTTCGTGTGATAAAAGTTGGA
RI-AT1G10890-XLOC_000523-8246-1
TTCATTGATCCCTTGTCTCGTTAGCTTCTCTTCGTGTGATAAAAGTTGGA
CONSENSUS
TTCATTGATCCCTTGTCTCGTTAGCTTCTCTTCGTGTGATAAAAGTTGGA

RI-AT1G10890-XLOC_000523-8246-0
GCTGATAATTCTTTACAGTGTATGAGGTAACTGCACATACTTTTGTTTTC
RI-AT1G10890-XLOC_000523-8246-1
GCTGATAATTCTTTACAGTGTATGAGGTAACTGCACATACTTTTGTTTTC
CONSENSUS
GCTGATAATTCTTTACAGTGTATGAGGTAACTGCACATACTTTTGTTTTC

RI-AT1G10890-XLOC_000523-8246-0
TTTATGTGTAACTGTGGGCTACCTGATGCCTAGTCTTCACGTGCGTGGTA
RI-AT1G10890-XLOC_000523-8246-1
TTTATGTGTAACTGTGGGCTACCTGATGCCTAGTCTTCACGTGCGTGGTA
CONSENSUS
TTTATGTGTAACTGTGGGCTACCTGATGCCTAGTCTTCACGTGCGTGGTA

RI-AT1G10890-XLOC_000523-8246-0
CTTCATTAGTTCCATCTTTTTGCTTACTACTTTATTTTGGGTAATGTTCA
RI-AT1G10890-XLOC_000523-8246-1
CTTCATTAGTTCCATCTTTTTGCTTACTACTTTATTTTGGGTAATGTTCA
CONSENSUS
CTTCATTAGTTCCATCTTTTTGCTTACTACTTTATTTTGGGTAATGTTCA

RI-AT1G10890-XLOC_000523-8246-0
GGCGAAAAAGTCGTTCTATTTCTCCTAGGCGCCATCGAAGTCGATCTGTT
RI-AT1G10890-XLOC_000523-8246-1
GGCGAAAAAGTCGTTCTATTTCTCCTAGGCGCCATCGAAGTCGATCTGTT
CONSENSUS
GGCGAAAAAGTCGTTCTATTTCTCCTAGGCGCCATCGAAGTCGATCTGTT

RI-AT1G10890-XLOC_000523-8246-0
ACTCCTAAGAGACGTTCTCCAACCCCAAAACGTTACAAAAGACAAAAGAG
RI-AT1G10890-XLOC_000523-8246-1
ACTCCTAAGAGACGTTCTCCAACCCCAAAACGTTACAAAAGACAAAAGAG
CONSENSUS
ACTCCTAAGAGACGTTCTCCAACCCCAAAACGTTACAAAAGACAAAAGAG

RI-AT1G10890-XLOC_000523-8246-0
TAGGAGTTCAACTCCATCTCCTGCAAAAAGATCTCCCGCCGCAACCCTTG
RI-AT1G10890-XLOC_000523-8246-1
TAGGAGTTCAACTCCATCTCCTGCAAAAAGATCTCCCGCCGCAACCCTTG
CONSENSUS
TAGGAGTTCAACTCCATCTCCTGCAAAAAGATCTCCCGCCGCAACCCTTG

RI-AT1G10890-XLOC_000523-8246-0
AGTCAGCCAAAAATAGGAATGGAGAAAAACTTAAAAGAGAAGAGGAAGAA
RI-AT1G10890-XLOC_000523-8246-1
AGTCAGCCAAAAATAGGAATGGAGAAAAACTTAAAAGAGAAGAGGAAGAA
CONSENSUS
AGTCAGCCAAAAATAGGAATGGAGAAAAACTTAAAAGAGAAGAGGAAGAA

RI-AT1G10890-XLOC_000523-8246-0
CGAAAAAGGTAAATCAATACAAAATTTTGAAGTAAGATGGATGTTATGGT
RI-AT1G10890-XLOC_000523-8246-1
CGAAAAAGGTAAATCAATACAAAATTTTGAAGTAAGATGGATGTTATGGT
CONSENSUS
CGAAAAAGGTAAATCAATACAAAATTTTGAAGTAAGATGGATGTTATGGT

RI-AT1G10890-XLOC_000523-8246-0
GAAATTAAGGTTTTTTGTTCTCAAATGTTATTTTAGTGTAGTGTGGAAGT
RI-AT1G10890-XLOC_000523-8246-1
GAAATTAAGGTTTTTTGTTCTCAAATGTTATTTTAGTGTAGTGTGGAAGT
CONSENSUS
GAAATTAAGGTTTTTTGTTCTCAAATGTTATTTTAGTGTAGTGTGGAAGT

RI-AT1G10890-XLOC_000523-8246-0
CCTTGATTGTTAGTCTCAAAAGTGCGACAGGTTTTATGGTATTCTCTCAC
RI-AT1G10890-XLOC_000523-8246-1
CCTTGATTGTTAGTCTCAAAAGTGCGACAGGTTTTATGGTATTCTCTCAC
CONSENSUS
CCTTGATTGTTAGTCTCAAAAGTGCGACAGGTTTTATGGTATTCTCTCAC

RI-AT1G10890-XLOC_000523-8246-0
AGGCTTTGCTTATGGAAGATAAGCTATATTTCGATTTGTTTATATTTGAT
RI-AT1G10890-XLOC_000523-8246-1
AGGCTTTGCTTATGGAAGATAAGCTATATTTCGATTTGTTTATATTTGAT
CONSENSUS
AGGCTTTGCTTATGGAAGATAAGCTATATTTCGATTTGTTTATATTTGAT

RI-AT1G10890-XLOC_000523-8246-0
CCTCTGGCACAAATGTTCGTACTATCTCGATTTGATGAGTTACCGTTTGT
RI-AT1G10890-XLOC_000523-8246-1
CCTCTGGCACAAATGTTCGTACTATCTCGATTTGATGAGTTACCGTTTGT
CONSENSUS
CCTCTGGCACAAATGTTCGTACTATCTCGATTTGATGAGTTACCGTTTGT

RI-AT1G10890-XLOC_000523-8246-0
CATTACATGGATCATGATTGACTTAGAGATGTAAATATGTCCAAAGCACA
RI-AT1G10890-XLOC_000523-8246-1
CATTACATGGATCATGATTGACTTAGAGATGTAAATATGTCCAAAGCACA
CONSENSUS
CATTACATGGATCATGATTGACTTAGAGATGTAAATATGTCCAAAGCACA

RI-AT1G10890-XLOC_000523-8246-0
AGAATAATCTTGGTGAAGGTTTGCTTGTGTTATGGTGACAAAAAAGAAAT
RI-AT1G10890-XLOC_000523-8246-1
AGAATAATCTTGGTGAAGGTTTGCTTGTGTTATGGTGACAAAAAAGAAAT
CONSENSUS
AGAATAATCTTGGTGAAGGTTTGCTTGTGTTATGGTGACAAAAAAGAAAT

RI-AT1G10890-XLOC_000523-8246-0
TTCTTCACGAAATTTACCATTGGTATTGGGAGGAGTGAAAATGGAAACGA
RI-AT1G10890-XLOC_000523-8246-1
TTCTTCACGAAATTTACCATTGGTATTGGGAGGAGTGAAAATGGAAACGA
CONSENSUS
TTCTTCACGAAATTTACCATTGGTATTGGGAGGAGTGAAAATGGAAACGA

RI-AT1G10890-XLOC_000523-8246-0
TCTCAACATTTTTTACGGATTTGCAATAAAATTTTGGTCTTTTGCTGTTC
RI-AT1G10890-XLOC_000523-8246-1
TCTCAACATTTTTTACGGATTTGCAATAAAATTTTGGTCTTTTGCTGTTC
CONSENSUS
TCTCAACATTTTTTACGGATTTGCAATAAAATTTTGGTCTTTTGCTGTTC

RI-AT1G10890-XLOC_000523-8246-0
AAGATTGAAGACATCAGGAAATGAGAAAGTTCAGGTGATATTCAATATTC
RI-AT1G10890-XLOC_000523-8246-1
AAGATTGAAGACATCAGGAAATGAGAAAGTTCAGGTGATATTCAATATTC
CONSENSUS
AAGATTGAAGACATCAGGAAATGAGAAAGTTCAGGTGATATTCAATATTC

RI-AT1G10890-XLOC_000523-8246-0
TGAACCTGCTGTGGATGTCCTCTAATTTTTCACTGTATTTGCTACCAGGC
RI-AT1G10890-XLOC_000523-8246-1
TGAACCTGCTGTGGATGTCCTCTAATTTTTCACTGTATTTGCTACCAGGC
CONSENSUS
TGAACCTGCTGTGGATGTCCTCTAATTTTTCACTGTATTTGCTACCAGGC

RI-AT1G10890-XLOC_000523-8246-0
GACAGCGTGAAGCAGAACTGAAGCTAATAGAGGAAGAAACTGTGAAACGG
RI-AT1G10890-XLOC_000523-8246-1
GACAGCGTGAAGCAGAACTGAAGCTAATAGAGGAAGAAACTGTGAAACGG
CONSENSUS
GACAGCGTGAAGCAGAACTGAAGCTAATAGAGGAAGAAACTGTGAAACGG

RI-AT1G10890-XLOC_000523-8246-0
GTTGAAGAAGCTATTCGAAAGAAGGTCGAAGAAAGCTTACAGTCTGAGAA
RI-AT1G10890-XLOC_000523-8246-1
GTTGAAGAAGCTATTCGAAAGAAGGTCGAAGAAAGCTTACAGTCTGAGAA
CONSENSUS
GTTGAAGAAGCTATTCGAAAGAAGGTCGAAGAAAGCTTACAGTCTGAGAA

RI-AT1G10890-XLOC_000523-8246-0
AATCAAAATGGAAATTCTAACGCTGTTGGAGGAAGGGCGAAAGAGACTTA
RI-AT1G10890-XLOC_000523-8246-1
AATCAAAATGGAAATTCTAACGCTGTTGGAGGAAGGGCGAAAGAGACTTA
CONSENSUS
AATCAAAATGGAAATTCTAACGCTGTTGGAGGAAGGGCGAAAGAGACTTA

RI-AT1G10890-XLOC_000523-8246-0
ATGAAGAAGTCGCGGCTCAACTTGAGGAGGAGAAAGAGGCTTCTCTTATT
RI-AT1G10890-XLOC_000523-8246-1
ATGAAGAAGTCGCGGCTCAACTTGAGGAGGAGAAAGAGGCTTCTCTTATT
CONSENSUS
ATGAAGAAGTCGCGGCTCAACTTGAGGAGGAGAAAGAGGCTTCTCTTATT

RI-AT1G10890-XLOC_000523-8246-0 GAGGCTAAAGAAAAAGAG
RI-AT1G10890-XLOC_000523-8246-1 GAGGCTAAAGAAAAAGAG
CONSENSUS GAGGCTAAAGAAAAAGAG

alignment for event: A5-AT1G63260-XLOC_007195-712

A5-AT1G63260-XLOC_007195-712-0
AGCTGGAGTTGCTCAGTACATGAAAACCGAGTGGCGACTTGTTGCGATCT
A5-AT1G63260-XLOC_007195-712-1
AGCTGGAGTTGCTCAGTACATGAAAACCGAGTGGCGACTTGTTGCGATCT
CONSENSUS
AGCTGGAGTTGCTCAGTACATGAAAACCGAGTGGCGACTTGTTGCGATCT

A5-AT1G63260-XLOC_007195-712-0
TCAATGTGGTCCTGTTTGTTGTCTTGATAAGCTCTCTTCTTAGCACGAGA
A5-AT1G63260-XLOC_007195-712-1
TCAATGTG--
CONSENSUS
TCAATGTG..

A5-AT1G63260-XLOC_007195-712-0
TTTGACTCTGAACAAAGTTTTGGCCTTTTAAACGTCAATGGTTTACTTTG
A5-AT1G63260-XLOC_007195-712-1
-----------------------------------TCAATGGTTTACTTTG
CONSENSUS
..................................TCAATGGTTTACTTTG

A5-AT1G63260-XLOC_007195-712-0
TTGGATGCTGTGCGAGAAGAAATGCTGCTAGTTACCGATCCAAAGCTTAG
A5-AT1G63260-XLOC_007195-712-1
TTGGATGCTGTGCGAGAAGAAATGCTGCTAGTTACCGATCCAAAGCTTAG
CONSENSUS
TTGGATGCTGTGCGAGAAGAAATGCTGCTAGTTACCGATCCAAAGCTTAG

A5-AT1G63260-XLOC_007195-712-0
AAACCGAAGCCCGGGTTAAAGAAAACACTTCACCGGATTCTCCCAAGCTA
A5-AT1G63260-XLOC_007195-712-1
AAACCGAAGCCCGGGTTAAAGAAAACACTTCACCGGATTCTCCCAAGCTA
CONSENSUS
AAACCGAAGCCCGGGTTAAAGAAAACACTTCACCGGATTCTCCCAAGCTA

A5-AT1G63260-XLOC_007195-712-0
CAGATGAATCACATTCTCTTCCATGTTGGTGAATGCTTCCAAGAGAGACA
A5-AT1G63260-XLOC_007195-712-1
CAGATGAATCACATTCTCTTCCATGTTGGTGAATGCTTCCAAGAGAGACA
CONSENSUS
CAGATGAATCACATTCTCTTCCATGTTGGTGAATGCTTCCAAGAGAGACA

A5-AT1G63260-XLOC_007195-712-0
CAAGAGCAACAACTTCTCATCGTTCTTTTTACTCTGTAATCTATATGTTG
A5-AT1G63260-XLOC_007195-712-1
CAAGAGCAACAACTTCTCATCGTTCTTTTTACTCTGTAATCTATATGTTG
CONSENSUS
CAAGAGCAACAACTTCTCATCGTTCTTTTTACTCTGTAATCTATATGTTG

A5-AT1G63260-XLOC_007195-712-0
TTGAAACAGAGCAAAAATTTGATCAGATTTTTCACACAGAAAACATATTT
A5-AT1G63260-XLOC_007195-712-1
TTGAAACAGAGCAAAAATTTGATCAGATTTTTCACACAGAAAACATATTT
CONSENSUS
TTGAAACAGAGCAAAAATTTGATCAGATTTTTCACACAGAAAACATATTT

A5-AT1G63260-XLOC_007195-712-0
GACATCAAAATCTACACTTTTTATCTTTTAAGAGTAAAAATTTAG
A5-AT1G63260-XLOC_007195-712-1
GACATCAAAATCTACACTTTTTATCTTTTAAGAGTAAAAATTTAG
CONSENSUS
GACATCAAAATCTACACTTTTTATCTTTTAAGAGTAAAAATTTAG

alignment for event: RI-AT1G02090-XLOC_004204-1143

RI-AT1G02090-XLOC_004204-1143-0
GTTGAACACGTCAGAGAATCTGCTCATTTCAATTCAAGACAAGATCAAAT
RI-AT1G02090-XLOC_004204-1143-1
GTTGAACACGTCAGAGAATCTGCTCATTTCAATTCAAGACAAGATCAAAT
CONSENSUS
GTTGAACACGTCAGAGAATCTGCTCATTTCAATTCAAGACAAGATCAAAT

RI-AT1G02090-XLOC_004204-1143-0
GGGCGGACAATATGAGTGAGATGGACAAAAAACATCGTAAGGAAGCAGAA
RI-AT1G02090-XLOC_004204-1143-1
GGGCGGACAATATGAGTGAGATGGACAAAAAACATCGTAAGGAAGCAGAA
CONSENSUS
GGGCGGACAATATGAGTGAGATGGACAAAAAACATCGTAAGGAAGCAGAA

RI-AT1G02090-XLOC_004204-1143-0
GAAGGGGTGGAAGAAGTGAAGAAGTCTCTGTCCATGAAGGTCAGTCGCGC
RI-AT1G02090-XLOC_004204-1143-1
GAAGGGGTGGAAGAAGTGAAGAAGTCTCTGTCCATGAAG-----------
CONSENSUS
GAAGGGGTGGAAGAAGTGAAGAAGTCTCTGTCCATGAAG...........

RI-AT1G02090-XLOC_004204-1143-0
TTGGAGTCCTATTGGCTTCACTTTTCCCTACTAAGTATATTATGTAACTA
RI-AT1G02090-XLOC_004204-1143-1

CONSENSUS
....

RI-AT1G02090-XLOC_004204-1143-0
ATATTGGTTACATTCTTTCTTGGTTACTGTCTTGTGACCATGAGTTAGAG
RI-AT1G02090-XLOC_004204-1143-1

CONSENSUS
....

RI-AT1G02090-XLOC_004204-1143-0
TCCAGTCAGCTATTCTGTCTTATATTTCTTCAGAGAATTGTAGTGGGTTT
RI-AT1G02090-XLOC_004204-1143-1

CONSENSUS
....

RI-AT1G02090-XLOC_004204-1143-0
CTTCTTATTAATAGCTTAGGTCTATGTAAGCGGGGATGGGAGAGACTTGA
RI-AT1G02090-XLOC_004204-1143-1

CONSENSUS
....

RI-AT1G02090-XLOC_004204-1143-0
TAAAAAAATCTTTCTAGGTCTTTGAGCTTTCCAAGTCCAGTCCTATCATA

RI-AT1G02090-XLOC_004204-1143-1

CONSENSUS
....

RI-AT1G02090-XLOC_004204-1143-0
GATTAAAAAGTTATGTTTCTTAATAACAAACGAATCGATCAGCCAACTGT
RI-AT1G02090-XLOC_004204-1143-1

CONSENSUS
....

RI-AT1G02090-XLOC_004204-1143-0
GGCGGGCTTTTTTAACCGTTCTTTCGTGGAACAAGAAATCTTACATGTTG
RI-AT1G02090-XLOC_004204-1143-1

CONSENSUS
....

RI-AT1G02090-XLOC_004204-1143-0
GATGTAAACATATATTATGAAGTTTTCTTTAATTAAAAACAAACAAGGAA
RI-AT1G02090-XLOC_004204-1143-1

CONSENSUS
....

RI-AT1G02090-XLOC_004204-1143-0
CGCAATCACAACGGCTGTTAGTGAAGTGTTAAAGTCATTAGAGGACATGA
RI-AT1G02090-XLOC_004204-1143-1

CONSENSUS
....

RI-AT1G02090-XLOC_004204-1143-0
ATTGTTTGGAATGGTAGTACTTGGTTATTGTCTGATTGTTAAATGAAATG
RI-AT1G02090-XLOC_004204-1143-1

CONSENSUS
....

RI-AT1G02090-XLOC_004204-1143-0
AAACAAGGTTCGTTTGGTTTTTTGTGTCTGAAATATGATTGGGAAGGGTG
RI-AT1G02090-XLOC_004204-1143-1

CONSENSUS
....

RI-AT1G02090-XLOC_004204-1143-0
GTGGTCGATATAGTCTTACTTACGTTGTGGGCAGGGGGATGTTGACATCA
RI-AT1G02090-XLOC_004204-1143-1

CONSENSUS
....

RI-AT1G02090-XLOC_004204-1143-0
GAGGGAATAAGGAGATGTTTGGGGAACCAAGTGGAGTGATGGACTACGAA

RI-AT1G02090-XLOC_004204-1143-1 -
AGGGAATAAGGAGATGTTTGGGGAACCAAGTGGAGTGATGGACTACGAA
CONSENSUS
      .AGGGAATAAGGAGATGTTTGGGGAACCAAGTGGAGTGATGGACTACGAA

RI-AT1G02090-XLOC_004204-1143-0 GAAGATGGGATCCGACCAAAGAG
RI-AT1G02090-XLOC_004204-1143-1 GAAGATGGGATCCGACCAAAGAG
CONSENSUS   GAAGATGGGATCCGACCAAAGAG

alignment for event: RI-AT1G77080-XLOC_003953-9825

RI-AT1G77080-XLOC_003953-9825-0
      GATCTTGAAGAAAAAATTCAGAATTATCTTCCACACAAGGAGTTACTAGA
RI-AT1G77080-XLOC_003953-9825-1
      GATCTTGAAGAAAAAATTCAGAATTATCTTCCACACAAGGAGTTACTAGA
CONSENSUS
      GATCTTGAAGAAAAAATTCAGAATTATCTTCCACACAAGGAGTTACTAGA

RI-AT1G77080-XLOC_003953-9825-0
      AACAGTCCAAAGGTTAGCAGTACGACACATTTTTCTCCCCTCTTCTTCTG
RI-AT1G77080-XLOC_003953-9825-1
      AACAGTCCAAAG--------------------------------------
CONSENSUS
      AACAGTCCAAAG......................................

RI-AT1G77080-XLOC_003953-9825-0
      ATAAAAAAAATGTTTTTTTTCTTTTGTCTACTTGTGAATACAGCAAGCTT
RI-AT1G77080-XLOC_003953-9825-1
      ---CAAGCTT
CONSENSUS
      ....CAAGCTT

RI-AT1G77080-XLOC_003953-9825-0
      GAAGAACCAAATGTCGATAATGTAAGTGTAGATTCTCTAATTTCTCTGGA
RI-AT1G77080-XLOC_003953-9825-1
      GAAGAACCAAATGTCGATAATGTAAGTGTAGATTCTCTAATTTCTCTGGA
CONSENSUS
      GAAGAACCAAATGTCGATAATGTAAGTGTAGATTCTCTAATTTCTCTGGA

RI-AT1G77080-XLOC_003953-9825-0
      GGAACAACTTGAGACTGCTCTGTCCGTAAGTAGAGCTAGGAAG
RI-AT1G77080-XLOC_003953-9825-1
      GGAACAACTTGAGACTGCTCTGTCCGTAAGTAGAGCTAGGAAG
CONSENSUS
      GGAACAACTTGAGACTGCTCTGTCCGTAAGTAGAGCTAGGAAG

alignment for event: A5-AT1G53800-XLOC_002700-12704

A5-AT1G53800-XLOC_002700-12704-0
      AGAAGAGTGTTTCAAAGTTTCTTCCTTTTTCGTTTTCCTGGTCTGAATGA
A5-AT1G53800-XLOC_002700-12704-1
      AGAAGAGTGTTTCAAAGTTTCTTCCTTTTTCGTTTTCCTGGTCTGAATGA

CONSENSUS
AGAAGAGTGTTTCAAAGTTTCTTCCTTTTTCGTTTTCCTGGTCTGAATGA

A5-AT1G53800-XLOC_002700-12704-0
GCTCCCAATGCCTTCTTTAG------------ATATTGCTACTATCCAAC
A5-AT1G53800-XLOC_002700-12704-1
GCTCCCAATGCCTTCTTTAGGTACCTTTTCTGATATTGCTACTATCCAAC
CONSENSUS
GCTCCCAATGCCTTCTTTAG............ATATTGCTACTATCCAAC

A5-AT1G53800-XLOC_002700-12704-0
CTTCATTTCAAGCTCATCTTGTTCCATTGGGGGCTCAGAGTATTATCCAT
A5-AT1G53800-XLOC_002700-12704-1
CTTCATTTCAAGCTCATCTTGTTCCATTGGGGGCTCAGAGTATTATCCAT
CONSENSUS
CTTCATTTCAAGCTCATCTTGTTCCATTGGGGGCTCAGAGTATTATCCAT

A5-AT1G53800-XLOC_002700-12704-0
GCTAAAAGTCTACCTAATCCATGGAGACAGTCTTGTTTCTCCAAGAATTT
A5-AT1G53800-XLOC_002700-12704-1
GCTAAAAGTCTACCTAATCCATGGAGACAGTCTTGTTTCTCCAAGAATTT
CONSENSUS
GCTAAAAGTCTACCTAATCCATGGAGACAGTCTTGTTTCTCCAAGAATTT

A5-AT1G53800-XLOC_002700-12704-0
GAAATTTTACACGGGTCATAGTCATGTCCGAAGAGGAAAAGTTCTTATTA
A5-AT1G53800-XLOC_002700-12704-1
GAAATTTTACACGGGTCATAGTCATGTCCGAAGAGGAAAAGTTCTTATTA
CONSENSUS
GAAATTTTACACGGGTCATAGTCATGTCCGAAGAGGAAAAGTTCTTATTA

A5-AT1G53800-XLOC_002700-12704-0
CTGCAGTGGCCACTCTTGAAACCAAGTATCCGGCTCAAAAAGAAAACGAA
A5-AT1G53800-XLOC_002700-12704-1
CTGCAGTGGCCACTCTTGAAACCAAGTATCCGGCTCAAAAAGAAAACGAA
CONSENSUS
CTGCAGTGGCCACTCTTGAAACCAAGTATCCGGCTCAAAAAGAAAACGAA

A5-AT1G53800-XLOC_002700-12704-0
CGGAGCTCTTCTTTGTCTTCTGCTTCTTCGAAATCCTCAAATGGAAGTGC
A5-AT1G53800-XLOC_002700-12704-1
CGGAGCTCTTCTTTGTCTTCTGCTTCTTCGAAATCCTCAAATGGAAGTGC
CONSENSUS
CGGAGCTCTTCTTTGTCTTCTGCTTCTTCGAAATCCTCAAATGGAAGTGC

A5-AT1G53800-XLOC_002700-12704-0
TGATGATGGCGAAGAACAAGTGGATGACAGAGAAAAGTTGAGAAGGATGA
A5-AT1G53800-XLOC_002700-12704-1
TGATGATGGCGAAGAACAAGTGGATGACAGAGAAAAGTTGAGAAGGATGA
CONSENSUS
TGATGATGGCGAAGAACAAGTGGATGACAGAGAAAAGTTGAGAAGGATGA

A5-AT1G53800-XLOC_002700-12704-0
GGATTTCTAAGGCAAATAGAGGGAACACTCCTTGGAACAAAGGCAGGAAG
A5-AT1G53800-XLOC_002700-12704-1
GGATTTCTAAGGCAAATAGAGGGAACACTCCTTGGAACAAAGGCAGGAAG

CONSENSUS
      GGATTTCTAAGGCAAATAGAGGGAACACTCCTTGGAACAAAGGCAGGAAG

A5-AT1G53800-XLOC_002700-12704-0 CATAGTCCAG
A5-AT1G53800-XLOC_002700-12704-1 CATAGTCCAG
CONSENSUS CATAGTCCAG

alignment for event: A3-AT1G71220-XLOC_003644-13374

A3-AT1G71220-XLOC_003644-13374-0
      GTCATTGCGCGGAAAAAGATCATGAGGCTCCTCGTGGCCTTCAGCTGATT
A3-AT1G71220-XLOC_003644-13374-1
      GTCATTGCGCGGAAAAAGATCATGAGGCTCCTCGTGGCCTTCAGCTGATT
CONSENSUS
      GTCATTGCGCGGAAAAAGATCATGAGGCTCCTCGTGGCCTTCAGCTGATT

A3-AT1G71220-XLOC_003644-13374-0
      CTGGGAACCAAAAATAGACCGCATTTGGTTGATACCCTTGTAATGGCCAA
A3-AT1G71220-XLOC_003644-13374-1
      CTGGGAACCAAAAATAGACCGCATTTGGTTGATACCCTTGTAATGGCCAA
CONSENSUS
      CTGGGAACCAAAAATAGACCGCATTTGGTTGATACCCTTGTAATGGCCAA

A3-AT1G71220-XLOC_003644-13374-0
      TTTGGGTTATTGGCAGATGAAAGTATCCCCAGGGGTTTGGTATTTGCAAC
A3-AT1G71220-XLOC_003644-13374-1
      TTTGGGTTATTGGCAGATGAAAGTATCCCCAGGGGTTTGGTATTTGCAAC
CONSENSUS
      TTTGGGTTATTGGCAGATGAAAGTATCCCCAGGGGTTTGGTATTTGCAAC

A3-AT1G71220-XLOC_003644-13374-0
      TTGCTCCGGGTAGAAGTTCGGAGCTATACGCTTTGAAAGGAGGAAATGAT
A3-AT1G71220-XLOC_003644-13374-1
      TTGCTCCGGGTAGAAGTTCGGAGCTATACGCTTTGAAAGGAGGAAATGAT
CONSENSUS
      TTGCTCCGGGTAGAAGTTCGGAGCTATACGCTTTGAAAGGAGGAAATGAT

A3-AT1G71220-XLOC_003644-13374-0
      GGGAGTCAAGATCAATCCTCGCTGAAACGTATAACTATCGATGATCTGCG
A3-AT1G71220-XLOC_003644-13374-1
      GGGAGTCAAGATCAATCCTCGCTGAAACGTATAACTATCGATGATCTGCG
CONSENSUS
      GGGAGTCAAGATCAATCCTCGCTGAAACGTATAACTATCGATGATCTGCG

A3-AT1G71220-XLOC_003644-13374-0
      TGGTAAAGTTGTTCATCTAGAAGTAGTTAAGAGAAAGGGTAAGGAGCATG
A3-AT1G71220-XLOC_003644-13374-1
      TGGTAAAGTTGTTCATCTAGAAGTAGTTAAGAGAAAGGGTAAGGAGCATG
CONSENSUS
      TGGTAAAGTTGTTCATCTAGAAGTAGTTAAGAGAAAGGGTAAGGAGCATG

A3-AT1G71220-XLOC_003644-13374-0
      AAAAGTTGCTAGTTCCTTCAGACGGTGATGATGCTGTGCAACAAAACAAG
A3-AT1G71220-XLOC_003644-13374-1

AAAAGTTGCTAGTTCCTTCAGACGGTGATGATGCTGTGCAACAAAACAAG
CONSENSUS
AAAAGTTGCTAGTTCCTTCAGACGGTGATGATGCTGTGCAACAAAACAAG

A3-AT1G71220-XLOC_003644-13374-0
GAACAGGGAAGCTGGAACTCAAATTTCTTGAAATGGGCGTCTGGTTTTGT
A3-AT1G71220-XLOC_003644-13374-1 GAA---
GGAAGCTGGAACTCAAATTTCTTGAAATGGGCGTCTGGTTTTGT
CONSENSUS
GAA...GGAAGCTGGAACTCAAATTTCTTGAAATGGGCGTCTGGTTTTGT

A3-AT1G71220-XLOC_003644-13374-0
TGGTGGTCGTCAACAATCAATGAAGGGAGGTCCTGACAAA
A3-AT1G71220-XLOC_003644-13374-1
TGGTGGTCGTCAACAATCAATGAAGGGAGGTCCTGACAAA
CONSENSUS
TGGTGGTCGTCAACAATCAATGAAGGGAGGTCCTGACAAA

alignment for event: RI-AT1G10390-XLOC_004682-4136

RI-AT1G10390-XLOC_004682-4136-0
AGAGAGAAGTCATCATATCATACCAACAAAAAGGGCCAATCGTTCGCTCT
RI-AT1G10390-XLOC_004682-4136-1
AGAGAGAAGTCATCATATCATACCAACAAAAAGGGCCAATCGTTCGCTCT
CONSENSUS
AGAGAGAAGTCATCATATCATACCAACAAAAAGGGCCAATCGTTCGCTCT

RI-AT1G10390-XLOC_004682-4136-0
TTGCAAGTTGCAACCGTCGCGCTTTCGATTCCGGCGAAATTGCTCCATCT
RI-AT1G10390-XLOC_004682-4136-1
TTGCAAGTTGCAACCGTCGCGCTTTCGATTCCGGCGAAATTGCTCCATCT
CONSENSUS
TTGCAAGTTGCAACCGTCGCGCTTTCGATTCCGGCGAAATTGCTCCATCT

RI-AT1G10390-XLOC_004682-4136-0
TCTAGTCGCTTTTCAGATCGAGCTCGTCGTTTTCAGGTTCCATTTCTCCT
RI-AT1G10390-XLOC_004682-4136-1
TCTAGTCGCTTTTCAGATCGAGCTCGTCGTTTTCAG--------------
CONSENSUS
TCTAGTCGCTTTTCAGATCGAGCTCGTCGTTTTCAG..............

RI-AT1G10390-XLOC_004682-4136-0
ACATCTTCTTGCTCCACATTTGATTCCACGCCGCCTATTCGTTTCTCGGC
RI-AT1G10390-XLOC_004682-4136-1

CONSENSUS
....

RI-AT1G10390-XLOC_004682-4136-0
GATTTCACTCCTGAACCCTAATTTCCTCCACGCAGATAATTTCGACGATC
RI-AT1G10390-XLOC_004682-4136-1
-----------------------------------ATAATTTCGACGATC
CONSENSUS
...................................ATAATTTCGACGATC

RI-AT1G10390-XLOC_004682-4136-0
      GGAGCTTCCGCTCTTGGTCTCTTGAGAGTCCTTTGATATCATATCTGATT
RI-AT1G10390-XLOC_004682-4136-1
      GGAGCTTCCGCTCTTGGTCTCTTGAGAGTCCTTTGATATCATATCTGATT
CONSENSUS
      GGAGCTTCCGCTCTTGGTCTCTTGAGAGTCCTTTGATATCATATCTGATT

RI-AT1G10390-XLOC_004682-4136-0
      GGTCGAAGAACGTGTGTCCTTGTTCTTCTCTTTTTGACATCGGATTCATA
RI-AT1G10390-XLOC_004682-4136-1
      GGTCGAAGAACGTGTGTCCTTGTTCTTCTCTTTTTGACATCGGATTCATA
CONSENSUS
      GGTCGAAGAACGTGTGTCCTTGTTCTTCTCTTTTTGACATCGGATTCATA

RI-AT1G10390-XLOC_004682-4136-0 G
RI-AT1G10390-XLOC_004682-4136-1 G
CONSENSUS   G

alignment for event: RI-AT1G10890-XLOC_000523-8248

RI-AT1G10890-XLOC_000523-8248-0
      GACCAATCCGTTCAGAGAGATTAAAGCTGAGCTGAGCTTCTCCTTATAAA
RI-AT1G10890-XLOC_000523-8248-1
      GACCAATCCGTTCAGAGAGATTAAAGCTGAGCTGAGCTTCTCCTTATAAA
CONSENSUS
      GACCAATCCGTTCAGAGAGATTAAAGCTGAGCTGAGCTTCTCCTTATAAA

RI-AT1G10890-XLOC_000523-8248-0
      GGAAGATACTGCTTGTTCTACGGAATTTTGAGAGATGCCTCGGGACTTGT
RI-AT1G10890-XLOC_000523-8248-1
      GGAAGATACTGCTTGTTCTACGGAATTTTGAGAGATGCCTCGGGACTTGT
CONSENSUS
      GGAAGATACTGCTTGTTCTACGGAATTTTGAGAGATGCCTCGGGACTTGT

RI-AT1G10890-XLOC_000523-8248-0
      CAAGATCGAGGTCACCGTCTCCATCACCTTCACGTCGTAGAAAGCACTCG
RI-AT1G10890-XLOC_000523-8248-1
      CAAGATCGAGGTCACCGTCTCCATCACCTTCACGTCGTAGAAAGCACTCG
CONSENSUS
      CAAGATCGAGGTCACCGTCTCCATCACCTTCACGTCGTAGAAAGCACTCG

RI-AT1G10890-XLOC_000523-8248-0
      AGGTCTCCCGTAAGGCAGAGGCATAGCAGGAGGAGTAGAAGAGACAGAAG
RI-AT1G10890-XLOC_000523-8248-1
      AGGTCTCCCGTAAGGCAGAGGCATAGCAGGAGGAGTAGAAGAGACAGAAG
CONSENSUS
      AGGTCTCCCGTAAGGCAGAGGCATAGCAGGAGGAGTAGAAGAGACAGAAG

RI-AT1G10890-XLOC_000523-8248-0
      CCCTTCTCCATACTCATCTCATTCGTATAGCAGGTGATACCCCTAGTGTT
RI-AT1G10890-XLOC_000523-8248-1
      CCCTTCTCCATACTCATCTCATTCGTATAGCAG-----------------
CONSENSUS

CCCTTCTCCATACTCATCTCATTCGTATAGCAG................

RI-AT1G10890-XLOC_000523-8248-0
TGTTTTTTCCGCTATTCGTTTGCCTTAGTTATCATTCGTTTATACGAACT
RI-AT1G10890-XLOC_000523-8248-1

CONSENSUS
....

RI-AT1G10890-XLOC_000523-8248-0
GTAAGAAACATTGGTGGAACACGGTTATAGATAGACATGTAAAGTTTACA
RI-AT1G10890-XLOC_000523-8248-1

CONSENSUS
....

RI-AT1G10890-XLOC_000523-8248-0
GACTCAAGAATTGCTTTTAGTAAAGTTCCTACTTATAGATATATTTTCTA
RI-AT1G10890-XLOC_000523-8248-1

CONSENSUS
....

RI-AT1G10890-XLOC_000523-8248-0
CCCTTCTCTAAAAGAGAGTGCAATGTCTGGATGCTGCTGGCTCTGTATCG
RI-AT1G10890-XLOC_000523-8248-1

CONSENSUS
....

RI-AT1G10890-XLOC_000523-8248-0
TAAGCGTTTTTTTGAGTGATGCTGCGTATTGGAAATATATCGTGTTTATT
RI-AT1G10890-XLOC_000523-8248-1

CONSENSUS
....

RI-AT1G10890-XLOC_000523-8248-0
TGGCTGTTGTGATTAAATTTGTTGCAAGAGAATATGTTTTATGACCTGAG
RI-AT1G10890-XLOC_000523-8248-1

CONSENSUS
....

RI-AT1G10890-XLOC_000523-8248-0
CTTTGTGATTTTTAGAATGAAACCTGTATATTCGGTAAAGACATAAGTTA
RI-AT1G10890-XLOC_000523-8248-1

CONSENSUS
....

RI-AT1G10890-XLOC_000523-8248-0
AGCTGGGATAATAGCACTACTGATAGTCTGATCTAGTTTATATTTTAATT
RI-AT1G10890-XLOC_000523-8248-1

CONSENSUS

....

RI-AT1G10890-XLOC_000523-8248-0
GTATCTGTAGATTATTAGTTAGAATCAGTTTTAAGAGCGACTATATTATG
RI-AT1G10890-XLOC_000523-8248-1

CONSENSUS
....

RI-AT1G10890-XLOC_000523-8248-0
TTCTTTTAAATGGAGTTTGTTAGCAATAGTGATACAAAACCACCACTATT
RI-AT1G10890-XLOC_000523-8248-1

CONSENSUS
....

RI-AT1G10890-XLOC_000523-8248-0
CCTTAGGTAAGATAATATAGTAATTTGTTGAGAACATGGCTGAGAAGCTA
RI-AT1G10890-XLOC_000523-8248-1

CONSENSUS
....

RI-AT1G10890-XLOC_000523-8248-0
GGAAAATGATACATGCGCTTACCATTATAAAATAGCTCAGTTTCTTCCCG
RI-AT1G10890-XLOC_000523-8248-1

CONSENSUS
....

RI-AT1G10890-XLOC_000523-8248-0
TTCCCACGGGAGGGGAAATGAGCAGCTGATGGATTGCTCTTAGGTGTTCT
RI-AT1G10890-XLOC_000523-8248-1

CONSENSUS
....

RI-AT1G10890-XLOC_000523-8248-0
TTCATTGATCCCTTGTCTCGTTAGCTTCTCTTCGTGTGATAAAAGTTGGA
RI-AT1G10890-XLOC_000523-8248-1

CONSENSUS
....

RI-AT1G10890-XLOC_000523-8248-0
GCTGATAATTCTTTACAGTGTATGAGGTAACTGCACATACTTTTGTTTTC
RI-AT1G10890-XLOC_000523-8248-1

CONSENSUS
....

RI-AT1G10890-XLOC_000523-8248-0
TTTATGTGTAACTGTGGGCTACCTGATGCCTAGTCTTCACGTGCGTGGTA
RI-AT1G10890-XLOC_000523-8248-1

CONSENSUS

....

RI-AT1G10890-XLOC_000523-8248-0
CTTCATTAGTTCCATCTTTTTGCTTACTACTTTATTTTGGGTAATGTTCA
RI-AT1G10890-XLOC_000523-8248-1

CONSENSUS
....

RI-AT1G10890-XLOC_000523-8248-0
GGCGAAAAAGTCGTTCTATTTCTCCTAGGCGCCATCGAAGTCGATCTGTT
RI-AT1G10890-XLOC_000523-8248-1 -
GCGAAAAAGTCGTTCTATTTCTCCTAGGCGCCATCGAAGTCGATCTGTT
CONSENSUS
.GCGAAAAAGTCGTTCTATTTCTCCTAGGCGCCATCGAAGTCGATCTGTT

RI-AT1G10890-XLOC_000523-8248-0
ACTCCTAAGAGACGTTCTCCAACCCCAAAACGTTACAAAAGACAAAAGAG
RI-AT1G10890-XLOC_000523-8248-1
ACTCCTAAGAGACGTTCTCCAACCCCAAAACGTTACAAAAGACAAAAGAG
CONSENSUS
ACTCCTAAGAGACGTTCTCCAACCCCAAAACGTTACAAAAGACAAAAGAG

RI-AT1G10890-XLOC_000523-8248-0
TAGGAGTTCAACTCCATCTCCTGCAAAAAGATCTCCCGCCGCAACCCTTG
RI-AT1G10890-XLOC_000523-8248-1
TAGGAGTTCAACTCCATCTCCTGCAAAAAGATCTCCCGCCGCAACCCTTG
CONSENSUS
TAGGAGTTCAACTCCATCTCCTGCAAAAAGATCTCCCGCCGCAACCCTTG

RI-AT1G10890-XLOC_000523-8248-0
AGTCAGCCAAAAATAGGAATGGAGAAAAACTTAAAAGAGAAGAGGAAGAA
RI-AT1G10890-XLOC_000523-8248-1
AGTCAGCCAAAAATAGGAATGGAGAAAAACTTAAAAGAGAAGAGGAAGAA
CONSENSUS
AGTCAGCCAAAAATAGGAATGGAGAAAAACTTAAAAGAGAAGAGGAAGAA

RI-AT1G10890-XLOC_000523-8248-0
CGAAAAAGGTAAATCAATACAAAATTTTGAAGTAAGATGGATGTTATGGT
RI-AT1G10890-XLOC_000523-8248-1
CGAAAAAGGTAAATCAATACAAAATTTTGAAGTAAGATGGATGTTATGGT
CONSENSUS
CGAAAAAGGTAAATCAATACAAAATTTTGAAGTAAGATGGATGTTATGGT

RI-AT1G10890-XLOC_000523-8248-0
GAAATTAAGGTTTTTTGTTCTCAAATGTTATTTTAGTGTAGTGTGGAAGT
RI-AT1G10890-XLOC_000523-8248-1
GAAATTAAGGTTTTTTGTTCTCAAATGTTATTTTAGTGTAGTGTGGAAGT
CONSENSUS
GAAATTAAGGTTTTTTGTTCTCAAATGTTATTTTAGTGTAGTGTGGAAGT

RI-AT1G10890-XLOC_000523-8248-0
CCTTGATTGTTAGTCTCAAAAGTGCGACAGGTTTTATGGTATTCTCTCAC
RI-AT1G10890-XLOC_000523-8248-1
CCTTGATTGTTAGTCTCAAAAGTGCGACAGGTTTTATGGTATTCTCTCAC
CONSENSUS

CCTTGATTGTTAGTCTCAAAAGTGCGACAGGTTTTATGGTATTCTCTCAC

RI-AT1G10890-XLOC_000523-8248-0
AGGCTTTGCTTATGGAAGATAAGCTATATTTCGATTTGTTTATATTTGAT
RI-AT1G10890-XLOC_000523-8248-1
AGGCTTTGCTTATGGAAGATAAGCTATATTTCGATTTGTTTATATTTGAT
CONSENSUS
AGGCTTTGCTTATGGAAGATAAGCTATATTTCGATTTGTTTATATTTGAT

RI-AT1G10890-XLOC_000523-8248-0
CCTCTGGCACAAATGTTCGTACTATCTCGATTTGATGAGTTACCGTTTGT
RI-AT1G10890-XLOC_000523-8248-1
CCTCTGGCACAAATGTTCGTACTATCTCGATTTGATGAGTTACCGTTTGT
CONSENSUS
CCTCTGGCACAAATGTTCGTACTATCTCGATTTGATGAGTTACCGTTTGT

RI-AT1G10890-XLOC_000523-8248-0
CATTACATGGATCATGATTGACTTAGAGATGTAAATATGTCCAAAGCACA
RI-AT1G10890-XLOC_000523-8248-1
CATTACATGGATCATGATTGACTTAGAGATGTAAATATGTCCAAAGCACA
CONSENSUS
CATTACATGGATCATGATTGACTTAGAGATGTAAATATGTCCAAAGCACA

RI-AT1G10890-XLOC_000523-8248-0
AGAATAATCTTGGTGAAGGTTTGCTTGTGTTATGGTGACAAAAAAGAAAT
RI-AT1G10890-XLOC_000523-8248-1
AGAATAATCTTGGTGAAGGTTTGCTTGTGTTATGGTGACAAAAAAGAAAT
CONSENSUS
AGAATAATCTTGGTGAAGGTTTGCTTGTGTTATGGTGACAAAAAAGAAAT

RI-AT1G10890-XLOC_000523-8248-0
TTCTTCACGAAATTTACCATTGGTATTGGGAGGAGTGAAAATGGAAACGA
RI-AT1G10890-XLOC_000523-8248-1
TTCTTCACGAAATTTACCATTGGTATTGGGAGGAGTGAAAATGGAAACGA
CONSENSUS
TTCTTCACGAAATTTACCATTGGTATTGGGAGGAGTGAAAATGGAAACGA

RI-AT1G10890-XLOC_000523-8248-0
TCTCAACATTTTTTACGGATTTGCAATAAAATTTTGGTCTTTTGCTGTTC
RI-AT1G10890-XLOC_000523-8248-1
TCTCAACATTTTTTACGGATTTGCAATAAAATTTTGGTCTTTTGCTGTTC
CONSENSUS
TCTCAACATTTTTTACGGATTTGCAATAAAATTTTGGTCTTTTGCTGTTC

RI-AT1G10890-XLOC_000523-8248-0
AAGATTGAAGACATCAGGAAATGAGAAAGTTCAGGTGATATTCAATATTC
RI-AT1G10890-XLOC_000523-8248-1
AAGATTGAAGACATCAGGAAATGAGAAAGTTCAGGTGATATTCAATATTC
CONSENSUS
AAGATTGAAGACATCAGGAAATGAGAAAGTTCAGGTGATATTCAATATTC

RI-AT1G10890-XLOC_000523-8248-0
TGAACCTGCTGTGGATGTCCTCTAATTTTTCACTGTATTTGCTACCAGGC
RI-AT1G10890-XLOC_000523-8248-1
TGAACCTGCTGTGGATGTCCTCTAATTTTTCACTGTATTTGCTACCAGGC
CONSENSUS

TGAACCTGCTGTGGATGTCCTCTAATTTTTCACTGTATTTGCTACCAGGC

RI-AT1G10890-XLOC_000523-8248-0
GACAGCGTGAAGCAGAACTGAAGCTAATAGAGGAAGAAACTGTGAAACGG
RI-AT1G10890-XLOC_000523-8248-1
GACAGCGTGAAGCAGAACTGAAGCTAATAGAGGAAGAAACTGTGAAACGG
CONSENSUS
GACAGCGTGAAGCAGAACTGAAGCTAATAGAGGAAGAAACTGTGAAACGG

RI-AT1G10890-XLOC_000523-8248-0
GTTGAAGAAGCTATTCGAAAGAAGGTCGAAGAAAGCTTACAGTCTGAGAA
RI-AT1G10890-XLOC_000523-8248-1
GTTGAAGAAGCTATTCGAAAGAAGGTCGAAGAAAGCTTACAGTCTGAGAA
CONSENSUS
GTTGAAGAAGCTATTCGAAAGAAGGTCGAAGAAAGCTTACAGTCTGAGAA

RI-AT1G10890-XLOC_000523-8248-0
AATCAAAATGGAAATTCTAACGCTGTTGGAGGAAGGGCGAAAGAGACTTA
RI-AT1G10890-XLOC_000523-8248-1
AATCAAAATGGAAATTCTAACGCTGTTGGAGGAAGGGCGAAAGAGACTTA
CONSENSUS
AATCAAAATGGAAATTCTAACGCTGTTGGAGGAAGGGCGAAAGAGACTTA

RI-AT1G10890-XLOC_000523-8248-0
ATGAAGAAGTCGCGGCTCAACTTGAGGAGGAGAAAGAGGCTTCTCTTATT
RI-AT1G10890-XLOC_000523-8248-1
ATGAAGAAGTCGCGGCTCAACTTGAGGAGGAGAAAGAGGCTTCTCTTATT
CONSENSUS
ATGAAGAAGTCGCGGCTCAACTTGAGGAGGAGAAAGAGGCTTCTCTTATT

RI-AT1G10890-XLOC_000523-8248-0 GAGGCTAAAGAAAAAGAG
RI-AT1G10890-XLOC_000523-8248-1 GAGGCTAAAGAAAAAGAG
CONSENSUS GAGGCTAAAGAAAAAGAG

alignment for event: A3-AT1G10890-XLOC_000523-8260

A3-AT1G10890-XLOC_000523-8260-0
GACCAATCCGTTCAGAGAGATTAAAGCTGAGCTGAGCTTCTCCTTATAAA
A3-AT1G10890-XLOC_000523-8260-1
GACCAATCCGTTCAGAGAGATTAAAGCTGAGCTGAGCTTCTCCTTATAAA
CONSENSUS
GACCAATCCGTTCAGAGAGATTAAAGCTGAGCTGAGCTTCTCCTTATAAA

A3-AT1G10890-XLOC_000523-8260-0
GGAAGATACTGCTTGTTCTACGGAATTTTGAGAGATGCCTCGGGACTTGT
A3-AT1G10890-XLOC_000523-8260-1
GGAAGATACTGCTTGTTCTACGGAATTTTGAGAGATGCCTCGGGACTTGT
CONSENSUS
GGAAGATACTGCTTGTTCTACGGAATTTTGAGAGATGCCTCGGGACTTGT

A3-AT1G10890-XLOC_000523-8260-0
CAAGATCGAGGTCACCGTCTCCATCACCTTCACGTCGTAGAAAGCACTCG
A3-AT1G10890-XLOC_000523-8260-1
CAAGATCGAGGTCACCGTCTCCATCACCTTCACGTCGTAGAAAGCACTCG

CONSENSUS
CAAGATCGAGGTCACCGTCTCCATCACCTTCACGTCGTAGAAAGCACTCG

A3-AT1G10890-XLOC_000523-8260-0
AGGTCTCCCGTAAGGCAGAGGCATAGCAGGAGGAGTAGAAGAGACAGAAG
A3-AT1G10890-XLOC_000523-8260-1
AGGTCTCCCGTAAGGCAGAGGCATAGCAGGAGGAGTAGAAGAGACAGAAG
CONSENSUS
AGGTCTCCCGTAAGGCAGAGGCATAGCAGGAGGAGTAGAAGAGACAGAAG

A3-AT1G10890-XLOC_000523-8260-0
CCCTTCTCCATACTCATCTCATTCGTATAGCAGGTGTTCTTTCATTGATC
A3-AT1G10890-XLOC_000523-8260-1
CCCTTCTCCATACTCATCTCATTCGTATAGCAG-----------------
CONSENSUS
CCCTTCTCCATACTCATCTCATTCGTATAGCAG.................

A3-AT1G10890-XLOC_000523-8260-0
CCTTGTCTCGTTAGCTTCTCTTCGTGTGATAAAAGTTGGAGCTGATAATT
A3-AT1G10890-XLOC_000523-8260-1

CONSENSUS
....

A3-AT1G10890-XLOC_000523-8260-0
CTTTACAGTGTATGAGGTAACTGCACATACTTTTGTTTTCTTTATGTGTA
A3-AT1G10890-XLOC_000523-8260-1

CONSENSUS
....

A3-AT1G10890-XLOC_000523-8260-0
ACTGTGGGCTACCTGATGCCTAGTCTTCACGTGCGTGGTACTTCATTAGT
A3-AT1G10890-XLOC_000523-8260-1

CONSENSUS
....

A3-AT1G10890-XLOC_000523-8260-0
TCCATCTTTTTGCTTACTACTTTATTTTGGGTAATGTTCAGGCGAAAAAG
A3-AT1G10890-XLOC_000523-8260-1
---GCGAAAAAG
CONSENSUS
....GCGAAAAAG

A3-AT1G10890-XLOC_000523-8260-0
TCGTTCTATTTCTCCTAGGCGCCATCGAAGTCGATCTGTTACTCCTAAGA
A3-AT1G10890-XLOC_000523-8260-1
TCGTTCTATTTCTCCTAGGCGCCATCGAAGTCGATCTGTTACTCCTAAGA
CONSENSUS
TCGTTCTATTTCTCCTAGGCGCCATCGAAGTCGATCTGTTACTCCTAAGA

A3-AT1G10890-XLOC_000523-8260-0
GACGTTCTCCAACCCCAAAACGTTACAAAAGACAAAAGAGTAGGAGTTCA
A3-AT1G10890-XLOC_000523-8260-1
GACGTTCTCCAACCCCAAAACGTTACAAAAGACAAAAGAGTAGGAGTTCA

CONSENSUS
GACGTTCTCCAACCCCAAAACGTTACAAAAGACAAAAGAGTAGGAGTTCA

A3-AT1G10890-XLOC_000523-8260-0
ACTCCATCTCCTGCAAAAAGATCTCCCGCCGCAACCCTTGAGTCAGCCAA
A3-AT1G10890-XLOC_000523-8260-1
ACTCCATCTCCTGCAAAAAGATCTCCCGCCGCAACCCTTGAGTCAGCCAA
CONSENSUS
ACTCCATCTCCTGCAAAAAGATCTCCCGCCGCAACCCTTGAGTCAGCCAA

A3-AT1G10890-XLOC_000523-8260-0
AAATAGGAATGGAGAAAAACTTAAAAGAGAAGAGGAAGAACGAAAAAGGT
A3-AT1G10890-XLOC_000523-8260-1
AAATAGGAATGGAGAAAAACTTAAAAGAGAAGAGGAAGAACGAAAAAGGT
CONSENSUS
AAATAGGAATGGAGAAAAACTTAAAAGAGAAGAGGAAGAACGAAAAAGGT

A3-AT1G10890-XLOC_000523-8260-0
AAATCAATACAAAATTTTGAAGTAAGATGGATGTTATGGTGAAATTAAGG
A3-AT1G10890-XLOC_000523-8260-1
AAATCAATACAAAATTTTGAAGTAAGATGGATGTTATGGTGAAATTAAGG
CONSENSUS
AAATCAATACAAAATTTTGAAGTAAGATGGATGTTATGGTGAAATTAAGG

A3-AT1G10890-XLOC_000523-8260-0
TTTTTTGTTCTCAAATGTTATTTTAGTGTAGTGTGGAAGTCCTTGATTGT
A3-AT1G10890-XLOC_000523-8260-1
TTTTTTGTTCTCAAATGTTATTTTAGTGTAGTGTGGAAGTCCTTGATTGT
CONSENSUS
TTTTTTGTTCTCAAATGTTATTTTAGTGTAGTGTGGAAGTCCTTGATTGT

A3-AT1G10890-XLOC_000523-8260-0
TAGTCTCAAAAGTGCGACAGGTTTTATGGTATTCTCTCACAGGCTTTGCT
A3-AT1G10890-XLOC_000523-8260-1
TAGTCTCAAAAGTGCGACAGGTTTTATGGTATTCTCTCACAGGCTTTGCT
CONSENSUS
TAGTCTCAAAAGTGCGACAGGTTTTATGGTATTCTCTCACAGGCTTTGCT

A3-AT1G10890-XLOC_000523-8260-0
TATGGAAGATAAGCTATATTTCGATTTGTTTATATTTGATCCTCTGGCAC
A3-AT1G10890-XLOC_000523-8260-1
TATGGAAGATAAGCTATATTTCGATTTGTTTATATTTGATCCTCTGGCAC
CONSENSUS
TATGGAAGATAAGCTATATTTCGATTTGTTTATATTTGATCCTCTGGCAC

A3-AT1G10890-XLOC_000523-8260-0
AAATGTTCGTACTATCTCGATTTGATGAGTTACCGTTTGTCATTACATGG
A3-AT1G10890-XLOC_000523-8260-1
AAATGTTCGTACTATCTCGATTTGATGAGTTACCGTTTGTCATTACATGG
CONSENSUS
AAATGTTCGTACTATCTCGATTTGATGAGTTACCGTTTGTCATTACATGG

A3-AT1G10890-XLOC_000523-8260-0
ATCATGATTGACTTAGAGATGTAAATATGTCCAAAGCACAAGAATAATCT
A3-AT1G10890-XLOC_000523-8260-1
ATCATGATTGACTTAGAGATGTAAATATGTCCAAAGCACAAGAATAATCT

CONSENSUS
ATCATGATTGACTTAGAGATGTAAATATGTCCAAAGCACAAGAATAATCT

A3-AT1G10890-XLOC_000523-8260-0
TGGTGAAGGTTTGCTTGTGTTATGGTGACAAAAAAGAAATTTCTTCACGA
A3-AT1G10890-XLOC_000523-8260-1
TGGTGAAGGTTTGCTTGTGTTATGGTGACAAAAAAGAAATTTCTTCACGA
CONSENSUS
TGGTGAAGGTTTGCTTGTGTTATGGTGACAAAAAAGAAATTTCTTCACGA

A3-AT1G10890-XLOC_000523-8260-0
AATTTACCATTGGTATTGGGAGGAGTGAAAATGGAAACGATCTCAACATT
A3-AT1G10890-XLOC_000523-8260-1
AATTTACCATTGGTATTGGGAGGAGTGAAAATGGAAACGATCTCAACATT
CONSENSUS
AATTTACCATTGGTATTGGGAGGAGTGAAAATGGAAACGATCTCAACATT

A3-AT1G10890-XLOC_000523-8260-0
TTTTACGGATTTGCAATAAAATTTTGGTCTTTTGCTGTTCAAGATTGAAG
A3-AT1G10890-XLOC_000523-8260-1
TTTTACGGATTTGCAATAAAATTTTGGTCTTTTGCTGTTCAAGATTGAAG
CONSENSUS
TTTTACGGATTTGCAATAAAATTTTGGTCTTTTGCTGTTCAAGATTGAAG

A3-AT1G10890-XLOC_000523-8260-0
ACATCAGGAAATGAGAAAGTTCAGGTGATATTCAATATTCTGAACCTGCT
A3-AT1G10890-XLOC_000523-8260-1
ACATCAGGAAATGAGAAAGTTCAGGTGATATTCAATATTCTGAACCTGCT
CONSENSUS
ACATCAGGAAATGAGAAAGTTCAGGTGATATTCAATATTCTGAACCTGCT

A3-AT1G10890-XLOC_000523-8260-0
GTGGATGTCCTCTAATTTTTCACTGTATTTGCTACCAGGCGACAGCGTGA
A3-AT1G10890-XLOC_000523-8260-1
GTGGATGTCCTCTAATTTTTCACTGTATTTGCTACCAGGCGACAGCGTGA
CONSENSUS
GTGGATGTCCTCTAATTTTTCACTGTATTTGCTACCAGGCGACAGCGTGA

A3-AT1G10890-XLOC_000523-8260-0
AGCAGAACTGAAGCTAATAGAGGAAGAAACTGTGAAACGGGTTGAAGAAG
A3-AT1G10890-XLOC_000523-8260-1
AGCAGAACTGAAGCTAATAGAGGAAGAAACTGTGAAACGGGTTGAAGAAG
CONSENSUS
AGCAGAACTGAAGCTAATAGAGGAAGAAACTGTGAAACGGGTTGAAGAAG

A3-AT1G10890-XLOC_000523-8260-0
CTATTCGAAAGAAGGTCGAAGAAAGCTTACAGTCTGAGAAAATCAAAATG
A3-AT1G10890-XLOC_000523-8260-1
CTATTCGAAAGAAGGTCGAAGAAAGCTTACAGTCTGAGAAAATCAAAATG
CONSENSUS
CTATTCGAAAGAAGGTCGAAGAAAGCTTACAGTCTGAGAAAATCAAAATG

A3-AT1G10890-XLOC_000523-8260-0
GAAATTCTAACGCTGTTGGAGGAAGGGCGAAAGAGACTTAATGAAGAAGT
A3-AT1G10890-XLOC_000523-8260-1
GAAATTCTAACGCTGTTGGAGGAAGGGCGAAAGAGACTTAATGAAGAAGT

```
CONSENSUS
      GAAATTCTAACGCTGTTGGAGGAAGGGCGAAAGAGACTTAATGAAGAAGT

A3-AT1G10890-XLOC_000523-8260-0
      CGCGGCTCAACTTGAGGAGGAGAAAGAGGCTTCTCTTATTGAGGCTAAAG
A3-AT1G10890-XLOC_000523-8260-1
      CGCGGCTCAACTTGAGGAGGAGAAAGAGGCTTCTCTTATTGAGGCTAAAG
CONSENSUS
      CGCGGCTCAACTTGAGGAGGAGAAAGAGGCTTCTCTTATTGAGGCTAAAG

A3-AT1G10890-XLOC_000523-8260-0  AAAAAGAG
A3-AT1G10890-XLOC_000523-8260-1  AAAAAGAG
CONSENSUS                        AAAAAGAG



alignment for event: A5-AT1G77080-XLOC_003953-9827

A5-AT1G77080-XLOC_003953-9827-0
      GAGAAATTGCTGAGAGAAGAGAACCAGGTTCTGGCTAGCCAG--------
A5-AT1G77080-XLOC_003953-9827-1
      GAGAAATTGCTGAGAGAAGAGAACCAGGTTCTGGCTAGCCAGGTAACAAT
CONSENSUS
      GAGAAATTGCTGAGAGAAGAGAACCAGGTTCTGGCTAGCCAG........

A5-AT1G77080-XLOC_003953-9827-0
      --------------------------------------------------
A5-AT1G77080-XLOC_003953-9827-1
      GACCACAATATCTTCTGCTCTTGAAGCTAATTAATCACTTTATACGTCCC
CONSENSUS
      ..................................................

A5-AT1G77080-XLOC_003953-9827-0
      --------------------------------------------------
A5-AT1G77080-XLOC_003953-9827-1
      CGTTATAGAGAGATACACATATACACGTACATGAAAACTAAAAGTTGAAG
CONSENSUS
      ..................................................

A5-AT1G77080-XLOC_003953-9827-0
      --------------------------------------------------
A5-AT1G77080-XLOC_003953-9827-1
      GACTTTGATGGATACTAGACAATTATAGTGAAACCCTAAATATGTGATAA
CONSENSUS
      ..................................................

A5-AT1G77080-XLOC_003953-9827-0
      --------------------------------------------------
A5-AT1G77080-XLOC_003953-9827-1
      GTGATAACAAAATGCTTTTAAAATCTATCTTTCTTGTTAATTTAGTAGCT
CONSENSUS
      ..................................................

A5-AT1G77080-XLOC_003953-9827-0
      ------------------------------------------------AT
A5-AT1G77080-XLOC_003953-9827-1
```

GTCAGAGAAGAAAGGTATGTCTCACCGATGAAAGATACTCAAAACCCGAT
CONSENSUS
....AT

A5-AT1G77080-XLOC_003953-9827-0
GGGAAAGAATACGTTGCTGGCAACAGATGATGAGAGAGGAATGTTTCCGG
A5-AT1G77080-XLOC_003953-9827-1
GGGAAAGAATACGTTGCTGGCAACAGATGATGAGAGAGGAATGTTTCCGG
CONSENSUS
GGGAAAGAATACGTTGCTGGCAACAGATGATGAGAGAGGAATGTTTCCGG

A5-AT1G77080-XLOC_003953-9827-0
GAAGTAGCTCCGGCAACAAAATACCGGAGACTCTCCCGCTGCTCAATTAG
A5-AT1G77080-XLOC_003953-9827-1
GAAGTAGCTCCGGCAACAAAATACCGGAGACTCTCCCGCTGCTCAATTAG
CONSENSUS
GAAGTAGCTCCGGCAACAAAATACCGGAGACTCTCCCGCTGCTCAATTAG

A5-AT1G77080-XLOC_003953-9827-0
CCACCATCATCAACGGCTGAGTTTTCACCTTAAACTCAAAGCCTGATTCA
A5-AT1G77080-XLOC_003953-9827-1
CCACCATCATCAACGGCTGAGTTTTCACCTTAAACTCAAAGCCTGATTCA
CONSENSUS
CCACCATCATCAACGGCTGAGTTTTCACCTTAAACTCAAAGCCTGATTCA

A5-AT1G77080-XLOC_003953-9827-0
TAATTAAGAGAATAAATTTGTATATTATAAAAAGCTGTGTAATCTCAAAC
A5-AT1G77080-XLOC_003953-9827-1
TAATTAAGAGAATAAATTTGTATATTATAAAAAGCTGTGTAATCTCAAAC
CONSENSUS
TAATTAAGAGAATAAATTTGTATATTATAAAAAGCTGTGTAATCTCAAAC

A5-AT1G77080-XLOC_003953-9827-0
CTTTTATCTTCCTCTAGTGTGGAATTTAAGGTCAAAAAGAAAACGAGAAA
A5-AT1G77080-XLOC_003953-9827-1
CTTTTATCTTCCTCTAGTGTGGAATTTAAGGTCAAAAAGAAAACGAGAAA
CONSENSUS
CTTTTATCTTCCTCTAGTGTGGAATTTAAGGTCAAAAAGAAAACGAGAAA

A5-AT1G77080-XLOC_003953-9827-0
GTATGGATCAGTGTTGTACCTCCTTCGGAGACAAGATCAGAGTTTGTGTG
A5-AT1G77080-XLOC_003953-9827-1
GTATGGATCAGTGTTGTACCTCCTTCGGAGACAAGATCAGAGTTTGTGTG
CONSENSUS
GTATGGATCAGTGTTGTACCTCCTTCGGAGACAAGATCAGAGTTTGTGTG

A5-AT1G77080-XLOC_003953-9827-0
TTTGTGTCTGAATGTACGGATTGGATTTTTAAAGTTGTGCTTTCTTTCTT
A5-AT1G77080-XLOC_003953-9827-1
TTTGTGTCTGAATGTACGGATTGGATTTTTAAAGTTGTGCTTTCTTTCTT
CONSENSUS
TTTGTGTCTGAATGTACGGATTGGATTTTTAAAGTTGTGCTTTCTTTCTT

A5-AT1G77080-XLOC_003953-9827-0 C
A5-AT1G77080-XLOC_003953-9827-1 C
CONSENSUS C

alignment for event: A3-AT1G48410-XLOC_006423-1560

A3-AT1G48410-XLOC_006423-1560-0
      ACGAGAAAGGGAATTTAAAGTTGTGATCAAGCTAGTTGCACGTGCTGATC
A3-AT1G48410-XLOC_006423-1560-1
      ACGAGAAAGGGAATTTAAAGTTGTGATCAAGCTAGTTGCACGTGCTGATC
CONSENSUS
      ACGAGAAAGGGAATTTAAAGTTGTGATCAAGCTAGTTGCACGTGCTGATC

A3-AT1G48410-XLOC_006423-1560-0
      TGCATCACCTAGGAATGTTTTTGGAGGGGAAACAATCAGATGCCCCACAG
A3-AT1G48410-XLOC_006423-1560-1
      TGCATCACCTAGGAATGTTTTTGGAGGGGAAACAATCAGATGCCCCACAG
CONSENSUS
      TGCATCACCTAGGAATGTTTTTGGAGGGGAAACAATCAGATGCCCCACAG

A3-AT1G48410-XLOC_006423-1560-0
      GAAGCTCTGCAGGTTCTTGACATTGTTCTTCGTGAGCTGCCGACCTCTAG
A3-AT1G48410-XLOC_006423-1560-1
      GAAGCTCTGCAGGTTCTTGACATTGTTCTTCGTGAGCTGCCGACCTCTAG
CONSENSUS
      GAAGCTCTGCAGGTTCTTGACATTGTTCTTCGTGAGCTGCCGACCTCTAG

A3-AT1G48410-XLOC_006423-1560-0 ------
GTATATTCCGGTGGGCCGGTCCTTTTATTCCCCTGATATAGGAA
A3-AT1G48410-XLOC_006423-1560-1
      AATCAGGTATATTCCGGTGGGCCGGTCCTTTTATTCCCCTGATATAGGAA
CONSENSUS
      ......GTATATTCCGGTGGGCCGGTCCTTTTATTCCCCTGATATAGGAA

A3-AT1G48410-XLOC_006423-1560-0
      AAAAACAATCATTGGGGGATGGCTTGGAGAGCTGGCGTGGATTCTACCAA
A3-AT1G48410-XLOC_006423-1560-1
      AAAAACAATCATTGGGGGATGGCTTGGAGAGCTGGCGTGGATTCTACCAA
CONSENSUS
      AAAAACAATCATTGGGGGATGGCTTGGAGAGCTGGCGTGGATTCTACCAA

A3-AT1G48410-XLOC_006423-1560-0
      AGCATTCGTCCTACACAGATGGGCTTATCACTCAATATTG
A3-AT1G48410-XLOC_006423-1560-1
      AGCATTCGTCCTACACAGATGGGCTTATCACTCAATATTG
CONSENSUS
      AGCATTCGTCCTACACAGATGGGCTTATCACTCAATATTG

alignment for event: A3-AT1G07350-XLOC_004507-10752

A3-AT1G07350-XLOC_004507-10752-0
      GTAACTGATGTTCACCTTGTCCTGGACCCATGGACTAGAGAATCTCGCGG
A3-AT1G07350-XLOC_004507-10752-1
      GTAACTGATGTTCACCTTGTCCTGGACCCATGGACTAGAGAATCTCGCGG
CONSENSUS

GTAACTGATGTTCACCTTGTCCTGGACCCATGGACTAGAGAATCTCGCGG

A3-AT1G07350-XLOC_004507-10752-0
ATTTGGTTTTATCTCTATGAAAAGTGTTGGTGATGCTAACCGTTGCATCA
A3-AT1G07350-XLOC_004507-10752-1
ATTTGGTTTTATCTCTATGAAAAGTGTTGGTGATGCTAACCGTTGCATCA
CONSENSUS
ATTTGGTTTTATCTCTATGAAAAGTGTTGGTGATGCTAACCGTTGCATCA

A3-AT1G07350-XLOC_004507-10752-0
GATCTCTAGATCACTCTGTTCTGCAGGGCCGCGTCATCACTGTTGAGAAG
A3-AT1G07350-XLOC_004507-10752-1
GATCTCTAGATCACTCTGTTCTGCAGGGCCGCGTCATCACTGTTGAGAAG
CONSENSUS
GATCTCTAGATCACTCTGTTCTGCAGGGCCGCGTCATCACTGTTGAGAAG

A3-AT1G07350-XLOC_004507-10752-0

A3-AT1G07350-XLOC_004507-10752-1
GTCTGCTGTTTGTAGCAGCAGTGCTTCACCAAATAGCAAACGTTGCAACA
CONSENSUS
....

A3-AT1G07350-XLOC_004507-10752-0

A3-AT1G07350-XLOC_004507-10752-1
GTTCAAACACATCAAGTTTCAGACATTTAGGGTTGTATGCCCTGCATTTC
CONSENSUS
....

A3-AT1G07350-XLOC_004507-10752-0

A3-AT1G07350-XLOC_004507-10752-1
TCTCTTTCTTGAACAATTTAATATTCCGTCCTTTAGTAGCTTCAATATAG
CONSENSUS
....

A3-AT1G07350-XLOC_004507-10752-0

A3-AT1G07350-XLOC_004507-10752-1
GAATGTTGTTTTTGTCGTGTTTCCTGTTTCATTGATGAATGTCTGACAGA
CONSENSUS
....

A3-AT1G07350-XLOC_004507-10752-0 ----------------------------
GCAAGACGTCGTAGAGGACGTA
A3-AT1G07350-XLOC_004507-10752-1
TCTGTGATATCTCTGGTTTTCTGTTCAGGCAAGACGTCGTAGAGGACGTA
CONSENSUS
............................GCAAGACGTCGTAGAGGACGTA

A3-AT1G07350-XLOC_004507-10752-0
CTCCAACTCCAGGAAAGTACTTGGGGCTGAGAACTGCTCGAG
A3-AT1G07350-XLOC_004507-10752-1
CTCCAACTCCAGGAAAGTACTTGGGGCTGAGAACTGCTCGAG
CONSENSUS

CTCCAACTCCAGGAAAGTACTTGGGGCTGAGAACTGCTCGAG

alignment for event: A5-AT1G10890-XLOC_000523-8251

A5-AT1G10890-XLOC_000523-8251-0
GACCAATCCGTTCAGAGAGATTAAAGCTGAGCTGAGCTTCTCCTTATAAA
A5-AT1G10890-XLOC_000523-8251-1
GACCAATCCGTTCAGAGAGATTAAAGCTGAGCTGAGCTTCTCCTTATAAA
CONSENSUS
GACCAATCCGTTCAGAGAGATTAAAGCTGAGCTGAGCTTCTCCTTATAAA

A5-AT1G10890-XLOC_000523-8251-0
GGAAGATACTGCTTGTTCTACGGAATTTTGAGAGATGCCTCGGGACTTGT
A5-AT1G10890-XLOC_000523-8251-1
GGAAGATACTGCTTGTTCTACGGAATTTTGAGAGATGCCTCGGGACTTGT
CONSENSUS
GGAAGATACTGCTTGTTCTACGGAATTTTGAGAGATGCCTCGGGACTTGT

A5-AT1G10890-XLOC_000523-8251-0
CAAGATCGAGGTCACCGTCTCCATCACCTTCACGTCGTAGAAAGCACTCG
A5-AT1G10890-XLOC_000523-8251-1
CAAGATCGAGGTCACCGTCTCCATCACCTTCACGTCGTAGAAAGCACTCG
CONSENSUS
CAAGATCGAGGTCACCGTCTCCATCACCTTCACGTCGTAGAAAGCACTCG

A5-AT1G10890-XLOC_000523-8251-0
AGGTCTCCCGTAAGGCAGAGGCATAGCAGGAGGAGTAGAAGAGACAGAAG
A5-AT1G10890-XLOC_000523-8251-1
AGGTCTCCCGTAAGGCAGAGGCATAGCAGGAGGAGTAGAAGAGACAGAAG
CONSENSUS
AGGTCTCCCGTAAGGCAGAGGCATAGCAGGAGGAGTAGAAGAGACAGAAG

A5-AT1G10890-XLOC_000523-8251-0
CCCTTCTCCATACTCATCTCATTCGTATAGCAG-----------------
A5-AT1G10890-XLOC_000523-8251-1
CCCTTCTCCATACTCATCTCATTCGTATAGCAGGTGATACCCCTAGTGTT
CONSENSUS
CCCTTCTCCATACTCATCTCATTCGTATAGCAG.................

A5-AT1G10890-XLOC_000523-8251-0

A5-AT1G10890-XLOC_000523-8251-1
TGTTTTTTCCGCTATTCGTTTGCCTTAGTTATCATTCGTTTATACGAACT
CONSENSUS
....

A5-AT1G10890-XLOC_000523-8251-0

A5-AT1G10890-XLOC_000523-8251-1
GTAAGAAACATTGGTGGAACACGGTTATAGATAGACATGTAAAGTTTACA
CONSENSUS
....

A5-AT1G10890-XLOC_000523-8251-0

A5-AT1G10890-XLOC_000523-8251-1
GACTCAAGAATTGCTTTTAGTAAAGTTCCTACTTATAGATATATTTTCTA
CONSENSUS
....

A5-AT1G10890-XLOC_000523-8251-0

A5-AT1G10890-XLOC_000523-8251-1
CCCTTCTCTAAAAGAGAGTGCAATGTCTGGATGCTGCTGGCTCTGTATCG
CONSENSUS
....

A5-AT1G10890-XLOC_000523-8251-0

A5-AT1G10890-XLOC_000523-8251-1
TAAGCGTTTTTTTGAGTGATGCTGCGTATTGGAAATATATCGTGTTTATT
CONSENSUS
....

A5-AT1G10890-XLOC_000523-8251-0

A5-AT1G10890-XLOC_000523-8251-1
TGGCTGTTGTGATTAAATTTGTTGCAAGAGAATATGTTTTATGACCTGAG
CONSENSUS
....

A5-AT1G10890-XLOC_000523-8251-0

A5-AT1G10890-XLOC_000523-8251-1
CTTTGTGATTTTTAGAATGAAACCTGTATATTCGGTAAAGACATAAGTTA
CONSENSUS
....

A5-AT1G10890-XLOC_000523-8251-0

A5-AT1G10890-XLOC_000523-8251-1
AGCTGGGATAATAGCACTACTGATAGTCTGATCTAGTTTATATTTTAATT
CONSENSUS
....

A5-AT1G10890-XLOC_000523-8251-0

A5-AT1G10890-XLOC_000523-8251-1
GTATCTGTAGATTATTAGTTAGAATCAGTTTTAAGAGCGACTATATTATG
CONSENSUS
....

A5-AT1G10890-XLOC_000523-8251-0

A5-AT1G10890-XLOC_000523-8251-1
TTCTTTTAAATGGAGTTTGTTAGCAATAGTGATACAAAACCACCACTATT
CONSENSUS
....

A5-AT1G10890-XLOC_000523-8251-0

A5-AT1G10890-XLOC_000523-8251-1
CCTTAGGTAAGATAATATAGTAATTTGTTGAGAACATGGCTGAGAAGCTA
CONSENSUS
....

A5-AT1G10890-XLOC_000523-8251-0

A5-AT1G10890-XLOC_000523-8251-1
GGAAAATGATACATGCGCTTACCATTATAAAATAGCTCAGTTTCTTCCCG
CONSENSUS
....

A5-AT1G10890-XLOC_000523-8251-0

A5-AT1G10890-XLOC_000523-8251-1
TTCCCACGGGAGGGGAAATGAGCAGCTGATGGATTGCTCTTAGGTGTTCT
CONSENSUS
....

A5-AT1G10890-XLOC_000523-8251-0

A5-AT1G10890-XLOC_000523-8251-1
TTCATTGATCCCTTGTCTCGTTAGCTTCTCTTCGTGTGATAAAAGTTGGA
CONSENSUS
....

A5-AT1G10890-XLOC_000523-8251-0 --------------------------
GCGAAAAAGTCGTTCTATTTCTCC
A5-AT1G10890-XLOC_000523-8251-1
GCTGATAATTCTTTACAGTGTATGAGGCGAAAAAGTCGTTCTATTTCTCC
CONSENSUS
..........................GCGAAAAAGTCGTTCTATTTCTCC

A5-AT1G10890-XLOC_000523-8251-0
TAGGCGCCATCGAAGTCGATCTGTTACTCCTAAGAGACGTTCTCCAACCC
A5-AT1G10890-XLOC_000523-8251-1
TAGGCGCCATCGAAGTCGATCTGTTACTCCTAAGAGACGTTCTCCAACCC
CONSENSUS
TAGGCGCCATCGAAGTCGATCTGTTACTCCTAAGAGACGTTCTCCAACCC

A5-AT1G10890-XLOC_000523-8251-0
CAAAACGTTACAAAAGACAAAAGAGTAGGAGTTCAACTCCATCTCCTGCA
A5-AT1G10890-XLOC_000523-8251-1
CAAAACGTTACAAAAGACAAAAGAGTAGGAGTTCAACTCCATCTCCTGCA
CONSENSUS
CAAAACGTTACAAAAGACAAAAGAGTAGGAGTTCAACTCCATCTCCTGCA

A5-AT1G10890-XLOC_000523-8251-0
AAAAGATCTCCCGCCGCAACCCTTGAGTCAGCCAAAAATAGGAATGGAGA
A5-AT1G10890-XLOC_000523-8251-1
AAAAGATCTCCCGCCGCAACCCTTGAGTCAGCCAAAAATAGGAATGGAGA
CONSENSUS
AAAAGATCTCCCGCCGCAACCCTTGAGTCAGCCAAAAATAGGAATGGAGA

A5-AT1G10890-XLOC_000523-8251-0

AAAACTTAAAAGAGAAGAGGAAGAACGAAAAAGGTAAATCAATACAAAAT
A5-AT1G10890-XLOC_000523-8251-1
AAAACTTAAAAGAGAAGAGGAAGAACGAAAAAGGTAAATCAATACAAAAT
CONSENSUS
AAAACTTAAAAGAGAAGAGGAAGAACGAAAAAGGTAAATCAATACAAAAT

A5-AT1G10890-XLOC_000523-8251-0
TTTGAAGTAAGATGGATGTTATGGTGAAATTAAGGTTTTTTGTTCTCAAA
A5-AT1G10890-XLOC_000523-8251-1
TTTGAAGTAAGATGGATGTTATGGTGAAATTAAGGTTTTTTGTTCTCAAA
CONSENSUS
TTTGAAGTAAGATGGATGTTATGGTGAAATTAAGGTTTTTTGTTCTCAAA

A5-AT1G10890-XLOC_000523-8251-0
TGTTATTTTAGTGTAGTGTGGAAGTCCTTGATTGTTAGTCTCAAAAGTGC
A5-AT1G10890-XLOC_000523-8251-1
TGTTATTTTAGTGTAGTGTGGAAGTCCTTGATTGTTAGTCTCAAAAGTGC
CONSENSUS
TGTTATTTTAGTGTAGTGTGGAAGTCCTTGATTGTTAGTCTCAAAAGTGC

A5-AT1G10890-XLOC_000523-8251-0
GACAGGTTTTATGGTATTCTCTCACAGGCTTTGCTTATGGAAGATAAGCT
A5-AT1G10890-XLOC_000523-8251-1
GACAGGTTTTATGGTATTCTCTCACAGGCTTTGCTTATGGAAGATAAGCT
CONSENSUS
GACAGGTTTTATGGTATTCTCTCACAGGCTTTGCTTATGGAAGATAAGCT

A5-AT1G10890-XLOC_000523-8251-0
ATATTTCGATTTGTTTATATTTGATCCTCTGGCACAAATGTTCGTACTAT
A5-AT1G10890-XLOC_000523-8251-1
ATATTTCGATTTGTTTATATTTGATCCTCTGGCACAAATGTTCGTACTAT
CONSENSUS
ATATTTCGATTTGTTTATATTTGATCCTCTGGCACAAATGTTCGTACTAT

A5-AT1G10890-XLOC_000523-8251-0
CTCGATTTGATGAGTTACCGTTTGTCATTACATGGATCATGATTGACTTA
A5-AT1G10890-XLOC_000523-8251-1
CTCGATTTGATGAGTTACCGTTTGTCATTACATGGATCATGATTGACTTA
CONSENSUS
CTCGATTTGATGAGTTACCGTTTGTCATTACATGGATCATGATTGACTTA

A5-AT1G10890-XLOC_000523-8251-0
GAGATGTAAATATGTCCAAAGCACAAGAATAATCTTGGTGAAGGTTTGCT
A5-AT1G10890-XLOC_000523-8251-1
GAGATGTAAATATGTCCAAAGCACAAGAATAATCTTGGTGAAGGTTTGCT
CONSENSUS
GAGATGTAAATATGTCCAAAGCACAAGAATAATCTTGGTGAAGGTTTGCT

A5-AT1G10890-XLOC_000523-8251-0
TGTGTTATGGTGACAAAAAAGAAATTTCTTCACGAAATTTACCATTGGTA
A5-AT1G10890-XLOC_000523-8251-1
TGTGTTATGGTGACAAAAAAGAAATTTCTTCACGAAATTTACCATTGGTA
CONSENSUS
TGTGTTATGGTGACAAAAAAGAAATTTCTTCACGAAATTTACCATTGGTA

A5-AT1G10890-XLOC_000523-8251-0

TTGGGAGGAGTGAAAATGGAAACGATCTCAACATTTTTTACGGATTTGCA
A5-AT1G10890-XLOC_000523-8251-1
TTGGGAGGAGTGAAAATGGAAACGATCTCAACATTTTTTACGGATTTGCA
CONSENSUS
TTGGGAGGAGTGAAAATGGAAACGATCTCAACATTTTTTACGGATTTGCA

A5-AT1G10890-XLOC_000523-8251-0
ATAAAATTTTGGTCTTTTGCTGTTCAAGATTGAAGACATCAGGAAATGAG
A5-AT1G10890-XLOC_000523-8251-1
ATAAAATTTTGGTCTTTTGCTGTTCAAGATTGAAGACATCAGGAAATGAG
CONSENSUS
ATAAAATTTTGGTCTTTTGCTGTTCAAGATTGAAGACATCAGGAAATGAG

A5-AT1G10890-XLOC_000523-8251-0
AAAGTTCAGGTGATATTCAATATTCTGAACCTGCTGTGGATGTCCTCTAA
A5-AT1G10890-XLOC_000523-8251-1
AAAGTTCAGGTGATATTCAATATTCTGAACCTGCTGTGGATGTCCTCTAA
CONSENSUS
AAAGTTCAGGTGATATTCAATATTCTGAACCTGCTGTGGATGTCCTCTAA

A5-AT1G10890-XLOC_000523-8251-0
TTTTTCACTGTATTTGCTACCAGGCGACAGCGTGAAGCAGAACTGAAGCT
A5-AT1G10890-XLOC_000523-8251-1
TTTTTCACTGTATTTGCTACCAGGCGACAGCGTGAAGCAGAACTGAAGCT
CONSENSUS
TTTTTCACTGTATTTGCTACCAGGCGACAGCGTGAAGCAGAACTGAAGCT

A5-AT1G10890-XLOC_000523-8251-0
AATAGAGGAAGAAACTGTGAAACGGGTTGAAGAAGCTATTCGAAAGAAGG
A5-AT1G10890-XLOC_000523-8251-1
AATAGAGGAAGAAACTGTGAAACGGGTTGAAGAAGCTATTCGAAAGAAGG
CONSENSUS
AATAGAGGAAGAAACTGTGAAACGGGTTGAAGAAGCTATTCGAAAGAAGG

A5-AT1G10890-XLOC_000523-8251-0
TCGAAGAAAGCTTACAGTCTGAGAAAATCAAAATGGAAATTCTAACGCTG
A5-AT1G10890-XLOC_000523-8251-1
TCGAAGAAAGCTTACAGTCTGAGAAAATCAAAATGGAAATTCTAACGCTG
CONSENSUS
TCGAAGAAAGCTTACAGTCTGAGAAAATCAAAATGGAAATTCTAACGCTG

A5-AT1G10890-XLOC_000523-8251-0
TTGGAGGAAGGGCGAAAGAGACTTAATGAAGAAGTCGCGGCTCAACTTGA
A5-AT1G10890-XLOC_000523-8251-1
TTGGAGGAAGGGCGAAAGAGACTTAATGAAGAAGTCGCGGCTCAACTTGA
CONSENSUS
TTGGAGGAAGGGCGAAAGAGACTTAATGAAGAAGTCGCGGCTCAACTTGA

A5-AT1G10890-XLOC_000523-8251-0
GGAGGAGAAAGAGGCTTCTCTTATTGAGGCTAAAGAAAAAGAG
A5-AT1G10890-XLOC_000523-8251-1
GGAGGAGAAAGAGGCTTCTCTTATTGAGGCTAAAGAAAAAGAG
CONSENSUS
GGAGGAGAAAGAGGCTTCTCTTATTGAGGCTAAAGAAAAAGAG

alignment for event: A3-AT1G73350-XLOC_007739-13202

A3-AT1G73350-XLOC_007739-13202-0
      ATAAGAAATCAGGGAAAGGTAAGATTGATGATGAAGACGTCACCTTTCAA
A3-AT1G73350-XLOC_007739-13202-1
      ATAAGAAATCAGGGAAAGGTAAGATTGATGATGAAGACGTCACCTTTCAA
CONSENSUS
      ATAAGAAATCAGGGAAAGGTAAGATTGATGATGAAGACGTCACCTTTCAA

A3-AT1G73350-XLOC_007739-13202-0
      CGAATGGTTGCAAAG-----------------------------------
A3-AT1G73350-XLOC_007739-13202-1
      CGAATGGTTGCAAAGGGATAACGGCCCCGCCACATTTTCTGGTTATTGCA
CONSENSUS
      CGAATGGTTGCAAAG...................................

A3-AT1G73350-XLOC_007739-13202-0 -----
ATGCAAGAGGTTGCTGGTGAACGTGGAGGCTATCTTCATGGACGA
A3-AT1G73350-XLOC_007739-13202-1
      TATAGATGCAAGAGGTTGCTGGTGAACGTGGAGGCTATCTTCATGGACGA
CONSENSUS
      .....ATGCAAGAGGTTGCTGGTGAACGTGGAGGCTATCTTCATGGACGA

A3-AT1G73350-XLOC_007739-13202-0 GGCG
A3-AT1G73350-XLOC_007739-13202-1 GGCG
CONSENSUS GGCG

alignment for event: RI-AT1G10890-XLOC_000523-8250

RI-AT1G10890-XLOC_000523-8250-0
      GACCAATCCGTTCAGAGAGATTAAAGCTGAGCTGAGCTTCTCCTTATAAA
RI-AT1G10890-XLOC_000523-8250-1
      GACCAATCCGTTCAGAGAGATTAAAGCTGAGCTGAGCTTCTCCTTATAAA
CONSENSUS
      GACCAATCCGTTCAGAGAGATTAAAGCTGAGCTGAGCTTCTCCTTATAAA

RI-AT1G10890-XLOC_000523-8250-0
      GGAAGATACTGCTTGTTCTACGGAATTTTGAGAGATGCCTCGGGACTTGT
RI-AT1G10890-XLOC_000523-8250-1
      GGAAGATACTGCTTGTTCTACGGAATTTTGAGAGATGCCTCGGGACTTGT
CONSENSUS
      GGAAGATACTGCTTGTTCTACGGAATTTTGAGAGATGCCTCGGGACTTGT

RI-AT1G10890-XLOC_000523-8250-0
      CAAGATCGAGGTCACCGTCTCCATCACCTTCACGTCGTAGAAAGCACTCG
RI-AT1G10890-XLOC_000523-8250-1
      CAAGATCGAGGTCACCGTCTCCATCACCTTCACGTCGTAGAAAGCACTCG
CONSENSUS
      CAAGATCGAGGTCACCGTCTCCATCACCTTCACGTCGTAGAAAGCACTCG

RI-AT1G10890-XLOC_000523-8250-0
      AGGTCTCCCGTAAGGCAGAGGCATAGCAGGAGGAGTAGAAGAGACAGAAG
RI-AT1G10890-XLOC_000523-8250-1

AGGTCTCCCGTAAGGCAGAGGCATAGCAGGAGGAGTAGAAGAGACAGAAG
CONSENSUS
AGGTCTCCCGTAAGGCAGAGGCATAGCAGGAGGAGTAGAAGAGACAGAAG

RI-AT1G10890-XLOC_000523-8250-0
CCCTTCTCCATACTCATCTCATTCGTATAGCAGGTGATACCCCTAGTGTT
RI-AT1G10890-XLOC_000523-8250-1
CCCTTCTCCATACTCATCTCATTCGTATAGCAGGTGATACCCCTAGTGTT
CONSENSUS
CCCTTCTCCATACTCATCTCATTCGTATAGCAGGTGATACCCCTAGTGTT

RI-AT1G10890-XLOC_000523-8250-0
TGTTTTTTCCGCTATTCGTTTGCCTTAGTTATCATTCGTTTATACGAACT
RI-AT1G10890-XLOC_000523-8250-1
TGTTTTTTCCGCTATTCGTTTGCCTTAGTTATCATTCGTTTATACGAACT
CONSENSUS
TGTTTTTTCCGCTATTCGTTTGCCTTAGTTATCATTCGTTTATACGAACT

RI-AT1G10890-XLOC_000523-8250-0
GTAAGAAACATTGGTGGAACACGGTTATAGATAGACATGTAAAGTTTACA
RI-AT1G10890-XLOC_000523-8250-1
GTAAGAAACATTGGTGGAACACGGTTATAGATAGACATGTAAAGTTTACA
CONSENSUS
GTAAGAAACATTGGTGGAACACGGTTATAGATAGACATGTAAAGTTTACA

RI-AT1G10890-XLOC_000523-8250-0
GACTCAAGAATTGCTTTTAGTAAAGTTCCTACTTATAGATATATTTTCTA
RI-AT1G10890-XLOC_000523-8250-1
GACTCAAGAATTGCTTTTAGTAAAGTTCCTACTTATAGATATATTTTCTA
CONSENSUS
GACTCAAGAATTGCTTTTAGTAAAGTTCCTACTTATAGATATATTTTCTA

RI-AT1G10890-XLOC_000523-8250-0
CCCTTCTCTAAAAGAGAGTGCAATGTCTGGATGCTGCTGGCTCTGTATCG
RI-AT1G10890-XLOC_000523-8250-1
CCCTTCTCTAAAAGAGAGTGCAATGTCTGGATGCTGCTGGCTCTGTATCG
CONSENSUS
CCCTTCTCTAAAAGAGAGTGCAATGTCTGGATGCTGCTGGCTCTGTATCG

RI-AT1G10890-XLOC_000523-8250-0
TAAGCGTTTTTTTGAGTGATGCTGCGTATTGGAAATATATCGTGTTTATT
RI-AT1G10890-XLOC_000523-8250-1
TAAGCGTTTTTTTGAGTGATGCTGCGTATTGGAAATATATCGTGTTTATT
CONSENSUS
TAAGCGTTTTTTTGAGTGATGCTGCGTATTGGAAATATATCGTGTTTATT

RI-AT1G10890-XLOC_000523-8250-0
TGGCTGTTGTGATTAAATTTGTTGCAAGAGAATATGTTTTATGACCTGAG
RI-AT1G10890-XLOC_000523-8250-1
TGGCTGTTGTGATTAAATTTGTTGCAAGAGAATATGTTTTATGACCTGAG
CONSENSUS
TGGCTGTTGTGATTAAATTTGTTGCAAGAGAATATGTTTTATGACCTGAG

RI-AT1G10890-XLOC_000523-8250-0
CTTTGTGATTTTTAGAATGAAACCTGTATATTCGGTAAAGACATAAGTTA
RI-AT1G10890-XLOC_000523-8250-1

CTTTGTGATTTTTAGAATGAAACCTGTATATTCGGTAAAGACATAAGTTA
CONSENSUS
CTTTGTGATTTTTAGAATGAAACCTGTATATTCGGTAAAGACATAAGTTA

RI-AT1G10890-XLOC_000523-8250-0
AGCTGGGATAATAGCACTACTGATAGTCTGATCTAGTTTATATTTTAATT
RI-AT1G10890-XLOC_000523-8250-1
AGCTGGGATAATAGCACTACTGATAGTCTGATCTAGTTTATATTTTAATT
CONSENSUS
AGCTGGGATAATAGCACTACTGATAGTCTGATCTAGTTTATATTTTAATT

RI-AT1G10890-XLOC_000523-8250-0
GTATCTGTAGATTATTAGTTAGAATCAGTTTTAAGAGCGACTATATTATG
RI-AT1G10890-XLOC_000523-8250-1
GTATCTGTAGATTATTAGTTAGAATCAGTTTTAAGAGCGACTATATTATG
CONSENSUS
GTATCTGTAGATTATTAGTTAGAATCAGTTTTAAGAGCGACTATATTATG

RI-AT1G10890-XLOC_000523-8250-0
TTCTTTTAAATGGAGTTTGTTAGCAATAGTGATACAAAACCACCACTATT
RI-AT1G10890-XLOC_000523-8250-1
TTCTTTTAAATGGAGTTTGTTAGCAATAGTGATACAAAACCACCACTATT
CONSENSUS
TTCTTTTAAATGGAGTTTGTTAGCAATAGTGATACAAAACCACCACTATT

RI-AT1G10890-XLOC_000523-8250-0
CCTTAGGTAAGATAATATAGTAATTTGTTGAGAACATGGCTGAGAAGCTA
RI-AT1G10890-XLOC_000523-8250-1
CCTTAGGTAAGATAATATAGTAATTTGTTGAGAACATGGCTGAGAAGCTA
CONSENSUS
CCTTAGGTAAGATAATATAGTAATTTGTTGAGAACATGGCTGAGAAGCTA

RI-AT1G10890-XLOC_000523-8250-0
GGAAAATGATACATGCGCTTACCATTATAAAATAGCTCAGTTTCTTCCCG
RI-AT1G10890-XLOC_000523-8250-1
GGAAAATGATACATGCGCTTACCATTATAAAATAGCTCAGTTTCTTCCCG
CONSENSUS
GGAAAATGATACATGCGCTTACCATTATAAAATAGCTCAGTTTCTTCCCG

RI-AT1G10890-XLOC_000523-8250-0
TTCCCACGGGAGGGGAAATGAGCAGCTGATGGATTGCTCTTAGGTGTTCT
RI-AT1G10890-XLOC_000523-8250-1
TTCCCACGGGAGGGGAAATGAGCAGCTGATGGATTGCTCTTAGGTGTTCT
CONSENSUS
TTCCCACGGGAGGGGAAATGAGCAGCTGATGGATTGCTCTTAGGTGTTCT

RI-AT1G10890-XLOC_000523-8250-0
TTCATTGATCCCTTGTCTCGTTAGCTTCTCTTCGTGTGATAAAAGTTGGA
RI-AT1G10890-XLOC_000523-8250-1
TTCATTGATCCCTTGTCTCGTTAGCTTCTCTTCGTGTGATAAAAGTTGGA
CONSENSUS
TTCATTGATCCCTTGTCTCGTTAGCTTCTCTTCGTGTGATAAAAGTTGGA

RI-AT1G10890-XLOC_000523-8250-0
GCTGATAATTCTTTACAGTGTATGAGGTAACTGCACATACTTTTGTTTTC
RI-AT1G10890-XLOC_000523-8250-1

GCTGATAATTCTTTACAGTGTATGAG-----------------------
CONSENSUS
GCTGATAATTCTTTACAGTGTATGAG........................

RI-AT1G10890-XLOC_000523-8250-0
TTTATGTGTAACTGTGGGCTACCTGATGCCTAGTCTTCACGTGCGTGGTA
RI-AT1G10890-XLOC_000523-8250-1

CONSENSUS
....

RI-AT1G10890-XLOC_000523-8250-0
CTTCATTAGTTCCATCTTTTTGCTTACTACTTTATTTTGGGTAATGTTCA
RI-AT1G10890-XLOC_000523-8250-1

CONSENSUS
....

RI-AT1G10890-XLOC_000523-8250-0
GGCGAAAAAGTCGTTCTATTTCTCCTAGGCGCCATCGAAGTCGATCTGTT
RI-AT1G10890-XLOC_000523-8250-1 -
GCGAAAAAGTCGTTCTATTTCTCCTAGGCGCCATCGAAGTCGATCTGTT
CONSENSUS
.GCGAAAAAGTCGTTCTATTTCTCCTAGGCGCCATCGAAGTCGATCTGTT

RI-AT1G10890-XLOC_000523-8250-0
ACTCCTAAGAGACGTTCTCCAACCCCAAAACGTTACAAAAGACAAAAGAG
RI-AT1G10890-XLOC_000523-8250-1
ACTCCTAAGAGACGTTCTCCAACCCCAAAACGTTACAAAAGACAAAAGAG
CONSENSUS
ACTCCTAAGAGACGTTCTCCAACCCCAAAACGTTACAAAAGACAAAAGAG

RI-AT1G10890-XLOC_000523-8250-0
TAGGAGTTCAACTCCATCTCCTGCAAAAAGATCTCCCGCCGCAACCCTTG
RI-AT1G10890-XLOC_000523-8250-1
TAGGAGTTCAACTCCATCTCCTGCAAAAAGATCTCCCGCCGCAACCCTTG
CONSENSUS
TAGGAGTTCAACTCCATCTCCTGCAAAAAGATCTCCCGCCGCAACCCTTG

RI-AT1G10890-XLOC_000523-8250-0
AGTCAGCCAAAAATAGGAATGGAGAAAAACTTAAAAGAGAAGAGGAAGAA
RI-AT1G10890-XLOC_000523-8250-1
AGTCAGCCAAAAATAGGAATGGAGAAAAACTTAAAAGAGAAGAGGAAGAA
CONSENSUS
AGTCAGCCAAAAATAGGAATGGAGAAAAACTTAAAAGAGAAGAGGAAGAA

RI-AT1G10890-XLOC_000523-8250-0
CGAAAAAGGTAAATCAATACAAAATTTTGAAGTAAGATGGATGTTATGGT
RI-AT1G10890-XLOC_000523-8250-1
CGAAAAAGGTAAATCAATACAAAATTTTGAAGTAAGATGGATGTTATGGT
CONSENSUS
CGAAAAAGGTAAATCAATACAAAATTTTGAAGTAAGATGGATGTTATGGT

RI-AT1G10890-XLOC_000523-8250-0
GAAATTAAGGTTTTTTGTTCTCAAATGTTATTTTAGTGTAGTGTGGAAGT
RI-AT1G10890-XLOC_000523-8250-1

GAAATTAAGGTTTTTTGTTCTCAAATGTTATTTTAGTGTAGTGTGGAAGT
CONSENSUS
GAAATTAAGGTTTTTTGTTCTCAAATGTTATTTTAGTGTAGTGTGGAAGT

RI-AT1G10890-XLOC_000523-8250-0
CCTTGATTGTTAGTCTCAAAAGTGCGACAGGTTTTATGGTATTCTCTCAC
RI-AT1G10890-XLOC_000523-8250-1
CCTTGATTGTTAGTCTCAAAAGTGCGACAGGTTTTATGGTATTCTCTCAC
CONSENSUS
CCTTGATTGTTAGTCTCAAAAGTGCGACAGGTTTTATGGTATTCTCTCAC

RI-AT1G10890-XLOC_000523-8250-0
AGGCTTTGCTTATGGAAGATAAGCTATATTTCGATTTGTTTATATTTGAT
RI-AT1G10890-XLOC_000523-8250-1
AGGCTTTGCTTATGGAAGATAAGCTATATTTCGATTTGTTTATATTTGAT
CONSENSUS
AGGCTTTGCTTATGGAAGATAAGCTATATTTCGATTTGTTTATATTTGAT

RI-AT1G10890-XLOC_000523-8250-0
CCTCTGGCACAAATGTTCGTACTATCTCGATTTGATGAGTTACCGTTTGT
RI-AT1G10890-XLOC_000523-8250-1
CCTCTGGCACAAATGTTCGTACTATCTCGATTTGATGAGTTACCGTTTGT
CONSENSUS
CCTCTGGCACAAATGTTCGTACTATCTCGATTTGATGAGTTACCGTTTGT

RI-AT1G10890-XLOC_000523-8250-0
CATTACATGGATCATGATTGACTTAGAGATGTAAATATGTCCAAAGCACA
RI-AT1G10890-XLOC_000523-8250-1
CATTACATGGATCATGATTGACTTAGAGATGTAAATATGTCCAAAGCACA
CONSENSUS
CATTACATGGATCATGATTGACTTAGAGATGTAAATATGTCCAAAGCACA

RI-AT1G10890-XLOC_000523-8250-0
AGAATAATCTTGGTGAAGGTTTGCTTGTGTTATGGTGACAAAAAAGAAAT
RI-AT1G10890-XLOC_000523-8250-1
AGAATAATCTTGGTGAAGGTTTGCTTGTGTTATGGTGACAAAAAAGAAAT
CONSENSUS
AGAATAATCTTGGTGAAGGTTTGCTTGTGTTATGGTGACAAAAAAGAAAT

RI-AT1G10890-XLOC_000523-8250-0
TTCTTCACGAAATTTACCATTGGTATTGGGAGGAGTGAAAATGGAAACGA
RI-AT1G10890-XLOC_000523-8250-1
TTCTTCACGAAATTTACCATTGGTATTGGGAGGAGTGAAAATGGAAACGA
CONSENSUS
TTCTTCACGAAATTTACCATTGGTATTGGGAGGAGTGAAAATGGAAACGA

RI-AT1G10890-XLOC_000523-8250-0
TCTCAACATTTTTTACGGATTTGCAATAAAATTTTGGTCTTTTGCTGTTC
RI-AT1G10890-XLOC_000523-8250-1
TCTCAACATTTTTTACGGATTTGCAATAAAATTTTGGTCTTTTGCTGTTC
CONSENSUS
TCTCAACATTTTTTACGGATTTGCAATAAAATTTTGGTCTTTTGCTGTTC

RI-AT1G10890-XLOC_000523-8250-0
AAGATTGAAGACATCAGGAAATGAGAAAGTTCAGGTGATATTCAATATTC
RI-AT1G10890-XLOC_000523-8250-1

AAGATTGAAGACATCAGGAAATGAGAAAGTTCAGGTGATATTCAATATTC
CONSENSUS
AAGATTGAAGACATCAGGAAATGAGAAAGTTCAGGTGATATTCAATATTC

RI-AT1G10890-XLOC_000523-8250-0
TGAACCTGCTGTGGATGTCCTCTAATTTTTCACTGTATTTGCTACCAGGC
RI-AT1G10890-XLOC_000523-8250-1
TGAACCTGCTGTGGATGTCCTCTAATTTTTCACTGTATTTGCTACCAGGC
CONSENSUS
TGAACCTGCTGTGGATGTCCTCTAATTTTTCACTGTATTTGCTACCAGGC

RI-AT1G10890-XLOC_000523-8250-0
GACAGCGTGAAGCAGAACTGAAGCTAATAGAGGAAGAAACTGTGAAACGG
RI-AT1G10890-XLOC_000523-8250-1
GACAGCGTGAAGCAGAACTGAAGCTAATAGAGGAAGAAACTGTGAAACGG
CONSENSUS
GACAGCGTGAAGCAGAACTGAAGCTAATAGAGGAAGAAACTGTGAAACGG

RI-AT1G10890-XLOC_000523-8250-0
GTTGAAGAAGCTATTCGAAAGAAGGTCGAAGAAAGCTTACAGTCTGAGAA
RI-AT1G10890-XLOC_000523-8250-1
GTTGAAGAAGCTATTCGAAAGAAGGTCGAAGAAAGCTTACAGTCTGAGAA
CONSENSUS
GTTGAAGAAGCTATTCGAAAGAAGGTCGAAGAAAGCTTACAGTCTGAGAA

RI-AT1G10890-XLOC_000523-8250-0
AATCAAAATGGAAATTCTAACGCTGTTGGAGGAAGGGCGAAAGAGACTTA
RI-AT1G10890-XLOC_000523-8250-1
AATCAAAATGGAAATTCTAACGCTGTTGGAGGAAGGGCGAAAGAGACTTA
CONSENSUS
AATCAAAATGGAAATTCTAACGCTGTTGGAGGAAGGGCGAAAGAGACTTA

RI-AT1G10890-XLOC_000523-8250-0
ATGAAGAAGTCGCGGCTCAACTTGAGGAGGAGAAAGAGGCTTCTCTTATT
RI-AT1G10890-XLOC_000523-8250-1
ATGAAGAAGTCGCGGCTCAACTTGAGGAGGAGAAAGAGGCTTCTCTTATT
CONSENSUS
ATGAAGAAGTCGCGGCTCAACTTGAGGAGGAGAAAGAGGCTTCTCTTATT

RI-AT1G10890-XLOC_000523-8250-0 GAGGCTAAAGAAAAAGAG
RI-AT1G10890-XLOC_000523-8250-1 GAGGCTAAAGAAAAAGAG
CONSENSUS   GAGGCTAAAGAAAAAGAG

alignment for event: A3-AT1G65540-XLOC_007309-7882

A3-AT1G65540-XLOC_007309-7882-0
AAACGGTCAAAATCAGCAGAAACACGTTGAATCTTCTTCTTCTTCTTCTT
A3-AT1G65540-XLOC_007309-7882-1
AAACGGTCAAAATCAGCAGAAACACGTTGAATCTTCTTCTTCTTCTTCTT
CONSENSUS
AAACGGTCAAAATCAGCAGAAACACGTTGAATCTTCTTCTTCTTCTTCTT

A3-AT1G65540-XLOC_007309-7882-0
CTTCTTCGGCTCAGTGACGACGACGATTCTTTTGACGGTCTTTTTCTCAA

A3-AT1G65540-XLOC_007309-7882-1
CTTCTTCGGCTCAGTGACGACGACGATTCTTTTGACGGTCTTTTTCTCAA
CONSENSUS
CTTCTTCGGCTCAGTGACGACGACGATTCTTTTGACGGTCTTTTTCTCAA

A3-AT1G65540-XLOC_007309-7882-0
AGTATTTTGGATTGTCTTCCACGTTTTGATCGATCCTTTCCTCTCCACTC
A3-AT1G65540-XLOC_007309-7882-1
AGTATTTTGGATTGTCTTCCACGTTTTGATCGATCCTTTCCTCTCCACTC
CONSENSUS
AGTATTTTGGATTGTCTTCCACGTTTTGATCGATCCTTTCCTCTCCACTC

A3-AT1G65540-XLOC_007309-7882-0
CTCCTCTTCTCTGTAGAT------------------------ATTGGTAT
A3-AT1G65540-XLOC_007309-7882-1
CTCCTCTTCTCTGTAGATATTTTTCATACAATCACTGTGCAGATTGGTAT
CONSENSUS
CTCCTCTTCTCTGTAGAT........................ATTGGTAT

A3-AT1G65540-XLOC_007309-7882-0
GTAGCTGGTATATATATACTGTATATAGCTACACAATTACTGCAATATGG
A3-AT1G65540-XLOC_007309-7882-1
GTAGCTGGTATATATATACTGTATATAGCTACACAATTACTGCAATATGG
CONSENSUS
GTAGCTGGTATATATATACTGTATATAGCTACACAATTACTGCAATATGG

A3-AT1G65540-XLOC_007309-7882-0
CTTCACGAGCGCTTCTTCGAAGGAGAAGTTACGACGTAGTGAAATCTCTA
A3-AT1G65540-XLOC_007309-7882-1
CTTCACGAGCGCTTCTTCGAAGGAGAAGTTACGACGTAGTGAAATCTCTA
CONSENSUS
CTTCACGAGCGCTTCTTCGAAGGAGAAGTTACGACGTAGTGAAATCTCTA

A3-AT1G65540-XLOC_007309-7882-0
AGTGAACATTTGCCTACGGTTCAATGTTTTTCAAGTATTGAGCGGCAAGG
A3-AT1G65540-XLOC_007309-7882-1
AGTGAACATTTGCCTACGGTTCAATGTTTTTCAAGTATTGAGCGGCAAGG
CONSENSUS
AGTGAACATTTGCCTACGGTTCAATGTTTTTCAAGTATTGAGCGGCAAGG

A3-AT1G65540-XLOC_007309-7882-0
ACAACGAGGATACCGTGATTTTAAAGATGTGAAGAAGAAAGAGTCGAGCG
A3-AT1G65540-XLOC_007309-7882-1
ACAACGAGGATACCGTGATTTTAAAGATGTGAAGAAGAAAGAGTCGAGCG
CONSENSUS
ACAACGAGGATACCGTGATTTTAAAGATGTGAAGAAGAAAGAGTCGAGCG

A3-AT1G65540-XLOC_007309-7882-0
TGTTAATGGAGGGGTTCTATAGAAGTAGATTACTTCAGTCTCCAAGTTTT
A3-AT1G65540-XLOC_007309-7882-1
TGTTAATGGAGGGGTTCTATAGAAGTAGATTACTTCAGTCTCCAAGTTTT
CONSENSUS
TGTTAATGGAGGGGTTCTATAGAAGTAGATTACTTCAGTCTCCAAGTTTT

A3-AT1G65540-XLOC_007309-7882-0
AGTAATGGAGTTGGAATATTAGAATTGCCTTATCCTTTGGGATATAGATT

A3-AT1G65540-XLOC_007309-7882-1
AGTAATGGAGTTGGAATATTAGAATTGCCTTATCCTTTGGGATATAGATT
CONSENSUS
AGTAATGGAGTTGGAATATTAGAATTGCCTTATCCTTTGGGATATAGATT

A3-AT1G65540-XLOC_007309-7882-0
GGTTTTTCAATCTATGTACTCGTCTTTGGCAACGGCTAATAAGCCTGACC
A3-AT1G65540-XLOC_007309-7882-1
GGTTTTTCAATCTATGTACTCGTCTTTGGCAACGGCTAATAAGCCTGACC
CONSENSUS
GGTTTTTCAATCTATGTACTCGTCTTTGGCAACGGCTAATAAGCCTGACC

A3-AT1G65540-XLOC_007309-7882-0
ATGATAAAAAGGGAGAAAAGGTTACCTCGCAAACTAAAGAAGCTTCTCCT
A3-AT1G65540-XLOC_007309-7882-1
ATGATAAAAAGGGAGAAAAGGTTACCTCGCAAACTAAAGAAGCTTCTCCT
CONSENSUS
ATGATAAAAAGGGAGAAAAGGTTACCTCGCAAACTAAAGAAGCTTCTCCT

A3-AT1G65540-XLOC_007309-7882-0
GAGGAGTGTGATGAGGCTGTTGAGGGGTTAAGTTTAGCTAAGGCTAAAGC
A3-AT1G65540-XLOC_007309-7882-1
GAGGAGTGTGATGAGGCTGTTGAGGGGTTAAGTTTAGCTAAGGCTAAAGC
CONSENSUS
GAGGAGTGTGATGAGGCTGTTGAGGGGTTAAGTTTAGCTAAGGCTAAAGC

A3-AT1G65540-XLOC_007309-7882-0
TAAAGCTATGAAATTGGAAGAATCACAGAAATCTGATATATCCATTATGC
A3-AT1G65540-XLOC_007309-7882-1
TAAAGCTATGAAATTGGAAGAATCACAGAAATCTGATATATCCATTATGC
CONSENSUS
TAAAGCTATGAAATTGGAAGAATCACAGAAATCTGATATATCCATTATGC

A3-AT1G65540-XLOC_007309-7882-0
AACGTGTGCGGTTGTTTCTTCTGGGGATAGGTCCTGCTTTGAGAGCTATT
A3-AT1G65540-XLOC_007309-7882-1
AACGTGTGCGGTTGTTTCTTCTGGGGATAGGTCCTGCTTTGAGAGCTATT
CONSENSUS
AACGTGTGCGGTTGTTTCTTCTGGGGATAGGTCCTGCTTTGAGAGCTATT

A3-AT1G65540-XLOC_007309-7882-0 GCATCAATGAGCAG
A3-AT1G65540-XLOC_007309-7882-1 GCATCAATGAGCAG
CONSENSUS GCATCAATGAGCAG

alignment for event: SE-AT1G07350-XLOC_004507-10753

SE-AT1G07350-XLOC_004507-10753-0
GTAACTGATGTTCACCTTGTCCTGGACCCATGGACTAGAGAATCTCGCGG
SE-AT1G07350-XLOC_004507-10753-1
GTAACTGATGTTCACCTTGTCCTGGACCCATGGACTAGAGAATCTCGCGG
CONSENSUS
GTAACTGATGTTCACCTTGTCCTGGACCCATGGACTAGAGAATCTCGCGG

SE-AT1G07350-XLOC_004507-10753-0

ATTTGGTTTTATCTCTATGAAAAGTGTTGGTGATGCTAACCGTTGCATCA
SE-AT1G07350-XLOC_004507-10753-1
ATTTGGTTTTATCTCTATGAAAAGTGTTGGTGATGCTAACCGTTGCATCA
CONSENSUS
ATTTGGTTTTATCTCTATGAAAAGTGTTGGTGATGCTAACCGTTGCATCA

SE-AT1G07350-XLOC_004507-10753-0
GATCTCTAGATCACTCTGTTCTGCAGGGCCGCGTCATCACTGTTGAGAAG
SE-AT1G07350-XLOC_004507-10753-1
GATCTCTAGATCACTCTGTTCTGCAGGGCCGCGTCATCACTGTTGAGAAG
CONSENSUS
GATCTCTAGATCACTCTGTTCTGCAGGGCCGCGTCATCACTGTTGAGAAG

SE-AT1G07350-XLOC_004507-10753-0

SE-AT1G07350-XLOC_004507-10753-1
TTTCTGTGGCAGCAGGTCTGCTGTTTGTAGCAGCAGTGCTTCACCAAATA
CONSENSUS
....

SE-AT1G07350-XLOC_004507-10753-0
--GCAA
SE-AT1G07350-XLOC_004507-10753-1
GCAAACGTTGCAACAGTTCAAACACATCAAGTTTCAGACATTTAGGGCAA
CONSENSUS
....GCAA

SE-AT1G07350-XLOC_004507-10753-0
GACGTCGTAGAGGACGTACTCCAACTCCAGGAAAGTACTTGGGGCTGAGA
SE-AT1G07350-XLOC_004507-10753-1
GACGTCGTAGAGGACGTACTCCAACTCCAGGAAAGTACTTGGGGCTGAGA
CONSENSUS
GACGTCGTAGAGGACGTACTCCAACTCCAGGAAAGTACTTGGGGCTGAGA

SE-AT1G07350-XLOC_004507-10753-0 ACTGCTCGAG
SE-AT1G07350-XLOC_004507-10753-1 ACTGCTCGAG
CONSENSUS ACTGCTCGAG

alignment for event: A3-AT1G19396-XLOC_001012-4564

A3-AT1G19396-XLOC_001012-4564-0
TGTTCGGTATCCTTTTTTTTTAGTTGCGATGATTGTTTAGCTGAGTTTCT
A3-AT1G19396-XLOC_001012-4564-1
TGTTCGGTATCCTTTTTTTTTAGTTGCGATGATTGTTTAGCTGAGTTTCT
CONSENSUS
TGTTCGGTATCCTTTTTTTTTAGTTGCGATGATTGTTTAGCTGAGTTTCT

A3-AT1G19396-XLOC_001012-4564-0
TATTGCTTATCTATATATTATGTATCAAATCTCCTTGTTTGATCTATGAT
A3-AT1G19396-XLOC_001012-4564-1
TATTGCTTATCTATATATTATGTATCAAATCTCCTTGTTTGATCTATGAT
CONSENSUS
TATTGCTTATCTATATATTATGTATCAAATCTCCTTGTTTGATCTATGAT

A3-AT1G19396-XLOC_001012-4564-0
      CATCTTTCTAGGGTTTTGCTCGATTTCCCGGATTCGGGTGGGAAATCATC
A3-AT1G19396-XLOC_001012-4564-1
      CATCTTTCTAGGGTTTTGCTCGATTTCCCGGATTCGGGTGGGAAATCATC
CONSENSUS
      CATCTTTCTAGGGTTTTGCTCGATTTCCCGGATTCGGGTGGGAAATCATC

A3-AT1G19396-XLOC_001012-4564-0
      ATCTGATCGATTATCGCGGCTGGACATGGAGGGATTTATACGGACAAAAC
A3-AT1G19396-XLOC_001012-4564-1
      ATCTGATCGATTATCGCGGCTGGACATGGAG-------------------
CONSENSUS
      ATCTGATCGATTATCGCGGCTGGACATGGAG...................

A3-AT1G19396-XLOC_001012-4564-0
      AACATCTCGTTGATCTTAACTAATCAACATCTCGTTGATCTCTATCTTAT
A3-AT1G19396-XLOC_001012-4564-1

CONSENSUS
      ....

A3-AT1G19396-XLOC_001012-4564-0
      CAACTCCTCGTTGATCTCTATCTTATCAACTCCTCGTTGATCTAACTGAT
A3-AT1G19396-XLOC_001012-4564-1

CONSENSUS
      ....

A3-AT1G19396-XLOC_001012-4564-0
      CAATTCTCGTTGATCTCTCGAATCAACATCTCGTTGATCTAACTGATCAA
A3-AT1G19396-XLOC_001012-4564-1

CONSENSUS
      ....

A3-AT1G19396-XLOC_001012-4564-0
      TTCTCGTTGATCTCTCGAATCAATCCCTCGTTGATCTTGTTAAATCAATT
A3-AT1G19396-XLOC_001012-4564-1

CONSENSUS
      ....

A3-AT1G19396-XLOC_001012-4564-0
      CGTTGCTCAGTAATTAGAATTCTGTGTACGCATGAGAAAAGAAAGGATTT
A3-AT1G19396-XLOC_001012-4564-1

CONSENSUS
      ....

A3-AT1G19396-XLOC_001012-4564-0
      TGGTCCGGTTATTTTCATGTTTCTAATTGCGATTAATTTATGGCTGTTGC
A3-AT1G19396-XLOC_001012-4564-1

CONSENSUS
      ....

A3-AT1G19396-XLOC_001012-4564-0
TCTCTTTTATTAACCTTTGAATCTCCTTATCTAGGGGAAAAAAACAACAT
A3-AT1G19396-XLOC_001012-4564-1

CONSENSUS
....

A3-AT1G19396-XLOC_001012-4564-0
CTGATCAACTCTCATTGATCCAATCATCAACTCTCGTTGATCATAGCCAC
A3-AT1G19396-XLOC_001012-4564-1

CONSENSUS
....

A3-AT1G19396-XLOC_001012-4564-0
TTATCATCAACTCTCGTTGATCATAACCACTTATCATCAACGCTTGTTGA
A3-AT1G19396-XLOC_001012-4564-1

CONSENSUS
....

A3-AT1G19396-XLOC_001012-4564-0
TCCAACCATCAACGCTCGTTGATCCAACCATCAACGCTCGTTGATCCAAC
A3-AT1G19396-XLOC_001012-4564-1

CONSENSUS
....

A3-AT1G19396-XLOC_001012-4564-0
CATCAACGCTCGTTGATCCAACCATCAACGCTCGTTGATCCAACCATCAA
A3-AT1G19396-XLOC_001012-4564-1

CONSENSUS
....

A3-AT1G19396-XLOC_001012-4564-0
CGCTCGTTGATCCAACCATCAACGCTCGTTGATCCAACCATCAACGCTCG
A3-AT1G19396-XLOC_001012-4564-1

CONSENSUS
....

A3-AT1G19396-XLOC_001012-4564-0
TTGATCCAACCATCAACGCTCGTTGATCCAACCATCAACGCTCGTTGATC
A3-AT1G19396-XLOC_001012-4564-1

CONSENSUS
....

A3-AT1G19396-XLOC_001012-4564-0
CAACCATCAACGCTCGTTGATCCAACCATCAACGCTCGTTGATCCAATCA
A3-AT1G19396-XLOC_001012-4564-1

CONSENSUS
....

A3-AT1G19396-XLOC_001012-4564-0
ACTCTCGTTGATCCAATCAACTCTCGTTGATCCAATCAACCCCTCGTTGA
A3-AT1G19396-XLOC_001012-4564-1

CONSENSUS
....

A3-AT1G19396-XLOC_001012-4564-0
TCATAACCTCTAATCATCAACGCTCGTTGATCCAGTTATCAACTCTCGTT
A3-AT1G19396-XLOC_001012-4564-1

CONSENSUS
....

A3-AT1G19396-XLOC_001012-4564-0
GATTTAAAAGAGAAGGAACCTTGATTTTAGTTAGTGTTATCTATCTCCAT
A3-AT1G19396-XLOC_001012-4564-1

CONSENSUS
....

A3-AT1G19396-XLOC_001012-4564-0
AGATTTTAACACTTGTACAGGTTTTTTGTTTGAGCTTTCAAACTGAAAAG
A3-AT1G19396-XLOC_001012-4564-1

CONSENSUS
....

A3-AT1G19396-XLOC_001012-4564-0
AAACAGATGTACCAAAAACACAGAGAGGGGATTTTGTACATGACTTTTGC
A3-AT1G19396-XLOC_001012-4564-1

CONSENSUS
....

A3-AT1G19396-XLOC_001012-4564-0
CATGACAGGTGCGCTTGCTTGGCAGGTCACAAACCCTTTAATAAAAAGAT
A3-AT1G19396-XLOC_001012-4564-1

CONSENSUS
....

A3-AT1G19396-XLOC_001012-4564-0
ACAGTTTTTGGTTTCGTGGAGGGATGAGGGAAGGTACGGGGTTGGTTAAT
A3-AT1G19396-XLOC_001012-4564-1

CONSENSUS
....

A3-AT1G19396-XLOC_001012-4564-0
AAACTTTCTTTGCCCGGCTCTCTCTCCCTCCTGAATGTGTGTCTACTGTC
A3-AT1G19396-XLOC_001012-4564-1

CONSENSUS
....

A3-AT1G19396-XLOC_001012-4564-0
      TTGTTTCATATGCTTCTTGCATACTACACAGACCACAATTATTGAGGCAT
A3-AT1G19396-XLOC_001012-4564-1

CONSENSUS
      ....

A3-AT1G19396-XLOC_001012-4564-0
      CACTTGTGCTCTTTAATATGCTTCTTGCATGTGCTATGATCGAAGAACAT
A3-AT1G19396-XLOC_001012-4564-1

CONSENSUS
      ....

A3-AT1G19396-XLOC_001012-4564-0
      TGAGTTTCTAATGACCAAGGTATTGATTATGTTTTCTTATCCTGTTGAGA
A3-AT1G19396-XLOC_001012-4564-1

CONSENSUS
      ....

A3-AT1G19396-XLOC_001012-4564-0
      TGTTTAATCTGTTTGCTCATATTTTTTCAGAAACAGAGTGAGCAGGGTAC
A3-AT1G19396-XLOC_001012-4564-1 ------------------------------
AAACAGAGTGAGCAGGGTAC
CONSENSUS
      ..............................AAACAGAGTGAGCAGGGTAC

A3-AT1G19396-XLOC_001012-4564-0
      GTTATAGTCTCATTCAAAGTAGGACAATGGAATATAGTCTAACCTGAAGA
A3-AT1G19396-XLOC_001012-4564-1
      GTTATAGTCTCATTCAAAGTAGGACAATGGAATATAGTCTAACCTGAAGA
CONSENSUS
      GTTATAGTCTCATTCAAAGTAGGACAATGGAATATAGTCTAACCTGAAGA

A3-AT1G19396-XLOC_001012-4564-0
      ATTGTTAGGAGGTTGGTCCCTGTGGAATTGCGCACGGATTGTTG
A3-AT1G19396-XLOC_001012-4564-1
      ATTGTTAGGAGGTTGGTCCCTGTGGAATTGCGCACGGATTGTTG
CONSENSUS
      ATTGTTAGGAGGTTGGTCCCTGTGGAATTGCGCACGGATTGTTG

alignment for event: A3-AT1G70610-XLOC_003612-9585

A3-AT1G70610-XLOC_003612-9585-0
      GTTACAGATGAATTATTTGATGAAATTGCAACCAAAGTCATTAACGAAGA
A3-AT1G70610-XLOC_003612-9585-1
      GTTACAGATGAATTATTTGATGAAATTGCAACCAAAGTCATTAACGAAGA
CONSENSUS
      GTTACAGATGAATTATTTGATGAAATTGCAACCAAAGTCATTAACGAAGA

A3-AT1G70610-XLOC_003612-9585-0
      TGAAGCAATACCTAAAGATGATAGTGTACAGCATAATCATAAATTGTCAT
A3-AT1G70610-XLOC_003612-9585-1

TGAAGCAATACCTAAAG---------------------------------
CONSENSUS
TGAAGCAATACCTAAAG.................................

A3-AT1G70610-XLOC_003612-9585-0
CATCCCTTCTCTCCACAGCTGATCCACTCCACAAGGCTTCGGCAAAGATT
A3-AT1G70610-XLOC_003612-9585-1 ------------------
CTGATCCACTCCACAAGGCTTCGGCAAAGATT
CONSENSUS
..................CTGATCCACTCCACAAGGCTTCGGCAAAGATT

A3-AT1G70610-XLOC_003612-9585-0
TTGGTCTCGGTAGAAGGTGCAAATACAAAAGCTAGTTCTGGCTCTCCAGC
A3-AT1G70610-XLOC_003612-9585-1
TTGGTCTCGGTAGAAGGTGCAAATACAAAAGCTAGTTCTGGCTCTCCAGC
CONSENSUS
TTGGTCTCGGTAGAAGGTGCAAATACAAAAGCTAGTTCTGGCTCTCCAGC

A3-AT1G70610-XLOC_003612-9585-0
AGATGTGCTAGGTCTTGCTAGCTATGCCTCTGATGATGATGATGCTGATA
A3-AT1G70610-XLOC_003612-9585-1
AGATGTGCTAGGTCTTGCTAGCTATGCCTCTGATGATGATGATGCTGATA
CONSENSUS
AGATGTGCTAGGTCTTGCTAGCTATGCCTCTGATGATGATGATGCTGATA

A3-AT1G70610-XLOC_003612-9585-0
CTGATGCTGCTTCTGACGCGAATGCTGATGAAAATGGAGTGGAGAGTCTT
A3-AT1G70610-XLOC_003612-9585-1
CTGATGCTGCTTCTGACGCGAATGCTGATGAAAATGGAGTGGAGAGTCTT
CONSENSUS
CTGATGCTGCTTCTGACGCGAATGCTGATGAAAATGGAGTGGAGAGTCTT

A3-AT1G70610-XLOC_003612-9585-0
GGTGTGGGGTCAAGACACAATGTTAGTCAGCAGCCAAGCACTGAGAAACT
A3-AT1G70610-XLOC_003612-9585-1
GGTGTGGGGTCAAGACACAATGTTAGTCAGCAGCCAAGCACTGAGAAACT
CONSENSUS
GGTGTGGGGTCAAGACACAATGTTAGTCAGCAGCCAAGCACTGAGAAACT

A3-AT1G70610-XLOC_003612-9585-0
TCCTGACCCTGAAGCAATGGCCAGTGCGAAATTGGATCCAGCAGTTGGAG
A3-AT1G70610-XLOC_003612-9585-1
TCCTGACCCTGAAGCAATGGCCAGTGCGAAATTGGATCCAGCAGTTGGAG
CONSENSUS
TCCTGACCCTGAAGCAATGGCCAGTGCGAAATTGGATCCAGCAGTTGGAG

A3-AT1G70610-XLOC_003612-9585-0
TCAATGCTAATTCTGGCAAGAATAGTAAGTCAGGCTTGGAGGATTATTCT
A3-AT1G70610-XLOC_003612-9585-1
TCAATGCTAATTCTGGCAAGAATAGTAAGTCAGGCTTGGAGGATTATTCT
CONSENSUS
TCAATGCTAATTCTGGCAAGAATAGTAAGTCAGGCTTGGAGGATTATTCT

A3-AT1G70610-XLOC_003612-9585-0
CAGATGCCAGGCTCCACGAGAAAAGATGATGAGGCGGGTAGTACCAAAAT
A3-AT1G70610-XLOC_003612-9585-1

CAGATGCCAGGCTCCACGAGAAAAGATGATGAGGCGGGTAGTACCAAAAT
CONSENSUS
CAGATGCCAGGCTCCACGAGAAAAGATGATGAGGCGGGTAGTACCAAAAT

A3-AT1G70610-XLOC_003612-9585-0
ATCTGATGTAAGCGCCAGCTCTGGACTTGATGATGATACTTCAGGAAGCA
A3-AT1G70610-XLOC_003612-9585-1
ATCTGATGTAAGCGCCAGCTCTGGACTTGATGATGATACTTCAGGAAGCA
CONSENSUS
ATCTGATGTAAGCGCCAGCTCTGGACTTGATGATGATACTTCAGGAAGCA

A3-AT1G70610-XLOC_003612-9585-0
GAAAAGAGCATCCTGACAGAACTGATAGTGATAAAGATGCCATACTAGAT
A3-AT1G70610-XLOC_003612-9585-1
GAAAAGAGCATCCTGACAGAACTGATAGTGATAAAGATGCCATACTAGAT
CONSENSUS
GAAAAGAGCATCCTGACAGAACTGATAGTGATAAAGATGCCATACTAGAT

A3-AT1G70610-XLOC_003612-9585-0
GAACCTCACGTGAAGAATTCTGGCGTGAAATCAGATTGCAACCTTCGTCA
A3-AT1G70610-XLOC_003612-9585-1
GAACCTCACGTGAAGAATTCTGGCGTGAAATCAGATTGCAACCTTCGTCA
CONSENSUS
GAACCTCACGTGAAGAATTCTGGCGTGAAATCAGATTGCAACCTTCGTCA

A3-AT1G70610-XLOC_003612-9585-0
GGATAGTAATAAACCTTATGGGAAAGATTTGAGTGACGAAGTGAGTACAG
A3-AT1G70610-XLOC_003612-9585-1
GGATAGTAATAAACCTTATGGGAAAGATTTGAGTGACGAAGTGAGTACAG
CONSENSUS
GGATAGTAATAAACCTTATGGGAAAGATTTGAGTGACGAAGTGAGTACAG

A3-AT1G70610-XLOC_003612-9585-0
ATAGAAGTAGAATAGTTGAAACGAAAGGTGGGAAAGAGAAAGGAGATTCT
A3-AT1G70610-XLOC_003612-9585-1
ATAGAAGTAGAATAGTTGAAACGAAAGGTGGGAAAGAGAAAGGAGATTCT
CONSENSUS
ATAGAAGTAGAATAGTTGAAACGAAAGGTGGGAAAGAGAAAGGAGATTCT

A3-AT1G70610-XLOC_003612-9585-0
CAGAATGACTCAAAAGATAGAATGAAGGAAAATGACTTAAAGTCAGCAGA
A3-AT1G70610-XLOC_003612-9585-1
CAGAATGACTCAAAAGATAGAATGAAGGAAAATGACTTAAAGTCAGCAGA
CONSENSUS
CAGAATGACTCAAAAGATAGAATGAAGGAAAATGACTTAAAGTCAGCAGA

A3-AT1G70610-XLOC_003612-9585-0
GAAAGTTAAAGGCGTTGAATCAAATAAAAAATCTACTGATCCCCATGTAA
A3-AT1G70610-XLOC_003612-9585-1
GAAAGTTAAAGGCGTTGAATCAAATAAAAAATCTACTGATCCCCATGTAA
CONSENSUS
GAAAGTTAAAGGCGTTGAATCAAATAAAAAATCTACTGATCCCCATGTAA

A3-AT1G70610-XLOC_003612-9585-0
AGAAAGACTCAAGGGATGTAGAGAGGCCTCACAGAACTAATTCTAAGGAA
A3-AT1G70610-XLOC_003612-9585-1

AGAAAGACTCAAGGGATGTAGAGAGGCCTCACAGAACTAATTCTAAGGAA
CONSENSUS
AGAAAGACTCAAGGGATGTAGAGAGGCCTCACAGAACTAATTCTAAGGAA

A3-AT1G70610-XLOC_003612-9585-0
GACCGGGGTAAAAGAAAAGAGAAGGAAAAGGAAGAAGAAAGGTCAAGACA
A3-AT1G70610-XLOC_003612-9585-1
GACCGGGGTAAAAGAAAAGAGAAGGAAAAGGAAGAAGAAAGGTCAAGACA
CONSENSUS
GACCGGGGTAAAAGAAAAGAGAAGGAAAAGGAAGAAGAAAGGTCAAGACA

A3-AT1G70610-XLOC_003612-9585-0
CAGGCGGGCTGAAAACTCGAGCAAGGACAAAAGAAGACGTTCTCCAACCA
A3-AT1G70610-XLOC_003612-9585-1
CAGGCGGGCTGAAAACTCGAGCAAGGACAAAAGAAGACGTTCTCCAACCA
CONSENSUS
CAGGCGGGCTGAAAACTCGAGCAAGGACAAAAGAAGACGTTCTCCAACCA

A3-AT1G70610-XLOC_003612-9585-0 GTAATGAATCTTCTGATGATTCGAAGAG
A3-AT1G70610-XLOC_003612-9585-1 GTAATGAATCTTCTGATGATTCGAAGAG
CONSENSUS GTAATGAATCTTCTGATGATTCGAAGAG

alignment for event: A5-AT1G01040-XLOC_000002-677

A5-AT1G01040-XLOC_000002-677-0
GGAGAATGGGTTTCATCTGGAAAGGAAGTTTGTGAGAGCTCAAAGCTATT
A5-AT1G01040-XLOC_000002-677-1
GGAGAATGGGTTTCATCTGGAAAGGAAGTTTGTGAGAGCTCAAAGCTATT
CONSENSUS
GGAGAATGGGTTTCATCTGGAAAGGAAGTTTGTGAGAGCTCAAAGCTATT

A5-AT1G01040-XLOC_000002-677-0
CCATTTATACATGTATAATGTCAGATGTGTAGATTTTGGCTCTTCAAAAG
A5-AT1G01040-XLOC_000002-677-1
CCATTTATACATGTATAATGTCAGATGTGTAGATTTTGGCTCTTCAAAAG
CONSENSUS
CCATTTATACATGTATAATGTCAGATGTGTAGATTTTGGCTCTTCAAAAG

A5-AT1G01040-XLOC_000002-677-0
ATCCATTCCTAAGCGAAGTTTCAGAGTTCGCGATTCTTTTTGGCAATGAG
A5-AT1G01040-XLOC_000002-677-1
ATCCATTCCTAAGCGAAGTTTCAGAGTTCGCGATTCTTTTTGGCAATGAG
CONSENSUS
ATCCATTCCTAAGCGAAGTTTCAGAGTTCGCGATTCTTTTTGGCAATGAG

A5-AT1G01040-XLOC_000002-677-0 CTGGATGCAGAG---
GTATTATCGATGTCTATGGATCTTTATGTTGCTCG
A5-AT1G01040-XLOC_000002-677-1
CTGGATGCAGAGGTTGTATTATCGATGTCTATGGATCTTTATGTTGCTCG
CONSENSUS
CTGGATGCAGAG...GTATTATCGATGTCTATGGATCTTTATGTTGCTCG

A5-AT1G01040-XLOC_000002-677-0
GGCCATGATCACTAAAGCATCTCTTGCTTTCAAGGGATCACTTGATATTA

A5-AT1G01040-XLOC_000002-677-1
      GGCCATGATCACTAAAGCATCTCTTGCTTTCAAGGGATCACTTGATATTA
CONSENSUS
      GGCCATGATCACTAAAGCATCTCTTGCTTTCAAGGGATCACTTGATATTA

A5-AT1G01040-XLOC_000002-677-0   CAGAAAACCAG
A5-AT1G01040-XLOC_000002-677-1   CAGAAAACCAG
CONSENSUS   CAGAAAACCAG

alignment for event: A3-AT1G52000-XLOC_006610-4604

A3-AT1G52000-XLOC_006610-4604-0
      TTGCAATTTACTCTCCTCCAATCATCCTCCACTCAG---ATATCAAACGG
A3-AT1G52000-XLOC_006610-4604-1
      TTGCAATTTACTCTCCTCCAATCATCCTCCACTCAGAAGATATCAAACGG
CONSENSUS
      TTGCAATTTACTCTCCTCCAATCATCCTCCACTCAG...ATATCAAACGG

A3-AT1G52000-XLOC_006610-4604-0
      AATTCAACGGCCACGATGTCTTGGGATGACGGAAAACACACGAAGGTGAA
A3-AT1G52000-XLOC_006610-4604-1
      AATTCAACGGCCACGATGTCTTGGGATGACGGAAAACACACGAAGGTGAA
CONSENSUS
      AATTCAACGGCCACGATGTCTTGGGATGACGGAAAACACACGAAGGTGAA

A3-AT1G52000-XLOC_006610-4604-0
      GAGAGTTCAGCTTACGTTCGATGATGTCATCCGATCTATCGAGGTCGAAT
A3-AT1G52000-XLOC_006610-4604-1
      GAGAGTTCAGCTTACGTTCGATGATGTCATCCGATCTATCGAGGTCGAAT
CONSENSUS
      GAGAGTTCAGCTTACGTTCGATGATGTCATCCGATCTATCGAGGTCGAAT

A3-AT1G52000-XLOC_006610-4604-0
      ACGACGGAACCTCCCTTAAGTCCCAGCCACGTGGCACCGCTGGCACCAAA
A3-AT1G52000-XLOC_006610-4604-1
      ACGACGGAACCTCCCTTAAGTCCCAGCCACGTGGCACCGCTGGCACCAAA
CONSENSUS
      ACGACGGAACCTCCCTTAAGTCCCAGCCACGTGGCACCGCTGGCACCAAA

A3-AT1G52000-XLOC_006610-4604-0 ATTGACGGA
A3-AT1G52000-XLOC_006610-4604-1 ATTGACGGA
CONSENSUS   ATTGACGGA

alignment for event: A3-AT1G50300-XLOC_006519-1346

A3-AT1G50300-XLOC_006519-1346-0
      GTATTGATAAAGATGGAAGAGAGAGAAGCAGAGACAGGCAAAGAGACCGT
A3-AT1G50300-XLOC_006519-1346-1
      GTATTGATAAAGATGGAAGAGAGAGAAGCAGAGACAGGCAAAGAGACCGT
CONSENSUS
      GTATTGATAAAGATGGAAGAGAGAGAAGCAGAGACAGGCAAAGAGACCGT

A3-AT1G50300-XLOC_006519-1346-0
GGTAGAGATCATCACTACGATAAGGATAGACGCAGAAGCAGAAGCCGAGA
A3-AT1G50300-XLOC_006519-1346-1
GGTAGAGATCATCACTACGATAAGGATAGACGCAGAAGCAGAAGCCGAGA
CONSENSUS
GGTAGAGATCATCACTACGATAAGGATAGACGCAGAAGCAGAAGCCGAGA

A3-AT1G50300-XLOC_006519-1346-0
GAGGGAAAGAGGCAAGGAGCGTGACTATGACTATGACCATGATCGGGACA
A3-AT1G50300-XLOC_006519-1346-1
GAGGGAAAGAGGCAAGGAGCGTGACTATGACTATGACCATGATCGGGACA
CONSENSUS
GAGGGAAAGAGGCAAGGAGCGTGACTATGACTATGACCATGATCGGGACA

A3-AT1G50300-XLOC_006519-1346-0
GAGACAGAGACTACGGTCGCGAACGTGGAAGCAG----------------
A3-AT1G50300-XLOC_006519-1346-1
GAGACAGAGACTACGGTCGCGAACGTGGAAGCAGTTTACCAGAATAATCC
CONSENSUS
GAGACAGAGACTACGGTCGCGAACGTGGAAGCAG................

A3-AT1G50300-XLOC_006519-1346-0

A3-AT1G50300-XLOC_006519-1346-1
GGTCGACAGAATTCAACAGTTCCTCTCTCTAAAAGATATGAAGGAGATTA
CONSENSUS
....

A3-AT1G50300-XLOC_006519-1346-0

A3-AT1G50300-XLOC_006519-1346-1
ATTTTGGCTTTGTTAGCAAAGATGGCCGGTCCTCTTCTGGCAAATCCGGC
CONSENSUS
....

A3-AT1G50300-XLOC_006519-1346-0

A3-AT1G50300-XLOC_006519-1346-1
TTTTACAAGCGGTTTTGGCCGGCTTTTATCCTTCCTCCGCCGTTCGATCG
CONSENSUS
....

A3-AT1G50300-XLOC_006519-1346-0

A3-AT1G50300-XLOC_006519-1346-1
GTTGTTCTTACCGATTGCTTTAGTTTCTTTTTCAGTTTTTCTTTCAATAA
CONSENSUS
....

A3-AT1G50300-XLOC_006519-1346-0

A3-AT1G50300-XLOC_006519-1346-1
TTTCTAAAGATTTCAATTGGAGATTAATTTTAGGTCATAATAGCCTAAGA
CONSENSUS
....

A3-AT1G50300-XLOC_006519-1346-0

A3-AT1G50300-XLOC_006519-1346-1
      TTAAGTTTTCGGATTTGTATCTTTAGAAAGTTTGATTTGCAGAATTACCT
CONSENSUS
      ....

A3-AT1G50300-XLOC_006519-1346-0

A3-AT1G50300-XLOC_006519-1346-1
      CAAGTATGAAAGGAAGTTTTGTTTCCAGCATATTGGGATTGCCTGTAGCC
CONSENSUS
      ....

A3-AT1G50300-XLOC_006519-1346-0

A3-AT1G50300-XLOC_006519-1346-1
      GTTTCTACATGTTTATGGAGCTTCGATCGATCAAATTTGAGGAATACTCA
CONSENSUS
      ....

A3-AT1G50300-XLOC_006519-1346-0

A3-AT1G50300-XLOC_006519-1346-1
      GATTTTTCTTTCCTCATTACCTGTTTTTGCCAAATCTCTTGATTGATGAT
CONSENSUS
      ....

A3-AT1G50300-XLOC_006519-1346-0
      --CAGT
A3-AT1G50300-XLOC_006519-1346-1
      TTTGGAATTGAATTCTCTGTTTGTGTATCCTCCCTTCTCTGTTTAGCAGT
CONSENSUS
      ....CAGT

A3-AT1G50300-XLOC_006519-1346-0
      TTCCGGTGAGAAAAAAACAAAAATCGATGTTTGCCCTTTTTCGACGTTGA
A3-AT1G50300-XLOC_006519-1346-1
      TTCCGGTGAGAAAAAAACAAAAATCGATGTTTGCCCTTTTTCGACGTTGA
CONSENSUS
      TTCCGGTGAGAAAAAAACAAAAATCGATGTTTGCCCTTTTTCGACGTTGA

A3-AT1G50300-XLOC_006519-1346-0
      TTGTGTTCACCGGATCGGAGTCTTTGACGGCACAGGATGACAGGAACGTT
A3-AT1G50300-XLOC_006519-1346-1
      TTGTGTTCACCGGATCGGAGTCTTTGACGGCACAGGATGACAGGAACGTT
CONSENSUS
      TTGTGTTCACCGGATCGGAGTCTTTGACGGCACAGGATGACAGGAACGTT

A3-AT1G50300-XLOC_006519-1346-0
      TCATCGCTTCTCTTGCAACACGTGTCTTGTTATACGATCGTGTTTTGTTA
A3-AT1G50300-XLOC_006519-1346-1
      TCATCGCTTCTCTTGCAACACGTGTCTTGTTATACGATCGTGTTTTGTTA
CONSENSUS
      TCATCGCTTCTCTTGCAACACGTGTCTTGTTATACGATCGTGTTTTGTTA

A3-AT1G50300-XLOC_006519-1346-0
      AAGAGATATCCTTCCTTTAAAGAAATGTAACTAAGTGGACCTTTGTTGGG
A3-AT1G50300-XLOC_006519-1346-1
      AAGAGATATCCTTCCTTTAAAGAAATGTAACTAAGTGGACCTTTGTTGGG
CONSENSUS
      AAGAGATATCCTTCCTTTAAAGAAATGTAACTAAGTGGACCTTTGTTGGG

A3-AT1G50300-XLOC_006519-1346-0
      CTGTATTCTGTGGATCATTAATAAACATAATCTTTCCAGTG
A3-AT1G50300-XLOC_006519-1346-1
      CTGTATTCTGTGGATCATTAATAAACATAATCTTTCCAGTG
CONSENSUS
      CTGTATTCTGTGGATCATTAATAAACATAATCTTTCCAGTG

alignment for event: RI-AT1G69250-XLOC_003532-4052

RI-AT1G69250-XLOC_003532-4052-0
      GCACTTCGATATTTGTTGCAAATCTGCCATTGAATGCAATGCCGCCTCAA
RI-AT1G69250-XLOC_003532-4052-1
      GCACTTCGATATTTGTTGCAAATCTGCCATTGAATGCAATGCCGCCTCAA
CONSENSUS
      GCACTTCGATATTTGTTGCAAATCTGCCATTGAATGCAATGCCGCCTCAA

RI-AT1G69250-XLOC_003532-4052-0
      CTCTTTGAACTGTTCAAGGATTTTGGTCCTATCAAAGAAAACGGGATTCA
RI-AT1G69250-XLOC_003532-4052-1
      CTCTTTGAACTGTTCAAGGATTTTGGTCCTATCAAAGAAAACGGGATTCA
CONSENSUS
      CTCTTTGAACTGTTCAAGGATTTTGGTCCTATCAAAGAAAACGGGATTCA

RI-AT1G69250-XLOC_003532-4052-0
      AGTCCGAAGCTCCAGGGTTTTAATCTTTGACCTATCATGTCTTTGTGCTA
RI-AT1G69250-XLOC_003532-4052-1
      AGTCCGAAGCTCCAGG----------------------------------
CONSENSUS
      AGTCCGAAGCTCCAGG..................................

RI-AT1G69250-XLOC_003532-4052-0
      TTTCTTTACTCATTACATGTCCTACTCTCGTTACACGAATTCCACACTTT
RI-AT1G69250-XLOC_003532-4052-1

CONSENSUS
      ....

RI-AT1G69250-XLOC_003532-4052-0
      TCTTATGTGTCCAGGGTAATGCTAATCCAGTTTGCTTCGGGTTTATCTCC
RI-AT1G69250-XLOC_003532-4052-1 --------------
GGTAATGCTAATCCAGTTTGCTTCGGGTTTATCTCC
CONSENSUS
      ..............GGTAATGCTAATCCAGTTTGCTTCGGGTTTATCTCC

RI-AT1G69250-XLOC_003532-4052-0
      TTTGAAACTGTTGCCTCAGTCCAGAGCGTGCTTCAG
RI-AT1G69250-XLOC_003532-4052-1

TTTGAAACTGTTGCCTCAGTCCAGAGCGTGCTTCAG
CONSENSUS
TTTGAAACTGTTGCCTCAGTCCAGAGCGTGCTTCAG

alignment for event: A3-AT1G62130-XLOC_007128-13595

A3-AT1G62130-XLOC_007128-13595-0
GGTGAGGAACTTCTTAAATCTTGGAAGTATCATTTGGATCGTGATGCTGA
A3-AT1G62130-XLOC_007128-13595-1
GGTGAGGAACTTCTTAAATCTTGGAAGTATCATTTGGATCGTGATGCTGA
CONSENSUS
GGTGAGGAACTTCTTAAATCTTGGAAGTATCATTTGGATCGTGATGCTGA

A3-AT1G62130-XLOC_007128-13595-0
GACTTTGAAAATGAAGGCTAATTACAATCACTTGCGCATG----------
A3-AT1G62130-XLOC_007128-13595-1
GACTTTGAAAATGAAGGCTAATTACAATCACTTGCGCATGGGGAATACAG
CONSENSUS
GACTTTGAAAATGAAGGCTAATTACAATCACTTGCGCATG..........

A3-AT1G62130-XLOC_007128-13595-0
--GTGTTGGGACG
A3-AT1G62130-XLOC_007128-13595-1
AAGCTTTAATAAAGAATTCATAATTGCATCTCAATTTAGGTGTTGGGACG
CONSENSUS
....GTGTTGGGACG

A3-AT1G62130-XLOC_007128-13595-0
ATGTGGTATAGAGTGTGAAGGCATTGAGACGCTGTGCATGAAGGATCTCA
A3-AT1G62130-XLOC_007128-13595-1
ATGTGGTATAGAGTGTGAAGGCATTGAGACGCTGTGCATGAAGGATCTCA
CONSENSUS
ATGTGGTATAGAGTGTGAAGGCATTGAGACGCTGTGCATGAAGGATCTCA

A3-AT1G62130-XLOC_007128-13595-0
CACTTCGAAGAGACAGTATGTTTTCCCTTTCTTCGGCATGATGACCAGAG
A3-AT1G62130-XLOC_007128-13595-1
CACTTCGAAGAGACAGTATGTTTTCCCTTTCTTCGGCATGATGACCAGAG
CONSENSUS
CACTTCGAAGAGACAGTATGTTTTCCCTTTCTTCGGCATGATGACCAGAG

A3-AT1G62130-XLOC_007128-13595-0
TTGACAATGGAATGCTTTTCGAAAAAGTTGACTTTGGATATCTGCATCAG
A3-AT1G62130-XLOC_007128-13595-1
TTGACAATGGAATGCTTTTCGAAAAAGTTGACTTTGGATATCTGCATCAG
CONSENSUS
TTGACAATGGAATGCTTTTCGAAAAAGTTGACTTTGGATATCTGCATCAG

A3-AT1G62130-XLOC_007128-13595-0
GTGCAGAAAAGATCATTGGATGGGCTCTTAGTCACCATATAAAGTCTAAT
A3-AT1G62130-XLOC_007128-13595-1
GTGCAGAAAAGATCATTGGATGGGCTCTTAGTCACCATATAAAGTCTAAT
CONSENSUS
GTGCAGAAAAGATCATTGGATGGGCTCTTAGTCACCATATAAAGTCTAAT

A3-AT1G62130-XLOC_007128-13595-0
      CCTGGTGCTGATCCAGATGTTAGGGTTATTTTGTCTCTTGAGAG
A3-AT1G62130-XLOC_007128-13595-1
      CCTGGTGCTGATCCAGATGTTAGGGTTATTTTGTCTCTTGAGAG
CONSENSUS
      CCTGGTGCTGATCCAGATGTTAGGGTTATTTTGTCTCTTGAGAG

alignment for event: A3-AT1G70620-XLOC_003612-848

A3-AT1G70620-XLOC_003612-848-0
      GTTACAGATGAATTATTTGATGAAATTGCAACCAAAGTCATTAACGAAGA
A3-AT1G70620-XLOC_003612-848-1
      GTTACAGATGAATTATTTGATGAAATTGCAACCAAAGTCATTAACGAAGA
CONSENSUS
      GTTACAGATGAATTATTTGATGAAATTGCAACCAAAGTCATTAACGAAGA

A3-AT1G70620-XLOC_003612-848-0
      TGAAGCAATACCTAAAGATGATAGTGTACAGCATAATCATAAATTGTCAT
A3-AT1G70620-XLOC_003612-848-1
      TGAAGCAATACCTAAAG---------------------------------
CONSENSUS
      TGAAGCAATACCTAAAG.................................

A3-AT1G70620-XLOC_003612-848-0
      CATCCCTTCTCTCCACAGCTGATCCACTCCACAAGGCTTCGGCAAAGATT
A3-AT1G70620-XLOC_003612-848-1   ------------------
CTGATCCACTCCACAAGGCTTCGGCAAAGATT
CONSENSUS
      ..................CTGATCCACTCCACAAGGCTTCGGCAAAGATT

A3-AT1G70620-XLOC_003612-848-0
      TTGGTCTCGGTAGAAGGTGCAAATACAAAAGCTAGTTCTGGCTCTCCAGC
A3-AT1G70620-XLOC_003612-848-1
      TTGGTCTCGGTAGAAGGTGCAAATACAAAAGCTAGTTCTGGCTCTCCAGC
CONSENSUS
      TTGGTCTCGGTAGAAGGTGCAAATACAAAAGCTAGTTCTGGCTCTCCAGC

A3-AT1G70620-XLOC_003612-848-0
      AGATGTGCTAGGTCTTGCTAGCTATGCCTCTGATGATGATGATGCTGATA
A3-AT1G70620-XLOC_003612-848-1
      AGATGTGCTAGGTCTTGCTAGCTATGCCTCTGATGATGATGATGCTGATA
CONSENSUS
      AGATGTGCTAGGTCTTGCTAGCTATGCCTCTGATGATGATGATGCTGATA

A3-AT1G70620-XLOC_003612-848-0
      CTGATGCTGCTTCTGACGCGAATGCTGATGAAAATGGAGTGGAGAGTCTT
A3-AT1G70620-XLOC_003612-848-1
      CTGATGCTGCTTCTGACGCGAATGCTGATGAAAATGGAGTGGAGAGTCTT
CONSENSUS
      CTGATGCTGCTTCTGACGCGAATGCTGATGAAAATGGAGTGGAGAGTCTT

A3-AT1G70620-XLOC_003612-848-0
      GGTGTGGGGTCAAGACACAATGTTAGTCAGCAGCCAAGCACTGAGAAACT

A3-AT1G70620-XLOC_003612-848-1
GGTGTGGGGTCAAGACACAATGTTAGTCAGCAGCCAAGCACTGAGAAACT
CONSENSUS
GGTGTGGGGTCAAGACACAATGTTAGTCAGCAGCCAAGCACTGAGAAACT

A3-AT1G70620-XLOC_003612-848-0
TCCTGACCCTGAAGCAATGGCCAGTGCGAAATTGGATCCAGCAGTTGGAG
A3-AT1G70620-XLOC_003612-848-1
TCCTGACCCTGAAGCAATGGCCAGTGCGAAATTGGATCCAGCAGTTGGAG
CONSENSUS
TCCTGACCCTGAAGCAATGGCCAGTGCGAAATTGGATCCAGCAGTTGGAG

A3-AT1G70620-XLOC_003612-848-0
TCAATGCTAATTCTGGCAAGAATAGTAAGTCAGGCTTGGAGGATTATTCT
A3-AT1G70620-XLOC_003612-848-1
TCAATGCTAATTCTGGCAAGAATAGTAAGTCAGGCTTGGAGGATTATTCT
CONSENSUS
TCAATGCTAATTCTGGCAAGAATAGTAAGTCAGGCTTGGAGGATTATTCT

A3-AT1G70620-XLOC_003612-848-0
CAGATGCCAGGCTCCACGAGAAAAGATGATGAGGCGGGTAGTACCAAAAT
A3-AT1G70620-XLOC_003612-848-1
CAGATGCCAGGCTCCACGAGAAAAGATGATGAGGCGGGTAGTACCAAAAT
CONSENSUS
CAGATGCCAGGCTCCACGAGAAAAGATGATGAGGCGGGTAGTACCAAAAT

A3-AT1G70620-XLOC_003612-848-0
ATCTGATGTAAGCGCCAGCTCTGGACTTGATGATGATACTTCAGGAAGCA
A3-AT1G70620-XLOC_003612-848-1
ATCTGATGTAAGCGCCAGCTCTGGACTTGATGATGATACTTCAGGAAGCA
CONSENSUS
ATCTGATGTAAGCGCCAGCTCTGGACTTGATGATGATACTTCAGGAAGCA

A3-AT1G70620-XLOC_003612-848-0
GAAAAGAGCATCCTGACAGAACTGATAGTGATAAAGATGCCATACTAGAT
A3-AT1G70620-XLOC_003612-848-1
GAAAAGAGCATCCTGACAGAACTGATAGTGATAAAGATGCCATACTAGAT
CONSENSUS
GAAAAGAGCATCCTGACAGAACTGATAGTGATAAAGATGCCATACTAGAT

A3-AT1G70620-XLOC_003612-848-0
GAACCTCACGTGAAGAATTCTGGCGTGAAATCAGATTGCAACCTTCGTCA
A3-AT1G70620-XLOC_003612-848-1
GAACCTCACGTGAAGAATTCTGGCGTGAAATCAGATTGCAACCTTCGTCA
CONSENSUS
GAACCTCACGTGAAGAATTCTGGCGTGAAATCAGATTGCAACCTTCGTCA

A3-AT1G70620-XLOC_003612-848-0
GGATAGTAATAAACCTTATGGGAAAGATTTGAGTGACGAAGTGAGTACAG
A3-AT1G70620-XLOC_003612-848-1
GGATAGTAATAAACCTTATGGGAAAGATTTGAGTGACGAAGTGAGTACAG
CONSENSUS
GGATAGTAATAAACCTTATGGGAAAGATTTGAGTGACGAAGTGAGTACAG

A3-AT1G70620-XLOC_003612-848-0
ATAGAAGTAGAATAGTTGAAACGAAAGGTGGGAAAGAGAAAGGAGATTCT

A3-AT1G70620-XLOC_003612-848-1
      ATAGAAGTAGAATAGTTGAAACGAAAGGTGGGAAAGAGAAAGGAGATTCT
CONSENSUS
      ATAGAAGTAGAATAGTTGAAACGAAAGGTGGGAAAGAGAAAGGAGATTCT

A3-AT1G70620-XLOC_003612-848-0
      CAGAATGACTCAAAAGATAGAATGAAGGAAAATGACTTAAAGTCAGCAGA
A3-AT1G70620-XLOC_003612-848-1
      CAGAATGACTCAAAAGATAGAATGAAGGAAAATGACTTAAAGTCAGCAGA
CONSENSUS
      CAGAATGACTCAAAAGATAGAATGAAGGAAAATGACTTAAAGTCAGCAGA

A3-AT1G70620-XLOC_003612-848-0
      GAAAGTTAAAGGCGTTGAATCAAATAAAAAATCTACTGATCCCCATGTAA
A3-AT1G70620-XLOC_003612-848-1
      GAAAGTTAAAGGCGTTGAATCAAATAAAAAATCTACTGATCCCCATGTAA
CONSENSUS
      GAAAGTTAAAGGCGTTGAATCAAATAAAAAATCTACTGATCCCCATGTAA

A3-AT1G70620-XLOC_003612-848-0
      AGAAAGACTCAAGGGATGTAGAGAGGCCTCACAGAACTAATTCTAAGGAA
A3-AT1G70620-XLOC_003612-848-1
      AGAAAGACTCAAGGGATGTAGAGAGGCCTCACAGAACTAATTCTAAGGAA
CONSENSUS
      AGAAAGACTCAAGGGATGTAGAGAGGCCTCACAGAACTAATTCTAAGGAA

A3-AT1G70620-XLOC_003612-848-0
      GACCGGGGTAAAAGAAAAGAGAAGGAAAAGGAAGAAGAAAGGTCAAGACA
A3-AT1G70620-XLOC_003612-848-1
      GACCGGGGTAAAAGAAAAGAGAAGGAAAAGGAAGAAGAAAGGTCAAGACA
CONSENSUS
      GACCGGGGTAAAAGAAAAGAGAAGGAAAAGGAAGAAGAAAGGTCAAGACA

A3-AT1G70620-XLOC_003612-848-0
      CAGGCGGGCTGAAAACTCGAGCAAGGACAAAAGAAGACGTTCTCCAACCA
A3-AT1G70620-XLOC_003612-848-1
      CAGGCGGGCTGAAAACTCGAGCAAGGACAAAAGAAGACGTTCTCCAACCA
CONSENSUS
      CAGGCGGGCTGAAAACTCGAGCAAGGACAAAAGAAGACGTTCTCCAACCA

A3-AT1G70620-XLOC_003612-848-0   GTAATGAATCTTCTGATGATTCGAAGAG
A3-AT1G70620-XLOC_003612-848-1   GTAATGAATCTTCTGATGATTCGAAGAG
CONSENSUS   GTAATGAATCTTCTGATGATTCGAAGAG

alignment for event: A3-AT1G31870-XLOC_001684-7674

A3-AT1G31870-XLOC_001684-7674-0
      TTAATTGCTTGAAATTTCAAATTCCGCCATAACTTAAGTTTTTCTCTATT
A3-AT1G31870-XLOC_001684-7674-1
      TTAATTGCTTGAAATTTCAAATTCCGCCATAACTTAAGTTTTTCTCTATT
CONSENSUS
      TTAATTGCTTGAAATTTCAAATTCCGCCATAACTTAAGTTTTTCTCTATT

A3-AT1G31870-XLOC_001684-7674-0

CTTCGTCTCCAGGAGATCCAAAAACCCTAGAAGAGCTAGCTCTCCGAGTT
A3-AT1G31870-XLOC_001684-7674-1
CTTCGTCTCCAGGAGATCCAAAAACCCTAGAAGAGCTAGCTCTCCGAGTT
CONSENSUS
CTTCGTCTCCAGGAGATCCAAAAACCCTAGAAGAGCTAGCTCTCCGAGTT

A3-AT1G31870-XLOC_001684-7674-0
ATCATTTTCAACTATTCGCGTTGGGTTACTGATATTTTCCCGGTCATCTC
A3-AT1G31870-XLOC_001684-7674-1
ATCATTTTCAACTATTCGCGTTGGGTTACTGATATTTTCCCGGTCATCTC
CONSENSUS
ATCATTTTCAACTATTCGCGTTGGGTTACTGATATTTTCCCGGTCATCTC

A3-AT1G31870-XLOC_001684-7674-0
CGGCGTTAATTTTCCGATCAGGCTCCAAATTCTCAAAGATGGCAGGAAAC
A3-AT1G31870-XLOC_001684-7674-1
CGGCGTTAATTTTCCGATCAG-----------------ATGGCAGGAAAC
CONSENSUS
CGGCGTTAATTTTCCGATCAG.................ATGGCAGGAAAC

A3-AT1G31870-XLOC_001684-7674-0
CAATCTTTAAAAGATTACTTGAAGAAGTATGAAAGTAGTGATGTTGTAGA
A3-AT1G31870-XLOC_001684-7674-1
CAATCTTTAAAAGATTACTTGAAGAAGTATGAAAGTAGTGATGTTGTAGA
CONSENSUS
CAATCTTTAAAAGATTACTTGAAGAAGTATGAAAGTAGTGATGTTGTAGA

A3-AT1G31870-XLOC_001684-7674-0
GAAGAAGAAGAAGAAGAAGAAGCAGAAGAAGCCATCTAAACCTGAACCAA
A3-AT1G31870-XLOC_001684-7674-1
GAAGAAGAAGAAGAAGAAGAAGCAGAAGAAGCCATCTAAACCTGAACCAA
CONSENSUS
GAAGAAGAAGAAGAAGAAGAAGCAGAAGAAGCCATCTAAACCTGAACCAA

A3-AT1G31870-XLOC_001684-7674-0
GAGGTGTTTTAGTTGTCGACGAAGATCCAGTTTGGCAAAAGCAGGTTGAT
A3-AT1G31870-XLOC_001684-7674-1
GAGGTGTTTTAGTTGTCGACGAAGATCCAGTTTGGCAAAAGCAGGTTGAT
CONSENSUS
GAGGTGTTTTAGTTGTCGACGAAGATCCAGTTTGGCAAAAGCAGGTTGAT

A3-AT1G31870-XLOC_001684-7674-0
CCTGAAGAGGACGAAAACGAAGATGATTCAGCAG
A3-AT1G31870-XLOC_001684-7674-1
CCTGAAGAGGACGAAAACGAAGATGATTCAGCAG
CONSENSUS
CCTGAAGAGGACGAAAACGAAGATGATTCAGCAG

alignment for event: RI-AT1G02090-XLOC_004204-1144

RI-AT1G02090-XLOC_004204-1144-0
GTTGAACACGTCAGAGAATCTGCTCATTTCAATTCAAGACAAGATCAAAT
RI-AT1G02090-XLOC_004204-1144-1
GTTGAACACGTCAGAGAATCTGCTCATTTCAATTCAAGACAAGATCAAAT

CONSENSUS
GTTGAACACGTCAGAGAATCTGCTCATTTCAATTCAAGACAAGATCAAAT

RI-AT1G02090-XLOC_004204-1144-0
GGGCGGACAATATGAGTGAGATGGACAAAAAACATCGTAAGGAAGCAGAA
RI-AT1G02090-XLOC_004204-1144-1
GGGCGGACAATATGAGTGAGATGGACAAAAAACATCGTAAGGAAGCAGAA
CONSENSUS
GGGCGGACAATATGAGTGAGATGGACAAAAAACATCGTAAGGAAGCAGAA

RI-AT1G02090-XLOC_004204-1144-0
GAAGGGGTGGAAGAAGTGAAGAAGTCTCTGTCCATGAAGGTCAGTCGCGC
RI-AT1G02090-XLOC_004204-1144-1
GAAGGGGTGGAAGAAGTGAAGAAGTCTCTGTCCATGAAG-----------
CONSENSUS
GAAGGGGTGGAAGAAGTGAAGAAGTCTCTGTCCATGAAG...........

RI-AT1G02090-XLOC_004204-1144-0
TTGGAGTCCTATTGGCTTCACTTTTCCCTACTAAGTATATTATGTAACTA
RI-AT1G02090-XLOC_004204-1144-1

CONSENSUS
....

RI-AT1G02090-XLOC_004204-1144-0
ATATTGGTTACATTCTTTCTTGGTTACTGTCTTGTGACCATGAGTTAGAG
RI-AT1G02090-XLOC_004204-1144-1

CONSENSUS
....

RI-AT1G02090-XLOC_004204-1144-0
TCCAGTCAGCTATTCTGTCTTATATTTCTTCAGAGAATTGTAGTGGGTTT
RI-AT1G02090-XLOC_004204-1144-1

CONSENSUS
....

RI-AT1G02090-XLOC_004204-1144-0
CTTCTTATTAATAGCTTAGGTCTATGTAAGCGGGGATGGGAGAGACTTGA
RI-AT1G02090-XLOC_004204-1144-1

CONSENSUS
....

RI-AT1G02090-XLOC_004204-1144-0
TAAAAAAATCTTTCTAGGTCTTTGAGCTTTCCAAGTCCAGTCCTATCATA
RI-AT1G02090-XLOC_004204-1144-1

CONSENSUS
....

RI-AT1G02090-XLOC_004204-1144-0
GATTAAAAAGTTATGTTTCTTAATAACAAACGAATCGATCAGCCAACTGT
RI-AT1G02090-XLOC_004204-1144-1

CONSENSUS
      ....

RI-AT1G02090-XLOC_004204-1144-0
      GGCGGGCTTTTTTAACCGTTCTTTCGTGGAACAAGAAATCTTACATGTTG
RI-AT1G02090-XLOC_004204-1144-1

CONSENSUS
      ....

RI-AT1G02090-XLOC_004204-1144-0
      GATGTAAACATATATTATGAAGTTTTCTTTAATTAAAAACAAACAAGGAA
RI-AT1G02090-XLOC_004204-1144-1

CONSENSUS
      ....

RI-AT1G02090-XLOC_004204-1144-0
      CGCAATCACAACGGCTGTTAGTGAAGTGTTAAAGTCATTAGAGGACATGA
RI-AT1G02090-XLOC_004204-1144-1

CONSENSUS
      ....

RI-AT1G02090-XLOC_004204-1144-0
      ATTGTTTGGAATGGTAGTACTTGGTTATTGTCTGATTGTTAAATGAAATG
RI-AT1G02090-XLOC_004204-1144-1

CONSENSUS
      ....

RI-AT1G02090-XLOC_004204-1144-0
      AAACAAGGTTCGTTTGGTTTTTTGTGTCTGAAATATGATTGGGAAGGGTG
RI-AT1G02090-XLOC_004204-1144-1

CONSENSUS
      ....

RI-AT1G02090-XLOC_004204-1144-0
      GTGGTCGATATAGTCTTACTTACGTTGTGGGCAGGGGGATGTTGACATCA
RI-AT1G02090-XLOC_004204-1144-1
      ----------------------------------GGGGATGTTGACATCA
CONSENSUS
      ..................................GGGGATGTTGACATCA

RI-AT1G02090-XLOC_004204-1144-0
      GAGGGAATAAGGAGATGTTTGGGGAACCAAGTGGAGTGATGGACTACGAA
RI-AT1G02090-XLOC_004204-1144-1
      GAGGGAATAAGGAGATGTTTGGGGAACCAAGTGGAGTGATGGACTACGAA
CONSENSUS
      GAGGGAATAAGGAGATGTTTGGGGAACCAAGTGGAGTGATGGACTACGAA

RI-AT1G02090-XLOC_004204-1144-0 GAAGATGGGATCCGACCAAAGAG
RI-AT1G02090-XLOC_004204-1144-1 GAAGATGGGATCCGACCAAAGAG
CONSENSUS   GAAGATGGGATCCGACCAAAGAG

alignment for event: RI-AT1G52370-XLOC_002615-12231

RI-AT1G52370-XLOC_002615-12231-0
CGACGCTCTTCTTCTATTAAACCTCTTCTTCGTCTCTGGCTCAGGGCGTT
RI-AT1G52370-XLOC_002615-12231-1
CGACGCTCTTCTTCTATTAAACCTCTTCTTCGTCTCTGGCTCAGGGCGTT
CONSENSUS
CGACGCTCTTCTTCTATTAAACCTCTTCTTCGTCTCTGGCTCAGGGCGTT

RI-AT1G52370-XLOC_002615-12231-0
AGTTTTTTTTTCCGCTTCTCTGCTACTGCTCCTTCGCGGTAAGTCTTTCC
RI-AT1G52370-XLOC_002615-12231-1
AGTTTTTTTTTCCGCTTCTCTGCTACTGCTCCTTCGCGGTAAGTCTTTCC
CONSENSUS
AGTTTTTTTTTCCGCTTCTCTGCTACTGCTCCTTCGCGGTAAGTCTTTCC

RI-AT1G52370-XLOC_002615-12231-0
CATTTTCTCCTCGCCGGGCATTTTCGTCTAAGCTTAGGTTATCTTCATCC
RI-AT1G52370-XLOC_002615-12231-1
CATTTTCTCCTCGCCGGGCATTTTCGTCTAAGCTTAG-------------
CONSENSUS
CATTTTCTCCTCGCCGGGCATTTTCGTCTAAGCTTAG.............

RI-AT1G52370-XLOC_002615-12231-0
CCAGTTACTTTGATTTTTTTTTTTTTTTTTCTTCTCGTCGTGTTGTTTGTT
RI-AT1G52370-XLOC_002615-12231-1

CONSENSUS
....

RI-AT1G52370-XLOC_002615-12231-0
GTGGAAATAGTTATCGGCCATTTCTTTATTTTATTCTTGACAGTTTGTTT
RI-AT1G52370-XLOC_002615-12231-1

CONSENSUS
....

RI-AT1G52370-XLOC_002615-12231-0
TGATGGTTAGGGTTTTTAGAATTTAACTCGTATTTGACCCATTTGGGTTG
RI-AT1G52370-XLOC_002615-12231-1

CONSENSUS
....

RI-AT1G52370-XLOC_002615-12231-0
TGATCGAATTTTAAATCTCTTCGAATAATGGGTGCGTTGCTTTGATTACT
RI-AT1G52370-XLOC_002615-12231-1

CONSENSUS
....

RI-AT1G52370-XLOC_002615-12231-0
CGGTTCTTATATTGTTGAAAGCTAGAATCAATTTGAATTTGAATCAACTC
RI-AT1G52370-XLOC_002615-12231-1

CONSENSUS
....

RI-AT1G52370-XLOC_002615-12231-0
CACTAGTGCATTGGTCCCTTTCGTGCAACTCCTTTGTCTGAATCTTATAC
RI-AT1G52370-XLOC_002615-12231-1

CONSENSUS
....

RI-AT1G52370-XLOC_002615-12231-0
ATAGATTTAGAATAGTAGATCCACTCATGTTGATTTTTTCATTACTTATT
RI-AT1G52370-XLOC_002615-12231-1

CONSENSUS
....

RI-AT1G52370-XLOC_002615-12231-0
AGTAAAGTCTCTAATGCATGTCTTACATCTAAGTTTAGCTCTACTTATTT
RI-AT1G52370-XLOC_002615-12231-1

CONSENSUS
....

RI-AT1G52370-XLOC_002615-12231-0
TGTTCAAGATTGTTTTTTTACCATCTTCTGCAGAAGAAGCATAGACTAAG
RI-AT1G52370-XLOC_002615-12231-1
---------------------------------AAGAAGCATAGACTAAG
CONSENSUS
.................................AAGAAGCATAGACTAAG

RI-AT1G52370-XLOC_002615-12231-0
GTTTAGGGTAGAATATAATGGCGGGTTGGCAGAAGAATTTACAGATTGTT
RI-AT1G52370-XLOC_002615-12231-1
GTTTAGGGTAGAATATAATGGCGGGTTGGCAGAAGAATTTACAGATTGTT
CONSENSUS
GTTTAGGGTAGAATATAATGGCGGGTTGGCAGAAGAATTTACAGATTGTT

RI-AT1G52370-XLOC_002615-12231-0
ATTCGTCAAGTTGGTAAAAGAGTTAAGGACAGTCACATTTCCACGGCAAA
RI-AT1G52370-XLOC_002615-12231-1
ATTCGTCAAGTTGGTAAAAGAGTTAAGGACAGTCACATTTCCACGGCAAA
CONSENSUS
ATTCGTCAAGTTGGTAAAAGAGTTAAGGACAGTCACATTTCCACGGCAAA

RI-AT1G52370-XLOC_002615-12231-0
TTACTCTTCCACACGGAATTTGGAATCCCCTTTCTCACAAG
RI-AT1G52370-XLOC_002615-12231-1
TTACTCTTCCACACGGAATTTGGAATCCCCTTTCTCACAAG
CONSENSUS
TTACTCTTCCACACGGAATTTGGAATCCCCTTTCTCACAAG

alignment for event: A3-AT1G60080-XLOC_006993-12260

A3-AT1G60080-XLOC_006993-12260-0
TCGTCACCGTCACCGTCTCCGTCGAAATCCCCATTACTTACGACACCGCG
A3-AT1G60080-XLOC_006993-12260-1
TCGTCACCGTCACCGTCTCCGTCGAAATCCCCATTACTTACGACACCGCG
CONSENSUS
TCGTCACCGTCACCGTCTCCGTCGAAATCCCCATTACTTACGACACCGCG

A3-AT1G60080-XLOC_006993-12260-0
AGCTCTCAATCTTCTTCTTACACTCTATTGCTTGTGTTTCCTCTTCACTA
A3-AT1G60080-XLOC_006993-12260-1
AGCTCTCAATCTTCTTCTTACACTCTATTGCTTGTGTTTCCTCTTCACTA
CONSENSUS
AGCTCTCAATCTTCTTCTTACACTCTATTGCTTGTGTTTCCTCTTCACTA

A3-AT1G60080-XLOC_006993-12260-0
CAACAGTTTCGTGTACCAAAGGCAGTTCTTTCTTTTGTCCCAAG---AGC
A3-AT1G60080-XLOC_006993-12260-1
CAACAGTTTCGTGTACCAAAGGCAGTTCTTTCTTTTGTCCCAAGCAGAGC
CONSENSUS
CAACAGTTTCGTGTACCAAAGGCAGTTCTTTCTTTTGTCCCAAG...AGC

A3-AT1G60080-XLOC_006993-12260-0
TAAAGAAAACATGGGAATACCAGATGCAGCTCAAGACTTGTCGACAGAGA
A3-AT1G60080-XLOC_006993-12260-1
TAAAGAAAACATGGGAATACCAGATGCAGCTCAAGACTTGTCGACAGAGA
CONSENSUS
TAAAGAAAACATGGGAATACCAGATGCAGCTCAAGACTTGTCGACAGAGA

A3-AT1G60080-XLOC_006993-12260-0
TGGAAGTTGATGCTTTTAGAAGAATCTTCCCTCTTCGTTTCTTTGAGCGT
A3-AT1G60080-XLOC_006993-12260-1
TGGAAGTTGATGCTTTTAGAAGAATCTTCCCTCTTCGTTTCTTTGAGCGT
CONSENSUS
TGGAAGTTGATGCTTTTAGAAGAATCTTCCCTCTTCGTTTCTTTGAGCGT

A3-AT1G60080-XLOC_006993-12260-0
CATCTCTCTGAATCTCTTCGTCCTGATGGAAGACAACTTGGAAAAGCTAG
A3-AT1G60080-XLOC_006993-12260-1
CATCTCTCTGAATCTCTTCGTCCTGATGGAAGACAACTTGGAAAAGCTAG
CONSENSUS
CATCTCTCTGAATCTCTTCGTCCTGATGGAAGACAACTTGGAAAAGCTAG

A3-AT1G60080-XLOC_006993-12260-0 AGACACCATTGTTAATCTTG
A3-AT1G60080-XLOC_006993-12260-1 AGACACCATTGTTAATCTTG
CONSENSUS AGACACCATTGTTAATCTTG

alignment for event: A5-AT1G17520-XLOC_005070-4382

A5-AT1G17520-XLOC_005070-4382-0
CAACGACAAGAAGTGCCACCAAATTTCAGGAGGATGCTTAGTTCAAGATT
A5-AT1G17520-XLOC_005070-4382-1
CAACGACAAGAAGTGCCACCAAATTTCAGGAGGATGCTTAGTTCAAGATT
CONSENSUS

CAACGACAAGAAGTGCCACCAAATTTCAGGAGGATGCTTAGTTCAAGATT

A5-AT1G17520-XLOC_005070-4382-0
GAGGAGGCTTGCAGCTCAGGGAAAGCTTGAAAAGGTTAGCCACTTAAAAT
A5-AT1G17520-XLOC_005070-4382-1
GAGGAGGCTTGCAGCTCAGGGAAAGCTTGAAAAG----------------
CONSENSUS
GAGGAGGCTTGCAGCTCAGGGAAAGCTTGAAAAG................

A5-AT1G17520-XLOC_005070-4382-0
CAACACAGAACTTCTATAAGATGAATGATAATAGCTTGGTACAAAGAACA
A5-AT1G17520-XLOC_005070-4382-1 --
ACACAGAACTTCTATAAGATGAATGATAATAGCTTGGTACAAAGAACA
CONSENSUS
..ACACAGAACTTCTATAAGATGAATGATAATAGCTTGGTACAAAGAACA

A5-AT1G17520-XLOC_005070-4382-0
CCACATGTAGCGAGACCAAAAGAGAGCAACACGAAATCCCGGCAGCAGAC
A5-AT1G17520-XLOC_005070-4382-1
CCACATGTAGCGAGACCAAAAGAGAGCAACACGAAATCCCGGCAGCAGAC
CONSENSUS
CCACATGTAGCGAGACCAAAAGAGAGCAACACGAAATCCCGGCAGCAGAC

A5-AT1G17520-XLOC_005070-4382-0
AAACAGTCAGGGACCCTCTATTTCACAGCAGATTGTTGAAGCTTCAATAA
A5-AT1G17520-XLOC_005070-4382-1
AAACAGTCAGGGACCCTCTATTTCACAGCAGATTGTTGAAGCTTCAATAA
CONSENSUS
AAACAGTCAGGGACCCTCTATTTCACAGCAGATTGTTGAAGCTTCAATAA

A5-AT1G17520-XLOC_005070-4382-0
CAGCAGCTTATAAGCTCGTAGAAGTAGAGAACAAATTAGATGTGTCGAAA
A5-AT1G17520-XLOC_005070-4382-1
CAGCAGCTTATAAGCTCGTAGAAGTAGAGAACAAATTAGATGTGTCGAAA
CONSENSUS
CAGCAGCTTATAAGCTCGTAGAAGTAGAGAACAAATTAGATGTGTCGAAA

A5-AT1G17520-XLOC_005070-4382-0
GGAGCTGCAGAAGAGATAGAGAGACTGATGAAACTGGCAGAAGAGGCTGA
A5-AT1G17520-XLOC_005070-4382-1
GGAGCTGCAGAAGAGATAGAGAGACTGATGAAACTGGCAGAAGAGGCTGA
CONSENSUS
GGAGCTGCAGAAGAGATAGAGAGACTGATGAAACTGGCAGAAGAGGCTGA

A5-AT1G17520-XLOC_005070-4382-0
TGAGATGCTAGTGATAGCGAGAGAGATGCACGAAGAAT
A5-AT1G17520-XLOC_005070-4382-1
TGAGATGCTAGTGATAGCGAGAGAGATGCACGAAGAAT
CONSENSUS
TGAGATGCTAGTGATAGCGAGAGAGATGCACGAAGAAT

alignment for event: A3-AT1G26440-XLOC_005521-3651

A3-AT1G26440-XLOC_005521-3651-0

GCACTACTGTCAATTACTTTTTGGACAACGGATTGAACAGAGCTGATATT
A3-AT1G26440-XLOC_005521-3651-1
GCACTACTGTCAATTACTTTTTGGACAACGGATTGAACAGAGCTGATATT
CONSENSUS
GCACTACTGTCAATTACTTTTTGGACAACGGATTGAACAGAGCTGATATT

A3-AT1G26440-XLOC_005521-3651-0
CTTTTCTCTGGTGTTGGTTGCTTCATGGTAGCTGTTTGTCTTGGTTCTGC
A3-AT1G26440-XLOC_005521-3651-1
CTTTTCTCTGGTGTTGGTTGCTTCATGGTAGCTGTTTGTCTTGGTTCTGC
CONSENSUS
CTTTTCTCTGGTGTTGGTTGCTTCATGGTAGCTGTTTGTCTTGGTTCTGC

A3-AT1G26440-XLOC_005521-3651-0
TGTTCACTCTTCCAATTCTGCTGATATCAAGGCGAAACTCGGAAAACTCT
A3-AT1G26440-XLOC_005521-3651-1
TGTTCACTCTTCCAATTCTGCTGATATCAAGGCGAAACTCGGAAAACTCT
CONSENSUS
TGTTCACTCTTCCAATTCTGCTGATATCAAGGCGAAACTCGGAAAACTCT

A3-AT1G26440-XLOC_005521-3651-0
CAGGGGATTGTGAAACTGTGACCCCTGAAGAGTGCCAGAGACTATTTGGA
A3-AT1G26440-XLOC_005521-3651-1
CAGGGGATTGTGAAACTGTGACCCCTGAAGAGTGCCAGAGACTATTTGGA
CONSENSUS
CAGGGGATTGTGAAACTGTGACCCCTGAAGAGTGCCAGAGACTATTTGGA

A3-AT1G26440-XLOC_005521-3651-0 G---
AAGAAGAAGAAGAAGAAGAAAAAGAGATGGAGAATGTAAAGGAAGG
A3-AT1G26440-XLOC_005521-3651-1
GTAGAAGAAGAAGAAGAAGAAGAAAAAGAGATGGAGAATGTAAAGGAAGG
CONSENSUS
G...AAGAAGAAGAAGAAGAAGAAAAAGAGATGGAGAATGTAAAGGAAGG

A3-AT1G26440-XLOC_005521-3651-0
GTCTGCAGCTTTTCTTATAGCACTCGAAAACAAAAGAGCAATCAAA
A3-AT1G26440-XLOC_005521-3651-1
GTCTGCAGCTTTTCTTATAGCACTCGAAAACAAAAGAGCAATCAAA
CONSENSUS
GTCTGCAGCTTTTCTTATAGCACTCGAAAACAAAAGAGCAATCAAA

alignment for event: SE-AT1G15200-XLOC_004959-8765

SE-AT1G15200-XLOC_004959-8765-0
ACTCTTCGGGCTCGTGTTGCTGCTAAAGCAGAACAAAAGAAATTGGAATT
SE-AT1G15200-XLOC_004959-8765-1
ACTCTTCGGGCTCGTGTTGCTGCTAAAGCAGAACAAAAGAAATTGGAATT
CONSENSUS
ACTCTTCGGGCTCGTGTTGCTGCTAAAGCAGAACAAAAGAAATTGGAATT

SE-AT1G15200-XLOC_004959-8765-0
GCTTTTCCTTCAATGGAGTGAGCACCAAAAGAAACTTAGCAATTTTATAA
SE-AT1G15200-XLOC_004959-8765-1
GCTTTTCCTTCAATGGAGTGAGCACCAAAAGAAACTTAGCAATTTTATAA

CONSENSUS
      GCTTTTCCTTCAATGGAGTGAGCACCAAAAGAAACTTAGCAATTTTATAA

SE-AT1G15200-XLOC_004959-8765-0
      G---
SE-AT1G15200-XLOC_004959-8765-1
      GGTTATTTTTTATTTCGGTCCAAAAAGTGTGCATATTGACAATTTTATAG
CONSENSUS
      G...

SE-AT1G15200-XLOC_004959-8765-0

SE-AT1G15200-XLOC_004959-8765-1
      AGGCAAAGATAAGTTTCCATTCACGTGCATACAAAGGCAATGTTTGGTGC
CONSENSUS
      ....

SE-AT1G15200-XLOC_004959-8765-0 -----
GACTAAAGCAGAGCCACGGATATACTATGCACCAGTGAAGCCTTT
SE-AT1G15200-XLOC_004959-8765-1
      TATAGGACTAAAGCAGAGCCACGGATATACTATGCACCAGTGAAGCCTTT
CONSENSUS
      .....GACTAAAGCAGAGCCACGGATATACTATGCACCAGTGAAGCCTTT

SE-AT1G15200-XLOC_004959-8765-0
      GGAAGAAGATACAAGTGAGGTAGAACAACAGAAAGAGCGG
SE-AT1G15200-XLOC_004959-8765-1
      GGAAGAAGATACAAGTGAGGTAGAACAACAGAAAGAGCGG
CONSENSUS
      GGAAGAAGATACAAGTGAGGTAGAACAACAGAAAGAGCGG

alignment for event: RI-AT1G78070-XLOC_004007-6423

RI-AT1G78070-XLOC_004007-6423-0
      GAATGCTTGCAAGTAGAGAAAGGAAAGAACTTTTATGACTTTCAGTTCAA
RI-AT1G78070-XLOC_004007-6423-1
      GAATGCTTGCAAGTAGAGAAAGGAAAGAACTTTTATGACTTTCAGTTCAA
CONSENSUS
      GAATGCTTGCAAGTAGAGAAAGGAAAGAACTTTTATGACTTTCAGTTCAA

RI-AT1G78070-XLOC_004007-6423-0
      TACAAGGCTTGTCAAGTCCACAATAGCGCATTTTCAGGTAGAGAACTTGA
RI-AT1G78070-XLOC_004007-6423-1
      TACAAGGCTTGTCAAGTCCACAATAGCGCATTTTCAGGTAGAGAACTTGA
CONSENSUS
      TACAAGGCTTGTCAAGTCCACAATAGCGCATTTTCAGGTAGAGAACTTGA

RI-AT1G78070-XLOC_004007-6423-0
      CCAACTATGTTTGCTATGTTTGGAATACTCTGTTTACTGATGCTTGGAGT
RI-AT1G78070-XLOC_004007-6423-1
      CCAACTATGTTTGCTATGTTTGGAATACTCTGTTTACTGATGCTTGGAGT
CONSENSUS
      CCAACTATGTTTGCTATGTTTGGAATACTCTGTTTACTGATGCTTGGAGT

RI-AT1G78070-XLOC_004007-6423-0
CATCTAGGTTTTCACTCTTTATCATATATAAATCCTCTGTGTTGCTCAGC
RI-AT1G78070-XLOC_004007-6423-1
CATCTAGGTTTTCACTCTTTATCATATATAAATCCTCTGTGTTGCTCAGC
CONSENSUS
CATCTAGGTTTTCACTCTTTATCATATATAAATCCTCTGTGTTGCTCAGC

RI-AT1G78070-XLOC_004007-6423-0
TCTTGTCTTTAGCAGTCGTGTCTGCAACTGCAAACAGTAGTTCCACAGTG
RI-AT1G78070-XLOC_004007-6423-1
TCTTGTCTTTAGCAGTCGTGTCTGCAACTGCAAACAGTAGTTCCACAGTG
CONSENSUS
TCTTGTCTTTAGCAGTCGTGTCTGCAACTGCAAACAGTAGTTCCACAGTG

RI-AT1G78070-XLOC_004007-6423-0
GCTTCTTATCACGCATGAGTTCTCTAGTTACATGGAAATATTTTATGAGC
RI-AT1G78070-XLOC_004007-6423-1
GCTTCTTATCACGCATGAGTTCTCTAGTTACATGGAAATATTTTATGAGC
CONSENSUS
GCTTCTTATCACGCATGAGTTCTCTAGTTACATGGAAATATTTTATGAGC

RI-AT1G78070-XLOC_004007-6423-0
TTAGAGATTGTAGTTCCATGCTGATTTCAGTGTCACAATTCTTTCACACC
RI-AT1G78070-XLOC_004007-6423-1
TTAGAGATTGTAGTTCCATGCTGATTTCAGTGTCACAATTCTTTCACACC
CONSENSUS
TTAGAGATTGTAGTTCCATGCTGATTTCAGTGTCACAATTCTTTCACACC

RI-AT1G78070-XLOC_004007-6423-0
GAGTCTGAAAACCCCAACTTTTAAGTGAGATCTGCCAAACTTATTTTGAA
RI-AT1G78070-XLOC_004007-6423-1
GAGTCTGAAAACCCCAACTTTTAAGTGAGATCTGCCAAACTTATTTTGAA
CONSENSUS
GAGTCTGAAAACCCCAACTTTTAAGTGAGATCTGCCAAACTTATTTTGAA

RI-AT1G78070-XLOC_004007-6423-0
GATGAAAAATGTATCTGTTGTCAGTGTTTATTCTTTTTGTTAATTATGTT
RI-AT1G78070-XLOC_004007-6423-1
GATGAAAAATGTATCTGTTGTCAGTGTTTATTCTTTTTGTTAATTATGTT
CONSENSUS
GATGAAAAATGTATCTGTTGTCAGTGTTTATTCTTTTTGTTAATTATGTT

RI-AT1G78070-XLOC_004007-6423-0
TGATCTTAGAATTTGCGATAACTCTTCCTGTTTTCTTATGTTGATCATTG
RI-AT1G78070-XLOC_004007-6423-1
TGATCTTAGAATTTGCGATAACTCTTCCTGTTTTCTTATGTTGATCATTG
CONSENSUS
TGATCTTAGAATTTGCGATAACTCTTCCTGTTTTCTTATGTTGATCATTG

RI-AT1G78070-XLOC_004007-6423-0
TTCCCACCTTCAGCGCTGTAGCTGGGGGTGTTGATTCACACAAAGTCCTT
RI-AT1G78070-XLOC_004007-6423-1
TTCCCACCTTCAGCGCTGTAGCTGGGGGTGTTGATTCACACAAAGTCCTT
CONSENSUS
TTCCCACCTTCAGCGCTGTAGCTGGGGGTGTTGATTCACACAAAGTCCTT

RI-AT1G78070-XLOC_004007-6423-0
TTCTTTTGCAGATAGTCTTCAAGGGTGCTTAATTTAACCTGCTCGTTTCT
RI-AT1G78070-XLOC_004007-6423-1
TTCTTTTGCAGATAGTCTTCAAGGGTGCTTAATTTAACCTGCTCGTTTCT
CONSENSUS
TTCTTTTGCAGATAGTCTTCAAGGGTGCTTAATTTAACCTGCTCGTTTCT

RI-AT1G78070-XLOC_004007-6423-0
AACTTCTCGAAATATAATAACTTAGGTTCGTTTTTTACTCAGGGACAATA
RI-AT1G78070-XLOC_004007-6423-1
AACTTCTCGAAATATAATAACTTAGGTTCGTTTTTTACTCAGGGACAATA
CONSENSUS
AACTTCTCGAAATATAATAACTTAGGTTCGTTTTTTACTCAGGGACAATA

RI-AT1G78070-XLOC_004007-6423-0
TTAAGGTTCTAACTGATTCAAAAAGAGTGTCATGATATGTTTCTAGGATG
RI-AT1G78070-XLOC_004007-6423-1
TTAAGGTTCTAACTGATTCAAAAAGAGTGTCATGATATGTTTCTAGGATG
CONSENSUS
TTAAGGTTCTAACTGATTCAAAAAGAGTGTCATGATATGTTTCTAGGATG

RI-AT1G78070-XLOC_004007-6423-0
ATCGAGGCAGAGTTGATATATCTAACTGGTACAGGCAACTTTTTAACATC
RI-AT1G78070-XLOC_004007-6423-1
ATCGAGGCAGAGTTGATATATCTAACTGGTACAGGCAACTTTTTAACATC
CONSENSUS
ATCGAGGCAGAGTTGATATATCTAACTGGTACAGGCAACTTTTTAACATC

RI-AT1G78070-XLOC_004007-6423-0
TTCATATACTTTCATCACCTCAAAAATTAGAGTCAGAGAGTGTATTAAGA
RI-AT1G78070-XLOC_004007-6423-1
TTCATATACTTTCATCACCTCAAAAATTAGAGTCAGAGAGTGTATTAAGA
CONSENSUS
TTCATATACTTTCATCACCTCAAAAATTAGAGTCAGAGAGTGTATTAAGA

RI-AT1G78070-XLOC_004007-6423-0
TTCCCTTGCACATTAGACGTCGCTCAATGTCCATTATTAGACCACGGGAA
RI-AT1G78070-XLOC_004007-6423-1
TTCCCTTGCACATTAGACGTCGCTCAATGTCCATTATTAGACCACGGGAA
CONSENSUS
TTCCCTTGCACATTAGACGTCGCTCAATGTCCATTATTAGACCACGGGAA

RI-AT1G78070-XLOC_004007-6423-0
CTGTATTGAAGATTTAACACTACCTCTGCTGCATTTTGAGAAACAGTTAC
RI-AT1G78070-XLOC_004007-6423-1
CTGTATTGAAGATTTAACACTACCTCTGCTGCATTTTGAGAAACAGTTAC
CONSENSUS
CTGTATTGAAGATTTAACACTACCTCTGCTGCATTTTGAGAAACAGTTAC

RI-AT1G78070-XLOC_004007-6423-0
TGTATGACTTAACCTTTCTTAAACAGGTTGGAAGAGCAGCTGGCGCCTTA
RI-AT1G78070-XLOC_004007-6423-1
TGTATGACTTAACCTTTCTTAAACAGGTTGGAAGAGCAGCTGGCGCCTTA
CONSENSUS
TGTATGACTTAACCTTTCTTAAACAGGTTGGAAGAGCAGCTGGCGCCTTA

RI-AT1G78070-XLOC_004007-6423-0
      AGGAAACCTTTGTTAATATGGTATGATAGGAAATGTTTGCTAATCTGCAA
RI-AT1G78070-XLOC_004007-6423-1
      AGGAAACCTTTGTTAATATG------------------------------
CONSENSUS
      AGGAAACCTTTGTTAATATG..............................

RI-AT1G78070-XLOC_004007-6423-0
      GGACTCTCTAACTTTGTCATCCTTATTTCTTACAGGAAATCTTTTTGTTC
RI-AT1G78070-XLOC_004007-6423-1

CONSENSUS
      ....

RI-AT1G78070-XLOC_004007-6423-0
      AAGCTTATATGAGTGCTTCAATATCATCTGCATTTTTTCGTGATACTATG
RI-AT1G78070-XLOC_004007-6423-1

CONSENSUS
      ....

RI-AT1G78070-XLOC_004007-6423-0
      CTCTTGTGCTTCTTATTATTTCTTTTGGTTTTGGTCTTCTCTTCTTTCTT
RI-AT1G78070-XLOC_004007-6423-1

CONSENSUS
      ....

RI-AT1G78070-XLOC_004007-6423-0
      TTTTTTCTGGCACAGTTGCTTCATAAACGATTAATGCTGAAGTTTTTAAA
RI-AT1G78070-XLOC_004007-6423-1

CONSENSUS
      ....

RI-AT1G78070-XLOC_004007-6423-0
      GTCAACATTTGATTACTGAAACGGTTGCTTTGTTTCCTTCTTGGCTTGTG
RI-AT1G78070-XLOC_004007-6423-1

CONSENSUS
      ....

RI-AT1G78070-XLOC_004007-6423-0
      TTTTGTGTGAGAACTTGCATGTTCTCTGCTTTTTTGTGTAAGATGACCAG
RI-AT1G78070-XLOC_004007-6423-1

CONSENSUS
      ....

RI-AT1G78070-XLOC_004007-6423-0
      TTGTATCCTATGCAACAATCCTAATCATTATTGTCTCATTTTACAGCTGA
RI-AT1G78070-XLOC_004007-6423-1
      --CTGA
CONSENSUS
      ....CTGA

RI-AT1G78070-XLOC_004007-6423-0
GGAACTTGGTATGGGCAACATCAAAGCACGATGTGTATTTCATGAACAAC
RI-AT1G78070-XLOC_004007-6423-1
GGAACTTGGTATGGGCAACATCAAAGCACGATGTGTATTTCATGAACAAC
CONSENSUS
GGAACTTGGTATGGGCAACATCAAAGCACGATGTGTATTTCATGAACAAC

RI-AT1G78070-XLOC_004007-6423-0
TACTCTCTCATGCACTGGTCATCTTTGTTGCAAAGGGGCAAAGAAGTACT
RI-AT1G78070-XLOC_004007-6423-1
TACTCTCTCATGCACTGGTCATCTTTGTTGCAAAGGGGCAAAGAAGTACT
CONSENSUS
TACTCTCTCATGCACTGGTCATCTTTGTTGCAAAGGGGCAAAGAAGTACT

RI-AT1G78070-XLOC_004007-6423-0
TAATGTGGCAAAGCCCATTGTTCCTTCAATG
RI-AT1G78070-XLOC_004007-6423-1
TAATGTGGCAAAGCCCATTGTTCCTTCAATG
CONSENSUS
TAATGTGGCAAAGCCCATTGTTCCTTCAATG

alignment for event: A5-AT1G55325-XLOC_002797-3382

A5-AT1G55325-XLOC_002797-3382-0
GTGGTGGCCAGAGCAGTACTGGTATGAGTTCCGTTAATTACATTGAAGGT
A5-AT1G55325-XLOC_002797-3382-1
GTGGTGGCCAGAGCAGTACTGGTATGAGTTCCGTTAATTACATTGAAGGT
CONSENSUS
GTGGTGGCCAGAGCAGTACTGGTATGAGTTCCGTTAATTACATTGAAGGT

A5-AT1G55325-XLOC_002797-3382-0
TTCACTCCTGTCAAGTCTCTTGGTTCTACCGCTTTCTCTTACATGATGAT
A5-AT1G55325-XLOC_002797-3382-1
TTCACTCCTGTCAAGTCTCTTGGTTCTACCGCTTTCTCTTACATGATGAT
CONSENSUS
TTCACTCCTGTCAAGTCTCTTGGTTCTACCGCTTTCTCTTACATGATGAT

A5-AT1G55325-XLOC_002797-3382-0
ACCATCACCCAACATGCGCTTTCTTCACCCAAGTCCTCTTCAGCTTCCGA
A5-AT1G55325-XLOC_002797-3382-1
ACCATCACCCAACATGCGCTTTCTTCACCCAAGTCCTCTTCAGCTTCCGA
CONSENSUS
ACCATCACCCAACATGCGCTTTCTTCACCCAAGTCCTCTTCAGCTTCCGA

A5-AT1G55325-XLOC_002797-3382-0
CATGTTTAACTGCCGAGTCACCTCCACTTGCTCACCTCCTTCACAGCAAA
A5-AT1G55325-XLOC_002797-3382-1
CATGTTTAACTGCCGAGTCACCTCCACTTGCTCACCTCCTTCACAGCAAA
CONSENSUS
CATGTTTAACTGCCGAGTCACCTCCACTTGCTCACCTCCTTCACAGCAAA

A5-AT1G55325-XLOC_002797-3382-0
GGCTATGCAATCCCCTTGTCTACTGGATTTGTTGTTTCAAAAGCTGTGCC
A5-AT1G55325-XLOC_002797-3382-1

GGCTATGCAATCCCCTTGTCTACTGGATTTGTTGTTTCAAAAGCTGTGCC
CONSENSUS
GGCTATGCAATCCCCTTGTCTACTGGATTTGTTGTTTCAAAAGCTGTGCC

A5-AT1G55325-XLOC_002797-3382-0
TTCCATGAGAAAAGACTCAAGGATAAACGTGAAAGAAGAATGGCCATCAG
A5-AT1G55325-XLOC_002797-3382-1
TTCCATGAGAAAAGACTCAAGGATAAACGTGAAAGAAGAATGGCCATCAG
CONSENSUS
TTCCATGAGAAAAGACTCAAGGATAAACGTGAAAGAAGAATGGCCATCAG

A5-AT1G55325-XLOC_002797-3382-0
TTCTTTCTGTAAGTCTCATAGACTATTATGGTGGTTATGACAACGCTCAT
A5-AT1G55325-XLOC_002797-3382-1
TTCTTTCTGTAAGTCTCATAGACTATTATGGTGGTTATGACAACGCTCAT
CONSENSUS
TTCTTTCTGTAAGTCTCATAGACTATTATGGTGGTTATGACAACGCTCAT

A5-AT1G55325-XLOC_002797-3382-0
GACAAAATTCTTCAGGGAATCGTGAAGCAGGGAGGAGGGACCAAAGAAAC
A5-AT1G55325-XLOC_002797-3382-1
GACAAAATTCTTCAGGGAATCGTGAAGCAGGGAGGAGGGACCAAAGAAAC
CONSENSUS
GACAAAATTCTTCAGGGAATCGTGAAGCAGGGAGGAGGGACCAAAGAAAC

A5-AT1G55325-XLOC_002797-3382-0
TAGAGATTTTGAGGTTGAAAGCCATCTTATCCTTGAGTCAATTGCGGCAG
A5-AT1G55325-XLOC_002797-3382-1
TAGAGATTTTGAGGTTGAAAGCCATCTTATCCTTGAGTCAATTGCGGCAG
CONSENSUS
TAGAGATTTTGAGGTTGAAAGCCATCTTATCCTTGAGTCAATTGCGGCAG

A5-AT1G55325-XLOC_002797-3382-0
AACTCCATGCTCTATCATGGATGACTGTTAGTCCAGCATATCTGGACAGG
A5-AT1G55325-XLOC_002797-3382-1
AACTCCATGCTCTATCATGGATGACTGTTAGTCCAGCATATCTGGACAGG
CONSENSUS
AACTCCATGCTCTATCATGGATGACTGTTAGTCCAGCATATCTGGACAGG

A5-AT1G55325-XLOC_002797-3382-0
CGGACGGCATTGCCGTTTCACTGTGATATGGTTCTGAGATTGAGACGTCT
A5-AT1G55325-XLOC_002797-3382-1
CGGACGGCATTGCCGTTTCACTGTGATATGGTTCTGAGATTGAGACGTCT
CONSENSUS
CGGACGGCATTGCCGTTTCACTGTGATATGGTTCTGAGATTGAGACGTCT

A5-AT1G55325-XLOC_002797-3382-0
TCTTCACTTTGCGGACAAAGAAGTCTCCAGGATACCTGATAAGACAGGAG
A5-AT1G55325-XLOC_002797-3382-1
TCTTCACTTTGCGGACAAAGAAGTCTCCAGGATACCTGATAAGACAGGAG
CONSENSUS
TCTTCACTTTGCGGACAAAGAAGTCTCCAGGATACCTGATAAGACAGGAG

A5-AT1G55325-XLOC_002797-3382-0
TTTAAAACACTGTAAAAGAACGCAGATCACTAAATTTCTCTATCAGAAGA
A5-AT1G55325-XLOC_002797-3382-1

TTTAAAACACTGTAAAAGAACGCAGATCACTAAATTTCTCTATCAGAAGA
CONSENSUS
TTTAAAACACTGTAAAAGAACGCAGATCACTAAATTTCTCTATCAGAAGA

A5-AT1G55325-XLOC_002797-3382-0
TTAGACGAATCTTTCAGCGAACTTCATCCTGCACCTCCCGAGTAGTCTTG
A5-AT1G55325-XLOC_002797-3382-1
TTAGACGAATCTTTCAGCGAACTTCATCCTGCACCTCCCGAGTAGTCTTG
CONSENSUS
TTAGACGAATCTTTCAGCGAACTTCATCCTGCACCTCCCGAGTAGTCTTG

A5-AT1G55325-XLOC_002797-3382-0
TCAAATATAAGGCAT-----------------------------------
A5-AT1G55325-XLOC_002797-3382-1
TCAAATATAAGGCATGTAGGTATCTCGGGGTAGTTCACCACTGCTATATG
CONSENSUS
TCAAATATAAGGCAT...................................

A5-AT1G55325-XLOC_002797-3382-0

A5-AT1G55325-XLOC_002797-3382-1
TTACAATAAAGTAGGGAGAAATGGTGTAGATGTAGTCGAGGTTTTGATTT
CONSENSUS
....

A5-AT1G55325-XLOC_002797-3382-0

A5-AT1G55325-XLOC_002797-3382-1
GGTGCTTATGAAATCTGTAAAAATTAGCATTTAAGGCTTGCTCTACTGGT
CONSENSUS
....

A5-AT1G55325-XLOC_002797-3382-0 ----------------------
TCACATAATGGATCCTGAATGTTGTATG
A5-AT1G55325-XLOC_002797-3382-1
TCTGATTCACTTAGGTGCATAGTCACATAATGGATCCTGAATGTTGTATG
CONSENSUS
......................TCACATAATGGATCCTGAATGTTGTATG

A5-AT1G55325-XLOC_002797-3382-0
AGTTGTGCAGCTGAAAGTTCTGACAACAGACTCGGGTTCTCAGAGTCTCT
A5-AT1G55325-XLOC_002797-3382-1
AGTTGTGCAGCTGAAAGTTCTGACAACAGACTCGGGTTCTCAGAGTCTCT
CONSENSUS
AGTTGTGCAGCTGAAAGTTCTGACAACAGACTCGGGTTCTCAGAGTCTCT

A5-AT1G55325-XLOC_002797-3382-0
CTATGTCTCTGCCTCGGGACCATCTTGATGGTATCTGTTTTCAGCATACA
A5-AT1G55325-XLOC_002797-3382-1
CTATGTCTCTGCCTCGGGACCATCTTGATGGTATCTGTTTTCAGCATACA
CONSENSUS
CTATGTCTCTGCCTCGGGACCATCTTGATGGTATCTGTTTTCAGCATACA

A5-AT1G55325-XLOC_002797-3382-0
GACTGCAACTTCCTCAGGTGTTGCTCCCCTTTCTTATCTG
A5-AT1G55325-XLOC_002797-3382-1

GACTGCAACTTCCTCAGGTGTTGCTCCCCTTTCTTATCTG
CONSENSUS
GACTGCAACTTCCTCAGGTGTTGCTCCCCTTTCTTATCTG

alignment for event: RI-AT1G60510-XLOC_003054-4428

RI-AT1G60510-XLOC_003054-4428-0
ATGGGAGGTAGTAAGAAGCATGTTGTCACTAGAACCTCTTCTCCTTCTCT
RI-AT1G60510-XLOC_003054-4428-1
ATGGGAGGTAGTAAGAAGCATGTTGTCACTAGAACCTCTTCTCCTTCTCT
CONSENSUS
ATGGGAGGTAGTAAGAAGCATGTTGTCACTAGAACCTCTTCTCCTTCTCT

RI-AT1G60510-XLOC_003054-4428-0
TGCAATTGTCCAGGCGAATCCTCATGACAACAGAGAAGTTGTTCCCATCG
RI-AT1G60510-XLOC_003054-4428-1
TGCAATTGTCCAGGCGAATCCTCATGACAACAGAGAAGTTGTTCCCATCG
CONSENSUS
TGCAATTGTCCAGGCGAATCCTCATGACAACAGAGAAGTTGTTCCCATCG

RI-AT1G60510-XLOC_003054-4428-0
AAGCACCGATTATATCTTCATATAACGATAGGATTAGGCCGTTGCTTGAC
RI-AT1G60510-XLOC_003054-4428-1
AAGCACCGATTATATCTTCATATAACGATAGGATTAGGCCGTTGCTTGAC
CONSENSUS
AAGCACCGATTATATCTTCATATAACGATAGGATTAGGCCGTTGCTTGAC

RI-AT1G60510-XLOC_003054-4428-0
ACGGTTGACCGTCTAAGGAACCTCAATGTGATGAGAGAAGGCATTCAGCT
RI-AT1G60510-XLOC_003054-4428-1
ACGGTTGACCGTCTAAGGAACCTCAATGTGATGAGAGAAGGCATTCAGCT
CONSENSUS
ACGGTTGACCGTCTAAGGAACCTCAATGTGATGAGAGAAGGCATTCAGCT

RI-AT1G60510-XLOC_003054-4428-0
TCCCACCATTGTCGTGGTTGGAGACCAGTCCTCGGGGAAGTCAAGTGTCC
RI-AT1G60510-XLOC_003054-4428-1
TCCCACCATTGTCGTGGTTGGAGACCAGTCCTCGGGGAAGTCAAGTGTCC
CONSENSUS
TCCCACCATTGTCGTGGTTGGAGACCAGTCCTCGGGGAAGTCAAGTGTCC

RI-AT1G60510-XLOC_003054-4428-0
TCGACTCGTTGGCAGGAATCAGTCTACCTCGTGGCCAAGGAATCTGCACT
RI-AT1G60510-XLOC_003054-4428-1
TCGACTCGTTGGCAGGAATCAGTCTACCTCGTGGCCAAGGAATCTGCACT
CONSENSUS
TCGACTCGTTGGCAGGAATCAGTCTACCTCGTGGCCAAGGAATCTGCACT

RI-AT1G60510-XLOC_003054-4428-0
AGGGTTCCTCTTGTCATGCGACTTCAGCGAAGCTCTAGCCCTGTACCTGA
RI-AT1G60510-XLOC_003054-4428-1
AGGGTTCCTCTTGTCATGCGACTTCAGCGAAGCTCTAGCCCTGTACCTGA
CONSENSUS
AGGGTTCCTCTTGTCATGCGACTTCAGCGAAGCTCTAGCCCTGTACCTGA

RI-AT1G60510-XLOC_003054-4428-0
GATATGGCTTGAGTACAGTGACAAGATTGTTCCCACGGATGAGGAGCACA
RI-AT1G60510-XLOC_003054-4428-1
GATATGGCTTGAGTACAGTGACAAGATTGTTCCCACGGATGAGGAGCACA
CONSENSUS
GATATGGCTTGAGTACAGTGACAAGATTGTTCCCACGGATGAGGAGCACA

RI-AT1G60510-XLOC_003054-4428-0
TCGCTGAAGCTATCTGTGCTGCAACAGATGTGATTGCTGGTAAGTTCACT
RI-AT1G60510-XLOC_003054-4428-1
TCGCTGAAGCTATCTGTGCTGCAACAGATGTGATTGCTGGTAAGTTCACT
CONSENSUS
TCGCTGAAGCTATCTGTGCTGCAACAGATGTGATTGCTGGTAAGTTCACT

RI-AT1G60510-XLOC_003054-4428-0
CTCTATCTTGGTATTAAGTGTGTTTAGCTGCAAGAGAGATTACTGAGAGT
RI-AT1G60510-XLOC_003054-4428-1
CTCTATCTTGGTATTAAGTGTGTTTAGCTGCAAGAGAGATTACTGAGAGT
CONSENSUS
CTCTATCTTGGTATTAAGTGTGTTTAGCTGCAAGAGAGATTACTGAGAGT

RI-AT1G60510-XLOC_003054-4428-0
GCGACCTTTAATCTAAAACATGAATATTTGAACGAAGTTGGTTTACTTAC
RI-AT1G60510-XLOC_003054-4428-1
GCGACCTTTAATCTAAAACATGAATATTTGAACGAAGTTGGTTTACTTAC
CONSENSUS
GCGACCTTTAATCTAAAACATGAATATTTGAACGAAGTTGGTTTACTTAC

RI-AT1G60510-XLOC_003054-4428-0
CATAGAATTCCTTGGAGTTAGATGTGTACATATACCTAAATGACAGTGCC
RI-AT1G60510-XLOC_003054-4428-1
CATAGAATTCCTTGGAGTTAGATGTGTACATATACCTAAATGACAGTGCC
CONSENSUS
CATAGAATTCCTTGGAGTTAGATGTGTACATATACCTAAATGACAGTGCC

RI-AT1G60510-XLOC_003054-4428-0
TAGTGATGTGTTTTATATACTCTGAAACTCCTAGTTGGATCTTAATGGTC
RI-AT1G60510-XLOC_003054-4428-1
TAGTGATGTGTTTTATATACTCTGAAACTCCTAGTTGGATCTTAATGGTC
CONSENSUS
TAGTGATGTGTTTTATATACTCTGAAACTCCTAGTTGGATCTTAATGGTC

RI-AT1G60510-XLOC_003054-4428-0
TGGTTTTTTTTCATGCTGTTAATTATACTTTAGCTGTTCACGTTACTTAC
RI-AT1G60510-XLOC_003054-4428-1
TGGTTTTTTTTCATGCTGTTAATTATACTTTAGCTGTTCACGTTACTTAC
CONSENSUS
TGGTTTTTTTTCATGCTGTTAATTATACTTTAGCTGTTCACGTTACTTAC

RI-AT1G60510-XLOC_003054-4428-0
TGTTGAAACTTCATAGTGTTACTTACTACCATTGATGTCCCTAAAATAAG
RI-AT1G60510-XLOC_003054-4428-1
TGTTGAAACTTCATAGTGTTACTTACTACCATTGATGTCCCTAAAATAAG
CONSENSUS
TGTTGAAACTTCATAGTGTTACTTACTACCATTGATGTCCCTAAAATAAG

RI-AT1G60510-XLOC_003054-4428-0
ATGTCTACATGAACCTGAATACAAGTACCTAGATTTGAAATATGATTATG
RI-AT1G60510-XLOC_003054-4428-1
ATGTCTACATGAACCTGAATACAAGTACCTAGATTTGAAATATGATTATG
CONSENSUS
ATGTCTACATGAACCTGAATACAAGTACCTAGATTTGAAATATGATTATG

RI-AT1G60510-XLOC_003054-4428-0
GAAAACCTAGTGTAGTCTGATAACTTTAGTGCTCTTTATACTACTAGTTT
RI-AT1G60510-XLOC_003054-4428-1
GAAAACCTAGTGTAGTCTGATAACTTTAGTGCTCTTTATACTACTAGTTT
CONSENSUS
GAAAACCTAGTGTAGTCTGATAACTTTAGTGCTCTTTATACTACTAGTTT

RI-AT1G60510-XLOC_003054-4428-0
TGCTATTTGAAGGAACTAAAAGTGTTGTGTGTGATGCTAACCAGTTTTGA
RI-AT1G60510-XLOC_003054-4428-1
TGCTATTTGAAGGAACTAAAAGTGTTGTGTGTGATGCTAACCAGTTTTGA
CONSENSUS
TGCTATTTGAAGGAACTAAAAGTGTTGTGTGTGATGCTAACCAGTTTTGA

RI-AT1G60510-XLOC_003054-4428-0
ACTTTTGACTACCTATACTGCAGGATCTGGCAAAGGGGTCTCAGACGCTC
RI-AT1G60510-XLOC_003054-4428-1
ACTTTTGACTACCTATACTGCAGGATCTGGCAAAGGGGTCTCAGACGCTC
CONSENSUS
ACTTTTGACTACCTATACTGCAGGATCTGGCAAAGGGGTCTCAGACGCTC

RI-AT1G60510-XLOC_003054-4428-0
CATTGACCCTCCATGTTAAGAAGGCTGGGGTCCCATATCTTACCATGGTC
RI-AT1G60510-XLOC_003054-4428-1
CATTGACCCTCCATGTTAAGAAGGCTGGGGTCCCATATCTTACCATGGTC
CONSENSUS
CATTGACCCTCCATGTTAAGAAGGCTGGGGTCCCATATCTTACCATGGTC

RI-AT1G60510-XLOC_003054-4428-0
GATCTTCCCGGTATAACTCGAGGCCAGTGAATGGACAACCGGAGAATATT
RI-AT1G60510-XLOC_003054-4428-1
GATCTTCCCGGTATAACTCGAGGCCAGTGAATGGACAACCGGAGAATATT
CONSENSUS
GATCTTCCCGGTATAACTCGAGGCCAGTGAATGGACAACCGGAGAATATT

RI-AT1G60510-XLOC_003054-4428-0
TACGAACAGATTTCTGGGATGATCATGAAGTACATCGAGCCACAAGAATC
RI-AT1G60510-XLOC_003054-4428-1
TACGAACAGATTTCTGGGATGATCATGAAGTACATCGAGCCACAAGAATC
CONSENSUS
TACGAACAGATTTCTGGGATGATCATGAAGTACATCGAGCCACAAGAATC

RI-AT1G60510-XLOC_003054-4428-0
AATAATCCTCAATGTTCTGTCAGCTACAGTCGACTTCACCACCTGTGAAT
RI-AT1G60510-XLOC_003054-4428-1
AATAATCCTCAATGTTCTGTCAGCTACAGTCGACTTCACCACCTGTGAAT
CONSENSUS
AATAATCCTCAATGTTCTGTCAGCTACAGTCGACTTCACCACCTGTGAAT

RI-AT1G60510-XLOC_003054-4428-0
CCATCCGTATGTGAACGGACTCTGGCCGTTGTCACCAAGGCAGACATGGC
RI-AT1G60510-XLOC_003054-4428-1
CCATCCGTATGTGAACGGACTCTGGCCGTTGTCACCAAGGCAGACATGGC
CONSENSUS
CCATCCGTATGTGAACGGACTCTGGCCGTTGTCACCAAGGCAGACATGGC

RI-AT1G60510-XLOC_003054-4428-0
TCCCGAAGGTCTCCTACAGAAAGTAACTGCGGATGATGTGAGTATTGGTC
RI-AT1G60510-XLOC_003054-4428-1
TCCCGAAGGTCTCCTACAGAAAGTAACTG---------------------
CONSENSUS
TCCCGAAGGTCTCCTACAGAAAGTAACTG.....................

RI-AT1G60510-XLOC_003054-4428-0
TAGGTTACGTCTGTGTCAGAAACCGCATTGGAGAAGAGCGTATGAACAAG
RI-AT1G60510-XLOC_003054-4428-1

CONSENSUS
....

RI-AT1G60510-XLOC_003054-4428-0
CTAGAATGCAAGAAGAGTTGCTCTTCAGGACTCACCCGATGCTGAGCTTG
RI-AT1G60510-XLOC_003054-4428-1
-----------------------------------CCGATGCTGAGCTTG
CONSENSUS
...................................CCGATGCTGAGCTTG

RI-AT1G60510-XLOC_003054-4428-0
ATTGATGAAGACATCGTGGGAATCCCTGTTATAGCTCAGAAGCTAATGCT
RI-AT1G60510-XLOC_003054-4428-1
ATTGATGAAGACATCGTGGGAATCCCTGTTATAGCTCAGAAGCTAATGCT
CONSENSUS
ATTGATGAAGACATCGTGGGAATCCCTGTTATAGCTCAGAAGCTAATGCT

RI-AT1G60510-XLOC_003054-4428-0
TATCCAAGCAACGATGATTTCCCGCTGTTTGCCTGAAATCGTTCGCAAGA
RI-AT1G60510-XLOC_003054-4428-1
TATCCAAGCAACGATGATTTCCCGCTGTTTGCCTGAAATCGTTCGCAAGA
CONSENSUS
TATCCAAGCAACGATGATTTCCCGCTGTTTGCCTGAAATCGTTCGCAAGA

RI-AT1G60510-XLOC_003054-4428-0
TCAATCACAAGATGGAAACTGCTGTCCTGGAGTTGAACAAGCTACCGATG
RI-AT1G60510-XLOC_003054-4428-1
TCAATCACAAGATGGAAACTGCTGTCCTGGAGTTGAACAAGCTACCGATG
CONSENSUS
TCAATCACAAGATGGAAACTGCTGTCCTGGAGTTGAACAAGCTACCGATG

RI-AT1G60510-XLOC_003054-4428-0
GTTATGGCTTCTACCGGAGAAGCATTGATGGCATTGATGGACATCATTGG
RI-AT1G60510-XLOC_003054-4428-1
GTTATGGCTTCTACCGGAGAAGCATTGATGGCATTGATGGACATCATTGG
CONSENSUS
GTTATGGCTTCTACCGGAGAAGCATTGATGGCATTGATGGACATCATTGG

RI-AT1G60510-XLOC_003054-4428-0
TTCTGCCAAGGAGTCCCTCTTCAGAATCCTTGTCCAAGGAGACTTCTCTG
RI-AT1G60510-XLOC_003054-4428-1
TTCTGCCAAGGAGTCCCTCTTCAGAATCCTTGTCCAAGGAGACTTCTCTG
CONSENSUS
TTCTGCCAAGGAGTCCCTCTTCAGAATCCTTGTCCAAGGAGACTTCTCTG

RI-AT1G60510-XLOC_003054-4428-0
AATTCCCGGATGACCAGAACATGCACTGTACTGCTCGTTTGGCTGACATG
RI-AT1G60510-XLOC_003054-4428-1
AATTCCCGGATGACCAGAACATGCACTGTACTGCTCGTTTGGCTGACATG
CONSENSUS
AATTCCCGGATGACCAGAACATGCACTGTACTGCTCGTTTGGCTGACATG

RI-AT1G60510-XLOC_003054-4428-0
TTAAGCCAATTCTCCGATAATCTGCAAGAGAAGCCAAAGGAAGTGACCGA
RI-AT1G60510-XLOC_003054-4428-1
TTAAGCCAATTCTCCGATAATCTGCAAGAGAAGCCAAAGGAAGTGACCGA
CONSENSUS
TTAAGCCAATTCTCCGATAATCTGCAAGAGAAGCCAAAGGAAGTGACCGA

RI-AT1G60510-XLOC_003054-4428-0
GTTCTTGATGAATGAGATCAAGATCCTTGACGAATGCAAGTGTGTTGGAC
RI-AT1G60510-XLOC_003054-4428-1
GTTCTTGATGAATGAGATCAAGATCCTTGACGAATGCAAGTGTGTTGGAC
CONSENSUS
GTTCTTGATGAATGAGATCAAGATCCTTGACGAATGCAAGTGTGTTGGAC

RI-AT1G60510-XLOC_003054-4428-0
TGCCCAATTTCATCCCCAGGTCAGCCTTCTTGGCTATACTATCACAACAC
RI-AT1G60510-XLOC_003054-4428-1
TGCCCAATTTCATCCCCAGGTCAGCCTTCTTGGCTATACTATCACAACAC
CONSENSUS
TGCCCAATTTCATCCCCAGGTCAGCCTTCTTGGCTATACTATCACAACAC

RI-AT1G60510-XLOC_003054-4428-0
GAAGATGCCATACATGTCAAGCCCGTGGAGTTCATCAAGAAGATATGGGA
RI-AT1G60510-XLOC_003054-4428-1
GAAGATGCCATACATGTCAAGCCCGTGGAGTTCATCAAGAAGATATGGGA
CONSENSUS
GAAGATGCCATACATGTCAAGCCCGTGGAGTTCATCAAGAAGATATGGGA

RI-AT1G60510-XLOC_003054-4428-0
CTACATTGAAGTTGTTCTCTCATCCGTCATTGCCAAGTATTCTGAAAACT
RI-AT1G60510-XLOC_003054-4428-1
CTACATTGAAGTTGTTCTCTCATCCGTCATTGCCAAGTATTCTGAAAACT
CONSENSUS
CTACATTGAAGTTGTTCTCTCATCCGTCATTGCCAAGTATTCTGAAAACT

RI-AT1G60510-XLOC_003054-4428-0
TCCCACAGATCCAATCTGCCATCAAACGTGCTGGTCGAAATCTAATCACC
RI-AT1G60510-XLOC_003054-4428-1
TCCCACAGATCCAATCTGCCATCAAACGTGCTGGTCGAAATCTAATCACC
CONSENSUS
TCCCACAGATCCAATCTGCCATCAAACGTGCTGGTCGAAATCTAATCACC

RI-AT1G60510-XLOC_003054-4428-0
AAGATCAAGGAACACTCTGTGGAGCGAGTGTTGGAGATTGTTGAAATGGA
RI-AT1G60510-XLOC_003054-4428-1
AAGATCAAGGAACACTCTGTGGAGCGAGTGTTGGAGATTGTTGAAATGGA
CONSENSUS
AAGATCAAGGAACACTCTGTGGAGCGAGTGTTGGAGATTGTTGAAATGGA

RI-AT1G60510-XLOC_003054-4428-0
GAAGCTGACTGATTACACATGTAACCCTGAGTACATGACGTCTTGGACTC
RI-AT1G60510-XLOC_003054-4428-1
GAAGCTGACTGATTACACATGTAACCCTGAGTACATGACGTCTTGGACTC
CONSENSUS
GAAGCTGACTGATTACACATGTAACCCTGAGTACATGACGTCTTGGACTC

RI-AT1G60510-XLOC_003054-4428-0
AGATGACTGCTGAACAACAAAACTTCATTTATGCTGTGTTGTCCGATGGG
RI-AT1G60510-XLOC_003054-4428-1
AGATGACTGCTGAACAACAAAACTTCATTTATGCTGTGTTGTCCGATGGG
CONSENSUS
AGATGACTGCTGAACAACAAAACTTCATTTATGCTGTGTTGTCCGATGGG

RI-AT1G60510-XLOC_003054-4428-0
AAAAAGCCTGAGCACTTCTCGTTGACTGGATTTGGTGGAATTGTGAAGAT
RI-AT1G60510-XLOC_003054-4428-1
AAAAAGCCTGAGCACTTCTCGTTGACTGGATTTGGTGGAATTGTGAAGAT
CONSENSUS
AAAAAGCCTGAGCACTTCTCGTTGACTGGATTTGGTGGAATTGTGAAGAT

RI-AT1G60510-XLOC_003054-4428-0
CTCTCACCTACGGAAGTATCATGCTCATCTCCTGCAGCAAGCCTTCGATA
RI-AT1G60510-XLOC_003054-4428-1
CTCTCACCTACGGAAGTATCATGCTCATCTCCTGCAGCAAGCCTTCGATA
CONSENSUS
CTCTCACCTACGGAAGTATCATGCTCATCTCCTGCAGCAAGCCTTCGATA

RI-AT1G60510-XLOC_003054-4428-0
TGAAGATGAGGATAGCATCTTACTGGACGATAGTCTTACGAAGGATTGTG
RI-AT1G60510-XLOC_003054-4428-1
TGAAGATGAGGATAGCATCTTACTGGACGATAGTCTTACGAAGGATTGTG
CONSENSUS
TGAAGATGAGGATAGCATCTTACTGGACGATAGTCTTACGAAGGATTGTG

RI-AT1G60510-XLOC_003054-4428-0
GACAGTCTTGCTCTGTATCTTCAGCTGTCAGTGAAGAATCTCGTGAATTA
RI-AT1G60510-XLOC_003054-4428-1
GACAGTCTTGCTCTGTATCTTCAGCTGTCAGTGAAGAATCTCGTGAATTA
CONSENSUS
GACAGTCTTGCTCTGTATCTTCAGCTGTCAGTGAAGAATCTCGTGAATTA

RI-AT1G60510-XLOC_003054-4428-0
TTAGTTTCAGAAGGAGATTGTGGCGGAGATGGTGGATCCAAGAGCTGGCG
RI-AT1G60510-XLOC_003054-4428-1
TTAGTTTCAGAAGGAGATTGTGGCGGAGATGGTGGATCCAAGAGCTGGCG
CONSENSUS
TTAGTTTCAGAAGGAGATTGTGGCGGAGATGGTGGATCCAAGAGCTGGCG

RI-AT1G60510-XLOC_003054-4428-0
      GAGGTCTTCAGAGGATGCTAGAGGAGTCGCCGTCGGTGGCGAGCAAGAGA
RI-AT1G60510-XLOC_003054-4428-1
      GAGGTCTTCAGAGGATGCTAGAGGAGTCGCCGTCGGTGGCGAGCAAGAGA
CONSENSUS
      GAGGTCTTCAGAGGATGCTAGAGGAGTCGCCGTCGGTGGCGAGCAAGAGA

RI-AT1G60510-XLOC_003054-4428-0 GAGAAGCTGAAGAACAGT
RI-AT1G60510-XLOC_003054-4428-1 GAGAAGCTGAAGAACAGT
CONSENSUS   GAGAAGCTGAAGAACAGT

alignment for event: A3-AT1G16540-XLOC_000846-7953

A3-AT1G16540-XLOC_000846-7953-0
      ACTTGAAAACCGTAATGAGGAAACTAGAATCAATCGTTGGTTCACCAATG
A3-AT1G16540-XLOC_000846-7953-1
      ACTTGAAAACCGTAATGAGGAAACTAGAATCAATCGTTGGTTCACCAATG
CONSENSUS
      ACTTGAAAACCGTAATGAGGAAACTAGAATCAATCGTTGGTTCACCAATG

A3-AT1G16540-XLOC_000846-7953-0
      CCATTGGTCGACAATGCAAGTTGCTACGGTATTCTAGCTCTACTTCCAAA
A3-AT1G16540-XLOC_000846-7953-1
      CCATTGGTCGACAATGCAAGTTGCTACGGTATTCTAGCTCTACTTCCAAA
CONSENSUS
      CCATTGGTCGACAATGCAAGTTGCTACGGTATTCTAGCTCTACTTCCAAA

A3-AT1G16540-XLOC_000846-7953-0
      GACTGCTTGAACAGAAACAAGAGTCCTGGTTTGTGCAGAGATTTGGAAAG
A3-AT1G16540-XLOC_000846-7953-1
      GACTGCTTGAACAGAAACAAGAGTCCTGGTTTGTGCAGAGATTTGGAAAG
CONSENSUS
      GACTGCTTGAACAGAAACAAGAGTCCTGGTTTGTGCAGAGATTTGGAAAG

A3-AT1G16540-XLOC_000846-7953-0
      CAATATCAACTTTGCTAATGAAGCTCAGTTCTTGTTAATCTCCGAGGAGA
A3-AT1G16540-XLOC_000846-7953-1
      CAATATCAACTTTGCTAATGAAGCTCAGTTCTTGTTAATCTCCGAGGAGA
CONSENSUS
      CAATATCAACTTTGCTAATGAAGCTCAGTTCTTGTTAATCTCCGAGGAGA

A3-AT1G16540-XLOC_000846-7953-0
      GTGTTGCTGACCTAAACAGAAGATTAGAAGCAAAAGACGAGGATTACAAA
A3-AT1G16540-XLOC_000846-7953-1
      GTGTTGCTGACCTAAACAGAAGATTAGAAGCAA---ACGAGGATTACAAA
CONSENSUS
      GTGTTGCTGACCTAAACAGAAGATTAGAAGCAA...ACGAGGATTACAAA

A3-AT1G16540-XLOC_000846-7953-0
      CGGGCTCATGAAAAACTCAATCCACATAGGTTCAGACCAAATCTGGTTAT
A3-AT1G16540-XLOC_000846-7953-1
      CGGGCTCATGAAAAACTCAATCCACATAGGTTCAGACCAAATCTGGTTAT
CONSENSUS

CGGGCTCATGAAAAACTCAATCCACATAGGTTCAGACCAAATCTGGTTAT

A3-AT1G16540-XLOC_000846-7953-0
ATCTGGAGGTGAACCATACGGGGAAGATAAATGGAAAACTGTCAAGATAG
A3-AT1G16540-XLOC_000846-7953-1
ATCTGGAGGTGAACCATACGGGGAAGATAAATGGAAAACTGTCAAGATAG
CONSENSUS
ATCTGGAGGTGAACCATACGGGGAAGATAAATGGAAAACTGTCAAGATAG

A3-AT1G16540-XLOC_000846-7953-0 GAGACAATCATTTCACA
A3-AT1G16540-XLOC_000846-7953-1 GAGACAATCATTTCACA
CONSENSUS GAGACAATCATTTCACA

alignment for event: A3-AT1G06150-XLOC_004443-2372

A3-AT1G06150-XLOC_004443-2372-0
TGCAGTATTGATTCCTTGCTAGAGTGCACGATCAAGCACATGCTCTTCCT
A3-AT1G06150-XLOC_004443-2372-1
TGCAGTATTGATTCCTTGCTAGAGTGCACGATCAAGCACATGCTCTTCCT
CONSENSUS
TGCAGTATTGATTCCTTGCTAGAGTGCACGATCAAGCACATGCTCTTCCT

A3-AT1G06150-XLOC_004443-2372-0
GCAGAGTGTCTCTCAGCATGCTGACAAGCTCACTAAAAGTGCAAGTTCAA
A3-AT1G06150-XLOC_004443-2372-1
GCAGAGTGTCTCTCAGCATGCTGACAAGCTCACTAAAAGTGCAAGTTCAA
CONSENSUS
GCAGAGTGTCTCTCAGCATGCTGACAAGCTCACTAAAAGTGCAAGTTCAA

A3-AT1G06150-XLOC_004443-2372-0 AG------
ATGCAACACAAGGATACCGGCACCCTAGGAATATCAAGCACT
A3-AT1G06150-XLOC_004443-2372-1
AGTTACAGATGCAACACAAGGATACCGGCACCCTAGGAATATCAAGCACT
CONSENSUS
AG......ATGCAACACAAGGATACCGGCACCCTAGGAATATCAAGCACT

A3-AT1G06150-XLOC_004443-2372-0
GAACAAGGTTCGAGCTGGGCAGTGGAGATTGGAGGCCATCTGCAAGTGTG
A3-AT1G06150-XLOC_004443-2372-1
GAACAAGGTTCGAGCTGGGCAGTGGAGATTGGAGGCCATCTGCAAGTGTG
CONSENSUS
GAACAAGGTTCGAGCTGGGCAGTGGAGATTGGAGGCCATCTGCAAGTGTG

A3-AT1G06150-XLOC_004443-2372-0
CTCAATCATGGTGGAGAATCTGGACAAAGAAGGAGTGATGCTTATTGAG
A3-AT1G06150-XLOC_004443-2372-1
CTCAATCATGGTGGAGAATCTGGACAAAGAAGGAGTGATGCTTATTGAG
CONSENSUS
CTCAATCATGGTGGAGAATCTGGACAAAGAAGGAGTGATGCTTATTGAG

alignment for event: A3-AT1G17480-XLOC_005067-2740

A3-AT1G17480-XLOC_005067-2740-0
CACAACCAAATTCCCAGAAACAATCTTTTTGCTTTTCCCACATTTCAAGC
A3-AT1G17480-XLOC_005067-2740-1
CACAACCAAATTCCCAGAAACAATCTTTTTGCTTTTCCCACATTTCAAGC
CONSENSUS
CACAACCAAATTCCCAGAAACAATCTTTTTGCTTTTCCCACATTTCAAGC

A3-AT1G17480-XLOC_005067-2740-0
TCCAAAGCTTCGAATTTTTGAATCAATAGCTTCCGAAGTTGTGATTTTTG
A3-AT1G17480-XLOC_005067-2740-1
TCCAAAGCTTCGAATTTTTGAATCAATAGCTTCCGAAGTTGTGATTTTTG
CONSENSUS
TCCAAAGCTTCGAATTTTTGAATCAATAGCTTCCGAAGTTGTGATTTTTG

A3-AT1G17480-XLOC_005067-2740-0
TAGAATTCGAAAACCAAGGTCTCGATTTGAGAAGATGGGTGGGTCAGGAA
A3-AT1G17480-XLOC_005067-2740-1
TAGAATTCGAAAACCAAGGTCTCGATTTGAGAAGATGGGTGGGTCAGGAA
CONSENSUS
TAGAATTCGAAAACCAAGGTCTCGATTTGAGAAGATGGGTGGGTCAGGAA

A3-AT1G17480-XLOC_005067-2740-0
ATTGGATTAGATCACTGATATCTAACAGAAAACCCGTAAATGATCAG---
A3-AT1G17480-XLOC_005067-2740-1
ATTGGATTAGATCACTGATATCTAACAGAAAACCCGTAAATGATCAGCAG
CONSENSUS
ATTGGATTAGATCACTGATATCTAACAGAAAACCCGTAAATGATCAG...

A3-AT1G17480-XLOC_005067-2740-0
GAGAAGTTAAGTGATAAGAGCAGCAAGAAGAAATGGAAGCTGTGGAGGAT
A3-AT1G17480-XLOC_005067-2740-1
GAGAAGTTAAGTGATAAGAGCAGCAAGAAGAAATGGAAGCTGTGGAGGAT
CONSENSUS
GAGAAGTTAAGTGATAAGAGCAGCAAGAAGAAATGGAAGCTGTGGAGGAT

A3-AT1G17480-XLOC_005067-2740-0
TTCATCGGAGAGCTTAGCATCTTCTTCTTTCAAGAGCCGAGGAAGCTATG
A3-AT1G17480-XLOC_005067-2740-1
TTCATCGGAGAGCTTAGCATCTTCTTCTTTCAAGAGCCGAGGAAGCTATG
CONSENSUS
TTCATCGGAGAGCTTAGCATCTTCTTCTTTCAAGAGCCGAGGAAGCTATG

A3-AT1G17480-XLOC_005067-2740-0
CAGCTTCTTCGTTAGGTTCAGAGCTACCGTCTTTCTCGGCTGACGAGGCT
A3-AT1G17480-XLOC_005067-2740-1
CAGCTTCTTCGTTAGGTTCAGAGCTACCGTCTTTCTCGGCTGACGAGGCT
CONSENSUS
CAGCTTCTTCGTTAGGTTCAGAGCTACCGTCTTTCTCGGCTGACGAGGCT

A3-AT1G17480-XLOC_005067-2740-0
TTTACTACGGCCATGGCAGCACTCATTCGAGCTCCTCCTAGGGATTTTTT
A3-AT1G17480-XLOC_005067-2740-1
TTTACTACGGCCATGGCAGCACTCATTCGAGCTCCTCCTAGGGATTTTTT
CONSENSUS
TTTACTACGGCCATGGCAGCACTCATTCGAGCTCCTCCTAGGGATTTTTT

A3-AT1G17480-XLOC_005067-2740-0
GATGGTTAAGAGAGAATGGGCTTCAACAAGAATCCAGGCTGCTTTTCGTG
A3-AT1G17480-XLOC_005067-2740-1
GATGGTTAAGAGAGAATGGGCTTCAACAAGAATCCAGGCTGCTTTTCGTG
CONSENSUS
GATGGTTAAGAGAGAATGGGCTTCAACAAGAATCCAGGCTGCTTTTCGTG

A3-AT1G17480-XLOC_005067-2740-0 CTTTCCTC
A3-AT1G17480-XLOC_005067-2740-1 CTTTCCTC
CONSENSUS CTTTCCTC

alignment for event: RI-AT1G28610-XLOC_005638-10387

RI-AT1G28610-XLOC_005638-10387-0
ATTGTAGAGATATGATTGGAAATGCTTTTATTATCATGGGAGAGATTGGA
RI-AT1G28610-XLOC_005638-10387-1
ATTGTAGAGATATGATTGGAAATGCTTTTATTATCATGGGAGAGATTGGA
CONSENSUS
ATTGTAGAGATATGATTGGAAATGCTTTTATTATCATGGGAGAGATTGGA

RI-AT1G28610-XLOC_005638-10387-0
GGGAATGATTTTAATTTCGCATTCTTTGTGAACAAAACCAGTGAGGTCAA
RI-AT1G28610-XLOC_005638-10387-1
GGGAATGATTTTAATTTCGCATTCTTTGTGAACAAAACCAGTGAGGTCAA
CONSENSUS
GGGAATGATTTTAATTTCGCATTCTTTGTGAACAAAACCAGTGAGGTCAA

RI-AT1G28610-XLOC_005638-10387-0
AGAGTTGGTTCCGTTGGTGATCACTAAAATTTCTTCTGCAATTGTGGTAA
RI-AT1G28610-XLOC_005638-10387-1
AGAGTTGGTTCCGTTGGTGATCACTAAAATTTCTTCTGCAATTGTG----
CONSENSUS
AGAGTTGGTTCCGTTGGTGATCACTAAAATTTCTTCTGCAATTGTG....

RI-AT1G28610-XLOC_005638-10387-0
ACATTCTTGATCAGCGAGTCCCTAGAACGAGTAATTTTTTCAAAAAACTG
RI-AT1G28610-XLOC_005638-10387-1

CONSENSUS
....

RI-AT1G28610-XLOC_005638-10387-0
TTATGTTTTTTGTTTTGTTTTTGTGTTTTGGTACTGAATGTTGAATCTAG
RI-AT1G28610-XLOC_005638-10387-1

CONSENSUS
....

RI-AT1G28610-XLOC_005638-10387-0
GAGTTGGTCGATATGGGAGGAAGAACATTCCTAGTTCCTGGAAACTTCCC
RI-AT1G28610-XLOC_005638-10387-1
GAGTTGGTCGATATGGGAGGAAGAACATTCCTAGTTCCTGGAAACTTCCC
CONSENSUS
GAGTTGGTCGATATGGGAGGAAGAACATTCCTAGTTCCTGGAAACTTCCC

RI-AT1G28610-XLOC_005638-10387-0
      GCTCGGATGCTCAGCAACGTATTTAACATTATACCAAACATCAAACAAGG
RI-AT1G28610-XLOC_005638-10387-1
      GCTCGGATGCTCAGCAACGTATTTAACATTATACCAAACATCAAACAAGG
CONSENSUS
      GCTCGGATGCTCAGCAACGTATTTAACATTATACCAAACATCAAACAAGG

RI-AT1G28610-XLOC_005638-10387-0
      AAGAGTATGATCCTCTAACAGGATGTTTGACATGGCTGAACGATTTTTCA
RI-AT1G28610-XLOC_005638-10387-1
      AAGAGTATGATCCTCTAACAGGATGTTTGACATGGCTGAACGATTTTTCA
CONSENSUS
      AAGAGTATGATCCTCTAACAGGATGTTTGACATGGCTGAACGATTTTTCA

RI-AT1G28610-XLOC_005638-10387-0
      GAATACTACAACGAGAAGCTTCAGGCAGAACTCAACAGACTCAGTAAACT
RI-AT1G28610-XLOC_005638-10387-1
      GAATACTACAACGAGAAGCTTCAGGCAGAACTCAACAGACTCAGTAAACT
CONSENSUS
      GAATACTACAACGAGAAGCTTCAGGCAGAACTCAACAGACTCAGTAAACT

RI-AT1G28610-XLOC_005638-10387-0
      TTACCCTCATGTCAACATCATATATGGTGACTACTTTAATGCTCTGTTGC
RI-AT1G28610-XLOC_005638-10387-1
      TTACCCTCATGTCAACATCATATATGGTGACTACTTTAATGCTCTGTTGC
CONSENSUS
      TTACCCTCATGTCAACATCATATATGGTGACTACTTTAATGCTCTGTTGC

RI-AT1G28610-XLOC_005638-10387-0 GCCTTTACCAAGAACCATCCAAATTTG
RI-AT1G28610-XLOC_005638-10387-1 GCCTTTACCAAGAACCATCCAAATTTG
CONSENSUS   GCCTTTACCAAGAACCATCCAAATTTG

alignment for event: RI-AT1G79880-XLOC_008100-12394

RI-AT1G79880-XLOC_008100-12394-0
      GTTGAGTTCTACTTCAGTGACAGTAATCTACCCACAGACGGATTTCTCAA
RI-AT1G79880-XLOC_008100-12394-1
      GTTGAGTTCTACTTCAGTGACAGTAATCTACCCACAGACGGATTTCTCAA
CONSENSUS
      GTTGAGTTCTACTTCAGTGACAGTAATCTACCCACAGACGGATTTCTCAA

RI-AT1G79880-XLOC_008100-12394-0
      CAGGGAAGTCACCAAGAGCAAAGATGGCCGTATCCTGTATACATATATAT
RI-AT1G79880-XLOC_008100-12394-1
      CAGGGAAGTCACCAAGAGCAAAGATGGCC---------------------
CONSENSUS
      CAGGGAAGTCACCAAGAGCAAAGATGGCC.....................

RI-AT1G79880-XLOC_008100-12394-0
      TGTTGTAGCATACAGATATAGTTGAAAAGTTATTCCTTAGATGCCCGAAA
RI-AT1G79880-XLOC_008100-12394-1

CONSENSUS

....

RI-AT1G79880-XLOC_008100-12394-0
      CTAGTGGTTAGTTTGCCTCTGGTTTGTTCCTTCTCTCGTATGAGGAACCT
RI-AT1G79880-XLOC_008100-12394-1 ----
TGGTTAGTTTGCCTCTGGTTTGTTCCTTCTCTCGTATGAGGAACCT
CONSENSUS
      ....TGGTTAGTTTGCCTCTGGTTTGTTCCTTCTCTCGTATGAGGAACCT

RI-AT1G79880-XLOC_008100-12394-0
      CCTTGGCCTCGGTAACATCAATAGAGAAGACATCCCTCCGAGGATTGTGG
RI-AT1G79880-XLOC_008100-12394-1
      CCTTGGCCTCGGTAACATCAATAGAGAAGACATCCCTCCGAGGATTGTGG
CONSENSUS
      CCTTGGCCTCGGTAACATCAATAGAGAAGACATCCCTCCGAGGATTGTGG

RI-AT1G79880-XLOC_008100-12394-0
      AGGAAGTTGCTAATCTCTTGCGCACCTCTGATTTTCTTAAAGTTTCCAAT
RI-AT1G79880-XLOC_008100-12394-1
      AGGAAGTTGCTAATCTCTTGCGCACCTCTGATTTTCTTAAAGTTTCCAAT
CONSENSUS
      AGGAAGTTGCTAATCTCTTGCGCACCTCTGATTTTCTTAAAGTTTCCAAT

RI-AT1G79880-XLOC_008100-12394-0 AATG
RI-AT1G79880-XLOC_008100-12394-1 AATG
CONSENSUS   AATG

alignment for event: RI-AT1G43620-XLOC_006255-9889

RI-AT1G43620-XLOC_006255-9889-0
      TGCAGCTAACTTTCTGTACCTAAGGATTTGACTGGTTGTTGCTTCAAGCT
RI-AT1G43620-XLOC_006255-9889-1
      TGCAGCTAACTTTCTGTACCTAAGGATTTGACTGGTTGTTGCTTCAAGCT
CONSENSUS
      TGCAGCTAACTTTCTGTACCTAAGGATTTGACTGGTTGTTGCTTCAAGCT

RI-AT1G43620-XLOC_006255-9889-0
      TTGATTCGTCTGTGTACAGCATTTGCCAGGAGAGCCATTAGCATCACTAT
RI-AT1G43620-XLOC_006255-9889-1
      TTGATTCGTCTGTGTACAGCATTTGCCAGGAGAGCCATTAGCATCACTAT
CONSENSUS
      TTGATTCGTCTGTGTACAGCATTTGCCAGGAGAGCCATTAGCATCACTAT

RI-AT1G43620-XLOC_006255-9889-0
      GGTTGAATAAATGTATAAGAATAGTGAAATTCTTTGAGTGTGTATAGATG
RI-AT1G43620-XLOC_006255-9889-1
      GGTTGAATAAAT--------------------------------------
CONSENSUS
      GGTTGAATAAAT......................................

RI-AT1G43620-XLOC_006255-9889-0
      TTCTTTTGTGGAGTTTGATCCATATGTAGGTGGTTTGTTGCTGCAGCTGA
RI-AT1G43620-XLOC_006255-9889-1
      --CTGA

CONSENSUS
      ....CTGA

RI-AT1G43620-XLOC_006255-9889-0
      TTGTGTGGGTTTAGGCAGTGAGAGCTGTTTAGTTGAATTCAGTTTTTAAA
RI-AT1G43620-XLOC_006255-9889-1
      TTGTGTGGGTTTAGGCAGTGAGAGCTGTTTAGTTGAATTCAGTTTTTAAA
CONSENSUS
      TTGTGTGGGTTTAGGCAGTGAGAGCTGTTTAGTTGAATTCAGTTTTTAAA

RI-AT1G43620-XLOC_006255-9889-0
      GGTGATGGCTAGTAATGTATTTGATCATCCACTTCAAGAATTAGAGGGTG
RI-AT1G43620-XLOC_006255-9889-1
      GGTGATGGCTAGTAATGTATTTGATCATCCACTTCAAGAATTAGAGGGTG
CONSENSUS
      GGTGATGGCTAGTAATGTATTTGATCATCCACTTCAAGAATTAGAGGGTG

RI-AT1G43620-XLOC_006255-9889-0
      AAGATAATGGTGTAAAGAGCGAGAAAGCGAGTTTATTGGAAACATCTGGT
RI-AT1G43620-XLOC_006255-9889-1
      AAGATAATGGTGTAAAGAGCGAGAAAGCGAGTTTATTGGAAACATCTGGT
CONSENSUS
      AAGATAATGGTGTAAAGAGCGAGAAAGCGAGTTTATTGGAAACATCTGGT

RI-AT1G43620-XLOC_006255-9889-0
      TCTGTAGATACTACTCCTGAGGATTCTGGTCATCGTTCTTCTGATGGGCA
RI-AT1G43620-XLOC_006255-9889-1
      TCTGTAGATACTACTCCTGAGGATTCTGGTCATCGTTCTTCTGATGGGCA
CONSENSUS
      TCTGTAGATACTACTCCTGAGGATTCTGGTCATCGTTCTTCTGATGGGCA

RI-AT1G43620-XLOC_006255-9889-0 TAGGG
RI-AT1G43620-XLOC_006255-9889-1 TAGGG
CONSENSUS   TAGGG

alignment for event: RI-AT1G74330-XLOC_007788-6881

RI-AT1G74330-XLOC_007788-6881-0
      GATGTGCGGCACCAAACTGAAACATTTCAGAAACGAATTGGTCATTTAGT
RI-AT1G74330-XLOC_007788-6881-1
      GATGTGCGGCACCAAACTGAAACATTTCAGAAACGAATTGGTCATTTAGT
CONSENSUS
      GATGTGCGGCACCAAACTGAAACATTTCAGAAACGAATTGGTCATTTAGT

RI-AT1G74330-XLOC_007788-6881-0
      TCATAGTTCGATTGAAAGTGATGCCAGGTTATGTGGGAAACTGCAAAACC
RI-AT1G74330-XLOC_007788-6881-1
      TCATAGTTCGATTGAAAGTGATGCCAGGTTATGTGGGAAACTGCAAAACC
CONSENSUS
      TCATAGTTCGATTGAAAGTGATGCCAGGTTATGTGGGAAACTGCAAAACC

RI-AT1G74330-XLOC_007788-6881-0
      CGTTAGATCACAAGAAAGACGAAGCTTCCCATGTGAAGCATGCATCACAA
RI-AT1G74330-XLOC_007788-6881-1

CGTTAGATCACAAGAAAGACGAAGCTTCCCATGTGAAGCATGCATCACAA
CONSENSUS
CGTTAGATCACAAGAAAGACGAAGCTTCCCATGTGAAGCATGCATCACAA

RI-AT1G74330-XLOC_007788-6881-0
GGAGATGTGCCTTTCTCAGGGCCGTTACAAGTCTCTAAATCAAACAGTTT
RI-AT1G74330-XLOC_007788-6881-1
GGAGATGTGCCTTTCTCAGGGCCGTTACAAGTCTCTAAATCAAACAGTTT
CONSENSUS
GGAGATGTGCCTTTCTCAGGGCCGTTACAAGTCTCTAAATCAAACAGTTT

RI-AT1G74330-XLOC_007788-6881-0
TGCTTGGGCGAAACGAGAAAAAGATGACGTATGTGTTAGGGTGCATAATC
RI-AT1G74330-XLOC_007788-6881-1
TGCTTGGGCGAAACGAGAAAAAGATGACGTATGTGTTAGGGTGCATAATC
CONSENSUS
TGCTTGGGCGAAACGAGAAAAAGATGACGTATGTGTTAGGGTGCATAATC

RI-AT1G74330-XLOC_007788-6881-0
GATCTCTCTCAAGAGGTTACATTCCTAGCTTGTCAGGACATTCCCCCGCA
RI-AT1G74330-XLOC_007788-6881-1
GATCTCTCTCAAGAGGTTACATTCCTAGCTTGTCAGGACATTCCCCCGCA
CONSENSUS
GATCTCTCTCAAGAGGTTACATTCCTAGCTTGTCAGGACATTCCCCCGCA

RI-AT1G74330-XLOC_007788-6881-0
TTCAATGGGAAAAGTGATGTTGAATCCAAGATAAATAAGGATGAAAAGGA
RI-AT1G74330-XLOC_007788-6881-1
TTCAATGGGAAAAGTGATGTTGAATCCAAGATAAATAAGGATGAAAAGGA
CONSENSUS
TTCAATGGGAAAAGTGATGTTGAATCCAAGATAAATAAGGATGAAAAGGA

RI-AT1G74330-XLOC_007788-6881-0
GGACAAGACAGATTCTCGAGGCGAGGAGTCATATGAGATGGTGAAGCGTT
RI-AT1G74330-XLOC_007788-6881-1
GGACAAGACAGATTCTCGAGGCGAGGAGTCATATGAGATGGTGAAGCGTT
CONSENSUS
GGACAAGACAGATTCTCGAGGCGAGGAGTCATATGAGATGGTGAAGCGTT

RI-AT1G74330-XLOC_007788-6881-0
CTATGCTAAAGCAGTGGAGACAACTTGAACGTCCAGATTCTTTTGGTGCA
RI-AT1G74330-XLOC_007788-6881-1
CTATGCTAAAGCAGTGGAGACAACTTGAACGTCCAGATTCTTTTGGTGCA
CONSENSUS
CTATGCTAAAGCAGTGGAGACAACTTGAACGTCCAGATTCTTTTGGTGCA

RI-AT1G74330-XLOC_007788-6881-0
TCTGACGAGTATCACTCGCAGGAACTGTCATTGGGACTCTATCAGAGAGA
RI-AT1G74330-XLOC_007788-6881-1
TCTGACGAGTATCACTCGCAGGAACTGTCATTGGGACTCTATCAGAGAGA
CONSENSUS
TCTGACGAGTATCACTCGCAGGAACTGTCATTGGGACTCTATCAGAGAGA

RI-AT1G74330-XLOC_007788-6881-0
TGAAATGGCAAAAAAGATGGGTAATAATTTGGTACGTCCATAGATTATAG
RI-AT1G74330-XLOC_007788-6881-1

TGAAATGGCAAAAAAGATGGGTAATAATTTG-------------------
CONSENSUS
TGAAATGGCAAAAAAGATGGGTAATAATTTG...................

RI-AT1G74330-XLOC_007788-6881-0
AAGCTCTAAAAGCAGCCTTATAGTTTATTTAATATCCAAATTCCATATAG
RI-AT1G74330-XLOC_007788-6881-1

CONSENSUS
....

RI-AT1G74330-XLOC_007788-6881-0
CAGATTGTTATTTGCATTGTTAGTGCTACTGATAGACATAGTAGAGACCA
RI-AT1G74330-XLOC_007788-6881-1

CONSENSUS
....

RI-AT1G74330-XLOC_007788-6881-0
AGTATGCTTTGTGCCAGACGTTCTTACATGTGAAACTTAGGATTTGGTTT
RI-AT1G74330-XLOC_007788-6881-1

CONSENSUS
....

RI-AT1G74330-XLOC_007788-6881-0
CATTCTTTCTTATGCTGTAATGCGAGTGTCAAATTTGATTCTGTGAAATA
RI-AT1G74330-XLOC_007788-6881-1

CONSENSUS
....

RI-AT1G74330-XLOC_007788-6881-0
AAATGAAAGTAATTCTAAAGATATGATTGTAGTAGTTATATATGCAACAT
RI-AT1G74330-XLOC_007788-6881-1

CONSENSUS
....

RI-AT1G74330-XLOC_007788-6881-0
ACATATAAGATACGGTGTAAATGTGATATACAGCTCAAAAAGTAGAAATA
RI-AT1G74330-XLOC_007788-6881-1

CONSENSUS
....

RI-AT1G74330-XLOC_007788-6881-0
TTGGACCAGATTACCGTTGGTAGTCAGATCATGTTTCCTCAGCTGTTTAA
RI-AT1G74330-XLOC_007788-6881-1

CONSENSUS
....

RI-AT1G74330-XLOC_007788-6881-0
TTTTTTTTTTAATCTTCTCTCTCTCTCTCTCCACGCTTTCACCTAAAGTT
RI-AT1G74330-XLOC_007788-6881-1

CONSENSUS
....

RI-AT1G74330-XLOC_007788-6881-0
CAGAGGAGAGAGGAAAAAATAGGCATGATTTAGTTTGTCTACTTTTTGTG
RI-AT1G74330-XLOC_007788-6881-1

CONSENSUS
....

RI-AT1G74330-XLOC_007788-6881-0
TTTAGTGACAAGCATGCTCAATGATACTGATGACTTAAGCCTTGACATGA
RI-AT1G74330-XLOC_007788-6881-1

CONSENSUS
....

RI-AT1G74330-XLOC_007788-6881-0
CAGGGTGATGGGGACAAGATCGAATTCTCGGGCCCTTTGTTGTCTCAATC
RI-AT1G74330-XLOC_007788-6881-1 ---
GGTGATGGGGACAAGATCGAATTCTCGGGCCCTTTGTTGTCTCAATC
CONSENSUS
...GGTGATGGGGACAAGATCGAATTCTCGGGCCCTTTGTTGTCTCAATC

RI-AT1G74330-XLOC_007788-6881-0
TTATGGAGTTGATGAACTGTTGGAACGCCATGAACGCAACATCCGCAAGC
RI-AT1G74330-XLOC_007788-6881-1
TTATGGAGTTGATGAACTGTTGGAACGCCATGAACGCAACATCCGCAAGC
CONSENSUS
TTATGGAGTTGATGAACTGTTGGAACGCCATGAACGCAACATCCGCAAGC

RI-AT1G74330-XLOC_007788-6881-0 TAATTCGAAAACCGTGGTTTCAAAAAG
RI-AT1G74330-XLOC_007788-6881-1 TAATTCGAAAACCGTGGTTTCAAAAAG
CONSENSUS TAATTCGAAAACCGTGGTTTCAAAAAG

alignment for event: A3-AT1G02840-XLOC_000103-11645

A3-AT1G02840-XLOC_000103-11645-0
GTTAGAGAATATGATTCAAGGAAGGATTCTAGGAGTCCTAGCCGGGGAAG
A3-AT1G02840-XLOC_000103-11645-1
GTTAGAGAATATGATTCAAGGAAGGATTCTAGGAGTCCTAGCCGGGGAAG
CONSENSUS
GTTAGAGAATATGATTCAAGGAAGGATTCTAGGAGTCCTAGCCGGGGAAG

A3-AT1G02840-XLOC_000103-11645-0
ATCCTATTCTAAGAGCCGCAGCCGCAGCCGTGGACGAAGCGTGAGCCGAA
A3-AT1G02840-XLOC_000103-11645-1
ATCCTATTCTAAGAGCCGCAGCCGCAGCCGTGGACGAAGCGTGAGCCGAA
CONSENSUS
ATCCTATTCTAAGAGCCGCAGCCGCAGCCGTGGACGAAGCGTGAGCCGAA

A3-AT1G02840-XLOC_000103-11645-0
GCAGGAGCAGAAGCAGGAGCAGGAGCAGAAGTCCCAAGGCAAAGTCTTCA

A3-AT1G02840-XLOC_000103-11645-1
      GCAGGAGCAGAAGCAGGAGCAGGAGCAGAAGTCCCAAGGCAAAGTCTTCA
CONSENSUS
      GCAGGAGCAGAAGCAGGAGCAGGAGCAGAAGTCCCAAGGCAAAGTCTTCA

A3-AT1G02840-XLOC_000103-11645-0
      CGTAGGTCCCCTGCAAAATCTACATCAAGATCTCCTGGCCCCCGCTCGAA
A3-AT1G02840-XLOC_000103-11645-1
      CGTAGGTCCCCTGCAAAATCTACATCAAGATCTCCTGGCCCCCGCTCGAA
CONSENSUS
      CGTAGGTCCCCTGCAAAATCTACATCAAGATCTCCTGGCCCCCGCTCGAA

A3-AT1G02840-XLOC_000103-11645-0
      GTCAAGGTCACCGTCTCCAAGAAGATGGATAACAGTGGAGACATTGGATC
A3-AT1G02840-XLOC_000103-11645-1
      GTCAAGGTCACCGTCTCCAAGAAG--------------------------
CONSENSUS
      GTCAAGGTCACCGTCTCCAAGAAG..........................

A3-AT1G02840-XLOC_000103-11645-0
      ACTTGGATCACAATATTATATCGGGATTTCTGTAAAACTATATTGGCTCG
A3-AT1G02840-XLOC_000103-11645-1

CONSENSUS
      ....

A3-AT1G02840-XLOC_000103-11645-0
      ATGGATTGACAATATGGAATCTGGGTTCTCTTGGGACGTCCGTGGCTCAT
A3-AT1G02840-XLOC_000103-11645-1

CONSENSUS
      ....

A3-AT1G02840-XLOC_000103-11645-0
      TTGGCAACACAAGTTTTTTTGGCCACATGGCTTATAAAACCTCTGTCCTA
A3-AT1G02840-XLOC_000103-11645-1

CONSENSUS
      ....

A3-AT1G02840-XLOC_000103-11645-0
      TCACCTATGTTTTAACTAAGTAGCAGAATAGTTTGGTTTATGTTTCTTTT
A3-AT1G02840-XLOC_000103-11645-1

CONSENSUS
      ....

A3-AT1G02840-XLOC_000103-11645-0
      TTTTTATTTGTTGCAACTTCTTAATCTCTGTGAGATAGAAGGAGAGGCTC
A3-AT1G02840-XLOC_000103-11645-1

CONSENSUS
      ....

A3-AT1G02840-XLOC_000103-11645-0
      CAGGACCTTGCTGAACAGTATAAAACACAACATGTTTGGATTTTTGAATC

A3-AT1G02840-XLOC_000103-11645-1

CONSENSUS
      ....

A3-AT1G02840-XLOC_000103-11645-0
      TGAGTTTCTTTTCTTGGACTTTTGCAGATCGCGTTCAAGATCAAGATCTC
A3-AT1G02840-XLOC_000103-11645-1 ---------------------------
ATCGCGTTCAAGATCAAGATCTC
CONSENSUS
      ...........................ATCGCGTTCAAGATCAAGATCTC

A3-AT1G02840-XLOC_000103-11645-0 CTCTACCTTCT
A3-AT1G02840-XLOC_000103-11645-1 CTCTACCTTCT
CONSENSUS CTCTACCTTCT

alignment for event: RI-AT1G22660-XLOC_001193-95

RI-AT1G22660-XLOC_001193-95-0
      GGTTTCTACTTAGAGATATTAAAGACTTCTGGCGTGTTTCTCTGTTAACA
RI-AT1G22660-XLOC_001193-95-1
      GGTTTCTACTTAGAGATATTAAAGACTTCTGGCGTGTTTCTCTGTTAACA
CONSENSUS
      GGTTTCTACTTAGAGATATTAAAGACTTCTGGCGTGTTTCTCTGTTAACA

RI-AT1G22660-XLOC_001193-95-0
      TCTTTGTTATTGTCTGCTACGGTTGACGGTAGCAACGATCATCAAGATAT
RI-AT1G22660-XLOC_001193-95-1
      TCTTTGTTATTGTCTGCTACGGTTGACGGTAGCAACGATCATCAAGATAT
CONSENSUS
      TCTTTGTTATTGTCTGCTACGGTTGACGGTAGCAACGATCATCAAGATAT

RI-AT1G22660-XLOC_001193-95-0
      CGGGCAGCTGGACTTCCAACTGGAAAGGATGCGAGAAACTTATCTGACAG
RI-AT1G22660-XLOC_001193-95-1
      CGGGCAGCTGGACTTCCAACTGGAAAGGATGCGAGAAACTTATCTGACAG
CONSENSUS
      CGGGCAGCTGGACTTCCAACTGGAAAGGATGCGAGAAACTTATCTGACAG

RI-AT1G22660-XLOC_001193-95-0
      TTGAGGCCACCATTCATGAACTGGGTAAGCGTTAGAATGTTTGATAAATG
RI-AT1G22660-XLOC_001193-95-1
      TTGAGGCCACCATTCATGAACTGG--------------------------
CONSENSUS
      TTGAGGCCACCATTCATGAACTGG..........................

RI-AT1G22660-XLOC_001193-95-0
      GTAGCATATAAGATAGAAATTGCTAAGAGGAGGTAGAAGAAGTAATGGCT
RI-AT1G22660-XLOC_001193-95-1

CONSENSUS
      ....

RI-AT1G22660-XLOC_001193-95-0

GTGACTAGTTACCAAGGCTCCCCACGTTTTTGTGCTTACCAGACTCACTA
RI-AT1G22660-XLOC_001193-95-1

CONSENSUS
....

RI-AT1G22660-XLOC_001193-95-0
GTAGAACTAAGAACATCAAAAGAACATGAGAAGCTGATTTCATAATTCCT
RI-AT1G22660-XLOC_001193-95-1

CONSENSUS
....

RI-AT1G22660-XLOC_001193-95-0
GTTTCTGGGCAATCTTTCTGACTTTGAAAAGTGATGCATGAGTAGGGTGG
RI-AT1G22660-XLOC_001193-95-1

CONSENSUS
....

RI-AT1G22660-XLOC_001193-95-0
AGAGAAAATCATCTTTAGAAAAGCTCATTTATTCTATCATTAGAACTGCA
RI-AT1G22660-XLOC_001193-95-1

CONSENSUS
....

RI-AT1G22660-XLOC_001193-95-0
TTATCAATATGCCTGGGGAACCATTCCACATACAGTGTTTCTTTCTTATT
RI-AT1G22660-XLOC_001193-95-1

CONSENSUS
....

RI-AT1G22660-XLOC_001193-95-0
CTATCATTAATATTCAGGTCTTGATAAGATCTGGGATGCAAAGCCTCTAG
RI-AT1G22660-XLOC_001193-95-1 -----------------
GTCTTGATAAGATCTGGGATGCAAAGCCTCTAG
CONSENSUS
.................GTCTTGATAAGATCTGGGATGCAAAGCCTCTAG

RI-AT1G22660-XLOC_001193-95-0
TCAATGGCAGGGAGATAATGCAAATTGCTGAGCTGAAGGGAGGATCCCGG
RI-AT1G22660-XLOC_001193-95-1
TCAATGGCAGGGAGATAATGCAAATTGCTGAGCTGAAGGGAGGATCCCGG
CONSENSUS
TCAATGGCAGGGAGATAATGCAAATTGCTGAGCTGAAGGGAGGATCCCGG

RI-AT1G22660-XLOC_001193-95-0 CTCATCCGTGAATGG
RI-AT1G22660-XLOC_001193-95-1 CTCATCCGTGAATGG
CONSENSUS CTCATCCGTGAATGG

alignment for event: A3-AT1G10890-XLOC_000523-8258

A3-AT1G10890-XLOC_000523-8258-0
GACCAATCCGTTCAGAGAGATTAAAGCTGAGCTGAGCTTCTCCTTATAAA
A3-AT1G10890-XLOC_000523-8258-1
GACCAATCCGTTCAGAGAGATTAAAGCTGAGCTGAGCTTCTCCTTATAAA
CONSENSUS
GACCAATCCGTTCAGAGAGATTAAAGCTGAGCTGAGCTTCTCCTTATAAA

A3-AT1G10890-XLOC_000523-8258-0
GGAAGATACTGCTTGTTCTACGGAATTTTGAGAGATGCCTCGGGACTTGT
A3-AT1G10890-XLOC_000523-8258-1
GGAAGATACTGCTTGTTCTACGGAATTTTGAGAGATGCCTCGGGACTTGT
CONSENSUS
GGAAGATACTGCTTGTTCTACGGAATTTTGAGAGATGCCTCGGGACTTGT

A3-AT1G10890-XLOC_000523-8258-0
CAAGATCGAGGTCACCGTCTCCATCACCTTCACGTCGTAGAAAGCACTCG
A3-AT1G10890-XLOC_000523-8258-1
CAAGATCGAGGTCACCGTCTCCATCACCTTCACGTCGTAGAAAGCACTCG
CONSENSUS
CAAGATCGAGGTCACCGTCTCCATCACCTTCACGTCGTAGAAAGCACTCG

A3-AT1G10890-XLOC_000523-8258-0
AGGTCTCCCGTAAGGCAGAGGCATAGCAGGAGGAGTAGAAGAGACAGAAG
A3-AT1G10890-XLOC_000523-8258-1
AGGTCTCCCGTAAGGCAGAGGCATAGCAGGAGGAGTAGAAGAGACAGAAG
CONSENSUS
AGGTCTCCCGTAAGGCAGAGGCATAGCAGGAGGAGTAGAAGAGACAGAAG

A3-AT1G10890-XLOC_000523-8258-0
CCCTTCTCCATACTCATCTCATTCGTATAGCAGTTTCTTCCCGTTCCCAC
A3-AT1G10890-XLOC_000523-8258-1
CCCTTCTCCATACTCATCTCATTCGTATAGCAG-----------------
CONSENSUS
CCCTTCTCCATACTCATCTCATTCGTATAGCAG.................

A3-AT1G10890-XLOC_000523-8258-0
GGGAGGGGAAATGAGCAGCTGATGGATTGCTCTTAGGTGTTCTTTCATTG
A3-AT1G10890-XLOC_000523-8258-1

CONSENSUS
....

A3-AT1G10890-XLOC_000523-8258-0
ATCCCTTGTCTCGTTAGCTTCTCTTCGTGTGATAAAAGTTGGAGCTGATA
A3-AT1G10890-XLOC_000523-8258-1

CONSENSUS
....

A3-AT1G10890-XLOC_000523-8258-0
ATTCTTTACAGTGTATGAGGTAACTGCACATACTTTTGTTTTCTTTATGT
A3-AT1G10890-XLOC_000523-8258-1

CONSENSUS
....

A3-AT1G10890-XLOC_000523-8258-0
GTAACTGTGGGCTACCTGATGCCTAGTCTTCACGTGCGTGGTACTTCATT
A3-AT1G10890-XLOC_000523-8258-1

CONSENSUS
....

A3-AT1G10890-XLOC_000523-8258-0
AGTTCCATCTTTTTGCTTACTACTTTATTTTGGGTAATGTTCAGGCGAAA
A3-AT1G10890-XLOC_000523-8258-1
--GCGAAA
CONSENSUS
....GCGAAA

A3-AT1G10890-XLOC_000523-8258-0
AAGTCGTTCTATTTCTCCTAGGCGCCATCGAAGTCGATCTGTTACTCCTA
A3-AT1G10890-XLOC_000523-8258-1
AAGTCGTTCTATTTCTCCTAGGCGCCATCGAAGTCGATCTGTTACTCCTA
CONSENSUS
AAGTCGTTCTATTTCTCCTAGGCGCCATCGAAGTCGATCTGTTACTCCTA

A3-AT1G10890-XLOC_000523-8258-0
AGAGACGTTCTCCAACCCCAAAACGTTACAAAAGACAAAAGAGTAGGAGT
A3-AT1G10890-XLOC_000523-8258-1
AGAGACGTTCTCCAACCCCAAAACGTTACAAAAGACAAAAGAGTAGGAGT
CONSENSUS
AGAGACGTTCTCCAACCCCAAAACGTTACAAAAGACAAAAGAGTAGGAGT

A3-AT1G10890-XLOC_000523-8258-0
TCAACTCCATCTCCTGCAAAAAGATCTCCCGCCGCAACCCTTGAGTCAGC
A3-AT1G10890-XLOC_000523-8258-1
TCAACTCCATCTCCTGCAAAAAGATCTCCCGCCGCAACCCTTGAGTCAGC
CONSENSUS
TCAACTCCATCTCCTGCAAAAAGATCTCCCGCCGCAACCCTTGAGTCAGC

A3-AT1G10890-XLOC_000523-8258-0
CAAAAATAGGAATGGAGAAAAACTTAAAAGAGAAGAGGAAGAACGAAAAA
A3-AT1G10890-XLOC_000523-8258-1
CAAAAATAGGAATGGAGAAAAACTTAAAAGAGAAGAGGAAGAACGAAAAA
CONSENSUS
CAAAAATAGGAATGGAGAAAAACTTAAAAGAGAAGAGGAAGAACGAAAAA

A3-AT1G10890-XLOC_000523-8258-0
GGTAAATCAATACAAAATTTTGAAGTAAGATGGATGTTATGGTGAAATTA
A3-AT1G10890-XLOC_000523-8258-1
GGTAAATCAATACAAAATTTTGAAGTAAGATGGATGTTATGGTGAAATTA
CONSENSUS
GGTAAATCAATACAAAATTTTGAAGTAAGATGGATGTTATGGTGAAATTA

A3-AT1G10890-XLOC_000523-8258-0
AGGTTTTTTGTTCTCAAATGTTATTTTAGTGTAGTGTGGAAGTCCTTGAT
A3-AT1G10890-XLOC_000523-8258-1
AGGTTTTTTGTTCTCAAATGTTATTTTAGTGTAGTGTGGAAGTCCTTGAT
CONSENSUS
AGGTTTTTTGTTCTCAAATGTTATTTTAGTGTAGTGTGGAAGTCCTTGAT

A3-AT1G10890-XLOC_000523-8258-0
TGTTAGTCTCAAAAGTGCGACAGGTTTTATGGTATTCTCTCACAGGCTTT
A3-AT1G10890-XLOC_000523-8258-1
TGTTAGTCTCAAAAGTGCGACAGGTTTTATGGTATTCTCTCACAGGCTTT
CONSENSUS
TGTTAGTCTCAAAAGTGCGACAGGTTTTATGGTATTCTCTCACAGGCTTT

A3-AT1G10890-XLOC_000523-8258-0
GCTTATGGAAGATAAGCTATATTTCGATTTGTTTATATTTGATCCTCTGG
A3-AT1G10890-XLOC_000523-8258-1
GCTTATGGAAGATAAGCTATATTTCGATTTGTTTATATTTGATCCTCTGG
CONSENSUS
GCTTATGGAAGATAAGCTATATTTCGATTTGTTTATATTTGATCCTCTGG

A3-AT1G10890-XLOC_000523-8258-0
CACAAATGTTCGTACTATCTCGATTTGATGAGTTACCGTTTGTCATTACA
A3-AT1G10890-XLOC_000523-8258-1
CACAAATGTTCGTACTATCTCGATTTGATGAGTTACCGTTTGTCATTACA
CONSENSUS
CACAAATGTTCGTACTATCTCGATTTGATGAGTTACCGTTTGTCATTACA

A3-AT1G10890-XLOC_000523-8258-0
TGGATCATGATTGACTTAGAGATGTAAATATGTCCAAAGCACAAGAATAA
A3-AT1G10890-XLOC_000523-8258-1
TGGATCATGATTGACTTAGAGATGTAAATATGTCCAAAGCACAAGAATAA
CONSENSUS
TGGATCATGATTGACTTAGAGATGTAAATATGTCCAAAGCACAAGAATAA

A3-AT1G10890-XLOC_000523-8258-0
TCTTGGTGAAGGTTTGCTTGTGTTATGGTGACAAAAAAGAAATTTCTTCA
A3-AT1G10890-XLOC_000523-8258-1
TCTTGGTGAAGGTTTGCTTGTGTTATGGTGACAAAAAAGAAATTTCTTCA
CONSENSUS
TCTTGGTGAAGGTTTGCTTGTGTTATGGTGACAAAAAAGAAATTTCTTCA

A3-AT1G10890-XLOC_000523-8258-0
CGAAATTTACCATTGGTATTGGGAGGAGTGAAAATGGAAACGATCTCAAC
A3-AT1G10890-XLOC_000523-8258-1
CGAAATTTACCATTGGTATTGGGAGGAGTGAAAATGGAAACGATCTCAAC
CONSENSUS
CGAAATTTACCATTGGTATTGGGAGGAGTGAAAATGGAAACGATCTCAAC

A3-AT1G10890-XLOC_000523-8258-0
ATTTTTTACGGATTTGCAATAAAATTTTGGTCTTTTGCTGTTCAAGATTG
A3-AT1G10890-XLOC_000523-8258-1
ATTTTTTACGGATTTGCAATAAAATTTTGGTCTTTTGCTGTTCAAGATTG
CONSENSUS
ATTTTTTACGGATTTGCAATAAAATTTTGGTCTTTTGCTGTTCAAGATTG

A3-AT1G10890-XLOC_000523-8258-0
AAGACATCAGGAAATGAGAAAGTTCAGGTGATATTCAATATTCTGAACCT
A3-AT1G10890-XLOC_000523-8258-1
AAGACATCAGGAAATGAGAAAGTTCAGGTGATATTCAATATTCTGAACCT
CONSENSUS
AAGACATCAGGAAATGAGAAAGTTCAGGTGATATTCAATATTCTGAACCT

A3-AT1G10890-XLOC_000523-8258-0
      GCTGTGGATGTCCTCTAATTTTTCACTGTATTTGCTACCAGGCGACAGCG
A3-AT1G10890-XLOC_000523-8258-1
      GCTGTGGATGTCCTCTAATTTTTCACTGTATTTGCTACCAGGCGACAGCG
CONSENSUS
      GCTGTGGATGTCCTCTAATTTTTCACTGTATTTGCTACCAGGCGACAGCG

A3-AT1G10890-XLOC_000523-8258-0
      TGAAGCAGAACTGAAGCTAATAGAGGAAGAAACTGTGAAACGGGTTGAAG
A3-AT1G10890-XLOC_000523-8258-1
      TGAAGCAGAACTGAAGCTAATAGAGGAAGAAACTGTGAAACGGGTTGAAG
CONSENSUS
      TGAAGCAGAACTGAAGCTAATAGAGGAAGAAACTGTGAAACGGGTTGAAG

A3-AT1G10890-XLOC_000523-8258-0
      AAGCTATTCGAAAGAAGGTCGAAGAAAGCTTACAGTCTGAGAAAATCAAA
A3-AT1G10890-XLOC_000523-8258-1
      AAGCTATTCGAAAGAAGGTCGAAGAAAGCTTACAGTCTGAGAAAATCAAA
CONSENSUS
      AAGCTATTCGAAAGAAGGTCGAAGAAAGCTTACAGTCTGAGAAAATCAAA

A3-AT1G10890-XLOC_000523-8258-0
      ATGGAAATTCTAACGCTGTTGGAGGAAGGGCGAAAGAGACTTAATGAAGA
A3-AT1G10890-XLOC_000523-8258-1
      ATGGAAATTCTAACGCTGTTGGAGGAAGGGCGAAAGAGACTTAATGAAGA
CONSENSUS
      ATGGAAATTCTAACGCTGTTGGAGGAAGGGCGAAAGAGACTTAATGAAGA

A3-AT1G10890-XLOC_000523-8258-0
      AGTCGCGGCTCAACTTGAGGAGGAGAAAGAGGCTTCTCTTATTGAGGCTA
A3-AT1G10890-XLOC_000523-8258-1
      AGTCGCGGCTCAACTTGAGGAGGAGAAAGAGGCTTCTCTTATTGAGGCTA
CONSENSUS
      AGTCGCGGCTCAACTTGAGGAGGAGAAAGAGGCTTCTCTTATTGAGGCTA

A3-AT1G10890-XLOC_000523-8258-0 AAGAAAAAGAG
A3-AT1G10890-XLOC_000523-8258-1 AAGAAAAAGAG
CONSENSUS   AAGAAAAAGAG

alignment for event: SE-AT1G55310-XLOC_002795-1799

SE-AT1G55310-XLOC_002795-1799-0
      GCAAGAAGATCTCAGGAAGTCGTTTGAGCAGTTTGGTCCTGTCAAGGACA
SE-AT1G55310-XLOC_002795-1799-1
      GCAAGAAGATCTCAGGAAGTCGTTTGAGCAGTTTGGTCCTGTCAAGGACA
CONSENSUS
      GCAAGAAGATCTCAGGAAGTCGTTTGAGCAGTTTGGTCCTGTCAAGGACA

SE-AT1G55310-XLOC_002795-1799-0
      TTTACCTGCCAAGGGATTATTATACCGG----------------------
SE-AT1G55310-XLOC_002795-1799-1
      TTTACCTGCCAAGGGATTATTATACCGGTGAATCTAAAGAATTGAAGACA
CONSENSUS
      TTTACCTGCCAAGGGATTATTATACCGG......................

SE-AT1G55310-XLOC_002795-1799-0

SE-AT1G55310-XLOC_002795-1799-1
      TCAAAGAAGTAATTAGAGTTCTTATGAAGATGTTCTATATGGTAGTGAAG
CONSENSUS
      ....

SE-AT1G55310-XLOC_002795-1799-0

SE-AT1G55310-XLOC_002795-1799-1
      AATTGAAGTGAAGTTGAGTTTGTATTCTATGTGAAGATGAATCAAGTCTT
CONSENSUS
      ....

SE-AT1G55310-XLOC_002795-1799-0
      --AGATCCGCGA
SE-AT1G55310-XLOC_002795-1799-1
      CAAGAAGTCATCTTTGTACTGACACTTGCAAGGCTAGCAGAGATCCGCGA
CONSENSUS
      ....AGATCCGCGA

SE-AT1G55310-XLOC_002795-1799-0
      GGGTTTGGGTTCGTTCAATTTATGGACCCTGCTGATGCTGCTGATGCAAA
SE-AT1G55310-XLOC_002795-1799-1
      GGGTTTGGGTTCGTTCAATTTATGGACCCTGCTGATGCTGCTGATGCAAA
CONSENSUS
      GGGTTTGGGTTCGTTCAATTTATGGACCCTGCTGATGCTGCTGATGCAAA

SE-AT1G55310-XLOC_002795-1799-0
      ACATCACATGGATGGTTATCTTCTTCTTGGCCGTGAGTTGACTGTCGTGT
SE-AT1G55310-XLOC_002795-1799-1
      ACATCACATGGATGGTTATCTTCTTCTTGGCCGTGAGTTGACTGTCGTGT
CONSENSUS
      ACATCACATGGATGGTTATCTTCTTCTTGGCCGTGAGTTGACTGTCGTGT

SE-AT1G55310-XLOC_002795-1799-0
      TTGCAGAAGAGAACAGAAAGAAACCGACTGAAATGAGAGCAAGGGAGCGT
SE-AT1G55310-XLOC_002795-1799-1
      TTGCAGAAGAGAACAGAAAGAAACCGACTGAAATGAGAGCAAGGGAGCGT
CONSENSUS
      TTGCAGAAGAGAACAGAAAGAAACCGACTGAAATGAGAGCAAGGGAGCGT

SE-AT1G55310-XLOC_002795-1799-0 GGTGGAGGAAG
SE-AT1G55310-XLOC_002795-1799-1 GGTGGAGGAAG
CONSENSUS   GGTGGAGGAAG

alignment for event: A5-AT1G17520-XLOC_005070-4381

A5-AT1G17520-XLOC_005070-4381-0
      CAGCAACGACAAGAAGTGCCACCAAATTTCAGGAGGATGCTTAGTTCAAG
A5-AT1G17520-XLOC_005070-4381-1
      CAGCAACGACAAGAAGTGCCACCAAATTTCAGGAGGATGCTTAGTTCAAG
CONSENSUS

CAGCAACGACAAGAAGTGCCACCAAATTTCAGGAGGATGCTTAGTTCAAG

A5-AT1G17520-XLOC_005070-4381-0
ATTGAGGAGGCTTGCAGCTCAGGGAAAGCTTGAAAAGGTTAGCCACTTAA
A5-AT1G17520-XLOC_005070-4381-1
ATTGAGGAGGCTTGCAGCTCAGGGAAAGCTTGAAAAG-------------
CONSENSUS
ATTGAGGAGGCTTGCAGCTCAGGGAAAGCTTGAAAAG.............

A5-AT1G17520-XLOC_005070-4381-0
AATCAACACAGAACTTCTATAAGATGAATGATAATAGCTTGGTACAAAGA
A5-AT1G17520-XLOC_005070-4381-1 -----
ACACAGAACTTCTATAAGATGAATGATAATAGCTTGGTACAAAGA
CONSENSUS
.....ACACAGAACTTCTATAAGATGAATGATAATAGCTTGGTACAAAGA

A5-AT1G17520-XLOC_005070-4381-0
ACACCACATGTAGCGAGACCAAAAGAGAGCAACACGAAATCCCGGCAGCA
A5-AT1G17520-XLOC_005070-4381-1
ACACCACATGTAGCGAGACCAAAAGAGAGCAACACGAAATCCCGGCAGCA
CONSENSUS
ACACCACATGTAGCGAGACCAAAAGAGAGCAACACGAAATCCCGGCAGCA

A5-AT1G17520-XLOC_005070-4381-0
GACAAACAGTCAGGGACCCTCTATTTCACAGCAGATTGTTGAAGCTTCAA
A5-AT1G17520-XLOC_005070-4381-1
GACAAACAGTCAGGGACCCTCTATTTCACAGCAGATTGTTGAAGCTTCAA
CONSENSUS
GACAAACAGTCAGGGACCCTCTATTTCACAGCAGATTGTTGAAGCTTCAA

A5-AT1G17520-XLOC_005070-4381-0
TAACAGCAGCTTATAAGCTCGTAGAAGTAGAGAACAAATTAGATGTGTCG
A5-AT1G17520-XLOC_005070-4381-1
TAACAGCAGCTTATAAGCTCGTAGAAGTAGAGAACAAATTAGATGTGTCG
CONSENSUS
TAACAGCAGCTTATAAGCTCGTAGAAGTAGAGAACAAATTAGATGTGTCG

A5-AT1G17520-XLOC_005070-4381-0
AAAGGAGCTGCAGAAGAGATAGAGAGACTGATGAAACTGGCAGAAGAGGC
A5-AT1G17520-XLOC_005070-4381-1
AAAGGAGCTGCAGAAGAGATAGAGAGACTGATGAAACTGGCAGAAGAGGC
CONSENSUS
AAAGGAGCTGCAGAAGAGATAGAGAGACTGATGAAACTGGCAGAAGAGGC

A5-AT1G17520-XLOC_005070-4381-0
TGATGAGATGCTAGTGATAGCGAGAGAGATGCACGAAGAAT
A5-AT1G17520-XLOC_005070-4381-1
TGATGAGATGCTAGTGATAGCGAGAGAGATGCACGAAGAAT
CONSENSUS
TGATGAGATGCTAGTGATAGCGAGAGAGATGCACGAAGAAT

alignment for event: SE-AT1G13350-XLOC_004843-11309

SE-AT1G13350-XLOC_004843-11309-0

CACGGATACACAAATTGAGAGAGAGCGCGCGACAAAATCGAACCTAACCC
SE-AT1G13350-XLOC_004843-11309-1
CACGGATACACAAATTGAGAGAGAGCGCGCGACAAAATCGAACCTAACCC
CONSENSUS
CACGGATACACAAATTGAGAGAGAGCGCGCGACAAAATCGAACCTAACCC

SE-AT1G13350-XLOC_004843-11309-0
TAATTTTGCGATTTCCCCCTAATTTCGACGATCTGATGGTGAGTGACAAG
SE-AT1G13350-XLOC_004843-11309-1
TAATTTTGCGATTTCCCCCTAATTTCGACGATCTGATGGTGAGTGACAAG
CONSENSUS
TAATTTTGCGATTTCCCCCTAATTTCGACGATCTGATGGTGAGTGACAAG

SE-AT1G13350-XLOC_004843-11309-0
CATGTAGAATCAAACCACCGCAAACACCGACGGTCGTTTTCGCCGTCCGA
SE-AT1G13350-XLOC_004843-11309-1
CATGTAGAATCAAACCACCGCAAACACCGACGGTCGTTTTCGCCGTCCGA
CONSENSUS
CATGTAGAATCAAACCACCGCAAACACCGACGGTCGTTTTCGCCGTCCGA

SE-AT1G13350-XLOC_004843-11309-0
CGAGGTCTTTAAATCTCCGAAGCGGCACAAGTCCCGTCATCACCATCGCA
SE-AT1G13350-XLOC_004843-11309-1
CGAGGTCTTTAAATCTCCGAAGCGGCACAAGTCCCGTCATCACCATCGCA
CONSENSUS
CGAGGTCTTTAAATCTCCGAAGCGGCACAAGTCCCGTCATCACCATCGCA

SE-AT1G13350-XLOC_004843-11309-0
GGCATGGCCACCGTCATCATCGTGATGAGGAAGTTCAATATAACGATGAT
SE-AT1G13350-XLOC_004843-11309-1
GGCATGGCCACCGTCATCATCGTGATGAGGAAGTTCAATATAACGATGAT
CONSENSUS
GGCATGGCCACCGTCATCATCGTGATGAGGAAGTTCAATATAACGATGAT

SE-AT1G13350-XLOC_004843-11309-0
GAGAATGTTAACGGTGGTGATCTTGATATGGAAGAAGGTGAGATATTAGG
SE-AT1G13350-XLOC_004843-11309-1
GAGAATGTTAACGGTGGTGATCTTGATATGGAAGAAGGTGAGATATTAGG
CONSENSUS
GAGAATGTTAACGGTGGTGATCTTGATATGGAAGAAGGTGAGATATTAGG

SE-AT1G13350-XLOC_004843-11309-0
AAAAGAAGGGATTGGGGAGACATTGAAGAAGAAATTAGAGTCCGTCGACG
SE-AT1G13350-XLOC_004843-11309-1
AAAAGAAGGGATTGGGGAGACATTGAAGAAGAAATTAGAGTCCGTCGACG
CONSENSUS
AAAAGAAGGGATTGGGGAGACATTGAAGAAGAAATTAGAGTCCGTCGACG

SE-AT1G13350-XLOC_004843-11309-0
AGTTTGGGGATATAAAATCTGGTCAATTCCGGGAGAATAATCTG------
SE-AT1G13350-XLOC_004843-11309-1
AGTTTGGGGATATAAAATCTGGTCAATTCCGGGAGAATAATCTGGCGTTC
CONSENSUS
AGTTTGGGGATATAAAATCTGGTCAATTCCGGGAGAATAATCTG......

SE-AT1G13350-XLOC_004843-11309-0

SE-AT1G13350-XLOC_004843-11309-1
AATGGAGATGATGGCTTTCCCTTGATTTGTAAGAGCTGGATACGCGTTTA
CONSENSUS
....

SE-AT1G13350-XLOC_004843-11309-0 ------------------------
GGGAGAAATCAGCGGAGGGAAAGAG
SE-AT1G13350-XLOC_004843-11309-1
TAACTATCTTTCATATAAATGTCATGGGAGAAATCAGCGGAGGGAAAGAG
CONSENSUS
........................GGGAGAAATCAGCGGAGGGAAAGAG

SE-AT1G13350-XLOC_004843-11309-0
AATGTGAGAAAAGAAAAGAGATAGAGCCTGACCGTGAAAGGAGAAAAGAG
SE-AT1G13350-XLOC_004843-11309-1
AATGTGAGAAAAGAAAAGAGATAGAGCCTGACCGTGAAAGGAGAAAAGAG
CONSENSUS
AATGTGAGAAAAGAAAAGAGATAGAGCCTGACCGTGAAAGGAGAAAAGAG

SE-AT1G13350-XLOC_004843-11309-0
AGGGGAAGCGTTGATAGAGATAGCAGGGGAGACAGGGAAAAAGATTACCT
SE-AT1G13350-XLOC_004843-11309-1
AGGGGAAGCGTTGATAGAGATAGCAGGGGAGACAGGGAAAAAGATTACCT
CONSENSUS
AGGGGAAGCGTTGATAGAGATAGCAGGGGAGACAGGGAAAAAGATTACCT

SE-AT1G13350-XLOC_004843-11309-0
ACGGGATAGAGACAACGACAGAGGTAGGAGTAGAGATAAAGCCAGGTATA
SE-AT1G13350-XLOC_004843-11309-1
ACGGGATAGAGACAACGACAGAGGTAGGAGTAGAGATAAAGCCAGGTATA
CONSENSUS
ACGGGATAGAGACAACGACAGAGGTAGGAGTAGAGATAAAGCCAGGTATA

SE-AT1G13350-XLOC_004843-11309-0
GTAGTAGAGAGAGGGGGAGGGAGAATGAAAGAGAGAGACGGAGTGAAAAA
SE-AT1G13350-XLOC_004843-11309-1
GTAGTAGAGAGAGGGGGAGGGAGAATGAAAGAGAGAGACGGAGTGAAAAA
CONSENSUS
GTAGTAGAGAGAGGGGGAGGGAGAATGAAAGAGAGAGACGGAGTGAAAAA

SE-AT1G13350-XLOC_004843-11309-0
GATAGGGATAAAGGACGAGAATTCCAGAGTGATAGAGAGAAGCATAAAAG
SE-AT1G13350-XLOC_004843-11309-1
GATAGGGATAAAGGACGAGAATTCCAGAGTGATAGAGAGAAGCATAAAAG
CONSENSUS
GATAGGGATAAAGGACGAGAATTCCAGAGTGATAGAGAGAAGCATAAAAG

SE-AT1G13350-XLOC_004843-11309-0
TCTTGATGATGGATATGGTGAAGTGAGGCATAAACATTCTGGACACTCAA
SE-AT1G13350-XLOC_004843-11309-1
TCTTGATGATGGATATGGTGAAGTGAGGCATAAACATTCTGGACACTCAA
CONSENSUS
TCTTGATGATGGATATGGTGAAGTGAGGCATAAACATTCTGGACACTCAA

SE-AT1G13350-XLOC_004843-11309-0

GACATGATGCGGAAGATGACTTAGAGTTAAGAAGCCCAACTTCTGTAAAT
SE-AT1G13350-XLOC_004843-11309-1
GACATGATGCGGAAGATGACTTAGAGTTAAGAAGCCCAACTTCTGTAAAT
CONSENSUS
GACATGATGCGGAAGATGACTTAGAGTTAAGAAGCCCAACTTCTGTAAAT

SE-AT1G13350-XLOC_004843-11309-0
GGCCATGATCCTAACAGTGGCGATGTCAAAGAAACTCGGGGAAATGTTGA
SE-AT1G13350-XLOC_004843-11309-1
GGCCATGATCCTAACAGTGGCGATGTCAAAGAAACTCGGGGAAATGTTGA
CONSENSUS
GGCCATGATCCTAACAGTGGCGATGTCAAAGAAACTCGGGGAAATGTTGA

SE-AT1G13350-XLOC_004843-11309-0 AAG
SE-AT1G13350-XLOC_004843-11309-1 AAG
CONSENSUS AAG

alignment for event: RI-AT1G35220-XLOC_001895-7072

RI-AT1G35220-XLOC_001895-7072-0
GGTTACGGCGCATTGTATAAAGAAAAGACTCCTATGGAATACGTGTTTTG
RI-AT1G35220-XLOC_001895-7072-1
GGTTACGGCGCATTGTATAAAGAAAAGACTCCTATGGAATACGTGTTTTG
CONSENSUS
GGTTACGGCGCATTGTATAAAGAAAAGACTCCTATGGAATACGTGTTTTG

RI-AT1G35220-XLOC_001895-7072-0
CTTGTAAAGTTTGCAAAGAAGGTGAATATTATGAAGATATGATGCGTTAC
RI-AT1G35220-XLOC_001895-7072-1
CTTGTAAAGTTTGCAAAGAAGGTGAATATTATGAAGATATGATGCGTTAC
CONSENSUS
CTTGTAAAGTTTGCAAAGAAGGTGAATATTATGAAGATATGATGCGTTAC

RI-AT1G35220-XLOC_001895-7072-0
TTGCGAAAGAATCTGGCGGTGCGTGTTGATGGATTACTCTTTAAGAGGTC
RI-AT1G35220-XLOC_001895-7072-1
TTGCGAAAGAATCTGGCG--------------------------------
CONSENSUS
TTGCGAAAGAATCTGGCG................................

RI-AT1G35220-XLOC_001895-7072-0
TTTTTGTTTTGATTGCATTCTAGTGGAGAGTTACTAGCAAATCATTTTTC
RI-AT1G35220-XLOC_001895-7072-1

CONSENSUS
....

RI-AT1G35220-XLOC_001895-7072-0
TGAAGTTTATTTTTGTTTGTGTTCGTTCCTCATTATATCTCATTTTCAAT
RI-AT1G35220-XLOC_001895-7072-1

CONSENSUS
....

RI-AT1G35220-XLOC_001895-7072-0
      GGCTTTCTCACTGCGAATTTTTATGTACTATAGCGACAATGCATAAGAAA
RI-AT1G35220-XLOC_001895-7072-1

CONSENSUS
      ....

RI-AT1G35220-XLOC_001895-7072-0
      AGGGTTTGGAATTGACAATAGTGGTTAACAAGGTTTATTTTAGATTTTAG
RI-AT1G35220-XLOC_001895-7072-1

CONSENSUS
      ....

RI-AT1G35220-XLOC_001895-7072-0
      ATACCATAGTCTGTGATGCCAATTTATGAATATGTTTGCTACTCTGAAAG
RI-AT1G35220-XLOC_001895-7072-1

CONSENSUS
      ....

RI-AT1G35220-XLOC_001895-7072-0
      ATAGCCGTGGGTTCAGATAGTCGATAGAAAGAGAGAAACTTGGTGATCAA
RI-AT1G35220-XLOC_001895-7072-1

CONSENSUS
      ....

RI-AT1G35220-XLOC_001895-7072-0
      GGACGAAATATAGGGCATTGTTGTCTGTTACTGTCAGAGGATCTCTAATC
RI-AT1G35220-XLOC_001895-7072-1

CONSENSUS
      ....

RI-AT1G35220-XLOC_001895-7072-0
      TTAATTGCTGTTAACAATTATCCGCAGCTCTTTCCGTATCACCTTGCAGA
RI-AT1G35220-XLOC_001895-7072-1 ---------------------------
CTCTTTCCGTATCACCTTGCAGA
CONSENSUS
      ...........................CTCTTTCCGTATCACCTTGCAGA

RI-AT1G35220-XLOC_001895-7072-0
      GTATGTTTGCCGTGTGATGAGGATATCACCTTTCAGATACTACTGTGATA
RI-AT1G35220-XLOC_001895-7072-1
      GTATGTTTGCCGTGTGATGAGGATATCACCTTTCAGATACTACTGTGATA
CONSENSUS
      GTATGTTTGCCGTGTGATGAGGATATCACCTTTCAGATACTACTGTGATA

RI-AT1G35220-XLOC_001895-7072-0 TGATATTTGAAGTCATGAGAAATG
RI-AT1G35220-XLOC_001895-7072-1 TGATATTTGAAGTCATGAGAAATG
CONSENSUS   TGATATTTGAAGTCATGAGAAATG

alignment for event: A3-AT1G10890-XLOC_000523-8259

A3-AT1G10890-XLOC_000523-8259-0
GCGACAGCGTGAAGCAGAACTGAAGCTAATAGAGGAAGAAACTGTGAAAC
A3-AT1G10890-XLOC_000523-8259-1
GCGACAGCGTGAAGCAGAACTGAAGCTAATAGAGGAAGAAACTGTGAAAC
CONSENSUS
GCGACAGCGTGAAGCAGAACTGAAGCTAATAGAGGAAGAAACTGTGAAAC

A3-AT1G10890-XLOC_000523-8259-0
GGGTTGAAGAAGCTATTCGAAAGAAGGTCGAAGAAAGCTTACAGTCTGAG
A3-AT1G10890-XLOC_000523-8259-1
GGGTTGAAGAAGCTATTCGAAAGAAGGTCGAAGAAAGCTTACAGTCTGAG
CONSENSUS
GGGTTGAAGAAGCTATTCGAAAGAAGGTCGAAGAAAGCTTACAGTCTGAG

A3-AT1G10890-XLOC_000523-8259-0
AAAATCAAAATGGAAATTCTAACGCTGTTGGAGGAAGGGCGAAAGAGACT
A3-AT1G10890-XLOC_000523-8259-1
AAAATCAAAATGGAAATTCTAACGCTGTTGGAGGAAGGGCGAAAGAGACT
CONSENSUS
AAAATCAAAATGGAAATTCTAACGCTGTTGGAGGAAGGGCGAAAGAGACT

A3-AT1G10890-XLOC_000523-8259-0
TAATGAAGAAGTCGCGGCTCAACTTGAGGAGGAGAAAGAGGCTTCTCTTA
A3-AT1G10890-XLOC_000523-8259-1
TAATGAAGAAGTCGCGGCTCAACTTGAGGAGGAGAAAGAGGCTTCTCTTA
CONSENSUS
TAATGAAGAAGTCGCGGCTCAACTTGAGGAGGAGAAAGAGGCTTCTCTTA

A3-AT1G10890-XLOC_000523-8259-0
TTGAGGCTAAAGAAAAAGAGGGTGTTATGCGGTGTTTGTCACAGGAAAGA
A3-AT1G10890-XLOC_000523-8259-1
TTGAGGCTAAAGAAAAAGAG------------------------GAAAGA
CONSENSUS
TTGAGGCTAAAGAAAAAGAG........................GAAAGA

A3-AT1G10890-XLOC_000523-8259-0
GAGCAACAAGAGAAAGAAGAGAGGGAGAGAATAGCAGAGGAGAACCTAAA
A3-AT1G10890-XLOC_000523-8259-1
GAGCAACAAGAGAAAGAAGAGAGGGAGAGAATAGCAGAGGAGAACCTAAA
CONSENSUS
GAGCAACAAGAGAAAGAAGAGAGGGAGAGAATAGCAGAGGAGAACCTAAA

A3-AT1G10890-XLOC_000523-8259-0
GAGAGTGGAAGAAGCTCAGAGAAAAGAAGCAATGGAGAGGCAAAGGAAAG
A3-AT1G10890-XLOC_000523-8259-1
GAGAGTGGAAGAAGCTCAGAGAAAAGAAGCAATGGAGAGGCAAAGGAAAG
CONSENSUS
GAGAGTGGAAGAAGCTCAGAGAAAAGAAGCAATGGAGAGGCAAAGGAAAG

A3-AT1G10890-XLOC_000523-8259-0
AGGAGGAACGGTATCGAGAGCTAGAGGAGCTGCAACGACAGAAAGAAGAA
A3-AT1G10890-XLOC_000523-8259-1
AGGAGGAACGGTATCGAGAGCTAGAGGAGCTGCAACGACAGAAAGAAGAA
CONSENSUS
AGGAGGAACGGTATCGAGAGCTAGAGGAGCTGCAACGACAGAAAGAAGAA

A3-AT1G10890-XLOC_000523-8259-0
GCGATGCGAAGGAAGAAAGCTGAAGAGGAAGAAGAACGTCTCAAACAGAT
A3-AT1G10890-XLOC_000523-8259-1
GCGATGCGAAGGAAGAAAGCTGAAGAGGAAGAAGAACGTCTCAAACAGAT
CONSENSUS
GCGATGCGAAGGAAGAAAGCTGAAGAGGAAGAAGAACGTCTCAAACAGAT

A3-AT1G10890-XLOC_000523-8259-0
GAAACTGTTGGGTAAAAACAAATCACGGCCTAAATTATCCTTTGCCTTAA
A3-AT1G10890-XLOC_000523-8259-1
GAAACTGTTGGGTAAAAACAAATCACGGCCTAAATTATCCTTTGCCTTAA
CONSENSUS
GAAACTGTTGGGTAAAAACAAATCACGGCCTAAATTATCCTTTGCCTTAA

A3-AT1G10890-XLOC_000523-8259-0
GCTCCAAGTAAATGCGTGCATGCATGAAGATAAAAGGATTGATGTGATGG
A3-AT1G10890-XLOC_000523-8259-1
GCTCCAAGTAAATGCGTGCATGCATGAAGATAAAAGGATTGATGTGATGG
CONSENSUS
GCTCCAAGTAAATGCGTGCATGCATGAAGATAAAAGGATTGATGTGATGG

A3-AT1G10890-XLOC_000523-8259-0
ATGATGATATGCATCTTCTTCCTCTCAAAGATGCTTTATGATTATTGTTA
A3-AT1G10890-XLOC_000523-8259-1
ATGATGATATGCATCTTCTTCCTCTCAAAGATGCTTTATGATTATTGTTA
CONSENSUS
ATGATGATATGCATCTTCTTCCTCTCAAAGATGCTTTATGATTATTGTTA

A3-AT1G10890-XLOC_000523-8259-0
TTAGTGCTTCTTGTTGGAGCTTAAACTCTTTTATGGCTTTTAATTTTTTG
A3-AT1G10890-XLOC_000523-8259-1
TTAGTGCTTCTTGTTGGAGCTTAAACTCTTTTATGGCTTTTAATTTTTTG
CONSENSUS
TTAGTGCTTCTTGTTGGAGCTTAAACTCTTTTATGGCTTTTAATTTTTTG

A3-AT1G10890-XLOC_000523-8259-0
TAATTCTATTTTTCTCGTTTTGTAATTTTACGTTAGGTTAATGGTGATGA
A3-AT1G10890-XLOC_000523-8259-1
TAATTCTATTTTTCTCGTTTTGTAATTTTACGTTAGGTTAATGGTGATGA
CONSENSUS
TAATTCTATTTTTCTCGTTTTGTAATTTTACGTTAGGTTAATGGTGATGA

A3-AT1G10890-XLOC_000523-8259-0
ATGATAATATAGCAATGATTCAGAAAATTTA
A3-AT1G10890-XLOC_000523-8259-1
ATGATAATATAGCAATGATTCAGAAAATTTA
CONSENSUS
ATGATAATATAGCAATGATTCAGAAAATTTA

alignment for event: RI-AT1G79880-XLOC_008100-12395

RI-AT1G79880-XLOC_008100-12395-0
TGACAGTAATCTACCCACAGACGGATTTCTCAACAGGGAAGTCACCAAGA

RI-AT1G79880-XLOC_008100-12395-1
TGACAGTAATCTACCCACAGACGGATTTCTCAACAGGGAAGTCACCAAGA
CONSENSUS
TGACAGTAATCTACCCACAGACGGATTTCTCAACAGGGAAGTCACCAAGA

RI-AT1G79880-XLOC_008100-12395-0
GCAAAGATGGCCGTATCCTGTATACATATATATTGTTGTAGCATACAGAT
RI-AT1G79880-XLOC_008100-12395-1
GCAAAGATGGCC--------------------------------------
CONSENSUS
GCAAAGATGGCC......................................

RI-AT1G79880-XLOC_008100-12395-0
ATAGTTGAAAAGTTATTCCTTAGATGCCCGAAACTAGTGGTTAGTTTGCC
RI-AT1G79880-XLOC_008100-12395-1
-------------------------------------TGGTTAGTTTGCC
CONSENSUS
.....................................TGGTTAGTTTGCC

RI-AT1G79880-XLOC_008100-12395-0
TCTGGTTTGTTCCTTCTCTCGTATGAGGAACCTCCTTGGCCTCGGTAACA
RI-AT1G79880-XLOC_008100-12395-1
TCTGGTTTGTTCCTTCTCTCGTATGAGGAACCTCCTTGGCCTCGGTAACA
CONSENSUS
TCTGGTTTGTTCCTTCTCTCGTATGAGGAACCTCCTTGGCCTCGGTAACA

RI-AT1G79880-XLOC_008100-12395-0
TCAATAGAGAAGACATCCCTCCGAGGATTGTGGAGGAAGTTGCTAATCTC
RI-AT1G79880-XLOC_008100-12395-1
TCAATAGAGAAGACATCCCTCCGAGGATTGTGGAGGAAGTTGCTAATCTC
CONSENSUS
TCAATAGAGAAGACATCCCTCCGAGGATTGTGGAGGAAGTTGCTAATCTC

RI-AT1G79880-XLOC_008100-12395-0
TTGCGCACCTCTGATTTTCTTAAAGTTTCCAATAATG
RI-AT1G79880-XLOC_008100-12395-1
TTGCGCACCTCTGATTTTCTTAAAGTTTCCAATAATG
CONSENSUS
TTGCGCACCTCTGATTTTCTTAAAGTTTCCAATAATG

alignment for event: A3-AT1G16650-XLOC_000851-6567

A3-AT1G16650-XLOC_000851-6567-0
GGTTATCTAGCGCAGGTTCTCTCTTTCCAATATAAGCACTCAGTGGTTGC
A3-AT1G16650-XLOC_000851-6567-1
GGTTATCTAGCGCAGGTTCTCTCTTTCCAATATAAGCACTCAGTGGTTGC
CONSENSUS
GGTTATCTAGCGCAGGTTCTCTCTTTCCAATATAAGCACTCAGTGGTTGC

A3-AT1G16650-XLOC_000851-6567-0
AATCGATTCTTCCTCTCATCATGGAAAGGTAACAGATGCACGCGCAGCAC
A3-AT1G16650-XLOC_000851-6567-1
AATCGATTCTTCCTCTCATCATGGAAAGGTAACAGATGCACGCGCAGCAC
CONSENSUS

AATCGATTCTTCCTCTCATCATGGAAAGGTAACAGATGCACGCGCAGCAC

A3-AT1G16650-XLOC_000851-6567-0
GCATAAAAAAGCATTTTGCAGCACAGATGCGTAAATCTGGTTCAGGAAAC
A3-AT1G16650-XLOC_000851-6567-1
GCATAAAAAAGCATTTTGCAGCACAGATGCGTAAATCTGG-----GAAAC
CONSENSUS
GCATAAAAAAGCATTTTGCAGCACAGATGCGTAAATCTGG.....GAAAC

A3-AT1G16650-XLOC_000851-6567-0
AAGTGCCCAGATGTTCCAATGACGATTACATGCCGTGTTTTATCCACAGA
A3-AT1G16650-XLOC_000851-6567-1
AAGTGCCCAGATGTTCCAATGACGATTACATGCCGTGTTTTATCCACAGA
CONSENSUS
AAGTGCCCAGATGTTCCAATGACGATTACATGCCGTGTTTTATCCACAGA

A3-AT1G16650-XLOC_000851-6567-0
AATGTTGAAGGCCTTGACTGATGTTCATCTGGAGAAGGATGAGACGGATT
A3-AT1G16650-XLOC_000851-6567-1
AATGTTGAAGGCCTTGACTGATGTTCATCTGGAGAAGGATGAGACGGATT
CONSENSUS
AATGTTGAAGGCCTTGACTGATGTTCATCTGGAGAAGGATGAGACGGATT

A3-AT1G16650-XLOC_000851-6567-0
CTAGTGGAAGTGCATTGAATGAAGAAGGTCAAAGCAGATCACAATCATCA
A3-AT1G16650-XLOC_000851-6567-1
CTAGTGGAAGTGCATTGAATGAAGAAGGTCAAAGCAGATCACAATCATCA
CONSENSUS
CTAGTGGAAGTGCATTGAATGAAGAAGGTCAAAGCAGATCACAATCATCA

A3-AT1G16650-XLOC_000851-6567-0
AGTGATGCAAACAGATCGTGTTCACTTGTTCTTGCTGGCCTTCATGCATG
A3-AT1G16650-XLOC_000851-6567-1
AGTGATGCAAACAGATCGTGTTCACTTGTTCTTGCTGGCCTTCATGCATG
CONSENSUS
AGTGATGCAAACAGATCGTGTTCACTTGTTCTTGCTGGCCTTCATGCATG

A3-AT1G16650-XLOC_000851-6567-0 TGGAGATCTATCAGTTACAATGCTAAG
A3-AT1G16650-XLOC_000851-6567-1 TGGAGATCTATCAGTTACAATGCTAAG
CONSENSUS   TGGAGATCTATCAGTTACAATGCTAAG

alignment for event: A3-AT1G80490-XLOC_008129-10788

A3-AT1G80490-XLOC_008129-10788-0
CCTTTTCAACCAACACCTTCTCCGGTTCCGACACCTCTTGCTGGTTGGAT
A3-AT1G80490-XLOC_008129-10788-1
CCTTTTCAACCAACACCTTCTCCGGTTCCGACACCTCTTGCTGGTTGGAT
CONSENSUS
CCTTTTCAACCAACACCTTCTCCGGTTCCGACACCTCTTGCTGGTTGGAT

A3-AT1G80490-XLOC_008129-10788-0
GTCTAGTCCTTCCTCTGTCCCACATCCAGCTGTGTCTGGAGGACCCATTG
A3-AT1G80490-XLOC_008129-10788-1
GTCTAGTCCTTCCTCTGTCCCACATCCAGCTGTGTCTGGAGGACCCATTG

CONSENSUS
GTCTAGTCCTTCCTCTGTCCCACATCCAGCTGTGTCTGGAGGACCCATTG

A3-AT1G80490-XLOC_008129-10788-0 CTCTTGGTGCTCCATCCATCCAAG---CCTTGAAACACCCGAGAACTCCT
A3-AT1G80490-XLOC_008129-10788-1
CTCTTGGTGCTCCATCCATCCAAGCAGCCTTGAAACACCCGAGAACTCCT
CONSENSUS
CTCTTGGTGCTCCATCCATCCAAG...CCTTGAAACACCCGAGAACTCCT

A3-AT1G80490-XLOC_008129-10788-0
CCTTCTAATTCCGCTGTAGACTATCCATCAGGTGACTCAGACCATGTCTC
A3-AT1G80490-XLOC_008129-10788-1
CCTTCTAATTCCGCTGTAGACTATCCATCAGGTGACTCAGACCATGTCTC
CONSENSUS
CCTTCTAATTCCGCTGTAGACTATCCATCAGGTGACTCAGACCATGTCTC

A3-AT1G80490-XLOC_008129-10788-0
AAAGCGAACCAGACCTATGGGAATCTCTGACGAG
A3-AT1G80490-XLOC_008129-10788-1
AAAGCGAACCAGACCTATGGGAATCTCTGACGAG
CONSENSUS
AAAGCGAACCAGACCTATGGGAATCTCTGACGAG

alignment for event: A3-AT1G10890-XLOC_000523-8256

A3-AT1G10890-XLOC_000523-8256-0
GCGAAAAAGTCGTTCTATTTCTCCTAGGCGCCATCGAAGTCGATCTGTTA
A3-AT1G10890-XLOC_000523-8256-1
GCGAAAAAGTCGTTCTATTTCTCCTAGGCGCCATCGAAGTCGATCTGTTA
CONSENSUS
GCGAAAAAGTCGTTCTATTTCTCCTAGGCGCCATCGAAGTCGATCTGTTA

A3-AT1G10890-XLOC_000523-8256-0
CTCCTAAGAGACGTTCTCCAACCCCAAAACGTTACAAAAGACAAAAGAGT
A3-AT1G10890-XLOC_000523-8256-1
CTCCTAAGAGACGTTCTCCAACCCCAAAACGTTACAAAAGACAAAAGAGT
CONSENSUS
CTCCTAAGAGACGTTCTCCAACCCCAAAACGTTACAAAAGACAAAAGAGT

A3-AT1G10890-XLOC_000523-8256-0
AGGAGTTCAACTCCATCTCCTGCAAAAAGATCTCCCGCCGCAACCCTTGA
A3-AT1G10890-XLOC_000523-8256-1
AGGAGTTCAACTCCATCTCCTGCAAAAAGATCTCCCGCCGCAACCCTTGA
CONSENSUS
AGGAGTTCAACTCCATCTCCTGCAAAAAGATCTCCCGCCGCAACCCTTGA

A3-AT1G10890-XLOC_000523-8256-0
GTCAGCCAAAAATAGGAATGGAGAAAAACTTAAAAGAGAAGAGGAAGAAC
A3-AT1G10890-XLOC_000523-8256-1
GTCAGCCAAAAATAGGAATGGAGAAAAACTTAAAAGAGAAGAGGAAGAAC
CONSENSUS
GTCAGCCAAAAATAGGAATGGAGAAAAACTTAAAAGAGAAGAGGAAGAAC

A3-AT1G10890-XLOC_000523-8256-0
GAAAAAGGTAAATCAATACAAAATTTTGAAGTAAGATGGATGTTATGGTG
A3-AT1G10890-XLOC_000523-8256-1
GAAAAAGGTAAATCAATACAAAATTTTGAAGTAAGATGGATGTTATGGTG
CONSENSUS
GAAAAAGGTAAATCAATACAAAATTTTGAAGTAAGATGGATGTTATGGTG

A3-AT1G10890-XLOC_000523-8256-0
AAATTAAGGTTTTTTGTTCTCAAATGTTATTTTAGTGTAGTGTGGAAGTC
A3-AT1G10890-XLOC_000523-8256-1
AAATTAAGGTTTTTTGTTCTCAAATGTTATTTTAGTGTAGTGTGGAAGTC
CONSENSUS
AAATTAAGGTTTTTTGTTCTCAAATGTTATTTTAGTGTAGTGTGGAAGTC

A3-AT1G10890-XLOC_000523-8256-0
CTTGATTGTTAGTCTCAAAAGTGCGACAGGTTTTATGGTATTCTCTCACA
A3-AT1G10890-XLOC_000523-8256-1
CTTGATTGTTAGTCTCAAAAGTGCGACAGGTTTTATGGTATTCTCTCACA
CONSENSUS
CTTGATTGTTAGTCTCAAAAGTGCGACAGGTTTTATGGTATTCTCTCACA

A3-AT1G10890-XLOC_000523-8256-0
GGCTTTGCTTATGGAAGATAAGCTATATTTCGATTTGTTTATATTTGATC
A3-AT1G10890-XLOC_000523-8256-1
GGCTTTGCTTATGGAAGATAAGCTATATTTCGATTTGTTTATATTTGATC
CONSENSUS
GGCTTTGCTTATGGAAGATAAGCTATATTTCGATTTGTTTATATTTGATC

A3-AT1G10890-XLOC_000523-8256-0
CTCTGGCACAAATGTTCGTACTATCTCGATTTGATGAGTTACCGTTTGTC
A3-AT1G10890-XLOC_000523-8256-1
CTCTGGCACAAATGTTCGTACTATCTCGATTTGATGAGTTACCGTTTGTC
CONSENSUS
CTCTGGCACAAATGTTCGTACTATCTCGATTTGATGAGTTACCGTTTGTC

A3-AT1G10890-XLOC_000523-8256-0
ATTACATGGATCATGATTGACTTAGAGATGTAAATATGTCCAAAGCACAA
A3-AT1G10890-XLOC_000523-8256-1
ATTACATGGATCATGATTGACTTAGAGATGTAAATATGTCCAAAGCACAA
CONSENSUS
ATTACATGGATCATGATTGACTTAGAGATGTAAATATGTCCAAAGCACAA

A3-AT1G10890-XLOC_000523-8256-0
GAATAATCTTGGTGAAGGTTTGCTTGTGTTATGGTGACAAAAAAGAAATT
A3-AT1G10890-XLOC_000523-8256-1
GAATAATCTTGGTGAAGGTTTGCTTGTGTTATGGTGACAAAAAAGAAATT
CONSENSUS
GAATAATCTTGGTGAAGGTTTGCTTGTGTTATGGTGACAAAAAAGAAATT

A3-AT1G10890-XLOC_000523-8256-0
TCTTCACGAAATTTACCATTGGTATTGGGAGGAGTGAAAATGGAAACGAT
A3-AT1G10890-XLOC_000523-8256-1
TCTTCACGAAATTTACCATTGGTATTGGGAGGAGTGAAAATGGAAACGAT
CONSENSUS
TCTTCACGAAATTTACCATTGGTATTGGGAGGAGTGAAAATGGAAACGAT

A3-AT1G10890-XLOC_000523-8256-0
      CTCAACATTTTTTACGGATTTGCAATAAAATTTTGGTCTTTTGCTGTTCA
A3-AT1G10890-XLOC_000523-8256-1
      CTCAACATTTTTTACGGATTTGCAATAAAATTTTGGTCTTTTGCTGTTCA
CONSENSUS
      CTCAACATTTTTTACGGATTTGCAATAAAATTTTGGTCTTTTGCTGTTCA

A3-AT1G10890-XLOC_000523-8256-0
      AGATTGAAGACATCAGGAAATGAGAAAGTTCAGGTGATATTCAATATTCT
A3-AT1G10890-XLOC_000523-8256-1
      AGATTGAAGACATCAGGAAATGAGAAAGTTCAGGTGATATTCAATATTCT
CONSENSUS
      AGATTGAAGACATCAGGAAATGAGAAAGTTCAGGTGATATTCAATATTCT

A3-AT1G10890-XLOC_000523-8256-0
      GAACCTGCTGTGGATGTCCTCTAATTTTTCACTGTATTTGCTACCAGGCG
A3-AT1G10890-XLOC_000523-8256-1
      GAACCTGCTGTGGATGTCCTCTAATTTTTCACTGTATTTGCTACCAGGCG
CONSENSUS
      GAACCTGCTGTGGATGTCCTCTAATTTTTCACTGTATTTGCTACCAGGCG

A3-AT1G10890-XLOC_000523-8256-0
      ACAGCGTGAAGCAGAACTGAAGCTAATAGAGGAAGAAACTGTGAAACGGG
A3-AT1G10890-XLOC_000523-8256-1
      ACAGCGTGAAGCAGAACTGAAGCTAATAGAGGAAGAAACTGTGAAACGGG
CONSENSUS
      ACAGCGTGAAGCAGAACTGAAGCTAATAGAGGAAGAAACTGTGAAACGGG

A3-AT1G10890-XLOC_000523-8256-0
      TTGAAGAAGCTATTCGAAAGAAGGTCGAAGAAAGCTTACAGTCTGAGAAA
A3-AT1G10890-XLOC_000523-8256-1
      TTGAAGAAGCTATTCGAAAGAAGGTCGAAGAAAGCTTACAGTCTGAGAAA
CONSENSUS
      TTGAAGAAGCTATTCGAAAGAAGGTCGAAGAAAGCTTACAGTCTGAGAAA

A3-AT1G10890-XLOC_000523-8256-0
      ATCAAAATGGAAATTCTAACGCTGTTGGAGGAAGGGCGAAAGAGACTTAA
A3-AT1G10890-XLOC_000523-8256-1
      ATCAAAATGGAAATTCTAACGCTGTTGGAGGAAGGGCGAAAGAGACTTAA
CONSENSUS
      ATCAAAATGGAAATTCTAACGCTGTTGGAGGAAGGGCGAAAGAGACTTAA

A3-AT1G10890-XLOC_000523-8256-0
      TGAAGAAGTCGCGGCTCAACTTGAGGAGGAGAAAGAGGCTTCTCTTATTG
A3-AT1G10890-XLOC_000523-8256-1
      TGAAGAAGTCGCGGCTCAACTTGAGGAGGAGAAAGAGGCTTCTCTTATTG
CONSENSUS
      TGAAGAAGTCGCGGCTCAACTTGAGGAGGAGAAAGAGGCTTCTCTTATTG

A3-AT1G10890-XLOC_000523-8256-0
      AGGCTAAAGAAAAAGAGGGTGTTATGCGGTGTTTGTCACAGGAAAGAGAG
A3-AT1G10890-XLOC_000523-8256-1
      AGGCTAAAGAAAAAGAG-----------------------GAAAGAGAG
CONSENSUS
      AGGCTAAAGAAAAAGAG.......................GAAAGAGAG

A3-AT1G10890-XLOC_000523-8256-0
CAACAAGAGAAAGAAGAGAGGGAGAGAATAGCAGAGGAGAACCTAAAGAG
A3-AT1G10890-XLOC_000523-8256-1
CAACAAGAGAAAGAAGAGAGGGAGAGAATAGCAGAGGAGAACCTAAAGAG
CONSENSUS
CAACAAGAGAAAGAAGAGAGGGAGAGAATAGCAGAGGAGAACCTAAAGAG

A3-AT1G10890-XLOC_000523-8256-0
AGTGGAAGAAGCTCAGAGAAAAGAAGCAATGGAGAGGCAAAGGAAAGAGG
A3-AT1G10890-XLOC_000523-8256-1
AGTGGAAGAAGCTCAGAGAAAAGAAGCAATGGAGAGGCAAAGGAAAGAGG
CONSENSUS
AGTGGAAGAAGCTCAGAGAAAAGAAGCAATGGAGAGGCAAAGGAAAGAGG

A3-AT1G10890-XLOC_000523-8256-0
AGGAACGGTATCGAGAGCTAGAGGAGCTGCAACGACAGAAAGAAGAAGCG
A3-AT1G10890-XLOC_000523-8256-1
AGGAACGGTATCGAGAGCTAGAGGAGCTGCAACGACAGAAAGAAGAAGCG
CONSENSUS
AGGAACGGTATCGAGAGCTAGAGGAGCTGCAACGACAGAAAGAAGAAGCG

A3-AT1G10890-XLOC_000523-8256-0
ATGCGAAGGAAGAAAGCTGAAGAGGAAGAAGAACGTCTCAAACAGATGAA
A3-AT1G10890-XLOC_000523-8256-1
ATGCGAAGGAAGAAAGCTGAAGAGGAAGAAGAACGTCTCAAACAGATGAA
CONSENSUS
ATGCGAAGGAAGAAAGCTGAAGAGGAAGAAGAACGTCTCAAACAGATGAA

A3-AT1G10890-XLOC_000523-8256-0
ACTGTTGGGTAAAAACAAATCACGGCCTAAATTATCCTTTGCCTTAAGCT
A3-AT1G10890-XLOC_000523-8256-1
ACTGTTGGGTAAAAACAAATCACGGCCTAAATTATCCTTTGCCTTAAGCT
CONSENSUS
ACTGTTGGGTAAAAACAAATCACGGCCTAAATTATCCTTTGCCTTAAGCT

A3-AT1G10890-XLOC_000523-8256-0
CCAAGTAAATGCGTGCATGCATGAAGATAAAAGGATTGATGTGATGGATG
A3-AT1G10890-XLOC_000523-8256-1
CCAAGTAAATGCGTGCATGCATGAAGATAAAAGGATTGATGTGATGGATG
CONSENSUS
CCAAGTAAATGCGTGCATGCATGAAGATAAAAGGATTGATGTGATGGATG

A3-AT1G10890-XLOC_000523-8256-0
ATGATATGCATCTTCTTCCTCTCAAAGATGCTTTATGATTATTGTTATTA
A3-AT1G10890-XLOC_000523-8256-1
ATGATATGCATCTTCTTCCTCTCAAAGATGCTTTATGATTATTGTTATTA
CONSENSUS
ATGATATGCATCTTCTTCCTCTCAAAGATGCTTTATGATTATTGTTATTA

A3-AT1G10890-XLOC_000523-8256-0
GTGCTTCTTGTTGGAGCTTAAACTCTTTTATGGCTTTTAATTTTTTGTAA
A3-AT1G10890-XLOC_000523-8256-1
GTGCTTCTTGTTGGAGCTTAAACTCTTTTATGGCTTTTAATTTTTTGTAA
CONSENSUS
GTGCTTCTTGTTGGAGCTTAAACTCTTTTATGGCTTTTAATTTTTTGTAA

A3-AT1G10890-XLOC_000523-8256-0
      TTCTATTTTTCTCGTTTTGTAATTTTACGTTAGGTTAATGGTGATGAATG
A3-AT1G10890-XLOC_000523-8256-1
      TTCTATTTTTCTCGTTTTGTAATTTTACGTTAGGTTAATGGTGATGAATG
CONSENSUS
      TTCTATTTTTCTCGTTTTGTAATTTTACGTTAGGTTAATGGTGATGAATG

A3-AT1G10890-XLOC_000523-8256-0 ATAATATAGCAATGATTCAGAAAATTTA
A3-AT1G10890-XLOC_000523-8256-1 ATAATATAGCAATGATTCAGAAAATTTA
CONSENSUS   ATAATATAGCAATGATTCAGAAAATTTA

alignment for event: RI-AT1G70590-XLOC_003611-3693

RI-AT1G70590-XLOC_003611-3693-0
      AGAAATTGGATTTGACTTGATGGATGAGTTTATTGCTGATGAATTTGTGG
RI-AT1G70590-XLOC_003611-3693-1
      AGAAATTGGATTTGACTTGATGGATGAGTTTATTGCTGATGAATTTGTGG
CONSENSUS
      AGAAATTGGATTTGACTTGATGGATGAGTTTATTGCTGATGAATTTGTGG

RI-AT1G70590-XLOC_003611-3693-0
      AGCCTTTTTCTGAGCAATTTGATTGCTTCTATCTGCAGTTCAACCTTCAA
RI-AT1G70590-XLOC_003611-3693-1
      AGCCTTTTTCTGAGCAATTTGATTGCTTCTATCTGCAGTTCAACCTTCAA
CONSENSUS
      AGCCTTTTTCTGAGCAATTTGATTGCTTCTATCTGCAGTTCAACCTTCAA

RI-AT1G70590-XLOC_003611-3693-0
      ATCCCAAGGAAGCGATGAAATGGTTGAAACAATCTGCAGAGAATGGTTAT
RI-AT1G70590-XLOC_003611-3693-1
      ATCCCAAGGAAGCGATGAAATGGTTGAAACAATCTGCAGAGAATGGTTAT
CONSENSUS
      ATCCCAAGGAAGCGATGAAATGGTTGAAACAATCTGCAGAGAATGGTTAT

RI-AT1G70590-XLOC_003611-3693-0
      GTTCGAGCACAGTATCAGTTAGCTCTTTGTTTACACCATGGTCGGGTCGT
RI-AT1G70590-XLOC_003611-3693-1
      GTTCGAGCACAGTATCAGTTAGCTCTTTGTTTACACCATGGTCGGGTCGT
CONSENSUS
      GTTCGAGCACAGTATCAGTTAGCTCTTTGTTTACACCATGGTCGGGTCGT

RI-AT1G70590-XLOC_003611-3693-0
      GCAAACCAATCTGCTTGAAGCTGTATGTGCCTTCTTCTGATTCTTTTCTT
RI-AT1G70590-XLOC_003611-3693-1
      GCAAACCAATCTGCTTGAAGCT----------------------------
CONSENSUS
      GCAAACCAATCTGCTTGAAGCT............................

RI-AT1G70590-XLOC_003611-3693-0
      CATGCTTCTAGTTGAGATTCTATATTGTTTTCATGAGGTGAAGCTGTTAT
RI-AT1G70590-XLOC_003611-3693-1

CONSENSUS
      ....

RI-AT1G70590-XLOC_003611-3693-0
CTAGATTGAAAGTAGTATCTAGTCGTTTATACTAATGGCTGCACTCTTGG
RI-AT1G70590-XLOC_003611-3693-1

CONSENSUS
....

RI-AT1G70590-XLOC_003611-3693-0
TGGAAACTGATAATCCTATGTATAGACATGTGCTTAGTTCGTTTGTGGAA
RI-AT1G70590-XLOC_003611-3693-1

CONSENSUS
....

RI-AT1G70590-XLOC_003611-3693-0
ACTGATACTGCTATGTTTGGATGGGTTATTGGTTCATTTGTGGAAGCTGA
RI-AT1G70590-XLOC_003611-3693-1

CONSENSUS
....

RI-AT1G70590-XLOC_003611-3693-0
TAATGCTATGTTTGGACATGTGCTTAGTTCATTTGTGGAAATGTGCGGTT
RI-AT1G70590-XLOC_003611-3693-1

CONSENSUS
....

RI-AT1G70590-XLOC_003611-3693-0
TCTGTTTTTCATCTTTCTAGAATCAGTAAAGTTTGTATCGATATGATGAT
RI-AT1G70590-XLOC_003611-3693-1

CONSENSUS
....

RI-AT1G70590-XLOC_003611-3693-0
TGGATTTTTTTTCTCTTTTTCGACAGACTAAATGGTACCTAAAGGCAGCA
RI-AT1G70590-XLOC_003611-3693-1 --------------------------
ACTAAATGGTACCTAAAGGCAGCA
CONSENSUS
..........................ACTAAATGGTACCTAAAGGCAGCA

RI-AT1G70590-XLOC_003611-3693-0
GAAGGTGGGTATGTACGAGCGATGTATAACATTTCGCTTTGTTATTCGGT
RI-AT1G70590-XLOC_003611-3693-1
GAAGGTGGGTATGTACGAGCGATGTATAACATTTCGCTTTGTTATTCGGT
CONSENSUS
GAAGGTGGGTATGTACGAGCGATGTATAACATTTCGCTTTGTTATTCGGT

RI-AT1G70590-XLOC_003611-3693-0
TGGAGAAGGCTTACCACAAAACCGCAAGCTAGCAAGAAAATGGATGAAAC
RI-AT1G70590-XLOC_003611-3693-1
TGGAGAAGGCTTACCACAAAACCGCAAGCTAGCAAGAAAATGGATGAAAC
CONSENSUS
TGGAGAAGGCTTACCACAAAACCGCAAGCTAGCAAGAAAATGGATGAAAC

RI-AT1G70590-XLOC_003611-3693-0
      GAGCAGCTGATCACGGCCACAGTAAAGCTCAGTTTGAACACGGCCTTGCT
RI-AT1G70590-XLOC_003611-3693-1
      GAGCAGCTGATCACGGCCACAGTAAAGCTCAGTTTGAACACGGCCTTGCT
CONSENSUS
      GAGCAGCTGATCACGGCCACAGTAAAGCTCAGTTTGAACACGGCCTTGCT

RI-AT1G70590-XLOC_003611-3693-0 CTATTTTCT
RI-AT1G70590-XLOC_003611-3693-1 CTATTTTCT
CONSENSUS   CTATTTTCT

alignment for event: RI-AT1G79020-XLOC_008053-9268

RI-AT1G79020-XLOC_008053-9268-0
      AGCCGCCGCAATTTTTCCCGGCAGAGGAAAAAAAACCCCTAGAATCTGGG
RI-AT1G79020-XLOC_008053-9268-1
      AGCCGCCGCAATTTTTCCCGGCAGAGGAAAAAAAACCCCTAGAATCTGGG
CONSENSUS
      AGCCGCCGCAATTTTTCCCGGCAGAGGAAAAAAAACCCCTAGAATCTGGG

RI-AT1G79020-XLOC_008053-9268-0
      AAATTGACGACGAAGAGGGATATAGGTTTGGAAATTTTGGGTATTTAATT
RI-AT1G79020-XLOC_008053-9268-1
      AAATTGACGACGAAGAGGGATATAGGTTTGGAAATTTTGGGTATTTAATT
CONSENSUS
      AAATTGACGACGAAGAGGGATATAGGTTTGGAAATTTTGGGTATTTAATT

RI-AT1G79020-XLOC_008053-9268-0
      TGATTTTTCTTTTTCTCGTTCCATTCAGATTCAAAAACAAAAACCCTAAC
RI-AT1G79020-XLOC_008053-9268-1
      TGATTTTTCTTTTTCTCGTTCCATTCAGATTCAAAAACAAAAACCCTAAC
CONSENSUS
      TGATTTTTCTTTTTCTCGTTCCATTCAGATTCAAAAACAAAAACCCTAAC

RI-AT1G79020-XLOC_008053-9268-0
      CCTAAAATTCGTGTTCGATTAATTTCGTTGTCTCTGTTTTGTTTTCTCTT
RI-AT1G79020-XLOC_008053-9268-1
      CCTAAAATTCGTGTTCGATTAATTTCGTTGTCTCTGTTTTGTTTTCTCTT
CONSENSUS
      CCTAAAATTCGTGTTCGATTAATTTCGTTGTCTCTGTTTTGTTTTCTCTT

RI-AT1G79020-XLOC_008053-9268-0
      CGTGTAAACCTTTTTTTTTCGAGTTTTGGCACTCTGAGCTATTTGAGTAT
RI-AT1G79020-XLOC_008053-9268-1
      CGTGTAAACCTTTTTTTTTCGAGTTTTGGCACTCTGAGCTATTTGAGTAT
CONSENSUS
      CGTGTAAACCTTTTTTTTTCGAGTTTTGGCACTCTGAGCTATTTGAGTAT

RI-AT1G79020-XLOC_008053-9268-0
      TACAGATTAAGCTTCGTCCTGTCTTATTGGAGCAACATACATATATATAT
RI-AT1G79020-XLOC_008053-9268-1
      TACAGATTAAGCTTCGTCCTGTCTTATTGGAGCAACATACATATATATAT
CONSENSUS

TACAGATTAAGCTTCGTCCTGTCTTATTGGAGCAACATACATATATATAT

RI-AT1G79020-XLOC_008053-9268-0
ATATATATATAGATATCTATCATCTGTATTTATAGGCACACTTGTAAGTA
RI-AT1G79020-XLOC_008053-9268-1
ATATATATATAGATATCTATCATCTGTATTTATAGGCACACTTGTAAGTA
CONSENSUS
ATATATATATAGATATCTATCATCTGTATTTATAGGCACACTTGTAAGTA

RI-AT1G79020-XLOC_008053-9268-0
GAATAGTGAGGAAGAAGGTAATATGAGTAGGCTCTCTTTCCGGCCACGGC
RI-AT1G79020-XLOC_008053-9268-1
GAATAGTGAGGAAGAAGGTAATATGAGTAGGCTCTCTTTCCGGCCACGGC
CONSENSUS
GAATAGTGAGGAAGAAGGTAATATGAGTAGGCTCTCTTTCCGGCCACGGC

RI-AT1G79020-XLOC_008053-9268-0
CATTAGACATTCATAAGAAGCTTCCCATTTTAAAATCCTTTAAAGACTTT
RI-AT1G79020-XLOC_008053-9268-1
CATTAGACATTCATAAGAAGCTTCCCATTTTAAAATCCTTTAAAGACTTT
CONSENSUS
CATTAGACATTCATAAGAAGCTTCCCATTTTAAAATCCTTTAAAGACTTT

RI-AT1G79020-XLOC_008053-9268-0
GAAGATGATGAGACTCCAACTTCTACCACTAGAAATTCTCAGTTGCTGCG
RI-AT1G79020-XLOC_008053-9268-1
GAAGATGATGAGACTCCAACTTCTACCACTAGAAATTCTCAGTTGCTGCG
CONSENSUS
GAAGATGATGAGACTCCAACTTCTACCACTAGAAATTCTCAGTTGCTGCG

RI-AT1G79020-XLOC_008053-9268-0
TATTGCTTCCGTAGAAGTTGACAATGAGGTGAATTTCCATTTTGTTCTAA
RI-AT1G79020-XLOC_008053-9268-1
TATTGCTTCCGTAGAAGTTGACAATGAG----------------------
CONSENSUS
TATTGCTTCCGTAGAAGTTGACAATGAG......................

RI-AT1G79020-XLOC_008053-9268-0
CTTCGAAACATTCGACCATTTTAACCATTGTTACTATCAGGTGGCTCCAG
RI-AT1G79020-XLOC_008053-9268-1
--GTGGCTCCAG
CONSENSUS
....GTGGCTCCAG

RI-AT1G79020-XLOC_008053-9268-0
TGCCAAGTAAGAAACCGGCTTCAGAAATACCAACACCTCAATTTGTTATT
RI-AT1G79020-XLOC_008053-9268-1
TGCCAAGTAAGAAACCGGCTTCAGAAATACCAACACCTCAATTTGTTATT
CONSENSUS
TGCCAAGTAAGAAACCGGCTTCAGAAATACCAACACCTCAATTTGTTATT

RI-AT1G79020-XLOC_008053-9268-0
GTGGATACATATGAAAGGGATTATTCTCCGACTTTTGGTCAGCCTGCTTC
RI-AT1G79020-XLOC_008053-9268-1
GTGGATACATATGAAAGGGATTATTCTCCGACTTTTGGTCAGCCTGCTTC
CONSENSUS

GTGGATACATATGAAAGGGATTATTCTCCGACTTTTGGTCAGCCTGCTTC

RI-AT1G79020-XLOC_008053-9268-0 TTATCTACGTGCAAGAGGAG
RI-AT1G79020-XLOC_008053-9268-1 TTATCTACGTGCAAGAGGAG
CONSENSUS   TTATCTACGTGCAAGAGGAG

alignment for event: RI-AT1G08510-XLOC_004583-11928

RI-AT1G08510-XLOC_004583-11928-0
GAAACGGCGCTTAATCATGTTAAGACTGCTGGATTGCTTGGAGATGGGTT
RI-AT1G08510-XLOC_004583-11928-1
GAAACGGCGCTTAATCATGTTAAGACTGCTGGATTGCTTGGAGATGGGTT
CONSENSUS
GAAACGGCGCTTAATCATGTTAAGACTGCTGGATTGCTTGGAGATGGGTT

RI-AT1G08510-XLOC_004583-11928-0
TGGCTCTACACCTGAGATGTTTAAGAAGAACTTGATATGGGTTGTCACTC
RI-AT1G08510-XLOC_004583-11928-1
TGGCTCTACACCTGAGATGTTTAAGAAGAACTTGATATGGGTTGTCACTC
CONSENSUS
TGGCTCTACACCTGAGATGTTTAAGAAGAACTTGATATGGGTTGTCACTC

RI-AT1G08510-XLOC_004583-11928-0
GTATGCAGGTTGTGGTTGATAAATATCCTACTTGGTAAGCTATCCTCTTG
RI-AT1G08510-XLOC_004583-11928-1
GTATGCAGGTTGTGGTTGATAAATATCCTACTTG----------------
CONSENSUS
GTATGCAGGTTGTGGTTGATAAATATCCTACTTG................

RI-AT1G08510-XLOC_004583-11928-0
CATAAACCTGGTTCTGCAGGTTCATGCTCTCACTCTTTTTAACCAGGTTT
RI-AT1G08510-XLOC_004583-11928-1

CONSENSUS
....

RI-AT1G08510-XLOC_004583-11928-0
GGGAAAAATGATGTGTATTTCGTTTTTTCAGTTGATACTGCTTTTACAGT
RI-AT1G08510-XLOC_004583-11928-1

CONSENSUS
....

RI-AT1G08510-XLOC_004583-11928-0
ACGAGATATATGCTCATATGACTAATGACTTCTTGCACCCTGAATTATAT
RI-AT1G08510-XLOC_004583-11928-1

CONSENSUS
....

RI-AT1G08510-XLOC_004583-11928-0
GCTCTGCATGCATATTATATTGCATCATAACTCATTTGCTTATTCAATAT
RI-AT1G08510-XLOC_004583-11928-1

CONSENSUS
      ....

RI-AT1G08510-XLOC_004583-11928-0
      ATGCCTCACAGGGGAGATGTTGTTGAAGTAGACACCTGGGTCAGTCAGTC
RI-AT1G08510-XLOC_004583-11928-1 -----------
GGGAGATGTTGTTGAAGTAGACACCTGGGTCAGTCAGTC
CONSENSUS
      ...........GGGAGATGTTGTTGAAGTAGACACCTGGGTCAGTCAGTC

RI-AT1G08510-XLOC_004583-11928-0
      TGGAAAGAATGGTATGCGTCGTGATTGGCTAGTTCGGGACTGTAATACTG
RI-AT1G08510-XLOC_004583-11928-1
      TGGAAAGAATGGTATGCGTCGTGATTGGCTAGTTCGGGACTGTAATACTG
CONSENSUS
      TGGAAAGAATGGTATGCGTCGTGATTGGCTAGTTCGGGACTGTAATACTG

RI-AT1G08510-XLOC_004583-11928-0 GAGAAACCTTAACACGAGCATCAAG
RI-AT1G08510-XLOC_004583-11928-1 GAGAAACCTTAACACGAGCATCAAG
CONSENSUS GAGAAACCTTAACACGAGCATCAAG

alignment for event: SE-AT1G55310-XLOC_002795-1800

SE-AT1G55310-XLOC_002795-1800-0
      GCAAGAAGATCTCAGGAAGTCGTTTGAGCAGTTTGGTCCTGTCAAGGACA
SE-AT1G55310-XLOC_002795-1800-1
      GCAAGAAGATCTCAGGAAGTCGTTTGAGCAGTTTGGTCCTGTCAAGGACA
CONSENSUS
      GCAAGAAGATCTCAGGAAGTCGTTTGAGCAGTTTGGTCCTGTCAAGGACA

SE-AT1G55310-XLOC_002795-1800-0
      TTTACCTGCCAAGGGATTATTATACCGG----------------------
SE-AT1G55310-XLOC_002795-1800-1
      TTTACCTGCCAAGGGATTATTATACCGGAAGTCATCTTTGTACTGACACT
CONSENSUS
      TTTACCTGCCAAGGGATTATTATACCGG......................

SE-AT1G55310-XLOC_002795-1800-0 --------------
AGATCCGCGAGGGTTTGGGTTCGTTCAATTTATGGA
SE-AT1G55310-XLOC_002795-1800-1
      TGCAAGGCTAGCAGAGATCCGCGAGGGTTTGGGTTCGTTCAATTTATGGA
CONSENSUS
      ..............AGATCCGCGAGGGTTTGGGTTCGTTCAATTTATGGA

SE-AT1G55310-XLOC_002795-1800-0
      CCCTGCTGATGCTGCTGATGCAAAACATCACATGGATGGTTATCTTCTTC
SE-AT1G55310-XLOC_002795-1800-1
      CCCTGCTGATGCTGCTGATGCAAAACATCACATGGATGGTTATCTTCTTC
CONSENSUS
      CCCTGCTGATGCTGCTGATGCAAAACATCACATGGATGGTTATCTTCTTC

SE-AT1G55310-XLOC_002795-1800-0
      TTGGCCGTGAGTTGACTGTCGTGTTTGCAGAAGAGAACAGAAAGAAACCG
SE-AT1G55310-XLOC_002795-1800-1

TTGGCCGTGAGTTGACTGTCGTGTTTGCAGAAGAGAACAGAAAGAAACCG
CONSENSUS
TTGGCCGTGAGTTGACTGTCGTGTTTGCAGAAGAGAACAGAAAGAAACCG

SE-AT1G55310-XLOC_002795-1800-0
ACTGAAATGAGAGCAAGGGAGCGTGGTGGAGGAAG
SE-AT1G55310-XLOC_002795-1800-1
ACTGAAATGAGAGCAAGGGAGCGTGGTGGAGGAAG
CONSENSUS
ACTGAAATGAGAGCAAGGGAGCGTGGTGGAGGAAG

alignment for event: A3-AT1G28320-XLOC_005621-6

A3-AT1G28320-XLOC_005621-6-0
GAATGGAAGGGGCTCCGGTGTTTGCTAAGAATGGGCACTTAATTGGCATT
A3-AT1G28320-XLOC_005621-6-1
GAATGGAAGGGGCTCCGGTGTTTGCTAAGAATGGGCACTTAATTGGCATT
CONSENSUS
GAATGGAAGGGGCTCCGGTGTTTGCTAAGAATGGGCACTTAATTGGCATT

A3-AT1G28320-XLOC_005621-6-0
TTGATTAGACCACTAAGGCAAAAGAATAGCGGCGTTGAAATTCAG-----
A3-AT1G28320-XLOC_005621-6-1
TTGATTAGACCACTAAGGCAAAAGAATAGCGGCGTTGAAATTCAGCCTTC
CONSENSUS
TTGATTAGACCACTAAGGCAAAAGAATAGCGGCGTTGAAATTCAG.....

A3-AT1G28320-XLOC_005621-6-0 --------
CTGGTGGTTCCATGGGGAGCAATCACAACTGCTTGCAGCCAC
A3-AT1G28320-XLOC_005621-6-1
GGTAGCAGCTGGTGGTTCCATGGGGAGCAATCACAACTGCTTGCAGCCAC
CONSENSUS
........CTGGTGGTTCCATGGGGAGCAATCACAACTGCTTGCAGCCAC

A3-AT1G28320-XLOC_005621-6-0
TTGCTGCTTGAGGAACCATCTGTAGAAGGAAAAGCAAGTCAGTGGGGGAG
A3-AT1G28320-XLOC_005621-6-1
TTGCTGCTTGAGGAACCATCTGTAGAAGGAAAAGCAAGTCAGTGGGGGAG
CONSENSUS
TTGCTGCTTGAGGAACCATCTGTAGAAGGAAAAGCAAGTCAGTGGGGGAG

A3-AT1G28320-XLOC_005621-6-0
CGAAGTCCTAAGTGTTAAATCAGATGCTAGTATTCCTGCACAAGTGGCTA
A3-AT1G28320-XLOC_005621-6-1
CGAAGTCCTAAGTGTTAAATCAGATGCTAGTATTCCTGCACAAGTGGCTA
CONSENSUS
CGAAGTCCTAAGTGTTAAATCAGATGCTAGTATTCCTGCACAAGTGGCTA

A3-AT1G28320-XLOC_005621-6-0
TTGAGAAGGCGATGGAATCAGTTTGTCTTATTACGGTCAATGATGGTGTT
A3-AT1G28320-XLOC_005621-6-1
TTGAGAAGGCGATGGAATCAGTTTGTCTTATTACGGTCAATGATGGTGTT
CONSENSUS
TTGAGAAGGCGATGGAATCAGTTTGTCTTATTACGGTCAATGATGGTGTT

A3-AT1G28320-XLOC_005621-6-0
TGGGCTTCTGGTATTATTCTTAACGAACATGGTCTCATACTAACAAATGC
A3-AT1G28320-XLOC_005621-6-1
TGGGCTTCTGGTATTATTCTTAACGAACATGGTCTCATACTAACAAATGC
CONSENSUS
TGGGCTTCTGGTATTATTCTTAACGAACATGGTCTCATACTAACAAATGC

A3-AT1G28320-XLOC_005621-6-0
TCACCTGCTTGAGCCGTGGAGGTATGGAAAAGGTGGTGTATATGGTGAAG
A3-AT1G28320-XLOC_005621-6-1
TCACCTGCTTGAGCCGTGGAGGTATGGAAAAGGTGGTGTATATGGTGAAG
CONSENSUS
TCACCTGCTTGAGCCGTGGAGGTATGGAAAAGGTGGTGTATATGGTGAAG

A3-AT1G28320-XLOC_005621-6-0
GATTTAAACCCTATGTCTTAGGAGCCGAGGAATTTTCTTCCACGGGAAGT
A3-AT1G28320-XLOC_005621-6-1
GATTTAAACCCTATGTCTTAGGAGCCGAGGAATTTTCTTCCACGGGAAGT
CONSENSUS
GATTTAAACCCTATGTCTTAGGAGCCGAGGAATTTTCTTCCACGGGAAGT

A3-AT1G28320-XLOC_005621-6-0
AAATTTTGGGAACAGAAGAGTCAAACATTGCCACGGAAAGCTCCACGAAA
A3-AT1G28320-XLOC_005621-6-1
AAATTTTGGGAACAGAAGAGTCAAACATTGCCACGGAAAGCTCCACGAAA
CONSENSUS
AAATTTTGGGAACAGAAGAGTCAAACATTGCCACGGAAAGCTCCACGAAA

A3-AT1G28320-XLOC_005621-6-0
TCATTATTCGTCTGTTGGAGAGAACATCAGGGAATACAAACACAATTTCC
A3-AT1G28320-XLOC_005621-6-1
TCATTATTCGTCTGTTGGAGAGAACATCAGGGAATACAAACACAATTTCC
CONSENSUS
TCATTATTCGTCTGTTGGAGAGAACATCAGGGAATACAAACACAATTTCC

A3-AT1G28320-XLOC_005621-6-0
TTCAGACTGGGCATAGAGACATACGTGTGCGTTTGTGTCACCTAGATTCT
A3-AT1G28320-XLOC_005621-6-1
TTCAGACTGGGCATAGAGACATACGTGTGCGTTTGTGTCACCTAGATTCT
CONSENSUS
TTCAGACTGGGCATAGAGACATACGTGTGCGTTTGTGTCACCTAGATTCT

A3-AT1G28320-XLOC_005621-6-0
TGGACTTGGTGTCCTGCAAACGTGGTCTATATTTGCAAGGAACAATTAGA
A3-AT1G28320-XLOC_005621-6-1
TGGACTTGGTGTCCTGCAAACGTGGTCTATATTTGCAAGGAACAATTAGA
CONSENSUS
TGGACTTGGTGTCCTGCAAACGTGGTCTATATTTGCAAGGAACAATTAGA

A3-AT1G28320-XLOC_005621-6-0
TATTGCCTTACTGCAGCTAGAATATGTCCCTGGAAAGCTCCAACCTATTA
A3-AT1G28320-XLOC_005621-6-1
TATTGCCTTACTGCAGCTAGAATATGTCCCTGGAAAGCTCCAACCTATTA
CONSENSUS
TATTGCCTTACTGCAGCTAGAATATGTCCCTGGAAAGCTCCAACCTATTA

A3-AT1G28320-XLOC_005621-6-0
CTGCCAATTTTTCTTCTCCTCCTTTGGGTACAACAGCGCATGTTGTTGGA
A3-AT1G28320-XLOC_005621-6-1
CTGCCAATTTTTCTTCTCCTCCTTTGGGTACAACAGCGCATGTTGTTGGA
CONSENSUS
CTGCCAATTTTTCTTCTCCTCCTTTGGGTACAACAGCGCATGTTGTTGGA

A3-AT1G28320-XLOC_005621-6-0 CATGGACTCTTCGGACCAAGATGTG
A3-AT1G28320-XLOC_005621-6-1 CATGGACTCTTCGGACCAAGATGTG
CONSENSUS CATGGACTCTTCGGACCAAGATGTG

alignment for event: RI-AT1G77800-XLOC_003990-10549

RI-AT1G77800-XLOC_003990-10549-0
CGAGAGTTGGCTATTTCCTCACATGAAATACTTGCTGCCAAAAGGGATCA
RI-AT1G77800-XLOC_003990-10549-1
CGAGAGTTGGCTATTTCCTCACATGAAATACTTGCTGCCAAAAGGGATCA
CONSENSUS
CGAGAGTTGGCTATTTCCTCACATGAAATACTTGCTGCCAAAAGGGATCA

RI-AT1G77800-XLOC_003990-10549-0
TGCTGCACGGCCATTACATGTCCGTAATCCATTTTCTCCTCCAGAAGTTT
RI-AT1G77800-XLOC_003990-10549-1
TGCTGCACGGCCATTACATGTCCGTAATCCATTTTCTCCTCCAGAAGTTT
CONSENSUS
TGCTGCACGGCCATTACATGTCCGTAATCCATTTTCTCCTCCAGAAGTTT

RI-AT1G77800-XLOC_003990-10549-0
CGTCGGACTCAGCTACAACATCAATAAAAGGCCATCCTGATAGTAATATA
RI-AT1G77800-XLOC_003990-10549-1
CGTCGGACTCAGCTACAACATCAATAAAAGGCCATCCTGATAGTAATATA
CONSENSUS
CGTCGGACTCAGCTACAACATCAATAAAAGGCCATCCTGATAGTAATATA

RI-AT1G77800-XLOC_003990-10549-0
TCTGGCAGTGAAGCAATACAGAGGTCAGATGATATCACTATTGACAGCAC
RI-AT1G77800-XLOC_003990-10549-1
TCTGGCAGTGAAGCAATACAGAGGTCAGATGATATCACTATTGACAGCAC
CONSENSUS
TCTGGCAGTGAAGCAATACAGAGGTCAGATGATATCACTATTGACAGCAC

RI-AT1G77800-XLOC_003990-10549-0
AGTCACTGATAAGCGCAGAGGCAAAGGTCCCTTATTAATGGACACGGATC
RI-AT1G77800-XLOC_003990-10549-1
AGTCACTGATAAGCGCAGAGGCAAAGGTCCCTTATTAATGGACACGGATC
CONSENSUS
AGTCACTGATAAGCGCAGAGGCAAAGGTCCCTTATTAATGGACACGGATC

RI-AT1G77800-XLOC_003990-10549-0
AGAAAACTGATGACAGTGCTACTTCCAAGAGTCGGTTTTCCCGTAAACTA
RI-AT1G77800-XLOC_003990-10549-1
AGAAAACTGATGACAGTGCTACTTCCAAGAGTCGGTTTTCCCGTAAACTA
CONSENSUS

AGAAAACTGATGACAGTGCTACTTCCAAGAGTCGGTTTTCCCGTAAACTA

RI-AT1G77800-XLOC_003990-10549-0
ACAGAAAGACAGATCTTATCTGGGAAAACTGTTCCCCGCAAACATTGTAT
RI-AT1G77800-XLOC_003990-10549-1
ACAGAAAGACAGATCTTATCTGGGAAAACTGTTCCCCGCAAACATTGTAT
CONSENSUS
ACAGAAAGACAGATCTTATCTGGGAAAACTGTTCCCCGCAAACATTGTAT

RI-AT1G77800-XLOC_003990-10549-0
AGTGTCACCTAGTGTTTCAGAGGATGGAGATAACGGGTCAAAGCCCAAGA
RI-AT1G77800-XLOC_003990-10549-1
AGTGTCACCTAGTGTTTCAGAGGATGGAGATAACGGGTCAAAGCCCAAGA
CONSENSUS
AGTGTCACCTAGTGTTTCAGAGGATGGAGATAACGGGTCAAAGCCCAAGA

RI-AT1G77800-XLOC_003990-10549-0
AGGTGAGAAGATTAACTTTGTCGTAAGTTACATGGATTGTTAAGTTCCGA
RI-AT1G77800-XLOC_003990-10549-1
AG--
CONSENSUS
AG..

RI-AT1G77800-XLOC_003990-10549-0
CTCTTTCTGTGCCCCCAACTCAGCACATTCATAGGATCTTACACTTGTTT
RI-AT1G77800-XLOC_003990-10549-1

CONSENSUS
....

RI-AT1G77800-XLOC_003990-10549-0
GTCTTGATAACTGATAATAGCAGCATGTAGAAACATTTGCAAAAGAGCTG
RI-AT1G77800-XLOC_003990-10549-1 -----------------------
CATGTAGAAACATTTGCAAAAGAGCTG
CONSENSUS
.......................CATGTAGAAACATTTGCAAAAGAGCTG

RI-AT1G77800-XLOC_003990-10549-0
GTGATGACATCAGATGAAGCTTCTTTCAAGAACCGGCGGCTCCCAAAGGG
RI-AT1G77800-XLOC_003990-10549-1
GTGATGACATCAGATGAAGCTTCTTTCAAGAACCGGCGGCTCCCAAAGGG
CONSENSUS
GTGATGACATCAGATGAAGCTTCTTTCAAGAACCGGCGGCTCCCAAAGGG

RI-AT1G77800-XLOC_003990-10549-0
ATACTTCTATGTTCCTGTTGATTGTCTTCAGGAAGACAAACCGGGAAACC
RI-AT1G77800-XLOC_003990-10549-1
ATACTTCTATGTTCCTGTTGATTGTCTTCAGGAAGACAAACCGGGAAACC
CONSENSUS
ATACTTCTATGTTCCTGTTGATTGTCTTCAGGAAGACAAACCGGGAAACC

RI-AT1G77800-XLOC_003990-10549-0
AGAAGCTGGCTTCATCTGACAAGCCAGCGAACCAGAAGACATCTTCAGGT
RI-AT1G77800-XLOC_003990-10549-1
AGAAGCTGGCTTCATCTGACAAGCCAGCGAACCAGAAGACATCTTCAGGT
CONSENSUS

AGAAGCTGGCTTCATCTGACAAGCCAGCGAACCAGAAGACATCTTCAGGT

RI-AT1G77800-XLOC_003990-10549-0
GATCAGTCAGGTAAAGACGACGGGTAAAAGCTGAGAACACACATTTTGGC
RI-AT1G77800-XLOC_003990-10549-1
GATCAGTCAGGTAAAGACGACGGGTAAAAGCTGAGAACACACATTTTGGC
CONSENSUS
GATCAGTCAGGTAAAGACGACGGGTAAAAGCTGAGAACACACATTTTGGC

RI-AT1G77800-XLOC_003990-10549-0
TCATTGCTCCTACCTTAATTAGATACTGGCTATTGGTGGACATTGTCTTC
RI-AT1G77800-XLOC_003990-10549-1
TCATTGCTCCTACCTTAATTAGATACTGGCTATTGGTGGACATTGTCTTC
CONSENSUS
TCATTGCTCCTACCTTAATTAGATACTGGCTATTGGTGGACATTGTCTTC

RI-AT1G77800-XLOC_003990-10549-0
TGCGCCCTAACCAGGATTCACCAACTGGCTTAAAGCAAAAGAACAGATGG
RI-AT1G77800-XLOC_003990-10549-1
TGCGCCCTAACCAGGATTCACCAACTGGCTTAAAGCAAAAGAACAGATGG
CONSENSUS
TGCGCCCTAACCAGGATTCACCAACTGGCTTAAAGCAAAAGAACAGATGG

RI-AT1G77800-XLOC_003990-10549-0
TATTGCGCTACAAAAGAGCTGTGGCTGCAACTGAGAATCAGCTAAAAAAA
RI-AT1G77800-XLOC_003990-10549-1
TATTGCGCTACAAAAGAGCTGTGGCTGCAACTGAGAATCAGCTAAAAAAA
CONSENSUS
TATTGCGCTACAAAAGAGCTGTGGCTGCAACTGAGAATCAGCTAAAAAAA

RI-AT1G77800-XLOC_003990-10549-0
GCTATTGTGAAGCTTTTGGGTACTCGATACAAACTGTGCCTATTGGTAAT
RI-AT1G77800-XLOC_003990-10549-1
GCTATTGTGAAGCTTTTGGGTACTCGATACAAACTGTGCCTATTGGTAAT
CONSENSUS
GCTATTGTGAAGCTTTTGGGTACTCGATACAAACTGTGCCTATTGGTAAT

RI-AT1G77800-XLOC_003990-10549-0
GTTTGAGATTTGAGGGTAACATCTCTATAGGAAAAAGGCTTCAGATTGGA
RI-AT1G77800-XLOC_003990-10549-1
GTTTGAGATTTGAGGGTAACATCTCTATAGGAAAAAGGCTTCAGATTGGA
CONSENSUS
GTTTGAGATTTGAGGGTAACATCTCTATAGGAAAAAGGCTTCAGATTGGA

RI-AT1G77800-XLOC_003990-10549-0
TTCAGGGAAGTGAGAAAAAAGCTCCCTATTACCATATAAAGAAGGATCGG
RI-AT1G77800-XLOC_003990-10549-1
TTCAGGGAAGTGAGAAAAAAGCTCCCTATTACCATATAAAGAAGGATCGG
CONSENSUS
TTCAGGGAAGTGAGAAAAAAGCTCCCTATTACCATATAAAGAAGGATCGG

RI-AT1G77800-XLOC_003990-10549-0
TCACGTGTAATTCTGTTTGTATAAAGAAGCGGAAAAAAAAGAAAAGGGTT
RI-AT1G77800-XLOC_003990-10549-1
TCACGTGTAATTCTGTTTGTATAAAGAAGCGGAAAAAAAAGAAAAGGGTT
CONSENSUS

TCACGTGTAATTCTGTTTGTATAAAGAAGCGGAAAAAAAAGAAAAGGGTT

RI-AT1G77800-XLOC_003990-10549-0
GCATAAGTGTATATATTCATCAATTGTGTCTAAAATCTGACCAAGGGAAT
RI-AT1G77800-XLOC_003990-10549-1
GCATAAGTGTATATATTCATCAATTGTGTCTAAAATCTGACCAAGGGAAT
CONSENSUS
GCATAAGTGTATATATTCATCAATTGTGTCTAAAATCTGACCAAGGGAAT

RI-AT1G77800-XLOC_003990-10549-0 AATAGTTGTCTGAAA
RI-AT1G77800-XLOC_003990-10549-1 AATAGTTGTCTGAAA
CONSENSUS AATAGTTGTCTGAAA

alignment for event: A3-AT1G14270-XLOC_004896-7016

A3-AT1G14270-XLOC_004896-7016-0
TTACAAGGAGGTAGCTCAGAGTGGCCGATACTTCAGAGATGGGAAGTTCC
A3-AT1G14270-XLOC_004896-7016-1
TTACAAGGAGGTAGCTCAGAGTGGCCGATACTTCAGAGATGGGAAGTTCC
CONSENSUS
TTACAAGGAGGTAGCTCAGAGTGGCCGATACTTCAGAGATGGGAAGTTCC

A3-AT1G14270-XLOC_004896-7016-0
ATGGGGATGGCAGACAGTTTCGTTAACTTCGTTTGCTTGTGCACTAAG--
A3-AT1G14270-XLOC_004896-7016-1
ATGGGGATGGCAGACAGTTTCGTTAACTTCGTTTGCTTGTGCACTAAGGC
CONSENSUS
ATGGGGATGGCAGACAGTTTCGTTAACTTCGTTTGCTTGTGCACTAAG..

A3-AT1G14270-XLOC_004896-7016-0 --
TTTTGTTTTGACAGGATTAACCGAGATGGCAGTCATACCGTTTTTAGG
A3-AT1G14270-XLOC_004896-7016-1
AGTTTTGTTTTGACAGGATTAACCGAGATGGCAGTCATACCGTTTTTAGG
CONSENSUS
..TTTTGTTTTGACAGGATTAACCGAGATGGCAGTCATACCGTTTTTAGG

A3-AT1G14270-XLOC_004896-7016-0
AATCGACGTTGAGAAACTGACCTTGGACGATAAGGCGGAGATTTTGTTCC
A3-AT1G14270-XLOC_004896-7016-1
AATCGACGTTGAGAAACTGACCTTGGACGATAAGGCGGAGATTTTGTTCC
CONSENSUS
AATCGACGTTGAGAAACTGACCTTGGACGATAAGGCGGAGATTTTGTTCC

A3-AT1G14270-XLOC_004896-7016-0 TGGATCAAGG
A3-AT1G14270-XLOC_004896-7016-1 TGGATCAAGG
CONSENSUS TGGATCAAGG

alignment for event: RI-AT1G28280-XLOC_005615-7520

RI-AT1G28280-XLOC_005615-7520-0
AACAAAACACAGTTTCTCTCTCTCTCTCTCTCTCTCTCTCTCTCTCTCTC
RI-AT1G28280-XLOC_005615-7520-1

AACAAAACACAGTTTCTCTCTCTCTCTCTCTCTCTCTCTCTCTCTCTCTC
CONSENSUS
AACAAAACACAGTTTCTCTCTCTCTCTCTCTCTCTCTCTCTCTCTCTCTC

RI-AT1G28280-XLOC_005615-7520-0
TCTCTCTCTCTCTAAAAGCTCTCTTCTCTTACAATGGAGAATTCACCGAG
RI-AT1G28280-XLOC_005615-7520-1
TCTCTCTCTCTCTAAAAGCTCTCTTCTCTTACAATGGAGAATTCACCGAG
CONSENSUS
TCTCTCTCTCTCTAAAAGCTCTCTTCTCTTACAATGGAGAATTCACCGAG

RI-AT1G28280-XLOC_005615-7520-0
ATACAGAGAAGCGACGAATCTGATTCCGTCGCCGAGATGTCACAACAGCA
RI-AT1G28280-XLOC_005615-7520-1
ATACAGAGAAGCGACGAATCTGATTCCGTCGCCGAGATGTCACAACAGCA
CONSENSUS
ATACAGAGAAGCGACGAATCTGATTCCGTCGCCGAGATGTCACAACAGCA

RI-AT1G28280-XLOC_005615-7520-0
ACAACAGTTGCGGTATGAGCAGTAGCAGTGAAAGTAACAAACCACCAACA
RI-AT1G28280-XLOC_005615-7520-1
ACAACAGTTGCGGTATGAGCAGTAGCAGTGAAAGTAACAAACCACCAACA
CONSENSUS
ACAACAGTTGCGGTATGAGCAGTAGCAGTGAAAGTAACAAACCACCAACA

RI-AT1G28280-XLOC_005615-7520-0
ACTCCGACCCGACACGTAACCACAAGATCCGAATCCGGTAACCCGTACCC
RI-AT1G28280-XLOC_005615-7520-1
ACTCCGACCCGACACGTAACCACAAGATCCGAATCCGGTAACCCGTACCC
CONSENSUS
ACTCCGACCCGACACGTAACCACAAGATCCGAATCCGGTAACCCGTACCC

RI-AT1G28280-XLOC_005615-7520-0
GACAACATTCGTCCAAGCAGATACTTCTTCCTTCAAACAAGTCGTCCAGA
RI-AT1G28280-XLOC_005615-7520-1
GACAACATTCGTCCAAGCAGATACTTCTTCCTTCAAACAAGTCGTCCAGA
CONSENSUS
GACAACATTCGTCCAAGCAGATACTTCTTCCTTCAAACAAGTCGTCCAGA

RI-AT1G28280-XLOC_005615-7520-0
TGCTAACCGGATCCGCCGAGAGACCCAAACACGGTTCATCTCTCAAACCA
RI-AT1G28280-XLOC_005615-7520-1
TGCTAACCGGATCCGCCGAGAGACCCAAACACGGTTCATCTCTCAAACCA
CONSENSUS
TGCTAACCGGATCCGCCGAGAGACCCAAACACGGTTCATCTCTCAAACCA

RI-AT1G28280-XLOC_005615-7520-0
AACCCGACCCATCATCAACCCGACCCAAGATCCACTCCTTCTTCTTTCTC
RI-AT1G28280-XLOC_005615-7520-1
AACCCGACCCATCATCAACCCGACCCAAGATCCACTCCTTCTTCTTTCTC
CONSENSUS
AACCCGACCCATCATCAACCCGACCCAAGATCCACTCCTTCTTCTTTCTC

RI-AT1G28280-XLOC_005615-7520-0
AATCCCACCAATCAAAGCCGTACCTAACAAGAAACAGTCTTCTTCTTCAG
RI-AT1G28280-XLOC_005615-7520-1

AATCCCACCAATCAAAGCCGTACCTAACAAGAAACAGTCTTCTTCTTCAG
CONSENSUS
AATCCCACCAATCAAAGCCGTACCTAACAAGAAACAGTCTTCTTCTTCAG

RI-AT1G28280-XLOC_005615-7520-0
CATCAGGGTTTCGTCTCTACGAGCGTCGTAATTCAATGAAGAATCTCAAA
RI-AT1G28280-XLOC_005615-7520-1
CATCAGGGTTTCGTCTCTACGAGCGTCGTAATTCAATGAAGAATCTCAAA
CONSENSUS
CATCAGGGTTTCGTCTCTACGAGCGTCGTAATTCAATGAAGAATCTCAAA

RI-AT1G28280-XLOC_005615-7520-0
ATCAATCCTTTAAACCCGGTTTTTAACCCGGTTAATTCAGCTTTCTCTCC
RI-AT1G28280-XLOC_005615-7520-1
ATCAATCCTTTAAACCCGGTTTTTAACCCGGTTAATTCAGCTTTCTCTCC
CONSENSUS
ATCAATCCTTTAAACCCGGTTTTTAACCCGGTTAATTCAGCTTTCTCTCC

RI-AT1G28280-XLOC_005615-7520-0
CCGTAAACCGGAGATCCTCTCTCCAAGCATCTTAGATTTCCCATCTCTCG
RI-AT1G28280-XLOC_005615-7520-1
CCGTAAACCGGAGATCCTCTCTCCAAGCATCTTAGATTTCCCATCTCTCG
CONSENSUS
CCGTAAACCGGAGATCCTCTCTCCAAGCATCTTAGATTTCCCATCTCTCG

RI-AT1G28280-XLOC_005615-7520-0
TTCTCAGCCCGGTTACGCCTCTTATACCCGACCCGTTTGATCGATCCGGG
RI-AT1G28280-XLOC_005615-7520-1
TTCTCAGCCCGGTTACGCCTCTTATACCCGACCCGTTTGATCGATCCGGG
CONSENSUS
TTCTCAGCCCGGTTACGCCTCTTATACCCGACCCGTTTGATCGATCCGGG

RI-AT1G28280-XLOC_005615-7520-0
TCATCAAACCAAAGCCCCAACGAGCTTGCTGCGGAGGAGAAAGCAATGAA
RI-AT1G28280-XLOC_005615-7520-1
TCATCAAACCAAAGCCCCAACGAGCTTGCTGCGGAGGAGAAAGCAATGAA
CONSENSUS
TCATCAAACCAAAGCCCCAACGAGCTTGCTGCGGAGGAGAAAGCAATGAA

RI-AT1G28280-XLOC_005615-7520-0
AGAGAGAGGCTTTTATTTGCATCCATCTCCGGCAACAACTCCGATGGATC
RI-AT1G28280-XLOC_005615-7520-1
AGAGAGAGGCTTTTATTTGCATCCATCTCCGGCAACAACTCCGATGGATC
CONSENSUS
AGAGAGAGGCTTTTATTTGCATCCATCTCCGGCAACAACTCCGATGGATC

RI-AT1G28280-XLOC_005615-7520-0
CAGAGCCTCGACTTCTTCCTCTATTCCCGGTGACTTCTCCTAGAGTCTCA
RI-AT1G28280-XLOC_005615-7520-1
CAGAGCCTCGACTTCTTCCTCTATTCCCGGTGACTTCTCCTAGAGTCTCA
CONSENSUS
CAGAGCCTCGACTTCTTCCTCTATTCCCGGTGACTTCTCCTAGAGTCTCA

RI-AT1G28280-XLOC_005615-7520-0
GGTTCTTCATCAGCTTCTACTTCTTGAAAAAAAGACCTAATTTTTCTTTT
RI-AT1G28280-XLOC_005615-7520-1

G---
CONSENSUS
G...

RI-AT1G28280-XLOC_005615-7520-0
TTACTCAAATTTTTATTATCCTTTTGTGATCTTAATTATATATTATAATT
RI-AT1G28280-XLOC_005615-7520-1

CONSENSUS
....

RI-AT1G28280-XLOC_005615-7520-0
GGGGAATTGAGATGAATCATGTTGAGAATTATACGAGAAAATTTGTAATG
RI-AT1G28280-XLOC_005615-7520-1

CONSENSUS
....

RI-AT1G28280-XLOC_005615-7520-0
TTGAGATGTTTGCTAAATCCAATTCGCGATTTCAATTGTATTAATGGTTT
RI-AT1G28280-XLOC_005615-7520-1

CONSENSUS
....

RI-AT1G28280-XLOC_005615-7520-0
AATATTGATGAGTTTATTCTGCAGAATTTGATAAATTAGATTAGTACTGA
RI-AT1G28280-XLOC_005615-7520-1

CONSENSUS
....

RI-AT1G28280-XLOC_005615-7520-0
TAATAATTCAGTGAGTTTTGTACAATTTTAGTGAAATGAGCAAATTGTTT
RI-AT1G28280-XLOC_005615-7520-1

CONSENSUS
....

RI-AT1G28280-XLOC_005615-7520-0
TTTGTTTCTATTCTATTTTATTTCCATAAACACACAATTAGTTGCATTCT
RI-AT1G28280-XLOC_005615-7520-1

CONSENSUS
....

RI-AT1G28280-XLOC_005615-7520-0
ACATTTGTTTTTTTTCAGTAAACAAAAGATGAAGAGATTGGTCTTTAGAG
RI-AT1G28280-XLOC_005615-7520-1 ------------------
TAAACAAAAGATGAAGAGATTGGTCTTTAGAG
CONSENSUS
..................TAAACAAAAGATGAAGAGATTGGTCTTTAGAG

RI-AT1G28280-XLOC_005615-7520-0
GGAACCAAATTGGTCCAACCTTTTTTTGCTAAGGATTGATGAAAGGGTAA
RI-AT1G28280-XLOC_005615-7520-1

GGAACCAAATTGGTCCAACCTTTTTTTGCTAAGGATTGATGAAAGGGTAA
CONSENSUS
GGAACCAAATTGGTCCAACCTTTTTTTGCTAAGGATTGATGAAAGGGTAA

RI-AT1G28280-XLOC_005615-7520-0
ACAAAAAGAAAAAGGAGTGTGGAAAATAAAAGAGATTTTTTTCTTGTTAT
RI-AT1G28280-XLOC_005615-7520-1
ACAAAAAGAAAAAGGAGTGTGGAAAATAAAAGAGATTTTTTTCTTGTTAT
CONSENSUS
ACAAAAAGAAAAAGGAGTGTGGAAAATAAAAGAGATTTTTTTCTTGTTAT

RI-AT1G28280-XLOC_005615-7520-0
TATTATTTTCATATTTTCTCCTTAGAAAAAGGAGAATACGAAATTGAAAA
RI-AT1G28280-XLOC_005615-7520-1
TATTATTTTCATATTTTCTCCTTAGAAAAAGGAGAATACGAAATTGAAAA
CONSENSUS
TATTATTTTCATATTTTCTCCTTAGAAAAAGGAGAATACGAAATTGAAAA

RI-AT1G28280-XLOC_005615-7520-0
AAAGAAAGAAAAGAGTAAATTGGATAGAAGGAAGATAAAGAGTGGGGCTT
RI-AT1G28280-XLOC_005615-7520-1
AAAGAAAGAAAAGAGTAAATTGGATAGAAGGAAGATAAAGAGTGGGGCTT
CONSENSUS
AAAGAAAGAAAAGAGTAAATTGGATAGAAGGAAGATAAAGAGTGGGGCTT

RI-AT1G28280-XLOC_005615-7520-0 AG
RI-AT1G28280-XLOC_005615-7520-1 AG
CONSENSUS   AG

alignment for event: RI-AT1G55310-XLOC_002795-1791

RI-AT1G55310-XLOC_002795-1791-0
TGAATCTAAAGAATTGAAGACATCAAAGAAGTAATTAGAGTTCTTATGAA
RI-AT1G55310-XLOC_002795-1791-1
TGAATCTAAAGAATTGAAGACATCAAAGAAGTAATTAGAGTTCTTATGAA
CONSENSUS
TGAATCTAAAGAATTGAAGACATCAAAGAAGTAATTAGAGTTCTTATGAA

RI-AT1G55310-XLOC_002795-1791-0
GATGTTCTATATGGTAGTGAAGAATTGAAGTGAAGTTGAGTTTGTATTCT
RI-AT1G55310-XLOC_002795-1791-1
GATGTTCTATATGGTAGTGAAGAATTGAAGTGAAGTTGAGTTTGTATTCT
CONSENSUS
GATGTTCTATATGGTAGTGAAGAATTGAAGTGAAGTTGAGTTTGTATTCT

RI-AT1G55310-XLOC_002795-1791-0
ATGTGAAGATGAATCAAGTCTTCAAGAAGTCATCTTTGTACTGACACTTG
RI-AT1G55310-XLOC_002795-1791-1
ATGTGAAGATGAATCAAGTCTTCAAGAAGTCATCTTTGTACTGACACTTG
CONSENSUS
ATGTGAAGATGAATCAAGTCTTCAAGAAGTCATCTTTGTACTGACACTTG

RI-AT1G55310-XLOC_002795-1791-0
CAAGGCTAGCAGGCAAGTGCTTGCTTTTTTCATGTTTACTGATATCTTCG

RI-AT1G55310-XLOC_002795-1791-1
CAAGGCTAGCAG--
CONSENSUS
CAAGGCTAGCAG..

RI-AT1G55310-XLOC_002795-1791-0
GTTGCAGGGGATCATATCGCCAGTTGTCCTTCAACAGGATTGTTATAAAG
RI-AT1G55310-XLOC_002795-1791-1

CONSENSUS
....

RI-AT1G55310-XLOC_002795-1791-0
TGGTACATCTTCCTCGCCATTTTGTATGTTTGGTTTCTAGTCTGAGATCT
RI-AT1G55310-XLOC_002795-1791-1

CONSENSUS
....

RI-AT1G55310-XLOC_002795-1791-0
TTTTGTTCTACATTTTGAATGCAGAGATCCGCGAGGGTTTGGGTTCGTTC
RI-AT1G55310-XLOC_002795-1791-1 ------------------------
AGATCCGCGAGGGTTTGGGTTCGTTC
CONSENSUS
........................AGATCCGCGAGGGTTTGGGTTCGTTC

RI-AT1G55310-XLOC_002795-1791-0
AATTTATGGACCCTGCTGATGCTGCTGATGCAAAACATCACATGGATGGT
RI-AT1G55310-XLOC_002795-1791-1
AATTTATGGACCCTGCTGATGCTGCTGATGCAAAACATCACATGGATGGT
CONSENSUS
AATTTATGGACCCTGCTGATGCTGCTGATGCAAAACATCACATGGATGGT

RI-AT1G55310-XLOC_002795-1791-0
TATCTTCTTCTTGGCCGTGAGTTGACTGTCGTGTTTGCAGAAGAGAACAG
RI-AT1G55310-XLOC_002795-1791-1
TATCTTCTTCTTGGCCGTGAGTTGACTGTCGTGTTTGCAGAAGAGAACAG
CONSENSUS
TATCTTCTTCTTGGCCGTGAGTTGACTGTCGTGTTTGCAGAAGAGAACAG

RI-AT1G55310-XLOC_002795-1791-0
AAAGAAACCGACTGAAATGAGAGCAAGGGAGCGTGGTGGAGGAAG
RI-AT1G55310-XLOC_002795-1791-1
AAAGAAACCGACTGAAATGAGAGCAAGGGAGCGTGGTGGAGGAAG
CONSENSUS
AAAGAAACCGACTGAAATGAGAGCAAGGGAGCGTGGTGGAGGAAG

alignment for event: A3-AT1G22750-XLOC_001197-3434

A3-AT1G22750-XLOC_001197-3434-0
CGTTGGCACGAGCGCAGTCATTGTCGTGCAGCTATTCCGTCTGTCTCCTC
A3-AT1G22750-XLOC_001197-3434-1
CGTTGGCACGAGCGCAGTCATTGTCGTGCAGCTATTCCGTCTGTCTCCTC
CONSENSUS

CGTTGGCACGAGCGCAGTCATTGTCGTGCAGCTATTCCGTCTGTCTCCTC

A3-AT1G22750-XLOC_001197-3434-0
TCGACCCGCTCTACCTCGTTCTAGTGAACAACAGCAATCGGAAGCAAAGT
A3-AT1G22750-XLOC_001197-3434-1
TCGACCCGCTCTACCTCGTTCTAGTGAACAACAGCAATCG----------
CONSENSUS
TCGACCCGCTCTACCTCGTTCTAGTGAACAACAGCAATCG..........

A3-AT1G22750-XLOC_001197-3434-0
TAAAAGTAAAGGCTAAGTTAGTGAACTCTCTCATGGGTCAATGGCAGGGC
A3-AT1G22750-XLOC_001197-3434-1
---GGC
CONSENSUS
....GGC

A3-AT1G22750-XLOC_001197-3434-0
TGGAGATATGTACGAGCGCACTCATTCTGCGGCTTTGCGCATAATGTGAA
A3-AT1G22750-XLOC_001197-3434-1
TGGAGATATGTACGAGCGCACTCATTCTGCGGCTTTGCGCATAATGTGAA
CONSENSUS
TGGAGATATGTACGAGCGCACTCATTCTGCGGCTTTGCGCATAATGTGAA

A3-AT1G22750-XLOC_001197-3434-0
GCTTTGTCATTTCATTTTTAACATTTTACCACAAAAGTTCAAAACATATT
A3-AT1G22750-XLOC_001197-3434-1
GCTTTGTCATTTCATTTTTAACATTTTACCACAAAAGTTCAAAACATATT
CONSENSUS
GCTTTGTCATTTCATTTTTAACATTTTACCACAAAAGTTCAAAACATATT

A3-AT1G22750-XLOC_001197-3434-0
TTTATCTACATTTTAAGTTTGTATGTTTCATAGTTTTCTTCTGTATATAC
A3-AT1G22750-XLOC_001197-3434-1
TTTATCTACATTTTAAGTTTGTATGTTTCATAGTTTTCTTCTGTATATAC
CONSENSUS
TTTATCTACATTTTAAGTTTGTATGTTTCATAGTTTTCTTCTGTATATAC

A3-AT1G22750-XLOC_001197-3434-0
TTTCTCTTATGTGACGACAATTACTTATGTACTTAACTTTTTTGGGTCAC
A3-AT1G22750-XLOC_001197-3434-1
TTTCTCTTATGTGACGACAATTACTTATGTACTTAACTTTTTTGGGTCAC
CONSENSUS
TTTCTCTTATGTGACGACAATTACTTATGTACTTAACTTTTTTGGGTCAC

A3-AT1G22750-XLOC_001197-3434-0
GACTTGAAAAACCACATATGGATTGAGACTTTTTGGGCT
A3-AT1G22750-XLOC_001197-3434-1
GACTTGAAAAACCACATATGGATTGAGACTTTTTGGGCT
CONSENSUS
GACTTGAAAAACCACATATGGATTGAGACTTTTTGGGCT

alignment for event: A3-AT1G01910-XLOC_004195-519

A3-AT1G01910-XLOC_004195-519-0

GTGACAGGGGTTGAAGCCTTAAAGGCGTTTTCACATAAGTTCTTGACGCC
A3-AT1G01910-XLOC_004195-519-1
GTGACAGGGGTTGAAGCCTTAAAGGCGTTTTCACATAAGTTCTTGACGCC
CONSENSUS
GTGACAGGGGTTGAAGCCTTAAAGGCGTTTTCACATAAGTTCTTGACGCC

A3-AT1G01910-XLOC_004195-519-0
GTACCATCCTACCACTAGCAGGAGCAATGTAGAGGAGCTGGAGAGGAAAG
A3-AT1G01910-XLOC_004195-519-1
GTACCATCCTACCACTAGCAGGAGCAATGTAGAGGAGCTGGAGAGGAAAG
CONSENSUS
GTACCATCCTACCACTAGCAGGAGCAATGTAGAGGAGCTGGAGAGGAAAG

A3-AT1G01910-XLOC_004195-519-0
TACACACATTGCGTTTGCAGTTAAAAACAGCTGAAGAAGAACTCGAACGG
A3-AT1G01910-XLOC_004195-519-1
TACACACATTGCGTTTGCAGTTAAAAACAGCTGAAGAAGAACTCGAACGG
CONSENSUS
TACACACATTGCGTTTGCAGTTAAAAACAGCTGAAGAAGAACTCGAACGG

A3-AT1G01910-XLOC_004195-519-0
GTCAAGAGTGGCTAAAGATAAGGCTCTGTTCTTCCCTTTAATT-------
A3-AT1G01910-XLOC_004195-519-1
GTCAAGAGTGGCTAAAGATAAGGCTCTGTTCTTCCCTTTAATTGTGGAAA
CONSENSUS
GTCAAGAGTGGCTAAAGATAAGGCTCTGTTCTTCCCTTTAATT.......

A3-AT1G01910-XLOC_004195-519-0 ---------------------------
TTCTTCATCTAGCAACAACTCTT
A3-AT1G01910-XLOC_004195-519-1
ATTAAAACTGAATCTTTTTCTCTCTAGTTCTTCATCTAGCAACAACTCTT
CONSENSUS
...........................TTCTTCATCTAGCAACAACTCTT

A3-AT1G01910-XLOC_004195-519-0
TATAGGACAGGAAAGAAGATCAAGCAGAATCTTCTTTTGCGTTGTTTTAT
A3-AT1G01910-XLOC_004195-519-1
TATAGGACAGGAAAGAAGATCAAGCAGAATCTTCTTTTGCGTTGTTTTAT
CONSENSUS
TATAGGACAGGAAAGAAGATCAAGCAGAATCTTCTTTTGCGTTGTTTTAT

A3-AT1G01910-XLOC_004195-519-0
TGTTATGCATTGGATTTGTTTTTATATTTGTAATAATCATGACTACATAG
A3-AT1G01910-XLOC_004195-519-1
TGTTATGCATTGGATTTGTTTTTATATTTGTAATAATCATGACTACATAG
CONSENSUS
TGTTATGCATTGGATTTGTTTTTATATTTGTAATAATCATGACTACATAG

A3-AT1G01910-XLOC_004195-519-0
ACTCATATCTACCAAGAAATCGTTGTTTTACGACTTAATCAGCTTTTTAT
A3-AT1G01910-XLOC_004195-519-1
ACTCATATCTACCAAGAAATCGTTGTTTTACGACTTAATCAGCTTTTTAT
CONSENSUS
ACTCATATCTACCAAGAAATCGTTGTTTTACGACTTAATCAGCTTTTTAT

A3-AT1G01910-XLOC_004195-519-0

ATATTAAGTTGAAGGGTATCTGTGACAAGCGAATGCTATGATCTCTTGTA
A3-AT1G01910-XLOC_004195-519-1
ATATTAAGTTGAAGGGTATCTGTGACAAGCGAATGCTATGATCTCTTGTA
CONSENSUS
ATATTAAGTTGAAGGGTATCTGTGACAAGCGAATGCTATGATCTCTTGTA

A3-AT1G01910-XLOC_004195-519-0
ACAGCTTTTTATATACTGTAGACTGGTCTTGCAACAGAGAAGGAAAATTC
A3-AT1G01910-XLOC_004195-519-1
ACAGCTTTTTATATACTGTAGACTGGTCTTGCAACAGAGAAGGAAAATTC
CONSENSUS
ACAGCTTTTTATATACTGTAGACTGGTCTTGCAACAGAGAAGGAAAATTC

A3-AT1G01910-XLOC_004195-519-0
ATGTAAAAAAATTATAGTAAATAAGAGATGTTTTGATTTATTGAC
A3-AT1G01910-XLOC_004195-519-1
ATGTAAAAAAATTATAGTAAATAAGAGATGTTTTGATTTATTGAC
CONSENSUS
ATGTAAAAAAATTATAGTAAATAAGAGATGTTTTGATTTATTGAC

alignment for event: RI-AT1G59560-XLOC_003009-11030

RI-AT1G59560-XLOC_003009-11030-0
GCTGTTAGGGATGGCATGGGGAATGTCAGGATTCAAAAACCTGAGCAAGG
RI-AT1G59560-XLOC_003009-11030-1
GCTGTTAGGGATGGCATGGGGAATGTCAGGATTCAAAAACCTGAGCAAGG
CONSENSUS
GCTGTTAGGGATGGCATGGGGAATGTCAGGATTCAAAAACCTGAGCAAGG

RI-AT1G59560-XLOC_003009-11030-0
ACCTTTCTACGTCACTTATATACCGCTAGATCAGCTCATCTCTAAATTGG
RI-AT1G59560-XLOC_003009-11030-1
ACCTTTCTACGTCACTTATATACCGCTAGATCAGCTCATCTCTAAATTGG
CONSENSUS
ACCTTTCTACGTCACTTATATACCGCTAGATCAGCTCATCTCTAAATTGG

RI-AT1G59560-XLOC_003009-11030-0
GAGATTTGTCAAGGTTGTTTCTTTTCTCTGTTTTTGCCTATTCTTTTCTT
RI-AT1G59560-XLOC_003009-11030-1
GAGATTTGTCAAG-------------------------------------
CONSENSUS
GAGATTTGTCAAG.....................................

RI-AT1G59560-XLOC_003009-11030-0
CGAAAACTATGATTGAAAAAAATTCTAAAAGATCTCTTTCTTTTGGCAGG
RI-AT1G59560-XLOC_003009-11030-1
---G
CONSENSUS
....G

RI-AT1G59560-XLOC_003009-11030-0
AGGTTCAAGTATGCCTCCATGGGTTTAACTGTTCTTGGTGTGATTCTTAT
RI-AT1G59560-XLOC_003009-11030-1
AGGTTCAAGTATGCCTCCATGGGTTTAACTGTTCTTGGTGTGATTCTTAT

CONSENSUS
AGGTTCAAGTATGCCTCCATGGGTTTAACTGTTCTTGGTGTGATTCTTAT

RI-AT1G59560-XLOC_003009-11030-0
TTCAAAGCCTGTGATTGAATATATTCTAAAGAGAATTGAAGATACTCTAG
RI-AT1G59560-XLOC_003009-11030-1
TTCAAAGCCTGTGATTGAATATATTCTAAAGAGAATTGAAGATACTCTAG
CONSENSUS
TTCAAAGCCTGTGATTGAATATATTCTAAAGAGAATTGAAGATACTCTAG

RI-AT1G59560-XLOC_003009-11030-0
AAAGAAGGCGGCGACAATTCGCACTGAAAAG
RI-AT1G59560-XLOC_003009-11030-1
AAAGAAGGCGGCGACAATTCGCACTGAAAAG
CONSENSUS
AAAGAAGGCGGCGACAATTCGCACTGAAAAG

alignment for event: RI-AT1G27120-XLOC_001404-9541

RI-AT1G27120-XLOC_001404-9541-0
ATTCCTAATAACTATTATACACATCGTGAATTTGAAACATTTTTAAGCGC
RI-AT1G27120-XLOC_001404-9541-1
ATTCCTAATAACTATTATACACATCGTGAATTTGAAACATTTTTAAGCGC
CONSENSUS
ATTCCTAATAACTATTATACACATCGTGAATTTGAAACATTTTTAAGCGC

RI-AT1G27120-XLOC_001404-9541-0
AAATTCTCAGCGAGATAAAAACGACGTTGTTTTAATAGTTATTGGCTTTA
RI-AT1G27120-XLOC_001404-9541-1
AAATTCTCAGCGAGATAAAAACGACGTTGTTTTAATAGTTATTGGCTTTA
CONSENSUS
AAATTCTCAGCGAGATAAAAACGACGTTGTTTTAATAGTTATTGGCTTTA

RI-AT1G27120-XLOC_001404-9541-0
CAACTTCGTCGTACACGCCGGTTACCAAATATCGCTAAGGCTCAGAATTA
RI-AT1G27120-XLOC_001404-9541-1
CAACTTCGTCGTACACGCCGGTTACCAAATATCGCTAAGGCTCAGAATTA
CONSENSUS
CAACTTCGTCGTACACGCCGGTTACCAAATATCGCTAAGGCTCAGAATTA

RI-AT1G27120-XLOC_001404-9541-0
TCTCTGTTCAAAGCTTTTGTTTTGTTCCCAATTCTGAAGTTTTGAATCAA
RI-AT1G27120-XLOC_001404-9541-1
TCTCTGTTCAAAGCTTTTGTTTTGTTCCCAATTCTGAAGTTTTGAATCAA
CONSENSUS
TCTCTGTTCAAAGCTTTTGTTTTGTTCCCAATTCTGAAGTTTTGAATCAA

RI-AT1G27120-XLOC_001404-9541-0
CAAATCTTCAATAAAGTTTTCTCCTTTCACTATCTTTCCATATTCTTCTT
RI-AT1G27120-XLOC_001404-9541-1
CAAATCTTCAATAAAGTTTTCTCCTTTCACTATCTTTCCATATTCTTCTT
CONSENSUS
CAAATCTTCAATAAAGTTTTCTCCTTTCACTATCTTTCCATATTCTTCTT

RI-AT1G27120-XLOC_001404-9541-0
CTTAGTAAAACTTTGCATTAATGGCTTACCCAAATTTGTAAGCTCTTTGT
RI-AT1G27120-XLOC_001404-9541-1
CTTAGTAAAACTTTGCATTAATGGCTTACCCAAATTT-------------
CONSENSUS
CTTAGTAAAACTTTGCATTAATGGCTTACCCAAATTT.............

RI-AT1G27120-XLOC_001404-9541-0
TTCCTCCCTTCTTCTTATTCTCTTCTTCATTCTCCAAATTTGTTTCTAGG
RI-AT1G27120-XLOC_001404-9541-1

CONSENSUS
....

RI-AT1G27120-XLOC_001404-9541-0
TTTCTTCAGTTTGAGCAATGAAGAAGTCTAAACTCGATAATTCTTCTTCA
RI-AT1G27120-XLOC_001404-9541-1

CONSENSUS
....

RI-AT1G27120-XLOC_001404-9541-0
CAGATTCGATTCGGGCTTGTTCAGTTCTTATTAGTTGTTCTGCTTTTTTA
RI-AT1G27120-XLOC_001404-9541-1 ---
ATTCGATTCGGGCTTGTTCAGTTCTTATTAGTTGTTCTGCTTTTTTA
CONSENSUS
...ATTCGATTCGGGCTTGTTCAGTTCTTATTAGTTGTTCTGCTTTTTTA

RI-AT1G27120-XLOC_001404-9541-0
CTTCCTCTGCATGAGCTTCGAGATCCCATTCATCTTCAGAACCGGGTCTG
RI-AT1G27120-XLOC_001404-9541-1
CTTCCTCTGCATGAGCTTCGAGATCCCATTCATCTTCAGAACCGGGTCTG
CONSENSUS
CTTCCTCTGCATGAGCTTCGAGATCCCATTCATCTTCAGAACCGGGTCTG

RI-AT1G27120-XLOC_001404-9541-0
GGTCCGGGTCTGATGATGTTTCATCTTCTTCTTTTGCTGACGCATTACCG
RI-AT1G27120-XLOC_001404-9541-1
GGTCCGGGTCTGATGATGTTTCATCTTCTTCTTTTGCTGACGCATTACCG
CONSENSUS
GGTCCGGGTCTGATGATGTTTCATCTTCTTCTTTTGCTGACGCATTACCG

RI-AT1G27120-XLOC_001404-9541-0
AGACCAATGGTTGTTGGTGGTGGTAGTAGGGAAGCTAATTGGGTTGTCGG
RI-AT1G27120-XLOC_001404-9541-1
AGACCAATGGTTGTTGGTGGTGGTAGTAGGGAAGCTAATTGGGTTGTCGG
CONSENSUS
AGACCAATGGTTGTTGGTGGTGGTAGTAGGGAAGCTAATTGGGTTGTCGG

RI-AT1G27120-XLOC_001404-9541-0
AGAAGAAGAAGAAGCAGACCCACATCGACATTTCAAGGATCCGGGTCGGG
RI-AT1G27120-XLOC_001404-9541-1
AGAAGAAGAAGAAGCAGACCCACATCGACATTTCAAGGATCCGGGTCGGG
CONSENSUS
AGAAGAAGAAGAAGCAGACCCACATCGACATTTCAAGGATCCGGGTCGGG

RI-AT1G27120-XLOC_001404-9541-0
TACAGCTTCGGTTACCGGAGCGGAAAATGAGGGAATTTAAGTCCGTCTCT
RI-AT1G27120-XLOC_001404-9541-1
TACAGCTTCGGTTACCGGAGCGGAAAATGAGGGAATTTAAGTCCGTCTCT
CONSENSUS
TACAGCTTCGGTTACCGGAGCGGAAAATGAGGGAATTTAAGTCCGTCTCT

RI-AT1G27120-XLOC_001404-9541-0
GAGATTTTCGTCAACGAGAGCTTCTTCGACAATGGCGGATTCAGCGATGA
RI-AT1G27120-XLOC_001404-9541-1
GAGATTTTCGTCAACGAGAGCTTCTTCGACAATGGCGGATTCAGCGATGA
CONSENSUS
GAGATTTTCGTCAACGAGAGCTTCTTCGACAATGGCGGATTCAGCGATGA

RI-AT1G27120-XLOC_001404-9541-0
ATTCTCAATCTTTCACAAAACAGCGAAGCATGCGATTTCAATGGGTCGAA
RI-AT1G27120-XLOC_001404-9541-1
ATTCTCAATCTTTCACAAAACAGCGAAGCATGCGATTTCAATGGGTCGAA
CONSENSUS
ATTCTCAATCTTTCACAAAACAGCGAAGCATGCGATTTCAATGGGTCGAA

RI-AT1G27120-XLOC_001404-9541-0
AAATGTGGGACGGACTCGATTCGGGTTTAATCAAACCCGATAAAGCTCCG
RI-AT1G27120-XLOC_001404-9541-1
AAATGTGGGACGGACTCGATTCGGGTTTAATCAAACCCGATAAAGCTCCG
CONSENSUS
AAATGTGGGACGGACTCGATTCGGGTTTAATCAAACCCGATAAAGCTCCG

RI-AT1G27120-XLOC_001404-9541-0
GTTAAGACCCGGATTGAGAAATGTCCGGATATGGTTTCGGTTTCTGAGTC
RI-AT1G27120-XLOC_001404-9541-1
GTTAAGACCCGGATTGAGAAATGTCCGGATATGGTTTCGGTTTCTGAGTC
CONSENSUS
GTTAAGACCCGGATTGAGAAATGTCCGGATATGGTTTCGGTTTCTGAGTC

RI-AT1G27120-XLOC_001404-9541-0
GGAGTTTGTGAACCGGAGTCGGATCTTGGTTTTGCCGTGTGGGTTAACGT
RI-AT1G27120-XLOC_001404-9541-1
GGAGTTTGTGAACCGGAGTCGGATCTTGGTTTTGCCGTGTGGGTTAACGT
CONSENSUS
GGAGTTTGTGAACCGGAGTCGGATCTTGGTTTTGCCGTGTGGGTTAACGT

RI-AT1G27120-XLOC_001404-9541-0
TAGGATCTCACATTACCGTCGTGGCTACGCCGCATTGGGCTCACGTTGAG
RI-AT1G27120-XLOC_001404-9541-1
TAGGATCTCACATTACCGTCGTGGCTACGCCGCATTGGGCTCACGTTGAG
CONSENSUS
TAGGATCTCACATTACCGTCGTGGCTACGCCGCATTGGGCTCACGTTGAG

RI-AT1G27120-XLOC_001404-9541-0
AAAGATGGTGATAAGACGGCGATGGTGAGTCAGTTCATGATGGAGTTACA
RI-AT1G27120-XLOC_001404-9541-1
AAAGATGGTGATAAGACGGCGATGGTGAGTCAGTTCATGATGGAGTTACA
CONSENSUS
AAAGATGGTGATAAGACGGCGATGGTGAGTCAGTTCATGATGGAGTTACA

RI-AT1G27120-XLOC_001404-9541-0
AGGATTAAAGGCGGTGGATGGTGAAGATCCGCCTCGGATACTTCATTTTA
RI-AT1G27120-XLOC_001404-9541-1
AGGATTAAAGGCGGTGGATGGTGAAGATCCGCCTCGGATACTTCATTTTA
CONSENSUS
AGGATTAAAGGCGGTGGATGGTGAAGATCCGCCTCGGATACTTCATTTTA

RI-AT1G27120-XLOC_001404-9541-0
ACCCGAGGATTAAAGGTGATTGGAGTGGAAGACCAGTGATTGAGCAAAAC
RI-AT1G27120-XLOC_001404-9541-1
ACCCGAGGATTAAAGGTGATTGGAGTGGAAGACCAGTGATTGAGCAAAAC
CONSENSUS
ACCCGAGGATTAAAGGTGATTGGAGTGGAAGACCAGTGATTGAGCAAAAC

RI-AT1G27120-XLOC_001404-9541-0
ACTTGTTATCGAATGCAATGGGGCTCAGGTTTACGTTGTGATGGTCGTGA
RI-AT1G27120-XLOC_001404-9541-1
ACTTGTTATCGAATGCAATGGGGCTCAGGTTTACGTTGTGATGGTCGTGA
CONSENSUS
ACTTGTTATCGAATGCAATGGGGCTCAGGTTTACGTTGTGATGGTCGTGA

RI-AT1G27120-XLOC_001404-9541-0 ATCTAGTGATGATGAAGAATATG
RI-AT1G27120-XLOC_001404-9541-1 ATCTAGTGATGATGAAGAATATG
CONSENSUS ATCTAGTGATGATGAAGAATATG

alignment for event: RI-AT1G60860-XLOC_007046-776

RI-AT1G60860-XLOC_007046-776-0
GCTGAAAATGGAGCTGATAGGATGGATTGGGTAAATAAGATCACAGCAGC
RI-AT1G60860-XLOC_007046-776-1
GCTGAAAATGGAGCTGATAGGATGGATTGGGTAAATAAGATCACAGCAGC
CONSENSUS
GCTGAAAATGGAGCTGATAGGATGGATTGGGTAAATAAGATCACAGCAGC

RI-AT1G60860-XLOC_007046-776-0
TATAACAATACGTCTGAATTCTCATTTTCTGCAGCAGGTTTACTCAATAT
RI-AT1G60860-XLOC_007046-776-1
TATAACAATACGTCTGAATTCTCATTTTCTGCAGCAG-------------
CONSENSUS
TATAACAATACGTCTGAATTCTCATTTTCTGCAGCAG.............

RI-AT1G60860-XLOC_007046-776-0
GAGTCTCATACTTTTCTTTTACCTTCAAATTGAAAATTTTGTGTTGTTAT
RI-AT1G60860-XLOC_007046-776-1

CONSENSUS
....

RI-AT1G60860-XLOC_007046-776-0
AGAATCTAATATCTTCTGCAGTCACCAGCAAGATACTTGGATAAAAAGAA
RI-AT1G60860-XLOC_007046-776-1 ---------------------
TCACCAGCAAGATACTTGGATAAAAAGAA
CONSENSUS
.....................TCACCAGCAAGATACTTGGATAAAAAGAA

RI-AT1G60860-XLOC_007046-776-0
TACTAGTTCTGGTCCTGCCACTGAAAACCTCACCCTGAATCAAAAAGAAG
RI-AT1G60860-XLOC_007046-776-1
TACTAGTTCTGGTCCTGCCACTGAAAACCTCACCCTGAATCAAAAAGAAG
CONSENSUS
TACTAGTTCTGGTCCTGCCACTGAAAACCTCACCCTGAATCAAAAAGAAG

RI-AT1G60860-XLOC_007046-776-0
ATTATAATCAAAGACTAAATGTGGGGGATGACGTCCTTACAATACTCAGA
RI-AT1G60860-XLOC_007046-776-1
ATTATAATCAAAGACTAAATGTGGGGGATGACGTCCTTACAATACTCAGA
CONSENSUS
ATTATAATCAAAGACTAAATGTGGGGGATGACGTCCTTACAATACTCAGA

RI-AT1G60860-XLOC_007046-776-0
GAAATTCCTGGAAATAACACTTGTGCAGAATGCAATGCACCTGACCCCGA
RI-AT1G60860-XLOC_007046-776-1
GAAATTCCTGGAAATAACACTTGTGCAGAATGCAATGCACCTGACCCCGA
CONSENSUS
GAAATTCCTGGAAATAACACTTGTGCAGAATGCAATGCACCTGACCCCGA

RI-AT1G60860-XLOC_007046-776-0
TTGGGCATCATTGAATCTTGGAGTTTTGATGTGCATTGAATGCTCTGGTG
RI-AT1G60860-XLOC_007046-776-1
TTGGGCATCATTGAATCTTGGAGTTTTGATGTGCATTGAATGCTCTGGTG
CONSENSUS
TTGGGCATCATTGAATCTTGGAGTTTTGATGTGCATTGAATGCTCTGGTG

RI-AT1G60860-XLOC_007046-776-0
TTCACAGGAATCTCGGCGTTCATATATCCAAG
RI-AT1G60860-XLOC_007046-776-1
TTCACAGGAATCTCGGCGTTCATATATCCAAG
CONSENSUS
TTCACAGGAATCTCGGCGTTCATATATCCAAG

alignment for event: RI-AT1G02880-XLOC_004248-2292

RI-AT1G02880-XLOC_004248-2292-0
CAAAACTTCGTCTCTGTGCTGATGGAGGCGCTAATCGCATCTACGACGAA
RI-AT1G02880-XLOC_004248-2292-1
CAAAACTTCGTCTCTGTGCTGATGGAGGCGCTAATCGCATCTACGACGAA
CONSENSUS
CAAAACTTCGTCTCTGTGCTGATGGAGGCGCTAATCGCATCTACGACGAA

RI-AT1G02880-XLOC_004248-2292-0
TTGCCTCTCTTCTTCCCTAATGAAGACGCTTTGGCCATTCGAAACAGGTC
RI-AT1G02880-XLOC_004248-2292-1
TTGCCTCTCTTCTTCCCTAATGAAGACGCTTTGGCCATTCGAAACAG---
CONSENSUS
TTGCCTCTCTTCTTCCCTAATGAAGACGCTTTGGCCATTCGAAACAG...

RI-AT1G02880-XLOC_004248-2292-0
CAACTTTCTGAAGCTGTATCTCTCGCTCTCTTACTCTTTCTTCGGATTTG

RI-AT1G02880-XLOC_004248-2292-1

CONSENSUS
      ....

RI-AT1G02880-XLOC_004248-2292-0
      AAGATTATTATTCAAATTTAGTGATGGTTTATTGGAATAATTAGTCTAAA
RI-AT1G02880-XLOC_004248-2292-1

CONSENSUS
      ....

RI-AT1G02880-XLOC_004248-2292-0
      TTTGAGCTGGAGATATTTTTTTTGTATGAGTTCATAGATTCACCTTCTTC
RI-AT1G02880-XLOC_004248-2292-1

CONSENSUS
      ....

RI-AT1G02880-XLOC_004248-2292-0
      TTATCTTTATGTATATTACATAGAGAAAGATTTCATGAAGAAGAACTTGT
RI-AT1G02880-XLOC_004248-2292-1

CONSENSUS
      ....

RI-AT1G02880-XLOC_004248-2292-0
      ATTTTAGGTATAAGCCGGATGTTATCAAAGGAGATATGGATTCTATACGT
RI-AT1G02880-XLOC_004248-2292-1 -------
GTATAAGCCGGATGTTATCAAAGGAGATATGGATTCTATACGT
CONSENSUS
      .......GTATAAGCCGGATGTTATCAAAGGAGATATGGATTCTATACGT

RI-AT1G02880-XLOC_004248-2292-0 CGTGACGTCCTCGACTTTTATATAAACTTG
RI-AT1G02880-XLOC_004248-2292-1 CGTGACGTCCTCGACTTTTATATAAACTTG
CONSENSUS   CGTGACGTCCTCGACTTTTATATAAACTTG

alignment for event: A3-AT1G20570-XLOC_005227-11821

A3-AT1G20570-XLOC_005227-11821-0
      GTATTGACTTCGAAGAAGAATGTGGGACACTTCCCTCATCTGGCTGATTT
A3-AT1G20570-XLOC_005227-11821-1
      GTATTGACTTCGAAGAAGAATGTGGGACACTTCCCTCATCTGGCTGATTT
CONSENSUS
      GTATTGACTTCGAAGAAGAATGTGGGACACTTCCCTCATCTGGCTGATTT

A3-AT1G20570-XLOC_005227-11821-0
      GGTGACCAGAATCAATTACAATTATCACTACATGTCTGACACTGGAAGCT
A3-AT1G20570-XLOC_005227-11821-1
      GGTGACCAGAATCAATTACAATTATCACTACATGTCTGACACTGGAAGCT
CONSENSUS
      GGTGACCAGAATCAATTACAATTATCACTACATGTCTGACACTGGAAGCT

A3-AT1G20570-XLOC_005227-11821-0

CGATGACTGCTTCTGGATCATAGACCAACTCTTCCAGGTCCATTGAACTA
A3-AT1G20570-XLOC_005227-11821-1
CGATGACTGCTTCTGGATCATAGACCAACTCTTCCAGGTCCATTGAACTA
CONSENSUS
CGATGACTGCTTCTGGATCATAGACCAACTCTTCCAGGTCCATTGAACTA

A3-AT1G20570-XLOC_005227-11821-0
AATCCCAAATGAATGTGTGCAAAAAAAAAAAACATAAAATTATTATTATG
A3-AT1G20570-XLOC_005227-11821-1
AATCCCAAATGAATGTGTGCAAAAAAAAAAAACATAAAATTATTATTATG
CONSENSUS
AATCCCAAATGAATGTGTGCAAAAAAAAAAAACATAAAATTATTATTATG

A3-AT1G20570-XLOC_005227-11821-0
ACATGTTTGAGCAAAAATAATATGTGGTCGGGACAGGTCACTACCAGTAG
A3-AT1G20570-XLOC_005227-11821-1
ACATGTTTGAGCAAAAATAATATGTGGTCGGGACAGGTCACTACCAGTAG
CONSENSUS
ACATGTTTGAGCAAAAATAATATGTGGTCGGGACAGGTCACTACCAGTAG

A3-AT1G20570-XLOC_005227-11821-0
TCGCCCACTAGTCTGATTGCAAAATTCAAATAGAAAAGATATATGATAAT
A3-AT1G20570-XLOC_005227-11821-1
TCGCCCACTAGTCTGATTGCAAAATTCAAATAGAAAAGATATATGATAAT
CONSENSUS
TCGCCCACTAGTCTGATTGCAAAATTCAAATAGAAAAGATATATGATAAT

A3-AT1G20570-XLOC_005227-11821-0
ACGACAACAAACTCAGGCTAGATTACAGAGTAGTTAACTTATATTATATA
A3-AT1G20570-XLOC_005227-11821-1
ACGACAACAAACTCAGGCTAGATTACAGAGTAGTTAACTTATATTATATA
CONSENSUS
ACGACAACAAACTCAGGCTAGATTACAGAGTAGTTAACTTATATTATATA

A3-AT1G20570-XLOC_005227-11821-0
CCTCCTCGATGCACGAGCGAGCAAAACTACTACTGTACTACTTTTCCGGC
A3-AT1G20570-XLOC_005227-11821-1
CCTCCTCGATGCACGAGCGAGCAAAACTACTACTGTACTACTTTTCCGGC
CONSENSUS
CCTCCTCGATGCACGAGCGAGCAAAACTACTACTGTACTACTTTTCCGGC

A3-AT1G20570-XLOC_005227-11821-0
GATAAAATCTATGAAAATGGAGGGAACTATCAAATCTCCGGCCAACTACG
A3-AT1G20570-XLOC_005227-11821-1
GATAAAATCTATGAAAATGGAGGGAACTATCAAATCTCCGGCCAACTACG
CONSENSUS
GATAAAATCTATGAAAATGGAGGGAACTATCAAATCTCCGGCCAACTACG

A3-AT1G20570-XLOC_005227-11821-0
TTCCTCTCACTCCGATCAGCTTCCTCGATAGATCTGCTGTCGTCTACGCT
A3-AT1G20570-XLOC_005227-11821-1
TTCCTCTCACTCCGATCAGCTTCCTCGATAGATCTGCTGTCGTCTACGCT
CONSENSUS
TTCCTCTCACTCCGATCAGCTTCCTCGATAGATCTGCTGTCGTCTACGCT

A3-AT1G20570-XLOC_005227-11821-0

GACAGAGTCTCCATCGTTTATGGCTCCGTCAAGTACACGTGGCGCCAGAC
A3-AT1G20570-XLOC_005227-11821-1
GACAGAGTCTCCATCGTTTATGGCTCCGTCAAGTACACGTGGCGCCAGAC
CONSENSUS
GACAGAGTCTCCATCGTTTATGGCTCCGTCAAGTACACGTGGCGCCAGAC

A3-AT1G20570-XLOC_005227-11821-0
TCGTGACCGCTGCGTCAGAATCGCCTCCGCTCTCTCCCAGCTCGGAATCT
A3-AT1G20570-XLOC_005227-11821-1
TCGTGACCGCTGCGTCAGAATCGCCTCCGCTCTCTCCCAGCTCGGAATCT
CONSENSUS
TCGTGACCGCTGCGTCAGAATCGCCTCCGCTCTCTCCCAGCTCGGAATCT

A3-AT1G20570-XLOC_005227-11821-0
CTACCGGAGATGTG------------------------------------
A3-AT1G20570-XLOC_005227-11821-1
CTACCGGAGATGTGTGTTGGCTCCAAACGTTCCAGCTATGGTTGAATTGC
CONSENSUS
CTACCGGAGATGTG....................................

A3-AT1G20570-XLOC_005227-11821-0

A3-AT1G20570-XLOC_005227-11821-1
ATTTTGGTGTTCCTATGGCTGGAGCTTTGCTCTGTACACTCAACATTCGT
CONSENSUS
....

A3-AT1G20570-XLOC_005227-11821-0

A3-AT1G20570-XLOC_005227-11821-1
CATGATTCATCACTTGTTGCTGTCTTGCTTAGACATTCAGGGACAAAAGT
CONSENSUS
....

A3-AT1G20570-XLOC_005227-11821-0 -----------
ATCATCAGTTTCTCCAAATAGCTGAAGGAGCTTGTGAAA
A3-AT1G20570-XLOC_005227-11821-1
GATTTTTGCAGATCATCAGTTTCTCCAAATAGCTGAAGGAGCTTGTGAAA
CONSENSUS
...........ATCATCAGTTTCTCCAAATAGCTGAAGGAGCTTGTGAAA

A3-AT1G20570-XLOC_005227-11821-0
TCCTCTCAAATAAAGGTGACAAGGTCCCGATTTTGGTCTTGATCCCAGAG
A3-AT1G20570-XLOC_005227-11821-1
TCCTCTCAAATAAAGGTGACAAGGTCCCGATTTTGGTCTTGATCCCAGAG
CONSENSUS
TCCTCTCAAATAAAGGTGACAAGGTCCCGATTTTGGTCTTGATCCCAGAG

A3-AT1G20570-XLOC_005227-11821-0
CCTCTTACTCAATCTGTTTCAAGGAAGAAGAGATCTGAGGAAATGATGGA
A3-AT1G20570-XLOC_005227-11821-1
CCTCTTACTCAATCTGTTTCAAGGAAGAAGAGATCTGAGGAAATGATGGA
CONSENSUS
CCTCTTACTCAATCTGTTTCAAGGAAGAAGAGATCTGAGGAAATGATGGA

A3-AT1G20570-XLOC_005227-11821-0

ATACGAAGATGTTGTAGCGATGGGGAAATCGGACTTCGAGGTTATACGAC
A3-AT1G20570-XLOC_005227-11821-1
ATACGAAGATGTTGTAGCGATGGGGAAATCGGACTTCGAGGTTATACGAC
CONSENSUS
ATACGAAGATGTTGTAGCGATGGGGAAATCGGACTTCGAGGTTATACGAC

A3-AT1G20570-XLOC_005227-11821-0
CAACAGATGAGTGTGATGCTATATCTGTTAATTACACATCAGGTACCACT
A3-AT1G20570-XLOC_005227-11821-1
CAACAGATGAGTGTGATGCTATATCTGTTAATTACACATCAGGTACCACT
CONSENSUS
CAACAGATGAGTGTGATGCTATATCTGTTAATTACACATCAGGTACCACT

A3-AT1G20570-XLOC_005227-11821-0
TCAAGCCCCAAAGGTGTTGTTTATAGTCACAGAGGTGCTTATTTGAATTC
A3-AT1G20570-XLOC_005227-11821-1
TCAAGCCCCAAAGGTGTTGTTTATAGTCACAGAGGTGCTTATTTGAATTC
CONSENSUS
TCAAGCCCCAAAGGTGTTGTTTATAGTCACAGAGGTGCTTATTTGAATTC

A3-AT1G20570-XLOC_005227-11821-0
TCTGGCTGCGGTTTTACTCAACGAAATGCACTCCTCGCCTACTTATCTAT
A3-AT1G20570-XLOC_005227-11821-1
TCTGGCTGCGGTTTTACTCAACGAAATGCACTCCTCGCCTACTTATCTAT
CONSENSUS
TCTGGCTGCGGTTTTACTCAACGAAATGCACTCCTCGCCTACTTATCTAT

A3-AT1G20570-XLOC_005227-11821-0
GGACTAATCCCATGTTTCACTGCAATGGCTGGTGCTTATTGTGGGGTGTT
A3-AT1G20570-XLOC_005227-11821-1
GGACTAATCCCATGTTTCACTGCAATGGCTGGTGCTTATTGTGGGGTGTT
CONSENSUS
GGACTAATCCCATGTTTCACTGCAATGGCTGGTGCTTATTGTGGGGTGTT

A3-AT1G20570-XLOC_005227-11821-0
ACTGCTATTGGTGGGACTAATATATGTTTGAGGAATGTTACGGCCAAGGC
A3-AT1G20570-XLOC_005227-11821-1
ACTGCTATTGGTGGGACTAATATATGTTTGAGGAATGTTACGGCCAAGGC
CONSENSUS
ACTGCTATTGGTGGGACTAATATATGTTTGAGGAATGTTACGGCCAAGGC

A3-AT1G20570-XLOC_005227-11821-0
TATATTTGATAATATTTCCCAGCATAAGGTGACTCATATGGGAGGTGCGC
A3-AT1G20570-XLOC_005227-11821-1
TATATTTGATAATATTTCCCAGCATAAGGTGACTCATATGGGAGGTGCGC
CONSENSUS
TATATTTGATAATATTTCCCAGCATAAGGTGACTCATATGGGAGGTGCGC

A3-AT1G20570-XLOC_005227-11821-0
CGACGATATTGAATATGATCATCAATGCGCCTGAATCTGAGCAGAAACCG
A3-AT1G20570-XLOC_005227-11821-1
CGACGATATTGAATATGATCATCAATGCGCCTGAATCTGAGCAGAAACCG
CONSENSUS
CGACGATATTGAATATGATCATCAATGCGCCTGAATCTGAGCAGAAACCG

A3-AT1G20570-XLOC_005227-11821-0

CTTCCCGGGAAGGTGTCTTTTATAACCGGTGCTGCACCGCCACCAGCTCA
A3-AT1G20570-XLOC_005227-11821-1
CTTCCCGGGAAGGTGTCTTTTATAACCGGTGCTGCACCGCCACCAGCTCA
CONSENSUS
CTTCCCGGGAAGGTGTCTTTTATAACCGGTGCTGCACCGCCACCAGCTCA

A3-AT1G20570-XLOC_005227-11821-0
TGTGATTTTCAAGATGGAAGAGTTGGGGTTTTCTATGTTTCATTCCTATG
A3-AT1G20570-XLOC_005227-11821-1
TGTGATTTTCAAGATGGAAGAGTTGGGGTTTTCTATGTTTCATTCCTATG
CONSENSUS
TGTGATTTTCAAGATGGAAGAGTTGGGGTTTTCTATGTTTCATTCCTATG

A3-AT1G20570-XLOC_005227-11821-0
GGTTAACTGAAACTTATGGACCAGGCACAATCTGTACATGGAAGCCTGAG
A3-AT1G20570-XLOC_005227-11821-1
GGTTAACTGAAACTTATGGACCAGGCACAATCTGTACATGGAAGCCTGAG
CONSENSUS
GGTTAACTGAAACTTATGGACCAGGCACAATCTGTACATGGAAGCCTGAG

A3-AT1G20570-XLOC_005227-11821-0
TGGGACTCTTTGCCTAGAGAAGAACAGGCGAAAATGAAAGCTCGACAAGG
A3-AT1G20570-XLOC_005227-11821-1
TGGGACTCTTTGCCTAGAGAAGAACAGGCGAAAATGAAAGCTCGACAAGG
CONSENSUS
TGGGACTCTTTGCCTAGAGAAGAACAGGCGAAAATGAAAGCTCGACAAGG

A3-AT1G20570-XLOC_005227-11821-0
CGTGAATCATTTAGGGCTCGAGGAAATACAAGTTAAAGACCCTGTAACCA
A3-AT1G20570-XLOC_005227-11821-1
CGTGAATCATTTAGGGCTCGAGGAAATACAAGTTAAAGACCCTGTAACCA
CONSENSUS
CGTGAATCATTTAGGGCTCGAGGAAATACAAGTTAAAGACCCTGTAACCA

A3-AT1G20570-XLOC_005227-11821-0
TGAGAACTTTGCCAGCTGATGGTGTGACTATGGGTGAAGTTGTCTTCAGA
A3-AT1G20570-XLOC_005227-11821-1
TGAGAACTTTGCCAGCTGATGGTGTGACTATGGGTGAAGTTGTCTTCAGA
CONSENSUS
TGAGAACTTTGCCAGCTGATGGTGTGACTATGGGTGAAGTTGTCTTCAGA

A3-AT1G20570-XLOC_005227-11821-0
GGAAACACGGTGATGAATGGTTACTTAAAGAACCCTGAAGCAACCAAGGA
A3-AT1G20570-XLOC_005227-11821-1
GGAAACACGGTGATGAATGGTTACTTAAAGAACCCTGAAGCAACCAAGGA
CONSENSUS
GGAAACACGGTGATGAATGGTTACTTAAAGAACCCTGAAGCAACCAAGGA

A3-AT1G20570-XLOC_005227-11821-0
AGCTTTTAAAGGAGGTTGGTTTTGGAGTGGCGACTTAGGTGTTAAACACC
A3-AT1G20570-XLOC_005227-11821-1
AGCTTTTAAAGGAGGTTGGTTTTGGAGTGGCGACTTAGGTGTTAAACACC
CONSENSUS
AGCTTTTAAAGGAGGTTGGTTTTGGAGTGGCGACTTAGGTGTTAAACACC

A3-AT1G20570-XLOC_005227-11821-0

CTGACGGATACATAGAGCTGAAAGACAGATCGAAAGACATTATAATCTCT
A3-AT1G20570-XLOC_005227-11821-1
CTGACGGATACATAGAGCTGAAAGACAGATCGAAAGACATTATAATCTCT
CONSENSUS
CTGACGGATACATAGAGCTGAAAGACAGATCGAAAGACATTATAATCTCT

A3-AT1G20570-XLOC_005227-11821-0
GGAGGAGAAAACATTAGCTCGATTGAAGTCGAGTCTACTCTGTTCACTCA
A3-AT1G20570-XLOC_005227-11821-1
GGAGGAGAAAACATTAGCTCGATTGAAGTCGAGTCTACTCTGTTCACTCA
CONSENSUS
GGAGGAGAAAACATTAGCTCGATTGAAGTCGAGTCTACTCTGTTCACTCA

A3-AT1G20570-XLOC_005227-11821-0
CCCTTGTGTTCTTGAAGCAGCTGTAGTTGCGAGGCCTGATGAGTATTGGG
A3-AT1G20570-XLOC_005227-11821-1
CCCTTGTGTTCTTGAAGCAGCTGTAGTTGCGAGGCCTGATGAGTATTGGG
CONSENSUS
CCCTTGTGTTCTTGAAGCAGCTGTAGTTGCGAGGCCTGATGAGTATTGGG

A3-AT1G20570-XLOC_005227-11821-0
GTGAGACTGCTTGTGCATTTGTGAAACTTAAAGACGGGTCTAAGGCCAGT
A3-AT1G20570-XLOC_005227-11821-1
GTGAGACTGCTTGTGCATTTGTGAAACTTAAAGACGGGTCTAAGGCCAGT
CONSENSUS
GTGAGACTGCTTGTGCATTTGTGAAACTTAAAGACGGGTCTAAGGCCAGT

A3-AT1G20570-XLOC_005227-11821-0
GCGGAGGAGCTTATTAGCTATTGCAGGGACCGGCTTCCACATTATATGGC
A3-AT1G20570-XLOC_005227-11821-1
GCGGAGGAGCTTATTAGCTATTGCAGGGACCGGCTTCCACATTATATGGC
CONSENSUS
GCGGAGGAGCTTATTAGCTATTGCAGGGACCGGCTTCCACATTATATGGC

A3-AT1G20570-XLOC_005227-11821-0
TCCGAGGAGTATTGTGTTTGAGGATCTTCCTAAAACATCGACTGGAAAAG
A3-AT1G20570-XLOC_005227-11821-1
TCCGAGGAGTATTGTGTTTGAGGATCTTCCTAAAACATCGACTGGAAAAG
CONSENSUS
TCCGAGGAGTATTGTGTTTGAGGATCTTCCTAAAACATCGACTGGAAAAG

A3-AT1G20570-XLOC_005227-11821-0
TCCAGAAGTTTGTTCTGAGGACCAAGGCTAAGGCTTTGGTAAGCTTATCA
A3-AT1G20570-XLOC_005227-11821-1
TCCAGAAGTTTGTTCTGAGGACCAAGGCTAAGGCTTTGGTAAGCTTATCA
CONSENSUS
TCCAGAAGTTTGTTCTGAGGACCAAGGCTAAGGCTTTGGTAAGCTTATCA

A3-AT1G20570-XLOC_005227-11821-0
AAGAAAGGCAGAAGCAAGTTATGAATTGGTAAGCTTTCTTTGTTACTTCC
A3-AT1G20570-XLOC_005227-11821-1
AAGAAAGGCAGAAGCAAGTTATGAATTGGTAAGCTTTCTTTGTTACTTCC
CONSENSUS
AAGAAAGGCAGAAGCAAGTTATGAATTGGTAAGCTTTCTTTGTTACTTCC

A3-AT1G20570-XLOC_005227-11821-0

AATCTCTACAAGATGTCTTATAAATTACTGTCTATAAGATCATGTTTGTG
A3-AT1G20570-XLOC_005227-11821-1
AATCTCTACAAGATGTCTTATAAATTACTGTCTATAAGATCATGTTTGTG
CONSENSUS
AATCTCTACAAGATGTCTTATAAATTACTGTCTATAAGATCATGTTTGTG

A3-AT1G20570-XLOC_005227-11821-0
TCTAAGCTAAACCAGAATGAGATACTAAAACCAATTTGTATGTTTCTGAA
A3-AT1G20570-XLOC_005227-11821-1
TCTAAGCTAAACCAGAATGAGATACTAAAACCAATTTGTATGTTTCTGAA
CONSENSUS
TCTAAGCTAAACCAGAATGAGATACTAAAACCAATTTGTATGTTTCTGAA

A3-AT1G20570-XLOC_005227-11821-0
ATAAATAAAACCGGGATGAAATTGTAATTCCGGAAGATTAGATCTATAGC
A3-AT1G20570-XLOC_005227-11821-1
ATAAATAAAACCGGGATGAAATTGTAATTCCGGAAGATTAGATCTATAGC
CONSENSUS
ATAAATAAAACCGGGATGAAATTGTAATTCCGGAAGATTAGATCTATAGC

A3-AT1G20570-XLOC_005227-11821-0
ATTCTGATTAGAGTTGAATCTGATGGCAAGTGGATATATTATTATCTTAA
A3-AT1G20570-XLOC_005227-11821-1
ATTCTGATTAGAGTTGAATCTGATGGCAAGTGGATATATTATTATCTTAA
CONSENSUS
ATTCTGATTAGAGTTGAATCTGATGGCAAGTGGATATATTATTATCTTAA

A3-AT1G20570-XLOC_005227-11821-0 GGGAATCAATCTTTGGATTGTGTCT
A3-AT1G20570-XLOC_005227-11821-1 GGGAATCAATCTTTGGATTGTGTCT
CONSENSUS GGGAATCAATCTTTGGATTGTGTCT

alignment for event: RI-AT1G30970-XLOC_005753-4663

RI-AT1G30970-XLOC_005753-4663-0
GAGGAAAGAAGAATGTCCTTACCAAAATACAAAGTGCACGATGAAACCAG
RI-AT1G30970-XLOC_005753-4663-1
GAGGAAAGAAGAATGTCCTTACCAAAATACAAAGTGCACGATGAAACCAG
CONSENSUS
GAGGAAAGAAGAATGTCCTTACCAAAATACAAAGTGCACGATGAAACCAG

RI-AT1G30970-XLOC_005753-4663-0
CCAGGTAAGTTCTGATTTACCCTTTTTAAAGTTAAGAAGAGTTTGCTTTT
RI-AT1G30970-XLOC_005753-4663-1
CCAG--
CONSENSUS
CCAG..

RI-AT1G30970-XLOC_005753-4663-0
GCAAGTCTAACCCATTCGGTAGGCTTTATTTATACATTTCTGTTCCAGAT
RI-AT1G30970-XLOC_005753-4663-1

CONSENSUS
....

RI-AT1G30970-XLOC_005753-4663-0
CCTATTGTTTCTGTGCTAAGAGGTACCACCGTATTATTCAGTTTATAATT
RI-AT1G30970-XLOC_005753-4663-1

CONSENSUS
....

RI-AT1G30970-XLOC_005753-4663-0
TCTTCCCTTAGAATATGATAAGAAGGACTTAGGCAAGAAACTTGAGCATT
RI-AT1G30970-XLOC_005753-4663-1

CONSENSUS
....

RI-AT1G30970-XLOC_005753-4663-0
TTGAATCAATGTTCGAAAGGTATCATTTGTAGCTTGCAATAGTAGGTAGT
RI-AT1G30970-XLOC_005753-4663-1

CONSENSUS
....

RI-AT1G30970-XLOC_005753-4663-0
GAGAAACATAGCGTTTGTATCTGTTTGTTTTTCATTATATTTCCTGTCTA
RI-AT1G30970-XLOC_005753-4663-1

CONSENSUS
....

RI-AT1G30970-XLOC_005753-4663-0
TTGTATTGTATTAAAAATGTGGTGATTGGTTCCACAAGATCATACTCTAC
RI-AT1G30970-XLOC_005753-4663-1

CONSENSUS
....

RI-AT1G30970-XLOC_005753-4663-0
AGGTTTGGTTTCAAACCCAGGTCAGCTCTTTTTATATTCTTAACTCTGCA
RI-AT1G30970-XLOC_005753-4663-1

CONSENSUS
....

RI-AT1G30970-XLOC_005753-4663-0
AAGTTTGGTACCTTTGCACAATCTCACTGCCTTGGGGAACCAAAACAATA
RI-AT1G30970-XLOC_005753-4663-1

CONSENSUS
....

RI-AT1G30970-XLOC_005753-4663-0
CGCACTTAACTCTTTCAAAATAAAGCATCCAACTTCTTAACTCAACACCA
RI-AT1G30970-XLOC_005753-4663-1

CONSENSUS
....

RI-AT1G30970-XLOC_005753-4663-0
AGTTTGTTTCTTTTGGTTTGCAGATGAACTCGATAAATGCAGCGATAGAC
RI-AT1G30970-XLOC_005753-4663-1 ----------------------
ATGAACTCGATAAATGCAGCGATAGAC
CONSENSUS
......................ATGAACTCGATAAATGCAGCGATAGAC

RI-AT1G30970-XLOC_005753-4663-0
AGACGAATCTCAGAGAGTAGGCTTGCTGGGCGGATGGCGTTTTAGAATTT
RI-AT1G30970-XLOC_005753-4663-1
AGACGAATCTCAGAGAGTAGGCTTGCTGGGCGGATGGCGTTTTAGAATTT
CONSENSUS
AGACGAATCTCAGAGAGTAGGCTTGCTGGGCGGATGGCGTTTTAGAATTT

RI-AT1G30970-XLOC_005753-4663-0
GGCACCAAACCAAACCAGCGTCAAAGAAAAGGCGATATGGATGAAGGCTT
RI-AT1G30970-XLOC_005753-4663-1
GGCACCAAACCAAACCAGCGTCAAAGAAAAGGCGATATGGATGAAGGCTT
CONSENSUS
GGCACCAAACCAAACCAGCGTCAAAGAAAAGGCGATATGGATGAAGGCTT

RI-AT1G30970-XLOC_005753-4663-0
TGTTACAACAACAAGTCTTCACTCACTTTTGTTCAGATACTGAATTTATG
RI-AT1G30970-XLOC_005753-4663-1
TGTTACAACAACAAGTCTTCACTCACTTTTGTTCAGATACTGAATTTATG
CONSENSUS
TGTTACAACAACAAGTCTTCACTCACTTTTGTTCAGATACTGAATTTATG

RI-AT1G30970-XLOC_005753-4663-0
ATCTGATTTTAAGTTATTAACTTTCCATCTGTTGTATTCTTTAGCCAGGT
RI-AT1G30970-XLOC_005753-4663-1
ATCTGATTTTAAGTTATTAACTTTCCATCTGTTGTATTCTTTAGCCAGGT
CONSENSUS
ATCTGATTTTAAGTTATTAACTTTCCATCTGTTGTATTCTTTAGCCAGGT

RI-AT1G30970-XLOC_005753-4663-0
GAGTTAGTTTACCATTACATTGTTTAGGGATCTCAGAGATGTTTTTTCTC
RI-AT1G30970-XLOC_005753-4663-1
GAGTTAGTTTACCATTACATTGTTTAGGGATCTCAGAGATGTTTTTTCTC
CONSENSUS
GAGTTAGTTTACCATTACATTGTTTAGGGATCTCAGAGATGTTTTTTCTC

RI-AT1G30970-XLOC_005753-4663-0
AGAAGTTTAGTCCTTTTTTTTTTTGGTCGTTGGAGACTTGGAGGAAGAAC
RI-AT1G30970-XLOC_005753-4663-1
AGAAGTTTAGTCCTTTTTTTTTTTGGTCGTTGGAGACTTGGAGGAAGAAC
CONSENSUS
AGAAGTTTAGTCCTTTTTTTTTTTGGTCGTTGGAGACTTGGAGGAAGAAC

RI-AT1G30970-XLOC_005753-4663-0
TTTGTCTCCTTTTCTTCTGAATTGATCAAAGTATTATATTATACAGATTC
RI-AT1G30970-XLOC_005753-4663-1
TTTGTCTCCTTTTCTTCTGAATTGATCAAAGTATTATATTATACAGATTC
CONSENSUS
TTTGTCTCCTTTTCTTCTGAATTGATCAAAGTATTATATTATACAGATTC

RI-AT1G30970-XLOC_005753-4663-0 ACCCAAAATGTTTGCACCATGTTGTTC
RI-AT1G30970-XLOC_005753-4663-1 ACCCAAAATGTTTGCACCATGTTGTTC
CONSENSUS   ACCCAAAATGTTTGCACCATGTTGTTC

alignment for event: SE-AT1G50140-XLOC_006512-8383

SE-AT1G50140-XLOC_006512-8383-0
GAGACCAAGTAAAGTATGTTGGGTCTCCTAAGAAGGACGAAGCAAAACAT
SE-AT1G50140-XLOC_006512-8383-1
GAGACCAAGTAAAGTATGTTGGGTCTCCTAAGAAGGACGAAGCAAAACAT
CONSENSUS
GAGACCAAGTAAAGTATGTTGGGTCTCCTAAGAAGGACGAAGCAAAACAT

SE-AT1G50140-XLOC_006512-8383-0
AG--
SE-AT1G50140-XLOC_006512-8383-1
AGGGTCGTATTGGGGAAGATTTCCACATCTGATGGTCAAAAGAGTGCTTT
CONSENSUS
AG..

SE-AT1G50140-XLOC_006512-8383-0 ------------------
GCCATTGTCTAGTGGACAACGTGGAGAGGTAT
SE-AT1G50140-XLOC_006512-8383-1
TACCGTTATTCCTGGCAGGCCATTGTCTAGTGGACAACGTGGAGAGGTAT
CONSENSUS
..................GCCATTGTCTAGTGGACAACGTGGAGAGGTAT

SE-AT1G50140-XLOC_006512-8383-0
ATGAGGTGATTGGGAACCGTGTTGCTGTCATATTCGAATACGGTGATGAT
SE-AT1G50140-XLOC_006512-8383-1
ATGAGGTGATTGGGAACCGTGTTGCTGTCATATTCGAATACGGTGATGAT
CONSENSUS
ATGAGGTGATTGGGAACCGTGTTGCTGTCATATTCGAATACGGTGATGAT

SE-AT1G50140-XLOC_006512-8383-0
AAAACATCAGAGGGAAGTGAGAAAAAGCCAGCAGAGCAGCCCCAGATGTT
SE-AT1G50140-XLOC_006512-8383-1
AAAACATCAGAGGGAAGTGAGAAAAAGCCAGCAGAGCAGCCCCAGATGTT
CONSENSUS
AAAACATCAGAGGGAAGTGAGAAAAAGCCAGCAGAGCAGCCCCAGATGTT

SE-AT1G50140-XLOC_006512-8383-0 ACCCATCCACTGGTTAGATG
SE-AT1G50140-XLOC_006512-8383-1 ACCCATCCACTGGTTAGATG
CONSENSUS   ACCCATCCACTGGTTAGATG

alignment for event: RI-AT1G55350-XLOC_006801-3513

RI-AT1G55350-XLOC_006801-3513-0
GGCGTAGGTTTCTCGAGAACAACTCCTGGATTTCGTAACTACCAATCAAG
RI-AT1G55350-XLOC_006801-3513-1
GGCGTAGGTTTCTCGAGAACAACTCCTGGATTTCGTAACTACCAATCAAG
CONSENSUS

GGCGTAGGTTTCTCGAGAACAACTCCTGGATTTCGTAACTACCAATCAAG

RI-AT1G55350-XLOC_006801-3513-0
CCATGATTCACAGTTGTTCTATATCGGATTGAGGATTCTTAAAACTCGTG
RI-AT1G55350-XLOC_006801-3513-1
CCATGATTCACAGTTGTTCTATATCGGATTGAGGATTCTTAAAACTCGTG
CONSENSUS
CCATGATTCACAGTTGTTCTATATCGGATTGAGGATTCTTAAAACTCGTG

RI-AT1G55350-XLOC_006801-3513-0
GACGTCGTGCTGCTTACAACATATTTCTTCATGAATCTGTTGGTGGAACA
RI-AT1G55350-XLOC_006801-3513-1
GACGTCGTGCTGCTTACAACATATTTCTTCATGAATCTGTTGGTGGAACA
CONSENSUS
GACGTCGTGCTGCTTACAACATATTTCTTCATGAATCTGTTGGTGGAACA

RI-AT1G55350-XLOC_006801-3513-0
GACTATGTGAATTCCCGTGAGATTTCATGTGAAATGGTTCTTGACCCTGA
RI-AT1G55350-XLOC_006801-3513-1
GACTATGTGAATTCCCGTGAGATTTCATGTGAAATGGTTCTTGACCCTGA
CONSENSUS
GACTATGTGAATTCCCGTGAGATTTCATGTGAAATGGTTCTTGACCCTGA

RI-AT1G55350-XLOC_006801-3513-0
TCCTAAGGGTTATACTATTGTCCCAACCACGATACACCCAGGGGAAGAAG
RI-AT1G55350-XLOC_006801-3513-1
TCCTAAGGGTTATACTATTGTCCCAACCACGATACACCCAGGGGAAGAAG
CONSENSUS
TCCTAAGGGTTATACTATTGTCCCAACCACGATACACCCAGGGGAAGAAG

RI-AT1G55350-XLOC_006801-3513-0
CACCTTTTGTCCTTTCAGTCTTCACAAAAGCATCCATTGTTCTTGAAGCT
RI-AT1G55350-XLOC_006801-3513-1
CACCTTTTGTCCTTTCAGTCTTCACAAAAGCATCCATTGTTCTTGAAGCT
CONSENSUS
CACCTTTTGTCCTTTCAGTCTTCACAAAAGCATCCATTGTTCTTGAAGCT

RI-AT1G55350-XLOC_006801-3513-0
TTGTAGTGCCCGTATTGTCAGATGGCTCTCTCAGCAACCTGCATGCCATG
RI-AT1G55350-XLOC_006801-3513-1
TTGTAGTGCCCGTATTGTCAGATGGCTCTCTCAGCAACCTGCATGCCATG
CONSENSUS
TTGTAGTGCCCGTATTGTCAGATGGCTCTCTCAGCAACCTGCATGCCATG

RI-AT1G55350-XLOC_006801-3513-0
AAATCATCCAAGTGCTTGTGTTGTTTAAGGAACCAGACGGCTTACGTCTC
RI-AT1G55350-XLOC_006801-3513-1
AAATCATCCAAGTGCTTGTGTTGTTTAAGGAACCAGACGGCTTACGTCTC
CONSENSUS
AAATCATCCAAGTGCTTGTGTTGTTTAAGGAACCAGACGGCTTACGTCTC

RI-AT1G55350-XLOC_006801-3513-0
AATGTTAAGACTTGTTTTGCCCACGATCCACGCAACATTAGAGAAGGTAA
RI-AT1G55350-XLOC_006801-3513-1
AATGTTAAGACTTGTTTTGCCCACGATCCACGCAACATTAGAGAAG----
CONSENSUS

AATGTTAAGACTTGTTTTGCCCACGATCCACGCAACATTAGAGAAG....

RI-AT1G55350-XLOC_006801-3513-0
ATTTCTTAACCTTTCTGCAAGGATTGCTCTTTTCCTATCCTCACTCACTG
RI-AT1G55350-XLOC_006801-3513-1

CONSENSUS
....

RI-AT1G55350-XLOC_006801-3513-0
TTCCTCATTCAGACTTAACTCCCTCCGCAACAGCGTCTGATCTGATAGCT
RI-AT1G55350-XLOC_006801-3513-1 ------------
ACTTAACTCCCTCCGCAACAGCGTCTGATCTGATAGCT
CONSENSUS
............ACTTAACTCCCTCCGCAACAGCGTCTGATCTGATAGCT

RI-AT1G55350-XLOC_006801-3513-0
GGCCATCACATTATTGGTGAATCTATGAAGTCTCGGGATAAAATGTTTTA
RI-AT1G55350-XLOC_006801-3513-1
GGCCATCACATTATTGGTGAATCTATGAAGTCTCGGGATAAAATGTTTTA
CONSENSUS
GGCCATCACATTATTGGTGAATCTATGAAGTCTCGGGATAAAATGTTTTA

RI-AT1G55350-XLOC_006801-3513-0
GTTATTGTCCTGATTTTGAACCACGAAGTTAACTGCTCAGAGGATAGATA
RI-AT1G55350-XLOC_006801-3513-1
GTTATTGTCCTGATTTTGAACCACGAAGTTAACTGCTCAGAGGATAGATA
CONSENSUS
GTTATTGTCCTGATTTTGAACCACGAAGTTAACTGCTCAGAGGATAGATA

RI-AT1G55350-XLOC_006801-3513-0
ATTTGCACTGGAGAAAGCACTTTTCAGGCATGGCTCACCGGCATTCCATC
RI-AT1G55350-XLOC_006801-3513-1
ATTTGCACTGGAGAAAGCACTTTTCAGGCATGGCTCACCGGCATTCCATC
CONSENSUS
ATTTGCACTGGAGAAAGCACTTTTCAGGCATGGCTCACCGGCATTCCATC

RI-AT1G55350-XLOC_006801-3513-0
TCGATCTTGAGACACGCATCAAATGTACATTTGTAGGTATTGCGACAAGT
RI-AT1G55350-XLOC_006801-3513-1
TCGATCTTGAGACACGCATCAAATGTACATTTGTAGGTATTGCGACAAGT
CONSENSUS
TCGATCTTGAGACACGCATCAAATGTACATTTGTAGGTATTGCGACAAGT

RI-AT1G55350-XLOC_006801-3513-0
ACAGATTTATTAAATGTAGAATAACTACTTCATAACTGAGGAAACAAGGA
RI-AT1G55350-XLOC_006801-3513-1
ACAGATTTATTAAATGTAGAATAACTACTTCATAACTGAGGAAACAAGGA
CONSENSUS
ACAGATTTATTAAATGTAGAATAACTACTTCATAACTGAGGAAACAAGGA

RI-AT1G55350-XLOC_006801-3513-0
GAGAAATAGGAGACTTGTGACTTGTTGGGTAAAGGTTTGGTATTCGGTAG
RI-AT1G55350-XLOC_006801-3513-1
GAGAAATAGGAGACTTGTGACTTGTTGGGTAAAGGTTTGGTATTCGGTAG
CONSENSUS

GAGAAATAGGAGACTTGTGACTTGTTGGGTAAAGGTTTGGTATTCGGTAG

RI-AT1G55350-XLOC_006801-3513-0
TGGTAGCTGCAGTTTTGGTTGGTGTGTTGTAATATTCAGACACTAATCTG
RI-AT1G55350-XLOC_006801-3513-1
TGGTAGCTGCAGTTTTGGTTGGTGTGTTGTAATATTCAGACACTAATCTG
CONSENSUS
TGGTAGCTGCAGTTTTGGTTGGTGTGTTGTAATATTCAGACACTAATCTG

RI-AT1G55350-XLOC_006801-3513-0
TGTGCAGATTTCGTTTCAACTCTAATATATGAGGGCTCTCTTTCTCTCT
RI-AT1G55350-XLOC_006801-3513-1
TGTGCAGATTTCGTTTCAACTCTAATATATGAGGGCTCTCTTTCTCTCT
CONSENSUS
TGTGCAGATTTCGTTTCAACTCTAATATATGAGGGCTCTCTTTCTCTCT

alignment for event: A3-AT1G11330-XLOC_000547-8537

A3-AT1G11330-XLOC_000547-8537-0
AAACACATAGCAATCTAGCAGTTATGATCGCAGCACCTGTGATAGGCGTT
A3-AT1G11330-XLOC_000547-8537-1
AAACACATAGCAATCTAGCAGTTATGATCGCAGCACCTGTGATAGGCGTT
CONSENSUS
AAACACATAGCAATCTAGCAGTTATGATCGCAGCACCTGTGATAGGCGTT

A3-AT1G11330-XLOC_000547-8537-0
ATGTTAATTGCTGCGGTCTGCGTTCTTTTAGCATGCCGGAAATACAAAAA
A3-AT1G11330-XLOC_000547-8537-1
ATGTTAATTGCTGCGGTCTGCGTTCTTTTAGCATGCCGGAAATACAAAAA
CONSENSUS
ATGTTAATTGCTGCGGTCTGCGTTCTTTTAGCATGCCGGAAATACAAAAA

A3-AT1G11330-XLOC_000547-8537-0
GCGTCCAGCAGCTCCAGCGAAAGATAGAAGTGCAGAGCTAATGTTTAAGA
A3-AT1G11330-XLOC_000547-8537-1 GCGTCCAG---------
CGAAAGATAGAAGTGCAGAGCTAATGTTTAAGA
CONSENSUS
GCGTCCAG.........CGAAAGATAGAAGTGCAGAGCTAATGTTTAAGA

A3-AT1G11330-XLOC_000547-8537-0
GAATGGAAGCACTTACAAGTGATAATGAGTCTGCTTCTAACCAAATCAAG
A3-AT1G11330-XLOC_000547-8537-1
GAATGGAAGCACTTACAAGTGATAATGAGTCTGCTTCTAACCAAATCAAG
CONSENSUS
GAATGGAAGCACTTACAAGTGATAATGAGTCTGCTTCTAACCAAATCAAG

A3-AT1G11330-XLOC_000547-8537-0
CTCAAGGAGCTTCCACTCTTTGAGTTTCAAGTGTTAGCTACATCAACTGA
A3-AT1G11330-XLOC_000547-8537-1
CTCAAGGAGCTTCCACTCTTTGAGTTTCAAGTGTTAGCTACATCAACTGA
CONSENSUS
CTCAAGGAGCTTCCACTCTTTGAGTTTCAAGTGTTAGCTACATCAACTGA

A3-AT1G11330-XLOC_000547-8537-0

TAGCTTCTCTCTAAGAAACAAGCTCGGGCAAGGCGGGTTTGGTCCTGTTT
A3-AT1G11330-XLOC_000547-8537-1
TAGCTTCTCTCTAAGAAACAAGCTCGGGCAAGGCGGGTTTGGTCCTGTTT
CONSENSUS
TAGCTTCTCTCTAAGAAACAAGCTCGGGCAAGGCGGGTTTGGTCCTGTTT

A3-AT1G11330-XLOC_000547-8537-0 ACAAG
A3-AT1G11330-XLOC_000547-8537-1 ACAAG
CONSENSUS ACAAG

alignment for event: MXE-AT1G77080-XLOC_003953-9829

MXE-AT1G77080-XLOC_003953-9829-0
AGGATTAAATTAGGGCATAACCCTTATCGGAGATTTGAAGCCATGGGAAG
MXE-AT1G77080-XLOC_003953-9829-1
AGGATTAAATTAGGGCATAACCCTTATCGGAGATTTGAAGCCATGGGAAG
CONSENSUS
AGGATTAAATTAGGGCATAACCCTTATCGGAGATTTGAAGCCATGGGAAG

MXE-AT1G77080-XLOC_003953-9829-0
AAGAAAAATCGAGATCAAGCGAATCGAGAACAAAAGCAGTCGACAAGTCA
MXE-AT1G77080-XLOC_003953-9829-1
AAGAAAAATCGAGATCAAGCGAATCGAGAACAAAAGCAGTCGACAAGTCA
CONSENSUS
AAGAAAAATCGAGATCAAGCGAATCGAGAACAAAAGCAGTCGACAAGTCA

MXE-AT1G77080-XLOC_003953-9829-0
CTTTCTCCAAACGACGCAATGGTCTCATCGACAAAGCTCGACAACTTTCG
MXE-AT1G77080-XLOC_003953-9829-1
CTTTCTCCAAACGACGCAATGGTCTCATCGACAAAGCTCGACAACTTTCG
CONSENSUS
CTTTCTCCAAACGACGCAATGGTCTCATCGACAAAGCTCGACAACTTTCG

MXE-AT1G77080-XLOC_003953-9829-0
ATTCTCTGTGAATCCTCCGTCGCTGTTGTCGTCGTATCTGCCTCCGGAAA
MXE-AT1G77080-XLOC_003953-9829-1
ATTCTCTGTGAATCCTCCGTCGCTGTTGTCGTCGTATCTGCCTCCGGAAA
CONSENSUS
ATTCTCTGTGAATCCTCCGTCGCTGTTGTCGTCGTATCTGCCTCCGGAAA

MXE-AT1G77080-XLOC_003953-9829-0
ACTCTATGACTCTTCCTCCGGTGACGACATTTCCAAGATCATTGATCGTT
MXE-AT1G77080-XLOC_003953-9829-1
ACTCTATGACTCTTCCTCCGGTGACGA-----------------------
CONSENSUS
ACTCTATGACTCTTCCTCCGGTGACGA.......................

MXE-AT1G77080-XLOC_003953-9829-0
ATGAAATACAACATGCTGATGAACTTAGAGCCTTA---------------
MXE-AT1G77080-XLOC_003953-9829-1
-----------------------------------GATAGAAGCGCTGTT
CONSENSUS
....

MXE-AT1G77080-XLOC_003953-9829-0
-------------------------------GATCTTGAAGAAAAAATTC
MXE-AT1G77080-XLOC_003953-9829-1
CAAGCCGGAGAAACCTCAATGTTTTGAACTCGATCTTGAAGAAAAAATTC
CONSENSUS
...............................GATCTTGAAGAAAAAATTC

MXE-AT1G77080-XLOC_003953-9829-0
AGAATTATCTTCCACACAAGGAGTTACTAGAAACAGTCCAAAG
MXE-AT1G77080-XLOC_003953-9829-1
AGAATTATCTTCCACACAAGGAGTTACTAGAAACAGTCCAAAG
CONSENSUS
AGAATTATCTTCCACACAAGGAGTTACTAGAAACAGTCCAAAG

alignment for event: A3-AT1G63640-XLOC_007213-9982

A3-AT1G63640-XLOC_007213-9982-0
GTATCGAACTTGAAGGATGTAATTGCTAAGAAAGATGAAGAGCTACAAAA
A3-AT1G63640-XLOC_007213-9982-1
GTATCGAACTTGAAGGATGTAATTGCTAAGAAAGATGAAGAGCTACAAAA
CONSENSUS
GTATCGAACTTGAAGGATGTAATTGCTAAGAAAGATGAAGAGCTACAAAA

A3-AT1G63640-XLOC_007213-9982-0
TTTTCAGAAGGTAAAAGGTAATAATGCAACATCCCTGAAACGTGGATTAA
A3-AT1G63640-XLOC_007213-9982-1
TTTTCAGAAGGTAAAAGGTAATAATGCAACATCCCTGAAACGTGGATTAA
CONSENSUS
TTTTCAGAAGGTAAAAGGTAATAATGCAACATCCCTGAAACGTGGATTAA

A3-AT1G63640-XLOC_007213-9982-0
GCAATTTAAGGTTGGTGGGGCCTACATCACCTAGAAGACATTCTATTGGA
A3-AT1G63640-XLOC_007213-9982-1
GCAATTTAAGGTTGGTGGGGCCTACATCACCTAGAAGACATTCTATTGGA
CONSENSUS
GCAATTTAAGGTTGGTGGGGCCTACATCACCTAGAAGACATTCTATTGGA

A3-AT1G63640-XLOC_007213-9982-0
GCGTCACCAAACGCTCGACGAGGAAAGGCCTCTGGTTTATTTGGAAGAGG
A3-AT1G63640-XLOC_007213-9982-1
GCGTCACCAAACGCTCGACGAGGAAAGGCCTCTGGTTTATTTGGAAGAGG
CONSENSUS
GCGTCACCAAACGCTCGACGAGGAAAGGCCTCTGGTTTATTTGGAAGAGG

A3-AT1G63640-XLOC_007213-9982-0
AACCTCAGATGTTGACAACTGCTCTGAATATAGCAGCAAGCATTCGGATT
A3-AT1G63640-XLOC_007213-9982-1
AACCTCAGATGTTGACAACTGCTCTGAATATAGCAGCAAGCATTCGGATT
CONSENSUS
AACCTCAGATGTTGACAACTGCTCTGAATATAGCAGCAAGCATTCGGATT

A3-AT1G63640-XLOC_007213-9982-0
CTGGTTCACAACAATCATCAGACGAACGTAAACATCAAAAAGATTATCAC
A3-AT1G63640-XLOC_007213-9982-1

CTGGTTCACAACAATCATCAGACGAACGTAAACATCAAAAAGATTATCAC
CONSENSUS
CTGGTTCACAACAATCATCAGACGAACGTAAACATCAAAAAGATTATCAC

A3-AT1G63640-XLOC_007213-9982-0
CAACCATCTAAATTTGCTGGTGCGGCGAAAGGAATCGATTTTGATGATGA
A3-AT1G63640-XLOC_007213-9982-1
CAACCATCTAAATTTGCTGGTGCGGCGAAAGGAATCGATTTTGATGATGA
CONSENSUS
CAACCATCTAAATTTGCTGGTGCGGCGAAAGGAATCGATTTTGATGATGA

A3-AT1G63640-XLOC_007213-9982-0
AGATGTTGAACTCGTAGGTCTTGCAGATGCAGATTCTGAGGACAGATTGA
A3-AT1G63640-XLOC_007213-9982-1
AGATGTTGAACTCGTAGGTCTTGCAGATGCAGATTCTGAGGACAGATTGA
CONSENSUS
AGATGTTGAACTCGTAGGTCTTGCAGATGCAGATTCTGAGGACAGATTGA

A3-AT1G63640-XLOC_007213-9982-0
GTGATATCTCTGATAGTTGTCTTTCAATGGGAACAGAAACTGATGGATCC
A3-AT1G63640-XLOC_007213-9982-1
GTGATATCTCTGATAGTTGTCTTTCAATGGGAACAGAAACTGATGGATCC
CONSENSUS
GTGATATCTCTGATAGTTGTCTTTCAATGGGAACAGAAACTGATGGATCC

A3-AT1G63640-XLOC_007213-9982-0
ATAAGCAGTGCAGTAGAGTTGACTCTATTCCCTGAAACTGCTAAGCCTCT
A3-AT1G63640-XLOC_007213-9982-1
ATAAGCAGTGCAGTAGAGTTGACTCTATTCCCTGAAACTGCTAAGCCTCT
CONSENSUS
ATAAGCAGTGCAGTAGAGTTGACTCTATTCCCTGAAACTGCTAAGCCTCT

A3-AT1G63640-XLOC_007213-9982-0
TGAACTAATAGAACGACCTGAAGCACGCATGACTTCTGAGAAACTCGAGA
A3-AT1G63640-XLOC_007213-9982-1
TGAACTAATAGAACGACCTGAAGCACGCATGACTTCTGAGAAACTCGAGA
CONSENSUS
TGAACTAATAGAACGACCTGAAGCACGCATGACTTCTGAGAAACTCGAGA

A3-AT1G63640-XLOC_007213-9982-0
AATCAGTGAAAATGGGGAAAACCGAGCCAAAAGACAG---AACTAATATT
A3-AT1G63640-XLOC_007213-9982-1
AATCAGTGAAAATGGGGAAAACCGAGCCAAAAGACAGCAGAACTAATATT
CONSENSUS
AATCAGTGAAAATGGGGAAAACCGAGCCAAAAGACAG...AACTAATATT

A3-AT1G63640-XLOC_007213-9982-0
CCATCGAAGATTCCAAAGCAGACCTTGAAACCACCAGGCCAAACCAGACC
A3-AT1G63640-XLOC_007213-9982-1
CCATCGAAGATTCCAAAGCAGACCTTGAAACCACCAGGCCAAACCAGACC
CONSENSUS
CCATCGAAGATTCCAAAGCAGACCTTGAAACCACCAGGCCAAACCAGACC

A3-AT1G63640-XLOC_007213-9982-0
GTCTCGTCTGTCAATTGCCACTAGCTCCTCCTCTAAGGCTTTAACAG
A3-AT1G63640-XLOC_007213-9982-1

GTCTCGTCTGTCAATTGCCACTAGCTCCTCCTCTAAGGCTTTAACAG
CONSENSUS
GTCTCGTCTGTCAATTGCCACTAGCTCCTCCTCTAAGGCTTTAACAG
