# Supplementary material for: Splicing-related genes are alternatively spliced upon changes in ambient temperatures in plants
Source: PLoS One. 2017 Mar 3;12(3):e0172950. doi: 10.1371/journal.pone.0172950 (PMC5336241; doi:10.1371/journal.pone.0172950)
Supplement: S6 Table — (PDF) [file pone.0172950.s006.pdf]

alignment for event: RI-AT4G38240-XLOC_025375-5152

RI-AT4G38240-XLOC_025375-5152-0
      GATGGTGTGCCTCGAACAGCATATAAAGGAGTAGTGGTGTTTCGAATCCA
RI-AT4G38240-XLOC_025375-5152-1
      GATGGTGTGCCTCGAACAGCATATAAAGGAGTAGTGGTGTTTCGAATCCA
CONSENSUS
      GATGGTGTGCCTCGAACAGCATATAAAGGAGTAGTGGTGTTTCGAATCCA

RI-AT4G38240-XLOC_025375-5152-0
      GACAACAAGACGTGTATTCCTGGTTGGGCCAGATTCTGTAATGCAGCTTG
RI-AT4G38240-XLOC_025375-5152-1
      GACAACAAGACGTGTATTCCTGGTTGGGCCAGATTCTGTAATGCAGCTTG
CONSENSUS
      GACAACAAGACGTGTATTCCTGGTTGGGCCAGATTCTGTAATGCAGCTTG

RI-AT4G38240-XLOC_025375-5152-0
      GAATTCGAAATTCCTGATGCAAAACATGTACTTCTCCAACCAGCATCATT
RI-AT4G38240-XLOC_025375-5152-1
      GAATTCGAAATTCCTGATGCAAAACAT-----------------------
CONSENSUS
      GAATTCGAAATTCCTGATGCAAAACAT.......................

RI-AT4G38240-XLOC_025375-5152-0
      TACATCTTAACCATTGCTTACAGTTCTGTGCTAACCGGGTTTCACAAGCA
RI-AT4G38240-XLOC_025375-5152-1

CONSENSUS
      ....

RI-AT4G38240-XLOC_025375-5152-0
      GATGAAAGGAAAAGAAGATTTTGGACCGCATGCAGCCTCCTTCTAGCAGC
RI-AT4G38240-XLOC_025375-5152-1 -
ATGAAAGGAAAAGAAGATTTTGGACCGCATGCAGCCTCCTTCTAGCAGC
CONSENSUS
      .ATGAAAGGAAAAGAAGATTTTGGACCGCATGCAGCCTCCTTCTAGCAGC

RI-AT4G38240-XLOC_025375-5152-0
      TGTTAGGTTGTATTGTTATTTATGGATGAGTTTGTCGAGCGGTGGGGTTA
RI-AT4G38240-XLOC_025375-5152-1
      TGTTAGGTTGTATTGTTATTTATGGATGAGTTTGTCGAGCGGTGGGGTTA
CONSENSUS
      TGTTAGGTTGTATTGTTATTTATGGATGAGTTTGTCGAGCGGTGGGGTTA

RI-AT4G38240-XLOC_025375-5152-0
      ACTTTAACAGCAAGGAAGCTCTGGTGACCTGGCTGATTGGCTTAGAAGTT
RI-AT4G38240-XLOC_025375-5152-1
      ACTTTAACAGCAAGGAAGCTCTGGTGACCTGGCTGATTGGCTTAGAAGTT
CONSENSUS
      ACTTTAACAGCAAGGAAGCTCTGGTGACCTGGCTGATTGGCTTAGAAGTT

RI-AT4G38240-XLOC_025375-5152-0
      ATGGGAACCCCTTGAAAGGGTCAGGGTTAAATATATTTCAGTTGTTTTAT
RI-AT4G38240-XLOC_025375-5152-1
      ATGGGAACCCCTTGAAAGGGTCAGGGTTAAATATATTTCAGTTGTTTTAT
CONSENSUS

```
      ATGGGAACCCCTTGAAAGGGTCAGGGTTAAATATATTTCAGTTGTTTTAT

RI-AT4G38240-XLOC_025375-5152-0
      TAGTGATTATCTTGTGGGTAACTTATACGAATGCAAATCATTCTATGCAG
RI-AT4G38240-XLOC_025375-5152-1
      TAGTGATTATCTTGTGGGTAACTTATACGAATGCAAATCATTCTATGCAG
CONSENSUS
      TAGTGATTATCTTGTGGGTAACTTATACGAATGCAAATCATTCTATGCAG

RI-AT4G38240-XLOC_025375-5152-0
      TTTTTCTTCGTCCCACTTGTTTTGGCTTCTCTATTGCTAGTGTACATATC
RI-AT4G38240-XLOC_025375-5152-1
      TTTTTCTTCGTCCCACTTGTTTTGGCTTCTCTATTGCTAGTGTACATATC
CONSENSUS
      TTTTTCTTCGTCCCACTTGTTTTGGCTTCTCTATTGCTAGTGTACATATC

RI-AT4G38240-XLOC_025375-5152-0
      TCTTCAAACATGTACTAAATAATGCGTGTTGCTTCAAAGAAGTAACTTTT
RI-AT4G38240-XLOC_025375-5152-1
      TCTTCAAACATGTACTAAATAATGCGTGTTGCTTCAAAGAAGTAACTTTT
CONSENSUS
      TCTTCAAACATGTACTAAATAATGCGTGTTGCTTCAAAGAAGTAACTTTT

RI-AT4G38240-XLOC_025375-5152-0  ATT
RI-AT4G38240-XLOC_025375-5152-1  ATT
CONSENSUS                        ATT


alignment for event: RI-AT4G06701-XLOC_023449-3034

RI-AT4G06701-XLOC_023449-3034-0
      CAGACAGGAAAGAGGCGTTAAAGATTTGGTGCAAGCTGCGACTCTGTGAT
RI-AT4G06701-XLOC_023449-3034-1
      CAGACAGGAAAGAGGCGTTAAAGATTTGGTGCAAGCTGCGACTCTGTGAT
CONSENSUS
      CAGACAGGAAAGAGGCGTTAAAGATTTGGTGCAAGCTGCGACTCTGTGAT

RI-AT4G06701-XLOC_023449-3034-0
      CACAACTCCACACCAAGTATTCTCTTACATCGCACTCGTCGCTCCTGCAC
RI-AT4G06701-XLOC_023449-3034-1
      CACAACTCCACACCAA----------------------------------
CONSENSUS
      CACAACTCCACACCAA..................................

RI-AT4G06701-XLOC_023449-3034-0
      TCGTAGTTTCTTTGAATAGCCAATGAAGAGTAAAGTTCAATTATGTGTGT
RI-AT4G06701-XLOC_023449-3034-1
      --------------------------------------------------
CONSENSUS
      ..................................................

RI-AT4G06701-XLOC_023449-3034-0
      CGTGTTTAGTAATTTGACTTGAAAAAAGAGTTTGCTAGGGTCTCTTGTGA
RI-AT4G06701-XLOC_023449-3034-1
      --------------------------------------------------
```

```
CONSENSUS
      ..................................................

RI-AT4G06701-XLOC_023449-3034-0
      ACAAGCTGTTATGTCTGCAATTCGTGAGTTTTGGTTAATTTTGGATTTGT
RI-AT4G06701-XLOC_023449-3034-1
      --------------------------------------------------
CONSENSUS
      ..................................................

RI-AT4G06701-XLOC_023449-3034-0
      ACTTCTTGCAATGGCTGCCCTCAAACCATTGATTAATGTATTATATAGGT
RI-AT4G06701-XLOC_023449-3034-1
      --------------------------------------------------
CONSENSUS
      ..................................................

RI-AT4G06701-XLOC_023449-3034-0
      GGTGTGGTTAATGATCCTCTTTAGTTTATTCGATTTTTTTTTATCATATT
RI-AT4G06701-XLOC_023449-3034-1
      --------------------------------------------------
CONSENSUS
      ..................................................

RI-AT4G06701-XLOC_023449-3034-0
      GCTTCTATTTTTGTATTGAATTGAAAAACTTGATTGCAGGAGCTCTGATG
RI-AT4G06701-XLOC_023449-3034-1
      ---------------------------------------GAGCTCTGATG
CONSENSUS
      .......................................GAGCTCTGATG

RI-AT4G06701-XLOC_023449-3034-0
      TCAAGCATCTCTACAAGAAAGAAGAATGGGTTCTTATCATATAAATATGT
RI-AT4G06701-XLOC_023449-3034-1
      TCAAGCATCTCTACAAGAAAGAAGAATGGGTTCTTATCATATAAATATGT
CONSENSUS
      TCAAGCATCTCTACAAGAAAGAAGAATGGGTTCTTATCATATAAATATGT

RI-AT4G06701-XLOC_023449-3034-0
      GCAATGGAGAAAATACAACACGTTTGGATGGTCAACTCTATGAATCTGAT
RI-AT4G06701-XLOC_023449-3034-1
      GCAATGGAGAAAATACAACACGTTTGGATGGTCAACTCTATGAATCTGAT
CONSENSUS
      GCAATGGAGAAAATACAACACGTTTGGATGGTCAACTCTATGAATCTGAT

RI-AT4G06701-XLOC_023449-3034-0
      AATCATATAAATTTCAAGCTTCTTAATTCTTTTAGTGTGATGGAATGTAA
RI-AT4G06701-XLOC_023449-3034-1
      AATCATATAAATTTCAAGCTTCTTAATTCTTTTAGTGTGATGGAATGTAA
CONSENSUS
      AATCATATAAATTTCAAGCTTCTTAATTCTTTTAGTGTGATGGAATGTAA

RI-AT4G06701-XLOC_023449-3034-0
      AATACTAATTAGTTAATTTCTTCTTGTTCGTGCAAATTTTTATGTGAAAC
RI-AT4G06701-XLOC_023449-3034-1
      AATACTAATTAGTTAATTTCTTCTTGTTCGTGCAAATTTTTATGTGAAAC
```

```
CONSENSUS
      AATACTAATTAGTTAATTTCTTCTTGTTCGTGCAAATTTTTATGTGAAAC

RI-AT4G06701-XLOC_023449-3034-0
      TCATCGGTTTGCAAACAAGTTTTATTATTATGATAAATAAATAAAACATT
RI-AT4G06701-XLOC_023449-3034-1
      TCATCGGTTTGCAAACAAGTTTTATTATTATGATAAATAAATAAAACATT
CONSENSUS
      TCATCGGTTTGCAAACAAGTTTTATTATTATGATAAATAAATAAAACATT

RI-AT4G06701-XLOC_023449-3034-0  ATTTTTTG
RI-AT4G06701-XLOC_023449-3034-1  ATTTTTTG
CONSENSUS                        ATTTTTTG



alignment for event: A3-AT4G16360-XLOC_021593-4984

A3-AT4G16360-XLOC_021593-4984-0
      GTTCCGGTGCTTCCACTTCAAAGACCTGATGAAATTCATATCCCTAATCC
A3-AT4G16360-XLOC_021593-4984-1
      GTTCCGGTGCTTCCACTTCAAAGACCTGATGAAATTCATATCCCTAATCC
CONSENSUS
      GTTCCGGTGCTTCCACTTCAAAGACCTGATGAAATTCATATCCCTAATCC

A3-AT4G16360-XLOC_021593-4984-0
      TTCGTGGATGCAATCGCCATCTTCGTTGTATGAAGAAGCTTCTAACGAAC
A3-AT4G16360-XLOC_021593-4984-1
      TTCGTGGATGCAATCGCCATCTTCGTTGTATGAAGAAGCTTCTAACGAAC
CONSENSUS
      TTCGTGGATGCAATCGCCATCTTCGTTGTATGAAGAAGCTTCTAACGAAC

A3-AT4G16360-XLOC_021593-4984-0
      AAGGAATCCCTACGATGATCACTTGGTGTCATGGAGGCAAGGAGATTGCT
A3-AT4G16360-XLOC_021593-4984-1
      AAGGAATCCCTACGATGATCACTTGGTGTCATGGAGGCAAGGAGATTGCT
CONSENSUS
      AAGGAATCCCTACGATGATCACTTGGTGTCATGGAGGCAAGGAGATTGCT

A3-AT4G16360-XLOC_021593-4984-0
      GTGGAGGGATCATGGGATAATTGGAAGACAAGAAGTCGGCTGCAGAGATC
A3-AT4G16360-XLOC_021593-4984-1
      GTGGAGGGATCATGGGATAATTGGAAGACAAG---TCGGCTGCAGAGATC
CONSENSUS
      GTGGAGGGATCATGGGATAATTGGAAGACAAG...TCGGCTGCAGAGATC

A3-AT4G16360-XLOC_021593-4984-0
      TGGGAAGGACTTCACTATCATGAAAGTGTTACCTTCAGGAGTCTATGAGT
A3-AT4G16360-XLOC_021593-4984-1
      TGGGAAGGACTTCACTATCATGAAAGTGTTACCTTCAGGAGTCTATGAGT
CONSENSUS
      TGGGAAGGACTTCACTATCATGAAAGTGTTACCTTCAGGAGTCTATGAGT

A3-AT4G16360-XLOC_021593-4984-0
      ACAGGTTCATTGTGGATGGACAGTGGAGGCATGCCCCTGAGCTCCCTTTA
A3-AT4G16360-XLOC_021593-4984-1
```

```
      ACAGGTTCATTGTGGATGGACAGTGGAGGCATGCCCCTGAGCTCCCTTTA
CONSENSUS
      ACAGGTTCATTGTGGATGGACAGTGGAGGCATGCCCCTGAGCTCCCTTTA

A3-AT4G16360-XLOC_021593-4984-0
      GCTAGAGATGATGCTGGGAACACTTTCAACATTTTGGATCTTCAG
A3-AT4G16360-XLOC_021593-4984-1
      GCTAGAGATGATGCTGGGAACACTTTCAACATTTTGGATCTTCAG
CONSENSUS
      GCTAGAGATGATGCTGGGAACACTTTCAACATTTTGGATCTTCAG



alignment for event: SE-AT4G25500-XLOC_022114-4172

SE-AT4G25500-XLOC_022114-4172-0
      GAAGCATGAAGCCAGTCTTCTGTGGGAACTTTGAGTATGATGCGCGCGAA
SE-AT4G25500-XLOC_022114-4172-1
      GAAGCATGAAGCCAGTCTTCTGTGGGAACTTTGAGTATGATGCGCGCGAA
CONSENSUS
      GAAGCATGAAGCCAGTCTTCTGTGGGAACTTTGAGTATGATGCGCGCGAA

SE-AT4G25500-XLOC_022114-4172-0
      GGTGACCTGGAACGACTATTCAGGAAATACGGCAAGGTTGAGAGGGTTGA
SE-AT4G25500-XLOC_022114-4172-1
      GGTGACCTGGAACGACTATTCAGGAAATACGGCAAGGTTGAGAGGGTTGA
CONSENSUS
      GGTGACCTGGAACGACTATTCAGGAAATACGGCAAGGTTGAGAGGGTTGA

SE-AT4G25500-XLOC_022114-4172-0
      TATGAAAGCTG---------------------------------------
SE-AT4G25500-XLOC_022114-4172-1
      TATGAAAGCTGGATGTGTTTGATAATCTTGGGACGCCTATACTCACACCT
CONSENSUS
      TATGAAAGCTG.......................................

SE-AT4G25500-XLOC_022114-4172-0
      --------------------------------------------------
SE-AT4G25500-XLOC_022114-4172-1
      GGCCATGAATGGACCATAGGGAAATCCATCTCTTATCCCTCAAAATCAGT
CONSENSUS
      ..................................................

SE-AT4G25500-XLOC_022114-4172-0
      --------------------------------------------------
SE-AT4G25500-XLOC_022114-4172-1
      CACTTTCCATCCGCATTAGCACTGCCATCTTAATTGCATTTCATTCCTCA
CONSENSUS
      ..................................................

SE-AT4G25500-XLOC_022114-4172-0
      --------------------------------------------------
SE-AT4G25500-XLOC_022114-4172-1
      TCACTTTGCACACTTGGACATGCCTCTGCAATGGAGAAGGCTCTCTATTT
CONSENSUS
      ..................................................
```

```
SE-AT4G25500-XLOC_022114-4172-0
      --------------------------------------------------
SE-AT4G25500-XLOC_022114-4172-1
      TCATTCATATCCCCGTCTGACTTCCACATTTTCAGTTGTTCCTTGACATA
CONSENSUS
      ..................................................

SE-AT4G25500-XLOC_022114-4172-0  ------------------
GGTTTGCTTTTGTATACATGGAAGATGAAAGG
SE-AT4G25500-XLOC_022114-4172-1
      TTAATTCCATAATGCAAGGGTTTGCTTTTGTATACATGGAAGATGAAAGG
CONSENSUS
      ..................GGTTTGCTTTTGTATACATGGAAGATGAAAGG

SE-AT4G25500-XLOC_022114-4172-0
      GATGCGGAAGATGCCATCCGAGCACTTGACCGCTTTGAATTTGGGCGTAA
SE-AT4G25500-XLOC_022114-4172-1
      GATGCGGAAGATGCCATCCGAGCACTTGACCGCTTTGAATTTGGGCGTAA
CONSENSUS
      GATGCGGAAGATGCCATCCGAGCACTTGACCGCTTTGAATTTGGGCGTAA

SE-AT4G25500-XLOC_022114-4172-0
      GGGACGCAGACTTCGTGTTGAATGGACAAAG
SE-AT4G25500-XLOC_022114-4172-1
      GGGACGCAGACTTCGTGTTGAATGGACAAAG
CONSENSUS
      GGGACGCAGACTTCGTGTTGAATGGACAAAG


alignment for event: RI-AT4G27040-XLOC_024742-1999

RI-AT4G27040-XLOC_024742-1999-0
      TGCAAGACGACAAGTGATGAAAAGATAGAGACAAAGAAAGGCTTCAAGTT
RI-AT4G27040-XLOC_024742-1999-1
      TGCAAGACGACAAGTGATGAAAAGATAGAGACAAAGAAAGGCTTCAAGTT
CONSENSUS
      TGCAAGACGACAAGTGATGAAAAGATAGAGACAAAGAAAGGCTTCAAGTT

RI-AT4G27040-XLOC_024742-1999-0
      TGTTGATAACTGCTGTCTAAAGAGTTCGTTCCCTTGAGAGATTCTGACTC
RI-AT4G27040-XLOC_024742-1999-1
      TGTTGATAACTGCTGTCTAAAGAGTTCGTTCCCTTGAGAGATTCTGACTC
CONSENSUS
      TGTTGATAACTGCTGTCTAAAGAGTTCGTTCCCTTGAGAGATTCTGACTC

RI-AT4G27040-XLOC_024742-1999-0
      TGACATAAGAGGTAAAAAAGTTCAAATACTCAACACTTACTTGGAATTTA
RI-AT4G27040-XLOC_024742-1999-1
      TGACATAAGAG---------------------------------------
CONSENSUS
      TGACATAAGAG.......................................

RI-AT4G27040-XLOC_024742-1999-0
      AAAGAACACTGATTTGTTGATATTATAAGGTATAAAAATATTTCGTCTTC
```

RI-AT4G27040-XLOC_024742-1999-1 ----------------------------
GTATAAAAATATTTCGTCTTC
CONSENSUS
.............................GTATAAAAATATTTCGTCTTC

RI-AT4G27040-XLOC_024742-1999-0
TCAAAATCCAATTCTTCATCATATTATAAGTTCCCCCAACCGGAAGAGAA
RI-AT4G27040-XLOC_024742-1999-1
TCAAAATCCAATTCTTCATCATATTATAAGTTCCCCCAACCGGAAGAGAA
CONSENSUS
TCAAAATCCAATTCTTCATCATATTATAAGTTCCCCCAACCGGAAGAGAA

RI-AT4G27040-XLOC_024742-1999-0
ATTCAAAGATCAGAAAACGGTGAAAGATGCGACGACGACCAGGAATTGGA
RI-AT4G27040-XLOC_024742-1999-1
ATTCAAAGATCAGAAAACGGTGAAAGATGCGACGACGACCAGGAATTGGA
CONSENSUS
ATTCAAAGATCAGAAAACGGTGAAAGATGCGACGACGACCAGGAATTGGA

RI-AT4G27040-XLOC_024742-1999-0 GGATTACAAAAGGCCGCAGCTGCTAGG
RI-AT4G27040-XLOC_024742-1999-1 GGATTACAAAAGGCCGCAGCTGCTAGG
CONSENSUS GGATTACAAAAGGCCGCAGCTGCTAGG

alignment for event: RI-AT4G35410-XLOC_022687-7145

RI-AT4G35410-XLOC_022687-7145-0
GTGTGTGAACTCGATTTGATTTTTAACTTCCACAAGGTAATAGCATGCTG
RI-AT4G35410-XLOC_022687-7145-1
GTGTGTGAACTCGATTTGATTTTTAACTTCCACAAG--------------
CONSENSUS
GTGTGTGAACTCGATTTGATTTTTAACTTCCACAAG..............

RI-AT4G35410-XLOC_022687-7145-0
CAAACACTCAAGGACTCTGCCATTCGTCACTTGATTCTCTATCCTCTGCA
RI-AT4G35410-XLOC_022687-7145-1

CONSENSUS
....

RI-AT4G35410-XLOC_022687-7145-0
TCTTTATATCTTGCTGACAGTTTCTGGATTTCTTGTTGCTTTCTTAGGCA
RI-AT4G35410-XLOC_022687-7145-1
---GCA
CONSENSUS
....GCA

RI-AT4G35410-XLOC_022687-7145-0
TATTACATATTGGATGAGCTCTTGATTGCTGGGGAGCTTCAAGAGTCAAG
RI-AT4G35410-XLOC_022687-7145-1
TATTACATATTGGATGAGCTCTTGATTGCTGGGGAGCTTCAAGAGTCAAG
CONSENSUS
TATTACATATTGGATGAGCTCTTGATTGCTGGGGAGCTTCAAGAGTCAAG

RI-AT4G35410-XLOC_022687-7145-0

```
      CAAGAAAACAGTAGCCAGGATTATATCCGCTCAG
RI-AT4G35410-XLOC_022687-7145-1
      CAAGAAAACAGTAGCCAGGATTATATCCGCTCAG
CONSENSUS
      CAAGAAAACAGTAGCCAGGATTATATCCGCTCAG



alignment for event: RI-AT4G12560-XLOC_021342-7181

RI-AT4G12560-XLOC_021342-7181-0
      GGATGATTTCCTGTCCAAAGGATTCAAGCTGGTCTTATAAGCCAAAAACA
RI-AT4G12560-XLOC_021342-7181-1
      GGATGATTTCCTGTCCAAAGGATTCAAGCTGGTCTTATAAGCCAAAAACA
CONSENSUS
      GGATGATTTCCTGTCCAAAGGATTCAAGCTGGTCTTATAAGCCAAAAACA

RI-AT4G12560-XLOC_021342-7181-0
      AGAGACAGGAGCTAAAGCTGCACAAGGTAAGTGAAAAAGTTGACCGTTCT
RI-AT4G12560-XLOC_021342-7181-1
      AGAGACAGGAGCTAAAGCTGCACAAGGTAAGTGAAAAAGTTGACCGTTCT
CONSENSUS
      AGAGACAGGAGCTAAAGCTGCACAAGGTAAGTGAAAAAGTTGACCGTTCT

RI-AT4G12560-XLOC_021342-7181-0
      TAATGGGTCTAGTTTGCTGCTGCATTCTTATTGCATTTCATTCTTTTCTG
RI-AT4G12560-XLOC_021342-7181-1
      TAATGGGTCTAGTTTGCTGCTGCATTCTTATTGCATTTCATTCTTTTCTG
CONSENSUS
      TAATGGGTCTAGTTTGCTGCTGCATTCTTATTGCATTTCATTCTTTTCTG

RI-AT4G12560-XLOC_021342-7181-0
      TCTCTGCTCTGTTTTGTCGCGGACGTTCATATCCGGTGTCTGTTTTGTGG
RI-AT4G12560-XLOC_021342-7181-1
      TCTCTGCTCTGTTTTGTCGCGGACGTTCATATCCGGTGTCTGTTTTGTGG
CONSENSUS
      TCTCTGCTCTGTTTTGTCGCGGACGTTCATATCCGGTGTCTGTTTTGTGG

RI-AT4G12560-XLOC_021342-7181-0
      CGGACGAGCAATTTTCATATTCGGGTTGGGACAAGAGTGAAAGTCACAGT
RI-AT4G12560-XLOC_021342-7181-1
      CGGACGAGCAATTTTCATATTCGGGTTGGGACAAGAGTGAAAGTCACAGT
CONSENSUS
      CGGACGAGCAATTTTCATATTCGGGTTGGGACAAGAGTGAAAGTCACAGT

RI-AT4G12560-XLOC_021342-7181-0
      ATGTCATAGGTATTGAGTGCTTAGAAGTTAGAACATTCCCTAGAAAATGT
RI-AT4G12560-XLOC_021342-7181-1
      ATGTCATAG-----------------------------------------
CONSENSUS
      ATGTCATAG.........................................

RI-AT4G12560-XLOC_021342-7181-0
      TATGGAGTCATCATCAGGATAATTACTTTAGCATATGACCATATTCAAGT
RI-AT4G12560-XLOC_021342-7181-1
      --------------------------------------------------
```

```
CONSENSUS
      ..................................................

RI-AT4G12560-XLOC_021342-7181-0
      GTGGTTTTTTCACATCAATCTATCTGCAAAACAATCATTGGGTATGATTT
RI-AT4G12560-XLOC_021342-7181-1
      --------------------------------------------------
CONSENSUS
      ..................................................

RI-AT4G12560-XLOC_021342-7181-0
      TTTCAGGGAGGAGAAACGAGAAGGGGAACAAACAATAACTTCAACCAAAA
RI-AT4G12560-XLOC_021342-7181-1  ------
GGAGGAGAAACGAGAAGGGGAACAAACAATAACTTCAACCAAAA
CONSENSUS
      ......GGAGGAGAAACGAGAAGGGGAACAAACAATAACTTCAACCAAAA

RI-AT4G12560-XLOC_021342-7181-0
      AGCTCTAATATACCAAAGGAGAATAATATACAACTTACCTACGTATATGT
RI-AT4G12560-XLOC_021342-7181-1
      AGCTCTAATATACCAAAGGAGAATAATATACAACTTACCTACGTATATGT
CONSENSUS
      AGCTCTAATATACCAAAGGAGAATAATATACAACTTACCTACGTATATGT

RI-AT4G12560-XLOC_021342-7181-0
      GCTTATAGAAGAGAGGACAAATGTTTTTGGTTTGTAAATAACTCTTTTTA
RI-AT4G12560-XLOC_021342-7181-1
      GCTTATAGAAGAGAGGACAAATGTTTTTGGTTTGTAAATAACTCTTTTTA
CONSENSUS
      GCTTATAGAAGAGAGGACAAATGTTTTTGGTTTGTAAATAACTCTTTTTA

RI-AT4G12560-XLOC_021342-7181-0
      AGTTTGATATATATAACTTACTAGTACTAACGCTTTGTATACTTGCTTTT
RI-AT4G12560-XLOC_021342-7181-1
      AGTTTGATATATATAACTTACTAGTACTAACGCTTTGTATACTTGCTTTT
CONSENSUS
      AGTTTGATATATATAACTTACTAGTACTAACGCTTTGTATACTTGCTTTT

RI-AT4G12560-XLOC_021342-7181-0
      TTAAGTTTCTGCATAATATGAGATGTTAAATAAATTA
RI-AT4G12560-XLOC_021342-7181-1
      TTAAGTTTCTGCATAATATGAGATGTTAAATAAATTA
CONSENSUS
      TTAAGTTTCTGCATAATATGAGATGTTAAATAAATTA



alignment for event: RI-AT4G11830-XLOC_023804-2901

RI-AT4G11830-XLOC_023804-2901-0
      TTCTTTCGAGCCTCGAGTTTCGTATCGTGGTGTTGCTGCTGGAATAGACG
RI-AT4G11830-XLOC_023804-2901-1
      TTCTTTCGAGCCTCGAGTTTCGTATCGTGGTGTTGCTGCTGGAATAGACG
CONSENSUS
      TTCTTTCGAGCCTCGAGTTTCGTATCGTGGTGTTGCTGCTGGAATAGACG
```

RI-AT4G11830-XLOC_023804-2901-0
ATGATTACCTTGGTGCTATACGGAAAATGTCTGCGGATGGAAACTTCTAA
RI-AT4G11830-XLOC_023804-2901-1
ATGATTACCTTGGTGCTATACGGAAAATGTCTGCGGATGGAAACTTCTAA
CONSENSUS
ATGATTACCTTGGTGCTATACGGAAAATGTCTGCGGATGGAAACTTCTAA

RI-AT4G11830-XLOC_023804-2901-0
AGGGTAACATCATCTTTAATATGATTTCATTTAAAGAAAATGTCATGCTT
RI-AT4G11830-XLOC_023804-2901-1
AGGGTAACATCATCTTTAATATGATTTCATTTAAAGAAAATGTCATGCTT
CONSENSUS
AGGGTAACATCATCTTTAATATGATTTCATTTAAAGAAAATGTCATGCTT

RI-AT4G11830-XLOC_023804-2901-0
TATTTATTAATTACACTACTAAATGATATAATTTAGTGATTATGTAGAAT
RI-AT4G11830-XLOC_023804-2901-1
TATTTATTAATTACACTACTAAATGATATAATTTAGTGATTATGTAGAAT
CONSENSUS
TATTTATTAATTACACTACTAAATGATATAATTTAGTGATTATGTAGAAT

RI-AT4G11830-XLOC_023804-2901-0
GTTTAATTTCTTGTTGATATATAAGAATAGTAATATCACCAATCAAAATG
RI-AT4G11830-XLOC_023804-2901-1
GTTTAATTTCTTGTTGATATATAAGAATAGTAATATCACCAATCAAAATG
CONSENSUS
GTTTAATTTCTTGTTGATATATAAGAATAGTAATATCACCAATCAAAATG

RI-AT4G11830-XLOC_023804-2901-0
AAGTTGCATCAAAAAAGGGCCAGAGAGAAAGAGCTTTGTGAGCAGCCAAA
RI-AT4G11830-XLOC_023804-2901-1
AAGTTGCATCAAAAAAGGGCCAGAGAGAAAGAGCTTTGTGAGCAGCCAAA
CONSENSUS
AAGTTGCATCAAAAAAGGGCCAGAGAGAAAGAGCTTTGTGAGCAGCCAAA

RI-AT4G11830-XLOC_023804-2901-0
AACCAAAGTGGGCAAGTCTTCTTTCTTAGTCGTCGTTACTAAAACACAAA
RI-AT4G11830-XLOC_023804-2901-1
AACCAAAGTGGGCAAGTCTTCTTTCTTAGTCGTCGTTACTAAAACACAAA
CONSENSUS
AACCAAAGTGGGCAAGTCTTCTTTCTTAGTCGTCGTTACTAAAACACAAA

RI-AT4G11830-XLOC_023804-2901-0
TTGTTTATTCGGCATTTGTGTTTAATTAAGTTCTTTAATTAATGATTAAA
RI-AT4G11830-XLOC_023804-2901-1
TTGTTTATTCGGCATTTGTGTTTAATTAAGTTCTTTAATTAATGATTAAA
CONSENSUS
TTGTTTATTCGGCATTTGTGTTTAATTAAGTTCTTTAATTAATGATTAAA

RI-AT4G11830-XLOC_023804-2901-0
GATCTTAAATTTGTGTTATTAATGGCTAAAGTTAAACGATTCAACAACGG
RI-AT4G11830-XLOC_023804-2901-1
GATCTTAAATTTGTGTTATTAATGGCTAAAGTTAAACGATTCAACAACGG
CONSENSUS
GATCTTAAATTTGTGTTATTAATGGCTAAAGTTAAACGATTCAACAACGG

RI-AT4G11830-XLOC_023804-2901-0
CATCCAAAATCTTTAAATTTTTCCGATTCTTCCGTTGCAAGTCAACGTTT
RI-AT4G11830-XLOC_023804-2901-1
CATCCAAAATCTTTAAATTTTTCCGATTCTTCCGTTGCAAGTCAACGTTT
CONSENSUS
CATCCAAAATCTTTAAATTTTTCCGATTCTTCCGTTGCAAGTCAACGTTT

RI-AT4G11830-XLOC_023804-2901-0
TCCTTCGCTTCCTCCTACCTTCTTCTTCTTCTTCACAAAAATCTGAACTT
RI-AT4G11830-XLOC_023804-2901-1
TCCTTCGCTTCCTCCTACCTTCTTCTTCTTCTTCACAAAAATCTGAACTT
CONSENSUS
TCCTTCGCTTCCTCCTACCTTCTTCTTCTTCTTCACAAAAATCTGAACTT

RI-AT4G11830-XLOC_023804-2901-0
TAAATTTGAATTTGCCCATATTTTTAGACCCACATTTATTCATATAGATT
RI-AT4G11830-XLOC_023804-2901-1
TAAATTTGAATTTGCCCATATTTTTAGACCCACATTTATTCATATAGATT
CONSENSUS
TAAATTTGAATTTGCCCATATTTTTAGACCCACATTTATTCATATAGATT

RI-AT4G11830-XLOC_023804-2901-0
TGATCTGAGTTTGTATTATCTCAAAGTTTTATCCTTTTGTTGAATCTTGA
RI-AT4G11830-XLOC_023804-2901-1
TGATCTGAGTTTGTATTATCTCAAAGTTTTATCCTTTTGTTGAATCTTGA
CONSENSUS
TGATCTGAGTTTGTATTATCTCAAAGTTTTATCCTTTTGTTGAATCTTGA

RI-AT4G11830-XLOC_023804-2901-0
TCTTTCGATTAGTCCCCACCAAGCACCAAGTGATCGATCTTTTGTCTCAC
RI-AT4G11830-XLOC_023804-2901-1
TCTTTCGATTAGTCCCCACCAAGCACCAAGTGATCGATCTTTTGTCTCAC
CONSENSUS
TCTTTCGATTAGTCCCCACCAAGCACCAAGTGATCGATCTTTTGTCTCAC

RI-AT4G11830-XLOC_023804-2901-0
TGAACTTGGTTATGCTTGAAATTTGATCTGACAACTAAAAGTTTCCTGCT
RI-AT4G11830-XLOC_023804-2901-1
TGAACTTGGTTATGCTTGAAATTTGATCTGACAACTAAAAGTTTCCTGCT
CONSENSUS
TGAACTTGGTTATGCTTGAAATTTGATCTGACAACTAAAAGTTTCCTGCT

RI-AT4G11830-XLOC_023804-2901-0
TTGGAGGTTTTTGATTTTGTATCATCCAGTTTATAACGAGACTATGTCAA
RI-AT4G11830-XLOC_023804-2901-1
TTGGAGGTTTTTGATTTTGTATCATCCAGTTTATAACGAGACTATGTCAA
CONSENSUS
TTGGAGGTTTTTGATTTTGTATCATCCAGTTTATAACGAGACTATGTCAA

RI-AT4G11830-XLOC_023804-2901-0
TGGGAGGAGGGTCAAACCACGAGTTTGGCCAGTGGCTTGACCAGCAACTC
RI-AT4G11830-XLOC_023804-2901-1
TGGGAGGAGGGTCAAACCACGAGTTTGGCCAGTGGCTTGACCAGCAACTC
CONSENSUS
TGGGAGGAGGGTCAAACCACGAGTTTGGCCAGTGGCTTGACCAGCAACTC

RI-AT4G11830-XLOC_023804-2901-0
GTTCCGTTAGCTACGAGTAGTGGCTCTTTGATGGTTGAATTGTTACATGG
RI-AT4G11830-XLOC_023804-2901-1
GTTCCGTTAGCTACGAGTAGTGGCTCTTTGATGGTTGAATTGTTACATG-
CONSENSUS
GTTCCGTTAGCTACGAGTAGTGGCTCTTTGATGGTTGAATTGTTACATG.

RI-AT4G11830-XLOC_023804-2901-0
TAACTTAGACATTTGGGTTAAGGAAGCTAAACATCTTCCTAACATGATAT
RI-AT4G11830-XLOC_023804-2901-1

CONSENSUS
....

RI-AT4G11830-XLOC_023804-2901-0
GTTACCGTAACAAGCTTGTTGGTGGGATTTCGTTTTCTGAGTTAGGTCGG
RI-AT4G11830-XLOC_023804-2901-1
---GTCGG
CONSENSUS
....GTCGG

RI-AT4G11830-XLOC_023804-2901-0
AGGATTCGTAAAGTGGATGGTGAGAAGTCTTCTAAGTTCACAAGTGATCC
RI-AT4G11830-XLOC_023804-2901-1
AGGATTCGTAAAGTGGATGGTGAGAAGTCTTCTAAGTTCACAAGTGATCC
CONSENSUS
AGGATTCGTAAAGTGGATGGTGAGAAGTCTTCTAAGTTCACAAGTGATCC

RI-AT4G11830-XLOC_023804-2901-0
TTATGTTACTGTCTCTATCTCTGGTGCTGTCATTGGTAGAACTTTTGTTA
RI-AT4G11830-XLOC_023804-2901-1
TTATGTTACTGTCTCTATCTCTGGTGCTGTCATTGGTAGAACTTTTGTTA
CONSENSUS
TTATGTTACTGTCTCTATCTCTGGTGCTGTCATTGGTAGAACTTTTGTTA

RI-AT4G11830-XLOC_023804-2901-0
TTAGCAATAGTGAGAATCCTGTGTGGATGCAGCATTTCGATGTACCCGTT
RI-AT4G11830-XLOC_023804-2901-1
TTAGCAATAGTGAGAATCCTGTGTGGATGCAGCATTTCGATGTACCCGTT
CONSENSUS
TTAGCAATAGTGAGAATCCTGTGTGGATGCAGCATTTCGATGTACCCGTT

RI-AT4G11830-XLOC_023804-2901-0
GCTCATAGTGCTGCTGAAGTACATTTTGTTGTGAAAGACAATGATCCTAT
RI-AT4G11830-XLOC_023804-2901-1
GCTCATAGTGCTGCTGAAGTACATTTTGTTGTGAAAGACAATGATCCTAT
CONSENSUS
GCTCATAGTGCTGCTGAAGTACATTTTGTTGTGAAAGACAATGATCCTAT

RI-AT4G11830-XLOC_023804-2901-0
TGGATCAAAGATCATAGGAGTTGTTGGAATACCAACCAAGCAGTTGTGTT
RI-AT4G11830-XLOC_023804-2901-1
TGGATCAAAGATCATAGGAGTTGTTGGAATACCAACCAAGCAGTTGTGTT
CONSENSUS
TGGATCAAAGATCATAGGAGTTGTTGGAATACCAACCAAGCAGTTGTGTT

RI-AT4G11830-XLOC_023804-2901-0
CCGGGAATAGAATCGAAGGGCTGTTTCCGATACTTAACAGTAGTGGGAAG
RI-AT4G11830-XLOC_023804-2901-1
CCGGGAATAGAATCGAAGGGCTGTTTCCGATACTTAACAGTAGTGGGAAG
CONSENSUS
CCGGGAATAGAATCGAAGGGCTGTTTCCGATACTTAACAGTAGTGGGAAG

RI-AT4G11830-XLOC_023804-2901-0
CCTTGTAGAAAAGGTGCTATGTTGAGTCTGTCTATTCAGTATACTCCAAT
RI-AT4G11830-XLOC_023804-2901-1
CCTTGTAGAAAAGGTGCTATGTTGAGTCTGTCTATTCAGTATACTCCAAT
CONSENSUS
CCTTGTAGAAAAGGTGCTATGTTGAGTCTGTCTATTCAGTATACTCCAAT

RI-AT4G11830-XLOC_023804-2901-0
GGAAAGAATGAGACTTTACCAAAAGGGTGTTGGTTCTGGTGTTGAGTGTG
RI-AT4G11830-XLOC_023804-2901-1
GGAAAGAATGAGACTTTACCAAAAGGGTGTTGGTTCTGGTGTTGAGTGTG
CONSENSUS
GGAAAGAATGAGACTTTACCAAAAGGGTGTTGGTTCTGGTGTTGAGTGTG

RI-AT4G11830-XLOC_023804-2901-0
TAGGAGTTCCCGGTACATACTTCCCTTTGAGGAAAGGCGGTAGGGTTACT
RI-AT4G11830-XLOC_023804-2901-1
TAGGAGTTCCCGGTACATACTTCCCTTTGAGGAAAGGCGGTAGGGTTACT
CONSENSUS
TAGGAGTTCCCGGTACATACTTCCCTTTGAGGAAAGGCGGTAGGGTTACT

RI-AT4G11830-XLOC_023804-2901-0
CTTTATCAGGATGCTCATGTCGATGACGGTACTCTTCCGAGTGTACATCT
RI-AT4G11830-XLOC_023804-2901-1
CTTTATCAGGATGCTCATGTCGATGACGGTACTCTTCCGAGTGTACATCT
CONSENSUS
CTTTATCAGGATGCTCATGTCGATGACGGTACTCTTCCGAGTGTACATCT

RI-AT4G11830-XLOC_023804-2901-0
TGATGGTGGGATTCAGTATAGACATGGAAAATGCTGGGAGGATATGGCTG
RI-AT4G11830-XLOC_023804-2901-1
TGATGGTGGGATTCAGTATAGACATGGAAAATGCTGGGAGGATATGGCTG
CONSENSUS
TGATGGTGGGATTCAGTATAGACATGGAAAATGCTGGGAGGATATGGCTG

RI-AT4G11830-XLOC_023804-2901-0
ATGCGATACGACGGGCAAGGAGGCTGATTTATATCACAGGTTGGTCAGTT
RI-AT4G11830-XLOC_023804-2901-1
ATGCGATACGACGGGCAAGGAGGCTGATTTATATCACAGGTTGGTCAGTT
CONSENSUS
ATGCGATACGACGGGCAAGGAGGCTGATTTATATCACAGGTTGGTCAGTT

RI-AT4G11830-XLOC_023804-2901-0
TTCCATCCGGTTAGGCTGGTTCGTCGTAACAATGATCCGACCGAAGGTAC
RI-AT4G11830-XLOC_023804-2901-1
TTCCATCCGGTTAGGCTGGTTCGTCGTAACAATGATCCGACCGAAGGTAC
CONSENSUS
TTCCATCCGGTTAGGCTGGTTCGTCGTAACAATGATCCGACCGAAGGTAC

```
RI-AT4G11830-XLOC_023804-2901-0
      ATTAGGGGAGTTACTTAAAGTCAAATCTCAAGAAGGTGTTAGAGTGTTGG
RI-AT4G11830-XLOC_023804-2901-1
      ATTAGGGGAGTTACTTAAAGTCAAATCTCAAGAAGGTGTTAGAGTGTTGG
CONSENSUS
      ATTAGGGGAGTTACTTAAAGTCAAATCTCAAGAAGGTGTTAGAGTGTTGG

RI-AT4G11830-XLOC_023804-2901-0
      TTTTGGTGTGGGATGATCCAACTTCAATGAGTTTTCCGGGATTCAGTACA
RI-AT4G11830-XLOC_023804-2901-1
      TTTTGGTGTGGGATGATCCAACTTCAATGAGTTTTCCGGGATTCAGTACA
CONSENSUS
      TTTTGGTGTGGGATGATCCAACTTCAATGAGTTTTCCGGGATTCAGTACA



alignment for event: RI-AT4G39270-XLOC_022886-2201

RI-AT4G39270-XLOC_022886-2201-0
      GTCTTCTCAAGAAAGTGTTCCTGGTGAGTTTATCTTCAAATGCATAAAAC
RI-AT4G39270-XLOC_022886-2201-1
      GTCTTCTCAAGAAAGTGTTCCTG---------------------------
CONSENSUS
      GTCTTCTCAAGAAAGTGTTCCTG...........................

RI-AT4G39270-XLOC_022886-2201-0
      AAGCTTGCTAATTTCTTAACTATTCTAACAATCCCTAAAATTCCCAGGGT
RI-AT4G39270-XLOC_022886-2201-1
      -----------------------------------------------GGT
CONSENSUS
      ...............................................GGT

RI-AT4G39270-XLOC_022886-2201-0
      CTGCAGCTACAGCAACATGTGCCTACGATGTGTACTGCTTCGGCAAGATA
RI-AT4G39270-XLOC_022886-2201-1
      CTGCAGCTACAGCAACATGTGCCTACGATGTGTACTGCTTCGGCAAGATA
CONSENSUS
      CTGCAGCTACAGCAACATGTGCCTACGATGTGTACTGCTTCGGCAAGATA

RI-AT4G39270-XLOC_022886-2201-0
      CTTCTGGAGCTTATCACAGGGAAACTCGGAATCAGCTCATGTAAAGAAAC
RI-AT4G39270-XLOC_022886-2201-1
      CTTCTGGAGCTTATCACAGGGAAACTCGGAATCAGCTCATGTAAAGAAAC
CONSENSUS
      CTTCTGGAGCTTATCACAGGGAAACTCGGAATCAGCTCATGTAAAGAAAC

RI-AT4G39270-XLOC_022886-2201-0
      CCAATTCAAAAAAATCCTAACCGAGATCATGCCTTACATTTCATCGCAAG
RI-AT4G39270-XLOC_022886-2201-1
      CCAATTCAAAAAAATCCTAACCGAGATCATGCCTTACATTTCATCGCAAG
CONSENSUS
      CCAATTCAAAAAAATCCTAACCGAGATCATGCCTTACATTTCATCGCAAG

RI-AT4G39270-XLOC_022886-2201-0
      AGAAAGAACCCGTAATGAACATTCTAGACCAATCTCTTCTGGTCGACGAA
RI-AT4G39270-XLOC_022886-2201-1
```

AGAAAGAACCCGTAATGAACATTCTAGACCAATCTCTTCTGGTCGACGAA
CONSENSUS
AGAAAGAACCCGTAATGAACATTCTAGACCAATCTCTTCTGGTCGACGAA

RI-AT4G39270-XLOC_022886-2201-0
GACCTCTTAGAAGAAGTCTGGGCAATGGCAATCGTCGCTAGATCTTGTCT
RI-AT4G39270-XLOC_022886-2201-1
GACCTCTTAGAAGAAGTCTGGGCAATGGCAATCGTCGCTAGATCTTGTCT
CONSENSUS
GACCTCTTAGAAGAAGTCTGGGCAATGGCAATCGTCGCTAGATCTTGTCT

RI-AT4G39270-XLOC_022886-2201-0
TAACCCAAAACCAACGAGACGGCCGCTTATGAGACACATAGTACAAGCCT
RI-AT4G39270-XLOC_022886-2201-1
TAACCCAAAACCAACGAGACGGCCGCTTATGAGACACATAGTACAAGCCT
CONSENSUS
TAACCCAAAACCAACGAGACGGCCGCTTATGAGACACATAGTACAAGCCT

RI-AT4G39270-XLOC_022886-2201-0
TGGAGAATCCACTGAGAGTTGTGAGAGAAGACAGCAGCGAATCTGAGAGA
RI-AT4G39270-XLOC_022886-2201-1
TGGAGAATCCACTGAGAGTTGTGAGAGAAGACAGCAGCGAATCTGAGAGA
CONSENSUS
TGGAGAATCCACTGAGAGTTGTGAGAGAAGACAGCAGCGAATCTGAGAGA

RI-AT4G39270-XLOC_022886-2201-0
TTCCGTACGACTGGATCTTCGAGAGGATCTTCGAGTAGCGGTCGAATATT
RI-AT4G39270-XLOC_022886-2201-1
TTCCGTACGACTGGATCTTCGAGAGGATCTTCGAGTAGCGGTCGAATATT
CONSENSUS
TTCCGTACGACTGGATCTTCGAGAGGATCTTCGAGTAGCGGTCGAATATT

RI-AT4G39270-XLOC_022886-2201-0
TGGTAGTTGGAGACAGAGCGTGTCTGATCCTGTCGCAGCGGGAACAAGTA
RI-AT4G39270-XLOC_022886-2201-1
TGGTAGTTGGAGACAGAGCGTGTCTGATCCTGTCGCAGCGGGAACAAGTA
CONSENSUS
TGGTAGTTGGAGACAGAGCGTGTCTGATCCTGTCGCAGCGGGAACAAGTA

RI-AT4G39270-XLOC_022886-2201-0
GCCTTCTGTCTCAGGCGGAGGGTTTAGCGACTGGATCGTCAGCGAGAGGA
RI-AT4G39270-XLOC_022886-2201-1
GCCTTCTGTCTCAGGCGGAGGGTTTAGCGACTGGATCGTCAGCGAGAGGA
CONSENSUS
GCCTTCTGTCTCAGGCGGAGGGTTTAGCGACTGGATCGTCAGCGAGAGGA

RI-AT4G39270-XLOC_022886-2201-0
AGTAGCCGTGGCGCGTCGAGTCGACGGAGTATGAAAGATGTATAGAAAGA
RI-AT4G39270-XLOC_022886-2201-1
AGTAGCCGTGGCGCGTCGAGTCGACGGAGTATGAAAGATGTATAGAAAGA
CONSENSUS
AGTAGCCGTGGCGCGTCGAGTCGACGGAGTATGAAAGATGTATAGAAAGA

RI-AT4G39270-XLOC_022886-2201-0
AAGACACACACACACACGATCAATATTTATTTTTTGACGTCAATTTTTAA
RI-AT4G39270-XLOC_022886-2201-1

AAGACACACACACACACGATCAATATTTATTTTTTGACGTCAATTTTTAA
CONSENSUS
AAGACACACACACACACGATCAATATTTATTTTTTGACGTCAATTTTTAA

RI-AT4G39270-XLOC_022886-2201-0
TTATTTTTTATGTGTGGTTATGATTTGATCTCTTGTTGATAAAAAATTCC
RI-AT4G39270-XLOC_022886-2201-1
TTATTTTTTATGTGTGGTTATGATTTGATCTCTTGTTGATAAAAAATTCC
CONSENSUS
TTATTTTTTATGTGTGGTTATGATTTGATCTCTTGTTGATAAAAAATTCC

RI-AT4G39270-XLOC_022886-2201-0
CGTTAATTTATAATATTTTTCGATGATCAAACC
RI-AT4G39270-XLOC_022886-2201-1
CGTTAATTTATAATATTTTTCGATGATCAAACC
CONSENSUS
CGTTAATTTATAATATTTTTCGATGATCAAACC

alignment for event: RI-AT4G20380-XLOC_021814-7367

RI-AT4G20380-XLOC_021814-7367-0
AAGTTTTTGGCTTTGAATTGGATTTGGGTTTCGTTCCAAAATCAGCTCTT
RI-AT4G20380-XLOC_021814-7367-1
AAGTTTTTGGCTTTGAATTGGATTTGGGTTTCGTTCCAAAATCAGCTCTT
CONSENSUS
AAGTTTTTGGCTTTGAATTGGATTTGGGTTTCGTTCCAAAATCAGCTCTT

RI-AT4G20380-XLOC_021814-7367-0
TTTGTTAATCAGGTGAGTTTTTAGGTATTTGAATCTCCAATTGCTTCCTT
RI-AT4G20380-XLOC_021814-7367-1
TTTGTTAATCAG--------------------------------------
CONSENSUS
TTTGTTAATCAG......................................

RI-AT4G20380-XLOC_021814-7367-0
GCAATGACTAAGTATTGTGAAATGTTTAGGGTTTCATCTGTGTGGGTCTT
RI-AT4G20380-XLOC_021814-7367-1

CONSENSUS
....

RI-AT4G20380-XLOC_021814-7367-0
GTTTTGAAGCAATTTGTGTGTGTTTGGATGAAAGTAGCAGATATGCAGGA
RI-AT4G20380-XLOC_021814-7367-1
-------------------------------------CAGATATGCAGGA
CONSENSUS
.....................................CAGATATGCAGGA

RI-AT4G20380-XLOC_021814-7367-0
CCAGCTGGTGTGTCATGGTTGTAGGAATTTATTGATGTATCCTAGAGGAG
RI-AT4G20380-XLOC_021814-7367-1
CCAGCTGGTGTGTCATGGTTGTAGGAATTTATTGATGTATCCTAGAGGAG
CONSENSUS
CCAGCTGGTGTGTCATGGTTGTAGGAATTTATTGATGTATCCTAGAGGAG

RI-AT4G20380-XLOC_021814-7367-0
CATCTAATGTGCGTTGTGCGTTATGTAACACTATCAACATGGTTCCTCCT
RI-AT4G20380-XLOC_021814-7367-1
CATCTAATGTGCGTTGTGCGTTATGTAACACTATCAACATGGTTCCTCCT
CONSENSUS
CATCTAATGTGCGTTGTGCGTTATGTAACACTATCAACATGGTTCCTCCT

RI-AT4G20380-XLOC_021814-7367-0 CCTCCTCCACCTCACG
RI-AT4G20380-XLOC_021814-7367-1 CCTCCTCCACCTCACG
CONSENSUS CCTCCTCCACCTCACG

alignment for event: A3-AT4G22890-XLOC_021968-1829

A3-AT4G22890-XLOC_021968-1829-0
ATCTCCTAATTTCTTCCCTCCAAATTTATAATAAAACCAAAAAAAAAAAA
A3-AT4G22890-XLOC_021968-1829-1
ATCTCCTAATTTCTTCCCTCCAAATTTATAATAAAACCAAAAAAAAAAAA
CONSENSUS
ATCTCCTAATTTCTTCCCTCCAAATTTATAATAAAACCAAAAAAAAAAAA

A3-AT4G22890-XLOC_021968-1829-0
AAAAAAAAAAATCAGAAGAAACCTGAGAAGCTCACAGTAAACACATCTTC
A3-AT4G22890-XLOC_021968-1829-1
AAAAAAAAAAATCAGAAGAAACCTGAGAAGCTCACAGTAAACACATCTTC
CONSENSUS
AAAAAAAAAAATCAGAAGAAACCTGAGAAGCTCACAGTAAACACATCTTC

A3-AT4G22890-XLOC_021968-1829-0
AACCACAGGTTCATACTTACTGAAAAAAACAGAGGAAAAAAAGGAGCTCC
A3-AT4G22890-XLOC_021968-1829-1
AACCACAGGTTCATACTTACTGAAAAAAACAGAGGAAAAAAAGGAGCTCC
CONSENSUS
AACCACAGGTTCATACTTACTGAAAAAAACAGAGGAAAAAAAGGAGCTCC

A3-AT4G22890-XLOC_021968-1829-0
CTTTTCTATCTCTAAGGGAAAATGGGTAGCAAGATGTTGTTTAGTTTGAC
A3-AT4G22890-XLOC_021968-1829-1
CTTTTCTATCTCTAAGGGAAAATGGGTAGCAAGATGTTGTTTAGTTTGAC
CONSENSUS
CTTTTCTATCTCTAAGGGAAAATGGGTAGCAAGATGTTGTTTAGTTTGAC

A3-AT4G22890-XLOC_021968-1829-0
AAGTCCTCGACTTTTCTCCGCCGTTTCTCGCAAACCTTCCTCTTCTTTCT
A3-AT4G22890-XLOC_021968-1829-1
AAGTCCTCGACTTTTCTCCGCCGTTTCTCGCAAACCTTCCTCTTCTTTCT
CONSENSUS
AAGTCCTCGACTTTTCTCCGCCGTTTCTCGCAAACCTTCCTCTTCTTTCT

A3-AT4G22890-XLOC_021968-1829-0
CTCCTTCTCCTCCGTCGCCGTCTTCGAGGACTCAATGGACTCAGCTCAGC
A3-AT4G22890-XLOC_021968-1829-1
CTCCTTCTCCTCCGTCGCCGTCTTCGAGGACTCAATGGACTCAGCTCAGC
CONSENSUS

CTCCTTCTCCTCCGTCGCCGTCTTCGAGGACTCAATGGACTCAGCTCAGC

A3-AT4G22890-XLOC_021968-1829-0
CCTGGAAAATCGATTTCTTTGAGAAGAAGAGTCTTCTTGTTGCCTGCTAA
A3-AT4G22890-XLOC_021968-1829-1
CCTGGAAAATCGATTTCTTTGAGAAGAAGAGTCTTCTTGTTGCCTGCTAA
CONSENSUS
CCTGGAAAATCGATTTCTTTGAGAAGAAGAGTCTTCTTGTTGCCTGCTAA

A3-AT4G22890-XLOC_021968-1829-0
AGCCACAACAGAGCAATCAGTAGGAGGAGACAACGTCGATAGCAATGTTT
A3-AT4G22890-XLOC_021968-1829-1 AGCCACAACAGAGCAATCAG---
GAGGAGACAACGTCGATAGCAATGTTT
CONSENSUS
AGCCACAACAGAGCAATCAG...GAGGAGACAACGTCGATAGCAATGTTT

A3-AT4G22890-XLOC_021968-1829-0
TGCCCTATTGTAGCATCAACAAGGCTGAGAAGAAAACAATTGGTGAAATG
A3-AT4G22890-XLOC_021968-1829-1
TGCCCTATTGTAGCATCAACAAGGCTGAGAAGAAAACAATTGGTGAAATG
CONSENSUS
TGCCCTATTGTAGCATCAACAAGGCTGAGAAGAAAACAATTGGTGAAATG

A3-AT4G22890-XLOC_021968-1829-0 GAACAAGAGTTTCTCCAAGCGTTGCAA
A3-AT4G22890-XLOC_021968-1829-1 GAACAAGAGTTTCTCCAAGCGTTGCAA
CONSENSUS GAACAAGAGTTTCTCCAAGCGTTGCAA

alignment for event: RI-AT4G21560-XLOC_024421-5576

RI-AT4G21560-XLOC_024421-5576-0
ACAGAGACAGAGTTCGTTGCCTTGCCTCCGTGTTGTTTTGTTCATACCGC
RI-AT4G21560-XLOC_024421-5576-1
ACAGAGACAGAGTTCGTTGCCTTGCCTCCGTGTTGTTTTGTTCATACCGC
CONSENSUS
ACAGAGACAGAGTTCGTTGCCTTGCCTCCGTGTTGTTTTGTTCATACCGC

RI-AT4G21560-XLOC_024421-5576-0
GTGAAAAGCTTTCATCATCAATTCATCATCATAAAGAGGTGTCGTGTGAG
RI-AT4G21560-XLOC_024421-5576-1
GTGAAAAGCTTTCATCATCAATTCATCATCATAAAGAGGTGTCGTGTGAG
CONSENSUS
GTGAAAAGCTTTCATCATCAATTCATCATCATAAAGAGGTGTCGTGTGAG

RI-AT4G21560-XLOC_024421-5576-0
CACACGCAATCTCCCAATCTCTCTTCTCTCTCTCTCTCTCCAATCTCCAT
RI-AT4G21560-XLOC_024421-5576-1
CACACGCAATCTCCCAATCTCTCTTCTCTCTCTCTCTCTCCAATCTCCAT
CONSENSUS
CACACGCAATCTCCCAATCTCTCTTCTCTCTCTCTCTCTCCAATCTCCAT

RI-AT4G21560-XLOC_024421-5576-0
ATCTTTGTCCTCTATAATCCGGAGAAGTAGCTCTACCATCGAAATCTCCC
RI-AT4G21560-XLOC_024421-5576-1
ATCTTTGTCCTCTATAATCCGGAGAAGTAGCTCTACCATCGAAATCTCCC

CONSENSUS
ATCTTTGTCCTCTATAATCCGGAGAAGTAGCTCTACCATCGAAATCTCCC

RI-AT4G21560-XLOC_024421-5576-0
AACTTGCTTCCTTGTGAGTTTCTCTCTCACTTCATAAATTATGGTTTTTG
RI-AT4G21560-XLOC_024421-5576-1
AACTTGCTTCCTT-------------------------------------
CONSENSUS
AACTTGCTTCCTT.....................................

RI-AT4G21560-XLOC_024421-5576-0
CTTCAGTAGAACGTAGAAATTTGGTATTTTGATTGTTGGGTTTCAATGAT
RI-AT4G21560-XLOC_024421-5576-1

CONSENSUS
....

RI-AT4G21560-XLOC_024421-5576-0
TTAGGGTAAAGATTCATTGGGTTTTCTCCATTTCTAGCTTTAGTGATTAT
RI-AT4G21560-XLOC_024421-5576-1

CONSENSUS
....

RI-AT4G21560-XLOC_024421-5576-0
GATCATGATTTCATATCATCCTCTCTCTCGGTTTTTGTTCATCAATGTCT
RI-AT4G21560-XLOC_024421-5576-1

CONSENSUS
....

RI-AT4G21560-XLOC_024421-5576-0
TCTGGTTCTTGAGATAACCTCCTAAGCTTTTTGGTTTAATGATTCCTCTT
RI-AT4G21560-XLOC_024421-5576-1

CONSENSUS
....

RI-AT4G21560-XLOC_024421-5576-0
TTCTATCTTCTATGATCACCTGCATTTCTCTTGTTTTGGTTAGATGTTAA
RI-AT4G21560-XLOC_024421-5576-1

CONSENSUS
....

RI-AT4G21560-XLOC_024421-5576-0
TCTCCTTAGTGTGATTTATTAACTTGGTACTATTGATTTTGATATCAGCT
RI-AT4G21560-XLOC_024421-5576-1
--CT
CONSENSUS
....CT

RI-AT4G21560-XLOC_024421-5576-0
AGGAAGCTTTTTGCTTTTTGTTTTCGTCTTTGCGCGTGTCCCATTCTCTT
RI-AT4G21560-XLOC_024421-5576-1
AGGAAGCTTTTTGCTTTTTGTTTTCGTCTTTGCGCGTGTCCCATTCTCTT

CONSENSUS
AGGAAGCTTTTTGCTTTTTGTTTTCGTCTTTGCGCGTGTCCCATTCTCTT

RI-AT4G21560-XLOC_024421-5576-0
CTCCAAAGTTCCCTATAATAGTGAAAGCTCATCAAGTTATTTAGCTCTCA
RI-AT4G21560-XLOC_024421-5576-1
CTCCAAAGTTCCCTATAATAGTGAAAGCTCATCAAGTTATTTAGCTCTCA
CONSENSUS
CTCCAAAGTTCCCTATAATAGTGAAAGCTCATCAAGTTATTTAGCTCTCA

RI-AT4G21560-XLOC_024421-5576-0
GTTTCTGAAAGATGGAGGTCAAGTTATGGAATGATAAGCGTGAGAGAGAA
RI-AT4G21560-XLOC_024421-5576-1
GTTTCTGAAAGATGGAGGTCAAGTTATGGAATGATAAGCGTGAGAGAGAA
CONSENSUS
GTTTCTGAAAGATGGAGGTCAAGTTATGGAATGATAAGCGTGAGAGAGAA

RI-AT4G21560-XLOC_024421-5576-0
ATGTATGAGAATTTCGCAGAGCTTTATGCTATCATCAAAGCTACTGAGAA
RI-AT4G21560-XLOC_024421-5576-1
ATGTATGAGAATTTCGCAGAGCTTTATGCTATCATCAAAGCTACTGAGAA
CONSENSUS
ATGTATGAGAATTTCGCAGAGCTTTATGCTATCATCAAAGCTACTGAGAA

RI-AT4G21560-XLOC_024421-5576-0
GCTCGAGAAGGCTTATATTCGTGATCTAATCTCTCCATCTGAATACGAAA
RI-AT4G21560-XLOC_024421-5576-1
GCTCGAGAAGGCTTATATTCGTGATCTAATCTCTCCATCTGAATACGAAA
CONSENSUS
GCTCGAGAAGGCTTATATTCGTGATCTAATCTCTCCATCTGAATACGAAA

RI-AT4G21560-XLOC_024421-5576-0
CCGAGTGCCAGAAACTCATTGTTCACTTCAAGACATTATCTGCATCTCTC
RI-AT4G21560-XLOC_024421-5576-1
CCGAGTGCCAGAAACTCATTGTTCACTTCAAGACATTATCTGCATCTCTC
CONSENSUS
CCGAGTGCCAGAAACTCATTGTTCACTTCAAGACATTATCTGCATCTCTC

RI-AT4G21560-XLOC_024421-5576-0
AAAGATATGGTCCCAAACATTGAGAGATTTGCAGAGACGTACAAGATGGA
RI-AT4G21560-XLOC_024421-5576-1
AAAGATATGGTCCCAAACATTGAGAGATTTGCAGAGACGTACAAGATGGA
CONSENSUS
AAAGATATGGTCCCAAACATTGAGAGATTTGCAGAGACGTACAAGATGGA

RI-AT4G21560-XLOC_024421-5576-0
TTGCTCAGCTGCAGTTTATCGCCTTGTGACTTCTGGTGTTCCAGCTACTG
RI-AT4G21560-XLOC_024421-5576-1
TTGCTCAGCTGCAGTTTATCGCCTTGTGACTTCTGGTGTTCCAGCTACTG
CONSENSUS
TTGCTCAGCTGCAGTTTATCGCCTTGTGACTTCTGGTGTTCCAGCTACTG

RI-AT4G21560-XLOC_024421-5576-0
TGGAGCATAGAGCTGCTGCTTCGGCTTCTACATCAAGCTCTGCTTCTGTT
RI-AT4G21560-XLOC_024421-5576-1
TGGAGCATAGAGCTGCTGCTTCGGCTTCTACATCAAGCTCTGCTTCTGTT

CONSENSUS
TGGAGCATAGAGCTGCTGCTTCGGCTTCTACATCAAGCTCTGCTTCTGTT

RI-AT4G21560-XLOC_024421-5576-0
GTTGCTGAGTGTGTTCAGAACTTCATCACTTCCATGGATTCTTTGAAACT
RI-AT4G21560-XLOC_024421-5576-1
GTTGCTGAGTGTGTTCAGAACTTCATCACTTCCATGGATTCTTTGAAACT
CONSENSUS
GTTGCTGAGTGTGTTCAGAACTTCATCACTTCCATGGATTCTTTGAAACT

RI-AT4G21560-XLOC_024421-5576-0
CAACATGGTTGCTGTTGATCAGGTGTATCCGTTGTTGTCTGATCTCTCGG
RI-AT4G21560-XLOC_024421-5576-1
CAACATGGTTGCTGTTGATCAGGTGTATCCGTTGTTGTCTGATCTCTCGG
CONSENSUS
CAACATGGTTGCTGTTGATCAGGTGTATCCGTTGTTGTCTGATCTCTCGG

RI-AT4G21560-XLOC_024421-5576-0
CTTCTCTTAACAAACTAAGTATATTGCCACCAGATTTCGAGGGGAAGATT
RI-AT4G21560-XLOC_024421-5576-1
CTTCTCTTAACAAACTAAGTATATTGCCACCAGATTTCGAGGGGAAGATT
CONSENSUS
CTTCTCTTAACAAACTAAGTATATTGCCACCAGATTTCGAGGGGAAGATT

RI-AT4G21560-XLOC_024421-5576-0
AAGATGAAGGAATGGCTTTTGAGGCTGTCAAAGATGGGAGCTTCAGATGA
RI-AT4G21560-XLOC_024421-5576-1
AAGATGAAGGAATGGCTTTTGAGGCTGTCAAAGATGGGAGCTTCAGATGA
CONSENSUS
AAGATGAAGGAATGGCTTTTGAGGCTGTCAAAGATGGGAGCTTCAGATGA

RI-AT4G21560-XLOC_024421-5576-0
ACTCACTGAGCAGCAGGCTCGGCAACTTCACTTTGATCTTGAGTCATCCT
RI-AT4G21560-XLOC_024421-5576-1
ACTCACTGAGCAGCAGGCTCGGCAACTTCACTTTGATCTTGAGTCATCCT
CONSENSUS
ACTCACTGAGCAGCAGGCTCGGCAACTTCACTTTGATCTTGAGTCATCCT

RI-AT4G21560-XLOC_024421-5576-0
ATAACTCGTTCATGGCTGCTTTGCCTAATGCTGGTAATTAACCGATGTGC
RI-AT4G21560-XLOC_024421-5576-1
ATAACTCGTTCATGGCTGCTTTGCCTAATGCTGGTAATTAACCGATGTGC
CONSENSUS
ATAACTCGTTCATGGCTGCTTTGCCTAATGCTGGTAATTAACCGATGTGC

RI-AT4G21560-XLOC_024421-5576-0
CACTAGAGACTGATGTGTGTTCGGTTTATTTTGTTTAGTTTGGCTTTATG
RI-AT4G21560-XLOC_024421-5576-1
CACTAGAGACTGATGTGTGTTCGGTTTATTTTGTTTAGTTTGGCTTTATG
CONSENSUS
CACTAGAGACTGATGTGTGTTCGGTTTATTTTGTTTAGTTTGGCTTTATG

RI-AT4G21560-XLOC_024421-5576-0
TAGTTATAATGATGGCTTTAGGAACAAAGATTTTTGTTTCATGACAATTT
RI-AT4G21560-XLOC_024421-5576-1
TAGTTATAATGATGGCTTTAGGAACAAAGATTTTTGTTTCATGACAATTT

```
CONSENSUS
      TAGTTATAATGATGGCTTTAGGAACAAAGATTTTTGTTTCATGACAATTT

RI-AT4G21560-XLOC_024421-5576-0
      GCTACATATATTTTGGGGATGAATGTTCGTAGACAAAACAAGCTTTTCCT
RI-AT4G21560-XLOC_024421-5576-1
      GCTACATATATTTTGGGGATGAATGTTCGTAGACAAAACAAGCTTTTCCT
CONSENSUS
      GCTACATATATTTTGGGGATGAATGTTCGTAGACAAAACAAGCTTTTCCT

RI-AT4G21560-XLOC_024421-5576-0
      TTTGTTGTGTAAAAGATGATGCCAATCCAGAAATTGTTTTTTTCATGAAG
RI-AT4G21560-XLOC_024421-5576-1
      TTTGTTGTGTAAAAGATGATGCCAATCCAGAAATTGTTTTTTTCATGAAG
CONSENSUS
      TTTGTTGTGTAAAAGATGATGCCAATCCAGAAATTGTTTTTTTCATGAAG

RI-AT4G21560-XLOC_024421-5576-0
      TGTAATGTATGAATCTAACTCTTTTGTGCTGATTATTGGCTTTATATCGT
RI-AT4G21560-XLOC_024421-5576-1
      TGTAATGTATGAATCTAACTCTTTTGTGCTGATTATTGGCTTTATATCGT
CONSENSUS
      TGTAATGTATGAATCTAACTCTTTTGTGCTGATTATTGGCTTTATATCGT

RI-AT4G21560-XLOC_024421-5576-0  TACATGGGTGGCTTTAT
RI-AT4G21560-XLOC_024421-5576-1  TACATGGGTGGCTTTAT
CONSENSUS                        TACATGGGTGGCTTTAT


alignment for event: A3-AT4G36050-XLOC_025242-5605

A3-AT4G36050-XLOC_025242-5605-0
      TTGTTTTCAATGTTTATGGGCCGCGAGCTGTAGCTGATGATGCTGATAGG
A3-AT4G36050-XLOC_025242-5605-1
      TTGTTTTCAATGTTTATGGGCCGCGAGCTGTAGCTGATGATGCTGATAGG
CONSENSUS
      TTGTTTTCAATGTTTATGGGCCGCGAGCTGTAGCTGATGATGCTGATAGG

A3-AT4G36050-XLOC_025242-5605-0
      ATCGAGTTTAAGCATCGGTTCTATGGTGTTTTAGAG-----ATGGGAGTG
A3-AT4G36050-XLOC_025242-5605-1
      ATCGAGTTTAAGCATCGGTTCTATGGTGTTTTAGAGAGAAGATGGGAGTG
CONSENSUS
      ATCGAGTTTAAGCATCGGTTCTATGGTGTTTTAGAG.....ATGGGAGTG

A3-AT4G36050-XLOC_025242-5605-0
      TCTTCTGCGTCAAGGAAGGAGGGTATTTGTTGTTGGGGATCTCAACATTG
A3-AT4G36050-XLOC_025242-5605-1
      TCTTCTGCGTCAAGGAAGGAGGGTATTTGTTGTTGGGGATCTCAACATTG
CONSENSUS
      TCTTCTGCGTCAAGGAAGGAGGGTATTTGTTGTTGGGGATCTCAACATTG

A3-AT4G36050-XLOC_025242-5605-0
      CTCCTTTTGCTATGGATCGATGTGAAGCTGGGCCTGATTTTGAGAAAAAC
A3-AT4G36050-XLOC_025242-5605-1
```

```
      CTCCTTTTGCTATGGATCGATGTGAAGCTGGGCCTGATTTTGAGAAAAAC
CONSENSUS
      CTCCTTTTGCTATGGATCGATGTGAAGCTGGGCCTGATTTTGAGAAAAAC

A3-AT4G36050-XLOC_025242-5605-0  GA
A3-AT4G36050-XLOC_025242-5605-1  GA
CONSENSUS                        GA



alignment for event: A3-AT4G18140-XLOC_024238-5530

A3-AT4G18140-XLOC_024238-5530-0
      GTAATAACTAATAAGCTTCTCTCATTCGTGGAATTGTCTTTCAGAAAGAA
A3-AT4G18140-XLOC_024238-5530-1
      GTAATAACTAATAAGCTTCTCTCATTCGTGGAATTGTCTTTCAGAAAGAA
CONSENSUS
      GTAATAACTAATAAGCTTCTCTCATTCGTGGAATTGTCTTTCAGAAAGAA

A3-AT4G18140-XLOC_024238-5530-0
      AAAAACCAGAGAGGAGGAAGAAAAAAAAAACTCTTGCTTCTTCAG-----
A3-AT4G18140-XLOC_024238-5530-1
      AAAAACCAGAGAGGAGGAAGAAAAAAAAAACTCTTGCTTCTTCAGTATCT
CONSENSUS
      AAAAACCAGAGAGGAGGAAGAAAAAAAAAACTCTTGCTTCTTCAG.....

A3-AT4G18140-XLOC_024238-5530-0
      ------------------------------------CTTGTTGTTGTATT
A3-AT4G18140-XLOC_024238-5530-1
      GCTGCTGGTTTGATCTTGAGACCATTTCTTTTGCAGCTTGTTGTTGTATT
CONSENSUS
      ....................................CTTGTTGTTGTATT

A3-AT4G18140-XLOC_024238-5530-0
      TCTAGAGACTATGCCAATGCCATTTTTGAAAATGAAGAGCAAGATAAGCA
A3-AT4G18140-XLOC_024238-5530-1
      TCTAGAGACTATGCCAATGCCATTTTTGAAAATGAAGAGCAAGATAAGCA
CONSENSUS
      TCTAGAGACTATGCCAATGCCATTTTTGAAAATGAAGAGCAAGATAAGCA

A3-AT4G18140-XLOC_024238-5530-0
      AGGACTCGTTAAGAGAGAAAAGAGTTCTTGGTGTTTGTAAGAAACCTCAT
A3-AT4G18140-XLOC_024238-5530-1
      AGGACTCGTTAAGAGAGAAAAGAGTTCTTGGTGTTTGTAAGAAACCTCAT
CONSENSUS
      AGGACTCGTTAAGAGAGAAAAGAGTTCTTGGTGTTTGTAAGAAACCTCAT

A3-AT4G18140-XLOC_024238-5530-0
      AAGGATGTTAAAAATTCATGCTTTGATCCCAAAATATCGGAGAGAATGGA
A3-AT4G18140-XLOC_024238-5530-1
      AAGGATGTTAAAAATTCATGCTTTGATCCCAAAATATCGGAGAGAATGGA
CONSENSUS
      AAGGATGTTAAAAATTCATGCTTTGATCCCAAAATATCGGAGAGAATGGA

A3-AT4G18140-XLOC_024238-5530-0
      ACTTTTGGTCACTGCTACTCAGAATTGCAACAACAACGAGGAACAGAATA
```

```
A3-AT4G18140-XLOC_024238-5530-1
      ACTTTTGGTCACTGCTACTCAGAATTGCAACAACAACGAGGAACAGAATA
CONSENSUS
      ACTTTTGGTCACTGCTACTCAGAATTGCAACAACAACGAGGAACAGAATA

A3-AT4G18140-XLOC_024238-5530-0  TAG
A3-AT4G18140-XLOC_024238-5530-1  TAG
CONSENSUS                        TAG



alignment for event: RI-AT4G21560-XLOC_024421-5577

RI-AT4G21560-XLOC_024421-5577-0
      AGACAGAGTTCGTTGCCTTGCCTCCGTGTTGTTTTGTTCATACCGCGTGA
RI-AT4G21560-XLOC_024421-5577-1
      AGACAGAGTTCGTTGCCTTGCCTCCGTGTTGTTTTGTTCATACCGCGTGA
CONSENSUS
      AGACAGAGTTCGTTGCCTTGCCTCCGTGTTGTTTTGTTCATACCGCGTGA

RI-AT4G21560-XLOC_024421-5577-0
      AAAGCTTTCATCATCAATTCATCATCATAAAGAGGTGTCGTGTGAGCACA
RI-AT4G21560-XLOC_024421-5577-1
      AAAGCTTTCATCATCAATTCATCATCATAAAGAGGTGTCGTGTGAGCACA
CONSENSUS
      AAAGCTTTCATCATCAATTCATCATCATAAAGAGGTGTCGTGTGAGCACA

RI-AT4G21560-XLOC_024421-5577-0
      CGCAATCTCCCAATCTCTCTTCTCTCTCTCTCTCTCCAATCTCCATATCT
RI-AT4G21560-XLOC_024421-5577-1
      CGCAATCTCCCAATCTCTCTTCTCTCTCTCTCTCTCCAATCTCCATATCT
CONSENSUS
      CGCAATCTCCCAATCTCTCTTCTCTCTCTCTCTCTCCAATCTCCATATCT

RI-AT4G21560-XLOC_024421-5577-0
      TTGTCCTCTATAATCCGGAGAAGTAGCTCTACCATCGAAATCTCCCAACT
RI-AT4G21560-XLOC_024421-5577-1
      TTGTCCTCTATAATCCGGAGAAGTAGCTCTACCATCGAAATCTCCCAACT
CONSENSUS
      TTGTCCTCTATAATCCGGAGAAGTAGCTCTACCATCGAAATCTCCCAACT

RI-AT4G21560-XLOC_024421-5577-0
      TGCTTCCTTGTGAGTTTCTCTCTCACTTCATAAATTATGGTTTTTGCTTC
RI-AT4G21560-XLOC_024421-5577-1
      TGCTTCCTT-----------------------------------------
CONSENSUS
      TGCTTCCTT.........................................

RI-AT4G21560-XLOC_024421-5577-0
      AGTAGAACGTAGAAATTTGGTATTTTGATTGTTGGGTTTCAATGATTTAG
RI-AT4G21560-XLOC_024421-5577-1
      --------------------------------------------------
CONSENSUS
      ..................................................

RI-AT4G21560-XLOC_024421-5577-0
```

GGTAAAGATTCATTGGGTTTTCTCCATTTCTAGCTTTAGTGATTATGATC
RI-AT4G21560-XLOC_024421-5577-1

CONSENSUS
....

RI-AT4G21560-XLOC_024421-5577-0
ATGATTTCATATCATCCTCTCTCTCGGTTTTTGTTCATCAATGTCTTCTG
RI-AT4G21560-XLOC_024421-5577-1

CONSENSUS
....

RI-AT4G21560-XLOC_024421-5577-0
GTTCTTGAGATAACCTCCTAAGCTTTTTGGTTTAATGATTCCTCTTTTCT
RI-AT4G21560-XLOC_024421-5577-1

CONSENSUS
....

RI-AT4G21560-XLOC_024421-5577-0
ATCTTCTATGATCACCTGCATTTCTCTTGTTTTGGTTAGATGTTAATCTC
RI-AT4G21560-XLOC_024421-5577-1

CONSENSUS
....

RI-AT4G21560-XLOC_024421-5577-0
CTTAGTGTGATTTATTAACTTGGTACTATTGATTTTGATATCAGCTAGGA
RI-AT4G21560-XLOC_024421-5577-1
--CTAGGA
CONSENSUS
....CTAGGA

RI-AT4G21560-XLOC_024421-5577-0
AGCTTTTTGCTTTTTGTTTTCGTCTTTGCGCGTGTCCCATTCTCTTCTCC
RI-AT4G21560-XLOC_024421-5577-1
AGCTTTTTGCTTTTTGTTTTCGTCTTTGCGCGTGTCCCATTCTCTTCTCC
CONSENSUS
AGCTTTTTGCTTTTTGTTTTCGTCTTTGCGCGTGTCCCATTCTCTTCTCC

RI-AT4G21560-XLOC_024421-5577-0
AAAGTTCCCTATAATAGTGAAAGCTCATCAAGTTATTTAGCTCTCAGTTT
RI-AT4G21560-XLOC_024421-5577-1
AAAGTTCCCTATAATAGTGAAAGCTCATCAAGTTATTTAGCTCTCAGTTT
CONSENSUS
AAAGTTCCCTATAATAGTGAAAGCTCATCAAGTTATTTAGCTCTCAGTTT

RI-AT4G21560-XLOC_024421-5577-0
CTGAAAGATGGAGGTCAAGTTATGGAATGATAAGCGTGAGAGAGAAATGT
RI-AT4G21560-XLOC_024421-5577-1
CTGAAAGATGGAGGTCAAGTTATGGAATGATAAGCGTGAGAGAGAAATGT
CONSENSUS
CTGAAAGATGGAGGTCAAGTTATGGAATGATAAGCGTGAGAGAGAAATGT

RI-AT4G21560-XLOC_024421-5577-0

ATGAGAATTTCGCAGAGCTTTATGCTATCATCAAAGCTACTGAGAAGCTC
RI-AT4G21560-XLOC_024421-5577-1
ATGAGAATTTCGCAGAGCTTTATGCTATCATCAAAGCTACTGAGAAGCTC
CONSENSUS
ATGAGAATTTCGCAGAGCTTTATGCTATCATCAAAGCTACTGAGAAGCTC

RI-AT4G21560-XLOC_024421-5577-0
GAGAAGGCTTATATTCGTGATCTAATCTCTCCATCTGAATACGAAACCGA
RI-AT4G21560-XLOC_024421-5577-1
GAGAAGGCTTATATTCGTGATCTAATCTCTCCATCTGAATACGAAACCGA
CONSENSUS
GAGAAGGCTTATATTCGTGATCTAATCTCTCCATCTGAATACGAAACCGA

RI-AT4G21560-XLOC_024421-5577-0
GTGCCAGAAACTCATTGTTCACTTCAAGACATTATCTGCATCTCTCAAAG
RI-AT4G21560-XLOC_024421-5577-1
GTGCCAGAAACTCATTGTTCACTTCAAGACATTATCTGCATCTCTCAAAG
CONSENSUS
GTGCCAGAAACTCATTGTTCACTTCAAGACATTATCTGCATCTCTCAAAG

RI-AT4G21560-XLOC_024421-5577-0
ATATGGTCCCAAACATTGAGAGATTTGCAGAGACGTACAAGATGGATTGC
RI-AT4G21560-XLOC_024421-5577-1
ATATGGTCCCAAACATTGAGAGATTTGCAGAGACGTACAAGATGGATTGC
CONSENSUS
ATATGGTCCCAAACATTGAGAGATTTGCAGAGACGTACAAGATGGATTGC

RI-AT4G21560-XLOC_024421-5577-0
TCAGCTGCAGTTTATCGCCTTGTGACTTCTGGTGTTCCAGCTACTGTGGA
RI-AT4G21560-XLOC_024421-5577-1
TCAGCTGCAGTTTATCGCCTTGTGACTTCTGGTGTTCCAGCTACTGTGGA
CONSENSUS
TCAGCTGCAGTTTATCGCCTTGTGACTTCTGGTGTTCCAGCTACTGTGGA

RI-AT4G21560-XLOC_024421-5577-0
GCATAGAGCTGCTGCTTCGGCTTCTACATCAAGCTCTGCTTCTGTTGTTG
RI-AT4G21560-XLOC_024421-5577-1
GCATAGAGCTGCTGCTTCGGCTTCTACATCAAGCTCTGCTTCTGTTGTTG
CONSENSUS
GCATAGAGCTGCTGCTTCGGCTTCTACATCAAGCTCTGCTTCTGTTGTTG

RI-AT4G21560-XLOC_024421-5577-0
CTGAGTGTGTTCAGAACTTCATCACTTCCATGGATTCTTTGAAACTCAAC
RI-AT4G21560-XLOC_024421-5577-1
CTGAGTGTGTTCAGAACTTCATCACTTCCATGGATTCTTTGAAACTCAAC
CONSENSUS
CTGAGTGTGTTCAGAACTTCATCACTTCCATGGATTCTTTGAAACTCAAC

RI-AT4G21560-XLOC_024421-5577-0
ATGGTTGCTGTTGATCAGGTGTATCCGTTGTTGTCTGATCTCTCGGCTTC
RI-AT4G21560-XLOC_024421-5577-1
ATGGTTGCTGTTGATCAGGTGTATCCGTTGTTGTCTGATCTCTCGGCTTC
CONSENSUS
ATGGTTGCTGTTGATCAGGTGTATCCGTTGTTGTCTGATCTCTCGGCTTC

RI-AT4G21560-XLOC_024421-5577-0

TCTTAACAAACTAAGTATATTGCCACCAGATTTCGAGGGGAAGATTAAGA
RI-AT4G21560-XLOC_024421-5577-1
TCTTAACAAACTAAGTATATTGCCACCAGATTTCGAGGGGAAGATTAAGA
CONSENSUS
TCTTAACAAACTAAGTATATTGCCACCAGATTTCGAGGGGAAGATTAAGA

RI-AT4G21560-XLOC_024421-5577-0
TGAAGGAATGGCTTTTGAGGCTGTCAAAGATGGGAGCTTCAGATGAACTC
RI-AT4G21560-XLOC_024421-5577-1
TGAAGGAATGGCTTTTGAGGCTGTCAAAGATGGGAGCTTCAGATGAACTC
CONSENSUS
TGAAGGAATGGCTTTTGAGGCTGTCAAAGATGGGAGCTTCAGATGAACTC

RI-AT4G21560-XLOC_024421-5577-0
ACTGAGCAGCAGGCTCGGCAACTTCACTTTGATCTTGAGTCATCCTATAA
RI-AT4G21560-XLOC_024421-5577-1
ACTGAGCAGCAGGCTCGGCAACTTCACTTTGATCTTGAGTCATCCTATAA
CONSENSUS
ACTGAGCAGCAGGCTCGGCAACTTCACTTTGATCTTGAGTCATCCTATAA

RI-AT4G21560-XLOC_024421-5577-0
CTCGTTCATGGCTGCTTTGCCTAATGCTGGTAATTAACCGATGTGCCACT
RI-AT4G21560-XLOC_024421-5577-1
CTCGTTCATGGCTGCTTTGCCTAATGCTGGTAATTAACCGATGTGCCACT
CONSENSUS
CTCGTTCATGGCTGCTTTGCCTAATGCTGGTAATTAACCGATGTGCCACT

RI-AT4G21560-XLOC_024421-5577-0
AGAGACTGATGTGTGTTCGGTTTATTTTGTTTAGTTTGGCTTTATGTAGT
RI-AT4G21560-XLOC_024421-5577-1
AGAGACTGATGTGTGTTCGGTTTATTTTGTTTAGTTTGGCTTTATGTAGT
CONSENSUS
AGAGACTGATGTGTGTTCGGTTTATTTTGTTTAGTTTGGCTTTATGTAGT

RI-AT4G21560-XLOC_024421-5577-0
TATAATGATGGCTTTAGGAACAAAGATTTTTGTTTCATGACAATTTGCTA
RI-AT4G21560-XLOC_024421-5577-1
TATAATGATGGCTTTAGGAACAAAGATTTTTGTTTCATGACAATTTGCTA
CONSENSUS
TATAATGATGGCTTTAGGAACAAAGATTTTTGTTTCATGACAATTTGCTA

RI-AT4G21560-XLOC_024421-5577-0
CATATATTTTGGGGATGAATGTTCGTAGACAAAACAAGCTTTTCCTTTTG
RI-AT4G21560-XLOC_024421-5577-1
CATATATTTTGGGGATGAATGTTCGTAGACAAAACAAGCTTTTCCTTTTG
CONSENSUS
CATATATTTTGGGGATGAATGTTCGTAGACAAAACAAGCTTTTCCTTTTG

RI-AT4G21560-XLOC_024421-5577-0
TTGTGTAAAAGATGATGCCAATCCAGAAATTGTTTTTTTCATGAAGTGTA
RI-AT4G21560-XLOC_024421-5577-1
TTGTGTAAAAGATGATGCCAATCCAGAAATTGTTTTTTTCATGAAGTGTA
CONSENSUS
TTGTGTAAAAGATGATGCCAATCCAGAAATTGTTTTTTTCATGAAGTGTA

RI-AT4G21560-XLOC_024421-5577-0

```
      ATGTATGAATCTAACTCTTTTGTGCTGATTATTGGCTTTATATCGTTACA
RI-AT4G21560-XLOC_024421-5577-1
      ATGTATGAATCTAACTCTTTTGTGCTGATTATTGGCTTTATATCGTTACA
CONSENSUS
      ATGTATGAATCTAACTCTTTTGTGCTGATTATTGGCTTTATATCGTTACA

RI-AT4G21560-XLOC_024421-5577-0  TGGGTGGCTTTAT
RI-AT4G21560-XLOC_024421-5577-1  TGGGTGGCTTTAT
CONSENSUS                        TGGGTGGCTTTAT
```

alignment for event: A5-AT4G12560-XLOC_021342-7183

```
A5-AT4G12560-XLOC_021342-7183-0
      GGATGATTTCCTGTCCAAAGGATTCAAGCTGGTCTTATAAGCCAAAAACA
A5-AT4G12560-XLOC_021342-7183-1
      GGATGATTTCCTGTCCAAAGGATTCAAGCTGGTCTTATAAGCCAAAAACA
CONSENSUS
      GGATGATTTCCTGTCCAAAGGATTCAAGCTGGTCTTATAAGCCAAAAACA

A5-AT4G12560-XLOC_021342-7183-0
      AGAGACAGGAGCTAAAGCTGCACAAG------------------------
A5-AT4G12560-XLOC_021342-7183-1
      AGAGACAGGAGCTAAAGCTGCACAAGGTAAGTGAAAAAGTTGACCGTTCT
CONSENSUS
      AGAGACAGGAGCTAAAGCTGCACAAG........................

A5-AT4G12560-XLOC_021342-7183-0
      --------------------------------------------------
A5-AT4G12560-XLOC_021342-7183-1
      TAATGGGTCTAGTTTGCTGCTGCATTCTTATTGCATTTCATTCTTTTCTG
CONSENSUS
      ..................................................

A5-AT4G12560-XLOC_021342-7183-0
      --------------------------------------------------
A5-AT4G12560-XLOC_021342-7183-1
      TCTCTGCTCTGTTTTGTCGCGGACGTTCATATCCGGTGTCTGTTTTGTGG
CONSENSUS
      ..................................................

A5-AT4G12560-XLOC_021342-7183-0
      --------------------------------------------------
A5-AT4G12560-XLOC_021342-7183-1
      CGGACGAGCAATTTTCATATTCGGGTTGGGACAAGAGTGAAAGTCACAGT
CONSENSUS
      ..................................................

A5-AT4G12560-XLOC_021342-7183-0  ---------
GGAGGAGAAACGAGAAGGGGAACAAACAATAACTTCAACCA
A5-AT4G12560-XLOC_021342-7183-1
      ATGTCATAGGGAGGAGAAACGAGAAGGGGAACAAACAATAACTTCAACCA
CONSENSUS
      .........GGAGGAGAAACGAGAAGGGGAACAAACAATAACTTCAACCA
```

A5-AT4G12560-XLOC_021342-7183-0
AAAAGCTCTAATATACCAAAGGAGAATAATATACAACTTACCTACGTATA
A5-AT4G12560-XLOC_021342-7183-1
AAAAGCTCTAATATACCAAAGGAGAATAATATACAACTTACCTACGTATA
CONSENSUS
AAAAGCTCTAATATACCAAAGGAGAATAATATACAACTTACCTACGTATA

A5-AT4G12560-XLOC_021342-7183-0
TGTGCTTATAGAAGAGAGGACAAATGTTTTTGGTTTGTAAATAACTCTTT
A5-AT4G12560-XLOC_021342-7183-1
TGTGCTTATAGAAGAGAGGACAAATGTTTTTGGTTTGTAAATAACTCTTT
CONSENSUS
TGTGCTTATAGAAGAGAGGACAAATGTTTTTGGTTTGTAAATAACTCTTT

A5-AT4G12560-XLOC_021342-7183-0
TTAAGTTTGATATATATAACTTACTAGTACTAACGCTTTGTATACTTGCT
A5-AT4G12560-XLOC_021342-7183-1
TTAAGTTTGATATATATAACTTACTAGTACTAACGCTTTGTATACTTGCT
CONSENSUS
TTAAGTTTGATATATATAACTTACTAGTACTAACGCTTTGTATACTTGCT

A5-AT4G12560-XLOC_021342-7183-0
TTTTTAAGTTTCTGCATAATATGAGATGTTAAATAAATTA
A5-AT4G12560-XLOC_021342-7183-1
TTTTTAAGTTTCTGCATAATATGAGATGTTAAATAAATTA
CONSENSUS
TTTTTAAGTTTCTGCATAATATGAGATGTTAAATAAATTA

alignment for event: SE-AT4G33060-XLOC_022558-2450

SE-AT4G33060-XLOC_022558-2450-0
GAAATTGAATTTACTGTCATTCGGAGAAGAAGCTGAAGAAGAGGAAAAGG
SE-AT4G33060-XLOC_022558-2450-1
GAAATTGAATTTACTGTCATTCGGAGAAGAAGCTGAAGAAGAGGAAAAGG
CONSENSUS
GAAATTGAATTTACTGTCATTCGGAGAAGAAGCTGAAGAAGAGGAAAAGG

SE-AT4G33060-XLOC_022558-2450-0
AACTGGCTGTTGTAAAGCAAAAGATTAAGAGCAGTCATGACGTATTGAAT
SE-AT4G33060-XLOC_022558-2450-1
AACTGGCTGTTGTAAAGCAAAAGATTAAGAGCAGTCATGACGTATTGAAT
CONSENSUS
AACTGGCTGTTGTAAAGCAAAAGATTAAGAGCAGTCATGACGTATTGAAT

SE-AT4G33060-XLOC_022558-2450-0
GATCCTCGACTTTTGAAGGCAGAAGCTTCAGATAAAGAGAGG--------
SE-AT4G33060-XLOC_022558-2450-1
GATCCTCGACTTTTGAAGGCAGAAGCTTCAGATAAAGAGAGGGCTAAGCT
CONSENSUS
GATCCTCGACTTTTGAAGGCAGAAGCTTCAGATAAAGAGAGG........

SE-AT4G33060-XLOC_022558-2450-0

SE-AT4G33060-XLOC_022558-2450-1

GTATTAAAAAAACTGAGGCGACTTATTTTATCTGCCATATTGTGGAATAC
CONSENSUS
....

SE-AT4G33060-XLOC_022558-2450-0 -----------------------------
AACGCATCTGAGTCCAAGGAG
SE-AT4G33060-XLOC_022558-2450-1
TAGTGGAGAGTTCCTCGTGGTTGCTATATAACGCATCTGAGTCCAAGGAG
CONSENSUS
.............................AACGCATCTGAGTCCAAGGAG

SE-AT4G33060-XLOC_022558-2450-0
GTACTATCTGTGCGAGAAGCTCTGAATGCTAAGAAAGAAGCGGCTCAAAA
SE-AT4G33060-XLOC_022558-2450-1
GTACTATCTGTGCGAGAAGCTCTGAATGCTAAGAAAGAAGCGGCTCAAAA
CONSENSUS
GTACTATCTGTGCGAGAAGCTCTGAATGCTAAGAAAGAAGCGGCTCAAAA

SE-AT4G33060-XLOC_022558-2450-0
AGATAAAAGCTTTTCTGTATCTGATACAGTTGGAAATAGTGATGATGACG
SE-AT4G33060-XLOC_022558-2450-1
AGATAAAAGCTTTTCTGTATCTGATACAGTTGGAAATAGTGATGATGACG
CONSENSUS
AGATAAAAGCTTTTCTGTATCTGATACAGTTGGAAATAGTGATGATGACG

SE-AT4G33060-XLOC_022558-2450-0
ATGATGGTGAAGATGAGACTAAATTTGATGCAAAGATGAGGAATCAAGTG
SE-AT4G33060-XLOC_022558-2450-1
ATGATGGTGAAGATGAGACTAAATTTGATGCAAAGATGAGGAATCAAGTG
CONSENSUS
ATGATGGTGAAGATGAGACTAAATTTGATGCAAAGATGAGGAATCAAGTG

SE-AT4G33060-XLOC_022558-2450-0
CTCAGCAGAAGGAAGGAGATTGGAGATACGCCTTCAAAGCCGACTCAGAA
SE-AT4G33060-XLOC_022558-2450-1
CTCAGCAGAAGGAAGGAGATTGGAGATACGCCTTCAAAGCCGACTCAGAA
CONSENSUS
CTCAGCAGAAGGAAGGAGATTGGAGATACGCCTTCAAAGCCGACTCAGAA

SE-AT4G33060-XLOC_022558-2450-0 AAAAA
SE-AT4G33060-XLOC_022558-2450-1 AAAAA
CONSENSUS AAAAA

alignment for event: A3-AT4G34900-XLOC_025182-172

A3-AT4G34900-XLOC_025182-172-0
GTTCCTAATGCGTCACCTACTGCTGCTTCTGCGAGTTCTGATATGTATGG
A3-AT4G34900-XLOC_025182-172-1
GTTCCTAATGCGTCACCTACTGCTGCTTCTGCGAGTTCTGATATGTATGG
CONSENSUS
GTTCCTAATGCGTCACCTACTGCTGCTTCTGCGAGTTCTGATATGTATGG

A3-AT4G34900-XLOC_025182-172-0
TGCTGCAGTTTTAGACGCTTGTGAGCAGATTATAGCAAGAATGGAGCCTG

A3-AT4G34900-XLOC_025182-172-1
      TGCTGCAGTTTTAGACGCTTGTGAGCAGATTATAGCAAGAATGGAGCCTG
CONSENSUS
      TGCTGCAGTTTTAGACGCTTGTGAGCAGATTATAGCAAGAATGGAGCCTG

A3-AT4G34900-XLOC_025182-172-0
      TTGCATCTAAACACAATTTCAACACATTCTCTGAG---------------
A3-AT4G34900-XLOC_025182-172-1
      TTGCATCTAAACACAATTTCAACACATTCTCTGAGCTAGCAAGTGCCTGC
CONSENSUS
      TTGCATCTAAACACAATTTCAACACATTCTCTGAG...............

A3-AT4G34900-XLOC_025182-172-0
      ---A
A3-AT4G34900-XLOC_025182-172-1
      TACTTTCAACGTATAGACCTATCAGCTCACGGTTTTCACATTGTTCCAGA
CONSENSUS
      ....A

A3-AT4G34900-XLOC_025182-172-0
      ACTTGAATTTGATTGGGTATCTGGAAAAGGGAACGCATATAGATATTACA
A3-AT4G34900-XLOC_025182-172-1
      ACTTGAATTTGATTGGGTATCTGGAAAAGGGAACGCATATAGATATTACA
CONSENSUS
      ACTTGAATTTGATTGGGTATCTGGAAAAGGGAACGCATATAGATATTACA

A3-AT4G34900-XLOC_025182-172-0
      CATATGGAGCTGCCTTTGCTGAAGTTGAGATAGATACATTGACTGGTGAT
A3-AT4G34900-XLOC_025182-172-1
      CATATGGAGCTGCCTTTGCTGAAGTTGAGATAGATACATTGACTGGTGAT
CONSENSUS
      CATATGGAGCTGCCTTTGCTGAAGTTGAGATAGATACATTGACTGGTGAT

A3-AT4G34900-XLOC_025182-172-0
      TTTCACACAAGAAAAGCAGACATAATGTTGGATCTCGGATATTCTCTTAA
A3-AT4G34900-XLOC_025182-172-1
      TTTCACACAAGAAAAGCAGACATAATGTTGGATCTCGGATATTCTCTTAA
CONSENSUS
      TTTCACACAAGAAAAGCAGACATAATGTTGGATCTCGGATATTCTCTTAA

A3-AT4G34900-XLOC_025182-172-0   CCCAACCATTGATATTGGACAA
A3-AT4G34900-XLOC_025182-172-1   CCCAACCATTGATATTGGACAA
CONSENSUS   CCCAACCATTGATATTGGACAA

alignment for event: A5-AT4G06701-XLOC_023449-3036

A5-AT4G06701-XLOC_023449-3036-0
      ACAGGAAAGAGGCGTTAAAGATTTGGTGCAAGCTGCGACTCTGTGATCAC
A5-AT4G06701-XLOC_023449-3036-1
      ACAGGAAAGAGGCGTTAAAGATTTGGTGCAAGCTGCGACTCTGTGATCAC
CONSENSUS
      ACAGGAAAGAGGCGTTAAAGATTTGGTGCAAGCTGCGACTCTGTGATCAC

A5-AT4G06701-XLOC_023449-3036-0

AACTCCACACCAAGTATTCTCTTACATCGCACTCGTCGCTCCTGCACTCG
A5-AT4G06701-XLOC_023449-3036-1
AACTCCACACCAA--------------------------------------
CONSENSUS
AACTCCACACCAA......................................

A5-AT4G06701-XLOC_023449-3036-0
TAGTTTCTTTGAATAGCCAATGAAGAGTAAAGTTCAATTATGTGTGTCGT
A5-AT4G06701-XLOC_023449-3036-1

CONSENSUS
....

A5-AT4G06701-XLOC_023449-3036-0
GTTTAGTAATTTGACTTGAAAAAAGAGTTTGCTAGGGTCTCTTGTGAACA
A5-AT4G06701-XLOC_023449-3036-1

CONSENSUS
....

A5-AT4G06701-XLOC_023449-3036-0
AGCTGTTATGTCTGCAATTCGAGCTCTGATGTCAAGCATCTCTACAAGAA
A5-AT4G06701-XLOC_023449-3036-1 --------------------
GAGCTCTGATGTCAAGCATCTCTACAAGAA
CONSENSUS
....................GAGCTCTGATGTCAAGCATCTCTACAAGAA

A5-AT4G06701-XLOC_023449-3036-0
AGAAGAATGGGTTCTTATCATATAAATATGTGCAATGGAGAAAATACAAC
A5-AT4G06701-XLOC_023449-3036-1
AGAAGAATGGGTTCTTATCATATAAATATGTGCAATGGAGAAAATACAAC
CONSENSUS
AGAAGAATGGGTTCTTATCATATAAATATGTGCAATGGAGAAAATACAAC

A5-AT4G06701-XLOC_023449-3036-0
ACGTTTGGATGGTCAACTCTATGAATCTGATAATCATATAAATTTCAAGC
A5-AT4G06701-XLOC_023449-3036-1
ACGTTTGGATGGTCAACTCTATGAATCTGATAATCATATAAATTTCAAGC
CONSENSUS
ACGTTTGGATGGTCAACTCTATGAATCTGATAATCATATAAATTTCAAGC

A5-AT4G06701-XLOC_023449-3036-0
TTCTTAATTCTTTTAGTGTGATGGAATGTAAAATACTAATTAGTTAATTT
A5-AT4G06701-XLOC_023449-3036-1
TTCTTAATTCTTTTAGTGTGATGGAATGTAAAATACTAATTAGTTAATTT
CONSENSUS
TTCTTAATTCTTTTAGTGTGATGGAATGTAAAATACTAATTAGTTAATTT

A5-AT4G06701-XLOC_023449-3036-0
CTTCTTGTTCGTGCAAATTTTTATGTGAAACTCATCGGTTTGCAAACAAG
A5-AT4G06701-XLOC_023449-3036-1
CTTCTTGTTCGTGCAAATTTTTATGTGAAACTCATCGGTTTGCAAACAAG
CONSENSUS
CTTCTTGTTCGTGCAAATTTTTATGTGAAACTCATCGGTTTGCAAACAAG

A5-AT4G06701-XLOC_023449-3036-0

TTTTATTATTATGATAAATAAATAAAACATTATTTTTTG
A5-AT4G06701-XLOC_023449-3036-1
TTTTATTATTATGATAAATAAATAAAACATTATTTTTTG
CONSENSUS
TTTTATTATTATGATAAATAAATAAAACATTATTTTTTG

alignment for event: RI-AT4G12560-XLOC_021342-7182

RI-AT4G12560-XLOC_021342-7182-0
GGATGATTTCCTGTCCAAAGGATTCAAGCTGGTCTTATAAGCCAAAAACA
RI-AT4G12560-XLOC_021342-7182-1
GGATGATTTCCTGTCCAAAGGATTCAAGCTGGTCTTATAAGCCAAAAACA
CONSENSUS
GGATGATTTCCTGTCCAAAGGATTCAAGCTGGTCTTATAAGCCAAAAACA

RI-AT4G12560-XLOC_021342-7182-0
AGAGACAGGAGCTAAAGCTGCACAAGGTAAGTGAAAAAGTTGACCGTTCT
RI-AT4G12560-XLOC_021342-7182-1
AGAGACAGGAGCTAAAGCTGCACAAG------------------------
CONSENSUS
AGAGACAGGAGCTAAAGCTGCACAAG........................

RI-AT4G12560-XLOC_021342-7182-0
TAATGGGTCTAGTTTGCTGCTGCATTCTTATTGCATTTCATTCTTTTCTG
RI-AT4G12560-XLOC_021342-7182-1

CONSENSUS
....

RI-AT4G12560-XLOC_021342-7182-0
TCTCTGCTCTGTTTTGTCGCGGACGTTCATATCCGGTGTCTGTTTTGTGG
RI-AT4G12560-XLOC_021342-7182-1

CONSENSUS
....

RI-AT4G12560-XLOC_021342-7182-0
CGGACGAGCAATTTTCATATTCGGGTTGGGACAAGAGTGAAAGTCACAGT
RI-AT4G12560-XLOC_021342-7182-1

CONSENSUS
....

RI-AT4G12560-XLOC_021342-7182-0
ATGTCATAGGTATTGAGTGCTTAGAAGTTAGAACATTCCCTAGAAAATGT
RI-AT4G12560-XLOC_021342-7182-1

CONSENSUS
....

RI-AT4G12560-XLOC_021342-7182-0
TATGGAGTCATCATCAGGATAATTACTTTAGCATATGACCATATTCAAGT
RI-AT4G12560-XLOC_021342-7182-1

CONSENSUS
      ....

RI-AT4G12560-XLOC_021342-7182-0
      GTGGTTTTTTCACATCAATCTATCTGCAAAACAATCATTGGGTATGATTT
RI-AT4G12560-XLOC_021342-7182-1

CONSENSUS
      ....

RI-AT4G12560-XLOC_021342-7182-0
      TTTCAGGGAGGAGAAACGAGAAGGGGAACAAACAATAACTTCAACCAAAA
RI-AT4G12560-XLOC_021342-7182-1 ------
GGAGGAGAAACGAGAAGGGGAACAAACAATAACTTCAACCAAAA
CONSENSUS
      ......GGAGGAGAAACGAGAAGGGGAACAAACAATAACTTCAACCAAAA

RI-AT4G12560-XLOC_021342-7182-0
      AGCTCTAATATACCAAAGGAGAATAATATACAACTTACCTACGTATATGT
RI-AT4G12560-XLOC_021342-7182-1
      AGCTCTAATATACCAAAGGAGAATAATATACAACTTACCTACGTATATGT
CONSENSUS
      AGCTCTAATATACCAAAGGAGAATAATATACAACTTACCTACGTATATGT

RI-AT4G12560-XLOC_021342-7182-0
      GCTTATAGAAGAGAGGACAAATGTTTTTGGTTTGTAAATAACTCTTTTTA
RI-AT4G12560-XLOC_021342-7182-1
      GCTTATAGAAGAGAGGACAAATGTTTTTGGTTTGTAAATAACTCTTTTTA
CONSENSUS
      GCTTATAGAAGAGAGGACAAATGTTTTTGGTTTGTAAATAACTCTTTTTA

RI-AT4G12560-XLOC_021342-7182-0
      AGTTTGATATATATAACTTACTAGTACTAACGCTTTGTATACTTGCTTTT
RI-AT4G12560-XLOC_021342-7182-1
      AGTTTGATATATATAACTTACTAGTACTAACGCTTTGTATACTTGCTTTT
CONSENSUS
      AGTTTGATATATATAACTTACTAGTACTAACGCTTTGTATACTTGCTTTT

RI-AT4G12560-XLOC_021342-7182-0
      TTAAGTTTCTGCATAATATGAGATGTTAAATAAATTA
RI-AT4G12560-XLOC_021342-7182-1
      TTAAGTTTCTGCATAATATGAGATGTTAAATAAATTA
CONSENSUS
      TTAAGTTTCTGCATAATATGAGATGTTAAATAAATTA

alignment for event: A3-AT4G32330-XLOC_022510-6719

A3-AT4G32330-XLOC_022510-6719-0
      GGAGAAGGTGAAACCAAAGTCTCAAAAGAAACAAGCCCATGAGACATCTG
A3-AT4G32330-XLOC_022510-6719-1
      GGAGAAGGTGAAACCAAAGTCTCAAAAGAAACAAGCCCATGAGACATCTG
CONSENSUS
      GGAGAAGGTGAAACCAAAGTCTCAAAAGAAACAAGCCCATGAGACATCTG

```
A3-AT4G32330-XLOC_022510-6719-0
      AAGATGATACTCAGTCTTCTAATAGTCCGAAAGCAGACGATGGAAAACCT
A3-AT4G32330-XLOC_022510-6719-1  AAGATGATACTCAGTCTTCTAA---
TCCGAAAGCAGACGATGGAAAACCT
CONSENSUS
      AAGATGATACTCAGTCTTCTAA...TCCGAAAGCAGACGATGGAAAACCT

A3-AT4G32330-XLOC_022510-6719-0
      CGTAAAGTTGGTGCACTTCCAAATTATGGATTCAGTTTCAAATGTGACCA
A3-AT4G32330-XLOC_022510-6719-1
      CGTAAAGTTGGTGCACTTCCAAATTATGGATTCAGTTTCAAATGTGACCA
CONSENSUS
      CGTAAAGTTGGTGCACTTCCAAATTATGGATTCAGTTTCAAATGTGACCA

A3-AT4G32330-XLOC_022510-6719-0  ACGGGCTGAAAAGAGAAAAGAG
A3-AT4G32330-XLOC_022510-6719-1  ACGGGCTGAAAAGAGAAAAGAG
CONSENSUS                        ACGGGCTGAAAAGAGAAAAGAG
```

alignment for event: SE-AT4G27820-XLOC_024787-3987

```
SE-AT4G27820-XLOC_024787-3987-0
      ATGAAACATTTCTCTCTACTTTTCATTTTTCTGGTCATCCTCTTGGCAAC
SE-AT4G27820-XLOC_024787-3987-1
      ATGAAACATTTCTCTCTACTTTTCATTTTTCTGGTCATCCTCTTGGCAAC
CONSENSUS
      ATGAAACATTTCTCTCTACTTTTCATTTTTCTGGTCATCCTCTTGGCAAC

SE-AT4G27820-XLOC_024787-3987-0
      AAGCTACAGTGATGCCTTTACCAGAAACAGTTTTCCAAAGGATTTCCTCT
SE-AT4G27820-XLOC_024787-3987-1
      AAGCTACAGTGATGCCTTTACCAGAAACAGTTTTCCAAAGGATTTCCTCT
CONSENSUS
      AAGCTACAGTGATGCCTTTACCAGAAACAGTTTTCCAAAGGATTTCCTCT

SE-AT4G27820-XLOC_024787-3987-0
      TCGGAGCCGCCACTTCTGCTTATCAG------------------------
SE-AT4G27820-XLOC_024787-3987-1
      TCGGAGCCGCCACTTCTGCTTATCAGTGGGAAGGAGCTGTTGCTGAAGAT
CONSENSUS
      TCGGAGCCGCCACTTCTGCTTATCAG........................

SE-AT4G27820-XLOC_024787-3987-0
      ----------------------------------------ACGATACAGG
SE-AT4G27820-XLOC_024787-3987-1
      GGAAGAACTCCTAGTGTCTGGGATACTTTCTCCAACTCTTACGATACAGG
CONSENSUS
      ........................................ACGATACAGG

SE-AT4G27820-XLOC_024787-3987-0
      TAATGGAGATGTAACATCTGATGGGTATCACAAATACAAG
SE-AT4G27820-XLOC_024787-3987-1
      TAATGGAGATGTAACATCTGATGGGTATCACAAATACAAG
CONSENSUS
      TAATGGAGATGTAACATCTGATGGGTATCACAAATACAAG
```

alignment for event: A3-AT4G25290-XLOC_024638-6256

A3-AT4G25290-XLOC_024638-6256-0
AATTTGACAGATCTACCACAATCTTGGGAGGAGTTTAAGAAACTGAAGCT
A3-AT4G25290-XLOC_024638-6256-1
AATTTGACAGATCTACCACAATCTTGGGAGGAGTTTAAGAAACTGAAGCT
CONSENSUS
AATTTGACAGATCTACCACAATCTTGGGAGGAGTTTAAGAAACTGAAGCT

A3-AT4G25290-XLOC_024638-6256-0
GCCTCTCACTTTGCCAGTTCCTGCAGCAAAATTTTCATCTCCAGGAAGTG
A3-AT4G25290-XLOC_024638-6256-1
GCCTCTCACTTTGCCAGTTCCTGCAGCAAAATTTTCATCTCCAGGAAGTG
CONSENSUS
GCCTCTCACTTTGCCAGTTCCTGCAGCAAAATTTTCATCTCCAGGAAGTG

A3-AT4G25290-XLOC_024638-6256-0
AATTGCAGTGGG----------------------------GTTCTGTGCC
A3-AT4G25290-XLOC_024638-6256-1
AATTGCAGTGGGGGGGAAATGGTAAATGAAACATGCTCCAGGTTCTGTGCC
CONSENSUS
AATTGCAGTGGG............................GTTCTGTGCC

A3-AT4G25290-XLOC_024638-6256-0
AACGCTCGATGACCTGAAGGACTATTTGAAGGAAAGCTTATGGGAAATAG
A3-AT4G25290-XLOC_024638-6256-1
AACGCTCGATGACCTGAAGGACTATTTGAAGGAAAGCTTATGGGAAATAG
CONSENSUS
AACGCTCGATGACCTGAAGGACTATTTGAAGGAAAGCTTATGGGAAATAG

A3-AT4G25290-XLOC_024638-6256-0
AAAATAGTTGGAGGGAAATGGCACAGGCATCTGCTGAAAGAGTATTAATG
A3-AT4G25290-XLOC_024638-6256-1
AAAATAGTTGGAGGGAAATGGCACAGGCATCTGCTGAAAGAGTATTAATG
CONSENSUS
AAAATAGTTGGAGGGAAATGGCACAGGCATCTGCTGAAAGAGTATTAATG

A3-AT4G25290-XLOC_024638-6256-0
GAAAGGCTTGGTAACTTGAAGGAAAGCAGTATGGAGCCAATTGTTGATGG
A3-AT4G25290-XLOC_024638-6256-1
GAAAGGCTTGGTAACTTGAAGGAAAGCAGTATGGAGCCAATTGTTGATGG
CONSENSUS
GAAAGGCTTGGTAACTTGAAGGAAAGCAGTATGGAGCCAATTGTTGATGG

A3-AT4G25290-XLOC_024638-6256-0
AAGTTTAGGGAAGAAGGTTGATAACTCTGTTTTTGTCACAAGTAAAAGAG
A3-AT4G25290-XLOC_024638-6256-1
AAGTTTAGGGAAGAAGGTTGATAACTCTGTTTTTGTCACAAGTAAAAGAG
CONSENSUS
AAGTTTAGGGAAGAAGGTTGATAACTCTGTTTTTGTCACAAGTAAAAGAG

A3-AT4G25290-XLOC_024638-6256-0
ATACTGTTGGAGGTGGAAATGAGGTTGTACTGAATGCTCTAGCAGGATAC

A3-AT4G25290-XLOC_024638-6256-1
ATACTGTTGGAGGTGGAAATGAGGTTGTACTGAATGCTCTAGCAGGATAC
CONSENSUS
ATACTGTTGGAGGTGGAAATGAGGTTGTACTGAATGCTCTAGCAGGATAC

A3-AT4G25290-XLOC_024638-6256-0
TTGAGGTACTTGGAGGGTACAAGTCGAGATGACTGGCAAGA
A3-AT4G25290-XLOC_024638-6256-1
TTGAGGTACTTGGAGGGTACAAGTCGAGATGACTGGCAAGA
CONSENSUS
TTGAGGTACTTGGAGGGTACAAGTCGAGATGACTGGCAAGA

alignment for event: SE-AT2G13650-XLOC_011796-691

SE-AT2G13650-XLOC_011796-691-0
TTTGAAATACATCAATGTAGCAATGGTCACTGTCCTGAAGAATGTCACTA
SE-AT2G13650-XLOC_011796-691-1
TTTGAAATACATCAATGTAGCAATGGTCACTGTCCTGAAGAATGTCACTA
CONSENSUS
TTTGAAATACATCAATGTAGCAATGGTCACTGTCCTGAAGAATGTCACTA

SE-AT2G13650-XLOC_011796-691-0
ATGTGATAACTGCAGTTGGTGAGATGTATCTGTTCAACAAGCAACATGAC
SE-AT2G13650-XLOC_011796-691-1
ATGTGATAACTGCAGTTGGTGAGATGTATCTGTTCAACAAGCAACATGAC
CONSENSUS
ATGTGATAACTGCAGTTGGTGAGATGTATCTGTTCAACAAGCAACATGAC

SE-AT2G13650-XLOC_011796-691-0
AACAGAGTGTGGGCTGCTCTCTTCTTAATG--------------------
SE-AT2G13650-XLOC_011796-691-1
AACAGAGTGTGGGCTGCTCTCTTCTTAATGTTACACTTGTCGTAGCGGCT
CONSENSUS
AACAGAGTGTGGGCTGCTCTCTTCTTAATG....................

SE-AT2G13650-XLOC_011796-691-0

SE-AT2G13650-XLOC_011796-691-1
TTCAGTGGTGTGGCATAGCCTAACCTTTCAATACTGTTGGAGATACATGT
CONSENSUS
....

SE-AT2G13650-XLOC_011796-691-0
------------------------------------ATAATTTCCGCAGT
SE-AT2G13650-XLOC_011796-691-1
TTTTAACTGCTTCCACAATTAGTCTGCCAGGGAAATATAATTTCCGCAGT
CONSENSUS
....................................ATAATTTCCGCAGT

SE-AT2G13650-XLOC_011796-691-0
TTCTGGAGGAATAACAGACCTATCATTCAATGCTGTTGGCTATGCTTGGC
SE-AT2G13650-XLOC_011796-691-1
TTCTGGAGGAATAACAGACCTATCATTCAATGCTGTTGGCTATGCTTGGC
CONSENSUS

TTCTGGAGGAATAACAGACCTATCATTCAATGCTGTTGGCTATGCTTGGC

SE-AT2G13650-XLOC_011796-691-0
AGATTGCTAATTGCTTCTTAACTGCATCGTACTCG
SE-AT2G13650-XLOC_011796-691-1
AGATTGCTAATTGCTTCTTAACTGCATCGTACTCG
CONSENSUS
AGATTGCTAATTGCTTCTTAACTGCATCGTACTCG

alignment for event: RI-AT2G44140-XLOC_013578-5612

RI-AT2G44140-XLOC_013578-5612-0
ATAAGGTTCTCAGGATTTAGGTTGCTTCTATGAGAAGGACACGTTGCAGT
RI-AT2G44140-XLOC_013578-5612-1
ATAAGGTTCTCAGGATTTAGGTTGCTTCTATGAGAAGGACACGTTGCAGT
CONSENSUS
ATAAGGTTCTCAGGATTTAGGTTGCTTCTATGAGAAGGACACGTTGCAGT

RI-AT2G44140-XLOC_013578-5612-0
TGGTGCTTCATGTCTAGGTAATGAAGGCTTTATGTGATAGATTTGTTCCT
RI-AT2G44140-XLOC_013578-5612-1
TGGTGCTTCATGTCTAG---------------------------------
CONSENSUS
TGGTGCTTCATGTCTAG.................................

RI-AT2G44140-XLOC_013578-5612-0
CAACAATGTTCTTCATCAAGCAAGAGTGATACGCATGATAAATCCCCTTT
RI-AT2G44140-XLOC_013578-5612-1

CONSENSUS
....

RI-AT2G44140-XLOC_013578-5612-0
AGTTTCAGATTCTGGACCTAGTGATAATAAGTCCAAGTTTACCTTATGGT
RI-AT2G44140-XLOC_013578-5612-1

CONSENSUS
....

RI-AT2G44140-XLOC_013578-5612-0
CAAACGTGTTTACGTCTTCTTCCTCAGTTTCTCAACCGTATAGGGAGTCT
RI-AT2G44140-XLOC_013578-5612-1 ---------------------------
TTTCTCAACCGTATAGGGAGTCT
CONSENSUS
...........................TTTCTCAACCGTATAGGGAGTCT

RI-AT2G44140-XLOC_013578-5612-0
TCAACTTCTGGGCATAAGCAAGTTTGCACCACTCGTAATGGTTGGACAGC
RI-AT2G44140-XLOC_013578-5612-1
TCAACTTCTGGGCATAAGCAAGTTTGCACCACTCGTAATGGTTGGACAGC
CONSENSUS
TCAACTTCTGGGCATAAGCAAGTTTGCACCACTCGTAATGGTTGGACAGC

RI-AT2G44140-XLOC_013578-5612-0

ATTTGTAAAAAGAGTCTCTATGGCTAGTGGAGCAATTAGGAGATTCCAGG
RI-AT2G44140-XLOC_013578-5612-1
ATTTGTAAAAAGAGTCTCTATGGCTAGTGGAGCAATTAGGAGATTCCAGG
CONSENSUS
ATTTGTAAAAAGAGTCTCTATGGCTAGTGGAGCAATTAGGAGATTCCAGG

RI-AT2G44140-XLOC_013578-5612-0
AGCGTGTTTTAGGGCCTAATAGGACCGGTCTTCCGAGCACAACTAGTGAC
RI-AT2G44140-XLOC_013578-5612-1
AGCGTGTTTTAGGGCCTAATAGGACCGGTCTTCCGAGCACAACTAGTGAC
CONSENSUS
AGCGTGTTTTAGGGCCTAATAGGACCGGTCTTCCGAGCACAACTAGTGAC

RI-AT2G44140-XLOC_013578-5612-0
GTATGGCTCTTGGGTGTCTGCTATAAAATATCTGCGGATGAGAACTCAGG
RI-AT2G44140-XLOC_013578-5612-1
GTATGGCTCTTGGGTGTCTGCTATAAAATATCTGCGGATGAGAACTCAGG
CONSENSUS
GTATGGCTCTTGGGTGTCTGCTATAAAATATCTGCGGATGAGAACTCAGG

RI-AT2G44140-XLOC_013578-5612-0
GGAAACCGATACTGGCACTGTATTGGCTGCATTGCAACTAGATTTTTCAT
RI-AT2G44140-XLOC_013578-5612-1
GGAAACCGATACTGGCACTGTATTGGCTGCATTGCAACTAGATTTTTCAT
CONSENSUS
GGAAACCGATACTGGCACTGTATTGGCTGCATTGCAACTAGATTTTTCAT

RI-AT2G44140-XLOC_013578-5612-0 CCAAAATACTGATGACATATCGTAAAG
RI-AT2G44140-XLOC_013578-5612-1 CCAAAATACTGATGACATATCGTAAAG
CONSENSUS CCAAAATACTGATGACATATCGTAAAG

alignment for event: A3-AT2G25910-XLOC_012517-799

A3-AT2G25910-XLOC_012517-799-0
ATAACCTGAAGTCAGAAGATCAATGTCTTGAAGAAGAGATCCTGTCAGTG
A3-AT2G25910-XLOC_012517-799-1
ATAACCTGAAGTCAGAAGATCAATGTCTTGAAGAAGAGATCCTGTCAGTG
CONSENSUS
ATAACCTGAAGTCAGAAGATCAATGTCTTGAAGAAGAGATCCTGTCAGTG

A3-AT2G25910-XLOC_012517-799-0
CTTGATGTTCCACCAGGAAAGATGGGACGTGTGATTGGAAGGAAAGGAGC
A3-AT2G25910-XLOC_012517-799-1
CTTGATGTTCCACCAGGAAAGATGGGACGTGTGATTGGAAGGAAAGGAGC
CONSENSUS
CTTGATGTTCCACCAGGAAAGATGGGACGTGTGATTGGAAGGAAAGGAGC

A3-AT2G25910-XLOC_012517-799-0
ATCGATCCTCGCCATTAAGGAAGCTTGCAA---CGCGGAAATTCTAATTG
A3-AT2G25910-XLOC_012517-799-1
ATCGATCCTCGCCATTAAGGAAGCTTGCAACAGCGCGGAAATTCTAATTG
CONSENSUS
ATCGATCCTCGCCATTAAGGAAGCTTGCAA...CGCGGAAATTCTAATTG

A3-AT2G25910-XLOC_012517-799-0   GAGGGGCAAAGGGTCCACCTGATAAG
A3-AT2G25910-XLOC_012517-799-1   GAGGGGCAAAGGGTCCACCTGATAAG
CONSENSUS   GAGGGGCAAAGGGTCCACCTGATAAG

alignment for event: A3-AT2G05520-XLOC_008599-1452

A3-AT2G05520-XLOC_008599-1452-0
CCACAGTGAATTCAGAGAGTAAGGAAACTGTGAAACCTGATCAACGTGGC
A3-AT2G05520-XLOC_008599-1452-1
CCACAGTGAATTCAGAGAGTAAGGAAACTGTGAAACCTGATCAACGTGGC
CONSENSUS
CCACAGTGAATTCAGAGAGTAAGGAAACTGTGAAACCTGATCAACGTGGC

A3-AT2G05520-XLOC_008599-1452-0
TACGGTGACAATGGAGGAAATTACAATAACGGAGGAGGTTACCAGGGAGG
A3-AT2G05520-XLOC_008599-1452-1
TACGGTGACAATGGAGGAAATTACAATAACGGAGGAGGTTACCAGGGAGG
CONSENSUS
TACGGTGACAATGGAGGAAATTACAATAACGGAGGAGGTTACCAGGGAGG

A3-AT2G05520-XLOC_008599-1452-0
AGGGGGACGGTACCAAGGAGGAGGAGGACGATACCAAGGAGGCGGTGGGC
A3-AT2G05520-XLOC_008599-1452-1 AGGGG---------------------
GACGATACCAAGGAGGCGGTGGGC
CONSENSUS
AGGGG.....................GACGATACCAAGGAGGCGGTGGGC

A3-AT2G05520-XLOC_008599-1452-0
GACAAGGAGGAGGGGGAAGTGGGGGAAGTTACTGCCGCCACGGCTGCTGC
A3-AT2G05520-XLOC_008599-1452-1
GACAAGGAGGAGGGGGAAGTGGGGGAAGTTACTGCCGCCACGGCTGCTGC
CONSENSUS
GACAAGGAGGAGGGGGAAGTGGGGGAAGTTACTGCCGCCACGGCTGCTGC

A3-AT2G05520-XLOC_008599-1452-0
TACAGAGGTTACAACGGCTGCTCAAGATGCTGCTCGTATGCCGGAGAAGC
A3-AT2G05520-XLOC_008599-1452-1
TACAGAGGTTACAACGGCTGCTCAAGATGCTGCTCGTATGCCGGAGAAGC
CONSENSUS
TACAGAGGTTACAACGGCTGCTCAAGATGCTGCTCGTATGCCGGAGAAGC

A3-AT2G05520-XLOC_008599-1452-0
TGTTCAGACTCAGCCCGGTCACTAAAACAATATATTAACCAATCACCACC
A3-AT2G05520-XLOC_008599-1452-1
TGTTCAGACTCAGCCCGGTCACTAAAACAATATATTAACCAATCACCACC
CONSENSUS
TGTTCAGACTCAGCCCGGTCACTAAAACAATATATTAACCAATCACCACC

A3-AT2G05520-XLOC_008599-1452-0
ATGCATGTATTGCATTATGTATGTATGATTTTAAGTAAACCATGGTGCGT
A3-AT2G05520-XLOC_008599-1452-1
ATGCATGTATTGCATTATGTATGTATGATTTTAAGTAAACCATGGTGCGT
CONSENSUS
ATGCATGTATTGCATTATGTATGTATGATTTTAAGTAAACCATGGTGCGT

A3-AT2G05520-XLOC_008599-1452-0
TTGTAATGAACTGCCTCAAGTTTTTGAGGCACTATAGATTAAGAAGAGAA
A3-AT2G05520-XLOC_008599-1452-1
TTGTAATGAACTGCCTCAAGTTTTTGAGGCACTATAGATTAAGAAGAGAA
CONSENSUS
TTGTAATGAACTGCCTCAAGTTTTTGAGGCACTATAGATTAAGAAGAGAA

A3-AT2G05520-XLOC_008599-1452-0
CTATGGGAATAAAGTTTGATTATGTAATGTTTTATGTGGTTTGAGTTGTA
A3-AT2G05520-XLOC_008599-1452-1
CTATGGGAATAAAGTTTGATTATGTAATGTTTTATGTGGTTTGAGTTGTA
CONSENSUS
CTATGGGAATAAAGTTTGATTATGTAATGTTTTATGTGGTTTGAGTTGTA

A3-AT2G05520-XLOC_008599-1452-0
ATACTTGCTGTTTGCATAATAAAATCGTTTGTAGTTTATGTTAATCTCTT
A3-AT2G05520-XLOC_008599-1452-1
ATACTTGCTGTTTGCATAATAAAATCGTTTGTAGTTTATGTTAATCTCTT
CONSENSUS
ATACTTGCTGTTTGCATAATAAAATCGTTTGTAGTTTATGTTAATCTCTT

A3-AT2G05520-XLOC_008599-1452-0 TCGTTTTTTT
A3-AT2G05520-XLOC_008599-1452-1 TCGTTTTTTT
CONSENSUS TCGTTTTTTT

alignment for event: A3-AT2G28290-XLOC_009942-279

A3-AT2G28290-XLOC_009942-279-0
CTCAAGAAGCCAATGTTCCTTCTCTTCCAGCAGCCTTGCCTGCTAAGAGG
A3-AT2G28290-XLOC_009942-279-1
CTCAAGAAGCCAATGTTCCTTCTCTTCCAGCAGCCTTGCCTGCTAAGAGG
CONSENSUS
CTCAAGAAGCCAATGTTCCTTCTCTTCCAGCAGCCTTGCCTGCTAAGAGG

A3-AT2G28290-XLOC_009942-279-0
CGAGTCCGCAATTTGCCAAGCAGAGGAGAAACTCCTAAACGCCAAGGAAA
A3-AT2G28290-XLOC_009942-279-1
CGAGTCCGCAATTTGCCAAGCAGAGGAGAAACTCCTAAACGCCAAGGAAA
CONSENSUS
CGAGTCCGCAATTTGCCAAGCAGAGGAGAAACTCCTAAACGCCAAGGAAA

A3-AT2G28290-XLOC_009942-279-0
GAGGCGTGGCCAACCTTTACCTGCAACCGATGCCTCTTCTGCAAGGAGTA
A3-AT2G28290-XLOC_009942-279-1
GAGGCGTGGCCAACCTTTACCTGCAACCGATGCCTCTTCTGCAAGGAGTA
CONSENSUS
GAGGCGTGGCCAACCTTTACCTGCAACCGATGCCTCTTCTGCAAGGAGTA

A3-AT2G28290-XLOC_009942-279-0
CAGGATTAACACCACAAATAGAGGTCAAGGTTGGTAATTTATCAGGCACC
A3-AT2G28290-XLOC_009942-279-1
CAGGATTAACACCACAAATAGAGGTCAAGGTTGGTAATTTATCAGGCACC
CONSENSUS

CAGGATTAACACCACAAATAGAGGTCAAGGTTGGTAATTTATCAGGCACC

A3-AT2G28290-XLOC_009942-279-0
AAAGCTAAGTTTGATGCTGTTGCCAAAGAACAACCCCACTTCAGCCAGTC
A3-AT2G28290-XLOC_009942-279-1
AAAGCTAAGTTTGATGCTGTTGCCAAAGAACAACCCCACTTCAGCCAGTC
CONSENSUS
AAAGCTAAGTTTGATGCTGTTGCCAAAGAACAACCCCACTTCAGCCAGTC

A3-AT2G28290-XLOC_009942-279-0
AGTTGCACCCGATATTCACTCTTCTGGTAGTTTGAGTCAGGAAATTAGAA
A3-AT2G28290-XLOC_009942-279-1
AGTTGCACCCGATATTCACTCTTCTGGTAGTTTGAGTCAGGAAATTAGAA
CONSENSUS
AGTTGCACCCGATATTCACTCTTCTGGTAGTTTGAGTCAGGAAATTAGAA

A3-AT2G28290-XLOC_009942-279-0
GAGACACCTCTGGTACTGGTGGTTCTGCTAGGAAACAAACTGCTGATGTA
A3-AT2G28290-XLOC_009942-279-1
GAGACACCTCTGGTACTGGTGGTTCTGCTAGGAAACAAACTGCTGATGTA
CONSENSUS
GAGACACCTCTGGTACTGGTGGTTCTGCTAGGAAACAAACTGCTGATGTA

A3-AT2G28290-XLOC_009942-279-0
ACTGATGTTGCTCGAGTCATGAAAGAGATCTTTTCAGAGACTTCCCTATT
A3-AT2G28290-XLOC_009942-279-1
ACTGATGTTGCTCGAGTCATGAAAGAGATCTTTTCAGAGACTTCCCTATT
CONSENSUS
ACTGATGTTGCTCGAGTCATGAAAGAGATCTTTTCAGAGACTTCCCTATT

A3-AT2G28290-XLOC_009942-279-0
AAAACATAAAGTTGGAGAGCCTTCTGCAACAACGAGAACAAATGTGCCTG
A3-AT2G28290-XLOC_009942-279-1
AAAACATAAAGTTGGAGAGCCTTCTGCAACAACGAGAACAAATGTGCCTG
CONSENSUS
AAAACATAAAGTTGGAGAGCCTTCTGCAACAACGAGAACAAATGTGCCTG

A3-AT2G28290-XLOC_009942-279-0
ACGCACAATCCCCTGGTGAGATGAATTTGCACACAGTTGAGACCCACAAG
A3-AT2G28290-XLOC_009942-279-1
ACGCACAATCCCCTGGTGAGATGAATTTGCACACAGTTGAGACCCACAAG
CONSENSUS
ACGCACAATCCCCTGGTGAGATGAATTTGCACACAGTTGAGACCCACAAG

A3-AT2G28290-XLOC_009942-279-0
GCAGAGGATTCTTCTGGTCTTAAGAATCAAGAAGCTTTATATAACCTGAG
A3-AT2G28290-XLOC_009942-279-1
GCAGAGGATTCTTCTGGTCTTAAGAATCAAGAAGCTTTATATAACCTGAG
CONSENSUS
GCAGAGGATTCTTCTGGTCTTAAGAATCAAGAAGCTTTATATAACCTGAG

A3-AT2G28290-XLOC_009942-279-0
CAAGGCAGATAAACTGGTATCAGATATTCCTCATCCTGTTCCTGGTGATC
A3-AT2G28290-XLOC_009942-279-1
CAAGGCAGATAAACTGGTATCAGATATTCCTCATCCTGTTCCTGGTGATC
CONSENSUS

CAAGGCAGATAAACTGGTATCAGATATTCCTCATCCTGTTCCTGGTGATC

A3-AT2G28290-XLOC_009942-279-0
TGACAACTTCAGGATCAGTTGCAAACAAAGATGTTGACATTGGGTCGTCT
A3-AT2G28290-XLOC_009942-279-1
TGACAACTTCAGGATCAGTTGCAAACAAAGATGTTGACATTGGGTCGTCT
CONSENSUS
TGACAACTTCAGGATCAGTTGCAAACAAAGATGTTGACATTGGGTCGTCT

A3-AT2G28290-XLOC_009942-279-0
AAGGTTGCTGCTGAAAATGAGCTTGTCAAAATTCCGGGTGGTGACGTAGA
A3-AT2G28290-XLOC_009942-279-1
AAGGTTGCTGCTGAAAATGAGCTTGTCAAAATTCCGGGTGGTGACGTAGA
CONSENSUS
AAGGTTGCTGCTGAAAATGAGCTTGTCAAAATTCCGGGTGGTGACGTAGA

A3-AT2G28290-XLOC_009942-279-0
TTCTTCTGTAATACAACTCTCTTTGGGAAATACTTTGACTGCTAAATCGT
A3-AT2G28290-XLOC_009942-279-1
TTCTTCTGTAATACAACTCTCTTTGGGAAATACTTTGACTGCTAAATCGT
CONSENSUS
TTCTTCTGTAATACAACTCTCTTTGGGAAATACTTTGACTGCTAAATCGT

A3-AT2G28290-XLOC_009942-279-0
CTTTGGAAAAGTGCACTGCAGATCAGCTTCTGGGAGAAAAACTGTCTCAA
A3-AT2G28290-XLOC_009942-279-1
CTTTGGAAAAGTGCACTGCAGATCAGCTTCTGGGAGAAAAACTGTCTCAA
CONSENSUS
CTTTGGAAAAGTGCACTGCAGATCAGCTTCTGGGAGAAAAACTGTCTCAA

A3-AT2G28290-XLOC_009942-279-0
GAAGGTGAAACCACACCTGCTAGTGATGGTGAAACATGTCACCTGGCAGA
A3-AT2G28290-XLOC_009942-279-1
GAAGGTGAAACCACACCTGCTAGTGATGGTGAAACATGTCACCTGGCAGA
CONSENSUS
GAAGGTGAAACCACACCTGCTAGTGATGGTGAAACATGTCACCTGGCAGA

A3-AT2G28290-XLOC_009942-279-0
AGAAACGGCATCTTCATTGAGTTATGTTCGATCTGAGCCTACTGCATCTG
A3-AT2G28290-XLOC_009942-279-1
AGAAACGGCATCTTCATTGAGTTATGTTCGATCTGAGCCTACTGCATCTG
CONSENSUS
AGAAACGGCATCTTCATTGAGTTATGTTCGATCTGAGCCTACTGCATCTG

A3-AT2G28290-XLOC_009942-279-0
CGTCGACAACTGCGGAACCTCTACCTACTGACAAGTTGGAAAAAAATATT
A3-AT2G28290-XLOC_009942-279-1
CGTCGACAACTGCGGAACCTCTACCTACTGACAAGTTGGAAAAAAATATT
CONSENSUS
CGTCGACAACTGCGGAACCTCTACCTACTGACAAGTTGGAAAAAAATATT

A3-AT2G28290-XLOC_009942-279-0
TCTTTTCAAGATGAAGTTAAAACTCTCAATGGTGATAAAAGAGAAGCTAT
A3-AT2G28290-XLOC_009942-279-1
TCTTTTCAAGATGAAGTTAAAACTCTCAATGGTGATAAAAGAGAAGCTAT
CONSENSUS

TCTTTTCAAGATGAAGTTAAAACTCTCAATGGTGATAAAAGAGAAGCTAT

A3-AT2G28290-XLOC_009942-279-0
CCTCCTAAGTTCGGAAGAGCAAACGAATGTTAACTCCAAGATTGAGACAA
A3-AT2G28290-XLOC_009942-279-1
CCTCCTAAGTTCGGAAGAGCAAACGAATGTTAACTCCAAGATTGAGACAA
CONSENSUS
CCTCCTAAGTTCGGAAGAGCAAACGAATGTTAACTCCAAGATTGAGACAA

A3-AT2G28290-XLOC_009942-279-0
ATTCTGAGGAACTTCAAGCCAGTAGAACAGATGAAGTTCCACATGTGGAT
A3-AT2G28290-XLOC_009942-279-1
ATTCTGAGGAACTTCAAGCCAGTAGAACAGATGAAGTTCCACATGTGGAT
CONSENSUS
ATTCTGAGGAACTTCAAGCCAGTAGAACAGATGAAGTTCCACATGTGGAT

A3-AT2G28290-XLOC_009942-279-0
GGAAAATCTGTTGATGTTGCAAATCAGACGGTGAAAGAAGATGAGGCAAA
A3-AT2G28290-XLOC_009942-279-1
GGAAAATCTGTTGATGTTGCAAATCAGACGGTGAAAGAAGATGAGGCAAA
CONSENSUS
GGAAAATCTGTTGATGTTGCAAATCAGACGGTGAAAGAAGATGAGGCAAA

A3-AT2G28290-XLOC_009942-279-0
ACATTCTGTTGAAATTCAATCGTCTATGCTGGAGCCTGATGAACTGCCAA
A3-AT2G28290-XLOC_009942-279-1
ACATTCTGTTGAAATTCAATCGTCTATGCTGGAGCCTGATGAACTGCCAA
CONSENSUS
ACATTCTGTTGAAATTCAATCGTCTATGCTGGAGCCTGATGAACTGCCAA

A3-AT2G28290-XLOC_009942-279-0
ATGCTGGACAAAAGGGTCACAGTAGCATTGACTTGCAGCCATTGGTTTTA
A3-AT2G28290-XLOC_009942-279-1
ATGCTGGACAAAAGGGTCACAGTAGCATTGACTTGCAGCCATTGGTTTTA
CONSENSUS
ATGCTGGACAAAAGGGTCACAGTAGCATTGACTTGCAGCCATTGGTTTTA

A3-AT2G28290-XLOC_009942-279-0
GTTACAAGCAATGAGAATGCTATGTCCCTTGACGATAAAGATTATGATCC
A3-AT2G28290-XLOC_009942-279-1
GTTACAAGCAATGAGAATGCTATGTCCCTTGACGATAAAGATTATGATCC
CONSENSUS
GTTACAAGCAATGAGAATGCTATGTCCCTTGACGATAAAGATTATGATCC

A3-AT2G28290-XLOC_009942-279-0
TATCTCTAAATCTGCTGATATAGAACAAGATCCTGAAGAATCTGTTTTTG
A3-AT2G28290-XLOC_009942-279-1
TATCTCTAAATCTGCTGATATAGAACAAGATCCTGAAGAATCTGTTTTTG
CONSENSUS
TATCTCTAAATCTGCTGATATAGAACAAGATCCTGAAGAATCTGTTTTTG

A3-AT2G28290-XLOC_009942-279-0
TTCAAGGTGTTGGTAGGCCTAAAGTTGGTACTGCTGATACACAGATGGAG
A3-AT2G28290-XLOC_009942-279-1
TTCAAGGTGTTGGTAGGCCTAAAGTTGGTACTGCTGATACACAGATGGAG
CONSENSUS

TTCAAGGTGTTGGTAGGCCTAAAGTTGGTACTGCTGATACACAGATGGAG

A3-AT2G28290-XLOC_009942-279-0
GATACCAATGATGCCAAACTTCTAGTGGGTTGTTCAGTTGAGAGTGAGGA
A3-AT2G28290-XLOC_009942-279-1
GATACCAATGATGCCAAACTTCTAGTGGGTTGTTCAGTTGAGAGTGAGGA
CONSENSUS
GATACCAATGATGCCAAACTTCTAGTGGGTTGTTCAGTTGAGAGTGAGGA

A3-AT2G28290-XLOC_009942-279-0
AAAAGAGAAAACTCTTCAATCCCTCATACCCGGTGATGATGCTGATACAG
A3-AT2G28290-XLOC_009942-279-1
AAAAGAGAAAACTCTTCAATCCCTCATACCCGGTGATGATGCTGATACAG
CONSENSUS
AAAAGAGAAAACTCTTCAATCCCTCATACCCGGTGATGATGCTGATACAG

A3-AT2G28290-XLOC_009942-279-0
AACAAGATCCTGAAGAATCTGTTTCGGATCAAAGGCCTAAAGTTGGTTCT
A3-AT2G28290-XLOC_009942-279-1
AACAAGATCCTGAAGAATCTGTTTCGGATCAAAGGCCTAAAGTTGGTTCT
CONSENSUS
AACAAGATCCTGAAGAATCTGTTTCGGATCAAAGGCCTAAAGTTGGTTCT

A3-AT2G28290-XLOC_009942-279-0
GCTTACACACAGATGGAGGATACGGATGAGGCGAAACTTCTAATGGGTTG
A3-AT2G28290-XLOC_009942-279-1
GCTTACACACAGATGGAGGATACGGATGAGGCGAAACTTCTAATGGGTTG
CONSENSUS
GCTTACACACAGATGGAGGATACGGATGAGGCGAAACTTCTAATGGGTTG

A3-AT2G28290-XLOC_009942-279-0
TTCAGTTGAGAGTGAGGAAAAAGAGAAAACTCTTCAATCCCATATACCCG
A3-AT2G28290-XLOC_009942-279-1
TTCAGTTGAGAGTGAGGAAAAAGAGAAAACTCTTCAATCCCATATACCCG
CONSENSUS
TTCAGTTGAGAGTGAGGAAAAAGAGAAAACTCTTCAATCCCATATACCCG

A3-AT2G28290-XLOC_009942-279-0
GTGATGATGCTGATACAGAAAAAAATCCTGAAGAATCTGTTTCCGTTCAA
A3-AT2G28290-XLOC_009942-279-1
GTGATGATGCTGATACAGAAAAAAATCCTGAAGAATCTGTTTCCGTTCAA
CONSENSUS
GTGATGATGCTGATACAGAAAAAAATCCTGAAGAATCTGTTTCCGTTCAA

A3-AT2G28290-XLOC_009942-279-0
GGTGTTGATAGGCCGAAAGTTGGTACTACTGACACACAGATGGAGGATAC
A3-AT2G28290-XLOC_009942-279-1
GGTGTTGATAGGCCGAAAGTTGGTACTACTGACACACAGATGGAGGATAC
CONSENSUS
GGTGTTGATAGGCCGAAAGTTGGTACTACTGACACACAGATGGAGGATAC

A3-AT2G28290-XLOC_009942-279-0
CAATGATGCCAAACTTCTAGTGGGTTGTTCAGTTGCGAGTGAGGAGAAAG
A3-AT2G28290-XLOC_009942-279-1
CAATGATGCCAAACTTCTAGTGGGTTGTTCAGTTGCGAGTGAGGAGAAAG
CONSENSUS

CAATGATGCCAAACTTCTAGTGGGTTGTTCAGTTGCGAGTGAGGAGAAAG

A3-AT2G28290-XLOC_009942-279-0
AGAAAACTCTTCAATCCCATATACCCGGTGATGATGCTGATACAGAACAA
A3-AT2G28290-XLOC_009942-279-1
AGAAAACTCTTCAATCCCATATACCCGGTGATGATGCTGATACAGAACAA
CONSENSUS
AGAAAACTCTTCAATCCCATATACCCGGTGATGATGCTGATACAGAACAA

A3-AT2G28290-XLOC_009942-279-0
AATCCTGAAGAATCTGTTTCAGTTCAAGGTGTTAATAGGCCTAAAGTTGG
A3-AT2G28290-XLOC_009942-279-1
AATCCTGAAGAATCTGTTTCAGTTCAAGGTGTTAATAGGCCTAAAGTTGG
CONSENSUS
AATCCTGAAGAATCTGTTTCAGTTCAAGGTGTTAATAGGCCTAAAGTTGG

A3-AT2G28290-XLOC_009942-279-0
TAATGCTAACACACAGATGGAGGATACGGATGAGGCCAAAGTTCTAGTGG
A3-AT2G28290-XLOC_009942-279-1
TAATGCTAACACACAGATGGAGGATACGGATGAGGCCAAAGTTCTAGTGG
CONSENSUS
TAATGCTAACACACAGATGGAGGATACGGATGAGGCCAAAGTTCTAGTGG

A3-AT2G28290-XLOC_009942-279-0
GTTGTTCAGTTGAGAGTGAGGAGAAAGAGAAAACTCTTCAATCCCACATA
A3-AT2G28290-XLOC_009942-279-1
GTTGTTCAGTTGAGAGTGAGGAGAAAGAGAAAACTCTTCAATCCCACATA
CONSENSUS
GTTGTTCAGTTGAGAGTGAGGAGAAAGAGAAAACTCTTCAATCCCACATA

A3-AT2G28290-XLOC_009942-279-0
CCTGGTGATGATGCTGATACAGAACAAAATCCTGAAGAATCTGTTTCGAA
A3-AT2G28290-XLOC_009942-279-1
CCTGGTGATGATGCTGATACAGAACAAAATCCTGAAGAATCTGTTTCGAA
CONSENSUS
CCTGGTGATGATGCTGATACAGAACAAAATCCTGAAGAATCTGTTTCGAA

A3-AT2G28290-XLOC_009942-279-0
TTTTGATAGGCCTAAAGATGGGACTGCTGACACACATATGGAGGATATCG
A3-AT2G28290-XLOC_009942-279-1
TTTTGATAGGCCTAAAGATGGGACTGCTGACACACATATGGAGGATATCG
CONSENSUS
TTTTGATAGGCCTAAAGATGGGACTGCTGACACACATATGGAGGATATCG

A3-AT2G28290-XLOC_009942-279-0
ATGATGCCAAACTTCTAGTGGGTTGTTCAGTTGAGAGTGAGGAGAAAGAG
A3-AT2G28290-XLOC_009942-279-1
ATGATGCCAAACTTCTAGTGGGTTGTTCAGTTGAGAGTGAGGAGAAAGAG
CONSENSUS
ATGATGCCAAACTTCTAGTGGGTTGTTCAGTTGAGAGTGAGGAGAAAGAG

A3-AT2G28290-XLOC_009942-279-0
AAAAGTCTTCAATCCCATATGCCCAGTGATGATGCTGTTCTCCATGCGCC
A3-AT2G28290-XLOC_009942-279-1
AAAAGTCTTCAATCCCATATGCCCAGTGATGATGCTGTTCTCCATGCGCC
CONSENSUS

AAAAGTCTTCAATCCCATATGCCCAGTGATGATGCTGTTCTCCATGCGCC

A3-AT2G28290-XLOC_009942-279-0
TTTTGAGAACACAAAAGACAGTAAAGGAGATGATTTACATGGAGAGTCTC
A3-AT2G28290-XLOC_009942-279-1
TTTTGAGAACACAAAAGACAGTAAAGGAGATGATTTACATGGAGAGTCTC
CONSENSUS
TTTTGAGAACACAAAAGACAGTAAAGGAGATGATTTACATGGAGAGTCTC

A3-AT2G28290-XLOC_009942-279-0
TTGTTTCCTGTCCAACAATGGAAGTGATGGAACAGAAGGGGTTTGAATCA
A3-AT2G28290-XLOC_009942-279-1
TTGTTTCCTGTCCAACAATGGAAGTGATGGAACAGAAGGGGTTTGAATCA
CONSENSUS
TTGTTTCCTGTCCAACAATGGAAGTGATGGAACAGAAGGGGTTTGAATCA

A3-AT2G28290-XLOC_009942-279-0
GAGACACATGCTCGTACAGATTCAGGTGGTATTGATAGGGGAAATGAGGT
A3-AT2G28290-XLOC_009942-279-1
GAGACACATGCTCGTACAGATTCAGGTGGTATTGATAGGGGAAATGAGGT
CONSENSUS
GAGACACATGCTCGTACAGATTCAGGTGGTATTGATAGGGGAAATGAGGT

A3-AT2G28290-XLOC_009942-279-0
ATCAGAAAATATGTCTGATGGCGTCAAAATGAATATTTCATCTGTGCAGG
A3-AT2G28290-XLOC_009942-279-1
ATCAGAAAATATGTCTGATGGCGTCAAAATGAATATTTCATCTGTGCAGG
CONSENSUS
ATCAGAAAATATGTCTGATGGCGTCAAAATGAATATTTCATCTGTGCAGG

A3-AT2G28290-XLOC_009942-279-0
TCCCGGATGCATCACATGATTTAAATGTATCACAGGATCAAACAGACATT
A3-AT2G28290-XLOC_009942-279-1
TCCCGGATGCATCACATGATTTAAATGTATCACAGGATCAAACAGACATT
CONSENSUS
TCCCGGATGCATCACATGATTTAAATGTATCACAGGATCAAACAGACATT

A3-AT2G28290-XLOC_009942-279-0
CCCCTAGTTGGTGGGATAGACCCTGAACACGTACAAGAGAATGTGGATGT
A3-AT2G28290-XLOC_009942-279-1
CCCCTAGTTGGTGGGATAGACCCTGAACACGTACAAGAGAATGTGGATGT
CONSENSUS
CCCCTAGTTGGTGGGATAGACCCTGAACACGTACAAGAGAATGTGGATGT

A3-AT2G28290-XLOC_009942-279-0
ACCTGCATCACCTCACGGAGCAGCGCCAAACATTGTGATTTTCCAGTCTG
A3-AT2G28290-XLOC_009942-279-1
ACCTGCATCACCTCACGGAGCAGCGCCAAACATTGTGATTTTCCAGTCTG
CONSENSUS
ACCTGCATCACCTCACGGAGCAGCGCCAAACATTGTGATTTTCCAGTCTG

A3-AT2G28290-XLOC_009942-279-0
AGGGACATCTGTCTCCAAGTATCTTACCGGACGATGTGGCAGGACAACTA
A3-AT2G28290-XLOC_009942-279-1
AGGGACATCTGTCTCCAAGTATCTTACCGGACGATGTGGCAGGACAACTA
CONSENSUS

AGGGACATCTGTCTCCAAGTATCTTACCGGACGATGTGGCAGGACAACTA

A3-AT2G28290-XLOC_009942-279-0
GAAAGCATGTCTAATGACGAAAAAACGAATATTTCATCTGAGCAGGTCCC
A3-AT2G28290-XLOC_009942-279-1
GAAAGCATGTCTAATGACGAAAAAACGAATATTTCATCTGAGCAGGTCCC
CONSENSUS
GAAAGCATGTCTAATGACGAAAAAACGAATATTTCATCTGAGCAGGTCCC

A3-AT2G28290-XLOC_009942-279-0
AGATGTATCACATGATTTGAAAGTGTCTCAGGATCAAACTGACATTCCCC
A3-AT2G28290-XLOC_009942-279-1
AGATGTATCACATGATTTGAAAGTGTCTCAGGATCAAACTGACATTCCCC
CONSENSUS
AGATGTATCACATGATTTGAAAGTGTCTCAGGATCAAACTGACATTCCCC

A3-AT2G28290-XLOC_009942-279-0
CAGTTGGTGGGATAGTGCCTGAAAATTTGCAAGAGATTGTGGATGTACCT
A3-AT2G28290-XLOC_009942-279-1
CAGTTGGTGGGATAGTGCCTGAAAATTTGCAAGAGATTGTGGATGTACCT
CONSENSUS
CAGTTGGTGGGATAGTGCCTGAAAATTTGCAAGAGATTGTGGATGTACCT

A3-AT2G28290-XLOC_009942-279-0
GCATCACCTCATGGAGTAGTGCCAGACGTTGTTGTTTCCCAGTCTGAGGA
A3-AT2G28290-XLOC_009942-279-1
GCATCACCTCATGGAGTAGTGCCAGACGTTGTTGTTTCCCAGTCTGAGGA
CONSENSUS
GCATCACCTCATGGAGTAGTGCCAGACGTTGTTGTTTCCCAGTCTGAGGA

A3-AT2G28290-XLOC_009942-279-0
AATTCAATCTCCAAGTATTTTGCCCGACGATGTACCAGGACAACCAGACG
A3-AT2G28290-XLOC_009942-279-1
AATTCAATCTCCAAGTATTTTGCCCGACGATGTACCAGGACAACCAGACG
CONSENSUS
AATTCAATCTCCAAGTATTTTGCCCGACGATGTACCAGGACAACCAGACG

A3-AT2G28290-XLOC_009942-279-0
ATGGCAACTGTGAGAAAATGGATACCATGCAGAACAATACCTCTATTGAT
A3-AT2G28290-XLOC_009942-279-1
ATGGCAACTGTGAGAAAATGGATACCATGCAGAACAATACCTCTATTGAT
CONSENSUS
ATGGCAACTGTGAGAAAATGGATACCATGCAGAACAATACCTCTATTGAT

A3-AT2G28290-XLOC_009942-279-0
ATTGGCATAACTTCAGGTAAGACATGTCAGCCTTCATCTTCTACCCAGCC
A3-AT2G28290-XLOC_009942-279-1
ATTGGCATAACTTCAGGTAAGACATGTCAGCCTTCATCTTCTACCCAGCC
CONSENSUS
ATTGGCATAACTTCAGGTAAGACATGTCAGCCTTCATCTTCTACCCAGCC

A3-AT2G28290-XLOC_009942-279-0
TGAGGATGAGAACAGAAATAGCTTATCACACTGTGAACCGTCAGAAGTAG
A3-AT2G28290-XLOC_009942-279-1
TGAGGATGAGAACAGAAATAGCTTATCACACTGTGAACCGTCAGAAGTAG
CONSENSUS

TGAGGATGAGAACAGAAATAGCTTATCACACTGTGAACCGTCAGAAGTAG

A3-AT2G28290-XLOC_009942-279-0
TTGAACAAAGGGATTCAAGAGATCAAGTTTGCATAGGGTCTGTGGAATCT
A3-AT2G28290-XLOC_009942-279-1
TTGAACAAAGGGATTCAAGAGATCAAGTTTGCATAGGGTCTGTGGAATCT
CONSENSUS
TTGAACAAAGGGATTCAAGAGATCAAGTTTGCATAGGGTCTGTGGAATCT

A3-AT2G28290-XLOC_009942-279-0
CAAGTAGAGATCAGCTCTGCTATACTGGAAAATAGATCAGCTGATATCCA
A3-AT2G28290-XLOC_009942-279-1
CAAGTAGAGATCAGCTCTGCTATACTGGAAAATAGATCAGCTGATATCCA
CONSENSUS
CAAGTAGAGATCAGCTCTGCTATACTGGAAAATAGATCAGCTGATATCCA

A3-AT2G28290-XLOC_009942-279-0
GCCCCCGCAATCCATTTTGGTTGATCAAAAGGATATTGAAGAATCCAAAG
A3-AT2G28290-XLOC_009942-279-1
GCCCCCGCAATCCATTTTGGTTGATCAAAAGGATATTGAAGAATCCAAAG
CONSENSUS
GCCCCCGCAATCCATTTTGGTTGATCAAAAGGATATTGAAGAATCCAAAG

A3-AT2G28290-XLOC_009942-279-0
AACCTGGTATCGAGAGTGCTGATGTGTCTTTACACCAATTAGCTGATATC
A3-AT2G28290-XLOC_009942-279-1
AACCTGGTATCGAGAGTGCTGATGTGTCTTTACACCAATTAGCTGATATC
CONSENSUS
AACCTGGTATCGAGAGTGCTGATGTGTCTTTACACCAATTAGCTGATATC

A3-AT2G28290-XLOC_009942-279-0
CAGGCCGAGCCATCCAATTTGGTTGATCAAATGGATATTGAAGAATCCAA
A3-AT2G28290-XLOC_009942-279-1
CAGGCCGAGCCATCCAATTTGGTTGATCAAATGGATATTGAAGAATCCAA
CONSENSUS
CAGGCCGAGCCATCCAATTTGGTTGATCAAATGGATATTGAAGAATCCAA

A3-AT2G28290-XLOC_009942-279-0
AGAACCTGGTACCGAGAGTGCTGATGTGTCTTTACACCAATTAGCTGATA
A3-AT2G28290-XLOC_009942-279-1
AGAACCTGGTACCGAGAGTGCTGATGTGTCTTTACACCAATTAGCTGATA
CONSENSUS
AGAACCTGGTACCGAGAGTGCTGATGTGTCTTTACACCAATTAGCTGATA

A3-AT2G28290-XLOC_009942-279-0
TCCAGCCCGGGCCATCCATTTTGGTTGATCAAATGGATACTGAAAAATCC
A3-AT2G28290-XLOC_009942-279-1
TCCAGCCCGGGCCATCCATTTTGGTTGATCAAATGGATACTGAAAAATCC
CONSENSUS
TCCAGCCCGGGCCATCCATTTTGGTTGATCAAATGGATACTGAAAAATCC

A3-AT2G28290-XLOC_009942-279-0
AAAGAACCTGGTACCGAGAGTGCTGATGTGTCTTTACACCAATTAGCTGA
A3-AT2G28290-XLOC_009942-279-1
AAAGAACCTGGTACCGAGAGTGCTGATGTGTCTTTACACCAATTAGCTGA
CONSENSUS

AAAGAACCTGGTACCGAGAGTGCTGATGTGTCTTTACACCAATTAGCTGA

A3-AT2G28290-XLOC_009942-279-0
TATCCAGCCCGGGCCATCCATTTTGGTTGATCAAATGGATACTGAAAAAT
A3-AT2G28290-XLOC_009942-279-1
TATCCAGCCCGGGCCATCCATTTTGGTTGATCAAATGGATACTGAAAAAT
CONSENSUS
TATCCAGCCCGGGCCATCCATTTTGGTTGATCAAATGGATACTGAAAAAT

A3-AT2G28290-XLOC_009942-279-0
CCAAAGAACCTGGTACCGAGAGTGCTGATGTGTCTTTACACCAATTAGCT
A3-AT2G28290-XLOC_009942-279-1
CCAAAGAACCTGGTACCGAGAGTGCTGATGTGTCTTTACACCAATTAGCT
CONSENSUS
CCAAAGAACCTGGTACCGAGAGTGCTGATGTGTCTTTACACCAATTAGCT

A3-AT2G28290-XLOC_009942-279-0
GATATCCAGCCCGGGCCATCCATTTTGGTTGATCAAATGGATACTGAAGA
A3-AT2G28290-XLOC_009942-279-1
GATATCCAGCCCGGGCCATCCATTTTGGTTGATCAAATGGATACTGAAGA
CONSENSUS
GATATCCAGCCCGGGCCATCCATTTTGGTTGATCAAATGGATACTGAAGA

A3-AT2G28290-XLOC_009942-279-0
ATTCAAAAATCCTGATGTGTCTTTACACCAATTAGCTGATATTGAGCCCT
A3-AT2G28290-XLOC_009942-279-1
ATTCAAAAATCCTGATGTGTCTTTACACCAATTAGCTGATATTGAGCCCT
CONSENSUS
ATTCAAAAATCCTGATGTGTCTTTACACCAATTAGCTGATATTGAGCCCT

A3-AT2G28290-XLOC_009942-279-0
CACTGTCTATTTCAGCTGTGCAAAAGAATATTGAGGATAAGGATCAAAGT
A3-AT2G28290-XLOC_009942-279-1
CACTGTCTATTTCAGCTGTGCAAAAGAATATTGAGGATAAGGATCAAAGT
CONSENSUS
CACTGTCTATTTCAGCTGTGCAAAAGAATATTGAGGATAAGGATCAAAGT

A3-AT2G28290-XLOC_009942-279-0
CACGTTGAAACTGCTGGATCTGAGTTAGTTGATGTCTCTGCCGAATGTTC
A3-AT2G28290-XLOC_009942-279-1
CACGTTGAAACTGCTGGATCTGAGTTAGTTGATGTCTCTGCCGAATGTTC
CONSENSUS
CACGTTGAAACTGCTGGATCTGAGTTAGTTGATGTCTCTGCCGAATGTTC

A3-AT2G28290-XLOC_009942-279-0
AACAGAACCTCAAGTTCAATTACCGCCATCTTCAGAGCCAGTGGGAGATA
A3-AT2G28290-XLOC_009942-279-1
AACAGAACCTCAAGTTCAATTACCGCCATCTTCAGAGCCAGTGGGAGATA
CONSENSUS
AACAGAACCTCAAGTTCAATTACCGCCATCTTCAGAGCCAGTGGGAGATA

A3-AT2G28290-XLOC_009942-279-0
TGCACGTTCATTTAGGGGCAAGCAAATCAGAAATAGTTGCCGAAGGTACT
A3-AT2G28290-XLOC_009942-279-1
TGCACGTTCATTTAGGGGCAAGCAAATCAGAAATAGTTGCCGAAGGTACT
CONSENSUS

TGCACGTTCATTTAGGGGCAAGCAAATCAGAAATAGTTGCCGAAGGTACT

A3-AT2G28290-XLOC_009942-279-0
GACTTCTCTTCATCTCTCCCGAAGACGGAGGAAGAAAATGCCAAGAGCCA
A3-AT2G28290-XLOC_009942-279-1
GACTTCTCTTCATCTCTCCCGAAGACGGAGGAAGAAAATGCCAAGAGCCA
CONSENSUS
GACTTCTCTTCATCTCTCCCGAAGACGGAGGAAGAAAATGCCAAGAGCCA

A3-AT2G28290-XLOC_009942-279-0
ATTAGCTGACACCGAGCCATCATCGTCTCTTACAGCTGTGCAAAAGAACA
A3-AT2G28290-XLOC_009942-279-1
ATTAGCTGACACCGAGCCATCATCGTCTCTTACAGCTGTGCAAAAGAACA
CONSENSUS
ATTAGCTGACACCGAGCCATCATCGTCTCTTACAGCTGTGCAAAAGAACA

A3-AT2G28290-XLOC_009942-279-0
TTGAAGATCAAGTTGAAACTGCTGGATGTGAATTTGTTGTTGTCTCTACC
A3-AT2G28290-XLOC_009942-279-1
TTGAAGATCAAGTTGAAACTGCTGGATGTGAATTTGTTGTTGTCTCTACC
CONSENSUS
TTGAAGATCAAGTTGAAACTGCTGGATGTGAATTTGTTGTTGTCTCTACC

A3-AT2G28290-XLOC_009942-279-0
GGATGTTCAACAGAACCACAAGTTCAATTACCGCCGTCCGCAGAGCCAGT
A3-AT2G28290-XLOC_009942-279-1
GGATGTTCAACAGAACCACAAGTTCAATTACCGCCGTCCGCAGAGCCAGT
CONSENSUS
GGATGTTCAACAGAACCACAAGTTCAATTACCGCCGTCCGCAGAGCCAGT

A3-AT2G28290-XLOC_009942-279-0
GGTTGCTGAAGGTACAGAATTCCCTTCTTCCCTCCTAATGACCGGGGTAG
A3-AT2G28290-XLOC_009942-279-1
GGTTGCTGAAGGTACAGAATTCCCTTCTTCCCTCCTAATGACCGGGGTAG
CONSENSUS
GGTTGCTGAAGGTACAGAATTCCCTTCTTCCCTCCTAATGACCGGGGTAG

A3-AT2G28290-XLOC_009942-279-0
ATAATTCTTCCCATCTAATGACCGGGGTAGATAATGCCAAGACCCATCTC
A3-AT2G28290-XLOC_009942-279-1
ATAATTCTTCCCATCTAATGACCGGGGTAGATAATGCCAAGACCCATCTC
CONSENSUS
ATAATTCTTCCCATCTAATGACCGGGGTAGATAATGCCAAGACCCATCTC

A3-AT2G28290-XLOC_009942-279-0
GCTGATGTTGTGCCTTCATCGTCACCTACAACTATGGAAAAGAACATTGA
A3-AT2G28290-XLOC_009942-279-1
GCTGATGTTGTGCCTTCATCGTCACCTACAACTATGGAAAAGAACATTGA
CONSENSUS
GCTGATGTTGTGCCTTCATCGTCACCTACAACTATGGAAAAGAACATTGA

A3-AT2G28290-XLOC_009942-279-0
AGCTCAAGATCAAGATCAAGTTACAACTGGTGGATGTGGTCTAGTTGATG
A3-AT2G28290-XLOC_009942-279-1
AGCTCAAGATCAAGATCAAGTTACAACTGGTGGATGTGGTCTAGTTGATG
CONSENSUS

AGCTCAAGATCAAGATCAAGTTACAACTGGTGGATGTGGTCTAGTTGATG

A3-AT2G28290-XLOC_009942-279-0
TCTTGACCGAATGTTCGTCAGAACCTCAACTTCAACTGCCGCCATCCGCA
A3-AT2G28290-XLOC_009942-279-1
TCTTGACCGAATGTTCGTCAGAACCTCAACTTCAACTGCCGCCATCCGCA
CONSENSUS
TCTTGACCGAATGTTCGTCAGAACCTCAACTTCAACTGCCGCCATCCGCA

A3-AT2G28290-XLOC_009942-279-0
GAACCAGTGATTTCTGAAGGTACAGAACTCGCTACACTCCCATTGACGGA
A3-AT2G28290-XLOC_009942-279-1
GAACCAGTGATTTCTGAAGGTACAGAACTCGCTACACTCCCATTGACGGA
CONSENSUS
GAACCAGTGATTTCTGAAGGTACAGAACTCGCTACACTCCCATTGACGGA

A3-AT2G28290-XLOC_009942-279-0
GGAAGAAAATGCTGATAGCCAATTAGCTAATATTGAGCCCTCATCGTCTC
A3-AT2G28290-XLOC_009942-279-1
GGAAGAAAATGCTGATAGCCAATTAGCTAATATTGAGCCCTCATCGTCTC
CONSENSUS
GGAAGAAAATGCTGATAGCCAATTAGCTAATATTGAGCCCTCATCGTCTC

A3-AT2G28290-XLOC_009942-279-0
CTTCAGTTGTGGAAAAGAACATTGAGGCTCAAGATCAAGATCAAGTTAAA
A3-AT2G28290-XLOC_009942-279-1
CTTCAGTTGTGGAAAAGAACATTGAGGCTCAAGATCAAGATCAAGTTAAA
CONSENSUS
CTTCAGTTGTGGAAAAGAACATTGAGGCTCAAGATCAAGATCAAGTTAAA

A3-AT2G28290-XLOC_009942-279-0
ACTGCTGGATGTGAGTTAGTCTCGACTGGATGTTCGTCAGAACCACAAGT
A3-AT2G28290-XLOC_009942-279-1
ACTGCTGGATGTGAGTTAGTCTCGACTGGATGTTCGTCAGAACCACAAGT
CONSENSUS
ACTGCTGGATGTGAGTTAGTCTCGACTGGATGTTCGTCAGAACCACAAGT

A3-AT2G28290-XLOC_009942-279-0
TCATTTACCGCCCTCCGCAGAGCCAGATGGAGATATACACGTTCACTTAA
A3-AT2G28290-XLOC_009942-279-1
TCATTTACCGCCCTCCGCAGAGCCAGATGGAGATATACACGTTCACTTAA
CONSENSUS
TCATTTACCGCCCTCCGCAGAGCCAGATGGAGATATACACGTTCACTTAA

A3-AT2G28290-XLOC_009942-279-0
AGGAAACAGAGAAATCTGAAAGCATGGTTGTGGTTGGCGAAGGTACAGCA
A3-AT2G28290-XLOC_009942-279-1
AGGAAACAGAGAAATCTGAAAGCATGGTTGTGGTTGGCGAAGGTACAGCA
CONSENSUS
AGGAAACAGAGAAATCTGAAAGCATGGTTGTGGTTGGCGAAGGTACAGCA

A3-AT2G28290-XLOC_009942-279-0
TTCCCTTCATCTCTCCCAGTGACAGAGGAAGGAAATGCTGAGAGCCAATT
A3-AT2G28290-XLOC_009942-279-1
TTCCCTTCATCTCTCCCAGTGACAGAGGAAGGAAATGCTGAGAGCCAATT
CONSENSUS

TTCCCTTCATCTCTCCCAGTGACAGAGGAAGGAAATGCTGAGAGCCAATT

A3-AT2G28290-XLOC_009942-279-0
AGCTGACACTGAGCCCTTTACGTCTCCTACAGTTGTGGAAAAGAACATTA
A3-AT2G28290-XLOC_009942-279-1
AGCTGACACTGAGCCCTTTACGTCTCCTACAGTTGTGGAAAAGAACATTA
CONSENSUS
AGCTGACACTGAGCCCTTTACGTCTCCTACAGTTGTGGAAAAGAACATTA

A3-AT2G28290-XLOC_009942-279-0
AGGATCAAGAACAAGTTGAAACTACTGGATGTGGGTTAGTTGATGATTCT
A3-AT2G28290-XLOC_009942-279-1
AGGATCAAGAACAAGTTGAAACTACTGGATGTGGGTTAGTTGATGATTCT
CONSENSUS
AGGATCAAGAACAAGTTGAAACTACTGGATGTGGGTTAGTTGATGATTCT

A3-AT2G28290-XLOC_009942-279-0
ACCGGATGTTCGTCAGAACCTCAAGTTCAATTACCGCCATCCGCAGAGCC
A3-AT2G28290-XLOC_009942-279-1
ACCGGATGTTCGTCAGAACCTCAAGTTCAATTACCGCCATCCGCAGAGCC
CONSENSUS
ACCGGATGTTCGTCAGAACCTCAAGTTCAATTACCGCCATCCGCAGAGCC

A3-AT2G28290-XLOC_009942-279-0
AATGGAAGTTGTGCAAACGAATATTGAGGATCAAGATCAAATTGAAACAG
A3-AT2G28290-XLOC_009942-279-1
AATGGAAG--
CONSENSUS
AATGGAAG..

A3-AT2G28290-XLOC_009942-279-0
GTGGATGTGATTTAATTAATGTCCCTTCCGGATGTTCAACAGAACCTCAA
A3-AT2G28290-XLOC_009942-279-1
GTGGATGTGATTTAATTAATGTCCCTTCCGGATGTTCAACAGAACCTCAA
CONSENSUS
GTGGATGTGATTTAATTAATGTCCCTTCCGGATGTTCAACAGAACCTCAA

A3-AT2G28290-XLOC_009942-279-0
ATTCAATTATCGTCATCCGCAGAGCCCGAGGAAGGTATGCACATTCACTT
A3-AT2G28290-XLOC_009942-279-1
ATTCAATTATCGTCATCCGCAGAGCCCGAGGAAGGTATGCACATTCACTT
CONSENSUS
ATTCAATTATCGTCATCCGCAGAGCCCGAGGAAGGTATGCACATTCACTT

A3-AT2G28290-XLOC_009942-279-0
AGAGGCAGCAATGAACTCTGAAACGGTGGTTACTGAAGGTTCAGAACTCC
A3-AT2G28290-XLOC_009942-279-1
AGAGGCAGCAATGAACTCTGAAACGGTGGTTACTGAAGGTTCAGAACTCC
CONSENSUS
AGAGGCAGCAATGAACTCTGAAACGGTGGTTACTGAAGGTTCAGAACTCC

A3-AT2G28290-XLOC_009942-279-0
CTTCATCTCTCCCAATGACGGAGGACGAAAATGCTGATGGCCAATTAGCT
A3-AT2G28290-XLOC_009942-279-1
CTTCATCTCTCCCAATGACGGAGGACGAAAATGCTGATGGCCAATTAGCT
CONSENSUS

CTTCATCTCTCCCAATGACGGAGGACGAAAATGCTGATGGCCAATTAGCT

A3-AT2G28290-XLOC_009942-279-0
GAAGTCGAGCCCTCAGTGTCTCTTACAGTTGAGCAAACTAACATTGAGGA
A3-AT2G28290-XLOC_009942-279-1
GAAGTCGAGCCCTCAGTGTCTCTTACAGTTGAGCAAACTAACATTGAGGA
CONSENSUS
GAAGTCGAGCCCTCAGTGTCTCTTACAGTTGAGCAAACTAACATTGAGGA

A3-AT2G28290-XLOC_009942-279-0
GAAAGATCACATTGAAACTGCCGAATGTGAGTTAGTTGATGTCTCTCCCG
A3-AT2G28290-XLOC_009942-279-1
GAAAGATCACATTGAAACTGCCGAATGTGAGTTAGTTGATGTCTCTCCCG
CONSENSUS
GAAAGATCACATTGAAACTGCCGAATGTGAGTTAGTTGATGTCTCTCCCG

A3-AT2G28290-XLOC_009942-279-0
GATGTTCATCACAACCTGAAGTTAAATTTCCGCCATCCCCAGATGCAGTG
A3-AT2G28290-XLOC_009942-279-1
GATGTTCATCACAACCTGAAGTTAAATTTCCGCCATCCCCAGATGCAGTG
CONSENSUS
GATGTTCATCACAACCTGAAGTTAAATTTCCGCCATCCCCAGATGCAGTG

A3-AT2G28290-XLOC_009942-279-0
GGAGGTATGGACGTTCACTTAGAAACCGTTGTTACTGAAGACACAGATTC
A3-AT2G28290-XLOC_009942-279-1
GGAGGTATGGACGTTCACTTAGAAACCGTTGTTACTGAAGACACAGATTC
CONSENSUS
GGAGGTATGGACGTTCACTTAGAAACCGTTGTTACTGAAGACACAGATTC

A3-AT2G28290-XLOC_009942-279-0
AAATTCATCCCTCCCGAAGACGGAGGAAAAAGATGCCGAGAATCCATCAG
A3-AT2G28290-XLOC_009942-279-1
AAATTCATCCCTCCCGAAGACGGAGGAAAAAGATGCCGAGAATCCATCAG
CONSENSUS
AAATTCATCCCTCCCGAAGACGGAGGAAAAAGATGCCGAGAATCCATCAG

A3-AT2G28290-XLOC_009942-279-0
ACAGGCTTGACGGTGAATCCGATGGTACAACTGTTGCTACTGTTGAAGGA
A3-AT2G28290-XLOC_009942-279-1
ACAGGCTTGACGGTGAATCCGATGGTACAACTGTTGCTACTGTTGAAGGA
CONSENSUS
ACAGGCTTGACGGTGAATCCGATGGTACAACTGTTGCTACTGTTGAAGGA

A3-AT2G28290-XLOC_009942-279-0
ACTTGTGTTGAGTCGAATTCATTGGTCGCCGAAGAGAGCAACATAGAAGT
A3-AT2G28290-XLOC_009942-279-1
ACTTGTGTTGAGTCGAATTCATTGGTCGCCGAAGAGAGCAACATAGAAGT
CONSENSUS
ACTTGTGTTGAGTCGAATTCATTGGTCGCCGAAGAGAGCAACATAGAAGT

A3-AT2G28290-XLOC_009942-279-0
GCCAAAAGACAATGAAGATGTGTAGTTCATTTCATCTAATATTAAGTTGT
A3-AT2G28290-XLOC_009942-279-1
GCCAAAAGACAATGAAGATGTGTAGTTCATTTCATCTAATATTAAGTTGT
CONSENSUS

```
      GCCAAAAGACAATGAAGATGTGTAGTTCATTTCATCTAATATTAAGTTGT

A3-AT2G28290-XLOC_009942-279-0
      AAAACTTTTAATATCTAATATAACAAACCCAATGTTGAAGATGTATTGAC
A3-AT2G28290-XLOC_009942-279-1
      AAAACTTTTAATATCTAATATAACAAACCCAATGTTGAAGATGTATTGAC
CONSENSUS
      AAAACTTTTAATATCTAATATAACAAACCCAATGTTGAAGATGTATTGAC

A3-AT2G28290-XLOC_009942-279-0
      TGCATCTGACTTATTTAACTTATCCTCTCATTTGGGAATATTAATAGGGC
A3-AT2G28290-XLOC_009942-279-1
      TGCATCTGACTTATTTAACTTATCCTCTCATTTGGGAATATTAATAGGGC
CONSENSUS
      TGCATCTGACTTATTTAACTTATCCTCTCATTTGGGAATATTAATAGGGC

A3-AT2G28290-XLOC_009942-279-0   TTAGAATC
A3-AT2G28290-XLOC_009942-279-1   TTAGAATC
CONSENSUS                        TTAGAATC



alignment for event: A3-AT2G37340-XLOC_013195-4336

A3-AT2G37340-XLOC_013195-4336-0
      CTTGACAATGCCTCGCTATGATGATCGCTATGGGAACACTCGTCTTTACG
A3-AT2G37340-XLOC_013195-4336-1
      CTTGACAATGCCTCGCTATGATGATCGCTATGGGAACACTCGTCTTTACG
CONSENSUS
      CTTGACAATGCCTCGCTATGATGATCGCTATGGGAACACTCGTCTTTACG

A3-AT2G37340-XLOC_013195-4336-0
      TTGGCCGATTATCATCGAGAACTAGGACCCGAGACCTTGAACGTCTTTTT
A3-AT2G37340-XLOC_013195-4336-1
      TTGGCCGATTATCATCGAGAACTAGGACCCGAGACCTTGAACGTCTTTTT
CONSENSUS
      TTGGCCGATTATCATCGAGAACTAGGACCCGAGACCTTGAACGTCTTTTT

A3-AT2G37340-XLOC_013195-4336-0
      AGCAGATACGGAAG------------------------------------
A3-AT2G37340-XLOC_013195-4336-1
      AGCAGATACGGAAGGTCTCTGGGATTTTGCCTAACCTGGAGGTGTTTGAT
CONSENSUS
      AGCAGATACGGAAG....................................

A3-AT2G37340-XLOC_013195-4336-0
      --------------------------------------------------
A3-AT2G37340-XLOC_013195-4336-1
      GAGTGGGTGGACTTGGTGGAAATTCTCGCCAAGTGTTTAGGGGACAGGGC
CONSENSUS
      ..................................................

A3-AT2G37340-XLOC_013195-4336-0
      --------------------------------------------------
A3-AT2G37340-XLOC_013195-4336-1
      GCCTTGGCGATTCTGTTTGTAGAATTTTTTAATGTGTTTTGATGGAAATG
```

```
CONSENSUS
      ..................................................

A3-AT2G37340-XLOC_013195-4336-0
      --------------------------------------------------
A3-AT2G37340-XLOC_013195-4336-1
      GAGACTCAGTCTCAAAAATGGTGTTTAGAAGTACCTTAAAGTAATGATGG
CONSENSUS
      ..................................................

A3-AT2G37340-XLOC_013195-4336-0
      --------------------------------AGTGCGAGATGTGGATAT
A3-AT2G37340-XLOC_013195-4336-1
      CCTTACTTTTCTGCCAACAATTTCTTCCGCAGAGTGCGAGATGTGGATAT
CONSENSUS
      ................................AGTGCGAGATGTGGATAT

A3-AT2G37340-XLOC_013195-4336-0  GAAGCGAGATTATGCTTTCGTT
A3-AT2G37340-XLOC_013195-4336-1  GAAGCGAGATTATGCTTTCGTT
CONSENSUS                        GAAGCGAGATTATGCTTTCGTT


alignment for event: RI-AT2G19250-XLOC_009416-6934

RI-AT2G19250-XLOC_009416-6934-0
      ATGAATGATGATGAAAATATGAATTTTGTCTGTTGCGTGAGAGATTCCAG
RI-AT2G19250-XLOC_009416-6934-1
      ATGAATGATGATGAAAATATGAATTTTGTCTGTTGCGTGAGAGATTCCAG
CONSENSUS
      ATGAATGATGATGAAAATATGAATTTTGTCTGTTGCGTGAGAGATTCCAG

RI-AT2G19250-XLOC_009416-6934-0
      GCGCGAAGAGCCGATTGATGAAGAAGAACAAGTAGCTAAATGCTAATTAA
RI-AT2G19250-XLOC_009416-6934-1
      GCGCGAAGAGCCGATTGATGAAGAAGAACAAGTAGCTAAATGCTAATTAA
CONSENSUS
      GCGCGAAGAGCCGATTGATGAAGAAGAACAAGTAGCTAAATGCTAATTAA

RI-AT2G19250-XLOC_009416-6934-0
      ATTAAAATTGAATTTTGTCTTGTTCTTTTTGTTAATTGTAACCTAAAAAA
RI-AT2G19250-XLOC_009416-6934-1
      ATTAAAATTGAATTTTGTCTTGTTCTTTTTGTTAATTGTAACCTAAAAAA
CONSENSUS
      ATTAAAATTGAATTTTGTCTTGTTCTTTTTGTTAATTGTAACCTAAAAAA

RI-AT2G19250-XLOC_009416-6934-0
      TGGGTATTAATTATTAAAATAATGTTGAGAAAAAAATCCAAAATTAATTA
RI-AT2G19250-XLOC_009416-6934-1
      TGGGTATTAATTATTAAAATAATGTTGAGAAAAAAATCCAAAATTAATTA
CONSENSUS
      TGGGTATTAATTATTAAAATAATGTTGAGAAAAAAATCCAAAATTAATTA

RI-AT2G19250-XLOC_009416-6934-0
      GAGCACAAAAGAGTCATTCCAGGAATGTTGAGAAAAAAGGTATTAAATAT
RI-AT2G19250-XLOC_009416-6934-1
```

GAGCACAAAAGAGTCATTCCAGGAATGTTGAGAAAAAAGGTATTAAATAT
CONSENSUS
GAGCACAAAAGAGTCATTCCAGGAATGTTGAGAAAAAAGGTATTAAATAT

RI-AT2G19250-XLOC_009416-6934-0
CAGTAATATATTATCACTGAAAAATAAATTATCACTTAATTGAAATACCC
RI-AT2G19250-XLOC_009416-6934-1
CAGTAATATATTATCACTGAAAAATAAATTATCACTTAATTGAAATACCC
CONSENSUS
CAGTAATATATTATCACTGAAAAATAAATTATCACTTAATTGAAATACCC

RI-AT2G19250-XLOC_009416-6934-0
TATAATCAGATTTTTCTTTGATAATTAATTAATTGACCATCTTTTTCTCT
RI-AT2G19250-XLOC_009416-6934-1
TATAATCAGATTTTTCTTTGATAATTAATTAATTGACCATCTTTTTCTCT
CONSENSUS
TATAATCAGATTTTTCTTTGATAATTAATTAATTGACCATCTTTTTCTCT

RI-AT2G19250-XLOC_009416-6934-0
TTTTGCATAATTAAATTGACCATCTGTTAATTGCCTATGGATTTATTTTT
RI-AT2G19250-XLOC_009416-6934-1
TTTTGCATAATTAAATTGACCATCTGTTAATTGCCTATGGATTTATTTTT
CONSENSUS
TTTTGCATAATTAAATTGACCATCTGTTAATTGCCTATGGATTTATTTTT

RI-AT2G19250-XLOC_009416-6934-0
ATTTTTATTTTTTTGGGAAACAGTTATTTTCTTTTTTCCAAAGTAAAACC
RI-AT2G19250-XLOC_009416-6934-1
ATTTTTATTTTTTTGGGAAACAGTTATTTTCTTTTTTCCAAAGTAAAACC
CONSENSUS
ATTTTTATTTTTTTGGGAAACAGTTATTTTCTTTTTTCCAAAGTAAAACC

RI-AT2G19250-XLOC_009416-6934-0
AACAAACAAATTTGAAATATGTCTCAAAAATATAGGGAATGGAATGTAAT
RI-AT2G19250-XLOC_009416-6934-1
AACAAACAAATTTGAAATATGTCTCAAAAATATAGGGAATGGAATGTAAT
CONSENSUS
AACAAACAAATTTGAAATATGTCTCAAAAATATAGGGAATGGAATGTAAT

RI-AT2G19250-XLOC_009416-6934-0
TTTCATTTCATGAACATAAAACAAATTGAATATAGATTGTTAAATAGAGG
RI-AT2G19250-XLOC_009416-6934-1
TTTCATTTCATGAACATAAAACAAATTGAATATAGATTGTTAAATAGAGG
CONSENSUS
TTTCATTTCATGAACATAAAACAAATTGAATATAGATTGTTAAATAGAGG

RI-AT2G19250-XLOC_009416-6934-0
GGCTCAAATGTAAAAACGTTAAGAGTTGAACAAAAAGTAAAGTTGAAGGT
RI-AT2G19250-XLOC_009416-6934-1
GGCTCAAATGTAAAAACGTTAAGAGTTGAACAAAAAGTAAAGTTGAAGGT
CONSENSUS
GGCTCAAATGTAAAAACGTTAAGAGTTGAACAAAAAGTAAAGTTGAAGGT

RI-AT2G19250-XLOC_009416-6934-0
GACGACGAACCCAAACCCCACAGCTTCGTCAGTCGCCGCCGGTGACTCAA
RI-AT2G19250-XLOC_009416-6934-1

GACGACGAACCCAAACCCCACAGCTTCGTCAGTCGCCGCCGGTGACTCAA
CONSENSUS
GACGACGAACCCAAACCCCACAGCTTCGTCAGTCGCCGCCGGTGACTCAA

RI-AT2G19250-XLOC_009416-6934-0
CCGACCACGCATGATCGGTAAGTAATTTATCGCAGTTCTGTATCAATTTT
RI-AT2G19250-XLOC_009416-6934-1
CCGACCACGCATGATCG---------------------------------
CONSENSUS
CCGACCACGCATGATCG.................................

RI-AT2G19250-XLOC_009416-6934-0
TCGATTCAGATGCGAAAAGACTTGATGGCTTCTGGCTTTGCATCAATTTT
RI-AT2G19250-XLOC_009416-6934-1

CONSENSUS
....

RI-AT2G19250-XLOC_009416-6934-0
TGCTCTTTATCATATACACTGCTCAACAACAGTTTCAATATGATCTTTGA
RI-AT2G19250-XLOC_009416-6934-1

CONSENSUS
....

RI-AT2G19250-XLOC_009416-6934-0
ACAGTTTACATTACTCTGTATATGTAATGTCTGTGTTTCGTTATATTTTT
RI-AT2G19250-XLOC_009416-6934-1

CONSENSUS
....

RI-AT2G19250-XLOC_009416-6934-0
CTAAATTGACTTGACTTCGTGTGTATCACTACTTTTAAAAAGAGGAATTT
RI-AT2G19250-XLOC_009416-6934-1

CONSENSUS
....

RI-AT2G19250-XLOC_009416-6934-0
GACGTGCGTCTCTGTGTCTGTTTCTCTATATGAGTCTGATATAGAGCTAC
RI-AT2G19250-XLOC_009416-6934-1

CONSENSUS
....

RI-AT2G19250-XLOC_009416-6934-0
TACAGATGTTTAATACTCAATATAGTTGAAAGAGAAGGAATCAGCTTTAG
RI-AT2G19250-XLOC_009416-6934-1 -----
ATGTTTAATACTCAATATAGTTGAAAGAGAAGGAATCAGCTTTAG
CONSENSUS
.....ATGTTTAATACTCAATATAGTTGAAAGAGAAGGAATCAGCTTTAG

RI-AT2G19250-XLOC_009416-6934-0
GAACCCATGCAAGCCACTTTTAGTTGCAATCTACCAACCTGAAGAAGGTG
RI-AT2G19250-XLOC_009416-6934-1

GAACCCATGCAAGCCACTTTTAGTTGCAATCTACCAACCTGAAGAAGGTG
CONSENSUS
GAACCCATGCAAGCCACTTTTAGTTGCAATCTACCAACCTGAAGAAGGTG

RI-AT2G19250-XLOC_009416-6934-0
CTATCCAAGAGTAACCGTGTTGCCATTAATTTAAG
RI-AT2G19250-XLOC_009416-6934-1
CTATCCAAGAGTAACCGTGTTGCCATTAATTTAAG
CONSENSUS
CTATCCAAGAGTAACCGTGTTGCCATTAATTTAAG

alignment for event: RI-AT2G28290-XLOC_009942-276

RI-AT2G28290-XLOC_009942-276-0
CTCAAGAAGCCAATGTTCCTTCTCTTCCAGCAGCCTTGCCTGCTAAGAGG
RI-AT2G28290-XLOC_009942-276-1
CTCAAGAAGCCAATGTTCCTTCTCTTCCAGCAGCCTTGCCTGCTAAGAGG
CONSENSUS
CTCAAGAAGCCAATGTTCCTTCTCTTCCAGCAGCCTTGCCTGCTAAGAGG

RI-AT2G28290-XLOC_009942-276-0
CGAGTCCGCAATTTGCCAAGCAGAGGAGAAACTCCTAAACGCCAAGGAAA
RI-AT2G28290-XLOC_009942-276-1
CGAGTCCGCAATTTGCCAAGCAGAGGAGAAACTCCTAAACGCCAAGGAAA
CONSENSUS
CGAGTCCGCAATTTGCCAAGCAGAGGAGAAACTCCTAAACGCCAAGGAAA

RI-AT2G28290-XLOC_009942-276-0
GAGGCGTGGCCAACCTTTACCTGCAACCGATGCCTCTTCTGCAAGGAGTA
RI-AT2G28290-XLOC_009942-276-1
GAGGCGTGGCCAACCTTTACCTGCAACCGATGCCTCTTCTGCAAGGAGTA
CONSENSUS
GAGGCGTGGCCAACCTTTACCTGCAACCGATGCCTCTTCTGCAAGGAGTA

RI-AT2G28290-XLOC_009942-276-0
CAGGATTAACACCACAAATAGAGGTCAAGGTTGGTAATTTATCAGGCACC
RI-AT2G28290-XLOC_009942-276-1
CAGGATTAACACCACAAATAGAGGTCAAGGTTGGTAATTTATCAGGCACC
CONSENSUS
CAGGATTAACACCACAAATAGAGGTCAAGGTTGGTAATTTATCAGGCACC

RI-AT2G28290-XLOC_009942-276-0
AAAGCTAAGTTTGATGCTGTTGCCAAAGAACAACCCCACTTCAGCCAGTC
RI-AT2G28290-XLOC_009942-276-1
AAAGCTAAGTTTGATGCTGTTGCCAAAGAACAACCCCACTTCAGCCAGTC
CONSENSUS
AAAGCTAAGTTTGATGCTGTTGCCAAAGAACAACCCCACTTCAGCCAGTC

RI-AT2G28290-XLOC_009942-276-0
AGTTGCACCCGATATTCACTCTTCTGGTAGTTTGAGTCAGGAAATTAGAA
RI-AT2G28290-XLOC_009942-276-1
AGTTGCACCCGATATTCACTCTTCTGGTAGTTTGAGTCAGGAAATTAGAA
CONSENSUS
AGTTGCACCCGATATTCACTCTTCTGGTAGTTTGAGTCAGGAAATTAGAA

RI-AT2G28290-XLOC_009942-276-0
GAGACACCTCTGGTACTGGTGGTTCTGCTAGGAAACAAACTGCTGATGTA
RI-AT2G28290-XLOC_009942-276-1
GAGACACCTCTGGTACTGGTGGTTCTGCTAGGAAACAAACTGCTGATGTA
CONSENSUS
GAGACACCTCTGGTACTGGTGGTTCTGCTAGGAAACAAACTGCTGATGTA

RI-AT2G28290-XLOC_009942-276-0
ACTGATGTTGCTCGAGTCATGAAAGAGATCTTTTCAGAGACTTCCCTATT
RI-AT2G28290-XLOC_009942-276-1
ACTGATGTTGCTCGAGTCATGAAAGAGATCTTTTCAGAGACTTCCCTATT
CONSENSUS
ACTGATGTTGCTCGAGTCATGAAAGAGATCTTTTCAGAGACTTCCCTATT

RI-AT2G28290-XLOC_009942-276-0
AAAACATAAAGTTGGAGAGCCTTCTGCAACAACGAGAACAAATGTGCCTG
RI-AT2G28290-XLOC_009942-276-1
AAAACATAAAGTTGGAGAGCCTTCTGCAACAACGAGAACAAATGTGCCTG
CONSENSUS
AAAACATAAAGTTGGAGAGCCTTCTGCAACAACGAGAACAAATGTGCCTG

RI-AT2G28290-XLOC_009942-276-0
ACGCACAATCCCCTGGTGAGATGAATTTGCACACAGTTGAGACCCACAAG
RI-AT2G28290-XLOC_009942-276-1
ACGCACAATCCCCTGGTGAGATGAATTTGCACACAGTTGAGACCCACAAG
CONSENSUS
ACGCACAATCCCCTGGTGAGATGAATTTGCACACAGTTGAGACCCACAAG

RI-AT2G28290-XLOC_009942-276-0
GCAGAGGATTCTTCTGGTCTTAAGAATCAAGAAGCTTTATATAACCTGAG
RI-AT2G28290-XLOC_009942-276-1
GCAGAGGATTCTTCTGGTCTTAAGAATCAAGAAGCTTTATATAACCTGAG
CONSENSUS
GCAGAGGATTCTTCTGGTCTTAAGAATCAAGAAGCTTTATATAACCTGAG

RI-AT2G28290-XLOC_009942-276-0
CAAGGCAGATAAACTGGTATCAGATATTCCTCATCCTGTTCCTGGTGATC
RI-AT2G28290-XLOC_009942-276-1
CAAGGCAGATAAACTGGTATCAGATATTCCTCATCCTGTTCCTGGTGATC
CONSENSUS
CAAGGCAGATAAACTGGTATCAGATATTCCTCATCCTGTTCCTGGTGATC

RI-AT2G28290-XLOC_009942-276-0
TGACAACTTCAGGATCAGTTGCAAACAAAGATGTTGACATTGGGTCGTCT
RI-AT2G28290-XLOC_009942-276-1
TGACAACTTCAGGATCAGTTGCAAACAAAGATGTTGACATTGGGTCGTCT
CONSENSUS
TGACAACTTCAGGATCAGTTGCAAACAAAGATGTTGACATTGGGTCGTCT

RI-AT2G28290-XLOC_009942-276-0
AAGGTTGCTGCTGAAAATGAGCTTGTCAAAATTCCGGGTGGTGACGTAGA
RI-AT2G28290-XLOC_009942-276-1
AAGGTTGCTGCTGAAAATGAGCTTGTCAAAATTCCGGGTGGTGACGTAGA
CONSENSUS
AAGGTTGCTGCTGAAAATGAGCTTGTCAAAATTCCGGGTGGTGACGTAGA

RI-AT2G28290-XLOC_009942-276-0
TTCTTCTGTAATACAACTCTCTTTGGGAAATACTTTGACTGCTAAATCGT
RI-AT2G28290-XLOC_009942-276-1
TTCTTCTGTAATACAACTCTCTTTGGGAAATACTTTGACTGCTAAATCGT
CONSENSUS
TTCTTCTGTAATACAACTCTCTTTGGGAAATACTTTGACTGCTAAATCGT

RI-AT2G28290-XLOC_009942-276-0
CTTTGGAAAAGTGCACTGCAGATCAGCTTCTGGGAGAAAAACTGTCTCAA
RI-AT2G28290-XLOC_009942-276-1
CTTTGGAAAAGTGCACTGCAGATCAGCTTCTGGGAGAAAAACTGTCTCAA
CONSENSUS
CTTTGGAAAAGTGCACTGCAGATCAGCTTCTGGGAGAAAAACTGTCTCAA

RI-AT2G28290-XLOC_009942-276-0
GAAGGTGAAACCACACCTGCTAGTGATGGTGAAACATGTCACCTGGCAGA
RI-AT2G28290-XLOC_009942-276-1
GAAGGTGAAACCACACCTGCTAGTGATGGTGAAACATGTCACCTGGCAGA
CONSENSUS
GAAGGTGAAACCACACCTGCTAGTGATGGTGAAACATGTCACCTGGCAGA

RI-AT2G28290-XLOC_009942-276-0
AGAAACGGCATCTTCATTGAGTTATGTTCGATCTGAGCCTACTGCATCTG
RI-AT2G28290-XLOC_009942-276-1
AGAAACGGCATCTTCATTGAGTTATGTTCGATCTGAGCCTACTGCATCTG
CONSENSUS
AGAAACGGCATCTTCATTGAGTTATGTTCGATCTGAGCCTACTGCATCTG

RI-AT2G28290-XLOC_009942-276-0
CGTCGACAACTGCGGAACCTCTACCTACTGACAAGTTGGAAAAAAATATT
RI-AT2G28290-XLOC_009942-276-1
CGTCGACAACTGCGGAACCTCTACCTACTGACAAGTTGGAAAAAAATATT
CONSENSUS
CGTCGACAACTGCGGAACCTCTACCTACTGACAAGTTGGAAAAAAATATT

RI-AT2G28290-XLOC_009942-276-0
TCTTTTCAAGATGAAGTTAAAACTCTCAATGGTGATAAAAGAGAAGCTAT
RI-AT2G28290-XLOC_009942-276-1
TCTTTTCAAGATGAAGTTAAAACTCTCAATGGTGATAAAAGAGAAGCTAT
CONSENSUS
TCTTTTCAAGATGAAGTTAAAACTCTCAATGGTGATAAAAGAGAAGCTAT

RI-AT2G28290-XLOC_009942-276-0
CCTCCTAAGTTCGGAAGAGCAAACGAATGTTAACTCCAAGATTGAGACAA
RI-AT2G28290-XLOC_009942-276-1
CCTCCTAAGTTCGGAAGAGCAAACGAATGTTAACTCCAAGATTGAGACAA
CONSENSUS
CCTCCTAAGTTCGGAAGAGCAAACGAATGTTAACTCCAAGATTGAGACAA

RI-AT2G28290-XLOC_009942-276-0
ATTCTGAGGAACTTCAAGCCAGTAGAACAGATGAAGTTCCACATGTGGAT
RI-AT2G28290-XLOC_009942-276-1
ATTCTGAGGAACTTCAAGCCAGTAGAACAGATGAAGTTCCACATGTGGAT
CONSENSUS
ATTCTGAGGAACTTCAAGCCAGTAGAACAGATGAAGTTCCACATGTGGAT

RI-AT2G28290-XLOC_009942-276-0
GGAAAATCTGTTGATGTTGCAAATCAGACGGTGAAAGAAGATGAGGCAAA
RI-AT2G28290-XLOC_009942-276-1
GGAAAATCTGTTGATGTTGCAAATCAGACGGTGAAAGAAGATGAGGCAAA
CONSENSUS
GGAAAATCTGTTGATGTTGCAAATCAGACGGTGAAAGAAGATGAGGCAAA

RI-AT2G28290-XLOC_009942-276-0
ACATTCTGTTGAAATTCAATCGTCTATGCTGGAGCCTGATGAACTGCCAA
RI-AT2G28290-XLOC_009942-276-1
ACATTCTGTTGAAATTCAATCGTCTATGCTGGAGCCTGATGAACTGCCAA
CONSENSUS
ACATTCTGTTGAAATTCAATCGTCTATGCTGGAGCCTGATGAACTGCCAA

RI-AT2G28290-XLOC_009942-276-0
ATGCTGGACAAAAGGGTCACAGTAGCATTGACTTGCAGCCATTGGTTTTA
RI-AT2G28290-XLOC_009942-276-1
ATGCTGGACAAAAGGGTCACAGTAGCATTGACTTGCAGCCATTGGTTTTA
CONSENSUS
ATGCTGGACAAAAGGGTCACAGTAGCATTGACTTGCAGCCATTGGTTTTA

RI-AT2G28290-XLOC_009942-276-0
GTTACAAGCAATGAGAATGCTATGTCCCTTGACGATAAAGATTATGATCC
RI-AT2G28290-XLOC_009942-276-1
GTTACAAGCAATGAGAATGCTATGTCCCTTGACGATAAAGATTATGATCC
CONSENSUS
GTTACAAGCAATGAGAATGCTATGTCCCTTGACGATAAAGATTATGATCC

RI-AT2G28290-XLOC_009942-276-0
TATCTCTAAATCTGCTGATATAGAACAAGATCCTGAAGAATCTGTTTTTG
RI-AT2G28290-XLOC_009942-276-1
TATCTCTAAATCTGCTGATATAGAACAAGATCCTGAAGAATCTGTTTTTG
CONSENSUS
TATCTCTAAATCTGCTGATATAGAACAAGATCCTGAAGAATCTGTTTTTG

RI-AT2G28290-XLOC_009942-276-0
TTCAAGGTGTTGGTAGGCCTAAAGTTGGTACTGCTGATACACAGATGGAG
RI-AT2G28290-XLOC_009942-276-1
TTCAAGGTGTTGGTAGGCCTAAAGTTGGTACTGCTGATACACAGATGGAG
CONSENSUS
TTCAAGGTGTTGGTAGGCCTAAAGTTGGTACTGCTGATACACAGATGGAG

RI-AT2G28290-XLOC_009942-276-0
GATACCAATGATGCCAAACTTCTAGTGGGTTGTTCAGTTGAGAGTGAGGA
RI-AT2G28290-XLOC_009942-276-1
GATACCAATGATGCCAAACTTCTAGTGGGTTGTTCAGTTGAGAGTGAGGA
CONSENSUS
GATACCAATGATGCCAAACTTCTAGTGGGTTGTTCAGTTGAGAGTGAGGA

RI-AT2G28290-XLOC_009942-276-0
AAAAGAGAAAACTCTTCAATCCCTCATACCCGGTGATGATGCTGATACAG
RI-AT2G28290-XLOC_009942-276-1
AAAAGAGAAAACTCTTCAATCCCTCATACCCGGTGATGATGCTGATACAG
CONSENSUS
AAAAGAGAAAACTCTTCAATCCCTCATACCCGGTGATGATGCTGATACAG

RI-AT2G28290-XLOC_009942-276-0
AACAAGATCCTGAAGAATCTGTTTCGGATCAAAGGCCTAAAGTTGGTTCT
RI-AT2G28290-XLOC_009942-276-1
AACAAGATCCTGAAGAATCTGTTTCGGATCAAAGGCCTAAAGTTGGTTCT
CONSENSUS
AACAAGATCCTGAAGAATCTGTTTCGGATCAAAGGCCTAAAGTTGGTTCT

RI-AT2G28290-XLOC_009942-276-0
GCTTACACACAGATGGAGGATACGGATGAGGCGAAACTTCTAATGGGTTG
RI-AT2G28290-XLOC_009942-276-1
GCTTACACACAGATGGAGGATACGGATGAGGCGAAACTTCTAATGGGTTG
CONSENSUS
GCTTACACACAGATGGAGGATACGGATGAGGCGAAACTTCTAATGGGTTG

RI-AT2G28290-XLOC_009942-276-0
TTCAGTTGAGAGTGAGGAAAAAGAGAAAACTCTTCAATCCCATATACCCG
RI-AT2G28290-XLOC_009942-276-1
TTCAGTTGAGAGTGAGGAAAAAGAGAAAACTCTTCAATCCCATATACCCG
CONSENSUS
TTCAGTTGAGAGTGAGGAAAAAGAGAAAACTCTTCAATCCCATATACCCG

RI-AT2G28290-XLOC_009942-276-0
GTGATGATGCTGATACAGAAAAAAATCCTGAAGAATCTGTTTCCGTTCAA
RI-AT2G28290-XLOC_009942-276-1
GTGATGATGCTGATACAGAAAAAAATCCTGAAGAATCTGTTTCCGTTCAA
CONSENSUS
GTGATGATGCTGATACAGAAAAAAATCCTGAAGAATCTGTTTCCGTTCAA

RI-AT2G28290-XLOC_009942-276-0
GGTGTTGATAGGCCGAAAGTTGGTACTACTGACACACAGATGGAGGATAC
RI-AT2G28290-XLOC_009942-276-1
GGTGTTGATAGGCCGAAAGTTGGTACTACTGACACACAGATGGAGGATAC
CONSENSUS
GGTGTTGATAGGCCGAAAGTTGGTACTACTGACACACAGATGGAGGATAC

RI-AT2G28290-XLOC_009942-276-0
CAATGATGCCAAACTTCTAGTGGGTTGTTCAGTTGCGAGTGAGGAGAAAG
RI-AT2G28290-XLOC_009942-276-1
CAATGATGCCAAACTTCTAGTGGGTTGTTCAGTTGCGAGTGAGGAGAAAG
CONSENSUS
CAATGATGCCAAACTTCTAGTGGGTTGTTCAGTTGCGAGTGAGGAGAAAG

RI-AT2G28290-XLOC_009942-276-0
AGAAAACTCTTCAATCCCATATACCCGGTGATGATGCTGATACAGAACAA
RI-AT2G28290-XLOC_009942-276-1
AGAAAACTCTTCAATCCCATATACCCGGTGATGATGCTGATACAGAACAA
CONSENSUS
AGAAAACTCTTCAATCCCATATACCCGGTGATGATGCTGATACAGAACAA

RI-AT2G28290-XLOC_009942-276-0
AATCCTGAAGAATCTGTTTCAGTTCAAGGTGTTAATAGGCCTAAAGTTGG
RI-AT2G28290-XLOC_009942-276-1
AATCCTGAAGAATCTGTTTCAGTTCAAGGTGTTAATAGGCCTAAAGTTGG
CONSENSUS
AATCCTGAAGAATCTGTTTCAGTTCAAGGTGTTAATAGGCCTAAAGTTGG

RI-AT2G28290-XLOC_009942-276-0
TAATGCTAACACACAGATGGAGGATACGGATGAGGCCAAAGTTCTAGTGG
RI-AT2G28290-XLOC_009942-276-1
TAATGCTAACACACAGATGGAGGATACGGATGAGGCCAAAGTTCTAGTGG
CONSENSUS
TAATGCTAACACACAGATGGAGGATACGGATGAGGCCAAAGTTCTAGTGG

RI-AT2G28290-XLOC_009942-276-0
GTTGTTCAGTTGAGAGTGAGGAGAAAGAGAAAACTCTTCAATCCCACATA
RI-AT2G28290-XLOC_009942-276-1
GTTGTTCAGTTGAGAGTGAGGAGAAAGAGAAAACTCTTCAATCCCACATA
CONSENSUS
GTTGTTCAGTTGAGAGTGAGGAGAAAGAGAAAACTCTTCAATCCCACATA

RI-AT2G28290-XLOC_009942-276-0
CCTGGTGATGATGCTGATACAGAACAAAATCCTGAAGAATCTGTTTCGAA
RI-AT2G28290-XLOC_009942-276-1
CCTGGTGATGATGCTGATACAGAACAAAATCCTGAAGAATCTGTTTCGAA
CONSENSUS
CCTGGTGATGATGCTGATACAGAACAAAATCCTGAAGAATCTGTTTCGAA

RI-AT2G28290-XLOC_009942-276-0
TTTTGATAGGCCTAAAGATGGGACTGCTGACACACATATGGAGGATATCG
RI-AT2G28290-XLOC_009942-276-1
TTTTGATAGGCCTAAAGATGGGACTGCTGACACACATATGGAGGATATCG
CONSENSUS
TTTTGATAGGCCTAAAGATGGGACTGCTGACACACATATGGAGGATATCG

RI-AT2G28290-XLOC_009942-276-0
ATGATGCCAAACTTCTAGTGGGTTGTTCAGTTGAGAGTGAGGAGAAAGAG
RI-AT2G28290-XLOC_009942-276-1
ATGATGCCAAACTTCTAGTGGGTTGTTCAGTTGAGAGTGAGGAGAAAGAG
CONSENSUS
ATGATGCCAAACTTCTAGTGGGTTGTTCAGTTGAGAGTGAGGAGAAAGAG

RI-AT2G28290-XLOC_009942-276-0
AAAAGTCTTCAATCCCATATGCCCAGTGATGATGCTGTTCTCCATGCGCC
RI-AT2G28290-XLOC_009942-276-1
AAAAGTCTTCAATCCCATATGCCCAGTGATGATGCTGTTCTCCATGCGCC
CONSENSUS
AAAAGTCTTCAATCCCATATGCCCAGTGATGATGCTGTTCTCCATGCGCC

RI-AT2G28290-XLOC_009942-276-0
TTTTGAGAACACAAAAGACAGTAAAGGAGATGATTTACATGGAGAGTCTC
RI-AT2G28290-XLOC_009942-276-1
TTTTGAGAACACAAAAGACAGTAAAGGAGATGATTTACATGGAGAGTCTC
CONSENSUS
TTTTGAGAACACAAAAGACAGTAAAGGAGATGATTTACATGGAGAGTCTC

RI-AT2G28290-XLOC_009942-276-0
TTGTTTCCTGTCCAACAATGGAAGTGATGGAACAGAAGGGGTTTGAATCA
RI-AT2G28290-XLOC_009942-276-1
TTGTTTCCTGTCCAACAATGGAAGTGATGGAACAGAAGGGGTTTGAATCA
CONSENSUS
TTGTTTCCTGTCCAACAATGGAAGTGATGGAACAGAAGGGGTTTGAATCA

RI-AT2G28290-XLOC_009942-276-0
GAGACACATGCTCGTACAGATTCAGGTGGTATTGATAGGGGAAATGAGGT
RI-AT2G28290-XLOC_009942-276-1
GAGACACATGCTCGTACAGATTCAGGTGGTATTGATAGGGGAAATGAGGT
CONSENSUS
GAGACACATGCTCGTACAGATTCAGGTGGTATTGATAGGGGAAATGAGGT

RI-AT2G28290-XLOC_009942-276-0
ATCAGAAAATATGTCTGATGGCGTCAAAATGAATATTTCATCTGTGCAGG
RI-AT2G28290-XLOC_009942-276-1
ATCAGAAAATATGTCTGATGGCGTCAAAATGAATATTTCATCTGTGCAGG
CONSENSUS
ATCAGAAAATATGTCTGATGGCGTCAAAATGAATATTTCATCTGTGCAGG

RI-AT2G28290-XLOC_009942-276-0
TCCCGGATGCATCACATGATTTAAATGTATCACAGGATCAAACAGACATT
RI-AT2G28290-XLOC_009942-276-1
TCCCGGATGCATCACATGATTTAAATGTATCACAGGATCAAACAGACATT
CONSENSUS
TCCCGGATGCATCACATGATTTAAATGTATCACAGGATCAAACAGACATT

RI-AT2G28290-XLOC_009942-276-0
CCCCTAGTTGGTGGGATAGACCCTGAACACGTACAAGAGAATGTGGATGT
RI-AT2G28290-XLOC_009942-276-1
CCCCTAGTTGGTGGGATAGACCCTGAACACGTACAAGAGAATGTGGATGT
CONSENSUS
CCCCTAGTTGGTGGGATAGACCCTGAACACGTACAAGAGAATGTGGATGT

RI-AT2G28290-XLOC_009942-276-0
ACCTGCATCACCTCACGGAGCAGCGCCAAACATTGTGATTTTCCAGTCTG
RI-AT2G28290-XLOC_009942-276-1
ACCTGCATCACCTCACGGAGCAGCGCCAAACATTGTGATTTTCCAGTCTG
CONSENSUS
ACCTGCATCACCTCACGGAGCAGCGCCAAACATTGTGATTTTCCAGTCTG

RI-AT2G28290-XLOC_009942-276-0
AGGGACATCTGTCTCCAAGTATCTTACCGGACGATGTGGCAGGACAACTA
RI-AT2G28290-XLOC_009942-276-1
AGGGACATCTGTCTCCAAGTATCTTACCGGACGATGTGGCAGGACAACTA
CONSENSUS
AGGGACATCTGTCTCCAAGTATCTTACCGGACGATGTGGCAGGACAACTA

RI-AT2G28290-XLOC_009942-276-0
GAAAGCATGTCTAATGACGAAAAAACGAATATTTCATCTGAGCAGGTCCC
RI-AT2G28290-XLOC_009942-276-1
GAAAGCATGTCTAATGACGAAAAAACGAATATTTCATCTGAGCAGGTCCC
CONSENSUS
GAAAGCATGTCTAATGACGAAAAAACGAATATTTCATCTGAGCAGGTCCC

RI-AT2G28290-XLOC_009942-276-0
AGATGTATCACATGATTTGAAAGTGTCTCAGGATCAAACTGACATTCCCC
RI-AT2G28290-XLOC_009942-276-1
AGATGTATCACATGATTTGAAAGTGTCTCAGGATCAAACTGACATTCCCC
CONSENSUS
AGATGTATCACATGATTTGAAAGTGTCTCAGGATCAAACTGACATTCCCC

RI-AT2G28290-XLOC_009942-276-0
CAGTTGGTGGGATAGTGCCTGAAAATTTGCAAGAGATTGTGGATGTACCT
RI-AT2G28290-XLOC_009942-276-1
CAGTTGGTGGGATAGTGCCTGAAAATTTGCAAGAGATTGTGGATGTACCT
CONSENSUS
CAGTTGGTGGGATAGTGCCTGAAAATTTGCAAGAGATTGTGGATGTACCT

RI-AT2G28290-XLOC_009942-276-0
GCATCACCTCATGGAGTAGTGCCAGACGTTGTTGTTTCCCAGTCTGAGGA
RI-AT2G28290-XLOC_009942-276-1
GCATCACCTCATGGAGTAGTGCCAGACGTTGTTGTTTCCCAGTCTGAGGA
CONSENSUS
GCATCACCTCATGGAGTAGTGCCAGACGTTGTTGTTTCCCAGTCTGAGGA

RI-AT2G28290-XLOC_009942-276-0
AATTCAATCTCCAAGTATTTTGCCCGACGATGTACCAGGACAACCAGACG
RI-AT2G28290-XLOC_009942-276-1
AATTCAATCTCCAAGTATTTTGCCCGACGATGTACCAGGACAACCAGACG
CONSENSUS
AATTCAATCTCCAAGTATTTTGCCCGACGATGTACCAGGACAACCAGACG

RI-AT2G28290-XLOC_009942-276-0
ATGGCAACTGTGAGAAAATGGATACCATGCAGAACAATACCTCTATTGAT
RI-AT2G28290-XLOC_009942-276-1
ATGGCAACTGTGAGAAAATGGATACCATGCAGAACAATACCTCTATTGAT
CONSENSUS
ATGGCAACTGTGAGAAAATGGATACCATGCAGAACAATACCTCTATTGAT

RI-AT2G28290-XLOC_009942-276-0
ATTGGCATAACTTCAGGTAAGACATGTCAGCCTTCATCTTCTACCCAGCC
RI-AT2G28290-XLOC_009942-276-1
ATTGGCATAACTTCAGGTAAGACATGTCAGCCTTCATCTTCTACCCAGCC
CONSENSUS
ATTGGCATAACTTCAGGTAAGACATGTCAGCCTTCATCTTCTACCCAGCC

RI-AT2G28290-XLOC_009942-276-0
TGAGGATGAGAACAGAAATAGCTTATCACACTGTGAACCGTCAGAAGTAG
RI-AT2G28290-XLOC_009942-276-1
TGAGGATGAGAACAGAAATAGCTTATCACACTGTGAACCGTCAGAAGTAG
CONSENSUS
TGAGGATGAGAACAGAAATAGCTTATCACACTGTGAACCGTCAGAAGTAG

RI-AT2G28290-XLOC_009942-276-0
TTGAACAAAGGGATTCAAGAGATCAAGTTTGCATAGGGTCTGTGGAATCT
RI-AT2G28290-XLOC_009942-276-1
TTGAACAAAGGGATTCAAGAGATCAAGTTTGCATAGGGTCTGTGGAATCT
CONSENSUS
TTGAACAAAGGGATTCAAGAGATCAAGTTTGCATAGGGTCTGTGGAATCT

RI-AT2G28290-XLOC_009942-276-0
CAAGTAGAGATCAGCTCTGCTATACTGGAAAATAGATCAGCTGATATCCA
RI-AT2G28290-XLOC_009942-276-1
CAAGTAGAGATCAGCTCTGCTATACTGGAAAATAGATCAGCTGATATCCA
CONSENSUS
CAAGTAGAGATCAGCTCTGCTATACTGGAAAATAGATCAGCTGATATCCA

RI-AT2G28290-XLOC_009942-276-0
GCCCCCGCAATCCATTTTGGTTGATCAAAAGGATATTGAAGAATCCAAAG
RI-AT2G28290-XLOC_009942-276-1
GCCCCCGCAATCCATTTTGGTTGATCAAAAGGATATTGAAGAATCCAAAG
CONSENSUS
GCCCCCGCAATCCATTTTGGTTGATCAAAAGGATATTGAAGAATCCAAAG

RI-AT2G28290-XLOC_009942-276-0
AACCTGGTATCGAGAGTGCTGATGTGTCTTTACACCAATTAGCTGATATC
RI-AT2G28290-XLOC_009942-276-1
AACCTGGTATCGAGAGTGCTGATGTGTCTTTACACCAATTAGCTGATATC
CONSENSUS
AACCTGGTATCGAGAGTGCTGATGTGTCTTTACACCAATTAGCTGATATC

RI-AT2G28290-XLOC_009942-276-0
CAGGCCGAGCCATCCAATTTGGTTGATCAAATGGATATTGAAGAATCCAA
RI-AT2G28290-XLOC_009942-276-1
CAGGCCGAGCCATCCAATTTGGTTGATCAAATGGATATTGAAGAATCCAA
CONSENSUS
CAGGCCGAGCCATCCAATTTGGTTGATCAAATGGATATTGAAGAATCCAA

RI-AT2G28290-XLOC_009942-276-0
AGAACCTGGTACCGAGAGTGCTGATGTGTCTTTACACCAATTAGCTGATA
RI-AT2G28290-XLOC_009942-276-1
AGAACCTGGTACCGAGAGTGCTGATGTGTCTTTACACCAATTAGCTGATA
CONSENSUS
AGAACCTGGTACCGAGAGTGCTGATGTGTCTTTACACCAATTAGCTGATA

RI-AT2G28290-XLOC_009942-276-0
TCCAGCCCGGGCCATCCATTTTGGTTGATCAAATGGATACTGAAAAATCC
RI-AT2G28290-XLOC_009942-276-1
TCCAGCCCGGGCCATCCATTTTGGTTGATCAAATGGATACTGAAAAATCC
CONSENSUS
TCCAGCCCGGGCCATCCATTTTGGTTGATCAAATGGATACTGAAAAATCC

RI-AT2G28290-XLOC_009942-276-0
AAAGAACCTGGTACCGAGAGTGCTGATGTGTCTTTACACCAATTAGCTGA
RI-AT2G28290-XLOC_009942-276-1
AAAGAACCTGGTACCGAGAGTGCTGATGTGTCTTTACACCAATTAGCTGA
CONSENSUS
AAAGAACCTGGTACCGAGAGTGCTGATGTGTCTTTACACCAATTAGCTGA

RI-AT2G28290-XLOC_009942-276-0
TATCCAGCCCGGGCCATCCATTTTGGTTGATCAAATGGATACTGAAAAAT
RI-AT2G28290-XLOC_009942-276-1
TATCCAGCCCGGGCCATCCATTTTGGTTGATCAAATGGATACTGAAAAAT
CONSENSUS
TATCCAGCCCGGGCCATCCATTTTGGTTGATCAAATGGATACTGAAAAAT

RI-AT2G28290-XLOC_009942-276-0
CCAAAGAACCTGGTACCGAGAGTGCTGATGTGTCTTTACACCAATTAGCT
RI-AT2G28290-XLOC_009942-276-1
CCAAAGAACCTGGTACCGAGAGTGCTGATGTGTCTTTACACCAATTAGCT
CONSENSUS
CCAAAGAACCTGGTACCGAGAGTGCTGATGTGTCTTTACACCAATTAGCT

RI-AT2G28290-XLOC_009942-276-0
GATATCCAGCCCGGGCCATCCATTTTGGTTGATCAAATGGATACTGAAGA
RI-AT2G28290-XLOC_009942-276-1
GATATCCAGCCCGGGCCATCCATTTTGGTTGATCAAATGGATACTGAAGA
CONSENSUS
GATATCCAGCCCGGGCCATCCATTTTGGTTGATCAAATGGATACTGAAGA

RI-AT2G28290-XLOC_009942-276-0
ATTCAAAAATCCTGATGTGTCTTTACACCAATTAGCTGATATTGAGCCCT
RI-AT2G28290-XLOC_009942-276-1
ATTCAAAAATCCTGATGTGTCTTTACACCAATTAGCTGATATTGAGCCCT
CONSENSUS
ATTCAAAAATCCTGATGTGTCTTTACACCAATTAGCTGATATTGAGCCCT

RI-AT2G28290-XLOC_009942-276-0
CACTGTCTATTTCAGCTGTGCAAAAGAATATTGAGGATAAGGATCAAAGT
RI-AT2G28290-XLOC_009942-276-1
CACTGTCTATTTCAGCTGTGCAAAAGAATATTGAGGATAAGGATCAAAGT
CONSENSUS
CACTGTCTATTTCAGCTGTGCAAAAGAATATTGAGGATAAGGATCAAAGT

RI-AT2G28290-XLOC_009942-276-0
CACGTTGAAACTGCTGGATCTGAGTTAGTTGATGTCTCTGCCGAATGTTC
RI-AT2G28290-XLOC_009942-276-1
CACGTTGAAACTGCTGGATCTGAGTTAGTTGATGTCTCTGCCGAATGTTC
CONSENSUS
CACGTTGAAACTGCTGGATCTGAGTTAGTTGATGTCTCTGCCGAATGTTC

RI-AT2G28290-XLOC_009942-276-0
AACAGAACCTCAAGTTCAATTACCGCCATCTTCAGAGCCAGTGGGAGATA
RI-AT2G28290-XLOC_009942-276-1
AACAGAACCTCAAGTTCAATTACCGCCATCTTCAGAGCCAGTGGGAGATA
CONSENSUS
AACAGAACCTCAAGTTCAATTACCGCCATCTTCAGAGCCAGTGGGAGATA

RI-AT2G28290-XLOC_009942-276-0
TGCACGTTCATTTAGGGGCAAGCAAATCAGAAATAGTTGCCGAAGGTACT
RI-AT2G28290-XLOC_009942-276-1
TGCACGTTCATTTAGGGGCAAGCAAATCAGAAATAGTTGCCGAAGGTACT
CONSENSUS
TGCACGTTCATTTAGGGGCAAGCAAATCAGAAATAGTTGCCGAAGGTACT

RI-AT2G28290-XLOC_009942-276-0
GACTTCTCTTCATCTCTCCCGAAGACGGAGGAAGAAAATGCCAAGAGCCA
RI-AT2G28290-XLOC_009942-276-1
GACTTCTCTTCATCTCTCCCGAAGACGGAGGAAGAAAATGCCAAGAGCCA
CONSENSUS
GACTTCTCTTCATCTCTCCCGAAGACGGAGGAAGAAAATGCCAAGAGCCA

RI-AT2G28290-XLOC_009942-276-0
ATTAGCTGACACCGAGCCATCATCGTCTCTTACAGCTGTGCAAAAGAACA
RI-AT2G28290-XLOC_009942-276-1
ATTAGCTGACACCGAGCCATCATCGTCTCTTACAGCTGTGCAAAAGAACA
CONSENSUS
ATTAGCTGACACCGAGCCATCATCGTCTCTTACAGCTGTGCAAAAGAACA

RI-AT2G28290-XLOC_009942-276-0
TTGAAGATCAAGTTGAAACTGCTGGATGTGAATTTGTTGTTGTCTCTACC
RI-AT2G28290-XLOC_009942-276-1
TTGAAGATCAAGTTGAAACTGCTGGATGTGAATTTGTTGTTGTCTCTACC
CONSENSUS
TTGAAGATCAAGTTGAAACTGCTGGATGTGAATTTGTTGTTGTCTCTACC

RI-AT2G28290-XLOC_009942-276-0
GGATGTTCAACAGAACCACAAGTTCAATTACCGCCGTCCGCAGAGCCAGT
RI-AT2G28290-XLOC_009942-276-1
GGATGTTCAACAGAACCACAAGTTCAATTACCGCCGTCCGCAGAGCCAGT
CONSENSUS
GGATGTTCAACAGAACCACAAGTTCAATTACCGCCGTCCGCAGAGCCAGT

RI-AT2G28290-XLOC_009942-276-0
GGTTGCTGAAGGTACAGAATTCCCTTCTTCCCTCCTAATGACCGGGGTAG
RI-AT2G28290-XLOC_009942-276-1
GGTTGCTGAAGGTACAGAATTCCCTTCTTCCCTCCTAATGACCGGGGTAG
CONSENSUS
GGTTGCTGAAGGTACAGAATTCCCTTCTTCCCTCCTAATGACCGGGGTAG

RI-AT2G28290-XLOC_009942-276-0
ATAATTCTTCCCATCTAATGACCGGGGTAGATAATGCCAAGACCCATCTC
RI-AT2G28290-XLOC_009942-276-1
ATAATTCTTCCCATCTAATGACCGGGGTAGATAATGCCAAGACCCATCTC
CONSENSUS
ATAATTCTTCCCATCTAATGACCGGGGTAGATAATGCCAAGACCCATCTC

RI-AT2G28290-XLOC_009942-276-0
GCTGATGTTGTGCCTTCATCGTCACCTACAACTATGGAAAAGAACATTGA
RI-AT2G28290-XLOC_009942-276-1
GCTGATGTTGTGCCTTCATCGTCACCTACAACTATGGAAAAGAACATTGA
CONSENSUS
GCTGATGTTGTGCCTTCATCGTCACCTACAACTATGGAAAAGAACATTGA

RI-AT2G28290-XLOC_009942-276-0
AGCTCAAGATCAAGATCAAGTTACAACTGGTGGATGTGGTCTAGTTGATG
RI-AT2G28290-XLOC_009942-276-1
AGCTCAAGATCAAGATCAAGTTACAACTGGTGGATGTGGTCTAGTTGATG
CONSENSUS
AGCTCAAGATCAAGATCAAGTTACAACTGGTGGATGTGGTCTAGTTGATG

RI-AT2G28290-XLOC_009942-276-0
TCTTGACCGAATGTTCGTCAGAACCTCAACTTCAACTGCCGCCATCCGCA
RI-AT2G28290-XLOC_009942-276-1
TCTTGACCGAATGTTCGTCAGAACCTCAACTTCAACTGCCGCCATCCGCA
CONSENSUS
TCTTGACCGAATGTTCGTCAGAACCTCAACTTCAACTGCCGCCATCCGCA

RI-AT2G28290-XLOC_009942-276-0
GAACCAGTGATTTCTGAAGGTACAGAACTCGCTACACTCCCATTGACGGA
RI-AT2G28290-XLOC_009942-276-1
GAACCAGTGATTTCTGAAGGTACAGAACTCGCTACACTCCCATTGACGGA
CONSENSUS
GAACCAGTGATTTCTGAAGGTACAGAACTCGCTACACTCCCATTGACGGA

RI-AT2G28290-XLOC_009942-276-0
GGAAGAAAATGCTGATAGCCAATTAGCTAATATTGAGCCCTCATCGTCTC
RI-AT2G28290-XLOC_009942-276-1
GGAAGAAAATGCTGATAGCCAATTAGCTAATATTGAGCCCTCATCGTCTC
CONSENSUS
GGAAGAAAATGCTGATAGCCAATTAGCTAATATTGAGCCCTCATCGTCTC

RI-AT2G28290-XLOC_009942-276-0
CTTCAGTTGTGGAAAAGAACATTGAGGCTCAAGATCAAGATCAAGTTAAA
RI-AT2G28290-XLOC_009942-276-1
CTTCAGTTGTGGAAAAGAACATTGAGGCTCAAGATCAAGATCAAGTTAAA
CONSENSUS
CTTCAGTTGTGGAAAAGAACATTGAGGCTCAAGATCAAGATCAAGTTAAA

RI-AT2G28290-XLOC_009942-276-0
ACTGCTGGATGTGAGTTAGTCTCGACTGGATGTTCGTCAGAACCACAAGT
RI-AT2G28290-XLOC_009942-276-1
ACTGCTGGATGTGAGTTAGTCTCGACTGGATGTTCGTCAGAACCACAAGT
CONSENSUS
ACTGCTGGATGTGAGTTAGTCTCGACTGGATGTTCGTCAGAACCACAAGT

RI-AT2G28290-XLOC_009942-276-0
TCATTTACCGCCCTCCGCAGAGCCAGATGGAGATATACACGTTCACTTAA
RI-AT2G28290-XLOC_009942-276-1
TCATTTACCGCCCTCCGCAGAGCCAGATGGAGATATACACGTTCACTTAA
CONSENSUS
TCATTTACCGCCCTCCGCAGAGCCAGATGGAGATATACACGTTCACTTAA

RI-AT2G28290-XLOC_009942-276-0
AGGAAACAGAGAAATCTGAAAGCATGGTTGTGGTTGGCGAAGGTACAGCA
RI-AT2G28290-XLOC_009942-276-1
AGGAAACAGAGAAATCTGAAAGCATGGTTGTGGTTGGCGAAGGTACAGCA
CONSENSUS
AGGAAACAGAGAAATCTGAAAGCATGGTTGTGGTTGGCGAAGGTACAGCA

RI-AT2G28290-XLOC_009942-276-0
TTCCCTTCATCTCTCCCAGTGACAGAGGAAGGAAATGCTGAGAGCCAATT
RI-AT2G28290-XLOC_009942-276-1
TTCCCTTCATCTCTCCCAGTGACAGAGGAAGGAAATGCTGAGAGCCAATT
CONSENSUS
TTCCCTTCATCTCTCCCAGTGACAGAGGAAGGAAATGCTGAGAGCCAATT

RI-AT2G28290-XLOC_009942-276-0
AGCTGACACTGAGCCCTTTACGTCTCCTACAGTTGTGGAAAAGAACATTA
RI-AT2G28290-XLOC_009942-276-1
AGCTGACACTGAGCCCTTTACGTCTCCTACAGTTGTGGAAAAGAACATTA
CONSENSUS
AGCTGACACTGAGCCCTTTACGTCTCCTACAGTTGTGGAAAAGAACATTA

RI-AT2G28290-XLOC_009942-276-0
AGGATCAAGAACAAGTTGAAACTACTGGATGTGGGTTAGTTGATGATTCT
RI-AT2G28290-XLOC_009942-276-1
AGGATCAAGAACAAGTTGAAACTACTGGATGTGGGTTAGTTGATGATTCT
CONSENSUS
AGGATCAAGAACAAGTTGAAACTACTGGATGTGGGTTAGTTGATGATTCT

RI-AT2G28290-XLOC_009942-276-0
ACCGGATGTTCGTCAGAACCTCAAGTTCAATTACCGCCATCCGCAGAGCC
RI-AT2G28290-XLOC_009942-276-1
ACCGGATGTTCGTCAGAACCTCAAGTTCAATTACCGCCATCCGCAGAGCC
CONSENSUS
ACCGGATGTTCGTCAGAACCTCAAGTTCAATTACCGCCATCCGCAGAGCC

RI-AT2G28290-XLOC_009942-276-0
AATGGAAGGTACACACATGCACTTAGAGGAAACAAAGAAATCTGAAACTG
RI-AT2G28290-XLOC_009942-276-1
AATGGAAG--
CONSENSUS
AATGGAAG..

RI-AT2G28290-XLOC_009942-276-0
TAGTTACCGAGATTCAATTAGCTGATATAGATCCCTCATTTTCTCTTATA
RI-AT2G28290-XLOC_009942-276-1

CONSENSUS
....

RI-AT2G28290-XLOC_009942-276-0
GTTGTGCAAACGAATATTGAGGATCAAGATCAAATTGAAACAGGTGGATG
RI-AT2G28290-XLOC_009942-276-1 -
TTGTGCAAACGAATATTGAGGATCAAGATCAAATTGAAACAGGTGGATG
CONSENSUS
.TTGTGCAAACGAATATTGAGGATCAAGATCAAATTGAAACAGGTGGATG

RI-AT2G28290-XLOC_009942-276-0
TGATTTAATTAATGTCCCTTCCGGATGTTCAACAGAACCTCAAATTCAAT
RI-AT2G28290-XLOC_009942-276-1
TGATTTAATTAATGTCCCTTCCGGATGTTCAACAGAACCTCAAATTCAAT
CONSENSUS
TGATTTAATTAATGTCCCTTCCGGATGTTCAACAGAACCTCAAATTCAAT

RI-AT2G28290-XLOC_009942-276-0
TATCGTCATCCGCAGAGCCCGAGGAAGGTATGCACATTCACTTAGAGGCA
RI-AT2G28290-XLOC_009942-276-1
TATCGTCATCCGCAGAGCCCGAGGAAGGTATGCACATTCACTTAGAGGCA
CONSENSUS
TATCGTCATCCGCAGAGCCCGAGGAAGGTATGCACATTCACTTAGAGGCA

RI-AT2G28290-XLOC_009942-276-0
GCAATGAACTCTGAAACGGTGGTTACTGAAGGTTCAGAACTCCCTTCATC
RI-AT2G28290-XLOC_009942-276-1
GCAATGAACTCTGAAACGGTGGTTACTGAAGGTTCAGAACTCCCTTCATC
CONSENSUS
GCAATGAACTCTGAAACGGTGGTTACTGAAGGTTCAGAACTCCCTTCATC

RI-AT2G28290-XLOC_009942-276-0
TCTCCCAATGACGGAGGACGAAAATGCTGATGGCCAATTAGCTGAAGTCG
RI-AT2G28290-XLOC_009942-276-1
TCTCCCAATGACGGAGGACGAAAATGCTGATGGCCAATTAGCTGAAGTCG
CONSENSUS
TCTCCCAATGACGGAGGACGAAAATGCTGATGGCCAATTAGCTGAAGTCG

RI-AT2G28290-XLOC_009942-276-0
AGCCCTCAGTGTCTCTTACAGTTGAGCAAACTAACATTGAGGAGAAAGAT
RI-AT2G28290-XLOC_009942-276-1
AGCCCTCAGTGTCTCTTACAGTTGAGCAAACTAACATTGAGGAGAAAGAT
CONSENSUS
AGCCCTCAGTGTCTCTTACAGTTGAGCAAACTAACATTGAGGAGAAAGAT

RI-AT2G28290-XLOC_009942-276-0
CACATTGAAACTGCCGAATGTGAGTTAGTTGATGTCTCTCCCGGATGTTC
RI-AT2G28290-XLOC_009942-276-1
CACATTGAAACTGCCGAATGTGAGTTAGTTGATGTCTCTCCCGGATGTTC
CONSENSUS
CACATTGAAACTGCCGAATGTGAGTTAGTTGATGTCTCTCCCGGATGTTC

RI-AT2G28290-XLOC_009942-276-0
ATCACAACCTGAAGTTAAATTTCCGCCATCCCCAGATGCAGTGGGAGGTA
RI-AT2G28290-XLOC_009942-276-1
ATCACAACCTGAAGTTAAATTTCCGCCATCCCCAGATGCAGTGGGAGGTA
CONSENSUS
ATCACAACCTGAAGTTAAATTTCCGCCATCCCCAGATGCAGTGGGAGGTA

RI-AT2G28290-XLOC_009942-276-0
TGGACGTTCACTTAGAAACCGTTGTTACTGAAGACACAGATTCAAATTCA
RI-AT2G28290-XLOC_009942-276-1
TGGACGTTCACTTAGAAACCGTTGTTACTGAAGACACAGATTCAAATTCA
CONSENSUS
TGGACGTTCACTTAGAAACCGTTGTTACTGAAGACACAGATTCAAATTCA

RI-AT2G28290-XLOC_009942-276-0
TCCCTCCCGAAGACGGAGGAAAAAGATGCCGAGAATCCATCAGACAGGCT
RI-AT2G28290-XLOC_009942-276-1
TCCCTCCCGAAGACGGAGGAAAAAGATGCCGAGAATCCATCAGACAGGCT
CONSENSUS
TCCCTCCCGAAGACGGAGGAAAAAGATGCCGAGAATCCATCAGACAGGCT

RI-AT2G28290-XLOC_009942-276-0
TGACGGTGAATCCGATGGTACAACTGTTGCTACTGTTGAAGGAACTTGTG
RI-AT2G28290-XLOC_009942-276-1
TGACGGTGAATCCGATGGTACAACTGTTGCTACTGTTGAAGGAACTTGTG
CONSENSUS
TGACGGTGAATCCGATGGTACAACTGTTGCTACTGTTGAAGGAACTTGTG

RI-AT2G28290-XLOC_009942-276-0
TTGAGTCGAATTCATTGGTCGCCGAAGAGAGCAACATAGAAGTGCCAAAA
RI-AT2G28290-XLOC_009942-276-1
TTGAGTCGAATTCATTGGTCGCCGAAGAGAGCAACATAGAAGTGCCAAAA
CONSENSUS
TTGAGTCGAATTCATTGGTCGCCGAAGAGAGCAACATAGAAGTGCCAAAA

RI-AT2G28290-XLOC_009942-276-0
GACAATGAAGATGTGTAGTTCATTTCATCTAATATTAAGTTGTAAAACTT
RI-AT2G28290-XLOC_009942-276-1
GACAATGAAGATGTGTAGTTCATTTCATCTAATATTAAGTTGTAAAACTT
CONSENSUS
GACAATGAAGATGTGTAGTTCATTTCATCTAATATTAAGTTGTAAAACTT

```
RI-AT2G28290-XLOC_009942-276-0
      TTAATATCTAATATAACAAACCCAATGTTGAAGATGTATTGACTGCATCT
RI-AT2G28290-XLOC_009942-276-1
      TTAATATCTAATATAACAAACCCAATGTTGAAGATGTATTGACTGCATCT
CONSENSUS
      TTAATATCTAATATAACAAACCCAATGTTGAAGATGTATTGACTGCATCT

RI-AT2G28290-XLOC_009942-276-0
      GACTTATTTAACTTATCCTCTCATTTGGGAATATTAATAGGGCTTAGAAT
RI-AT2G28290-XLOC_009942-276-1
      GACTTATTTAACTTATCCTCTCATTTGGGAATATTAATAGGGCTTAGAAT
CONSENSUS
      GACTTATTTAACTTATCCTCTCATTTGGGAATATTAATAGGGCTTAGAAT

RI-AT2G28290-XLOC_009942-276-0   C
RI-AT2G28290-XLOC_009942-276-1   C
CONSENSUS                        C



alignment for event: RI-AT2G41520-XLOC_010701-4018

RI-AT2G41520-XLOC_010701-4018-0
      AACGCAGGTAATGAAGCTGTTCGGGACAGAAAGTATATGGAAGCAGTAGA
RI-AT2G41520-XLOC_010701-4018-1
      AACGCAG-------------------------------------------
CONSENSUS
      AACGCAG...........................................

RI-AT2G41520-XLOC_010701-4018-0
      GCAGTATACTGCTGCACTATCAAGAAATGTTGACTCACGCCCTTTTGCAG
RI-AT2G41520-XLOC_010701-4018-1
      --------------------------------------------------
CONSENSUS
      ..................................................

RI-AT2G41520-XLOC_010701-4018-0
      CAATTTGCTTCTGCAATCGTGCGGCTGCTAATCAGGCCCTAGTTCAAATT
RI-AT2G41520-XLOC_010701-4018-1
      CAATTTGCTTCTGCAATCGTGCGGCTGCTAATCAGGCCCTAGTTCAAATT
CONSENSUS
      CAATTTGCTTCTGCAATCGTGCGGCTGCTAATCAGGCCCTAGTTCAAATT

RI-AT2G41520-XLOC_010701-4018-0
      GCTGATGCAATTGCCGACTGTAGTCTTGCCATGGCTCTTGATGAAAACTA
RI-AT2G41520-XLOC_010701-4018-1
      GCTGATGCAATTGCCGACTGTAGTCTTGCCATGGCTCTTGATGAAAACTA
CONSENSUS
      GCTGATGCAATTGCCGACTGTAGTCTTGCCATGGCTCTTGATGAAAACTA

RI-AT2G41520-XLOC_010701-4018-0  CACAAAG
RI-AT2G41520-XLOC_010701-4018-1  CACAAAG
CONSENSUS                        CACAAAG
```

alignment for event: RI-AT2G32840-XLOC_012922-5929

RI-AT2G32840-XLOC_012922-5929-0
CCGCAGCGAATTGATATGATGACATGTTTGCCAAAGCCTTTACCTGTGGA
RI-AT2G32840-XLOC_012922-5929-1
CCGCAGCGAATTGATATGATGACATGTTTGCCAAAGCCTTTACCTGTGGA
CONSENSUS
CCGCAGCGAATTGATATGATGACATGTTTGCCAAAGCCTTTACCTGTGGA

RI-AT2G32840-XLOC_012922-5929-0
CAAGACGGAGACAAGTTTGCCAAAAGATTTAGTTGAAGAAGCAATTTGCG
RI-AT2G32840-XLOC_012922-5929-1
CAAGACGGAGACAAGTTTGCCAAAAGATTTAGTTGAAGAAGCAATTTGCG
CONSENSUS
CAAGACGGAGACAAGTTTGCCAAAAGATTTAGTTGAAGAAGCAATTTGCG

RI-AT2G32840-XLOC_012922-5929-0
AAGAAGACAAAGAGGTTTCGTGTTTCTCTGATTTCTTTCTAAAATGGTAA
RI-AT2G32840-XLOC_012922-5929-1
AAGAAGACAAAGAG------------------------------------
CONSENSUS
AAGAAGACAAAGAG....................................

RI-AT2G32840-XLOC_012922-5929-0
TTGTGAAGGGTAAAGCTAAGTTTGATTATTTACTTCAACTCTCTTGATGA
RI-AT2G32840-XLOC_012922-5929-1

CONSENSUS
....

RI-AT2G32840-XLOC_012922-5929-0
GCTTCAGGACGAGGAATCTGTGAAACATTTGTCAGAATCTGATCTTTTGA
RI-AT2G32840-XLOC_012922-5929-1 -------
GACGAGGAATCTGTGAAACATTTGTCAGAATCTGATCTTTTGA
CONSENSUS
.......GACGAGGAATCTGTGAAACATTTGTCAGAATCTGATCTTTTGA

RI-AT2G32840-XLOC_012922-5929-0
AAAGACATATAGACCGAGCTAAGAAGGTCCGCGCTCG
RI-AT2G32840-XLOC_012922-5929-1
AAAGACATATAGACCGAGCTAAGAAGGTCCGCGCTCG
CONSENSUS
AAAGACATATAGACCGAGCTAAGAAGGTCCGCGCTCG

alignment for event: A5-AT2G36060-XLOC_013116-3514

A5-AT2G36060-XLOC_013116-3514-0
TTCCGAGGAATTTCCGGTTGCTGGAGGAGCTTGAACGTGGAGAGAAAGGT
A5-AT2G36060-XLOC_013116-3514-1
TTCCGAGGAATTTCCGGTTGCTGGAGGAGCTTGAACGTGGAGAGAAAGGT
CONSENSUS
TTCCGAGGAATTTCCGGTTGCTGGAGGAGCTTGAACGTGGAGAGAAAGGT

A5-AT2G36060-XLOC_013116-3514-0
ATTGGAGATGGAACTGTGAGCTATGGAATGGATGATGGAGATGACATTTA
A5-AT2G36060-XLOC_013116-3514-1
ATTGGAGATGGAACTGTGAGCTATGGAATGGATGATGGAGATGACATTTA
CONSENSUS
ATTGGAGATGGAACTGTGAGCTATGGAATGGATGATGGAGATGACATTTA

A5-AT2G36060-XLOC_013116-3514-0
TATGCGCTCTTGGACTGGCACTATCATCGGTCCTCACAACGTAACTGTAC
A5-AT2G36060-XLOC_013116-3514-1
TATGCGCTCTTGGACTGGCACTATCATCGGTCCTCACAAC---ACTGTAC
CONSENSUS
TATGCGCTCTTGGACTGGCACTATCATCGGTCCTCACAAC...ACTGTAC

A5-AT2G36060-XLOC_013116-3514-0
ATGAGGGTCGGATTTATCAGTTGAAGCTCTTCTGTGACAAAGATTACCCT
A5-AT2G36060-XLOC_013116-3514-1
ATGAGGGTCGGATTTATCAGTTGAAGCTCTTCTGTGACAAAGATTACCCT
CONSENSUS
ATGAGGGTCGGATTTATCAGTTGAAGCTCTTCTGTGACAAAGATTACCCT

A5-AT2G36060-XLOC_013116-3514-0
GAGAAACCTCCGACTGTTCGGTTCCATTCGCGTATCAACATGACTTGTGT
A5-AT2G36060-XLOC_013116-3514-1
GAGAAACCTCCGACTGTTCGGTTCCATTCGCGTATCAACATGACTTGTGT
CONSENSUS
GAGAAACCTCCGACTGTTCGGTTCCATTCGCGTATCAACATGACTTGTGT

A5-AT2G36060-XLOC_013116-3514-0 CAACCATGATACCGGCGTG
A5-AT2G36060-XLOC_013116-3514-1 CAACCATGATACCGGCGTG
CONSENSUS CAACCATGATACCGGCGTG

alignment for event: A5-AT2G43920-XLOC_013565-4517

A5-AT2G43920-XLOC_013565-4517-0
GCGGATGGGACAAATGTTGGGAAGACGGGGTAACACCATGGGACCAAGGA
A5-AT2G43920-XLOC_013565-4517-1
GCGGATGGGACAAATGTTGGGAAGACGGGGTAACACCATGGGACCAAGGA
CONSENSUS
GCGGATGGGACAAATGTTGGGAAGACGGGGTAACACCATGGGACCAAGGA

A5-AT2G43920-XLOC_013565-4517-0
AGAGCCACACCTCTCATTCTTCATCTTCTCGACTCTTCAGCTCTCCCTCT
A5-AT2G43920-XLOC_013565-4517-1
AGAGCCACACCTCTCATTCTTCATCTTCTCGACTCTTCAGCTCTCCCTCT
CONSENSUS
AGAGCCACACCTCTCATTCTTCATCTTCTCGACTCTTCAGCTCTCCCTCT

A5-AT2G43920-XLOC_013565-4517-0
TGGCCGTACCCTTGTCCCCGGCTGTGGCGGAGTTAGTCTTCTCTCTTTCC
A5-AT2G43920-XLOC_013565-4517-1
TGGCCGTACCCTTGTCCCCGGCTGTGGCGGA-------------------
CONSENSUS
TGGCCGTACCCTTGTCCCCGGCTGTGGCGGA...................

A5-AT2G43920-XLOC_013565-4517-0
ATTTTGGTTTGGTTTGAGTTCGAGCTTTTTTTTTAGTTTTTATATCTTTG
A5-AT2G43920-XLOC_013565-4517-1

CONSENSUS
....

A5-AT2G43920-XLOC_013565-4517-0
TAGTTTAGAACATATTTAAGGATTTGGAGATTACAACCGAATCTAGTTTG
A5-AT2G43920-XLOC_013565-4517-1

CONSENSUS
....

A5-AT2G43920-XLOC_013565-4517-0
AGTTGGTAACTTGGGACACGACGTCGTTGCGATGGCAAGCCCTGAACGTT
A5-AT2G43920-XLOC_013565-4517-1 -------------
GGACACGACGTCGTTGCGATGGCAAGCCCTGAACGTT
CONSENSUS
.............GGACACGACGTCGTTGCGATGGCAAGCCCTGAACGTT

A5-AT2G43920-XLOC_013565-4517-0
TCGTAGTTGGATTGGATATTTCTGACAAGGCTCTCAACAAAGCTAATGAG
A5-AT2G43920-XLOC_013565-4517-1
TCGTAGTTGGATTGGATATTTCTGACAAGGCTCTCAACAAAGCTAATGAG
CONSENSUS
TCGTAGTTGGATTGGATATTTCTGACAAGGCTCTCAACAAAGCTAATGAG

alignment for event: A3-AT2G05520-XLOC_008599-1450

A3-AT2G05520-XLOC_008599-1450-0
CCACAGTGAATTCAGAGAGTAAGGAAACTGTGAAACCTGATCAACGTGGC
A3-AT2G05520-XLOC_008599-1450-1
CCACAGTGAATTCAGAGAGTAAGGAAACTGTGAAACCTGATCAACGTGGC
CONSENSUS
CCACAGTGAATTCAGAGAGTAAGGAAACTGTGAAACCTGATCAACGTGGC

A3-AT2G05520-XLOC_008599-1450-0
TACGGTGACAATGGAGGAAATTACAATAACGGAGGAGGTTACCAGGGAGG
A3-AT2G05520-XLOC_008599-1450-1
TACGGTGACAATGGAGGAAATTACAATAACGGAGGAGGTTACCAGGGAGG
CONSENSUS
TACGGTGACAATGGAGGAAATTACAATAACGGAGGAGGTTACCAGGGAGG

A3-AT2G05520-XLOC_008599-1450-0
AGGGGGTAATTACCAAGGAGGAGGAGGACGGTACCAAGGAGGAGGAGGAC
A3-AT2G05520-XLOC_008599-1450-1
AGGGGGTAATTACCAAGGAGGAGGAG---------------------GAC
CONSENSUS
AGGGGGTAATTACCAAGGAGGAGGAG.....................GAC

A3-AT2G05520-XLOC_008599-1450-0
GATACCAAGGAGGCGGTGGGCGACAAGGAGGAGGGGGAAGTGGGGGAAGT

A3-AT2G05520-XLOC_008599-1450-1
GATACCAAGGAGGCGGTGGGCGACAAGGAGGAGGGGGAAGTGGGGGAAGT
CONSENSUS
GATACCAAGGAGGCGGTGGGCGACAAGGAGGAGGGGGAAGTGGGGGAAGT

A3-AT2G05520-XLOC_008599-1450-0
TACTGCCGCCACGGCTGCTGCTACAGAGGTTACAACGGCTGCTCAAGATG
A3-AT2G05520-XLOC_008599-1450-1
TACTGCCGCCACGGCTGCTGCTACAGAGGTTACAACGGCTGCTCAAGATG
CONSENSUS
TACTGCCGCCACGGCTGCTGCTACAGAGGTTACAACGGCTGCTCAAGATG

A3-AT2G05520-XLOC_008599-1450-0
CTGCTCGTATGCCGGAGAAGCTGTTCAGACTCAGCCCGGTCACTAAAACA
A3-AT2G05520-XLOC_008599-1450-1
CTGCTCGTATGCCGGAGAAGCTGTTCAGACTCAGCCCGGTCACTAAAACA
CONSENSUS
CTGCTCGTATGCCGGAGAAGCTGTTCAGACTCAGCCCGGTCACTAAAACA

A3-AT2G05520-XLOC_008599-1450-0
ATATATTAACCAATCACCACCATGCATGTATTGCATTATGTATGTATGAT
A3-AT2G05520-XLOC_008599-1450-1
ATATATTAACCAATCACCACCATGCATGTATTGCATTATGTATGTATGAT
CONSENSUS
ATATATTAACCAATCACCACCATGCATGTATTGCATTATGTATGTATGAT

A3-AT2G05520-XLOC_008599-1450-0
TTTAAGTAAACCATGGTGCGTTTGTAATGAACTGCCTCAAGTTTTTGAGG
A3-AT2G05520-XLOC_008599-1450-1
TTTAAGTAAACCATGGTGCGTTTGTAATGAACTGCCTCAAGTTTTTGAGG
CONSENSUS
TTTAAGTAAACCATGGTGCGTTTGTAATGAACTGCCTCAAGTTTTTGAGG

A3-AT2G05520-XLOC_008599-1450-0
CACTATAGATTAAGAAGAGAACTATGGGAATAAAGTTTGATTATGTAATG
A3-AT2G05520-XLOC_008599-1450-1
CACTATAGATTAAGAAGAGAACTATGGGAATAAAGTTTGATTATGTAATG
CONSENSUS
CACTATAGATTAAGAAGAGAACTATGGGAATAAAGTTTGATTATGTAATG

A3-AT2G05520-XLOC_008599-1450-0
TTTTATGTGGTTTGAGTTGTAATACTTGCTGTTTGCATAATAAAATCGTT
A3-AT2G05520-XLOC_008599-1450-1
TTTTATGTGGTTTGAGTTGTAATACTTGCTGTTTGCATAATAAAATCGTT
CONSENSUS
TTTTATGTGGTTTGAGTTGTAATACTTGCTGTTTGCATAATAAAATCGTT

A3-AT2G05520-XLOC_008599-1450-0
TGTAGTTTATGTTAATCTCTTTCGTTTTTTT
A3-AT2G05520-XLOC_008599-1450-1
TGTAGTTTATGTTAATCTCTTTCGTTTTTTT
CONSENSUS
TGTAGTTTATGTTAATCTCTTTCGTTTTTTT

alignment for event: A5-AT2G21630-XLOC_009549-7474

A5-AT2G21630-XLOC_009549-7474-0
GTAGGCATTGCTGAGCTCAAAGTTGCAGTTGAGCAGACTGGTGGATTTGT
A5-AT2G21630-XLOC_009549-7474-1
GTAGGCATTGCTGAGCTCAAAGTTGCAGTTGAGCAGACTGGTGGATTTGT
CONSENSUS
GTAGGCATTGCTGAGCTCAAAGTTGCAGTTGAGCAGACTGGTGGATTTGT

A5-AT2G21630-XLOC_009549-7474-0
CGTGCTTGCTGAAAGTTTTGGCCACTCAGTATTTAGAGATTCTCTCAAAC
A5-AT2G21630-XLOC_009549-7474-1
CGTGCTTGCTGAAAGTTTTGGCCACTCAGTATTTAGAGATTCTCTCAAAC
CONSENSUS
CGTGCTTGCTGAAAGTTTTGGCCACTCAGTATTTAGAGATTCTCTCAAAC

A5-AT2G21630-XLOC_009549-7474-0
GCGTGTGTCAGTCAGGTGAAAATGATCTAGGCTTGTCCTCATG-------
A5-AT2G21630-XLOC_009549-7474-1
GCGTGTGTCAGTCAGGTGAAAATGATCTAGGCTTGTCCTCATGGTAATGG
CONSENSUS
GCGTGTGTCAGTCAGGTGAAAATGATCTAGGCTTGTCCTCATG.......

A5-AT2G21630-XLOC_009549-7474-0 ----------------
TGGTATATTTGAAATTAACTGCTCAAAGGATATC
A5-AT2G21630-XLOC_009549-7474-1
CTTACCTAACTTTCATTGGTATATTTGAAATTAACTGCTCAAAGGATATC
CONSENSUS
................TGGTATATTTGAAATTAACTGCTCAAAGGATATC

A5-AT2G21630-XLOC_009549-7474-0
AAAGTTCAGGGAATTATTGGCCCTTGCGCATCTCTCGAAAAG
A5-AT2G21630-XLOC_009549-7474-1
AAAGTTCAGGGAATTATTGGCCCTTGCGCATCTCTCGAAAAG
CONSENSUS
AAAGTTCAGGGAATTATTGGCCCTTGCGCATCTCTCGAAAAG

alignment for event: A3-AT2G41710-XLOC_013437-3931

A3-AT2G41710-XLOC_013437-3931-0
GTGACTGATTATACCAGGGATTTAGAAGAAATGCAAAATCTCTCAAGGGA
A3-AT2G41710-XLOC_013437-3931-1
GTGACTGATTATACCAGGGATTTAGAAGAAATGCAAAATCTCTCAAGGGA
CONSENSUS
GTGACTGATTATACCAGGGATTTAGAAGAAATGCAAAATCTCTCAAGGGA

A3-AT2G41710-XLOC_013437-3931-0
AGAATACCTTGCATCTTTACGTAG---------------AAAAAGCAGCG
A3-AT2G41710-XLOC_013437-3931-1
AGAATACCTTGCATCTTTACGTAGATATCCCTTTGGCAGAAAAAGCAGCG
CONSENSUS
AGAATACCTTGCATCTTTACGTAG...............AAAAAGCAGCG

A3-AT2G41710-XLOC_013437-3931-0

GTTTCTCTAGGGGAATAGCGAAATATCGTGGACTTCAAAG
A3-AT2G41710-XLOC_013437-3931-1
GTTTCTCTAGGGGAATAGCGAAATATCGTGGACTTCAAAG
CONSENSUS
GTTTCTCTAGGGGAATAGCGAAATATCGTGGACTTCAAAG

alignment for event: A3-AT2G46920-XLOC_013718-7039

A3-AT2G46920-XLOC_013718-7039-0
GCTCACTCTCACCTATGAGCTTAAGGAAATTAAAAGAAAAAGGTGAAATC
A3-AT2G46920-XLOC_013718-7039-1
GCTCACTCTCACCTATGAGCTTAAGGAAATTAAAAGAAAAAGGTGAAATC
CONSENSUS
GCTCACTCTCACCTATGAGCTTAAGGAAATTAAAAGAAAAAGGTGAAATC

A3-AT2G46920-XLOC_013718-7039-0
ATACATAATTCTTGACCTGCTTAAATCAAAATCAAACCTGTTGCAGCAGC
A3-AT2G46920-XLOC_013718-7039-1
ATACATAATTCTTGACCTGCTTAAATCAAAATCAAACCTGTTGCAGCAGC
CONSENSUS
ATACATAATTCTTGACCTGCTTAAATCAAAATCAAACCTGTTGCAGCAGC

A3-AT2G46920-XLOC_013718-7039-0
TTCAAGCTTCTTCTTTTTCCCCTCTTCTTCAGCTCTATTTGGATTGTGAC
A3-AT2G46920-XLOC_013718-7039-1
TTCAAGCTTCTTCTTTTTCCCCTCTTCTTCAGCTCTATTTGGATTGTGAC
CONSENSUS
TTCAAGCTTCTTCTTTTTCCCCTCTTCTTCAGCTCTATTTGGATTGTGAC

A3-AT2G46920-XLOC_013718-7039-0
TCTACTAATTTTCGATTTTGAATCTTGGTTTCTCCGTAACATATCTGAAA
A3-AT2G46920-XLOC_013718-7039-1
TCTACTAATTTTCGATTTTGAATCTTGGTTTCTCCGTAACATATCTGAAA
CONSENSUS
TCTACTAATTTTCGATTTTGAATCTTGGTTTCTCCGTAACATATCTGAAA

A3-AT2G46920-XLOC_013718-7039-0
GAGGGGTTTCTGGGTTTTAGCTTCTTTTTTTTCCCCTGAAAAGCTTGGAT
A3-AT2G46920-XLOC_013718-7039-1
GAGGGGTTTCTGGGTTTTAGCTTCTTTTTTTTCCCCTGAAAAGCTTGGAT
CONSENSUS
GAGGGGTTTCTGGGTTTTAGCTTCTTTTTTTTCCCCTGAAAAGCTTGGAT

A3-AT2G46920-XLOC_013718-7039-0
CTTTAACAGCTGGTCGTCTCTGCATGGTTCAATCTCAATCTGGGTCTTCT
A3-AT2G46920-XLOC_013718-7039-1
CTTTAACAGCTGGTCGTCTCTGCATGGTTCAATCTCAATCTGGGTCTTCT
CONSENSUS
CTTTAACAGCTGGTCGTCTCTGCATGGTTCAATCTCAATCTGGGTCTTCT

A3-AT2G46920-XLOC_013718-7039-0
TCCTTTTTTTTTTTCTTTTTTAAAAAATGGGTATAATTGTTTTTTTTTGT
A3-AT2G46920-XLOC_013718-7039-1
TCCTTTTTTTTTTTCTTTTTTAAAAAATGGGTATAATTGTTTTTTTTTGT

CONSENSUS
TCCTTTTTTTTTTTCTTTTTTAAAAAATGGGTATAATTGTTTTTTTTTGT

A3-AT2G46920-XLOC_013718-7039-0
TTTTGTATAATTAGTTTTTAAAAATGGGTACTTTTTGTCAGATTCCCATA
A3-AT2G46920-XLOC_013718-7039-1
TTTTGTATAATTAGTTTTTAAAAATGGGTACTTTTTGTCAGATTCCCATA
CONSENSUS
TTTTGTATAATTAGTTTTTAAAAATGGGTACTTTTTGTCAGATTCCCATA

A3-AT2G46920-XLOC_013718-7039-0
GAAGAAATCTAAAGGGGTGTTTTTTCCCCTTGTTTACTGTCTCTATTTTG
A3-AT2G46920-XLOC_013718-7039-1
GAAGAAATCTAAAGGGGTGTTTTTTCCCCTTGTTTACTGTCTCTATTTTG
CONSENSUS
GAAGAAATCTAAAGGGGTGTTTTTTCCCCTTGTTTACTGTCTCTATTTTG

A3-AT2G46920-XLOC_013718-7039-0
CTTCCGATGTGTTCAATACAACACTGTCTTCTCTTTGCCGCTCCTTTAGA
A3-AT2G46920-XLOC_013718-7039-1
CTTCCGATGTGTTCAATACAACACTGTCTTCTCTTTGCCGCTCCTTTAGA
CONSENSUS
CTTCCGATGTGTTCAATACAACACTGTCTTCTCTTTGCCGCTCCTTTAGA

A3-AT2G46920-XLOC_013718-7039-0
TCTGTTTCCAGTTTCTGCTTGATGATCCATCTCCTTCTTCAACAG-----
A3-AT2G46920-XLOC_013718-7039-1
TCTGTTTCCAGTTTCTGCTTGATGATCCATCTCCTTCTTCAACAGGTGCT
CONSENSUS
TCTGTTTCCAGTTTCTGCTTGATGATCCATCTCCTTCTTCAACAG.....

A3-AT2G46920-XLOC_013718-7039-0 ---------------------
AGATTGAGCTTCAGATGATGCTTGGATTC
A3-AT2G46920-XLOC_013718-7039-1
TGTTTGCGTTTTAATGTTCAGAGATTGAGCTTCAGATGATGCTTGGATTC
CONSENSUS
.....................AGATTGAGCTTCAGATGATGCTTGGATTC

A3-AT2G46920-XLOC_013718-7039-0
TGCATAACGCGTCGAATCTTTGTAGGCTTTATAAGAATTTCGCCTCTTGT
A3-AT2G46920-XLOC_013718-7039-1
TGCATAACGCGTCGAATCTTTGTAGGCTTTATAAGAATTTCGCCTCTTGT
CONSENSUS
TGCATAACGCGTCGAATCTTTGTAGGCTTTATAAGAATTTCGCCTCTTGT

A3-AT2G46920-XLOC_013718-7039-0
TAAGTGTTGTGGTGCTGGAAATTGCGGAAACTCTTTAGAGGTTTATGATG
A3-AT2G46920-XLOC_013718-7039-1
TAAGTGTTGTGGTGCTGGAAATTGCGGAAACTCTTTAGAGGTTTATGATG
CONSENSUS
TAAGTGTTGTGGTGCTGGAAATTGCGGAAACTCTTTAGAGGTTTATGATG

A3-AT2G46920-XLOC_013718-7039-0
AGATTGTGCCTCCAAGGTGTTTGTAGTATTGCCTGAGTGATGGGAAACGG
A3-AT2G46920-XLOC_013718-7039-1
AGATTGTGCCTCCAAGGTGTTTGTAGTATTGCCTGAGTGATGGGAAACGG

CONSENSUS
AGATTGTGCCTCCAAGGTGTTTGTAGTATTGCCTGAGTGATGGGAAACGG

A3-AT2G46920-XLOC_013718-7039-0
GACTTCCCGTGTTGTTGGTTGTTTCGTGCCGTCTAATGACAAAAACGGTG
A3-AT2G46920-XLOC_013718-7039-1
GACTTCCCGTGTTGTTGGTTGTTTCGTGCCGTCTAATGACAAAAACGGTG
CONSENSUS
GACTTCCCGTGTTGTTGGTTGTTTCGTGCCGTCTAATGACAAAAACGGTG

A3-AT2G46920-XLOC_013718-7039-0
TTGATTTGGAGTTTCTGGAACCTTTAGATGAAGGTTTAGGCCATTCCTTT
A3-AT2G46920-XLOC_013718-7039-1
TTGATTTGGAGTTTCTGGAACCTTTAGATGAAGGTTTAGGCCATTCCTTT
CONSENSUS
TTGATTTGGAGTTTCTGGAACCTTTAGATGAAGGTTTAGGCCATTCCTTT

A3-AT2G46920-XLOC_013718-7039-0
TGCTACGTTAGGCCAAGCATCTTTGAGTCTCCTGATATTACTCCGTCTAA
A3-AT2G46920-XLOC_013718-7039-1
TGCTACGTTAGGCCAAGCATCTTTGAGTCTCCTGATATTACTCCGTCTAA
CONSENSUS
TGCTACGTTAGGCCAAGCATCTTTGAGTCTCCTGATATTACTCCGTCTAA

A3-AT2G46920-XLOC_013718-7039-0
CTCGGAGAGGTTCACCATTGATTCAAGCACTATTGATTCTGAGACACTCA
A3-AT2G46920-XLOC_013718-7039-1
CTCGGAGAGGTTCACCATTGATTCAAGCACTATTGATTCTGAGACACTCA
CONSENSUS
CTCGGAGAGGTTCACCATTGATTCAAGCACTATTGATTCTGAGACACTCA

A3-AT2G46920-XLOC_013718-7039-0
CTGGGTCTTTTCGAAACGATATCGTTGATGATCCTTCTTTCTTGAATAGA
A3-AT2G46920-XLOC_013718-7039-1
CTGGGTCTTTTCGAAACGATATCGTTGATGATCCTTCTTTCTTGAATAGA
CONSENSUS
CTGGGTCTTTTCGAAACGATATCGTTGATGATCCTTCTTTCTTGAATAGA

A3-AT2G46920-XLOC_013718-7039-0
CACAACAGTAAAGGTTTGGCGGAAACTACGTTTAAGGCAATCTCAGGTGC
A3-AT2G46920-XLOC_013718-7039-1
CACAACAGTAAAGGTTTGGCGGAAACTACGTTTAAGGCAATCTCAGGTGC
CONSENSUS
CACAACAGTAAAGGTTTGGCGGAAACTACGTTTAAGGCAATCTCAGGTGC

A3-AT2G46920-XLOC_013718-7039-0
TTCGGTTAGTGCCAATGTTTCCACGGCTAGGACTGGGAATCAAATGGCAT
A3-AT2G46920-XLOC_013718-7039-1
TTCGGTTAGTGCCAATGTTTCCACGGCTAGGACTGGGAATCAAATGGCAT
CONSENSUS
TTCGGTTAGTGCCAATGTTTCCACGGCTAGGACTGGGAATCAAATGGCAT

A3-AT2G46920-XLOC_013718-7039-0
TGTGTTCAAGTGACGTTTTGGAGCCTGCTGCTTCATTTGAGAGCACTTCC
A3-AT2G46920-XLOC_013718-7039-1
TGTGTTCAAGTGACGTTTTGGAGCCTGCTGCTTCATTTGAGAGCACTTCC

CONSENSUS
TGTGTTCAAGTGACGTTTTGGAGCCTGCTGCTTCATTTGAGAGCACTTCC

A3-AT2G46920-XLOC_013718-7039-0
TCGTTTGCTTCTATTCCTTTGCAGCCGCTCCCTCGTGGTGGTTCGGGACC
A3-AT2G46920-XLOC_013718-7039-1
TCGTTTGCTTCTATTCCTTTGCAGCCGCTCCCTCGTGGTGGTTCGGGACC
CONSENSUS
TCGTTTGCTTCTATTCCTTTGCAGCCGCTCCCTCGTGGTGGTTCGGGACC

A3-AT2G46920-XLOC_013718-7039-0
CTTAAACGGATTCATGTCCGGGCCTCTAGAGAGAGGTTTTGCATCTGGTC
A3-AT2G46920-XLOC_013718-7039-1
CTTAAACGGATTCATGTCCGGGCCTCTAGAGAGAGGTTTTGCATCTGGTC
CONSENSUS
CTTAAACGGATTCATGTCCGGGCCTCTAGAGAGAGGTTTTGCATCTGGTC

A3-AT2G46920-XLOC_013718-7039-0
CTTTGGATAGAAACAACGGTTTCATGTCTGGGCCTATTGAAAAAGGAGTA
A3-AT2G46920-XLOC_013718-7039-1
CTTTGGATAGAAACAACGGTTTCATGTCTGGGCCTATTGAAAAAGGAGTA
CONSENSUS
CTTTGGATAGAAACAACGGTTTCATGTCTGGGCCTATTGAAAAAGGAGTA

A3-AT2G46920-XLOC_013718-7039-0
ATGTCTGGACCCCTTGATGTATCTGATAGATCTAATTTCTCTGCGCCTCT
A3-AT2G46920-XLOC_013718-7039-1
ATGTCTGGACCCCTTGATGTATCTGATAGATCTAATTTCTCTGCGCCTCT
CONSENSUS
ATGTCTGGACCCCTTGATGTATCTGATAGATCTAATTTCTCTGCGCCTCT

A3-AT2G46920-XLOC_013718-7039-0
TTCTTTTAGACGTAAAAAGCCTCGGTTTCAGCGTTTTATGAGGAGTGTGA
A3-AT2G46920-XLOC_013718-7039-1
TTCTTTTAGACGTAAAAAGCCTCGGTTTCAGCGTTTTATGAGGAGTGTGA
CONSENSUS
TTCTTTTAGACGTAAAAAGCCTCGGTTTCAGCGTTTTATGAGGAGTGTGA

A3-AT2G46920-XLOC_013718-7039-0
GCGGACCAATGAAAAGTACATTAGCAAGGACATTTTCTAGACGATCTGGA
A3-AT2G46920-XLOC_013718-7039-1
GCGGACCAATGAAAAGTACATTAGCAAGGACATTTTCTAGACGATCTGGA
CONSENSUS
GCGGACCAATGAAAAGTACATTAGCAAGGACATTTTCTAGACGATCTGGA

A3-AT2G46920-XLOC_013718-7039-0
GGGTTAAGTTGGATGCATCGCTTCTTCTTGCATCCAGAGACTAGGGTTTC
A3-AT2G46920-XLOC_013718-7039-1
GGGTTAAGTTGGATGCATCGCTTCTTCTTGCATCCAGAGACTAGGGTTTC
CONSENSUS
GGGTTAAGTTGGATGCATCGCTTCTTCTTGCATCCAGAGACTAGGGTTTC

A3-AT2G46920-XLOC_013718-7039-0
CTGGGCTGTTGGAAAGGACGGTAAGCTACATGGTGAAGACCCTGAAAGTT
A3-AT2G46920-XLOC_013718-7039-1
CTGGGCTGTTGGAAAGGACGGTAAGCTACATGGTGAAGACCCTGAAAGTT

CONSENSUS
CTGGGCTGTTGGAAAGGACGGTAAGCTACATGGTGAAGACCCTGAAAGTT

A3-AT2G46920-XLOC_013718-7039-0
GTCTAGAGAGCAACCGTAACTTGCAATGGGCTCATGGGAAAGCTGGAGAA
A3-AT2G46920-XLOC_013718-7039-1
GTCTAGAGAGCAACCGTAACTTGCAATGGGCTCATGGGAAAGCTGGAGAA
CONSENSUS
GTCTAGAGAGCAACCGTAACTTGCAATGGGCTCATGGGAAAGCTGGAGAA

A3-AT2G46920-XLOC_013718-7039-0
GATAGGGTTCACGTTGTGCTTTCGGAGGAACAAGGATGGCTCTTTATCGG
A3-AT2G46920-XLOC_013718-7039-1
GATAGGGTTCACGTTGTGCTTTCGGAGGAACAAGGATGGCTCTTTATCGG
CONSENSUS
GATAGGGTTCACGTTGTGCTTTCGGAGGAACAAGGATGGCTCTTTATCGG

A3-AT2G46920-XLOC_013718-7039-0
GATATATGATGGGTTTAGTGGACCGGATGCTCCAGATTTCGTAATGAGTC
A3-AT2G46920-XLOC_013718-7039-1
GATATATGATGGGTTTAGTGGACCGGATGCTCCAGATTTCGTAATGAGTC
CONSENSUS
GATATATGATGGGTTTAGTGGACCGGATGCTCCAGATTTCGTAATGAGTC

A3-AT2G46920-XLOC_013718-7039-0
ATCTTTATAAAGCTATTGACAAGGAATTGGAAGGTCTTCTTTGGGATTAT
A3-AT2G46920-XLOC_013718-7039-1
ATCTTTATAAAGCTATTGACAAGGAATTGGAAGGTCTTCTTTGGGATTAT
CONSENSUS
ATCTTTATAAAGCTATTGACAAGGAATTGGAAGGTCTTCTTTGGGATTAT

A3-AT2G46920-XLOC_013718-7039-0
GAAGAGCCATCTGAAGATAATCAATTGCAGCCTGACCAGGAACCTCCTAC
A3-AT2G46920-XLOC_013718-7039-1
GAAGAGCCATCTGAAGATAATCAATTGCAGCCTGACCAGGAACCTCCTAC
CONSENSUS
GAAGAGCCATCTGAAGATAATCAATTGCAGCCTGACCAGGAACCTCCTAC

A3-AT2G46920-XLOC_013718-7039-0
AGAAGAGAACATGTGTGATCCAGAATCTATCAGTGAGCAGCATTCAAAGT
A3-AT2G46920-XLOC_013718-7039-1
AGAAGAGAACATGTGTGATCCAGAATCTATCAGTGAGCAGCATTCAAAGT
CONSENSUS
AGAAGAGAACATGTGTGATCCAGAATCTATCAGTGAGCAGCATTCAAAGT

A3-AT2G46920-XLOC_013718-7039-0
CAGTAGTGGCAGAAAGTGAGGAGGTTATGATTGATGATATCAGTAGCCTT
A3-AT2G46920-XLOC_013718-7039-1
CAGTAGTGGCAGAAAGTGAGGAGGTTATGATTGATGATATCAGTAGCCTT
CONSENSUS
CAGTAGTGGCAGAAAGTGAGGAGGTTATGATTGATGATATCAGTAGCCTT

A3-AT2G46920-XLOC_013718-7039-0
GGAAATACCGATACTCAGATTGCTGATGGTCCACCTGGAGACTCAGCTGG
A3-AT2G46920-XLOC_013718-7039-1
GGAAATACCGATACTCAGATTGCTGATGGTCCACCTGGAGACTCAGCTGG

CONSENSUS
GGAAATACCGATACTCAGATTGCTGATGGTCCACCTGGAGACTCAGCTGG

A3-AT2G46920-XLOC_013718-7039-0
TCCTGGCAAGAAAAGCATGAGACTTTACGAGCTACTTCAATTGGAACAGT
A3-AT2G46920-XLOC_013718-7039-1
TCCTGGCAAGAAAAGCATGAGACTTTACGAGCTACTTCAATTGGAACAGT
CONSENSUS
TCCTGGCAAGAAAAGCATGAGACTTTACGAGCTACTTCAATTGGAACAGT

A3-AT2G46920-XLOC_013718-7039-0
GGGAAGGAGAAGAAATCGGGCTTAAACGATATGGAGGGAATGTCGCTCTA
A3-AT2G46920-XLOC_013718-7039-1
GGGAAGGAGAAGAAATCGGGCTTAAACGATATGGAGGGAATGTCGCTCTA
CONSENSUS
GGGAAGGAGAAGAAATCGGGCTTAAACGATATGGAGGGAATGTCGCTCTA

A3-AT2G46920-XLOC_013718-7039-0
AACAATATGACCAATCAGGTTGAAAATCCATCTACTTCAGGTGGAGGAGC
A3-AT2G46920-XLOC_013718-7039-1
AACAATATGACCAATCAGGTTGAAAATCCATCTACTTCAGGTGGAGGAGC
CONSENSUS
AACAATATGACCAATCAGGTTGAAAATCCATCTACTTCAGGTGGAGGAGC

A3-AT2G46920-XLOC_013718-7039-0
TGGAAATGATCCTTGTACCACCGACCGTAGTGCTCTTGATGGGATTCCGA
A3-AT2G46920-XLOC_013718-7039-1
TGGAAATGATCCTTGTACCACCGACCGTAGTGCTCTTGATGGGATTCCGA
CONSENSUS
TGGAAATGATCCTTGTACCACCGACCGTAGTGCTCTTGATGGGATTCCGA

A3-AT2G46920-XLOC_013718-7039-0
ACTCGGGACAAAGGCATGGGACTAAAAAATCACAGATAAGCTCTAAGATA
A3-AT2G46920-XLOC_013718-7039-1
ACTCGGGACAAAGGCATGGGACTAAAAAATCACAGATAAGCTCTAAGATA
CONSENSUS
ACTCGGGACAAAGGCATGGGACTAAAAAATCACAGATAAGCTCTAAGATA

A3-AT2G46920-XLOC_013718-7039-0
AGAAGAATGTATCAAAAACAGAAGTCTTTGCGGAAAAAACTGTTTCCATG
A3-AT2G46920-XLOC_013718-7039-1
AGAAGAATGTATCAAAAACAGAAGTCTTTGCGGAAAAAACTGTTTCCATG
CONSENSUS
AGAAGAATGTATCAAAAACAGAAGTCTTTGCGGAAAAAACTGTTTCCATG

A3-AT2G46920-XLOC_013718-7039-0
GAGTTATGATTGGCACAGGGAAGAAGGGATTTGCGTTGAAGAGAAGATAG
A3-AT2G46920-XLOC_013718-7039-1
GAGTTATGATTGGCACAGGGAAGAAGGGATTTGCGTTGAAGAGAAGATAG
CONSENSUS
GAGTTATGATTGGCACAGGGAAGAAGGGATTTGCGTTGAAGAGAAGATAG

A3-AT2G46920-XLOC_013718-7039-0
TGGAGTCATCGGGACCGATTAGGAGACGCTGGTCAGGGACTGTGGACCAT
A3-AT2G46920-XLOC_013718-7039-1
TGGAGTCATCGGGACCGATTAGGAGACGCTGGTCAGGGACTGTGGACCAT

CONSENSUS
TGGAGTCATCGGGACCGATTAGGAGACGCTGGTCAGGGACTGTGGACCAT

A3-AT2G46920-XLOC_013718-7039-0
GATGCTGTTCTAAGAGCAATGGCTAGAGCACTAGAGAGTACTGAAGAGGC
A3-AT2G46920-XLOC_013718-7039-1
GATGCTGTTCTAAGAGCAATGGCTAGAGCACTAGAGAGTACTGAAGAGGC
CONSENSUS
GATGCTGTTCTAAGAGCAATGGCTAGAGCACTAGAGAGTACTGAAGAGGC

A3-AT2G46920-XLOC_013718-7039-0
GTACATGGATATGGTAGAGAAGTCTCTTGACATAAACCCAGAGCTCGCGC
A3-AT2G46920-XLOC_013718-7039-1
GTACATGGATATGGTAGAGAAGTCTCTTGACATAAACCCAGAGCTCGCGC
CONSENSUS
GTACATGGATATGGTAGAGAAGTCTCTTGACATAAACCCAGAGCTCGCGC

A3-AT2G46920-XLOC_013718-7039-0
TAATGGGTTCATGTGTTCTTGTAATGCTAATGAAAGATCAAGATGTTTAC
A3-AT2G46920-XLOC_013718-7039-1
TAATGGGTTCATGTGTTCTTGTAATGCTAATGAAAGATCAAGATGTTTAC
CONSENSUS
TAATGGGTTCATGTGTTCTTGTAATGCTAATGAAAGATCAAGATGTTTAC

A3-AT2G46920-XLOC_013718-7039-0
GTGATGAACGTTGGGGATAGTCGGGCTATCTTAGCCCAAGAAAGGCTCCA
A3-AT2G46920-XLOC_013718-7039-1
GTGATGAACGTTGGGGATAGTCGGGCTATCTTAGCCCAAGAAAGGCTCCA
CONSENSUS
GTGATGAACGTTGGGGATAGTCGGGCTATCTTAGCCCAAGAAAGGCTCCA

A3-AT2G46920-XLOC_013718-7039-0
TGATCGTCACTCTAATCCTGGTTTTGGGAATGATGAGGGTATCGGGCATA
A3-AT2G46920-XLOC_013718-7039-1
TGATCGTCACTCTAATCCTGGTTTTGGGAATGATGAGGGTATCGGGCATA
CONSENSUS
TGATCGTCACTCTAATCCTGGTTTTGGGAATGATGAGGGTATCGGGCATA

A3-AT2G46920-XLOC_013718-7039-0
AGAGTAGGTCTCGAGAATCACTTGTGCGTATTGAACTGGATAGAATATCA
A3-AT2G46920-XLOC_013718-7039-1
AGAGTAGGTCTCGAGAATCACTTGTGCGTATTGAACTGGATAGAATATCA
CONSENSUS
AGAGTAGGTCTCGAGAATCACTTGTGCGTATTGAACTGGATAGAATATCA

A3-AT2G46920-XLOC_013718-7039-0
GAGGAATCTCCAATACACAATCAAGCAACTCCAATCAGTGTGTCAAACAA
A3-AT2G46920-XLOC_013718-7039-1
GAGGAATCTCCAATACACAATCAAGCAACTCCAATCAGTGTGTCAAACAA
CONSENSUS
GAGGAATCTCCAATACACAATCAAGCAACTCCAATCAGTGTGTCAAACAA

A3-AT2G46920-XLOC_013718-7039-0
AAACAGAGATGTGACCTCTTACCGGTTAAAGATGAGAGCAGTTCAACTAT
A3-AT2G46920-XLOC_013718-7039-1
AAACAGAGATGTGACCTCTTACCGGTTAAAGATGAGAGCAGTTCAACTAT

CONSENSUS
      AAACAGAGATGTGACCTCTTACCGGTTAAAGATGAGAGCAGTTCAACTAT

A3-AT2G46920-XLOC_013718-7039-0 CAAGTGACCATAGCACAAGTGTGGAAGAG
A3-AT2G46920-XLOC_013718-7039-1 CAAGTGACCATAGCACAAGTGTGGAAGAG
CONSENSUS   CAAGTGACCATAGCACAAGTGTGGAAGAG

alignment for event: A3-AT2G05520-XLOC_008599-1449

A3-AT2G05520-XLOC_008599-1449-0
      CCACAGTGAATTCAGAGAGTAAGGAAACTGTGAAACCTGATCAACGTGGC
A3-AT2G05520-XLOC_008599-1449-1
      CCACAGTGAATTCAGAGAGTAAGGAAACTGTGAAACCTGATCAACGTGGC
CONSENSUS
      CCACAGTGAATTCAGAGAGTAAGGAAACTGTGAAACCTGATCAACGTGGC

A3-AT2G05520-XLOC_008599-1449-0
      TACGGTGACAATGGAGGAAATTACAATAACGGAGGAGGTAATTACCAAGG
A3-AT2G05520-XLOC_008599-1449-1
      TACGGTGACAATGGAGGAAATTACAATAACGGAGGAG-------------
CONSENSUS
      TACGGTGACAATGGAGGAAATTACAATAACGGAGGAG.............

A3-AT2G05520-XLOC_008599-1449-0
      AGGAGGAGGAAGATACCAAGGAGGAGGAGGACGGTACCAAGGAGGAGGAG
A3-AT2G05520-XLOC_008599-1449-1 -----------------------------
GACGGTACCAAGGAGGAGGAG
CONSENSUS
      .............................GACGGTACCAAGGAGGAGGAG

A3-AT2G05520-XLOC_008599-1449-0
      GACGATACCAAGGAGGCGGTGGGCGACAAGGAGGAGGGGGAAGTGGGGGA
A3-AT2G05520-XLOC_008599-1449-1
      GACGATACCAAGGAGGCGGTGGGCGACAAGGAGGAGGGGGAAGTGGGGGA
CONSENSUS
      GACGATACCAAGGAGGCGGTGGGCGACAAGGAGGAGGGGGAAGTGGGGGA

A3-AT2G05520-XLOC_008599-1449-0
      AGTTACTGCCGCCACGGCTGCTGCTACAGAGGTTACAACGGCTGCTCAAG
A3-AT2G05520-XLOC_008599-1449-1
      AGTTACTGCCGCCACGGCTGCTGCTACAGAGGTTACAACGGCTGCTCAAG
CONSENSUS
      AGTTACTGCCGCCACGGCTGCTGCTACAGAGGTTACAACGGCTGCTCAAG

A3-AT2G05520-XLOC_008599-1449-0
      ATGCTGCTCGTATGCCGGAGAAGCTGTTCAGACTCAGCCCGGTCACTAAA
A3-AT2G05520-XLOC_008599-1449-1
      ATGCTGCTCGTATGCCGGAGAAGCTGTTCAGACTCAGCCCGGTCACTAAA
CONSENSUS
      ATGCTGCTCGTATGCCGGAGAAGCTGTTCAGACTCAGCCCGGTCACTAAA

A3-AT2G05520-XLOC_008599-1449-0
      ACAATATATTAACCAATCACCACCATGCATGTATTGCATTATGTATGTAT
A3-AT2G05520-XLOC_008599-1449-1

ACAATATATTAACCAATCACCACCATGCATGTATTGCATTATGTATGTAT
CONSENSUS
ACAATATATTAACCAATCACCACCATGCATGTATTGCATTATGTATGTAT

A3-AT2G05520-XLOC_008599-1449-0
GATTTTAAGTAAACCATGGTGCGTTTGTAATGAACTGCCTCAAGTTTTTG
A3-AT2G05520-XLOC_008599-1449-1
GATTTTAAGTAAACCATGGTGCGTTTGTAATGAACTGCCTCAAGTTTTTG
CONSENSUS
GATTTTAAGTAAACCATGGTGCGTTTGTAATGAACTGCCTCAAGTTTTTG

A3-AT2G05520-XLOC_008599-1449-0
AGGCACTATAGATTAAGAAGAGAACTATGGGAATAAAGTTTGATTATGTA
A3-AT2G05520-XLOC_008599-1449-1
AGGCACTATAGATTAAGAAGAGAACTATGGGAATAAAGTTTGATTATGTA
CONSENSUS
AGGCACTATAGATTAAGAAGAGAACTATGGGAATAAAGTTTGATTATGTA

A3-AT2G05520-XLOC_008599-1449-0
ATGTTTTATGTGGTTTGAGTTGTAATACTTGCTGTTTGCATAATAAAATC
A3-AT2G05520-XLOC_008599-1449-1
ATGTTTTATGTGGTTTGAGTTGTAATACTTGCTGTTTGCATAATAAAATC
CONSENSUS
ATGTTTTATGTGGTTTGAGTTGTAATACTTGCTGTTTGCATAATAAAATC

A3-AT2G05520-XLOC_008599-1449-0
GTTTGTAGTTTATGTTAATCTCTTTCGTTTTTTT
A3-AT2G05520-XLOC_008599-1449-1
GTTTGTAGTTTATGTTAATCTCTTTCGTTTTTTT
CONSENSUS
GTTTGTAGTTTATGTTAATCTCTTTCGTTTTTTT

alignment for event: A5-AT2G46270-XLOC_010973-3210

A5-AT2G46270-XLOC_010973-3210-0
ATGGGAAACAATTGGTTCAAGCTAGCTCATTTCATTCTGTTTCTCCGTCA
A5-AT2G46270-XLOC_010973-3210-1
ATGGGAAACAATTGGTTCAAGCTAGCTCATTTCATTCTGTTTCTCCGTCA
CONSENSUS
ATGGGAAACAATTGGTTCAAGCTAGCTCATTTCATTCTGTTTCTCCGTCA

A5-AT2G46270-XLOC_010973-3210-0
AGTGGTGATACCGGCGTAAAACTCATTCAAGGATCTGGAGCTATACTCTC
A5-AT2G46270-XLOC_010973-3210-1
AGTGGTGATACCGGCGTAAAACTCATTCAAGGATCTGGAGCTATACTCTC
CONSENSUS
AGTGGTGATACCGGCGTAAAACTCATTCAAGGATCTGGAGCTATACTCTC

A5-AT2G46270-XLOC_010973-3210-0
TCCTGGT---
A5-AT2G46270-XLOC_010973-3210-1
TCCTGGTGTAAGTGCAAATTCCAACCCCTTCATGTCACAATCTTTAGCCA
CONSENSUS
TCCTGGT...

A5-AT2G46270-XLOC_010973-3210-0 -------------------------
AACGAGAGAGAACTGAAACGGGAG
A5-AT2G46270-XLOC_010973-3210-1
TGGTTCCTCCTGAAACTTGGCTTCAGAACGAGAGAGAACTGAAACGGGAG
CONSENSUS
..........................AACGAGAGAGAACTGAAACGGGAG

A5-AT2G46270-XLOC_010973-3210-0
CGAAGGAAACAGTCTAATAGAGAATCTGCTAGAAGGTCAAGATTAAGGAA
A5-AT2G46270-XLOC_010973-3210-1
CGAAGGAAACAGTCTAATAGAGAATCTGCTAGAAGGTCAAGATTAAGGAA
CONSENSUS
CGAAGGAAACAGTCTAATAGAGAATCTGCTAGAAGGTCAAGATTAAGGAA

A5-AT2G46270-XLOC_010973-3210-0 ACAG
A5-AT2G46270-XLOC_010973-3210-1 ACAG
CONSENSUS ACAG

alignment for event: RI-AT2G27340-XLOC_009895-5177

RI-AT2G27340-XLOC_009895-5177-0
TTTTCATGGGCTTCTACAGCCATCACGTCCTTCCTCATTCTTGCCATCGA
RI-AT2G27340-XLOC_009895-5177-1
TTTTCATGGGCTTCTACAGCCATCACGTCCTTCCTCATTCTTGCCATCGA
CONSENSUS
TTTTCATGGGCTTCTACAGCCATCACGTCCTTCCTCATTCTTGCCATCGA

RI-AT2G27340-XLOC_009895-5177-0
TCAAGCTCTTACTTTCTCAGTTCTCAGTGTCGATAGATTCTCTTCTTTGG
RI-AT2G27340-XLOC_009895-5177-1
TCAAGCTCTTACTTTCTCAGTTCTCAGTGTCGATAGATTCTCTTCTTTGG
CONSENSUS
TCAAGCTCTTACTTTCTCAGTTCTCAGTGTCGATAGATTCTCTTCTTTGG

RI-AT2G27340-XLOC_009895-5177-0
TTTAGCTGTTCCACTCTCTGATTTTACTTAGCTTGATCCTTCCGAATGTG
RI-AT2G27340-XLOC_009895-5177-1
TTTAGCTGTTCCACTCTCTGATTTTACTTAGCTTGATCCTTCCGAATGTG
CONSENSUS
TTTAGCTGTTCCACTCTCTGATTTTACTTAGCTTGATCCTTCCGAATGTG

RI-AT2G27340-XLOC_009895-5177-0
AAAATGTTTCTGTTATGTTTATCTGAAATGTGGACTCATGAATCTGGAAT
RI-AT2G27340-XLOC_009895-5177-1
AAAATGTTTCTGTTATGTTTATCTGAAATGTGGACTCATGAATCTGGAAT
CONSENSUS
AAAATGTTTCTGTTATGTTTATCTGAAATGTGGACTCATGAATCTGGAAT

RI-AT2G27340-XLOC_009895-5177-0
TTGAGCAAACAAGGGTTTCTTCCGAATGTGAGCTTGAATGCTTACAAATT
RI-AT2G27340-XLOC_009895-5177-1
TTGAGCAAACAAGGGTTTCTTCCGAATG----------------------
CONSENSUS

```
      TTGAGCAAACAAGGGTTTCTTCCGAAT.......................

RI-AT2G27340-XLOC_009895-5177-0
      TTGAATCTTTTTGTTTATTTGTTTGCTCTCTTCATTTTTTAATTGATCTG
RI-AT2G27340-XLOC_009895-5177-1
      --------------------------------------------------
CONSENSUS
      ..................................................

RI-AT2G27340-XLOC_009895-5177-0
      GTCACCTGAAATGAGTTAAGTTCGACTTATATAGAAGCTATGTTCTGCAA
RI-AT2G27340-XLOC_009895-5177-1
      --------------------------------------------------
CONSENSUS
      ..................................................

RI-AT2G27340-XLOC_009895-5177-0
      ATCCACATTGATGATTTTACCATTTTGTTAATTTGGATGAAGTTGATCCA
RI-AT2G27340-XLOC_009895-5177-1
      --------------------------------------------------
CONSENSUS
      ..................................................

RI-AT2G27340-XLOC_009895-5177-0
      TCAAGAAAACACTTTAAGACTTATAATTGTTGGACTTGATCTGACTTTCTT
RI-AT2G27340-XLOC_009895-5177-1
      --------------------------------------------------
CONSENSUS
      ..................................................

RI-AT2G27340-XLOC_009895-5177-0
      TTTGGGTACTGACCATTTATGCTGACAATTTTGTATTGATTCAATATCAT
RI-AT2G27340-XLOC_009895-5177-1
      --------------------------------------------------
CONSENSUS
      ..................................................

RI-AT2G27340-XLOC_009895-5177-0
      TTATGGTGTCAGATGGTTGTTGTTTTTCTATCTCTGATTGTTGTTATATG
RI-AT2G27340-XLOC_009895-5177-1  ------------
ATGGTTGTTGTTTTTCTATCTCTGATTGTTGTTATATG
CONSENSUS
      ............ATGGTTGTTGTTTTTCTATCTCTGATTGTTGTTATATG

RI-AT2G27340-XLOC_009895-5177-0
      GGTGGCTTCTTTCTTCAAAATTTTCTTCCGAGCTACATCTATCTCCAGAG
RI-AT2G27340-XLOC_009895-5177-1
      GGTGGCTTCTTTCTTCAAAATTTTCTTCCGAGCTACATCTATCTCCAGAG
CONSENSUS
      GGTGGCTTCTTTCTTCAAAATTTTCTTCCGAGCTACATCTATCTCCAGAG

RI-AT2G27340-XLOC_009895-5177-0  CCACTATTCTTGATGATG
RI-AT2G27340-XLOC_009895-5177-1  CCACTATTCTTGATGATG
CONSENSUS                        CCACTATTCTTGATGATG
```

alignment for event: A3-AT2G02390-XLOC_008392-6817

A3-AT2G02390-XLOC_008392-6817-0
GGCTTGATTATGAGTATATACCAGTGAATTTGCTCAAGGGTGATCAATTC
A3-AT2G02390-XLOC_008392-6817-1
GGCTTGATTATGAGTATATACCAGTGAATTTGCTCAAGGGTGATCAATTC
CONSENSUS
GGCTTGATTATGAGTATATACCAGTGAATTTGCTCAAGGGTGATCAATTC

A3-AT2G02390-XLOC_008392-6817-0
GATTCAGTTTATCGTTTTGATCTTCAAGATTTCAAGAAGATCAATCCAAT
A3-AT2G02390-XLOC_008392-6817-1 GATTCAG---------------------
ATTTCAAGAAGATCAATCCAAT
CONSENSUS
GATTCAG.....................ATTTCAAGAAGATCAATCCAAT

A3-AT2G02390-XLOC_008392-6817-0
GGGAACTGTACCAGCTCTGGTGGATGGAGATGTTGTGATTAATGATTCTT
A3-AT2G02390-XLOC_008392-6817-1
GGGAACTGTACCAGCTCTGGTGGATGGAGATGTTGTGATTAATGATTCTT
CONSENSUS
GGGAACTGTACCAGCTCTGGTGGATGGAGATGTTGTGATTAATGATTCTT

A3-AT2G02390-XLOC_008392-6817-0 TTGCGATAATAATG
A3-AT2G02390-XLOC_008392-6817-1 TTGCGATAATAATG
CONSENSUS TTGCGATAATAATG

alignment for event: A3-AT3G54500-XLOC_019882-2374

A3-AT3G54500-XLOC_019882-2374-0
CTGGCGATGTGTGGAGCAATCAGGGAGCTTGATTTTGGGTCTGTGATTCG
A3-AT3G54500-XLOC_019882-2374-1
CTGGCGATGTGTGGAGCAATCAGGGAGCTTGATTTTGGGTCTGTGATTCG
CONSENSUS
CTGGCGATGTGTGGAGCAATCAGGGAGCTTGATTTTGGGTCTGTGATTCG

A3-AT3G54500-XLOC_019882-2374-0
ATTTTGTTTCTCCAGTCATGTTTGATTGGGAAGAAGAAGAG---------
A3-AT3G54500-XLOC_019882-2374-1
ATTTTGTTTCTCCAGTCATGTTTGATTGGGAAGAAGAAGAGCTTACTAAT
CONSENSUS
ATTTTGTTTCTCCAGTCATGTTTGATTGGGAAGAAGAAGAG.........

A3-AT3G54500-XLOC_019882-2374-0 ----------------------------
GCGACCATATTGTGCCTTTTAA
A3-AT3G54500-XLOC_019882-2374-1
ATGATATGGGGTGATGATGCTGAGACAGGCGACCATATTGTGCCTTTTAA
CONSENSUS
............................GCGACCATATTGTGCCTTTTAA

A3-AT3G54500-XLOC_019882-2374-0
AGTCAGAAGTGAACAACTTAACAAAAAGGAACAGATTGAGGAATCTAAGA
A3-AT3G54500-XLOC_019882-2374-1

```
      AGTCAGAAGTGAACAACTTAACAAAAAGGAACAGATTGAGGAATCTAAGA
CONSENSUS
      AGTCAGAAGTGAACAACTTAACAAAAAGGAACAGATTGAGGAATCTAAGA

A3-AT3G54500-XLOC_019882-2374-0
      CAGCTGAGCAAAAGATAACTGGGACTAAAATTGACCTCCATGATAAAAAT
A3-AT3G54500-XLOC_019882-2374-1
      CAGCTGAGCAAAAGATAACTGGGACTAAAATTGACCTCCATGATAAAAAT
CONSENSUS
      CAGCTGAGCAAAAGATAACTGGGACTAAAATTGACCTCCATGATAAAAAT

A3-AT3G54500-XLOC_019882-2374-0
      TTGGGGAGCAGTTCGAGCCATAATGTTGATGAGGGGCTTCCTCAGCCAGA
A3-AT3G54500-XLOC_019882-2374-1
      TTGGGGAGCAGTTCGAGCCATAATGTTGATGAGGGGCTTCCTCAGCCAGA
CONSENSUS
      TTGGGGAGCAGTTCGAGCCATAATGTTGATGAGGGGCTTCCTCAGCCAGA

A3-AT3G54500-XLOC_019882-2374-0
      TTTCTGTATGAGCTCATGGCCTGACACGTCGCTAACTAATGCTACAAAGG
A3-AT3G54500-XLOC_019882-2374-1
      TTTCTGTATGAGCTCATGGCCTGACACGTCGCTAACTAATGCTACAAAGG
CONSENSUS
      TTTCTGTATGAGCTCATGGCCTGACACGTCGCTAACTAATGCTACAAAGG

A3-AT3G54500-XLOC_019882-2374-0
      TTGATCAAGATTTGAGTGCGACTGAACTTTCAAAATGCTTAGCTGAGCCA
A3-AT3G54500-XLOC_019882-2374-1
      TTGATCAAGATTTGAGTGCGACTGAACTTTCAAAATGCTTAGCTGAGCCA
CONSENSUS
      TTGATCAAGATTTGAGTGCGACTGAACTTTCAAAATGCTTAGCTGAGCCA

A3-AT3G54500-XLOC_019882-2374-0  GTCAGATATGATTCAACAAGAGGTG
A3-AT3G54500-XLOC_019882-2374-1  GTCAGATATGATTCAACAAGAGGTG
CONSENSUS                        GTCAGATATGATTCAACAAGAGGTG


alignment for event: RI-AT3G54500-XLOC_019882-2370

RI-AT3G54500-XLOC_019882-2370-0
      AAAAAAAACTCTTTTTTCTTTTTTCCACTGTAACTCCTTTCTCTTCCCAT
RI-AT3G54500-XLOC_019882-2370-1
      AAAAAAAACTCTTTTTTCTTTTTTCCACTGTAACTCCTTTCTCTTCCCAT
CONSENSUS
      AAAAAAAACTCTTTTTTCTTTTTTCCACTGTAACTCCTTTCTCTTCCCAT

RI-AT3G54500-XLOC_019882-2370-0
      ATCCTTTGAGCACGAAGGAGGATTCTGCTGAATCAAGGTTTTTCAAAGCT
RI-AT3G54500-XLOC_019882-2370-1
      ATCCTTTGAGCACGAAGGAGGATTCTGCTGAATCAAG-------------
CONSENSUS
      ATCCTTTGAGCACGAAGGAGGATTCTGCTGAATCAAG.............

RI-AT3G54500-XLOC_019882-2370-0
      TGTTGCTTTTTCAGTTTAATATTCTTTTATTTATCGTTTTTTCCACTTTA
```

RI-AT3G54500-XLOC_019882-2370-1

CONSENSUS
      ....

RI-AT3G54500-XLOC_019882-2370-0
      TTATTGTGTGTGGATTTACGTTATCCGATTTGTTGATCTTGTTGTTTTTT
RI-AT3G54500-XLOC_019882-2370-1

CONSENSUS
      ....

RI-AT3G54500-XLOC_019882-2370-0
      CCTGGATTTTTTTCGTTGTGGAGCTGGCGATGTGTGGAGCAATCAGGGAG
RI-AT3G54500-XLOC_019882-2370-1 ----------------------
CTGGCGATGTGTGGAGCAATCAGGGAG
CONSENSUS
      ......................CTGGCGATGTGTGGAGCAATCAGGGAG

RI-AT3G54500-XLOC_019882-2370-0
      CTTGATTTTGGGTCTGTGATTCGATTTTGTTTCTCCAGTCATGTTTGATT
RI-AT3G54500-XLOC_019882-2370-1
      CTTGATTTTGGGTCTGTGATTCGATTTTGTTTCTCCAGTCATGTTTGATT
CONSENSUS
      CTTGATTTTGGGTCTGTGATTCGATTTTGTTTCTCCAGTCATGTTTGATT

RI-AT3G54500-XLOC_019882-2370-0 GGGAAGAAGAAGAG
RI-AT3G54500-XLOC_019882-2370-1 GGGAAGAAGAAGAG
CONSENSUS   GGGAAGAAGAAGAG

alignment for event: RI-AT3G52180-XLOC_019747-92

RI-AT3G52180-XLOC_019747-92-0
      CTTACAGGACTCAAGAGGAAGACTGTTACTCTGACACTGAAAGATAAGGG
RI-AT3G52180-XLOC_019747-92-1
      CTTACAGGACTCAAGAGGAAGACTGTTACTCTGACACTGAAAGATAAGGG
CONSENSUS
      CTTACAGGACTCAAGAGGAAGACTGTTACTCTGACACTGAAAGATAAGGG

RI-AT3G52180-XLOC_019747-92-0
      GTTCTCCAGAGTAGAAATTTCTGGCCTTGACATTGGATGGGGACAGGTAA
RI-AT3G52180-XLOC_019747-92-1
      GTTCTCCAGAGTAGAAATTTCTGGCCTTGACATTGGATGGGGACAG----
CONSENSUS
      GTTCTCCAGAGTAGAAATTTCTGGCCTTGACATTGGATGGGGACAG....

RI-AT3G52180-XLOC_019747-92-0
      ATATATTTTATCTTAAACCAATTTATTTATTATGACACCTTGCTCTTAAA
RI-AT3G52180-XLOC_019747-92-1

CONSENSUS
      ....

RI-AT3G52180-XLOC_019747-92-0

```
      TCTATGGTGTGCCATATAAGCTTTAAAGCCAGGATGCACTTTTGTCACTG
RI-AT3G52180-XLOC_019747-92-1
      --------------------------------------------------
CONSENSUS
      ..................................................

RI-AT3G52180-XLOC_019747-92-0
      CTATTAAAAGAATGGCCTCTTTGATAAGAAGTGGAAGAGATGAGTGTTGG
RI-AT3G52180-XLOC_019747-92-1
      --------------------------------------------------
CONSENSUS
      ..................................................

RI-AT3G52180-XLOC_019747-92-0
      ATTTCTGAAAATTATTGTCTTATGATTGTGCAAAACTATTTGGACGTATA
RI-AT3G52180-XLOC_019747-92-1
      --------------------------------------------------
CONSENSUS
      ..................................................

RI-AT3G52180-XLOC_019747-92-0
      TATATAAAAGGTAATGTATTTCTTTAGCTCTTTTCGTTTTTACCATTTTT
RI-AT3G52180-XLOC_019747-92-1
      --------------------------------------------------
CONSENSUS
      ..................................................

RI-AT3G52180-XLOC_019747-92-0
      CTCTTTATGTAGAGGATACCTCTAACACTGGACAAGGGAACAGGATTCTG
RI-AT3G52180-XLOC_019747-92-1    ------------
AGGATACCTCTAACACTGGACAAGGGAACAGGATTCTG
CONSENSUS
      ............AGGATACCTCTAACACTGGACAAGGGAACAGGATTCTG

RI-AT3G52180-XLOC_019747-92-0    GATCCTAAAGAGAGAACTGCCT
RI-AT3G52180-XLOC_019747-92-1    GATCCTAAAGAGAGAACTGCCT
CONSENSUS                        GATCCTAAAGAGAGAACTGCCT


alignment for event: RI-AT3G13420-XLOC_014430-6298

RI-AT3G13420-XLOC_014430-6298-0
      GAACATTTCATCTGATATGTATCAAGTCTTTTACATCTTCTCTGTGAAAC
RI-AT3G13420-XLOC_014430-6298-1
      GAACATTTCATCTGATATGTATCAAGTCTTTTACATCTTCTCTGTGAAAC
CONSENSUS
      GAACATTTCATCTGATATGTATCAAGTCTTTTACATCTTCTCTGTGAAAC

RI-AT3G13420-XLOC_014430-6298-0
      TCACCCTGGCTATGGAAATTGCTGGGTTCGTTGTGAGGCTATCGTCCTCT
RI-AT3G13420-XLOC_014430-6298-1
      TCACCCTGGCTATGGAAATTGCTGGGTTCGTTGTGAGGCTATCGTCCTCT
CONSENSUS
      TCACCCTGGCTATGGAAATTGCTGGGTTCGTTGTGAGGCTATCGTCCTCT
```

RI-AT3G13420-XLOC_014430-6298-0
CTGTTATGGTTTCAGATCTATAGGCTGGGAGCTTCTATTATAGACTCTCC
RI-AT3G13420-XLOC_014430-6298-1
CTGTTATGGTTTCAGATCTATAGGCTGGGAGCTTCTATTATAGACTCTCC
CONSENSUS
CTGTTATGGTTTCAGATCTATAGGCTGGGAGCTTCTATTATAGACTCTCC

RI-AT3G13420-XLOC_014430-6298-0
ATTTCCCCGTCAATCAGATTCAGATTTACGGAATAGCTTCTTAGAACCTC
RI-AT3G13420-XLOC_014430-6298-1
ATTTCCCCGTCAATCAGATTCAGATTTACGGAATAGCTTCTTAGAACCTC
CONSENSUS
ATTTCCCCGTCAATCAGATTCAGATTTACGGAATAGCTTCTTAGAACCTC

RI-AT3G13420-XLOC_014430-6298-0
CTCTTTTAGCTAGGCAACGTTCACGTGATCCAGAATTACGAAATAGCTTC
RI-AT3G13420-XLOC_014430-6298-1
CTCTTTTAGCTAGGCAACGTTCACGTGATCCAGAATTACGAAATAGCTTC
CONSENSUS
CTCTTTTAGCTAGGCAACGTTCACGTGATCCAGAATTACGAAATAGCTTC

RI-AT3G13420-XLOC_014430-6298-0
TTACAGGCTCCTGCTATAGCTAAGCAACGTTCACGTTCTGACGAAATCTT
RI-AT3G13420-XLOC_014430-6298-1
TTACAGGCTCCTGCTATAGCTAAGCAACGTTCACGTTCTGACGAAATCTT
CONSENSUS
TTACAGGCTCCTGCTATAGCTAAGCAACGTTCACGTTCTGACGAAATCTT

RI-AT3G13420-XLOC_014430-6298-0
GGAAGATTCTATTGACGAACCTGCCTCCTACACTCCTCTGTTAGATGGCG
RI-AT3G13420-XLOC_014430-6298-1
GGAAGATTCTATTGACGAACCTGCCTCCTACACTCCTCTGTTAGATGGCG
CONSENSUS
GGAAGATTCTATTGACGAACCTGCCTCCTACACTCCTCTGTTAGATGGCG

RI-AT3G13420-XLOC_014430-6298-0
GCCTAAGCAATATCACTTTGCCCAATGCAACACAGGTCTTTTTATTTCTA
RI-AT3G13420-XLOC_014430-6298-1
GCCTAAGCAATATCACTTTGCCCAATGCAACACAG---------------
CONSENSUS
GCCTAAGCAATATCACTTTGCCCAATGCAACACAG...............

RI-AT3G13420-XLOC_014430-6298-0
CCTATTCTTCTCTTAAGTTGTTTGTGTACTCATTGTAGTTGAGGTTTTTA
RI-AT3G13420-XLOC_014430-6298-1

CONSENSUS
....

RI-AT3G13420-XLOC_014430-6298-0
TACTTGGTTTGTCTATGGTTTTACCAATTGGGTTTCCAGATGCTTCTTTT
RI-AT3G13420-XLOC_014430-6298-1

CONSENSUS
....

```
RI-AT3G13420-XLOC_014430-6298-0
      CGGACCTTTCTTTTGAAACTGTATGCAATATAGTGAGAATTGGATTGAAC
RI-AT3G13420-XLOC_014430-6298-1
      --------------------------------------------------
CONSENSUS
      ..................................................

RI-AT3G13420-XLOC_014430-6298-0
      TATCTAGATCACCCAACCATGCTTTTTAGCTCTTTGCCACTCTAGGTTAG
RI-AT3G13420-XLOC_014430-6298-1
      --------------------------------------------------
CONSENSUS
      ..................................................

RI-AT3G13420-XLOC_014430-6298-0
      GTGAAACTTGTTTCTCACTAACAAACAAAGGCCACATAGTATTGATATTA
RI-AT3G13420-XLOC_014430-6298-1
      --------------------------------------------------
CONSENSUS
      ..................................................

RI-AT3G13420-XLOC_014430-6298-0
      CATCATAGTATGCTGCTTTTCTTGTCTCAGTACTCCTCTAAAACTTACAC
RI-AT3G13420-XLOC_014430-6298-1
      --------------------------------------------------
CONSENSUS
      ..................................................

RI-AT3G13420-XLOC_014430-6298-0
      AGACATAGCAAGTGGGTTTTCAACCTTTTGTAGGTGATTAAGCACCATTC
RI-AT3G13420-XLOC_014430-6298-1
      ---------------------------------GTGATTAAGCACCATTC
CONSENSUS
      .................................GTGATTAAGCACCATTC

RI-AT3G13420-XLOC_014430-6298-0
      AGCTGGAGACATTTACAAAGGATCCCAATCATCTGCTGCAGAAGCCTCTC
RI-AT3G13420-XLOC_014430-6298-1
      AGCTGGAGACATTTACAAAGGATCCCAATCATCTGCTGCAGAAGCCTCTC
CONSENSUS
      AGCTGGAGACATTTACAAAGGATCCCAATCATCTGCTGCAGAAGCCTCTC

RI-AT3G13420-XLOC_014430-6298-0
      GAAAGAAGTCTCCTCTATCCAGATCCTTAGAAGCACTTCATTCTAGTCTC
RI-AT3G13420-XLOC_014430-6298-1
      GAAAGAAGTCTCCTCTATCCAGATCCTTAGAAGCACTTCATTCTAGTCTC
CONSENSUS
      GAAAGAAGTCTCCTCTATCCAGATCCTTAGAAGCACTTCATTCTAGTCTC

RI-AT3G13420-XLOC_014430-6298-0  TGTTGATAGAACATCTAATGATTGTGGACG
RI-AT3G13420-XLOC_014430-6298-1  TGTTGATAGAACATCTAATGATTGTGGACG
CONSENSUS                        TGTTGATAGAACATCTAATGATTGTGGACG


alignment for event: RI-AT3G03380-XLOC_013914-6613
```

RI-AT3G03380-XLOC_013914-6613-0
TCTGTGTTAGTCACGATTGATCATCATGAATGGTACGCTCCTCCTCAGTT
RI-AT3G03380-XLOC_013914-6613-1
TCTGTGTTAGTCACGATTGATCATCATGAATGGTACGCTCCTCCTCAGTT
CONSENSUS
TCTGTGTTAGTCACGATTGATCATCATGAATGGTACGCTCCTCCTCAGTT

RI-AT3G03380-XLOC_013914-6613-0
GTATACCCGTAATGACAGTTCCGGTTTATGGGATGCGAAACCTGCAATTG
RI-AT3G03380-XLOC_013914-6613-1
GTATACCCGTAATGACAGTTCCGGTTTATGGGATGCGAAACCTGCAATTG
CONSENSUS
GTATACCCGTAATGACAGTTCCGGTTTATGGGATGCGAAACCTGCAATTG

RI-AT3G03380-XLOC_013914-6613-0
AACCTGCTTCTGTTTCACCATCTATTGGTAATAATGGCTTTCCTATAAGC
RI-AT3G03380-XLOC_013914-6613-1
AACCTGCTTCTGTTTCACCATCTATTG-----------------------
CONSENSUS
AACCTGCTTCTGTTTCACCATCTATTG.......................

RI-AT3G03380-XLOC_013914-6613-0
CAAGATATTTCTCTATGCCATCACGATACGGAACCCATGCATGAAGTGAA
RI-AT3G03380-XLOC_013914-6613-1

CONSENSUS
....

RI-AT3G03380-XLOC_013914-6613-0
TGTTCGTGGGGTTACTGATATTGCAGCGATTATGGAAACTTCTAGTGGGG
RI-AT3G03380-XLOC_013914-6613-1 -------------------------
CGATTATGGAAACTTCTAGTGGGG
CONSENSUS
..........................CGATTATGGAAACTTCTAGTGGGG

RI-AT3G03380-XLOC_013914-6613-0
ATGGTTCTCAGAATGATTTTGGCTCAGAAGCAAAGAAACAAAGAGTTGAC
RI-AT3G03380-XLOC_013914-6613-1
ATGGTTCTCAGAATGATTTTGGCTCAGAAGCAAAGAAACAAAGAGTTGAC
CONSENSUS
ATGGTTCTCAGAATGATTTTGGCTCAGAAGCAAAGAAACAAAGAGTTGAC

RI-AT3G03380-XLOC_013914-6613-0
GAGGATTCTTCAGATGGGATTGCTGCAAACGGTTCCTTATATGGCAGTGA
RI-AT3G03380-XLOC_013914-6613-1
GAGGATTCTTCAGATGGGATTGCTGCAAACGGTTCCTTATATGGCAGTGA
CONSENSUS
GAGGATTCTTCAGATGGGATTGCTGCAAACGGTTCCTTATATGGCAGTGA

RI-AT3G03380-XLOC_013914-6613-0
ATTTAAATCTGATGATGCAATGGAAACAGATACTACAGTTTTAAGAGACT
RI-AT3G03380-XLOC_013914-6613-1
ATTTAAATCTGATGATGCAATGGAAACAGATACTACAGTTTTAAGAGACT
CONSENSUS
ATTTAAATCTGATGATGCAATGGAAACAGATACTACAGTTTTAAGAGACT

```
RI-AT3G03380-XLOC_013914-6613-0
      TCGAAGGTGCAACAGCACTGTCTGCTAATGCTTCATTGGCTGAGCGTGCT
RI-AT3G03380-XLOC_013914-6613-1
      TCGAAGGTGCAACAGCACTGTCTGCTAATGCTTCATTGGCTGAGCGTGCT
CONSENSUS
      TCGAAGGTGCAACAGCACTGTCTGCTAATGCTTCATTGGCTGAGCGTGCT

RI-AT3G03380-XLOC_013914-6613-0  ATTGAGCCTGCACTTGTCATGTTTGAG
RI-AT3G03380-XLOC_013914-6613-1  ATTGAGCCTGCACTTGTCATGTTTGAG
CONSENSUS                        ATTGAGCCTGCACTTGTCATGTTTGAG
```

alignment for event: RI-AT3G48050-XLOC_019497-7330

```
RI-AT3G48050-XLOC_019497-7330-0
      TTACTAAGAGATGAGAGTGAATGTGATGCCACAAGGCTTCATTGGCTGGC
RI-AT3G48050-XLOC_019497-7330-1
      TTACTAAGAGATGAGAGTGAATGTGATGCCACAAGGCTTCATTGGCTGGC
CONSENSUS
      TTACTAAGAGATGAGAGTGAATGTGATGCCACAAGGCTTCATTGGCTGGC

RI-AT3G48050-XLOC_019497-7330-0
      CTGCTCTGTACAAATGAGGCATGGGTGAGTCAATTTGCGACTTCACAGTG
RI-AT3G48050-XLOC_019497-7330-1
      CTGCTCTGTACAAATGAGGCATGG--------------------------
CONSENSUS
      CTGCTCTGTACAAATGAGGCATGG..........................

RI-AT3G48050-XLOC_019497-7330-0
      GTCTCAACCTTTTTAAGAACACAAACCTGACCTCTTGGTTTCACCATCAT
RI-AT3G48050-XLOC_019497-7330-1
      --------------------------------------------------
CONSENSUS
      ..................................................

RI-AT3G48050-XLOC_019497-7330-0
      GTAGATCTTCCAAAAACAGTCCTAAATTCACCGGAGTGGCCATTCTCTTT
RI-AT3G48050-XLOC_019497-7330-1  ----
ATCTTCCAAAAACAGTCCTAAATTCACCGGAGTGGCCATTCTCTTT
CONSENSUS
      ....ATCTTCCAAAAACAGTCCTAAATTCACCGGAGTGGCCATTCTCTTT

RI-AT3G48050-XLOC_019497-7330-0
      CAGAAGAGTCATCAACAAGGTTCTCAGCGAGAGACCCGCATCACCACCTC
RI-AT3G48050-XLOC_019497-7330-1
      CAGAAGAGTCATCAACAAGGTTCTCAGCGAGAGACCCGCATCACCACCTC
CONSENSUS
      CAGAAGAGTCATCAACAAGGTTCTCAGCGAGAGACCCGCATCACCACCTC

RI-AT3G48050-XLOC_019497-7330-0
      CCCACAAAGAGTTTTAAAAGGTGAAAACACACTTTTAGGTCATTTTTCAT
RI-AT3G48050-XLOC_019497-7330-1
      CCCACAAAGAGTTTTAAAAGGTGAAAACACACTTTTAGGTCATTTTTCAT
CONSENSUS
```

CCCACAAAGAGTTTTAAAAGGTGAAAACACACTTTTAGGTCATTTTTCAT

RI-AT3G48050-XLOC_019497-7330-0
GCATGTGTGTATCTCTATACACCGGCTCAAAAAACTAGCGATAAAACAAA
RI-AT3G48050-XLOC_019497-7330-1
GCATGTGTGTATCTCTATACACCGGCTCAAAAAACTAGCGATAAAACAAA
CONSENSUS
GCATGTGTGTATCTCTATACACCGGCTCAAAAAACTAGCGATAAAACAAA

RI-AT3G48050-XLOC_019497-7330-0
TGTTGGTGGCTTTTGAGTGTGTTCAAAATGATAAAAAAAAAAGTCTTTCG
RI-AT3G48050-XLOC_019497-7330-1
TGTTGGTGGCTTTTGAGTGTGTTCAAAATGATAAAAAAAAAAGTCTTTCG
CONSENSUS
TGTTGGTGGCTTTTGAGTGTGTTCAAAATGATAAAAAAAAAAGTCTTTCG

RI-AT3G48050-XLOC_019497-7330-0
CATTGTGGCTTTACATGTGTGCGTTAGGTGATGGGGGTTGTATTATAGAC
RI-AT3G48050-XLOC_019497-7330-1
CATTGTGGCTTTACATGTGTGCGTTAGGTGATGGGGGTTGTATTATAGAC
CONSENSUS
CATTGTGGCTTTACATGTGTGCGTTAGGTGATGGGGGTTGTATTATAGAC

RI-AT3G48050-XLOC_019497-7330-0
ACAAGATTAGTTACTTCTTTTTATCTTTCTTTTTCCAGTTTGTAGATTTA
RI-AT3G48050-XLOC_019497-7330-1
ACAAGATTAGTTACTTCTTTTTATCTTTCTTTTTCCAGTTTGTAGATTTA
CONSENSUS
ACAAGATTAGTTACTTCTTTTTATCTTTCTTTTTCCAGTTTGTAGATTTA

RI-AT3G48050-XLOC_019497-7330-0
TTACTTCTTGTGCTGGTTCTGTTTGAAATTTACAGGCTTCTTCTTACTTG
RI-AT3G48050-XLOC_019497-7330-1
TTACTTCTTGTGCTGGTTCTGTTTGAAATTTACAGGCTTCTTCTTACTTG
CONSENSUS
TTACTTCTTGTGCTGGTTCTGTTTGAAATTTACAGGCTTCTTCTTACTTG

RI-AT3G48050-XLOC_019497-7330-0
TCACGCTTTTGTCGTTTGGTCTTGGATCTCATAATAGGACCAAATAAATT
RI-AT3G48050-XLOC_019497-7330-1
TCACGCTTTTGTCGTTTGGTCTTGGATCTCATAATAGGACCAAATAAATT
CONSENSUS
TCACGCTTTTGTCGTTTGGTCTTGGATCTCATAATAGGACCAAATAAATT

RI-AT3G48050-XLOC_019497-7330-0 CGAAT
RI-AT3G48050-XLOC_019497-7330-1 CGAAT
CONSENSUS CGAAT

alignment for event: A3-AT3G18930-XLOC_018093-3385

A3-AT3G18930-XLOC_018093-3385-0
CTGCATTAAAAAAACACTGACGTGTAAATTTTATTTATCCCAATACCGTT
A3-AT3G18930-XLOC_018093-3385-1
CTGCATTAAAAAAACACTGACGTGTAAATTTTATTTATCCCAATACCGTT

CONSENSUS
CTGCATTAAAAAAACACTGACGTGTAAATTTTATTTATCCCAATACCGTT

A3-AT3G18930-XLOC_018093-3385-0
TACCTTTAGTCAACGATTTTTTTCCCACCAAAGTCCCCAAATACACTCGT
A3-AT3G18930-XLOC_018093-3385-1
TACCTTTAGTCAACGATTTTTTTCCCACCAAAGTCCCCAAATACACTCGT
CONSENSUS
TACCTTTAGTCAACGATTTTTTTCCCACCAAAGTCCCCAAATACACTCGT

A3-AT3G18930-XLOC_018093-3385-0
TTGCGTTTAAACCTTTCACTTGAAACTCTGTTTTAGACTCTTTTATTTGT
A3-AT3G18930-XLOC_018093-3385-1
TTGCGTTTAAACCTTTCACTTGAAACTCTGTTTTAGACTCTTTTATTTGT
CONSENSUS
TTGCGTTTAAACCTTTCACTTGAAACTCTGTTTTAGACTCTTTTATTTGT

A3-AT3G18930-XLOC_018093-3385-0
TAGACTTTTTTGTTTGTTTCGTCGTTGTTGTTTTTTGACCAAACCACCAT
A3-AT3G18930-XLOC_018093-3385-1
TAGACTTTTTTGTTTGTTTCGTCGTTGTTGTTTTTTGACCAAACCACCAT
CONSENSUS
TAGACTTTTTTGTTTGTTTCGTCGTTGTTGTTTTTTGACCAAACCACCAT

A3-AT3G18930-XLOC_018093-3385-0
GCGATTCGTCGCGCCACCGCCTCGTTCCGGCGATAACTCACCGTCACCTT
A3-AT3G18930-XLOC_018093-3385-1
GCGATTCGTCGCGCCACCGCCTCGTTCCGGCGATAACTCACCGTCACCTT
CONSENSUS
GCGATTCGTCGCGCCACCGCCTCGTTCCGGCGATAACTCACCGTCACCTT

A3-AT3G18930-XLOC_018093-3385-0
CACCATCTTCCGGTATATCCGAAGAGATTCTCTCCCGTTCTTCGGATCCT
A3-AT3G18930-XLOC_018093-3385-1
CACCATCTTCCGGTATATCCGAAGAGATTCTCTCCCGTTCTTCGGATCCT
CONSENSUS
CACCATCTTCCGGTATATCCGAAGAGATTCTCTCCCGTTCTTCGGATCCT

A3-AT3G18930-XLOC_018093-3385-0
CCTTTAGAGTTTAGTCCTCCATTGATCGCCATGGTGGTGGTTCTAGCCGC
A3-AT3G18930-XLOC_018093-3385-1
CCTTTAGAGTTTAGTCCTCCATTGATCGCCATGGTGGTGGTTCTAGCCGC
CONSENSUS
CCTTTAGAGTTTAGTCCTCCATTGATCGCCATGGTGGTGGTTCTAGCCGC

A3-AT3G18930-XLOC_018093-3385-0
CGCTTTTCTATTCGTAACTTACTCTCGTCTCATCTCCCGCCGTTTTCTTT
A3-AT3G18930-XLOC_018093-3385-1
CGCTTTTCTATTCGTAACTTACTCTCGTCTCATCTCCCGCCGTTTTCTTT
CONSENSUS
CGCTTTTCTATTCGTAACTTACTCTCGTCTCATCTCCCGCCGTTTTCTTT

A3-AT3G18930-XLOC_018093-3385-0
CACCTCTATTCCGTCGTTTTAGAAGGTGGCGATGCCGTCGACGCCGTCTC
A3-AT3G18930-XLOC_018093-3385-1
CACCTCTATTCCGTCGTTTTAGAAGGTGGCGATGCCGTCGACGCCGTCTC

CONSENSUS
CACCTCTATTCCGTCGTTTTAGAAGGTGGCGATGCCGTCGACGCCGTCTC

A3-AT3G18930-XLOC_018093-3385-0
CTTCACTTATCTTCAGCTTCCTCTGCTTCAACTTCATCCTCCGATCTCCG
A3-AT3G18930-XLOC_018093-3385-1
CTTCACTTATCTTCAGCTTCCTCTGCTTCAACTTCATCCTCCGATCTCCG
CONSENSUS
CTTCACTTATCTTCAGCTTCCTCTGCTTCAACTTCATCCTCCGATCTCCG

A3-AT3G18930-XLOC_018093-3385-0
ATCATTTTCACCTTTCCCTTTCGACTCTTTTCATTACTCTTCTTACTCAC
A3-AT3G18930-XLOC_018093-3385-1
ATCATTTTCACCTTTCCCTTTCGACTCTTTTCATTACTCTTCTTACTCAC
CONSENSUS
ATCATTTTCACCTTTCCCTTTCGACTCTTTTCATTACTCTTCTTACTCAC

A3-AT3G18930-XLOC_018093-3385-0
CTTACGGATTAGACGATTCCGTTATCAAAACTTTACCGCTGTTTCTCTAC
A3-AT3G18930-XLOC_018093-3385-1
CTTACGGATTAGACGATTCCGTTATCAAAACTTTACCGCTGTTTCTCTAC
CONSENSUS
CTTACGGATTAGACGATTCCGTTATCAAAACTTTACCGCTGTTTCTCTAC

A3-AT3G18930-XLOC_018093-3385-0
TCCGCCGCTGCTTGCACCGGAAAACCCGCGGTGGGGAAAACCTCCGCCGC
A3-AT3G18930-XLOC_018093-3385-1
TCCGCCGCTGCTTGCACCGGAAAACCCGCGGTGGGGAAAACCTCCGCCGC
CONSENSUS
TCCGCCGCTGCTTGCACCGGAAAACCCGCGGTGGGGAAAACCTCCGCCGC

A3-AT3G18930-XLOC_018093-3385-0
CAATTGCAGAGATTGTGCTGTTTGTTTGTTGGAATTCGAAGAAGGTGATT
A3-AT3G18930-XLOC_018093-3385-1
CAATTGCAGAGATTGTGCTGTTTGTTTGTTGGAATTCGAAGAAGGTGATT
CONSENSUS
CAATTGCAGAGATTGTGCTGTTTGTTTGTTGGAATTCGAAGAAGGTGATT

A3-AT3G18930-XLOC_018093-3385-0
ATGTACGAACACTTCCGTTATGCTTCCATGCTTTTCACCTTGAATGCATC
A3-AT3G18930-XLOC_018093-3385-1
ATGTACGAACACTTCCGTTATGCTTCCATGCTTTTCACCTTGAATGCATC
CONSENSUS
ATGTACGAACACTTCCGTTATGCTTCCATGCTTTTCACCTTGAATGCATC

A3-AT3G18930-XLOC_018093-3385-0
GATGAATGGCTCCGATCTCACCCTAACTGTCCGTTATGCCGTACGGCGAT
A3-AT3G18930-XLOC_018093-3385-1
GATGAATGGCTCCGATCTCACCCTAACTGTCCGTTATGCCGTACGGCGAT
CONSENSUS
GATGAATGGCTCCGATCTCACCCTAACTGTCCGTTATGCCGTACGGCGAT

A3-AT3G18930-XLOC_018093-3385-0
TCTCGGATCCGCCGGAGTTTTAACGCCTATGTCGCCGTTCGTTCCGTTAA
A3-AT3G18930-XLOC_018093-3385-1
TCTCGGATCCGCCGGAGTTTTAACGCCTATGTCGCCGTTCGTTCCGTTAA

CONSENSUS
TCTCGGATCCGCCGGAGTTTTAACGCCTATGTCGCCGTTCGTTCCGTTAA

A3-AT3G18930-XLOC_018093-3385-0
TGGCTCCTCGAATTCGTCCGAGTCTCGATGATGAAGAGAATAACGCGATC
A3-AT3G18930-XLOC_018093-3385-1
TGGCTCCTCGAATTCGTCCGAGTCTCGATGATGAAGAGAATAACGCGATC
CONSENSUS
TGGCTCCTCGAATTCGTCCGAGTCTCGATGATGAAGAGAATAACGCGATC

A3-AT3G18930-XLOC_018093-3385-0
ATCATCCGCGGCGAAATCACACCGTCGCGGAGCAATTGGAACACAATCGC
A3-AT3G18930-XLOC_018093-3385-1
ATCATCCGCGGCGAAATCACACCGTCGCGGAGCAATTGGAACACAATCGC
CONSENSUS
ATCATCCGCGGCGAAATCACACCGTCGCGGAGCAATTGGAACACAATCGC

A3-AT3G18930-XLOC_018093-3385-0
TGCCGATACGACGAACGATCAGGAGATCAGAGCGTCGGTGGAAGAGCAAT
A3-AT3G18930-XLOC_018093-3385-1
TGCCGATACGACGAACGATCAGGAGATCAGAGCGTCGGTGGAAGAGCAAT
CONSENSUS
TGCCGATACGACGAACGATCAGGAGATCAGAGCGTCGGTGGAAGAGCAAT

A3-AT3G18930-XLOC_018093-3385-0
CATCACCGGCGATTTCTCGGTTCCGGGAGCTGAAGAGATCGTACTCGTTC
A3-AT3G18930-XLOC_018093-3385-1
CATCACCGGCGATTTCTCGGTTCCGGGAGCTGAAGAGATCGTACTCGTTC
CONSENSUS
CATCACCGGCGATTTCTCGGTTCCGGGAGCTGAAGAGATCGTACTCGTTC

A3-AT3G18930-XLOC_018093-3385-0
GAATGCGAGAGAGAATCTGAATCAGAGAGAGTAACGATGGAGCCAGCGAC
A3-AT3G18930-XLOC_018093-3385-1
GAATGCGAGAGAGAATCTGAATCAGAGAGAGTAACGATGGAGCCAGCGAC
CONSENSUS
GAATGCGAGAGAGAATCTGAATCAGAGAGAGTAACGATGGAGCCAGCGAC

A3-AT3G18930-XLOC_018093-3385-0
GGTGTCTCCATGGAGATACCGGAGATCTACATGGAACAAACGTCAATCAC
A3-AT3G18930-XLOC_018093-3385-1
GGTGTCTCCATGGAGATACCGGAGATCTACATGGAACAAACGTCAATCAC
CONSENSUS
GGTGTCTCCATGGAGATACCGGAGATCTACATGGAACAAACGTCAATCAC

A3-AT3G18930-XLOC_018093-3385-0
CGTTCGGAAACCTAATATCGAAATCAAGAGTGTTCTCGTTCCGTTACTAC
A3-AT3G18930-XLOC_018093-3385-1
CGTTCGGAAACCTAATATCGAAATCAAGAGTGTTCTCGTTCCGTTACTAC
CONSENSUS
CGTTCGGAAACCTAATATCGAAATCAAGAGTGTTCTCGTTCCGTTACTAC

A3-AT3G18930-XLOC_018093-3385-0
AGAAGCACGAAATCACCGTTCTTCCGACGGAGATCATCGGCGGGAGTGTT
A3-AT3G18930-XLOC_018093-3385-1
AGAAGCACGAAATCACCGTTCTTCCGACGGAGATCATCGGCGGGAGTGTT

CONSENSUS
AGAAGCACGAAATCACCGTTCTTCCGACGGAGATCATCGGCGGGAGTGTT

A3-AT3G18930-XLOC_018093-3385-0
TTACCCGATATCAGAACGGATTCCAGCGACGGGAAGCTCATCGCGGCGAA
A3-AT3G18930-XLOC_018093-3385-1
TTACCCGATATCAGAACGGATTCCAGCGACGGGAAGCTCATCGCGGCGAA
CONSENSUS
TTACCCGATATCAGAACGGATTCCAGCGACGGGAAGCTCATCGCGGCGAA

A3-AT3G18930-XLOC_018093-3385-0
CGAAATCGATGACGAGTCCGATGTTCTTCAGAACAGCACCGCACTCATCG
A3-AT3G18930-XLOC_018093-3385-1
CGAAATCGATGACGAGTCCGATGTTCTTCAGAACAGCACCGCACTCATCG
CONSENSUS
CGAAATCGATGACGAGTCCGATGTTCTTCAGAACAGCACCGCACTCATCG

A3-AT3G18930-XLOC_018093-3385-0
AGCCGGTTAAGATGCGGAGATCCAGAGGCACTGTTATCGCCGGAGAGATG
A3-AT3G18930-XLOC_018093-3385-1
AGCCGGTTAAGATGCGGAGATCCAGAGGCACTGTTATCGCCGGAGAGATG
CONSENSUS
AGCCGGTTAAGATGCGGAGATCCAGAGGCACTGTTATCGCCGGAGAGATG

A3-AT3G18930-XLOC_018093-3385-0
GAGAAGAAGAGACACGTGTAGGGCAGAGATGTAAGATGAAATTTGGACGG
A3-AT3G18930-XLOC_018093-3385-1
GAGAAGAAGAGACACGTGTAGGGCAGAGATGTAAGATGAAATTTGGACGG
CONSENSUS
GAGAAGAAGAGACACGTGTAGGGCAGAGATGTAAGATGAAATTTGGACGG

A3-AT3G18930-XLOC_018093-3385-0
TGGTGATGATAAAAAGAAATATTGAAGTGGAAGGAGAGAGCATTAATGGA
A3-AT3G18930-XLOC_018093-3385-1
TGGTGATGATAAAAAGAAATATTGAAGTGGAAGGAGAGAGCATTAATGGA
CONSENSUS
TGGTGATGATAAAAAGAAATATTGAAGTGGAAGGAGAGAGCATTAATGGA

A3-AT3G18930-XLOC_018093-3385-0
ATGTGGGGTTTAAGCATTTAATGGAATTAAAATGAACGAAACAGAAGAAG
A3-AT3G18930-XLOC_018093-3385-1
ATGTGGGGTTTAAGCATTTAATGGAATTAAAATGAACGAAACAGAAGAAG
CONSENSUS
ATGTGGGGTTTAAGCATTTAATGGAATTAAAATGAACGAAACAGAAGAAG

A3-AT3G18930-XLOC_018093-3385-0
ATAGAGAGATTCAGAAATTGCTCAGCTGCTCACATTCACAACAAG-----
A3-AT3G18930-XLOC_018093-3385-1
ATAGAGAGATTCAGAAATTGCTCAGCTGCTCACATTCACAACAAGGATAC
CONSENSUS
ATAGAGAGATTCAGAAATTGCTCAGCTGCTCACATTCACAACAAG.....

A3-AT3G18930-XLOC_018093-3385-0 -----------------------------
ACAAAAGACGCCAAGACGATG
A3-AT3G18930-XLOC_018093-3385-1
ACACTTTCTTATATTACTATGGGTCTCAGACAAAAGACGCCAAGACGATG

CONSENSUS
............................ACAAAAGACGCCAAGACGATG

A3-AT3G18930-XLOC_018093-3385-0
AAGGTTTTGGAGCCTGTACGACGGCGTTTTGGCTGATGATTCGTTTTTTC
A3-AT3G18930-XLOC_018093-3385-1
AAGGTTTTGGAGCCTGTACGACGGCGTTTTGGCTGATGATTCGTTTTTTC
CONSENSUS
AAGGTTTTGGAGCCTGTACGACGGCGTTTTGGCTGATGATTCGTTTTTTC

A3-AT3G18930-XLOC_018093-3385-0
CTCTCTTTGGAGTTATCAAAATGCATTAACTTCAAAAGCTGCCAAAGTTA
A3-AT3G18930-XLOC_018093-3385-1
CTCTCTTTGGAGTTATCAAAATGCATTAACTTCAAAAGCTGCCAAAGTTA
CONSENSUS
CTCTCTTTGGAGTTATCAAAATGCATTAACTTCAAAAGCTGCCAAAGTTA

A3-AT3G18930-XLOC_018093-3385-0
TCATAGAAACGAAAGTCAACAACACATTGAATTTCGTTTCTATAATAACT
A3-AT3G18930-XLOC_018093-3385-1
TCATAGAAACGAAAGTCAACAACACATTGAATTTCGTTTCTATAATAACT
CONSENSUS
TCATAGAAACGAAAGTCAACAACACATTGAATTTCGTTTCTATAATAACT

A3-AT3G18930-XLOC_018093-3385-0
TTACATCGTCGGTGGCTTTACTGTTTTCTGTTACCGAATTTTATGTTCAA
A3-AT3G18930-XLOC_018093-3385-1
TTACATCGTCGGTGGCTTTACTGTTTTCTGTTACCGAATTTTATGTTCAA
CONSENSUS
TTACATCGTCGGTGGCTTTACTGTTTTCTGTTACCGAATTTTATGTTCAA

A3-AT3G18930-XLOC_018093-3385-0
ACTTTTAGCTGTCGATTTAATTTTTGACCAAATTTTTTTGATGATTTATT
A3-AT3G18930-XLOC_018093-3385-1
ACTTTTAGCTGTCGATTTAATTTTTGACCAAATTTTTTTGATGATTTATT
CONSENSUS
ACTTTTAGCTGTCGATTTAATTTTTGACCAAATTTTTTTGATGATTTATT

A3-AT3G18930-XLOC_018093-3385-0
ACATATTGATGAATAACGTTAACCAACTGAGATTGAGAAGATCTGGATCA
A3-AT3G18930-XLOC_018093-3385-1
ACATATTGATGAATAACGTTAACCAACTGAGATTGAGAAGATCTGGATCA
CONSENSUS
ACATATTGATGAATAACGTTAACCAACTGAGATTGAGAAGATCTGGATCA

A3-AT3G18930-XLOC_018093-3385-0
ATTGCGGCCCTGTAAGAATAAATATTATGGGCTTTTTAGTATA
A3-AT3G18930-XLOC_018093-3385-1
ATTGCGGCCCTGTAAGAATAAATATTATGGGCTTTTTAGTATA
CONSENSUS
ATTGCGGCCCTGTAAGAATAAATATTATGGGCTTTTTAGTATA

alignment for event: A3-AT3G13990-XLOC_017828-941

A3-AT3G13990-XLOC_017828-941-0
      TTCGCCAGTTACTTGTCCTGAGGCTTCAGCAGATGGTCCGGGCGTTCTGC
A3-AT3G13990-XLOC_017828-941-1
      TTCGCCAGTTACTTGTCCTGAGGCTTCAGCAGATGGTCCGGGCGTTCTGC
CONSENSUS
      TTCGCCAGTTACTTGTCCTGAGGCTTCAGCAGATGGTCCGGGCGTTCTGC

A3-AT3G13990-XLOC_017828-941-0
      CTATCACTATTGATGATCATGTCAGAGTTTCTAGATCTGACTCACCCGTC
A3-AT3G13990-XLOC_017828-941-1
      CTATCACTATTGATGATCATGTCAGAGTTTCTAGATCTGACTCACCCGTC
CONSENSUS
      CTATCACTATTGATGATCATGTCAGAGTTTCTAGATCTGACTCACCCGTC

A3-AT3G13990-XLOC_017828-941-0
      TCGGCAGTATCTGAGCCACAGTTACCCGAACAGAAAGAAACTCATAGATC
A3-AT3G13990-XLOC_017828-941-1
      TCGGCAGTATCTGAGCCACAGTTACCCGAACAGAAAGAAACTCATAGATC
CONSENSUS
      TCGGCAGTATCTGAGCCACAGTTACCCGAACAGAAAGAAACTCATAGATC

A3-AT3G13990-XLOC_017828-941-0
      TGACAATTTGGATGCGTACTCGGGGATTGAATTACTCAATAGGAATGGTC
A3-AT3G13990-XLOC_017828-941-1
      TGACAATTTGGATGCGTACTCGGGGATTGAATTACTCAATAGGAATGGTC
CONSENSUS
      TGACAATTTGGATGCGTACTCGGGGATTGAATTACTCAATAGGAATGGTC

A3-AT3G13990-XLOC_017828-941-0
      CACCTTATACTCCTTTGGAGTTTGAACAGCAGCAAGATCCTCCGGAGTTG
A3-AT3G13990-XLOC_017828-941-1
      CACCTTATACTCCTTTGGAGTTTGAACAGCAGCAAGATCCTCCGGAGTTG
CONSENSUS
      CACCTTATACTCCTTTGGAGTTTGAACAGCAGCAAGATCCTCCGGAGTTG

A3-AT3G13990-XLOC_017828-941-0   CAAAAATTTTCT---
GCTTATGACAATCATGGTAGTTATGAATTTCCGTA
A3-AT3G13990-XLOC_017828-941-1
      CAAAAATTTTCTCAGGCTTATGACAATCATGGTAGTTATGAATTTCCGTA
CONSENSUS
      CAAAAATTTTCT...GCTTATGACAATCATGGTAGTTATGAATTTCCGTA

A3-AT3G13990-XLOC_017828-941-0
      CTTTAGTCCTGCAATGGATGAAACTGTACGAGTTCAAGGATTACCATCTC
A3-AT3G13990-XLOC_017828-941-1
      CTTTAGTCCTGCAATGGATGAAACTGTACGAGTTCAAGGATTACCATCTC
CONSENSUS
      CTTTAGTCCTGCAATGGATGAAACTGTACGAGTTCAAGGATTACCATCTC

A3-AT3G13990-XLOC_017828-941-0   CACACGAG
A3-AT3G13990-XLOC_017828-941-1   CACACGAG
CONSENSUS   CACACGAG

alignment for event: A3-AT3G62840-XLOC_020367-6423

A3-AT3G62840-XLOC_020367-6423-0 TAAACCAATGGAAGAGGATACCAAC---
GGAAAGACTGAGGAGGAGGAGT
A3-AT3G62840-XLOC_020367-6423-1
      TAAACCAATGGAAGAGGATACCAACCAGGGAAAGACTGAGGAGGAGGAGT
CONSENSUS
      TAAACCAATGGAAGAGGATACCAAC...GGAAAGACTGAGGAGGAGGAGT

A3-AT3G62840-XLOC_020367-6423-0
      TCAACACTGGACCACTCTCTGTTTTGATGATGAGTGTTAAGAATAACACT
A3-AT3G62840-XLOC_020367-6423-1
      TCAACACTGGACCACTCTCTGTTTTGATGATGAGTGTTAAGAATAACACT
CONSENSUS
      TCAACACTGGACCACTCTCTGTTTTGATGATGAGTGTTAAGAATAACACT

A3-AT3G62840-XLOC_020367-6423-0
      CAGGTGTTGATCAATTGCCGTAACAACAGGAAACTCCTTGGCCGAGTTAG
A3-AT3G62840-XLOC_020367-6423-1
      CAGGTGTTGATCAATTGCCGTAACAACAGGAAACTCCTTGGCCGAGTTAG
CONSENSUS
      CAGGTGTTGATCAATTGCCGTAACAACAGGAAACTCCTTGGCCGAGTTAG

A3-AT3G62840-XLOC_020367-6423-0
      GGCTTTTGACAGGCACTGCAACATGGTTCTTGAAAATGTCAGAGAAATGT
A3-AT3G62840-XLOC_020367-6423-1
      GGCTTTTGACAGGCACTGCAACATGGTTCTTGAAAATGTCAGAGAAATGT
CONSENSUS
      GGCTTTTGACAGGCACTGCAACATGGTTCTTGAAAATGTCAGAGAAATGT

A3-AT3G62840-XLOC_020367-6423-0 GGACTGAG
A3-AT3G62840-XLOC_020367-6423-1 GGACTGAG
CONSENSUS   GGACTGAG

alignment for event: RI-AT3G26690-XLOC_018534-6334

RI-AT3G26690-XLOC_018534-6334-0
      CAACAAACCAAAACCGTTCCTTTCTTTTTCCATTTTTCTTGTTTTTTTTT
RI-AT3G26690-XLOC_018534-6334-1
      CAACAAACCAAAACCGTTCCTTTCTTTTTCCATTTTTCTTGTTTTTTTTT
CONSENSUS
      CAACAAACCAAAACCGTTCCTTTCTTTTTCCATTTTTCTTGTTTTTTTTT

RI-AT3G26690-XLOC_018534-6334-0
      GTTTTGTTTTTGGAGTTGTGTTTCACCTGTTGTTGGTTCTTTAGTTCGTC
RI-AT3G26690-XLOC_018534-6334-1
      GTTTTGTTTTTGGAGTTGTGTTTCACCTGTTGTTGGTTCTTTAGTTCGTC
CONSENSUS
      GTTTTGTTTTTGGAGTTGTGTTTCACCTGTTGTTGGTTCTTTAGTTCGTC

RI-AT3G26690-XLOC_018534-6334-0
      TTCTGGGTTAAACAAAGAAGATCGGTTCTGTTTTTATTTTTATTTTGAAA
RI-AT3G26690-XLOC_018534-6334-1
      TTCTGGGTTAAACAAAGAAGATCGGTTCTGTTTTTATTTTTATTTTGAAA
CONSENSUS

TTCTGGGTTAAACAAAGAAGATCGGTTCTGTTTTTATTTTTATTTTGAAA

RI-AT3G26690-XLOC_018534-6334-0
CAAGAAGAAGAAAGCAAGCTACGATGATGTTTCTTGCTGACGCCAATAGA
RI-AT3G26690-XLOC_018534-6334-1
CAAGAAGAAGAAAGCAAGCTACGATGATGTTTCTTGCTGACGCCAATAGA
CONSENSUS
CAAGAAGAAGAAAGCAAGCTACGATGATGTTTCTTGCTGACGCCAATAGA

RI-AT3G26690-XLOC_018534-6334-0
TAAAGACACGACTTTTACAAAGCTTAGGACCTGCATTCTCACCGTAATTA
RI-AT3G26690-XLOC_018534-6334-1
TAAAGACACGACTTTTACAAAGCTTAGGACCTGCATTCTCACC-------
CONSENSUS
TAAAGACACGACTTTTACAAAGCTTAGGACCTGCATTCTCACC.......

RI-AT3G26690-XLOC_018534-6334-0
GCTCTTCTTCTCAATTTCTCCAAATAAATTTGTGTGTTTTTAATGTGTGT
RI-AT3G26690-XLOC_018534-6334-1

CONSENSUS
....

RI-AT3G26690-XLOC_018534-6334-0
GTTGCAGGTTATCAAAAATGTCGAATCTTTCTGCAAGAACAGGACGAGAC
RI-AT3G26690-XLOC_018534-6334-1 -------
GTTATCAAAAATGTCGAATCTTTCTGCAAGAACAGGACGAGAC
CONSENSUS
.......GTTATCAAAAATGTCGAATCTTTCTGCAAGAACAGGACGAGAC

RI-AT3G26690-XLOC_018534-6334-0
CATCAACGTTATGACAACAACTTCCGTCTTGTTTCTGG
RI-AT3G26690-XLOC_018534-6334-1
CATCAACGTTATGACAACAACTTCCGTCTTGTTTCTGG
CONSENSUS
CATCAACGTTATGACAACAACTTCCGTCTTGTTTCTGG

alignment for event: A5-AT3G60250-XLOC_020207-1076

A5-AT3G60250-XLOC_020207-1076-0
ACTGAGAAGTACAAGAACTGTGATTTCGGGAGATGCCCGAGAGTTTTCTG
A5-AT3G60250-XLOC_020207-1076-1
ACTGAGAAGTACAAGAACTGTGATTTCGGGAGATGCCCGAGAGTTTTCTG
CONSENSUS
ACTGAGAAGTACAAGAACTGTGATTTCGGGAGATGCCCGAGAGTTTTCTG

A5-AT3G60250-XLOC_020207-1076-0
TTGCGGTCAGTCTTGTCTTCCAGTTGGACAATCCGATATCCCGAGATCGA
A5-AT3G60250-XLOC_020207-1076-1
TTGCGGTCAGTCTTGTCTTCCAGTTGGACAATCCGATATCCCGAGATCGA
CONSENSUS
TTGCGGTCAGTCTTGTCTTCCAGTTGGACAATCCGATATCCCGAGATCGA

A5-AT3G60250-XLOC_020207-1076-0

GTACTGTGAAGATATACTGCCCTAAATGCGAGGATATATCTTACCCGCGA
A5-AT3G60250-XLOC_020207-1076-1
GTACTGTGAAGATATACTGCCCTAAATGCGAGGATATATCTTACCCGCGA
CONSENSUS
GTACTGTGAAGATATACTGCCCTAAATGCGAGGATATATCTTACCCGCGA

A5-AT3G60250-XLOC_020207-1076-0
TCTAAATTCCAAGGCAATATTGATGGAGCGTACTTTGGAACCACATTCCC
A5-AT3G60250-XLOC_020207-1076-1 TCTAAATTCCAAG---
ATATTGATGGAGCGTACTTTGGAACCACATTCCC
CONSENSUS
TCTAAATTCCAAG...ATATTGATGGAGCGTACTTTGGAACCACATTCCC

A5-AT3G60250-XLOC_020207-1076-0
TCACTTGTTCTTGATGACTTACGGGAACTTAAAGCCGCAGAAGCCTACTC
A5-AT3G60250-XLOC_020207-1076-1
TCACTTGTTCTTGATGACTTACGGGAACTTAAAGCCGCAGAAGCCTACTC
CONSENSUS
TCACTTGTTCTTGATGACTTACGGGAACTTAAAGCCGCAGAAGCCTACTC

A5-AT3G60250-XLOC_020207-1076-0
AAAGCTATGTCCCAAAAATCTTTGGCTTCAAGGTACACAAACCATGATAC
A5-AT3G60250-XLOC_020207-1076-1
AAAGCTATGTCCCAAAAATCTTTGGCTTCAAGGTACACAAACCATGATAC
CONSENSUS
AAAGCTATGTCCCAAAAATCTTTGGCTTCAAGGTACACAAACCATGATAC

A5-AT3G60250-XLOC_020207-1076-0
TAGTGCTCTGCATTCTCAATGGTGATACATTTAGTGGCTCTGTAATTGCA
A5-AT3G60250-XLOC_020207-1076-1
TAGTGCTCTGCATTCTCAATGGTGATACATTTAGTGGCTCTGTAATTGCA
CONSENSUS
TAGTGCTCTGCATTCTCAATGGTGATACATTTAGTGGCTCTGTAATTGCA

A5-AT3G60250-XLOC_020207-1076-0
TCCGGATGAGCAACTGAAACGATAGCTGCGGTGACTGGAGCATACATCAA
A5-AT3G60250-XLOC_020207-1076-1
TCCGGATGAGCAACTGAAACGATAGCTGCGGTGACTGGAGCATACATCAA
CONSENSUS
TCCGGATGAGCAACTGAAACGATAGCTGCGGTGACTGGAGCATACATCAA

A5-AT3G60250-XLOC_020207-1076-0
CCATTGGGTTAGAGGATGAGAGCTGAAGTTGAAAGATGGAATCTTGAAAG
A5-AT3G60250-XLOC_020207-1076-1
CCATTGGGTTAGAGGATGAGAGCTGAAGTTGAAAGATGGAATCTTGAAAG
CONSENSUS
CCATTGGGTTAGAGGATGAGAGCTGAAGTTGAAAGATGGAATCTTGAAAG

A5-AT3G60250-XLOC_020207-1076-0
AATCTCATGAAGTTCCAAGAAAACTTAGAAGTGCAACAGCCTGGCTCATT
A5-AT3G60250-XLOC_020207-1076-1
AATCTCATGAAGTTCCAAGAAAACTTAGAAGTGCAACAGCCTGGCTCATT
CONSENSUS
AATCTCATGAAGTTCCAAGAAAACTTAGAAGTGCAACAGCCTGGCTCATT

A5-AT3G60250-XLOC_020207-1076-0

GGGGGAAAAGAACGACACACTTGGGTCTTGTTGTTGTCTCCGAAAAGATT
A5-AT3G60250-XLOC_020207-1076-1
GGGGGAAAAGAACGACACACTTGGGTCTTGTTGTTGTCTCCGAAAAGATT
CONSENSUS
GGGGGAAAAGAACGACACACTTGGGTCTTGTTGTTGTCTCCGAAAAGATT

A5-AT3G60250-XLOC_020207-1076-0
TTCCCCAGTTAGTTGGATTTTGTTTTGAACTGTTGGATTATTATTCGTTT
A5-AT3G60250-XLOC_020207-1076-1
TTCCCCAGTTAGTTGGATTTTGTTTTGAACTGTTGGATTATTATTCGTTT
CONSENSUS
TTCCCCAGTTAGTTGGATTTTGTTTTGAACTGTTGGATTATTATTCGTTT

A5-AT3G60250-XLOC_020207-1076-0
TTCTAATTTCATGTAAAACTCATAAATTTCAACTGAAGTATAACTTAGAT
A5-AT3G60250-XLOC_020207-1076-1
TTCTAATTTCATGTAAAACTCATAAATTTCAACTGAAGTATAACTTAGAT
CONSENSUS
TTCTAATTTCATGTAAAACTCATAAATTTCAACTGAAGTATAACTTAGAT

A5-AT3G60250-XLOC_020207-1076-0 CAATCAATTTCATCAAAGATCATA
A5-AT3G60250-XLOC_020207-1076-1 CAATCAATTTCATCAAAGATCATA
CONSENSUS CAATCAATTTCATCAAAGATCATA

alignment for event: A5-AT3G23340-XLOC_015072-3660

A5-AT3G23340-XLOC_015072-3660-0
AAATAAATAACATCCGTCAAAACCATCTGTCTCTGTTTTCTCATTCATCT
A5-AT3G23340-XLOC_015072-3660-1
AAATAAATAACATCCGTCAAAACCATCTGTCTCTGTTTTCTCATTCATCT
CONSENSUS
AAATAAATAACATCCGTCAAAACCATCTGTCTCTGTTTTCTCATTCATCT

A5-AT3G23340-XLOC_015072-3660-0
CCTCTACTTGTTTAGTCGGTCATCAAACTATCTCTTCAAGTTTATCCTCA
A5-AT3G23340-XLOC_015072-3660-1
CCTCTACTTGTTTAGTCGGTCATCAAACTATCTCTTCAAGTTTATCCTCA
CONSENSUS
CCTCTACTTGTTTAGTCGGTCATCAAACTATCTCTTCAAGTTTATCCTCA

A5-AT3G23340-XLOC_015072-3660-0
GAAATATACAAACTCCTCAGAAAATTCTTTCGTTTGGTTTGCTTCGTTCT
A5-AT3G23340-XLOC_015072-3660-1
GAAATATACAAACTCCTCAGAAAATTCTTTCGTTTGGTTTGCTTCGTTCT
CONSENSUS
GAAATATACAAACTCCTCAGAAAATTCTTTCGTTTGGTTTGCTTCGTTCT

A5-AT3G23340-XLOC_015072-3660-0
ACAATTTTGTATTGATGAGAATGTTATATTGAGTCATTAACCGTTTGATA
A5-AT3G23340-XLOC_015072-3660-1
ACAATTTTGTATTGATGAGAATGTTATATTGAGTCATTAACCGTTTGATA
CONSENSUS
ACAATTTTGTATTGATGAGAATGTTATATTGAGTCATTAACCGTTTGATA

```
A5-AT3G23340-XLOC_015072-3660-0
      AATCGTATTTGGCTCTGTAAGTAGTATTTATTGGTATTGAAACGATGGAT
A5-AT3G23340-XLOC_015072-3660-1
      AATCGTATTTGGCTCTGTAAGTAGTATTTATTGGTATTGAAACGATGGAT
CONSENSUS
      AATCGTATTTGGCTCTGTAAGTAGTATTTATTGGTATTGAAACGATGGAT

A5-AT3G23340-XLOC_015072-3660-0
      CATGTAATCGGTGGCAAGTTCAAGTTAG----------------------
A5-AT3G23340-XLOC_015072-3660-1
      CATGTAATCGGTGGCAAGTTCAAGTTAGGTAGGAAGATTGGTAGTGGATC
CONSENSUS
      CATGTAATCGGTGGCAAGTTCAAGTTAG......................

A5-AT3G23340-XLOC_015072-3660-0  --------------------
GGATTAATGTTCAAACTGGAGAAGAAGTTG
A5-AT3G23340-XLOC_015072-3660-1
      ATTCGGTGAACTTTATATTGGGATTAATGTTCAAACTGGAGAAGAAGTTG
CONSENSUS
      ....................GGATTAATGTTCAAACTGGAGAAGAAGTTG

A5-AT3G23340-XLOC_015072-3660-0  CTCTTAAGCTG
A5-AT3G23340-XLOC_015072-3660-1  CTCTTAAGCTG
CONSENSUS                        CTCTTAAGCTG


alignment for event: A5-AT3G01310-XLOC_017062-6381

A5-AT3G01310-XLOC_017062-6381-0
      AGAGAAGAACACTGCGACGTCGTTAAAAGTAGGTTGAGAGCGAGAAAGGC
A5-AT3G01310-XLOC_017062-6381-1
      AGAGAAGAACACTGCGACGTCGTTAAAAGTAGGTTGAGAGCGAGAAAGGC
CONSENSUS
      AGAGAAGAACACTGCGACGTCGTTAAAAGTAGGTTGAGAGCGAGAAAGGC

A5-AT3G01310-XLOC_017062-6381-0
      CGAGAGAATGGAGATGGAAGAAGGAGCAAGTGGTGTTGGTGAGAAGATAA
A5-AT3G01310-XLOC_017062-6381-1
      CGAGAGAATGGAGATGGAAGAAGGAGCAAGTGGTGTTGGTGAGAAGATAA
CONSENSUS
      CGAGAGAATGGAGATGGAAGAAGGAGCAAGTGGTGTTGGTGAGAAGATAA

A5-AT3G01310-XLOC_017062-6381-0
      AGATTGGAGTCTGCGTCATGGAAAAGAAGGTGAAATGCGGCTCCGAGGTT
A5-AT3G01310-XLOC_017062-6381-1
      AGATTGGAGTCTGCGTCATGGAAAAGAAG------------------GTT
CONSENSUS
      AGATTGGAGTCTGCGTCATGGAAAAGAAG..................GTT

A5-AT3G01310-XLOC_017062-6381-0
      TTCTCAGCTCCCATGGGGGAAATTCTCGACAGACTCCAGTCTTTTGGTGA
A5-AT3G01310-XLOC_017062-6381-1
      TTCTCAGCTCCCATGGGGGAAATTCTCGACAGACTCCAGTCTTTTGGTGA
CONSENSUS
      TTCTCAGCTCCCATGGGGGAAATTCTCGACAGACTCCAGTCTTTTGGTGA
```

A5-AT3G01310-XLOC_017062-6381-0 ATTTGAG
A5-AT3G01310-XLOC_017062-6381-1 ATTTGAG
CONSENSUS   ATTTGAG

alignment for event: RI-AT3G61600-XLOC_016947-1033

RI-AT3G61600-XLOC_016947-1033-0
GTTTCATGAGGAGGTAATGTCTCTACCTTTGGCTGGAATTGAGGCAATTC
RI-AT3G61600-XLOC_016947-1033-1
GTTTCATGAGGAGGTAATGTCTCTACCTTTGGCTGGAATTGAGGCAATTC
CONSENSUS
GTTTCATGAGGAGGTAATGTCTCTACCTTTGGCTGGAATTGAGGCAATTC

RI-AT3G61600-XLOC_016947-1033-0
TATCAAGCGATGAACTCCAAATTGCATCAGAGGATGCAGTTTATGATTTT
RI-AT3G61600-XLOC_016947-1033-1
TATCAAGCGATGAACTCCAAATTGCATCAGAGGATGCAGTTTATGATTTT
CONSENSUS
TATCAAGCGATGAACTCCAAATTGCATCAGAGGATGCAGTTTATGATTTT

RI-AT3G61600-XLOC_016947-1033-0
ATCTTGAAGTGGGCAAGGGCGCAATACCCTTGTTTGGAAGAGCGAAGAGA
RI-AT3G61600-XLOC_016947-1033-1
ATCTTGAAGTGGGCAAGGGCGCAATACCCTTGTTTGGAAGAGCGAAGAGA
CONSENSUS
ATCTTGAAGTGGGCAAGGGCGCAATACCCTTGTTTGGAAGAGCGAAGAGA

RI-AT3G61600-XLOC_016947-1033-0
GATTCTCGGGTCACGCCTTGCACTCTCCATCCGCTTCCCATTCATGACAT
RI-AT3G61600-XLOC_016947-1033-1
GATTCTCGGGTCACGCCTTGCACTCTCCATCCGCTTCCCATTCATGACAT
CONSENSUS
GATTCTCGGGTCACGCCTTGCACTCTCCATCCGCTTCCCATTCATGACAT

RI-AT3G61600-XLOC_016947-1033-0
GCCGAAAGCTGAAGAAAGTGCTGACTTGCAGTGACTTTGAGCATGAAATA
RI-AT3G61600-XLOC_016947-1033-1
GCCGAAAGCTGAAGAAAGTGCTGACTTGCAGTGACTTTGAGCATGAAATA
CONSENSUS
GCCGAAAGCTGAAGAAAGTGCTGACTTGCAGTGACTTTGAGCATGAAATA

RI-AT3G61600-XLOC_016947-1033-0
GCATCAAAGCTTGTTCTAGAAGCTCTTTTCTTCAAAGCAGAAGCCCCACA
RI-AT3G61600-XLOC_016947-1033-1
GCATCAAAGCTTGTTCTAGAAGCTCTTTTCTTCAAAGCAGAAGCCCCACA
CONSENSUS
GCATCAAAGCTTGTTCTAGAAGCTCTTTTCTTCAAAGCAGAAGCCCCACA

RI-AT3G61600-XLOC_016947-1033-0
CAGACAACGTAGCCTAGCCTCCGAAGAATCTGCATCCCTGAACCGCCGCC
RI-AT3G61600-XLOC_016947-1033-1
CAGACAACGTAGCCTAGCCTCCGAAGAATCTGCATCCCTGAACCGCCGCC
CONSENSUS

CAGACAACGTAGCCTAGCCTCCGAAGAATCTGCATCCCTGAACCGCCGCC

RI-AT3G61600-XLOC_016947-1033-0
TGATAGAGAGGGCTTACAAATACAGACCCGTCAAAGTGGTCGAGTTTGAG
RI-AT3G61600-XLOC_016947-1033-1
TGATAGAGAGGGCTTACAAATACAGACCCGTCAAAGTGGTCGAGTTTGAG
CONSENSUS
TGATAGAGAGGGCTTACAAATACAGACCCGTCAAAGTGGTCGAGTTTGAG

RI-AT3G61600-XLOC_016947-1033-0
CTTCCTAGACCGCAGTGTGTAGTCTACCTAGACTTGAAAAGAGAAGAATG
RI-AT3G61600-XLOC_016947-1033-1
CTTCCTAGACCGCAGTGTGTAGTCTACCTAGACTTGAAAAGAGAAGAATG
CONSENSUS
CTTCCTAGACCGCAGTGTGTAGTCTACCTAGACTTGAAAAGAGAAGAATG

RI-AT3G61600-XLOC_016947-1033-0
TGGGGGACTGTTCCCGTCGGGTAGAGTGTATTCGCAGGCCTTTCACTTGG
RI-AT3G61600-XLOC_016947-1033-1
TGGGGGACTGTTCCCGTCGGGTAGAGTGTATTCGCAGGCCTTTCACTTGG
CONSENSUS
TGGGGGACTGTTCCCGTCGGGTAGAGTGTATTCGCAGGCCTTTCACTTGG

RI-AT3G61600-XLOC_016947-1033-0
GAGGTCAAGGGTTTTTCCTGTCAGCTCACTGCAACATGGACCAACAGAGC
RI-AT3G61600-XLOC_016947-1033-1
GAGGTCAAGGGTTTTTCCTGTCAGCTCACTGCAACATGGACCAACAGAGC
CONSENSUS
GAGGTCAAGGGTTTTTCCTGTCAGCTCACTGCAACATGGACCAACAGAGC

RI-AT3G61600-XLOC_016947-1033-0
TCGTTCCACTGTTTCGGGCTGTTCCTAGGGATGCAGGAGAAAGGGTCGGT
RI-AT3G61600-XLOC_016947-1033-1
TCGTTCCACTGTTTCGGGCTGTTCCTAGGGATGCAGGAGAAAGGGTCGGT
CONSENSUS
TCGTTCCACTGTTTCGGGCTGTTCCTAGGGATGCAGGAGAAAGGGTCGGT

RI-AT3G61600-XLOC_016947-1033-0
GAGTTTCGGAGTGGACTATGAATTCTCGGCAAGGTCAAAGCCCGCAGAGG
RI-AT3G61600-XLOC_016947-1033-1
GAGTTTCGGAGTGGACTATGAATTCTCGGCAAGGTCAAAGCCCGCAGAGG
CONSENSUS
GAGTTTCGGAGTGGACTATGAATTCTCGGCAAGGTCAAAGCCCGCAGAGG

RI-AT3G61600-XLOC_016947-1033-0
ATTTCATAAGCAAATACAAAGGGAACTACACATTCACAGGAGGGAAAGCA
RI-AT3G61600-XLOC_016947-1033-1
ATTTCATAAGCAAATACAAAGGGAACTACACATTCACAGGAGGGAAAGCA
CONSENSUS
ATTTCATAAGCAAATACAAAGGGAACTACACATTCACAGGAGGGAAAGCA

RI-AT3G61600-XLOC_016947-1033-0
GTAGGTTACAGAAACCTGTTTGGGGTCCCATGGACGTCTTTTATAGCGGA
RI-AT3G61600-XLOC_016947-1033-1
GTAGGTTACAGAAACCTGTTTGGGGTCCCATGGACGTCTTTTATAGCGGA
CONSENSUS

GTAGGTTACAGAAACCTGTTTGGGGTCCCATGGACGTCTTTTATAGCGGA

RI-AT3G61600-XLOC_016947-1033-0
AGATAGTCAATACTTCATCAATGGCATTCTCCATCTCAGAGCAGAGCTTA
RI-AT3G61600-XLOC_016947-1033-1
AGATAGTCAATACTTCATCAATGGCATTCTCCATCTCAGAGCAGAGCTTA
CONSENSUS
AGATAGTCAATACTTCATCAATGGCATTCTCCATCTCAGAGCAGAGCTTA

RI-AT3G61600-XLOC_016947-1033-0
CCATCAAAAGGTCTACAGATCCTTAGTGACCGTTTACTTCTCAATTGTCA
RI-AT3G61600-XLOC_016947-1033-1
CCATCAAAAG--
CONSENSUS
CCATCAAAAG..

RI-AT3G61600-XLOC_016947-1033-0
CTGTCTCTCTTTCCTTTACCTGCTGCTAAATCTCTCGCTCTCTCTAACTT
RI-AT3G61600-XLOC_016947-1033-1

CONSENSUS
....

RI-AT3G61600-XLOC_016947-1033-0
GCCTTTTTTTTCTTTTCTTTTATAAGGCTTGAAGAAGACTAATAAAGCTT
RI-AT3G61600-XLOC_016947-1033-1 --------------------------
GCTTGAAGAAGACTAATAAAGCTT
CONSENSUS
..........................GCTTGAAGAAGACTAATAAAGCTT

RI-AT3G61600-XLOC_016947-1033-0
ATAAAAGTAAGGTTAAAAACGTTTCGACGACAACGTTATAAGAAGAATGT
RI-AT3G61600-XLOC_016947-1033-1
ATAAAAGTAAGGTTAAAAACGTTTCGACGACAACGTTATAAGAAGAATGT
CONSENSUS
ATAAAAGTAAGGTTAAAAACGTTTCGACGACAACGTTATAAGAAGAATGT

RI-AT3G61600-XLOC_016947-1033-0
AAATGCGTTCTTTTTTTCTATCTACCCTTACTCTTTTGGCTTTGGATTAT
RI-AT3G61600-XLOC_016947-1033-1
AAATGCGTTCTTTTTTTCTATCTACCCTTACTCTTTTGGCTTTGGATTAT
CONSENSUS
AAATGCGTTCTTTTTTTCTATCTACCCTTACTCTTTTGGCTTTGGATTAT

RI-AT3G61600-XLOC_016947-1033-0
AAGTTTAACTCTTCAATGAAATTCTTAGCGTAGAGATTCTTGTATTATGA
RI-AT3G61600-XLOC_016947-1033-1
AAGTTTAACTCTTCAATGAAATTCTTAGCGTAGAGATTCTTGTATTATGA
CONSENSUS
AAGTTTAACTCTTCAATGAAATTCTTAGCGTAGAGATTCTTGTATTATGA

RI-AT3G61600-XLOC_016947-1033-0
ACGAAGTTGTGAACAGAAAAGAACGAACGTAACTCTCTCGTCTCATTTAA
RI-AT3G61600-XLOC_016947-1033-1
ACGAAGTTGTGAACAGAAAAGAACGAACGTAACTCTCTCGTCTCATTTAA
CONSENSUS

ACGAAGTTGTGAACAGAAAAGAACGAACGTAACTCTCTCGTCTCATTTAA

RI-AT3G61600-XLOC_016947-1033-0
CTAATTTATCTAGTTGGATTGAAATAGACATGACTTTTCGATT
RI-AT3G61600-XLOC_016947-1033-1
CTAATTTATCTAGTTGGATTGAAATAGACATGACTTTTCGATT
CONSENSUS
CTAATTTATCTAGTTGGATTGAAATAGACATGACTTTTCGATT

alignment for event: RI-AT3G26100-XLOC_015240-1460

RI-AT3G26100-XLOC_015240-1460-0
ATAATCTGTTTCAAAAATGGATGCTACGAGTGGAACTCCGAGTTTACAGT
RI-AT3G26100-XLOC_015240-1460-1
ATAATCTGTTTCAAAAATGGATGCTACGAGTGGAACTCCGAGTTTACAGT
CONSENSUS
ATAATCTGTTTCAAAAATGGATGCTACGAGTGGAACTCCGAGTTTACAGT

RI-AT3G26100-XLOC_015240-1460-0
ATATTAACTTGCCGGAACAATCTGTTTCGACTACTTCTCCTCCTGTGTCA
RI-AT3G26100-XLOC_015240-1460-1
ATATTAACTTGCCGGAACAATCTGTTTCGACTACTTCTCCTCCTGTGTCA
CONSENSUS
ATATTAACTTGCCGGAACAATCTGTTTCGACTACTTCTCCTCCTGTGTCA

RI-AT3G26100-XLOC_015240-1460-0
CCATTTCAGAGGCCAAAACGACATTGCTTTGGTGACACAACTCCAGGAGA
RI-AT3G26100-XLOC_015240-1460-1
CCATTTCAGAGGCCAAAACGACATTGCTTTGGTGACACAACTCCAGGAGA
CONSENSUS
CCATTTCAGAGGCCAAAACGACATTGCTTTGGTGACACAACTCCAGGAGA

RI-AT3G26100-XLOC_015240-1460-0
GTTTCCTTTAGCAGCTAACCCTTCCATTGTCCTACATGTTCTCACTGAAT
RI-AT3G26100-XLOC_015240-1460-1
GTTTCCTTTAGCAGCTAACCCTTCCATTGTCCTACATGTTCTCACTGAAT
CONSENSUS
GTTTCCTTTAGCAGCTAACCCTTCCATTGTCCTACATGTTCTCACTGAAT

RI-AT3G26100-XLOC_015240-1460-0
GTAGATTGGATCCTCGTGACCTCGCTAATCTCGAGGTTTTGTTTCGAAAA
RI-AT3G26100-XLOC_015240-1460-1
GTAGATTGGATCCTCGTGACCTCGCTAATCTCGAG---------------
CONSENSUS
GTAGATTGGATCCTCGTGACCTCGCTAATCTCGAG...............

RI-AT3G26100-XLOC_015240-1460-0
TGTCGGAAATGTTTTACTGAAATTTTCGAGGATTGATTGTTCTGCTGATT
RI-AT3G26100-XLOC_015240-1460-1

CONSENSUS
....

RI-AT3G26100-XLOC_015240-1460-0

TGATTGTTGTTGTTGTTGTTGTTGTTTTAGGCAACATGCTCGTTCTTTAG
RI-AT3G26100-XLOC_015240-1460-1 ------------------------------
GCAACATGCTCGTTCTTTAG
CONSENSUS
.............................GCAACATGCTCGTTCTTTAG

RI-AT3G26100-XLOC_015240-1460-0
CCAGCCAGCAAACTTTGCCCCGGACATTAACCTATCACTATCGGAGCTCG
RI-AT3G26100-XLOC_015240-1460-1
CCAGCCAGCAAACTTTGCCCCGGACATTAACCTATCACTATCGGAGCTCG
CONSENSUS
CCAGCCAGCAAACTTTGCCCCGGACATTAACCTATCACTATCGGAGCTCG

RI-AT3G26100-XLOC_015240-1460-0
CTGCTCTCGACATGTGTAATAAAAGGGTGATTTTCAAGCCGATGAATGAA
RI-AT3G26100-XLOC_015240-1460-1
CTGCTCTCGACATGTGTAATAAAAGGGTGATTTTCAAGCCGATGAATGAA
CONSENSUS
CTGCTCTCGACATGTGTAATAAAAGGGTGATTTTCAAGCCGATGAATGAA

RI-AT3G26100-XLOC_015240-1460-0
GAAGAACGTCAAGAGATGAAACGTAGGTGCGGAGGATCATGGAAATTAGT
RI-AT3G26100-XLOC_015240-1460-1
GAAGAACGTCAAGAGATGAAACGTAGGTGCGGAGGATCATGGAAATTAGT
CONSENSUS
GAAGAACGTCAAGAGATGAAACGTAGGTGCGGAGGATCATGGAAATTAGT

RI-AT3G26100-XLOC_015240-1460-0
CCTTCGGTTTTTGCTGGCTGGTGAAGCGTGTTGTCGAAGAGAGAAATCTC
RI-AT3G26100-XLOC_015240-1460-1
CCTTCGGTTTTTGCTGGCTGGTGAAGCGTGTTGTCGAAGAGAGAAATCTC
CONSENSUS
CCTTCGGTTTTTGCTGGCTGGTGAAGCGTGTTGTCGAAGAGAGAAATCTC

RI-AT3G26100-XLOC_015240-1460-0
AAGCTGTTGCTGGTCCTGGTCATAGTGTAGCAGTCACATCGAAAGGAGAA
RI-AT3G26100-XLOC_015240-1460-1
AAGCTGTTGCTGGTCCTGGTCATAGTGTAGCAGTCACATCGAAAGGAGAA
CONSENSUS
AAGCTGTTGCTGGTCCTGGTCATAGTGTAGCAGTCACATCGAAAGGAGAA

RI-AT3G26100-XLOC_015240-1460-0
GTTTATACTTTCGGATATAATAACTCTGGACAGCTAGGACATGGTCATAC
RI-AT3G26100-XLOC_015240-1460-1
GTTTATACTTTCGGATATAATAACTCTGGACAGCTAGGACATGGTCATAC
CONSENSUS
GTTTATACTTTCGGATATAATAACTCTGGACAGCTAGGACATGGTCATAC

RI-AT3G26100-XLOC_015240-1460-0 CGAGGACGAAGCTCGAATTCAACCTGTTAG
RI-AT3G26100-XLOC_015240-1460-1 CGAGGACGAAGCTCGAATTCAACCTGTTAG
CONSENSUS CGAGGACGAAGCTCGAATTCAACCTGTTAG

alignment for event: A5-AT3G45850-XLOC_019367-174

A5-AT3G45850-XLOC_019367-174-0
CATCTCTTCTTCTGCTTTGAGAAGAAGGAACAATAAATGCAGTTGAAGTC
A5-AT3G45850-XLOC_019367-174-1
CATCTCTTCTTCTGCTTTGAGAAGAAGGAACAATAAATGCAGTTGAAGTC
CONSENSUS
CATCTCTTCTTCTGCTTTGAGAAGAAGGAACAATAAATGCAGTTGAAGTC

A5-AT3G45850-XLOC_019367-174-0
ACACTCTCTCTCTCTCTCTCCAAGTCGCTCTCTTTCGAATCCACCATTGC
A5-AT3G45850-XLOC_019367-174-1
ACACTCTCTCTCTCTCTCTCCAAGTCGCTCTCTTTCGAATCCACCATTGC
CONSENSUS
ACACTCTCTCTCTCTCTCTCCAAGTCGCTCTCTTTCGAATCCACCATTGC

A5-AT3G45850-XLOC_019367-174-0
TGAAACTTGATCCTTCTCTGTAAGTCTTCAAATCTCCAGAGTGGGTTTTG
A5-AT3G45850-XLOC_019367-174-1
TGAAACTTGATCCTTCTCT-------------------------------
CONSENSUS
TGAAACTTGATCCTTCTCT...............................

A5-AT3G45850-XLOC_019367-174-0
CTTCCGGGAAAAGTACTACTCGCTGAATCATCTTCCAAGATTCTTCATTT
A5-AT3G45850-XLOC_019367-174-1

CONSENSUS
....

A5-AT3G45850-XLOC_019367-174-0
TGTTACTCGATCGTTTGATCCGATTTCCTGTCATCGGTATTAAGAGAAGG
A5-AT3G45850-XLOC_019367-174-1

CONSENSUS
....

A5-AT3G45850-XLOC_019367-174-0
ATCTAGATATAGCTTGAGAATCTAAATGGTAGATAATTTTAGCAGAATGG
A5-AT3G45850-XLOC_019367-174-1 ----------------
AGAATCTAAATGGTAGATAATTTTAGCAGAATGG
CONSENSUS
................AGAATCTAAATGGTAGATAATTTTAGCAGAATGG

A5-AT3G45850-XLOC_019367-174-0
ATTCGATTCAGCAACGAAGAGGTGGGATTGTATCTCTCTCTCCAGCTCAG
A5-AT3G45850-XLOC_019367-174-1
ATTCGATTCAGCAACGAAGAGGTGGGATTGTATCTCTCTCTCCAGCTCAG
CONSENSUS
ATTCGATTCAGCAACGAAGAGGTGGGATTGTATCTCTCTCTCCAGCTCAG

A5-AT3G45850-XLOC_019367-174-0
ACACCACGATCAAGTGACAAATCAGCAAGAGAATCGAGATCTTCTGAATC
A5-AT3G45850-XLOC_019367-174-1
ACACCACGATCAAGTGACAAATCAGCAAGAGAATCGAGATCTTCTGAATC
CONSENSUS
ACACCACGATCAAGTGACAAATCAGCAAGAGAATCGAGATCTTCTGAATC

```
A5-AT3G45850-XLOC_019367-174-0
      AAATTCAACCAATAGAAATGATAAAGAAAAGGGTGTGAATGTGCAGGTCA
A5-AT3G45850-XLOC_019367-174-1
      AAATTCAACCAATAGAAATGATAAAGAAAAGGGTGTGAATGTGCAGGTCA
CONSENSUS
      AAATTCAACCAATAGAAATGATAAAGAAAAGGGTGTGAATGTGCAGGTCA

A5-AT3G45850-XLOC_019367-174-0   TATTGCGTTGCAG
A5-AT3G45850-XLOC_019367-174-1   TATTGCGTTGCAG
CONSENSUS                        TATTGCGTTGCAG



alignment for event: A3-AT5G16520-XLOC_030024-7065

A3-AT5G16520-XLOC_030024-7065-0
      TGTGAGTACAGGATCAAGAAGCTGCGAAATGAAGATACTGTTTTCAGGAA
A3-AT5G16520-XLOC_030024-7065-1
      TGTGAGTACAGGATCAAGAAGCTGCGAAATGAAGATACTGTTTTCAGGAA
CONSENSUS
      TGTGAGTACAGGATCAAGAAGCTGCGAAATGAAGATACTGTTTTCAGGAA

A3-AT5G16520-XLOC_030024-7065-0
      GATAAGGAATCGTCGTAAAGCTCTAGATCACTGGGATAAA----------
A3-AT5G16520-XLOC_030024-7065-1
      GATAAGGAATCGTCGTAAAGCTCTAGATCACTGGGATAAACTGAGAAGAT
CONSENSUS
      GATAAGGAATCGTCGTAAAGCTCTAGATCACTGGGATAAA..........

A3-AT5G16520-XLOC_030024-7065-0  -------------------------
CATATTGGTCGATGATGCCATCAAA
A3-AT5G16520-XLOC_030024-7065-1
      ATGTTGCATACACATGGGATTGCAGCATATTGGTCGATGATGCCATCAAA
CONSENSUS
      .........................CATATTGGTCGATGATGCCATCAAA

A3-AT5G16520-XLOC_030024-7065-0
      ACGACAAAGGTATCAGGAATGTGTGGTTTAACGAGACCTCCAACATCTTC
A3-AT5G16520-XLOC_030024-7065-1
      ACGACAAAGGTATCAGGAATGTGTGGTTTAACGAGACCTCCAACATCTTC
CONSENSUS
      ACGACAAAGGTATCAGGAATGTGTGGTTTAACGAGACCTCCAACATCTTC

A3-AT5G16520-XLOC_030024-7065-0
      TAATAGCTCGGAACACGGGGAATCGATCATGGCGAACAAGAAGCCTAACT
A3-AT5G16520-XLOC_030024-7065-1
      TAATAGCTCGGAACACGGGGAATCGATCATGGCGAACAAGAAGCCTAACT
CONSENSUS
      TAATAGCTCGGAACACGGGGAATCGATCATGGCGAACAAGAAGCCTAACT

A3-AT5G16520-XLOC_030024-7065-0  TAGGTATTGAGAAG
A3-AT5G16520-XLOC_030024-7065-1  TAGGTATTGAGAAG
CONSENSUS                        TAGGTATTGAGAAG
```

alignment for event: A3-AT5G15020-XLOC_029939-7601

A3-AT5G15020-XLOC_029939-7601-0
GGGAGAGACCTCATAGGCAAATTCCGTCCTCGTTTCTTCTTCTTCTTCAT
A3-AT5G15020-XLOC_029939-7601-1
GGGAGAGACCTCATAGGCAAATTCCGTCCTCGTTTCTTCTTCTTCTTCAT
CONSENSUS
GGGAGAGACCTCATAGGCAAATTCCGTCCTCGTTTCTTCTTCTTCTTCAT

A3-AT5G15020-XLOC_029939-7601-0
TATTCGAAAATTACTTTCTCTCTCTTGCTTCTCAGTCGTAGCAACTCCAA
A3-AT5G15020-XLOC_029939-7601-1
TATTCGAAAATTACTTTCTCTCTCTTGCTTCTCAGTCGTAGCAACTCCAA
CONSENSUS
TATTCGAAAATTACTTTCTCTCTCTTGCTTCTCAGTCGTAGCAACTCCAA

A3-AT5G15020-XLOC_029939-7601-0
TTCAAAACATTTCTCTCTCATTTGTTTTTAATCTTCTTCTGAATTCCAC
A3-AT5G15020-XLOC_029939-7601-1
TTCAAAACATTTCTCTCTCATTTGTTTTTAATCTTCTTCTGAATTCCAC
CONSENSUS
TTCAAAACATTTCTCTCTCATTTGTTTTTAATCTTCTTCTGAATTCCAC

A3-AT5G15020-XLOC_029939-7601-0
TCTGTTCTCATCGGAATTGTTCTTGGGGTTTGGCACAATTCGGGAAATTT
A3-AT5G15020-XLOC_029939-7601-1
TCTGTTCTCATCGGAATTGTTCTTGGGGTTTGGCACAATTCGGGAAATTT
CONSENSUS
TCTGTTCTCATCGGAATTGTTCTTGGGGTTTGGCACAATTCGGGAAATTT

A3-AT5G15020-XLOC_029939-7601-0
GAAGTTTTCGCCGGAATTTGATCGGAAGTGTTTCTTATCGATTCCGCTCT
A3-AT5G15020-XLOC_029939-7601-1
GAAGTTTTCGCCGGAATTTGATCGGAAGTGTTTCTTATCGATTCCGCTCT
CONSENSUS
GAAGTTTTCGCCGGAATTTGATCGGAAGTGTTTCTTATCGATTCCGCTCT

A3-AT5G15020-XLOC_029939-7601-0
TACTCGAGAGATCTGATTAATCTCTTCCCGGAGTTATATCTCTGGGAAAT
A3-AT5G15020-XLOC_029939-7601-1
TACTCGAGAGATCTGATTAATCTCTTCCCGGAGTTATATCTCTGGGAAAT
CONSENSUS
TACTCGAGAGATCTGATTAATCTCTTCCCGGAGTTATATCTCTGGGAAAT

A3-AT5G15020-XLOC_029939-7601-0 CTGGAGAACGAGAAA-----
CAATGAAGCGAATTAGGGATGATATTTACG
A3-AT5G15020-XLOC_029939-7601-1
CTGGAGAACGAGAAAAGCAGCAATGAAGCGAATTAGGGATGATATTTACG
CONSENSUS
CTGGAGAACGAGAAA.....CAATGAAGCGAATTAGGGATGATATTTACG

A3-AT5G15020-XLOC_029939-7601-0
CAACCGGGTCTCAATTTAAACGTCCTTTGGGCTCTTCTCGTGGCGAATC
A3-AT5G15020-XLOC_029939-7601-1
CAACCGGGTCTCAATTTAAACGTCCTTTGGGCTCTTCTCGTGGCGAATC
CONSENSUS

CAACCGGGTCTCAATTTAAACGTCCTTTGGGCTCTTCTCGTGGCGAATC

alignment for event: A3-AT5G14440-XLOC_029908-804

A3-AT5G14440-XLOC_029908-804-0
GTGAATTTGATGAAGATGGAGAGATTAGTATGGAAGAGTCCATACTAATA
A3-AT5G14440-XLOC_029908-804-1
GTGAATTTGATGAAGATGGAGAGATTAGTATGGAAGAGTCCATACTAATA
CONSENSUS
GTGAATTTGATGAAGATGGAGAGATTAGTATGGAAGAGTCCATACTAATA

A3-AT5G14440-XLOC_029908-804-0
GGTGAAGTTGATGAAGATGGCAAGATTGTTTTGGATGACACTCATGCCAG
A3-AT5G14440-XLOC_029908-804-1
GGTGAAGTTGATGAAGATGGCAAGATTGTTTTGGATGACACTCATGCCAG
CONSENSUS
GGTGAAGTTGATGAAGATGGCAAGATTGTTTTGGATGACACTCATGCCAG

A3-AT5G14440-XLOC_029908-804-0 ---
CAACAAGAGGAAACATGAAGAACTCGGTTCCAGTGACCTTCCTTCGA
A3-AT5G14440-XLOC_029908-804-1
CAGCAACAAGAGGAAACATGAAGAACTCGGTTCCAGTGACCTTCCTTCGA
CONSENSUS
...CAACAAGAGGAAACATGAAGAACTCGGTTCCAGTGACCTTCCTTCGA

A3-AT5G14440-XLOC_029908-804-0
AGAAGAAGAATAAGAAGAAGAAAAAGAAGAAGAATGTCGAAGCAACATCG
A3-AT5G14440-XLOC_029908-804-1
AGAAGAAGAATAAGAAGAAGAAAAAGAAGAAGAATGTCGAAGCAACATCG
CONSENSUS
AGAAGAAGAATAAGAAGAAGAAAAAGAAGAAGAATGTCGAAGCAACATCG

A3-AT5G14440-XLOC_029908-804-0
TCGTCATATTAAGTCTGATATGTTTTGTATTTCACTTTGTACTTGCTCTA
A3-AT5G14440-XLOC_029908-804-1
TCGTCATATTAAGTCTGATATGTTTTGTATTTCACTTTGTACTTGCTCTA
CONSENSUS
TCGTCATATTAAGTCTGATATGTTTTGTATTTCACTTTGTACTTGCTCTA

A3-AT5G14440-XLOC_029908-804-0
TTTCAGCTTGTGTGCTCAGTTTTGTTATAATTGGTGATGGAAAATGGTGA
A3-AT5G14440-XLOC_029908-804-1
TTTCAGCTTGTGTGCTCAGTTTTGTTATAATTGGTGATGGAAAATGGTGA
CONSENSUS
TTTCAGCTTGTGTGCTCAGTTTTGTTATAATTGGTGATGGAAAATGGTGA

A3-AT5G14440-XLOC_029908-804-0
TTTACATATTAATGAAGCAATTTATTTTTCTCACAAAAGCAGATTTTAAT
A3-AT5G14440-XLOC_029908-804-1
TTTACATATTAATGAAGCAATTTATTTTTCTCACAAAAGCAGATTTTAAT
CONSENSUS
TTTACATATTAATGAAGCAATTTATTTTTCTCACAAAAGCAGATTTTAAT

A3-AT5G14440-XLOC_029908-804-0 TATAAAGAGGAACATGAAAATCGC

A3-AT5G14440-XLOC_029908-804-1   TATAAAGAGGAACATGAAAATCGC
CONSENSUS   TATAAAGAGGAACATGAAAATCGC

alignment for event: A3-AT5G64600-XLOC_029036-5724

A3-AT5G64600-XLOC_029036-5724-0
GTCATCCATGTCGCAAAATCAGATTCTCGCCTTGCAAATAATGACCTGCC
A3-AT5G64600-XLOC_029036-5724-1
GTCATCCATGTCGCAAAATCAGATTCTCGCCTTGCAAATAATGACCTGCC
CONSENSUS
GTCATCCATGTCGCAAAATCAGATTCTCGCCTTGCAAATAATGACCTGCC

A3-AT5G64600-XLOC_029036-5724-0
TATCGACGTTCAAAGACTGAGATGTCGTGTACTATATCGTGGTCTCTGCT
A3-AT5G64600-XLOC_029036-5724-1
TATCGACGTTCAAAGACTGAGATGTCGTGTACTATATCGTGGTCTCTGCT
CONSENSUS
TATCGACGTTCAAAGACTGAGATGTCGTGTACTATATCGTGGTCTCTGCT

A3-AT5G64600-XLOC_029036-5724-0
TCTCTCCTGCAATTGAAAGCCTTGGACAGACTGTGGGTTCTGAGCAATCA
A3-AT5G64600-XLOC_029036-5724-1
TCTCTCCTGCAATTGAAAGCCTTGGACAG---------------------
CONSENSUS
TCTCTCCTGCAATTGAAAGCCTTGGACAG.....................

A3-AT5G64600-XLOC_029036-5724-0
TGACGGTGACACCACAGAAGCTGGTTGAGAGACTCAAGTCACGTGCTGGG
A3-AT5G64600-XLOC_029036-5724-1 -----------------
AAGCTGGTTGAGAGACTCAAGTCACGTGCTGGG
CONSENSUS
.................AAGCTGGTTGAGAGACTCAAGTCACGTGCTGGG

A3-AT5G64600-XLOC_029036-5724-0
AGATATATTGCATTGCACCTGAGATATGAGAAAGATATGTTGGCTTTCAC
A3-AT5G64600-XLOC_029036-5724-1
AGATATATTGCATTGCACCTGAGATATGAGAAAGATATGTTGGCTTTCAC
CONSENSUS
AGATATATTGCATTGCACCTGAGATATGAGAAAGATATGTTGGCTTTCAC

A3-AT5G64600-XLOC_029036-5724-0
TGGTTGCACCTATGGTCTCACTGATGCTGAATCCGAAGAACTGAGAGTAA
A3-AT5G64600-XLOC_029036-5724-1
TGGTTGCACCTATGGTCTCACTGATGCTGAATCCGAAGAACTGAGAGTAA
CONSENSUS
TGGTTGCACCTATGGTCTCACTGATGCTGAATCCGAAGAACTGAGAGTAA

A3-AT5G64600-XLOC_029036-5724-0 TGCG
A3-AT5G64600-XLOC_029036-5724-1 TGCG
CONSENSUS   TGCG

alignment for event: SE-AT5G52040-XLOC_028317-447

SE-AT5G52040-XLOC_028317-447-0
GAATCATGAAGCCTGTCTTTTGCGGAAACTTTGAGTATGATGCTCGTGAG
SE-AT5G52040-XLOC_028317-447-1
GAATCATGAAGCCTGTCTTTTGCGGAAACTTTGAGTATGATGCTCGTGAG
CONSENSUS
GAATCATGAAGCCTGTCTTTTGCGGAAACTTTGAGTATGATGCTCGTGAG

SE-AT5G52040-XLOC_028317-447-0
AGTGATCTTGAGCGGCTTTTCAGAAAGTACGGCAAGGTTGAGAGGGTTGA
SE-AT5G52040-XLOC_028317-447-1
AGTGATCTTGAGCGGCTTTTCAGAAAGTACGGCAAGGTTGAGAGGGTTGA
CONSENSUS
AGTGATCTTGAGCGGCTTTTCAGAAAGTACGGCAAGGTTGAGAGGGTTGA

SE-AT5G52040-XLOC_028317-447-0
TATGAAAGCTG---------------------------------------
SE-AT5G52040-XLOC_028317-447-1
TATGAAAGCTGGATTTGTTTCGATCTTGGGATGCCCATTCTCCCACACAG
CONSENSUS
TATGAAAGCTG.......................................

SE-AT5G52040-XLOC_028317-447-0

SE-AT5G52040-XLOC_028317-447-1
CCCATTGATGGACAATATGGAAAATCCATTTCCTGCCCAAAAGAGTCAAC
CONSENSUS
....

SE-AT5G52040-XLOC_028317-447-0

SE-AT5G52040-XLOC_028317-447-1
CTCATCCACGCAATGGCACTACGTTCCCATAACCCCGACTTTAGTTGCAT
CONSENSUS
....

SE-AT5G52040-XLOC_028317-447-0

SE-AT5G52040-XLOC_028317-447-1
TTCATTCCTCTTGACTTTTCTCCTCTTCGAGATGCACATGCCTCTTTGGT
CONSENSUS
....

SE-AT5G52040-XLOC_028317-447-0

SE-AT5G52040-XLOC_028317-447-1
AGACTTATACCAACCTCTCACAGCAGAGGCAGCAAATCAAATGGAGTTTG
CONSENSUS
....

SE-AT5G52040-XLOC_028317-447-0

SE-AT5G52040-XLOC_028317-447-1
CGCCACCACGTTTCTGTTCACAATTCCGTCTCATCTCCTCCTCATTTTCA
CONSENSUS
....

```
SE-AT5G52040-XLOC_028317-447-0
      ------------------------------------GGTTTGCTTTTGT
SE-AT5G52040-XLOC_028317-447-1
      GTTCTTCTTCTTTGACATATTAATTCCATAATGCAAGGGTTTGCTTTTGT
CONSENSUS
      ....................................GGTTTGCTTTTGT

SE-AT5G52040-XLOC_028317-447-0
      CTATATGGAAGATGAAAGGGATGCTGAAGATGCAATTCGAGCGCTTGATC
SE-AT5G52040-XLOC_028317-447-1
      CTATATGGAAGATGAAAGGGATGCTGAAGATGCAATTCGAGCGCTTGATC
CONSENSUS
      CTATATGGAAGATGAAAGGGATGCTGAAGATGCAATTCGAGCGCTTGATC

SE-AT5G52040-XLOC_028317-447-0
      GCTTTGAATATGGTCGCACAGGACGCAGACTCCGTGTTGAGTGGACAAAG
SE-AT5G52040-XLOC_028317-447-1
      GCTTTGAATATGGTCGCACAGGACGCAGACTCCGTGTTGAGTGGACAAAG
CONSENSUS
      GCTTTGAATATGGTCGCACAGGACGCAGACTCCGTGTTGAGTGGACAAAG
```

alignment for event: A3-AT5G20380-XLOC_030236-839

```
A3-AT5G20380-XLOC_030236-839-0
      AAAAGTGCTTGAGATTGGTGTCTTTACATGGTCTTTCGCTACAGCTCTTG
A3-AT5G20380-XLOC_030236-839-1
      AAAAGTGCTTGAGATTGGTGTCTTTACATGGTCTTTCGCTACAGCTCTTG
CONSENSUS
      AAAAGTGCTTGAGATTGGTGTCTTTACATGGTCTTTCGCTACAGCTCTTG

A3-AT5G20380-XLOC_030236-839-0
      TTCCACTACTTGCTGGATTTATGCCCGGTTTGATCTTTTCTCGAATTTTG
A3-AT5G20380-XLOC_030236-839-1
      TTCCACTACTTGCTGGATTTATGCCCGGTTTGATCTTTTCTCGAATTTTG
CONSENSUS
      TTCCACTACTTGCTGGATTTATGCCCGGTTTGATCTTTTCTCGAATTTTG

A3-AT5G20380-XLOC_030236-839-0   ----------------
GTGGGAATAGGAGAAGGTGTTTCCCCATCGGCTG
A3-AT5G20380-XLOC_030236-839-1
      AGATTTGTAACTCCAGGTGGGAATAGGAGAAGGTGTTTCCCCATCGGCTG
CONSENSUS
      ................GTGGGAATAGGAGAAGGTGTTTCCCCATCGGCTG

A3-AT5G20380-XLOC_030236-839-0   CGACAGACCTTATTGCCAG
A3-AT5G20380-XLOC_030236-839-1   CGACAGACCTTATTGCCAG
CONSENSUS                        CGACAGACCTTATTGCCAG
```

alignment for event: SE-AT5G53850-XLOC_032121-4982

SE-AT5G53850-XLOC_032121-4982-0

GTGTTCAAAAGGAGAGGATGCAACCTGAGGATATGTACATCTTATCTGCT
SE-AT5G53850-XLOC_032121-4982-1
GTGTTCAAAAGGAGAGGATGCAACCTGAGGATATGTACATCTTATCTGCT
CONSENSUS
GTGTTCAAAAGGAGAGGATGCAACCTGAGGATATGTACATCTTATCTGCT

SE-AT5G53850-XLOC_032121-4982-0
AATGGATCCATCATATCTACACCCTCTCCAAAGCCATACCCAAATAAGCC
SE-AT5G53850-XLOC_032121-4982-1
AATGGATCCATCATATCTACACCCTCTCCAAAGCCATACCCAAATAAGCC
CONSENSUS
AATGGATCCATCATATCTACACCCTCTCCAAAGCCATACCCAAATAAGCC

SE-AT5G53850-XLOC_032121-4982-0
TCCCAAGTGTACCGATTGTGCTCCACTTTTCATGAAG-------------
SE-AT5G53850-XLOC_032121-4982-1
TCCCAAGTGTACCGATTGTGCTCCACTTTTCATGAAGTGTTGAGCAGCAG
CONSENSUS
TCCCAAGTGTACCGATTGTGCTCCACTTTTCATGAAG.............

SE-AT5G53850-XLOC_032121-4982-0

SE-AT5G53850-XLOC_032121-4982-1
TTACGGATTTCAAGTTATAATGTTTTCTGTTAAGAAGGATTTTTCTTAGA
CONSENSUS
....

SE-AT5G53850-XLOC_032121-4982-0

SE-AT5G53850-XLOC_032121-4982-1
GCACATGGTTATGAACGAAATATGTTAAGAGCTCGTCGCTCTTTTCTAGT
CONSENSUS
....

SE-AT5G53850-XLOC_032121-4982-0 -
AACAGGCGACTTTCTTTGGGGAAACGCTTGCGAAAACTCGGTTACAAG
SE-AT5G53850-XLOC_032121-4982-1
GAACAGGCGACTTTCTTTGGGGAAACGCTTGCGAAAACTCGGTTACAAG
CONSENSUS
.AACAGGCGACTTTCTTTGGGGAAACGCTTGCGAAAACTCGGTTACAAG

alignment for event: A3-AT5G42560-XLOC_027757-992

A3-AT5G42560-XLOC_027757-992-0
ATAATTTTCTAAGAGGCGTGACTTTAAAAATCTGTAAAAAAGCAAATCCG
A3-AT5G42560-XLOC_027757-992-1
ATAATTTTCTAAGAGGCGTGACTTTAAAAATCTGTAAAAAAGCAAATCCG
CONSENSUS
ATAATTTTCTAAGAGGCGTGACTTTAAAAATCTGTAAAAAAGCAAATCCG

A3-AT5G42560-XLOC_027757-992-0
AAGAAAGAGAGAGAGAGAAGGTGATTTTGAAATTTCAGATAACGCAGAAT
A3-AT5G42560-XLOC_027757-992-1
AAGAAAGAGAGAGAGAGAAGGTGATTTTGAAATTTCAGATAACGCAGAAT

CONSENSUS
      AAGAAAGAGAGAGAGAGAAGGTGATTTTGAAATTTCAGATAACGCAGAAT

A3-AT5G42560-XLOC_027757-992-0
      TTTTTTATTCTCGCCGGAGTTTTCTCCTCCCACCGTCTCTGATTCTCGTC
A3-AT5G42560-XLOC_027757-992-1
      TTTTTTATTCTCGCCGGAGTTTTCTCCTCCCACCGTCTCTGATTCTCGTC
CONSENSUS
      TTTTTTATTCTCGCCGGAGTTTTCTCCTCCCACCGTCTCTGATTCTCGTC

A3-AT5G42560-XLOC_027757-992-0
      GCCGCCGTACACAGTTGTTGTTCTCTGCCGTTTCTGCAAGATGATTGGAT
A3-AT5G42560-XLOC_027757-992-1
      GCCGCCGTACACAGTTGTTGTTCTCTGCCG--------------------
CONSENSUS
      GCCGCCGTACACAGTTGTTGTTCTCTGCCG....................

A3-AT5G42560-XLOC_027757-992-0
      CGTTTCTTACAAGAGGATTGGTGTACGAAAATCATATTCTCATTTGTTTG
A3-AT5G42560-XLOC_027757-992-1

CONSENSUS
      ....

A3-AT5G42560-XLOC_027757-992-0
      CTAAACGATATATGATGTGGTAGTTACTGTTGATTAGTTCTCCTTAATTT
A3-AT5G42560-XLOC_027757-992-1

CONSENSUS
      ....

A3-AT5G42560-XLOC_027757-992-0
      ACTACTATATTTGTTATAGGATGGTGTTGGGCTATGCTTACCCTGCGTAT
A3-AT5G42560-XLOC_027757-992-1   -------------------
GATGGTGTTGGGCTATGCTTACCCTGCGTAT
CONSENSUS
      ...................GATGGTGTTGGGCTATGCTTACCCTGCGTAT

A3-AT5G42560-XLOC_027757-992-0
      GAATGTTACAAGACTGTTGAAAAGAATAGGCCTGAAATCGAGCAACTTCG
A3-AT5G42560-XLOC_027757-992-1
      GAATGTTACAAGACTGTTGAAAAGAATAGGCCTGAAATCGAGCAACTTCG
CONSENSUS
      GAATGTTACAAGACTGTTGAAAAGAATAGGCCTGAAATCGAGCAACTTCG

A3-AT5G42560-XLOC_027757-992-0   CTTTTGGTGTCAGTACTG
A3-AT5G42560-XLOC_027757-992-1   CTTTTGGTGTCAGTACTG
CONSENSUS   CTTTTGGTGTCAGTACTG

alignment for event: A5-AT5G24750-XLOC_030460-2302

A5-AT5G24750-XLOC_030460-2302-0
      ATTACTGGCCATTGAGCGTTCGAGTCTGCGGCTTTTGGTTTCTGCCCAAT
A5-AT5G24750-XLOC_030460-2302-1

```
      ATTACTGGCCATTGAGCGTTCGAGTCTGCGGCTTTTGGTTTCTGCCCAAT
CONSENSUS
      ATTACTGGCCATTGAGCGTTCGAGTCTGCGGCTTTTGGTTTCTGCCCAAT

A5-AT5G24750-XLOC_030460-2302-0
      GAATGGCAGTTTTCATGCAACGAGTGTGGAGACAATCCTTTTGCAGGGCG
A5-AT5G24750-XLOC_030460-2302-1
      GAATGGCAGTTTTCATGCAACGAGTGTGGAGACAATCCTTTTGCAGGGCG
CONSENSUS
      GAATGGCAGTTTTCATGCAACGAGTGTGGAGACAATCCTTTTGCAGGGCG

A5-AT5G24750-XLOC_030460-2302-0
      CCTGGGTACAGACGATTCACATACATGCTCAAACCACACTGAGTTATATA
A5-AT5G24750-XLOC_030460-2302-1
      CCTGG---------------------------------------------
CONSENSUS
      CCTGG.............................................

A5-AT5G24750-XLOC_030460-2302-0
      CATTTATATCGTCTTGTGAGCCAGCTCTGCCTATCTTTGTAGGGTTGAGT
A5-AT5G24750-XLOC_030460-2302-1
      --------------------------------------------------
CONSENSUS
      ..................................................

A5-AT5G24750-XLOC_030460-2302-0
      TCTGTTGGAAGCATGGGCTTTGTAAGGGATCCCATAGCCTTTCTTCGAGT
A5-AT5G24750-XLOC_030460-2302-1  -----------
CATGGGCTTTGTAAGGGATCCCATAGCCTTTCTTCGAGT
CONSENSUS
      ...........CATGGGCTTTGTAAGGGATCCCATAGCCTTTCTTCGAGT

A5-AT5G24750-XLOC_030460-2302-0
      TCTCCAATCTGTTATTCAGATTACAGGTTACAGATTTATCATTTTCACAG
A5-AT5G24750-XLOC_030460-2302-1
      TCTCCAATCTGTTATTCAGATTACAGGTTACAGATTTATCATTTTCACAG
CONSENSUS
      TCTCCAATCTGTTATTCAGATTACAGGTTACAGATTTATCATTTTCACAG

A5-AT5G24750-XLOC_030460-2302-0
      CTAGTTATGGACCTTTAGATGCAGCAATTAGGACCATTGCTAATGGATCA
A5-AT5G24750-XLOC_030460-2302-1
      CTAGTTATGGACCTTTAGATGCAGCAATTAGGACCATTGCTAATGGATCA
CONSENSUS
      CTAGTTATGGACCTTTAGATGCAGCAATTAGGACCATTGCTAATGGATCA

A5-AT5G24750-XLOC_030460-2302-0
      GATTCAAGTGAGAAACAACCGTTACATGCGGGAATCTCTATTTTCAACGG
A5-AT5G24750-XLOC_030460-2302-1
      GATTCAAGTGAGAAACAACCGTTACATGCGGGAATCTCTATTTTCAACGG
CONSENSUS
      GATTCAAGTGAGAAACAACCGTTACATGCGGGAATCTCTATTTTCAACGG

A5-AT5G24750-XLOC_030460-2302-0  CAAGCTTTTCTGCTTCTCTGG
A5-AT5G24750-XLOC_030460-2302-1  CAAGCTTTTCTGCTTCTCTGG
CONSENSUS                        CAAGCTTTTCTGCTTCTCTGG
```

```
alignment for event: RI-AT5G43920-XLOC_027844-1991

RI-AT5G43920-XLOC_027844-1991-0
          GTACTGGATGACAACAAAGTCGAACTGAAGCACACACTTGAGAGCCACCA
RI-AT5G43920-XLOC_027844-1991-1
          GTACTGGATGACAACAAAGTCGAACTGAAGCACACACTTGAGAGCCACCA
CONSENSUS
          GTACTGGATGACAACAAAGTCGAACTGAAGCACACACTTGAGAGCCACCA

RI-AT5G43920-XLOC_027844-1991-0
          AAATCCAGTTTCTTTTGTCTCGTGGAGTCCTGACGATACTAAACTGCTTA
RI-AT5G43920-XLOC_027844-1991-1
          AAATCCAGTTTCTTTTGTCTCGTGGAGTCCTGACGATACTAAACTGCTTA
CONSENSUS
          AAATCCAGTTTCTTTTGTCTCGTGGAGTCCTGACGATACTAAACTGCTTA

RI-AT5G43920-XLOC_027844-1991-0
          CATGCGGAAACGCTGAGGTTCTTAAGCTATGGGATGTTGACACAGGTGTG
RI-AT5G43920-XLOC_027844-1991-1
          CATGCGGAAACGCTGAGGTTCTTAAGCTATGGGATGTTGACACAG-----
CONSENSUS
          CATGCGGAAACGCTGAGGTTCTTAAGCTATGGGATGTTGACACAG.....

RI-AT5G43920-XLOC_027844-1991-0
          TTGAGACACACATTTGGAAACAACAATACTGGATTCACTGTCAGCTCTTG
RI-AT5G43920-XLOC_027844-1991-1
          --------------------------------------------------
CONSENSUS
          ..................................................

RI-AT5G43920-XLOC_027844-1991-0
          CGCATGGTTCCCTGACTCAACTCGGCTTGTCTGTGGCAGTTCTGACCCAG
RI-AT5G43920-XLOC_027844-1991-1
          ---------------------------------------TTCTGACCCAG
CONSENSUS
          .......................................TTCTGACCCAG

RI-AT5G43920-XLOC_027844-1991-0
          AAAGAGGGATTGTAATGTGGGACACTGATGGAAACGAGATCAAAGCCTGG
RI-AT5G43920-XLOC_027844-1991-1
          AAAGAGGGATTGTAATGTGGGACACTGATGGAAACGAGATCAAAGCCTGG
CONSENSUS
          AAAGAGGGATTGTAATGTGGGACACTGATGGAAACGAGATCAAAGCCTGG

RI-AT5G43920-XLOC_027844-1991-0  AGAGGAACGAGAATTCCAAAG
RI-AT5G43920-XLOC_027844-1991-1  AGAGGAACGAGAATTCCAAAG
CONSENSUS                        AGAGGAACGAGAATTCCAAAG


alignment for event: A5-AT5G48610-XLOC_028128-2118

A5-AT5G48610-XLOC_028128-2118-0
```

GACAAGTACAAGGAAAAGAAGCATAAGAAGGATAAAGAAAAGAGAGAGGG
A5-AT5G48610-XLOC_028128-2118-1
GACAAGTACAAGGAAAAGAAGCATAAGAAGGATAAAGAAAAGAGAGAGGG
CONSENSUS
GACAAGTACAAGGAAAAGAAGCATAAGAAGGATAAAGAAAAGAGAGAGGG

A5-AT5G48610-XLOC_028128-2118-0
TAAAGAGAAAAAGAGTAAAGATAGAAGCAAAGACAAACAGAAGGAAAGAA
A5-AT5G48610-XLOC_028128-2118-1
TAAAGAGAAAAAGAGTAAAGATAGAAGCAAAGACAAACAGAAGGAAAGAA
CONSENSUS
TAAAGAGAAAAAGAGTAAAGATAGAAGCAAAGACAAACAGAAGGAAAGAA

A5-AT5G48610-XLOC_028128-2118-0
AGGAGAAAAAAGACAAACATAAAGATCAGAAAGACAAGGAGAAAGGTAAA
A5-AT5G48610-XLOC_028128-2118-1
AGGAGAAAAAAGACAAACATAAAGATCAGAAAGACAAGGAGAAAGGTAAA
CONSENSUS
AGGAGAAAAAAGACAAACATAAAGATCAGAAAGACAAGGAGAAAGGTAAA

A5-AT5G48610-XLOC_028128-2118-0
GAAAAAGGCAAACCTTTGGAGGAGAAGAAAGCTGAGCTGCTGACAAATGC
A5-AT5G48610-XLOC_028128-2118-1
GAAAAAGGCAAACCTTTGGAGGAGAAGAAAGCTGAGCTGCTGACAAATGC
CONSENSUS
GAAAAAGGCAAACCTTTGGAGGAGAAGAAAGCTGAGCTGCTGACAAATGC

A5-AT5G48610-XLOC_028128-2118-0
CGGGCACAGAGAGAATCGTGTAACAGATACGGTGCAGAATAATAGTAATG
A5-AT5G48610-XLOC_028128-2118-1
CGGGCACAGAGAGAATCGTGTAACAGATACGGTGCAGAATAATAGTAATG
CONSENSUS
CGGGCACAGAGAGAATCGTGTAACAGATACGGTGCAGAATAATAGTAATG

A5-AT5G48610-XLOC_028128-2118-0
GAGAGTCAAAGTATGTACAGGACCTGGCAAGAAGGATCAGATATGACGAA
A5-AT5G48610-XLOC_028128-2118-1
GAGAGTCAAAGTATGTACAGGACCTGGCAAGAAGGATCAGATATGACGAA
CONSENSUS
GAGAGTCAAAGTATGTACAGGACCTGGCAAGAAGGATCAGATATGACGAA

A5-AT5G48610-XLOC_028128-2118-0
GAAGCAACAGGAAGTCAGAGTGCACAAAGGATTGATCATCCTAACCAAAA
A5-AT5G48610-XLOC_028128-2118-1
GAAGCAACAGGAAGTCAGAGTGCACAAAGGATTGATCATCCTAACCAAAA
CONSENSUS
GAAGCAACAGGAAGTCAGAGTGCACAAAGGATTGATCATCCTAACCAAAA

A5-AT5G48610-XLOC_028128-2118-0
AAATGTAGGAATAACAGAAAAAGCATTTGAGAACAGCCCAATTGAAGAAA
A5-AT5G48610-XLOC_028128-2118-1
AAATGTAGGAATAACAGAAAAAGCATTTGAGAACAGCCCAATTGAAGAAA
CONSENSUS
AAATGTAGGAATAACAGAAAAAGCATTTGAGAACAGCCCAATTGAAGAAA

A5-AT5G48610-XLOC_028128-2118-0

CAAGCCATAGGGTAGATGACAATAAGAGAATCAATAACCAGAAGAACTTT
A5-AT5G48610-XLOC_028128-2118-1
CAAGCCATAGGGTAGATGACAATAAGAGAATCAATAACCAGAAGAACTTT
CONSENSUS
CAAGCCATAGGGTAGATGACAATAAGAGAATCAATAACCAGAAGAACTTT

A5-AT5G48610-XLOC_028128-2118-0
ACAGCGGCAAAAAGTTCTGAAAATGCAGTTTCTCGAGTATCATTTGGTGC
A5-AT5G48610-XLOC_028128-2118-1
ACAGCGGCAAAAAGTTCTGAAAATGCAGTTTCTCGAGTATCATTTGGTGC
CONSENSUS
ACAGCGGCAAAAAGTTCTGAAAATGCAGTTTCTCGAGTATCATTTGGTGC

A5-AT5G48610-XLOC_028128-2118-0
AGATCATAAAAGAGCTGAGGTGATGGGTAAACCAATGGAGAACAGAGACC
A5-AT5G48610-XLOC_028128-2118-1
AGATCATAAAAGAGCTGAGGTGATGGGTAAACCAATGGAGAACAGAGACC
CONSENSUS
AGATCATAAAAGAGCTGAGGTGATGGGTAAACCAATGGAGAACAGAGACC

A5-AT5G48610-XLOC_028128-2118-0
AGGTGAGGCAAACAGAATCAGCTGAGAAGAGTCACCGCAAGGAAAATGTG
A5-AT5G48610-XLOC_028128-2118-1
AGGTGAGGCAAACAGAATCAGCTGAGAAGAGTCACCGCAAGGAAAATGTG
CONSENSUS
AGGTGAGGCAAACAGAATCAGCTGAGAAGAGTCACCGCAAGGAAAATGTG

A5-AT5G48610-XLOC_028128-2118-0
ACCAAGAGTGAGAAACCAAGGGATCAAGAAGGGGTAAAGAAGACTGAAGC
A5-AT5G48610-XLOC_028128-2118-1
ACCAAGAGTGAGAAACCAAGGGATCAAGAAGGGGTAAAGAAGACTGAAGC
CONSENSUS
ACCAAGAGTGAGAAACCAAGGGATCAAGAAGGGGTAAAGAAGACTGAAGC

A5-AT5G48610-XLOC_028128-2118-0
AAAGGATAAAGACAGAAATAAAGAGAAGAAAGAGGAAAAAACAGAGTCGA
A5-AT5G48610-XLOC_028128-2118-1
AAAGGATAAAGACAGAAATAAAGAGAAGAAAGAGGAAAAAACAGAGTCGA
CONSENSUS
AAAGGATAAAGACAGAAATAAAGAGAAGAAAGAGGAAAAAACAGAGTCGA

A5-AT5G48610-XLOC_028128-2118-0
TAAATAAAACTCGTCAGGAGAAACCAAAATTGATACGAGGGCCCAAGTTA
A5-AT5G48610-XLOC_028128-2118-1
TAAATAAAACTCGTCAGGAGAAACCAAAATTGATACGAGGGCCCAAGTTA
CONSENSUS
TAAATAAAACTCGTCAGGAGAAACCAAAATTGATACGAGGGCCCAAGTTA

A5-AT5G48610-XLOC_028128-2118-0
GAGGAAAGAGAAAAGGACTCTCCGGATTTAAGAAATTGCAAGCTGCCTGA
A5-AT5G48610-XLOC_028128-2118-1
GAGGAAAGAGAAAAGGACTCTCCGGATTTAAGAAATTGCAAGCTGCCTGA
CONSENSUS
GAGGAAAGAGAAAAGGACTCTCCGGATTTAAGAAATTGCAAGCTGCCTGA

A5-AT5G48610-XLOC_028128-2118-0

CGTTTCACGGACAAGCATCAAGAACCTTCACACCGAGGGAAATCTTGGCA
A5-AT5G48610-XLOC_028128-2118-1
CGTTTCACGGACAAGCATCAAGAACCTTCACACCGAGGGAAATCTTGGCA
CONSENSUS
CGTTTCACGGACAAGCATCAAGAACCTTCACACCGAGGGAAATCTTGGCA

A5-AT5G48610-XLOC_028128-2118-0
AACGGAAGGATCATATGACAAATGGATTCCTGTATG--------------
A5-AT5G48610-XLOC_028128-2118-1
AACGGAAGGATCATATGACAAATGGATTCCTGTATGGTGAGTTATTCTCT
CONSENSUS
AACGGAAGGATCATATGACAAATGGATTCCTGTATG..............

A5-AT5G48610-XLOC_028128-2118-0

A5-AT5G48610-XLOC_028128-2118-1
GATCCAATTCTCTGTCTGGTGAATGAGGAGTGTAGTTGCCATATTAACCA
CONSENSUS
....

A5-AT5G48610-XLOC_028128-2118-0

A5-AT5G48610-XLOC_028128-2118-1
TCGTATGATATATGGTTTGGTACTTAGAAGCATCTAATAGTTTTATCCAA
CONSENSUS
....

A5-AT5G48610-XLOC_028128-2118-0 -----
AAAATGGAACCACGCCACACAAGTTACAGAAGCTGAGTGCTTCTG
A5-AT5G48610-XLOC_028128-2118-1
TCATGAAAATGGAACCACGCCACACAAGTTACAGAAGCTGAGTGCTTCTG
CONSENSUS
.....AAAATGGAACCACGCCACACAAGTTACAGAAGCTGAGTGCTTCTG

A5-AT5G48610-XLOC_028128-2118-0
TACCATCTGTGGAAAATGGAAGAACATTAGGTGCACCCCGAACTCCTCCA
A5-AT5G48610-XLOC_028128-2118-1
TACCATCTGTGGAAAATGGAAGAACATTAGGTGCACCCCGAACTCCTCCA
CONSENSUS
TACCATCTGTGGAAAATGGAAGAACATTAGGTGCACCCCGAACTCCTCCA

A5-AT5G48610-XLOC_028128-2118-0
ATGCCTGCTTCTGAGGTGCAAGGAACAACTTGCAAGCCACAAGTCAAAGA
A5-AT5G48610-XLOC_028128-2118-1
ATGCCTGCTTCTGAGGTGCAAGGAACAACTTGCAAGCCACAAGTCAAAGA
CONSENSUS
ATGCCTGCTTCTGAGGTGCAAGGAACAACTTGCAAGCCACAAGTCAAAGA

A5-AT5G48610-XLOC_028128-2118-0
AGTCAGAATTAATGGTTTTGCTGTCTCTGGAGAAAAACGTAAAGTCTGTC
A5-AT5G48610-XLOC_028128-2118-1
AGTCAGAATTAATGGTTTTGCTGTCTCTGGAGAAAAACGTAAAGTCTGTC
CONSENSUS
AGTCAGAATTAATGGTTTTGCTGTCTCTGGAGAAAAACGTAAAGTCTGTC

A5-AT5G48610-XLOC_028128-2118-0

CACCAAGCCCTTTGGCTGCAACAATGAAAGTGAAAGTCAAGGAAAATGGT
A5-AT5G48610-XLOC_028128-2118-1
CACCAAGCCCTTTGGCTGCAACAATGAAAGTGAAAGTCAAGGAAAATGGT
CONSENSUS
CACCAAGCCCTTTGGCTGCAACAATGAAAGTGAAAGTCAAGGAAAATGGT

A5-AT5G48610-XLOC_028128-2118-0
GAAGCATCCGCAAAGCCGCCTCATCCTGACCTAAAGTATCTCAATCAGAT
A5-AT5G48610-XLOC_028128-2118-1
GAAGCATCCGCAAAGCCGCCTCATCCTGACCTAAAGTATCTCAATCAGAT
CONSENSUS
GAAGCATCCGCAAAGCCGCCTCATCCTGACCTAAAGTATCTCAATCAGAT

A5-AT5G48610-XLOC_028128-2118-0
ACTCCATGTACCTACAAGGGAGCTGTTGCCAGAGATTGATGATGACCAAG
A5-AT5G48610-XLOC_028128-2118-1
ACTCCATGTACCTACAAGGGAGCTGTTGCCAGAGATTGATGATGACCAAG
CONSENSUS
ACTCCATGTACCTACAAGGGAGCTGTTGCCAGAGATTGATGATGACCAAG

A5-AT5G48610-XLOC_028128-2118-0
AATGGCTACTTGGTCAGTCGGGTATCAAATTAAAAAGGGCGAGAACAGAT
A5-AT5G48610-XLOC_028128-2118-1
AATGGCTACTTGGTCAGTCGGGTATCAAATTAAAAAGGGCGAGAACAGAT
CONSENSUS
AATGGCTACTTGGTCAGTCGGGTATCAAATTAAAAAGGGCGAGAACAGAT

A5-AT5G48610-XLOC_028128-2118-0
CCTCCAGATTCTGGCGAATCCTTGCAGGTCTGGAACCAAGCTTTCAGAAT
A5-AT5G48610-XLOC_028128-2118-1
CCTCCAGATTCTGGCGAATCCTTGCAGGTCTGGAACCAAGCTTTCAGAAT
CONSENSUS
CCTCCAGATTCTGGCGAATCCTTGCAGGTCTGGAACCAAGCTTTCAGAAT

A5-AT5G48610-XLOC_028128-2118-0
AGAGTCTGCGGATATCACAGCTCTTCCTTATGTAGTTCCATTTTAGCTAT
A5-AT5G48610-XLOC_028128-2118-1
AGAGTCTGCGGATATCACAGCTCTTCCTTATGTAGTTCCATTTTAGCTAT
CONSENSUS
AGAGTCTGCGGATATCACAGCTCTTCCTTATGTAGTTCCATTTTAGCTAT

A5-AT5G48610-XLOC_028128-2118-0
GACTATATTCCTACGCTACAATCCCTGAGGAGAAGAACTATCCCTCGCAC
A5-AT5G48610-XLOC_028128-2118-1
GACTATATTCCTACGCTACAATCCCTGAGGAGAAGAACTATCCCTCGCAC
CONSENSUS
GACTATATTCCTACGCTACAATCCCTGAGGAGAAGAACTATCCCTCGCAC

A5-AT5G48610-XLOC_028128-2118-0
AACGCCACTTTGCAGCAGCATCTATTAAAGAAGACCGACAACAGCAGTCC
A5-AT5G48610-XLOC_028128-2118-1
AACGCCACTTTGCAGCAGCATCTATTAAAGAAGACCGACAACAGCAGTCC
CONSENSUS
AACGCCACTTTGCAGCAGCATCTATTAAAGAAGACCGACAACAGCAGTCC

A5-AT5G48610-XLOC_028128-2118-0

ATGGCTCCCCAAAGTTTATGAGCAGGGATCAAATATGTTATACACGCCCA
A5-AT5G48610-XLOC_028128-2118-1
ATGGCTCCCCAAAGTTTATGAGCAGGGATCAAATATGTTATACACGCCCA
CONSENSUS
ATGGCTCCCCAAAGTTTATGAGCAGGGATCAAATATGTTATACACGCCCA

A5-AT5G48610-XLOC_028128-2118-0
TAATATGAACACCCACAAAGCTCTGCATGGGTGGGGGAAAAGAATGAATG
A5-AT5G48610-XLOC_028128-2118-1
TAATATGAACACCCACAAAGCTCTGCATGGGTGGGGGAAAAGAATGAATG
CONSENSUS
TAATATGAACACCCACAAAGCTCTGCATGGGTGGGGGAAAAGAATGAATG

A5-AT5G48610-XLOC_028128-2118-0
AGGAAGATGTAAGTAGAGTAAGTTTATAGAGAAGTTGATTGTTGTTTGAC
A5-AT5G48610-XLOC_028128-2118-1
AGGAAGATGTAAGTAGAGTAAGTTTATAGAGAAGTTGATTGTTGTTTGAC
CONSENSUS
AGGAAGATGTAAGTAGAGTAAGTTTATAGAGAAGTTGATTGTTGTTTGAC

A5-AT5G48610-XLOC_028128-2118-0
ACCATGTTGTGTTATAAGGACAAGGATATGTGTGGCATTTTGTTCTGCAC
A5-AT5G48610-XLOC_028128-2118-1
ACCATGTTGTGTTATAAGGACAAGGATATGTGTGGCATTTTGTTCTGCAC
CONSENSUS
ACCATGTTGTGTTATAAGGACAAGGATATGTGTGGCATTTTGTTCTGCAC

A5-AT5G48610-XLOC_028128-2118-0
GCAATTTTGATTCCCTGCTTGCATCATCAACATTAGTGGATAAAGTTTGT
A5-AT5G48610-XLOC_028128-2118-1
GCAATTTTGATTCCCTGCTTGCATCATCAACATTAGTGGATAAAGTTTGT
CONSENSUS
GCAATTTTGATTCCCTGCTTGCATCATCAACATTAGTGGATAAAGTTTGT

A5-AT5G48610-XLOC_028128-2118-0 ATATTACAGAGAA
A5-AT5G48610-XLOC_028128-2118-1 ATATTACAGAGAA
CONSENSUS ATATTACAGAGAA

alignment for event: SE-AT5G64170-XLOC_032687-6762

SE-AT5G64170-XLOC_032687-6762-0
GTCAAAAAAAAAAAAATAGGAAGCCTTCATCGTCTTCTGCCGAACATCTC
SE-AT5G64170-XLOC_032687-6762-1
GTCAAAAAAAAAAAAATAGGAAGCCTTCATCGTCTTCTGCCGAACATCTC
CONSENSUS
GTCAAAAAAAAAAAAATAGGAAGCCTTCATCGTCTTCTGCCGAACATCTC

SE-AT5G64170-XLOC_032687-6762-0
TAGAAAGTCCTACACAGTTTTTTTTTTCGATCTAGCTTCGATGAATTGAT
SE-AT5G64170-XLOC_032687-6762-1
TAGAAAGTCCTACACAGTTTTTTTTTTCGATCTAGCTTCGATGAATTGAT
CONSENSUS
TAGAAAGTCCTACACAGTTTTTTTTTTCGATCTAGCTTCGATGAATTGAT

SE-AT5G64170-XLOC_032687-6762-0
ACAATCTCTTCTATCTGCTGAGTTTTTTGTTTTCTGGTAAACAATTTTAT
SE-AT5G64170-XLOC_032687-6762-1
ACAATCTCTTCTATCTGCTGAGTTTTTTGTTTTCTGGTAAACAATTTTAT
CONSENSUS
ACAATCTCTTCTATCTGCTGAGTTTTTTGTTTTCTGGTAAACAATTTTAT

SE-AT5G64170-XLOC_032687-6762-0
CTGTGGAGTTTTCTGGAGTTGCTAGTGGAAGAATCTGTCTCACAG-----
SE-AT5G64170-XLOC_032687-6762-1
CTGTGGAGTTTTCTGGAGTTGCTAGTGGAAGAATCTGTCTCACAGAACCA
CONSENSUS
CTGTGGAGTTTTCTGGAGTTGCTAGTGGAAGAATCTGTCTCACAG.....

SE-AT5G64170-XLOC_032687-6762-0

SE-AT5G64170-XLOC_032687-6762-1
GAAATAGTGTTTGTGGGAGTTACATGTTTTGCAATAGAGATGACATATCG
CONSENSUS
....

SE-AT5G64170-XLOC_032687-6762-0

SE-AT5G64170-XLOC_032687-6762-1
GGGTAATGTGCATGATTTTAGTGGTGATTAAAGAGTCTGAAGTTGGGAGG
CONSENSUS
....

SE-AT5G64170-XLOC_032687-6762-0 -------------------------
CTAGGCGATTATCTTTCGGATGAAT
SE-AT5G64170-XLOC_032687-6762-1
AATGTCGGACTTGTACATTCATGAGCTAGGCGATTATCTTTCGGATGAAT
CONSENSUS
.........................CTAGGCGATTATCTTTCGGATGAAT

SE-AT5G64170-XLOC_032687-6762-0
TTCATGGGAACGATGATGGTATAGTGCCAGACTCAGCGTATGAGGATGGA
SE-AT5G64170-XLOC_032687-6762-1
TTCATGGGAACGATGATGGTATAGTGCCAGACTCAGCGTATGAGGATGGA
CONSENSUS
TTCATGGGAACGATGATGGTATAGTGCCAGACTCAGCGTATGAGGATGGA

SE-AT5G64170-XLOC_032687-6762-0
GGTCAGTTTCCAATTCTAGTTAGTAACAGGAAGAAACGAAGAAATGATGA
SE-AT5G64170-XLOC_032687-6762-1
GGTCAGTTTCCAATTCTAGTTAGTAACAGGAAGAAACGAAGAAATGATGA
CONSENSUS
GGTCAGTTTCCAATTCTAGTTAGTAACAGGAAGAAACGAAGAAATGATGA

SE-AT5G64170-XLOC_032687-6762-0
TATGGGTAGTGGAACAAACCATCTAAAGAGTAATACTTTTATCAAGAGAG
SE-AT5G64170-XLOC_032687-6762-1
TATGGGTAGTGGAACAAACCATCTAAAGAGTAATACTTTTATCAAGAGAG
CONSENSUS
TATGGGTAGTGGAACAAACCATCTAAAGAGTAATACTTTTATCAAGAGAG

SE-AT5G64170-XLOC_032687-6762-0
AGGCAAACATGTTAGGAAAAAATCCATGGCCTGAGAAAGATAGTGGTGGC
SE-AT5G64170-XLOC_032687-6762-1
AGGCAAACATGTTAGGAAAAAATCCATGGCCTGAGAAAGATAGTGGTGGC
CONSENSUS
AGGCAAACATGTTAGGAAAAAATCCATGGCCTGAGAAAGATAGTGGTGGC

SE-AT5G64170-XLOC_032687-6762-0
TCTTCGGTTTCTCGTGATACGGGAACAGGAAAAGATGTTCAGGATATGAC
SE-AT5G64170-XLOC_032687-6762-1
TCTTCGGTTTCTCGTGATACGGGAACAGGAAAAGATGTTCAGGATATGAC
CONSENSUS
TCTTCGGTTTCTCGTGATACGGGAACAGGAAAAGATGTTCAGGATATGAC

SE-AT5G64170-XLOC_032687-6762-0
ATTGGAGGATACAAATACTTCAGATCATGGTTTCAATGGTGGCCACGTAG
SE-AT5G64170-XLOC_032687-6762-1
ATTGGAGGATACAAATACTTCAGATCATGGTTTCAATGGTGGCCACGTAG
CONSENSUS
ATTGGAGGATACAAATACTTCAGATCATGGTTTCAATGGTGGCCACGTAG

SE-AT5G64170-XLOC_032687-6762-0
ATGTGGTTGAAAATTTCTCCACTGGGGATCCCATGTTGTGTGACACTTCC
SE-AT5G64170-XLOC_032687-6762-1
ATGTGGTTGAAAATTTCTCCACTGGGGATCCCATGTTGTGTGACACTTCC
CONSENSUS
ATGTGGTTGAAAATTTCTCCACTGGGGATCCCATGTTGTGTGACACTTCC

SE-AT5G64170-XLOC_032687-6762-0
GCTGCAACGAATGATGGCGTATATAATTATTCCCTCAACAGCATTCCGGA
SE-AT5G64170-XLOC_032687-6762-1
GCTGCAACGAATGATGGCGTATATAATTATTCCCTCAACAGCATTCCGGA
CONSENSUS
GCTGCAACGAATGATGGCGTATATAATTATTCCCTCAACAGCATTCCGGA

SE-AT5G64170-XLOC_032687-6762-0
TGCTGAAAATGATCTTAGCTTTTTCGACAATGGAGATAAAGAAAAAAATG
SE-AT5G64170-XLOC_032687-6762-1
TGCTGAAAATGATCTTAGCTTTTTCGACAATGGAGATAAAGAAAAAAATG
CONSENSUS
TGCTGAAAATGATCTTAGCTTTTTCGACAATGGAGATAAAGAAAAAAATG

SE-AT5G64170-XLOC_032687-6762-0
ATCTCTTCTATGGCTGGGGTGACATAGGAAATTTTGAGGATGTGGACAAC
SE-AT5G64170-XLOC_032687-6762-1
ATCTCTTCTATGGCTGGGGTGACATAGGAAATTTTGAGGATGTGGACAAC
CONSENSUS
ATCTCTTCTATGGCTGGGGTGACATAGGAAATTTTGAGGATGTGGACAAC

SE-AT5G64170-XLOC_032687-6762-0 ATGCTTAG
SE-AT5G64170-XLOC_032687-6762-1 ATGCTTAG
CONSENSUS ATGCTTAG

alignment for event: RI-AT5G38590-XLOC_031240-6860

RI-AT5G38590-XLOC_031240-6860-0
GGGAAGACAAGATTCGGGTATCTGTAACAGTAACTTGAATCTGGTAAGAT
RI-AT5G38590-XLOC_031240-6860-1
GGGAAGACAAGATTCGGGTATCTGTAACAGTAACTTGAATCTGGTAAGAT
CONSENSUS
GGGAAGACAAGATTCGGGTATCTGTAACAGTAACTTGAATCTGGTAAGAT

RI-AT5G38590-XLOC_031240-6860-0
ATGGACAAAATCAATGGATTGCCTGATGATTTGCTGGTGAAGATTTTATC
RI-AT5G38590-XLOC_031240-6860-1
ATGGACAAAATCAATGGATTGCCTGATGATTTGCTGGTGAAGATTTTATC
CONSENSUS
ATGGACAAAATCAATGGATTGCCTGATGATTTGCTGGTGAAGATTTTATC

RI-AT5G38590-XLOC_031240-6860-0
GTATGTTCCAACGGATATTGCTGTATCCACTAGCATTTTGTCTAAGCGAT
RI-AT5G38590-XLOC_031240-6860-1
GTATGTTCCAACGGATATTGCTGTATCCACTAGCATTTTGTCTAAGCGAT
CONSENSUS
GTATGTTCCAACGGATATTGCTGTATCCACTAGCATTTTGTCTAAGCGAT

RI-AT5G38590-XLOC_031240-6860-0
GGGAGTTTCTTTGGATGTGGTTGCCTAATCTCGACTACACTTCTCGGTGG
RI-AT5G38590-XLOC_031240-6860-1
GGGAGTTTCTTTGGATGTGGTTGCCTAATCTCGACTACACTTCTCGGTGG
CONSENSUS
GGGAGTTTCTTTGGATGTGGTTGCCTAATCTCGACTACACTTCTCGGTGG

RI-AT5G38590-XLOC_031240-6860-0
TGTAGAAAGCCCGGTGACGTTGGACTTAGGGACTTTATCCACAAGAATCT
RI-AT5G38590-XLOC_031240-6860-1
TGTAGAAAGCCCGGTGACGTTGGACTTAGGGACTTTATCCACAAGAATCT
CONSENSUS
TGTAGAAAGCCCGGTGACGTTGGACTTAGGGACTTTATCCACAAGAATCT

RI-AT5G38590-XLOC_031240-6860-0
GCCATTACATAGAGCTCCCGTCATAGAAAGCTTGCGTTTTCATTCTAATA
RI-AT5G38590-XLOC_031240-6860-1
GCCATTACATAGAGCTCCCGTCATAGAAAGCTTGCGTTTTCATTCTAATA
CONSENSUS
GCCATTACATAGAGCTCCCGTCATAGAAAGCTTGCGTTTTCATTCTAATA

RI-AT5G38590-XLOC_031240-6860-0
GCCCTGATATTAAACCTGAAGATATCAGACGATGGATTGAAATTGCAGTT
RI-AT5G38590-XLOC_031240-6860-1
GCCCTGATATTAAACCTGAAGATATCAGACGATGGATTGAAATTGCAGTT
CONSENSUS
GCCCTGATATTAAACCTGAAGATATCAGACGATGGATTGAAATTGCAGTT

RI-AT5G38590-XLOC_031240-6860-0
TCACGCCATGTACATGATCTTGATATTGATCATTTTTCGGAGAATGAAAA
RI-AT5G38590-XLOC_031240-6860-1
TCACGCCATGTACATGATCTTGATATTGATCATTTTTCGGAGAATGAAAA
CONSENSUS
TCACGCCATGTACATGATCTTGATATTGATCATTTTTCGGAGAATGAAAA

RI-AT5G38590-XLOC_031240-6860-0
CATATTCCTGAGTAGCTTTTTTGCCTGCAAATCGCTCGTGACCTTGAAAC
RI-AT5G38590-XLOC_031240-6860-1
CATATTCCTGAGTAGCTTTTTTGCCTGCAAATCGCTCGTGACCTTGAAAC
CONSENSUS
CATATTCCTGAGTAGCTTTTTTGCCTGCAAATCGCTCGTGACCTTGAAAC

RI-AT5G38590-XLOC_031240-6860-0
TAAGAAGCGTGACTCTCAGGGATATTCCCTCTATGGTTTGTCTCCCTTCT
RI-AT5G38590-XLOC_031240-6860-1
TAAGAAGCGTGACTCTCAGGGATATTCCCTCTATG---------------
CONSENSUS
TAAGAAGCGTGACTCTCAGGGATATTCCCTCTATG...............

RI-AT5G38590-XLOC_031240-6860-0
CTGAAAACTTTGCTACTTGACAATGTGTCATTTGTAGAAGGAAAATCTCT
RI-AT5G38590-XLOC_031240-6860-1

CONSENSUS
....

RI-AT5G38590-XLOC_031240-6860-0
TCAGGAGCTTCTATCTATTTGTCCTGTTCTTGAAGATCTATCGGTGTATT
RI-AT5G38590-XLOC_031240-6860-1

CONSENSUS
....

RI-AT5G38590-XLOC_031240-6860-0
GTGATGACTATGAAAATACAAAAGAGTTAACTATTGTTGTCCCGTCTTTG
RI-AT5G38590-XLOC_031240-6860-1

CONSENSUS
....

RI-AT5G38590-XLOC_031240-6860-0
CTGAGTTTATCACTCTATATACCTGATGAATGGTTGTTAGATGGGTATTG
RI-AT5G38590-XLOC_031240-6860-1

CONSENSUS
....

RI-AT5G38590-XLOC_031240-6860-0
GATAGATACTCCTTCTTTGGAATATTTGAAACTTGAGGATTGGAATTCTT
RI-AT5G38590-XLOC_031240-6860-1

CONSENSUS
....

RI-AT5G38590-XLOC_031240-6860-0
GTGATCACTTATCTTTGATTAAGAATATGCCTAAGCTGAGGGAAGCATAT
RI-AT5G38590-XLOC_031240-6860-1

CONSENSUS
....

RI-AT5G38590-XLOC_031240-6860-0
      GTTGATGCAAAATGCTTTCTTCCCAAGAGTGTCATTGAATCAATCACATC
RI-AT5G38590-XLOC_031240-6860-1

CONSENSUS
      ....

RI-AT5G38590-XLOC_031240-6860-0
      TGTCAAGCATCTTACAATATGTTCAAAGGTTCCATTTCTATCAACTGCAG
RI-AT5G38590-XLOC_031240-6860-1

CONSENSUS
      ....

RI-AT5G38590-XLOC_031240-6860-0
      GTTGTCAATCTAAATTATAGAACTCTTTGTTAATAATTTTGGTATTGTGG
RI-AT5G38590-XLOC_031240-6860-1

CONSENSUS
      ....

RI-AT5G38590-XLOC_031240-6860-0
      CCAGGATGGGTATGGTGATGGTTTTGTTTTCAACCAGCTTGAACATCTGA
RI-AT5G38590-XLOC_031240-6860-1 ----
GATGGGTATGGTGATGGTTTTGTTTTCAACCAGCTTGAACATCTGA
CONSENSUS
      ....GATGGGTATGGTGATGGTTTTGTTTTCAACCAGCTTGAACATCTGA

RI-AT5G38590-XLOC_031240-6860-0
      CCCTATGTGTGTGTAGAGGGGATTCGCCGAGTCTCCTTGGCCAGTTACTC
RI-AT5G38590-XLOC_031240-6860-1
      CCCTATGTGTGTGTAGAGGGGATTCGCCGAGTCTCCTTGGCCAGTTACTC
CONSENSUS
      CCCTATGTGTGTGTAGAGGGGATTCGCCGAGTCTCCTTGGCCAGTTACTC

RI-AT5G38590-XLOC_031240-6860-0
      AAGGATTCTCCTAACTTACGAATATTAGAAATCTCTGTAATGGAA
RI-AT5G38590-XLOC_031240-6860-1
      AAGGATTCTCCTAACTTACGAATATTAGAAATCTCTGTAATGGAA
CONSENSUS
      AAGGATTCTCCTAACTTACGAATATTAGAAATCTCTGTAATGGAA

alignment for event: A3-AT5G47690-XLOC_028073-1737

A3-AT5G47690-XLOC_028073-1737-0
      GTGATCCCCCAAACTTCAGAAAATGAGGTTATGATTGATGGGGAAAGTGA
A3-AT5G47690-XLOC_028073-1737-1
      GTGATCCCCCAAACTTCAGAAAATGAGGTTATGATTGATGGGGAAAGTGA
CONSENSUS
      GTGATCCCCCAAACTTCAGAAAATGAGGTTATGATTGATGGGGAAAGTGA

A3-AT5G47690-XLOC_028073-1737-0
      TGGAAATGAAATTCCTCTGGGTAAAATAGTAGAGCGTTTGAGAGCTCAGG

A3-AT5G47690-XLOC_028073-1737-1
TGGAAATGAAATTCCTCTGGGTAAAATAGTAGAGCGTTTGAGAGCTCAGG
CONSENSUS
TGGAAATGAAATTCCTCTGGGTAAAATAGTAGAGCGTTTGAGAGCTCAGG

A3-AT5G47690-XLOC_028073-1737-0
GAACCAAGACTAGAAAGGGCAAAAAGAATAAATCTGTACCAGCTGAAGAT
A3-AT5G47690-XLOC_028073-1737-1
GAACCAAGACTAGAAAGGGCAAAAAGAATAAATCTGTACCAGCTGAAGAT
CONSENSUS
GAACCAAGACTAGAAAGGGCAAAAAGAATAAATCTGTACCAGCTGAAGAT

A3-AT5G47690-XLOC_028073-1737-0
GAGAATGGTAAAAATGATGTCGATGTTTTGAAGATGGTGAGGGAGATAAA
A3-AT5G47690-XLOC_028073-1737-1
GAGAATGGTAAAAATGATGTCGATGTTTTGAAGATGGTGAGGGAGATAAA
CONSENSUS
GAGAATGGTAAAAATGATGTCGATGTTTTGAAGATGGTGAGGGAGATAAA

A3-AT5G47690-XLOC_028073-1737-0
TCTAGATCACTTACAAATGCTGGATAAATTCGAATCCAGTAATGGACACA
A3-AT5G47690-XLOC_028073-1737-1
TCTAGATCACTTACAAATGCTGGATAAATTCGAATCCAGTAATGGACACA
CONSENSUS
TCTAGATCACTTACAAATGCTGGATAAATTCGAATCCAGTAATGGACACA

A3-AT5G47690-XLOC_028073-1737-0
AACATTCCCCTAGTGAGAGAGCAGAGATATGTCAAAGAGATCAGAAGGGT
A3-AT5G47690-XLOC_028073-1737-1
AACATTCCCCTAGTGAGAGAGCAGAGATATGTCAAAGAGATCAGAAGGGT
CONSENSUS
AACATTCCCCTAGTGAGAGAGCAGAGATATGTCAAAGAGATCAGAAGGGT

A3-AT5G47690-XLOC_028073-1737-0
AACAAAAGGAATGTTGGTGATGCAACATCAGTAGTTTCAGTCCCTAAGCG
A3-AT5G47690-XLOC_028073-1737-1
AACAAAAGGAATGTTGGTGATGCAACATCAGTAGTTTCAGTCCCTAAGCG
CONSENSUS
AACAAAAGGAATGTTGGTGATGCAACATCAGTAGTTTCAGTCCCTAAGCG

A3-AT5G47690-XLOC_028073-1737-0
CCGGAGATCATCTTCTGGCCATAGTCCTTACAAGTTCTCAAACAGTGGTC
A3-AT5G47690-XLOC_028073-1737-1
CCGGAGATCATCTTCTGGCCATAGTCCTTACAAGTTCTCAAACAGTGGTC
CONSENSUS
CCGGAGATCATCTTCTGGCCATAGTCCTTACAAGTTCTCAAACAGTGGTC

A3-AT5G47690-XLOC_028073-1737-0
CTAAAGTTCAACTGAAAGCTTCTGAAGATGAGTTGCATCTAGAGAGTGAC
A3-AT5G47690-XLOC_028073-1737-1
CTAAAGTTCAACTGAAAGCTTCTGAAGATGAGTTGCATCTAGAGAGTGAC
CONSENSUS
CTAAAGTTCAACTGAAAGCTTCTGAAGATGAGTTGCATCTAGAGAGTGAC

A3-AT5G47690-XLOC_028073-1737-0
ATGGACAAAAATGTCTCTTTGGACTCGCACGATGAAAATTCTGACCAAGA

A3-AT5G47690-XLOC_028073-1737-1
ATGGACAAAAATGTCTCTTTGGACTCGCACGATGAAAATTCTGACCAAGA
CONSENSUS
ATGGACAAAAATGTCTCTTTGGACTCGCACGATGAAAATTCTGACCAAGA

A3-AT5G47690-XLOC_028073-1737-0
GAAAATGTTAGAAAGCATCTCTCCCCGGAAGAGAAAGAAAAGCTTATCAT
A3-AT5G47690-XLOC_028073-1737-1
GAAAATGTTAGAAAGCATCTCTCCCCGGAAGAGAAAGAAAAGCTTATCAT
CONSENSUS
GAAAATGTTAGAAAGCATCTCTCCCCGGAAGAGAAAGAAAAGCTTATCAT

A3-AT5G47690-XLOC_028073-1737-0
CAAAATTAAAGATTACAGAATCTGATTGGGCTCTCACCGATGTGGAGAGG
A3-AT5G47690-XLOC_028073-1737-1
CAAAATTAAAGATTACAGAATCTGATTGGGCTCTCACCGATGTGGAGAGG
CONSENSUS
CAAAATTAAAGATTACAGAATCTGATTGGGCTCTCACCGATGTGGAGAGG

A3-AT5G47690-XLOC_028073-1737-0
CAGAGTAGGTCAGCAGGAGGTGGTGATAGCAAATTAAAGTCTGCCTCTGG
A3-AT5G47690-XLOC_028073-1737-1 ---
AGTAGGTCAGCAGGAGGTGGTGATAGCAAATTAAAGTCTGCCTCTGG
CONSENSUS
...AGTAGGTCAGCAGGAGGTGGTGATAGCAAATTAAAGTCTGCCTCTGG

A3-AT5G47690-XLOC_028073-1737-0
ATCAATGAAAAAGCGAAAAAACGTGTCAGGACTGGCGAAG
A3-AT5G47690-XLOC_028073-1737-1
ATCAATGAAAAAGCGAAAAAACGTGTCAGGACTGGCGAAG
CONSENSUS
ATCAATGAAAAAGCGAAAAAACGTGTCAGGACTGGCGAAG

alignment for event: A3-AT5G19210-XLOC_026455-6086

A3-AT5G19210-XLOC_026455-6086-0
AATGGAGGAGATTGGATTTGTCTTCCCCACGGATATTCAAAGAGAAGCTC
A3-AT5G19210-XLOC_026455-6086-1
AATGGAGGAGATTGGATTTGTCTTCCCCACGGATATTCAAAGAGAAGCTC
CONSENSUS
AATGGAGGAGATTGGATTTGTCTTCCCCACGGATATTCAAAGAGAAGCTC

A3-AT5G19210-XLOC_026455-6086-0
TTCCTACTTTGTTTACAGGCCGTGATTGCATCCTCCATGCTCAAACAGGT
A3-AT5G19210-XLOC_026455-6086-1
TTCCTACTTTGTTTACAGGCCGTGATTGCATCCTCCATGCTCAA----GT
CONSENSUS
TTCCTACTTTGTTTACAGGCCGTGATTGCATCCTCCATGCTCAA....GT

A3-AT5G19210-XLOC_026455-6086-0
TCAGGAAAGACATTGACTTACCTCTTACTCATATTCTCTCTTATAAACCC
A3-AT5G19210-XLOC_026455-6086-1
TCAGGAAAGACATTGACTTACCTCTTACTCATATTCTCTCTTATAAACCC
CONSENSUS

TCAGGAAAGACATTGACTTACCTCTTACTCATATTCTCTCTTATAAACCC

A3-AT5G19210-XLOC_026455-6086-0
TCAACGATCTTCTGTGCAAGCTGTTATTGTTGTTCCCACTCGAGAGCTCG
A3-AT5G19210-XLOC_026455-6086-1
TCAACGATCTTCTGTGCAAGCTGTTATTGTTGTTCCCACTCGAGAGCTCG
CONSENSUS
TCAACGATCTTCTGTGCAAGCTGTTATTGTTGTTCCCACTCGAGAGCTCG

A3-AT5G19210-XLOC_026455-6086-0 GTATGCAA
A3-AT5G19210-XLOC_026455-6086-1 GTATGCAA
CONSENSUS GTATGCAA

alignment for event: A3-AT5G26742-XLOC_030556-3182

A3-AT5G26742-XLOC_030556-3182-0
TTACCTCCACTTCAAGATGACGGACCATCTAGTGATAACTACGGACGGTT
A3-AT5G26742-XLOC_030556-3182-1
TTACCTCCACTTCAAGATGACGGACCATCTAGTGATAACTACGGACGGTT
CONSENSUS
TTACCTCCACTTCAAGATGACGGACCATCTAGTGATAACTACGGACGGTT

A3-AT5G26742-XLOC_030556-3182-0
CTCTAGCAGAGACAGGATGCCTAGAGGAGGAGGAGGTTCTAGAGGGTCAA
A3-AT5G26742-XLOC_030556-3182-1
CTCTAGCAGAGACAGGATGCCTAGAGGAGGAGGAGGTTCTAGAGGGTCAA
CONSENSUS
CTCTAGCAGAGACAGGATGCCTAGAGGAGGAGGAGGTTCTAGAGGGTCAA

A3-AT5G26742-XLOC_030556-3182-0
GAGGCGGTAGAGGAGGATCATCACGAGGCCGTGATAGTTGGGGAGGTGAT
A3-AT5G26742-XLOC_030556-3182-1
GAGGCGGTAGAGGAGGATCATCACGAGGCCGTGATAGTTGGGGAGGTGAT
CONSENSUS
GAGGCGGTAGAGGAGGATCATCACGAGGCCGTGATAGTTGGGGAGGTGAT

A3-AT5G26742-XLOC_030556-3182-0
GATGACAGAGGTAGTAGAAGGAGCAGTGGTGGAGGAAGCAGCTGGTCCCG
A3-AT5G26742-XLOC_030556-3182-1
GATGACAGAGGTAGTAGAAGGAGCAGTGGTGGAGGAAGCAGCTGGTCCCG
CONSENSUS
GATGACAGAGGTAGTAGAAGGAGCAGTGGTGGAGGAAGCAGCTGGTCCCG

A3-AT5G26742-XLOC_030556-3182-0
AGGTGGTAGTAGTTCCAGAGGAAGTTCTGATGATTGGTTGATCGGTGGCA
A3-AT5G26742-XLOC_030556-3182-1
AGGTGGTAGTAGTTCCAGAGGAAGTTCTGATGATTGGTTGATCGGTGGCA
CONSENSUS
AGGTGGTAGTAGTTCCAGAGGAAGTTCTGATGATTGGTTGATCGGTGGCA

A3-AT5G26742-XLOC_030556-3182-0
GAAGTTCATCAAGCAGCAGAGCTCCTTCGCGGGAGAG---TTTTGGAGGT
A3-AT5G26742-XLOC_030556-3182-1
GAAGTTCATCAAGCAGCAGAGCTCCTTCGCGGGAGAGAAGTTTTGGAGGT

CONSENSUS
GAAGTTCATCAAGCAGCAGAGCTCCTTCGCGGGAGAG...TTTTGGAGGT

A3-AT5G26742-XLOC_030556-3182-0
TCATGCTTCATTTGTGGGAAATCTGGACACAGGGCAACAGATTGTCCTGA
A3-AT5G26742-XLOC_030556-3182-1
TCATGCTTCATTTGTGGGAAATCTGGACACAGGGCAACAGATTGTCCTGA
CONSENSUS
TCATGCTTCATTTGTGGGAAATCTGGACACAGGGCAACAGATTGTCCTGA

A3-AT5G26742-XLOC_030556-3182-0
TAAGAGAGGATTTTAGAGTTATCGTAGCAAATGGTTTCTGCTCTTGGGAG
A3-AT5G26742-XLOC_030556-3182-1
TAAGAGAGGATTTTAGAGTTATCGTAGCAAATGGTTTCTGCTCTTGGGAG
CONSENSUS
TAAGAGAGGATTTTAGAGTTATCGTAGCAAATGGTTTCTGCTCTTGGGAG

A3-AT5G26742-XLOC_030556-3182-0
AAAGATCCTTGTCCTTTTTGGTGTTCTTAATGCGAATCTTTTCGTGCTTG
A3-AT5G26742-XLOC_030556-3182-1
AAAGATCCTTGTCCTTTTTGGTGTTCTTAATGCGAATCTTTTCGTGCTTG
CONSENSUS
AAAGATCCTTGTCCTTTTTGGTGTTCTTAATGCGAATCTTTTCGTGCTTG

A3-AT5G26742-XLOC_030556-3182-0
CTTCAACGGTTTGTTTCAGACGGTAGAGAAGCAATCAGGGAGGAGGATAC
A3-AT5G26742-XLOC_030556-3182-1
CTTCAACGGTTTGTTTCAGACGGTAGAGAAGCAATCAGGGAGGAGGATAC
CONSENSUS
CTTCAACGGTTTGTTTCAGACGGTAGAGAAGCAATCAGGGAGGAGGATAC

A3-AT5G26742-XLOC_030556-3182-0
ATGCTTCGGACAAGGTAGAGCAGAAATAAATAAGAGAGAGGCAATAGCAA
A3-AT5G26742-XLOC_030556-3182-1
ATGCTTCGGACAAGGTAGAGCAGAAATAAATAAGAGAGAGGCAATAGCAA
CONSENSUS
ATGCTTCGGACAAGGTAGAGCAGAAATAAATAAGAGAGAGGCAATAGCAA

A3-AT5G26742-XLOC_030556-3182-0
AAGTAAAGGAGAAGGTTTTCCTCAAATTGGCATTTTCAGAGTTTGCGTGA
A3-AT5G26742-XLOC_030556-3182-1
AAGTAAAGGAGAAGGTTTTCCTCAAATTGGCATTTTCAGAGTTTGCGTGA
CONSENSUS
AAGTAAAGGAGAAGGTTTTCCTCAAATTGGCATTTTCAGAGTTTGCGTGA

A3-AT5G26742-XLOC_030556-3182-0
GAAACAAGGGAAAGCTGATGGATCTTTTTATGTTAGGGTTATTATAATAG
A3-AT5G26742-XLOC_030556-3182-1
GAAACAAGGGAAAGCTGATGGATCTTTTTATGTTAGGGTTATTATAATAG
CONSENSUS
GAAACAAGGGAAAGCTGATGGATCTTTTTATGTTAGGGTTATTATAATAG

A3-AT5G26742-XLOC_030556-3182-0
TCTTAATGATCAGTTTCCATGTTTCTAAGGTTTTGTTTGATTTAAGTGGT
A3-AT5G26742-XLOC_030556-3182-1
TCTTAATGATCAGTTTCCATGTTTCTAAGGTTTTGTTTGATTTAAGTGGT

CONSENSUS
      TCTTAATGATCAGTTTCCATGTTTCTAAGGTTTTGTTTGATTTAAGTGGT

A3-AT5G26742-XLOC_030556-3182-0
      TATTAGTCAGTCTACTTACACTACTTTTATTTTCATTTTTGTACGTTTTC
A3-AT5G26742-XLOC_030556-3182-1
      TATTAGTCAGTCTACTTACACTACTTTTATTTTCATTTTTGTACGTTTTC
CONSENSUS
      TATTAGTCAGTCTACTTACACTACTTTTATTTTCATTTTTGTACGTTTTC

A3-AT5G26742-XLOC_030556-3182-0
      TATTGTTATATGTGAATTCGGATCACATGTGTTTGGACAGAA
A3-AT5G26742-XLOC_030556-3182-1
      TATTGTTATATGTGAATTCGGATCACATGTGTTTGGACAGAA
CONSENSUS
      TATTGTTATATGTGAATTCGGATCACATGTGTTTGGACAGAA

alignment for event: A3-AT5G13530-XLOC_026146-1720

A3-AT5G13530-XLOC_026146-1720-0
      ACCGTTACATATGTGTGTAGCAACATGGAATGTTGCTGTTATCAAGAGAT
A3-AT5G13530-XLOC_026146-1720-1
      ACCGTTACATATGTGTGTAGCAACATGGAATGTTGCTGTTATCAAGAGAT
CONSENSUS
      ACCGTTACATATGTGTGTAGCAACATGGAATGTTGCTGTTATCAAGAGAT

A3-AT5G13530-XLOC_026146-1720-0
      GGGTGGAAGTTTCCAGTCCAGAAGAAATTTCTCAGGCAATTAATATCCCA
A3-AT5G13530-XLOC_026146-1720-1
      GGGTGGAAGTTTCCAGTCCAGAAGAAATTTCTCAGGCAATTAATATCCCA
CONSENSUS
      GGGTGGAAGTTTCCAGTCCAGAAGAAATTTCTCAGGCAATTAATATCCCA

A3-AT5G13530-XLOC_026146-1720-0
      AGCCCAGTTGGCACTGCTCTGTGTATGGCTGCTTCTATAAGGAAAGATCA
A3-AT5G13530-XLOC_026146-1720-1
      AGCCCAGTTGGCACTGCTCTGTGTATGGCTGCTTCTATAAGGAAAGATCA
CONSENSUS
      AGCCCAGTTGGCACTGCTCTGTGTATGGCTGCTTCTATAAGGAAAGATCA

A3-AT5G13530-XLOC_026146-1720-0
      TGAAAAGGAAGGTAGAGAGCTGGTTCAGATATTGCTTGCAGCTGGGGCAG
A3-AT5G13530-XLOC_026146-1720-1 TGAAAAGG---
GTAGAGAGCTGGTTCAGATATTGCTTGCAGCTGGGGCAG
CONSENSUS
      TGAAAAGG...GTAGAGAGCTGGTTCAGATATTGCTTGCAGCTGGGGCAG

A3-AT5G13530-XLOC_026146-1720-0
      ATCCGACTGCGCAAGATGCTCAGCACGGGCGAACAGCGTTGCATACTGCT
A3-AT5G13530-XLOC_026146-1720-1
      ATCCGACTGCGCAAGATGCTCAGCACGGGCGAACAGCGTTGCATACTGCT
CONSENSUS
      ATCCGACTGCGCAAGATGCTCAGCACGGGCGAACAGCGTTGCATACTGCT

A3-AT5G13530-XLOC_026146-1720-0 GCTATGGCTAACAATGTGGAACTTGTGAGA
A3-AT5G13530-XLOC_026146-1720-1 GCTATGGCTAACAATGTGGAACTTGTGAGA
CONSENSUS GCTATGGCTAACAATGTGGAACTTGTGAGA

alignment for event: RI-AT5G43920-XLOC_027844-1990

RI-AT5G43920-XLOC_027844-1990-0
AGGGATTAGCGATAGGACTTAACGATATAAACACATATGTATATGGTTTT
RI-AT5G43920-XLOC_027844-1990-1
AGGGATTAGCGATAGGACTTAACGATATAAACACATATGTATATGGTTTT
CONSENSUS
AGGGATTAGCGATAGGACTTAACGATATAAACACATATGTATATGGTTTT

RI-AT5G43920-XLOC_027844-1990-0
CGACCTCTCTTCTCCAAAACCATCACAGAGAGAGTCGCCGAAATAAATTG
RI-AT5G43920-XLOC_027844-1990-1
CGACCTCTCTTCTCCAAAACCATCACAGAGAGAGTCGCCGAAATAAATTG
CONSENSUS
CGACCTCTCTTCTCCAAAACCATCACAGAGAGAGTCGCCGAAATAAATTG

RI-AT5G43920-XLOC_027844-1990-0
AAATCCGGTGAGAAGATTTTGGATTCACTCCTTCCTTCTTTCTTCTTCTT
RI-AT5G43920-XLOC_027844-1990-1
AAATCCG---
CONSENSUS
AAATCCG...

RI-AT5G43920-XLOC_027844-1990-0
TATCATCATTTGAGCTGGTTTTGGTATCTCTCCTGACGAATTTGAATCTC
RI-AT5G43920-XLOC_027844-1990-1

CONSENSUS
....

RI-AT5G43920-XLOC_027844-1990-0
TCAGCCATTTTCTCTTCTCGCATCTGTGTTTTCGTGCTTGGAGCTGTACA
RI-AT5G43920-XLOC_027844-1990-1

CONSENSUS
....

RI-AT5G43920-XLOC_027844-1990-0
CGTTTCTTTAATTTGAATAGTGATTTTGCGTTCGTTAATTAGCCTTCTCG
RI-AT5G43920-XLOC_027844-1990-1

CONSENSUS
....

RI-AT5G43920-XLOC_027844-1990-0
TACGAGTGGGCTATTTTGGGAATGATTTAGGTGGTTTTAAGTTACTGGGT
RI-AT5G43920-XLOC_027844-1990-1

CONSENSUS
....

RI-AT5G43920-XLOC_027844-1990-0
AGTAGTCACTGCTGAGCGATTTCTGTTTCTCCTGTTGAATGATTGATTCA
RI-AT5G43920-XLOC_027844-1990-1

CONSENSUS
....

RI-AT5G43920-XLOC_027844-1990-0
GAAATTTGAAATTCACATCGAAACCAAATCTTAAATCATATTGGTAGCTT
RI-AT5G43920-XLOC_027844-1990-1

CONSENSUS
....

RI-AT5G43920-XLOC_027844-1990-0
TTTTTTTGTTTTGTGATTAGATACTGTTTTTTTATAGTATTAAATTCGTT
RI-AT5G43920-XLOC_027844-1990-1

CONSENSUS
....

RI-AT5G43920-XLOC_027844-1990-0
TTGGTGTGAGTGATTTAGTGTTTGGGAAATTATTTAATTTAGTGGTTATT
RI-AT5G43920-XLOC_027844-1990-1

CONSENSUS
....

RI-AT5G43920-XLOC_027844-1990-0
TTCTGATTTAGTGATGATTTGAGAATTTTGTGTTGTTTATATTCTTGGTA
RI-AT5G43920-XLOC_027844-1990-1

CONSENSUS
....

RI-AT5G43920-XLOC_027844-1990-0
GCGGGAAATTTGTACTTTCCTTTGTGGACCTTTCAACTATTGAATGTGGA
RI-AT5G43920-XLOC_027844-1990-1 -
CGGGAAATTTGTACTTTCCTTTGTGGACCTTTCAACTATTGAATGTGGA
CONSENSUS
.CGGGAAATTTGTACTTTCCTTTGTGGACCTTTCAACTATTGAATGTGGA

RI-AT5G43920-XLOC_027844-1990-0
AGATTTGCTGAGAATCTTAGAAAGTCATCATCATCCATACGTAAGAGAAC
RI-AT5G43920-XLOC_027844-1990-1
AGATTTGCTGAGAATCTTAGAAAGTCATCATCATCCATACGTAAGAGAAC
CONSENSUS
AGATTTGCTGAGAATCTTAGAAAGTCATCATCATCCATACGTAAGAGAAC

RI-AT5G43920-XLOC_027844-1990-0
TTTTTTTTAGCTTGAGAGTGATTTTCAGATGGAGAATGGGTTATGGGAGG
RI-AT5G43920-XLOC_027844-1990-1
TTTTTTTTAGCTTGAGAGTGATTTTCAGATGGAGAATGGGTTATGGGAGG
CONSENSUS
TTTTTTTTAGCTTGAGAGTGATTTTCAGATGGAGAATGGGTTATGGGAGG

RI-AT5G43920-XLOC_027844-1990-0
TTTTAGGTTCAAAAGGGTTGTTGAAGAAACATGAATTCATTAGGATTTTG
RI-AT5G43920-XLOC_027844-1990-1
TTTTAGGTTCAAAAGGGTTGTTGAAGAAACATGAATTCATTAGGATTTTG
CONSENSUS
TTTTAGGTTCAAAAGGGTTGTTGAAGAAACATGAATTCATTAGGATTTTG

RI-AT5G43920-XLOC_027844-1990-0
GTTCAATGCTTATACTCGTTAGGATTCAAGAACTCTGCTTCTTGTCTGGA
RI-AT5G43920-XLOC_027844-1990-1
GTTCAATGCTTATACTCGTTAGGATTCAAGAACTCTGCTTCTTGTCTGGA
CONSENSUS
GTTCAATGCTTATACTCGTTAGGATTCAAGAACTCTGCTTCTTGTCTGGA

RI-AT5G43920-XLOC_027844-1990-0
ATTCGAGTCAAAGATCTTGTATAAAACAGCTGATTCTGAGTTTCTTGAAA
RI-AT5G43920-XLOC_027844-1990-1
ATTCGAGTCAAAGATCTTGTATAAAACAGCTGATTCTGAGTTTCTTGAAA
CONSENSUS
ATTCGAGTCAAAGATCTTGTATAAAACAGCTGATTCTGAGTTTCTTGAAA

RI-AT5G43920-XLOC_027844-1990-0
AGCAAGTTTTGAGTGGGAACTGGGATAGTTGCGTACAGGTTCTAGACAGA
RI-AT5G43920-XLOC_027844-1990-1
AGCAAGTTTTGAGTGGGAACTGGGATAGTTGCGTACAGGTTCTAGACAGA
CONSENSUS
AGCAAGTTTTGAGTGGGAACTGGGATAGTTGCGTACAGGTTCTAGACAGA

RI-AT5G43920-XLOC_027844-1990-0
ATTTTTGATAATTCCATGGATGATACGAGGAACACGGCTTTATATCTAGT
RI-AT5G43920-XLOC_027844-1990-1
ATTTTTGATAATTCCATGGATGATACGAGGAACACGGCTTTATATCTAGT
CONSENSUS
ATTTTTGATAATTCCATGGATGATACGAGGAACACGGCTTTATATCTAGT

RI-AT5G43920-XLOC_027844-1990-0
GTTCAAGCAATGTTTGTTGGAGTATTTGAAACGTGGGGATGTTTCTTTGG
RI-AT5G43920-XLOC_027844-1990-1
GTTCAAGCAATGTTTGTTGGAGTATTTGAAACGTGGGGATGTTTCTTTGG
CONSENSUS
GTTCAAGCAATGTTTGTTGGAGTATTTGAAACGTGGGGATGTTTCTTTGG

RI-AT5G43920-XLOC_027844-1990-0
CCTTGAATGTGTTACGGAAGCAAGCTCCGTTGTTACGGATGGGAAAAGAG
RI-AT5G43920-XLOC_027844-1990-1
CCTTGAATGTGTTACGGAAGCAAGCTCCGTTGTTACGGATGGGAAAAGAG
CONSENSUS
CCTTGAATGTGTTACGGAAGCAAGCTCCGTTGTTACGGATGGGAAAAGAG

RI-AT5G43920-XLOC_027844-1990-0
AAGATTCATAGGCTTGCTTGTGATATTGTTTATTCGAAAGAGATGGAATC
RI-AT5G43920-XLOC_027844-1990-1
AAGATTCATAGGCTTGCTTGTGATATTGTTTATTCGAAAGAGATGGAATC
CONSENSUS
AAGATTCATAGGCTTGCTTGTGATATTGTTTATTCGAAAGAGATGGAATC

RI-AT5G43920-XLOC_027844-1990-0
CGGTGAAGTAGACAACTGTTTAGTTCTAGATTTGAGGAGAAAGTTGTTGG
RI-AT5G43920-XLOC_027844-1990-1
CGGTGAAGTAGACAACTGTTTAGTTCTAGATTTGAGGAGAAAGTTGTTGG
CONSENSUS
CGGTGAAGTAGACAACTGTTTAGTTCTAGATTTGAGGAGAAAGTTGTTGG

RI-AT5G43920-XLOC_027844-1990-0
TTGAACTGGAGAAGTTGATTCCCTTGCCAATTGTTATTCCTGAGAGAAGG
RI-AT5G43920-XLOC_027844-1990-1
TTGAACTGGAGAAGTTGATTCCCTTGCCAATTGTTATTCCTGAGAGAAGG
CONSENSUS
TTGAACTGGAGAAGTTGATTCCCTTGCCAATTGTTATTCCTGAGAGAAGG

RI-AT5G43920-XLOC_027844-1990-0
TTGGAACATTTGGTTGAGACTGCTGTGATGGACCAGATTGATACGTGTAT
RI-AT5G43920-XLOC_027844-1990-1
TTGGAACATTTGGTTGAGACTGCTGTGATGGACCAGATTGATACGTGTAT
CONSENSUS
TTGGAACATTTGGTTGAGACTGCTGTGATGGACCAGATTGATACGTGTAT

RI-AT5G43920-XLOC_027844-1990-0
GTATCATAACTCATGTGATGCAGTATCGCTTTACAAGGATCACTGTTGCG
RI-AT5G43920-XLOC_027844-1990-1
GTATCATAACTCATGTGATGCAGTATCGCTTTACAAGGATCACTGTTGCG
CONSENSUS
GTATCATAACTCATGTGATGCAGTATCGCTTTACAAGGATCACTGTTGCG

RI-AT5G43920-XLOC_027844-1990-0
GTAGAGACCAAATTCCTTCAGAGACAGTCCAG
RI-AT5G43920-XLOC_027844-1990-1
GTAGAGACCAAATTCCTTCAGAGACAGTCCAG
CONSENSUS
GTAGAGACCAAATTCCTTCAGAGACAGTCCAG

alignment for event: A3-AT1G34418-XLOC_001897-1625

A3-AT1G34418-XLOC_001897-1625-0
AGTCTCAATTTTTTGGATCTGCGCTGCCTCTTACGAAACTCAAATAGAGA
A3-AT1G34418-XLOC_001897-1625-1
AGTCTCAATTTTTTGGATCTGCGCTGCCTCTTACGAAACTCAAATAGAGA
CONSENSUS
AGTCTCAATTTTTTGGATCTGCGCTGCCTCTTACGAAACTCAAATAGAGA

A3-AT1G34418-XLOC_001897-1625-0
GTTCTTCTTCTTCCTTTACCAAATCCATAATCAGTCCATAACCAGCCTAA
A3-AT1G34418-XLOC_001897-1625-1
GTTCTTCTTCTTCCTTTACCAAATCCATAATCAGTCCATAACCAGCCTAA
CONSENSUS
GTTCTTCTTCTTCCTTTACCAAATCCATAATCAGTCCATAACCAGCCTAA

A3-AT1G34418-XLOC_001897-1625-0
CGTTTCTTCTCCATCATGAATTCATCATCTTCAACTTGCTCTTTCATTCT

A3-AT1G34418-XLOC_001897-1625-1
      CGTTTCTTCTCCATCATGAATTCATCATCTTCAACTTGCTCTTTCATTCT
CONSENSUS
      CGTTTCTTCTCCATCATGAATTCATCATCTTCAACTTGCTCTTTCATTCT

A3-AT1G34418-XLOC_001897-1625-0
      CTCAAATCTCAATCTTTGTTAGCTCTTCTCTATTCTCGTTAATCGTATCT
A3-AT1G34418-XLOC_001897-1625-1
      CTCAAATCTCAATCTTTGTTAGCTCTTCTCTATTCTCGTTAATCGTATCT
CONSENSUS
      CTCAAATCTCAATCTTTGTTAGCTCTTCTCTATTCTCGTTAATCGTATCT

A3-AT1G34418-XLOC_001897-1625-0
      TCGTCTGATTCTTTGATGATTGCTCGCTAAGCTTTCCTTATCACCAAAAT
A3-AT1G34418-XLOC_001897-1625-1
      TCGTCTGATTCTTTGATGATTGCTCGCTAAGCTTTCCTTATCACCAAAAT
CONSENSUS
      TCGTCTGATTCTTTGATGATTGCTCGCTAAGCTTTCCTTATCACCAAAAT

A3-AT1G34418-XLOC_001897-1625-0
      CATGGATTCATTGATTGCATGTGGTTATGATTATGATCTCGTTGGTGGTA
A3-AT1G34418-XLOC_001897-1625-1
      CATGGATTCATTG-------------------------------------
CONSENSUS
      CATGGATTCATTG.....................................

A3-AT1G34418-XLOC_001897-1625-0
      GTTTTTTTTGAGGTTAGAGAGATTGGCCGTCGCCGTCCGTCGCTGCCTCA
A3-AT1G34418-XLOC_001897-1625-1 -
TTTTTTTTGAGGTTAGAGAGATTGGCCGTCGCCGTCCGTCGCTGCCTCA
CONSENSUS
      .TTTTTTTTGAGGTTAGAGAGATTGGCCGTCGCCGTCCGTCGCTGCCTCA

A3-AT1G34418-XLOC_001897-1625-0
      CCGTCAACAAATGTATCGAAAACCCTAATTGTTAATGGGTTTGGGCTTTG
A3-AT1G34418-XLOC_001897-1625-1
      CCGTCAACAAATGTATCGAAAACCCTAATTGTTAATGGGTTTGGGCTTTG
CONSENSUS
      CCGTCAACAAATGTATCGAAAACCCTAATTGTTAATGGGTTTGGGCTTTG

A3-AT1G34418-XLOC_001897-1625-0
      TAAACTTTTTATTGGGTATGTAAATGTTAATGGGTTTGGGCTTTGTAAAT
A3-AT1G34418-XLOC_001897-1625-1
      TAAACTTTTTATTGGGTATGTAAATGTTAATGGGTTTGGGCTTTGTAAAT
CONSENSUS
      TAAACTTTTTATTGGGTATGTAAATGTTAATGGGTTTGGGCTTTGTAAAT

A3-AT1G34418-XLOC_001897-1625-0
      GTTTTGTTGGGTATGTAAATGCAGTTTAATAATAATGAGTTTGGGCTTTA
A3-AT1G34418-XLOC_001897-1625-1
      GTTTTGTTGGGTATGTAAATGCAGTTTAATAATAATGAGTTTGGGCTTTA
CONSENSUS
      GTTTTGTTGGGTATGTAAATGCAGTTTAATAATAATGAGTTTGGGCTTTA

A3-AT1G34418-XLOC_001897-1625-0 TTTAAGCCTATTATCC
A3-AT1G34418-XLOC_001897-1625-1 TTTAAGCCTATTATCC

CONSENSUS   TTTAAGCCTATTATCC

alignment for event: RI-AT1G73320-XLOC_007901-6586

RI-AT1G73320-XLOC_007901-6586-0
TTGTATAGCAGCACTTTTGGGAGGCAATGCTGTCCTCACTGATCTTCCAG
RI-AT1G73320-XLOC_007901-6586-1
TTGTATAGCAGCACTTTTGGGAGGCAATGCTGTCCTCACTGATCTTCCAG
CONSENSUS
TTGTATAGCAGCACTTTTGGGAGGCAATGCTGTCCTCACTGATCTTCCAG

RI-AT1G73320-XLOC_007901-6586-0
ATAGACTGAGGCTACTCAAAAAGAACATCCAAACCAATTTGCACCGTGGG
RI-AT1G73320-XLOC_007901-6586-1
ATAGACTGAGGCTACTCAAAAAGAACATCCAAACCAATTTGCACCGTGGG
CONSENSUS
ATAGACTGAGGCTACTCAAAAAGAACATCCAAACCAATTTGCACCGTGGG

RI-AT1G73320-XLOC_007901-6586-0
AACACGCGTGGATCTGCTATTGTGCAGGAACTTGTTTGGGGAGATGACCC
RI-AT1G73320-XLOC_007901-6586-1
AACACGCGTGGATCTGCTATTGTGCAGGAACTTGTTTGGGGAGATGACCC
CONSENSUS
AACACGCGTGGATCTGCTATTGTGCAGGAACTTGTTTGGGGAGATGACCC

RI-AT1G73320-XLOC_007901-6586-0
TGATCCAGATTTGATCGAACCATTCCCTGATTACGGTAATCATCTCGGTT
RI-AT1G73320-XLOC_007901-6586-1
TGATCCAGATTTGATCGAACCATTCCCTGATTACG---------------
CONSENSUS
TGATCCAGATTTGATCGAACCATTCCCTGATTACG...............

RI-AT1G73320-XLOC_007901-6586-0
TCATTTTTTCTCCTTTTGCCTTGTACATACCATTCTCACACCTTTTATTT
RI-AT1G73320-XLOC_007901-6586-1

CONSENSUS
....

RI-AT1G73320-XLOC_007901-6586-0
GCTATGTACTTGTAGTATTAGGCTCAGATGTTATATACAGCGAAGAAGCT
RI-AT1G73320-XLOC_007901-6586-1 ---------------
TATTAGGCTCAGATGTTATATACAGCGAAGAAGCT
CONSENSUS
...............TATTAGGCTCAGATGTTATATACAGCGAAGAAGCT

RI-AT1G73320-XLOC_007901-6586-0
GTTCACCATTTGGTAAAAACGCTTTTGCAACTTTGCAGCGATCAAACTAC
RI-AT1G73320-XLOC_007901-6586-1
GTTCACCATTTGGTAAAAACGCTTTTGCAACTTTGCAGCGATCAAACTAC
CONSENSUS
GTTCACCATTTGGTAAAAACGCTTTTGCAACTTTGCAGCGATCAAACTAC

RI-AT1G73320-XLOC_007901-6586-0 AATCTTCCTATCAGGAGAACTACGAAATG

RI-AT1G73320-XLOC_007901-6586-1 AATCTTCCTATCAGGAGAACTACGAAATG
CONSENSUS   AATCTTCCTATCAGGAGAACTACGAAATG

alignment for event: A3-AT1G77810-XLOC_008153-5184

A3-AT1G77810-XLOC_008153-5184-0
AAAGCTACACAAGAAAAAGATGTAACTGGCGAAGTTTTGAGAACTCATGA
A3-AT1G77810-XLOC_008153-5184-1
AAAGCTACACAAGAAAAAGATGTAACTGGCGAAGTTTTGAGAACTCATGA
CONSENSUS
AAAGCTACACAAGAAAAAGATGTAACTGGCGAAGTTTTGAGAACTCATGA

A3-AT1G77810-XLOC_008153-5184-0 AGCTATACA---------
GTCGTTGGATAAGTCAGTTTCTACACTTTCTT
A3-AT1G77810-XLOC_008153-5184-1
AGCTATACAGGATGACAGGTCGTTGGATAAGTCAGTTTCTACACTTTCTT
CONSENSUS
AGCTATACA.........GTCGTTGGATAAGTCAGTTTCTACACTTTCTT

A3-AT1G77810-XLOC_008153-5184-0
CTACAAGGAGCTCTCAAGAGATGGTTGATGGCTCGGAAACAAACCCGAGG
A3-AT1G77810-XLOC_008153-5184-1
CTACAAGGAGCTCTCAAGAGATGGTTGATGGCTCGGAAACAAACCCGAGG
CONSENSUS
CTACAAGGAGCTCTCAAGAGATGGTTGATGGCTCGGAAACAAACCCGAGG

A3-AT1G77810-XLOC_008153-5184-0
AAGAAAGTGTTTATGGTAATGGGAATCAATACCGCATTTAGCAGTAGAAA
A3-AT1G77810-XLOC_008153-5184-1
AAGAAAGTGTTTATGGTAATGGGAATCAATACCGCATTTAGCAGTAGAAA
CONSENSUS
AAGAAAGTGTTTATGGTAATGGGAATCAATACCGCATTTAGCAGTAGAAA

A3-AT1G77810-XLOC_008153-5184-0
ACGGCGTGATTCAGTTAGAGAAACATGGATGCCTCAAG
A3-AT1G77810-XLOC_008153-5184-1
ACGGCGTGATTCAGTTAGAGAAACATGGATGCCTCAAG
CONSENSUS
ACGGCGTGATTCAGTTAGAGAAACATGGATGCCTCAAG

alignment for event: RI-AT1G77080-XLOC_004046-1247

RI-AT1G77080-XLOC_004046-1247-0
GATCTTGAAGAAAAAATTCAGAATTATCTTCCACACAAGGAGTTACTAGA
RI-AT1G77080-XLOC_004046-1247-1
GATCTTGAAGAAAAAATTCAGAATTATCTTCCACACAAGGAGTTACTAGA
CONSENSUS
GATCTTGAAGAAAAAATTCAGAATTATCTTCCACACAAGGAGTTACTAGA

RI-AT1G77080-XLOC_004046-1247-0
AACAGTCCAAAGGTTAGCAGTACGACACATTTTTCTCCCCTCTTCTTCTG
RI-AT1G77080-XLOC_004046-1247-1

AACAGTCCAAAG------------------------------------
CONSENSUS
AACAGTCCAAAG....................................

RI-AT1G77080-XLOC_004046-1247-0
ATAAAAAAAATGTTTTTTTTCTTTTGTCTACTTGTGAATACAGCAAGCTT
RI-AT1G77080-XLOC_004046-1247-1
---CAAGCTT
CONSENSUS
....CAAGCTT

RI-AT1G77080-XLOC_004046-1247-0
GAAGAACCAAATGTCGATAATGTAAGTGTAGATTCTCTAATTTCTCTGGA
RI-AT1G77080-XLOC_004046-1247-1
GAAGAACCAAATGTCGATAATGTAAGTGTAGATTCTCTAATTTCTCTGGA
CONSENSUS
GAAGAACCAAATGTCGATAATGTAAGTGTAGATTCTCTAATTTCTCTGGA

RI-AT1G77080-XLOC_004046-1247-0
GGAACAACTTGAGACTGCTCTGTCCGTAAGTAGAGCTAGGAAG
RI-AT1G77080-XLOC_004046-1247-1
GGAACAACTTGAGACTGCTCTGTCCGTAAGTAGAGCTAGGAAG
CONSENSUS
GGAACAACTTGAGACTGCTCTGTCCGTAAGTAGAGCTAGGAAG

alignment for event: RI-AT1G02960-XLOC_004358-4362

RI-AT1G02960-XLOC_004358-4362-0
TCATGTCAAGACACTTGGAGGGAAAGCTTGTCGTCTGCAAATGCTCTGTC
RI-AT1G02960-XLOC_004358-4362-1
TCATGTCAAGACACTTGGAGGGAAAGCTTGTCGTCTGCAAATGCTCTGTC
CONSENSUS
TCATGTCAAGACACTTGGAGGGAAAGCTTGTCGTCTGCAAATGCTCTGTC

RI-AT1G02960-XLOC_004358-4362-0
ATTGACCTCTCTGGGGTTTATCTCTCCATCTCTTTGACGCATCTGATAAA
RI-AT1G02960-XLOC_004358-4362-1
ATTGACCTCTCTGGG-----------------------------------
CONSENSUS
ATTGACCTCTCTGGG...................................

RI-AT1G02960-XLOC_004358-4362-0
CCTGCTCCTTTAATTAGACCCTTAAGCAATTGATTTAGTCACATTCGATT
RI-AT1G02960-XLOC_004358-4362-1

CONSENSUS
....

RI-AT1G02960-XLOC_004358-4362-0
TTGTAGGACTCCCTATTACTCAAGAACACACAAGCTCTTGCGGCAAAAGA
RI-AT1G02960-XLOC_004358-4362-1 ------
GACTCCCTATTACTCAAGAACACACAAGCTCTTGCGGCAAAAGA
CONSENSUS
......GACTCCCTATTACTCAAGAACACACAAGCTCTTGCGGCAAAAGA

RI-AT1G02960-XLOC_004358-4362-0
      AACAGAAACAACAATTATTTTTAGTCCGAAAGTTTGTGCGGATGTTGATA
RI-AT1G02960-XLOC_004358-4362-1
      AACAGAAACAACAATTATTTTTAGTCCGAAAGTTTGTGCGGATGTTGATA
CONSENSUS
      AACAGAAACAACAATTATTTTTAGTCCGAAAGTTTGTGCGGATGTTGATA

RI-AT1G02960-XLOC_004358-4362-0
      TCGAAATAGGGAACTTTATCCGAGTATATGCGCCATG
RI-AT1G02960-XLOC_004358-4362-1
      TCGAAATAGGGAACTTTATCCGAGTATATGCGCCATG
CONSENSUS
      TCGAAATAGGGAACTTTATCCGAGTATATGCGCCATG

alignment for event: A5-AT1G19080-XLOC_001019-4800

A5-AT1G19080-XLOC_001019-4800-0
      AATTTGATTGGGGTAATTTGGTCAAAGGATGGATAGGATCAATGGTCTAT
A5-AT1G19080-XLOC_001019-4800-1
      AATTTGATTGGGGTAATTTGGTCAAAGGATGGATAGGATCAATGGTCTAT
CONSENSUS
      AATTTGATTGGGGTAATTTGGTCAAAGGATGGATAGGATCAATGGTCTAT

A5-AT1G19080-XLOC_001019-4800-0
      CAGATGAGATAATCTGCCACATTCTATCTTTCCTTCCGACAAAAGAGTCT
A5-AT1G19080-XLOC_001019-4800-1
      CAGATGAGATAATCTGCCACATTCTATCTTTCCTTCCGACAAAAGAGTCT
CONSENSUS
      CAGATGAGATAATCTGCCACATTCTATCTTTCCTTCCGACAAAAGAGTCT

A5-AT1G19080-XLOC_001019-4800-0
      GCTTTAACATCTGTCCTGTCAAAACGATGGC-------------------
A5-AT1G19080-XLOC_001019-4800-1
      GCTTTAACATCTGTCCTGTCAAAACGATGGCGTAATCTGTTTGCCTTGAC
CONSENSUS
      GCTTTAACATCTGTCCTGTCAAAACGATGGC...................

A5-AT1G19080-XLOC_001019-4800-0

A5-AT1G19080-XLOC_001019-4800-1
      ACCGAATCTTCATTTAGTTGATGATGATCTAGAAGTGGGTGGTTGTGGAC
CONSENSUS
      ....

A5-AT1G19080-XLOC_001019-4800-0

A5-AT1G19080-XLOC_001019-4800-1
      AAACCCTCATCGATTTTGTGGATAGAGTATTGGCTGTGTCAAGCAATTTG
CONSENSUS
      ....

A5-AT1G19080-XLOC_001019-4800-0

A5-AT1G19080-XLOC_001019-4800-1
CCCATTAGGGAGTTTTCAATAAAATGTTGAACACAATTTGAATTTTCATA
CONSENSUS
....

A5-AT1G19080-XLOC_001019-4800-0 --
ACCGCGAATTTAATCTCAAGGTTAAGGTGGTGTGGAATCAGCCTTAGG
A5-AT1G19080-XLOC_001019-4800-1
GGACCGCGAATTTAATCTCAAGGTTAAGGTGGTGTGGAATCAGCCTTAGG
CONSENSUS
..ACCGCGAATTTAATCTCAAGGTTAAGGTGGTGTGGAATCAGCCTTAGG

A5-AT1G19080-XLOC_001019-4800-0
GGATGGCGAAGTACTTTGACATTGATGACATACTTATAGAGGAAGAG
A5-AT1G19080-XLOC_001019-4800-1
GGATGGCGAAGTACTTTGACATTGATGACATACTTATAGAGGAAGAG
CONSENSUS
GGATGGCGAAGTACTTTGACATTGATGACATACTTATAGAGGAAGAG

alignment for event: A3-AT1G80490-XLOC_008302-4823

A3-AT1G80490-XLOC_008302-4823-0
CCTTTTCAACCAACACCTTCTCCGGTTCCGACACCTCTTGCTGGTTGGAT
A3-AT1G80490-XLOC_008302-4823-1
CCTTTTCAACCAACACCTTCTCCGGTTCCGACACCTCTTGCTGGTTGGAT
CONSENSUS
CCTTTTCAACCAACACCTTCTCCGGTTCCGACACCTCTTGCTGGTTGGAT

A3-AT1G80490-XLOC_008302-4823-0
GTCTAGTCCTTCCTCTGTCCCACATCCAGCTGTGTCTGGAGGACCCATTG
A3-AT1G80490-XLOC_008302-4823-1
GTCTAGTCCTTCCTCTGTCCCACATCCAGCTGTGTCTGGAGGACCCATTG
CONSENSUS
GTCTAGTCCTTCCTCTGTCCCACATCCAGCTGTGTCTGGAGGACCCATTG

A3-AT1G80490-XLOC_008302-4823-0 CTCTTGGTGCTCCATCCATCCAAG---
CCTTGAAACACCCGAGAACTCCT
A3-AT1G80490-XLOC_008302-4823-1
CTCTTGGTGCTCCATCCATCCAAGCAGCCTTGAAACACCCGAGAACTCCT
CONSENSUS
CTCTTGGTGCTCCATCCATCCAAG...CCTTGAAACACCCGAGAACTCCT

A3-AT1G80490-XLOC_008302-4823-0
CCTTCTAATTCCGCTGTAGACTATCCATCAGGTGACTCAGACCATGTCTC
A3-AT1G80490-XLOC_008302-4823-1
CCTTCTAATTCCGCTGTAGACTATCCATCAGGTGACTCAGACCATGTCTC
CONSENSUS
CCTTCTAATTCCGCTGTAGACTATCCATCAGGTGACTCAGACCATGTCTC

A3-AT1G80490-XLOC_008302-4823-0
AAAGCGAACCAGACCTATGGGAATCTCTGACGAG
A3-AT1G80490-XLOC_008302-4823-1
AAAGCGAACCAGACCTATGGGAATCTCTGACGAG
CONSENSUS

AAAGCGAACCAGACCTATGGGAATCTCTGACGAG

alignment for event: A5-AT1G08610-XLOC_004694-5966

A5-AT1G08610-XLOC_004694-5966-0
CATCTCAAAACCCGCGTCGGCTTCGAACAACGAACAGTGAGTTTCTCACA
A5-AT1G08610-XLOC_004694-5966-1
CATCTCAAAACCCGCGTCGGCTTCGAACAACGAACA--------------
CONSENSUS
CATCTCAAAACCCGCGTCGGCTTCGAACAACGAACA..............

A5-AT1G08610-XLOC_004694-5966-0
CTCATCATTTCTCCAGATTTGTTAGGGTTTCTCAAACTTAACCGCAACTT
A5-AT1G08610-XLOC_004694-5966-1

CONSENSUS
....

A5-AT1G08610-XLOC_004694-5966-0
GAAGGTTTGGTTTCTCCATTCACTCAAGAATAGGTTATGAGATAAAGCAG
A5-AT1G08610-XLOC_004694-5966-1 ----
GTTTGGTTTCTCCATTCACTCAAGAATAGGTTATGAGATAAAGCAG
CONSENSUS
....GTTTGGTTTCTCCATTCACTCAAGAATAGGTTATGAGATAAAGCAG

A5-AT1G08610-XLOC_004694-5966-0
CAGAGACTGAACATCATACCAGCAAATAATTATTGGTCTAACTTCTTCTT
A5-AT1G08610-XLOC_004694-5966-1
CAGAGACTGAACATCATACCAGCAAATAATTATTGGTCTAACTTCTTCTT
CONSENSUS
CAGAGACTGAACATCATACCAGCAAATAATTATTGGTCTAACTTCTTCTT

A5-AT1G08610-XLOC_004694-5966-0
CATTGAGTTTGATAAGCTTTTGTAAAAGGAAAGATCTCTGTTTTGTTAAA
A5-AT1G08610-XLOC_004694-5966-1
CATTGAGTTTGATAAGCTTTTGTAAAAGGAAAGATCTCTGTTTTGTTAAA
CONSENSUS
CATTGAGTTTGATAAGCTTTTGTAAAAGGAAAGATCTCTGTTTTGTTAAA

A5-AT1G08610-XLOC_004694-5966-0
ATAGCTTCTGGATTCTGAGAAATGGCTTCGTCGAATTTGCTACTTGAGGT
A5-AT1G08610-XLOC_004694-5966-1
ATAGCTTCTGGATTCTGAGAAATGGCTTCGTCGAATTTGCTACTTGAGGT
CONSENSUS
ATAGCTTCTGGATTCTGAGAAATGGCTTCGTCGAATTTGCTACTTGAGGT

A5-AT1G08610-XLOC_004694-5966-0
TCAGTTCTTGCATATTCCTAGTCTGTCTCAAAAGCAGGCGAATCCTTTAT
A5-AT1G08610-XLOC_004694-5966-1
TCAGTTCTTGCATATTCCTAGTCTGTCTCAAAAGCAGGCGAATCCTTTAT
CONSENSUS
TCAGTTCTTGCATATTCCTAGTCTGTCTCAAAAGCAGGCGAATCCTTTAT

A5-AT1G08610-XLOC_004694-5966-0

CTTGCAGAAAGTTTTCTTCTTTAGATTGGAAGCAAGAAATTGGGCTGAAA
A5-AT1G08610-XLOC_004694-5966-1
CTTGCAGAAAGTTTTCTTCTTTAGATTGGAAGCAAGAAATTGGGCTGAAA
CONSENSUS
CTTGCAGAAAGTTTTCTTCTTTAGATTGGAAGCAAGAAATTGGGCTGAAA

A5-AT1G08610-XLOC_004694-5966-0
AAGGATGTTTTTTTTAGATGTCACGGCTTGTTAAGTAGTGTGTGCATTGA
A5-AT1G08610-XLOC_004694-5966-1
AAGGATGTTTTTTTTAGATGTCACGGCTTGTTAAGTAGTGTGTGCATTGA
CONSENSUS
AAGGATGTTTTTTTTAGATGTCACGGCTTGTTAAGTAGTGTGTGCATTGA

A5-AT1G08610-XLOC_004694-5966-0
CAATGTAAATGATCATGCTGAGAGAAGCTCAGAGTTCCATCACTATGGTG
A5-AT1G08610-XLOC_004694-5966-1
CAATGTAAATGATCATGCTGAGAGAAGCTCAGAGTTCCATCACTATGGTG
CONSENSUS
CAATGTAAATGATCATGCTGAGAGAAGCTCAGAGTTCCATCACTATGGTG

A5-AT1G08610-XLOC_004694-5966-0
TCGGTACTAACTTGAGAGCCAGAGTGAAACCTATGAAGCAATTTGGTTTA
A5-AT1G08610-XLOC_004694-5966-1
TCGGTACTAACTTGAGAGCCAGAGTGAAACCTATGAAGCAATTTGGTTTA
CONSENSUS
TCGGTACTAACTTGAGAGCCAGAGTGAAACCTATGAAGCAATTTGGTTTA

A5-AT1G08610-XLOC_004694-5966-0
TCATCTGATGGACCAATTACAGAGAACGACGAAGAGACCAACAATGAGAT
A5-AT1G08610-XLOC_004694-5966-1
TCATCTGATGGACCAATTACAGAGAACGACGAAGAGACCAACAATGAGAT
CONSENSUS
TCATCTGATGGACCAATTACAGAGAACGACGAAGAGACCAACAATGAGAT

A5-AT1G08610-XLOC_004694-5966-0
CCTTCATAACCTGTGCAGCAATGGGAAGTTAACAGATGCGTGCAAACTTG
A5-AT1G08610-XLOC_004694-5966-1
CCTTCATAACCTGTGCAGCAATGGGAAGTTAACAGATGCGTGCAAACTTG
CONSENSUS
CCTTCATAACCTGTGCAGCAATGGGAAGTTAACAGATGCGTGCAAACTTG

A5-AT1G08610-XLOC_004694-5966-0
TCGAGGTGATGGCACGACATAATCAGGTTCCTCATTTTCCTTCTTGCTCA
A5-AT1G08610-XLOC_004694-5966-1
TCGAGGTGATGGCACGACATAATCAGGTTCCTCATTTTCCTTCTTGCTCA
CONSENSUS
TCGAGGTGATGGCACGACATAATCAGGTTCCTCATTTTCCTTCTTGCTCA

A5-AT1G08610-XLOC_004694-5966-0
AACTTAGTCCGTGGTCTAGCCAGAATTGATCAACTGGATAAAGCAATGTG
A5-AT1G08610-XLOC_004694-5966-1
AACTTAGTCCGTGGTCTAGCCAGAATTGATCAACTGGATAAAGCAATGTG
CONSENSUS
AACTTAGTCCGTGGTCTAGCCAGAATTGATCAACTGGATAAAGCAATGTG

A5-AT1G08610-XLOC_004694-5966-0

TATCTTGAGAGTAATGGTAATGTCTGGTGGGGTTCCAGATACTATTACCT
A5-AT1G08610-XLOC_004694-5966-1
TATCTTGAGAGTAATGGTAATGTCTGGTGGGGTTCCAGATACTATTACCT
CONSENSUS
TATCTTGAGAGTAATGGTAATGTCTGGTGGGGTTCCAGATACTATTACCT

A5-AT1G08610-XLOC_004694-5966-0
ATAACATGATCATCGGAAATCTTTGCAAGAAAGGGCATATAAGAACTGCT
A5-AT1G08610-XLOC_004694-5966-1
ATAACATGATCATCGGAAATCTTTGCAAGAAAGGGCATATAAGAACTGCT
CONSENSUS
ATAACATGATCATCGGAAATCTTTGCAAGAAAGGGCATATAAGAACTGCT

A5-AT1G08610-XLOC_004694-5966-0
TTGGTCCTCTTAGAAGATATGAGTTTGAGTGGGAGCCCTCCTGATGTGAT
A5-AT1G08610-XLOC_004694-5966-1
TTGGTCCTCTTAGAAGATATGAGTTTGAGTGGGAGCCCTCCTGATGTGAT
CONSENSUS
TTGGTCCTCTTAGAAGATATGAGTTTGAGTGGGAGCCCTCCTGATGTGAT

A5-AT1G08610-XLOC_004694-5966-0
CACGTATAACACAGTGATTCGGTGTATGTTTGATTATGGGAATGCTGAGC
A5-AT1G08610-XLOC_004694-5966-1
CACGTATAACACAGTGATTCGGTGTATGTTTGATTATGGGAATGCTGAGC
CONSENSUS
CACGTATAACACAGTGATTCGGTGTATGTTTGATTATGGGAATGCTGAGC

A5-AT1G08610-XLOC_004694-5966-0
AAGCCATCAGGTTTTGGAAGGATCAACTGCAGAATGGTTGCCCTCCATTT
A5-AT1G08610-XLOC_004694-5966-1
AAGCCATCAGGTTTTGGAAGGATCAACTGCAGAATGGTTGCCCTCCATTT
CONSENSUS
AAGCCATCAGGTTTTGGAAGGATCAACTGCAGAATGGTTGCCCTCCATTT

A5-AT1G08610-XLOC_004694-5966-0
ATGATTACATACACGGTTTTGGTCGAACTCGTGTGCAGGTACTGTGGAAG
A5-AT1G08610-XLOC_004694-5966-1
ATGATTACATACACGGTTTTGGTCGAACTCGTGTGCAGGTACTGTGGAAG
CONSENSUS
ATGATTACATACACGGTTTTGGTCGAACTCGTGTGCAGGTACTGTGGAAG

A5-AT1G08610-XLOC_004694-5966-0
CGCTCGAGCTATAGAAGTTTTAGAAGACATGGCAGTGGAAGGTTGCTATC
A5-AT1G08610-XLOC_004694-5966-1
CGCTCGAGCTATAGAAGTTTTAGAAGACATGGCAGTGGAAGGTTGCTATC
CONSENSUS
CGCTCGAGCTATAGAAGTTTTAGAAGACATGGCAGTGGAAGGTTGCTATC

A5-AT1G08610-XLOC_004694-5966-0
CTGACATTGTCACATATAATTCTCTGGTTAACTATAACTGCAGGCGAGGG
A5-AT1G08610-XLOC_004694-5966-1
CTGACATTGTCACATATAATTCTCTGGTTAACTATAACTGCAGGCGAGGG
CONSENSUS
CTGACATTGTCACATATAATTCTCTGGTTAACTATAACTGCAGGCGAGGG

A5-AT1G08610-XLOC_004694-5966-0

AATTTGGAAGAGGTGGCTTCGGTTATTCAGCATATCTTATCTCATGGATT
A5-AT1G08610-XLOC_004694-5966-1
AATTTGGAAGAGGTGGCTTCGGTTATTCAGCATATCTTATCTCATGGATT
CONSENSUS
AATTTGGAAGAGGTGGCTTCGGTTATTCAGCATATCTTATCTCATGGATT

A5-AT1G08610-XLOC_004694-5966-0
GGAACTGAACACTGTAACTTACAACACTCTTCTACATTCCCTCTGTTCCC
A5-AT1G08610-XLOC_004694-5966-1
GGAACTGAACACTGTAACTTACAACACTCTTCTACATTCCCTCTGTTCCC
CONSENSUS
GGAACTGAACACTGTAACTTACAACACTCTTCTACATTCCCTCTGTTCCC

A5-AT1G08610-XLOC_004694-5966-0
ACGAGTACTGGGATGAAGTGGAGGAGATTCTAAACATCATGTACCAGACG
A5-AT1G08610-XLOC_004694-5966-1
ACGAGTACTGGGATGAAGTGGAGGAGATTCTAAACATCATGTACCAGACG
CONSENSUS
ACGAGTACTGGGATGAAGTGGAGGAGATTCTAAACATCATGTACCAGACG

A5-AT1G08610-XLOC_004694-5966-0
TCCTACTGTCCAACTGTTATTACTTACAACATCCTGATTAATGGTTTATG
A5-AT1G08610-XLOC_004694-5966-1
TCCTACTGTCCAACTGTTATTACTTACAACATCCTGATTAATGGTTTATG
CONSENSUS
TCCTACTGTCCAACTGTTATTACTTACAACATCCTGATTAATGGTTTATG

A5-AT1G08610-XLOC_004694-5966-0
CAAAGCCAGGCTTTTGAGTCGTGCAATTGATTTCTTCTACCAAATGCTGG
A5-AT1G08610-XLOC_004694-5966-1
CAAAGCCAGGCTTTTGAGTCGTGCAATTGATTTCTTCTACCAAATGCTGG
CONSENSUS
CAAAGCCAGGCTTTTGAGTCGTGCAATTGATTTCTTCTACCAAATGCTGG

A5-AT1G08610-XLOC_004694-5966-0
AACAGAAGTGTTTGCCTGATATAGTCACTTACAACACAGTTCTCGGAGCC
A5-AT1G08610-XLOC_004694-5966-1
AACAGAAGTGTTTGCCTGATATAGTCACTTACAACACAGTTCTCGGAGCC
CONSENSUS
AACAGAAGTGTTTGCCTGATATAGTCACTTACAACACAGTTCTCGGAGCC

A5-AT1G08610-XLOC_004694-5966-0
ATGTCCAAAGAGGGCATGGTAGATGATGCGATTGAGTTGCTTGGATTGCT
A5-AT1G08610-XLOC_004694-5966-1
ATGTCCAAAGAGGGCATGGTAGATGATGCGATTGAGTTGCTTGGATTGCT
CONSENSUS
ATGTCCAAAGAGGGCATGGTAGATGATGCGATTGAGTTGCTTGGATTGCT

A5-AT1G08610-XLOC_004694-5966-0
GAAAAACACATGTTGTCCGCCGGGCTTGATCACTTACAACTCTGTAATCG
A5-AT1G08610-XLOC_004694-5966-1
GAAAAACACATGTTGTCCGCCGGGCTTGATCACTTACAACTCTGTAATCG
CONSENSUS
GAAAAACACATGTTGTCCGCCGGGCTTGATCACTTACAACTCTGTAATCG

A5-AT1G08610-XLOC_004694-5966-0

ATGGATTGGCTAAAAAGGGCCTGATGAAGAAAGCATTGGAGCTGTACCAT
A5-AT1G08610-XLOC_004694-5966-1
ATGGATTGGCTAAAAAGGGCCTGATGAAGAAAGCATTGGAGCTGTACCAT
CONSENSUS
ATGGATTGGCTAAAAAGGGCCTGATGAAGAAAGCATTGGAGCTGTACCAT

A5-AT1G08610-XLOC_004694-5966-0
CAGATGCTGGATGCTGGAATCTTTCCGGATGATATAACTCGTCGGTCTTT
A5-AT1G08610-XLOC_004694-5966-1
CAGATGCTGGATGCTGGAATCTTTCCGGATGATATAACTCGTCGGTCTTT
CONSENSUS
CAGATGCTGGATGCTGGAATCTTTCCGGATGATATAACTCGTCGGTCTTT

A5-AT1G08610-XLOC_004694-5966-0
AATATATGGTTTCTGCCGGGCAAACCTAGTTGAAGAAGCTGGCCAAGTTT
A5-AT1G08610-XLOC_004694-5966-1
AATATATGGTTTCTGCCGGGCAAACCTAGTTGAAGAAGCTGGCCAAGTTT
CONSENSUS
AATATATGGTTTCTGCCGGGCAAACCTAGTTGAAGAAGCTGGCCAAGTTT

A5-AT1G08610-XLOC_004694-5966-0
TAAAGGAGACGAGCAATAGAGGGAATGGGATCAGAGGTAGCACTTACAGG
A5-AT1G08610-XLOC_004694-5966-1
TAAAGGAGACGAGCAATAGAGGGAATGGGATCAGAGGTAGCACTTACAGG
CONSENSUS
TAAAGGAGACGAGCAATAGAGGGAATGGGATCAGAGGTAGCACTTACAGG

A5-AT1G08610-XLOC_004694-5966-0
TTGGTGATCCAAGGGCTGTGCAAGAAGAAAGAGATAGAAATGGCGATAGA
A5-AT1G08610-XLOC_004694-5966-1
TTGGTGATCCAAGGGCTGTGCAAGAAGAAAGAGATAGAAATGGCGATAGA
CONSENSUS
TTGGTGATCCAAGGGCTGTGCAAGAAGAAAGAGATAGAAATGGCGATAGA

A5-AT1G08610-XLOC_004694-5966-0
GGTTGTTGAGATAATGCTAACAGGTGGGTGTAAGCCAGATGAGACAATAT
A5-AT1G08610-XLOC_004694-5966-1
GGTTGTTGAGATAATGCTAACAGGTGGGTGTAAGCCAGATGAGACAATAT
CONSENSUS
GGTTGTTGAGATAATGCTAACAGGTGGGTGTAAGCCAGATGAGACAATAT

A5-AT1G08610-XLOC_004694-5966-0
ACACCGCAATAGTTAAAGGCGTGGAAGAGATGGGAATGGGAAGCGAGGCT
A5-AT1G08610-XLOC_004694-5966-1
ACACCGCAATAGTTAAAGGCGTGGAAGAGATGGGAATGGGAAGCGAGGCT
CONSENSUS
ACACCGCAATAGTTAAAGGCGTGGAAGAGATGGGAATGGGAAGCGAGGCT

A5-AT1G08610-XLOC_004694-5966-0
GTTCAGTTGCAGAAGAAACTGAAGCAATGGAAACTCTTGAAAGAAGTGTA
A5-AT1G08610-XLOC_004694-5966-1
GTTCAGTTGCAGAAGAAACTGAAGCAATGGAAACTCTTGAAAGAAGTGTA
CONSENSUS
GTTCAGTTGCAGAAGAAACTGAAGCAATGGAAACTCTTGAAAGAAGTGTA

A5-AT1G08610-XLOC_004694-5966-0

GGAAACGTAATTGAAGCAGACTCTTTTTTTGCTTTTTCTATTAAGCTTTT
A5-AT1G08610-XLOC_004694-5966-1
GGAAACGTAATTGAAGCAGACTCTTTTTTTGCTTTTTCTATTAAGCTTTT
CONSENSUS
GGAAACGTAATTGAAGCAGACTCTTTTTTTGCTTTTTCTATTAAGCTTTT

A5-AT1G08610-XLOC_004694-5966-0
TTGTACAGGAAATCAGTCGTATCCGGCAATGGTTGAATCAGAAAGCTTAG
A5-AT1G08610-XLOC_004694-5966-1
TTGTACAGGAAATCAGTCGTATCCGGCAATGGTTGAATCAGAAAGCTTAG
CONSENSUS
TTGTACAGGAAATCAGTCGTATCCGGCAATGGTTGAATCAGAAAGCTTAG

A5-AT1G08610-XLOC_004694-5966-0
GCCTCCATTTGTTCAGTGACTGCTTTCTGCTA
A5-AT1G08610-XLOC_004694-5966-1
GCCTCCATTTGTTCAGTGACTGCTTTCTGCTA
CONSENSUS
GCCTCCATTTGTTCAGTGACTGCTTTCTGCTA

alignment for event: RI-AT1G32540-XLOC_001775-7401

RI-AT1G32540-XLOC_001775-7401-0
ATTGATGAAAGTGAGGAAATGGGAGAAGAAATGAAGAACACAGGATGTGT
RI-AT1G32540-XLOC_001775-7401-1
ATTGATGAAAGTGAGGAAATGGGAGAAGAAATGAAGAACACAGGATGTGT
CONSENSUS
ATTGATGAAAGTGAGGAAATGGGAGAAGAAATGAAGAACACAGGATGTGT

RI-AT1G32540-XLOC_001775-7401-0
CTTCTTCTTCTAAGTCACTAACAAAATCAACAAAGAGGAGAAGCCATTAT
RI-AT1G32540-XLOC_001775-7401-1
CTTCTTCTTCTAAGTCACTAACAAAATCAACAAAGAGGAGAAGCCATTAT
CONSENSUS
CTTCTTCTTCTAAGTCACTAACAAAATCAACAAAGAGGAGAAGCCATTAT

RI-AT1G32540-XLOC_001775-7401-0
TATATAATAGAGAGATTGAGAGAAGAGATTTATCCAAAAAAATATTGCAA
RI-AT1G32540-XLOC_001775-7401-1
TATATAATAGAGAGATTGAGAGAAGAGATTTATCCAAAAAAATATTGCAA
CONSENSUS
TATATAATAGAGAGATTGAGAGAAGAGATTTATCCAAAAAAATATTGCAA

RI-AT1G32540-XLOC_001775-7401-0
TTCTTCTTGGAGTGAATAATGCCAGTCCCTCTTGCACCATATCCAACACC
RI-AT1G32540-XLOC_001775-7401-1
TTCTTCTTGGAGTGAATAATGCCAGTCCCTCTTGCACCATATCCAACACC
CONSENSUS
TTCTTCTTGGAGTGAATAATGCCAGTCCCTCTTGCACCATATCCAACACC

RI-AT1G32540-XLOC_001775-7401-0
TCCGGCACCGGCACTGGCTCCGTCGTACAACACTCCTCCGGCAAATGGTA
RI-AT1G32540-XLOC_001775-7401-1
TCCGGCACCGGCACTGGCTCCGTCGTACAACACTCCTCCGGCAAATG---

CONSENSUS
      TCCGGCACCGGCACTGGCTCCGTCGTACAACACTCCTCCGGCAAATG...

RI-AT1G32540-XLOC_001775-7401-0
      AAATCTTTCTTAAATGGACAAGCTATTTTTAATCCAAAAAGATCCTCTGT
RI-AT1G32540-XLOC_001775-7401-1

CONSENSUS
      ....

RI-AT1G32540-XLOC_001775-7401-0
      TTTCATATTCTTTTACAAAAGTTTTAGTTTCTTATAAGATTGATTTTTTG
RI-AT1G32540-XLOC_001775-7401-1

CONSENSUS
      ....

RI-AT1G32540-XLOC_001775-7401-0
      GCTTTATTAAACAAGATTGTTTTTGGTCATGCATACTTGGAAAAATCAAA
RI-AT1G32540-XLOC_001775-7401-1

CONSENSUS
      ....

RI-AT1G32540-XLOC_001775-7401-0
      TATTTTCAGATTCCCAATTTGTTTTACTCTTCATGGGAAAAAATTGCTCA
RI-AT1G32540-XLOC_001775-7401-1

CONSENSUS
      ....

RI-AT1G32540-XLOC_001775-7401-0
      ATTATTTTCCCAGAACCACATTTATTTATTTCACATGTGTTCATAATCCA
RI-AT1G32540-XLOC_001775-7401-1

CONSENSUS
      ....

RI-AT1G32540-XLOC_001775-7401-0
      TTTGAATGTACTTTTTTTGGTTAAAAAAATAAATAAAAACAACTTGAGAT
RI-AT1G32540-XLOC_001775-7401-1

CONSENSUS
      ....

RI-AT1G32540-XLOC_001775-7401-0
      TTGTAGGAAGTACAAGTGGGCAGAGCCAGTTAGTGTGTTCAGGTTGCAGA
RI-AT1G32540-XLOC_001775-7401-1 ------
GAAGTACAAGTGGGCAGAGCCAGTTAGTGTGTTCAGGTTGCAGA
CONSENSUS
      ......GAAGTACAAGTGGGCAGAGCCAGTTAGTGTGTTCAGGTTGCAGA

RI-AT1G32540-XLOC_001775-7401-0
      AACCTTCTGATGTATCCCGTCGGAGCAACCTCCGTCTGTTGCGCCGTCTG
RI-AT1G32540-XLOC_001775-7401-1
      AACCTTCTGATGTATCCCGTCGGAGCAACCTCCGTCTGTTGCGCCGTCTG

CONSENSUS
AACCTTCTGATGTATCCCGTCGGAGCAACCTCCGTCTGTTGCGCCGTCTG

RI-AT1G32540-XLOC_001775-7401-0 TAACGCCGTCACGGCCGTTCCTCCGCCCG
RI-AT1G32540-XLOC_001775-7401-1 TAACGCCGTCACGGCCGTTCCTCCGCCCG
CONSENSUS TAACGCCGTCACGGCCGTTCCTCCGCCCG

alignment for event: A3-AT1G22750-XLOC_001229-6747

A3-AT1G22750-XLOC_001229-6747-0
CGTTGGCACGAGCGCAGTCATTGTCGTGCAGCTATTCCGTCTGTCTCCTC
A3-AT1G22750-XLOC_001229-6747-1
CGTTGGCACGAGCGCAGTCATTGTCGTGCAGCTATTCCGTCTGTCTCCTC
CONSENSUS
CGTTGGCACGAGCGCAGTCATTGTCGTGCAGCTATTCCGTCTGTCTCCTC

A3-AT1G22750-XLOC_001229-6747-0
TCGACCCGCTCTACCTCGTTCTAGTGAACAACAGCAATCGTTAAAAGTAA
A3-AT1G22750-XLOC_001229-6747-1
TCGACCCGCTCTACCTCGTTCTAGTGAACAACAGCAATCG----------
CONSENSUS
TCGACCCGCTCTACCTCGTTCTAGTGAACAACAGCAATCG..........

A3-AT1G22750-XLOC_001229-6747-0
AGGCTAAGTTAGTGAACTCTCTCATGGGTCAATGGCAGGGCTGGAGATAT
A3-AT1G22750-XLOC_001229-6747-1
--------------------------------------GGCTGGAGATAT
CONSENSUS
......................................GGCTGGAGATAT

A3-AT1G22750-XLOC_001229-6747-0
GTACGAGCGCACTCATTCTGCGGCTTTGCGCATAATGTGAAGCTTTGTCA
A3-AT1G22750-XLOC_001229-6747-1
GTACGAGCGCACTCATTCTGCGGCTTTGCGCATAATGTGAAGCTTTGTCA
CONSENSUS
GTACGAGCGCACTCATTCTGCGGCTTTGCGCATAATGTGAAGCTTTGTCA

A3-AT1G22750-XLOC_001229-6747-0
TTTCATTTTTAACATTTTACCACAAAAGTTCAAAACATATTTTTATCTAC
A3-AT1G22750-XLOC_001229-6747-1
TTTCATTTTTAACATTTTACCACAAAAGTTCAAAACATATTTTTATCTAC
CONSENSUS
TTTCATTTTTAACATTTTACCACAAAAGTTCAAAACATATTTTTATCTAC

A3-AT1G22750-XLOC_001229-6747-0
ATTTTAAGTTTGTATGTTTCATAGTTTTCTTCTGTATATACTTTCTCTTA
A3-AT1G22750-XLOC_001229-6747-1
ATTTTAAGTTTGTATGTTTCATAGTTTTCTTCTGTATATACTTTCTCTTA
CONSENSUS
ATTTTAAGTTTGTATGTTTCATAGTTTTCTTCTGTATATACTTTCTCTTA

A3-AT1G22750-XLOC_001229-6747-0
TGTGACGACAATTACTTATGTACTTAACTTTTTTGGGTCACGACTTGAAA
A3-AT1G22750-XLOC_001229-6747-1

```
      TGTGACGACAATTACTTATGTACTTAACTTTTTTGGGTCACGACTTGAAA
CONSENSUS
      TGTGACGACAATTACTTATGTACTTAACTTTTTTGGGTCACGACTTGAAA

A3-AT1G22750-XLOC_001229-6747-0  AACCACATATGGATTGAGACTTTTTGGGCT
A3-AT1G22750-XLOC_001229-6747-1  AACCACATATGGATTGAGACTTTTTGGGCT
CONSENSUS                        AACCACATATGGATTGAGACTTTTTGGGCT
```

alignment for event: A3-AT1G22750-XLOC_001229-6745

```
A3-AT1G22750-XLOC_001229-6745-0
      CGTTGGCACGAGCGCAGTCATTGTCGTGCAGCTATTCCGTCTGTCTCCTC
A3-AT1G22750-XLOC_001229-6745-1
      CGTTGGCACGAGCGCAGTCATTGTCGTGCAGCTATTCCGTCTGTCTCCTC
CONSENSUS
      CGTTGGCACGAGCGCAGTCATTGTCGTGCAGCTATTCCGTCTGTCTCCTC

A3-AT1G22750-XLOC_001229-6745-0
      TCGACCCGCTCTACCTCGTTCTAGTGAACAACAGCAATCGGAAGCAAAGT
A3-AT1G22750-XLOC_001229-6745-1
      TCGACCCGCTCTACCTCGTTCTAGTGAACAACAGCAATCG----------
CONSENSUS
      TCGACCCGCTCTACCTCGTTCTAGTGAACAACAGCAATCG..........

A3-AT1G22750-XLOC_001229-6745-0
      TAAAAGTAAAGGCTAAGTTAGTGAACTCTCTCATGGGTCAATGGCAGGGC
A3-AT1G22750-XLOC_001229-6745-1
      -----------------------------------------------GGC
CONSENSUS
      ...............................................GGC

A3-AT1G22750-XLOC_001229-6745-0
      TGGAGATATGTACGAGCGCACTCATTCTGCGGCTTTGCGCATAATGTGAA
A3-AT1G22750-XLOC_001229-6745-1
      TGGAGATATGTACGAGCGCACTCATTCTGCGGCTTTGCGCATAATGTGAA
CONSENSUS
      TGGAGATATGTACGAGCGCACTCATTCTGCGGCTTTGCGCATAATGTGAA

A3-AT1G22750-XLOC_001229-6745-0
      GCTTTGTCATTTCATTTTTAACATTTTACCACAAAAGTTCAAAACATATT
A3-AT1G22750-XLOC_001229-6745-1
      GCTTTGTCATTTCATTTTTAACATTTTACCACAAAAGTTCAAAACATATT
CONSENSUS
      GCTTTGTCATTTCATTTTTAACATTTTACCACAAAAGTTCAAAACATATT

A3-AT1G22750-XLOC_001229-6745-0
      TTTATCTACATTTTAAGTTTGTATGTTTCATAGTTTTCTTCTGTATATAC
A3-AT1G22750-XLOC_001229-6745-1
      TTTATCTACATTTTAAGTTTGTATGTTTCATAGTTTTCTTCTGTATATAC
CONSENSUS
      TTTATCTACATTTTAAGTTTGTATGTTTCATAGTTTTCTTCTGTATATAC

A3-AT1G22750-XLOC_001229-6745-0
      TTTCTCTTATGTGACGACAATTACTTATGTACTTAACTTTTTTGGGTCAC
```

A3-AT1G22750-XLOC_001229-6745-1
TTTCTCTTATGTGACGACAATTACTTATGTACTTAACTTTTTTGGGTCAC
CONSENSUS
TTTCTCTTATGTGACGACAATTACTTATGTACTTAACTTTTTTGGGTCAC

A3-AT1G22750-XLOC_001229-6745-0
GACTTGAAAAACCACATATGGATTGAGACTTTTTGGGCT
A3-AT1G22750-XLOC_001229-6745-1
GACTTGAAAAACCACATATGGATTGAGACTTTTTGGGCT
CONSENSUS
GACTTGAAAAACCACATATGGATTGAGACTTTTTGGGCT

alignment for event: RI-AT1G54730-XLOC_002819-2052

RI-AT1G54730-XLOC_002819-2052-0
GTGTACACCGGATCATTTTCATTAGGGATGGGTGGGATTCCTTGGGTCAT
RI-AT1G54730-XLOC_002819-2052-1
GTGTACACCGGATCATTTTCATTAGGGATGGGTGGGATTCCTTGGGTCAT
CONSENSUS
GTGTACACCGGATCATTTTCATTAGGGATGGGTGGGATTCCTTGGGTCAT

RI-AT1G54730-XLOC_002819-2052-0
TATGTCAGAGGTAAGTAACATTCATAAAAATTCTTTGGTTTTAAACATAC
RI-AT1G54730-XLOC_002819-2052-1
TATGTCAGAGGTAAGTAACATTCATAAAAATTCTTTGGTTTTAAACATAC
CONSENSUS
TATGTCAGAGGTAAGTAACATTCATAAAAATTCTTTGGTTTTAAACATAC

RI-AT1G54730-XLOC_002819-2052-0
TCTTATACTTAATAATCCGATCGATGTCTTAAGAAATATTAAACTTTATA
RI-AT1G54730-XLOC_002819-2052-1
TCTTATACTTAATAATCCGATCGATGTCTTAAGAAATATTAAACTTTATA
CONSENSUS
TCTTATACTTAATAATCCGATCGATGTCTTAAGAAATATTAAACTTTATA

RI-AT1G54730-XLOC_002819-2052-0
TCATAAACCTACGGTTTAGTATTAAAATTAAATAATAAATTATTTAACTC
RI-AT1G54730-XLOC_002819-2052-1
TCATAAACCTACGGTTTAGTATTAAAATTAAATAATAAATTATTTAACTC
CONSENSUS
TCATAAACCTACGGTTTAGTATTAAAATTAAATAATAAATTATTTAACTC

RI-AT1G54730-XLOC_002819-2052-0
TCATATGGTTGTATGTGGTATGATTTTGATCAGTGTATATAAATTATGTT
RI-AT1G54730-XLOC_002819-2052-1
TCATATGGTTGTATGTGGTATGATTTTGATCAGTGTATATAAATTATGTT
CONSENSUS
TCATATGGTTGTATGTGGTATGATTTTGATCAGTGTATATAAATTATGTT

RI-AT1G54730-XLOC_002819-2052-0
CCTTTATGATTTATTTATTGATTCTTTGTTTATATAACATTTTAATTCAA
RI-AT1G54730-XLOC_002819-2052-1
CCTTTATGATTTATTTATTGATTCTTTGTTTATATAACATTTTAATTCAA
CONSENSUS

CCTTTATGATTTATTTATTGATTCTTTGTTTATATAACATTTTAATTCAA

RI-AT1G54730-XLOC_002819-2052-0
TTTAGTTGGTGCGTAGATATTAATTTTATGTTTTATATGATAAAGATATT
RI-AT1G54730-XLOC_002819-2052-1
TTTAGTTGGTGCGTAGATATTAATTTTATGTTTTATATGATAAAGATATT
CONSENSUS
TTTAGTTGGTGCGTAGATATTAATTTTATGTTTTATATGATAAAGATATT

RI-AT1G54730-XLOC_002819-2052-0
TCCAATAGACATAAAAGGATCAGCCGGAAGCCTCGTGACTGTTGTTAGCT
RI-AT1G54730-XLOC_002819-2052-1
TCCAATAGACATAAAAGGATCAGCCGGAAGCCTCGTGACTGTTGTTAGCT
CONSENSUS
TCCAATAGACATAAAAGGATCAGCCGGAAGCCTCGTGACTGTTGTTAGCT

RI-AT1G54730-XLOC_002819-2052-0
GGGTCGGATCATGGATTATATCTTTTACATTTAACTTTCTAATGAATTGG
RI-AT1G54730-XLOC_002819-2052-1
GGGTCGGATCATGGATTATATCTTTTACATTTAACTTTCTAATGAATTGG
CONSENSUS
GGGTCGGATCATGGATTATATCTTTTACATTTAACTTTCTAATGAATTGG

RI-AT1G54730-XLOC_002819-2052-0
AATCCGGCAGGTATAAAATTTGTTTAAATTTTGCTTTTATCAATAAATTT
RI-AT1G54730-XLOC_002819-2052-1
AATCCGGCAG--
CONSENSUS
AATCCGGCAG..

RI-AT1G54730-XLOC_002819-2052-0
TGTTCTTTATCTAATAGTGGCACAAGTTTTAAATTTTCAGGAACGTTTTA
RI-AT1G54730-XLOC_002819-2052-1
--GAACGTTTTA
CONSENSUS
....GAACGTTTTA

RI-AT1G54730-XLOC_002819-2052-0
TGTTTTCGCTACTGTTTGTGGGGCTACTGTTATTTTTGTAGCAAAACTCG
RI-AT1G54730-XLOC_002819-2052-1
TGTTTTCGCTACTGTTTGTGGGGCTACTGTTATTTTTGTAGCAAAACTCG
CONSENSUS
TGTTTTCGCTACTGTTTGTGGGGCTACTGTTATTTTTGTAGCAAAACTCG

RI-AT1G54730-XLOC_002819-2052-0
TACCAGAAACCAAAGGTCGCACACTTGAGGAAATCCAATATTCGATTGGT
RI-AT1G54730-XLOC_002819-2052-1
TACCAGAAACCAAAGGTCGCACACTTGAGGAAATCCAATATTCGATTGGT
CONSENSUS
TACCAGAAACCAAAGGTCGCACACTTGAGGAAATCCAATATTCGATTGGT

RI-AT1G54730-XLOC_002819-2052-0
TATGTAGAATTGTAATGTATAGTAGTTGATTACGAGAATATCAGTCCCTA
RI-AT1G54730-XLOC_002819-2052-1
TATGTAGAATTGTAATGTATAGTAGTTGATTACGAGAATATCAGTCCCTA
CONSENSUS

TATGTAGAATTGTAATGTATAGTAGTTGATTACGAGAATATCAGTCCCTA

RI-AT1G54730-XLOC_002819-2052-0
ATTATAACGATTAAAAGTAAGATTTTTTATTTTTCTTCTTTCCTTTTGGT
RI-AT1G54730-XLOC_002819-2052-1
ATTATAACGATTAAAAGTAAGATTTTTTATTTTTCTTCTTTCCTTTTGGT
CONSENSUS
ATTATAACGATTAAAAGTAAGATTTTTTATTTTTCTTCTTTCCTTTTGGT

RI-AT1G54730-XLOC_002819-2052-0
ATAATCGTATCATCCTTTTTGCTTAGTGAATTTGAATAAATGGTGTTGTT
RI-AT1G54730-XLOC_002819-2052-1
ATAATCGTATCATCCTTTTTGCTTAGTGAATTTGAATAAATGGTGTTGTT
CONSENSUS
ATAATCGTATCATCCTTTTTGCTTAGTGAATTTGAATAAATGGTGTTGTT

RI-AT1G54730-XLOC_002819-2052-0 TTTGGTTACC
RI-AT1G54730-XLOC_002819-2052-1 TTTGGTTACC
CONSENSUS TTTGGTTACC

alignment for event: A3-AT1G77180-XLOC_008117-4874

A3-AT1G77180-XLOC_008117-4874-0
CCTTTTTTTTGTTGTTAATCCTATCAACCCTAGAAAAGGCTTCACGCCCG
A3-AT1G77180-XLOC_008117-4874-1
CCTTTTTTTTGTTGTTAATCCTATCAACCCTAGAAAAGGCTTCACGCCCG
CONSENSUS
CCTTTTTTTTGTTGTTAATCCTATCAACCCTAGAAAAGGCTTCACGCCCG

A3-AT1G77180-XLOC_008117-4874-0
ATTTCTTTTTTCCCCAGAAAAAGGCTTCACTTCCGTTCGTTGTTCTTCAT
A3-AT1G77180-XLOC_008117-4874-1
ATTTCTTTTTTCCCCAGAAAAAGGCTTCACTTCCGTTCGTTGTTCTTCAT
CONSENSUS
ATTTCTTTTTTCCCCAGAAAAAGGCTTCACTTCCGTTCGTTGTTCTTCAT

A3-AT1G77180-XLOC_008117-4874-0
CGGTCTGCGCCGCCTCGCACATCGTTTCACCTTTCTCAGAATCTCTCTTC
A3-AT1G77180-XLOC_008117-4874-1
CGGTCTGCGCCGCCTCGCACATCGTTTCACCTTTCTCAGAATCTCTCTTC
CONSENSUS
CGGTCTGCGCCGCCTCGCACATCGTTTCACCTTTCTCAGAATCTCTCTTC

A3-AT1G77180-XLOC_008117-4874-0
CCGTCGATTAGTTTCCTCAGAG----------------------------
A3-AT1G77180-XLOC_008117-4874-1
CCGTCGATTAGTTTCCTCAGAGTGAATCCAACCCAGATTTTTCAGATTTT
CONSENSUS
CCGTCGATTAGTTTCCTCAGAG............................

A3-AT1G77180-XLOC_008117-4874-0

A3-AT1G77180-XLOC_008117-4874-1
AATCGGAATTATTCGGGATCTAATGCTCGCGTCGAGTTTATCGCTAGAAA

```
CONSENSUS
      ..................................................

A3-AT1G77180-XLOC_008117-4874-0
      --------------------------------------------------
A3-AT1G77180-XLOC_008117-4874-1
      ACCCTAATTTCGGAGGATCTGTCACTTTTATTATCAGAAGCTTGTTGTTG
CONSENSUS
      ..................................................

A3-AT1G77180-XLOC_008117-4874-0
      ------------------------------------------------GT
A3-AT1G77180-XLOC_008117-4874-1
      TAATTACGGATTAGGTATGTCCTGATCGTTATTGTTCTTTGTTTGCAGGT
CONSENSUS
      ................................................GT

A3-AT1G77180-XLOC_008117-4874-0
      TGCGATTTGTTGAAAACCTTGATTTAGACTATGAAGTCTCTTAATGATCT
A3-AT1G77180-XLOC_008117-4874-1
      TGCGATTTGTTGAAAACCTTGATTTAGACTATGAAGTCTCTTAATGATCT
CONSENSUS
      TGCGATTTGTTGAAAACCTTGATTTAGACTATGAAGTCTCTTAATGATCT

A3-AT1G77180-XLOC_008117-4874-0
      TCCTGCGCCCAAATCAACCACTACCACGTATTATGATCATTCTAATGATG
A3-AT1G77180-XLOC_008117-4874-1
      TCCTGCGCCCAAATCAACCACTACCACGTATTATGATCATTCTAATGATG
CONSENSUS
      TCCTGCGCCCAAATCAACCACTACCACGTATTATGATCATTCTAATGATG

A3-AT1G77180-XLOC_008117-4874-0
      CTTGGTTCAAAAACCGAGTCACTGAATCCGAGACGGTGAAATCCTCCAGT
A3-AT1G77180-XLOC_008117-4874-1
      CTTGGTTCAAAAACCGAGTCACTGAATCCGAGACGGTGAAATCCTCCAGT
CONSENSUS
      CTTGGTTCAAAAACCGAGTCACTGAATCCGAGACGGTGAAATCCTCCAGT

A3-AT1G77180-XLOC_008117-4874-0
      ATCAAATTTAAAGTGGTGCCTGCTTACCTGAATCGTCAAGGTTTACGTCC
A3-AT1G77180-XLOC_008117-4874-1
      ATCAAATTTAAAGTGGTGCCTGCTTACCTGAATCGTCAAGGTTTACGTCC
CONSENSUS
      ATCAAATTTAAAGTGGTGCCTGCTTACCTGAATCGTCAAGGTTTACGTCC

A3-AT1G77180-XLOC_008117-4874-0
      GAAGAATCCAGAGGATTTTGGCGATGGTGGTGCTTTCCCGGAGATTCATC
A3-AT1G77180-XLOC_008117-4874-1
      GAAGAATCCAGAGGATTTTGGCGATGGTGGTGCTTTCCCGGAGATTCATC
CONSENSUS
      GAAGAATCCAGAGGATTTTGGCGATGGTGGTGCTTTCCCGGAGATTCATC

A3-AT1G77180-XLOC_008117-4874-0
      TTCCTCAGTATCCTCTTCTTATGGGTAAGAATAAATCCAATAAGCCTGGA
A3-AT1G77180-XLOC_008117-4874-1
      TTCCTCAGTATCCTCTTCTTATGGGTAAGAATAAATCCAATAAGCCTGGA
```

CONSENSUS
TTCCTCAGTATCCTCTTCTTATGGGTAAGAATAAATCCAATAAGCCTGGA

A3-AT1G77180-XLOC_008117-4874-0
GCTAAGACTCTCCCTGTTACTGTCGATGCTCAGGGAAACGTGGTCTTTGA
A3-AT1G77180-XLOC_008117-4874-1
GCTAAGACTCTCCCTGTTACTGTCGATGCTCAGGGAAACGTGGTCTTTGA
CONSENSUS
GCTAAGACTCTCCCTGTTACTGTCGATGCTCAGGGAAACGTGGTCTTTGA

A3-AT1G77180-XLOC_008117-4874-0
CGCTATAGTGAGGCAGAATGAGAACTCGAGGAAGATTGTTTATTCTCAGC
A3-AT1G77180-XLOC_008117-4874-1
CGCTATAGTGAGGCAGAATGAGAACTCGAGGAAGATTGTTTATTCTCAGC
CONSENSUS
CGCTATAGTGAGGCAGAATGAGAACTCGAGGAAGATTGTTTATTCTCAGC

A3-AT1G77180-XLOC_008117-4874-0
ATAAGGATATTATTCCAAAGTTTCTGAAAAACGAGGGAGACTTGGGTACT
A3-AT1G77180-XLOC_008117-4874-1
ATAAGGATATTATTCCAAAGTTTCTGAAAAACGAGGGAGACTTGGGTACT
CONSENSUS
ATAAGGATATTATTCCAAAGTTTCTGAAAAACGAGGGAGACTTGGGTACT

A3-AT1G77180-XLOC_008117-4874-0
GTTGTTGATGAAGAGGAGGAATTGCAGAAGGAGATTCAGGAGACTGCTGA
A3-AT1G77180-XLOC_008117-4874-1
GTTGTTGATGAAGAGGAGGAATTGCAGAAGGAGATTCAGGAGACTGCTGA
CONSENSUS
GTTGTTGATGAAGAGGAGGAATTGCAGAAGGAGATTCAGGAGACTGCTGA

A3-AT1G77180-XLOC_008117-4874-0
AGAAACGAAAGCTGCCATTGAGAAGATTGTGAATGTGAGACTGAGTGCAG
A3-AT1G77180-XLOC_008117-4874-1
AGAAACGAAAGCTGCCATTGAGAAGATTGTGAATGTGAGACTGAGTGCAG
CONSENSUS
AGAAACGAAAGCTGCCATTGAGAAGATTGTGAATGTGAGACTGAGTGCAG

A3-AT1G77180-XLOC_008117-4874-0
CGCAGCCTAGTAATATAGCAAGGCAATCGGGAGATTCACAGTACATTAAA
A3-AT1G77180-XLOC_008117-4874-1
CGCAGCCTAGTAATATAGCAAGGCAATCGGGAGATTCACAGTACATTAAA
CONSENSUS
CGCAGCCTAGTAATATAGCAAGGCAATCGGGAGATTCACAGTACATTAAA

A3-AT1G77180-XLOC_008117-4874-0
TATAAGCCATCCCAGCAATCTTCTGCCTTCAATTCTGGTGCTAAAGAGAG
A3-AT1G77180-XLOC_008117-4874-1
TATAAGCCATCCCAGCAATCTTCTGCCTTCAATTCTGGTGCTAAAGAGAG
CONSENSUS
TATAAGCCATCCCAGCAATCTTCTGCCTTCAATTCTGGTGCTAAAGAGAG

A3-AT1G77180-XLOC_008117-4874-0
GATCATTAGGATGGTGGAGATGCCTGTAGATCCACTTGATCCACCAAAGT
A3-AT1G77180-XLOC_008117-4874-1
GATCATTAGGATGGTGGAGATGCCTGTAGATCCACTTGATCCACCAAAGT

CONSENSUS
GATCATTAGGATGGTGGAGATGCCTGTAGATCCACTTGATCCACCAAAGT

A3-AT1G77180-XLOC_008117-4874-0
TCAAGCACAAGAGAGTCCCAAGGGCTTCTGGTTCTCCGCCTGTTCCGGTC
A3-AT1G77180-XLOC_008117-4874-1
TCAAGCACAAGAGAGTCCCAAGGGCTTCTGGTTCTCCGCCTGTTCCGGTC
CONSENSUS
TCAAGCACAAGAGAGTCCCAAGGGCTTCTGGTTCTCCGCCTGTTCCGGTC

A3-AT1G77180-XLOC_008117-4874-0
ATGCATTCACCGCCCAGGCCTGTTACCGTCAAGGACCAGCAAGACTGGAA
A3-AT1G77180-XLOC_008117-4874-1
ATGCATTCACCGCCCAGGCCTGTTACCGTCAAGGACCAGCAAGACTGGAA
CONSENSUS
ATGCATTCACCGCCCAGGCCTGTTACCGTCAAGGACCAGCAAGACTGGAA

A3-AT1G77180-XLOC_008117-4874-0
AATCCCTCCTTGTATCTCCAATTGGAAGAATCCGAAAGGTTACACAATCC
A3-AT1G77180-XLOC_008117-4874-1
AATCCCTCCTTGTATCTCCAATTGGAAGAATCCGAAAGGTTACACAATCC
CONSENSUS
AATCCCTCCTTGTATCTCCAATTGGAAGAATCCGAAAGGTTACACAATCC

A3-AT1G77180-XLOC_008117-4874-0
CTCTTGACAAACGTCTAGCTGCTGATGGAAGGGGCCTACAAGACGTTCAG
A3-AT1G77180-XLOC_008117-4874-1
CTCTTGACAAACGTCTAGCTGCTGATGGAAGGGGCCTACAAGACGTTCAG
CONSENSUS
CTCTTGACAAACGTCTAGCTGCTGATGGAAGGGGCCTACAAGACGTTCAG

A3-AT1G77180-XLOC_008117-4874-0
ATCAATGATAACTTTGCCAAGTTATCAGAGGCCCTCTATGTAGCTGAGCA
A3-AT1G77180-XLOC_008117-4874-1
ATCAATGATAACTTTGCCAAGTTATCAGAGGCCCTCTATGTAGCTGAGCA
CONSENSUS
ATCAATGATAACTTTGCCAAGTTATCAGAGGCCCTCTATGTAGCTGAGCA

A3-AT1G77180-XLOC_008117-4874-0
GAAAGCTAGAGAGGCTGTTTCAATGCGTTCCAAGGTGCAAAAAGAGATGG
A3-AT1G77180-XLOC_008117-4874-1
GAAAGCTAGAGAGGCTGTTTCAATGCGTTCCAAGGTGCAAAAAGAGATGG
CONSENSUS
GAAAGCTAGAGAGGCTGTTTCAATGCGTTCCAAGGTGCAAAAAGAGATGG

A3-AT1G77180-XLOC_008117-4874-0
TGATGAAGGACAAGGAAAGGAAAGAGCAGGAATTGAGGGCTCTTGCCCAA
A3-AT1G77180-XLOC_008117-4874-1
TGATGAAGGACAAGGAAAGGAAAGAGCAGGAATTGAGGGCTCTTGCCCAA
CONSENSUS
TGATGAAGGACAAGGAAAGGAAAGAGCAGGAATTGAGGGCTCTTGCCCAA

A3-AT1G77180-XLOC_008117-4874-0
AAAGCTCGCTCCGAAAGAACCGGTGCTGCTATGAGTATGCCTGTCTCATC
A3-AT1G77180-XLOC_008117-4874-1
AAAGCTCGCTCCGAAAGAACCGGTGCTGCTATGAGTATGCCTGTCTCATC

CONSENSUS
AAAGCTCGCTCCGAAAGAACCGGTGCTGCTATGAGTATGCCTGTCTCATC

A3-AT1G77180-XLOC_008117-4874-0
TGACAGGGGTAGGAGTGAAAGTGTTGACCCAAGAGGTGATTATGACAACT
A3-AT1G77180-XLOC_008117-4874-1
TGACAGGGGTAGGAGTGAAAGTGTTGACCCAAGAGGTGATTATGACAACT
CONSENSUS
TGACAGGGGTAGGAGTGAAAGTGTTGACCCAAGAGGTGATTATGACAACT

A3-AT1G77180-XLOC_008117-4874-0
ATGACCAGGATAGAGGAAGGGAAAGAGAAAGGGAAGAGCCTCAAGAGACG
A3-AT1G77180-XLOC_008117-4874-1
ATGACCAGGATAGAGGAAGGGAAAGAGAAAGGGAAGAGCCTCAAGAGACG
CONSENSUS
ATGACCAGGATAGAGGAAGGGAAAGAGAAAGGGAAGAGCCTCAAGAGACG

A3-AT1G77180-XLOC_008117-4874-0
AGGGAGGAGAGGGAAAAAAGGATTCAGAGAGAAAAGATACGAGAAGAGCG
A3-AT1G77180-XLOC_008117-4874-1
AGGGAGGAGAGGGAAAAAAGGATTCAGAGAGAAAAGATACGAGAAGAGCG
CONSENSUS
AGGGAGGAGAGGGAAAAAAGGATTCAGAGAGAAAAGATACGAGAAGAGCG

A3-AT1G77180-XLOC_008117-4874-0
TCGCAGGGAAAGAGAGAGGGAGAGAAGGTTGGATGCCAAAGATGCTGCCA
A3-AT1G77180-XLOC_008117-4874-1
TCGCAGGGAAAGAGAGAGGGAGAGAAGGTTGGATGCCAAAGATGCTGCCA
CONSENSUS
TCGCAGGGAAAGAGAGAGGGAGAGAAGGTTGGATGCCAAAGATGCTGCCA

A3-AT1G77180-XLOC_008117-4874-0
TGGGGAAGAAGAGCAAGATCACCAGAGACAGAGACCGTGACATCAGTGAG
A3-AT1G77180-XLOC_008117-4874-1
TGGGGAAGAAGAGCAAGATCACCAGAGACAGAGACCGTGACATCAGTGAG
CONSENSUS
TGGGGAAGAAGAGCAAGATCACCAGAGACAGAGACCGTGACATCAGTGAG

A3-AT1G77180-XLOC_008117-4874-0
AAAGTTGCTCTTGGAATGGCATCTACTGGAGGAAAAGGTGGTGGTGAAGT
A3-AT1G77180-XLOC_008117-4874-1
AAAGTTGCTCTTGGAATGGCATCTACTGGAGGAAAAGGTGGTGGTGAAGT
CONSENSUS
AAAGTTGCTCTTGGAATGGCATCTACTGGAGGAAAAGGTGGTGGTGAAGT

A3-AT1G77180-XLOC_008117-4874-0
TATGTATGATCAACGTCTGTTTAACCAAGACAAAGGAATGGACTCTGGTT
A3-AT1G77180-XLOC_008117-4874-1
TATGTATGATCAACGTCTGTTTAACCAAGACAAAGGAATGGACTCTGGTT
CONSENSUS
TATGTATGATCAACGTCTGTTTAACCAAGACAAAGGAATGGACTCTGGTT

A3-AT1G77180-XLOC_008117-4874-0
TCGCTGCTGATGACCAATACAACTTATACGACAAAGGCTTGTTCACTGCA
A3-AT1G77180-XLOC_008117-4874-1
TCGCTGCTGATGACCAATACAACTTATACGACAAAGGCTTGTTCACTGCA

CONSENSUS
TCGCTGCTGATGACCAATACAACTTATACGACAAAGGCTTGTTCACTGCA

A3-AT1G77180-XLOC_008117-4874-0
CAACCAACTCTTTCGACTTTGTACAAGCCAAAGAAGGATAACGATGAAGA
A3-AT1G77180-XLOC_008117-4874-1
CAACCAACTCTTTCGACTTTGTACAAGCCAAAGAAGGATAACGATGAAGA
CONSENSUS
CAACCAACTCTTTCGACTTTGTACAAGCCAAAGAAGGATAACGATGAAGA

A3-AT1G77180-XLOC_008117-4874-0
AATGTATGGAAACGCTGATGAGCAGCTCGATAAGATCAAGAATACTGAGA
A3-AT1G77180-XLOC_008117-4874-1
AATGTATGGAAACGCTGATGAGCAGCTCGATAAGATCAAGAATACTGAGA
CONSENSUS
AATGTATGGAAACGCTGATGAGCAGCTCGATAAGATCAAGAATACTGAGA

A3-AT1G77180-XLOC_008117-4874-0
GGTTTAAGCCTGACAAAGCTTTCACAGGGGCTTCAGAGAGGGTGGGTAGT
A3-AT1G77180-XLOC_008117-4874-1
GGTTTAAGCCTGACAAAGCTTTCACAGGGGCTTCAGAGAGGGTGGGTAGT
CONSENSUS
GGTTTAAGCCTGACAAAGCTTTCACAGGGGCTTCAGAGAGGGTGGGTAGT

A3-AT1G77180-XLOC_008117-4874-0
AAGAGAGACCGACCTGTTGAGTTTGAGAAGGAAGAGGAACAAGATCCTTT
A3-AT1G77180-XLOC_008117-4874-1
AAGAGAGACCGACCTGTTGAGTTTGAGAAGGAAGAGGAACAAGATCCTTT
CONSENSUS
AAGAGAGACCGACCTGTTGAGTTTGAGAAGGAAGAGGAACAAGATCCTTT

A3-AT1G77180-XLOC_008117-4874-0
CGGTCTTGAGAAGTGGGTTTCTGATTTAAAGAAGGGTAAGAAACCTTTGG
A3-AT1G77180-XLOC_008117-4874-1
CGGTCTTGAGAAGTGGGTTTCTGATTTAAAGAAGGGTAAGAAACCTTTGG
CONSENSUS
CGGTCTTGAGAAGTGGGTTTCTGATTTAAAGAAGGGTAAGAAACCTTTGG

A3-AT1G77180-XLOC_008117-4874-0
ACAAGATCGGGTCTGGTGGAACTATGAGAGCAAGTGGCGGTGGTGGTAGC
A3-AT1G77180-XLOC_008117-4874-1
ACAAGATCGGGTCTGGTGGAACTATGAGAGCAAGTGGCGGTGGTGGTAGC
CONSENSUS
ACAAGATCGGGTCTGGTGGAACTATGAGAGCAAGTGGCGGTGGTGGTAGC

A3-AT1G77180-XLOC_008117-4874-0
TCTTCAAGGGACGATGACCACGGCGGTTCTGGTCGAACCAAGATCAATTT
A3-AT1G77180-XLOC_008117-4874-1
TCTTCAAGGGACGATGACCACGGCGGTTCTGGTCGAACCAAGATCAATTT
CONSENSUS
TCTTCAAGGGACGATGACCACGGCGGTTCTGGTCGAACCAAGATCAATTT

A3-AT1G77180-XLOC_008117-4874-0
CGAACGCAGTGACCGGCGTTAAAAGTCAAGACAAAGAAAAAGCTCAGTAA
A3-AT1G77180-XLOC_008117-4874-1
CGAACGCAGTGACCGGCGTTAAAAGTCAAGACAAAGAAAAAGCTCAGTAA

```
CONSENSUS
      CGAACGCAGTGACCGGCGTTAAAAGTCAAGACAAAGAAAAAGCTCAGTAA

A3-AT1G77180-XLOC_008117-4874-0
      CTGATCTTTGATTTCAATATATAACTTGTTATTTTGGATCTGTTAAAACA
A3-AT1G77180-XLOC_008117-4874-1
      CTGATCTTTGATTTCAATATATAACTTGTTATTTTGGATCTGTTAAAACA
CONSENSUS
      CTGATCTTTGATTTCAATATATAACTTGTTATTTTGGATCTGTTAAAACA

A3-AT1G77180-XLOC_008117-4874-0
      GTTTGCTTTGGATTATCAGTATTTATGTTGTTGTGAGTCTCTTAAACTAC
A3-AT1G77180-XLOC_008117-4874-1
      GTTTGCTTTGGATTATCAGTATTTATGTTGTTGTGAGTCTCTTAAACTAC
CONSENSUS
      GTTTGCTTTGGATTATCAGTATTTATGTTGTTGTGAGTCTCTTAAACTAC

A3-AT1G77180-XLOC_008117-4874-0
      TCGAATACAATCTTTCAGCTTCATAATATCAGATCTTTTGAGCTTATTTT
A3-AT1G77180-XLOC_008117-4874-1
      TCGAATACAATCTTTCAGCTTCATAATATCAGATCTTTTGAGCTTATTTT
CONSENSUS
      TCGAATACAATCTTTCAGCTTCATAATATCAGATCTTTTGAGCTTATTTT

A3-AT1G77180-XLOC_008117-4874-0  AGTTT
A3-AT1G77180-XLOC_008117-4874-1  AGTTT
CONSENSUS                        AGTTT


alignment for event: SE-AT1G06220-XLOC_000280-1975

SE-AT1G06220-XLOC_000280-1975-0
      AGAGTCTTCCAATCAAAACCTAAATCTAAATTTTTCATTCCCTTGTTCCG
SE-AT1G06220-XLOC_000280-1975-1
      AGAGTCTTCCAATCAAAACCTAAATCTAAATTTTTCATTCCCTTGTTCCG
CONSENSUS
      AGAGTCTTCCAATCAAAACCTAAATCTAAATTTTTCATTCCCTTGTTCCG

SE-AT1G06220-XLOC_000280-1975-0
      GCGATAACAGTCTCAGTGGCGACAAACCCTATTGCCGAGCCAACTCGCAT
SE-AT1G06220-XLOC_000280-1975-1
      GCGATAACAGTCTCAGTGGCGACAAACCCTATTGCCGAGCCAACTCGCAT
CONSENSUS
      GCGATAACAGTCTCAGTGGCGACAAACCCTATTGCCGAGCCAACTCGCAT

SE-AT1G06220-XLOC_000280-1975-0
      TTTCCTCTAGCTATCGCAACGCCTCTCGCTCTCTCTCCCCACCG------
SE-AT1G06220-XLOC_000280-1975-1
      TTTCCTCTAGCTATCGCAACGCCTCTCGCTCTCTCTCCCCACCGCTTTTT
CONSENSUS
      TTTCCTCTAGCTATCGCAACGCCTCTCGCTCTCTCTCCCCACCG......

SE-AT1G06220-XLOC_000280-1975-0
      --------------------------------------------------
SE-AT1G06220-XLOC_000280-1975-1
```

CATGGTGTTCTCTTGGACTTGGTGTTGAGCATTAGAGGAGATTACTCGCC
CONSENSUS
....

SE-AT1G06220-XLOC_000280-1975-0

SE-AT1G06220-XLOC_000280-1975-1
AATGGGTTAAATAAAATATGTTGCAGAATTGCTATAGTAAATAACCCAGA
CONSENSUS
....

SE-AT1G06220-XLOC_000280-1975-0 ---------------------------
GTTTACATAAAGATGGAAAGTAG
SE-AT1G06220-XLOC_000280-1975-1
TAGATCTATCAAAAGCTTGATTTTGTGGTTTACATAAAGATGGAAAGTAG
CONSENSUS
...........................GTTTACATAAAGATGGAAAGTAG

SE-AT1G06220-XLOC_000280-1975-0
CTTGTATGATGAGTTTGGTAACTATGTTGGACCTGAGATTGAGTCTGACA
SE-AT1G06220-XLOC_000280-1975-1
CTTGTATGATGAGTTTGGTAACTATGTTGGACCTGAGATTGAGTCTGACA
CONSENSUS
CTTGTATGATGAGTTTGGTAACTATGTTGGACCTGAGATTGAGTCTGACA

SE-AT1G06220-XLOC_000280-1975-0
GAGATAGTGATGATGAAGTAGAAGATGAAGATCTCCAAGATAAGCATCTT
SE-AT1G06220-XLOC_000280-1975-1
GAGATAGTGATGATGAAGTAGAAGATGAAGATCTCCAAGATAAGCATCTT
CONSENSUS
GAGATAGTGATGATGAAGTAGAAGATGAAGATCTCCAAGATAAGCATCTT

SE-AT1G06220-XLOC_000280-1975-0
GAAGAAAATGGTTCCGATGGCGAACAAGGGCCTGGGGGTTCTAATGGCTG
SE-AT1G06220-XLOC_000280-1975-1
GAAGAAAATGGTTCCGATGGCGAACAAGGGCCTGGGGGTTCTAATGGCTG
CONSENSUS
GAAGAAAATGGTTCCGATGGCGAACAAGGGCCTGGGGGTTCTAATGGCTG

SE-AT1G06220-XLOC_000280-1975-0
GATTACCACTATCAATGATGTTGAGATGGAGAACCAAATTGTTCTTCCTG
SE-AT1G06220-XLOC_000280-1975-1
GATTACCACTATCAATGATGTTGAGATGGAGAACCAAATTGTTCTTCCTG
CONSENSUS
GATTACCACTATCAATGATGTTGAGATGGAGAACCAAATTGTTCTTCCTG

SE-AT1G06220-XLOC_000280-1975-0
AGGATAAGAAGTACTACCCTACTGCTGAGGAGGTTTATGGTGAGGATGTT
SE-AT1G06220-XLOC_000280-1975-1
AGGATAAGAAGTACTACCCTACTGCTGAGGAGGTTTATGGTGAGGATGTT
CONSENSUS
AGGATAAGAAGTACTACCCTACTGCTGAGGAGGTTTATGGTGAGGATGTT

SE-AT1G06220-XLOC_000280-1975-0
GAGACCTTGGTTATGGATGAAGATGAGCAGCCTCTTGAGCAACCCATTAT
SE-AT1G06220-XLOC_000280-1975-1

GAGACCTTGGTTATGGATGAAGATGAGCAGCCTCTTGAGCAACCCATTAT
CONSENSUS
GAGACCTTGGTTATGGATGAAGATGAGCAGCCTCTTGAGCAACCCATTAT

SE-AT1G06220-XLOC_000280-1975-0
CAAACCTGTTAGAGATATTAGATTTGAGGTGGGGGTCAAAGATCAAGCAA
SE-AT1G06220-XLOC_000280-1975-1
CAAACCTGTTAGAGATATTAGATTTGAGGTGGGGGTCAAAGATCAAGCAA
CONSENSUS
CAAACCTGTTAGAGATATTAGATTTGAGGTGGGGGTCAAAGATCAAGCAA

SE-AT1G06220-XLOC_000280-1975-0
CGTATGTGTCAACACAGTTTCTTATCGGCCTCATGTCTAATCCTGCGCTT
SE-AT1G06220-XLOC_000280-1975-1
CGTATGTGTCAACACAGTTTCTTATCGGCCTCATGTCTAATCCTGCGCTT
CONSENSUS
CGTATGTGTCAACACAGTTTCTTATCGGCCTCATGTCTAATCCTGCGCTT

SE-AT1G06220-XLOC_000280-1975-0
GTGAGGAATGTTGCTCTTGTGGGGCATCTACAGCACGGCAAAACTGTCTT
SE-AT1G06220-XLOC_000280-1975-1
GTGAGGAATGTTGCTCTTGTGGGGCATCTACAGCACGGCAAAACTGTCTT
CONSENSUS
GTGAGGAATGTTGCTCTTGTGGGGCATCTACAGCACGGCAAAACTGTCTT

SE-AT1G06220-XLOC_000280-1975-0
CATGGATATGTTGGTAGAGCAGACGCATCATATGTCTACTTTTAATGCTA
SE-AT1G06220-XLOC_000280-1975-1
CATGGATATGTTGGTAGAGCAGACGCATCATATGTCTACTTTTAATGCTA
CONSENSUS
CATGGATATGTTGGTAGAGCAGACGCATCATATGTCTACTTTTAATGCTA

SE-AT1G06220-XLOC_000280-1975-0
AAAACGAGAAGCATATGAAATATACAGACACACGAGTCGATGAGCAGGAG
SE-AT1G06220-XLOC_000280-1975-1
AAAACGAGAAGCATATGAAATATACAGACACACGAGTCGATGAGCAGGAG
CONSENSUS
AAAACGAGAAGCATATGAAATATACAGACACACGAGTCGATGAGCAGGAG

SE-AT1G06220-XLOC_000280-1975-0
AGAAATATATCAATCAAGGCGGTTCCAATGTCTCTTGTCCTTGAGGACAG
SE-AT1G06220-XLOC_000280-1975-1
AGAAATATATCAATCAAGGCGGTTCCAATGTCTCTTGTCCTTGAGGACAG
CONSENSUS
AGAAATATATCAATCAAGGCGGTTCCAATGTCTCTTGTCCTTGAGGACAG

SE-AT1G06220-XLOC_000280-1975-0
TAGATCCAAATCATACCTGTGCAATATCATGGATACCCCTGGACATGTCA
SE-AT1G06220-XLOC_000280-1975-1
TAGATCCAAATCATACCTGTGCAATATCATGGATACCCCTGGACATGTCA
CONSENSUS
TAGATCCAAATCATACCTGTGCAATATCATGGATACCCCTGGACATGTCA

SE-AT1G06220-XLOC_000280-1975-0
ATTTCTCTGACGAAATGACTGCTTCTTTAAGACTTGCTGATGGTGCTGTT
SE-AT1G06220-XLOC_000280-1975-1

ATTTCTCTGACGAAATGACTGCTTCTTTAAGACTTGCTGATGGTGCTGTT
CONSENSUS
ATTTCTCTGACGAAATGACTGCTTCTTTAAGACTTGCTGATGGTGCTGTT

SE-AT1G06220-XLOC_000280-1975-0 CTGATTGTTGATGCTGCTGAAGGAGTGATG
SE-AT1G06220-XLOC_000280-1975-1 CTGATTGTTGATGCTGCTGAAGGAGTGATG
CONSENSUS CTGATTGTTGATGCTGCTGAAGGAGTGATG

alignment for event: RI-AT1G79245-XLOC_008236-227

RI-AT1G79245-XLOC_008236-227-0
ACCTTTCAACCACACATCTTCTTCCTTTCCTGAATAAGTTTTCTCTACGC
RI-AT1G79245-XLOC_008236-227-1
ACCTTTCAACCACACATCTTCTTCCTTTCCTGAATAAGTTTTCTCTACGC
CONSENSUS
ACCTTTCAACCACACATCTTCTTCCTTTCCTGAATAAGTTTTCTCTACGC

RI-AT1G79245-XLOC_008236-227-0
GTTCGCTGGTGATATTCTTCTAAACGCGTCTCTGATTTGTTTGAATCGAT
RI-AT1G79245-XLOC_008236-227-1
GTTCGCTGGTGATATTCTTCTAAACGCGTCTCTGATTTGTTTGAATCGAT
CONSENSUS
GTTCGCTGGTGATATTCTTCTAAACGCGTCTCTGATTTGTTTGAATCGAT

RI-AT1G79245-XLOC_008236-227-0
CATCGTTCCTTCTTCTGGATTTTCCATCGATATTTGATTTTTCTAGGGTT
RI-AT1G79245-XLOC_008236-227-1
CATCGTTCCTTCTTCTGGATTTTCCATCGATATTTGATTTTTCTAGGGTT
CONSENSUS
CATCGTTCCTTCTTCTGGATTTTCCATCGATATTTGATTTTTCTAGGGTT

RI-AT1G79245-XLOC_008236-227-0
TGTTTCTAAATTGCATCGATGGCTTTTTATGTTCATGCGATTTACTTTTC
RI-AT1G79245-XLOC_008236-227-1
TGTTTCTAAATTGCATCGATGGCTTTTTATGTTCATGCGATTTACTTTTC
CONSENSUS
TGTTTCTAAATTGCATCGATGGCTTTTTATGTTCATGCGATTTACTTTTC

RI-AT1G79245-XLOC_008236-227-0
CCTCTGTTATTCTAGATTGTTGTTCGATGATTTGATCGTTCAGGGATGGA
RI-AT1G79245-XLOC_008236-227-1
CCTCTGTTATTCTAGATTGTTGTTCGATGATTTGATCGTTCAGGGATGGA
CONSENSUS
CCTCTGTTATTCTAGATTGTTGTTCGATGATTTGATCGTTCAGGGATGGA

RI-AT1G79245-XLOC_008236-227-0
TTTGATGGATCTTATGTTTTCACGGGGCTTCTCTATGCTGCTACGTCTTC
RI-AT1G79245-XLOC_008236-227-1
TTTGATGGATCTTATGTTTTCACGGGGCTTCTCTATGCTGCTACGTCTTC
CONSENSUS
TTTGATGGATCTTATGTTTTCACGGGGCTTCTCTATGCTGCTACGTCTTC

RI-AT1G79245-XLOC_008236-227-0
AGGGGGTGTTCTTCTTCCTTACGCGTCTCTGATTTTTGTTTTCTTCTCTA

RI-AT1G79245-XLOC_008236-227-1
AGGGGGTGTTCTTCTTCCTTACGCGTCTCTGATTTTTGTTTTCTTCTCTA
CONSENSUS
AGGGGGTGTTCTTCTTCCTTACGCGTCTCTGATTTTTGTTTTCTTCTCTA

RI-AT1G79245-XLOC_008236-227-0
CGTTTCTTATTCGTCTCGTCTCGCAGTTTTTTTGGAAGCTAATTATGATC
RI-AT1G79245-XLOC_008236-227-1
CGTTTCTTATTCGTCTCGTCTCGCAGTTTTTTTGGAAGCTAATTATGATC
CONSENSUS
CGTTTCTTATTCGTCTCGTCTCGCAGTTTTTTTGGAAGCTAATTATGATC

RI-AT1G79245-XLOC_008236-227-0
CGATCATCGCTTTCGATTCTGTTGTTGTAGAGTGTGGATGAGGCTATGAT
RI-AT1G79245-XLOC_008236-227-1
CGATCATCGCTTTCGATTCTGTTGTTGTAGAGTGTGGATGAGGCTATGAT
CONSENSUS
CGATCATCGCTTTCGATTCTGTTGTTGTAGAGTGTGGATGAGGCTATGAT

RI-AT1G79245-XLOC_008236-227-0
CGATCGGTGATTGTTTTTTTCTGGATTTTTGTCACGCGTTTTCTCTAGGA
RI-AT1G79245-XLOC_008236-227-1
CGATCGGTGATTGTTTTTTTCTGGATTTTTGTCACGCGTTTTCTCTAGGA
CONSENSUS
CGATCGGTGATTGTTTTTTTCTGGATTTTTGTCACGCGTTTTCTCTAGGA

RI-AT1G79245-XLOC_008236-227-0
TACGTCTTCAAGCGAATTCTGATCCGATCTTCGTCCTCGCTTCTGAGTTT
RI-AT1G79245-XLOC_008236-227-1
TACGTCTTCAAGCGAATTCTGATCCGATCTTCGTCCTCGCTTCTGAGTTT
CONSENSUS
TACGTCTTCAAGCGAATTCTGATCCGATCTTCGTCCTCGCTTCTGAGTTT

RI-AT1G79245-XLOC_008236-227-0
TCTCTTCTCACTGATTAATTGATTTTCTAGGTTTTGGTTTATAAACTGCA
RI-AT1G79245-XLOC_008236-227-1
TCTCTTCTCACTGATTAATTGATTTTCTAGGTTTTGGTTTATAAACTGCA
CONSENSUS
TCTCTTCTCACTGATTAATTGATTTTCTAGGTTTTGGTTTATAAACTGCA

RI-AT1G79245-XLOC_008236-227-0
TCAAGTGGTCTTTCTAATTCATGTTATTTGCTCCTCGTTTTATTGTTGAT
RI-AT1G79245-XLOC_008236-227-1
TCAAGTGGTCTTTCTAATTCATGTTATTTGCTCCTCGTTTTATTGTTGAT
CONSENSUS
TCAAGTGGTCTTTCTAATTCATGTTATTTGCTCCTCGTTTTATTGTTGAT

RI-AT1G79245-XLOC_008236-227-0
TTAGATTGTGGAGGATGCTATGATTGATCGGTGATGGCTTTGATTGATCC
RI-AT1G79245-XLOC_008236-227-1
TTAGATTGTGGAGGATGCTATGATTGATCGGTGATGGCTTTGATTGATCC
CONSENSUS
TTAGATTGTGGAGGATGCTATGATTGATCGGTGATGGCTTTGATTGATCC

RI-AT1G79245-XLOC_008236-227-0
TTGTCACGCGGTTTCTTAGGATCTTTTGTCCCTTCTATTCCAAATTTTTA

RI-AT1G79245-XLOC_008236-227-1
TTGTCACGCGGTTTCTTAGGATCTTTTGTCCCTTCTATTCCAAATTTTTA
CONSENSUS
TTGTCACGCGGTTTCTTAGGATCTTTTGTCCCTTCTATTCCAAATTTTTA

RI-AT1G79245-XLOC_008236-227-0
ATTCCTATGCCGTTTTTTTCTTCTATATAAGCGGTAAATATCATCTCTAT
RI-AT1G79245-XLOC_008236-227-1
ATTCCTATGCCGTTTTTTTCTTCTATATAAGCGGTAAATATCATCTCTAT
CONSENSUS
ATTCCTATGCCGTTTTTTTCTTCTATATAAGCGGTAAATATCATCTCTAT

RI-AT1G79245-XLOC_008236-227-0
TGGTTGCAGATCGTCGAGGCACCGGAATTGCTCCGATAAATCTCTCATTC
RI-AT1G79245-XLOC_008236-227-1
TGGTTGCAGATCGTCGAGGCACCGGAATTGCTCCGATAAATCTCTCATTC
CONSENSUS
TGGTTGCAGATCGTCGAGGCACCGGAATTGCTCCGATAAATCTCTCATTC

RI-AT1G79245-XLOC_008236-227-0
TTCTCTGTCATCGATTTAGGTTTCGTCTGATTTCTCTTGTTACATCTGTT
RI-AT1G79245-XLOC_008236-227-1
TTCTCTGTCATCGATTTAGGTTTCGTCTGATTTCTCTTGTTACATCTGTT
CONSENSUS
TTCTCTGTCATCGATTTAGGTTTCGTCTGATTTCTCTTGTTACATCTGTT

RI-AT1G79245-XLOC_008236-227-0
TTATCTAACCACAATTACCTCTGTTCTATTTCGTCTCTGCTAATTGAAGG
RI-AT1G79245-XLOC_008236-227-1
TTATCTAACCACAATTACCTCTGTTCTATTTCGTCTCTGCTAATTGAAGG
CONSENSUS
TTATCTAACCACAATTACCTCTGTTCTATTTCGTCTCTGCTAATTGAAGG

RI-AT1G79245-XLOC_008236-227-0
ATGTGGTTGATCTGTCGTCGCTCCCTTTTACTTTCTGGCGGTTTCTTCTT
RI-AT1G79245-XLOC_008236-227-1
ATGTGGTTGATCTGTCGTCGCTCCCTTTTACTTTCTGGCGGTTTCTTCTT
CONSENSUS
ATGTGGTTGATCTGTCGTCGCTCCCTTTTACTTTCTGGCGGTTTCTTCTT

RI-AT1G79245-XLOC_008236-227-0
TCCCTTTGCTTCCTTTTTGGAGTTCTTAGATTACCTGATTTCTTTTCTTT
RI-AT1G79245-XLOC_008236-227-1
TCCCTTTGCTTCCTTTTTGGAGTTCTTAGATTACCTGATTTCTTTTCTTT
CONSENSUS
TCCCTTTGCTTCCTTTTTGGAGTTCTTAGATTACCTGATTTCTTTTCTTT

RI-AT1G79245-XLOC_008236-227-0
TCTGAATTTCTGGAGAAATTTCTACGTGTTAGATTGTGTAGATGTTGAAG
RI-AT1G79245-XLOC_008236-227-1
TCTGAATTTCTGGAGAAATTTCTACGTGTTAGATTGTGTAGATGTTGAAG
CONSENSUS
TCTGAATTTCTGGAGAAATTTCTACGTGTTAGATTGTGTAGATGTTGAAG

RI-AT1G79245-XLOC_008236-227-0
GCATTGTTGGAGGGAGGATGGTTTACGTATGAGTTGGTGTTTTTGGTTTC

RI-AT1G79245-XLOC_008236-227-1
GCATTGTTGGAGGGAGGATGGTTTACGTATGAGTTGGTGTTTTTGGTTTC
CONSENSUS
GCATTGTTGGAGGGAGGATGGTTTACGTATGAGTTGGTGTTTTTGGTTTC

RI-AT1G79245-XLOC_008236-227-0
TTCTCTTCTATACAACTTTTGGTCTAATTATTGTTTTATTTAATATTGGC
RI-AT1G79245-XLOC_008236-227-1
TTCTCTTCTATACAACTTTTGGTCTAATTATTGTTTTATTTAATATTGGC
CONSENSUS
TTCTCTTCTATACAACTTTTGGTCTAATTATTGTTTTATTTAATATTGGC

RI-AT1G79245-XLOC_008236-227-0
TAATGTTGGTGAGGTATGATTTCGTTCTCTGTGTCTTGTTGTGTTTTCCA
RI-AT1G79245-XLOC_008236-227-1
TAATGTTGGTGAGGTATGATTTCGTTCTCTGTGTCTTGTTGTGTTTTCCA
CONSENSUS
TAATGTTGGTGAGGTATGATTTCGTTCTCTGTGTCTTGTTGTGTTTTCCA

RI-AT1G79245-XLOC_008236-227-0
GATTCTCTTCATCAATCAATTCAGTCTGTTTCTCTTCTGTGTACACATTC
RI-AT1G79245-XLOC_008236-227-1
GATTCTCTTCATCAATCAATTCAGTCTGTTTCTCTTCTGTGTACACATTC
CONSENSUS
GATTCTCTTCATCAATCAATTCAGTCTGTTTCTCTTCTGTGTACACATTC

RI-AT1G79245-XLOC_008236-227-0
AGATGAAAGATGAACATATATTCCTTGCAGACGAGTTTTTTCCTATCTCT
RI-AT1G79245-XLOC_008236-227-1
AGATGAAAGATGAACATATATTCCTTGCAGACGAGTTTTTTCCTATCTCT
CONSENSUS
AGATGAAAGATGAACATATATTCCTTGCAGACGAGTTTTTTCCTATCTCT

RI-AT1G79245-XLOC_008236-227-0
TGTCCTTTTTATAAGTAGTTATTCTATTGAAGTCTTCAAGTTTCTCAGTA
RI-AT1G79245-XLOC_008236-227-1
TGTCCTTTTTATAAGTAGTTATTCTATTGAAGTCTTCAAGTTTCTCAGTA
CONSENSUS
TGTCCTTTTTATAAGTAGTTATTCTATTGAAGTCTTCAAGTTTCTCAGTA

RI-AT1G79245-XLOC_008236-227-0
ATGCTATGTCTTAGCTCCTCTCATGTGGTTTATTCAGGTTTTGTTCCTCT
RI-AT1G79245-XLOC_008236-227-1
ATGCTATGTCTTAGCTCCTCTCATGTGGTTTATTCAGGTTTTGTTCCTCT
CONSENSUS
ATGCTATGTCTTAGCTCCTCTCATGTGGTTTATTCAGGTTTTGTTCCTCT

RI-AT1G79245-XLOC_008236-227-0
TTGCCTCCGGGATCTGTTGCAATCTATTTGTTCTAACATACGCCCTGGTT
RI-AT1G79245-XLOC_008236-227-1
TTGCCTCCGGGATCTGTTGCAATCTATTTGTTCTAACATACGCCCTGGTT
CONSENSUS
TTGCCTCCGGGATCTGTTGCAATCTATTTGTTCTAACATACGCCCTGGTT

RI-AT1G79245-XLOC_008236-227-0
AACTGGTCTATTCCTCTTTCTCTCTATTTATAGTGTTTGATCCTACTCTC

RI-AT1G79245-XLOC_008236-227-1
AACTGGTCTATTCCTCTTTCTCTCTATTTATAGTGTTTGATCCTACTCTC
CONSENSUS
AACTGGTCTATTCCTCTTTCTCTCTATTTATAGTGTTTGATCCTACTCTC

RI-AT1G79245-XLOC_008236-227-0
TTATGCTTGTGACGGTGTTTTTTTTTATGTTAAGTGTCCCATTCTTTTAG
RI-AT1G79245-XLOC_008236-227-1
TTATGCTTGTGACGGTGTTTTTTTTTATGTTAAGTGTCCCATTCTTTTAG
CONSENSUS
TTATGCTTGTGACGGTGTTTTTTTTTATGTTAAGTGTCCCATTCTTTTAG

RI-AT1G79245-XLOC_008236-227-0
GTTTTTTGAAGATGCAAAGAAGGCTAGTTCTGTCTCAGTGTATTCTGAAA
RI-AT1G79245-XLOC_008236-227-1
GTTTTTTGAAGATGCAAAGAAGGCTAGTTCTGTCTCAGTGTATTCTGAAA
CONSENSUS
GTTTTTTGAAGATGCAAAGAAGGCTAGTTCTGTCTCAGTGTATTCTGAAA

RI-AT1G79245-XLOC_008236-227-0
GATATCTTTGATCTGTCGTGGTTCCTGTTACCTTTCTGGCGGTGTCTTAT
RI-AT1G79245-XLOC_008236-227-1
GATATCTTTGATCTGTCGTGGTTCCTGTTACCTTTCTGGCGGTGTCTTAT
CONSENSUS
GATATCTTTGATCTGTCGTGGTTCCTGTTACCTTTCTGGCGGTGTCTTAT

RI-AT1G79245-XLOC_008236-227-0
CTCCCTTTGTTTTCTTTTTAAGGTTTTTAATTTCCCTATTACCTGATGTT
RI-AT1G79245-XLOC_008236-227-1
CTCCCTTTGTTTTCTTTTTAAGGTTTTTAATTTCCCTATTACCTGATGTT
CONSENSUS
CTCCCTTTGTTTTCTTTTTAAGGTTTTTAATTTCCCTATTACCTGATGTT

RI-AT1G79245-XLOC_008236-227-0
TTTGTCGTTTTAAATCGTAGAGGTCAAGGCATTGTTGGAGGGAAGGATGG
RI-AT1G79245-XLOC_008236-227-1
TTTGTCGTTTTAAATCGTAGAGGTCAAGGCATTGTTGGAGGGAAGGATGG
CONSENSUS
TTTGTCGTTTTAAATCGTAGAGGTCAAGGCATTGTTGGAGGGAAGGATGG

RI-AT1G79245-XLOC_008236-227-0
TATGAGTTTAATTTTGTGTTTCTGGTTACTTCTCATCTACACACTTTTTT
RI-AT1G79245-XLOC_008236-227-1
TATGAGTTTAATTTTGTGTTTCTGGTTACTTCTCATCTACACACTTTTTT
CONSENSUS
TATGAGTTTAATTTTGTGTTTCTGGTTACTTCTCATCTACACACTTTTTT

RI-AT1G79245-XLOC_008236-227-0
CCCCCTAATTGTTTTATCTAATATTGGCTATTATTGTCAGTGAGGTATGA
RI-AT1G79245-XLOC_008236-227-1
CCCCCTAATTGTTTTATCTAATATTGGCTATTATTGTCAGTGAGGTATGA
CONSENSUS
CCCCCTAATTGTTTTATCTAATATTGGCTATTATTGTCAGTGAGGTATGA

RI-AT1G79245-XLOC_008236-227-0
TTTGTCCTCTGTGTCTTATGTTTCCCAGAATTTTCTTCACCAATCACTTC

RI-AT1G79245-XLOC_008236-227-1
TTTGTCCTCTGTGTCTTATGTTTCCCAGAATTTTCTTCACCAATCACTTC
CONSENSUS
TTTGTCCTCTGTGTCTTATGTTTCCCAGAATTTTCTTCACCAATCACTTC

RI-AT1G79245-XLOC_008236-227-0
ACTACGTTTCTCTGCTGTGTACACACTTGAAAGATGATCATATATTCCTT
RI-AT1G79245-XLOC_008236-227-1
ACTACGTTTCTCTGCTGTGTACACACTTGAAAGATGATCATATATTCCTT
CONSENSUS
ACTACGTTTCTCTGCTGTGTACACACTTGAAAGATGATCATATATTCCTT

RI-AT1G79245-XLOC_008236-227-0
CCAAACGAGTTTGTTCTTATACCTTGTCCTTTTAAGTAGTTTTTGTACTG
RI-AT1G79245-XLOC_008236-227-1
CCAAACGAGTTTGTTCTTATACCTTGTCCTTTTAAGTAGTTTTTGTACTG
CONSENSUS
CCAAACGAGTTTGTTCTTATACCTTGTCCTTTTAAGTAGTTTTTGTACTG

RI-AT1G79245-XLOC_008236-227-0
AGCATTTTCAAATATCTTAGGATATCTATCTCTGAGCTCCTCTGATGGAG
RI-AT1G79245-XLOC_008236-227-1
AGCATTTTCAAATATCTTAGGATATCTATCTCTGAGCTCCTCTGATGGAG
CONSENSUS
AGCATTTTCAAATATCTTAGGATATCTATCTCTGAGCTCCTCTGATGGAG

RI-AT1G79245-XLOC_008236-227-0
AAGGCTTGGCCTGAGGTCTGGACTGTACCATCAGGGAGTTCTCAACATGA
RI-AT1G79245-XLOC_008236-227-1
AAGGCTTGGCCTGAGGTCTGGACTGTACCATCAGGGAGTTCTCAACATGA
CONSENSUS
AAGGCTTGGCCTGAGGTCTGGACTGTACCATCAGGGAGTTCTCAACATGA

RI-AT1G79245-XLOC_008236-227-0
CTTGATTTTTGATTTTTCAGGTTAAAGGTAGATTTTCGTAGCGATGTTGG
RI-AT1G79245-XLOC_008236-227-1
CTTGATTTTTGATTTTTCAGGTTAAAGGTAGATTTTCGTAGCGATGTTGG
CONSENSUS
CTTGATTTTTGATTTTTCAGGTTAAAGGTAGATTTTCGTAGCGATGTTGG

RI-AT1G79245-XLOC_008236-227-0
CATTTCTGTTTTAAGCCTAAGGCCCTGTCTTTTGTTATAGATATTCAAGC
RI-AT1G79245-XLOC_008236-227-1
CATTTCTGTTTTAAGCCTAAGGCCCTGTCTTTTGTTATAGATATTCAAGC
CONSENSUS
CATTTCTGTTTTAAGCCTAAGGCCCTGTCTTTTGTTATAGATATTCAAGC

RI-AT1G79245-XLOC_008236-227-0
TTATAAAAGGGAAAAGGTTCAGGTTTACTAAATGGATTCTTTCCCCTTTA
RI-AT1G79245-XLOC_008236-227-1
TTATAAAAGGGAAAAGGTTCAGGTTTACTAAATGGATTCTTTCCCCTTTA
CONSENSUS
TTATAAAAGGGAAAAGGTTCAGGTTTACTAAATGGATTCTTTCCCCTTTA

RI-AT1G79245-XLOC_008236-227-0
TCTCTGTTGGTGTTATCTCTCATGCTACATCTATATTAGTGATGGCTTGT

RI-AT1G79245-XLOC_008236-227-1
TCTCTGTTGGTGTTATCTCTCATGCTACATCTATATTAGTGATGGCTTGT
CONSENSUS
TCTCTGTTGGTGTTATCTCTCATGCTACATCTATATTAGTGATGGCTTGT

RI-AT1G79245-XLOC_008236-227-0
TTTTATGTATGTGAAGTGCTCCGCTCCCCTATATAGGTATGTTGAGGATG
RI-AT1G79245-XLOC_008236-227-1
TTTTATGTATGTGAAGTGCTCCGCTCCCCTATATAGGTATGTTGAGGATG
CONSENSUS
TTTTATGTATGTGAAGTGCTCCGCTCCCCTATATAGGTATGTTGAGGATG

RI-AT1G79245-XLOC_008236-227-0
CAAAGGTGGCAAGTTCTACTTTGATCTATTTTTCTCGAAATTTTCATCTA
RI-AT1G79245-XLOC_008236-227-1
CAAAGGTGGCAAGTTCTACTTTGATCTATTTTTCTCGAAATTTTCATCTA
CONSENSUS
CAAAGGTGGCAAGTTCTACTTTGATCTATTTTTCTCGAAATTTTCATCTA

RI-AT1G79245-XLOC_008236-227-0
CAGTCCTCTTTTCATTGGAGATGTCTCTATTTCTGGTTCTATGCTCTGCA
RI-AT1G79245-XLOC_008236-227-1
CAGTCCTCTTTTCATTGGAGATGTCTCTATTTCTGGTTCTATGCTCTGCA
CONSENSUS
CAGTCCTCTTTTCATTGGAGATGTCTCTATTTCTGGTTCTATGCTCTGCA

RI-AT1G79245-XLOC_008236-227-0
TTTTCCTATGGTTTAAATGCTAATCACTTCGGCTCGAGATCTACTCTTTC
RI-AT1G79245-XLOC_008236-227-1
TTTTCCTATGGTTTAAATGCTAATCACTTCGGCTCGAGATCTACTCTTTC
CONSENSUS
TTTTCCTATGGTTTAAATGCTAATCACTTCGGCTCGAGATCTACTCTTTC

RI-AT1G79245-XLOC_008236-227-0
TACATGAATTCATTATCTTCATATAGGAATTTGGATGATGCTAGGACGAT
RI-AT1G79245-XLOC_008236-227-1
TACATGAATTCATTATCTTCATATAGGAATTTGGATGATGCTAGGACGAT
CONSENSUS
TACATGAATTCATTATCTTCATATAGGAATTTGGATGATGCTAGGACGAT

RI-AT1G79245-XLOC_008236-227-0
TCTGTTTCGACCTGTTCGCCTAGATTTATTGGCAACGAGGGATGGATGTA
RI-AT1G79245-XLOC_008236-227-1
TCTGTTTCGACCTGTTCGCCTAGATTTATTGGCAACGAGGGATGGATGTA
CONSENSUS
TCTGTTTCGACCTGTTCGCCTAGATTTATTGGCAACGAGGGATGGATGTA

RI-AT1G79245-XLOC_008236-227-0
ATCAGCTTTTCTTCCCTTATCTGTTTTCCCTTTCTGGTTATGTAATCTGC
RI-AT1G79245-XLOC_008236-227-1
ATCAGCTTTTCTTCCCTTATCTGTTTTCCCTTTCTGGTTATGTAATCTGC
CONSENSUS
ATCAGCTTTTCTTCCCTTATCTGTTTTCCCTTTCTGGTTATGTAATCTGC

RI-AT1G79245-XLOC_008236-227-0
ATCTTCTCTATGGTAATCTCTTCTGCTTCAGATGAAATCATTATCTTCAC

RI-AT1G79245-XLOC_008236-227-1
ATCTTCTCTATGGTAATCTCTTCTGCTTCAGATGAAATCATTATCTTCAC
CONSENSUS
ATCTTCTCTATGGTAATCTCTTCTGCTTCAGATGAAATCATTATCTTCAC

RI-AT1G79245-XLOC_008236-227-0
ATAGGAGTTTGTCTTCACATAGGAGTTTGGATGTTGCAGTGGAAGTTCTG
RI-AT1G79245-XLOC_008236-227-1
ATAGGAGTTTGTCTTCACATAGGAGTTTGGATGTTGCAGTGGAAGTTCTG
CONSENSUS
ATAGGAGTTTGTCTTCACATAGGAGTTTGGATGTTGCAGTGGAAGTTCTG

RI-AT1G79245-XLOC_008236-227-0
TTTCGGCCTGCTTGCCTACATCGCCCTATATCTGTTACATCATGAGTTTA
RI-AT1G79245-XLOC_008236-227-1
TTTCGGCCTGCTTGCCTACATCGCCCTATATCTGTTACATCATGAGTTTA
CONSENSUS
TTTCGGCCTGCTTGCCTACATCGCCCTATATCTGTTACATCATGAGTTTA

RI-AT1G79245-XLOC_008236-227-0
TGAGGTGGCTTATTTCTATTGATATCTATAGTGTTGATTGCCGAGGAAGG
RI-AT1G79245-XLOC_008236-227-1
TGAGGTGGCTTATTTCTATTGATATCTATAGTGTTGATTGCCGAGGAAGG
CONSENSUS
TGAGGTGGCTTATTTCTATTGATATCTATAGTGTTGATTGCCGAGGAAGG

RI-AT1G79245-XLOC_008236-227-0
AAAAACAAGTTTCAACCGTGGACCGTGGTTGTCTCAACAGGGATGAACTT
RI-AT1G79245-XLOC_008236-227-1
AAAAACAAGTTTCAACCGTGGACCGTGGTTGTCTCAACAGGGATGAACTT
CONSENSUS
AAAAACAAGTTTCAACCGTGGACCGTGGTTGTCTCAACAGGGATGAACTT

RI-AT1G79245-XLOC_008236-227-0
GGAGGTTCCATCTCTGGTTGGATTCAGGTGTTTTGAGCTAATCTTCTTCT
RI-AT1G79245-XLOC_008236-227-1
GGAGGTTCCATCTCTGGTTGGATTCAGGTGTTTTGAGCTAATCTTCTTCT
CONSENSUS
GGAGGTTCCATCTCTGGTTGGATTCAGGTGTTTTGAGCTAATCTTCTTCT

RI-AT1G79245-XLOC_008236-227-0
GGGTTTACTTCTCTACCATACTTTCTACCGATTTGAAGATTTGTCTTCTG
RI-AT1G79245-XLOC_008236-227-1
GGGTTTACTTCTCTACCATACTTTCTACCGATTTGAAGATTTGTCTTCTG
CONSENSUS
GGGTTTACTTCTCTACCATACTTTCTACCGATTTGAAGATTTGTCTTCTG

RI-AT1G79245-XLOC_008236-227-0
TACCTTTGATGATGATTCTCAGTTTTGCAGCAATGACTCAGTTTTTCGCC
RI-AT1G79245-XLOC_008236-227-1
TACCTTTGATGATGATTCTCAGTTTTGCAGCAATGACTCAGTTTTTCGCC
CONSENSUS
TACCTTTGATGATGATTCTCAGTTTTGCAGCAATGACTCAGTTTTTCGCC

RI-AT1G79245-XLOC_008236-227-0
TTCTAGTCATTAAACTACTTTCCCACAATGAGATACTTAGTGTTTTTGTA

RI-AT1G79245-XLOC_008236-227-1
TTCTAGTCATTAAACTACTTTCCCACAATGAGATACTTAGTGTTTTTGTA
CONSENSUS
TTCTAGTCATTAAACTACTTTCCCACAATGAGATACTTAGTGTTTTTGTA

RI-AT1G79245-XLOC_008236-227-0
GGTTCTTTCACACTTGTTAGCAAGGGTATATGTATGTTGCCGTTCTCAGA
RI-AT1G79245-XLOC_008236-227-1
GGTTCTTTCACACTTGTTAGCAAGGGTATATGTATGTTGCCGTTCTCAGA
CONSENSUS
GGTTCTTTCACACTTGTTAGCAAGGGTATATGTATGTTGCCGTTCTCAGA

RI-AT1G79245-XLOC_008236-227-0
ATTTGGTAGGTATGAATCACAGATGTCATTGTTTATTATTGATAACATAA
RI-AT1G79245-XLOC_008236-227-1
ATTTGGTAGGTATGAATCACAGATGTCATTGTTTATTATTGATAACATAA
CONSENSUS
ATTTGGTAGGTATGAATCACAGATGTCATTGTTTATTATTGATAACATAA

RI-AT1G79245-XLOC_008236-227-0
GTGATTCTTGCTGGTAGTGTGACCTCTTGAGGGCAGGCTCATAGAAGATG
RI-AT1G79245-XLOC_008236-227-1
GTGATTCTTGCTGGTAGTGTGACCTCTTGAGGGCAGGCTCATAGAAGATG
CONSENSUS
GTGATTCTTGCTGGTAGTGTGACCTCTTGAGGGCAGGCTCATAGAAGATG

RI-AT1G79245-XLOC_008236-227-0
AAGAGCCTCTTGGTTTTGATGATAATGCTCCCTACTTCTGAGTTCTGAGG
RI-AT1G79245-XLOC_008236-227-1
AAGAGCCTCTTGGTTTTGATGATAATGCTCCCTACTTCTGAGTTCTGAGG
CONSENSUS
AAGAGCCTCTTGGTTTTGATGATAATGCTCCCTACTTCTGAGTTCTGAGG

RI-AT1G79245-XLOC_008236-227-0
TTTCTTTATTCCTGGGTTGTAGCTGGTTTAATGGTTCTGTAGACATTTGA
RI-AT1G79245-XLOC_008236-227-1
TTTCTTTATTCCTGGGTTGTAGCTGGTTTAATGGTTCTGTAGACATTTGA
CONSENSUS
TTTCTTTATTCCTGGGTTGTAGCTGGTTTAATGGTTCTGTAGACATTTGA

RI-AT1G79245-XLOC_008236-227-0
TAATGAAGTCTCTCACTTCATTTTGTTTGGGTGATGTAAAATGTTGTCAG
RI-AT1G79245-XLOC_008236-227-1
TAATGAAGTCTCTCACTTCATTTTGTTTGGGTGATGTAAAATGTTGTCAG
CONSENSUS
TAATGAAGTCTCTCACTTCATTTTGTTTGGGTGATGTAAAATGTTGTCAG

RI-AT1G79245-XLOC_008236-227-0
TGGATTTTAGGTGAGGCTTAATCTCATGGATTATTCTTTTGAAATCCCCT
RI-AT1G79245-XLOC_008236-227-1
TGGATTTTAGGTGAGGCTTAATCTCATGGATTATTCTTTTGAAATCCCCT
CONSENSUS
TGGATTTTAGGTGAGGCTTAATCTCATGGATTATTCTTTTGAAATCCCCT

RI-AT1G79245-XLOC_008236-227-0
CAGGTGAATCAGTGAAAATTGAGAAAGTGATGTTCAACTTCTTATCTCTT

RI-AT1G79245-XLOC_008236-227-1
CAGGTGAATCAGTGAAAATTGAGAAAGTGATGTTCAACTTCTTATCTCTT
CONSENSUS
CAGGTGAATCAGTGAAAATTGAGAAAGTGATGTTCAACTTCTTATCTCTT

RI-AT1G79245-XLOC_008236-227-0
AATCCTTCATTTGCTCTGTCACACATTGAAGCCTATTTTCGGTTGCAGTT
RI-AT1G79245-XLOC_008236-227-1
AATCCTTCATTTGCTCTGTCACACATTGAAGCCTATTTTCGGTTGCAGTT
CONSENSUS
AATCCTTCATTTGCTCTGTCACACATTGAAGCCTATTTTCGGTTGCAGTT

RI-AT1G79245-XLOC_008236-227-0
TGTTAAGTTTTCATGGGAAGCGTTGTCTTTGGAGTGTTCTGAAGTTGACT
RI-AT1G79245-XLOC_008236-227-1
TGTTAAGTTTTCATGGGAAGCGTTGTCTTTGGAGTGTTCTGAAGTTGACT
CONSENSUS
TGTTAAGTTTTCATGGGAAGCGTTGTCTTTGGAGTGTTCTGAAGTTGACT

RI-AT1G79245-XLOC_008236-227-0
TTAATCCTGAAAGAAGAAGGATATGCTTTTACTGAAGTTATCATGGGAAG
RI-AT1G79245-XLOC_008236-227-1
TTAATCCTGAAAGAAGAAGGATATGCTTTTACTGAAGTTATCATGGGAAG
CONSENSUS
TTAATCCTGAAAGAAGAAGGATATGCTTTTACTGAAGTTATCATGGGAAG

RI-AT1G79245-XLOC_008236-227-0
CACTTTTTGTGGAGTTTACAGGAGTGTCTATGCCGTTGCAGTATTCGAGG
RI-AT1G79245-XLOC_008236-227-1
CACTTTTTGTGGAGTTTACAGGAGTGTCTATGCCGTTGCAGTATTCGAGG
CONSENSUS
CACTTTTTGTGGAGTTTACAGGAGTGTCTATGCCGTTGCAGTATTCGAGG

RI-AT1G79245-XLOC_008236-227-0
ATCGCTTTGATCTGCCAGTCGTTCCTTGTTACCTTTTTCGCTTTTGCAGT
RI-AT1G79245-XLOC_008236-227-1
ATCGCTTTGATCTGCCAGTCGTTCCTTGTTACCTTTTTCGCTTTTGCAGT
CONSENSUS
ATCGCTTTGATCTGCCAGTCGTTCCTTGTTACCTTTTTCGCTTTTGCAGT

RI-AT1G79245-XLOC_008236-227-0
TCTTTCTTACTTGCCTTGCTTTTCTCTCGTTCATGTATTACTTGATGCAA
RI-AT1G79245-XLOC_008236-227-1
TCTTTCTTACTTGCCTTGCTTTTCTCTCGTTCATGTATTACTTGATGCAA
CONSENSUS
TCTTTCTTACTTGCCTTGCTTTTCTCTCGTTCATGTATTACTTGATGCAA

RI-AT1G79245-XLOC_008236-227-0
GTCTTGAAACTTTCCCGATATTGGACCAAACTCTTTTTCATTCATGCCTT
RI-AT1G79245-XLOC_008236-227-1
GTCTTGAAACTTTCCCGATATTGGACCAAACTCTTTTTCATTCATGCCTT
CONSENSUS
GTCTTGAAACTTTCCCGATATTGGACCAAACTCTTTTTCATTCATGCCTT

RI-AT1G79245-XLOC_008236-227-0
TTAGGACTACTAAGTATGAACTCCTCTGATGTAGAAGGTTCGATATGTGC

RI-AT1G79245-XLOC_008236-227-1
TTAGGACTACTAAGTATGAACTCCTCTGATGTAGAAGGTTCGATATGTGC
CONSENSUS
TTAGGACTACTAAGTATGAACTCCTCTGATGTAGAAGGTTCGATATGTGC

RI-AT1G79245-XLOC_008236-227-0
TAATCAGTCTACTGGTAATCCTAGTAACGCTGGACTTCTATCTATGCTAA
RI-AT1G79245-XLOC_008236-227-1
TAATCAGTCTACTGGTAATCCTAGTAACGCTGGACTTCTATCTATGCTAA
CONSENSUS
TAATCAGTCTACTGGTAATCCTAGTAACGCTGGACTTCTATCTATGCTAA

RI-AT1G79245-XLOC_008236-227-0
ATATTGTCTTGGTGTTATAGAAAACCATAAGGTGGCTATTTGCAATTTCA
RI-AT1G79245-XLOC_008236-227-1
ATATTGTCTTGGTGTTATAGAAAACCATAAGGTGGCTATTTGCAATTTCA
CONSENSUS
ATATTGTCTTGGTGTTATAGAAAACCATAAGGTGGCTATTTGCAATTTCA

RI-AT1G79245-XLOC_008236-227-0
GGGCCACAACTTGTGCAGATTCCAGGCCTTTATCTTGTGTTGGTGATTAA
RI-AT1G79245-XLOC_008236-227-1
GGGCCACAACTTGTGCAGATTCCAGGCCTTTATCTTGTGTTGGTGATTAA
CONSENSUS
GGGCCACAACTTGTGCAGATTCCAGGCCTTTATCTTGTGTTGGTGATTAA

RI-AT1G79245-XLOC_008236-227-0
TGTTACTCATATTATACCTACGGAAGGGAGTTCACTGCAGTAAGCAACAC
RI-AT1G79245-XLOC_008236-227-1
TGTTACTCATATTATACCTACGGAAGGGAGTTCACTGCAGTAAGCAACAC
CONSENSUS
TGTTACTCATATTATACCTACGGAAGGGAGTTCACTGCAGTAAGCAACAC

RI-AT1G79245-XLOC_008236-227-0
AAAAGAGAGCTTTAGTGATGCAAATTACATGATTTCTTGTTTGAAGTTAT
RI-AT1G79245-XLOC_008236-227-1
AAAAGAGAGCTTTAGTGATGCAAATTACATGATTTCTTGTTTGAAGTTAT
CONSENSUS
AAAAGAGAGCTTTAGTGATGCAAATTACATGATTTCTTGTTTGAAGTTAT

RI-AT1G79245-XLOC_008236-227-0
GTTGAGTACATTCTACAAGAAGATACAATGTCGTGGATGATCCATAATTG
RI-AT1G79245-XLOC_008236-227-1
GTTGAGTACATTCTACAAGAAGATACAATGTCGTGGATGATCCATAATTG
CONSENSUS
GTTGAGTACATTCTACAAGAAGATACAATGTCGTGGATGATCCATAATTG

RI-AT1G79245-XLOC_008236-227-0
GATCCAATTATCTCGTGGAGCAAAAGCCACAATAGCTTCATCGTTGGAAT
RI-AT1G79245-XLOC_008236-227-1
GATCCAATTATCTCGTGGAGCAAAAGCCACAATAGCTTCATCGTTGGAAT
CONSENSUS
GATCCAATTATCTCGTGGAGCAAAAGCCACAATAGCTTCATCGTTGGAAT

RI-AT1G79245-XLOC_008236-227-0
CTGGAAGCGCTTCACCGAGAAGTTCTGCCGAAATCGATAGAATTTGGCAG

RI-AT1G79245-XLOC_008236-227-1
CTGGAAGCGCTTCACCGAGAAGTTCTGCCGAAATCGATAGAATTTGGCAG
CONSENSUS
CTGGAAGCGCTTCACCGAGAAGTTCTGCCGAAATCGATAGAATTTGGCAG

RI-AT1G79245-XLOC_008236-227-0
ACATTACTTGGAGTTTATGACCGAACTCAACTTACATGTAAGTTTTATTG
RI-AT1G79245-XLOC_008236-227-1
ACATTACTTGGAGTTTATGACCGAACTCAACTTACAT-------------
CONSENSUS
ACATTACTTGGAGTTTATGACCGAACTCAACTTACAT.............

RI-AT1G79245-XLOC_008236-227-0
TATGTATATATATACATACAAAGATTATATCTCCGAGTTTGGAATCGTCT
RI-AT1G79245-XLOC_008236-227-1

CONSENSUS
....

RI-AT1G79245-XLOC_008236-227-0
CTCATCTTGTTTTTGTTGTTGACGACTGGGCTTTAGAAGAATTAAGGAGT
RI-AT1G79245-XLOC_008236-227-1
--AATTAAGGAGT
CONSENSUS
....AATTAAGGAGT

RI-AT1G79245-XLOC_008236-227-0
CAGAGCAATGAGAATGTGGACATGAGTATTTTGTGAGAGGCCAACCTGAG
RI-AT1G79245-XLOC_008236-227-1
CAGAGCAATGAGAATGTGGACATGAGTATTTTGTGAGAGGCCAACCTGAG
CONSENSUS
CAGAGCAATGAGAATGTGGACATGAGTATTTTGTGAGAGGCCAACCTGAG

RI-AT1G79245-XLOC_008236-227-0
CTTTTGGTGAAGATGATGTTCAAAACTGGGATGAAGAGGTTTGAGGAACT
RI-AT1G79245-XLOC_008236-227-1
CTTTTGGTGAAGATGATGTTCAAAACTGGGATGAAGAGGTTTGAGGAACT
CONSENSUS
CTTTTGGTGAAGATGATGTTCAAAACTGGGATGAAGAGGTTTGAGGAACT

RI-AT1G79245-XLOC_008236-227-0
TGCAGTCAAACGCCGAGCAAAGAAAAAGAAAGCTAAAGCTGAAGCGGAAG
RI-AT1G79245-XLOC_008236-227-1
TGCAGTCAAACGCCGAGCAAAGAAAAAGAAAGCTAAAGCTGAAGCGGAAG
CONSENSUS
TGCAGTCAAACGCCGAGCAAAGAAAAAGAAAGCTAAAGCTGAAGCGGAAG

RI-AT1G79245-XLOC_008236-227-0
TAAAGGATCGCTTGCAACACTTGAGAATCTGATCAATAAATCTTTTCGAT
RI-AT1G79245-XLOC_008236-227-1
TAAAGGATCGCTTGCAACACTTGAGAATCTGATCAATAAATCTTTTCGAT
CONSENSUS
TAAAGGATCGCTTGCAACACTTGAGAATCTGATCAATAAATCTTTTCGAT

RI-AT1G79245-XLOC_008236-227-0
TTAATGAATTAAATAAGCTCTTGTATTGTTTGTATCAATAACAATTTAGT

RI-AT1G79245-XLOC_008236-227-1
TTAATGAATTAAATAAGCTCTTGTATTGTTTGTATCAATAACAATTTAGT
CONSENSUS
TTAATGAATTAAATAAGCTCTTGTATTGTTTGTATCAATAACAATTTAGT

RI-AT1G79245-XLOC_008236-227-0 GTGGCAATAAAATTGATGGGTTTTATTTTG
RI-AT1G79245-XLOC_008236-227-1 GTGGCAATAAAATTGATGGGTTTTATTTTG
CONSENSUS GTGGCAATAAAATTGATGGGTTTTATTTTG

alignment for event: A5-AT1G23950-XLOC_005542-1750

A5-AT1G23950-XLOC_005542-1750-0
GGGACAAGCTTCCAGCTCGCTACCCTAATGAAATTCAACATGTTACCGAA
A5-AT1G23950-XLOC_005542-1750-1
GGGACAAGCTTCCAGCTCGCTACCCTAATGAAATTCAACATGTTACCGAA
CONSENSUS
GGGACAAGCTTCCAGCTCGCTACCCTAATGAAATTCAACATGTTACCGAA

A5-AT1G23950-XLOC_005542-1750-0
CTATATTTCCTCTTTCTACATGACTCTGCTTGCACATGATCCCGATCCAG
A5-AT1G23950-XLOC_005542-1750-1
CTATATTTCCTCTTTCTACATGACTCTGCTTGCACATGATCCCGATCCAG
CONSENSUS
CTATATTTCCTCTTTCTACATGACTCTGCTTGCACATGATCCCGATCCAG

A5-AT1G23950-XLOC_005542-1750-0
CTGCTGGCTCATCGCAGAAAACCTTTCAGGTTCGAGTTGATGAACAACAA
A5-AT1G23950-XLOC_005542-1750-1
CTGCTGGCTCATCGCAGAAAACCTTTCAGGTTCGAGTTGATGAACAACAA
CONSENSUS
CTGCTGGCTCATCGCAGAAAACCTTTCAGGTTCGAGTTGATGAACAACAA

A5-AT1G23950-XLOC_005542-1750-0
TTTGGCAGTTTGGATATAAACTGCTCCATTGCTAGACCTAAACACGAAGG
A5-AT1G23950-XLOC_005542-1750-1
TTTGGCAGTTTGGATATAAACTGCTCCATTGCTAGACCTAAACACGAAG-
CONSENSUS
TTTGGCAGTTTGGATATAAACTGCTCCATTGCTAGACCTAAACACGAAG.

A5-AT1G23950-XLOC_005542-1750-0
TGACTTACTTGAAGTGTCCACCGAGACGCCCTTCATGCCTCACTTTCATG
A5-AT1G23950-XLOC_005542-1750-1 --------------
TGTCCACCGAGACGCCCTTCATGCCTCACTTTCATG
CONSENSUS
..............TGTCCACCGAGACGCCCTTCATGCCTCACTTTCATG

A5-AT1G23950-XLOC_005542-1750-0
GTGGCGCACTGGGCGATGGTATCTTCAAAGTTGAATTGCCTGATTGTCTC
A5-AT1G23950-XLOC_005542-1750-1
GTGGCGCACTGGGCGATGGTATCTTCAAAGTTGAATTGCCTGATTGTCTC
CONSENSUS
GTGGCGCACTGGGCGATGGTATCTTCAAAGTTGAATTGCCTGATTGTCTC

A5-AT1G23950-XLOC_005542-1750-0

TCAGATACTGCTTTGAATGAACTGGCCGGTGCTGTCCTCAGAGGTGAATT
A5-AT1G23950-XLOC_005542-1750-1
TCAGATACTGCTTTGAATGAACTGGCCGGTGCTGTCCTCAGAGGTGAATT
CONSENSUS
TCAGATACTGCTTTGAATGAACTGGCCGGTGCTGTCCTCAGAGGTGAATT

A5-AT1G23950-XLOC_005542-1750-0
GCCTGAGCATGTCTTTGATGATGCTTTGTATGCACGGGCCGGTGGTATCT
A5-AT1G23950-XLOC_005542-1750-1
GCCTGAGCATGTCTTTGATGATGCTTTGTATGCACGGGCCGGTGGTATCT
CONSENSUS
GCCTGAGCATGTCTTTGATGATGCTTTGTATGCACGGGCCGGTGGTATCT

A5-AT1G23950-XLOC_005542-1750-0
TCCAAGGTGAATTGCCTGATTGGCCCTCAGATGATGTTTTGAATGACGGA
A5-AT1G23950-XLOC_005542-1750-1
TCCAAGGTGAATTGCCTGATTGGCCCTCAGATGATGTTTTGAATGACGGA
CONSENSUS
TCCAAGGTGAATTGCCTGATTGGCCCTCAGATGATGTTTTGAATGACGGA

A5-AT1G23950-XLOC_005542-1750-0 AAACGATTTTACATG
A5-AT1G23950-XLOC_005542-1750-1 AAACGATTTTACATG
CONSENSUS AAACGATTTTACATG

alignment for event: RI-AT1G23860-XLOC_005538-1644

RI-AT1G23860-XLOC_005538-1644-0
GGTGTGAAACTGGACCCTGAAGAGAAAGAAGCTATTACTAAGCTATGATG
RI-AT1G23860-XLOC_005538-1644-1
GGTGTGAAACTGGACCCTGAAGAGAAAGAAGCTATTACTAAGCTATGATG
CONSENSUS
GGTGTGAAACTGGACCCTGAAGAGAAAGAAGCTATTACTAAGCTATGATG

RI-AT1G23860-XLOC_005538-1644-0
CTATATCTACTCTGTGGCGCCTTTTAGGATAGATCTTTATATCTGCTATT
RI-AT1G23860-XLOC_005538-1644-1
CTATATCTACTCTGTGGCGCCTTTTAGGATAGATCTTTATATCTGCTATT
CONSENSUS
CTATATCTACTCTGTGGCGCCTTTTAGGATAGATCTTTATATCTGCTATT

RI-AT1G23860-XLOC_005538-1644-0
ATGGTATTAGCTCTTCTAGGTTTGGTTGTTGAGCATGGACATGTCATGGT
RI-AT1G23860-XLOC_005538-1644-1
ATGGTATTAGCTCTTCTAGGTTTGGTTGTTGAGCATGGACATGTCATGGT
CONSENSUS
ATGGTATTAGCTCTTCTAGGTTTGGTTGTTGAGCATGGACATGTCATGGT

RI-AT1G23860-XLOC_005538-1644-0
ATGTTCGAGTTTTATTAGACAATTGGATTTGGTGAGTTCTCCTTCATGCT
RI-AT1G23860-XLOC_005538-1644-1
ATGTTCGAGTTTTATTAGACAATTGGATTTGGTGAGTTCTCCTTCATGCT
CONSENSUS
ATGTTCGAGTTTTATTAGACAATTGGATTTGGTGAGTTCTCCTTCATGCT

RI-AT1G23860-XLOC_005538-1644-0
GTTTTCCAACAGCCGTCTTGACTTATTCTCTATAACACGCACTGGCCTGC
RI-AT1G23860-XLOC_005538-1644-1
GTTTTCCAACAGCCGTCTTGACTTATTCTCTATAACACGCACTGGCCTGC
CONSENSUS
GTTTTCCAACAGCCGTCTTGACTTATTCTCTATAACACGCACTGGCCTGC

RI-AT1G23860-XLOC_005538-1644-0
TGTCTTCACACAGCGTCCTTGAACCCCTACACTTGAGGTCGTTCTTTCGT
RI-AT1G23860-XLOC_005538-1644-1
TGTCTTCACACAGCGTCCTTGAACCCCTACACTTGAG-------------
CONSENSUS
TGTCTTCACACAGCGTCCTTGAACCCCTACACTTGAG.............

RI-AT1G23860-XLOC_005538-1644-0
ATCCATAAATGTTTTGTCTCTTGTTGAATGCACTTTCCTTATTATATAGA
RI-AT1G23860-XLOC_005538-1644-1

CONSENSUS
....

RI-AT1G23860-XLOC_005538-1644-0
CATATCTAAAAAGCTCAACTGTTTCCACTCTTTTTCCCTTGTGAGGTATG
RI-AT1G23860-XLOC_005538-1644-1

CONSENSUS
....

RI-AT1G23860-XLOC_005538-1644-0
ATTTATCGGGGATCTCTTGGCAGCACAGCTGCATCTTACTCTCCCCACAA
RI-AT1G23860-XLOC_005538-1644-1 ----------------------
CACAGCTGCATCTTACTCTCCCCACAA
CONSENSUS
.......................CACAGCTGCATCTTACTCTCCCCACAA

RI-AT1G23860-XLOC_005538-1644-0
CTTAGTTTTATCATGAATCTGCATCCTCAACTCGACCAACATCAGCTAGA
RI-AT1G23860-XLOC_005538-1644-1
CTTAGTTTTATCATGAATCTGCATCCTCAACTCGACCAACATCAGCTAGA
CONSENSUS
CTTAGTTTTATCATGAATCTGCATCCTCAACTCGACCAACATCAGCTAGA

RI-AT1G23860-XLOC_005538-1644-0
ATTTGCTTGTTACATTGTGTGACTTTTAGATTGCCCTGCTCTTTACTCAC
RI-AT1G23860-XLOC_005538-1644-1
ATTTGCTTGTTACATTGTGTGACTTTTAGATTGCCCTGCTCTTTACTCAC
CONSENSUS
ATTTGCTTGTTACATTGTGTGACTTTTAGATTGCCCTGCTCTTTACTCAC

RI-AT1G23860-XLOC_005538-1644-0
CACTCCTACCCCAAGTCTGCAACCAAGCTTCTATTGAGAAAGACGAATCT
RI-AT1G23860-XLOC_005538-1644-1
CACTCCTACCCCAAGTCTGCAACCAAGCTTCTATTGAGAAAGACGAATCT
CONSENSUS
CACTCCTACCCCAAGTCTGCAACCAAGCTTCTATTGAGAAAGACGAATCT

RI-AT1G23860-XLOC_005538-1644-0
GTTTCTTCACCCCGTCCTCTTCCTAGCTCTCGGCTGCTATATCACCACTT
RI-AT1G23860-XLOC_005538-1644-1
GTTTCTTCACCCCGTCCTCTTCCTAGCTCTCGGCTGCTATATCACCACTT
CONSENSUS
GTTTCTTCACCCCGTCCTCTTCCTAGCTCTCGGCTGCTATATCACCACTT

RI-AT1G23860-XLOC_005538-1644-0
GGTTTCTCTACTCACTTTTGTTTTTCGGTCAACTTTGTTACACATCTGTT
RI-AT1G23860-XLOC_005538-1644-1
GGTTTCTCTACTCACTTTTGTTTTTCGGTCAACTTTGTTACACATCTGTT
CONSENSUS
GGTTTCTCTACTCACTTTTGTTTTTCGGTCAACTTTGTTACACATCTGTT

RI-AT1G23860-XLOC_005538-1644-0
GGTGGATCACACACTTACATTAAATTAAACCCATTTGTGGAT
RI-AT1G23860-XLOC_005538-1644-1
GGTGGATCACACACTTACATTAAATTAAACCCATTTGTGGAT
CONSENSUS
GGTGGATCACACACTTACATTAAATTAAACCCATTTGTGGAT

alignment for event: A3-AT1G63640-XLOC_007374-2621

A3-AT1G63640-XLOC_007374-2621-0
GTATCGAACTTGAAGGATGTAATTGCTAAGAAAGATGAAGAGCTACAAAA
A3-AT1G63640-XLOC_007374-2621-1
GTATCGAACTTGAAGGATGTAATTGCTAAGAAAGATGAAGAGCTACAAAA
CONSENSUS
GTATCGAACTTGAAGGATGTAATTGCTAAGAAAGATGAAGAGCTACAAAA

A3-AT1G63640-XLOC_007374-2621-0
TTTTCAGAAGGTAAAAGGTAATAATGCAACATCCCTGAAACGTGGATTAA
A3-AT1G63640-XLOC_007374-2621-1
TTTTCAGAAGGTAAAAGGTAATAATGCAACATCCCTGAAACGTGGATTAA
CONSENSUS
TTTTCAGAAGGTAAAAGGTAATAATGCAACATCCCTGAAACGTGGATTAA

A3-AT1G63640-XLOC_007374-2621-0
GCAATTTAAGGTTGGTGGGGCCTACATCACCTAGAAGACATTCTATTGGA
A3-AT1G63640-XLOC_007374-2621-1
GCAATTTAAGGTTGGTGGGGCCTACATCACCTAGAAGACATTCTATTGGA
CONSENSUS
GCAATTTAAGGTTGGTGGGGCCTACATCACCTAGAAGACATTCTATTGGA

A3-AT1G63640-XLOC_007374-2621-0
GCGTCACCAAACGCTCGACGAGGAAAGGCCTCTGGTTTATTTGGAAGAGG
A3-AT1G63640-XLOC_007374-2621-1
GCGTCACCAAACGCTCGACGAGGAAAGGCCTCTGGTTTATTTGGAAGAGG
CONSENSUS
GCGTCACCAAACGCTCGACGAGGAAAGGCCTCTGGTTTATTTGGAAGAGG

A3-AT1G63640-XLOC_007374-2621-0
AACCTCAGATGTTGACAACTGCTCTGAATATAGCAGCAAGCATTCGGATT
A3-AT1G63640-XLOC_007374-2621-1

AACCTCAGATGTTGACAACTGCTCTGAATATAGCAGCAAGCATTCGGATT
CONSENSUS
AACCTCAGATGTTGACAACTGCTCTGAATATAGCAGCAAGCATTCGGATT

A3-AT1G63640-XLOC_007374-2621-0
CTGGTTCACAACAATCATCAGACGAACGTAAACATCAAAAAGATTATCAC
A3-AT1G63640-XLOC_007374-2621-1
CTGGTTCACAACAATCATCAGACGAACGTAAACATCAAAAAGATTATCAC
CONSENSUS
CTGGTTCACAACAATCATCAGACGAACGTAAACATCAAAAAGATTATCAC

A3-AT1G63640-XLOC_007374-2621-0
CAACCATCTAAATTTGCTGGTGCGGCGAAAGGAATCGATTTTGATGATGA
A3-AT1G63640-XLOC_007374-2621-1
CAACCATCTAAATTTGCTGGTGCGGCGAAAGGAATCGATTTTGATGATGA
CONSENSUS
CAACCATCTAAATTTGCTGGTGCGGCGAAAGGAATCGATTTTGATGATGA

A3-AT1G63640-XLOC_007374-2621-0
AGATGTTGAACTCGTAGGTCTTGCAGATGCAGATTCTGAGGACAGATTGA
A3-AT1G63640-XLOC_007374-2621-1
AGATGTTGAACTCGTAGGTCTTGCAGATGCAGATTCTGAGGACAGATTGA
CONSENSUS
AGATGTTGAACTCGTAGGTCTTGCAGATGCAGATTCTGAGGACAGATTGA

A3-AT1G63640-XLOC_007374-2621-0
GTGATATCTCTGATAGTTGTCTTTCAATGGGAACAGAAACTGATGGATCC
A3-AT1G63640-XLOC_007374-2621-1
GTGATATCTCTGATAGTTGTCTTTCAATGGGAACAGAAACTGATGGATCC
CONSENSUS
GTGATATCTCTGATAGTTGTCTTTCAATGGGAACAGAAACTGATGGATCC

A3-AT1G63640-XLOC_007374-2621-0
ATAAGCAGTGCAGTAGAGTTGACTCTATTCCCTGAAACTGCTAAGCCTCT
A3-AT1G63640-XLOC_007374-2621-1
ATAAGCAGTGCAGTAGAGTTGACTCTATTCCCTGAAACTGCTAAGCCTCT
CONSENSUS
ATAAGCAGTGCAGTAGAGTTGACTCTATTCCCTGAAACTGCTAAGCCTCT

A3-AT1G63640-XLOC_007374-2621-0
TGAACTAATAGAACGACCTGAAGCACGCATGACTTCTGAGAAACTCGAGA
A3-AT1G63640-XLOC_007374-2621-1
TGAACTAATAGAACGACCTGAAGCACGCATGACTTCTGAGAAACTCGAGA
CONSENSUS
TGAACTAATAGAACGACCTGAAGCACGCATGACTTCTGAGAAACTCGAGA

A3-AT1G63640-XLOC_007374-2621-0
AATCAGTGAAAATGGGGAAAACCGAGCCAAAAGACAG---AACTAATATT
A3-AT1G63640-XLOC_007374-2621-1
AATCAGTGAAAATGGGGAAAACCGAGCCAAAAGACAGCAGAACTAATATT
CONSENSUS
AATCAGTGAAAATGGGGAAAACCGAGCCAAAAGACAG...AACTAATATT

A3-AT1G63640-XLOC_007374-2621-0
CCATCGAAGATTCCAAAGCAGACCTTGAAACCACCAGGCCAAACCAGACC
A3-AT1G63640-XLOC_007374-2621-1

CCATCGAAGATTCCAAAGCAGACCTTGAAACCACCAGGCCAAACCAGACC
CONSENSUS
CCATCGAAGATTCCAAAGCAGACCTTGAAACCACCAGGCCAAACCAGACC

A3-AT1G63640-XLOC_007374-2621-0
GTCTCGTCTGTCAATTGCCACTAGCTCCTCCTCTAAGGCTTTAACAG
A3-AT1G63640-XLOC_007374-2621-1
GTCTCGTCTGTCAATTGCCACTAGCTCCTCCTCTAAGGCTTTAACAG
CONSENSUS
GTCTCGTCTGTCAATTGCCACTAGCTCCTCCTCTAAGGCTTTAACAG

alignment for event: A5-AT1G11360-XLOC_004857-3743

A5-AT1G11360-XLOC_004857-3743-0
ATTGGAGAATATCTATGGTTTTGGTTATATATTTTTACAGCCGTTAGATC
A5-AT1G11360-XLOC_004857-3743-1
ATTGGAGAATATCTATGGTTTTGGTTATATATTTTTACAGCCGTTAGATC
CONSENSUS
ATTGGAGAATATCTATGGTTTTGGTTATATATTTTTACAGCCGTTAGATC

A5-AT1G11360-XLOC_004857-3743-0
TTCAAACCGATCAATCTAACGGTTCTTAACAATATCCAATTCTCATAAGA
A5-AT1G11360-XLOC_004857-3743-1
TTCAAACCGATCAATCTAACGGTTCTTAACAATATCCAATTCTCATAAGA
CONSENSUS
TTCAAACCGATCAATCTAACGGTTCTTAACAATATCCAATTCTCATAAGA

A5-AT1G11360-XLOC_004857-3743-0
AAAGAGTACAAAAATAAAAAGAGTCGCTGTCACTATACCGTACCGTACAG
A5-AT1G11360-XLOC_004857-3743-1
AAAGAGTACAAAAATAAAAAGAGTCGCTGTCACTATACCGTACCGTACAG
CONSENSUS
AAAGAGTACAAAAATAAAAAGAGTCGCTGTCACTATACCGTACCGTACAG

A5-AT1G11360-XLOC_004857-3743-0
AGTCAATTGAAGAAACCGAGAGAGAGAGTGAGCGGAATCATGACTTCTCC
A5-AT1G11360-XLOC_004857-3743-1
AGTCAATTGAAGAAACCGAGAGAGAGAGTGAGCGGAATCATGACTTCTCC
CONSENSUS
AGTCAATTGAAGAAACCGAGAGAGAGAGTGAGCGGAATCATGACTTCTCC

A5-AT1G11360-XLOC_004857-3743-0
GGGAAAATCTCCGAGATCCGACCGGAAATCACCGACGGTTGTCACCGTCC
A5-AT1G11360-XLOC_004857-3743-1
GGGAAAATCTCCGAGATCCGACCGGAAATCACCGACGGTTGTCACCGTCC
CONSENSUS
GGGAAAATCTCCGAGATCCGACCGGAAATCACCGACGGTTGTCACCGTCC

A5-AT1G11360-XLOC_004857-3743-0
AGCCGTCTTCTCCCAGATTCCCGATCAGCACACCTACTGCTGGAGCTCAA
A5-AT1G11360-XLOC_004857-3743-1
AGCCGTCTTCTCCCAGATTCCCGATCAGCACACCTACTGCTGGAGCTCAA
CONSENSUS
AGCCGTCTTCTCCCAGATTCCCGATCAGCACACCTACTGCTGGAGCTCAA

A5-AT1G11360-XLOC_004857-3743-0
CGTAAGATCGGGATCGCTGTCGATCTAAGCGACGAGAGTGCGTACGCAGT
A5-AT1G11360-XLOC_004857-3743-1
CGTAAGATCGGGATCGCTGTCGATCTAAGCGACGAGAGTGCGTACGCAGT
CONSENSUS
CGTAAGATCGGGATCGCTGTCGATCTAAGCGACGAGAGTGCGTACGCAGT

A5-AT1G11360-XLOC_004857-3743-0
TCAATGGGCTGTTCAGAACTATCTCCGATCAGGTGACGCCGTTGTTCTCC
A5-AT1G11360-XLOC_004857-3743-1
TCAATGGGCTGTTCAGAACTATCTCCGATCAGGTGACGCCGTTGTTCTCC
CONSENSUS
TCAATGGGCTGTTCAGAACTATCTCCGATCAGGTGACGCCGTTGTTCTCC

A5-AT1G11360-XLOC_004857-3743-0
TTCATGTTCAACCGACGAGTGTACTTTACGGCGCCGATTGGGGCGCTATG
A5-AT1G11360-XLOC_004857-3743-1
TTCATGTTCAACCGACGAGTGTACTTTACGGCGCCGATTGGGGCGCTATG
CONSENSUS
TTCATGTTCAACCGACGAGTGTACTTTACGGCGCCGATTGGGGCGCTATG

A5-AT1G11360-XLOC_004857-3743-0
GATTTGTCGCCGCAGTGGGATCCAAATAACGAAGAGTCGCAGAGGAAGCT
A5-AT1G11360-XLOC_004857-3743-1
GATTTGTCGCCGCAGTGGGATCCAAATAACGAAGAGTCGCAGAGGAAGCT
CONSENSUS
GATTTGTCGCCGCAGTGGGATCCAAATAACGAAGAGTCGCAGAGGAAGCT

A5-AT1G11360-XLOC_004857-3743-0
TGAGGACGATTTCGACATTGTCACTAATAAGAAAGCGAGCGACGTTGCTC
A5-AT1G11360-XLOC_004857-3743-1
TGAGGACGATTTCGACATTGTCACTAATAAGAAAGCGAGCGACGTTGCTC
CONSENSUS
TGAGGACGATTTCGACATTGTCACTAATAAGAAAGCGAGCGACGTTGCTC

A5-AT1G11360-XLOC_004857-3743-0
AGCCTTTGGTTGAGGCGGATATTCCGTTTAAGATCCATATCGTCAAGGAT
A5-AT1G11360-XLOC_004857-3743-1
AGCCTTTGGTTGAGGCGGATATTCCGTTTAAGATCCATATCGTCAAGGAT
CONSENSUS
AGCCTTTGGTTGAGGCGGATATTCCGTTTAAGATCCATATCGTCAAGGAT

A5-AT1G11360-XLOC_004857-3743-0
CACGATATGAAGGAGAGGCTTTGTTTGGAGGTGGAGAGGTTAGGGCTAAG
A5-AT1G11360-XLOC_004857-3743-1
CACGATATGAAGGAGAGGCTTTGTTTGGAGGTGGAGAGGTTAGGGCTAAG
CONSENSUS
CACGATATGAAGGAGAGGCTTTGTTTGGAGGTGGAGAGGTTAGGGCTAAG

A5-AT1G11360-XLOC_004857-3743-0
TACTTTGATTATGGGTAGTAGAGGATTTGGTGCTACGAAGAGGAGTAGTA
A5-AT1G11360-XLOC_004857-3743-1
TACTTTGATTATGGGTAGTAGAGGATTTGGTGCTACGAAGAGGAGTAGTA
CONSENSUS
TACTTTGATTATGGGTAGTAGAGGATTTGGTGCTACGAAGAGGAGTAGTA

A5-AT1G11360-XLOC_004857-3743-0
AAGGGAGATTAGGGAGTGTCAGTGATTACTCAGTTCACCATTGTGCTTGT
A5-AT1G11360-XLOC_004857-3743-1
AAGGGAGATTAGGGAGTGTCAGTGATTACTCAGTTCACCATTGTGCTTGT
CONSENSUS
AAGGGAGATTAGGGAGTGTCAGTGATTACTCAGTTCACCATTGTGCTTGT

A5-AT1G11360-XLOC_004857-3743-0
CCGGTGGTTGTTGTTAGATTTCCTGATGATAAGGATGGAGAAGATGAGAA
A5-AT1G11360-XLOC_004857-3743-1
CCGGTGGTTGTTGTTAGATTTCCTGATGATAAGGATGGAGAAGATGAGAA
CONSENSUS
CCGGTGGTTGTTGTTAGATTTCCTGATGATAAGGATGGAGAAGATGAGAA

A5-AT1G11360-XLOC_004857-3743-0
ATCCGGCGACAGTGGTGGGGAGAATCTGATGGACAGTGATAAACTTCATA
A5-AT1G11360-XLOC_004857-3743-1
ATCCGGCGACAGTGGTGGGGAGAATCTGATGGACAGTGATAAACTTCATA
CONSENSUS
ATCCGGCGACAGTGGTGGGGAGAATCTGATGGACAGTGATAAACTTCATA

A5-AT1G11360-XLOC_004857-3743-0
CAGTGCCTGAGGTGGCTGAAGAAGAGGGAGATAAGGATGAGTACCATGAT
A5-AT1G11360-XLOC_004857-3743-1
CAGTGCCTGAGGTGGCTGAAGAAGAGGGAGATAAGGATGAGTACCATGAT
CONSENSUS
CAGTGCCTGAGGTGGCTGAAGAAGAGGGAGATAAGGATGAGTACCATGAT

A5-AT1G11360-XLOC_004857-3743-0
GCTTCGGATAAGCAGTAGCAGCAAGGTTGACTTTGAATGCATGCTTTCAT
A5-AT1G11360-XLOC_004857-3743-1
GCTTCGGATAAGCAGTAGCAGCAAG-----------------GCTTTCAT
CONSENSUS
GCTTCGGATAAGCAGTAGCAGCAAG.................GCTTTCAT

A5-AT1G11360-XLOC_004857-3743-0
GTTGCAGAAGGAGACCTCCCTAAGGAGACTTAGAAAGAAAAGAAAGCGTT
A5-AT1G11360-XLOC_004857-3743-1
GTTGCAGAAGGAGACCTCCCTAAGGAGACTTAGAAAGAAAAGAAAGCGTT
CONSENSUS
GTTGCAGAAGGAGACCTCCCTAAGGAGACTTAGAAAGAAAAGAAAGCGTT

A5-AT1G11360-XLOC_004857-3743-0
CCTTTGCCCTTGGTTTGTGTTCTTGAGTGTGCTTAATTGTGTTGTTTTTG
A5-AT1G11360-XLOC_004857-3743-1
CCTTTGCCCTTGGTTTGTGTTCTTGAGTGTGCTTAATTGTGTTGTTTTTG
CONSENSUS
CCTTTGCCCTTGGTTTGTGTTCTTGAGTGTGCTTAATTGTGTTGTTTTTG

A5-AT1G11360-XLOC_004857-3743-0
GGGTTTGGGGTATTCTAGATCTCTTTTGTAAGTATTCTCTTGTTTCTTGG
A5-AT1G11360-XLOC_004857-3743-1
GGGTTTGGGGTATTCTAGATCTCTTTTGTAAGTATTCTCTTGTTTCTTGG
CONSENSUS
GGGTTTGGGGTATTCTAGATCTCTTTTGTAAGTATTCTCTTGTTTCTTGG

A5-AT1G11360-XLOC_004857-3743-0
TTTTGTTTTAAACTCTATGGAATTTGGGATGAGTGGATTCGATGTCTTTT
A5-AT1G11360-XLOC_004857-3743-1
TTTTGTTTTAAACTCTATGGAATTTGGGATGAGTGGATTCGATGTCTTTT
CONSENSUS
TTTTGTTTTAAACTCTATGGAATTTGGGATGAGTGGATTCGATGTCTTTT

A5-AT1G11360-XLOC_004857-3743-0
GTTTGTCTTTGAAAAAGCAGTGAAAAGAATTTCAGTGGTCTTGTATATCT
A5-AT1G11360-XLOC_004857-3743-1
GTTTGTCTTTGAAAAAGCAGTGAAAAGAATTTCAGTGGTCTTGTATATCT
CONSENSUS
GTTTGTCTTTGAAAAAGCAGTGAAAAGAATTTCAGTGGTCTTGTATATCT

A5-AT1G11360-XLOC_004857-3743-0
CTGCAGTTGAATAGTTGGTACAAAATAACATCTTTGACGGTTCCAAATAA
A5-AT1G11360-XLOC_004857-3743-1
CTGCAGTTGAATAGTTGGTACAAAATAACATCTTTGACGGTTCCAAATAA
CONSENSUS
CTGCAGTTGAATAGTTGGTACAAAATAACATCTTTGACGGTTCCAAATAA

A5-AT1G11360-XLOC_004857-3743-0
TATATCTTGTCAGTAATGACCATTAAATAAACTTTACCATGAAAT
A5-AT1G11360-XLOC_004857-3743-1
TATATCTTGTCAGTAATGACCATTAAATAAACTTTACCATGAAAT
CONSENSUS
TATATCTTGTCAGTAATGACCATTAAATAAACTTTACCATGAAAT

alignment for event: A3-AT1G03457-XLOC_004381-42

A3-AT1G03457-XLOC_004381-42-0
GCATCTAGTCCGTTGCAAGTTAAGTATGCAGATGGCGAGTTGGAAAGACT
A3-AT1G03457-XLOC_004381-42-1
GCATCTAGTCCGTTGCAAGTTAAGTATGCAGATGGCGAGTTGGAAAGACT
CONSENSUS
GCATCTAGTCCGTTGCAAGTTAAGTATGCAGATGGCGAGTTGGAAAGACT

A3-AT1G03457-XLOC_004381-42-0 AG---------------------------
AGCACAAACTTTTCGTCGGTA
A3-AT1G03457-XLOC_004381-42-1
AGATGTTCTTGACTGTTCGTGCAATCCAGAGCACAAACTTTTCGTCGGTA
CONSENSUS
AG...........................AGCACAAACTTTTCGTCGGTA

A3-AT1G03457-XLOC_004381-42-0
TGCTTCCAAAGAATGTCTCTGAAACTGAAGTCCAGTCCTTATTCTCTGAA
A3-AT1G03457-XLOC_004381-42-1
TGCTTCCAAAGAATGTCTCTGAAACTGAAGTCCAGTCCTTATTCTCTGAA
CONSENSUS
TGCTTCCAAAGAATGTCTCTGAAACTGAAGTCCAGTCCTTATTCTCTGAA

A3-AT1G03457-XLOC_004381-42-0
TACGGAACCATTAAGGATCTACAGATTCTAAGAGGGTCTCTACAAACTAG

A3-AT1G03457-XLOC_004381-42-1
      TACGGAACCATTAAGGATCTACAGATTCTAAGAGGGTCTCTACAAACTAG
CONSENSUS
      TACGGAACCATTAAGGATCTACAGATTCTAAGAGGGTCTCTACAAACTAG

A3-AT1G03457-XLOC_004381-42-0   CAAAG
A3-AT1G03457-XLOC_004381-42-1   CAAAG
CONSENSUS   CAAAG

alignment for event: A3-AT1G54217-XLOC_006891-6279

A3-AT1G54217-XLOC_006891-6279-0
      GTTACTTGGTGAGAGGAAGTTTAATCGGCGCTACAAAATTGCACTTCTTT
A3-AT1G54217-XLOC_006891-6279-1
      GTTACTTGGTGAGAGGAAGTTTAATCGGCGCTACAAAATTGCACTTCTTT
CONSENSUS
      GTTACTTGGTGAGAGGAAGTTTAATCGGCGCTACAAAATTGCACTTCTTT

A3-AT1G54217-XLOC_006891-6279-0 CGCCATGAATTCGA---
ATTTAGAGGAACAAAAGAAAGGAAAGTATGTTC
A3-AT1G54217-XLOC_006891-6279-1
      CGCCATGAATTCGACAGATTTAGAGGAACAAAAGAAAGGAAAGTATGTTC
CONSENSUS
      CGCCATGAATTCGA...ATTTAGAGGAACAAAAGAAAGGAAAGTATGTTC

A3-AT1G54217-XLOC_006891-6279-0
      TAATCCGTGATGATGCTGAGGACTCTGAGTTAGGACAATTCTATAAGCCC
A3-AT1G54217-XLOC_006891-6279-1
      TAATCCGTGATGATGCTGAGGACTCTGAGTTAGGACAATTCTATAAGCCC
CONSENSUS
      TAATCCGTGATGATGCTGAGGACTCTGAGTTAGGACAATTCTATAAGCCC

A3-AT1G54217-XLOC_006891-6279-0
      CTTCCTTGCTTCGGTTTTGGAATCGGATGGTTCTC
A3-AT1G54217-XLOC_006891-6279-1
      CTTCCTTGCTTCGGTTTTGGAATCGGATGGTTCTC
CONSENSUS
      CTTCCTTGCTTCGGTTTTGGAATCGGATGGTTCTC

alignment for event: A3-AT1G07110-XLOC_004600-330

A3-AT1G07110-XLOC_004600-330-0
      AGTGCTGATTTCTTCCGAGCTGATAATCCTGAAGGTGTAGAGGCACGGAC
A3-AT1G07110-XLOC_004600-330-1
      AGTGCTGATTTCTTCCGAGCTGATAATCCTGAAGGTGTAGAGGCACGGAC
CONSENSUS
      AGTGCTGATTTCTTCCGAGCTGATAATCCTGAAGGTGTAGAGGCACGGAC

A3-AT1G07110-XLOC_004600-330-0   TGAG-----------------------
GTAGCAGCACTTGCTATGGAGG
A3-AT1G07110-XLOC_004600-330-1
      TGAGTTTTAATGTATCACATGCCACTAGGTAGCAGCACTTGCTATGGAGG

CONSENSUS
      TGAG........................GTAGCAGCACTTGCTATGGAGG

A3-AT1G07110-XLOC_004600-330-0
      ACATGATAGCTTGGATGCAAGAAGGTGGTCAA
A3-AT1G07110-XLOC_004600-330-1
      ACATGATAGCTTGGATGCAAGAAGGTGGTCAA
CONSENSUS
      ACATGATAGCTTGGATGCAAGAAGGTGGTCAA

alignment for event: A3-AT1G73350-XLOC_007904-176

A3-AT1G73350-XLOC_007904-176-0
      ATAAGAAATCAGGGAAAGGTAAGATTGATGATGAAGACGTCACCTTTCAA
A3-AT1G73350-XLOC_007904-176-1
      ATAAGAAATCAGGGAAAGGTAAGATTGATGATGAAGACGTCACCTTTCAA
CONSENSUS
      ATAAGAAATCAGGGAAAGGTAAGATTGATGATGAAGACGTCACCTTTCAA

A3-AT1G73350-XLOC_007904-176-0
      CGAATGGTTGCAAAG-----------------------------------
A3-AT1G73350-XLOC_007904-176-1
      CGAATGGTTGCAAAGGGATAACGGCCCCGCCACATTTTCTGGTTATTGCA
CONSENSUS
      CGAATGGTTGCAAAG...................................

A3-AT1G73350-XLOC_007904-176-0   -----
ATGCAAGAGGTTGCTGGTGAACGTGGAGGCTATCTTCATGGACGA
A3-AT1G73350-XLOC_007904-176-1
      TATAGATGCAAGAGGTTGCTGGTGAACGTGGAGGCTATCTTCATGGACGA
CONSENSUS
      .....ATGCAAGAGGTTGCTGGTGAACGTGGAGGCTATCTTCATGGACGA

A3-AT1G73350-XLOC_007904-176-0   GGCG
A3-AT1G73350-XLOC_007904-176-1   GGCG
CONSENSUS   GGCG

alignment for event: SE-AT1G13350-XLOC_004964-2584

SE-AT1G13350-XLOC_004964-2584-0
      CACGGATACACAAATTGAGAGAGAGCGCGCGACAAAATCGAACCTAACCC
SE-AT1G13350-XLOC_004964-2584-1
      CACGGATACACAAATTGAGAGAGAGCGCGCGACAAAATCGAACCTAACCC
CONSENSUS
      CACGGATACACAAATTGAGAGAGAGCGCGCGACAAAATCGAACCTAACCC

SE-AT1G13350-XLOC_004964-2584-0
      TAATTTTGCGATTTCCCCCTAATTTCGACGATCTGATGGTGAGTGACAAG
SE-AT1G13350-XLOC_004964-2584-1
      TAATTTTGCGATTTCCCCCTAATTTCGACGATCTGATGGTGAGTGACAAG
CONSENSUS
      TAATTTTGCGATTTCCCCCTAATTTCGACGATCTGATGGTGAGTGACAAG

SE-AT1G13350-XLOC_004964-2584-0
CATGTAGAATCAAACCACCGCAAACACCGACGGTCGTTTTCGCCGTCCGA
SE-AT1G13350-XLOC_004964-2584-1
CATGTAGAATCAAACCACCGCAAACACCGACGGTCGTTTTCGCCGTCCGA
CONSENSUS
CATGTAGAATCAAACCACCGCAAACACCGACGGTCGTTTTCGCCGTCCGA

SE-AT1G13350-XLOC_004964-2584-0
CGAGGTCTTTAAATCTCCGAAGCGGCACAAGTCCCGTCATCACCATCGCA
SE-AT1G13350-XLOC_004964-2584-1
CGAGGTCTTTAAATCTCCGAAGCGGCACAAGTCCCGTCATCACCATCGCA
CONSENSUS
CGAGGTCTTTAAATCTCCGAAGCGGCACAAGTCCCGTCATCACCATCGCA

SE-AT1G13350-XLOC_004964-2584-0
GGCATGGCCACCGTCATCATCGTGATGAGGAAGTTCAATATAACGATGAT
SE-AT1G13350-XLOC_004964-2584-1
GGCATGGCCACCGTCATCATCGTGATGAGGAAGTTCAATATAACGATGAT
CONSENSUS
GGCATGGCCACCGTCATCATCGTGATGAGGAAGTTCAATATAACGATGAT

SE-AT1G13350-XLOC_004964-2584-0
GAGAATGTTAACGGTGGTGATCTTGATATGGAAGAAGGTGAGATATTAGG
SE-AT1G13350-XLOC_004964-2584-1
GAGAATGTTAACGGTGGTGATCTTGATATGGAAGAAGGTGAGATATTAGG
CONSENSUS
GAGAATGTTAACGGTGGTGATCTTGATATGGAAGAAGGTGAGATATTAGG

SE-AT1G13350-XLOC_004964-2584-0
AAAAGAAGGGATTGGGGAGACATTGAAGAAGAAATTAGAGTCCGTCGACG
SE-AT1G13350-XLOC_004964-2584-1
AAAAGAAGGGATTGGGGAGACATTGAAGAAGAAATTAGAGTCCGTCGACG
CONSENSUS
AAAAGAAGGGATTGGGGAGACATTGAAGAAGAAATTAGAGTCCGTCGACG

SE-AT1G13350-XLOC_004964-2584-0
AGTTTGGGGATATAAAATCTGGTCAATTCCGGGAGAATAATCTG------
SE-AT1G13350-XLOC_004964-2584-1
AGTTTGGGGATATAAAATCTGGTCAATTCCGGGAGAATAATCTGGCGTTC
CONSENSUS
AGTTTGGGGATATAAAATCTGGTCAATTCCGGGAGAATAATCTG......

SE-AT1G13350-XLOC_004964-2584-0

SE-AT1G13350-XLOC_004964-2584-1
AATGGAGATGATGGCTTTCCCTTGATTTGTAAGAGCTGGATACGCGTTTA
CONSENSUS
....

SE-AT1G13350-XLOC_004964-2584-0 ------------------------
GGGAGAAATCAGCGGAGGGAAAGAG
SE-AT1G13350-XLOC_004964-2584-1
TAACTATCTTTCATATAAATGTCATGGGAGAAATCAGCGGAGGGAAAGAG
CONSENSUS
.........................GGGAGAAATCAGCGGAGGGAAAGAG

SE-AT1G13350-XLOC_004964-2584-0
AATGTGAGAAAAGAAAAGAGATAGAGCCTGACCGTGAAAGGAGAAAAGAG
SE-AT1G13350-XLOC_004964-2584-1
AATGTGAGAAAAGAAAAGAGATAGAGCCTGACCGTGAAAGGAGAAAAGAG
CONSENSUS
AATGTGAGAAAAGAAAAGAGATAGAGCCTGACCGTGAAAGGAGAAAAGAG

SE-AT1G13350-XLOC_004964-2584-0
AGGGGAAGCGTTGATAGAGATAGCAGGGGAGACAGGGAAAAAGATTACCT
SE-AT1G13350-XLOC_004964-2584-1
AGGGGAAGCGTTGATAGAGATAGCAGGGGAGACAGGGAAAAAGATTACCT
CONSENSUS
AGGGGAAGCGTTGATAGAGATAGCAGGGGAGACAGGGAAAAAGATTACCT

SE-AT1G13350-XLOC_004964-2584-0
ACGGGATAGAGACAACGACAGAGGTAGGAGTAGAGATAAAGCCAGGTATA
SE-AT1G13350-XLOC_004964-2584-1
ACGGGATAGAGACAACGACAGAGGTAGGAGTAGAGATAAAGCCAGGTATA
CONSENSUS
ACGGGATAGAGACAACGACAGAGGTAGGAGTAGAGATAAAGCCAGGTATA

SE-AT1G13350-XLOC_004964-2584-0
GTAGTAGAGAGAGGGGGAGGGAGAATGAAAGAGAGAGACGGAGTGAAAAA
SE-AT1G13350-XLOC_004964-2584-1
GTAGTAGAGAGAGGGGGAGGGAGAATGAAAGAGAGAGACGGAGTGAAAAA
CONSENSUS
GTAGTAGAGAGAGGGGGAGGGAGAATGAAAGAGAGAGACGGAGTGAAAAA

SE-AT1G13350-XLOC_004964-2584-0
GATAGGGATAAAGGACGAGAATTCCAGAGTGATAGAGAGAAGCATAAAAG
SE-AT1G13350-XLOC_004964-2584-1
GATAGGGATAAAGGACGAGAATTCCAGAGTGATAGAGAGAAGCATAAAAG
CONSENSUS
GATAGGGATAAAGGACGAGAATTCCAGAGTGATAGAGAGAAGCATAAAAG

SE-AT1G13350-XLOC_004964-2584-0
TCTTGATGATGGATATGGTGAAGTGAGGCATAAACATTCTGGACACTCAA
SE-AT1G13350-XLOC_004964-2584-1
TCTTGATGATGGATATGGTGAAGTGAGGCATAAACATTCTGGACACTCAA
CONSENSUS
TCTTGATGATGGATATGGTGAAGTGAGGCATAAACATTCTGGACACTCAA

SE-AT1G13350-XLOC_004964-2584-0
GACATGATGCGGAAGATGACTTAGAGTTAAGAAGCCCAACTTCTGTAAAT
SE-AT1G13350-XLOC_004964-2584-1
GACATGATGCGGAAGATGACTTAGAGTTAAGAAGCCCAACTTCTGTAAAT
CONSENSUS
GACATGATGCGGAAGATGACTTAGAGTTAAGAAGCCCAACTTCTGTAAAT

SE-AT1G13350-XLOC_004964-2584-0
GGCCATGATCCTAACAGTGGCGATGTCAAAGAAACTCGGGGAAATGTTGA
SE-AT1G13350-XLOC_004964-2584-1
GGCCATGATCCTAACAGTGGCGATGTCAAAGAAACTCGGGGAAATGTTGA
CONSENSUS
GGCCATGATCCTAACAGTGGCGATGTCAAAGAAACTCGGGGAAATGTTGA

SE-AT1G13350-XLOC_004964-2584-0 AAG
SE-AT1G13350-XLOC_004964-2584-1 AAG
CONSENSUS AAG

alignment for event: A3-AT1G63080-XLOC_007343-4115

A3-AT1G63080-XLOC_007343-4115-0
CTCTCCATCTTCAGGTTAAGCAACGACGAACTGTGTTCATTTTAGAGTTT
A3-AT1G63080-XLOC_007343-4115-1
CTCTCCATCTTCAGGTTAAGCAACGACGAACTGTGTTCATTTTAGAGTTT
CONSENSUS
CTCTCCATCTTCAGGTTAAGCAACGACGAACTGTGTTCATTTTAGAGTTT

A3-AT1G63080-XLOC_007343-4115-0
GTTAAAAGATCCAGAGAAGTATCACAATTGGAAAGAAGATGAGTTTGGCG
A3-AT1G63080-XLOC_007343-4115-1
GTTAAAAGATCCAGAGAAGTATCACAATTGGAAAGAAGATGAGTTTGGCG
CONSENSUS
GTTAAAAGATCCAGAGAAGTATCACAATTGGAAAGAAGATGAGTTTGGCG

A3-AT1G63080-XLOC_007343-4115-0
AAGAGATTTGTTCACCTGAGGAAAGCTTCTCCATCGTTTTGCCTTCGAGG
A3-AT1G63080-XLOC_007343-4115-1
AAGAGATTTGTTCACCTGAGGAAAGCTTCTCCATCGTTTTGCCTTCGAGG
CONSENSUS
AAGAGATTTGTTCACCTGAGGAAAGCTTCTCCATCGTTTTGCCTTCGAGG

A3-AT1G63080-XLOC_007343-4115-0
TATCTATTTCTCTGGTTTGAGTTATGATGGCTACAGAGAGAAATTGAGTA
A3-AT1G63080-XLOC_007343-4115-1
TATCTATTTCTCTGGTTTGAGTTATGATGGCTACAGAGAGAAATTGAGTA
CONSENSUS
TATCTATTTCTCTGGTTTGAGTTATGATGGCTACAGAGAGAAATTGAGTA

A3-AT1G63080-XLOC_007343-4115-0
GAAATGCGTTACTCCATTTGAAGTTAGATGAAGCTGTTGATCTGTTCGGT
A3-AT1G63080-XLOC_007343-4115-1
GAAATGCGTTACTCCATTTGAAGTTAGATGAAGCTGTTGATCTGTTCGGT
CONSENSUS
GAAATGCGTTACTCCATTTGAAGTTAGATGAAGCTGTTGATCTGTTCGGT

A3-AT1G63080-XLOC_007343-4115-0
GAAATGGTCAAATCTCGCCCCTTTCCTTCCATTGTTGAGTTCAGTAAATT
A3-AT1G63080-XLOC_007343-4115-1
GAAATGGTCAAATCTCGCCCCTTTCCTTCCATTGTTGAGTTCAGTAAATT
CONSENSUS
GAAATGGTCAAATCTCGCCCCTTTCCTTCCATTGTTGAGTTCAGTAAATT

A3-AT1G63080-XLOC_007343-4115-0
GTTGAGTGCTATTGCTAAGATGAAGAAGTTCGATCTTGTCATCTCCTTCG
A3-AT1G63080-XLOC_007343-4115-1
GTTGAGTGCTATTGCTAAGATGAAGAAGTTCGATCTTGTCATCTCCTTCG
CONSENSUS

GTTGAGTGCTATTGCTAAGATGAAGAAGTTCGATCTTGTCATCTCCTTCG

A3-AT1G63080-XLOC_007343-4115-0
GGGAGAAGATGGAGATTTTAGGAGTTTCACATAATCTCTATACTTACAAT
A3-AT1G63080-XLOC_007343-4115-1
GGGAGAAGATGGAGATTTTAGGAGTTTCACATAATCTCTATACTTACAAT
CONSENSUS
GGGAGAAGATGGAGATTTTAGGAGTTTCACATAATCTCTATACTTACAAT

A3-AT1G63080-XLOC_007343-4115-0
ATTATGATTAACTGTTTATGCCGCCGCTCTCAACTCTCATTTGCTTTAGC
A3-AT1G63080-XLOC_007343-4115-1
ATTATGATTAACTGTTTATGCCGCCGCTCTCAACTCTCATTTGCTTTAGC
CONSENSUS
ATTATGATTAACTGTTTATGCCGCCGCTCTCAACTCTCATTTGCTTTAGC

A3-AT1G63080-XLOC_007343-4115-0
AATTCTTGGCAAGATGATGAAACTTGGCTATGGGCCCAGCATTGTTACCC
A3-AT1G63080-XLOC_007343-4115-1
AATTCTTGGCAAGATGATGAAACTTGGCTATGGGCCCAGCATTGTTACCC
CONSENSUS
AATTCTTGGCAAGATGATGAAACTTGGCTATGGGCCCAGCATTGTTACCC

A3-AT1G63080-XLOC_007343-4115-0
TGAACTCGCTTCTCAATGGTTTCTGTCACGGGAATAGGATATCCGAGGCT
A3-AT1G63080-XLOC_007343-4115-1
TGAACTCGCTTCTCAATGGTTTCTGTCACGGGAATAGGATATCCGAGGCT
CONSENSUS
TGAACTCGCTTCTCAATGGTTTCTGTCACGGGAATAGGATATCCGAGGCT

A3-AT1G63080-XLOC_007343-4115-0
GTTGCTTTGGTTGATCAGATGGTGGAAATGGGATATCAACCTGATACCGT
A3-AT1G63080-XLOC_007343-4115-1
GTTGCTTTGGTTGATCAGATGGTGGAAATGGGATATCAACCTGATACCGT
CONSENSUS
GTTGCTTTGGTTGATCAGATGGTGGAAATGGGATATCAACCTGATACCGT

A3-AT1G63080-XLOC_007343-4115-0
CACATTTACAACTTTGGTTCATGGGCTTTTTCAACACAACAAAGCTTCCG
A3-AT1G63080-XLOC_007343-4115-1
CACATTTACAACTTTGGTTCATGGGCTTTTTCAACACAACAAAGCTTCCG
CONSENSUS
CACATTTACAACTTTGGTTCATGGGCTTTTTCAACACAACAAAGCTTCCG

A3-AT1G63080-XLOC_007343-4115-0
AAGCTGTGGCTTTAGTTGAACGGATGGTTGTGAAAGGTTGTCAACCAGAT
A3-AT1G63080-XLOC_007343-4115-1
AAGCTGTGGCTTTAGTTGAACGGATGGTTGTGAAAGGTTGTCAACCAGAT
CONSENSUS
AAGCTGTGGCTTTAGTTGAACGGATGGTTGTGAAAGGTTGTCAACCAGAT

A3-AT1G63080-XLOC_007343-4115-0
CTGGTTACTTATGGTGCGGTAATTAATGGATTATGTAAGAGAGGTGAACC
A3-AT1G63080-XLOC_007343-4115-1
CTGGTTACTTATGGTGCGGTAATTAATGGATTATGTAAGAGAGGTGAACC
CONSENSUS

CTGGTTACTTATGGTGCGGTAATTAATGGATTATGTAAGAGAGGTGAACC

A3-AT1G63080-XLOC_007343-4115-0
TGATTTGGCTTTAAATCTGCTCAACAAGATGGAGAAAGGGAAAATAGAGG
A3-AT1G63080-XLOC_007343-4115-1
TGATTTGGCTTTAAATCTGCTCAACAAGATGGAGAAAGGGAAAATAGAGG
CONSENSUS
TGATTTGGCTTTAAATCTGCTCAACAAGATGGAGAAAGGGAAAATAGAGG

A3-AT1G63080-XLOC_007343-4115-0
CTGATGTTGTGATCTACAGCACAGTCATAGATAGTCTCTGCAAATATAGA
A3-AT1G63080-XLOC_007343-4115-1
CTGATGTTGTGATCTACAGCACAGTCATAGATAGTCTCTGCAAATATAGA
CONSENSUS
CTGATGTTGTGATCTACAGCACAGTCATAGATAGTCTCTGCAAATATAGA

A3-AT1G63080-XLOC_007343-4115-0
CATGTGGATGATGCACTCAATCTGTTCACTGAAATGGATAACAAAGGTAT
A3-AT1G63080-XLOC_007343-4115-1
CATGTGGATGATGCACTCAATCTGTTCACTGAAATGGATAACAAAGGTAT
CONSENSUS
CATGTGGATGATGCACTCAATCTGTTCACTGAAATGGATAACAAAGGTAT

A3-AT1G63080-XLOC_007343-4115-0
TAGACCAGATGTTTTTACCTACAGCTCCCTCATAAGTTGCCTTTGTAATT
A3-AT1G63080-XLOC_007343-4115-1
TAGACCAGATGTTTTTACCTACAGCTCCCTCATAAGTTGCCTTTGTAATT
CONSENSUS
TAGACCAGATGTTTTTACCTACAGCTCCCTCATAAGTTGCCTTTGTAATT

A3-AT1G63080-XLOC_007343-4115-0
ACGGAAGATGGAGTGATGCTTCTCGACTACTCAGTGATATGCTTGAGAGG
A3-AT1G63080-XLOC_007343-4115-1
ACGGAAGATGGAGTGATGCTTCTCGACTACTCAGTGATATGCTTGAGAGG
CONSENSUS
ACGGAAGATGGAGTGATGCTTCTCGACTACTCAGTGATATGCTTGAGAGG

A3-AT1G63080-XLOC_007343-4115-0
AAAATCAATCCAAATGTAGTCACTTTCAACTCATTGATCGATGCGTTTGC
A3-AT1G63080-XLOC_007343-4115-1
AAAATCAATCCAAATGTAGTCACTTTCAACTCATTGATCGATGCGTTTGC
CONSENSUS
AAAATCAATCCAAATGTAGTCACTTTCAACTCATTGATCGATGCGTTTGC

A3-AT1G63080-XLOC_007343-4115-0
GAAGGAGGGGAAGCTTATAGAGGCTGAGAAATTGTTTGATGAGATGATCC
A3-AT1G63080-XLOC_007343-4115-1
GAAGGAGGGGAAGCTTATAGAGGCTGAGAAATTGTTTGATGAGATGATCC
CONSENSUS
GAAGGAGGGGAAGCTTATAGAGGCTGAGAAATTGTTTGATGAGATGATCC

A3-AT1G63080-XLOC_007343-4115-0
AAAGGTCTATAGATCCTAATATTGTCACTTACAATTCATTGATCAACGGG
A3-AT1G63080-XLOC_007343-4115-1
AAAGGTCTATAGATCCTAATATTGTCACTTACAATTCATTGATCAACGGG
CONSENSUS

AAAGGTCTATAGATCCTAATATTGTCACTTACAATTCATTGATCAACGGG

A3-AT1G63080-XLOC_007343-4115-0
TTTTGTATGCATGATCGTCTAGATGAGGCCCAGCAGATTTTTACTTTGAT
A3-AT1G63080-XLOC_007343-4115-1
TTTTGTATGCATGATCGTCTAGATGAGGCCCAGCAGATTTTTACTTTGAT
CONSENSUS
TTTTGTATGCATGATCGTCTAGATGAGGCCCAGCAGATTTTTACTTTGAT

A3-AT1G63080-XLOC_007343-4115-0
GGTTAGCAAAGATTGTCTGCCAGACGTAGTAACTTATAATACACTTATAA
A3-AT1G63080-XLOC_007343-4115-1
GGTTAGCAAAGATTGTCTGCCAGACGTAGTAACTTATAATACACTTATAA
CONSENSUS
GGTTAGCAAAGATTGTCTGCCAGACGTAGTAACTTATAATACACTTATAA

A3-AT1G63080-XLOC_007343-4115-0
ATGGATTTTGTAAGGCTAAGAAAGTGGTGGATGGTATGGAACTTTTCCGG
A3-AT1G63080-XLOC_007343-4115-1
ATGGATTTTGTAAGGCTAAGAAAGTGGTGGATGGTATGGAACTTTTCCGG
CONSENSUS
ATGGATTTTGTAAGGCTAAGAAAGTGGTGGATGGTATGGAACTTTTCCGG

A3-AT1G63080-XLOC_007343-4115-0
GACATGTCTCGAAGAGGATTGGTTGGCAACACAGTCACTTACACCACTCT
A3-AT1G63080-XLOC_007343-4115-1
GACATGTCTCGAAGAGGATTGGTTGGCAACACAGTCACTTACACCACTCT
CONSENSUS
GACATGTCTCGAAGAGGATTGGTTGGCAACACAGTCACTTACACCACTCT

A3-AT1G63080-XLOC_007343-4115-0
TATCCATGGATTTTTTCAAGCTAGTGATTGTGATAATGCCCAAATGGTTT
A3-AT1G63080-XLOC_007343-4115-1
TATCCATGGATTTTTTCAAGCTAGTGATTGTGATAATGCCCAAATGGTTT
CONSENSUS
TATCCATGGATTTTTTCAAGCTAGTGATTGTGATAATGCCCAAATGGTTT

A3-AT1G63080-XLOC_007343-4115-0
TCAAACAGATGGTTTCTGATGGTGTGCATCCCAATATTATGACATACAAC
A3-AT1G63080-XLOC_007343-4115-1
TCAAACAGATGGTTTCTGATGGTGTGCATCCCAATATTATGACATACAAC
CONSENSUS
TCAAACAGATGGTTTCTGATGGTGTGCATCCCAATATTATGACATACAAC

A3-AT1G63080-XLOC_007343-4115-0
ACTCTGTTAGATGGACTTTGTAAAAATGGGAAGCTAGAGAAAGCAATGGT
A3-AT1G63080-XLOC_007343-4115-1
ACTCTGTTAGATGGACTTTGTAAAAATGGGAAGCTAGAGAAAGCAATGGT
CONSENSUS
ACTCTGTTAGATGGACTTTGTAAAAATGGGAAGCTAGAGAAAGCAATGGT

A3-AT1G63080-XLOC_007343-4115-0
TGTATTCGAGTATCTGCAAAAGAGTAAAATGGAACCTGATATTTACACAT
A3-AT1G63080-XLOC_007343-4115-1
TGTATTCGAGTATCTGCAAAAGAGTAAAATGGAACCTGATATTTACACAT
CONSENSUS

TGTATTCGAGTATCTGCAAAAGAGTAAAATGGAACCTGATATTTACACAT

A3-AT1G63080-XLOC_007343-4115-0
ATAATATTATGAGTGAAGGGATGTGCAAGGCAGGGAAGGTGGAAGATGGG
A3-AT1G63080-XLOC_007343-4115-1
ATAATATTATGAGTGAAGGGATGTGCAAGGCAGGGAAGGTGGAAGATGGG
CONSENSUS
ATAATATTATGAGTGAAGGGATGTGCAAGGCAGGGAAGGTGGAAGATGGG

A3-AT1G63080-XLOC_007343-4115-0
TGGGATCTATTTTGTAGCCTCAGCCTTAAAGGAGTGAAACCTGATGTTAT
A3-AT1G63080-XLOC_007343-4115-1
TGGGATCTATTTTGTAGCCTCAGCCTTAAAGGAGTGAAACCTGATGTTAT
CONSENSUS
TGGGATCTATTTTGTAGCCTCAGCCTTAAAGGAGTGAAACCTGATGTTAT

A3-AT1G63080-XLOC_007343-4115-0
AGCCTACAATACAATGATCTCAGGATTCTGTAAGAAAGGTTTAAAGGAGG
A3-AT1G63080-XLOC_007343-4115-1
AGCCTACAATACAATGATCTCAGGATTCTGTAAGAAAGGTTTAAAGGAGG
CONSENSUS
AGCCTACAATACAATGATCTCAGGATTCTGTAAGAAAGGTTTAAAGGAGG

A3-AT1G63080-XLOC_007343-4115-0
AAGCATATACCTTGTTCATAAAGATGAAAGAAGACGGGCCTCTTCCAGAT
A3-AT1G63080-XLOC_007343-4115-1
AAGCATATACCTTGTTCATAAAGATGAAAGAAGACGGGCCTCTTCCAGAT
CONSENSUS
AAGCATATACCTTGTTCATAAAGATGAAAGAAGACGGGCCTCTTCCAGAT

A3-AT1G63080-XLOC_007343-4115-0
AGTGGTACATATAATACGCTTATCAGGGCACATCTGAGAGATGGTGACAA
A3-AT1G63080-XLOC_007343-4115-1
AGTGGTACATATAATACGCTTATCAGGGCACATCTGAGAGATGGTGACAA
CONSENSUS
AGTGGTACATATAATACGCTTATCAGGGCACATCTGAGAGATGGTGACAA

A3-AT1G63080-XLOC_007343-4115-0
AGCAGCATCAGCTGAACTCATCAAAGAAATGAGGAGTTGCAGGTTTGCTG
A3-AT1G63080-XLOC_007343-4115-1
AGCAGCATCAGCTGAACTCATCAAAGAAATGAGGAGTTGCAGGTTTGCTG
CONSENSUS
AGCAGCATCAGCTGAACTCATCAAAGAAATGAGGAGTTGCAGGTTTGCTG

A3-AT1G63080-XLOC_007343-4115-0
GCGATGCTTCAACTTATGGCTTGGTCACAGATATGTTGCATGATGGGAGA
A3-AT1G63080-XLOC_007343-4115-1
GCGATGCTTCAACTTATGGCTTGGTCACAGATATGTTGCATGATGGGAGA
CONSENSUS
GCGATGCTTCAACTTATGGCTTGGTCACAGATATGTTGCATGATGGGAGA

A3-AT1G63080-XLOC_007343-4115-0
TTGGACAAAGGATTTTTGGAAGTGCTTTCTTAAG----------------
A3-AT1G63080-XLOC_007343-4115-1
TTGGACAAAGGATTTTTGGAAGTGCTTTCTTAAGGTTGCAAACATGAGTT
CONSENSUS

TTGGACAAAGGATTTTTGGAAGTGCTTTCTTAAG................

A3-AT1G63080-XLOC_007343-4115-0

A3-AT1G63080-XLOC_007343-4115-1
GATCTAAACGCTCATTGATGCAAGTTCAAGGTTAATTCCCGGCCGGGATT
CONSENSUS
....

A3-AT1G63080-XLOC_007343-4115-0

A3-AT1G63080-XLOC_007343-4115-1
TGTATTTTTGTATGTTCATTGCATTTGCACACGGAAACATACAAGAAGAC
CONSENSUS
....

A3-AT1G63080-XLOC_007343-4115-0

A3-AT1G63080-XLOC_007343-4115-1
AGGGATATTGTGGGTTTAGTTTCATTTTAGAAGGTTACATATGTGTTTCC
CONSENSUS
....

A3-AT1G63080-XLOC_007343-4115-0

A3-AT1G63080-XLOC_007343-4115-1
TTAGCTGACAGATACTATATTGCAGCAAATTTGGTGACATTCCAAAGAAA
CONSENSUS
....

A3-AT1G63080-XLOC_007343-4115-0

A3-AT1G63080-XLOC_007343-4115-1
ATATGACAAAGAATCTGTCAGTTTTCGGAGTAATTAGTCAATGTTTGTCT
CONSENSUS
....

A3-AT1G63080-XLOC_007343-4115-0

A3-AT1G63080-XLOC_007343-4115-1
CATTTCTATGTTGTTCTTGATCTACACAAAAGAATCAACTTTCATGGATA
CONSENSUS
....

A3-AT1G63080-XLOC_007343-4115-0

A3-AT1G63080-XLOC_007343-4115-1
GAAGAAAGTCTTTCAAAATAATCACGGAAGTCGGGAAGAAAGAGATCTAG
CONSENSUS
....

A3-AT1G63080-XLOC_007343-4115-0

A3-AT1G63080-XLOC_007343-4115-1
TACAGTAACTCATCCTCGGAAATTCCGGTCACTGGAAAAAGTTTGGGATT
CONSENSUS

....

A3-AT1G63080-XLOC_007343-4115-0

A3-AT1G63080-XLOC_007343-4115-1
GGTACAATGCCTTCTGAAACTTTCTTCAAAGACTGAAAGTTCCGAGATTG
CONSENSUS
....

A3-AT1G63080-XLOC_007343-4115-0

A3-AT1G63080-XLOC_007343-4115-1
GGTTGCTATGTTGGATCTCTGTCTTCTCAACTTCATGTGATCATTTTATA
CONSENSUS
....

A3-AT1G63080-XLOC_007343-4115-0

A3-AT1G63080-XLOC_007343-4115-1
CCTCACAAGCATTGAGAAATATGAAAGATATATACTTACTATTTTCTTCT
CONSENSUS
....

A3-AT1G63080-XLOC_007343-4115-0

A3-AT1G63080-XLOC_007343-4115-1
GTTTTCATGAAAGTTATTGGTTTTTGTGTTGACTAAGAACATTGAGGCAA
CONSENSUS
....

A3-AT1G63080-XLOC_007343-4115-0

A3-AT1G63080-XLOC_007343-4115-1
CCATTGCCTGATATTAAAGACAGATAGATTCTTTTTTCTCCTTTTTTGAC
CONSENSUS
....

A3-AT1G63080-XLOC_007343-4115-0

A3-AT1G63080-XLOC_007343-4115-1
CGAACACGCTTCAGAAGTTTTAGTAGCAGCAACAACGGAGGATTCTTTTT
CONSENSUS
....

A3-AT1G63080-XLOC_007343-4115-0

A3-AT1G63080-XLOC_007343-4115-1
GATGTAGCTGACAACTTTGTTTCTTTTAGTATTGAGCTCTTTCGTTTATG
CONSENSUS
....

A3-AT1G63080-XLOC_007343-4115-0

A3-AT1G63080-XLOC_007343-4115-1
TCTTTGTTTTCTTATTGATGGGTTTTTGATGGTACAGTTCTCCTCCCTTT
CONSENSUS

       ....

A3-AT1G63080-XLOC_007343-4115-0

A3-AT1G63080-XLOC_007343-4115-1
       CCTTCCACTATTGATTGATTTCAGTATACATACGGTTGCTGTTCTGTGTA
CONSENSUS
       ....

A3-AT1G63080-XLOC_007343-4115-0
       ---G
A3-AT1G63080-XLOC_007343-4115-1
       AGCTTCATGAAGATGACTAATATTGTGCATTTGTGTATTTCATTGTCAGG
CONSENSUS
       ....G

A3-AT1G63080-XLOC_007343-4115-0
       TTAACACAAGATCAGTGGTGAGCATCATAAGAAGCTCTTCCCTTCATGAA
A3-AT1G63080-XLOC_007343-4115-1
       TTAACACAAGATCAGTGGTGAGCATCATAAGAAGCTCTTCCCTTCATGAA
CONSENSUS
       TTAACACAAGATCAGTGGTGAGCATCATAAGAAGCTCTTCCCTTCATGAA

A3-AT1G63080-XLOC_007343-4115-0
       GATCTCAGACTTGGTTTGACATATGATTATTGTTCTACAGTAGATTTGTT
A3-AT1G63080-XLOC_007343-4115-1
       GATCTCAGACTTGGTTTGACATATGATTATTGTTCTACAGTAGATTTGTT
CONSENSUS
       GATCTCAGACTTGGTTTGACATATGATTATTGTTCTACAGTAGATTTGTT

A3-AT1G63080-XLOC_007343-4115-0
       TTTTAACCACAGTTGGTTCTGTTTCTTGTACACAAACTTTTTGCATCTGT
A3-AT1G63080-XLOC_007343-4115-1
       TTTTAACCACAGTTGGTTCTGTTTCTTGTACACAAACTTTTTGCATCTGT
CONSENSUS
       TTTTAACCACAGTTGGTTCTGTTTCTTGTACACAAACTTTTTGCATCTGT

A3-AT1G63080-XLOC_007343-4115-0
       TTCTAGTAGTAATATAAATGAAACATGTAAACC
A3-AT1G63080-XLOC_007343-4115-1
       TTCTAGTAGTAATATAAATGAAACATGTAAACC
CONSENSUS
       TTCTAGTAGTAATATAAATGAAACATGTAAACC

alignment for event: A3-AT1G10890-XLOC_000531-1571

A3-AT1G10890-XLOC_000531-1571-0
       GCGACAGCGTGAAGCAGAACTGAAGCTAATAGAGGAAGAAACTGTGAAAC
A3-AT1G10890-XLOC_000531-1571-1
       GCGACAGCGTGAAGCAGAACTGAAGCTAATAGAGGAAGAAACTGTGAAAC
CONSENSUS
       GCGACAGCGTGAAGCAGAACTGAAGCTAATAGAGGAAGAAACTGTGAAAC

A3-AT1G10890-XLOC_000531-1571-0

GGGTTGAAGAAGCTATTCGAAAGAAGGTCGAAGAAAGCTTACAGTCTGAG
A3-AT1G10890-XLOC_000531-1571-1
GGGTTGAAGAAGCTATTCGAAAGAAGGTCGAAGAAAGCTTACAGTCTGAG
CONSENSUS
GGGTTGAAGAAGCTATTCGAAAGAAGGTCGAAGAAAGCTTACAGTCTGAG

A3-AT1G10890-XLOC_000531-1571-0
AAAATCAAAATGGAAATTCTAACGCTGTTGGAGGAAGGGCGAAAGAGACT
A3-AT1G10890-XLOC_000531-1571-1
AAAATCAAAATGGAAATTCTAACGCTGTTGGAGGAAGGGCGAAAGAGACT
CONSENSUS
AAAATCAAAATGGAAATTCTAACGCTGTTGGAGGAAGGGCGAAAGAGACT

A3-AT1G10890-XLOC_000531-1571-0
TAATGAAGAAGTCGCGGCTCAACTTGAGGAGGAGAAAGAGGCTTCTCTTA
A3-AT1G10890-XLOC_000531-1571-1
TAATGAAGAAGTCGCGGCTCAACTTGAGGAGGAGAAAGAGGCTTCTCTTA
CONSENSUS
TAATGAAGAAGTCGCGGCTCAACTTGAGGAGGAGAAAGAGGCTTCTCTTA

A3-AT1G10890-XLOC_000531-1571-0
TTGAGGCTAAAGAAAAAGAGGGTGTTATGCGGTGTTTGTCACAGGAAAGA
A3-AT1G10890-XLOC_000531-1571-1
TTGAGGCTAAAGAAAAAGAG------------------------GAAAGA
CONSENSUS
TTGAGGCTAAAGAAAAAGAG........................GAAAGA

A3-AT1G10890-XLOC_000531-1571-0
GAGCAACAAGAGAAAGAAGAGAGGGAGAGAATAGCAGAGGAGAACCTAAA
A3-AT1G10890-XLOC_000531-1571-1
GAGCAACAAGAGAAAGAAGAGAGGGAGAGAATAGCAGAGGAGAACCTAAA
CONSENSUS
GAGCAACAAGAGAAAGAAGAGAGGGAGAGAATAGCAGAGGAGAACCTAAA

A3-AT1G10890-XLOC_000531-1571-0
GAGAGTGGAAGAAGCTCAGAGAAAAGAAGCAATGGAGAGGCAAAGGAAAG
A3-AT1G10890-XLOC_000531-1571-1
GAGAGTGGAAGAAGCTCAGAGAAAAGAAGCAATGGAGAGGCAAAGGAAAG
CONSENSUS
GAGAGTGGAAGAAGCTCAGAGAAAAGAAGCAATGGAGAGGCAAAGGAAAG

A3-AT1G10890-XLOC_000531-1571-0
AGGAGGAACGGTATCGAGAGCTAGAGGAGCTGCAACGACAGAAAGAAGAA
A3-AT1G10890-XLOC_000531-1571-1
AGGAGGAACGGTATCGAGAGCTAGAGGAGCTGCAACGACAGAAAGAAGAA
CONSENSUS
AGGAGGAACGGTATCGAGAGCTAGAGGAGCTGCAACGACAGAAAGAAGAA

A3-AT1G10890-XLOC_000531-1571-0
GCGATGCGAAGGAAGAAAGCTGAAGAGGAAGAAGAACGTCTCAAACAGAT
A3-AT1G10890-XLOC_000531-1571-1
GCGATGCGAAGGAAGAAAGCTGAAGAGGAAGAAGAACGTCTCAAACAGAT
CONSENSUS
GCGATGCGAAGGAAGAAAGCTGAAGAGGAAGAAGAACGTCTCAAACAGAT

A3-AT1G10890-XLOC_000531-1571-0

GAAACTGTTGGGTAAAAACAAATCACGGCCTAAATTATCCTTTGCCTTAA
A3-AT1G10890-XLOC_000531-1571-1
GAAACTGTTGGGTAAAAACAAATCACGGCCTAAATTATCCTTTGCCTTAA
CONSENSUS
GAAACTGTTGGGTAAAAACAAATCACGGCCTAAATTATCCTTTGCCTTAA

A3-AT1G10890-XLOC_000531-1571-0
GCTCCAAGTAAATGCGTGCATGCATGAAGATAAAAGGATTGATGTGATGG
A3-AT1G10890-XLOC_000531-1571-1
GCTCCAAGTAAATGCGTGCATGCATGAAGATAAAAGGATTGATGTGATGG
CONSENSUS
GCTCCAAGTAAATGCGTGCATGCATGAAGATAAAAGGATTGATGTGATGG

A3-AT1G10890-XLOC_000531-1571-0
ATGATGATATGCATCTTCTTCCTCTCAAAGATGCTTTATGATTATTGTTA
A3-AT1G10890-XLOC_000531-1571-1
ATGATGATATGCATCTTCTTCCTCTCAAAGATGCTTTATGATTATTGTTA
CONSENSUS
ATGATGATATGCATCTTCTTCCTCTCAAAGATGCTTTATGATTATTGTTA

A3-AT1G10890-XLOC_000531-1571-0
TTAGTGCTTCTTGTTGGAGCTTAAACTCTTTTATGGCTTTTAATTTTTTG
A3-AT1G10890-XLOC_000531-1571-1
TTAGTGCTTCTTGTTGGAGCTTAAACTCTTTTATGGCTTTTAATTTTTTG
CONSENSUS
TTAGTGCTTCTTGTTGGAGCTTAAACTCTTTTATGGCTTTTAATTTTTTG

A3-AT1G10890-XLOC_000531-1571-0
TAATTCTATTTTTCTCGTTTTGTAATTTTACGTTAGGTTAATGGTGATGA
A3-AT1G10890-XLOC_000531-1571-1
TAATTCTATTTTTCTCGTTTTGTAATTTTACGTTAGGTTAATGGTGATGA
CONSENSUS
TAATTCTATTTTTCTCGTTTTGTAATTTTACGTTAGGTTAATGGTGATGA

A3-AT1G10890-XLOC_000531-1571-0
ATGATAATATAGCAATGATTCAGAAAATTTA
A3-AT1G10890-XLOC_000531-1571-1
ATGATAATATAGCAATGATTCAGAAAATTTA
CONSENSUS
ATGATAATATAGCAATGATTCAGAAAATTTA

alignment for event: SE-AT1G07350-XLOC_004615-3568

SE-AT1G07350-XLOC_004615-3568-0
GTAACTGATGTTCACCTTGTCCTGGACCCATGGACTAGAGAATCTCGCGG
SE-AT1G07350-XLOC_004615-3568-1
GTAACTGATGTTCACCTTGTCCTGGACCCATGGACTAGAGAATCTCGCGG
CONSENSUS
GTAACTGATGTTCACCTTGTCCTGGACCCATGGACTAGAGAATCTCGCGG

SE-AT1G07350-XLOC_004615-3568-0
ATTTGGTTTTATCTCTATGAAAAGTGTTGGTGATGCTAACCGTTGCATCA
SE-AT1G07350-XLOC_004615-3568-1
ATTTGGTTTTATCTCTATGAAAAGTGTTGGTGATGCTAACCGTTGCATCA

```
CONSENSUS
      ATTTGGTTTTATCTCTATGAAAAGTGTTGGTGATGCTAACCGTTGCATCA

SE-AT1G07350-XLOC_004615-3568-0
      GATCTCTAGATCACTCTGTTCTGCAGGGCCGCGTCATCACTGTTGAGAAG
SE-AT1G07350-XLOC_004615-3568-1
      GATCTCTAGATCACTCTGTTCTGCAGGGCCGCGTCATCACTGTTGAGAAG
CONSENSUS
      GATCTCTAGATCACTCTGTTCTGCAGGGCCGCGTCATCACTGTTGAGAAG

SE-AT1G07350-XLOC_004615-3568-0
      --------------------------------------------------
SE-AT1G07350-XLOC_004615-3568-1
      TTTCTGTGGCAGCAGGTCTGCTGTTTGTAGCAGCAGTGCTTCACCAAATA
CONSENSUS
      ..................................................

SE-AT1G07350-XLOC_004615-3568-0
      ----------------------------------------------GCAA
SE-AT1G07350-XLOC_004615-3568-1
      GCAAACGTTGCAACAGTTCAAACACATCAAGTTTCAGACATTTAGGGCAA
CONSENSUS
      ..............................................GCAA

SE-AT1G07350-XLOC_004615-3568-0
      GACGTCGTAGAGGACGTACTCCAACTCCAGGAAAGTACTTGGGGCTGAGA
SE-AT1G07350-XLOC_004615-3568-1
      GACGTCGTAGAGGACGTACTCCAACTCCAGGAAAGTACTTGGGGCTGAGA
CONSENSUS
      GACGTCGTAGAGGACGTACTCCAACTCCAGGAAAGTACTTGGGGCTGAGA

SE-AT1G07350-XLOC_004615-3568-0  ACTGCTCGAG
SE-AT1G07350-XLOC_004615-3568-1  ACTGCTCGAG
CONSENSUS                        ACTGCTCGAG


alignment for event: A3-AT1G70620-XLOC_003695-84

A3-AT1G70620-XLOC_003695-84-0
      GTTACAGATGAATTATTTGATGAAATTGCAACCAAAGTCATTAACGAAGA
A3-AT1G70620-XLOC_003695-84-1
      GTTACAGATGAATTATTTGATGAAATTGCAACCAAAGTCATTAACGAAGA
CONSENSUS
      GTTACAGATGAATTATTTGATGAAATTGCAACCAAAGTCATTAACGAAGA

A3-AT1G70620-XLOC_003695-84-0
      TGAAGCAATACCTAAAGATGATAGTGTACAGCATAATCATAAATTGTCAT
A3-AT1G70620-XLOC_003695-84-1
      TGAAGCAATACCTAAAG---------------------------------
CONSENSUS
      TGAAGCAATACCTAAAG.................................

A3-AT1G70620-XLOC_003695-84-0
      CATCCCTTCTCTCCACAGCTGATCCACTCCACAAGGCTTCGGCAAAGATT
A3-AT1G70620-XLOC_003695-84-1    ------------------
```

CTGATCCACTCCACAAGGCTTCGGCAAAGATT
CONSENSUS
..................CTGATCCACTCCACAAGGCTTCGGCAAAGATT

A3-AT1G70620-XLOC_003695-84-0
TTGGTCTCGGTAGAAGGTGCAAATACAAAAGCTAGTTCTGGCTCTCCAGC
A3-AT1G70620-XLOC_003695-84-1
TTGGTCTCGGTAGAAGGTGCAAATACAAAAGCTAGTTCTGGCTCTCCAGC
CONSENSUS
TTGGTCTCGGTAGAAGGTGCAAATACAAAAGCTAGTTCTGGCTCTCCAGC

A3-AT1G70620-XLOC_003695-84-0
AGATGTGCTAGGTCTTGCTAGCTATGCCTCTGATGATGATGATGCTGATA
A3-AT1G70620-XLOC_003695-84-1
AGATGTGCTAGGTCTTGCTAGCTATGCCTCTGATGATGATGATGCTGATA
CONSENSUS
AGATGTGCTAGGTCTTGCTAGCTATGCCTCTGATGATGATGATGCTGATA

A3-AT1G70620-XLOC_003695-84-0
CTGATGCTGCTTCTGACGCGAATGCTGATGAAAATGGAGTGGAGAGTCTT
A3-AT1G70620-XLOC_003695-84-1
CTGATGCTGCTTCTGACGCGAATGCTGATGAAAATGGAGTGGAGAGTCTT
CONSENSUS
CTGATGCTGCTTCTGACGCGAATGCTGATGAAAATGGAGTGGAGAGTCTT

A3-AT1G70620-XLOC_003695-84-0
GGTGTGGGGTCAAGACACAATGTTAGTCAGCAGCCAAGCACTGAGAAACT
A3-AT1G70620-XLOC_003695-84-1
GGTGTGGGGTCAAGACACAATGTTAGTCAGCAGCCAAGCACTGAGAAACT
CONSENSUS
GGTGTGGGGTCAAGACACAATGTTAGTCAGCAGCCAAGCACTGAGAAACT

A3-AT1G70620-XLOC_003695-84-0
TCCTGACCCTGAAGCAATGGCCAGTGCGAAATTGGATCCAGCAGTTGGAG
A3-AT1G70620-XLOC_003695-84-1
TCCTGACCCTGAAGCAATGGCCAGTGCGAAATTGGATCCAGCAGTTGGAG
CONSENSUS
TCCTGACCCTGAAGCAATGGCCAGTGCGAAATTGGATCCAGCAGTTGGAG

A3-AT1G70620-XLOC_003695-84-0
TCAATGCTAATTCTGGCAAGAATAGTAAGTCAGGCTTGGAGGATTATTCT
A3-AT1G70620-XLOC_003695-84-1
TCAATGCTAATTCTGGCAAGAATAGTAAGTCAGGCTTGGAGGATTATTCT
CONSENSUS
TCAATGCTAATTCTGGCAAGAATAGTAAGTCAGGCTTGGAGGATTATTCT

A3-AT1G70620-XLOC_003695-84-0
CAGATGCCAGGCTCCACGAGAAAAGATGATGAGGCGGGTAGTACCAAAAT
A3-AT1G70620-XLOC_003695-84-1
CAGATGCCAGGCTCCACGAGAAAAGATGATGAGGCGGGTAGTACCAAAAT
CONSENSUS
CAGATGCCAGGCTCCACGAGAAAAGATGATGAGGCGGGTAGTACCAAAAT

A3-AT1G70620-XLOC_003695-84-0
ATCTGATGTAAGCGCCAGCTCTGGACTTGATGATGATACTTCAGGAAGCA
A3-AT1G70620-XLOC_003695-84-1

ATCTGATGTAAGCGCCAGCTCTGGACTTGATGATGATACTTCAGGAAGCA
CONSENSUS
ATCTGATGTAAGCGCCAGCTCTGGACTTGATGATGATACTTCAGGAAGCA

A3-AT1G70620-XLOC_003695-84-0
GAAAAGAGCATCCTGACAGAACTGATAGTGATAAAGATGCCATACTAGAT
A3-AT1G70620-XLOC_003695-84-1
GAAAAGAGCATCCTGACAGAACTGATAGTGATAAAGATGCCATACTAGAT
CONSENSUS
GAAAAGAGCATCCTGACAGAACTGATAGTGATAAAGATGCCATACTAGAT

A3-AT1G70620-XLOC_003695-84-0
GAACCTCACGTGAAGAATTCTGGCGTGAAATCAGATTGCAACCTTCGTCA
A3-AT1G70620-XLOC_003695-84-1
GAACCTCACGTGAAGAATTCTGGCGTGAAATCAGATTGCAACCTTCGTCA
CONSENSUS
GAACCTCACGTGAAGAATTCTGGCGTGAAATCAGATTGCAACCTTCGTCA

A3-AT1G70620-XLOC_003695-84-0
GGATAGTAATAAACCTTATGGGAAAGATTTGAGTGACGAAGTGAGTACAG
A3-AT1G70620-XLOC_003695-84-1
GGATAGTAATAAACCTTATGGGAAAGATTTGAGTGACGAAGTGAGTACAG
CONSENSUS
GGATAGTAATAAACCTTATGGGAAAGATTTGAGTGACGAAGTGAGTACAG

A3-AT1G70620-XLOC_003695-84-0
ATAGAAGTAGAATAGTTGAAACGAAAGGTGGGAAAGAGAAAGGAGATTCT
A3-AT1G70620-XLOC_003695-84-1
ATAGAAGTAGAATAGTTGAAACGAAAGGTGGGAAAGAGAAAGGAGATTCT
CONSENSUS
ATAGAAGTAGAATAGTTGAAACGAAAGGTGGGAAAGAGAAAGGAGATTCT

A3-AT1G70620-XLOC_003695-84-0
CAGAATGACTCAAAAGATAGAATGAAGGAAAATGACTTAAAGTCAGCAGA
A3-AT1G70620-XLOC_003695-84-1
CAGAATGACTCAAAAGATAGAATGAAGGAAAATGACTTAAAGTCAGCAGA
CONSENSUS
CAGAATGACTCAAAAGATAGAATGAAGGAAAATGACTTAAAGTCAGCAGA

A3-AT1G70620-XLOC_003695-84-0
GAAAGTTAAAGGCGTTGAATCAAATAAAAAATCTACTGATCCCCATGTAA
A3-AT1G70620-XLOC_003695-84-1
GAAAGTTAAAGGCGTTGAATCAAATAAAAAATCTACTGATCCCCATGTAA
CONSENSUS
GAAAGTTAAAGGCGTTGAATCAAATAAAAAATCTACTGATCCCCATGTAA

A3-AT1G70620-XLOC_003695-84-0
AGAAAGACTCAAGGGATGTAGAGAGGCCTCACAGAACTAATTCTAAGGAA
A3-AT1G70620-XLOC_003695-84-1
AGAAAGACTCAAGGGATGTAGAGAGGCCTCACAGAACTAATTCTAAGGAA
CONSENSUS
AGAAAGACTCAAGGGATGTAGAGAGGCCTCACAGAACTAATTCTAAGGAA

A3-AT1G70620-XLOC_003695-84-0
GACCGGGGTAAAAGAAAAGAGAAGGAAAAGGAAGAAGAAAGGTCAAGACA
A3-AT1G70620-XLOC_003695-84-1

```
      GACCGGGGTAAAAGAAAAGAGAAGGAAAAGGAAGAAGAAAGGTCAAGACA
CONSENSUS
      GACCGGGGTAAAAGAAAAGAGAAGGAAAAGGAAGAAGAAAGGTCAAGACA

A3-AT1G70620-XLOC_003695-84-0
      CAGGCGGGCTGAAAACTCGAGCAAGGACAAAAGAAGACGTTCTCCAACCA
A3-AT1G70620-XLOC_003695-84-1
      CAGGCGGGCTGAAAACTCGAGCAAGGACAAAAGAAGACGTTCTCCAACCA
CONSENSUS
      CAGGCGGGCTGAAAACTCGAGCAAGGACAAAAGAAGACGTTCTCCAACCA

A3-AT1G70620-XLOC_003695-84-0    GTAATGAATCTTCTGATGATTCGAAGAG
A3-AT1G70620-XLOC_003695-84-1    GTAATGAATCTTCTGATGATTCGAAGAG
CONSENSUS                        GTAATGAATCTTCTGATGATTCGAAGAG


alignment for event: RI-AT1G22630-XLOC_001222-2010

RI-AT1G22630-XLOC_001222-2010-0
      ATCCTTGTCAACATATTCCCTCCACATGATCCGTTTCAATATCCACAAAC
RI-AT1G22630-XLOC_001222-2010-1
      ATCCTTGTCAACATATTCCCTCCACATGATCCGTTTCAATATCCACAAAC
CONSENSUS
      ATCCTTGTCAACATATTCCCTCCACATGATCCGTTTCAATATCCACAAAC

RI-AT1G22630-XLOC_001222-2010-0
      CACAACCAAGCAAATTCTTTAGCTTTCTCACACCATCCATATTCACTATC
RI-AT1G22630-XLOC_001222-2010-1
      CACAACCAAGCAAATTCTTTAGCTTTCTCACACCATCCATATTCACTATC
CONSENSUS
      CACAACCAAGCAAATTCTTTAGCTTTCTCACACCATCCATATTCACTATC

RI-AT1G22630-XLOC_001222-2010-0
      ACTTGTATGCATAGATATGCATATAGATGTACATAGATATCTTACTTCCT
RI-AT1G22630-XLOC_001222-2010-1
      ACTT----------------------------------------------
CONSENSUS
      ACTT..............................................

RI-AT1G22630-XLOC_001222-2010-0
      AGTTCTAATTTGAGTTACAAAAATGTTGAAATTTCCACTTTGTTGCAGTG
RI-AT1G22630-XLOC_001222-2010-1
      ------------------------------------------------TG
CONSENSUS
      ................................................TG

RI-AT1G22630-XLOC_001222-2010-0
      AGTTATCTTTAGAACCGAAGATTCTAGCTTTTGAAAGTCAGCTTGTTTCG
RI-AT1G22630-XLOC_001222-2010-1
      AGTTATCTTTAGAACCGAAGATTCTAGCTTTTGAAAGTCAGCTTGTTTCG
CONSENSUS
      AGTTATCTTTAGAACCGAAGATTCTAGCTTTTGAAAGTCAGCTTGTTTCG

RI-AT1G22630-XLOC_001222-2010-0
      GGGCGACGCGATACAATCAAAGTTTCTGCGGGAAAAATCGGAAATTTCTC
```

```
RI-AT1G22630-XLOC_001222-2010-1
      GGGCGACGCGATACAATCAAAGTTTCTGCGGGAAAAATCGGAAATTTCTC
CONSENSUS
      GGGCGACGCGATACAATCAAAGTTTCTGCGGGAAAAATCGGAAATTTCTC

RI-AT1G22630-XLOC_001222-2010-0  TCTTGGTTCG
RI-AT1G22630-XLOC_001222-2010-1  TCTTGGTTCG
CONSENSUS                        TCTTGGTTCG



alignment for event: RI-AT1G63010-XLOC_007338-5558

RI-AT1G63010-XLOC_007338-5558-0
      AATGATTGGGTCCGAGAGTAAATGCGATTGATAAATGATCACATTACTCG
RI-AT1G63010-XLOC_007338-5558-1
      AATGATTGGGTCCGAGAGTAAATGCGATTGATAAATGATCACATTACTCG
CONSENSUS
      AATGATTGGGTCCGAGAGTAAATGCGATTGATAAATGATCACATTACTCG

RI-AT1G63010-XLOC_007338-5558-0
      CGATAAGAGTCTGCGATCTACTATAAGCAAACTAGTCGTCTTTTGATTCT
RI-AT1G63010-XLOC_007338-5558-1
      CGATAAGAGTCTGCGATCTACTATAAGCAAACTAGTCGTCTTTTGATTCT
CONSENSUS
      CGATAAGAGTCTGCGATCTACTATAAGCAAACTAGTCGTCTTTTGATTCT

RI-AT1G63010-XLOC_007338-5558-0
      CCTCTTCTTCACCTTTTTACCAATTTCCTCGCTTACAAGATTCAAAGGGT
RI-AT1G63010-XLOC_007338-5558-1
      CCTCTTCTTCACCTTTTTACCAATTTCCTCGCTTACAAGATTCAAAGG--
CONSENSUS
      CCTCTTCTTCACCTTTTTACCAATTTCCTCGCTTACAAGATTCAAAGG..

RI-AT1G63010-XLOC_007338-5558-0
      TTGTGATTGAGAGAAGCTTTAAAAAACCCATCTTTGGTGTTTGATTCTCT
RI-AT1G63010-XLOC_007338-5558-1
      --------------------------------------------------
CONSENSUS
      ..................................................

RI-AT1G63010-XLOC_007338-5558-0
      GTTTATTTAATTCATAAAGGAAGGCTCTTTTTTTTTTGTTTATTTCAGGC
RI-AT1G63010-XLOC_007338-5558-1
      ------------------------------------------------GC
CONSENSUS
      ................................................GC

RI-AT1G63010-XLOC_007338-5558-0
      TCATTACGTTGATGGTCATCTAAATCTCAAGAAGACAAAGTTGCGATTTT
RI-AT1G63010-XLOC_007338-5558-1
      TCATTACGTTGATGGTCATCTAAATCTCAAGAAGACAAAGTTGCGATTTT
CONSENSUS
      TCATTACGTTGATGGTCATCTAAATCTCAAGAAGACAAAGTTGCGATTTT

RI-AT1G63010-XLOC_007338-5558-0  TGCAACTGGGTTTCTCTCTTCAG
```

RI-AT1G63010-XLOC_007338-5558-1 TGCAACTGGGTTTCTCTCTTCAG
CONSENSUS   TGCAACTGGGTTTCTCTCTTCAG

alignment for event: A3-AT1G30510-XLOC_005854-6547

A3-AT1G30510-XLOC_005854-6547-0
      GCTGGTGCTGTCTCAGTTTCAATTGAAAACCAACGTTCTCTTAGAAGATC
A3-AT1G30510-XLOC_005854-6547-1
      GCTGGTGCTGTCTCAGTTTCAATTGAAAACCAACGTTCTCTTAGAAGATC
CONSENSUS
      GCTGGTGCTGTCTCAGTTTCAATTGAAAACCAACGTTCTCTTAGAAGATC

A3-AT1G30510-XLOC_005854-6547-0 CGTCTTCAAG---
AACAATAGCATAAGCTTCAACAGCAAGTCATGGTCAT
A3-AT1G30510-XLOC_005854-6547-1
      CGTCTTCAAGCAGAACAATAGCATAAGCTTCAACAGCAAGTCATGGTCAT
CONSENSUS
      CGTCTTCAAG...AACAATAGCATAAGCTTCAACAGCAAGTCATGGTCAT

A3-AT1G30510-XLOC_005854-6547-0
      CTTCTTTAGCATTGAACCAGAAGACAACAAGCATAAGAGATGGGAAACGG
A3-AT1G30510-XLOC_005854-6547-1
      CTTCTTTAGCATTGAACCAGAAGACAACAAGCATAAGAGATGGGAAACGG
CONSENSUS
      CTTCTTTAGCATTGAACCAGAAGACAACAAGCATAAGAGATGGGAAACGG

A3-AT1G30510-XLOC_005854-6547-0
      TACCCGAGCACGACAATATGTATGTCGGTTCAACAAACAAGTAGTTCCAA
A3-AT1G30510-XLOC_005854-6547-1
      TACCCGAGCACGACAATATGTATGTCGGTTCAACAAACAAGTAGTTCCAA
CONSENSUS
      TACCCGAGCACGACAATATGTATGTCGGTTCAACAAACAAGTAGTTCCAA

A3-AT1G30510-XLOC_005854-6547-0
      GGTTACTGTCTCTCCTATAGAGTTGGAAGACCCTAAGGATCCTCCTTTGA
A3-AT1G30510-XLOC_005854-6547-1
      GGTTACTGTCTCTCCTATAGAGTTGGAAGACCCTAAGGATCCTCCTTTGA
CONSENSUS
      GGTTACTGTCTCTCCTATAGAGTTGGAAGACCCTAAGGATCCTCCTTTGA

A3-AT1G30510-XLOC_005854-6547-0
      ACTTGTACAAACCCAAGGAGTCTTACACCGCTAAGATTGTCTCTGTGGAG
A3-AT1G30510-XLOC_005854-6547-1
      ACTTGTACAAACCCAAGGAGTCTTACACCGCTAAGATTGTCTCTGTGGAG
CONSENSUS
      ACTTGTACAAACCCAAGGAGTCTTACACCGCTAAGATTGTCTCTGTGGAG

A3-AT1G30510-XLOC_005854-6547-0
      CGAGTAGTTGGCCCGAAAGCCCCTGGAGAAACTTGTCATATCGTCATCGA
A3-AT1G30510-XLOC_005854-6547-1
      CGAGTAGTTGGCCCGAAAGCCCCTGGAGAAACTTGTCATATCGTCATCGA
CONSENSUS
      CGAGTAGTTGGCCCGAAAGCCCCTGGAGAAACTTGTCATATCGTCATCGA

```
A3-AT1G30510-XLOC_005854-6547-0
      TCATGATGGTAACCTTCCTTACTGGGAAGGACAGAGTTACGGTGTGATTC
A3-AT1G30510-XLOC_005854-6547-1
      TCATGATGGTAACCTTCCTTACTGGGAAGGACAGAGTTACGGTGTGATTC
CONSENSUS
      TCATGATGGTAACCTTCCTTACTGGGAAGGACAGAGTTACGGTGTGATTC

A3-AT1G30510-XLOC_005854-6547-0  CTCCA
A3-AT1G30510-XLOC_005854-6547-1  CTCCA
CONSENSUS                        CTCCA



alignment for event: A5-AT1G72740-XLOC_007870-6005

A5-AT1G72740-XLOC_007870-6005-0
      CCCAGGCATGAAGTACCGCCAAATTTTAGAAGAATCCTTAGTACAAGACT
A5-AT1G72740-XLOC_007870-6005-1
      CCCAGGCATGAAGTACCGCCAAATTTTAGAAGAATCCTTAGTACAAGACT
CONSENSUS
      CCCAGGCATGAAGTACCGCCAAATTTTAGAAGAATCCTTAGTACAAGACT

A5-AT1G72740-XLOC_007870-6005-0
      GAGGAGGCTTGCAGCTCAGAGCAAACTTGAAAAGGTTAGCACCTTCAAAT
A5-AT1G72740-XLOC_007870-6005-1
      GAGGAGGCTTGCAGCTCAGAGCAAACTTGAAAAG----------------
CONSENSUS
      GAGGAGGCTTGCAGCTCAGAGCAAACTTGAAAAG................

A5-AT1G72740-XLOC_007870-6005-0
      CTATACAGAACTTCTATAAGATACCCGATCCATCAGGAACAAAAATAGGT
A5-AT1G72740-XLOC_007870-6005-1  --
ATACAGAACTTCTATAAGATACCCGATCCATCAGGAACAAAAATAGGT
CONSENSUS
      ..ATACAGAACTTCTATAAGATACCCGATCCATCAGGAACAAAAATAGGT

A5-AT1G72740-XLOC_007870-6005-0
      GTCCCAAAACCAAAGGAGACACATACGAAGCTGCGCCAAGCAAACAATCA
A5-AT1G72740-XLOC_007870-6005-1
      GTCCCAAAACCAAAGGAGACACATACGAAGCTGCGCCAAGCAAACAATCA
CONSENSUS
      GTCCCAAAACCAAAGGAGACACATACGAAGCTGCGCCAAGCAAACAATCA

A5-AT1G72740-XLOC_007870-6005-0
      GACCTCTGCTGATTCACAACAGATGATCGAGGAAGCTGCAATAACCGCAG
A5-AT1G72740-XLOC_007870-6005-1
      GACCTCTGCTGATTCACAACAGATGATCGAGGAAGCTGCAATAACCGCAG
CONSENSUS
      GACCTCTGCTGATTCACAACAGATGATCGAGGAAGCTGCAATAACCGCAG

A5-AT1G72740-XLOC_007870-6005-0
      CCTGTAAAGTCGTAGAGGCGGAGAATAAAATAGACGTTGCCAAACTAGCA
A5-AT1G72740-XLOC_007870-6005-1
      CCTGTAAAGTCGTAGAGGCGGAGAATAAAATAGACGTTGCCAAACTAGCA
CONSENSUS
      CCTGTAAAGTCGTAGAGGCGGAGAATAAAATAGACGTTGCCAAACTAGCA
```

A5-AT1G72740-XLOC_007870-6005-0
      GCAGAAGAGTTTGAGAAGATGACAAAGATTGCAGAAGAAAATAGAAAACT
A5-AT1G72740-XLOC_007870-6005-1
      GCAGAAGAGTTTGAGAAGATGACAAAGATTGCAGAAGAAAATAGAAAACT
CONSENSUS
      GCAGAAGAGTTTGAGAAGATGACAAAGATTGCAGAAGAAAATAGAAAACT

A5-AT1G72740-XLOC_007870-6005-0
      CCTGGTCATAGCTACTGAGATGCATGAACTAT
A5-AT1G72740-XLOC_007870-6005-1
      CCTGGTCATAGCTACTGAGATGCATGAACTAT
CONSENSUS
      CCTGGTCATAGCTACTGAGATGCATGAACTAT

alignment for event: A5-AT1G11360-XLOC_004857-3741

A5-AT1G11360-XLOC_004857-3741-0
      ATTGGAGAATATCTATGGTTTTGGTTATATATTTTTACAGCCGTTAGATC
A5-AT1G11360-XLOC_004857-3741-1
      ATTGGAGAATATCTATGGTTTTGGTTATATATTTTTACAGCCGTTAGATC
CONSENSUS
      ATTGGAGAATATCTATGGTTTTGGTTATATATTTTTACAGCCGTTAGATC

A5-AT1G11360-XLOC_004857-3741-0
      TTCAAACCGATCAATCTAACGGTTCTTAACAATATCCAATTCTCATAAGA
A5-AT1G11360-XLOC_004857-3741-1
      TTCAAACCGATCAATCTAACGGTTCTTAACAATATCCAATTCTCATAAGA
CONSENSUS
      TTCAAACCGATCAATCTAACGGTTCTTAACAATATCCAATTCTCATAAGA

A5-AT1G11360-XLOC_004857-3741-0
      AAAGAGTACAAAAATAAAAAGAGTCGCTGTCACTATACCGTACCGTACAG
A5-AT1G11360-XLOC_004857-3741-1
      AAAGAGTACAAAAATAAAAAGAGTCGCTGTCACTATACCGTACCGTACAG
CONSENSUS
      AAAGAGTACAAAAATAAAAAGAGTCGCTGTCACTATACCGTACCGTACAG

A5-AT1G11360-XLOC_004857-3741-0
      AGTCAATTGAAGAAACCGAGAGAGAGAGTGAGCGGAATCATGACTTCTCC
A5-AT1G11360-XLOC_004857-3741-1
      AGTCAATTGAAGAAACCGAGAGAGAGAGTGAGCGGAATCATGACTTCTCC
CONSENSUS
      AGTCAATTGAAGAAACCGAGAGAGAGAGTGAGCGGAATCATGACTTCTCC

A5-AT1G11360-XLOC_004857-3741-0
      GGGAAAATCTCCGAGATCCGACCGGAAATCACCGACGGTTGTCACCGTCC
A5-AT1G11360-XLOC_004857-3741-1
      GGGAAAATCTCCGAGATCCGACCGGAAATCACCGACGGTTGTCACCGTCC
CONSENSUS
      GGGAAAATCTCCGAGATCCGACCGGAAATCACCGACGGTTGTCACCGTCC

A5-AT1G11360-XLOC_004857-3741-0
      AGCCGTCTTCTCCCAGATTCCCGATCAGCACACCTACTGCTGGAGCTCAA

A5-AT1G11360-XLOC_004857-3741-1
AGCCGTCTTCTCCCAGATTCCCGATCAGCACACCTACTGCTGGAGCTCAA
CONSENSUS
AGCCGTCTTCTCCCAGATTCCCGATCAGCACACCTACTGCTGGAGCTCAA

A5-AT1G11360-XLOC_004857-3741-0
CGTAAGATCGGGATCGCTGTCGATCTAAGCGACGAGAGTGCGTACGCAGT
A5-AT1G11360-XLOC_004857-3741-1
CGTAAGATCGGGATCGCTGTCGATCTAAGCGACGAGAGTGCGTACGCAGT
CONSENSUS
CGTAAGATCGGGATCGCTGTCGATCTAAGCGACGAGAGTGCGTACGCAGT

A5-AT1G11360-XLOC_004857-3741-0
TCAATGGGCTGTTCAGAACTATCTCCGATCAGGTGACGCCGTTGTTCTCC
A5-AT1G11360-XLOC_004857-3741-1
TCAATGGGCTGTTCAGAACTATCTCCGATCAGGTGACGCCGTTGTTCTCC
CONSENSUS
TCAATGGGCTGTTCAGAACTATCTCCGATCAGGTGACGCCGTTGTTCTCC

A5-AT1G11360-XLOC_004857-3741-0
TTCATGTTCAACCGACGAGTGTACTTTACGGCGCCGATTGGGGCGCTATG
A5-AT1G11360-XLOC_004857-3741-1
TTCATGTTCAACCGACGAGTGTACTTTACGGCGCCGATTGGGGCGCTATG
CONSENSUS
TTCATGTTCAACCGACGAGTGTACTTTACGGCGCCGATTGGGGCGCTATG

A5-AT1G11360-XLOC_004857-3741-0
GATTTGTCGCCGCAGTGGGATCCAAATAACGAAGAGTCGCAGAGGAAGCT
A5-AT1G11360-XLOC_004857-3741-1
GATTTGTCGCCGCAGTGGGATCCAAATAACGAAGAGTCGCAGAGGAAGCT
CONSENSUS
GATTTGTCGCCGCAGTGGGATCCAAATAACGAAGAGTCGCAGAGGAAGCT

A5-AT1G11360-XLOC_004857-3741-0
TGAGGACGATTTCGACATTGTCACTAATAAGAAAGCGAGCGACGTTGCTC
A5-AT1G11360-XLOC_004857-3741-1
TGAGGACGATTTCGACATTGTCACTAATAAGAAAGCGAGCGACGTTGCTC
CONSENSUS
TGAGGACGATTTCGACATTGTCACTAATAAGAAAGCGAGCGACGTTGCTC

A5-AT1G11360-XLOC_004857-3741-0
AGCCTTTGGTTGAGGCGGATATTCCGTTTAAGATCCATATCGTCAAGGAT
A5-AT1G11360-XLOC_004857-3741-1
AGCCTTTGGTTGAGGCGGATATTCCGTTTAAGATCCATATCGTCAAGGAT
CONSENSUS
AGCCTTTGGTTGAGGCGGATATTCCGTTTAAGATCCATATCGTCAAGGAT

A5-AT1G11360-XLOC_004857-3741-0
CACGATATGAAGGAGAGGCTTTGTTTGGAGGTGGAGAGGTTAGGGCTAAG
A5-AT1G11360-XLOC_004857-3741-1
CACGATATGAAGGAGAGGCTTTGTTTGGAGGTGGAGAGGTTAGGGCTAAG
CONSENSUS
CACGATATGAAGGAGAGGCTTTGTTTGGAGGTGGAGAGGTTAGGGCTAAG

A5-AT1G11360-XLOC_004857-3741-0
TACTTTGATTATGGGTAGTAGAGGATTTGGTGCTACGAAGAGGAGTAGTA

A5-AT1G11360-XLOC_004857-3741-1
TACTTTGATTATGGGTAGTAGAGGATTTGGTGCTACGAAGAGGAGTAGTA
CONSENSUS
TACTTTGATTATGGGTAGTAGAGGATTTGGTGCTACGAAGAGGAGTAGTA

A5-AT1G11360-XLOC_004857-3741-0
AAGGGAGATTAGGGAGTGTCAGTGATTACTCAGTTCACCATTGTGCTTGT
A5-AT1G11360-XLOC_004857-3741-1
AAGGGAGATTAGGGAGTGTCAGTGATTACTCAGTTCACCATTGTGCTTGT
CONSENSUS
AAGGGAGATTAGGGAGTGTCAGTGATTACTCAGTTCACCATTGTGCTTGT

A5-AT1G11360-XLOC_004857-3741-0
CCGGTGGTTGTTGTTAGATTTCCTGATGATAAGGATGGAGAAGATGAGAA
A5-AT1G11360-XLOC_004857-3741-1
CCGGTGGTTGTTGTTAGATTTCCTGATGATAAGGATGGAGAAGATGAGAA
CONSENSUS
CCGGTGGTTGTTGTTAGATTTCCTGATGATAAGGATGGAGAAGATGAGAA

A5-AT1G11360-XLOC_004857-3741-0
ATCCGGCGACAGTGGTGGGGAGAATCTGATGGACAGTGATAAACTTCATA
A5-AT1G11360-XLOC_004857-3741-1
ATCCGGCGACAGTGGTGGGGAGAATCTGATGGACAGTGATAAACTTCATA
CONSENSUS
ATCCGGCGACAGTGGTGGGGAGAATCTGATGGACAGTGATAAACTTCATA

A5-AT1G11360-XLOC_004857-3741-0
CAGTGCCTGAGGTGGCTGAAGAAGAGGGAGATAAGGATGAGTACCATGAT
A5-AT1G11360-XLOC_004857-3741-1
CAGTGCCTGAGGTGGCTGAAGAAGAGGGAGATAAGGATGAGTACCATGAT
CONSENSUS
CAGTGCCTGAGGTGGCTGAAGAAGAGGGAGATAAGGATGAGTACCATGAT

A5-AT1G11360-XLOC_004857-3741-0
GCTTCGGATAAGCAGTAGCAGCAAGGTTGACTTTGAATGCATGTCAGTTT
A5-AT1G11360-XLOC_004857-3741-1
GCTTCGGATAAGCAGTAGCAGCAAG-------------------------
CONSENSUS
GCTTCGGATAAGCAGTAGCAGCAAG.........................

A5-AT1G11360-XLOC_004857-3741-0
CAATTTACTTAGGAATATTATATACAATCAGTAATACTCTGCTCGGATCA
A5-AT1G11360-XLOC_004857-3741-1

CONSENSUS
....

A5-AT1G11360-XLOC_004857-3741-0
AGCTTTGACCTTTAGAATGATTGGATATTAGGAAGATCCACTAAGATGCT
A5-AT1G11360-XLOC_004857-3741-1

CONSENSUS
....

A5-AT1G11360-XLOC_004857-3741-0
ATTTTATTCCTGATGCTTGAGGCTTTCATGTTGCAGAAGGAGACCTCCCT

A5-AT1G11360-XLOC_004857-3741-1 --------------------
GCTTTCATGTTGCAGAAGGAGACCTCCCT
CONSENSUS
    ....................GCTTTCATGTTGCAGAAGGAGACCTCCCT

A5-AT1G11360-XLOC_004857-3741-0
    AAGGAGACTTAGAAAGAAAAGAAAGCGTTCCTTTGCCCTTGGTTTGTGTT
A5-AT1G11360-XLOC_004857-3741-1
    AAGGAGACTTAGAAAGAAAAGAAAGCGTTCCTTTGCCCTTGGTTTGTGTT
CONSENSUS
    AAGGAGACTTAGAAAGAAAAGAAAGCGTTCCTTTGCCCTTGGTTTGTGTT

A5-AT1G11360-XLOC_004857-3741-0
    CTTGAGTGTGCTTAATTGTGTTGTTTTTGGGGTTTGGGGTATTCTAGATC
A5-AT1G11360-XLOC_004857-3741-1
    CTTGAGTGTGCTTAATTGTGTTGTTTTTGGGGTTTGGGGTATTCTAGATC
CONSENSUS
    CTTGAGTGTGCTTAATTGTGTTGTTTTTGGGGTTTGGGGTATTCTAGATC

A5-AT1G11360-XLOC_004857-3741-0
    TCTTTTGTAAGTATTCTCTTGTTTCTTGGTTTTGTTTTAAACTCTATGGA
A5-AT1G11360-XLOC_004857-3741-1
    TCTTTTGTAAGTATTCTCTTGTTTCTTGGTTTTGTTTTAAACTCTATGGA
CONSENSUS
    TCTTTTGTAAGTATTCTCTTGTTTCTTGGTTTTGTTTTAAACTCTATGGA

A5-AT1G11360-XLOC_004857-3741-0
    ATTTGGGATGAGTGGATTCGATGTCTTTTGTTTGTCTTTGAAAAAGCAGT
A5-AT1G11360-XLOC_004857-3741-1
    ATTTGGGATGAGTGGATTCGATGTCTTTTGTTTGTCTTTGAAAAAGCAGT
CONSENSUS
    ATTTGGGATGAGTGGATTCGATGTCTTTTGTTTGTCTTTGAAAAAGCAGT

A5-AT1G11360-XLOC_004857-3741-0
    GAAAAGAATTTCAGTGGTCTTGTATATCTCTGCAGTTGAATAGTTGGTAC
A5-AT1G11360-XLOC_004857-3741-1
    GAAAAGAATTTCAGTGGTCTTGTATATCTCTGCAGTTGAATAGTTGGTAC
CONSENSUS
    GAAAAGAATTTCAGTGGTCTTGTATATCTCTGCAGTTGAATAGTTGGTAC

A5-AT1G11360-XLOC_004857-3741-0
    AAAATAACATCTTTGACGGTTCCAAATAATATATCTTGTCAGTAATGACC
A5-AT1G11360-XLOC_004857-3741-1
    AAAATAACATCTTTGACGGTTCCAAATAATATATCTTGTCAGTAATGACC
CONSENSUS
    AAAATAACATCTTTGACGGTTCCAAATAATATATCTTGTCAGTAATGACC

A5-AT1G11360-XLOC_004857-3741-0 ATTAAATAAACTTTACCATGAAAT
A5-AT1G11360-XLOC_004857-3741-1 ATTAAATAAACTTTACCATGAAAT
CONSENSUS   ATTAAATAAACTTTACCATGAAAT

alignment for event: RI-AT1G71310-XLOC_007808-3859

RI-AT1G71310-XLOC_007808-3859-0

AATGGTCAGGAGAAGTTCGAAGTGTCACTTACTCTCCTGATGGCAACACT
RI-AT1G71310-XLOC_007808-3859-1
AATGGTCAGGAGAAGTTCGAAGTGTCACTTACTCTCCTGATGGCAACACT
CONSENSUS
AATGGTCAGGAGAAGTTCGAAGTGTCACTTACTCTCCTGATGGCAACACT

RI-AT1G71310-XLOC_007808-3859-0
GTTACTGTGGCTTATCGTGTGACTCTCTACGGCACTGATGCTGAGGTCTT
RI-AT1G71310-XLOC_007808-3859-1
GTTACTGTGGCTTATCGTGTGACTCTCTACGGCACTGATGCTGAG-----
CONSENSUS
GTTACTGTGGCTTATCGTGTGACTCTCTACGGCACTGATGCTGAG.....

RI-AT1G71310-XLOC_007808-3859-0
TCCCTTCCTCTTGTTTCAGTGTTATGGTTTGATTTGGTTAGCCTTTAGCT
RI-AT1G71310-XLOC_007808-3859-1

CONSENSUS
....

RI-AT1G71310-XLOC_007808-3859-0
CAAGTCGTCTCCTGGTAGAGTTCTTTGCCTTCTTGCCTCAGAAGCTGCAA
RI-AT1G71310-XLOC_007808-3859-1

CONSENSUS
....

RI-AT1G71310-XLOC_007808-3859-0
ATATAGAAACCTAGTTGTTTTCTTTTGGGTTGTGTCCATTTTGAATTGTA
RI-AT1G71310-XLOC_007808-3859-1

CONSENSUS
....

RI-AT1G71310-XLOC_007808-3859-0
TGATGAAACCCGTCAAGGAATACAGCTTAGAGAGTGTATGTTTATCAACT
RI-AT1G71310-XLOC_007808-3859-1

CONSENSUS
....

RI-AT1G71310-XLOC_007808-3859-0
CTGGAGAAACACCAACTCTGGGTTATAGATTCTGTTATCAGTACTTACAA
RI-AT1G71310-XLOC_007808-3859-1

CONSENSUS
....

RI-AT1G71310-XLOC_007808-3859-0
ACCTTAGAGATGATATCACTTGTGAATAGGGAGGTTTGACCTGTTTTCGT
RI-AT1G71310-XLOC_007808-3859-1

CONSENSUS
....

RI-AT1G71310-XLOC_007808-3859-0

TAGTAAATATGCTAGTGGTTTGCTTCCTTGCATACTTTCTTGAGCATATT
RI-AT1G71310-XLOC_007808-3859-1

CONSENSUS
....

RI-AT1G71310-XLOC_007808-3859-0
ATGTTTTCATTGTTTTGCTTGTAACTTGTACGCTGAAATCATTTGCTAAT
RI-AT1G71310-XLOC_007808-3859-1

CONSENSUS
....

RI-AT1G71310-XLOC_007808-3859-0
AATCATTTTTTTGGTGTTGTCAAGATATTTAGGGAATCAACAGGTACCAC
RI-AT1G71310-XLOC_007808-3859-1 ------------------------
ATATTTAGGGAATCAACAGGTACCAC
CONSENSUS
........................ATATTTAGGGAATCAACAGGTACCAC

RI-AT1G71310-XLOC_007808-3859-0
ATCTGTAGATGACAAAGGCTATGGTGATGCTGTGCAAAAGGCGGAAGCAA
RI-AT1G71310-XLOC_007808-3859-1
ATCTGTAGATGACAAAGGCTATGGTGATGCTGTGCAAAAGGCGGAAGCAA
CONSENSUS
ATCTGTAGATGACAAAGGCTATGGTGATGCTGTGCAAAAGGCGGAAGCAA

RI-AT1G71310-XLOC_007808-3859-0
TGGCATTTCGCCGTGCGTGTGCAAGATTTGGATTAGGGCTTCATCTTTAT
RI-AT1G71310-XLOC_007808-3859-1
TGGCATTTCGCCGTGCGTGTGCAAGATTTGGATTAGGGCTTCATCTTTAT
CONSENSUS
TGGCATTTCGCCGTGCGTGTGCAAGATTTGGATTAGGGCTTCATCTTTAT

RI-AT1G71310-XLOC_007808-3859-0 CACGAGGATGCTTTGTAAAGG
RI-AT1G71310-XLOC_007808-3859-1 CACGAGGATGCTTTGTAAAGG
CONSENSUS CACGAGGATGCTTTGTAAAGG

alignment for event: A3-AT1G01910-XLOC_004298-3292

A3-AT1G01910-XLOC_004298-3292-0
GTGACAGGGGTTGAAGCCTTAAAGGCGTTTTCACATAAGTTCTTGACGCC
A3-AT1G01910-XLOC_004298-3292-1
GTGACAGGGGTTGAAGCCTTAAAGGCGTTTTCACATAAGTTCTTGACGCC
CONSENSUS
GTGACAGGGGTTGAAGCCTTAAAGGCGTTTTCACATAAGTTCTTGACGCC

A3-AT1G01910-XLOC_004298-3292-0
GTACCATCCTACCACTAGCAGGAGCAATGTAGAGGAGCTGGAGAGGAAAG
A3-AT1G01910-XLOC_004298-3292-1
GTACCATCCTACCACTAGCAGGAGCAATGTAGAGGAGCTGGAGAGGAAAG
CONSENSUS
GTACCATCCTACCACTAGCAGGAGCAATGTAGAGGAGCTGGAGAGGAAAG

A3-AT1G01910-XLOC_004298-3292-0
TACACACATTGCGTTTGCAGTTAAAAACAGCTGAAGAAGAACTCGAACGG
A3-AT1G01910-XLOC_004298-3292-1
TACACACATTGCGTTTGCAGTTAAAAACAGCTGAAGAAGAACTCGAACGG
CONSENSUS
TACACACATTGCGTTTGCAGTTAAAAACAGCTGAAGAAGAACTCGAACGG

A3-AT1G01910-XLOC_004298-3292-0
GTCAAGAGTGGCTAAAGATAAGGCTCTGTTCTTCCCTTTAATT-------
A3-AT1G01910-XLOC_004298-3292-1
GTCAAGAGTGGCTAAAGATAAGGCTCTGTTCTTCCCTTTAATTGTGGAAA
CONSENSUS
GTCAAGAGTGGCTAAAGATAAGGCTCTGTTCTTCCCTTTAATT.......

A3-AT1G01910-XLOC_004298-3292-0 ---------------------------
TTCTTCATCTAGCAACAACTCTT
A3-AT1G01910-XLOC_004298-3292-1
ATTAAAACTGAATCTTTTTCTCTCTAGTTCTTCATCTAGCAACAACTCTT
CONSENSUS
...........................TTCTTCATCTAGCAACAACTCTT

A3-AT1G01910-XLOC_004298-3292-0
TATAGGACAGGAAAGAAGATCAAGCAGAATCTTCTTTTGCGTTGTTTTAT
A3-AT1G01910-XLOC_004298-3292-1
TATAGGACAGGAAAGAAGATCAAGCAGAATCTTCTTTTGCGTTGTTTTAT
CONSENSUS
TATAGGACAGGAAAGAAGATCAAGCAGAATCTTCTTTTGCGTTGTTTTAT

A3-AT1G01910-XLOC_004298-3292-0
TGTTATGCATTGGATTTGTTTTTATATTTGTAATAATCATGACTACATAG
A3-AT1G01910-XLOC_004298-3292-1
TGTTATGCATTGGATTTGTTTTTATATTTGTAATAATCATGACTACATAG
CONSENSUS
TGTTATGCATTGGATTTGTTTTTATATTTGTAATAATCATGACTACATAG

A3-AT1G01910-XLOC_004298-3292-0
ACTCATATCTACCAAGAAATCGTTGTTTTACGACTTAATCAGCTTTTTAT
A3-AT1G01910-XLOC_004298-3292-1
ACTCATATCTACCAAGAAATCGTTGTTTTACGACTTAATCAGCTTTTTAT
CONSENSUS
ACTCATATCTACCAAGAAATCGTTGTTTTACGACTTAATCAGCTTTTTAT

A3-AT1G01910-XLOC_004298-3292-0
ATATTAAGTTGAAGGGTATCTGTGACAAGCGAATGCTATGATCTCTTGTA
A3-AT1G01910-XLOC_004298-3292-1
ATATTAAGTTGAAGGGTATCTGTGACAAGCGAATGCTATGATCTCTTGTA
CONSENSUS
ATATTAAGTTGAAGGGTATCTGTGACAAGCGAATGCTATGATCTCTTGTA

A3-AT1G01910-XLOC_004298-3292-0
ACAGCTTTTTATATACTGTAGACTGGTCTTGCAACAGAGAAGGAAAATTC
A3-AT1G01910-XLOC_004298-3292-1
ACAGCTTTTTATATACTGTAGACTGGTCTTGCAACAGAGAAGGAAAATTC
CONSENSUS
ACAGCTTTTTATATACTGTAGACTGGTCTTGCAACAGAGAAGGAAAATTC

A3-AT1G01910-XLOC_004298-3292-0
      ATGTAAAAAAATTATAGTAAATAAGAGATGTTTTGATTTATTGAC
A3-AT1G01910-XLOC_004298-3292-1
      ATGTAAAAAAATTATAGTAAATAAGAGATGTTTTGATTTATTGAC
CONSENSUS
      ATGTAAAAAAATTATAGTAAATAAGAGATGTTTTGATTTATTGAC

alignment for event: A3-AT1G02840-XLOC_000102-6319

A3-AT1G02840-XLOC_000102-6319-0
      GTTAGAGAATATGATTCAAGGAAGGATTCTAGGAGTCCTAGCCGGGGAAG
A3-AT1G02840-XLOC_000102-6319-1
      GTTAGAGAATATGATTCAAGGAAGGATTCTAGGAGTCCTAGCCGGGGAAG
CONSENSUS
      GTTAGAGAATATGATTCAAGGAAGGATTCTAGGAGTCCTAGCCGGGGAAG

A3-AT1G02840-XLOC_000102-6319-0
      ATCCTATTCTAAGAGCCGCAGCCGCAGCCGTGGACGAAGCGTGAGCCGAA
A3-AT1G02840-XLOC_000102-6319-1
      ATCCTATTCTAAGAGCCGCAGCCGCAGCCGTGGACGAAGCGTGAGCCGAA
CONSENSUS
      ATCCTATTCTAAGAGCCGCAGCCGCAGCCGTGGACGAAGCGTGAGCCGAA

A3-AT1G02840-XLOC_000102-6319-0
      GCAGGAGCAGAAGCAGGAGCAGGAGCAGAAGTCCCAAGGCAAAGTCTTCA
A3-AT1G02840-XLOC_000102-6319-1
      GCAGGAGCAGAAGCAGGAGCAGGAGCAGAAGTCCCAAGGCAAAGTCTTCA
CONSENSUS
      GCAGGAGCAGAAGCAGGAGCAGGAGCAGAAGTCCCAAGGCAAAGTCTTCA

A3-AT1G02840-XLOC_000102-6319-0
      CGTAGGTCCCCTGCAAAATCTACATCAAGATCTCCTGGCCCCCGCTCGAA
A3-AT1G02840-XLOC_000102-6319-1
      CGTAGGTCCCCTGCAAAATCTACATCAAGATCTCCTGGCCCCCGCTCGAA
CONSENSUS
      CGTAGGTCCCCTGCAAAATCTACATCAAGATCTCCTGGCCCCCGCTCGAA

A3-AT1G02840-XLOC_000102-6319-0
      GTCAAGGTCACCGTCTCCAAGAAGATGGATAACAGTGGAGACATTGGATC
A3-AT1G02840-XLOC_000102-6319-1
      GTCAAGGTCACCGTCTCCAAGAAG--------------------------
CONSENSUS
      GTCAAGGTCACCGTCTCCAAGAAG..........................

A3-AT1G02840-XLOC_000102-6319-0
      ACTTGGATCACAATATTATATCGGGATTTCTGTAAAACTATATTGGCTCG
A3-AT1G02840-XLOC_000102-6319-1

CONSENSUS
      ....

A3-AT1G02840-XLOC_000102-6319-0
      ATGGATTGACAATATGGAATCTGGGTTCTCTTGGGACGTCCGTGGCTCAT
A3-AT1G02840-XLOC_000102-6319-1

CONSENSUS
....

A3-AT1G02840-XLOC_000102-6319-0
TTGGCAACACAAGTTTTTTTGGCCACATGGCTTATAAAACCTCTGTCCTA
A3-AT1G02840-XLOC_000102-6319-1

CONSENSUS
....

A3-AT1G02840-XLOC_000102-6319-0
TCACCTATGTTTTAACTAAGTAGCAGAATAGTTTGGTTTATGTTTCTTTT
A3-AT1G02840-XLOC_000102-6319-1

CONSENSUS
....

A3-AT1G02840-XLOC_000102-6319-0
TTTTTATTTGTTGCAACTTCTTAATCTCTGTGAGATAGAAGGAGAGGCTC
A3-AT1G02840-XLOC_000102-6319-1

CONSENSUS
....

A3-AT1G02840-XLOC_000102-6319-0
CAGGACCTTGCTGAACAGTATAAAACACAACATGTTTGGATTTTTGAATC
A3-AT1G02840-XLOC_000102-6319-1

CONSENSUS
....

A3-AT1G02840-XLOC_000102-6319-0
TGAGTTTCTTTTCTTGGACTTTTGCAGATCGCGTTCAAGATCAAGATCTC
A3-AT1G02840-XLOC_000102-6319-1 ---------------------------
ATCGCGTTCAAGATCAAGATCTC
CONSENSUS
...........................ATCGCGTTCAAGATCAAGATCTC

A3-AT1G02840-XLOC_000102-6319-0 CTCTACCTTCT
A3-AT1G02840-XLOC_000102-6319-1 CTCTACCTTCT
CONSENSUS CTCTACCTTCT

alignment for event: RI-AT1G49730-XLOC_006631-6008

RI-AT1G49730-XLOC_006631-6008-0
GTCACTTGTCAGTGAGTATACAAAACAATACACAGAGCCTCGCATGTCAT
RI-AT1G49730-XLOC_006631-6008-1
GTCACTTGTCAGTGAGTATACAAAACAATACACAGAGCCTCGCATGTCAT
CONSENSUS
GTCACTTGTCAGTGAGTATACAAAACAATACACAGAGCCTCGCATGTCAT

RI-AT1G49730-XLOC_006631-6008-0
ATTCACACAGGGACCTTCCGTTAAACTCCTTTTTTATAATCTCGTTCTCT

RI-AT1G49730-XLOC_006631-6008-1
ATTCACACAGGGACCTTCCGTTAAACTCCTTTTTTATAATCTCGTTCTCT
CONSENSUS
ATTCACACAGGGACCTTCCGTTAAACTCCTTTTTTATAATCTCGTTCTCT

RI-AT1G49730-XLOC_006631-6008-0
CTTCTCTCTTTCTCTCTCTGGTTGCTCTTGGCCATGGTTGTGAATAGTCA
RI-AT1G49730-XLOC_006631-6008-1
CTTCTCTCTTTCTCTCTCTGGTTGCTCTTGGCCATGGTTGTGAATAGTCA
CONSENSUS
CTTCTCTCTTTCTCTCTCTGGTTGCTCTTGGCCATGGTTGTGAATAGTCA

RI-AT1G49730-XLOC_006631-6008-0
AGCTTTCTTGTTGGCATTAATTGCATTACTTGCTACCCAATTGCCTTCAC
RI-AT1G49730-XLOC_006631-6008-1
AGCTTTCTTGTTGGCATTAATTGCATTACTTGCTACCCAATTGCCTTCAC
CONSENSUS
AGCTTTCTTGTTGGCATTAATTGCATTACTTGCTACCCAATTGCCTTCAC

RI-AT1G49730-XLOC_006631-6008-0
TAATGGCTGCAGGTTTCTCTTTATCGATTTTCCTCTGTTTCTTTCTCTTT
RI-AT1G49730-XLOC_006631-6008-1
TAATGGCTGCAG--------------------------------------
CONSENSUS
TAATGGCTGCAG......................................

RI-AT1G49730-XLOC_006631-6008-0
TAAAGCTTCATTATTTAATCCTGTTCTTGCCTAAATCAGGATAAGATTAT
RI-AT1G49730-XLOC_006631-6008-1

CONSENSUS
....

RI-AT1G49730-XLOC_006631-6008-0
GAATGTTGGATTTTGAGCTTCTGGATTTTGCAAATTTTGTTGCTTTCTTT
RI-AT1G49730-XLOC_006631-6008-1

CONSENSUS
....

RI-AT1G49730-XLOC_006631-6008-0
GACTAGATTTGAGTTTCGATGGAAATTCGCATAATTTTGGTTCATTAGTT
RI-AT1G49730-XLOC_006631-6008-1

CONSENSUS
....

RI-AT1G49730-XLOC_006631-6008-0
GCTTACATGCAAAAATACTCTCTTTTTAAATTGAAATCAGATCTTGAGAA
RI-AT1G49730-XLOC_006631-6008-1

CONSENSUS
....

RI-AT1G49730-XLOC_006631-6008-0
ACCATAAAAATTCAAACTTTGTTTGTTCTTTTCAAGCTATTGTGGTTAGA

RI-AT1G49730-XLOC_006631-6008-1

CONSENSUS
      ....

RI-AT1G49730-XLOC_006631-6008-0
      AAGTTAAAAGCTTGAAACTTGCGGACCAATCTTTATAGGTTACCAGACAC
RI-AT1G49730-XLOC_006631-6008-1

CONSENSUS
      ....

RI-AT1G49730-XLOC_006631-6008-0
      TAATGCTGCATTGTCATGTGTGTTTGCAGATTGTCCATTAGATTTCAGTG
RI-AT1G49730-XLOC_006631-6008-1 -----------------------------
ATTGTCCATTAGATTTCAGTG
CONSENSUS
      .............................ATTGTCCATTAGATTTCAGTG

RI-AT1G49730-XLOC_006631-6008-0
      GATCAAATTTCACTCTAGTAGCTACTGTGTGCTCCAACATAACCAACAGA
RI-AT1G49730-XLOC_006631-6008-1
      GATCAAATTTCACTCTAGTAGCTACTGTGTGCTCCAACATAACCAACAGA
CONSENSUS
      GATCAAATTTCACTCTAGTAGCTACTGTGTGCTCCAACATAACCAACAGA

RI-AT1G49730-XLOC_006631-6008-0
      GGCAAATGTTGCCGCTACATGAACGCTTTTGTTGCGGTGTCCGTGGCTCG
RI-AT1G49730-XLOC_006631-6008-1
      GGCAAATGTTGCCGCTACATGAACGCTTTTGTTGCGGTGTCCGTGGCTCG
CONSENSUS
      GGCAAATGTTGCCGCTACATGAACGCTTTTGTTGCGGTGTCCGTGGCTCG

RI-AT1G49730-XLOC_006631-6008-0
      TTACGCGAACTTGTCAACTAATCTTGGAGTCACATCAGATTTATCTGAAA
RI-AT1G49730-XLOC_006631-6008-1
      TTACGCGAACTTGTCAACTAATCTTGGAGTCACATCAGATTTATCTGAAA
CONSENSUS
      TTACGCGAACTTGTCAACTAATCTTGGAGTCACATCAGATTTATCTGAAA

RI-AT1G49730-XLOC_006631-6008-0
      CCTGCATTGCTTCCATCTCTAGAGCCATGGAGGGCTACGGAGTCTCGAGA
RI-AT1G49730-XLOC_006631-6008-1
      CCTGCATTGCTTCCATCTCTAGAGCCATGGAGGGCTACGGAGTCTCGAGA
CONSENSUS
      CCTGCATTGCTTCCATCTCTAGAGCCATGGAGGGCTACGGAGTCTCGAGA

RI-AT1G49730-XLOC_006631-6008-0
      AACGCCACTAGTTTCTGCGGTTTGGGAACCAAGATCCTTGTTAAGTATGA
RI-AT1G49730-XLOC_006631-6008-1
      AACGCCACTAGTTTCTGCGGTTTGGGAACCAAGATCCTTGTTAAGTATGA
CONSENSUS
      AACGCCACTAGTTTCTGCGGTTTGGGAACCAAGATCCTTGTTAAGTATGA

RI-AT1G49730-XLOC_006631-6008-0
      CTGTGACGGTCGAACCACTGTTACACAAATGCATCAGTCTCCGGGATTTG

RI-AT1G49730-XLOC_006631-6008-1
      CTGTGACGGTCGAACCACTGTTACACAAATGCATCAGTCTCCGGGATTTG
CONSENSUS
      CTGTGACGGTCGAACCACTGTTACACAAATGCATCAGTCTCCGGGATTTG

RI-AT1G49730-XLOC_006631-6008-0
      GACATGTTTCAAGAAACTGCAGACTCCCATTTTCGCCGGGACACCAATGC
RI-AT1G49730-XLOC_006631-6008-1
      GACATGTTTCAAGAAACTGCAGACTCCCATTTTCGCCGGGACACCAATGC
CONSENSUS
      GACATGTTTCAAGAAACTGCAGACTCCCATTTTCGCCGGGACACCAATGC

RI-AT1G49730-XLOC_006631-6008-0
      AGAAAGTGTTTGAACTCTGGCATCACTTACCTTCGTAATCTCATAGGTGC
RI-AT1G49730-XLOC_006631-6008-1
      AGAAAGTGTTTGAACTCTGGCATCACTTACCTTCGTAATCTCATAGGTGC
CONSENSUS
      AGAAAGTGTTTGAACTCTGGCATCACTTACCTTCGTAATCTCATAGGTGC

RI-AT1G49730-XLOC_006631-6008-0
      AGAGACTAATAACATTACTCTGTGTACTTGTCGTGATGCGACTTATGCTA
RI-AT1G49730-XLOC_006631-6008-1
      AGAGACTAATAACATTACTCTGTGTACTTGTCGTGATGCGACTTATGCTA
CONSENSUS
      AGAGACTAATAACATTACTCTGTGTACTTGTCGTGATGCGACTTATGCTA

RI-AT1G49730-XLOC_006631-6008-0
      CATTAGCAAGCCGAATAGACGATACATCAGCTCTTGAACTCCTTAGTTGT
RI-AT1G49730-XLOC_006631-6008-1
      CATTAGCAAGCCGAATAGACGATACATCAGCTCTTGAACTCCTTAGTTGT
CONSENSUS
      CATTAGCAAGCCGAATAGACGATACATCAGCTCTTGAACTCCTTAGTTGT

RI-AT1G49730-XLOC_006631-6008-0
      TTCTTTCAAGTGACAGAGCTTAACATTCCTTCAG
RI-AT1G49730-XLOC_006631-6008-1
      TTCTTTCAAGTGACAGAGCTTAACATTCCTTCAG
CONSENSUS
      TTCTTTCAAGTGACAGAGCTTAACATTCCTTCAG

alignment for event: RI-AT1G64430-XLOC_003343-6584

RI-AT1G64430-XLOC_003343-6584-0
      AAATGGAAAATACAGGCTGAAGCAAACGACGAGGCCGAAAGACTACTTAG
RI-AT1G64430-XLOC_003343-6584-1
      AAATGGAAAATACAGGCTGAAGCAAACGACGAGGCCGAAAGACTACTTAG
CONSENSUS
      AAATGGAAAATACAGGCTGAAGCAAACGACGAGGCCGAAAGACTACTTAG

RI-AT1G64430-XLOC_003343-6584-0
      CTCCCAACCTTAAGCACTCACACACTTGTCAACAAACCCGGTTAGTTAAT
RI-AT1G64430-XLOC_003343-6584-1
      CTCCCAACCTTAAGCACTCACACACTTGTCAACAAACCCG----------
CONSENSUS

CTCCCAACCTTAAGCACTCACACACTTGTCAACAAACCCG..........

RI-AT1G64430-XLOC_003343-6584-0
CATTATACATCACACATCATACATCATCATCATCATTAATTCATTATCAT
RI-AT1G64430-XLOC_003343-6584-1

CONSENSUS
....

RI-AT1G64430-XLOC_003343-6584-0
CATCATGTAAAGCAATTATCTTTCGGCGGCATATGTATGACATTAACAAT
RI-AT1G64430-XLOC_003343-6584-1

CONSENSUS
....

RI-AT1G64430-XLOC_003343-6584-0
GTCTGCTGTCAAAACTGCAACGTAGATTCGTCAATGATATGAAAATGTGG
RI-AT1G64430-XLOC_003343-6584-1

CONSENSUS
....

RI-AT1G64430-XLOC_003343-6584-0
ACGACTGTTCTGGCAATTACACTTTTAAGTACAATATTTATTGAATTTTA
RI-AT1G64430-XLOC_003343-6584-1

CONSENSUS
....

RI-AT1G64430-XLOC_003343-6584-0
GTTCCCTTTTTAAGAATCTTTTTCTTTCTCCAGAGCTGCATTGGGATTTT
RI-AT1G64430-XLOC_003343-6584-1

CONSENSUS
....

RI-AT1G64430-XLOC_003343-6584-0
TGACACTGTAAACATAATACTTTTTGAAAATTTTAGTAATGGAAATTGTG
RI-AT1G64430-XLOC_003343-6584-1

CONSENSUS
....

RI-AT1G64430-XLOC_003343-6584-0
ATCATCTTGTATCTGGTACAGGTCAAAAACTCTGGAGCATCGCTAAAAGT
RI-AT1G64430-XLOC_003343-6584-1 ---------------------
GTCAAAAACTCTGGAGCATCGCTAAAAGT
CONSENSUS
.....................GTCAAAAACTCTGGAGCATCGCTAAAAGT

RI-AT1G64430-XLOC_003343-6584-0
TAAGTTTCTTTTGTCGGTCGCTATAAGTTAATGTTGATAAATTTGTTTGT
RI-AT1G64430-XLOC_003343-6584-1
TAAGTTTCTTTTGTCGGTCGCTATAAGTTAATGTTGATAAATTTGTTTGT
CONSENSUS

TAAGTTTCTTTTGTCGGTCGCTATAAGTTAATGTTGATAAATTTGTTTGT

RI-AT1G64430-XLOC_003343-6584-0
AATACTATAGTTTAATAATCACCGAAATCATTAATTTCTTGTTTCTTGTC
RI-AT1G64430-XLOC_003343-6584-1
AATACTATAGTTTAATAATCACCGAAATCATTAATTTCTTGTTTCTTGTC
CONSENSUS
AATACTATAGTTTAATAATCACCGAAATCATTAATTTCTTGTTTCTTGTC

RI-AT1G64430-XLOC_003343-6584-0
CATTTGTAATCTTTTTTTCTTAAATGCAAGCATTTGTTAGTTAA
RI-AT1G64430-XLOC_003343-6584-1
CATTTGTAATCTTTTTTTCTTAAATGCAAGCATTTGTTAGTTAA
CONSENSUS
CATTTGTAATCTTTTTTTCTTAAATGCAAGCATTTGTTAGTTAA
