# Supplementary material for: Splicing-related genes are alternatively spliced upon changes in ambient temperatures in plants
Source: PLoS One. 2017 Mar 3;12(3):e0172950. doi: 10.1371/journal.pone.0172950 (PMC5336241; doi:10.1371/journal.pone.0172950)
Supplement: S7 Table — (PDF) [file pone.0172950.s007.pdf]

alignment for event: A3-gBol025462-XLOC_023011-14729

A3-gBol025462-XLOC_023011-14729-0
      CCGATATCTAGACTTGTAGGAGGGTAAGTGTGAACAGAGCCAGGCTGTAA
A3-gBol025462-XLOC_023011-14729-1
      CCGATATCTAGACTTGTAGGAGGGTAAGTGTGAACAGAGCCAGGCTGTAA
CONSENSUS
      CCGATATCTAGACTTGTAGGAGGGTAAGTGTGAACAGAGCCAGGCTGTAA

A3-gBol025462-XLOC_023011-14729-0
      TGTCAAGTTGGCGTTTGGTGGCTCCACGTTTCTCTCTGATGACTTTTCGA
A3-gBol025462-XLOC_023011-14729-1
      TGTCAAGTTGGCGTTTGGTGGCTCCACGTTTCTCTCTGATGACTTTTCGA
CONSENSUS
      TGTCAAGTTGGCGTTTGGTGGCTCCACGTTTCTCTCTGATGACTTTTCGA

A3-gBol025462-XLOC_023011-14729-0
      ATTAAGTCAGCGACTCTCCTTCCTTTCTTTTCTTCTTTTTCCTCGAAACC
A3-gBol025462-XLOC_023011-14729-1
      ATTAAGTCAGCGACTCTCCTTCCTTTCTTTTCTTCTTTTTCCTCGAAACC
CONSENSUS
      ATTAAGTCAGCGACTCTCCTTCCTTTCTTTTCTTCTTTTTCCTCGAAACC

A3-gBol025462-XLOC_023011-14729-0
      CTTCTCTCTTCTTCATTCCTTCCCTTTAAGCTTTTTTTGCCTCTCTCTCT
A3-gBol025462-XLOC_023011-14729-1
      CTTCTCTCTTCTTCATTCCTTCCCTTTAAGCTTTTTTTGCCTCTCTCTCT
CONSENSUS
      CTTCTCTCTTCTTCATTCCTTCCCTTTAAGCTTTTTTTGCCTCTCTCTCT

A3-gBol025462-XLOC_023011-14729-0
      CTCTCTCTCTCTCGTTCTCTGTCCCACGCACGAGGAGGCTGTTGAATCAA
A3-gBol025462-XLOC_023011-14729-1
      CTCTCTCTCTCTCGTTCTCTGTCCCACGCACGAGGAGGCTGTTGAATCAA
CONSENSUS
      CTCTCTCTCTCTCGTTCTCTGTCCCACGCACGAGGAGGCTGTTGAATCAA

A3-gBol025462-XLOC_023011-14729-0
      GCTCGTGGAGCAAGCTCGATTGCTTTAAATTGGATCCATGGAATACGAGG
A3-gBol025462-XLOC_023011-14729-1
      GCTCGTGGAGCAAGCTCGATTGCTTTAAATTGGATCCATGGAATACGAGG
CONSENSUS
      GCTCGTGGAGCAAGCTCGATTGCTTTAAATTGGATCCATGGAATACGAGG

A3-gBol025462-XLOC_023011-14729-0
      AAGCTATCTTCTTCTTCTACTCGAGTTTGTCAACTTAGAAGACTAGCGCC
A3-gBol025462-XLOC_023011-14729-1
      AAGCTATCTTCTTCTTCTACTCGAGTTTGTCAACTTAGAAGACTAGCGCC
CONSENSUS
      AAGCTATCTTCTTCTTCTACTCGAGTTTGTCAACTTAGAAGACTAGCGCC

A3-gBol025462-XLOC_023011-14729-0
      TTTTTGAATCCGTTCCTTCTCTGTAGCTCTAAGTCGCTTCTGCGATCGAG
A3-gBol025462-XLOC_023011-14729-1
      TTTTTGAATCCGTTCCTTCTCTGTAGCTCTAAGTCGCTTCTGCGATCGAG
CONSENSUS

TTTTTGAATCCGTTCCTTCTCTGTAGCTCTAAGTCGCTTCTGCGATCGAG

A3-gBol025462-XLOC_023011-14729-0
ATATTTGCTTGTGTGAGTGTGCAGGACTAGCTGAAAATGGTTGGAAGCTG
A3-gBol025462-XLOC_023011-14729-1 ATATTTGCTTG------------
GACTAGCTGAAAATGGTTGGAAGCTG
CONSENSUS
ATATTTGCTTG............GACTAGCTGAAAATGGTTGGAAGCTG

A3-gBol025462-XLOC_023011-14729-0
GGATGGAAGCCAGTCTGATGACAGCTCTCACTTCGAGCGAGGCGTCGATC
A3-gBol025462-XLOC_023011-14729-1
GGATGGAAGCCAGTCTGATGACAGCTCTCACTTCGAGCGAGGCGTCGATC
CONSENSUS
GGATGGAAGCCAGTCTGATGACAGCTCTCACTTCGAGCGAGGCGTCGATC

A3-gBol025462-XLOC_023011-14729-0
ACATCTACGAAGCATTCATCTGCCCCTTGACTAAAGAAGTCATGCACGAT
A3-gBol025462-XLOC_023011-14729-1
ACATCTACGAAGCATTCATCTGCCCCTTGACTAAAGAAGTCATGCACGAT
CONSENSUS
ACATCTACGAAGCATTCATCTGCCCCTTGACTAAAGAAGTCATGCACGAT

A3-gBol025462-XLOC_023011-14729-0
CCCGTCACTCTAGAGAACGGTCGAACGTTCGAGCGTGAAGCCATTGAGAA
A3-gBol025462-XLOC_023011-14729-1
CCCGTCACTCTAGAGAACGGTCGAACGTTCGAGCGTGAAGCCATTGAGAA
CONSENSUS
CCCGTCACTCTAGAGAACGGTCGAACGTTCGAGCGTGAAGCCATTGAGAA

A3-gBol025462-XLOC_023011-14729-0
ATATTTCAAGGAATGTTCAGACACTGGTAAACCTCCTTCTTGCCCGTTAA
A3-gBol025462-XLOC_023011-14729-1
ATATTTCAAGGAATGTTCAGACACTGGTAAACCTCCTTCTTGCCCGTTAA
CONSENSUS
ATATTTCAAGGAATGTTCAGACACTGGTAAACCTCCTTCTTGCCCGTTAA

A3-gBol025462-XLOC_023011-14729-0
CTTCTCAGACGCTGAGTAGCACTGATGTCAGCCCCAGTATTGCTCTGCGC
A3-gBol025462-XLOC_023011-14729-1
CTTCTCAGACGCTGAGTAGCACTGATGTCAGCCCCAGTATTGCTCTGCGC
CONSENSUS
CTTCTCAGACGCTGAGTAGCACTGATGTCAGCCCCAGTATTGCTCTGCGC

A3-gBol025462-XLOC_023011-14729-0
AACACTATCCAAGAGTGGAGATCTAGGAACGACGCTGCGAAGCTCGATAT
A3-gBol025462-XLOC_023011-14729-1
AACACTATCCAAGAGTGGAGATCTAGGAACGACGCTGCGAAGCTCGATAT
CONSENSUS
AACACTATCCAAGAGTGGAGATCTAGGAACGACGCTGCGAAGCTCGATAT

A3-gBol025462-XLOC_023011-14729-0
TGCTCGTCAGGCGCTGTTTATAGGAAACGATGAGAATGATGTCTTGCAAG
A3-gBol025462-XLOC_023011-14729-1
TGCTCGTCAGGCGCTGTTTATAGGAAACGATGAGAATGATGTCTTGCAAG
CONSENSUS

TGCTCGTCAGGCGCTGTTTATAGGAAACGATGAGAATGATGTCTTGCAAG

A3-gBol025462-XLOC_023011-14729-0
CTTTGATGCATGTGCGGCAGATTTGCAGGAGTATCAGATCGAATAGGCAG
A3-gBol025462-XLOC_023011-14729-1
CTTTGATGCATGTGCGGCAGATTTGCAGGAGTATCAGATCGAATAGGCAG
CONSENSUS
CTTTGATGCATGTGCGGCAGATTTGCAGGAGTATCAGATCGAATAGGCAG

A3-gBol025462-XLOC_023011-14729-0
GGTGTGCGTAACTTTCAGCTTATTCGTATGATTATTGATATGTTGAAAAG
A3-gBol025462-XLOC_023011-14729-1
GGTGTGCGTAACTTTCAGCTTATTCGTATGATTATTGATATGTTGAAAAG
CONSENSUS
GGTGTGCGTAACTTTCAGCTTATTCGTATGATTATTGATATGTTGAAAAG

A3-gBol025462-XLOC_023011-14729-0
TAATAGTCATAAAGTGAGGTATAAGGCTTTGCAGACTCTTCAAGTTGTTG
A3-gBol025462-XLOC_023011-14729-1
TAATAGTCATAAAGTGAGGTATAAGGCTTTGCAGACTCTTCAAGTTGTTG
CONSENSUS
TAATAGTCATAAAGTGAGGTATAAGGCTTTGCAGACTCTTCAAGTTGTTG

A3-gBol025462-XLOC_023011-14729-0 TAGAAGGAGATGAGGAGAGCAAG
A3-gBol025462-XLOC_023011-14729-1 TAGAAGGAGATGAGGAGAGCAAG
CONSENSUS TAGAAGGAGATGAGGAGAGCAAG

alignment for event: RI-X-XLOC_023059-5095

RI-X-XLOC_023059-5095-0
GAAACACTTTTGAATCTCTTCCTTCGCAACTACCTGCCCTACAACCTTTA
RI-X-XLOC_023059-5095-1
GAAACACTTTTGAATCTCTTCCTTCGCAACTACCTGCCCTACAACCTTTA
CONSENSUS
GAAACACTTTTGAATCTCTTCCTTCGCAACTACCTGCCCTACAACCTTTA

RI-X-XLOC_023059-5095-0
TGACAAAGCAGAGAGGTTGAGATCAAAAGCACCAAGATTTGAGGCACATT
RI-X-XLOC_023059-5095-1
TGACAAAGCAGAGAGGTTGAGATCAAAAGCACCAAGATTTGAGGCACATT
CONSENSUS
TGACAAAGCAGAGAGGTTGAGATCAAAAGCACCAAGATTTGAGGCACATT

RI-X-XLOC_023059-5095-0
TTAACCAACAGGTTTATCTTTTATTATTCTTTCATAGAACTTGGTTATTG
RI-X-XLOC_023059-5095-1
TTAACCAACAG---------------------------------------
CONSENSUS
TTAACCAACAG.......................................

RI-X-XLOC_023059-5095-0
TAGCTTTCTAGATTATAGTTTATATCGTTTCTCTGATTTCTACAATTAGG
RI-X-XLOC_023059-5095-1

CONSENSUS
....

RI-X-XLOC_023059-5095-0
CGTACCAGAACACATTTTATTTGGCTAGCAATACACAATATCCCATAATA
RI-X-XLOC_023059-5095-1

CONSENSUS
....

RI-X-XLOC_023059-5095-0
AAAGACTACCAACCTCTCTCCGGCTCCCACCTTTATTGTTAAGATATATG
RI-X-XLOC_023059-5095-1

CONSENSUS
....

RI-X-XLOC_023059-5095-0
AGCACAATTCTTGCTTTTTGCAACAGATAGCTCATCCGCTCTGATCTATC
RI-X-XLOC_023059-5095-1

CONSENSUS
....

RI-X-XLOC_023059-5095-0
ACCATTGCTAACCTTTTAACAGTCAATTTTTTTTAAAAAAATACTGAGTT
RI-X-XLOC_023059-5095-1

CONSENSUS
....

RI-X-XLOC_023059-5095-0
TATATGTATCTCCTGCTAGGCTTTGCTCTCCCATTATGTTCTTATATTTA
RI-X-XLOC_023059-5095-1

CONSENSUS
....

RI-X-XLOC_023059-5095-0
CATTTTGTTTCTTTTCCCTGCCTTACTTGTGCCTTCTTAGACCTCCATAT
RI-X-XLOC_023059-5095-1

CONSENSUS
....

RI-X-XLOC_023059-5095-0
CTACAAACGTAATATAGTGTGTATATATATACACATCTCACAGAATATGT
RI-X-XLOC_023059-5095-1

CONSENSUS
....

RI-X-XLOC_023059-5095-0
TCTTACAGTTGTTGATATTTGTCCAACTCGGTAAAATCTAGACGATCCAA
RI-X-XLOC_023059-5095-1 --------
TTGTTGATATTTGTCCAACTCGGTAAAATCTAGACGATCCAA

CONSENSUS
........TTGTTGATATTTGTCCAACTCGGTAAAATCTAGACGATCCAA

RI-X-XLOC_023059-5095-0
CTAGAGTAAACTGATGCAAAAGAGAGCCTCCTCCAGGCGGCTAGAAAAGC
RI-X-XLOC_023059-5095-1
CTAGAGTAAACTGATGCAAAAGAGAGCCTCCTCCAGGCGGCTAGAAAAGC
CONSENSUS
CTAGAGTAAACTGATGCAAAAGAGAGCCTCCTCCAGGCGGCTAGAAAAGC

RI-X-XLOC_023059-5095-0
ACTTGTCGCTACTCTAGGCTTCAGAGTCCAATGCGTCAAGTGGGCTATTT
RI-X-XLOC_023059-5095-1
ACTTGTCGCTACTCTAGGCTTCAGAGTCCAATGCGTCAAGTGGGCTATTT
CONSENSUS
ACTTGTCGCTACTCTAGGCTTCAGAGTCCAATGCGTCAAGTGGGCTATTT

RI-X-XLOC_023059-5095-0
TAGTTCGACTCCTCTTGGGTGAAATCCCATAGCGATCCATCTTCACTCAA
RI-X-XLOC_023059-5095-1
TAGTTCGACTCCTCTTGGGTGAAATCCCATAGCGATCCATCTTCACTCAA
CONSENSUS
TAGTTCGACTCCTCTTGGGTGAAATCCCATAGCGATCCATCTTCACTCAA

RI-X-XLOC_023059-5095-0
AAAGGAATGAGAAAGCTCTTACGCCATATTTGGAACTTACCAAT
RI-X-XLOC_023059-5095-1
AAAGGAATGAGAAAGCTCTTACGCCATATTTGGAACTTACCAAT
CONSENSUS
AAAGGAATGAGAAAGCTCTTACGCCATATTTGGAACTTACCAAT

alignment for event: RI-X-XLOC_023059-5094

RI-X-XLOC_023059-5094-0
GAAACACTTTTGAATCTCTTCCTTCGCAACTACCTGCCCTACAACCTTTA
RI-X-XLOC_023059-5094-1
GAAACACTTTTGAATCTCTTCCTTCGCAACTACCTGCCCTACAACCTTTA
CONSENSUS
GAAACACTTTTGAATCTCTTCCTTCGCAACTACCTGCCCTACAACCTTTA

RI-X-XLOC_023059-5094-0
TGACAAAGCAGAGAGGTTGAGATCAAAAGCACCAAGATTTGAGGCACATT
RI-X-XLOC_023059-5094-1
TGACAAAGCAGAGAGGTTGAGATCAAAAGCACCAAGATTTGAGGCACATT
CONSENSUS
TGACAAAGCAGAGAGGTTGAGATCAAAAGCACCAAGATTTGAGGCACATT

RI-X-XLOC_023059-5094-0
TTAACCAACAGGTTTATCTTTTATTATTCTTTCATAGAACTTGGTTATTG
RI-X-XLOC_023059-5094-1
TTAACCAACAG---------------------------------------
CONSENSUS
TTAACCAACAG.......................................

```
RI-X-XLOC_023059-5094-0
      TAGCTTTCTAGATTATAGTTTATATCGTTTCTCTGATTTCTACAATTAGG
RI-X-XLOC_023059-5094-1
      --------------------------------------------------
CONSENSUS
      ..................................................

RI-X-XLOC_023059-5094-0
      CGTACCAGAACACATTTTATTTGGCTAGCAATACACAATATCCCATAATA
RI-X-XLOC_023059-5094-1
      --------------------------------------------------
CONSENSUS
      ..................................................

RI-X-XLOC_023059-5094-0
      AAAGACTACCAACCTCTCTCCGGCTCCCACCTTTATTGTTAAGATATATG
RI-X-XLOC_023059-5094-1
      --------------------------------------------------
CONSENSUS
      ..................................................

RI-X-XLOC_023059-5094-0
      AGCACAATTCTTGCTTTTTGCAACAGATAGCTCATCCGCTCTGATCTATC
RI-X-XLOC_023059-5094-1
      --------------------------------------------------
CONSENSUS
      ..................................................

RI-X-XLOC_023059-5094-0
      ACCATTGCTAACCTTTTAACAGTCAATTTTTTTTAAAAAAATACTGAGTT
RI-X-XLOC_023059-5094-1
      --------------------------------------------------
CONSENSUS
      ..................................................

RI-X-XLOC_023059-5094-0
      TATATGTATCTCCTGCTAGGCTTTGCTCTCCCATTATGTTCTTATATTTA
RI-X-XLOC_023059-5094-1
      --------------------------------------------------
CONSENSUS
      ..................................................

RI-X-XLOC_023059-5094-0
      CATTTTGTTTCTTTTCCCTGCCTTACTTGTGCCTTCTTAGACCTCCATAT
RI-X-XLOC_023059-5094-1
      --------------------------------------------------
CONSENSUS
      ..................................................

RI-X-XLOC_023059-5094-0
      CTACAAACGTAATATAGTGTGTATATATATACACATCTCACAGAATATGT
RI-X-XLOC_023059-5094-1
      --------------------------------------------------
CONSENSUS
      ..................................................
```

RI-X-XLOC_023059-5094-0
      TCTTACAGTTGTTGATATTTGTCCAACTCGGTAAAATCTAGACGATCCAA
RI-X-XLOC_023059-5094-1
      ---ACGATCCAA
CONSENSUS
      ....ACGATCCAA

RI-X-XLOC_023059-5094-0
      CTAGAGTAAACTGATGCAAAAGAGAGCCTCCTCCAGGCGGCTAGAAAAGC
RI-X-XLOC_023059-5094-1
      CTAGAGTAAACTGATGCAAAAGAGAGCCTCCTCCAGGCGGCTAGAAAAGC
CONSENSUS
      CTAGAGTAAACTGATGCAAAAGAGAGCCTCCTCCAGGCGGCTAGAAAAGC

RI-X-XLOC_023059-5094-0
      ACTTGTCGCTACTCTAGGCTTCAGAGTCCAATGCGTCAAGTGGGCTATTT
RI-X-XLOC_023059-5094-1
      ACTTGTCGCTACTCTAGGCTTCAGAGTCCAATGCGTCAAGTGGGCTATTT
CONSENSUS
      ACTTGTCGCTACTCTAGGCTTCAGAGTCCAATGCGTCAAGTGGGCTATTT

RI-X-XLOC_023059-5094-0
      TAGTTCGACTCCTCTTGGGTGAAATCCCATAGCGATCCATCTTCACTCAA
RI-X-XLOC_023059-5094-1
      TAGTTCGACTCCTCTTGGGTGAAATCCCATAGCGATCCATCTTCACTCAA
CONSENSUS
      TAGTTCGACTCCTCTTGGGTGAAATCCCATAGCGATCCATCTTCACTCAA

RI-X-XLOC_023059-5094-0
      AAAGGAATGAGAAAGCTCTTACGCCATATTTGGAACTTACCAAT
RI-X-XLOC_023059-5094-1
      AAAGGAATGAGAAAGCTCTTACGCCATATTTGGAACTTACCAAT
CONSENSUS
      AAAGGAATGAGAAAGCTCTTACGCCATATTTGGAACTTACCAAT

alignment for event: RI-gBol041810-XLOC_004829-10109

RI-gBol041810-XLOC_004829-10109-0
      TGAGGATGCAGAATCATAATGATGCAGTGAAGGATGATGAAGCGGTTAAA
RI-gBol041810-XLOC_004829-10109-1
      TGAGGATGCAGAATCATAATGATGCAGTGAAGGATGATGAAGCGGTTAAA
CONSENSUS
      TGAGGATGCAGAATCATAATGATGCAGTGAAGGATGATGAAGCGGTTAAA

RI-gBol041810-XLOC_004829-10109-0
      GTTGGGAATTGTTCTGTGGAAAATGTTCAATCTTTTCAAGAAGAAGCTGC
RI-gBol041810-XLOC_004829-10109-1
      GTTGGGAATTGTTCTGTGGAAAATGTTCAATCTTTTCAAGAAGAAGCTGC
CONSENSUS
      GTTGGGAATTGTTCTGTGGAAAATGTTCAATCTTTTCAAGAAGAAGCTGC

RI-gBol041810-XLOC_004829-10109-0
      TAAATTTCCTTATGTTGGTGATAAGGTTAAAACTTAGATGGAATCAACTT
RI-gBol041810-XLOC_004829-10109-1

TAAATTTCCTTATGTTGGTGATAAG------------------------
CONSENSUS
TAAATTTCCTTATGTTGGTGATAAG........................

RI-gBol041810-XLOC_004829-10109-0
CTCCCTTATCACATCTTCTTATCTATGGGTTTCATGGAATCTACATGATG
RI-gBol041810-XLOC_004829-10109-1

CONSENSUS
....

RI-gBol041810-XLOC_004829-10109-0
TTGCTCTCTCTCTCTCTCTACTATCTTCTTTTTGGTTCAATCACTGTGCA
RI-gBol041810-XLOC_004829-10109-1

CONSENSUS
....

RI-gBol041810-XLOC_004829-10109-0
GGAACCTCTCTCTAGTTTAGCTGCAGAGTACCAAGCAGGGAGTCCCATTC
RI-gBol041810-XLOC_004829-10109-1 -
GAACCTCTCTCTAGTTTAGCTGCAGAGTACCAAGCAGGGAGTCCCATTC
CONSENSUS
.GAACCTCTCTCTAGTTTAGCTGCAGAGTACCAAGCAGGGAGTCCCATTC

RI-gBol041810-XLOC_004829-10109-0 TGCTCGAGAAGATCAAG
RI-gBol041810-XLOC_004829-10109-1 TGCTCGAGAAGATCAAG
CONSENSUS TGCTCGAGAAGATCAAG

alignment for event: RI-gBol041826-XLOC_004840-10248

RI-gBol041826-XLOC_004840-10248-0
GCGAGTATGCAGAGCAAGAACATGATTGTGGCTCCGTCTCATAAGCAGCA
RI-gBol041826-XLOC_004840-10248-1
GCGAGTATGCAGAGCAAGAACATGATTGTGGCTCCGTCTCATAAGCAGCA
CONSENSUS
GCGAGTATGCAGAGCAAGAACATGATTGTGGCTCCGTCTCATAAGCAGCA

RI-gBol041826-XLOC_004840-10248-0
GCAACAACAGCAAGCGCATCAACCGCAACCACAACTTAAATGCCCTCGTT
RI-gBol041826-XLOC_004840-10248-1
GCAACAACAGCAAGCGCATCAACCGCAACCACAACTTAAATGCCCTCGTT
CONSENSUS
GCAACAACAGCAAGCGCATCAACCGCAACCACAACTTAAATGCCCTCGTT

RI-gBol041826-XLOC_004840-10248-0
GTGATTCTTCTAACACAAAGTTCTGTTACTACAACAACTACAGCCTCTCT
RI-gBol041826-XLOC_004840-10248-1
GTGATTCTTCTAACACAAAGTTCTGTTACTACAACAACTACAGCCTCTCT
CONSENSUS
GTGATTCTTCTAACACAAAGTTCTGTTACTACAACAACTACAGCCTCTCT

RI-gBol041826-XLOC_004840-10248-0
CAGCCGCGGCACTTTTGCAAGGCTTGCAAGAGGTATTGGACACGAGGTGG

RI-gBol041826-XLOC_004840-10248-1
CAGCCGCGGCACTTTTGCAAGGCTTGCAAGAGGTATTGGACACGAGGTGG
CONSENSUS
CAGCCGCGGCACTTTTGCAAGGCTTGCAAGAGGTATTGGACACGAGGTGG

RI-gBol041826-XLOC_004840-10248-0
GACCCTCCGTAACGTTCCCGTAGGCGGTAGCTACCGGAAAAATAAACGTG
RI-gBol041826-XLOC_004840-10248-1
GACCCTCCGTAACGTTCCCGTAGGCGGTAGCTACCGGAAAAATAAACGTG
CONSENSUS
GACCCTCCGTAACGTTCCCGTAGGCGGTAGCTACCGGAAAAATAAACGTG

RI-gBol041826-XLOC_004840-10248-0
TGAAGCGGCCAGCAACCGCTGCCTCCACGGTCTCGAAGACTACTTCTTCA
RI-gBol041826-XLOC_004840-10248-1
TGAAGCGGCCAGCAACCGCTGCCTCCACGGTCTCGAAGACTACTTCTTCA
CONSENSUS
TGAAGCGGCCAGCAACCGCTGCCTCCACGGTCTCGAAGACTACTTCTTCA

RI-gBol041826-XLOC_004840-10248-0
TCCCCTAATCATCAGATCTCCCATTTCTCTTCCATGAATCATCATCCCAT
RI-gBol041826-XLOC_004840-10248-1
TCCCCTAATCATCAGATCTCCCATTTCTCTTCCATGAATCATCATCCCAT
CONSENSUS
TCCCCTAATCATCAGATCTCCCATTTCTCTTCCATGAATCATCATCCCAT

RI-gBol041826-XLOC_004840-10248-0
GCTCTATGGTTTATCAGATCATGTGAACAGTTGTAATAGTCTTCCAATGA
RI-gBol041826-XLOC_004840-10248-1
GCTCTATGGTTTATCAGATCATGTGAACAGTTGTAATAGTCTTCCAATGA
CONSENSUS
GCTCTATGGTTTATCAGATCATGTGAACAGTTGTAATAGTCTTCCAATGA

RI-gBol041826-XLOC_004840-10248-0
TTCCAAGCCGGTTCAATGATTCTTCAAAGACTTCATCAAGTGGTTTAGAG
RI-gBol041826-XLOC_004840-10248-1
TTCCAAGCCGGTTCAATGATTCTTCAAAGACTTCATCAAGTGGTTTAGAG
CONSENSUS
TTCCAAGCCGGTTCAATGATTCTTCAAAGACTTCATCAAGTGGTTTAGAG

RI-gBol041826-XLOC_004840-10248-0
AGTGAGTTTTTCTCATCTGGTTTTAGTGGCCTAGGTGCTCTTGGTCTAGG
RI-gBol041826-XLOC_004840-10248-1
AGTGAGTTTTTCTCATCTGGTTTTAGTGGCCTAGGTGCTCTTGGTCTAGG
CONSENSUS
AGTGAGTTTTTCTCATCTGGTTTTAGTGGCCTAGGTGCTCTTGGTCTAGG

RI-gBol041826-XLOC_004840-10248-0
GTTTCCACATCATATGAGTCATGACCACACCGCCATTAACGGTGGCTTCA
RI-gBol041826-XLOC_004840-10248-1
GTTTCCACATCATATGAGTCATGACCACACCGCCATTAACGGTGGCTTCA
CONSENSUS
GTTTCCACATCATATGAGTCATGACCACACCGCCATTAACGGTGGCTTCA

RI-gBol041826-XLOC_004840-10248-0
TCGACAACTCTACCGCAAACAAACCCTTTCTTCTCTCGGGGCTATTTGGG

RI-gBol041826-XLOC_004840-10248-1
TCGACAACTCTACCGCAAACAAACCCTTTCTTCTCTCGGGGCTATTTGGG
CONSENSUS
TCGACAACTCTACCGCAAACAAACCCTTTCTTCTCTCGGGGCTATTTGGG

RI-gBol041826-XLOC_004840-10248-0
TCCTCGGTATCTTCATCTTCCTCTAATCTTGTCCAGCATCCACACAAACC
RI-gBol041826-XLOC_004840-10248-1
TCCTCGGTATCTTCATCTTCCTCTAATCTTGTCCAGCATCCACACAAACC
CONSENSUS
TCCTCGGTATCTTCATCTTCCTCTAATCTTGTCCAGCATCCACACAAACC

RI-gBol041826-XLOC_004840-10248-0
CATGAACAATGGTGGTGAGATGATGGGACAATCCCATCTCCAAACCCTAG
RI-gBol041826-XLOC_004840-10248-1
CATGAACAATGGTGGTGAGATGATGGGACAATCCCATCTCCAAACCCTAG
CONSENSUS
CATGAACAATGGTGGTGAGATGATGGGACAATCCCATCTCCAAACCCTAG

RI-gBol041826-XLOC_004840-10248-0
CATCCCTTCAAGATCTGCATGTTGGAGGTAACAATGAAGATGTGAACAAA
RI-gBol041826-XLOC_004840-10248-1
CATCCCTTCAAGATCTGCATGTTGGAGGTAACAATGAAGATGTGAACAAA
CONSENSUS
CATCCCTTCAAGATCTGCATGTTGGAGGTAACAATGAAGATGTGAACAAA

RI-gBol041826-XLOC_004840-10248-0
GAAGGGAAGCTTGGTCAGATCTCAGGGACCATTAGTGGATTCATGTCACC
RI-gBol041826-XLOC_004840-10248-1
GAAGGGAAGCTTGGTCAGATCTCAGGGACCATTAGTGGATTCATGTCACC
CONSENSUS
GAAGGGAAGCTTGGTCAGATCTCAGGGACCATTAGTGGATTCATGTCACC

RI-gBol041826-XLOC_004840-10248-0
ATCCTCTTTAGATCCTTCAAACTACAACAACATATGGAATAATGCGAGTG
RI-gBol041826-XLOC_004840-10248-1
ATCCTCTTTAGATCCTTCAAACTACAACAACATATGGAATAATGCGAGTG
CONSENSUS
ATCCTCTTTAGATCCTTCAAACTACAACAACATATGGAATAATGCGAGTG

RI-gBol041826-XLOC_004840-10248-0
TTGTCAATGGAGAATGGCTTGATTCGACAAATAATAACGTTGGATCCTCC
RI-gBol041826-XLOC_004840-10248-1
TTGTCAATGGAGAATGGCTTGATTCGACAAATAATAACGTTGGATCCTCC
CONSENSUS
TTGTCAATGGAGAATGGCTTGATTCGACAAATAATAACGTTGGATCCTCC

RI-gBol041826-XLOC_004840-10248-0
CTTACCTCCTTGATATAAACCAAAATTAACTTATTGCTTATAGAATCTAT
RI-gBol041826-XLOC_004840-10248-1
CTTACCTCCTTGATATAAACCAAAATTAACTTATTGCTTATAGAATCTAT
CONSENSUS
CTTACCTCCTTGATATAAACCAAAATTAACTTATTGCTTATAGAATCTAT

RI-gBol041826-XLOC_004840-10248-0
TACTAGCTGGGGTTTCATCTTCATCACTTTTTTTTCTTTGCGTTTTGAGA

RI-gBol041826-XLOC_004840-10248-1
TACTAGCTGGGGTTTCATCTTCATCACTTTTTTTTCTTTGCGTTTTGAGA
CONSENSUS
TACTAGCTGGGGTTTCATCTTCATCACTTTTTTTTCTTTGCGTTTTGAGA

RI-gBol041826-XLOC_004840-10248-0
TGAGAGAAGAAGCTGGGATCAGAGAGATAAAATCAGGGGAAATGTTGATG
RI-gBol041826-XLOC_004840-10248-1
TGAGAGAAGAAGCTGGGATCAGAGAGATAAAATCAGGGGAAATGTTGATG
CONSENSUS
TGAGAGAAGAAGCTGGGATCAGAGAGATAAAATCAGGGGAAATGTTGATG

RI-gBol041826-XLOC_004840-10248-0
TTAGCTTCTATAAACTCCCTCGGATCCTCTTCATCTCAACATCTTCATCC
RI-gBol041826-XLOC_004840-10248-1
TTAGCTTCTATAAACTCCCTCGGATCCTCTTCATCTCAACATCTTCATCC
CONSENSUS
TTAGCTTCTATAAACTCCCTCGGATCCTCTTCATCTCAACATCTTCATCC

RI-gBol041826-XLOC_004840-10248-0
CGCGTTGTTCAAGAAAAACAAAAGAGCGCTCAAGATTGGTTGAAGGTTAA
RI-gBol041826-XLOC_004840-10248-1
CGCGTTGTTCAAGAAAAACAAAAGAGCGCTCAAGATTGGTTGAAGGTTAA
CONSENSUS
CGCGTTGTTCAAGAAAAACAAAAGAGCGCTCAAGATTGGTTGAAGGTTAA

RI-gBol041826-XLOC_004840-10248-0
CTATGTTCGACTGTTATGTTCACTCGAGGTAAATGATATGTGTACGTAAA
RI-gBol041826-XLOC_004840-10248-1
CTATGTTCGACTGTTATGTTCACTCGAG----------------------
CONSENSUS
CTATGTTCGACTGTTATGTTCACTCGAG......................

RI-gBol041826-XLOC_004840-10248-0
TAATATGGTCCTGACTTTCATCATCTTTTTTTCATCTATGTAATTCGAAG
RI-gBol041826-XLOC_004840-10248-1

CONSENSUS
....

RI-gBol041826-XLOC_004840-10248-0
GTCCACCACATGGGTTGGTGTTTCTTTTCATTGGAACTAGAAGGGTAAAT
RI-gBol041826-XLOC_004840-10248-1

CONSENSUS
....

RI-gBol041826-XLOC_004840-10248-0
TTGGGTTAAATTGAGTTTCTCATGGGGTCAGAGAACTCATTTCACGTTTG
RI-gBol041826-XLOC_004840-10248-1

CONSENSUS
....

RI-gBol041826-XLOC_004840-10248-0
ATTTCTTTTTAGTTTCACATTTCTTCTTTGGTGTGGCTTTCATTGGGTAT

RI-gBol041826-XLOC_004840-10248-1

CONSENSUS
....

RI-gBol041826-XLOC_004840-10248-0
ACAAACAGAGGGAAGGGTGTGGTTTTGTATTGTTAAAACTTTGGTTATTC
RI-gBol041826-XLOC_004840-10248-1

CONSENSUS
....

RI-gBol041826-XLOC_004840-10248-0
ATGTAAGAGTTATGTTATTCAGGTTCTTTTGGATACAAGTCTATGTATAT
RI-gBol041826-XLOC_004840-10248-1

CONSENSUS
....

RI-gBol041826-XLOC_004840-10248-0
TTATGTTATATATATCTACATTATAATCACGATAGATATGGGACGAAGGT
RI-gBol041826-XLOC_004840-10248-1

CONSENSUS
....

RI-gBol041826-XLOC_004840-10248-0
ACATGTGGATGACTTGATTAATTAATTGTCCAAAAGGAAAGCGTCCTTTA
RI-gBol041826-XLOC_004840-10248-1

CONSENSUS
....

RI-gBol041826-XLOC_004840-10248-0
GCTAATGTCAAATGGGAGATTCATGAATATGAGAAACTTCGCAGAAACGA
RI-gBol041826-XLOC_004840-10248-1

CONSENSUS
....

RI-gBol041826-XLOC_004840-10248-0
CGTTAATGGATACAAATTAGTTGATACATATATATACAATATACATGTAA
RI-gBol041826-XLOC_004840-10248-1

CONSENSUS
....

RI-gBol041826-XLOC_004840-10248-0
TATACTAAGACCGACAAAGTGAAAGCGTGGTTAGACATCTTATTTATATA
RI-gBol041826-XLOC_004840-10248-1

CONSENSUS
....

RI-gBol041826-XLOC_004840-10248-0
AACATTCTGCTCGTAGAGTCGTAGTTATGTCATATTAATGAATAATGATT

RI-gBol041826-XLOC_004840-10248-1

CONSENSUS
    ....

RI-gBol041826-XLOC_004840-10248-0
    TTGTTCTTTACCGTTTTGGCTACAAGCCTACAACTCATGGTTTCATGTAT
RI-gBol041826-XLOC_004840-10248-1

CONSENSUS
    ....

RI-gBol041826-XLOC_004840-10248-0
    ATATGAACACAAGTTTTGGTCCAGGGCTTGGAGTTTATTGATGCGTCATT
RI-gBol041826-XLOC_004840-10248-1 -------------
TTTTGGTCCAGGGCTTGGAGTTTATTGATGCGTCATT
CONSENSUS
    .............TTTTGGTCCAGGGCTTGGAGTTTATTGATGCGTCATT

RI-gBol041826-XLOC_004840-10248-0
    ATAAAGTGGGCACGAGTTCGTTACCAGCTTCTGCATGTCATTTTCTCTGC
RI-gBol041826-XLOC_004840-10248-1
    ATAAAGTGGGCACGAGTTCGTTACCAGCTTCTGCATGTCATTTTCTCTGC
CONSENSUS
    ATAAAGTGGGCACGAGTTCGTTACCAGCTTCTGCATGTCATTTTCTCTGC

RI-gBol041826-XLOC_004840-10248-0
    AATGATTCTTTTCGTGCAAGTATTGGAGATAGATTTATTCGTTAAAGATT
RI-gBol041826-XLOC_004840-10248-1
    AATGATTCTTTTCGTGCAAGTATTGGAGATAGATTTATTCGTTAAAGATT
CONSENSUS
    AATGATTCTTTTCGTGCAAGTATTGGAGATAGATTTATTCGTTAAAGATT

RI-gBol041826-XLOC_004840-10248-0
    TGGGTCGGCAGACTCGGTACCGTGTGGACCAAGAACTTTATTACCTTTGT
RI-gBol041826-XLOC_004840-10248-1
    TGGGTCGGCAGACTCGGTACCGTGTGGACCAAGAACTTTATTACCTTTGT
CONSENSUS
    TGGGTCGGCAGACTCGGTACCGTGTGGACCAAGAACTTTATTACCTTTGT

RI-gBol041826-XLOC_004840-10248-0
    GTGGATAATTTATGGATCAAAATCAATTATCAAACGAAAGAATGTTATTA
RI-gBol041826-XLOC_004840-10248-1
    GTGGATAATTTATGGATCAAAATCAATTATCAAACGAAAGAATGTTATTA
CONSENSUS
    GTGGATAATTTATGGATCAAAATCAATTATCAAACGAAAGAATGTTATTA

RI-gBol041826-XLOC_004840-10248-0
    ACACGTATACTGTCGTCTGACATTCTAGCTTATGGACACATCAGACGAGA
RI-gBol041826-XLOC_004840-10248-1
    ACACGTATACTGTCGTCTGACATTCTAGCTTATGGACACATCAGACGAGA
CONSENSUS
    ACACGTATACTGTCGTCTGACATTCTAGCTTATGGACACATCAGACGAGA

RI-gBol041826-XLOC_004840-10248-0
    ACTTGACCTGATTATTTTCAGCAATCTGCATATACGTTTTCATTGTTGAT

RI-gBol041826-XLOC_004840-10248-1
      ACTTGACCTGATTATTTTCAGCAATCTGCATATACGTTTTCATTGTTGAT
CONSENSUS
      ACTTGACCTGATTATTTTCAGCAATCTGCATATACGTTTTCATTGTTGAT

RI-gBol041826-XLOC_004840-10248-0
      ATTATGTATTTATATTTATTATGACCTCTTTAACCAATCAAATTGCAAAC
RI-gBol041826-XLOC_004840-10248-1
      ATTATGTATTTATATTTATTATGACCTCTTTAACCAATCAAATTGCAAAC
CONSENSUS
      ATTATGTATTTATATTTATTATGACCTCTTTAACCAATCAAATTGCAAAC

RI-gBol041826-XLOC_004840-10248-0   GATTTAGGTATTTAAC
RI-gBol041826-XLOC_004840-10248-1   GATTTAGGTATTTAAC
CONSENSUS   GATTTAGGTATTTAAC

alignment for event: A3-gBol028014-XLOC_020410-14196

A3-gBol028014-XLOC_020410-14196-0
      CGATCGCACTATCAGATGACCATAAGCCAAACAGAAGCGATGAAAGAAAG
A3-gBol028014-XLOC_020410-14196-1
      CGATCGCACTATCAGATGACCATAAGCCAAACAGAAGCGATGAAAGAAAG
CONSENSUS
      CGATCGCACTATCAGATGACCATAAGCCAAACAGAAGCGATGAAAGAAAG

A3-gBol028014-XLOC_020410-14196-0
      CGAATTGAAAGCGCTGGTGGTGTTATCATGTGGGCAG-------------
A3-gBol028014-XLOC_020410-14196-1
      CGAATTGAAAGCGCTGGTGGTGTTATCATGTGGGCAGGCTTTAAACAATC
CONSENSUS
      CGAATTGAAAGCGCTGGTGGTGTTATCATGTGGGCAG.............

A3-gBol028014-XLOC_020410-14196-0

A3-gBol028014-XLOC_020410-14196-1
      ACCGTCAGGGACTGAACTAGTAGTCTTGAGGCCTGCCCTTTTAATATCTT
CONSENSUS
      ....

A3-gBol028014-XLOC_020410-14196-0

A3-gBol028014-XLOC_020410-14196-1
      CCCCTAGATCTCTTTGTCAGCCTGAGTTTTGAAGTAATATCATCTGGATT
CONSENSUS
      ....

A3-gBol028014-XLOC_020410-14196-0   ----------------
GAACATGGAGGGTAGGTGGAGTCCTGGCAATGTC
A3-gBol028014-XLOC_020410-14196-1
      TGCCTTGTCTTGTCAGGAACATGGAGGGTAGGTGGAGTCCTGGCAATGTC
CONSENSUS
      ................GAACATGGAGGGTAGGTGGAGTCCTGGCAATGTC

A3-gBol028014-XLOC_020410-14196-0

```
      CCGGGCCTTTGGAAACAGAATGCTGAAGCAATTCGTTATTGCTGAACCCG
A3-gBol028014-XLOC_020410-14196-1
      CCGGGCCTTTGGAAACAGAATGCTGAAGCAATTCGTTATTGCTGAACCCG
CONSENSUS
      CCGGGCCTTTGGAAACAGAATGCTGAAGCAATTCGTTATTGCTGAACCCG

A3-gBol028014-XLOC_020410-14196-0      AGATTCAA
A3-gBol028014-XLOC_020410-14196-1      AGATTCAA
CONSENSUS                              AGATTCAA
```

alignment for event: A3-gBol028014-XLOC_020410-14194

```
A3-gBol028014-XLOC_020410-14194-0
      CGATCGCACTATCAGATGACCATAAGCCAAACAGAAGCGATGAAAGAAAG
A3-gBol028014-XLOC_020410-14194-1
      CGATCGCACTATCAGATGACCATAAGCCAAACAGAAGCGATGAAAGAAAG
CONSENSUS
      CGATCGCACTATCAGATGACCATAAGCCAAACAGAAGCGATGAAAGAAAG

A3-gBol028014-XLOC_020410-14194-0
      CGAATTGAAAGCGCTGGTGGTGTTATCATGTGGGCAG-------------
A3-gBol028014-XLOC_020410-14194-1
      CGAATTGAAAGCGCTGGTGGTGTTATCATGTGGGCAGGTCTAATACTGTT
CONSENSUS
      CGAATTGAAAGCGCTGGTGGTGTTATCATGTGGGCAG.............

A3-gBol028014-XLOC_020410-14194-0
      --------------------------------------------------
A3-gBol028014-XLOC_020410-14194-1
      ACTAAGTTCGAGGGAACACTGACCCACAAACCATTTTCTTATGGAGGCAT
CONSENSUS
      ..................................................

A3-gBol028014-XLOC_020410-14194-0
      --------------------------------------------------
A3-gBol028014-XLOC_020410-14194-1
      TCTATATCTCCGGTTATTCAGGCTTTAAACAATCACCGTCAGGGACTGAA
CONSENSUS
      ..................................................

A3-gBol028014-XLOC_020410-14194-0
      --------------------------------------------------
A3-gBol028014-XLOC_020410-14194-1
      CTAGTAGTCTTGAGGCCTGCCCTTTTAATATCTTCCCCTAGATCTCTTTG
CONSENSUS
      ..................................................

A3-gBol028014-XLOC_020410-14194-0
      --------------------------------------------------
A3-gBol028014-XLOC_020410-14194-1
      TCAGCCTGAGTTTTGAAGTAATATCATCTGGATTTGCCTTGTCTTGTCAG
CONSENSUS
      ..................................................
```

```
A3-gBol028014-XLOC_020410-14194-0
      GAACATGGAGGGTAGGTGGAGTCCTGGCAATGTCCCGGGCCTTTGGAAAC
A3-gBol028014-XLOC_020410-14194-1
      GAACATGGAGGGTAGGTGGAGTCCTGGCAATGTCCCGGGCCTTTGGAAAC
CONSENSUS
      GAACATGGAGGGTAGGTGGAGTCCTGGCAATGTCCCGGGCCTTTGGAAAC

A3-gBol028014-XLOC_020410-14194-0
      AGAATGCTGAAGCAATTCGTTATTGCTGAACCCGAGATTCAA
A3-gBol028014-XLOC_020410-14194-1
      AGAATGCTGAAGCAATTCGTTATTGCTGAACCCGAGATTCAA
CONSENSUS
      AGAATGCTGAAGCAATTCGTTATTGCTGAACCCGAGATTCAA



alignment for event: RI-gBol015732-XLOC_034003-15103

RI-gBol015732-XLOC_034003-15103-0
      GAGAAGGAAGCAACAAGGTGATGCATAGGTCGTTGACTATGCCGACGGTA
RI-gBol015732-XLOC_034003-15103-1
      GAGAAGGAAGCAACAAGGTGATGCATAGGTCGTTGACTATGCCGACGGTA
CONSENSUS
      GAGAAGGAAGCAACAAGGTGATGCATAGGTCGTTGACTATGCCGACGGTA

RI-gBol015732-XLOC_034003-15103-0
      GTGAGCCCCGGAACTCCAACTACTCCGACCACTCCGACAACGCCACATAA
RI-gBol015732-XLOC_034003-15103-1
      GTGAGCCCCGGAACTCCAACTACTCCGACCACTCCGACAACGCCACATAA
CONSENSUS
      GTGAGCCCCGGAACTCCAACTACTCCGACCACTCCGACAACGCCACATAA

RI-gBol015732-XLOC_034003-15103-0
      GGATAACGTGTGGAGGAGCGTCTTTAATCCTGGAAGCAACCTCGCCACGA
RI-gBol015732-XLOC_034003-15103-1
      GGATAACGTGTGGAGGAGCGTCTTTAATCCTGGAAGCAACCTCGCCACGA
CONSENSUS
      GGATAACGTGTGGAGGAGCGTCTTTAATCCTGGAAGCAACCTCGCCACGA

RI-gBol015732-XLOC_034003-15103-0
      GAGCCATCGGCTCCAACATCTTTGATAAACCAGCCCACCCAAATTCTCCA
RI-gBol015732-XLOC_034003-15103-1
      GAGCCATCGGCTCCAACATCTTTGATAAACCAGCCCACCCAAATTCTCCA
CONSENSUS
      GAGCCATCGGCTCCAACATCTTTGATAAACCAGCCCACCCAAATTCTCCA

RI-gBol015732-XLOC_034003-15103-0
      TCCGTCTACGACTGGTGAGTTCATTAATTATATGTTATTTTCTGTGAAGT
RI-gBol015732-XLOC_034003-15103-1
      TCCGTCTACGACTG------------------------------------
CONSENSUS
      TCCGTCTACGACTG....................................

RI-gBol015732-XLOC_034003-15103-0
      CATATGCTTAAGACTTTAAATCTTAGATGTCATGATCATAAATCATAATA
RI-gBol015732-XLOC_034003-15103-1
```

CONSENSUS
....

RI-gBol015732-XLOC_034003-15103-0
TAGAGGAACAAGTCATGTGATCTGGTCAGCTTTTATGTTCTGACTTTTGA
RI-gBol015732-XLOC_034003-15103-1

CONSENSUS
....

RI-gBol015732-XLOC_034003-15103-0
AACTAGTTAAAGTACCGAACCGGATAAATCTAGGAATGGTTTGGTTCGTT
RI-gBol015732-XLOC_034003-15103-1

CONSENSUS
....

RI-gBol015732-XLOC_034003-15103-0
TTAATTTACTGATATCTTGGTATGAGTTATTTATGTGTCAGCGATGATAA
RI-gBol015732-XLOC_034003-15103-1
---CGATGATAA
CONSENSUS
....CGATGATAA

RI-gBol015732-XLOC_034003-15103-0
CGAAGCTCAAAGGAAGGAACACGTGGCACTGTGTTTAGTAGGCGCGTGGA
RI-gBol015732-XLOC_034003-15103-1
CGAAGCTCAAAGGAAGGAACACGTGGCACTGTGTTTAGTAGGCGCGTGGA
CONSENSUS
CGAAGCTCAAAGGAAGGAACACGTGGCACTGTGTTTAGTAGGCGCGTGGA

RI-gBol015732-XLOC_034003-15103-0
TTAAATGAAAACCAATAAAACATTATTCTGTTTTACCATATCAACCACCT
RI-gBol015732-XLOC_034003-15103-1
TTAAATGAAAACCAATAAAACATTATTCTGTTTTACCATATCAACCACCT
CONSENSUS
TTAAATGAAAACCAATAAAACATTATTCTGTTTTACCATATCAACCACCT

RI-gBol015732-XLOC_034003-15103-0 CCTTTATTGTGCATATG
RI-gBol015732-XLOC_034003-15103-1 CCTTTATTGTGCATATG
CONSENSUS CCTTTATTGTGCATATG

alignment for event: A3-gBol024345-XLOC_024343-10940

A3-gBol024345-XLOC_024343-10940-0
ATCCGTGGGAGCATGTGGTGGGCATGGGGCTCGGTGCTGTGTTCGCGAAC
A3-gBol024345-XLOC_024343-10940-1
ATCCGTGGGAGCATGTGGTGGGCATGGGGCTCGGTGCTGTGTTCGCGAAC
CONSENSUS
ATCCGTGGGAGCATGTGGTGGGCATGGGGCTCGGTGCTGTGTTCGCGAAC

A3-gBol024345-XLOC_024343-10940-0
CAGCTCGTGAAATGGGATGTGAAGCTCAAGGAAGATCTCGAGGTGATGCT

A3-gBol024345-XLOC_024343-10940-1
      CAGCTCGTGAAATGGGATGTGAAGCTCAAGGAAGATCTCGAGGTGATGCT
CONSENSUS
      CAGCTCGTGAAATGGGATGTGAAGCTCAAGGAAGATCTCGAGGTGATGCT

A3-gBol024345-XLOC_024343-10940-0
      CGATAAGGCTAGAGCTGCCAACGAGCGCCGTTACTTTGGCAGATGAAGAT
A3-gBol024345-XLOC_024343-10940-1
      CGATAAGGCTAGAGCTGCCAACGAGCGCCGTTACTTTG----ATGAAGAT
CONSENSUS
      CGATAAGGCTAGAGCTGCCAACGAGCGCCGTTACTTTG....ATGAAGAT

A3-gBol024345-XLOC_024343-10940-0
      CGGGATTAATTAATTGAGTGATCTTTGAAGTCGTCCTCTGAAGTCATCTC
A3-gBol024345-XLOC_024343-10940-1
      CGGGATTAATTAATTGAGTGATCTTTGAAGTCGTCCTCTGAAGTCATCTC
CONSENSUS
      CGGGATTAATTAATTGAGTGATCTTTGAAGTCGTCCTCTGAAGTCATCTC

A3-gBol024345-XLOC_024343-10940-0
      AAGTGCTTGTTTGTTTTGTTTGATCTTCCCTGTAAAAGCTTTTCTTAGAG
A3-gBol024345-XLOC_024343-10940-1
      AAGTGCTTGTTTGTTTTGTTTGATCTTCCCTGTAAAAGCTTTTCTTAGAG
CONSENSUS
      AAGTGCTTGTTTGTTTTGTTTGATCTTCCCTGTAAAAGCTTTTCTTAGAG

A3-gBol024345-XLOC_024343-10940-0
      CTGGTTATCTTTTGCTTAATGAACCTTAAGATTTGTGTTCTACAATCTGA
A3-gBol024345-XLOC_024343-10940-1
      CTGGTTATCTTTTGCTTAATGAACCTTAAGATTTGTGTTCTACAATCTGA
CONSENSUS
      CTGGTTATCTTTTGCTTAATGAACCTTAAGATTTGTGTTCTACAATCTGA

A3-gBol024345-XLOC_024343-10940-0
      ATCTGGCTAATAATTTCGAATGAGAA
A3-gBol024345-XLOC_024343-10940-1
      ATCTGGCTAATAATTTCGAATGAGAA
CONSENSUS
      ATCTGGCTAATAATTTCGAATGAGAA

alignment for event: A5-X-XLOC_024472-8981

A5-X-XLOC_024472-8981-0
      AAAGTAGATTTCTTGCAACTGAAAGTACTTTTCTTGAGCTTCTCTCTTTG
A5-X-XLOC_024472-8981-1
      AAAGTAGATTTCTTGCAACTGAAAGTACTTTTCTTGAGCTTCTCTCTTTG
CONSENSUS
      AAAGTAGATTTCTTGCAACTGAAAGTACTTTTCTTGAGCTTCTCTCTTTG

A5-X-XLOC_024472-8981-0
      GTTTCTTCTCAGTCTAATCGAAAACATCATTCTTCGATATTCATTTACTG
A5-X-XLOC_024472-8981-1
      GTTTCTTCTCAGTCTAATCGAAAACATCATTCTTCGATATTCATTTACTG
CONSENSUS

GTTTCTTCTCAGTCTAATCGAAAACATCATTCTTCGATATTCATTTACTG

A5-X-XLOC_024472-8981-0
AAGTTTTGAGTGAAGAAAAACCCTAGAAATAAGTACAATTTTTCTTTAAT
A5-X-XLOC_024472-8981-1
AAGTTTTGAGTGAAGAAAAACCCTAGAAATAAGTACAATTTTTCTTTAAT
CONSENSUS
AAGTTTTGAGTGAAGAAAAACCCTAGAAATAAGTACAATTTTTCTTTAAT

A5-X-XLOC_024472-8981-0
AGGTTGAAAATCTATGGCTGTTGAGTCCTCGCCGCCTTCTGCGACAGACT
A5-X-XLOC_024472-8981-1
AGGTTGAAAATCTATGGCTGTTGAGTCCTCGCCGCCTTCTGCGACAGACT
CONSENSUS
AGGTTGAAAATCTATGGCTGTTGAGTCCTCGCCGCCTTCTGCGACAGACT

A5-X-XLOC_024472-8981-0
TCGCTTTCTCCACGGTTTCGCTTTCATCCGACTGACTAGGAAGTCGTAAG
A5-X-XLOC_024472-8981-1
TCGCTTTCTCCACGGTTTCGCTTTCATCCGACTGACTAGGAAGTCGTAAG
CONSENSUS
TCGCTTTCTCCACGGTTTCGCTTTCATCCGACTGACTAGGAAGTCGTAAG

A5-X-XLOC_024472-8981-0
CTATAACTTGAAGATAAACGTTCTGGGTAAACCCGTACACTTCGATGCAA
A5-X-XLOC_024472-8981-1
CTATAACTTGAAGATAAACGTTCTGGGTAAACCCGTACACTTCGATGCAA
CONSENSUS
CTATAACTTGAAGATAAACGTTCTGGGTAAACCCGTACACTTCGATGCAA

A5-X-XLOC_024472-8981-0
TTGGGGAGGTTGATATCTACAAGCATGAGCCTTGGGACTTAGAAGGTTTG
A5-X-XLOC_024472-8981-1
TTGGGGAGGTTGATATCTACAAGCATGAGCCTTGGGACTTAGAAG-----
CONSENSUS
TTGGGGAGGTTGATATCTACAAGCATGAGCCTTGGGACTTAGAAG.....

A5-X-XLOC_024472-8981-0
TTGGGTTTCTTTGTCTCTTTTTTTTTGTTTACGTATATGTATGATTTGGT
A5-X-XLOC_024472-8981-1

CONSENSUS
....

A5-X-XLOC_024472-8981-0
TTCTCTGGTAGACCTGTGAGTTTGTTTCTTCTGATTTTGGATTCTTTGCA
A5-X-XLOC_024472-8981-1

CONSENSUS
....

A5-X-XLOC_024472-8981-0
GTTTGGTCTCTTAACTCAATTATGGGAAAAGAGCTTAGCTTTATGGACTG
A5-X-XLOC_024472-8981-1

CONSENSUS

....

A5-X-XLOC_024472-8981-0
AATTCCTTTTCTGGAGAAGTTTCGTTAAAGATTTACCCTTTTGCTATGTT
A5-X-XLOC_024472-8981-1

CONSENSUS
....

A5-X-XLOC_024472-8981-0
TAGACTATAAAACTTTGACGTAAATTGACTTATTAATTGTATAAATCTAG
A5-X-XLOC_024472-8981-1

CONSENSUS
....

A5-X-XLOC_024472-8981-0
TAATTTTTGGCAGATTCCTTAAGAGTTTCTTGCTATGTTTGCTTGTGTTT
A5-X-XLOC_024472-8981-1

CONSENSUS
....

A5-X-XLOC_024472-8981-0
CAGGGAATGAAGCTTTGTTGCTTTGTGGTTAGCCATTACTCTGAGGTTCA
A5-X-XLOC_024472-8981-1

CONSENSUS
....

A5-X-XLOC_024472-8981-0
TTCCAATTCTAGTCTATTGATTGCTTTTTCAATTATATTGATCTAAGAAC
A5-X-XLOC_024472-8981-1

CONSENSUS
....

A5-X-XLOC_024472-8981-0
TGTTTTCTTTGTTGTTACTTCGTGTTAGTGTTAATAACAATTTGACTTGA
A5-X-XLOC_024472-8981-1

CONSENSUS
....

A5-X-XLOC_024472-8981-0
ATAGCAGATAATTGTTACAAACATTAAATCAGTTTTTCGGATAGTTTGTC
A5-X-XLOC_024472-8981-1

CONSENSUS
....

A5-X-XLOC_024472-8981-0
AACATTACACGGCAATTAGAATGTTAACCATCTCCAGCATTACTTGACCA
A5-X-XLOC_024472-8981-1

CONSENSUS

....

A5-X-XLOC_024472-8981-0
TATTGCCGTTGTAGCTGGGGTATCAAAATTATTGTCTAGATAATATTATA
A5-X-XLOC_024472-8981-1

CONSENSUS
....

A5-X-XLOC_024472-8981-0
TTTACCATTTAGCAATACCGAAAAGAATTACTTTTCAGGAACAAATTCCA
A5-X-XLOC_024472-8981-1

CONSENSUS
....

A5-X-XLOC_024472-8981-0
TCAATATAGTCCTGCTTAATTGTTAATTGCATATTTTGTCTGAAAATAAG
A5-X-XLOC_024472-8981-1

CONSENSUS
....

A5-X-XLOC_024472-8981-0
AACATGTTTTTCGTTTCGCAAAATGTTGTTGAAGCTTTTGATGAAATGGA
A5-X-XLOC_024472-8981-1

CONSENSUS
....

A5-X-XLOC_024472-8981-0
TAGTCTCGTTAGAATGCATCCTTGTCTCTTTCAATCTCCATTAGCTACAA
A5-X-XLOC_024472-8981-1

CONSENSUS
....

A5-X-XLOC_024472-8981-0
ACATTCTTTAAAACACTTTTAAAGGGCTCACCTATGATCTCTGCACAAAG
A5-X-XLOC_024472-8981-1

CONSENSUS
....

A5-X-XLOC_024472-8981-0
AATGTGGTTTCTTTGTATTAAGTTCTTGAAAACGATAGGTGTAGCTTATG
A5-X-XLOC_024472-8981-1

CONSENSUS
....

A5-X-XLOC_024472-8981-0
CTTCAGCTGTTTAGTTATCTCGAGAGTGGGTGTTGTGATTTGCTCGGAGA
A5-X-XLOC_024472-8981-1

CONSENSUS

....

A5-X-XLOC_024472-8981-0
TCATGGTCTCAACCATTTTCTTCTGATACCATCACCACATAGAGACGAAG
A5-X-XLOC_024472-8981-1 -------------------------
ATACCATCACCACATAGAGACGAAG
CONSENSUS
.........................ATACCATCACCACATAGAGACGAAG

A5-X-XLOC_024472-8981-0
ATGATCAAGAAAGGAATGTGTATTTTTATAACACGGAGTGTGTCTTTAAA
A5-X-XLOC_024472-8981-1
ATGATCAAGAAAGGAATGTGTATTTTTATAACACGGAGTGTGTCTTTAAA
CONSENSUS
ATGATCAAGAAAGGAATGTGTATTTTTATAACACGGAGTGTGTCTTTAAA

A5-X-XLOC_024472-8981-0
GGCATACATTATTAGCTCAAAAGTAGTAACTGAGGAAGAGCTTTAAATCA
A5-X-XLOC_024472-8981-1
GGCATACATTATTAGCTCAAAAGTAGTAACTGAGGAAGAGCTTTAAATCA
CONSENSUS
GGCATACATTATTAGCTCAAAAGTAGTAACTGAGGAAGAGCTTTAAATCA

A5-X-XLOC_024472-8981-0
CTCTTCGAGCTGCAAAGGAAGGAATATATTCGCTCCAACGGAATTTAGAT
A5-X-XLOC_024472-8981-1
CTCTTCGAGCTGCAAAGGAAGGAATATATTCGCTCCAACGGAATTTAGAT
CONSENSUS
CTCTTCGAGCTGCAAAGGAAGGAATATATTCGCTCCAACGGAATTTAGAT

A5-X-XLOC_024472-8981-0
GCATCAAGCGATTTGTATTTATGTGTTAAAATTTTGATTAATTTTCTAAA
A5-X-XLOC_024472-8981-1
GCATCAAGCGATTTGTATTTATGTGTTAAAATTTTGATTAATTTTCTAAA
CONSENSUS
GCATCAAGCGATTTGTATTTATGTGTTAAAATTTTGATTAATTTTCTAAA

A5-X-XLOC_024472-8981-0
AAGTTGTTTAGCGCGATAAACAAATAGTTGAAAGTCACTTACAGATTTAT
A5-X-XLOC_024472-8981-1
AAGTTGTTTAGCGCGATAAACAAATAGTTGAAAGTCACTTACAGATTTAT
CONSENSUS
AAGTTGTTTAGCGCGATAAACAAATAGTTGAAAGTCACTTACAGATTTAT

A5-X-XLOC_024472-8981-0
TCTAGACAATAAAAGTTTTAGCCAGATATTATCTACATATAAATTCCAAA
A5-X-XLOC_024472-8981-1
TCTAGACAATAAAAGTTTTAGCCAGATATTATCTACATATAAATTCCAAA
CONSENSUS
TCTAGACAATAAAAGTTTTAGCCAGATATTATCTACATATAAATTCCAAA

A5-X-XLOC_024472-8981-0 TGAAGTTTAAAATTCAT
A5-X-XLOC_024472-8981-1 TGAAGTTTAAAATTCAT
CONSENSUS TGAAGTTTAAAATTCAT

```
alignment for event: RI-gBol038279-XLOC_008914-11648

RI-gBol038279-XLOC_008914-11648-0
      GTCTTCTGTGGTTTGGACAAAAAGCTTGCACCGAGAGAGCATTTCCCTTC
RI-gBol038279-XLOC_008914-11648-1
      GTCTTCTGTGGTTTGGACAAAAAGCTTGCACCGAGAGAGCATTTCCCTTC
CONSENSUS
      GTCTTCTGTGGTTTGGACAAAAAGCTTGCACCGAGAGAGCATTTCCCTTC

RI-gBol038279-XLOC_008914-11648-0
      TGTTAATTGTTTGATTTCCTACTCAAAGTATTCAACGGTAAGCTTACGCA
RI-gBol038279-XLOC_008914-11648-1
      TGTTAATTGTTTGATTTCCTACTCAAAGTATTCAACG-------------
CONSENSUS
      TGTTAATTGTTTGATTTCCTACTCAAAGTATTCAACG.............

RI-gBol038279-XLOC_008914-11648-0
      TCACCGTCCTTCTCCAGACCCGTCTTGGTATACTATCTCTAAGTTCTTGG
RI-gBol038279-XLOC_008914-11648-1
      --------------------------------------------------
CONSENSUS
      ..................................................

RI-gBol038279-XLOC_008914-11648-0
      TATATATATTTTTTTCTGTAATTGGAATCTTTCTATGAGAAGTTCGATCC
RI-gBol038279-XLOC_008914-11648-1
      ------------------------------------------TTCGATCC
CONSENSUS
      ..........................................TTCGATCC

RI-gBol038279-XLOC_008914-11648-0
      AGATTTCATCAACATCAGGACAAAGGCCAGAGAGGTGTTGCAGAGGGAAG
RI-gBol038279-XLOC_008914-11648-1
      AGATTTCATCAACATCAGGACAAAGGCCAGAGAGGTGTTGCAGAGGGAAG
CONSENSUS
      AGATTTCATCAACATCAGGACAAAGGCCAGAGAGGTGTTGCAGAGGGAAG

RI-gBol038279-XLOC_008914-11648-0     ATGATCTTAGTGAAATTGTCCAG
RI-gBol038279-XLOC_008914-11648-1     ATGATCTTAGTGAAATTGTCCAG
CONSENSUS                             ATGATCTTAGTGAAATTGTCCAG


alignment for event: RI-gBol026169-XLOC_022374-7567

RI-gBol026169-XLOC_022374-7567-0
      AAAATTGATTATTGGTTTCTAGATATCGAGTGGAGTTGGGCATAGCTGAT
RI-gBol026169-XLOC_022374-7567-1
      AAAATTGATTATTGGTTTCTAGATATCGAGTGGAGTTGGGCATAGCTGAT
CONSENSUS
      AAAATTGATTATTGGTTTCTAGATATCGAGTGGAGTTGGGCATAGCTGAT

RI-gBol026169-XLOC_022374-7567-0
      GATACTGCTGAGGGCGTTTTCGTTTGTTTTGATGGGGTGATGACGAAACT
RI-gBol026169-XLOC_022374-7567-1
```

GATACTGCTGAGGGCGTTTTCGTTTGTTTTGATGGGGTGATGACGAAACT
CONSENSUS
GATACTGCTGAGGGCGTTTTCGTTTGTTTTGATGGGGTGATGACGAAACT

RI-gBol026169-XLOC_022374-7567-0
TCACAACCTCAGAGCAAATGAAGCTGGTCATATGTTGGTAAGTTACATTA
RI-gBol026169-XLOC_022374-7567-1
TCACAACCTCAGAGCAAATGAAGCTGGTCATATGTTGGTAAGTTACATTA
CONSENSUS
TCACAACCTCAGAGCAAATGAAGCTGGTCATATGTTGGTAAGTTACATTA

RI-gBol026169-XLOC_022374-7567-0
TAGCTCTCAAGAGTCGTCCTCCCCGTGTAAGTTCTACCCAAGCGATCTTG
RI-gBol026169-XLOC_022374-7567-1
TAGCTCTCAAGAGTCGTCCTCCCCGTGTAAGTTCTACCCAAGCGATCTTG
CONSENSUS
TAGCTCTCAAGAGTCGTCCTCCCCGTGTAAGTTCTACCCAAGCGATCTTG

RI-gBol026169-XLOC_022374-7567-0
ATTTAACAACTAGGCTGACAGCTCCACGGCAAGTCATCAGACTTACACCA
RI-gBol026169-XLOC_022374-7567-1
ATTTAACAACTAGGCTGACAGCTCCACGGCAAGTCATCAGACTTACACCA
CONSENSUS
ATTTAACAACTAGGCTGACAGCTCCACGGCAAGTCATCAGACTTACACCA

RI-gBol026169-XLOC_022374-7567-0
TCATACGGATCCTTAACTGACGTGATCGTGCACCACTCCCTGATTTCGTT
RI-gBol026169-XLOC_022374-7567-1
TCATACGGATCCTTAACTGACGTGATCGTGCACCACTCCCTGATTTCGTT
CONSENSUS
TCATACGGATCCTTAACTGACGTGATCGTGCACCACTCCCTGATTTCGTT

RI-gBol026169-XLOC_022374-7567-0
GATAGTGTGAGTCTTATGATTGTTCTGTTGTGTCACTTTCATGTACTGTA
RI-gBol026169-XLOC_022374-7567-1
GATAGT--
CONSENSUS
GATAGT..

RI-gBol026169-XLOC_022374-7567-0
AATTAATATAGATTATTATACATGATCAGGGAAACAACGTGGACAAGGGG
RI-gBol026169-XLOC_022374-7567-1 -----------------------------
GGAAACAACGTGGACAAGGGG
CONSENSUS
.............................GGAAACAACGTGGACAAGGGG

RI-gBol026169-XLOC_022374-7567-0
AACGACATATAGAACCCATTACTCTACAATTCATCCTTACTTTAGTGGTT
RI-gBol026169-XLOC_022374-7567-1
AACGACATATAGAACCCATTACTCTACAATTCATCCTTACTTTAGTGGTT
CONSENSUS
AACGACATATAGAACCCATTACTCTACAATTCATCCTTACTTTAGTGGTT

RI-gBol026169-XLOC_022374-7567-0
TCAAGCCATGCATATTAATTAAGATTTTTGAGTGACCGTTTTAAGATATG
RI-gBol026169-XLOC_022374-7567-1

TCAAGCCATGCATATTAATTAAGATTTTTGAGTGACCGTTTTAAGATATG
CONSENSUS
TCAAGCCATGCATATTAATTAAGATTTTTGAGTGACCGTTTTAAGATATG

RI-gBol026169-XLOC_022374-7567-0
TTTTTTGGTCAATGTGTTTAGAGAAGCTAAATTAGTTTTGTTAGATTTAA
RI-gBol026169-XLOC_022374-7567-1
TTTTTTGGTCAATGTGTTTAGAGAAGCTAAATTAGTTTTGTTAGATTTAA
CONSENSUS
TTTTTTGGTCAATGTGTTTAGAGAAGCTAAATTAGTTTTGTTAGATTTAA

RI-gBol026169-XLOC_022374-7567-0
GAAAAGAATATAAAGATATATAATTGAATTTGCATTTTTCTTTGTTCTAC
RI-gBol026169-XLOC_022374-7567-1
GAAAAGAATATAAAGATATATAATTGAATTTGCATTTTTCTTTGTTCTAC
CONSENSUS
GAAAAGAATATAAAGATATATAATTGAATTTGCATTTTTCTTTGTTCTAC

RI-gBol026169-XLOC_022374-7567-0
AAGAGAGAATGATCTCTACTATATATAAGATTTTTGTAAGGA
RI-gBol026169-XLOC_022374-7567-1
AAGAGAGAATGATCTCTACTATATATAAGATTTTTGTAAGGA
CONSENSUS
AAGAGAGAATGATCTCTACTATATATAAGATTTTTGTAAGGA

alignment for event: RI-X-XLOC_026433-8813

RI-X-XLOC_026433-8813-0
CGAAGACATATATTAGCCTGCGATTTGAGAAACCAGCAAAAGGTGCGGCT
RI-X-XLOC_026433-8813-1
CGAAGACATATATTAGCCTGCGATTTGAGAAACCAGCAAAAGGTGCGGCT
CONSENSUS
CGAAGACATATATTAGCCTGCGATTTGAGAAACCAGCAAAAGGTGCGGCT

RI-X-XLOC_026433-8813-0
TCTCCACTGTACTTGATCTGCTTTCAATATTGTAAAAGGGTATGTGCAGG
RI-X-XLOC_026433-8813-1
TCTCCACTGTACTTGATCTGCTTTCAATATTGTAAAAGG-----------
CONSENSUS
TCTCCACTGTACTTGATCTGCTTTCAATATTGTAAAAGG...........

RI-X-XLOC_026433-8813-0
ACAAGAGAATCTAATTTGGCTATTAATCTATCTTGCATGTTCAGTTAATT
RI-X-XLOC_026433-8813-1

CONSENSUS
....

RI-X-XLOC_026433-8813-0
TATTAACTCCATTTTGGATTTTAGAAACTGTAGTCCTTTTTTGCAGTTTG
RI-X-XLOC_026433-8813-1

CONSENSUS
....

RI-X-XLOC_026433-8813-0
ATTTTTATTTTATTTTCTCTGTGGAGTTTGATTCCTATGATGAGTTTTTG
RI-X-XLOC_026433-8813-1

CONSENSUS
....

RI-X-XLOC_026433-8813-0
GTAGTCAATGGGATATCCAAGCTCATTCGTTCTACATATGTAGTAAAACG
RI-X-XLOC_026433-8813-1

CONSENSUS
....

RI-X-XLOC_026433-8813-0
CATCACTTTATTCCGTTTGATTTCTCCATAATTATATTTTTTTTTGTTTT
RI-X-XLOC_026433-8813-1

CONSENSUS
....

RI-X-XLOC_026433-8813-0
TGGTGTTTATTAACGGTATTTTTGTTGTTGAATTGACTAGATTTTAGTTG
RI-X-XLOC_026433-8813-1

CONSENSUS
....

RI-X-XLOC_026433-8813-0
TTCTTACGAGGTTCTGACTGCATCCAAGAAAAAAAAACTCAAGTTTTCAGA
RI-X-XLOC_026433-8813-1

CONSENSUS
....

RI-X-XLOC_026433-8813-0
TAAACTAACATGTTTCTTTGTTAGTATTTTGCTATGACTTATTTTCCATC
RI-X-XLOC_026433-8813-1

CONSENSUS
....

RI-X-XLOC_026433-8813-0
ACATGATCTTAGTCGTTTTTTGTTTACGGTGAGCTGATTGATTCATGACT
RI-X-XLOC_026433-8813-1

CONSENSUS
....

RI-X-XLOC_026433-8813-0
ATCCTACTTTGGTTCACATGATCTTAGTCGTACACAGGTCGCGCACAATA
RI-X-XLOC_026433-8813-1

CONSENSUS
....

```
RI-X-XLOC_026433-8813-0
      TACATCGTTTACATGACATCTAGCTTACATCTTCAGGTTTAGTTAACAGG
RI-X-XLOC_026433-8813-1
      --------------------------------------------------
CONSENSUS
      ..................................................

RI-X-XLOC_026433-8813-0
      CTTCCCTGAAGTACTAAGTCTGTGTGTGGTTAAGGAAAATGTTCATTCTC
RI-X-XLOC_026433-8813-1
      --------------------------------------------------
CONSENSUS
      ..................................................

RI-X-XLOC_026433-8813-0
      TGTGTTGTTGACTACTTCCTGCTATTCTGAGATATCTAATTTTAGGAACT
RI-X-XLOC_026433-8813-1
      --------------------------------------------------
CONSENSUS
      ..................................................

RI-X-XLOC_026433-8813-0
      GTGGCTCCAATTTAATATATACAACTTTATTTATCTTTGGTACAGAGCAG
RI-X-XLOC_026433-8813-1
      ---------------------------------------------AGCAG
CONSENSUS
      .............................................AGCAG

RI-X-XLOC_026433-8813-0
      AGGAAGAAGCTTGGTAAAGGAAAACACTTTCTCTTAACCCTTAGTTTTCT
RI-X-XLOC_026433-8813-1
      AGGAAGAAGCTTGGTAAAGGAAAACACTTTCTCTTAACCCTTAGTTTTCT
CONSENSUS
      AGGAAGAAGCTTGGTAAAGGAAAACACTTTCTCTTAACCCTTAGTTTTCT

RI-X-XLOC_026433-8813-0
      CTGTATCAAACTTTAAAAACATTTTATTATGTGATGAATGTCATGATAAA
RI-X-XLOC_026433-8813-1
      CTGTATCAAACTTTAAAAACATTTTATTATGTGATGAATGTCATGATAAA
CONSENSUS
      CTGTATCAAACTTTAAAAACATTTTATTATGTGATGAATGTCATGATAAA

RI-X-XLOC_026433-8813-0
      AATCTATAACTTTGTGTAGTGGTATTAATTTTTATTCAATTTTCTCTACA
RI-X-XLOC_026433-8813-1
      AATCTATAACTTTGTGTAGTGGTATTAATTTTTATTCAATTTTCTCTACA
CONSENSUS
      AATCTATAACTTTGTGTAGTGGTATTAATTTTTATTCAATTTTCTCTACA

RI-X-XLOC_026433-8813-0
      TTTTTTGGTATTTTCATCTGTGGAAAGGTGGAAGCTTTAAGGTTGCAAAA
RI-X-XLOC_026433-8813-1
      TTTTTTGGTATTTTCATCTGTGGAAAGGTGGAAGCTTTAAGGTTGCAAAA
CONSENSUS
      TTTTTTGGTATTTTCATCTGTGGAAAGGTGGAAGCTTTAAGGTTGCAAAA
```

```
RI-X-XLOC_026433-8813-0
      TGACATTTAATTTTTAACATCCGACTGTGACAGAATCGCATGCATAAGTG
RI-X-XLOC_026433-8813-1
      TGACATTTAATTTTTAACATCCGACTGTGACAGAATCGCATGCATAAGTG
CONSENSUS
      TGACATTTAATTTTTAACATCCGACTGTGACAGAATCGCATGCATAAGTG

RI-X-XLOC_026433-8813-0
      TATTCGACAACCTTGCGGAGCTCCGATGAGGAATTAGCAATGTTTTAAAA
RI-X-XLOC_026433-8813-1
      TATTCGACAACCTTGCGGAGCTCCGATGAGGAATTAGCAATGTTTTAAAA
CONSENSUS
      TATTCGACAACCTTGCGGAGCTCCGATGAGGAATTAGCAATGTTTTAAAA

RI-X-XLOC_026433-8813-0    ACCGGACCGGATACCGACTCGGCG
RI-X-XLOC_026433-8813-1    ACCGGACCGGATACCGACTCGGCG
CONSENSUS                  ACCGGACCGGATACCGACTCGGCG



alignment for event: RI-gBol021865-XLOC_027113-431

RI-gBol021865-XLOC_027113-431-0
      TTTGTAACAAAGAAGCTAGACAAAAGATTACTAGATCTTGGGGCCACAAC
RI-gBol021865-XLOC_027113-431-1
      TTTGTAACAAAGAAGCTAGACAAAAGATTACTAGATCTTGGGGCCACAAC
CONSENSUS
      TTTGTAACAAAGAAGCTAGACAAAAGATTACTAGATCTTGGGGCCACAAC

RI-gBol021865-XLOC_027113-431-0
      CATCATCGAGAAAGGTCTAGGAGATGATCAACACCCATCATGGTATGGAA
RI-gBol021865-XLOC_027113-431-1
      CATCATCGAGAAAGGTCTAGGAGATGATCAACACCCATCATG--------
CONSENSUS
      CATCATCGAGAAAGGTCTAGGAGATGATCAACACCCATCATG........

RI-gBol021865-XLOC_027113-431-0
      CGGTATATTATTGAGGAAAATAATGATTCTGTAAAATTTGATCTTCCCTT
RI-gBol021865-XLOC_027113-431-1
      --------------------------------------------------
CONSENSUS
      ..................................................

RI-gBol021865-XLOC_027113-431-0
      TATTCTTTTTTAGCACATGAACCATTTGATCAGTGGAAAATATAGTATCC
RI-gBol021865-XLOC_027113-431-1
      --------------------------------------------------
CONSENSUS
      ..................................................

RI-gBol021865-XLOC_027113-431-0
      ACGTTTTTTCCTGCTGCTTGTTTTGTACTATATGTCTAAGTGTTTGAATA
RI-gBol021865-XLOC_027113-431-1
      --------------------------------------------------
CONSENSUS
```

```
      .......................................

RI-gBol021865-XLOC_027113-431-0
      CTAAATTTTTGGTTTTGGTGTTTTCTTTATTTTTGAGGTATGAAGCAATA
RI-gBol021865-XLOC_027113-431-1
      ---------------------------------------------CAATA
CONSENSUS
      .............................................CAATA

RI-gBol021865-XLOC_027113-431-0
      TGTTGTCTTTGTGGAGTATTTTATATCAGATCAAGCCCATATACTTTCCT
RI-gBol021865-XLOC_027113-431-1
      TGTTGTCTTTGTGGAGTATTTTATATCAGATCAAGCCCATATACTTTCCT
CONSENSUS
      TGTTGTCTTTGTGGAGTATTTTATATCAGATCAAGCCCATATACTTTCCT

RI-gBol021865-XLOC_027113-431-0
      AATGGTCCAGAAGTGGTGATTCTTGACATATTTGTTGATTGGTGTTATTT
RI-gBol021865-XLOC_027113-431-1
      AATGGTCCAGAAGTGGTGATTCTTGACATATTTGTTGATTGGTGTTATTT
CONSENSUS
      AATGGTCCAGAAGTGGTGATTCTTGACATATTTGTTGATTGGTGTTATTT

RI-gBol021865-XLOC_027113-431-0
      GAGAAGGATCATTGATTACTCCCTGAAAAAAAAACTCGAGCTTTCAGATT
RI-gBol021865-XLOC_027113-431-1
      GAGAAGGATCATTGATTACTCCCTGAAAAAAAAACTCGAGCTTTCAGATT
CONSENSUS
      GAGAAGGATCATTGATTACTCCCTGAAAAAAAAACTCGAGCTTTCAGATT

RI-gBol021865-XLOC_027113-431-0
      ACCGGAATTAATACATCGTTTTACTTCAATCGATGGG
RI-gBol021865-XLOC_027113-431-1
      ACCGGAATTAATACATCGTTTTACTTCAATCGATGGG
CONSENSUS
      ACCGGAATTAATACATCGTTTTACTTCAATCGATGGG



alignment for event: A3-gBol021865-XLOC_027113-433

A3-gBol021865-XLOC_027113-433-0
      TTTGTAACAAAGAAGCTAGACAAAAGATTACTAGATCTTGGGGCCACAAC
A3-gBol021865-XLOC_027113-433-1
      TTTGTAACAAAGAAGCTAGACAAAAGATTACTAGATCTTGGGGCCACAAC
CONSENSUS
      TTTGTAACAAAGAAGCTAGACAAAAGATTACTAGATCTTGGGGCCACAAC

A3-gBol021865-XLOC_027113-433-0
      CATCATCGAGAAAGGTCTAGGAGATGATCAACACCCATCATGGTATGAAG
A3-gBol021865-XLOC_027113-433-1
      CATCATCGAGAAAGGTCTAGGAGATGATCAACACCCATCATG--------
CONSENSUS
      CATCATCGAGAAAGGTCTAGGAGATGATCAACACCCATCATG........

A3-gBol021865-XLOC_027113-433-0
```

CAATATGTTGTCTTTGTGGAGTATTTTATATCAGATCAAGCCCATATACT
A3-gBol021865-XLOC_027113-433-1
CAATATGTTGTCTTTGTGGAGTATTTTATATCAGATCAAGCCCATATACT
CONSENSUS
CAATATGTTGTCTTTGTGGAGTATTTTATATCAGATCAAGCCCATATACT

A3-gBol021865-XLOC_027113-433-0
TTCCTAATGGTCCAGAAGTGGTGATTCTTGACATATTTGTTGATTGGTGT
A3-gBol021865-XLOC_027113-433-1
TTCCTAATGGTCCAGAAGTGGTGATTCTTGACATATTTGTTGATTGGTGT
CONSENSUS
TTCCTAATGGTCCAGAAGTGGTGATTCTTGACATATTTGTTGATTGGTGT

A3-gBol021865-XLOC_027113-433-0
TATTTGAGAAGGATCATTGATTACTCCCTGAAAAAAAAACTCGAGCTTTC
A3-gBol021865-XLOC_027113-433-1
TATTTGAGAAGGATCATTGATTACTCCCTGAAAAAAAAACTCGAGCTTTC
CONSENSUS
TATTTGAGAAGGATCATTGATTACTCCCTGAAAAAAAAACTCGAGCTTTC

A3-gBol021865-XLOC_027113-433-0
AGATTACCGGAATTAATACATCGTTTTACTTCAATCGATGGG
A3-gBol021865-XLOC_027113-433-1
AGATTACCGGAATTAATACATCGTTTTACTTCAATCGATGGG
CONSENSUS
AGATTACCGGAATTAATACATCGTTTTACTTCAATCGATGGG

alignment for event: A5-gBol045301-XLOC_000367-46

A5-gBol045301-XLOC_000367-46-0
AATTCACGAAAGTTGCTGTTCGTACAGCGATTGGGTTCGTTGTGATGGGA
A5-gBol045301-XLOC_000367-46-1
AATTCACGAAAGTTGCTGTTCGTACAGCGATTGGGTTCGTTGTGATGGGA
CONSENSUS
AATTCACGAAAGTTGCTGTTCGTACAGCGATTGGGTTCGTTGTGATGGGA

A5-gBol045301-XLOC_000367-46-0
TTCGTTGGCTTCTTTGTGAAGCTCATCTTTATTCCAATCAACAACATAAT
A5-gBol045301-XLOC_000367-46-1
TTCGTTGGCTTCTTTGTGAAGCTCATCTTTATTCCAATCAACAACATAAT
CONSENSUS
TTCGTTGGCTTCTTTGTGAAGCTCATCTTTATTCCAATCAACAACATAAT

A5-gBol045301-XLOC_000367-46-0
CGTCGGTGCCACTTA-----------------------------------
A5-gBol045301-XLOC_000367-46-1
CGTCGGTGCCACTTAGTTATGTGAATGATGATGATTTTCCTATTCATCAC
CONSENSUS
CGTCGGTGCCACTTA...................................

A5-gBol045301-XLOC_000367-46-0 ----
GGAGCAAAGAACAAAATGGGAGAAAGGCAAAAACTAGTTTTGAGAA
A5-gBol045301-XLOC_000367-46-1
AATAGGAGCAAAGAACAAAATGGGAGAAAGGCAAAAACTAGTTTTGAGAA

```
CONSENSUS
      ....GGAGCAAAGAACAAAATGGGAGAAAGGCAAAAACTAGTTTTGAGAA

A5-gBol045301-XLOC_000367-46-0
      ATGAAAGCTAGAGCTTTGCTGTTTTCAACAATTCGCACGTTATGTTTATG
A5-gBol045301-XLOC_000367-46-1
      ATGAAAGCTAGAGCTTTGCTGTTTTCAACAATTCGCACGTTATGTTTATG
CONSENSUS
      ATGAAAGCTAGAGCTTTGCTGTTTTCAACAATTCGCACGTTATGTTTATG

A5-gBol045301-XLOC_000367-46-0
      TAATCGTATTCATTTGTTTCTTTCTCTTTTTTTTTTCTTTCATTTTGTTC
A5-gBol045301-XLOC_000367-46-1
      TAATCGTATTCATTTGTTTCTTTCTCTTTTTTTTTTCTTTCATTTTGTTC
CONSENSUS
      TAATCGTATTCATTTGTTTCTTTCTCTTTTTTTTTTCTTTCATTTTGTTC

A5-gBol045301-XLOC_000367-46-0
      ACGACTGAAAAACTGGACTCATCTTGAATGTAGATCAAACTAATTTAGAA
A5-gBol045301-XLOC_000367-46-1
      ACGACTGAAAAACTGGACTCATCTTGAATGTAGATCAAACTAATTTAGAA
CONSENSUS
      ACGACTGAAAAACTGGACTCATCTTGAATGTAGATCAAACTAATTTAGAA

A5-gBol045301-XLOC_000367-46-0   ACTTGT
A5-gBol045301-XLOC_000367-46-1   ACTTGT
CONSENSUS                        ACTTGT
```

alignment for event: RI-gBol016246-XLOC_033569-1018

```
RI-gBol016246-XLOC_033569-1018-0
      ATCTTGCGTCGGAAAGGATCGTTCAGAGCAATGTCTCCGGCTAAAGGTAC
RI-gBol016246-XLOC_033569-1018-1
      ATCTTGCGTCGGAAAGGATCGTTCAGAGCAATGTCTCCGGCTAAAG----
CONSENSUS
      ATCTTGCGTCGGAAAGGATCGTTCAGAGCAATGTCTCCGGCTAAAG....

RI-gBol016246-XLOC_033569-1018-0
      GATCTTTGGCGTTGTTAATGAGATTCAATACGTTCTTCTTGTTTGATTTG
RI-gBol016246-XLOC_033569-1018-1
      --------------------------------------------------
CONSENSUS
      ..................................................

RI-gBol016246-XLOC_033569-1018-0
      AATCTCTTACAAAAGACTTATGTTGAATGCAAACGGTAGTAACTAGTCTT
RI-gBol016246-XLOC_033569-1018-1
      --------------------------------------------------
CONSENSUS
      ..................................................

RI-gBol016246-XLOC_033569-1018-0
      GTATTGATGCTTTGTAACCACGTTTTGTAAAGTACTTAGCTTCACTTCTA
RI-gBol016246-XLOC_033569-1018-1
```

```
      --------------------------------------------------
CONSENSUS
      ..................................................

RI-gBol016246-XLOC_033569-1018-0
      GATGGTGTTCAAATGATCTGTAAGTATTATAACTCTCCGCCGCTGAGAAA
RI-gBol016246-XLOC_033569-1018-1
      --------------------------------------------------
CONSENSUS
      ..................................................

RI-gBol016246-XLOC_033569-1018-0
      TGAACTAGGGTTTCAGCTGCTTCACCTCTCCAGGTTCGTTCTGTCTCTTG
RI-gBol016246-XLOC_033569-1018-1
      --------------------------------------------------
CONSENSUS
      ..................................................

RI-gBol016246-XLOC_033569-1018-0
      CCTTCTCACTGCTACGTCCTGTAGCTGGAGCTGTTGCAGGAGATAGAAAG
RI-gBol016246-XLOC_033569-1018-1
      --------------------------------------------------
CONSENSUS
      ..................................................

RI-gBol016246-XLOC_033569-1018-0
      GGCTTTTGTGCATCTTGATTATGAGTACACTCACAAGCCTGAGCATGCTA
RI-gBol016246-XLOC_033569-1018-1
      --------------------------------------------------
CONSENSUS
      ..................................................

RI-gBol016246-XLOC_033569-1018-0
      GATCCCACTGTTAGCTGTTTGTTTGCTTCTTGTGTCGTTTAAAGCAGTGA
RI-gBol016246-XLOC_033569-1018-1
      --------------------------------------------------
CONSENSUS
      ..................................................

RI-gBol016246-XLOC_033569-1018-0
      AGAGATCTCTCTCTACGTCTAAATTCTTAAGAGCATCAAATCGTTTAGTA
RI-gBol016246-XLOC_033569-1018-1
      --------------------------------------------------
CONSENSUS
      ..................................................

RI-gBol016246-XLOC_033569-1018-0
      GACAATCACCTTTGTAAGTTGTAACTATATTTACTTTTTTCTTGTGCTTA
RI-gBol016246-XLOC_033569-1018-1
      --------------------------------------------------
CONSENSUS
      ..................................................

RI-gBol016246-XLOC_033569-1018-0
      AATTAGATAGTAGTAAAACTACAAAGATCATTTCAGTTTAGTTTGATTAT
RI-gBol016246-XLOC_033569-1018-1
```

CONSENSUS
....

RI-gBol016246-XLOC_033569-1018-0
GGTAGAAACTACAAATTATTGTAGTAACTAAAAATCAACTTTGAAATCAA
RI-gBol016246-XLOC_033569-1018-1

CONSENSUS
....

RI-gBol016246-XLOC_033569-1018-0
ATTCATAAATAAATTGGTAAAATTGAATAACCCCCAATTTTGACAAATAT
RI-gBol016246-XLOC_033569-1018-1

CONSENSUS
....

RI-gBol016246-XLOC_033569-1018-0
TTTAATATCACTAATTCAATAGTACATAATTTTAGGAAGAAAAAATAAAA
RI-gBol016246-XLOC_033569-1018-1

CONSENSUS
....

RI-gBol016246-XLOC_033569-1018-0
TCAAGAATCCCATAACAATTTTTTCGGCTTAGATTTATAATTTCATTAAA
RI-gBol016246-XLOC_033569-1018-1

CONSENSUS
....

RI-gBol016246-XLOC_033569-1018-0
ATGCAATAGCTTGATTGTGGTAAAAAACTACAAATAAAACAGAATTGAAA
RI-gBol016246-XLOC_033569-1018-1

CONSENSUS
....

RI-gBol016246-XLOC_033569-1018-0
ATTATGTCAGGTCTGGTTCTATTCAATTCACCGGTTTGGATAGATTAGTT
RI-gBol016246-XLOC_033569-1018-1

CONSENSUS
....

RI-gBol016246-XLOC_033569-1018-0
CAATTATAAGAGAACCGAAAACGAACCGAATCCTAATCGGTTTGGAATGA
RI-gBol016246-XLOC_033569-1018-1

CONSENSUS
....

RI-gBol016246-XLOC_033569-1018-0
AAAAAAGCGCGTGTGGTGGAATATGTTTGTTTTCATCCCACATCGGGAAC
RI-gBol016246-XLOC_033569-1018-1

```
      --------------------------------------------------
CONSENSUS
      ..................................................

RI-gBol016246-XLOC_033569-1018-0
      TTGAGGCGCATGAGTCATGCGGATGCTGCTATAAAAGGTCAAGGATCATC
RI-gBol016246-XLOC_033569-1018-1
      --------------------------------------------------
CONSENSUS
      ..................................................

RI-gBol016246-XLOC_033569-1018-0
      AACTATAAAAAATTGCCTTAAACTAATGAGTAAGGAAAACAAAGCGAGCG
RI-gBol016246-XLOC_033569-1018-1
      --------------------------------------------------
CONSENSUS
      ..................................................

RI-gBol016246-XLOC_033569-1018-0
      GTGAGAGCCGGTCGCAGCGCTAACGAAAAACATAAAAAGTGGCGCAAGAA
RI-gBol016246-XLOC_033569-1018-1
      --------------------------------------------------
CONSENSUS
      ..................................................

RI-gBol016246-XLOC_033569-1018-0
      TTTATGGAAGGGAAATAGGCTGGCCGAGTCGTTGACGCTGCTTACTTTTG
RI-gBol016246-XLOC_033569-1018-1
      --------------------------------------------------
CONSENSUS
      ..................................................

RI-gBol016246-XLOC_033569-1018-0
      CAGAACTCGACTCGCGCGGGCCTCCCTCATCCCCCAAATCTATTATACCT
RI-gBol016246-XLOC_033569-1018-1
      --------------------------------------------------
CONSENSUS
      ..................................................

RI-gBol016246-XLOC_033569-1018-0
      AAAATTATTTAAACTTTAGAGTACAAATGCAAACAAATGAATTTTGATTG
RI-gBol016246-XLOC_033569-1018-1
      --------------------------------------------------
CONSENSUS
      ..................................................

RI-gBol016246-XLOC_033569-1018-0
      ATCTCTGTCTTTGTGATTCACGTTTCTTCCTTTTTTTTTTTTTTTTTTTT
RI-gBol016246-XLOC_033569-1018-1
      --------------------------------------------------
CONSENSUS
      ..................................................

RI-gBol016246-XLOC_033569-1018-0
      TTTTTTTTATTGATTTGATTTTTACATGACTACGAATTCGTTAGCTGGGT
RI-gBol016246-XLOC_033569-1018-1
```

CONSENSUS
....

RI-gBol016246-XLOC_033569-1018-0
TTTTGGGTATTTTGTCGAATATGATCAAGCTTCATCCCTAACTTAGAGTT
RI-gBol016246-XLOC_033569-1018-1

CONSENSUS
....

RI-gBol016246-XLOC_033569-1018-0
AGGCCAAATTCGATCGTTTATAATTGAAATTGTGCTTCGGTATGTTTAGA
RI-gBol016246-XLOC_033569-1018-1

CONSENSUS
....

RI-gBol016246-XLOC_033569-1018-0
GATTTGGTTTCTGGAGTCATAATCTGCGATATTTGGCTTTGGTTGTTACA
RI-gBol016246-XLOC_033569-1018-1

CONSENSUS
....

RI-gBol016246-XLOC_033569-1018-0
CTTTGTCGTTTATGTTCTCTTTCGTTTCGTTTCACTGGATCTAGTTTCAG
RI-gBol016246-XLOC_033569-1018-1

CONSENSUS
....

RI-gBol016246-XLOC_033569-1018-0
CTGTTTCTTGTATAGATGTTTAATCTGAATTGATGATTCTTTGTAAGATT
RI-gBol016246-XLOC_033569-1018-1

CONSENSUS
....

RI-gBol016246-XLOC_033569-1018-0
TGGAGAAAGACAACTTTAGAGTGAATCTAGTGAGAAAACATAATAATTGA
RI-gBol016246-XLOC_033569-1018-1

CONSENSUS
....

RI-gBol016246-XLOC_033569-1018-0
TAGTTGGTTTTAACTACTAGAACTTATATCGACAGACTGATTTCAAGATA
RI-gBol016246-XLOC_033569-1018-1

CONSENSUS
....

RI-gBol016246-XLOC_033569-1018-0
GATATTTCACATTCTAGTCTGGGAATTTAAAAAGGTTTATACTTTGGTTG
RI-gBol016246-XLOC_033569-1018-1

CONSENSUS
....

RI-gBol016246-XLOC_033569-1018-0
TCTCCTTTTTCGTTAGCCTGATGATATTGGCATTTCCTTCTCTCATGTGT
RI-gBol016246-XLOC_033569-1018-1

CONSENSUS
....

RI-gBol016246-XLOC_033569-1018-0
AAGCTTGTTGTTTTAGCAACTGCTAGATTTTGAGAAAAAAAAAGATTTGA
RI-gBol016246-XLOC_033569-1018-1

CONSENSUS
....

RI-gBol016246-XLOC_033569-1018-0
TCTTTGTGTTGTACCAACCATTGTTCCTAGAGTTTTGAGGAAGCTTAGAT
RI-gBol016246-XLOC_033569-1018-1

CONSENSUS
....

RI-gBol016246-XLOC_033569-1018-0
TTTTATCCACAGATCTTTCTCTATTCTGATAATACGCTCTCAAATCTCTG
RI-gBol016246-XLOC_033569-1018-1

CONSENSUS
....

RI-gBol016246-XLOC_033569-1018-0
CCACTTGCCTGCTTTTAATTTGTCTACTTGTTAGTCTTTCCTCATATTTA
RI-gBol016246-XLOC_033569-1018-1

CONSENSUS
....

RI-gBol016246-XLOC_033569-1018-0
TAGAATCTATATACAAGGTTCTAATTTGATATCTAGTCATACCAAAAACC
RI-gBol016246-XLOC_033569-1018-1

CONSENSUS
....

RI-gBol016246-XLOC_033569-1018-0
ATTCACTGATGTTCATTCTTTAAATTTATCAGTTGATTTCTCAGCGGCAT
RI-gBol016246-XLOC_033569-1018-1
--------------------------------TTGATTTCTCAGCGGCAT
CONSENSUS
................................TTGATTTCTCAGCGGCAT

RI-gBol016246-XLOC_033569-1018-0
CCTTTGTCCGAAACAATGGAAGAAGCTCATTCAGTGAACAGTCCAAGGCG
RI-gBol016246-XLOC_033569-1018-1

CCTTTGTCCGAAACAATGGAAGAAGCTCATTCAGTGAACAGTCCAAGGCG
CONSENSUS
CCTTTGTCCGAAACAATGGAAGAAGCTCATTCAGTGAACAGTCCAAGGCG

RI-gBol016246-XLOC_033569-1018-0
GGTTCTTAGCTTCTCCAAGAGAAAGAAGCAAAAGCCCTTCGGTTTCCAGG
RI-gBol016246-XLOC_033569-1018-1
GGTTCTTAGCTTCTCCAAGAGAAAGAAGCAAAAGCCCTTCGGTTTCCAGG
CONSENSUS
GGTTCTTAGCTTCTCCAAGAGAAAGAAGCAAAAGCCCTTCGGTTTCCAGG

RI-gBol016246-XLOC_033569-1018-0
ATTCAGACAGCACAAGGTCATCTCCGTTCAGAGCATCACATGTTCAGGGA
RI-gBol016246-XLOC_033569-1018-1
ATTCAGACAGCACAAGGTCATCTCCGTTCAGAGCATCACATGTTCAGGGA
CONSENSUS
ATTCAGACAGCACAAGGTCATCTCCGTTCAGAGCATCACATGTTCAGGGA

RI-gBol016246-XLOC_033569-1018-0
CCTAAACCCTCTGAGGTGTATGGTTTTGTAGGCTCCATCTCTACCGTTGT
RI-gBol016246-XLOC_033569-1018-1
CCTAAACCCTCTGAGGTGTATGGTTTTGTAGGCTCCATCTCTACCGTTGT
CONSENSUS
CCTAAACCCTCTGAGGTGTATGGTTTTGTAGGCTCCATCTCTACCGTTGT

RI-gBol016246-XLOC_033569-1018-0
TGCCACATTGATCTTCTTGATATGGGCTTATGTACCTGATAAACTGTTGG
RI-gBol016246-XLOC_033569-1018-1
TGCCACATTGATCTTCTTGATATGGGCTTATGTACCTGATAAACTGTTGG
CONSENSUS
TGCCACATTGATCTTCTTGATATGGGCTTATGTACCTGATAAACTGTTGG

RI-gBol016246-XLOC_033569-1018-0
AGTCTATAGGGATCCATTACTACCCAAGCAGGTACTGGGTGTTGGCTATG
RI-gBol016246-XLOC_033569-1018-1
AGTCTATAGGGATCCATTACTACCCAAGCAGGTACTGGGTGTTGGCTATG
CONSENSUS
AGTCTATAGGGATCCATTACTACCCAAGCAGGTACTGGGTGTTGGCTATG

RI-gBol016246-XLOC_033569-1018-0
CCCACGTACCTGATGGTGACTTTGCTGCTAGGTTTGGCGTTTTACATTGG
RI-gBol016246-XLOC_033569-1018-1
CCCACGTACCTGATGGTGACTTTGCTGCTAGGTTTGGCGTTTTACATTGG
CONSENSUS
CCCACGTACCTGATGGTGACTTTGCTGCTAGGTTTGGCGTTTTACATTGG

RI-gBol016246-XLOC_033569-1018-0
TCTCAACTTCATCGCCACTCCACACCCTACCTCTCTCAATACCTTGTTTG
RI-gBol016246-XLOC_033569-1018-1
TCTCAACTTCATCGCCACTCCACACCCTACCTCTCTCAATACCTTGTTTG
CONSENSUS
TCTCAACTTCATCGCCACTCCACACCCTACCTCTCTCAATACCTTGTTTG

alignment for event: SE-gBol003749-XLOC_047704-3700

SE-gBol003749-XLOC_047704-3700-0
      TTGGAGCTCTTAGAAGATTCAAGGAAGCTATCTATGAGGACCCTTTGCTC
SE-gBol003749-XLOC_047704-3700-1
      TTGGAGCTCTTAGAAGATTCAAGGAAGCTATCTATGAGGACCCTTTGCTC
CONSENSUS
      TTGGAGCTCTTAGAAGATTCAAGGAAGCTATCTATGAGGACCCTTTGCTC

SE-gBol003749-XLOC_047704-3700-0
      GTTATGTCTAACTGGAATGACCACCCCAGTTCAGATCCTTGTGCTTGGAC
SE-gBol003749-XLOC_047704-3700-1
      GTTATGTCTAACTGGAATGACCACCCCAGTTCAGATCCTTGTGCTTGGAC
CONSENSUS
      GTTATGTCTAACTGGAATGACCACCCCAGTTCAGATCCTTGTGCTTGGAC

SE-gBol003749-XLOC_047704-3700-0
      CGGCATTGCTTGCTCTCCCTCTAAAGACCATGTTATCAACAT--------
SE-gBol003749-XLOC_047704-3700-1
      CGGCATTGCTTGCTCTCCCTCTAAAGACCATGTTATCAACATTATATCTG
CONSENSUS
      CGGCATTGCTTGCTCTCCCTCTAAAGACCATGTTATCAACAT........

SE-gBol003749-XLOC_047704-3700-0

SE-gBol003749-XLOC_047704-3700-1
      CTTCATCTATAAAAGGGTTTCTCGCTCCTGAGTTATGTCAAATCACCTAC
CONSENSUS
      ....

SE-gBol003749-XLOC_047704-3700-0 -----------
AATCCTACATGGGAACCTTCTACTGGGGACAATACCAAA
SE-gBol003749-XLOC_047704-3700-1
      CTTCAGGAACTAATCCTACATGGGAACCTTCTACTGGGGACAATACCAAA
CONSENSUS
      ...........AATCCTACATGGGAACCTTCTACTGGGGACAATACCAAA

SE-gBol003749-XLOC_047704-3700-0
      GGAGATTGGGAAGTGGAAGAATCTCAAGATCTTGGACTTGGGAAACAATC
SE-gBol003749-XLOC_047704-3700-1
      GGAGATTGGGAAGTGGAAGAATCTCAAGATCTTGGACTTGGGAAACAATC
CONSENSUS
      GGAGATTGGGAAGTGGAAGAATCTCAAGATCTTGGACTTGGGAAACAATC

SE-gBol003749-XLOC_047704-3700-0
      ATCTCATGGGACCTATTCCTGCTGAGATCGGGAGCTTGTCCAGCATTACG
SE-gBol003749-XLOC_047704-3700-1
      ATCTCATGGGACCTATTCCTGCTGAGATCGGGAGCTTGTCCAGCATTACG
CONSENSUS
      ATCTCATGGGACCTATTCCTGCTGAGATCGGGAGCTTGTCCAGCATTACG

SE-gBol003749-XLOC_047704-3700-0 ATAAT
SE-gBol003749-XLOC_047704-3700-1 ATAAT
CONSENSUS   ATAAT

alignment for event: SE-gBol045874-XLOC_001159-8322

SE-gBol045874-XLOC_001159-8322-0
GTGGCAAACGAGTAGTAAATGAGAAGATCGAAAACATCACTAAAATTTAA
SE-gBol045874-XLOC_001159-8322-1
GTGGCAAACGAGTAGTAAATGAGAAGATCGAAAACATCACTAAAATTTAA
CONSENSUS
GTGGCAAACGAGTAGTAAATGAGAAGATCGAAAACATCACTAAAATTTAA

SE-gBol045874-XLOC_001159-8322-0
GGAAGAAAAAGAAAAGCGAACGGCCACTGCTTTTAATGCACGTGGCAGCT
SE-gBol045874-XLOC_001159-8322-1
GGAAGAAAAAGAAAAGCGAACGGCCACTGCTTTTAATGCACGTGGCAGCT
CONSENSUS
GGAAGAAAAAGAAAAGCGAACGGCCACTGCTTTTAATGCACGTGGCAGCT

SE-gBol045874-XLOC_001159-8322-0
TTCTCAGATTCCCGACAACGTTCCAGGGGTAAAACTGGAATTTCAATGGT
SE-gBol045874-XLOC_001159-8322-1
TTCTCAGATTCCCGACAACGTTCCAGGGGTAAAACTGGAATTTCAATGGT
CONSENSUS
TTCTCAGATTCCCGACAACGTTCCAGGGGTAAAACTGGAATTTCAATGGT

SE-gBol045874-XLOC_001159-8322-0
ATCGTTCTTCCACCGCACTCTCGGCCCGGCGAATAGAAAAAAGGAGTGTG
SE-gBol045874-XLOC_001159-8322-1
ATCGTTCTTCCACCGCACTCTCGGCCCGGCGAATAGAAAAAAGGAGTGTG
CONSENSUS
ATCGTTCTTCCACCGCACTCTCGGCCCGGCGAATAGAAAAAAGGAGTGTG

SE-gBol045874-XLOC_001159-8322-0
AAAGAGAGAGAAAGAGAGAGAGGCGCTTCCCTACGAGATTCGCGGGACCA
SE-gBol045874-XLOC_001159-8322-1
AAAGAGAGAGAAAGAGAGAGAGGCGCTTCCCTACGAGATTCGCGGGACCA
CONSENSUS
AAAGAGAGAGAAAGAGAGAGAGGCGCTTCCCTACGAGATTCGCGGGACCA

SE-gBol045874-XLOC_001159-8322-0
AATCAGTGTCCCCATTGGTCCCTCCCATGGCGATTTTTTAGAGAGCAGCA
SE-gBol045874-XLOC_001159-8322-1
AATCAGTGTCCCCATTGGTCCCTCCCATGGCGATTTTTTAGAGAGCAGCA
CONSENSUS
AATCAGTGTCCCCATTGGTCCCTCCCATGGCGATTTTTTAGAGAGCAGCA

SE-gBol045874-XLOC_001159-8322-0
GAGGAAGAAGAAGACGACGAAGCCGACGAAGAGGAAGAAGAAGGAGGAG-
SE-gBol045874-XLOC_001159-8322-1
GAGGAAGAAGAAGACGACGAAGCCGACGAAGAGGAAGAAGAAGGAGGAGG
CONSENSUS
GAGGAAGAAGAAGACGACGAAGCCGACGAAGAGGAAGAAGAAGGAGGAG.

SE-gBol045874-XLOC_001159-8322-0

SE-gBol045874-XLOC_001159-8322-1
TTCCTCATATTCCACCTTCACCGTGCTAATCACTTTTCGCCTAATTTTAT
CONSENSUS

```
      ..........................................

SE-gBol045874-XLOC_001159-8322-0 ------------
TCTTTCATATCAGAGCTTCTGGAATCACTTTGGGGAAG
SE-gBol045874-XLOC_001159-8322-1
      AGATCGATTTTGTCTTTCATATCAGAGCTTCTGGAATCACTTTGGGGAAG
CONSENSUS
      ............TCTTTCATATCAGAGCTTCTGGAATCACTTTGGGGAAG

SE-gBol045874-XLOC_001159-8322-0 AAGACAAAACCACTCATTAG
SE-gBol045874-XLOC_001159-8322-1 AAGACAAAACCACTCATTAG
CONSENSUS                        AAGACAAAACCACTCATTAG



alignment for event: A5-X-XLOC_000860-4840

A5-X-XLOC_000860-4840-0
      GTACCGTGTGGAACTGTCGTTGTCATACCACGCAAATATTGTATCAG---
A5-X-XLOC_000860-4840-1
      GTACCGTGTGGAACTGTCGTTGTCATACCACGCAAATATTGTATCAGGTC
CONSENSUS
      GTACCGTGTGGAACTGTCGTTGTCATACCACGCAAATATTGTATCAG...

A5-X-XLOC_000860-4840-0     --
GTAACAGCTAGGGACAGCGCTCCAGTTTTTAAAAATAACGGCCAAATC
A5-X-XLOC_000860-4840-1
      AAGTAACAGCTAGGGACAGCGCTCCAGTTTTTAAAAATAACGGCCAAATC
CONSENSUS
      ..GTAACAGCTAGGGACAGCGCTCCAGTTTTTAAAAATAACGGCCAAATC

A5-X-XLOC_000860-4840-0
      AACTGACTGGAGACAAATCTTAAAAGGACAGCAACATTGCTAGGAATAGC
A5-X-XLOC_000860-4840-1
      AACTGACTGGAGACAAATCTTAAAAGGACAGCAACATTGCTAGGAATAGC
CONSENSUS
      AACTGACTGGAGACAAATCTTAAAAGGACAGCAACATTGCTAGGAATAGC

A5-X-XLOC_000860-4840-0
      TATCAAAAAGGTCGACACAAAATACATAGATTGCTTTGTTTTTGAATTAA
A5-X-XLOC_000860-4840-1
      TATCAAAAAGGTCGACACAAAATACATAGATTGCTTTGTTTTTGAATTAA
CONSENSUS
      TATCAAAAAGGTCGACACAAAATACATAGATTGCTTTGTTTTTGAATTAA

A5-X-XLOC_000860-4840-0
      TATCTAAAGAGAAAGAGGTTCATCCTCCCACGTTTCTTAAAACAACAAGA
A5-X-XLOC_000860-4840-1
      TATCTAAAGAGAAAGAGGTTCATCCTCCCACGTTTCTTAAAACAACAAGA
CONSENSUS
      TATCTAAAGAGAAAGAGGTTCATCCTCCCACGTTTCTTAAAACAACAAGA

A5-X-XLOC_000860-4840-0
      ACAAATTCGTTTAGTACACATCTTGGTTCATAGAGTCAAAGCTTTGGTAC
A5-X-XLOC_000860-4840-1
      ACAAATTCGTTTAGTACACATCTTGGTTCATAGAGTCAAAGCTTTGGTAC
```

CONSENSUS
ACAAATTCGTTTAGTACACATCTTGGTTCATAGAGTCAAAGCTTTGGTAC

A5-X-XLOC_000860-4840-0
ATGAATTTCTTCAAACTGTTTCTCTTCATAGGTCTTAATTCCACTATCAC
A5-X-XLOC_000860-4840-1
ATGAATTTCTTCAAACTGTTTCTCTTCATAGGTCTTAATTCCACTATCAC
CONSENSUS
ATGAATTTCTTCAAACTGTTTCTCTTCATAGGTCTTAATTCCACTATCAC

A5-X-XLOC_000860-4840-0 TTGAAACAACGAG
A5-X-XLOC_000860-4840-1 TTGAAACAACGAG
CONSENSUS TTGAAACAACGAG

alignment for event: A3-X-XLOC_000860-4846

A3-X-XLOC_000860-4846-0
GTTCTATCGGGCTTGGATGTTTTCCTCTGAGATATGGAGCAACTTTTTGT
A3-X-XLOC_000860-4846-1
GTTCTATCGGGCTTGGATGTTTTCCTCTGAGATATGGAGCAACTTTTTGT
CONSENSUS
GTTCTATCGGGCTTGGATGTTTTCCTCTGAGATATGGAGCAACTTTTTGT

A3-X-XLOC_000860-4846-0
CTGGCCAAGCTTTTACCATTGTCGGCCTCACTCATTCCAAGAAGCAATCC
A3-X-XLOC_000860-4846-1
CTGGCCAAGCTTTTACCATTGTCGGCCTCACTCATTCCAAGAAGCAATCC
CONSENSUS
CTGGCCAAGCTTTTACCATTGTCGGCCTCACTCATTCCAAGAAGCAATCC

A3-X-XLOC_000860-4846-0
ACTAGGTGTTTCTTAATGTTGGTTTGCTCTTATCGTGCATGTAGATATGA
A3-X-XLOC_000860-4846-1
ACTAGGTGTTTCTTAATGTTGGTTTGCTCTTATCGTGCATGTAGATATGA
CONSENSUS
ACTAGGTGTTTCTTAATGTTGGTTTGCTCTTATCGTGCATGTAGATATGA

A3-X-XLOC_000860-4846-0
ACAAGAGCAGTTTGACAATTTAACAGGTGATGGAACAGAGAAAACAGCAT
A3-X-XLOC_000860-4846-1 ACAAGAGCAGTTTGACAA--------
GTGATGGAACAGAGAAAACAGCAT
CONSENSUS
ACAAGAGCAGTTTGACAA........GTGATGGAACAGAGAAAACAGCAT

A3-X-XLOC_000860-4846-0
CTTCATCAAAGGTGGTTCATGCCCAGAAAATTGAACAACCGTTTCTAGCT
A3-X-XLOC_000860-4846-1
CTTCATCAAAGGTGGTTCATGCCCAGAAAATTGAACAACCGTTTCTAGCT
CONSENSUS
CTTCATCAAAGGTGGTTCATGCCCAGAAAATTGAACAACCGTTTCTAGCT

A3-X-XLOC_000860-4846-0 AAATGAATATGTTATCACTGCACACCCTCAG
A3-X-XLOC_000860-4846-1 AAATGAATATGTTATCACTGCACACCCTCAG
CONSENSUS AAATGAATATGTTATCACTGCACACCCTCAG

alignment for event: RI-X-XLOC_000860-4833

RI-X-XLOC_000860-4833-0
      GTTCTATCGGGCTTGGATGTTTTCCTCTGAGATATGGAGCAACTTTTTGT
RI-X-XLOC_000860-4833-1
      GTTCTATCGGGCTTGGATGTTTTCCTCTGAGATATGGAGCAACTTTTTGT
CONSENSUS
      GTTCTATCGGGCTTGGATGTTTTCCTCTGAGATATGGAGCAACTTTTTGT

RI-X-XLOC_000860-4833-0
      CTGGCCAAGCTTTTACCATTGTCGGCCTCACTCATTCCAAGAAGCAATCC
RI-X-XLOC_000860-4833-1
      CTGGCCAAGCTTTTACCATTGTCGGCCTCACTCATTCCAAGAAGCAATCC
CONSENSUS
      CTGGCCAAGCTTTTACCATTGTCGGCCTCACTCATTCCAAGAAGCAATCC

RI-X-XLOC_000860-4833-0
      ACTAGGTGTTTCTTAATGTTGGTTTGCTCTTATCGTGCATGTAGATATGA
RI-X-XLOC_000860-4833-1
      ACTAGGTGTTTCTTAATGTTGGTTTGCTCTTATCGTGCATGTAGATATGA
CONSENSUS
      ACTAGGTGTTTCTTAATGTTGGTTTGCTCTTATCGTGCATGTAGATATGA

RI-X-XLOC_000860-4833-0
      ACAAGAGCAGTTTGACAAGCAAGTTCTCCTTATTCTAATTAGATAAACTC
RI-X-XLOC_000860-4833-1
      ACAAGAGCAGTTTGACAA--------------------------------
CONSENSUS
      ACAAGAGCAGTTTGACAA................................

RI-X-XLOC_000860-4833-0
      CACCTGATATATTGGCATCTTCTTCCCTCTTATTTTGACTGCCGTTTGAC
RI-X-XLOC_000860-4833-1

CONSENSUS
      ....

RI-X-XLOC_000860-4833-0
      ACTTATCCTCTTTCAGTTTAACAGGTGATGGAACAGAGAAAACAGCATCT
RI-X-XLOC_000860-4833-1   ------------------------
GTGATGGAACAGAGAAAACAGCATCT
CONSENSUS
      ........................GTGATGGAACAGAGAAAACAGCATCT

RI-X-XLOC_000860-4833-0
      TCATCAAAGGTGGTTCATGCCCAGAAAATTGAACAACCGTTTCTAGCTAA
RI-X-XLOC_000860-4833-1
      TCATCAAAGGTGGTTCATGCCCAGAAAATTGAACAACCGTTTCTAGCTAA
CONSENSUS
      TCATCAAAGGTGGTTCATGCCCAGAAAATTGAACAACCGTTTCTAGCTAA

RI-X-XLOC_000860-4833-0   ATGAATATGTTATCACTGCACACCCTCAG
RI-X-XLOC_000860-4833-1   ATGAATATGTTATCACTGCACACCCTCAG

```
CONSENSUS                    ATGAATATGTTATCACTGCACACCCTCAG


alignment for event: SE-gBol020565-XLOC_028660-7621

SE-gBol020565-XLOC_028660-7621-0
      TGATGGAAAGACGGTTGGAGCCTGCCTTGACCGCAATGGTCTTCGGCCTG
SE-gBol020565-XLOC_028660-7621-1
      TGATGGAAAGACGGTTGGAGCCTGCCTTGACCGCAATGGTCTTCGGCCTG
CONSENSUS
      TGATGGAAAGACGGTTGGAGCCTGCCTTGACCGCAATGGTCTTCGGCCTG

SE-gBol020565-XLOC_028660-7621-0
      CTAGATATTGGCGGACAAGTGATAATGTTGTCTATGTAGCCTCAGAG---
SE-gBol020565-XLOC_028660-7621-1
      CTAGATATTGGCGGACAAGTGATAATGTTGTCTATGTAGCCTCAGAGATG
CONSENSUS
      CTAGATATTGGCGGACAAGTGATAATGTTGTCTATGTAGCCTCAGAG...

SE-gBol020565-XLOC_028660-7621-0
      --------------------------------------------GTCGGT
SE-gBol020565-XLOC_028660-7621-1
      ACTGGGCTTATGCCTGAATCTTAAAGCTTTGGTCCTGACAGGTCGTCGGT
CONSENSUS
      ............................................GTCGGT

SE-gBol020565-XLOC_028660-7621-0
      GTTCTTCCAATGGATGAATCAAAAGTCACCATGAAGGGTCGTCTAGGACC
SE-gBol020565-XLOC_028660-7621-1
      GTTCTTCCAATGGATGAATCAAAAGTCACCATGAAGGGTCGTCTAGGACC
CONSENSUS
      GTTCTTCCAATGGATGAATCAAAAGTCACCATGAAGGGTCGTCTAGGACC

SE-gBol020565-XLOC_028660-7621-0
      TGGCATGATGATATCTGTCGACTTGGAAAGTGGACAG
SE-gBol020565-XLOC_028660-7621-1
      TGGCATGATGATATCTGTCGACTTGGAAAGTGGACAG
CONSENSUS
      TGGCATGATGATATCTGTCGACTTGGAAAGTGGACAG



alignment for event: A5-gBol013748-XLOC_036311-1358

A5-gBol013748-XLOC_036311-1358-0
      AGGATATATGGCTCCTGAGTATGCGTGGACTGGTACGTTCTCAGAGAAGT
A5-gBol013748-XLOC_036311-1358-1
      AGGATATATGGCTCCTGAGTATGCGTGGACTGGTACGTTCTCAGAGAAGT
CONSENSUS
      AGGATATATGGCTCCTGAGTATGCGTGGACTGGTACGTTCTCAGAGAAGT

A5-gBol013748-XLOC_036311-1358-0
      CAGACATTTACAGCTTCGGAGTTCTATTGTTAGAAATCATCAGCGGAGAG
A5-gBol013748-XLOC_036311-1358-1
      CAGACATTTACAGCTTCGGAGTTCTATTGTTAGAAATCATCAGCGGAGAG
```

CONSENSUS
      CAGACATTTACAGCTTCGGAGTTCTATTGTTAGAAATCATCAGCGGAGAG

A5-gBol013748-XLOC_036311-1358-0
      AAAATCTCAAGATTTAGCTATGGCGAAGAAGGAAAAACCCTTCTTGCATA
A5-gBol013748-XLOC_036311-1358-1
      AAAATCTCAAGATTTAGCTATGGCGAAGAAGGAAAAACCCTTCTTGCATA
CONSENSUS
      AAAATCTCAAGATTTAGCTATGGCGAAGAAGGAAAAACCCTTCTTGCATA

A5-gBol013748-XLOC_036311-1358-0
      TGTAAGCATGGGAATCTTGGTATGAAAATGGAGGAATCGATCTTTTGGAT
A5-gBol013748-XLOC_036311-1358-1 T----
GCATGGGAATCTTGGTATGAAAATGGAGGAATCGATCTTTTGGAT
CONSENSUS
      T....GCATGGGAATCTTGGTATGAAAATGGAGGAATCGATCTTTTGGAT

A5-gBol013748-XLOC_036311-1358-0
      AAAGATGTTGCTAACTCATGTCAGCCATTAGAAGTTAAGAGATGTGTTCA
A5-gBol013748-XLOC_036311-1358-1
      AAAGATGTTGCTAACTCATGTCAGCCATTAGAAGTTAAGAGATGTGTTCA
CONSENSUS
      AAAGATGTTGCTAACTCATGTCAGCCATTAGAAGTTAAGAGATGTGTTCA

A5-gBol013748-XLOC_036311-1358-0
      GATTGGTCTGCTCTGTGTTCAACATCAACCTGCAGACAGACCCAATACAC
A5-gBol013748-XLOC_036311-1358-1
      GATTGGTCTGCTCTGTGTTCAACATCAACCTGCAGACAGACCCAATACAC
CONSENSUS
      GATTGGTCTGCTCTGTGTTCAACATCAACCTGCAGACAGACCCAATACAC

A5-gBol013748-XLOC_036311-1358-0
      TTGAGTTGATGTCTCTGCTCACCACTACATCAGACCTTCAATCACCAGAA
A5-gBol013748-XLOC_036311-1358-1
      TTGAGTTGATGTCTCTGCTCACCACTACATCAGACCTTCAATCACCAGAA
CONSENSUS
      TTGAGTTGATGTCTCTGCTCACCACTACATCAGACCTTCAATCACCAGAA

A5-gBol013748-XLOC_036311-1358-0
      CAACCCACATTTTCAGTGCACAAGAGAGATGACAGATACTTGTGTAAGGG
A5-gBol013748-XLOC_036311-1358-1
      CAACCCACATTTTCAGTGCACAAGAGAGATGACAGATACTTGTGTAAGGG
CONSENSUS
      CAACCCACATTTTCAGTGCACAAGAGAGATGACAGATACTTGTGTAAGGG

A5-gBol013748-XLOC_036311-1358-0
      TTTGAGCACTGTCAATGAGATAACACAATCTGCTATCCTTGGGCGTTAAG
A5-gBol013748-XLOC_036311-1358-1
      TTTGAGCACTGTCAATGAGATAACACAATCTGCTATCCTTGGGCGTTAAG
CONSENSUS
      TTTGAGCACTGTCAATGAGATAACACAATCTGCTATCCTTGGGCGTTAAG

A5-gBol013748-XLOC_036311-1358-0
      AAACGATGTGTAATGGAAGTTGTTGAGTTAAATTGATTTTTTTTCTTTCA
A5-gBol013748-XLOC_036311-1358-1
      AAACGATGTGTAATGGAAGTTGTTGAGTTAAATTGATTTTTTTTCTTTCA

```
CONSENSUS
      AAACGATGTGTAATGGAAGTTGTTGAGTTAAATTGATTTTTTTTCTTTCA

A5-gBol013748-XLOC_036311-1358-0
      ATGTTGATAACTTCAACAACATCACGCTCTTTCATTTTGATACATTTTGG
A5-gBol013748-XLOC_036311-1358-1
      ATGTTGATAACTTCAACAACATCACGCTCTTTCATTTTGATACATTTTGG
CONSENSUS
      ATGTTGATAACTTCAACAACATCACGCTCTTTCATTTTGATACATTTTGG

A5-gBol013748-XLOC_036311-1358-0
      AACAAAATGGTTCTTAAGTTAGTGTTTATTTTATTTTTGAAAAATTCACT
A5-gBol013748-XLOC_036311-1358-1
      AACAAAATGGTTCTTAAGTTAGTGTTTATTTTATTTTTGAAAAATTCACT
CONSENSUS
      AACAAAATGGTTCTTAAGTTAGTGTTTATTTTATTTTTGAAAAATTCACT

A5-gBol013748-XLOC_036311-1358-0 AGGATCTCAAGGTTAAGA
A5-gBol013748-XLOC_036311-1358-1 AGGATCTCAAGGTTAAGA
CONSENSUS                        AGGATCTCAAGGTTAAGA



alignment for event: RI-gBol028200-XLOC_020195-6635

RI-gBol028200-XLOC_020195-6635-0
      ATCTGCTCTTGGATTTCGGATTCTGCTGCAAACGAAGAAAGCCAAAGTGT
RI-gBol028200-XLOC_020195-6635-1
      ATCTGCTCTTGGATTTCGGATTCTGCTGCAAACGAAGAAAGCCAAAGTGT
CONSENSUS
      ATCTGCTCTTGGATTTCGGATTCTGCTGCAAACGAAGAAAGCCAAAGTGT

RI-gBol028200-XLOC_020195-6635-0
      TCATCTGTTCAGTGTAAGAAACTCTAGAAATTTTCTCAGGAAACTATGGT
RI-gBol028200-XLOC_020195-6635-1
      TCATCTGTTCAGT-------------------------------------
CONSENSUS
      TCATCTGTTCAGT.....................................

RI-gBol028200-XLOC_020195-6635-0
      TTTCTTTGAAGCTGAGAATAGTAAAATGAGTCCGAATTAAAAGGTTAGCA
RI-gBol028200-XLOC_020195-6635-1
      --------------------------------------------------
CONSENSUS
      ..................................................

RI-gBol028200-XLOC_020195-6635-0
      AAATTCATTCTTTTAATAGCAAGATTTTAATTTTTGAATGTTTTTTCATG
RI-gBol028200-XLOC_020195-6635-1
      --------------------------------------------------
CONSENSUS
      ..................................................

RI-gBol028200-XLOC_020195-6635-0
      GAAATTTTTTTTTCTCGGGAAGATTTCATCTAGGGGTTGATATACTGATT
RI-gBol028200-XLOC_020195-6635-1
```

CONSENSUS
....

RI-gBol028200-XLOC_020195-6635-0
CTTTTAGCTTCGATTTCAGTTTCCTAGATAGCTGAGTTAGGGAAGCTGAG
RI-gBol028200-XLOC_020195-6635-1

CONSENSUS
....

RI-gBol028200-XLOC_020195-6635-0
ATTACTAAGTTTATTCCACTCGTTGATTATCCCGAGAAAATCTTCGATTT
RI-gBol028200-XLOC_020195-6635-1

CONSENSUS
....

RI-gBol028200-XLOC_020195-6635-0
TGTTTTTAACGCTATTTTTCGTGGTCTTTCAGTTCACAATCTCATTCTAT
RI-gBol028200-XLOC_020195-6635-1
-------------------------------TTCACAATCTCATTCTAT
CONSENSUS
...............................TTCACAATCTCATTCTAT

RI-gBol028200-XLOC_020195-6635-0
ATTCTCTCTTGACTCCGTAAAAGATGATGCAAATGGACGGTGGTGATAGG
RI-gBol028200-XLOC_020195-6635-1
ATTCTCTCTTGACTCCGTAAAAGATGATGCAAATGGACGGTGGTGATAGG
CONSENSUS
ATTCTCTCTTGACTCCGTAAAAGATGATGCAAATGGACGGTGGTGATAGG

RI-gBol028200-XLOC_020195-6635-0
CTGAGAGTGACGTTACTAGATTGTATGGAGACTGGCCGGAGCTCGTTACC
RI-gBol028200-XLOC_020195-6635-1
CTGAGAGTGACGTTACTAGATTGTATGGAGACTGGCCGGAGCTCGTTACC
CONSENSUS
CTGAGAGTGACGTTACTAGATTGTATGGAGACTGGCCGGAGCTCGTTACC

RI-gBol028200-XLOC_020195-6635-0
GGGGTTGACTCTCGAAGCTATTCTAATGGCCGACAGAAACGGAACCTCGC
RI-gBol028200-XLOC_020195-6635-1
GGGGTTGACTCTCGAAGCTATTCTAATGGCCGACAGAAACGGAACCTCGC
CONSENSUS
GGGGTTGACTCTCGAAGCTATTCTAATGGCCGACAGAAACGGAACCTCGC

RI-gBol028200-XLOC_020195-6635-0
CGCAGCCTCTGCCGTCTAGGAACCAATCAAATCGGACACTTCTTGATGTA
RI-gBol028200-XLOC_020195-6635-1
CGCAGCCTCTGCCGTCTAGGAACCAATCAAATCGGACACTTCTTGATGTA
CONSENSUS
CGCAGCCTCTGCCGTCTAGGAACCAATCAAATCGGACACTTCTTGATGTA

RI-gBol028200-XLOC_020195-6635-0
ATGCAGAGAGAGCACCGACACGACTACAGCAGCCACCGAGACAAGACCGC
RI-gBol028200-XLOC_020195-6635-1

ATGCAGAGAGAGCACCGACACGACTACAGCAGCCACCGAGACAAGACCGC
CONSENSUS
ATGCAGAGAGAGCACCGACACGACTACAGCAGCCACCGAGACAAGACCGC

RI-gBol028200-XLOC_020195-6635-0
TTGGAAGTCTCTACGCGAAAAGCTCCGCCTCAAACGCAATGCTACCGTTT
RI-gBol028200-XLOC_020195-6635-1
TTGGAAGTCTCTACGCGAAAAGCTCCGCCTCAAACGCAATGCTACCGTTT
CONSENSUS
TTGGAAGTCTCTACGCGAAAAGCTCCGCCTCAAACGCAATGCTACCGTTT

RI-gBol028200-XLOC_020195-6635-0
GGATCTCGTCGAATCCTATCCCGAGTTTGAATACCCCTGTCCTTAACCGG
RI-gBol028200-XLOC_020195-6635-1
GGATCTCGTCGAATCCTATCCCGAGTTTGAATACCCCTGTCCTTAACCGG
CONSENSUS
GGATCTCGTCGAATCCTATCCCGAGTTTGAATACCCCTGTCCTTAACCGG

RI-gBol028200-XLOC_020195-6635-0
GATAGTGATAGTCACCAGCTCGGTTCTCTACTCTCCAACTCCAGAAACGA
RI-gBol028200-XLOC_020195-6635-1
GATAGTGATAGTCACCAGCTCGGTTCTCTACTCTCCAACTCCAGAAACGA
CONSENSUS
GATAGTGATAGTCACCAGCTCGGTTCTCTACTCTCCAACTCCAGAAACGA

RI-gBol028200-XLOC_020195-6635-0
AGGAGGACAATCGCAACCGGAGTCTGCTTCCAGCGGCGGAGTAACGGCGG
RI-gBol028200-XLOC_020195-6635-1
AGGAGGACAATCGCAACCGGAGTCTGCTTCCAGCGGCGGAGTAACGGCGG
CONSENSUS
AGGAGGACAATCGCAACCGGAGTCTGCTTCCAGCGGCGGAGTAACGGCGG

RI-gBol028200-XLOC_020195-6635-0
AGGGCAGGTTACAGTTAGGAGTGGTGTTGGCGGAGGAGAGAGCGTTATCA
RI-gBol028200-XLOC_020195-6635-1
AGGGCAGGTTACAGTTAGGAGTGGTGTTGGCGGAGGAGAGAGCGTTATCA
CONSENSUS
AGGGCAGGTTACAGTTAGGAGTGGTGTTGGCGGAGGAGAGAGCGTTATCA

RI-gBol028200-XLOC_020195-6635-0
GCGAGGGAAGAGGAAACGCCGCCGGTGACGACAGACATGCAGCCGGCGAG
RI-gBol028200-XLOC_020195-6635-1
GCGAGGGAAGAGGAAACGCCGCCGGTGACGACAGACATGCAGCCGGCGAG
CONSENSUS
GCGAGGGAAGAGGAAACGCCGCCGGTGACGACAGACATGCAGCCGGCGAG

RI-gBol028200-XLOC_020195-6635-0
GATGTCGTTGATGGAGTTGTTGGATGAGAACGAAGGGCAAATGAGCTTGG
RI-gBol028200-XLOC_020195-6635-1
GATGTCGTTGATGGAGTTGTTGGATGAGAACGAAGGGCAAATGAGCTTGG
CONSENSUS
GATGTCGTTGATGGAGTTGTTGGATGAGAACGAAGGGCAAATGAGCTTGG

RI-gBol028200-XLOC_020195-6635-0
TAGAAGTAGGAGGAGACGGGGAGGAAGAGGAGAGAGGAGTGGTGGAGGAG
RI-gBol028200-XLOC_020195-6635-1

TAGAAGTAGGAGGAGACGGGGAGGAAGAGGAGAGAGGAGTGGTGGAGGAG
CONSENSUS
TAGAAGTAGGAGGAGACGGGGAGGAAGAGGAGAGAGGAGTGGTGGAGGAG

RI-gBol028200-XLOC_020195-6635-0
ACTGCGGCGGCGGCGGAGATAAGTTGTTGTGTTTGTATGGTAAGAAGCAA
RI-gBol028200-XLOC_020195-6635-1
ACTGCGGCGGCGGCGGAGATAAGTTGTTGTGTTTGTATGGTAAGAAGCAA
CONSENSUS
ACTGCGGCGGCGGCGGAGATAAGTTGTTGTGTTTGTATGGTAAGAAGCAA

RI-gBol028200-XLOC_020195-6635-0
AGGAGCAGCGTTTATACCGTGTGGTCATACGTTCTGCAGGTTGTGTTCGA
RI-gBol028200-XLOC_020195-6635-1
AGGAGCAGCGTTTATACCGTGTGGTCATACGTTCTGCAGGTTGTGTTCGA
CONSENSUS
AGGAGCAGCGTTTATACCGTGTGGTCATACGTTCTGCAGGTTGTGTTCGA

RI-gBol028200-XLOC_020195-6635-0
GAGAGCTTTGGGTTCAAAGAGGAAACTGTCCTCTATGCAACACCTCCATT
RI-gBol028200-XLOC_020195-6635-1
GAGAGCTTTGGGTTCAAAGAGGAAACTGTCCTCTATGCAACACCTCCATT
CONSENSUS
GAGAGCTTTGGGTTCAAAGAGGAAACTGTCCTCTATGCAACACCTCCATT

RI-gBol028200-XLOC_020195-6635-0
TCAGAAATCCTTGATCTCTTTTAGTCATTTTCTTTTCACTTTTAATTATC
RI-gBol028200-XLOC_020195-6635-1
TCAGAAATCCTTGATCTCTTTTAGTCATTTTCTTTTCACTTTTAATTATC
CONSENSUS
TCAGAAATCCTTGATCTCTTTTAGTCATTTTCTTTTCACTTTTAATTATC

RI-gBol028200-XLOC_020195-6635-0
ATATTTAGGTTGTTTTATGTCTTGTTGTAATGTGTTGTTGAAAACTGAAG
RI-gBol028200-XLOC_020195-6635-1
ATATTTAGGTTGTTTTATGTCTTGTTGTAATGTGTTGTTGAAAACTGAAG
CONSENSUS
ATATTTAGGTTGTTTTATGTCTTGTTGTAATGTGTTGTTGAAAACTGAAG

RI-gBol028200-XLOC_020195-6635-0
AGAAACTCTTTGTCGAATTCTCTCTCTCTCAGATTCTTGGAAGTGTTAAC
RI-gBol028200-XLOC_020195-6635-1
AGAAACTCTTTGTCGAATTCTCTCTCTCTCAGATTCTTGGAAGTGTTAAC
CONSENSUS
AGAAACTCTTTGTCGAATTCTCTCTCTCTCAGATTCTTGGAAGTGTTAAC

alignment for event: A5-gBol028200-XLOC_020195-6636

A5-gBol028200-XLOC_020195-6636-0
ATCTGCTCTTGGATTTCGGATTCTGCTGCAAACGAAGAAAGCCAAAGTGT
A5-gBol028200-XLOC_020195-6636-1
ATCTGCTCTTGGATTTCGGATTCTGCTGCAAACGAAGAAAGCCAAAGTGT
CONSENSUS
ATCTGCTCTTGGATTTCGGATTCTGCTGCAAACGAAGAAAGCCAAAGTGT

```
A5-gBol028200-XLOC_020195-6636-0
      TCATCTGTTCAGTGTAAGAAACTCTAGAAATTTTCTCAGGAAACTATGGT
A5-gBol028200-XLOC_020195-6636-1
      TCATCTGTTCAGT-------------------------------------
CONSENSUS
      TCATCTGTTCAGT.....................................

A5-gBol028200-XLOC_020195-6636-0
      TTTCTTTGAAGCTGAGAATAGTAAAATGAGTCCGAATTAAAAGTTCACAA
A5-gBol028200-XLOC_020195-6636-1
      -------------------------------------------TTCACAA
CONSENSUS
      ...........................................TTCACAA

A5-gBol028200-XLOC_020195-6636-0
      TCTCATTCTATATTCTCTCTTGACTCCGTAAAAGATGATGCAAATGGACG
A5-gBol028200-XLOC_020195-6636-1
      TCTCATTCTATATTCTCTCTTGACTCCGTAAAAGATGATGCAAATGGACG
CONSENSUS
      TCTCATTCTATATTCTCTCTTGACTCCGTAAAAGATGATGCAAATGGACG

A5-gBol028200-XLOC_020195-6636-0
      GTGGTGATAGGCTGAGAGTGACGTTACTAGATTGTATGGAGACTGGCCGG
A5-gBol028200-XLOC_020195-6636-1
      GTGGTGATAGGCTGAGAGTGACGTTACTAGATTGTATGGAGACTGGCCGG
CONSENSUS
      GTGGTGATAGGCTGAGAGTGACGTTACTAGATTGTATGGAGACTGGCCGG

A5-gBol028200-XLOC_020195-6636-0
      AGCTCGTTACCGGGGTTGACTCTCGAAGCTATTCTAATGGCCGACAGAAA
A5-gBol028200-XLOC_020195-6636-1
      AGCTCGTTACCGGGGTTGACTCTCGAAGCTATTCTAATGGCCGACAGAAA
CONSENSUS
      AGCTCGTTACCGGGGTTGACTCTCGAAGCTATTCTAATGGCCGACAGAAA

A5-gBol028200-XLOC_020195-6636-0
      CGGAACCTCGCCGCAGCCTCTGCCGTCTAGGAACCAATCAAATCGGACAC
A5-gBol028200-XLOC_020195-6636-1
      CGGAACCTCGCCGCAGCCTCTGCCGTCTAGGAACCAATCAAATCGGACAC
CONSENSUS
      CGGAACCTCGCCGCAGCCTCTGCCGTCTAGGAACCAATCAAATCGGACAC

A5-gBol028200-XLOC_020195-6636-0
      TTCTTGATGTAATGCAGAGAGAGCACCGACACGACTACAGCAGCCACCGA
A5-gBol028200-XLOC_020195-6636-1
      TTCTTGATGTAATGCAGAGAGAGCACCGACACGACTACAGCAGCCACCGA
CONSENSUS
      TTCTTGATGTAATGCAGAGAGAGCACCGACACGACTACAGCAGCCACCGA

A5-gBol028200-XLOC_020195-6636-0
      GACAAGACCGCTTGGAAGTCTCTACGCGAAAAGCTCCGCCTCAAACGCAA
A5-gBol028200-XLOC_020195-6636-1
      GACAAGACCGCTTGGAAGTCTCTACGCGAAAAGCTCCGCCTCAAACGCAA
CONSENSUS
      GACAAGACCGCTTGGAAGTCTCTACGCGAAAAGCTCCGCCTCAAACGCAA
```

A5-gBol028200-XLOC_020195-6636-0
TGCTACCGTTTGGATCTCGTCGAATCCTATCCCGAGTTTGAATACCCCTG
A5-gBol028200-XLOC_020195-6636-1
TGCTACCGTTTGGATCTCGTCGAATCCTATCCCGAGTTTGAATACCCCTG
CONSENSUS
TGCTACCGTTTGGATCTCGTCGAATCCTATCCCGAGTTTGAATACCCCTG

A5-gBol028200-XLOC_020195-6636-0
TCCTTAACCGGGATAGTGATAGTCACCAGCTCGGTTCTCTACTCTCCAAC
A5-gBol028200-XLOC_020195-6636-1
TCCTTAACCGGGATAGTGATAGTCACCAGCTCGGTTCTCTACTCTCCAAC
CONSENSUS
TCCTTAACCGGGATAGTGATAGTCACCAGCTCGGTTCTCTACTCTCCAAC

A5-gBol028200-XLOC_020195-6636-0
TCCAGAAACGAAGGAGGACAATCGCAACCGGAGTCTGCTTCCAGCGGCGG
A5-gBol028200-XLOC_020195-6636-1
TCCAGAAACGAAGGAGGACAATCGCAACCGGAGTCTGCTTCCAGCGGCGG
CONSENSUS
TCCAGAAACGAAGGAGGACAATCGCAACCGGAGTCTGCTTCCAGCGGCGG

A5-gBol028200-XLOC_020195-6636-0
AGTAACGGCGGAGGGCAGGTTACAGTTAGGAGTGGTGTTGGCGGAGGAGA
A5-gBol028200-XLOC_020195-6636-1
AGTAACGGCGGAGGGCAGGTTACAGTTAGGAGTGGTGTTGGCGGAGGAGA
CONSENSUS
AGTAACGGCGGAGGGCAGGTTACAGTTAGGAGTGGTGTTGGCGGAGGAGA

A5-gBol028200-XLOC_020195-6636-0
GAGCGTTATCAGCGAGGGAAGAGGAAACGCCGCCGGTGACGACAGACATG
A5-gBol028200-XLOC_020195-6636-1
GAGCGTTATCAGCGAGGGAAGAGGAAACGCCGCCGGTGACGACAGACATG
CONSENSUS
GAGCGTTATCAGCGAGGGAAGAGGAAACGCCGCCGGTGACGACAGACATG

A5-gBol028200-XLOC_020195-6636-0
CAGCCGGCGAGGATGTCGTTGATGGAGTTGTTGGATGAGAACGAAGGGCA
A5-gBol028200-XLOC_020195-6636-1
CAGCCGGCGAGGATGTCGTTGATGGAGTTGTTGGATGAGAACGAAGGGCA
CONSENSUS
CAGCCGGCGAGGATGTCGTTGATGGAGTTGTTGGATGAGAACGAAGGGCA

A5-gBol028200-XLOC_020195-6636-0
AATGAGCTTGGTAGAAGTAGGAGGAGACGGGGAGGAAGAGGAGAGAGGAG
A5-gBol028200-XLOC_020195-6636-1
AATGAGCTTGGTAGAAGTAGGAGGAGACGGGGAGGAAGAGGAGAGAGGAG
CONSENSUS
AATGAGCTTGGTAGAAGTAGGAGGAGACGGGGAGGAAGAGGAGAGAGGAG

A5-gBol028200-XLOC_020195-6636-0
TGGTGGAGGAGACTGCGGCGGCGGCGGAGATAAGTTGTTGTGTTTGTATG
A5-gBol028200-XLOC_020195-6636-1
TGGTGGAGGAGACTGCGGCGGCGGCGGAGATAAGTTGTTGTGTTTGTATG
CONSENSUS
TGGTGGAGGAGACTGCGGCGGCGGCGGAGATAAGTTGTTGTGTTTGTATG

A5-gBol028200-XLOC_020195-6636-0
      GTAAGAAGCAAAGGAGCAGCGTTTATACCGTGTGGTCATACGTTCTGCAG
A5-gBol028200-XLOC_020195-6636-1
      GTAAGAAGCAAAGGAGCAGCGTTTATACCGTGTGGTCATACGTTCTGCAG
CONSENSUS
      GTAAGAAGCAAAGGAGCAGCGTTTATACCGTGTGGTCATACGTTCTGCAG

A5-gBol028200-XLOC_020195-6636-0
      GTTGTGTTCGAGAGAGCTTTGGGTTCAAAGAGGAAACTGTCCTCTATGCA
A5-gBol028200-XLOC_020195-6636-1
      GTTGTGTTCGAGAGAGCTTTGGGTTCAAAGAGGAAACTGTCCTCTATGCA
CONSENSUS
      GTTGTGTTCGAGAGAGCTTTGGGTTCAAAGAGGAAACTGTCCTCTATGCA

A5-gBol028200-XLOC_020195-6636-0
      ACACCTCCATTTCAGAAATCCTTGATCTCTTTTAGTCATTTTCTTTTCAC
A5-gBol028200-XLOC_020195-6636-1
      ACACCTCCATTTCAGAAATCCTTGATCTCTTTTAGTCATTTTCTTTTCAC
CONSENSUS
      ACACCTCCATTTCAGAAATCCTTGATCTCTTTTAGTCATTTTCTTTTCAC

A5-gBol028200-XLOC_020195-6636-0
      TTTTAATTATCATATTTAGGTTGTTTTATGTCTTGTTGTAATGTGTTGTT
A5-gBol028200-XLOC_020195-6636-1
      TTTTAATTATCATATTTAGGTTGTTTTATGTCTTGTTGTAATGTGTTGTT
CONSENSUS
      TTTTAATTATCATATTTAGGTTGTTTTATGTCTTGTTGTAATGTGTTGTT

A5-gBol028200-XLOC_020195-6636-0
      GAAAACTGAAGAGAAACTCTTTGTCGAATTCTCTCTCTCTCAGATTCTTG
A5-gBol028200-XLOC_020195-6636-1
      GAAAACTGAAGAGAAACTCTTTGTCGAATTCTCTCTCTCTCAGATTCTTG
CONSENSUS
      GAAAACTGAAGAGAAACTCTTTGTCGAATTCTCTCTCTCTCAGATTCTTG

A5-gBol028200-XLOC_020195-6636-0 GAAGTGTTAAC
A5-gBol028200-XLOC_020195-6636-1 GAAGTGTTAAC
CONSENSUS GAAGTGTTAAC

alignment for event: RI-X-XLOC_020183-12413

RI-X-XLOC_020183-12413-0
      TATCATTGAATGTATAAACCGCAAAGCAAAGCACAATATATACACAAGAA
RI-X-XLOC_020183-12413-1
      TATCATTGAATGTATAAACCGCAAAGCAAAGCACAATATATACACAAGAA
CONSENSUS
      TATCATTGAATGTATAAACCGCAAAGCAAAGCACAATATATACACAAGAA

RI-X-XLOC_020183-12413-0
      CCCGGAGGAATGGAGTAAGGGATTCTCAGTCATGTGTTATGTAAGCTCTT
RI-X-XLOC_020183-12413-1
      CCCGGAGGAATGGAGTAAGGGATTCTCAGTCATGTGTTATGTAAGCTCTT
CONSENSUS

CCCGGAGGAATGGAGTAAGGGATTCTCAGTCATGTGTTATGTAAGCTCTT

RI-X-XLOC_020183-12413-0
CTATCAACAAGAGACTGTGAAGATCAATATAATCAAACCAAGTAGTCAAT
RI-X-XLOC_020183-12413-1
CTATCAACAAGAGACTGTGAAGATCAATATAATCAAACCAAGTAGTCAAT
CONSENSUS
CTATCAACAAGAGACTGTGAAGATCAATATAATCAAACCAAGTAGTCAAT

RI-X-XLOC_020183-12413-0
TTTGGATTGTTGGAAGGGGGAGTGACCACCTGAATCTAGATATGATGGAA
RI-X-XLOC_020183-12413-1
TTTGGATTGTTGGAAGGGGGAGTGACCACCTGAATCTAGATATGATGGAA
CONSENSUS
TTTGGATTGTTGGAAGGGGGAGTGACCACCTGAATCTAGATATGATGGAA

RI-X-XLOC_020183-12413-0
CATTGATGAATATGTTTCGTGCAAGTCTATATTCTATTATAGTTTCTCTT
RI-X-XLOC_020183-12413-1
CATTGATGAATATGTTTCGTGCAAGTCTATATTCTATTATAGTTTCTCTT
CONSENSUS
CATTGATGAATATGTTTCGTGCAAGTCTATATTCTATTATAGTTTCTCTT

RI-X-XLOC_020183-12413-0
ATTTACTGCTCCAAGTCCCACGATATACATGAGCTTAGTAACAAAAGTAT
RI-X-XLOC_020183-12413-1
ATTTACTGCTCCAAGTCCCACGATATACATGAGCTTAGTAACAAAAGTAT
CONSENSUS
ATTTACTGCTCCAAGTCCCACGATATACATGAGCTTAGTAACAAAAGTAT

RI-X-XLOC_020183-12413-0
TCATTCATGGAGAAGGTTATAGTATGCTAACTTATATTCAGGTTTGTTAT
RI-X-XLOC_020183-12413-1
TCATTCATGGAGAAGGTTATAGTATGCTAACTTATATTCAGGTTTGTTAT
CONSENSUS
TCATTCATGGAGAAGGTTATAGTATGCTAACTTATATTCAGGTTTGTTAT

RI-X-XLOC_020183-12413-0
GATGATTGCTAAATTTCTTAAAACTGATTTAACAGGATGGAGAGTCTGGG
RI-X-XLOC_020183-12413-1
GATGATTGCTAAATTTCTTAAAACTGATTTAACAGGATGGAGAGTCTGGG
CONSENSUS
GATGATTGCTAAATTTCTTAAAACTGATTTAACAGGATGGAGAGTCTGGG

RI-X-XLOC_020183-12413-0
ATGACAAGGTGATACTGTAGGTTCCATTGCCCGCCACAGTTTACCAATAA
RI-X-XLOC_020183-12413-1
ATGACAAGGTGATACTGTAGGTTCCATTGCCCGCCACAGTTTACCAATAA
CONSENSUS
ATGACAAGGTGATACTGTAGGTTCCATTGCCCGCCACAGTTTACCAATAA

RI-X-XLOC_020183-12413-0
CTGGTTAGATTACATAGCACATCTACTTTATTTTCACTATTAAATTAGTT
RI-X-XLOC_020183-12413-1
CTG---
CONSENSUS

CTG...

RI-X-XLOC_020183-12413-0
TTTCTTCACCGCAGTAGCTCTTGGAGGCTCAAGTGCTCAACCTTTCTTTT
RI-X-XLOC_020183-12413-1 --------------
TAGCTCTTGGAGGCTCAAGTGCTCAACCTTTCTTTT
CONSENSUS
..............TAGCTCTTGGAGGCTCAAGTGCTCAACCTTTCTTTT

RI-X-XLOC_020183-12413-0
GGTCGCTAATGCGTGGAAAACTGGTATGCCTACAAGGTTTCAAGCAATGG
RI-X-XLOC_020183-12413-1
GGTCGCTAATGCGTGGAAAACTGGTATGCCTACAAGGTTTCAAGCAATGG
CONSENSUS
GGTCGCTAATGCGTGGAAAACTGGTATGCCTACAAGGTTTCAAGCAATGG

RI-X-XLOC_020183-12413-0
ACTTCTGTTTCTTTTTTATGTCCTTCAGTAAGAGAACATTATCAGTTTAG
RI-X-XLOC_020183-12413-1
ACTTCTGTTTCTTTTTTATGTCCTTCAGTAAGAGAACATTATCAGTTTAG
CONSENSUS
ACTTCTGTTTCTTTTTTATGTCCTTCAGTAAGAGAACATTATCAGTTTAG

RI-X-XLOC_020183-12413-0
GAGATCTCTTCAAAAATTACAATTTTGAAGTCCATTGCAAATTAGTAGAC
RI-X-XLOC_020183-12413-1
GAGATCTCTTCAAAAATTACAATTTTGAAGTCCATTGCAAATTAGTAGAC
CONSENSUS
GAGATCTCTTCAAAAATTACAATTTTGAAGTCCATTGCAAATTAGTAGAC

RI-X-XLOC_020183-12413-0
ATTAATTAGCTAGGCAAAAATCAGATACTTATATTGGAGAATAGGTCCTA
RI-X-XLOC_020183-12413-1
ATTAATTAGCTAGGCAAAAATCAGATACTTATATTGGAGAATAGGTCCTA
CONSENSUS
ATTAATTAGCTAGGCAAAAATCAGATACTTATATTGGAGAATAGGTCCTA

RI-X-XLOC_020183-12413-0
AGTAAATATTTAATTGATTTTCTGCAAGATGCTTTGTGAAAGGAGCTGAG
RI-X-XLOC_020183-12413-1
AGTAAATATTTAATTGATTTTCTGCAAGATGCTTTGTGAAAGGAGCTGAG
CONSENSUS
AGTAAATATTTAATTGATTTTCTGCAAGATGCTTTGTGAAAGGAGCTGAG

RI-X-XLOC_020183-12413-0
CTTTGGGAAATATAGGGGAAGGGAAACAACGTTTGTAATTTGATGGAAAA
RI-X-XLOC_020183-12413-1
CTTTGGGAAATATAGGGGAAGGGAAACAACGTTTGTAATTTGATGGAAAA
CONSENSUS
CTTTGGGAAATATAGGGGAAGGGAAACAACGTTTGTAATTTGATGGAAAA

RI-X-XLOC_020183-12413-0
TGAGATCTTGATGTGAATGCATTGTTTTAACAAAATAATTGTTTTGTTAT
RI-X-XLOC_020183-12413-1
TGAGATCTTGATGTGAATGCATTGTTTTAACAAAATAATTGTTTTGTTAT
CONSENSUS

TGAGATCTTGATGTGAATGCATTGTTTTAACAAAATAATTGTTTTGTTAT

RI-X-XLOC_020183-12413-0
TGTATTAAAAGATCTAAAATTTTTAGAAACATTAGTTTGCAGAAATAAAT
RI-X-XLOC_020183-12413-1
TGTATTAAAAGATCTAAAATTTTTAGAAACATTAGTTTGCAGAAATAAAT
CONSENSUS
TGTATTAAAAGATCTAAAATTTTTAGAAACATTAGTTTGCAGAAATAAAT

RI-X-XLOC_020183-12413-0 CAATTAATTTGATTCAATATAAG
RI-X-XLOC_020183-12413-1 CAATTAATTTGATTCAATATAAG
CONSENSUS CAATTAATTTGATTCAATATAAG

alignment for event: RI-gBol025401-XLOC_023392-10467

RI-gBol025401-XLOC_023392-10467-0
GCCGTTGCGGTTAACAACTTGGGAAAGTGGAAAACCGCCACGCAGATGAC
RI-gBol025401-XLOC_023392-10467-1
GCCGTTGCGGTTAACAACTTGGGAAAGTGGAAAACCGCCACGCAGATGAC
CONSENSUS
GCCGTTGCGGTTAACAACTTGGGAAAGTGGAAAACCGCCACGCAGATGAC

RI-gBol025401-XLOC_023392-10467-0
AGCACTAACCATACTTCTCGCAAGCCGGGACAGCAGCGTTGGATGGCTCG
RI-gBol025401-XLOC_023392-10467-1
AGCACTAACCATACTTCTCGCAAGCCGGGACAGCAGCGTTGGATGGCTCG
CONSENSUS
AGCACTAACCATACTTCTCGCAAGCCGGGACAGCAGCGTTGGATGGCTCG

RI-gBol025401-XLOC_023392-10467-0
TAGCTTCCGGTGCTGGGTTGCTTTATGTATCAGCAGGACTATCGGTTTGG
RI-gBol025401-XLOC_023392-10467-1
TAGCTTCCGGTGCTGGGTTGCTTTATGTATCAGCAGGACTATCGGTTTGG
CONSENSUS
TAGCTTCCGGTGCTGGGTTGCTTTATGTATCAGCAGGACTATCGGTTTGG

RI-gBol025401-XLOC_023392-10467-0
TCTTTAGTCGTTTATGTGAGGAAGATATGGAAAGTAAGTAGGTTGTAATC
RI-gBol025401-XLOC_023392-10467-1
TCTTTAGTCGTTTATGTGAGGAAGATATGGAAAGTAAGTAG---------
CONSENSUS
TCTTTAGTCGTTTATGTGAGGAAGATATGGAAAGTAAGTAG.........

RI-gBol025401-XLOC_023392-10467-0
TATCTATCTATCGGTTTTGTTTTGCTTTAAACCTGCATTTGCATATAAGC
RI-gBol025401-XLOC_023392-10467-1

CONSENSUS
....

RI-gBol025401-XLOC_023392-10467-0
ATTGATGGAGTGTCTCGGTCATAGTCACAAGATTTGAATATGCTGTTGAG
RI-gBol025401-XLOC_023392-10467-1

CONSENSUS
....

RI-gBol025401-XLOC_023392-10467-0
CAGGAATCTTGTGGGGGAAATGATTGAACGGCAGGTAGAAATGAAGCAGT
RI-gBol025401-XLOC_023392-10467-1 ---
GAATCTTGTGGGGGAAATGATTGAACGGCAGGTAGAAATGAAGCAGT
CONSENSUS
...GAATCTTGTGGGGGAAATGATTGAACGGCAGGTAGAAATGAAGCAGT

RI-gBol025401-XLOC_023392-10467-0
TGTAGTAGTTTGAGAATATGCTAGATGGTTTTGCTCTGCTCTGCACTTAG
RI-gBol025401-XLOC_023392-10467-1
TGTAGTAGTTTGAGAATATGCTAGATGGTTTTGCTCTGCTCTGCACTTAG
CONSENSUS
TGTAGTAGTTTGAGAATATGCTAGATGGTTTTGCTCTGCTCTGCACTTAG

RI-gBol025401-XLOC_023392-10467-0
CAGCTTCTTGTTTAATTAATAATATTTTATTGAAAAAAAAGTTCTTGTAT
RI-gBol025401-XLOC_023392-10467-1
CAGCTTCTTGTTTAATTAATAATATTTTATTGAAAAAAAAGTTCTTGTAT
CONSENSUS
CAGCTTCTTGTTTAATTAATAATATTTTATTGAAAAAAAAGTTCTTGTAT

RI-gBol025401-XLOC_023392-10467-0
GATGTTTTTGTTACTTTAGTGTTTGCTCTTTTATTAAAAAGAGAATAGAA
RI-gBol025401-XLOC_023392-10467-1
GATGTTTTTGTTACTTTAGTGTTTGCTCTTTTATTAAAAAGAGAATAGAA
CONSENSUS
GATGTTTTTGTTACTTTAGTGTTTGCTCTTTTATTAAAAAGAGAATAGAA

RI-gBol025401-XLOC_023392-10467-0
GATTATTGTACACAGAATATGGGTAATTTTGTGTGATATTTCTGTAACAA
RI-gBol025401-XLOC_023392-10467-1
GATTATTGTACACAGAATATGGGTAATTTTGTGTGATATTTCTGTAACAA
CONSENSUS
GATTATTGTACACAGAATATGGGTAATTTTGTGTGATATTTCTGTAACAA

RI-gBol025401-XLOC_023392-10467-0
AATTATTAGTGTTTGTTTTTTGTTAAAAT
RI-gBol025401-XLOC_023392-10467-1
AATTATTAGTGTTTGTTTTTTGTTAAAAT
CONSENSUS
AATTATTAGTGTTTGTTTTTTGTTAAAAT

alignment for event: A5-gBol025401-XLOC_023392-10468

A5-gBol025401-XLOC_023392-10468-0
GCCGTTGCGGTTAACAACTTGGGAAAGTGGAAAACCGCCACGCAGATGAC
A5-gBol025401-XLOC_023392-10468-1
GCCGTTGCGGTTAACAACTTGGGAAAGTGGAAAACCGCCACGCAGATGAC
CONSENSUS
GCCGTTGCGGTTAACAACTTGGGAAAGTGGAAAACCGCCACGCAGATGAC

A5-gBol025401-XLOC_023392-10468-0
AGCACTAACCATACTTCTCGCAAGCCGGGACAGCAGCGTTGGATGGCTCG
A5-gBol025401-XLOC_023392-10468-1
AGCACTAACCATACTTCTCGCAAGCCGGGACAGCAGCGTTGGATGGCTCG
CONSENSUS
AGCACTAACCATACTTCTCGCAAGCCGGGACAGCAGCGTTGGATGGCTCG

A5-gBol025401-XLOC_023392-10468-0
TAGCTTCCGGTGCTGGGTTGCTTTATGTATCAGCAGGACTATCGGTTTGG
A5-gBol025401-XLOC_023392-10468-1
TAGCTTCCGGTGCTGGGTTGCTTTATGTATCAGCAGGACTATCGGTTTGG
CONSENSUS
TAGCTTCCGGTGCTGGGTTGCTTTATGTATCAGCAGGACTATCGGTTTGG

A5-gBol025401-XLOC_023392-10468-0
TCTTTAGTCGTTTATGTGAGGAAGATATGGAAAGTAAGTAGGAATCTTGT
A5-gBol025401-XLOC_023392-10468-1
TCTTTAGTCGTTTATGTGAGGAAGATATGGAAA--------GAATCTTGT
CONSENSUS
TCTTTAGTCGTTTATGTGAGGAAGATATGGAAA........GAATCTTGT

A5-gBol025401-XLOC_023392-10468-0
GGGGGAAATGATTGAACGGCAGGTAGAAATGAAGCAGTTGTAGTAGTTTG
A5-gBol025401-XLOC_023392-10468-1
GGGGGAAATGATTGAACGGCAGGTAGAAATGAAGCAGTTGTAGTAGTTTG
CONSENSUS
GGGGGAAATGATTGAACGGCAGGTAGAAATGAAGCAGTTGTAGTAGTTTG

A5-gBol025401-XLOC_023392-10468-0
AGAATATGCTAGATGGTTTTGCTCTGCTCTGCACTTAGCAGCTTCTTGTT
A5-gBol025401-XLOC_023392-10468-1
AGAATATGCTAGATGGTTTTGCTCTGCTCTGCACTTAGCAGCTTCTTGTT
CONSENSUS
AGAATATGCTAGATGGTTTTGCTCTGCTCTGCACTTAGCAGCTTCTTGTT

A5-gBol025401-XLOC_023392-10468-0
TAATTAATAATATTTTATTGAAAAAAAAGTTCTTGTATGATGTTTTTGTT
A5-gBol025401-XLOC_023392-10468-1
TAATTAATAATATTTTATTGAAAAAAAAGTTCTTGTATGATGTTTTTGTT
CONSENSUS
TAATTAATAATATTTTATTGAAAAAAAAGTTCTTGTATGATGTTTTTGTT

A5-gBol025401-XLOC_023392-10468-0
ACTTTAGTGTTTGCTCTTTTATTAAAAAGAGAATAGAAGATTATTGTACA
A5-gBol025401-XLOC_023392-10468-1
ACTTTAGTGTTTGCTCTTTTATTAAAAAGAGAATAGAAGATTATTGTACA
CONSENSUS
ACTTTAGTGTTTGCTCTTTTATTAAAAAGAGAATAGAAGATTATTGTACA

A5-gBol025401-XLOC_023392-10468-0
CAGAATATGGGTAATTTTGTGTGATATTTCTGTAACAAAATTATTAGTGT
A5-gBol025401-XLOC_023392-10468-1
CAGAATATGGGTAATTTTGTGTGATATTTCTGTAACAAAATTATTAGTGT
CONSENSUS
CAGAATATGGGTAATTTTGTGTGATATTTCTGTAACAAAATTATTAGTGT

```
A5-gBol025401-XLOC_023392-10468-0      TTGTTTTTTGTTAAAAT
A5-gBol025401-XLOC_023392-10468-1      TTGTTTTTTGTTAAAAT
CONSENSUS                              TTGTTTTTTGTTAAAAT


alignment for event: RI-X-XLOC_027378-12869

RI-X-XLOC_027378-12869-0
     GCTGTAAGGCACTAATATTTCAGACAACGTTGGTCCAAGGTTTATTGGGG
RI-X-XLOC_027378-12869-1
     GCTGTAAGGCACTAATATTTCAGACAACGTTGGTCCAAGGTTTATTGGGG
CONSENSUS
     GCTGTAAGGCACTAATATTTCAGACAACGTTGGTCCAAGGTTTATTGGGG

RI-X-XLOC_027378-12869-0
     ATCATAGACTCAACACTTTCAAGCGCCTGCTGAGTTGTTGAGGGAAGAGA
RI-X-XLOC_027378-12869-1
     ATCATAGACTCAACACTTTCAAGCGCCTGCTGAGTTGTTGAGGGAAGAGA
CONSENSUS
     ATCATAGACTCAACACTTTCAAGCGCCTGCTGAGTTGTTGAGGGAAGAGA

RI-X-XLOC_027378-12869-0
     GAATCTACTACTAATAATGACGTGGATTTGCAGGGTATGTCTGTCTACTC
RI-X-XLOC_027378-12869-1
     GAATCTACTACTAATAATGACGTGGATTTGCAGG----------------
CONSENSUS
     GAATCTACTACTAATAATGACGTGGATTTGCAGG................

RI-X-XLOC_027378-12869-0
     ATCCTTTTCTCAAGCTCTAACTTTTAAGCTGAAATTTAGAGCTTAATAAT
RI-X-XLOC_027378-12869-1
     --------------------------------------------------
CONSENSUS
     ..................................................

RI-X-XLOC_027378-12869-0
     GTTTTCAGCGGCTGTTACACATGTCTTCTGCGGATGTTTTTGGTGTTGTT
RI-X-XLOC_027378-12869-1
     --------------------------------------------------
CONSENSUS
     ..................................................

RI-X-XLOC_027378-12869-0
     TGTGGCATCAAGGTCACCTTTAACAATGAAACTTAGAGAATACAACGTGT
RI-X-XLOC_027378-12869-1
     --------------------------------------------------
CONSENSUS
     ..................................................

RI-X-XLOC_027378-12869-0
     TATGATAACAATCTGAGTTGAAAAGTAGGCTAGCTTAACGCTTATGTTAT
RI-X-XLOC_027378-12869-1
     --------------------------------------------------
CONSENSUS
     ..................................................
```

```
RI-X-XLOC_027378-12869-0
      ATAGTTTTTTTTTATAATTTTTGTTGATTTAGTTATTTTGGGGCATTTTA
RI-X-XLOC_027378-12869-1
      --------------------------------------------------
CONSENSUS
      ..................................................

RI-X-XLOC_027378-12869-0
      CGATTCCGTTGCATTTTATTTACAATTTATGTGTCTGTTTATATAAAAGT
RI-X-XLOC_027378-12869-1
      --------------------------------------------------
CONSENSUS
      ..................................................

RI-X-XLOC_027378-12869-0
      TGTTGCATTTTACGATTCTGTTTCTTACTTTGATTTTTTTTGTTTGTGTA
RI-X-XLOC_027378-12869-1
      --------------------------------------------------
CONSENSUS
      ..................................................

RI-X-XLOC_027378-12869-0
      TATTCTTAGTATGATTTCTTCATCGTTTGATTGATGCTGATTTGTATTGT
RI-X-XLOC_027378-12869-1
      --------------------------------------------------
CONSENSUS
      ..................................................

RI-X-XLOC_027378-12869-0
      GTTCTTTGTGTTGCAGTTTTAGCAGATGTCCCTGTGTCTCTTCCTATTTG
RI-X-XLOC_027378-12869-1
      --------------------------------------------------
CONSENSUS
      ..................................................

RI-X-XLOC_027378-12869-0
      AATTTCAGTTTACATCTTAGATGTCTGAATCTCTGTTGATATCTGATTAT
RI-X-XLOC_027378-12869-1
      --------------------------------------------------
CONSENSUS
      ..................................................

RI-X-XLOC_027378-12869-0
      ATGTAACTGGATTTGAAATACATTATGTTGCCAGATGAAATGAGCAACTG
RI-X-XLOC_027378-12869-1
      --------------------------------------------------
CONSENSUS
      ..................................................

RI-X-XLOC_027378-12869-0
      AGTGTTTCTAAGATCTGATTCATTGATCGAGTTTGTTTTTTCTGAAGGAG
RI-X-XLOC_027378-12869-1
      -----------------------------------------------GAG
CONSENSUS
      ...............................................GAG
```

RI-X-XLOC_027378-12869-0
AAAAAAGTGGAATCTTTGGATGATTAGGGTGACACCAGTAGCTTATGGTC
RI-X-XLOC_027378-12869-1
AAAAAAGTGGAATCTTTGGATGATTAGGGTGACACCAGTAGCTTATGGTC
CONSENSUS
AAAAAAGTGGAATCTTTGGATGATTAGGGTGACACCAGTAGCTTATGGTC

RI-X-XLOC_027378-12869-0
CTGGAATAGTGCATCGTGTCGAGCCTGTGAGCTTAGAAGAACTTGGACGG
RI-X-XLOC_027378-12869-1
CTGGAATAGTGCATCGTGTCGAGCCTGTGAGCTTAGAAGAACTTGGACGG
CONSENSUS
CTGGAATAGTGCATCGTGTCGAGCCTGTGAGCTTAGAAGAACTTGGACGG

RI-X-XLOC_027378-12869-0
CTGAGGTTTGATGTCATAATTGCTTTGTGTTTAATCACCAGTACATGTTT
RI-X-XLOC_027378-12869-1
CTGAGGTTTGATGTCATAATTGCTTTGTGTTTAATCACCAGTACATGTTT
CONSENSUS
CTGAGGTTTGATGTCATAATTGCTTTGTGTTTAATCACCAGTACATGTTT

RI-X-XLOC_027378-12869-0
TTGAGCCGTAAATCGTTTGTGTTTGAAAGTGATGCTCCTGAAGCAAAAAA
RI-X-XLOC_027378-12869-1
TTGAGCCGTAAATCGTTTGTGTTTGAAAGTGATGCTCCTGAAGCAAAAAA
CONSENSUS
TTGAGCCGTAAATCGTTTGTGTTTGAAAGTGATGCTCCTGAAGCAAAAAA

RI-X-XLOC_027378-12869-0
GGCTGAGAAGAAGAGCAAATCTTTAAGATGGCTCTTAGCACTGCAACAGT
RI-X-XLOC_027378-12869-1
GGCTGAGAAGAAGAGCAAATCTTTAAGATGGCTCTTAGCACTGCAACAGT
CONSENSUS
GGCTGAGAAGAAGAGCAAATCTTTAAGATGGCTCTTAGCACTGCAACAGT

RI-X-XLOC_027378-12869-0
CACGTTAGAGAACCAAGCTGCTGAATCCAAAGTGATACCAAAGGATGACG
RI-X-XLOC_027378-12869-1
CACGTTAGAGAACCAAGCTGCTGAATCCAAAGTGATACCAAAGGATGACG
CONSENSUS
CACGTTAGAGAACCAAGCTGCTGAATCCAAAGTGATACCAAAGGATGACG

RI-X-XLOC_027378-12869-0
TCAAGTGGATACGAAGGAGGAGGTCGTGAGATATAGAAGAGGCTCACCAA
RI-X-XLOC_027378-12869-1
TCAAGTGGATACGAAGGAGGAGGTCGTGAGATATAGAAGAGGCTCACCAA
CONSENSUS
TCAAGTGGATACGAAGGAGGAGGTCGTGAGATATAGAAGAGGCTCACCAA

RI-X-XLOC_027378-12869-0
CATTGTAGCCAAGAGATGTACACAAAAAGTTCTATTGAGTCTAGCCCATC
RI-X-XLOC_027378-12869-1
CATTGTAGCCAAGAGATGTACACAAAAAGTTCTATTGAGTCTAGCCCATC
CONSENSUS
CATTGTAGCCAAGAGATGTACACAAAAAGTTCTATTGAGTCTAGCCCATC

```
RI-X-XLOC_027378-12869-0   ATCTACTTCAGACTTCTTCCCTAAA
RI-X-XLOC_027378-12869-1   ATCTACTTCAGACTTCTTCCCTAAA
CONSENSUS                  ATCTACTTCAGACTTCTTCCCTAAA


alignment for event: A3-gBol031053-XLOC_016655-2908

A3-gBol031053-XLOC_016655-2908-0
     ATTTGTTATATGAAAATAAGGTCAATACTTTCCAAGGAGGATAGCTTCTG
A3-gBol031053-XLOC_016655-2908-1
     ATTTGTTATATGAAAATAAGGTCAATACTTTCCAAGGAGGATAGCTTCTG
CONSENSUS
     ATTTGTTATATGAAAATAAGGTCAATACTTTCCAAGGAGGATAGCTTCTG

A3-gBol031053-XLOC_016655-2908-0
     AAGCCCTATCAAGCAATGATGAATTAAAGGGATATAAAGAAGATGGCTAT
A3-gBol031053-XLOC_016655-2908-1
     AAGCCCTATCAAGCAATGATGAATTAAAGGGATATAAAGAAGAT------
CONSENSUS
     AAGCCCTATCAAGCAATGATGAATTAAAGGGATATAAAGAAGAT......

A3-gBol031053-XLOC_016655-2908-0
     GAGTGTTGGTCTAGGAGTTTCAACTGATTCTATTGGTGAATCAGGTGGAT
A3-gBol031053-XLOC_016655-2908-1
     --------------------------------------------GTGGAT
CONSENSUS
     ............................................GTGGAT

A3-gBol031053-XLOC_016655-2908-0
     GGATGGTTCGGAAGGGAATGCACACTCGCCAGTTCCTGCAACAAAGAACG
A3-gBol031053-XLOC_016655-2908-1
     GGATGGTTCGGAAGGGAATGCACACTCGCCAGTTCCTGCAACAAAGAACG
CONSENSUS
     GGATGGTTCGGAAGGGAATGCACACTCGCCAGTTCCTGCAACAAAGAACG

A3-gBol031053-XLOC_016655-2908-0 GACACTGACGATCAAACAGAGACAG
A3-gBol031053-XLOC_016655-2908-1 GACACTGACGATCAAACAGAGACAG
CONSENSUS                        GACACTGACGATCAAACAGAGACAG


alignment for event: A5-gBol031053-XLOC_016655-2905

A5-gBol031053-XLOC_016655-2905-0
     GTGGATGGATGGTTCGGAAGGGAATGCACACTCGCCAGTTCCTGCAACAA
A5-gBol031053-XLOC_016655-2905-1
     GTGGATGGATGGTTCGGAAGGGAATGCACACTCGCCAGTTCCTGCAACAA
CONSENSUS
     GTGGATGGATGGTTCGGAAGGGAATGCACACTCGCCAGTTCCTGCAACAA

A5-gBol031053-XLOC_016655-2905-0
     AGAACGGACACTGACGATCAAACAGAGACAG-------------------
A5-gBol031053-XLOC_016655-2905-1
     AGAACGGACACTGACGATCAAACAGAGACAGGTGAAACATGGACTCCACT
```

CONSENSUS
AGAACGGACACTGACGATCAAACAGAGACAG...................

A5-gBol031053-XLOC_016655-2905-0
-------------------------------GCAAGATACTTTTTCAAG
A5-gBol031053-XLOC_016655-2905-1
TCTGAACATGTACAAAGCCCAAATGCAATTCTGCAAGATACTTTTTCAAG
CONSENSUS
...............................GCAAGATACTTTTTCAAG

A5-gBol031053-XLOC_016655-2905-0
CAGATTATATCAAGAGCGAGTGATTGCCATGATGTAGTAAGGAATGACTT
A5-gBol031053-XLOC_016655-2905-1
CAGATTATATCAAGAGCGAGTGATTGCCATGATGTAGTAAGGAATGACTT
CONSENSUS
CAGATTATATCAAGAGCGAGTGATTGCCATGATGTAGTAAGGAATGACTT

A5-gBol031053-XLOC_016655-2905-0
ACGAAGGTCTACTCAACTCCCACAGACATACTAAAGCTTTGTTTATCATC
A5-gBol031053-XLOC_016655-2905-1
ACGAAGGTCTACTCAACTCCCACAGACATACTAAAGCTTTGTTTATCATC
CONSENSUS
ACGAAGGTCTACTCAACTCCCACAGACATACTAAAGCTTTGTTTATCATC

A5-gBol031053-XLOC_016655-2905-0
CCTGTAGTCACATTATTTTCAATATTCTTATAGCAAATA
A5-gBol031053-XLOC_016655-2905-1
CCTGTAGTCACATTATTTTCAATATTCTTATAGCAAATA
CONSENSUS
CCTGTAGTCACATTATTTTCAATATTCTTATAGCAAATA

alignment for event: A5-gBol023002-XLOC_025887-308

A5-gBol023002-XLOC_025887-308-0
GTAGAAGAAGAAAATGGTCATGTAGAGAATGAAGGAGGTGAAGAGGAAGA
A5-gBol023002-XLOC_025887-308-1
GTAGAAGAAGAAAATGGTCATGTAGAGAATGAAGGAGGTGAAGAGGAAGA
CONSENSUS
GTAGAAGAAGAAAATGGTCATGTAGAGAATGAAGGAGGTGAAGAGGAAGA

A5-gBol023002-XLOC_025887-308-0
GAATGAAGAAGAGGAAATGAATCAAGAAGAGGGAGTTGTTGATGAAGACT
A5-gBol023002-XLOC_025887-308-1
GAATGAAGAAGAGGAAATGAATCAAGAAGAGGGAGTTGTTGATGAAGACT
CONSENSUS
GAATGAAGAAGAGGAAATGAATCAAGAAGAGGGAGTTGTTGATGAAGACT

A5-gBol023002-XLOC_025887-308-0
CTACAGATG---
A5-gBol023002-XLOC_025887-308-1
CTACAGATGGTAGTGCTGTACTTGTTAATAGAAGTGAGGGTGAGGAAGAG
CONSENSUS
CTACAGATG...

```
A5-gBol023002-XLOC_025887-308-0
      -------------------------------------------TGCTTA
A5-gBol023002-XLOC_025887-308-1
      TGGGGTACGAATAGTAAAGACAACCAGTCAGCCTAATAAAGCAATGCTTA
CONSENSUS
      ............................................TGCTTA

A5-gBol023002-XLOC_025887-308-0
      CGCCACATCAGTAGCCAGTAAGAGTACTTGGATCCATTTTTGGATAAGGA
A5-gBol023002-XLOC_025887-308-1
      CGCCACATCAGTAGCCAGTAAGAGTACTTGGATCCATTTTTGGATAAGGA
CONSENSUS
      CGCCACATCAGTAGCCAGTAAGAGTACTTGGATCCATTTTTGGATAAGGA

A5-gBol023002-XLOC_025887-308-0  GCAGGGGAATCAGTTCTTGG
A5-gBol023002-XLOC_025887-308-1  GCAGGGGAATCAGTTCTTGG
CONSENSUS                        GCAGGGGAATCAGTTCTTGG



alignment for event: SE-X-XLOC_035024-2385

SE-X-XLOC_035024-2385-0
      TTTTGAATTTTAAATTGATAAAATCACTCTTTCTCTCTCTCTTTGTGTTC
SE-X-XLOC_035024-2385-1
      TTTTGAATTTTAAATTGATAAAATCACTCTTTCTCTCTCTCTTTGTGTTC
CONSENSUS
      TTTTGAATTTTAAATTGATAAAATCACTCTTTCTCTCTCTCTTTGTGTTC

SE-X-XLOC_035024-2385-0
      TCTATTTTCCTCAATTTCTCAGATCTGTGTTAAACTAAATGTCGAACATT
SE-X-XLOC_035024-2385-1
      TCTATTTTCCTCAATTTCTCAGATCTGTGTTAAACTAAATGTCGAACATT
CONSENSUS
      TCTATTTTCCTCAATTTCTCAGATCTGTGTTAAACTAAATGTCGAACATT

SE-X-XLOC_035024-2385-0
      TTTTTGGCGAGAAACTTGAAGGTTTAATAAGTTATACAAATGGTTAAGTT
SE-X-XLOC_035024-2385-1
      TTTTTGGCGAGAAACTTGAAGGTTTAATAAGTTATACAAATGGTTAAGTT
CONSENSUS
      TTTTTGGCGAGAAACTTGAAGGTTTAATAAGTTATACAAATGGTTAAGTT

SE-X-XLOC_035024-2385-0
      TTATGGAATCTTAGGTTTGATGTTGCCCAGATGTGTAAAATACTATGGAA
SE-X-XLOC_035024-2385-1
      TTATGGAATCTTAGGTTTGATGTTGCCCAGATGTGTAAAATACTATGGAA
CONSENSUS
      TTATGGAATCTTAGGTTTGATGTTGCCCAGATGTGTAAAATACTATGGAA

SE-X-XLOC_035024-2385-0
      CCGATTGACACAAGATAAGAAGAATAAGATGACGTTTTACATTCGTCTCG
SE-X-XLOC_035024-2385-1
      CCGATTGACACAAGATAAGAAGAATAAGATGACGTTTTACATTCGTCTCG
CONSENSUS
      CCGATTGACACAAGATAAGAAGAATAAGATGACGTTTTACATTCGTCTCG
```

```
SE-X-XLOC_035024-2385-0
      AATCTTCCAAG---------------------------------------
SE-X-XLOC_035024-2385-1
      AATCTTCCAAGTATATGTACGTCGAAAACTTAATCCACGTGCACACATTT
CONSENSUS
      AATCTTCCAAG.......................................

SE-X-XLOC_035024-2385-0
      --------------------------------------------------
SE-X-XLOC_035024-2385-1
      TCAAAGTCTACATGTACACCAGATCACAAAGCTTCACTATCGTATCCACA
CONSENSUS
      ..................................................

SE-X-XLOC_035024-2385-0
      --------------------------------------------------
SE-X-XLOC_035024-2385-1
      TGTACACCGGCTGGCAAGATCTATGTGTACACCACAATTATCACTTGCGT
CONSENSUS
      ..................................................

SE-X-XLOC_035024-2385-0
      --------------------------------------------------
SE-X-XLOC_035024-2385-1
      TAATTGCTTATTTTTATTGATTCCTGAGTGATTGAGTTTTGTTGAATTTG
CONSENSUS
      ..................................................

SE-X-XLOC_035024-2385-0
      --------------------------------------------------
SE-X-XLOC_035024-2385-1
      CATATAATGCCTCGGAAAGCCTTTGTCACCACGAGTGCTTATGTGATTAG
CONSENSUS
      ..................................................

SE-X-XLOC_035024-2385-0     ------------
GGCTATATAGGTTCTGTTCGAAAAAGAAAAAAGAGAAT
SE-X-XLOC_035024-2385-1
      CTAAATCCATAGGGCTATATAGGTTCTGTTCGAAAAAGAAAAAAGAGAAT
CONSENSUS
      ............GGCTATATAGGTTCTGTTCGAAAAAGAAAAAAGAGAAT

SE-X-XLOC_035024-2385-0     CTATGGACTACAAAAGAGGAG
SE-X-XLOC_035024-2385-1     CTATGGACTACAAAAGAGGAG
CONSENSUS                   CTATGGACTACAAAAGAGGAG


alignment for event: RI-gBol036789-XLOC_010394-13026

RI-gBol036789-XLOC_010394-13026-0
      GCCAAAGTGGTGACCCCTCACCTCAAGCATGCTTTTGACAACCTCTGCAA
RI-gBol036789-XLOC_010394-13026-1
      GCCAAAGTGGTGACCCCTCACCTCAAGCATGCTTTTGACAACCTCTGCAA
CONSENSUS
```

GCCAAAGTGGTGACCCCTCACCTCAAGCATGCTTTTGACAACCTCTGCAA

RI-gBol036789-XLOC_010394-13026-0
TGCTAATCTAGATGACCCTAAACAGAACCATCCATCAGGTAAGTTATCTT
RI-gBol036789-XLOC_010394-13026-1
TGCTAATCTAGATGACCCTAAACAGAACCATCCATCAG------------
CONSENSUS
TGCTAATCTAGATGACCCTAAACAGAACCATCCATCAG............

RI-gBol036789-XLOC_010394-13026-0
TTTGGAGCTGGCTGCTTGGGTCGAGCCAAAAAACCGAGCTCTAATTACTT
RI-gBol036789-XLOC_010394-13026-1

CONSENSUS
....

RI-gBol036789-XLOC_010394-13026-0
CATAACTTTTGTTCTTAAACTCTGGCTGTGTCCAGTTTCAAAACCTGAAA
RI-gBol036789-XLOC_010394-13026-1

CONSENSUS
....

RI-gBol036789-XLOC_010394-13026-0
TCAGATTGAGCATTATTGTCTGTTTTTTGTTTGTTTGTCCCCTTGCCACA
RI-gBol036789-XLOC_010394-13026-1

CONSENSUS
....

RI-gBol036789-XLOC_010394-13026-0
GTTTAAGCTAAGTTCAATCCGCTAATCTGAAATGGTTCGAGAGGCATCTC
RI-gBol036789-XLOC_010394-13026-1 -
TTTAAGCTAAGTTCAATCCGCTAATCTGAAATGGTTCGAGAGGCATCTC
CONSENSUS
.TTTAAGCTAAGTTCAATCCGCTAATCTGAAATGGTTCGAGAGGCATCTC

RI-gBol036789-XLOC_010394-13026-0
ACTCAAGCTTGATTTGATCTCTGTTTCTCTTTACATGCTTGGTAGAAACC
RI-gBol036789-XLOC_010394-13026-1
ACTCAAGCTTGATTTGATCTCTGTTTCTCTTTACATGCTTGGTAGAAACC
CONSENSUS
ACTCAAGCTTGATTTGATCTCTGTTTCTCTTTACATGCTTGGTAGAAACC

RI-gBol036789-XLOC_010394-13026-0
AAAAGCTCTATATTACAGATCTTAAGGACGTGTAGTATGTGTGTTCATTT
RI-gBol036789-XLOC_010394-13026-1
AAAAGCTCTATATTACAGATCTTAAGGACGTGTAGTATGTGTGTTCATTT
CONSENSUS
AAAAGCTCTATATTACAGATCTTAAGGACGTGTAGTATGTGTGTTCATTT

RI-gBol036789-XLOC_010394-13026-0
TGTATAACTGAGAAAAGAATATGTATATTAATCAACTTATAAGATAGAGA
RI-gBol036789-XLOC_010394-13026-1
TGTATAACTGAGAAAAGAATATGTATATTAATCAACTTATAAGATAGAGA
CONSENSUS

TGTATAACTGAGAAAAGAATATGTATATTAATCAACTTATAAGATAGAGA

RI-gBol036789-XLOC_010394-13026-0
CTGAAAACAAATCTAAGTGAGATGACTGATGGAATCAGGACACTCGAGAC
RI-gBol036789-XLOC_010394-13026-1
CTGAAAACAAATCTAAGTGAGATGACTGATGGAATCAGGACACTCGAGAC
CONSENSUS
CTGAAAACAAATCTAAGTGAGATGACTGATGGAATCAGGACACTCGAGAC

RI-gBol036789-XLOC_010394-13026-0 GAATATAGTCATACATGAG
RI-gBol036789-XLOC_010394-13026-1 GAATATAGTCATACATGAG
CONSENSUS GAATATAGTCATACATGAG

alignment for event: RI-gBol036658-XLOC_010328-13490

RI-gBol036658-XLOC_010328-13490-0
GAACGTGGGATTTTGATAATAATAGCGACATGACGTTGAGGAAGAAGGGA
RI-gBol036658-XLOC_010328-13490-1
GAACGTGGGATTTTGATAATAATAGCGACATGACGTTGAGGAAGAAGGGA
CONSENSUS
GAACGTGGGATTTTGATAATAATAGCGACATGACGTTGAGGAAGAAGGGA

RI-gBol036658-XLOC_010328-13490-0
TTAGGTGCCGTGAAATCATCATCAAACACAAATCGAAAAGTAGTTGGTGA
RI-gBol036658-XLOC_010328-13490-1
TTAGGTGCCGTGAAATCATCATCAAACACAAATCGAAAAGTAGTTGGTGA
CONSENSUS
TTAGGTGCCGTGAAATCATCATCAAACACAAATCGAAAAGTAGTTGGTGA

RI-gBol036658-XLOC_010328-13490-0
TGGAGGATTAGGGTTAGGGTTAGACTGTGATAAGTATAAGAAAGGATTTG
RI-gBol036658-XLOC_010328-13490-1
TGGAGGATTAGGGTTAGGGTTAGACTGTGATAAGTATAAGAAAGGATTTG
CONSENSUS
TGGAGGATTAGGGTTAGGGTTAGACTGTGATAAGTATAAGAAAGGATTTG

RI-gBol036658-XLOC_010328-13490-0
GGAGGAAGAGGGTTTTGATTCCCATTGGCAAGAACCGAGAGCTTGAAGCT
RI-gBol036658-XLOC_010328-13490-1
GGAGGAAGAGGGTTTTGATTCCCATTGGCAAGAACCGAGAGCTTGAAGCT
CONSENSUS
GGAGGAAGAGGGTTTTGATTCCCATTGGCAAGAACCGAGAGCTTGAAGCT

RI-gBol036658-XLOC_010328-13490-0
CTTCCTCAAGACATCCTGGTTTGTCTTCTTTTAAACCAATCTCACAAACT
RI-gBol036658-XLOC_010328-13490-1
CTTCCTCAAGACATCCTG--------------------------------
CONSENSUS
CTTCCTCAAGACATCCTG................................

RI-gBol036658-XLOC_010328-13490-0
CGTTTTAATAGGTTTTCTATTATATGTGGGTTCTTTGGATTAGTTGTTTT
RI-gBol036658-XLOC_010328-13490-1

```
CONSENSUS
      ..................................................

RI-gBol036658-XLOC_010328-13490-0
      TAGTGTTCTTGTTCTGATGATTACTCACATAGAGATAAATCTTGAATTAT
RI-gBol036658-XLOC_010328-13490-1
      --------------------------------------------------
CONSENSUS
      ..................................................

RI-gBol036658-XLOC_010328-13490-0
      TTATTGTCTCTAGGGACAGAGTCAATACTGAAAATGGAAAATCTTCAGTT
RI-gBol036658-XLOC_010328-13490-1
      --------------------------------------------------
CONSENSUS
      ..................................................

RI-gBol036658-XLOC_010328-13490-0
      TCCCCTGTGAAATGCTTCTACTTTTGGCCAGAGAAAAAAAAATTATTGGG
RI-gBol036658-XLOC_010328-13490-1
      --------------------------------------------------
CONSENSUS
      ..................................................

RI-gBol036658-XLOC_010328-13490-0
      TGAGACTGAGATTACTGAAAATTTTCATTGGATTATTTGCAGATAAGAGT
RI-gBol036658-XLOC_010328-13490-1
      ------------------------------------------ATAAGAGT
CONSENSUS
      ..........................................ATAAGAGT

RI-gBol036658-XLOC_010328-13490-0
      TATATGTGGAGTGGAGCATGGAGATTTAAAGCAGTTGTTTAATGTGTCAA
RI-gBol036658-XLOC_010328-13490-1
      TATATGTGGAGTGGAGCATGGAGATTTAAAGCAGTTGTTTAATGTGTCAA
CONSENSUS
      TATATGTGGAGTGGAGCATGGAGATTTAAAGCAGTTGTTTAATGTGTCAA

RI-gBol036658-XLOC_010328-13490-0     AGACTATACGTGAAGCT
RI-gBol036658-XLOC_010328-13490-1     AGACTATACGTGAAGCT
CONSENSUS                             AGACTATACGTGAAGCT


alignment for event: RI-gBol023630-XLOC_025103-2662

RI-gBol023630-XLOC_025103-2662-0
      GCCAAGTGGAAACCTTCTAAAAAGAGTTTGGCTAGCATCACACTGCTGGG
RI-gBol023630-XLOC_025103-2662-1
      GCCAAGTGGAAACCTTCTAAAAAGAGTTTGGCTAGCATCACACTGCTGGG
CONSENSUS
      GCCAAGTGGAAACCTTCTAAAAAGAGTTTGGCTAGCATCACACTGCTGGG

RI-gBol023630-XLOC_025103-2662-0
      TGTTTGTTAGAGGGTGAGGATCTCTGAAAATATTGTCTAGGTATGGTTCA
RI-gBol023630-XLOC_025103-2662-1
```

TGTTTGTTAGAGGGTGAGGATCTCTGAAAATATTGTCTAG----------
CONSENSUS
TGTTTGTTAGAGGGTGAGGATCTCTGAAAATATTGTCTAG..........

RI-gBol023630-XLOC_025103-2662-0
TTGTTTATTGATTCTCTGAAATATCCTATTTCATCACTGTTTCAATTGCA
RI-gBol023630-XLOC_025103-2662-1

CONSENSUS
....

RI-gBol023630-XLOC_025103-2662-0
AGTGTATATGAATGTTAGAAGACTTCCTACAGTTCAATACCTCATATGTT
RI-gBol023630-XLOC_025103-2662-1

CONSENSUS
....

RI-gBol023630-XLOC_025103-2662-0
ACTTTGCCAAGCAGCTCTATAGCCATGTATTAGAAAACCCTTCTAGCTAA
RI-gBol023630-XLOC_025103-2662-1

CONSENSUS
....

RI-gBol023630-XLOC_025103-2662-0
GGTTGTACTGGTTGCTCAGATTGGAGTTACATGGGGTCTGTCATTCCCTA
RI-gBol023630-XLOC_025103-2662-1 -------------------
ATTGGAGTTACATGGGGTCTGTCATTCCCTA
CONSENSUS
...................ATTGGAGTTACATGGGGTCTGTCATTCCCTA

RI-gBol023630-XLOC_025103-2662-0
TCCCTTCTTTCACATTCAGTGGTTTCAAACGAACAGAAACACAAAACTTC
RI-gBol023630-XLOC_025103-2662-1
TCCCTTCTTTCACATTCAGTGGTTTCAAACGAACAGAAACACAAAACTTC
CONSENSUS
TCCCTTCTTTCACATTCAGTGGTTTCAAACGAACAGAAACACAAAACTTC

RI-gBol023630-XLOC_025103-2662-0
TCTTCCCTCCGGGAAGATCCTTGCATCTGATCCTCTCCAGCAGCCATTTT
RI-gBol023630-XLOC_025103-2662-1
TCTTCCCTCCGGGAAGATCCTTGCATCTGATCCTCTCCAGCAGCCATTTT
CONSENSUS
TCTTCCCTCCGGGAAGATCCTTGCATCTGATCCTCTCCAGCAGCCATTTT

RI-gBol023630-XLOC_025103-2662-0
CATCCCACACCTCCCTTCCATCATCCTAGCTTAAGAAAGAAGAAAAACAA
RI-gBol023630-XLOC_025103-2662-1
CATCCCACACCTCCCTTCCATCATCCTAGCTTAAGAAAGAAGAAAAACAA
CONSENSUS
CATCCCACACCTCCCTTCCATCATCCTAGCTTAAGAAAGAAGAAAAACAA

RI-gBol023630-XLOC_025103-2662-0
CATCAATGTCATACATATGTAAAGGAAGAGAAAGAATAATAAGTTTAAGA
RI-gBol023630-XLOC_025103-2662-1

CATCAATGTCATACATATGTAAAGGAAGAGAAAGAATAATAAGTTTAAGA
CONSENSUS
CATCAATGTCATACATATGTAAAGGAAGAGAAAGAATAATAAGTTTAAGA

RI-gBol023630-XLOC_025103-2662-0
CAGCTCAGTCATTGTAAATTGTTTTCTTTTACCGGTGAGCACGAAGATGG
RI-gBol023630-XLOC_025103-2662-1
CAGCTCAGTCATTGTAAATTGTTTTCTTTTACCGGTGAGCACGAAGATGG
CONSENSUS
CAGCTCAGTCATTGTAAATTGTTTTCTTTTACCGGTGAGCACGAAGATGG

RI-gBol023630-XLOC_025103-2662-0
GAATTTCATATTTGGTTATATAACTGCTGTAGAAGTTTTTTTTATTTCAT
RI-gBol023630-XLOC_025103-2662-1
GAATTTCATATTTGGTTATATAACTGCTGTAGAAGTTTTTTTTATTTCAT
CONSENSUS
GAATTTCATATTTGGTTATATAACTGCTGTAGAAGTTTTTTTTATTTCAT

RI-gBol023630-XLOC_025103-2662-0
TAATCAATCGTAGTTGAACATATTAATTTAAGAACTTTTGGTTTATTCTT
RI-gBol023630-XLOC_025103-2662-1
TAATCAATCGTAGTTGAACATATTAATTTAAGAACTTTTGGTTTATTCTT
CONSENSUS
TAATCAATCGTAGTTGAACATATTAATTTAAGAACTTTTGGTTTATTCTT

RI-gBol023630-XLOC_025103-2662-0
TATGTCTCTCTGAATTGCAGTGTAGAAAATGTCTACTTAGCTCATTTGTA
RI-gBol023630-XLOC_025103-2662-1
TATGTCTCTCTGAATTGCAGTGTAGAAAATGTCTACTTAGCTCATTTGTA
CONSENSUS
TATGTCTCTCTGAATTGCAGTGTAGAAAATGTCTACTTAGCTCATTTGTA

RI-gBol023630-XLOC_025103-2662-0
CCTTTTCAGATTATTCAATATTTTGTATTATGTTGTTGAATAATGTCACT
RI-gBol023630-XLOC_025103-2662-1
CCTTTTCAGATTATTCAATATTTTGTATTATGTTGTTGAATAATGTCACT
CONSENSUS
CCTTTTCAGATTATTCAATATTTTGTATTATGTTGTTGAATAATGTCACT

RI-gBol023630-XLOC_025103-2662-0 ATATAACG
RI-gBol023630-XLOC_025103-2662-1 ATATAACG
CONSENSUS ATATAACG

alignment for event: A3-X-XLOC_019998-6449

A3-X-XLOC_019998-6449-0
GTTTGCGTGTGTGGACCGTGGTCAAACAGAGAATAGCTGTTGTAGTCAGA
A3-X-XLOC_019998-6449-1
GTTTGCGTGTGTGGACCGTGGTCAAACAGAGAATAGCTGTTGTAGTCAGA
CONSENSUS
GTTTGCGTGTGTGGACCGTGGTCAAACAGAGAATAGCTGTTGTAGTCAGA

A3-X-XLOC_019998-6449-0
CAGAGAATAGCTGTTGTGTGAAGTGTCCATTCTGTGAGAGGTTTGCGTGT

A3-X-XLOC_019998-6449-1
      CAGAGAATAGCTGTTGTGTGAAGTGTCCATTCTGTGAGAGGTTTGCGTGT
CONSENSUS
      CAGAGAATAGCTGTTGTGTGAAGTGTCCATTCTGTGAGAGGTTTGCGTGT

A3-X-XLOC_019998-6449-0
      GTGGACCGTAATGTTCCTTGGCATGAATCTATGAGCAGTGAAGAGTTTCA
A3-X-XLOC_019998-6449-1
      GTGGACCGTAATGTTCCTTGGCATGAATCTATGAGCAGTGAAGAGTTTCA
CONSENSUS
      GTGGACCGTAATGTTCCTTGGCATGAATCTATGAGCAGTGAAGAGTTTCA

A3-X-XLOC_019998-6449-0
      GATTCTTCAGGTTCATTAAATATATCAAGATGATATCATATTGCATCGCT
A3-X-XLOC_019998-6449-1
      GATTCTTCAGGTTCATTAAATATATCAAGATGATATCATATTGCATCGCT
CONSENSUS
      GATTCTTCAGGTTCATTAAATATATCAAGATGATATCATATTGCATCGCT

A3-X-XLOC_019998-6449-0
      TGGCTAGGTATAAGAGGTGGAGACGATGTCAGCAATGTCGTGTAATGATT
A3-X-XLOC_019998-6449-1
      TGGCTAGGTATAAGAGGTGGAGACGATGTCAGCAATGTCGTGTAATGATT
CONSENSUS
      TGGCTAGGTATAAGAGGTGGAGACGATGTCAGCAATGTCGTGTAATGATT

A3-X-XLOC_019998-6449-0
      TTAATTGCTCAAGGCTGCAACCACATGACTTGCCGAGTACAGTGAAAGAC
A3-X-XLOC_019998-6449-1
      TTAATTGCTCAAGGCTGCAACCACATGACTTGCCG---------------
CONSENSUS
      TTAATTGCTCAAGGCTGCAACCACATGACTTGCCG...............

A3-X-XLOC_019998-6449-0
      AACAGAGTTACACTTGTGCTTTGTGGTGGATTGTTGCAGGTAGTTTACAG
A3-X-XLOC_019998-6449-1
      ---------------------------------------GTAGTTTACAG
CONSENSUS
      ....GTAGTTTACAG

A3-X-XLOC_019998-6449-0
      AGGAAAGCAACTCCATCATCTTGTCTACAAGAGGAAGGCTAAACAAGTTG
A3-X-XLOC_019998-6449-1
      AGGAAAGCAACTCCATCATCTTGTCTACAAGAGGAAGGCTAAACAAGTTG
CONSENSUS
      AGGAAAGCAACTCCATCATCTTGTCTACAAGAGGAAGGCTAAACAAGTTG

A3-X-XLOC_019998-6449-0   AAGACTTTAGCAATTGTGAGCAAATGATATAG
A3-X-XLOC_019998-6449-1   AAGACTTTAGCAATTGTGAGCAAATGATATAG
CONSENSUS   AAGACTTTAGCAATTGTGAGCAAATGATATAG

alignment for event: SE-gBol016445-XLOC_033306-16640

SE-gBol016445-XLOC_033306-16640-0

GTTATAAATCATGGTGTTAGCAAAGCAACTATAGTTGAGATGGTGAGTGT
SE-gBol016445-XLOC_033306-16640-1
GTTATAAATCATGGTGTTAGCAAAGCAACTATAGTTGAGATGGTGAGTGT
CONSENSUS
GTTATAAATCATGGTGTTAGCAAAGCAACTATAGTTGAGATGGTGAGTGT

SE-gBol016445-XLOC_033306-16640-0
TGCGCATGAGTTTTTTGGCATGCCTATGGACGAAAAAATGAAGCTATACT
SE-gBol016445-XLOC_033306-16640-1
TGCGCATGAGTTTTTTGGCATGCCTATGGACGAAAAAATGAAGCTATACT
CONSENSUS
TGCGCATGAGTTTTTTGGCATGCCTATGGACGAAAAAATGAAGCTATACT

SE-gBol016445-XLOC_033306-16640-0
CGGACGATCCAACAAAGACACCGAGATTATCGACGAGCTTTAATGTGAAG
SE-gBol016445-XLOC_033306-16640-1
CGGACGATCCAACAAAGACACCGAGATTATCGACGAGCTTTAATGTGAAG
CONSENSUS
CGGACGATCCAACAAAGACACCGAGATTATCGACGAGCTTTAATGTGAAG

SE-gBol016445-XLOC_033306-16640-0
GAAGAAGAGGTTAATAATTGGAGAGACTATCTAAGACTTCATTGTTATCC
SE-gBol016445-XLOC_033306-16640-1
GAAGAAGAGGTTAATAATTGGAGAGACTATCTAAGACTTCATTGTTATCC
CONSENSUS
GAAGAAGAGGTTAATAATTGGAGAGACTATCTAAGACTTCATTGTTATCC

SE-gBol016445-XLOC_033306-16640-0
AATCGACAAGTATGTTCATGAGTGGCCTTCCAACCCACCTTCTTTCAA--
SE-gBol016445-XLOC_033306-16640-1
AATCGACAAGTATGTTCATGAGTGGCCTTCCAACCCACCTTCTTTCAAGC
CONSENSUS
AATCGACAAGTATGTTCATGAGTGGCCTTCCAACCCACCTTCTTTCAA..

SE-gBol016445-XLOC_033306-16640-0

SE-gBol016445-XLOC_033306-16640-1
CCGGTCTGAGGGGGCATACACATGAACCATTGGAATTAGCCCTCAGACGG
CONSENSUS
....

SE-gBol016445-XLOC_033306-16640-0 --------------
GGAAGTTGTGAGTAAATACAGTAGAGAAATAAGAGA
SE-gBol016445-XLOC_033306-16640-1
ATCGGATATCCGGGGGAAGTTGTGAGTAAATACAGTAGAGAAATAAGAGA
CONSENSUS
..............GGAAGTTGTGAGTAAATACAGTAGAGAAATAAGAGA

SE-gBol016445-XLOC_033306-16640-0
ATTGGGATTTAAAATAGAGGAATTGATATCAGAGAGCTTAGGTTTAGAAA
SE-gBol016445-XLOC_033306-16640-1
ATTGGGATTTAAAATAGAGGAATTGATATCAGAGAGCTTAGGTTTAGAAA
CONSENSUS
ATTGGGATTTAAAATAGAGGAATTGATATCAGAGAGCTTAGGTTTAGAAA

SE-gBol016445-XLOC_033306-16640-0

```
      AAGATTACATGAAGAAAGTGCTTGGTGAACAAGGTCAACACATGGCAGTC
SE-gBol016445-XLOC_033306-16640-1
      AAGATTACATGAAGAAAGTGCTTGGTGAACAAGGTCAACACATGGCAGTC
CONSENSUS
      AAGATTACATGAAGAAAGTGCTTGGTGAACAAGGTCAACACATGGCAGTC

SE-gBol016445-XLOC_033306-16640-0
      AACTATTATCCTCCATGTCCTGAGCCTGAGCTCACCTATGGTTTACCTGC
SE-gBol016445-XLOC_033306-16640-1
      AACTATTATCCTCCATGTCCTGAGCCTGAGCTCACCTATGGTTTACCTGC
CONSENSUS
      AACTATTATCCTCCATGTCCTGAGCCTGAGCTCACCTATGGTTTACCTGC

SE-gBol016445-XLOC_033306-16640-0
      TCATACCGACCCAAATGCCCTCACCATTCTTCTCCAAGATGCTACCGTTT
SE-gBol016445-XLOC_033306-16640-1
      TCATACCGACCCAAATGCCCTCACCATTCTTCTCCAAGATGCTACCGTTT
CONSENSUS
      TCATACCGACCCAAATGCCCTCACCATTCTTCTCCAAGATGCTACCGTTT

SE-gBol016445-XLOC_033306-16640-0
      GCGGTCTCCAGATCTTGATCGACGGTCACTGGTTTGCTGTTAATCCTCGT
SE-gBol016445-XLOC_033306-16640-1
      GCGGTCTCCAGATCTTGATCGACGGTCACTGGTTTGCTGTTAATCCTCGT
CONSENSUS
      GCGGTCTCCAGATCTTGATCGACGGTCACTGGTTTGCTGTTAATCCTCGT

SE-gBol016445-XLOC_033306-16640-0
      CCTGATGCTTTTGTCATCAACATTGGTGACCAGTTGCAG
SE-gBol016445-XLOC_033306-16640-1
      CCTGATGCTTTTGTCATCAACATTGGTGACCAGTTGCAG
CONSENSUS
      CCTGATGCTTTTGTCATCAACATTGGTGACCAGTTGCAG



alignment for event: MXE-gBol035972-XLOC_011249-13701

MXE-gBol035972-XLOC_011249-13701-0
      CTATATTAGCGTATAGCAGAGAGACGTCTGTCTTCTACATCTACTATGTT
MXE-gBol035972-XLOC_011249-13701-1
      CTATATTAGCGTATAGCAGAGAGACGTCTGTCTTCTACATCTACTATGTT
CONSENSUS
      CTATATTAGCGTATAGCAGAGAGACGTCTGTCTTCTACATCTACTATGTT

MXE-gBol035972-XLOC_011249-13701-0
      CTCAAGACTTTCACGTCCATATTTTGTGAAGGAAGTGTGTTCTGTCTTGC
MXE-gBol035972-XLOC_011249-13701-1
      CTCAAGACTTTCACGTCCATATTTTGTGAAGGAAGTGTGTTCTGTCTTGC
CONSENSUS
      CTCAAGACTTTCACGTCCATATTTTGTGAAGGAAGTGTGTTCTGTCTTGC

MXE-gBol035972-XLOC_011249-13701-0
      CCTTGCTTATGTGGCTGATAATGTATCCGAAAATCAACGAGCCTCTGCAT
MXE-gBol035972-XLOC_011249-13701-1
      CCTTGCTTATGTG-------------------------------------
```

CONSENSUS
CCTTGCTTATGTG.....................................

MXE-gBol035972-XLOC_011249-13701-0
TTGGAATACTTACCGGAATTGGATCTTGCGCATTTGTATGTGCAAATTGT
MXE-gBol035972-XLOC_011249-13701-1

CONSENSUS
....

MXE-gBol035972-XLOC_011249-13701-0
TGTGCTCGATTCTTATCCACCACCGCTACATTTCAG--------------
MXE-gBol035972-XLOC_011249-13701-1
------------------------------------GTTTCCTTTCCATT
CONSENSUS
....

MXE-gBol035972-XLOC_011249-13701-0
--------------------------------GTCGCGACAACGGTAGCA
MXE-gBol035972-XLOC_011249-13701-1
CATATATGTAGACATGTGCATCTACATTTCAGGTCGCGACAACGGTAGCA
CONSENSUS
................................GTCGCGACAACGGTAGCA

MXE-gBol035972-XLOC_011249-13701-0
ATTTTTTCAACAGTGTACATGAGAATTTTTCTACCTGATTCTATCCGAGA
MXE-gBol035972-XLOC_011249-13701-1
ATTTTTTCAACAGTGTACATGAGAATTTTTCTACCTGATTCTATCCGAGA
CONSENSUS
ATTTTTTCAACAGTGTACATGAGAATTTTTCTACCTGATTCTATCCGAGA

MXE-gBol035972-XLOC_011249-13701-0
CAATAGCTTGGTTACTTCCATTGTTTCAACCGAGAAATTGAGTTACGTGT
MXE-gBol035972-XLOC_011249-13701-1
CAATAGCTTGGTTACTTCCATTGTTTCAACCGAGAAATTGAGTTACGTGT
CONSENSUS
CAATAGCTTGGTTACTTCCATTGTTTCAACCGAGAAATTGAGTTACGTGT

MXE-gBol035972-XLOC_011249-13701-0
TACTTGAAGATTACCCTGGACATAGAAATCAGATATCTAGAACAGTACGT
MXE-gBol035972-XLOC_011249-13701-1
TACTTGAAGATTACCCTGGACATAGAAATCAGATATCTAGAACAGTACGT
CONSENSUS
TACTTGAAGATTACCCTGGACATAGAAATCAGATATCTAGAACAGTACGT

MXE-gBol035972-XLOC_011249-13701-0
TCTGTACGTGAAATGGCCTCTCTAATGAGGAGCAG
MXE-gBol035972-XLOC_011249-13701-1
TCTGTACGTGAAATGGCCTCTCTAATGAGGAGCAG
CONSENSUS
TCTGTACGTGAAATGGCCTCTCTAATGAGGAGCAG

alignment for event: A3-X-XLOC_012986-8469

A3-X-XLOC_012986-8469-0
CGATCTTTCTTTCCGACCTGAAGACCGGACGATGCTATACCACCGTCCAA
A3-X-XLOC_012986-8469-1
CGATCTTTCTTTCCGACCTGAAGACCGGACGATGCTATACCACCGTCCAA
CONSENSUS
CGATCTTTCTTTCCGACCTGAAGACCGGACGATGCTATACCACCGTCCAA

A3-X-XLOC_012986-8469-0
ATGTCAAAGGAAGACGAAGAGATGTTGAGGACAGATTTGCCGGCTTTCAG
A3-X-XLOC_012986-8469-1
ATGTCAAAGGAAGACGAAGAGATGTTGAGGACAGATTTGCCGGCTTTCAG
CONSENSUS
ATGTCAAAGGAAGACGAAGAGATGTTGAGGACAGATTTGCCGGCTTTCAG

A3-X-XLOC_012986-8469-0
GGCTAAATAAGAGGAGATCGAGAAGAAGAAGATGGAGGTTTGGAAAAAGG
A3-X-XLOC_012986-8469-1
GGCTAAATAAGAGGAGATCGAGAAGAAGAAGATGGAGGTTTGGAAAAAGG
CONSENSUS
GGCTAAATAAGAGGAGATCGAGAAGAAGAAGATGGAGGTTTGGAAAAAGG

A3-X-XLOC_012986-8469-0
ATTTAAGCTCAACTTTGTTGCGTCAAAGAAGAGACCGAGCGAGTGGATCG
A3-X-XLOC_012986-8469-1
ATTTAAGCTCAACTTTGTTGCGTCAAAGAAGAGACCGAGCGAGTG-----
CONSENSUS
ATTTAAGCTCAACTTTGTTGCGTCAAAGAAGAGACCGAGCGAGTG.....

A3-X-XLOC_012986-8469-0
GACTTCAATGTTAGGCCTACAACTAAAGTGATCAGTATCACGAAAGACAT
A3-X-XLOC_012986-8469-1

CONSENSUS
....

A3-X-XLOC_012986-8469-0
GATATAGTATATGGTTTTGGGGGCTTATATGAGTTATGTGGATCAAAGCT
A3-X-XLOC_012986-8469-1

CONSENSUS
....

A3-X-XLOC_012986-8469-0
CTTTCTTTTTTATGAGAAAGACCATTTCTTCTTCTCGGTAATGTTATACT
A3-X-XLOC_012986-8469-1

CONSENSUS
....

A3-X-XLOC_012986-8469-0
TGATGACTAAGAAGCTGAGTGGTGGAATTACATATCAGGAACTTGAAGGT
A3-X-XLOC_012986-8469-1
---------------------------------------GAACTTGAAGGT
CONSENSUS
......................................GAACTTGAAGGT

A3-X-XLOC_012986-8469-0
      TTAGCTGACCCCATGAGGAAAGAAGTTGATTTGGCCAGAAAGGAGATTGA
A3-X-XLOC_012986-8469-1
      TTAGCTGACCCCATGAGGAAAGAAGTTGATTTGGCCAGAAAGGAGATTGA
CONSENSUS
      TTAGCTGACCCCATGAGGAAAGAAGTTGATTTGGCCAGAAAGGAGATTGA

A3-X-XLOC_012986-8469-0   TTCTGTCAATAAATTATTAAAGCCATTGGCAAC
A3-X-XLOC_012986-8469-1   TTCTGTCAATAAATTATTAAAGCCATTGGCAAC
CONSENSUS   TTCTGTCAATAAATTATTAAAGCCATTGGCAAC

alignment for event: A5-X-XLOC_043557-15884

A5-X-XLOC_043557-15884-0
      TTTTCAATTTTCAGAAAATGATCATGCTGTTGGAAGCTCGATAGAGAGCT
A5-X-XLOC_043557-15884-1
      TTTTCAATTTTCAGAAAATGATCATGCTGTTGGAAGCTCGATAGAGAGCT
CONSENSUS
      TTTTCAATTTTCAGAAAATGATCATGCTGTTGGAAGCTCGATAGAGAGCT

A5-X-XLOC_043557-15884-0
      GGAAGATGCAGAGCAAAAGGTCACAAAATTAGGAAGAACGATAACAACGT
A5-X-XLOC_043557-15884-1
      GGAAGATGCAGAGCAAAAGGTCACAAAATTAGGAAGAACGATAACAACGT
CONSENSUS
      GGAAGATGCAGAGCAAAAGGTCACAAAATTAGGAAGAACGATAACAACGT

A5-X-XLOC_043557-15884-0   GT----
ATTATCCTGGTGACGATATTCCTGGAGCTGGAGCTGTGACAACA
A5-X-XLOC_043557-15884-1
      GTGTGAATTATCCTGGTGACGATATTCCTGGAGCTGGAGCTGTGACAACA
CONSENSUS
      GT....ATTATCCTGGTGACGATATTCCTGGAGCTGGAGCTGTGACAACA

A5-X-XLOC_043557-15884-0
      AAGCTCTCTTAAGGTGTGACCAGGGACCTTTTGTATACCAAGCATATACG
A5-X-XLOC_043557-15884-1
      AAGCTCTCTTAAGGTGTGACCAGGGACCTTTTGTATACCAAGCATATACG
CONSENSUS
      AAGCTCTCTTAAGGTGTGACCAGGGACCTTTTGTATACCAAGCATATACG

A5-X-XLOC_043557-15884-0
      GAGAATCACATCATCCATGGCACTAGGTCCATAAATACAAAGCACCCGGT
A5-X-XLOC_043557-15884-1
      GAGAATCACATCATCCATGGCACTAGGTCCATAAATACAAAGCACCCGGT
CONSENSUS
      GAGAATCACATCATCCATGGCACTAGGTCCATAAATACAAAGCACCCGGT

A5-X-XLOC_043557-15884-0
      TGTGGTCGGTGGTTCAGACACGGAAGAGACTTCTAGGTTATTTAATTTTT
A5-X-XLOC_043557-15884-1
      TGTGGTCGGTGGTTCAGACACGGAAGAGACTTCTAGGTTATTTAATTTTT
CONSENSUS
      TGTGGTCGGTGGTTCAGACACGGAAGAGACTTCTAGGTTATTTAATTTTT

A5-X-XLOC_043557-15884-0
TTGTGAGATGATCTACCCGGGAAGACATCATGCCGTATCATTTTATTAGA
A5-X-XLOC_043557-15884-1
TTGTGAGATGATCTACCCGGGAAGACATCATGCCGTATCATTTTATTAGA
CONSENSUS
TTGTGAGATGATCTACCCGGGAAGACATCATGCCGTATCATTTTATTAGA

A5-X-XLOC_043557-15884-0
ACAACACAAATTTTCTTTTGCTGCCTTTTTGTTTGAATTCGCTTTTGACC
A5-X-XLOC_043557-15884-1
ACAACACAAATTTTCTTTTGCTGCCTTTTTGTTTGAATTCGCTTTTGACC
CONSENSUS
ACAACACAAATTTTCTTTTGCTGCCTTTTTGTTTGAATTCGCTTTTGACC

A5-X-XLOC_043557-15884-0
ATTACCTTATGTGCATGTTCTCCCAACATATCAACGATATTTTTTAATTG
A5-X-XLOC_043557-15884-1
ATTACCTTATGTGCATGTTCTCCCAACATATCAACGATATTTTTTAATTG
CONSENSUS
ATTACCTTATGTGCATGTTCTCCCAACATATCAACGATATTTTTTAATTG

A5-X-XLOC_043557-15884-0
TATAGAGATCTTTACTCTTGTGGTGTTATCAAATTCTTTTGTTAGTTGCC
A5-X-XLOC_043557-15884-1
TATAGAGATCTTTACTCTTGTGGTGTTATCAAATTCTTTTGTTAGTTGCC
CONSENSUS
TATAGAGATCTTTACTCTTGTGGTGTTATCAAATTCTTTTGTTAGTTGCC

A5-X-XLOC_043557-15884-0 A
A5-X-XLOC_043557-15884-1 A
CONSENSUS A

alignment for event: A3-gBol040565-XLOC_006059-577

A3-gBol040565-XLOC_006059-577-0
GATTCCGTGATCTACAGATTCATCCCGGCTGGTCTTGCCAATCACTACCG
A3-gBol040565-XLOC_006059-577-1
GATTCCGTGATCTACAGATTCATCCCGGCTGGTCTTGCCAATCACTACCG
CONSENSUS
GATTCCGTGATCTACAGATTCATCCCGGCTGGTCTTGCCAATCACTACCG

A3-gBol040565-XLOC_006059-577-0
TCCCTCTTTGCTTGCTGGTTCCATTGCGAAAGTCGATCGGTTTGAAGATT
A3-gBol040565-XLOC_006059-577-1
TCCCTCTTTGCTTGCTGGTTCCATTGCGAAAGTCGATCGGTTTGAAGATT
CONSENSUS
TCCCTCTTTGCTTGCTGGTTCCATTGCGAAAGTCGATCGGTTTGAAGATT

A3-gBol040565-XLOC_006059-577-0
CATCCAATCATAGGATTGGCCAGGCCTAATTCAACCATATGATGCAAAAG
A3-gBol040565-XLOC_006059-577-1
CATCCAATCATAGGATTGGCCAG---------------------------
CONSENSUS

```
      CATCCAATCATAGGATTGGCCAG...........................

A3-gBol040565-XLOC_006059-577-0
      AACTTTGCTATGTTCTGGAAACAGAGAAATGAATGAAGGGTCAGAAGCTT
A3-gBol040565-XLOC_006059-577-1  ------------------------
AGAAATGAATGAAGGGTCAGAAGCTT
CONSENSUS
      ........................AGAAATGAATGAAGGGTCAGAAGCTT

A3-gBol040565-XLOC_006059-577-0
      TGATGCAGAGGTCAAAGCATTGAGAGAAAGAAAGAAAACGGGAGGCTGAC
A3-gBol040565-XLOC_006059-577-1
      TGATGCAGAGGTCAAAGCATTGAGAGAAAGAAAGAAAACGGGAGGCTGAC
CONSENSUS
      TGATGCAGAGGTCAAAGCATTGAGAGAAAGAAAGAAAACGGGAGGCTGAC

A3-gBol040565-XLOC_006059-577-0
      ATGAAAACAGAAAATCAGTGAAGAAAGATCTTGATACAAAACTGCACACA
A3-gBol040565-XLOC_006059-577-1
      ATGAAAACAGAAAATCAGTGAAGAAAGATCTTGATACAAAACTGCACACA
CONSENSUS
      ATGAAAACAGAAAATCAGTGAAGAAAGATCTTGATACAAAACTGCACACA

A3-gBol040565-XLOC_006059-577-0
      GAACAATGTAATATGTAGAGATAGTGGACATGATTCTTTTTTTTCATTGT
A3-gBol040565-XLOC_006059-577-1
      GAACAATGTAATATGTAGAGATAGTGGACATGATTCTTTTTTTTCATTGT
CONSENSUS
      GAACAATGTAATATGTAGAGATAGTGGACATGATTCTTTTTTTTCATTGT

A3-gBol040565-XLOC_006059-577-0  GCAAAGATGTAGAAAACCATCTTTGCAAC
A3-gBol040565-XLOC_006059-577-1  GCAAAGATGTAGAAAACCATCTTTGCAAC
CONSENSUS                        GCAAAGATGTAGAAAACCATCTTTGCAAC


alignment for event: A3-gBol002198-XLOC_049457-1519

A3-gBol002198-XLOC_049457-1519-0
      AAGATTATGCTACAACAGGGAACAGCAGCAGCAGCAGCCACCAAAGTCGT
A3-gBol002198-XLOC_049457-1519-1
      AAGATTATGCTACAACAGGGAACAGCAGCAGCAGCAGCCACCAAAGTCGT
CONSENSUS
      AAGATTATGCTACAACAGGGAACAGCAGCAGCAGCAGCCACCAAAGTCGT

A3-gBol002198-XLOC_049457-1519-0
      GTGCCTGACTCAAGTAGTTACCGAGGACGAGCTTAAAAATGATGAGGAGT
A3-gBol002198-XLOC_049457-1519-1
      GTGCCTGACTCAAGTAGTTACCGAGGACGAGCTTAAAAATGATGAGGAGT
CONSENSUS
      GTGCCTGACTCAAGTAGTTACCGAGGACGAGCTTAAAAATGATGAGGAGT

A3-gBol002198-XLOC_049457-1519-0
      ACGAAGACATTGTTGTAGACATGAGAGATGAAGGCGGAAAGTTTGCCTTT
A3-gBol002198-XLOC_049457-1519-1
      ACGAAGACATTGTTGTAGACATGAGAGATGAAGGCGGAAAGTTTG-----
```

CONSENSUS
ACGAAGACATTGTTGTAGACATGAGAGATGAAGGCGGAAAGTTTG.....

A3-gBol002198-XLOC_049457-1519-0
TGTTGTCCCACATTCTGCTATAGAGAATCAGGCCATAACAGGAGACTGCA
A3-gBol002198-XLOC_049457-1519-1

CONSENSUS
....

A3-gBol002198-XLOC_049457-1519-0
TAACTTCCTTATCTATATATCATCCAAGACACATCCTACTTAGAAGGAAC
A3-gBol002198-XLOC_049457-1519-1

CONSENSUS
....

A3-gBol002198-XLOC_049457-1519-0
AACTTGTTGTTTTGTGTTTTTTTTGCTCTTGGATTCGAATATGTTTGCTA
A3-gBol002198-XLOC_049457-1519-1

CONSENSUS
....

A3-gBol002198-XLOC_049457-1519-0
TTAAAATGCAGGTGCGTTAACTAAAGTCGTGATCCCTCGCCCCAGCTCCA
A3-gBol002198-XLOC_049457-1519-1 -----------
GTGCGTTAACTAAAGTCGTGATCCCTCGCCCCAGCTCCA
CONSENSUS
...........GTGCGTTAACTAAAGTCGTGATCCCTCGCCCCAGCTCCA

A3-gBol002198-XLOC_049457-1519-0 ACGGTGAGCCAGTTCAGGGCGTTGGCAAG
A3-gBol002198-XLOC_049457-1519-1 ACGGTGAGCCAGTTCAGGGCGTTGGCAAG
CONSENSUS ACGGTGAGCCAGTTCAGGGCGTTGGCAAG

alignment for event: RI-gBol041431-XLOC_004895-3098

RI-gBol041431-XLOC_004895-3098-0
CCGTCTCCAAAAGTGTGTCTGCAACATTACTGAAACGAAACCTTCCCATT
RI-gBol041431-XLOC_004895-3098-1
CCGTCTCCAAAAGTGTGTCTGCAACATTACTGAAACGAAACCTTCCCATT
CONSENSUS
CCGTCTCCAAAAGTGTGTCTGCAACATTACTGAAACGAAACCTTCCCATT

RI-gBol041431-XLOC_004895-3098-0
GGATTCTCACTACCTTTTGTTTTGTTTTTTAAATTAAATCTTTTGAGCAG
RI-gBol041431-XLOC_004895-3098-1
GGATTCTCACTACCTTTTGTTTTGTTTTTTAAATTAAATCTTTTGAGCAG
CONSENSUS
GGATTCTCACTACCTTTTGTTTTGTTTTTTAAATTAAATCTTTTGAGCAG

RI-gBol041431-XLOC_004895-3098-0
AAACAGAGAAAGAAAATTGTGTCGTAGTGGTTCTTTGTTTTAGTTCGAGA
RI-gBol041431-XLOC_004895-3098-1

AAACAGAGAAAGAAAATTGTGTCGTAGTGGTTCTTTGTTTTAGTTCGAGA
CONSENSUS
AAACAGAGAAAGAAAATTGTGTCGTAGTGGTTCTTTGTTTTAGTTCGAGA

RI-gBol041431-XLOC_004895-3098-0
TGGAAAGAGATTTTCTTGGTTTGAGCGACAAGAAGTATCTCAGCAACGTT
RI-gBol041431-XLOC_004895-3098-1
TGGAAAGAGATTTTCTTGGTTTGAGCGACAAGAAGTATCTCAGCAACGTT
CONSENSUS
TGGAAAGAGATTTTCTTGGTTTGAGCGACAAGAAGTATCTCAGCAACGTT

RI-gBol041431-XLOC_004895-3098-0
AAACGCGAGGCCAACGATGATCGTGTCGGAGAACGAGGTTTGTGTTCTTG
RI-gBol041431-XLOC_004895-3098-1
AAACGCGAGGCCAACGATGATCGTGTCGGAGAACGAG-------------
CONSENSUS
AAACGCGAGGCCAACGATGATCGTGTCGGAGAACGAG.............

RI-gBol041431-XLOC_004895-3098-0
TCTCTCTACCGTATAAGCATGTTTACTATAGTCCAAAGTTGTTGTTTTTA
RI-gBol041431-XLOC_004895-3098-1

CONSENSUS
....

RI-gBol041431-XLOC_004895-3098-0
AATAGAATTAACTTAGATTTGATCAACTGCTTAGATTCCAAAACGTTGTT
RI-gBol041431-XLOC_004895-3098-1

CONSENSUS
....

RI-gBol041431-XLOC_004895-3098-0
GTAAAAGTAGATAATAATAATAATATTTGATCAAATGATGCATGAAAAAT
RI-gBol041431-XLOC_004895-3098-1

CONSENSUS
....

RI-gBol041431-XLOC_004895-3098-0
GAGAATTGGAGTTTACAAAATGAGTAATTTTATTTGATGTCAATAAAAAT
RI-gBol041431-XLOC_004895-3098-1

CONSENSUS
....

RI-gBol041431-XLOC_004895-3098-0
TGGATAATTTTAGTATTGAGCAAGAAGGCAGCTATACAATGGGGAAAGGC
RI-gBol041431-XLOC_004895-3098-1 -------------
TATTGAGCAAGAAGGCAGCTATACAATGGGGAAAGGC
CONSENSUS
.............TATTGAGCAAGAAGGCAGCTATACAATGGGGAAAGGC

RI-gBol041431-XLOC_004895-3098-0
AAAGCTCTTGCCTAATTCAAATTTCATGCCTGATTTTCAGGTTTGTTCAA
RI-gBol041431-XLOC_004895-3098-1

AAAGCTCTTGCCTAATTCAAATTTCATGCCTGATTTTCAGGTTTGTTCAA
CONSENSUS
AAAGCTCTTGCCTAATTCAAATTTCATGCCTGATTTTCAGGTTTGTTCAA

RI-gBol041431-XLOC_004895-3098-0
CTGCTTTTAAGATTCAATTCAACTTCTTTTAATCACTAGTTTGGTTACAG
RI-gBol041431-XLOC_004895-3098-1
CTGCTTTTAAGATTCAATTCAACTTCTTTTAATCACTAGTTTGGTTACAG
CONSENSUS
CTGCTTTTAAGATTCAATTCAACTTCTTTTAATCACTAGTTTGGTTACAG

RI-gBol041431-XLOC_004895-3098-0
AACTTTCACAATTTCACCATCTTATGGCTGGGGCTGATTTAATGGTTACA
RI-gBol041431-XLOC_004895-3098-1
AACTTTCACAATTTCACCATCTTATGGCTGGGGCTGATTTAATGGTTACA
CONSENSUS
AACTTTCACAATTTCACCATCTTATGGCTGGGGCTGATTTAATGGTTACA

RI-gBol041431-XLOC_004895-3098-0
TTAACTACTTTCCCGGACAACTGTCAGCGGAAAGCTCCGGGAGAATGGTC
RI-gBol041431-XLOC_004895-3098-1
TTAACTACTTTCCCGGACAACTGTCAGCGGAAAGCTCCGGGAGAATGGTC
CONSENSUS
TTAACTACTTTCCCGGACAACTGTCAGCGGAAAGCTCCGGGAGAATGGTC

RI-gBol041431-XLOC_004895-3098-0
GGAAAACCTGTCGGAGTTTTAATTTTATTTCTTGAACTTTTATTGATTTG
RI-gBol041431-XLOC_004895-3098-1
GGAAAACCTGTCGGAGTTTTAATTTTATTTCTTGAACTTTTATTGATTTG
CONSENSUS
GGAAAACCTGTCGGAGTTTTAATTTTATTTCTTGAACTTTTATTGATTTG

RI-gBol041431-XLOC_004895-3098-0
TTAACAAGATTTTTAATTGATCTATCAAAAGAAAAAAAACTATTGTGTGT
RI-gBol041431-XLOC_004895-3098-1
TTAACAAGATTTTTAATTGATCTATCAAAAGAAAAAAAACTATTGTGTGT
CONSENSUS
TTAACAAGATTTTTAATTGATCTATCAAAAGAAAAAAAACTATTGTGTGT

RI-gBol041431-XLOC_004895-3098-0
GGCCAATCATTGATGGAAATTGTAAAAGGAAAAAAATAGTTTTCTTTATA
RI-gBol041431-XLOC_004895-3098-1
GGCCAATCATTGATGGAAATTGTAAAAGGAAAAAAATAGTTTTCTTTATA
CONSENSUS
GGCCAATCATTGATGGAAATTGTAAAAGGAAAAAAATAGTTTTCTTTATA

RI-gBol041431-XLOC_004895-3098-0
AGTGAGATCTAAGAGTCGCTAGCCAGCCGCAAAATTAGAAAACAATAATA
RI-gBol041431-XLOC_004895-3098-1
AGTGAGATCTAAGAGTCGCTAGCCAGCCGCAAAATTAGAAAACAATAATA
CONSENSUS
AGTGAGATCTAAGAGTCGCTAGCCAGCCGCAAAATTAGAAAACAATAATA

RI-gBol041431-XLOC_004895-3098-0
AAAGATGTGGCGACTGGCCTTTGAAGTTTTAGATAAACGCATATGTCGGC
RI-gBol041431-XLOC_004895-3098-1

AAAGATGTGGCGACTGGCCTTTGAAGTTTTAGATAAACGCATATGTCGGC
CONSENSUS
AAAGATGTGGCGACTGGCCTTTGAAGTTTTAGATAAACGCATATGTCGGC

RI-gBol041431-XLOC_004895-3098-0
ACTTTTTGTATTAAAAGACATAGTGAAAAAAAAATGTTAAGATGTTGACG
RI-gBol041431-XLOC_004895-3098-1
ACTTTTTGTATTAAAAGACATAGTGAAAAAAAAATGTTAAGATGTTGACG
CONSENSUS
ACTTTTTGTATTAAAAGACATAGTGAAAAAAAAATGTTAAGATGTTGACG

RI-gBol041431-XLOC_004895-3098-0
TTTCAGGCGGGCTCTTACCAGCGGGGCCCAGTTTCTGCGGCCAGCAATCT
RI-gBol041431-XLOC_004895-3098-1
TTTCAGGCGGGCTCTTACCAGCGGGGCCCAGTTTCTGCGGCCAGCAATCT
CONSENSUS
TTTCAGGCGGGCTCTTACCAGCGGGGCCCAGTTTCTGCGGCCAGCAATCT

RI-gBol041431-XLOC_004895-3098-0
CCGCAGAAGCCAATTTAGCGGTGGTGCGTTTCAGAACGCGAATCCGCCTT
RI-gBol041431-XLOC_004895-3098-1
CCGCAGAAGCCAATTTAGCGGTGGTGCGTTTCAGAACGCGAATCCGCCTT
CONSENSUS
CCGCAGAAGCCAATTTAGCGGTGGTGCGTTTCAGAACGCGAATCCGCCTT

RI-gBol041431-XLOC_004895-3098-0
TACTTGGCGGTTCAGTTCCGTTAACTAATCATTCTTCTTTCCGTCCTGCT
RI-gBol041431-XLOC_004895-3098-1
TACTTGGCGGTTCAGTTCCGTTAACTAATCATTCTTCTTTCCGTCCTGCT
CONSENSUS
TACTTGGCGGTTCAGTTCCGTTAACTAATCATTCTTCTTTCCGTCCTGCT

RI-gBol041431-XLOC_004895-3098-0 TTTAACTTGTCGGCGGATGCTCG
RI-gBol041431-XLOC_004895-3098-1 TTTAACTTGTCGGCGGATGCTCG
CONSENSUS TTTAACTTGTCGGCGGATGCTCG

alignment for event: A3-gBol041496-XLOC_004939-9120

A3-gBol041496-XLOC_004939-9120-0
AGGTATGATGGAAATGATAAGCCTAGCAGCAGTAATTCACCTCAAGGAGA
A3-gBol041496-XLOC_004939-9120-1
AGGTATGATGGAAATGATAAGCCTAGCAGCAGTAATTCACCTCAAGGAGA
CONSENSUS
AGGTATGATGGAAATGATAAGCCTAGCAGCAGTAATTCACCTCAAGGAGA

A3-gBol041496-XLOC_004939-9120-0
TGAAAATACGGCTCAGACGTGAAGCTTAGACCAGCCTCTAATATCTTGCA
A3-gBol041496-XLOC_004939-9120-1
TGAAAATACGGCTCAGACGTGAAGCTTAGACCAG----------------
CONSENSUS
TGAAAATACGGCTCAGACGTGAAGCTTAGACCAG................

A3-gBol041496-XLOC_004939-9120-0
ATGCAGACCATGGCTTGGTGCGTTTGGAGTAATACGCCAAATCACTGATC

A3-gBol041496-XLOC_004939-9120-1 ------
ACCATGGCTTGGTGCGTTTGGAGTAATACGCCAAATCACTGATC
CONSENSUS
      ......ACCATGGCTTGGTGCGTTTGGAGTAATACGCCAAATCACTGATC

A3-gBol041496-XLOC_004939-9120-0
      CGAATCACCTCACTGAAGCAGTAGAAGTGGATAATAATATGCCATGTTAC
A3-gBol041496-XLOC_004939-9120-1
      CGAATCACCTCACTGAAGCAGTAGAAGTGGATAATAATATGCCATGTTAC
CONSENSUS
      CGAATCACCTCACTGAAGCAGTAGAAGTGGATAATAATATGCCATGTTAC

A3-gBol041496-XLOC_004939-9120-0
      TCGAATACTATATATTCTAGAGCTTGCTTTGATTACTTGTCACCAGATCT
A3-gBol041496-XLOC_004939-9120-1
      TCGAATACTATATATTCTAGAGCTTGCTTTGATTACTTGTCACCAGATCT
CONSENSUS
      TCGAATACTATATATTCTAGAGCTTGCTTTGATTACTTGTCACCAGATCT

A3-gBol041496-XLOC_004939-9120-0
      GTAAAACTGCTCCTGGTTTTGTCTCTCTGTTAACTCAGATCAGGACTGAA
A3-gBol041496-XLOC_004939-9120-1
      GTAAAACTGCTCCTGGTTTTGTCTCTCTGTTAACTCAGATCAGGACTGAA
CONSENSUS
      GTAAAACTGCTCCTGGTTTTGTCTCTCTGTTAACTCAGATCAGGACTGAA

A3-gBol041496-XLOC_004939-9120-0
      TGTAACTTGTCCTTTATTGAATAGATCAAAACATGTTGAAGGATTTATGT
A3-gBol041496-XLOC_004939-9120-1
      TGTAACTTGTCCTTTATTGAATAGATCAAAACATGTTGAAGGATTTATGT
CONSENSUS
      TGTAACTTGTCCTTTATTGAATAGATCAAAACATGTTGAAGGATTTATGT

A3-gBol041496-XLOC_004939-9120-0 TCTCTTCCATTCACATACATTCATCAAG
A3-gBol041496-XLOC_004939-9120-1 TCTCTTCCATTCACATACATTCATCAAG
CONSENSUS   TCTCTTCCATTCACATACATTCATCAAG

alignment for event: RI-gBol041431-XLOC_004895-3095

RI-gBol041431-XLOC_004895-3095-0
      GCTCAAGCTATTATGTTATGTGCTCGGAACGGTTTGAAAGGAGAAACTGG
RI-gBol041431-XLOC_004895-3095-1
      GCTCAAGCTATTATGTTATGTGCTCGGAACGGTTTGAAAGGAGAAACTGG
CONSENSUS
      GCTCAAGCTATTATGTTATGTGCTCGGAACGGTTTGAAAGGAGAAACTGG

RI-gBol041431-XLOC_004895-3095-0
      AGAGAGCAGTTTGAAGAAACCAGTTCAAGAAACTGAAAGAGTTTATGGAA
RI-gBol041431-XLOC_004895-3095-1
      AGAGAGCAGTTTGAAGAAACCAGTTCAAGAAACTGAAAGAGTTTATGGAA
CONSENSUS
      AGAGAGCAGTTTGAAGAAACCAGTTCAAGAAACTGAAAGAGTTTATGGAA

RI-gBol041431-XLOC_004895-3095-0

AACAAGTCCATAACGCTGCTGCTGCTGCGGCATCATCAAGCTCTGCCACT
RI-gBol041431-XLOC_004895-3095-1
AACAAGTCCATAACGCTGCTGCTGCTGCGGCATCATCAAGCTCTGCCACT
CONSENSUS
AACAAGTCCATAACGCTGCTGCTGCTGCGGCATCATCAAGCTCTGCCACT

RI-gBol041431-XLOC_004895-3095-0
TACGCTGATAGTTTCTCTAGGTGTAGAGACAGACCCGTTGGTGCGACTAA
RI-gBol041431-XLOC_004895-3095-1
TACGCTGATAGTTTCTCTAGGTGTAGAGACAGACCCGTTGGTGCGACTAA
CONSENSUS
TACGCTGATAGTTTCTCTAGGTGTAGAGACAGACCCGTTGGTGCGACTAA

RI-gBol041431-XLOC_004895-3095-0
TGCAATGAGCATGATCGAATCATTCAACGCAGCAGGTCCTGGAAATATGA
RI-gBol041431-XLOC_004895-3095-1
TGCAATGAGCATGATCGAATCATTCAACGCAGCAGGTCCTGGAAATATGA
CONSENSUS
TGCAATGAGCATGATCGAATCATTCAACGCAGCAGGTCCTGGAAATATGA

RI-gBol041431-XLOC_004895-3095-0
TTCCTTCAGGTATATATATCGACATGAAATCAAATTTTTGTGTGTTCAAA
RI-gBol041431-XLOC_004895-3095-1
TTCCTTCAG---
CONSENSUS
TTCCTTCAG...

RI-gBol041431-XLOC_004895-3095-0
ATTTTCATCGCATAAATCATGTCTAATTGCTAAATTTAAATTTCTGATAC
RI-gBol041431-XLOC_004895-3095-1

CONSENSUS
....

RI-gBol041431-XLOC_004895-3095-0
ACTTCCAACTTAAAACCAGTATTGATAGATCATAACGTTTCACATATTAG
RI-gBol041431-XLOC_004895-3095-1

CONSENSUS
....

RI-gBol041431-XLOC_004895-3095-0
TTCATTAATACTCCACCTAGCGATGCTAGACTTTTATTCTTTAACACTAA
RI-gBol041431-XLOC_004895-3095-1

CONSENSUS
....

RI-gBol041431-XLOC_004895-3095-0
TCTAAAAGATGTTTCAATCTCAGTTCCTCAAGCTCGTAAAGCCTCATTGG
RI-gBol041431-XLOC_004895-3095-1
--------------------------------CTCGTAAAGCCTCATTGG
CONSENSUS
................................CTCGTAAAGCCTCATTGG

RI-gBol041431-XLOC_004895-3095-0 CTCGGTTCTTGGAGAAGCGCAAAGAGAG

RI-gBol041431-XLOC_004895-3095-1 CTCGGTTCTTGGAGAAGCGCAAAGAGAG
CONSENSUS CTCGGTTCTTGGAGAAGCGCAAAGAGAG

alignment for event: A5-gBol041443-XLOC_004903-12264

A5-gBol041443-XLOC_004903-12264-0
ATGTAGTTGGCCAAATCCTTTCCGTTCAAGGCTCTGATCTGAACAACTCT
A5-gBol041443-XLOC_004903-12264-1
ATGTAGTTGGCCAAATCCTTTCCGTTCAAGGCTCTGATCTGAACAACTCT
CONSENSUS
ATGTAGTTGGCCAAATCCTTTCCGTTCAAGGCTCTGATCTGAACAACTCT

A5-gBol041443-XLOC_004903-12264-0
GCAGCAACAACTCGACTTGCCGTCCGTTTCCTCACTGACCC---------
A5-gBol041443-XLOC_004903-12264-1
GCAGCAACAACTCGACTTGCCGTCCGTTTCCTCACTGACCCGTAAGCCAT
CONSENSUS
GCAGCAACAACTCGACTTGCCGTCCGTTTCCTCACTGACCC.........

A5-gBol041443-XLOC_004903-12264-0

A5-gBol041443-XLOC_004903-12264-1
GAATTCATCTTTAAATAGTTTTGATCCTTATCTCATTTAGTTCTTTCTTT
CONSENSUS
....

A5-gBol041443-XLOC_004903-12264-0

A5-gBol041443-XLOC_004903-12264-1
CAAATAATTCCGTTGTGGTGTAATGGAATGGAATTCACCACGGTGAATCC
CONSENSUS
....

A5-gBol041443-XLOC_004903-12264-0 --------------
GTAATCTCTATGACTCTATCTAAACTCAACACCAGC
A5-gBol041443-XLOC_004903-12264-1
AACAATATTTGGAGGTAATCTCTATGACTCTATCTAAACTCAACACCAGC
CONSENSUS
..............GTAATCTCTATGACTCTATCTAAACTCAACACCAGC

A5-gBol041443-XLOC_004903-12264-0
AACAAAATTTTACTTTGACAACACCATTCATGCTACCAAAGAGTTTACAA
A5-gBol041443-XLOC_004903-12264-1
AACAAAATTTTACTTTGACAACACCATTCATGCTACCAAAGAGTTTACAA
CONSENSUS
AACAAAATTTTACTTTGACAACACCATTCATGCTACCAAAGAGTTTACAA

A5-gBol041443-XLOC_004903-12264-0 ACAG
A5-gBol041443-XLOC_004903-12264-1 ACAG
CONSENSUS ACAG

alignment for event: RI-X-XLOC_001962-11632

```
RI-X-XLOC_001962-11632-0
      GTGAGTTGCAAATCACCAACGCGTATGATGCTTCACTTTTGATACTCAAC
RI-X-XLOC_001962-11632-1
      GTGAGTTGCAAATCACCAACGCGTATGATGCTTCACTTTTGATACTCAAC
CONSENSUS
      GTGAGTTGCAAATCACCAACGCGTATGATGCTTCACTTTTGATACTCAAC

RI-X-XLOC_001962-11632-0
      CCAACAATGGAGGAAGCTATTTATTTTAAACAGAAGTAAGTTGCTTTAGT
RI-X-XLOC_001962-11632-1
      CCAACAATGGAGGAAGCTATTTATTTTAAACAGAA---------------
CONSENSUS
      CCAACAATGGAGGAAGCTATTTATTTTAAACAGAA...............

RI-X-XLOC_001962-11632-0
      TTGATCTAATCGAAGTTGTAATGATTTTAAACCGAAGTAAATTATTTTGC
RI-X-XLOC_001962-11632-1
      --------------------------------------------------
CONSENSUS
      ..................................................

RI-X-XLOC_001962-11632-0
      TTTAATCTGAAACAAGTTCTTTTCTATTTTTACACAAATAAACGTGACTA
RI-X-XLOC_001962-11632-1
      --------------------------------------------------
CONSENSUS
      ..................................................

RI-X-XLOC_001962-11632-0
      TATTCTTAAGGTAAACTTCGTATGTTGTTTTCAAAGAGGTTTTTAATAAC
RI-X-XLOC_001962-11632-1
      --------------------------------------------------
CONSENSUS
      ..................................................

RI-X-XLOC_001962-11632-0
      CTCGAAGAGTAGTAGTGATAAGGTATTCTTAAGGTAAACTTCTTATAATC
RI-X-XLOC_001962-11632-1
      --------------------------------------------------
CONSENSUS
      ..................................................

RI-X-XLOC_001962-11632-0
      CACATTTATGTTTTTTTTTTCTGTTTTCTATTGTTGTAGATATAAAAATA
RI-X-XLOC_001962-11632-1
      --------------------------------------------------
CONSENSUS
      ..................................................

RI-X-XLOC_001962-11632-0
      TTTTAACCCTTGGAACTATTTTTGGTACATGCAGGTTCAACTATCGTTGA
RI-X-XLOC_001962-11632-1    ----------------------------------
GTTCAACTATCGTTGA
CONSENSUS
      ..................................GTTCAACTATCGTTGA
```

RI-X-XLOC_001962-11632-0
TTTTAAAGTGTCAGATATTTCCTATTTTGAAGTGAAAGAGTGATGTGGAT
RI-X-XLOC_001962-11632-1
TTTTAAAGTGTCAGATATTTCCTATTTTGAAGTGAAAGAGTGATGTGGAT
CONSENSUS
TTTTAAAGTGTCAGATATTTCCTATTTTGAAGTGAAAGAGTGATGTGGAT

RI-X-XLOC_001962-11632-0 TGTCTTTTACAATCTGTAAAG
RI-X-XLOC_001962-11632-1 TGTCTTTTACAATCTGTAAAG
CONSENSUS TGTCTTTTACAATCTGTAAAG

alignment for event: RI-X-XLOC_002051-79

RI-X-XLOC_002051-79-0
GTTTTCAGTTCCAATTTCCTCGTATCTCGCCAGCGAAAGGAAGTGATTAT
RI-X-XLOC_002051-79-1
GTTTTCAGTTCCAATTTCCTCGTATCTCGCCAGCGAAAGGAAGTGATTAT
CONSENSUS
GTTTTCAGTTCCAATTTCCTCGTATCTCGCCAGCGAAAGGAAGTGATTAT

RI-X-XLOC_002051-79-0
CATTACGATAATCAGTACGGATTATGGTTGTGTCTCCAAGGATGGTCATT
RI-X-XLOC_002051-79-1
CATTACGATAATCAGTACGGATTATGGTTGTGTCTCCAAGGATGGTCATT
CONSENSUS
CATTACGATAATCAGTACGGATTATGGTTGTGTCTCCAAGGATGGTCATT

RI-X-XLOC_002051-79-0
TTGTATTGAAGTTTTGTTGGTCTGTATTGACCTCCAGTACCGCTTATCTT
RI-X-XLOC_002051-79-1
TTGTATTGAAGTTTTGTTGGTCTGTATTGACCTCCAGTACCGCTTATCTT
CONSENSUS
TTGTATTGAAGTTTTGTTGGTCTGTATTGACCTCCAGTACCGCTTATCTT

RI-X-XLOC_002051-79-0
AAAATTTTCAACCACTCTCCATTCTCCTACAGGTAGATCTCGTTGAACAC
RI-X-XLOC_002051-79-1
AAAATTTTCAACCACTCTCCATTCTCCTACAG------------------
CONSENSUS
AAAATTTTCAACCACTCTCCATTCTCCTACAG..................

RI-X-XLOC_002051-79-0
GATACATCAGTGATCTTCTGCAAGATGCATGAATTTTTTGACCCTGTAAA
RI-X-XLOC_002051-79-1

CONSENSUS
....

RI-X-XLOC_002051-79-0
AAAGTAGTATTAGATTCAGCATAGTTGACATCACACATATCTATAAATTA
RI-X-XLOC_002051-79-1

CONSENSUS

```
      .................................................

RI-X-XLOC_002051-79-0
      TAAAACTTAAGCTCTTATTGTCTCGTCTGCAAGGATTAACTCAAGAGTCT
RI-X-XLOC_002051-79-1 ---------------------------------
GATTAACTCAAGAGTCT
CONSENSUS
      .................................GATTAACTCAAGAGTCT

RI-X-XLOC_002051-79-0
      CCCCGCCGAAATTGGTTTTCGATGGTTTCCACGAATGAAGAAGTTTGACA
RI-X-XLOC_002051-79-1
      CCCCGCCGAAATTGGTTTTCGATGGTTTCCACGAATGAAGAAGTTTGACA
CONSENSUS
      CCCCGCCGAAATTGGTTTTCGATGGTTTCCACGAATGAAGAAGTTTGACA

RI-X-XLOC_002051-79-0 CGGACTCGCCATTCGTCTTTGAAAG
RI-X-XLOC_002051-79-1 CGGACTCGCCATTCGTCTTTGAAAG
CONSENSUS             CGGACTCGCCATTCGTCTTTGAAAG



alignment for event: A3-gBol044180-XLOC_001970-935

A3-gBol044180-XLOC_001970-935-0
      TTCTTTTTTTTTTTTTTTTTTTGGTTATTCGGCAAATAAACCCTAAATAAA
A3-gBol044180-XLOC_001970-935-1
      TTCTTTTTTTTTTTTTTTTTTTGGTTATTCGGCAAATAAACCCTAAATAAA
CONSENSUS
      TTCTTTTTTTTTTTTTTTTTTTGGTTATTCGGCAAATAAACCCTAAATAAA

A3-gBol044180-XLOC_001970-935-0
      GAGCGAGGCTTTAAAATAAAACTCGAAAATCTTTGGCAAAGTCAAAATCA
A3-gBol044180-XLOC_001970-935-1
      GAGCGAGGCTTTAAAATAAAACTCGAAAATCTTTGGCAAAGTCAAAATCA
CONSENSUS
      GAGCGAGGCTTTAAAATAAAACTCGAAAATCTTTGGCAAAGTCAAAATCA

A3-gBol044180-XLOC_001970-935-0
      CCGGACGTTCTTCTTCTCCGATTCGGTGCCATCAGCGCCCAGAACTTGGA
A3-gBol044180-XLOC_001970-935-1
      CCGGACGTTCTTCTTCTCCGATTCGGTGCCATCAGCGCCCAGAACTTGGA
CONSENSUS
      CCGGACGTTCTTCTTCTCCGATTCGGTGCCATCAGCGCCCAGAACTTGGA

A3-gBol044180-XLOC_001970-935-0
      TACGATCTCTCATCTCTATACGCTATGGGATGTGCTGCTTCTCTTCCAGA
A3-gBol044180-XLOC_001970-935-1
      TACGATCTCTCATCTCTATACGCTATGGGATGTGCTGCTTCTCTTCCAG-
CONSENSUS
      TACGATCTCTCATCTCTATACGCTATGGGATGTGCTGCTTCTCTTCCAG.

A3-gBol044180-XLOC_001970-935-0
      AAAGGCGAAACCTTTGGGGTTTAATTAGAATATTCACGAAATTTAGGGGT
A3-gBol044180-XLOC_001970-935-1
      --------------------------------------------------
```

CONSENSUS
      ....

A3-gBol044180-XLOC_001970-935-0
      CATTTGCGTTAATTTCGGGTTTCCGTGTGAAGAACAAATGGGGTTTTGAA
A3-gBol044180-XLOC_001970-935-1

CONSENSUS
      ....

A3-gBol044180-XLOC_001970-935-0
      GATGATGTGTGTGTTTCAGACAGGAACTCTGGAGCCTTAAACGGTCTTAG
A3-gBol044180-XLOC_001970-935-1 ------------------
ACAGGAACTCTGGAGCCTTAAACGGTCTTAG
CONSENSUS
      ..................ACAGGAACTCTGGAGCCTTAAACGGTCTTAG

A3-gBol044180-XLOC_001970-935-0
      CAGCTCAGAGAATGCAGATGCTAAGAATCTACGCGTAAAG
A3-gBol044180-XLOC_001970-935-1
      CAGCTCAGAGAATGCAGATGCTAAGAATCTACGCGTAAAG
CONSENSUS
      CAGCTCAGAGAATGCAGATGCTAAGAATCTACGCGTAAAG

alignment for event: RI-gBol016324-XLOC_033410-16354

RI-gBol016324-XLOC_033410-16354-0
      ATGAATATGTTCCAGCCGGGTCCAGTGGCAACTACAGTTGTATGTTTGAC
RI-gBol016324-XLOC_033410-16354-1
      ATGAATATGTTCCAGCCGGGTCCAGTGGCAACTACAGTTGTATGTTTGAC
CONSENSUS
      ATGAATATGTTCCAGCCGGGTCCAGTGGCAACTACAGTTGTATGTTTGAC

RI-gBol016324-XLOC_033410-16354-0
      TCAAGTTGTTACTGAGGATGAGCTTAAAGACGATGAGGAGTTTGAAGATA
RI-gBol016324-XLOC_033410-16354-1
      TCAAGTTGTTACTGAGGATGAGCTTAAAGACGATGAGGAGTTTGAAGATA
CONSENSUS
      TCAAGTTGTTACTGAGGATGAGCTTAAAGACGATGAGGAGTTTGAAGATA

RI-gBol016324-XLOC_033410-16354-0
      TAATGGAAGACATGAGACAAGAAGGCGGAAAGTTTGGTAAGAGAATGTCT
RI-gBol016324-XLOC_033410-16354-1
      TAATGGAAGACATGAGACAAGAAGGCGGAAAGTTTG--------------
CONSENSUS
      TAATGGAAGACATGAGACAAGAAGGCGGAAAGTTTG..............

RI-gBol016324-XLOC_033410-16354-0
      TTTATTTGCATTTTATCATATATCCAATTTTGGAGATATATAAAGATTAA
RI-gBol016324-XLOC_033410-16354-1

CONSENSUS
      ....

RI-gBol016324-XLOC_033410-16354-0
AAAAAAGCATATTGATTTGTGTCTCTGTTTTCTGTGGTTGTATATGTCTG
RI-gBol016324-XLOC_033410-16354-1

CONSENSUS
....

RI-gBol016324-XLOC_033410-16354-0
TTTTACAGCCTTTTGCTATAAAGAATCAGCCCCTAACATACTCAGACTGG
RI-gBol016324-XLOC_033410-16354-1

CONSENSUS
....

RI-gBol016324-XLOC_033410-16354-0
AGACTGCATATCTTATTTTTTTCACAAATGGCTACTCTTTAAAAACTAAC
RI-gBol016324-XLOC_033410-16354-1

CONSENSUS
....

RI-gBol016324-XLOC_033410-16354-0
GTTTTTGTCTCTGTATGTCTCGTGTATTTGGAATGTTTGTTGTTTAACTC
RI-gBol016324-XLOC_033410-16354-1

CONSENSUS
....

RI-gBol016324-XLOC_033410-16354-0
TTAAAATGCAGGTATGTTGACCAGCGTTGTGATTCCGCGTCCTAGCCCCA
RI-gBol016324-XLOC_033410-16354-1 -----------
GTATGTTGACCAGCGTTGTGATTCCGCGTCCTAGCCCCA
CONSENSUS
...........GTATGTTGACCAGCGTTGTGATTCCGCGTCCTAGCCCCA

RI-gBol016324-XLOC_033410-16354-0
GCGGTGAGCCAGTGCCAGGCCTTGGCAAG
RI-gBol016324-XLOC_033410-16354-1
GCGGTGAGCCAGTGCCAGGCCTTGGCAAG
CONSENSUS
GCGGTGAGCCAGTGCCAGGCCTTGGCAAG

alignment for event: A3-gBol036955-XLOC_009974-5009

A3-gBol036955-XLOC_009974-5009-0
GGGAGCTTTGTGAGTGGAGGAGGAGGAACCAAAAGTTTTTTCTTTGCGAA
A3-gBol036955-XLOC_009974-5009-1
GGGAGCTTTGTGAGTGGAGGAGGAGGAACCAAAAGTTTTTTCTTTGCGAA
CONSENSUS
GGGAGCTTTGTGAGTGGAGGAGGAGGAACCAAAAGTTTTTTCTTTGCGAA

A3-gBol036955-XLOC_009974-5009-0
CCAG--
A3-gBol036955-XLOC_009974-5009-1

CCAGGAAATGTAAAACCTTAAAACACGCACAGGTGTCATCATCAGGTGTC
CONSENSUS
CCAG..

A3-gBol036955-XLOC_009974-5009-0

A3-gBol036955-XLOC_009974-5009-1
TATGAGATCATCTAAGGCTGAGAATGGATTTGACAGCTCGATCATATGCG
CONSENSUS
....

A3-gBol036955-XLOC_009974-5009-0

A3-gBol036955-XLOC_009974-5009-1
CTCCTTTAGTCGTACAAGTTTTCTTTCATAGGAGTATTTCTAACATATTA
CONSENSUS
....

A3-gBol036955-XLOC_009974-5009-0

A3-gBol036955-XLOC_009974-5009-1
TTATATTTTCTCAAATGTCTCTTTTTGAAAAATAATTTTGTTAATGTAAT
CONSENSUS
....

A3-gBol036955-XLOC_009974-5009-0

A3-gBol036955-XLOC_009974-5009-1
CCTATAAAATGAAAGAATATTTCTATCTCTCTTTAAAAATTCTTATCTTT
CONSENSUS
....

A3-gBol036955-XLOC_009974-5009-0

A3-gBol036955-XLOC_009974-5009-1
TTGAGAAAAAAAAAAATAGAAGTGGTTGGAAATAGTCTAACAAAAAAAAAT
CONSENSUS
....

A3-gBol036955-XLOC_009974-5009-0
--------------------------------GTAACAGACGTAAGACAA
A3-gBol036955-XLOC_009974-5009-1
ATATACGCTGTTGTATATAATATATCAACAAGGTAACAGACGTAAGACAA
CONSENSUS
................................GTAACAGACGTAAGACAA

A3-gBol036955-XLOC_009974-5009-0
GAATCAGAGGGGTATTTTCGTAAATGACATAGGTGAGCTGGTAAAGTGCT
A3-gBol036955-XLOC_009974-5009-1
GAATCAGAGGGGTATTTTCGTAAATGACATAGGTGAGCTGGTAAAGTGCT
CONSENSUS
GAATCAGAGGGGTATTTTCGTAAATGACATAGGTGAGCTGGTAAAGTGCT

A3-gBol036955-XLOC_009974-5009-0
CATCCTTCTCGAGTCTCCCGCTCCGCTCAGCGTGTAGCCCTCTTTCTCCT
A3-gBol036955-XLOC_009974-5009-1

```
      CATCCTTCTCGAGTCTCCCGCTCCGCTCAGCGTGTAGCCCTCTTTCTCCT
CONSENSUS
      CATCCTTCTCGAGTCTCCCGCTCCGCTCAGCGTGTAGCCCTCTTTCTCCT

A3-gBol036955-XLOC_009974-5009-0
      TTCTCTATGCGTTTTCTTCGGAAACCCTAAAACGCTGTGAGAAGAAGACT
A3-gBol036955-XLOC_009974-5009-1
      TTCTCTATGCGTTTTCTTCGGAAACCCTAAAACGCTGTGAGAAGAAGACT
CONSENSUS
      TTCTCTATGCGTTTTCTTCGGAAACCCTAAAACGCTGTGAGAAGAAGACT

A3-gBol036955-XLOC_009974-5009-0
      CTCGCTTGAAAATCGAAGCTGTAGCAAGCTCTCTCGTAGAAAGTTTCCTT
A3-gBol036955-XLOC_009974-5009-1
      CTCGCTTGAAAATCGAAGCTGTAGCAAGCTCTCTCGTAGAAAGTTTCCTT
CONSENSUS
      CTCGCTTGAAAATCGAAGCTGTAGCAAGCTCTCTCGTAGAAAGTTTCCTT

A3-gBol036955-XLOC_009974-5009-0 CACTTTCCCCGGAGAAACTCTCAGCTAG
A3-gBol036955-XLOC_009974-5009-1 CACTTTCCCCGGAGAAACTCTCAGCTAG
CONSENSUS                        CACTTTCCCCGGAGAAACTCTCAGCTAG



alignment for event: A3-gBol036955-XLOC_009974-5007

A3-gBol036955-XLOC_009974-5007-0
      GGGAGCTTTGTGAGTGGAGGAGGAGGAACCAAAAGTTTTTTCTTTGCGAA
A3-gBol036955-XLOC_009974-5007-1
      GGGAGCTTTGTGAGTGGAGGAGGAGGAACCAAAAGTTTTTTCTTTGCGAA
CONSENSUS
      GGGAGCTTTGTGAGTGGAGGAGGAGGAACCAAAAGTTTTTTCTTTGCGAA

A3-gBol036955-XLOC_009974-5007-0
      CCAG----------------------------------------------
A3-gBol036955-XLOC_009974-5007-1
      CCAGGAAATGTAAAACCTTAAAACACGCACAGGTGTCATCATCAGGTGTC
CONSENSUS
      CCAG..............................................

A3-gBol036955-XLOC_009974-5007-0
      --------------------------------------------------
A3-gBol036955-XLOC_009974-5007-1
      TATGAGATCATCTAAGGCTGAGAATGGATTTGACAGCTCGATCATATGCG
CONSENSUS
      ..................................................

A3-gBol036955-XLOC_009974-5007-0
      --------------------------------------------------
A3-gBol036955-XLOC_009974-5007-1
      CTCCTTTAGTCGTACAAGTTTTCTTTCATAGGAGTATTTCTAACATATTA
CONSENSUS
      ..................................................

A3-gBol036955-XLOC_009974-5007-0
      --------------------------------------------------
```

A3-gBol036955-XLOC_009974-5007-1
      TTATATTTTCTCAAATGTCTCTTTTTGAAAAATAATTTTGTTAATGTAAT
CONSENSUS
      ....

A3-gBol036955-XLOC_009974-5007-0

A3-gBol036955-XLOC_009974-5007-1
      CCTATAAAATGAAAGAATATTTCTATCTCTCTTTAAAAATTCTTATCTTT
CONSENSUS
      ....

A3-gBol036955-XLOC_009974-5007-0

A3-gBol036955-XLOC_009974-5007-1
      TTGAGAAAAAAAAAAATAGAAGTGGTTGGAAATAGTCTAACAAAAAAAAAT
CONSENSUS
      ....

A3-gBol036955-XLOC_009974-5007-0
      ---------------------------------------ACGTAAGACAA
A3-gBol036955-XLOC_009974-5007-1
      ATATACGCTGTTGTATATAATATATCAACAAGGTAACAGACGTAAGACAA
CONSENSUS
      ....ACGTAAGACAA

A3-gBol036955-XLOC_009974-5007-0
      GAATCAGAGGGGTATTTTCGTAAATGACATAGGTGAGCTGGTAAAGTGCT
A3-gBol036955-XLOC_009974-5007-1
      GAATCAGAGGGGTATTTTCGTAAATGACATAGGTGAGCTGGTAAAGTGCT
CONSENSUS
      GAATCAGAGGGGTATTTTCGTAAATGACATAGGTGAGCTGGTAAAGTGCT

A3-gBol036955-XLOC_009974-5007-0
      CATCCTTCTCGAGTCTCCCGCTCCGCTCAGCGTGTAGCCCTCTTTCTCCT
A3-gBol036955-XLOC_009974-5007-1
      CATCCTTCTCGAGTCTCCCGCTCCGCTCAGCGTGTAGCCCTCTTTCTCCT
CONSENSUS
      CATCCTTCTCGAGTCTCCCGCTCCGCTCAGCGTGTAGCCCTCTTTCTCCT

A3-gBol036955-XLOC_009974-5007-0
      TTCTCTATGCGTTTTCTTCGGAAACCCTAAAACGCTGTGAGAAGAAGACT
A3-gBol036955-XLOC_009974-5007-1
      TTCTCTATGCGTTTTCTTCGGAAACCCTAAAACGCTGTGAGAAGAAGACT
CONSENSUS
      TTCTCTATGCGTTTTCTTCGGAAACCCTAAAACGCTGTGAGAAGAAGACT

A3-gBol036955-XLOC_009974-5007-0
      CTCGCTTGAAAATCGAAGCTGTAGCAAGCTCTCTCGTAGAAAGTTTCCTT
A3-gBol036955-XLOC_009974-5007-1
      CTCGCTTGAAAATCGAAGCTGTAGCAAGCTCTCTCGTAGAAAGTTTCCTT
CONSENSUS
      CTCGCTTGAAAATCGAAGCTGTAGCAAGCTCTCTCGTAGAAAGTTTCCTT

A3-gBol036955-XLOC_009974-5007-0 CACTTTCCCCGGAGAAACTCTCAGCTAG
A3-gBol036955-XLOC_009974-5007-1 CACTTTCCCCGGAGAAACTCTCAGCTAG

CONSENSUS   CACTTTCCCCGGAGAAACTCTCAGCTAG

alignment for event: A3-gBol002304-XLOC_049378-9600

A3-gBol002304-XLOC_049378-9600-0
ATTAAATTGACTGATGTTACTGATCCCATGGTCAATGAGAAGGTTGCTGA
A3-gBol002304-XLOC_049378-9600-1
ATTAAATTGACTGATGTTACTGATCCCATGGTCAATGAGAAGGTTGCTGA
CONSENSUS
ATTAAATTGACTGATGTTACTGATCCCATGGTCAATGAGAAGGTTGCTGA

A3-gBol002304-XLOC_049378-9600-0
TCGG--
A3-gBol002304-XLOC_049378-9600-1
TCGGGTAGGAGAGGATTTAGAAGGAGTTACCCACAAAGTTTACTTTGATA
CONSENSUS
TCGG..

A3-gBol002304-XLOC_049378-9600-0

A3-gBol002304-XLOC_049378-9600-1
TTCAGATCAATGGTTCACCTGAATGAGTTACCCACAAAGTTTACTTTGTT
CONSENSUS
....

A3-gBol002304-XLOC_049378-9600-0

A3-gBol002304-XLOC_049378-9600-1
CCCTCTTCTTCATTGGGTTTGTAAGCTCTGTGTTTTTTCCAATTTGGTTT
CONSENSUS
....

A3-gBol002304-XLOC_049378-9600-0

A3-gBol002304-XLOC_049378-9600-1
TAGCTTAATCAGTTCATATTTATTTTGCTATGTTACTGGGTGAGATGGTA
CONSENSUS
....

A3-gBol002304-XLOC_049378-9600-0

A3-gBol002304-XLOC_049378-9600-1
GGAGAGTGAGCTCCCGAGAGAATCCGACAGACACTTGACAACTAGTTCAT
CONSENSUS
....

A3-gBol002304-XLOC_049378-9600-0

A3-gBol002304-XLOC_049378-9600-1
GTTCTTGTCTAAACCAAGCTTTTTGTTAAAAGATTCAAGCTTTCTTCTTT
CONSENSUS
....

A3-gBol002304-XLOC_049378-9600-0

A3-gBol002304-XLOC_049378-9600-1
GTGTTTTGCTGAAATTCGAACTTGAAAAGATCCTTCCTTTTTCTTTGTAC
CONSENSUS
....

A3-gBol002304-XLOC_049378-9600-0

A3-gBol002304-XLOC_049378-9600-1
TTGAATCATGAACCCGAGCCGCTGATTCCTCAGTTTATAACTCATATGCA
CONSENSUS
....

A3-gBol002304-XLOC_049378-9600-0

A3-gBol002304-XLOC_049378-9600-1
ATGTGGGTTGTTTGGAGAGACAGTAAAGAATTAATTAGGCTAATTCAATT
CONSENSUS
....

A3-gBol002304-XLOC_049378-9600-0 ---------------------------
CGGAGGACTTCAGTGCTAGTTAA
A3-gBol002304-XLOC_049378-9600-1
TTTGATTTTGTATCTTCCTTGTTGCAGCGGAGGACTTCAGTGCTAGTTAA
CONSENSUS
...........................CGGAGGACTTCAGTGCTAGTTAA

A3-gBol002304-XLOC_049378-9600-0
CTTACTGAAACACTTAATGGGTTTATACAGGATGAGCTGCGTGATGTTGT
A3-gBol002304-XLOC_049378-9600-1
CTTACTGAAACACTTAATGGGTTTATACAGGATGAGCTGCGTGATGTTGT
CONSENSUS
CTTACTGAAACACTTAATGGGTTTATACAGGATGAGCTGCGTGATGTTGT

A3-gBol002304-XLOC_049378-9600-0
TATGCTCGTGTTTGCTAACAAACAGGATCTTCCAAATGCCATGAACGAAG
A3-gBol002304-XLOC_049378-9600-1
TATGCTCGTGTTTGCTAACAAACAGGATCTTCCAAATGCCATGAACGAAG
CONSENSUS
TATGCTCGTGTTTGCTAACAAACAGGATCTTCCAAATGCCATGAACGAAG

A3-gBol002304-XLOC_049378-9600-0
CTGAGATCACCGATAAGCTTGGTCTCCACTCTCTCCGTCAGCGTCACTGG
A3-gBol002304-XLOC_049378-9600-1
CTGAGATCACCGATAAGCTTGGTCTCCACTCTCTCCGTCAGCGTCACTGG
CONSENSUS
CTGAGATCACCGATAAGCTTGGTCTCCACTCTCTCCGTCAGCGTCACTGG

A3-gBol002304-XLOC_049378-9600-0
CTCAGTCTTCGTTTTGATTTGTTCTCTTGCTTATAACGTTTCTCTGATCA
A3-gBol002304-XLOC_049378-9600-1
CTCAGTCTTCGTTTTGATTTGTTCTCTTGCTTATAACGTTTCTCTGATCA
CONSENSUS
CTCAGTCTTCGTTTTGATTTGTTCTCTTGCTTATAACGTTTCTCTGATCA

A3-gBol002304-XLOC_049378-9600-0

ATTTGTTTCTCTTGCTTATAACGTTTCTCTTGCCAAACCGCAATTTGTTT
A3-gBol002304-XLOC_049378-9600-1
ATTTGTTTCTCTTGCTTATAACGTTTCTCTTGCCAAACCGCAATTTGTTT
CONSENSUS
ATTTGTTTCTCTTGCTTATAACGTTTCTCTTGCCAAACCGCAATTTGTTT

A3-gBol002304-XLOC_049378-9600-0
AATTTATTTTCTTCTTATATGTGTGCAAGTTGTAATACTTTCTTATGTGT
A3-gBol002304-XLOC_049378-9600-1
AATTTATTTTCTTCTTATATGTGTGCAAGTTGTAATACTTTCTTATGTGT
CONSENSUS
AATTTATTTTCTTCTTATATGTGTGCAAGTTGTAATACTTTCTTATGTGT

A3-gBol002304-XLOC_049378-9600-0
TCTTGTTGCTTGATTCTTGCAGGCGTAGAGGAAAAGATTGTGGTTGAGCT
A3-gBol002304-XLOC_049378-9600-1
TCTTGTTGCTTGATTCTTGCAGGCGTAGAGGAAAAGATTGTGGTTGAGCT
CONSENSUS
TCTTGTTGCTTGATTCTTGCAGGCGTAGAGGAAAAGATTGTGGTTGAGCT

A3-gBol002304-XLOC_049378-9600-0
GATTTTGCTGGGCAAATCTGGATGCAGGGGCAAACATTATCTGATTTTTT
A3-gBol002304-XLOC_049378-9600-1
GATTTTGCTGGGCAAATCTGGATGCAGGGGCAAACATTATCTGATTTTTT
CONSENSUS
GATTTTGCTGGGCAAATCTGGATGCAGGGGCAAACATTATCTGATTTTTT

A3-gBol002304-XLOC_049378-9600-0
TCTTTATTGTTCTGTTCTTTTTTTTTGGTTTCGGGTATGTGGATTTGATT
A3-gBol002304-XLOC_049378-9600-1
TCTTTATTGTTCTGTTCTTTTTTTTTGGTTTCGGGTATGTGGATTTGATT
CONSENSUS
TCTTTATTGTTCTGTTCTTTTTTTTTGGTTTCGGGTATGTGGATTTGATT

A3-gBol002304-XLOC_049378-9600-0
TTGTGATTGTTGTTCTGATTGTGCAAGGGGGAGTCTCTTGCACGTAGAAC
A3-gBol002304-XLOC_049378-9600-1
TTGTGATTGTTGTTCTGATTGTGCAAGGGGGAGTCTCTTGCACGTAGAAC
CONSENSUS
TTGTGATTGTTGTTCTGATTGTGCAAGGGGGAGTCTCTTGCACGTAGAAC

A3-gBol002304-XLOC_049378-9600-0
ATAGAAACTACCTTTTTATATAGTTTGTAGTCAAAGATTTACTCTACTTA
A3-gBol002304-XLOC_049378-9600-1
ATAGAAACTACCTTTTTATATAGTTTGTAGTCAAAGATTTACTCTACTTA
CONSENSUS
ATAGAAACTACCTTTTTATATAGTTTGTAGTCAAAGATTTACTCTACTTA

A3-gBol002304-XLOC_049378-9600-0
TTTCTGCTTCACGTTTCATTGTTGAGATGATCTTGTTCCCTG
A3-gBol002304-XLOC_049378-9600-1
TTTCTGCTTCACGTTTCATTGTTGAGATGATCTTGTTCCCTG
CONSENSUS
TTTCTGCTTCACGTTTCATTGTTGAGATGATCTTGTTCCCTG

alignment for event: RI-gBol004448-XLOC_046918-8035

RI-gBol004448-XLOC_046918-8035-0
GTTGAGACATTGTCAAAAGATGATTTAGCTTCCTGGTTAACTTGACAGTA
RI-gBol004448-XLOC_046918-8035-1
GTTGAGACATTGTCAAAAGATGATTTAGCTTCCTGGTTAACTTGACAGTA
CONSENSUS
GTTGAGACATTGTCAAAAGATGATTTAGCTTCCTGGTTAACTTGACAGTA

RI-gBol004448-XLOC_046918-8035-0
GACACTGGTCTGTGGGATCTCTACTACTTTCTGATACACTCATCTCTGTA
RI-gBol004448-XLOC_046918-8035-1
GACACTG---
CONSENSUS
GACACTG...

RI-gBol004448-XLOC_046918-8035-0
ATAGATGGTACGTTTTGACTAGCCAAATGAAGCAGCAGAGGATGAAGAAG
RI-gBol004448-XLOC_046918-8035-1
---------------------------------AGCAGAGGATGAAGAAG
CONSENSUS
.................................AGCAGAGGATGAAGAAG

RI-gBol004448-XLOC_046918-8035-0
TTGTCGGAATTGAGAGGGTTGAAAGAAAAGTTATCGCTGAAATGAGTGAG
RI-gBol004448-XLOC_046918-8035-1
TTGTCGGAATTGAGAGGGTTGAAAGAAAAGTTATCGCTGAAATGAGTGAG
CONSENSUS
TTGTCGGAATTGAGAGGGTTGAAAGAAAAGTTATCGCTGAAATGAGTGAG

RI-gBol004448-XLOC_046918-8035-0
AGATCTGAAGATGATATACCTGGTAATAATGCAGAAGCAAACCATTCCGA
RI-gBol004448-XLOC_046918-8035-1
AGATCTGAAGATGATATACCTGGTAATAATGCAGAAGCAAACCATTCCGA
CONSENSUS
AGATCTGAAGATGATATACCTGGTAATAATGCAGAAGCAAACCATTCCGA

RI-gBol004448-XLOC_046918-8035-0
CATAGACTCAAAAGATTACAACGGACCGTTTCATGAAATGAGATCTAGAG
RI-gBol004448-XLOC_046918-8035-1
CATAGACTCAAAAGATTACAACGGACCGTTTCATGAAATGAGATCTAGAG
CONSENSUS
CATAGACTCAAAAGATTACAACGGACCGTTTCATGAAATGAGATCTAGAG

RI-gBol004448-XLOC_046918-8035-0
GGAAGAAGATAACATTCTTGAAACCTAAGAGTAGCAATAGAAGAACATTT
RI-gBol004448-XLOC_046918-8035-1
GGAAGAAGATAACATTCTTGAAACCTAAGAGTAGCAATAGAAGAACATTT
CONSENSUS
GGAAGAAGATAACATTCTTGAAACCTAAGAGTAGCAATAGAAGAACATTT

RI-gBol004448-XLOC_046918-8035-0
CTGATTTCTTTGGTAAATTCAAGCGAAGCCAATCCAAGGCATTAGCAAAT
RI-gBol004448-XLOC_046918-8035-1
CTGATTTCTTTGGTAAATTCAAGCGAAGCCAATCCAAGGCATTAGCAAAT

CONSENSUS
    CTGATTTCTTTGGTAAATTCAAGCGAAGCCAATCCAAGGCATTAGCAAAT

RI-gBol004448-XLOC_046918-8035-0
    TCCTTTGTTATCATAAATAACTTCATTGATTTGTGGAGTATAAGTTAATG
RI-gBol004448-XLOC_046918-8035-1
    TCCTTTGTTATCATAAATAACTTCATTGATTTGTGGAGTATAAGTTAATG
CONSENSUS
    TCCTTTGTTATCATAAATAACTTCATTGATTTGTGGAGTATAAGTTAATG

RI-gBol004448-XLOC_046918-8035-0
    ATACTTTAAAATCTCCAAGGTTTAAGGATCTTTCTGTAGTCTGTTGTCCT
RI-gBol004448-XLOC_046918-8035-1
    ATACTTTAAAATCTCCAAGGTTTAAGGATCTTTCTGTAGTCTGTTGTCCT
CONSENSUS
    ATACTTTAAAATCTCCAAGGTTTAAGGATCTTTCTGTAGTCTGTTGTCCT

RI-gBol004448-XLOC_046918-8035-0
    GGTCTAAAAAGGTTCTTGACAAACAAGTACCGAACAAGAAATGATGCTCA
RI-gBol004448-XLOC_046918-8035-1
    GGTCTAAAAAGGTTCTTGACAAACAAGTACCGAACAAGAAATGATGCTCA
CONSENSUS
    GGTCTAAAAAGGTTCTTGACAAACAAGTACCGAACAAGAAATGATGCTCA

alignment for event: RI-gBol004448-XLOC_046918-8034

RI-gBol004448-XLOC_046918-8034-0
    GTTGAGACATTGTCAAAAGATGATTTAGCTTCCTGGTTAACTTGACAGTA
RI-gBol004448-XLOC_046918-8034-1
    GTTGAGACATTGTCAAAAGATGATTTAGCTTCCTGGTTAACTTGACAGTA
CONSENSUS
    GTTGAGACATTGTCAAAAGATGATTTAGCTTCCTGGTTAACTTGACAGTA

RI-gBol004448-XLOC_046918-8034-0
    GACACTGGTCTGTGGGATCTCTACTACTTTCTGATACACTCATCTCTGTA
RI-gBol004448-XLOC_046918-8034-1
    GACACTG---
CONSENSUS
    GACACTG...

RI-gBol004448-XLOC_046918-8034-0
    ATAGATGGTACGTTTTGACTAGCCAAATGAAGCAGCAGAGGATGAAGAAG
RI-gBol004448-XLOC_046918-8034-1
    -----------------------------------CAGAGGATGAAGAAG
CONSENSUS
    ...................................CAGAGGATGAAGAAG

RI-gBol004448-XLOC_046918-8034-0
    TTGTCGGAATTGAGAGGGTTGAAAGAAAAGTTATCGCTGAAATGAGTGAG
RI-gBol004448-XLOC_046918-8034-1
    TTGTCGGAATTGAGAGGGTTGAAAGAAAAGTTATCGCTGAAATGAGTGAG
CONSENSUS
    TTGTCGGAATTGAGAGGGTTGAAAGAAAAGTTATCGCTGAAATGAGTGAG

RI-gBol004448-XLOC_046918-8034-0
AGATCTGAAGATGATATACCTGGTAATAATGCAGAAGCAAACCATTCCGA
RI-gBol004448-XLOC_046918-8034-1
AGATCTGAAGATGATATACCTGGTAATAATGCAGAAGCAAACCATTCCGA
CONSENSUS
AGATCTGAAGATGATATACCTGGTAATAATGCAGAAGCAAACCATTCCGA

RI-gBol004448-XLOC_046918-8034-0
CATAGACTCAAAAGATTACAACGGACCGTTTCATGAAATGAGATCTAGAG
RI-gBol004448-XLOC_046918-8034-1
CATAGACTCAAAAGATTACAACGGACCGTTTCATGAAATGAGATCTAGAG
CONSENSUS
CATAGACTCAAAAGATTACAACGGACCGTTTCATGAAATGAGATCTAGAG

RI-gBol004448-XLOC_046918-8034-0
GGAAGAAGATAACATTCTTGAAACCTAAGAGTAGCAATAGAAGAACATTT
RI-gBol004448-XLOC_046918-8034-1
GGAAGAAGATAACATTCTTGAAACCTAAGAGTAGCAATAGAAGAACATTT
CONSENSUS
GGAAGAAGATAACATTCTTGAAACCTAAGAGTAGCAATAGAAGAACATTT

RI-gBol004448-XLOC_046918-8034-0
CTGATTTCTTTGGTAAATTCAAGCGAAGCCAATCCAAGGCATTAGCAAAT
RI-gBol004448-XLOC_046918-8034-1
CTGATTTCTTTGGTAAATTCAAGCGAAGCCAATCCAAGGCATTAGCAAAT
CONSENSUS
CTGATTTCTTTGGTAAATTCAAGCGAAGCCAATCCAAGGCATTAGCAAAT

RI-gBol004448-XLOC_046918-8034-0
TCCTTTGTTATCATAAATAACTTCATTGATTTGTGGAGTATAAGTTAATG
RI-gBol004448-XLOC_046918-8034-1
TCCTTTGTTATCATAAATAACTTCATTGATTTGTGGAGTATAAGTTAATG
CONSENSUS
TCCTTTGTTATCATAAATAACTTCATTGATTTGTGGAGTATAAGTTAATG

RI-gBol004448-XLOC_046918-8034-0
ATACTTTAAAATCTCCAAGGTTTAAGGATCTTTCTGTAGTCTGTTGTCCT
RI-gBol004448-XLOC_046918-8034-1
ATACTTTAAAATCTCCAAGGTTTAAGGATCTTTCTGTAGTCTGTTGTCCT
CONSENSUS
ATACTTTAAAATCTCCAAGGTTTAAGGATCTTTCTGTAGTCTGTTGTCCT

RI-gBol004448-XLOC_046918-8034-0
GGTCTAAAAAGGTTCTTGACAAACAAGTACCGAACAAGAAATGATGCTCA
RI-gBol004448-XLOC_046918-8034-1
GGTCTAAAAAGGTTCTTGACAAACAAGTACCGAACAAGAAATGATGCTCA
CONSENSUS
GGTCTAAAAAGGTTCTTGACAAACAAGTACCGAACAAGAAATGATGCTCA

alignment for event: A3-gBol010123-XLOC_040404-12648

A3-gBol010123-XLOC_040404-12648-0
AATCATTTCATCGAGATCTTGGATCTTATGTCTGGGACTTCAAAAAAAAG
A3-gBol010123-XLOC_040404-12648-1

```
      AATCATTTCATCGAGATCTTGGATCTTATGTCTGGGACTTCAAAAAAAAG
CONSENSUS
      AATCATTTCATCGAGATCTTGGATCTTATGTCTGGGACTTCAAAAAAAAG

A3-gBol010123-XLOC_040404-12648-0
      TGTTAACATGCATCTCCTGAGCATGGGTACTAG-----------------
A3-gBol010123-XLOC_040404-12648-1
      TGTTAACATGCATCTCCTGAGCATGGGTACTAGGAATGGCTGCATATGTG
CONSENSUS
      TGTTAACATGCATCTCCTGAGCATGGGTACTAG.................

A3-gBol010123-XLOC_040404-12648-0
      --------------------------------------------------
A3-gBol010123-XLOC_040404-12648-1
      ATTCAACAACCGTGCAATTGCTTTTTGAAGTTTCTCAGGGTCTTTATGAT
CONSENSUS
      ..................................................

A3-gBol010123-XLOC_040404-12648-0      -------
ATTTCGTTAACATAAAGGATGATGATAATCGACAGACATCACA
A3-gBol010123-XLOC_040404-12648-1
      GCTACAGATTTCGTTAACATAAAGGATGATGATAATCGACAGACATCACA
CONSENSUS
      .......ATTTCGTTAACATAAAGGATGATGATAATCGACAGACATCACA

A3-gBol010123-XLOC_040404-12648-0
      TTTAATTTCTCGCTTTGTTGAGATG
A3-gBol010123-XLOC_040404-12648-1
      TTTAATTTCTCGCTTTGTTGAGATG
CONSENSUS
      TTTAATTTCTCGCTTTGTTGAGATG



alignment for event: RI-X-XLOC_034132-12643

RI-X-XLOC_034132-12643-0
      CTTTTCCAAATAACACTTTGAACCGCGAAGTCTTACCAAATTTTGAATTT
RI-X-XLOC_034132-12643-1
      CTTTTCCAAATAACACTTTGAACCGCGAAGTCTTACCAAATTTTGAATTT
CONSENSUS
      CTTTTCCAAATAACACTTTGAACCGCGAAGTCTTACCAAATTTTGAATTT

RI-X-XLOC_034132-12643-0
      TGACACAAACCTTTTGTTTTGGAGACTTTTTTTTCCTTTGCTAAGTCCTT
RI-X-XLOC_034132-12643-1
      TGACACAAACCTTTTGTTTTGGAGACTTTTTTTTCCTTTGCTAAGTCCTT
CONSENSUS
      TGACACAAACCTTTTGTTTTGGAGACTTTTTTTTCCTTTGCTAAGTCCTT

RI-X-XLOC_034132-12643-0
      TTTTCCTCTCAACAAATTTTTTTCATTAATTCGTGTCTAGGACAAACAAT
RI-X-XLOC_034132-12643-1
      TTTTCCTCTCAACAAATTTTTTTCATTAATTCGTGTCTAGGACAAACAAT
CONSENSUS
      TTTTCCTCTCAACAAATTTTTTTCATTAATTCGTGTCTAGGACAAACAAT
```

RI-X-XLOC_034132-12643-0
CAATTATTTTCCACTCAATGATCTCAAACCCAGATTCGTCACAACTCCGG
RI-X-XLOC_034132-12643-1
CAATTATTTTCCACTCAATGATCTCAAACCCAGATTCGTCACAACTCCGG
CONSENSUS
CAATTATTTTCCACTCAATGATCTCAAACCCAGATTCGTCACAACTCCGG

RI-X-XLOC_034132-12643-0
CGAGTTTCCCCACCTCTTCCGCCAACTCTTAGTCTCCAACTCTCAATCGG
RI-X-XLOC_034132-12643-1
CGAGTTTCCCCACCTCTTCCGCCAACTCTTAGTCTCCAACTCTCAATCGG
CONSENSUS
CGAGTTTCCCCACCTCTTCCGCCAACTCTTAGTCTCCAACTCTCAATCGG

RI-X-XLOC_034132-12643-0
GACATAGTTTCTTTTACTTCTTACCACAAGAAAAAAAACAATGCTTCATT
RI-X-XLOC_034132-12643-1
GACATAGTTTCTTTTACTTCTTACCACAAGAAAAAAAACAATGCTTCATT
CONSENSUS
GACATAGTTTCTTTTACTTCTTACCACAAGAAAAAAAACAATGCTTCATT

RI-X-XLOC_034132-12643-0
ATTAGGGTTCTTTATCACTTTCTCTTCGATCCTCTCGATCTTCTTCCCGA
RI-X-XLOC_034132-12643-1
ATTAGGGTTCTTTATCACTTTCTCTTCGATCCTCTCGATCTTCTTCCCGA
CONSENSUS
ATTAGGGTTCTTTATCACTTTCTCTTCGATCCTCTCGATCTTCTTCCCGA

RI-X-XLOC_034132-12643-0
CGGTTCCATGGAATCCAGTTTCAGTCACCAACTACCTCTCTCTCGCCTCA
RI-X-XLOC_034132-12643-1
CGGTTCCATGGAATCCAGTTTCAGTCACCAACTACCTCTCTCTCGCCTCA
CONSENSUS
CGGTTCCATGGAATCCAGTTTCAGTCACCAACTACCTCTCTCTCGCCTCA

RI-X-XLOC_034132-12643-0
GAGGCCTTTGGCTCTTGCTGTGATTCACTCTGCTCTCTCTATCACCATCT
RI-X-XLOC_034132-12643-1
GAGGCCTTTGGCTCTTGCTGTGATTCACTCTGCTCTCTCTATCACCATCT
CONSENSUS
GAGGCCTTTGGCTCTTGCTGTGATTCACTCTGCTCTCTCTATCACCATCT

RI-X-XLOC_034132-12643-0
GAACCTCCAGGGCCACCAGAACCGCCGGATATGTCATCTCCTTTACGCTT
RI-X-XLOC_034132-12643-1
GAACCTCCAGGGCCACCAGAACCGCCGGATATGTCATCTCCTTTACGCTT
CONSENSUS
GAACCTCCAGGGCCACCAGAACCGCCGGATATGTCATCTCCTTTACGCTT

RI-X-XLOC_034132-12643-0
CCCCTCCTCCCATCTGTTTCGTACACCTTGTAGTTTTGGATCTCAACCCA
RI-X-XLOC_034132-12643-1
CCCCTCCTCCCATCTGTTTCGTACACCTTGTAGTTTTGGATCTCAACCCA
CONSENSUS
CCCCTCCTCCCATCTGTTTCGTACACCTTGTAGTTTTGGATCTCAACCCA

RI-X-XLOC_034132-12643-0
TGTGTTGTCGACTCTGTTCCAACTCCGAAGGTGGCTCCTAACCTGACTTG
RI-X-XLOC_034132-12643-1
TGTGTTGTCGACTCTGTTCCAACTCCGAAG--------------------
CONSENSUS
TGTGTTGTCGACTCTGTTCCAACTCCGAAG....................

RI-X-XLOC_034132-12643-0
TTTACTCCGTGTCTCTCAGTCGGTGGCTCCTCCTTCTCCGACGAATTCCC
RI-X-XLOC_034132-12643-1

CONSENSUS
....

RI-X-XLOC_034132-12643-0
TAGTCTCGTGTAGTCGCAGAGACTGTACTGATCAATTGGTTAAGAAAGCT
RI-X-XLOC_034132-12643-1

CONSENSUS
....

RI-X-XLOC_034132-12643-0
AGGAAGATGTGTACCGAATCCTCAATGAAAGATGCTTTCTCTACTTACTC
RI-X-XLOC_034132-12643-1

CONSENSUS
....

RI-X-XLOC_034132-12643-0
TGATTATCTCAACAACTTCGTGAGTTTTTTTCCTCTCTTCCCTGTAGAAA
RI-X-XLOC_034132-12643-1

CONSENSUS
....

RI-X-XLOC_034132-12643-0
AGGACGATTCTTTCTGTTTTTTTTTTGTTTCCAGTTAAGAAAGATTCAAC
RI-X-XLOC_034132-12643-1

CONSENSUS
....

RI-X-XLOC_034132-12643-0
CTTTGCGTCTTGGGATTGAGTTTTCTATGAGGTAGAACTGTAGAAGGCTA
RI-X-XLOC_034132-12643-1

CONSENSUS
....

RI-X-XLOC_034132-12643-0
AAGGAAGGGTCTTGGACTGTTGTTGGAGAGAGCTGTTTATCCAGAAAGCA
RI-X-XLOC_034132-12643-1

CONSENSUS
....

```
RI-X-XLOC_034132-12643-0
      TCTTGTTTTGATATGTTGCTGGAATCTATAGTTTATCCTCCCAAAGACTA
RI-X-XLOC_034132-12643-1
      --------------------------------------------------
CONSENSUS
      ..................................................

RI-X-XLOC_034132-12643-0
      GTTTTGTTTTCAGTCTGTTTTGCTTGTTTGTCTCCTTGTCCTTCAATCAT
RI-X-XLOC_034132-12643-1
      --------------------------------------------------
CONSENSUS
      ..................................................

RI-X-XLOC_034132-12643-0
      CATTTGTAACTCTAGAACTCTGCTTGTTCTTTTTTTTTAACCTGCAGAAT
RI-X-XLOC_034132-12643-1
      --------------------------------------------------
CONSENSUS
      ..................................................

RI-X-XLOC_034132-12643-0
      CTATCAATCATCATGTGGAGATTTTCTCTAGCCATCTCTTACTTTCTTTT
RI-X-XLOC_034132-12643-1
      --------------------------------------------------
CONSENSUS
      ..................................................

RI-X-XLOC_034132-12643-0
      CATCTCAATGATTGTTAGAGCCACCGATTGTCCAGTACTGCGCTATGTCT
RI-X-XLOC_034132-12643-1
      --------------------------------------------------
CONSENSUS
      ..................................................

RI-X-XLOC_034132-12643-0
      GGTCTCCCAAAAGCTTACTGTGTGCTTCATTGGTTCTTGATTGCCAGATG
RI-X-XLOC_034132-12643-1
      --------------------------------------------------
CONSENSUS
      ..................................................

RI-X-XLOC_034132-12643-0
      TTTGATTACAGTGTCTTGATCGAGTTATTTAATCTTATTAAAGATGATTT
RI-X-XLOC_034132-12643-1
      --------------------------------------------------
CONSENSUS
      ..................................................

RI-X-XLOC_034132-12643-0
      TGAATCTTGATCTGATTTGTTATGTGGTTGGTTCCAGATGACAACGAAGA
RI-X-XLOC_034132-12643-1
      -------------------------------------ATGACAACGAAGA
CONSENSUS
      .....................................ATGACAACGAAGA
```

RI-X-XLOC_034132-12643-0
    GTATGAAGCAAAGGTCCAAGGAATTGATATATCGCCTTATCCTACAGTTA
RI-X-XLOC_034132-12643-1
    GTATGAAGCAAAGGTCCAAGGAATTGATATATCGCCTTATCCTACAGTTA
CONSENSUS
    GTATGAAGCAAAGGTCCAAGGAATTGATATATCGCCTTATCCTACAGTTA

RI-X-XLOC_034132-12643-0
    GAGGCGAACCAGCCACTTTCAGCATTTCTGCTAACAAAG
RI-X-XLOC_034132-12643-1
    GAGGCGAACCAGCCACTTTCAGCATTTCTGCTAACAAAG
CONSENSUS
    GAGGCGAACCAGCCACTTTCAGCATTTCTGCTAACAAAG

alignment for event: A5-gBol014989-XLOC_034933-10804

A5-gBol014989-XLOC_034933-10804-0
    GTGTTGGTAGCGCTTCAGGGAGCACTAACTGTGATTGGTAATATTACTCT
A5-gBol014989-XLOC_034933-10804-1
    GTGTTGGTAGCGCTTCAGGGAGCACTAACTGTGATTGGTAATATTACTCT
CONSENSUS
    GTGTTGGTAGCGCTTCAGGGAGCACTAACTGTGATTGGTAATATTACTCT

A5-gBol014989-XLOC_034933-10804-0
    GTGCATTGCTGCCTTTCGAATCAACAAGTCTGCATCCAAAATGGAAACCT
A5-gBol014989-XLOC_034933-10804-1
    GTGCATTGCTGCCTTTCGAATCAACAAGTCTGCATCCAAAATGGAAACCT
CONSENSUS
    GTGCATTGCTGCCTTTCGAATCAACAAGTCTGCATCCAAAATGGAAACCT

A5-gBol014989-XLOC_034933-10804-0
    CTGAAGAGCCGTGAGGGCTAAATACACATCTATGTGTTTAAAATAAAGGT
A5-gBol014989-XLOC_034933-10804-1
    CTGAAGAGCCGTGAGGGCTAAATACACATCTATGTGTTTAAAATAAAGGT
CONSENSUS
    CTGAAGAGCCGTGAGGGCTAAATACACATCTATGTGTTTAAAATAAAGGT

A5-gBol014989-XLOC_034933-10804-0
    CAAAGGAATGATTTGTTTTCAGTAATTTCTACAG----GAATCTGAGGTG
A5-gBol014989-XLOC_034933-10804-1
    CAAAGGAATGATTTGTTTTCAGTAATTTCTACAGGCAGGAATCTGAGGTG
CONSENSUS
    CAAAGGAATGATTTGTTTTCAGTAATTTCTACAG....GAATCTGAGGTG

A5-gBol014989-XLOC_034933-10804-0
    TAGCCTTTTTGGAGAGTGGTGATGGTTAGAGAAGCAACTGGATCTTTGGA
A5-gBol014989-XLOC_034933-10804-1
    TAGCCTTTTTGGAGAGTGGTGATGGTTAGAGAAGCAACTGGATCTTTGGA
CONSENSUS
    TAGCCTTTTTGGAGAGTGGTGATGGTTAGAGAAGCAACTGGATCTTTGGA

A5-gBol014989-XLOC_034933-10804-0
    TGTGGTATTAGCGGAAAGATCTTTTTGCTTTATATCTCTAGTGATTGCAC

A5-gBol014989-XLOC_034933-10804-1
TGTGGTATTAGCGGAAAGATCTTTTTGCTTTATATCTCTAGTGATTGCAC
CONSENSUS
TGTGGTATTAGCGGAAAGATCTTTTTGCTTTATATCTCTAGTGATTGCAC

A5-gBol014989-XLOC_034933-10804-0
TAAACATGTTTCTTGAGCGTGTGTGTGTATATACTTTTATATTGTAGTGT
A5-gBol014989-XLOC_034933-10804-1
TAAACATGTTTCTTGAGCGTGTGTGTGTATATACTTTTATATTGTAGTGT
CONSENSUS
TAAACATGTTTCTTGAGCGTGTGTGTGTATATACTTTTATATTGTAGTGT

A5-gBol014989-XLOC_034933-10804-0
ATTGTATTGTATTATCCTGATTATATTCCTGGTGAGT
A5-gBol014989-XLOC_034933-10804-1
ATTGTATTGTATTATCCTGATTATATTCCTGGTGAGT
CONSENSUS
ATTGTATTGTATTATCCTGATTATATTCCTGGTGAGT

alignment for event: RI-gBol008367-XLOC_042451-14532

RI-gBol008367-XLOC_042451-14532-0
GTGGAGCCAAGACACACATGTGACAACTAAAAAGACAGTAGCATGTGACA
RI-gBol008367-XLOC_042451-14532-1
GTGGAGCCAAGACACACATGTGACAACTAAAAAGACAGTAGCATGTGACA
CONSENSUS
GTGGAGCCAAGACACACATGTGACAACTAAAAAGACAGTAGCATGTGACA

RI-gBol008367-XLOC_042451-14532-0
CAAATGAACAACAAAAAGACAGTAGCATGTGACAACAAACGCAAGTGGAA
RI-gBol008367-XLOC_042451-14532-1
CAAATGAACAACAAAAAGACAGTAGCATGTGACAACAAACGCAAGTGGAA
CONSENSUS
CAAATGAACAACAAAAAGACAGTAGCATGTGACAACAAACGCAAGTGGAA

RI-gBol008367-XLOC_042451-14532-0
GACAGTAGCATTTCTTTATCAGCTTGTTTCCTTCTCCTTTTCGAAACAGA
RI-gBol008367-XLOC_042451-14532-1
GACAGTAGCATTTCTTTATCAGCTTGTTTCCTTCTCCTTTTCGAAACAGA
CONSENSUS
GACAGTAGCATTTCTTTATCAGCTTGTTTCCTTCTCCTTTTCGAAACAGA

RI-gBol008367-XLOC_042451-14532-0
GAACACATGTTACGTGACATAAAGCTTGTGTAGTTCTCTTAGCTGTGTTT
RI-gBol008367-XLOC_042451-14532-1
GAACACATGTTACGTGACATAAAGCTTGTGTAGTTCTCTTAGCTGTGTTT
CONSENSUS
GAACACATGTTACGTGACATAAAGCTTGTGTAGTTCTCTTAGCTGTGTTT

RI-gBol008367-XLOC_042451-14532-0
CAGAAATATTTTCTTTATAAGTAAATCGTATGGCCATTTGTTCCAGCAAA
RI-gBol008367-XLOC_042451-14532-1
CAGAAATATTTTCTTTATAAGTAAATCGTATGGCCATTTGTTCCAGCAAA
CONSENSUS

CAGAAATATTTTCTTTATAAGTAAATCGTATGGCCATTTGTTCCAGCAAA

RI-gBol008367-XLOC_042451-14532-0
CCCCATTTGCTTCTCTTCATCTTCTGAAATTTTGTATCTTTTAAGTTTTT
RI-gBol008367-XLOC_042451-14532-1
CCCCATTTGCTTCTCTTCATCTTCTGAAATTTTGTATCTTTTAAGTTTTT
CONSENSUS
CCCCATTTGCTTCTCTTCATCTTCTGAAATTTTGTATCTTTTAAGTTTTT

RI-gBol008367-XLOC_042451-14532-0
TTGTATCTCTTCATCTTCTCAAATCATTTTATCTCACCACATTTCTTTCT
RI-gBol008367-XLOC_042451-14532-1
TTGTATCTCTTCATCTTCTCAAATCATTTTATCTCACCACATTTCTTTCT
CONSENSUS
TTGTATCTCTTCATCTTCTCAAATCATTTTATCTCACCACATTTCTTTCT

RI-gBol008367-XLOC_042451-14532-0
TTAAATTCAGCATAATACCAAGATTAAATGGCTTCTTTTTCTGATAATAA
RI-gBol008367-XLOC_042451-14532-1
TTAAATTCAGCATAATACCAAGATTAAATGGCTTCTTTTTCTGATAATAA
CONSENSUS
TTAAATTCAGCATAATACCAAGATTAAATGGCTTCTTTTTCTGATAATAA

RI-gBol008367-XLOC_042451-14532-0
TATTAAAGACACGATGGATGAAAAATTCGACCAAGTTTTCGATCAACAAT
RI-gBol008367-XLOC_042451-14532-1
TATTAAAGACACGATGGATGAAAAATTCGACCAAGTTTTCGATCAACAAT
CONSENSUS
TATTAAAGACACGATGGATGAAAAATTCGACCAAGTTTTCGATCAACAAT

RI-gBol008367-XLOC_042451-14532-0
TCGAAAATTTTTTCATTCATCGTGATGATCATCCTTAAATGGATTTTTTT
RI-gBol008367-XLOC_042451-14532-1
TCGAAAATTTTTTCATTCATCGTGATGATCATCCTTAAATGGATTTTTTT
CONSENSUS
TCGAAAATTTTTTCATTCATCGTGATGATCATCCTTAAATGGATTTTTTT

RI-gBol008367-XLOC_042451-14532-0
CGTTTACGATTTGTATTTGTATTTTTAATATGTTTTTATGTCATTTTTAT
RI-gBol008367-XLOC_042451-14532-1
CGTTTACGATTTGTATTTGTATTTTTAATATGTTTTTATGTCATTTTTAT
CONSENSUS
CGTTTACGATTTGTATTTGTATTTTTAATATGTTTTTATGTCATTTTTAT

RI-gBol008367-XLOC_042451-14532-0
TTAAATTTTTATGTATGTTTTCTTTCATGTTATAAAATCCAATCATTCAA
RI-gBol008367-XLOC_042451-14532-1
TTAAATTTTTATGTATGTTTTCTTTCATGTTATAAAATCCAATCATTCAA
CONSENSUS
TTAAATTTTTATGTATGTTTTCTTTCATGTTATAAAATCCAATCATTCAA

RI-gBol008367-XLOC_042451-14532-0
TTTAAAAAAAAAAAAAATTTAAGACAAACAAGGGTTCTAAACTTTTCAAA
RI-gBol008367-XLOC_042451-14532-1
TTTAAAAAAAAAAAAAATTTAAGACAAACAAGGGTTCTAAACTTTTCAAA
CONSENSUS

TTTAAAAAAAAAAAAAAATTTAAGACAAACAAGGGTTCTAAACTTTTCAAA

RI-gBol008367-XLOC_042451-14532-0
TCACAAAATTAATTACTAATTTATTTATTAGAACCGATAAAGGTTTCTAA
RI-gBol008367-XLOC_042451-14532-1
TCACAAAATTAATTACTAATTTATTTATTAGAACCGATAAAGGTTTCTAA
CONSENSUS
TCACAAAATTAATTACTAATTTATTTATTAGAACCGATAAAGGTTTCTAA

RI-gBol008367-XLOC_042451-14532-0
CCAATGGTGTCATCTTTATTTATCTTGAAAACTTTAGTGTGCCATGTTTT
RI-gBol008367-XLOC_042451-14532-1
CCAATGGTGTCATCTTTATTTATCTTGAAAACTTTAGTGTGCCATGTTTT
CONSENSUS
CCAATGGTGTCATCTTTATTTATCTTGAAAACTTTAGTGTGCCATGTTTT

RI-gBol008367-XLOC_042451-14532-0
TGCGCATATTGTGATATCGAGAATCTACGTGCATTTATGATTTGCATGAT
RI-gBol008367-XLOC_042451-14532-1
TGCGCATATTGTGATATCGAGAATCTACGTGCATTTATGATTTGCATGAT
CONSENSUS
TGCGCATATTGTGATATCGAGAATCTACGTGCATTTATGATTTGCATGAT

RI-gBol008367-XLOC_042451-14532-0
TAAATATGAAACCAACTGTGTAATGTCATAATGTTTTTTTTTTTTTTGAT
RI-gBol008367-XLOC_042451-14532-1
TAAATATGAAACCAACTGTGTAATGTCATAATGTTTTTTTTTTTTTTGAT
CONSENSUS
TAAATATGAAACCAACTGTGTAATGTCATAATGTTTTTTTTTTTTTTGAT

RI-gBol008367-XLOC_042451-14532-0
ATCAGTGGGGTTTTCCAGTCGGTAAACCCAAACTAATCCCCACGAAATCT
RI-gBol008367-XLOC_042451-14532-1
ATCAGTGGGGTTTTCCAGTCGGTAAACCCAAACTAATCCCCACGAAATCT
CONSENSUS
ATCAGTGGGGTTTTCCAGTCGGTAAACCCAAACTAATCCCCACGAAATCT

RI-gBol008367-XLOC_042451-14532-0
TCCATCCGAGCACTCACGGTTATAGGCGGGAAGGTGGCCGAGGTGACTCG
RI-gBol008367-XLOC_042451-14532-1
TCCATCCGAGCACTCACGGTTATAGGCGGGAAGGTGGCCGAGGTGACTCG
CONSENSUS
TCCATCCGAGCACTCACGGTTATAGGCGGGAAGGTGGCCGAGGTGACTCG

RI-gBol008367-XLOC_042451-14532-0
AGGCTTTCCATTCGGGCACGCACGGATATGAGGTTCATTGGATGAATTAT
RI-gBol008367-XLOC_042451-14532-1
AGGCTTTCCATTCGGGCACGCACGGATATGAGGTTCATTGGATGAATTAT
CONSENSUS
AGGCTTTCCATTCGGGCACGCACGGATATGAGGTTCATTGGATGAATTAT

RI-gBol008367-XLOC_042451-14532-0
TGGCAGAGAGCTGCTCTCATACTGGCAAGCTCGAGATTATTTTCAATTTA
RI-gBol008367-XLOC_042451-14532-1
TGGCAGAGAGCTGCTCTCATACTGGCAAGCTCGAGATTATTTTCAATTTA
CONSENSUS

TGGCAGAGAGCTGCTCTCATACTGGCAAGCTCGAGATTATTTTCAATTTA

RI-gBol008367-XLOC_042451-14532-0
TTCACCATTAGCATTTTATTACGTTACCAACTTGTCATTCATAACATAGA
RI-gBol008367-XLOC_042451-14532-1
TTCACCATTAGCATTTTATTACGTTACCAACTTGTCATTCATAACATAGA
CONSENSUS
TTCACCATTAGCATTTTATTACGTTACCAACTTGTCATTCATAACATAGA

RI-gBol008367-XLOC_042451-14532-0
TGCCATAAAACCTACGAGCGTTAGGATCCATTGCCTGACTTTTGAATAAT
RI-gBol008367-XLOC_042451-14532-1
TGCCATAAAACCTACGAGCGTTAGGATCCATTGCCTGACTTTTGAATAAT
CONSENSUS
TGCCATAAAACCTACGAGCGTTAGGATCCATTGCCTGACTTTTGAATAAT

RI-gBol008367-XLOC_042451-14532-0
TGTTAGAGCATCTCCAGCGTAAAACTCTATTTTTTTTTCCAAAATAAAGT
RI-gBol008367-XLOC_042451-14532-1
TGTTAGAGCATCTCCAGCGTAAAACTCTATTTTTTTTTCCAAAATAAAGT
CONSENSUS
TGTTAGAGCATCTCCAGCGTAAAACTCTATTTTTTTTTCCAAAATAAAGT

RI-gBol008367-XLOC_042451-14532-0
AAAAATGCTTGAATCCTACTTCATTTTTCACTTCATAATAGAGTGATGCT
RI-gBol008367-XLOC_042451-14532-1
AAAAATGCTTGAATCCTACTTCATTTTTCACTTCATAATAGAGTGATGCT
CONSENSUS
AAAAATGCTTGAATCCTACTTCATTTTTCACTTCATAATAGAGTGATGCT

RI-gBol008367-XLOC_042451-14532-0
CCAAAATAGAGGAAAAAATGGTGTTGGGTTGGAGATGCCCTTAGCACAAT
RI-gBol008367-XLOC_042451-14532-1
CCAAAATAGAGGAAAAAATGGTGTTGGGTTGGAGATGCCCTTAGCACAAT
CONSENSUS
CCAAAATAGAGGAAAAAATGGTGTTGGGTTGGAGATGCCCTTAGCACAAT

RI-gBol008367-XLOC_042451-14532-0
TTTAGTCTAACATATATACGCTATACATAGCAAAAAGAGTTTGATGAAAC
RI-gBol008367-XLOC_042451-14532-1
TTTAGTCTAACATATATACGCTATACATAGCAAAAAGAGTTTGATGAAAC
CONSENSUS
TTTAGTCTAACATATATACGCTATACATAGCAAAAAGAGTTTGATGAAAC

RI-gBol008367-XLOC_042451-14532-0
GGTGTTCTACAATTATCACTTGCTATACCTTTTCCCCAATGTCTAGATGG
RI-gBol008367-XLOC_042451-14532-1
GGTGTTCTACAATTATCACTTGCTATACCTTTTCCCCAATGTCTAGATGG
CONSENSUS
GGTGTTCTACAATTATCACTTGCTATACCTTTTCCCCAATGTCTAGATGG

RI-gBol008367-XLOC_042451-14532-0
AATATTGCCTAGAGATACATTTTCCCCATTTCCACATTCCTTCTGTTTTT
RI-gBol008367-XLOC_042451-14532-1
AATATTGCCTAGAGATACATTTTCCCCATTTCCACATTCCTTCTGTTTTT
CONSENSUS

AATATTGCCTAGAGATACATTTTCCCCATTTCCACATTCCTTCTGTTTTT

RI-gBol008367-XLOC_042451-14532-0
CATTTCATAATTTATGGTTCGTTGATGTATTATTAAAGAGATTGTTGAAA
RI-gBol008367-XLOC_042451-14532-1
CATTTCATAATTTATGGTTCGTTGATGTATTATTAAAGAGATTGTTGAAA
CONSENSUS
CATTTCATAATTTATGGTTCGTTGATGTATTATTAAAGAGATTGTTGAAA

RI-gBol008367-XLOC_042451-14532-0
TGTGAGTTGGAGTCTTGGAGAAGAATGTGACCATAGGGAGGAAGTGAAGA
RI-gBol008367-XLOC_042451-14532-1
TGTGAGTTGGAGTCTTGGAGAAGAATGTGACCATAGGGAGGAAGTGAAGA
CONSENSUS
TGTGAGTTGGAGTCTTGGAGAAGAATGTGACCATAGGGAGGAAGTGAAGA

RI-gBol008367-XLOC_042451-14532-0
AGATCCAAAAATAAAATAAGATGTGAGAGTGAAATTTTCTCAAGTCATCT
RI-gBol008367-XLOC_042451-14532-1
AGATCCAAAAATAAAATAAGATGTGAGAGTGAAATTTTCTCAAGTCATCT
CONSENSUS
AGATCCAAAAATAAAATAAGATGTGAGAGTGAAATTTTCTCAAGTCATCT

RI-gBol008367-XLOC_042451-14532-0
CAACTTCCACCAACCGAATGTGGTTTAAGAGATTAGGTATTAATCTTTAA
RI-gBol008367-XLOC_042451-14532-1
CAACTTCCACCAACCGAATGTGGTTTAAGAGATTAG--------------
CONSENSUS
CAACTTCCACCAACCGAATGTGGTTTAAGAGATTAG..............

RI-gBol008367-XLOC_042451-14532-0
TTACTATTATTTAAATGCTTTGGCTCACACCTTCTGCATGCGTTTATTCG
RI-gBol008367-XLOC_042451-14532-1

CONSENSUS
....

RI-gBol008367-XLOC_042451-14532-0
CTTAAAAACTTTAGTTTTCTTATTCCTTATTTAATTCAATTCTCTCTCTC
RI-gBol008367-XLOC_042451-14532-1

CONSENSUS
....

RI-gBol008367-XLOC_042451-14532-0
CCACAAAGTTAGGTGTACATATTTATTGTGTATTTATACATGTCTCCGGA
RI-gBol008367-XLOC_042451-14532-1

CONSENSUS
....

RI-gBol008367-XLOC_042451-14532-0
ACATGACTTCCTAATTTATGTATTACTCCTTTCTGTAAATAGTAGGCGTG
RI-gBol008367-XLOC_042451-14532-1
--TAGGCGTG
CONSENSUS

.....................................TAGGCGTG

RI-gBol008367-XLOC_042451-14532-0
GGCGTTTTTAACTCAACCCGAAGTACCGACCTTAACCCGAACAGGAAAAA
RI-gBol008367-XLOC_042451-14532-1
GGCGTTTTTAACTCAACCCGAAGTACCGACCTTAACCCGAACAGGAAAAA
CONSENSUS
GGCGTTTTTAACTCAACCCGAAGTACCGACCTTAACCCGAACAGGAAAAA

RI-gBol008367-XLOC_042451-14532-0
TCGAATCCGAATCCGAACTGCTTTACAAAAATATCCGAATGAAACTTATG
RI-gBol008367-XLOC_042451-14532-1
TCGAATCCGAATCCGAACTGCTTTACAAAAATATCCGAATGAAACTTATG
CONSENSUS
TCGAATCCGAATCCGAACTGCTTTACAAAAATATCCGAATGAAACTTATG

RI-gBol008367-XLOC_042451-14532-0
AGCTTACTACTTTGGACTTTGGTTATAACCCGAACTGAACCGAAATTCGA
RI-gBol008367-XLOC_042451-14532-1
AGCTTACTACTTTGGACTTTGGTTATAACCCGAACTGAACCGAAATTCGA
CONSENSUS
AGCTTACTACTTTGGACTTTGGTTATAACCCGAACTGAACCGAAATTCGA

RI-gBol008367-XLOC_042451-14532-0
ATTAGGATCCGAAGATATCCGAAATTAGTTAAATATGTTAATGTTTTTTA
RI-gBol008367-XLOC_042451-14532-1
ATTAGGATCCGAAGATATCCGAAATTAGTTAAATATGTTAATGTTTTTTA
CONSENSUS
ATTAGGATCCGAAGATATCCGAAATTAGTTAAATATGTTAATGTTTTTTA

RI-gBol008367-XLOC_042451-14532-0 TATATA
RI-gBol008367-XLOC_042451-14532-1 TATATA
CONSENSUS TATATA

alignment for event: RI-gBol014689-XLOC_035325-11888

RI-gBol014689-XLOC_035325-11888-0
GCGAAAATAAAGAGGATTTCTCAATAAGATTAGCCAAGAATTTAGAGGAT
RI-gBol014689-XLOC_035325-11888-1
GCGAAAATAAAGAGGATTTCTCAATAAGATTAGCCAAGAATTTAGAGGAT
CONSENSUS
GCGAAAATAAAGAGGATTTCTCAATAAGATTAGCCAAGAATTTAGAGGAT

RI-gBol014689-XLOC_035325-11888-0
CTTATCATTACAGAGGGACCAGAAACTGTGAGCAATCTTCTATAATCACA
RI-gBol014689-XLOC_035325-11888-1
CTTATCATTACAGAGGGACCAGAAACTGTGAGCAATCTTCTATAATCACA
CONSENSUS
CTTATCATTACAGAGGGACCAGAAACTGTGAGCAATCTTCTATAATCACA

RI-gBol014689-XLOC_035325-11888-0
CTCTTGTCTTGGACCAGTGAGTGTTGTGCTTTCTATTTCTGACCGTAAGC
RI-gBol014689-XLOC_035325-11888-1
CTCTTGTCTTGGACCAGTGAGTGTTGTGCTTTCTATTTCTGACC------

CONSENSUS
CTCTTGTCTTGGACCAGTGAGTGTTGTGCTTTCTATTTCTGACC......

RI-gBol014689-XLOC_035325-11888-0
TTTCTTTCAAACTTGTGTTATGATCTCAAATTGTGAAGCATGTTGGTAAA
RI-gBol014689-XLOC_035325-11888-1

CONSENSUS
....

RI-gBol014689-XLOC_035325-11888-0
GTTCTACACCGCGCTTTTTATGTTAGCTTTTGCCTGAAAATTGACTCAAG
RI-gBol014689-XLOC_035325-11888-1

CONSENSUS
....

RI-gBol014689-XLOC_035325-11888-0
TCTGCTGCTTGATAGCTGATTAGTTCAATGTTGGGTTTTGCTTTTATGAT
RI-gBol014689-XLOC_035325-11888-1

CONSENSUS
....

RI-gBol014689-XLOC_035325-11888-0
TGAGCTGCAGAAGTAAATTTCATAACTTTTTTTTTGTTGCTCTGTTCAGT
RI-gBol014689-XLOC_035325-11888-1
---T
CONSENSUS
....T

RI-gBol014689-XLOC_035325-11888-0
TGATTTTGCATAATGAGAACTGCAGTTTCATGGATACCAAAAGGGGCTTC
RI-gBol014689-XLOC_035325-11888-1
TGATTTTGCATAATGAGAACTGCAGTTTCATGGATACCAAAAGGGGCTTC
CONSENSUS
TGATTTTGCATAATGAGAACTGCAGTTTCATGGATACCAAAAGGGGCTTC

RI-gBol014689-XLOC_035325-11888-0
AAAGGCCATGCTGATCCTCCTTCTAAGATCAAAGAGCTGATCGAGAATGG
RI-gBol014689-XLOC_035325-11888-1
AAAGGCCATGCTGATCCTCCTTCTAAGATCAAAGAGCTGATCGAGAATGG
CONSENSUS
AAAGGCCATGCTGATCCTCCTTCTAAGATCAAAGAGCTGATCGAGAATGG

RI-gBol014689-XLOC_035325-11888-0
CACTCAAGAAAAGGTGAATTATTTTCCCTTTCCATCTTCTACTGGACTTT
RI-gBol014689-XLOC_035325-11888-1
CACTCAAGAAAAGGTGAATTATTTTCCCTTTCCATCTTCTACTGGACTTT
CONSENSUS
CACTCAAGAAAAGGTGAATTATTTTCCCTTTCCATCTTCTACTGGACTTT

RI-gBol014689-XLOC_035325-11888-0
TGTAAAACTCACCCTTTGATTTGTGGTTACATGTACAACATATGCTATCT
RI-gBol014689-XLOC_035325-11888-1
TGTAAAACTCACCCTTTGATTTGTGGTTACATGTACAACATATGCTATCT

CONSENSUS
      TGTAAAACTCACCCTTTGATTTGTGGTTACATGTACAACATATGCTATCT

RI-gBol014689-XLOC_035325-11888-0
      GAGTGTGACATGGATGGAGCGTGAAATACATAGTGGTGAAGTTCCTCATG
RI-gBol014689-XLOC_035325-11888-1
      GAGTGTGACATGGATGGAGCGTGAAATACATAGTGGTGAAGTTCCTCATG
CONSENSUS
      GAGTGTGACATGGATGGAGCGTGAAATACATAGTGGTGAAGTTCCTCATG

RI-gBol014689-XLOC_035325-11888-0
      CCAAGGCTGTAGCCAAATATTTTGAAAAACCATGTGCCAAAAGCAAGTTT
RI-gBol014689-XLOC_035325-11888-1
      CCAAGGCTGTAGCCAAATATTTTGAAAAACCATGTGCCAAAAGCAAGTTT
CONSENSUS
      CCAAGGCTGTAGCCAAATATTTTGAAAAACCATGTGCCAAAAGCAAGTTT

RI-gBol014689-XLOC_035325-11888-0   ACAATTCTAAGCACT
RI-gBol014689-XLOC_035325-11888-1   ACAATTCTAAGCACT
CONSENSUS   ACAATTCTAAGCACT

alignment for event: A5-gBol009977-XLOC_040581-5327

A5-gBol009977-XLOC_040581-5327-0
      ATTAGATTCTTGCATGATCCGTCCAAAGATATAGGGTATGTTGGGAGTGC
A5-gBol009977-XLOC_040581-5327-1
      ATTAGATTCTTGCATGATCCGTCCAAAGATATAGGGTATGTTGGGAGTGC
CONSENSUS
      ATTAGATTCTTGCATGATCCGTCCAAAGATATAGGGTATGTTGGGAGTGC

A5-gBol009977-XLOC_040581-5327-0
      TTTGTCCAGTAATATGATAAGATTTTTCAGGAACAGTGATGACACATGGA
A5-gBol009977-XLOC_040581-5327-1
      TTTGTCCAGTAATATGATAAGATTTTTCAGGAACAGTGATGACACATGGA
CONSENSUS
      TTTGTCCAGTAATATGATAAGATTTTTCAGGAACAGTGATGACACATGGA

A5-gBol009977-XLOC_040581-5327-0 GCCATGAG---------
GTGGTTATATCTGTGAAACCATTGAAAGTAGAA
A5-gBol009977-XLOC_040581-5327-1
      GCCATGAGGCAAGAAGAGTGGTTATATCTGTGAAACCATTGAAAGTAGAA
CONSENSUS
      GCCATGAG.........GTGGTTATATCTGTGAAACCATTGAAAGTAGAA

A5-gBol009977-XLOC_040581-5327-0
      AACTGGATTCTTCCAGAAATGCCAGGGCTTATCACTGACTTCTTGATCTC
A5-gBol009977-XLOC_040581-5327-1
      AACTGGATTCTTCCAGAAATGCCAGGGCTTATCACTGACTTCTTGATCTC
CONSENSUS
      AACTGGATTCTTCCAGAAATGCCAGGGCTTATCACTGACTTCTTGATCTC

A5-gBol009977-XLOC_040581-5327-0
      CCTCGATGACCGGTTCATGTACTTTGTGAACTGGCTTCATGGAGACGTTC
A5-gBol009977-XLOC_040581-5327-1

CCTCGATGACCGGTTCATGTACTTTGTGAACTGGCTTCATGGAGACGTTC
CONSENSUS
CCTCGATGACCGGTTCATGTACTTTGTGAACTGGCTTCATGGAGACGTTC

A5-gBol009977-XLOC_040581-5327-0
GCCAGTACAACATCGAAGACCCTAAAAACCCTGTCTTAACCGGACAGATT
A5-gBol009977-XLOC_040581-5327-1
GCCAGTACAACATCGAAGACCCTAAAAACCCTGTCTTAACCGGACAGATT
CONSENSUS
GCCAGTACAACATCGAAGACCCTAAAAACCCTGTCTTAACCGGACAGATT

A5-gBol009977-XLOC_040581-5327-0
TGGGTGGGAGGACTGCTACAGAAGGGCAGTCCTGTTAAGGCTGTTAGAGA
A5-gBol009977-XLOC_040581-5327-1
TGGGTGGGAGGACTGCTACAGAAGGGCAGTCCTGTTAAGGCTGTTAGAGA
CONSENSUS
TGGGTGGGAGGACTGCTACAGAAGGGCAGTCCTGTTAAGGCTGTTAGAGA

A5-gBol009977-XLOC_040581-5327-0
AGATGGTAGCACTTACCAGTTTGATGTTCCTCAGATTAAG
A5-gBol009977-XLOC_040581-5327-1
AGATGGTAGCACTTACCAGTTTGATGTTCCTCAGATTAAG
CONSENSUS
AGATGGTAGCACTTACCAGTTTGATGTTCCTCAGATTAAG

alignment for event: SE-gBol006555-XLOC_044525-7413

SE-gBol006555-XLOC_044525-7413-0
CAAAAAAAGACCTGAATTAAAAAAATAAATATAACTGAGAAAGAAAAGAA
SE-gBol006555-XLOC_044525-7413-1
CAAAAAAAGACCTGAATTAAAAAAATAAATATAACTGAGAAAGAAAAGAA
CONSENSUS
CAAAAAAAGACCTGAATTAAAAAAATAAATATAACTGAGAAAGAAAAGAA

SE-gBol006555-XLOC_044525-7413-0
AATACAGAGAGACAAATTTAAACAAAAGGAAAGGGAGATCGAGAGAGGCA
SE-gBol006555-XLOC_044525-7413-1
AATACAGAGAGACAAATTTAAACAAAAGGAAAGGGAGATCGAGAGAGGCA
CONSENSUS
AATACAGAGAGACAAATTTAAACAAAAGGAAAGGGAGATCGAGAGAGGCA

SE-gBol006555-XLOC_044525-7413-0
CACACACACAAAGGAGAATTTTAGGGTTTGGGGAGACTCCGAAGAGATTG
SE-gBol006555-XLOC_044525-7413-1
CACACACACAAAGGAGAATTTTAGGGTTTGGGGAGACTCCGAAGAGATTG
CONSENSUS
CACACACACAAAGGAGAATTTTAGGGTTTGGGGAGACTCCGAAGAGATTG

SE-gBol006555-XLOC_044525-7413-0
GCGTAACCTTCATTGTACACTTC---------------------------
SE-gBol006555-XLOC_044525-7413-1
GCGTAACCTTCATTGTACACTTCCTGAGAGTATTGTGAACCTGCAGCTGT
CONSENSUS
GCGTAACCTTCATTGTACACTTC...........................

SE-gBol006555-XLOC_044525-7413-0

SE-gBol006555-XLOC_044525-7413-1
GGTTTGGATTGTTTATAGCTCAATGGTTGAAACTTGATCATTCTTACACA
CONSENSUS
....

SE-gBol006555-XLOC_044525-7413-0
----------------------------------TGTCTTTGAAAGCTCA
SE-gBol006555-XLOC_044525-7413-1
TAAAAATTGTTCCTTTACTTCCGTTGATTACTTGTGTCTTTGAAAGCTCA
CONSENSUS
..................................TGTCTTTGAAAGCTCA

SE-gBol006555-XLOC_044525-7413-0
TCTTCCTCGTCATGGTGGCTACTTCCGCTACGTCGTCGTTTTTTCATGTT
SE-gBol006555-XLOC_044525-7413-1
TCTTCCTCGTCATGGTGGCTACTTCCGCTACGTCGTCGTTTTTTCATGTT
CONSENSUS
TCTTCCTCGTCATGGTGGCTACTTCCGCTACGTCGTCGTTTTTTCATGTT

SE-gBol006555-XLOC_044525-7413-0
CCATCTTCCTCCTCTCTTGATACTAATGGGAAGGGGAACAGAGTTGCGTC
SE-gBol006555-XLOC_044525-7413-1
CCATCTTCCTCCTCTCTTGATACTAATGGGAAGGGGAACAGAGTTGCGTC
CONSENSUS
CCATCTTCCTCCTCTCTTGATACTAATGGGAAGGGGAACAGAGTTGCGTC

SE-gBol006555-XLOC_044525-7413-0
CACGAACTTCGCTGGACTTAACTCAACGCCAAGCTCTGGGAGGATGAAGG
SE-gBol006555-XLOC_044525-7413-1
CACGAACTTCGCTGGACTTAACTCAACGCCAAGCTCTGGGAGGATGAAGG
CONSENSUS
CACGAACTTCGCTGGACTTAACTCAACGCCAAGCTCTGGGAGGATGAAGG

SE-gBol006555-XLOC_044525-7413-0
TTAAACCAAACGCTCAGGCTCCACCCAAGATCAACGGGAAGAAAGCTAAC
SE-gBol006555-XLOC_044525-7413-1
TTAAACCAAACGCTCAGGCTCCACCCAAGATCAACGGGAAGAAAGCTAAC
CONSENSUS
TTAAACCAAACGCTCAGGCTCCACCCAAGATCAACGGGAAGAAAGCTAAC

SE-gBol006555-XLOC_044525-7413-0
TTGCCTGGTTCTGTAGAGATATCAAAGTCTGACAACGAGACTTCGCAACC
SE-gBol006555-XLOC_044525-7413-1
TTGCCTGGTTCTGTAGAGATATCAAAGTCTGACAACGAGACTTCGCAACC
CONSENSUS
TTGCCTGGTTCTGTAGAGATATCAAAGTCTGACAACGAGACTTCGCAACC

SE-gBol006555-XLOC_044525-7413-0
CGCACCCGCACCGAGGACGTTTATCAACCAGCTGCCTGACTGGAGCATGC
SE-gBol006555-XLOC_044525-7413-1
CGCACCCGCACCGAGGACGTTTATCAACCAGCTGCCTGACTGGAGCATGC
CONSENSUS
CGCACCCGCACCGAGGACGTTTATCAACCAGCTGCCTGACTGGAGCATGC

```
SE-gBol006555-XLOC_044525-7413-0
      TTCTCGCTGCCATAACAACTATTTTCTTAGCGGCTGAGAAACAGTGGATG
SE-gBol006555-XLOC_044525-7413-1
      TTCTCGCTGCCATAACAACTATTTTCTTAGCGGCTGAGAAACAGTGGATG
CONSENSUS
      TTCTCGCTGCCATAACAACTATTTTCTTAGCGGCTGAGAAACAGTGGATG

SE-gBol006555-XLOC_044525-7413-0
      ATGCTTGACTGGAAACCCAGGCGTTCTGATATGATAATGGATCCTTTCGG
SE-gBol006555-XLOC_044525-7413-1
      ATGCTTGACTGGAAACCCAGGCGTTCTGATATGATAATGGATCCTTTCGG
CONSENSUS
      ATGCTTGACTGGAAACCCAGGCGTTCTGATATGATAATGGATCCTTTCGG

SE-gBol006555-XLOC_044525-7413-0
      TTTAGGGAGAATCGTTCAGGATGGTCTTGTGTTTCGTCAGAATTTCTCCA
SE-gBol006555-XLOC_044525-7413-1
      TTTAGGGAGAATCGTTCAGGATGGTCTTGTGTTTCGTCAGAATTTCTCCA
CONSENSUS
      TTTAGGGAGAATCGTTCAGGATGGTCTTGTGTTTCGTCAGAATTTCTCCA

SE-gBol006555-XLOC_044525-7413-0
      TTAGGTCTTATGAGATAGGTGCTGATCGCTCTGCGTCTATAGAAACTGTT
SE-gBol006555-XLOC_044525-7413-1
      TTAGGTCTTATGAGATAGGTGCTGATCGCTCTGCGTCTATAGAAACTGTT
CONSENSUS
      TTAGGTCTTATGAGATAGGTGCTGATCGCTCTGCGTCTATAGAAACTGTT

SE-gBol006555-XLOC_044525-7413-0 ATGAATCATTTACAG
SE-gBol006555-XLOC_044525-7413-1 ATGAATCATTTACAG
CONSENSUS                        ATGAATCATTTACAG



alignment for event: RI-gBol041964-XLOC_004096-7384

RI-gBol041964-XLOC_004096-7384-0
      GAAATCAAACTTCTCAGTAATCTTCAGCATCCAAACATTCGAGTATATAT
RI-gBol041964-XLOC_004096-7384-1
      GAAATCAAACTTCTCAGTAATCTTCAGCATCCAAACATTCGA--------
CONSENSUS
      GAAATCAAACTTCTCAGTAATCTTCAGCATCCAAACATTCGA........

RI-gBol041964-XLOC_004096-7384-0
      ATCCTCTCTTTACATTCATATAATCTAACTATGGAGTTCACTCTGGCTGT
RI-gBol041964-XLOC_004096-7384-1
      --------------------------------------------------
CONSENSUS
      ..................................................

RI-gBol041964-XLOC_004096-7384-0
      ATTGAGTAATTGGAGTATGTTGGTATGTAACATGTATATGATCGTTTCTT
RI-gBol041964-XLOC_004096-7384-1
      --------------------------------------------------
CONSENSUS
```

....

RI-gBol041964-XLOC_004096-7384-0
TATATACCTGGAATATGTTCACCCGGGTTCTATCAACAAATATATCCAAG
RI-gBol041964-XLOC_004096-7384-1

CONSENSUS
....

RI-gBol041964-XLOC_004096-7384-0
ACCATTGCGGCGGTACCATGACCGAATCTGTTGTTCGCAATTTTACTCGT
RI-gBol041964-XLOC_004096-7384-1
ACCATTGCGGCGGTACCATGACCGAATCTGTTGTTCGCAATTTTACTCGT
CONSENSUS
ACCATTGCGGCGGTACCATGACCGAATCTGTTGTTCGCAATTTTACTCGT

RI-gBol041964-XLOC_004096-7384-0
CATATCTTGTCCGGGCTGGCTTATTTGCACAGCAAAAAGACAGTACATAG
RI-gBol041964-XLOC_004096-7384-1
CATATCTTGTCCGGGCTGGCTTATTTGCACAGCAAAAAGACAGTACATAG
CONSENSUS
CATATCTTGTCCGGGCTGGCTTATTTGCACAGCAAAAAGACAGTACATAG

alignment for event: RI-X-XLOC_016067-9586

RI-X-XLOC_016067-9586-0
GGGAGTATAGGTGAATGGAGGCGATGTAATTATGATGTCATTCCGTATAA
RI-X-XLOC_016067-9586-1
GGGAGTATAGGTGAATGGAGGCGATGTAATTATGATGTCATTCCGTATAA
CONSENSUS
GGGAGTATAGGTGAATGGAGGCGATGTAATTATGATGTCATTCCGTATAA

RI-X-XLOC_016067-9586-0
CTACGACATTCAAAGCACCGTACCATACCATATGAATAACAGCATTAGTG
RI-X-XLOC_016067-9586-1
CTACGACATTCAAAGCACCGTACCATACCATATGAATAACAGCATTAGTG
CONSENSUS
CTACGACATTCAAAGCACCGTACCATACCATATGAATAACAGCATTAGTG

RI-X-XLOC_016067-9586-0
GCTTTCGATCTCTTATCTACAGGTAAGAGTTAAATCTGTTTTCTTCACTC
RI-X-XLOC_016067-9586-1
GCTTTCGATCTCTTATCTACAG----------------------------
CONSENSUS
GCTTTCGATCTCTTATCTACAG............................

RI-X-XLOC_016067-9586-0
CATTCACTCTTACGTACAAAATATATACCAGAGATTATAAGATTTGTGCG
RI-X-XLOC_016067-9586-1

CONSENSUS
....

RI-X-XLOC_016067-9586-0

GTTTATGGTGATAAGTGGTGATCATGATATGTTGGTGCCTTTCATTGCAA
RI-X-XLOC_016067-9586-1

CONSENSUS
....

RI-X-XLOC_016067-9586-0
CTCAAGCATGGATAAGGTCTCTCAATTACTCCATCGTCGATGACTGGAGA
RI-X-XLOC_016067-9586-1 ------
CATGGATAAGGTCTCTCAATTACTCCATCGTCGATGACTGGAGA
CONSENSUS
......CATGGATAAGGTCTCTCAATTACTCCATCGTCGATGACTGGAGA

RI-X-XLOC_016067-9586-0 CCTTGGATGGTAAACGATCAAATCGCTGG
RI-X-XLOC_016067-9586-1 CCTTGGATGGTAAACGATCAAATCGCTGG
CONSENSUS CCTTGGATGGTAAACGATCAAATCGCTGG

alignment for event: A5-gBol005916-XLOC_045196-15721

A5-gBol005916-XLOC_045196-15721-0
GGAATGGGTGATTTCTTGAACGAAATGGCAGCAATGATGAATCAATCCAA
A5-gBol005916-XLOC_045196-15721-1
GGAATGGGTGATTTCTTGAACGAAATGGCAGCAATGATGAATCAATCCAA
CONSENSUS
GGAATGGGTGATTTCTTGAACGAAATGGCAGCAATGATGAATCAATCCAA

A5-gBol005916-XLOC_045196-15721-0
GCCTAAT------------------GAGAATAGTTCAGGGGACAGTTTTG
A5-gBol005916-XLOC_045196-15721-1
GCCTAATGCAAGACTCTCTTCACTAGAGAATAGTTCAGGGGACAGTTTTG
CONSENSUS
GCCTAAT..................GAGAATAGTTCAGGGGACAGTTTTG

A5-gBol005916-XLOC_045196-15721-0
AACAGCTACAAGATCTGTTTAATGAGATGTTTCAAGGAGACGCCACAGCA
A5-gBol005916-XLOC_045196-15721-1
AACAGCTACAAGATCTGTTTAATGAGATGTTTCAAGGAGACGCCACAGCA
CONSENSUS
AACAGCTACAAGATCTGTTTAATGAGATGTTTCAAGGAGACGCCACAGCA

A5-gBol005916-XLOC_045196-15721-0
TTCCCACCCTCATCATCGTCTTTCCCTGCTTCAACTTTCACTTCCTCTTG
A5-gBol005916-XLOC_045196-15721-1
TTCCCACCCTCATCATCGTCTTTCCCTGCTTCAACTTTCACTTCCTCTTG
CONSENSUS
TTCCCACCCTCATCATCGTCTTTCCCTGCTTCAACTTTCACTTCCTCTTG

A5-gBol005916-XLOC_045196-15721-0
TGACTTTGTCTTTGATACAAACTATCAGCAGTCACCGTTTGGGATGAGCT
A5-gBol005916-XLOC_045196-15721-1
TGACTTTGTCTTTGATACAAACTATCAGCAGTCACCGTTTGGGATGAGCT
CONSENSUS
TGACTTTGTCTTTGATACAAACTATCAGCAGTCACCGTTTGGGATGAGCT

A5-gBol005916-XLOC_045196-15721-0
CGATGGGGACTAGTGATCCTTTCGGATTTGACTCAAGATCTCACACCTTC
A5-gBol005916-XLOC_045196-15721-1
CGATGGGGACTAGTGATCCTTTCGGATTTGACTCAAGATCTCACACCTTC
CONSENSUS
CGATGGGGACTAGTGATCCTTTCGGATTTGACTCAAGATCTCACACCTTC

A5-gBol005916-XLOC_045196-15721-0 TCTTTAGGG
A5-gBol005916-XLOC_045196-15721-1 TCTTTAGGG
CONSENSUS TCTTTAGGG

alignment for event: RI-gBol028518-XLOC_019836-16543

RI-gBol028518-XLOC_019836-16543-0
GTCCTGAAGGTTCCAAGAGTGTTCAAAAGGGTGTGACAGAGAATGCTCTT
RI-gBol028518-XLOC_019836-16543-1
GTCCTGAAGGTTCCAAGAGTGTTCAAAAGGGTGTGACAGAGAATGCTCTT
CONSENSUS
GTCCTGAAGGTTCCAAGAGTGTTCAAAAGGGTGTGACAGAGAATGCTCTT

RI-gBol028518-XLOC_019836-16543-0
GTTGTTGGTCCAACTCTACAGCCAAAACTCTTTCAGGTGTTGTTAACCTT
RI-gBol028518-XLOC_019836-16543-1
GTTGTTGGTCCAACTCTACAGCCAAAACTCTTTCAG--------------
CONSENSUS
GTTGTTGGTCCAACTCTACAGCCAAAACTCTTTCAG..............

RI-gBol028518-XLOC_019836-16543-0
TGCTGTTTATTCGAGTAGTGTTTTTGACTCGTGCTTAATTATTATTTTCA
RI-gBol028518-XLOC_019836-16543-1

CONSENSUS
....

RI-gBol028518-XLOC_019836-16543-0
CAGCTCATCCAGGTGCACTTGGGTTGGGTTACTGGAGCATAAATTGTGCT
RI-gBol028518-XLOC_019836-16543-1 ---
CTCATCCAGGTGCACTTGGGTTGGGTTACTGGAGCATAAATTGTGCT
CONSENSUS
...CTCATCCAGGTGCACTTGGGTTGGGTTACTGGAGCATAAATTGTGCT

RI-gBol028518-XLOC_019836-16543-0
TTAGCTCTTGAGAAAGAAAGTATGAG
RI-gBol028518-XLOC_019836-16543-1
TTAGCTCTTGAGAAAGAAAGTATGAG
CONSENSUS
TTAGCTCTTGAGAAAGAAAGTATGAG

alignment for event: RI-gBol028441-XLOC_019799-16513

RI-gBol028441-XLOC_019799-16513-0
CTCGTGTCCCCCCCCTTTGGGCTTTCATCTCTTCCGCGACTTCTCTTCTT

RI-gBol028441-XLOC_019799-16513-1
CTCGTGTCCCCCCCCTTTGGGCTTTCATCTCTTCCGCGACTTCTCTTCTT
CONSENSUS
CTCGTGTCCCCCCCCTTTGGGCTTTCATCTCTTCCGCGACTTCTCTTCTT

RI-gBol028441-XLOC_019799-16513-0
CTTCTGCGATGGCGATGTTGCTCTCTTGGGTTTGAAGGAGGAGCTTGGCT
RI-gBol028441-XLOC_019799-16513-1
CTTCTGCGATGGCGATGTTGCTCTCTTGGGTTTGAAGGAGGAGCTTGGCT
CONSENSUS
CTTCTGCGATGGCGATGTTGCTCTCTTGGGTTTGAAGGAGGAGCTTGGCT

RI-gBol028441-XLOC_019799-16513-0
CTCTCGATTGGGGCCACGATCGTGTAGCCGACGCCTCCCATCACGGCTCC
RI-gBol028441-XLOC_019799-16513-1
CTCTCGATTGGGGCCACGATCGTGTAGCCGACGCCTCCCATCACGGCTCC
CONSENSUS
CTCTCGATTGGGGCCACGATCGTGTAGCCGACGCCTCCCATCACGGCTCC

RI-gBol028441-XLOC_019799-16513-0
GGCGAGTAGCCGTTCGTCCTCTCTAGATTCTTCCATCTCTCCTCCTGTCA
RI-gBol028441-XLOC_019799-16513-1
GGCGAGTAGCCGTTCGTCCTCTCTAGATTCTTCCATCTCTCCTCCTGTCA
CONSENSUS
GGCGAGTAGCCGTTCGTCCTCTCTAGATTCTTCCATCTCTCCTCCTGTCA

RI-gBol028441-XLOC_019799-16513-0
TTTTCTCTACTCACCGCTCTCTAAAAGGTGTAACCTTTTTCACTTTTACA
RI-gBol028441-XLOC_019799-16513-1
TTTTCTCTACTCACCGCTCTCTAAAAGGTGTAACCTTTTTCACTTTTACA
CONSENSUS
TTTTCTCTACTCACCGCTCTCTAAAAGGTGTAACCTTTTTCACTTTTACA

RI-gBol028441-XLOC_019799-16513-0
CGACAACAATGGCGGATTGGGCTCCGATTATCGTCGGCGTCATCCTCTTC
RI-gBol028441-XLOC_019799-16513-1
CGACAACAATGGCGGATTGGGCTCCGATTATCGTCGGCGTCATCCTCTTC
CONSENSUS
CGACAACAATGGCGGATTGGGCTCCGATTATCGTCGGCGTCATCCTCTTC

RI-gBol028441-XLOC_019799-16513-0
GTGATTCTCTCCTGGACTTCTCTTCTCACTGCCCCGAACCAACCGAGGAG
RI-gBol028441-XLOC_019799-16513-1
GTGATTCTCTCCTGGACTTCTCTTCTCACTGCCCCGAACCAACCGAGGAG
CONSENSUS
GTGATTCTCTCCTGGACTTCTCTTCTCACTGCCCCGAACCAACCGAGGAG

RI-gBol028441-XLOC_019799-16513-0
TAGACTTCGGTAATCACAAAACCAACGGAAAAGCCATAGCTGTTCACACT
RI-gBol028441-XLOC_019799-16513-1
TAGACTTCGGTAATCACAAAACCAACGGAAAAGCCATAGCTGTTCACACT
CONSENSUS
TAGACTTCGGTAATCACAAAACCAACGGAAAAGCCATAGCTGTTCACACT

RI-gBol028441-XLOC_019799-16513-0
CTCATCTTCTTCGCCATTTACTCTATTTTGATCATCGCCGTCAATCTACA

RI-gBol028441-XLOC_019799-16513-1
CTCATCTTCTTCGCCATTTACTCTATTTTGATCATCGCCGTCAATCTACA
CONSENSUS
CTCATCTTCTTCGCCATTTACTCTATTTTGATCATCGCCGTCAATCTACA

RI-gBol028441-XLOC_019799-16513-0
CATCTACAGCGGTTGATTCTCTTCTGGGTATCTCTCTGTTTTTGTTGTTT
RI-gBol028441-XLOC_019799-16513-1
CATCTACAGCGGTTGATTCTCTTCTGGGTATCTCTCTGTTTTTGTTGTTT
CONSENSUS
CATCTACAGCGGTTGATTCTCTTCTGGGTATCTCTCTGTTTTTGTTGTTT

RI-gBol028441-XLOC_019799-16513-0
GTATGAATTAAAGGTACCTAGAAACTCTATAAATTATGGGTTTTATATAC
RI-gBol028441-XLOC_019799-16513-1
GTATGAATTAAAG-------------------------------------
CONSENSUS
GTATGAATTAAAG.....................................

RI-gBol028441-XLOC_019799-16513-0
CTTAAACCTGTAGTTTCTTTTCTTCTTGTTGATGTTGTAACAGACATATA
RI-gBol028441-XLOC_019799-16513-1

CONSENSUS
....

RI-gBol028441-XLOC_019799-16513-0
TGGTGAAAGTTCTGTTTCGTACTTGTATTACTTCAGTTTGCTTTTTACTT
RI-gBol028441-XLOC_019799-16513-1

CONSENSUS
....

RI-gBol028441-XLOC_019799-16513-0
TAAAGTCTCTTTAGCTCTGTTTATTTGCTCAAATTCAGACAAAAATTGCT
RI-gBol028441-XLOC_019799-16513-1

CONSENSUS
....

RI-gBol028441-XLOC_019799-16513-0
TACTTTCTGTCATCTTTCTTTAAACATGTTGAACGAGAGATTTGTACATT
RI-gBol028441-XLOC_019799-16513-1

CONSENSUS
....

RI-gBol028441-XLOC_019799-16513-0
ACATTGGAGCTTTTGAAGCATATGAGTTTTTATTATGTCAAGCTTTGGGT
RI-gBol028441-XLOC_019799-16513-1

CONSENSUS
....

RI-gBol028441-XLOC_019799-16513-0
TGTGTGTGTTGTGATTGATACATAGCTTGTCTTTGGGAGCAGCATTTAGT

RI-gBol028441-XLOC_019799-16513-1

CONSENSUS
      ....

RI-gBol028441-XLOC_019799-16513-0
      TCTCCGACGTCTCCCATGATCAAGCGTCCAAAGAGAAGAGTAATGTTTTC
RI-gBol028441-XLOC_019799-16513-1

CONSENSUS
      ....

RI-gBol028441-XLOC_019799-16513-0
      TCTTCTTCTAGGATTATCAGGAGAGATCATCAACCTGAGGTTGAGGCAAA
RI-gBol028441-XLOC_019799-16513-1   -----------
GATTATCAGGAGAGATCATCAACCTGAGGTTGAGGCAAA
CONSENSUS
      ...........GATTATCAGGAGAGATCATCAACCTGAGGTTGAGGCAAA

RI-gBol028441-XLOC_019799-16513-0
      CTCATGGGATATCTCATCAACCTGAGGTTGAACAAGCTTCAAACAGCTAA
RI-gBol028441-XLOC_019799-16513-1
      CTCATGGGATATCTCATCAACCTGAGGTTGAACAAGCTTCAAACAGCTAA
CONSENSUS
      CTCATGGGATATCTCATCAACCTGAGGTTGAACAAGCTTCAAACAGCTAA

RI-gBol028441-XLOC_019799-16513-0
      GGTGAAAGCACAGAAGATCATTGAGAAAGCCCCACAG
RI-gBol028441-XLOC_019799-16513-1
      GGTGAAAGCACAGAAGATCATTGAGAAAGCCCCACAG
CONSENSUS
      GGTGAAAGCACAGAAGATCATTGAGAAAGCCCCACAG

alignment for event: A5-gBol006720-XLOC_044257-5526

A5-gBol006720-XLOC_044257-5526-0
      TGCTTTGCCAGAAGATGCTAACCGTGCCATTGAGTTGAAGAACGGTTCCA
A5-gBol006720-XLOC_044257-5526-1
      TGCTTTGCCAGAAGATGCTAACCGTGCCATTGAGTTGAAGAACGGTTCCA
CONSENSUS
      TGCTTTGCCAGAAGATGCTAACCGTGCCATTGAGTTGAAGAACGGTTCCA

A5-gBol006720-XLOC_044257-5526-0
      CTGTTGGTGGCCGTAGGATTACAGTCAAACAGGCCACCCATCGCCCTTCT
A5-gBol006720-XLOC_044257-5526-1
      CTGTTGGTGGCCGTAGGATTACAGTCAAACAGGCCACCCATCGCCCTTCT
CONSENSUS
      CTGTTGGTGGCCGTAGGATTACAGTCAAACAGGCCACCCATCGCCCTTCT

A5-gBol006720-XLOC_044257-5526-0
      CTTAAAGAGCGTCGTTCAAAAGCAGCCCAAG---GGATCTCAGTACCTGA
A5-gBol006720-XLOC_044257-5526-1
      CTTAAAGAGCGTCGTTCAAAAGCAGCCCAAGGTGGGATCTCAGTACCTGA
CONSENSUS

CTTAAAGAGCGTCGTTCAAAAGCAGCCCAAG...GGATCTCAGTACCTGA

A5-gBol006720-XLOC_044257-5526-0
TAATTTCCCTGAGACAGACACGCAAGGTATAAGTCTGCCTAGATTTGGTT
A5-gBol006720-XLOC_044257-5526-1
TAATTTCCCTGAGACAGACACGCAAGGTATAAGTCTGCCTAGATTTGGTT
CONSENSUS
TAATTTCCCTGAGACAGACACGCAAGGTATAAGTCTGCCTAGATTTGGTT

A5-gBol006720-XLOC_044257-5526-0
TCTTAGTCTAAGTAAGTTGCATCAGTTCCTCCACCCGAGGAAAAAATAGA
A5-gBol006720-XLOC_044257-5526-1
TCTTAGTCTAAGTAAGTTGCATCAGTTCCTCCACCCGAGGAAAAAATAGA
CONSENSUS
TCTTAGTCTAAGTAAGTTGCATCAGTTCCTCCACCCGAGGAAAAAATAGA

A5-gBol006720-XLOC_044257-5526-0
GAAGAAGCCAATTGAGCGCAAAAAACCAACTAAGCTTCATGTTGATTTAG
A5-gBol006720-XLOC_044257-5526-1
GAAGAAGCCAATTGAGCGCAAAAAACCAACTAAGCTTCATGTTGATTTAG
CONSENSUS
GAAGAAGCCAATTGAGCGCAAAAAACCAACTAAGCTTCATGTTGATTTAG

A5-gBol006720-XLOC_044257-5526-0
CTGATAAAGAAACATGTTCAGATAAGCAAAGGTATGAGTCTATAATAACG
A5-gBol006720-XLOC_044257-5526-1
CTGATAAAGAAACATGTTCAGATAAGCAAAGGTATGAGTCTATAATAACG
CONSENSUS
CTGATAAAGAAACATGTTCAGATAAGCAAAGGTATGAGTCTATAATAACG

A5-gBol006720-XLOC_044257-5526-0
TGCTGTCCATTTGTAAGGAAGACTCTCTAATTGAAATTAAGCAAGTTGTT
A5-gBol006720-XLOC_044257-5526-1
TGCTGTCCATTTGTAAGGAAGACTCTCTAATTGAAATTAAGCAAGTTGTT
CONSENSUS
TGCTGTCCATTTGTAAGGAAGACTCTCTAATTGAAATTAAGCAAGTTGTT

A5-gBol006720-XLOC_044257-5526-0
CTATACGTATCATTAAAAATATATATCTATGTATTTTTCGTTCTGTGAAG
A5-gBol006720-XLOC_044257-5526-1
CTATACGTATCATTAAAAATATATATCTATGTATTTTTCGTTCTGTGAAG
CONSENSUS
CTATACGTATCATTAAAAATATATATCTATGTATTTTTCGTTCTGTGAAG

A5-gBol006720-XLOC_044257-5526-0
AGTGGCAAGAACTGTAATCTTTGGTGGCCTTGTTAATGCTGACATGGCAG
A5-gBol006720-XLOC_044257-5526-1
AGTGGCAAGAACTGTAATCTTTGGTGGCCTTGTTAATGCTGACATGGCAG
CONSENSUS
AGTGGCAAGAACTGTAATCTTTGGTGGCCTTGTTAATGCTGACATGGCAG

A5-gBol006720-XLOC_044257-5526-0
AGGCAGTCCATAGTCGTGTCAAAGAGATTGGCACTGTGTGCTCTGTCAGA
A5-gBol006720-XLOC_044257-5526-1
AGGCAGTCCATAGTCGTGTCAAAGAGATTGGCACTGTGTGCTCTGTCAGA
CONSENSUS

AGGCAGTCCATAGTCGTGTCAAAGAGATTGGCACTGTGTGCTCTGTCAGA

A5-gBol006720-XLOC_044257-5526-0
TATCCCCTCCCCAAAGAAGAGCTTCAACAAAATG
A5-gBol006720-XLOC_044257-5526-1
TATCCCCTCCCCAAAGAAGAGCTTCAACAAAATG
CONSENSUS
TATCCCCTCCCCAAAGAAGAGCTTCAACAAAATG

alignment for event: A5-gBol035368-XLOC_012007-7685

A5-gBol035368-XLOC_012007-7685-0
GGAGATTGATTTTGTCAACGAGAGAGTAGAGACTCTGGCTGGTAATGGAA
A5-gBol035368-XLOC_012007-7685-1
GGAGATTGATTTTGTCAACGAGAGAGTAGAGACTCTGGCTGGTAATGGAA
CONSENSUS
GGAGATTGATTTTGTCAACGAGAGAGTAGAGACTCTGGCTGGTAATGGAA

A5-gBol035368-XLOC_012007-7685-0
CTAAAGGATCAGATTACCAAGGTGGAAGAAAAGGAACCTCACAG------
A5-gBol035368-XLOC_012007-7685-1
CTAAAGGATCAGATTACCAAGGTGGAAGAAAAGGAACCTCACAGGCAAGA
CONSENSUS
CTAAAGGATCAGATTACCAAGGTGGAAGAAAAGGAACCTCACAG......

A5-gBol035368-XLOC_012007-7685-0
---CTTCT
A5-gBol035368-XLOC_012007-7685-1
AACTTGATTCCGCGTATGTTTTTAATTATTCTGAGAGATCAAAGACTTCT
CONSENSUS
....CTTCT

A5-gBol035368-XLOC_012007-7685-0
GAATTCTCCCTGGGATATATGCTATGAGCCAGTGAAAGAGAAGGTCTACG
A5-gBol035368-XLOC_012007-7685-1
GAATTCTCCCTGGGATATATGCTATGAGCCAGTGAAAGAGAAGGTCTACG
CONSENSUS
GAATTCTCCCTGGGATATATGCTATGAGCCAGTGAAAGAGAAGGTCTACG

A5-gBol035368-XLOC_012007-7685-0
TTGCAATGGCAGGTCAGCACCAGATTTGGGAATACAATGTGCTTGATGGT
A5-gBol035368-XLOC_012007-7685-1
TTGCAATGGCAGGTCAGCACCAGATTTGGGAATACAATGTGCTTGATGGT
CONSENSUS
TTGCAATGGCAGGTCAGCACCAGATTTGGGAATACAATGTGCTTGATGGT

A5-gBol035368-XLOC_012007-7685-0
GTTACTAAAGTTTTCAGTGGAAATGGCTATGAAAGAAACCTTAACGGTTC
A5-gBol035368-XLOC_012007-7685-1
GTTACTAAAGTTTTCAGTGGAAATGGCTATGAAAGAAACCTTAACGGTTC
CONSENSUS
GTTACTAAAGTTTTCAGTGGAAATGGCTATGAAAGAAACCTTAACGGTTC

A5-gBol035368-XLOC_012007-7685-0 TAC

A5-gBol035368-XLOC_012007-7685-1 TAC
CONSENSUS TAC

alignment for event: SE-gBol031847-XLOC_015867-6859

SE-gBol031847-XLOC_015867-6859-0
CAATCTGTTCCGTGTGTTTGGTGTTTCCCCGGGAAGTACTGAGACTGCAA
SE-gBol031847-XLOC_015867-6859-1
CAATCTGTTCCGTGTGTTTGGTGTTTCCCCGGGAAGTACTGAGACTGCAA
CONSENSUS
CAATCTGTTCCGTGTGTTTGGTGTTTCCCCGGGAAGTACTGAGACTGCAA

SE-gBol031847-XLOC_015867-6859-0
CCTTAGAAGCAAGCAGAAATCCAATGAG----------------------
SE-gBol031847-XLOC_015867-6859-1
CCTTAGAAGCAAGCAGAAATCCAATGAGGAGACAGGTTCCAGTTCCCTCG
CONSENSUS
CCTTAGAAGCAAGCAGAAATCCAATGAG......................

SE-gBol031847-XLOC_015867-6859-0 -----------------------------
TTTGAACCTCTCGGGGAAAGA
SE-gBol031847-XLOC_015867-6859-1
AGTCCATATCTAAAGAGTAGGACACGAAATTTGAACCTCTCGGGGAAAGA
CONSENSUS
.............................TTTGAACCTCTCGGGGAAAGA

SE-gBol031847-XLOC_015867-6859-0
AAACGAAATTGGCAAACATGAAACCAGACAAGAGAATTATGACGGTACTT
SE-gBol031847-XLOC_015867-6859-1
AAACGAAATTGGCAAACATGAAACCAGACAAGAGAATTATGACGGTACTT
CONSENSUS
AAACGAAATTGGCAAACATGAAACCAGACAAGAGAATTATGACGGTACTT

SE-gBol031847-XLOC_015867-6859-0
GAGACTTTGTCATTTTAGGCCTTGCAAGGATGCTTAAAATGCATTTTACA
SE-gBol031847-XLOC_015867-6859-1
GAGACTTTGTCATTTTAGGCCTTGCAAGGATGCTTAAAATGCATTTTACA
CONSENSUS
GAGACTTTGTCATTTTAGGCCTTGCAAGGATGCTTAAAATGCATTTTACA

SE-gBol031847-XLOC_015867-6859-0
TGTTATACCATATCTTTATGAACACTCACTAATTTTTTTCTCAAAACTTT
SE-gBol031847-XLOC_015867-6859-1
TGTTATACCATATCTTTATGAACACTCACTAATTTTTTTCTCAAAACTTT
CONSENSUS
TGTTATACCATATCTTTATGAACACTCACTAATTTTTTTCTCAAAACTTT

SE-gBol031847-XLOC_015867-6859-0
GTGTTTTAATGATTTTCTCTGACTCATATTGGACAGTAAAAAGTCTGCAA
SE-gBol031847-XLOC_015867-6859-1
GTGTTTTAATGATTTTCTCTGACTCATATTGGACAGTAAAAAGTCTGCAA
CONSENSUS
GTGTTTTAATGATTTTCTCTGACTCATATTGGACAGTAAAAAGTCTGCAA

```
SE-gBol031847-XLOC_015867-6859-0
      AGTTTTATTTTTCAATCAACTGAATGACTCAGGTCATGTACAACCTTTAT
SE-gBol031847-XLOC_015867-6859-1
      AGTTTTATTTTTCAATCAACTGAATGACTCAGGTCATGTACAACCTTTAT
CONSENSUS
      AGTTTTATTTTTCAATCAACTGAATGACTCAGGTCATGTACAACCTTTAT

SE-gBol031847-XLOC_015867-6859-0 TACTGTATGACTGTATCAAAC
SE-gBol031847-XLOC_015867-6859-1 TACTGTATGACTGTATCAAAC
CONSENSUS                        TACTGTATGACTGTATCAAAC


alignment for event: A3-gBol031847-XLOC_015867-6857

A3-gBol031847-XLOC_015867-6857-0
      CAATCTGTTCCGTGTGTTTGGTGTTTCCCCGGGAAGTACTGAGACTGCAA
A3-gBol031847-XLOC_015867-6857-1
      CAATCTGTTCCGTGTGTTTGGTGTTTCCCCGGGAAGTACTGAGACTGCAA
CONSENSUS
      CAATCTGTTCCGTGTGTTTGGTGTTTCCCCGGGAAGTACTGAGACTGCAA

A3-gBol031847-XLOC_015867-6857-0
      CCTTAGAAGCAAGCAGAAATCCAATGAG----------------------
A3-gBol031847-XLOC_015867-6857-1
      CCTTAGAAGCAAGCAGAAATCCAATGAGGAGACAGGTTCCAGTTCCCTCG
CONSENSUS
      CCTTAGAAGCAAGCAGAAATCCAATGAG......................

A3-gBol031847-XLOC_015867-6857-0
      --------------------------------------------------
A3-gBol031847-XLOC_015867-6857-1
      AGTCCATATCTAAAGAGTAGGACACGAAAGTAATGAGATTGTCGATTGTA
CONSENSUS
      ..................................................

A3-gBol031847-XLOC_015867-6857-0
      --------------------------------------------------
A3-gBol031847-XLOC_015867-6857-1
      TATATATTAATACAACTCAAGATGTCTACACCGCGCACATCTTCGTATTT
CONSENSUS
      ..................................................

A3-gBol031847-XLOC_015867-6857-0
      --------------------------------------------------
A3-gBol031847-XLOC_015867-6857-1
      TTTTAAGATTTGATTGATCAAAAAAGAAACTGGCTTTGTTTGTCTGCTAA
CONSENSUS
      ..................................................

A3-gBol031847-XLOC_015867-6857-0
      --------------------------------------------------
A3-gBol031847-XLOC_015867-6857-1
      AACTTTTGAACTTGATGTTCTGTGTTCACTTACATAAGAATTCTTTCATT
CONSENSUS
      ..................................................
```

A3-gBol031847-XLOC_015867-6857-0 -------------
TTTGAACCTCTCGGGGAAAGAAAACGAAATTGGCAAA
A3-gBol031847-XLOC_015867-6857-1
TTGCAATACGCAGTTTGAACCTCTCGGGGAAAGAAAACGAAATTGGCAAA
CONSENSUS
.............TTTGAACCTCTCGGGGAAAGAAAACGAAATTGGCAAA

A3-gBol031847-XLOC_015867-6857-0
CATGAAACCAGACAAGAGAATTATGACGGTACTTGAGACTTTGTCATTTT
A3-gBol031847-XLOC_015867-6857-1
CATGAAACCAGACAAGAGAATTATGACGGTACTTGAGACTTTGTCATTTT
CONSENSUS
CATGAAACCAGACAAGAGAATTATGACGGTACTTGAGACTTTGTCATTTT

A3-gBol031847-XLOC_015867-6857-0
AGGCCTTGCAAGGATGCTTAAAATGCATTTTACATGTTATACCATATCTT
A3-gBol031847-XLOC_015867-6857-1
AGGCCTTGCAAGGATGCTTAAAATGCATTTTACATGTTATACCATATCTT
CONSENSUS
AGGCCTTGCAAGGATGCTTAAAATGCATTTTACATGTTATACCATATCTT

A3-gBol031847-XLOC_015867-6857-0
TATGAACACTCACTAATTTTTTTCTCAAAACTTTGTGTTTTAATGATTTT
A3-gBol031847-XLOC_015867-6857-1
TATGAACACTCACTAATTTTTTTCTCAAAACTTTGTGTTTTAATGATTTT
CONSENSUS
TATGAACACTCACTAATTTTTTTCTCAAAACTTTGTGTTTTAATGATTTT

A3-gBol031847-XLOC_015867-6857-0
CTCTGACTCATATTGGACAGTAAAAAGTCTGCAAAGTTTTATTTTTCAAT
A3-gBol031847-XLOC_015867-6857-1
CTCTGACTCATATTGGACAGTAAAAAGTCTGCAAAGTTTTATTTTTCAAT
CONSENSUS
CTCTGACTCATATTGGACAGTAAAAAGTCTGCAAAGTTTTATTTTTCAAT

A3-gBol031847-XLOC_015867-6857-0
CAACTGAATGACTCAGGTCATGTACAACCTTTATTACTGTATGACTGTAT
A3-gBol031847-XLOC_015867-6857-1
CAACTGAATGACTCAGGTCATGTACAACCTTTATTACTGTATGACTGTAT
CONSENSUS
CAACTGAATGACTCAGGTCATGTACAACCTTTATTACTGTATGACTGTAT

A3-gBol031847-XLOC_015867-6857-0 CAAAC
A3-gBol031847-XLOC_015867-6857-1 CAAAC
CONSENSUS CAAAC

alignment for event: RI-X-XLOC_036012-11777

RI-X-XLOC_036012-11777-0
GAGGAGGAGGTGATTTCACCACCTGATGAGAATCTCTTGATCTACTGTAA
RI-X-XLOC_036012-11777-1
GAGGAGGAGGTGATTTCACCACCTGATGAGAATCTCTTGATCTACTGTAA
CONSENSUS

GAGGAGGAGGTGATTTCACCACCTGATGAGAATCTCTTGATCTACTGTAA

RI-X-XLOC_036012-11777-0
ACCTGTTCGTCTCTACAACATTCTTCGCATTCGCTCTCTATTCAACGTAA
RI-X-XLOC_036012-11777-1
ACCTGTTCGTCTCTACAACATTCTTCGCATTCGCTCTCTATTCAACGTAA
CONSENSUS
ACCTGTTCGTCTCTACAACATTCTTCGCATTCGCTCTCTATTCAACGTAA

RI-X-XLOC_036012-11777-0
ATCAATTGCGTCTCTCTCTCTCTGAATCATTCTTTTAGCCCAGCTGATAC
RI-X-XLOC_036012-11777-1
ATCAATTGCGTCTCTCTCTCTCTGAATCATTCTTTTAGCCCAGCTGATAC
CONSENSUS
ATCAATTGCGTCTCTCTCTCTCTGAATCATTCTTTTAGCCCAGCTGATAC

RI-X-XLOC_036012-11777-0
TGCTCGTCTCTCACTCATGGTCTTTGTTTTTTTCATGCAGCCATCGTTTC
RI-X-XLOC_036012-11777-1
TGCTCGTCTCTCACTCATGGTCTTTGTTTTTTTCATGCAGCCATCGTTTC
CONSENSUS
TGCTCGTCTCTCACTCATGGTCTTTGTTTTTTTCATGCAGCCATCGTTTC

RI-X-XLOC_036012-11777-0
TTCCTAGATGCTTTCTTCTTTTTATCCTTTTCTCGGGTCTAATTATGAAA
RI-X-XLOC_036012-11777-1
TTCCTAGATGCTTTCTTCTTTTTATCCTTTTCTCGGGTCTAATTATGAAA
CONSENSUS
TTCCTAGATGCTTTCTTCTTTTTATCCTTTTCTCGGGTCTAATTATGAAA

RI-X-XLOC_036012-11777-0
ACTCATGAATGGTTTCTTCTCTGTTGTGTGTGTTTTTTGGTTTAAGTCCG
RI-X-XLOC_036012-11777-1
ACTCATGAATGGTTTCTTCTCTGTTGTGTGTGTTTTTTGGTTTAAGTCCG
CONSENSUS
ACTCATGAATGGTTTCTTCTCTGTTGTGTGTGTTTTTTGGTTTAAGTCCG

RI-X-XLOC_036012-11777-0
GATCTGCTGGGATTGTAGTTTTTAATTATAAGGATTGTAATAACACGTTG
RI-X-XLOC_036012-11777-1
GATCTGCTGGGATTGTAGTTTTTAATTATAAGGATTGTAATAACACGTTG
CONSENSUS
GATCTGCTGGGATTGTAGTTTTTAATTATAAGGATTGTAATAACACGTTG

RI-X-XLOC_036012-11777-0
CAGAAAACTGAAGCTGTGTTTTATTATTTTCTCTTCTTCCGGCAATTTTG
RI-X-XLOC_036012-11777-1
CAGAAAACTGAAGCTGTGTTTTATTATTTTCTCTTCTTCCGGCAATTTTG
CONSENSUS
CAGAAAACTGAAGCTGTGTTTTATTATTTTCTCTTCTTCCGGCAATTTTG

RI-X-XLOC_036012-11777-0
TTTCTGATGTTTTTGTGGATTTAGGTGAAGTGGTGACGGATGGTGAGAAG
RI-X-XLOC_036012-11777-1
TTTCTGATGTTTTTGTGGATTTAGGTGAAGTGGTGACGGATGGTGAGAAG
CONSENSUS

TTTCTGATGTTTTTGTGGATTTAGGTGAAGTGGTGACGGATGGTGAGAAG

RI-X-XLOC_036012-11777-0
TCGAAGACGGGGAAGAAGGGAGCTAAGGCTCCATGGGCGAAGCCACTTTC
RI-X-XLOC_036012-11777-1
TCGAAGACGGGGAAGAAGGGAGCTAAGGCTCCATGGGCGAAGCCACTTTC
CONSENSUS
TCGAAGACGGGGAAGAAGGGAGCTAAGGCTCCATGGGCGAAGCCACTTTC

RI-X-XLOC_036012-11777-0
TCAGTATTCTCAGGTGGGTCAAGTTTCATCAGTGATTCTTTTTTTTATTG
RI-X-XLOC_036012-11777-1
TCAGTATTCTCAG-------------------------------------
CONSENSUS
TCAGTATTCTCAG.....................................

RI-X-XLOC_036012-11777-0
GTTTGATCTAATAGTCTGTGAATTGCGCCTTTAGGGTTTAGCAGTGTGCA
RI-X-XLOC_036012-11777-1

CONSENSUS
....

RI-X-XLOC_036012-11777-0
GTTCGTGTAACATTTTTAGTAAAGGTTTGGTCTTTCAGTTCATGGGGAAC
RI-X-XLOC_036012-11777-1

CONSENSUS
....

RI-X-XLOC_036012-11777-0
GAGTTTAGTTGTCTGCATGTCCGGTTCTTTTATACTACACTCATAAGTAT
RI-X-XLOC_036012-11777-1

CONSENSUS
....

RI-X-XLOC_036012-11777-0
GTTGTTATGTATGTGTCTTTGTGACTTTCTTTGGTCTGCAAGTTACGCAT
RI-X-XLOC_036012-11777-1

CONSENSUS
....

RI-X-XLOC_036012-11777-0
TGAGGGTTTAGCAGTGTGCTGTTGAAGGTTTGGTCTTTCAGTTCATGTCC
RI-X-XLOC_036012-11777-1

CONSENSUS
....

RI-X-XLOC_036012-11777-0
AACGAGTTTAGTTGGCCTCATGTTTGGTTCTTTTATATTTTTCTGACAGA
RI-X-XLOC_036012-11777-1

CONSENSUS

```
      ..............................................

RI-X-XLOC_036012-11777-0
      CCACACGATGTGATCTAATGAACAATATTTGGGTCTGTTGCAGGCATACT
RI-X-XLOC_036012-11777-1
      -------------------------------------------GCATACT
CONSENSUS
      ...........................................GCATACT

RI-X-XLOC_036012-11777-0
      TTGATGCGCCCGGGGTTTAGTAAATTTAAGTTTTTTATATTGGCCAATTT
RI-X-XLOC_036012-11777-1
      TTGATGCGCCCGGGGTTTAGTAAATTTAAGTTTTTTATATTGGCCAATTT
CONSENSUS
      TTGATGCGCCCGGGGTTTAGTAAATTTAAGTTTTTTATATTGGCCAATTT

RI-X-XLOC_036012-11777-0
      GTAACCACTGTTATTGTTTTCCATATTCATATTCATGAATGGAGTTTTAG
RI-X-XLOC_036012-11777-1
      GTAACCACTGTTATTGTTTTCCATATTCATATTCATGAATGGAGTTTTAG
CONSENSUS
      GTAACCACTGTTATTGTTTTCCATATTCATATTCATGAATGGAGTTTTAG

RI-X-XLOC_036012-11777-0
      TGAAGGTGATTTCCTTGAAATAAGCAGATGATGCTTAATCACCAAAAGCT
RI-X-XLOC_036012-11777-1
      TGAAGGTGATTTCCTTGAAATAAGCAGATGATGCTTAATCACCAAAAGCT
CONSENSUS
      TGAAGGTGATTTCCTTGAAATAAGCAGATGATGCTTAATCACCAAAAGCT

RI-X-XLOC_036012-11777-0
      TACACAACCGAGGATTACCTCAAGATCACTGAAGAGCAGCTCAAAGCTTC
RI-X-XLOC_036012-11777-1
      TACACAACCGAGGATTACCTCAAGATCACTGAAGAGCAGCTCAAAGCTTC
CONSENSUS
      TACACAACCGAGGATTACCTCAAGATCACTGAAGAGCAGCTCAAAGCTTC

RI-X-XLOC_036012-11777-0
      GTCTCCTGGTAAAAACCAAACAGATGATCAAATTCAGACTCAAGAACCTG
RI-X-XLOC_036012-11777-1
      GTCTCCTGGTAAAAACCAAACAGATGATCAAATTCAGACTCAAGAACCTG
CONSENSUS
      GTCTCCTGGTAAAAACCAAACAGATGATCAAATTCAGACTCAAGAACCTG

RI-X-XLOC_036012-11777-0   CTGTACAAAGCCAGCCTGAAG
RI-X-XLOC_036012-11777-1   CTGTACAAAGCCAGCCTGAAG
CONSENSUS                  CTGTACAAAGCCAGCCTGAAG



alignment for event: RI-gBol007208-XLOC_043748-10287

RI-gBol007208-XLOC_043748-10287-0
      ATTCGAAATTCGAGGACTTTTAATTCAAGCCGAGCGAGATTGATTTGAAT
RI-gBol007208-XLOC_043748-10287-1
      ATTCGAAATTCGAGGACTTTTAATTCAAGCCGAGCGAGATTGATTTGAAT
```

CONSENSUS
ATTCGAAATTCGAGGACTTTTAATTCAAGCCGAGCGAGATTGATTTGAAT

RI-gBol007208-XLOC_043748-10287-0
CGTAGCTTTTTTTGAAGACATGTTGGAGAATCGATCACCAGATTCGTGTT
RI-gBol007208-XLOC_043748-10287-1
CGTAGCTTTTTTTGAAGACATGTTGGAGAATCGATCACCAGATTCGTGTT
CONSENSUS
CGTAGCTTTTTTTGAAGACATGTTGGAGAATCGATCACCAGATTCGTGTT

RI-gBol007208-XLOC_043748-10287-0
TGAGTTCAAGGCTTTTCTCCGAATCAACCTGGTCCAAGTCTTTCATGTTT
RI-gBol007208-XLOC_043748-10287-1
TGAGTTCAAGGCTTTTCTCCGAATCAACCTGGTCCAAGTCTTTCATGTTT
CONSENSUS
TGAGTTCAAGGCTTTTCTCCGAATCAACCTGGTCCAAGTCTTTCATGTTT

RI-gBol007208-XLOC_043748-10287-0
CCACAGGATGACGACGACAATAAGCTCAGCAACGGCAAGAAGAGAGCTTT
RI-gBol007208-XLOC_043748-10287-1
CCACAGGATGACGACGACAATAAGCTCAGCAACGGCAAGAAGAGAGCTTT
CONSENSUS
CCACAGGATGACGACGACAATAAGCTCAGCAACGGCAAGAAGAGAGCTTT

RI-gBol007208-XLOC_043748-10287-0
GGAGGTTGTTGGTGAGATCAGAGGCACCAAGTCGCTTAAACTAATGGGTT
RI-gBol007208-XLOC_043748-10287-1
GGAGGTTGTTGGTGAGATCAGAGGCACCAAGTCGCTTAAACTAATGGGTT
CONSENSUS
GGAGGTTGTTGGTGAGATCAGAGGCACCAAGTCGCTTAAACTAATGGGTT

RI-gBol007208-XLOC_043748-10287-0
TCTCTATTACATACGATAGCGATTCTTCTGACTATTCTGAAGATGGGTCT
RI-gBol007208-XLOC_043748-10287-1
TCTCTATTACATACGATAGCGATTCTTCTGACTATTCTGAAGATGGGTCT
CONSENSUS
TCTCTATTACATACGATAGCGATTCTTCTGACTATTCTGAAGATGGGTCT

RI-gBol007208-XLOC_043748-10287-0
ATTCAGGAGCAAGAGCAAGGTGATTCCAACAACAACGGTGGTGATTCGTC
RI-gBol007208-XLOC_043748-10287-1
ATTCAGGAGCAAGAGCAAGGTGATTCCAACAACAACGGTGGTGATTCGTC
CONSENSUS
ATTCAGGAGCAAGAGCAAGGTGATTCCAACAACAACGGTGGTGATTCGTC

RI-gBol007208-XLOC_043748-10287-0
TGATTCACATTCTCTTATCAACGAGATTGGTCGGGACAACTCCATCGACT
RI-gBol007208-XLOC_043748-10287-1
TGATTCACATTCTCTTATCAACGAGATTGGTCGGGACAACTCCATCGACT
CONSENSUS
TGATTCACATTCTCTTATCAACGAGATTGGTCGGGACAACTCCATCGACT

RI-gBol007208-XLOC_043748-10287-0
GTCTGATCCGCTGCTCGCGGTCTGATTACGGCTCCATCGCTTCCTTGAGT
RI-gBol007208-XLOC_043748-10287-1
GTCTGATCCGCTGCTCGCGGTCTGATTACGGCTCCATCGCTTCCTTGAGT

CONSENSUS
GTCTGATCCGCTGCTCGCGGTCTGATTACGGCTCCATCGCTTCCTTGAGT

RI-gBol007208-XLOC_043748-10287-0
AGAAACTTCCGTTCTTTGGTGAAGAGTGGAGATATCTATAAGCTAAGACG
RI-gBol007208-XLOC_043748-10287-1
AGAAACTTCCGTTCTTTGGTGAAGAGTGGAGATATCTATAAGCTAAGACG
CONSENSUS
AGAAACTTCCGTTCTTTGGTGAAGAGTGGAGATATCTATAAGCTAAGACG

RI-gBol007208-XLOC_043748-10287-0
GCAAAACGGGTTCGTGGAGCATTGGGTTTACTTCTCTTGCCAGCTCTTGG
RI-gBol007208-XLOC_043748-10287-1
GCAAAACGGGTTCGTGGAGCATTGGGTTTACTTCTCTTGCCAGCTCTTGG
CONSENSUS
GCAAAACGGGTTCGTGGAGCATTGGGTTTACTTCTCTTGCCAGCTCTTGG

RI-gBol007208-XLOC_043748-10287-0
AGTGGGTTGCCTTCGACCCTGTAGAGAGAAGGTGGATGCAGCTGCCAACG
RI-gBol007208-XLOC_043748-10287-1
AGTGGGTTGCCTTCGACCCTGTAGAGAGAAGGTGGATGCAGCTGCCAACG
CONSENSUS
AGTGGGTTGCCTTCGACCCTGTAGAGAGAAGGTGGATGCAGCTGCCAACG

RI-gBol007208-XLOC_043748-10287-0
ATGCCTTCTAGTGTCACCTTCATGTGTGCAGACAAGGAGTCTTTAGCCGT
RI-gBol007208-XLOC_043748-10287-1
ATGCCTTCTAGTGTCACCTTCATGTGTGCAGACAAGGAGTCTTTAGCCGT
CONSENSUS
ATGCCTTCTAGTGTCACCTTCATGTGTGCAGACAAGGAGTCTTTAGCCGT

RI-gBol007208-XLOC_043748-10287-0
CGGCACGGATCTTCTCGTCTTAGGAAAAGATGATTTCTCTTCTCATGTAA
RI-gBol007208-XLOC_043748-10287-1
CGGCACGGATCTTCTCGTCTTAGGAAAAGATGATTTCTCTTCTCATGTAA
CONSENSUS
CGGCACGGATCTTCTCGTCTTAGGAAAAGATGATTTCTCTTCTCATGTAA

RI-gBol007208-XLOC_043748-10287-0
TATACAGATACAGCCTTCTAACTAATTCTTGGTCCTCTGGTATGAAGATG
RI-gBol007208-XLOC_043748-10287-1
TATACAGATACAGCCTTCTAACTAATTCTTGGTCCTCTGGTATGAAGATG
CONSENSUS
TATACAGATACAGCCTTCTAACTAATTCTTGGTCCTCTGGTATGAAGATG

RI-gBol007208-XLOC_043748-10287-0
AACTCTCCGAGGTGTTTGTTCGGTTCCGCTAGTCTCGGAGAGATTGCTAT
RI-gBol007208-XLOC_043748-10287-1
AACTCTCCGAGGTGTTTGTTCGGTTCCGCTAGTCTCGGAGAGATTGCTAT
CONSENSUS
AACTCTCCGAGGTGTTTGTTCGGTTCCGCTAGTCTCGGAGAGATTGCTAT

RI-gBol007208-XLOC_043748-10287-0
ATTCGCTGGCGGTTGTGATTCTCAGGGGAAGATTCTTGACTTCGCTGAGA
RI-gBol007208-XLOC_043748-10287-1
ATTCGCTGGCGGTTGTGATTCTCAGGGGAAGATTCTTGACTTCGCTGAGA

CONSENSUS
ATTCGCTGGCGGTTGTGATTCTCAGGGGAAGATTCTTGACTTCGCTGAGA

RI-gBol007208-XLOC_043748-10287-0
TGTACAACTCCGAGCTTCAGACTTGGGTGACTCTTCCTCGGATGAACAAT
RI-gBol007208-XLOC_043748-10287-1
TGTACAACTCCGAGCTTCAGACTTGGGTGACTCTTCCTCGGATGAACAAT
CONSENSUS
TGTACAACTCCGAGCTTCAGACTTGGGTGACTCTTCCTCGGATGAACAAT

RI-gBol007208-XLOC_043748-10287-0
CCGAGGAAGATGTGTTCAGGGGTCTTCATGGATGGGAGGTTCTATGTCAT
RI-gBol007208-XLOC_043748-10287-1
CCGAGGAAGATGTGTTCAGGGGTCTTCATGGATGGGAGGTTCTATGTCAT
CONSENSUS
CCGAGGAAGATGTGTTCAGGGGTCTTCATGGATGGGAGGTTCTATGTCAT

RI-gBol007208-XLOC_043748-10287-0
TGGTGGAATAGGCGGTGCTGATGCCAAAGCCTTGACGTGCGGCGAAGAGT
RI-gBol007208-XLOC_043748-10287-1
TGGTGGAATAGGCGGTGCTGATGCCAAAGCCTTGACGTGCGGCGAAGAGT
CONSENSUS
TGGTGGAATAGGCGGTGCTGATGCCAAAGCCTTGACGTGCGGCGAAGAGT

RI-gBol007208-XLOC_043748-10287-0
ATGACCTTGAGGCCAAGAAATGGACTGAAGTCCCTGACTTGTCGCCTCCA
RI-gBol007208-XLOC_043748-10287-1
ATGACCTTGAGGCCAAGAAATGGACTGAAGTCCCTGACTTGTCGCCTCCA
CONSENSUS
ATGACCTTGAGGCCAAGAAATGGACTGAAGTCCCTGACTTGTCGCCTCCA

RI-gBol007208-XLOC_043748-10287-0
AGAAGCCGTGCTGATCAGGCTGATGCGCGGCCAGCGGCTGAAGCACCGCC
RI-gBol007208-XLOC_043748-10287-1
AGAAGCCGTGCTGATCAGGCTGATGCGCGGCCAGCGGCTGAAGCACCGCC
CONSENSUS
AGAAGCCGTGCTGATCAGGCTGATGCGCGGCCAGCGGCTGAAGCACCGCC

RI-gBol007208-XLOC_043748-10287-0
TCTTGTTGCGGTTGTGGATAACCAATTGTACGCTGCTGATCACGCGGACA
RI-gBol007208-XLOC_043748-10287-1
TCTTGTTGCGGTTGTGGATAACCAATTGTACGCTGCTGATCACGCGGACA
CONSENSUS
TCTTGTTGCGGTTGTGGATAACCAATTGTACGCTGCTGATCACGCGGACA

RI-gBol007208-XLOC_043748-10287-0
TGGAGGTGAGGAAGTATGATAAGGAGAAGAAGAAATGGTTGACTATTGGG
RI-gBol007208-XLOC_043748-10287-1
TGGAGGTGAGGAAGTATGATAAGGAGAAGAAGAAATGGTTGACTATTGGG
CONSENSUS
TGGAGGTGAGGAAGTATGATAAGGAGAAGAAGAAATGGTTGACTATTGGG

RI-gBol007208-XLOC_043748-10287-0
AGGTTGCCTGAGAGAGCAGGCTCGGTTAACGGATGGGGACTTGCTTTTAG
RI-gBol007208-XLOC_043748-10287-1
AGGTTGCCTGAGAGAGCAGGCTCGGTTAACGGATGGGGACTTGCTTTTAG

CONSENSUS
AGGTTGCCTGAGAGAGCAGGCTCGGTTAACGGATGGGGACTTGCTTTTAG

RI-gBol007208-XLOC_043748-10287-0
AGCTTGTGGGGAGAGGTTGATAGTTATAGGTGGACCAAAGTACTCGGGAG
RI-gBol007208-XLOC_043748-10287-1
AGCTTGTGGGGAGAGGTTGATAGTTATAGGTGGACCAAAGTACTCGGGAG
CONSENSUS
AGCTTGTGGGGAGAGGTTGATAGTTATAGGTGGACCAAAGTACTCGGGAG

RI-gBol007208-XLOC_043748-10287-0
GTGGGTTTATAGAGCTGAATTCATGGATACCGAGGGACGGTGATCCACCG
RI-gBol007208-XLOC_043748-10287-1
GTGGGTTTATAGAGCTGAATTCATGGATACCGAGGGACGGTGATCCACCG
CONSENSUS
GTGGGTTTATAGAGCTGAATTCATGGATACCGAGGGACGGTGATCCACCG

RI-gBol007208-XLOC_043748-10287-0
CAGTGGACGTTGCTTGATAGGAAACATTCTCCTAACTTCGTGTACAATTG
RI-gBol007208-XLOC_043748-10287-1
CAGTGGACGTTGCTTGATAGGAAACATTCTCCTAACTTCGTGTACAATTG
CONSENSUS
CAGTGGACGTTGCTTGATAGGAAACATTCTCCTAACTTCGTGTACAATTG

RI-gBol007208-XLOC_043748-10287-0
TGCAGTGATGGGTTGCTGAAACAGCATTTGAAGACCCTCCCAGATTCTTG
RI-gBol007208-XLOC_043748-10287-1
TGCAGTGATGGGTTGCTGAAACAGCATTTGAAGACCCTCCCAGATTCTTG
CONSENSUS
TGCAGTGATGGGTTGCTGAAACAGCATTTGAAGACCCTCCCAGATTCTTG

RI-gBol007208-XLOC_043748-10287-0
TTGATGGGTCTAAAAGACTCCATTGAAGGTTCCAAGCTCTCTCTCCAACC
RI-gBol007208-XLOC_043748-10287-1
TTGATGGGTCTAAAAGACTCCATTGAAG----------------------
CONSENSUS
TTGATGGGTCTAAAAGACTCCATTGAAG......................

RI-gBol007208-XLOC_043748-10287-0
CCTTTATGCTTCTTCTTCCGCTTCCGTATTTGTTTTTCCTTACAAAAAAA
RI-gBol007208-XLOC_043748-10287-1

CONSENSUS
....

RI-gBol007208-XLOC_043748-10287-0
TTTCAATTTGAATACAATCATCTCTGTATTGGTAGAGGACCAGAAAAAGG
RI-gBol007208-XLOC_043748-10287-1

CONSENSUS
....

RI-gBol007208-XLOC_043748-10287-0
AAACAGGTTATAATAAGTTGGTTAAAAGTTAAAAAGCTTTTTAGAGTTTG
RI-gBol007208-XLOC_043748-10287-1

CONSENSUS
....

RI-gBol007208-XLOC_043748-10287-0
CCAGTATCATCTGGTTAACATTGTTGTATGTGTTTTTAATCCATTTGTT
RI-gBol007208-XLOC_043748-10287-1

CONSENSUS
....

RI-gBol007208-XLOC_043748-10287-0
TCCTAATAAGTTGTTATGTTTGTAGAGCCCATCTTTTGATCTTTGTTCAC
RI-gBol007208-XLOC_043748-10287-1

CONSENSUS
....

RI-gBol007208-XLOC_043748-10287-0
AGTTTTGCTTTTGTGTTCTTTCTTTTTATTTTTACAAGTCGAATAATCGT
RI-gBol007208-XLOC_043748-10287-1

CONSENSUS
....

RI-gBol007208-XLOC_043748-10287-0
TAGATTCACTTTACAGCGGTGGTTTGGAGTGTGTGTCATTTTTATGAAGA
RI-gBol007208-XLOC_043748-10287-1 ----------------
CGGTGGTTTGGAGTGTGTGTCATTTTTATGAAGA
CONSENSUS
................CGGTGGTTTGGAGTGTGTGTCATTTTTATGAAGA

RI-gBol007208-XLOC_043748-10287-0
CAGAAAAGAAGCGTCACTTTTGTTGGGGGGTAAGGAGGGGCAAATGTGAT
RI-gBol007208-XLOC_043748-10287-1
CAGAAAAGAAGCGTCACTTTTGTTGGGGGGTAAGGAGGGGCAAATGTGAT
CONSENSUS
CAGAAAAGAAGCGTCACTTTTGTTGGGGGGTAAGGAGGGGCAAATGTGAT

RI-gBol007208-XLOC_043748-10287-0
GGTCTTTAAGGATGTGTCCTTTAAGATATCTCAATCTGTTGCTTGCAAGT
RI-gBol007208-XLOC_043748-10287-1
GGTCTTTAAGGATGTGTCCTTTAAGATATCTCAATCTGTTGCTTGCAAGT
CONSENSUS
GGTCTTTAAGGATGTGTCCTTTAAGATATCTCAATCTGTTGCTTGCAAGT

RI-gBol007208-XLOC_043748-10287-0
TACTGTTCTGTTAAGATTGTTTTTGGACTTTTTGCTTTTAGTTCTTTGGC
RI-gBol007208-XLOC_043748-10287-1
TACTGTTCTGTTAAGATTGTTTTTGGACTTTTTGCTTTTAGTTCTTTGGC
CONSENSUS
TACTGTTCTGTTAAGATTGTTTTTGGACTTTTTGCTTTTAGTTCTTTGGC

RI-gBol007208-XLOC_043748-10287-0
TTTGCGTATTTTCGTGTAGAAAACAAATTCC
RI-gBol007208-XLOC_043748-10287-1
TTTGCGTATTTTCGTGTAGAAAACAAATTCC

CONSENSUS
      TTTGCGTATTTTCGTGTAGAAAACAAATTCC

alignment for event: SE-X-XLOC_020686-14025

SE-X-XLOC_020686-14025-0
      AAGGACATGGGAGATAAGGATTTATAGAAAAGCCCAGGGAGAGTGATGGT
SE-X-XLOC_020686-14025-1
      AAGGACATGGGAGATAAGGATTTATAGAAAAGCCCAGGGAGAGTGATGGT
CONSENSUS
      AAGGACATGGGAGATAAGGATTTATAGAAAAGCCCAGGGAGAGTGATGGT

SE-X-XLOC_020686-14025-0
      GTCGGATAGAGATGATCATCAATTGTAAAATGACATAGCAAAGGGAAGGC
SE-X-XLOC_020686-14025-1
      GTCGGATAGAGATGATCATCAATTGTAAAATGACATAGCAAAGGGAAGGC
CONSENSUS
      GTCGGATAGAGATGATCATCAATTGTAAAATGACATAGCAAAGGGAAGGC

SE-X-XLOC_020686-14025-0
      ACAATGATCATTTAGTGGAAAGTATCAAGTACCAGTAGTGAAGATGAAAA
SE-X-XLOC_020686-14025-1
      ACAATGATCATTTAGTGGAAAGTATCAAGTACCAGTAGTGAAGATGAAAA
CONSENSUS
      ACAATGATCATTTAGTGGAAAGTATCAAGTACCAGTAGTGAAGATGAAAA

SE-X-XLOC_020686-14025-0
      AGAAGACAACAACCAAGACATGACAACAGACTTACTACACG---------
SE-X-XLOC_020686-14025-1
      AGAAGACAACAACCAAGACATGACAACAGACTTACTACACGTTATTCTTG
CONSENSUS
      AGAAGACAACAACCAAGACATGACAACAGACTTACTACACG.........

SE-X-XLOC_020686-14025-0

SE-X-XLOC_020686-14025-1
      AAAGGCCTGAGTTTTGTGAGATGATGGAAAGTTGTAATTGCAGACATTTT
CONSENSUS
      ....

SE-X-XLOC_020686-14025-0
      --AC
SE-X-XLOC_020686-14025-1
      TGGAGGATTTGCTTTGAATGATACGATGTTGAAGCTATAGAGGAAGAGAC
CONSENSUS
      ....AC

SE-X-XLOC_020686-14025-0
      CAAAACTACTAATCTGATGCACTGAAGTAAGGGAGTATGACAATGATATA
SE-X-XLOC_020686-14025-1
      CAAAACTACTAATCTGATGCACTGAAGTAAGGGAGTATGACAATGATATA
CONSENSUS
      CAAAACTACTAATCTGATGCACTGAAGTAAGGGAGTATGACAATGATATA

SE-X-XLOC_020686-14025-0
      CAACGGTATGAAATAGGTAAACAGCAATTGTATTTTTATTGTTTTGTGAT
SE-X-XLOC_020686-14025-1
      CAACGGTATGAAATAGGTAAACAGCAATTGTATTTTTATTGTTTTGTGAT
CONSENSUS
      CAACGGTATGAAATAGGTAAACAGCAATTGTATTTTTATTGTTTTGTGAT

SE-X-XLOC_020686-14025-0
      GTAGATGTTTAGTAATGGTTTGTAATAGATGGTTTAGTAGTAAGATAAAA
SE-X-XLOC_020686-14025-1
      GTAGATGTTTAGTAATGGTTTGTAATAGATGGTTTAGTAGTAAGATAAAA
CONSENSUS
      GTAGATGTTTAGTAATGGTTTGTAATAGATGGTTTAGTAGTAAGATAAAA

SE-X-XLOC_020686-14025-0
      CGTCTGTGTTTAATGTAATGCATAGAACGATATGTTAGGAAGTGGTAGGA
SE-X-XLOC_020686-14025-1
      CGTCTGTGTTTAATGTAATGCATAGAACGATATGTTAGGAAGTGGTAGGA
CONSENSUS
      CGTCTGTGTTTAATGTAATGCATAGAACGATATGTTAGGAAGTGGTAGGA

SE-X-XLOC_020686-14025-0
      TGTGGTTTAATTTAATGGTACGATAAGTAAGGAAACATGTTTCTCGGTTT
SE-X-XLOC_020686-14025-1
      TGTGGTTTAATTTAATGGTACGATAAGTAAGGAAACATGTTTCTCGGTTT
CONSENSUS
      TGTGGTTTAATTTAATGGTACGATAAGTAAGGAAACATGTTTCTCGGTTT

SE-X-XLOC_020686-14025-0   AATAATAAACGTA
SE-X-XLOC_020686-14025-1   AATAATAAACGTA
CONSENSUS   AATAATAAACGTA

alignment for event: A3-gBol013381-XLOC_036845-5425

A3-gBol013381-XLOC_036845-5425-0
      TTCTGGGTTTCACTTGCTATAGATGGCCACTCAACATTGATTTCTTTAGA
A3-gBol013381-XLOC_036845-5425-1
      TTCTGGGTTTCACTTGCTATAGATGGCCACTCAACATTGATTTCTTTAGA
CONSENSUS
      TTCTGGGTTTCACTTGCTATAGATGGCCACTCAACATTGATTTCTTTAGA

A3-gBol013381-XLOC_036845-5425-0
      AACATCAATGGCAAACCCTTCAGATTCTTATTTGCATATCATACTTACCT
A3-gBol013381-XLOC_036845-5425-1
      AACATCAATGGCAAACCCTTCAGATTCTTATTTGCATATCATACTTACCT
CONSENSUS
      AACATCAATGGCAAACCCTTCAGATTCTTATTTGCATATCATACTTACCT

A3-gBol013381-XLOC_036845-5425-0 CGCTGTCTCAGATATAAG------
AAACATTTGAGCACTTGGAGAACCTC
A3-gBol013381-XLOC_036845-5425-1
      CGCTGTCTCAGATATAAGGGTTAGAAACATTTGAGCACTTGGAGAACCTC
CONSENSUS
      CGCTGTCTCAGATATAAG......AAACATTTGAGCACTTGGAGAACCTC

A3-gBol013381-XLOC_036845-5425-0
AGCAAAAAGGAGCTTTTAACAGAACAAGGCGACATCAATAACCTTTAAAT
A3-gBol013381-XLOC_036845-5425-1
AGCAAAAAGGAGCTTTTAACAGAACAAGGCGACATCAATAACCTTTAAAT
CONSENSUS
AGCAAAAAGGAGCTTTTAACAGAACAAGGCGACATCAATAACCTTTAAAT

A3-gBol013381-XLOC_036845-5425-0 CTGAG
A3-gBol013381-XLOC_036845-5425-1 CTGAG
CONSENSUS CTGAG

alignment for event: A5-gBol008310-XLOC_042479-13064

A5-gBol008310-XLOC_042479-13064-0
ATCTTACTTACAAATCCCGGCATGACGATGTATCCGCTGCTTTTACTTCA
A5-gBol008310-XLOC_042479-13064-1
ATCTTACTTACAAATCCCGGCATGACGATGTATCCGCTGCTTTTACTTCA
CONSENSUS
ATCTTACTTACAAATCCCGGCATGACGATGTATCCGCTGCTTTTACTTCA

A5-gBol008310-XLOC_042479-13064-0
CCCGTATCTAGTGATGAACCCATGTATGAAGGTGAGGAATGCGTAAACGC
A5-gBol008310-XLOC_042479-13064-1
CCCGTATCTAGTGATGAACCCATGTATGAAGGTGAGGAATGCGTAAACGC
CONSENSUS
CCCGTATCTAGTGATGAACCCATGTATGAAGGTGAGGAATGCGTAAACGC

A5-gBol008310-XLOC_042479-13064-0
TGTGGCTCCACCAGTTTGTGCAGACAAAGAACCCGTTTATGAAGGCGAAT
A5-gBol008310-XLOC_042479-13064-1
TGTGGCTCCACCAGTTTGTGCAGACAAAGAACCCGTTTATGAAGGCGAAT
CONSENSUS
TGTGGCTCCACCAGTTTGTGCAGACAAAGAACCCGTTTATGAAGGCGAAT

A5-gBol008310-XLOC_042479-13064-0
CACTTCTTGGGAAAAGGGCTGAGAAGAATGTTGATGATTGTTCTTCTGAA
A5-gBol008310-XLOC_042479-13064-1
CACTTCTTGGGAAAAGGGCTGAGAAGAATGTTGATGATTGTTCTTCTGAA
CONSENSUS
CACTTCTTGGGAAAAGGGCTGAGAAGAATGTTGATGATTGTTCTTCTGAA

A5-gBol008310-XLOC_042479-13064-0
G------------------------GTGAAGTAATCAGGAACTTCCTGAA
A5-gBol008310-XLOC_042479-13064-1
GGTAACGGGCTTACAGCAAAAGAGGGTGAAGTAATCAGGAACTTCCTGAA
CONSENSUS
G........................GTGAAGTAATCAGGAACTTCCTGAA

A5-gBol008310-XLOC_042479-13064-0
GAACAGTGCCAGTCAATTAACCTTTTGCGG
A5-gBol008310-XLOC_042479-13064-1
GAACAGTGCCAGTCAATTAACCTTTTGCGG
CONSENSUS

GAACAGTGCCAGTCAATTAACCTTTTGCGG

alignment for event: A3-gBol041217-XLOC_005874-15151

```
A3-gBol041217-XLOC_005874-15151-0
      CATCTTCTCTTCCTATTGAAGAGATTGTGGTCTGGGCGCATGATGTTGGA
A3-gBol041217-XLOC_005874-15151-1
      CATCTTCTCTTCCTATTGAAGAGATTGTGGTCTGGGCGCATGATGTTGGA
CONSENSUS
      CATCTTCTCTTCCTATTGAAGAGATTGTGGTCTGGGCGCATGATGTTGGA

A3-gBol041217-XLOC_005874-15151-0
      GCGAAGGTTCTTGTGGATGCTTGTCAAAGTGTTCCACACATGGTGGTTGA
A3-gBol041217-XLOC_005874-15151-1
      GCGAAGGTTCTTGTGGATGCTTGTCAAAGTGTTCCACACATGGTGGTTGA
CONSENSUS
      GCGAAGGTTCTTGTGGATGCTTGTCAAAGTGTTCCACACATGGTGGTTGA

A3-gBol041217-XLOC_005874-15151-0
      TGTACAGAAGCTAAATGCTGATTTCTTAGTTGCGTCTTCTCACAAG----
A3-gBol041217-XLOC_005874-15151-1
      TGTACAGAAGCTAAATGCTGATTTCTTAGTTGCGTCTTCTCACAAGATGT
CONSENSUS
      TGTACAGAAGCTAAATGCTGATTTCTTAGTTGCGTCTTCTCACAAG....

A3-gBol041217-XLOC_005874-15151-0     ------------
GCATTGGTTTCTTATATGGTAAGAGTGATCTTCTACTT
A3-gBol041217-XLOC_005874-15151-1
      GTGGACCTACAGGCATTGGTTTCTTATATGGTAAGAGTGATCTTCTACTT
CONSENSUS
      ............GCATTGGTTTCTTATATGGTAAGAGTGATCTTCTACTT

A3-gBol041217-XLOC_005874-15151-0     GCCATGCCTCCATTCTTAG
A3-gBol041217-XLOC_005874-15151-1     GCCATGCCTCCATTCTTAG
CONSENSUS                             GCCATGCCTCCATTCTTAG
```

alignment for event: A3-gBol041064-XLOC_005787-7341

```
A3-gBol041064-XLOC_005787-7341-0
      CTTAAATAAACCTGGTCGGAAGTGCGGAACTAATAATGGTCAAAAAGACA
A3-gBol041064-XLOC_005787-7341-1
      CTTAAATAAACCTGGTCGGAAGTGCGGAACTAATAATGGTCAAAAAGACA
CONSENSUS
      CTTAAATAAACCTGGTCGGAAGTGCGGAACTAATAATGGTCAAAAAGACA

A3-gBol041064-XLOC_005787-7341-0
      AGACTTTTCCAGCTTTGATGCCTCCGTCTAATTTTGATTCTTCTCTTCGA
A3-gBol041064-XLOC_005787-7341-1
      AGACTTTTCCAGCTTTGATGCCTCCGTCTAATTTTGATTCTTCTCTTCGA
CONSENSUS
      AGACTTTTCCAGCTTTGATGCCTCCGTCTAATTTTGATTCTTCTCTTCGA
```

A3-gBol041064-XLOC_005787-7341-0 TACTTGAGACTCTGAG---------
ATATGAACTGGGTTCAACGCAAAAT
A3-gBol041064-XLOC_005787-7341-1
TACTTGAGACTCTGAGTGGGATCAGATATGAACTGGGTTCAACGCAAAAT
CONSENSUS
TACTTGAGACTCTGAG.........ATATGAACTGGGTTCAACGCAAAAT

A3-gBol041064-XLOC_005787-7341-0
CTACCTTTACAATGTCACGTTCGGGCTCTACATGCTTGATTGGTGGGAAC
A3-gBol041064-XLOC_005787-7341-1
CTACCTTTACAATGTCACGTTCGGGCTCTACATGCTTGATTGGTGGGAAC
CONSENSUS
CTACCTTTACAATGTCACGTTCGGGCTCTACATGCTTGATTGGTGGGAAC

A3-gBol041064-XLOC_005787-7341-0 GATACCTCTTCA
A3-gBol041064-XLOC_005787-7341-1 GATACCTCTTCA
CONSENSUS GATACCTCTTCA

alignment for event: A5-gBol021237-XLOC_027802-7693

A5-gBol021237-XLOC_027802-7693-0
GTAGAAGAAATGAGCCGTGAAGAAGGAGAGGTGCAAGTGCTTCCTTATGT
A5-gBol021237-XLOC_027802-7693-1
GTAGAAGAAATGAGCCGTGAAGAAGGAGAGGTGCAAGTGCTTCCTTATGT
CONSENSUS
GTAGAAGAAATGAGCCGTGAAGAAGGAGAGGTGCAAGTGCTTCCTTATGT

A5-gBol021237-XLOC_027802-7693-0
ACTTCTTTTGCATGCTTTCTGTCTGGTGGATCCCGGGCTGTGTACACCGT
A5-gBol021237-XLOC_027802-7693-1
ACTTCTTTTGCATGCTTTCTGTCTGGTGGATCCCGGGCTGTGTACACCGT
CONSENSUS
ACTTCTTTTGCATGCTTTCTGTCTGGTGGATCCCGGGCTGTGTACACCGT

A5-gBol021237-XLOC_027802-7693-0
CTTCAGACCCTACTAAATTTGTTATCACCCTACAACCGTATCTGAAGAGT
A5-gBol021237-XLOC_027802-7693-1
CTTCAGACCCTACTAAATTTGTTATCACCCTACAACCGTATCTGAAGAGT
CONSENSUS
CTTCAGACCCTACTAAATTTGTTATCACCCTACAACCGTATCTGAAGAGT

A5-gBol021237-XLOC_027802-7693-0
CAGGTTATATATAGCCAATCTGCTCAAGATTCTGTTAAGTTTTGTAAAGG
A5-gBol021237-XLOC_027802-7693-1
CAG---
CONSENSUS
CAG...

A5-gBol021237-XLOC_027802-7693-0
AATAATTTTTTAGGAGCTACGCTTGTTTCCTTTGAAAAATTGTGGTACTA
A5-gBol021237-XLOC_027802-7693-1

CONSENSUS
....

A5-gBol021237-XLOC_027802-7693-0
ATGCGGAGAAGAAATTATCTGAGTGAGAGGTTGATAGTAGAATAGGAGCA
A5-gBol021237-XLOC_027802-7693-1 -----------------------------
GTTGATAGTAGAATAGGAGCA
CONSENSUS
.............................GTTGATAGTAGAATAGGAGCA

A5-gBol021237-XLOC_027802-7693-0
CAATTACTAGAGAGTATTATTTTTATAATCGATTCTGTCCTGCCCTTGAT
A5-gBol021237-XLOC_027802-7693-1
CAATTACTAGAGAGTATTATTTTTATAATCGATTCTGTCCTGCCCTTGAT
CONSENSUS
CAATTACTAGAGAGTATTATTTTTATAATCGATTCTGTCCTGCCCTTGAT

A5-gBol021237-XLOC_027802-7693-0
CCGCAAGCTGCCTCTTAGTGTCACTGAAGACCTGGAGCAAGACCTAAAGC
A5-gBol021237-XLOC_027802-7693-1
CCGCAAGCTGCCTCTTAGTGTCACTGAAGACCTGGAGCAAGACCTAAAGC
CONSENSUS
CCGCAAGCTGCCTCTTAGTGTCACTGAAGACCTGGAGCAAGACCTAAAGC

A5-gBol021237-XLOC_027802-7693-0
ATATGATTGTTCGGCATTCATTTCTAACCGTTGTTCATGCTTGCGTTAG
A5-gBol021237-XLOC_027802-7693-1
ATATGATTGTTCGGCATTCATTTCTAACCGTTGTTCATGCTTGCGTTAG
CONSENSUS
ATATGATTGTTCGGCATTCATTTCTAACCGTTGTTCATGCTTGCGTTAG

alignment for event: SE-gBol021198-XLOC_027675-10188

SE-gBol021198-XLOC_027675-10188-0
CGACGCCGTCTCGAGCCACACTACATGATGAATACGACTCGTGAAAGTGC
SE-gBol021198-XLOC_027675-10188-1
CGACGCCGTCTCGAGCCACACTACATGATGAATACGACTCGTGAAAGTGC
CONSENSUS
CGACGCCGTCTCGAGCCACACTACATGATGAATACGACTCGTGAAAGTGC

SE-gBol021198-XLOC_027675-10188-0
ATCGCCGGTTAAATAATTTTTTTCGTCTCTCTCTTCACTCCATTTTCTCT
SE-gBol021198-XLOC_027675-10188-1
ATCGCCGGTTAAATAATTTTTTTCGTCTCTCTCTTCACTCCATTTTCTCT
CONSENSUS
ATCGCCGGTTAAATAATTTTTTTCGTCTCTCTCTTCACTCCATTTTCTCT

SE-gBol021198-XLOC_027675-10188-0
CGCATTTCAAATTTGATTTCCTTATTTAGATTCTTAAGGTCGGCGAGGCG
SE-gBol021198-XLOC_027675-10188-1
CGCATTTCAAATTTGATTTCCTTATTTAGATTCTTAAGGTCGGCGAGGCG
CONSENSUS
CGCATTTCAAATTTGATTTCCTTATTTAGATTCTTAAGGTCGGCGAGGCG

SE-gBol021198-XLOC_027675-10188-0
ATTCTCGAGTCCACGGCGATTCTGCTTACTCCGCCTTTCAGTTTCTCTAC

SE-gBol021198-XLOC_027675-10188-1
      ATTCTCGAGTCCACGGCGATTCTGCTTACTCCGCCTTTCAGTTTCTCTAC
CONSENSUS
      ATTCTCGAGTCCACGGCGATTCTGCTTACTCCGCCTTTCAGTTTCTCTAC

SE-gBol021198-XLOC_027675-10188-0
      TCAGATTCTGCTACGTTTCGCCACCGCTCGATTCGCCG------------
SE-gBol021198-XLOC_027675-10188-1
      TCAGATTCTGCTACGTTTCGCCACCGCTCGATTCGCCGTCAGCCGGTGAA
CONSENSUS
      TCAGATTCTGCTACGTTTCGCCACCGCTCGATTCGCCG............

SE-gBol021198-XLOC_027675-10188-0

SE-gBol021198-XLOC_027675-10188-1
      TCGCTTTTGTAATCGGATTCGGAATCGCGAGGACTTGCTTGGTATCGAGT
CONSENSUS
      ....

SE-gBol021198-XLOC_027675-10188-0

SE-gBol021198-XLOC_027675-10188-1
      CGAGATCTTCCGAGTGTTATTGAGCGAGATCGTGTTAATCTGCGAGTTCG
CONSENSUS
      ....

SE-gBol021198-XLOC_027675-10188-0

SE-gBol021198-XLOC_027675-10188-1
      AAATGCAGATCTCATCGCTGTTGAATTGGTCTGCTGTGATCTTATTTGAA
CONSENSUS
      ....

SE-gBol021198-XLOC_027675-10188-0

SE-gBol021198-XLOC_027675-10188-1
      CAGTCTGTGGAGTTTGTTGACTGATTCAAGCTTTATAGATTTGAATTCAT
CONSENSUS
      ....

SE-gBol021198-XLOC_027675-10188-0   ----------
CTCCTTCACGCATGGCGAGCAATACTTTAAAGGATATGAA
SE-gBol021198-XLOC_027675-10188-1
      TGAGAGATCTCTCCTTCACGCATGGCGAGCAATACTTTAAAGGATATGAA
CONSENSUS
      ..........CTCCTTCACGCATGGCGAGCAATACTTTAAAGGATATGAA

SE-gBol021198-XLOC_027675-10188-0
      TACTCTTCCTGTAACTGAGAAGATGAGTGAATGTAAACCAAGCTTAGCCA
SE-gBol021198-XLOC_027675-10188-1
      TACTCTTCCTGTAACTGAGAAGATGAGTGAATGTAAACCAAGCTTAGCCA
CONSENSUS
      TACTCTTCCTGTAACTGAGAAGATGAGTGAATGTAAACCAAGCTTAGCCA

SE-gBol021198-XLOC_027675-10188-0
      AGCCTTGTGTTGGGAAAATGAATGGAAAGTCTGAAGATAGACCACTAGCG

SE-gBol021198-XLOC_027675-10188-1
AGCCTTGTGTTGGGAAAATGAATGGAAAGTCTGAAGATAGACCACTAGCG
CONSENSUS
AGCCTTGTGTTGGGAAAATGAATGGAAAGTCTGAAGATAGACCACTAGCG

SE-gBol021198-XLOC_027675-10188-0
AGCTCTGCTGCTGCTTTGGATCCTAGTGTTGTGGAAACTGAGAAACCTGA
SE-gBol021198-XLOC_027675-10188-1
AGCTCTGCTGCTGCTTTGGATCCTAGTGTTGTGGAAACTGAGAAACCTGA
CONSENSUS
AGCTCTGCTGCTGCTTTGGATCCTAGTGTTGTGGAAACTGAGAAACCTGA

SE-gBol021198-XLOC_027675-10188-0
GGCAGAGAAAGCTGCCGTGGAAGTTGAGTATATTGAATCTGAGAACTTGG
SE-gBol021198-XLOC_027675-10188-1
GGCAGAGAAAGCTGCCGTGGAAGTTGAGTATATTGAATCTGAGAACTTGG
CONSENSUS
GGCAGAGAAAGCTGCCGTGGAAGTTGAGTATATTGAATCTGAGAACTTGG

SE-gBol021198-XLOC_027675-10188-0
ATAATGTTGACGATGCTGATGCAGTTCTCAAG
SE-gBol021198-XLOC_027675-10188-1
ATAATGTTGACGATGCTGATGCAGTTCTCAAG
CONSENSUS
ATAATGTTGACGATGCTGATGCAGTTCTCAAG

alignment for event: A3-gBol023312-XLOC_025548-15245

A3-gBol023312-XLOC_025548-15245-0
AATGACGTCTAATTGTCGAGGGAACGCAAGTGCTTCGGGACAAAGAGGTC
A3-gBol023312-XLOC_025548-15245-1
AATGACGTCTAATTGTCGAGGGAACGCAAGTGCTTCGGGACAAAGAGGTC
CONSENSUS
AATGACGTCTAATTGTCGAGGGAACGCAAGTGCTTCGGGACAAAGAGGTC

A3-gBol023312-XLOC_025548-15245-0
GGAATGCTTCTAATGAAACTAGAACTAGAACTACAGAGAGATTTAAATGG
A3-gBol023312-XLOC_025548-15245-1
GGAATGCTTCTAATGAAACTAGAACTAGA---------------------
CONSENSUS
GGAATGCTTCTAATGAAACTAGAACTAGA.....................

A3-gBol023312-XLOC_025548-15245-0
ACGTACGAACAAGAGAAAAAATTGATTGAATTGTTTGATGAGGCAATTTC
A3-gBol023312-XLOC_025548-15245-1

CONSENSUS
....

A3-gBol023312-XLOC_025548-15245-0
CATGTCAAATTATAGTCTCAAAGATCCTAAGGCTCATGGCAGAGTATATG
A3-gBol023312-XLOC_025548-15245-1

CONSENSUS

....

A3-gBol023312-XLOC_025548-15245-0
GTTGAAAATTCAATCTTGCATTTAACATGAATATAACTTACGCATTTTTC
A3-gBol023312-XLOC_025548-15245-1

CONSENSUS
....

A3-gBol023312-XLOC_025548-15245-0
AAAAATAAGCTTGATGAGTTCAAAAAATCATACAAGATGTAGAAGTTTCT
A3-gBol023312-XLOC_025548-15245-1

CONSENSUS
....

A3-gBol023312-XLOC_025548-15245-0
GATGAAATCCACCGGCATTTCGGTGGATCCTGAAACATCTATGATTTACG
A3-gBol023312-XLOC_025548-15245-1

CONSENSUS
....

A3-gBol023312-XLOC_025548-15245-0
CATCTCCGGAGTGGTGGGATAATCACGAGGCGGTAATAATTAATAAATAT
A3-gBol023312-XLOC_025548-15245-1

CONSENSUS
....

A3-gBol023312-XLOC_025548-15245-0
TATATTTATATTATCAACTATTAAGACACATATAACTAAATACCGAGAGA
A3-gBol023312-XLOC_025548-15245-1

CONSENSUS
....

A3-gBol023312-XLOC_025548-15245-0
CACCAAAATTTTGGGATGTAATGGTACGGTATTTTGCATTACATGATGTA
A3-gBol023312-XLOC_025548-15245-1

CONSENSUS
....

A3-gBol023312-XLOC_025548-15245-0
TATTCGCAACCACAATATTCTGCGCGACAAAGAAGAAAAGAGATAATGAA
A3-gBol023312-XLOC_025548-15245-1

CONSENSUS
....

A3-gBol023312-XLOC_025548-15245-0
TGAGGGACGAGCTGATGATTCCACACATGGAAACTCATATTTCAAAGGGG
A3-gBol023312-XLOC_025548-15245-1

CONSENSUS

....

A3-gBol023312-XLOC_025548-15245-0
GTGACATGCCAGACACTGAAGTTCTAGAGATTAAAGAAAATGAAGAAGTA
A3-gBol023312-XLOC_025548-15245-1

CONSENSUS
....

A3-gBol023312-XLOC_025548-15245-0
TATCGCGTCAAGATATGAATGAGAGGTATATGGCAGAGATCAAGTCACAA
A3-gBol023312-XLOC_025548-15245-1

CONSENSUS
....

A3-gBol023312-XLOC_025548-15245-0
GATCCTGGAAATTCAATTGTGGGACATAAGATGAGTGTGGTCCCATATTG
A3-gBol023312-XLOC_025548-15245-1

CONSENSUS
....

A3-gBol023312-XLOC_025548-15245-0
GAAATTTAGACAAAGAGTGTCTGATATACAAAGAGATGTCAAACTCTAAT
A3-gBol023312-XLOC_025548-15245-1

CONSENSUS
....

A3-gBol023312-XLOC_025548-15245-0
TAGTACGAATGCCTTTTGGGAAAGATCCCAAAAGTAAATTCATGCGGACT
A3-gBol023312-XLOC_025548-15245-1

CONSENSUS
....

A3-gBol023312-XLOC_025548-15245-0
TGTCTCTTATGCCCAAAGTGGAAAATATCGTACTAATCAGACAATAAAGA
A3-gBol023312-XLOC_025548-15245-1

CONSENSUS
....

A3-gBol023312-XLOC_025548-15245-0
ATTAGACATGAGCTTAATAAATCCCAACACCCTCAACGTCAGACCGCATC
A3-gBol023312-XLOC_025548-15245-1

CONSENSUS
....

A3-gBol023312-XLOC_025548-15245-0
TTTTGTCCTTTGTCTCGAACAGAACCAGGATTGATCGATCTGGTTGGTCG
A3-gBol023312-XLOC_025548-15245-1

CONSENSUS

....

A3-gBol023312-XLOC_025548-15245-0
GCTCGTTTCCAACCAACTTTCTGGATATTTTCAGAACAGGTACCTGGAAT
A3-gBol023312-XLOC_025548-15245-1

CONSENSUS
....

A3-gBol023312-XLOC_025548-15245-0
TTTAGCTATTTTCATGTTGACCCTAAATTTCTTTAATTTGCACATTGTCC
A3-gBol023312-XLOC_025548-15245-1

CONSENSUS
....

A3-gBol023312-XLOC_025548-15245-0
TTTTTATTTTATATCAAAGAAAATGGACACATATAAGCAATGTAGACACC
A3-gBol023312-XLOC_025548-15245-1

CONSENSUS
....

A3-gBol023312-XLOC_025548-15245-0
TAAATACACATCTAAACAGCGTAAAACAACATATATCATTGTATAGAAAT
A3-gBol023312-XLOC_025548-15245-1

CONSENSUS
....

A3-gBol023312-XLOC_025548-15245-0
TTTAAAATATGTATTTAGAAATGTAAGAAGACAAATCAAATTGATATCAC
A3-gBol023312-XLOC_025548-15245-1

CONSENSUS
....

A3-gBol023312-XLOC_025548-15245-0
TCAATTACTCTAAGGAGTGATTTACTCTCTCAAATAAGAGGTTCAACTGT
A3-gBol023312-XLOC_025548-15245-1

CONSENSUS
....

A3-gBol023312-XLOC_025548-15245-0
TGTATTTATGGATCTAATCCACAAGGAACTAGGACACACAATAGATCTAA
A3-gBol023312-XLOC_025548-15245-1

CONSENSUS
....

A3-gBol023312-XLOC_025548-15245-0
ACAGTTTATGATTAAGCTAGGTAAATTATGAAAATAGTAAATTGCAAGTA
A3-gBol023312-XLOC_025548-15245-1

CONSENSUS

....

A3-gBol023312-XLOC_025548-15245-0
AGCGAACAAGGTAGTTGTTCAGTTGATTGGTTTAAGGTTTTGTAAACAAT
A3-gBol023312-XLOC_025548-15245-1

CONSENSUS
....

A3-gBol023312-XLOC_025548-15245-0
TATTAGAAAGCATTAGATCTAGGGTTTCTATTTAGGTAGTTGGCATTATA
A3-gBol023312-XLOC_025548-15245-1

CONSENSUS
....

A3-gBol023312-XLOC_025548-15245-0
GAGGTATATAATACTTAAATGTCAATCCTAGAACTCGACTTATAGTGTAA
A3-gBol023312-XLOC_025548-15245-1

CONSENSUS
....

A3-gBol023312-XLOC_025548-15245-0
AGATATCCAGCTTTTGCATGTGAGTCTTATGTTTTGACTAAGTCCAATAT
A3-gBol023312-XLOC_025548-15245-1

CONSENSUS
....

A3-gBol023312-XLOC_025548-15245-0
CTTAGATGCCGCTTGTTGATACGAACTGAACGTCGATCGATGTTATGGTA
A3-gBol023312-XLOC_025548-15245-1

CONSENSUS
....

A3-gBol023312-XLOC_025548-15245-0
AGAATGTCGATCGATGTTCTCTTAGACGTGCGTTAGCGAGTTGATCGATA
A3-gBol023312-XLOC_025548-15245-1

CONSENSUS
....

A3-gBol023312-XLOC_025548-15245-0
GGTCACTAGTATTATCTAAATCAGCTTCCGCTTGTTTCTAGCAATAATAG
A3-gBol023312-XLOC_025548-15245-1

CONSENSUS
....

A3-gBol023312-XLOC_025548-15245-0
CTCAAATGAATTAGTTTTGGTGCAGAACCTACCGACGTAGCAGAACCTAC
A3-gBol023312-XLOC_025548-15245-1

CONSENSUS

....

A3-gBol023312-XLOC_025548-15245-0
CGTCGTAGTTGTCCACAATCCTAAGTTCAAGGTTCTATTAATTAACCATA
A3-gBol023312-XLOC_025548-15245-1

CONSENSUS
....

A3-gBol023312-XLOC_025548-15245-0
ATTCTTTGAAGGATATTTATCACCTTAGCAATTCTATATTTTGGGCTAAT
A3-gBol023312-XLOC_025548-15245-1

CONSENSUS
....

A3-gBol023312-XLOC_025548-15245-0
CCCTCTTAACCGATTTGGACCCTAAATCTAGCAAGTGAATTACTCATACA
A3-gBol023312-XLOC_025548-15245-1

CONSENSUS
....

A3-gBol023312-XLOC_025548-15245-0
TAGCAAAACAATTCATAAGCATAAACTGAATAAAATGCATAAACAGATTA
A3-gBol023312-XLOC_025548-15245-1

CONSENSUS
....

A3-gBol023312-XLOC_025548-15245-0
AAGTTAGAAATACAGGAGTTCCAATACAAATCTCTGAAGGAGTCTTGGAT
A3-gBol023312-XLOC_025548-15245-1

CONSENSUS
....

A3-gBol023312-XLOC_025548-15245-0
ATTCTCTCCAAATCTACTAAAAACCCTAAGTGTTGTTTGATGTGAAAAGT
A3-gBol023312-XLOC_025548-15245-1

CONSENSUS
....

A3-gBol023312-XLOC_025548-15245-0
AGAAAGTAAAAAAGCGTGTTTTGCCTAGAACAATGACAGAGCACATAAAT
A3-gBol023312-XLOC_025548-15245-1

CONSENSUS
....

A3-gBol023312-XLOC_025548-15245-0
ATTAGGTTAAAAGTCGGAAAGGGGTATTTCTTTAAACATGTCGTGACTTG
A3-gBol023312-XLOC_025548-15245-1

CONSENSUS

```
      ..................................................

A3-gBol023312-XLOC_025548-15245-0
      GGATTTTAGCTTGCTGGAAACCTAGCTGAGTTTTGCGCGGTTTCGCTGTC
A3-gBol023312-XLOC_025548-15245-1
      --------------------------------------------------
CONSENSUS
      ..................................................

A3-gBol023312-XLOC_025548-15245-0
      GATCGACGGTACAGGGTGTGCGTCGATCAATGTTTGTCTCTGATCGTCTA
A3-gBol023312-XLOC_025548-15245-1
      --------------------------------------------------
CONSENSUS
      ..................................................

A3-gBol023312-XLOC_025548-15245-0
      CATTCAGACATGTTCGATGGTCAGCTACGAGCTTCCTCTCATTTTATCTC
A3-gBol023312-XLOC_025548-15245-1
      --------------------------------------------------
CONSENSUS
      ..................................................

A3-gBol023312-XLOC_025548-15245-0
      TAAAATGCATCCAAATCATCGCTTTTTACAAAACACTCATGAACATGTAC
A3-gBol023312-XLOC_025548-15245-1
      --------------------------------------------------
CONSENSUS
      ..................................................

A3-gBol023312-XLOC_025548-15245-0
      ATGTGTTAAAAAGACTTCAAAACATAATAAATAGTTCCTAAAATACCTAT
A3-gBol023312-XLOC_025548-15245-1
      --------------------------------------------------
CONSENSUS
      ..................................................

A3-gBol023312-XLOC_025548-15245-0
      ATACCATGGCTAAAAATGGGTAAAATCAATGGTATATCAAAAACCTAATG
A3-gBol023312-XLOC_025548-15245-1
      --------------------------------------------------
CONSENSUS
      ..................................................

A3-gBol023312-XLOC_025548-15245-0
      AAAAAAAAAATCCATATTTTAGCCTAACTAGATCTAAAAAACTAGCTAAT
A3-gBol023312-XLOC_025548-15245-1
      --------------------------------------------------
CONSENSUS
      ..................................................

A3-gBol023312-XLOC_025548-15245-0
      TCAATTTAGAGTAACTGTTGTAATTTGATATCTCATGATTTTAATAAGTT
A3-gBol023312-XLOC_025548-15245-1
      --------------------------------------------------
CONSENSUS
```

....

A3-gBol023312-XLOC_025548-15245-0
TTAGATTCATCTTTTACCTTATTATGATCATTTTAGGTTGCATTTAGGTC
A3-gBol023312-XLOC_025548-15245-1
------------------------------------GTTGCATTTAGGTC
CONSENSUS
....................................GTTGCATTTAGGTC

A3-gBol023312-XLOC_025548-15245-0
TTTGTGTTGCATATGCATACTCATTTGCATATCATAAGGTTTGGAATGCT
A3-gBol023312-XLOC_025548-15245-1
TTTGTGTTGCATATGCATACTCATTTGCATATCATAAGGTTTGGAATGCT
CONSENSUS
TTTGTGTTGCATATGCATACTCATTTGCATATCATAAGGTTTGGAATGCT

A3-gBol023312-XLOC_025548-15245-0
CGTTTGAGAAGTTTGGGGCTAAACCGTGGGTTATTCTTGCTATTTACAAA
A3-gBol023312-XLOC_025548-15245-1
CGTTTGAGAAGTTTGGGGCTAAACCGTGGGTTATTCTTGCTATTTACAAA
CONSENSUS
CGTTTGAGAAGTTTGGGGCTAAACCGTGGGTTATTCTTGCTATTTACAAA

A3-gBol023312-XLOC_025548-15245-0
GTTTGGGTCCGAATTGAAAACATCCAGAAGTTCAAGGTCCATCTCTGCAA
A3-gBol023312-XLOC_025548-15245-1
GTTTGGGTCCGAATTGAAAACATCCAGAAGTTCAAGGTCCATCTCTGCAA
CONSENSUS
GTTTGGGTCCGAATTGAAAACATCCAGAAGTTCAAGGTCCATCTCTGCAA

A3-gBol023312-XLOC_025548-15245-0
ATTTAGAATACTCGCTTGGGTTATTTTTCACAATCAAAAGATTCGGGGCC
A3-gBol023312-XLOC_025548-15245-1
ATTTAGAATACTCGCTTGGGTTATTTTTCACAATCAAAAGATTCGGGGCC
CONSENSUS
ATTTAGAATACTCGCTTGGGTTATTTTTCACAATCAAAAGATTCGGGGCC

A3-gBol023312-XLOC_025548-15245-0
AATTCGAGAGGTCCTTTATGCAACTATAAGAGTTTGGGGCGAATGTGTAA
A3-gBol023312-XLOC_025548-15245-1
AATTCGAGAGGTCCTTTATGCAACTATAAGAGTTTGGGGCGAATGTGTAA
CONSENSUS
AATTCGAGAGGTCCTTTATGCAACTATAAGAGTTTGGGGCGAATGTGTAA

A3-gBol023312-XLOC_025548-15245-0
ATAATCAAGATTCCGGGGACCTGTTTTGCAATTACTGAAGAAATCACCAT
A3-gBol023312-XLOC_025548-15245-1
ATAATCAAGATTCCGGGGACCTGTTTTGCAATTACTGAAGAAATCACCAT
CONSENSUS
ATAATCAAGATTCCGGGGACCTGTTTTGCAATTACTGAAGAAATCACCAT

A3-gBol023312-XLOC_025548-15245-0
TGGAGAGAACGAGCTTTCTCTTGGTCGATCTGTAGGCGACGGCGTGGCTG
A3-gBol023312-XLOC_025548-15245-1
TGGAGAGAACGAGCTTTCTCTTGGTCGATCTGTAGGCGACGGCGTGGCTG
CONSENSUS

TGGAGAGAACGAGCTTTCTCTTGGTCGATCTGTAGGCGACGGCGTGGCTG

A3-gBol023312-XLOC_025548-15245-0
AGAGCACGACGAAGACGTGGAGATAGAGAGGCCGTGAGCCAGTGACGACG
A3-gBol023312-XLOC_025548-15245-1
AGAGCACGACGAAGACGTGGAGATAGAGAGGCCGTGAGCCAGTGACGACG
CONSENSUS
AGAGCACGACGAAGACGTGGAGATAGAGAGGCCGTGAGCCAGTGACGACG

A3-gBol023312-XLOC_025548-15245-0
AAGCCTGAAGATAAGACCTCCACGAGCGCCAACGGCTATGGCTGGCTCCA
A3-gBol023312-XLOC_025548-15245-1
AAGCCTGAAGATAAGACCTCCACGAGCGCCAACGGCTATGGCTGGCTCCA
CONSENSUS
AAGCCTGAAGATAAGACCTCCACGAGCGCCAACGGCTATGGCTGGCTCCA

A3-gBol023312-XLOC_025548-15245-0
GCGACGTTGCGACGAAGGAGAAGCACTCCAGGGCTTCAGGACAAGCTGTC
A3-gBol023312-XLOC_025548-15245-1
GCGACGTTGCGACGAAGGAGAAGCACTCCAGGGCTTCAGGACAAGCTGTC
CONSENSUS
GCGACGTTGCGACGAAGGAGAAGCACTCCAGGGCTTCAGGACAAGCTGTC

A3-gBol023312-XLOC_025548-15245-0
ACGGCTCGATCTGAGACCAAGACGGTGATTCTGAGCACAGCCACAGAATG
A3-gBol023312-XLOC_025548-15245-1
ACGGCTCGATCTGAGACCAAGACGGTGATTCTGAGCACAGCCACAGAATG
CONSENSUS
ACGGCTCGATCTGAGACCAAGACGGTGATTCTGAGCACAGCCACAGAATG

A3-gBol023312-XLOC_025548-15245-0
ATGATGATGACGGCATGAATCGTGGAGGCGCGTCGTGACAACGATTGAAG
A3-gBol023312-XLOC_025548-15245-1
ATGATGATGACGGCATGAATCGTGGAGGCGCGTCGTGACAACGATTGAAG
CONSENSUS
ATGATGATGACGGCATGAATCGTGGAGGCGCGTCGTGACAACGATTGAAG

A3-gBol023312-XLOC_025548-15245-0
TCTCCGCCATGACGGAGGATATGAAGCTCGACGACGCAGGTTGAAGGCTC
A3-gBol023312-XLOC_025548-15245-1
TCTCCGCCATGACGGAGGATATGAAGCTCGACGACGCAGGTTGAAGGCTC
CONSENSUS
TCTCCGCCATGACGGAGGATATGAAGCTCGACGACGCAGGTTGAAGGCTC

A3-gBol023312-XLOC_025548-15245-0
GCGGGATCGTGAACCCACGCGCACGAAGAGGAAGATGCGTCGACGTCTCG
A3-gBol023312-XLOC_025548-15245-1
GCGGGATCGTGAACCCACGCGCACGAAGAGGAAGATGCGTCGACGTCTCG
CONSENSUS
GCGGGATCGTGAACCCACGCGCACGAAGAGGAAGATGCGTCGACGTCTCG

A3-gBol023312-XLOC_025548-15245-0
ACTCGGGTTGAAGGCACGCGGGACAGCCTTACCGCGCGCACGAAGAGGAG
A3-gBol023312-XLOC_025548-15245-1
ACTCGGGTTGAAGGCACGCGGGACAGCCTTACCGCGCGCACGAAGAGGAG
CONSENSUS

ACTCGGGTTGAAGGCACGCGGGACAGCCTTACCGCGCGCACGAAGAGGAG

A3-gBol023312-XLOC_025548-15245-0
CGATCGACGGCCTAAAGCGGGTTGGGGCACGCGGGTTCGCCTACCCGCGC
A3-gBol023312-XLOC_025548-15245-1
CGATCGACGGCCTAAAGCGGGTTGGGGCACGCGGGTTCGCCTACCCGCGC
CONSENSUS
CGATCGACGGCCTAAAGCGGGTTGGGGCACGCGGGTTCGCCTACCCGCGC

A3-gBol023312-XLOC_025548-15245-0
GCATGACAAAAGAGTTTCAACGACGGTCCAACGCGGGAGAGTGGCTCGGG
A3-gBol023312-XLOC_025548-15245-1
GCATGACAAAAGAGTTTCAACGACGGTCCAACGCGGGAGAGTGGCTCGGG
CONSENSUS
GCATGACAAAAGAGTTTCAACGACGGTCCAACGCGGGAGAGTGGCTCGGG

A3-gBol023312-XLOC_025548-15245-0
TGGGAGTTGGAGATTTCAACCCGGGTCGAATCTTTGCCTTAGTCGAATTT
A3-gBol023312-XLOC_025548-15245-1
TGGGAGTTGGAGATTTCAACCCGGGTCGAATCTTTGCCTTAGTCGAATTT
CONSENSUS
TGGGAGTTGGAGATTTCAACCCGGGTCGAATCTTTGCCTTAGTCGAATTT

A3-gBol023312-XLOC_025548-15245-0
TAATGTTTTTAAAGGGCTTTTTAGATTTTTTTTAATAGAAACAATTAGAT
A3-gBol023312-XLOC_025548-15245-1
TAATGTTTTTAAAGGGCTTTTTAGATTTTTTTTAATAGAAACAATTAGAT
CONSENSUS
TAATGTTTTTAAAGGGCTTTTTAGATTTTTTTTAATAGAAACAATTAGAT

A3-gBol023312-XLOC_025548-15245-0
TTACCAAAGCCATATAAATACTTTTGTAATCCGAAGAGGGGAGATCATCT
A3-gBol023312-XLOC_025548-15245-1
TTACCAAAGCCATATAAATACTTTTGTAATCCGAAGAGGGGAGATCATCT
CONSENSUS
TTACCAAAGCCATATAAATACTTTTGTAATCCGAAGAGGGGAGATCATCT

A3-gBol023312-XLOC_025548-15245-0
TATTTTCTATCTAATCACAAAGAATTTTGAGTCGAAAGATC
A3-gBol023312-XLOC_025548-15245-1
TATTTTCTATCTAATCACAAAGAATTTTGAGTCGAAAGATC
CONSENSUS
TATTTTCTATCTAATCACAAAGAATTTTGAGTCGAAAGATC

alignment for event: SE-gBol009865-XLOC_040757-15003

SE-gBol009865-XLOC_040757-15003-0
AATGGAGATGCGAGGAATATGGTGGATGTGAAGCCAGTGATAACAGAGGA
SE-gBol009865-XLOC_040757-15003-1
AATGGAGATGCGAGGAATATGGTGGATGTGAAGCCAGTGATAACAGAGGA
CONSENSUS
AATGGAGATGCGAGGAATATGGTGGATGTGAAGCCAGTGATAACAGAGGA

SE-gBol009865-XLOC_040757-15003-0

ATCAAATGATAAGTCTAAGATATGGAAGCTTACTGAAGTCAGCGAGCCTT
SE-gBol009865-XLOC_040757-15003-1
ATCAAATGATAAGTCTAAGATATGGAAGCTTACTGAAGTCAGCGAGCCTT
CONSENSUS
ATCAAATGATAAGTCTAAGATATGGAAGCTTACTGAAGTCAGCGAGCCTT

SE-gBol009865-XLOC_040757-15003-0
CTCAATGCAGGTCACTGAGACTCCCCGAGAATCTGAGAGTAGCTAAG---
SE-gBol009865-XLOC_040757-15003-1
CTCAATGCAGGTCACTGAGACTCCCCGAGAATCTGAGAGTAGCTAAGATA
CONSENSUS
CTCAATGCAGGTCACTGAGACTCCCCGAGAATCTGAGAGTAGCTAAG...

SE-gBol009865-XLOC_040757-15003-0

SE-gBol009865-XLOC_040757-15003-1
TCAAGATTGATTTTCACCAATTCCGGAAACGCTATCTTGGCTTTGGCGTC
CONSENSUS
....

SE-gBol009865-XLOC_040757-15003-0

SE-gBol009865-XLOC_040757-15003-1
AAACGCTATTCATCTTCTATGGAAATGGCAGAGGAATGAGCGCAACGCAA
CONSENSUS
....

SE-gBol009865-XLOC_040757-15003-0 --------
GCTACAGCTTCTTTGCCACCTCAGCAGTGGCAACCAGCAAGT
SE-gBol009865-XLOC_040757-15003-1
CTGGAAAGGCTACAGCTTCTTTGCCACCTCAGCAGTGGCAACCAGCAAGT
CONSENSUS
........GCTACAGCTTCTTTGCCACCTCAGCAGTGGCAACCAGCAAGT

SE-gBol009865-XLOC_040757-15003-0
GGAATCCTCATGACTAACGACGCAGCTGAAAACAACCCTGAGGAAGCTGT
SE-gBol009865-XLOC_040757-15003-1
GGAATCCTCATGACTAACGACGCAGCTGAAAACAACCCTGAGGAAGCTGT
CONSENSUS
GGAATCCTCATGACTAACGACGCAGCTGAAAACAACCCTGAGGAAGCTGT

SE-gBol009865-XLOC_040757-15003-0
ACCTTGTTTTGCTTTATCCAAGAATGATTCGTATGTAATGTCAGCCTCTG
SE-gBol009865-XLOC_040757-15003-1
ACCTTGTTTTGCTTTATCCAAGAATGATTCGTATGTAATGTCAGCCTCTG
CONSENSUS
ACCTTGTTTTGCTTTATCCAAGAATGATTCGTATGTAATGTCAGCCTCTG

SE-gBol009865-XLOC_040757-15003-0
GAGGAAAGATCTCCTTATTTAACATGATGACGTTTAAG
SE-gBol009865-XLOC_040757-15003-1
GAGGAAAGATCTCCTTATTTAACATGATGACGTTTAAG
CONSENSUS
GAGGAAAGATCTCCTTATTTAACATGATGACGTTTAAG

alignment for event: RI-gBol019892-XLOC_029466-16389

RI-gBol019892-XLOC_029466-16389-0
CGGCAATACTGCAGTCACACCAATGTCTTTTCAAGAGCTACCGACAAGCC
RI-gBol019892-XLOC_029466-16389-1
CGGCAATACTGCAGTCACACCAATGTCTTTTCAAGAGCTACCGACAAGCC
CONSENSUS
CGGCAATACTGCAGTCACACCAATGTCTTTTCAAGAGCTACCGACAAGCC

RI-gBol019892-XLOC_029466-16389-0
CTCATGTTTTGGAGACCAAATTATCTCCAAACATGTACGAGCCTGACCTA
RI-gBol019892-XLOC_029466-16389-1
CTCATGTTTTGGAGACCAAATTATCTCCAAACATGTACGAGCCTGACCTA
CONSENSUS
CTCATGTTTTGGAGACCAAATTATCTCCAAACATGTACGAGCCTGACCTA

RI-gBol019892-XLOC_029466-16389-0
AACAAACCAGTTCCAGTTTCGGGGTATCCACAATTTCTGAACCAAGCTCA
RI-gBol019892-XLOC_029466-16389-1
AACAAACCAGTTCCAGTTTCGGGGTATCCACAATTTCTGAACCAAGCTCA
CONSENSUS
AACAAACCAGTTCCAGTTTCGGGGTATCCACAATTTCTGAACCAAGCTCA

RI-gBol019892-XLOC_029466-16389-0
ACAACAACAACCTATCTACGTGGTTTACACTGGACAGCCGCCTTACATGA
RI-gBol019892-XLOC_029466-16389-1
ACAACAACAACCTATCTACGTGGTTTACACTGGACAGCCGCCTTACATGA
CONSENSUS
ACAACAACAACCTATCTACGTGGTTTACACTGGACAGCCGCCTTACATGA

RI-gBol019892-XLOC_029466-16389-0
CCGGAAACTCACCTATGGCATTGCCTGCGTACCAGCACATGAACCAGATA
RI-gBol019892-XLOC_029466-16389-1
CCGGAAACTCACCTATGGCATTGCCTGCGTACCAGCACATGAACCAGATA
CONSENSUS
CCGGAAACTCACCTATGGCATTGCCTGCGTACCAGCACATGAACCAGATA

RI-gBol019892-XLOC_029466-16389-0
CATTATCAGCTCCAGCTCCAGCCTCAGCCATACCCAGTCTATTATCCAGT
RI-gBol019892-XLOC_029466-16389-1
CATTATCAGCTCCAGCTCCAGCCTCAGCCATACCCAGTCTATTATCCAGT
CONSENSUS
CATTATCAGCTCCAGCTCCAGCCTCAGCCATACCCAGTCTATTATCCAGT

RI-gBol019892-XLOC_029466-16389-0
TGAGCAGTATAATTCGAGGTATGTTCAAGCTCCGCCTGTGAGACACAACA
RI-gBol019892-XLOC_029466-16389-1
TGAGCAGTATAATTCGAGGTATGTTCAAGCTCCGCCTGTGAGACACAACA
CONSENSUS
TGAGCAGTATAATTCGAGGTATGTTCAAGCTCCGCCTGTGAGACACAACA

RI-gBol019892-XLOC_029466-16389-0
CCGCTCTAAACACCCACCATGTTGAATCCCCAGTGGCTCGCACCATCAGC
RI-gBol019892-XLOC_029466-16389-1
CCGCTCTAAACACCCACCATGTTGAATCCCCAGTGGCTCGCACCATCAGC

CONSENSUS
CCGCTCTAAACACCCACCATGTTGAATCCCCAGTGGCTCGCACCATCAGC

RI-gBol019892-XLOC_029466-16389-0
CCTCTAGCTCCAGAGTTTTCCTCGCAGGTTTACCTTACACCCAAACCCGA
RI-gBol019892-XLOC_029466-16389-1
CCTCTAGCTCCAGAGTTTTCCTCGCAGGTTTACCTTACACCCAAACCCGA
CONSENSUS
CCTCTAGCTCCAGAGTTTTCCTCGCAGGTTTACCTTACACCCAAACCCGA

RI-gBol019892-XLOC_029466-16389-0
TTCATCGGTGCAAACATCAAGTGAAGCTGGTGTTAGTACTACATGCAAAG
RI-gBol019892-XLOC_029466-16389-1
TTCATCGGTGCAAACATCAAGTGAAGCTGGTGTTAGTACTACATGCAAAG
CONSENSUS
TTCATCGGTGCAAACATCAAGTGAAGCTGGTGTTAGTACTACATGCAAAG

RI-gBol019892-XLOC_029466-16389-0
ATGATTTCATCTACAACACTGACCTCGATGACGATACAGCTCGTGCTCAG
RI-gBol019892-XLOC_029466-16389-1
ATGATTTCATCTACAACACTGACCTCGATGACGATACAGCTCGTGCTCAG
CONSENSUS
ATGATTTCATCTACAACACTGACCTCGATGACGATACAGCTCGTGCTCAG

RI-gBol019892-XLOC_029466-16389-0
CTTTACAAATCACAGCCACCCGCTCCAATAGTATCGTCTGAGTTACAAAC
RI-gBol019892-XLOC_029466-16389-1
CTTTACAAATCACAGCCACCCGCTCCAATAGTATCGTCTGAGTTACAAAC
CONSENSUS
CTTTACAAATCACAGCCACCCGCTCCAATAGTATCGTCTGAGTTACAAAC

RI-gBol019892-XLOC_029466-16389-0
AATGATGCTGGCTGAAGCTCTTGGTCAGCTAAACACCCACAAAGGTTAAT
RI-gBol019892-XLOC_029466-16389-1
AATGATGCTGGCTGAAGCTCTTGGTCAGCTAAACACCCACAAAGGTTAAT
CONSENSUS
AATGATGCTGGCTGAAGCTCTTGGTCAGCTAAACACCCACAAAGGTTAAT

RI-gBol019892-XLOC_029466-16389-0
AAGACTTGAGAGCATTAAAATCGGTTTTCATGCTGCTGAGGTTCGTTTCA
RI-gBol019892-XLOC_029466-16389-1
AAGACTTGAGAGCATTAAAATCGGTTTTCATGCTGCTGAGGTTCGTTTCA
CONSENSUS
AAGACTTGAGAGCATTAAAATCGGTTTTCATGCTGCTGAGGTTCGTTTCA

RI-gBol019892-XLOC_029466-16389-0
GGTATTTGGTTTTTACAGTTTTGTTTTCGTTTTCTTACATATATATATAC
RI-gBol019892-XLOC_029466-16389-1
GGTATTTGGTTTTTACAGTTTTGTTTTCGTTTTCTTACATATATATATAC
CONSENSUS
GGTATTTGGTTTTTACAGTTTTGTTTTCGTTTTCTTACATATATATATAC

RI-gBol019892-XLOC_029466-16389-0
ATACGTCTGCTTTTCTTGTCTCCTTGGTGATCCTTGTCCCACTTCTTTCA
RI-gBol019892-XLOC_029466-16389-1
ATACGTCTGCTTTTCTTGTCTCCTTGGTGATCCTTGTCCCACTTCTTTCA

CONSENSUS
ATACGTCTGCTTTTCTTGTCTCCTTGGTGATCCTTGTCCCACTTCTTTCA

RI-gBol019892-XLOC_029466-16389-0
CACACTTGGTTGCTGTAATATAATGATGATCCTATTTTATTTCCTTTATG
RI-gBol019892-XLOC_029466-16389-1
CACACTTGGTTGCTGTAATATAATGATGATCCTATTTTATTTCCTTTATG
CONSENSUS
CACACTTGGTTGCTGTAATATAATGATGATCCTATTTTATTTCCTTTATG

RI-gBol019892-XLOC_029466-16389-0
AAGTTTACAGGTTACATTTTATAAAGAAATATGTTAGGATATGATTACTT
RI-gBol019892-XLOC_029466-16389-1
AAGTTTACAGGTTACATTTTATAAAGAAATATGTTAGGATATGATTACTT
CONSENSUS
AAGTTTACAGGTTACATTTTATAAAGAAATATGTTAGGATATGATTACTT

RI-gBol019892-XLOC_029466-16389-0
TTTTCATTTTGGCCCAGTAAAAAGTCATTAATGAATTAAATATATGTCTC
RI-gBol019892-XLOC_029466-16389-1
TTTTCATTTTGGCCCAGTAAAAAGTCATTAATGAATTAAATATATGTCTC
CONSENSUS
TTTTCATTTTGGCCCAGTAAAAAGTCATTAATGAATTAAATATATGTCTC

RI-gBol019892-XLOC_029466-16389-0
GCTGTATTTATTGTTCTTCAAATTTGCCAATTATGTATCTTGAGATCTGC
RI-gBol019892-XLOC_029466-16389-1
GCTGTATTTATTGTTCTTCAAATTTGCCAATTATGTATCTTGAGATCTGC
CONSENSUS
GCTGTATTTATTGTTCTTCAAATTTGCCAATTATGTATCTTGAGATCTGC

RI-gBol019892-XLOC_029466-16389-0
ACCGTCCACAGGCTAACGAACCACTACAGTGTAGCACCTGATTCATACCA
RI-gBol019892-XLOC_029466-16389-1
ACCGTCCACAGGCTAACGAACCACTACAGTGTAGCACCTGATTCATACCA
CONSENSUS
ACCGTCCACAGGCTAACGAACCACTACAGTGTAGCACCTGATTCATACCA

RI-gBol019892-XLOC_029466-16389-0
CCGTCACTCCCTTCCGGAGAGATTAGTCGGCGAGTACTCGGACACTCGAT
RI-gBol019892-XLOC_029466-16389-1
CCGTCACTCCCTTCCGGAGAGATTAGTCGGCGAGTACTCGGACACTCGAT
CONSENSUS
CCGTCACTCCCTTCCGGAGAGATTAGTCGGCGAGTACTCGGACACTCGAT

RI-gBol019892-XLOC_029466-16389-0
CTGGTGAATCGAAACCGAGGTGCATCAGATCAAATCGTGAGATAAAATTT
RI-gBol019892-XLOC_029466-16389-1
CTGGTGAATCGAAACCGAGGTGCATCAGATCAAATCGTGAGATAAAATTT
CONSENSUS
CTGGTGAATCGAAACCGAGGTGCATCAGATCAAATCGTGAGATAAAATTT

RI-gBol019892-XLOC_029466-16389-0
TGAATTCACTTTCCGAATGGAGGATTGACCTGTTATTGGGTTTTAGGGAT
RI-gBol019892-XLOC_029466-16389-1
TGAATTCACTTTCCGAATGGAGGATTGACCTGTTATTGGGTTTTAGGGAT

CONSENSUS
TGAATTCACTTTCCGAATGGAGGATTGACCTGTTATTGGGTTTTAGGGAT

RI-gBol019892-XLOC_029466-16389-0
CCCTTGATAAGAAACATAAAAAAAATGAACGCGGAGCGATCGTCGTTGTG
RI-gBol019892-XLOC_029466-16389-1
CCCTTGATAAGAAACATAAAAAAAATGAACGCGGAGCGATCGTCGTTGTG
CONSENSUS
CCCTTGATAAGAAACATAAAAAAAATGAACGCGGAGCGATCGTCGTTGTG

RI-gBol019892-XLOC_029466-16389-0
AACTTCCTGATGGAGGAGAACTACCTCTTGACGGCGTTTGAGCTTCTTCA
RI-gBol019892-XLOC_029466-16389-1
AACTTCCTGATGGAGGAGAACTACCTCTTGACGGCGTTTGAGCTTCTTCA
CONSENSUS
AACTTCCTGATGGAGGAGAACTACCTCTTGACGGCGTTTGAGCTTCTTCA

RI-gBol019892-XLOC_029466-16389-0
CGAGCTCCTCGACGATGGCCGCGACGCCTAAACAATTCGTCTTAAGGAGT
RI-gBol019892-XLOC_029466-16389-1
CGAGCTCCTCGACGATGGCCGCGACGCCTAAACAATTCGTCTTAAGGAGT
CONSENSUS
CGAGCTCCTCGACGATGGCCGCGACGCCTAAACAATTCGTCTTAAGGAGT

RI-gBol019892-XLOC_029466-16389-0
TCTTCTCCGATCCTTCCCGTTCCCCTCCTGATCAGATTTCTCGGTATAGT
RI-gBol019892-XLOC_029466-16389-1
TCTTCTCCGATCCTTCCCGTTCCCCTCCTGATCAGATTTCTCGGTATAGT
CONSENSUS
TCTTCTCCGATCCTTCCCGTTCCCCTCCTGATCAGATTTCTCGGTATAGT

RI-gBol019892-XLOC_029466-16389-0
TCCATTAGAGGTTCGCCAATTCTTCGCCTCAGCGTTTGTATTGTATACTC
RI-gBol019892-XLOC_029466-16389-1
TCCATTAGAG--
CONSENSUS
TCCATTAGAG..

RI-gBol019892-XLOC_029466-16389-0
GCTGGTGAATATCCGCCTCCTCACATTGTTATTTTGTCACTTAAATTATC
RI-gBol019892-XLOC_029466-16389-1

CONSENSUS
....

RI-gBol019892-XLOC_029466-16389-0
AGATTGTGCTTGTAAGTAGGTTGAGTTGCGTTAAGTTTAGGGATCTTATC
RI-gBol019892-XLOC_029466-16389-1

CONSENSUS
....

RI-gBol019892-XLOC_029466-16389-0
TGTTGAAAGTGTAATGGTTGGGTCATGAACATGTTCTCCATGCAATTTGA
RI-gBol019892-XLOC_029466-16389-1

CONSENSUS
      ....

RI-gBol019892-XLOC_029466-16389-0
      TTGAAGGTTTGCACATGTATCCATAGTCTTTTAAGTTTGCAAAATAATTC
RI-gBol019892-XLOC_029466-16389-1

CONSENSUS
      ....

RI-gBol019892-XLOC_029466-16389-0
      ATGATTAAAGCATTTGTTGAATGGGGTGATGTTATAACCAAGAACAACCC
RI-gBol019892-XLOC_029466-16389-1

CONSENSUS
      ....

RI-gBol019892-XLOC_029466-16389-0
      GTGTCGCATTATGAGGTTTTGATTAATGTAGTTTTGGTTCAGCTTCAGGC
RI-gBol019892-XLOC_029466-16389-1

CONSENSUS
      ....

RI-gBol019892-XLOC_029466-16389-0
      TTTCTTCAACATCATGGTATTGGTTGGTAACTACTAGCTAGTAATTGTTA
RI-gBol019892-XLOC_029466-16389-1

CONSENSUS
      ....

RI-gBol019892-XLOC_029466-16389-0
      CCTTGCAATGCAGTTGTGGACCCACAAAGTTTACTGCAGGAGAAAGAAGC
RI-gBol019892-XLOC_029466-16389-1   -------------
TTGTGGACCCACAAAGTTTACTGCAGGAGAAAGAAGC
CONSENSUS
      .............TTGTGGACCCACAAAGTTTACTGCAGGAGAAAGAAGC

RI-gBol019892-XLOC_029466-16389-0
      GTTATCGGAGAAGCTAGCTATCAGTGAATATGAATTTCGTCTAGCACAGG
RI-gBol019892-XLOC_029466-16389-1
      GTTATCGGAGAAGCTAGCTATCAGTGAATATGAATTTCGTCTAGCACAGG
CONSENSUS
      GTTATCGGAGAAGCTAGCTATCAGTGAATATGAATTTCGTCTAGCACAGG

RI-gBol019892-XLOC_029466-16389-0
      AGGATATTACAAGACTGAAAACTGAAGGACAGACGAGATCTGATTGTTCG
RI-gBol019892-XLOC_029466-16389-1
      AGGATATTACAAGACTGAAAACTGAAGGACAGACGAGATCTGATTGTTCG
CONSENSUS
      AGGATATTACAAGACTGAAAACTGAAGGACAGACGAGATCTGATTGTTCG

RI-gBol019892-XLOC_029466-16389-0   ATTGACAAGCTAAAAG
RI-gBol019892-XLOC_029466-16389-1   ATTGACAAGCTAAAAG
CONSENSUS   ATTGACAAGCTAAAAG

alignment for event: A3-gBol002735-XLOC_048856-3405

```
A3-gBol002735-XLOC_048856-3405-0
      CGGATACATCAGCCTCAAAGAGCACAAAGCGATGCCTGATCAGGAGAAAG
A3-gBol002735-XLOC_048856-3405-1
      CGGATACATCAGCCTCAAAGAGCACAAAGCGATGCCTGATCAGGAGAAAG
CONSENSUS
      CGGATACATCAGCCTCAAAGAGCACAAAGCGATGCCTGATCAGGAGAAAG

A3-gBol002735-XLOC_048856-3405-0
      AGACTTGTGTACAAAATCTATTCCAGGATGGTTGCTTCTACTTGTCAACT
A3-gBol002735-XLOC_048856-3405-1
      AGACTTGTGTACAAAATCTATTCCAGGATGGTTGCTTCTACTTGTCAACT
CONSENSUS
      AGACTTGTGTACAAAATCTATTCCAGGATGGTTGCTTCTACTTGTCAACT

A3-gBol002735-XLOC_048856-3405-0
      AAACAACATTTGGCGTCTCTTCTTCAGAGCGAGTTCTCATTTTCAAAGAT
A3-gBol002735-XLOC_048856-3405-1
      AAACAACATTTGGCGTCTCTTCTTCAG-----------------------
CONSENSUS
      AAACAACATTTGGCGTCTCTTCTTCAG.......................

A3-gBol002735-XLOC_048856-3405-0
      GAGATATAGATAGAATGATCTCGTCCTACTTTTTTTTCCAGCGCACGTTA
A3-gBol002735-XLOC_048856-3405-1
      -----------------------------------------CGCACGTTA
CONSENSUS
      .........................................CGCACGTTA

A3-gBol002735-XLOC_048856-3405-0
      CACCTAGTCTCATACCAGCATCAGCAACAGAGTTGGGAAATACACAGCAG
A3-gBol002735-XLOC_048856-3405-1
      CACCTAGTCTCATACCAGCATCAGCAACAGAGTTGGGAAATACACAGCAG
CONSENSUS
      CACCTAGTCTCATACCAGCATCAGCAACAGAGTTGGGAAATACACAGCAG

A3-gBol002735-XLOC_048856-3405-0
      TCAGTCTATAACCAAAAAAGCGAAAGAGTCATGCTCCTTCTGTTTCTAAT
A3-gBol002735-XLOC_048856-3405-1
      TCAGTCTATAACCAAAAAAGCGAAAGAGTCATGCTCCTTCTGTTTCTAAT
CONSENSUS
      TCAGTCTATAACCAAAAAAGCGAAAGAGTCATGCTCCTTCTGTTTCTAAT

A3-gBol002735-XLOC_048856-3405-0 AAAG
A3-gBol002735-XLOC_048856-3405-1 AAAG
CONSENSUS                        AAAG
```

alignment for event: RI-gBol010754-XLOC_039688-15224

```
RI-gBol010754-XLOC_039688-15224-0
      CTAGTGGTATCATTGACGCAAGAAGCATTGCTAAAAGAAGCGGTGACGAT
```

RI-gBol010754-XLOC_039688-15224-1
CTAGTGGTATCATTGACGCAAGAAGCATTGCTAAAAGAAGCGGTGACGAT
CONSENSUS
CTAGTGGTATCATTGACGCAAGAAGCATTGCTAAAAGAAGCGGTGACGAT

RI-gBol010754-XLOC_039688-15224-0
TAAGCCCATCTTCGGCGCCAAGCTCTAAGTAACACACACACACACACACC
RI-gBol010754-XLOC_039688-15224-1
TAAGCCCATCTTCGGCGCCAAGCTCTAA----------------------
CONSENSUS
TAAGCCCATCTTCGGCGCCAAGCTCTAA......................

RI-gBol010754-XLOC_039688-15224-0
ATATAATATCAGAATATATTCAAGAAGAGTTTTTGCAATCTGTTACACCT
RI-gBol010754-XLOC_039688-15224-1

CONSENSUS
....

RI-gBol010754-XLOC_039688-15224-0
CGCCAGTCACACTGTGATGCTTAAATTATTTTTCCCATTTATATTATTAT
RI-gBol010754-XLOC_039688-15224-1

CONSENSUS
....

RI-gBol010754-XLOC_039688-15224-0
GACATGTTTGTATGTAAATTGTGACACCGGCGGTCATTTATTGTGTGTAC
RI-gBol010754-XLOC_039688-15224-1

CONSENSUS
....

RI-gBol010754-XLOC_039688-15224-0
ATGACAAACGGCGTCGTTTAGTGAAAAAAACACATTATTATTACAGGAGG
RI-gBol010754-XLOC_039688-15224-1
--GAGG
CONSENSUS
....GAGG

RI-gBol010754-XLOC_039688-15224-0
AGACGCATTGGTTAATCGTTAATACGCTCTCGCTCTCCCTCTCCCTCTCC
RI-gBol010754-XLOC_039688-15224-1
AGACGCATTGGTTAATCGTTAATACGCTCTCGCTCTCCCTCTCCCTCTCC
CONSENSUS
AGACGCATTGGTTAATCGTTAATACGCTCTCGCTCTCCCTCTCCCTCTCC

RI-gBol010754-XLOC_039688-15224-0
CTCTCCCTCTCCCTCTCCTCCCTCTCCCTCTCCCTCTCCCTCACCCTTTT
RI-gBol010754-XLOC_039688-15224-1
CTCTCCCTCTCCCTCTCCTCCCTCTCCCTCTCCCTCTCCCTCACCCTTTT
CONSENSUS
CTCTCCCTCTCCCTCTCCTCCCTCTCCCTCTCCCTCTCCCTCACCCTTTT

RI-gBol010754-XLOC_039688-15224-0
CCATAATCAAATCTCAACGGAAAAATTGGAAAGGAATCAATGGCGGAGTC

RI-gBol010754-XLOC_039688-15224-1
      CCATAATCAAATCTCAACGGAAAAATTGGAAAGGAATCAATGGCGGAGTC
CONSENSUS
      CCATAATCAAATCTCAACGGAAAAATTGGAAAGGAATCAATGGCGGAGTC

RI-gBol010754-XLOC_039688-15224-0
      GGAATCGGAGCATGAGCTCATCGATGAAGGAGAATTGGAGAAAATGGAGT
RI-gBol010754-XLOC_039688-15224-1
      GGAATCGGAGCATGAGCTCATCGATGAAGGAGAATTGGAGAAAATGGAGT
CONSENSUS
      GGAATCGGAGCATGAGCTCATCGATGAAGGAGAATTGGAGAAAATGGAGT

RI-gBol010754-XLOC_039688-15224-0
      GGGAAGTGAGGGAGATGGCGAAGAAGATAACAGAGTACAGGAAAACCCTA
RI-gBol010754-XLOC_039688-15224-1
      GGGAAGTGAGGGAGATGGCGAAGAAGATAACAGAGTACAGGAAAACCCTA
CONSENSUS
      GGGAAGTGAGGGAGATGGCGAAGAAGATAACAGAGTACAGGAAAACCCTA

RI-gBol010754-XLOC_039688-15224-0
      CCGGATAACCTCAGGAACACCCTTGATTCCGCGCTTTCCTCCTCTCATAG
RI-gBol010754-XLOC_039688-15224-1
      CCGGATAACCTCAGGAACACCCTTGATTCCGCGCTTTCCTCCTCTCATAG
CONSENSUS
      CCGGATAACCTCAGGAACACCCTTGATTCCGCGCTTTCCTCCTCTCATAG

RI-gBol010754-XLOC_039688-15224-0
      CTTCTTCCCCAGCATCTCGGATCCTCTTCCCTCTTCTTCTGAACGCCTCA
RI-gBol010754-XLOC_039688-15224-1
      CTTCTTCCCCAGCATCTCGGATCCTCTTCCCTCTTCTTCTGAACGCCTCA
CONSENSUS
      CTTCTTCCCCAGCATCTCGGATCCTCTTCCCTCTTCTTCTGAACGCCTCA

RI-gBol010754-XLOC_039688-15224-0   CCATCGCACCAG
RI-gBol010754-XLOC_039688-15224-1   CCATCGCACCAG
CONSENSUS   CCATCGCACCAG

alignment for event: A3-gBol014725-XLOC_035137-363

A3-gBol014725-XLOC_035137-363-0
      AAGCGGAGGGTCAGGCGAACAGAAAAGTGTCGTTGCAAAGATATCGTGAA
A3-gBol014725-XLOC_035137-363-1
      AAGCGGAGGGTCAGGCGAACAGAAAAGTGTCGTTGCAAAGATATCGTGAA
CONSENSUS
      AAGCGGAGGGTCAGGCGAACAGAAAAGTGTCGTTGCAAAGATATCGTGAA

A3-gBol014725-XLOC_035137-363-0
      AAGCGGAACGAGAGGAGAAGATTGTTTAAGACCAGAAAGGCTCCAGGAGT
A3-gBol014725-XLOC_035137-363-1 AAGCGGAACGAGAG------
ATTGTTTAAGACCAGAAAGGCTCCAGGAGT
CONSENSUS
      AAGCGGAACGAGAG......ATTGTTTAAGACCAGAAAGGCTCCAGGAGT

A3-gBol014725-XLOC_035137-363-0

GGGATCATCTAGCTCGGAGATGTATCTGAACCGTAGTCAGCCACTGATGA
A3-gBol014725-XLOC_035137-363-1
GGGATCATCTAGCTCGGAGATGTATCTGAACCGTAGTCAGCCACTGATGA
CONSENSUS
GGGATCATCTAGCTCGGAGATGTATCTGAACCGTAGTCAGCCACTGATGA

A3-gBol014725-XLOC_035137-363-0
ACGCTGCTGCATATTCACAAAACCCTAGTGGCGGCACAGGAGGAGAGCAC
A3-gBol014725-XLOC_035137-363-1
ACGCTGCTGCATATTCACAAAACCCTAGTGGCGGCACAGGAGGAGAGCAC
CONSENSUS
ACGCTGCTGCATATTCACAAAACCCTAGTGGCGGCACAGGAGGAGAGCAC

A3-gBol014725-XLOC_035137-363-0
CAGTCACCTCAAAACCAGACAAGAAGCCCCAATATTTCAGTTGATCTGAA
A3-gBol014725-XLOC_035137-363-1
CAGTCACCTCAAAACCAGACAAGAAGCCCCAATATTTCAGTTGATCTGAA
CONSENSUS
CAGTCACCTCAAAACCAGACAAGAAGCCCCAATATTTCAGTTGATCTGAA

A3-gBol014725-XLOC_035137-363-0 CTGTGATCTAAACAGTGAAG
A3-gBol014725-XLOC_035137-363-1 CTGTGATCTAAACAGTGAAG
CONSENSUS CTGTGATCTAAACAGTGAAG

alignment for event: RI-gBol044099-XLOC_001852-13538

RI-gBol044099-XLOC_001852-13538-0
ACTTGAGCAGAAATGGGTTGAAGTCACAGTAGATACACAAGTTGATGCTC
RI-gBol044099-XLOC_001852-13538-1
ACTTGAGCAGAAATGGGTTGAAGTCACAGTAGATACACAAGTTGATGCTC
CONSENSUS
ACTTGAGCAGAAATGGGTTGAAGTCACAGTAGATACACAAGTTGATGCTC

RI-gBol044099-XLOC_001852-13538-0
TCATAGATGCTGTAAACAACAACAACAACAGCACAGACAGGAGGACGATG
RI-gBol044099-XLOC_001852-13538-1
TCATAGATGCTGTAAACAACAACAACAACAGCACAGACAGGAGGACGATG
CONSENSUS
TCATAGATGCTGTAAACAACAACAACAACAGCACAGACAGGAGGACGATG

RI-gBol044099-XLOC_001852-13538-0
GTGTTTGCAAATACTGTTGAGGCGGTTGAAGCAGTAGCTGATATATTGGA
RI-gBol044099-XLOC_001852-13538-1
GTGTTTGCAAATACTGTTGAGGCGGTTGAAGCAGTAGCTGATATATTGGA
CONSENSUS
GTGTTTGCAAATACTGTTGAGGCGGTTGAAGCAGTAGCTGATATATTGGA

RI-gBol044099-XLOC_001852-13538-0
GAAAGCCAGTATCCAATGTTATCGTTATCACAAGAACCATACACTTGAAG
RI-gBol044099-XLOC_001852-13538-1
GAAAGCCAGTATCCAATGTTATCGTTATCACAAGAACCATACACTTGAAG
CONSENSUS
GAAAGCCAGTATCCAATGTTATCGTTATCACAAGAACCATACACTTGAAG

RI-gBol044099-XLOC_001852-13538-0
AACGTGCTAATATATTAGCTGATTTCAGAGAAAACGGTGGTGTATTTGTC
RI-gBol044099-XLOC_001852-13538-1
AACGTGCTAATATATTAGCTGATTTCAGAGAAAACGGTGGTGTATTTGTC
CONSENSUS
AACGTGCTAATATATTAGCTGATTTCAGAGAAAACGGTGGTGTATTTGTC

RI-gBol044099-XLOC_001852-13538-0
TGTACTGATGCAGCTGCACGTGGAGTTGATGTTCCTAATGTCTCGCATGT
RI-gBol044099-XLOC_001852-13538-1
TGTACTGATGCAGCTGCACGTGGAGTTGATGTTCCTAATGTCTCGCATGT
CONSENSUS
TGTACTGATGCAGCTGCACGTGGAGTTGATGTTCCTAATGTCTCGCATGT

RI-gBol044099-XLOC_001852-13538-0
TATTCAGGTTTTTTGTTTCCATCTCACACACAGTTTGAAGTACTCACCTC
RI-gBol044099-XLOC_001852-13538-1
TATTCAG---
CONSENSUS
TATTCAG...

RI-gBol044099-XLOC_001852-13538-0
TCTAAAAACTATTGTGTGATTTCCAGGCGGATTTTTCTAGTTGTGTGGTG
RI-gBol044099-XLOC_001852-13538-1
--TTGTGTGGTG
CONSENSUS
....TTGTGTGGTG

RI-gBol044099-XLOC_001852-13538-0
GATTTTCTTCACAGGATAGGTCGAACAGCTAGAGCTGGACAATATGGAAC
RI-gBol044099-XLOC_001852-13538-1
GATTTTCTTCACAGGATAGGTCGAACAGCTAGAGCTGGACAATATGGAAC
CONSENSUS
GATTTTCTTCACAGGATAGGTCGAACAGCTAGAGCTGGACAATATGGAAC

RI-gBol044099-XLOC_001852-13538-0
GGTGACAAGTCTATACACCGAGGCTAGTCGTGATTTAGTGGAAGCAATCC
RI-gBol044099-XLOC_001852-13538-1
GGTGACAAGTCTATACACCGAGGCTAGTCGTGATTTAGTGGAAGCAATCC
CONSENSUS
GGTGACAAGTCTATACACCGAGGCTAGTCGTGATTTAGTGGAAGCAATCC

RI-gBol044099-XLOC_001852-13538-0
GTGAAGCAGTGAAAGACGGGTCAGCCAGTGGTATACATTCTGTCAAACTT
RI-gBol044099-XLOC_001852-13538-1
GTGAAGCAGTGAAAGACGGGTCAGCCAGTGGTATACATTCTGTCAAACTT
CONSENSUS
GTGAAGCAGTGAAAGACGGGTCAGCCAGTGGTATACATTCTGTCAAACTT

RI-gBol044099-XLOC_001852-13538-0 ATTAATTTG
RI-gBol044099-XLOC_001852-13538-1 ATTAATTTG
CONSENSUS ATTAATTTG

alignment for event: A3-gBol044099-XLOC_001852-13543

A3-gBol044099-XLOC_001852-13543-0
ACTTGAGCAGAAATGGGTTGAAGTCACAGTAGATACACAAGTTGATGCTC
A3-gBol044099-XLOC_001852-13543-1
ACTTGAGCAGAAATGGGTTGAAGTCACAGTAGATACACAAGTTGATGCTC
CONSENSUS
ACTTGAGCAGAAATGGGTTGAAGTCACAGTAGATACACAAGTTGATGCTC

A3-gBol044099-XLOC_001852-13543-0
TCATAGATGCTGTAAACAACAACAACAACAGCACAGACAGGAGGACGATG
A3-gBol044099-XLOC_001852-13543-1
TCATAGATGCTGTAAACAACAACAACAACAGCACAGACAGGAGGACGATG
CONSENSUS
TCATAGATGCTGTAAACAACAACAACAACAGCACAGACAGGAGGACGATG

A3-gBol044099-XLOC_001852-13543-0
GTGTTTGCAAATACTGTTGAGGCGGTTGAAGCAGTAGCTGATATATTGGA
A3-gBol044099-XLOC_001852-13543-1
GTGTTTGCAAATACTGTTGAGGCGGTTGAAGCAGTAGCTGATATATTGGA
CONSENSUS
GTGTTTGCAAATACTGTTGAGGCGGTTGAAGCAGTAGCTGATATATTGGA

A3-gBol044099-XLOC_001852-13543-0
GAAAGCCAGTATCCAATGTTATCGTTATCACAAGAACCATACACTTGAAG
A3-gBol044099-XLOC_001852-13543-1
GAAAGCCAGTATCCAATGTTATCGTTATCACAAGAACCATACACTTGAAG
CONSENSUS
GAAAGCCAGTATCCAATGTTATCGTTATCACAAGAACCATACACTTGAAG

A3-gBol044099-XLOC_001852-13543-0
AACGTGCTAATATATTAGCTGATTTCAGAGAAAACGGTGGTGTATTTGTC
A3-gBol044099-XLOC_001852-13543-1
AACGTGCTAATATATTAGCTGATTTCAGAGAAAACGGTGGTGTATTTGTC
CONSENSUS
AACGTGCTAATATATTAGCTGATTTCAGAGAAAACGGTGGTGTATTTGTC

A3-gBol044099-XLOC_001852-13543-0
TGTACTGATGCAGCTGCACGTGGAGTTGATGTTCCTAATGTCTCGCATGT
A3-gBol044099-XLOC_001852-13543-1
TGTACTGATGCAGCTGCACGTGGAGTTGATGTTCCTAATGTCTCGCATGT
CONSENSUS
TGTACTGATGCAGCTGCACGTGGAGTTGATGTTCCTAATGTCTCGCATGT

A3-gBol044099-XLOC_001852-13543-0
TATTCAGGCGGATTTTTCTAGTTGTGTGGTGGATTTTCTTCACAGGATAG
A3-gBol044099-XLOC_001852-13543-1 TATTCAG-------------
TTGTGTGGTGGATTTTCTTCACAGGATAG
CONSENSUS
TATTCAG.............TTGTGTGGTGGATTTTCTTCACAGGATAG

A3-gBol044099-XLOC_001852-13543-0
GTCGAACAGCTAGAGCTGGACAATATGGAACGGTGACAAGTCTATACACC
A3-gBol044099-XLOC_001852-13543-1
GTCGAACAGCTAGAGCTGGACAATATGGAACGGTGACAAGTCTATACACC
CONSENSUS
GTCGAACAGCTAGAGCTGGACAATATGGAACGGTGACAAGTCTATACACC

A3-gBol044099-XLOC_001852-13543-0
      GAGGCTAGTCGTGATTTAGTGGAAGCAATCCGTGAAGCAGTGAAAGACGG
A3-gBol044099-XLOC_001852-13543-1
      GAGGCTAGTCGTGATTTAGTGGAAGCAATCCGTGAAGCAGTGAAAGACGG
CONSENSUS
      GAGGCTAGTCGTGATTTAGTGGAAGCAATCCGTGAAGCAGTGAAAGACGG

A3-gBol044099-XLOC_001852-13543-0
      GTCAGCCAGTGGTATACATTCTGTCAAACTTATTAATTTG
A3-gBol044099-XLOC_001852-13543-1
      GTCAGCCAGTGGTATACATTCTGTCAAACTTATTAATTTG
CONSENSUS
      GTCAGCCAGTGGTATACATTCTGTCAAACTTATTAATTTG

alignment for event: A3-gBol021100-XLOC_027974-628

A3-gBol021100-XLOC_027974-628-0
      TTACAAGGAGACAGAAGTTGCTTATTTCCCGAGGACCCTGGTCAG-----
A3-gBol021100-XLOC_027974-628-1
      TTACAAGGAGACAGAAGTTGCTTATTTCCCGAGGACCCTGGTCAGCTCGT
CONSENSUS
      TTACAAGGAGACAGAAGTTGCTTATTTCCCGAGGACCCTGGTCAG.....

A3-gBol021100-XLOC_027974-628-0 ----------
TGCATGAAGAGAAGAGGAGCTTCGTCTTCGTCCACGGAAA
A3-gBol021100-XLOC_027974-628-1
      GAATAATCAGTGCATGAAGAGAAGAGGAGCTTCGTCTTCGTCCACGGAAA
CONSENSUS
      ..........TGCATGAAGAGAAGAGGAGCTTCGTCTTCGTCCACGGAAA

A3-gBol021100-XLOC_027974-628-0
      ATCAAAATGCAAACGAAGAACCTAAGAAAGACTTGAGAAGTCGAGGTTTA
A3-gBol021100-XLOC_027974-628-1
      ATCAAAATGCAAACGAAGAACCTAAGAAAGACTTGAGAAGTCGAGGTTTA
CONSENSUS
      ATCAAAATGCAAACGAAGAACCTAAGAAAGACTTGAGAAGTCGAGGTTTA

A3-gBol021100-XLOC_027974-628-0
      TGTCTTGTTCCAATCTCCTGCACACTCCAAGTCGGCAGCGACAACGGCGC
A3-gBol021100-XLOC_027974-628-1
      TGTCTTGTTCCAATCTCCTGCACACTCCAAGTCGGCAGCGACAACGGCGC
CONSENSUS
      TGTCTTGTTCCAATCTCCTGCACACTCCAAGTCGGCAGCGACAACGGCGC

A3-gBol021100-XLOC_027974-628-0
      CAACTATTGGGCTTCGGCGCTCGGCTCGGCCGGTTTCCAGTGAAAGCCAA
A3-gBol021100-XLOC_027974-628-1
      CAACTATTGGGCTTCGGCGCTCGGCTCGGCCGGTTTCCAGTGAAAGCCAA
CONSENSUS
      CAACTATTGGGCTTCGGCGCTCGGCTCGGCCGGTTTCCAGTGAAAGCCAA

A3-gBol021100-XLOC_027974-628-0
      TGAAACTATGCGACGCGGAATTCCAACCTTAGAGAAAATGATATAACATC

A3-gBol021100-XLOC_027974-628-1
      TGAAACTATGCGACGCGGAATTCCAACCTTAGAGAAAATGATATAACATC
CONSENSUS
      TGAAACTATGCGACGCGGAATTCCAACCTTAGAGAAAATGATATAACATC

A3-gBol021100-XLOC_027974-628-0
      ACGAGCAGTCAGTCTACTGAGAAACCAACAAATTGAGTTTCCAAGGCTGA
A3-gBol021100-XLOC_027974-628-1
      ACGAGCAGTCAGTCTACTGAGAAACCAACAAATTGAGTTTCCAAGGCTGA
CONSENSUS
      ACGAGCAGTCAGTCTACTGAGAAACCAACAAATTGAGTTTCCAAGGCTGA

A3-gBol021100-XLOC_027974-628-0
      TTGCTCATGATGCTATATACAAAGTACTTCTTTTATTAATACTTTGCTAG
A3-gBol021100-XLOC_027974-628-1
      TTGCTCATGATGCTATATACAAAGTACTTCTTTTATTAATACTTTGCTAG
CONSENSUS
      TTGCTCATGATGCTATATACAAAGTACTTCTTTTATTAATACTTTGCTAG

A3-gBol021100-XLOC_027974-628-0
      AGAATTTGCGTTTAGCATTTGGTGAATATGCGTTTAGGCTTTCATCATTC
A3-gBol021100-XLOC_027974-628-1
      AGAATTTGCGTTTAGCATTTGGTGAATATGCGTTTAGGCTTTCATCATTC
CONSENSUS
      AGAATTTGCGTTTAGCATTTGGTGAATATGCGTTTAGGCTTTCATCATTC

A3-gBol021100-XLOC_027974-628-0
      GTTGTGTTATCAGATGATCAAGCGTTATGTCCTTGCTTTAGGAAACTGTT
A3-gBol021100-XLOC_027974-628-1
      GTTGTGTTATCAGATGATCAAGCGTTATGTCCTTGCTTTAGGAAACTGTT
CONSENSUS
      GTTGTGTTATCAGATGATCAAGCGTTATGTCCTTGCTTTAGGAAACTGTT

A3-gBol021100-XLOC_027974-628-0
      TGTTATATTATCGGTTGTAACTTGTAATTTGTCGTTACTTAGTTTTTTTT
A3-gBol021100-XLOC_027974-628-1
      TGTTATATTATCGGTTGTAACTTGTAATTTGTCGTTACTTAGTTTTTTTT
CONSENSUS
      TGTTATATTATCGGTTGTAACTTGTAATTTGTCGTTACTTAGTTTTTTTT

A3-gBol021100-XLOC_027974-628-0
      CTTTTCATTACTTCCATGTGATTTGTGTCGTCACATTTGCTAATATGATT
A3-gBol021100-XLOC_027974-628-1
      CTTTTCATTACTTCCATGTGATTTGTGTCGTCACATTTGCTAATATGATT
CONSENSUS
      CTTTTCATTACTTCCATGTGATTTGTGTCGTCACATTTGCTAATATGATT

A3-gBol021100-XLOC_027974-628-0 ATACGTACTG
A3-gBol021100-XLOC_027974-628-1 ATACGTACTG
CONSENSUS   ATACGTACTG

alignment for event: A5-X-XLOC_027911-15070

A5-X-XLOC_027911-15070-0

GTAGAAGAAGCGGAGACGGTGTCAACAATGTCGTGTAATGATTTTAATTG
A5-X-XLOC_027911-15070-1
GTAGAAGAAGCGGAGACGGTGTCAACAATGTCGTGTAATGATTTTAATTG
CONSENSUS
GTAGAAGAAGCGGAGACGGTGTCAACAATGTCGTGTAATGATTTTAATTG

A5-X-XLOC_027911-15070-0
CTCAAGGCTGCAACCACATGACTTGCCGGTAAGAAATACACCCATAGAAC
A5-X-XLOC_027911-15070-1
CTCAAGGCTGCAACCACATGACTTGCCGGTAAGAAATACACCCATAGAAC
CONSENSUS
CTCAAGGCTGCAACCACATGACTTGCCGGTAAGAAATACACCCATAGAAC

A5-X-XLOC_027911-15070-0
GTTTCATATCTATATAATGTTTGGCTTTGTGGTTTCTTATATCAATGTTG
A5-X-XLOC_027911-15070-1
GTTTCATATCTATATAATGTTTGGCTTTGTGGTTTCTTATATCAATGTTG
CONSENSUS
GTTTCATATCTATATAATGTTTGGCTTTGTGGTTTCTTATATCAATGTTG

A5-X-XLOC_027911-15070-0
AGATATCTCTTTGTGTTCTTTGTATGTGTGGGCACGAGTTTTGCTACTGT
A5-X-XLOC_027911-15070-1
AGATATCTCTTTGTGTTCTTTGTATGTGTGGGCACGAGTTTTGCTACTGT
CONSENSUS
AGATATCTCTTTGTGTTCTTTGTATGTGTGGGCACGAGTTTTGCTACTGT

A5-X-XLOC_027911-15070-0
TGCGGAGCAGAGTACAGTGAAGGACAACAGAGTTGCACATGTGCTTTGTG
A5-X-XLOC_027911-15070-1
TGCGGAGCAGAGTACAGTGAAGGACAACAGAGTTGCACATGTGCTTTGTG
CONSENSUS
TGCGGAGCAGAGTACAGTGAAGGACAACAGAGTTGCACATGTGCTTTGTG

A5-X-XLOC_027911-15070-0
GTGGATTGTTGCAGGAAGTTTACAGAGGAAAGCAAAATATG---------
A5-X-XLOC_027911-15070-1
GTGGATTGTTGCAGGAAGTTTACAGAGGAAAGCAAAATATGGTATGCTTG
CONSENSUS
GTGGATTGTTGCAGGAAGTTTACAGAGGAAAGCAAAATATG.........

A5-X-XLOC_027911-15070-0 ------------------------
GTTTTGAGATATCAGAAGGAGAATCA
A5-X-XLOC_027911-15070-1
GAGCAAATATGCTGAGCTAGAGAGGTTTTGAGATATCAGAAGGAGAATCA
CONSENSUS
........................GTTTTGAGATATCAGAAGGAGAATCA

A5-X-XLOC_027911-15070-0
TAGAGGACATGAGCTTTATATGAGCGACTATTGGACCGCACAAAGCATTA
A5-X-XLOC_027911-15070-1
TAGAGGACATGAGCTTTATATGAGCGACTATTGGACCGCACAAAGCATTA
CONSENSUS
TAGAGGACATGAGCTTTATATGAGCGACTATTGGACCGCACAAAGCATTA

A5-X-XLOC_027911-15070-0

CAAGGTGTGGCGGTGTAGGTTAGTTTTGCAAAGTTTGAAGCTTATGCTGC
A5-X-XLOC_027911-15070-1
CAAGGTGTGGCGGTGTAGGTTAGTTTTGCAAAGTTTGAAGCTTATGCTGC
CONSENSUS
CAAGGTGTGGCGGTGTAGGTTAGTTTTGCAAAGTTTGAAGCTTATGCTGC

A5-X-XLOC_027911-15070-0
GGATCAAGAAGAAGACGAGGAAGAGGAAGACCAAGAAGAAATAAATGCTA
A5-X-XLOC_027911-15070-1
GGATCAAGAAGAAGACGAGGAAGAGGAAGACCAAGAAGAAATAAATGCTA
CONSENSUS
GGATCAAGAAGAAGACGAGGAAGAGGAAGACCAAGAAGAAATAAATGCTA

A5-X-XLOC_027911-15070-0
TTGAACTCAAGAAAGAGTGCATCAGACGTGCCTGAG
A5-X-XLOC_027911-15070-1
TTGAACTCAAGAAAGAGTGCATCAGACGTGCCTGAG
CONSENSUS
TTGAACTCAAGAAAGAGTGCATCAGACGTGCCTGAG

alignment for event: A3-gBol008838-XLOC_041898-7232

A3-gBol008838-XLOC_041898-7232-0
AAGCGAGCGAATAAATCAAAAAGAAGATTATAAATTTATTATAATATATA
A3-gBol008838-XLOC_041898-7232-1
AAGCGAGCGAATAAATCAAAAAGAAGATTATAAATTTATTATAATATATA
CONSENSUS
AAGCGAGCGAATAAATCAAAAAGAAGATTATAAATTTATTATAATATATA

A3-gBol008838-XLOC_041898-7232-0
AACTCGACTCGAGACAGCATCAATCAATCTGCTTTTCAAAAAATCAAAAT
A3-gBol008838-XLOC_041898-7232-1
AACTCGACTCGAGACAGCATCAATCAATCTGCTTTTCAAAAAATCAAAAT
CONSENSUS
AACTCGACTCGAGACAGCATCAATCAATCTGCTTTTCAAAAAATCAAAAT

A3-gBol008838-XLOC_041898-7232-0
TTGGATTTGGGAATTTTTCCGGACGAGTAAAAGCTTCGTGCTTGGTGCGT
A3-gBol008838-XLOC_041898-7232-1
TTGGATTTGGGAATTTTTCCGGACGAGTAAAAGCTTCGTGCTTGGTGCGT
CONSENSUS
TTGGATTTGGGAATTTTTCCGGACGAGTAAAAGCTTCGTGCTTGGTGCGT

A3-gBol008838-XLOC_041898-7232-0
CGATTGTGTGTGTTTCCTTCTCTCCTTTCGCCTGCTCGATCTCTAGGGGA
A3-gBol008838-XLOC_041898-7232-1
CGATTGTGTGTGTTTCCTTCTCTCCTTTCGCCTGCTCGATCTCTAGGGGA
CONSENSUS
CGATTGTGTGTGTTTCCTTCTCTCCTTTCGCCTGCTCGATCTCTAGGGGA

A3-gBol008838-XLOC_041898-7232-0
TTTTTCATCTGATCATCTTCTTCTCTGTCGCTTTTTGAAGTGCAGAAGTT
A3-gBol008838-XLOC_041898-7232-1
TTTTTCATCTGATCATCTTCTTCTCTGTCGCTTTTTGAAG-----AAGTT

CONSENSUS
TTTTTCATCTGATCATCTTCTTCTCTGTCGCTTTTTGAAG.....AAGTT

A3-gBol008838-XLOC_041898-7232-0
TTTGTCTTTTATAGTTGAAGTGGGTTTTGAACAAAACTTATTCAGATGGA
A3-gBol008838-XLOC_041898-7232-1
TTTGTCTTTTATAGTTGAAGTGGGTTTTGAACAAAACTTATTCAGATGGA
CONSENSUS
TTTGTCTTTTATAGTTGAAGTGGGTTTTGAACAAAACTTATTCAGATGGA

A3-gBol008838-XLOC_041898-7232-0
TAATGGATGATGAAAGATATGGACTTTGAATAAATAGTACACGAATTTTC
A3-gBol008838-XLOC_041898-7232-1
TAATGGATGATGAAAGATATGGACTTTGAATAAATAGTACACGAATTTTC
CONSENSUS
TAATGGATGATGAAAGATATGGACTTTGAATAAATAGTACACGAATTTTC

A3-gBol008838-XLOC_041898-7232-0
TTTGTGGGGGCGTCTGATTGGCTCGAGCTTCTGGGATTATCATCACACGT
A3-gBol008838-XLOC_041898-7232-1
TTTGTGGGGGCGTCTGATTGGCTCGAGCTTCTGGGATTATCATCACACGT
CONSENSUS
TTTGTGGGGGCGTCTGATTGGCTCGAGCTTCTGGGATTATCATCACACGT

A3-gBol008838-XLOC_041898-7232-0
GTCGAGTTGCAGCATAGACAAATGGAGGACGATGGAGAAATGGGGATGTT
A3-gBol008838-XLOC_041898-7232-1
GTCGAGTTGCAGCATAGACAAATGGAGGACGATGGAGAAATGGGGATGTT
CONSENSUS
GTCGAGTTGCAGCATAGACAAATGGAGGACGATGGAGAAATGGGGATGTT

A3-gBol008838-XLOC_041898-7232-0
CAGCAAGAATCCACTTGAAATGGTTGAGTCTTGCGGGTTCATCTTCACTT
A3-gBol008838-XLOC_041898-7232-1
CAGCAAGAATCCACTTGAAATGGTTGAGTCTTGCGGGTTCATCTTCACTT
CONSENSUS
CAGCAAGAATCCACTTGAAATGGTTGAGTCTTGCGGGTTCATCTTCACTT

A3-gBol008838-XLOC_041898-7232-0
GCTTTGTTGCTGCCCTTATTGGTATTCTCACCATAGCCTACACTGCTTCT
A3-gBol008838-XLOC_041898-7232-1
GCTTTGTTGCTGCCCTTATTGGTATTCTCACCATAGCCTACACTGCTTCT
CONSENSUS
GCTTTGTTGCTGCCCTTATTGGTATTCTCACCATAGCCTACACTGCTTCT

A3-gBol008838-XLOC_041898-7232-0
CAGTGGCGAAGGAATATCAACTTAAGCTGGACGAAAGCCATCGCCAGGTC
A3-gBol008838-XLOC_041898-7232-1
CAGTGGCGAAGGAATATCAACTTAAGCTGGACGAAAGCCATCGCCAGGTC
CONSENSUS
CAGTGGCGAAGGAATATCAACTTAAGCTGGACGAAAGCCATCGCCAGGTC

A3-gBol008838-XLOC_041898-7232-0
GAAGAAAAACCCAAAGGCACGGCACAAGACTCCTGTTGCCCCGCATAGCT
A3-gBol008838-XLOC_041898-7232-1
GAAGAAAAACCCAAAGGCACGGCACAAGACTCCTGTTGCCCCGCATAGCT

CONSENSUS
GAAGAAAAACCCAAAGGCACGGCACAAGACTCCTGTTGCCCCGCATAGCT

A3-gBol008838-XLOC_041898-7232-0
GGGAACGCGACTCTGTATCCCGTGCCAAGAACTTGAACTGCTCTTTGTGC
A3-gBol008838-XLOC_041898-7232-1
GGGAACGCGACTCTGTATCCCGTGCCAAGAACTTGAACTGCTCTTTGTGC
CONSENSUS
GGGAACGCGACTCTGTATCCCGTGCCAAGAACTTGAACTGCTCTTTGTGC

A3-gBol008838-XLOC_041898-7232-0
TTGAAGTCGATGTCGCCGTCTCAGACAATTGTAGCTTCAGAAAGTGTTAT
A3-gBol008838-XLOC_041898-7232-1
TTGAAGTCGATGTCGCCGTCTCAGACAATTGTAGCTTCAGAAAGTGTTAT
CONSENSUS
TTGAAGTCGATGTCGCCGTCTCAGACAATTGTAGCTTCAGAAAGTGTTAT

A3-gBol008838-XLOC_041898-7232-0
CCACAGGTGCACGATCTGTGGAGCGGCAGCTCATTTTAGTTGCTCTTCAA
A3-gBol008838-XLOC_041898-7232-1
CCACAGGTGCACGATCTGTGGAGCGGCAGCTCATTTTAGTTGCTCTTCAA
CONSENSUS
CCACAGGTGCACGATCTGTGGAGCGGCAGCTCATTTTAGTTGCTCTTCAA

A3-gBol008838-XLOC_041898-7232-0
GTGCCCCTAAAGATTGCAAATGCGTCTCCATGGTTGGATACGAGCATGTG
A3-gBol008838-XLOC_041898-7232-1
GTGCCCCTAAAGATTGCAAATGCGTCTCCATGGTTGGATACGAGCATGTG
CONSENSUS
GTGCCCCTAAAGATTGCAAATGCGTCTCCATGGTTGGATACGAGCATGTG

A3-gBol008838-XLOC_041898-7232-0
GTGCACCAGTGGGCGGTGCGGTGGACGGAAGGTGCTGACCAGTCTGACGA
A3-gBol008838-XLOC_041898-7232-1
GTGCACCAGTGGGCGGTGCGGTGGACGGAAGGTGCTGACCAGTCTGACGA
CONSENSUS
GTGCACCAGTGGGCGGTGCGGTGGACGGAAGGTGCTGACCAGTCTGACGA

A3-gBol008838-XLOC_041898-7232-0
ATCCTCGTTTTGTAGCTACTGTGACGAGTCGTGTAGTAGCTCCTTTCTTG
A3-gBol008838-XLOC_041898-7232-1
ATCCTCGTTTTGTAGCTACTGTGACGAGTCGTGTAGTAGCTCCTTTCTTG
CONSENSUS
ATCCTCGTTTTGTAGCTACTGTGACGAGTCGTGTAGTAGCTCCTTTCTTG

A3-gBol008838-XLOC_041898-7232-0
GGGGTTCTCCTGTATGGTGCTGCTTGTGGTGTCAACGTCTTGTCCATGTC
A3-gBol008838-XLOC_041898-7232-1
GGGGTTCTCCTGTATGGTGCTGCTTGTGGTGTCAACGTCTTGTCCATGTC
CONSENSUS
GGGGTTCTCCTGTATGGTGCTGCTTGTGGTGTCAACGTCTTGTCCATGTC

A3-gBol008838-XLOC_041898-7232-0
GACTGTCACAGTAATATGTCAAATGAAACAGGTGACGTTTGTGATCTAGG
A3-gBol008838-XLOC_041898-7232-1
GACTGTCACAGTAATATGTCAAATGAAACAGGTGACGTTTGTGATCTAGG

CONSENSUS
GACTGTCACAGTAATATGTCAAATGAAACAGGTGACGTTTGTGATCTAGG

A3-gBol008838-XLOC_041898-7232-0
CCCTCTTAGAAGGCTAGTCTTGTGTCCTCTCTATGTTAAGGAGTTAACAC
A3-gBol008838-XLOC_041898-7232-1
CCCTCTTAGAAGGCTAGTCTTGTGTCCTCTCTATGTTAAGGAGTTAACAC
CONSENSUS
CCCTCTTAGAAGGCTAGTCTTGTGTCCTCTCTATGTTAAGGAGTTAACAC

A3-gBol008838-XLOC_041898-7232-0
GGAATCCTTCTGGAGGGTTTTTGAGCACGATCACGCATGGTGCTAACGAA
A3-gBol008838-XLOC_041898-7232-1
GGAATCCTTCTGGAGGGTTTTTGAGCACGATCACGCATGGTGCTAACGAA
CONSENSUS
GGAATCCTTCTGGAGGGTTTTTGAGCACGATCACGCATGGTGCTAACGAA

A3-gBol008838-XLOC_041898-7232-0
CTTGCATCTACGGTCCGTGCCAGTATCAGGATTCAAAGCAAAAAATACAA
A3-gBol008838-XLOC_041898-7232-1
CTTGCATCTACGGTCCGTGCCAGTATCAGGATTCAAAGCAAAAAATACAA
CONSENSUS
CTTGCATCTACGGTCCGTGCCAGTATCAGGATTCAAAGCAAAAAATACAA

A3-gBol008838-XLOC_041898-7232-0
GCAAGGTAATGAAACTTCGGTTGACTCGGATAATAGTGGTAGCAATTGTG
A3-gBol008838-XLOC_041898-7232-1
GCAAGGTAATGAAACTTCGGTTGACTCGGATAATAGTGGTAGCAATTGTG
CONSENSUS
GCAAGGTAATGAAACTTCGGTTGACTCGGATAATAGTGGTAGCAATTGTG

A3-gBol008838-XLOC_041898-7232-0
ATGAATCGACGGAAAGCACAGCTGATACAAGTCCAGCTGTTGTTAATGGC
A3-gBol008838-XLOC_041898-7232-1
ATGAATCGACGGAAAGCACAGCTGATACAAGTCCAGCTGTTGTTAATGGC
CONSENSUS
ATGAATCGACGGAAAGCACAGCTGATACAAGTCCAGCTGTTGTTAATGGC

A3-gBol008838-XLOC_041898-7232-0
ACCCATTCCGCGTTGGAAAACTCAAGCAGCGTTGTGAATGGAGGTTCCTC
A3-gBol008838-XLOC_041898-7232-1
ACCCATTCCGCGTTGGAAAACTCAAGCAGCGTTGTGAATGGAGGTTCCTC
CONSENSUS
ACCCATTCCGCGTTGGAAAACTCAAGCAGCGTTGTGAATGGAGGTTCCTC

A3-gBol008838-XLOC_041898-7232-0
TAACGAGGACAGCGATGAGAAGAAGCCTAGTGTTAAAAGAAGCGGTTCCT
A3-gBol008838-XLOC_041898-7232-1
TAACGAGGACAGCGATGAGAAGAAGCCTAGTGTTAAAAGAAGCGGTTCCT
CONSENSUS
TAACGAGGACAGCGATGAGAAGAAGCCTAGTGTTAAAAGAAGCGGTTCCT

A3-gBol008838-XLOC_041898-7232-0
TTGGTAAGAAAGATAAATATCAGGGACTAAGGTCCAAACTTAAGTATGAG
A3-gBol008838-XLOC_041898-7232-1
TTGGTAAGAAAGATAAATATCAGGGACTAAGGTCCAAACTTAAGTATGAG

CONSENSUS
TTGGTAAGAAAGATAAATATCAGGGACTAAGGTCCAAACTTAAGTATGAG

A3-gBol008838-XLOC_041898-7232-0
CTAGCTGATTTGCCTCCAGATGCAAGACCGTTGTTGGTTTTCATTAACAA
A3-gBol008838-XLOC_041898-7232-1
CTAGCTGATTTGCCTCCAGATGCAAGACCGTTGTTGGTTTTCATTAACAA
CONSENSUS
CTAGCTGATTTGCCTCCAGATGCAAGACCGTTGTTGGTTTTCATTAACAA

A3-gBol008838-XLOC_041898-7232-0
AAAGAGTGGGGCTCAACGAGGTGATTCTCTTCGGCAGCGTCTTAATCTTC
A3-gBol008838-XLOC_041898-7232-1
AAAGAGTGGGGCTCAACGAGGTGATTCTCTTCGGCAGCGTCTTAATCTTC
CONSENSUS
AAAGAGTGGGGCTCAACGAGGTGATTCTCTTCGGCAGCGTCTTAATCTTC

A3-gBol008838-XLOC_041898-7232-0 TTCTAAATCCCGTGCAG
A3-gBol008838-XLOC_041898-7232-1 TTCTAAATCCCGTGCAG
CONSENSUS TTCTAAATCCCGTGCAG

alignment for event: A5-gBol031377-XLOC_016347-16369

A5-gBol031377-XLOC_016347-16369-0
CTAAGCGGGAAGCGAGTTGGGATACTTGGACTAGGGAGCATAGGGTCTTT
A5-gBol031377-XLOC_016347-16369-1
CTAAGCGGGAAGCGAGTTGGGATACTTGGACTAGGGAGCATAGGGTCTTT
CONSENSUS
CTAAGCGGGAAGCGAGTTGGGATACTTGGACTAGGGAGCATAGGGTCTTT

A5-gBol031377-XLOC_016347-16369-0
TATTGCTAAAAGACTCGAACCCTTTGGCTGCATCATCTCTTACAACTCGA
A5-gBol031377-XLOC_016347-16369-1
TATTGCTAAAAGACTCGAACCCTTTGGCTGCATCATCTCTTACAACTCGA
CONSENSUS
TATTGCTAAAAGACTCGAACCCTTTGGCTGCATCATCTCTTACAACTCGA

A5-gBol031377-XLOC_016347-16369-0
CGATTCAGAAACAGAGCATCACTTACCCGTACTACCCGGACGTTCTCTCC
A5-gBol031377-XLOC_016347-16369-1
CGATTCAGAAACAGAGCATCACTTACCCGTACTACCCGGACGTTCTCTCC
CONSENSUS
CGATTCAGAAACAGAGCATCACTTACCCGTACTACCCGGACGTTCTCTCC

A5-gBol031377-XLOC_016347-16369-0
TTAGCAGCAAACAACGAAGTCATCATCCTCTGCTGCGCTCTGAATGATCA
A5-gBol031377-XLOC_016347-16369-1
TTAGCAGCAAACAACGAAGTCATCATCCTCTGCTGCGCTCTGAATGATCA
CONSENSUS
TTAGCAGCAAACAACGAAGTCATCATCCTCTGCTGCGCTCTGAATGATCA

A5-gBol031377-XLOC_016347-16369-0
GATGCGCCACATTGTGAACAGAGAAGTGATGGAGTCGCTTGGGAAGAACG
A5-gBol031377-XLOC_016347-16369-1

```
      GATGCGCCACATTGTGAACAGAGAAGTGATGGAGTCGCTTGGGAAGAACG
CONSENSUS
      GATGCGCCACATTGTGAACAGAGAAGTGATGGAGTCGCTTGGGAAGAACG

A5-gBol031377-XLOC_016347-16369-0
      GGGTTATAATCAATGTGGGACGGGGAGGGCTGATTGATGAGAAGGAGATG
A5-gBol031377-XLOC_016347-16369-1
      GGGTTATAATCAATGTGGGACGGGGAGGGCTGATTGATGAGAAGGAGATG
CONSENSUS
      GGGTTATAATCAATGTGGGACGGGGAGGGCTGATTGATGAGAAGGAGATG

A5-gBol031377-XLOC_016347-16369-0
      GTTAAGTGTCTTGTCGATGGTGTGATTGGTGGTGCTGGTTTAGATGTGTT
A5-gBol031377-XLOC_016347-16369-1
      GTTAAGTGTCTTGTCGATGGTGTGATTGGTGGTGCTGGTTTAGATGTGTT
CONSENSUS
      GTTAAGTGTCTTGTCGATGGTGTGATTGGTGGTGCTGGTTTAGATGTGTT

A5-gBol031377-XLOC_016347-16369-0
      TGAGAAAGAACCGGGAGGTCCTGAGGAGTTGTTTGGTTTGGACAATGTTG
A5-gBol031377-XLOC_016347-16369-1
      TGAGAAAGAACCGGGAGGTCCTGAGGAGTTGTTTGGTTTGGACAATGTTG
CONSENSUS
      TGAGAAAGAACCGGGAGGTCCTGAGGAGTTGTTTGGTTTGGACAATGTTG

A5-gBol031377-XLOC_016347-16369-0
      TCTTGTCTCCACATTGTGCGATAGCCACGCCAGGGTCTTTGAACAATATT
A5-gBol031377-XLOC_016347-16369-1
      TCTTGTCTCCACATTGTGCGATAGCCACGCCAGGGTCTTTGAACAATATT
CONSENSUS
      TCTTGTCTCCACATTGTGCGATAGCCACGCCAGGGTCTTTGAACAATATT

A5-gBol031377-XLOC_016347-16369-0
      GCGCAGCTTACTTTGGCTAACTTGAAGGCGTTTTTCTCGAACCAGCCTTT
A5-gBol031377-XLOC_016347-16369-1
      GCGCAGCTTACTTTGGCTAACTTGAAGGCGTTTTTCTCGAACCAGCCTTT
CONSENSUS
      GCGCAGCTTACTTTGGCTAACTTGAAGGCGTTTTTCTCGAACCAGCCTTT

A5-gBol031377-XLOC_016347-16369-0
      GATTTCTCCG----------------------------------------
A5-gBol031377-XLOC_016347-16369-1
      GATTTCTCCGGTTCGGTTAGATTGAGAGATCGTCCGGTTTGATTAAAAGG
CONSENSUS
      GATTTCTCCG........................................

A5-gBol031377-XLOC_016347-16369-0
      --------------------------------------------------
A5-gBol031377-XLOC_016347-16369-1
      GCTATAATAATTTAGTTATTGAACAATATGATTGAGTAAGGGGTGTATTG
CONSENSUS
      ..................................................

A5-gBol031377-XLOC_016347-16369-0
      --------------------------------------------------
A5-gBol031377-XLOC_016347-16369-1
```

TTGATCATTGACTCCCCTATATAGGAGAAGACAACATACAAAAATCAAGA
CONSENSUS
....

A5-gBol031377-XLOC_016347-16369-0

A5-gBol031377-XLOC_016347-16369-1
TGCTAATGTGTAAATTGTTGGAGCGACCTGTGGAGGTTTTAAATAAAAAT
CONSENSUS
....

A5-gBol031377-XLOC_016347-16369-0 ------
GAGAGGAAATGCGAGGTTGGGACCGGCGAGGTAAGCGGACAGAG
A5-gBol031377-XLOC_016347-16369-1
TTAAAAGAGAGGAAATGCGAGGTTGGGACCGGCGAGGTAAGCGGACAGAG
CONSENSUS
......GAGAGGAAATGCGAGGTTGGGACCGGCGAGGTAAGCGGACAGAG

A5-gBol031377-XLOC_016347-16369-0
AAGGGTGAAGCCGTGACTCAGAGGCTCATCTATAAAATCTATAGCCGGAG
A5-gBol031377-XLOC_016347-16369-1
AAGGGTGAAGCCGTGACTCAGAGGCTCATCTATAAAATCTATAGCCGGAG
CONSENSUS
AAGGGTGAAGCCGTGACTCAGAGGCTCATCTATAAAATCTATAGCCGGAG

A5-gBol031377-XLOC_016347-16369-0
GACGGAGAAACTAAAGACTCCTCTTCCATATCAGAAACCTAACTCTTTGT
A5-gBol031377-XLOC_016347-16369-1
GACGGAGAAACTAAAGACTCCTCTTCCATATCAGAAACCTAACTCTTTGT
CONSENSUS
GACGGAGAAACTAAAGACTCCTCTTCCATATCAGAAACCTAACTCTTTGT

A5-gBol031377-XLOC_016347-16369-0 TTCTGTTTTTGTAACTTC
A5-gBol031377-XLOC_016347-16369-1 TTCTGTTTTTGTAACTTC
CONSENSUS TTCTGTTTTTGTAACTTC

alignment for event: A3-gBol015218-XLOC_034598-6841

A3-gBol015218-XLOC_034598-6841-0
AATCTCAGCCATTCATTGGAGTAAACTATGGCCAAACGGCCGACAATCTT
A3-gBol015218-XLOC_034598-6841-1
AATCTCAGCCATTCATTGGAGTAAACTATGGCCAAACGGCCGACAATCTT
CONSENSUS
AATCTCAGCCATTCATTGGAGTAAACTATGGCCAAACGGCCGACAATCTT

A3-gBol015218-XLOC_034598-6841-0
CCTCCACCGTCAGCCACCGCAAAGCTCCTGCGGTCCACAACTATCCAAAA
A3-gBol015218-XLOC_034598-6841-1
CCTCCACCGTCAGCCACCGCAAAGCTCCTGCGGTCCACAACTATCCAAAA
CONSENSUS
CCTCCACCGTCAGCCACCGCAAAGCTCCTGCGGTCCACAACTATCCAAAA

A3-gBol015218-XLOC_034598-6841-0
AGTCCGTCTTTACGGATCTGACCCCGCGATAATCAAGGCCCTAGCAAACA

A3-gBol015218-XLOC_034598-6841-1
AGTCCGTCTTTACGGATCTGACCCCGCGATAATCAAGGCCCTAGCAAACA
CONSENSUS
AGTCCGTCTTTACGGATCTGACCCCGCGATAATCAAGGCCCTAGCAAACA

A3-gBol015218-XLOC_034598-6841-0
CTGGTATCGAGATCGTCATCGGAACAGGCGATGTACCCGGTTTAGCATCC
A3-gBol015218-XLOC_034598-6841-1
CTGGTATCGAGATCGTCATCGGAACAGGCGATGTACCCGGTTTAGCATCC
CONSENSUS
CTGGTATCGAGATCGTCATCGGAACAGGCGATGTACCCGGTTTAGCATCC

A3-gBol015218-XLOC_034598-6841-0
GACCCCAGTTTCGCTCGGAGCTGGGTGGAAACCAACGTGGTCCCTTATTA
A3-gBol015218-XLOC_034598-6841-1
GACCCCAGTTTCGCTCGGAGCTGGGTGGAAACCAACGTGGTCCCTTATTA
CONSENSUS
GACCCCAGTTTCGCTCGGAGCTGGGTGGAAACCAACGTGGTCCCTTATTA

A3-gBol015218-XLOC_034598-6841-0
CCCGGCGAGCAAGATCACTCTCATCGACGTCGGAAACGAGGCAACAACCT
A3-gBol015218-XLOC_034598-6841-1
CCCGGCGAGCAAGATCACTCTCATCGACGTCGGAAACGAGGCAACAACCT
CONSENSUS
CCCGGCGAGCAAGATCACTCTCATCGACGTCGGAAACGAGGCAACAACCT

A3-gBol015218-XLOC_034598-6841-0
TTGGCGACCGCAATTTCATGTTGCAGCTCTTGCCGGCTATGAAAAATGTC
A3-gBol015218-XLOC_034598-6841-1
TTGGCGACCGCAATTTCATGTTGCAGCTCTTGCCGGCTATGAAAAATGTC
CONSENSUS
TTGGCGACCGCAATTTCATGTTGCAGCTCTTGCCGGCTATGAAAAATGTC

A3-gBol015218-XLOC_034598-6841-0
CAAAGTGCACTCGAGGCAGCCTCTCTCGGTGGCAAAATTAAGAGTTTAAT
A3-gBol015218-XLOC_034598-6841-1
CAAAGTGCACTCGAGGCAGCCTCTCTCGGTGGCAAAATTAAG--------
CONSENSUS
CAAAGTGCACTCGAGGCAGCCTCTCTCGGTGGCAAAATTAAG........

A3-gBol015218-XLOC_034598-6841-0
AGGGAGACGGGGTCGCCTTTTGCGGTTAACCCGTACCCGTTTTTCGCTTA
A3-gBol015218-XLOC_034598-6841-1

CONSENSUS
....

A3-gBol015218-XLOC_034598-6841-0
CCAGAGCGATCCTAGACCCGAGACATTGGCCTTTTGCTTGTTTCAGCCTA
A3-gBol015218-XLOC_034598-6841-1
--CCTA
CONSENSUS
....CCTA

A3-gBol015218-XLOC_034598-6841-0
ATCCTGGCCGTGTTGACGCAAACAGCAAGATCAAGTATATGAATATGTTC

A3-gBol015218-XLOC_034598-6841-1
ATCCTGGCCGTGTTGACGCAAACAGCAAGATCAAGTATATGAATATGTTC
CONSENSUS
ATCCTGGCCGTGTTGACGCAAACAGCAAGATCAAGTATATGAATATGTTC

A3-gBol015218-XLOC_034598-6841-0 GATGCTCAG
A3-gBol015218-XLOC_034598-6841-1 GATGCTCAG
CONSENSUS GATGCTCAG

alignment for event: A3-X-XLOC_033884-417

A3-X-XLOC_033884-417-0
ACGACCACCGTCTCTTGAAAGTCCAGCCGGTAACACCTCTGAGCGACTTC
A3-X-XLOC_033884-417-1
ACGACCACCGTCTCTTGAAAGTCCAGCCGGTAACACCTCTGAGCGACTTC
CONSENSUS
ACGACCACCGTCTCTTGAAAGTCCAGCCGGTAACACCTCTGAGCGACTTC

A3-X-XLOC_033884-417-0
AATTGATACAGTTACCGCTGTAAATCAGTCGGAGCTCTCGCTACTTAGTC
A3-X-XLOC_033884-417-1
AATTGATACAGTTACCGCTGTAAATCAGTCGGAGCTCTCGCTACTTAGTC
CONSENSUS
AATTGATACAGTTACCGCTGTAAATCAGTCGGAGCTCTCGCTACTTAGTC

A3-X-XLOC_033884-417-0
TTCAAAACGGCTGAAGCTTTGGAAGCGAGCACAGATCTGGTTTCTTCCTC
A3-X-XLOC_033884-417-1
TTCAAAACGGCTGAAGCTTTGGAAGCGAGCACAGATCTGGTTTCTTCCTC
CONSENSUS
TTCAAAACGGCTGAAGCTTTGGAAGCGAGCACAGATCTGGTTTCTTCCTC

A3-X-XLOC_033884-417-0
TTCACGATTTTGCTAGAGGACGAAGTGGTGGTTTTTAGATCTGTTTCGAG
A3-X-XLOC_033884-417-1
TTCACGATTTTGCTAGAGGACGAAGTGGTGGTTTTTAGATCTGTTTCGAG
CONSENSUS
TTCACGATTTTGCTAGAGGACGAAGTGGTGGTTTTTAGATCTGTTTCGAG

A3-X-XLOC_033884-417-0
CGGAAGCGGTGGAGGTCGTGATGAGCAGCTTCGGGTATAGATCGGCATGG
A3-X-XLOC_033884-417-1
CGGAAGCGGTGGAGGTCGTGATGAGCAGCTTCGGGTATAGATCGGCATGG
CONSENSUS
CGGAAGCGGTGGAGGTCGTGATGAGCAGCTTCGGGTATAGATCGGCATGG

A3-X-XLOC_033884-417-0
AGGGAAACACGGCGAGTCAAGATTGGCCCGGCGAGATCCACAGAGAAATC
A3-X-XLOC_033884-417-1
AGGGAAACACGGCGAGTCAAGATTGGCCCGGCGAGATCCACAGAGAAATC
CONSENSUS
AGGGAAACACGGCGAGTCAAGATTGGCCCGGCGAGATCCACAGAGAAATC

A3-X-XLOC_033884-417-0

AGAAACAGAACTAAAGCTGATACAAAAAAACATTCGTTATAACAACAATG
A3-X-XLOC_033884-417-1
AGAAACAGAACTAAAGCTGATACAAAAAAACATTCGTTATAACAACAATG
CONSENSUS
AGAAACAGAACTAAAGCTGATACAAAAAAACATTCGTTATAACAACAATG

A3-X-XLOC_033884-417-0
ATGATAATGAAGTTATGGGATCCACATTCGTCTTTTACTCGAGCATCCTC
A3-X-XLOC_033884-417-1
ATGATAATGAAGTTATGGGATCCACATTCGTCTTTTACTCGAGCATCCTC
CONSENSUS
ATGATAATGAAGTTATGGGATCCACATTCGTCTTTTACTCGAGCATCCTC

A3-X-XLOC_033884-417-0
TTTGAGAAACGCCAAATGAAAGACTGTGAAAGGATCATCTATACAATGTA
A3-X-XLOC_033884-417-1
TTTGAGAAACGCCAAATGAAAGACTGTGAAAG------------------
CONSENSUS
TTTGAGAAACGCCAAATGAAAGACTGTGAAAG..................

A3-X-XLOC_033884-417-0
CACACATGTTGGTGACTAAGTTTGCAACATCTTAGAAACAATGAAATAGC
A3-X-XLOC_033884-417-1

CONSENSUS
....

A3-X-XLOC_033884-417-0
CGGTGAGGAGATTCGTTTATGAAAATTTGATTCTCACTTGATGTATGTTC
A3-X-XLOC_033884-417-1

CONSENSUS
....

A3-X-XLOC_033884-417-0
CCTATGATTATAGATAAATAAGCGACATTAATGAGCTCTGATTTCCAGTG
A3-X-XLOC_033884-417-1 -------------
ATAAATAAGCGACATTAATGAGCTCTGATTTCCAGTG
CONSENSUS
.............ATAAATAAGCGACATTAATGAGCTCTGATTTCCAGTG

A3-X-XLOC_033884-417-0
GAAAAAGGTTGAAGAACCAAAAGAGTTCACCGGCTTGAGCTGTTCTTTGA
A3-X-XLOC_033884-417-1
GAAAAAGGTTGAAGAACCAAAAGAGTTCACCGGCTTGAGCTGTTCTTTGA
CONSENSUS
GAAAAAGGTTGAAGAACCAAAAGAGTTCACCGGCTTGAGCTGTTCTTTGA

A3-X-XLOC_033884-417-0
GGCTTAAATTAAGAACAATGGGATTGCCGGTTTGGAG
A3-X-XLOC_033884-417-1
GGCTTAAATTAAGAACAATGGGATTGCCGGTTTGGAG
CONSENSUS
GGCTTAAATTAAGAACAATGGGATTGCCGGTTTGGAG

alignment for event: RI-X-XLOC_033955-4298

RI-X-XLOC_033955-4298-0
      CATAGAAAGCAAGCCAGTAGGGAGCAGAGCTGTCGACATACTGTGGGTTG
RI-X-XLOC_033955-4298-1
      CATAGAAAGCAAGCCAGTAGGGAGCAGAGCTGTCGACATACTGTGGGTTG
CONSENSUS
      CATAGAAAGCAAGCCAGTAGGGAGCAGAGCTGTCGACATACTGTGGGTTG

RI-X-XLOC_033955-4298-0
      ATACCTCGTGATCTTCCACCTTTAGCAAGGCTGTCTGCACGGACGTTCCA
RI-X-XLOC_033955-4298-1
      ATACCTCGTGATCTTCCACCTTTAGCAAGGCTGTCTGCACGGACGTTCCA
CONSENSUS
      ATACCTCGTGATCTTCCACCTTTAGCAAGGCTGTCTGCACGGACGTTCCA

RI-X-XLOC_033955-4298-0
      GGATCTTGGAATATGAATGATGGAAATTTCCAGAAATTTGATCTCATCAA
RI-X-XLOC_033955-4298-1
      GGATCTTGGAATATGAATGATGGAAATTTCCAGAAATTTGATCTCATCAA
CONSENSUS
      GGATCTTGGAATATGAATGATGGAAATTTCCAGAAATTTGATCTCATCAA

RI-X-XLOC_033955-4298-0
      TTTCAGCTGCCATGGAGGGCCAATCTTCTTCACTTTGTATCAGCTTAACG
RI-X-XLOC_033955-4298-1
      TTTCAGCTGCCATGGAGGGCCAATCTTCTTCACTTTGTATCAGCTTAACG
CONSENSUS
      TTTCAGCTGCCATGGAGGGCCAATCTTCTTCACTTTGTATCAGCTTAACG

RI-X-XLOC_033955-4298-0
      AGTTGCTTGCAGTCTATTTCAAATTGTACGCTTCTGTTCCTTGTTTTTAA
RI-X-XLOC_033955-4298-1
      AGTTGCTTGCAGTCTATTTCAAATTGTACGCTTCTGTTCCTTGTTTTTAA
CONSENSUS
      AGTTGCTTGCAGTCTATTTCAAATTGTACGCTTCTGTTCCTTGTTTTTAA

RI-X-XLOC_033955-4298-0
      AACTTCCTGCATTGCCCATATAAGACCTTCGGCTTCTGCGTGAAGGGAGG
RI-X-XLOC_033955-4298-1
      AACTTCCTGCATTGCCCATATAAGACCTTCGGCTTCTGCGTGAAGGGAGG
CONSENSUS
      AACTTCCTGCATTGCCCATATAAGACCTTCGGCTTCTGCGTGAAGGGAGG

RI-X-XLOC_033955-4298-0
      ATGTCTCTCTGGATAGTTCTCTCGCTCCATACAGCATCGGTTTACCTGCA
RI-X-XLOC_033955-4298-1
      ATGTCTCTCTGGATAGTTCTCTCGCTCCATACAGCATCGGTTTACCTGCA
CONSENSUS
      ATGTCTCTCTGGATAGTTCTCTCGCTCCATACAGCATCGGTTTACCTGCA

RI-X-XLOC_033955-4298-0
      TTCATAACCACAAAACCCAGGTCTGCTCTCTCGTGTTTGTTTATCCATGA
RI-X-XLOC_033955-4298-1
      TTCATAACCACAAAACCCAG------------------------------

CONSENSUS
      TTCATAACCACAAAACCCAG..............................

RI-X-XLOC_033955-4298-0
      TGCGTCGGTTTGACAACTCCACAGTGGAGGACAGCTTGTGTGTGGCTGAG
RI-X-XLOC_033955-4298-1

CONSENSUS
      ....

RI-X-XLOC_033955-4298-0
      GCTGTTCGTCGGTTATGCAGCTCCTCTTCCATCGGGTCTTCCACGAGTTC
RI-X-XLOC_033955-4298-1   --------------------
CTCCTCTTCCATCGGGTCTTCCACGAGTTC
CONSENSUS
      ....................CTCCTCTTCCATCGGGTCTTCCACGAGTTC

RI-X-XLOC_033955-4298-0
      CGGGATTCGTTGGGCCAATATCCAGCTTTTGGCTTCCTTTACCGCGAACT
RI-X-XLOC_033955-4298-1
      CGGGATTCGTTGGGCCAATATCCAGCTTTTGGCTTCCTTTACCGCGAACT
CONSENSUS
      CGGGATTCGTTGGGCCAATATCCAGCTTTTGGCTTCCTTTACCGCGAACT

RI-X-XLOC_033955-4298-0
      GTATGGTCTCTAGAGGCAATACGTCTTTGTTAGAAAATATTTTCTCGTTT
RI-X-XLOC_033955-4298-1
      GTATGGTCTCTAGAGGCAATACGTCTTTGTTAGAAAATATTTTCTCGTTT
CONSENSUS
      GTATGGTCTCTAGAGGCAATACGTCTTTGTTAGAAAATATTTTCTCGTTT

RI-X-XLOC_033955-4298-0
      CTAGCCTTCCATATGTACCATATTTTCCACGGAATAGCATCCAACACATC
RI-X-XLOC_033955-4298-1
      CTAGCCTTCCATATGTACCATATTTTCCACGGAATAGCATCCAACACATC
CONSENSUS
      CTAGCCTTCCATATGTACCATATTTTCCACGGAATAGCATCCAACACATC

RI-X-XLOC_033955-4298-0
      AGCGTGAATCCCTTGTTCCTTGATACGTAGGAGAAGGTAGTCGATGTTCA
RI-X-XLOC_033955-4298-1
      AGCGTGAATCCCTTGTTCCTTGATACGTAGGAGAAGGTAGTCGATGTTCA
CONSENSUS
      AGCGTGAATCCCTTGTTCCTTGATACGTAGGAGAAGGTAGTCGATGTTCA

RI-X-XLOC_033955-4298-0
      CGTATAGTGATGTACACAGGAATCATCCCGGAGAGGTTGGAATAAGGGAT
RI-X-XLOC_033955-4298-1
      CGTATAGTGATGTACACAGGAATCATCCCGGAGAGGTTGGAATAAGGGAT
CONSENSUS
      CGTATAGTGATGTACACAGGAATCATCCCGGAGAGGTTGGAATAAGGGAT

RI-X-XLOC_033955-4298-0
      AGAGCCCAAGTTTGGAAGGCCGGAGGGCATTCAAACAGCGTGTGGTTTAT
RI-X-XLOC_033955-4298-1
      AGAGCCCAAGTTTGGAAGGCCGGAGGGCATTCAAACAGCGTGTGGTTTAT

CONSENSUS
AGAGCCCAAGTTTGGAAGGCCGGAGGGCATTCAAACAGCGTGTGGTTTAT

RI-X-XLOC_033955-4298-0
GGACTCCGTCTCTGCGCCGCTTCTCATACATATACTTTCTCTGGCGCAAT
RI-X-XLOC_033955-4298-1
GGACTCCGTCTCTGCGCCGCTTCTCATACATATACTTTCTCTGGCGCAAT
CONSENSUS
GGACTCCGTCTCTGCGCCGCTTCTCATACATATACTTTCTCTGGCGCAAT

RI-X-XLOC_033955-4298-0
GTCTCTCTTTTAATTTCTCAGCTGTGGCTATGTAACCAGCCGTAGCCTGT
RI-X-XLOC_033955-4298-1
GTCTCTCTTTTAATTTCTCAGCTGTGGCTATGTAACCAGCCGTAGCCTGT
CONSENSUS
GTCTCTCTTTTAATTTCTCAGCTGTGGCTATGTAACCAGCCGTAGCCTGT

RI-X-XLOC_033955-4298-0
CAGAGGAAGTGTTTGAGTTTTCGTGGGGCTTGGATCTTCCGGATTATTTT
RI-X-XLOC_033955-4298-1
CAGAGGAAGTGTTTGAGTTTTCGTGGGGCTTGGATCTTCCGGATTATTTT
CONSENSUS
CAGAGGAAGTGTTTGAGTTTTCGTGGGGCTTGGATCTTCCGGATTATTTT

RI-X-XLOC_033955-4298-0
CTTCAGCCCTATCGTACTCGGCTCCGAAACCAGTGTGCTATGGTAAGTAG
RI-X-XLOC_033955-4298-1
CTTCAGCCCTATCGTACTCGGCTCCGAAACCAGTGTGCTATGGTAAGTAG
CONSENSUS
CTTCAGCCCTATCGTACTCGGCTCCGAAACCAGTGTGCTATGGTAAGTAG

RI-X-XLOC_033955-4298-0
TTTGTAGTTTCCGTGCAATGGAGTAGCCGGACTTTACGGAGTATAACCCG
RI-X-XLOC_033955-4298-1
TTTGTAGTTTCCGTGCAATGGAGTAGCCGGACTTTACGGAGTATAACCCG
CONSENSUS
TTTGTAGTTTCCGTGCAATGGAGTAGCCGGACTTTACGGAGTATAACCCG

RI-X-XLOC_033955-4298-0
GACTTGGTGAAGTCCCAACTGTAGCAATCGGGTCGACCTGTTCTACTGAC
RI-X-XLOC_033955-4298-1
GACTTGGTGAAGTCCCAACTGTAGCAATCGGGTCGACCTGTTCTACTGAC
CONSENSUS
GACTTGGTGAAGTCCCAACTGTAGCAATCGGGTCGACCTGTTCTACTGAC

RI-X-XLOC_033955-4298-0
CCTGATCGAGGTAATATGAGGGATGTCCTCCGGTGCAATCATCTCATTTA
RI-X-XLOC_033955-4298-1
CCTGATCGAGGTAATATGAGGGATGTCCTCCGGTGCAATCATCTCATTTA
CONSENSUS
CCTGATCGAGGTAATATGAGGGATGTCCTCCGGTGCAATCATCTCATTTA

RI-X-XLOC_033955-4298-0
AAAGATCAAGGTTCCATTCCTGGCTCCCAAGATCGATCAGATGATGCACT
RI-X-XLOC_033955-4298-1
AAAGATCAAGGTTCCATTCCTGGCTCCCAAGATCGATCAGATGATGCACT

CONSENSUS
AAAGATCAAGGTTCCATTCCTGGCTCCCAAGATCGATCAGATGATGCACT

RI-X-XLOC_033955-4298-0
CGGAGGACCTCATCTCTAGGGGTTGCCCTATGTAAGGGTGATCTAGCAGG
RI-X-XLOC_033955-4298-1
CGGAGGACCTCATCTCTAGGGGTTGCCCTATGTAAGGGTGATCTAGCAGG
CONSENSUS
CGGAGGACCTCATCTCTAGGGGTTGCCCTATGTAAGGGTGATCTAGCAGG

RI-X-XLOC_033955-4298-0
GTTTGTTTGAAGCCAAGGTTCCTCCCAAGCTCTTGTGTCGTGCCCGTTTC
RI-X-XLOC_033955-4298-1
GTTTGTTTGAAGCCAAGGTTCCTCCCAAGCTCTTGTGTCGTGCCCGTTTC
CONSENSUS
GTTTGTTTGAAGCCAAGGTTCCTCCCAAGCTCTTGTGTCGTGCCCGTTTC

RI-X-XLOC_033955-4298-0
CGATCTTCCTGAGACCTTGCTGTAGAATCTGTCGCGAAGCGACTATACTG
RI-X-XLOC_033955-4298-1
CGATCTTCCTGAGACCTTGCTGTAGAATCTGTCGCGAAGCGACTATACTG
CONSENSUS
CGATCTTCCTGAGACCTTGCTGTAGAATCTGTCGCGAAGCGACTATACTG

RI-X-XLOC_033955-4298-0
CGCCAGCCGTACGATGGGTTGTTGTTCGTTCGTTTTATCATCATCGGGTT
RI-X-XLOC_033955-4298-1
CGCCAGCCGTACGATGGGTTGTTGTTCGTTCGTTTTATCATCATCGGGTT
CONSENSUS
CGCCAGCCGTACGATGGGTTGTTGTTCGTTCGTTTTATCATCATCGGGTT

RI-X-XLOC_033955-4298-0
GGAGTGTCGGTAATATCTTCCTTTCAGAACCCGAGCAAGAAGCGAACGGG
RI-X-XLOC_033955-4298-1
GGAGTGTCGGTAATATCTTCCTTTCAGAACCCGAGCAAGAAGCGAACGGG
CONSENSUS
GGAGTGTCGGTAATATCTTCCTTTCAGAACCCGAGCAAGAAGCGAACGGG

RI-X-XLOC_033955-4298-0
GGTACTTGAGTAGTCTCCACATCTGTTTCGTCAGTAAAGCGAGATTAAAA
RI-X-XLOC_033955-4298-1
GGTACTTGAGTAGTCTCCACATCTGTTTCGTCAGTAAAGCGAGATTAAAA
CONSENSUS
GGTACTTGAGTAGTCTCCACATCTGTTTCGTCAGTAAAGCGAGATTAAAA

RI-X-XLOC_033955-4298-0
TCGTGAAAGTCACGAAACCTCAATCCTCCTTCTTCTGTTGGGGCGCAAAT
RI-X-XLOC_033955-4298-1
TCGTGAAAGTCACGAAACCTCAATCCTCCTTCTTCTGTTGGGGCGCAAAT
CONSENSUS
TCGTGAAAGTCACGAAACCTCAATCCTCCTTCTTCTGTTGGGGCGCAAAT

RI-X-XLOC_033955-4298-0
CTTATCCCATGCCACCCAACGTAGACCCCTATTGTTTTCCTTGGTACTCC
RI-X-XLOC_033955-4298-1
CTTATCCCATGCCACCCAACGTAGACCCCTATTGTTTTCCTTGGTACTCC

CONSENSUS
      CTTATCCCATGCCACCCAACGTAGACCCCTATTGTTTTCCTTGGTACTCC

RI-X-XLOC_033955-4298-0
      ACCAGAATCTCGAAACTGCACTAGTAAGCTTTTTTGTTACTCCTTGTGGT
RI-X-XLOC_033955-4298-1
      ACCAGAATCTCGAAACTGCACTAGTAAGCTTTTTTGTTACTCCTTGTGGT
CONSENSUS
      ACCAGAATCTCGAAACTGCACTAGTAAGCTTTTTTGTTACTCCTTGTGGT

RI-X-XLOC_033955-4298-0
      AAGAGATAGCACGACATAACGTGGGAAGGGACTGCCTGTGCTACCGCTTT
RI-X-XLOC_033955-4298-1
      AAGAGATAGCACGACATAACGTGGGAAGGGACTGCCTGTGCTACCGCTTT
CONSENSUS
      AAGAGATAGCACGACATAACGTGGGAAGGGACTGCCTGTGCTACCGCTTT

RI-X-XLOC_033955-4298-0
      GATCTGGATTTCTTTGCCTCCCTTTGACAGAAGCTTTGTTGACCATGAGT
RI-X-XLOC_033955-4298-1
      GATCTGGATTTCTTTGCCTCCCTTTGACAGAAGCTTTGTTGACCATGAGT
CONSENSUS
      GATCTGGATTTCTTTGCCTCCCTTTGACAGAAGCTTTGTTGACCATGAGT

RI-X-XLOC_033955-4298-0
      TTGTTCGGTTGTTTAATCTATCTTGAACAAAGCTAAACACTTGTTTCTTA
RI-X-XLOC_033955-4298-1
      TTGTTCGGTTGTTTAATCTATCTTGAACAAAGCTAAACACTTGTTTCTTA
CONSENSUS
      TTGTTCGGTTGTTTAATCTATCTTGAACAAAGCTAAACACTTGTTTCTTA

RI-X-XLOC_033955-4298-0
      GAGCCACAAATCTTCTCGGGAATTCCGAGATACATACCCATTCCACCTTC
RI-X-XLOC_033955-4298-1
      GAGCCACAAATCTTCTCGGGAATTCCGAGATACATACCCATTCCACCTTC
CONSENSUS
      GAGCCACAAATCTTCTCGGGAATTCCGAGATACATACCCATTCCACCTTC

RI-X-XLOC_033955-4298-0
      TTGAGTGATATTGAGTGACCTTTTGAGGTCTGTTTTAAAGGACGCTACGA
RI-X-XLOC_033955-4298-1
      TTGAGTGATATTGAGTGACCTTTTGAGGTCTGTTTTAAAGGACGCTACGA
CONSENSUS
      TTGAGTGATATTGAGTGACCTTTTGAGGTCTGTTTTAAAGGACGCTACGA

RI-X-XLOC_033955-4298-0
      CCTTAGAACCGAACAGAGCCGAAGATTTTGACTTATTCAGTTGTTGTCCT
RI-X-XLOC_033955-4298-1
      CCTTAGAACCGAACAGAGCCGAAGATTTTGACTTATTCAGTTGTTGTCCT
CONSENSUS
      CCTTAGAACCGAACAGAGCCGAAGATTTTGACTTATTCAGTTGTTGTCCT

RI-X-XLOC_033955-4298-0
      GAGGAATTGCTGTAGACGTCAATGATCCGCCTGAGTTCGCTACGTTCTTC
RI-X-XLOC_033955-4298-1
      GAGGAATTGCTGTAGACGTCAATGATCCGCCTGAGTTCGCTACGTTCTTC

CONSENSUS
GAGGAATTGCTGTAGACGTCAATGATCCGCCTGAGTTCGCTACGTTCTTC

RI-X-XLOC_033955-4298-0
CTGAGTTGCATTACAGAAGAATAAACTATCGTCCGCGAATAGGAGGTGTG
RI-X-XLOC_033955-4298-1
CTGAGTTGCATTACAGAAGAATAAACTATCGTCCGCGAATAGGAGGTGTG
CONSENSUS
CTGAGTTGCATTACAGAAGAATAAACTATCGTCCGCGAATAGGAGGTGTG

RI-X-XLOC_033955-4298-0
AGATGGGTGGACATTGTCTCGCTATCTTTAGGCCAGAGAGCTTTTTGTCT
RI-X-XLOC_033955-4298-1
AGATGGGTGGACATTGTCTCGCTATCTTTAGGCCAGAGAGCTTTTTGTCT
CONSENSUS
AGATGGGTGGACATTGTCTCGCTATCTTTAGGCCAGAGAGCTTTTTGTCT

RI-X-XLOC_033955-4298-0
CTTTCGGCATGCTGGAGTTGAGATATAAGAACTTTCGTGCATAGACGAAT
RI-X-XLOC_033955-4298-1
CTTTCGGCATGCTGGAGTTGAGATATAAGAACTTTCGTGCATAGACGAAT
CONSENSUS
CTTTCGGCATGCTGGAGTTGAGATATAAGAACTTTCGTGCATAGACGAAT

RI-X-XLOC_033955-4298-0
AAGAAAGGTGACAACGGGTCGCCTTGTCGCAGTCCACGGGTTGGGATTAT
RI-X-XLOC_033955-4298-1
AAGAAAGGTGACAACGGGTCGCCTTGTCGCAGTCCACGGGTTGGGATTAT
CONSENSUS
AAGAAAGGTGACAACGGGTCGCCTTGTCGCAGTCCACGGGTTGGGATTAT

RI-X-XLOC_033955-4298-0
CCTACCTTTAGCTTCTCCGTTTATGAGAACTTGGTAGGAGACAGTCGAAA
RI-X-XLOC_033955-4298-1
CCTACCTTTAGCTTCTCCGTTTATGAGAACTTGGTAGGAGACAGTCGAAA
CONSENSUS
CCTACCTTTAGCTTCTCCGTTTATGAGAACTTGGTAGGAGACAGTCGAAA

RI-X-XLOC_033955-4298-0
CACATCTCATGATCAGGTGGATCCATCTCTCGTCAAAGCCCATTTTTCCA
RI-X-XLOC_033955-4298-1
CACATCTCATGATCAGGTGGATCCATCTCTCGTCAAAGCCCATTTTTCCA
CONSENSUS
CACATCTCATGATCAGGTGGATCCATCTCTCGTCAAAGCCCATTTTTCCA

RI-X-XLOC_033955-4298-0
TTAGAGTTTCCAAGAAACTCCATTCTACCCGGTCATATGCCTTGCTCATG
RI-X-XLOC_033955-4298-1
TTAGAGTTTCCAAGAAACTCCATTCTACCCGGTCATATGCCTTGCTCATG
CONSENSUS
TTAGAGTTTCCAAGAAACTCCATTCTACCCGGTCATATGCCTTGCTCATG

RI-X-XLOC_033955-4298-0
TTTGTCTTTATCGCCACATATTTGCTTTGGCAGCTAGGTATCGTCCTGAG
RI-X-XLOC_033955-4298-1
TTTGTCTTTATCGCCACATATTTGCTTTGGCAGCTAGGTATCGTCCTGAG

CONSENSUS
TTTGTCTTTATCGCCACATATTTGCTTTGGCAGCTAGGTATCGTCCTGAG

RI-X-XLOC_033955-4298-0
CGCATGAAACATCTATTGTGCAATCAAGATGTTATCTGAGATGAGTCTAC
RI-X-XLOC_033955-4298-1
CGCATGAAACATCTATTGTGCAATCAAGATGTTATCTGAGATGAGTCTAC
CONSENSUS
CGCATGAAACATCTATTGTGCAATCAAGATGTTATCTGAGATGAGTCTAC

RI-X-XLOC_033955-4298-0
GAGCCACAAAGGCCAATTGGGTTTCCAAAAATAAATTCGGAAGCACATGT
RI-X-XLOC_033955-4298-1
GAGCCACAAAGGCCAATTGGGTTTCCAAAAATAAATTCGGAAGCACATGT
CONSENSUS
GAGCCACAAAGGCCAATTGGGTTTCCAAAAATAAATTCGGAAGCACATGT

RI-X-XLOC_033955-4298-0
TTTAGTCTTGCAGACATGGCCTTGGAGATGACTTTATAGCTAACATTACA
RI-X-XLOC_033955-4298-1
TTTAGTCTTGCAGACATGGCCTTGGAGATGACTTTATAGCTAACATTACA
CONSENSUS
TTTAGTCTTGCAGACATGGCCTTGGAGATGACTTTATAGCTAACATTACA

RI-X-XLOC_033955-4298-0
TAAGCTGATAGGTCGAAATTCTGTCATAGATTTCGATCTCTCTGTCTTCG
RI-X-XLOC_033955-4298-1
TAAGCTGATAGGTCGAAATTCTGTCATAGATTTCGATCTCTCTGTCTTCG
CONSENSUS
TAAGCTGATAGGTCGAAATTCTGTCATAGATTTCGATCTCTCTGTCTTCG

RI-X-XLOC_033955-4298-0
GTATCAGACATATATTCGTCTGATTAATCCTTTCATCTAGGTCCCTGAGT
RI-X-XLOC_033955-4298-1
GTATCAGACATATATTCGTCTGATTAATCCTTTCATCTAGGTCCCTGAGT
CONSENSUS
GTATCAGACATATATTCGTCTGATTAATCCTTTCATCTAGGTCCCTGAGT

RI-X-XLOC_033955-4298-0
TGAAGAAATCTTTAACCATATGCACTATGTCTGGGCCTATGAGTCTCCAA
RI-X-XLOC_033955-4298-1
TGAAGAAATCTTTAACCATATGCACTATGTCTGGGCCTATGAGTCTCCAA
CONSENSUS
TGAAGAAATCTTTAACCATATGCACTATGTCTGGGCCTATGAGTCTCCAA

RI-X-XLOC_033955-4298-0
AATCTTTGATAGAATAGGCTGGTCATACCATCGGGATCCGGGGCTTTTTC
RI-X-XLOC_033955-4298-1
AATCTTTGATAGAATAGGCTGGTCATACCATCGGGATCCGGGGCTTTTTC
CONSENSUS
AATCTTTGATAGAATAGGCTGGTCATACCATCGGGATCCGGGGCTTTTTC

RI-X-XLOC_033955-4298-0
TGGATGGATCGAGAATATTGCTTTCTTTATCTCTTCCTCTGTGGGTTCTC
RI-X-XLOC_033955-4298-1
TGGATGGATCGAGAATATTGCTTTCTTTATCTCTTCCTCTGTGGGTTCTC

CONSENSUS
TGGATGGATCGAGAATATTGCTTTCTTTATCTCTTCCTCTGTGGGTTCTC

RI-X-XLOC_033955-4298-0
TTATTAGCATTTCGTTCATTTCATGGGAAACCTTTGCGGTAGTAAACCAG
RI-X-XLOC_033955-4298-1
TTATTAGCATTTCGTTCATTTCATGGGAAACCTTTGCGGTAGTAAACCAG
CONSENSUS
TTATTAGCATTTCGTTCATTTCATGGGAAACCTTTGCGGTAGTAAACCAG

RI-X-XLOC_033955-4298-0
AAGGTTTCATCACGGTCATACGGTTCTGATGCAGTGAAAAGGTTCGCGAA
RI-X-XLOC_033955-4298-1
AAGGTTTCATCACGGTCATACGGTTCTGATGCAGTGAAAAGGTTCGCGAA
CONSENSUS
AAGGTTTCATCACGGTCATACGGTTCTGATGCAGTGAAAAGGTTCGCGAA

RI-X-XLOC_033955-4298-0
ATACTCGGATGCCAGCGCTTCTATGCCTTCCTCCGACCCCACCCAATTGC
RI-X-XLOC_033955-4298-1
ATACTCGGATGCCAGCGCTTCTATGCCTTCCTCCGACCCCACCCAATTGC
CONSENSUS
ATACTCGGATGCCAGCGCTTCTATGCCTTCCTCCGACCCCACCCAATTGC

RI-X-XLOC_033955-4298-0
CCATGGAGTCTAGTAACTTTGTTATGCGGTTGCGCGCTCTCTGTTGTTTA
RI-X-XLOC_033955-4298-1
CCATGGAGTCTAGTAACTTTGTTATGCGGTTGCGCGCTCTCTGTTGTTTA
CONSENSUS
CCATGGAGTCTAGTAACTTTGTTATGCGGTTGCGCGCTCTCTGTTGTTTA

RI-X-XLOC_033955-4298-0
GTCTTTGCACGGAAGAACTTTGTGTTTCGATCGCCTTCTCTGAGCCAGAT
RI-X-XLOC_033955-4298-1
GTCTTTGCACGGAAGAACTTTGTGTTTCGATCGCCTTCTCTGAGCCAGAT
CONSENSUS
GTCTTTGCACGGAAGAACTTTGTGTTTCGATCGCCTTCTCTGAGCCAGAT

RI-X-XLOC_033955-4298-0
TGTTCTACTTTTATGCTTCCAGAACAGTTCCTCCTCCCTATAAGCTTCGC
RI-X-XLOC_033955-4298-1
TGTTCTACTTTTATGCTTCCAGAACAGTTCCTCCTCCCTATAAGCTTCGC
CONSENSUS
TGTTCTACTTTTATGCTTCCAGAACAGTTCCTCCTCCCTATAAGCTTCGC

RI-X-XLOC_033955-4298-0
ACAGCTTCCATTTTAGCTCAAGCTCCTCTTCTGTTGATATACTATCATCA
RI-X-XLOC_033955-4298-1
ACAGCTTCCATTTTAGCTCAAGCTCCTCTTCTGTTGATATACTATCATCA
CONSENSUS
ACAGCTTCCATTTTAGCTCAAGCTCCTCTTCTGTTGATATACTATCATCA

RI-X-XLOC_033955-4298-0
TTCTGGGCTCTATCAATTTCATGCTTCATCCTCTCAATCAGTTTTTGGTT
RI-X-XLOC_033955-4298-1
TTCTGGGCTCTATCAATTTCATGCTTCATCCTCTCAATCAGTTTTTGGTT

CONSENSUS
TTCTGGGCTCTATCAATTTCATGCTTCATCCTCTCAATCAGTTTTTGGTT

RI-X-XLOC_033955-4298-0
GCTGGTGGGGTTTCTTTTCTTCCAACGTGAGATGGCTTTCCTGCATCTAC
RI-X-XLOC_033955-4298-1
GCTGGTGGGGTTTCTTTTCTTCCAACGTGAGATGGCTTTCCTGCATCTAC
CONSENSUS
GCTGGTGGGGTTTCTTTTCTTCCAACGTGAGATGGCTTTCCTGCATCTAC

RI-X-XLOC_033955-4298-0
CAATTTTTTTCGTGCAGACATGTTGTTTCCGCAGGATCAAATCGTCCCCA
RI-X-XLOC_033955-4298-1
CAATTTTTTTCGTGCAGACATGTTGTTTCCGCAGGATCAAATCGTCCCCA
CONSENSUS
CAATTTTTTTCGTGCAGACATGTTGTTTCCGCAGGATCAAATCGTCCCCA

RI-X-XLOC_033955-4298-0
TCCCTGCTTGACTGTTTCGTATAGGCCTTCCTTCCCTAGCCAGCGCTTAT
RI-X-XLOC_033955-4298-1
TCCCTGCTTGACTGTTTCGTATAGGCCTTCCTTCCCTAGCCAGCGCTTAT
CONSENSUS
TCCCTGCTTGACTGTTTCGTATAGGCCTTCCTTCCCTAGCCAGCGCTTAT

RI-X-XLOC_033955-4298-0
CAAACTTGAAGCCTCTGCGTCCACCAGCTTCTTTAGACTTAATCTTTACA
RI-X-XLOC_033955-4298-1
CAAACTTGAAGCCTCTGCGTCCACCAGCTTCTTTAGACTTAATCTTTACA
CONSENSUS
CAAACTTGAAGCCTCTGCGTCCACCAGCTTCTTTAGACTTAATCTTTACA

RI-X-XLOC_033955-4298-0
AGAACCGGCCGGTGGTCGGATCCCCATCTTAGAAGGTACTCCACATCTGT
RI-X-XLOC_033955-4298-1
AGAACCGGCCGGTGGTCGGATCCCCATCTTAGAAGGTACTCCACATCTGT
CONSENSUS
AGAACCGGCCGGTGGTCGGATCCCCATCTTAGAAGGTACTCCACATCTGT

RI-X-XLOC_033955-4298-0
GTGGGAGAACACTTGGTGCCAGTCCTCATTCCCAAGTGCTCGATCTAATC
RI-X-XLOC_033955-4298-1
GTGGGAGAACACTTGGTGCCAGTCCTCATTCCCAAGTGCTCGATCTAATC
CONSENSUS
GTGGGAGAACACTTGGTGCCAGTCCTCATTCCCAAGTGCTCGATCTAATC

RI-X-XLOC_033955-4298-0
GACATTGGACTCTCCCGGATTTCCTTCTCCCTGCCCAAGAGAAAAAAATT
RI-X-XLOC_033955-4298-1
GACATTGGACTCTCCCGGATTTCCTTCTCCCTGCCCAAGAGAAAAAAATT
CONSENSUS
GACATTGGACTCTCCCGGATTTCCTTCTCCCTGCCCAAGAGAAAAAAATT

RI-X-XLOC_033955-4298-0
CCAGAGTGGTGGAATTCGATCATTCCGCATGCTGAGATCATGTTCTTGAA
RI-X-XLOC_033955-4298-1
CCAGAGTGGTGGAATTCGATCATTCCGCATGCTGAGATCATGTTCTTGAA

CONSENSUS
      CCAGAGTGGTGGAATTCGATCATTCCGCATGCTGAGATCATGTTCTTGAA

RI-X-XLOC_033955-4298-0
      ATTAGAAAGGAAGAATCTGGGCGTTTCCTGCCTCCTTTTTTTCTCTAGGT
RI-X-XLOC_033955-4298-1
      ATTAGAAAGGAAGAATCTGGGCGTTTCCTGCCTCCTTTTTTTCTCTAGGT
CONSENSUS
      ATTAGAAAGGAAGAATCTGGGCGTTTCCTGCCTCCTTTTTTTCTCTAGGT

RI-X-XLOC_033955-4298-0
      TGCTTGTGATTTCATCGAAGTCTCCGACCATTAGCCAAGCCCCTGATCTT
RI-X-XLOC_033955-4298-1
      TGCTTGTGATTTCATCGAAGTCTCCGACCATTAGCCAAGCCCCTGATCTT
CONSENSUS
      TGCTTGTGATTTCATCGAAGTCTCCGACCATTAGCCAAGCCCCTGATCTT

RI-X-XLOC_033955-4298-0
      TGTAGACTAAATCTCATTAGTCTCTTCCATACTTGTTCGCGGTACTCAAC
RI-X-XLOC_033955-4298-1
      TGTAGACTAAATCTCATTAGTCTCTTCCATACTTGTTCGCGGTACTCAAC
CONSENSUS
      TGTAGACTAAATCTCATTAGTCTCTTCCATACTTGTTCGCGGTACTCAAC

RI-X-XLOC_033955-4298-0   TACCGGATCTCCATACACAAAG
RI-X-XLOC_033955-4298-1   TACCGGATCTCCATACACAAAG
CONSENSUS   TACCGGATCTCCATACACAAAG

alignment for event: A3-gBol009095-XLOC_041594-4319

A3-gBol009095-XLOC_041594-4319-0
      GGATATAGACAAGCGCCTTGTCTCTCTTCCGGAGATCCGTGAAACGGGAG
A3-gBol009095-XLOC_041594-4319-1
      GGATATAGACAAGCGCCTTGTCTCTCTTCCGGAGATCCGTGAAACGGGAG
CONSENSUS
      GGATATAGACAAGCGCCTTGTCTCTCTTCCGGAGATCCGTGAAACGGGAG

A3-gBol009095-XLOC_041594-4319-0
      AATACATAGCCGAACTCAAACTTCACCCTGATGTCACTGCTCGCGTTAGG
A3-gBol009095-XLOC_041594-4319-1
      AATACATAGCCGAACTCAAACTTCACCCTGATGTCACTGCTCGCGTTAGG
CONSENSUS
      AATACATAGCCGAACTCAAACTTCACCCTGATGTCACTGCTCGCGTTAGG

A3-gBol009095-XLOC_041594-4319-0
      CTCAACGTTTTCGCCAACTAACAACCTTTCCAATAGTCAAAGACTGCTAC
A3-gBol009095-XLOC_041594-4319-1
      CTCAACGTTTTCGCCAACTAACAACCTTTCCAATAGTCAAAGACTGCTAC
CONSENSUS
      CTCAACGTTTTCGCCAACTAACAACCTTTCCAATAGTCAAAGACTGCTAC

A3-gBol009095-XLOC_041594-4319-0
      TGCTGCATCCCCAAGGTTGCAGGAACATAGAGAAAGGGGGGAAGAGATTG
A3-gBol009095-XLOC_041594-4319-1 TGCTGCATCCCCAAG-------

GAACATAGAGAAAGGGGGGAAGAGATTG
CONSENSUS
TGCTGCATCCCCAAG.......GAACATAGAGAAAGGGGGGAAGAGATTG

A3-gBol009095-XLOC_041594-4319-0
GCCAGGATCAAAGTTTGTGAAGTTTTTCTTGCTGCATTTGTCTTTTTTGC
A3-gBol009095-XLOC_041594-4319-1
GCCAGGATCAAAGTTTGTGAAGTTTTTCTTGCTGCATTTGTCTTTTTTGC
CONSENSUS
GCCAGGATCAAAGTTTGTGAAGTTTTTCTTGCTGCATTTGTCTTTTTTGC

A3-gBol009095-XLOC_041594-4319-0
TATTTAAAAAAAATATTTTGGAATCTTGAAAATGTTATAAATATTTTCTT
A3-gBol009095-XLOC_041594-4319-1
TATTTAAAAAAAATATTTTGGAATCTTGAAAATGTTATAAATATTTTCTT
CONSENSUS
TATTTAAAAAAAATATTTTGGAATCTTGAAAATGTTATAAATATTTTCTT

A3-gBol009095-XLOC_041594-4319-0 GAAGCTATTTTCTAAT
A3-gBol009095-XLOC_041594-4319-1 GAAGCTATTTTCTAAT
CONSENSUS GAAGCTATTTTCTAAT

alignment for event: A3-gBol008008-XLOC_042847-9329

A3-gBol008008-XLOC_042847-9329-0
GCTCTGCAGAGCAGAGAGTGGTTCCTCTGGTATATCTTCCAAAATATGGT
A3-gBol008008-XLOC_042847-9329-1
GCTCTGCAGAGCAGAGAGTGGTTCCTCTGGTATATCTTCCAAAATATGGT
CONSENSUS
GCTCTGCAGAGCAGAGAGTGGTTCCTCTGGTATATCTTCCAAAATATGGT

A3-gBol008008-XLOC_042847-9329-0
GGTGTTAAGAAACTTGAATCTGTCAGACTCTCTGAACTTAATATCTACGT
A3-gBol008008-XLOC_042847-9329-1
GGTGTTAAGAAACTTGAATCTGTCAGACTCTCTGAACTTAATATCTACGT
CONSENSUS
GGTGTTAAGAAACTTGAATCTGTCAGACTCTCTGAACTTAATATCTACGT

A3-gBol008008-XLOC_042847-9329-0
TCTGAATTCACCCCTACAGCACCACACAAGCGATGATATGTCTATAGCTG
A3-gBol008008-XLOC_042847-9329-1
TCTGAATTCACCCCTACAG-------------------------------
CONSENSUS
TCTGAATTCACCCCTACAG...............................

A3-gBol008008-XLOC_042847-9329-0
TTAAACTTTCTATGGTCCCAAATCCAGTGGTGAAAAGCAGTTGTTCGTAT
A3-gBol008008-XLOC_042847-9329-1 ---------------------------
TGGTGAAAAGCAGTTGTTCGTAT
CONSENSUS
...........................TGGTGAAAAGCAGTTGTTCGTAT

A3-gBol008008-XLOC_042847-9329-0
CACACAATCGGGCCAGATGATGTTCCAATTATTGAAGGATGTCCAGCCAG

A3-gBol008008-XLOC_042847-9329-1
CACACAATCGGGCCAGATGATGTTCCAATTATTGAAGGATGTCCAGCCAG
CONSENSUS
CACACAATCGGGCCAGATGATGTTCCAATTATTGAAGGATGTCCAGCCAG

A3-gBol008008-XLOC_042847-9329-0
CACTGACCCAGAGCCTGCACATACTGATTCAGTCCTGACCTCCACCGAGA
A3-gBol008008-XLOC_042847-9329-1
CACTGACCCAGAGCCTGCACATACTGATTCAGTCCTGACCTCCACCGAGA
CONSENSUS
CACTGACCCAGAGCCTGCACATACTGATTCAGTCCTGACCTCCACCGAGA

A3-gBol008008-XLOC_042847-9329-0
CTCCCATTGATGATGAAAAGGCCCCCGGTTCAAAGTAA
A3-gBol008008-XLOC_042847-9329-1
CTCCCATTGATGATGAAAAGGCCCCCGGTTCAAAGTAA
CONSENSUS
CTCCCATTGATGATGAAAAGGCCCCCGGTTCAAAGTAA

alignment for event: RI-X-XLOC_042869-5982

RI-X-XLOC_042869-5982-0
GTAACATGTTGAGCTAGCAGTGGCAGATGACACTTCTTATAAATGTAAGA
RI-X-XLOC_042869-5982-1
GTAACATGTTGAGCTAGCAGTGGCAGATGACACTTCTTATAAATGTAAGA
CONSENSUS
GTAACATGTTGAGCTAGCAGTGGCAGATGACACTTCTTATAAATGTAAGA

RI-X-XLOC_042869-5982-0
GACGTGTTTACATGCCATATAACATTATGGGGACATGAATGTTTTCACAT
RI-X-XLOC_042869-5982-1
GACGTGTTTACATGCCATATAACATTATGGGGACATGAATGTTTTCACAT
CONSENSUS
GACGTGTTTACATGCCATATAACATTATGGGGACATGAATGTTTTCACAT

RI-X-XLOC_042869-5982-0
CAACCACCAATAAAGTTCTTGCTGCTCATATGTGGAGAGATGATTCACCA
RI-X-XLOC_042869-5982-1
CAACCACCAATAAAGTTCTTGCTGCTCATATGTGGAGAGATGATTCACCA
CONSENSUS
CAACCACCAATAAAGTTCTTGCTGCTCATATGTGGAGAGATGATTCACCA

RI-X-XLOC_042869-5982-0
GAATAATGCAGGTTTCACTTTTAAAATGGTCAATCTTCTCTGAAAAATAA
RI-X-XLOC_042869-5982-1
GAATAATGCAG---------------------------------------
CONSENSUS
GAATAATGCAG.......................................

RI-X-XLOC_042869-5982-0
GCACATAAGACATTATACGATTGTAGATAGGTGAATTTTTTCTAATCTTA
RI-X-XLOC_042869-5982-1

CONSENSUS

....

RI-X-XLOC_042869-5982-0
ATGGTACGGTAATAGACCGCAAAGTGAGTTAAGGGAAGAGCTAACAAAGT
RI-X-XLOC_042869-5982-1 ---------------
ACCGCAAAGTGAGTTAAGGGAAGAGCTAACAAAGT
CONSENSUS
...............ACCGCAAAGTGAGTTAAGGGAAGAGCTAACAAAGT

RI-X-XLOC_042869-5982-0
TAACTCAGAGAAGAAGAAGCTAACAGAGATGTAGCAACAGTCTACGAGAA
RI-X-XLOC_042869-5982-1
TAACTCAGAGAAGAAGAAGCTAACAGAGATGTAGCAACAGTCTACGAGAA
CONSENSUS
TAACTCAGAGAAGAAGAAGCTAACAGAGATGTAGCAACAGTCTACGAGAA

RI-X-XLOC_042869-5982-0
CTACTACGCATTACATCATCATCTGGAGAAGCTTCAGAGTCGAAGTAGAC
RI-X-XLOC_042869-5982-1
CTACTACGCATTACATCATCATCTGGAGAAGCTTCAGAGTCGAAGTAGAC
CONSENSUS
CTACTACGCATTACATCATCATCTGGAGAAGCTTCAGAGTCGAAGTAGAC

RI-X-XLOC_042869-5982-0
ATGAAATAGGTATGCTCAAACAACTAGAAAAGAAAATAAAACAAGATCCA
RI-X-XLOC_042869-5982-1
ATGAAATAGGTATGCTCAAACAACTAGAAAAGAAAATAAAACAAGATCCA
CONSENSUS
ATGAAATAGGTATGCTCAAACAACTAGAAAAGAAAATAAAACAAGATCCA

RI-X-XLOC_042869-5982-0
TAGGAGTTCTTAGGGTTTCCTAATGGAGTCAGTAGTAGAGAACAACTCCA
RI-X-XLOC_042869-5982-1
TAGGAGTTCTTAGGGTTTCCTAATGGAGTCAGTAGTAGAGAACAACTCCA
CONSENSUS
TAGGAGTTCTTAGGGTTTCCTAATGGAGTCAGTAGTAGAGAACAACTCCA

RI-X-XLOC_042869-5982-0
GCTACCAAGATCATCATCAGCTGCATCAACATGAGCAGAAAAAAAAACAG
RI-X-XLOC_042869-5982-1
GCTACCAAGATCATCATCAGCTGCATCAACATGAGCAGAAAAAAAAACAG
CONSENSUS
GCTACCAAGATCATCATCAGCTGCATCAACATGAGCAGAAAAAAAAACAG

RI-X-XLOC_042869-5982-0
CTTCTTTCAGGCAAAAGACCAGTCATTGATAGCTTCAACAAAGCAAAGGT
RI-X-XLOC_042869-5982-1
CTTCTTTCAGGCAAAAGACCAGTCATTGATAGCTTCAACAAAGCAAAGGT
CONSENSUS
CTTCTTTCAGGCAAAAGACCAGTCATTGATAGCTTCAACAAAGCAAAGGT

RI-X-XLOC_042869-5982-0
TTCAACAGTATTTGTGCCTACTGATACTCCTGACACGAGCTTG
RI-X-XLOC_042869-5982-1
TTCAACAGTATTTGTGCCTACTGATACTCCTGACACGAGCTTG
CONSENSUS

TTCAACAGTATTTGTGCCTACTGATACTCCTGACACGAGCTTG

alignment for event: A3-X-XLOC_042877-16254

A3-X-XLOC_042877-16254-0
CTCACGACAGATCTCATCTCATCTCCTTCTCTGCTTTTTTTTCCCCCGAT
A3-X-XLOC_042877-16254-1
CTCACGACAGATCTCATCTCATCTCCTTCTCTGCTTTTTTTTCCCCCGAT
CONSENSUS
CTCACGACAGATCTCATCTCATCTCCTTCTCTGCTTTTTTTTCCCCCGAT

A3-X-XLOC_042877-16254-0
TCAGTCTTCTCTCTTCACTCTAACCTCTCATTCTCATATCTCATATCTCT
A3-X-XLOC_042877-16254-1
TCAGTCTTCTCTCTTCACTCTAACCTCTCATTCTCATATCTCATATCTCT
CONSENSUS
TCAGTCTTCTCTCTTCACTCTAACCTCTCATTCTCATATCTCATATCTCT

A3-X-XLOC_042877-16254-0
CTTCTCTCTCATCTCTCTCAACGCCTCTCTCATCTCGCCCATCTCTCTCA
A3-X-XLOC_042877-16254-1
CTTCTCTCTCATCTCTCTCAACGCCTCTCTCATCTCGCCCATCTCTCTCA
CONSENSUS
CTTCTCTCTCATCTCTCTCAACGCCTCTCTCATCTCGCCCATCTCTCTCA

A3-X-XLOC_042877-16254-0
ACGTCTCTCTCATCTTACCACCACCGTGTATCCTCCTCGTCTCACCACCA
A3-X-XLOC_042877-16254-1
ACGTCTCTCTCATCTTACCACCACCGTGTATCCTCCTCGTCTCACCACCA
CONSENSUS
ACGTCTCTCTCATCTTACCACCACCGTGTATCCTCCTCGTCTCACCACCA

A3-X-XLOC_042877-16254-0 CCGTTTTCCCCCTTGTCTCACCACCACCACG----
GTGGAGGAGCCAAGG
A3-X-XLOC_042877-16254-1
CCGTTTTCCCCCTTGTCTCACCACCACCACGATAGGTGGAGGAGCCAAGG
CONSENSUS
CCGTTTTCCCCCTTGTCTCACCACCACCACG....GTGGAGGAGCCAAGG

A3-X-XLOC_042877-16254-0
TTCATGGTGGCTGGAGGAAGCAAGGTTCATGGCTGCTGGAAGAGGGACAA
A3-X-XLOC_042877-16254-1
TTCATGGTGGCTGGAGGAAGCAAGGTTCATGGCTGCTGGAAGAGGGACAA
CONSENSUS
TTCATGGTGGCTGGAGGAAGCAAGGTTCATGGCTGCTGGAAGAGGGACAA

A3-X-XLOC_042877-16254-0
GGCTATGGAGGAGGCGGCATGAGTTTTTTCTTTAGGTTTAGGTTAGGTCT
A3-X-XLOC_042877-16254-1
GGCTATGGAGGAGGCGGCATGAGTTTTTTCTTTAGGTTTAGGTTAGGTCT
CONSENSUS
GGCTATGGAGGAGGCGGCATGAGTTTTTTCTTTAGGTTTAGGTTAGGTCT

A3-X-XLOC_042877-16254-0

GGTTAAGTGTAAACCGAGTTAGGAAAATTGTAGTGGTCTGATTTTATTTT
A3-X-XLOC_042877-16254-1
GGTTAAGTGTAAACCGAGTTAGGAAAATTGTAGTGGTCTGATTTTATTTT
CONSENSUS
GGTTAAGTGTAAACCGAGTTAGGAAAATTGTAGTGGTCTGATTTTATTTT

A3-X-XLOC_042877-16254-0
TTAATTTCTGGTTTAGTTTTGTAAACCAGAATTAATCTGTAAACCAAATT
A3-X-XLOC_042877-16254-1
TTAATTTCTGGTTTAGTTTTGTAAACCAGAATTAATCTGTAAACCAAATT
CONSENSUS
TTAATTTCTGGTTTAGTTTTGTAAACCAGAATTAATCTGTAAACCAAATT

A3-X-XLOC_042877-16254-0
TAATTTATTAATAAAATTTATTTTATTTTCAGTTACAAAATAA
A3-X-XLOC_042877-16254-1
TAATTTATTAATAAAATTTATTTTATTTTCAGTTACAAAATAA
CONSENSUS
TAATTTATTAATAAAATTTATTTTATTTTCAGTTACAAAATAA

alignment for event: RI-X-XLOC_049959-2670

RI-X-XLOC_049959-2670-0
ATGTTGGAGGTTGAGAAGACAATACAGGAGGCTAAGAAGGCAAGTCTCTA
RI-X-XLOC_049959-2670-1
ATGTTGGAGGTTGAGAAGACAATACAGGAGGCTAAGAAGGCAAGTCTCTA
CONSENSUS
ATGTTGGAGGTTGAGAAGACAATACAGGAGGCTAAGAAGGCAAGTCTCTA

RI-X-XLOC_049959-2670-0
TCTTCATAGGATCCCTCATAATGTTCCTTCTCAAGAGTTAAAAGGAGTTA
RI-X-XLOC_049959-2670-1
TCTTCATAGGATCCCTCATAATGTTCCTTCTCAAGAGTTAAAAGGAGTTA
CONSENSUS
TCTTCATAGGATCCCTCATAATGTTCCTTCTCAAGAGTTAAAAGGAGTTA

RI-X-XLOC_049959-2670-0
TTATGGGAGATTTCAAAGTCGAGGTTAAGGTAATGTTTTTGTAAACCCCA
RI-X-XLOC_049959-2670-1
TTATGGGAGATTTCAAAGTCGAGGTTAAGGTAATGTTTTTGTAAACCCCA
CONSENSUS
TTATGGGAGATTTCAAAGTCGAGGTTAAGGTAATGTTTTTGTAAACCCCA

RI-X-XLOC_049959-2670-0
CCCCAATATGCTAATGTTTTCCTTCTCACTAGGAAACTGAAGTATCATGT
RI-X-XLOC_049959-2670-1
CCCCAATATGCTAATGTTTTCCTTCTCACTAGGAAACTGAAGTATCATGT
CONSENSUS
CCCCAATATGCTAATGTTTTCCTTCTCACTAGGAAACTGAAGTATCATGT

RI-X-XLOC_049959-2670-0
TTTGTTTTGTTTTTGGGCAGCCACCAAAGAAGCTAGGACCTTATTATAGC
RI-X-XLOC_049959-2670-1
TTTGTTTTGTTTTTGGGCAGCCACCAAAGAAGCTAGGACCTTATTATAGC

CONSENSUS
TTTGTTTTGTTTTTGGGCAGCCACCAAAGAAGCTAGGACCTTATTATAGC

RI-X-XLOC_049959-2670-0
GCATAAGTTGTTTTTAGTAGTCATGAGGAAGCAAATCAAGCATTTGATAA
RI-X-XLOC_049959-2670-1
GCATAAGTTGTTTTTAGTAGTCATGAGGAAGCAAATCAAGCATTTGATAA
CONSENSUS
GCATAAGTTGTTTTTAGTAGTCATGAGGAAGCAAATCAAGCATTTGATAA

RI-X-XLOC_049959-2670-0
CGTTAATGGAGATATAGTTAAGGTACTTGGGATCATCTCTCTCACTGTGG
RI-X-XLOC_049959-2670-1
CGTTAATGGAGATATAGTTAAG----------------------------
CONSENSUS
CGTTAATGGAGATATAGTTAAG............................

RI-X-XLOC_049959-2670-0
TCATGTTCATGATCTGTTGCTATCTTTAGAAAATCTATAGGATGTGTATG
RI-X-XLOC_049959-2670-1

CONSENSUS
....

RI-X-XLOC_049959-2670-0
CCATCTCTTTCTTTCAGGATAAAATGGGTTTGTCACAAAAAAATGGTTGA
RI-X-XLOC_049959-2670-1 -----------------
GATAAAATGGGTTTGTCACAAAAAAATGGTTGA
CONSENSUS
.................GATAAAATGGGTTTGTCACAAAAAAATGGTTGA

RI-X-XLOC_049959-2670-0
GTTTAAGATGATGAGTTCTGGATCAGTATCTAGACTATACGTTTGTAAAA
RI-X-XLOC_049959-2670-1
GTTTAAGATGATGAGTTCTGGATCAGTATCTAGACTATACGTTTGTAAAA
CONSENSUS
GTTTAAGATGATGAGTTCTGGATCAGTATCTAGACTATACGTTTGTAAAA

RI-X-XLOC_049959-2670-0
ATGTTCAAGACAGACAGTGAAGTCTCTGCCAAGAAGAGAAGTAACACAGA
RI-X-XLOC_049959-2670-1
ATGTTCAAGACAGACAGTGAAGTCTCTGCCAAGAAGAGAAGTAACACAGA
CONSENSUS
ATGTTCAAGACAGACAGTGAAGTCTCTGCCAAGAAGAGAAGTAACACAGA

RI-X-XLOC_049959-2670-0
GGAGATCAAATTGAGTTCTAAGAGACACAGGAGGGAGAATGATGCTGAGG
RI-X-XLOC_049959-2670-1
GGAGATCAAATTGAGTTCTAAGAGACACAGGAGGGAGAATGATGCTGAGG
CONSENSUS
GGAGATCAAATTGAGTTCTAAGAGACACAGGAGGGAGAATGATGCTGAGG

RI-X-XLOC_049959-2670-0
AGACAAGAGAAGACAATGTAAACCATGGCAGCAGCCAAGAGAGGTAAATG
RI-X-XLOC_049959-2670-1
AGACAAGAGAAGACAATGTAAACCATGGCAGCAGCCAAGAGAGGTAAATG

CONSENSUS
      AGACAAGAGAAGACAATGTAAACCATGGCAGCAGCCAAGAGAGGTAAATG

RI-X-XLOC_049959-2670-0
      TGAAAATCATATAAAGGAAATTGAAGAACTGAAGGAGAATCATAG
RI-X-XLOC_049959-2670-1
      TGAAAATCATATAAAGGAAATTGAAGAACTGAAGGAGAATCATAG
CONSENSUS
      TGAAAATCATATAAAGGAAATTGAAGAACTGAAGGAGAATCATAG

alignment for event: RI-gBol036122-XLOC_011033-14755

RI-gBol036122-XLOC_011033-14755-0
      GTAAAAAAGTTAGTTGAAGAGGGAACTGGGAAAACCACTTTGGCATAGTT
RI-gBol036122-XLOC_011033-14755-1
      GTAAAAAAGTTAGTTGAAGAGGGAACTGGGAAAACCACTTTGGCATAGTT
CONSENSUS
      GTAAAAAAGTTAGTTGAAGAGGGAACTGGGAAAACCACTTTGGCATAGTT

RI-gBol036122-XLOC_011033-14755-0
      GATGATGCAAATGATGTTGGAATGATTCAAGAAGCTGACATTGGTGTTGG
RI-gBol036122-XLOC_011033-14755-1
      GATGATGCAAATGATGTTGGAATGATTCAAGAAGCTGACATTGGTGTTGG
CONSENSUS
      GATGATGCAAATGATGTTGGAATGATTCAAGAAGCTGACATTGGTGTTGG

RI-gBol036122-XLOC_011033-14755-0
      AATTAGTGGAGTCCAAAGAATGCAGGTTAATGTGATTTTTTATTGAATGT
RI-gBol036122-XLOC_011033-14755-1
      AATTAGTGGAGTCCAAAGAATGCAG-------------------------
CONSENSUS
      AATTAGTGGAGTCCAAAGAATGCAG.........................

RI-gBol036122-XLOC_011033-14755-0
      ATTTTCGTGTCAAGTTGTAGCAAATAACTGTTTTTTTTTTTTTGGTGGGT
RI-gBol036122-XLOC_011033-14755-1

CONSENSUS
      ....

RI-gBol036122-XLOC_011033-14755-0
      GCAGTTTATGACTGCCCAGTTCAGGTCTCTGGAGAGATTACTCATGTTCA
RI-gBol036122-XLOC_011033-14755-1   ----
TTTATGACTGCCCAGTTCAGGTCTCTGGAGAGATTACTCATGTTCA
CONSENSUS
      ....TTTATGACTGCCCAGTTCAGGTCTCTGGAGAGATTACTCATGTTCA

RI-gBol036122-XLOC_011033-14755-0
      TGGAAACTGGGGCTATAAATGGATAGCTCAAATG
RI-gBol036122-XLOC_011033-14755-1
      TGGAAACTGGGGCTATAAATGGATAGCTCAAATG
CONSENSUS
      TGGAAACTGGGGCTATAAATGGATAGCTCAAATG

alignment for event: RI-X-XLOC_007509-10638

RI-X-XLOC_007509-10638-0
      GCCACTCTCACCTAAGCCACCGTGAATGTCTACCGCTTAAACACATTCAG
RI-X-XLOC_007509-10638-1
      GCCACTCTCACCTAAGCCACCGTGAATGTCTACCGCTTAAACACATTCAG
CONSENSUS
      GCCACTCTCACCTAAGCCACCGTGAATGTCTACCGCTTAAACACATTCAG

RI-X-XLOC_007509-10638-0
      GCATCTTTTCACTGTTGGTTAAGTGTACTCTGTTAGCGGCTTTGAGCTGT
RI-X-XLOC_007509-10638-1
      GCATCTTTTCACTGTTGGTTAAGTGTACTCTGTTAGCGGCTTTGAGCTGT
CONSENSUS
      GCATCTTTTCACTGTTGGTTAAGTGTACTCTGTTAGCGGCTTTGAGCTGT

RI-X-XLOC_007509-10638-0
      CCGGACTTCAAGCTGTCCGACTCTCCTGTGTCGATCCGGCTCAATATTGA
RI-X-XLOC_007509-10638-1
      CCGGACTTCAAGCTGTCCGACTCTCCTGTGTCGATCCGGCTCAATATTGA
CONSENSUS
      CCGGACTTCAAGCTGTCCGACTCTCCTGTGTCGATCCGGCTCAATATTGA

RI-X-XLOC_007509-10638-0
      GACGGGTTTGAAAGACTTAACCGATCCAGGTTCAAATTCCTCAAGAACGT
RI-X-XLOC_007509-10638-1
      GACGGGTTTGAAAGACTTAACCGATCCAGGTTCAAATTCCTCAAGAACGT
CONSENSUS
      GACGGGTTTGAAAGACTTAACCGATCCAGGTTCAAATTCCTCAAGAACGT

RI-X-XLOC_007509-10638-0
      TTCAGGTTCTGCAAAGATGAGTTGCTTGGTCTAGCAAACACCAACACCCA
RI-X-XLOC_007509-10638-1
      TTCAGGTTCTGCAAAGATGAGTTGCTTGGTCTAGCAAACACCAACACCCA
CONSENSUS
      TTCAGGTTCTGCAAAGATGAGTTGCTTGGTCTAGCAAACACCAACACCCA

RI-X-XLOC_007509-10638-0
      TCTTCCAGGTAACCCTATTTTTTTTTTTTGCAAATAAGTCATTGATAAAT
RI-X-XLOC_007509-10638-1
      TCTTCCAG--
CONSENSUS
      TCTTCCAG..

RI-X-XLOC_007509-10638-0
      CGATCCACACCGGAATCTTGGATGAGAATCAAGGTATTTTGTAATTTATA
RI-X-XLOC_007509-10638-1

CONSENSUS
      ....

RI-X-XLOC_007509-10638-0
      TGTAAAGGCTTATATAGTTTATTTCTTAATAAACATCATCCTCACAACTC
RI-X-XLOC_007509-10638-1

CONSENSUS
....

RI-X-XLOC_007509-10638-0
CTACTAACACCAGAAACTTCTTTTCTCATATATCCTTCTAGATATGAGTT
RI-X-XLOC_007509-10638-1

CONSENSUS
....

RI-X-XLOC_007509-10638-0
GGTTGATAAATTTGTGCAGGAGCATGGGCTAACTGAATCGTTCCTCTGTG
RI-X-XLOC_007509-10638-1 -------------------
GAGCATGGGCTAACTGAATCGTTCCTCTGTG
CONSENSUS
...................GAGCATGGGCTAACTGAATCGTTCCTCTGTG

RI-X-XLOC_007509-10638-0 CCTGTCCTATTGGTATAATGCCTGATGGATCAG
RI-X-XLOC_007509-10638-1 CCTGTCCTATTGGTATAATGCCTGATGGATCAG
CONSENSUS CCTGTCCTATTGGTATAATGCCTGATGGATCAG

alignment for event: RI-X-XLOC_007509-10636

RI-X-XLOC_007509-10636-0
GCCACTCTCACCTAAGCCACCGTGAATGTCTACCGCTTAAACACATTCAG
RI-X-XLOC_007509-10636-1
GCCACTCTCACCTAAGCCACCGTGAATGTCTACCGCTTAAACACATTCAG
CONSENSUS
GCCACTCTCACCTAAGCCACCGTGAATGTCTACCGCTTAAACACATTCAG

RI-X-XLOC_007509-10636-0
GCATCTTTTCACTGTTGGTTAAGTGTACTCTGTTAGCGGCTTTGAGCTGT
RI-X-XLOC_007509-10636-1
GCATCTTTTCACTGTTGGTTAAGTGTACTCTGTTAGCGGCTTTGAGCTGT
CONSENSUS
GCATCTTTTCACTGTTGGTTAAGTGTACTCTGTTAGCGGCTTTGAGCTGT

RI-X-XLOC_007509-10636-0
CCGGACTTCAAGCTGTCCGACTCTCCTGTGTCGATCCGGCTCAATATTGA
RI-X-XLOC_007509-10636-1
CCGGACTTCAAGCTGTCCGACTCTCCTGTGTCGATCCGGCTCAATATTGA
CONSENSUS
CCGGACTTCAAGCTGTCCGACTCTCCTGTGTCGATCCGGCTCAATATTGA

RI-X-XLOC_007509-10636-0
GACGGGTTTGAAAGACTTAACCGATCCAGGTTCAAATTCCTCAAGAACGT
RI-X-XLOC_007509-10636-1
GACGGGTTTGAAAGACTTAACCGATCCAGGTTCAAATTCCTCAAGAACGT
CONSENSUS
GACGGGTTTGAAAGACTTAACCGATCCAGGTTCAAATTCCTCAAGAACGT

RI-X-XLOC_007509-10636-0
TTCAGGTTCTGCAAAGATGAGTTGCTTGGTCTAGCAAACACCAACACCCA

RI-X-XLOC_007509-10636-1
TTCAGGTTCTGCAAAGATGAGTTGCTTGGTCTAGCAAACACCAACACCCA
CONSENSUS
TTCAGGTTCTGCAAAGATGAGTTGCTTGGTCTAGCAAACACCAACACCCA

RI-X-XLOC_007509-10636-0
TCTTCCAGGTAACCCTATTTTTTTTTTTTGCAAATAAGTCATTGATAAAT
RI-X-XLOC_007509-10636-1
TCTTCCAGGTAACCCTATTTTTTTTTTTTGCAAATAAGTCATTGATAAAT
CONSENSUS
TCTTCCAGGTAACCCTATTTTTTTTTTTTGCAAATAAGTCATTGATAAAT

RI-X-XLOC_007509-10636-0
CGATCCACACCGGAATCTTGGATGAGAATCAAGGTATTTTGTAATTTATA
RI-X-XLOC_007509-10636-1
CGATCCACACCGGAATCTTGGATGAGAATCAAG-----------------
CONSENSUS
CGATCCACACCGGAATCTTGGATGAGAATCAAG.................

RI-X-XLOC_007509-10636-0
TGTAAAGGCTTATATAGTTTATTTCTTAATAAACATCATCCTCACAACTC
RI-X-XLOC_007509-10636-1

CONSENSUS
....

RI-X-XLOC_007509-10636-0
CTACTAACACCAGAAACTTCTTTTCTCATATATCCTTCTAGATATGAGTT
RI-X-XLOC_007509-10636-1

CONSENSUS
....

RI-X-XLOC_007509-10636-0
GGTTGATAAATTTGTGCAGGAGCATGGGCTAACTGAATCGTTCCTCTGTG
RI-X-XLOC_007509-10636-1 -------------------
GAGCATGGGCTAACTGAATCGTTCCTCTGTG
CONSENSUS
...................GAGCATGGGCTAACTGAATCGTTCCTCTGTG

RI-X-XLOC_007509-10636-0 CCTGTCCTATTGGTATAATGCCTGATGGATCAG
RI-X-XLOC_007509-10636-1 CCTGTCCTATTGGTATAATGCCTGATGGATCAG
CONSENSUS CCTGTCCTATTGGTATAATGCCTGATGGATCAG

alignment for event: A3-X-XLOC_007376-5765

A3-X-XLOC_007376-5765-0
CCAGGCTATACGTGCTTTAAAAGAAGCCACTTGCTGTGGAAGTTTGAGCT
A3-X-XLOC_007376-5765-1
CCAGGCTATACGTGCTTTAAAAGAAGCCACTTGCTGTGGAAGTTTGAGCT
CONSENSUS
CCAGGCTATACGTGCTTTAAAAGAAGCCACTTGCTGTGGAAGTTTGAGCT

A3-X-XLOC_007376-5765-0

CACGCTGGCTGGATGCACATTTAATCATCTCAAATATATATTTTTCTCTG
A3-X-XLOC_007376-5765-1
CACGCTGGCTGGATGCACATTTAATCATCTCAAATATATATTTTTCTCTG
CONSENSUS
CACGCTGGCTGGATGCACATTTAATCATCTCAAATATATATTTTTCTCTG

A3-X-XLOC_007376-5765-0
CAGGGAAAGGGTTTCTTTAGCTCATGGTTCTGCATCGACGGTTCTGTATT
A3-X-XLOC_007376-5765-1
CAGGGAAAGGGTTTCTTTAGCTCATGGTTCTGCATCGACGGTTCTGTATT
CONSENSUS
CAGGGAAAGGGTTTCTTTAGCTCATGGTTCTGCATCGACGGTTCTGTATT

A3-X-XLOC_007376-5765-0
GGGTGCATGAGAATATGATCAAGATAGATGAAAAGAGTGTCTCTTTCTGC
A3-X-XLOC_007376-5765-1
GGGTGCATGAGAATATGATCAAGATAGATGAAAAGAGTGTCTCTTTCTGC
CONSENSUS
GGGTGCATGAGAATATGATCAAGATAGATGAAAAGAGTGTCTCTTTCTGC

A3-X-XLOC_007376-5765-0
AGCCGGAGATGGTTATATTGCAAAGAGTTTTGGCTACCAGCCTAAGGAAC
A3-X-XLOC_007376-5765-1
AGCCGGAGATGGTTATATTGCAAAG-------------------------
CONSENSUS
AGCCGGAGATGGTTATATTGCAAAG.........................

A3-X-XLOC_007376-5765-0
GGATTTCGGTTCACCGCCTTTCTCCAGTATCATTGCTGGTATCCTTCTTA
A3-X-XLOC_007376-5765-1

CONSENSUS
....

A3-X-XLOC_007376-5765-0
AAATCTTTATTAACTCTTCAAGCTTAGTGTTGAGAAACCAACATGATCAA
A3-X-XLOC_007376-5765-1

CONSENSUS
....

A3-X-XLOC_007376-5765-0
CATCAACCACATATGTCTGAGTACAGACTTTGACAATCACAGCTTTTAAC
A3-X-XLOC_007376-5765-1

CONSENSUS
....

A3-X-XLOC_007376-5765-0
CACCAACTGTGAACAGGAACCAGTTTTTAGCATTAGCTTATTTCAAACCC
A3-X-XLOC_007376-5765-1

CONSENSUS
....

A3-X-XLOC_007376-5765-0

TGAAACTGCAAAGTTTTTCTTTTGTCTTGGACTTAATTTTTCATGTAATG
A3-X-XLOC_007376-5765-1

CONSENSUS
....

A3-X-XLOC_007376-5765-0
ACAGTCTTAGCAATGTAGATTGCAGCTGCACTTTTGGAACTGTTAAGTTT
A3-X-XLOC_007376-5765-1 ------------------
ATTGCAGCTGCACTTTTGGAACTGTTAAGTTT
CONSENSUS
..................ATTGCAGCTGCACTTTTGGAACTGTTAAGTTT

A3-X-XLOC_007376-5765-0 GCGTAAAGATCAAGAAGCTGCTCATAAG
A3-X-XLOC_007376-5765-1 GCGTAAAGATCAAGAAGCTGCTCATAAG
CONSENSUS GCGTAAAGATCAAGAAGCTGCTCATAAG

alignment for event: RI-gBol009399-XLOC_041212-15274

RI-gBol009399-XLOC_041212-15274-0
GGCTCGTCTCAGTTCTCTAACTGATGACATACCTACCACTTCTCAGAGTA
RI-gBol009399-XLOC_041212-15274-1
GGCTCGTCTCAGTTCTCTAACTGATGACATACCTACCACTTCTCAGAGTA
CONSENSUS
GGCTCGTCTCAGTTCTCTAACTGATGACATACCTACCACTTCTCAGAGTA

RI-gBol009399-XLOC_041212-15274-0
CTTCCACAGTTGACATGCCCCGTAGCTCTAATATGCAGGATTTATCTGGC
RI-gBol009399-XLOC_041212-15274-1
CTTCCACAGTTGACATGCCCCGTAGCTCTAATATGCAGGATTTATCTGGC
CONSENSUS
CTTCCACAGTTGACATGCCCCGTAGCTCTAATATGCAGGATTTATCTGGC

RI-gBol009399-XLOC_041212-15274-0
AGTATAGACCAATCTCATTTGCAAGAGCAGTCAAATTCGCAAGAACAAAG
RI-gBol009399-XLOC_041212-15274-1
AGTATAGACCAATCTCATTTGCAAGAGCAGTCAAATTCGCAAGAACAAAG
CONSENSUS
AGTATAGACCAATCTCATTTGCAAGAGCAGTCAAATTCGCAAGAACAAAG

RI-gBol009399-XLOC_041212-15274-0
TGAAGCACAAGTACAATCAGATTTACAAGAGTCATCGAGACCTCGTGATT
RI-gBol009399-XLOC_041212-15274-1
TGAAGCACAAGTACAATCAGATTTACAAGAGTCATCGAGACCTCGTGATT
CONSENSUS
TGAAGCACAAGTACAATCAGATTTACAAGAGTCATCGAGACCTCGTGATT

RI-gBol009399-XLOC_041212-15274-0
CTATAACTGGTTCAGGTTATATAGAGTATGTCATCAATCACAGTCCTGTG
RI-gBol009399-XLOC_041212-15274-1
CTATAACTGGTTCAGGTTATATAGAGTATGTCATCAATCACAGTCCTGTG
CONSENSUS
CTATAACTGGTTCAGGTTATATAGAGTATGTCATCAATCACAGTCCTGTG

RI-gBol009399-XLOC_041212-15274-0
AATAAGCCTGACGTGAAGCTATTAGAGGGATCTCTGCCTAGTTCCTTACA
RI-gBol009399-XLOC_041212-15274-1
AATAAGCCTGACGTGAAGCTATTAGAGGGATCTCTGCCTAGTTCCTTACA
CONSENSUS
AATAAGCCTGACGTGAAGCTATTAGAGGGATCTCTGCCTAGTTCCTTACA

RI-gBol009399-XLOC_041212-15274-0
ACCTGCGGATAGAATAGGTTCAACTGTTCCTCAAGGCCGCGTTGAAGTTG
RI-gBol009399-XLOC_041212-15274-1
ACCTGCGGATAGAATAGGTTCAACTGTTCCTCAAGGCCGCGTTGAAGTTG
CONSENSUS
ACCTGCGGATAGAATAGGTTCAACTGTTCCTCAAGGCCGCGTTGAAGTTG

RI-gBol009399-XLOC_041212-15274-0
TAGATGACAATATTCGATATAGTCCCTCAAAAGAGATACCTGTGCCTTAT
RI-gBol009399-XLOC_041212-15274-1
TAGATGACAATATTCGATATAGTCCCTCAAAAGAGATACCTGTGCCTTAT
CONSENSUS
TAGATGACAATATTCGATATAGTCCCTCAAAAGAGATACCTGTGCCTTAT

RI-gBol009399-XLOC_041212-15274-0
GCTTCTATTGTTTGTGATGATAAAAAAGAAGCACTTGAATCAAGGGGTGA
RI-gBol009399-XLOC_041212-15274-1
GCTTCTATTGTTTGTGATGATAAAAAAGAAGCACTTGAATCAAGGGGTGA
CONSENSUS
GCTTCTATTGTTTGTGATGATAAAAAAGAAGCACTTGAATCAAGGGGTGA

RI-gBol009399-XLOC_041212-15274-0
GAAAAGTAACAAAGACGAAGAAGCGTCAGAGATACACGAGCCAAAAATTG
RI-gBol009399-XLOC_041212-15274-1
GAAAAGTAACAAAGACGAAGAAGCGTCAGAGATACACGAGCCAAAAATTG
CONSENSUS
GAAAAGTAACAAAGACGAAGAAGCGTCAGAGATACACGAGCCAAAAATTG

RI-gBol009399-XLOC_041212-15274-0
GTCCAGGAACACCAGACAGAGTGAAGCAAAATCAAAGGGACTTTGATAGG
RI-gBol009399-XLOC_041212-15274-1
GTCCAGGAACACCAGACAGAGTGAAGCAAAATCAAAGGGACTTTGATAGG
CONSENSUS
GTCCAGGAACACCAGACAGAGTGAAGCAAAATCAAAGGGACTTTGATAGG

RI-gBol009399-XLOC_041212-15274-0
ACGTACGATTTTTTCGACGAGGTATGTATTGTGGGAGAAAAACAAAGTAA
RI-gBol009399-XLOC_041212-15274-1
ACGTACGATTTTTTCGACGAGGTATGTATTGTGGGAGAAAAACAAAGTAA
CONSENSUS
ACGTACGATTTTTTCGACGAGGTATGTATTGTGGGAGAAAAACAAAGTAA

RI-gBol009399-XLOC_041212-15274-0
GAGCCAGGCCAATAGTATTGATGAAAAACCAAGAATTGAGAGCGAAGAAG
RI-gBol009399-XLOC_041212-15274-1
GAGCCAGGCCAATAGTATTGATGAAAAACCAAGAATTGAGAGCGAAGAAG
CONSENSUS
GAGCCAGGCCAATAGTATTGATGAAAAACCAAGAATTGAGAGCGAAGAAG

RI-gBol009399-XLOC_041212-15274-0
AAAATACGAGTGAAGCAGATGAGTTTGTAGATGCACGTAATACAATTGAA
RI-gBol009399-XLOC_041212-15274-1
AAAATACGAGTGAAGCAGATGAGTTTGTAGATGCACGTAATACAATTGAA
CONSENSUS
AAAATACGAGTGAAGCAGATGAGTTTGTAGATGCACGTAATACAATTGAA

RI-gBol009399-XLOC_041212-15274-0
TCTGAATCAGAAAGTGAGTTTGATGGAATACCAAAACCAAAACTGGAGCA
RI-gBol009399-XLOC_041212-15274-1
TCTGAATCAGAAAGTGAGTTTGATGGAATACCAAAACCAAAACTGGAGCA
CONSENSUS
TCTGAATCAGAAAGTGAGTTTGATGGAATACCAAAACCAAAACTGGAGCA

RI-gBol009399-XLOC_041212-15274-0
TTATTTTGGGGATATCAGCACTTACTGTTCTGAAGATGCTAGCAGTGACA
RI-gBol009399-XLOC_041212-15274-1
TTATTTTGGGGATATCAGCACTTACTGTTCTGAAGATGCTAGCAGTGACA
CONSENSUS
TTATTTTGGGGATATCAGCACTTACTGTTCTGAAGATGCTAGCAGTGACA

RI-gBol009399-XLOC_041212-15274-0
ACAATGGTGGATCAGAAGATATACCATATGAAGAAATGGTAGAAGATCCA
RI-gBol009399-XLOC_041212-15274-1
ACAATGGTGGATCAGAAGATATACCATATGAAGAAATGGTAGAAGATCCA
CONSENSUS
ACAATGGTGGATCAGAAGATATACCATATGAAGAAATGGTAGAAGATCCA

RI-gBol009399-XLOC_041212-15274-0
CGCCATGAAAACTCTTTAGATGAATCTTGTTCAGTTTCCTACCTTTCTGA
RI-gBol009399-XLOC_041212-15274-1
CGCCATGAAAACTCTTTAGATGAATCTTGTTCAGTTTCCTACCTTTCTGA
CONSENSUS
CGCCATGAAAACTCTTTAGATGAATCTTGTTCAGTTTCCTACCTTTCTGA

RI-gBol009399-XLOC_041212-15274-0
TGATGCAAGTGTTTCTTGTTGCCAGTCAGATCCAGTGTGTGGGAAAGTTC
RI-gBol009399-XLOC_041212-15274-1
TGATGCAAGTGTTTCTTGTTGCCAGTCAGATCCAGTGTGTGGGAAAGTTC
CONSENSUS
TGATGCAAGTGTTTCTTGTTGCCAGTCAGATCCAGTGTGTGGGAAAGTTC

RI-gBol009399-XLOC_041212-15274-0
TGTCTCATGATGAAACTTTTCAAAATCCAAGGGATTTTTCTGCCATGCGT
RI-gBol009399-XLOC_041212-15274-1
TGTCTCATGATGAAACTTTTCAAAATCCAAGGGATTTTTCTGCCATGCGT
CONSENSUS
TGTCTCATGATGAAACTTTTCAAAATCCAAGGGATTTTTCTGCCATGCGT

RI-gBol009399-XLOC_041212-15274-0
CCTTCATTATTGGCTGAAGCAGCTTTTCCGGATGAAACAATTTTGCGAGA
RI-gBol009399-XLOC_041212-15274-1
CCTTCATTATTGGCTGAAGCAGCTTTTCCGGATGAAACAATTTTGCGAGA
CONSENSUS
CCTTCATTATTGGCTGAAGCAGCTTTTCCGGATGAAACAATTTTGCGAGA

RI-gBol009399-XLOC_041212-15274-0
ACCTGTTGCCGCACATACCTTGTTGGCAGGAGATTGTGCTAATGAAAAGA
RI-gBol009399-XLOC_041212-15274-1
ACCTGTTGCCGCACATACCTTGTTGGCAGGAGATTGTGCTAATGAAAAGA
CONSENSUS
ACCTGTTGCCGCACATACCTTGTTGGCAGGAGATTGTGCTAATGAAAAGA

RI-gBol009399-XLOC_041212-15274-0
TCTCATTTGAAGAACGTATTTCCTCAGGCATGTTTCTGAAAGATGCTATT
RI-gBol009399-XLOC_041212-15274-1
TCTCATTTGAAGAACGTATTTCCTCAGGCATGTTTCTGAAAGATGCTATT
CONSENSUS
TCTCATTTGAAGAACGTATTTCCTCAGGCATGTTTCTGAAAGATGCTATT

RI-gBol009399-XLOC_041212-15274-0
CCTGCTGAAAAGATCTTGCCAGAAGAACATTTGGCCAACTATCCATCTCT
RI-gBol009399-XLOC_041212-15274-1
CCTGCTGAAAAGATCTTGCCAGAAGAACATTTGGCCAACTATCCATCTCT
CONSENSUS
CCTGCTGAAAAGATCTTGCCAGAAGAACATTTGGCCAACTATCCATCTCT

RI-gBol009399-XLOC_041212-15274-0
TGCAGAAGCTGTTCCTCATGAAAAGAGCTTGCCAGGAGAGTCTGTTGCCA
RI-gBol009399-XLOC_041212-15274-1
TGCAGAAGCTGTTCCTCATGAAAAGAGCTTGCCAGGAGAGTCTGTTGCCA
CONSENSUS
TGCAGAAGCTGTTCCTCATGAAAAGAGCTTGCCAGGAGAGTCTGTTGCCA

RI-gBol009399-XLOC_041212-15274-0
AATATCCGTCTTTTGAAGAAATTGCACCCTCTGAAAGGATCTTGCCCGAA
RI-gBol009399-XLOC_041212-15274-1
AATATCCGTCTTTTGAAGAAATTGCACCCTCTGAAAGGATCTTGCCCGAA
CONSENSUS
AATATCCGTCTTTTGAAGAAATTGCACCCTCTGAAAGGATCTTGCCCGAA

RI-gBol009399-XLOC_041212-15274-0
AACTCTCTTTCCAAAATTCGGTCTTTGGCAGAAGCTGTGCCTGACAATAT
RI-gBol009399-XLOC_041212-15274-1
AACTCTCTTTCCAAAATTCGGTCTTTGGCAGAAGCTGTGCCTGACAATAT
CONSENSUS
AACTCTCTTTCCAAAATTCGGTCTTTGGCAGAAGCTGTGCCTGACAATAT

RI-gBol009399-XLOC_041212-15274-0
GGCCCTAGCAGAAGAACCAGGTGCTGCTCATCCATCTTTTCCCAAAGCTG
RI-gBol009399-XLOC_041212-15274-1
GGCCCTAGCAGAAGAACCAGGTGCTGCTCATCCATCTTTTCCCAAAGCTG
CONSENSUS
GGCCCTAGCAGAAGAACCAGGTGCTGCTCATCCATCTTTTCCCAAAGCTG

RI-gBol009399-XLOC_041212-15274-0
TTCAGGAAAATAAAATTTCACCAGAAGTTCTTGATTCCAAAAATTTGTCT
RI-gBol009399-XLOC_041212-15274-1
TTCAGGAAAATAAAATTTCACCAGAAGTTCTTGATTCCAAAAATTTGTCT
CONSENSUS
TTCAGGAAAATAAAATTTCACCAGAAGTTCTTGATTCCAAAAATTTGTCT

RI-gBol009399-XLOC_041212-15274-0
GACACAGTGCCAGAAGCTGTTTCAGAAGAGCAAATATCAGTTGAAGGATT
RI-gBol009399-XLOC_041212-15274-1
GACACAGTGCCAGAAGCTGTTTCAGAAGAGCAAATATCAGTTGAAGGATT
CONSENSUS
GACACAGTGCCAGAAGCTGTTTCAGAAGAGCAAATATCAGTTGAAGGATT

RI-gBol009399-XLOC_041212-15274-0
TGTTGGTGTATCTCGGTGTTTGGCAGAAGTTGTTCCTGATGAAAGGTTCT
RI-gBol009399-XLOC_041212-15274-1
TGTTGGTGTATCTCGGTGTTTGGCAGAAGTTGTTCCTGATGAAAGGTTCT
CONSENSUS
TGTTGGTGTATCTCGGTGTTTGGCAGAAGTTGTTCCTGATGAAAGGTTCT

RI-gBol009399-XLOC_041212-15274-0
TGACAGAAGAACCAGAAGAAGCTGCCACAGCATGTACGTCTTTGTCAAAG
RI-gBol009399-XLOC_041212-15274-1
TGACAGAAGAACCAGAAGAAGCTGCCACAGCATGTACGTCTTTGTCAAAG
CONSENSUS
TGACAGAAGAACCAGAAGAAGCTGCCACAGCATGTACGTCTTTGTCAAAG

RI-gBol009399-XLOC_041212-15274-0
GTCATGCCTACAGAAAAATTATTCCCAGAAAAAACTTTGGAAGCGCCTCA
RI-gBol009399-XLOC_041212-15274-1
GTCATGCCTACAGAAAAATTATTCCCAGAAAAAACTTTGGAAGCGCCTCA
CONSENSUS
GTCATGCCTACAGAAAAATTATTCCCAGAAAAAACTTTGGAAGCGCCTCA

RI-gBol009399-XLOC_041212-15274-0
GGATTTGTCAGAATTGCCTGAAGAAAATATCTTGCGAGAAAAAATCTGTTG
RI-gBol009399-XLOC_041212-15274-1
GGATTTGTCAGAATTGCCTGAAGAAAATATCTTGCGAGAAAAAATCTGTTG
CONSENSUS
GGATTTGTCAGAATTGCCTGAAGAAAATATCTTGCGAGAAAAAATCTGTTG

RI-gBol009399-XLOC_041212-15274-0
ATTATACACATCCATCTTGTGCCAAAGCTGTTTCTCCAAAAGAAAATCTA
RI-gBol009399-XLOC_041212-15274-1
ATTATACACATCCATCTTGTGCCAAAGCTGTTTCTCCAAAAGAAAATCTA
CONSENSUS
ATTATACACATCCATCTTGTGCCAAAGCTGTTTCTCCAAAAGAAAATCTA

RI-gBol009399-XLOC_041212-15274-0
TCACCACAAGTTCTTGATTCCGCAAATTTCTCTGTGGCAGAAGCTGTTTC
RI-gBol009399-XLOC_041212-15274-1
TCACCACAAGTTCTTGATTCCGCAAATTTCTCTGTGGCAGAAGCTGTTTC
CONSENSUS
TCACCACAAGTTCTTGATTCCGCAAATTTCTCTGTGGCAGAAGCTGTTTC

RI-gBol009399-XLOC_041212-15274-0
ACATGAGCAGATCAGACTTGAAGAATCTGGTGGCAAAAATCCGTGTTTGG
RI-gBol009399-XLOC_041212-15274-1
ACATGAGCAGATCAGACTTGAAGAATCTGGTGGCAAAAATCCGTGTTTGG
CONSENSUS
ACATGAGCAGATCAGACTTGAAGAATCTGGTGGCAAAAATCCGTGTTTGG

RI-gBol009399-XLOC_041212-15274-0
CAGAAGCTGTTCCTGATGAAAATTTCTTGACAGGAGAACGAGAAGAAGCT
RI-gBol009399-XLOC_041212-15274-1
CAGAAGCTGTTCCTGATGAAAATTTCTTGACAGGAGAACGAGAAGAAGCT
CONSENSUS
CAGAAGCTGTTCCTGATGAAAATTTCTTGACAGGAGAACGAGAAGAAGCT

RI-gBol009399-XLOC_041212-15274-0
GCCACCACATGCATGCCTGTCGAAAAGTTATTGCCAGAAAAACCTTTAGA
RI-gBol009399-XLOC_041212-15274-1
GCCACCACATGCATGCCTGTCGAAAAGTTATTGCCAGAAAAACCTTTAGA
CONSENSUS
GCCACCACATGCATGCCTGTCGAAAAGTTATTGCCAGAAAAACCTTTAGA

RI-gBol009399-XLOC_041212-15274-0
AGCGCATCATTATTTGCCAGAATTGCCTCAGGAAAATATCTTGCCAGAAA
RI-gBol009399-XLOC_041212-15274-1
AGCGCATCATTATTTGCCAGAATTGCCTCAGGAAAATATCTTGCCAGAAA
CONSENSUS
AGCGCATCATTATTTGCCAGAATTGCCTCAGGAAAATATCTTGCCAGAAA

RI-gBol009399-XLOC_041212-15274-0
AATCTGTTGGTTCTGCTCCTCCAGACGAATATCTACCACCGGAAGTTCTT
RI-gBol009399-XLOC_041212-15274-1
AATCTGTTGGTTCTGCTCCTCCAGACGAATATCTACCACCGGAAGTTCTT
CONSENSUS
AATCTGTTGGTTCTGCTCCTCCAGACGAATATCTACCACCGGAAGTTCTT

RI-gBol009399-XLOC_041212-15274-0
GATTCAACAAATGTGTCTGTGGCAGAAGCTGTTCCACAAGAGCAAATCGG
RI-gBol009399-XLOC_041212-15274-1
GATTCAACAAATGTGTCTGTGGCAGAAGCTGTTCCACAAGAGCAAATCGG
CONSENSUS
GATTCAACAAATGTGTCTGTGGCAGAAGCTGTTCCACAAGAGCAAATCGG

RI-gBol009399-XLOC_041212-15274-0
ACTTGGAGAATTTCTTGGCATAGATGCGTGTTTGGCGAAAGCTGTTCCTG
RI-gBol009399-XLOC_041212-15274-1
ACTTGGAGAATTTCTTGGCATAGATGCGTGTTTGGCGAAAGCTGTTCCTG
CONSENSUS
ACTTGGAGAATTTCTTGGCATAGATGCGTGTTTGGCGAAAGCTGTTCCTG

RI-gBol009399-XLOC_041212-15274-0
ATGAAAGGGGTTTGCCAGAAGAACCTGTCACCACATGTGTGTCTTTGACA
RI-gBol009399-XLOC_041212-15274-1
ATGAAAGGGGTTTGCCAGAAGAACCTGTCACCACATGTGTGTCTTTGACA
CONSENSUS
ATGAAAGGGGTTTGCCAGAAGAACCTGTCACCACATGTGTGTCTTTGACA

RI-gBol009399-XLOC_041212-15274-0
AAAGCCGGGGCCAATGAAAAGACCTTGCCAGAAGAGCCTTTGGAAACATA
RI-gBol009399-XLOC_041212-15274-1
AAAGCCGGGGCCAATGAAAAGACCTTGCCAGAAGAGCCTTTGGAAACATA
CONSENSUS
AAAGCCGGGGCCAATGAAAAGACCTTGCCAGAAGAGCCTTTGGAAACATA

RI-gBol009399-XLOC_041212-15274-0
TCCTTTGGCAGAATTGCCTGAAGAAAAGATTTCTCCTCAAGAAACTGTTG
RI-gBol009399-XLOC_041212-15274-1
TCCTTTGGCAGAATTGCCTGAAGAAAAGATTTCTCCTCAAGAAACTGTTG
CONSENSUS
TCCTTTGGCAGAATTGCCTGAAGAAAAGATTTCTCCTCAAGAAACTGTTG

RI-gBol009399-XLOC_041212-15274-0
ATGCCACACATCCATTTGAATCTGTTATTGATGAAGAAAGCTCACCAGAA
RI-gBol009399-XLOC_041212-15274-1
ATGCCACACATCCATTTGAATCTGTTATTGATGAAGAAAGCTCACCAGAA
CONSENSUS
ATGCCACACATCCATTTGAATCTGTTATTGATGAAGAAAGCTCACCAGAA

RI-gBol009399-XLOC_041212-15274-0
GTTTCTTGTTTGTCTTTGGAAGAAGCTCTTCCACAAGAGCAAATCTCACT
RI-gBol009399-XLOC_041212-15274-1
GTTTCTTGTTTGTCTTTGGAAGAAGCTCTTCCACAAGAGCAAATCTCACT
CONSENSUS
GTTTCTTGTTTGTCTTTGGAAGAAGCTCTTCCACAAGAGCAAATCTCACT

RI-gBol009399-XLOC_041212-15274-0
TGAAGAATTTGTTGGCATAGATCCGTGCTTGGCAGAAGCTGTTCCTGATG
RI-gBol009399-XLOC_041212-15274-1
TGAAGAATTTGTTGGCATAGATCCGTGCTTGGCAGAAGCTGTTCCTGATG
CONSENSUS
TGAAGAATTTGTTGGCATAGATCCGTGCTTGGCAGAAGCTGTTCCTGATG

RI-gBol009399-XLOC_041212-15274-0
AATGGCTCTCGCCAGAAGAAGCTGTCACCACATGCCTCTCTTTGGAAAAA
RI-gBol009399-XLOC_041212-15274-1
AATGGCTCTCGCCAGAAGAAGCTGTCACCACATGCCTCTCTTTGGAAAAA
CONSENSUS
AATGGCTCTCGCCAGAAGAAGCTGTCACCACATGCCTCTCTTTGGAAAAA

RI-gBol009399-XLOC_041212-15274-0
GCTGCCACAATAGAAGAAGTCTTTCCAGAAAAATCTTTGGAAACATATCC
RI-gBol009399-XLOC_041212-15274-1
GCTGCCACAATAGAAGAAGTCTTTCCAGAAAAATCTTTGGAAACATATCC
CONSENSUS
GCTGCCACAATAGAAGAAGTCTTTCCAGAAAAATCTTTGGAAACATATCC

RI-gBol009399-XLOC_041212-15274-0
CTTTTTCGCAGAATTGCCGGAAGAAAAGATTGTGCACGAAGAAGCTGATG
RI-gBol009399-XLOC_041212-15274-1
CTTTTTCGCAGAATTGCCGGAAGAAAAGATTGTGCACGAAGAAGCTGATG
CONSENSUS
CTTTTTCGCAGAATTGCCGGAAGAAAAGATTGTGCACGAAGAAGCTGATG

RI-gBol009399-XLOC_041212-15274-0
ATGCCACTCATCCATCTGTTTCTGAATCTGTTAGTGATGAAAAAATACTA
RI-gBol009399-XLOC_041212-15274-1
ATGCCACTCATCCATCTGTTTCTGAATCTGTTAGTGATGAAAAAATACTA
CONSENSUS
ATGCCACTCATCCATCTGTTTCTGAATCTGTTAGTGATGAAAAAATACTA

RI-gBol009399-XLOC_041212-15274-0
CCAGAAATTCTTGAGTCCACAAATTTTCCTGTTGCAACAGCTCTTCCGCT
RI-gBol009399-XLOC_041212-15274-1
CCAGAAATTCTTGAGTCCACAAATTTTCCTGTTGCAACAGCTCTTCCGCT
CONSENSUS
CCAGAAATTCTTGAGTCCACAAATTTTCCTGTTGCAACAGCTCTTCCGCT

RI-gBol009399-XLOC_041212-15274-0
AGAGCAAACCTTGCTTGAAGAATTTGTTGGCAATCCATATCTGACAGAAG
RI-gBol009399-XLOC_041212-15274-1
AGAGCAAACCTTGCTTGAAGAATTTGTTGGCAATCCATATCTGACAGAAG
CONSENSUS
AGAGCAAACCTTGCTTGAAGAATTTGTTGGCAATCCATATCTGACAGAAG

RI-gBol009399-XLOC_041212-15274-0
CTGTTGTTGATAAGAGGGTTTTGCCAGAAGAACCTGTTACCACATGTCTC
RI-gBol009399-XLOC_041212-15274-1
CTGTTGTTGATAAGAGGGTTTTGCCAGAAGAACCTGTTACCACATGTCTC
CONSENSUS
CTGTTGTTGATAAGAGGGTTTTGCCAGAAGAACCTGTTACCACATGTCTC

RI-gBol009399-XLOC_041212-15274-0
TCTTTGAAAAAGGCTGCGACAGTTGGAGTCTTGCCAGAAAAACCTTCGGA
RI-gBol009399-XLOC_041212-15274-1
TCTTTGAAAAAGGCTGCGACAGTTGGAGTCTTGCCAGAAAAACCTTCGGA
CONSENSUS
TCTTTGAAAAAGGCTGCGACAGTTGGAGTCTTGCCAGAAAAACCTTCGGA

RI-gBol009399-XLOC_041212-15274-0
AACATATCCTTCTTTCGGAGAATTTCCTGATGAAAAGATCGTGCTCCAAG
RI-gBol009399-XLOC_041212-15274-1
AACATATCCTTCTTTCGGAGAATTTCCTGATGAAAAGATCGTGCTCCAAG
CONSENSUS
AACATATCCTTCTTTCGGAGAATTTCCTGATGAAAAGATCGTGCTCCAAG

RI-gBol009399-XLOC_041212-15274-0
AAGCTGATGATGCCATCCATCTATCTGTTTCTGAAGCTGTTGGTGATGAA
RI-gBol009399-XLOC_041212-15274-1
AAGCTGATGATGCCATCCATCTATCTGTTTCTGAAGCTGTTGGTGATGAA
CONSENSUS
AAGCTGATGATGCCATCCATCTATCTGTTTCTGAAGCTGTTGGTGATGAA

RI-gBol009399-XLOC_041212-15274-0
AAAATCTCACCAGAAGTTCCTGATTCCACAAATTTCCCTGCTGCTGCAGC
RI-gBol009399-XLOC_041212-15274-1
AAAATCTCACCAGAAGTTCCTGATTCCACAAATTTCCCTGCTGCTGCAGC
CONSENSUS
AAAATCTCACCAGAAGTTCCTGATTCCACAAATTTCCCTGCTGCTGCAGC

RI-gBol009399-XLOC_041212-15274-0
TCTTCCGCAGGAGCAAATAGTAGAAGAATTTGTTGGCAATCCATGTATGA
RI-gBol009399-XLOC_041212-15274-1
TCTTCCGCAGGAGCAAATAGTAGAAGAATTTGTTGGCAATCCATGTATGA
CONSENSUS
TCTTCCGCAGGAGCAAATAGTAGAAGAATTTGTTGGCAATCCATGTATGA

RI-gBol009399-XLOC_041212-15274-0
CGGAAGCTGTTCCTGATAAGACGATTTTGCCAGGAGAACCTGTCACTACA
RI-gBol009399-XLOC_041212-15274-1
CGGAAGCTGTTCCTGATAAGACGATTTTGCCAGGAGAACCTGTCACTACA
CONSENSUS
CGGAAGCTGTTCCTGATAAGACGATTTTGCCAGGAGAACCTGTCACTACA

RI-gBol009399-XLOC_041212-15274-0
TGTCTCTCTTTGACAAAAGCTGCGACAATTGAAGGAGCCTTGCCAGAAAA
RI-gBol009399-XLOC_041212-15274-1
TGTCTCTCTTTGACAAAAGCTGCGACAATTGAAGGAGCCTTGCCAGAAAA
CONSENSUS
TGTCTCTCTTTGACAAAAGCTGCGACAATTGAAGGAGCCTTGCCAGAAAA

RI-gBol009399-XLOC_041212-15274-0
GCCTTTGGAAACATATCCTCCTTTCGCAGAATTGCCTAAAGAAGAGATCG
RI-gBol009399-XLOC_041212-15274-1
GCCTTTGGAAACATATCCTCCTTTCGCAGAATTGCCTAAAGAAGAGATCG
CONSENSUS
GCCTTTGGAAACATATCCTCCTTTCGCAGAATTGCCTAAAGAAGAGATCG

RI-gBol009399-XLOC_041212-15274-0
TGTTCAAAGAAGCTGATGATGCCACCCATCCATCTGTTTCTGAAGCTATT
RI-gBol009399-XLOC_041212-15274-1
TGTTCAAAGAAGCTGATGATGCCACCCATCCATCTGTTTCTGAAGCTATT
CONSENSUS
TGTTCAAAGAAGCTGATGATGCCACCCATCCATCTGTTTCTGAAGCTATT

RI-gBol009399-XLOC_041212-15274-0
AGTGATGAACAAATCTCACCAGTGGTTCTTCATTCCGAAAAGTTTCCTGC
RI-gBol009399-XLOC_041212-15274-1
AGTGATGAACAAATCTCACCAGTGGTTCTTCATTCCGAAAAGTTTCCTGC
CONSENSUS
AGTGATGAACAAATCTCACCAGTGGTTCTTCATTCCGAAAAGTTTCCTGC

RI-gBol009399-XLOC_041212-15274-0
TGCAGCAGCTCCTCCGCAGGAGCAAATCGTACTTGAAGAATTTGTTGCCA
RI-gBol009399-XLOC_041212-15274-1
TGCAGCAGCTCCTCCGCAGGAGCAAATCGTACTTGAAGAATTTGTTGCCA
CONSENSUS
TGCAGCAGCTCCTCCGCAGGAGCAAATCGTACTTGAAGAATTTGTTGCCA

RI-gBol009399-XLOC_041212-15274-0
ATCTTTGTCTGACAGAAGCAGTTCTTGATAAGAGGGTTTTGCCAGAAGAA
RI-gBol009399-XLOC_041212-15274-1
ATCTTTGTCTGACAGAAGCAGTTCTTGATAAGAGGGTTTTGCCAGAAGAA
CONSENSUS
ATCTTTGTCTGACAGAAGCAGTTCTTGATAAGAGGGTTTTGCCAGAAGAA

RI-gBol009399-XLOC_041212-15274-0
CCAGTCACTACATGTCTCTCTTTGACAAAAGCTGCGACAATTGAAGAAGT
RI-gBol009399-XLOC_041212-15274-1
CCAGTCACTACATGTCTCTCTTTGACAAAAGCTGCGACAATTGAAGAAGT
CONSENSUS
CCAGTCACTACATGTCTCTCTTTGACAAAAGCTGCGACAATTGAAGAAGT

RI-gBol009399-XLOC_041212-15274-0
CTTAGCAGAAAAACCTTTGGAAGCATATTCTTCTTTCGCAGAATTGCCTG
RI-gBol009399-XLOC_041212-15274-1
CTTAGCAGAAAAACCTTTGGAAGCATATTCTTCTTTCGCAGAATTGCCTG
CONSENSUS
CTTAGCAGAAAAACCTTTGGAAGCATATTCTTCTTTCGCAGAATTGCCTG

RI-gBol009399-XLOC_041212-15274-0
AAGAAATTATTGTGCAAGACGAAGCTGATTTTACCACTCATCCATCTGTT
RI-gBol009399-XLOC_041212-15274-1
AAGAAATTATTGTGCAAGACGAAGCTGATTTTACCACTCATCCATCTGTT
CONSENSUS
AAGAAATTATTGTGCAAGACGAAGCTGATTTTACCACTCATCCATCTGTT

RI-gBol009399-XLOC_041212-15274-0
TCTGGAGCTGGTATTGATGAAAAGTTCTCACCAGAAGTTCTTGATTCAAC
RI-gBol009399-XLOC_041212-15274-1
TCTGGAGCTGGTATTGATGAAAAGTTCTCACCAGAAGTTCTTGATTCAAC
CONSENSUS
TCTGGAGCTGGTATTGATGAAAAGTTCTCACCAGAAGTTCTTGATTCAAC

RI-gBol009399-XLOC_041212-15274-0
ATTTTTTCCTGTTACAGCAGCTCTTCAGCAAGAGAAAATCTTACTTGAAG
RI-gBol009399-XLOC_041212-15274-1
ATTTTTTCCTGTTACAGCAGCTCTTCAGCAAGAGAAAATCTTACTTGAAG
CONSENSUS
ATTTTTTCCTGTTACAGCAGCTCTTCAGCAAGAGAAAATCTTACTTGAAG

RI-gBol009399-XLOC_041212-15274-0
AATTTGTTGGCAATCCATATCTGGCAGAAGCTGCTCCTAATAGTAAGGTT
RI-gBol009399-XLOC_041212-15274-1
AATTTGTTGGCAATCCATATCTGGCAGAAGCTGCTCCTAATAGTAAGGTT
CONSENSUS
AATTTGTTGGCAATCCATATCTGGCAGAAGCTGCTCCTAATAGTAAGGTT

RI-gBol009399-XLOC_041212-15274-0
TTGCCAGAAGAGCCTGTCACCACAGGTATGTCTTTAACGAAACCGGAGCC
RI-gBol009399-XLOC_041212-15274-1
TTGCCAGAAGAGCCTGTCACCACAGGTATGTCTTTAACGAAACCGGAGCC
CONSENSUS
TTGCCAGAAGAGCCTGTCACCACAGGTATGTCTTTAACGAAACCGGAGCC

RI-gBol009399-XLOC_041212-15274-0
TACTGAAGAACTCTTGCCAGAAGAACCTTTAGAAGCGTATGCTTCTTTGG
RI-gBol009399-XLOC_041212-15274-1
TACTGAAGAACTCTTGCCAGAAGAACCTTTAGAAGCGTATGCTTCTTTGG
CONSENSUS
TACTGAAGAACTCTTGCCAGAAGAACCTTTAGAAGCGTATGCTTCTTTGG

RI-gBol009399-XLOC_041212-15274-0
AAAAATCTGATGATGACATACATCCATCATGTGCCGAAGCTGAAAGTGAT
RI-gBol009399-XLOC_041212-15274-1
AAAAATCTGATGATGACATACATCCATCATGTGCCGAAGCTGAAAGTGAT
CONSENSUS
AAAAATCTGATGATGACATACATCCATCATGTGCCGAAGCTGAAAGTGAT

RI-gBol009399-XLOC_041212-15274-0
AAAAACGTCGTACCAGATGTTCTTGATTCCAAAAATTTGTCCTTATCAGA
RI-gBol009399-XLOC_041212-15274-1
AAAAACGTCGTACCAGATGTTCTTGATTCCAAAAATTTGTCCTTATCAGA
CONSENSUS
AAAAACGTCGTACCAGATGTTCTTGATTCCAAAAATTTGTCCTTATCAGA

RI-gBol009399-XLOC_041212-15274-0
AGCTATTACACAAGAGCAAATTGAACTTGAAGAATCTGTTGGCGTAGATC
RI-gBol009399-XLOC_041212-15274-1
AGCTATTACACAAGAGCAAATTGAACTTGAAGAATCTGTTGGCGTAGATC
CONSENSUS
AGCTATTACACAAGAGCAAATTGAACTTGAAGAATCTGTTGGCGTAGATC

RI-gBol009399-XLOC_041212-15274-0
CTTGTTCTGCAGAAGCTGTTCCTGATGAAAGAGTTTTGCCAGAAGAAGCT
RI-gBol009399-XLOC_041212-15274-1
CTTGTTCTGCAGAAGCTGTTCCTGATGAAAGAGTTTTGCCAGAAGAAGCT
CONSENSUS
CTTGTTCTGCAGAAGCTGTTCCTGATGAAAGAGTTTTGCCAGAAGAAGCT

RI-gBol009399-XLOC_041212-15274-0
GTTCCTGATGAAAGGATTTTGCGAGAAGAAGCTGTCACCGCAAGTTTGTC
RI-gBol009399-XLOC_041212-15274-1
GTTCCTGATGAAAGGATTTTGCGAGAAGAAGCTGTCACCGCAAGTTTGTC
CONSENSUS
GTTCCTGATGAAAGGATTTTGCGAGAAGAAGCTGTCACCGCAAGTTTGTC

RI-gBol009399-XLOC_041212-15274-0
TTTGACAAAGGTAGCGCCCATCGAAAAGATCTTGCCAGAAGAACCTATGG
RI-gBol009399-XLOC_041212-15274-1
TTTGACAAAGGTAGCGCCCATCGAAAAGATCTTGCCAGAAGAACCTATGG
CONSENSUS
TTTGACAAAGGTAGCGCCCATCGAAAAGATCTTGCCAGAAGAACCTATGG

RI-gBol009399-XLOC_041212-15274-0
ATATGTATCCTTCTCTAGAAGAGTTGGCAGAGGAAAAGATCTCACAAGAA
RI-gBol009399-XLOC_041212-15274-1
ATATGTATCCTTCTCTAGAAGAGTTGGCAGAGGAAAAGATCTCACAAGAA
CONSENSUS
ATATGTATCCTTCTCTAGAAGAGTTGGCAGAGGAAAAGATCTCACAAGAA

RI-gBol009399-XLOC_041212-15274-0
GAAGAACCTGATGATGACACACATCCATGTTTTTCTGAAGCTGTTGATGA
RI-gBol009399-XLOC_041212-15274-1
GAAGAACCTGATGATGACACACATCCATGTTTTTCTGAAGCTGTTGATGA
CONSENSUS
GAAGAACCTGATGATGACACACATCCATGTTTTTCTGAAGCTGTTGATGA

RI-gBol009399-XLOC_041212-15274-0
TGAAAATATCTCGCTACTAGAACATCCTTGTTCCATATATCCATCTTTGG
RI-gBol009399-XLOC_041212-15274-1
TGAAAATATCTCGCTACTAGAACATCCTTGTTCCATATATCCATCTTTGG
CONSENSUS
TGAAAATATCTCGCTACTAGAACATCCTTGTTCCATATATCCATCTTTGG

RI-gBol009399-XLOC_041212-15274-0
AAGTGTCTGTTCCGCACGAGAAAACCTCAGATGAAGAAATTGTTGGCACA
RI-gBol009399-XLOC_041212-15274-1
AAGTGTCTGTTCCGCACGAGAAAACCTCAGATGAAGAAATTGTTGGCACA
CONSENSUS
AAGTGTCTGTTCCGCACGAGAAAACCTCAGATGAAGAAATTGTTGGCACA

RI-gBol009399-XLOC_041212-15274-0
AACCCATTTTTGGAACTAGCCGTTCCCAACGTAAGCAGTTTTCCAGACAC
RI-gBol009399-XLOC_041212-15274-1
AACCCATTTTTGGAACTAGCCGTTCCCAACGTAAGCAGTTTTCCAGACAC
CONSENSUS
AACCCATTTTTGGAACTAGCCGTTCCCAACGTAAGCAGTTTTCCAGACAC

RI-gBol009399-XLOC_041212-15274-0
ACATGGCATTACGTATCAGACTTCAGCAGAAGCTGTTTCTAAGGAAAAGA
RI-gBol009399-XLOC_041212-15274-1
ACATGGCATTACGTATCAGACTTCAGCAGAAGCTGTTTCTAAGGAAAAGA
CONSENSUS
ACATGGCATTACGTATCAGACTTCAGCAGAAGCTGTTTCTAAGGAAAAGA

RI-gBol009399-XLOC_041212-15274-0
AGTTGCCAGAAGAATCCTTACTTACATATCCGTCTTTGGCAGAAGATCTT
RI-gBol009399-XLOC_041212-15274-1
AGTTGCCAGAAGAATCCTTACTTACATATCCGTCTTTGGCAGAAGATCTT
CONSENSUS
AGTTGCCAGAAGAATCCTTACTTACATATCCGTCTTTGGCAGAAGATCTT

RI-gBol009399-XLOC_041212-15274-0
TTTGATGAAAAGGCCTCAGGCTCAGAAGCTCCTGGTTACACAACAGAAGC
RI-gBol009399-XLOC_041212-15274-1
TTTGATGAAAAGGCCTCAGGCTCAGAAGCTCCTGGTTACACAACAGAAGC
CONSENSUS
TTTGATGAAAAGGCCTCAGGCTCAGAAGCTCCTGGTTACACAACAGAAGC

RI-gBol009399-XLOC_041212-15274-0
TGGTCCACGCAACACTGAAGAACCTGTTGCCTCGGATCTGTCATTGACCG
RI-gBol009399-XLOC_041212-15274-1
TGGTCCACGCAACACTGAAGAACCTGTTGC--------------------
CONSENSUS
TGGTCCACGCAACACTGAAGAACCTGTTGC....................

RI-gBol009399-XLOC_041212-15274-0
AATCTATTTTAGATGAAGAGATCCCCGGCTTAGAAGCTCCTGCTTCCACA
RI-gBol009399-XLOC_041212-15274-1

CONSENSUS
....

RI-gBol009399-XLOC_041212-15274-0
ACAGAAACTGGCCCACACAACAAATCTTTCCCTGAAGAGCCTGTTGCCAC
RI-gBol009399-XLOC_041212-15274-1

CONSENSUS
....

RI-gBol009399-XLOC_041212-15274-0
CTGTCTGTCTTTGGCAGAAGCTGTACCTGAAGAAAAGATCTCTCTAGAAG
RI-gBol009399-XLOC_041212-15274-1

CONSENSUS
....

RI-gBol009399-XLOC_041212-15274-0
AAACCGATACCACATGTCCACCGTCTGCAGAAGCTGCTTTTGATGAAGAG
RI-gBol009399-XLOC_041212-15274-1

CONSENSUS
....

RI-gBol009399-XLOC_041212-15274-0
ATCTCAGGCTCAGAATCTCGTGGGGACACAACAGAAGCTGGTCCACACAA
RI-gBol009399-XLOC_041212-15274-1

CONSENSUS
....

RI-gBol009399-XLOC_041212-15274-0
CAATGAAGAACCTCTTACCACGGATCTGTCATTGACGGAATCCAGTTTTG
RI-gBol009399-XLOC_041212-15274-1 ------------------
CACGGATCTGTCATTGACGGAATCCAGTTTTG
CONSENSUS
..................CACGGATCTGTCATTGACGGAATCCAGTTTTG

RI-gBol009399-XLOC_041212-15274-0
ATGAAAAGATCCCCGGCTTAGAAGCTCCTGCTTCCACAACAGAAACTGAT
RI-gBol009399-XLOC_041212-15274-1
ATGAAAAGATCCCCGGCTTAGAAGCTCCTGCTTCCACAACAGAAACTGAT
CONSENSUS
ATGAAAAGATCCCCGGCTTAGAAGCTCCTGCTTCCACAACAGAAACTGAT

RI-gBol009399-XLOC_041212-15274-0
CCACACGGCAAAACTTTCCCTGAAGAACCAGTTGCCACGTATCTGTCCTT
RI-gBol009399-XLOC_041212-15274-1
CCACACGGCAAAACTTTCCCTGAAGAACCAGTTGCCACGTATCTGTCCTT
CONSENSUS
CCACACGGCAAAACTTTCCCTGAAGAACCAGTTGCCACGTATCTGTCCTT

RI-gBol009399-XLOC_041212-15274-0
GGAAGAACCTGTTCGGGACGACAAAGTTCTGCCAAAAGAACCTGCCGCTG
RI-gBol009399-XLOC_041212-15274-1
GGAAGAACCTGTTCGGGACGACAAAGTTCTGCCAAAAGAACCTGCCGCTG
CONSENSUS
GGAAGAACCTGTTCGGGACGACAAAGTTCTGCCAAAAGAACCTGCCGCTG

RI-gBol009399-XLOC_041212-15274-0
CATTTCTGGATTTGTCGGAAGGTATTCCTGACCAACAAGTGTTTTTGGAC
RI-gBol009399-XLOC_041212-15274-1
CATTTCTGGATTTGTCGGAAGGTATTCCTGACCAACAAGTGTTTTTGGAC
CONSENSUS
CATTTCTGGATTTGTCGGAAGGTATTCCTGACCAACAAGTGTTTTTGGAC

RI-gBol009399-XLOC_041212-15274-0
GATGCAGCATTTTTATCTTTTGCAGAAGCAATTTTCGATCAGAAGTTCTC
RI-gBol009399-XLOC_041212-15274-1
GATGCAGCATTTTTATCTTTTGCAGAAGCAATTTTCGATCAGAAGTTCTC
CONSENSUS
GATGCAGCATTTTTATCTTTTGCAGAAGCAATTTTCGATCAGAAGTTCTC

RI-gBol009399-XLOC_041212-15274-0
ACCAGAAGTTCCTGATTCCATGGATCTTCCTGCAAAAAACACATTGGAAA
RI-gBol009399-XLOC_041212-15274-1
ACCAGAAGTTCCTGATTCCATGGATCTTCCTGCAAAAAACACATTGGAAA
CONSENSUS
ACCAGAAGTTCCTGATTCCATGGATCTTCCTGCAAAAAACACATTGGAAA

RI-gBol009399-XLOC_041212-15274-0
AAGAAGTTGAAACATCTGATGGTACAATTGTTGAACCAGTAAATATATGG
RI-gBol009399-XLOC_041212-15274-1
AAGAAGTTGAAACATCTGATGGTACAATTGTTGAACCAGTAAATATATGG
CONSENSUS
AAGAAGTTGAAACATCTGATGGTACAATTGTTGAACCAGTAAATATATGG

RI-gBol009399-XLOC_041212-15274-0
AGCAACGGAGGACTCCTTGGACTTGCACCATCGAAACCTCCAGTTTTTGC
RI-gBol009399-XLOC_041212-15274-1
AGCAACGGAGGACTCCTTGGACTTGCACCATCGAAACCTCCAGTTTTTGC
CONSENSUS
AGCAACGGAGGACTCCTTGGACTTGCACCATCGAAACCTCCAGTTTTTGC

RI-gBol009399-XLOC_041212-15274-0
TGAGCCTAAGTCTGTAAGCGAACATATACAGAACGAGTTCAACGAAGCTA
RI-gBol009399-XLOC_041212-15274-1
TGAGCCTAAGTCTGTAAGCGAACATATACAGAACGAGTTCAACGAAGCTA
CONSENSUS
TGAGCCTAAGTCTGTAAGCGAACATATACAGAACGAGTTCAACGAAGCTA

RI-gBol009399-XLOC_041212-15274-0
GTGTTATTGCTACAAAGAAGCAGGGATTATCGAGCAGACCAGTTGAAGAT
RI-gBol009399-XLOC_041212-15274-1
GTGTTATTGCTACAAAGAAGCAGGGATTATCGAGCAGACCAGTTGAAGAT
CONSENSUS
GTGTTATTGCTACAAAGAAGCAGGGATTATCGAGCAGACCAGTTGAAGAT

RI-gBol009399-XLOC_041212-15274-0
ACTGAGAAGAGTTCACTTCCTTTAGTTGTTTCAGATCCCACGTCACAACA
RI-gBol009399-XLOC_041212-15274-1
ACTGAGAAGAGTTCACTTCCTTTAGTTGTTTCAGATCCCACGTCACAACA
CONSENSUS
ACTGAGAAGAGTTCACTTCCTTTAGTTGTTTCAGATCCCACGTCACAACA

RI-gBol009399-XLOC_041212-15274-0
ACAAAGCAACATGTTGAGTCATTCTAATGGATCTCTTTCCCCTCTGCAGA
RI-gBol009399-XLOC_041212-15274-1
ACAAAGCAACATGTTGAGTCATTCTAATGGATCTCTTTCCCCTCTGCAGA
CONSENSUS
ACAAAGCAACATGTTGAGTCATTCTAATGGATCTCTTTCCCCTCTGCAGA

RI-gBol009399-XLOC_041212-15274-0
GCACTGCAACATCATTCAAAGTATTTGGCTTAAGTCATAGATTACTCATG
RI-gBol009399-XLOC_041212-15274-1
GCACTGCAACATCATTCAAAGTATTTGGCTTAAGTCATAGATTACTCATG
CONSENSUS
GCACTGCAACATCATTCAAAGTATTTGGCTTAAGTCATAGATTACTCATG

RI-gBol009399-XLOC_041212-15274-0
GCTGGGTTTCGTGGAAACACTTCCTCAACCTACAAGTTTGAATCCATACC
RI-gBol009399-XLOC_041212-15274-1
GCTGGGTTTCGTGGAAACACTTCCTCAACCTACAAGTTTGAATCCATACC
CONSENSUS
GCTGGGTTTCGTGGAAACACTTCCTCAACCTACAAGTTTGAATCCATACC

RI-gBol009399-XLOC_041212-15274-0
TACTACCAGCTATGATACCAAAGCAGCAGCTATCGAAGACAAGACTCAAC
RI-gBol009399-XLOC_041212-15274-1
TACTACCAGCTATGATACCAAAGCAGCAGCTATCGAAGACAAGACTCAAC
CONSENSUS
TACTACCAGCTATGATACCAAAGCAGCAGCTATCGAAGACAAGACTCAAC

RI-gBol009399-XLOC_041212-15274-0
AAACTCCTCCCCGTGGCCCTAGCTTTGAGGAACAATTGGCTTATGAATCA
RI-gBol009399-XLOC_041212-15274-1
AAACTCCTCCCCGTGGCCCTAGCTTTGAGGAACAATTGGCTTATGAATCA
CONSENSUS
AAACTCCTCCCCGTGGCCCTAGCTTTGAGGAACAATTGGCTTATGAATCA

RI-gBol009399-XLOC_041212-15274-0
TCTATCTTTGGCTCGCCTACTTCATCACCACCGGTTGAACATATGAAAAT
RI-gBol009399-XLOC_041212-15274-1
TCTATCTTTGGCTCGCCTACTTCATCACCACCGGTTGAACATATGAAAAT
CONSENSUS
TCTATCTTTGGCTCGCCTACTTCATCACCACCGGTTGAACATATGAAAAT

RI-gBol009399-XLOC_041212-15274-0
ATCATTCAGTCCGATAGATCCTTCCCCAGTTTCCAAATTGAAACTGAGAA
RI-gBol009399-XLOC_041212-15274-1
ATCATTCAGTCCGATAGATCCTTCCCCAGTTTCCAAATTGAAACTGAGAA
CONSENSUS
ATCATTCAGTCCGATAGATCCTTCCCCAGTTTCCAAATTGAAACTGAGAA

RI-gBol009399-XLOC_041212-15274-0
TCCCATGCCAACCACAGTACAACGGTGAAAATGTGGACACTTTCCCTTCT
RI-gBol009399-XLOC_041212-15274-1
TCCCATGCCAACCACAGTACAACGGTGAAAATGTGGACACTTTCCCTTCT
CONSENSUS
TCCCATGCCAACCACAGTACAACGGTGAAAATGTGGACACTTTCCCTTCT

RI-gBol009399-XLOC_041212-15274-0
TTTCAGTTGGTCCCAGAGGCTAGTAACTCCGATAATGAGGATGACAACAG
RI-gBol009399-XLOC_041212-15274-1
TTTCAGTTGGTCCCAGAGGCTAGTAACTCCGATAATGAGGATGACAACAG
CONSENSUS
TTTCAGTTGGTCCCAGAGGCTAGTAACTCCGATAATGAGGATGACAACAG

RI-gBol009399-XLOC_041212-15274-0
    CGACATCTTTTGTCAGTCATCCCCTGGTGTTTCAGACAATTGCTTGTCGG
RI-gBol009399-XLOC_041212-15274-1
    CGACATCTTTTGTCAGTCATCCCCTGGTGTTTCAGACAATTGCTTGTCGG
CONSENSUS
    CGACATCTTTTGTCAGTCATCCCCTGGTGTTTCAGACAATTGCTTGTCGG

RI-gBol009399-XLOC_041212-15274-0
    ATTCTGAGCTGTGGGAATCTGATGAAAGCCCTAGAGAATCTGCATCAAGC
RI-gBol009399-XLOC_041212-15274-1
    ATTCTGAGCTGTGGGAATCTGATGAAAGCCCTAGAGAATCTGCATCAAGC
CONSENSUS
    ATTCTGAGCTGTGGGAATCTGATGAAAGCCCTAGAGAATCTGCATCAAGC

RI-gBol009399-XLOC_041212-15274-0
    TTGAAGCAAGGTGGAGAGAGAAGTACACATGGCGATATGGGTTCTTTTTC
RI-gBol009399-XLOC_041212-15274-1
    TTGAAGCAAGGTGGAGAGAGAAGTACACATGGCGATATGGGTTCTTTTTC
CONSENSUS
    TTGAAGCAAGGTGGAGAGAGAAGTACACATGGCGATATGGGTTCTTTTTC

RI-gBol009399-XLOC_041212-15274-0
    CAGTTTGTTTCTTGATCTTCCATGTTACGACTCTGTAGATCATCACTCCA
RI-gBol009399-XLOC_041212-15274-1
    CAGTTTGTTTCTTGATCTTCCATGTTACGACTCTGTAGATCATCACTCCA
CONSENSUS
    CAGTTTGTTTCTTGATCTTCCATGTTACGACTCTGTAGATCATCACTCCA

RI-gBol009399-XLOC_041212-15274-0
    CCTCACCAAGACTAGAGCAAGAGCATGAGCGAGAACAAGTTCCAGAATAT
RI-gBol009399-XLOC_041212-15274-1
    CCTCACCAAGACTAGAGCAAGAGCATGAGCGAGAACAAGTTCCAGAATAT
CONSENSUS
    CCTCACCAAGACTAGAGCAAGAGCATGAGCGAGAACAAGTTCCAGAATAT

RI-gBol009399-XLOC_041212-15274-0
    AAACCATCTGTTTCAGAGATCATCCGTGATTGGCCGCCAAACGCAAACGC
RI-gBol009399-XLOC_041212-15274-1
    AAACCATCTGTTTCAGAGATCATCCGTGATTGGCCGCCAAACGCAAACGC
CONSENSUS
    AAACCATCTGTTTCAGAGATCATCCGTGATTGGCCGCCAAACGCAAACGC

RI-gBol009399-XLOC_041212-15274-0
    TGTTCTAAAGAAAACGCAAACTTAATCCGCCAG
RI-gBol009399-XLOC_041212-15274-1
    TGTTCTAAAGAAAACGCAAACTTAATCCGCCAG
CONSENSUS
    TGTTCTAAAGAAAACGCAAACTTAATCCGCCAG

alignment for event: RI-gBol039453-XLOC_006869-16547

RI-gBol039453-XLOC_006869-16547-0
    AAATAAAAATAAAAATAAAAAGAAAAATAAAATAAAAAATCTGGAGGAAA
RI-gBol039453-XLOC_006869-16547-1

AAATAAAAATAAAAATAAAAAGAAAAATAAAATAAAAAATCTGGAGGAAA
CONSENSUS
AAATAAAAATAAAAATAAAAAGAAAAATAAAATAAAAAATCTGGAGGAAA

RI-gBol039453-XLOC_006869-16547-0
CCGTGCAGCCCAGAACTTCGACGCATTCTCGTCTTTGAACCAATCCGAGC
RI-gBol039453-XLOC_006869-16547-1
CCGTGCAGCCCAGAACTTCGACGCATTCTCGTCTTTGAACCAATCCGAGC
CONSENSUS
CCGTGCAGCCCAGAACTTCGACGCATTCTCGTCTTTGAACCAATCCGAGC

RI-gBol039453-XLOC_006869-16547-0
TCTCATCCCATCACTTGAATTCGTTTCACGACAGATCCTTTAGGTTTCCT
RI-gBol039453-XLOC_006869-16547-1
TCTCATCCCATCACTTGAATTCGTTTCACGACAGATCCTTTAGGTTTCCT
CONSENSUS
TCTCATCCCATCACTTGAATTCGTTTCACGACAGATCCTTTAGGTTTCCT

RI-gBol039453-XLOC_006869-16547-0
CCTTCATCTTCCTTGTTCGATTCCATCCATCACCCACTAGAACATCCGAT
RI-gBol039453-XLOC_006869-16547-1
CCTTCATCTTCCTTGTTCGATTCCATCCATCACCCACTAGAACATCCGAT
CONSENSUS
CCTTCATCTTCCTTGTTCGATTCCATCCATCACCCACTAGAACATCCGAT

RI-gBol039453-XLOC_006869-16547-0
CTGCTCCCCTCCCCACCTCCCTCCTGTTCGATTCTCACCATCCATATCTA
RI-gBol039453-XLOC_006869-16547-1
CTGCTCCCCTCCCCACCTCCCTCCTGTTCGATTCTCACCATCCATATCTA
CONSENSUS
CTGCTCCCCTCCCCACCTCCCTCCTGTTCGATTCTCACCATCCATATCTA

RI-gBol039453-XLOC_006869-16547-0
GGGTTTCCGAGGAGCTCCCTCAAAGGATTTCCCTTTGGTCAGTTATTTCT
RI-gBol039453-XLOC_006869-16547-1
GGGTTTCCGAGGAGCTCCCTCAAAGGATTTCCCTTTGGTCAGTTATTTCT
CONSENSUS
GGGTTTCCGAGGAGCTCCCTCAAAGGATTTCCCTTTGGTCAGTTATTTCT

RI-gBol039453-XLOC_006869-16547-0
CTTCATCGCCCCCGCGAGCTAGCAATCTCGTGCCGTATTGAATGCGACGA
RI-gBol039453-XLOC_006869-16547-1
CTTCATCGCCCCCGCGAGCTAGCAATCTCGTGCCGTATTGAATGCGACGA
CONSENSUS
CTTCATCGCCCCCGCGAGCTAGCAATCTCGTGCCGTATTGAATGCGACGA

RI-gBol039453-XLOC_006869-16547-0
GTGACGGGTTTATAGAATTATTATCATCATCTGACTAAAAGTTGGGTCCT
RI-gBol039453-XLOC_006869-16547-1
GTGACGGGTTTATAGAATTATTATCATCATCTGACTAAAA----------
CONSENSUS
GTGACGGGTTTATAGAATTATTATCATCATCTGACTAAAA..........

RI-gBol039453-XLOC_006869-16547-0
TTCGTTGATTGATTGATTGATTACTGGGAACACGTAATCGGGTTTCTCTT
RI-gBol039453-XLOC_006869-16547-1

CONSENSUS
....

RI-gBol039453-XLOC_006869-16547-0
ATCAATTTGATTGTCGAGCATTGTCTGTTTCTTCTATTTGGCCAAAAAAG
RI-gBol039453-XLOC_006869-16547-1

CONSENSUS
....

RI-gBol039453-XLOC_006869-16547-0
AAAAAAAAAACATTGTGTTGTCTGACAATTGATTTTGGTATTATTTGTAA
RI-gBol039453-XLOC_006869-16547-1

CONSENSUS
....

RI-gBol039453-XLOC_006869-16547-0
TGGATTTTTCTTGAATTTGTTTGCTTTCTTTTATAAATTGGTAGTTCGAC
RI-gBol039453-XLOC_006869-16547-1

CONSENSUS
....

RI-gBol039453-XLOC_006869-16547-0
CTTCTAATTTACACTAAAGAACTGGTCAGTTTGATTACTGTTATCATATC
RI-gBol039453-XLOC_006869-16547-1

CONSENSUS
....

RI-gBol039453-XLOC_006869-16547-0
AATTGTTCTTTAGAGTTAAGCTTGGTTTGAATTGATTCTTTGGGATTTGA
RI-gBol039453-XLOC_006869-16547-1

CONSENSUS
....

RI-gBol039453-XLOC_006869-16547-0
GTTTCATTTTGTGAGCCTAATCGTGCACTTGCCTTACAGGTTTCATAAGG
RI-gBol039453-XLOC_006869-16547-1
---------------------------------------GTTTCATAAGG
CONSENSUS
....GTTTCATAAGG

RI-gBol039453-XLOC_006869-16547-0
GGTGGTTGGATCCTGTAGCTTCTTAGTTATGACCTCTAGCACGACAGGGG
RI-gBol039453-XLOC_006869-16547-1
GGTGGTTGGATCCTGTAGCTTCTTAGTTATGACCTCTAGCACGACAGGGG
CONSENSUS
GGTGGTTGGATCCTGTAGCTTCTTAGTTATGACCTCTAGCACGACAGGGG

RI-gBol039453-XLOC_006869-16547-0 ATCGGAG
RI-gBol039453-XLOC_006869-16547-1 ATCGGAG
CONSENSUS ATCGGAG

alignment for event: RI-gBol039533-XLOC_006908-1952

RI-gBol039533-XLOC_006908-1952-0
GAATCATGAAGCCAGTCTTCTGTGGGAACTTTGAGTATGATGCTCGTGAG
RI-gBol039533-XLOC_006908-1952-1
GAATCATGAAGCCAGTCTTCTGTGGGAACTTTGAGTATGATGCTCGTGAG
CONSENSUS
GAATCATGAAGCCAGTCTTCTGTGGGAACTTTGAGTATGATGCTCGTGAG

RI-gBol039533-XLOC_006908-1952-0
GGTGATCTTGAACGCCTCTTCAGGAAATACGGCAGAGTTGAGAGGGTCGA
RI-gBol039533-XLOC_006908-1952-1
GGTGATCTTGAACGCCTCTTCAGGAAATACGGCAGAGTTGAGAGGGTCGA
CONSENSUS
GGTGATCTTGAACGCCTCTTCAGGAAATACGGCAGAGTTGAGAGGGTCGA

RI-gBol039533-XLOC_006908-1952-0
TATGAAAGCTGGTAAGCAAGTCCCCCCACTTTTCTTTCCTTCTTCTTCCT
RI-gBol039533-XLOC_006908-1952-1
TATGAAAGCTG---------------------------------------
CONSENSUS
TATGAAAGCTG.......................................

RI-gBol039533-XLOC_006908-1952-0
TTCACGTGATTTGACCTCAATGCGCAAGCAGTATCCGTAAACCACTAAGT
RI-gBol039533-XLOC_006908-1952-1

CONSENSUS
....

RI-gBol039533-XLOC_006908-1952-0
GCGATGTGTCAAAGCAAAATTCAAATGCATGTCATGAAATCCATTTCCAC
RI-gBol039533-XLOC_006908-1952-1

CONSENSUS
....

RI-gBol039533-XLOC_006908-1952-0
GACATGGCACTGGAACACTCATTCCCTCCCTCCCTGCCTCCACACAGACG
RI-gBol039533-XLOC_006908-1952-1

CONSENSUS
....

RI-gBol039533-XLOC_006908-1952-0
CACTCTTTTGCACAACATCTTCCTGGCCATGTCAAGGCAGCATGCCGCAA
RI-gBol039533-XLOC_006908-1952-1

CONSENSUS
....

RI-gBol039533-XLOC_006908-1952-0
AGTACCTCAGAGGATAGAGATTAAAAAAAAAAAATAGAGTAAAACCGCCA

RI-gBol039533-XLOC_006908-1952-1

CONSENSUS
....

RI-gBol039533-XLOC_006908-1952-0
TACTCAGCATTTTCTTTGGCGCAGGATTTTTAATCTTGGGATGCCTATAC
RI-gBol039533-XLOC_006908-1952-1

CONSENSUS
....

RI-gBol039533-XLOC_006908-1952-0
TCCCACCAGGCCATGAATGGACCGTAAGGAAATCTATCACACTGTCCCCC
RI-gBol039533-XLOC_006908-1952-1

CONSENSUS
....

RI-gBol039533-XLOC_006908-1952-0
TAAAACGAGTCACTTTCCTTCCGCAAATGGCACTGCCATCTTAATTACAT
RI-gBol039533-XLOC_006908-1952-1

CONSENSUS
....

RI-gBol039533-XLOC_006908-1952-0
TTCATTCCTCATGACTTTGGTTGGACACATTCCTCTCCCATGGAGCACTC
RI-gBol039533-XLOC_006908-1952-1

CONSENSUS
....

RI-gBol039533-XLOC_006908-1952-0
TGTTTTCATTCACATTCCGTCTTGACTACCACATTTTCAGTTGTTCCTTG
RI-gBol039533-XLOC_006908-1952-1

CONSENSUS
....

RI-gBol039533-XLOC_006908-1952-0
ACATATATTATTCCACAATACAAGGTCTGTTACTGCTTCACTGGTTATTT
RI-gBol039533-XLOC_006908-1952-1

CONSENSUS
....

RI-gBol039533-XLOC_006908-1952-0
AGTTTGTAAAGGCTGTTTGATGCCTCTGAAGGAGAAAGGATTTGTCTTCT
RI-gBol039533-XLOC_006908-1952-1

CONSENSUS
....

RI-gBol039533-XLOC_006908-1952-0
TGTTTGCTGGAGGTTATTAACTTGTTTTACATTGCTCACTGCTCGTGGAC

RI-gBol039533-XLOC_006908-1952-1

CONSENSUS
....

RI-gBol039533-XLOC_006908-1952-0
TCAGTTCTTTTATATTTGTGGCTTCTTCTCCAGGGTTTGCTTTCGTCTAC
RI-gBol039533-XLOC_006908-1952-1
---------------------------------GGTTTGCTTTCGTCTAC
CONSENSUS
.................................GGTTTGCTTTCGTCTAC

RI-gBol039533-XLOC_006908-1952-0
ATGGAAAACGAAAGGGATGCTGACGATGCAATCCGAGGGCTTGACCGCAT
RI-gBol039533-XLOC_006908-1952-1
ATGGAAAACGAAAGGGATGCTGACGATGCAATCCGAGGGCTTGACCGCAT
CONSENSUS
ATGGAAAACGAAAGGGATGCTGACGATGCAATCCGAGGGCTTGACCGCAT

RI-gBol039533-XLOC_006908-1952-0
TGAATTTGGGCGTAAGGGACGCAGACTTCGTGTTGAGTGGACAAAG
RI-gBol039533-XLOC_006908-1952-1
TGAATTTGGGCGTAAGGGACGCAGACTTCGTGTTGAGTGGACAAAG
CONSENSUS
TGAATTTGGGCGTAAGGGACGCAGACTTCGTGTTGAGTGGACAAAG

alignment for event: A3-gBol039533-XLOC_006908-1954

A3-gBol039533-XLOC_006908-1954-0
GAATCATGAAGCCAGTCTTCTGTGGGAACTTTGAGTATGATGCTCGTGAG
A3-gBol039533-XLOC_006908-1954-1
GAATCATGAAGCCAGTCTTCTGTGGGAACTTTGAGTATGATGCTCGTGAG
CONSENSUS
GAATCATGAAGCCAGTCTTCTGTGGGAACTTTGAGTATGATGCTCGTGAG

A3-gBol039533-XLOC_006908-1954-0
GGTGATCTTGAACGCCTCTTCAGGAAATACGGCAGAGTTGAGAGGGTCGA
A3-gBol039533-XLOC_006908-1954-1
GGTGATCTTGAACGCCTCTTCAGGAAATACGGCAGAGTTGAGAGGGTCGA
CONSENSUS
GGTGATCTTGAACGCCTCTTCAGGAAATACGGCAGAGTTGAGAGGGTCGA

A3-gBol039533-XLOC_006908-1954-0
TATGAAAGCTGGATTTTTAATCTTGGGATGCCTATACTCCCACCAGGCCA
A3-gBol039533-XLOC_006908-1954-1
TATGAAAGCTG---------------------------------------
CONSENSUS
TATGAAAGCTG.......................................

A3-gBol039533-XLOC_006908-1954-0
TGAATGGACCGTAAGGAAATCTATCACACTGTCCCCCTAAAACGAGTCAC
A3-gBol039533-XLOC_006908-1954-1

CONSENSUS

....

A3-gBol039533-XLOC_006908-1954-0
TTTCCTTCCGCAAATGGCACTGCCATCTTAATTACATTTCATTCCTCATG
A3-gBol039533-XLOC_006908-1954-1

CONSENSUS
....

A3-gBol039533-XLOC_006908-1954-0
ACTTTGGTTGGACACATTCCTCTCCCATGGAGCACTCTGTTTTCATTCAC
A3-gBol039533-XLOC_006908-1954-1

CONSENSUS
....

A3-gBol039533-XLOC_006908-1954-0
ATTCCGTCTTGACTACCACATTTTCAGTTGTTCCTTGACATATATTATTC
A3-gBol039533-XLOC_006908-1954-1

CONSENSUS
....

A3-gBol039533-XLOC_006908-1954-0
CACAATACAAGGTCTGTTACTGCTTCACTGGTTATTTAGTTTGTAAAGGC
A3-gBol039533-XLOC_006908-1954-1

CONSENSUS
....

A3-gBol039533-XLOC_006908-1954-0
TGTTTGATGCCTCTGAAGGAGAAAGGATTTGTCTTCTTGTTTGCTGGAGG
A3-gBol039533-XLOC_006908-1954-1

CONSENSUS
....

A3-gBol039533-XLOC_006908-1954-0
TTATTAACTTGTTTTACATTGCTCACTGCTCGTGGACTCAGTTCTTTTAT
A3-gBol039533-XLOC_006908-1954-1

CONSENSUS
....

A3-gBol039533-XLOC_006908-1954-0
ATTTGTGGCTTCTTCTCCAGGGTTTGCTTTCGTCTACATGGAAAACGAAA
A3-gBol039533-XLOC_006908-1954-1 --------------------
GGTTTGCTTTCGTCTACATGGAAAACGAAA
CONSENSUS
....................GGTTTGCTTTCGTCTACATGGAAAACGAAA

A3-gBol039533-XLOC_006908-1954-0
GGGATGCTGACGATGCAATCCGAGGGCTTGACCGCATTGAATTTGGGCGT
A3-gBol039533-XLOC_006908-1954-1
GGGATGCTGACGATGCAATCCGAGGGCTTGACCGCATTGAATTTGGGCGT
CONSENSUS

GGGATGCTGACGATGCAATCCGAGGGCTTGACCGCATTGAATTTGGGCGT

A3-gBol039533-XLOC_006908-1954-0
AAGGGACGCAGACTTCGTGTTGAGTGGACAAAG
A3-gBol039533-XLOC_006908-1954-1
AAGGGACGCAGACTTCGTGTTGAGTGGACAAAG
CONSENSUS
AAGGGACGCAGACTTCGTGTTGAGTGGACAAAG

alignment for event: A5-gBol043066-XLOC_003549-8539

A5-gBol043066-XLOC_003549-8539-0
TTGCTTAACTCTGGCACCTTGGATATTGATTACCTTGGAAAGATGCTTGA
A5-gBol043066-XLOC_003549-8539-1
TTGCTTAACTCTGGCACCTTGGATATTGATTACCTTGGAAAGATGCTTGA
CONSENSUS
TTGCTTAACTCTGGCACCTTGGATATTGATTACCTTGGAAAGATGCTTGA

A5-gBol043066-XLOC_003549-8539-0
GTTTGCATTAGCTACTCTTCGGAAACTCTCGGCTCCAGCCAATGACCGTG
A5-gBol043066-XLOC_003549-8539-1
GTTTGCATTAGCTACTCTTCGGAAACTCTCGGCTCCAGCCAATGACCGTG
CONSENSUS
GTTTGCATTAGCTACTCTTCGGAAACTCTCGGCTCCAGCCAATGACCGTG

A5-gBol043066-XLOC_003549-8539-0
AGAATGAAAGCACCCACCAGAGTTTACTGGAGGAATTTCACAGGTTGTGT
A5-gBol043066-XLOC_003549-8539-1
AGAATGAAAGCACCCACCAGAGTTTACTGGAGGAATTTCACAGGTTGTGT
CONSENSUS
AGAATGAAAGCACCCACCAGAGTTTACTGGAGGAATTTCACAGGTTGTGT

A5-gBol043066-XLOC_003549-8539-0
CAAGCTAAAGATGAGTCTGGTAGCCTCCATGCTGTTGCAATTGTCAAGGG
A5-gBol043066-XLOC_003549-8539-1
CAAGCTAAAGATGAGTCTGGTAGCCTCCATGCTGTTGCAATTGTCAAGGG
CONSENSUS
CAAGCTAAAGATGAGTCTGGTAGCCTCCATGCTGTTGCAATTGTCAAGGG

A5-gBol043066-XLOC_003549-8539-0
GATCCGCTTCATTCTTGAGCAGATTCACGTTGACCTTAAGCGAGAGATAG
A5-gBol043066-XLOC_003549-8539-1
GATCCGCTTCATTCTTGAGCAGATTCAC---GACCTTAAGCGAGAGATAG
CONSENSUS
GATCCGCTTCATTCTTGAGCAGATTCAC...GACCTTAAGCGAGAGATAG

A5-gBol043066-XLOC_003549-8539-0
GCATTGGGCACATTTTATAGGAGTTCTCCTGGTGATGCTGAAGCGTGAGT
A5-gBol043066-XLOC_003549-8539-1
GCATTGGGCACATTTTATAGGAGTTCTCCTGGTGATGCTGAAGCGTGAGT
CONSENSUS
GCATTGGGCACATTTTATAGGAGTTCTCCTGGTGATGCTGAAGCGTGAGT

A5-gBol043066-XLOC_003549-8539-0

TTGCTGTCAGTGTCTTTAATCTTAACATCCTCTTGGAGGGTTTATGGAAG
A5-gBol043066-XLOC_003549-8539-1
TTGCTGTCAGTGTCTTTAATCTTAACATCCTCTTGGAGGGTTTATGGAAG
CONSENSUS
TTGCTGTCAGTGTCTTTAATCTTAACATCCTCTTGGAGGGTTTATGGAAG

A5-gBol043066-XLOC_003549-8539-0
AATCTGGAATACGGCAAGAATGTTACTTTGCTACCCAATAGAGTGAGCGT
A5-gBol043066-XLOC_003549-8539-1
AATCTGGAATACGGCAAGAATGTTACTTTGCTACCCAATAGAGTGAGCGT
CONSENSUS
AATCTGGAATACGGCAAGAATGTTACTTTGCTACCCAATAGAGTGAGCGT

A5-gBol043066-XLOC_003549-8539-0
TGCTATGTAAATGCAAGCTGAAGCTAAACGTAAAAAGAGAACCCAAACTC
A5-gBol043066-XLOC_003549-8539-1
TGCTATGTAAATGCAAGCTGAAGCTAAACGTAAAAAGAGAACCCAAACTC
CONSENSUS
TGCTATGTAAATGCAAGCTGAAGCTAAACGTAAAAAGAGAACCCAAACTC

A5-gBol043066-XLOC_003549-8539-0 TTGAGTCTTAAG
A5-gBol043066-XLOC_003549-8539-1 TTGAGTCTTAAG
CONSENSUS TTGAGTCTTAAG

alignment for event: RI-gBol043066-XLOC_003549-8537

RI-gBol043066-XLOC_003549-8537-0
TTGCTTAACTCTGGCACCTTGGATATTGATTACCTTGGAAAGATGCTTGA
RI-gBol043066-XLOC_003549-8537-1
TTGCTTAACTCTGGCACCTTGGATATTGATTACCTTGGAAAGATGCTTGA
CONSENSUS
TTGCTTAACTCTGGCACCTTGGATATTGATTACCTTGGAAAGATGCTTGA

RI-gBol043066-XLOC_003549-8537-0
GTTTGCATTAGCTACTCTTCGGAAACTCTCGGCTCCAGCCAATGACCGTG
RI-gBol043066-XLOC_003549-8537-1
GTTTGCATTAGCTACTCTTCGGAAACTCTCGGCTCCAGCCAATGACCGTG
CONSENSUS
GTTTGCATTAGCTACTCTTCGGAAACTCTCGGCTCCAGCCAATGACCGTG

RI-gBol043066-XLOC_003549-8537-0
AGAATGAAAGCACCCACCAGAGTTTACTGGAGGAATTTCACAGGTTGTGT
RI-gBol043066-XLOC_003549-8537-1
AGAATGAAAGCACCCACCAGAGTTTACTGGAGGAATTTCACAGGTTGTGT
CONSENSUS
AGAATGAAAGCACCCACCAGAGTTTACTGGAGGAATTTCACAGGTTGTGT

RI-gBol043066-XLOC_003549-8537-0
CAAGCTAAAGATGAGTCTGGTAGCCTCCATGCTGTTGCAATTGTCAAGGG
RI-gBol043066-XLOC_003549-8537-1
CAAGCTAAAGATGAGTCTGGTAGCCTCCATGCTGTTGCAATTGTCAAGGG
CONSENSUS
CAAGCTAAAGATGAGTCTGGTAGCCTCCATGCTGTTGCAATTGTCAAGGG

RI-gBol043066-XLOC_003549-8537-0
      GATCCGCTTCATTCTTGAGCAGATTCACGTTGTATCCACTATAGCTTTAT
RI-gBol043066-XLOC_003549-8537-1
      GATCCGCTTCATTCTTGAGCAGATTCAC----------------------
CONSENSUS
      GATCCGCTTCATTCTTGAGCAGATTCAC......................

RI-gBol043066-XLOC_003549-8537-0
      TTCTCTCTCTCTTCATCAACATTAGCTTTGGAAAGAAAAATAATCTTTTC
RI-gBol043066-XLOC_003549-8537-1

CONSENSUS
      ....

RI-gBol043066-XLOC_003549-8537-0
      GTTTCCTTTGTCAACTTGACAGGACCTTAAGCGAGAGATAGGCATTGGGC
RI-gBol043066-XLOC_003549-8537-1 ----------------------
GACCTTAAGCGAGAGATAGGCATTGGGC
CONSENSUS
      ......................GACCTTAAGCGAGAGATAGGCATTGGGC

RI-gBol043066-XLOC_003549-8537-0
      ACATTTTATAGGAGTTCTCCTGGTGATGCTGAAGCGTGAGTTTGCTGTCA
RI-gBol043066-XLOC_003549-8537-1
      ACATTTTATAGGAGTTCTCCTGGTGATGCTGAAGCGTGAGTTTGCTGTCA
CONSENSUS
      ACATTTTATAGGAGTTCTCCTGGTGATGCTGAAGCGTGAGTTTGCTGTCA

RI-gBol043066-XLOC_003549-8537-0
      GTGTCTTTAATCTTAACATCCTCTTGGAGGGTTTATGGAAGAATCTGGAA
RI-gBol043066-XLOC_003549-8537-1
      GTGTCTTTAATCTTAACATCCTCTTGGAGGGTTTATGGAAGAATCTGGAA
CONSENSUS
      GTGTCTTTAATCTTAACATCCTCTTGGAGGGTTTATGGAAGAATCTGGAA

RI-gBol043066-XLOC_003549-8537-0
      TACGGCAAGAATGTTACTTTGCTACCCAATAGAGTGAGCGTTGCTATGTA
RI-gBol043066-XLOC_003549-8537-1
      TACGGCAAGAATGTTACTTTGCTACCCAATAGAGTGAGCGTTGCTATGTA
CONSENSUS
      TACGGCAAGAATGTTACTTTGCTACCCAATAGAGTGAGCGTTGCTATGTA

RI-gBol043066-XLOC_003549-8537-0
      AATGCAAGCTGAAGCTAAACGTAAAAAGAGAACCCAAACTCTTGAGTCTT
RI-gBol043066-XLOC_003549-8537-1
      AATGCAAGCTGAAGCTAAACGTAAAAAGAGAACCCAAACTCTTGAGTCTT
CONSENSUS
      AATGCAAGCTGAAGCTAAACGTAAAAAGAGAACCCAAACTCTTGAGTCTT

RI-gBol043066-XLOC_003549-8537-0 AAG
RI-gBol043066-XLOC_003549-8537-1 AAG
CONSENSUS   AAG

alignment for event: A5-gBol033464-XLOC_014131-1490

A5-gBol033464-XLOC_014131-1490-0
TGAAATCCCATTTTAGAAGATGATTAATAATAAATTTATATAAGAAGAAA
A5-gBol033464-XLOC_014131-1490-1
TGAAATCCCATTTTAGAAGATGATTAATAATAAATTTATATAAGAAGAAA
CONSENSUS
TGAAATCCCATTTTAGAAGATGATTAATAATAAATTTATATAAGAAGAAA

A5-gBol033464-XLOC_014131-1490-0
AATCGAAAATGATGAAGAAAGCAAATGGGTTAGGTTTGAGCTGTCAGTGA
A5-gBol033464-XLOC_014131-1490-1
AATCGAAAATGATGAAGAAAGCAAATGGGTTAGGTTTGAGCTGTCAGTGA
CONSENSUS
AATCGAAAATGATGAAGAAAGCAAATGGGTTAGGTTTGAGCTGTCAGTGA

A5-gBol033464-XLOC_014131-1490-0
GCTGTCTGTCTTTCATTTATATTCTCACCTCGCTCTCCTTCCACACAACA
A5-gBol033464-XLOC_014131-1490-1
GCTGTCTGTCTTTCATTTATATTCTCACCTCGCTCTCCTTCCACACAACA
CONSENSUS
GCTGTCTGTCTTTCATTTATATTCTCACCTCGCTCTCCTTCCACACAACA

A5-gBol033464-XLOC_014131-1490-0
CAAACAACGCTCCTTCTCTTACTCGCATCTCCCCATATTTTATATTTCAT
A5-gBol033464-XLOC_014131-1490-1
CAAACAACGCTCCTTCTCTTACTCGCATCTCCCCATATTTTATATTTCAT
CONSENSUS
CAAACAACGCTCCTTCTCTTACTCGCATCTCCCCATATTTTATATTTCAT

A5-gBol033464-XLOC_014131-1490-0
ATTTTACCTTGCTTCTTCTTCGAAGTCTCACCACTATTGCCACTCTACAC
A5-gBol033464-XLOC_014131-1490-1
ATTTTACCTTGCTTCTTCTTCGAAGTCTCACCACTATTGCCACTCTACAC
CONSENSUS
ATTTTACCTTGCTTCTTCTTCGAAGTCTCACCACTATTGCCACTCTACAC

A5-gBol033464-XLOC_014131-1490-0
TCAGATGTCTTGTGCAGTGAAATGAAGAAAACTATTCAAATCATCCTCCT
A5-gBol033464-XLOC_014131-1490-1
TCAGATGTCTTGTGCAGTGAAATGAAGAAAACTATTCAAATCATCCTCCT
CONSENSUS
TCAGATGTCTTGTGCAGTGAAATGAAGAAAACTATTCAAATCATCCTCCT

A5-gBol033464-XLOC_014131-1490-0
CTTACTTCTCCTCAATCTCTCACGTTCTCTCTCCATCTCTTCTCACGGTA
A5-gBol033464-XLOC_014131-1490-1
CTTACTTCTCCTCAATCTCTCACGTTCTCTCTCCATCTCTTCTCACGGTA
CONSENSUS
CTTACTTCTCCTCAATCTCTCACGTTCTCTCTCCATCTCTTCTCACGGTA

A5-gBol033464-XLOC_014131-1490-0
CTCTCTCCGACAGCGAAGTCCGTCACATTCAACGCCGTCAGCTACTTGAG
A5-gBol033464-XLOC_014131-1490-1
CTCTCTCCGACAGCGAAGTCCGTCACATTCAACGCCGTCAGCTACTTGAG
CONSENSUS
CTCTCTCCGACAGCGAAGTCCGTCACATTCAACGCCGTCAGCTACTTGAG

A5-gBol033464-XLOC_014131-1490-0
TTCGCCGAGCGCAGCCAGAATGTCATAGTCGATCCATCCCTCGTCTTTGA
A5-gBol033464-XLOC_014131-1490-1
TTCGCCGAGCGCAGCCAGAATGTCATAGTCGATCCATCCCTCGTCTTTGA
CONSENSUS
TTCGCCGAGCGCAGCCAGAATGTCATAGTCGATCCATCCCTCGTCTTTGA

A5-gBol033464-XLOC_014131-1490-0
AAACCCCAGATTACGCAATGCTTACATAGCTCTCCAAGCTTGGAAACAAG
A5-gBol033464-XLOC_014131-1490-1
AAACCCCAGATTACGCAATGCTTACATAGCTCTCCAAGCTTGGAAACAAG
CONSENSUS
AAACCCCAGATTACGCAATGCTTACATAGCTCTCCAAGCTTGGAAACAAG

A5-gBol033464-XLOC_014131-1490-0
CAATATTCTCCGATCCTTTCAATTTCACCGCGAATTGGATCGGATCCAAT
A5-gBol033464-XLOC_014131-1490-1
CAATATTCTCCGATCCTTTCAATTTCACCGCGAATTGGATCGGATCCAAT
CONSENSUS
CAATATTCTCCGATCCTTTCAATTTCACCGCGAATTGGATCGGATCCAAT

A5-gBol033464-XLOC_014131-1490-0
GTCTGTAACTACACTGGAGTCTTTTGTTCGCCGGCGCTCGATAACCGGAG
A5-gBol033464-XLOC_014131-1490-1
GTCTGTAACTACACTGGAGTCTTTTGTTCGCCGGCGCTCGATAACCGGAG
CONSENSUS
GTCTGTAACTACACTGGAGTCTTTTGTTCGCCGGCGCTCGATAACCGGAG

A5-gBol033464-XLOC_014131-1490-0
AATCCCTACTGTCGCCGGAATCAATCTCAATCACGCCGATATCGCCGGGT
A5-gBol033464-XLOC_014131-1490-1
AATCCCTACTGTCGCCGGAATCAATCTCAATCACGCCGATATCGCCGGGT
CONSENSUS
AATCCCTACTGTCGCCGGAATCAATCTCAATCACGCCGATATCGCCGGGT

A5-gBol033464-XLOC_014131-1490-0
ATTTACCTGAAGAGCTTGGTTTGTTATCAGATTTAGGGTCCGATTGGTAA
A5-gBol033464-XLOC_014131-1490-1
ATTTACCTGAAGAGCTTGGTTTGTTATCAGATTTAGGGTCCGATTGGTAA
CONSENSUS
ATTTACCTGAAGAGCTTGGTTTGTTATCAGATTTAGGGTCCGATTGGTAA

A5-gBol033464-XLOC_014131-1490-0
CAATTTTTGGTAACGACTTTCAGTTTTAGGCTTTGGTTTTAGAAATATGG
A5-gBol033464-XLOC_014131-1490-1
CAATTTTTGGTAACGACTTTCAGTTTTAGGCTTTGGTTTTAGAAATATGG
CONSENSUS
CAATTTTTGGTAACGACTTTCAGTTTTAGGCTTTGGTTTTAGAAATATGG

A5-gBol033464-XLOC_014131-1490-0
CTGTAAAAAATTTGGCTGCAAATTTGGCTTTAAAGATTGCGACTGTTAGA
A5-gBol033464-XLOC_014131-1490-1
CTGTAAAAAATTTGGCTGCAAATTTGGCTTTAAAGATTGCGACTGTTAGA
CONSENSUS
CTGTAAAAAATTTGGCTGCAAATTTGGCTTTAAAGATTGCGACTGTTAGA

A5-gBol033464-XLOC_014131-1490-0
GCATCATTATCGCGGATGCCAAGTCCGTCTCTTACGTTTTTAAAAAAAAA
A5-gBol033464-XLOC_014131-1490-1
GCATCATTATCGCGGATGCCAAGTCCGTCTCTTACGTTTTTAAAAAAAAA
CONSENSUS
GCATCATTATCGCGGATGCCAAGTCCGTCTCTTACGTTTTTAAAAAAAAA

A5-gBol033464-XLOC_014131-1490-0
AAAAAAAAAAACCTTTAATAATGCAAAAGACGTCGTTTAATTAAGCGCTA
A5-gBol033464-XLOC_014131-1490-1
AAAAAAAAAAACCTTTAATAATGCAAAAGACGTCGTTTAATTAAGCGCTA
CONSENSUS
AAAAAAAAAAACCTTTAATAATGCAAAAGACGTCGTTTAATTAAGCGCTA

A5-gBol033464-XLOC_014131-1490-0
CAAGAGACGTCCCTTACGGGACACGCGTCCTAACACGACATCTCAGTCTC
A5-gBol033464-XLOC_014131-1490-1
CAAGAGACGTCCCTTACGGGACACGCGTCCTAACACGACATCTCAGTCTC
CONSENSUS
CAAGAGACGTCCCTTACGGGACACGCGTCCTAACACGACATCTCAGTCTC

A5-gBol033464-XLOC_014131-1490-0
TCTCTCATCTCTCTTTCTCGACTGATTTGGTTTGATTCTTAAACTTCCAC
A5-gBol033464-XLOC_014131-1490-1
TCTCTCATCTCTCTTTCTCGACTGATTTGGTTTGATTCTTAAACTTCCAC
CONSENSUS
TCTCTCATCTCTCTTTCTCGACTGATTTGGTTTGATTCTTAAACTTCCAC

A5-gBol033464-XLOC_014131-1490-0
CACTGGAATCCCCGCCGTCTCAGCCGCTTCACCGCCTCCTCCGTTGACAA
A5-gBol033464-XLOC_014131-1490-1
CACTGGAATCCCCGCCGTCTCAGCCGCTTCACCGCCTCCTCCGTTGACAA
CONSENSUS
CACTGGAATCCCCGCCGTCTCAGCCGCTTCACCGCCTCCTCCGTTGACAA

A5-gBol033464-XLOC_014131-1490-0
ATTGAAGAAAGTGGTAGAGGAAGGTAATGCCTACGGAGCTTTGCAGATGT
A5-gBol033464-XLOC_014131-1490-1
ATTGAAGAAAGTGGTAGAGGAAGGTAATGCCTACGGAGCTTTGCAGATGT
CONSENSUS
ATTGAAGAAAGTGGTAGAGGAAGGTAATGCCTACGGAGCTTTGCAGATGT

A5-gBol033464-XLOC_014131-1490-0
ACAAATCCTTCAGTGCCAGGTTCCTTCATTACCCAATTCATCAAGTATCG
A5-gBol033464-XLOC_014131-1490-1
ACAAATCCTTCAGTGCCAG-------------------------------
CONSENSUS
ACAAATCCTTCAGTGCCAG...............................

A5-gBol033464-XLOC_014131-1490-0
AAATACCTTTTTTTGACTCTGTTAATCGGTTAAAGTTTGGATCTTTGATG
A5-gBol033464-XLOC_014131-1490-1

CONSENSUS
....

A5-gBol033464-XLOC_014131-1490-0
TTGTTTCTTACTTTTGAATATGAGATTAAAANNNNTTTTTTTTTGTTTGC
A5-gBol033464-XLOC_014131-1490-1

CONSENSUS
....

A5-gBol033464-XLOC_014131-1490-0
TCGGCAGATATGTTACGGGAAGAGGAAAGATCTGCAGAGAAGAAGAAGAG
A5-gBol033464-XLOC_014131-1490-1 ----------------------
AGGAAAGATCTGCAGAGAAGAAGAAGAG
CONSENSUS
......................AGGAAAGATCTGCAGAGAAGAAGAAGAG

A5-gBol033464-XLOC_014131-1490-0
AAAGAACAAGGAGACAAAATCAGAAACAAAAGAAGAGAAGAGAGCAGAGA
A5-gBol033464-XLOC_014131-1490-1
AAAGAACAAGGAGACAAAATCAGAAACAAAAGAAGAGAAGAGAGCAGAGA
CONSENSUS
AAAGAACAAGGAGACAAAATCAGAAACAAAAGAAGAGAAGAGAGCAGAGA

A5-gBol033464-XLOC_014131-1490-0
AGGATGAGAAGATCTTGCCAGAGGAGAAGAACACAACTTCTGTTGCGCTT
A5-gBol033464-XLOC_014131-1490-1
AGGATGAGAAGATCTTGCCAGAGGAGAAGAACACAACTTCTGTTGCGCTT
CONSENSUS
AGGATGAGAAGATCTTGCCAGAGGAGAAGAACACAACTTCTGTTGCGCTT

A5-gBol033464-XLOC_014131-1490-0
CCTTGCATTGATAAGCTTCGAGACGAGTTGTCTTGTGCA
A5-gBol033464-XLOC_014131-1490-1
CCTTGCATTGATAAGCTTCGAGACGAGTTGTCTTGTGCA
CONSENSUS
CCTTGCATTGATAAGCTTCGAGACGAGTTGTCTTGTGCA

alignment for event: A3-gBol001202-XLOC_050590-3349

A3-gBol001202-XLOC_050590-3349-0
AGGGACAACTTGGTTATACATCTGTGGATACTCAGGCAACACCACGCAAA
A3-gBol001202-XLOC_050590-3349-1
AGGGACAACTTGGTTATACATCTGTGGATACTCAGGCAACACCACGCAAA
CONSENSUS
AGGGACAACTTGGTTATACATCTGTGGATACTCAGGCAACACCACGCAAA

A3-gBol001202-XLOC_050590-3349-0
GTGACTTCTTTGAAAGCGAAGATTGTAGCTGTTTCTGCGGCAAACAAACA
A3-gBol001202-XLOC_050590-3349-1
GTGACTTCTTTGAAAGCGAAGATTGTAGCTGTTTCTGCGGCAAACAAACA
CONSENSUS
GTGACTTCTTTGAAAGCGAAGATTGTAGCTGTTTCTGCGGCAAACAAACA

A3-gBol001202-XLOC_050590-3349-0
CACCGCTGTAGTTTCTGACTGCGGTGAAGTTTTCACTTGGGGATGCAACA

A3-gBol001202-XLOC_050590-3349-1
      CACCGCTGTAGTTTCTGACTGCGGTGAAGTTTTCACTTGGGGATGCAACA
CONSENSUS
      CACCGCTGTAGTTTCTGACTGCGGTGAAGTTTTCACTTGGGGATGCAACA

A3-gBol001202-XLOC_050590-3349-0
      AGGAAGGTCAGCTTGGTTATGGAACCTCCAACTCAGCTTCAAACTATTTC
A3-gBol001202-XLOC_050590-3349-1
      AGGAAGGTCAGCTTGGTTATGGAACCTCCAACTCAGCTTCAAACTATTTC
CONSENSUS
      AGGAAGGTCAGCTTGGTTATGGAACCTCCAACTCAGCTTCAAACTATTTC

A3-gBol001202-XLOC_050590-3349-0
      CCTAGATTGGTTGATTACTTGAAAGGAAAAGTTTTCACGGCTATCTCATC
A3-gBol001202-XLOC_050590-3349-1
      CCTAGATTGGTTGATTACTTGAAAGGAAAAGTTTTCACGGCTATCTCATC
CONSENSUS
      CCTAGATTGGTTGATTACTTGAAAGGAAAAGTTTTCACGGCTATCTCATC

A3-gBol001202-XLOC_050590-3349-0
      TGCAAAATACCACACACTCGTCTTGAGAGAAGATGGAGAGTATTTCTTTC
A3-gBol001202-XLOC_050590-3349-1
      TGCAAAATACCACACACTCGTCTTGAGAGAAGATGGAGAG----------
CONSENSUS
      TGCAAAATACCACACACTCGTCTTGAGAGAAGATGGAGAG..........

A3-gBol001202-XLOC_050590-3349-0
      TTGCTTCTGTGACCAGGTGTACACTTGGGGTCATCGGCTGGTGACTCCGA
A3-gBol001202-XLOC_050590-3349-1 ----------------
GTGTACACTTGGGGTCATCGGCTGGTGACTCCGA
CONSENSUS
      ................GTGTACACTTGGGGTCATCGGCTGGTGACTCCGA

A3-gBol001202-XLOC_050590-3349-0
      GACGTGTTGTTGTTTCCCGGAATCTAAAGAAAGCTGGGAACACGTTGTTG
A3-gBol001202-XLOC_050590-3349-1
      GACGTGTTGTTGTTTCCCGGAATCTAAAGAAAGCTGGGAACACGTTGTTG
CONSENSUS
      GACGTGTTGTTGTTTCCCGGAATCTAAAGAAAGCTGGGAACACGTTGTTG

A3-gBol001202-XLOC_050590-3349-0
      AATTTTCATCGAAGGAGACCTCTTCGTGTGACTGCTATAGCTGCAGGGAT
A3-gBol001202-XLOC_050590-3349-1
      AATTTTCATCGAAGGAGACCTCTTCGTGTGACTGCTATAGCTGCAGGGAT
CONSENSUS
      AATTTTCATCGAAGGAGACCTCTTCGTGTGACTGCTATAGCTGCAGGGAT

A3-gBol001202-XLOC_050590-3349-0
      GGTACACAGCATGGCTCTAGCAGAAGATGGTGCATTGTTTTATTGGGTTT
A3-gBol001202-XLOC_050590-3349-1
      GGTACACAGCATGGCTCTAGCAGAAGATGGTGCATTGTTTTATTGGGTTT
CONSENSUS
      GGTACACAGCATGGCTCTAGCAGAAGATGGTGCATTGTTTTATTGGGTTT

A3-gBol001202-XLOC_050590-3349-0 CTTCTGACTCCAATCTCAAAGGCCAACAG
A3-gBol001202-XLOC_050590-3349-1 CTTCTGACTCCAATCTCAAAGGCCAACAG

CONSENSUS CTTCTGACTCCAATCTCAAAGGCCAACAG

alignment for event: RI-gBol001191-XLOC_050592-13348

RI-gBol001191-XLOC_050592-13348-0
TGAGCTTGAATTGTACATGAGGGATGGTTGTAAAGATACATAGCAACCTT
RI-gBol001191-XLOC_050592-13348-1
TGAGCTTGAATTGTACATGAGGGATGGTTGTAAAGATACATAGCAACCTT
CONSENSUS
TGAGCTTGAATTGTACATGAGGGATGGTTGTAAAGATACATAGCAACCTT

RI-gBol001191-XLOC_050592-13348-0
AGATTTTTCCCACACCAGCCATTACTTCTCCATGATCCATTGACATCAGA
RI-gBol001191-XLOC_050592-13348-1
AGATTTTTCCCACACCAGCCATTACTTCTCCATGATCCATTGACATCAGA
CONSENSUS
AGATTTTTCCCACACCAGCCATTACTTCTCCATGATCCATTGACATCAGA

RI-gBol001191-XLOC_050592-13348-0
GACTCCAAGAGACCCATACTCATTCCAATCGTCCCTGAACTCGTGATTTA
RI-gBol001191-XLOC_050592-13348-1
GACTCCAAGAGACCCATACTCATTCCAATCGTCCCTGAACTCGTGATTTA
CONSENSUS
GACTCCAAGAGACCCATACTCATTCCAATCGTCCCTGAACTCGTGATTTA

RI-gBol001191-XLOC_050592-13348-0
AGCTCACAGAAGAGATGTGACTCGAGCAGGAAGTTTGAGAACTTCCCCCC
RI-gBol001191-XLOC_050592-13348-1
AGCTCACAGAAGAGATGTGACTCGAGCAGGAAGTTTGAGAACTTCCCCCC
CONSENSUS
AGCTCACAGAAGAGATGTGACTCGAGCAGGAAGTTTGAGAACTTCCCCCC

RI-gBol001191-XLOC_050592-13348-0
CTGATAGATTTGAAAGTTGAAATCTTTGGTATCAAAGAGGCCAAGTTTCA
RI-gBol001191-XLOC_050592-13348-1
CTGATAGATTTGAAAGTTGAAATCTTTGGTATCAAAGAGGCCAAGTTTCA
CONSENSUS
CTGATAGATTTGAAAGTTGAAATCTTTGGTATCAAAGAGGCCAAGTTTCA

RI-gBol001191-XLOC_050592-13348-0
TATCTTAGAAAGCGACACGTGGGAGAGAGAGTCAAAGGTCAAACCTTTTT
RI-gBol001191-XLOC_050592-13348-1
TATCTTAGAAAGCGACACGTGGGAGAGAGAGTCAAAG-------------
CONSENSUS
TATCTTAGAAAGCGACACGTGGGAGAGAGAGTCAAAG.............

RI-gBol001191-XLOC_050592-13348-0
TCCCCCAAATACTTTCTTCAATAAATTCTCCAACATCACACAGTACACTT
RI-gBol001191-XLOC_050592-13348-1

CONSENSUS
....

RI-gBol001191-XLOC_050592-13348-0

CTCTGTCACTAAGCGAGAGATGTCTTCCCTTGCAGCTAGGGTTTTCAGAT
RI-gBol001191-XLOC_050592-13348-1
----------------------------------CTAGGGTTTTCAGAT
CONSENSUS
..................................CTAGGGTTTTCAGAT

RI-gBol001191-XLOC_050592-13348-0
CGCTTCTAGCTCCTGCATCTCGAGCCGCCACCTCTTCTTCCCGAAGTGCA
RI-gBol001191-XLOC_050592-13348-1
CGCTTCTAGCTCCTGCATCTCGAGCCGCCACCTCTTCTTCCCGAAGTGCA
CONSENSUS
CGCTTCTAGCTCCTGCATCTCGAGCCGCCACCTCTTCTTCCCGAAGTGCA

RI-gBol001191-XLOC_050592-13348-0
AGCACTGTCGCCGCTGGAGGCACCAAGAAGTCGGCGGCGAAACCGAAGGC
RI-gBol001191-XLOC_050592-13348-1
AGCACTGTCGCCGCTGGAGGCACCAAGAAGTCGGCGGCGAAACCGAAGGC
CONSENSUS
AGCACTGTCGCCGCTGGAGGCACCAAGAAGTCGGCGGCGAAACCGAAGGC

RI-gBol001191-XLOC_050592-13348-0
GAAACCTAAGCCAAAGTCGAAACCAGACTCTCTGGCGAAGAAGAAGACGC
RI-gBol001191-XLOC_050592-13348-1
GAAACCTAAGCCAAAGTCGAAACCAGACTCTCTGGCGAAGAAGAAGACGC
CONSENSUS
GAAACCTAAGCCAAAGTCGAAACCAGACTCTCTGGCGAAGAAGAAGACGC

RI-gBol001191-XLOC_050592-13348-0
CGAGGACTACGGGAATCTTCAAGGCGACGACTGTCTCTCCAGCTCTCGCT
RI-gBol001191-XLOC_050592-13348-1
CGAGGACTACGGGAATCTTCAAGGCGACGACTGTCTCTCCAGCTCTCGCT
CONSENSUS
CGAGGACTACGGGAATCTTCAAGGCGACGACTGTCTCTCCAGCTCTCGCT

RI-gBol001191-XLOC_050592-13348-0
CAGTTCCTTGGTACCGGTGAAACCACACGTACCGACGCCGTCAAAGAGAT
RI-gBol001191-XLOC_050592-13348-1
CAGTTCCTTGGTACCGGTGAAACCACACGTACCGACGCCGTCAAAGAGAT
CONSENSUS
CAGTTCCTTGGTACCGGTGAAACCACACGTACCGACGCCGTCAAAGAGAT

RI-gBol001191-XLOC_050592-13348-0
TTGGACCTATGTCAAGTCCCACGATCTTCAG
RI-gBol001191-XLOC_050592-13348-1
TTGGACCTATGTCAAGTCCCACGATCTTCAG
CONSENSUS
TTGGACCTATGTCAAGTCCCACGATCTTCAG

alignment for event: RI-gBol010009-XLOC_040497-2076

RI-gBol010009-XLOC_040497-2076-0
GTTAGGAAACATGACGACTAACGAAACAGGTGATTTACGTTCAGCTGGGG
RI-gBol010009-XLOC_040497-2076-1
GTTAGGAAACATGACGACTAACGAAACAG---------------------

CONSENSUS
      GTTAGGAAACATGACGACTAACGAAACAG.....................

RI-gBol010009-XLOC_040497-2076-0
      TTGAGCAAGTTCTTGATGTTGGATGTGGAGTTGCTAGCTTTGCAGCTTAC
RI-gBol010009-XLOC_040497-2076-1

CONSENSUS
      ....

RI-gBol010009-XLOC_040497-2076-0
      CTTCTTCCTTTGGGTATACAGACGATGTCCTTTGCCCCTAAAGACGGGCA
RI-gBol010009-XLOC_040497-2076-1 ---------------------
ACGATGTCCTTTGCCCCTAAAGACGGGCA
CONSENSUS
      .....................ACGATGTCCTTTGCCCCTAAAGACGGGCA

RI-gBol010009-XLOC_040497-2076-0
      TGAGAACCAAATCCAGTTTGCGTTGGAGAGAGGAATAGGCGCAATGATCT
RI-gBol010009-XLOC_040497-2076-1
      TGAGAACCAAATCCAGTTTGCGTTGGAGAGAGGAATAGGCGCAATGATCT
CONSENSUS
      TGAGAACCAAATCCAGTTTGCGTTGGAGAGAGGAATAGGCGCAATGATCT

RI-gBol010009-XLOC_040497-2076-0
      CTGCCATTGCCACCAAACAAATGCCATATCCCTCATCCTCCTTTGACATG
RI-gBol010009-XLOC_040497-2076-1
      CTGCCATTGCCACCAAACAAATGCCATATCCCTCATCCTCCTTTGACATG
CONSENSUS
      CTGCCATTGCCACCAAACAAATGCCATATCCCTCATCCTCCTTTGACATG

RI-gBol010009-XLOC_040497-2076-0
      GTTCATTGCTCAAGATGTCGTGTTGATTGGCATGAAAATG
RI-gBol010009-XLOC_040497-2076-1
      GTTCATTGCTCAAGATGTCGTGTTGATTGGCATGAAAATG
CONSENSUS
      GTTCATTGCTCAAGATGTCGTGTTGATTGGCATGAAAATG

alignment for event: A5-X-XLOC_040441-881

A5-X-XLOC_040441-881-0
      AACGGAATCAAGCCTCACTAAATCCCGGTTAAAGCGAAAGCTCAATGGAT
A5-X-XLOC_040441-881-1
      AACGGAATCAAGCCTCACTAAATCCCGGTTAAAGCGAAAGCTCAATGGAT
CONSENSUS
      AACGGAATCAAGCCTCACTAAATCCCGGTTAAAGCGAAAGCTCAATGGAT

A5-X-XLOC_040441-881-0
      CCTGATCCGGGTCTGGATGCGAGTCCGGGTCCTTCTATACATGTCGACAT
A5-X-XLOC_040441-881-1
      CCTGATCCGGGTCTGGATGCGAGTCCGGGTCCTTCTATACATGTCGACAT
CONSENSUS
      CCTGATCCGGGTCTGGATGCGAGTCCGGGTCCTTCTATACATGTCGACAT

A5-X-XLOC_040441-881-0
CATGTCACTGCGAGGTAGTCGTCTTACAAAGCTTTCTTCTCCACACTACG
A5-X-XLOC_040441-881-1
CATGTCACTGCGAGGTAGTCGTCTTACAAAGCTTTCTTCTCCACACTACG
CONSENSUS
CATGTCACTGCGAGGTAGTCGTCTTACAAAGCTTTCTTCTCCACACTACG

A5-X-XLOC_040441-881-0
TCTTCCCTCAAG--------------------------------------
A5-X-XLOC_040441-881-1
TCTTCCCTCAAGGTCTCTCCCTCTCTCTCTCTCACTCTTCGGAATCCT
CONSENSUS
TCTTCCCTCAAG......................................

A5-X-XLOC_040441-881-0

A5-X-XLOC_040441-881-1
TTGTTAATCATTCAAATGTAACTTCGCGATTAGCGTTTTTGACGATTCCA
CONSENSUS
....

A5-X-XLOC_040441-881-0 -----------------
TCTTTTTCAATGTCTTGTTCTGTCAGGGCGAAA
A5-X-XLOC_040441-881-1
CTCTTCCTTTTTGTTGGTCTTTTTCAATGTCTTGTTCTGTCAGGGCGAAA
CONSENSUS
.................TCTTTTTCAATGTCTTGTTCTGTCAGGGCGAAA

A5-X-XLOC_040441-881-0
ACAAGATTGGGAATGGAGTCTAACCCCAGCGTTCACGAGGTTCTCAACAC
A5-X-XLOC_040441-881-1
ACAAGATTGGGAATGGAGTCTAACCCCAGCGTTCACGAGGTTCTCAACAC
CONSENSUS
ACAAGATTGGGAATGGAGTCTAACCCCAGCGTTCACGAGGTTCTCAACAC

A5-X-XLOC_040441-881-0
GTTCGACACTCTCTCCGAGATCCTCCTCCTCTTACCACCCGAGGAGACAT
A5-X-XLOC_040441-881-1
GTTCGACACTCTCTCCGAGATCCTCCTCCTCTTACCACCCGAGGAGACAT
CONSENSUS
GTTCGACACTCTCTCCGAGATCCTCCTCCTCTTACCACCCGAGGAGACAT

A5-X-XLOC_040441-881-0
ACAAGCTCATCCTCGTCTCTAAGCGCTGGCTCCAAATCATCTCCAGCCCT
A5-X-XLOC_040441-881-1
ACAAGCTCATCCTCGTCTCTAAGCGCTGGCTCCAAATCATCTCCAGCCCT
CONSENSUS
ACAAGCTCATCCTCGTCTCTAAGCGCTGGCTCCAAATCATCTCCAGCCCT

A5-X-XLOC_040441-881-0
TTCTTCCGCCACGCCTACCTCGCCAGATGGAAACCGAACTTCCACCTCAT
A5-X-XLOC_040441-881-1
TTCTTCCGCCACGCCTACCTCGCCAGATGGAAACCGAACTTCCACCTCAT
CONSENSUS
TTCTTCCGCCACGCCTACCTCGCCAGATGGAAACCGAACTTCCACCTCAT

A5-X-XLOC_040441-881-0
CGGCTTCTTCATCTCCAACACCTCCTACGTCGGCAAGAAGCACGTGGAGC
A5-X-XLOC_040441-881-1
CGGCTTCTTCATCTCCAACACCTCCTACGTCGGCAAGAAGCACGTGGAGC
CONSENSUS
CGGCTTCTTCATCTCCAACACCTCCTACGTCGGCAAGAAGCACGTGGAGC

A5-X-XLOC_040441-881-0
GTGTCCGCCGCCCTCGCTCCGAATCCTCCATGCCCTTGCTCTCTACCAGC
A5-X-XLOC_040441-881-1
GTGTCCGCCGCCCTCGCTCCGAATCCTCCATGCCCTTGCTCTCTACCAGC
CONSENSUS
GTGTCCGCCGCCCTCGCTCCGAATCCTCCATGCCCTTGCTCTCTACCAGC

A5-X-XLOC_040441-881-0
AGTTTAGGCGACGAGGTGGAAACCTCCGGAGCCCTCAAGAAGCTTGGTTA
A5-X-XLOC_040441-881-1
AGTTTAGGCGACGAGGTGGAAACCTCCGGAGCCCTCAAGAAGCTTGGTTA
CONSENSUS
AGTTTAGGCGACGAGGTGGAAACCTCCGGAGCCCTCAAGAAGCTTGGTTA

A5-X-XLOC_040441-881-0
CTACATTGACTCTTCCAACGGTGTGCTTCTCTGTGGACGCCACCCGAAAG
A5-X-XLOC_040441-881-1
CTACATTGACTCTTCCAACGGTGTGCTTCTCTGTGGACGCCACCCGAAAG
CONSENSUS
CTACATTGACTCTTCCAACGGTGTGCTTCTCTGTGGACGCCACCCGAAAG

A5-X-XLOC_040441-881-0
CTTACTACTTGTGGGATCCTGTTACGAGGAAGCAGCATAAGATCCCGAGG
A5-X-XLOC_040441-881-1
CTTACTACTTGTGGGATCCTGTTACGAGGAAGCAGCATAAGATCCCGAGG
CONSENSUS
CTTACTACTTGTGGGATCCTGTTACGAGGAAGCAGCATAAGATCCCGAGG

A5-X-XLOC_040441-881-0
CATAGGGTTCATTTCGAGGAGGTTTGTATGTCTTTGATCACCGAGGATTG
A5-X-XLOC_040441-881-1
CATAGGGTTCATTTCGAGGAGGTTTGTATGTCTTTGATCACCGAGGATTG
CONSENSUS
CATAGGGTTCATTTCGAGGAGGTTTGTATGTCTTTGATCACCGAGGATTG

A5-X-XLOC_040441-881-0
TCCTGTTGAGGGGTTTAGCTACAAGGTGGTGCGTGGGGAGTGTGTTTCTT
A5-X-XLOC_040441-881-1
TCCTGTTGAGGGGTTTAGCTACAAGGTGGTGCGTGGGGAGTGTGTTTCTT
CONSENSUS
TCCTGTTGAGGGGTTTAGCTACAAGGTGGTGCGTGGGGAGTGTGTTTCTT

A5-X-XLOC_040441-881-0
ATGCGGCGCATAGTAATAAGGTGAGAGTCGAGATTTATTCTTCTAAGACC
A5-X-XLOC_040441-881-1
ATGCGGCGCATAGTAATAAGGTGAGAGTCGAGATTTATTCTTCTAAGACC
CONSENSUS
ATGCGGCGCATAGTAATAAGGTGAGAGTCGAGATTTATTCTTCTAAGACC

A5-X-XLOC_040441-881-0
ACCACGTGGAGCTACTCTGAGCTGGTTTGCAATGAGGCGGTGTCTCTCAC
A5-X-XLOC_040441-881-1
ACCACGTGGAGCTACTCTGAGCTGGTTTGCAATGAGGCGGTGTCTCTCAC
CONSENSUS
ACCACGTGGAGCTACTCTGAGCTGGTTTGCAATGAGGCGGTGTCTCTCAC

A5-X-XLOC_040441-881-0
TCCTTGGACTGCAGGGAGAGTGATTAAAGGTGTGGTGTATTGGCACGCCA
A5-X-XLOC_040441-881-1
TCCTTGGACTGCAGGGAGAGTGATTAAAGGTGTGGTGTATTGGCACGCCA
CONSENSUS
TCCTTGGACTGCAGGGAGAGTGATTAAAGGTGTGGTGTATTGGCACGCCA

A5-X-XLOC_040441-881-0
CGGGAGGTAAAGTTGCCATCTACGACACAGAAGATGAGGAGAAAAAGATC
A5-X-XLOC_040441-881-1
CGGGAGGTAAAGTTGCCATCTACGACACAGAAGATGAGGAGAAAAAGATC
CONSENSUS
CGGGAGGTAAAGTTGCCATCTACGACACAGAAGATGAGGAGAAAAAGATC

A5-X-XLOC_040441-881-0
GATGTGATAAAGCTTCCCAAGACGTTTAACTACGATGAGCAGGTTCTGGG
A5-X-XLOC_040441-881-1
GATGTGATAAAGCTTCCCAAGACGTTTAACTACGATGAGCAGGTTCTGGG
CONSENSUS
GATGTGATAAAGCTTCCCAAGACGTTTAACTACGATGAGCAGGTTCTGGG

A5-X-XLOC_040441-881-0
AGAATCCTCTGATGGGTGTCTGCAGTACGGATGGAGTAATAAGTCTGTGA
A5-X-XLOC_040441-881-1
AGAATCCTCTGATGGGTGTCTGCAGTACGGATGGAGTAATAAGTCTGTGA
CONSENSUS
AGAATCCTCTGATGGGTGTCTGCAGTACGGATGGAGTAATAAGTCTGTGA

A5-X-XLOC_040441-881-0
TGGAGATATGGAAGCTGGAGAAGGTTGGCGAGGTTCTTGAGTGGAGCATT
A5-X-XLOC_040441-881-1
TGGAGATATGGAAGCTGGAGAAGGTTGGCGAGGTTCTTGAGTGGAGCATT
CONSENSUS
TGGAGATATGGAAGCTGGAGAAGGTTGGCGAGGTTCTTGAGTGGAGCATT

A5-X-XLOC_040441-881-0
CAGTTTAAGGTGAATTTCAAGGCCATGTGGAGGTTGAACCCGGTGGAGTC
A5-X-XLOC_040441-881-1
CAGTTTAAGGTGAATTTCAAGGCCATGTGGAGGTTGAACCCGGTGGAGTC
CONSENSUS
CAGTTTAAGGTGAATTTCAAGGCCATGTGGAGGTTGAACCCGGTGGAGTC

A5-X-XLOC_040441-881-0
TGCGAGGTTCAGCACTAGGACTAAAGAGACGCAGCTGCTTGCATTCTTTA
A5-X-XLOC_040441-881-1
TGCGAGGTTCAGCACTAGGACTAAAGAGACGCAGCTGCTTGCATTCTTTA
CONSENSUS
TGCGAGGTTCAGCACTAGGACTAAAGAGACGCAGCTGCTTGCATTCTTTA

A5-X-XLOC_040441-881-0
ACCAGAACTCGGACTCGGTTTTCATCAGATGCGACTCGCACATCTGCGTG
A5-X-XLOC_040441-881-1
ACCAGAACTCGGACTCGGTTTTCATCAGATGCGACTCGCACATCTGCGTG
CONSENSUS
ACCAGAACTCGGACTCGGTTTTCATCAGATGCGACTCGCACATCTGCGTG

A5-X-XLOC_040441-881-0
TTTGACACGAAGACTCAGAGGGTTGAAGAGGTTCAGTACCAAGGACGAGG
A5-X-XLOC_040441-881-1
TTTGACACGAAGACTCAGAGGGTTGAAGAGGTTCAGTACCAAGGACGAGG
CONSENSUS
TTTGACACGAAGACTCAGAGGGTTGAAGAGGTTCAGTACCAAGGACGAGG

A5-X-XLOC_040441-881-0
GTCTTCGTTCGTTTGGGATTACTGCAAGGTCTTGCCTTACTTTCAGCTAT
A5-X-XLOC_040441-881-1
GTCTTCGTTCGTTTGGGATTACTGCAAGGTCTTGCCTTACTTTCAGCTAT
CONSENSUS
GTCTTCGTTCGTTTGGGATTACTGCAAGGTCTTGCCTTACTTTCAGCTAT

A5-X-XLOC_040441-881-0
CTTGGCCTTGTTCTTCTTCTTCTCTGCTGGAAGAAGGAAACATCTGAAGA
A5-X-XLOC_040441-881-1
CTTGGCCTTGTTCTTCTTCTTCTCTGCTGGAAGAAGGAAACATCTGAAGA
CONSENSUS
CTTGGCCTTGTTCTTCTTCTTCTCTGCTGGAAGAAGGAAACATCTGAAGA

A5-X-XLOC_040441-881-0
TCCAAACTGTGATGTCCCTTTCCGCAGTAATAAGTAGCTTATGATAATAA
A5-X-XLOC_040441-881-1
TCCAAACTGTGATGTCCCTTTCCGCAGTAATAAGTAGCTTATGATAATAA
CONSENSUS
TCCAAACTGTGATGTCCCTTTCCGCAGTAATAAGTAGCTTATGATAATAA

A5-X-XLOC_040441-881-0
TAATAATAATCAGTTCGTGTGTAAGCCTCTAATATCATTTTGTTGTGTTA
A5-X-XLOC_040441-881-1
TAATAATAATCAGTTCGTGTGTAAGCCTCTAATATCATTTTGTTGTGTTA
CONSENSUS
TAATAATAATCAGTTCGTGTGTAAGCCTCTAATATCATTTTGTTGTGTTA

A5-X-XLOC_040441-881-0
TGGCCATTGGATTATATGGGCTTTGATGTTTTTTGTTTGACCATTGATGA
A5-X-XLOC_040441-881-1
TGGCCATTGGATTATATGGGCTTTGATGTTTTTTGTTTGACCATTGATGA
CONSENSUS
TGGCCATTGGATTATATGGGCTTTGATGTTTTTTGTTTGACCATTGATGA

A5-X-XLOC_040441-881-0
GCCTCTCATGGCCATTGAATTACTGTTTAGTTATTAAAAGATTGTGCATT
A5-X-XLOC_040441-881-1
GCCTCTCATGGCCATTGAATTACTGTTTAGTTATTAAAAGATTGTGCATT
CONSENSUS
GCCTCTCATGGCCATTGAATTACTGTTTAGTTATTAAAAGATTGTGCATT

A5-X-XLOC_040441-881-0
      TTGTAATGACAACAAGTTTTTGCGTTTGTGTTTTTCATTGACTTGGTTTG
A5-X-XLOC_040441-881-1
      TTGTAATGACAACAAGTTTTTGCGTTTGTGTTTTTCATTGACTTGGTTTG
CONSENSUS
      TTGTAATGACAACAAGTTTTTGCGTTTGTGTTTTTCATTGACTTGGTTTG

A5-X-XLOC_040441-881-0   TGGAAAGATTCCGATCG
A5-X-XLOC_040441-881-1   TGGAAAGATTCCGATCG
CONSENSUS   TGGAAAGATTCCGATCG

alignment for event: A5-X-XLOC_042115-9448

A5-X-XLOC_042115-9448-0
      ATTCTTCAACTGTTTATCTTCCTCCACCTCTCTCTTTCTCTCTCCCTATC
A5-X-XLOC_042115-9448-1
      ATTCTTCAACTGTTTATCTTCCTCCACCTCTCTCTTTCTCTCTCCCTATC
CONSENSUS
      ATTCTTCAACTGTTTATCTTCCTCCACCTCTCTCTTTCTCTCTCCCTATC

A5-X-XLOC_042115-9448-0
      TCATAAAAGCCCTTCTTCGCCGCAACAGACGTGGTCCTTGAAACCGTTGC
A5-X-XLOC_042115-9448-1
      TCATAAAAGCCCTTCTTCGCCGCAACAGACGTGGTCCTTGAAACCGTTGC
CONSENSUS
      TCATAAAAGCCCTTCTTCGCCGCAACAGACGTGGTCCTTGAAACCGTTGC

A5-X-XLOC_042115-9448-0
      AATTCCCTTCCCTTTTCACCCTATAGAAGCACATCAAGCAATGGCTTCCA
A5-X-XLOC_042115-9448-1
      AATTCCCTTCCCTTTTCACCCTATAGAAGCACATCAAGCAATGGCTTCCA
CONSENSUS
      AATTCCCTTCCCTTTTCACCCTATAGAAGCACATCAAGCAATGGCTTCCA

A5-X-XLOC_042115-9448-0
      GAACTGCAAAATCCACCTAGAGAAATAAATCTACAGTCTCATTAACCAAA
A5-X-XLOC_042115-9448-1
      GAACTGCAAAATCCACCTAGAGAAATAAATCTACAGTCTCATTAACCAAA
CONSENSUS
      GAACTGCAAAATCCACCTAGAGAAATAAATCTACAGTCTCATTAACCAAA

A5-X-XLOC_042115-9448-0
      GAAATCTACCGAGTTGCTGATCACTCTCGAATCATTTGTTTCACTCACAT
A5-X-XLOC_042115-9448-1
      GAAATCTACCGAGTTGCTGATCACTCTCGAATCATTTGTTTCACTCACAT
CONSENSUS
      GAAATCTACCGAGTTGCTGATCACTCTCGAATCATTTGTTTCACTCACAT

A5-X-XLOC_042115-9448-0
      CTCTGACCTCCCCGAGTTGGAAGACACCCGTCTGTTCATCGACGCAGATG
A5-X-XLOC_042115-9448-1
      CTCTGACCTCCCCGAGTTGGAAGACACCCGTCTGTTCATCGACGCAGATG
CONSENSUS
      CTCTGACCTCCCCGAGTTGGAAGACACCCGTCTGTTCATCGACGCAGATG

A5-X-XLOC_042115-9448-0
      GATTCAATTCCACTTATTCAAAGAGTTTGAAGTTAATCTT----------
A5-X-XLOC_042115-9448-1
      GATTCAATTCCACTTATTCAAAGAGTTTGAAGTTAATCTTGTGCGCAAAG
CONSENSUS
      GATTCAATTCCACTTATTCAAAGAGTTTGAAGTTAATCTT..........

A5-X-XLOC_042115-9448-0   --------------
ATGGAGGCAGCAAGCTACTCTTGGAAGAACAGACAA
A5-X-XLOC_042115-9448-1
      GCGATCTCTATTTGATGGAGGCAGCAAGCTACTCTTGGAAGAACAGACAA
CONSENSUS
      ..............ATGGAGGCAGCAAGCTACTCTTGGAAGAACAGACAA

A5-X-XLOC_042115-9448-0
      CCAATGTAGTTTTTTTCAGATTACATCTCATCTGAGAAATGAGAATATCA
A5-X-XLOC_042115-9448-1
      CCAATGTAGTTTTTTTCAGATTACATCTCATCTGAGAAATGAGAATATCA
CONSENSUS
      CCAATGTAGTTTTTTTCAGATTACATCTCATCTGAGAAATGAGAATATCA

A5-X-XLOC_042115-9448-0   CAGATGTTGATGATCATCTTCATGATATCTCCAA
A5-X-XLOC_042115-9448-1   CAGATGTTGATGATCATCTTCATGATATCTCCAA
CONSENSUS   CAGATGTTGATGATCATCTTCATGATATCTCCAA

alignment for event: A3-gBol019011-XLOC_030395-3534

A3-gBol019011-XLOC_030395-3534-0
      AACAAGGCTAAGTCTCATACTCGAAATACTTTGGAACAAACGTTCATGGA
A3-gBol019011-XLOC_030395-3534-1
      AACAAGGCTAAGTCTCATACTCGAAATACTTTGGAACAAACGTTCATGGA
CONSENSUS
      AACAAGGCTAAGTCTCATACTCGAAATACTTTGGAACAAACGTTCATGGA

A3-gBol019011-XLOC_030395-3534-0
      AAGAGTAGTTCATATATTCCGATCACAGATCTCTCTGCATGATCAGAAGT
A3-gBol019011-XLOC_030395-3534-1
      AAGAGTAGTTCATATATTCCGATCACAGATCTCTCTG---------AAGT
CONSENSUS
      AAGAGTAGTTCATATATTCCGATCACAGATCTCTCTG.........AAGT

A3-gBol019011-XLOC_030395-3534-0
      GTCAAGAAGATACCCCAGCAGTTGTTTTGGAGGATGGAGATTTGCCTCTA
A3-gBol019011-XLOC_030395-3534-1
      GTCAAGAAGATACCCCAGCAGTTGTTTTGGAGGATGGAGATTTGCCTCTA
CONSENSUS
      GTCAAGAAGATACCCCAGCAGTTGTTTTGGAGGATGGAGATTTGCCTCTA

A3-gBol019011-XLOC_030395-3534-0
      TTATTGGGAAATCAAATCGAAACCTCAATGACTGGTTCCACTGAAGCCAG
A3-gBol019011-XLOC_030395-3534-1
      TTATTGGGAAATCAAATCGAAACCTCAATGACTGGTTCCACTGAAGCCAG
CONSENSUS

TTATTGGGAAATCAAATCGAAACCTCAATGACTGGTTCCACTGAAGCCAG

A3-gBol019011-XLOC_030395-3534-0
CAAAAACAACACTAGGTGTAGCAAAAAGCGGAGTCATTTGGGTATGCCTG
A3-gBol019011-XLOC_030395-3534-1
CAAAAACAACACTAGGTGTAGCAAAAAGCGGAGTCATTTGGGTATGCCTG
CONSENSUS
CAAAAACAACACTAGGTGTAGCAAAAAGCGGAGTCATTTGGGTATGCCTG

A3-gBol019011-XLOC_030395-3534-0
AAAAAAGTACTACTACTTCCAGAGAGATGAAGAATGTTACTTCCAAGAAG
A3-gBol019011-XLOC_030395-3534-1
AAAAAAGTACTACTACTTCCAGAGAGATGAAGAATGTTACTTCCAAGAAG
CONSENSUS
AAAAAAGTACTACTACTTCCAGAGAGATGAAGAATGTTACTTCCAAGAAG

A3-gBol019011-XLOC_030395-3534-0
TGTAAAACAGTAGAAGGGAGAGGACACATTTCTCGTGATTCCACCGTATC
A3-gBol019011-XLOC_030395-3534-1
TGTAAAACAGTAGAAGGGAGAGGACACATTTCTCGTGATTCCACCGTATC
CONSENSUS
TGTAAAACAGTAGAAGGGAGAGGACACATTTCTCGTGATTCCACCGTATC

A3-gBol019011-XLOC_030395-3534-0
TTTGAGAACAGTTGAGTCGGAAATTCGTAAAAAGAAGCTAGAGAGACACA
A3-gBol019011-XLOC_030395-3534-1
TTTGAGAACAGTTGAGTCGGAAATTCGTAAAAAGAAGCTAGAGAGACACA
CONSENSUS
TTTGAGAACAGTTGAGTCGGAAATTCGTAAAAAGAAGCTAGAGAGACACA

A3-gBol019011-XLOC_030395-3534-0
GTACTTGTTCAAAGGAGAGTGCTGGAGCAAGTGTCAGCAACAATGTTACA
A3-gBol019011-XLOC_030395-3534-1
GTACTTGTTCAAAGGAGAGTGCTGGAGCAAGTGTCAGCAACAATGTTACA
CONSENSUS
GTACTTGTTCAAAGGAGAGTGCTGGAGCAAGTGTCAGCAACAATGTTACA

A3-gBol019011-XLOC_030395-3534-0
TCTTCCAAACCTTCCGGGGTAGTATCTACGTTAAAG
A3-gBol019011-XLOC_030395-3534-1
TCTTCCAAACCTTCCGGGGTAGTATCTACGTTAAAG
CONSENSUS
TCTTCCAAACCTTCCGGGGTAGTATCTACGTTAAAG

alignment for event: A5-gBol018983-XLOC_030427-9896

A5-gBol018983-XLOC_030427-9896-0
AGACAGAAAAACAGAACCAGAAAATTGCCAAATGTCAAAAACCCAAGTTT
A5-gBol018983-XLOC_030427-9896-1
AGACAGAAAAACAGAACCAGAAAATTGCCAAATGTCAAAAACCCAAGTTT
CONSENSUS
AGACAGAAAAACAGAACCAGAAAATTGCCAAATGTCAAAAACCCAAGTTT

A5-gBol018983-XLOC_030427-9896-0

GGTCGCCGTCAGAAAGAAAAACTTTCCAATTCGTAGAAGAAATAAAGGAG
A5-gBol018983-XLOC_030427-9896-1
GGTCGCCGTCAGAAAGAAAAACTTTCCAATTCGTAGAAGAAATAAAGGAG
CONSENSUS
GGTCGCCGTCAGAAAGAAAAACTTTCCAATTCGTAGAAGAAATAAAGGAG

A5-gBol018983-XLOC_030427-9896-0
AGAGTCAAATAAAAACCCTAATCATTTCAATTGATCCCACACACACACAC
A5-gBol018983-XLOC_030427-9896-1
AGAGTCAAATAAAAACCCTAATCATTTCAATTGATCCCACACACACACAC
CONSENSUS
AGAGTCAAATAAAAACCCTAATCATTTCAATTGATCCCACACACACACAC

A5-gBol018983-XLOC_030427-9896-0
TACCTTTTACAACCCCTGGCGTCGTCGTCTTCCCATTAACTTCTCTCGTC
A5-gBol018983-XLOC_030427-9896-1
TACCTTTTACAACCCCTGGCGTCGTCGTCTTCCCATTAACTTCTCTCGTC
CONSENSUS
TACCTTTTACAACCCCTGGCGTCGTCGTCTTCCCATTAACTTCTCTCGTC

A5-gBol018983-XLOC_030427-9896-0
CTCATATAAAGATTCAAAACGTTCGGTCTAAGGTGTAGTAATTTCATCTA
A5-gBol018983-XLOC_030427-9896-1
CTCATATAAAGATTCAAAACGTTCGGTCTAAG------------------
CONSENSUS
CTCATATAAAGATTCAAAACGTTCGGTCTAAG..................

A5-gBol018983-XLOC_030427-9896-0
TTGTTCTATTGTGCTTCTCCTCTCCATCCTTGGTTTCATCATCGTCACAG
A5-gBol018983-XLOC_030427-9896-1

CONSENSUS
....

A5-gBol018983-XLOC_030427-9896-0
CCTCTTAGCTGTTTTACAGAAATGGCTACGGTTGTGTCTCACCAAGCAAT
A5-gBol018983-XLOC_030427-9896-1 -----------
TTTTACAGAAATGGCTACGGTTGTGTCTCACCAAGCAAT
CONSENSUS
...........TTTTACAGAAATGGCTACGGTTGTGTCTCACCAAGCAAT

A5-gBol018983-XLOC_030427-9896-0
TACTTCTCAAAGACCTTCTTCTAACTTCAAAGCTTCTCACTTTCTTAAGG
A5-gBol018983-XLOC_030427-9896-1
TACTTCTCAAAGACCTTCTTCTAACTTCAAAGCTTCTCACTTTCTTAAGG
CONSENSUS
TACTTCTCAAAGACCTTCTTCTAACTTCAAAGCTTCTCACTTTCTTAAGG

A5-gBol018983-XLOC_030427-9896-0
AACCTCTCAATGTTCCCATCAAACTCAGACAAAAACGTTTCAAGATCCAA
A5-gBol018983-XLOC_030427-9896-1
AACCTCTCAATGTTCCCATCAAACTCAGACAAAAACGTTTCAAGATCCAA
CONSENSUS
AACCTCTCAATGTTCCCATCAAACTCAGACAAAAACGTTTCAAGATCCAA

A5-gBol018983-XLOC_030427-9896-0

GCAACTGCATCTCAAGCTCCACTTCTTGACCCTGTCTTGTCTCCTTCCAA
A5-gBol018983-XLOC_030427-9896-1
GCAACTGCATCTCAAGCTCCACTTCTTGACCCTGTCTTGTCTCCTTCCAA
CONSENSUS
GCAACTGCATCTCAAGCTCCACTTCTTGACCCTGTCTTGTCTCCTTCCAA

A5-gBol018983-XLOC_030427-9896-0
GACCATCCCTCAATCTTACAAGAAAAAATCAA
A5-gBol018983-XLOC_030427-9896-1
GACCATCCCTCAATCTTACAAGAAAAAATCAA
CONSENSUS
GACCATCCCTCAATCTTACAAGAAAAAATCAA

alignment for event: A5-gBol041324-XLOC_005227-10343

A5-gBol041324-XLOC_005227-10343-0
CAGATTGATTTCATCTGCACCGGGAAGGTCGCTGGTATCAAGCTGGACAA
A5-gBol041324-XLOC_005227-10343-1
CAGATTGATTTCATCTGCACCGGGAAGGTCGCTGGTATCAAGCTGGACAA
CONSENSUS
CAGATTGATTTCATCTGCACCGGGAAGGTCGCTGGTATCAAGCTGGACAA

A5-gBol041324-XLOC_005227-10343-0
GGGATTGTGCTACGTTTCTTGCTCCAACTGCACCAAAAGCTCCAACGCAT
A5-gBol041324-XLOC_005227-10343-1
GGGATTGTGCTACGTTTCTTGCTCCAACTGCACCAAAAGCTCCAACGCAT
CONSENSUS
GGGATTGTGCTACGTTTCTTGCTCCAACTGCACCAAAAGCTCCAACGCAT

A5-gBol041324-XLOC_005227-10343-0
TGTCTCAGCTTTCACCTGGCTGCATTGTACTAATACTAATGCTGTTGGGG
A5-gBol041324-XLOC_005227-10343-1
TGTCTCAGCTTTCACCTGGCTGCATT------------------------
CONSENSUS
TGTCTCAGCTTTCACCTGGCTGCATT........................

A5-gBol041324-XLOC_005227-10343-0
TCTAACCATTTATATCGGGTGGAGATGTCCATTGTTGTTTGTTTTAATGG
A5-gBol041324-XLOC_005227-10343-1 -----------
ATATCGGGTGGAGATGTCCATTGTTGTTTGTTTTAATGG
CONSENSUS
...........ATATCGGGTGGAGATGTCCATTGTTGTTTGTTTTAATGG

A5-gBol041324-XLOC_005227-10343-0
GGTTATGACAAAACTGCATAATATGAGAGCCTATGAAACTGGCCATCTTT
A5-gBol041324-XLOC_005227-10343-1
GGTTATGACAAAACTGCATAATATGAGAGCCTATGAAACTGGCCATCTTT
CONSENSUS
GGTTATGACAAAACTGCATAATATGAGAGCCTATGAAACTGGCCATCTTT

A5-gBol041324-XLOC_005227-10343-0 TG
A5-gBol041324-XLOC_005227-10343-1 TG
CONSENSUS TG

alignment for event: RI-X-XLOC_005163-12201

RI-X-XLOC_005163-12201-0
      AAACTTCTTTTGTCATGTTTTCTTTAGTTTGAAATATTTCTTTTGTTGTT
RI-X-XLOC_005163-12201-1
      AAACTTCTTTTGTCATGTTTTCTTTAGTTTGAAATATTTCTTTTGTTGTT
CONSENSUS
      AAACTTCTTTTGTCATGTTTTCTTTAGTTTGAAATATTTCTTTTGTTGTT

RI-X-XLOC_005163-12201-0
      AGTTACTAAAAAACCCAAGGCGAGATTGTGGGTCGAAGATGACGATTCGA
RI-X-XLOC_005163-12201-1
      AGTTACTAAAAAACCCAAGGCGAGATTGTGGGTCGAAGATGACGATTCGA
CONSENSUS
      AGTTACTAAAAAACCCAAGGCGAGATTGTGGGTCGAAGATGACGATTCGA

RI-X-XLOC_005163-12201-0
      ATGAATCTCGGTTTTTAAGAATCCGTGAGGCCCGGATTGATGGGGTTTTT
RI-X-XLOC_005163-12201-1
      ATGAATCTCGGTTTTTAAGAATCCGTGAGGCCCGGATTGATGGGGTTTTT
CONSENSUS
      ATGAATCTCGGTTTTTAAGAATCCGTGAGGCCCGGATTGATGGGGTTTTT

RI-X-XLOC_005163-12201-0
      AACTTTCAGGGTATTTTAGCGGATCCTCAGTGTATCTTCATTGATCTGCC
RI-X-XLOC_005163-12201-1
      AACTTTCAGGGTATTTTAGCGGATCCTCAGTGTATCTTCATTGATCTGCC
CONSENSUS
      AACTTTCAGGGTATTTTAGCGGATCCTCAGTGTATCTTCATTGATCTGCC

RI-X-XLOC_005163-12201-0
      ACAGATTGATTAGATCTCGGTTGGCGTTTTGAGGTTGGAAAGTTTTCACT
RI-X-XLOC_005163-12201-1
      ACAGATTGATTAGATCTCGGTTGGCGTTTTGAGGTTGGAAAGTTTTCACT
CONSENSUS
      ACAGATTGATTAGATCTCGGTTGGCGTTTTGAGGTTGGAAAGTTTTCACT

RI-X-XLOC_005163-12201-0
      CTGGCGGATCCTTTAAGGTTCTCGGAGATCTGGTACCAGATAGAGGTGAA
RI-X-XLOC_005163-12201-1
      CTGGCGGATCCTTTAAGGTTCTCGGAGATCTGGTACCAGATAGAGGTGAA
CONSENSUS
      CTGGCGGATCCTTTAAGGTTCTCGGAGATCTGGTACCAGATAGAGGTGAA

RI-X-XLOC_005163-12201-0
      AAGAGATGATTGGGTTGTGAGTTATAAGGAAAAAATTGATTGGGGAATAT
RI-X-XLOC_005163-12201-1
      AAGAGATGATTGGGTTGTGAGTTATAAGGAAAAAATTGATTGGGGAATAT
CONSENSUS
      AAGAGATGATTGGGTTGTGAGTTATAAGGAAAAAATTGATTGGGGAATAT

RI-X-XLOC_005163-12201-0
      ACATGATTCGATGTTGAGATCTCCTTGCAGCCAATTATTACGGTATGTTT
RI-X-XLOC_005163-12201-1

ACATGATTCGATGTTGAGATCTCCTTGCAGCCAATTATTACGGTATGTTT
CONSENSUS
ACATGATTCGATGTTGAGATCTCCTTGCAGCCAATTATTACGGTATGTTT

RI-X-XLOC_005163-12201-0
CTTTTTTTGGATTGGTCTTAGGTGGTGTATATCGGTGGATGCTTCTAAAA
RI-X-XLOC_005163-12201-1
CTTTTTTTGGATTGGTCTTAGGTGGTGTATATCGGTGGATGCTTCTAAAA
CONSENSUS
CTTTTTTTGGATTGGTCTTAGGTGGTGTATATCGGTGGATGCTTCTAAAA

RI-X-XLOC_005163-12201-0
GATCTGAGAGATTATTTAGAGAGCAGTGAGAGCAAAGAAAGGATCTTGGT
RI-X-XLOC_005163-12201-1
GATCTGAGAGATTATTTAGAGAGCAGTGAGAGCAAAGAAAGGATCTTGGT
CONSENSUS
GATCTGAGAGATTATTTAGAGAGCAGTGAGAGCAAAGAAAGGATCTTGGT

RI-X-XLOC_005163-12201-0
TTTCAGAAATATTTTTCGTAATAGAGATATTGAAACAAATCATCAAAGGT
RI-X-XLOC_005163-12201-1
TTTCAGAAATATTTTTCGTAATAGAGATATTGAAACAAATCATCAAAGGT
CONSENSUS
TTTCAGAAATATTTTTCGTAATAGAGATATTGAAACAAATCATCAAAGGT

RI-X-XLOC_005163-12201-0
TTCCTAGGACTGAGATTGAGAATTTAATGGTTCTACTCATTCAATTCAGT
RI-X-XLOC_005163-12201-1
TTCCTAGGACTGAGATTGAGAATTTAATGGTTCTACTCATTCAATTCAGT
CONSENSUS
TTCCTAGGACTGAGATTGAGAATTTAATGGTTCTACTCATTCAATTCAGT

RI-X-XLOC_005163-12201-0
ATCACTATGGATTTGATTGAAGAACATATTGCGGTCACGAATCTCTGGTT
RI-X-XLOC_005163-12201-1
ATCACTATGGATTTGATTGAAGAACATATTGCGGTCACGAATCTCTGGTT
CONSENSUS
ATCACTATGGATTTGATTGAAGAACATATTGCGGTCACGAATCTCTGGTT

RI-X-XLOC_005163-12201-0
TTACTTGATAGGAACAGCTAGGATGAATGGGATCCTGCATCACAGCATCA
RI-X-XLOC_005163-12201-1
TTACTTGATAGGAACAGCTAGGATGAATGGGATCCTGCATCACAGCATCA
CONSENSUS
TTACTTGATAGGAACAGCTAGGATGAATGGGATCCTGCATCACAGCATCA

RI-X-XLOC_005163-12201-0
TTCTCTCACCACTTCTCTATTGTTTCTAACATACGGTTCTGGTTTTTACA
RI-X-XLOC_005163-12201-1
TTCTCTCACCACTTCTCTATTGTTTCTAACATACGGTTCTGGTTTTTACA
CONSENSUS
TTCTCTCACCACTTCTCTATTGTTTCTAACATACGGTTCTGGTTTTTACA

RI-X-XLOC_005163-12201-0
TTGGTCTCTTTGGTTTCATAATGATTCAAGGGAAGTGGGTCGCTAAATCG
RI-X-XLOC_005163-12201-1

TTGGTCTCTTTGGTTTCATAATGATTCAAGGGAAGTGGGTCGCTAAATCG
CONSENSUS
TTGGTCTCTTTGGTTTCATAATGATTCAAGGGAAGTGGGTCGCTAAATCG

RI-X-XLOC_005163-12201-0
GGAGGTGACCATGCCAGGTCTAAAGATTACGGTTCCCCGTTTTAATAACT
RI-X-XLOC_005163-12201-1
GGAGGTGACCATGCCAGGTCTAAAGATTACGGTTCCCCGTTTTAATAACT
CONSENSUS
GGAGGTGACCATGCCAGGTCTAAAGATTACGGTTCCCCGTTTTAATAACT

RI-X-XLOC_005163-12201-0
CGGAACTCATAACGAGTTATGCTAAAACCCTAATAGGCCAGGTCTAAAGA
RI-X-XLOC_005163-12201-1
CGGAACTCATAACGAGTTATGCTAAAACCCTAATAGGCCAGGTCTAAAGA
CONSENSUS
CGGAACTCATAACGAGTTATGCTAAAACCCTAATAGGCCAGGTCTAAAGA

RI-X-XLOC_005163-12201-0
TTTTTCTCTTCATGCTTCCCAAGATATGGCAAGCTGAGAGGCGTGCGGTT
RI-X-XLOC_005163-12201-1
TTTTTCTCTTCATGCTTCCCAAGATATGGCAAGCTGAGAGGCGTGCGGTT
CONSENSUS
TTTTTCTCTTCATGCTTCCCAAGATATGGCAAGCTGAGAGGCGTGCGGTT

RI-X-XLOC_005163-12201-0
GGTGCAGATCTTGGGTTGGGAAGATTTCAGTTTGACTTTGATTTGGAGGA
RI-X-XLOC_005163-12201-1
GGTGCAGATCTTGGGTTGGGAAGATTTCAGTTTGACTTTGATTTGGAGGA
CONSENSUS
GGTGCAGATCTTGGGTTGGGAAGATTTCAGTTTGACTTTGATTTGGAGGA

RI-X-XLOC_005163-12201-0
AGATATAATTGAGGTCCTGAAGATGGAACCTTTTCATTTCGATTACTGGA
RI-X-XLOC_005163-12201-1
AGATATAATTGAGGTCCTGAAGATGGAACCTTTTCATTTCGATTACTGGA
CONSENSUS
AGATATAATTGAGGTCCTGAAGATGGAACCTTTTCATTTCGATTACTGGA

RI-X-XLOC_005163-12201-0
TGGTGTCACTTGTTAGGTGGAAGCCGGTTTTTGAAGCTAATTATCCATCA
RI-X-XLOC_005163-12201-1
TGGTGTCACTTGTTAGGTGGAAGCCGGTTTTTGAAGCTAATTATCCATCA
CONSENSUS
TGGTGTCACTTGTTAGGTGGAAGCCGGTTTTTGAAGCTAATTATCCATCA

RI-X-XLOC_005163-12201-0
AAAATTACATTTTGGGTTCTAGTGCTAGATATCCCTCTTCAGTTTTTGGG
RI-X-XLOC_005163-12201-1
AAAATTACATTTTGGGTTCTAGTGCTAGATATCCCTCTTCAGTTTTTGGG
CONSENSUS
AAAATTACATTTTGGGTTCTAGTGCTAGATATCCCTCTTCAGTTTTTGGG

RI-X-XLOC_005163-12201-0
CTGCTCCGACCTTCCAAAGTGTTGGAGATGCTATAGGAAAGATTCAGGGT
RI-X-XLOC_005163-12201-1

CTGCTCCGACCTTCCAAAGTGTTGGAGATGCTATAGGAAAGATTCAGGGT
CONSENSUS
CTGCTCCGACCTTCCAAAGTGTTGGAGATGCTATAGGAAAGATTCAGGGT

RI-X-XLOC_005163-12201-0
GACGTTGATCTCATCTAGGGAAAAGTATGGGTGGAGCTTGATGGTTTCAA
RI-X-XLOC_005163-12201-1
GACGTTGATCTCATCTAGGGAAAAGTATGGGTGGAGCTTGATGGTTTCAA
CONSENSUS
GACGTTGATCTCATCTAGGGAAAAGTATGGGTGGAGCTTGATGGTTTCAA

RI-X-XLOC_005163-12201-0
GCCCTTGGTGTTTCCATGGAAGTAAAACTCGATGAGGGAGTGGAGATCAA
RI-X-XLOC_005163-12201-1
GCCCTTGGTGTTTCCATGGAAGTAAAACTCGATGAGGGAGTGGAGATCAA
CONSENSUS
GCCCTTGGTGTTTCCATGGAAGTAAAACTCGATGAGGGAGTGGAGATCAA

RI-X-XLOC_005163-12201-0
GGTGTCTCTCAGGTATGAGAAGCTCTTTGGTTTTTTTCAGAGAGTGCTTC
RI-X-XLOC_005163-12201-1
GGTGTCTCTCAGGTATGAGAAGCTCTTTGGTTTTTTTCAGAGAGTGCTTC
CONSENSUS
GGTGTCTCTCAGGTATGAGAAGCTCTTTGGTTTTTTTCAGAGAGTGCTTC

RI-X-XLOC_005163-12201-0
AGTTTGGCACCTGATCAGTCTAGGTGCCCGAGCTTGCACAAGGAGGAGAC
RI-X-XLOC_005163-12201-1
AGTTTGGCACCTGATCAGTCTAGGTGCCCGAGCTTGCACAAGGAGGAGAC
CONSENSUS
AGTTTGGCACCTGATCAGTCTAGGTGCCCGAGCTTGCACAAGGAGGAGAC

RI-X-XLOC_005163-12201-0
CGTAAGTGATAGTGTGGGGATCAAAAGTGACAATGGCTCTCATGTGACAA
RI-X-XLOC_005163-12201-1
CGTAAGTGATAGTGTGGGGATCAAAAGTGACAATGGCTCTCATGTGACAA
CONSENSUS
CGTAAGTGATAGTGTGGGGATCAAAAGTGACAATGGCTCTCATGTGACAA

RI-X-XLOC_005163-12201-0
GTTATAAGGCAGCTGTCTCTAATGGTAGAGAGAGAGCACAATGGTGAACA
RI-X-XLOC_005163-12201-1
GTTATAAGGCAGCTGTCTCTAATGGTAGAGAGAGAGCACAATGGTGAACA
CONSENSUS
GTTATAAGGCAGCTGTCTCTAATGGTAGAGAGAGAGCACAATGGTGAACA

RI-X-XLOC_005163-12201-0
TATAGAGGGACAACTCTAAGGCGTTCAGAGACGGAGACAAGGGTAAAAGT
RI-X-XLOC_005163-12201-1
TATAGAGGGACAACTCTAAGGCGTTCAGAGACGGAGACAAGGGTAAAAGT
CONSENSUS
TATAGAGGGACAACTCTAAGGCGTTCAGAGACGGAGACAAGGGTAAAAGT

RI-X-XLOC_005163-12201-0
ATTGCTTGTGAGAAGCAAGGGCTTTACAGACATGAGGTACCAAGTGGCAT
RI-X-XLOC_005163-12201-1

ATTGCTTGTGAGAAGCAAGGGCTTTACAGACATGAGGTACCAAGTGGCAT
CONSENSUS
ATTGCTTGTGAGAAGCAAGGGCTTTACAGACATGAGGTACCAAGTGGCAT

RI-X-XLOC_005163-12201-0
ACATGTGGAGAAAGAAATACAAAACTCTATCACGCATTGACGAAGCAAAG
RI-X-XLOC_005163-12201-1
ACATGTGGAGAAAGAAATACAAAACTCTATCACGCATTGACGAAGCAAAG
CONSENSUS
ACATGTGGAGAAAGAAATACAAAACTCTATCACGCATTGACGAAGCAAAG

RI-X-XLOC_005163-12201-0
GCGGATTCAAAATAGGATCATTGGATTATACAATGAGGAGGGGTACTGGA
RI-X-XLOC_005163-12201-1
GCGGATTCAAAATAGGATCATTGGATTATACAATGAGGAGGGGTACTGGA
CONSENSUS
GCGGATTCAAAATAGGATCATTGGATTATACAATGAGGAGGGGTACTGGA

RI-X-XLOC_005163-12201-0
TTAATTTGGAAGCAAAGGTCGAAGGAGTTGCAGTCAGATATTTTTGTGAC
RI-X-XLOC_005163-12201-1
TTAATTTGGAAGCAAAGGTCGAAGGAGTTGCAGTCAGATATTTTTGTGAC
CONSENSUS
TTAATTTGGAAGCAAAGGTCGAAGGAGTTGCAGTCAGATATTTTTGTGAC

RI-X-XLOC_005163-12201-0
GACCCGGTTTTCCTTAACCAATTTTGGTTTAATAAAATAATTAAATCAAA
RI-X-XLOC_005163-12201-1
GACCCGGTTTTCCTTAACCAATTTTGGTTTAATAAAATAATTAAATCAAA
CONSENSUS
GACCCGGTTTTCCTTAACCAATTTTGGTTTAATAAAATAATTAAATCAAA

RI-X-XLOC_005163-12201-0
CCCAGTTTCCTATTTTACCTTAACCTAAACGTGTTAGGTATGTGTATACA
RI-X-XLOC_005163-12201-1
CCCAGTTTCCTATTTTACCTTAACCTAAACGTGTTAG-------------
CONSENSUS
CCCAGTTTCCTATTTTACCTTAACCTAAACGTGTTAG.............

RI-X-XLOC_005163-12201-0
TATCCTTTTCTTCCAACCTAGCCGTCATTGTTATTAGAGCAACATTATCA
RI-X-XLOC_005163-12201-1

CONSENSUS
....

RI-X-XLOC_005163-12201-0
AGACATTTGAACACTTCTTATGGGCGATCCTTACCCAATATTGGCCCAAA
RI-X-XLOC_005163-12201-1

CONSENSUS
....

RI-X-XLOC_005163-12201-0
ATTAAATATAAAGTCCAGTTAAATGAAGGAGACGATTTTTAGGTAAGAAG
RI-X-XLOC_005163-12201-1

CONSENSUS
....

RI-X-XLOC_005163-12201-0
TTTTTCGACTTCGTTCTTCTGTCACGTGTCATTCTATGATACGTTTTGTG
RI-X-XLOC_005163-12201-1

CONSENSUS
....

RI-X-XLOC_005163-12201-0
AAGGGGAAAGGGTCTCGTCGTTTTGCTCTGGATATATTCTCTCCGCCTCC
RI-X-XLOC_005163-12201-1 ---
GGGAAAGGGTCTCGTCGTTTTGCTCTGGATATATTCTCTCCGCCTCC
CONSENSUS
...GGGAAAGGGTCTCGTCGTTTTGCTCTGGATATATTCTCTCCGCCTCC

RI-X-XLOC_005163-12201-0
CTCTTCTCTCTCTCTCTCGATCCGTAGATGGCGATTCGCTGGAAGTGAAG
RI-X-XLOC_005163-12201-1
CTCTTCTCTCTCTCTCTCGATCCGTAGATGGCGATTCGCTGGAAGTGAAG
CONSENSUS
CTCTTCTCTCTCTCTCTCGATCCGTAGATGGCGATTCGCTGGAAGTGAAG

RI-X-XLOC_005163-12201-0
AATCATCGGTCCACATCGATCGATCGTTTAATCTCGGCATCGAATCTGCA
RI-X-XLOC_005163-12201-1
AATCATCGGTCCACATCGATCGATCGTTTAATCTCGGCATCGAATCTGCA
CONSENSUS
AATCATCGGTCCACATCGATCGATCGTTTAATCTCGGCATCGAATCTGCA

RI-X-XLOC_005163-12201-0 CCAG
RI-X-XLOC_005163-12201-1 CCAG
CONSENSUS CCAG

alignment for event: RI-gBol041324-XLOC_005227-10341

RI-gBol041324-XLOC_005227-10341-0
CAGATTGATTTCATCTGCACCGGGAAGGTCGCTGGTATCAAGCTGGACAA
RI-gBol041324-XLOC_005227-10341-1
CAGATTGATTTCATCTGCACCGGGAAGGTCGCTGGTATCAAGCTGGACAA
CONSENSUS
CAGATTGATTTCATCTGCACCGGGAAGGTCGCTGGTATCAAGCTGGACAA

RI-gBol041324-XLOC_005227-10341-0
GGGATTGTGCTACGTTTCTTGCTCCAACTGCACCAAAAGCTCCAACGCAT
RI-gBol041324-XLOC_005227-10341-1
GGGATTGTGCTACGTTTCTTGCTCCAACTGCACCAAAAGCTCCAACGCAT
CONSENSUS
GGGATTGTGCTACGTTTCTTGCTCCAACTGCACCAAAAGCTCCAACGCAT

RI-gBol041324-XLOC_005227-10341-0
TGTCTCAGCTTTCACCTGGCTGCATTGTACTAATACTAATGCTGTTGGGG

RI-gBol041324-XLOC_005227-10341-1
      TGTCTCAGCTTTCACCTGGCTGCATT------------------------
CONSENSUS
      TGTCTCAGCTTTCACCTGGCTGCATT........................

RI-gBol041324-XLOC_005227-10341-0
      TCTAACCATTTGTACAAATCTTATTTGCATTCATTAATGTTGGTGTCCAC
RI-gBol041324-XLOC_005227-10341-1

CONSENSUS
      ....

RI-gBol041324-XLOC_005227-10341-0
      GCACATATTCAACTTTGAGATTTGATACCTGTTAACTTCCTGCTAGATAT
RI-gBol041324-XLOC_005227-10341-1
      --ATAT
CONSENSUS
      ....ATAT

RI-gBol041324-XLOC_005227-10341-0
      CGGGTGGAGATGTCCATTGTTGTTTGTTTTAATGGGGTTATGACAAAACT
RI-gBol041324-XLOC_005227-10341-1
      CGGGTGGAGATGTCCATTGTTGTTTGTTTTAATGGGGTTATGACAAAACT
CONSENSUS
      CGGGTGGAGATGTCCATTGTTGTTTGTTTTAATGGGGTTATGACAAAACT

RI-gBol041324-XLOC_005227-10341-0
      GCATAATATGAGAGCCTATGAAACTGGCCATCTTTTG
RI-gBol041324-XLOC_005227-10341-1
      GCATAATATGAGAGCCTATGAAACTGGCCATCTTTTG
CONSENSUS
      GCATAATATGAGAGCCTATGAAACTGGCCATCTTTTG

alignment for event: A3-X-XLOC_029602-14427

A3-X-XLOC_029602-14427-0
      AAATCTACAGGCAGAAGCTTCAACAATTTGTTTTGATGGAGAGCCGAGCG
A3-X-XLOC_029602-14427-1
      AAATCTACAGGCAGAAGCTTCAACAATTTGTTTTGATGGAGAGCCGAGCG
CONSENSUS
      AAATCTACAGGCAGAAGCTTCAACAATTTGTTTTGATGGAGAGCCGAGCG

A3-X-XLOC_029602-14427-0
      ATGACAATGTCGCTGCTTTGTTAAGGTCAAGAGTCATGCTCTGAACTATT
A3-X-XLOC_029602-14427-1
      ATGACAATGTCGCTGCTTTGTTAAGGTCAAGAGTCATGCTCTGAACTATT
CONSENSUS
      ATGACAATGTCGCTGCTTTGTTAAGGTCAAGAGTCATGCTCTGAACTATT

A3-X-XLOC_029602-14427-0
      GAGGCAGCTCATGCAGAAATCTGTGGAAGCAAATTTGAAAAGTTTGGCCG
A3-X-XLOC_029602-14427-1
      GAGGCAGCTCATGCAGAAATCTGTGGAAGCAAATTTGAAAAG--------
CONSENSUS

GAGGCAGCTCATGCAGAAATCTGTGGAAGCAAATTTGAAAAG........

A3-X-XLOC_029602-14427-0
GCAGGTTATTTCTTCTGGAGTGAGATGGTTACGATGCTGAAACTACAAAT
A3-X-XLOC_029602-14427-1 ----
GTTATTTCTTCTGGAGTGAGATGGTTACGATGCTGAAACTACAAAT
CONSENSUS
....GTTATTTCTTCTGGAGTGAGATGGTTACGATGCTGAAACTACAAAT

A3-X-XLOC_029602-14427-0
CAGTAAGAAAGGCTACTCATGGTCTTCGGTTATCCAGAGAGACTGCCATG
A3-X-XLOC_029602-14427-1
CAGTAAGAAAGGCTACTCATGGTCTTCGGTTATCCAGAGAGACTGCCATG
CONSENSUS
CAGTAAGAAAGGCTACTCATGGTCTTCGGTTATCCAGAGAGACTGCCATG

A3-X-XLOC_029602-14427-0 TCTATTGCTAGCAAGGCT
A3-X-XLOC_029602-14427-1 TCTATTGCTAGCAAGGCT
CONSENSUS TCTATTGCTAGCAAGGCT

alignment for event: RI-gBol028809-XLOC_019284-13926

RI-gBol028809-XLOC_019284-13926-0
ATCATTAACGATCGTGAGACTGGGAGATCGAGGGGATTCGGATTCGTGAC
RI-gBol028809-XLOC_019284-13926-1
ATCATTAACGATCGTGAGACTGGGAGATCGAGGGGATTCGGATTCGTGAC
CONSENSUS
ATCATTAACGATCGTGAGACTGGGAGATCGAGGGGATTCGGATTCGTGAC

RI-gBol028809-XLOC_019284-13926-0
CTTCAAGGATGAGAAGTCCATGAAGGATGCCATCGATGAGATGAACGGGA
RI-gBol028809-XLOC_019284-13926-1
CTTCAAGGATGAGAAGTCCATGAAGGATGCCATCGATGAGATGAACGGGA
CONSENSUS
CTTCAAGGATGAGAAGTCCATGAAGGATGCCATCGATGAGATGAACGGGA

RI-gBol028809-XLOC_019284-13926-0
AGGAGCTCGACGGTCGTACCATCACCGTCAACGAGGCTCAGTCCAGAGGA
RI-gBol028809-XLOC_019284-13926-1
AGGAGCTCGACGGTCGTACCATCACCGTCAACGAGGCTCAGTCCAGAGGA
CONSENSUS
AGGAGCTCGACGGTCGTACCATCACCGTCAACGAGGCTCAGTCCAGAGGA

RI-gBol028809-XLOC_019284-13926-0
GGCGGCGGTGGAGGAGGCCGTGGTGGAGGTGGATACGGTGGCCGTGGAGG
RI-gBol028809-XLOC_019284-13926-1
G---
CONSENSUS
G...

RI-gBol028809-XLOC_019284-13926-0
TGGTGGATACGGTGGAGGCGGCGGTGGATACGGTGACAGACGTGGAGGCG
RI-gBol028809-XLOC_019284-13926-1

CONSENSUS
      ....

RI-gBol028809-XLOC_019284-13926-0
      GCGGTTACGGATCTGGAGGTGGCGGCCGTGGAGGCGGCGGTTACGGATCT
RI-gBol028809-XLOC_019284-13926-1

CONSENSUS
      ....

RI-gBol028809-XLOC_019284-13926-0
      GGAGGTGGAGGATACGGTGGTGGTGGTGGCAGACGTGATGGAGGTGGCTA
RI-gBol028809-XLOC_019284-13926-1
      ---GTGGCTA
CONSENSUS
      ....GTGGCTA

RI-gBol028809-XLOC_019284-13926-0
      CGGAGGTGGTGACGGTGGCTACGGAGGAGGAAGTGGAGGCGGTGGATGGT
RI-gBol028809-XLOC_019284-13926-1
      CGGAGGTGGTGACGGTGGCTACGGAGGAGGAAGTGGAGGCGGTGGATGGT
CONSENSUS
      CGGAGGTGGTGACGGTGGCTACGGAGGAGGAAGTGGAGGCGGTGGATGGT

RI-gBol028809-XLOC_019284-13926-0
      AATCGATGATGAAGTGGTTGTTTTGCTGCTCTGTTTTCGGTTTAAACTTG
RI-gBol028809-XLOC_019284-13926-1
      AATCGATGATGAAGTGGTTGTTTTGCTGCTCTGTTTTCGGTTTAAACTTG
CONSENSUS
      AATCGATGATGAAGTGGTTGTTTTGCTGCTCTGTTTTCGGTTTAAACTTG

RI-gBol028809-XLOC_019284-13926-0
      CTTCCGTATGAATGTTTCTCCGGTTTGGTTTGGTTCTGTATTTCTTGTTT
RI-gBol028809-XLOC_019284-13926-1
      CTTCCGTATGAATGTTTCTCCGGTTTGGTTTGGTTCTGTATTTCTTGTTT
CONSENSUS
      CTTCCGTATGAATGTTTCTCCGGTTTGGTTTGGTTCTGTATTTCTTGTTT

RI-gBol028809-XLOC_019284-13926-0
      ACTTTTTTGTTGTAACGGATCGTTAAAGTCTTTGCTTGCCTGAAACGAAA
RI-gBol028809-XLOC_019284-13926-1
      ACTTTTTTGTTGTAACGGATCGTTAAAGTCTTTGCTTGCCTGAAACGAAA
CONSENSUS
      ACTTTTTTGTTGTAACGGATCGTTAAAGTCTTTGCTTGCCTGAAACGAAA

RI-gBol028809-XLOC_019284-13926-0
      TGTTAAATCGCATCTTGTTCTCTAAATTATAAGCTTCTCTTGATTTCTTT
RI-gBol028809-XLOC_019284-13926-1
      TGTTAAATCGCATCTTGTTCTCTAAATTATAAGCTTCTCTTGATTTCTTT
CONSENSUS
      TGTTAAATCGCATCTTGTTCTCTAAATTATAAGCTTCTCTTGATTTCTTT

RI-gBol028809-XLOC_019284-13926-0
      TTTTTTCACCGTCGCATTATACTCGTTCGTCGCCCGATCAGCTATTATGA
RI-gBol028809-XLOC_019284-13926-1
      TTTTTTCACCGTCGCATTATACTCGTTCGTCGCCCGATCAGCTATTATGA

```
CONSENSUS
      TTTTTTCACCGTCGCATTATACTCGTTCGTCGCCCGATCAGCTATTATGA

RI-gBol028809-XLOC_019284-13926-0     ACTCATTTATA
RI-gBol028809-XLOC_019284-13926-1     ACTCATTTATA
CONSENSUS                             ACTCATTTATA
```

alignment for event: A5-X-XLOC_027229-9606

```
A5-X-XLOC_027229-9606-0
      GAAGGTGGGTCGTGAGCAGAGTTTGTTGTCGTGAGCTTGAAGGCGGCATG
A5-X-XLOC_027229-9606-1
      GAAGGTGGGTCGTGAGCAGAGTTTGTTGTCGTGAGCTTGAAGGCGGCATG
CONSENSUS
      GAAGGTGGGTCGTGAGCAGAGTTTGTTGTCGTGAGCTTGAAGGCGGCATG

A5-X-XLOC_027229-9606-0
      ACAAGGCAGAGCAGCGTCGAAGGCGGCGGCAGTAAATCCTCTGATCTGAG
A5-X-XLOC_027229-9606-1
      ACAAGGCAGAGCAGCGTCGAAGGCGGCGGCAGTAAATCCTCTGATCTGAG
CONSENSUS
      ACAAGGCAGAGCAGCGTCGAAGGCGGCGGCAGTAAATCCTCTGATCTGAG

A5-X-XLOC_027229-9606-0
      GACGAGGACTATGTTTTTGTCCTTTCCCGATCTGCAAATGTAAAGCTCGT
A5-X-XLOC_027229-9606-1
      GACGAGGACTATGTTTTTGTCCTTTCCCGATCTGCAAAT-----------
CONSENSUS
      GACGAGGACTATGTTTTTGTCCTTTCCCGATCTGCAAAT...........

A5-X-XLOC_027229-9606-0
      ACTTGTTCTTAACTTTGTAGAGCTGTGATTTGGTGGAGATAGTGTTTACT
A5-X-XLOC_027229-9606-1
      --------------------------------------------------
CONSENSUS
      ..................................................

A5-X-XLOC_027229-9606-0
      GAATCATTAGATTAGTGAATGGAAGGCCAGAACATCAGGAAGCTTGTGAA
A5-X-XLOC_027229-9606-1     -------------------------
GCCAGAACATCAGGAAGCTTGTGAA
CONSENSUS
      .........................GCCAGAACATCAGGAAGCTTGTGAA

A5-X-XLOC_027229-9606-0     GGATGGTTTATGCTTGAACATCAG
A5-X-XLOC_027229-9606-1     GGATGGTTTATGCTTGAACATCAG
CONSENSUS                   GGATGGTTTATGCTTGAACATCAG
```

alignment for event: A5-gBol001807-XLOC_049914-3561

```
A5-gBol001807-XLOC_049914-3561-0
      GCTATAATGAACAAGGCCAACTTGGTAGAGGAGTCACTTGTGAAGGACTA
```

A5-gBol001807-XLOC_049914-3561-1
GCTATAATGAACAAGGCCAACTTGGTAGAGGAGTCACTTGTGAAGGACTA
CONSENSUS
GCTATAATGAACAAGGCCAACTTGGTAGAGGAGTCACTTGTGAAGGACTA

A5-gBol001807-XLOC_049914-3561-0
CAAGCACCTCGTGTGATAACTGCTTATGCGAAGTTCCTTGACGAAGCACC
A5-gBol001807-XLOC_049914-3561-1
CAAGCACCTCGTGTGATAACTGCTTATGCGAAGTTCCTTGACGAAGCACC
CONSENSUS
CAAGCACCTCGTGTGATAACTGCTTATGCGAAGTTCCTTGACGAAGCACC

A5-gBol001807-XLOC_049914-3561-0
CGAGCTTGTGAAGATTACGCAAGTCTCATGTGGAGAATACCATACTGCTG
A5-gBol001807-XLOC_049914-3561-1
CGAGCTTGTGAAGATTACGCAAGTCTCATGTGGAGAATACCATACTGCTG
CONSENSUS
CGAGCTTGTGAAGATTACGCAAGTCTCATGTGGAGAATACCATACTGCTG

A5-gBol001807-XLOC_049914-3561-0
CTGTATCAGAAACGGGCGAGGTGTAAGTTTTCTAAAAGACCTTTTCTCTT
A5-gBol001807-XLOC_049914-3561-1
CTGTATCAGAAACGGGCGAGGT----------------------------
CONSENSUS
CTGTATCAGAAACGGGCGAGGT............................

A5-gBol001807-XLOC_049914-3561-0
TGAAAACTTTGTGGTTATTCACATACCAATTCTCTGCAAGGGTCCTGGGA
A5-gBol001807-XLOC_049914-3561-1

CONSENSUS
....

A5-gBol001807-XLOC_049914-3561-0
AAGGAGATATCCATATCTCATCACCATCCTACCAGATGGGGAATGCTTGA
A5-gBol001807-XLOC_049914-3561-1

CONSENSUS
....

A5-gBol001807-XLOC_049914-3561-0
GGAAGCAAATTTACACTTGGGGACTAGGAAGCATGGGCCAACTCGGCCAT
A5-gBol001807-XLOC_049914-3561-1 ----------
TTACACTTGGGGACTAGGAAGCATGGGCCAACTCGGCCAT
CONSENSUS
..........TTACACTTGGGGACTAGGAAGCATGGGCCAACTCGGCCAT

A5-gBol001807-XLOC_049914-3561-0
GTTTCTCTCCAATCCGGGGATAAAGAGTTGATACCAAGGCGAGTCGCTGG
A5-gBol001807-XLOC_049914-3561-1
GTTTCTCTCCAATCCGGGGATAAAGAGTTGATACCAAGGCGAGTCGCTGG
CONSENSUS
GTTTCTCTCCAATCCGGGGATAAAGAGTTGATACCAAGGCGAGTCGCTGG

A5-gBol001807-XLOC_049914-3561-0
TCTCGATGGTGTGTCCATGAAGGAAGTCGCTTGTGGCGGTGTACACACTT

A5-gBol001807-XLOC_049914-3561-1
TCTCGATGGTGTGTCCATGAAGGAAGTCGCTTGTGGCGGTGTACACACTT
CONSENSUS
TCTCGATGGTGTGTCCATGAAGGAAGTCGCTTGTGGCGGTGTACACACTT

A5-gBol001807-XLOC_049914-3561-0
GTGCTTTATCTCTGGAAGGAGCGCTTTACGCTTGGGGCGGTGGCCAAGCG
A5-gBol001807-XLOC_049914-3561-1
GTGCTTTATCTCTGGAAGGAGCGCTTTACGCTTGGGGCGGTGGCCAAGCG
CONSENSUS
GTGCTTTATCTCTGGAAGGAGCGCTTTACGCTTGGGGCGGTGGCCAAGCG

A5-gBol001807-XLOC_049914-3561-0
GGACAGCTAGGTCTTGGTCCTCAGTCTGGTTTCTTGTTTTCTGTCTCTAA
A5-gBol001807-XLOC_049914-3561-1
GGACAGCTAGGTCTTGGTCCTCAGTCTGGTTTCTTGTTTTCTGTCTCTAA
CONSENSUS
GGACAGCTAGGTCTTGGTCCTCAGTCTGGTTTCTTGTTTTCTGTCTCTAA

A5-gBol001807-XLOC_049914-3561-0
TGGAAGCGAAATGCTTCTGCGGAACGTCCCGGTTCTAGTCATCCCGACCG
A5-gBol001807-XLOC_049914-3561-1
TGGAAGCGAAATGCTTCTGCGGAACGTCCCGGTTCTAGTCATCCCGACCG
CONSENSUS
TGGAAGCGAAATGCTTCTGCGGAACGTCCCGGTTCTAGTCATCCCGACCG

A5-gBol001807-XLOC_049914-3561-0
ATGTCAGGCTTGTTGCTTGTGGACATTCACACACTCTGGTTTATATGAGA
A5-gBol001807-XLOC_049914-3561-1
ATGTCAGGCTTGTTGCTTGTGGACATTCACACACTCTGGTTTATATGAGA
CONSENSUS
ATGTCAGGCTTGTTGCTTGTGGACATTCACACACTCTGGTTTATATGAGA

A5-gBol001807-XLOC_049914-3561-0
GAGGGACGGATCTGTGGTTGGGGATACAATAGCTATGGTCAAGCGGCTAA
A5-gBol001807-XLOC_049914-3561-1
GAGGGACGGATCTGTGGTTGGGGATACAATAGCTATGGTCAAGCGGCTAA
CONSENSUS
GAGGGACGGATCTGTGGTTGGGGATACAATAGCTATGGTCAAGCGGCTAA

A5-gBol001807-XLOC_049914-3561-0
TGAGAAATCATCGTATGCTTGGTACCCTTCGCCTGTAGACTG
A5-gBol001807-XLOC_049914-3561-1
TGAGAAATCATCGTATGCTTGGTACCCTTCGCCTGTAGACTG
CONSENSUS
TGAGAAATCATCGTATGCTTGGTACCCTTCGCCTGTAGACTG

alignment for event: RI-X-XLOC_006729-6001

RI-X-XLOC_006729-6001-0
ATCATGTAGATGGGTGAGGAGCCAGCAATACTGACTTAGAGGGGAAGATC
RI-X-XLOC_006729-6001-1
ATCATGTAGATGGGTGAGGAGCCAGCAATACTGACTTAGAGGGGAAGATC
CONSENSUS

ATCATGTAGATGGGTGAGGAGCCAGCAATACTGACTTAGAGGGGAAGATC

RI-X-XLOC_006729-6001-0
CACATGAATACTGTTGAATAAAGTTGAGTCTATTGTTATTGTTAAGATTA
RI-X-XLOC_006729-6001-1
CACATGAATACTGTTGAATAAA----------------------------
CONSENSUS
CACATGAATACTGTTGAATAAA............................

RI-X-XLOC_006729-6001-0
TGTATTTCCCTTGTGATTCTTTTTGTGTTATTGCTTATTGGTAAATGTTT
RI-X-XLOC_006729-6001-1

CONSENSUS
....

RI-X-XLOC_006729-6001-0
GATTACCTCTTTCTTTTGTTATGGCTATGTGGCAGTGTTATGCTTTATTG
RI-X-XLOC_006729-6001-1

CONSENSUS
....

RI-X-XLOC_006729-6001-0
TAAGTTTAGTAATTGTGTTTTTTGTTTTCTGGTTTCTTAGGCAGTCAAGC
RI-X-XLOC_006729-6001-1
--GCAGTCAAGC
CONSENSUS
....GCAGTCAAGC

RI-X-XLOC_006729-6001-0
CACACAAAAGATGTTTGTTTGGACGTGGAACAACACAAATGGAGAACAAT
RI-X-XLOC_006729-6001-1
CACACAAAAGATGTTTGTTTGGACGTGGAACAACACAAATGGAGAACAAT
CONSENSUS
CACACAAAAGATGTTTGTTTGGACGTGGAACAACACAAATGGAGAACAAT

RI-X-XLOC_006729-6001-0
TATCACATTGATCCACCAGGCCGTCGTTCTTACTCGTCAATCTCGTTTAG
RI-X-XLOC_006729-6001-1
TATCACATTGATCCACCAGGCCGTCGTTCTTACTCGTCAATCTCGTTTAG
CONSENSUS
TATCACATTGATCCACCAGGCCGTCGTTCTTACTCGTCAATCTCGTTTAG

RI-X-XLOC_006729-6001-0
AGAAGGAGTTGATTAGTTTGACTGAAACTGTCTACTTGATGAGCTAGCAG
RI-X-XLOC_006729-6001-1
AGAAGGAGTTGATTAGTTTGACTGAAACTGTCTACTTGATGAGCTAGCAG
CONSENSUS
AGAAGGAGTTGATTAGTTTGACTGAAACTGTCTACTTGATGAGCTAGCAG

RI-X-XLOC_006729-6001-0
CTCACTGCTTTGTGTGAAGCTAGAGGAGTGGCAAGCACTGATGCAACTAC
RI-X-XLOC_006729-6001-1
CTCACTGCTTTGTGTGAAGCTAGAGGAGTGGCAAGCACTGATGCAACTAC
CONSENSUS

CTCACTGCTTTGTGTGAAGCTAGAGGAGTGGCAAGCACTGATGCAACTAC

RI-X-XLOC_006729-6001-0
CAACCCTTCTCCATCCTCACCTCCTAGCACCAATGAGCAGAGAATCTAGA
RI-X-XLOC_006729-6001-1
CAACCCTTCTCCATCCTCACCTCCTAGCACCAATGAGCAGAGAATCTAGA
CONSENSUS
CAACCCTTCTCCATCCTCACCTCCTAGCACCAATGAGCAGAGAATCTAGA

RI-X-XLOC_006729-6001-0
TGATGTTGATGACTTTCTACAATCAATTGAAGACATAGCAACTGGAATCT
RI-X-XLOC_006729-6001-1
TGATGTTGATGACTTTCTACAATCAATTGAAGACATAGCAACTGGAATCT
CONSENSUS
TGATGTTGATGACTTTCTACAATCAATTGAAGACATAGCAACTGGAATCT

RI-X-XLOC_006729-6001-0
TTTGTGTTTAGTTTGAACTTTTATTATGTCATTAGTTTCCACCTATTGGG
RI-X-XLOC_006729-6001-1
TTTGTGTTTAGTTTGAACTTTTATTATGTCATTAGTTTCCACCTATTGGG
CONSENSUS
TTTGTGTTTAGTTTGAACTTTTATTATGTCATTAGTTTCCACCTATTGGG

RI-X-XLOC_006729-6001-0
TTTAATATTTTGTTTTCTGCTAGCAACGAATGCAATGTTTCTATTCTGTT
RI-X-XLOC_006729-6001-1
TTTAATATTTTGTTTTCTGCTAGCAACGAATGCAATGTTTCTATTCTGTT
CONSENSUS
TTTAATATTTTGTTTTCTGCTAGCAACGAATGCAATGTTTCTATTCTGTT

RI-X-XLOC_006729-6001-0 TTGGGAATTTCAGATTTTTTTT
RI-X-XLOC_006729-6001-1 TTGGGAATTTCAGATTTTTTTT
CONSENSUS TTGGGAATTTCAGATTTTTTTT

alignment for event: A3-gBol039925-XLOC_006599-12515

A3-gBol039925-XLOC_006599-12515-0
GGGACTCGATAACTACTTGAGTGTGAAGCAAGTGACTCTCTACACATCAA
A3-gBol039925-XLOC_006599-12515-1
GGGACTCGATAACTACTTGAGTGTGAAGCAAGTGACTCTCTACACATCAA
CONSENSUS
GGGACTCGATAACTACTTGAGTGTGAAGCAAGTGACTCTCTACACATCAA

A3-gBol039925-XLOC_006599-12515-0
ACGACCCGTGGGGATGGTACAAACCCCCTTGCTAAGTAATGATGCATACG
A3-gBol039925-XLOC_006599-12515-1
ACGACCCGTGGGGATGGTACAAACCCCCTTGCTAAGTAATGATGCATACG
CONSENSUS
ACGACCCGTGGGGATGGTACAAACCCCCTTGCTAAGTAATGATGCATACG

A3-gBol039925-XLOC_006599-12515-0
CTGTCATGAAAACGGAAAACCAAATGCTGTCTGTGTTGTCCATCTGTGTA
A3-gBol039925-XLOC_006599-12515-1
CTGTCATGAAAACGGAAAACCAAATGCTGTCTGTGTTGTCCATCTGTGTA

CONSENSUS
CTGTCATGAAAACGGAAAACCAAATGCTGTCTGTGTTGTCCATCTGTGTA

A3-gBol039925-XLOC_006599-12515-0
CTTTTTTATCAGTCTGCTATTTGTGGAACCTTTTGAGTATAAGTAAAGAC
A3-gBol039925-XLOC_006599-12515-1
CTTTTTTATCAGTCTGCTATTTGTGGAACCTTTTGAGTATAAGTAAAGAC
CONSENSUS
CTTTTTTATCAGTCTGCTATTTGTGGAACCTTTTGAGTATAAGTAAAGAC

A3-gBol039925-XLOC_006599-12515-0
GATATGAATAAAGTCAGGTTTGGTGATTCGCTTGTTGTGCATTGAGGCAC
A3-gBol039925-XLOC_006599-12515-1
GATATGAATAAAGTCAGGTTTGGTGATTCGCTTGTTGTGCATTGAGGCAC
CONSENSUS
GATATGAATAAAGTCAGGTTTGGTGATTCGCTTGTTGTGCATTGAGGCAC

A3-gBol039925-XLOC_006599-12515-0
CATTTTAGGGATCTGGTGCAGAGATATCATATATCAAGAACATGCACCAT
A3-gBol039925-XLOC_006599-12515-1
CATTTTAGGGATCTGGTGCAGAGATATCATATATCAAGAACATGCACCAT
CONSENSUS
CATTTTAGGGATCTGGTGCAGAGATATCATATATCAAGAACATGCACCAT

A3-gBol039925-XLOC_006599-12515-0
TTTATGTAACCTTCAAGTCGCAACAAAGTTTGAAACTCACATGGCATATT
A3-gBol039925-XLOC_006599-12515-1
TTTATGTAACCTTCAAGTCGCAACAAAGTTTGAAACTCACATGGCATATT
CONSENSUS
TTTATGTAACCTTCAAGTCGCAACAAAGTTTGAAACTCACATGGCATATT

A3-gBol039925-XLOC_006599-12515-0
TAATTCAAAGTCGGACTATTTGCTTAGAAAAAGCGTTTTATATAAACAAA
A3-gBol039925-XLOC_006599-12515-1
TAATTCAAAGTCGGACTATTTGCTTAGAAAAAGCGTTTTATATAAACAAA
CONSENSUS
TAATTCAAAGTCGGACTATTTGCTTAGAAAAAGCGTTTTATATAAACAAA

A3-gBol039925-XLOC_006599-12515-0
TCTGAAAGGGGAAGAAGGTAAAAAAGGATAAAGAAGAGAGAGAGGTGTGT
A3-gBol039925-XLOC_006599-12515-1
TCTGAAAGGGGAAGAAGGTAAAAAAGGATAAAGAAGAGAGAGAGGTGTGT
CONSENSUS
TCTGAAAGGGGAAGAAGGTAAAAAAGGATAAAGAAGAGAGAGAGGTGTGT

A3-gBol039925-XLOC_006599-12515-0
GGGGTTTTTCTGGTGCTTATCCTTTTAGAGTGTGACTTGGACCCATTACC
A3-gBol039925-XLOC_006599-12515-1
GGGGTTTTTCTGGTGCTTATCCTTTTAGAGTGTGACTTGGACCCATTACC
CONSENSUS
GGGGTTTTTCTGGTGCTTATCCTTTTAGAGTGTGACTTGGACCCATTACC

A3-gBol039925-XLOC_006599-12515-0
ATAACAGTACAGCTCTCCTTAGATCTATCTCATCTCGAGATAGACCACTA
A3-gBol039925-XLOC_006599-12515-1
ATAACAGTACAGCTCTCCTTAGATCTATCTCATCTCGAGATAGACCACTA

CONSENSUS
      ATAACAGTACAGCTCTCCTTAGATCTATCTCATCTCGAGATAGACCACTA

A3-gBol039925-XLOC_006599-12515-0
      CTTACTTACTAGAACATTAGTGATATTAGTGTTTAAGAAAGAACTCTGAG
A3-gBol039925-XLOC_006599-12515-1
      CTTACTTACTAG--------------------------------------
CONSENSUS
      CTTACTTACTAG......................................

A3-gBol039925-XLOC_006599-12515-0
      GTTTTGTGGGTCTCTCTCTGCTTAAAGATCTGTGCGTTTGGGGATTCGTT
A3-gBol039925-XLOC_006599-12515-1
      ---------------------------ATCTGTGCGTTTGGGGATTCGTT
CONSENSUS
      ...........................ATCTGTGCGTTTGGGGATTCGTT

A3-gBol039925-XLOC_006599-12515-0
      ATTGTCTGAATTTTGAGAAAAATGGGGAGCGCAGTGGAGGAGAAAGTGGT
A3-gBol039925-XLOC_006599-12515-1
      ATTGTCTGAATTTTGAGAAAAATGGGGAGCGCAGTGGAGGAGAAAGTGGT
CONSENSUS
      ATTGTCTGAATTTTGAGAAAAATGGGGAGCGCAGTGGAGGAGAAAGTGGT

A3-gBol039925-XLOC_006599-12515-0
      GGCTGTGATCATGGTCGGTGGTCCAACCAAAG
A3-gBol039925-XLOC_006599-12515-1
      GGCTGTGATCATGGTCGGTGGTCCAACCAAAG
CONSENSUS
      GGCTGTGATCATGGTCGGTGGTCCAACCAAAG

alignment for event: A3-X-XLOC_029001-11301

A3-X-XLOC_029001-11301-0
      CACTTGAAAGTTGAGCCTACAGATCCAAAACAAGGCTTTCTTGATTTTTG
A3-X-XLOC_029001-11301-1
      CACTTGAAAGTTGAGCCTACAGATCCAAAACAAGGCTTTCTTGATTTTTG
CONSENSUS
      CACTTGAAAGTTGAGCCTACAGATCCAAAACAAGGCTTTCTTGATTTTTG

A3-X-XLOC_029001-11301-0
      TTATCCCCCACGACAAAGAGACATTTCTTACG----------------CT
A3-X-XLOC_029001-11301-1
      TTATCCCCCACGACAAAGAGACATTTCTTACGAAACTCGGACTTACAGCT
CONSENSUS
      TTATCCCCCACGACAAAGAGACATTTCTTACG................CT

A3-X-XLOC_029001-11301-0
      CGGGCTTGCATCTCTTGAAAAAGTGTTTCTGAACATCGCTAGACGGGCTA
A3-X-XLOC_029001-11301-1
      CGGGCTTGCATCTCTTGAAAAAGTGTTTCTGAACATCGCTAGACGGGCTA
CONSENSUS
      CGGGCTTGCATCTCTTGAAAAAGTGTTTCTGAACATCGCTAGACGGGCTA

A3-X-XLOC_029001-11301-0
AACTAGAAAGTGCAACCGCTGCAAGAACCATGGTGACCCTCTATTTAGAA
A3-X-XLOC_029001-11301-1
AACTAGAAAGTGCAACCGCTGCAAGAACCATGGTGACCCTCTATTTAGAA
CONSENSUS
AACTAGAAAGTGCAACCGCTGCAAGAACCATGGTGACCCTCTATTTAGAA

A3-X-XLOC_029001-11301-0
TCTGGCATCTCACTTAAAGGGAAATCCAATAGCAGATATAAATATAAAAT
A3-X-XLOC_029001-11301-1
TCTGGCATCTCACTTAAAGGGAAATCCAATAGCAGATATAAATATAAAAT
CONSENSUS
TCTGGCATCTCACTTAAAGGGAAATCCAATAGCAGATATAAATATAAAAT

A3-X-XLOC_029001-11301-0
GCAACAAAGATGAGGAATTCCACTAAATTATATGATCATTGTAGATATAG
A3-X-XLOC_029001-11301-1
GCAACAAAGATGAGGAATTCCACTAAATTATATGATCATTGTAGATATAG
CONSENSUS
GCAACAAAGATGAGGAATTCCACTAAATTATATGATCATTGTAGATATAG

A3-X-XLOC_029001-11301-0
ATATGGATACCAATGGGAGCCAGATTTGGTGGGATCCTTGACACCAAACG
A3-X-XLOC_029001-11301-1
ATATGGATACCAATGGGAGCCAGATTTGGTGGGATCCTTGACACCAAACG
CONSENSUS
ATATGGATACCAATGGGAGCCAGATTTGGTGGGATCCTTGACACCAAACG

A3-X-XLOC_029001-11301-0
CAGAAAATTCAATTGGAGTAATGGTTGAAGTTTACTGGCAACAAGATGGG
A3-X-XLOC_029001-11301-1
CAGAAAATTCAATTGGAGTAATGGTTGAAGTTTACTGGCAACAAGATGGG
CONSENSUS
CAGAAAATTCAATTGGAGTAATGGTTGAAGTTTACTGGCAACAAGATGGG

A3-X-XLOC_029001-11301-0
TCGGGGTAGATGGCAATAGTCCACATCTTTTTTTTTTGGTAGAGATGTTA
A3-X-XLOC_029001-11301-1
TCGGGGTAGATGGCAATAGTCCACATCTTTTTTTTTTGGTAGAGATGTTA
CONSENSUS
TCGGGGTAGATGGCAATAGTCCACATCTTTTTTTTTTGGTAGAGATGTTA

A3-X-XLOC_029001-11301-0
AATATTATAGTTCACATCTTCACATTGAGTTTTGTGATTGTTCAATGTGA
A3-X-XLOC_029001-11301-1
AATATTATAGTTCACATCTTCACATTGAGTTTTGTGATTGTTCAATGTGA
CONSENSUS
AATATTATAGTTCACATCTTCACATTGAGTTTTGTGATTGTTCAATGTGA

A3-X-XLOC_029001-11301-0
CAAAAATATCGCAATGTATATGCATGTTACAGACACAAGTTCGTGACTGA
A3-X-XLOC_029001-11301-1
CAAAAATATCGCAATGTATATGCATGTTACAGACACAAGTTCGTGACTGA
CONSENSUS
CAAAAATATCGCAATGTATATGCATGTTACAGACACAAGTTCGTGACTGA

A3-X-XLOC_029001-11301-0
CTTTTTATGATAGGTTTTGTCTTTCTCAACAGTTATATG
A3-X-XLOC_029001-11301-1
CTTTTTATGATAGGTTTTGTCTTTCTCAACAGTTATATG
CONSENSUS
CTTTTTATGATAGGTTTTGTCTTTCTCAACAGTTATATG

alignment for event: A5-gBol028542-XLOC_019631-7070

A5-gBol028542-XLOC_019631-7070-0
GACCTGCGAGATGTTACCGTCAGATCAAGGGGAAGCCATACCCTAAATCA
A5-gBol028542-XLOC_019631-7070-1
GACCTGCGAGATGTTACCGTCAGATCAAGGGGAAGCCATACCCTAAATCA
CONSENSUS
GACCTGCGAGATGTTACCGTCAGATCAAGGGGAAGCCATACCCTAAATCA

A5-gBol028542-XLOC_019631-7070-0
CGATACTGCCGTGGTGTCCCCGATCCCAAGATCAGGATCTACGACGTCGG
A5-gBol028542-XLOC_019631-7070-1
CGATACTGCCGTGGTGTCCCCGATCCCAAGATCAGGATCTACGACGTCGG
CONSENSUS
CGATACTGCCGTGGTGTCCCCGATCCCAAGATCAGGATCTACGACGTCGG

A5-gBol028542-XLOC_019631-7070-0
GATGAAGAAGAAAGGAGTCGACGAGTTCCCATTCTGCGTCCATCTCGTCT
A5-gBol028542-XLOC_019631-7070-1
GATGAAGAAGAAAGGAGTCGACGAGTTCCCATTCTGCGTCCATCTCGTCT
CONSENSUS
GATGAAGAAGAAAGGAGTCGACGAGTTCCCATTCTGCGTCCATCTCGTCT

A5-gBol028542-XLOC_019631-7070-0
CATGGGAGAAAGAAAACGTCTCCAGCGAAGCTCTCGAGGCTGCGCGTATC
A5-gBol028542-XLOC_019631-7070-1
CATGGGAGAAAGAAAACGTCTCCAGCGAAGCTCTCGAGGCTGCGCGTATC
CONSENSUS
CATGGGAGAAAGAAAACGTCTCCAGCGAAGCTCTCGAGGCTGCGCGTATC

A5-gBol028542-XLOC_019631-7070-0
GCTTGCAACAAGTACATGGTGAAATCAGCTGGGAAAGATGCTTTTCATTT
A5-gBol028542-XLOC_019631-7070-1
GCTTGCAACAAGTACATGGTGAAATCAGCTGGGAAAGATGCTTTTCATTT
CONSENSUS
GCTTGCAACAAGTACATGGTGAAATCAGCTGGGAAAGATGCTTTTCATTT

A5-gBol028542-XLOC_019631-7070-0
GAGGATTAGGGTTCATCCTTTCCATGTTCTGAGGATTAACAAGATGCTTT
A5-gBol028542-XLOC_019631-7070-1
GAGGATTAGGGTTCATCCTTTCCATGTTCTGAGGATTAACAAGATGCTTT
CONSENSUS
GAGGATTAGGGTTCATCCTTTCCATGTTCTGAGGATTAACAAGATGCTTT

A5-gBol028542-XLOC_019631-7070-0
CGTGCGCTGGAGCTGATAGGCTTCAGACTGGTATGAGAGGTGCTTTTGGA
A5-gBol028542-XLOC_019631-7070-1

```
      CGTGCGCTGGAGCTGATAGGCTTCAGACTGGTATGAGAG---------GA
CONSENSUS
      CGTGCGCTGGAGCTGATAGGCTTCAGACTGGTATGAGAG.........GA

A5-gBol028542-XLOC_019631-7070-0
      GGGCTAAGTTTAAGTTCCCTGGTCGTCAGAAGATCATTGTTAGCAGGAAA
A5-gBol028542-XLOC_019631-7070-1
      GGGCTAAGTTTAAGTTCCCTGGTCGTCAGAAGATCATTGTTAGCAGGAAA
CONSENSUS
      GGGCTAAGTTTAAGTTCCCTGGTCGTCAGAAGATCATTGTTAGCAGGAAA

A5-gBol028542-XLOC_019631-7070-0 TG
A5-gBol028542-XLOC_019631-7070-1 TG
CONSENSUS                        TG



alignment for event: RI-gBol044934-XLOC_000225-10318

RI-gBol044934-XLOC_000225-10318-0
      GTGTCTCTTGGATGTGTTACTCCTTTTGCAGTCGTAAATGAATCAGCAAG
RI-gBol044934-XLOC_000225-10318-1
      GTGTCTCTTGGATGTGTTACTCCTTTTGCAGTCGTAAATGAATCAGCAAG
CONSENSUS
      GTGTCTCTTGGATGTGTTACTCCTTTTGCAGTCGTAAATGAATCAGCAAG

RI-gBol044934-XLOC_000225-10318-0
      GTATCGTTTTCCTTGACATTGGTTCATAGATCCGTTGACTGACATTTCTT
RI-gBol044934-XLOC_000225-10318-1
      --------------------------------------------------
CONSENSUS
      ..................................................

RI-gBol044934-XLOC_000225-10318-0
      TTTGAAAACTTTCTTTGTTTAAGAGATGTATCGCTCTTGTTAGACCAAAA
RI-gBol044934-XLOC_000225-10318-1      ----------------------
AGATGTATCGCTCTTGTTAGACCAAAA
CONSENSUS
      ......................AGATGTATCGCTCTTGTTAGACCAAAA

RI-gBol044934-XLOC_000225-10318-0
      ATTCAAGAACCAAACACGCTGCATCTTCCACCCGTTGTCTAACAACGTCT
RI-gBol044934-XLOC_000225-10318-1
      ATTCAAGAACCAAACACGCTGCATCTTCCACCCGTTGTCTAACAACGTCT
CONSENSUS
      ATTCAAGAACCAAACACGCTGCATCTTCCACCCGTTGTCTAACAACGTCT

RI-gBol044934-XLOC_000225-10318-0      CAATCT
RI-gBol044934-XLOC_000225-10318-1      CAATCT
CONSENSUS                              CAATCT



alignment for event: A5-gBol024534-XLOC_023998-16347

A5-gBol024534-XLOC_023998-16347-0
```

AGAGTTCGGGTTGCAGTCTGCAACCATCACAGATGAGGTTGATATCTGTG
A5-gBol024534-XLOC_023998-16347-1
AGAGTTCGGGTTGCAGTCTGCAACCATCACAGATGAGGTTGATATCTGTG
CONSENSUS
AGAGTTCGGGTTGCAGTCTGCAACCATCACAGATGAGGTTGATATCTGTG

A5-gBol024534-XLOC_023998-16347-0
TGGTTTGCCTGGAAAGAACATGCACTGTTGCTGCTGAAG-----------
A5-gBol024534-XLOC_023998-16347-1
TGGTTTGCCTGGAAAGAACATGCACTGTTGCTGCTGAAGGTCTGTCTAAA
CONSENSUS
TGGTTTGCCTGGAAAGAACATGCACTGTTGCTGCTGAAG...........

A5-gBol024534-XLOC_023998-16347-0

A5-gBol024534-XLOC_023998-16347-1
TTTTTGTCACACCCTTTAACGGATCTAGGCATGCGGGTTTGGTTACTTCG
CONSENSUS
....

A5-gBol024534-XLOC_023998-16347-0 -
GTTGTGACCATCAGCTATGTGTGAGATGCGCGTTATACCTTTGCTCATC
A5-gBol024534-XLOC_023998-16347-1
GGTTGTGACCATCAGCTATGTGTGAGATGCGCGTTATACCTTTGCTCATC
CONSENSUS
.GTTGTGACCATCAGCTATGTGTGAGATGCGCGTTATACCTTTGCTCATC

A5-gBol024534-XLOC_023998-16347-0
AAGCAATGTTCCCTCAGTGACAGTAGATCCACCCGGTTCAATCCCTTGTC
A5-gBol024534-XLOC_023998-16347-1
AAGCAATGTTCCCTCAGTGACAGTAGATCCACCCGGTTCAATCCCTTGTC
CONSENSUS
AAGCAATGTTCCCTCAGTGACAGTAGATCCACCCGGTTCAATCCCTTGTC

A5-gBol024534-XLOC_023998-16347-0
CTCTCTGCAGACACGGAATAGTGTCCTTTAAACGTCTCCCAAGCTCCCTG
A5-gBol024534-XLOC_023998-16347-1
CTCTCTGCAGACACGGAATAGTGTCCTTTAAACGTCTCCCAAGCTCCCTG
CONSENSUS
CTCTCTGCAGACACGGAATAGTGTCCTTTAAACGTCTCCCAAGCTCCCTG

A5-gBol024534-XLOC_023998-16347-0
ACCAAAGAAACGAAGTTACCTATGTCACTCGGGTTATGTGCGCCGTGTAT
A5-gBol024534-XLOC_023998-16347-1
ACCAAAGAAACGAAGTTACCTATGTCACTCGGGTTATGTGCGCCGTGTAT
CONSENSUS
ACCAAAGAAACGAAGTTACCTATGTCACTCGGGTTATGTGCGCCGTGTAT

A5-gBol024534-XLOC_023998-16347-0
GCTTCACACAAGCGACGCAACAGATCAATCTTCACCGACAACAGAACAGC
A5-gBol024534-XLOC_023998-16347-1
GCTTCACACAAGCGACGCAACAGATCAATCTTCACCGACAACAGAACAGC
CONSENSUS
GCTTCACACAAGCGACGCAACAGATCAATCTTCACCGACAACAGAACAGC

A5-gBol024534-XLOC_023998-16347-0

AACAGCGTAGTAGCAAGACTCGGACATCCTCGGTTTCGTCTGATATGTTC
A5-gBol024534-XLOC_023998-16347-1
AACAGCGTAGTAGCAAGACTCGGACATCCTCGGTTTCGTCTGATATGTTC
CONSENSUS
AACAGCGTAGTAGCAAGACTCGGACATCCTCGGTTTCGTCTGATATGTTC

A5-gBol024534-XLOC_023998-16347-0
TGTCCTGTAACATGTAGCCCATTCCCTTCGGTTAATATACCAATGTGCAC
A5-gBol024534-XLOC_023998-16347-1
TGTCCTGTAACATGTAGCCCATTCCCTTCGGTTAATATACCAATGTGCAC
CONSENSUS
TGTCCTGTAACATGTAGCCCATTCCCTTCGGTTAATATACCAATGTGCAC

A5-gBol024534-XLOC_023998-16347-0
ATGCAACGATGGAACGTGTCCGAACTTTGAGACGCATGGAACAGAGAGAC
A5-gBol024534-XLOC_023998-16347-1
ATGCAACGATGGAACGTGTCCGAACTTTGAGACGCATGGAACAGAGAGAC
CONSENSUS
ATGCAACGATGGAACGTGTCCGAACTTTGAGACGCATGGAACAGAGAGAC

A5-gBol024534-XLOC_023998-16347-0
ATAGTGAAGAAGATGACGAGTCTTCTTCGCCTCCTCGGGGGACGAGTGAG
A5-gBol024534-XLOC_023998-16347-1
ATAGTGAAGAAGATGACGAGTCTTCTTCGCCTCCTCGGGGGACGAGTGAG
CONSENSUS
ATAGTGAAGAAGATGACGAGTCTTCTTCGCCTCCTCGGGGGACGAGTGAG

A5-gBol024534-XLOC_023998-16347-0
CAGGAGAAGATAGGAGAAGGGCAAAGACTTGGGAAGGCAACAACTTGTTC
A5-gBol024534-XLOC_023998-16347-1
CAGGAGAAGATAGGAGAAGGGCAAAGACTTGGGAAGGCAACAACTTGTTC
CONSENSUS
CAGGAGAAGATAGGAGAAGGGCAAAGACTTGGGAAGGCAACAACTTGTTC

A5-gBol024534-XLOC_023998-16347-0
AAGCATGTTCTGGGGGAGAAGAAGCTGTAGCAGAGAGAACCAGTGCAACG
A5-gBol024534-XLOC_023998-16347-1
AAGCATGTTCTGGGGGAGAAGAAGCTGTAGCAGAGAGAACCAGTGCAACG
CONSENSUS
AAGCATGTTCTGGGGGAGAAGAAGCTGTAGCAGAGAGAACCAGTGCAACG

A5-gBol024534-XLOC_023998-16347-0
CTGAGATCAATGCCTGATCACAAACATTTTTATGTACAGTGTGTGAGAGA
A5-gBol024534-XLOC_023998-16347-1
CTGAGATCAATGCCTGATCACAAACATTTTTATGTACAGTGTGTGAGAGA
CONSENSUS
CTGAGATCAATGCCTGATCACAAACATTTTTATGTACAGTGTGTGAGAGA

A5-gBol024534-XLOC_023998-16347-0
GGGAGGGAGAGAATCAATGTGTCCTACAAAGTCTTGTTTGTTTCGACTTT
A5-gBol024534-XLOC_023998-16347-1
GGGAGGGAGAGAATCAATGTGTCCTACAAAGTCTTGTTTGTTTCGACTTT
CONSENSUS
GGGAGGGAGAGAATCAATGTGTCCTACAAAGTCTTGTTTGTTTCGACTTT

A5-gBol024534-XLOC_023998-16347-0

```
      TGAGAGGCTTCAGATATAAATTATGGTGGAAGATTTGTAATTAACTTTAT
A5-gBol024534-XLOC_023998-16347-1
      TGAGAGGCTTCAGATATAAATTATGGTGGAAGATTTGTAATTAACTTTAT
CONSENSUS
      TGAGAGGCTTCAGATATAAATTATGGTGGAAGATTTGTAATTAACTTTAT

A5-gBol024534-XLOC_023998-16347-0
      AATGATGGGGTCATTTTATTTCCCAGCTTTATTCTAATACTATTTGTAGA
A5-gBol024534-XLOC_023998-16347-1
      AATGATGGGGTCATTTTATTTCCCAGCTTTATTCTAATACTATTTGTAGA
CONSENSUS
      AATGATGGGGTCATTTTATTTCCCAGCTTTATTCTAATACTATTTGTAGA

A5-gBol024534-XLOC_023998-16347-0      TTGATTTATTATTCAG
A5-gBol024534-XLOC_023998-16347-1      TTGATTTATTATTCAG
CONSENSUS                              TTGATTTATTATTCAG
```

alignment for event: A3-gBol018340-XLOC_031022-8215

```
A3-gBol018340-XLOC_031022-8215-0
      GGCGTTGAAGGAAGAGAAGTATGCTGCTCGTAGAGCTATACTTCCCATTC
A3-gBol018340-XLOC_031022-8215-1
      GGCGTTGAAGGAAGAGAAGTATGCTGCTCGTAGAGCTATACTTCCCATTC
CONSENSUS
      GGCGTTGAAGGAAGAGAAGTATGCTGCTCGTAGAGCTATACTTCCCATTC

A3-gBol018340-XLOC_031022-8215-0
      TTCAAGCTGAGGAGGATGAAAGATGTACTGTCCTGATGGCGTTCGAGCCT
A3-gBol018340-XLOC_031022-8215-1
      TTCAAGCTGAGGAGGATGAAAG----------------------------
CONSENSUS
      TTCAAGCTGAGGAGGATGAAAG............................

A3-gBol018340-XLOC_031022-8215-0
      TGGCCACAACGGATTTAACATCAAAAAAAAAAATAAAAAAAAAAAAAAAA
A3-gBol018340-XLOC_031022-8215-1
      --------------------------------------------------
CONSENSUS
      ..................................................

A3-gBol018340-XLOC_031022-8215-0
      AAAAAAAAAAAGGAACGATATTTGTGTTAAATAGGTTTGTGTCTGAGTGG
A3-gBol018340-XLOC_031022-8215-1
      --------------------------------------------------
CONSENSUS
      ..................................................

A3-gBol018340-XLOC_031022-8215-0
      AAAAAGTATTTAGACTACGAGGCTGATGTAATGAAGGGATGTTCCGGGAT
A3-gBol018340-XLOC_031022-8215-1
      -------------------------------------GATGTTCCGGGAT
CONSENSUS
      .....................................GATGTTCCGGGAT
```

A3-gBol018340-XLOC_031022-8215-0
GGAAAGTTGGCGAGAATGTTTACAATTCTGGTCGTTGGATGCCACCGGCT
A3-gBol018340-XLOC_031022-8215-1
GGAAAGTTGGCGAGAATGTTTACAATTCTGGTCGTTGGATGCCACCGGCT
CONSENSUS
GGAAAGTTGGCGAGAATGTTTACAATTCTGGTCGTTGGATGCCACCGGCT

A3-gBol018340-XLOC_031022-8215-0
ACTGGTGAGCTCCGTCCTGATGTTTGGTGATTCTCTCCTAGTTTCTCACA
A3-gBol018340-XLOC_031022-8215-1
ACTGGTGAGCTCCGTCCTGATGTTTGGTGATTCTCTCCTAGTTTCTCACA
CONSENSUS
ACTGGTGAGCTCCGTCCTGATGTTTGGTGATTCTCTCCTAGTTTCTCACA

A3-gBol018340-XLOC_031022-8215-0
AATGCATACGATGATGATTATGAATGAATGTTTCAGTGAGGATTAATATG
A3-gBol018340-XLOC_031022-8215-1
AATGCATACGATGATGATTATGAATGAATGTTTCAGTGAGGATTAATATG
CONSENSUS
AATGCATACGATGATGATTATGAATGAATGTTTCAGTGAGGATTAATATG

A3-gBol018340-XLOC_031022-8215-0
TTTTTGTTTTATCTTTTTCACTCGTTAATAAGAGAGAGAATGATTCAACA
A3-gBol018340-XLOC_031022-8215-1
TTTTTGTTTTATCTTTTTCACTCGTTAATAAGAGAGAGAATGATTCAACA
CONSENSUS
TTTTTGTTTTATCTTTTTCACTCGTTAATAAGAGAGAGAATGATTCAACA

A3-gBol018340-XLOC_031022-8215-0
TACAGTTGTTCTTGTATTGGTTTTGCTGAAACAATAATCTGTGGTTTTGA
A3-gBol018340-XLOC_031022-8215-1
TACAGTTGTTCTTGTATTGGTTTTGCTGAAACAATAATCTGTGGTTTTGA
CONSENSUS
TACAGTTGTTCTTGTATTGGTTTTGCTGAAACAATAATCTGTGGTTTTGA

A3-gBol018340-XLOC_031022-8215-0
GTAATTGTTTCAATGGTAAAGAATACTTTCATCCCATC
A3-gBol018340-XLOC_031022-8215-1
GTAATTGTTTCAATGGTAAAGAATACTTTCATCCCATC
CONSENSUS
GTAATTGTTTCAATGGTAAAGAATACTTTCATCCCATC

alignment for event: A5-gBol018309-XLOC_030997-776

A5-gBol018309-XLOC_030997-776-0
GTTGGTTTTCTCAAATCAGCAATCTGGTCTCTTTGTGTCATTGTAAGAGA
A5-gBol018309-XLOC_030997-776-1
GTTGGTTTTCTCAAATCAGCAATCTGGTCTCTTTGTGTCATTGTAAGAGA
CONSENSUS
GTTGGTTTTCTCAAATCAGCAATCTGGTCTCTTTGTGTCATTGTAAGAGA

A5-gBol018309-XLOC_030997-776-0
GGTGGCATGGAGGTTGAATTCAAAGCAATGCTCGACGATCTCGATGTTCT
A5-gBol018309-XLOC_030997-776-1

GGTGGCATGGAGGTTGAATTCAAAGCAATGCTCGACGATCTCGATGTTCT
CONSENSUS
GGTGGCATGGAGGTTGAATTCAAAGCAATGCTCGACGATCTCGATGTTCT

A5-gBol018309-XLOC_030997-776-0
CGAGAAATCTCTCTCCGATCCGGCTCCGATCCACAAG-------------
A5-gBol018309-XLOC_030997-776-1
CGAGAAATCTCTCTCCGATCCGGCTCCGATCCACAAGGTGTTTGTCCTTT
CONSENSUS
CGAGAAATCTCTCTCCGATCCGGCTCCGATCCACAAG.............

A5-gBol018309-XLOC_030997-776-0

A5-gBol018309-XLOC_030997-776-1
TGCTTCGGATCTCGTTTCATTAAACTATGGAATTGCGGTCTTTAATTCGA
CONSENSUS
....

A5-gBol018309-XLOC_030997-776-0
---CTGCG
A5-gBol018309-XLOC_030997-776-1
AAGAAACGAGCTCTCACATGCATTTGGTGTTCAAGTGATATAATTCTGCG
CONSENSUS
....CTGCG

A5-gBol018309-XLOC_030997-776-0
ATCACATGTTGAGAATCTAGCGGCTCTGTCTAAGTGTAACCCACAACGGC
A5-gBol018309-XLOC_030997-776-1
ATCACATGTTGAGAATCTAGCGGCTCTGTCTAAGTGTAACCCACAACGGC
CONSENSUS
ATCACATGTTGAGAATCTAGCGGCTCTGTCTAAGTGTAACCCACAACGGC

A5-gBol018309-XLOC_030997-776-0 GTTCCAAAGTGAAG
A5-gBol018309-XLOC_030997-776-1 GTTCCAAAGTGAAG
CONSENSUS   GTTCCAAAGTGAAG

alignment for event: A3-gBol018499-XLOC_031212-13948

A3-gBol018499-XLOC_031212-13948-0
GTTGAACACATCAGAGAATCTTCTCGGTTCAATTCAAGACAAGATTAAAT
A3-gBol018499-XLOC_031212-13948-1
GTTGAACACATCAGAGAATCTTCTCGGTTCAATTCAAGACAAGATTAAAT
CONSENSUS
GTTGAACACATCAGAGAATCTTCTCGGTTCAATTCAAGACAAGATTAAAT

A3-gBol018499-XLOC_031212-13948-0
GGGCGGACAATATGAGTGAGATTGACAAAAAACATCGTAAGGAAGCAGAA
A3-gBol018499-XLOC_031212-13948-1
GGGCGGACAATATGAGTGAGATTGACAAAAAACATCGTAAGGAAGCAGAA
CONSENSUS
GGGCGGACAATATGAGTGAGATTGACAAAAAACATCGTAAGGAAGCAGAA

A3-gBol018499-XLOC_031212-13948-0
GAAGGAGTGGAAGAAGTCAAGAAGTCTCTGTCCATGAAG-----------

A3-gBol018499-XLOC_031212-13948-1
      GAAGGAGTGGAAGAAGTCAAGAAGTCTCTGTCCATGAAGGGGGACATTGA
CONSENSUS
      GAAGGAGTGGAAGAAGTCAAGAAGTCTCTGTCCATGAAG...........

A3-gBol018499-XLOC_031212-13948-0   ------
AGGGCATAAAGAGATGTTTGGGGAACCAAGTGGAGTGATGGACT
A3-gBol018499-XLOC_031212-13948-1
      CAGCAGAGGGCATAAAGAGATGTTTGGGGAACCAAGTGGAGTGATGGACT
CONSENSUS
      ......AGGGCATAAAGAGATGTTTGGGGAACCAAGTGGAGTGATGGACT

A3-gBol018499-XLOC_031212-13948-0
      ATGAAGAAGATAGAATCCGACCAAAGAG
A3-gBol018499-XLOC_031212-13948-1
      ATGAAGAAGATAGAATCCGACCAAAGAG
CONSENSUS
      ATGAAGAAGATAGAATCCGACCAAAGAG

alignment for event: RI-gBol031616-XLOC_016220-15377

RI-gBol031616-XLOC_016220-15377-0
      TGGGACATTAAGGGAGATGCTCATGGCATCTTGTCCTCTCATCCCATCGC
RI-gBol031616-XLOC_016220-15377-1
      TGGGACATTAAGGGAGATGCTCATGGCATCTTGTCCTCTCATCCCATCGC
CONSENSUS
      TGGGACATTAAGGGAGATGCTCATGGCATCTTGTCCTCTCATCCCATCGC

RI-gBol031616-XLOC_016220-15377-0
      ACCTTTTGTCTCTATTCATCACGTTGAAGCTGTTAATCCTCTCTATCCGG
RI-gBol031616-XLOC_016220-15377-1
      ACCTTTTGTCTCTATTCATCACGTTGAAGCTGTTAATCCTCTCTATCCGG
CONSENSUS
      ACCTTTTGTCTCTATTCATCACGTTGAAGCTGTTAATCCTCTCTATCCGG

RI-gBol031616-XLOC_016220-15377-0
      GCTTGTCCACATTAGATAGTTTGAAACTCTTCACAGAAGCTATGGATTTG
RI-gBol031616-XLOC_016220-15377-1
      GCTTGTCCACATTAGATAGTTTGAAACTCTTCACAGAAGCTATGGATTTG
CONSENSUS
      GCTTGTCCACATTAGATAGTTTGAAACTCTTCACAGAAGCTATGGATTTG

RI-gBol031616-XLOC_016220-15377-0
      AGCCCCAGGAGTGTTTTGCAGCGATCCATCTGTTACGATCATACTCACAA
RI-gBol031616-XLOC_016220-15377-1
      AGCCCCAGGAGTGTTTTGCAGCGATCCATCTGTTACGATCATACTCACAA
CONSENSUS
      AGCCCCAGGAGTGTTTTGCAGCGATCCATCTGTTACGATCATACTCACAA

RI-gBol031616-XLOC_016220-15377-0
      GCTCACCTTTTCAATCTCTCTTGGCTATGTTGTACAAGTTTTCCCGAACG
RI-gBol031616-XLOC_016220-15377-1
      GCTCACCTTTTCAATCTCTCTTGGCTATGTTGTACAAGTTTTCCCGAACG
CONSENSUS

GCTCACCTTTTCAATCTCTCTTGGCTATGTTGTACAAGTTTTCCCGAACG

RI-gBol031616-XLOC_016220-15377-0
TTCTCTTACCACGGGATCTTGAGCGAGCTGAGCTTACCTTCTCTGCCTGG
RI-gBol031616-XLOC_016220-15377-1
TTCTCTTACCACGGGATCTTGAGCGAGCTGAGCTTACCTTCTCTGCCTGG
CONSENSUS
TTCTCTTACCACGGGATCTTGAGCGAGCTGAGCTTACCTTCTCTGCCTGG

RI-gBol031616-XLOC_016220-15377-0
AACGGGATAAGACATCCGAGTGAGTTTGACCTTGACATAAAGATTCCTGT
RI-gBol031616-XLOC_016220-15377-1
AACGGGATAAGACATCCGA-------------------------------
CONSENSUS
AACGGGATAAGACATCCGA...............................

RI-gBol031616-XLOC_016220-15377-0
TTCATCTCTCTGTAAGAAGCCTATACTCTTCTTCTTAAACGAGGTAGGAC
RI-gBol031616-XLOC_016220-15377-1
---GAC
CONSENSUS
....GAC

RI-gBol031616-XLOC_016220-15377-0
GTGAAGGCAATGCGACTCTTGGAACCTACTCTAGATCATTGGTAAAATTT
RI-gBol031616-XLOC_016220-15377-1
GTGAAGGCAATGCGACTCTTGGAACCTACTCTAGATCATTGGTAAAATTT
CONSENSUS
GTGAAGGCAATGCGACTCTTGGAACCTACTCTAGATCATTGGTAAAATTT

RI-gBol031616-XLOC_016220-15377-0
GATATGAAGAGGAAGCTTCTATGTTTTCCAAGCTCTCCTCCTCTCCCTAA
RI-gBol031616-XLOC_016220-15377-1
GATATGAAGAGGAAGCTTCTATGTTTTCCAAGCTCTCCTCCTCTCCCTAA
CONSENSUS
GATATGAAGAGGAAGCTTCTATGTTTTCCAAGCTCTCCTCCTCTCCCTAA

RI-gBol031616-XLOC_016220-15377-0
TGTGGAAAAGATCCAGGTCCTAGGGCTTCCTCTGAGCAAAAATTGGCACT
RI-gBol031616-XLOC_016220-15377-1
TGTGGAAAAGATCCAGGTCCTAGGGCTTCCTCTGAGCAAAAATTGGCACT
CONSENSUS
TGTGGAAAAGATCCAGGTCCTAGGGCTTCCTCTGAGCAAAAATTGGCACT

RI-gBol031616-XLOC_016220-15377-0 TG
RI-gBol031616-XLOC_016220-15377-1 TG
CONSENSUS TG

alignment for event: A3-gBol022248-XLOC_026777-12564

A3-gBol022248-XLOC_026777-12564-0
TGCGAGGTACTGTGATATTGACGAAAATGGAGTACGGTACATGGTTTTGT
A3-gBol022248-XLOC_026777-12564-1
TGCGAGGTACTGTGATATTGACGAAAATGGAGTACGGTACATGGTTTTGT

CONSENSUS
      TGCGAGGTACTGTGATATTGACGAAAATGGAGTACGGTACATGGTTTTGT

A3-gBol022248-XLOC_026777-12564-0
      GCCGTGTAATAATGGGGAACATGGAGCTTCTTCGTGGTGATAAAGCACAG
A3-gBol022248-XLOC_026777-12564-1
      GCCGTGTAATAATGGGGAACATGGAGCTTCTTCGTGGTGATAAAGCACAG
CONSENSUS
      GCCGTGTAATAATGGGGAACATGGAGCTTCTTCGTGGTGATAAAGCACAG

A3-gBol022248-XLOC_026777-12564-0
      TTTTTCTCTGGTGGAGAAGAGTATGACAATGGAGTTGATGATGTCGAGAA
A3-gBol022248-XLOC_026777-12564-1
      TTTTTCTCTGGTGGAGAAGAGTATGACAATGGAGTTGATGATGTCGAGAA
CONSENSUS
      TTTTTCTCTGGTGGAGAAGAGTATGACAATGGAGTTGATGATGTCGAGAA

A3-gBol022248-XLOC_026777-12564-0
      TCCGAAAAATTACATTGTCTGGAACATCAATATGAATACCCATATATTCC
A3-gBol022248-XLOC_026777-12564-1
      TCCGAAAAATTACATTGTCTGGAACATCAATATGAATACCCATATATTCC
CONSENSUS
      TCCGAAAAATTACATTGTCTGGAACATCAATATGAATACCCATATATTCC

A3-gBol022248-XLOC_026777-12564-0
      CTGAATTTGTTGTTAGGTTCAAGCTGTCTGTTCCCCACAATGCTGAAG--
A3-gBol022248-XLOC_026777-12564-1
      CTGAATTTGTTGTTAGGTTCAAGCTGTCTGTTCCCCACAATGCTGAAGGG
CONSENSUS
      CTGAATTTGTTGTTAGGTTCAAGCTGTCTGTTCCCCACAATGCTGAAG..

A3-gBol022248-XLOC_026777-12564-0

A3-gBol022248-XLOC_026777-12564-1
      AGACAGCTTAGAGATCTGGCTGAAGCTCAGGTCTCAACTGATAAATTTAT
CONSENSUS
      ....

A3-gBol022248-XLOC_026777-12564-0

A3-gBol022248-XLOC_026777-12564-1
      AAAGAAAAAAAAAGGATCCATGTGTGATAAAACCTAACGACCTCCGGTGT
CONSENSUS
      ....

A3-gBol022248-XLOC_026777-12564-0

A3-gBol022248-XLOC_026777-12564-1
      TATTGAACACGTTTGGTTTCAAGAGACACGTCTTTGATTGAATCATCCGG
CONSENSUS
      ....

A3-gBol022248-XLOC_026777-12564-0

A3-gBol022248-XLOC_026777-12564-1
      GGGTCGGATGGAGATACTCACGGTTTAATGGGTATTTCGATCCATTAAAT

CONSENSUS
      ....

A3-gBol022248-XLOC_026777-12564-0
      --GT
A3-gBol022248-XLOC_026777-12564-1
      TCTGTTTTGTGCATATAAGAAGTCGATGACATACACTGGTTCCTGCAGGT
CONSENSUS
      ....GT

A3-gBol022248-XLOC_026777-12564-0
      AATATGGTTGCTAGGCATGATAACTCGGGTGTCACTTTGGAAGGACCCAA
A3-gBol022248-XLOC_026777-12564-1
      AATATGGTTGCTAGGCATGATAACTCGGGTGTCACTTTGGAAGGACCCAA
CONSENSUS
      AATATGGTTGCTAGGCATGATAACTCGGGTGTCACTTTGGAAGGACCCAA

A3-gBol022248-XLOC_026777-12564-0   GGATCTTCCTCCTCCGCAG
A3-gBol022248-XLOC_026777-12564-1   GGATCTTCCTCCTCCGCAG
CONSENSUS   GGATCTTCCTCCTCCGCAG

alignment for event: A3-gBol040199-XLOC_006286-6966

A3-gBol040199-XLOC_006286-6966-0
      GGCACAGCTGAAGTGGTCTATCCACGAAGAAGTGATGCAATTCAAGCTCT
A3-gBol040199-XLOC_006286-6966-1
      GGCACAGCTGAAGTGGTCTATCCACGAAGAAGTGATGCAATTCAAGCTCT
CONSENSUS
      GGCACAGCTGAAGTGGTCTATCCACGAAGAAGTGATGCAATTCAAGCTCT

A3-gBol040199-XLOC_006286-6966-0
      GAAGAAATACAACAATGTGTTGTTGGATGGAAGGCCAATGAGACTTGAGA
A3-gBol040199-XLOC_006286-6966-1
      GAAGAAATACAACAATGTGTTGTTGGATGGAAGGCCAATGAGACTTGAGA
CONSENSUS
      GAAGAAATACAACAATGTGTTGTTGGATGGAAGGCCAATGAGACTTGAGA

A3-gBol040199-XLOC_006286-6966-0
      TTTTGGGTGGAAACAACTATGAGGCTCCTCCTTTATCTGGTCGTGTGAAT
A3-gBol040199-XLOC_006286-6966-1
      TTTTGGGTGGAAACAACTATGAGGCTCCTCCTTTATCTGGTCGTGTGAAT
CONSENSUS
      TTTTGGGTGGAAACAACTATGAGGCTCCTCCTTTATCTGGTCGTGTGAAT

A3-gBol040199-XLOC_006286-6966-0
      GTGAATGTCTCTGGACTCAATGGAAGGCTGAAGAGGACCGTCGTTATCCA
A3-gBol040199-XLOC_006286-6966-1
      GTGAATGTCTCTGGACTCAATGGAAGGCTGAAGAGGACCGTCGTTATCCA
CONSENSUS
      GTGAATGTCTCTGGACTCAATGGAAGGCTGAAGAGGACCGTCGTTATCCA

A3-gBol040199-XLOC_006286-6966-0
      GCAAGGAGGAGGGAGAGCTATTGGTAGATTGAGAGGGAGAGGGAGAGGAG
A3-gBol040199-XLOC_006286-6966-1

-------------------------------------AGGGAGAGGAG
CONSENSUS
.....................................AGGGAGAGGAG

A3-gBol040199-XLOC_006286-6966-0
GAAGAGGTCCAGCTCCTACTGTCAATCGCCTTCCAAT
A3-gBol040199-XLOC_006286-6966-1
GAAGAGGTCCAGCTCCTACTGTCAATCGCCTTCCAAT
CONSENSUS
GAAGAGGTCCAGCTCCTACTGTCAATCGCCTTCCAAT

alignment for event: A5-X-XLOC_006468-817

A5-X-XLOC_006468-817-0
CTTTGTACAAACCCGACCTTTCATATTTTCTCTCTTTAGCCGACTGCAAA
A5-X-XLOC_006468-817-1
CTTTGTACAAACCCGACCTTTCATATTTTCTCTCTTTAGCCGACTGCAAA
CONSENSUS
CTTTGTACAAACCCGACCTTTCATATTTTCTCTCTTTAGCCGACTGCAAA

A5-X-XLOC_006468-817-0
ATTAGATCGTCACCAGTTACAAATCAAAGACAACGCCCACTTCTTCCACT
A5-X-XLOC_006468-817-1
ATTAGATCGTCACCAGTTACAAATCAAAGACAACGCCCACTTCTTCCACT
CONSENSUS
ATTAGATCGTCACCAGTTACAAATCAAAGACAACGCCCACTTCTTCCACT

A5-X-XLOC_006468-817-0
ATCTTATCATTTACTCTCCTCTTCAAATTAGTAATCGATCAATATTCTCA
A5-X-XLOC_006468-817-1
ATCTTATCATTTACTCTCCTCTTCAAATTAGTAATCGATCAATATTCTCA
CONSENSUS
ATCTTATCATTTACTCTCCTCTTCAAATTAGTAATCGATCAATATTCTCA

A5-X-XLOC_006468-817-0
TTTCTATATATCTTCTGCCTCTTCAAAATTTCTATAAGAGTAGTTGAGGG
A5-X-XLOC_006468-817-1
TTTCTATATATCTTCTGCCTCTTCAAAATTTCTATAAGAGTAGTTGAGGG
CONSENSUS
TTTCTATATATCTTCTGCCTCTTCAAAATTTCTATAAGAGTAGTTGAGGG

A5-X-XLOC_006468-817-0
ATTTGATTGTTAAGATCCTGAAGAATCCTGAAGAACCTAAAAGGATTTTT
A5-X-XLOC_006468-817-1
ATTTGATTGTTAAGATCCTGAAGAATCCTGAAGAACCTAAAAGGATTTTT
CONSENSUS
ATTTGATTGTTAAGATCCTGAAGAATCCTGAAGAACCTAAAAGGATTTTT

A5-X-XLOC_006468-817-0
TTGACTACGACGGCACTAATCCAAAGGCAGATGATTGTTAGGAGATGGAA
A5-X-XLOC_006468-817-1
TTGACTACGACGGCACTAATCCAAAGGCAGATGATTGTTAGGAGATGGAA
CONSENSUS
TTGACTACGACGGCACTAATCCAAAGGCAGATGATTGTTAGGAGATGGAA

A5-X-XLOC_006468-817-0
GAAACATGGGGAAAAAGATCCAATTTTTATTTTTGCTGTTACTAAACGCC
A5-X-XLOC_006468-817-1
GAAACATGGGGAAAAAGATCCAATTTTTATTTTTGCTGTTACTAAACGCC
CONSENSUS
GAAACATGGGGAAAAAGATCCAATTTTTATTTTTGCTGTTACTAAACGCC

A5-X-XLOC_006468-817-0
AAGTTGTTTGGTGGTAGAGGAGGACATCTACTCTGCTTTGCTGTTTAGAA
A5-X-XLOC_006468-817-1
AAGTTGTTTGGTGGTAGAGGAGGACATCTACTCTGCTTTGCTGTTTAGAA
CONSENSUS
AAGTTGTTTGGTGGTAGAGGAGGACATCTACTCTGCTTTGCTGTTTAGAA

A5-X-XLOC_006468-817-0
TCAATTTGAATCTGAATCTCCTCACGTCTGCTAGCAGGTTTTTTTTTGGC
A5-X-XLOC_006468-817-1
TCAATTTGAATCTGAATCTCCTCACGTCTGCTAGCAGGTTTTTTTTTGGC
CONSENSUS
TCAATTTGAATCTGAATCTCCTCACGTCTGCTAGCAGGTTTTTTTTTGGC

A5-X-XLOC_006468-817-0
CTCTTCAATTCTATTCTAATCTTCAGTTTGGTCAATGCTTCGATAGATTT
A5-X-XLOC_006468-817-1
CTCTTCAATTCTATTCTAATCTTCAGTTTGGTCAATGCTTCGATAGATTT
CONSENSUS
CTCTTCAATTCTATTCTAATCTTCAGTTTGGTCAATGCTTCGATAGATTT

A5-X-XLOC_006468-817-0
ACCAGTTAAGAAACCCTCCAATGAAGTTGTATGGACAGTTTTCTTTTTTA
A5-X-XLOC_006468-817-1
ACCA--
CONSENSUS
ACCA..

A5-X-XLOC_006468-817-0
TTACATAACCGAAGTTGCAAGCTTCTGTTTCTGTTTGTATATCTTGCAAT
A5-X-XLOC_006468-817-1

CONSENSUS
....

A5-X-XLOC_006468-817-0
TACCATCATTTTATTCATGTAGGCCCTCTTAGGCTATACACTTAGATGAT
A5-X-XLOC_006468-817-1

CONSENSUS
....

A5-X-XLOC_006468-817-0
TTCCTCTCCAATTGATTCCGAAGGCTTTAGGATTGAGAGCCTGAAGTTGT
A5-X-XLOC_006468-817-1

CONSENSUS
....

A5-X-XLOC_006468-817-0
      GATTGAACCAAATTTGCAGCTTTAGATGTTGTTATCTCCGTTCTTTAAAT
A5-X-XLOC_006468-817-1

CONSENSUS
      ....

A5-X-XLOC_006468-817-0
      ATGAGAGGTTAGCTTTCAGGATAATGGTGGAGGAGAGACACCATTGATGT
A5-X-XLOC_006468-817-1   ---
AGAGGTTAGCTTTCAGGATAATGGTGGAGGAGAGACACCATTGATGT
CONSENSUS
      ...AGAGGTTAGCTTTCAGGATAATGGTGGAGGAGAGACACCATTGATGT

A5-X-XLOC_006468-817-0
      GGCTTATAAAGTTTCAGACATGGAGACTTAATAGCATCTGTTGTGGTCGG
A5-X-XLOC_006468-817-1
      GGCTTATAAAGTTTCAGACATGGAGACTTAATAGCATCTGTTGTGGTCGG
CONSENSUS
      GGCTTATAAAGTTTCAGACATGGAGACTTAATAGCATCTGTTGTGGTCGG

A5-X-XLOC_006468-817-0
      CTCTGAAATCTGGTCGTGCTTCTCAGTCGGTGGTGATGCAAACGTCAATA
A5-X-XLOC_006468-817-1
      CTCTGAAATCTGGTCGTGCTTCTCAGTCGGTGGTGATGCAAACGTCAATA
CONSENSUS
      CTCTGAAATCTGGTCGTGCTTCTCAGTCGGTGGTGATGCAAACGTCAATA

A5-X-XLOC_006468-817-0
      AAGGAGGAGAGCTCATGGGGCTCCTACTTGATGAAAAG
A5-X-XLOC_006468-817-1
      AAGGAGGAGAGCTCATGGGGCTCCTACTTGATGAAAAG
CONSENSUS
      AAGGAGGAGAGCTCATGGGGCTCCTACTTGATGAAAAG

alignment for event: A5-X-XLOC_006468-818

A5-X-XLOC_006468-818-0
      ACAAACCCGACCTTTCATATTTTCTCTCTTTAGCCGACTGCAAAATTAGA
A5-X-XLOC_006468-818-1
      ACAAACCCGACCTTTCATATTTTCTCTCTTTAGCCGACTGCAAAATTAGA
CONSENSUS
      ACAAACCCGACCTTTCATATTTTCTCTCTTTAGCCGACTGCAAAATTAGA

A5-X-XLOC_006468-818-0
      TCGTCACCAGTTACAAATCAAAGACAACGCCCACTTCTTCCACTATCTTA
A5-X-XLOC_006468-818-1
      TCGTCACCAGTTACAAATCAAAGACAACGCCCACTTCTTCCACTATCTTA
CONSENSUS
      TCGTCACCAGTTACAAATCAAAGACAACGCCCACTTCTTCCACTATCTTA

A5-X-XLOC_006468-818-0
      TCATTTACTCTCCTCTTCAAATTAGTAATCGATCAATATTCTCATTTCTA

A5-X-XLOC_006468-818-1
TCATTTACTCTCCTCTTCAAATTAGTAATCGATCAATATTCTCATTTCTA
CONSENSUS
TCATTTACTCTCCTCTTCAAATTAGTAATCGATCAATATTCTCATTTCTA

A5-X-XLOC_006468-818-0
TATATCTTCTGCCTCTTCAAAATTTCTATAAGAGTAGTTGAGGGATTTGA
A5-X-XLOC_006468-818-1
TATATCTTCTGCCTCTTCAAAATTTCTATAAGAGTAGTTGAGGGATTTGA
CONSENSUS
TATATCTTCTGCCTCTTCAAAATTTCTATAAGAGTAGTTGAGGGATTTGA

A5-X-XLOC_006468-818-0
TTGTTAAGATCCTGAAGAATCCTGAAGAACCTAAAAGGATTTTTTTGACT
A5-X-XLOC_006468-818-1
TTGTTAAGATCCTGAAGAATCCTGAAGAACCTAAAAGGATTTTTTTGACT
CONSENSUS
TTGTTAAGATCCTGAAGAATCCTGAAGAACCTAAAAGGATTTTTTTGACT

A5-X-XLOC_006468-818-0
ACGACGGCACTAATCCAAAGGCAGATGATTGTTAGGAGATGGAAGAAACA
A5-X-XLOC_006468-818-1
ACGACGGCACTAATCCAAAGGCAGATGATTGTTAGGAGATGGAAGAAACA
CONSENSUS
ACGACGGCACTAATCCAAAGGCAGATGATTGTTAGGAGATGGAAGAAACA

A5-X-XLOC_006468-818-0
TGGGGAAAAAGATCCAATTTTTATTTTTGCTGTTACTAAACGCCAAGTTG
A5-X-XLOC_006468-818-1
TGGGGAAAAAGATCCAATTTTTATTTTTGCTGTTACTAAACGCCAAGTTG
CONSENSUS
TGGGGAAAAAGATCCAATTTTTATTTTTGCTGTTACTAAACGCCAAGTTG

A5-X-XLOC_006468-818-0
TTTGGTGGTAGAGGAGGACATCTACTCTGCTTTGCTGTTTAGAATCAATT
A5-X-XLOC_006468-818-1
TTTGGTGGTAGAGGAGGACATCTACTCTGCTTTGCTGTTTAGAATCAATT
CONSENSUS
TTTGGTGGTAGAGGAGGACATCTACTCTGCTTTGCTGTTTAGAATCAATT

A5-X-XLOC_006468-818-0
TGAATCTGAATCTCCTCACGTCTGCTAGCAGGTTTTTTTTTGGCCTCTTC
A5-X-XLOC_006468-818-1
TGAATCTGAATCTCCTCACGTCTGCTAGCAGGTTTTTTTTTGGCCTCTTC
CONSENSUS
TGAATCTGAATCTCCTCACGTCTGCTAGCAGGTTTTTTTTTGGCCTCTTC

A5-X-XLOC_006468-818-0
AATTCTATTCTAATCTTCAGTTTGGTCAATGCTTCGATAGATTTACCAGT
A5-X-XLOC_006468-818-1
AATTCTATTCTAATCTTCAGTTTGGTCAATGCTTCGATAGATTTACCA--
CONSENSUS
AATTCTATTCTAATCTTCAGTTTGGTCAATGCTTCGATAGATTTACCA..

A5-X-XLOC_006468-818-0
TAAGAAACCCTCCAATGAAGTTGTATGGACAGTTTTCTTTTTTATTACAT

A5-X-XLOC_006468-818-1

CONSENSUS
    ....

A5-X-XLOC_006468-818-0
    AACCGAAGTTGCAAGCTTCTGTTTCTGTTTGTATATCTTGCAATTACCAT
A5-X-XLOC_006468-818-1

CONSENSUS
    ....

A5-X-XLOC_006468-818-0
    CATTTTATTCATGTAGGCCCTCTTAGGCTATACACTTAGATGATTTCCTC
A5-X-XLOC_006468-818-1

CONSENSUS
    ....

A5-X-XLOC_006468-818-0
    TCCAATTGATTCCGAAGGCTTTAGGATTGAGAGCCTGAAGTTGTGATTGA
A5-X-XLOC_006468-818-1

CONSENSUS
    ....

A5-X-XLOC_006468-818-0
    ACCAAATTTGCAGCTTTAGATGTTGTTATCTCCGTTCTTTAAATATGAGA
A5-X-XLOC_006468-818-1
    ---AGA
CONSENSUS
    ....AGA

A5-X-XLOC_006468-818-0
    GGTTAGCTTTCAGGATAATGGTGGAGGAGAGACACCATTGATGTGGCTTA
A5-X-XLOC_006468-818-1
    GGTTAGCTTTCAGGATAATGGTGGAGGAGAGACACCATTGATGTGGCTTA
CONSENSUS
    GGTTAGCTTTCAGGATAATGGTGGAGGAGAGACACCATTGATGTGGCTTA

A5-X-XLOC_006468-818-0
    TAAAGTTTCAGACATGGAGACTTAATAGCATCTGTTGTGGTCGGCTCTGA
A5-X-XLOC_006468-818-1
    TAAAGTTTCAGACATGGAGACTTAATAGCATCTGTTGTGGTCGGCTCTGA
CONSENSUS
    TAAAGTTTCAGACATGGAGACTTAATAGCATCTGTTGTGGTCGGCTCTGA

A5-X-XLOC_006468-818-0
    AATCTGGTCGTGCTTCTCAGTCGGTGGTGATGCAAACGTCAATAAAGGAG
A5-X-XLOC_006468-818-1
    AATCTGGTCGTGCTTCTCAGTCGGTGGTGATGCAAACGTCAATAAAGGAG
CONSENSUS
    AATCTGGTCGTGCTTCTCAGTCGGTGGTGATGCAAACGTCAATAAAGGAG

A5-X-XLOC_006468-818-0   GAGAGCTCATGGGGCTCCTACTTGATGAAAAG
A5-X-XLOC_006468-818-1   GAGAGCTCATGGGGCTCCTACTTGATGAAAAG

CONSENSUS GAGAGCTCATGGGGCTCCTACTTGATGAAAAG

alignment for event: A5-X-XLOC_006421-9463

A5-X-XLOC_006421-9463-0
GCGCAACATGAGAGAGCAGATAACAATGCACTAAAGGCAGAGAACGACAA
A5-X-XLOC_006421-9463-1
GCGCAACATGAGAGAGCAGATAACAATGCACTAAAGGCAGAGAACGACAA
CONSENSUS
GCGCAACATGAGAGAGCAGATAACAATGCACTAAAGGCAGAGAACGACAA

A5-X-XLOC_006421-9463-0
GATTCGATGTGAGAACATAGCCATTAGAGAAGCACTCAAGCATGCTATAT
A5-X-XLOC_006421-9463-1
GATTCGATGTGAGAACATAGCCATTAGAGAAGCACTCAAGCATGCTATAT
CONSENSUS
GATTCGATGTGAGAACATAGCCATTAGAGAAGCACTCAAGCATGCTATAT

A5-X-XLOC_006421-9463-0
GCCCTAACTGTGGATGTCCTCCCGTTCGTGAAGATCCTTACTTCGATGAG
A5-X-XLOC_006421-9463-1
GCCCTAACTGTGGATGTCCTCCCGTTCGTGAAGATCCTTACTTCGATGAG
CONSENSUS
GCCCTAACTGTGGATGTCCTCCCGTTCGTGAAGATCCTTACTTCGATGAG

A5-X-XLOC_006421-9463-0
CGTAAGCTCCGGATCGAAAATGTACATCTTAGAGACGAGCTTGAAAGAAT
A5-X-XLOC_006421-9463-1
CGTAAGCTCCGGATCGAAAAT------------------CTTGAAAGAAT
CONSENSUS
CGTAAGCTCCGGATCGAAAAT..................CTTGAAAGAAT

A5-X-XLOC_006421-9463-0
GTCAACGGTTGCATCAAAGTACATGGGAAGACCAATCTCCTCCCACCTTG
A5-X-XLOC_006421-9463-1
GTCAACGGTTGCATCAAAGTACATGGGAAGACCAATCTCCTCCCACCTTG
CONSENSUS
GTCAACGGTTGCATCAAAGTACATGGGAAGACCAATCTCCTCCCACCTTG

A5-X-XLOC_006421-9463-0
CAACGCTACACCCAATGCGCATGTCACCGTTGGATCTGTCCATGATTGGT
A5-X-XLOC_006421-9463-1
CAACGCTACACCCAATGCGCATGTCACCGTTGGATCTGTCCATGATTGGT
CONSENSUS
CAACGCTACACCCAATGCGCATGTCACCGTTGGATCTGTCCATGATTGGT

A5-X-XLOC_006421-9463-0
CCTTCACTGGATTTTGATCTTCTTCCAGCGAGTTCTATGCATTCTCAGCC
A5-X-XLOC_006421-9463-1
CCTTCACTGGATTTTGATCTTCTTCCAGCGAGTTCTATGCATTCTCAGCC
CONSENSUS
CCTTCACTGGATTTTGATCTTCTTCCAGCGAGTTCTATGCATTCTCAGCC

A5-X-XLOC_006421-9463-0

TAATAACTTGGCTACTATATCAGACATGGACAAGCCTCTCATGAACGACA
A5-X-XLOC_006421-9463-1
TAATAACTTGGCTACTATATCAGACATGGACAAGCCTCTCATGAACGACA
CONSENSUS
TAATAACTTGGCTACTATATCAGACATGGACAAGCCTCTCATGAACGACA

A5-X-XLOC_006421-9463-0
TTGCTTTGACTGCGATGGAAGAATTGCTTAGGCTTTTTAACACAAACGAA
A5-X-XLOC_006421-9463-1
TTGCTTTGACTGCGATGGAAGAATTGCTTAGGCTTTTTAACACAAACGAA
CONSENSUS
TTGCTTTGACTGCGATGGAAGAATTGCTTAGGCTTTTTAACACAAACGAA

A5-X-XLOC_006421-9463-0
CCTCTCTGGACTAGAGCTGATGGTGGCGGTGGCAGAGACATTCTCTATTT
A5-X-XLOC_006421-9463-1
CCTCTCTGGACTAGAGCTGATGGTGGCGGTGGCAGAGACATTCTCTATTT
CONSENSUS
CCTCTCTGGACTAGAGCTGATGGTGGCGGTGGCAGAGACATTCTCTATTT

A5-X-XLOC_006421-9463-0
GATGCTCAGGTTTATATGTTTGCAGAGTTTGACTATGAAACCAGTGCTGG
A5-X-XLOC_006421-9463-1
GATGCTCAGGTTTATATGTTTGCAGAGTTTGACTATGAAACCAGTGCTGG
CONSENSUS
GATGCTCAGGTTTATATGTTTGCAGAGTTTGACTATGAAACCAGTGCTGG

A5-X-XLOC_006421-9463-0
TCAATGCTTTCACAGTAAGTAAGATGCTCAAATAATAGTCTGTTAGTTAC
A5-X-XLOC_006421-9463-1
TCAATGCTTTCACAGTAAGTAAGATGCTCAAATAATAGTCTGTTAGTTAC
CONSENSUS
TCAATGCTTTCACAGTAAGTAAGATGCTCAAATAATAGTCTGTTAGTTAC

A5-X-XLOC_006421-9463-0
GAGCATGAAAAGCAACAAAATCGTGGTCTTTAGATTCTTAATGCACTGCA
A5-X-XLOC_006421-9463-1
GAGCATGAAAAGCAACAAAATCGTGGTCTTTAGATTCTTAATGCACTGCA
CONSENSUS
GAGCATGAAAAGCAACAAAATCGTGGTCTTTAGATTCTTAATGCACTGCA

A5-X-XLOC_006421-9463-0
TATATATTTAACTTCAGAAGAGTATACAGAGACTGTCTCAGATTTTTGAT
A5-X-XLOC_006421-9463-1
TATATATTTAACTTCAGAAGAGTATACAGAGACTGTCTCAGATTTTTGAT
CONSENSUS
TATATATTTAACTTCAGAAGAGTATACAGAGACTGTCTCAGATTTTTGAT

A5-X-XLOC_006421-9463-0
GAATGATACAATCAGTTTATGGGTACATGGTTTGAACCTCTTGCACTGAT
A5-X-XLOC_006421-9463-1
GAATGATACAATCAGTTTATGGGTACATGGTTTGAACCTCTTGCACTGAT
CONSENSUS
GAATGATACAATCAGTTTATGGGTACATGGTTTGAACCTCTTGCACTGAT

A5-X-XLOC_006421-9463-0

TATATCTTTGTCCACCATGCTCACTTGCACGGAAATCTATAATCCTTTTT
A5-X-XLOC_006421-9463-1
TATATCTTTGTCCACCATGCTCACTTGCACGGAAATCTATAATCCTTTTT
CONSENSUS
TATATCTTTGTCCACCATGCTCACTTGCACGGAAATCTATAATCCTTTTT

A5-X-XLOC_006421-9463-0
GTCGCTATTATTTGTTATATCAATGTACAGATAAAAGGTCA
A5-X-XLOC_006421-9463-1
GTCGCTATTATTTGTTATATCAATGTACAGATAAAAGGTCA
CONSENSUS
GTCGCTATTATTTGTTATATCAATGTACAGATAAAAGGTCA

alignment for event: A3-gBol027004-XLOC_021542-2284

A3-gBol027004-XLOC_021542-2284-0
TTTCCCTTTACAATAACCAGACTTTTTTTTTATAGTGAAGGCTCCACAAT
A3-gBol027004-XLOC_021542-2284-1
TTTCCCTTTACAATAACCAGACTTTTTTTTTATAGTGAAGGCTCCACAAT
CONSENSUS
TTTCCCTTTACAATAACCAGACTTTTTTTTTATAGTGAAGGCTCCACAAT

A3-gBol027004-XLOC_021542-2284-0
AGGGTTCCGACGCTCCGGTCTATCTCGATTATTCTCGTAGCTTCGTCTTC
A3-gBol027004-XLOC_021542-2284-1
AGGGTTCCGACGCTCCGGTCTATCTCGATTATTCTCGTAGCTTCGTCTTC
CONSENSUS
AGGGTTCCGACGCTCCGGTCTATCTCGATTATTCTCGTAGCTTCGTCTTC

A3-gBol027004-XLOC_021542-2284-0
GCTTTCTCCTCCTATTATCTCTGGCAGTTCGCGGAATCTGGGGTGAATTC
A3-gBol027004-XLOC_021542-2284-1 GCTTTCTCCTCCTATTATCTCT-----
TTCGCGGAATCTGGGGTGAATTC
CONSENSUS
GCTTTCTCCTCCTATTATCTCT.....TTCGCGGAATCTGGGGTGAATTC

A3-gBol027004-XLOC_021542-2284-0
GATTGATTTCTCATCGAATCCGTGAAATCGAGCTCCTGTTGCTGAAGATA
A3-gBol027004-XLOC_021542-2284-1
GATTGATTTCTCATCGAATCCGTGAAATCGAGCTCCTGTTGCTGAAGATA
CONSENSUS
GATTGATTTCTCATCGAATCCGTGAAATCGAGCTCCTGTTGCTGAAGATA

A3-gBol027004-XLOC_021542-2284-0
GATATGGAGGTCGTTAAATCCAAGTATAGGGACGAGGATTTGGAGGTGGA
A3-gBol027004-XLOC_021542-2284-1
GATATGGAGGTCGTTAAATCCAAGTATAGGGACGAGGATTTGGAGGTGGA
CONSENSUS
GATATGGAGGTCGTTAAATCCAAGTATAGGGACGAGGATTTGGAGGTGGA

A3-gBol027004-XLOC_021542-2284-0
TGATTTGAAGAGCAGCCGGAGGGACCGTGATCGGAGTAAGGAGAGGAAGA
A3-gBol027004-XLOC_021542-2284-1
TGATTTGAAGAGCAGCCGGAGGGACCGTGATCGGAGTAAGGAGAGGAAGA

CONSENSUS
TGATTTGAAGAGCAGCCGGAGGGACCGTGATCGGAGTAAGGAGAGGAAGA

A3-gBol027004-XLOC_021542-2284-0
AGGAGAGGAGTTCTGATAAGCGCCGGGAGAAGGATAGACGGAAGAGGCGG
A3-gBol027004-XLOC_021542-2284-1
AGGAGAGGAGTTCTGATAAGCGCCGGGAGAAGGATAGACGGAAGAGGCGG
CONSENSUS
AGGAGAGGAGTTCTGATAAGCGCCGGGAGAAGGATAGACGGAAGAGGCGG

A3-gBol027004-XLOC_021542-2284-0
TCTGAGAGAGTTAAGAGTAGTGATGATTCTGAAGATGAGTACGATAGAGG
A3-gBol027004-XLOC_021542-2284-1
TCTGAGAGAGTTAAGAGTAGTGATGATTCTGAAGATGAGTACGATAGAGG
CONSENSUS
TCTGAGAGAGTTAAGAGTAGTGATGATTCTGAAGATGAGTACGATAGAGG

A3-gBol027004-XLOC_021542-2284-0
GGATGATGATGATGAAGAGAGGGAGAGGCGTAAAGAAAAGGAGAGGAGGC
A3-gBol027004-XLOC_021542-2284-1
GGATGATGATGATGAAGAGAGGGAGAGGCGTAAAGAAAAGGAGAGGAGGC
CONSENSUS
GGATGATGATGATGAAGAGAGGGAGAGGCGTAAAGAAAAGGAGAGGAGGC

A3-gBol027004-XLOC_021542-2284-0
GGAGGGATAAAGAGAGGGGGAAGAGACGGTCTGAGAGAAGGAAGAGTAGT
A3-gBol027004-XLOC_021542-2284-1
GGAGGGATAAAGAGAGGGGGAAGAGACGGTCTGAGAGAAGGAAGAGTAGT
CONSENSUS
GGAGGGATAAAGAGAGGGGGAAGAGACGGTCTGAGAGAAGGAAGAGTAGT

A3-gBol027004-XLOC_021542-2284-0
GATTCTGAAGATGAGGATGAAGAAGATGGCGGGAGAGATAAACGCCGAGT
A3-gBol027004-XLOC_021542-2284-1
GATTCTGAAGATGAGGATGAAGAAGATGGCGGGAGAGATAAACGCCGAGT
CONSENSUS
GATTCTGAAGATGAGGATGAAGAAGATGGCGGGAGAGATAAACGCCGAGT

A3-gBol027004-XLOC_021542-2284-0
GAAGGACAAGGAGAGAGGGCACAGAGAACATAGGGACAAAGATCGGAAAA
A3-gBol027004-XLOC_021542-2284-1
GAAGGACAAGGAGAGAGGGCACAGAGAACATAGGGACAAAGATCGGAAAA
CONSENSUS
GAAGGACAAGGAGAGAGGGCACAGAGAACATAGGGACAAAGATCGGAAAA

A3-gBol027004-XLOC_021542-2284-0
GGGATAGAGAGAGGGAAGAGAGGAAGGAGAAAGAAAGAGAACGAGAAAGG
A3-gBol027004-XLOC_021542-2284-1
GGGATAGAGAGAGGGAAGAGAGGAAGGAGAAAGAAAGAGAACGAGAAAGG
CONSENSUS
GGGATAGAGAGAGGGAAGAGAGGAAGGAGAAAGAAAGAGAACGAGAAAGG

A3-gBol027004-XLOC_021542-2284-0
GAGAAGGATAGAGTTAGGAGAGAACGGGAACGAGAGGAGCGTGAGAAGGA
A3-gBol027004-XLOC_021542-2284-1
GAGAAGGATAGAGTTAGGAGAGAACGGGAACGAGAGGAGCGTGAGAAGGA

CONSENSUS
GAGAAGGATAGAGTTAGGAGAGAACGGGAACGAGAGGAGCGTGAGAAGGA

A3-gBol027004-XLOC_021542-2284-0
GAGACTGAAAGAAAGAGAGAGGCGGGAGCGTGAACGGGAAGATGGAGAGA
A3-gBol027004-XLOC_021542-2284-1
GAGACTGAAAGAAAGAGAGAGGCGGGAGCGTGAACGGGAAGATGGAGAGA
CONSENSUS
GAGACTGAAAGAAAGAGAGAGGCGGGAGCGTGAACGGGAAGATGGAGAGA

A3-gBol027004-XLOC_021542-2284-0
GGGATAGGAGGGAACGTGAGAAAGAAAGGAGTAGGAGGAACCGGGAAAGG
A3-gBol027004-XLOC_021542-2284-1
GGGATAGGAGGGAACGTGAGAAAGAAAGGAGTAGGAGGAACCGGGAAAGG
CONSENSUS
GGGATAGGAGGGAACGTGAGAAAGAAAGGAGTAGGAGGAACCGGGAAAGG

A3-gBol027004-XLOC_021542-2284-0
GGGAGGTCAAGGGAGGATGGACATGAAGAGAGTGATGATGATGTCAAGCG
A3-gBol027004-XLOC_021542-2284-1
GGGAGGTCAAGGGAGGATGGACATGAAGAGAGTGATGATGATGTCAAGCG
CONSENSUS
GGGAGGTCAAGGGAGGATGGACATGAAGAGAGTGATGATGATGTCAAGCG

A3-gBol027004-XLOC_021542-2284-0
TGAACTGAAACGTAGAAGAAGAGAGAGTGGAGAACGGAAGGAGAAGGAGC
A3-gBol027004-XLOC_021542-2284-1
TGAACTGAAACGTAGAAGAAGAGAGAGTGGAGAACGGAAGGAGAAGGAGC
CONSENSUS
TGAACTGAAACGTAGAAGAAGAGAGAGTGGAGAACGGAAGGAGAAGGAGC

A3-gBol027004-XLOC_021542-2284-0
GTGAGAAGAGTGTTGGTAGATCTAGCAGGCATGGAGATGACAATGGAGAT
A3-gBol027004-XLOC_021542-2284-1
GTGAGAAGAGTGTTGGTAGATCTAGCAGGCATGGAGATGACAATGGAGAT
CONSENSUS
GTGAGAAGAGTGTTGGTAGATCTAGCAGGCATGGAGATGACAATGGAGAT

A3-gBol027004-XLOC_021542-2284-0
AGTCCAAGGAGAAAGAGTGTCGAGGAGGATGATGAAAAGAAAGAGAAGAA
A3-gBol027004-XLOC_021542-2284-1
AGTCCAAGGAGAAAGAGTGTCGAGGAGGATGATGAAAAGAAAGAGAAGAA
CONSENSUS
AGTCCAAGGAGAAAGAGTGTCGAGGAGGATGATGAAAAGAAAGAGAAGAA

A3-gBol027004-XLOC_021542-2284-0
AACGAGGGAAGAAGAACTAGAGGAGGAGCAGAAGAAGTTGGATGAGGAGG
A3-gBol027004-XLOC_021542-2284-1
AACGAGGGAAGAAGAACTAGAGGAGGAGCAGAAGAAGTTGGATGAGGAGG
CONSENSUS
AACGAGGGAAGAAGAACTAGAGGAGGAGCAGAAGAAGTTGGATGAGGAGG

A3-gBol027004-XLOC_021542-2284-0
TTGAGAAACGAAGGAGAAGAGTTCAGGAGTGGCAAGAGTTGAAGAGGAAA
A3-gBol027004-XLOC_021542-2284-1
TTGAGAAACGAAGGAGAAGAGTTCAGGAGTGGCAAGAGTTGAAGAGGAAA

CONSENSUS
TTGAGAAACGAAGGAGAAGAGTTCAGGAGTGGCAAGAGTTGAAGAGGAAA

A3-gBol027004-XLOC_021542-2284-0
AAAGAGGAAGCTGAAAGTGAAAGTAAGGGTGATACGGATGATAAAGAGCT
A3-gBol027004-XLOC_021542-2284-1
AAAGAGGAAGCTGAAAGTGAAAGTAAGGGTGATACGGATGATAAAGAGCT
CONSENSUS
AAAGAGGAAGCTGAAAGTGAAAGTAAGGGTGATACGGATGATAAAGAGCT

A3-gBol027004-XLOC_021542-2284-0
AAAGGCCGGCAAGGCTTGGACTCTTGATGGGGAATCTGATGATGAAGAAG
A3-gBol027004-XLOC_021542-2284-1
AAAGGCCGGCAAGGCTTGGACTCTTGATGGGGAATCTGATGATGAAGAAG
CONSENSUS
AAAGGCCGGCAAGGCTTGGACTCTTGATGGGGAATCTGATGATGAAGAAG

A3-gBol027004-XLOC_021542-2284-0
GGCATCCGGAGGAAAACCCAGAAACAGAGATGGATGTTGATGGAGAAACT
A3-gBol027004-XLOC_021542-2284-1
GGCATCCGGAGGAAAACCCAGAAACAGAGATGGATGTTGATGGAGAAACT
CONSENSUS
GGCATCCGGAGGAAAACCCAGAAACAGAGATGGATGTTGATGGAGAAACT

A3-gBol027004-XLOC_021542-2284-0
AAACCCGGAAATGGTGCAGATGCCAAGATGGTAGAAGCGGAGAACGAGGT
A3-gBol027004-XLOC_021542-2284-1
AAACCCGGAAATGGTGCAGATGCCAAGATGGTAGAAGCGGAGAACGAGGT
CONSENSUS
AAACCCGGAAATGGTGCAGATGCCAAGATGGTAGAAGCGGAGAACGAGGT

A3-gBol027004-XLOC_021542-2284-0
GGCTGTTACTGTCTCTGAAGTTGGAGGTGATGGGGCTGCAGATGAAGAGG
A3-gBol027004-XLOC_021542-2284-1
GGCTGTTACTGTCTCTGAAGTTGGAGGTGATGGGGCTGCAGATGAAGAGG
CONSENSUS
GGCTGTTACTGTCTCTGAAGTTGGAGGTGATGGGGCTGCAGATGAAGAGG

A3-gBol027004-XLOC_021542-2284-0
AAATTGATCCTTTAGATGCTTTTATGAATGCGATGGTATTACCTGAGGTT
A3-gBol027004-XLOC_021542-2284-1
AAATTGATCCTTTAGATGCTTTTATGAATGCGATGGTATTACCTGAGGTT
CONSENSUS
AAATTGATCCTTTAGATGCTTTTATGAATGCGATGGTATTACCTGAGGTT

A3-gBol027004-XLOC_021542-2284-0
GAGAAGCTTAGCAGCAGTACTCCTCCAGCAATTGAAGATAGTATTTTGGT
A3-gBol027004-XLOC_021542-2284-1
GAGAAGCTTAGCAGCAGTACTCCTCCAGCAATTGAAGATAGTATTTTGGT
CONSENSUS
GAGAAGCTTAGCAGCAGTACTCCTCCAGCAATTGAAGATAGTATTTTGGT

A3-gBol027004-XLOC_021542-2284-0
AACTAAGAATAATGGGAAGAAAAGTGATCACCAGCCGAAGAAAGGTTTTA
A3-gBol027004-XLOC_021542-2284-1
AACTAAGAATAATGGGAAGAAAAGTGATCACCAGCCGAAGAAAGGTTTTA

CONSENSUS
AACTAAGAATAATGGGAAGAAAAGTGATCACCAGCCGAAGAAAGGTTTTA

A3-gBol027004-XLOC_021542-2284-0
ACAAATCCCTTGGTAGGATAATGCAAGGTGAAGATTCTGATTCTGATTAT
A3-gBol027004-XLOC_021542-2284-1
ACAAATCCCTTGGTAGGATAATGCAAGGTGAAGATTCTGATTCTGATTAT
CONSENSUS
ACAAATCCCTTGGTAGGATAATGCAAGGTGAAGATTCTGATTCTGATTAT

A3-gBol027004-XLOC_021542-2284-0
TCAGAACCGAAGGATGATGATGATCCAAGTTTAGAAGAAGATGATGAGGA
A3-gBol027004-XLOC_021542-2284-1
TCAGAACCGAAGGATGATGATGATCCAAGTTTAGAAGAAGATGATGAGGA
CONSENSUS
TCAGAACCGAAGGATGATGATGATCCAAGTTTAGAAGAAGATGATGAGGA

A3-gBol027004-XLOC_021542-2284-0
GTTCATGAAGAGAGTAAAGAAGACAAAAGCAGAAAAATTGGCTCTTGTTG
A3-gBol027004-XLOC_021542-2284-1
GTTCATGAAGAGAGTAAAGAAGACAAAAGCAGAAAAATTGGCTCTTGTTG
CONSENSUS
GTTCATGAAGAGAGTAAAGAAGACAAAAGCAGAAAAATTGGCTCTTGTTG

A3-gBol027004-XLOC_021542-2284-0
ACCACTCAAAAATAGAGTATGAACCTTTCCGGAAGAACTTCTATATTGAA
A3-gBol027004-XLOC_021542-2284-1
ACCACTCAAAAATAGAGTATGAACCTTTCCGGAAGAACTTCTATATTGAA
CONSENSUS
ACCACTCAAAAATAGAGTATGAACCTTTCCGGAAGAACTTCTATATTGAA

A3-gBol027004-XLOC_021542-2284-0
GTGAAGGAGATCTCAAGGATGACACAAGAAGAAGTTAACGCTTACAGAAA
A3-gBol027004-XLOC_021542-2284-1
GTGAAGGAGATCTCAAGGATGACACAAGAAGAAGTTAACGCTTACAGAAA
CONSENSUS
GTGAAGGAGATCTCAAGGATGACACAAGAAGAAGTTAACGCTTACAGAAA

A3-gBol027004-XLOC_021542-2284-0
GGAATTTGAGCTGAAAGTCCATGGAAAGGATGTACCGAGACCCATAAAAT
A3-gBol027004-XLOC_021542-2284-1
GGAATTTGAGCTGAAAGTCCATGGAAAGGATGTACCGAGACCCATAAAAT
CONSENSUS
GGAATTTGAGCTGAAAGTCCATGGAAAGGATGTACCGAGACCCATAAAAT

A3-gBol027004-XLOC_021542-2284-0
CTTGGCACCAGACTGGACTAACCAGCAAAATTTTGGATACCATGAACAAG
A3-gBol027004-XLOC_021542-2284-1
CTTGGCACCAGACTGGACTAACCAGCAAAATTTTGGATACCATGAACAAG
CONSENSUS
CTTGGCACCAGACTGGACTAACCAGCAAAATTTTGGATACCATGAACAAG

A3-gBol027004-XLOC_021542-2284-0
CTCAAGTATGAAAAGCCAATGCCTATCCAAACGCAAGCACTGCCAATCAT
A3-gBol027004-XLOC_021542-2284-1
CTCAAGTATGAAAAGCCAATGCCTATCCAAACGCAAGCACTGCCAATCAT

CONSENSUS
CTCAAGTATGAAAAGCCAATGCCTATCCAAACGCAAGCACTGCCAATCAT

A3-gBol027004-XLOC_021542-2284-0
CATGAGCGGTCGAGATTGCATTGGAGTTGCAAAAACCGGATCAGGTAAAA
A3-gBol027004-XLOC_021542-2284-1
CATGAGCGGTCGAGATTGCATTGGAGTTGCAAAAACCGGATCAGGTAAAA
CONSENSUS
CATGAGCGGTCGAGATTGCATTGGAGTTGCAAAAACCGGATCAGGTAAAA

A3-gBol027004-XLOC_021542-2284-0
CGCTAGGTTTTGTTTTGCCTATGTTGAGGCATATCAAGGATCAGCCTCCC
A3-gBol027004-XLOC_021542-2284-1
CGCTAGGTTTTGTTTTGCCTATGTTGAGGCATATCAAGGATCAGCCTCCC
CONSENSUS
CGCTAGGTTTTGTTTTGCCTATGTTGAGGCATATCAAGGATCAGCCTCCC

A3-gBol027004-XLOC_021542-2284-0
GTTGAAGCTGGCGAGGGGCCAATTGGGCTTGTAATGGCACCTACTAGGGA
A3-gBol027004-XLOC_021542-2284-1
GTTGAAGCTGGCGAGGGGCCAATTGGGCTTGTAATGGCACCTACTAGGGA
CONSENSUS
GTTGAAGCTGGCGAGGGGCCAATTGGGCTTGTAATGGCACCTACTAGGGA

A3-gBol027004-XLOC_021542-2284-0
GCTTGTTCAGCAGATTCACAGCGATATCAAAAAGTTTTCAAAGGCATTGG
A3-gBol027004-XLOC_021542-2284-1
GCTTGTTCAGCAGATTCACAGCGATATCAAAAAGTTTTCAAAGGCATTGG
CONSENSUS
GCTTGTTCAGCAGATTCACAGCGATATCAAAAAGTTTTCAAAGGCATTGG

A3-gBol027004-XLOC_021542-2284-0
GTATAAGATGTGTTCCTGTGTATGGAGGATCAGGAGTTGCGCAGCAAATT
A3-gBol027004-XLOC_021542-2284-1
GTATAAGATGTGTTCCTGTGTATGGAGGATCAGGAGTTGCGCAGCAAATT
CONSENSUS
GTATAAGATGTGTTCCTGTGTATGGAGGATCAGGAGTTGCGCAGCAAATT

A3-gBol027004-XLOC_021542-2284-0
AGTGAGCTAAAGCGAGGGACAGAGATTGTTGTGTGCACTCCTGGAAGGAT
A3-gBol027004-XLOC_021542-2284-1
AGTGAGCTAAAGCGAGGGACAGAGATTGTTGTGTGCACTCCTGGAAGGAT
CONSENSUS
AGTGAGCTAAAGCGAGGGACAGAGATTGTTGTGTGCACTCCTGGAAGGAT

A3-gBol027004-XLOC_021542-2284-0
GATTGACATTCTTTGCACAAGCAGTGGGAAAATCACCAATCTGCGGAGAG
A3-gBol027004-XLOC_021542-2284-1
GATTGACATTCTTTGCACAAGCAGTGGGAAAATCACCAATCTGCGGAGAG
CONSENSUS
GATTGACATTCTTTGCACAAGCAGTGGGAAAATCACCAATCTGCGGAGAG

A3-gBol027004-XLOC_021542-2284-0
TCACATTTTTGGTAATGGATGAAGCTGATCGTATGTTTGACATGGGTTTT
A3-gBol027004-XLOC_021542-2284-1
TCACATTTTTGGTAATGGATGAAGCTGATCGTATGTTTGACATGGGTTTT

CONSENSUS
TCACATTTTTGGTAATGGATGAAGCTGATCGTATGTTTGACATGGGTTTT

A3-gBol027004-XLOC_021542-2284-0
GAGCCTCAAATTACTCGTATTATTCAAAATATTCGACCTGATCGGCAGAC
A3-gBol027004-XLOC_021542-2284-1
GAGCCTCAAATTACTCGTATTATTCAAAATATTCGACCTGATCGGCAGAC
CONSENSUS
GAGCCTCAAATTACTCGTATTATTCAAAATATTCGACCTGATCGGCAGAC

A3-gBol027004-XLOC_021542-2284-0
TGTGCTCTTTTCTGCCACTTTTCCACGTCAAGTTGAAACATTGGCACGTA
A3-gBol027004-XLOC_021542-2284-1
TGTGCTCTTTTCTGCCACTTTTCCACGTCAAGTTGAAACATTGGCACGTA
CONSENSUS
TGTGCTCTTTTCTGCCACTTTTCCACGTCAAGTTGAAACATTGGCACGTA

A3-gBol027004-XLOC_021542-2284-0
AAGTCTTGAACAAGCCTATTGAGATACAGGTTGGTGGAAGGAGTGTTGTG
A3-gBol027004-XLOC_021542-2284-1
AAGTCTTGAACAAGCCTATTGAGATACAGGTTGGTGGAAGGAGTGTTGTG
CONSENSUS
AAGTCTTGAACAAGCCTATTGAGATACAGGTTGGTGGAAGGAGTGTTGTG

A3-gBol027004-XLOC_021542-2284-0
AATAAGGATATAACACAGTTAGTTGAAGTCAGACCGGAGAGTGAGAGGTT
A3-gBol027004-XLOC_021542-2284-1
AATAAGGATATAACACAGTTAGTTGAAGTCAGACCGGAGAGTGAGAGGTT
CONSENSUS
AATAAGGATATAACACAGTTAGTTGAAGTCAGACCGGAGAGTGAGAGGTT

A3-gBol027004-XLOC_021542-2284-0
CTTAAGACTGCTGGAACTTCTTGGAGAATGGTATGAGAAAGGAAAGATAT
A3-gBol027004-XLOC_021542-2284-1
CTTAAGACTGCTGGAACTTCTTGGAGAATGGTATGAGAAAGGAAAGATAT
CONSENSUS
CTTAAGACTGCTGGAACTTCTTGGAGAATGGTATGAGAAAGGAAAGATAT

A3-gBol027004-XLOC_021542-2284-0
TGGTTTTTGTGCAGTCGCAGGAAAAATGTGATGCCTTGTTGAAGGATTTG
A3-gBol027004-XLOC_021542-2284-1
TGGTTTTTGTGCAGTCGCAGGAAAAATGTGATGCCTTGTTGAAGGATTTG
CONSENSUS
TGGTTTTTGTGCAGTCGCAGGAAAAATGTGATGCCTTGTTGAAGGATTTG

A3-gBol027004-XLOC_021542-2284-0
TTTAAAAGGAGTTATCCATGTCTATCTCTTCACGGTGGTAAAGAGCAGTC
A3-gBol027004-XLOC_021542-2284-1
TTTAAAAGGAGTTATCCATGTCTATCTCTTCACGGTGGTAAAGAGCAGTC
CONSENSUS
TTTAAAAGGAGTTATCCATGTCTATCTCTTCACGGTGGTAAAGAGCAGTC

A3-gBol027004-XLOC_021542-2284-0
TGATCGTGAATCAACAATATCTGATTTTAAGAGCAATGTGTGCAATATAT
A3-gBol027004-XLOC_021542-2284-1
TGATCGTGAATCAACAATATCTGATTTTAAGAGCAATGTGTGCAATATAT

CONSENSUS
TGATCGTGAATCAACAATATCTGATTTTAAGAGCAATGTGTGCAATATAT

A3-gBol027004-XLOC_021542-2284-0
TGATTGCCACAAGTGTTGCAGCTAGGGGTCTAGATGTGAAAGACCTTGAG
A3-gBol027004-XLOC_021542-2284-1
TGATTGCCACAAGTGTTGCAGCTAGGGGTCTAGATGTGAAAGACCTTGAG
CONSENSUS
TGATTGCCACAAGTGTTGCAGCTAGGGGTCTAGATGTGAAAGACCTTGAG

A3-gBol027004-XLOC_021542-2284-0
TTGGTTGTAAACTATGATGCTCCGAACCACTATGAAGATTATGTGCATCG
A3-gBol027004-XLOC_021542-2284-1
TTGGTTGTAAACTATGATGCTCCGAACCACTATGAAGATTATGTGCATCG
CONSENSUS
TTGGTTGTAAACTATGATGCTCCGAACCACTATGAAGATTATGTGCATCG

A3-gBol027004-XLOC_021542-2284-0
CGTTGGCAGGACAGGAAGGGCAGGGCGGAAAGGCTGTGCCGTGACATTTA
A3-gBol027004-XLOC_021542-2284-1
CGTTGGCAGGACAGGAAGGGCAGGGCGGAAAGGCTGTGCCGTGACATTTA
CONSENSUS
CGTTGGCAGGACAGGAAGGGCAGGGCGGAAAGGCTGTGCCGTGACATTTA

A3-gBol027004-XLOC_021542-2284-0
TCTCTGAAGATGATGCAAAATATGCACCGGATTTAGTAAAGGCCCTGGAA
A3-gBol027004-XLOC_021542-2284-1
TCTCTGAAGATGATGCAAAATATGCACCGGATTTAGTAAAGGCCCTGGAA
CONSENSUS
TCTCTGAAGATGATGCAAAATATGCACCGGATTTAGTAAAGGCCCTGGAA

A3-gBol027004-XLOC_021542-2284-0
CTTTCTGAGCAGCCAATCCCCGATGATCTGAAAGCAATCGCTGACGGTTT
A3-gBol027004-XLOC_021542-2284-1
CTTTCTGAGCAGCCAATCCCCGATGATCTGAAAGCAATCGCTGACGGTTT
CONSENSUS
CTTTCTGAGCAGCCAATCCCCGATGATCTGAAAGCAATCGCTGACGGTTT

A3-gBol027004-XLOC_021542-2284-0
CATGGTAAAGGTTAGACAGGGTACTGAGCAAGCTCATGGAACTGGCTATG
A3-gBol027004-XLOC_021542-2284-1
CATGGTAAAGGTTAGACAGGGTACTGAGCAAGCTCATGGAACTGGCTATG
CONSENSUS
CATGGTAAAGGTTAGACAGGGTACTGAGCAAGCTCATGGAACTGGCTATG

A3-gBol027004-XLOC_021542-2284-0
GAGGTAGTGGCTTTAAATTCAACGAAGAGGAGGAAGAAGTTAGGAAAGCA
A3-gBol027004-XLOC_021542-2284-1
GAGGTAGTGGCTTTAAATTCAACGAAGAGGAGGAAGAAGTTAGGAAAGCA
CONSENSUS
GAGGTAGTGGCTTTAAATTCAACGAAGAGGAGGAAGAAGTTAGGAAAGCA

A3-gBol027004-XLOC_021542-2284-0
GCAAAGAAAGCACAAGCCAAGGAGTATGGGTTTGAGGAAGATAAGTCTGA
A3-gBol027004-XLOC_021542-2284-1
GCAAAGAAAGCACAAGCCAAGGAGTATGGGTTTGAGGAAGATAAGTCTGA

CONSENSUS
      GCAAAGAAAGCACAAGCCAAGGAGTATGGGTTTGAGGAAGATAAGTCTGA

A3-gBol027004-XLOC_021542-2284-0
      CTCAGAAGATGAGAATGATGTTGTAAGAAAGGCAGGTACTGTTGGTGGTG
A3-gBol027004-XLOC_021542-2284-1
      CTCAGAAGATGAGAATGATGTTGTAAGAAAGGCAGGTACTGTTGGTGGTG
CONSENSUS
      CTCAGAAGATGAGAATGATGTTGTAAGAAAGGCAGGTACTGTTGGTGGTG

A3-gBol027004-XLOC_021542-2284-0
      ATACCTCACAACACCAGGCTGCTCTTGCTCATATAGCCGCCATTGCTGCT
A3-gBol027004-XLOC_021542-2284-1
      ATACCTCACAACACCAGGCTGCTCTTGCTCATATAGCCGCCATTGCTGCT
CONSENSUS
      ATACCTCACAACACCAGGCTGCTCTTGCTCATATAGCCGCCATTGCTGCT

A3-gBol027004-XLOC_021542-2284-0
      GCTGCTAAAGCTAATGCTGCTGCAATGAATCCTCCTGTGACTACAAACCA
A3-gBol027004-XLOC_021542-2284-1
      GCTGCTAAAGCTAATGCTGCTGCAATGAATCCTCCTGTGACTACAAACCA
CONSENSUS
      GCTGCTAAAGCTAATGCTGCTGCAATGAATCCTCCTGTGACTACAAACCA

A3-gBol027004-XLOC_021542-2284-0
      GTTGCTGCCGAATGGTGGTGGGCTTACCTCTCTGCCAGGTACCCTTCCGG
A3-gBol027004-XLOC_021542-2284-1
      GTTGCTGCCGAATGGTGGTGGGCTTACCTCTCTGCCAGGTACCCTTCCGG
CONSENSUS
      GTTGCTGCCGAATGGTGGTGGGCTTACCTCTCTGCCAGGTACCCTTCCGG

A3-gBol027004-XLOC_021542-2284-0
      TTACTATCCCTGTCCTTCCTAATGATGGGGCAGGCCGTGCTGCAGCCATG
A3-gBol027004-XLOC_021542-2284-1
      TTACTATCCCTGTCCTTCCTAATGATGGGGCAGGCCGTGCTGCAGCCATG
CONSENSUS
      TTACTATCCCTGTCCTTCCTAATGATGGGGCAGGCCGTGCTGCAGCCATG

A3-gBol027004-XLOC_021542-2284-0
      GTTGCTGCCATTAACCTGCAACATAACCTGGCAAAGATTCAAGCTGATGC
A3-gBol027004-XLOC_021542-2284-1
      GTTGCTGCCATTAACCTGCAACATAACCTGGCAAAGATTCAAGCTGATGC
CONSENSUS
      GTTGCTGCCATTAACCTGCAACATAACCTGGCAAAGATTCAAGCTGATGC

A3-gBol027004-XLOC_021542-2284-0
      AATGCCTGAACACTATGAAGCAGAACTGGAGATCAATGATTTCCCGCAAA
A3-gBol027004-XLOC_021542-2284-1
      AATGCCTGAACACTATGAAGCAGAACTGGAGATCAATGATTTCCCGCAAA
CONSENSUS
      AATGCCTGAACACTATGAAGCAGAACTGGAGATCAATGATTTCCCGCAAA

A3-gBol027004-XLOC_021542-2284-0
      ACGCTCGTTGGAAGGTCACCCACAAAGAAACATTGGGTCCAATATCAGAC
A3-gBol027004-XLOC_021542-2284-1
      ACGCTCGTTGGAAGGTCACCCACAAAGAAACATTGGGTCCAATATCAGAC

CONSENSUS
ACGCTCGTTGGAAGGTCACCCACAAAGAAACATTGGGTCCAATATCAGAC

A3-gBol027004-XLOC_021542-2284-0
TGGACTGGAGCCGCAATTACCACTAGAGGTCAGTTTTATCCTCCTGGACG
A3-gBol027004-XLOC_021542-2284-1
TGGACTGGAGCCGCAATTACCACTAGAGGTCAGTTTTATCCTCCTGGACG
CONSENSUS
TGGACTGGAGCCGCAATTACCACTAGAGGTCAGTTTTATCCTCCTGGACG

A3-gBol027004-XLOC_021542-2284-0
TATCCCTGGACCTGGGGAACGCAAGCTCTACTTGTTCATCGAAGGGCCTA
A3-gBol027004-XLOC_021542-2284-1
TATCCCTGGACCTGGGGAACGCAAGCTCTACTTGTTCATCGAAGGGCCTA
CONSENSUS
TATCCCTGGACCTGGGGAACGCAAGCTCTACTTGTTCATCGAAGGGCCTA

A3-gBol027004-XLOC_021542-2284-0
CCGAAAAATCCGTGAAGACGGCGAAAGTTGAACTCAAGCGTGTTCTTGAA
A3-gBol027004-XLOC_021542-2284-1
CCGAAAAATCCGTGAAGACGGCGAAAGTTGAACTCAAGCGTGTTCTTGAA
CONSENSUS
CCGAAAAATCCGTGAAGACGGCGAAAGTTGAACTCAAGCGTGTTCTTGAA

A3-gBol027004-XLOC_021542-2284-0
GACATTACTAATCAGGCTATGTCACTTCCTGGAGGATCGCAAGCTGGAAA
A3-gBol027004-XLOC_021542-2284-1
GACATTACTAATCAGGCTATGTCACTTCCTGGAGGATCGCAAGCTGGAAA
CONSENSUS
GACATTACTAATCAGGCTATGTCACTTCCTGGAGGATCGCAAGCTGGAAA

A3-gBol027004-XLOC_021542-2284-0 GTACTCCGTCATATAAT
A3-gBol027004-XLOC_021542-2284-1 GTACTCCGTCATATAAT
CONSENSUS GTACTCCGTCATATAAT

alignment for event: RI-gBol029761-XLOC_018304-3677

RI-gBol029761-XLOC_018304-3677-0
CCTTCTAGGTTTTCCAAGGAGAGGACAGAGAGAAGAACGTTTCTTGATTC
RI-gBol029761-XLOC_018304-3677-1
CCTTCTAGGTTTTCCAAGGAGAGGACAGAGAGAAGAACGTTTCTTGATTC
CONSENSUS
CCTTCTAGGTTTTCCAAGGAGAGGACAGAGAGAAGAACGTTTCTTGATTC

RI-gBol029761-XLOC_018304-3677-0
TCCTGGATCCAAGTTTCTCAAGAGGTCCCATGAAAGCTATACAGAAGTTG
RI-gBol029761-XLOC_018304-3677-1
TCCTGGATCCAAGTTTCTCAAGAGGTCCCATGAAAGCTATACAGAAGTTG
CONSENSUS
TCCTGGATCCAAGTTTCTCAAGAGGTCCCATGAAAGCTATACAGAAGTTG

RI-gBol029761-XLOC_018304-3677-0
ACCATGGTCTGCAAATTGAGAAGAAAAGGGCCATATCTTGGCCGGAAAAT
RI-gBol029761-XLOC_018304-3677-1

ACCATGGTCTGCAAATTGAGAAGAAAAGGGCCATATCTTGGCCGGAAAAT
CONSENSUS
ACCATGGTCTGCAAATTGAGAAGAAAAGGGCCATATCTTGGCCGGAAAAT

RI-gBol029761-XLOC_018304-3677-0
AGCATTGCAACGAATCATGTCCACTACAGTTGCATCCCAAGAGATCCCCA
RI-gBol029761-XLOC_018304-3677-1
AGCATTGCAACGAATCATGTCCACTACAGTTGCATCCCAAGAGATCCCCA
CONSENSUS
AGCATTGCAACGAATCATGTCCACTACAGTTGCATCCCAAGAGATCCCCA

RI-gBol029761-XLOC_018304-3677-0
AGACTTTAACTTTCCCTTCTCATATCCTAAGGAACCGGTTTATAGACCTC
RI-gBol029761-XLOC_018304-3677-1
AGACTTTAACTTTCCCTTCTCATATCCTAAGGAACCGGTTTATAGACCTC
CONSENSUS
AGACTTTAACTTTCCCTTCTCATATCCTAAGGAACCGGTTTATAGACCTC

RI-gBol029761-XLOC_018304-3677-0
CACTGTTGACACAGAAGACATTCATCTCGTTTCCTGTTGAAGAAACTCTG
RI-gBol029761-XLOC_018304-3677-1
CACTGTTGACACAGAAGACATTCATCTCGTTTCCTGTTGAAGAAACTCTG
CONSENSUS
CACTGTTGACACAGAAGACATTCATCTCGTTTCCTGTTGAAGAAACTCTG

RI-gBol029761-XLOC_018304-3677-0
GACTCCAGCCTCCATTTCAGAAACTACAACTCTCCTTCACTGGGTTATCA
RI-gBol029761-XLOC_018304-3677-1
GACTCCAGCCTCCATTTCAGAAACTACAACTCTCCTTCACTGGGTTATCA
CONSENSUS
GACTCCAGCCTCCATTTCAGAAACTACAACTCTCCTTCACTGGGTTATCA

RI-gBol029761-XLOC_018304-3677-0
TGGAGAAGACATTGGATACAGCTCAGAGGCCTTACTACCACATGATCATA
RI-gBol029761-XLOC_018304-3677-1
TGGAGAAGACATTGGATACAGCTCAGAGGCCTTACTACCACATGATCATA
CONSENSUS
TGGAGAAGACATTGGATACAGCTCAGAGGCCTTACTACCACATGATCATA

RI-gBol029761-XLOC_018304-3677-0
GGGAGCCTTCATCTGCATTACTTCTCGAGTGGAACACTGAGAACGCAAGC
RI-gBol029761-XLOC_018304-3677-1
GGGAGCCTTCATCTGCATTACTTCTCGAGTGGAACACTGAGAACGCAAGC
CONSENSUS
GGGAGCCTTCATCTGCATTACTTCTCGAGTGGAACACTGAGAACGCAAGC

RI-gBol029761-XLOC_018304-3677-0
ACCAGAAAGACTGATGATTTGCAACCAAGTAATCACACAGAACTCATCAC
RI-gBol029761-XLOC_018304-3677-1
ACCAGAAAGACTGATGATTTGCAACCAAGTAATCACACAGAACTCATCAC
CONSENSUS
ACCAGAAAGACTGATGATTTGCAACCAAGTAATCACACAGAACTCATCAC

RI-gBol029761-XLOC_018304-3677-0
GTGTCCAAATGCATTATCATCTCTGGCTGATAATCCATGGAGGTCTGACT
RI-gBol029761-XLOC_018304-3677-1

GTGTCCAAATGCATTATCATCTCTGGCTGATAATCCATGGAGGTCTGACT
CONSENSUS
GTGTCCAAATGCATTATCATCTCTGGCTGATAATCCATGGAGGTCTGACT

RI-gBol029761-XLOC_018304-3677-0
ATTCTTCATCCCATGATGTTGTTACGAGGGAGCTGTACCCTTTACCTCTG
RI-gBol029761-XLOC_018304-3677-1
ATTCTTCATCCCATGATGTTGTTACGAGGGAGCTGTACCCTTTACCTCTG
CONSENSUS
ATTCTTCATCCCATGATGTTGTTACGAGGGAGCTGTACCCTTTACCTCTG

RI-gBol029761-XLOC_018304-3677-0
CTCTCTCACTACACTTCAGGCAGTTTCCTCTTACCAGCAACAAACCAAAC
RI-gBol029761-XLOC_018304-3677-1
CTCTCTCACTACACTTCAGGCAGTTTCCTCTTACCAGCAACAAACCAAAC
CONSENSUS
CTCTCTCACTACACTTCAGGCAGTTTCCTCTTACCAGCAACAAACCAAAC

RI-gBol029761-XLOC_018304-3677-0
CCGCCATTTTGAGCATGAATTGGAGAGGCATATGATTGATGATGAAGATG
RI-gBol029761-XLOC_018304-3677-1
CCGCCATTTTGAGCATGAATTGGAGAGGCATATGATTGATGATGAAGATG
CONSENSUS
CCGCCATTTTGAGCATGAATTGGAGAGGCATATGATTGATGATGAAGATG

RI-gBol029761-XLOC_018304-3677-0
TAGTTGCAGCAAACCAGAATCTCCAGACATTTCATCAAGCGACAAGCTTG
RI-gBol029761-XLOC_018304-3677-1
TAGTTGCAGCAAACCAGAATCTCCAGACATTTCATCAAGCGACAAGCTTG
CONSENSUS
TAGTTGCAGCAAACCAGAATCTCCAGACATTTCATCAAGCGACAAGCTTG

RI-gBol029761-XLOC_018304-3677-0
TCAGATTGTTTAACCAGAGGCCACACATACTATCATAGTCCTTCCAACTC
RI-gBol029761-XLOC_018304-3677-1
TCAGATTGTTTAACCAGAGGCCACACATACTATCATAGTCCTTCCAACTC
CONSENSUS
TCAGATTGTTTAACCAGAGGCCACACATACTATCATAGTCCTTCCAACTC

RI-gBol029761-XLOC_018304-3677-0
TCCATTGGATCATTCCCCTTTTAAATCCCCTGGACGTGAAATGGTATCCT
RI-gBol029761-XLOC_018304-3677-1
TCCATTGGATCATTCCCCTTTTAAATCCCCTGGACGTGAAATGGTATCCT
CONSENSUS
TCCATTGGATCATTCCCCTTTTAAATCCCCTGGACGTGAAATGGTATCCT

RI-gBol029761-XLOC_018304-3677-0
TTCCGTTTTCCAGCATCAGCAATTCAGATTTGTTGGAAGAGTCAAGTCCA
RI-gBol029761-XLOC_018304-3677-1
TTCCGTTTTCCAGCATCAGCAATTCAGATTTGTTGGAAGAGTCAAGTCCA
CONSENSUS
TTCCGTTTTCCAGCATCAGCAATTCAGATTTGTTGGAAGAGTCAAGTCCA

RI-gBol029761-XLOC_018304-3677-0
ACAACACAAAGCGATCGGTGGTGGATATGAGCTTTACTGAAGAACTACTT
RI-gBol029761-XLOC_018304-3677-1

ACAACACAAAGCGATCGGTGGTGGATATGAGCTTTACTGAAGAACTACTT
CONSENSUS
ACAACACAAAGCGATCGGTGGTGGATATGAGCTTTACTGAAGAACTACTT

RI-gBol029761-XLOC_018304-3677-0
GAAACGAGTTTGGTTGCCACCAGGTCAGTAACTCTTCTTACTCGCACCGT
RI-gBol029761-XLOC_018304-3677-1
GAAACGAGTTTGGTTGCCACCAG---------------------------
CONSENSUS
GAAACGAGTTTGGTTGCCACCAG...........................

RI-gBol029761-XLOC_018304-3677-0
TCTCATAATTTGGTGTCATTTGTTGTTTCTTGCATTCTTTTTAGATAAGA
RI-gBol029761-XLOC_018304-3677-1

CONSENSUS
....

RI-gBol029761-XLOC_018304-3677-0
CATTTAAGTTCAGAAGACATAAGAATCATCGATTTTAGTTCTTGTCCTAA
RI-gBol029761-XLOC_018304-3677-1

CONSENSUS
....

RI-gBol029761-XLOC_018304-3677-0
CCGCATTATTGATGAATTTACTTGAAGGAGATGTCGATAAAATTCCATCC
RI-gBol029761-XLOC_018304-3677-1 ---------------------------
GAGATGTCGATAAAATTCCATCC
CONSENSUS
...........................GAGATGTCGATAAAATTCCATCC

RI-gBol029761-XLOC_018304-3677-0
GACATAGACTGATACATGGTTGAGATGGATGTTAAAAGATCTTTCTCATT
RI-gBol029761-XLOC_018304-3677-1
GACATAGACTGATACATGGTTGAGATGGATGTTAAAAGATCTTTCTCATT
CONSENSUS
GACATAGACTGATACATGGTTGAGATGGATGTTAAAAGATCTTTCTCATT

RI-gBol029761-XLOC_018304-3677-0
GTAAGTAAAAGATTTTTAGATAATCGCTTCTTTCTTAATTCCCATCAATC
RI-gBol029761-XLOC_018304-3677-1
GTAAGTAAAAGATTTTTAGATAATCGCTTCTTTCTTAATTCCCATCAATC
CONSENSUS
GTAAGTAAAAGATTTTTAGATAATCGCTTCTTTCTTAATTCCCATCAATC

RI-gBol029761-XLOC_018304-3677-0
ATCATTACTGTCCCCAATAAAATTTTGTAATCATGAGTGTTTTGTGTGTA
RI-gBol029761-XLOC_018304-3677-1
ATCATTACTGTCCCCAATAAAATTTTGTAATCATGAGTGTTTTGTGTGTA
CONSENSUS
ATCATTACTGTCCCCAATAAAATTTTGTAATCATGAGTGTTTTGTGTGTA

RI-gBol029761-XLOC_018304-3677-0
TAGAGTCAGCTAAAAACTGTAATGTTCAAAATGTTATAAGACATTTATAC
RI-gBol029761-XLOC_018304-3677-1

TAGAGTCAGCTAAAAACTGTAATGTTCAAAATGTTATAAGACATTTATAC
CONSENSUS
TAGAGTCAGCTAAAAACTGTAATGTTCAAAATGTTATAAGACATTTATAC

RI-gBol029761-XLOC_018304-3677-0
ATCTTTACTGTTTATTATTCTGTCACAACATATCTTTTATGTAAGATAAA
RI-gBol029761-XLOC_018304-3677-1
ATCTTTACTGTTTATTATTCTGTCACAACATATCTTTTATGTAAGATAAA
CONSENSUS
ATCTTTACTGTTTATTATTCTGTCACAACATATCTTTTATGTAAGATAAA

RI-gBol029761-XLOC_018304-3677-0 AGTGTATATTTAGAAATAAAAAGTGC
RI-gBol029761-XLOC_018304-3677-1 AGTGTATATTTAGAAATAAAAAGTGC
CONSENSUS AGTGTATATTTAGAAATAAAAAGTGC

alignment for event: A3-gBol003054-XLOC_048482-16380

A3-gBol003054-XLOC_048482-16380-0
ATTTAAAAATTATACATTGGCGCTCCCGACGTTTCCCTCTTCTCACTCAC
A3-gBol003054-XLOC_048482-16380-1
ATTTAAAAATTATACATTGGCGCTCCCGACGTTTCCCTCTTCTCACTCAC
CONSENSUS
ATTTAAAAATTATACATTGGCGCTCCCGACGTTTCCCTCTTCTCACTCAC

A3-gBol003054-XLOC_048482-16380-0
GTCTGAACCTTCTTCGCCTTCTCCACTCATCGGTGTTTCCTCCATCTGCA
A3-gBol003054-XLOC_048482-16380-1
GTCTGAACCTTCTTCGCCTTCTCCACTCATCGGTGTTTCCTCCATCTGCA
CONSENSUS
GTCTGAACCTTCTTCGCCTTCTCCACTCATCGGTGTTTCCTCCATCTGCA

A3-gBol003054-XLOC_048482-16380-0
TCGTCGATTTGAAATGATCTCCCACTCTCTGCTCTAAACCA---------
A3-gBol003054-XLOC_048482-16380-1
TCGTCGATTTGAAATGATCTCCCACTCTCTGCTCTAAACCACGTAGATCA
CONSENSUS
TCGTCGATTTGAAATGATCTCCCACTCTCTGCTCTAAACCA.........

A3-gBol003054-XLOC_048482-16380-0

A3-gBol003054-XLOC_048482-16380-1
GGCGTTCGATAAAATGTCTGAATGAAAAATTGAAGTGTGGTTGTGTTCTC
CONSENSUS
....

A3-gBol003054-XLOC_048482-16380-0

A3-gBol003054-XLOC_048482-16380-1
CGTTAGCTTCTTTTTTGAACCAGTGTTAGAGTCCTCCCTGTTCCAACTTC
CONSENSUS
....

A3-gBol003054-XLOC_048482-16380-0 ---------
GAGATTCTATCCTAGCGGTTTCCGACAAGATGCACTTGTTT

A3-gBol003054-XLOC_048482-16380-1
CAATTTCAGGAGATTCTATCCTAGCGGTTTCCGACAAGATGCACTTGTTT
CONSENSUS
.........GAGATTCTATCCTAGCGGTTTCCGACAAGATGCACTTGTTT

A3-gBol003054-XLOC_048482-16380-0
CTCAAGGACATAGTTCCGGCGGCACAGAACAACATAAACACGCGTTTCAT
A3-gBol003054-XLOC_048482-16380-1
CTCAAGGACATAGTTCCGGCGGCACAGAACAACATAAACACGCGTTTCAT
CONSENSUS
CTCAAGGACATAGTTCCGGCGGCACAGAACAACATAAACACGCGTTTCAT

A3-gBol003054-XLOC_048482-16380-0
CATCCTCGACAAAGCAAGATCACCCGCCTCGGCGAGCGGAAAGAGTTGTA
A3-gBol003054-XLOC_048482-16380-1
CATCCTCGACAAAGCAAGATCACCCGCCTCGGCGAGCGGAAAGAGTTGTA
CONSENSUS
CATCCTCGACAAAGCAAGATCACCCGCCTCGGCGAGCGGAAAGAGTTGTA

A3-gBol003054-XLOC_048482-16380-0
TCGCGTTAGCTGCGGATGAAACGGCGGCGGTTCACATACAGCTGTGGGGA
A3-gBol003054-XLOC_048482-16380-1
TCGCGTTAGCTGCGGATGAAACGGCGGCGGTTCACATACAGCTGTGGGGA
CONSENSUS
TCGCGTTAGCTGCGGATGAAACGGCGGCGGTTCACATACAGCTGTGGGGA

A3-gBol003054-XLOC_048482-16380-0
GACGAGTGCGAGGCCTTCGAAGCAGGGGATATCGTGAAGCTCACCAATGG
A3-gBol003054-XLOC_048482-16380-1
GACGAGTGCGAGGCCTTCGAAGCAGGGGATATCGTGAAGCTCACCAATGG
CONSENSUS
GACGAGTGCGAGGCCTTCGAAGCAGGGGATATCGTGAAGCTCACCAATGG

A3-gBol003054-XLOC_048482-16380-0
GATATTCTCGTACGTTAGGAACAGTGGGCTCCTTCTCAGAGCTGGGAAAC
A3-gBol003054-XLOC_048482-16380-1
GATATTCTCGTACGTTAGGAACAGTGGGCTCCTTCTCAGAGCTGGGAAAC
CONSENSUS
GATATTCTCGTACGTTAGGAACAGTGGGCTCCTTCTCAGAGCTGGGAAAC

A3-gBol003054-XLOC_048482-16380-0
GTGGGAAGATGGAGAAGATGGGTGAGTTCACGGTTGCGTTTGTCGAAACG
A3-gBol003054-XLOC_048482-16380-1
GTGGGAAGATGGAGAAGATGGGTGAGTTCACGGTTGCGTTTGTCGAAACG
CONSENSUS
GTGGGAAGATGGAGAAGATGGGTGAGTTCACGGTTGCGTTTGTCGAAACG

A3-gBol003054-XLOC_048482-16380-0
CCCAACGTTAGCGAGATCCAGTGGAATCCTGATCCTGAGAATCCCAAACG
A3-gBol003054-XLOC_048482-16380-1
CCCAACGTTAGCGAGATCCAGTGGAATCCTGATCCTGAGAATCCCAAACG
CONSENSUS
CCCAACGTTAGCGAGATCCAGTGGAATCCTGATCCTGAGAATCCCAAACG

A3-gBol003054-XLOC_048482-16380-0
CTATATTCAGAACGGGGTCGTTTCAGCGCATTCCCGTATTTTCCCTCCTT

A3-gBol003054-XLOC_048482-16380-1
      CTATATTCAGAACGGGGTCGTTTCAGCGCATTCCCGTATTTTCCCTCCTT
CONSENSUS
      CTATATTCAGAACGGGGTCGTTTCAGCGCATTCCCGTATTTTCCCTCCTT

A3-gBol003054-XLOC_048482-16380-0
      TGCCTTGATAAGGTTTGTAAACAACACATGGAGAATGGTAACAAACTTGT
A3-gBol003054-XLOC_048482-16380-1
      TGCCTTGATAAGGTTTGTAAACAACACATGGAGAATGGTAACAAACTTGT
CONSENSUS
      TGCCTTGATAAGGTTTGTAAACAACACATGGAGAATGGTAACAAACTTGT

A3-gBol003054-XLOC_048482-16380-0   TTTTTTTTTTT
A3-gBol003054-XLOC_048482-16380-1   TTTTTTTTTTT
CONSENSUS   TTTTTTTTTTT

alignment for event: A3-X-XLOC_020547-7176

A3-X-XLOC_020547-7176-0
      CTAGTATATTGATGGTATAACAATGTTAGATTCGTCCTTCTTTCTATGAC
A3-X-XLOC_020547-7176-1
      CTAGTATATTGATGGTATAACAATGTTAGATTCGTCCTTCTTTCTATGAC
CONSENSUS
      CTAGTATATTGATGGTATAACAATGTTAGATTCGTCCTTCTTTCTATGAC

A3-X-XLOC_020547-7176-0
      TGATTTCTCCATTTTTCCAATTTCTCTTAACAGTACTCTATATGTTCGAT
A3-X-XLOC_020547-7176-1
      TGATTTCTCCATTTTTCCAATTTCTCTTAACAGTACTCTATATGTTCGAT
CONSENSUS
      TGATTTCTCCATTTTTCCAATTTCTCTTAACAGTACTCTATATGTTCGAT

A3-X-XLOC_020547-7176-0
      TTTGAAAAACTACCCATGGTGCGATTCGGTTTCGGTTTCCATGTTGGAAG
A3-X-XLOC_020547-7176-1
      TTTGAAAAACTACCCATGGTGCGATTCGGTTTCGGTTTCCATGTTGGAAG
CONSENSUS
      TTTGAAAAACTACCCATGGTGCGATTCGGTTTCGGTTTCCATGTTGGAAG

A3-X-XLOC_020547-7176-0
      ACGAACATGTTTCTGATTTTTGTAATCAAAAGTTTTAACCCTTATCATTT
A3-X-XLOC_020547-7176-1
      ACGAACATGTTTCTGATTTTTGTAATCAAAAGTTTTAACCCTTATCATTT
CONSENSUS
      ACGAACATGTTTCTGATTTTTGTAATCAAAAGTTTTAACCCTTATCATTT

A3-X-XLOC_020547-7176-0
      GTGGTCTCGTCGTTAGGGTTCAAAGACAAAGATATGCATCAAAGTTTAAA
A3-X-XLOC_020547-7176-1
      GTGGTCTCGTCGTTAGGGTTCAAAGACAAAGATATGCATCAAAGTTTAAA
CONSENSUS
      GTGGTCTCGTCGTTAGGGTTCAAAGACAAAGATATGCATCAAAGTTTAAA

A3-X-XLOC_020547-7176-0

GTAATGACATAAGCGAAGAAAGGAGTCGGTGGTGACTTGATACGGTAATT
A3-X-XLOC_020547-7176-1
GTAATGACATAAGCGAAGAAAGGAGTCGGTGGTGACTTGATACGGTAATT
CONSENSUS
GTAATGACATAAGCGAAGAAAGGAGTCGGTGGTGACTTGATACGGTAATT

A3-X-XLOC_020547-7176-0
ATCTCCCTCACCACAGCGTCTCCAGCCACATCCCATCGATTTCCCCAGTT
A3-X-XLOC_020547-7176-1
ATCTCCCTCACCACAGCGTCTCCAGCCACATCCCATCGATTTCCCCAGTT
CONSENSUS
ATCTCCCTCACCACAGCGTCTCCAGCCACATCCCATCGATTTCCCCAGTT

A3-X-XLOC_020547-7176-0
CTAAACAAGATGTTAAGGTGATTGAATAATGCTACCTATATTATTTAGCG
A3-X-XLOC_020547-7176-1
CTAAACAAGATGTTAAGGTGATTGAATAATGCTACCTATATTATTTAGCG
CONSENSUS
CTAAACAAGATGTTAAGGTGATTGAATAATGCTACCTATATTATTTAGCG

A3-X-XLOC_020547-7176-0
GCGATAGCGATGGCGACGTTCTAGGGTTCCATGAATCCACGACGGTGGAA
A3-X-XLOC_020547-7176-1
GCGATAGCGATGGCGACGTTCTAGGGTTCCATGAATCCACGACGGTGGAA
CONSENSUS
GCGATAGCGATGGCGACGTTCTAGGGTTCCATGAATCCACGACGGTGGAA

A3-X-XLOC_020547-7176-0
TCCCCGAATTGAACGGGAGTTTCAGATCGAGACGGTATTCGTTCAAGAGA
A3-X-XLOC_020547-7176-1
TCCCCGAATTGAACGGGAGTTTCAGATCGAGACGGTATTCGTTCAAGAGA
CONSENSUS
TCCCCGAATTGAACGGGAGTTTCAGATCGAGACGGTATTCGTTCAAGAGA

A3-X-XLOC_020547-7176-0
GTAAAGATGGAAAGTTTGGGATCTGTTGGAGGGATCGGGGAAGAAGATCT
A3-X-XLOC_020547-7176-1
GTAAAGATGGAAAGTTTGGGATCTGTTGGAGGGATCGGGGAAGAAGATCT
CONSENSUS
GTAAAGATGGAAAGTTTGGGATCTGTTGGAGGGATCGGGGAAGAAGATCT

A3-X-XLOC_020547-7176-0
CGATCGGATCTTCGACGGGTTTCGGGAAAATCATCACAATATGGAAAGCG
A3-X-XLOC_020547-7176-1
CGATCGGATCTTCGACGGGTTTCGGGAAAATCATCACAATATGGAAAGCG
CONSENSUS
CGATCGGATCTTCGACGGGTTTCGGGAAAATCATCACAATATGGAAAGCG

A3-X-XLOC_020547-7176-0
AATAGGGATATCAATCTTCTCTCAGTCAAAATCCCCGCAAACCGAAG---
A3-X-XLOC_020547-7176-1
AATAGGGATATCAATCTTCTCTCAGTCAAAATCCCCGCAAACCGAAGGAA
CONSENSUS
AATAGGGATATCAATCTTCTCTCAGTCAAAATCCCCGCAAACCGAAG...

A3-X-XLOC_020547-7176-0

A3-X-XLOC_020547-7176-1
AAAAAGAATCTTGGAGACTACTATAAAAAGGGCCTTGGTTTCTGCTTTTT
CONSENSUS
....

A3-X-XLOC_020547-7176-0
--TC
A3-X-XLOC_020547-7176-1
CTACATCACATTTGAAAGTTTTCATACATACGAATTCTTCTTTCTCAGTC
CONSENSUS
....TC

A3-X-XLOC_020547-7176-0
GTTCGTTTGTCAGCTCATTGGAAGTTCTGGAGAGATGAAAAATGGCGAAG
A3-X-XLOC_020547-7176-1
GTTCGTTTGTCAGCTCATTGGAAGTTCTGGAGAGATGAAAAATGGCGAAG
CONSENSUS
GTTCGTTTGTCAGCTCATTGGAAGTTCTGGAGAGATGAAAAATGGCGAAG

A3-X-XLOC_020547-7176-0
GGGCTCGTAAGCGATTGAAGATATCGGTCCCTCACTTCAACAACTCGGAT
A3-X-XLOC_020547-7176-1
GGGCTCGTAAGCGATTGAAGATATCGGTCCCTCACTTCAACAACTCGGAT
CONSENSUS
GGGCTCGTAAGCGATTGAAGATATCGGTCCCTCACTTCAACAACTCGGAT

A3-X-XLOC_020547-7176-0
CTCATCAAGGGCTATTCCAAAACATTAATTGGAAGATGCATGAACCCGGA
A3-X-XLOC_020547-7176-1
CTCATCAAGGGCTATTCCAAAACATTAATTGGAAGATGCATGAACCCGGA
CONSENSUS
CTCATCAAGGGCTATTCCAAAACATTAATTGGAAGATGCATGAACCCGGA

A3-X-XLOC_020547-7176-0
GGAGCAGAATGTCAAGTTTCTGGTGGTGACGCTTCCTAAAATTTGGAATT
A3-X-XLOC_020547-7176-1
GGAGCAGAATGTCAAGTTTCTGGTGGTGACGCTTCCTAAAATTTGGAATT
CONSENSUS
GGAGCAGAATGTCAAGTTTCTGGTGGTGACGCTTCCTAAAATTTGGAATT

A3-X-XLOC_020547-7176-0
TGGAAGAAAAAGTTATGGGTACTGATTTGGGGCTTGGGAGGCTTCAGTTT
A3-X-XLOC_020547-7176-1
TGGAAGAAAAAGTTATGGGTACTGATTTGGGGCTTGGGAGGCTTCAGTTT
CONSENSUS
TGGAAGAAAAAGTTATGGGTACTGATTTGGGGCTTGGGAGGCTTCAGTTT

A3-X-XLOC_020547-7176-0
GACTTTGATGCCGAGGAAGACATCGAAACGGTGCTGAAGATGCAACCATG
A3-X-XLOC_020547-7176-1
GACTTTGATGCCGAGGAAGACATCGAAACGGTGCTGAAGATGCAACCATG
CONSENSUS
GACTTTGATGCCGAGGAAGACATCGAAACGGTGCTGAAGATGCAACCATG

A3-X-XLOC_020547-7176-0

TCATTTCGACTATTGGATGATTTCTCTGGTTCGTTGGCAACCAAAGAAGT
A3-X-XLOC_020547-7176-1
TCATTTCGACTATTGGATGATTTCTCTGGTTCGTTGGCAACCAAAGAAGT
CONSENSUS
TCATTTCGACTATTGGATGATTTCTCTGGTTCGTTGGCAACCAAAGAAGT

A3-X-XLOC_020547-7176-0
CCAAAAACTACCCCTCGGAGATCAATTTCTGGATTAAGGTTTTGGGAGTG
A3-X-XLOC_020547-7176-1
CCAAAAACTACCCCTCGGAGATCAATTTCTGGATTAAGGTTTTGGGAGTG
CONSENSUS
CCAAAAACTACCCCTCGGAGATCAATTTCTGGATTAAGGTTTTGGGAGTG

A3-X-XLOC_020547-7176-0
CCTTTGGAGTTTTGGGAGTCTCCTACTTTCCGGAGCATTGGCGATGCAAT
A3-X-XLOC_020547-7176-1
CCTTTGGAGTTTTGGGAGTCTCCTACTTTCCGGAGCATTGGCGATGCAAT
CONSENSUS
CCTTTGGAGTTTTGGGAGTCTCCTACTTTCCGGAGCATTGGCGATGCAAT

A3-X-XLOC_020547-7176-0
TGTTGTAACAAAAGCAGTTGATTTGGATTATGGTCGAGTGCAAGTGGCGG
A3-X-XLOC_020547-7176-1
TGTTGTAACAAAAGCAGTTGATTTGGATTATGGTCGAGTGCAAGTGGCGG
CONSENSUS
TGTTGTAACAAAAGCAGTTGATTTGGATTATGGTCGAGTGCAAGTGGCGG

A3-X-XLOC_020547-7176-0
TCGATGGGTACAAGGAGCTGACGTTTGAGACGACAGTTGACTTTAAGGCA
A3-X-XLOC_020547-7176-1
TCGATGGGTACAAGGAGCTGACGTTTGAGACGACAGTTGACTTTAAGGCA
CONSENSUS
TCGATGGGTACAAGGAGCTGACGTTTGAGACGACAGTTGACTTTAAGGCA

A3-X-XLOC_020547-7176-0
GGAGAGTACTATGAAGAAGAAGAAGCTCCAGTCTCTTTGCGGTACGAAAA
A3-X-XLOC_020547-7176-1
GGAGAGTACTATGAAGAAGAAGAAGCTCCAGTCTCTTTGCGGTACGAAAA
CONSENSUS
GGAGAGTACTATGAAGAAGAAGAAGCTCCAGTCTCTTTGCGGTACGAAAA

A3-X-XLOC_020547-7176-0
ACTGTTTGGTTTTTGTAAAACATGCCTCAGCATTTGCCACAAGATGGAGA
A3-X-XLOC_020547-7176-1
ACTGTTTGGTTTTTGTAAAACATGCCTCAGCATTTGCCACAAGATGGAGA
CONSENSUS
ACTGTTTGGTTTTTGTAAAACATGCCTCAGCATTTGCCACAAGATGGAGA

A3-X-XLOC_020547-7176-0
AGTGCCCGCTGAACTCAAACAACTCTGACAAGACGATGGAGATAAGGGAT
A3-X-XLOC_020547-7176-1
AGTGCCCGCTGAACTCAAACAACTCTGACAAGACGATGGAGATAAGGGAT
CONSENSUS
AGTGCCCGCTGAACTCAAACAACTCTGACAAGACGATGGAGATAAGGGAT

A3-X-XLOC_020547-7176-0

GAACCCGAAGCAGGTTTTGATGATCGGGCTCGAAGTTACAAAGGTGTGGT
A3-X-XLOC_020547-7176-1
GAACCCGAAGCAGGTTTTGATGATCGGGCTCGAAGTTACAAAGGTGTGGT
CONSENSUS
GAACCCGAAGCAGGTTTTGATGATCGGGCTCGAAGTTACAAAGGTGTGGT

A3-X-XLOC_020547-7176-0
GATTAACGGAAATGGAGGGCAACAAGATAGGGGAAGAGAGAAACGTGAGC
A3-X-XLOC_020547-7176-1
GATTAACGGAAATGGAGGGCAACAAGATAGGGGAAGAGAGAAACGTGAGC
CONSENSUS
GATTAACGGAAATGGAGGGCAACAAGATAGGGGAAGAGAGAAACGTGAGC

A3-X-XLOC_020547-7176-0
ACCAGGGGAAAGGCAAAGAAAAAATGTTTGAGGAAACAGACTCAAAGTGG
A3-X-XLOC_020547-7176-1
ACCAGGGGAAAGGCAAAGAAAAAATGTTTGAGGAAACAGACTCAAAGTGG
CONSENSUS
ACCAGGGGAAAGGCAAAGAAAAAATGTTTGAGGAAACAGACTCAAAGTGG

A3-X-XLOC_020547-7176-0
GCCAGGGCTGCAGATAGGGAGCATAAATCTTACAATAACAGAAACCATCG
A3-X-XLOC_020547-7176-1
GCCAGGGCTGCAGATAGGGAGCATAAATCTTACAATAACAGAAACCATCG
CONSENSUS
GCCAGGGCTGCAGATAGGGAGCATAAATCTTACAATAACAGAAACCATCG

A3-X-XLOC_020547-7176-0
GAGTGGTCACTGTGGAGAGGAGGAAAGTTCTCGTCATAGGAACTCCAGAA
A3-X-XLOC_020547-7176-1
GAGTGGTCACTGTGGAGAGGAGGAAAGTTCTCGTCATAGGAACTCCAGAA
CONSENSUS
GAGTGGTCACTGTGGAGAGGAGGAAAGTTCTCGTCATAGGAACTCCAGAA

A3-X-XLOC_020547-7176-0
GGGAGCAGACAAGGACTCATTATCAAGATGAACGCGCAAGAGTTCCTACA
A3-X-XLOC_020547-7176-1
GGGAGCAGACAAGGACTCATTATCAAGATGAACGCGCAAGAGTTCCTACA
CONSENSUS
GGGAGCAGACAAGGACTCATTATCAAGATGAACGCGCAAGAGTTCCTACA

A3-X-XLOC_020547-7176-0
GGCCCGAGAGGTGAAAGAGCAGCTCGCAGTGAGGCTCGAACGGAAGGGAA
A3-X-XLOC_020547-7176-1
GGCCCGAGAGGTGAAAGAGCAGCTCGCAGTGAGGCTCGAACGGAAGGGAA
CONSENSUS
GGCCCGAGAGGTGAAAGAGCAGCTCGCAGTGAGGCTCGAACGGAAGGGAA

A3-X-XLOC_020547-7176-0
GGAAGAAGGCGAGATCAAAGAGAAAGAGATGGAGCGGCCAAACCATAAGG
A3-X-XLOC_020547-7176-1
GGAAGAAGGCGAGATCAAAGAGAAAGAGATGGAGCGGCCAAACCATAAGG
CONSENSUS
GGAAGAAGGCGAGATCAAAGAGAAAGAGATGGAGCGGCCAAACCATAAGG

A3-X-XLOC_020547-7176-0

TGGAGAAGGTACATGATCAGGCACAACCTTCTCAAGCTTTCCTTGTTGAG
A3-X-XLOC_020547-7176-1
TGGAGAAGGTACATGATCAGGCACAACCTTCTCAAGCTTTCCTTGTTGAG
CONSENSUS
TGGAGAAGGTACATGATCAGGCACAACCTTCTCAAGCTTTCCTTGTTGAG

A3-X-XLOC_020547-7176-0
GTAATGGAGACACAAGGAGAATTATCTAAGGTAATATCGAATCCTTCTGG
A3-X-XLOC_020547-7176-1
GTAATGGAGACACAAGGAGAATTATCTAAGGTAATATCGAATCCTTCTGG
CONSENSUS
GTAATGGAGACACAAGGAGAATTATCTAAGGTAATATCGAATCCTTCTGG

A3-X-XLOC_020547-7176-0
TGGAGAACAAGAGCCAGACTTGGAAAATATGGATTTGGGTTTAGTGGAAG
A3-X-XLOC_020547-7176-1
TGGAGAACAAGAGCCAGACTTGGAAAATATGGATTTGGGTTTAGTGGAAG
CONSENSUS
TGGAGAACAAGAGCCAGACTTGGAAAATATGGATTTGGGTTTAGTGGAAG

A3-X-XLOC_020547-7176-0
GGAATAATTTAGAATCTGATGTGGGTACGGGATTGGAAGAACATCATATT
A3-X-XLOC_020547-7176-1
GGAATAATTTAGAATCTGATGTGGGTACGGGATTGGAAGAACATCATATT
CONSENSUS
GGAATAATTTAGAATCTGATGTGGGTACGGGATTGGAAGAACATCATATT

A3-X-XLOC_020547-7176-0
GGTAATGCTAGTATCGGGAATCAGGAGAGTTTGTGGGCTGAATCTCACGT
A3-X-XLOC_020547-7176-1
GGTAATGCTAGTATCGGGAATCAGGAGAGTTTGTGGGCTGAATCTCACGT
CONSENSUS
GGTAATGCTAGTATCGGGAATCAGGAGAGTTTGTGGGCTGAATCTCACGT

A3-X-XLOC_020547-7176-0
AATTTTTGAGGAAGAGATAAGGAAGATGTAAGAATGCAGGAGGTTGCGAC
A3-X-XLOC_020547-7176-1
AATTTTTGAGGAAGAGATAAGGAAGATGTAAGAATGCAGGAGGTTGCGAC
CONSENSUS
AATTTTTGAGGAAGAGATAAGGAAGATGTAAGAATGCAGGAGGTTGCGAC

A3-X-XLOC_020547-7176-0
TGGGGATCTCGAGGAAAAGAAGCATATGGAGGATATGGAAGGGAAATACC
A3-X-XLOC_020547-7176-1
TGGGGATCTCGAGGAAAAGAAGCATATGGAGGATATGGAAGGGAAATACC
CONSENSUS
TGGGGATCTCGAGGAAAAGAAGCATATGGAGGATATGGAAGGGAAATACC

A3-X-XLOC_020547-7176-0
GGGTTACTGGGGAGGTGGAGAAGAGGCAGAGCACACGTAAAAAGGCGGTT
A3-X-XLOC_020547-7176-1
GGGTTACTGGGGAGGTGGAGAAGAGGCAGAGCACACGTAAAAAGGCGGTT
CONSENSUS
GGGTTACTGGGGAGGTGGAGAAGAGGCAGAGCACACGTAAAAAGGCGGTT

A3-X-XLOC_020547-7176-0

AAGCCGTCTCTGGGTGCAGCAGCTTCCAACAAGTTGAAGATGGCTCAGCT
A3-X-XLOC_020547-7176-1
AAGCCGTCTCTGGGTGCAGCAGCTTCCAACAAGTTGAAGATGGCTCAGCT
CONSENSUS
AAGCCGTCTCTGGGTGCAGCAGCTTCCAACAAGTTGAAGATGGCTCAGCT

A3-X-XLOC_020547-7176-0
GGTGGCGGCCAAACGCACGGTAGCCAAACCAGGCATTCGTCATGGTGATT
A3-X-XLOC_020547-7176-1
GGTGGCGGCCAAACGCACGGTAGCCAAACCAGGCATTCGTCATGGTGATT
CONSENSUS
GGTGGCGGCCAAACGCACGGTAGCCAAACCAGGCATTCGTCATGGTGATT

A3-X-XLOC_020547-7176-0
ACTCTAAGCAGGGAGAGGAAAAGGGCACATCAGGTCCAAGACATGATCCT
A3-X-XLOC_020547-7176-1
ACTCTAAGCAGGGAGAGGAAAAGGGCACATCAGGTCCAAGACATGATCCT
CONSENSUS
ACTCTAAGCAGGGAGAGGAAAAGGGCACATCAGGTCCAAGACATGATCCT

A3-X-XLOC_020547-7176-0
GCAAAGCAAGCAAAAGATCCATGAATCAAAGCGTCATGATAGAGAAGTTA
A3-X-XLOC_020547-7176-1
GCAAAGCAAGCAAAAGATCCATGAATCAAAGCGTCATGATAGAGAAGTTA
CONSENSUS
GCAAAGCAAGCAAAAGATCCATGAATCAAAGCGTCATGATAGAGAAGTTA

A3-X-XLOC_020547-7176-0
CGTGGGCATGTTATGGTTTATGTTTTTTTTTACAGTTTCAATAAGCTCTT
A3-X-XLOC_020547-7176-1
CGTGGGCATGTTATGGTTTATGTTTTTTTTTACAGTTTCAATAAGCTCTT
CONSENSUS
CGTGGGCATGTTATGGTTTATGTTTTTTTTTACAGTTTCAATAAGCTCTT

A3-X-XLOC_020547-7176-0
TGAGCATTTGTACTTGTTTTTTCTTTGATTTTCTGTTGGTTTATGGTTGT
A3-X-XLOC_020547-7176-1
TGAGCATTTGTACTTGTTTTTTCTTTGATTTTCTGTTGGTTTATGGTTGT
CONSENSUS
TGAGCATTTGTACTTGTTTTTTCTTTGATTTTCTGTTGGTTTATGGTTGT

A3-X-XLOC_020547-7176-0
TTCTTGTTATCTTGGGCTGTGGAATGGTCCTTCAATAAGGTTTCTAAAAG
A3-X-XLOC_020547-7176-1
TTCTTGTTATCTTGGGCTGTGGAATGGTCCTTCAATAAGGTTTCTAAAAG
CONSENSUS
TTCTTGTTATCTTGGGCTGTGGAATGGTCCTTCAATAAGGTTTCTAAAAG

A3-X-XLOC_020547-7176-0
GAATAAAAACGGTTTGTTAGTGAGACATTGGTGGTATCATTTTCTGTTAC
A3-X-XLOC_020547-7176-1
GAATAAAAACGGTTTGTTAGTGAGACATTGGTGGTATCATTTTCTGTTAC
CONSENSUS
GAATAAAAACGGTTTGTTAGTGAGACATTGGTGGTATCATTTTCTGTTAC

A3-X-XLOC_020547-7176-0

GAGTTGTATACATCAGAATGTATGGGATGAGTTTTTCGCCTTGC
A3-X-XLOC_020547-7176-1
GAGTTGTATACATCAGAATGTATGGGATGAGTTTTTCGCCTTGC
CONSENSUS
GAGTTGTATACATCAGAATGTATGGGATGAGTTTTTCGCCTTGC

alignment for event: RI-X-XLOC_020545-7169

RI-X-XLOC_020545-7169-0
ATATGTGATTCGAAACACTCGGATCTTATACGGTGTTTGGATAGAGATCT
RI-X-XLOC_020545-7169-1
ATATGTGATTCGAAACACTCGGATCTTATACGGTGTTTGGATAGAGATCT
CONSENSUS
ATATGTGATTCGAAACACTCGGATCTTATACGGTGTTTGGATAGAGATCT

RI-X-XLOC_020545-7169-0
TCACCACCAGTTGAAACTGAGACTCAACTTATCTCTTTGTTTTCTCTCTT
RI-X-XLOC_020545-7169-1
TCACCACCAGTTGAAACTGAGACTCAACTTATCTCTTTGTTTTCTCTCTT
CONSENSUS
TCACCACCAGTTGAAACTGAGACTCAACTTATCTCTTTGTTTTCTCTCTT

RI-X-XLOC_020545-7169-0
CTTGAGTTTATAGGATACTGCATATTTTCAGTGGCTTTGTTTGTTTATTG
RI-X-XLOC_020545-7169-1
CTTGAGTTTATAGGATACTGCATATTTTCAGTGGCTTTGTTTGTTTATTG
CONSENSUS
CTTGAGTTTATAGGATACTGCATATTTTCAGTGGCTTTGTTTGTTTATTG

RI-X-XLOC_020545-7169-0
AGTTCTAAAGGTTGTAGGTTTAATGGTGTTTGTTGTGCAGGAGAATGATT
RI-X-XLOC_020545-7169-1
AGTTCTAAAGGTTGTAGGTTTAATGGTGTTTGTTGTGCAGGAGAATGATT
CONSENSUS
AGTTCTAAAGGTTGTAGGTTTAATGGTGTTTGTTGTGCAGGAGAATGATT

RI-X-XLOC_020545-7169-0
TACTCCTTTTGGAGGTGATTTGATGCGAGAGTTTAAGAATGCACTTTTGA
RI-X-XLOC_020545-7169-1
TACTCCTTTTGGAGGTGATTTGATGCGAGAGTTTAAGAATGCACTTTTGA
CONSENSUS
TACTCCTTTTGGAGGTGATTTGATGCGAGAGTTTAAGAATGCACTTTTGA

RI-X-XLOC_020545-7169-0
AGCAGAGAACGAACAGAGCATCAGGCTCTCGATTGGCAAAATTGCAGGAT
RI-X-XLOC_020545-7169-1
AGCAGAGAACGAACAGAGCATCAGGCTCTCGATTGGCAAAATTGCAGGAT
CONSENSUS
AGCAGAGAACGAACAGAGCATCAGGCTCTCGATTGGCAAAATTGCAGGAT

RI-X-XLOC_020545-7169-0
AATCAGAATAAGAGTCTTATCCAACGCCGCTTATCCGAACTTGAAGGTGA
RI-X-XLOC_020545-7169-1
AATCAGAATAAGAGTCTTATCCAACGCCGCTTATCCGAACTTGAAG----

CONSENSUS
AATCAGAATAAGAGTCTTATCCAACGCCGCTTATCCGAACTTGAAG....

RI-X-XLOC_020545-7169-0
CATTCCATATTTATTCTCTTAATTTACTTAAACCATTCACAAGTTATAAT
RI-X-XLOC_020545-7169-1

CONSENSUS
....

RI-X-XLOC_020545-7169-0
CTTTCTTTTTGCAAAACAATTTTCAACTATCTTTGTTCAAGAGGAATGCA
RI-X-XLOC_020545-7169-1

CONSENSUS
....

RI-X-XLOC_020545-7169-0
CCTGCTGATATTGTTGTCGTGTGTTTATATCAGAACTACCTTCCACCAAA
RI-X-XLOC_020545-7169-1

CONSENSUS
....

RI-X-XLOC_020545-7169-0
ATCACTTCTCATCAATTGCTTATACTCTCCTCACTGTCTTTCACCGTCTT
RI-X-XLOC_020545-7169-1

CONSENSUS
....

RI-X-XLOC_020545-7169-0
TTACTTGCTCATTTATTTTTTTCTTAGTTGGTCTCGGTGAACATACATGA
RI-X-XLOC_020545-7169-1

CONSENSUS
....

RI-X-XLOC_020545-7169-0
TTTAACATGTGGAACTTATCTGAATACTGAGTTGCTGTTGCCTAAAAGGA
RI-X-XLOC_020545-7169-1

CONSENSUS
....

RI-X-XLOC_020545-7169-0
GCAATGAAGTCCCATTAAATTGTGATATATAACTTTTTTATTTGGTTCTA
RI-X-XLOC_020545-7169-1

CONSENSUS
....

RI-X-XLOC_020545-7169-0
AATTCAGGCGGAAAGGTAAAGTCAAGTCTTAATTAGCAACACTGAGTTGC
RI-X-XLOC_020545-7169-1 -------
GCGGAAAGGTAAAGTCAAGTCTTAATTAGCAACACTGAGTTGC

CONSENSUS
.......GCGGAAAGGTAAAGTCAAGTCTTAATTAGCAACACTGAGTTGC

RI-X-XLOC_020545-7169-0
AGTTTGAAAGAAGGTTGGCTTGATGGTGGAAGCTTTGCTTTTTCTGTTTT
RI-X-XLOC_020545-7169-1
AGTTTGAAAGAAGGTTGGCTTGATGGTGGAAGCTTTGCTTTTTCTGTTTT
CONSENSUS
AGTTTGAAAGAAGGTTGGCTTGATGGTGGAAGCTTTGCTTTTTCTGTTTT

RI-X-XLOC_020545-7169-0 ACTTGTCATTGTTTTTACAG
RI-X-XLOC_020545-7169-1 ACTTGTCATTGTTTTTACAG
CONSENSUS ACTTGTCATTGTTTTTACAG

alignment for event: A3-gBol027778-XLOC_020550-7206

A3-gBol027778-XLOC_020550-7206-0
GTACGTATCTTGCGTGGTCGGGTGTCCAGTGGAAGGAGCGGTTCCACCCT
A3-gBol027778-XLOC_020550-7206-1
GTACGTATCTTGCGTGGTCGGGTGTCCAGTGGAAGGAGCGGTTCCACCCT
CONSENSUS
GTACGTATCTTGCGTGGTCGGGTGTCCAGTGGAAGGAGCGGTTCCACCCT

A3-gBol027778-XLOC_020550-7206-0
CAAAAGTAGCGCATGTCGTTAAAGAGCTCTTTGACATGGGCTGCTTTGAG
A3-gBol027778-XLOC_020550-7206-1
CAAAAGTAGCGCATGTCGTTAAAGAGCTCTTTGACATGGGCTGCTTTGAG
CONSENSUS
CAAAAGTAGCGCATGTCGTTAAAGAGCTCTTTGACATGGGCTGCTTTGAG

A3-gBol027778-XLOC_020550-7206-0
ATTTCTCTTGGTGATACAATTGGAATCGGCACTCCTG-------------
A3-gBol027778-XLOC_020550-7206-1
ATTTCTCTTGGTGATACAATTGGAATCGGCACTCCTGATTCTGTTCTTAT
CONSENSUS
ATTTCTCTTGGTGATACAATTGGAATCGGCACTCCTG.............

A3-gBol027778-XLOC_020550-7206-0 ------------
GTTCTGTGGTTCCCATGCTTGAAGCCGTGATGGCAGCT
A3-gBol027778-XLOC_020550-7206-1
AAAATGAATCAGGTTCTGTGGTTCCCATGCTTGAAGCCGTGATGGCAGCT
CONSENSUS
............GTTCTGTGGTTCCCATGCTTGAAGCCGTGATGGCAGCT

A3-gBol027778-XLOC_020550-7206-0
GTGCCAGCGGAAAAGCTGGCTGTTCACTTCCACGATACCTATGGACAAGC
A3-gBol027778-XLOC_020550-7206-1
GTGCCAGCGGAAAAGCTGGCTGTTCACTTCCACGATACCTATGGACAAGC
CONSENSUS
GTGCCAGCGGAAAAGCTGGCTGTTCACTTCCACGATACCTATGGACAAGC

A3-gBol027778-XLOC_020550-7206-0 TCTTGCAAACATATTGGTATCTCTCCAA
A3-gBol027778-XLOC_020550-7206-1 TCTTGCAAACATATTGGTATCTCTCCAA
CONSENSUS TCTTGCAAACATATTGGTATCTCTCCAA

alignment for event: RI-X-XLOC_020545-7165

RI-X-XLOC_020545-7165-0
GAGAATGATTTACTCCTTTTGGAGGTGATTTGATGCGAGAGTTTAAGAAT
RI-X-XLOC_020545-7165-1
GAGAATGATTTACTCCTTTTGGAGGTGATTTGATGCGAGAGTTTAAGAAT
CONSENSUS
GAGAATGATTTACTCCTTTTGGAGGTGATTTGATGCGAGAGTTTAAGAAT

RI-X-XLOC_020545-7165-0
GCACTTTTGAAGCAGAGAACGAACAGAGCATCAGGCTCTCGATTGGCAAA
RI-X-XLOC_020545-7165-1
GCACTTTTGAAGCAGAGAACGAACAGAGCATCAGGCTCTCGATTGGCAAA
CONSENSUS
GCACTTTTGAAGCAGAGAACGAACAGAGCATCAGGCTCTCGATTGGCAAA

RI-X-XLOC_020545-7165-0
ATTGCAGGATAATCAGAATAAGAGTCTTATCCAACGCCGCTTATCCGAAC
RI-X-XLOC_020545-7165-1
ATTGCAGGATAATCAGAATAAGAGTCTTATCCAACGCCGCTTATCCGAAC
CONSENSUS
ATTGCAGGATAATCAGAATAAGAGTCTTATCCAACGCCGCTTATCCGAAC

RI-X-XLOC_020545-7165-0
TTGAAGGTGACATTCCATATTTATTCTCTTAATTTACTTAAACCATTCAC
RI-X-XLOC_020545-7165-1
TTGAAG--
CONSENSUS
TTGAAG..

RI-X-XLOC_020545-7165-0
AAGTTATAATCTTTCTTTTTGCAAAACAATTTTCAACTATCTTTGTTCAA
RI-X-XLOC_020545-7165-1

CONSENSUS
....

RI-X-XLOC_020545-7165-0
GAGGAATGCACCTGCTGATATTGTTGTCGTGTGTTTATATCAGAACTACC
RI-X-XLOC_020545-7165-1

CONSENSUS
....

RI-X-XLOC_020545-7165-0
TTCCACCAAAATCACTTCTCATCAATTGCTTATACTCTCCTCACTGTCTT
RI-X-XLOC_020545-7165-1

CONSENSUS
....

RI-X-XLOC_020545-7165-0
TCACCGTCTTTTACTTGCTCATTTATTTTTTTCTTAGTTGGTCTCGGTGA

RI-X-XLOC_020545-7165-1

CONSENSUS
      ....

RI-X-XLOC_020545-7165-0
      ACATACATGATTTAACATGTGGAACTTATCTGAATACTGAGTTGCTGTTG
RI-X-XLOC_020545-7165-1

CONSENSUS
      ....

RI-X-XLOC_020545-7165-0
      CCTAAAAGGAGCAATGAAGTCCCATTAAATTGTGATATATAACTTTTTTA
RI-X-XLOC_020545-7165-1

CONSENSUS
      ....

RI-X-XLOC_020545-7165-0
      TTTGGTTCTAAATTCAGGCGGAAAGGTAAAGTCAAGTCTTAATTAGCAAC
RI-X-XLOC_020545-7165-1   -----------------
GCGGAAAGGTAAAGTCAAGTCTTAATTAGCAAC
CONSENSUS
      .................GCGGAAAGGTAAAGTCAAGTCTTAATTAGCAAC

RI-X-XLOC_020545-7165-0
      ACTGAGTTGCAGTTTGAAAGAAGGTTGGCTTGATGGTGGAAGCTTTGCTT
RI-X-XLOC_020545-7165-1
      ACTGAGTTGCAGTTTGAAAGAAGGTTGGCTTGATGGTGGAAGCTTTGCTT
CONSENSUS
      ACTGAGTTGCAGTTTGAAAGAAGGTTGGCTTGATGGTGGAAGCTTTGCTT

RI-X-XLOC_020545-7165-0
      TTTCTGTTTTACTTGTCATTGTTTTTACAGGTATGACCATGCTTTATTTA
RI-X-XLOC_020545-7165-1
      TTTCTGTTTTACTTGTCATTGTTTTTACAGGTATGACCATGCTTTATTTA
CONSENSUS
      TTTCTGTTTTACTTGTCATTGTTTTTACAGGTATGACCATGCTTTATTTA

RI-X-XLOC_020545-7165-0
      TGTTCACTGTCAAATTCTTAATCCATTGAAGATGACTTTTCTAATGTCTG
RI-X-XLOC_020545-7165-1
      TGTTCACTGTCAAATTCTTAATCCATTGAAGATGACTTTTCTAATGTCTG
CONSENSUS
      TGTTCACTGTCAAATTCTTAATCCATTGAAGATGACTTTTCTAATGTCTG

RI-X-XLOC_020545-7165-0
      TTTGTAAAGATGACTTTTTTAATGTCTGTCCGTAATATGCTGGTATGTCT
RI-X-XLOC_020545-7165-1
      TTTGTAAAGATGACTTTTTTAATGTCTGTCCGTAATATGCTGGTATGTCT
CONSENSUS
      TTTGTAAAGATGACTTTTTTAATGTCTGTCCGTAATATGCTGGTATGTCT

RI-X-XLOC_020545-7165-0
      TTCGTCCTATTCTGACCTCTGATATTATGTGATCTTTGCTGAGTTATGGT

RI-X-XLOC_020545-7165-1
TTCGTCCTATTCTGACCTCTGATATTATGTGATCTTTGCTGAGTTATGGT
CONSENSUS
TTCGTCCTATTCTGACCTCTGATATTATGTGATCTTTGCTGAGTTATGGT

RI-X-XLOC_020545-7165-0
TTGGTTGGAGTTAACATTTGAATGTTGGCTCATTGTGGATGTTCTCTGAA
RI-X-XLOC_020545-7165-1
TTGGTTGGAGTTAACATTTGAATGTTGGCTCATTGTGGATGTTCTCTGAA
CONSENSUS
TTGGTTGGAGTTAACATTTGAATGTTGGCTCATTGTGGATGTTCTCTGAA

RI-X-XLOC_020545-7165-0
TGTGTTTTAGGTTAACTTTCTCTCTCTCTCTCTCTCTCTTAGTGTTCTGA
RI-X-XLOC_020545-7165-1
TGTGTTTTAGGTTAACTTTCTCTCTCTCTCTCTCTCTCTTAGTGTTCTGA
CONSENSUS
TGTGTTTTAGGTTAACTTTCTCTCTCTCTCTCTCTCTCTTAGTGTTCTGA

RI-X-XLOC_020545-7165-0
TTCTGGTTCTGTTTTTGTGTTCTTAGTTAAACAAGGTGGAGGAGGCTGAA
RI-X-XLOC_020545-7165-1
TTCTGGTTCTGTTTTTGTGTTCTTAGTTAAACAAGGTGGAGGAGGCTGAA
CONSENSUS
TTCTGGTTCTGTTTTTGTGTTCTTAGTTAAACAAGGTGGAGGAGGCTGAA

RI-X-XLOC_020545-7165-0
GAAGCTTGATGGTGGTGGTCTCGCTACCGACGCCATATCAGAAGAAGCAT
RI-X-XLOC_020545-7165-1
GAAGCTTGATGGTGGTGGTCTCGCTACCGACGCCATATCAGAAGAAGCAT
CONSENSUS
GAAGCTTGATGGTGGTGGTCTCGCTACCGACGCCATATCAGAAGAAGCAT

RI-X-XLOC_020545-7165-0
CGCTCGAGATGAGAGAGAAGACAAAGGGCTGGTGATTCAAGATTCAAGCG
RI-X-XLOC_020545-7165-1
CGCTCGAGATGAGAGAGAAGACAAAGGGCTGGTGATTCAAGATTCAAGCG
CONSENSUS
CGCTCGAGATGAGAGAGAAGACAAAGGGCTGGTGATTCAAGATTCAAGCG

RI-X-XLOC_020545-7165-0
ATTCAAGAACATAAAATAACTCATGCTCTGTTTTTTTCTGTGTGTGTGTT
RI-X-XLOC_020545-7165-1
ATTCAAGAACATAAAATAACTCATGCTCTGTTTTTTTCTGTGTGTGTGTT
CONSENSUS
ATTCAAGAACATAAAATAACTCATGCTCTGTTTTTTTCTGTGTGTGTGTT

RI-X-XLOC_020545-7165-0
TTTTATTTACTTGGGACAAAGTTTAATGAAATACCATTACAAGATATTAA
RI-X-XLOC_020545-7165-1
TTTTATTTACTTGGGACAAAGTTTAATGAAATACCATTACAAGATATTAA
CONSENSUS
TTTTATTTACTTGGGACAAAGTTTAATGAAATACCATTACAAGATATTAA

RI-X-XLOC_020545-7165-0
TAACATTATGTATTTTTGGATAACTTTATGGAATAGAAAATTTTGTATTT

RI-X-XLOC_020545-7165-1
      TAACATTATGTATTTTTGGATAACTTTATGGAATAGAAAATTTTGTATTT
CONSENSUS
      TAACATTATGTATTTTTGGATAACTTTATGGAATAGAAAATTTTGTATTT

RI-X-XLOC_020545-7165-0   TGTAATTTA
RI-X-XLOC_020545-7165-1   TGTAATTTA
CONSENSUS   TGTAATTTA

alignment for event: RI-X-XLOC_020545-7168

RI-X-XLOC_020545-7168-0
      ATATGTGATTCGAAACACTCGGATCTTATACGGTGTTTGGATAGAGATCT
RI-X-XLOC_020545-7168-1
      ATATGTGATTCGAAACACTCGGATCTTATACGGTGTTTGGATAGAGATCT
CONSENSUS
      ATATGTGATTCGAAACACTCGGATCTTATACGGTGTTTGGATAGAGATCT

RI-X-XLOC_020545-7168-0
      TCACCACCAGTTGAAACTGAGACTCAACTTATCTCTTTGTTTTCTCTCTT
RI-X-XLOC_020545-7168-1
      TCACCACCAGTTGAAACTGAGACTCAACTTATCTCTTTGTTTTCTCTCTT
CONSENSUS
      TCACCACCAGTTGAAACTGAGACTCAACTTATCTCTTTGTTTTCTCTCTT

RI-X-XLOC_020545-7168-0
      CTTGAGTTTATAGGATACTGCATATTTTCAGTGGCTTTGTTTGTTTATTG
RI-X-XLOC_020545-7168-1
      CTTGAGTTTATAGGATACTGCATATTTTCAGTGGCTTTGTTTGTTTATTG
CONSENSUS
      CTTGAGTTTATAGGATACTGCATATTTTCAGTGGCTTTGTTTGTTTATTG

RI-X-XLOC_020545-7168-0
      AGTTCTAAAGGTTGTAGGTTTAATGGTGTTTGTTGTGCAGGAGAATGATT
RI-X-XLOC_020545-7168-1
      AGTTCTAAAGGTTGTAGGTTTAATGGTGTTTGTTGTGCAGGAGAATGATT
CONSENSUS
      AGTTCTAAAGGTTGTAGGTTTAATGGTGTTTGTTGTGCAGGAGAATGATT

RI-X-XLOC_020545-7168-0
      TACTCCTTTTGGAGGTGATTTGATGCGAGAGTTTAAGAATGCACTTTTGA
RI-X-XLOC_020545-7168-1
      TACTCCTTTTGGAGGTGATTTGATGCGAGAGTTTAAGAATGCACTTTTGA
CONSENSUS
      TACTCCTTTTGGAGGTGATTTGATGCGAGAGTTTAAGAATGCACTTTTGA

RI-X-XLOC_020545-7168-0
      AGCAGAGAACGAACAGAGCATCAGGCTCTCGATTGGCAAAATTGCAGGAT
RI-X-XLOC_020545-7168-1
      AGCAGAGAACGAACAGAGCATCAGGCTCTCGATTGGCAAAATTGCAGGAT
CONSENSUS
      AGCAGAGAACGAACAGAGCATCAGGCTCTCGATTGGCAAAATTGCAGGAT

RI-X-XLOC_020545-7168-0

AATCAGAATAAGAGTCTTATCCAACGCCGCTTATCCGAACTTGAAGGTGA
RI-X-XLOC_020545-7168-1
AATCAGAATAAGAGTCTTATCCAACGCCGCTTATCCGAACTTGAAG----
CONSENSUS
AATCAGAATAAGAGTCTTATCCAACGCCGCTTATCCGAACTTGAAG....

RI-X-XLOC_020545-7168-0
CATTCCATATTTATTCTCTTAATTTACTTAAACCATTCACAAGTTATAAT
RI-X-XLOC_020545-7168-1

CONSENSUS
....

RI-X-XLOC_020545-7168-0
CTTTCTTTTTGCAAAACAATTTTCAACTATCTTTGTTCAAGAGGAATGCA
RI-X-XLOC_020545-7168-1

CONSENSUS
....

RI-X-XLOC_020545-7168-0
CCTGCTGATATTGTTGTCGTGTGTTTATATCAGAACTACCTTCCACCAAA
RI-X-XLOC_020545-7168-1

CONSENSUS
....

RI-X-XLOC_020545-7168-0
ATCACTTCTCATCAATTGCTTATACTCTCCTCACTGTCTTTCACCGTCTT
RI-X-XLOC_020545-7168-1

CONSENSUS
....

RI-X-XLOC_020545-7168-0
TTACTTGCTCATTTATTTTTTTCTTAGTTGGTCTCGGTGAACATACATGA
RI-X-XLOC_020545-7168-1

CONSENSUS
....

RI-X-XLOC_020545-7168-0
TTTAACATGTGGAACTTATCTGAATACTGAGTTGCTGTTGCCTAAAAGGA
RI-X-XLOC_020545-7168-1

CONSENSUS
....

RI-X-XLOC_020545-7168-0
GCAATGAAGTCCCATTAAATTGTGATATATAACTTTTTTATTTGGTTCTA
RI-X-XLOC_020545-7168-1

CONSENSUS
....

RI-X-XLOC_020545-7168-0

AATTCAGGCGGAAAGGTAAAGTCAAGTCTTAATTAGCAACACTGAGTTGC
RI-X-XLOC_020545-7168-1 -------
GCGGAAAGGTAAAGTCAAGTCTTAATTAGCAACACTGAGTTGC
CONSENSUS
.......GCGGAAAGGTAAAGTCAAGTCTTAATTAGCAACACTGAGTTGC

RI-X-XLOC_020545-7168-0
AGTTTGAAAGAAGGTTGGCTTGATGGTGGAAGCTTTGCTTTTTCTGTTTT
RI-X-XLOC_020545-7168-1
AGTTTGAAAGAAGGTTGGCTTGATGGTGGAAGCTTTGCTTTTTCTGTTTT
CONSENSUS
AGTTTGAAAGAAGGTTGGCTTGATGGTGGAAGCTTTGCTTTTTCTGTTTT

RI-X-XLOC_020545-7168-0
ACTTGTCATTGTTTTTACAGGTATGACCATGCTTTATTTATGTTCACTGT
RI-X-XLOC_020545-7168-1
ACTTGTCATTGTTTTTACAGGTATGACCATGCTTTATTTATGTTCACTGT
CONSENSUS
ACTTGTCATTGTTTTTACAGGTATGACCATGCTTTATTTATGTTCACTGT

RI-X-XLOC_020545-7168-0
CAAATTCTTAATCCATTGAAGATGACTTTTCTAATGTCTGTTTGTAAAGA
RI-X-XLOC_020545-7168-1
CAAATTCTTAATCCATTGAAGATGACTTTTCTAATGTCTGTTTGTAAAGA
CONSENSUS
CAAATTCTTAATCCATTGAAGATGACTTTTCTAATGTCTGTTTGTAAAGA

RI-X-XLOC_020545-7168-0
TGACTTTTTTAATGTCTGTCCGTAATATGCTGGTATGTCTTTCGTCCTAT
RI-X-XLOC_020545-7168-1
TGACTTTTTTAATGTCTGTCCGTAATATGCTGGTATGTCTTTCGTCCTAT
CONSENSUS
TGACTTTTTTAATGTCTGTCCGTAATATGCTGGTATGTCTTTCGTCCTAT

RI-X-XLOC_020545-7168-0
TCTGACCTCTGATATTATGTGATCTTTGCTGAGTTATGGTTTGGTTGGAG
RI-X-XLOC_020545-7168-1
TCTGACCTCTGATATTATGTGATCTTTGCTGAGTTATGGTTTGGTTGGAG
CONSENSUS
TCTGACCTCTGATATTATGTGATCTTTGCTGAGTTATGGTTTGGTTGGAG

RI-X-XLOC_020545-7168-0
TTAACATTTGAATGTTGGCTCATTGTGGATGTTCTCTGAATGTGTTTTAG
RI-X-XLOC_020545-7168-1
TTAACATTTGAATGTTGGCTCATTGTGGATGTTCTCTGAATGTGTTTTAG
CONSENSUS
TTAACATTTGAATGTTGGCTCATTGTGGATGTTCTCTGAATGTGTTTTAG

RI-X-XLOC_020545-7168-0
GTTAACTTTCTCTCTCTCTCTCTCTCTCTTAGTGTTCTGATTCTGGTTCT
RI-X-XLOC_020545-7168-1
GTTAACTTTCTCTCTCTCTCTCTCTCTCTTAGTGTTCTGATTCTGGTTCT
CONSENSUS
GTTAACTTTCTCTCTCTCTCTCTCTCTCTTAGTGTTCTGATTCTGGTTCT

RI-X-XLOC_020545-7168-0

GTTTTTGTGTTCTTAGTTAAACAAGGTGGAGGAGGCTGAAGAAGCTTGAT
RI-X-XLOC_020545-7168-1
GTTTTTGTGTTCTTAGTTAAACAAGGTGGAGGAGGCTGAAGAAGCTTGAT
CONSENSUS
GTTTTTGTGTTCTTAGTTAAACAAGGTGGAGGAGGCTGAAGAAGCTTGAT

RI-X-XLOC_020545-7168-0
GGTGGTGGTCTCGCTACCGACGCCATATCAGAAGAAGCATCGCTCGAGAT
RI-X-XLOC_020545-7168-1
GGTGGTGGTCTCGCTACCGACGCCATATCAGAAGAAGCATCGCTCGAGAT
CONSENSUS
GGTGGTGGTCTCGCTACCGACGCCATATCAGAAGAAGCATCGCTCGAGAT

RI-X-XLOC_020545-7168-0
GAGAGAGAAGACAAAGGGCTGGTGATTCAAGATTCAAGCGATTCAAGAAC
RI-X-XLOC_020545-7168-1
GAGAGAGAAGACAAAGGGCTGGTGATTCAAGATTCAAGCGATTCAAGAAC
CONSENSUS
GAGAGAGAAGACAAAGGGCTGGTGATTCAAGATTCAAGCGATTCAAGAAC

RI-X-XLOC_020545-7168-0
ATAAAATAACTCATGCTCTGTTTTTTTCTGTGTGTGTGTTTTTTATTTAC
RI-X-XLOC_020545-7168-1
ATAAAATAACTCATGCTCTGTTTTTTTCTGTGTGTGTGTTTTTTATTTAC
CONSENSUS
ATAAAATAACTCATGCTCTGTTTTTTTCTGTGTGTGTGTTTTTTATTTAC

RI-X-XLOC_020545-7168-0
TTGGGACAAAGTTTAATGAAATACCATTACAAGATATTAATAACATTATG
RI-X-XLOC_020545-7168-1
TTGGGACAAAGTTTAATGAAATACCATTACAAGATATTAATAACATTATG
CONSENSUS
TTGGGACAAAGTTTAATGAAATACCATTACAAGATATTAATAACATTATG

RI-X-XLOC_020545-7168-0
TATTTTTGGATAACTTTATGGAATAGAAAATTTTGTATTTTGTAATTTA
RI-X-XLOC_020545-7168-1
TATTTTTGGATAACTTTATGGAATAGAAAATTTTGTATTTTGTAATTTA
CONSENSUS
TATTTTTGGATAACTTTATGGAATAGAAAATTTTGTATTTTGTAATTTA

alignment for event: SE-X-XLOC_036860-12524

SE-X-XLOC_036860-12524-0
ATTGGGACTGAACGAATTGGACTTGATGCTGGTGACAGTCTTGGTGCTCT
SE-X-XLOC_036860-12524-1
ATTGGGACTGAACGAATTGGACTTGATGCTGGTGACAGTCTTGGTGCTCT
CONSENSUS
ATTGGGACTGAACGAATTGGACTTGATGCTGGTGACAGTCTTGGTGCTCT

SE-X-XLOC_036860-12524-0
TGGGTTGAACCTCGAATCCACTTGGCCTGGTGTATTTGATGCTCTGTTTT
SE-X-XLOC_036860-12524-1
TGGGTTGAACCTCGAATCCACTTGGCCTGGTGTATTTGATGCTCTGTTTT

CONSENSUS
      TGGGTTGAACCTCGAATCCACTTGGCCTGGTGTATTTGATGCTCTGTTTT

SE-X-XLOC_036860-12524-0
      CGAGTTTCTCCATGATTCTTGTGACAGAG---------------------
SE-X-XLOC_036860-12524-1
      CGAGTTTCTCCATGATTCTTGTGACAGAGGACGTTGATGAAAGTGTTTTC
CONSENSUS
      CGAGTTTCTCCATGATTCTTGTGACAGAG.....................

SE-X-XLOC_036860-12524-0   --
AAAGACCAGTGATGTATATGGCGGAGATGTGTTGAGCGAGACTAAACC
SE-X-XLOC_036860-12524-1
      TGAAAGACCAGTGATGTATATGGCGGAGATGTGTTGAGCGAGACTAAACC
CONSENSUS
      ..AAAGACCAGTGATGTATATGGCGGAGATGTGTTGAGCGAGACTAAACC

SE-X-XLOC_036860-12524-0
      ATTACAGATCCAGGCTTCAGCCAAGAAAGACATTGCCTCCACCAAAGTTG
SE-X-XLOC_036860-12524-1
      ATTACAGATCCAGGCTTCAGCCAAGAAAGACATTGCCTCCACCAAAGTTG
CONSENSUS
      ATTACAGATCCAGGCTTCAGCCAAGAAAGACATTGCCTCCACCAAAGTTG

SE-X-XLOC_036860-12524-0
      CCAAGTGATCACATAACCAAGCTCTGTCGTGGGGATTGGATGATTAAAAG
SE-X-XLOC_036860-12524-1
      CCAAGTGATCACATAACCAAGCTCTGTCGTGGGGATTGGATGATTAAAAG
CONSENSUS
      CCAAGTGATCACATAACCAAGCTCTGTCGTGGGGATTGGATGATTAAAAG

SE-X-XLOC_036860-12524-0
      GTCGTCAAGTTCTACCGTAGTGCAGCATCAACGTCCATTACAAAAGGTTG
SE-X-XLOC_036860-12524-1
      GTCGTCAAGTTCTACCGTAGTGCAGCATCAACGTCCATTACAAAAGGTTG
CONSENSUS
      GTCGTCAAGTTCTACCGTAGTGCAGCATCAACGTCCATTACAAAAGGTTG

SE-X-XLOC_036860-12524-0
      TATTATTAGAAACTTGTATAAATGATATGGATAATGAAAGTGTATTCACA
SE-X-XLOC_036860-12524-1
      TATTATTAGAAACTTGTATAAATGATATGGATAATGAAAGTGTATTCACA
CONSENSUS
      TATTATTAGAAACTTGTATAAATGATATGGATAATGAAAGTGTATTCACA

SE-X-XLOC_036860-12524-0
      AGATCAAATATTTCTTTTCAAGTTCTTTCTTCCTTTTCTAAGTTCTTTCA
SE-X-XLOC_036860-12524-1
      AGATCAAATATTTCTTTTCAAGTTCTTTCTTCCTTTTCTAAGTTCTTTCA
CONSENSUS
      AGATCAAATATTTCTTTTCAAGTTCTTTCTTCCTTTTCTAAGTTCTTTCA

SE-X-XLOC_036860-12524-0
      ATTTTTTTTGTAAGAACTGTTGCTTAAAAAAATTGCAATGGAAGAAAAAA
SE-X-XLOC_036860-12524-1
      ATTTTTTTTGTAAGAACTGTTGCTTAAAAAAATTGCAATGGAAGAAAAAA

CONSENSUS
ATTTTTTTTGTAAGAACTGTTGCTTAAAAAAATTGCAATGGAAGAAAAAA

SE-X-XLOC_036860-12524-0 ATTA
SE-X-XLOC_036860-12524-1 ATTA
CONSENSUS ATTA

alignment for event: RI-gBol038420-XLOC_008537-6755

RI-gBol038420-XLOC_008537-6755-0
ATCAGGAGGAGCAGGACAGTTTGGGGATGAGGGTTACTGGTTTGGTAGTC
RI-gBol038420-XLOC_008537-6755-1
ATCAGGAGGAGCAGGACAGTTTGGGGATGAGGGTTACTGGTTTGGTAGTC
CONSENSUS
ATCAGGAGGAGCAGGACAGTTTGGGGATGAGGGTTACTGGTTTGGTAGTC

RI-gBol038420-XLOC_008537-6755-0
CAGGGCGACATGAAAGAGAGGAGTTTATGGGGATGGGAGATAAATCAAAC
RI-gBol038420-XLOC_008537-6755-1
CAGGGCGACATGAAAGAGAGGAGTTTATGGGGATGGGAGATAAATCAAAC
CONSENSUS
CAGGGCGACATGAAAGAGAGGAGTTTATGGGGATGGGAGATAAATCAAAC

RI-gBol038420-XLOC_008537-6755-0
TCTGCATCTAAGCAGATTTACTTGACATTTCCAGCTGACAGCTCCTTCAC
RI-gBol038420-XLOC_008537-6755-1
TCTGCATCTAAGCAGATTTACTTGACATTTCCAGCTGACAGCTCCTTCAC
CONSENSUS
TCTGCATCTAAGCAGATTTACTTGACATTTCCAGCTGACAGCTCCTTCAC

RI-gBol038420-XLOC_008537-6755-0
AGATGAAGATGTCTCCAATTACTTCGGGTCTCCTTCAAAAACACAACTGC
RI-gBol038420-XLOC_008537-6755-1
AGATGAAGATGTCTCCAATTACTTCGG-----------------------
CONSENSUS
AGATGAAGATGTCTCCAATTACTTCGG.......................

RI-gBol038420-XLOC_008537-6755-0
ATCAAAAGCTGTTATGCGTTTGCATCATCTAAACTTTGGTTGATATTTAC
RI-gBol038420-XLOC_008537-6755-1

CONSENSUS
....

RI-gBol038420-XLOC_008537-6755-0
TTACATTTGTTCTTTTTGTTGTAGCAATTTTGGACCAGTGCAGGATGTTA
RI-gBol038420-XLOC_008537-6755-1 -----------------------
CAATTTTGGACCAGTGCAGGATGTTA
CONSENSUS
........................CAATTTTGGACCAGTGCAGGATGTTA

RI-gBol038420-XLOC_008537-6755-0
GGATTCCATACCAGCAGAAACGGATGTTTGGGTTTGTCACTTTTGTCCAC
RI-gBol038420-XLOC_008537-6755-1

GGATTCCATACCAGCAGAAACGGATGTTTGGGTTTGTCACTTTTGTCCAC
CONSENSUS
GGATTCCATACCAGCAGAAACGGATGTTTGGGTTTGTCACTTTTGTCCAC

RI-gBol038420-XLOC_008537-6755-0
TCTGAGACTGTGAGAATCATATTGGCCAGAGGAAACCCACATTTCATCTG
RI-gBol038420-XLOC_008537-6755-1
TCTGAGACTGTGAGAATCATATTGGCCAGAGGAAACCCACATTTCATCTG
CONSENSUS
TCTGAGACTGTGAGAATCATATTGGCCAGAGGAAACCCACATTTCATCTG

RI-gBol038420-XLOC_008537-6755-0
CGACTCACGCGTGCTTGTTAAACCCTACAAAGAGAAAGGAAGAATCCTTG
RI-gBol038420-XLOC_008537-6755-1
CGACTCACGCGTGCTTGTTAAACCCTACAAAGAGAAAGGAAGAATCCTTG
CONSENSUS
CGACTCACGCGTGCTTGTTAAACCCTACAAAGAGAAAGGAAGAATCCTTG

RI-gBol038420-XLOC_008537-6755-0 AAAA
RI-gBol038420-XLOC_008537-6755-1 AAAA
CONSENSUS AAAA

alignment for event: SE-gBol003791-XLOC_047596-2308

SE-gBol003791-XLOC_047596-2308-0
GAAAGTTTATAAATATAAGAAGCAGAGTAAAAGCTTAATTGCAGAACTTA
SE-gBol003791-XLOC_047596-2308-1
GAAAGTTTATAAATATAAGAAGCAGAGTAAAAGCTTAATTGCAGAACTTA
CONSENSUS
GAAAGTTTATAAATATAAGAAGCAGAGTAAAAGCTTAATTGCAGAACTTA

SE-gBol003791-XLOC_047596-2308-0
AAATAAATAAAAGTGTACAACAAGAAGAACCTCTCCCTCTCTCTCTCTCT
SE-gBol003791-XLOC_047596-2308-1
AAATAAATAAAAGTGTACAACAAGAAGAACCTCTCCCTCTCTCTCTCTCT
CONSENSUS
AAATAAATAAAAGTGTACAACAAGAAGAACCTCTCCCTCTCTCTCTCTCT

SE-gBol003791-XLOC_047596-2308-0
TTGTCGATAAAAACATCTTTTGGGATTAGAAATGGGATCGGTTAGCGAAT
SE-gBol003791-XLOC_047596-2308-1
TTGTCGATAAAAACATCTTTTGGGATTAGAAATGGGATCGGTTAGCGAAT
CONSENSUS
TTGTCGATAAAAACATCTTTTGGGATTAGAAATGGGATCGGTTAGCGAAT

SE-gBol003791-XLOC_047596-2308-0
CTGTACCTACGGCGAACTCTTCTACGACGGTGGTGATGAGCTCTATTCCC
SE-gBol003791-XLOC_047596-2308-1
CTGTACCTACGGCGAACTCTTCTACGACGGTGGTGATGAGCTCTATTCCC
CONSENSUS
CTGTACCTACGGCGAACTCTTCTACGACGGTGGTGATGAGCTCTATTCCC

SE-gBol003791-XLOC_047596-2308-0
CCGTTTCTGAGCAAGACATACGACATGGTTGACGATCCATCGACTGATGA

SE-gBol003791-XLOC_047596-2308-1
CCGTTTCTGAGCAAGACATACGACATGGTTGACGATCCATCGACTGATGA
CONSENSUS
CCGTTTCTGAGCAAGACATACGACATGGTTGACGATCCATCGACTGATGA

SE-gBol003791-XLOC_047596-2308-0
GGTGGTGTCTTGGAGCAGCGGCAGCAACAGCTTCGTCGTGTGGAACGTGC
SE-gBol003791-XLOC_047596-2308-1
GGTGGTGTCTTGGAGCAGCGGCAGCAACAGCTTCGTCGTGTGGAACGTGC
CONSENSUS
GGTGGTGTCTTGGAGCAGCGGCAGCAACAGCTTCGTCGTGTGGAACGTGC

SE-gBol003791-XLOC_047596-2308-0
CTGAGTTCTCTAAGCAGTTCCTACCGAAGTACTTCAAGCACAACAACTTC
SE-gBol003791-XLOC_047596-2308-1
CTGAGTTCTCTAAGCAGTTCCTACCGAAGTACTTCAAGCACAACAACTTC
CONSENSUS
CTGAGTTCTCTAAGCAGTTCCTACCGAAGTACTTCAAGCACAACAACTTC

SE-gBol003791-XLOC_047596-2308-0
TCCAGCTTTGTCAGACAGCTCAACACTTAT--------------------
SE-gBol003791-XLOC_047596-2308-1
TCCAGCTTTGTCAGACAGCTCAACACTTATGCTTATTTGGACATGTCCCC
CONSENSUS
TCCAGCTTTGTCAGACAGCTCAACACTTAT....................

SE-gBol003791-XLOC_047596-2308-0

SE-gBol003791-XLOC_047596-2308-1
ATATGTGTCTGAAGATTTGGTTTCCTCAGCAGTTCCATTTCTACTCTTAA
CONSENSUS
....

SE-gBol003791-XLOC_047596-2308-0

SE-gBol003791-XLOC_047596-2308-1
GTCGGAAAACAAAATTTCTAATCAGTTCTCTCAAGTTAACTTTCCATTCA
CONSENSUS
....

SE-gBol003791-XLOC_047596-2308-0 --------------------
GGCTTCAGAAAAGTTGATCCAGACCGCTGG
SE-gBol003791-XLOC_047596-2308-1
TTTGTATTTTTACCGTCCAGGGCTTCAGAAAAGTTGATCCAGACCGCTGG
CONSENSUS
....................GGCTTCAGAAAAGTTGATCCAGACCGCTGG

SE-gBol003791-XLOC_047596-2308-0
GAATTCGCAAACGAGGGATTTCTAAAAGGCCAAAAACAGTTGCTAAAGAG
SE-gBol003791-XLOC_047596-2308-1
GAATTCGCAAACGAGGGATTTCTAAAAGGCCAAAAACAGTTGCTAAAGAG
CONSENSUS
GAATTCGCAAACGAGGGATTTCTAAAAGGCCAAAAACAGTTGCTAAAGAG

SE-gBol003791-XLOC_047596-2308-0
CATTATCCGTCGCAAGCCTTCACAGGTGCAGCCTCCACAACAGCCTCAAG

SE-gBol003791-XLOC_047596-2308-1
CATTATCCGTCGCAAGCCTTCACAGGTGCAGCCTCCACAACAGCCTCAAG
CONSENSUS
CATTATCCGTCGCAAGCCTTCACAGGTGCAGCCTCCACAACAGCCTCAAG

SE-gBol003791-XLOC_047596-2308-0
TCCAGCACTCATCCGTCGGCGCCTGCGTTGAAGTGGGCAAGTTCGGACTC
SE-gBol003791-XLOC_047596-2308-1
TCCAGCACTCATCCGTCGGCGCCTGCGTTGAAGTGGGCAAGTTCGGACTC
CONSENSUS
TCCAGCACTCATCCGTCGGCGCCTGCGTTGAAGTGGGCAAGTTCGGACTC

SE-gBol003791-XLOC_047596-2308-0
GAAGAAGAAGTCGAAAGACTCCAGCGGGACAAGAACGTCCTCATGCAGGA
SE-gBol003791-XLOC_047596-2308-1
GAAGAAGAAGTCGAAAGACTCCAGCGGGACAAGAACGTCCTCATGCAGGA
CONSENSUS
GAAGAAGAAGTCGAAAGACTCCAGCGGGACAAGAACGTCCTCATGCAGGA

SE-gBol003791-XLOC_047596-2308-0
ACTTGTTCGGTTAAGGCAGCAACAGCAAGTCACAGAAAATCATCTTCAGC
SE-gBol003791-XLOC_047596-2308-1
ACTTGTTCGGTTAAGGCAGCAACAGCAAGTCACAGAAAATCATCTTCAGC
CONSENSUS
ACTTGTTCGGTTAAGGCAGCAACAGCAAGTCACAGAAAATCATCTTCAGC

SE-gBol003791-XLOC_047596-2308-0
ACGTGGGGCAGAAGGTTCACGTGATGGAGCAGAGGCAGCAGCAAATGATG
SE-gBol003791-XLOC_047596-2308-1
ACGTGGGGCAGAAGGTTCACGTGATGGAGCAGAGGCAGCAGCAAATGATG
CONSENSUS
ACGTGGGGCAGAAGGTTCACGTGATGGAGCAGAGGCAGCAGCAAATGATG

SE-gBol003791-XLOC_047596-2308-0
TCGTTCTTAGCGAAGGCTGTACAGAGTCCAGGCTTCTTAAACCAGTTCTC
SE-gBol003791-XLOC_047596-2308-1
TCGTTCTTAGCGAAGGCTGTACAGAGTCCAGGCTTCTTAAACCAGTTCTC
CONSENSUS
TCGTTCTTAGCGAAGGCTGTACAGAGTCCAGGCTTCTTAAACCAGTTCTC

SE-gBol003791-XLOC_047596-2308-0
ACAGCAGAGTAATAACGAGGGGAGCCAGCACATGTCTGAGAGCAACAAGA
SE-gBol003791-XLOC_047596-2308-1
ACAGCAGAGTAATAACGAGGGGAGCCAGCACATGTCTGAGAGCAACAAGA
CONSENSUS
ACAGCAGAGTAATAACGAGGGGAGCCAGCACATGTCTGAGAGCAACAAGA

SE-gBol003791-XLOC_047596-2308-0
AGAGGAGGCTACCCGTTGAGGATCAGAAGAATAGTGGTGGTAGCCAGGGG
SE-gBol003791-XLOC_047596-2308-1
AGAGGAGGCTACCCGTTGAGGATCAGAAGAATAGTGGTGGTAGCCAGGGG
CONSENSUS
AGAGGAGGCTACCCGTTGAGGATCAGAAGAATAGTGGTGGTAGCCAGGGG

SE-gBol003791-XLOC_047596-2308-0
CTTAACGGTCTTAGCCGCCAGATTGTGAGGTACCAGTCGTCGATGAACGA

SE-gBol003791-XLOC_047596-2308-1
CTTAACGGTCTTAGCCGCCAGATTGTGAGGTACCAGTCGTCGATGAACGA
CONSENSUS
CTTAACGGTCTTAGCCGCCAGATTGTGAGGTACCAGTCGTCGATGAACGA

SE-gBol003791-XLOC_047596-2308-0
GTCATCAAACAGTATGCTCCAGCAGATAAACAACATGAGTAACACGCATA
SE-gBol003791-XLOC_047596-2308-1
GTCATCAAACAGTATGCTCCAGCAGATAAACAACATGAGTAACACGCATA
CONSENSUS
GTCATCAAACAGTATGCTCCAGCAGATAAACAACATGAGTAACACGCATA

SE-gBol003791-XLOC_047596-2308-0
CCAACAATCACGGTAGCTTTCTTTTGGGAGATGTTCCTAATCCTAACCTT
SE-gBol003791-XLOC_047596-2308-1
CCAACAATCACGGTAGCTTTCTTTTGGGAGATGTTCCTAATCCTAACCTT
CONSENSUS
CCAACAATCACGGTAGCTTTCTTTTGGGAGATGTTCCTAATCCTAACCTT

SE-gBol003791-XLOC_047596-2308-0
TCAGACAACGGGAGCTCCTCGAATGGACCATCTGGAGTTGTTGCATTCAC
SE-gBol003791-XLOC_047596-2308-1
TCAGACAACGGGAGCTCCTCGAATGGACCATCTGGAGTTGTTGCATTCAC
CONSENSUS
TCAGACAACGGGAGCTCCTCGAATGGACCATCTGGAGTTGTTGCATTCAC

SE-gBol003791-XLOC_047596-2308-0
TGATGTTTCATCCAATACAACAAACCAAGTCTTGGAGACCAATTTGCCTT
SE-gBol003791-XLOC_047596-2308-1
TGATGTTTCATCCAATACAACAAACCAAGTCTTGGAGACCAATTTGCCTT
CONSENSUS
TGATGTTTCATCCAATACAACAAACCAAGTCTTGGAGACCAATTTGCCTT

SE-gBol003791-XLOC_047596-2308-0
ATCCTCAACCTCAAGCTGATCTGTTAGCTCCAAAGCAAGGAGCAGAAGGA
SE-gBol003791-XLOC_047596-2308-1
ATCCTCAACCTCAAGCTGATCTGTTAGCTCCAAAGCAAGGAGCAGAAGGA
CONSENSUS
ATCCTCAACCTCAAGCTGATCTGTTAGCTCCAAAGCAAGGAGCAGAAGGA

SE-gBol003791-XLOC_047596-2308-0
GGTTCTGGGAGCCCAAGTCCGGATCTAGTTGGATGCGAGAGAGATACTGG
SE-gBol003791-XLOC_047596-2308-1
GGTTCTGGGAGCCCAAGTCCGGATCTAGTTGGATGCGAGAGAGATACTGG
CONSENSUS
GGTTCTGGGAGCCCAAGTCCGGATCTAGTTGGATGCGAGAGAGATACTGG

SE-gBol003791-XLOC_047596-2308-0
AGAGTGTCTGGATCCAATAATGGCTGTTTTGGATGGCTCAATGATGCTGG
SE-gBol003791-XLOC_047596-2308-1
AGAGTGTCTGGATCCAATAATGGCTGTTTTGGATGGCTCAATGATGCTGG
CONSENSUS
AGAGTGTCTGGATCCAATAATGGCTGTTTTGGATGGCTCAATGATGCTGG

SE-gBol003791-XLOC_047596-2308-0
AAACCAATGAGTTGCTTCCTGGAGTACAAGATTCATTGTGGGAACAGTTC

SE-gBol003791-XLOC_047596-2308-1
AAACCAATGAGTTGCTTCCTGGAGTACAAGATTCATTGTGGGAACAGTTC
CONSENSUS
AAACCAATGAGTTGCTTCCTGGAGTACAAGATTCATTGTGGGAACAGTTC

SE-gBol003791-XLOC_047596-2308-0
TTTGGTGAGAGCTCAGGGATTGGGGACTCTGACGAGCTAGTGCCAGGGTC
SE-gBol003791-XLOC_047596-2308-1
TTTGGTGAGAGCTCAGGGATTGGGGACTCTGACGAGCTAGTGCCAGGGTC
CONSENSUS
TTTGGTGAGAGCTCAGGGATTGGGGACTCTGACGAGCTAGTGCCAGGGTC

SE-gBol003791-XLOC_047596-2308-0
GGTGGACAATGAGTTGATAATGGAGCAGCTGGAGTTACAACCCAACCTGA
SE-gBol003791-XLOC_047596-2308-1
GGTGGACAATGAGTTGATAATGGAGCAGCTGGAGTTACAACCCAACCTGA
CONSENSUS
GGTGGACAATGAGTTGATAATGGAGCAGCTGGAGTTACAACCCAACCTGA

SE-gBol003791-XLOC_047596-2308-0
GGAATGTGTTGAGCAACAATCAACAAATGAACCATCTTACTGAACAGATG
SE-gBol003791-XLOC_047596-2308-1
GGAATGTGTTGAGCAACAATCAACAAATGAACCATCTTACTGAACAGATG
CONSENSUS
GGAATGTGTTGAGCAACAATCAACAAATGAACCATCTTACTGAACAGATG

SE-gBol003791-XLOC_047596-2308-0
GGACTTCTCACATCAGATGCTCTCAGGAAATGAATTCGCCATAATCTTTT
SE-gBol003791-XLOC_047596-2308-1
GGACTTCTCACATCAGATGCTCTCAGGAAATGAATTCGCCATAATCTTTT
CONSENSUS
GGACTTCTCACATCAGATGCTCTCAGGAAATGAATTCGCCATAATCTTTT

SE-gBol003791-XLOC_047596-2308-0
ACTCAAGAGGTAGTAAGCCCAATCTTTCTTCATTGATGAATTTATAATAT
SE-gBol003791-XLOC_047596-2308-1
ACTCAAGAGGTAGTAAGCCCAATCTTTCTTCATTGATGAATTTATAATAT
CONSENSUS
ACTCAAGAGGTAGTAAGCCCAATCTTTCTTCATTGATGAATTTATAATAT

SE-gBol003791-XLOC_047596-2308-0
CAACACACACTTTCCAGAATCTTGACTTTTTCTCGCAGATGTAGAAGATG
SE-gBol003791-XLOC_047596-2308-1
CAACACACACTTTCCAGAATCTTGACTTTTTCTCGCAGATGTAGAAGATG
CONSENSUS
CAACACACACTTTCCAGAATCTTGACTTTTTCTCGCAGATGTAGAAGATG

SE-gBol003791-XLOC_047596-2308-0
GGTTGAGATTGCAGCAACAAAGGGATAATGGCCAAAGGAAGTGGTGTTAT
SE-gBol003791-XLOC_047596-2308-1
GGTTGAGATTGCAGCAACAAAGGGATAATGGCCAAAGGAAGTGGTGTTAT
CONSENSUS
GGTTGAGATTGCAGCAACAAAGGGATAATGGCCAAAGGAAGTGGTGTTAT

SE-gBol003791-XLOC_047596-2308-0
GTATCTATGTATCAAAACTTGTGTTACTCTCATTCTTGACGTTGTGTAAT

SE-gBol003791-XLOC_047596-2308-1
      GTATCTATGTATCAAAACTTGTGTTACTCTCATTCTTGACGTTGTGTAAT
CONSENSUS
      GTATCTATGTATCAAAACTTGTGTTACTCTCATTCTTGACGTTGTGTAAT

SE-gBol003791-XLOC_047596-2308-0 ATAATAAGAGAAAAGAGAAATAAT
SE-gBol003791-XLOC_047596-2308-1 ATAATAAGAGAAAAGAGAAATAAT
CONSENSUS ATAATAAGAGAAAAGAGAAATAAT

alignment for event: RI-gBol026736-XLOC_021675-15352

RI-gBol026736-XLOC_021675-15352-0
      GTGCTGGCTTTTTCTTACCAGTCTTACAAGAAAGGCCCAGTTCCAAGAAG
RI-gBol026736-XLOC_021675-15352-1
      GTGCTGGCTTTTTCTTACCAGTCTTACAAGAAAGGCCCAGTTCCAAGAAG
CONSENSUS
      GTGCTGGCTTTTTCTTACCAGTCTTACAAGAAAGGCCCAGTTCCAAGAAG

RI-gBol026736-XLOC_021675-15352-0
      CTGAGCTGGAAAAACAGCCAAGCAGCTCAAAGAGGTGTTTACAATCTCTA
RI-gBol026736-XLOC_021675-15352-1
      CTGAGCTGGAAAAACAGCCAAGCAGCTCAAAGAGGTGTTTACAATCTCTA
CONSENSUS
      CTGAGCTGGAAAAACAGCCAAGCAGCTCAAAGAGGTGTTTACAATCTCTA

RI-gBol026736-XLOC_021675-15352-0
      GCGAAGAAACGACCAGATGTGCGGCAACAAAAAAAGGTTATCATATCTCT
RI-gBol026736-XLOC_021675-15352-1
      GCGAAGAAACGACCAGATGTGCGGCAACAAAAAAAG--------------
CONSENSUS
      GCGAAGAAACGACCAGATGTGCGGCAACAAAAAAAG..............

RI-gBol026736-XLOC_021675-15352-0
      TACCGCATAAGTAATTTCAAAGATCAAAATTAGTAATATGCTTTATACAA
RI-gBol026736-XLOC_021675-15352-1

CONSENSUS
      ....

RI-gBol026736-XLOC_021675-15352-0
      GTACGAGTGATTTCTTATGACTCCGTTATCAGCATCACTGAAATTATGTA
RI-gBol026736-XLOC_021675-15352-1

CONSENSUS
      ....

RI-gBol026736-XLOC_021675-15352-0
      GGTGAAATACATGGCTGAAATATTACCCGTTCGATCAGCTTGAACTGTGA
RI-gBol026736-XLOC_021675-15352-1 -
GTGAAATACATGGCTGAAATATTACCCGTTCGATCAGCTTGAACTGTGA
CONSENSUS
      .GTGAAATACATGGCTGAAATATTACCCGTTCGATCAGCTTGAACTGTGA

RI-gBol026736-XLOC_021675-15352-0

AGTCTCCTCCGTCCCTTGATTCATTTCCTTCCAGTCCAGGCCGCATTGAC
RI-gBol026736-XLOC_021675-15352-1
AGTCTCCTCCGTCCCTTGATTCATTTCCTTCCAGTCCAGGCCGCATTGAC
CONSENSUS
AGTCTCCTCCGTCCCTTGATTCATTTCCTTCCAGTCCAGGCCGCATTGAC

RI-gBol026736-XLOC_021675-15352-0
CGTTATAATATCTTAAACCAACCAAATGGGAACGCAGGCAAAATGGTGTA
RI-gBol026736-XLOC_021675-15352-1
CGTTATAATATCTTAAACCAACCAAATGGGAACGCAGGCAAAATGGTGTA
CONSENSUS
CGTTATAATATCTTAAACCAACCAAATGGGAACGCAGGCAAAATGGTGTA

RI-gBol026736-XLOC_021675-15352-0
AATACCACAATGTTTTCTAATTGGACCATTACACATGTACTTGGAAATGG
RI-gBol026736-XLOC_021675-15352-1
AATACCACAATGTTTTCTAATTGGACCATTACACATGTACTTGGAAATGG
CONSENSUS
AATACCACAATGTTTTCTAATTGGACCATTACACATGTACTTGGAAATGG

RI-gBol026736-XLOC_021675-15352-0
AGAAGCATCGACTGAAGCACATAACAAGAATGAATGGGTTGAGAAAGATG
RI-gBol026736-XLOC_021675-15352-1
AGAAGCATCGACTGAAGCACATAACAAGAATGAATGGGTTGAGAAAGATG
CONSENSUS
AGAAGCATCGACTGAAGCACATAACAAGAATGAATGGGTTGAGAAAGATG

RI-gBol026736-XLOC_021675-15352-0
CGCCTGCCGTCTCCATCATTCTTCCAGCCTTAGCCAGTGGTGTTACCGAC
RI-gBol026736-XLOC_021675-15352-1
CGCCTGCCGTCTCCATCATTCTTCCAGCCTTAGCCAGTGGTGTTACCGAC
CONSENSUS
CGCCTGCCGTCTCCATCATTCTTCCAGCCTTAGCCAGTGGTGTTACCGAC

RI-gBol026736-XLOC_021675-15352-0 CTCAAACGTGTCCGTTTCAG
RI-gBol026736-XLOC_021675-15352-1 CTCAAACGTGTCCGTTTCAG
CONSENSUS CTCAAACGTGTCCGTTTCAG

alignment for event: RI-gBol003215-XLOC_048312-14694

RI-gBol003215-XLOC_048312-14694-0
ATGACGACATGCGACGGCAATGGCGATTTTGATTTTGCGATTAAGGCTCG
RI-gBol003215-XLOC_048312-14694-1
ATGACGACATGCGACGGCAATGGCGATTTTGATTTTGCGATTAAGGCTCG
CONSENSUS
ATGACGACATGCGACGGCAATGGCGATTTTGATTTTGCGATTAAGGCTCG

RI-gBol003215-XLOC_048312-14694-0
AGGGGTTGTTGAGGTTTTTAGGGTCAGAATGGATCGGAATGAGGAGATCT
RI-gBol003215-XLOC_048312-14694-1
AGGGGTTGTTGAGGTTTTTAGGGTCAGAATGGATCGGAATGAGGAGATCT
CONSENSUS
AGGGGTTGTTGAGGTTTTTAGGGTCAGAATGGATCGGAATGAGGAGATCT

RI-gBol003215-XLOC_048312-14694-0
CTCCTCCTATTGGTATTATTATGGGGTATCGAGGTTTTTTCGCGAATCAA
RI-gBol003215-XLOC_048312-14694-1
CTCCTCCTATTG--------------------------------------
CONSENSUS
CTCCTCCTATTG......................................

RI-gBol003215-XLOC_048312-14694-0
GGGATTGATGCGATCTACGTCTACGTAACGGAAATAGATGGTCGCATAGG
RI-gBol003215-XLOC_048312-14694-1

CONSENSUS
....

RI-gBol003215-XLOC_048312-14694-0
GGTAAAATCGGAGAGGATCTCTGGATCTGGGTTGTTTTAGGAAGAAAGGA
RI-gBol003215-XLOC_048312-14694-1

CONSENSUS
....

RI-gBol003215-XLOC_048312-14694-0
GGAGGTTTTTTCATTTTTCTACATGATTGACGCAGGATTTGGGATCCCTC
RI-gBol003215-XLOC_048312-14694-1

CONSENSUS
....

RI-gBol003215-XLOC_048312-14694-0
TGATCTTGATGTCTCAGGTCAAAGAGAAAATACGGTATTTTTTCTTGATT
RI-gBol003215-XLOC_048312-14694-1

CONSENSUS
....

RI-gBol003215-XLOC_048312-14694-0
GCTTTGACTCTCTGTTATGAGATGATGAAATTGATCGATAGAGATTTACT
RI-gBol003215-XLOC_048312-14694-1

CONSENSUS
....

RI-gBol003215-XLOC_048312-14694-0
TTCTTCTTTTAAGACTTGTTTGGTTTGTGGGATGTTCTTTGATTTGGTAA
RI-gBol003215-XLOC_048312-14694-1

CONSENSUS
....

RI-gBol003215-XLOC_048312-14694-0
TTGGGGCTGGGATTTGGATCTCTCAATGGGGCTATGACGGATATCTCGTT
RI-gBol003215-XLOC_048312-14694-1

CONSENSUS
....

RI-gBol003215-XLOC_048312-14694-0
TCTATGTTTGGATTGCTTTCGTGTATCTGTGGTACTTTCTATAATTCTAA
RI-gBol003215-XLOC_048312-14694-1

CONSENSUS
....

RI-gBol003215-XLOC_048312-14694-0
AGAGGTGATTGCGGGTGTGATCTGAGATTGGTATCTTATATGTTATTTGG
RI-gBol003215-XLOC_048312-14694-1

CONSENSUS
....

RI-gBol003215-XLOC_048312-14694-0
TCTTTAACTCTTGTTTGCGGGAGGTTTCACTATATATTGTGGTTCTTGTT
RI-gBol003215-XLOC_048312-14694-1

CONSENSUS
....

RI-gBol003215-XLOC_048312-14694-0
TCAGTCAAGTTTCATACCTTTCTTCGATTCCAGTCTTCGCAAGTTATTTA
RI-gBol003215-XLOC_048312-14694-1

CONSENSUS
....

RI-gBol003215-XLOC_048312-14694-0
CAACAGTTTGTTTCTCTTGTTTCCTTTTCGTGTTAACATGTCTCAGCAAA
RI-gBol003215-XLOC_048312-14694-1
--CAAA
CONSENSUS
....CAAA

RI-gBol003215-XLOC_048312-14694-0
GTCATTGGATCACTAAATCAGGAGAGAAGAAGGCGGAGGTTGTTAGGTCG
RI-gBol003215-XLOC_048312-14694-1
GTCATTGGATCACTAAATCAGGAGAGAAGAAGGCGGAGGTTGTTAGGTCG
CONSENSUS
GTCATTGGATCACTAAATCAGGAGAGAAGAAGGCGGAGGTTGTTAGGTCG

RI-gBol003215-XLOC_048312-14694-0
GGGCTAAGGATCTCGATCCCCCGGTTTGACAACTCGGAGATCATTGCGCG
RI-gBol003215-XLOC_048312-14694-1
GGGCTAAGGATCTCGATCCCCCGGTTTGACAACTCGGAGATCATTGCGCG
CONSENSUS
GGGCTAAGGATCTCGATCCCCCGGTTTGACAACTCGGAGATCATTGCGCG

RI-gBol003215-XLOC_048312-14694-0
ATATGCACGAACCCTCATCGGAAGGTGTATGAACCCTCCAAAACAAGACA
RI-gBol003215-XLOC_048312-14694-1
ATATGCACGAACCCTCATCGGAAGGTGTATGAACCCTCCAAAACAAGACA
CONSENSUS
ATATGCACGAACCCTCATCGGAAGGTGTATGAACCCTCCAAAACAAGACA

RI-gBol003215-XLOC_048312-14694-0
TGAAAGTTCTCTTGTTTGTGTTTCCGAGGATTTGGAACATGGAGGGCCGT
RI-gBol003215-XLOC_048312-14694-1
TGAAAGTTCTCTTGTTTGTGTTTCCGAGGATTTGGAACATGGAGGGCCGT
CONSENSUS
TGAAAGTTCTCTTGTTTGTGTTTCCGAGGATTTGGAACATGGAGGGCCGT

RI-gBol003215-XLOC_048312-14694-0
GTGGTGGGTACGGATCTTGGTCTGGGGAGGTTCCAGCTCGAGGAGGATAT
RI-gBol003215-XLOC_048312-14694-1
GTGGTGGGTACGGATCTTGGTCTGGGGAGGTTCCAGCTCGAGGAGGATAT
CONSENSUS
GTGGTGGGTACGGATCTTGGTCTGGGGAGGTTCCAGCTCGAGGAGGATAT

RI-gBol003215-XLOC_048312-14694-0
CACGGAGGTCCTTAAGATGGTGCCTTATCACTTTGATTTCTGGATGGTGT
RI-gBol003215-XLOC_048312-14694-1
CACGGAGGTCCTTAAGATGGTGCCTTATCACTTTGATTTCTGGATGGTGT
CONSENSUS
CACGGAGGTCCTTAAGATGGTGCCTTATCACTTTGATTTCTGGATGGTGT

RI-gBol003215-XLOC_048312-14694-0
CTTTAGTGAGGTGGAAGCCGGTTTTAGAACCAAATTATCCCACTAAGATC
RI-gBol003215-XLOC_048312-14694-1
CTTTAGTGAGGTGGAAGCCGGTTTTAGAACCAAATTATCCCACTAAGATC
CONSENSUS
CTTTAGTGAGGTGGAAGCCGGTTTTAGAACCAAATTATCCCACTAAGATC

RI-gBol003215-XLOC_048312-14694-0
ACGTTTTGGGTGTGAGTCTTAGATATCCCTCTTCAGTTCAGGGCAGCTCA
RI-gBol003215-XLOC_048312-14694-1
ACGTTTTGGGTGTGAGTCTTAGATATCCCTCTTCAGTTCAGGGCAGCTCA
CONSENSUS
ACGTTTTGGGTGTGAGTCTTAGATATCCCTCTTCAGTTCAGGGCAGCTCA

RI-gBol003215-XLOC_048312-14694-0
GATCTTCCAGAGCGTGGGGGAAGCAATTGGCCAGGTCCAGGGCCAGGTTG
RI-gBol003215-XLOC_048312-14694-1
GATCTTCCAGAGCGTGGGGGAAGCAATTGGCCAGGTCCAGGGCCAGGTTG
CONSENSUS
GATCTTCCAGAGCGTGGGGGAAGCAATTGGCCAGGTCCAGGGCCAGGTTG

RI-gBol003215-XLOC_048312-14694-0
ATATCGTGGAGGGACGGGTTCGGGTGGAGATAGATGGATTTAAACCTTTG
RI-gBol003215-XLOC_048312-14694-1
ATATCGTGGAGGGACGGGTTCGGGTGGAGATAGATGGATTTAAACCTTTG
CONSENSUS
ATATCGTGGAGGGACGGGTTCGGGTGGAGATAGATGGATTTAAACCTTTG

RI-gBol003215-XLOC_048312-14694-0
GTGTTCTCAATGGACATTGAGTTTGAGGAAGGTGTGGAAATCAAGGTGGC
RI-gBol003215-XLOC_048312-14694-1
GTGTTCTCAATGGACATTGAGTTTGAGGAAGGTGTGGAAATCAAGGTGGC
CONSENSUS
GTGTTCTCAATGGACATTGAGTTTGAGGAAGGTGTGGAAATCAAGGTGGC

RI-gBol003215-XLOC_048312-14694-0
TCTCAGATATGAAAAGCTTTAAGGGTTTTGCACTGAATGTTTCTGCTTGA
RI-gBol003215-XLOC_048312-14694-1
TCTCAGATATGAAAAGCTTTAAGGGTTTTGCACTGAATGTTTCTGCTTGA
CONSENSUS
TCTCAGATATGAAAAGCTTTAAGGGTTTTGCACTGAATGTTTCTGCTTGA

RI-gBol003215-XLOC_048312-14694-0
CTCATGAATGCTCTCGTTGCCCGAGGTTACACAAGGAGGAGCTTTTGGGT
RI-gBol003215-XLOC_048312-14694-1
CTCATGAATGCTCTCGTTGCCCGAGGTTACACAAGGAGGAGCTTTTGGGT
CONSENSUS
CTCATGAATGCTCTCGTTGCCCGAGGTTACACAAGGAGGAGCTTTTGGGT

RI-gBol003215-XLOC_048312-14694-0
GTAGCTGGGGGAGGACCGGGTAGTGATGGGGCTCAGGCAACAAGCTATAA
RI-gBol003215-XLOC_048312-14694-1
GTAGCTGGGGGAGGACCGGGTAGTGATGGGGCTCAGGCAACAAGCTATAA
CONSENSUS
GTAGCTGGGGGAGGACCGGGTAGTGATGGGGCTCAGGCAACAAGCTATAA

RI-gBol003215-XLOC_048312-14694-0
GACGGTGGTGGCCCACGGTAGCAGTCAGAGCGGTGAACGTAGAGCGTATC
RI-gBol003215-XLOC_048312-14694-1
GACGGTGGTGGCCCACGGTAGCAGTCAGAGCGGTGAACGTAGAGCGTATC
CONSENSUS
GACGGTGGTGGCCCACGGTAGCAGTCAGAGCGGTGAACGTAGAGCGTATC

RI-gBol003215-XLOC_048312-14694-0
AGCAAACTCGGTCGCAGAACAACAGAGTGGGTGATAAGGGCAAAGGCATT
RI-gBol003215-XLOC_048312-14694-1
AGCAAACTCGGTCGCAGAACAACAGAGTGGGTGATAAGGGCAAAGGCATT
CONSENSUS
AGCAAACTCGGTCGCAGAACAACAGAGTGGGTGATAAGGGCAAAGGCATT

RI-gBol003215-XLOC_048312-14694-0
GCGTATGAGAAGCAAGGTAGTTCCAAGCAGGACGGGTCTTTTCATCCTTA
RI-gBol003215-XLOC_048312-14694-1
GCGTATGAGAAGCAAGGTAGTTCCAAGCAGGACGGGTCTTTTCATCCTTA
CONSENSUS
GCGTATGAGAAGCAAGGTAGTTCCAAGCAGGACGGGTCTTTTCATCCTTA

RI-gBol003215-XLOC_048312-14694-0
CAAAGGCAAGCACACTAGAGGCTATGGTGATGGTTCCTCAATGAATGGCA
RI-gBol003215-XLOC_048312-14694-1
CAAAGGCAAGCACACTAGAGGCTATGGTGATGGTTCCTCAATGAATGGCA
CONSENSUS
CAAAGGCAAGCACACTAGAGGCTATGGTGATGGTTCCTCAATGAATGGCA

RI-gBol003215-XLOC_048312-14694-0
GGGACTCGGGATATGGAGAAAGGAGACGTGGTATGCAGTCTCGTGGTACT
RI-gBol003215-XLOC_048312-14694-1
GGGACTCGGGATATGGAGAAAGGAGACGTGGTATGCAGTCTCGTGGTACT
CONSENSUS
GGGACTCGGGATATGGAGAAAGGAGACGTGGTATGCAGTCTCGTGGTACT

RI-gBol003215-XLOC_048312-14694-0
AAGCCTAGGGGAGTGGTACCTCGGGGTCAGCATCTTGCTACTGTTGCTGA
RI-gBol003215-XLOC_048312-14694-1
AAGCCTAGGGGAGTGGTACCTCGGGGTCAGCATCTTGCTACTGTTGCTGA
CONSENSUS
AAGCCTAGGGGAGTGGTACCTCGGGGTCAGCATCTTGCTACTGTTGCTGA

RI-gBol003215-XLOC_048312-14694-0
AGGGGAACAACAGCTACCAGATCCCTCCAAGCTTATGCTCGATGCCTTTA
RI-gBol003215-XLOC_048312-14694-1
AGGGGAACAACAGCTACCAGATCCCTCCAAGCTTATGCTCGATGCCTTTA
CONSENSUS
AGGGGAACAACAGCTACCAGATCCCTCCAAGCTTATGCTCGATGCCTTTA

RI-gBol003215-XLOC_048312-14694-0
AAGGGGCTGGAAAGTCTTCAGAAGAGGAAGCTCCAATAGGGGTGGAGGCG
RI-gBol003215-XLOC_048312-14694-1
AAGGGGCTGGAAAGTCTTCAGAAGAGGAAGCTCCAATAGGGGTGGAGGCG
CONSENSUS
AAGGGGCTGGAAAGTCTTCAGAAGAGGAAGCTCCAATAGGGGTGGAGGCG

RI-gBol003215-XLOC_048312-14694-0
GAAGGAAGTGGTGGACATAACAAAGCACGTAAGGCGCTTATGTTTGAAGA
RI-gBol003215-XLOC_048312-14694-1
GAAGGAAGTGGTGGACATAACAAAGCACGTAAGGCGCTTATGTTTGAAGA
CONSENSUS
GAAGGAAGTGGTGGACATAACAAAGCACGTAAGGCGCTTATGTTTGAAGA

RI-gBol003215-XLOC_048312-14694-0
ATCTAGTTCTGTTGGGCATGATCTTGTGTCGACAGGAGAGGGAGTGGAGC
RI-gBol003215-XLOC_048312-14694-1
ATCTAGTTCTGTTGGGCATGATCTTGTGTCGACAGGAGAGGGAGTGGAGC
CONSENSUS
ATCTAGTTCTGTTGGGCATGATCTTGTGTCGACAGGAGAGGGAGTGGAGC

RI-gBol003215-XLOC_048312-14694-0
TTGCTGGATCTCTGGCTTTGAAGATGCAGAGCGAGGGTGATGATGCCGCG
RI-gBol003215-XLOC_048312-14694-1
TTGCTGGATCTCTGGCTTTGAAGATGCAGAGCGAGGGTGATGATGCCGCG
CONSENSUS
TTGCTGGATCTCTGGCTTTGAAGATGCAGAGCGAGGGTGATGATGCCGCG

RI-gBol003215-XLOC_048312-14694-0
CAGAAGCTTGATGAGAGGGCATTGCATACACAGGCTTTGGATGATGCTAA
RI-gBol003215-XLOC_048312-14694-1
CAGAAGCTTGATGAGAGGGCATTGCATACACAGGCTTTGGATGATGCTAA
CONSENSUS
CAGAAGCTTGATGAGAGGGCATTGCATACACAGGCTTTGGATGATGCTAA

RI-gBol003215-XLOC_048312-14694-0
TATGATGTTAGAGGGCGAGCTTCTCTCCGACTCGGAGCTTCTGCTAGAGG
RI-gBol003215-XLOC_048312-14694-1
TATGATGTTAGAGGGCGAGCTTCTCTCCGACTCGGAGCTTCTGCTAGAGG
CONSENSUS
TATGATGTTAGAGGGCGAGCTTCTCTCCGACTCGGAGCTTCTGCTAGAGG

RI-gBol003215-XLOC_048312-14694-0
AGGGAGAGGAGTTAGAGGATTGGGAGCATGGGGAGCTCACTGATGTTATG
RI-gBol003215-XLOC_048312-14694-1
AGGGAGAGGAGTTAGAGGATTGGGAGCATGGGGAGCTCACTGATGTTATG
CONSENSUS
AGGGAGAGGAGTTAGAGGATTGGGAGCATGGGGAGCTCACTGATGTTATG

RI-gBol003215-XLOC_048312-14694-0
GAAGAGGAAGATCAGGTTGTTGAAGAATTAGAGGGGGGAGATCATGTTGG
RI-gBol003215-XLOC_048312-14694-1
GAAGAGGAAGATCAGGTTGTTGAAGAATTAGAGGGGGGAGATCATGTTGG
CONSENSUS
GAAGAGGAAGATCAGGTTGTTGAAGAATTAGAGGGGGGAGATCATGTTGG

RI-gBol003215-XLOC_048312-14694-0
TATGGAGGCTAATGCTGGCGAGACGGATGTGGTTGATGAAGGAAAGGCTC
RI-gBol003215-XLOC_048312-14694-1
TATGGAGGCTAATGCTGGCGAGACGGATGTGGTTGATGAAGGAAAGGCTC
CONSENSUS
TATGGAGGCTAATGCTGGCGAGACGGATGTGGTTGATGAAGGAAAGGCTC

RI-gBol003215-XLOC_048312-14694-0
CATTGAAGAAAGGTGCTAAGGTGGGGATTGGGGGAACCTCCAAGAAGCGA
RI-gBol003215-XLOC_048312-14694-1
CATTGAAGAAAGGTGCTAAGGTGGGGATTGGGGGAACCTCCAAGAAGCGA
CONSENSUS
CATTGAAGAAAGGTGCTAAGGTGGGGATTGGGGGAACCTCCAAGAAGCGA

RI-gBol003215-XLOC_048312-14694-0
TTAGGACCAGGGTTTGTCTCTCCACGTAAGAAGCTCCTGGCTAAGGTGGC
RI-gBol003215-XLOC_048312-14694-1
TTAGGACCAGGGTTTGTCTCTCCACGTAAGAAGCTCCTGGCTAAGGTGGC
CONSENSUS
TTAGGACCAGGGTTTGTCTCTCCACGTAAGAAGCTCCTGGCTAAGGTGGC

RI-gBol003215-XLOC_048312-14694-0
AGCTAAGGCTGGAGATAAAGGCGCGAAGAGGGCCGCTAGTAAGTCATAGA
RI-gBol003215-XLOC_048312-14694-1
AGCTAAGGCTGGAGATAAAGGCGCGAAGAGGGCCGCTAGTAAGTCATAGA
CONSENSUS
AGCTAAGGCTGGAGATAAAGGCGCGAAGAGGGCCGCTAGTAAGTCATAGA

RI-gBol003215-XLOC_048312-14694-0
GCTTAACGGAGTAAAGGGGTTTGCAGGCCACGTTTTGTTTTTTTGGTTTT
RI-gBol003215-XLOC_048312-14694-1
GCTTAACGGAGTAAAGGGGTTTGCAGGCCACGTTTTGTTTTTTTGGTTTT
CONSENSUS
GCTTAACGGAGTAAAGGGGTTTGCAGGCCACGTTTTGTTTTTTTGGTTTT

RI-gBol003215-XLOC_048312-14694-0
GAATAATGTCGGATTCTGATATGAAATCCTTTGAAGTTGGGTGTTGGTTT
RI-gBol003215-XLOC_048312-14694-1
GAATAATGTCGGATTCTGATATGAAATCCTTTGAAGTTGGGTGTTGGTTT
CONSENSUS
GAATAATGTCGGATTCTGATATGAAATCCTTTGAAGTTGGGTGTTGGTTT

RI-gBol003215-XLOC_048312-14694-0
AGTACTTTGTCTTGCTTCTTGGCTAGTCCTCTGGGTGTTACGAGGGTTTT
RI-gBol003215-XLOC_048312-14694-1
AGTACTTTGTCTTGCTTCTTGGCTAGTCCTCTGGGTGTTACGAGGGTTTT
CONSENSUS
AGTACTTTGTCTTGCTTCTTGGCTAGTCCTCTGGGTGTTACGAGGGTTTT

RI-gBol003215-XLOC_048312-14694-0
GTTTTCCTGTGTTGATTTTGTACCGCTCTTGGTTTGGGTGG
RI-gBol003215-XLOC_048312-14694-1
GTTTTCCTGTGTTGATTTTGTACCGCTCTTGGTTTGGGTGG
CONSENSUS
GTTTTCCTGTGTTGATTTTGTACCGCTCTTGGTTTGGGTGG

alignment for event: RI-gBol003215-XLOC_048312-14693

RI-gBol003215-XLOC_048312-14693-0
ATGACGACATGCGACGGCAATGGCGATTTTGATTTTGCGATTAAGGCTCG
RI-gBol003215-XLOC_048312-14693-1
ATGACGACATGCGACGGCAATGGCGATTTTGATTTTGCGATTAAGGCTCG
CONSENSUS
ATGACGACATGCGACGGCAATGGCGATTTTGATTTTGCGATTAAGGCTCG

RI-gBol003215-XLOC_048312-14693-0
AGGGGTTGTTGAGGTTTTTAGGGTCAGAATGGATCGGAATGAGGAGATCT
RI-gBol003215-XLOC_048312-14693-1
AGGGGTTGTTGAGGTTTTTAGGGTCAGAATGGATCGGAATGAGGAGATCT
CONSENSUS
AGGGGTTGTTGAGGTTTTTAGGGTCAGAATGGATCGGAATGAGGAGATCT

RI-gBol003215-XLOC_048312-14693-0
CTCCTCCTATTGGTATTATTATGGGGTATCGAGGTTTTTTCGCGAATCAA
RI-gBol003215-XLOC_048312-14693-1
CTCCTCCTATTGGTATTATTATGGGGTATCGAGGTTTTTTCGCGAATCAA
CONSENSUS
CTCCTCCTATTGGTATTATTATGGGGTATCGAGGTTTTTTCGCGAATCAA

RI-gBol003215-XLOC_048312-14693-0
GGGATTGATGCGATCTACGTCTACGTAACGGAAATAGATGGTCGCATAGG
RI-gBol003215-XLOC_048312-14693-1
GGGATTGATGCGATCTACGTCTACGTAACGGAAATAGATGGTCGCATAGG
CONSENSUS
GGGATTGATGCGATCTACGTCTACGTAACGGAAATAGATGGTCGCATAGG

RI-gBol003215-XLOC_048312-14693-0
GGTAAAATCGGAGAGGATCTCTGGATCTGGGTTGTTTTAGGAAGAAAGGA
RI-gBol003215-XLOC_048312-14693-1
GGTAAAATCGGAGAGGATCTCTGGATCTGGGTTGTTTTAGGAAGAAAGGA
CONSENSUS
GGTAAAATCGGAGAGGATCTCTGGATCTGGGTTGTTTTAGGAAGAAAGGA

RI-gBol003215-XLOC_048312-14693-0
GGAGGTTTTTTCATTTTTCTACATGATTGACGCAGGATTTGGGATCCCTC
RI-gBol003215-XLOC_048312-14693-1

GGAGGTTTTTTCATTTTTCTACATGATTGACGCAGGATTTGGGATCCCTC
CONSENSUS
GGAGGTTTTTTCATTTTTCTACATGATTGACGCAGGATTTGGGATCCCTC

RI-gBol003215-XLOC_048312-14693-0
TGATCTTGATGTCTCAGGTCAAAGAGAAAATACGGTATTTTTTCTTGATT
RI-gBol003215-XLOC_048312-14693-1
TGATCTTGATGTCTCAGGTCAAAGAGAAAATACG----------------
CONSENSUS
TGATCTTGATGTCTCAGGTCAAAGAGAAAATACG................

RI-gBol003215-XLOC_048312-14693-0
GCTTTGACTCTCTGTTATGAGATGATGAAATTGATCGATAGAGATTTACT
RI-gBol003215-XLOC_048312-14693-1

CONSENSUS
....

RI-gBol003215-XLOC_048312-14693-0
TTCTTCTTTTAAGACTTGTTTGGTTTGTGGGATGTTCTTTGATTTGGTAA
RI-gBol003215-XLOC_048312-14693-1

CONSENSUS
....

RI-gBol003215-XLOC_048312-14693-0
TTGGGGCTGGGATTTGGATCTCTCAATGGGGCTATGACGGATATCTCGTT
RI-gBol003215-XLOC_048312-14693-1

CONSENSUS
....

RI-gBol003215-XLOC_048312-14693-0
TCTATGTTTGGATTGCTTTCGTGTATCTGTGGTACTTTCTATAATTCTAA
RI-gBol003215-XLOC_048312-14693-1

CONSENSUS
....

RI-gBol003215-XLOC_048312-14693-0
AGAGGTGATTGCGGGTGTGATCTGAGATTGGTATCTTATATGTTATTTGG
RI-gBol003215-XLOC_048312-14693-1

CONSENSUS
....

RI-gBol003215-XLOC_048312-14693-0
TCTTTAACTCTTGTTTGCGGGAGGTTTCACTATATATTGTGGTTCTTGTT
RI-gBol003215-XLOC_048312-14693-1

CONSENSUS
....

RI-gBol003215-XLOC_048312-14693-0
TCAGTCAAGTTTCATACCTTTCTTCGATTCCAGTCTTCGCAAGTTATTTA
RI-gBol003215-XLOC_048312-14693-1

CONSENSUS
....

RI-gBol003215-XLOC_048312-14693-0
CAACAGTTTGTTTCTCTTGTTTCCTTTTCGTGTTAACATGTCTCAGCAAA
RI-gBol003215-XLOC_048312-14693-1
--CAAA
CONSENSUS
....CAAA

RI-gBol003215-XLOC_048312-14693-0
GTCATTGGATCACTAAATCAGGAGAGAAGAAGGCGGAGGTTGTTAGGTCG
RI-gBol003215-XLOC_048312-14693-1
GTCATTGGATCACTAAATCAGGAGAGAAGAAGGCGGAGGTTGTTAGGTCG
CONSENSUS
GTCATTGGATCACTAAATCAGGAGAGAAGAAGGCGGAGGTTGTTAGGTCG

RI-gBol003215-XLOC_048312-14693-0
GGGCTAAGGATCTCGATCCCCCGGTTTGACAACTCGGAGATCATTGCGCG
RI-gBol003215-XLOC_048312-14693-1
GGGCTAAGGATCTCGATCCCCCGGTTTGACAACTCGGAGATCATTGCGCG
CONSENSUS
GGGCTAAGGATCTCGATCCCCCGGTTTGACAACTCGGAGATCATTGCGCG

RI-gBol003215-XLOC_048312-14693-0
ATATGCACGAACCCTCATCGGAAGGTGTATGAACCCTCCAAAACAAGACA
RI-gBol003215-XLOC_048312-14693-1
ATATGCACGAACCCTCATCGGAAGGTGTATGAACCCTCCAAAACAAGACA
CONSENSUS
ATATGCACGAACCCTCATCGGAAGGTGTATGAACCCTCCAAAACAAGACA

RI-gBol003215-XLOC_048312-14693-0
TGAAAGTTCTCTTGTTTGTGTTTCCGAGGATTTGGAACATGGAGGGCCGT
RI-gBol003215-XLOC_048312-14693-1
TGAAAGTTCTCTTGTTTGTGTTTCCGAGGATTTGGAACATGGAGGGCCGT
CONSENSUS
TGAAAGTTCTCTTGTTTGTGTTTCCGAGGATTTGGAACATGGAGGGCCGT

RI-gBol003215-XLOC_048312-14693-0
GTGGTGGGTACGGATCTTGGTCTGGGGAGGTTCCAGCTCGAGGAGGATAT
RI-gBol003215-XLOC_048312-14693-1
GTGGTGGGTACGGATCTTGGTCTGGGGAGGTTCCAGCTCGAGGAGGATAT
CONSENSUS
GTGGTGGGTACGGATCTTGGTCTGGGGAGGTTCCAGCTCGAGGAGGATAT

RI-gBol003215-XLOC_048312-14693-0
CACGGAGGTCCTTAAGATGGTGCCTTATCACTTTGATTTCTGGATGGTGT
RI-gBol003215-XLOC_048312-14693-1
CACGGAGGTCCTTAAGATGGTGCCTTATCACTTTGATTTCTGGATGGTGT
CONSENSUS
CACGGAGGTCCTTAAGATGGTGCCTTATCACTTTGATTTCTGGATGGTGT

RI-gBol003215-XLOC_048312-14693-0
CTTTAGTGAGGTGGAAGCCGGTTTTAGAACCAAATTATCCCACTAAGATC
RI-gBol003215-XLOC_048312-14693-1

CTTTAGTGAGGTGGAAGCCGGTTTTAGAACCAAATTATCCCACTAAGATC
CONSENSUS
CTTTAGTGAGGTGGAAGCCGGTTTTAGAACCAAATTATCCCACTAAGATC

RI-gBol003215-XLOC_048312-14693-0
ACGTTTTGGGTGTGAGTCTTAGATATCCCTCTTCAGTTCAGGGCAGCTCA
RI-gBol003215-XLOC_048312-14693-1
ACGTTTTGGGTGTGAGTCTTAGATATCCCTCTTCAGTTCAGGGCAGCTCA
CONSENSUS
ACGTTTTGGGTGTGAGTCTTAGATATCCCTCTTCAGTTCAGGGCAGCTCA

RI-gBol003215-XLOC_048312-14693-0
GATCTTCCAGAGCGTGGGGGAAGCAATTGGCCAGGTCCAGGGCCAGGTTG
RI-gBol003215-XLOC_048312-14693-1
GATCTTCCAGAGCGTGGGGGAAGCAATTGGCCAGGTCCAGGGCCAGGTTG
CONSENSUS
GATCTTCCAGAGCGTGGGGGAAGCAATTGGCCAGGTCCAGGGCCAGGTTG

RI-gBol003215-XLOC_048312-14693-0
ATATCGTGGAGGGACGGGTTCGGGTGGAGATAGATGGATTTAAACCTTTG
RI-gBol003215-XLOC_048312-14693-1
ATATCGTGGAGGGACGGGTTCGGGTGGAGATAGATGGATTTAAACCTTTG
CONSENSUS
ATATCGTGGAGGGACGGGTTCGGGTGGAGATAGATGGATTTAAACCTTTG

RI-gBol003215-XLOC_048312-14693-0
GTGTTCTCAATGGACATTGAGTTTGAGGAAGGTGTGGAAATCAAGGTGGC
RI-gBol003215-XLOC_048312-14693-1
GTGTTCTCAATGGACATTGAGTTTGAGGAAGGTGTGGAAATCAAGGTGGC
CONSENSUS
GTGTTCTCAATGGACATTGAGTTTGAGGAAGGTGTGGAAATCAAGGTGGC

RI-gBol003215-XLOC_048312-14693-0
TCTCAGATATGAAAAGCTTTAAGGGTTTTGCACTGAATGTTTCTGCTTGA
RI-gBol003215-XLOC_048312-14693-1
TCTCAGATATGAAAAGCTTTAAGGGTTTTGCACTGAATGTTTCTGCTTGA
CONSENSUS
TCTCAGATATGAAAAGCTTTAAGGGTTTTGCACTGAATGTTTCTGCTTGA

RI-gBol003215-XLOC_048312-14693-0
CTCATGAATGCTCTCGTTGCCCGAGGTTACACAAGGAGGAGCTTTTGGGT
RI-gBol003215-XLOC_048312-14693-1
CTCATGAATGCTCTCGTTGCCCGAGGTTACACAAGGAGGAGCTTTTGGGT
CONSENSUS
CTCATGAATGCTCTCGTTGCCCGAGGTTACACAAGGAGGAGCTTTTGGGT

RI-gBol003215-XLOC_048312-14693-0
GTAGCTGGGGGAGGACCGGGTAGTGATGGGGCTCAGGCAACAAGCTATAA
RI-gBol003215-XLOC_048312-14693-1
GTAGCTGGGGGAGGACCGGGTAGTGATGGGGCTCAGGCAACAAGCTATAA
CONSENSUS
GTAGCTGGGGGAGGACCGGGTAGTGATGGGGCTCAGGCAACAAGCTATAA

RI-gBol003215-XLOC_048312-14693-0
GACGGTGGTGGCCCACGGTAGCAGTCAGAGCGGTGAACGTAGAGCGTATC
RI-gBol003215-XLOC_048312-14693-1

GACGGTGGTGGCCCACGGTAGCAGTCAGAGCGGTGAACGTAGAGCGTATC
CONSENSUS
GACGGTGGTGGCCCACGGTAGCAGTCAGAGCGGTGAACGTAGAGCGTATC

RI-gBol003215-XLOC_048312-14693-0
AGCAAACTCGGTCGCAGAACAACAGAGTGGGTGATAAGGGCAAAGGCATT
RI-gBol003215-XLOC_048312-14693-1
AGCAAACTCGGTCGCAGAACAACAGAGTGGGTGATAAGGGCAAAGGCATT
CONSENSUS
AGCAAACTCGGTCGCAGAACAACAGAGTGGGTGATAAGGGCAAAGGCATT

RI-gBol003215-XLOC_048312-14693-0
GCGTATGAGAAGCAAGGTAGTTCCAAGCAGGACGGGTCTTTTCATCCTTA
RI-gBol003215-XLOC_048312-14693-1
GCGTATGAGAAGCAAGGTAGTTCCAAGCAGGACGGGTCTTTTCATCCTTA
CONSENSUS
GCGTATGAGAAGCAAGGTAGTTCCAAGCAGGACGGGTCTTTTCATCCTTA

RI-gBol003215-XLOC_048312-14693-0
CAAAGGCAAGCACACTAGAGGCTATGGTGATGGTTCCTCAATGAATGGCA
RI-gBol003215-XLOC_048312-14693-1
CAAAGGCAAGCACACTAGAGGCTATGGTGATGGTTCCTCAATGAATGGCA
CONSENSUS
CAAAGGCAAGCACACTAGAGGCTATGGTGATGGTTCCTCAATGAATGGCA

RI-gBol003215-XLOC_048312-14693-0
GGGACTCGGGATATGGAGAAAGGAGACGTGGTATGCAGTCTCGTGGTACT
RI-gBol003215-XLOC_048312-14693-1
GGGACTCGGGATATGGAGAAAGGAGACGTGGTATGCAGTCTCGTGGTACT
CONSENSUS
GGGACTCGGGATATGGAGAAAGGAGACGTGGTATGCAGTCTCGTGGTACT

RI-gBol003215-XLOC_048312-14693-0
AAGCCTAGGGGAGTGGTACCTCGGGGTCAGCATCTTGCTACTGTTGCTGA
RI-gBol003215-XLOC_048312-14693-1
AAGCCTAGGGGAGTGGTACCTCGGGGTCAGCATCTTGCTACTGTTGCTGA
CONSENSUS
AAGCCTAGGGGAGTGGTACCTCGGGGTCAGCATCTTGCTACTGTTGCTGA

RI-gBol003215-XLOC_048312-14693-0
AGGGGAACAACAGCTACCAGATCCCTCCAAGCTTATGCTCGATGCCTTTA
RI-gBol003215-XLOC_048312-14693-1
AGGGGAACAACAGCTACCAGATCCCTCCAAGCTTATGCTCGATGCCTTTA
CONSENSUS
AGGGGAACAACAGCTACCAGATCCCTCCAAGCTTATGCTCGATGCCTTTA

RI-gBol003215-XLOC_048312-14693-0
AAGGGGCTGGAAAGTCTTCAGAAGAGGAAGCTCCAATAGGGGTGGAGGCG
RI-gBol003215-XLOC_048312-14693-1
AAGGGGCTGGAAAGTCTTCAGAAGAGGAAGCTCCAATAGGGGTGGAGGCG
CONSENSUS
AAGGGGCTGGAAAGTCTTCAGAAGAGGAAGCTCCAATAGGGGTGGAGGCG

RI-gBol003215-XLOC_048312-14693-0
GAAGGAAGTGGTGGACATAACAAAGCACGTAAGGCGCTTATGTTTGAAGA
RI-gBol003215-XLOC_048312-14693-1

GAAGGAAGTGGTGGACATAACAAAGCACGTAAGGCGCTTATGTTTGAAGA
CONSENSUS
GAAGGAAGTGGTGGACATAACAAAGCACGTAAGGCGCTTATGTTTGAAGA

RI-gBol003215-XLOC_048312-14693-0
ATCTAGTTCTGTTGGGCATGATCTTGTGTCGACAGGAGAGGGAGTGGAGC
RI-gBol003215-XLOC_048312-14693-1
ATCTAGTTCTGTTGGGCATGATCTTGTGTCGACAGGAGAGGGAGTGGAGC
CONSENSUS
ATCTAGTTCTGTTGGGCATGATCTTGTGTCGACAGGAGAGGGAGTGGAGC

RI-gBol003215-XLOC_048312-14693-0
TTGCTGGATCTCTGGCTTTGAAGATGCAGAGCGAGGGTGATGATGCCGCG
RI-gBol003215-XLOC_048312-14693-1
TTGCTGGATCTCTGGCTTTGAAGATGCAGAGCGAGGGTGATGATGCCGCG
CONSENSUS
TTGCTGGATCTCTGGCTTTGAAGATGCAGAGCGAGGGTGATGATGCCGCG

RI-gBol003215-XLOC_048312-14693-0
CAGAAGCTTGATGAGAGGGCATTGCATACACAGGCTTTGGATGATGCTAA
RI-gBol003215-XLOC_048312-14693-1
CAGAAGCTTGATGAGAGGGCATTGCATACACAGGCTTTGGATGATGCTAA
CONSENSUS
CAGAAGCTTGATGAGAGGGCATTGCATACACAGGCTTTGGATGATGCTAA

RI-gBol003215-XLOC_048312-14693-0
TATGATGTTAGAGGGCGAGCTTCTCTCCGACTCGGAGCTTCTGCTAGAGG
RI-gBol003215-XLOC_048312-14693-1
TATGATGTTAGAGGGCGAGCTTCTCTCCGACTCGGAGCTTCTGCTAGAGG
CONSENSUS
TATGATGTTAGAGGGCGAGCTTCTCTCCGACTCGGAGCTTCTGCTAGAGG

RI-gBol003215-XLOC_048312-14693-0
AGGGAGAGGAGTTAGAGGATTGGGAGCATGGGGAGCTCACTGATGTTATG
RI-gBol003215-XLOC_048312-14693-1
AGGGAGAGGAGTTAGAGGATTGGGAGCATGGGGAGCTCACTGATGTTATG
CONSENSUS
AGGGAGAGGAGTTAGAGGATTGGGAGCATGGGGAGCTCACTGATGTTATG

RI-gBol003215-XLOC_048312-14693-0
GAAGAGGAAGATCAGGTTGTTGAAGAATTAGAGGGGGGAGATCATGTTGG
RI-gBol003215-XLOC_048312-14693-1
GAAGAGGAAGATCAGGTTGTTGAAGAATTAGAGGGGGGAGATCATGTTGG
CONSENSUS
GAAGAGGAAGATCAGGTTGTTGAAGAATTAGAGGGGGGAGATCATGTTGG

RI-gBol003215-XLOC_048312-14693-0
TATGGAGGCTAATGCTGGCGAGACGGATGTGGTTGATGAAGGAAAGGCTC
RI-gBol003215-XLOC_048312-14693-1
TATGGAGGCTAATGCTGGCGAGACGGATGTGGTTGATGAAGGAAAGGCTC
CONSENSUS
TATGGAGGCTAATGCTGGCGAGACGGATGTGGTTGATGAAGGAAAGGCTC

RI-gBol003215-XLOC_048312-14693-0
CATTGAAGAAAGGTGCTAAGGTGGGGATTGGGGGAACCTCCAAGAAGCGA
RI-gBol003215-XLOC_048312-14693-1

CATTGAAGAAAGGTGCTAAGGTGGGGATTGGGGGAACCTCCAAGAAGCGA
CONSENSUS
CATTGAAGAAAGGTGCTAAGGTGGGGATTGGGGGAACCTCCAAGAAGCGA

RI-gBol003215-XLOC_048312-14693-0
TTAGGACCAGGGTTTGTCTCTCCACGTAAGAAGCTCCTGGCTAAGGTGGC
RI-gBol003215-XLOC_048312-14693-1
TTAGGACCAGGGTTTGTCTCTCCACGTAAGAAGCTCCTGGCTAAGGTGGC
CONSENSUS
TTAGGACCAGGGTTTGTCTCTCCACGTAAGAAGCTCCTGGCTAAGGTGGC

RI-gBol003215-XLOC_048312-14693-0
AGCTAAGGCTGGAGATAAAGGCGCGAAGAGGGCCGCTAGTAAGTCATAGA
RI-gBol003215-XLOC_048312-14693-1
AGCTAAGGCTGGAGATAAAGGCGCGAAGAGGGCCGCTAGTAAGTCATAGA
CONSENSUS
AGCTAAGGCTGGAGATAAAGGCGCGAAGAGGGCCGCTAGTAAGTCATAGA

RI-gBol003215-XLOC_048312-14693-0
GCTTAACGGAGTAAAGGGGTTTGCAGGCCACGTTTTGTTTTTTTGGTTTT
RI-gBol003215-XLOC_048312-14693-1
GCTTAACGGAGTAAAGGGGTTTGCAGGCCACGTTTTGTTTTTTTGGTTTT
CONSENSUS
GCTTAACGGAGTAAAGGGGTTTGCAGGCCACGTTTTGTTTTTTTGGTTTT

RI-gBol003215-XLOC_048312-14693-0
GAATAATGTCGGATTCTGATATGAAATCCTTTGAAGTTGGGTGTTGGTTT
RI-gBol003215-XLOC_048312-14693-1
GAATAATGTCGGATTCTGATATGAAATCCTTTGAAGTTGGGTGTTGGTTT
CONSENSUS
GAATAATGTCGGATTCTGATATGAAATCCTTTGAAGTTGGGTGTTGGTTT

RI-gBol003215-XLOC_048312-14693-0
AGTACTTTGTCTTGCTTCTTGGCTAGTCCTCTGGGTGTTACGAGGGTTTT
RI-gBol003215-XLOC_048312-14693-1
AGTACTTTGTCTTGCTTCTTGGCTAGTCCTCTGGGTGTTACGAGGGTTTT
CONSENSUS
AGTACTTTGTCTTGCTTCTTGGCTAGTCCTCTGGGTGTTACGAGGGTTTT

RI-gBol003215-XLOC_048312-14693-0
GTTTTCCTGTGTTGATTTTGTACCGCTCTTGGTTTGGGTGG
RI-gBol003215-XLOC_048312-14693-1
GTTTTCCTGTGTTGATTTTGTACCGCTCTTGGTTTGGGTGG
CONSENSUS
GTTTTCCTGTGTTGATTTTGTACCGCTCTTGGTTTGGGTGG

alignment for event: A5-gBol024048-XLOC_024719-7185

A5-gBol024048-XLOC_024719-7185-0
AATGGGCGGGTTCCACGAGGTAGAGCTCAAAGTTCGTGATTATGAATTGG
A5-gBol024048-XLOC_024719-7185-1
AATGGGCGGGTTCCACGAGGTAGAGCTCAAAGTTCGTGATTATGAATTGG
CONSENSUS
AATGGGCGGGTTCCACGAGGTAGAGCTCAAAGTTCGTGATTATGAATTGG

A5-gBol024048-XLOC_024719-7185-0
ATCAGTTTGGCGTTGTCAACAATGCTGTCTACGCTAACTACTGCCAACAC
A5-gBol024048-XLOC_024719-7185-1
ATCAGTTTGGCGTTGTCAACAATGCTGTCTACGCTAACTACTGCCAACAC
CONSENSUS
ATCAGTTTGGCGTTGTCAACAATGCTGTCTACGCTAACTACTGCCAACAC

A5-gBol024048-XLOC_024719-7185-0
G---
A5-gBol024048-XLOC_024719-7185-1
GGTAACAATATTGCTTGCTTGCTTGCTTCTTTCGAACATTTCTTATATCA
CONSENSUS
G...

A5-gBol024048-XLOC_024719-7185-0

A5-gBol024048-XLOC_024719-7185-1
ACAAGTGTATTGAACCGAACCTCCAGCTCTTGTGGTTAAGGGCTCACAGC
CONSENSUS
....

A5-gBol024048-XLOC_024719-7185-0

A5-gBol024048-XLOC_024719-7185-1
TGTGAGTACCGCCACTTGCGTTCGAGTCCAGCCACCCGGGACGCCTTAAA
CONSENSUS
....

A5-gBol024048-XLOC_024719-7185-0 --
GCCGACACGAGTTTCTGGAGAGTATCGGTATCAACTGTGATGAAGTCG
A5-gBol024048-XLOC_024719-7185-1
TGGCCGACACGAGTTTCTGGAGAGTATCGGTATCAACTGTGATGAAGTCG
CONSENSUS
..GCCGACACGAGTTTCTGGAGAGTATCGGTATCAACTGTGATGAAGTCG

A5-gBol024048-XLOC_024719-7185-0
CACGTTCTGGCCAATCCTTAGCTATTTCTGAGTTGACTATAAAGTTTCTT
A5-gBol024048-XLOC_024719-7185-1
CACGTTCTGGCCAATCCTTAGCTATTTCTGAGTTGACTATAAAGTTTCTT
CONSENSUS
CACGTTCTGGCCAATCCTTAGCTATTTCTGAGTTGACTATAAAGTTTCTT

A5-gBol024048-XLOC_024719-7185-0 GCACCTTTACGC
A5-gBol024048-XLOC_024719-7185-1 GCACCTTTACGC
CONSENSUS GCACCTTTACGC

alignment for event: RI-X-XLOC_027084-13406

RI-X-XLOC_027084-13406-0
GTTGAGGATGCAGAGGTAAGACAAAAGATTAAGACAATGGAAACACTTGA
RI-X-XLOC_027084-13406-1
GTTGAGGATGCAGAGGTAAGACAAAAGATTAAGACAATGGAAACACTTGA
CONSENSUS

GTTGAGGATGCAGAGGTAAGACAAAAGATTAAGACAATGGAAACACTTGA

RI-X-XLOC_027084-13406-0
AAAGAAACTGGAATTCCTCCACCGTCAGAGAGCCGTTGTGTCTGAACAAG
RI-X-XLOC_027084-13406-1
AAAGAAACTGGAATTCCTCCACCGTCAGAGAGCCGTTGTGTCTGAACAAG
CONSENSUS
AAAGAAACTGGAATTCCTCCACCGTCAGAGAGCCGTTGTGTCTGAACAAG

RI-X-XLOC_027084-13406-0
CCACCGCCAATATGAATGGCAAAAGACAAAACCCTGGTAGCATCTGAGGA
RI-X-XLOC_027084-13406-1
CCACCGCCAATATGAATGGCAAAAGACAAAACCCTGGTAGCATCTGAGGA
CONSENSUS
CCACCGCCAATATGAATGGCAAAAGACAAAACCCTGGTAGCATCTGAGGA

RI-X-XLOC_027084-13406-0
TGGCTTGCTGGAACTCTTCCGCCAAAAGCATGAATCCCCCATGAAGTTAC
RI-X-XLOC_027084-13406-1
TGGCTTGCTGGAACTCTTCCGCCAAAAGCATGAATCCCCCATGAA-----
CONSENSUS
TGGCTTGCTGGAACTCTTCCGCCAAAAGCATGAATCCCCCATGAA.....

RI-X-XLOC_027084-13406-0
GTTTTTTTTTCGGTATTTCTCTTTTTCATTACCAGTCTCATCTTTTATAT
RI-X-XLOC_027084-13406-1

CONSENSUS
....

RI-X-XLOC_027084-13406-0
AATGGATTCTATAAGTATTCATTTATGAAACCTCACTGTTGGTCTTTAGT
RI-X-XLOC_027084-13406-1

CONSENSUS
....

RI-X-XLOC_027084-13406-0
ATAGTTTATAAATCCACAGGAGCCAATTTATAGGTAATGTACTGGATATG
RI-X-XLOC_027084-13406-1

CONSENSUS
....

RI-X-XLOC_027084-13406-0
GTCTTTGTAAACAGGTGAGTGGAAGTGGACAAAGCTACTCAAGAGAACCG
RI-X-XLOC_027084-13406-1 --------------
GTGAGTGGAAGTGGACAAAGCTACTCAAGAGAACCG
CONSENSUS
..............GTGAGTGGAAGTGGACAAAGCTACTCAAGAGAACCG

RI-X-XLOC_027084-13406-0
AAGAGATCAACAAGGCCTTTGCCAATGAAGTCTCATGGGACCGAGCTTCC
RI-X-XLOC_027084-13406-1
AAGAGATCAACAAGGCCTTTGCCAATGAAGTCTCATGGGACCGAGCTTCC
CONSENSUS

AAGAGATCAACAAGGCCTTTGCCAATGAAGTCTCATGGGACCGAGCTTCC

RI-X-XLOC_027084-13406-0
ACGTCTTCTACTCAAGCACCAACACATAGACCAAGTGAAAGAACATATGG
RI-X-XLOC_027084-13406-1
ACGTCTTCTACTCAAGCACCAACACATAGACCAAGTGAAAGAACATATGG
CONSENSUS
ACGTCTTCTACTCAAGCACCAACACATAGACCAAGTGAAAGAACATATGG

RI-X-XLOC_027084-13406-0
TGCGATCTAAACCGTTAGTTGTCTTTGCTTAAGGTTCTGTCTCAGTCAAT
RI-X-XLOC_027084-13406-1
TGCGATCTAAACCGTTAGTTGTCTTTGCTTAAGGTTCTGTCTCAGTCAAT
CONSENSUS
TGCGATCTAAACCGTTAGTTGTCTTTGCTTAAGGTTCTGTCTCAGTCAAT

RI-X-XLOC_027084-13406-0
GTTATGGATTGCAGTTGAACTGTGGTATTCAGGTTTTTTTTTTTTTTACA
RI-X-XLOC_027084-13406-1
GTTATGGATTGCAGTTGAACTGTGGTATTCAGGTTTTTTTTTTTTTTACA
CONSENSUS
GTTATGGATTGCAGTTGAACTGTGGTATTCAGGTTTTTTTTTTTTTTACA

RI-X-XLOC_027084-13406-0
TTCATGTTTAGTTTCTAGAATGAGGAAAAACAAAATTGTGAAATTAAGAG
RI-X-XLOC_027084-13406-1
TTCATGTTTAGTTTCTAGAATGAGGAAAAACAAAATTGTGAAATTAAGAG
CONSENSUS
TTCATGTTTAGTTTCTAGAATGAGGAAAAACAAAATTGTGAAATTAAGAG

RI-X-XLOC_027084-13406-0
TCAAGAAAGTAGAAAGCTTCACCTTGAGATTGCTTTTACCTGCAGATTCA
RI-X-XLOC_027084-13406-1
TCAAGAAAGTAGAAAGCTTCACCTTGAGATTGCTTTTACCTGCAGATTCA
CONSENSUS
TCAAGAAAGTAGAAAGCTTCACCTTGAGATTGCTTTTACCTGCAGATTCA

RI-X-XLOC_027084-13406-0
TGTGTTATGGTAGATAACGACAAACAAAGTTTACTTTCTTTGCTTCAGTT
RI-X-XLOC_027084-13406-1
TGTGTTATGGTAGATAACGACAAACAAAGTTTACTTTCTTTGCTTCAGTT
CONSENSUS
TGTGTTATGGTAGATAACGACAAACAAAGTTTACTTTCTTTGCTTCAGTT

RI-X-XLOC_027084-13406-0
ACAAAAGAACAAGAGCGTGTTCACAAGTCGATAAAGATTGACACTTGAAA
RI-X-XLOC_027084-13406-1
ACAAAAGAACAAGAGCGTGTTCACAAGTCGATAAAGATTGACACTTGAAA
CONSENSUS
ACAAAAGAACAAGAGCGTGTTCACAAGTCGATAAAGATTGACACTTGAAA

RI-X-XLOC_027084-13406-0
GAAATTTTATTTTTTTTCAAAGAGTAGTTAGGAATCTTTCTACTCTCTTC
RI-X-XLOC_027084-13406-1
GAAATTTTATTTTTTTTCAAAGAGTAGTTAGGAATCTTTCTACTCTCTTC
CONSENSUS

GAAATTTTATTTTTTTTCAAAGAGTAGTTAGGAATCTTTCTACTCTCTTC

RI-X-XLOC_027084-13406-0   TTTTTC
RI-X-XLOC_027084-13406-1   TTTTTC
CONSENSUS   TTTTTC

alignment for event: A5-gBol006989-XLOC_043957-16288

A5-gBol006989-XLOC_043957-16288-0
GCACTTGGTCTGCTTGCTTCTGCTGACAAGAAAATGGTGTCATTCGTTAA
A5-gBol006989-XLOC_043957-16288-1
GCACTTGGTCTGCTTGCTTCTGCTGACAAGAAAATGGTGTCATTCGTTAA
CONSENSUS
GCACTTGGTCTGCTTGCTTCTGCTGACAAGAAAATGGTGTCATTCGTTAA

A5-gBol006989-XLOC_043957-16288-0
AGCATTACTTGGAGCACGCGACATTCTACTCGAGGAAATGACAAGACTTG
A5-gBol006989-XLOC_043957-16288-1
AGCATTACTTGGAGCACGCGACATTCTACTCGAGGAAATGACAAGACTTG
CONSENSUS
AGCATTACTTGGAGCACGCGACATTCTACTCGAGGAAATGACAAGACTTG

A5-gBol006989-XLOC_043957-16288-0
GCGAGGCAATTGGAAAATCAATTGATTTGTCTGACTTTGTATCAGATATG
A5-gBol006989-XLOC_043957-16288-1
GCGAGGCAATTGGAAAATCAATTGATTTGTCTGACTTTGTATCAGATATG
CONSENSUS
GCGAGGCAATTGGAAAATCAATTGATTTGTCTGACTTTGTATCAGATATG

A5-gBol006989-XLOC_043957-16288-0
AACAATGTTCCTTTATCTCAATCAGCAGTTCCCGGTTCAGGACAAG----
A5-gBol006989-XLOC_043957-16288-1
AACAATGTTCCTTTATCTCAATCAGCAGTTCCCGGTTCAGGACAAGGCAA
CONSENSUS
AACAATGTTCCTTTATCTCAATCAGCAGTTCCCGGTTCAGGACAAG....

A5-gBol006989-XLOC_043957-16288-0   ----------------------
AAGCTGCAGATTACATTTGATCTAACAA
A5-gBol006989-XLOC_043957-16288-1
GGAACAAAACAGTCCTCTTGAGAAGCTGCAGATTACATTTGATCTAACAA
CONSENSUS
......................AAGCTGCAGATTACATTTGATCTAACAA

A5-gBol006989-XLOC_043957-16288-0
GTGATGACTGGCTTCATGAACTCTCCAAGGATCATCTAACTCGTTTATTC
A5-gBol006989-XLOC_043957-16288-1
GTGATGACTGGCTTCATGAACTCTCCAAGGATCATCTAACTCGTTTATTC
CONSENSUS
GTGATGACTGGCTTCATGAACTCTCCAAGGATCATCTAACTCGTTTATTC

A5-gBol006989-XLOC_043957-16288-0
CACTTACTTGGCACCCAGCTTCATTATCTTTGGAACACGTTTCTTGGATT
A5-gBol006989-XLOC_043957-16288-1
CACTTACTTGGCACCCAGCTTCATTATCTTTGGAACACGTTTCTTGGATT

CONSENSUS
      CACTTACTTGGCACCCAGCTTCATTATCTTTGGAACACGTTTCTTGGATT

A5-gBol006989-XLOC_043957-16288-0   TCACCG
A5-gBol006989-XLOC_043957-16288-1   TCACCG
CONSENSUS   TCACCG

alignment for event: RI-gBol001654-XLOC_050102-9285

RI-gBol001654-XLOC_050102-9285-0
      TTACTGAAGCCAGACCCCATGGTAGTGGCGACAAAATTATTAGCAAGAAG
RI-gBol001654-XLOC_050102-9285-1
      TTACTGAAGCCAGACCCCATGGTAGTGGCGACAAAATTATTAGCAAGAAG
CONSENSUS
      TTACTGAAGCCAGACCCCATGGTAGTGGCGACAAAATTATTAGCAAGAAG

RI-gBol001654-XLOC_050102-9285-0
      AAAGTTGATCGACACGGGAAAACAATTTAATATGATTGCAGCTTCATGGA
RI-gBol001654-XLOC_050102-9285-1
      AAAGTTGATCGACACGGGAAAACAATTTAATATGATTGCAGCTTCATGGA
CONSENSUS
      AAAGTTGATCGACACGGGAAAACAATTTAATATGATTGCAGCTTCATGGA

RI-gBol001654-XLOC_050102-9285-0
      TACAATTCATGATTCATGATTGGGTTGATCATCTTGAAGAAACTAATCAG
RI-gBol001654-XLOC_050102-9285-1
      TACAATTCATGATTCATGATTGGGTTGATCATCTTGAAGAAACTAATCAG
CONSENSUS
      TACAATTCATGATTCATGATTGGGTTGATCATCTTGAAGAAACTAATCAG

RI-gBol001654-XLOC_050102-9285-0
      GTCGTTCATTAATCTTACATGCCCTACATCTACGATAATACTAAATTACT
RI-gBol001654-XLOC_050102-9285-1

CONSENSUS
      ....

RI-gBol001654-XLOC_050102-9285-0
      AATATGCTAAATAAGTAAAAATTGTAGCGGCAAAGAAAGGGTGATTTGTT
RI-gBol001654-XLOC_050102-9285-1

CONSENSUS
      ....

RI-gBol001654-XLOC_050102-9285-0
      TACTATTTATGGTTTCCTATAGTGAGTTTAATGTCCGGAGTTTTTTTTAC
RI-gBol001654-XLOC_050102-9285-1

CONSENSUS
      ....

RI-gBol001654-XLOC_050102-9285-0
      TAACTTTTTATAAAACAAACAATTGAATTAGTTAGTCGTGTTATTAATTC
RI-gBol001654-XLOC_050102-9285-1

CONSENSUS
....

RI-gBol001654-XLOC_050102-9285-0
GTAAACTTAGATCGGGCTCGTGGCTCCAAAAGAAGTAGCGAACGAGTGTC
RI-gBol001654-XLOC_050102-9285-1 ----------
ATCGGGCTCGTGGCTCCAAAAGAAGTAGCGAACGAGTGTC
CONSENSUS
..........ATCGGGCTCGTGGCTCCAAAAGAAGTAGCGAACGAGTGTC

RI-gBol001654-XLOC_050102-9285-0
CCTTAAGCTCCTTCAGATTCTTCAAGACAAAGGAAGTTCCTACCGGTTTC
RI-gBol001654-XLOC_050102-9285-1
CCTTAAGCTCCTTCAGATTCTTCAAGACAAAGGAAGTTCCTACCGGTTTC
CONSENSUS
CCTTAAGCTCCTTCAGATTCTTCAAGACAAAGGAAGTTCCTACCGGTTTC

RI-gBol001654-XLOC_050102-9285-0
TTCGAGATCAAGACCGGTTCGTTAAATTCCCGTACACCTTGGTGGTAAGT
RI-gBol001654-XLOC_050102-9285-1
TTCGAGATCAAGACCGGTTCGTTAAATTCCCGTACACCTTGGTGGTAAGT
CONSENSUS
TTCGAGATCAAGACCGGTTCGTTAAATTCCCGTACACCTTGGTGGTAAGT

RI-gBol001654-XLOC_050102-9285-0
TATAGTATAGCCTTATAGGTTATAAGGTGTACATGTAATTAAGAGATCAA
RI-gBol001654-XLOC_050102-9285-1
TATAGTATAGCCTTATAGGTTATAAGGTGTACATGTAATTAAGAGATCAA
CONSENSUS
TATAGTATAGCCTTATAGGTTATAAGGTGTACATGTAATTAAGAGATCAA

RI-gBol001654-XLOC_050102-9285-0
CAAAAATGTAGTACATTAATACGAGTGAGTATTATGAAATTAGGGATTCG
RI-gBol001654-XLOC_050102-9285-1
CAAAAATGTAGTACATTAATACGAGTGAGTATTATGAAATTAGGGATTCG
CONSENSUS
CAAAAATGTAGTACATTAATACGAGTGAGTATTATGAAATTAGGGATTCG

RI-gBol001654-XLOC_050102-9285-0
AGCGCCATCTATGGAAGCAACTCGAAAGCGATGGCGAGAGTGAGAACTTA
RI-gBol001654-XLOC_050102-9285-1
AGCGCCATCTATGGAAGCAACTCGAAAGCGATGGCGAGAGTGAGAACTTA
CONSENSUS
AGCGCCATCTATGGAAGCAACTCGAAAGCGATGGCGAGAGTGAGAACTTA

RI-gBol001654-XLOC_050102-9285-0
CAAAGACGGAAAACTAAAGATATCGGAGGAGACGGGTCTCTTACTCCAAG
RI-gBol001654-XLOC_050102-9285-1
CAAAGACGGAAAACTAAAGATATCGGAGGAGACGGGTCTCTTACTCCAAG
CONSENSUS
CAAAGACGGAAAACTAAAGATATCGGAGGAGACGGGTCTCTTACTCCAAG

RI-gBol001654-XLOC_050102-9285-0
ACCAAGACGGTTTAGCAATTTCCGGCGACATACGTAACAGTTGGGTCGGT
RI-gBol001654-XLOC_050102-9285-1

ACCAAGACGGTTTAGCAATTTCCGGCGACATACGTAACAGTTGGGTCGGT
CONSENSUS
ACCAAGACGGTTTAGCAATTTCCGGCGACATACGTAACAGTTGGGTCGGT

RI-gBol001654-XLOC_050102-9285-0
GTCTCCGCCTTGCAAGCTCTCTTCATAAAAGAGCACAACGCTGTATGCGA
RI-gBol001654-XLOC_050102-9285-1
GTCTCCGCCTTGCAAGCTCTCTTCATAAAAGAGCACAACGCTGTATGCGA
CONSENSUS
GTCTCCGCCTTGCAAGCTCTCTTCATAAAAGAGCACAACGCTGTATGCGA

RI-gBol001654-XLOC_050102-9285-0 CGTACTCAAG
RI-gBol001654-XLOC_050102-9285-1 CGTACTCAAG
CONSENSUS CGTACTCAAG

alignment for event: A3-gBol034852-XLOC_012365-3020

A3-gBol034852-XLOC_012365-3020-0
CTAGAAAGTGGGATAGGCACAGAGACTTTATTCTGTGTGTTTGGGTAAAA
A3-gBol034852-XLOC_012365-3020-1
CTAGAAAGTGGGATAGGCACAGAGACTTTATTCTGTGTGTTTGGGTAAAA
CONSENSUS
CTAGAAAGTGGGATAGGCACAGAGACTTTATTCTGTGTGTTTGGGTAAAA

A3-gBol034852-XLOC_012365-3020-0
GTGAAACAATTAAATAATTAAGCGCTCTGAAATGAATAATTAAACCACTT
A3-gBol034852-XLOC_012365-3020-1
GTGAAACAATTAAATAATTAAGCGCTCTGAAATGAATAATTAAACCACTT
CONSENSUS
GTGAAACAATTAAATAATTAAGCGCTCTGAAATGAATAATTAAACCACTT

A3-gBol034852-XLOC_012365-3020-0
AACGACAATAATGAACCACAAGGAGTGGTAATGGTGGCCATGGCGATGGT
A3-gBol034852-XLOC_012365-3020-1
AACGACAATAATGAACCACAAGGAGTGGTAATGGTGGCCATGGCGATGGT
CONSENSUS
AACGACAATAATGAACCACAAGGAGTGGTAATGGTGGCCATGGCGATGGT

A3-gBol034852-XLOC_012365-3020-0
GGTTTCCTAGAGAGAGAGAGAGAGAGAGAGAGAGAGAGAGAAGAAGACCA
A3-gBol034852-XLOC_012365-3020-1
GGTTTCCTAGAGAGAGAGAGAGAGAGAGAGAGAGAGAGAGAAGAAGACCA
CONSENSUS
GGTTTCCTAGAGAGAGAGAGAGAGAGAGAGAGAGAGAGAGAAGAAGACCA

A3-gBol034852-XLOC_012365-3020-0
AGAACCAGAAAAAGAATCACACAAGTCCAACACGACACACACACACTCTG
A3-gBol034852-XLOC_012365-3020-1
AGAACCAGAAAAAGAATCACACAAGTCCAACACGACACACACACACTCTG
CONSENSUS
AGAACCAGAAAAAGAATCACACAAGTCCAACACGACACACACACACTCTG

A3-gBol034852-XLOC_012365-3020-0
ATTCACCGAGTTTCCATATAAAGAGACAAGAAGCGTTTACTTCGGACAAG

A3-gBol034852-XLOC_012365-3020-1
ATTCACCGAGTTTCCATATAAAGAGACAAGAAGCGTTTACTTCGGACAAG
CONSENSUS
ATTCACCGAGTTTCCATATAAAGAGACAAGAAGCGTTTACTTCGGACAAG

A3-gBol034852-XLOC_012365-3020-0
GAGACATGCAAAACTAATCTGGTGTCCTGTCTTCTTCACCGTCGGTCCAT
A3-gBol034852-XLOC_012365-3020-1
GAGACATGCAAAACTAATCTGGTGTCCTGTCTTCTTCACCGTCGGTCCAT
CONSENSUS
GAGACATGCAAAACTAATCTGGTGTCCTGTCTTCTTCACCGTCGGTCCAT

A3-gBol034852-XLOC_012365-3020-0
CTAATTCTCAAAAAAGGGTTTTCTTCATCTTGTTAGACGTTATGCATCAG
A3-gBol034852-XLOC_012365-3020-1
CTAATTCTCAAAAAAGG------------------ACGTTATGCATCAG
CONSENSUS
CTAATTCTCAAAAAAGG..................ACGTTATGCATCAG

A3-gBol034852-XLOC_012365-3020-0
ATGAATAAGAAAGATTCAACTTTGCCGTACCTGAACACTAGCATCTCATG
A3-gBol034852-XLOC_012365-3020-1
ATGAATAAGAAAGATTCAACTTTGCCGTACCTGAACACTAGCATCTCATG
CONSENSUS
ATGAATAAGAAAGATTCAACTTTGCCGTACCTGAACACTAGCATCTCATG

A3-gBol034852-XLOC_012365-3020-0
GGGAGTGGTTCCAGCTGAACCCCTAAGCATGAAAGTGGTTGATGCAAGGC
A3-gBol034852-XLOC_012365-3020-1
GGGAGTGGTTCCAGCTGAACCCCTAAGCATGAAAGTGGTTGATGCAAGGC
CONSENSUS
GGGAGTGGTTCCAGCTGAACCCCTAAGCATGAAAGTGGTTGATGCAAGGC

A3-gBol034852-XLOC_012365-3020-0
CTGAACATACCACAAAGCAAATCAGTTTCCAGGACCAGGATTCATCTTCA
A3-gBol034852-XLOC_012365-3020-1
CTGAACATACCACAAAGCAAATCAGTTTCCAGGACCAGGATTCATCTTCA
CONSENSUS
CTGAACATACCACAAAGCAAATCAGTTTCCAGGACCAGGATTCATCTTCA

A3-gBol034852-XLOC_012365-3020-0
ACTCAGTCCACTGGTCAGTCTTACACTGATATTGCTAGTAGTGGTGATGA
A3-gBol034852-XLOC_012365-3020-1
ACTCAGTCCACTGGTCAGTCTTACACTGATATTGCTAGTAGTGGTGATGA
CONSENSUS
ACTCAGTCCACTGGTCAGTCTTACACTGATATTGCTAGTAGTGGTGATGA

A3-gBol034852-XLOC_012365-3020-0
TGATAATCCTTCCAGACAAATCTCTTTTTCAACAAAATCAG
A3-gBol034852-XLOC_012365-3020-1
TGATAATCCTTCCAGACAAATCTCTTTTTCAACAAAATCAG
CONSENSUS
TGATAATCCTTCCAGACAAATCTCTTTTTCAACAAAATCAG

alignment for event: RI-gBol034914-XLOC_012490-9164

RI-gBol034914-XLOC_012490-9164-0
GTGGAAGTTGAAATGGAAAGAGATGAAGAAGCTTGGGAGGCTGATGAAGA
RI-gBol034914-XLOC_012490-9164-1
GTGGAAGTTGAAATGGAAAGAGATGAAGAAGCTTGGGAGGCTGATGAAGA
CONSENSUS
GTGGAAGTTGAAATGGAAAGAGATGAAGAAGCTTGGGAGGCTGATGAAGA

RI-gBol034914-XLOC_012490-9164-0
AGATGTCTCTGGTAAACACACTTGCCAGGCGTGTGGTGCTGAGTTCAAGA
RI-gBol034914-XLOC_012490-9164-1
AGATGTCTCTGGTAAACACACTTGCCAGGCGTGTGGTGCTGAGTTCAAGA
CONSENSUS
AGATGTCTCTGGTAAACACACTTGCCAGGCGTGTGGTGCTGAGTTCAAGA

RI-gBol034914-XLOC_012490-9164-0
AACCTGCTCACTTGAAGCAGCATATGCAGAGCCACTCGCTCGAGGTATGC
RI-gBol034914-XLOC_012490-9164-1
AACCTGCTCACTTGAAGCAGCATATGCAGAGCCACTCGCTCGAG------
CONSENSUS
AACCTGCTCACTTGAAGCAGCATATGCAGAGCCACTCGCTCGAG......

RI-gBol034914-XLOC_012490-9164-0
ATCCTCGTGGTAGCTTCTTTATATAATTGAATCCTCGTGGAGACAGAGAA
RI-gBol034914-XLOC_012490-9164-1

CONSENSUS
....

RI-gBol034914-XLOC_012490-9164-0
GCTCGTGGGCTTCCTTAGTTCTGAAACTTGATCTTATTGTGCGCTAAAAG
RI-gBol034914-XLOC_012490-9164-1

CONSENSUS
....

RI-gBol034914-XLOC_012490-9164-0
TTTACACCTTTGCAGGAAATGAATGTCCTTTTTTTGTTTGATAACAAAAG
RI-gBol034914-XLOC_012490-9164-1 ---------------
GAAATGAATGTCCTTTTTTTGTTTGATAACAAAAG
CONSENSUS
...............GAAATGAATGTCCTTTTTTTGTTTGATAACAAAAG

RI-gBol034914-XLOC_012490-9164-0
GTCTTACTTATGTTATATTGCGTGAGGCATTGGATCCCAAAAGAGCGAAC
RI-gBol034914-XLOC_012490-9164-1
GTCTTACTTATGTTATATTGCGTGAGGCATTGGATCCCAAAAGAGCGAAC
CONSENSUS
GTCTTACTTATGTTATATTGCGTGAGGCATTGGATCCCAAAAGAGCGAAC

RI-gBol034914-XLOC_012490-9164-0
TCCAAAATGCGAGGCTACATGTTATGGACTAGTATCAGGCTGGGAGACCT
RI-gBol034914-XLOC_012490-9164-1
TCCAAAATGCGAGGCTACATGTTATGGACTAGTATCAGGCTGGGAGACCT
CONSENSUS

TCCAAAATGCGAGGCTACATGTTATGGACTAGTATCAGGCTGGGAGACCT

RI-gBol034914-XLOC_012490-9164-0
CCTGAGAAGCTCCTGCTAGTTTGCCTCCGTCACGCTAAAG
RI-gBol034914-XLOC_012490-9164-1
CCTGAGAAGCTCCTGCTAGTTTGCCTCCGTCACGCTAAAG
CONSENSUS
CCTGAGAAGCTCCTGCTAGTTTGCCTCCGTCACGCTAAAG

alignment for event: SE-gBol008404-XLOC_042334-6129

SE-gBol008404-XLOC_042334-6129-0
GTCTCCGGTGGAATTGAGCTGCTGCGTGGAGACGATGCTCCCTTACCACA
SE-gBol008404-XLOC_042334-6129-1
GTCTCCGGTGGAATTGAGCTGCTGCGTGGAGACGATGCTCCCTTACCACA
CONSENSUS
GTCTCCGGTGGAATTGAGCTGCTGCGTGGAGACGATGCTCCCTTACCACA

SE-gBol008404-XLOC_042334-6129-0
CGGCCACGGCTTCCGCTCTGCTCAATTCGATGCTCTCCGCTTCTCGCCGT
SE-gBol008404-XLOC_042334-6129-1
CGGCCACGGCTTCCGCTCTGCTCAATTCGATGCTCTCCGCTTCTCGCCGT
CONSENSUS
CGGCCACGGCTTCCGCTCTGCTCAATTCGATGCTCTCCGCTTCTCGCCGT

SE-gBol008404-XLOC_042334-6129-0
GGTTGGATCGTCGAAG----------------------------------
SE-gBol008404-XLOC_042334-6129-1
GGTTGGATCGTCGAAGATTGCAATGATGATGTATGATGAATTACTGAGGA
CONSENSUS
GGTTGGATCGTCGAAG..................................

SE-gBol008404-XLOC_042334-6129-0
--------------------------------ATGGATGACTCTCCTTCT
SE-gBol008404-XLOC_042334-6129-1
GAGGTCAGCGATGCATGCAGTCTTAAGCCAAGATGGATGACTCTCCTTCT
CONSENSUS
................................ATGGATGACTCTCCTTCT

SE-gBol008404-XLOC_042334-6129-0
CACCATTGCAGTAAACAGGAAGATTTGTTCAAGAGTTTTAAAACCACTTG
SE-gBol008404-XLOC_042334-6129-1
CACCATTGCAGTAAACAGGAAGATTTGTTCAAGAGTTTTAAAACCACTTG
CONSENSUS
CACCATTGCAGTAAACAGGAAGATTTGTTCAAGAGTTTTAAAACCACTTG

SE-gBol008404-XLOC_042334-6129-0
TGTTGATTCTTAAACCATAACAACAGATGTTTCTCCATGTTTTTTTTTTT
SE-gBol008404-XLOC_042334-6129-1
TGTTGATTCTTAAACCATAACAACAGATGTTTCTCCATGTTTTTTTTTTT
CONSENSUS
TGTTGATTCTTAAACCATAACAACAGATGTTTCTCCATGTTTTTTTTTTT

SE-gBol008404-XLOC_042334-6129-0

```
      TGCTGTTACTTTATAAAAGATCCAAGTGAATTGTTTAATCAGTCTCCTTC
SE-gBol008404-XLOC_042334-6129-1
      TGCTGTTACTTTATAAAAGATCCAAGTGAATTGTTTAATCAGTCTCCTTC
CONSENSUS
      TGCTGTTACTTTATAAAAGATCCAAGTGAATTGTTTAATCAGTCTCCTTC

SE-gBol008404-XLOC_042334-6129-0
      ATTCCATCGCCTTGTATTGTAGGTTGTTGTAGTTTGCTGGTATCTGAATT
SE-gBol008404-XLOC_042334-6129-1
      ATTCCATCGCCTTGTATTGTAGGTTGTTGTAGTTTGCTGGTATCTGAATT
CONSENSUS
      ATTCCATCGCCTTGTATTGTAGGTTGTTGTAGTTTGCTGGTATCTGAATT

SE-gBol008404-XLOC_042334-6129-0
      TATTTATCATTGATGAATAATTAATAGCTTCAATCGTAATAATTCATTTC
SE-gBol008404-XLOC_042334-6129-1
      TATTTATCATTGATGAATAATTAATAGCTTCAATCGTAATAATTCATTTC
CONSENSUS
      TATTTATCATTGATGAATAATTAATAGCTTCAATCGTAATAATTCATTTC


alignment for event: RI-gBol036377-XLOC_010692-9025

RI-gBol036377-XLOC_010692-9025-0
      CGGCTATTTGTTGTCCAGACAGAAAACATAGACACGTCCGCCTGTATTAT
RI-gBol036377-XLOC_010692-9025-1
      CGGCTATTTGTTGTCCAGACAGAAAACATAGACACGTCCGCCTGTATTAT
CONSENSUS
      CGGCTATTTGTTGTCCAGACAGAAAACATAGACACGTCCGCCTGTATTAT

RI-gBol036377-XLOC_010692-9025-0
      AAATCCTCCAGAAGTCAGGTGTAAAGAATTTGCTCTTTTTCATCGCTGAC
RI-gBol036377-XLOC_010692-9025-1
      AAATCCTCCAGAAGTCAG--------------------------------
CONSENSUS
      AAATCCTCCAGAAGTCAG................................

RI-gBol036377-XLOC_010692-9025-0
      ACGAAATATGATCCAATTTTAACCTCCTGGCCTTTGTATCAAAATTGCAG
RI-gBol036377-XLOC_010692-9025-1
      --------------------------------------------------
CONSENSUS
      ..................................................

RI-gBol036377-XLOC_010692-9025-0
      CTTCATTTTAAATGGAAAGGTGGTCGAGAAGAGGGTTAATATCTCAATG
RI-gBol036377-XLOC_010692-9025-1
      CTTCATTTTAAATGGAAAGGTGGTCGAGAAGAGGGTTAATATCTCAATG
CONSENSUS
      CTTCATTTTAAATGGAAAGGTGGTCGAGAAGAGGGTTAATATCTCAATG


alignment for event: RI-gBol016025-XLOC_033627-5838
```

RI-gBol016025-XLOC_033627-5838-0
ATCATTAACGATCGCGAGACTGGAAGGTCGAGGGGATTCGGATTCGTGAC
RI-gBol016025-XLOC_033627-5838-1
ATCATTAACGATCGCGAGACTGGAAGGTCGAGGGGATTCGGATTCGTGAC
CONSENSUS
ATCATTAACGATCGCGAGACTGGAAGGTCGAGGGGATTCGGATTCGTGAC

RI-gBol016025-XLOC_033627-5838-0
CTTCAAGGATGACAAGTCAATGAGGGATGCTATTGAAGAGATGAACGGGA
RI-gBol016025-XLOC_033627-5838-1
CTTCAAGGATGACAAGTCAATGAGGGATGCTATTGAAGAGATGAACGGGA
CONSENSUS
CTTCAAGGATGACAAGTCAATGAGGGATGCTATTGAAGAGATGAACGGGA

RI-gBol016025-XLOC_033627-5838-0
AAGAGCTCGATGGACGTACGATTACCGTCAACGAGGCTCAGTCTAGAGGA
RI-gBol016025-XLOC_033627-5838-1
AAGAGCTCGATGGACGTACGATTACCGTCAACGAGGCTCAGTCTAGAGGA
CONSENSUS
AAGAGCTCGATGGACGTACGATTACCGTCAACGAGGCTCAGTCTAGAGGA

RI-gBol016025-XLOC_033627-5838-0
GGCGGTGGAGGAGGCCGTGGTGGTGGTGGTGGTTACAACAGAGGTGGCGG
RI-gBol016025-XLOC_033627-5838-1
GGCGGTGGAGGAGGCCGTGGTGGTGGTGGTGGTTACAACAGAGGTGGCGG
CONSENSUS
GGCGGTGGAGGAGGCCGTGGTGGTGGTGGTGGTTACAACAGAGGTGGCGG

RI-gBol016025-XLOC_033627-5838-0
CGGCGGAGGATATGGTGGAGGCGGTGGTTACGGAGGAGGCGGTGGTGGAT
RI-gBol016025-XLOC_033627-5838-1
CGGCGGAGGATATGGTGGAGGCGGTGGTTACGGAGGAGGCGGTGGTGGAT
CONSENSUS
CGGCGGAGGATATGGTGGAGGCGGTGGTTACGGAGGAGGCGGTGGTGGAT

RI-gBol016025-XLOC_033627-5838-0
ATGGAAGACGTGATGGAGGTGGTTACGGATCTGGTGGTGGCGGAGGATAC
RI-gBol016025-XLOC_033627-5838-1
ATGGAAGACGTGATGGAG--------------------------------
CONSENSUS
ATGGAAGACGTGATGGAG................................

RI-gBol016025-XLOC_033627-5838-0
GGTGGTGGTGGAAGACGTGATGGAGGTGGTTACGGAGGAGGTGACGGTGG
RI-gBol016025-XLOC_033627-5838-1 ------------------------
GTGGTTACGGAGGAGGTGACGGTGG
CONSENSUS
.........................GTGGTTACGGAGGAGGTGACGGTGG

RI-gBol016025-XLOC_033627-5838-0
TTACGGAGGAAACAGCGGCGGTGGTGGTGGTGGCTGGTAATCAAAGATGA
RI-gBol016025-XLOC_033627-5838-1
TTACGGAGGAAACAGCGGCGGTGGTGGTGGTGGCTGGTAATCAAAGATGA
CONSENSUS
TTACGGAGGAAACAGCGGCGGTGGTGGTGGTGGCTGGTAATCAAAGATGA

RI-gBol016025-XLOC_033627-5838-0
TGTGGTTGTCTGCTTCTTCTGCTATGTGTTTGGTTTAGATTTGGTTTCGT
RI-gBol016025-XLOC_033627-5838-1
TGTGGTTGTCTGCTTCTTCTGCTATGTGTTTGGTTTAGATTTGGTTTCGT
CONSENSUS
TGTGGTTGTCTGCTTCTTCTGCTATGTGTTTGGTTTAGATTTGGTTTCGT

RI-gBol016025-XLOC_033627-5838-0
ATCAATGTTTCTCTGGTTTTTTTTATGTTTCTGGTTTGGGGTTCTATGTA
RI-gBol016025-XLOC_033627-5838-1
ATCAATGTTTCTCTGGTTTTTTTTATGTTTCTGGTTTGGGGTTCTATGTA
CONSENSUS
ATCAATGTTTCTCTGGTTTTTTTTATGTTTCTGGTTTGGGGTTCTATGTA

RI-gBol016025-XLOC_033627-5838-0 AC
RI-gBol016025-XLOC_033627-5838-1 AC
CONSENSUS AC

alignment for event: RI-gBol000543-XLOC_051292-16309

RI-gBol000543-XLOC_051292-16309-0
CGAGGGAGAAAGCAATTGTTGTGAACTTAGAAGTCATAAAGCTAATAGTT
RI-gBol000543-XLOC_051292-16309-1
CGAGGGAGAAAGCAATTGTTGTGAACTTAGAAGTCATAAAGCTAATAGTT
CONSENSUS
CGAGGGAGAAAGCAATTGTTGTGAACTTAGAAGTCATAAAGCTAATAGTT

RI-gBol000543-XLOC_051292-16309-0
ACCGCTGAAGAAGTGCTGCTCTTGGATCCTCTCCGTCCTGAAGTTCTTCC
RI-gBol000543-XLOC_051292-16309-1
ACCGCTGAAGAAGTGCTGCTCTTGGATCCTCTCCGTCCTGAAGTTCTTCC
CONSENSUS
ACCGCTGAAGAAGTGCTGCTCTTGGATCCTCTCCGTCCTGAAGTTCTTCC

RI-gBol000543-XLOC_051292-16309-0
ATTCGTTGAACGACTCAAGCTACAGTTTCCTCAGATTACCAATGCTTCTG
RI-gBol000543-XLOC_051292-16309-1
ATTCGTTGAACGACTCAAGCTACAGTTTCCTCAGATTACCAATGCTTCTG
CONSENSUS
ATTCGTTGAACGACTCAAGCTACAGTTTCCTCAGATTACCAATGCTTCTG

RI-gBol000543-XLOC_051292-16309-0
GAAACGTTGTGCAATCCTCTTCTCTTCTTGATCAAGAAGCTGGAGAGGGT
RI-gBol000543-XLOC_051292-16309-1
GAAACGTTGTGCAATCCTCTTCTCTTCTTGATCAAGAAGCTGGAGAGGGT
CONSENSUS
GAAACGTTGTGCAATCCTCTTCTCTTCTTGATCAAGAAGCTGGAGAGGGT

RI-gBol000543-XLOC_051292-16309-0
TTGCAGAGTGAGCTTCCTTTTGAGTTCCAGGTTCTCGAGATTGCGCTGGA
RI-gBol000543-XLOC_051292-16309-1
TTGCAGAGTGAGCTTCCTTTTGAGTTCCAGGTTCTCGAGATTGCGCTGGA
CONSENSUS
TTGCAGAGTGAGCTTCCTTTTGAGTTCCAGGTTCTCGAGATTGCGCTGGA

RI-gBol000543-XLOC_051292-16309-0
GGTCGTCTGCTCGCTTCTTGATACGAGCGTTGATGCACTTGAGACAGAAG
RI-gBol000543-XLOC_051292-16309-1
GGTCGTCTGCTCGCTTCTTGATACGAGCGTTGATGCACTTGAGACAGAAG
CONSENSUS
GGTCGTCTGCTCGCTTCTTGATACGAGCGTTGATGCACTTGAGACAGAAG

RI-gBol000543-XLOC_051292-16309-0
CTTGGCCTGTTCTTGATGAACTAACCAAGAATGTCAGCACTGAGAATCTT
RI-gBol000543-XLOC_051292-16309-1
CTTGGCCTGTTCTTGATGAACTAACCAAGAATGTCAGCACTGAGAATCTT
CONSENSUS
CTTGGCCTGTTCTTGATGAACTAACCAAGAATGTCAGCACTGAGAATCTT

RI-gBol000543-XLOC_051292-16309-0
GAATACGTTCGAAGCTTGAAAAGTAATCTCACCAGGTTGCTAGCCCGTGT
RI-gBol000543-XLOC_051292-16309-1
GAATACGTTCGAAGCTTGAAAAGTAATCTCACCAGGTTGCTA--------
CONSENSUS
GAATACGTTCGAAGCTTGAAAAGTAATCTCACCAGGTTGCTA........

RI-gBol000543-XLOC_051292-16309-0
ACAAAAGGTTTGATCATTTCGTCATGATTCTATATAATGTTTGTTGTGAT
RI-gBol000543-XLOC_051292-16309-1

CONSENSUS
....

RI-gBol000543-XLOC_051292-16309-0
GGAGGAGAGCCTAAAACTTCAAAGCAAAATCCATCTTACCTCTCCGAGAT
RI-gBol000543-XLOC_051292-16309-1

CONSENSUS
....

RI-gBol000543-XLOC_051292-16309-0
AATGTTATTTTTAGCTATTTCCATGGAATTTAGTTTTTTTTTTTTGGGTT
RI-gBol000543-XLOC_051292-16309-1

CONSENSUS
....

RI-gBol000543-XLOC_051292-16309-0
GATAGGATGATCATGTCATTTTTCGGGTAAGATTAATTAATGGGTAGAGT
RI-gBol000543-XLOC_051292-16309-1

CONSENSUS
....

RI-gBol000543-XLOC_051292-16309-0
ATGGGTTCGTTCTGATACCATATTAAATTTTTTGAACTTCTAACTTAAAA
RI-gBol000543-XLOC_051292-16309-1

CONSENSUS
....

RI-gBol000543-XLOC_051292-16309-0
TTAATTAACGGTAAATCTTTTGGATATTACTTAATTCATTTTTTTATTTC
RI-gBol000543-XLOC_051292-16309-1

CONSENSUS
....

RI-gBol000543-XLOC_051292-16309-0
CGATGCGAGAACTTTTCTTCTGCAATTGATGCAGGTGAGAGATGAAATCG
RI-gBol000543-XLOC_051292-16309-1
----------------------------------GTGAGAGATGAAATCG
CONSENSUS
..................................GTGAGAGATGAAATCG

RI-gBol000543-XLOC_051292-16309-0
AACACTTGTTAGATGACAACGAAGACATGGCAGACTTGTACTTAACAAGG
RI-gBol000543-XLOC_051292-16309-1
AACACTTGTTAGATGACAACGAAGACATGGCAGACTTGTACTTAACAAGG
CONSENSUS
AACACTTGTTAGATGACAACGAAGACATGGCAGACTTGTACTTAACAAGG

RI-gBol000543-XLOC_051292-16309-0
AAATGGATCCAACAGAACCAGCAATCCGAAGCAGAACTTGGTGAGACCGC
RI-gBol000543-XLOC_051292-16309-1
AAATGGATCCAACAGAACCAGCAATCCGAAGCAGAACTTGGTGAGACCGC
CONSENSUS
AAATGGATCCAACAGAACCAGCAATCCGAAGCAGAACTTGGTGAGACCGC

RI-gBol000543-XLOC_051292-16309-0
ATCAAATCTTCTCAGTCGGGCCACATCAAACAGAAGTTCCAGTATGGTGA
RI-gBol000543-XLOC_051292-16309-1
ATCAAATCTTCTCAGTCGGGCCACATCAAACAGAAGTTCCAGTATGGTGA
CONSENSUS
ATCAAATCTTCTCAGTCGGGCCACATCAAACAGAAGTTCCAGTATGGTGA

RI-gBol000543-XLOC_051292-16309-0
CGAGTAGTACGAAGGGCGACGACGTGGAGGATCTAGAGATGTTGCTGGAG
RI-gBol000543-XLOC_051292-16309-1
CGAGTAGTACGAAGGGCGACGACGTGGAGGATCTAGAGATGTTGCTGGAG
CONSENSUS
CGAGTAGTACGAAGGGCGACGACGTGGAGGATCTAGAGATGTTGCTGGAG

RI-gBol000543-XLOC_051292-16309-0
GCATACTTCATGCAACTAGAGGGGATGCGAAACAGGATTCTTACGGTGAG
RI-gBol000543-XLOC_051292-16309-1
GCATACTTCATGCAACTAGAGGGGATGCGAAACAGGATTCTTACGGTGAG
CONSENSUS
GCATACTTCATGCAACTAGAGGGGATGCGAAACAGGATTCTTACGGTGAG

RI-gBol000543-XLOC_051292-16309-0
GGAGTACATTGACGACACAGAAGACTACGTGAACATACAACTTGATAATC
RI-gBol000543-XLOC_051292-16309-1
GGAGTACATTGACGACACAGAAGACTACGTGAACATACAACTTGATAATC
CONSENSUS
GGAGTACATTGACGACACAGAAGACTACGTGAACATACAACTTGATAATC

RI-gBol000543-XLOC_051292-16309-0
AACGTAACGAAATGATCCAACTGCAACTGACGCTGACCATAGCCTCGTTT
RI-gBol000543-XLOC_051292-16309-1
AACGTAACGAAATGATCCAACTGCAACTGACGCTGACCATAGCCTCGTTT
CONSENSUS
AACGTAACGAAATGATCCAACTGCAACTGACGCTGACCATAGCCTCGTTT

RI-gBol000543-XLOC_051292-16309-0
GCTATAACTGCAAACACATTGTTGGTAAGCTTGTTCGGAATGAACATACC
RI-gBol000543-XLOC_051292-16309-1
GCTATAACTGCAAACACATTGTTGGTAAGCTTGTTCGGAATGAACATACC
CONSENSUS
GCTATAACTGCAAACACATTGTTGGTAAGCTTGTTCGGAATGAACATACC

RI-gBol000543-XLOC_051292-16309-0
GTGTCCATTGTATAAAATGGATGATGTGTTTGGTTACTTTCTCTCGAGTG
RI-gBol000543-XLOC_051292-16309-1
GTGTCCATTGTATAAAATGGATGATGTGTTTGGTTACTTTCTCTCGAGTG
CONSENSUS
GTGTCCATTGTATAAAATGGATGATGTGTTTGGTTACTTTCTCTCGAGTG

RI-gBol000543-XLOC_051292-16309-0
TTACTGCGGTTTGCGTTGTGCTATTCTTGCTCACCCTTGGTTACGCATGG
RI-gBol000543-XLOC_051292-16309-1
TTACTGCGGTTTGCGTTGTGCTATTCTTGCTCACCCTTGGTTACGCATGG
CONSENSUS
TTACTGCGGTTTGCGTTGTGCTATTCTTGCTCACCCTTGGTTACGCATGG

RI-gBol000543-XLOC_051292-16309-0
TGGAAGAAGCTGCTTGGCTCATAATCACTCTTTCTCACTTGTGTAGTAAA
RI-gBol000543-XLOC_051292-16309-1
TGGAAGAAGCTGCTTGGCTCATAATCACTCTTTCTCACTTGTGTAGTAAA
CONSENSUS
TGGAAGAAGCTGCTTGGCTCATAATCACTCTTTCTCACTTGTGTAGTAAA

RI-gBol000543-XLOC_051292-16309-0
AACCCATTGCCACATGTTTAATACATATTGTTGTAATTAGGCTTCCTTGA
RI-gBol000543-XLOC_051292-16309-1
AACCCATTGCCACATGTTTAATACATATTGTTGTAATTAGGCTTCCTTGA
CONSENSUS
AACCCATTGCCACATGTTTAATACATATTGTTGTAATTAGGCTTCCTTGA

RI-gBol000543-XLOC_051292-16309-0
TTTATCTCTTGATTATGCTGATGACAAATTGACAAGGTTAAAGTAAATGC
RI-gBol000543-XLOC_051292-16309-1
TTTATCTCTTGATTATGCTGATGACAAATTGACAAGGTTAAAGTAAATGC
CONSENSUS
TTTATCTCTTGATTATGCTGATGACAAATTGACAAGGTTAAAGTAAATGC

RI-gBol000543-XLOC_051292-16309-0 AATGAACATGTATACCAG
RI-gBol000543-XLOC_051292-16309-1 AATGAACATGTATACCAG
CONSENSUS AATGAACATGTATACCAG

alignment for event: A3-gBol028721-XLOC_019502-10568

A3-gBol028721-XLOC_019502-10568-0
TGTCACTGTAGTTGTTGCTTACCTCATGAAGAAACAGGGAATGACTTTAA
A3-gBol028721-XLOC_019502-10568-1
TGTCACTGTAGTTGTTGCTTACCTCATGAAGAAACAGGGAATGACTTTAA
CONSENSUS
TGTCACTGTAGTTGTTGCTTACCTCATGAAGAAACAGGGAATGACTTTAA

A3-gBol028721-XLOC_019502-10568-0
CCCAAGCATTGCAGCATGTTCAGAGCATTAGACCCGTGGCAAATCCTAAC
A3-gBol028721-XLOC_019502-10568-1
CCCAAGCATTGCAGCATGTTCAGAGCATTAGACCCGTGGCAAATCCTAAC
CONSENSUS
CCCAAGCATTGCAGCATGTTCAGAGCATTAGACCCGTGGCAAATCCTAAC

A3-gBol028721-XLOC_019502-10568-0
ACCGGTTTCATCAGGCAATTGCAGGACCTTGAAAAGTCTTTACAAG----
A3-gBol028721-XLOC_019502-10568-1
ACCGGTTTCATCAGGCAATTGCAGGACCTTGAAAAGTCTTTACAAGACCT
CONSENSUS
ACCGGTTTCATCAGGCAATTGCAGGACCTTGAAAAGTCTTTACAAG....

A3-gBol028721-XLOC_019502-10568-0

A3-gBol028721-XLOC_019502-10568-1
CTAAATTAGAGGTTCTTTACTTTGTCTGTCTGGGGCAATAGTAAATGAAT
CONSENSUS
....

A3-gBol028721-XLOC_019502-10568-0

A3-gBol028721-XLOC_019502-10568-1
ACCAGAACTCGTGGGACGGAAAGCAAAGTGTTGTGGAGGAACAAGACCGG
CONSENSUS
....

A3-gBol028721-XLOC_019502-10568-0

A3-gBol028721-XLOC_019502-10568-1
TTCATCTTAATTTACTCTTACTTGCACCTTCCTCTCTCTCTCTAAAACAG
CONSENSUS
....

A3-gBol028721-XLOC_019502-10568-0
GGAAGCAAGAACCACTTGCCCACTGTCAAGTTTGAAGACCCCACCAAGAT
A3-gBol028721-XLOC_019502-10568-1
GGAAGCAAGAACCACTTGCCCACTGTCAAGTTTGAAGACCCCACCAAGAT
CONSENSUS
GGAAGCAAGAACCACTTGCCCACTGTCAAGTTTGAAGACCCCACCAAGAT

A3-gBol028721-XLOC_019502-10568-0
CATACCTTTTGGAGGGAACTTGTAAATCTCAAACGAAGCATATCATATGT
A3-gBol028721-XLOC_019502-10568-1
CATACCTTTTGGAGGGAACTTGTAAATCTCAAACGAAGCATATCATATGT
CONSENSUS

CATACCTTTTGGAGGGAACTTGTAAATCTCAAACGAAGCATATCATATGT

A3-gBol028721-XLOC_019502-10568-0
TTTTGTTTTTTTGTTTCTCCTTTAACAACTTTGACTAAAAAAATGTAAAC
A3-gBol028721-XLOC_019502-10568-1
TTTTGTTTTTTTGTTTCTCCTTTAACAACTTTGACTAAAAAAATGTAAAC
CONSENSUS
TTTTGTTTTTTTGTTTCTCCTTTAACAACTTTGACTAAAAAAATGTAAAC

A3-gBol028721-XLOC_019502-10568-0
GATTCTGTGAAGTTTCACTGTTTCATGGAAGCATAGCTATACGGCCAACT
A3-gBol028721-XLOC_019502-10568-1
GATTCTGTGAAGTTTCACTGTTTCATGGAAGCATAGCTATACGGCCAACT
CONSENSUS
GATTCTGTGAAGTTTCACTGTTTCATGGAAGCATAGCTATACGGCCAACT

A3-gBol028721-XLOC_019502-10568-0
AAAAGCAGAAGATAACACAATCATATACATCTTCATATTATCA
A3-gBol028721-XLOC_019502-10568-1
AAAAGCAGAAGATAACACAATCATATACATCTTCATATTATCA
CONSENSUS
AAAAGCAGAAGATAACACAATCATATACATCTTCATATTATCA

alignment for event: A3-gBol028721-XLOC_019502-10567

A3-gBol028721-XLOC_019502-10567-0
TTGTGGATAAGGAAGACACAAATTTGGAAATATACTTCGATGAGTGTATT
A3-gBol028721-XLOC_019502-10567-1
TTGTGGATAAGGAAGACACAAATTTGGAAATATACTTCGATGAGTGTATT
CONSENSUS
TTGTGGATAAGGAAGACACAAATTTGGAAATATACTTCGATGAGTGTATT

A3-gBol028721-XLOC_019502-10567-0
GATTTCATTGATGAAGCCAAAAAGCAAGGTGGTAGCGTTCTTGTCCATTG
A3-gBol028721-XLOC_019502-10567-1
GATTTCATTGATGAAGCCAAAAAGCAAGGTGGTAGCGTTCTTGTCCATTG
CONSENSUS
GATTTCATTGATGAAGCCAAAAAGCAAGGTGGTAGCGTTCTTGTCCATTG

A3-gBol028721-XLOC_019502-10567-0
CTTCGTTGGAAAGTCACGCAGGTTTCTTATATTCTTTCACCCGTTTATGT
A3-gBol028721-XLOC_019502-10567-1
CTTCGTTGGAAAGTCACGCAGGTTTCTTATATTCTTTCACCCGTTTATGT
CONSENSUS
CTTCGTTGGAAAGTCACGCAGGTTTCTTATATTCTTTCACCCGTTTATGT

A3-gBol028721-XLOC_019502-10567-0
TCTTTACTTAATTGACCTTGTTCCCTGAATGTTTTGCTCCTTTTTATACT
A3-gBol028721-XLOC_019502-10567-1
TCTTTACTTAATTGACCTTGTTCCCTGAATGTTTTGCTCCTTTTTATACT
CONSENSUS
TCTTTACTTAATTGACCTTGTTCCCTGAATGTTTTGCTCCTTTTTATACT

A3-gBol028721-XLOC_019502-10567-0

TACTTGCAGTGTCACTGTAGTTGTTGCTTACCTCATGAAGAAACAGGGAA
A3-gBol028721-XLOC_019502-10567-1
TACTTGCAGTGTCACTGTAGTTGTTGCTTACCTCATGAAGAAACAGGGAA
CONSENSUS
TACTTGCAGTGTCACTGTAGTTGTTGCTTACCTCATGAAGAAACAGGGAA

A3-gBol028721-XLOC_019502-10567-0
TGACTTTAACCCAAGCATTGCAGCATGTTCAGAGCATTAGACCCGTGGCA
A3-gBol028721-XLOC_019502-10567-1
TGACTTTAACCCAAGCATTGCAGCATGTTCAGAGCATTAGACCCGTGGCA
CONSENSUS
TGACTTTAACCCAAGCATTGCAGCATGTTCAGAGCATTAGACCCGTGGCA

A3-gBol028721-XLOC_019502-10567-0
AATCCTAACACCGGTTTCATCAGGCAATTGCAGGACCTTGAAAAGTCTTT
A3-gBol028721-XLOC_019502-10567-1
AATCCTAACACCGGTTTCATCAGGCAATTGCAGGACCTTGAAAAGTCTTT
CONSENSUS
AATCCTAACACCGGTTTCATCAGGCAATTGCAGGACCTTGAAAAGTCTTT

A3-gBol028721-XLOC_019502-10567-0
ACAAG---
A3-gBol028721-XLOC_019502-10567-1
ACAAGTAAATGAATACCAGAACTCGTGGGACGGAAAGCAAAGTGTTGTGG
CONSENSUS
ACAAG...

A3-gBol028721-XLOC_019502-10567-0

A3-gBol028721-XLOC_019502-10567-1
AGGAACAAGACCGGTTCATCTTAATTTACTCTTACTTGCACCTTCCTCTC
CONSENSUS
....

A3-gBol028721-XLOC_019502-10567-0 --------------
GGAAGCAAGAACCACTTGCCCACTGTCAAGTTTGAA
A3-gBol028721-XLOC_019502-10567-1
TCTCTCTAAAACAGGGAAGCAAGAACCACTTGCCCACTGTCAAGTTTGAA
CONSENSUS
..............GGAAGCAAGAACCACTTGCCCACTGTCAAGTTTGAA

A3-gBol028721-XLOC_019502-10567-0
GACCCCACCAAGATCATACCTTTTGGAGGGAACTTGTAAATCTCAAACGA
A3-gBol028721-XLOC_019502-10567-1
GACCCCACCAAGATCATACCTTTTGGAGGGAACTTGTAAATCTCAAACGA
CONSENSUS
GACCCCACCAAGATCATACCTTTTGGAGGGAACTTGTAAATCTCAAACGA

A3-gBol028721-XLOC_019502-10567-0
AGCATATCATATGTTTTTGTTTTTTTGTTTCTCCTTTAACAACTTTGACT
A3-gBol028721-XLOC_019502-10567-1
AGCATATCATATGTTTTTGTTTTTTTGTTTCTCCTTTAACAACTTTGACT
CONSENSUS
AGCATATCATATGTTTTTGTTTTTTTGTTTCTCCTTTAACAACTTTGACT

A3-gBol028721-XLOC_019502-10567-0

AAAAAAATGTAAACGATTCTGTGAAGTTTCACTGTTTCATGGAAGCATAG
A3-gBol028721-XLOC_019502-10567-1
AAAAAAATGTAAACGATTCTGTGAAGTTTCACTGTTTCATGGAAGCATAG
CONSENSUS
AAAAAAATGTAAACGATTCTGTGAAGTTTCACTGTTTCATGGAAGCATAG

A3-gBol028721-XLOC_019502-10567-0
CTATACGGCCAACTAAAAGCAGAAGATAACACAATCATATACATCTTCAT
A3-gBol028721-XLOC_019502-10567-1
CTATACGGCCAACTAAAAGCAGAAGATAACACAATCATATACATCTTCAT
CONSENSUS
CTATACGGCCAACTAAAAGCAGAAGATAACACAATCATATACATCTTCAT

A3-gBol028721-XLOC_019502-10567-0 ATTATCA
A3-gBol028721-XLOC_019502-10567-1 ATTATCA
CONSENSUS ATTATCA

alignment for event: A3-gBol028721-XLOC_019502-10569

A3-gBol028721-XLOC_019502-10569-0
TGTCACTGTAGTTGTTGCTTACCTCATGAAGAAACAGGGAATGACTTTAA
A3-gBol028721-XLOC_019502-10569-1
TGTCACTGTAGTTGTTGCTTACCTCATGAAGAAACAGGGAATGACTTTAA
CONSENSUS
TGTCACTGTAGTTGTTGCTTACCTCATGAAGAAACAGGGAATGACTTTAA

A3-gBol028721-XLOC_019502-10569-0
CCCAAGCATTGCAGCATGTTCAGAGCATTAGACCCGTGGCAAATCCTAAC
A3-gBol028721-XLOC_019502-10569-1
CCCAAGCATTGCAGCATGTTCAGAGCATTAGACCCGTGGCAAATCCTAAC
CONSENSUS
CCCAAGCATTGCAGCATGTTCAGAGCATTAGACCCGTGGCAAATCCTAAC

A3-gBol028721-XLOC_019502-10569-0
ACCGGTTTCATCAGGCAATTGCAGGACCTTGAAAAGTCTTTACAAG----
A3-gBol028721-XLOC_019502-10569-1
ACCGGTTTCATCAGGCAATTGCAGGACCTTGAAAAGTCTTTACAAGTAAA
CONSENSUS
ACCGGTTTCATCAGGCAATTGCAGGACCTTGAAAAGTCTTTACAAG....

A3-gBol028721-XLOC_019502-10569-0

A3-gBol028721-XLOC_019502-10569-1
TGAATACCAGAACTCGTGGGACGGAAAGCAAAGTGTTGTGGAGGAACAAG
CONSENSUS
....

A3-gBol028721-XLOC_019502-10569-0

A3-gBol028721-XLOC_019502-10569-1
ACCGGTTCATCTTAATTTACTCTTACTTGCACCTTCCTCTCTCTCTCTAA
CONSENSUS
....

A3-gBol028721-XLOC_019502-10569-0   -----
GGAAGCAAGAACCACTTGCCCACTGTCAAGTTTGAAGACCCCACC
A3-gBol028721-XLOC_019502-10569-1
AACAGGGAAGCAAGAACCACTTGCCCACTGTCAAGTTTGAAGACCCCACC
CONSENSUS
.....GGAAGCAAGAACCACTTGCCCACTGTCAAGTTTGAAGACCCCACC

A3-gBol028721-XLOC_019502-10569-0
AAGATCATACCTTTTGGAGGGAACTTGTAAATCTCAAACGAAGCATATCA
A3-gBol028721-XLOC_019502-10569-1
AAGATCATACCTTTTGGAGGGAACTTGTAAATCTCAAACGAAGCATATCA
CONSENSUS
AAGATCATACCTTTTGGAGGGAACTTGTAAATCTCAAACGAAGCATATCA

A3-gBol028721-XLOC_019502-10569-0
TATGTTTTTGTTTTTTTGTTTCTCCTTTAACAACTTTGACTAAAAAAATG
A3-gBol028721-XLOC_019502-10569-1
TATGTTTTTGTTTTTTTGTTTCTCCTTTAACAACTTTGACTAAAAAAATG
CONSENSUS
TATGTTTTTGTTTTTTTGTTTCTCCTTTAACAACTTTGACTAAAAAAATG

A3-gBol028721-XLOC_019502-10569-0
TAAACGATTCTGTGAAGTTTCACTGTTTCATGGAAGCATAGCTATACGGC
A3-gBol028721-XLOC_019502-10569-1
TAAACGATTCTGTGAAGTTTCACTGTTTCATGGAAGCATAGCTATACGGC
CONSENSUS
TAAACGATTCTGTGAAGTTTCACTGTTTCATGGAAGCATAGCTATACGGC

A3-gBol028721-XLOC_019502-10569-0
CAACTAAAAGCAGAAGATAACACAATCATATACATCTTCATATTATCA
A3-gBol028721-XLOC_019502-10569-1
CAACTAAAAGCAGAAGATAACACAATCATATACATCTTCATATTATCA
CONSENSUS
CAACTAAAAGCAGAAGATAACACAATCATATACATCTTCATATTATCA

alignment for event: A5-gBol010337-XLOC_040275-13994

A5-gBol010337-XLOC_040275-13994-0
GCCAGATGATTTTCGTAGACCATTTGGGCGGTTTGGTCGTCTCAAGGATA
A5-gBol010337-XLOC_040275-13994-1
GCCAGATGATTTTCGTAGACCATTTGGGCGGTTTGGTCGTCTCAAGGATA
CONSENSUS
GCCAGATGATTTTCGTAGACCATTTGGGCGGTTTGGTCGTCTCAAGGATA

A5-gBol010337-XLOC_040275-13994-0
TCTACATACCTCGTAACTATTACACTGGGTGAGTAAAGGCGATTTTTTTT
A5-gBol010337-XLOC_040275-13994-1
TCTACATACCTCGTAACTATTACACTGG----------------------
CONSENSUS
TCTACATACCTCGTAACTATTACACTGG......................

A5-gBol010337-XLOC_040275-13994-0
CCTACTAATTATGTCCATTTGCGTGACCTCTTACGGGCCTACCTTTGTGT
A5-gBol010337-XLOC_040275-13994-1

CONSENSUS
....

A5-gBol010337-XLOC_040275-13994-0
AAGTGTAATTATGATAAACTGTGCTGGTTTATCAAAGTTTGGTTTGTCTT
A5-gBol010337-XLOC_040275-13994-1

CONSENSUS
....

A5-gBol010337-XLOC_040275-13994-0
ATTGGAAGTGTTGAAGATGAAGAAGTAGGAAGATTAATTTGCATGAAGTA
A5-gBol010337-XLOC_040275-13994-1

CONSENSUS
....

A5-gBol010337-XLOC_040275-13994-0
GTAAGAATACTTTGTTTGAGACTACAGAGGGTAAGTGAGATTAGTGTTGA
A5-gBol010337-XLOC_040275-13994-1

CONSENSUS
....

A5-gBol010337-XLOC_040275-13994-0
TGACTGGTTAATACTTCGAGATTGAGTTTCATTTTGAAGATGTTCTGGTC
A5-gBol010337-XLOC_040275-13994-1

CONSENSUS
....

A5-gBol010337-XLOC_040275-13994-0
AAAGTCGAAGTTAGTACAGTGGCTTCGAAAAGGGAGCCTCGTGGGTTTGG
A5-gBol010337-XLOC_040275-13994-1
--------------------------------GGAGCCTCGTGGGTTTGG
CONSENSUS
................................GGAGCCTCGTGGGTTTGG

A5-gBol010337-XLOC_040275-13994-0
CTTTGTGCAGTACTATGATCCTGATGATGCTGCAGATGCAAAGTATCATT
A5-gBol010337-XLOC_040275-13994-1
CTTTGTGCAGTACTATGATCCTGATGATGCTGCAGATGCAAAGTATCATT
CONSENSUS
CTTTGTGCAGTACTATGATCCTGATGATGCTGCAGATGCAAAGTATCATT

A5-gBol010337-XLOC_040275-13994-0
TAGATGGCTACGTTCTTCTAGGCCGCGAAATCACTGTGGTGTTTGCAGAG
A5-gBol010337-XLOC_040275-13994-1
TAGATGGCTACGTTCTTCTAGGCCGCGAAATCACTGTGGTGTTTGCAGAG
CONSENSUS
TAGATGGCTACGTTCTTCTAGGCCGCGAAATCACTGTGGTGTTTGCAGAG

A5-gBol010337-XLOC_040275-13994-0
GAGAACAGAAAGAAGCCTTCCGAAATGAGAGAACGATCAAG
A5-gBol010337-XLOC_040275-13994-1

GAGAACAGAAAGAAGCCTTCCGAAATGAGAGAACGATCAAG
CONSENSUS
GAGAACAGAAAGAAGCCTTCCGAAATGAGAGAACGATCAAG

alignment for event: A5-X-XLOC_040169-11873

A5-X-XLOC_040169-11873-0
GGACTGAGATGTTTCGATATGGCCTAGGGGGAACTCAGACAACAAGAGAA
A5-X-XLOC_040169-11873-1
GGACTGAGATGTTTCGATATGGCCTAGGGGGAACTCAGACAACAAGAGAA
CONSENSUS
GGACTGAGATGTTTCGATATGGCCTAGGGGGAACTCAGACAACAAGAGAA

A5-X-XLOC_040169-11873-0 AGCAACTATTTCTTAAC-----
CTCATATGCCGATTGTTTTAGTAAGTGT
A5-X-XLOC_040169-11873-1
AGCAACTATTTCTTAACGTAAGCTCATATGCCGATTGTTTTAGTAAGTGT
CONSENSUS
AGCAACTATTTCTTAAC.....CTCATATGCCGATTGTTTTAGTAAGTGT

A5-X-XLOC_040169-11873-0
TGGAGACGGGCCATGGGCAGACATGAGGAAGATGGGTGACCTTATCCCTA
A5-X-XLOC_040169-11873-1
TGGAGACGGGCCATGGGCAGACATGAGGAAGATGGGTGACCTTATCCCTA
CONSENSUS
TGGAGACGGGCCATGGGCAGACATGAGGAAGATGGGTGACCTTATCCCTA

A5-X-XLOC_040169-11873-0 AGCGTGTCGATCTTTCAG
A5-X-XLOC_040169-11873-1 AGCGTGTCGATCTTTCAG
CONSENSUS AGCGTGTCGATCTTTCAG

alignment for event: A5-gBol010281-XLOC_040179-15710

A5-gBol010281-XLOC_040179-15710-0
CATTCACTCCCCAAGCTGCTGGGATAAATTATCCTCTAGTGTCCCCGACC
A5-gBol010281-XLOC_040179-15710-1
CATTCACTCCCCAAGCTGCTGGGATAAATTATCCTCTAGTGTCCCCGACC
CONSENSUS
CATTCACTCCCCAAGCTGCTGGGATAAATTATCCTCTAGTGTCCCCGACC

A5-gBol010281-XLOC_040179-15710-0
AGTGCGAGTGTAAACCTTGGGTTGATTAACTCAGCTGCCTCCCTCTATCA
A5-gBol010281-XLOC_040179-15710-1
AGTGCGAGTGTAAACCTTGGGTTGATTAACTCAGCTGCCTCCCTCTATCA
CONSENSUS
AGTGCGAGTGTAAACCTTGGGTTGATTAACTCAGCTGCCTCCCTCTATCA

A5-gBol010281-XLOC_040179-15710-0
GACTCTTGCTCAACCCACG--------------------CTAGGAGCGC
A5-gBol010281-XLOC_040179-15710-1
GACTCTTGCTCAACCCACGGTAGATGTCTACAATCATGCTCTAGGAGCGC
CONSENSUS

GACTCTTGCTCAACCCACG.....................CTAGGAGCGC

A5-gBol010281-XLOC_040179-15710-0
TTACAGCAACGTATCCCCAAAGACCTGGACAACCAGAATGTGAC
A5-gBol010281-XLOC_040179-15710-1
TTACAGCAACGTATCCCCAAAGACCTGGACAACCAGAATGTGAC
CONSENSUS
TTACAGCAACGTATCCCCAAAGACCTGGACAACCAGAATGTGAC

alignment for event: A3-gBol027284-XLOC_021038-13677

A3-gBol027284-XLOC_021038-13677-0
TGGGAACATTCAGCCGAAACAATCGTGAACCAAAGTACACCATCGAGTAT
A3-gBol027284-XLOC_021038-13677-1
TGGGAACATTCAGCCGAAACAATCGTGAACCAAAGTACACCATCGAGTAT
CONSENSUS
TGGGAACATTCAGCCGAAACAATCGTGAACCAAAGTACACCATCGAGTAT

A3-gBol027284-XLOC_021038-13677-0
CTCCCCGAAGATGCACCGTTTCACCCG-----------------------
A3-gBol027284-XLOC_021038-13677-1
CTCCCCGAAGATGCACCGTTTCACCCGGGTGATAATCTGGAATCTCTGGT
CONSENSUS
CTCCCCGAAGATGCACCGTTTCACCCG.......................

A3-gBol027284-XLOC_021038-13677-0 -------------
CAAGGACGCAGATTCTTATGTTTCTTACCAAAGGAGG
A3-gBol027284-XLOC_021038-13677-1
GATGCTTGACAAGCAAGGACGCAGATTCTTATGTTTCTTACCAAAGGAGG
CONSENSUS
.............CAAGGACGCAGATTCTTATGTTTCTTACCAAAGGAGG

A3-gBol027284-XLOC_021038-13677-0
AAGAATCTCCTACCGGATGGACCTCTACGCAGCAAAACATCAGCACTGTG
A3-gBol027284-XLOC_021038-13677-1
AAGAATCTCCTACCGGATGGACCTCTACGCAGCAAAACATCAGCACTGTG
CONSENSUS
AAGAATCTCCTACCGGATGGACCTCTACGCAGCAAAACATCAGCACTGTG

A3-gBol027284-XLOC_021038-13677-0
TTGATGGAAACCGATAAACAGCTCAAGATCAAGACTCCTGACGAGCTGCT
A3-gBol027284-XLOC_021038-13677-1
TTGATGGAAACCGATAAACAGCTCAAGATCAAGACTCCTGACGAGCTGCT
CONSENSUS
TTGATGGAAACCGATAAACAGCTCAAGATCAAGACTCCTGACGAGCTGCT

A3-gBol027284-XLOC_021038-13677-0
TCAGCCACTCAACGACCAATGCCTTGTCAGG
A3-gBol027284-XLOC_021038-13677-1
TCAGCCACTCAACGACCAATGCCTTGTCAGG
CONSENSUS
TCAGCCACTCAACGACCAATGCCTTGTCAGG

alignment for event: A5-X-XLOC_021046-11665

A5-X-XLOC_021046-11665-0
      TCCGGCCCGCTCCTGATTTCATAACCCTAAACCCGTTAATTTTTGTTTGG
A5-X-XLOC_021046-11665-1
      TCCGGCCCGCTCCTGATTTCATAACCCTAAACCCGTTAATTTTTGTTTGG
CONSENSUS
      TCCGGCCCGCTCCTGATTTCATAACCCTAAACCCGTTAATTTTTGTTTGG

A5-X-XLOC_021046-11665-0
      GTTAAATTAGGGTCGTCTAAAATGAAACGCGGGTTAACCCTAAACCCGTT
A5-X-XLOC_021046-11665-1
      GTTAAATTAGGGTCGTCTAAAATGAAACGCGGGTTAACCCTAAACCCGTT
CONSENSUS
      GTTAAATTAGGGTCGTCTAAAATGAAACGCGGGTTAACCCTAAACCCGTT

A5-X-XLOC_021046-11665-0
      AATTTTTTTTAAGAAAACAAAAGTAAACAAAGCGACGTCGTTTCTTCTGT
A5-X-XLOC_021046-11665-1
      AATTTTTTTTAAGAAAACAAAAGTAAACAAAGCGACGTCGTTTCTTCTGT
CONSENSUS
      AATTTTTTTTAAGAAAACAAAAGTAAACAAAGCGACGTCGTTTCTTCTGT

A5-X-XLOC_021046-11665-0
      CAACTCTCACCATCTCATCATCGTTGTTTCTTCTCTCGACTCTCACCATC
A5-X-XLOC_021046-11665-1
      CAACTCTCACCATCTCATCATCGTTGTTTCTTCTCTCGACTCTCACCATC
CONSENSUS
      CAACTCTCACCATCTCATCATCGTTGTTTCTTCTCTCGACTCTCACCATC

A5-X-XLOC_021046-11665-0
      TCAACCAGAGACGAGATCCCAATGGCCTCACCATCTCATCTCTCGCCGCC
A5-X-XLOC_021046-11665-1
      TCAACCAGAGACGAGATCCCAATGGCCTCACCATCTCATCTCTCGCCGCC
CONSENSUS
      TCAACCAGAGACGAGATCCCAATGGCCTCACCATCTCATCTCTCGCCGCC

A5-X-XLOC_021046-11665-0
      TTAACGCCTCACCATCTCATCTCATCTCGTCGTCGTCACTCGTCATCATC
A5-X-XLOC_021046-11665-1
      TTAACGCCTCACCATCTCATCTCATCTCGTCGTCGTCACTCGTCATCATC
CONSENSUS
      TTAACGCCTCACCATCTCATCTCATCTCGTCGTCGTCACTCGTCATCATC

A5-X-XLOC_021046-11665-0
      ATTACTTCAGAGGGCAAACCAGAGACGAGATCCCAATCATCTCATCTCAT
A5-X-XLOC_021046-11665-1
      ATTACTTCAGAGGGCAAACCAGAGACGAGATCCCAATCATCTCATCTCAT
CONSENSUS
      ATTACTTCAGAGGGCAAACCAGAGACGAGATCCCAATCATCTCATCTCAT

A5-X-XLOC_021046-11665-0
      CTCATCACGGTCACAATCAGATCAGCAAGCATACCATTTTGTAAACAGAG
A5-X-XLOC_021046-11665-1
      CTCATCACGGTCACAATCAGATCAGCAAGCATACCATTTT--------AG

```
CONSENSUS
      CTCATCACGGTCACAATCAGATCAGCAAGCATACCATTTT........AG

A5-X-XLOC_021046-11665-0
      CAACGAGTGTTTGAAGAGACTCAAGAGTCAAGAGACTTGGAGAACAACAA
A5-X-XLOC_021046-11665-1
      CAACGAGTGTTTGAAGAGACTCAAGAGTCAAGAGACTTGGAGAACAACAA
CONSENSUS
      CAACGAGTGTTTGAAGAGACTCAAGAGTCAAGAGACTTGGAGAACAACAA

A5-X-XLOC_021046-11665-0
      GTGTTGGCTGACTTGAGAGTTTGAGTAAAGTTCTATTTATTAAAGTAGCT
A5-X-XLOC_021046-11665-1
      GTGTTGGCTGACTTGAGAGTTTGAGTAAAGTTCTATTTATTAAAGTAGCT
CONSENSUS
      GTGTTGGCTGACTTGAGAGTTTGAGTAAAGTTCTATTTATTAAAGTAGCT

A5-X-XLOC_021046-11665-0
      ACAAAGTTGTGCCCAAGAGTTATATTTATACTTTGCATTTACATTACATG
A5-X-XLOC_021046-11665-1
      ACAAAGTTGTGCCCAAGAGTTATATTTATACTTTGCATTTACATTACATG
CONSENSUS
      ACAAAGTTGTGCCCAAGAGTTATATTTATACTTTGCATTTACATTACATG

A5-X-XLOC_021046-11665-0
      AATGTTAACTTTGGTTTTTAATTTCAGTGAAAGACAAAAATGAATAAAAG
A5-X-XLOC_021046-11665-1
      AATGTTAACTTTGGTTTTTAATTTCAGTGAAAGACAAAAATGAATAAAAG
CONSENSUS
      AATGTTAACTTTGGTTTTTAATTTCAGTGAAAGACAAAAATGAATAAAAG

A5-X-XLOC_021046-11665-0    ACAC
A5-X-XLOC_021046-11665-1    ACAC
CONSENSUS                   ACAC


alignment for event: RI-gBol001843-XLOC_049887-14796

RI-gBol001843-XLOC_049887-14796-0
      ATTTTAGGAACTCTAATTCTGGGAAACTAGGGCACTTCTATGCACAGGTT
RI-gBol001843-XLOC_049887-14796-1
      ATTTTAGGAACTCTAATTCTGGGAAACTAGGGCACTTCTATGCACAG---
CONSENSUS
      ATTTTAGGAACTCTAATTCTGGGAAACTAGGGCACTTCTATGCACAG...

RI-gBol001843-XLOC_049887-14796-0
      CGTAAGATTTTTTACCCTCTACTTTCTTGCAGTCCTCCAACATGAAAAAT
RI-gBol001843-XLOC_049887-14796-1
      --------------------------------------------------
CONSENSUS
      ..................................................

RI-gBol001843-XLOC_049887-14796-0
      ATTAATTTTTAATGATGGGTCAATAATTAACTGAAGTGGTCAAATACTAT
RI-gBol001843-XLOC_049887-14796-1
```

CONSENSUS
....

RI-gBol001843-XLOC_049887-14796-0
GTTTCATATATATATATATATATATATCTAAGCATAACCAACCACATAAA
RI-gBol001843-XLOC_049887-14796-1

CONSENSUS
....

RI-gBol001843-XLOC_049887-14796-0
GTCCAAAAAGCTTAGTATAATCTTCTCCTGATTCAAGTATCTTTGAAGGA
RI-gBol001843-XLOC_049887-14796-1
--GA
CONSENSUS
....GA

RI-gBol001843-XLOC_049887-14796-0
TAAGACTGACGTTGATTCATCATCATCAATGACATTTTAAATAAGCTTAT
RI-gBol001843-XLOC_049887-14796-1
TAAGACTGACGTTGATTCATCATCATCAATGACATTTTAAATAAGCTTAT
CONSENSUS
TAAGACTGACGTTGATTCATCATCATCAATGACATTTTAAATAAGCTTAT

RI-gBol001843-XLOC_049887-14796-0
AATGTGGGGGTCTCTAAAAGGACTTCACAACAAAAGAAGGGGGGTGGGGG
RI-gBol001843-XLOC_049887-14796-1
AATGTGGGGGTCTCTAAAAGGACTTCACAACAAAAGAAGGGGGGTGGGGG
CONSENSUS
AATGTGGGGGTCTCTAAAAGGACTTCACAACAAAAGAAGGGGGGTGGGGG

RI-gBol001843-XLOC_049887-14796-0
GTGTTGCTAAAATACGCATGAACAGTCAGCGATTGTGACGGCATGTTGAG
RI-gBol001843-XLOC_049887-14796-1
GTGTTGCTAAAATACGCATGAACAGTCAGCGATTGTGACGGCATGTTGAG
CONSENSUS
GTGTTGCTAAAATACGCATGAACAGTCAGCGATTGTGACGGCATGTTGAG

RI-gBol001843-XLOC_049887-14796-0
GCGGACTGAGCAGCAGAGATGACATGTACTCCTGGGTGTTGCTAAAATAC
RI-gBol001843-XLOC_049887-14796-1
GCGGACTGAGCAGCAGAGATGACATGTACTCCTGGGTGTTGCTAAAATAC
CONSENSUS
GCGGACTGAGCAGCAGAGATGACATGTACTCCTGGGTGTTGCTAAAATAC

RI-gBol001843-XLOC_049887-14796-0
GCATGAACAGTCGGCGACTGTGACGGCATGTTGAGACGGACTGAGCAGCA
RI-gBol001843-XLOC_049887-14796-1
GCATGAACAGTCGGCGACTGTGACGGCATGTTGAGACGGACTGAGCAGCA
CONSENSUS
GCATGAACAGTCGGCGACTGTGACGGCATGTTGAGACGGACTGAGCAGCA

RI-gBol001843-XLOC_049887-14796-0
GAGATGCCATGTACTCCTGAAAAGGATCTCAGCCGTTCGATTCGCTTCAG
RI-gBol001843-XLOC_049887-14796-1

GAGATGCCATGTACTCCTGAAAAGGATCTCAGCCGTTCGATTCGCTTCAG
CONSENSUS
GAGATGCCATGTACTCCTGAAAAGGATCTCAGCCGTTCGATTCGCTTCAG

RI-gBol001843-XLOC_049887-14796-0
CACACGAGGACAAGTACAGTAATCGACTTTGGTCCGTTCACCACATATGG
RI-gBol001843-XLOC_049887-14796-1
CACACGAGGACAAGTACAGTAATCGACTTTGGTCCGTTCACCACATATGG
CONSENSUS
CACACGAGGACAAGTACAGTAATCGACTTTGGTCCGTTCACCACATATGG

RI-gBol001843-XLOC_049887-14796-0
GTCCCTGCCTTGCGTTTGTCTGTGTTTCAATCACGACCGAGTATTAAATT
RI-gBol001843-XLOC_049887-14796-1
GTCCCTGCCTTGCGTTTGTCTGTGTTTCAATCACGACCGAGTATTAAATT
CONSENSUS
GTCCCTGCCTTGCGTTTGTCTGTGTTTCAATCACGACCGAGTATTAAATT

RI-gBol001843-XLOC_049887-14796-0
GGTAATATTATCAGTTCACTCGCCCCCACGGCGCACGTTACCGTATGACA
RI-gBol001843-XLOC_049887-14796-1
GGTAATATTATCAGTTCACTCGCCCCCACGGCGCACGTTACCGTATGACA
CONSENSUS
GGTAATATTATCAGTTCACTCGCCCCCACGGCGCACGTTACCGTATGACA

RI-gBol001843-XLOC_049887-14796-0
AGCTTCTTGGAACTTCTCAATCTCTCTGGCTTTGAATATTTTAATCCGTC
RI-gBol001843-XLOC_049887-14796-1
AGCTTCTTGGAACTTCTCAATCTCTCTGGCTTTGAATATTTTAATCCGTC
CONSENSUS
AGCTTCTTGGAACTTCTCAATCTCTCTGGCTTTGAATATTTTAATCCGTC

RI-gBol001843-XLOC_049887-14796-0
CCGCACCGCAGTAACAGTAACAAAAATCTTTACATATATACCATATATTT
RI-gBol001843-XLOC_049887-14796-1
CCGCACCGCAGTAACAGTAACAAAAATCTTTACATATATACCATATATTT
CONSENSUS
CCGCACCGCAGTAACAGTAACAAAAATCTTTACATATATACCATATATTT

RI-gBol001843-XLOC_049887-14796-0
ATAAGTTTTTATAACTGTTAGAACCGCATCGCAGTTGTACCGCTTGTCCC
RI-gBol001843-XLOC_049887-14796-1
ATAAGTTTTTATAACTGTTAGAACCGCATCGCAGTTGTACCGCTTGTCCC
CONSENSUS
ATAAGTTTTTATAACTGTTAGAACCGCATCGCAGTTGTACCGCTTGTCCC

RI-gBol001843-XLOC_049887-14796-0
GCATTGCTCAATCCGCTGTTACCATTCGAAACCTCTAATTTTTTTGGTAA
RI-gBol001843-XLOC_049887-14796-1
GCATTGCTCAATCCGCTGTTACCATTCGAAACCTCTAATTTTTTTGGTAA
CONSENSUS
GCATTGCTCAATCCGCTGTTACCATTCGAAACCTCTAATTTTTTTGGTAA

RI-gBol001843-XLOC_049887-14796-0
TGTGCGTAGCGTTTTTTCGGAGCAGTAATCACCGTTCTTATCTCACATCC
RI-gBol001843-XLOC_049887-14796-1

TGTGCGTAGCGTTTTTTCGGAGCAGTAATCACCGTTCTTATCTCACATCC
CONSENSUS
TGTGCGTAGCGTTTTTTCGGAGCAGTAATCACCGTTCTTATCTCACATCC

RI-gBol001843-XLOC_049887-14796-0
CCAGCCCTATCTTCAAATTTTTTTCTTAGCTAAAATGCGCATTTAAACAA
RI-gBol001843-XLOC_049887-14796-1
CCAGCCCTATCTTCAAATTTTTTTCTTAGCTAAAATGCGCATTTAAACAA
CONSENSUS
CCAGCCCTATCTTCAAATTTTTTTCTTAGCTAAAATGCGCATTTAAACAA

RI-gBol001843-XLOC_049887-14796-0
GCACACGCGCATTTGAGCTCAATCGTGTACATTTTTGCTAAAATGTGTAC
RI-gBol001843-XLOC_049887-14796-1
GCACACGCGCATTTGAGCTCAATCGTGTACATTTTTGCTAAAATGTGTAC
CONSENSUS
GCACACGCGCATTTGAGCTCAATCGTGTACATTTTTGCTAAAATGTGTAC

RI-gBol001843-XLOC_049887-14796-0 TTGAGTAACATAGTTCACTCG
RI-gBol001843-XLOC_049887-14796-1 TTGAGTAACATAGTTCACTCG
CONSENSUS TTGAGTAACATAGTTCACTCG

alignment for event: RI-gBol007738-XLOC_043156-16038

RI-gBol007738-XLOC_043156-16038-0
CCGCCGATTGGGGAGAAGAGGGTTGAAGAGATGGGGAAATGGGTGAGGAA
RI-gBol007738-XLOC_043156-16038-1
CCGCCGATTGGGGAGAAGAGGGTTGAAGAGATGGGGAAATGGGTGAGGAA
CONSENSUS
CCGCCGATTGGGGAGAAGAGGGTTGAAGAGATGGGGAAATGGGTGAGGAA

RI-gBol007738-XLOC_043156-16038-0
GGAGTTCCGAGTGAGTGGGAGCAGTTGGGACGCAAGTTCAGTGGATATTG
RI-gBol007738-XLOC_043156-16038-1
GGAGTTCCGAGTGAGTGGGAGCAGTTGGGACGCAAGTTCAGTGGATATTG
CONSENSUS
GGAGTTCCGAGTGAGTGGGAGCAGTTGGGACGCAAGTTCAGTGGATATTG

RI-gBol007738-XLOC_043156-16038-0
CTGTTTCGGGTCTCGGTTGGTTTGCTATAGGGCTCAAAGGAAACGCGGTA
RI-gBol007738-XLOC_043156-16038-1
CTGTTTCGGGTCTCGGTTGGTTTGCTATAGGGCTCAAAGGAAACGCGGTA
CONSENSUS
CTGTTTCGGGTCTCGGTTGGTTTGCTATAGGGCTCAAAGGAAACGCGGTA

RI-gBol007738-XLOC_043156-16038-0
TTAGGTGTGTGGACTCACGAAGGGATTGATGTCTTTCTCCGTGACTCATT
RI-gBol007738-XLOC_043156-16038-1
TTAGGTGTGTGGACTCACGAAGGGATTGATGTCTTTCTCCGTGACTCATT
CONSENSUS
TTAGGTGTGTGGACTCACGAAGGGATTGATGTCTTTCTCCGTGACTCATT

RI-gBol007738-XLOC_043156-16038-0
GGTTCCACAACGAGCGCGCACTTTCGAAGACTCTGGTTTCACTGTCTCGA

RI-gBol007738-XLOC_043156-16038-1
GGTTCCACAACGAGCGCGCACTTTCGAAGACTCTGGTTTCACTGTCTCGA
CONSENSUS
GGTTCCACAACGAGCGCGCACTTTCGAAGACTCTGGTTTCACTGTCTCGA

RI-gBol007738-XLOC_043156-16038-0
AGATCGTTGCCAAAGCTGATAGAGATTTTAATATAAATCACAAGGCGAAA
RI-gBol007738-XLOC_043156-16038-1
AGATCGTTGCCAAAGCTGATAGAGATTTTAATATAAATCACAAGGCGAAA
CONSENSUS
AGATCGTTGCCAAAGCTGATAGAGATTTTAATATAAATCACAAGGCGAAA

RI-gBol007738-XLOC_043156-16038-0
CGGAGACTCAACAAGAAGTCCACTTCTTCAGATTCTGTGTCGGACATAGA
RI-gBol007738-XLOC_043156-16038-1
CGGAGACTCAACAAGAAGTCCACTTCTTCAGATTCTGTGTCGGACATAGA
CONSENSUS
CGGAGACTCAACAAGAAGTCCACTTCTTCAGATTCTGTGTCGGACATAGA

RI-gBol007738-XLOC_043156-16038-0
AAATTGCCAAGAGGTGTCCTTGTAGTAAAGTCACAGCCTTTCAGATCTAA
RI-gBol007738-XLOC_043156-16038-1
AAATTGCCAAGAGGTGTCCTTGTAGTAAAGTCACAGCCTTTCAGATCTAA
CONSENSUS
AAATTGCCAAGAGGTGTCCTTGTAGTAAAGTCACAGCCTTTCAGATCTAA

RI-gBol007738-XLOC_043156-16038-0
GTTAAGTTGTTTTATTTTCTTTGCTTGTTGAATTTACTTGGTTCTGTGAA
RI-gBol007738-XLOC_043156-16038-1

CONSENSUS
....

RI-gBol007738-XLOC_043156-16038-0
TTTCACCAAGGCTGATGTTTCTGTTTTCCTCCTAGCTTTTTTTTTTATTC
RI-gBol007738-XLOC_043156-16038-1

CONSENSUS
....

RI-gBol007738-XLOC_043156-16038-0
ATTAAAAAGGTTCGTTTCCCACCACGTGTTTTACGAAAAACTGTTTCTGA
RI-gBol007738-XLOC_043156-16038-1

CONSENSUS
....

RI-gBol007738-XLOC_043156-16038-0
ACGCTAGAGTTGAATAATCCTTTGATTGTAGCATCTACAAATTTATTCCA
RI-gBol007738-XLOC_043156-16038-1

CONSENSUS
....

RI-gBol007738-XLOC_043156-16038-0
GTGACAGACTGTTGAGTGACCGATCGATATGATATTCCACAGGTTTGCTT

RI-gBol007738-XLOC_043156-16038-1
---------------------------------------GTTTGCTT
CONSENSUS
....GTTTGCTT

RI-gBol007738-XLOC_043156-16038-0
GTTTGAGTCCATATTGGGCCGGAATGTTGAATCGACGGGTAAACAAGAGA
RI-gBol007738-XLOC_043156-16038-1
GTTTGAGTCCATATTGGGCCGGAATGTTGAATCGACGGGTAAACAAGAGA
CONSENSUS
GTTTGAGTCCATATTGGGCCGGAATGTTGAATCGACGGGTAAACAAGAGA

RI-gBol007738-XLOC_043156-16038-0
GTTACCTTCCCCTCGACTCACTTGATGCATCAGGATGATGCCATCTGTAC
RI-gBol007738-XLOC_043156-16038-1
GTTACCTTCCCCTCGACTCACTTGATGCATCAGGATGATGCCATCTGTAC
CONSENSUS
GTTACCTTCCCCTCGACTCACTTGATGCATCAGGATGATGCCATCTGTAC

RI-gBol007738-XLOC_043156-16038-0
AAAGGACGTTTGTAAGAACTAAGAACTGAGTGAATAGAAATCAAGAAAGT
RI-gBol007738-XLOC_043156-16038-1
AAAGGACGTTTGTAAGAACTAAGAACTGAGTGAATAGAAATCAAGAAAGT
CONSENSUS
AAAGGACGTTTGTAAGAACTAAGAACTGAGTGAATAGAAATCAAGAAAGT

RI-gBol007738-XLOC_043156-16038-0
TCTTCATTGTCCTCCAGGTTTTGGTTCTATTCTATTATCAAAACAAACAT
RI-gBol007738-XLOC_043156-16038-1
TCTTCATTGTCCTCCAGGTTTTGGTTCTATTCTATTATCAAAACAAACAT
CONSENSUS
TCTTCATTGTCCTCCAGGTTTTGGTTCTATTCTATTATCAAAACAAACAT

RI-gBol007738-XLOC_043156-16038-0
TTATATTTTTCTGTAGAAAAACAAAATTTAAAAACGCAAGAACACATAAA
RI-gBol007738-XLOC_043156-16038-1
TTATATTTTTCTGTAGAAAAACAAAATTTAAAAACGCAAGAACACATAAA
CONSENSUS
TTATATTTTTCTGTAGAAAAACAAAATTTAAAAACGCAAGAACACATAAA

RI-gBol007738-XLOC_043156-16038-0
AGGATGATCAATGATGCAACGTCCTTGATGAAACATATGTAAACATTCAG
RI-gBol007738-XLOC_043156-16038-1
AGGATGATCAATGATGCAACGTCCTTGATGAAACATATGTAAACATTCAG
CONSENSUS
AGGATGATCAATGATGCAACGTCCTTGATGAAACATATGTAAACATTCAG

RI-gBol007738-XLOC_043156-16038-0
GCATCTTAATGATGTTTCCATACAAACTGAACATGCTGTCGCTTTTTTTT
RI-gBol007738-XLOC_043156-16038-1
GCATCTTAATGATGTTTCCATACAAACTGAACATGCTGTCGCTTTTTTTT
CONSENSUS
GCATCTTAATGATGTTTCCATACAAACTGAACATGCTGTCGCTTTTTTTT

RI-gBol007738-XLOC_043156-16038-0
TCTTCTTCTTCCAAATCAATTGATTCCACTCTCTGTTTGTCCACCAGTCT

RI-gBol007738-XLOC_043156-16038-1
TCTTCTTCTTCCAAATCAATTGATTCCACTCTCTGTTTGTCCACCAGTCT
CONSENSUS
TCTTCTTCTTCCAAATCAATTGATTCCACTCTCTGTTTGTCCACCAGTCT

RI-gBol007738-XLOC_043156-16038-0
TTGTAACACACGATCAGAAGCAGATCTTTACGAAACAAATAACAGACGGC
RI-gBol007738-XLOC_043156-16038-1
TTGTAACACACGATCAGAAGCAGATCTTTACGAAACAAATAACAGACGGC
CONSENSUS
TTGTAACACACGATCAGAAGCAGATCTTTACGAAACAAATAACAGACGGC

RI-gBol007738-XLOC_043156-16038-0
CGTAATAAAAACCAAACCTTTCTGATCAACAAGACGTGGACCAACAAGGG
RI-gBol007738-XLOC_043156-16038-1
CGTAATAAAAACCAAACCTTTCTGATCAACAAGACGTGGACCAACAAGGG
CONSENSUS
CGTAATAAAAACCAAACCTTTCTGATCAACAAGACGTGGACCAACAAGGG

RI-gBol007738-XLOC_043156-16038-0
AGCTGTAAATGGACGAGTCGATCTCGGATCAGCCAGAAATGAGATGGAAC
RI-gBol007738-XLOC_043156-16038-1
AGCTGTAAATGGACGAGTCGATCTCGGATCAGCCAGAAATGAGATGGAAC
CONSENSUS
AGCTGTAAATGGACGAGTCGATCTCGGATCAGCCAGAAATGAGATGGAAC

RI-gBol007738-XLOC_043156-16038-0
TATGCAATTTGGTGGGGAAATGAAGAATGAGAGTGATGAAGATGATGAGC
RI-gBol007738-XLOC_043156-16038-1
TATGCAATTTGGTGGGGAAATGAAGAATGAGAGTGATGAAGATGATGAGC
CONSENSUS
TATGCAATTTGGTGGGGAAATGAAGAATGAGAGTGATGAAGATGATGAGC

RI-gBol007738-XLOC_043156-16038-0
TGGAAATGACTATGGAAGGACATTTTGTTCGACCAAATGAGAGCAAAAAA
RI-gBol007738-XLOC_043156-16038-1
TGGAAATGACTATGGAAGGACATTTTGTTCGACCAAATGAGAGCAAAAAA
CONSENSUS
TGGAAATGACTATGGAAGGACATTTTGTTCGACCAAATGAGAGCAAAAAA

RI-gBol007738-XLOC_043156-16038-0
CATCATAGATGCATCAAGAGAAACTTGGAACGGGGAGATGGACGAAATGG
RI-gBol007738-XLOC_043156-16038-1
CATCATAGATGCATCAAGAGAAACTTGGAACGGGGAGATGGACGAAATGG
CONSENSUS
CATCATAGATGCATCAAGAGAAACTTGGAACGGGGAGATGGACGAAATGG

RI-gBol007738-XLOC_043156-16038-0
GCTATCGACGGGATAAATGGAGTTTATCGATCAAGTTAAAGATCCACTTG
RI-gBol007738-XLOC_043156-16038-1
GCTATCGACGGGATAAATGGAGTTTATCGATCAAGTTAAAGATCCACTTG
CONSENSUS
GCTATCGACGGGATAAATGGAGTTTATCGATCAAGTTAAAGATCCACTTG

RI-gBol007738-XLOC_043156-16038-0
TGTTTACACTTGGGCCAATAACTAAATTACGATGAGCGTAGGGGCTTTGG

```
RI-gBol007738-XLOC_043156-16038-1
      TGTTTACACTTGGGCCAATAACTAAATTACGATGAGCGTAGGGGCTTTGG
CONSENSUS
      TGTTTACACTTGGGCCAATAACTAAATTACGATGAGCGTAGGGGCTTTGG

RI-gBol007738-XLOC_043156-16038-0
      TTCGACTTGTACAAGATGGGAAGGCCGGCTTGACATAAGAGTCGATCCAT
RI-gBol007738-XLOC_043156-16038-1
      TTCGACTTGTACAAGATGGGAAGGCCGGCTTGACATAAGAGTCGATCCAT
CONSENSUS
      TTCGACTTGTACAAGATGGGAAGGCCGGCTTGACATAAGAGTCGATCCAT

RI-gBol007738-XLOC_043156-16038-0      TGCTACCATTACTCGG
RI-gBol007738-XLOC_043156-16038-1      TGCTACCATTACTCGG
CONSENSUS                              TGCTACCATTACTCGG



alignment for event: SE-gBol015821-XLOC_034082-13273

SE-gBol015821-XLOC_034082-13273-0
      GCAAGAGGACCTTAGGAGGCCATTTGAGCAGTTCGGTCCTGTCAAGGACA
SE-gBol015821-XLOC_034082-13273-1
      GCAAGAGGACCTTAGGAGGCCATTTGAGCAGTTCGGTCCTGTCAAGGACA
CONSENSUS
      GCAAGAGGACCTTAGGAGGCCATTTGAGCAGTTCGGTCCTGTCAAGGACA

SE-gBol015821-XLOC_034082-13273-0
      TCTACCTTCCAAGGGATTATTATACCGG----------------------
SE-gBol015821-XLOC_034082-13273-1
      TCTACCTTCCAAGGGATTATTATACCGGTGAAGAGACCAATGAAGTAATT
CONSENSUS
      TCTACCTTCCAAGGGATTATTATACCGG......................

SE-gBol015821-XLOC_034082-13273-0
      --------------------------------------------------
SE-gBol015821-XLOC_034082-13273-1
      AGAGTTCTTTGGGAAGATGTTATGGTAGTGAAGGTTTTGAAGGAAGTTGA
CONSENSUS
      ..................................................

SE-gBol015821-XLOC_034082-13273-0
      --------------------------------------------------
SE-gBol015821-XLOC_034082-13273-1
      GTTTGAGTTCAATTTCAAGATATATCAAGTCTTGGAGAAGTCTTCTTTAT
CONSENSUS
      ..................................................

SE-gBol015821-XLOC_034082-13273-0      ---------------
AGATCCAAGGGGGTTTGGTTTCATTCAGTATGTGG
SE-gBol015821-XLOC_034082-13273-1
      TCTGAAAATTTGCAGAGATCCAAGGGGGTTTGGTTTCATTCAGTATGTGG
CONSENSUS
      ...............AGATCCAAGGGGGTTTGGTTTCATTCAGTATGTGG

SE-gBol015821-XLOC_034082-13273-0
```

ACCCTGCTGATGCTGCGGAGGCAAAACATCACATGGAAGGCTATCTTCTT
SE-gBol015821-XLOC_034082-13273-1
ACCCTGCTGATGCTGCGGAGGCAAAACATCACATGGAAGGCTATCTTCTT
CONSENSUS
ACCCTGCTGATGCTGCGGAGGCAAAACATCACATGGAAGGCTATCTTCTT

SE-gBol015821-XLOC_034082-13273-0
CTTGGTCGCGAGCTGACCGTTGTATTTGCAGAAGAGAACAGGAAGAAGCC
SE-gBol015821-XLOC_034082-13273-1
CTTGGTCGCGAGCTGACCGTTGTATTTGCAGAAGAGAACAGGAAGAAGCC
CONSENSUS
CTTGGTCGCGAGCTGACCGTTGTATTTGCAGAAGAGAACAGGAAGAAGCC

SE-gBol015821-XLOC_034082-13273-0
AACTGAAATGAGAACAAGGGATCGAGGTGGTAGGAGCAACAGATTCAATG
SE-gBol015821-XLOC_034082-13273-1
AACTGAAATGAGAACAAGGGATCGAGGTGGTAGGAGCAACAGATTCAATG
CONSENSUS
AACTGAAATGAGAACAAGGGATCGAGGTGGTAGGAGCAACAGATTCAATG

SE-gBol015821-XLOC_034082-13273-0
ACAGAAGACGTTCTCCTCCTCGCTACTCTCGTTCTCCACCCCCTCGACGT
SE-gBol015821-XLOC_034082-13273-1
ACAGAAGACGTTCTCCTCCTCGCTACTCTCGTTCTCCACCCCCTCGACGT
CONSENSUS
ACAGAAGACGTTCTCCTCCTCGCTACTCTCGTTCTCCACCCCCTCGACGT

SE-gBol015821-XLOC_034082-13273-0
GGTGGTAGAACGCGATCACGTAGCCGCGAGTATAATTCTCCTCCTCCTAA
SE-gBol015821-XLOC_034082-13273-1
GGTGGTAGAACGCGATCACGTAGCCGCGAGTATAATTCTCCTCCTCCTAA
CONSENSUS
GGTGGTAGAACGCGATCACGTAGCCGCGAGTATAATTCTCCTCCTCCTAA

SE-gBol015821-XLOC_034082-13273-0 AAGACATCAGTCTAG
SE-gBol015821-XLOC_034082-13273-1 AAGACATCAGTCTAG
CONSENSUS AAGACATCAGTCTAG

alignment for event: A5-X-XLOC_037182-6646

A5-X-XLOC_037182-6646-0
GTCTTAGCGTAAAGATGATACGACAAAATCTGTCATGAGAGTGCTTCAGT
A5-X-XLOC_037182-6646-1
GTCTTAGCGTAAAGATGATACGACAAAATCTGTCATGAGAGTGCTTCAGT
CONSENSUS
GTCTTAGCGTAAAGATGATACGACAAAATCTGTCATGAGAGTGCTTCAGT

A5-X-XLOC_037182-6646-0
GATCCAATCCTCAGAGACTCAGAAACAGCAGAAGGTATGATCATGTGGCC
A5-X-XLOC_037182-6646-1
GATCCAATCCTCAGAGACTCAGAAACAGCAGAAG----------------
CONSENSUS
GATCCAATCCTCAGAGACTCAGAAACAGCAGAAG................

A5-X-XLOC_037182-6646-0
ATTCTTCTCATTTTACAGCAGCAAAAGTTAGGGGAATCATAACATAAGAA
A5-X-XLOC_037182-6646-1 -----------------------------
AGGGGAATCATAACATAAGAA
CONSENSUS
.............................AGGGGAATCATAACATAAGAA

A5-X-XLOC_037182-6646-0
GCAAGGTGGTGTTTGGAAAAGGTGTTCTCGATATAATCAAGTACACTATA
A5-X-XLOC_037182-6646-1
GCAAGGTGGTGTTTGGAAAAGGTGTTCTCGATATAATCAAGTACACTATA
CONSENSUS
GCAAGGTGGTGTTTGGAAAAGGTGTTCTCGATATAATCAAGTACACTATA

A5-X-XLOC_037182-6646-0
TAACAAACATTCTAGATGGGTATAATATTTTGTCCTTGGTTTAAATTTAG
A5-X-XLOC_037182-6646-1
TAACAAACATTCTAGATGGGTATAATATTTTGTCCTTGGTTTAAATTTAG
CONSENSUS
TAACAAACATTCTAGATGGGTATAATATTTTGTCCTTGGTTTAAATTTAG

A5-X-XLOC_037182-6646-0
GGTTCATGAAAGTGATTAATTAGTAGACATGATTTTGTTTTGAACTAATG
A5-X-XLOC_037182-6646-1
GGTTCATGAAAGTGATTAATTAGTAGACATGATTTTGTTTTGAACTAATG
CONSENSUS
GGTTCATGAAAGTGATTAATTAGTAGACATGATTTTGTTTTGAACTAATG

A5-X-XLOC_037182-6646-0
CTCCTGAAAGTAGTTAGTCATCCACTAGCTGGACACAAAATAGTTGGTGT
A5-X-XLOC_037182-6646-1
CTCCTGAAAGTAGTTAGTCATCCACTAGCTGGACACAAAATAGTTGGTGT
CONSENSUS
CTCCTGAAAGTAGTTAGTCATCCACTAGCTGGACACAAAATAGTTGGTGT

A5-X-XLOC_037182-6646-0
GTTCTCATCATGTAGGATTTTTTTTTTGCCTTACATTTTCCGAGTTTAGT
A5-X-XLOC_037182-6646-1
GTTCTCATCATGTAGGATTTTTTTTTTGCCTTACATTTTCCGAGTTTAGT
CONSENSUS
GTTCTCATCATGTAGGATTTTTTTTTTGCCTTACATTTTCCGAGTTTAGT

A5-X-XLOC_037182-6646-0
TCCTCTTATAGAATATGGATTTGTGGATTGCTGTTACAGATAAATCTTCA
A5-X-XLOC_037182-6646-1
TCCTCTTATAGAATATGGATTTGTGGATTGCTGTTACAGATAAATCTTCA
CONSENSUS
TCCTCTTATAGAATATGGATTTGTGGATTGCTGTTACAGATAAATCTTCA

A5-X-XLOC_037182-6646-0
AAACGAGATATTAGGTGTTGAATTAAACAAATAACCTCCAAACTAGATGG
A5-X-XLOC_037182-6646-1
AAACGAGATATTAGGTGTTGAATTAAACAAATAACCTCCAAACTAGATGG
CONSENSUS
AAACGAGATATTAGGTGTTGAATTAAACAAATAACCTCCAAACTAGATGG

A5-X-XLOC_037182-6646-0
TTAATCTGTATGTTTGCTTATAGAACCGACTCATCTTAAATCCACCAGAT
A5-X-XLOC_037182-6646-1
TTAATCTGTATGTTTGCTTATAGAACCGACTCATCTTAAATCCACCAGAT
CONSENSUS
TTAATCTGTATGTTTGCTTATAGAACCGACTCATCTTAAATCCACCAGAT

A5-X-XLOC_037182-6646-0
ACAACGCGGACTAGTCAACCAATTAGGCTAGTTGACCAGTTTTTAGTGTA
A5-X-XLOC_037182-6646-1
ACAACGCGGACTAGTCAACCAATTAGGCTAGTTGACCAGTTTTTAGTGTA
CONSENSUS
ACAACGCGGACTAGTCAACCAATTAGGCTAGTTGACCAGTTTTTAGTGTA

A5-X-XLOC_037182-6646-0
TTTTTTTTAATTGTGTTGTGTTTGCGCCAATTTAAGGTACTTGAAGAAAT
A5-X-XLOC_037182-6646-1
TTTTTTTTAATTGTGTTGTGTTTGCGCCAATTTAAGGTACTTGAAGAAAT
CONSENSUS
TTTTTTTTAATTGTGTTGTGTTTGCGCCAATTTAAGGTACTTGAAGAAAT

A5-X-XLOC_037182-6646-0
GGTCCATTTAAGGTTTGTATAAAAATATACAGACCTTCCAGCAACAGGGC
A5-X-XLOC_037182-6646-1
GGTCCATTTAAGGTTTGTATAAAAATATACAGACCTTCCAGCAACAGGGC
CONSENSUS
GGTCCATTTAAGGTTTGTATAAAAATATACAGACCTTCCAGCAACAGGGC

A5-X-XLOC_037182-6646-0
GTCTTAGAAACACCTAGGGATGGGCGTTCGGGTACCCATTTGGGTTCGGT
A5-X-XLOC_037182-6646-1
GTCTTAGAAACACCTAGGGATGGGCGTTCGGGTACCCATTTGGGTTCGGT
CONSENSUS
GTCTTAGAAACACCTAGGGATGGGCGTTCGGGTACCCATTTGGGTTCGGT

A5-X-XLOC_037182-6646-0
TCGGATCTATCCGGGTTTCGGGTTTTCGGGTTTAAAGATTTCAGCTTCGT
A5-X-XLOC_037182-6646-1
TCGGATCTATCCGGGTTTCGGGTTTTCGGGTTTAAAGATTTCAGCTTCGT
CONSENSUS
TCGGATCTATCCGGGTTTCGGGTTTTCGGGTTTAAAGATTTCAGCTTCGT

A5-X-XLOC_037182-6646-0 TCGGG
A5-X-XLOC_037182-6646-1 TCGGG
CONSENSUS TCGGG

alignment for event: A3-gBol011582-XLOC_038823-14605

A3-gBol011582-XLOC_038823-14605-0
CAATGACGAAGTTGACGCTTGCAAAAAGCTACTAGCAAAAGTGGACCTCA
A3-gBol011582-XLOC_038823-14605-1
CAATGACGAAGTTGACGCTTGCAAAAAGCTACTAGCAAAAGTGGACCTCA
CONSENSUS
CAATGACGAAGTTGACGCTTGCAAAAAGCTACTAGCAAAAGTGGACCTCA

A3-gBol011582-XLOC_038823-14605-0
AAGGTTTTCAG-----------------------------GTCAAATG
A3-gBol011582-XLOC_038823-14605-1
AAGGTTTTCAGATCGGGAAAACAAAGGTGTTTCTTAGAGCAGGTCAAATG
CONSENSUS
AAGGTTTTCAG.............................GTCAAATG

A3-gBol011582-XLOC_038823-14605-0
GCAGAACTGGATGCTCACCGAGCTGAGGTTCTTGGTCGTTCAGCACGGAT
A3-gBol011582-XLOC_038823-14605-1
GCAGAACTGGATGCTCACCGAGCTGAGGTTCTTGGTCGTTCAGCACGGAT
CONSENSUS
GCAGAACTGGATGCTCACCGAGCTGAGGTTCTTGGTCGTTCAGCACGGAT

A3-gBol011582-XLOC_038823-14605-0
AATACAGAGGAAAGTCCTTTCTTATCAGTCCCGCAAAAAGTTTCTGTTGT
A3-gBol011582-XLOC_038823-14605-1
AATACAGAGGAAAGTCCTTTCTTATCAGTCCCGCAAAAAGTTTCTGTTGT
CONSENSUS
AATACAGAGGAAAGTCCTTTCTTATCAGTCCCGCAAAAAGTTTCTGTTGT

A3-gBol011582-XLOC_038823-14605-0
TGCAGGCTGCTTCAACAGATATCCAGGCCTTGTGTAGAG
A3-gBol011582-XLOC_038823-14605-1
TGCAGGCTGCTTCAACAGATATCCAGGCCTTGTGTAGAG
CONSENSUS
TGCAGGCTGCTTCAACAGATATCCAGGCCTTGTGTAGAG

alignment for event: RI-gBol011582-XLOC_038823-14601

RI-gBol011582-XLOC_038823-14601-0
ATGGAGATCGAGGAAGCAAAATCTCAAGAAATCGAAGCATTACAGTCAGC
RI-gBol011582-XLOC_038823-14601-1
ATGGAGATCGAGGAAGCAAAATCTCAAGAAATCGAAGCATTACAGTCAGC
CONSENSUS
ATGGAGATCGAGGAAGCAAAATCTCAAGAAATCGAAGCATTACAGTCAGC

RI-gBol011582-XLOC_038823-14601-0
TTTAACTGACATAAAGCTTAAGCTCAAGGAAACTCAAGAAACCAAAAGCG
RI-gBol011582-XLOC_038823-14601-1
TTTAACTGACATAAAGCTTAAGCTCAAGGAAACTCAAGAAACCAAAAGCG
CONSENSUS
TTTAACTGACATAAAGCTTAAGCTCAAGGAAACTCAAGAAACCAAAAGCG

RI-gBol011582-XLOC_038823-14601-0
CGGAGATCTCAAGATTGCAGTCTGCTTTACAAGACATGCAACTTGAGATT
RI-gBol011582-XLOC_038823-14601-1
CGGAGATCTCAAGATTGCAGTCTGCTTTACAAGACATGCAACTTGAGATT
CONSENSUS
CGGAGATCTCAAGATTGCAGTCTGCTTTACAAGACATGCAACTTGAGATT

RI-gBol011582-XLOC_038823-14601-0
GAAGAACTCTCAAAGGGACTTGAGATGAGTAACGATCTTTCTGCTGAAAA

RI-gBol011582-XLOC_038823-14601-1
      GAAGAACTCTCAAAGGGACTTGAGATGAGTAACGATCTTTCTGCTGAAAA
CONSENSUS
      GAAGAACTCTCAAAGGGACTTGAGATGAGTAACGATCTTTCTGCTGAAAA

RI-gBol011582-XLOC_038823-14601-0
      TGAACAGCTTAAAGTTAGTCCGTTAAAAAGTCTATGTGTTGAAGATAATG
RI-gBol011582-XLOC_038823-14601-1
      TGAACAGCTTAAA-------------------------------------
CONSENSUS
      TGAACAGCTTAAA.....................................

RI-gBol011582-XLOC_038823-14601-0
      TTTAAGATTTTTACATGTATTATCCTTGTATTTTTGTCTGATGCTCTTTC
RI-gBol011582-XLOC_038823-14601-1

CONSENSUS
      ....

RI-gBol011582-XLOC_038823-14601-0
      TTTATAAGGAGTTGGTGAGTTCTTTGCAAAACAAGAATGATGGAGACGTA
RI-gBol011582-XLOC_038823-14601-1   --------
GAGTTGGTGAGTTCTTTGCAAAACAAGAATGATGGAGACGTA
CONSENSUS
      ........GAGTTGGTGAGTTCTTTGCAAAACAAGAATGATGGAGACGTA

RI-gBol011582-XLOC_038823-14601-0
      AGCAAGCTCAGTGAAGAGCAGATAAAACAAGAAGTTCCTGTAATTGATCA
RI-gBol011582-XLOC_038823-14601-1
      AGCAAGCTCAGTGAAGAGCAGATAAAACAAGAAGTTCCTGTAATTGATCA
CONSENSUS
      AGCAAGCTCAGTGAAGAGCAGATAAAACAAGAAGTTCCTGTAATTGATCA

RI-gBol011582-XLOC_038823-14601-0
      GACCGCGATCATTAAACTTGAAGCTGAAAATCAGCAGCTGAAG
RI-gBol011582-XLOC_038823-14601-1
      GACCGCGATCATTAAACTTGAAGCTGAAAATCAGCAGCTGAAG
CONSENSUS
      GACCGCGATCATTAAACTTGAAGCTGAAAATCAGCAGCTGAAG
