# Supplementary material for: Splicing-related genes are alternatively spliced upon changes in ambient temperatures in plants
Source: PLoS One. 2017 Mar 3;12(3):e0172950. doi: 10.1371/journal.pone.0172950 (PMC5336241; doi:10.1371/journal.pone.0172950)
Supplement: S1 Fig — (DOCX) [file pone.0172950.s008.docx]

**A**

S1 Fig. Validation of RNAseq results by qPCR on 5 different genes.
For each gene, we amplified a region only present in transcripts undergoing a splicing event, and a region included in all isoforms. We calculated the fold change in transcripts undergoing the splicing event relative to the total amount of transcripts, and compared this to the RNAseq results. (A) Comparison of RNAseq and qPCR fold change (N=2 (16 plants per sample), mean ±SD). (B) Correlation of fold change in RNAseq and qPCR.

**B**
