# Supplementary material for: Splicing-related genes are alternatively spliced upon changes in ambient temperatures in plants
Source: PLoS One. 2017 Mar 3;12(3):e0172950. doi: 10.1371/journal.pone.0172950 (PMC5336241; doi:10.1371/journal.pone.0172950)
Supplement: S2 Fig — (DOCX) [file pone.0172950.s009.docx]

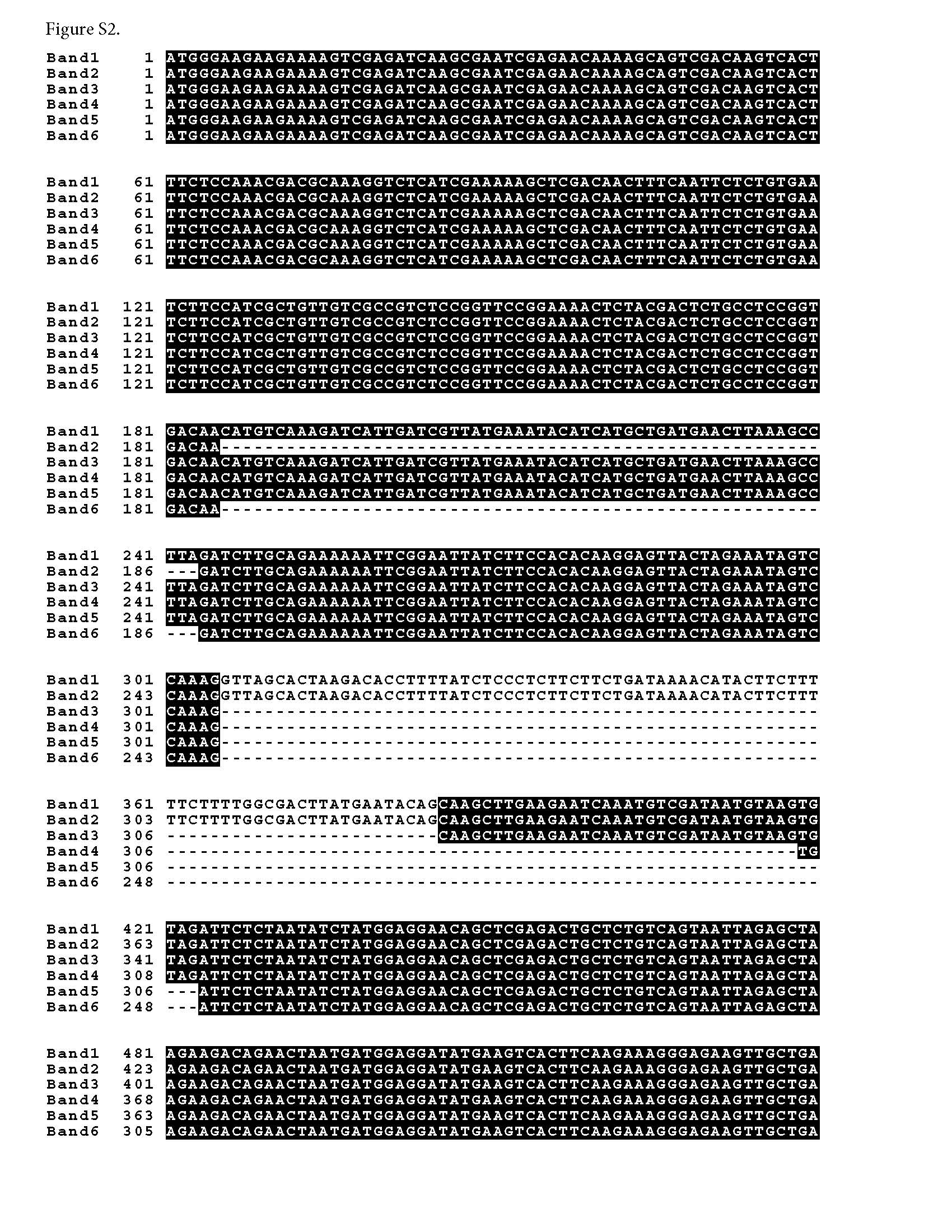

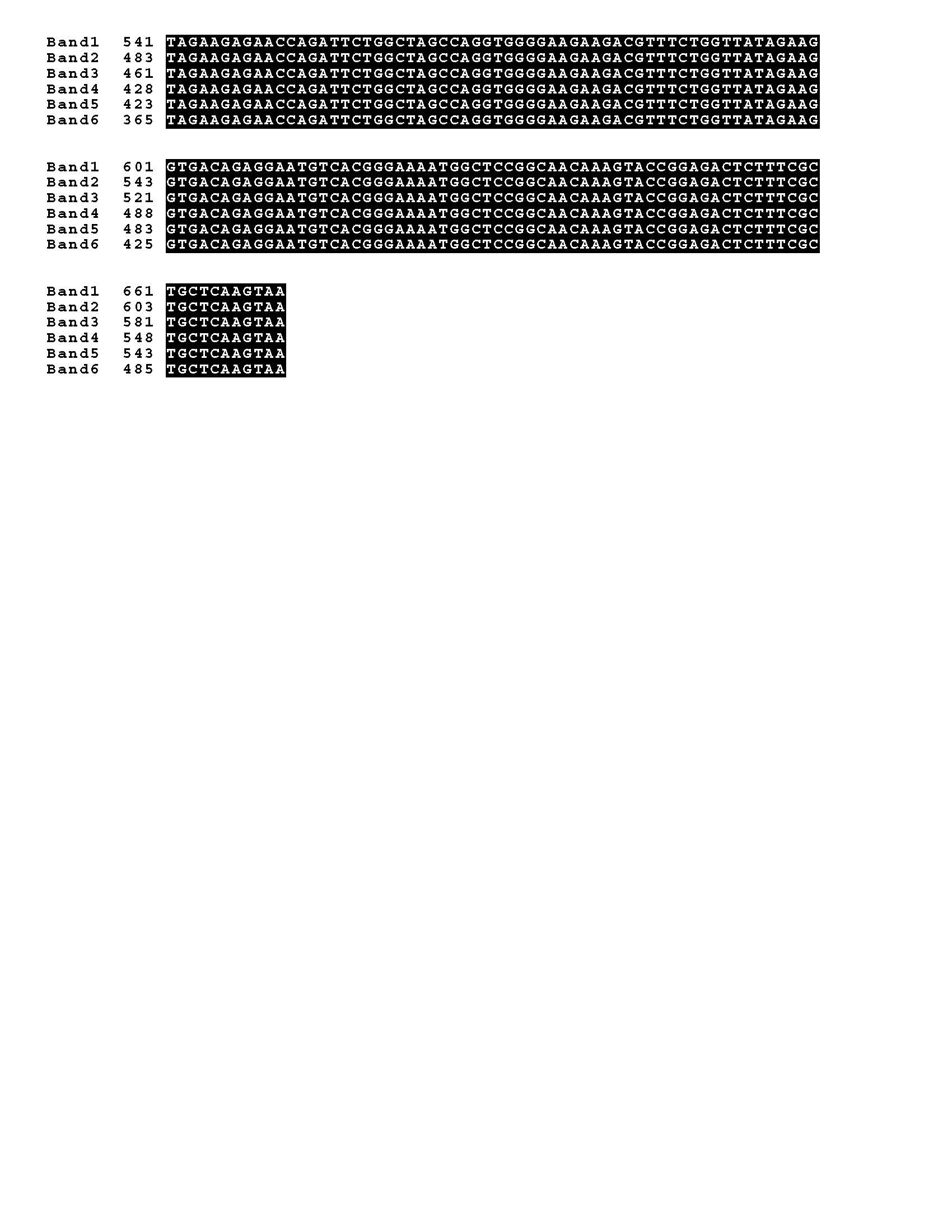


**S2 Fig. Sequence alignment of MAF3 isoforms detected by RT-PCR**

Sequences obtained by Sanger sequencing after RT-PCR on total RNA with *MAF3* primers at the start and stop codons.
