# Supplementary material for: Splicing-related genes are alternatively spliced upon changes in ambient temperatures in plants
Source: PLoS One. 2017 Mar 3;12(3):e0172950. doi: 10.1371/journal.pone.0172950 (PMC5336241; doi:10.1371/journal.pone.0172950)
Supplement: S3 Fig — (DOCX) [file pone.0172950.s010.docx]

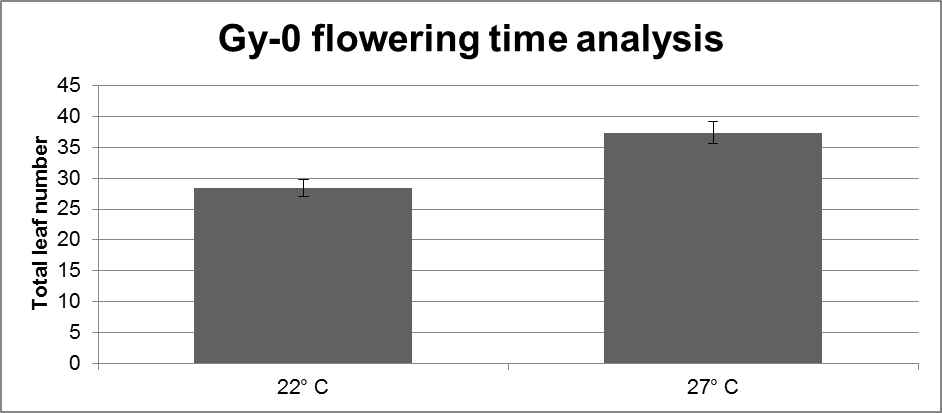


**S3 Fig.** Flowering time analysis of *A. thaliana* Gy-0. Plants were grown under 22°C and 27°C. Flowering time was determined by counting rosette leafs at the moment of flower induction. N=10, mean ±SD.
